# Classification of divorce causes during the COVID-19 pandemic using convolutional neural networks

**DOI:** 10.7717/peerj-cs.998

**Published:** 2022-06-30

**Authors:** Arif Bramantoro, Inge Virdyna

**Affiliations:** 1 School of Computing and Informatics, Universiti Teknologi Brunei, Bandar Seri Begawan, Brunei Darussalam; 2 Faculty of Information Technology Universitas Budi Luhur, Jakarta, Indonesia

**Keywords:** Convolutional neural network, COVID-19 pandemic, Classification, Divorce rate

## Abstract

The COVID-19 pandemic has affected day-to-day activities. Some families experienced a positive impact, such as an increase of bonding between family members. However, there are families that experienced a negative effect, such as the emergence of various conflicts that lead to a divorce. Based on the literature, it can be stated that the COVID-19 pandemic contributed to the increasing number of divorce rates. This paper proposes a convolutional neural network (CNN) classification algorithm in determining the dominant causes of the increase in divorce rate during the COVID-19 pandemic. CNN is considered suitable for classifying large amounts of data. The data used as research materials are available on the official website of the Indonesian Supreme Court. This research utilizes Supreme Court divorce decisions from March 2020 to July 2021, which constitutes 15,997 datasets. The proposed number of layers implemented during the classification is four. The results indicate that the classification using CNN is able to provide an accuracy value of 96% at the 100^th^ epoch. To provide a baseline comparison, the classical support vector machine (SVM) method was performed. The result confirms that CNN outweighs SVM. It is expected that the results will help any parties to provide a suitable anticipation based on the classified dominant causes of the divorce during the COVID-19 pandemic.

## Introduction

The COVID-19 pandemic remains a hot topic amongst multidisciplinary researchers. COVID-19 is one of the viruses that infects the respiratory system. The first case appeared in the city of Wuhan on December 30, 2019. The virus developed into the one that globally impacted in almost every aspect of modern human life. In domestic life, the COVID-19 pandemic has triggered a significant impact as well. Most governments have declared large scale social restriction followed by the enforcement of various other restrictions on community activities to stop the spread of the virus. This policy requires any members of the family to carry out their activities from home. Activities at home require that all family member interact more often. There are families who experienced a positive impact from this condition, but there are families who experienced a negative impact (Tristanto, 2020). This is because the family member gets to know each other more deeply and the incompatibility becomes more obvious, so that it can lead to a divorce (Apriasari & Al-jannah, 2021).

In the middle of 2020, there was a viral video on social media on both Facebook and Instagram, which showed a long queue of residents at the Soreang Religious Court, Bandung Regency, Indonesia. The queue was mainly made up of women who came to file divorce suits (Pribadi, 2021). Based on data from online services of West Java Religious High Court (Registrar of the Supreme Court, 2021), there was a total of 51,646 divorce cases that has been submitted and 17,397 processed between January and September 2020.

The divorce or dissolution of marriage is defined as a termination of the marital bond between a man and a woman (Fauziah, Fauzi & Ainayah, 2020). Previously, there has been a research on manually gathering the divorce data from the religious courts for specific areas, such as from Jambi religious court (Yanti, 2021). There is also a study that summarizes existing researches, so that the required data not only cover specific regional information, but also attempt to encompass national wide information (Bakhtiar, 2020). However, the data from previous works are not complete and real time enough to conduct the analysis, because they are manually obtained in each city or province. In this study, the authors propose to collect data from a dedicated website of the Supreme Court which contains a data bank of court decisions on various cases. It is expected that this data bank is the most valid and legal source of information regarding the divorce since it has the final and official decisions.

The Supreme Court decision directory site is an effort of Supreme Court to implement an open court. Openness is the key to the birth of accountability. The development of the electronic directory of decisions is one of the implementations of the Supreme Court head’s decree, with the aim of ensuring that complete information is rapidly available to the public. The head’s decree regulates the types of information that must be proactively announced by the court as well as its mechanism. The information is the one that is considered important by justice seekers and the public, such as court decisions (Registrar of the Supreme Court, 2021).

Recent advances in the field of machine learning and the explosion of data have affected in almost all aspect of modern life today, including the court decision. The text classification for complex court decision requires the latest machine learning techniques, hence, the use of convolutional neural network (CNN) method is considered suitable. The data collection is carried out for the retrieval of raw data that are ready to be processed (Can, Ezen-Can & Can, 2018). Hence, data preprocessing is required to process the raw data scrapped from the Supreme Court’s website. The results of the data preprocessing are datasets that are ready to be further analyzed by the classification method. The research contribution of this paper is mainly on which methods or techniques that are most capable of providing interesting information on the dominant causes of divorce during the COVID-19 pandemic. The suitable classification method is required to seamlessly perform advanced calculations on the available data.

The text classification carried out in this study is the convolutional neural network (CNN). This is because CNN is currently popular as the technique that has a high accuracy for any level of features, although this method is mostly applied in the field of image recognition (Shi, Jianping & Jie, 2018). CNN is considered one of the most representative neural network structures in deep learning. Consequently, CNN is also applicable in the field of natural language processing (NLP). To perform text processing tasks with CNN, the most important task that must be initially carried out is the digitization process of expressing the meaning of the text accurately. Hence, the biggest portion of this research is on this task. In addition to CNN, a traditional machine learning method is required to complement the work. The support vector machine (SVM) method is used to provide a fair comparison in accuracy values. The research discussion focuses on the method that is best able to provide an accurate information on the dominant causes of divorce during the COVID-19 pandemic.

This study aims to provide a comparative study for the performance of the two previously mentioned techniques to classify the causes of divorce cases that occurred during the COVID-19 pandemic. It seeks the factors that are the most dominant causes of divorce based on data scrapped from the decision directory site of the Supreme Court. This study uses data on divorce cases that were decided in March 2020 to September 2021. It is assumed that during this period of COVID-19, the divorce cases were significantly affected, hence, it requires dedicated research on finding their dominant factors although it is acknowledged that there might be other non-dominant factors. Other non-dominant factors are not covered to this research due to the insignificant result that it might occur at the end of the work.

## Theoretical background

Text mining is an artificial intelligence technique that processes unstructured text data in the form of documents or databases into more structured text data to extract useful information or interesting words that can represent the essence of the text data (Pandey, Garg & Rajput, 2019). Because natural language texts are generally inconsistent, the mining of unstructured content carried out by NLP techniques, statistical modeling, and machine learning is often challenging (Pandey, Garg & Rajput, 2019).

In its implementation in the real world, text mining usually uses large amounts of data and consists of several stages of data processing before it can eventually be understood by the machine. The first stage carried out in the text mining process is removing words that contain unimportant meaning. The purpose of this stage is to reduce the size of the dataset and the processing time in the data training stage (Rifano et al., 2020). In detail, there are several essential stages of text mining, including as follows:

1. Tokenizing: this stage is required for separating phrases, sentences, paragraphs, or entire text documents into smaller units called tokens. Tokens can be words, numbers, symbols, or punctuation marks.
2. Case Folding: this stage changes each token into uppercase or lowercase letters to generalize the characteristics of the tokens from in terms of capitalization.
3. Filtering: this stage is required for taking important words from text data that are produced from the tokenizing stage.
4. Punctuation Removal: this process removes punctuation marks in the text by replacing each predefined punctuation mark into an empty string (“”), for example: !”#$%&\’()*+,-./:;<=>?@[\\]^_‘{|}~.
5. Number Removal: this process is required to remove most numbers in the text by replacing each number into an empty string (“”).

Once the data preparation stages are completed, the next stage is text classification. Text classification is a machine learning technique that categorizes text data into a class based on the content of the text. Text classification can be used to process, organize, and categorize text types in the form of articles available on the web, documents, and other types of files (Sahami, 2009). The NLP application of text classification are sentiment analysis, data labeling, spam detection in the messages and the detection of hate speech (Hana et al., 2020).

Text classification in NLP has a big intersection in the area of machine learning. Machine learning is a field of computer science which has a rapid development on the study of pattern recognition and computational learning theory under umbrella of artificial intelligence. It teaches any computerized systems on how to make accurate predictions for inputted data through built-in algorithms. The algorithm operates by building a model from the data that has been entered to provide useful predictions or even informed decisions (Wolfewicz, 2021).

There are several branches of machine learning, one of which is deep learning, which is considered a sophisticated and complex algorithm. Deep learning uses a layered algorithmic structure or commonly known as an artificial neural network (ANN) which is inspired by the biological neural network of the human brain. Deep learning algorithms can be applied to different types of machine learning techniques based on the nature of the data or feedback inputted to the learning system.

Convolutional neural network (CNN) is a deep learning technique inspired by artificial neural network (ANN), which develops of multi-layer perceptron (MLP) with a special structure and fast network depth. Unlike other ANN variants CNN allows a feature extraction and studies feature representations of text and image data with various levels of abstraction (Ishaq, Asghar & Gillani, 2020). CNN consists of two components, namely:

1. Feature extraction, which consists of convolutional layer and pooling layer. The main purpose of convolution is to extract features from text input data. The convolution layers are arranged in a feature map to obtain special information from the input data. After the convolution layer operates, this pooling is carried out to reduce the dimensions or data size as well as the number of parameters of the text data, hence, it eventually the training time and reduces over fitting.
2. Fully connected layer, which is the last layer taking all the neurons in the previous layer and connects them to every neuron that it contains (Tseng, Chou & Tsai, 2018). Furthermore, a SoftMax layer is added to the end of the network to accommodate the classification.

SVM is one of the traditional classification algorithms that was first introduced in 1992 as a proportionate sequence of outstanding concepts in the field of pattern recognition. It is part of the supervised learning a type of learning that requires a training stage to introduce the model. The model is consecutively analyzed to lead to the testing stage. The concept of SVM is to find the best hyperplane that functions as a separator of two data classes (Hidayat, Garno & Ridha, 2021).

The Cross Industry Standard Process for Data Mining (CRISP-DM) methodology is described in terms of a sequential process. This process consists of a series of tasks described at four levels of abstraction, namely: the mining process, the common function stage, the concrete function, and the selection level (Lopez, 2021). In practice, multiple levels can be performed in different sequences. This often requires a numerous repetition for a particular action. Several procedural models and attempts to standardize the data mining process have been abandoned over the last few years. CRISP-DM is one very broad approach for standardizing various industrial processes related to data mining. Although widely accepted in industrial data mining as *de facto* standard, CRISP-DM and other process models are unable to cover the domains of specific complexities in acquiring and processing data and furthermore the knowledge involved in engineering contexts (Huber et al., 2019).

**Figure 1:**
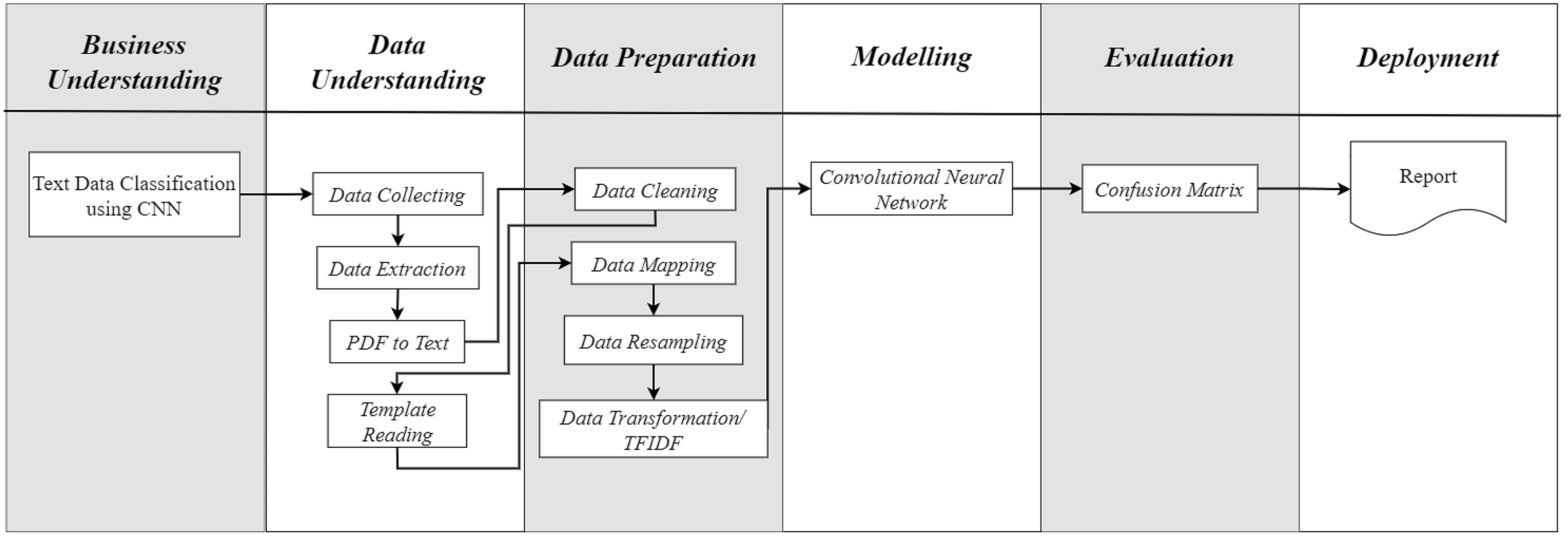
CRISP-DM with additional tasks.

Based on the required research steps in this study, the authors slightly modified the CRISP-DM method to accommodate the specific tasks as illustrated in Fig. 1. At the data understanding stage, there are several functions that must be carried out in the data preparation stage. This modification was proposed to adopt the CRISP-DM process flow to the research requirements, without complicating the research process.

In Indonesia, divorce or dissolution of marriage is the severance of the marital bond between a man and a woman. Other severances of the bond are caused by the death of one spouse, or that one spouse has left the residence for a specified period of time, so that the court assumes the person dead (Fauziah, Fauzi & Ainayah, 2020). The act of divorce or dissolution of marriage and its consequences, contains Article 38 that confirms that a marriage can be terminated due to death, divorce, and on the decision of the court. Furthermore, Article 39 paragraph 1 of this act affirms that divorce can only be carried out in when the court conducts a hearing and eventually fail to reconcile the two parties. One of the government’s efforts in suppressing the divorce rate is the issuance of regional act number 9 in 2014 concerning the family resilience and the formation of counselling as an extraordinary government commitment. However, it is found that a divorce cannot be intervened and resolved by regulation. Statistically, it is not fully effective in reducing the rate of increase in divorce (Pribadi, 2021). There are numerous impacts of divorce, not only experienced by the divorced couples, but also their children and the community in the area where the divorce occurs, because it involves emotional, economic, and social aspects. It also requires an official acknowledgement by the community (Yanti, 2021).

## Methods

**Figure 2:**
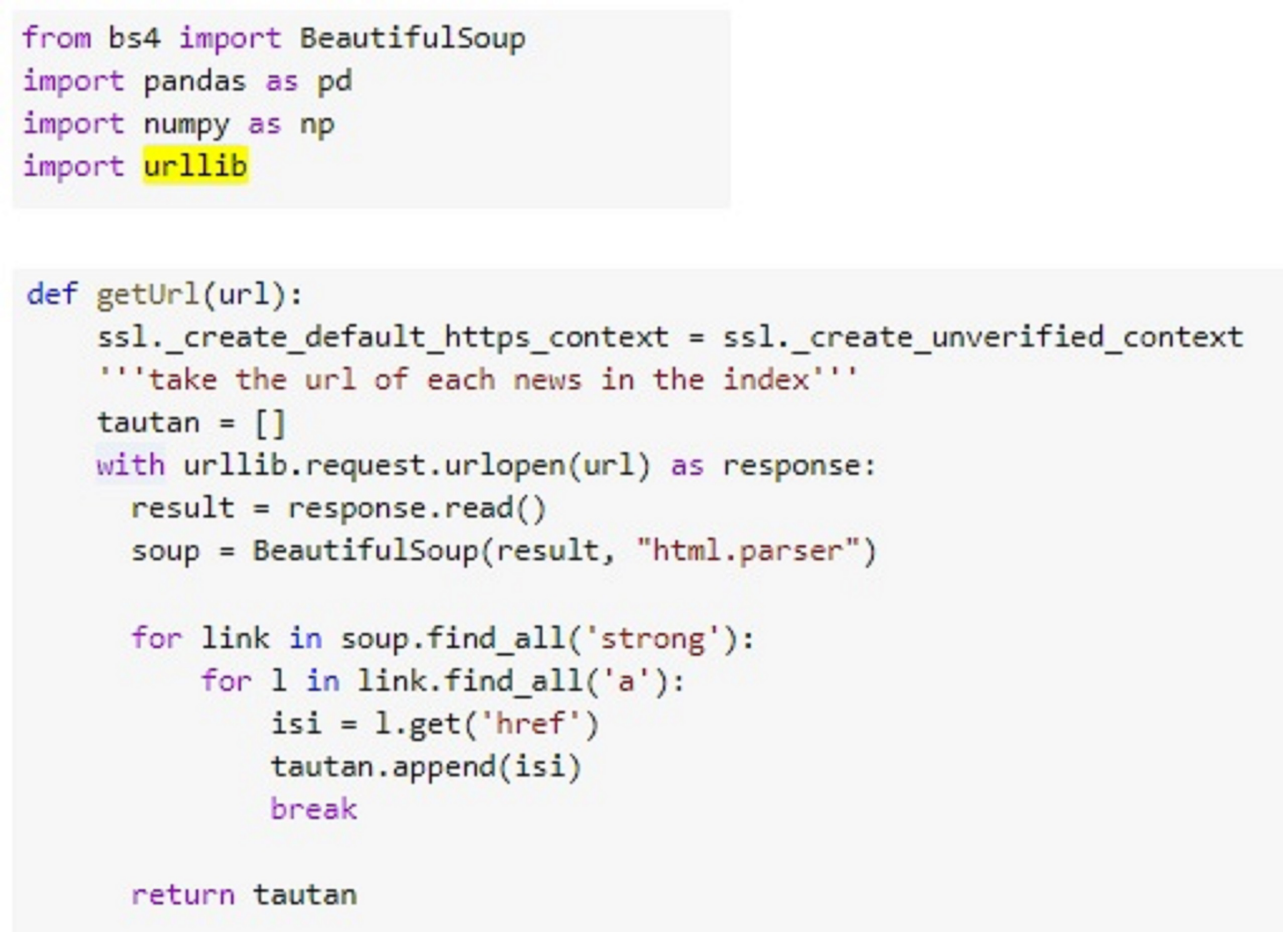
Data collection code.

A series of research steps are carried out using Scikit Learn library in Python. The development is inside Google Collab environment. To understand the data, the proposed system carries out three steps, namely data collection by utilizing the data crawling method, data conversion from PDF to text data, and document structure reading. Data collection is performed automatically within Python syntax, in contrast to the previous studies that relied on manual data collection. Figure 2 illustrates the syntax detail for retrieving data in Python. The system accesses the Supreme Court Decision Directory by providing the web page address to the URL variable. Then, the data are retrieved by adjusting the HTML tag structure and stored accordingly in the content variable.

The result of the data collection is shown in Table 1. It has five criteria for the divorce case decision from each religious court page from March 2020 to July 2021. The files downloaded from the website of Supreme Court decision directory are presented in PDF form. This produces the need for a file type conversion method before the calculation process is carried out. Moreover, the method of converting PDF files into data in the text form is nontrivial even before the data cleaning process is carried out. Initially, the system reads the document template. This task can be also carried out after the data preparation process has been completed. Then, the task is to read the data file from the beginning to the end.

**Table 1:** Data collection.

#	Class	Data number
1	Domestic violence	1.325
2	Conflict	3.580
3	Reconciliation	81
4	Economic problems	1.631
5	Negligence	104
Total		6.721

The system considers the contents of the document as a whole. It investigates the detail causes of the divorce decision. It searches for and mark the part of the document that has the title of the case for further data cleaning, and eventually, CNN classification is carried out. To ease the data preprocessing, any contents of documents that are not part of the case are removed. The data preparation consists of four stages, namely data cleaning, data mapping (including the data labeling method), data resampling, and the conversion of letters into numbers through Term Frequency-Inverse Document Frequency (TF-IDF).

The data cleaning process is processed seamlessly in Python function. This is in contrast to the previous related studies, that carried out data cleaning manually (Wijayanti, 2021). However, there are also previous study that obtained the data in a ready-to-process condition from the religious court where the research is conducted (Andari & Buulolo, 2020). The data cleaning process that is implemented in this study consists of five stages, namely tokenization, case folding, stop word removal, punctuation removal, and number removal. A more detailed discussion of each process is explained as follows.

1. Tokenization
The files that have been converted to text are further processed through the tokenization process. This process considers each sentence to be tokens, which are stored in array variables. The results of the tokenization process can be seen in Table 2.

**Table 2:** Tokenization stage.

#	Text
Tokenization result	[‘Halaman’, ‘1’, ‘P’, ‘E’, ‘N’, ‘E’, ‘T’, ‘A’, ‘P’, ‘A’, ‘NNomor’, ‘XXXX/Pdt.G/2020/PA.DmkDEMI’, ‘KEADILAN’, ‘BERDASARKAN’, ‘KETUHANAN’, ‘YANG’, ‘MAHA’, ‘ESA’, ‘Pengadilan’, ‘Agama’, ‘Demak’, ‘yang’, ‘memeriksa’, ‘dan’, ‘mengadili’, ‘perkara-perkara’, ‘tertentu’, ‘pada’, ‘tingkat’, ‘pertama’, ‘,’, ‘dalam’, ‘persidangan’, ‘Majelis’, ‘Hakim’, ‘telahmenjatuhkan’, ‘putusan’, ‘dalam’, ‘perkara’, ‘cerai’, ‘gugat’, ‘antara’, ‘:’, ‘Penggugat’, ‘,’, ‘umur’, ‘47’, ‘tahun’, ‘,’, ‘agama’, ‘Islam’, ‘,’, ‘Pekerjaan’, ‘Mengurus’, ‘RumahTangga’, ‘,’, ‘Pendidikan’, ‘Sekolah’, ‘Lanjutan’, ‘Tingkat’, ‘Pertama’, ‘,’, ‘tempat’, ‘kediaman’, ‘di’, ‘XXXXKabupaten’, ‘Demak’, ‘,’, ‘sekarangtinggal’, ‘dirumah’, ‘orang’, ‘tuanya’, ‘di’, ‘XXXXKabupaten’, ‘Demak’, ‘,’, ‘dalam’, ‘hal’, ‘ini’]

2. Case folding
The case folding process serves to generalize the typeface. In this study, the letters in the document are converted to lowercase letters. This is performed so that the system is not mistaken in recognizing a word that has the same meaning but uses a different typeface. The results of the case folding process can be seen in Table 3.

**Table 3:** Case folding stage.

#	Text
Case folding results	[‘halaman’, ‘1’, ‘p’, ‘e’, ‘n’, ‘e’, ‘t’, ‘a’, ‘p’, ‘a’, ‘nnomor’, ‘xxxx/pdt.g/2020/pa.dmkdemi’, ‘keadilan’, ‘berdasarkan’, ‘ketuhanan’, ‘yang’, ‘maha’, ‘esa’, ‘pengadilan’, ‘agama’, ‘demak’, ‘yang’, ‘memeriksa’, ‘dan’, ‘mengadili’, ‘perkara-perkara’, ‘tertentu’, ‘pada’, ‘tingkat’, ‘pertama’, ‘,’, ‘dalam’, ‘persidangan’, ‘majelis’, ‘hakim’, ‘telahmenjatuhkan’, ‘putusan’, ‘dalam’, ‘perkara’, ‘cerai’, ‘gugat’, ‘antara’, ‘:’, ‘penggugat’, ‘,’, ‘umur’, ‘47’, ‘tahun’, ‘,’, ‘agama’, ‘islam’, ‘,’, ‘pekerjaan’, ‘mengurus’, ‘rumahtangga’, ‘,’, ‘pendidikan’, ‘sekolah’, ‘lanjutan’, ‘tingkat’, ‘pertama’, ‘,’, ‘tempat’, ‘kediaman’, ‘di’, ‘xxxxkabupaten’, ‘demak’, ‘,’, ‘sekarangtinggal’, ‘dirumah’, ‘orang’, ‘tuanya’, ‘di’, ‘xxxxkabupaten’, ‘demak’, ‘,’, ‘dalam’, ‘hal’, ‘ini’]

3. Stop words removal
This function serves to eliminate words that are not required in the classification using the CNN method. Another advantage obtained from the implementation of the stop words removal function is that the classification becomes faster, because the calculation only involves the words that play an important role. The results of the stop words removal process are detailed in Table 4.

**Table 4:** Stop words removal stage.

#	Text
Stop words removal result	halaman 1 p e n e t a p a nnomor xxxx/pdt.g/2020/pa.dmkdemi keadilan berdasarkan ketuhanan maha esa pengadilan agama demak memeriksa mengadili perkara-perkara tingkat, persidangan majelis hakim telahmenjatuhkan putusan perkara cerai gugat : penggugat, umur 47, agama islam, pekerjaan mengurus rumahtangga, pendidikan sekolah lanjutan tingkat, kediaman xxxxkabupaten demak, sekarangtinggal dirumah orang tuanya xxxxkabupaten demak

4. Punctuation removal
The presence of punctuation is not required in the calculation process for this research. Therefore, a punctuation removal method is performed to remove the existing punctuation marks in a sentence. The results of the punctuation removal process can be seen in Table 5.

**Table 5:** Punctuation removal stage.

#	Text
Punctuation removal result	halaman 1 p e n e t a p a nnomor xxxxpdtg2020padmkdemi keadilan berdasarkan ketuhanan maha esa pengadilan agama demak memeriksa mengadili perkaraperkara tingkat persidangan majelis hakim telahmenjatuhkan putusan perkara cerai gugat penggugat umur 47 agama islam pekerjaan mengurus rumahtangga pendidikan sekolah lanjutan tingkat kediaman xxxxkabupaten demak sekarangtinggal dirumah orang tuanya xxxxkabupaten

5. Number removal
Based on preliminary analysis, the classification using the CNN method in this research do not require numerical data. To improve the system’s performance in conducting the classification process, the number removal method is performed to eliminate the numerical data in the input data. The results of the number removal process can be seen in Table 6.

**Table 6:** Number removal stage.

#	Text
Number removal result	halaman p e n e t a p a nnomor xxxxpdtgpadmkdemi keadilan berdasarkan ketuhanan maha esa pengadilan agama demak memeriksa mengadili perkaraperkara tingkat persidangan majelis hakim telahmenjatuhkan putusan perkara cerai gugat penggugat umur agama islam pekerjaan mengurus rumahtangga pendidikan sekolah lanjutan tingkat kediaman xxxxkabupaten demak sekarangtinggal dirumah orang tuanya xxxxkabupaten

**Figure 3:**
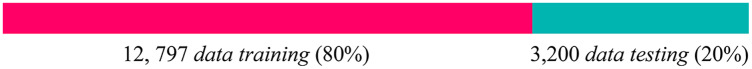
Weighted value of training data and testing data.

At the stage of data mapping, the available data are divided into two, namely as training data and testing data. The amount of data that has been collected from March 2020 to July 2021 is represented in 15,997 documents. This research follows the common practice in dividing the data that are not in a big size into 80% and 20% (Nguyen et al., 2021). Hence, the data splitting method from the above data resulted in 12,797 training data and 3,200 testing data. The data training aims to teach machine learning algorithm by changing existing parameters to match the data provided so that it can understand the information in the data. This type of machine learning technique is known as supervised learning. Consequently, the data testing aims to test the accuracy of the algorithm that has been trained. To grasp the idea of data mapping, the visualized proportion of each data can be seen in Fig. 3.

In the data mapping process, the system performs the data labeling as well. This process labels data that have gone through the data cleaning process. Based on the act number 1 of 1974 Article 39 paragraph (2) regarding the reasons that can be used as a basis for divorce, this research focuses on several keywords for calculating the classification model with CNN, which can be seen in Table 7.

**Table 7:** Data labeling keywords.

Class	Keyword
Domestic violence	[violence’, ‘hardness’, ‘persecution’, ‘beating’, ‘hit’, ‘rough’, ‘rude’, ‘wicked’]
Conflict	[‘difference’, ‘argue’, ‘quarrel’, ‘debate’, ‘sparring’, ‘controversy’ ‘communication’, ‘incompatibility’]
Reconciliation	[‘Negligence’, ‘pax’, ‘revoke’]
Economic problems	[‘economic’]
Negligence	Default Value

Based on the list of keywords above, the data labeling process searches for the input data from the results of the data cleaning process. The outcome of the data labeling process is a dataset that is ready to be included in the classification using the CNN method. An example of a dataset can be seen in Table 8.

**Table 8:** Dataset labels.

#	Document	Label
0	Data no-1.txt	Domestic violence
1	Data no-2.txt	Reconciliation
2	Data no-3.txt	Economic problems
3	Data no-4.txt	Negligence
4	Data no-5.txt	Conflict
5	Data no-6.txt	Conflict
6	Data no-7.txt	Conflict
7	Data no-8.txt	Conflict
8	Data no-9.txt	Economic problems
9	Data no-10.txt	Conflict
10	Data no-11.txt	Conflict
11	Data no-12.txt	Conflict
12	Data no-13.txt	Conflict
13	Data no-14.txt	Conflict
14	Data no-15.txt	Conflict

The results of the data labeling significantly show an imbalanced data. To overcome this problem, it requires a method of unbalanced datasets, which is called data resampling. The majority of machine learning algorithms assume that the data are evenly distributed. Therefore, when the data are imbalanced, the machine learning classifier tends to be more biased towards the majority class. This leads to an inappropriate minority class classification (Volpi, 2019). Therefore, resampling data is necessary in this research.

**Figure 4:**
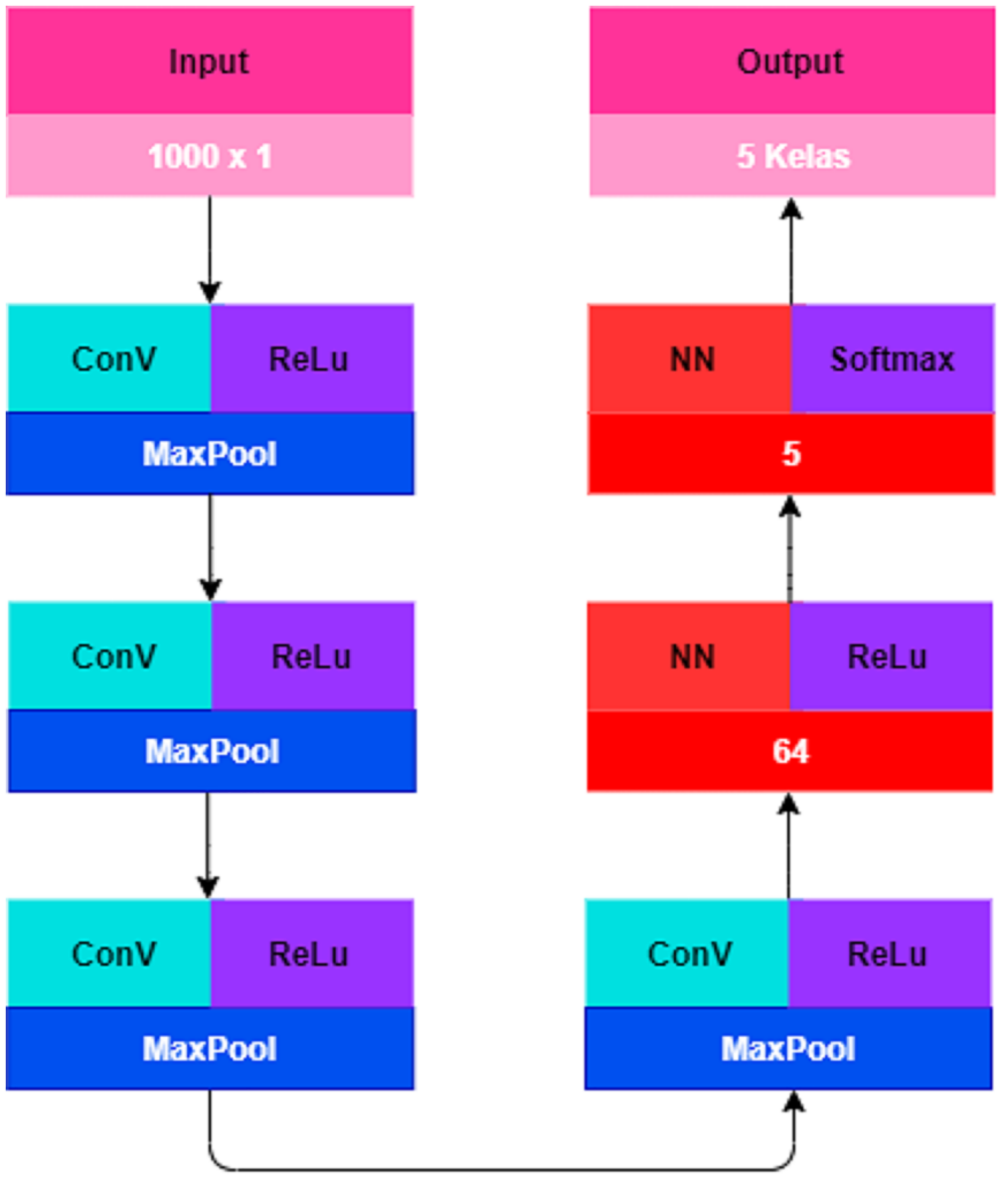
CNN architectural design.

The next process that must be carried out before starting the classification with the CNN method is data transformation. In data transformation, the dataset that is already produced must go through the TF-IDF process. This process is performed to convert text data into numeric data (Rahman, Wiranto & Doewes, 2017). The weighting for each term is seen from the number of occurrences of a term in each document. The results of the conversion of text data into numeric data by utilizing the TF-IDF technique can be seen in Table 9. The initial stage in a classification system using CNN starts from the CNN architectural design. The design of the CNN architecture in this study is shown in Fig. 4.

**Table 9:** TF-IDF result.

Data no-1.txt	Data no-2.txt	Data no-3.txt	Data no-4.txt	Data no-5.txt	Data no-6.txt	Data no-7.txt	Data no-8.txt	Data no-9.txt	Data no-10.txt
8.167.416.401.841.850	10.357.273.644.970.000	17.885.682.339.724.000	6.555.580.280.661.140	678.235.033.020.678	10.528.649.266.733.400	9.017.429.500.828.210	78.013.183.781.787.200	8.976.284.567.380.770	5.916.745.454.409.350
8.167.416.401.841.850	6.214.364.186.982.000	8.942.841.169.862.030	5.900.022.252.595.020	1.356.470.066.041.350	10.528.649.266.733.400	2.705.228.850.248.460	78.013.183.781.787.200	6.283.399.197.166.540	5.916.745.454.409.350
8.167.416.401.841.850	3.728.618.512.189.200	5.365.704.701.917.220	3.540.013.351.557.010	6.104.115.297.186.100	10.528.649.266.733.400	32.462.746.202.981.500	42.907.251.079.983.000	40.393.280.553.213.500	5.916.745.454.409.350
4.900.449.841.105.110	20.714.547.289.940.000	8.942.841.169.862.030	1.966.674.084.198.340	3.662.469.178.311.660	7.370.054.486.713.410	1.803.485.900.165.640	3.861.652.597.198.470	26.928.853.702.142.300	5.916.745.454.409.350
8.167.416.401.841.850	20.714.547.289.940.000	4.024.278.526.437.910	19.666.740.841.983.400	2.712.940.132.082.710	379.031.373.602.404	1.803.485.900.165.640	21.453.625.539.991.500	22.440.711.418.451.900	5.916.745.454.409.350
36.753.373.808.288.300	10.357.273.644.970.000	2.235.710.292.465.500	19.666.740.841.983.400	20.347.050.990.620.300	10.528.649.266.733.400	36.069.718.003.312.800	21.453.625.539.991.500	22.440.711.418.451.900	5.916.745.454.409.350
20.418.541.004.604.600	10.357.273.644.970.000	2.235.710.292.465.500	6.555.580.280.661.140	20.347.050.990.620.300	21.057.298.533.466.800	36.069.718.003.312.800	39.006.591.890.893.600	17.952.569.134.761.500	2.366.698.181.763.740
20.418.541.004.604.600	10.357.273.644.970.000	17.885.682.339.724.000	13.111.160.561.322.200	678.235.033.020.678	21.057.298.533.466.800	36.069.718.003.312.800	78.013.183.781.787.200	17.952.569.134.761.500	5.916.745.454.409.350
16.334.832.803.683.700	10.357.273.644.970.000	44.714.205.849.310.100	3.933.348.168.396.680	1.356.470.066.041.350	10.528.649.266.733.400	36.069.718.003.312.800	11.701.977.567.268.000	448.814.228.369.039	37.275.496.362.778.900
16.334.832.803.683.700	41.429.094.579.880.000	44.714.205.849.310.100	3.933.348.168.396.680	40.694.101.981.240.600	2.105.729.853.346.680	9.017.429.500.828.210	42.907.251.079.983.000	448.814.228.369.039	20.708.609.090.432.700
4.083.708.200.920.920	41.429.094.579.880.000	44.714.205.849.310.100	3.933.348.168.396.680	40.694.101.981.240.600	4.211.459.706.693.370	15.329.630.151.407.900	42.907.251.079.983.000	448.814.228.369.039	20.708.609.090.432.700
4.083.708.200.920.920	41.429.094.579.880.000	8.942.841.169.862.030	6.555.580.280.661.140	40.694.101.981.240.600	4.211.459.706.693.370	36.069.718.003.312.800	42.907.251.079.983.000	35.905.138.269.523.100	5.916.745.454.409.350
4.083.708.200.920.920	10.357.273.644.970.000	8.942.841.169.862.030	6.555.580.280.661.140	678.235.033.020.678	4.211.459.706.693.370	9.017.429.500.828.210	39.006.591.890.893.600	35.905.138.269.523.100	5.916.745.454.409.350
24.502.249.205.525.500	10.357.273.644.970.000	2.682.852.350.958.610	6.555.580.280.661.140	678.235.033.020.678	10.528.649.266.733.400	18.034.859.001.656.400	15.602.636.756.357.400	8.976.284.567.380.770	5.916.745.454.409.350
16.334.832.803.683.700	10.357.273.644.970.000	8.942.841.169.862.030	6.555.580.280.661.140	1.356.470.066.041.350	10.528.649.266.733.400	2.705.228.850.248.460	39.006.591.890.893.600	8.976.284.567.380.770	5.916.745.454.409.350

CNN was originally designed to handle two-dimensional data (Sharma, Chaurasia & Srivastava, 2020). Since text data are one-dimensional data, it is necessary to adjust the structure of the CNN model to implement text data using the CNN method. The CNN model requires an optimization to perform text classification. The CNN model in this study consists of an input layer, a convolution layer, a pooling layer, a fully connected layer, and an output layer. Table 10 details the CNN architectural composition optimized for this study.

**Table 10:** CNN architectural composition.

Layer		Size and feature	Kernel size	Stride	Activation
Input	Teks	1,000 × 1	–	–	–
1	Convolution	1,000 × 32	3 × 3	1	Relu
	Max pooling	500 × 32	2 × 2	2	Relu
2	Convolution	498 × 64	3 × 3	1	Relu
	Max pooling	248 × 64	2 × 2	2	Relu
3	Convolution	246 × 32	3 × 3	1	Relu
	Max pooling	122 × 64	2 × 2	2	Relu
4	Convolution	120 × 64	3 × 3	1	Relu
	Max pooling	59 × 64	2 × 2	2	Relu
5	Flatten	59 × 64	–	–	–
6	Fully connected	3,776	–	–	–
Output	Dense	5	–	–	Softmax

The input layer is textual data input that has run through the data understanding and data preparation. The data are stored in an array in the form of a one-dimensional data frame with a total of 6,721 data and data length 1,000 × 1 for each text. After the CNN architecture is properly designed, the next step is the learning process which is repeated for 100 epochs with a sample batch size of 128. At this stage the optimizer used is Adam (Adaptive Moment Estimation) which is the most popular optimizer algorithm in updating weights, minimizing the loss function, and calculating the individual adaptive learning speed for each parameter.

**Figure 5:**
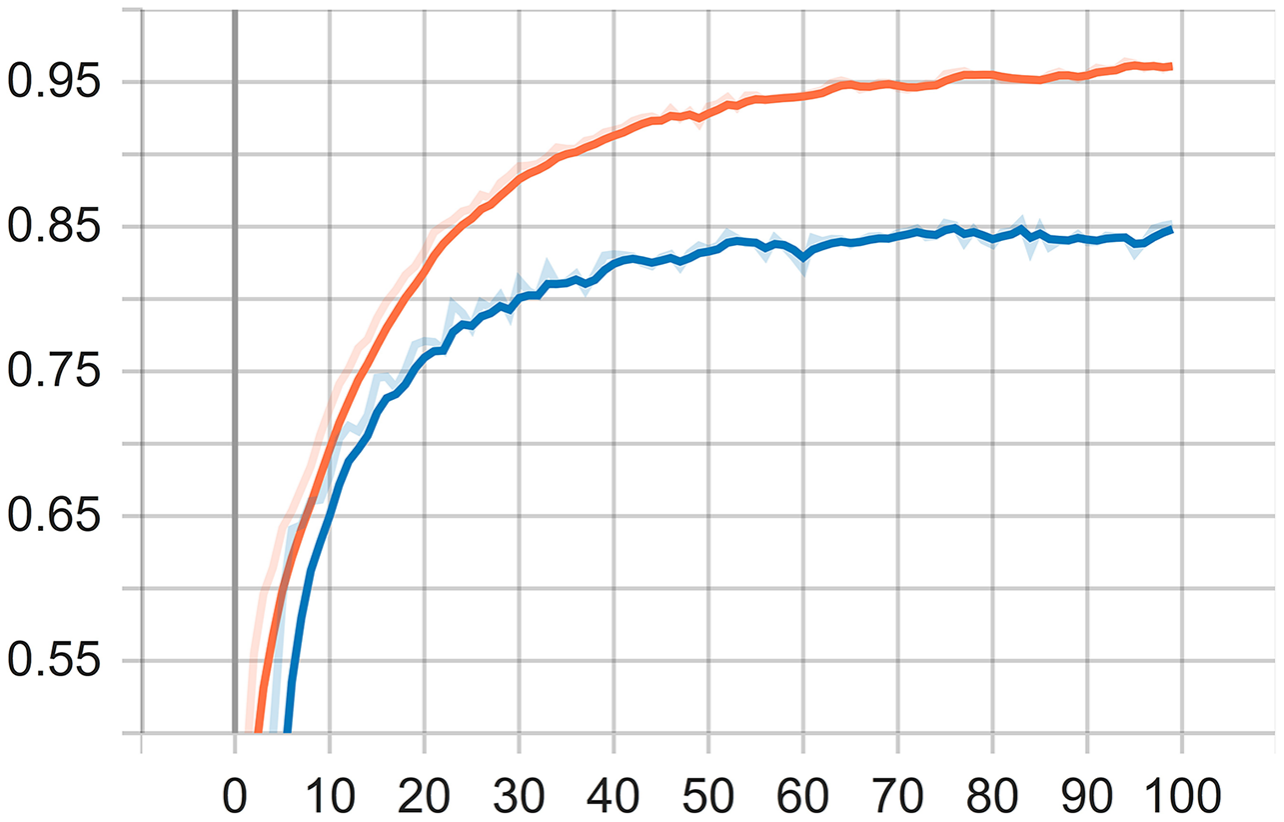
Accuracy graph visualization.

The learning process is carried out after the desired optimum value is achieved. It means that the CNN model meets the target. The reference variable for the desired target in this learning process is 0.97 for the accuracy and 0.7 for the loss value. After the reference variable is satisfied at epochs 100, the weight of the model is saved with the .h5 file extension. Loss value is a value returned by a function that estimates how close the distribution of predictions made by a model is based on the distribution of target variables in the training data. The loss function used in this study is sparse categorical cross entropy. The function is considered suitable for data that has more than two classes or labels in the form of integers like in this research. The testing is the last process of the CNN implementation. The weight of the CNN model that has been built on the previous learning process is then tested to determine the accuracy performance and classification results of the dominant causes of an increase in divorce during the COVID-19 pandemic from text data. The process of learning models on text data can be seen through a TensorFlow visualization graph is called a tensorboard. An accuracy graph visualization in tensorboard is shown in Fig. 5.

## Results and Discussion

During the visualization of testing, it can be inferred that the accuracy of the training data reaches 96% and the accuracy of the data testing reaches 85%. It is important to remind that the text data used in the training are 12,797 and data testing 3,200. Each data consists of five classes, *i.e*., Economic Problems, Domestic Violence, Conflict, Negligence, and Reconciliation. The output of the training is the model used for the testing, while the output of the testing is the classification of the dominant causes of divorce from the PDF file.

**Figure 6:**
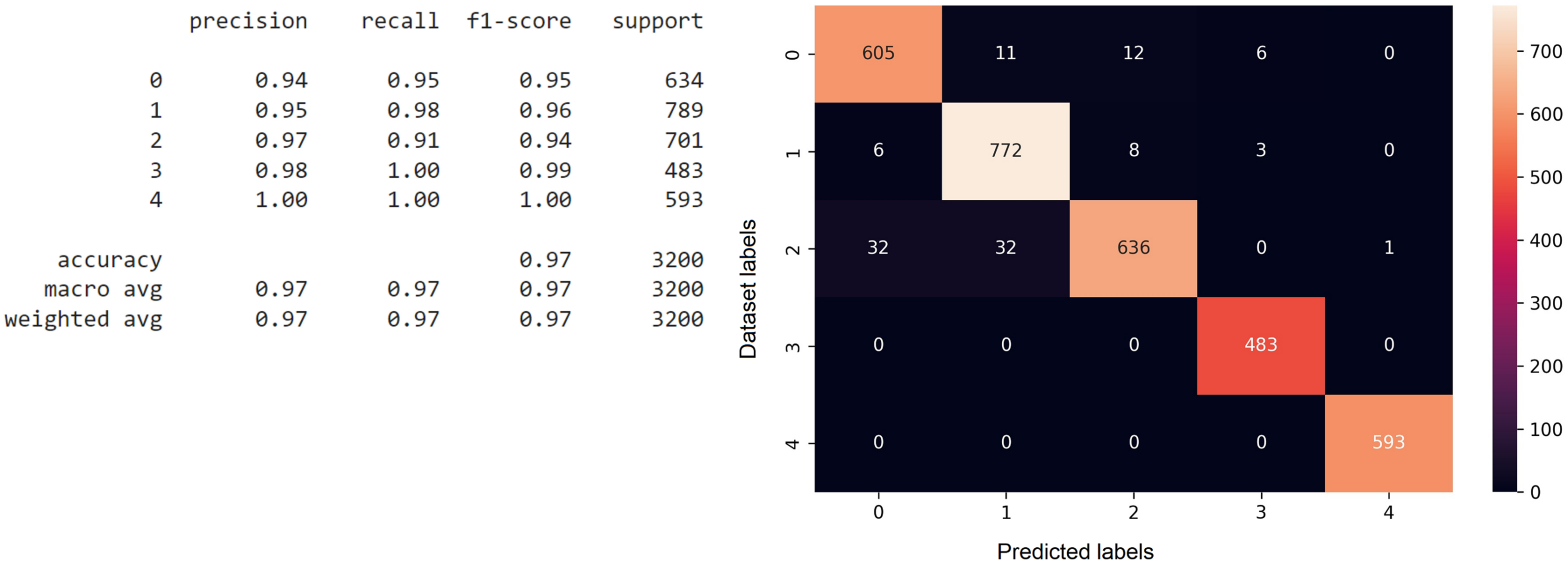
The implementation of the confusion matrix on the CNN model.

The confusion matrix function is executed after predicting the testing data on the model that has been previously trained, and the results are stored in one variable (Volpi, 2019). The confusion matrix function is called with the first parameter, which is multi-label test data, and the second parameter, which is a variable that stores the predicted results of the testing data against the previously trained model. The analysis results in the form of the confusion matrix are shown in Fig. 6. From the confusion matrix for the CNN model, it can be inferred that the accuracy reaches 96%, based on the precision, recall, and F1 scores for each class category index. The confusion matrix also explains the actual and predicted values of the testing data. The 0^th^ index is for the testing data for the Economic Problems class, the 1^st^ index is for the Domestic Violence class, the 2^nd^ index is for the Conflict class, the 3^rd^ index is for the Reconciliation class, and the 4^th^ index is for the Negligence class.

**Figure 7:**
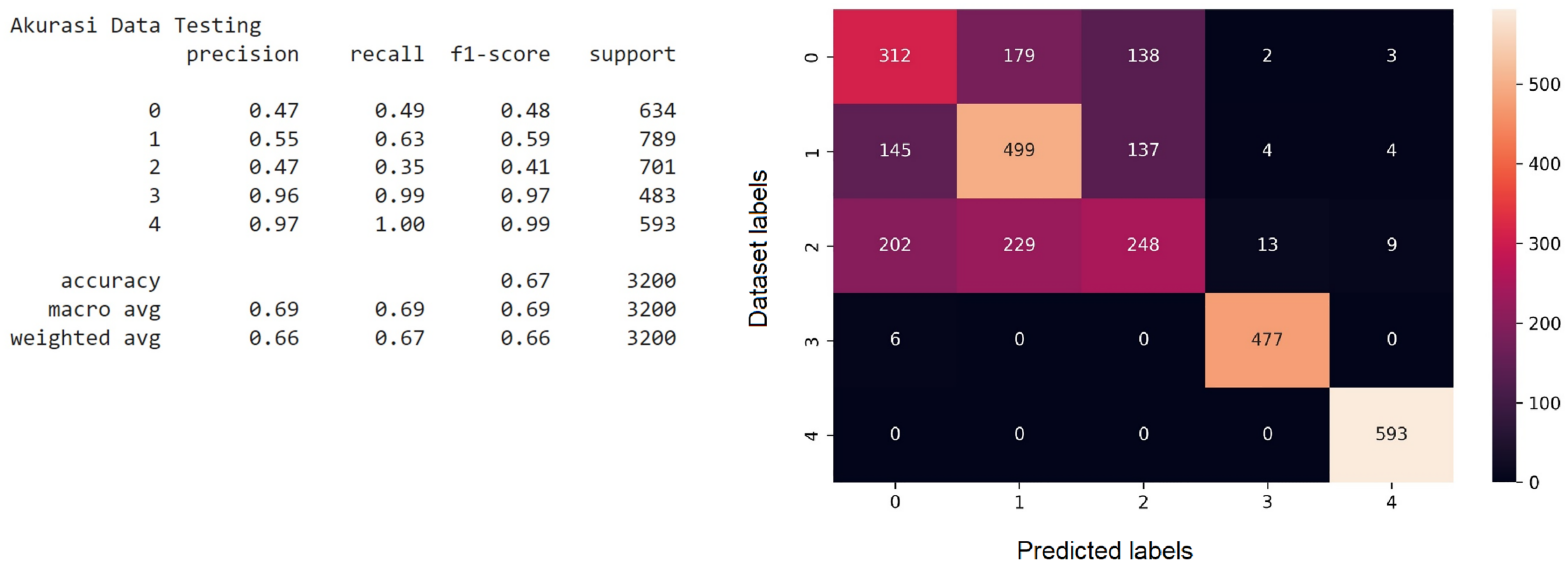
The implementation of the confusion matrix on the SVM model.

The objective of comparing CNN with the classical text classification method is to confirm that the CNN method has a better accuracy. Both CNN and SVM methods process the datasets with Python programming language. Similar to the CNN classification model, to check the accuracy of the model that has been achieved, a confusion matrix is used to describe the performance of the model against the test data. Figure 7 illustrates the confusion matrix of the SVM model.

It can be seen from the confusion matrix for the SVM model that the accuracy value reaches 67%. Overall, it shows that the calculation is implemented automatically as a whole to provide accurate results. However, it is interesting to note that the result is similar to the researches that are implemented manually in term of data collection process (Wijayanti, 2021), and data cleaning (Andari & Buulolo, 2020). Processing data with Python in an automated manner facilitates and speeds up the calculation process. Hence, it shows that the accuracy level of the CNN method is better than SVM in classifying divorce data from the Supreme Court directory site within the time span of the COVID-19 pandemic.

During the deployment, the results are presented as application with a user interface to ease the interaction with end users. The implementation of the frontend subsystem is based on html, CSS and JavaScript technology to present the classification results visualization. The python-based implementation acts as a backend system that performs the overall classification process according to the process previously described. The flask library available in python is utilized to integrate or the front end and the back end subsystems.

The user interface of the system contains three options, namely, classification by PDF file, classification by web page number, and classification by date. The division of classification is considered important to end user who needs more flexibility in classifying the dominant causes. In the option of classification by PDF file, users can upload files of the divorce court decisions as input for the classification. Users are allowed to upload files with a maximum number of 10 PDF files due to the scalability problem of the interface system. However, there is no limitation in the processing without user interface. This option is also capable of handling file types in the form of .zip and .rar. If the user wants to classify the uploaded data, they can press the Process Classification button, that leads the system to process the input data and provide the results of the classification process.

**Figure 8:**
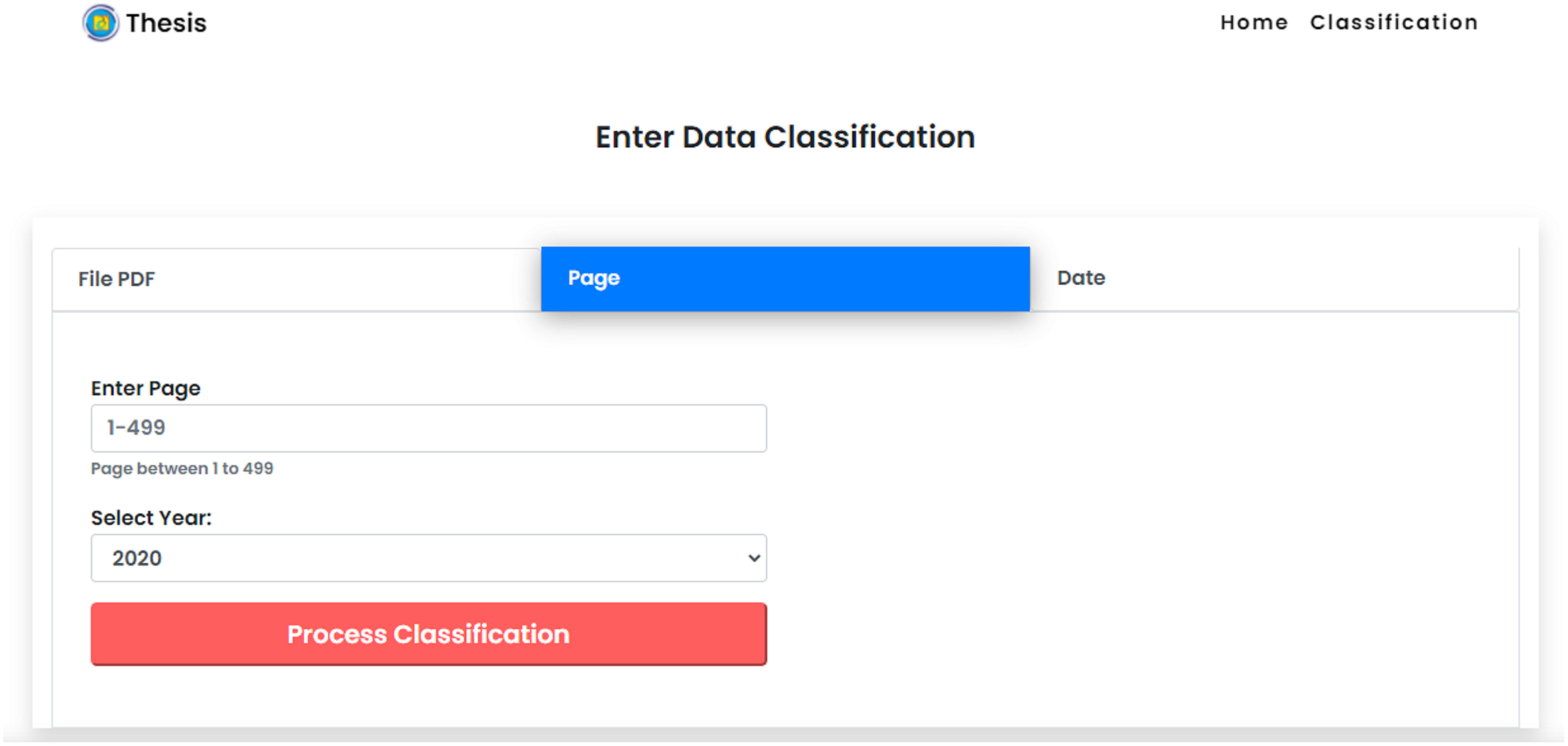
Classification by web page.

The Supreme Court’s decision directory website consists of a collection of data on court decisions presented on a page-by-page basis. This allows users to choose page values as input data based on their personal requirement for the dominant cause of divorce using the CNN method in the classification system. In the option of classification by page number, users can provide an input in the form of web page number values that are required by the classification. Users are allowed to enter single or multiple values. Figure. 8 shows the display of manual data input for calculating data based on web pages. Each dataset on the website of the Supreme Court’s decision directory consists of a collection of data based on the results of the court which explained in detail the time of the decision made. This allows users to classify the dominant cause of divorce using the CNN method in the classification system based on the value of the date of decision. Users are allowed to enter single or multiple values.

**Figure 9:**
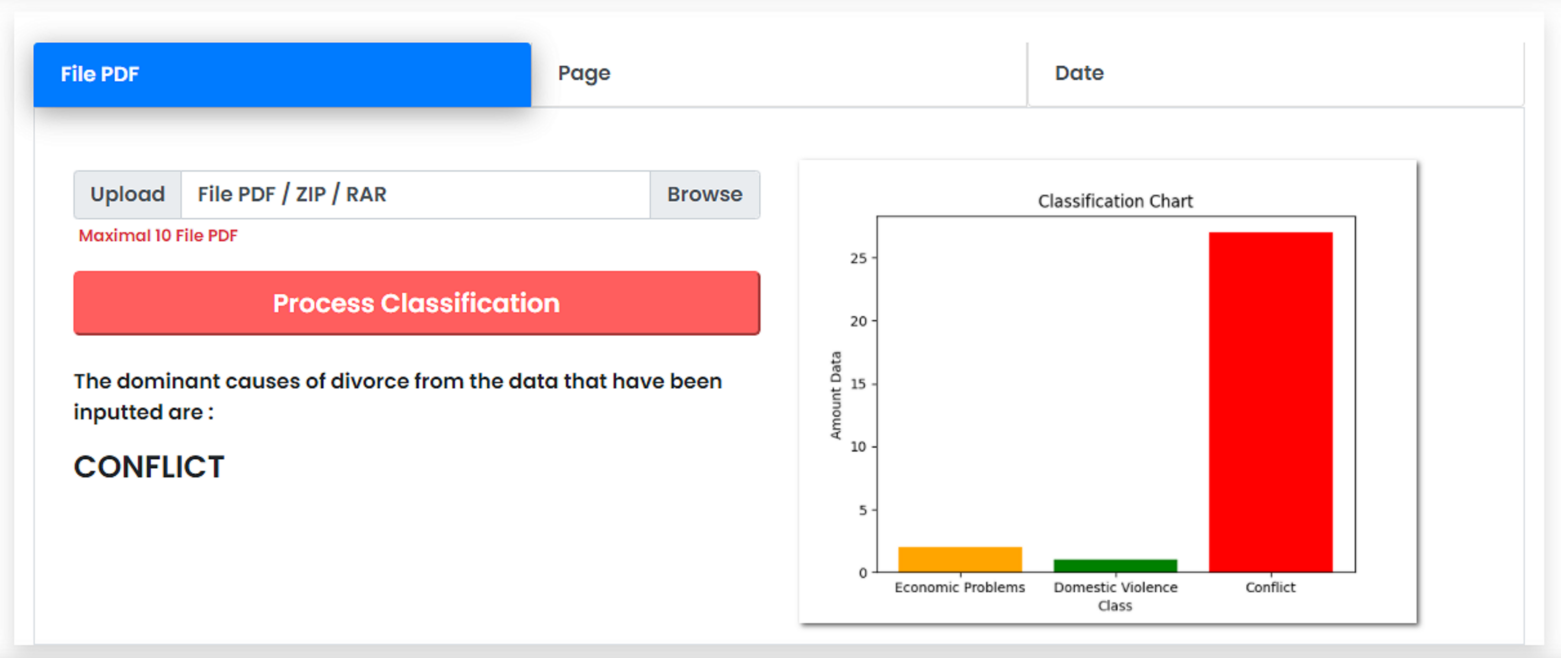
Classification results interface.

The classification results are presented in the form of text and graphics. The system displays the results in the form of text to provide the dominant causes of divorce within the period of data that is previously entered. The system displays the calculation results in graphical form to provide the percentage of each cause of divorce from the implemented classification. Figure 9 presents the display of the classification results using the CNN method. The dynamic display of classification is non-trivial especially for a layman user who are not familiar with the classification techniques and back-end processing.

### Related work

Based on the literature review conducted in this research, there is no previous research that uses the CNN classification method that processes data in pdf form as input data to be classified. As identified in the review paper (Widiastuti, 2019) that is most related to this research, further research is required to focus on new cases, such as themes taken and data with different formats. Hence, to have a classification system with an automated input with the dynamically published pdf is inevitable.

For the research dataset, there is no previous studies discussing divorce data with the web pages as the basis for collecting research data. The dataset is obtained from both social media, such as Twitter and Facebook, and the official web pages belonging to specific court bodies. To the best of the authors’ knowledge, all research in the literature review analyze divorce during the COVID-19 pandemic using manual methods to obtain research dataset. This is achieved by directly observing the religious court on place for data collection or at most using the focus group discussion method. For example, the data collection was conducted manually at the Jambi City Religious Court (Yanti, 2021).

Concurrent research on the cause of divorce during the COVID-19 pandemic was conducted in the Banyumas area (Wijayanti, 2021). The cross-sectional study with simple random sampling method in the study, showed in general that the divorce plaintiffs were from the wife who had the characteristics of young age, low education, not working, marriage age less than 5 years, and having only one child. The reason for filing a divorce is mainly due to the economic factors. Therefore, it is expected that any related institutions in Banyumas perform an intensive socialization regarding the preparation of family life for future married couples in terms of economy, family functions and maturation of marriage age (Wijayanti, 2021). The main advantage obtained by data crawling is that the authors are able to analyze the trend not only for certain regions, but also for throughout Indonesia.

Most current research uses input data in the form of ready-to-use datasets such as the research conducted in (Polignano et al., 2019). The datasets are taken from ISEAR and SemEval datasets which are ready-to-use for conducting research. The study was conducted using the BI-LSTM and self-attention CNN methods and the results of the study show that the effectiveness of the approach on three different current data sets. In addition, it is possible to measure the influence that the choice of textual input encoding technique through word insertion affects the result of the whole system. It has been shown that for this architecture, the FastText vector space allows obtaining the best results for emotion identification.

Previous studies carried out data labeling before the data cleaning process was also conducted for short text classification research using the CNN technique (Haitao et al., 2020). The proposed *N* gram based ACNN model uses word vectors as input models trained by the word2vec tool, skipping, and interval methods to get more effective feature expressions in the sliding window. The focus was on key property features by combining two different pooling operations with concentration mechanisms. The experiment was proposed on different models with different data sets, and the experimental results showed that the model was suitable and feasible for the short text classification task. It also argued that the classification effect increased rapidly within a certain limit. Our research using a different approach by implementing the labeling process once the data cleaning process is completed.

## Conclusions

This research applies the preprocessing testing stage, classification modeling, and evaluation on divorce court decisions to obtain the dominant causes of divorce during the COVID-19 pandemic using the convolutional neural network (CNN). The CNN method in the data mining classification is able to classify the divorce decision data during the COVID-19 pandemic period quite well. The data training uses 12,797 data that delivers the accuracy with 96%, and the data testing process uses 3,200 data that reaches 85% accuracy. Based on data processing and CNN classification that have been intensively carried out, it was found that the main cause of an increase in divorce during the COVID-19 pandemic, in the period March 2020 to July 2021, was unresolved conflict. The result of the system is implemented as an application that receives input from the user with three options, namely classification by pdf file, by webpage number, and by date. The results of the classification are presented in text form to display the dominant causes of divorce based on the time that has been determined in the input data. The results of the classification are also presented in graphical form to obtain an insight based on the percentage of each category causing divorce. The results showed that the CNN classification technique is capable of classifying text data with multiple data scales. Moreover, the CNN classification is compared to the classical classification method, *i.e.*, SVM, to show its superiority in handling the divorce dataset. In general, the classification result will be useful for any parties to investigate the dominant causes of divorce during the COVID-19 pandemic in all provinces in Indonesia, and eventually, to anticipate through suitable actions.

## Supplemental Information

10.7717/peerj-cs.998/supp-1 Supplemental Information 1 Source codes of Python programming language to analyze the datasets.

10.7717/peerj-cs.998/supp-2 Supplemental Information 2 Negligence factor decision files.

10.7717/peerj-cs.998/supp-3 Supplemental Information 3 Violence factor decision files.

10.7717/peerj-cs.998/supp-4 Supplemental Information 4 Conflict factor decision files.

10.7717/peerj-cs.998/supp-5 Supplemental Information 5 Economy factor decision files.

10.7717/peerj-cs.998/supp-6 Supplemental Information 6 Reconciliation.

## Additional Information and Declarations

### Data Availability

The following information was supplied regarding data availability: The source codes and the datasets are available in the Supplemental Files.
