# Supplementary material for: Classification of divorce causes during the COVID-19 pandemic using convolutional neural networks
Source: PeerJ Comput Sci. 2022 Jun 30;8:e998. doi: 10.7717/peerj-cs.998 (PMC9299239; doi:10.7717/peerj-cs.998)
Supplement: Supplemental Information 2 [file peerj-cs-08-998-s002.zip › Kelalaian Dataset/Data ke-1.pdf]

P E N E T A P A N

Nomor XXXX/Pdt.G/2020/PA.Dmk

بسم الله الرحمن الرحيم

DEMI KEADILAN BERDASARKAN KETUHANAN YANG MAHA ESA

Pengadilan Agama Demak yang memeriksa dan mengadili perkara-perkara tertentu pada tingkat pertama, dalam persidangan Majelis Hakim telah menjatuhkan putusan dalam perkara cerai talak antara :

Pemohon, umur 31 tahun, agama Islam, Pekerjaan Tukang Jahit, Pendidikan Sekolah Lanjutan Tingkat Pertama, tempat kediaman di XXXXKabupaten Demak, dalam hal ini memberikan kuasa kepada Mustain, SAg, SH, MH, Advokat yang berkantor di Advokat/Pengacara pada kantor Lembaga Konsultasi dan Bantuan Hukum (LKBH) Sultan Fatah Demak di Jl. Sultan Trenggono Km. 22, Semarang Demak Jawa Tengah Telp: (0291) 682495 berdasarkan surat kuasa khusus tanggal 05 Agustus 2020, sebagai **Pemohon;**

Melawan

Termohon, umur 23 tahun, agama Islam, Pekerjaan Mengurus Rumah Tangga, Pendidikan Sekolah Lanjutan Tingkat Pertama, tempat kediaman di dulu di XXXXKabupaten Demak, sekaang tidak diketahui alamat dan keberadaannya di Seluruh Wilayah Negara Republik Indonesia., sebagai Termohon;

Pengadilan Agama tersebut;
Telah membaca surat-surat perkara;
Telah mendengar keterangan Pemohon;

DUDUK PERKARA

Bahwa Pemohon dalam surat permohonan tertanggal 13 Juli 2020, terdaftar di Kepaniteraan Demak, Nomor XXXX/Pdt.G/2020/PA.Dmk. Tanggal 05 Agustus 2020, telah mengemukakan selengkapnya sebagaimana termuat dalam permohonan Pemohon.

Bahwa pada hari sidang yang telah ditentukan Pemohon yang diwakilkan oleh Kuasa Hukumnya hadir dalam sidang, sedangkan Termohon tidak hadir

Disclaimer
Kepaniteraan Mahkamah Agung Republik Indonesia berusaha untuk selalu mencantumkan informasi paling kini dan akurat sebagai bentuk komitmen Mahkamah Agung untuk pelayanan publik, transparansi dan akuntabilitas pelaksanaan fungsi peradilan. Namun dalam hal-hal tertentu masih dimungkinkan terjadi permasalahan teknis terkait dengan akurasi dan keterkinian informasi yang kami sajikan, hal mana akan terus kami perbaiki dari waktu kewaktu.
Dalam hal Anda menemukan inakurasi informasi yang termuat pada situs ini atau informasi yang seharusnya ada, namun belum tersedia, maka harap segera hubungi Kepaniteraan Mahkamah Agung RI melalui :
Email : kepaniteraan@mahkamahagung.go.id   Telp : 021-384 3348 (ext.318)

dan tidak pula mengutus wakil atau kuasa hukum meskipun telah dipanggil secara resmi dan patut dengan relaas untuk hadir di persidangan.

Bahwa Majelis Hakim telah memberi nasihat kepada Pemohon agar rukun kembali dengan Termohon dalam membina rumah tangga.

Bahwa Pemohon telah menyatakan maksud mencabut permohonan Pemohon dan berkesimpulan memohon agar Majelis Hakim mengabulkan permohonan pencabutan Pemohon karena akan mencoba rukun kembali dengan Pemohon dan memberi kesempatan Termohon untuk memperbaiki diri.

Bahwa tentang jalannya pemeriksaan selengkapnya telah dicatat dalam Berita Acara Persidangan perkara ini.

PERTIMBANGAN HUKUM

Menimbang, bahwa maksud dan tujuan permohonan Pemohon adalah sebagaimana telah diuraikan di atas;

Menimbang, bahwa Pemohon dan Termohon telah dipanggil secara resmi dan patut untuk hadir menghadap di persidangan sebagaimana ketentuan Pasal 145 RBg., jo. Pasal 26 ayat (1) Peraturan Pemerintah No. 9 Tahun 1975 dan atas panggilan tersebut Pemohon yang diwakilkan oleh Kuasa Hukumnya telah hadir datang menghadap sendiri di persidangan, sedangkan Termohon tidak datang menghadap di muka sidang dan tidak pula menyuruh orang lain sebagai wakil/kuasanya yang sah untuk datang menghadap, dan tidak ternyata tidak datangnya itu disebabkan suatu halangan yang sah.

Menimbang, bahwa sesuai dengan Pasal 82 ayat (1) dan (4) Undang-Undang Nomor 7 Tahun 1989 tentang Peradilan Agama yang telah diubah dengan Undang-Undang Nomor 3 Tahun 2006 dan perubahan kedua dengan Undang-Undang Nomor 50 Tahun 2009, Majelis Hakim telah mendamaikan/memberi nasihat Pemohon agar rukun kembali dengan Termohon dan karena upaya tersebut berhasil, maka Pemohon menyatakan mencabut perkara a quo.

Menimbang, bahwa Pemohon berkesimpulan memohon agar Pengadilan mengabulkan permohonan pencabutan permohonan Pemohon.

Menimbang, bahwa permohonan pencabutan tersebut diajukan oleh Pemohon sebelum perkara tersebut diputus, sehingga permohonan pencabutan tersebut tidak bertentangan dengan ketentuan Pasal 271 RV (*Reglement op de burgerlijke recht vordering*), oleh karenanya permohonan pencabutan

Disclaimer
Kepaniteraan Mahkamah Agung Republik Indonesia berusaha untuk selalu mencantumkan informasi paling kini dan akurat sebagai bentuk komitmen Mahkamah Agung untuk pelayanan publik, transparansi dan akuntabilitas pelaksanaan fungsi peradilan. Namun dalam hal-hal tertentu masih dimungkinkan terjadi permasalahan teknis terkait dengan akurasi dan keterkinian informasi yang kami sajikan, hal mana akan terus kami perbaiki dari waktu kewaktu.
Dalam hal Anda menemukan inakurasi informasi yang termuat pada situs ini atau informasi yang seharusnya ada, namun belum tersedia, maka harap segera hubungi Kepaniteraan Mahkamah Agung RI melalui :
Email : kepaniteraan@mahkamahagung.go.id Telp : 021-384 3348 (ext.318)

permohonan dapat dibenarkan, maka Majelis Hakim mengabulkan permohonan pencabutan permohonan Pemohon Nomor XXXX/Pdt.G/2020/PA.Dmk dan selanjutnya memerintahkan Panitera Pengadilan Agama Demak untuk mencatat pencabutan perkara tersebut dalam register perkara.

Menimbang, bahwa dengan dikabulkannya permohonan Pemohon tersebut maka pemeriksaan perkara ini dinyatakan selesai.

Menimbang, bahwa bahwa berdasarkan ketentuan Pasal 89 ayat (1) Undang-Undang Nomor 7 Tahun 1989, yang telah diubah dan ditambah dengan perubahan pertama menjadi Undang-Undang Nomor 3 Tahun 2006 dan perubahan kedua menjadi Undang-Undang Nomor 50 Tahun 2009, maka seluruh biaya perkara dibebankan kepada Pemohon.

Memperhatikan segala peraturan dan kaidah hukum yang berhubungan dengan perkara ini.

MENETAPKAN

1. Mengabulkan permohonan pencabutan perkara Nomor XXXX/Pdt.G/2020/PA.Dmk dari Pemohon;
2. Memerintahkan Panitera untuk mencatat pencabutan perkara tersebut dalam register perkara;
3. Membebankan kepada Pemohon untuk membayar biaya perkara sejumlah Rp461.000,00 (empat ratus enam puluh satu ribu rupiah);

Demikianlah putusan ini dijatuhkan dalam musyawarah Majelis Hakim di Demak pada hari Selasa tanggal 22 Desember 2020 Masehi, bertepatan dengan tanggal 7 Jumadil Awwal 1442 Hijriyah, oleh Kami **Drs. Makali** sebagai Ketua Majelis Hakim, dan **Drs. Ali Irfan, S.H., M.H.** serta **Dra. Nur Immawati** masing-masing sebagai Hakim Anggota. Putusan mana diucapkan oleh Ketua Majelis Hakim tersebut pada hari itu juga dalam sidang terbuka untuk umum dengan dihadiri oleh para Hakim Anggota dan **Hj. Siti Hajar Zulaikha, SH.** sebagai Panitera Pengganti dan dihadiri pula oleh Pemohon tanpa hadirnya Termohon;

Ketua Majelis

Drs. Makali

Disclaimer
Kepaniteraan Mahkamah Agung Republik Indonesia berusaha untuk selalu mencantumkan informasi paling kini dan akurat sebagai bentuk komitmen Mahkamah Agung untuk pelayanan publik, transparansi dan akuntabilitas pelaksanaan fungsi peradilan. Namun dalam hal-hal tertentu masih dimungkinkan terjadi permasalahan teknis terkait dengan akurasi dan keterkinian informasi yang kami sajikan, hal mana akan terus kami perbaiki dari waktu kewaktu.
Dalam hal Anda menemukan inakurasi informasi yang termuat pada situs ini atau informasi yang seharusnya ada, namun belum tersedia, maka harap segera hubungi Kepaniteraan Mahkamah Agung RI melalui :
Email : kepaniteraan@mahkamahagung.go.id Telp : 021-384 3348 (ext.318)

Hakim Anggota
Hakim Anggota

Drs. Ali Irfan, S.H., M.H.

Dra. Nur Immawati

Panitera Pengganti

Hj. Siti Hajar Zulaikha, SH.

Perincian Biaya :

1. Biaya Pendaftaran : Rp 30.000,00

2. Biaya Proses : Rp 75.000,00

3. Biaya Pemanggilan : Rp 320.000,00

Disclaimer
Kepaniteraan Mahkamah Agung Republik Indonesia berusaha untuk selalu mencantumkan informasi paling kini dan akurat sebagai bentuk komitmen Mahkamah Agung untuk pelayanan publik, transparansi dan akuntabilitas pelaksanaan fungsi peradilan. Namun dalam hal-hal tertentu masih dimungkinkan terjadi permasalahan teknis terkait dengan akurasi dan keterkinian informasi yang kami sajikan, hal mana akan terus kami perbaiki dari waktu kewaktu.
Dalam hal Anda menemukan inakurasi informasi yang termuat pada situs ini atau informasi yang seharusnya ada, namun belum tersedia, maka harap segera hubungi Kepaniteraan Mahkamah Agung RI melalui :
Email : kepaniteraan@mahkamahagung.go.id Telp : 021-384 3348 (ext.318)

4. PNBP Panggilan : Rp 20.000,00

5. Biaya Redaksi : Rp 10.000,00

6. Biaya Meterai : Rp 6.000,00

Jumlah : Rp 461.000,00

Disclaimer
Kepaniteraan Mahkamah Agung Republik Indonesia berusaha untuk selalu mencantumkan informasi paling kini dan akurat sebagai bentuk komitmen Mahkamah Agung untuk pelayanan publik, transparansi dan akuntabilitas pelaksanaan fungsi peradilan. Namun dalam hal-hal tertentu masih dimungkinkan terjadi permasalahan teknis terkait dengan akurasi dan keterkinian informasi yang kami sajikan, hal mana akan terus kami perbaiki dari waktu kewaktu.
Dalam hal Anda menemukan inakurasi informasi yang termuat pada situs ini atau informasi yang seharusnya ada, namun belum tersedia, maka harap segera hubungi Kepaniteraan Mahkamah Agung RI melalui :
Email : kepaniteraan@mahkamahagung.go.id   Telp : 021-384 3348 (ext.318)

Mahkamah Agung Republik Indonesia
