# Supplementary material for: Classification of divorce causes during the COVID-19 pandemic using convolutional neural networks
Source: PeerJ Comput Sci. 2022 Jun 30;8:e998. doi: 10.7717/peerj-cs.998 (PMC9299239; doi:10.7717/peerj-cs.998)
Supplement: Supplemental Information 2 [file peerj-cs-08-998-s002.zip › Kelalaian Dataset/Data ke-10.pdf]

PUTUSAN

Nomor 5485/Pdt.G/2020/PA.Cms

بسم الله الرحمن الرحيم

DEMI KEADILAN BERDASARKAN KETUHANAN YANG MAHA ESA

Pengadilan Agama Ciamis yang memeriksa dan mengadili perkara-perkara tertentu pada peradilan tingkat pertama, dalam sidang majelis telah menjatuhkan putusan perkara Cerai Talak antara pihak-pihak sebagai berikut :

Pemohon, umur 73 tahun, agama Islam, pekerjaan Petani, tempat kediaman di Kab. Ciamis, Jawa Barat, dalam hal ini memberikan kuasa khusus kepada Edi Kurniadi,SH.,MH Dan Uus Setianingsih, SH, yang berkantor di Lingk. Cibitung Girang RT. 002 RW. 007 Kelurahan Kertasari, Kecamatan Ciamis, Kabupaten Ciamis., berdasarkan surat kuasa khusus tanggal 10 Desember 2020 yang terdaftar dalam register kuasa Pengadilan Agama Ciamis Nomor 6722/XII/K/2020 tertanggal 11 Desember 2020, sebagai **Pemohon**;

melawan

Termohon, umur 55 tahun, agama Islam, pekerjaan Mengurus Rumah Tangga, tempat kediaman Kab. Ciamis, Jawa Barat, sebagai **Termohon**;

Pengadilan Agama tersebut ;
Setelah mempelajari berkas perkara yang bersangkutan;
Telah mendengar keterangan Pemohon dan saksi-saksi di depan persidangan ;

DUDUK PERKARA

Bahwa Pemohon dengan surat permohonannya tanggal 11 Desember 2020 yang terdaftar dalam register perkara di Kepaniteraan Pengadilan Agama

Disclaimer
Kepaniteraan Mahkamah Agung Republik Indonesia berusaha untuk selalu mencantumkan informasi paling kini dan akurat sebagai bentuk komitmen Mahkamah Agung untuk pelayanan publik, transparansi dan akuntabilitas pelaksanaan fungsi peradilan. Namun dalam hal-hal tertentu masih dimungkinkan terjadi permasalahan teknis terkait dengan akurasi dan keterkinian informasi yang kami sajikan, hal mana akan terus kami perbaiki dari waktu kewaktu.
Dalam hal Anda menemukan inakurasi informasi yang termuat pada situs ini atau informasi yang seharusnya ada, namun belum tersedia, maka harap segera hubungi Kepaniteraan Mahkamah Agung RI melalui :
Email : kepaniteraan@mahkamahagung.go.id Telp : 021-384 3348 (ext.318)

Ciamis dengan Nomor 5485/Pdt.G/2020/PA.Cms tertanggal 11 Desember 2020 mengajukan permohonan izin Cerai Talak yang posita dan petitumnya sebagai berikut :

1 Bahwa Pemohon telah menikah dengan Termohon pada tanggal 16 Oktober 2019 di hadapan Pejabat Kantor Urusan Agama Kecamatan Panawangan Kabupaten Ciamis, dengan Kutipan Akta Nikah Nomor : xxxx , tertanggal 17 Oktober 2019;

2 Bahwa setelah menikah Pemohon dengan Termohon bertempat tinggal di Kabupaten Ciamis;

3 Bahwa antara Pemohon dengan Termohon telah bergaul sebagai suami isteri yang baik dan hidup tentram serta belum dikaruniai anak;

4 Bahwa sejak bulan Juli 2020 rumah tangga Pemohon dengan Termohon mulai goyah serta sudah tidak melakukan hubungan badan/ senggama dengan Termohon yang mengakibatkan pada bulan Agustus 2020 Pemohon dengan Termohon berpisah rumah sampai dengan sekarang yang mana Pemohon tinggal di Kabupaten Ciamis dan Termohon tinggal di Kabupaten Ciamis;

5 Bahwa pertengkaran dan perselisihan tersebut sering terjadi dari bulan Juli 2020 sampai dengan bulan Agustus 2020;

6 Bahwa peristiwa tersebut terjadi akibat Termohon tidak mau lagi diajak berumah tangga ditempat tinggal Pemohon begitu juga sebaliknya;

7 Bahwa oleh karena hal tersebut Pemohon merasa sudah tidak tentram berumah tangga dengan Termohon yang setiap hari selalu mencari-cari alasan untuk bertengkar dan mengungkit-ungkit persoalan terdahulu;

8 Bahwa sampai sekarang dan telah berjalan 4 bulan Pemohon dan Termohon sulit melakukan komunikasi yang wajar layaknya suami isteri dan Pemohon merasa berat melanjutkan perkawinan dengan Termohon;

9 Bahwa Pemohon telah berusaha meminta bantuan baik kepada kerabat dekat, maupun pihak lain berusaha untuk mencari penyelesaian kemelut rumah tangga Pemohon dengan Termohon tetapi usaha tersebut tidak berhasil;

Disclaimer
Kepaniteraan Mahkamah Agung Republik Indonesia berusaha untuk selalu mencantumkan informasi paling kini dan akurat sebagai bentuk komitmen Mahkamah Agung untuk pelayanan publik, transparansi dan akuntabilitas pelaksanaan fungsi peradilan. Namun dalam hal-hal tertentu masih dimungkinkan terjadi permasalahan teknis terkait dengan akurasi dan keterkinian informasi yang kami sajikan, hal mana akan terus kami perbaiki dari waktu kewaktu.
Dalam hal Anda menemukan inakurasi informasi yang termuat pada situs ini atau informasi yang seharusnya ada, namun belum tersedia, maka harap segera hubungi Kepaniteraan Mahkamah Agung RI melalui :
Email : kepaniteraan@mahkamahagung.go.id Telp : 021-384 3348 (ext.318)

10 Bahwa atas dasar hal-hal tersebut di atas, dengan ini Pemohon mengajukan cerai talak dengan alasan sering terjadi perselisihan dan pertengkaran yang terus menerus disebabkan : Termohon tidak mau lagi diajak berumah tangga ditempat tinggal Pemohon begitu juga sebaliknya;

11 Berdasarkan uraian-uraian di atas, dengan ini Pemohon mohon agar Ketua Pengadilan Agama Ciamis, menerima, memeriksa, mengadili dan memutus perkara ini dengan putusan :

Berdasarkan dalil dan alasan-alasan tersebut diatas, maka dengan ini Pemohon memohon kepada Ketua Pengadilan Agama Ciamis cq. Majelis Hakim yang menangani perkara ini untuk menerima, memeriksa, mengadili dan memutus perkara ini dengan putusan sebagai berikut:

1. Mengabulkan Permohonan Pemohon;
2. Memberi ijin kepada Pemohon (Pemohon) untuk menjatuhkan talak satu Raj'i terhadap Termohon (Termohon) di depan Sidang Pengadilan Agama Ciamis;
3. Menetapkan biaya perkara ini menurut hukum;

Bahwa pada hari persidangan yang telah ditentukan, Pemohon didampingi Kuasanya datang menghadap di persidangan, sedangkan Termohon sekalipun telah dipanggil secara resmi dan patut tidak datang menghadap di persidangan, tidak pula orang lain sebagia kuasanya dan ketidakhadirannya tanpa suatu alasan serta Termohon tidak pula menyampaikan suatu eksepsi kompetensi ;

Bahwa oleh karena Termohon tidak pernah datang menghadap di persidangan, maka Majelis Hakim tidak mengupayakan damai dan tidak pula memerintahkan Pemohon untuk menempuh mediasi;

Bahwa karena Pemohon tetap dengan sikapnya bercerai dengan Termohon, maka surat permohonan Pemohon dibacakan yang isi dan maksudnya tetap dipertahankan Pemohon;

Bahwa untuk membuktikan dalil-dalil permohonannya, Pemohon telah mengajukan alat bukti surat berupa:

Disclaimer
Kepaniteraan Mahkamah Agung Republik Indonesia berusaha untuk selalu mencantumkan informasi paling kini dan akurat sebagai bentuk komitmen Mahkamah Agung untuk pelayanan publik, transparansi dan akuntabilitas pelaksanaan fungsi peradilan. Namun dalam hal-hal tertentu masih dimungkinkan terjadi permasalahan teknis terkait dengan akurasi dan keterkinian informasi yang kami sajikan, hal mana akan terus kami perbaiki dari waktu kewaktu.
Dalam hal Anda menemukan inakurasi informasi yang termuat pada situs ini atau informasi yang seharusnya ada, namun belum tersedia, maka harap segera hubungi Kepaniteraan Mahkamah Agung RI melalui :
Email : kepaniteraan@mahkamahagung.go.id   Telp : 021-384 3348 (ext.318)

- Fotokopi Kutipan Akta Nikah dari KUA Kecamatan Panawangan Kabupaten Ciamis,, Nomor xxx Kecamatan Panawangan Kabupaten Ciamis, Tanggal 16 Oktober 2020, bukti surat tersebut telah diberi meterai cukup, telah dicap pos dan telah dicocokkan dengan aslinya yang ternyata sesuai, lalu oleh Ketua Majelis diberi tanda P;;

Bahwa selain alat bukti surat tersebut, Pemohon juga telah mengajukan alat bukti saksi yaitu:

1. Saksi I umur 37 tahun, Agama Islam, pekerjaan Wiraswasta, tempat tinggal di Kabupaten Ciamis, di bawah sumpah telah memberikan keterangan yang pada pokoknya sebagai berikut:
   - Bahwa saksi kenal dengan Pemohon dan Termohon, karena Saksi adalah Tetangga Pemohon ;
   - Bahwa Pemohon dan Termohon adalah suami isteri yang menikah tahun 2019**,** di Panawangan ;
   - Bahwa selama berumah tangga Pemohon dan Termohon tinggal di rumah Kabupaten Ciamis dan belum dikaruniai anak;
   - Bahwa semula Pemohon dan Termohon hidup rukun, namun sejak awal bulan Juli 2020 tidak harmonis lagi dan sering terjadi perselisihan dan pertengkaran;
   - Bahwa penyebabnya, karena Termohon tidak mau lagi diajak berumah tangga ditempat tinggal Pemohon begitu juga sebaliknya;
   - Bahwa Saksi sering melihat dan mendengar Pemohon dan Termohon bertengkar di rumah tempat kediaman Pemohon dan Termohon ;
   - Bahwa Saksi sering merukunkan Pemohon dengan Termohon, namun tidak berhasil;
   - Bahwa Pemohon dan Termohon telah berpisah tempat tinggal sejak bulan Agustus 2020 dan hingga sekarang tidak pernah bersatu lagi dalam rumah tangga;
   - Bahwa Saksi tidak ada kesanggupan lagi mendamaikan Pemohon dengan Termohon ;

Disclaimer
Kepaniteraan Mahkamah Agung Republik Indonesia berusaha untuk selalu mencantumkan informasi paling kini dan akurat sebagai bentuk komitmen Mahkamah Agung untuk pelayanan publik, transparansi dan akuntabilitas pelaksanaan fungsi peradilan. Namun dalam hal-hal tertentu masih dimungkinkan terjadi permasalahan teknis terkait dengan akurasi dan keterkinian informasi yang kami sajikan, hal mana akan terus kami perbaiki dari waktu kewaktu.
Dalam hal Anda menemukan inakurasi informasi yang termuat pada situs ini atau informasi yang seharusnya ada, namun belum tersedia, maka harap segera hubungi Kepaniteraan Mahkamah Agung RI melalui :
Email : kepaniteraan@mahkamahagung.go.id Telp : 021-384 3348 (ext.318)

2. Saksi II umur 47 tahun, Agama Islam, pekerjaan Buruh Tani, tempat tinggal di Kabupaten Ciamis, di bawah sumpah telah memberikan keterangan yang pada pokoknya sebagai berikut :

- Bahwa saksi kenal Pemohon dan Termohon, karena saksi Tetangga Pemohon;
- Bahwa Pemohon Termohon adalah suami isteri, menikah tahun 2019 di Panawangan;
- Bahwa semula rumah tangga Pemohon dan Termohon rukun dan harmonis, namun sejak bulan Juli 2020 tidak harmonis lagi, dan sering terjadi perselisihan dan pertengkaran;
- Bahwa saksi sering melihat dan mendengar perselisihan dan pertengkaran Pemohon dengan Termohon;
- Bahwa penyebabnya adalah karena Termohon tidak mau lagi diajak berumah tangga ditempat tinggal Pemohon begitu juga sebaliknya;
- Bahwa sejak bulan Agustus 2020 Pemohon dengan Termohon telah berpisah tempat tinggal dan tidak pernah bersatu lagi;
- Bahwa Saksi telah berusaha mendamaikan, namun tidak berhasil;
- Bahwa Saksi tidak sanggup lagi untuk mendamaikan Pemohon dengan Termohon ;

Bahwa Pemohon menyatakan keterangan saksi-saksinya sudah cukup dan tidak mengajukan saksi-saksi lainnya ;

Bahwa Pemohon dalam kesimpulannya secara lisan, menyatakan tetap bercerai dengan Termohon dan memohon kepada Majelis Hakim agar menjatuhkan putusan dan mengabulkan permohonannya ;

Bahwa tentang jalannya pemeriksaan, telah dicatat dalam berita acara sidang dan selanjutnya untuk mempersingkat uraian pertimbangan ini, majelis menunjuk berita acara sidang perkara ini sebagai satu kesatuan yang tidak terpisahkan dari Putusan ;

Disclaimer
Kepaniteraan Mahkamah Agung Republik Indonesia berusaha untuk selalu mencantumkan informasi paling kini dan akurat sebagai bentuk komitmen Mahkamah Agung untuk pelayanan publik, transparansi dan akuntabilitas pelaksanaan fungsi peradilan. Namun dalam hal-hal tertentu masih dimungkinkan terjadi permasalahan teknis terkait dengan akurasi dan keterkinian informasi yang kami sajikan, hal mana akan terus kami perbaiki dari waktu kewaktu.
Dalam hal Anda menemukan inakurasi informasi yang termuat pada situs ini atau informasi yang seharusnya ada, namun belum tersedia, maka harap segera hubungi Kepaniteraan Mahkamah Agung RI melalui :
Email : kepaniteraan@mahkamahagung.go.id Telp : 021-384 3348 (ext.318)

PERTIMBANGAN HUKUM

Menimbang, bahwa maksud dan tujuan permohonan Pemohon adalah sebagaimana terurai di atas ;

Menimbang, bahwa Pemohon telah menguasakan kepada kuasa hukumnya : Edi Kurniadi,SH.,MH Dan Uus Setianingsih, SH, Advokat, dan pelimpahan Kuasa tersebut, menurut majelis telah memenuhi syarat formil pemberian Kuasa, dengan demikian Penerima Kuasa dipandang berwenang untuk bertindak mewakili kepentingan hukum Pemberi Kuasa ;

Menimbang, bahwa Termohon telah dipanggil secara resmi dan patut tidak datang menghadap dan tidak menyuruh orang lain sebagai kuasanya serta tidak menyampaikan suatu eksepsi, dan tidak ternyata tidak datangnya Termohon disebabkan suatu alasan yang sah, maka Termohon yang telah dipanggil tersebut harus dinyatakan tidak hadir dan sesuai dengan ketentuan Pasal 125 ayat (1) HIR perkara yang diajukan Pemohon dapat diperiksa dan diputus dengan verstek ;

Menimbang, bahwa terhadap perkara ini tidak dilakukan proses mediasi sebagaimana perintah PERMA Nomor 1 Tahun 2016, demikian pula upaya perdamaian sebagaimana ketentuan Pasal 82 Undang-Undang Nomor 7 Tahun 1989, disebabkan Termohon tidak pernah datang menghadap di persidangan ;

Menimbang, bahwa pada pokoknya Pemohon telah mendasarkan permohonan talaknya dengan alasan-alasan sejak bulan Juli 2020 antara Pemohon dengan Termohon terjadi perselisihan dan pertengkaran sehingga tidak harmonis lagi disebabkan faktor Termohon tidak mau lagi diajak berumah tangga ditempat tinggal Pemohon begitu juga sebaliknya, kemudian ketidakharmonisan rumah tangganya mencapai puncaknya pada bulan Agustus 2020 di mana sejak saat itu Pemohon dan Termohon telah berpisah rumah dan tidak pernah bersatu lagi dalam rumah tangga ;

Menimbang, bahwa guna meneguhkan dalil dan alasan permohonannya, Pemohon ke hadapan sidang telah mengajukan alat bukti berupa P serta 2 orang saksi ;

Disclaimer
Kepaniteraan Mahkamah Agung Republik Indonesia berusaha untuk selalu mencantumkan informasi paling kini dan akurat sebagai bentuk komitmen Mahkamah Agung untuk pelayanan publik, transparansi dan akuntabilitas pelaksanaan fungsi peradilan. Namun dalam hal-hal tertentu masih dimungkinkan terjadi permasalahan teknis terkait dengan akurasi dan keterkinian informasi yang kami sajikan, hal mana akan terus kami perbaiki dari waktu kewaktu.
Dalam hal Anda menemukan inakurasi informasi yang termuat pada situs ini atau informasi yang seharusnya ada, namun belum tersedia, maka harap segera hubungi Kepaniteraan Mahkamah Agung RI melalui :
Email : kepaniteraan@mahkamahagung.go.id Telp : 021-384 3348 (ext.318)

Menimbang, bahwa baik alat bukti surat (P) maupun alat bukti 2 (dua) orang saksi, secara formil telah memenuhi syarat sebagai bukti surat dan saksi sehingga bukti-bukti tersebut dapat diterima dan secara materil akan dipertimbangkan lebih lanjut ;

Menimbang, bahwa alat bukti (P) berupa Kutipan Akta Nikah adalah akta otentik yang isinya menerangkan adanya peristiwa hukum pernikahan antara Pemohon dan Termohon yang terjadi pada tanggal 16 Oktober 2020. Oleh karena itu, bukti P dimaksud secara materil mendukung kebenaran dalil gugatan Pemohon dan karenanya mempunyai kekuatan pembuktian sempurna dan mengikat, sehingga berdasarkan bukti tersebut, maka terbukti antara Pemohon dengan Termohon terikat dalam perkawinan yang sah sebagaimana diatur dalam ketentuan Pasal 2 Undang-Undang Nomor 1 Tahun 1974. Dengan demikian, Pemohon berhak serta mempunyai kepentingan hukum mengajukan perkara ini dan oleh karenanya patut dikualifisir sebagai ***Legitima Persona Standi in Judicio*** *;*

Menimbang, bahwa apa yang telah diterangkan Saksi Pertama adalah suatu fakta yang dilihat dan didengar sendiri oleh Saksi serta isi keterangannya sejalan dengan dalil dan alasan permohonan Pemohon, oleh karenanya keterangan Saksi tersebut secara materil mendukung kebenaran dalil dan alasan permohonan Pemohon ;

Menimbang, bahwa apa yang telah diterangkan Saksi Kedua adalah suatu fakta yang dilihat dan didengar sendiri oleh Saksi serta isi keterangannya sejalan dengan dalil dan alasan permohonan Pemohon, oleh karenanya keterangan Saksi tersebut secara materil mendukung kebenaran dalil dan alasan permohonan Pemohon ;

Menimbang, bahwa keterangan antara saksi pertama dengan saksi kedua satu sama lain saling bersesuaian serta relevan dengan dalil dan alasan permohonan Pemohon, sehingga dengan demikian pembuktian dari Pemohon telah memenuhi syarat minimal pembuktian dan karenanya Pemohon telah dapat membuktikan kebenaran dalil dan alasan permohonan Pemohon ;

Menimbang, bahwa berdasarkan hasil penilaian terhadap bukti-bukti sebagaimana terurai di atas dihubungkan dengan keterangan Pemohon,

Disclaimer
Kepaniteraan Mahkamah Agung Republik Indonesia berusaha untuk selalu mencantumkan informasi paling kini dan akurat sebagai bentuk komitmen Mahkamah Agung untuk pelayanan publik, transparansi dan akuntabilitas pelaksanaan fungsi peradilan. Namun dalam hal-hal tertentu masih dimungkinkan terjadi permasalahan teknis terkait dengan akurasi dan keterkinian informasi yang kami sajikan, hal mana akan terus kami perbaiki dari waktu kewaktu.
Dalam hal Anda menemukan inakurasi informasi yang termuat pada situs ini atau informasi yang seharusnya ada, namun belum tersedia, maka harap segera hubungi Kepaniteraan Mahkamah Agung RI melalui :
Email : kepaniteraan@mahkamahagung.go.id   Telp : 021-384 3348 (ext.318)

Majelis Hakim telah menemukan fakta-fakta tetap di persidangan sebagai berikut :

- Bahwa Pemohon dengan Termohon adalah suami isteri sah dan belum pernah bercerai ;
- Bahwa sejak awal bulan Juli 2020, antara Pemohon dengan Termohon terjadi perselisihan dan pertengkaran yang mengakibatkan telah terjadinya ketidak harmonisan dalam rumah tangga Pemohon dan Termohon yang dipicu oleh faktor Termohon tidak mau lagi diajak berumah tangga ditempat tinggal Pemohon begitu juga sebaliknya ;
- Bahwa keluarga kedua belah pihak sudah mendamaikan, akan tetapi tidak berhasil ;
- Bahwa sejak bulan Agustus 2020**,** antara Pemohon dengan Termohon sudah berpisah tempat tinggal dan tidak ada harapan bersatu lagi dalam rumah tangga Pemohon dan Termohon ;

Menimbang, bahwa berdasarkan fakta-fakta tersebut di atas, telah terbukti adanya fakta hukum bahwa Pemohon dan Termohon sebagai suami isteri telah terjadi perselisihan dan pertengkaran terus menerus dan tidak ada harapan akan hidup rukun lagi dalam rumah tangga ;

Menimbang, bahwa sehubungan dengan kondisi rumah tangga seperti terurai di atas, Majelis berpendapat bahwa konsep membangun rumah tangga dengan prinsip ***mu'asyarah bil-ma'ruf*** telah gagal diperankan Pemohon dan Termohon, sehingga apa yang menjadi tujuan perkawinan untuk mewujudkan keluarga yang sakinah, mawaddah dan rahmah telah gagal diwujudkan Pemohon dan Termohon, dan ketika kondisi suatu rumah tangga sudah dalam keadaan demikian, maka perceraian adalah solusi yang tepat daripada tetap harus mempertahankan perkawinannya yang diyakini akan mendatangkan madarat yang berkepanjangan bagi Pemohon dan Termohon ;

Menimbang, bahwa berdasarkan pertimbangan tersebut di atas, maka permohonan Pemohon telah memenuhi alasan perceraian sebagaimana diatur dalam ketentuan Pasal 39 ayat (2) Undang-undang Nomor 1 Tahun 1974, jo. Pasal 19 huruf (f) Peraturan Pemerintahan Nomor 9 Tahun 1975, jo. pasal 116 huruf (f) Kompilasi Hukum Islam ;

Disclaimer
Kepaniteraan Mahkamah Agung Republik Indonesia berusaha untuk selalu mencantumkan informasi paling kini dan akurat sebagai bentuk komitmen Mahkamah Agung untuk pelayanan publik, transparansi dan akuntabilitas pelaksanaan fungsi peradilan. Namun dalam hal-hal tertentu masih dimungkinkan terjadi permasalahan teknis terkait dengan akurasi dan keterkinian informasi yang kami sajikan, hal mana akan terus kami perbaiki dari waktu kewaktu.
Dalam hal Anda menemukan inakurasi informasi yang termuat pada situs ini atau informasi yang seharusnya ada, namun belum tersedia, maka harap segera hubungi Kepaniteraan Mahkamah Agung RI melalui :
Email : kepaniteraan@mahkamahagung.go.id   Telp : 021-384 3348 (ext.318)

Menimbang, bahwa oleh karena permohonan Pemohon terbukti beralasan serta Termohon tidak pernah hadir di persidangan, maka Majelis berkesimpulan bahwa petitum angka 2 permohonan Pemohon tentang permohonan izin ikrar talak patut dikabulkan dengan verstek ;

Menimbang, bahwa Pemohon dalam perkawinannya dengan Termohon, belum pernah menjatuhkan talaknya kepada Termohon dan permohonan izin talak dalam perkara ini adalah merupakan talaknya yang pertama, sehingga oleh karena itu maka talak yang dijatuhkan Pemohon terhadap Termohon adalah talak satu raj'i yang pertama ;

Menimbang, bahwa Majelis Hakim perlu mengetengahkan firman Allah dalam Al-Quran surat Al-Baqarah ayat 227, yang berbunyi sebagai berikut :

وان عزمواالطـلاق فان الله سمـيع عـليـم

Artinya : *"Dan jika mereka berazam (berketetapan hati) untuk menjatuhkan talak, maka sesungguhnya Allah Maha Mendengar lagi Maha Mengetahui";*

-----Menimbang, bahwa perkara ini termasuk bidang perkawinan, oleh karenanya berdasarkan ketentuan Pasal 89 ayat (1) Undang-Undang Nomor 7 Tahun 1989 sebagaimana telah diubah terakhir dengan Undang-Undang Nomor 50 Tahun 2009 tentang Perubahan Kedua Atas Undang-Undang Nomor 7 Tahun 1989, maka seluruh biaya perkara ini dibebankan kepada Pemohon ;

Mengingat, segala peraturan perundang-undangan yang berlaku beserta ketentuan hukum syar'i yang berkaitan dengan perkara ini ;

MENGADILI

1. Menyatakan, Termohon yang telah dipanggil secara resmi dan patut untuk datang menghadap di persidangan, tidak hadir;
2. Mengabulkan pemohonan Pemohon dengan verstek;

Disclaimer
Kepaniteraan Mahkamah Agung Republik Indonesia berusaha untuk selalu mencantumkan informasi paling kini dan akurat sebagai bentuk komitmen Mahkamah Agung untuk pelayanan publik, transparansi dan akuntabilitas pelaksanaan fungsi peradilan. Namun dalam hal-hal tertentu masih dimungkinkan terjadi permasalahan teknis terkait dengan akurasi dan keterkinian informasi yang kami sajikan, hal mana akan terus kami perbaiki dari waktu kewaktu.
Dalam hal Anda menemukan inakurasi informasi yang termuat pada situs ini atau informasi yang seharusnya ada, namun belum tersedia, maka harap segera hubungi Kepaniteraan Mahkamah Agung RI melalui :
Email : kepaniteraan@mahkamahagung.go.id Telp : 021-384 3348 (ext.318)

3. Memberi izin kepada Pemohon (Pemohon) untuk menjatuhkan talak satu raj'i terhadap Termohon (Termohon) di depan sidang Pengadilan Agama Ciamis;

4. Membebankan biaya perkara kepada Pemohon sejumlah Rp. 236.000,00 (dua ratus tiga puluh enam ribu rupiah)

Demikian, dijatuhkan dalam rapat musyawarah majelis Pengadilan Agama Ciamis pada hari Senin tanggal 28 Desember 2020 Masehi bertepatan dengan tanggal 13 Jumadil Awwal 1442 Hijriyah, oleh kami Drs. H. Muhlis Budiman, M.H sebagai Hakim Ketua Majelis serta Drs. H. Omay Mansur, M.Ag dan Drs. Damanhuri Aly, MH, masing-masing sebagai Hakim Anggota. Putusan tersebut pada hari dan tanggal itu juga diucapkan dalam sidang terbuka untuk umum oleh Ketua Majelis tersebut dengan didampingi para Hakim Anggota dan dibantu oleh Wahyudin, S.Ag, sebagai Panitera Pengganti serta dihadiri oleh Pemohon dan Kuasanya tanpa hadirnya Termohon.

Ketua Majelis,

Drs. H. Muhlis Budiman, M.H

Hakim Anggota,

Drs. H. Omay Mansur, M.Ag

Hakim Anggota,

Drs. Damanhuri Aly, MH

Panitera Pengganti,

Wahyudin, S.Ag

Disclaimer
Kepaniteraan Mahkamah Agung Republik Indonesia berusaha untuk selalu mencantumkan informasi paling kini dan akurat sebagai bentuk komitmen Mahkamah Agung untuk pelayanan publik, transparansi dan akuntabilitas pelaksanaan fungsi peradilan. Namun dalam hal-hal tertentu masih dimungkinkan terjadi permasalahan teknis terkait dengan akurasi dan keterkinian informasi yang kami sajikan, hal mana akan terus kami perbaiki dari waktu kewaktu.
Dalam hal Anda menemukan inakurasi informasi yang termuat pada situs ini atau informasi yang seharusnya ada, namun belum tersedia, maka harap segera hubungi Kepaniteraan Mahkamah Agung RI melalui :
Email : kepaniteraan@mahkamahagung.go.id Telp : 021-384 3348 (ext.318)

Perincian Biaya Perkara :

1. Biaya Pendaftaran	:	Rp.	30.000,-
2. Biaya Proses	:	Rp.	60.000,-
3. Biaya Panggilan	:	Rp.	100.000,-
4. Biaya PNBP Surat Kuasa	:	Rp.	10.000,-
5. Biaya PNBP Panggilan	:	Rp.	20.000,-
6. Biaya Redaksi	:	Rp.	10.000,-
7. Biaya Materai	:	Rp.	6.000,-
Jumlah	:	Rp.	236.000,-

(dua ratus dua puluh enam ribu rupiah)

Disclaimer
Kepaniteraan Mahkamah Agung Republik Indonesia berusaha untuk selalu mencantumkan informasi paling kini dan akurat sebagai bentuk komitmen Mahkamah Agung untuk pelayanan publik, transparansi dan akuntabilitas pelaksanaan fungsi peradilan. Namun dalam hal-hal tertentu masih dimungkinkan terjadi permasalahan teknis terkait dengan akurasi dan keterkinian informasi yang kami sajikan, hal mana akan terus kami perbaiki dari waktu kewaktu.
Dalam hal Anda menemukan inakurasi informasi yang termuat pada situs ini atau informasi yang seharusnya ada, namun belum tersedia, maka harap segera hubungi Kepaniteraan Mahkamah Agung RI melalui :
Email : kepaniteraan@mahkamahagung.go.id   Telp : 021-384 3348 (ext.318)
