# Supplementary material for: Classification of divorce causes during the COVID-19 pandemic using convolutional neural networks
Source: PeerJ Comput Sci. 2022 Jun 30;8:e998. doi: 10.7717/peerj-cs.998 (PMC9299239; doi:10.7717/peerj-cs.998)
Supplement: Supplemental Information 2 [file peerj-cs-08-998-s002.zip › Kelalaian Dataset/Data ke-11.pdf]

P U T U S A N

Nomor 4059/Pdt.G/2020/PA.Bks.

بِسْمِ اللهِ الرَّحْمَنِ الرَّحِيْمِ

DEMI KEADILAN BERDASARKAN KETUHANAN YANG MAHA ESA

Pengadilan Agama Bekasi yang memeriksa dan mengadili perkara pada tingkat pertama dalam persidangan majelis telah menjatuhkan putusan sebagaimana di bawah ini dalam perkara cerai gugat antara:

XXX, lahir di Bekasi, 12 Mei 1978 (umur 42 tahun), agama Islam, pendidikan terakhir SMA, pekerjaan Karyawan Swasta, tempat tinggal di XXX. Untuk selanjutnya disebut sebagai : **Penggugat**;

M e l a w a n

XXX, lahir di Purwokerto, 12 Maret 1967 (umur 53 tahun), agama Islam, pendidikan terakhir SMP, Pekerjaan Sopir, tempat tinggal di XXX. Untuk selanjutnya disebut sebagai : **Tergugat;**

Pengadilan Agama tersebut;

Setelah mempelajari dan meneliti surat-surat perkara;

Setelah mendengar keterangan Penggugat dan Tergugat serta memeriksa bukti surat dan saksi-saksi di persidangan;

DUDUK PERKARA

Bahwa, Penggugat telah mengajukan gugatan pada tanggal 4 Desember 2020 yang terdaftar di Kepaniteraan Pengadilan Agama Bekasi pada tanggal 4 Desember 2020 dengan register Nomor 4059/Pdt.G/2020/PA.Bks., dengan dalil-dalil yang pada pokoknya sebagai berikut:

Disclaimer
Kepaniteraan Mahkamah Agung Republik Indonesia berusaha untuk selalu mencantumkan informasi paling kini dan akurat sebagai bentuk komitmen Mahkamah Agung untuk pelayanan publik, transparansi dan akuntabilitas pelaksanaan fungsi peradilan. Namun dalam hal-hal tertentu masih dimungkinkan terjadi permasalahan teknis terkait dengan akurasi dan keterkinian informasi yang kami sajikan, hal mana akan terus kami perbaiki dari waktu kewaktu.
Dalam hal Anda menemukan inakurasi informasi yang termuat pada situs ini atau informasi yang seharusnya ada, namun belum tersedia, maka harap segera hubungi Kepaniteraan Mahkamah Agung RI melalui :
Email : kepaniteraan@mahkamahagung.go.id Telp : 021-384 3348 (ext.318)

1. Bahwa pada tanggal 1 Agustus 2010, Penggugat dengan Tergugat melangsungkan pernikahan yang dicatat oleh Pegawai Pencatat Nikah Kantor Urusan Agama Kecamatan Taruma Jaya, Kabupaten Bekasi, sesuai Kutipan Akta Nikah Nomor: XXX, tertanggal 1 Agustus 2010;

2. Bahwa setelah pernikahan tersebut Penggugat dengan Tergugat bertempat tinggal terakhir di XXX;

3. Bahwa dalam perkawinan tersebut Penggugat dengan Tergugat telah behubungan sebagaimana layaknya suami dan istri (ba'da dukhul), dan telah dikarunia keturunan seorang anak, yang bernama : XXX, perempuan, lahir di Bekasi, 9 Maret 2012;

4. Bahwa pada awalnya rumah tangga Penggugat dan Tergugat rukun dan harmonis, akan tetapi sejak tahun 2014 rumah tangga Penggugat dengan Tergugat sering terjadi pertengkaran, yang penyebabnya antara lain :
   a. Tergugat memiliki wanita idaman lain, yang diketahui Penggugat dari HP milik Tergugat dan sudah diakui oleh Tergugat;
   b. Tergugat diketahui sudah menikah lagi dengan wanita selingkuhannya, yang bernama Ikah tanpa izin dan sepengetahuan Penggugat;
   c. Tergugat kurang bertanggung jawab dalam pemenuhan kebutuhan rumah tangga, karena sejak tahun 2014 Tergugat sudah tidak memberikan nafkah kepada Penggugat;

5. Bahwa puncaknya terjadi pada tanggal 24 Pebruari 2018, dimana terjadi perselisihan dan pertengkaran antara Penggugat dengan Tergugat, lalu Penggugat keluar dari rumah tempat tinggal bersama, karena diusir oleh Tergugat dan sejak saat itu antara Penggugat dengan Tergugat sudah tidak terjalin hubungan sebagaimana layaknya suami dan istri hingga saat ini;

6. Bahwa pihak keluarga sudah berusaha mendamaikan, akan tetapi tidak berhasil;

7. Bahwa ikatan perkawinan antara Penggugat dan Tergugat sebagaimana diuraikan di atas sudah sulit dibina untuk membentuk suatu rumah tangga yang sakinah, mawaddah wa rahmah sebagaimana maksud dan tujuan dari suatu perkawinan, sehingga lebih baik diputus karena perceraian;

Disclaimer
Kepaniteraan Mahkamah Agung Republik Indonesia berusaha untuk selalu mencantumkan informasi paling kini dan akurat sebagai bentuk komitmen Mahkamah Agung untuk pelayanan publik, transparansi dan akuntabilitas pelaksanaan fungsi peradilan. Namun dalam hal-hal tertentu masih dimungkinkan terjadi permasalahan teknis terkait dengan akurasi dan keterkinian informasi yang kami sajikan, hal mana akan terus kami perbaiki dari waktu kewaktu.
Dalam hal Anda menemukan inakurasi informasi yang termuat pada situs ini atau informasi yang seharusnya ada, namun belum tersedia, maka harap segera hubungi Kepaniteraan Mahkamah Agung RI melalui :
Email : kepaniteraan@mahkamahagung.go.id   Telp : 021-384 3348 (ext.318)

8. Bahwa, dengan fakta-fakta tersebut diatas gugatan Penggugat telah memenuhi alasan perceraian sebagaimana diatur dalam Pasal 19 PP No. 9 Tahun 1975 jo. Pasal 116 Kompilasi Hukum Islam;

9. Bahwa, terhadap biaya yang timbul akibat perkara ini agar dibebankan menurut Peraturan Perundang-Undangan yang berlaku;

Berdasarkan hal-hal tersebut di atas, Pemohon memohon agar Ketua Pengadilan Agama Bekasi Bekasi C.q. Majelis Hakim yang memeriksa dan mengadili perkara ini, menjatuhkan putusan yang amarnya berbunyi sebagai berikut :

PRIMAIR:

1. Mengabulkan gugatan Penggugat;
2. Menjatuhkan talak 1 (satu) ba'in sugra Tergugat (XXX) terhadap Penggugat (XXX);
3. Membebankan biaya perkara kepada Penggugat sesuai dengan hukum yang berlaku;

SUBSIDAIR:

Atau apabila Majelis Hakim berpendapat lain mohon untuk menjatuhkan putusan yang seadil-adilnya (ex aequo et bono);

Bahwa, pada hari-hari sidang yang telah ditetapkan, Penggugat dan Tergugat dipanggil secara resmi dan patut dan atas panggilan tersebut Penggugat dan Tergugat masing-masing hadir di depan sidang;

Bahwa, Majelis Hakim telah berusaha menasihati Penggugat dan Tergugat agar bersabar dan mengurungkan niat bercerai tetapi tidak berhasil, karena Penggugat tetap bersikukuh dengan keinginannya untuk bercerai dengan Tergugat, sedang Tergugat tetap ingin melanjutkan rumah tangganya dengan Penggugat, kemudian Majelis Hakim memerintahkan Penggugat dan Tergugat untuk menempuh upaya mediasi dengan menunjuk mediator Drs. HM. Fadjri Rivai, S.H.,MH., dan berdasarkan laporan mediator, tertanggal 14 Desember 2020, yang melaporkan bahwa terhadap perkara a quo tidak layak untuk di mediasi, karena Tergugat tidak beritikad baik, dengan meninggalkan proses mediasi dan menyerahkan segala sesuatunya kepada Penggugat;

Disclaimer
Kepaniteraan Mahkamah Agung Republik Indonesia berusaha untuk selalu mencantumkan informasi paling kini dan akurat sebagai bentuk komitmen Mahkamah Agung untuk pelayanan publik, transparansi dan akuntabilitas pelaksanaan fungsi peradilan. Namun dalam hal-hal tertentu masih dimungkinkan terjadi permasalahan teknis terkait dengan akurasi dan keterkinian informasi yang kami sajikan, hal mana akan terus kami perbaiki dari waktu kewaktu.
Dalam hal Anda menemukan inakurasi informasi yang termuat pada situs ini atau informasi yang seharusnya ada, namun belum tersedia, maka harap segera hubungi Kepaniteraan Mahkamah Agung RI melalui :
Email : kepaniteraan@mahkamahagung.go.id Telp : 021-384 3348 (ext.318)

Bahwa oleh karena upaya damai tidak berhasil, selanjutnya dibacakan surat gugatan Penggugat, Nomor 4059/Pdt.G/2020/PA.Bks., tanggal 4 Desember 2020, yang isinya tetap dipertahankan oleh Penggugat;

Bahwa terhadap gugatan Penggugat seperti tersebut diatas, setelah proses mediasi Tergugat pernah dating menghadap di persidangan, sehingga Majelis Hakim di persidangan tidak dapat mendengar jawaban Tergugat;

Bahwa untuk memperkuat dalil-dalil gugatannya, di persidangan Penggugat mengajukan alat bukti berupa ك

1.Fotokopi Surat Keterangan Domisili, Atas Nama Penggugat, Nomor : XXX., tanggal 27 November 2020, yang dikeluarkan oleh Kantor Kelurahan Medan Satria, Kecamatan Medan Satria, Kota Bekasi, yang telah bermeterai cukup dan telah dinazegelen serta telah dicocokkan dengan aslinya, lalu oleh Ketua Majelis diberi tanda P.1.;

2.Fotokopi Kutipan Akta Nikah, Atas Nama Penggugat, Nomor : XXX, tanggal 1 Agustus 2010, yang dikeluarkan oleh Kantor Urusan Agama Kecamatan Taruma Jaya, Kabupaten Bekasi, yang telah bermeterai cukup dan telah dinazegelen serta telah dicocokkan dengan aslinya, lalu oleh Ketua Majelis diberi tanda P.2.;

Bahwa disamping mengajukan bukti surat seperti tersebut diatas, di persidangan Penggugat juga mengajukan dua orang saksi keluarga atau orang yang dekat dengan Penggugat, yang masing-masing bernama:

1.---XXX, Umur 40 Tahun, Agama Islam, Pekerjaan Ibu rumah tangga, Bertempat tinggal di XXX. Dibawah sumpahnya saksi memberikan keterangan sebagai berikut:

---Bahwa saksi kenal dengan Penggugat dan Tergugat karena saksi adalah Sepupu Penggugat;

Disclaimer
Kepaniteraan Mahkamah Agung Republik Indonesia berusaha untuk selalu mencantumkan informasi paling kini dan akurat sebagai bentuk komitmen Mahkamah Agung untuk pelayanan publik, transparansi dan akuntabilitas pelaksanaan fungsi peradilan. Namun dalam hal-hal tertentu masih dimungkinkan terjadi permasalahan teknis terkait dengan akurasi dan keterkinian informasi yang kami sajikan, hal mana akan terus kami perbaiki dari waktu kewaktu.
Dalam hal Anda menemukan inakurasi informasi yang termuat pada situs ini atau informasi yang seharusnya ada, namun belum tersedia, maka harap segera hubungi Kepaniteraan Mahkamah Agung RI melalui :
Email : kepaniteraan@mahkamahagung.go.id Telp : 021-384 3348 (ext.318)

---Bahwa hubungan Penggugat dengan Tergugat adalah sebagai suami istri yang menikah pada tahun 2010 di Kecamatan Taruma Jaya, Kota Bekasi;

--- Bahwa setelah menikah Penggugat dengan Tergugat terakhir tinggal bersama di XXX;

---Bahwa selama berumah tangga Penggugat dengan Tergugat telah hidup bersama sebagaimana layaknya suami istri dan telah dikaruniai 1 (satu) orang anak;

---Bahwa setahu saksi awalnya rumah tangga Penggugat dengan Tergugat berjalan rukun dan harmonis, selanjutnya sejak tahun 2014 sudah tidak harmonis, antara Penggugat dengan Tergugat sering terjadi perselisihan;

---Bahwa sepengetahuan saksi penyebabnya adalah karena Tergugat tidak bertanggung jawab dalam memberikan nafkah untuk Penggugat dan anak dan Tergugat tidak memperhatikan Penggugat dan rumah tangga, Tergugat juga telah menjalin hubungan dengan wanita idaman lain dan saksi mendengar kabar bahwa Tergugat telah menikah siri dengan perempan selingkuhannya tersebut;

---Bahwa saksi mengetahui masalah rumah tangga Penggugat dengan Tergugat dari cerita Penggugat kepada saksi dan saksi pernah melihat dan mendengar sendiri pertengkaran antara Penggugat dengan Tergugat;

---Bahwa setahu saksi sejak bulan Pebruari 2018 Penggugat dengan Tergugat bertengkar hebat, kemudian Tergugat mengusir Penggugat, sehingga Penggugat terpaksa pergi meninggalkan rumah dan tinggal di rumah kontrakan sampai sekarang dan antara Penggugat dengan Tergugat sudah tidak memperdulikan satu sama lainnya;

---Bahwa setahu saksi selama berpisah antara Penggugat dengan Tergugat

Disclaimer
Kepaniteraan Mahkamah Agung Republik Indonesia berusaha untuk selalu mencantumkan informasi paling kini dan akurat sebagai bentuk komitmen Mahkamah Agung untuk pelayanan publik, transparansi dan akuntabilitas pelaksanaan fungsi peradilan. Namun dalam hal-hal tertentu masih dimungkinkan terjadi permasalahan teknis terkait dengan akurasi dan keterkinian informasi yang kami sajikan, hal mana akan terus kami perbaiki dari waktu kewaktu.
Dalam hal Anda menemukan inakurasi informasi yang termuat pada situs ini atau informasi yang seharusnya ada, namun belum tersedia, maka harap segera hubungi Kepaniteraan Mahkamah Agung RI melalui :
Email : kepaniteraan@mahkamahagung.go.id Telp : 021-384 3348 (ext.318)

sudah tidak ada komunikasi layaknya suami istri lagi dan Tergugat sudah tidak memberikan nafkah untuk Penggugat dan anaknya;

--Bahwa upaya untuk merukunkan Penggugat dengan Tergugat pernah dilakukan oleh pihak keluarga kedua belah pihak, namun tidak berhasil;

--Bahwa saksi sudah tidak sanggup lagi untuk merukunkan Penggugat dengan Tergugat

2.--**XXX**, Umur 29 Tahun, Agama Islam, Pekerjaan Karyawan Swasta, Bertempat tinggal di XXX. Dibawah sumpahnya saksi memberikan keterangan sebagai berikut :

--Bahwa saksi kenal dengan Penggugat dan Tergugat karena saksi adalah Tetangga Penggugat;

--BBahwa Penggugat dengan Tergugat adalah sebagai suami istri yang menikah pada tahun 2010 di Kecamatan Taruma Jaya, Kota Bekasi;

--Bahwa sepengetahuan saksi setelah menikah Penggugat dengan Tergugat terakhir tinggal bersama di XXX;

--Bahwa selama berumah tangga Penggugat dengan Tergugat telah hidup bersama sebagaimana layaknya suami istri dan telah dikaruniai 1 (satu) orang anak;

--Bahwa sepengetahuan saksi awalnya rumah tangga Penggugat dengan Tergugat berjalan rukun dan harmonis, selanjutnya sejak tahun 2014 sudah tidak harmonis, antara Penggugat dengan Tergugat sering terjadi perselisihan;

--Bahwa setahu saksi penyebabnya adalah karena Tergugat tidak bertanggung jawab dalam memberikan nafkah untuk Penggugat dan anak dan Tergugat

Disclaimer
Kepaniteraan Mahkamah Agung Republik Indonesia berusaha untuk selalu mencantumkan informasi paling kini dan akurat sebagai bentuk komitmen Mahkamah Agung untuk pelayanan publik, transparansi dan akuntabilitas pelaksanaan fungsi peradilan. Namun dalam hal-hal tertentu masih dimungkinkan terjadi permasalahan teknis terkait dengan akurasi dan keterkinian informasi yang kami sajikan, hal mana akan terus kami perbaiki dari waktu kewaktu.
Dalam hal Anda menemukan inakurasi informasi yang termuat pada situs ini atau informasi yang seharusnya ada, namun belum tersedia, maka harap segera hubungi Kepaniteraan Mahkamah Agung RI melalui :
Email : kepaniteraan@mahkamahagung.go.id Telp : 021-384 3348 (ext.318)

tidak memperhatikan Penggugat dan rumah tangga, Tergugat juga telah menjalin hubungan dengan wanita idaman lain dan saksi mendengar kabar bahwa Tergugat telah menikah siri dengan perempan selingkuhannya tersebut;

--Bahwa saksi mengetahui masalah rumah tangga Penggugat dengan Tergugat dari cerita Penggugat kepada saksi dan saksi pernah melihat dan mendengar sendiri pertengkaran antara Penggugat dengan Tergugat;

--Bahwa sejak bulan Pebruari 2018 Penggugat dengan Tergugat bertengkar hebat, kemudian Tergugat mengusir Penggugat, sehingga Penggugat terpaksa pergi meninggalkan rumah dan tinggal di rumah kontrakan sampai sekarang dan antara Penggugat dengan Tergugat sudah tidak memperdulikan satu sama lainnya;

--Bahwa setahu saksi selama berpisah antara Penggugat dengan Tergugat sudah tidak ada komunikasi layaknya suami istri lagi dan Tergugat sudah tidak memberikan nafkah untuk Penggugat dan anaknya;

--Bahwa upaya untuk merukunkan Penggugat dengan Tergugat pernah dilakukan oleh pihak keluarga kedua belah pihak, namun tidak berhasil;

--Bahwa saksi sudah tidak sanggup lagi untuk merukunkan Penggugat dengan Tergugat;

Bahwa terhadap keterangan kedua orang saksi seperti tersebut diatas Penggugat membenarkan;

Bahwa kemudian atas pertanyaan Ketua Majelis, Penggugat mengajukan kesimpulan yang disampaikan secara lisan, yang pada pokoknya tetap pada pendiriannya untuk bercerai dengan Tergugat dan mohon putusan;

Bahwa untuk mempersingkat uraian putusan ini, ditunjuk hal-hal sebagaimana tercatat dalam berita acara sidang perkara ini sebagai bagian yang tidak terpisahkan dari putusan ini;

Disclaimer
Kepaniteraan Mahkamah Agung Republik Indonesia berusaha untuk selalu mencantumkan informasi paling kini dan akurat sebagai bentuk komitmen Mahkamah Agung untuk pelayanan publik, transparansi dan akuntabilitas pelaksanaan fungsi peradilan. Namun dalam hal-hal tertentu masih dimungkinkan terjadi permasalahan teknis terkait dengan akurasi dan keterkinian informasi yang kami sajikan, hal mana akan terus kami perbaiki dari waktu kewaktu.
Dalam hal Anda menemukan inakurasi informasi yang termuat pada situs ini atau informasi yang seharusnya ada, namun belum tersedia, maka harap segera hubungi Kepaniteraan Mahkamah Agung RI melalui :
Email : kepaniteraan@mahkamahagung.go.id Telp : 021-384 3348 (ext.318)

TENTANG HUKUMNYA

Menimbang, bahwa maksud dan tujuan gugatan Penggugat adalah sebagaimana diuraikan diatas;

Menimbang, bahwa perkara Cerai Gugat adalah termasuk bidang perkawinan, maka sesuai dengan ketentuan Pasal 49 ayat 1 Undang-Undang Nomor 7 Tahun 1989, Tentang Peradilan Agama, sebagaimana telah diubah dengan Undang-Undang Nomor 3 Tahun 2006 dan perubahan kedua dengan Undang-Undang Nomor 50 Tahun 2009, maka perkara a quo termasuk dalam kompetensi absolut Pengadilan Agama;

Menimbang, bahwa berdasarkan Surat Gugatan Penggugat yang tidak dibantah oleh Tergugat, bahwa Penggugat bertempat tinggal di Pondok Ungu, Gang H. Rimban, No. 149, (Kontrakan milik Bapak Sarifudin), RT. 006, RW. 005, Kelurahan Medan Satria, Kecamatan Medan Satria, Kota Bekasi, (Bukti P.1), maka sesuai dengan ketentuan Pasal 4 ayat 1 dan Pasal 73 ayat 1 Undang-Undang Nomor 7 Tahun 1989, Tentang Peradilan Agama, sebagaimana telah diubah dengan Undang-Undang Nomor 3 Tahun 2006 dan perubahan kedua dengan Undang-Undang Nomor 50 Tahun 2009, wilayah tersebut merupakan wilayah hukum (yurisdiksi) Pengadilan Agama Subang, sehingga Pengadilan Agama Bekasi berwenang memeriksa dan mengadili perkara ini;

Menimbang, bahwa sesuai dengan ketentuan Pasal 7 ayat 1 Kompilasi Hukum Islam, (Inpres. No. 1 Tahun 1991), perkawinan hanya dapat dibuktikan dengan Buku Kutipan Akta Nikah, yang dibuat oleh Pegawai Pencatat Nikah, sehingga berdasarkan Buku Kutipan Akta Nikah, Nomor : XXX, tanggal 1 Agustus 2010, yang dikeluarkan oleh Kepala Kantor Urusan Agama Kecamatan Taruma Jaya, Kabupaten Bekasi, (bukti P.2), maka telah terbukti secara hukum bahwa antara Penggugat dengan Tergugat telah terikat dalam ikatan pernikahan yang sah dan selama berumah tangga belum pernah bercerai, sehingga harus dinyatakan Penggugat dengan Tergugat berkualitas hukum untuk bertindak sebagai pihak-pihak dalam perkara ini, (persona standi in judicio);

Disclaimer
Kepaniteraan Mahkamah Agung Republik Indonesia berusaha untuk selalu mencantumkan informasi paling kini dan akurat sebagai bentuk komitmen Mahkamah Agung untuk pelayanan publik, transparansi dan akuntabilitas pelaksanaan fungsi peradilan. Namun dalam hal-hal tertentu masih dimungkinkan terjadi permasalahan teknis terkait dengan akurasi dan keterkinian informasi yang kami sajikan, hal mana akan terus kami perbaiki dari waktu kewaktu.
Dalam hal Anda menemukan inakurasi informasi yang termuat pada situs ini atau informasi yang seharusnya ada, namun belum tersedia, maka harap segera hubungi Kepaniteraan Mahkamah Agung RI melalui :
Email : kepaniteraan@mahkamahagung.go.id Telp : 021-384 3348 (ext.318)

Menimbang, bahwa untuk memenuhi maksud ketentuan pasal 65 dan 82 ayat 1, Undang-Undang Nomor 7 Tahun 1989, Tentang Peradilan Agama, sebagaimana telah diubah dengan Undang-Undang Nomor 3 Tahun 2006 dan perubahan kedua dengan Undang-Undang Nomor 50 Tahun 2009, Jo. Pasal 130 ayat 1 HIR, Jo. Pasal 39 ayat 1 Undang-Undang Nomor 1 Tahun 1974, Tentang Perkawinan, Majelis Hakim di persidangan telah berupaya menasehati Penggugat dan Tergugat agar mau rukun kembali dalam membina rumah tangganya, akan tetapi upaya tersebut tidak berhasil, karena Penggugat tetap menghendaki perceraian, sedang Tergugat ketika proses mediasi meninggalkan proses mediasi dan menyerahkan sepenuhnya kepada kehendak Penggugat;

Menimbang, bahwa selanjutnya untuk memenuhi kehendak Pasal 130 HIR, Jo. Pasal 2 Peraturan Mahkamah Agung RI, Nomor 1 Tahun 2016, Tentang Prosedur Mediasi di Pengadilan, Majelis Hakim telah memerintahkan Penggugat dan Tergugat untuk mengikuti mediasi dan mediasi tersebut telah dilaksanakan pada tanggal 14 Desember 2020, oleh Mediator, Drs. HM. Fadjri Rivai, SH., MH., Mediator Pengadilan Agama Bekasi dan berdasarkan laporan mediator, tanggal 14 Desember 2020, yang menyatakan bahwa terhadap perkara aquo tidak layak untuk di mediasi, karena Tergugat meninggalkan acara proses mediasi dan menyerahkan segala sesuatunya kepada Penggugat;

Menimbang, bahwa untuk melakukan perceraian harus ada cukup alasan, bahwa suami istri tidak dapat hidup rukun dalam rumah tangganya, sebagaimana dimaksud dalam ketentuan pasal 39 ayat 2 Undang-Undang Nomor 1 Tahun 1974, Tentang Perkawinan;

Menimbang, bahwa Penggugat mengajukan gugatan cerainya terhadap Tergugat dengan alasan yang pada pokoknya adalah, bahwa dalam rumah tangganya dengan Tergugat sejak tahun 2014 mulai tidak harmonis dan terjadi perselisihan dan pertengkaran. Penyebabnya adalah karena Tergugat memiliki wanita idaman lain, yang diketahui Penggugat dari HP milik Tergugat dan sudah diakui oleh Tergugat, Tergugat diketahui sudah menikah lagi dengan wanita selingkuhannya, yang bernama Ikah tanpa izin dan sepengetahuan Penggugat dan Tergugat kurang bertanggung jawab dalam pemenuhan kebutuhan rumah tangga,

Disclaimer
Kepaniteraan Mahkamah Agung Republik Indonesia berusaha untuk selalu mencantumkan informasi paling kini dan akurat sebagai bentuk komitmen Mahkamah Agung untuk pelayanan publik, transparansi dan akuntabilitas pelaksanaan fungsi peradilan. Namun dalam hal-hal tertentu masih dimungkinkan terjadi permasalahan teknis terkait dengan akurasi dan keterkinian informasi yang kami sajikan, hal mana akan terus kami perbaiki dari waktu kewaktu.
Dalam hal Anda menemukan inakurasi informasi yang termuat pada situs ini atau informasi yang seharusnya ada, namun belum tersedia, maka harap segera hubungi Kepaniteraan Mahkamah Agung RI melalui :
Email : kepaniteraan@mahkamahagung.go.id Telp : 021-384 3348 (ext.318)

karena sejak tahun 2014 Tergugat sudah tidak memberikan nafkah kepada Penggugat;

Menimbang, bahwa akibat dari adanya perselisihan dan pertengkaran tersebut sejak tanggal 24 Pebruari 2018 antara Penggugat dengan Tergugat pisah tempat tinggal, Penggugat keluar dari rumah kediaman bersama, karena diusir oleh Tergugat dan antara keduanya sampai saat ini tidak pernah rukun kembali;

Menimbang, bahwa terhadap Gugatan Penggugat seperti tersebut diatas, di persidangan Majelis Hakim tidak dapat mendengarkan Jawaban Tergugat, karena pada proses mediasi Tergugat keluar dari acara mediasi dengan menyatakan menyerahkan sepenuhnya kepada kehendak Penggugat;

Menimbang, bahwa meskipun berdasarkan ketentuan Pasal 174 dan 176 HIR, pengakuan merupakan bukti sempurna dan mengikat, akan tetapi oleh karena perkara ini adalah perkara perceraian yang merupakan perkara khusus yang acaranya pun diatur secara khusus, antara lain dengan Pasal 76 ayat 1 Undang-Undang Nomor 1 Tahun 1974, Tentang Perkawinan, Jo. Pasal 22 Peraturan Pemerintah Nomor 9 Tahun 1975, Tentang Pelaksanaan Undang-Undang Nomor 1 Tahun 1974, Tentang Perkawinan, yang mengatur, bahwa dalam hal perceraian didasarkan atas alasan perselisihan dan pertengkaran terus menerus, maka pengadilan terlebih dahulu harus mendengar keterangan pihak keluarga dan orang-orang yang dekat dengan suami istri tersebut;

Menimbang, bahwa di persidangan Penggugat telah mengajukan bukti surat, (bukti P.2), yang merupakan akta otentik, bermeterai cukup dan telah dicocokkan dengan aslinya, isi bukti tersebut menjelaskan mengenai perkawinan Penggugat dengan Tergugat, sehingga bukti tersebut telah memenuhi syarat formil dan materiil, oleh karena itu bukti tersebut mempunyai kekuatan pembuktian yang sempurna dan mengikat;

Menimbang, bahwa berdasarkan bukti P. tersebut, maka telah terbukti menurut hukum bahwa antara Penggugat dengan Tergugat sejak tanggal 1 Agustus 2010 telah terikat dalam ikatan tali perkawinan yang sah, sesuai dengan ketentuan Pasal 2 Undang-Undang Nomor 1 Tahun 1974, Tentang Perkawinan, Jo. Pasal 4 Kompilasi Hukum Islam, (Inpres Nomor 1 Tahun 1991);

Disclaimer
Kepaniteraan Mahkamah Agung Republik Indonesia berusaha untuk selalu mencantumkan informasi paling kini dan akurat sebagai bentuk komitmen Mahkamah Agung untuk pelayanan publik, transparansi dan akuntabilitas pelaksanaan fungsi peradilan. Namun dalam hal-hal tertentu masih dimungkinkan terjadi permasalahan teknis terkait dengan akurasi dan keterkinian informasi yang kami sajikan, hal mana akan terus kami perbaiki dari waktu kewaktu.
Dalam hal Anda menemukan inakurasi informasi yang termuat pada situs ini atau informasi yang seharusnya ada, namun belum tersedia, maka harap segera hubungi Kepaniteraan Mahkamah Agung RI melalui :
Email : kepaniteraan@mahkamahagung.go.id Telp : 021-384 3348 (ext.318)

Menimbang, bahwa selain mengajukan bukti tertulis seperti tersebut diatas, di persidangan Penggugat juga mengajukan 2 orang saksi, yang telah memenuhi syarat formil dan materiil kesaksian, sehingga patut untuk dipertimbangkan;

Menimbang, bahwa berdasarkan keterangan para saksi Penggugat yang saling berkesesuaian satu sama lainnya yang membenarkan bahwa dalam rumah tangga Penggugat dengan Tergugat sering terjadi perselisihan dan pertengkaran dan membenarkan pula bahwa dalam rumah tangga Penggugat dengan Tergugat sejak bulan Pebruari 2018 sampai dengan sekarang telah pisah tempat tinggal, Penggugat keluar dari rumah kediaman bersama, karena diusir oleh Penggugat dan selama itu pula antara Penggugat dengan Tergugat tidak pernah rukun kembali;

Menimbang, bahwa di persidangan Majelis Hakim tidak dapat mendengarkan Jawaban Tergugat, karena pada acara mediasi Tergugat meninggalkan acara mediasi dan menyerahkan sepenuhnya kepada Penggugat;

Menimbang, bahwa Majelis Hakim dalam telah berusaha merukunkan Penggugat dengan Tergugat, bahkan dengan melibatkan Mediator, akan tetapi upaya tersebut tidak berhasil, karena Penggugat tetap teguh dengan pendiriannya ingin bercerai dengan Tergugat;

Menimbang, bahwa pihak keluarga Penggugat di persidangan menyatakan ketidaksanggupannya untuk merukunkan kembali Penggugat dengan Tergugat, fakta tersebut merupakan indikasi bahwa permasalahan rumah tangga yang sedang dihadapi oleh Penggugat dengan Tergugat sudah pada taraf tingkatan yang sulit untuk dirukunkan lagi, karena Penggugat tetap bersikeras sudah tidak mau rukun dengan Tergugat;

Menimbang, bahwa dalam Yurisprudensi Mahkamah Agung RI. Nomor : 534 K/Pdt./1996, tanggal 18 Juni 1996, ditentukan kaidah hukum bahwa dalam hal perceraian yang perlu dilihat adalah perkawinan itu sendiri apakah masih dapat dipertahankan atau tidak, tanpa mempersoalkan apa dan siapa yang menjadi penyebab terjadinya perselisihan dan pertengkaran dalam rumah tangga tersebut;

Disclaimer
Kepaniteraan Mahkamah Agung Republik Indonesia berusaha untuk selalu mencantumkan informasi paling kini dan akurat sebagai bentuk komitmen Mahkamah Agung untuk pelayanan publik, transparansi dan akuntabilitas pelaksanaan fungsi peradilan. Namun dalam hal-hal tertentu masih dimungkinkan terjadi permasalahan teknis terkait dengan akurasi dan keterkinian informasi yang kami sajikan, hal mana akan terus kami perbaiki dari waktu kewaktu.
Dalam hal Anda menemukan inakurasi informasi yang termuat pada situs ini atau informasi yang seharusnya ada, namun belum tersedia, maka harap segera hubungi Kepaniteraan Mahkamah Agung RI melalui :
Email : kepaniteraan@mahkamahagung.go.id Telp : 021-384 3348 (ext.318)

Menimbang, bahwa yang dimaksud dengan perselisihan dan pertengkaran dalam rumah tangga tidaklah identik dengan pertengkaran mulut, rumah tangga dapat dinyatakan telah terjadi perselisihan dan pertengkaran jika hubungan antara pasangan suami istri sudah tidak lagi selaras, tidak saling percaya dan tidak saling melindungi;

Menimbang, bahwa dengan ditemukannya fakta bahwa antara Penggugat dengan Tergugat telah pisah rumah, Penggugat keluar dari rumah kediaman bersama, karena diusir oleh Tergugat, menunjukkan bahwa antara Penggugat dengan Tergugat sudah tidak lagi saling percaya, tidak saling melindungi dan sudah tidak ada lagi komunikasi antara suami istri yang harmonis yang merupakan bagian dari gejala perselisihan dan pertengkaran dalam rumah tangga, sebab kalau rumah tangga Penggugat dengan Tergugat rukun-rukun saja, tentunya Penggugat dengan Tergugat tidak pisah rumah yang sampai sekarang sudah berjalan selama lebih kurang 2 tahun 10 bulan lamanya;

Menimbang, bahwa dari keterangan Penggugat dan keterangan para saksi bila dihubungkan dengan gugatan Penggugat, maka ditemukan fakta-fakta hukum yang dapat disimpulkan sebagai berikut;

---Bahwa Penggugat dengan Tergugat adalah suami istri sah, yang menikah pada tanggal 1 Agustus 2010 dan dari pernikahannya telah dikaruniai seorang anak;

---Bahwa setelah menikah Penggugat dengan Tergugat membina rumah tangga dan tinggal bersama di rumah kontrakan Ibu XXX;

---Bahwa antara Penggugat dengan Tergugat sejak tahun 2014 sering terjadi perselisihan dan pertengkaran terus menerus yang sudah sulit untuk didamaikan kembali, yang berakibat sejak bulan Pebruari 2018 Penggugat dengan Tergugat pisah tempat tinggal, Penggugat keluar dari rumah kediaman bersama, karena diusir oleh Penggugat;

---Bahwa selama Penggugat dengan Tergugat pisah rumah dan selama itu pula

Disclaimer
Kepaniteraan Mahkamah Agung Republik Indonesia berusaha untuk selalu mencantumkan informasi paling kini dan akurat sebagai bentuk komitmen Mahkamah Agung untuk pelayanan publik, transparansi dan akuntabilitas pelaksanaan fungsi peradilan. Namun dalam hal-hal tertentu masih dimungkinkan terjadi permasalahan teknis terkait dengan akurasi dan keterkinian informasi yang kami sajikan, hal mana akan terus kami perbaiki dari waktu kewaktu.
Dalam hal Anda menemukan inakurasi informasi yang termuat pada situs ini atau informasi yang seharusnya ada, namun belum tersedia, maka harap segera hubungi Kepaniteraan Mahkamah Agung RI melalui :
Email : kepaniteraan@mahkamahagung.go.id   Telp : 021-384 3348 (ext.318)

antara keduanya sudah tidak saling melaksanakan hubungan sebagaimana layaknya suami istri;

---Bahwa Majelis Hakim, Mediator dan keluarga Penggugat telah berusaha mendamaikan Penggugat dengan Tergugat, namun tidak berhasil;

---Bahwa di persidangan Penggugat menyatakan tekadnya untuk tetap bercerai dengan Tergugat;

Menimbang, bahwa dengan telah pisah tempat tinggalnya Penggugat dengan Tergugat dan sudah tidak adanya saling melaksanakan hak dan kewajibannya sebagai suami istri selama lebih kurang 2 tahun 10 bulan lamanya, menunjukkan bahwa rumah tangga Penggugat dengan Tergugat sudah pecah, sehingga tidak ada harapan bagi keduanya untuk dapat hidup rukun dan damai lagi sebagai suami istri dalam rumah tangga, sesuai dengan Yurisprudensi Mahkamah Agung RI. Nomor : 379 K/AG/1995, tanggal 26 Maret 1997, bahwa suami istri yang tidak berdiam serumah lagi dan tidak ada harapan untuk hidup rukun kembali, maka rumah tangga tersebut terbukti telah retak dan pecah;

Menimbang, bahwa tujuan perkawinan adalah dalam rangka untuk membentuk keluarga (rumah tangga) yang bahagia dan kekal berdasarkan Ketuhanan Yang Maha Esa serta untuk mewujudkan rumah tangga yang sakinah, mawaddah dan rahmah yang diliputi dengan rasa saling cinta mencintai, hormat menghormati, setia dan saling memberi bantuan lahir dan batin yang satu kepada yang lain, sebagaiman dikehendaki Pasal 1 Undang-Undang Nomor 1 Tahun 1974, Tentang Perkawinan, Jo. Pasal 3 Kompilasi Hukum Islam, (Inpres. Nomor 1 Tahun 1991), hal ini sejalan dengan firman Allah dalam Surat Ar-Ruum ayat 21 yang artinya ; Dan diantara tanda-tanda kekuasaan-Nya ialah Dia menciptakan untukmu istri-istri dari jenismu sendiri, supaya kamu cenderung dan merasa tenteram kepadanya, dan dijadikan-Nya dinataramu rasa kasih dan sayang. Sesungguhnya pada yang demikian itu bernar-benar terdapat tanda-tanda bagi kaum yang berfikir;

Menimbang, bahwa oleh karena Penggugat dengan Tergugat sudah tidak mungkin lagi dapat mewujudkan tujuan hidup berumah tangga tersebut dengan

Disclaimer
Kepaniteraan Mahkamah Agung Republik Indonesia berusaha untuk selalu mencantumkan informasi paling kini dan akurat sebagai bentuk komitmen Mahkamah Agung untuk pelayanan publik, transparansi dan akuntabilitas pelaksanaan fungsi peradilan. Namun dalam hal-hal tertentu masih dimungkinkan terjadi permasalahan teknis terkait dengan akurasi dan keterkinian informasi yang kami sajikan, hal mana akan terus kami perbaiki dari waktu kewaktu.
Dalam hal Anda menemukan inakurasi informasi yang termuat pada situs ini atau informasi yang seharusnya ada, namun belum tersedia, maka harap segera hubungi Kepaniteraan Mahkamah Agung RI melalui :
Email : kepaniteraan@mahkamahagung.go.id Telp : 021-384 3348 (ext.318)

tidak ada harapan akan hidup rukun kembali dalam membina rumah tangga, maka Majelis Hakim berpendapat, kalau sekiranya keduanya dipaksa untuk tetap bersatu dalam rumah tangga, maka patut diduga bukan keharmonisan dan kedamaian yang akan muncul, malah keduanya akan selalu dalam kegelisahan. Membiarkan keduanya dalam keadaan seperti itu merupakan aniaya yang besar, oleh karena itu perceraian adalah jalan keluarnya agar keduanya terlepas dari perselisihan dan penderitaan batin yang bekepanjangan, hal ini sesuai dengan kaidah ushul fiqh, yang artinya : “Menolak kemudharatan lebih utama dari pada menarik (mempertahankan) kemaslahatan”;

Menimbang, bahwa Majelis Hakim berpendapat dan mengambil alih sebagai pendapat majelis terhadap pendapat pakar hukum islam dalam Kitab Ghoyatul Marom, yang artinya : “Apabila si istri telah menunjukkan sikap sangat benci kepada suaminya, maka Hakim boleh menjatuhkan talak satu suaminya kepada istrinya”;

Menimbang, bahwa berdasarkan ketentuan Pasal 33 Undang-Undang Nomor 1 Tahun 1974, Tentang Perkawinan : *“Suami istri wajib saling cinta mencintai, hormat menghormati, setia dan memberi bantuan lahir batin yang satu kepada yang lainnya”,* namun ternyata hal itu sudah tidak terwujud lagi dalam rumah tangga Penggugat dengan Tergugat, karena keduanya sudah hidup berpisah dan Penggugat telah menyatakan tidak ingin bersatu dan rukun lagi dengan Tergugat sebagai suami istri;

Menimbang, bahwa berdasarkan fakta-fakta dan pertimbangan-pertimbangan seperti tersebut diatas, Majelis Hakim memandang dalil gugatan Penggugat telah memenuhi maksud Pasal 19 huruf f Peraturan Pemerintah Nomor 9 Tahun 1975, Tentang Pelaksanaan Undang-Undang Nomor 1 Tahun 1974, Tentang Perkawinan, Jo. Pasal 116 huruf f Kompilasi Hukum Islam (Instruksi Presiden Nomor 1 Tahun 1991);

Mengingat semua ketentuan perundang-undangan yang berlaku dan hukum syara’ yang berkaitan dengan perkara ini;

M E N G A D I L I

Disclaimer
Kepaniteraan Mahkamah Agung Republik Indonesia berusaha untuk selalu mencantumkan informasi paling kini dan akurat sebagai bentuk komitmen Mahkamah Agung untuk pelayanan publik, transparansi dan akuntabilitas pelaksanaan fungsi peradilan. Namun dalam hal-hal tertentu masih dimungkinkan terjadi permasalahan teknis terkait dengan akurasi dan keterkinian informasi yang kami sajikan, hal mana akan terus kami perbaiki dari waktu kewaktu.
Dalam hal Anda menemukan inakurasi informasi yang termuat pada situs ini atau informasi yang seharusnya ada, namun belum tersedia, maka harap segera hubungi Kepaniteraan Mahkamah Agung RI melalui :
Email : kepaniteraan@mahkamahagung.go.id Telp : 021-384 3348 (ext.318)

1. Mengabulkan gugatan Penggugat;
2. Menjatuhkan talak satu ba'in sughra Tergugat (XXX) terhadap Penggugat (XXX);
3. Membebankan biaya perkara ini kepada Penggugat yang hingga kini dihitung sejumlah Rp.626.000,- (enam ratus dua puluh enam ribu rupiah) ;

Demikian putusan ini dijatuhkan di Bekasi dalam sidang permusyawaratan Majelis Hakim pada hari **Senin**, tanggal **28 Desember 2020 Masehi,** yang bertepatan dengan tanggal **13 Jumadil Awwal 1442 Hijriyyah,** oleh kami **Drs. H. Sirojuddin, S.H.** sebagai Ketua Majelis, serta **Drs. H. Syarif Hidayat, S.H.,** dan **Hj. Susilawati, S.E.I.,** masing-masing sebagai Hakim Anggota, dibantu oleh **Hj. Eva Zulva Wardiyanti, SHI.,MH.**, sebagai Panitera Pengganti, Putusan tersebut pada hari itu juga diucapkan Ketua Majelis dalam sidang yang dinyatakan terbuka untuk umum dengan dihadiri Hakim Anggota dan Panitera Pengganti serta Penggugat, diluar hadirnya Tergugat;

Ketua Majelis,

Drs. H. Sirojuddin, S.H.

Hakim Anggota I,

Drs. H.Syarif Hidayat, S.H.,

Hakim Anggota II,

Hj. Susilawati,S.E.I

Panitera Pengganti,

Disclaimer
Kepaniteraan Mahkamah Agung Republik Indonesia berusaha untuk selalu mencantumkan informasi paling kini dan akurat sebagai bentuk komitmen Mahkamah Agung untuk pelayanan publik, transparansi dan akuntabilitas pelaksanaan fungsi peradilan. Namun dalam hal-hal tertentu masih dimungkinkan terjadi permasalahan teknis terkait dengan akurasi dan keterkinian informasi yang kami sajikan, hal mana akan terus kami perbaiki dari waktu kewaktu.
Dalam hal Anda menemukan inakurasi informasi yang termuat pada situs ini atau informasi yang seharusnya ada, namun belum tersedia, maka harap segera hubungi Kepaniteraan Mahkamah Agung RI melalui :
Email : kepaniteraan@mahkamahagung.go.id Telp : 021-384 3348 (ext.318)

Hj, Eva Zulva Wardiyanti, SHI., MH.

Perincian Biaya Perkara :

- Biaya Pendaftaran-- Rp. 30.000,-
- Biaya Proses--- Rp. 50.000,-
- Biaya Panggilan--- Rp. 510.000,-
- BIaya PNBP Panggilan Rp. 20.000,-
- Biaya Redaksi-- Rp. 10.000,-
- Biaya Meterai-- Rp. 6.000,- +

Jumlah Rp. 626.000,-

(enam ratus dua puluh enam ribu rupiah);

Halaman 16 dari 15 halaman Putusan Nomor 4059/Pdt.G/2020/PA.Bks.

Disclaimer
Kepaniteraan Mahkamah Agung Republik Indonesia berusaha untuk selalu mencantumkan informasi paling kini dan akurat sebagai bentuk komitmen Mahkamah Agung untuk pelayanan publik, transparansi dan akuntabilitas pelaksanaan fungsi peradilan. Namun dalam hal-hal tertentu masih dimungkinkan terjadi permasalahan teknis terkait dengan akurasi dan keterkinian informasi yang kami sajikan, hal mana akan terus kami perbaiki dari waktu kewaktu.
Dalam hal Anda menemukan inakurasi informasi yang termuat pada situs ini atau informasi yang seharusnya ada, namun belum tersedia, maka harap segera hubungi Kepaniteraan Mahkamah Agung RI melalui :
Email : kepaniteraan@mahkamahagung.go.id Telp : 021-384 3348 (ext.318)

Disclaimer
Kepaniteraan Mahkamah Agung Republik Indonesia berusaha untuk selalu mencantumkan informasi paling kini dan akurat sebagai bentuk komitmen Mahkamah Agung untuk pelayanan publik, transparansi dan akuntabilitas pelaksanaan fungsi peradilan. Namun dalam hal-hal tertentu masih dimungkinkan terjadi permasalahan teknis terkait dengan akurasi dan keterkinian informasi yang kami sajikan, hal mana akan terus kami perbaiki dari waktu kewaktu.
Dalam hal Anda menemukan inakurasi informasi yang termuat pada situs ini atau informasi yang seharusnya ada, namun belum tersedia, maka harap segera hubungi Kepaniteraan Mahkamah Agung RI melalui :
Email : kepaniteraan@mahkamahagung.go.id   Telp : 021-384 3348 (ext.318)
