# Supplementary material for: Classification of divorce causes during the COVID-19 pandemic using convolutional neural networks
Source: PeerJ Comput Sci. 2022 Jun 30;8:e998. doi: 10.7717/peerj-cs.998 (PMC9299239; doi:10.7717/peerj-cs.998)
Supplement: Supplemental Information 2 [file peerj-cs-08-998-s002.zip › Kelalaian Dataset/Data ke-12.pdf]

P U T U S A N

Nomor 3712/Pdt.G/2020/PA.Bks

بِسْمِ اللهِ الرَّحْمٰنِ الرَّحِيْمِ

DEMI KEADILAN BERDASARKAN KETUHANAN YANG MAHA ESA

PENGADILAN AGAMA BEKASI

Memeriksa dan mengadili perkara perdata agama pada tingkat pertama telah menjatuhkan putusan dalam perkara Cerai Talak antara:

XXXX, tempat dan tanggal lahir Jakarta 16 Desember 1978, umur 41 tahun, agama Islam, pendidikan S1, pekerjaan GURU, tempat tinggal di XXXX, sebagai **Pemohon**;

melawan

XXXX, tempat dan tanggal lahir Jakarta 30 Juni 1975, umur 45 tahun, agama Islam, pendidikan S1, pekerjaan GURU, tempat tinggal di XXXX, sebagai **Termohon**;

Pengadilan Agama tersebut;

Telah mempelajari berkas perkara ini;

DUDUK PERKARA

Bahwa Pemohon telah mengajukan permohonan Cerai Talak pada tanggal 05 November 2020 yang telah terdaftar di Kepaniteraan Pengadilan Agama Bekasi pada tanggal yang sama tercatat dalam register perkara Nomor 3712/Pdt.G/2020/PA.Bks telah mengemukakan hal-hal sebagai berikut:

1. Bahwa Pemohon dengan Termohon telah melangsungkan pernikahan pada hari Minggu tanggal 18 Juni 2006, yang dicatat oleh Pegawai Pencatat Nikah Kantor Urusan Agama Kecamatan XXXX, sesuai Kutipan Akta Nikah Nomor XXXX, tanggal 19 Juni 2006 dan setelah akad nikah Termohon sebagaimana dalam Kutipan Akta Nikah tersebut;
2. Bahwa setelah menikah, Pemohon dengan Termohon XXXX;

Disclaimer
Kepaniteraan Mahkamah Agung Republik Indonesia berusaha untuk selalu mencantumkan informasi paling kini dan akurat sebagai bentuk komitmen Mahkamah Agung untuk pelayanan publik, transparansi dan akuntabilitas pelaksanaan fungsi peradilan. Namun dalam hal-hal tertentu masih dimungkinkan terjadi permasalahan teknis terkait dengan akurasi dan keterkinian informasi yang kami sajikan, hal mana akan terus kami perbaiki dari waktu kewaktu.
Dalam hal Anda menemukan inakurasi informasi yang termuat pada situs ini atau informasi yang seharusnya ada, namun belum tersedia, maka harap segera hubungi Kepaniteraan Mahkamah Agung RI melalui :
Email : kepaniteraan@mahkamahagung.go.id   Telp : 021-384 3348 (ext.318)

3. Bahwa selama pernikahan tersebut Pemohon dengan Termohon telah hidup bersama sebagaimana layaknya suami istri dan ...
4. Bahwa rumah tangga Pemohon dengan Termohon awalnya rukun-rukun saja selama tahun 2009 namun setelah itu dalam rumah tangga Pemohon dengan Termohon terus menerus terjadi perselisihan dan pertengkaran disebabkan antara lain:

   Termohon sering lepas control dan melakukan kekerasan fisik terhadap Pemohon dan Pemohon beberapa kali diusir dan diminta dengan paksa keluar dari rumah dan menjauh dari Termohon;
5. Bahwa puncak perselisihan dan pertengkaran antara Pemohon dan Termohon terjadi pada Februari 2020 disebabkan pisah rumah, sampai saat ini Pemohon dan Termohon telah berpisah kurang lebih 9 (sembilan) bulan lamanya lamanya dan selama berpisah antara Pemohon dan Termohon sudah tidak ada lagi hubungan baik lahir maupun batin dan Termohon tidak pernah memberikan nafkah wajib kepada Pemohon;
6. Bahwa pihak keluarga Pemohon dan Termohon telah berupaya mengatasi masalah tersebut dengan jalan musyawarah namun tidak berhasil;
7. Bahwa, dengan sikap dan perbuatan Termohon tersebut, Pemohon merasa tersiksa baik lahir maupun batin sehingga Pemohon tidak sanggup lagi berumah tangga dengan Termohon, oleh karenanya Pemohon bermaksud bercerai dengan Termohon di depan sidang Pengadilan Agama Bekasi;
8. Bahwa Pemohon bersedia membayar seluruh biaya yang timbul dalam perkara ini;

Bahwa berdasarkan dalil-dalil tersebut di atas, maka Pemohon mohon kepada Ketua Pengadilan Agama Bekasi cq. Majelis Hakim untuk memeriksa dan mengadili perkara ini, dan selanjutnya dapat memutuskan sebagai berikut :

PRIMAIR:

1. Mengabulkan permohonan Pemohon;
2. Memberikan izin kepada Pemohon (**XXXX**) untuk menjatuhkan talak satu raj'i terhadap Termohon (**XXXX**) di hadapan sidang Pengadilan Agama Bekasi;

Disclaimer
Kepaniteraan Mahkamah Agung Republik Indonesia berusaha untuk selalu mencantumkan informasi paling kini dan akurat sebagai bentuk komitmen Mahkamah Agung untuk pelayanan publik, transparansi dan akuntabilitas pelaksanaan fungsi peradilan. Namun dalam hal-hal tertentu masih dimungkinkan terjadi permasalahan teknis terkait dengan akurasi dan keterkinian informasi yang kami sajikan, hal mana akan terus kami perbaiki dari waktu kewaktu.
Dalam hal Anda menemukan inakurasi informasi yang termuat pada situs ini atau informasi yang seharusnya ada, namun belum tersedia, maka harap segera hubungi Kepaniteraan Mahkamah Agung RI melalui :
Email : kepaniteraan@mahkamahagung.go.id   Telp : 021-384 3348 (ext.318)

3. Membebankan biaya perkara kepada Pemohon sesuai dengan hukum yang berlaku;

SUBSIDAIR:

Dan, atau apabila Majelis Hakim berpendapat lain, mohon untuk menjatuhkan putusan yang seadil-adilnya (ex aequo et bono);

Bahwa pada hari sidang yang telah ditetapkan, Pemohon datang menghadap sendiri di persidangan, sedangkan Termohon tidak datang menghadap dan tidak menyuruh orang lain untuk menghadap sebagai kuasanya, meskipun menurut relaas panggilan Nomor 3712/Pdt.G/2020/PA.Bks tanggal 08 Desember 2020, dan tanggal 16 Desember 2020. Termohon telah dipanggil secara sah dan patut sedangkan tidak ternyata bahwa ketidak hadiran Termohon tersebut disebabkan oleh suatu halangan yang sah ;

Bahwa Majelis Hakim telah berusaha menasehati Pemohon untuk bersabar dan hidup rukun lagi dengan Termohon, akan tetapi tidak berhasil, lalu dibacakan surat gugatan Pemohon yang isinya tetap dipertahankan oleh Pemohon;

Bahwa untuk memperkuat dalil-dalilnya, Pemohon telah mengajukan bukti-bukti surat berupa :

1.Fotokopi Kartu Tanda Penduduk (KTP), 3275091612780010 atas nama Pemohon yang dikeluarkan oleh pemerintah kota Bekasi, bukti surat tersebut telah diberi meterai cukup dan telah dicocokkan dengan aslinya yang ternyata sesuai, lalu oleh Ketua Majelis diberi tanda P1;

2.Fotokopi Kutipan Akta Nikah Nomor 649/66/VI/2006 tanggal 19 Juni 2006 yang dikeluarkan oleh Kantor Urusan Agama Kecamatan Kantor Urusan Agama (KUA) Kecamatan Jatiasih, Kota Bekasi., bukti surat tersebut telah diberi meterai cukup dan telah dicocokkan dengan aslinya yang ternyata sesuai, lalu oleh Ketua Majelis diberi tanda P.2;

Bahwa selain itu Pemohon mengajukan bukti saksi dari keluarga dan tetangga dekatnya sebagai berikut :

Disclaimer
Kepaniteraan Mahkamah Agung Republik Indonesia berusaha untuk selalu mencantumkan informasi paling kini dan akurat sebagai bentuk komitmen Mahkamah Agung untuk pelayanan publik, transparansi dan akuntabilitas pelaksanaan fungsi peradilan. Namun dalam hal-hal tertentu masih dimungkinkan terjadi permasalahan teknis terkait dengan akurasi dan keterkinian informasi yang kami sajikan, hal mana akan terus kami perbaiki dari waktu kewaktu.
Dalam hal Anda menemukan inakurasi informasi yang termuat pada situs ini atau informasi yang seharusnya ada, namun belum tersedia, maka harap segera hubungi Kepaniteraan Mahkamah Agung RI melalui :
Email : kepaniteraan@mahkamahagung.go.id   Telp : 021-384 3348 (ext.318)

1. **XXXX**, tempat tinggal di XXXX, di hadapan sidang saksi tersebut memberikan keterangan di bawah sumpah yang pada pokoknya sebagai berikut :

4. B ahwa saksi kenal dengan Pemohon dengan Termohon karena saya adalah ibu Pemohon;
5. Bahwa benar Pemohon dan Termohon adalah suami isteri yang telah menikah pada tanggal 18 Juni 2006 di XXXX;
6. Bahwa Pemohon dan Termohon tinggal bersama diXXXX;
7. Bahwa saksi mengetahui Pemohon dengan Termohon adalah suami istri dan ...;
8. Bahwa sejak tahun 2009 rumah tangga Pemohon dan Termohon sering terjadi perselisihan dan pertengkaran terus menerus yang penyebabnya karena Termohon sering lepas control dan melakukan kekerasan fisik terhadap Pemohon dan Pemohon beberapa kali diusir dan diminta dengan paksa keluar dari rumah dan menjauh dari Termohon;;
9. Bahwa Saksi mendapat cerita dari Pemohon terkait pertengkaran dalam rumah tangga Pemohon dan Termohon;
10. Bahwa Pemohon dan Termohon sudah pisah rumah sejak Februari 2020 sampai sekarang;
11. Bahwa selama berpisah rumah, mereka tidak pernah berkomunikasi layaknya suami istri;
12. Bahwa, pihak dari keluarga kedua belah pihak telah berupaya untuk merukunkan kembali dan saksi sudah beberapa kali memberi saran agar rukun lagi, akan tetapi tidak berhasil;

2. **XXXX**, bertempat tinggal di XXXX, dihadapan sidang saksi tersebut memberikan keterangan di bawah sumpah yang pada pokoknya sebagai berikut :

13. B ahwa saksi kenal dengan Pemohon dan Termohon karena saksi kakak Pemohon;

Disclaimer
Kepaniteraan Mahkamah Agung Republik Indonesia berusaha untuk selalu mencantumkan informasi paling kini dan akurat sebagai bentuk komitmen Mahkamah Agung untuk pelayanan publik, transparansi dan akuntabilitas pelaksanaan fungsi peradilan. Namun dalam hal-hal tertentu masih dimungkinkan terjadi permasalahan teknis terkait dengan akurasi dan keterkinian informasi yang kami sajikan, hal mana akan terus kami perbaiki dari waktu kewaktu.
Dalam hal Anda menemukan inakurasi informasi yang termuat pada situs ini atau informasi yang seharusnya ada, namun belum tersedia, maka harap segera hubungi Kepaniteraan Mahkamah Agung RI melalui :
Email : kepaniteraan@mahkamahagung.go.id Telp : 021-384 3348 (ext.318)

14. Bahwa benar Pemohon dan Termohon adalah suami isteri yang telah menikah pada tanggal 18 Juni 2006 di XXXX;
15. Bahwa setelah menikah Pemohon dan Termohon bertempat tinggal terakhir diXXXX;
16. Bahwa setelah menikah Pemohon dan Termohon hidup rukun dan harmonis sebagaimana layaknya suami isteri dan ...;
17. Bahwa sejak tahun 2009 rumah tangga Pemohon dan Termohon sering terjadi perselisihan dan pertengkaran terus menerus yang penyebabnya karena Termohon sering lepas control dan melakukan kekerasan fisik terhadap Pemohon dan Pemohon beberapa kali diusir dan diminta dengan paksa keluar dari rumah dan menjauh dari Termohon;;
18. Bahwa, Saksi mendapat cerita dari Pemohon terkait pertengkaran dalam rumah tangga Pemohon dan Termohon;
19. Bahwa, Pemohon dan Termohon sudah pisah rumah sejak Februari 2020 sampai sekarang dan sudah tidak pernah lagi berkomunikasi yang baik layaknya suami isteri;
20. Bahwa, pihak dari keluarga kedua belah pihak telah berupaya untuk merukunkan kembali dan saksi sudah beberapa kali memberi saran agar rukun lagi, akan tetapi tidak berhasil;

Bahwa Pemohon menyampaikan kesimpulan yang isinya sebagaimana tertuang dalam berita acara sidang;

Selanjutnya untuk singkatnya uraian putusan ini, maka semua yang termuat dalam berita acara sidang merupakan bagian yang tidak terpisahkan dari putusan ini;

PERTIMBANGAN HUKUM

Menimbang, bahwa maksud dan tujuan permohonan Pemohon pada pokoknya adalah sebagaimana tersebut di atas ;

Menimbang, bahwa ternyata Termohon telah dipanggil secara resmi dan patut untuk menghadap di persidangan akan tetapi Termohon tidak hadir dan tidak ternyata bahwa tidak hadirnya itu disebabkan oleh suatu halangan yang sah, oleh karena itu Termohon harus dinyatakan tidak hadir dan karenanya

Disclaimer
Kepaniteraan Mahkamah Agung Republik Indonesia berusaha untuk selalu mencantumkan informasi paling kini dan akurat sebagai bentuk komitmen Mahkamah Agung untuk pelayanan publik, transparansi dan akuntabilitas pelaksanaan fungsi peradilan. Namun dalam hal-hal tertentu masih dimungkinkan terjadi permasalahan teknis terkait dengan akurasi dan keterkinian informasi yang kami sajikan, hal mana akan terus kami perbaiki dari waktu kewaktu.
Dalam hal Anda menemukan inakurasi informasi yang termuat pada situs ini atau informasi yang seharusnya ada, namun belum tersedia, maka harap segera hubungi Kepaniteraan Mahkamah Agung RI melalui :
Email : kepaniteraan@mahkamahagung.go.id Telp : 021-384 3348 (ext.318)

gugatan Pemohon tersebut dapat diputus dengan verstek, sesuai dengan pasal 125 ayat (1) HIR ;

Dalam Pokok Perkara

Menimbang, bahwa inti permohonan Pemohon adalah permohonan cerai talak terhadap Termohon dengan alasan pasal 19 huruf f Peraturan Pemerintah Nomor 9 Tahun 1975 jo Pasal 116 huruf (f) Kompilasi Hukum Islam yaitu : "*antara suami dan isteri terus menerus terjadi perselisihan dan pertengkaran dan tidak ada harapan akan hidup rukun lagi dalam rumah tangga"*, oleh karena itu yang harus dibuktikan oleh Pemohon adalah kejadian yang dijadikan alasan tersebut.

Analisis Pembuktian

Menimbang, bahwa Pemohon meskipun tidak ada bantahan telah mengajukan bukti surat P.1 dan P.2 serta dua orang saksi;

Menimbang, bahwa bukti P.1 (Foto Copy Kartu Tanda Penduduk atas nama Pemohon) telah memenuhi syarat formil dan materiil sebagai akta otentik, oleh karena itu mempunyai kekuatan pembuktian yang sempurna dan mengikat sesuai Pasal 165 HIR juncto Pasal 1870 KUHPerdata, membuktikan bahwa identitas Pemohon benar sebagaimana tercantum dalam gugatannya.

Menimbang, bahwa bukti P.2 (Foto Copy Kutipan Akta Nikah atas nama Pemohon dan Termohon) telah memenuhi syarat formil dan materiil sebagai akta otentik, oleh karena itu mempunyai kekuatan pembuktian yang sempurna dan mengikat sesuai Pasal 165 HIR juncto Pasal 1870 KUHPerdata, membuktikan bahwa Pemohon dengan Termohon menikah sah di hadapan Pegawai Pencatat Nikah di XXXX;

Menimbang, bahwa saksi 1 dan 2 dari Pemohon memenuhi syarat formil dan materiil sebagai saksi sesuai Pasal 145 ayat (1) HIR dan Pasal 171 dan 172 HIR. sehingga membuktikan bahwa Pemohon dan Termohon bertempat tinggal terakhir di XXXX, ..., sejak tahun 2009 rumah tangga Pemohon dan Termohon sering terjadi pertengkaran terus menerus yang penyebabnya karena Termohon sering lepas control dan melakukan kekerasan fisik terhadap Pemohon dan Pemohon beberapa kali diusir dan diminta dengan paksa keluar dari rumah dan menjauh dari Termohon; dan sejak saat itu antara

Disclaimer
Kepaniteraan Mahkamah Agung Republik Indonesia berusaha untuk selalu mencantumkan informasi paling kini dan akurat sebagai bentuk komitmen Mahkamah Agung untuk pelayanan publik, transparansi dan akuntabilitas pelaksanaan fungsi peradilan. Namun dalam hal-hal tertentu masih dimungkinkan terjadi permasalahan teknis terkait dengan akurasi dan keterkinian informasi yang kami sajikan, hal mana akan terus kami perbaiki dari waktu kewaktu.
Dalam hal Anda menemukan inakurasi informasi yang termuat pada situs ini atau informasi yang seharusnya ada, namun belum tersedia, maka harap segera hubungi Kepaniteraan Mahkamah Agung RI melalui :
Email : kepaniteraan@mahkamahagung.go.id Telp : 021-384 3348 (ext.318)

Pemohon dan Termohon sudah tidak terjalin hubungan sebagaimana layaknya suami dan istri hingga saat ini;

Bahwa saksi sudah beberapa kali memberi saran kepada Pemohon dan Termohon agar rukun lagi, tetapi tidak berhasil;

Fakta Hukum

Menimbang, bahwa berdasarkan analisis bukti-bukti dari kedua belah pihak ditemukan fakta-fakta sebagai berikut:

1. Bahwa Pemohon dan Termohon telah menikah sah di hadapan Pegawai Pencatat Nikah pada tanggal 18 Juni 2006 di XXXX;
2. Bahwa Pemohon dan Termohon telah tinggal bersama terakhir diXXXX dan dari pernikahan tersebut mereka ...;
3. Bahwa sejak tahun 2009 rumah tangga Pemohon dan Termohon sering terjadi perselisihan dan pertengkaran terus menerus yang penyebabnya karena Termohon sering lepas control dan melakukan kekerasan fisik terhadap Pemohon dan Pemohon beberapa kali diusir dan diminta dengan paksa keluar dari rumah dan menjauh dari Termohon;;
4. Bahwa puncak perselisihan dan pertengkaran antara Pemohon dengan Termohon tersebut sejak Februari 2020 dimana antara Pemohon dan Termohon telah pisah rumah sampai sekarang;
5. Bahwa kaluarga Pemohon dan Termohon sudah menasihati Pemohon supaya rukun lagi dengan Termohon, namun tidak berhasil.

Pertimbangan Petitum Perceraian

Menimbang, bahwa berdasarkan petitum gugatan Pemohon dan fakta hukum tersebut di atas, Majelis Hakim memberi pertimbangan sebagai berikut:

Bahwa terhadap fakta hukum tersebut, Majelis Hakim mempertimbangkan penerapan hukumnya (*mengkonstitutir*) sesuai alasan gugatan Pemohon yaitu Pasal 19 huruf (f) Peraturan Pemerintah Nomor 9 Tahun 1975 jo. Pasal 116 huruf (f) Kompilasi Hukum Islam yang unsur-unsurnya yaitu:

1. *“Antara suami dan isteri“* : fakta hukum pada angka 1 menunjukkan Pemohon dan Termohon adalah suami isteri sah dan belum pernah bercerai ;

Disclaimer
Kepaniteraan Mahkamah Agung Republik Indonesia berusaha untuk selalu mencantumkan informasi paling kini dan akurat sebagai bentuk komitmen Mahkamah Agung untuk pelayanan publik, transparansi dan akuntabilitas pelaksanaan fungsi peradilan. Namun dalam hal-hal tertentu masih dimungkinkan terjadi permasalahan teknis terkait dengan akurasi dan keterkinian informasi yang kami sajikan, hal mana akan terus kami perbaiki dari waktu kewaktu.
Dalam hal Anda menemukan inakurasi informasi yang termuat pada situs ini atau informasi yang seharusnya ada, namun belum tersedia, maka harap segera hubungi Kepaniteraan Mahkamah Agung RI melalui :
Email : kepaniteraan@mahkamahagung.go.id Telp : 021-384 3348 (ext.318)

2. *"Terus menerus terjadi perselisihan dan pertengkaran"* : fakta hukum pada angka 3 dan 4 tersebut telah menunjukkan sejak tahun 2009 rumah tangga Pemohon dan Termohon sering terjadi pertengkaran terus menerus yang penyebabnya karena Termohon sering lepas control dan melakukan kekerasan fisik terhadap Pemohon dan Pemohon beberapa kali diusir dan diminta dengan paksa keluar dari rumah dan menjauh dari Termohon; yang puncaknya sejak Februari 2020 antara Pemohon dan Termohon telah pisah rumah sampai sekarang.
3. *"Tidak ada harapan untuk hidup rukun lagi dalam rumah tangga".* Unsur ini secara nyata dan meyakinkan telah terpenuhi oleh fakta hukum pada angka 5 dimana semua usaha perdamaian telah dilakukan tetapi akhirnya tidak berhasil merukunkan Pemohon dengan Termohon ;-

Menimbang, bahwa ternyata dari fakta hukum di atas telah terpenuhi pula maksud pasal 22 Peraturan Pemerintah Nomor 9 Tahun 1975 jo Pasal 134 KHI yaitu telah jelas sebab akar permasalahan pertengkaran antara Pemohon dengan Termohon dari saksi-saksi orang dekat dan mereka menyatakan sudah berusaha mendamaikan Pemohon dan Termohon akan tetapi tidak berhasil;

Menimbang, bahwa di samping itu ternyata dari fakta hukum di atas gugatan Pemohon juga memenuhi alasan perceraian Pasal 19 huruf f Peraturan Pemerintah Nomor 9 Tahun 1975 jo. Pasal 116 huruf f Kompilasi Hukum Islam yaitu: "Antara suami dan isteri terus-menerus terjadi perselisihan dan pertengkaran dan tidak ada harapan akan hidup rukun lagi dalam rumah tangga".

Menimbang, bahwa dalam suatu rumah tangga jika suami istri telah terjadi pertengkaran dan perselisihan yang telah berlangsung lama disebabkan karena Termohon sering lepas control dan melakukan kekerasan fisik terhadap Pemohon dan Pemohon beberapa kali diusir dan diminta dengan paksa keluar dari rumah dan menjauh dari Termohon; kemudian telah pisah rumah dan keduanya telah diusahakan perdamaian akan tetapi tidak berhasil, maka keadaan tersebut menurut Majelis Hakim merupakan bukti rumah tangga yang telah pecah atau *broken marriage* dan tidak mungkin dapat dipertahankan lagi

Disclaimer
Kepaniteraan Mahkamah Agung Republik Indonesia berusaha untuk selalu mencantumkan informasi paling kini dan akurat sebagai bentuk komitmen Mahkamah Agung untuk pelayanan publik, transparansi dan akuntabilitas pelaksanaan fungsi peradilan. Namun dalam hal-hal tertentu masih dimungkinkan terjadi permasalahan teknis terkait dengan akurasi dan keterkinian informasi yang kami sajikan, hal mana akan terus kami perbaiki dari waktu kewaktu.
Dalam hal Anda menemukan inakurasi informasi yang termuat pada situs ini atau informasi yang seharusnya ada, namun belum tersedia, maka harap segera hubungi Kepaniteraan Mahkamah Agung RI melalui :
Email : kepaniteraan@mahkamahagung.go.id Telp : 021-384 3348 (ext.318)

karenanya tidak akan bisa mencapai tujuan perkawinan sebagaimana Al-qur'an surat Ar-Rum ayat 21 jo pasal 1 Undang-Undang No. 1 / 1974, yaitu terbentuknya keluarga sakinah, mawaddah warahmah ;

Menimbang, bahwa di samping itu keadaan sebagaimana fakta hukum tersebut diatas bila tidak segera diselesaikan, maka berpotensi menimbulkan pelanggaran Undang-Undang Republik Indonesia Nomor 23 Tahun 2004 Tentang Penghapusan Kekerasan Dalam Rumah Tangga, pasal 5 dan 9 ayat (1) yaitu :

Pasal 5 : *“Setiap orang dilarang melakukan kekerasan dalam rumah tangga terhadap orang dalam lingkup rumah tangganya, dengan cara: 1.kekerasan fisik; 2.kekerasan psikis; 3. kekerasan seksual; atau 4. penelantaran rumah tangga”.*

Pasal 9 : (1). *“Setiap orang dilarang menelantarkan orang dalam lingkup rumah tangganya, padahal menurut hukum yang berlaku baginya atau karena persetujuan atau perjanjian ia wajib memberikan kehidupan, perawatan, atau pemeliharaan kepada orang tersebut”.-*

Menimbang, bahwa fakta hukum tersebut juga telah memenuhi norma hukum Islam yang diambil sebagai pendapat Majelis, yaitu ketentuan dalam Fiqhussunnah juz II yang berbunyi:

Artinya : “Dan jika dakwaan isteri telah terbukti di hadapan hakim (tentang kamadlorotan dari suami) dengan bukti-bukti yang diajukan oleh isteri atau pengakuan suami, dan dakwaan isteri menunjukkan ketidakmampuan kedua belah pihak untuk melanjutkan hidup bersama sebagai suami istri sedang hakim tidak berhasil mendamaikan keduanya, maka hakim menjatuhkan talak bain”;.

Menimbang, bahwa atas dasar pertimbangan tersebut di atas, Majelis Hakim berpendapat bahwa gugatan Pemohon telah memenuhi ketentuan Pasal 39 ayat (2) Undang-undang Nomor 1 Tahun 1974 jo. Pasal 19 huruf f Peraturan Pemerintah Nomor 9 Tahun 1975, jo. Pasal 116 huruf f Kompilasi Hukum Islam;

Menimbang, bahwa berdasarkan hal-hal tersebut di atas, maka Gugatan Pemohon dinyatakan telah cukup alasan sebagaimana ketentuan Pasal 19

Disclaimer
Kepaniteraan Mahkamah Agung Republik Indonesia berusaha untuk selalu mencantumkan informasi paling kini dan akurat sebagai bentuk komitmen Mahkamah Agung untuk pelayanan publik, transparansi dan akuntabilitas pelaksanaan fungsi peradilan. Namun dalam hal-hal tertentu masih dimungkinkan terjadi permasalahan teknis terkait dengan akurasi dan keterkinian informasi yang kami sajikan, hal mana akan terus kami perbaiki dari waktu kewaktu.
Dalam hal Anda menemukan inakurasi informasi yang termuat pada situs ini atau informasi yang seharusnya ada, namun belum tersedia, maka harap segera hubungi Kepaniteraan Mahkamah Agung RI melalui :
Email : kepaniteraan@mahkamahagung.go.id Telp : 021-384 3348 (ext.318)

huruf (f) Peraturan Pemerintah Nomor 9 Tahun 1975 jo. Pasal 116 huruf (f) Kompilasi Hukum Islam, sehingga telah memenuhi ketentuan Pasal 39 Undang-Undang Nomor 1 Tahun 1974 tentang Perkawinan, oleh karena itu gugatan Pemohon dapat dikabulkan dengan jatuh talah satu bain sughra dari Termohon kepada Pemohon;

Biaya Perkara

Menimbang, bahwa karena perkara *a quo* masuk dalam bidang perkawinan, berdasarkan Pasal 89 ayat (1) Undang-Undang Nomor 7 Tahun 1989 tentang Peradilan Agama sebagaimana telah diubah dengan Undang-Undang Nomor 3 Tahun 2006 dan perubahan kedua dengan Undang-Undang Nomor 50 Tahun 2009, maka biaya perkara ini dibebankan kepada Pemohon;

Mengingat, semua peraturan perundang-undangan yang berlaku dan hukum *syara'* yang berkaitan dengan perkara ini;

Amar putusan

MENGADILI

1. Menyatakan, Termohon telah dipanggil dengan patut untuk datang menghadap di persidangan tidak hadir;
2. Mengabulkan permohonan Pemohon dengan verstek;
3. Memberi izin kepada Pemohon (XXXX) untuk menjatuhkan talak satu raj'i terhadap Termohon (XXXX), di depan sidang Pengadilan Agama Bekasi;
4. Membebankan kepada Pemohon untuk membayar biaya perkara sebesar Rp. 626.000 ,- (enam ratus dua puluh enam ribu)

Penutup

Demikian putusan ini dijatuhkan dalam rapat permusyawaratan Majelis Hakim yang dilangsungkan pada hari Senin tanggal 28 Desember 2020 Masehi bertepatan dengan tanggal 13 Jumadil Awwal 1442 Hijriah oleh kami Drs. H. Sirojuddin, S.H. sebagai Ketua Majelis, Drs. H. Syarif Hidayat, S.H. dan Hj. Susilawati, S.E.I., masing-masing sebagai Hakim Anggota, putusan tersebut diucapkan dalam sidang terbuka untuk umum pada hari itu juga oleh Ketua Majelis tersebut dengan didampingi oleh Hakim Anggota dan didampingi oleh

Disclaimer
Kepaniteraan Mahkamah Agung Republik Indonesia berusaha untuk selalu mencantumkan informasi paling kini dan akurat sebagai bentuk komitmen Mahkamah Agung untuk pelayanan publik, transparansi dan akuntabilitas pelaksanaan fungsi peradilan. Namun dalam hal-hal tertentu masih dimungkinkan terjadi permasalahan teknis terkait dengan akurasi dan keterkinian informasi yang kami sajikan, hal mana akan terus kami perbaiki dari waktu kewaktu.
Dalam hal Anda menemukan inakurasi informasi yang termuat pada situs ini atau informasi yang seharusnya ada, namun belum tersedia, maka harap segera hubungi Kepaniteraan Mahkamah Agung RI melalui :
Email : kepaniteraan@mahkamahagung.go.id Telp : 021-384 3348 (ext.318)

Eva Zulva Wardiyanti, S.H.I, M.H. sebagai Panitera Pengganti, dengan dihadiri oleh Pemohon tanpa hadirnya Termohon.

Ketua Majelis,

Drs. H. Sirojuddin, S.H.

Hakim Anggota, Hakim Anggota,

Drs. H. Syarif Hidayat, S.H. **Hj. Susilawati, S.E.I.**

Panitera Pengganti,

Eva Zulva Wardiyanti, S.H.I, M.H.

Rincian biaya:

1.	Biaya Pendaftaran	: Rp	30.000,00
2.	Biaya Proses	: Rp	60.000,00
3.	PNBP Panggilan	: Rp	20.000,00
4.	Biaya Panggilan	: Rp	500.000,00
5.	Biaya Redaksi	: Rp	10.000,00
6.	Biaya Meterai	: Rp	6.000,00
	**JUMLAH**	**: Rp**	**626.000,00**

(enam ratus dua puluh enam ribu rupiah).

Disclaimer
Kepaniteraan Mahkamah Agung Republik Indonesia berusaha untuk selalu mencantumkan informasi paling kini dan akurat sebagai bentuk komitmen Mahkamah Agung untuk pelayanan publik, transparansi dan akuntabilitas pelaksanaan fungsi peradilan. Namun dalam hal-hal tertentu masih dimungkinkan terjadi permasalahan teknis terkait dengan akurasi dan keterkinian informasi yang kami sajikan, hal mana akan terus kami perbaiki dari waktu kewaktu.
Dalam hal Anda menemukan inakurasi informasi yang termuat pada situs ini atau informasi yang seharusnya ada, namun belum tersedia, maka harap segera hubungi Kepaniteraan Mahkamah Agung RI melalui :
Email : kepaniteraan@mahkamahagung.go.id Telp : 021-384 3348 (ext.318)
