# Supplementary material for: Classification of divorce causes during the COVID-19 pandemic using convolutional neural networks
Source: PeerJ Comput Sci. 2022 Jun 30;8:e998. doi: 10.7717/peerj-cs.998 (PMC9299239; doi:10.7717/peerj-cs.998)
Supplement: Supplemental Information 2 [file peerj-cs-08-998-s002.zip › Kelalaian Dataset/Data ke-13.pdf]

P U T U S A N

Nomor 2663/Pdt.G/2020/PA.Pt

بِسْــــــــــــــمِ اللهِ الرَّحْمَنِ الرَّحِيْمِ

DEMI KEADILAN BERDASARKAN KETUHANAN YANG MAHA ESA

PENGADILAN AGAMA PATI

Mengadili perkara-perkara tertentu pada tingkat pertama dalam persidangan Majelis, menjatuhkan putusan sebagai berikut dalam perkara permohonan izin ikrar talak antara:

PEMOHON, umur 34 tahun, agama Islam, Pekerjaan Makelar, Pendidikan Sekolah Lanjutan Tingkat Pertama, tempat kediaman di alamat KTP: RT.01 RW.05, Desa Tunjungrejo, Kecamatan Margoyoso, Kabupaten Pati, sekarang bertempat tinggal di orang tua Dukuh Mojo RT.01 RW.03, Desa Langenharjo, Kecamatan Margorejo, Kabupaten Pati, Jawa Tengah, sebagai Pemohon;

Melawan

TERMOHON, umur 32 tahun, agama Islam, Pekerjaan Karyawan Pabrik, Pendidikan Sekolah Lanjutan Tingkat Atas, tempat kediaman di RT.01 RW.05, Desa Tunjungrejo, Kecamatan Margoyoso, Kabupaten Pati, Jawa Tengah, sebagai Termohon;

Pengadilan Agama tersebut;

Telah mempelajari berkas perkara;

Telah mendengar Pemohon dan memeriksa bukti-bukti;

DUDUK PERKARA

Bahwa, Pemohon telah mengajukan permohonan izin ikrar talak tertanggal 01 Desember 2020 yang telah didaftar dalam register perkara nomor: 2663/Pdt.G/2020/PA.Pt, tanggal 01 Desember 2020, mengemukakan hal-hal sebagai berikut:

1. Bahwa, Pemohon dan Termohon telah menikah pada tanggal 23 April 2009 tercatat di Kantor Urusan Agama Kecamatan Margorejo

Disclaimer
Kepaniteraan Mahkamah Agung Republik Indonesia berusaha untuk selalu mencantumkan informasi paling kini dan akurat sebagai bentuk komitmen Mahkamah Agung untuk pelayanan publik, transparansi dan akuntabilitas pelaksanaan fungsi peradilan. Namun dalam hal-hal tertentu masih dimungkinkan terjadi permasalahan teknis terkait dengan akurasi dan keterkinian informasi yang kami sajikan, hal mana akan terus kami perbaiki dari waktu kewaktu.
Dalam hal Anda menemukan inakurasi informasi yang termuat pada situs ini atau informasi yang seharusnya ada, namun belum tersedia, maka harap segera hubungi Kepaniteraan Mahkamah Agung RI melalui :
Email : kepaniteraan@mahkamahagung.go.id   Telp : 021-384 3348 (ext.318)

Kabupaten Pati sesuai dengan Kutipan Akta Nikah Nomor 34/13/IV/2009 tanggal 23 April 2009;

2. Bahwa, sebelum menikah Pemohon adalah jejaka dan Termohon adalah perawan;

3. Bahwa, setelah menikah Pemohon dan Termohon tinggal bersama di rumah orang tua Termohon yang beralamat di Desa Tunjungrejo Kecamatan Margoyoso Kabupaten Pati selama 9 tahun 10 bulan;

4. Bahwa, Pemohon dan Termohon telah pernah hidup rukun dan telah berhubungan sebagimana layaknya suami istri (ba'daddukhul) dan telah dikaruniai 2 orang anak bernama :

   a. ANAK I , lahir 30 Oktober 2015,
   b. ANAK II , lahir 24 Juni 2019, sekarang semuanya diasuh Termohon;

5. Bahwa, setelah perkawinan Pemohon dan Termohon berlangsung selama ± 7 Tahun 4 bulan atau sejak bulan Agustus tahun 2016 rumah tangga Pemohon dan Termohon mulai tidak harmonis, sering terjadi perselisihan dan pertengkaran disebabkan masalah Termohon kurang bersyukur atas uang hasil kerja Pemohon yang diberikan kepada Termohon. Setelah Pemohon bekerja sebagai makelar, Termohon kurang bisa menerima jika Pemohon bekerja sebagai makelar sehingga Termohon berharap Pemohon dapat bekerja di pabrik seperti Termohon namun Pemohon tetap memilih untuk meneruskan pekerjaa Pemohon karena Pemohon merasa penghasilan yang didapatkan sudah cukup untuk memenuhi kebutuhan rumah tangga.;

6. Bahwa, puncaknya pada bulan Februari tahun 2019 setelah terjadi pertengkaran kemudian Pemohon dijemput oleh Kepolisian karena terlibat dalam kasus pidana. Sejak saat itu Pemohon dan Termohon pisah rumah:

7. Bahwa, sebagai puncak perselisihan tersebut, sejak bulan Februari tahun 2019 atau hingga saat ini selama 1 tahun 9 bulan Pemohon dan Termohon telah berpisah hingga sekarang;

8. sesuai pasal 39 Undang-undang Nomor 1 Tahun 1974, jo pasal 19 huruf (f) PP No.9 tahun 1975 jo Pasal 116 huruf (f) KHI, kiranya cukup

Disclaimer
Kepaniteraan Mahkamah Agung Republik Indonesia berusaha untuk selalu mencantumkan informasi paling kini dan akurat sebagai bentuk komitmen Mahkamah Agung untuk pelayanan publik, transparansi dan akuntabilitas pelaksanaan fungsi peradilan. Namun dalam hal-hal tertentu masih dimungkinkan terjadi permasalahan teknis terkait dengan akurasi dan keterkinian informasi yang kami sajikan, hal mana akan terus kami perbaiki dari waktu kewaktu.
Dalam hal Anda menemukan inakurasi informasi yang termuat pada situs ini atau informasi yang seharusnya ada, namun belum tersedia, maka harap segera hubungi Kepaniteraan Mahkamah Agung RI melalui :
Email : kepaniteraan@mahkamahagung.go.id Telp : 021-384 3348 (ext.318)

beralasan bagi Pemohon untuk mengajukan permohonan cerai talak di Pengadilan Agama Pati.;

9. Bahwa, atas peristiwa-peristiwa tersebut, Pemohon mengajukan permohonan cerai talak dengan alasan: antara Pemohon dan Termohon terus menerus terjadi perselisihan dan pertengkaran dan tidak ada harapan akan hidup rukun dalam perkawinan dengan Termohon;

10. Bahwa Pemohon sanggup membayar biaya perkara ini;

Berdasarkan alasan-alasan tersebut, Pemohon mohon kepada Ketua Pengadilan Agama Pati cq Majelis Hakim yang memeriksa dan mengadili perkara ini, berkenan untuk menjatuhkan putusan sebagai berikut:

1. Mengabulkan permohonan Pemohon;

2. Memberi izin kepada Pemohon (PEMOHON) untuk menjatuhkan talak satu roj'i terhadap Termohon (TERMOHON) di depan sidang Pengadilan Agama Pati;

3. Menetapkan biaya perkara ini menurut hukum;

Apabila Pengadilan Agama Pati berpendapat lain, mohon putusan yang seadil-adilnya;

Bahwa, pada hari sidang yang telah ditetapkan Pemohon telah datang menghadap sidang, sedangkan Termohon tidak datang dan tidak menyuruh orang lain untuk menghadap sebagai wakil/kuasa hukumnya meskipun telah dipanggil secara resmi dan patut yang relaas panggilannya telah dibacakan di dalam sidang, sedangkan tidak ternyata bahwa tidak datangnya itu disebabkan suatu halangan yang sah;

Bahwa, Majelis Hakim telah menasehati Pemohon agar berpikir untuk tidak bercerai dengan Termohon, tetapi Pemohon tetap pada dalil permohonannya untuk bercerai dengan Termohon;

Bahwa, perkara ini tidak dapat di mediasi karena Termohon tidak pernah datang menghadap sidang meskipun telah dipanggil secara resmi dan patut, selanjutnya dimulai pemeriksaan dengan membacakan surat permohonan Pemohon yang maksud dan isinya tetap dipertahankan oleh Pemohon;

Bahwa, untuk meneguhkan dalil permohonannya Pemohon telah mengajukan bukti-bukti berupa:

Disclaimer
Kepaniteraan Mahkamah Agung Republik Indonesia berusaha untuk selalu mencantumkan informasi paling kini dan akurat sebagai bentuk komitmen Mahkamah Agung untuk pelayanan publik, transparansi dan akuntabilitas pelaksanaan fungsi peradilan. Namun dalam hal-hal tertentu masih dimungkinkan terjadi permasalahan teknis terkait dengan akurasi dan keterkinian informasi yang kami sajikan, hal mana akan terus kami perbaiki dari waktu kewaktu.
Dalam hal Anda menemukan inakurasi informasi yang termuat pada situs ini atau informasi yang seharusnya ada, namun belum tersedia, maka harap segera hubungi Kepaniteraan Mahkamah Agung RI melalui :
Email : kepaniteraan@mahkamahagung.go.id Telp : 021-384 3348 (ext.318)

A. Surat:

1. Fotokopi Kartu Tanda Penduduk Nomor X, telah dinasegelen dan bermeterai cukup serta cocok dengan aslinya (Bukti P.1);
2. Fotokopi Buku Kutipan Akta Nikah dari Kantor Urusan Agama Kecamatan Margorejo Kabupaten Pati Nomor 34/13/IV/2009 tanggal 23 April 2009, telah dinasegelen dan bermeterai cukup serta cocok dengan aslinya (Bukti P.2);

B. Saksi:

1. SAKSI I , umur 35 tahun, Agama Islam, pendidikan S1, pekerjaan Guru TK., tempat tinggal di RT.04 RW.02, Desa Sumurtawang, Kecamatan Kragan, Kabupaten Rembang, Jawa Tengah, dibawah sumpah memberikan keterangan sebagai berikut:
   - Bahwa saksi kenal dengan Pemohon dan Termohon karena saksi kakak Pemohon;
   - Bahwa saksi tahu Pemohon dengan Termohon adalah suami istri sah dan sudah dikaruniai 2 orang anak;
   - Bahwa saksi tahu setelah menikah Pemohon dengan Termohon tinggal di rumah orangtua Termohon;
   - Bahwa saksi tahu rumah tangga Pemohon dengan Termohon semula rukun dan harmonis, namun sejak bulan Agustus tahun 2016 sering terjadi perselisihan dan pertengkaran;
   - Bahwa saksi tahu perselisihan dan pertengkaran disebabkan Termohon kurang bersyukur atas uang hasil kerja Pemohon;
   - Bahwa saksi tahu akibat perselisihan dan pertengkaran Pemohon dengan Termohon berpisah tempat tinggal selama 1 tahun 9 bulan;
   - Bahwa saksi sudah menasehati Pemohon untuk tidak bercerai dengan Pemohon tetapi tidak berhasil;
2. SAKSI II , umur 39 tahun, Agama Islam, pendidikan SMP., pekerjaan Karyawan pabrik tahu, tempat tinggal di RT.02 RW.01, Desa Sendangmulyo, Kecamatan Sluke, Kabupaten Pati, Jawa Tengah, dibawah sumpah memberikan keterangan sebagai berikut:

Disclaimer
Kepaniteraan Mahkamah Agung Republik Indonesia berusaha untuk selalu mencantumkan informasi paling kini dan akurat sebagai bentuk komitmen Mahkamah Agung untuk pelayanan publik, transparansi dan akuntabilitas pelaksanaan fungsi peradilan. Namun dalam hal-hal tertentu masih dimungkinkan terjadi permasalahan teknis terkait dengan akurasi dan keterkinian informasi yang kami sajikan, hal mana akan terus kami perbaiki dari waktu kewaktu.
Dalam hal Anda menemukan inakurasi informasi yang termuat pada situs ini atau informasi yang seharusnya ada, namun belum tersedia, maka harap segera hubungi Kepaniteraan Mahkamah Agung RI melalui :
Email : kepaniteraan@mahkamahagung.go.id Telp : 021-384 3348 (ext.318)

- Bahwa saksi kenal dengan Pemohon dan Termohon karena saksi teman Pemohon;
- Bahwa saksi tahu Pemohon dengan Termohon adalah suami istri sah dan sudah dikaruniai 2 orang anak;
- Bahwa saksi tahu setelah menikah Pemohon dengan Termohon tinggal di rumah orangtua Termohon;
- Bahwa saksi tahu rumah tangga Pemohon dengan Termohon semula rukun dan harmonis, namun sejak bulan Agustus tahun 2016 sering terjadi perselisihan dan pertengkaran;
- Bahwa saksi tahu perselisihan dan pertengkaran disebabkan Termohon kurang bersyukur atas uang hasil kerja Pemohon;
- Bahwa saksi tahu akibat perselisihan dan pertengkaran Pemohon dengan Termohon berpisah tempat tinggal selama 1 tahun 9 bulan;
- Bahwa saksi sudah menasehati Pemohon untuk tidak bercerai dengan Pemohon tetapi tidak berhasil;

Bahwa, selanjutnya Pemohon menyampaikan kesimpulan yang pada pokoknya tetap sebagaimana dengan permohonannya dan mohon putusan;

Bahwa, untuk singkatnya uraian putusan ini, maka semua hal yang termuat dalam Berita Acara Sidang ini merupakan bagian yang tak terpisahkan dari putusan ini;

PERTIMBANGAN HUKUM

Menimbang, bahwa maksud dan tujuan permohonan Pemohon adalah sebagaimana terurai di atas;

Menimbang, bahwa ternyata Termohon meskipun dipanggil secara resmi dan patut, tidak datang menghadap sidang dan pula tidak ternyata bahwa tidak datangnya itu disebabkan suatu halangan yang sah. Oleh karena itu, Termohon harus dinyatakan tidak hadir dan permohonan tersebut harus diperiksa secara verstek. Sebagaimana ketentuan Pasal 125 ayat (1) HIR dan dalil syar'i:

من دعي إلى حاكم من حكام المسلمين فلم يجب فهو ظالم لا حق له

Halaman 5 dari 11 hal.aman Putusan No 2663/Pdt.G/2020/PA.Pt

Disclaimer
Kepaniteraan Mahkamah Agung Republik Indonesia berusaha untuk selalu mencantumkan informasi paling kini dan akurat sebagai bentuk komitmen Mahkamah Agung untuk pelayanan publik, transparansi dan akuntabilitas pelaksanaan fungsi peradilan. Namun dalam hal-hal tertentu masih dimungkinkan terjadi permasalahan teknis terkait dengan akurasi dan keterkinian informasi yang kami sajikan, hal mana akan terus kami perbaiki dari waktu kewaktu.
Dalam hal Anda menemukan inakurasi informasi yang termuat pada situs ini atau informasi yang seharusnya ada, namun belum tersedia, maka harap segera hubungi Kepaniteraan Mahkamah Agung RI melalui :
Email : kepaniteraan@mahkamahagung.go.id Telp : 021-384 3348 (ext.318)

Artinya: " Seseorang yang dipanggil Hakim (Pengadilan) tidak datang, ia dipandang sebagai dhalim dan tidak ada hak baginya. " (Ahkamul Qur-an II : 405) ;

Menimbang, bahwa dalam perkara ini Majelis Hakim tidak menempuh prosedur mediasi karena salah satu pihak (Termohon) tidak datang menghadap sidang, sehingga tidak dapat dilakukan perundingan, dimana Pasal 1 ayat (1) Peraturan Mahkamah Agung R.I. Nomor 1 Tahun 2016 tentang Prosedur Mediasi di Pengadilan;

Menimbang, bahwa Majelis Hakim telah berupaya menasehati Pemohon agar rukun kembali dengan Termohon, sebagaimana dikehendaki oleh ketentuan Pasal 130 HIR jo Pasal 82 Undang Undang Nomor 7 Tahun 1989 Tentang Peradilan Agama sebagaimana yang telah diubah terakhir dengan Undang Undang Nomor 50 Tahun 2009 jo Pasal 39 ayat (1) Undang Undang Nomor 1 Tahun 1974 jo Pasal 31 ayat (2) Peraturan Pemerintah Nomor 9 Tahun 1975 jo Pasal 143 Kompilasi Hukum Islam, tetapi tidak berhasil;

Menimbang, bahwa perkara ini adalah perkara cerai talak yang didasarkan atas dalil dan alasan antara Pemohon dengan Termohon sebagai suami istri terus menerus terjadi perselisihan dan pertengkaran, dan tidak bisa dirukunkan kembali dalam rumah tangga dan antara Pemonon dengan Termohon telah berpisah tempat tinggal, selengkapnya sebagaimana dalam posita permohonan a quo, oleh karena itu Pemohon mohon agar Majelis Hakim menjatuhkan talak satu raj'i Pemohon kepada Termohon;

Menimbang, bahwa berdasarkan ketentuan Surat Edaran Mahkamah Agung RI Nomor 3 Tahun 2015 huruf C angka 3 yaitu putusan yang dijatuhkan tanpa hadirnya Termohon dapat dikabulkan sepanjang berdasarkan hukum dan beralasan, maka walaupun pemeriksaan perkara ini tanpa dihadiri Termohon didalam sidang, akan tetapi karena terkait dalam bidang perkawinan, maka sesuai prinsip dan asas yang terdapat dalam penjelasan Undang undang Nomor 1 tahun 1974 angka (4) huruf (e), maka alasan-alasan yang menjadi dasar permohonan Pemohon harus dibuktikan, oleh karena itu Majelis membebani Pemohon untuk membuktikan dalil-dalil permohonannya;

Disclaimer
Kepaniteraan Mahkamah Agung Republik Indonesia berusaha untuk selalu mencantumkan informasi paling kini dan akurat sebagai bentuk komitmen Mahkamah Agung untuk pelayanan publik, transparansi dan akuntabilitas pelaksanaan fungsi peradilan. Namun dalam hal-hal tertentu masih dimungkinkan terjadi permasalahan teknis terkait dengan akurasi dan keterkinian informasi yang kami sajikan, hal mana akan terus kami perbaiki dari waktu kewaktu.
Dalam hal Anda menemukan inakurasi informasi yang termuat pada situs ini atau informasi yang seharusnya ada, namun belum tersedia, maka harap segera hubungi Kepaniteraan Mahkamah Agung RI melalui :
Email : kepaniteraan@mahkamahagung.go.id Telp : 021-384 3348 (ext.318)

Menimbang, bahwa untuk meneguhkan kebenaran dalil-dalil permohonannya, Pemohon telah mengajukan bukti surat tertanda P-1, P-2, dan saksi-saksi ;

Menimbang, bahwa bukti surat tersebut berupa Fotocopy yang ternyata cocok sesuai dengan aslinya dan telah dibubuhi meterai cukup, maka bukti surat tersebut telah memenuhi syarat formal sebagai alat bukti ;

Menimbang, bahwa saksi-saksi yang diajukan didalam sidang sudah dewasa dan sudah bersumpah menurut tatacara agamanya, saksi-saksi tersebut terdiri dari orang-orang dekat Pemohon, dan bukanlah orang yang dilarang untuk menjadi saksi dalam perkara ini, kemudian telah menyampaikan keterangan secara terpisah satu persatu didalam sidang, oleh karena itu saksi-saksi tersebut telah memenuhi syarat formil sebagai saksi sebagaimana diatur dalam Pasal 145 ayat 1 angka 3e HIR;

Menimbang, bahwa keterangan saksi-saksi didalam sidang atas dasar pengetahuan/penglihatan saksi-saksi sendiri, dimana satu dengan yang lain saling bersesuaian dan berhubungan, mendukung dan relevan dengan dalil-dalil Pemohon (mutual conformity), maka sesuai Pasal 170,171 dan pasal 172 HIR. kesaksian tersebut telah memenuhi syarat materiil saksi, oleh karena itu kesaksian tersebut dapat diterima dan telah mempunyai nilai kekuatan pembuktian yang sah;

Menimbang, bahwa perkara ini termasuk sengketa di bidang perkawinan dan berdasarkan Bukti P-1 Pemohon berdomisili di wilayah hukum Pengadilan Agama Pati, sedangkan berdasarkan relas panggilan Termohon, telah terbukti Termohon tinggal di wilayah Kabupaten Pati pula maka berdasarkan ketentuan Pasal 49 ayat (1) huruf a dan Pasal 66 ayat (2) Undang-undang Nomor 7 Tahun 1989 Tentang Peradilan Agama sebagaimana telah diubah dengan Undang-undang Nomor 3 Tahun 2006 dan perubahan kedua dengan Undang-undang Nomor 50 Tahun 2009, Pengadilan Agama Pati berwenang untuk memeriksa, memutus, dan menyelesaikan Perkara ini;

Menimbang, bahwa bukti P-2 (Fotokopi Kutipan Akta Nikah) yang merupakan akta otentik dan telah bermeterai cukup dan cocok dengan aslinya, isi bukti tersebut menjelaskan antara Pemohon dengan Termohon telah terikat

Disclaimer
Kepaniteraan Mahkamah Agung Republik Indonesia berusaha untuk selalu mencantumkan informasi paling kini dan akurat sebagai bentuk komitmen Mahkamah Agung untuk pelayanan publik, transparansi dan akuntabilitas pelaksanaan fungsi peradilan. Namun dalam hal-hal tertentu masih dimungkinkan terjadi permasalahan teknis terkait dengan akurasi dan keterkinian informasi yang kami sajikan, hal mana akan terus kami perbaiki dari waktu kewaktu.
Dalam hal Anda menemukan inakurasi informasi yang termuat pada situs ini atau informasi yang seharusnya ada, namun belum tersedia, maka harap segera hubungi Kepaniteraan Mahkamah Agung RI melalui :
Email : kepaniteraan@mahkamahagung.go.id Telp : 021-384 3348 (ext.318)

dalam perkawinan yang sah, sehingga bukti tersebut telah memenuhi syarat formal dan meteriil, serta mempunyai kekuatan pembuktian yang sempurna dan mengikat;

Menimbang, bahwa berdasarkan bukti P-2 tersebut, maka harus dinyatakan telah terbukti menurut hukum bahwa Pemohon dengan Termohon telah terikat dalam perkawinan yang sah;

Menimbang, bahwa oleh karena Pemohon dan Termohon telah terikat sebagai suami istri yang sah, maka Pemohon sebagai *persona standi in judicio* memiliki *legal standing* sehingga Pemohon dapat dinyatakan sebagai pihak yang mempunyai hak dan berkepentingan dalam perkara ini;

Menimbang, bahwa atas keterangan Pemohon dalam surat permohonannya bahwa Pemohon dan Termohon beragama Islam dan telah melakukan perkawinan secara islami, maka perkara ini merupakan *kompetensi absolut* Pengadilan Agama untuk memeriksa dan menyelesaikannya (vide Pasal 2 jo Pasal 49 ayat (1) Undang Undang Nomor 7 Tahun 1989 Tentang Peradilan Agama sebagai mana yang telah diubah yang kedua dengan Undang Undang Nomor 50 Tahun 2009) ;

Menimbang, bahwa keterangan saksi saksi Pemohon mengenai dalil permohonan tentang telah terjadinya perselisihan dan pertengkaran terus menerus dan antara Pemohon dengan Termohon telah berpisah tempat tinggal adalah fakta yang dilihat/didengar/dialami sendiri dan relevan dengan dalil yang harus dibuktikan oleh Pemohon, oleh karena itu keterangan saksi tersebut telah memenuhi syarat materiil sebagaimana telah diatur dalam Pasal 171 HIR dan Pasal 172 HIR, sehingga keterangan saksi tersebut memiliki kekuatan pembuktian dan dapat diterima sebagai alat bukti;

Menimbang, bahwa berdasarkan saksi 1 dan saksi 2, terbukti fakta kejadian sebagai berikut:

1. Antara Pemohon dengan Termohon terus menerus terjadi perselisihan dan pertengkaran yang disebabkan Termohon kurang bersyukur atas uang hasil kerja Pemohon;
2. Antara Pemohon dengan Termohon telah pisah tempat tinggal selama 1 tahun 9 bulan;

Disclaimer
Kepaniteraan Mahkamah Agung Republik Indonesia berusaha untuk selalu mencantumkan informasi paling kini dan akurat sebagai bentuk komitmen Mahkamah Agung untuk pelayanan publik, transparansi dan akuntabilitas pelaksanaan fungsi peradilan. Namun dalam hal-hal tertentu masih dimungkinkan terjadi permasalahan teknis terkait dengan akurasi dan keterkinian informasi yang kami sajikan, hal mana akan terus kami perbaiki dari waktu kewaktu.
Dalam hal Anda menemukan inakurasi informasi yang termuat pada situs ini atau informasi yang seharusnya ada, namun belum tersedia, maka harap segera hubungi Kepaniteraan Mahkamah Agung RI melalui :
Email : kepaniteraan@mahkamahagung.go.id Telp : 021-384 3348 (ext.318)

Menimbang, bahwa berdasarkan fakta-fakta tersebut di atas dapat disimpulkan fakta hukum sebagai berikut:

1. Antara Pemohon dengan Termohon terus menerus terjadi perselisihan dan pertengkaran yang sulit untuk didamaikan;
2. Antara Pemohon dengan Termohon telah pisah tempat tinggal tinggal selama 1 tahun 9 bulan;
3. Antara Pemohon dengan Termohon tidak ada harapan untuk hidup rukun kembali;

Menimbang, bahwa berdasarkan fakta tersebut Pemohon telah dapat membuktikan dalil-dalil permohonannya dan telah memenuhi norma hukum Islam yang terkandung dalam kitab suci Al Qur'an sebagai berikut:

وَإِنۡ عَزَمُواْ ٱلطَّلَٰقَ فَإِنَّ ٱللَّهَ سَمِيعٌ عَلِيمٞ

Artinya: *" Dan jika mereka ber'azam (untuk) talak, maka sesungguhnya Allah Maha mendengar lagi Maha mengetahui"*. (Q.S. Al Baqarah: 227);

Menimbang, bahwa bukti tersebut telah memenuhi Pasal 39 ayat 2 Undang-undang Nomor 1 tahun 1974 Tentang Perkawinan dan Pasal 19 huruf (f) Peraturan Pemerintah Nomor 9 Tahun 1975 Tentang Pelaksanaan Undang-undang Nomor 1 tahun 1974 Tentang Perkawinan jo. Pasal 116 huruf (f) Kompilasi Hukum Islam, karena itu Permohonan Pemohon telah memenuhi alasan hukum;

Menimbang, bahwa berdasarkan pertimbangan tersebut diatas, maka Majelis Hakim berpendapat permohonan Pemohon telah cukup alasan dan tidak dapat dirukunkan lagidengan memberi izin kepada Pemohon untuk menjatuhkan talak satu raj'i terhadap Termohon;

Menimbang, bahwa oleh karena perkara ini menyangkut bidang perkawinan, maka sesuai ketentuan Pasal 89 ayat (1) Undang-undang Nomor 7 Tahun 1989 Tentang Peradilan Agama sebagaimana telah dirubah dengan Undang-undang Nomor 3 Tahun 2006 dan Undang-undang Nomor 50 Tahun 2009, biaya perkara sepenuhnya dibebankan kepada Pemohon;

Mengingat semua pasal dalam peraturan perundang-undangan dan hukum Islam yang berkaitan dengan perkara ini;

Disclaimer
Kepaniteraan Mahkamah Agung Republik Indonesia berusaha untuk selalu mencantumkan informasi paling kini dan akurat sebagai bentuk komitmen Mahkamah Agung untuk pelayanan publik, transparansi dan akuntabilitas pelaksanaan fungsi peradilan. Namun dalam hal-hal tertentu masih dimungkinkan terjadi permasalahan teknis terkait dengan akurasi dan keterkinian informasi yang kami sajikan, hal mana akan terus kami perbaiki dari waktu kewaktu.
Dalam hal Anda menemukan inakurasi informasi yang termuat pada situs ini atau informasi yang seharusnya ada, namun belum tersedia, maka harap segera hubungi Kepaniteraan Mahkamah Agung RI melalui :
Email : kepaniteraan@mahkamahagung.go.id Telp : 021-384 3348 (ext.318)

M E N G A D I L I

1. Menyatakan Termohon yang telah dipanggil secara resmi dan patut untuk menghadap sidang tidak hadir;
2. Mengabulkan pemohonan Pemohon secara verstek;
3. Memberi izin kepada Pemohon (PEMOHON) untuk menjatuhkan talak satu raj'i terhadap Termohon (TERMOHON) di depan sidang Pengadilan Agama Pati;
4. Membebankan kepada Pemohon untuk membayar biaya perkara ini sejumlah Rp. 371.000,00 (tiga ratus tujuh puluh satu ribu rupiah);

Demikian diputuskan dalam rapat permusyawaratan Majelis yang dilangsungkan pada hari Senin tanggal 28 Desember 2020 *Masehi*. bertepatan dengan tanggal 13 Jumadil Awal 1442 *Hijriyah.* oleh Miftahorrahman, S.H., M.H. sebagai Hakim Ketua Majelis, Drs. Sutiyo, M.H. dan Drs. Rizal Pasi, M.H. masing-masing sebagai Hakim Anggota Majelis, putusan tersebut diucapkan dalam sidang terbuka untuk umum pada hari dan tanggal tersebut dengan didampingi oleh Hakim-Hakim Anggota Majelis dan dibantu oleh Drs. Slamet Abadi sebagai Panitera Pengganti serta dihadiri oleh Pemohon tanpa dihadiri oleh Termohon.

Hakim Ketua Majelis,

Miftahorrahman, S.H., M.H.

Hakim Anggota Majelis, Hakim Anggota Majelis,

Drs. Sutiyo, M.H. **Drs. Rizal Pasi, M.H.**

Panitera Pengganti,

Drs. Slamet Abadi

Perincian Biaya:

Disclaimer
Kepaniteraan Mahkamah Agung Republik Indonesia berusaha untuk selalu mencantumkan informasi paling kini dan akurat sebagai bentuk komitmen Mahkamah Agung untuk pelayanan publik, transparansi dan akuntabilitas pelaksanaan fungsi peradilan. Namun dalam hal-hal tertentu masih dimungkinkan terjadi permasalahan teknis terkait dengan akurasi dan keterkinian informasi yang kami sajikan, hal mana akan terus kami perbaiki dari waktu kewaktu.
Dalam hal Anda menemukan inakurasi informasi yang termuat pada situs ini atau informasi yang seharusnya ada, namun belum tersedia, maka harap segera hubungi Kepaniteraan Mahkamah Agung RI melalui :
Email : kepaniteraan@mahkamahagung.go.id   Telp : 021-384 3348 (ext.318)

Biaya Pendaftaran	:	Rp	30.000,00
Biaya Proses	:	Rp	75.000,00
Biaya Pemanggilan	:	Rp	250.000,00
Biaya Redaksi	:	Rp	10.000,00
Biaya Materai	:	Rp	6.000,00
Jumlah	:	Rp	371.000,00

Disclaimer
Kepaniteraan Mahkamah Agung Republik Indonesia berusaha untuk selalu mencantumkan informasi paling kini dan akurat sebagai bentuk komitmen Mahkamah Agung untuk pelayanan publik, transparansi dan akuntabilitas pelaksanaan fungsi peradilan. Namun dalam hal-hal tertentu masih dimungkinkan terjadi permasalahan teknis terkait dengan akurasi dan keterkinian informasi yang kami sajikan, hal mana akan terus kami perbaiki dari waktu kewaktu.
Dalam hal Anda menemukan inakurasi informasi yang termuat pada situs ini atau informasi yang seharusnya ada, namun belum tersedia, maka harap segera hubungi Kepaniteraan Mahkamah Agung RI melalui :
Email : kepaniteraan@mahkamahagung.go.id   Telp : 021-384 3348 (ext.318)
