# Supplementary material for: Classification of divorce causes during the COVID-19 pandemic using convolutional neural networks
Source: PeerJ Comput Sci. 2022 Jun 30;8:e998. doi: 10.7717/peerj-cs.998 (PMC9299239; doi:10.7717/peerj-cs.998)
Supplement: Supplemental Information 2 [file peerj-cs-08-998-s002.zip › Kelalaian Dataset/Data ke-14.pdf]

PUTUSAN

Nomor 1585/Pdt.G/2020/PA.JP

بِسْمِ اللَّهِ الرَّحْمَنِ الرَّحِيمِ

DEMI KEADILAN BERDASARKAN KETUHANAN YANG MAHA ESA

PENGADILAN AGAMA JAKARTA PUSAT

Memeriksa dan mengadili perkara perdata agama pada tingkat pertama telah menjatuhkan putusan dalam perkara cerai gugat antara:

XXXXXXXXXX, tempat tanggal lahir Tanjung karang, 28 Oktober 1978, NIK 3171056810780001, umur 42 tahun, agama Islam, Pendidikan SLTA, pekerjaan Mengurus rumah tangga, tempat kediaman di XXXXXXXXXX

Penggugat;

Lawan

XXXXXXXXXX, tempat tanggal lahir Tanjung karang, 08 Agustus 1974, umur 46 tahun, agama Islam, Pendidikan SLTA, pekerjaan Pedagang, tempat kediaman di XXXXXXXXXX;

Tergugat;

Pengadilan Agama tersebut;

Telah mempelajari berkas perkara;

DUDUK PERKARANYA

Bahwa Penggugat dalam surat gugatannya tanggal 02 Desember 2020 yang didaftarkan di Kepaniteraan Pengadilan Agama Jakarta Pusat, Nomor 1585/Pdt.G/2020/PA.JP, tanggal 02 Desember 2020,dengan dalil-dalil pada pokoknya sebagai berikut:

1. Bahwa Penggugat dan Tergugat telah melangsungkan pernikahan pada tanggal 20 Juli 2003 yang dicatat oleh Pegawai Pencatat Nikah Kantor Urusan Agama Kecamatan Tanjung Karang Barat Kota Bandar Lampung Provinsi Lampung, sesuai dengan Kutipan Akta Nikah Nomor: 475/27/VII/2003 tertanggal 21 Juli 2003;
2. Bahwa, setelah pernikahan tersebut Penggugat dengan Tergugat bertempat tinggal di rumah Tergugat di XXXXXXXXXX dan terakhir

Disclaimer
Kepaniteraan Mahkamah Agung Republik Indonesia berusaha untuk selalu mencantumkan informasi paling kini dan akurat sebagai bentuk komitmen Mahkamah Agung untuk pelayanan publik, transparansi dan akuntabilitas pelaksanaan fungsi peradilan. Namun dalam hal-hal tertentu masih dimungkinkan terjadi permasalahan teknis terkait dengan akurasi dan keterkinian informasi yang kami sajikan, hal mana akan terus kami perbaiki dari waktu kewaktu.
Dalam hal Anda menemukan inakurasi informasi yang termuat pada situs ini atau informasi yang seharusnya ada, namun belum tersedia, maka harap segera hubungi Kepaniteraan Mahkamah Agung RI melalui :
Email : kepaniteraan@mahkamahagung.go.id   Telp : 021-384 3348 (ext.318)

masing-masing bertempat tinggal sebagaimana alamat tersebut diatas;

3. Bahwa, dalam perkawinan tersebut Penggugat dengan telah bergaul sebagaimana layaknya suami istri (ba'da dukhul) dan telah dikaruniai 3 (tiga) orang anak yang masing-masing bernama :
    a. XXXXXXXXXX, laki-laki, lahir di Jakarta tanggal 22 Juni 2005;
    b. XXXXXXXXXX, laki-laki, lahi di Jakarta tanggal 19 Maret 2009;
    c. XXXXXXXXXX, perempuan, lahir di Jakarta tanggal 31 Agustus 2011;
4. Bahwa, semula kehidupan rumah tangga Penggugat dan Tergugat berjalan dengan rukun dan harmonis, namun sejak bulan November tahun 2019 pernikahan rumah tangga Penggugat dan Tergugat mulai tidak rukun dan terjadi pertengkaran dan perselisihan terus menerus yang disebabkan:
    a. Tergugat kerap curiga dan cemburu berlebih kepada Penggugat tanpa alasan yang jelas;
    b. Tergugat sulit untuk diajak berkomunikasi mengenai masalah rumahtangga sehingga komunikasi yang terjalin antara Penggugat dan Tergugat menjadi tidak baik;
    c. Tergugat kerap bertutur kata kasar yang menyakitkan hati Penggugat;
    d. Tergugat kurang mencukupi dalam memberikan nafkah lahir kepada Penggugat;
5. Bahwa, pada puncak perselisihan terjadi pada bulan September tahun 2020 akibatnya Penggugat dan Tergugat sudah pisah rumah dikarenakan Tergugat mengusir Penggugat. Sejak saat itu antara Penggugat dan Tergugat sudah tidak ada hubungan baik lahir maupun batin;
6. Bahwa, Penggugat telah berupaya mengatasi masalah tersebut dengan jalan musyawarah namun tidak berhasil;

Disclaimer
Kepaniteraan Mahkamah Agung Republik Indonesia berusaha untuk selalu mencantumkan informasi paling kini dan akurat sebagai bentuk komitmen Mahkamah Agung untuk pelayanan publik, transparansi dan akuntabilitas pelaksanaan fungsi peradilan. Namun dalam hal-hal tertentu masih dimungkinkan terjadi permasalahan teknis terkait dengan akurasi dan keterkinian informasi yang kami sajikan, hal mana akan terus kami perbaiki dari waktu kewaktu.
Dalam hal Anda menemukan inakurasi informasi yang termuat pada situs ini atau informasi yang seharusnya ada, namun belum tersedia, maka harap segera hubungi Kepaniteraan Mahkamah Agung RI melalui :
Email : kepaniteraan@mahkamahagung.go.id   Telp : 021-384 3348 (ext.318)

7. Bahwa, karena sebab-sebab tersebut di atas, rumah tangga Penggugat dan Tergugat sudah tidak dapat untuk dirukunkan kembali;
8. Bahwa, akibat tindakan tersebut di atas Penggugat telah menderita lahir bathin dan Penggugat tidak ridho atas perlakuan Tergugat terhadap Penggugat serta Penggugat merasa tidak sanggup lagi untuk melanjutkan rumah tangga dengan Tergugat oleh karenanya Penggugat berkesimpulan satu-satunya jalan keluar yang terbaik bagi Penggugat adalah bercerai dengan Tergugat;
9. Bahwa Penggugat sanggup membayar biaya yang timbul dalam perkara ini.

Berdasarkan alasan/dalil-dalil di atas, Penggugat mohon agar Ketua Pengadilan Agama Jakarta Pusat segera memeriksa dan mengadili perkara ini, selanjutnya menjatuhkan putusan sebagai berikut:

Primer:

1. Mengabulkan gugatan Penggugat;
2. Menjatuhkan talak satu bain sughra Tergugat (XXXXXXXXXX) terhadap Penggugat (XXXXXXXXXX);
3. Membebankan biaya perkara sesuai dengan ketentuan hukum yang berlaku.

Subsider:

Dan atau apabila Majelis Hakim berpendapat lain, mohon untuk menjatuhkan putusan yang seadil-adilnya.

Bahwa, dalam Surat Penetapan tertanggal 03 Desember 2020 Ketua Majelis Hakim telah memerintahkan kepada Jurusita Pengganti Pengadilan Agama Jakarta Pusat untuk memanggil kedua belah pihak yang berperkara agar menghadiri persidangan;

Bahwa, pada hari persidangan yang telah ditetapkan, Penggugat hadir pada pesidangan pertama saja tertanggal 14 Desember 2020 setelah itu Penggugat tidak pernah datang lagi menghadap dan tidak menyuruh orang lain untuk hadir sebagai wakilnya, meskipun menurut

Disclaimer
Kepaniteraan Mahkamah Agung Republik Indonesia berusaha untuk selalu mencantumkan informasi paling kini dan akurat sebagai bentuk komitmen Mahkamah Agung untuk pelayanan publik, transparansi dan akuntabilitas pelaksanaan fungsi peradilan. Namun dalam hal-hal tertentu masih dimungkinkan terjadi permasalahan teknis terkait dengan akurasi dan keterkinian informasi yang kami sajikan, hal mana akan terus kami perbaiki dari waktu kewaktu.
Dalam hal Anda menemukan inakurasi informasi yang termuat pada situs ini atau informasi yang seharusnya ada, namun belum tersedia, maka harap segera hubungi Kepaniteraan Mahkamah Agung RI melalui :
Email : kepaniteraan@mahkamahagung.go.id Telp : 021-384 3348 (ext.318)

Berita Acara (Relaas) panggilan Pengadilan Agama Jakarta Pusat, Penggugat telah dipanggil secara sah dan patut untuk menghadap sidang pada tanggal 22 Desember 2020 dan tanggal 28 Desember 2020, sedang tidak ternyata tidak hadirnya itu disebabkan oleh suatu halangan yang sah menurut hukum;

Bahwa untuk mempersingkat uraian putusan ini, maka semua hal yang termuat dalam berita acara sidang ini merupakan bagian yang tidak terpisahkan dari putusan ini;

PERTIMBANGAN HUKUMNYA

Menimbang, bahwa maksud dan tujuan gugatan Penggugat sebagaimana diuraikan di atas;

Menimbang, bahwa karena ternyata Penggugat, meskipun telah dipanggil secara resmi dan patut berturut-turut untuk persidangan tanggal 21 Desember 2020 dan tanggal 28 Desember 2020, namun tidak datang ke muka sidang, sedang tidak ternyata bahwa tidak datangannya itu disebabkan sesuatu halangan yang sah. Oleh karena itu majelis hakim sepakat berpendapat bahwa pihak Penggugat tidak bersungguh-sungguh dalam mengajukan gugatannya, karenanya berdasarkan Pasal 124 HIR. Gugatan Penggugat harus dinyatakan gugur;

Menimbang, bahwa oleh karena perkara *a quo* dalam bidang perkawinan, maka berdasarkan Pasal 89 ayat (1) Undang-Undang Nomor 7 Tahun 1989 tentang Peradilan Agama, yang telah diubah dengan Undang-Undang Nomor 3 Tahun 2006 dan perubahan kedua dengan Undang-Undang Nomor 50 Tahun 2009, maka biaya perkara ini dibebankan kepada Penggugat;

Mengingat, peraturan perundang-undangan yang berlaku dan hukum *syar'i* yang berkaitan dengan perkara ini;

MENGADILI:

1. Menyatakan gugatan Penggugat gugur;

Disclaimer
Kepaniteraan Mahkamah Agung Republik Indonesia berusaha untuk selalu mencantumkan informasi paling kini dan akurat sebagai bentuk komitmen Mahkamah Agung untuk pelayanan publik, transparansi dan akuntabilitas pelaksanaan fungsi peradilan. Namun dalam hal-hal tertentu masih dimungkinkan terjadi permasalahan teknis terkait dengan akurasi dan keterkinian informasi yang kami sajikan, hal mana akan terus kami perbaiki dari waktu kewaktu.
Dalam hal Anda menemukan inakurasi informasi yang termuat pada situs ini atau informasi yang seharusnya ada, namun belum tersedia, maka harap segera hubungi Kepaniteraan Mahkamah Agung RI melalui :
Email : kepaniteraan@mahkamahagung.go.id Telp : 021-384 3348 (ext.318)

2. Membebankan kepada Penggugat untuk membayar biaya perkara sejumlah Rp. 641.000,00 (enam ratus empat puluh satu ribu rupiah)

Demikian perkara ini diputus dalam permusyawaratan Majelis Hakim Pengadilan Agama Jakarta Pusat pada hari Senin, tanggal 28 Desember 2020 Masehi bertepatan dengan tanggal 13 Jumadil Awal 1442 Hijriyah, oleh **H. M. Arief, S.H., M.H.** sebagai Ketua Majelis, **Dra. Hj. Hafsah, S.H.** dan **Dr. H. Jarkasih, M.H.**, masing-masing sebagai hakim anggota, putusan mana diucapkan dalam sidang terbuka untuk umum pada hari dan tanggal tersebut di atas, oleh Ketua Majelis dengan dihadiri oleh hakim-hakim anggota tersebut dan dengan dibantu oleh **Dika Andrian, S.Kom., S.H.**, sebagai Panitera Pengganti dengan diluar hadirnya Penggugat dan tanpa hadirnya Tergugat;

Ketua Majelis,

H. M. Arief, S.H., M.H.

Hakim Anggota,	Hakim Anggota,
Dra. Hj. Hafsah, S.H.	Dr. H. Jarkasih, M.H.

Panitera Pengganti,

Dika Andrian, S.Kom., S.H.

Perincian Biaya Perkara

Biaya Pendaftaran	:	Rp.	30.000
Biaya Proses/ATK	:	Rp.	75.000

Disclaimer
Kepaniteraan Mahkamah Agung Republik Indonesia berusaha untuk selalu mencantumkan informasi paling kini dan akurat sebagai bentuk komitmen Mahkamah Agung untuk pelayanan publik, transparansi dan akuntabilitas pelaksanaan fungsi peradilan. Namun dalam hal-hal tertentu masih dimungkinkan terjadi permasalahan teknis terkait dengan akurasi dan keterkinian informasi yang kami sajikan, hal mana akan terus kami perbaiki dari waktu kewaktu.
Dalam hal Anda menemukan inakurasi informasi yang termuat pada situs ini atau informasi yang seharusnya ada, namun belum tersedia, maka harap segera hubungi Kepaniteraan Mahkamah Agung RI melalui :
Email : kepaniteraan@mahkamahagung.go.id Telp : 021-384 3348 (ext.318)

Biaya Panggilan	:	Rp.	500.000
Biaya PNBP Penyerahan Panggilan Pertama Penggugat	:	Rp.	10.000
Biaya PNBP Penyerahan Panggilan Pertama Tergugat	:	Rp.	10.000
Biaya Redaksi	:	Rp.	10.000
Biaya Meterai	:	Rp.	6.000
Jumlah		Rp.	641.000

(enam ratus empat puluh satu ribu rupiah)

Disclaimer
Kepaniteraan Mahkamah Agung Republik Indonesia berusaha untuk selalu mencantumkan informasi paling kini dan akurat sebagai bentuk komitmen Mahkamah Agung untuk pelayanan publik, transparansi dan akuntabilitas pelaksanaan fungsi peradilan. Namun dalam hal-hal tertentu masih dimungkinkan terjadi permasalahan teknis terkait dengan akurasi dan keterkinian informasi yang kami sajikan, hal mana akan terus kami perbaiki dari waktu kewaktu.
Dalam hal Anda menemukan inakurasi informasi yang termuat pada situs ini atau informasi yang seharusnya ada, namun belum tersedia, maka harap segera hubungi Kepaniteraan Mahkamah Agung RI melalui :
Email : kepaniteraan@mahkamahagung.go.id   Telp : 021-384 3348 (ext.318)
