# Supplementary material for: Classification of divorce causes during the COVID-19 pandemic using convolutional neural networks
Source: PeerJ Comput Sci. 2022 Jun 30;8:e998. doi: 10.7717/peerj-cs.998 (PMC9299239; doi:10.7717/peerj-cs.998)
Supplement: Supplemental Information 2 [file peerj-cs-08-998-s002.zip › Kelalaian Dataset/Data ke-15.pdf]

PUTUSAN

Nomor 1841/Pdt.G/2020/PA.Bi

بِسْمِ اللهِ الرَّحْمٰنِ الرَّحِيْمِ

DEMI KEADILAN BERDASARKAN KETUHANAN YANG MAHA ESA

Pengadilan Agama Boyolali yang memeriksa dan mengadili perkara tertentu pada tingkat pertama dalam sidang Majelis telah menjatuhkan putusan dalam perkara Cerai Gugat antara :

P, umur 65 tahun (tempat tanggal lahir Boyolali, 01-07-1955), agama Islam, pekerjaan Petani, pendidikan SD, tempat tinggal Kecamatan Andong, Kabupaten Boyolali, sebagai Pemohon;

Melawan

T, umur 51 tahun (Tempat tanggal lahir Sragen, 08-08-1969), agama Islam, pekerjaan Petani, pendidikan tidak tamat SD, bertempat tinggal di Kecamatan Andong, Kabupaten Boyolali, sebagai Termohon;

Pengadilan Agama tersebut;

Telah membaca dan mempelajari berkas perkara;

Telah mendengar keterangan Pemohon dan para saksi di muka sidang;

DUDUK PERKARA

Menimbang, bahwa Pemohon dalam surat permohonannya tertanggal 03 Desember 2020 yang didaftarkan di Kepaniteraan Pengadilan Agama Boyolali dengan Nomor 1841/Pdt.G/2020/PA.Bi, tanggal 03 Desember 2020, telah mengajukan permohonan cerai talak dengan mengemukakan dalil-dalil sebagai berikut:

1. Bahwa Pemohon dan Termohon telah menikah pada tanggal 06 Januari 2017 tercatat di Kantor Urusan Agama Kecamatan Andong Kabupaten Boyolali sebagaimana yang tercantum dalam Kutipan Akta Nikah Nomor : XXX tanggal 06-01-2017;
2. Bahwa sebelum menikah Pemohon berstatus duda mempunyai empat orang anak dan Termohon berstatus janda;

Disclaimer
Kepaniteraan Mahkamah Agung Republik Indonesia berusaha untuk selalu mencantumkan informasi paling kini dan akurat sebagai bentuk komitmen Mahkamah Agung untuk pelayanan publik, transparansi dan akuntabilitas pelaksanaan fungsi peradilan. Namun dalam hal-hal tertentu masih dimungkinkan terjadi permasalahan teknis terkait dengan akurasi dan keterkinian informasi yang kami sajikan, hal mana akan terus kami perbaiki dari waktu kewaktu.
Dalam hal Anda menemukan inakurasi informasi yang termuat pada situs ini atau informasi yang seharusnya ada, namun belum tersedia, maka harap segera hubungi Kepaniteraan Mahkamah Agung RI melalui :
Email : kepaniteraan@mahkamahagung.go.id Telp : 021-384 3348 (ext.318)

3. Bahwa setelah menikah Pemohon dan Termohon hidup bersama di rumah Pemohon di Dukuh Tegalrejo RT.026 RW.010, Desa Andong, Kecamatan Andong, Kabupaten Boyolali selama 1 tahun, sudah melakukan hubungan layaknya suami istri (*ba'da dukhul*) namun belum dikaruniai keturunan;

4. Bahwa sejak pertengahan tahun 2017 ketentraman rumah tangga antara Pemohon dan Termohon mulai goyah, yang disebabkan karena:

    a. Bahwa Termohon mudah marah karena masalah sepele, yakni jika Pemohon pulang terlambat Termohon sudah marah sehingga Pemohon dan Termohon sering bertengkar;

    b. Bahwa Termohon terlalu berani dan membantah nasehat Pemohon bahkan Termohon berani berkata kasar dan menyakitkan hati Pemohon sehingga Pemohon merasa tidak dihargai sebagai seorang istri yang syah;

5. Bahwa puncak keretakan rumah tangga antara Pemohon dan Termohon terjadi pada bulan Januari 2018 Termohon pergi meninggalkan Pemohon tanpa pamit kepada Pemohon, Termohon tinggal di saudara (jawa:*paklik*) Termohon yang bernama Dukuh Tegalrejo RT.025 RW.010, Desa Andong, Kecamatan Andong, Kabupaten Boyolali;

6. Bahwa Pemohon sudah berkali-kali menjemput Termohon namun Termohon tidak mau hidup bersama lagi dengan Pemohon dan meminta Pemohon segera mengurus perceraian;

7. Bahwa sejak Pemohon dan Termohon berpisah atau sejak bulan Januari 2018 sampai sekarang atau selama 2 tahun 11 bulan, Pemohon dan Termohon sudah tidak pernah berkumpul lagi;

8. Bahwa atas dasar keadaan tersebut, maka Pemohon merasa sudah tidak ada harapan untuk hidup bersama lagi dengan Termohon;

9. Bahwa dengan demikian Pemohon telah cukup alasan untuk mengajukan permohonan cerai talak sebagaimana yang diatur dalam peraturan perundang undangan yang berlaku;

Disclaimer
Kepaniteraan Mahkamah Agung Republik Indonesia berusaha untuk selalu mencantumkan informasi paling kini dan akurat sebagai bentuk komitmen Mahkamah Agung untuk pelayanan publik, transparansi dan akuntabilitas pelaksanaan fungsi peradilan. Namun dalam hal-hal tertentu masih dimungkinkan terjadi permasalahan teknis terkait dengan akurasi dan keterkinian informasi yang kami sajikan, hal mana akan terus kami perbaiki dari waktu kewaktu.
Dalam hal Anda menemukan inakurasi informasi yang termuat pada situs ini atau informasi yang seharusnya ada, namun belum tersedia, maka harap segera hubungi Kepaniteraan Mahkamah Agung RI melalui :
Email : kepaniteraan@mahkamahagung.go.id Telp : 021-384 3348 (ext.318)

Berdasarkan alasan/dalil-dalil tersebut diatas, Pemohon mohon agar Ketua Pengadilan Agama Boyolali Cq Majelis Hakim untuk segera memeriksa dan mengadili perkara ini, selanjutnya menjatuhkan putusan yang amarnya berbunyi:

1. Mengabulkan permohonan Pemohon;
2. Menetapkan memberi ijin kepada Pemohon (P) untuk menjatuhkan talak satu roji terhadap Termohon (T) di depan sidang Pengadilan Agama Boyolali;
3. Membebankan biaya perkara kepada Pemohon;
4. Menjatuhkan putusan dengan seadil-adilnya;

Menimbang, bahwa pada hari sidang yang telah ditetapkan Pemohon telah datang menghadap di persidangan, sedangkan Termohon tidak datang menghadap dan tidak menyuruh orang lain untuk menghadap sebagai wakil/kuasa hukumnya meskipun Termohon telah dipanggil sebanyak 1 kali berdasarkan relaas panggilan tanggal 07 Desember 2020;

Menimbang, Bahwa Majelis Hakim telah berusaha menasehati Pemohon untuk bersabar dan hidup rukun lagi dengan Termohon, akan tetapi tidak berhasil, lalu dibacakan surat permohonan Pemohon yang isinya tetap dipertahankan oleh Pemohon ;

Menimbang, bahwa untuk menguatkan dalil-dalil permohonannya, Pemohon telah mengajukan bukti surat berupa :

1. Fotokopi Kartu Tanda Penduduk Nomor 3309160107559012 yang dikeluarkan oleh kantor Dinas Kependudukan dan Catatan Sipil Kabupaten Boyolali, bermeterai cukup dan telah sesuai dengan aslinya (Bukti P.1);
2. Fotokopi Buku Kutipan Akta Nikah dari KUA Kecamatan Andong Kabupaten Boyolali Nomor 0023/23/I/2017 tanggal 06 Januari 2017, yang bermeterai cukup dan telah sesuai dengan aslinya (Bukti ;P.2);

Menimbang, bahwa bukti surat-surat tersebut bermeterai cukup yang oleh Ketua Majelis telah dicocokkan dengan aslinya dan telah sesuai yang masing-masing diberi tanda P.1 dan P.2;

Disclaimer
Kepaniteraan Mahkamah Agung Republik Indonesia berusaha untuk selalu mencantumkan informasi paling kini dan akurat sebagai bentuk komitmen Mahkamah Agung untuk pelayanan publik, transparansi dan akuntabilitas pelaksanaan fungsi peradilan. Namun dalam hal-hal tertentu masih dimungkinkan terjadi permasalahan teknis terkait dengan akurasi dan keterkinian informasi yang kami sajikan, hal mana akan terus kami perbaiki dari waktu kewaktu.
Dalam hal Anda menemukan inakurasi informasi yang termuat pada situs ini atau informasi yang seharusnya ada, namun belum tersedia, maka harap segera hubungi Kepaniteraan Mahkamah Agung RI melalui :
Email : kepaniteraan@mahkamahagung.go.id Telp : 021-384 3348 (ext.318)

Menimbang, bahwa selain bukti surat surat tersebut Pemohon telah mengajukan bukti saksi saksi :

1. **SAKSI**, umur 47 tahun, agama Islam, pekerjaan Swasta, tempat tinggal Kecamatan Andong, Kabupaten Boyolali, telah memberikan keterangan di bawah sumpah pada pokoknya sebagai berikut :
   - Bahwa saksi kenal dengan Pemohon dan Termohon karena sebagai tetangga dekat;
   - Bahwa saksi tahu Pemohon dan Termohon menikah pada 06 Januari 2017;
   - Bahwa setelah menikah Pemohon dan Termohon hidup bersama di rumah Pemohon di Dukuh Tegalrejo RT.026 RW.010, Desa Andong, Kecamatan Andong, Kabupaten Boyolali selama 1 tahun, sudah melakukan hubungan layaknya suami istri (*ba'da dukhul*) namun belum dikaruniai keturunan,
   - Bahwa Pemohon sehat jasmani dan rohani, berkelakuan baik
   - Bahwa saksi tahu sejak tahun Januari 2018, Termohon pergi meninggalkan Pemohon tanpa pamit kepada Pemohon, Termohon tinggal di saudara (jawa:*paklik*) Termohon di Dukuh Tegalrejo RT.025 RW.010, Desa Andong, Kecamatan Andong, Kabupaten Boyolali sampai sekarang tidak pernah kembali ;
   - Bahwa sebelum Termohon pergi, saksi sering mendengar Pemohon dan Termohon bertengkar, dalam pertengkaran tersebut Termohon mudah marah karena masalah sepele yakni jika Pemohon pulang terlambat Termohon marah dan Termohon berani dan membantah nasehat Pemohon;
   - Bahwa selama pisah tersebut Pemohon dan Termohon sudah tidak pernah kumpul lagi;
   - Bahwa akibat dari perselisihan dan pertengkaran tersebut, sekarang Pemohon dan Termohon sudah pisah tempat tinggal sejak Januari 2018 Termohon pergi tanpa pamit Pemohon Termohon tinggal di saudara (jawa:*paklik*) Termohon di Dukuh Tegalrejo RT.025 RW.010, Desa Andong, Kecamatan Andong, Kabupaten Boyolali ;

Disclaimer
Kepaniteraan Mahkamah Agung Republik Indonesia berusaha untuk selalu mencantumkan informasi paling kini dan akurat sebagai bentuk komitmen Mahkamah Agung untuk pelayanan publik, transparansi dan akuntabilitas pelaksanaan fungsi peradilan. Namun dalam hal-hal tertentu masih dimungkinkan terjadi permasalahan teknis terkait dengan akurasi dan keterkinian informasi yang kami sajikan, hal mana akan terus kami perbaiki dari waktu kewaktu.
Dalam hal Anda menemukan inakurasi informasi yang termuat pada situs ini atau informasi yang seharusnya ada, namun belum tersedia, maka harap segera hubungi Kepaniteraan Mahkamah Agung RI melalui :
Email : kepaniteraan@mahkamahagung.go.id Telp : 021-384 3348 (ext.318)

- Bahwa selama pisah tempat tinggal tersebut Pemohon dan Termohon sudah tidak pernah kumpul lagi;
- Bahwa pihak keluarga sudah berusaha mendamaikan Pemohon dan Termohon tetapi tidak berhasil

2. **SAKSI**, agama Islam, pekerjaan Swasta, tempat tinggal Kecamatan Andong, Kabupaten Boyolali, telah memberikan keterangan di bawah sumpah yang pada pokoknya sebagai berikut :
   - Bahwa saksi kenal dengan Pemohon dan Termohon karena sebagai tetangga dekat;
   - Bahwa saksi tahu Pemohon dan Termohon menikah pada 06 Januari 2017;
   - Bahwa setelah menikah Pemohon dan Termohon hidup bersama di rumah Pemohon di Dukuh Tegalrejo RT.026 RW.010, Desa Andong, Kecamatan Andong, Kabupaten Boyolali selama 1 tahun, sudah melakukan hubungan layaknya suami istri (*ba'da dukhul*) namun belum dikaruniai keturunan,
   - Bahwa Pemohon sehat jasmani dan rohani, berkelakuan baik
   - Bahwa saksi tahu sejak tahun Januari 2018, Termohon pergi meninggalkan Pemohon tanpa pamit kepada Pemohon, Termohon tinggal di saudara (jawa:*paklik*) Termohon di Dukuh Tegalrejo RT.025 RW.010, Desa Andong, Kecamatan Andong, Kabupaten Boyolali sampai sekarang tidak pernah kembali ;
   - Bahwa sebelum Termohon pergi, saksi sering mendengar Pemohon dan Termohon bertengkar, dalam pertengkaran tersebut Termohon mudah marah karena masalah sepele yakni jika Pemohon pulang terlambat Termohon marah dan Termohon berani dan membantah nasehat Pemohon;
   - Bahwa selama pisah tersebut Pemohon dan Termohon sudah tidak pernah kumpul lagi;
   - Bahwa akibat dari perselisihan dan pertengkaran tersebut, sekarang Pemohon dan Termohon sudah pisah tempat tinggal sejak Januari 2018 Termohon pergi tanpa pamit Pemohon Termohon tinggal di

Disclaimer
Kepaniteraan Mahkamah Agung Republik Indonesia berusaha untuk selalu mencantumkan informasi paling kini dan akurat sebagai bentuk komitmen Mahkamah Agung untuk pelayanan publik, transparansi dan akuntabilitas pelaksanaan fungsi peradilan. Namun dalam hal-hal tertentu masih dimungkinkan terjadi permasalahan teknis terkait dengan akurasi dan keterkinian informasi yang kami sajikan, hal mana akan terus kami perbaiki dari waktu kewaktu.
Dalam hal Anda menemukan inakurasi informasi yang termuat pada situs ini atau informasi yang seharusnya ada, namun belum tersedia, maka harap segera hubungi Kepaniteraan Mahkamah Agung RI melalui :
Email : kepaniteraan@mahkamahagung.go.id Telp : 021-384 3348 (ext.318)

saudara (jawa:*paklik*) Termohon di Dukuh Tegalrejo RT.025 RW.010, Desa Andong, Kecamatan Andong, Kabupaten Boyolali ;

- Bahwa selama pisah tempat tinggal tersebut Pemohon dan Termohon sudah tidak pernah kumpul lagi;
- Bahwa pihak keluarga sudah berusaha mendamaikan Pemohon dan Termohon tetapi tidak berhasil

Menimbang, bahwa Pemohon telah memberikan kesimpulan, bahwa Pemohon tetap pada permohonannya dan sudah tidak akan mengajukan suatu apapun lagi melainkan hanya mohon agar Majelis Hakim segera menjatuhkan putusan ;

Selanjutnya untuk singkatnya uraian putusan ini, maka semua hal yang termuat dalam Berita Acara Sidang ini merupakan bagian yang tak terpisahkan dari putusan ini;

PERTIMBANGAN HUKUM

Menimbang, bahwa maksud dan tujuan permohonan Pemohon adalah sebagaimana terurai di atas;

Menimbang, bahwa sebelum Majelis mempertimbangkan lebih lanjut mengenai materi pokok permohonan Pemohon, terlebih dahulu Majelis akan mempertimbangkan relaas panggilan mengenai ketidak hadiran Termohon di persidangan ;

Menimbang bahwa berdasarkan surat panggilan (relaas) Nomor: 1841/Pdt.G/2020/PA.Bi tanggal 07 Desember 2020 ternyata Termohon telah dipanggil secara resmi dan patut menurut hukum sesuai dengan ketentuan pasal 26 Peraturan Pemerintah nomor : 9 tahun 1975, akan tetapi tidak hadir dan tidak mewakilkan kepada orang lain sebagai kuasanya sedang ternyata tidak hadirnya Termohon tersebut bukan disebabkan oleh halangan yang sah, oleh karena itu harus dinyatakan Termohon yang telah dipanggil secara resmi dan patut untuk hadir di persidangan, tidak hadir ;

Menimbang, bahwa oleh karena Termohon tidak hadir di persidangan, perkara *a quo* diperiksa tanpa kehadiran Termohon dan Majelis akan menjatuhkan putusan dengan putusan verstek, sesuai dengan Pasal 125 HIR dan dalil syar'i

Disclaimer
Kepaniteraan Mahkamah Agung Republik Indonesia berusaha untuk selalu mencantumkan informasi paling kini dan akurat sebagai bentuk komitmen Mahkamah Agung untuk pelayanan publik, transparansi dan akuntabilitas pelaksanaan fungsi peradilan. Namun dalam hal-hal tertentu masih dimungkinkan terjadi permasalahan teknis terkait dengan akurasi dan keterkinian informasi yang kami sajikan, hal mana akan terus kami perbaiki dari waktu kewaktu.
Dalam hal Anda menemukan inakurasi informasi yang termuat pada situs ini atau informasi yang seharusnya ada, namun belum tersedia, maka harap segera hubungi Kepaniteraan Mahkamah Agung RI melalui :
Email : kepaniteraan@mahkamahagung.go.id   Telp : 021-384 3348 (ext.318)

من دعي إلى حاكم من حكام المسلمين فلم يجب فهو ظالم لا حق له

Artinya : " Seseorang yang dipanggil Hakim (Pengadilan) tidak datang, ia dipandang sebagai dhalim dan tidak ada hak baginya. " (Ahkamul Qur-an II : 405) ;

Menimbang, bahwa karena Termohon telah tidak hadir di persidangan, maka dianggap Termohon mengakui dalil dalil permohonan Pemohon;

Menimbang, bahwa gugatan Pemohon pada pokoknya adalah mohon agar diberikan izin untuk menceraikan Termohon karena sejak Januari 2018 antara Pemohon dan Termohon sering terjadi perselisihan dan pertengkaran yang disebabkan karena Termohon mudah marah karena masalah sepele yakni jika Pemohon pulang terlambat Termohon marah dan Termohon berani dan membantah nasehat Pemohon, Bahwa sejak Januari 2018 Termohon pergi meninggalkan Pemohon tanpa pamit kepada Pemohon, Termohon tinggal di saudara (jawa:*paklik*) Termohon di Dukuh Tegalrejo RT.025 RW.010, Desa Andong, Kecamatan Andong, Kabupaten Boyolali sampai sekarang tidak pernah kembali. Sehingga oleh karenanya Pemohon tidak ingin lagi mempertahankan rumah tangganya dengan Termohon;

Menimbang, bahwa untuk mengukuhkan dalil-dalil gugatannya, Pemohon telah mengajukan alat bukti berupa surat dan saksi-saksi, menurut Majelis kesemua alat bukti tersebut, telah memenuhi syarat formil dan materil;

Menimbang, bahwa berdasarkan keterangan saksi-saksi di persidangan ditemukan fakta-fakta sebagai berikut :

1. Bahwa Pemohon dan Termohon adalah suami isteri yang sah, yang menikah di hadapan Pegawai Pencatat Nikah Kantor Urusan Agama Kecamatan Andong Kabupaten Boyolali pada tanggal 06 Januari 2017;

2. Bahwa setelah menikah Pemohon dan Termohon hidup rukun harmonis, tinggal bersama di rumah Pemohon di Dukuh Tegalrejo RT.026 RW.010, Desa Andong, Kecamatan Andong, Kabupaten Boyolali

Disclaimer
Kepaniteraan Mahkamah Agung Republik Indonesia berusaha untuk selalu mencantumkan informasi paling kini dan akurat sebagai bentuk komitmen Mahkamah Agung untuk pelayanan publik, transparansi dan akuntabilitas pelaksanaan fungsi peradilan. Namun dalam hal-hal tertentu masih dimungkinkan terjadi permasalahan teknis terkait dengan akurasi dan keterkinian informasi yang kami sajikan, hal mana akan terus kami perbaiki dari waktu kewaktu.
Dalam hal Anda menemukan inakurasi informasi yang termuat pada situs ini atau informasi yang seharusnya ada, namun belum tersedia, maka harap segera hubungi Kepaniteraan Mahkamah Agung RI melalui :
Email : kepaniteraan@mahkamahagung.go.id Telp : 021-384 3348 (ext.318)

selama 1 tahun, sudah melakukan hubungan layaknya suami istri (*ba'da dukhul*) namun belum dikaruniai keturunan;

3. Bahwa rumah tangga Pemohon dan Termohon sudah tidak harmonis, sering terjadi perselisihan dan pertengkaran yang disebabkan Termohon mudah marah karena masalah sepele yakni jika Pemohon pulang terlambat Termohon marah dan Termohon berani dan membantah nasehat Pemohon;

4. Bahwa Termohon telah pergi meninggalkan Pemohon tanpa pamit Pemohon, Termohon tinggal di saudara (jawa:*paklik*) Termohon di Dukuh Tegalrejo RT.025 RW.010, Desa Andong, Kecamatan Andong, Kabupaten Boyolali sampai sekarang tidak pernah kembali;

5. Bahwa pihak keluarga sudah berusaha mendamaikan dan merukunkan Pemohon dan Termohon, akan tetap tidak berhasil;

Menimbang, bahwa berdasarkan fakta-fakta tersebut di atas di atas, Majelis berpendapat bahwa alasan Pemohon untuk bercerai dengan Termohon telah beralasan hukum serta memenuhi unsur-unsur yang terkandung dalam pasal 19 huruf (f) Peraturan Pemerintah No. 9 Tahun 1975 jo pasal 116 huruf (f) Kompilasi Hukum Islam, dan sejalan pula dengan maksud pasal 39 ayat (2) Undang-undang No.1 Tahun 1974 jo pasal 22 ayat (2) PP No.9 Tahun 1975 dan pasal 134 Kompilasi Hukum Islam.

Menimbang, bahwa sebagaimana terungkap di persidangan Pemohon dan Termohon sudah berpisah tempat tinggal lebih kurang 2 (dua) tahun 11 (sebelas) bulan lamanya hingga saat ini, dengan kondisi objektif rumah tangga seperti ini, Majelis Hakim menilai rumah tangga Pemohon dengan Termohon tidak lagi mencerminkan rumah tangga yang harmonis, karena masing-masing telah hidup terpisah serta tidak lagi memperdulikan satu sama lain;

Menimbang, bahwa salah satu unsur terpenting dalam membina rumah tangga yang bahagia dan harmonis adalah adanya kemesraan hubungan sebagaimana kehidupan berumah tangga, jika faktor penting ini terabaikan maka majelis hakim menilai kehidupan rumah tangga Pemohon dengan Termohon telah kehilangan makna sebuah perkawinan;

Disclaimer
Kepaniteraan Mahkamah Agung Republik Indonesia berusaha untuk selalu mencantumkan informasi paling kini dan akurat sebagai bentuk komitmen Mahkamah Agung untuk pelayanan publik, transparansi dan akuntabilitas pelaksanaan fungsi peradilan. Namun dalam hal-hal tertentu masih dimungkinkan terjadi permasalahan teknis terkait dengan akurasi dan keterkinian informasi yang kami sajikan, hal mana akan terus kami perbaiki dari waktu kewaktu.
Dalam hal Anda menemukan inakurasi informasi yang termuat pada situs ini atau informasi yang seharusnya ada, namun belum tersedia, maka harap segera hubungi Kepaniteraan Mahkamah Agung RI melalui :
Email : kepaniteraan@mahkamahagung.go.id Telp : 021-384 3348 (ext.318)

Menimbang, bahwa rumah tangga yang bahagia dan kekal akan terwujud manakala antara suami dan isteri saling mencintai dan menyayangi satu sama lain, sebagaimana diisyaratkan dalam al-Qur'an surat ar-Rum ayat 21:

ومن أيته أن خلق لكم من أنفسكم أزواجا لتسكنوا اليها وجعل بينكم مودة ورحمة ان فى ذلك لا يت لقوم يتفكرون.

"Dan di antara tanda-tanda kekuasaan-Nya ialah, Dia menciptakan untukmu isteri-isteri dari jenismu sendiri, supaya kamu cenderung dan merasa tentram kepadanya, dan dijadikan-Nya diantaramu rasa kasih dan sayang, sesungguhnya pada yang demikian itu benar-benar terdapat tanda-tanda bagi kaum yang berfikir.

Menimbang, bahwa sebagaimana ternyata Pemohon telah berketetapan hati untuk bercerai dengan Termohon, hal ini menjadi petunjuk bahwa rumah tangga Pemohon dengan Termohon telah pecah dan sulit dirukunkan kembali. Mempertahankan ikatan perkawinan dan melanjutkan rumah tangga yang seperti ini akan menimbulkan mudharat yang lebih besar dari pada maslahatnya karena tujuan perkawinan untuk membentuk keluarga yang bahagia dan kekal sebagaimana maksud pasal 1 Undang-undang No.1 Tahun 1974 jo pasal 3 Kompilasi Hukum Islam tidak mungkin lagi terwujud dalam rumah tangga Pemohon dan Termohon;

Menimbang, bahwa sesuai dengan Yurisprudensi Mahkamah Agung RI Nomor 273/K/AG/1998 tanggal 17 Maret 1999 yang menyatakan "Bahwa cekcok, hidup berpisah tidak dalam satu tempat kediaman bersama/berpisah tempat tidur, salah satu pihak tidak berniat untuk meneruskan kehidupan bersama dengan pihak lain merupakan fakta yang cukup sesuai alasan perceraian;

Menimbang, bahwa berdasarkan pertimbangan-pertimbangan tersebut di atas Majelis Hakim patut mengabulkan permohonan Pemohon;

Menimbang, bahwa oleh karena Termohon tidak hadir sedangkan Pemohon tetap hadir di persidangan dan menurut Majelis permohonan Pemohon telah cukup beralasan dan tidak melawan hukum, maka sesuai

Disclaimer
Kepaniteraan Mahkamah Agung Republik Indonesia berusaha untuk selalu mencantumkan informasi paling kini dan akurat sebagai bentuk komitmen Mahkamah Agung untuk pelayanan publik, transparansi dan akuntabilitas pelaksanaan fungsi peradilan. Namun dalam hal-hal tertentu masih dimungkinkan terjadi permasalahan teknis terkait dengan akurasi dan keterkinian informasi yang kami sajikan, hal mana akan terus kami perbaiki dari waktu kewaktu.
Dalam hal Anda menemukan inakurasi informasi yang termuat pada situs ini atau informasi yang seharusnya ada, namun belum tersedia, maka harap segera hubungi Kepaniteraan Mahkamah Agung RI melalui :
Email : kepaniteraan@mahkamahagung.go.id Telp : 021-384 3348 (ext.318)

dengan ketentuan pasal 125 HIR jo pasal 39 ayat 1 Undang-Undang nomor 1 tahun 1974, permohonan Pemohon dikabulkan dengan Verstek;

Menimbang bahwa perkara ini termasuk dalam bidang perkawinan, maka berdasarkan ketentuan Pasal 89 ayat (1) Undang-undang Nomor 7 Tahun 1989 tentang Peradilan Agama sebagaimana telah diubah dengan Undang-undang Nomor 3 Tahun 2006 dan perubahan kedua dengan Undang-undang Nomor 50 Tahun 2009 maka kepada Pemohon dibebankan untuk membayar semua biaya yang timbul dalam perkara ini;

Mengingat semua peraturan perundang-undangan yang berlaku dan hukum Islam yang berkaitan dengan perkara ini

M E N G A D I L I

1. Menyatakan Termohon yang telah dipanggil dengan resmi dan patut untuk menghadap di persidangan, tidak hadir;
2. Mengabulkan permohonan Pemohon dengan verstek;
3. Memberi izin kepada Pemohon (P) untuk menjatuhkan talak satu raj'i terhadap Termohon (T) di depan sidang Pengadilan Agama Boyolali;
4. Membebankan kepada Pemohon untuk membayar biaya perkara ini sejumlah Rp. 391.000,- (Tiga ratus sembilan puluh satu ribu rupiah);

Demikian diputuskan dalam rapat permusyawaratan Majelis Hakim yang dilangsungkan pada hari Rabu tanggal 23 Desember 2020 *Masehi*, bertepatan dengan tanggal 8 Jumadil Awal 1442 *Hijriyah,* oleh kami **Fakhrurazi, S.Ag., M.H.I.** sebagai Ketua Majelis, dan **Dra. Hj. Emi Suyati** serta **Syahruddin, S.H.I.,** M.H. masing-masing sebagai Hakim Anggota. Putusan tersebut diucapkan oleh Ketua Majelis pada hari itu juga dalam sidang terbuka untuk umum dengan dihadiri oleh Hakim-Hakim Anggota tersebut dan dibantu oleh **Drs. Arief Rokhman** sebagai Panitera Pengganti serta dihadiri Pemohon tanpa hadirnya Termohon;

Ketua Majelis,

Disclaimer
Kepaniteraan Mahkamah Agung Republik Indonesia berusaha untuk selalu mencantumkan informasi paling kini dan akurat sebagai bentuk komitmen Mahkamah Agung untuk pelayanan publik, transparansi dan akuntabilitas pelaksanaan fungsi peradilan. Namun dalam hal-hal tertentu masih dimungkinkan terjadi permasalahan teknis terkait dengan akurasi dan keterkinian informasi yang kami sajikan, hal mana akan terus kami perbaiki dari waktu kewaktu.
Dalam hal Anda menemukan inakurasi informasi yang termuat pada situs ini atau informasi yang seharusnya ada, namun belum tersedia, maka harap segera hubungi Kepaniteraan Mahkamah Agung RI melalui :
Email : kepaniteraan@mahkamahagung.go.id   Telp : 021-384 3348 (ext.318)

Fakhrurazi, S.Ag., M.H.I.

Hakim Anggota, Hakim Anggota,

Dra. Hj. Emi Suyati **Syahruddin, S.H.I., M.H.**

Panitera Pengganti,

Drs. Arief Rokhman

Perincian Biaya :

Biaya Pendaftaran	:	Rp	30.000,00,-
Biaya Proses	:	Rp	75.000,00,-
Biaya Pemanggilan	:	Rp	250.000,00,-
Biaya Redaksi	:	Rp	10.000,00,-
Biaya Materai	:	Rp	6.000,00,-
Biaya PNBP	:	Rp	20.000,00,-
Jumlah	:	Rp	391.000,00,-

Disclaimer
Kepaniteraan Mahkamah Agung Republik Indonesia berusaha untuk selalu mencantumkan informasi paling kini dan akurat sebagai bentuk komitmen Mahkamah Agung untuk pelayanan publik, transparansi dan akuntabilitas pelaksanaan fungsi peradilan. Namun dalam hal-hal tertentu masih dimungkinkan terjadi permasalahan teknis terkait dengan akurasi dan keterkinian informasi yang kami sajikan, hal mana akan terus kami perbaiki dari waktu kewaktu.
Dalam hal Anda menemukan inakurasi informasi yang termuat pada situs ini atau informasi yang seharusnya ada, namun belum tersedia, maka harap segera hubungi Kepaniteraan Mahkamah Agung RI melalui :
Email : kepaniteraan@mahkamahagung.go.id Telp : 021-384 3348 (ext.318)
