# Supplementary material for: Classification of divorce causes during the COVID-19 pandemic using convolutional neural networks
Source: PeerJ Comput Sci. 2022 Jun 30;8:e998. doi: 10.7717/peerj-cs.998 (PMC9299239; doi:10.7717/peerj-cs.998)
Supplement: Supplemental Information 2 [file peerj-cs-08-998-s002.zip › Kelalaian Dataset/Data ke-16.pdf]

P U T U S A N

Nomor 1302/Pdt.G/2020/PA.Amb

بِسْــــــــــــــمِ اللهِ الرَّحْمَنِ الرَّحِيْمِ

DEMI KEADILAN BERDASARKAN KETUHANAN YANG MAHA ESA

Pengadilan Agama Ambarawa yang memeriksa dan mengadili perkara tertentu pada tingkat pertama dalam sidang, Hakim telah menjatuhkan putusan perkara Gugatan Perceraian antara;

[redacted], umur 26 tahun, agama Islam, Pekerjaan Dosen, Pendidikan Strata II, tempat kediaman di [redacted] Kabupaten Semarang, sebagai Penggugat

Melawan

[redacted] umur 30 tahun, agama Islam, Pekerjaan Karyawan Swasta, Pendidikan Strata I, tempat kediaman di [redacted] Kabupten Bogor, Jawa Barat, sebagai Tergugat

Pengadilan Agama tersebut;

Telah mempelajari surat-surat yang berkaitan dengan perkara ini;

Telah mendengar keterangan Penggugat dan para saksi di muka sidang;

DUDUK PERKARA

Bahwa, Penggugat dalam surat gugatannya tanggal 01 Desember 2020 telah mengajukan gugatan perceraian yang telah didaftar di Kepaniteraan Pengadilan Agama Ambarawa dengan Nomor 1302/Pdt.G/2020/PA.Amb, tanggal 01 Desember 2020, dengan dalil-dalil sebagai berikut:

1. Bahwa pada tanggal 14 April 2019 Penggugat telah melangsungkan perkawinan dengan Tergugat dihadapan Pejabat KUA (Kantor Urusan Agama) Kecamatan Ungaran Timur, Kabupaten Semarang sebagaimana dalam Kutipan Akta Nikah Nomor: [redacted] yang dikeluarkan KUA tersebut tertanggal 14 April

Disclaimer
Kepaniteraan Mahkamah Agung Republik Indonesia berusaha untuk selalu mencantumkan informasi paling kini dan akurat sebagai bentuk komitmen Mahkamah Agung untuk pelayanan publik, transparansi dan akuntabilitas pelaksanaan fungsi peradilan. Namun dalam hal-hal tertentu masih dimungkinkan terjadi permasalahan teknis terkait dengan akurasi dan keterkinian informasi yang kami sajikan, hal mana akan terus kami perbaiki dari waktu kewaktu.
Dalam hal Anda menemukan inakurasi informasi yang termuat pada situs ini atau informasi yang seharusnya ada, namun belum tersedia, maka harap segera hubungi Kepaniteraan Mahkamah Agung RI melalui :
Email : kepaniteraan@mahkamahagung.go.id   Telp : 021-384 3348 (ext.318)

2019;

2. Bahwa setelah Akad Nikah tersebut Penggugat dengan Tergugat hidup bersama dirumah Tergugat di [redacted] selama 11 bulan, selama pernikahan tersebut Penggugat dengan Tergugat telah hidup rukun sebagaimana layaknya suami istri (ba'daddukhul), namun dalam perkawinan tersebut antara Penggugat dan Tergugat belum dikaruniai seorang anak;

3. bahwa rumah tangga Penggugat dan Tergugat semula dalam keadaan harmonis, namun sejak bulan September 2019 antara Penggugat dan Tergugat sering terjadi Pertengkaran dan perselisihan yang di sebabkan karena:

- Penggugat tidak bisa akur dengan ibu Tergugat;
- Tergugat tidak bisa mengambil sikap tentang perselisihan Penggugat dengan ibu Tergugat;
- Tergugat tidak bisa bersikap dan berlaku selayaknya kepala rumah tangga, tidak bisa mengayomi Penggugat selaku isteri;
- Tergugat menyepelekan dan tidak menganggap Penggugat sebagai Isteri dan tidak pernah mengajak berembug istri terkait dengan permasalahan rumah tangga;
- Antara Penggugat dengan Tergugat sudah tidak ada lagi kecocokan dalam membina rumah tangga;

4. Bahwa puncak perselisihan dan Pertengkaran antara Penggugat dan tergugat terjadi pada bulan Maret tahun 2020, Tergugat pergi meninggalkan Penggugat dan saat ini berada [redacted] Kabupten Bogor, Jawa Barat, bahkan pada bulan Agustus 2020, Tergugat kembali untuk menjatuhkan talak terhadap Penggugat dan mengembalikan Penggugat kepada orangtua Penggugat, sehingga antara Penggugat dengan Tergugat telah pisah rumah selama 8 bulan dan selama itu keduanya sudah tidak saling mempedulikan satu

Disclaimer
Kepaniteraan Mahkamah Agung Republik Indonesia berusaha untuk selalu mencantumkan informasi paling kini dan akurat sebagai bentuk komitmen Mahkamah Agung untuk pelayanan publik, transparansi dan akuntabilitas pelaksanaan fungsi peradilan. Namun dalam hal-hal tertentu masih dimungkinkan terjadi permasalahan teknis terkait dengan akurasi dan keterkinian informasi yang kami sajikan, hal mana akan terus kami perbaiki dari waktu kewaktu.
Dalam hal Anda menemukan inakurasi informasi yang termuat pada situs ini atau informasi yang seharusnya ada, namun belum tersedia, maka harap segera hubungi Kepaniteraan Mahkamah Agung RI melalui :
Email : kepaniteraan@mahkamahagung.go.id Telp : 021-384 3348 (ext.318)

sama lain;

5. Bahwa terhadap permasalahan rumah tangga yang terjadi antara Penggugat dengan Tergugat tersebut, pihak keluarga telah berusaha mendamaikan, namun tidak berhasil dan keduanya tetap tidak bisa rukun kembali;

6. Bahwa berdasarkan alasan dan dalil-dalil tersebut di atas, cukup alasan bagi Penggugat untuk mengajukan gugatan perceraian melalui Pengadilan Agama Ambarawa;

Bahwa, berdasarkan alasan /dalil-dalil di atas Pemohon mohon agar Ketua Pengadilan Agama Ambarawa segera memeriksa dan mengadili perkara ini, selanjutnya menjatuhkan putusan yang amarnya berbunyi:

PRIMAIR;

1. Mengabulkan gugatan Penggugat;
2. Menjatuhkan jatuh talak satu Bain Sughro Tergugat ([redacted]) terhadap Penggugat ([redacted]);
3. Menetapkan biaya perkara menurut hukum;

SUBSIDER;

Mohon putusan yang seadil adilnya;

Demikian atas terkabulnya gugatan ini, Penggugat menyampaikan terimakasih.

Bahwa, pada hari sidang yang telah ditetapkan Penggugat telah datang menghadap ke muka sidang, sedangkan Tergugat tidak datang menghadap ke muka sidang dan tidak menyuruh orang lain untuk menghadap sebagai wakil/kuasa hukumnya meskipun telah dipanggil secara resmi dan patut yang relaas panggilannya telah dibacakan di dalam sidang, sedangkan tidak ternyata bahwa tidak datangnya itu disebabkan suatu halangan yang sah;

Bahwa, Hakim telah menasehati Penggugat agar berpikir untuk tidak bercerai dengan Tergugat, tetapi Penggugat tetap pada dalil-dalil gugatannya untuk bercerai dengan Tergugat;

Bahwa, perkara ini tidak dapat di mediasi karena Tergugat tidak pernah datang menghadap meskipun telah dipanggil secara resmi dan patut, selanjutnya dimulai pemeriksaan dengan membacakan surat gugatan Penggugat yang maksud dan isinya tetap dipertahankan oleh Penggugat;

Disclaimer
Kepaniteraan Mahkamah Agung Republik Indonesia berusaha untuk selalu mencantumkan informasi paling kini dan akurat sebagai bentuk komitmen Mahkamah Agung untuk pelayanan publik, transparansi dan akuntabilitas pelaksanaan fungsi peradilan. Namun dalam hal-hal tertentu masih dimungkinkan terjadi permasalahan teknis terkait dengan akurasi dan keterkinian informasi yang kami sajikan, hal mana akan terus kami perbaiki dari waktu kewaktu.
Dalam hal Anda menemukan inakurasi informasi yang termuat pada situs ini atau informasi yang seharusnya ada, namun belum tersedia, maka harap segera hubungi Kepaniteraan Mahkamah Agung RI melalui :
Email : kepaniteraan@mahkamahagung.go.id Telp : 021-384 3348 (ext.318)

Bahwa, untuk menguatkan dalil-dalil gugatannya, Penggugat telah mengajukan bukti-bukti berupa :

A. Surat :

1. Fotokopi Kartu Tanda Penduduk Nomor [redacted] tanggal 05-03-2020, yang bermeterai cukup dan telah sesuai dengan aslinya (Bukti P.1);
2. Fotokopi Buku Kutipan Akta Nikah dari KUA (Kantor Urusan Agama) Kecamatan Ungaran Timur, Kabupaten Semarang Nomor [redacted] tanggal 14 April 2019, yang bermeterai cukup dan telah sesuai dengan aslinya (Bukti P.2);

B. Saksi :

1. [redacted] umur 55 tahun, Agama Islam, Pendidikan Sekolah Lanjutan Tingkat Atas, Pekerjaan Wiraswasta, tempat tinggal di [redacted] Kabupaten Semarang, menerangkan dibawah sumpah yang pada pokoknya :
   - Bahwa saksi kenal dengan Penggugat dan Tergugat karena sebagai ibu Penggugat;
   - Bahwa Penggugat dan Tergugat menikah pada tahun 2019;
   - Bahwa Penggugat dan Tergugat belum dikaruniai anak;
   - Bahwa setelah menikah Penggugat dan Tergugat hidup bersama dan tinggal bersama di rumah Penggugat;
   - Bahwa sepengetahuan saksi, rumah tangga Penggugat dan Tergugat pada awalnya harmonis namun sejak akhir tahun 2019 sering terjadi pertengkaran;
   - Bahwa saksi pernah mendengar sendiri Penggugat dan Tergugat bertengkar karena sering terjadi kesalah pahaman antara Penggugat dan orangtua Tergugat;
   - Bahwa sekarang Penggugat dan Tergugat sudah pisah rumah;
   - Bahwa sepengetahuan saksi pada bulan Maret tahun 2020 Tergugat pergi meninggalkan Penggugat;
   - Bahwa saksi menyatakan sudah tidak ada yang akan disampaikan;

Disclaimer
Kepaniteraan Mahkamah Agung Republik Indonesia berusaha untuk selalu mencantumkan informasi paling kini dan akurat sebagai bentuk komitmen Mahkamah Agung untuk pelayanan publik, transparansi dan akuntabilitas pelaksanaan fungsi peradilan. Namun dalam hal-hal tertentu masih dimungkinkan terjadi permasalahan teknis terkait dengan akurasi dan keterkinian informasi yang kami sajikan, hal mana akan terus kami perbaiki dari waktu kewaktu.
Dalam hal Anda menemukan inakurasi informasi yang termuat pada situs ini atau informasi yang seharusnya ada, namun belum tersedia, maka harap segera hubungi Kepaniteraan Mahkamah Agung RI melalui :
Email : kepaniteraan@mahkamahagung.go.id   Telp : 021-384 3348 (ext.318)

- Bahwa saksi pernah mendamaikan Penggugat dan Tergugat namun tidak berhasil;

2. [redacted], umur 49 tahun, Agama Islam, Pendidikan Strata I, Pekerjaan Swasta, tempat tinggal di [redacted] Kabupaten Kendal, menerangkan dibawah sumpah yang pada pokoknya :
   - Bahwa saksi kenal dengan Penggugat dan Tergugat karena saksi sebagai sepupu Penggugat;
   - Bahwa setelah menikah Penggugat dan Tergugat hidup bersama dan tinggal bersama di rumah Penggugat;
   - Bahwa rumah tangga Penggugat dan Tergugat tidak rukun dan harmonis sejak akhir tahun 2019;
   - Bahwa tidak tahu pasti penyebabnya yang saksi ketahui yakni pernah melihat dan mendengar secara langsung Penggugat dan Tergugat bertengkar;
   - Bahwa Penggugat dan Tergugat berpisah sejak bulan Maret tahun 2020 Tergugat pergi meninggalkan Penggugat;
   - Bahwa saksi maupun keluarga pernah berusaha menasehati atau mendamaikan Penggugat dan Tergugat tetapi tidak berhasil dan saksi sudah tidak sanggup lagi mendamaikannya

Selanjutnya untuk singkatnya uraian putusan ini, maka semua hal yang termuat dalam Berita Acara Sidang ini merupakan bagian yang tak terpisahkan dari putusan ini;

PERTIMBANGAN HUKUM

Menimbang, bahwa maksud dan tujuan gugatan Penggugat adalah sebagaimana terurai di atas;

Menimbang, bahwa berdasarkan bukti P.2, maka harus dinyatakan telah terbukti menurut hukum bahwa Penggugat dan Tergugat telah terikat dalam perkawinan yang sah sejak tanggal 14 April 2019.

Menimbang, bahwa oleh karena Penggugat dan Tergugat masih terikat sebagai suami istri yang sah, maka Penggugat sebagai *persona standi in judicio* memiliki *legal standing* sehingga Penggugat dapat dinyatakan sebagai

Disclaimer
Kepaniteraan Mahkamah Agung Republik Indonesia berusaha untuk selalu mencantumkan informasi paling kini dan akurat sebagai bentuk komitmen Mahkamah Agung untuk pelayanan publik, transparansi dan akuntabilitas pelaksanaan fungsi peradilan. Namun dalam hal-hal tertentu masih dimungkinkan terjadi permasalahan teknis terkait dengan akurasi dan keterkinian informasi yang kami sajikan, hal mana akan terus kami perbaiki dari waktu kewaktu.
Dalam hal Anda menemukan inakurasi informasi yang termuat pada situs ini atau informasi yang seharusnya ada, namun belum tersedia, maka harap segera hubungi Kepaniteraan Mahkamah Agung RI melalui :
Email : kepaniteraan@mahkamahagung.go.id Telp : 021-384 3348 (ext.318)

pihak yang mempunyai hak dan berkepentingan dalam perkara ini ;

Menimbang, bahwa atas keterangan Penggugat dalam surat gugatannya bahwa Penggugat dan Tergugat beragama Islam dan telah melakukan perkawinan secara islami, maka perkara ini merupakan kompetensi absolut Pengadilan Agama untuk memeriksa dan menyelesaikannya (vide Pasal 2 jo Pasal 49 ayat (1) Undang-undang Nomor 7 Tahun 1989 Tentang Peradilan Agama sebagai mana yang telah diubah yang kedua dengan Undang-undang Nomor 50 Tahun 2009) ;

Menimbang, bahwa berdasarkan surat gugatan dan bukti P.1, Penggugat bertempat tinggal wilayah yurisdiksi Pengadilan Agama Ambarawa, maka perkara ini secara relatif juga merupakan kompetensi Pengadilan Agama Ambarawa untuk memeriksanya (vide Pasal 73 Undang-undang Nomor 7 Tahun 1989 Tentang Peradilan Agama sebagai mana yang telah diubah yang kedua dengan Undang-undang Nomor 50 Tahun 2009) jo Pasal 132 Kompilasi Hukum Islam) ;

Menimbang, bahwa Hakim telah berupaya mendamaikan pihak Penggugat agar rukun kembali dengan Tergugat, sebagaimana dikehendaki oleh ketentuan Pasal 130 HIR jo Pasal 82 Undang-undang Nomor 7 Tahun 1989 Tentang Peradilan Agama sebagaimana yang telah diubah terakhir dengan Undang-undang Nomor 50 Tahun 2009 jo Pasal 39 ayat (1) Undang-undang Nomor 1 Tahun 1974 jo Pasal 31 ayat (2) Peraturan Pemerintah Nomor 9 Tahun 1975 jo Pasal 143 Kompilasi Hukum Islam, tetapi tidak berhasil ;

Menimbang, bahwa ternyata Tergugat meskipun dipanggil secara resmi dan patut, tidak datang menghadap di muka sidang dan pula tidak ternyata bahwa tidak datangnya itu disebabkan suatu halangan yang sah. Oleh karena itu, Tergugat harus dinyatakan tidak hadir dan gugatan tersebut harus diperiksa secara verstek. Sebagaimana ketentuan Pasal 125 ayat (1) HIR dan dalil syar'i :

من دعي إلى حاكم من حكام المسلمين فلم يجب فهو ظالم لا حق له

Artinya : “ *Seseorang yang dipanggil Hakim (Pengadilan) tidak datang, ia dipandang sebagai dhalim dan tidak ada hak baginya. “ (Ahkamul Qur-an II : 405) ;*

Hal 6 dari 11 hal Put. No 1302/Pdt.G/2020/PA.Amb

Disclaimer
Kepaniteraan Mahkamah Agung Republik Indonesia berusaha untuk selalu mencantumkan informasi paling kini dan akurat sebagai bentuk komitmen Mahkamah Agung untuk pelayanan publik, transparansi dan akuntabilitas pelaksanaan fungsi peradilan. Namun dalam hal-hal tertentu masih dimungkinkan terjadi permasalahan teknis terkait dengan akurasi dan keterkinian informasi yang kami sajikan, hal mana akan terus kami perbaiki dari waktu kewaktu.
Dalam hal Anda menemukan inakurasi informasi yang termuat pada situs ini atau informasi yang seharusnya ada, namun belum tersedia, maka harap segera hubungi Kepaniteraan Mahkamah Agung RI melalui :
Email : kepaniteraan@mahkamahagung.go.id   Telp : 021-384 3348 (ext.318)

Menimbang, bahwa dalam perkara ini Hakim tidak menempuh prosedur mediasi karena salah satu pihak (Tergugat) tidak hadir sehingga tidak dapat dilakukan perundingan, dimana Pasal 1 ayat (1) Peraturan Mahkamah Agung R.I. Nomor 1 Tahun 2016 tentang Prosedur Mediasi di Pengadilan menyatakan, bahwa mediasi adalah cara penyelesaian sengketa melalui proses perundingan untuk memperoleh kesepakatan para pihak dengan dibantu oleh mediator. Perundingan dapat terlaksana apabila kedua belah pihak hadir ;

Menimbang, bahwa berdasarkan ketentuan Surat Edaran Mahkamah Agung RI Nomor 3 Tahun 2015 huruf C angka 3 yaitu putusan yang dijatuhkan tanpa hadirnya Tergugat dapat dikabulkan sepanjang berdasarkan hukum dan beralasan, oleh karena itu Hakim membebani Penggugat untuk membuktikan dalil-dalil gugatannya;

Menimbang, bahwa untuk membuktikan dalil-dalil gugatannya, Penggugat telah mengajukan alat bukti surat serta 2 orang saksi;

Menimbang, bahwa alat bukti surat secara prosedural memenuhi syarat pengajuan bukti tertulis, berupa fotokopi yang telah di nazegelen cukup dan setelah dicocokkan dengan aslinya ternyata sesuai, maka berdasarkan ketentuan Pasal 1888 KUH Perdata jo. 165 ayat (1) *HetHerziene Indonesisch Reglement* (HIR) jo. Pasal 2 ayat (1) huruf (a), ayat (2), dan ayat (3), Pasal 10, dan Pasal 11 ayat (1) huruf (a) Undang-undang Republik Indonesia Nomor 13 Tahun 1985 tentang Bea Meterai jo. Pasal 1 huruf (a) dan huruf (f), serta Pasal 2 ayat (1) Peraturan Pemerintah Republik Indonesia Nomor 24 Tahun 2000 tentang Perubahan Tarif Bea Meterai dan Besarnya Batas Pengenaan Harga Nominal yang Dikenakan Bea Meterai, alat bukti surat tersebut secara formil telah memenuhi syarat sebagai alat bukti dan selanjutnya akan dipertimbangkan;

Menimbang, bahwa perkara ini termasuk sengketa di bidang perkawinan, dan berdasarkan Bukti P-1 Penggugat berdomisili di wilayah hukum Pengadilan Agama Ambarawa, maka berdasarkan ketentuan Pasal 49 ayat (1) huruf (a) dan Pasal 73 ayat (1) Undang-undang Nomor 7 Tahun 1989 Tentang Peradilan Agama sebagaimana telah diubah dengan Undang-undang Nomor 3 Tahun 2006 dan perubahan kedua dengan Undang-undang Nomor 50

Disclaimer
Kepaniteraan Mahkamah Agung Republik Indonesia berusaha untuk selalu mencantumkan informasi paling kini dan akurat sebagai bentuk komitmen Mahkamah Agung untuk pelayanan publik, transparansi dan akuntabilitas pelaksanaan fungsi peradilan. Namun dalam hal-hal tertentu masih dimungkinkan terjadi permasalahan teknis terkait dengan akurasi dan keterkinian informasi yang kami sajikan, hal mana akan terus kami perbaiki dari waktu kewaktu.
Dalam hal Anda menemukan inakurasi informasi yang termuat pada situs ini atau informasi yang seharusnya ada, namun belum tersedia, maka harap segera hubungi Kepaniteraan Mahkamah Agung RI melalui :
Email : kepaniteraan@mahkamahagung.go.id   Telp : 021-384 3348 (ext.318)

Tahun 2009, Pengadilan Agama Ambarawa berwenang untuk memeriksa, memutus, dan menyelesaikan Perkara ini;

Menimbang, bahwa bukti P-2 (Fotokopi Kutipan Akta Nikah) yang merupakan akta otentik dan telah bermeterai cukup dan cocok dengan aslinya, isi bukti tersebut menjelaskan antara Penggugat dan Tergugat telah terikat dalam perkawinan yang sah, sehingga bukti tersebut telah memenuhi syarat formal dan meteriil, serta mempunyai kekuatan pembuktian yang sempurna dan mengikat;

Menimbang, bahwa saksi 1 Penggugat, sudah dewasa dan sudah bersumpah, sehingga memenuhi syarat formal sebagaimana diatur dalam Pasal 145 ayat (1) angka (3e) HIR;

Menimbang, bahwa keterangan saksi 1 Penggugat mengenai dalil gugatan angka 3 dan 4, adalah fakta yang dilihat/didengar/dialami sendiri dan relevan dengan dalil yang harus dibuktikan oleh Penggugat, oleh karena itu keterangan saksi tersebut telah memenuhi syarat materiil sebagaimana telah diatur dalam Pasal 171 HIR, sehingga keterangan saksi tersebut memiliki kekuatan pembuktian dan dapat diterima sebagai alat bukti;

Menimbang, bahwa saksi 2 Penggugat, sudah dewasa dan sudah bersumpah, sehingga memenuhi syarat formal sebagaimana diatur dalam Pasal 145 ayat (1) angka (3e) HIR;

Menimbang, bahwa keterangan saksi 2 Penggugat mengenai dalil gugatan angka 3 dan 4, adalah fakta yang dilihat/didengar/dialami sendiri dan relevan dengan dalil yang harus dibuktikan oleh Penggugat, oleh karena itu keterangan saksi tersebut telah memenuhi syarat materiil sebagaimana telah diatur dalam Pasal 171 HIR, sehingga keterangan saksi tersebut memiliki kekuatan pembuktian dan dapat diterima sebagai alat bukti;

Menimbang, bahwa keterangan saksi 1 dan saksi 2 Penggugat bersesuaian dan cocok antara satu dengan yang lain oleh karena itu keterangan dua orang saksi tersebut memenuhi Pasal 171 dan Pasal 172 HIR;

Menimbang, bahwa berdasarkan saksi 1 dan saksi 2, terbukti fakta kejadian sebagai berikut;

1. Antara Penggugat dan Tergugat terus menerus terjadi perselisihan dan

Disclaimer
Kepaniteraan Mahkamah Agung Republik Indonesia berusaha untuk selalu mencantumkan informasi paling kini dan akurat sebagai bentuk komitmen Mahkamah Agung untuk pelayanan publik, transparansi dan akuntabilitas pelaksanaan fungsi peradilan. Namun dalam hal-hal tertentu masih dimungkinkan terjadi permasalahan teknis terkait dengan akurasi dan keterkinian informasi yang kami sajikan, hal mana akan terus kami perbaiki dari waktu kewaktu.
Dalam hal Anda menemukan inakurasi informasi yang termuat pada situs ini atau informasi yang seharusnya ada, namun belum tersedia, maka harap segera hubungi Kepaniteraan Mahkamah Agung RI melalui :
Email : kepaniteraan@mahkamahagung.go.id   Telp : 021-384 3348 (ext.318)

pertengkaran;
2. Penggugat dan Tergugat pisah rumah selama 9 bulan;

Menimbang, bahwa berdasarkan fakta-fakta tersebut di atas dapat disimpulkan fakta hukum sebagai berikut;

1. Antara Penggugat dan Tergugat terus menerus terjadi perselisihan dan pertengkaran yang sulit untuk didamaikan;
2. Penggugat dan Tergugat telah pisah rumah;
3. Antara Penggugat dan Tergugat tidak ada harapan hidup rukun kembali;

Menimbang, bahwa fakta hukum tersebut telah memenuhi norma hukum Islam yang terkandung dalam Kitab Fikih Sunah Juz II halaman 290;

أنّ للزوجة أن تطلب من القاضي التفريق إذا ادعت إضرار الزوج بها إضرارا لا يستطاع معه
دوام العشرة بين أمثالها

Artinya : "*bahwa istri boleh menuntut talak kepada hakim apabila dia mengaku selalu mendapat perlakuan yang menyakitkan dari suaminya sehingga hal tersebut dapat menghalangi keberlasungan hubungan suami istri antara mereka berdua";*

Menimbang, bahwa fakta hukum tersebut telah juga memenuhi Pasal 39 ayat 2 Undang-undang Nomor 1 tahun 1974 dan Pasal 19 huruf (f) Peraturan Pemerintah Nomor 9 Tahun 1975 jo. Pasal 116 huruf (f) Kompilasi Hukum Islam, oleh karena itu dapat dikabulkan;

Menimbang, bahwa berdasarkan ketentuan Pasal 84 ayat (1) dan (2) Undang-undang No. 7 tahun 1989 sebagaimana telah diubah dengan Undang-undang No. 3 tahun 2006 Jo Undang-undang No. 50 tahun 2009, maka diperintahkan kepada Panitera Pengadilan Agama Ambarawa untuk mengirimkan salinan putusan ini setelah mempunyai kekuatan hukum tetap kepada Pegawai Pencatat Nikah di wilayah tempat tinggal Penggugat dan Tergugat serta kepada Pegawai Pencatat Nikah ditempat perkawinan Penggugat dengan Tergugat dilangsungkan untuk dicatat dalam daftar yang disediakan untuk itu;

Menimbang, bahwa oleh karena perkara ini termasuk sengketa bidang perkawinan, maka sesuai Pasal 89 ayat 1 Undang-undang Republik Indonesia

Disclaimer
Kepaniteraan Mahkamah Agung Republik Indonesia berusaha untuk selalu mencantumkan informasi paling kini dan akurat sebagai bentuk komitmen Mahkamah Agung untuk pelayanan publik, transparansi dan akuntabilitas pelaksanaan fungsi peradilan. Namun dalam hal-hal tertentu masih dimungkinkan terjadi permasalahan teknis terkait dengan akurasi dan keterkinian informasi yang kami sajikan, hal mana akan terus kami perbaiki dari waktu kewaktu.
Dalam hal Anda menemukan inakurasi informasi yang termuat pada situs ini atau informasi yang seharusnya ada, namun belum tersedia, maka harap segera hubungi Kepaniteraan Mahkamah Agung RI melalui :
Email : kepaniteraan@mahkamahagung.go.id Telp : 021-384 3348 (ext.318)

Nomor 7 Tahun 1989 yang telah diubah dengan Undang-undang Republik Indonesia Nomor 3 Tahun 2006 jo. Undang-undang Republik Indonesia Nomor 50 Tahun 2009 tentang Perubahan Kedua Atas Undang-undang Republik Indonesia Nomor 7 Tahun 1989 tentang Peradilan Agama jo. Peraturan Pemerintah Republik Indonesia Nomor 5 Tahun 2019 tentang Jenis dan Tarif Atas Jenis Penerimaan Negara Bukan Pajak yang Berlaku pada Mahkamah Agung dan Badan Peradilan yang Berada di Bawahnya jo. Keputusan Ketua Mahkamah Agung Republik Indonesia Nomor 57/MA/SK/III/2019 tentang Biaya Proses Penyelesaian Perkara dan Pengelolaannya pada Mahkamah Agung dan Badan Peradilan yang Berada di Bawahnya, semua biaya perkara yang timbul dalam perkara ini dibebankan kepada Penggugat yang jumlahnya sebagaimana tersebut dalam amar putusan ini;

Mengingat semua pasal dalam peraturan perundang-undangan dan hukum Islam yang berkaitan dengan perkara ini;

M E N G A D I L I

1. Menyatakan Tergugat yang telah dipanggil secara resmi dan patut untuk menghadap dipersidangan, tidak hadir;
2. Mengabulkan gugatan Penggugat dengan verstek;
3. Menjatuhkan talak satu ba'in sughro Tergugat ([redacted]) terhadap Penggugat ([redacted]);
4. Membebankan kepada Penggugat untuk membayar biaya perkara sejumlah Rp.641.000,00 (enam ratus empat puluh satu ribu rupiah);

Demikian putusan ini dijatuhkan dan diucapkan pada hari Senin tanggal 28 Desember 2020 *Masehi*. bertepatan dengan tanggal 13 Jumadil Awwal 1442 *Hijriyah.* Oleh kami Siti Khoiriyah, S.H.I., M.H. sebagai Hakim Tunggal, dan putusan tersebut diucapkan dalam sidang terbuka untuk umum, dibantu oleh Sukarna, S.H.I. sebagai Panitera Pengganti serta dihadiri Penggugat tanpa hadirnya Tergugat;

Hakim,

Disclaimer
Kepaniteraan Mahkamah Agung Republik Indonesia berusaha untuk selalu mencantumkan informasi paling kini dan akurat sebagai bentuk komitmen Mahkamah Agung untuk pelayanan publik, transparansi dan akuntabilitas pelaksanaan fungsi peradilan. Namun dalam hal-hal tertentu masih dimungkinkan terjadi permasalahan teknis terkait dengan akurasi dan keterkinian informasi yang kami sajikan, hal mana akan terus kami perbaiki dari waktu kewaktu.
Dalam hal Anda menemukan inakurasi informasi yang termuat pada situs ini atau informasi yang seharusnya ada, namun belum tersedia, maka harap segera hubungi Kepaniteraan Mahkamah Agung RI melalui :
Email : kepaniteraan@mahkamahagung.go.id   Telp : 021-384 3348 (ext.318)

Siti Khoiriyah, S.H.I., M.H.
Panitera Pengganti,

Sukarna, S.H.I.

Perincian Biaya :

Biaya Pendaftaran	:	Rp	30.000,00
Biaya Proses	:	Rp	75.000,00
Biaya Pemanggilan	:	Rp	500.000,00
Biaya PNBP	:	Rp	20.000,00
Biaya Redaksi	:	Rp	10.000,00
Biaya Materai	:	Rp	6.000,00
Jumlah	:	Rp	641.000,00

Disclaimer
Kepaniteraan Mahkamah Agung Republik Indonesia berusaha untuk selalu mencantumkan informasi paling kini dan akurat sebagai bentuk komitmen Mahkamah Agung untuk pelayanan publik, transparansi dan akuntabilitas pelaksanaan fungsi peradilan. Namun dalam hal-hal tertentu masih dimungkinkan terjadi permasalahan teknis terkait dengan akurasi dan keterkinian informasi yang kami sajikan, hal mana akan terus kami perbaiki dari waktu kewaktu.
Dalam hal Anda menemukan inakurasi informasi yang termuat pada situs ini atau informasi yang seharusnya ada, namun belum tersedia, maka harap segera hubungi Kepaniteraan Mahkamah Agung RI melalui :
Email : kepaniteraan@mahkamahagung.go.id   Telp : 021-384 3348 (ext.318)
