# Supplementary material for: Classification of divorce causes during the COVID-19 pandemic using convolutional neural networks
Source: PeerJ Comput Sci. 2022 Jun 30;8:e998. doi: 10.7717/peerj-cs.998 (PMC9299239; doi:10.7717/peerj-cs.998)
Supplement: Supplemental Information 2 [file peerj-cs-08-998-s002.zip › Kelalaian Dataset/Data ke-18.pdf]

SALINAN

PUTUSAN

Nomor 1512/Pdt.G/2020/PA.Bi

بِسْمِ اللهِ الرَّحْمٰنِ الرَّحِيْمِ

DEMI KEADILAN BERDASARKAN KETUHANAN YANG MAHA ESA

Pengadilan Agama Boyolali yang memeriksa dan mengadili perkara tertentu pada tingkat pertama dalam sidang majelis hakim telah menjatuhkan putusan perkara Cerai Gugat antara:

Rika Kurniahati binti Sulardi, umur 22 tahun, agama Islam, Pekerjaan Karyawan swasta, Pendidikan Sekolah Lanjutan Tingkat Pertama, tempat kediaman di Dukuh Pakem RT. 06/ RW. 03, Desa Pakang, Kecamatan Andong, Kabupaten Boyolali, sebagai Penggugat;

melawan

M Nur Cholis bin Haryanto, umur 24 tahun, agama Islam, Pekerjaan Buruh harian lepas, Pendidikan Sekolah Dasar, tempat kediaman di Dukuh Ngrembes RT. 02/RW. 07, Desa Kauman, Kecamatan Kemusu, Kabupaten Boyolali, sebagai Tergugat;

Pengadilan Agama tersebut;

Telah mempelajari surat-surat yang berkaitan dengan perkara ini;

Telah mendengar keterangan Penggugat dan saksi-saksi di persidangan;

DUDUK PERKARA

Bahwa Penggugat dalam surat gugatannya tanggal 07 Oktober 2020 yang terdaftar di Kepaniteraan Pengadilan Agama Boyolali dengan register Nomor 1512/Pdt.G/2020/PA.Bi tanggal 07 Oktober 2020 mengemukakan hal-hal sebagai berikut:

1. Bahwa pada tanggal 06 Juli 2018 Penggugat dengan Tergugat telah

Disclaimer
Kepaniteraan Mahkamah Agung Republik Indonesia berusaha untuk selalu mencantumkan informasi paling kini dan akurat sebagai bentuk komitmen Mahkamah Agung untuk pelayanan publik, transparansi dan akuntabilitas pelaksanaan fungsi peradilan. Namun dalam hal-hal tertentu masih dimungkinkan terjadi permasalahan teknis terkait dengan akurasi dan keterkinian informasi yang kami sajikan, hal mana akan terus kami perbaiki dari waktu kewaktu.
Dalam hal Anda menemukan inakurasi informasi yang termuat pada situs ini atau informasi yang seharusnya ada, namun belum tersedia, maka harap segera hubungi Kepaniteraan Mahkamah Agung RI melalui :
Email : kepaniteraan@mahkamahagung.go.id Telp : 021-384 3348 (ext.318)

melangsungkan pernikahan yang sah dan dicatat oleh Pegawai Pencatat Nikah Kantor Urusan Agama Kecamatan Andong, Kabupaten Boyolali sebagaimana dalam Kutipan Akta Nikah Nomor : 0289/19/VII/2018 tertanggal 06 Juli 2018. Saat menikah Penggugat berstatus Perawan dan Tergugat berstatus Jejaka;

2. Bahwa setelah pernikahan tersebut Penggugat dan Tergugat bertempat tinggal di rumah kediaman orangtua Penggugat yang beralamat di Dukuh Pakem RT. 06/ RW. 03, Desa Pakang, Kecamatan Andong, Kabupaten Boyolali kurang lebih selama 2 (dua) tahun;

3. Bahwa selama pernikahan tersebut Penggugat dengan Tergugat telah hidup rukun sebagaimana layaknya suami istri (bada dukhul), namun belum dikaruniai anak;

4. Bahwa rumah tangga Penggugat dan Tergugat awal mulanya berjalan rukun dan harmonis namun pada bulan November 2018 rumah tangga Penggugat dan Tergugat mulai goyah, sering terjadi perselisihan dan pertengkaran disebabkan Tergugat tidak bertanggungjawab sebagai seorang suami, tidak mau bekerja dan tidak memberi uang belanja/nafkah kepada Penggugat;

5. Bahwa sebagai puncak dari permasalahan rumah tangga Penggugat dan Tergugat terjadi pada tanggal 26 Juni 2020 yaitu Tergugat pergi meninggalkan rumah kediaman orangtua Penggugat dan pulang ke rumah kediaman orangtua Tergugat yang beralamat di Dukuh Ngrembes RT. 02/RW. 07, Desa Kauman, Kecamatan Kemusu, Kabupaten Boyolali. Sejak kepergiannya Tergugat tidak pernah kembali pulang ke rumah kediaman orangtua Penggugat, tidak memberi kabar dan tidak pula memberi nafkah lahir batin kepada Penggugat;

6. Bahwa Penggugat dan Tergugat sudah pisah rumah kurang lebih selama 3 (tiga) bulan dan sudah tidak ada hubungan lahir batin lagi;

7. Bahwa pihak keluarga Penggugat sudah berusaha menasihati dan mendamaikan Penggugat dan Tergugat agar rukun dan harmonis lagi, namun tidak berhasil;

8. Bahwa rumah tangga Penggugat dan Tergugat sudah tidak ada

Disclaimer
Kepaniteraan Mahkamah Agung Republik Indonesia berusaha untuk selalu mencantumkan informasi paling kini dan akurat sebagai bentuk komitmen Mahkamah Agung untuk pelayanan publik, transparansi dan akuntabilitas pelaksanaan fungsi peradilan. Namun dalam hal-hal tertentu masih dimungkinkan terjadi permasalahan teknis terkait dengan akurasi dan keterkinian informasi yang kami sajikan, hal mana akan terus kami perbaiki dari waktu kewaktu.
Dalam hal Anda menemukan inakurasi informasi yang termuat pada situs ini atau informasi yang seharusnya ada, namun belum tersedia, maka harap segera hubungi Kepaniteraan Mahkamah Agung RI melalui :
Email : kepaniteraan@mahkamahagung.go.id   Telp : 021-384 3348 (ext.318)

kebahagiaan lahir dan batin serta tidak ada harapan untuk kembali membina rumah tangga yang sakinah mawaddah wa rahmah;

9. Bahwa berdasarkan hal-hal tersebut di atas, maka dalil-dalil permohonan cerai gugat telah berdasarkan atas hukum yang berlaku dan telah sesuai dengan ketentuan Pasal 39 ayat (2) Undang-undang Nomor 1 Tahun 1974 serta telah sesuai dengan alasan perceraian sebagaimana diatur dalam Pasal 19 huruf (f) PP No. 9 Tahun 1975 Jo Pasal 116 huruf (f) Kompilasi Hukum Islam;

10. Bahwa Penggugat sanggup membayar seluruh biaya yang timbul akibat perkara ini;

Berdasarkan alasan/dalil-dalil tersebut di atas, Penggugat mohon agar Ketua Pengadilan Agama Boyolali Cq Majelis Hakim untuk segera memeriksa dan mengadili perkara ini, selanjutnya menjatuhkan putusan yang amarnya berbunyi:

PRIMAIR :;

1. Mengabulkan gugatan Penggugat;
2. Menjatuhkan talak satu bain sughro Tergugat (M Nur Cholis bin Haryanto) kepada Penggugat (Rika Kurniahati binti Sulardi);
3. Membebankan biaya perkara menurut hukum kepada Penggugat;

SUBSIDAIR :;

Apabila Majelis Hakim berkehendak lain, maka Penggugat mohon menjatuhkan putusan lain yang seadil-adilnya.

Bahwa pada hari sidang yang telah ditetapkan, Penggugat telah datang menghadap ke persidangan, sedangkan Tergugat tidak datang menghadap ke persidangan dan tidak menyuruh orang lain untuk menghadap sebagai wakil/kuasanya yang sah, meskipun berdasarkan surat panggilan relaas Nomor 1512/Pdt.G/2020/PA.Bi tanggal 01 Desember 2020 dan tanggal 16 Desember 2020 yang dibacakan di persidangan, Tergugat telah dipanggil secara resmi dan patut, sedangkan tidak ternyata bahwa tidak datangnya Tergugat tersebut disebabkan oleh suatu alasan yang sah;

Disclaimer
Kepaniteraan Mahkamah Agung Republik Indonesia berusaha untuk selalu mencantumkan informasi paling kini dan akurat sebagai bentuk komitmen Mahkamah Agung untuk pelayanan publik, transparansi dan akuntabilitas pelaksanaan fungsi peradilan. Namun dalam hal-hal tertentu masih dimungkinkan terjadi permasalahan teknis terkait dengan akurasi dan keterkinian informasi yang kami sajikan, hal mana akan terus kami perbaiki dari waktu kewaktu.
Dalam hal Anda menemukan inakurasi informasi yang termuat pada situs ini atau informasi yang seharusnya ada, namun belum tersedia, maka harap segera hubungi Kepaniteraan Mahkamah Agung RI melalui :
Email : kepaniteraan@mahkamahagung.go.id Telp : 021-384 3348 (ext.318)

Bahwa Majelis Hakim telah menasehati Penggugat agar berpikir untuk tidak bercerai dengan Tergugat, tetapi Penggugat tetap pada dalil-dalil gugatannya untuk bercerai dengan Tergugat;

Bahwa perkara ini tidak dapat dimediasi karena Tergugat tidak pernah datang menghadap meskipun telah dipanggil secara resmi dan patut, selanjutnya dimulai pemeriksaan dengan membacakan surat gugatan Penggugat yang maksud dan isinya tetap dipertahankan oleh Penggugat;

Bahwa untuk menguatkan dalil-dalil gugatannya, Penggugat telah mengajukan bukti berupa:

I. Surat :

1. Fotokopi Kartu Tanda Penduduk Nomor 3309165308939003, yang dikeluarkan oleh kantor Dinas Kependudukan dan Catatan Sipil Kabupaten Boyolali, ;bermeterai cukup dan telah sesuai dengan aslinya (Bukti P.1);
2. Fotokopi Buku Kutipan Akta Nikah dari KUA Kecamatan Andong Kabupaten Boyolali Nomor 0289/19/VII/2018 tanggal 6 Juli 2018, yang bermeterai cukup dan telah sesuai dengan aslinya (Bukti P.2);

II. Saksi :

1. Saiful Ahmad bin Jumali, umur 32 tahun, agama Islam, pekerjaan swasta, alamat kediaman Pakem RT.05.RW.03, Keluarahan Pakang, Kecamatan Andong, Kabupaten Boyolali;, yang menerangkan di bawah sumpah pada pokoknya sebagai berikut:
   - Bahwa saksi kenal dengan Penggugat dan Tergugat karena bertetangga;
   - Bahwa Penggugat dan Tergugat adalah pasangan suami isteri;
   - Bahwa setelah menikah Penggugat dan Tergugat bertempat tinggal di Dukuh Pakem RT. 06/ RW. 03, Desa Pakang, Kecamatan Andong, Kabupaten Boyolali dan belum dikaruniai keturunan / anak;

Disclaimer
Kepaniteraan Mahkamah Agung Republik Indonesia berusaha untuk selalu mencantumkan informasi paling kini dan akurat sebagai bentuk komitmen Mahkamah Agung untuk pelayanan publik, transparansi dan akuntabilitas pelaksanaan fungsi peradilan. Namun dalam hal-hal tertentu masih dimungkinkan terjadi permasalahan teknis terkait dengan akurasi dan keterkinian informasi yang kami sajikan, hal mana akan terus kami perbaiki dari waktu kewaktu.
Dalam hal Anda menemukan inakurasi informasi yang termuat pada situs ini atau informasi yang seharusnya ada, namun belum tersedia, maka harap segera hubungi Kepaniteraan Mahkamah Agung RI melalui :
Email : kepaniteraan@mahkamahagung.go.id Telp : 021-384 3348 (ext.318)

- Bahwa semula rumah tangga Penggugat dan Tergugat kelihatan harmonis, namun sejak Nopember 2018 antara Penggugat dan Tergugat sering berselisih dan bertengkar karena sering terjadi perselisihan dan pertengkaran disebabkan Tergugat tidak bertanggungjawab sebagai seorang suami, tidak mau bekerja dan tidak memberi uang belanja/nafkah kepada Penggugat.;
- Bahwa pada Juni 2020 Penggugat dan Tergugat kembali bertengkar, kemudian Tergugat pergi meninggalkan Penggugat, sampai sekarang tidak pernah kembali dan tidak ada komunikasi lagi;
- Bahwa keluarga sudah berusaha mendamaikan Penggugat dan Tergugat, namun tidak berhasil;

2. Muntianah binti Giran, umur 32 tahun, agama Islam, pekerjaan ibu rumah tangga, alamat kediaman di Dukuh Pakem RT.05.RW.03, Keluarahan Pakang, Kecamatan Andong, Kabupaten Boyolali;, yang menerangkan di bawah sumpah pada pokoknya sebagai berikut:
   - Bahwa saksi kenal dengan Penggugat dan Tergugat karena bertetangga;
   - Bahwa Penggugat dan Tergugat adalah pasangan suami isteri;
   - Bahwa setelah menikah Penggugat dan Tergugat bertempat tinggal di Dukuh Pakem RT. 06/ RW. 03, Desa Pakang, Kecamatan Andong, Kabupaten Boyolali dan belum dikaruniai keturunan / anak;
   - Bahwa semula rumah tangga Penggugat dan Tergugat kelihatan harmonis, namun sejak Nopember 2018 antara Penggugat dan Tergugat sering berselisih dan bertengkar karena sering terjadi perselisihan dan pertengkaran disebabkan Tergugat tidak bertanggungjawab sebagai seorang suami, tidak mau bekerja dan tidak memberi uang belanja/nafkah kepada Penggugat.;
   - Bahwa pada Juni 2020 Penggugat dan Tergugat kembali bertengkar, kemudian Tergugat pergi meninggalkan Penggugat, sampai sekarang tidak pernah kembali dan tidak ada komunikasi lagi;

Disclaimer
Kepaniteraan Mahkamah Agung Republik Indonesia berusaha untuk selalu mencantumkan informasi paling kini dan akurat sebagai bentuk komitmen Mahkamah Agung untuk pelayanan publik, transparansi dan akuntabilitas pelaksanaan fungsi peradilan. Namun dalam hal-hal tertentu masih dimungkinkan terjadi permasalahan teknis terkait dengan akurasi dan keterkinian informasi yang kami sajikan, hal mana akan terus kami perbaiki dari waktu kewaktu.
Dalam hal Anda menemukan inakurasi informasi yang termuat pada situs ini atau informasi yang seharusnya ada, namun belum tersedia, maka harap segera hubungi Kepaniteraan Mahkamah Agung RI melalui :
Email : kepaniteraan@mahkamahagung.go.id   Telp : 021-384 3348 (ext.318)

- Bahwa keluarga sudah berusaha mendamaikan Penggugat dan Tergugat, namun tidak berhasil;

3. Fauzin bin Tugiran, umur 40 tahun, agama Islam, pekerjaan supir, alamat Duku hwates Timur RT 02 RW 02 Desa Bade Kecamatan Klego Kabupaten Boyolali, yang menerangkan di bawah sumpah pada pokoknya sebagai berikut:;
   - Bahwa saksi kenal dengan Penggugat dan Tergugat karena bertetangga;
   - Bahwa Penggugat dan Tergugat adalah pasangan suami isteri;
   - Bahwa setelah menikah Penggugat dan Tergugat bertempat tinggal di Dukuh Pakem RT. 06/ RW. 03, Desa Pakang, Kecamatan Andong, Kabupaten Boyolali dan belum dikaruniai keturunan / anak;
   - Bahwa semula rumah tangga Penggugat dan Tergugat kelihatan harmonis, namun sejak Nopember 2018 antara Penggugat dan Tergugat sering berselisih dan bertengkar karena sering terjadi perselisihan dan pertengkaran disebabkan Tergugat tidak bertanggungjawab sebagai seorang suami, tidak mau bekerja dan tidak memberi uang belanja/nafkah kepada Penggugat.;
   - Bahwa pada Juni 2020 Penggugat dan Tergugat kembali bertengkar, kemudian Tergugat pergi meninggalkan Penggugat, sampai sekarang tidak pernah kembali dan tidak ada komunikasi lagi;
   - Bahwa keluarga sudah berusaha mendamaikan Penggugat dan Tergugat, namun tidak berhasil;

4. Saiful Ahmad bin Jumali, umur 32 tahun, agama Islam, pekerjaan swasta, alamat kediaman Pakem RT.05.RW.03, Keluarahan Pakang, Kecamatan Andong, Kabupaten Boyolali yang menerangkan di bawah sumpah pada pokoknya sebagai berikut:;

Disclaimer
Kepaniteraan Mahkamah Agung Republik Indonesia berusaha untuk selalu mencantumkan informasi paling kini dan akurat sebagai bentuk komitmen Mahkamah Agung untuk pelayanan publik, transparansi dan akuntabilitas pelaksanaan fungsi peradilan. Namun dalam hal-hal tertentu masih dimungkinkan terjadi permasalahan teknis terkait dengan akurasi dan keterkinian informasi yang kami sajikan, hal mana akan terus kami perbaiki dari waktu kewaktu.
Dalam hal Anda menemukan inakurasi informasi yang termuat pada situs ini atau informasi yang seharusnya ada, namun belum tersedia, maka harap segera hubungi Kepaniteraan Mahkamah Agung RI melalui :
Email : kepaniteraan@mahkamahagung.go.id Telp : 021-384 3348 (ext.318)

- Bahwa saksi kenal dengan Penggugat dan Tergugat karena bertetangga;
- Bahwa Penggugat dan Tergugat adalah pasangan suami isteri;
- Bahwa setelah menikah Penggugat dan Tergugat bertempat tinggal di Dukuh Pakem RT. 06/ RW. 03, Desa Pakang, Kecamatan Andong, Kabupaten Boyolali dan belum dikaruniai keturunan / anak;
- Bahwa semula rumah tangga Penggugat dan Tergugat kelihatan harmonis, namun sejak Nopember 2018 antara Penggugat dan Tergugat sering berselisih dan bertengkar karena sering terjadi perselisihan dan pertengkaran disebabkan Tergugat tidak bertanggungjawab sebagai seorang suami, tidak mau bekerja dan tidak memberi uang belanja/nafkah kepada Penggugat.;
- Bahwa pada Juni 2020 Penggugat dan Tergugat kembali bertengkar, kemudian Tergugat pergi meninggalkan Penggugat, sampai sekarang tidak pernah kembali dan tidak ada komunikasi lagi;
- Bahwa keluarga sudah berusaha mendamaikan Penggugat dan Tergugat, namun tidak berhasil;

5. Sulardi bin Ngatirin, umur 53 tahun, agama Islam, pekerjaan buruh tani, tempat tinggal Dukuh Pakem RT 06 RW 03 Desa Pakang Kec. Andong Kab. Boyolali ayah kandung yang menerangkan di bawah sumpah pada pokoknya sebagai berikut:;
   - Bahwa saksi kenal dengan Penggugat dan Tergugat;
   - Bahwa Penggugat dan Tergugat adalah pasangan suami isteri;
   - Bahwa setelah menikah Penggugat dan Tergugat bertempat tinggal di Dukuh Pakem RT. 06/ RW. 03, Desa Pakang, Kecamatan Andong, Kabupaten Boyolali dan belum dikaruniai keturunan / anak;
   - Bahwa semula rumah tangga Penggugat dan Tergugat kelihatan harmonis, namun sejak Nopember 2018 antara Penggugat dan Tergugat sering berselisih dan bertengkar karena sering terjadi

Disclaimer
Kepaniteraan Mahkamah Agung Republik Indonesia berusaha untuk selalu mencantumkan informasi paling kini dan akurat sebagai bentuk komitmen Mahkamah Agung untuk pelayanan publik, transparansi dan akuntabilitas pelaksanaan fungsi peradilan. Namun dalam hal-hal tertentu masih dimungkinkan terjadi permasalahan teknis terkait dengan akurasi dan keterkinian informasi yang kami sajikan, hal mana akan terus kami perbaiki dari waktu kewaktu.
Dalam hal Anda menemukan inakurasi informasi yang termuat pada situs ini atau informasi yang seharusnya ada, namun belum tersedia, maka harap segera hubungi Kepaniteraan Mahkamah Agung RI melalui :
Email : kepaniteraan@mahkamahagung.go.id Telp : 021-384 3348 (ext.318)

perselisihan dan pertengkaran disebabkan Tergugat tidak bertanggungjawab sebagai seorang suami, tidak mau bekerja dan tidak memberi uang belanja/nafkah kepada Penggugat.;

- Bahwa pada Juni 2020 Penggugat dan Tergugat kembali bertengkar, kemudian Tergugat pergi meninggalkan Penggugat, sampai sekarang tidak pernah kembali dan tidak ada komunikasi lagi;
- Bahwa keluarga sudah berusaha mendamaikan Penggugat dan Tergugat, namun tidak berhasil;

Bahwa kemudian Penggugat menyatakan sudah cukup dan tidak akan mengajukan pembuktian lagi;

Bahwa selanjutnya Penggugat menyampaikan kesimpulan secara lisan yang pada pokoknya tetap pada dalil gugatannya dan mohon putusan;

Bahwa segala hal-ihwal sebagaimana tertuang dalam berita acara sidang perkara ini ditunjuk sebagai bagian tak terpisahkan dari putusan ini;

PERTIMBANGAN HUKUM

Menimbang, bahwa maksud dan tujuan gugatan Penggugat adalah sebagaimana tersebut di atas;

Menimbang, bahwa Tergugat tidak datang menghadap ke persidangan dan tidak menyuruh orang lain untuk menghadap sebagai wakil/kuasanya yang sah, meskipun berdasarkan surat panggilan relaas Nomor 1512/Pdt.G/2020/PA.Bi tanggal 01 Desember 2020 dan tanggal 16 Desember 2020 yang dibacakan di persidangan, Tergugat telah dipanggil secara resmi dan patut, sedangkan tidak ternyata bahwa tidak datangnya Tergugat tersebut disebabkan oleh suatu alasan yang sah;

Menimbang, bahwa meskipun Tergugat tidak pernah hadir di persidangan, Majelis Hakim tetap berusaha menasihati Penggugat agar berpikir untuk tidak bercerai dengan Tergugat, sesuai dengan maksud Pasal 82

Disclaimer
Kepaniteraan Mahkamah Agung Republik Indonesia berusaha untuk selalu mencantumkan informasi paling kini dan akurat sebagai bentuk komitmen Mahkamah Agung untuk pelayanan publik, transparansi dan akuntabilitas pelaksanaan fungsi peradilan. Namun dalam hal-hal tertentu masih dimungkinkan terjadi permasalahan teknis terkait dengan akurasi dan keterkinian informasi yang kami sajikan, hal mana akan terus kami perbaiki dari waktu kewaktu.
Dalam hal Anda menemukan inakurasi informasi yang termuat pada situs ini atau informasi yang seharusnya ada, namun belum tersedia, maka harap segera hubungi Kepaniteraan Mahkamah Agung RI melalui :
Email : kepaniteraan@mahkamahagung.go.id   Telp : 021-384 3348 (ext.318)

Undang-Undang Nomor 7 Tahun 1989 tentang Peradilan Agama, akan tetapi tidak berhasil;

Menimbang, bahwa oleh karena Tergugat atau kuasanya tidak pernah hadir di muka sidang, maka proses mediasi sebagaimana yang dikehendaki oleh Peraturan Mahkamah Agung Republik Indonesia Nomor 1 Tahun 2016 tidak dapat dilaksanakan;

Menimbang, bahwa Tergugat yang dipanggil secara resmi dan patut akan tetapi tidak datang menghadap harus dinyatakan tidak hadir dan gugatan tersebut harus diperiksa secara verstek;

Menimbang, bahwa berdasarkan ketentuan Pasal 125 ayat (1) HIR, yaitu putusan yang dijatuhkan tanpa hadirnya Tergugat dapat dikabulkan sepanjang berdasarkan hukum dan beralasan, oleh karena itu majelis hakim membebani Penggugat untuk membuktikan dalil gugatannya;

Menimbang, bahwa untuk membuktikan kebenaran dalil-dalil gugatannya, Penggugat telah mengajukan bukti surat (P-1, P-2) dan 2 (dua) orang saksi sebagaimana akan dipertimbangkan berikut ini;

Menimbang, bahwa dari posita gugatan Penggugat telah menunjukkan perkara ini termasuk sengketa di bidang perkawinan, dan Penggugat juga telah mengajukan bukti P-1 (Fotokopi Kartu Tanda Penduduk) yang merupakan akta otentik dan telah bermeterai cukup dan cocok dengan aslinya, menerangkan bahwa Penggugat bertempat tinggal di wilayah hukum (yurisdiksi) Pengadilan Agama Boyolali oleh karenanya bukti tersebut telah memenuhi syarat formal dan materiil serta mempunyai kekuatan pembuktian yang sempurna dan mengikat, maka berdasarkan ketentuan Pasal 49 ayat (1) huruf a dan pasal 73 ayat (1) Undang-undang Nomor 7 Tahun 1989 sebagaimana telah diubah dengan Undang-Undang Nomor 3 Tahun 2006 dan perubahan kedua dengan Undang-Undang Nomor 50 Tahun 2009, Pengadilan Agama Boyolali berwenang memeriksa, memutus dan menyelesaikan gugatan Penggugat;

Menimbang, bahwa Penggugat telah mengajukan bukti P-2 (Fotokopi Kutipan Akta Nikah) yang merupakan akta otentik dan telah bermeterai cukup dan cocok dengan aslinya, menerangkan bahwa pada tanggal 06 Juli 2018

Disclaimer
Kepaniteraan Mahkamah Agung Republik Indonesia berusaha untuk selalu mencantumkan informasi paling kini dan akurat sebagai bentuk komitmen Mahkamah Agung untuk pelayanan publik, transparansi dan akuntabilitas pelaksanaan fungsi peradilan. Namun dalam hal-hal tertentu masih dimungkinkan terjadi permasalahan teknis terkait dengan akurasi dan keterkinian informasi yang kami sajikan, hal mana akan terus kami perbaiki dari waktu kewaktu.
Dalam hal Anda menemukan inakurasi informasi yang termuat pada situs ini atau informasi yang seharusnya ada, namun belum tersedia, maka harap segera hubungi Kepaniteraan Mahkamah Agung RI melalui :
Email : kepaniteraan@mahkamahagung.go.id Telp : 021-384 3348 (ext.318)

tercatat di KUA Kecamatan Andong Kabupaten Boyolali, telah dilangsungkan akad nikah antara Penggugat dengan Tergugat, oleh karenanya bukti tersebut telah memenuhi syarat formal dan materiil serta mempunyai kekuatan pembuktian yang sempurna dan mengikat, sehingga dapat diterima sebagai bukti sepanjang mengenai hubungan hukum (perkawinan) antara Penggugat dengan Tergugat;

Menimbang, bahwa oleh karena Penggugat adalah isteri sah Tergugat, maka Penggugat dan Tergugat dipandang sebagai orang yang berkepentingan (*persona standi in judicio*) dan patut menjadi pihak (*legal standing*) dalam perkara ini, sehingga Majelis Hakim telah dapat mempertimbangkan lebih jauh mengenai dalil pokok gugatan Penggugat;

Menimbang, untuk mengetahui lebih jelas mengenai sebab-sebab perselisihan dan pertengkaran sebagaimana dimaksud Pasal 22 ayat (2) Peraturan Pemerintah Nomor 9 Tahun 1975, Majelis Hakim membebankan kepada Penggugat untuk menyempurnakan pembuktian dengan keterangan saksi-saksi yang berasal dari keluarga serta orang-orang yang dekat dengan Penggugat dan Tergugat;

Menimbang, bahwa kedua orang saksi yang diajukan oleh Penggugat ternyata sudah dewasa, tidak terlarang menjadi saksi, masing-masing memberikan keterangan di bawah sumpah, dengan demikian saksi-saksi tersebut telah memenuhi syarat formal sebagaimana diatur dalam pasal 145 ayat (1) HIR, sehingga telah dapat dipertimbangkan lebih jauh dalam perkara ini;

Menimbang, bahwa keterangan saksi pertama dan saksi kedua Penggugat mengenai pertengkaran Penggugat dan Tergugat sejak Nopember 2018 karena sering terjadi perselisihan dan pertengkaran disebabkan Tergugat tidak bertanggungjawab sebagai seorang suami, tidak mau bekerja dan tidak memberi uang belanja/nafkah kepada Penggugat. dan berpisahnya Penggugat dan Tergugat sejak Juni 2020 sampai sekarang tidak pernah hidup bersama kembali serta usaha keluarga yang sudah mendamaikan Penggugat dan Tergugat namun tidak berhasil, adalah fakta yang dilihat dan didengar sendiri

Disclaimer
Kepaniteraan Mahkamah Agung Republik Indonesia berusaha untuk selalu mencantumkan informasi paling kini dan akurat sebagai bentuk komitmen Mahkamah Agung untuk pelayanan publik, transparansi dan akuntabilitas pelaksanaan fungsi peradilan. Namun dalam hal-hal tertentu masih dimungkinkan terjadi permasalahan teknis terkait dengan akurasi dan keterkinian informasi yang kami sajikan, hal mana akan terus kami perbaiki dari waktu kewaktu.
Dalam hal Anda menemukan inakurasi informasi yang termuat pada situs ini atau informasi yang seharusnya ada, namun belum tersedia, maka harap segera hubungi Kepaniteraan Mahkamah Agung RI melalui :
Email : kepaniteraan@mahkamahagung.go.id Telp : 021-384 3348 (ext.318)

oleh saksi dan *relevan* dengan dalil yang harus dibuktikan oleh Penggugat, oleh karena itu keterangan saksi tersebut telah memenuhi syarat materil sebagaimana telah diatur dalam Pasal 171 HIR, sehingga memiliki kekuatan pembuktian dan dapat diterima sebagai bukti;

Menimbang, bahwa oleh karena keterangan saksi pertama dan saksi kedua Penggugat ternyata bersesuaian dan cocok antara satu dengan yang lainnya, maka kedua orang saksi tersebut dipandang telah memenuhi ketentuan Pasal 172 HIR sehingga telah dapat diterima sebagai bukti yang cukup dalam perkara ini;

Menimbang, bahwa berdasarkan bukti-bukti tersebut di atas ditemukan fakta sebagai berikut:

1. Bahwa Penggugat adalah isteri sah Tergugat yang menikah pada tanggal 06 Juli 2018, tercatat di KUA Kecamatan Andong Kabupaten Boyolali;
2. Bahwa sejak bulan Nopember 2018 , antara Penggugat dan Tergugat sering terjadi perselisihan dan pertengkaran yang disebabkan sering terjadi perselisihan dan pertengkaran disebabkan Tergugat tidak bertanggungjawab sebagai seorang suami, tidak mau bekerja dan tidak memberi uang belanja/nafkah kepada Penggugat.;
3. Bahwa sejak Juni 2020 Penggugat dan Tergugat telah berpisah tempat tinggal sampai sekarang tidak pernah hidup bersama kembali dan keduanya tidak ada komunikasi lagi;
4. Bahwa upaya perdamaian telah dilakukan keluarga Penggugat dan Tergugat, namun tidak berhasil;

Menimbang, bahwa berdasarkan fakta tersebut di atas, Majelis Hakim berpendapat dalil gugatan Penggugat telah terbukti kebenarannya, dengan demikian perkawinan Penggugat dengan Tergugat telah dapat dikwalifikasi sebagai “telah pecah” (*broken marriage*) yang sudah sangat sukar untuk disatukan serta tidak ada harapan akan hidup rukun lagi sebagai suami isteri sebagaimana dimaksud Pasal 39 ayat (2) Undang-Undang Nomor 1 Tahun 1974;

Disclaimer
Kepaniteraan Mahkamah Agung Republik Indonesia berusaha untuk selalu mencantumkan informasi paling kini dan akurat sebagai bentuk komitmen Mahkamah Agung untuk pelayanan publik, transparansi dan akuntabilitas pelaksanaan fungsi peradilan. Namun dalam hal-hal tertentu masih dimungkinkan terjadi permasalahan teknis terkait dengan akurasi dan keterkinian informasi yang kami sajikan, hal mana akan terus kami perbaiki dari waktu kewaktu.
Dalam hal Anda menemukan inakurasi informasi yang termuat pada situs ini atau informasi yang seharusnya ada, namun belum tersedia, maka harap segera hubungi Kepaniteraan Mahkamah Agung RI melalui :
Email : kepaniteraan@mahkamahagung.go.id Telp : 021-384 3348 (ext.318)

Menimbang, bahwa pecahnya perkawinan Penggugat dan Tergugat dapat dilihat dari fakta antara Penggugat dan Tergugat sering berselisih dan bertengkar sejak Nopember 2018 karena sering terjadi perselisihan dan pertengkaran disebabkan Tergugat tidak bertanggungjawab sebagai seorang suami, tidak mau bekerja dan tidak memberi uang belanja/nafkah kepada Penggugat. dan sejak Juni 2020 antara Penggugat dan Tergugat sudah tidak pernah lagi bersatu begitupun dengan upaya pihak keluarga yang telah mendamaikan Penggugat dan Tergugat namun tidak berhasil. Hal ini merupakan indikasi bahwa antara Penggugat dengan Tergugat sudah sangat sukar untuk disatukan serta tidak ada harapan akan hidup rukun lagi sebagai suami isteri;

Menimbang, bahwa oleh karena perkawinan Penggugat dengan Tergugat telah pecah, dalam kondisi tersebut patut diyakini sudah tidak akan dapat lagi mewujudkan tujuan perkawinan *a quo* sebagaimana disebutkan dalam Pasal 1 Undang-Undang Nomor 1 Tahun 1974 jo Pasal 3 Kompilasi Hukum Islam, yaitu untuk mewujudkan kehidupan rumah tangga yang sakinah mawaddah dan rahmah, bahkan patut diduga dalam perkawinan dengan kondisi demikian akan mendatangkan keburukan (*mafsadat*) yang lebih besar ketimbang kebaikan (*maslahat*) yang akan dicapai, diantaranya penderitaan batin yang berkepanjangan bagi kedua belah pihak terutama kepada Penggugat sendiri, sedangkan menghindari *mafsadat* harus lebih diutamakan dari pada mengharap *maslahat* sebagaimana *qaidah fiqhiyah* yang diambil alih menjadi pertimbangan Majelis yang berbunyi sebagai berikut:

درء المفاسد مقدّم على جلب المصالح

Artinya : *Mencegah kemudharatan harus lebih diutamakan daripada menarik kemashlahatan;*

Oleh karenanya jalan terbaik adalah memutuskan ikatan perkawinan Penggugat dengan Tergugat (bercerai);

Menimbang, bahwa berdasarkan pertimbangan di atas, Majelis Hakim berpendapat dengan tidak mempertimbangkan lagi lebih jauh mengenai apa dan dari pihak mana penyebab atau pemicu terjadinya perselisihan dan

Disclaimer
Kepaniteraan Mahkamah Agung Republik Indonesia berusaha untuk selalu mencantumkan informasi paling kini dan akurat sebagai bentuk komitmen Mahkamah Agung untuk pelayanan publik, transparansi dan akuntabilitas pelaksanaan fungsi peradilan. Namun dalam hal-hal tertentu masih dimungkinkan terjadi permasalahan teknis terkait dengan akurasi dan keterkinian informasi yang kami sajikan, hal mana akan terus kami perbaiki dari waktu kewaktu.
Dalam hal Anda menemukan inakurasi informasi yang termuat pada situs ini atau informasi yang seharusnya ada, namun belum tersedia, maka harap segera hubungi Kepaniteraan Mahkamah Agung RI melalui :
Email : kepaniteraan@mahkamahagung.go.id Telp : 021-384 3348 (ext.318)

pertengkaran a quo, gugatan Penggugat dipandang telah memenuhi alasan hukum sebagaimana ditentukan dalam Pasal 39 ayat (2) Undang-Undang Nomor 1 Tahun 1974 *Jo.* Pasal 19 huruf f Peraturan Pemerintah Nomor 9 Tahun 1975 dan Pasal 116 huruf f Kompilasi Hukum Islam, dan tidak melawan hukum;

Menimbang, bahwa oleh karena gugatan Penggugat telah beralasan dan tidak melawan hukum, dan karena ternyata di dalam bukti P-2 tidak terdapat catatan yang menyatakan bahwa antara Penggugat dengan Tergugat telah pernah bercerai, maka berdasarkan Pasal 119 ayat (2) huruf c Kompilasi Hukum Islam, Majelis Hakim berpendapat petitum gugatan Penggugat point 2 (dua) agar Majelis Hakim menjatuhkan talak satu *ba'in shugra* Tergugat terhadap Penggugat, sudah sepatutnya dikabulkan;

Menimbang, bahwa perkara ini termasuk bidang perkawinan maka berdasarkan pasal 89 ayat (1) Undang-Undang Nomor 7 Tahun 1989 tentang Peradilan Agama, biaya perkara dibebankan kepada Penggugat;

Mengingat dan memperhatikan semua peraturan perundang-undangan dan ketentuan hukum Islam yang berkaitan dengan perkara ini;

MENGADILI

1. Menyatakan Tergugat yang telah dipanggil secara resmi dan patut untuk menghadap di persidangan, tidak hadir;
2. Mengabulkan gugatan Penggugat secara verstek;
3. Menjatuhkan talak satu bain shugra Tergugat (M Nur Cholis bin Haryanto) terhadap Penggugat (Rika Kurniahati binti Sulardi);
4. Membebankan kepada Penggugat untuk membayar biaya perkara ini sejumlah Rp. 1.141.000,- (satu juta seratus empat puluh satu ribu rupiah);

Demikian diputuskan dalam rapat permusyawaratan Majelis Hakim yang dilangsungkan pada hari Senin tanggal 28 Desember 2020 *Masehi* bertepatan dengan tanggal 13 Jumadil Awwal 1442 *Hijriyah,* oleh kami Dra. Dzakiyatun

Disclaimer
Kepaniteraan Mahkamah Agung Republik Indonesia berusaha untuk selalu mencantumkan informasi paling kini dan akurat sebagai bentuk komitmen Mahkamah Agung untuk pelayanan publik, transparansi dan akuntabilitas pelaksanaan fungsi peradilan. Namun dalam hal-hal tertentu masih dimungkinkan terjadi permasalahan teknis terkait dengan akurasi dan keterkinian informasi yang kami sajikan, hal mana akan terus kami perbaiki dari waktu kewaktu.
Dalam hal Anda menemukan inakurasi informasi yang termuat pada situs ini atau informasi yang seharusnya ada, namun belum tersedia, maka harap segera hubungi Kepaniteraan Mahkamah Agung RI melalui :
Email : kepaniteraan@mahkamahagung.go.id Telp : 021-384 3348 (ext.318)

SF,SH. sebagai Ketua Majelis serta Dra. Hj. Emi Suyati dan Syahruddin, S.H.I., M.H. sebagai Hakim Anggota, putusan tersebut diucapkan pada hari itu juga dalam sidang terbuka untuk umum oleh Ketua Majelis tersebut, dengan didampingi oleh para Hakim Anggota, dibantu oleh Mubarok, SH, sebagai Panitera Pengganti dengan dihadiri oleh Penggugat tanpa hadirnya Tergugat.

Ketua Majelis,

Ttd.

Dra. Dzakiyatun SF,SH.

Hakim Anggota,	Hakim Anggota,
Ttd.	Ttd.
**Dra. Hj. Emi Suyati**	**Syahruddin, S.H.I., M.H.**

Panitera Pengganti,

Ttd.

Mubarok, SH

Rincian Biaya Perkara:

1. Biaya Pendaftaran	: Rp.	30.000,00
2. Biaya Proses	: Rp.	75.000,00
3. Biaya Panggilan	: Rp.	1.020.000,00
4. Biaya Redaksi	: Rp.	10.000,00
5. Biaya Meterai	: Rp.	6.000,00
**Jumlah**	**: Rp.**	**1.141.000,00**

Putusan tersebut telah mempunyai kekuatan hukum tetap sejak tanggal …………………………………..;

Untuk salinan yang sama bunyinya oleh
Panitera Pengadilan Agama Boyolali

Mochammad Fauzi, S.Ag

Hal. 14 dari 14 halaman Putusan Nomor 1512/Pdt.G/2020/PA.Bi

Disclaimer
Kepaniteraan Mahkamah Agung Republik Indonesia berusaha untuk selalu mencantumkan informasi paling kini dan akurat sebagai bentuk komitmen Mahkamah Agung untuk pelayanan publik, transparansi dan akuntabilitas pelaksanaan fungsi peradilan. Namun dalam hal-hal tertentu masih dimungkinkan terjadi permasalahan teknis terkait dengan akurasi dan keterkinian informasi yang kami sajikan, hal mana akan terus kami perbaiki dari waktu kewaktu.
Dalam hal Anda menemukan inakurasi informasi yang termuat pada situs ini atau informasi yang seharusnya ada, namun belum tersedia, maka harap segera hubungi Kepaniteraan Mahkamah Agung RI melalui :
Email : kepaniteraan@mahkamahagung.go.id Telp : 021-384 3348 (ext.318)
