# Supplementary material for: Classification of divorce causes during the COVID-19 pandemic using convolutional neural networks
Source: PeerJ Comput Sci. 2022 Jun 30;8:e998. doi: 10.7717/peerj-cs.998 (PMC9299239; doi:10.7717/peerj-cs.998)
Supplement: Supplemental Information 2 [file peerj-cs-08-998-s002.zip › Kelalaian Dataset/Data ke-19.pdf]

P U T U S A N

Nomor 1318/Pdt.G/2020/PA.Amb

بِسْمِ اللهِ الرَّحْمٰنِ الرَّحِيْمِ

DEMI KEADILAN BERDASARKAN KETUHANAN YANG MAHA ESA

Pengadilan Agama Ambarawa yang memeriksa dan mengadili perkara tertentu pada tingkat pertama dalam sidang, Hakim telah menjatuhkan putusan perkara Gugatan Perceraian antara;

[redacted] umur 38 tahun, agama Islam, Pekerjaan tidak bekerja, Pendidikan Sekolah Lanjutan Tingkat Atas, tempat kediaman di [redacted] Kabupaten Semarang, dalam hal ini memberikan kuasa kepada RONY ADHI WARDHANA, SH., MH., Advokat yang berkantor di Jalan Kenanga, RT 006, RW 007, Kelurahan : Bergas Kidul Kec. Bergas, Kab. Semarang berdasarkan surat kuasa khusus tanggal 02 Desember 2020, sebagai Penggugat

Melawan

[redacted], umur 44 tahun, agama Islam, Pekerjaan Karyawan Swasta, Pendidikan Sekolah Lanjutan Tingkat Atas, tempat kediaman di [redacted] Kabupaten Semarang, sebagai Tergugat

Pengadilan Agama tersebut;

Telah mempelajari surat-surat yang berkaitan dengan perkara ini;

Telah mendengar keterangan Penggugat dan para saksi di muka sidang;

DUDUK PERKARA

Bahwa, Penggugat dalam surat gugatannya tanggal 02 Desember 2020 telah mengajukan gugatan perceraian yang telah didaftar di Kepaniteraan Pengadilan Agama Ambarawa dengan Nomor 1318/Pdt.G/2020/PA.Amb,

Disclaimer
Kepaniteraan Mahkamah Agung Republik Indonesia berusaha untuk selalu mencantumkan informasi paling kini dan akurat sebagai bentuk komitmen Mahkamah Agung untuk pelayanan publik, transparansi dan akuntabilitas pelaksanaan fungsi peradilan. Namun dalam hal-hal tertentu masih dimungkinkan terjadi permasalahan teknis terkait dengan akurasi dan keterkinian informasi yang kami sajikan, hal mana akan terus kami perbaiki dari waktu kewaktu.
Dalam hal Anda menemukan inakurasi informasi yang termuat pada situs ini atau informasi yang seharusnya ada, namun belum tersedia, maka harap segera hubungi Kepaniteraan Mahkamah Agung RI melalui :
Email : kepaniteraan@mahkamahagung.go.id Telp : 021-384 3348 (ext.318)

tanggal 02 Desember 2020, dengan dalil-dalil sebagai berikut:

1. Bahwa pada tanggal 02 Agustus 2004 telah dilangsungkan perkawinan antara Penggugat dengan Tergugat yang dilaksanakan menurut hukum dan sesuai dengan tuntunan ajaran agama Islam. Perkawinan tersebut telah dicatatkan di Kantor Urusan Agama (KUA) Kecamatan Banjar, Kabupaten Pandeglang, Propinsi Banten, sebagaimana tercatat dalam Akta Nikah No [redacted] tertanggal 02 Agustus 2004;
2. Bahwa perkawinan antara Penggugat dan Tergugat ilangsungkan berdasarkan kehendak kedua belah pihak dengan tujuan membentuk rumah tangga yang sakinah, mawaddah, warahmah yang diridhoi oleh Allah Swt;
3. Bahwa setelah menikah, Penggugat dan Tergugat tinggal di Jakarta sampai dengan tahun 2015;
4. Bahwa setelah 2015 Penggugat dan Tergugat pulang ke Rumah Bersama [redacted] Kabupaten Semarang dikarenakan Tergugat terkena Pemutusan Hubungan Kerja;
5. Bahwa selama masa perkawinan, Penggugat dan Tergugat telah berkumpul sebagaimana layaknya suami-isteri dan dikaruniai tiga orang anak bernama;
    - [redacted], Laki-laki, Semarang, 02-03-2005, sekarang ikut Tergugat;
    - [redacted] Laki-Laki, Bekasi, 27-07-2009, sekarang ikut Tergugat;
    - [redacted], Perempuan, Pandeglang, 30-06-2018, sekarang ikut Tergugat;
6. Bahwa kebahagiaan yang dirasakan Penggugat setelah berumah tangga dengan Tergugat, ketentraman rumah tangga Penggugat dengan Tergugat mulai goyah setelah antara Penggugat dengan Tergugat terjadi perselisihan dan pertengkaran secara terus menerus kurang lebih sejak bulan Januari tahun 2015 sampai dengan saat ini, yang penyebabnya

Disclaimer
Kepaniteraan Mahkamah Agung Republik Indonesia berusaha untuk selalu mencantumkan informasi paling kini dan akurat sebagai bentuk komitmen Mahkamah Agung untuk pelayanan publik, transparansi dan akuntabilitas pelaksanaan fungsi peradilan. Namun dalam hal-hal tertentu masih dimungkinkan terjadi permasalahan teknis terkait dengan akurasi dan keterkinian informasi yang kami sajikan, hal mana akan terus kami perbaiki dari waktu kewaktu.
Dalam hal Anda menemukan inakurasi informasi yang termuat pada situs ini atau informasi yang seharusnya ada, namun belum tersedia, maka harap segera hubungi Kepaniteraan Mahkamah Agung RI melalui :
Email : kepaniteraan@mahkamahagung.go.id Telp : 021-384 3348 (ext.318)

antara lain;

- Bahwa sering timbul percekcokan antara Penggugat dan Tergugat yang disebabkan karena Tergugat kurang dalam memberikan nafkah, sehingga Tergugat harus bekerja untuk memenuhi kebutuhan sehari - hari;
- Bahwa atas masalah tersebut di atas sehingga sulit untuk membentuk suatu keluarga yang sakinah, mawaddah wa rahmah antara Penggugat dan Tergugat sebagaimana maksud dan tujuan dari suatu perkawinan;

7. Bahwa puncak dari percekcokan antara Penggugat dan Tergugat terjadi pada kurang lebih bulan Juni, tahun 2017 antara Penggugat dan Tergugat telah pisah rumah, dimana Penggugat pergi dan berdomisili di [redacted] Kabupaten Semarangrumah orang tua Penggugat. Sehingga sejak saat itu Penggugat dan Tergugat sudah tidak pernah lagi menjalin hubungan sebagaimana layaknya suami istri;

8. Bahwa atas permasalahan dan kemelut rumah tangga yang dihadapi, Penggugat telah mencoba memusyawarahkan dengan keluarga Penggugat dan Tergugat untuk mencari penyelesaian dan demi menyelamatkan perkawinan, namun usaha tersebut juga tidak membuahkan hasil;

9. Bahwa ikatan perkawinan antara Penggugat dan Tergugat sebagaimana yang diuraikan diatas sudah sulit dibina untuk membentuk suatu rumah tangga yang sakinah, mawaddah wa rahmah sebagaimana maksud dan tujuan dari suatu perkawinan, sehingga lebih baik diputus karena perceraian;

10. Bahwa berdasarkan hal-hal tersebut diatas, permohonan Penggugat untuk mengajukan gugatan perceraian terhadap Tergugat atas dasar pertengkaran yang terjadi terus menerus dan tidak mungkin hidup rukun dalam suatu ikatan perkawinan, telah memenuhi unsur Pasal 19 huruf (f) Peraturan Pemerintah No. 9 Tahun 1975 Jo. Pasal 116

Disclaimer
Kepaniteraan Mahkamah Agung Republik Indonesia berusaha untuk selalu mencantumkan informasi paling kini dan akurat sebagai bentuk komitmen Mahkamah Agung untuk pelayanan publik, transparansi dan akuntabilitas pelaksanaan fungsi peradilan. Namun dalam hal-hal tertentu masih dimungkinkan terjadi permasalahan teknis terkait dengan akurasi dan keterkinian informasi yang kami sajikan, hal mana akan terus kami perbaiki dari waktu kewaktu.
Dalam hal Anda menemukan inakurasi informasi yang termuat pada situs ini atau informasi yang seharusnya ada, namun belum tersedia, maka harap segera hubungi Kepaniteraan Mahkamah Agung RI melalui :
Email : kepaniteraan@mahkamahagung.go.id Telp : 021-384 3348 (ext.318)

huruf (f) Kompilasi Hukum Islam, sehingga berdasar hukum untuk menyatakan gugatan cerai ini dikabulkan;

Berdasarkan dalil dan alasan-alasan tersebut diatas, maka dengan ini Penggugat memohon kepada Ketua Pengadilan Agama Ambarawa cq. Majelis Hakim yang memeriksa dan mengadili perkara ini untuk dapat menentukan hari persidangan, kemudian memanggil Penggugat dan Tergugatuntuk diperiksa dan diadili, selanjutnya memberikan putusan yang amarnya sebagai berikut:

PRIMER:

1. Mengabulkan gugatan Penggugat untuk seluruhnya;
2. Menjatuhkan talak satu bain sughraa Tergugat [redacted] Terhadap Penggugat [redacted];
3. Membebankan biaya perkara sesuai hukum;

SUBSIDER:

Atau apabila Pengadilan Agama berpendapat lain mohon putusan yang seadil-adilnya (ex aequo et bono);

Bahwa, pada hari sidang yang telah ditetapkan Penggugat telah datang menghadap ke muka sidang, sedangkan Tergugat tidak datang menghadap ke muka sidang dan tidak menyuruh orang lain untuk menghadap sebagai wakil/kuasa hukumnya meskipun telah dipanggil secara resmi dan patut yang relaas panggilannya telah dibacakan di dalam sidang, sedangkan tidak ternyata bahwa tidak datangnya itu disebabkan suatu halangan yang sah;

Bahwa, Hakim telah menasehati Penggugat agar berpikir untuk tidak bercerai dengan Tergugat, tetapi Penggugat tetap pada dalil-dalil gugatannya untuk bercerai dengan Tergugat;

Bahwa, perkara ini tidak dapat di mediasi karena Tergugat tidak pernah datang menghadap meskipun telah dipanggil secara resmi dan patut, selanjutnya dimulai pemeriksaan dengan membacakan surat gugatan Penggugat yang maksud dan isinya tetap dipertahankan oleh Penggugat;

Bahwa, untuk menguatkan dalil-dalil gugatannya, Penggugat telah mengajukan bukti-bukti berupa :

A. Surat :

Disclaimer
Kepaniteraan Mahkamah Agung Republik Indonesia berusaha untuk selalu mencantumkan informasi paling kini dan akurat sebagai bentuk komitmen Mahkamah Agung untuk pelayanan publik, transparansi dan akuntabilitas pelaksanaan fungsi peradilan. Namun dalam hal-hal tertentu masih dimungkinkan terjadi permasalahan teknis terkait dengan akurasi dan keterkinian informasi yang kami sajikan, hal mana akan terus kami perbaiki dari waktu kewaktu.
Dalam hal Anda menemukan inakurasi informasi yang termuat pada situs ini atau informasi yang seharusnya ada, namun belum tersedia, maka harap segera hubungi Kepaniteraan Mahkamah Agung RI melalui :
Email : kepaniteraan@mahkamahagung.go.id   Telp : 021-384 3348 (ext.318)

1. Fotokopi Kartu Tanda Penduduk Penggugat, Nomor: [redacted], tanggal 20 Nopember 2020, yang bermeterai cukup dan telah sesuai dengan aslinya (bukti P.1);
2. Fotokopi Kutipan Akta Nikah dari Kantor Urusan Agama Kecamatan Banjar, Kabupaten Pandeglang, Nomor: [redacted], tanggal 2 Agustus 2004, yang bermeterai cukup dan telah sesuai dengan aslinya (bukti P.2);
3. Fotokopi Kartu Keluarga, Nomor: [redacted] tanggal 18 Nopember 2020, yang bermeterai cukup dan telah sesuai dengan aslinya (bukti P.3);

B. Saksi :

1. [redacted], umur 49 tahun, Agama Islam, Pendidikan Sekolah Lanjutan Tingkat Atas, Pekerjaan Swasta, tempat tinggal di [redacted] Kabupaten Semarang, menerangkan dibawah sumpah yang pada pokoknya :
   - Bahwa saksi kenal dengan Penggugat dan Tergugat sebagai kakak ipar Penggugat;
   - Bahwa Penggugat sudah menikah dengan Joko Prasojo bin Damsuki;
   - Bahwa Penggugat dan Tergugat menikah pada tahun 2004;
   - Bahwa selama pernikahan Penggugat dan Tergugat sudah dikaruniai 3 anak, anak yang pertama dan yang kedua dalam asuhan Tergugat, sedangkan anak yang ketiga sekarang dalam asuhan Penggugat;
   - Bahwa sepengetahuan saksi, Penggugat dan Tergugat setelah menikah tinggal dan hidup bersama membina rumah tangga dirumah tinggal di Jakarta, sedangkan yang terakhir tinggal bersama di rumah bersama, semula rumah tangga mereka rukun dan bahagia, namun kemudian rumah tangga mereka sering diwarnai perselisihan dan pertengkaran yang pada puncaknya sejak tahun 2017 mereka pisah rumah sampai dengan sekarang

Disclaimer
Kepaniteraan Mahkamah Agung Republik Indonesia berusaha untuk selalu mencantumkan informasi paling kini dan akurat sebagai bentuk komitmen Mahkamah Agung untuk pelayanan publik, transparansi dan akuntabilitas pelaksanaan fungsi peradilan. Namun dalam hal-hal tertentu masih dimungkinkan terjadi permasalahan teknis terkait dengan akurasi dan keterkinian informasi yang kami sajikan, hal mana akan terus kami perbaiki dari waktu kewaktu.
Dalam hal Anda menemukan inakurasi informasi yang termuat pada situs ini atau informasi yang seharusnya ada, namun belum tersedia, maka harap segera hubungi Kepaniteraan Mahkamah Agung RI melalui :
Email : kepaniteraan@mahkamahagung.go.id Telp : 021-384 3348 (ext.318)

tidak pernah kembali hidup bersama lagi;

- Bahwa saksi sering mendengar antara Penggugat dan Tergugat bertengkar;
- Bahwa Penggugat dan Tergugat sering bertengkar masalah ekonomi, Tergugat tidak pernah memberi nafkah pada Penggugat secara layak pada waktu Tergugat tidak bekerja, selain itu juga Penggugat selalu merasa kurang atas nafkah yang diberikan Tergugat;
- Bahwa Penggugat dan Tergugat pisah rumah, sepengetahuan saksi sejak tahun 2017, hingga sekarang kurang lebih 3 tahun;
- Bahwa, selama pisah tersebut, saksi tidak pernah melihat Penggugat datang ke tempat kediaman Tergugat, begitu juga sebaliknya;
- Bahwa, selama pisah rumah tersebut, saksi tidak pernah mendengar Penggugat dan Tergugat saling komunikasi satu sama lain;
- Bahwa, selama pisah rumah tersebut, saksi tidak pernah mendengar Tergugat kirim uang atau barang lainnya sebagai nafkah pada Penggugat;
- Bahwa, selama pisah rumah tersebut, Tergugat tidak pernah mengurusi pada Penggugat;
- Bahwa, selama pisah rumah tersebut, saksi tidak melihat Tergugat meninggalkan barang sesuatu yang dapat dijadikan sebagai pengganti nafkah pada Penggugat;
- Bahwa selama pisah rumah tersebut, Penggugat dengan Tergugat pernah dirukunkan, namun tidak berhasil;
- Bahwa pernah dinasehati, namun Penggugat tetap pada pendiriannya untuk pisah dengan Tergugat;
- Bahwa saksi tidak bersedia mendamaikan;

2. [redacted] umur 47 tahun, Agama Islam, Pendidikan Sekolah Lanjutan Tingkat Atas, Pekerjaan Wiraswasta, tempat tinggal di [redacted]

Disclaimer
Kepaniteraan Mahkamah Agung Republik Indonesia berusaha untuk selalu mencantumkan informasi paling kini dan akurat sebagai bentuk komitmen Mahkamah Agung untuk pelayanan publik, transparansi dan akuntabilitas pelaksanaan fungsi peradilan. Namun dalam hal-hal tertentu masih dimungkinkan terjadi permasalahan teknis terkait dengan akurasi dan keterkinian informasi yang kami sajikan, hal mana akan terus kami perbaiki dari waktu kewaktu.
Dalam hal Anda menemukan inakurasi informasi yang termuat pada situs ini atau informasi yang seharusnya ada, namun belum tersedia, maka harap segera hubungi Kepaniteraan Mahkamah Agung RI melalui :
Email : kepaniteraan@mahkamahagung.go.id Telp : 021-384 3348 (ext.318)

[redacted] Kabupaten Semarang, menerangkan dibawah sumpah yang pada pokoknya :

- Bahwa saksi kenal dengan Penggugat dan Tergugat sebagai teman Penggugat;
- Bahwa Penggugat sudah menikah dengan [redacted]
- Bahwa Penggugat dan Tergugat menikah pada tahun 2004;
- Bahwa selama pernikahan Penggugat dan Tergugat sudah dikaruniai 3 anak, anak yang pertama dan yang kedua dalam asuhan Tergugat, sedangkan anak yang ketiga sekarang dalam asuhan Penggugat;
- Bahwa Penggugat dan Tergugat setelah menikah tinggal dan hidup bersama membina rumah tangga di Jakarta dan yang terakhir tinggal bersama di rumah tinggal bersama, pada awalnya rumah tangga mereka rukun dan damai, namun kemudian sejak tahun 2017 sampai dengan sekarang mereka pisah rumah dan tidak pernah kembali hidup bersama lagi;
- Bahwa saksi pernah mendengar antara Penggugat dan Tergugat bertengkar;
- Bahwa Penggugat dan Tergugat sering bertengkar masalah ekonomi, Tergugat tidak pernah memberi nafkah pada Penggugat secara layak pada waktu Tergugat tidak bekerja, selain itu juga Penggugat selalu merasa kurang atas nafkah yang diberikan Tergugat;
- Bahwa Penggugat dan Tergugat pisah rumah sepengetahuan saksi sejak tahun 2017, hingga sekarang kurang lebih 3 tahun;
- Bahwa, selama pisah tersebut, saksi tidak pernah melihat Tergugat datang ke tempat kediaman Penggugat untuk rukun kembali;
- Bahwa, selama pisah rumah tersebut, saksi tidak pernah mendengar antara Penggugat dan Tergugat saling komunikasi satu sama lain;
- Bahwa, selama pisah rumah tersebut, saksi tidak pernah

Disclaimer
Kepaniteraan Mahkamah Agung Republik Indonesia berusaha untuk selalu mencantumkan informasi paling kini dan akurat sebagai bentuk komitmen Mahkamah Agung untuk pelayanan publik, transparansi dan akuntabilitas pelaksanaan fungsi peradilan. Namun dalam hal-hal tertentu masih dimungkinkan terjadi permasalahan teknis terkait dengan akurasi dan keterkinian informasi yang kami sajikan, hal mana akan terus kami perbaiki dari waktu kewaktu.
Dalam hal Anda menemukan inakurasi informasi yang termuat pada situs ini atau informasi yang seharusnya ada, namun belum tersedia, maka harap segera hubungi Kepaniteraan Mahkamah Agung RI melalui :
Email : kepaniteraan@mahkamahagung.go.id Telp : 021-384 3348 (ext.318)

mendengar Tergugat barang sesuatu apapun sebagai nafkah pada Penggugat;

- Bahwa, selama pisah rumah tersebut, Tergugat tidak pernah mengurusi pada Penggugat;
- Bahwa, selama pisah rumah tersebut, saksi tidak melihat Tergugat meninggalkan barang sesuatu yang dapat dijadikan sebagai pengganti nafkah pada Penggugat;
- Bahwa selama pisah rumah tersebut, Penggugat dengan Tergugat pernah dirukunkan, namun tidak berhasil;
- Bahwa saksi sudah berusaha untuk menasehati Penggugat agar hidup rukun lagi dengan Tergugat, namun Penggugat menyatakan sudah tidak sanggup lagi hidup bersama Tergugat;
- Bahwa saksi tidak bersedia mendamaikan;

Selanjutnya untuk singkatnya uraian putusan ini, maka semua hal yang termuat dalam Berita Acara Sidang ini merupakan bagian yang tak terpisahkan dari putusan ini;

PERTIMBANGAN HUKUM

Menimbang, bahwa maksud dan tujuan gugatan Penggugat adalah sebagaimana terurai di atas;

Menimbang, bahwa berdasarkan bukti P.2, maka harus dinyatakan telah terbukti menurut hukum bahwa Penggugat dan Tergugat telah terikat dalam perkawinan yang sah sejak tanggal 02 Agustus 2004.

Menimbang, bahwa oleh karena Penggugat dan Tergugat masih terikat sebagai suami istri yang sah, maka Penggugat sebagai *persona standi in judicio* memiliki *legal standing* sehingga Penggugat dapat dinyatakan sebagai pihak yang mempunyai hak dan berkepentingan dalam perkara ini ;

Menimbang, bahwa selanjutnya berdasarkan surat kuasa khusus yang didaftarkan di Kepaniteraan PA Ambarawa dalam hal ini Penggugat telah memberikan kuasa kepada Advokat/Pengacara sebagaimana tersebut di atas, Oleh karenanya sebagiamana ketentuan **Pasal 123 HIR** Jo. Pasal 1 dan 4 Undang-undang Nomor 18 Tahun 2003 tentang Advokat, dengan demikian Kuasa Hukum Penggugat sebagai kuasa hukum telah sah dan berhak untuk

Disclaimer
Kepaniteraan Mahkamah Agung Republik Indonesia berusaha untuk selalu mencantumkan informasi paling kini dan akurat sebagai bentuk komitmen Mahkamah Agung untuk pelayanan publik, transparansi dan akuntabilitas pelaksanaan fungsi peradilan. Namun dalam hal-hal tertentu masih dimungkinkan terjadi permasalahan teknis terkait dengan akurasi dan keterkinian informasi yang kami sajikan, hal mana akan terus kami perbaiki dari waktu kewaktu.
Dalam hal Anda menemukan inakurasi informasi yang termuat pada situs ini atau informasi yang seharusnya ada, namun belum tersedia, maka harap segera hubungi Kepaniteraan Mahkamah Agung RI melalui :
Email : kepaniteraan@mahkamahagung.go.id Telp : 021-384 3348 (ext.318)

mendampingi dan/atau mewakili Penggugat in person dalam setiap persidangan;

Menimbang, bahwa atas keterangan Penggugat dalam surat gugatannya bahwa Penggugat dan Tergugat beragama Islam dan telah melakukan perkawinan secara islami, maka perkara ini merupakan kompetensi absolut Pengadilan Agama untuk memeriksa dan menyelesaikannya (vide Pasal 2 jo Pasal 49 ayat (1) Undang-undang Nomor 7 Tahun 1989 Tentang Peradilan Agama sebagai mana yang telah diubah yang kedua dengan Undang-undang Nomor 50 Tahun 2009) ;

Menimbang, bahwa berdasarkan surat gugatan dan bukti P.1 dan P.3, Penggugat bertempat tinggal wilayah yurisdiksi Pengadilan Agama Ambarawa, maka perkara ini secara relatif juga merupakan kompetensi Pengadilan Agama Ambarawa untuk memeriksanya (vide Pasal 73 Undang-undang Nomor 7 Tahun 1989 Tentang Peradilan Agama sebagai mana yang telah diubah yang kedua dengan Undang-undang Nomor 50 Tahun 2009) jo Pasal 132 Kompilasi Hukum Islam) ;

Menimbang, bahwa Hakim telah berupaya mendamaikan pihak Penggugat agar rukun kembali dengan Tergugat, sebagaimana dikehendaki oleh ketentuan Pasal 130 HIR jo Pasal 82 Undang-undang Nomor 7 Tahun 1989 Tentang Peradilan Agama sebagaimana yang telah diubah terakhir dengan Undang-undang Nomor 50 Tahun 2009 jo Pasal 39 ayat (1) Undang-undang Nomor 1 Tahun 1974 jo Pasal 31 ayat (2) Peraturan Pemerintah Nomor 9 Tahun 1975 jo Pasal 143 Kompilasi Hukum Islam, tetapi tidak berhasil ;

Menimbang, bahwa ternyata Tergugat meskipun dipanggil secara resmi dan patut, tidak datang menghadap di muka sidang dan pula tidak ternyata bahwa tidak datangnya itu disebabkan suatu halangan yang sah. Oleh karena itu, Tergugat harus dinyatakan tidak hadir dan gugatan tersebut harus diperiksa secara verstek. Sebagaimana ketentuan Pasal 125 ayat (1) HIR dan dalil syar'i :

من دعي إلى حاكم من حكام المسلمين فلم يجب فهو ظالم لا حق له

Artinya : “ *Seseorang yang dipanggil Hakim (Pengadilan) tidak datang, ia dipandang sebagai dhalim dan tidak ada hak baginya. “ (Ahkamul Qur-an II : 405) ;*

Disclaimer
Kepaniteraan Mahkamah Agung Republik Indonesia berusaha untuk selalu mencantumkan informasi paling kini dan akurat sebagai bentuk komitmen Mahkamah Agung untuk pelayanan publik, transparansi dan akuntabilitas pelaksanaan fungsi peradilan. Namun dalam hal-hal tertentu masih dimungkinkan terjadi permasalahan teknis terkait dengan akurasi dan keterkinian informasi yang kami sajikan, hal mana akan terus kami perbaiki dari waktu kewaktu.
Dalam hal Anda menemukan inakurasi informasi yang termuat pada situs ini atau informasi yang seharusnya ada, namun belum tersedia, maka harap segera hubungi Kepaniteraan Mahkamah Agung RI melalui :
Email : kepaniteraan@mahkamahagung.go.id   Telp : 021-384 3348 (ext.318)

Menimbang, bahwa dalam perkara ini Hakim tidak menempuh prosedur mediasi karena salah satu pihak (Tergugat) tidak hadir sehingga tidak dapat dilakukan perundingan, dimana Pasal 1 ayat (1) Peraturan Mahkamah Agung R.I. Nomor 1 Tahun 2016 tentang Prosedur Mediasi di Pengadilan menyatakan, bahwa mediasi adalah cara penyelesaian sengketa melalui proses perundingan untuk memperoleh kesepakatan para pihak dengan dibantu oleh mediator. Perundingan dapat terlaksana apabila kedua belah pihak hadir ;

Menimbang, bahwa berdasarkan ketentuan Surat Edaran Mahkamah Agung RI Nomor 3 Tahun 2015 huruf C angka 3 yaitu putusan yang dijatuhkan tanpa hadirnya Tergugat dapat dikabulkan sepanjang berdasarkan hukum dan beralasan, oleh karena itu Hakim membebani Penggugat untuk membuktikan dalil-dalil gugatannya;

Menimbang, bahwa untuk membuktikan dalil-dalil gugatannya, Penggugat telah mengajukan alat bukti surat serta 2 orang saksi;

Menimbang, bahwa alat bukti surat secara prosedural memenuhi syarat pengajuan bukti tertulis, berupa fotokopi yang telah di nazegelen cukup dan setelah dicocokkan dengan aslinya ternyata sesuai, maka berdasarkan ketentuan Pasal 1888 KUH Perdata jo. 165 ayat (1) *HetHerziene Indonesisch Reglement* (HIR) jo. Pasal 2 ayat (1) huruf (a), ayat (2), dan ayat (3), Pasal 10, dan Pasal 11 ayat (1) huruf (a) Undang-undang Republik Indonesia Nomor 13 Tahun 1985 tentang Bea Meterai jo. Pasal 1 huruf (a) dan huruf (f), serta Pasal 2 ayat (1) Peraturan Pemerintah Republik Indonesia Nomor 24 Tahun 2000 tentang Perubahan Tarif Bea Meterai dan Besarnya Batas Pengenaan Harga Nominal yang Dikenakan Bea Meterai, alat bukti surat tersebut secara formil telah memenuhi syarat sebagai alat bukti dan selanjutnya akan dipertimbangkan;

Menimbang, bahwa perkara ini termasuk sengketa di bidang perkawinan, dan berdasarkan Bukti P-1 dan P-3 Penggugat berdomisili di wilayah hukum Pengadilan Agama Ambarawa, maka berdasarkan ketentuan Pasal 49 ayat (1) huruf (a) dan Pasal 73 ayat (1) Undang-undang Nomor 7 Tahun 1989 Tentang Peradilan Agama sebagaimana telah diubah dengan Undang-undang Nomor 3 Tahun 2006 dan perubahan kedua dengan Undang-

Disclaimer
Kepaniteraan Mahkamah Agung Republik Indonesia berusaha untuk selalu mencantumkan informasi paling kini dan akurat sebagai bentuk komitmen Mahkamah Agung untuk pelayanan publik, transparansi dan akuntabilitas pelaksanaan fungsi peradilan. Namun dalam hal-hal tertentu masih dimungkinkan terjadi permasalahan teknis terkait dengan akurasi dan keterkinian informasi yang kami sajikan, hal mana akan terus kami perbaiki dari waktu kewaktu.
Dalam hal Anda menemukan inakurasi informasi yang termuat pada situs ini atau informasi yang seharusnya ada, namun belum tersedia, maka harap segera hubungi Kepaniteraan Mahkamah Agung RI melalui :
Email : kepaniteraan@mahkamahagung.go.id   Telp : 021-384 3348 (ext.318)

undang Nomor 50 Tahun 2009, Pengadilan Agama Ambarawa berwenang untuk memeriksa, memutus, dan menyelesaikan Perkara ini;

Menimbang, bahwa bukti P-2 (Fotokopi Kutipan Akta Nikah) yang merupakan akta otentik dan telah bermeterai cukup dan cocok dengan aslinya, isi bukti tersebut menjelaskan antara Penggugat dan Tergugat telah terikat dalam perkawinan yang sah, sehingga bukti tersebut telah memenuhi syarat formal dan meteriil, serta mempunyai kekuatan pembuktian yang sempurna dan mengikat;

Menimbang, bahwa saksi 1 Penggugat, sudah dewasa dan sudah bersumpah, sehingga memenuhi syarat formal sebagaimana diatur dalam Pasal 145 ayat (1) angka (3e) HIR;

Menimbang, bahwa keterangan saksi 1 Penggugat mengenai dalil gugatan angka 6 dan 7, adalah fakta yang dilihat/didengar/dialami sendiri dan relevan dengan dalil yang harus dibuktikan oleh Penggugat, oleh karena itu keterangan saksi tersebut telah memenuhi syarat materiil sebagaimana telah diatur dalam Pasal 171 HIR, sehingga keterangan saksi tersebut memiliki kekuatan pembuktian dan dapat diterima sebagai alat bukti;

Menimbang, bahwa saksi 2 Penggugat, sudah dewasa dan sudah bersumpah, sehingga memenuhi syarat formal sebagaimana diatur dalam Pasal 145 ayat (1) angka (3e) HIR;

Menimbang, bahwa keterangan saksi 2 Penggugat mengenai dalil gugatan angka 6 dan 7, adalah fakta yang dilihat/didengar/dialami sendiri dan relevan dengan dalil yang harus dibuktikan oleh Penggugat, oleh karena itu keterangan saksi tersebut telah memenuhi syarat materiil sebagaimana telah diatur dalam Pasal 171 HIR, sehingga keterangan saksi tersebut memiliki kekuatan pembuktian dan dapat diterima sebagai alat bukti;

Menimbang, bahwa keterangan saksi 1 dan saksi 2 Penggugat bersesuaian dan cocok antara satu dengan yang lain oleh karena itu keterangan dua orang saksi tersebut memenuhi Pasal 171 dan Pasal 172 HIR;

Menimbang, bahwa berdasarkan saksi 1 dan saksi 2, terbukti fakta kejadian sebagai berikut;

1. Antara Penggugat dan Tergugat terus menerus terjadi perselisihan dan

Disclaimer
Kepaniteraan Mahkamah Agung Republik Indonesia berusaha untuk selalu mencantumkan informasi paling kini dan akurat sebagai bentuk komitmen Mahkamah Agung untuk pelayanan publik, transparansi dan akuntabilitas pelaksanaan fungsi peradilan. Namun dalam hal-hal tertentu masih dimungkinkan terjadi permasalahan teknis terkait dengan akurasi dan keterkinian informasi yang kami sajikan, hal mana akan terus kami perbaiki dari waktu kewaktu.
Dalam hal Anda menemukan inakurasi informasi yang termuat pada situs ini atau informasi yang seharusnya ada, namun belum tersedia, maka harap segera hubungi Kepaniteraan Mahkamah Agung RI melalui :
Email : kepaniteraan@mahkamahagung.go.id   Telp : 021-384 3348 (ext.318)

Direktori Putusan Mahkamah Agung Republik Indonesia
putusan.mahkamahagung.go.id

pertengkaran disebabkan karena Tergugat tidak layak dalam memberikan nafkah kepada Penggugat;

2. Penggugat dan Tergugat pisah rumah selama 3 tahun;

Menimbang, bahwa berdasarkan fakta-fakta tersebut di atas dapat disimpulkan fakta hukum sebagai berikut;

1. Antara Penggugat dan Tergugat terus menerus terjadi perselisihan dan pertengkaran yang sulit untuk didamaikan;
2. Penggugat dan Tergugat telah pisah rumah;
3. Antara Penggugat dan Tergugat tidak ada harapan hidup rukun kembali;

Menimbang, bahwa fakta hukum tersebut telah memenuhi norma hukum Islam yang terkandung dalam Kitab Fikih Sunah Juz II halaman 290;

أنّ للزوجة أن تطلب من القاضي التفريق إذا ادعت إضرار الزوج بها إضرارا لا يستطاع معه دوام العشرة بين أمثالها

Artinya : "*bahwa istri boleh menuntut talak kepada hakim apabila dia mengaku selalu mendapat perlakuan yang menyakitkan dari suaminya sehingga hal tersebut dapat menghalangi keberlasungan hubungan suami istri antara mereka berdua";*

Menimbang, bahwa fakta hukum tersebut telah juga memenuhi Pasal 39 ayat 2 Undang-undang Nomor 1 tahun 1974 dan Pasal 19 huruf (f) Peraturan Pemerintah Nomor 9 Tahun 1975 jo. Pasal 116 huruf (f) Kompilasi Hukum Islam, oleh karena itu dapat dikabulkan;

Menimbang, bahwa berdasarkan ketentuan Pasal 84 ayat (1) dan (2) Undang-undang No. 7 tahun 1989 sebagaimana telah diubah dengan Undang-undang No. 3 tahun 2006 Jo Undang-undang No. 50 tahun 2009, maka diperintahkan kepada Panitera Pengadilan Agama Ambarawa untuk mengirimkan salinan putusan ini setelah mempunyai kekuatan hukum tetap kepada Pegawai Pencatat Nikah di wilayah tempat tinggal Penggugat dan Tergugat serta kepada Pegawai Pencatat Nikah ditempat perkawinan Penggugat dengan Tergugat dilangsungkan untuk dicatat dalam daftar yang disediakan untuk itu;

Menimbang, bahwa oleh karena perkara ini termasuk sengketa bidang

Disclaimer
Kepaniteraan Mahkamah Agung Republik Indonesia berusaha untuk selalu mencantumkan informasi paling kini dan akurat sebagai bentuk komitmen Mahkamah Agung untuk pelayanan publik, transparansi dan akuntabilitas pelaksanaan fungsi peradilan. Namun dalam hal-hal tertentu masih dimungkinkan terjadi permasalahan teknis terkait dengan akurasi dan keterkinian informasi yang kami sajikan, hal mana akan terus kami perbaiki dari waktu kewaktu.
Dalam hal Anda menemukan inakurasi informasi yang termuat pada situs ini atau informasi yang seharusnya ada, namun belum tersedia, maka harap segera hubungi Kepaniteraan Mahkamah Agung RI melalui :
Email : kepaniteraan@mahkamahagung.go.id Telp : 021-384 3348 (ext.318)

perkawinan, maka sesuai Pasal 89 ayat 1 Undang-undang Republik Indonesia Nomor 7 Tahun 1989 yang telah diubah dengan Undang-undang Republik Indonesia Nomor 3 Tahun 2006 jo. Undang-undang Republik Indonesia Nomor 50 Tahun 2009 tentang Perubahan Kedua Atas Undang-undang Republik Indonesia Nomor 7 Tahun 1989 tentang Peradilan Agama jo. Peraturan Pemerintah Republik Indonesia Nomor 5 Tahun 2019 tentang Jenis dan Tarif Atas Jenis Penerimaan Negara Bukan Pajak yang Berlaku pada Mahkamah Agung dan Badan Peradilan yang Berada di Bawahnya jo. Keputusan Ketua Mahkamah Agung Republik Indonesia Nomor 57/MA/SK/III/2019 tentang Biaya Proses Penyelesaian Perkara dan Pengelolaannya pada Mahkamah Agung dan Badan Peradilan yang Berada di Bawahnya, semua biaya perkara yang timbul dalam perkara ini dibebankan kepada Penggugat yang jumlahnya sebagaimana tersebut dalam amar putusan ini;

Mengingat semua pasal dalam peraturan perundang-undangan dan hukum Islam yang berkaitan dengan perkara ini;

M E N G A D I L I

1. Menyatakan Tergugat yang telah dipanggil secara resmi dan patut untuk menghadap dipersidangan, tidak hadir;
2. Mengabulkan gugatan Penggugat dengan verstek;
3. Menjatuhkan talak satu ba'in sughro Tergugat ([redacted]) terhadap Penggugat ([redacted]);
4. Membebankan kepada Penggugat untuk membayar biaya perkara sejumlah Rp.341.000,00 (tiga ratus empat puluh satu ribu rupiah);

Demikian putusan ini dijatuhkan dan diucapkan pada hari Senin tanggal 28 Desember 2020 *Masehi*. bertepatan dengan tanggal 13 Jumadil Awwal 1442 *Hijriyah.* Oleh kami Rashif Imany, S.H.I., M.S.I. sebagai Hakim Tunggal, dan putusan tersebut diucapkan dalam sidang terbuka untuk umum, dibantu oleh Khalim Mudrik Masruhan, S.Sy. sebagai Panitera Pengganti serta dihadiri Penggugat tanpa hadirnya Tergugat;

Hakim,

Disclaimer
Kepaniteraan Mahkamah Agung Republik Indonesia berusaha untuk selalu mencantumkan informasi paling kini dan akurat sebagai bentuk komitmen Mahkamah Agung untuk pelayanan publik, transparansi dan akuntabilitas pelaksanaan fungsi peradilan. Namun dalam hal-hal tertentu masih dimungkinkan terjadi permasalahan teknis terkait dengan akurasi dan keterkinian informasi yang kami sajikan, hal mana akan terus kami perbaiki dari waktu kewaktu.
Dalam hal Anda menemukan inakurasi informasi yang termuat pada situs ini atau informasi yang seharusnya ada, namun belum tersedia, maka harap segera hubungi Kepaniteraan Mahkamah Agung RI melalui :
Email : kepaniteraan@mahkamahagung.go.id Telp : 021-384 3348 (ext.318)

Rashif Imany, S.H.I., M.S.I.
Panitera Pengganti,

Khalim Mudrik Masruhan, S.Sy.

Perincian Biaya :

Biaya Pendaftaran	:	Rp	30.000,00
Biaya Proses	:	Rp	75.000,00
Biaya Pemanggilan	:	Rp	200.000,00
Biaya PNBP	:	Rp	20.000,00
Biaya Redaksi	:	Rp	10.000,00
Biaya Materai	:	Rp	6.000,00
Jumlah	:	Rp	341.000,00

Disclaimer
Kepaniteraan Mahkamah Agung Republik Indonesia berusaha untuk selalu mencantumkan informasi paling kini dan akurat sebagai bentuk komitmen Mahkamah Agung untuk pelayanan publik, transparansi dan akuntabilitas pelaksanaan fungsi peradilan. Namun dalam hal-hal tertentu masih dimungkinkan terjadi permasalahan teknis terkait dengan akurasi dan keterkinian informasi yang kami sajikan, hal mana akan terus kami perbaiki dari waktu kewaktu.
Dalam hal Anda menemukan inakurasi informasi yang termuat pada situs ini atau informasi yang seharusnya ada, namun belum tersedia, maka harap segera hubungi Kepaniteraan Mahkamah Agung RI melalui :
Email : kepaniteraan@mahkamahagung.go.id   Telp : 021-384 3348 (ext.318)
