# Supplementary material for: Classification of divorce causes during the COVID-19 pandemic using convolutional neural networks
Source: PeerJ Comput Sci. 2022 Jun 30;8:e998. doi: 10.7717/peerj-cs.998 (PMC9299239; doi:10.7717/peerj-cs.998)
Supplement: Supplemental Information 2 [file peerj-cs-08-998-s002.zip › Kelalaian Dataset/Data ke-20.pdf]

Direktori Putusan Mahkamah Agung Republik Indonesia
putusan.mahkamahagung.go.id

PENETAPAN

Nomor 1303/Pdt.G/2020/PA.Smn

بِسْمِ اللَّهِ الرَّحْمَنِ الرَّحِيمِ

DEMI KEADILAN BERDASARKAN KETUHANAN YANG MAHA ESA

Pengadilan Agama Sleman yang memeriksa dan mengadili perkara tertentu pada tingkat pertama dalam sidang majelis hakim telah menjatuhkan putusan dalam perkara Cerai Gugat antara:

PENGGUGAT, tempat dan tanggal lahir Sleman, 03 Juni 1999, agama Islam, pekerjaan Mahasiswa, Pendidikan Sekolah Lanjutan Tingkat Atas, tempat kediaman di ---, Kabupaten Sleman; sebagai Penggugat**;**

melawan

TERGUGAT, tempat dan tanggal lahir Sleman, 09 April 2000, agama Islam, pekerjaan Karyawan Toko, Pendidikan Sekolah Lanjutan Tingkat Pertama, tempat kediaman di --- Kabupaten Sleman; sebagai Tergugat;

Pengadilan Agama tersebut;

Telah mempelajari surat-surat yang berkaitan dengan perkara ini;

Telah mendengar keterangan Penggugat dan Penggugat di muka sidang;

DUDUK PERKARA

Bahwa Penggugat dalam surat Gugatannya tanggal 28 September 2020 telah mengajukan gugatan Cerai Gugat, yang telah terdaftar di Kepaniteraan Pengadilan Agama Sleman, dengan Nomor 1303/Pdt.G/2020/PA.Smn, tanggal 28 September 2020, dengan dalil-dalil pada pokoknya sebagai berikut:

Disclaimer
Kepaniteraan Mahkamah Agung Republik Indonesia berusaha untuk selalu mencantumkan informasi paling kini dan akurat sebagai bentuk komitmen Mahkamah Agung untuk pelayanan publik, transparansi dan akuntabilitas pelaksanaan fungsi peradilan. Namun dalam hal-hal tertentu masih dimungkinkan terjadi permasalahan teknis terkait dengan akurasi dan keterkinian informasi yang kami sajikan, hal mana akan terus kami perbaiki dari waktu kewaktu.
Dalam hal Anda menemukan inakurasi informasi yang termuat pada situs ini atau informasi yang seharusnya ada, namun belum tersedia, maka harap segera hubungi Kepaniteraan Mahkamah Agung RI melalui :
Email : kepaniteraan@mahkamahagung.go.id Telp : 021-384 3348 (ext.318)

1. Bahwa pada tanggal 26 April 2019 telah dilangsungkan perkawinan antara Penggugat dengan Tergugat yang dilaksanakan menurut hukum dan sesuai dengan tuntunan ajaran agama Islam. Perkawinan tersebut telah dicatatkan di Kantor Urusan Agama (KUA) Kecamatan Pakem, Kabupaten Sleman sebagaimana tercatat dalam Kutipan Akta Nikah No: ---/028/IV/2019 tertanggal 26 April 2019 dan ketika menikah Penggugat berstatus Perawan dan Tergugat berstatus Jejaka;
2. Bahwa setelah menikah Penggugat dengan Tergugat tinggal bersama di rumah orang tua Tergugat di ---Kabupaten Sleman;
3. Bahwa selama masa perkawinan, Penggugat dengan Tergugat **sudah pernah berkumpul sebagaimana layaknya suami-isteri** (**ba'da dukhul)** dan **sudah** dikaruniai 1 (satu) orang anak yaitu :
   a. ANAK, jenis kelamin laki-laki, lahir di Sleman tanggal 15 Juni 2019, usia 1 tahun;

4. Bahwa pada awalnya rumah tangga Penggugat dan Tergugat berjalan harmonis, akan tetapi pada bulan Juli 2019 mulai tidak harmonis, antara Penggugat dan Tergugat terjadi pertengkaran terus menerus yang disebabkan hal sebagai berikut:
   a. Bahwa Tergugat tidak pernah memberi nafkah kepada Penggugat dan anak-anak Penggugat;
   b. Bahwa Tergugat sering berbohong kepada Penggugat ;
5. Bahwa puncak dari permasalahan antara Penggugat dengan Tergugat terjadi pada bulan September 2019 di mana Penggugat pergi meninggalkan tempat kediaman bersama ;
6. Bahwa atas permasalahan rumah tangga tersebut, Penggugat telah berupaya untuk bermusyawarah dengan keluarga Penggugat dan Tergugat agar dapat mencari penyelesaian untuk menyelamatkan perkawinan, akan tetapi upaya tersebut tidak berhasil;
7. Bahwa ikatan perkawinan antara Penggugat dengan Tergugat sebagaimana yang diuraikan diatas sudah sulit dibina untuk membentuk sebuah rumah tangga yang *sakinah, mawaddah wa rahmah*, sehingga lebih baik diputuskan karena perceraian;

Disclaimer
Kepaniteraan Mahkamah Agung Republik Indonesia berusaha untuk selalu mencantumkan informasi paling kini dan akurat sebagai bentuk komitmen Mahkamah Agung untuk pelayanan publik, transparansi dan akuntabilitas pelaksanaan fungsi peradilan. Namun dalam hal-hal tertentu masih dimungkinkan terjadi permasalahan teknis terkait dengan akurasi dan keterkinian informasi yang kami sajikan, hal mana akan terus kami perbaiki dari waktu kewaktu.
Dalam hal Anda menemukan inakurasi informasi yang termuat pada situs ini atau informasi yang seharusnya ada, namun belum tersedia, maka harap segera hubungi Kepaniteraan Mahkamah Agung RI melalui :
Email : kepaniteraan@mahkamahagung.go.id Telp : 021-384 3348 (ext.318)

8. Bahwa berdasarkan hal-hal tersebut diatas, maka gugatan perceraian Penggugat terhadap Tergugat atas dasar pertengkaran yang terjadi terus menerus dan tidak mungkin hidup rukun dalam suatu ikatan perkawinan, telah memenuhi unsur Pasal 19 huruf (f) Peraturan Pemerintah No. 9 Tahun 1975 Jo. Pasal 116 huruf (f) Kompilasi Hukum Islam, mohon agar dapat dikabulkan;
9. Bahwa Penggugat Sanggup membayar biaya perkara;

Berdasarkan dalil dan alasan-alasan tersebut diatas, maka dengan ini Penggugat memohon agar Ketua Pengadilan Agama Sleman cq. Majelis Hakim dapat memeriksa dan mengadili perkara ini, selanjutnya menjatuhkan putusan yang amarnya sebagai berikut :

PRIMAIR:

1. Mengabulkan gugatan Penggugat ;
2. Menjatuhkan talak satu ba'in sughra Tergugat (**TERGUGAT**) terhadap Penggugat (**PENGGUGAT (alm)**);
3. Membebankan biaya perkara menurut Hukum;

SUBSIDAIR:

Atau apabila Pengadilan Agama Sleman berpendapat lain mohon putusan yang seadil adilnya *(ex aequo et bono);c*

Bahwa pada persidangan yang telah ditetapkan Penggugat dan Tergugat telah datang menghadap di persidangan;

Bahwa majelis hakim telah mendamaikan Penggugat dan Tergugat, agar Penggugat rukun kembali dalam membina rumahtangganya;

Bahwa dalam persidangan tanggal 28 Desember 2020 Penggugat menyatakan hendak rukun kembali dengan Tergugat, oleh karenanya memohon kepada majelis untuk mencabut gugatannya;

Bahwa atas pernyataan pencabutan perkara oleh Penggugat tersebut, Tergugat menyatakan menyetujuinya;

Disclaimer
Kepaniteraan Mahkamah Agung Republik Indonesia berusaha untuk selalu mencantumkan informasi paling kini dan akurat sebagai bentuk komitmen Mahkamah Agung untuk pelayanan publik, transparansi dan akuntabilitas pelaksanaan fungsi peradilan. Namun dalam hal-hal tertentu masih dimungkinkan terjadi permasalahan teknis terkait dengan akurasi dan keterkinian informasi yang kami sajikan, hal mana akan terus kami perbaiki dari waktu kewaktu.
Dalam hal Anda menemukan inakurasi informasi yang termuat pada situs ini atau informasi yang seharusnya ada, namun belum tersedia, maka harap segera hubungi Kepaniteraan Mahkamah Agung RI melalui :
Email : kepaniteraan@mahkamahagung.go.id Telp : 021-384 3348 (ext.318)

Selanjutnya untuk singkatnya uraian putusan ini, maka semua hal yang termuat dalam berita acara sidang ini merupakan bagian yang tidak terpisahkan dari putusan ini;

PERTIMBANGAN HUKUM

Menimbang, bahwa maksud dan tujuan Permohonan Penggugat adalah sebagaimana terurai di atas;

Menimbang, bahwa Majelis Hakim telah berusaha mendamaikan kedua belah pihak baik secara langsung dalam persidangan sebagaimana ketentuan pasal 130 (1) HIR jo. Pasal 31 Peraturan Pemerintah Nomor 9 Tahun 1975 jo pasal 65 dan pasal 82 Undang-undang Nomor 7 Tahun 1989;

Menimbang, bahwa Penggugat dalam persidangan tanggal 28 Desember 2020 menyatakan dan mohon kepada Majelis untuk mencabut perkaranya, dengan alasan telah rukun kembali dengan Tergugat;

Menimbang, bahwa Tergugat menyatakan setuju atas pernyataan pencabutan perkara oleh Penggugat tersebut;

Menimbang, bahwa berdasarkan pertimbangan tersebut di atas, maka pencabutan perkara oleh Penggugat tersebut dapat dikabulkan;

Menimbang, bahwa meskipun gugatan Penggugat dicabut, oleh karena perkara ini telah didaftar dalam register perkara dan termasuk dalam bidang perkawinan, maka sesuai Pasal 89 ayat (1) Undang-undang Nomor 7 Tahun 1989 sebagaimana telah diubah dengan Undang-undang Nomor 3 Tahun 2006 dan perubahan kedua dengan Undang-undang Nomor 50 Tahun 2009, biaya perkara harus dibebankan kepada Penggugat;

Mengingat, semua pasal dalam peraturan perundang-undangan dan hukum Islam yang berkaitan dengan perkara ini;

MENETAPKAN

1. Mengabulkan permohonan Penggugat untuk mencabut perkaranya;

Disclaimer
Kepaniteraan Mahkamah Agung Republik Indonesia berusaha untuk selalu mencantumkan informasi paling kini dan akurat sebagai bentuk komitmen Mahkamah Agung untuk pelayanan publik, transparansi dan akuntabilitas pelaksanaan fungsi peradilan. Namun dalam hal-hal tertentu masih dimungkinkan terjadi permasalahan teknis terkait dengan akurasi dan keterkinian informasi yang kami sajikan, hal mana akan terus kami perbaiki dari waktu kewaktu.
Dalam hal Anda menemukan inakurasi informasi yang termuat pada situs ini atau informasi yang seharusnya ada, namun belum tersedia, maka harap segera hubungi Kepaniteraan Mahkamah Agung RI melalui :
Email : kepaniteraan@mahkamahagung.go.id Telp : 021-384 3348 (ext.318)

2. Menyatakan perkara Nomor 1303/Pdt.G/2020/PA.Smn dicabut ;
3. Membebankan kepada Penggugat untuk membayar biaya perkara sejumlah Rp631.000,00 (enam ratus tiga puluh satu ribu rupiah).

Demikian penetapan ini dijatuhkan dalam rapat permusyawaratan Majelis Hakim yang dilangsungkan pada hari Senin tanggal 28 Desember 2020 Masehi, bertepatan dengan tanggal 13 Jumadil Awwal 1442 Hijriah, oleh kami Drs. Yusuf, S.H., M.S.I. sebagai Ketua Majelis, Drs. H. Muhamad Dihan, M.H. dan Drs. H. Arif Irfan, S.H., M.Hum. masing-masing sebagai Hakim Anggota, putusan tersebut diucapkan dalam sidang terbuka untuk umum pada hari itu juga oleh Ketua Majelis tersebut dengan didampingi oleh Hakim Anggota dan dibantu oleh Nathalina Sri Ariyanti, S.H. sebagai Panitera Pengganti serta dihadiri oleh Penggugat dan Tergugat;

Hakim Anggota,	Ketua Majelis,
Drs. H. Muhamad Dihan, M.H.	Drs. Yusuf, S.H., M.S.I
Hakim Anggota,	
Drs. H. Arif Irfan, S.H., M.Hum	
	Panitera Pengganti,
	Nathalina Sri Ariyanti, S.H.

Perincian biaya :

1.	Pendaftaran	Rp30.000,00
2.	Proses	Rp75.000,00

Disclaimer
Kepaniteraan Mahkamah Agung Republik Indonesia berusaha untuk selalu mencantumkan informasi paling kini dan akurat sebagai bentuk komitmen Mahkamah Agung untuk pelayanan publik, transparansi dan akuntabilitas pelaksanaan fungsi peradilan. Namun dalam hal-hal tertentu masih dimungkinkan terjadi permasalahan teknis terkait dengan akurasi dan keterkinian informasi yang kami sajikan, hal mana akan terus kami perbaiki dari waktu kewaktu.
Dalam hal Anda menemukan inakurasi informasi yang termuat pada situs ini atau informasi yang seharusnya ada, namun belum tersedia, maka harap segera hubungi Kepaniteraan Mahkamah Agung RI melalui :
Email : kepaniteraan@mahkamahagung.go.id Telp : 021-384 3348 (ext.318)

3.	Panggilan	Rp490.000,00
4.	PNBP Panggilan	Rp20.000,00
5.	Redaksi	Rp10.000,00
6.	Meterai	Rp6.000,00
	**Jumlah**	**Rp631.000,00**

(enam ratus tiga puluh satu ribu rupiah)

Disclaimer
Kepaniteraan Mahkamah Agung Republik Indonesia berusaha untuk selalu mencantumkan informasi paling kini dan akurat sebagai bentuk komitmen Mahkamah Agung untuk pelayanan publik, transparansi dan akuntabilitas pelaksanaan fungsi peradilan. Namun dalam hal-hal tertentu masih dimungkinkan terjadi permasalahan teknis terkait dengan akurasi dan keterkinian informasi yang kami sajikan, hal mana akan terus kami perbaiki dari waktu kewaktu.
Dalam hal Anda menemukan inakurasi informasi yang termuat pada situs ini atau informasi yang seharusnya ada, namun belum tersedia, maka harap segera hubungi Kepaniteraan Mahkamah Agung RI melalui :
Email : kepaniteraan@mahkamahagung.go.id   Telp : 021-384 3348 (ext.318)
