# Supplementary material for: Classification of divorce causes during the COVID-19 pandemic using convolutional neural networks
Source: PeerJ Comput Sci. 2022 Jun 30;8:e998. doi: 10.7717/peerj-cs.998 (PMC9299239; doi:10.7717/peerj-cs.998)
Supplement: Supplemental Information 2 [file peerj-cs-08-998-s002.zip › Kelalaian Dataset/Data ke-21.pdf]

P U T U S A N

Nomor 1104/Pdt.G/2020/PA.Amb

بسم الله الرحمن الرحيم

DEMI KEADILAN BERDASARKAN KETUHANAN YANG MAHA ESA

Pengadilan Agama Ambarawa yang mengadili perkara-perkara tertentu pada tingkat pertama dalam persidangan, menjatuhkan putusan sebagai berikut dalam perkara Gugatan Perceraian antara;

[redacted], Tempat Tanggal lahir: Kab. Semarang 03 September 1991, umur 29 tahun, agama Islam, Pekerjaan Karyawan Swasta, Pendidikan Sekolah Dasar, tempat kediaman di [redacted] Kab. Semarang, dalam hal ini memberikan kuasa kepada Chusaeni Rafsanjani Assadami, S.Sy, Advokat yang berkantor di Dusun Krajan RT 02 RW 01, Desa Banyukuning, Kecamatan Bandungan, Kabupaten Semarang. berdasarkan surat kuasa khusus tanggal terdaftar 12 Oktober 2020, sebagai Penggugat;

Melawan

[redacted], Tempat Tanggal lahir: Kab. Semarang 03 September 1987, umur 33 tahun, agama Islam, Pekerjaan Swasta, Pendidikan Sekolah Lanjutan Tingkat Pertama, tempat kediaman di [redacted] Kab. Semarang, sebagai Tergugat;

Pengadilan Agama tersebut;

Telah mempelajari berkas perkara;

Telah mendengar para pihak dan memeriksa bukti-bukti;

DUDUK PERKARA

Disclaimer
Kepaniteraan Mahkamah Agung Republik Indonesia berusaha untuk selalu mencantumkan informasi paling kini dan akurat sebagai bentuk komitmen Mahkamah Agung untuk pelayanan publik, transparansi dan akuntabilitas pelaksanaan fungsi peradilan. Namun dalam hal-hal tertentu masih dimungkinkan terjadi permasalahan teknis terkait dengan akurasi dan keterkinian informasi yang kami sajikan, hal mana akan terus kami perbaiki dari waktu kewaktu.
Dalam hal Anda menemukan inakurasi informasi yang termuat pada situs ini atau informasi yang seharusnya ada, namun belum tersedia, maka harap segera hubungi Kepaniteraan Mahkamah Agung RI melalui :
Email : kepaniteraan@mahkamahagung.go.id   Telp : 021-384 3348 (ext.318)

Bahwa, Penggugat telah mengajukan gugatan perceraian tertanggal 12 Oktober 2020 yang telah didaftar dalam register perkara nomor: 1104/Pdt.G/2020/PA.Amb, tanggal 12 Oktober 2020, mengemukakan hal-hal sebagai berikut:

1. Bahwa Penggugat telah menikah dengan Tergugat pada tanggal 27 Maret 2007 oleh Pejabat Kantor Urusan Agama Kecamatan [redacted] Kabupaten Semarang sebagaimana kutipan [redacted];

2. Bahwa sesudah aqad nikah Tergugat mengucapkan sighat ta'lik talak yang berbunyi sebagai berikut;

Sewaktu-waktu saya:

1) Meninggalkan istri saya dua tahun berturut-turut,

2) Atau saya tidak memeberi nafkah wajib kepadanya tiga bulan lamanya,

3) Atau saya menyakiti badan/jasmani istri saya,

4) Atau saya membiarkan (tidak mempedulikan) istri saya enam bulan lamanya, kemudian itri saya tidak ridha dan mengadukan halnya kepada pengadilan Agama dan pengaduanya dibenarkan serta diterima oleh pengadilan tersebut, dan istri saya membayar uang sebesar Rp. 10.000,00 (sepuluh ribu rupiah) sebagai iwadh (pengganti) kepada maka jatuhlah talak saya satu kepadanya.;

3. Bahwa setelah akad nikah Penggugat dan Tergugat hidup dan bekerja bersama di [redacted];

4. Bahwa pada bulan Juli 2018 Pengguat dan Tergugat pulang ke rumah orang tua Penggugat Karena Sudah Tidak Bekerja di Villa;

5. Bahwa selama pernikahan tersebut Penggugat dengan Tergugat telah hidup rukun sebagaimana layaknya suami istri (Ba'daDhukul) dan sudah dikaruniai seorang anak yang bernama :

- [redacted], Perempuan lahir pada tanggal 01 Agustus 2007 (13 Tahun) yang sampai saat ini dalam asuhan Penggugat;

Disclaimer
Kepaniteraan Mahkamah Agung Republik Indonesia berusaha untuk selalu mencantumkan informasi paling kini dan akurat sebagai bentuk komitmen Mahkamah Agung untuk pelayanan publik, transparansi dan akuntabilitas pelaksanaan fungsi peradilan. Namun dalam hal-hal tertentu masih dimungkinkan terjadi permasalahan teknis terkait dengan akurasi dan keterkinian informasi yang kami sajikan, hal mana akan terus kami perbaiki dari waktu kewaktu.
Dalam hal Anda menemukan inakurasi informasi yang termuat pada situs ini atau informasi yang seharusnya ada, namun belum tersedia, maka harap segera hubungi Kepaniteraan Mahkamah Agung RI melalui :
Email : kepaniteraan@mahkamahagung.go.id Telp : 021-384 3348 (ext.318)

6. Bahwa kehidupan rumah tangga antara Penggugat dan Tergugat awalnya dalam keadaan harmonis, akan tetapi lebih kurang usia pernikahan 1 tahun (± Tahun 2008) keluarga Penggugat dan Tergugat mulai tidak harmonis, rumah tangga selalu terjadi perselisihan dan pertengkaran terus menerus yang disebabkan:

a. Tergugat kurang dalam memberi Nafkah;

b. Pada saat terjadi perselisihan dan pertengkaran Tergugat sering menyakiti badan/jasmani Penggugat;

7. Bahwa puncak terjadinya perselisihan dan pertengkaran pada bulan Maret 2020 tanpa sebab dan alasan yang jelas Tergugat pulang ke rumah orang tuanya dengan alamat sesuai yang tertera di atas, dan tidak pernah kembali lagi sampai saat ini, dan sejak saat itu antara Penggugat dengan Tergugat telah berpisah tempat tinggal lebih kurang 6 bulan lamanya dan antara Penggugat dan Tergugat sudah tidak pernah berhubungan layaknya suami istri;

8. Bahwa atas permasalahan dan kemelut rumah tangga yang dihadapi, Penggugat telah mencoba memusyawarahkan dengan keluarga Penggugat dan Tergugat untuk mencari penyelesaian dan demi menyelamatkan perkawinan, namun usaha tersebut juga tidak membuahkan hasil;

9. Bahwa selama kepergiannya tersebut, Tergugat tidak pernah memberi nafkah wajib dan Tergugat tidak meninggalkan sesuatu yang dapat Penggugat manfaatkan sebagai nafkah, kemudian Penggugat pun berusaha untuk mencari Nafkah sendiri;

10. Bahwa Penggugat dan Tergugat dalam perkawinan belum pernah bercerai;

11. Bahwa, Tergugat telah melanggar sighat talik talak yang pernah diucapkan Tergugat waktu pernikahan dahulu yaitu pada poin 2 dan poin 4;

12. Bahwa sekarang Penggugat tidak sabar dan ridha lagi atas perlakuan Tergugat tersebut, karena Penggugat telah menderita lahir dan bathin dan Penggugat bersedia membayar uang iwadh sebesar Rp. 10.000,- (sepuluh ribu rupiah) atas shighat taklik talak yang pernah diucapkan Tergugat dahulu;

Disclaimer
Kepaniteraan Mahkamah Agung Republik Indonesia berusaha untuk selalu mencantumkan informasi paling kini dan akurat sebagai bentuk komitmen Mahkamah Agung untuk pelayanan publik, transparansi dan akuntabilitas pelaksanaan fungsi peradilan. Namun dalam hal-hal tertentu masih dimungkinkan terjadi permasalahan teknis terkait dengan akurasi dan keterkinian informasi yang kami sajikan, hal mana akan terus kami perbaiki dari waktu kewaktu.
Dalam hal Anda menemukan inakurasi informasi yang termuat pada situs ini atau informasi yang seharusnya ada, namun belum tersedia, maka harap segera hubungi Kepaniteraan Mahkamah Agung RI melalui :
Email : kepaniteraan@mahkamahagung.go.id   Telp : 021-384 3348 (ext.318)

Berdasarkan alasan/dalil-dalil di atas Penggugat mohon Pengadilan Agama Ambarawa Cq. Majlis Hakim Segera memeriksa dan Mengadili Perkara ini, selanjutnya memberikan putusan yang amarnya berbunyi:

A. PRIMAIR

1. Mengabulkan gugatan Penggugat seluruhnya;
2. Menyatakan syarat taklik talak talak telah terpenuhi;
3. Menjatuhkan talak Satu Khul'i Tergugat [redacted] terhadap Penggugat [redacted] dengan iwadh berupa uang sejumlah Rp. 10.000.- (sepuluh ribu rupiah)
4. Membebankan biaya perkara menurut hukum.

B. SUBSIDAIR

Jika Hakim berpendapat lain, mohon putusan yang seadil-adilnya (*ex. Aequoet Bono*)

Bahwa, pada hari persidangan yang telah ditetapkan Penggugat dan Tergugat datang sendiri menghadap di persidangan;

Bahwa, kedua pihak berperkara telah menempuh prosedur mediasi dengan bantuan mediator bernama Ishak Lubis, S.Ag., namun mediasi tersebut tidak berhasil;

Bahwa, Hakim telah berusaha mendamaikan namun tidak berhasil, kemudian dibacakanlah surat gugatan Penggugat, atas pertanyaan Hakim Penggugat menyatakan perbaikan dalam identitas Tergugat (ada penambahan huruf "O"), dalam gugatan nama Tergugat tertulis Dwi Saptno bin Suyono. Adapun nama Tergugat yang betul adalah [redacted] , selanjutya mengenai isi serta maksudnya tetap dipertahankan oleh Penggugat;

Bahwa, atas Gugatan Penggugat, Tergugat memberikan jawaban secara tertulis sebagaimana selengkapnya dalam Berita Acara Sidang tanggal 18 November 2020 sebagai berikut;

1. Bahwa Tergugat menolak dalil-dalll penggugat seluruhnya kecuali yang tegas diikuti.
2. Bahwa benar saya (Tergugat) dan penggugat telah menikah tanggal 27 Maret .2007 oleh pejabat Kantor Urusan Agama [redacted]

Disclaimer
Kepaniteraan Mahkamah Agung Republik Indonesia berusaha untuk selalu mencantumkan informasi paling kini dan akurat sebagai bentuk komitmen Mahkamah Agung untuk pelayanan publik, transparansi dan akuntabilitas pelaksanaan fungsi peradilan. Namun dalam hal-hal tertentu masih dimungkinkan terjadi permasalahan teknis terkait dengan akurasi dan keterkinian informasi yang kami sajikan, hal mana akan terus kami perbaiki dari waktu kewaktu.
Dalam hal Anda menemukan inakurasi informasi yang termuat pada situs ini atau informasi yang seharusnya ada, namun belum tersedia, maka harap segera hubungi Kepaniteraan Mahkamah Agung RI melalui :
Email : kepaniteraan@mahkamahagung.go.id Telp : 021-384 3348 (ext.318)

[redacted] Kabupaten Semarang sebagaimana kutipan akta nikah nomor : [redacted]

3. Bahwa benar setelah akad nikah saya (Tergugat) dan Penggugat hidup dalam bekerja bersama di sebuah vila di [redacted] sampai dengan bulan Juni 2018.
4. Bahwa benar pada bulan Jull 2018 saya (tergugat) dan penggugat pulang ke rumah orang tua.

   Penggugat karena sudah tidak bekerja di villa, tetapi setelah pulang ke rumah orang tua penggugat, saya (Tergugat) langsung bekerja lagi sebagai satpam (security) di Perumahan Villa tersebut.
5. Bahwa benar selama pernikahan tersebut saya (tergugat) dengan penggugat telah hidup rukun sebagaimana layaknya suami istri dan dikaruniai seorang anak yang bernama : [redacted], perempuan lahir pada tanggal 01 Agustus 2007 (13 tahun) yang sampai saat ini dalam asuhan penggugat.
6. Bahwa tidak benar dalil nomor 6 kehidupan rumah tangga antara saya (Tergugat) dengan penggugat awalnya dalam keadaan harmonis akan tetapi lebih kurang usia pernikhan lebih kurang 1 tahun mulai tidak harmonis rumah tangga selalu terjadi perselisihan dan pertengkaran akan tetapi perselisihan dan pertengkaran tersebut selalu berakhir dengan perdamaian, mengenai memberi nafkah bukannya saya (Tergugat) kurang memberi nafkah akan tetapi istri saya (Penggugat) kurang hati-hati dalam mengelola keuangan (boros). Kejadian sebenarnya adalati pada saat terjadi perselislhan dan pertengkaran saya (Tergugat) sering menyakiti badan jasmani penggugat dikarenakan istri saya (Penggugat) kalau dikasih tau atau dinasehati selalu berani kepada saya tetapi semua itu semua berakhir dengan perdamaian seiring berjalannya waktu kurang lebih 3 tahun setelah saya bekerja keras untuk mencari nafkah untuk keluarga saya ternyata istri saya selingkuh di vila tempat saya dan istri saya bekerja dan ketahuan sama saya itu jelas tidak menghargai saya sebagai suaminya.

Disclaimer
Kepaniteraan Mahkamah Agung Republik Indonesia berusaha untuk selalu mencantumkan informasi paling kini dan akurat sebagai bentuk komitmen Mahkamah Agung untuk pelayanan publik, transparansi dan akuntabilitas pelaksanaan fungsi peradilan. Namun dalam hal-hal tertentu masih dimungkinkan terjadi permasalahan teknis terkait dengan akurasi dan keterkinian informasi yang kami sajikan, hal mana akan terus kami perbaiki dari waktu kewaktu.
Dalam hal Anda menemukan inakurasi informasi yang termuat pada situs ini atau informasi yang seharusnya ada, namun belum tersedia, maka harap segera hubungi Kepaniteraan Mahkamah Agung RI melalui :
Email : kepaniteraan@mahkamahagung.go.id Telp : 021-384 3348 (ext.318)

7. Bahwa tidak benar dalil nomor 7 terjadinya perselisihan dan pertengkaran pada bulan Maret
2020 saya masih pulang ke rumah istri saya (pisah ranjang) (Penggugat) sampai bulan September, selama itu saya masih mencoba mempertahankan keutuhan rumah tangga saya karena kasihan kepada anak saya setelah itu saya sudah merasa malu dan akhirnya saya pulang ke rumah orang tua saya karena sudah tidak tahan selalu disuruh pergi (ditundung) dari rumah istri saya akhirnya saya pulang ke rumah orang tua saya. Jadi bukan enam bulan lamanya saya tidak berhubungan dengan istri saya tetapi 3 bulan itu pun masih bisa berhubungan lewat HP.

8. Bahwa tidak benar daHl nomor 9 selama kepergian saya (Tergugat) masih memberi nafkah wajib dan meninggalkan sejumlah uang untuk kebutuhan anak dan istri saya sampai saat ini tetepi untuk bulan November ini belum saya kasih nafkah karena saya juga belum mendapat gaji dari tempat saya bekerja.

Maka berdasarkan segala yang terurai diatas saya (Tergugat) mohon dengan hormat sudilah kiranya Pengadilan Agama Amabrawa berkenan memutuskan menolak gugatan penggugat seluruhnya atau setidak-tidaknya tidak dapat diterima.

Bahwa atas jawaban Tergugat tersebut, Penggugat menyampaikan replik secara lisan yang pada pokoknya adalah tetap pada gugatannya;

Bahwa atas Replik Penggugat tersebut, Tergugat menyampaikan duplik secara lisan yang pada pokoknya tetap sebagaimana dalam jawaban;

Bahwa, untuk meneguhkan dalil-dalil gugatannya, Penggugat telah mengajukan bukti-bukti tertulis berupa

1. Fotocopy Kartu Tanda Penduduk NIK: [redacted] yang dikeluarkan oleh Kepala Dinas Kependudukan dan Catatan Sipil Kabupaten Semarang, Provinsi Jawa Tengah, bermeterai cukup, telah dicocokkan dan sesuai dengan aslinya, selanjutnya diberi kode P.1;
2. Fotocopy Buku Kutipan Akta Nikah Nomor: [redacted] yang

Disclaimer
Kepaniteraan Mahkamah Agung Republik Indonesia berusaha untuk selalu mencantumkan informasi paling kini dan akurat sebagai bentuk komitmen Mahkamah Agung untuk pelayanan publik, transparansi dan akuntabilitas pelaksanaan fungsi peradilan. Namun dalam hal-hal tertentu masih dimungkinkan terjadi permasalahan teknis terkait dengan akurasi dan keterkinian informasi yang kami sajikan, hal mana akan terus kami perbaiki dari waktu kewaktu.
Dalam hal Anda menemukan inakurasi informasi yang termuat pada situs ini atau informasi yang seharusnya ada, namun belum tersedia, maka harap segera hubungi Kepaniteraan Mahkamah Agung RI melalui :
Email : kepaniteraan@mahkamahagung.go.id Telp : 021-384 3348 (ext.318)

dikeluarkan oleh Pegawai Pencatat Nikah Kantor Urusan Agama Kecamatan [redacted] Kabupaten Semarang, Propinsi Jawa Tengah tanggal 27 Maret 2007, bermeterai cukup, telah dicocokkan dan sesuai dengan aslinya, selanjutnya diberi kode P.2;

Bahwa, Penggugat juga telah mengajukan bukti saksi dibawah sumpah yang keterangannya sebagai berikut;

1. [redacted], umur 63 tahun, Agama Islam, Pendidikan Sekolah Dasar, Pekerjaan Petani, tempat tinggal di L[redacted] Kabupaten Semarang,
   - Bahwa Saksi kenal dengan Penggugat dan Tergugat karena sebagai ayah kandung Penggugat;
   - Bahwa Penggugat sudah menikah dengan Tergugat pada maret tahun 2007, sudah dikaruniai seorang anak yang sekarang dalam asuhan Penggugat;
   - Bahwa Setelah menikah Penggugat dan Tergugat tinggal di sebuah villa di daerah G[redacted] Kabupaten Semarang. Sampai tahun 2018. Kemudian setelah itu Penggugat dan Tergugat pindah ke rumah saksi di [redacted] Kabupaten Semarang;
   - Bahwa Keadaan rumah tangga Penggugat dengan Tergugat pada awal rukun, namun sejak tahun 2008, antara Penggugat dan Tergugat sering terjadi percekcokan karena masalah ekonomi. Tergugat kurang dalam memberikan nafkah kepada Penggugat;
   - Bahwa Selain masalah ekonomi, penyebab lain yang menjadi penyebab percekcokan antara Penggugat dan Tergugat yakni karena Tergugat juga pernah melakukan Tindakan Kekerasan Dalam Rumah tangga (KDRT) yakni dengan cara memukul Penggugat. Saksi pernah melihat bekas pukulan yang dilakukan oleh Tergugat kepada Penggugat tersebut;
   - Bahwa Saat ini Penggugat dan Tergugat sudah pisah rumah, mereka telah pisah sekitar 4 bulan yang lalu. Tergugat pulang ke rumah orang tuanya;
   - Bahwa Selama pisah tersebut, saksi tidak pernah melihat Tergugat datang ke tempat Penggugat untuk mengajak rukun kembali Penggugat;

Disclaimer
Kepaniteraan Mahkamah Agung Republik Indonesia berusaha untuk selalu mencantumkan informasi paling kini dan akurat sebagai bentuk komitmen Mahkamah Agung untuk pelayanan publik, transparansi dan akuntabilitas pelaksanaan fungsi peradilan. Namun dalam hal-hal tertentu masih dimungkinkan terjadi permasalahan teknis terkait dengan akurasi dan keterkinian informasi yang kami sajikan, hal mana akan terus kami perbaiki dari waktu kewaktu.
Dalam hal Anda menemukan inakurasi informasi yang termuat pada situs ini atau informasi yang seharusnya ada, namun belum tersedia, maka harap segera hubungi Kepaniteraan Mahkamah Agung RI melalui :
Email : kepaniteraan@mahkamahagung.go.id Telp : 021-384 3348 (ext.318)

- Bahwa Pihak keluarga sudah pernah berupaya untuk menasehati Penggugat agar hidup rukun lagi dengan Tergugat, namun Penggugat menyatakan sudah tidak sanggup lagi hidup bersama Tergugat;
- Bahwa Selama pisah rumah tersebut, saksi tidak melihat Tergugat meninggalkan barang sesuatu yang dapat dijadikan sebagai pengganti nafkah pada Penggugat;

2. [redacted], umur 49 tahun, Agama Islam, Pendidikan Sekolah Dasar, Pekerjaan Wiraswasta, tempat tinggal di [redacted] Kabupaten Semarang,

- Bahwa Saksi kenal dengan Penggugat dan Tergugat karena sebagai sebagai tetangga Penggugat;
- Bahwa Penggugat sudah menikah dengan Tergugat dan dikaruniai seorang anak yang sekarang dalam asuhan Penggugat;
- Bahwa Setelah menikah Penggugat dan Tergugat tinggal di sebuah villa di [redacted] Kabupaten Semarang;
- Bahwa sepengetahuan Saksi Pada awal keadaan rumah tangga Penggugat dengan Tergugat rukun rukun saja, namun sejak 1 tahun setelah menikah, antara Penggugat dan Tergugat mulai ada pertengkaran disebabkan Tergugat kurang bisa memenuhi nafkah Penggugat;
- Bahwa Selain masalah ekonomi, penyebab lain yang menjadi penyebab percekcokan antara Penggugat dan Tergugat yakni karena Tergugat juga pernah melakukan Tindakan Kekerasan Dalam Rumah tangga (KDRT) yakni dengan cara memukul Penggugat. Saksi pernah melihat bekas pukulan yang dilakukan oleh Tergugat kepada Penggugat tersebut;
- Bahwa Saksi menyatakan demikian karena Untuk mencukupi kebutuhan sehari-hari, Penggugat saat ini harus dibantu oleh keluarga agar bisa mencukupi kebutuhan hidup sehari-harinya;
- Bahwa Saat ini Penggugat dan Tergugat sudah pisah rumah, Tergugat pulang ke rumah orang tua Tergugat sekitar 4 bulan yang lalu;
- Bahwa Selama pisah tersebut, saksi tidak pernah melihat Tergugat datang ke tempat Penggugat untuk mengajak rukun kembali Penggugat;

Disclaimer
Kepaniteraan Mahkamah Agung Republik Indonesia berusaha untuk selalu mencantumkan informasi paling kini dan akurat sebagai bentuk komitmen Mahkamah Agung untuk pelayanan publik, transparansi dan akuntabilitas pelaksanaan fungsi peradilan. Namun dalam hal-hal tertentu masih dimungkinkan terjadi permasalahan teknis terkait dengan akurasi dan keterkinian informasi yang kami sajikan, hal mana akan terus kami perbaiki dari waktu kewaktu.
Dalam hal Anda menemukan inakurasi informasi yang termuat pada situs ini atau informasi yang seharusnya ada, namun belum tersedia, maka harap segera hubungi Kepaniteraan Mahkamah Agung RI melalui :
Email : kepaniteraan@mahkamahagung.go.id Telp : 021-384 3348 (ext.318)

- Bahwa Pihak keluarga sudah berusaha untuk merukunkan Penggugat dan Tergugat agar kembali hidup rukun, namun tidak berhasil. Dan Penggugat menyatakan sudah tidak sanggup lagi hidup bersama Tergugat;

Bahwa, selanjutnya Hakim memberikan kesempatan yang sama kepada Tergugat untuk mengajukan bukti, Tergugat juga telah mengajukan 2 orang saksi di bawah sumpah yang keterangannya sebagai berikut;

1. [redacted], Tempat Tanggal lahir Kab Semarang 11 Agustus. 1977, Agama Islam, Pendidikan Sekolah Lanjutan Tingkat Pertama, Pekerjaan Wiraswasta, tempat tinggal di [redacted] Kabupaten Semarang,

- Bahwa Saksi kenal dengan Penggugat dan Tergugat karena sebagai kakak ipar Tergugat;
- Bahwa Penggugat telah dikaruniai seorang anak yang saat ini dalam asuhan Penggugat;
- Bahwa Setelah menikah Penggugat dan Tergugat tinggal dan hidup bersama di rumah orang tua Penggugat yang mana rumahnya orang tua Penggugat tersebut bertetangga dengan Saksi;
- Bahwa Sepengetahuan saksi, rumah tangga Penggugat dengan Tergugat semula memang ada masalah kecil, namun selanjutnya rumah tangga mereka baik lagi;
- Bahwa Saat ini Penggugat dan Tergugat sudah pisah rumah, Penggugat tinggal di rumah orang tua Penggugat, sementara Tergugat tinggal di rumah orang tua Tergugat;
- Bahwa Saksi kurang tahu persis apa yang menjadi penyebab Penggugat dan Tergugat pisah tempat tinggal, Saksi hanya mengetahui bahwa mereka sudah pisah tempat tinggal;
- Bahwa Sepengetahuan saksi, Penggugat dan Tergugat mulai pisah tempat tinggal sekitar 6 bulan yang lalu;
- Bahwa Selama pisah tempat tinggal tersebut, sepengetahuan saksi, Tergugat pernah berusaha mendekati Penggugat lagi untuk mengajak hidup rukun, namun Penggugat sudah tidak mau lagi dengan Tergugat;

Disclaimer
Kepaniteraan Mahkamah Agung Republik Indonesia berusaha untuk selalu mencantumkan informasi paling kini dan akurat sebagai bentuk komitmen Mahkamah Agung untuk pelayanan publik, transparansi dan akuntabilitas pelaksanaan fungsi peradilan. Namun dalam hal-hal tertentu masih dimungkinkan terjadi permasalahan teknis terkait dengan akurasi dan keterkinian informasi yang kami sajikan, hal mana akan terus kami perbaiki dari waktu kewaktu.
Dalam hal Anda menemukan inakurasi informasi yang termuat pada situs ini atau informasi yang seharusnya ada, namun belum tersedia, maka harap segera hubungi Kepaniteraan Mahkamah Agung RI melalui :
Email : kepaniteraan@mahkamahagung.go.id Telp : 021-384 3348 (ext.318)

- Bahwa Pihak keluarga sudah pernah berupaya untuk menasehati Penggugat dan Tergugat, namun hasilnya nihil;

2. [redacted], Tempat Tanggal lahir Kabupaten Semarang 16 Desember 1995, Agama Islam, Pendidikan Sekolah Lanjutan Tingkat Atas, Pekerjaan Pelajar/Mahasiswa, tempat tinggal di [redacted] Kabupaten Semarang,

- Bahwa Saksi kenal dengan Penggugat dan Tergugat sebagai tetangga Tergugat;
- Bahwa Penggugat dengan Tergugat adalah suami isteri yang telah memiliki seorang anak yang saat ini di asuh oleh Penggugat;
- Bahwa Setelah menikah Penggugat dan Tergugat tinggal dan hidup bersama di sebuah Villa di Bandungan Semarang. Kemudian pada pertengahan 2008 mereka pindah tempat tingagl di rumah orang tua Pengugat;
- Bahwa Sepengetahuan saksi sejak awal menikah sampai sekarang rumah tangga Penggugat dan Tergugat tidak ada permasalahan serius di antara mereka. Cuma kadang Penggugat pergi sendirian;
- Bahwa Saat ini Penggugat dan Tergugat sudah pisah rumah, Penggugat masih tinggal di rumah orang tua Penggugat, sementara Tergugat pulang ke rumah orang tua Tergugat;
- Bahwa Saksi tidak tahu apa yang menjadi penyebab Penggugat dan Tergugat pisah tempat tinggal, saksi hanya mengetahui bahwa mereka sudah pisah tempat tinggal;
- Bahwa Penggugat dan Tergugat Sepengetahuan saksi mulai pisah tempat tinggal sekitar 3 bulan yang lalu;
- Bahwa Selama pisah tempat tinggal tersebut, sepengetahuan saksi antara Penggugat dan Tergugat belum pernah saling mengunjungi;
- Bahwa Saksi kurang tahu persis apakah pihak keluarga sudah pernah berupaya merukunkan Penggugat dan Tergugat atau belum;

Bahwa, pada sidang selanjutnya pihak keluarga yang diberi waktu untuk

Disclaimer
Kepaniteraan Mahkamah Agung Republik Indonesia berusaha untuk selalu mencantumkan informasi paling kini dan akurat sebagai bentuk komitmen Mahkamah Agung untuk pelayanan publik, transparansi dan akuntabilitas pelaksanaan fungsi peradilan. Namun dalam hal-hal tertentu masih dimungkinkan terjadi permasalahan teknis terkait dengan akurasi dan keterkinian informasi yang kami sajikan, hal mana akan terus kami perbaiki dari waktu kewaktu.
Dalam hal Anda menemukan inakurasi informasi yang termuat pada situs ini atau informasi yang seharusnya ada, namun belum tersedia, maka harap segera hubungi Kepaniteraan Mahkamah Agung RI melalui :
Email : kepaniteraan@mahkamahagung.go.id   Telp : 021-384 3348 (ext.318)

melakukan usaha damai ternyata hasilnya tidak berhasil mendamaikan Penggugat dan Tergugat, selanjutnya Penggugat dalam kesimpulan tetap ingin bercerai, sedangkan Tergugat keberatan bercerai;

Bahwa, untuk menyingkat Putusan ini maka segala sesuatu yang terjadi di persidangan sebagaimana tercantum dalam berita acara sidang dianggap telah termuat seluruhnya dalam bagian tak terpisahkan dari Putusan ini;

PERTIMBANGAN HUKUM

Menimbang, bahwa maksud dan tujuan Cerai Gugat Penggugat adalah seperti tersebut diatas;

Menimbang, bahwa berdasarkan bukti P.2, maka harus dinyatakan telah terbukti menurut hukum bahwa Penggugat dan Tergugat telah terikat dalam perkawinan yang sah sejak [redacted]

Menimbang, bahwa oleh karena Penggugat dan Tergugat masih terikat sebagai suami istri yang sah, maka Penggugat sebagai *persona standi in judicio* memiliki *legal standing* sehingga Penggugat dapat dinyatakan sebagai pihak yang mempunyai hak dan berkepentingan dalam perkara ini ;

Menimbang, bahwa selanjutnya berdasarkan surat kuasa khusus yang didaftarkan di Kepaniteraan PA Ambarawa dalam hal ini Penggugat d telah memberikan kuasa kepada Advokat/Pengacara sebagaimana tersebut di atas, Oleh karenanya sebagiamana ketentuan Pasal 123 HIR Jo. Pasal 1 dan 4 Undang-undang Nomor 18 Tahun 2003 tentang Advokat, dengan demikian, Kuasa Hukum Penggugat sebagai kuasa hukum telah sah dan berhak untuk mendampingi dan/atau mewakili pemberi kuasa/ pihak in person dalam setiap persidangan;

Menimbang, bahwa atas keterangan Penggugat dalam surat gugatannya bahwa Penggugat dan Tergugat beragama Islam dan telah melakukan perkawinan secara islami, maka perkara ini merupakan kompetensi absolut Pengadilan Agama untuk memeriksa dan menyelesaikannya (vide Pasal 2 jo Pasal 49 ayat (1) Undang Undang Nomor 7 Tahun 1989 Tentang Peradilan Agama sebagai mana yang telah diubah yang kedua dengan Undang Undang Nomor 50 Tahun 2009) ;

Menimbang, bahwa berdasarkan surat gugatan dan bukti P.1, Penggugat

Disclaimer
Kepaniteraan Mahkamah Agung Republik Indonesia berusaha untuk selalu mencantumkan informasi paling kini dan akurat sebagai bentuk komitmen Mahkamah Agung untuk pelayanan publik, transparansi dan akuntabilitas pelaksanaan fungsi peradilan. Namun dalam hal-hal tertentu masih dimungkinkan terjadi permasalahan teknis terkait dengan akurasi dan keterkinian informasi yang kami sajikan, hal mana akan terus kami perbaiki dari waktu kewaktu.
Dalam hal Anda menemukan inakurasi informasi yang termuat pada situs ini atau informasi yang seharusnya ada, namun belum tersedia, maka harap segera hubungi Kepaniteraan Mahkamah Agung RI melalui :
Email : kepaniteraan@mahkamahagung.go.id Telp : 021-384 3348 (ext.318)

bertempat tinggal wilayah yurisdiksi Pengadilan Agama Ambarawa,maka perkara ini secara relatif juga merupakan kompetensi Pengadilan Agama Ambarawa untuk memeriksanya (vide Pasal 73 Undang Undang Nomor 7 Tahun 1989 Tentang Peradilan Agama sebagai mana yang telah diubah yang kedua dengan Undang Undang Nomor 50 Tahun 2009) jo Pasal 132 Kompilasi Hukum Islam) ;

Menimbang, bahwa Hakim telah berupaya mendamaikan pihak Penggugat agar rukun kembali dengan Tergugat, sebagaimana dikehendaki oleh ketentuan Pasal 130 HIR jo Pasal 82 Undang Undang Nomor 7 Tahun 1989 Tentang Peradilan Agama sebagaimana yang telah diubah terakhir dengan Undang Undang Nomor 50 Tahun 2009 jo Pasal 39 ayat (1) Undang Undang Nomor 1 Tahun 1974 jo Pasal 31 ayat (2) Peraturan Pemerintah Nomor 9 Tahun 1975 jo Pasal 143 Kompilasi Hukum Islam, tetapi tidak berhasil;

Menimbang, bahwa proses perdamaian selanjutnya diupayakan melalui Hakim Mediator bernama Ishak Lubis, S.Ag., sebagaimana yang dikehendaki Peraturan Mahkamah Agung Nomor 1 Tahun 2016, Tentang Prosedur Mediasi di Pengadilan, namun berdasarkan laporan hasil mediasi, mediator tidak berhasil mendamaikan;

Menimbang bahwa dalil yang diakui adalah pada posita angka 1, 2, 3 dan 5 mengenai pernikahan dan tempat tinggal setelah pernikahan, seta memiliki seorang anak dari hasil pernikahan;

Menimbang bahwa dalil yang diakui sebagian dengan sebab hal itu terjadi adalah pada posita angka 6, Tergugat menyatakan Penggugat kurang hati-hati dalam mengelola keuangan (boros). Adapun menyakiti badan jasmani penggugat dikarenakan Penggugat kalau dikasih tau atau dinasehati selalu berani namun semua berakhir dengan perdamaian seiring berjalannya waktu kurang lebih 3 tahun setelah Tergugat bekerja keras untuk mencari nafkah untuk keluarga, namun Penggugat selingkuh di vila tempat Penggugat dan Tergugat bekerja;

Menimbang bahwa selanjutnya Tergugat membantah dalil-dalil Penggugat mengenai Tergugat tidak memberi nafkah dan akibat pertengkaran pada posita angka 7 dan posita angka 9 sebagimana dalam duduk perkara dan

Disclaimer
Kepaniteraan Mahkamah Agung Republik Indonesia berusaha untuk selalu mencantumkan informasi paling kini dan akurat sebagai bentuk komitmen Mahkamah Agung untuk pelayanan publik, transparansi dan akuntabilitas pelaksanaan fungsi peradilan. Namun dalam hal-hal tertentu masih dimungkinkan terjadi permasalahan teknis terkait dengan akurasi dan keterkinian informasi yang kami sajikan, hal mana akan terus kami perbaiki dari waktu kewaktu.
Dalam hal Anda menemukan inakurasi informasi yang termuat pada situs ini atau informasi yang seharusnya ada, namun belum tersedia, maka harap segera hubungi Kepaniteraan Mahkamah Agung RI melalui :
Email : kepaniteraan@mahkamahagung.go.id   Telp : 021-384 3348 (ext.318)

selanjtnya Tergugat menyatakan keberatan bercerai;

Menimbang, terhadap jawaban Tergugat Tersebut, Penggugat telah mengajukan replik, selengkapnya termaktub dalam duduk perkara, *mutatis-mutandis* dianggap terulang utuh dalam pertimbangan ini, yang pada pokoknya tetap mempertahankan gugatan Penggugat serta menolak seluruh dalil-dalil bantahan Tergugat;

Menimbang, bahwa terhadap replik tersebut, Tergugat telah mengajukan duplik lisan, selengkapnya termaktub dalam duduk perkara, *mutatis-mutandis* dianggap terulang utuh dalam pertimbangan ini, yang pada pokoknya memohon kepada Hakim untuk menolak gugatan Penggugat untuk seluruhnya;

Menimbang, bahwa sebelum menentukan pokok permasalahan dalam gugatan Penggugat, perlu dikemukakan hal-hal sebagai berikut:

- Bahwa jawaban Tergugat yang membenarkan dalil-dalil Penggugat merupakan pengakuan murni, karenanya pengakuan atas dalil-dalil dimaksud menyebabkan dalil-dalil tersebut telah terbukti;
- Bahwa terhadap dalil-dalil yang tidak dijawab atau tidak diingkari secara tegas oleh Tergugat, maka hal tersebut dipandang sebagai pembenaran (pengakuan) secara diam-diam dan dalil-dalil dimaksud dianggap telah terbukti pula;
- Bahwa terhadap dalil-dalil yang dibantah secara tegas, maka dalil-dalil dimaksud harus dibuktikan oleh Penggugat dan sebaliknya Tergugat membuktikan bantahannya;

Menimbang, berdasarkan jawab menjawab (serta replik dan duplik) Pengadilan berpendapat bahwa pokok permasalahan utama dalam gugatan yang diajukan Penggugat dengan dalil sebagaimana tersebut di atas adalah : apakah rumah tangga Penggugat dan Tergugat telah terjadi pertengkaran yang wajar ataukah sudah tidak rukun dan tidak bisa didamaikan kembali?

Menimbang, bahwa oleh karena dalil-dalil Penggugat telah dibantah oleh Tergugat, maka sesuai ketentuan Pasal 163 HIR yang menyatakan "*Barangsiapa beranggapan mempunyai suatu hak atau suatu keadaan untuk menguatkan haknya atau menyangkal hak seseorang lain, harus membuktikan hak atau keadaan itu* ", maka selanjutnya kepada Penggugat dan Tergugat

Disclaimer
Kepaniteraan Mahkamah Agung Republik Indonesia berusaha untuk selalu mencantumkan informasi paling kini dan akurat sebagai bentuk komitmen Mahkamah Agung untuk pelayanan publik, transparansi dan akuntabilitas pelaksanaan fungsi peradilan. Namun dalam hal-hal tertentu masih dimungkinkan terjadi permasalahan teknis terkait dengan akurasi dan keterkinian informasi yang kami sajikan, hal mana akan terus kami perbaiki dari waktu kewaktu.
Dalam hal Anda menemukan inakurasi informasi yang termuat pada situs ini atau informasi yang seharusnya ada, namun belum tersedia, maka harap segera hubungi Kepaniteraan Mahkamah Agung RI melalui :
Email : kepaniteraan@mahkamahagung.go.id Telp : 021-384 3348 (ext.318)

diberi kesempatan untuk membuktikan dalil gugatan dan bantahan masing-masing dengan pembebanan pembuktian secara berimbang;

Menimbang, bahwa untuk membuktikan dalil-dalil gugatannya, Penggugat telah mengajukan alat bukti surat P.1, P.2, serta 2 orang saksi, sebagaimana selengkapnya dalam Duduk Perkara;

Menimbang, bahwa selanjutnya Hakim memberikan kesempatan yang sama kepada Tergugat untuk mengajukan bukti, dan di persidangan Tergugat mengajukan 2 orang saksi untuk menguatkan bantahannya;

Menimbang, bahwa alat bukti surat Penggugat secara prosedural memenuhi syarat pengajuan bukti tertulis, berupa fotokopi yang telah di nazegelen cukup dan setelah dicocokkan dengan aslinya ternyata sesuai, maka berdasarkan ketentuan Pasal 1888 KUH Perdata jo. 165 ayat (1) HetHerziene Indonesisch Reglement (HIR) jo. Pasal 2 ayat (1) huruf a, ayat (2), dan ayat (3), Pasal 10, dan Pasal 11 ayat (1) huruf a Undang-Undang Republik Indonesia Nomor 13 Tahun 1985 tentang Bea Meterai jo. Pasal 1 huruf a dan huruf f, serta Pasal 2 ayat (1) Peraturan Pemerintah Republik Indonesia Nomor 24 Tahun 2000 tentang Perubahan Tarif Bea Meterai dan Besarnya Batas Pengenaan Harga Nominal yang Dikenakan Bea Meterai, alat bukti surat tersebut secara formil telah memenuhi syarat sebagai alat bukti dan selanjutnya akan dipertimbangkan;

Menimbang, bahwa saksi-saksi Penggugat maupun saksi-saksi Tergugat tersebut diatas yang telah memberikan keterangan di bawah sumpah di muka persidangan secara terpisah dan saling bersesuaian dan saling melengkapi satu sama lain, Hakim mempertimbangkan sebagai berikut;

Menimbang, bahwa terhadap saksi-saksi Penggugat yang pada pokoknya dapat disimpulkan bahwa rumah tangga Penggugat dengan Tergugat terjadi pertengkaran dan perselisihan karena masalah ekonomi (dan Tergugat penah melakukan Tindakan Kekerasan Dalam Rumah tangga (KDRT). Saksi I Penggugat pernah melihat bekas pukulan yang dilakukan oleh Tergugat kepada Penggugat tersebut. Bahwa Akibat pertengkaran tersebut keduanya pisah rumah 4 bulan, Tergugat pulang ke rumah Orangtuanya;

Menimbang, terhadap saksi-saksi Tergugat yang pada pokoknya dapat

Disclaimer
Kepaniteraan Mahkamah Agung Republik Indonesia berusaha untuk selalu mencantumkan informasi paling kini dan akurat sebagai bentuk komitmen Mahkamah Agung untuk pelayanan publik, transparansi dan akuntabilitas pelaksanaan fungsi peradilan. Namun dalam hal-hal tertentu masih dimungkinkan terjadi permasalahan teknis terkait dengan akurasi dan keterkinian informasi yang kami sajikan, hal mana akan terus kami perbaiki dari waktu kewaktu.
Dalam hal Anda menemukan inakurasi informasi yang termuat pada situs ini atau informasi yang seharusnya ada, namun belum tersedia, maka harap segera hubungi Kepaniteraan Mahkamah Agung RI melalui :
Email : kepaniteraan@mahkamahagung.go.id   Telp : 021-384 3348 (ext.318)

disimpulkan bahwa Saat ini Penggugat dan Tergugat sudah pisah rumah, Penggugat tinggal di rumah orang tua Penggugat, sementara Tergugat tinggal di rumah Tergugat. Para Saksi Tergugat tidak tahu apa yang menjadi penyebab keduanya pisah tempat tinggal tersebut, namun Selama pisah tempat tinggal tersebut, sepengetahuan para Saksi, antara Tergugat pernah mengunjungi dan mengajak rukun Penggugat namun tidak berhasil;

Menimbang, bahwa terhadap saksi Tergugat Hakim menilai bahwa ketidaktahuan saksi Tergugat mengenai adanya pertengkaran antara Penggugat dengan Tergugat tidak serta merta menafikan secara mutlak terjadinya pertengkaran antara Penggugat dengan Tergugat, dikarenakan keterangan saksi-saksi Penggugat menyatakan sebaliknya, yang dalam hal ini hakim mengambil alih sebuah kaidah ushul sebagai bahan pertimbangan sebagaimana dinukil oleh Kamal bin as-Sayyid Salim dalam *Shahih Fiqh as-Sunnah wa Adillatuhu wa Taudhih Madzahib al-A'immah* (Juz ke-1, Kairo: Dar at-Taufiqiyyah li at-Turats, 2010 M, hal. 82) sebagai berikut:

أنَّ المُثْبِت مُقَدَّمٌ عَلى النَّافِى. أنَّ عَدَمَ العِلْمِ ليس عِلْماً بالعَدَم, وأنّ مَنْ عَلِمَ حُجَّةٌ عَلَى مَنْ لَمْ يَعْلَمْ

Artinya: "*Sesungguhnya ketetapan atas adanya sesuatu didahulukan daripada ketiadaannya. Bahwa ketidaktahuan tentang adanya sesuatu tidak serta merta menjadi fakta mengenai ketiadaannya, dan sesungguhnya orang yang tahu adalah hujjah atas orang yang tidak tahu*."

Menimbang, bahwa berdasarkan gugatan, jawaban, replik, duplik, serta bukti-bukti tertulis maupun saksi-saksi yang diajukan oleh Penggugat maupun Tergugat, Hakim telah menemukan fakta-fakta bahwa:

- Bahwa Penggugat telah melangsungkan pernikahan dengan Tergugat pada tanggal 27 Maret 2007 oleh Pejabat Kantor Urusan Agama Kecamatan [redacted] Kabupaten Semarang sebagaimana kutipan Akta Nikah Nomor: [redacted]
- Bahwa setelah menikah Penggugat dengan Tergugat bertempat tinggal di sebuah villa di [redacted] Kabupaten Semarang. Sampai tahun 2018. Kemudian setelah

Disclaimer
Kepaniteraan Mahkamah Agung Republik Indonesia berusaha untuk selalu mencantumkan informasi paling kini dan akurat sebagai bentuk komitmen Mahkamah Agung untuk pelayanan publik, transparansi dan akuntabilitas pelaksanaan fungsi peradilan. Namun dalam hal-hal tertentu masih dimungkinkan terjadi permasalahan teknis terkait dengan akurasi dan keterkinian informasi yang kami sajikan, hal mana akan terus kami perbaiki dari waktu kewaktu.
Dalam hal Anda menemukan inakurasi informasi yang termuat pada situs ini atau informasi yang seharusnya ada, namun belum tersedia, maka harap segera hubungi Kepaniteraan Mahkamah Agung RI melalui :
Email : kepaniteraan@mahkamahagung.go.id Telp : 021-384 3348 (ext.318)

itu Penggugat dan Tergugat pindah ke rumah saksi di [redacted] Kabupaten Semarang;

- Bahwa telah terjadi pertengkaran antara Penggugat dan Tergugat yang berakibat keduanya pisah tempat tinggal 3-4 bulan (sighat ta'lik talak angka 3) ;
- Bahwa pertengkaran Penggugat dan Tergugat disebabkan masalah ekonomi dan Tergugat penah melakukan Tindakan Kekerasan Dalam Rumah tangga (KDRT) sehingga menyakiti jasmani Penggugat (diakui Tergugat dengan sudah [redacted] atas hal tersebut) dalam hal ini hakim menilai sighat ta'lik talak angka 2 terpenuhi;
- Bahwa Penggugat dan Tergugat Pernah dirukunkan pihak keluarga, namun tidak berhasil;

Menimbang, bahwa berdasar uraian di atas Hakim menilai terlepas dari asal penyebab pertengkaran tersebut, dampak yang secara nyata ditimbulkan adalah bahwa Penggugat dengan Tergugat telah berpisah tempat tinggal dan tidak rukun sebagaimana layaknya suami istri rukun, saling menjalankan hak maupun kewajiban;

Menimbang, bahwa Hakim dan mediator juga telah berupaya mendamaikannya dalam setiap persidangan, akan tetapi tidak berhasil, maka cukup bagi pengadilan untuk memperoleh kesimpulan bahwa rumah tangga antara Penggugat dengan Tergugat telah retak (*broken marriage*) yang sulit diharapkan untuk bisa hidup rukun kembali. Keharmonisan rumah tangga hanya dapat terwujud apabila suami istri mempunyai komitmen yang sama untuk membina rumah tangga dengan baik. Dalam hal ini Penggugat sudah bersikukuh untuk bercerai dengan sikap yang ditunjukkan dari awal persidangan hingga tahap kesimpulan, maka Hakim menilai keadaan seperti ini sulit untuk hidup rukun dalam rumah tangganya, sehingga alternatif yang terbaik adalah memutuskan ikatan perkawinan guna menghindarkan dampak negatif yang lebih besar;

Menimbang, bahwa berdasarkan Yurisprudensi Mahkamah Agung RI No. 534 K/Pdt/1996, tanggal 18 Juni 1996 juga menyatakan bahwa dalam hal perceraian, tidak perlu dilihat dari siapa penyebab percekcokan atau salah satu

Disclaimer
Kepaniteraan Mahkamah Agung Republik Indonesia berusaha untuk selalu mencantumkan informasi paling kini dan akurat sebagai bentuk komitmen Mahkamah Agung untuk pelayanan publik, transparansi dan akuntabilitas pelaksanaan fungsi peradilan. Namun dalam hal-hal tertentu masih dimungkinkan terjadi permasalahan teknis terkait dengan akurasi dan keterkinian informasi yang kami sajikan, hal mana akan terus kami perbaiki dari waktu kewaktu.
Dalam hal Anda menemukan inakurasi informasi yang termuat pada situs ini atau informasi yang seharusnya ada, namun belum tersedia, maka harap segera hubungi Kepaniteraan Mahkamah Agung RI melalui :
Email : kepaniteraan@mahkamahagung.go.id Telp : 021-384 3348 (ext.318)

pihak telah meninggalkan pihak lain, tetapi yang perlu dilihat adalah perkawinan itu sendiri apakah perkawinan itu masih dapat dipertahankan lagi atau tidak.

Menimbang, bahwa pertimbangan di atas sejalan dengan pendapat pakar hukum Islam Dr. Musthofa As-Sibaiy sebagaimana termuat dalam kitabnya “*Al-Mar’ah Bainal Fiqh Wal Qanun*” halaman 100 yang menyatakan:

ولاخيرفي اجتماع بين متباغضين ومهما يكن اسباب هذا النزاع خطيرا كان اوتافها فان من الخير ان تنتهي العلاقة الزوجية بين هذين الزوجين

Artinya: *“Dan tidak ada kebaikan yang dapat diharapkan dalam mengumpulkan dua orang (suami-isteri) yang saling berselisih terlepas apakah sebab terjadinya perselisihan itu serius atau sepele namun kebaikan hanya dapat diterapkan dengan mengakhiri kehidupan rumah tangga antara suami-istri itu”*;

Menimbang, bahwa dengan adanya fakta-fakta tersebut diatas telah merupakan cukup bukti Tergugat telah melanggar sighat ta'lik talak nomor,2 dan 3 yang diucapkan sesudah akad nikah dan Penggugat telah menyatakan tidak rela atas sikap dan perlakuan Tergugat tersebut, dan untuk itu ia telah membayar iwadl sebesar Rp. 10.000,- (sepuluh ribu rupiah) dan dengan demikian syarat jatuhnya talak satu khul'i Tergugat terhadap Penggugat telah terwujud;

Menimbang, bahwa fakta hukum tersebut telah memenuhi norma hukum Islam yang terkandung dalam Kitab Kitab Syarqowi `alat Tahrir juz II halaman 302;

من علق طلاقا بصفة وقع بوجودها عملا بمقتضى اللفظ

Artinya: *“Barang siapa yang menggantungkan talak dengan suatu sifat, maka jatuhlah talak itu dengan terwujudnya sifat tersebut”;*

Menimbang, bahwa bukti tersebut telah memenuhi pasal 39 ayat 2 Undang-undang Nomor 1 tahun 1974 dan pasal 116 huruf (g) Kompilasi Hukum Islam, karena itu gugatan Penggugat telah memenuhi alasan hukum;

Disclaimer
Kepaniteraan Mahkamah Agung Republik Indonesia berusaha untuk selalu mencantumkan informasi paling kini dan akurat sebagai bentuk komitmen Mahkamah Agung untuk pelayanan publik, transparansi dan akuntabilitas pelaksanaan fungsi peradilan. Namun dalam hal-hal tertentu masih dimungkinkan terjadi permasalahan teknis terkait dengan akurasi dan keterkinian informasi yang kami sajikan, hal mana akan terus kami perbaiki dari waktu kewaktu.
Dalam hal Anda menemukan inakurasi informasi yang termuat pada situs ini atau informasi yang seharusnya ada, namun belum tersedia, maka harap segera hubungi Kepaniteraan Mahkamah Agung RI melalui :
Email : kepaniteraan@mahkamahagung.go.id Telp : 021-384 3348 (ext.318)

Menimbang, bahwa oleh karena perkara ini menyangkut bidang perkawinan, maka sesuai ketentuan pasal 89 ayat (1) Undang-undang Nomor 7 Tahun 1989 tentang Peradilan Agama sebagaimana telah dirubah dengan Undang-undang Nomor 3 Tahun 2006 dan Undang-undang Nomor 50 Tahun 2009, biaya perkara sepenuhnya dibebankan kepada Penggugat;

Mengingat semua peraturan perundang-undangan yang berlaku serta dalil syara' yang berkaitan dengan perkara ini;

M E N G A D I L I

1. Mengabulkan gugatan Penggugat;
2. Menyatakan syarat ta'lik talak terpenuhi;
3. Menjatuhkan talak satu khul'i Tergugat ([redacted]) terhadap Penggugat ([redacted]) dengan iwadl sejumlah Rp10.000,00(sepuluh ribu rupiah);
4. Membebankan kepada Penggugat untuk membayar biaya perkara sejumlah Rp341.000,00 (tiga ratus empat puluh satu ribu rupiah);

Demikian putusan tersebut diucapkan dalam sidang terbuka untuk umum pada hari Senin tanggal 28 Desember 2020 Masehi. bertepatan dengan tanggal 13 Jumadil Awwal 1442 Hijriyah. Oleh kami Rashif Imany, S.H.I., M.S.I. sebagai Hakim tunggal, dengan dibantu oleh Dr. M. Nur Hasan Latief, S.H.I., S.H., .M.M. sebagai Panitera Pengganti serta dihadiri Kuasa Penggugat dan Tergugat in person;

Hakim,

Rashif Imany, S.H.I., M.S.I.

Panitera Pengganti,

Dr. M. Nur Hasan Latief, S.H.I., S.H., .M.M.

Disclaimer
Kepaniteraan Mahkamah Agung Republik Indonesia berusaha untuk selalu mencantumkan informasi paling kini dan akurat sebagai bentuk komitmen Mahkamah Agung untuk pelayanan publik, transparansi dan akuntabilitas pelaksanaan fungsi peradilan. Namun dalam hal-hal tertentu masih dimungkinkan terjadi permasalahan teknis terkait dengan akurasi dan keterkinian informasi yang kami sajikan, hal mana akan terus kami perbaiki dari waktu kewaktu.
Dalam hal Anda menemukan inakurasi informasi yang termuat pada situs ini atau informasi yang seharusnya ada, namun belum tersedia, maka harap segera hubungi Kepaniteraan Mahkamah Agung RI melalui :
Email : kepaniteraan@mahkamahagung.go.id   Telp : 021-384 3348 (ext.318)

Perincian Biaya :

Biaya Pendaftaran	:	Rp	30.000,00
Biaya Proses	:	Rp	75.000,00
Biaya Pemanggilan	:	Rp	
Biaya PNBP	:	Rp	
Biaya Redaksi	:	Rp	
Biaya Materai	:	Rp	
Jumlah	:	Rp	

Disclaimer
Kepaniteraan Mahkamah Agung Republik Indonesia berusaha untuk selalu mencantumkan informasi paling kini dan akurat sebagai bentuk komitmen Mahkamah Agung untuk pelayanan publik, transparansi dan akuntabilitas pelaksanaan fungsi peradilan. Namun dalam hal-hal tertentu masih dimungkinkan terjadi permasalahan teknis terkait dengan akurasi dan keterkinian informasi yang kami sajikan, hal mana akan terus kami perbaiki dari waktu kewaktu.
Dalam hal Anda menemukan inakurasi informasi yang termuat pada situs ini atau informasi yang seharusnya ada, namun belum tersedia, maka harap segera hubungi Kepaniteraan Mahkamah Agung RI melalui :
Email : kepaniteraan@mahkamahagung.go.id   Telp : 021-384 3348 (ext.318)
