# Supplementary material for: Classification of divorce causes during the COVID-19 pandemic using convolutional neural networks
Source: PeerJ Comput Sci. 2022 Jun 30;8:e998. doi: 10.7717/peerj-cs.998 (PMC9299239; doi:10.7717/peerj-cs.998)
Supplement: Supplemental Information 2 [file peerj-cs-08-998-s002.zip › Kelalaian Dataset/Data ke-22.pdf]

PENETAPAN

Nomor 1606/Pdt.G/2020/PA.Bgr.

بسم الله الرحمن الرحيم

DEMI KEADILAN BERDASARKAN KETUHANAN YANG MAHA ESA

Pengadilan Agama Bogor yang memeriksa dan mengadili perkara tertentu pada tingkat pertama dalam sidang Majelis Hakim telah menjatuhkan penetepan sebagai berikut dalam perkara cerai gugat antara:

PENGGUGAT, lahir di Bogor, tanggal 19 Maret 1977 (umur 43 tahun), agama Islam, pendidikan D3, pekerjaan Mengurus Rumah Tangga, tempat tinggal di [redacted] Kelurahan Mulyaharja xxxxxxxxx Bogor Selatan, xxxx xxxxx, xxxxxxxx xxxx xxxxx, disebut sebagai **Penggugat**;

melawan

TERGUGAT, lahir di Bogor, tanggal 12 April 1975 (umur 45 tahun), agama Islam, pendidikan D3, pekerjaan xxxxxxxx xxxxxx, tempat tinggal di [redacted]xxxxxxxxx Ciasarua Kabupaten Bogor, xxxxxxxx xxxx xxxxx, disebut sebagai **Tergugat**;

Pengadilan Agama tersebut;
Telah membaca dan mempelajari berkas perkara;
Telah mendengar keterangan Penggugat di persidangan;

DUDUK PERKARA

Bahwa para Penggugat dengan surat gugatannya yang telah terdaftar di Kepaniteraan Pengadilan Agama Bogor Nomor 1606/Pdt.G/2020/PA.Bgr tanggal 02 Desember 2020 mengemukakan hal-hal sebagai berikut :

1. Bahwa Penggugat adalah istri sah dari Tergugat yang telah melangsungkan pernikahan pada hari Sabtu tanggal 03 September 2005 di hadapan Pejabat Pencatat Nikah Kantor Urusan Agama xxxxxxxxx Bogor

Disclaimer
Kepaniteraan Mahkamah Agung Republik Indonesia berusaha untuk selalu mencantumkan informasi paling kini dan akurat sebagai bentuk komitmen Mahkamah Agung untuk pelayanan publik, transparansi dan akuntabilitas pelaksanaan fungsi peradilan. Namun dalam hal-hal tertentu masih dimungkinkan terjadi permasalahan teknis terkait dengan akurasi dan keterkinian informasi yang kami sajikan, hal mana akan terus kami perbaiki dari waktu kewaktu.
Dalam hal Anda menemukan inakurasi informasi yang termuat pada situs ini atau informasi yang seharusnya ada, namun belum tersedia, maka harap segera hubungi Kepaniteraan Mahkamah Agung RI melalui :
Email : kepaniteraan@mahkamahagung.go.id Telp : 021-384 3348 (ext.318)

Barat , xxxx xxxxx, xxxxxxxx xxxx xxxxx, sebagaimana terbukti dalam Buku Kutipan Akta Nikah Nomor [redacted] tertanggal 5 September 2005;

2. Bahwa pada saat menikah Penggugat berstatus perawan dan Tergugat berstatus perjaka;

3. Bahwa setelah menikah Penggugat dengan Tergugat tinggal bersama terakhir di Komp. [redacted] Kelurahan Mulyaharja xxxxxxxxx Bogor Selatan, xxxx xxxxx, xxxxxxxx xxxx xxxxx;

4. Bahwa selama hidup berumah tangga antara Penggugat dengan Tergugat sudah berhubungan sebagaimana layaknya suami isteri dan sudah dikaruniai 2 orang anak yang bernama:

4.1. [redacted], perempuan, lahir di Bogor, 16 Mei 2007;

4.2. [redacted], perempuan, lahir di Bogor, 28 November 2009;

5. Bahwa semula rumah tangga antara Penggugat dengan Tergugat berjalan rukun dan harmonis namun sekitar awal tahun 2011 rumah tangga dirasakan mulai goyah disebabkan oleh hal-hal sebagai berikut:

5.1. Tergugat memiliki hubungan khusus dengan wanita idaman lain, yang diketahui langsung oleh Penggugat;

5.2. Tergugat sering mengucapkan kata-kata kasar kepada Penggugat, seperti kata hinaan;

5.3. Tergugat pernah dua kali melakukan kekerasan fisik kepada Penggugat, seperti dipukul

6. Bahwa puncak perselisihan antara Penggugat dengan Tergugat terjadi sekitar petengahan bulan November tahun 2020 yang mana Tergugat pergi meninggalkan kediaman terakhir, sehingga sejak itu antara Penggugat dengan Tergugat sudah pisah rumah dan tidak ada hubungan seperti layaknya suami istri lagi;

7. Bahwa Penggugat yakin tidak ada lagi harapan untuk meneruskan rumah tangga dengan Tergugat karena tujuan perkawinan untuk membina rumah tangga yang Sakinah, Mawaddah wa Rahmah sudah tidak terwujud

Disclaimer
Kepaniteraan Mahkamah Agung Republik Indonesia berusaha untuk selalu mencantumkan informasi paling kini dan akurat sebagai bentuk komitmen Mahkamah Agung untuk pelayanan publik, transparansi dan akuntabilitas pelaksanaan fungsi peradilan. Namun dalam hal-hal tertentu masih dimungkinkan terjadi permasalahan teknis terkait dengan akurasi dan keterkinian informasi yang kami sajikan, hal mana akan terus kami perbaiki dari waktu kewaktu.
Dalam hal Anda menemukan inakurasi informasi yang termuat pada situs ini atau informasi yang seharusnya ada, namun belum tersedia, maka harap segera hubungi Kepaniteraan Mahkamah Agung RI melalui :
Email : kepaniteraan@mahkamahagung.go.id Telp : 021-384 3348 (ext.318)

lagi sebagaimana yang diamanatkan oleh Undang-undang Nomor 1 Tahun 1974 tentang Perkawinan dan Instruksi Presiden Nomor 1 Tahun 1991 tentang Kompilasi Hukum Islam, sehingga sangat beralasan apabila gugatan ini dikabulkan;

8. Bahwa Penggugat sanggup membayar biaya yang timbul dari perkara ini;

Berdasarkan alasan-alasan tersebut, Penggugat mohon kepada Ketua Pengadilan Agama Bogor Cq. Majelis Hakim yang memeriksa dan mengadili perkara ini untuk menjatuhkan putusan sebagai berikut:

Primer:

1. Mengabulkan gugatan Penggugat;

2. Menjatuhkan talak satu ba'in shugro Tergugat (**TERGUGAT**) kepada Penggugat (**PENGGUGAT**);

3. Menetapkan biaya perkara menurut hukum;

Subsider:

Apabila Majelis Hakim berpendapat lain mohon keputusan yang seadil-adilnya;

Bahwa pada hari persidangan yang telah ditetapkan Penggugat dan Tergugat hadir di persidangan;

Bahwa Majelis Hakim telah berupaya keras mendamaikan Penggugat dan Tergugat dengan memberikan nasehat dan pandangan agar Penggugat dan Tergugat mau berdamai dan rukun kembali dalam rumah tangga mereka, akan tetapi tidak berhasil, Penggugat tetap pada pendirian ingin bercerai dengan Tergugat, walaupun Tergugat masih berkeinginan untuk mempertahankan keutuhan rumah tangga dengan Penggugat;

Bahwa untuk memaksimalkan upaya perdamaian tersebut, maka kedua belah pihak diwajibkan menempuh mediasi dan Ketua Majelis telah memerintahkan Penggugat dan Tergugat untuk menempuh upaya mediasi Untuk itu, Ketua Majelis telah menunjuk salah seorang mediator pilihan Penggugat dan Tergugat yang bernama H. Mumu, S.H., M.H. dengan Penetapan Nomor 1606/Pdt.G/2020/PA.Bgr., tanggal 14 Desember 2020. dan berdasarkan laporan Mediator pada tanggal 14 Desember 2020, mediasi dinyatakan berhasil;

Disclaimer
Kepaniteraan Mahkamah Agung Republik Indonesia berusaha untuk selalu mencantumkan informasi paling kini dan akurat sebagai bentuk komitmen Mahkamah Agung untuk pelayanan publik, transparansi dan akuntabilitas pelaksanaan fungsi peradilan. Namun dalam hal-hal tertentu masih dimungkinkan terjadi permasalahan teknis terkait dengan akurasi dan keterkinian informasi yang kami sajikan, hal mana akan terus kami perbaiki dari waktu kewaktu.
Dalam hal Anda menemukan inakurasi informasi yang termuat pada situs ini atau informasi yang seharusnya ada, namun belum tersedia, maka harap segera hubungi Kepaniteraan Mahkamah Agung RI melalui :
Email : kepaniteraan@mahkamahagung.go.id Telp : 021-384 3348 (ext.318)

Bahwa di muka persidangan Penggugat menyatakan ingin mencabut gugatannya;

Bahwa karena pencabutan perkara tersebut dilakukan Penggugat sebelum adanya pemeriksaan perkara ini dan atas kesadarannya sendiri, maka pencabutan perkara tersebut dapat dipertimbangkan;

Bahwa untuk mempersingkat uraian Penetapan ini ditunjuk kepada hal-hal sebagaimana tercantum dalam berita acara persidangan perkara ini;

PERTIMBANGAN HUKUM

Menimbang, bahwa maksud dan tujuan gugatan Penggugat adalah seperti diuraikan tersebut di atas;

Menimbang, bahwa Penggugat di muka persidangan menyatakan mencabut gugatan Penggugat;

Menimbang, bahwa pernyataan Penggugat mencabut gugatannya sebelum gugatan tersebut dibacakan dipersidangan oleh karena itu pencabutan perkara ini dapat dikabulkan sebagaimana maksud Pasal 54 Undang-Undang Nomor 7 Tahun 1989 Tentang Peradilan Agama, sebagaimana dalam Pasal 271 dan Pasal 272 Rv. dapat diberlakukan di lingkungan Pengadilan Agama;

Menimbang bahwa berdasarkan uraian di atas, maka perkara ini harus ditetapkan telah selesai karena dicabut oleh Penggugat;

Menimbang, bahwa gugatan ini termasuk bidang perkawinan, sesuai dengan Pasal 89 Ayat (1) Undang-Undang Nomor 7 Tahun 1989 Tentang Peradilan Agama sebagaimana telah diubah dan ditambah oleh Undang Undang Nomor 3 Tahun 2006 dan sebagaimana diubah kedua dengan Undang-Undang Nomor 50 Tahun 2009, maka biaya perkara dibebankan kepada Penggugat;

Mengingat, segala ketentuan perundang-undangan yang berlaku dan hukum Islam yang berkaitan dengan perkara ini;

MENETAPKAN

1. Mengabulkan permohonan pencabutan perkara Nomor 1606/Pdt.G/2020/ PA.Bgr., dari Penggugat;

Disclaimer
Kepaniteraan Mahkamah Agung Republik Indonesia berusaha untuk selalu mencantumkan informasi paling kini dan akurat sebagai bentuk komitmen Mahkamah Agung untuk pelayanan publik, transparansi dan akuntabilitas pelaksanaan fungsi peradilan. Namun dalam hal-hal tertentu masih dimungkinkan terjadi permasalahan teknis terkait dengan akurasi dan keterkinian informasi yang kami sajikan, hal mana akan terus kami perbaiki dari waktu kewaktu.
Dalam hal Anda menemukan inakurasi informasi yang termuat pada situs ini atau informasi yang seharusnya ada, namun belum tersedia, maka harap segera hubungi Kepaniteraan Mahkamah Agung RI melalui :
Email : kepaniteraan@mahkamahagung.go.id   Telp : 021-384 3348 (ext.318)

2. Memerintahkan Panitera untuk mencatat pencabutan perkara tersebut dalam register perkara;

3. Membebankan Penggugat untuk membayar biaya perkara sejumlah Rp. 416.000,- (empat ratus enam belas ribu rupiah);

Demikian ditetapkan dalam permusyawaratan Majelis Hakim Pengadilan Agama Bogor pada hari Senin tanggal 28 Desember 2020 Miladiyah bertepatan dengan tanggal 13 Jumadil Awwal 1442 Hijriyah, oleh Dra. Sulfita Netti, S.H., M.H., sebagai Ketua Majelis, Drs. Sangidin, S.H., M.H., dan Dra. Hj. Andi Bungawali, M.H., masing-masing sebagai Hakim Anggota, penetapan tersebut pada hari itu juga diucapkan dalam sidang terbuka untuk umum oleh Ketua Majelis di dampingi oleh Hakim Anggota tersebut dan dibantu oleh Raisul Wadhifuddin, S.H. sebagai Panitera Pengganti, dihadiri oleh Penggugat dan Tergugat;

Ketua Majelis,

ttd

Dra. Sulfita Netti, S.H., M.H.

Hakim Anggota	Hakim Anggota
ttd	ttd
**Drs. Sangidin, S.H., M.H.**	**Dra. Hj. Andi Bungawali, M.H.**

Panitera Pengganti,

ttd

Raisul Wadhifuddin, S.H.

Perincian biaya :

1. Pendaftaran : Rp 30.000,-
2. Biaya Proses : Rp 50.000,-
3. Panggilan : Rp 290.000,-
4. PNBP Panggilan : Rp. 30.000,-
5. Redaksi : Rp 10.000,-

Disclaimer
Kepaniteraan Mahkamah Agung Republik Indonesia berusaha untuk selalu mencantumkan informasi paling kini dan akurat sebagai bentuk komitmen Mahkamah Agung untuk pelayanan publik, transparansi dan akuntabilitas pelaksanaan fungsi peradilan. Namun dalam hal-hal tertentu masih dimungkinkan terjadi permasalahan teknis terkait dengan akurasi dan keterkinian informasi yang kami sajikan, hal mana akan terus kami perbaiki dari waktu kewaktu.
Dalam hal Anda menemukan inakurasi informasi yang termuat pada situs ini atau informasi yang seharusnya ada, namun belum tersedia, maka harap segera hubungi Kepaniteraan Mahkamah Agung RI melalui :
Email : kepaniteraan@mahkamahagung.go.id Telp : 021-384 3348 (ext.318)

6. Meterai : Rp 6.000,- +

J u m l a h : Rp 416.000,- (empat ratus tiga puluh enam ribu rupiah).

Disclaimer
Kepaniteraan Mahkamah Agung Republik Indonesia berusaha untuk selalu mencantumkan informasi paling kini dan akurat sebagai bentuk komitmen Mahkamah Agung untuk pelayanan publik, transparansi dan akuntabilitas pelaksanaan fungsi peradilan. Namun dalam hal-hal tertentu masih dimungkinkan terjadi permasalahan teknis terkait dengan akurasi dan keterkinian informasi yang kami sajikan, hal mana akan terus kami perbaiki dari waktu kewaktu.
Dalam hal Anda menemukan inakurasi informasi yang termuat pada situs ini atau informasi yang seharusnya ada, namun belum tersedia, maka harap segera hubungi Kepaniteraan Mahkamah Agung RI melalui :
Email : kepaniteraan@mahkamahagung.go.id Telp : 021-384 3348 (ext.318)
