# Supplementary material for: Classification of divorce causes during the COVID-19 pandemic using convolutional neural networks
Source: PeerJ Comput Sci. 2022 Jun 30;8:e998. doi: 10.7717/peerj-cs.998 (PMC9299239; doi:10.7717/peerj-cs.998)
Supplement: Supplemental Information 2 [file peerj-cs-08-998-s002.zip › Kelalaian Dataset/Data ke-23.pdf]

P U T U S A N

Nomor 4253/Pdt.G/2020/PA.Smdg

بِسْــــــــــــــــــمِ اللّٰهِ الرَّحْمٰنِ الرَّحِيْمِ

DEMI KEADILAN BERDASARKAN KETUHANAN YANG MAHA ESA

Pengadilan Agama Sumedang yang memeriksa dan mengadili perkara tertentu pada tingkat pertama dalam sidang majelis telah menjatuhkan putusan perkara Cerai Talak antara:

Aep Itang bin Rukma, umur 42 tahun, agama Islam, pekerjaan Wiraswasta, pendidikan SD, tempat kediaman di Dusun Bakom RT 06 RW 07 Desa Linggajaya Kecamatan Paseh Kabupaten Sumedang sebagai **Pemohon**;

melawan

Siti Rodiah binti Cahya, umur 36 tahun, agama Islam, pendidikan SLTP, pekerjaan Ibu Rumah Tangga, tempat kediaman di di rumah orangtuanya a.n Bapak Cahya / ibu Eros di Dusun Cileuksa RT 01 RW 04 Desa Legok Kaler Kecamatan Paseh Kabupaten Sumedang; sebagai **Termohon**;

Pengadilan Agama tersebut;

Telah mempelajari surat-surat yang berkaitan dengan perkara ini;

Telah mendengar keterangan Pemohon serta para saksi di muka sidang;

DUDUK PERKARA

Bahwa Pemohon dalam surat permohonannya tanggal 10 Desember 2020 telah mengajukan permohonan, yang telah didaftar di Kepaniteraan Pengadilan Agama Sumedang, dengan Nomor 4253/Pdt.G/2020/PA.Smdg, tanggal 10 Desember 2020, dengan dalil-dalil sebagai berikut:

1. Bahwa Pemohon dan Termohon adalah suami isteri sah menikah pada tanggal 20 Juli 2005 berdasarkan Kutipan Akta Nikah dari KUA KUA Kecamatan Paseh Kabupaten Sumedang, No: 181/30/VII/2005. Tertanggal 20 Juli 2005;

Disclaimer
Kepaniteraan Mahkamah Agung Republik Indonesia berusaha untuk selalu mencantumkan informasi paling kini dan akurat sebagai bentuk komitmen Mahkamah Agung untuk pelayanan publik, transparansi dan akuntabilitas pelaksanaan fungsi peradilan. Namun dalam hal-hal tertentu masih dimungkinkan terjadi permasalahan teknis terkait dengan akurasi dan keterkinian informasi yang kami sajikan, hal mana akan terus kami perbaiki dari waktu kewaktu.
Dalam hal Anda menemukan inakurasi informasi yang termuat pada situs ini atau informasi yang seharusnya ada, namun belum tersedia, maka harap segera hubungi Kepaniteraan Mahkamah Agung RI melalui :
Email : kepaniteraan@mahkamahagung.go.id Telp : 021-384 3348 (ext.318)

2. Bahwa sebelum membina rumahtangga, Pemohon berstatus Jejaka begitupun Termohon berstatus Perawan;
3. Bahwa antara Pemohon dan Termohon terakhir berumah tangga tinggal di rumah kediaman orangtua Penggugat di Dusun Cileuksa RT 01 RW 04 Desa Legok Kaler Kecamatan Paseh Kabupaten Sumedang dan telah bergaul baik sebagaimana layaknya suami isteri, dan telah dikaruniai 3 (tiga) anak yang masing-masing bernama: Nita Nur Oktavia (berusia 14 tahun), Ropik Tajud Pajari (berusia 6 tahun), dan Deden Rehan Al Parez (berusia 5 bulan);
4. Bahwa antara Pemohon dan Termohon sejak **bulan September 2018** sudah tidak harmonis lagi dalam menjalani hidup berumah tangga dikarenakan antara Pemohon dengan Termohon sering terjadi perselisihan dan pertengkaran terus-menerus yang disebabkan:
5. Bahwa Termohon sering berhutang kepada orang lain tanpa bermusyawarah dengan Pemohon dan penggunaan uangnya bukan digunakan untuk kepentingan bersama, sehingga banyak orang yang menagih hutang kepada Pemohon, dan hal tersebut menjadi pemicu rumah tangga sering terlibat pertengkaran.
6. Bahwa Termohon kurang menghargai Pemohon sebagai seorang suami yang sah, yakni Termohon apabila diberi nasihat sering tidak menerimanya, bahkan sering membantah dan melawan perkataan Pemohon dalam rangka membina rumah tangga yang lebih baik , sehingga keadaan rumah tangga dirasa Pemohon sudah tidak nyaman/tentram.
7. Bahwa keretakan rumah tangga antara Pemohon dan Termohon telah berlangsung lama. Pemohon berusaha sabar, tetapi Termohon tidak berusaha berubah dan merubah sifatnya. Oleh karena itu Pemohon merasa tidak nyaman berumah tangga dengan Termohon. Puncaknya pada **bulan Februari 2020**, antara Pemohon dan Termohon terjadi pertengkaran besar dan **Pemohon pergi meninggalkan rumah kediaman orangtua Termohon**, sejak itu Pemohon dengan Termohon pisah tempat tinggal dan tidak lagi bergaul layaknya suami isteri. **Dimana**

Disclaimer
Kepaniteraan Mahkamah Agung Republik Indonesia berusaha untuk selalu mencantumkan informasi paling kini dan akurat sebagai bentuk komitmen Mahkamah Agung untuk pelayanan publik, transparansi dan akuntabilitas pelaksanaan fungsi peradilan. Namun dalam hal-hal tertentu masih dimungkinkan terjadi permasalahan teknis terkait dengan akurasi dan keterkinian informasi yang kami sajikan, hal mana akan terus kami perbaiki dari waktu kewaktu.
Dalam hal Anda menemukan inakurasi informasi yang termuat pada situs ini atau informasi yang seharusnya ada, namun belum tersedia, maka harap segera hubungi Kepaniteraan Mahkamah Agung RI melalui :
Email : kepaniteraan@mahkamahagung.go.id Telp : 021-384 3348 (ext.318)

sekarang ini Pemohon tinggal di kediaman orangtua Pemohon dan **Termohon tinggal di rumah kediaman orangtua Termohon;**

8. Bahwa keretakan rumah tangga Pemohon dan Termohon sudah pernah didamaikan oleh keluarga, tetapi tidak berhasil.
9. Bahwa Permohonan Pemohon tersebut telah memenuhi syarat sesuai ketentuan Pasal 19 huruf (f) Peraturan Pemerintah Nomor 9 Tahun 1975 jo. Pasal 116 huruf (f) Kompilasi Hukum Islam.
10. Bahwa atas permasalahan tersebut di atas Pemohon sudah tidak sanggup lagi untuk mempertahankan perkawinan ini, oleh karena itu Pemohon telah berketetapan hati untuk bercerai dengan Termohon.
11.

Berdasarkan uraian tersebut diatas, Pemohon mohon kepada Ketua Pengadilan Agama Sumedang untuk memeriksa perkara ini, dan menjatuhkan putusan sebagai berikut :

1. Menerima dan mengabulkan Permohonan Ikrar Talak Pemohon.
2. Memberikan izin kepada Pemohon (Aep Itang bin Rukma) untuk mengikrarkan Talak Satu Raj'i kepada Termohon (Siti Rodiah binti Cahya) di depan sidang Pengadilan Agama Kelas IA Sumedang.
3. Menetapkan biaya perkara menurut hukum.
4.

Atau Majelis Hakim berpendapat lain mohon putusan yang seadil-adilnya (ex aequo et bono);

Bahwa pada persidangan yang telah ditetapkan Pemohon telah datang menghadap ke muka sidang, sedangkan Termohon tidak datang menghadap di persidangan dan tidak menyuruh orang lain untuk menghadap sebagai wakil/kuasa yang sah, meskipun berdasarkan surat panggilan (relass) Nomor 4253/Pdt.G/2020/PA.Smdg, **tanggal ...** dan **tanggal ...**, yang dibacakan di persidangan, Termohon telah dipanggil secara resmi dan patut, sedangkan tidak ternyata bahwa tidak datangnya itu disebabkan suatu alasan yang sah;

Bahwa majelis hakim telah menasehati Pemohon agar berpikir untuk tidak bercerai dengan Termohon, tetapi Pemohon tetap pada dalil-dalil

Disclaimer
Kepaniteraan Mahkamah Agung Republik Indonesia berusaha untuk selalu mencantumkan informasi paling kini dan akurat sebagai bentuk komitmen Mahkamah Agung untuk pelayanan publik, transparansi dan akuntabilitas pelaksanaan fungsi peradilan. Namun dalam hal-hal tertentu masih dimungkinkan terjadi permasalahan teknis terkait dengan akurasi dan keterkinian informasi yang kami sajikan, hal mana akan terus kami perbaiki dari waktu kewaktu.
Dalam hal Anda menemukan inakurasi informasi yang termuat pada situs ini atau informasi yang seharusnya ada, namun belum tersedia, maka harap segera hubungi Kepaniteraan Mahkamah Agung RI melalui :
Email : kepaniteraan@mahkamahagung.go.id   Telp : 021-384 3348 (ext.318)

permohonannya untuk bercerai dengan Termohon ;

Bahwa perkara ini tidak dapat dimediasi karena Termohon tidak pernah datang menghadap meskipun telah dipanggil secara resmi dan patut, selanjutnya dimulai pemeriksaan dengan membacakan surat permohonan Pemohon yang maksud dan isinya tetap dipertahankan oleh Pemohon ;

Bahwa karena Termohon tidak hadir maka pemeriksaan dilanjutkan tanpa jawaban dari Termohon;

Bahwa untuk menguatkan dalil-dalil permohonannya, Pemohon telah mengajukan alat bukti tertulis berupa: Fotocopi Kutipan Akta Nikah, Nomor **181/30/VII/2005, tertanggal 20 Juli 2005** yang diterbitkan oleh Kantor Urusan Agama KUA Kecamatan Paseh Kabupaten Sumedang (Bukti P.1);

Bahwa selain alat bukti tertulis, Pemohon juga telah menguatkan dalil-dalil permohonannya dengan bukti saksi sebagai berikut :

Saksi I

Tata bin Lili, umur 51 tahun, agama Islam, pekerjaan Pedagang, tempat tinggal di Dusun Galudra RT 01 RW 04 Desa Galudra Kecamatan Cimalaka Kabupaten Sumedang;

Saksi telah memberikan keterangan di bawah sumpahnya yang pada pokoknya sebagai berikut:

- Bahwa saksi kenal kepada Pemohon dan Termohon karena saksi adalah **Paman Pemohon** Pemohon;
- Bahwa antara Pemohon dengan Termohon telah hidup rukun sebagaimana layaknya suami istri dan telah dikaruniai 3 (tiga) anak yang masing-masing bernama: Nita Nur Oktavia (berusia 14 tahun), Ropik Tajud Pajari (berusia 6 tahun), dan Deden Rehan Al Parez (berusia 5 bulan) ... ;
- Bahwa sepengetahuan saksi hubungan rumah tangga Pemohon dengan Termohon mulai tidak harmonis sejak **bulan September 2018**, sering terjadi perselisihan dan pertengkaran;
- Bahwa saksi ... ;
- Bahwa Termohon sering meminjam uang kepada orang lain tanpa bermusyawarah dengan Pemohon dan penggunaanya bukan untuk

Disclaimer
Kepaniteraan Mahkamah Agung Republik Indonesia berusaha untuk selalu mencantumkan informasi paling kini dan akurat sebagai bentuk komitmen Mahkamah Agung untuk pelayanan publik, transparansi dan akuntabilitas pelaksanaan fungsi peradilan. Namun dalam hal-hal tertentu masih dimungkinkan terjadi permasalahan teknis terkait dengan akurasi dan keterkinian informasi yang kami sajikan, hal mana akan terus kami perbaiki dari waktu kewaktu.
Dalam hal Anda menemukan inakurasi informasi yang termuat pada situs ini atau informasi yang seharusnya ada, namun belum tersedia, maka harap segera hubungi Kepaniteraan Mahkamah Agung RI melalui :
Email : kepaniteraan@mahkamahagung.go.id Telp : 021-384 3348 (ext.318)

kepentingan bersama, tahu tahu banyak yang menagih utang kepada Pemohon;

- Bahwa Pemohon dan Termohon saat ini telah berpisah tempat tinggal sejak bulan September 2018 sampai dengan sekarang;
- Bahwa setahu saksi pihak keluarga Pemohon telah cukup menasihati Pemohon agar tetap bersabar dan dapat mempertahankan keutuhan rumah tangganya dengan Termohon namun Pemohon tetap bersikeras ingin bercerai dengan Termohon;

Saksi II

Imih binti Jasam, umur 64 tahun, agama Islam, pekerjaan Buruh, tempat tinggal di Dusun Cibunut RT 01 RW 04 Desa Cikole Kecamatan Cimalaka Kabupaten Sumedang;

Saksi telah memberikan keterangan di bawah sumpahnya yang pada pokoknya sebagai berikut:

- Bahwa saksi kenal kepada Pemohon dan Termohon karena saksi adalah **Uwa Pemohon** Pemohon;
- Bahwa antara Pemohon dengan Termohon telah hidup rukun sebagaimana layaknya suami istri dan telah dikaruniai 3 (tiga) anak yang masing-masing bernama: Nita Nur Oktavia (berusia 14 tahun), Ropik Tajud Pajari (berusia 6 tahun), dan Deden Rehan Al Parez (berusia 5 bulan) ... ;
- Bahwa sepengetahuan saksi hubungan rumah tangga Pemohon dengan Termohon mulai tidak lagi harmonis sejak bulan September 2018, sering terjadi perselisihan dan pertengkaran;
- Bahwa saksi tidak pernah melihat langsung antara Pemohon dengan Termohon bertengkar, hanya saja Pemohon bercerita kepada saya perihal masalah rumah tangganya ;
- Bahwa saksi Termohonn sering meminjam uang kepada oranglain tanpa seizin Pemohon, sehingga banyak yang menagih utang yang tidak jelas peruntukannya;
- Bahwa Pemohon dan Termohon saat ini telah berpisah tempat tinggal sejak bulan September 2018 sampai dengan sekarang;
- Bahwa pihak keluarga Pemohon telah cukup menasihati Pemohon agar

Disclaimer
Kepaniteraan Mahkamah Agung Republik Indonesia berusaha untuk selalu mencantumkan informasi paling kini dan akurat sebagai bentuk komitmen Mahkamah Agung untuk pelayanan publik, transparansi dan akuntabilitas pelaksanaan fungsi peradilan. Namun dalam hal-hal tertentu masih dimungkinkan terjadi permasalahan teknis terkait dengan akurasi dan keterkinian informasi yang kami sajikan, hal mana akan terus kami perbaiki dari waktu kewaktu.
Dalam hal Anda menemukan inakurasi informasi yang termuat pada situs ini atau informasi yang seharusnya ada, namun belum tersedia, maka harap segera hubungi Kepaniteraan Mahkamah Agung RI melalui :
Email : kepaniteraan@mahkamahagung.go.id Telp : 021-384 3348 (ext.318)

tetap bersabar dan dapat mempertahankan keutuhan rumah tangganya dengan Termohon namun Pemohon tetap bersikeras ingin bercerai dengan Termohon;

Atas keterangan para saksi tersebut Pemohon tidak lagi menyampaikan tanggapannya, dan Pemohon menyatakan tidak akan mengajukan bukti lain lagi;

Bahwa Pemohon dalam kesimpulannya secara lisan menyatakan tetap pada permohonannya dan mohon putusan;

Selanjutnya untuk singkatnya uraian putusan ini, maka semua hal yang termuat dalam berita acara sidang ini merupakan bagian yang tidak terpisahkan dari putusan ini;

PERTIMBANGAN HUKUM

Menimbang, bahwa maksud dan tujuan permohonan Pemohon permohonan Pemohon adalah sebagaimana terurai di atas;

Menimbang, bahwa yang menjadi pokok masalah dalam permohonan Pemohon, pada intinya Pemohon mohon agar diizinkan untuk berikrar menjatuhkan talak satu terhadap Termohon di depan sidang Pengadilan Agama Sumedang dengan alasan karena dalam rumah tangga antara Pemohon dengan Termohon telah terjadi perselisihan dalam rumah tangga yang disebabkan ...;

Menimbang, bahwa ternyata Termohon, meskipun dipanggil secara resmi dan patut, tidak datang menghadap di muka sidang dan pula tidak ternyata bahwa tidak datangnya itu disebabkan suatu halangan yang sah;

Menimbang, bahwa Termohon yang dipanggil secara resmi dan patut akan tetapi tidak datang menghadap harus dinyatakan tidak hadir dan permohonan tersebut diperiksa secara verstek;

Menimbang, bahwa oleh karena itu, maka berdasarkan ketentuan Pasal 125 ayat (1) HIR putusan atas perkara ini dapat dijatuhkan tanpa hadirnya Termohon (verstek);

Menimbang, bahwa untuk membuktikan dalil-dalil permohonannya, Pemohon telah mengajukan alat bukti surat serta 2 (dua) orang saksi;

Menimbang, bahwa berdasarkan bukti P.1 yang merupakan akta autentik

Disclaimer
Kepaniteraan Mahkamah Agung Republik Indonesia berusaha untuk selalu mencantumkan informasi paling kini dan akurat sebagai bentuk komitmen Mahkamah Agung untuk pelayanan publik, transparansi dan akuntabilitas pelaksanaan fungsi peradilan. Namun dalam hal-hal tertentu masih dimungkinkan terjadi permasalahan teknis terkait dengan akurasi dan keterkinian informasi yang kami sajikan, hal mana akan terus kami perbaiki dari waktu kewaktu.
Dalam hal Anda menemukan inakurasi informasi yang termuat pada situs ini atau informasi yang seharusnya ada, namun belum tersedia, maka harap segera hubungi Kepaniteraan Mahkamah Agung RI melalui :
Email : kepaniteraan@mahkamahagung.go.id Telp : 021-384 3348 (ext.318)

dimana berdasarkan pasal 7 ayat (1) Kompilasi Hukum Islam akta nikah adalah merupakan satu-satunya bukti pernikahan, maka harus dinyatakan terbukti bahwa antara Pemohon dengan Termohon telah terikat dalam perkawinan yang sah, oleh karenanya Pemohon dan Termohon adalah pihak yang berkepentingan langsung dalam perkara ini;

Pertimbangan saksi I yang melihat Pertengkaran

Menimbang, bahwa saksi Pemohon yang pertama **(Tata bin Lili, umur 51 tahun, agama Islam, pekerjaan Pedagang, tempat tinggal di Dusun Galudra RT 01 RW 04 Desa Galudra Kecamatan Cimalaka Kabupaten Sumedang)** adalah orang yang tidak terhalang untuk menjadi saksi dalam perkara ini serta telah memberikan keterangan di bawah sumpahnya sebagaimana diatur dalam Pasal 145 ayat 1 angka 3e HIR dengan demikian telah memenuhi syarat formil pembuktian, adapun keterangan saksi menyangkut dalil-dalil permohonan Pemohon, telah didasarkan kepada penglihatan saksi yang melihat adanya pertengkaran antara Pemohon dengan Termohon, yang penyebabnya karena Termohon sering meminjam uang kepada orang lain tanpa bermusyawarah dengan Pemohon dan penggunaanya bukan untuk kepentingan bersama, tahu tahu banyak yang menagih utang kepada Pemohon, serta saksi melihat saat ini antara Pemohon dengan Termohon telah berpisah tempat tinggal kurang lebih bulan September 2018 yang keterangan selengkapnya sebagaimana diuraikan dalam duduk perkara, dengan demikian patut diduga bahwa saksi sebagai Paman Pemohon Pemohon mengetahui hal tersebut, dengan demikian keterangan saksi tersebut telah memenuhi syarat materiil pembuktian, oleh karenanya dapat diterima sebagai bukti dalam perkara ini;

Pertimbangan saksi I yang tidak melihat Pertengkaran

Menimbang, bahwa keterangan saksi Pemohon yang kedua (Imih binti Jasam, umur 64 tahun, agama Islam, pekerjaan Buruh, tempat tinggal di Dusun Cibunut RT 01 RW 04 Desa Cikole Kecamatan Cimalaka Kabupaten Sumedang) adalah orang yang tidak terhalang untuk menjadi saksi dalam perkara ini serta telah memberikan keterangan di bawah sumpahnya sebagaimana diatur dalam Pasal 145 ayat 1 angka 3e HIR dengan demikian

Disclaimer
Kepaniteraan Mahkamah Agung Republik Indonesia berusaha untuk selalu mencantumkan informasi paling kini dan akurat sebagai bentuk komitmen Mahkamah Agung untuk pelayanan publik, transparansi dan akuntabilitas pelaksanaan fungsi peradilan. Namun dalam hal-hal tertentu masih dimungkinkan terjadi permasalahan teknis terkait dengan akurasi dan keterkinian informasi yang kami sajikan, hal mana akan terus kami perbaiki dari waktu kewaktu.
Dalam hal Anda menemukan inakurasi informasi yang termuat pada situs ini atau informasi yang seharusnya ada, namun belum tersedia, maka harap segera hubungi Kepaniteraan Mahkamah Agung RI melalui :
Email : kepaniteraan@mahkamahagung.go.id Telp : 021-384 3348 (ext.318)

telah memenuhi syarat formil pembuktian, adapun keterangan saksi menyangkut dalil-dalil permohonan Pemohon meskipun saksi tidak pernah melihat langsung pertengkaran Pemohon dengan Termohon akan tetapi saksi melihat bahwa saat ini antara Pemohon dengan Termohon telah berpisah tempat tinggal yang keterangan selengkapnya sebagaimana diuraikan dalam duduk perkara dengan demikian patut diduga bahwa saksi sebagai Uwa Pemohon Pemohon mengetahui peristiwa *a quo*, maka Majelis menilai keterangan saksi tersebut telah memenuhi syarat materiil pembuktian, oleh karenanya dapat diterima sebagai bukti dalam perkara ini;

Pertimbangan saksi II yang melihat Pertengkaran

Menimbang, bahwa keterangan saksi Pemohon yang kedua **(Imih binti Jasam, umur 64 tahun, agama Islam, pekerjaan Buruh, tempat tinggal di Dusun Cibunut RT 01 RW 04 Desa Cikole Kecamatan Cimalaka Kabupaten Sumedang)** adalah orang yang tidak terhalang untuk menjadi saksi dalam perkara ini serta telah memberikan keterangan di bawah sumpahnya sebagaimana diatur dalam Pasal 145 ayat 1 angka 3e HIR dengan demikian telah memenuhi syarat formil pembuktian, adapun keterangan saksi menyangkut dalil-dalil permohoan Pemohon telah didasarkan kepada penglihatan saksi yang melihat adanya pertengkaran antara Pemohon dengan Termohon yang bertengkar cekcok mulut yang kejadiannya bulan September 2018 di rumah tempat tinggal Pemohon dan Termohon, yang penyebabnya karena Termohonn sering meminjam uang kepada oranglain tanpa seizin Pemohon, sehingga banyak yang menagih utang yang tidak jelas peruntukannya, serta saksi melihat saat ini antara Pemohon dengan Termohon telah berpisah tempat tinggal kurang lebih bulan September 2018, yang keterangan selengkapnya sebagaimana diuraikan dalam duduk perkara, dengan demikian patut diduga bahwa saksi sebagai **Uwa Pemohon** mengetahui hal tersebut, dengan demikian keterangan saksi tersebut telah memenuhi syarat materiil pembuktian, oleh karenanya dapat diterima sebagai bukti dalam perkara ini;

Pertimbangan saksi II yang tidak melihat Pertengkaran

Disclaimer
Kepaniteraan Mahkamah Agung Republik Indonesia berusaha untuk selalu mencantumkan informasi paling kini dan akurat sebagai bentuk komitmen Mahkamah Agung untuk pelayanan publik, transparansi dan akuntabilitas pelaksanaan fungsi peradilan. Namun dalam hal-hal tertentu masih dimungkinkan terjadi permasalahan teknis terkait dengan akurasi dan keterkinian informasi yang kami sajikan, hal mana akan terus kami perbaiki dari waktu kewaktu.
Dalam hal Anda menemukan inakurasi informasi yang termuat pada situs ini atau informasi yang seharusnya ada, namun belum tersedia, maka harap segera hubungi Kepaniteraan Mahkamah Agung RI melalui :
Email : kepaniteraan@mahkamahagung.go.id Telp : 021-384 3348 (ext.318)

Menimbang, bahwa keterangan saksi Pemohon yang kedua (Imih binti Jasam, umur 64 tahun, agama Islam, pekerjaan Buruh, tempat tinggal di Dusun Cibunut RT 01 RW 04 Desa Cikole Kecamatan Cimalaka Kabupaten Sumedang) adalah orang yang tidak terhalang untuk menjadi saksi dalam perkara ini serta telah memberikan keterangan di bawah sumpahnya sebagaimana diatur dalam Pasal 145 ayat 1 angka 3e HIR dengan demikian telah memenuhi syarat formil pembuktian, adapun keterangan saksi menyangkut dalil-dalil permohonan Pemohon meskipun saksi tidak pernah melihat langsung pertengkaran Pemohon dengan Termohon akan tetapi saksi melihat bahwa saat ini antara Pemohon dengan Termohon telah berpisah tempat tinggal yang keterangan selengkapnya sebagaimana diuraikan dalam duduk perkara dengan demikian patut diduga bahwa saksi **yang pernah bertetangga** dengan Pemohon dan Termohon mengetahui peristiwa *a quo*, maka Majelis menilai keterangan saksi tersebut telah memenuhi syarat materiil pembuktian, oleh karenanya dapat diterima sebagai bukti dalam perkara ini;

Menimbang, bahwa keterangan saksi I dan saksi II Pemohon bersesuaian dan cocok antara satu dengan yang lain oleh karena itu keterangan dua orang saksi tersebut memenuhi Pasal 171 dan Pasal 172 HIR;

Menimbang, bahwa berdasarkan bukti P.1 yang dihubungkan dengan keterangan dua orang saksi yang saling bersesuaian, Maka Majelis menemukan fakta-fakta sebagai berikut:

- bahwa Pemohon dan Termohon adalah suami isteri dan masih terikat dalam perkawinan yang sah;
- bahwa rumah tangga Pemohon dan Termohon tidak rukun dan tidak harmonis sering terjadi pertengkaran dan perselisihan;
- bahwa penyebab Pertengkaran tersebut adalah karena masalah **Termohonn sering meminjam uang kepada oranglain tanpa seizin Pemohon, sehingga banyak yang menagih utang yang tidak jelas peruntukannya**;
- bahwa antara Pemohon dengan Termohon telah berpisah tempat tinggal sejak **bulan September 2018** sampai sekarang tidak bersatu lagi dalam rumah tangga;

Disclaimer
Kepaniteraan Mahkamah Agung Republik Indonesia berusaha untuk selalu mencantumkan informasi paling kini dan akurat sebagai bentuk komitmen Mahkamah Agung untuk pelayanan publik, transparansi dan akuntabilitas pelaksanaan fungsi peradilan. Namun dalam hal-hal tertentu masih dimungkinkan terjadi permasalahan teknis terkait dengan akurasi dan keterkinian informasi yang kami sajikan, hal mana akan terus kami perbaiki dari waktu kewaktu.
Dalam hal Anda menemukan inakurasi informasi yang termuat pada situs ini atau informasi yang seharusnya ada, namun belum tersedia, maka harap segera hubungi Kepaniteraan Mahkamah Agung RI melalui :
Email : kepaniteraan@mahkamahagung.go.id Telp : 021-384 3348 (ext.318)

– bahwa upaya perdamaian yang dilakukan pihak keluarga tidak berhasil, karena Penggugat tetap bersiteguh kepada pendiriannya untuk bercerai dengan Tergugat;

Menimbang, bahwa berdasarkan fakta-fakta tersebut di atas, Majelis Hakim berpendapat bahwa keadaan rumah tangga Pemohon dan Termohon sudah tidak rukun dan sudah benar-benar pecah dan sudah sulit untuk didamaikan kembali untuk melanjutkan rumah tangganya;

Menimbang, bahwa berdasarkan fakta-fakta kejadian tersebut di atas, majelis berkesimpulan bahwa fakta-fakta tersebut sesuai dengan fakta-fakta hukum sebagai berikut:

1. Salahsatu yang dapat dijadikan alasan perceraian adalah apabila dalam kehidupan suami isteri telah terjadi perselisihan yang terus menerus yang sulit untuk dirukunkan lagi sebagaimana tertuang dalam pasal 19 hurup f Peraturan Pemerintah Nomor 9 Tahun 1975 tentang Pelaksanaan Undng-undang Nomor 1 tahun 1974 tentang Perkawinan sejalan dengan ketentuan Hukum Islam sebagaimana tersurat pada pasal 116 huruf (f) Kompilasi Hukum Islam;
2. Perceraian dapat dilakukan di depan Sidang Pengadilan setelah Pengadilan yang bersangkutan berusaha dan tidak berhasil mendamaikan kedua belah pihak sesuai dengan ketentuan pasal 39 aya1 1 Undang-undang nomor 1 Tahun 1974 tentang Perkawinan sejalan dengan ketentuan Hukum Islam sebagaimana tersurat pada pasal 115 Kompilasi Hukum Islam;
3. Perceraian dapat dilakukan setelah ditemukan cukup alasan bahwa antara suami istri itu tidak akan dapat rukun sebagai suami isteri sesuai dengan ketentuan pasal 39 ayat 1 Undang-undang nomor 1 Tahun 1974 tentang Perkawinan;

Menimbang, bahwa dengan telah ditemukannya fakta kejadian yang selanjutnya telah memenuhi fakta hukum dan atau memenuhi ketentuan peraturan perundang-undangan dan hukum Islam yang berlaku, maka majelis berpendapat cukup alasan permohonan Pemohon untuk diterima dan dikabulkan;

Menimbang, bahwa berdasarkan ketentuan hukum Islam yang tersirat

Disclaimer
Kepaniteraan Mahkamah Agung Republik Indonesia berusaha untuk selalu mencantumkan informasi paling kini dan akurat sebagai bentuk komitmen Mahkamah Agung untuk pelayanan publik, transparansi dan akuntabilitas pelaksanaan fungsi peradilan. Namun dalam hal-hal tertentu masih dimungkinkan terjadi permasalahan teknis terkait dengan akurasi dan keterkinian informasi yang kami sajikan, hal mana akan terus kami perbaiki dari waktu kewaktu.
Dalam hal Anda menemukan inakurasi informasi yang termuat pada situs ini atau informasi yang seharusnya ada, namun belum tersedia, maka harap segera hubungi Kepaniteraan Mahkamah Agung RI melalui :
Email : kepaniteraan@mahkamahagung.go.id Telp : 021-384 3348 (ext.318)

dalam surat Ar-Rum ayat 21 dan juga ketentuan pasal 1 Undang-undang nomor 1 tahun 1974 dinyatakan bahwa tujuan perkawinan adalah untuk membentuk rumah tangga yang sakinah, mawaddah dan rahmah dan jika Pemohon dan Termohon selaku pasangan suami isteri telah ternyata sudah tidak lagi timbul sikap saling mencintai, saling pengertian dan saling melindungi dan bahkan Pemohon tetap sudah tidak berkeinginan berkeinginan untuk meneruskan rumah tangganya dengan Termohon, maka agar kedua belah pihak berperkara tidak lagi lebih jauh melanggar norma agama dan norma hukum maka perceraian dapat dijadikan salahsatu alternatif untuk menyelesaikan sengketa rumah tangga antara Pemohon dengan Termohon;

Menimbang, bahwa fakta hukum tersebut telah memenuhi norma hukum Islam yang terkandung dalam Al-Qur'an, Surat Al-Baqoroh, ayat 227 yang berbunyi :

وإن عزموا الطلاق فإن الله سميع عليم

Artinya :

"Apabila mereka berazam (bertetap hati) untuk thalak, maka sesunguhnya Allah Maha Mendengar lagi Maha Mengetahui"

Menimbang, bahwa berdasarkan pertimbangan-pertimbangan tersebut di atas, maka petitum permohonan Pemohon agar diizinkan untuk berikrar menjatuhkan talak terhadap Termohon di depan sidang Pengadilan Agama Sumedang telah memenuhi pada pasal 116 huruf (f) Kompilasi Hukum Islam, oleh karena itu dapat dikabulkan;

Menimbang, bahwa karena perkara a quo masuk bidang perkawinan, maka berdasarkan Pasal 89 ayat (1) Undang-Undang Nomor 7 Tahun 1989 sebagaimana telah diubah dengan Undang-Undang Nomor 3 Tahun 2006 dan perubahan kedua dengan Undang-Undang Nomor 50 Tahun 2009, biaya perkara harus dibebankan kepada Pemohon;

Mengingat, semua pasal dalam peraturan perundang-undangan dan hukum Islam yang berkaitan dengan perkara ini;

MENGADILI

1. Menyatakan Termohon yang telah dipanggil secara resmi dan patut untuk menghadap di persidangan, tidak hadir;

Disclaimer
Kepaniteraan Mahkamah Agung Republik Indonesia berusaha untuk selalu mencantumkan informasi paling kini dan akurat sebagai bentuk komitmen Mahkamah Agung untuk pelayanan publik, transparansi dan akuntabilitas pelaksanaan fungsi peradilan. Namun dalam hal-hal tertentu masih dimungkinkan terjadi permasalahan teknis terkait dengan akurasi dan keterkinian informasi yang kami sajikan, hal mana akan terus kami perbaiki dari waktu kewaktu.
Dalam hal Anda menemukan inakurasi informasi yang termuat pada situs ini atau informasi yang seharusnya ada, namun belum tersedia, maka harap segera hubungi Kepaniteraan Mahkamah Agung RI melalui :
Email : kepaniteraan@mahkamahagung.go.id Telp : 021-384 3348 (ext.318)

2. Mengabulkan Permohonan Pemohon dengan verstek;
3. Memberi izin kepada Pemohon (Aep Itang bin Rukma) untuk menjatuhkan talak satu raj'i terhadap Termohon (Siti Rodiah binti Cahya) di depan sidang Pengadilan Agama Sumedang;
4. Membebankan kepada Pemohon untuk membayar biaya perkara ini sejumlah Rp446000,00 (empat ratus empat puluh enam ribu rupiah).
5.

Demikian diputuskan dalam rapat permusyawaratan Majelis yang dilangsungkan pada hari Senin tanggal 28 Desember 2020 Masehi, bertepatan dengan tanggal 13 Jumadil Awwal 1442 Hijriyah, oleh kami Drs. Erik Sumarna, S.H., M.A. sebagai Ketua Majelis, Drs. Solihudin, S.H dan Drs. H. Syamsul Falah, M.H. masing-masing sebagai Hakim Anggota, putusan tersebut diucapkan dalam sidang terbuka untuk umum pada hari itu juga, oleh Ketua Majelis tersebut dengan didampingi oleh Hakim Anggota dan dibantu oleh H. Asep Suryana, SHI sebagai Panitera Pengganti serta dihadiri oleh Pemohon tanpa kehadiran Termohon;

Ketua Majelis

Drs. Erik Sumarna, S.H., M.A.

Hakim Anggota	Hakim Anggota
Drs. Solihudin, S.H	Drs. H. Syamsul Falah, M.H.

Panitera Pengganti

H. Asep Suryana, SHI

Perincian Biaya Perkara :

1. Pendaftaran : Rp. 30.000,-

Disclaimer
Kepaniteraan Mahkamah Agung Republik Indonesia berusaha untuk selalu mencantumkan informasi paling kini dan akurat sebagai bentuk komitmen Mahkamah Agung untuk pelayanan publik, transparansi dan akuntabilitas pelaksanaan fungsi peradilan. Namun dalam hal-hal tertentu masih dimungkinkan terjadi permasalahan teknis terkait dengan akurasi dan keterkinian informasi yang kami sajikan, hal mana akan terus kami perbaiki dari waktu kewaktu.
Dalam hal Anda menemukan inakurasi informasi yang termuat pada situs ini atau informasi yang seharusnya ada, namun belum tersedia, maka harap segera hubungi Kepaniteraan Mahkamah Agung RI melalui :
Email : kepaniteraan@mahkamahagung.go.id Telp : 021-384 3348 (ext.318)

2. Proses	: Rp.	50.000,-
3. Panggilan Pemohon	: Rp.	110.000,-
4. Panggilan Termohon	: Rp.	220.000,-
5. PNBP Panggilan	: Rp.	20.000,-
6. Redaksi	: Rp.	10.000,-
7. Materai	: Rp.	6.000,-

Jumlah : Rp. 426.000,-

13

Disclaimer
Kepaniteraan Mahkamah Agung Republik Indonesia berusaha untuk selalu mencantumkan informasi paling kini dan akurat sebagai bentuk komitmen Mahkamah Agung untuk pelayanan publik, transparansi dan akuntabilitas pelaksanaan fungsi peradilan. Namun dalam hal-hal tertentu masih dimungkinkan terjadi permasalahan teknis terkait dengan akurasi dan keterkinian informasi yang kami sajikan, hal mana akan terus kami perbaiki dari waktu kewaktu.
Dalam hal Anda menemukan inakurasi informasi yang termuat pada situs ini atau informasi yang seharusnya ada, namun belum tersedia, maka harap segera hubungi Kepaniteraan Mahkamah Agung RI melalui :
Email : kepaniteraan@mahkamahagung.go.id Telp : 021-384 3348 (ext.318)
