# Supplementary material for: Classification of divorce causes during the COVID-19 pandemic using convolutional neural networks
Source: PeerJ Comput Sci. 2022 Jun 30;8:e998. doi: 10.7717/peerj-cs.998 (PMC9299239; doi:10.7717/peerj-cs.998)
Supplement: Supplemental Information 2 [file peerj-cs-08-998-s002.zip › Kelalaian Dataset/Data ke-24.pdf]

P U T U S A N

Nomor 4210/Pdt.G/2020/PA.Smdg

بِسْمِ اللهِ الرَّحْمٰنِ الرَّحِيْمِ

DEMI KEADILAN BERDASARKAN KETUHANAN YANG MAHA ESA

Pengadilan Agama Sumedang yang memeriksa dan mengadili perkara tertentu pada tingkat pertama dalam sidang majelis hakim telah menjatuhkan putusan dalam perkara Cerai Gugat antara:

Apipih Nurhasanah Binti Suin, tempat dan tanggal lahir Sumedang, 25 Desember 1995, agama Islam, pekerjaan Ibu Rumah Tangga, Pendidikan Sekolah Lanjutan Tingkat Pertama, tempat kediaman di Dusun Babakan Calangcang Rt 33 Rw 10 Desa Legok Kidul Kecamatan Paseh Kabupaten Sumedang sebagai Penggugat**;**

melawan

M Dodi Suherman Bin Sarifudin, tempat dan tanggal lahir Bekasi, 22 November 1989, agama Islam, pekerjaan Buruh Harian Lepas, Pendidikan Sekolah Lanjutan Tingkat Pertama, tempat kediaman di Dengan Alamat Di Rumah Pamannya A.N Ibu Nani Di Dusun Babakan Calangcang Rt 33 Rw 10 Desa Legok Kidul Kecamatan Paseh Kabupaten Sumedang sebagai Tergugat;

Pengadilan Agama tersebut;

Telah mempelajari surat-surat yang berkaitan dengan perkara ini;

Telah mendengar keterangan Pemohon dan para saksi di muka sidang;

DUDUK PERKARA

Bahwa Pemohon dalam surat #permohonan/gugatan# tanggal 07 Desember 2020 telah mengajukan permohonan Cerai Gugat, yang telah terdaftar di Kepaniteraan Pengadilan Agama Sumedang, dengan Nomor

Disclaimer
Kepaniteraan Mahkamah Agung Republik Indonesia berusaha untuk selalu mencantumkan informasi paling kini dan akurat sebagai bentuk komitmen Mahkamah Agung untuk pelayanan publik, transparansi dan akuntabilitas pelaksanaan fungsi peradilan. Namun dalam hal-hal tertentu masih dimungkinkan terjadi permasalahan teknis terkait dengan akurasi dan keterkinian informasi yang kami sajikan, hal mana akan terus kami perbaiki dari waktu kewaktu.
Dalam hal Anda menemukan inakurasi informasi yang termuat pada situs ini atau informasi yang seharusnya ada, namun belum tersedia, maka harap segera hubungi Kepaniteraan Mahkamah Agung RI melalui :
Email : kepaniteraan@mahkamahagung.go.id   Telp : 021-384 3348 (ext.318)

4210/Pdt.G/2020/PA.Smdg, tanggal 07 Desember 2020, dengan dalil-dalil pada pokoknya sebagai berikut:

1. Bahwa Penggugat dan Tergugat adalah suami isteri sah menikah pada tanggal 17 November 2013 berdasarkan Kutipan Akta Nikah dari KUA Kecamatan Paseh Kabupaten Sumedang, No: 0409/023/XI/2013. Tertanggal 17 November 2013;
2. Bahwa sebelum membina rumahtangga, Penggugat berstatus Perawan begitupun Tergugat berstatus Jejaka;
3. Bahwa antara Penggugat dan Tergugat terakhir berumah tangga tinggal di rumah kediaman orangtua Penggugat di Dusun Babakan Calangcang RT 33 RW 10 Desa Legok Kidul Kecamatan Paseh Kabupaten Sumedang dan telah bergaul baik sebagaimana layaknya suami isteri, dan telah dikaruniai seorang anak bernama Muhammad Nurdiansyah (berusia 6 tahun);
4. Bahwa semula rumah tangga Penggugat dengan Tergugat berjalan dengan harmonis layaknya rumah tangga bahagia, namun seiring berjalannya waktu pada bulan **Juni 2018** rumah tangga mulai goyah dikarenakan Tergugat mulai menunjukan sikap yang berbeda yakni Tergugat kurang memberikan perhatian dan kurang menunjukan sikap kasih sayang terhadap Penggugat dan anak, sehingga dalam menjalani rumah tanggga menjadi tidak nyaman dan harmonis;
5. Bahwa keretakan rumah tangga antara Penggugat dan Tergugat telah berlangsung lama. Penggugat berusaha sabar, tetapi Tergugat tidak berusaha berubah dan merubah sifatnya. Oleh karena itu Penggugat merasa tidak nyaman berumah tangga dengan Tergugat. Puncaknya pada bulan **Desember 2018**, **Tergugat pergi meningalkan rumah kediaman orangtua Penggugat** terhitung sudah 2 (dua) tahun Tergugat pergi meninggalkan Penggugat tanpa alasan yang jelas, dan sejak itu antara Penggugat dan Tergugat sudah pisah tempat tinggal dan tidak lagi bergaul layaknya suami isteri bahkan sampai saat ini Tergugat tidak pernah memberikan nafkah terhadap Penggugat. Dimana sekarang ini **Penggugat tinggal di rumah kediaman orangtua Penggugat** sedangkan **Tergugat tinggal di rumah kediaman paman Tergugat;**

Disclaimer
Kepaniteraan Mahkamah Agung Republik Indonesia berusaha untuk selalu mencantumkan informasi paling kini dan akurat sebagai bentuk komitmen Mahkamah Agung untuk pelayanan publik, transparansi dan akuntabilitas pelaksanaan fungsi peradilan. Namun dalam hal-hal tertentu masih dimungkinkan terjadi permasalahan teknis terkait dengan akurasi dan keterkinian informasi yang kami sajikan, hal mana akan terus kami perbaiki dari waktu kewaktu.
Dalam hal Anda menemukan inakurasi informasi yang termuat pada situs ini atau informasi yang seharusnya ada, namun belum tersedia, maka harap segera hubungi Kepaniteraan Mahkamah Agung RI melalui :
Email : kepaniteraan@mahkamahagung.go.id Telp : 021-384 3348 (ext.318)

6. Bahwa keretakan rumah tangga Penggugat dan Tergugat sudah pernah didamaikan oleh keluarga, tetapi tidak berhasil;
7. Bahwa gugatan Penggugat tersebut telah memenuhi syarat sesuai ketentuan Pasal 19 huruf (b) Peraturan Pemerintah Nomor 9 Tahun 1975 jo. Pasal 116 huruf (b) Kompilasi Hukum Islam.;
8. Bahwa atas permasalahan tersebut di atas Penggugat sudah tidak sanggup lagi untuk mempertahankan perkawinan ini, oleh karena itu Penggugat telah berketetapan hati untuk menggugat cerai dari Tergugat;

Bahwa berdasarkan dalil-dalil tersebut, Pemohon memohon kepada Pengadilan Agama Sumedang, agar menjatuhkan putusan yang amarnya sebagai berikut:

1. Menerima dan mengabulkan gugatan Penggugat.
2. Menjatuhkan talak satu ba'in sughro dari Tergugat (M Dodi Suherman bin Sarifudin) kepada Penggugat (Apipih Nurhasanah binti Suin).
3. Menetapkan biaya perkara menurut hukum.

Bahwa pada persidangan yang telah ditetapkan Penggugat telah datang menghadap di persidangan, sedangkan Tergugat tidak datang menghadap di persidangan dan tidak menyuruh orang lain untuk menghadap sebagai wakil/kuasanya yang sah, meskipun berdasarkan surat panggilan (relaas) yang dibacakan di persidangan, Tergugat telah dipanggil secara resmi dan patut, sedangkan tidak ternyata bahwa tidak datangnya Tergugat tersebut disebabkan oleh suatu alasan yang sah;

Bahwa majelis hakim telah menasehati Penggugat agar berpikir untuk tidak bercerai dengan Tergugat, tetapi Penggugat tetap pada dalil-dalil Penggugatannya untuk bercerai dengan Tergugat;

Bahwa perkara ini tidak dapat dimediasi karena Tergugat tidak pernah datang menghadap meskipun telah dipanggil secara resmi dan patut, selanjutnya dimulai pemeriksaan dengan membacakan surat Penggugatan Penggugat yang maksud dan tujuannya tetap dipertahankan oleh Penggugat;

Disclaimer
Kepaniteraan Mahkamah Agung Republik Indonesia berusaha untuk selalu mencantumkan informasi paling kini dan akurat sebagai bentuk komitmen Mahkamah Agung untuk pelayanan publik, transparansi dan akuntabilitas pelaksanaan fungsi peradilan. Namun dalam hal-hal tertentu masih dimungkinkan terjadi permasalahan teknis terkait dengan akurasi dan keterkinian informasi yang kami sajikan, hal mana akan terus kami perbaiki dari waktu kewaktu.
Dalam hal Anda menemukan inakurasi informasi yang termuat pada situs ini atau informasi yang seharusnya ada, namun belum tersedia, maka harap segera hubungi Kepaniteraan Mahkamah Agung RI melalui :
Email : kepaniteraan@mahkamahagung.go.id Telp : 021-384 3348 (ext.318)

Bahwa untuk menguatkan dalil-dalil Penggugatannya, Penggugat telah mengajukan bukti berupa :

A. Surat :

Fotocopy Kutipan Akta Nikah Nomor : 0409/023/XI/2013. Tertanggal 17 November 2013 yang dikeluarkan oleh Kantor Urusan Agama Kecamatan Paseh, Kabupaten Sumedang, telah bermeterai cukup dan dicocokan dengan aslinya, kemudian diberi tanda alat bukti (P) ;

B. Saksi :

1. Kokom Komala binti Didi, umur 45 tahun, agama Islam, pekerjaan ibu rumah tangga, tempat kediaman di Dusun Babakan Calangcang Rt 33 Rw 10 Desa Legok Kidul Kecamatan Paseh Kabupaten Sumedang, di bawah sumpahnya memberikan keterangan sebagai berikut :
   - Bahwa saksi kenal kepada Penggugat dan Tergugat karena saksi sebagai ibu kandung Penggugat;
   - Bahwa saksi mengetahui Penggugat dan Tergugat setelah menikah bertempat tinggal bersama terakhir tinggal bersama di rumah kediaman orangtua Penggugat di Dusun Babakan Calangcang RT 33 RW 10 Desa Legok Kidul Kecamatan Paseh Kabupaten Sumedang ;
   - Bahwa saksi mengetahui rumah tangga Penggugat dan Tergugat semula rukun dan harmonis serta telah dikaruniai 1 (satu) orang anak, akan tetapi sejak bulan Juni 2018 rumah tangga Penggugat dan Tergugat mulai terjadi perselisihan dan pertengkaran;
   - Bahwa saksi melihat antara Penggugat dan Tergugat telah berselisih dan bertengkar karena Tergugat kurang memberikan perhatian dan kurang menunjukan sikap kasih sayang terhadap Penggugat dan anak ;
   - Bahwa pada bulan Desember 2018 Tergugat pergi meninggalkan rumah tempat kediaman bersama sampai sekarang, sehingga sejak saat itu antara Penggugat dan Tergugat telah pisah rumah hingga sekarang;

Disclaimer
Kepaniteraan Mahkamah Agung Republik Indonesia berusaha untuk selalu mencantumkan informasi paling kini dan akurat sebagai bentuk komitmen Mahkamah Agung untuk pelayanan publik, transparansi dan akuntabilitas pelaksanaan fungsi peradilan. Namun dalam hal-hal tertentu masih dimungkinkan terjadi permasalahan teknis terkait dengan akurasi dan keterkinian informasi yang kami sajikan, hal mana akan terus kami perbaiki dari waktu kewaktu.
Dalam hal Anda menemukan inakurasi informasi yang termuat pada situs ini atau informasi yang seharusnya ada, namun belum tersedia, maka harap segera hubungi Kepaniteraan Mahkamah Agung RI melalui :
Email : kepaniteraan@mahkamahagung.go.id Telp : 021-384 3348 (ext.318)

- Bahwa pihak keluarga Penggugat dan Tergugat telah mendamaikan dan menasehati Penggugat dan Tergugat akan tetapi tidak berhasil ;
- Bahwa saksi sudah tidak ada kesanggupan merukunkan Penggugat dan Tergugat ;

2. Wida Ningsih binti Ukar, umur 27 tahun, agama Islam, pekerjaan ibu rumah tangga, tempat kediaman di Dusun Babakan Calangcang Rt 33 Rw 10 Desa Legok Kidul Kecamatan Paseh Kabupaten Sumedang, di bawah sumpahnya memberikan keterangan sebagai berikut :
   - Bahwa saksi kenal kepada Penggugat dan Tergugat karena saksi sebagai tetangga Penggugat;
   - Bahwa saksi mengetahui semula rumah tangga Penggugat dan Tergugat rukun dan harmonis serta telah dikaruniai 1 (satu) orang anak, namun sejak bulan Juni 2018 rumah tangga Penggugat dan Tergugat mulai berselisih dan bertengkar ;
   - Bahwa saksi pernah mendengar antara Penggugat dan Tergugat berselisih dan bertengkar, yaitu pada bulan Juni 2018 disebabkan karena Tergugat yang seringkali kurang memperlihatkan tanggung jawab dalam hal pemenuhan nafkah kepada Penggugat ;
   - Bahwa puncak pertengkaran terjadi pada bulan Desember 2018 dimana Tergugat pergi meninggalkan rumah tempat kediaman bersama sampai sekarang, maka sejak saat itu antara Penggugat dan Tergugat telah pisah rumah hingga sekarang ;
   - Bahwa saksi dan keluarga Penggugat dan keluarga Tergugat telah mendamaikan dan menasehati Penggugat dan Tergugat supaya rukun kembali membina rumah tangga akan tetapi tidak berhasil ;
   - Bahwa saksi sudah tidak kesanggupan lagi merukunkan Penggugat dan Tergugat;

Bahwa Penggugat menyampaikan kesimpulan yang isinya sebagaimana tertuang dalam berita acara sidang;

Disclaimer
Kepaniteraan Mahkamah Agung Republik Indonesia berusaha untuk selalu mencantumkan informasi paling kini dan akurat sebagai bentuk komitmen Mahkamah Agung untuk pelayanan publik, transparansi dan akuntabilitas pelaksanaan fungsi peradilan. Namun dalam hal-hal tertentu masih dimungkinkan terjadi permasalahan teknis terkait dengan akurasi dan keterkinian informasi yang kami sajikan, hal mana akan terus kami perbaiki dari waktu kewaktu.
Dalam hal Anda menemukan inakurasi informasi yang termuat pada situs ini atau informasi yang seharusnya ada, namun belum tersedia, maka harap segera hubungi Kepaniteraan Mahkamah Agung RI melalui :
Email : kepaniteraan@mahkamahagung.go.id   Telp : 021-384 3348 (ext.318)

Selanjutnya untuk singkatnya uraian putusan ini, maka semua hal yang termuat dalam berita acara sidang merupakan bagian yang tidak terpisahkan dari putusan ini ;

PERTIMBANGAN HUKUM

Menimbang, bahwa maksud dan tujuan Penggugatan Penggugat adalah sebagaimana terurai di atas;

Menimbang, bahwa ternyata Tergugat tidak datang menghadap di persidangan dan tidak pula menyuruh orang lain untuk menghadap sebagai wakil/kuasanya yang sah, meskipun berdasarkan Surat Panggilan (relaas) yang dibacakan di persidangan, Tergugat telah dipanggil secara resmi dan patut, sedangkan tidak ternyata bahwa tidak datangnya Tergugat tersebut disebabkan oleh suatu alasan yang sah;

Menimbang, bahwa Tergugat yang dipanggil secara resmi dan patut akan tetapi tidak menghadap harus dinyatakan tidak hadir dan Penggugatan tersebut harus diperiksa secara verstek;

Menimbang, bahwa berdasarkan ketentuan Pasal 125 ayat (1) HIR/ Pasal 149 ayat (1) R.Bg, yaitu putusan yang dijatuhkan tanpa hadirnya Tergugat dapat dikabulkan sepanjang berdasarkan hukum dan beralasan, oleh karena itu majelis hakim membebani Penggugat untuk membuktikan dalil-dalil Penggugatannya;

Menimbang, bahwa untuk menguatkan perkara tersebut berdasarkan hokum dan beralasan, maka majelis membebani Penggugat untuk membuktikan dalil-dalil gugatannya ;

Menimbang, bahwa untuk membuktikan dalil permohonannya, Penggugat telah mengajukan alat bukti surat dan 2 orang saksi;

Menimbang, bahwa bukti P (Fotokopi Kutipan Akta Nikah) yang merupakan akta otentik dan telah bermeterai cukup dan cocok dengan aslinya, isi bukti tersebut menjelaskan mengenai hubungan suami isteri antara Penggugat dengan Termohon, sehingga bukti tersebut telah memenuhi syarat

Disclaimer
Kepaniteraan Mahkamah Agung Republik Indonesia berusaha untuk selalu mencantumkan informasi paling kini dan akurat sebagai bentuk komitmen Mahkamah Agung untuk pelayanan publik, transparansi dan akuntabilitas pelaksanaan fungsi peradilan. Namun dalam hal-hal tertentu masih dimungkinkan terjadi permasalahan teknis terkait dengan akurasi dan keterkinian informasi yang kami sajikan, hal mana akan terus kami perbaiki dari waktu kewaktu.
Dalam hal Anda menemukan inakurasi informasi yang termuat pada situs ini atau informasi yang seharusnya ada, namun belum tersedia, maka harap segera hubungi Kepaniteraan Mahkamah Agung RI melalui :
Email : kepaniteraan@mahkamahagung.go.id   Telp : 021-384 3348 (ext.318)

formal dan materiil, serta mempunyai kekuatan pembuktian yang sempurna dan mengikat;

Menimbang, bahwa berdasarkan bukti P.1 tentang kompetensi absolut dalam perkara ini, Majelis Hakim menyatakan bahwa perkara ini adalah wewenang Pengadilan Agama Sumedang ;

Menimbang, bahwa saksi 1 Penggugat, sudah dewasa dan sudah bersumpah, sehingga memenuhi syarat formal sebagaimana diatur dalam Pasal 145 ayat 1 angka 3e HIR;

Menimbang, bahwa keterangan saksi 1 Penggugat mengenai pernikahan Penggugat dengan Tergugat, adalah fakta yang dilihat sendiri dan relevan dengan dalil yang harus dibuktikan oleh Penggugat, oleh karena itu keterangan saksi tersebut telah memenuhi syarat materiil sebagaimana telah diatur dalam Pasal 171 HIR. sehingga keterangan saksi tersebut memiliki kekuatan pembuktian dan dapat diterima sebagai alat bukti;

Menimbang, bahwa saksi 2 Penggugat, sudah dewasa dan sudah bersumpah, sehingga memenuhi syarat formal sebagaimana diatur dalam Pasal 145 ayat 1 angka 3e HIR;

Menimbang, bahwa keterangan saksi 2 Penggugat mengenai terjadinya pernikahan Penggugat dengan Tergugat, adalah fakta yang didengar sendiri dan relevan dengan dalil yang harus dibuktikan oleh Penggugat, oleh karena itu keterangan saksi tersebut telah memenuhi syarat materiil sebagaimana telah diatur dalam Pasal 171 HIR. sehingga keterangan saksi tersebut memiliki kekuatan pembuktian dan dapat diterima sebagai alat bukti;

Menimbang, bahwa keterangan saksi 1 dan saksi 2 Penggugat telah terbukti bahwa Penggugat dengan Tergugat telah menikah secara sah berdasarkan syariat Islam ;

Menimbang, bahwa keterangan saksi 1 Penggugat mengenai perselisihan dan pertengkaran, adalah fakta yang dilihat sendiri dan relevan

Disclaimer
Kepaniteraan Mahkamah Agung Republik Indonesia berusaha untuk selalu mencantumkan informasi paling kini dan akurat sebagai bentuk komitmen Mahkamah Agung untuk pelayanan publik, transparansi dan akuntabilitas pelaksanaan fungsi peradilan. Namun dalam hal-hal tertentu masih dimungkinkan terjadi permasalahan teknis terkait dengan akurasi dan keterkinian informasi yang kami sajikan, hal mana akan terus kami perbaiki dari waktu kewaktu.
Dalam hal Anda menemukan inakurasi informasi yang termuat pada situs ini atau informasi yang seharusnya ada, namun belum tersedia, maka harap segera hubungi Kepaniteraan Mahkamah Agung RI melalui :
Email : kepaniteraan@mahkamahagung.go.id   Telp : 021-384 3348 (ext.318)

dengan dalil yang harus dibuktikan oleh Penggugat, oleh karena itu keterangan saksi tersebut telah memenuhi syarat materiil sebagaimana telah diatur dalam Pasal 171 HIR sehingga keterangan saksi tersebut memiliki kekuatan pembuktian dan dapat diterima sebagai alat bukti;

Menimbang, bahwa keterangan saksi 2 Penggugat mengenai perselisihan dan pertengkaran, adalah fakta yang didengar sendiri dan relevan dengan dalil yang harus dibuktikan oleh Penggugat, oleh karena itu keterangan saksi tersebut telah memenuhi syarat materiil sebagaimana telah diatur dalam Pasal 171 HIR sehingga keterangan saksi tersebut memiliki kekuatan pembuktian dan dapat diterima sebagai alat bukti;

Menimbang, bahwa keterangan saksi 1 dan saksi 2 Penggugat bersesuaian dan cocok antara satu dengan yang lain oleh karena itu keterangan dua orang saksi tersebut memenuhi Pasal 171 dan Pasal 172 HIR ;

Menimbang, bahwa berdasarkan bukti P, Saksi 1 dan Saksi 2 terbukti fakta kejadian sebagai berikut:

1. Bahwa Penggugat telah menikah dengan Tergugat dan belum bercerai sampai dengan sekarang ;
2. Bahwa Penggugat dan Tergugat sering terjadi perselisihan dan pertengkaran ;
3. Bahwa perselisihan dan pertengkaran antara Penggugat dan Tergugat telah didamaikan oleh pihak keluarga dan tokoh masyarakat setempat ;
4. Bahwa antara Penggugat dan Tergugat sudah pisah rumah tempat tinggal ;

Menimbang, bahwa berdasarkan fakta-fakta tersebut di atas dapat disimpulkan fakta hukum sebagai berikut:

1. Bahwa perkara dimaksud adalah kewenangan absolut Pengadilan Agama Sumedang ;
2. Bahwa Penggugat dan Tergugat adalah suami isteri yang sah dan tidak pernah berceri ;

Disclaimer
Kepaniteraan Mahkamah Agung Republik Indonesia berusaha untuk selalu mencantumkan informasi paling kini dan akurat sebagai bentuk komitmen Mahkamah Agung untuk pelayanan publik, transparansi dan akuntabilitas pelaksanaan fungsi peradilan. Namun dalam hal-hal tertentu masih dimungkinkan terjadi permasalahan teknis terkait dengan akurasi dan keterkinian informasi yang kami sajikan, hal mana akan terus kami perbaiki dari waktu kewaktu.
Dalam hal Anda menemukan inakurasi informasi yang termuat pada situs ini atau informasi yang seharusnya ada, namun belum tersedia, maka harap segera hubungi Kepaniteraan Mahkamah Agung RI melalui :
Email : kepaniteraan@mahkamahagung.go.id Telp : 021-384 3348 (ext.318)

3. Bahwa antara Penggugat dan Tergugat terus-menerus terjadi perselisihan dan pertengkaran dan tidak ada harapan akan hidup rukun lagi dalam rumah tangga ;
4. Bahwa antara Penggugat dan Tergugat telah pisah rumah;

Menimbang, bahwa fakta hukum tersebut telah pula memenuhi doktrin hukum Islam sebagai berikut :

إذا اشتدّ رغبة الزوجة لزوجها طلق عليها القاضى طلقة

Artinya: "Disaat istri telah memuncak kebencian terhadap suaminya, maka hakim diperkenankan menjatuhkan talak (suami) nya dengan talak satu";

Menimbang, bahwa fakta hukum tersebut telah juga memenuhi Pasal 22 jo Pasal 19 huruf (b) Peraturan Pemerintah Nomor 9 Tahun 1975 jo. Pasal 19 huruf (b) Kompilasi Hukum Islam ;

Menimbang, bahwa berdasarkan pertimbangan-pertimbangan tersebut di atas dan Penggugat belum pernah cerai dengan Tergugat, maka petitum permohonan Penggugat mengenai cerai gugat tersebut memenuhi Pasal 119 Kompilasi Hukum Islam, oleh karena itu dapat dikabulkan;

Menimbang, bahwa oleh karena perkara ini masuk dalam bidang perkawinan, maka sesuai Pasal 89 ayat (1) Undang-undang Nomor 7 Tahun 1989 sebagaimana telah diubah dengan Undang-undang Nomor 3 Tahun 2006 dan perubahan kedua dengan Undang-undang Nomor 50 Tahun 2009, biaya perkara dibebankan kepada Penggugat ;

Mengingat, semua pasal dalam peraturan perundang-undangan dan hukum Islam yang berkaitan dengan perkara ini;

MENGADILI:

1. Menyatakan Tergugat yang telah dipanggil secara resmi dan patut untuk menghadap di persidangan tidak hadir ;
2. Mengabulkan gugatan Penggugat dengan verstek ;

Disclaimer
Kepaniteraan Mahkamah Agung Republik Indonesia berusaha untuk selalu mencantumkan informasi paling kini dan akurat sebagai bentuk komitmen Mahkamah Agung untuk pelayanan publik, transparansi dan akuntabilitas pelaksanaan fungsi peradilan. Namun dalam hal-hal tertentu masih dimungkinkan terjadi permasalahan teknis terkait dengan akurasi dan keterkinian informasi yang kami sajikan, hal mana akan terus kami perbaiki dari waktu kewaktu.
Dalam hal Anda menemukan inakurasi informasi yang termuat pada situs ini atau informasi yang seharusnya ada, namun belum tersedia, maka harap segera hubungi Kepaniteraan Mahkamah Agung RI melalui :
Email : kepaniteraan@mahkamahagung.go.id   Telp : 021-384 3348 (ext.318)

3. Menjatuhkan talak satu ba'in sughra dari Tergugat **(M Dodi Suherman bin Sarifudin**) kepada Penggugat (**Apipih Nurhasanah binti Suin)** ;
4. Membebankan kepada Penggugat untuk membayar biaya perkara ini sejumlah Rp. 446.000,00 (empat ratus empat puluh enam ribu rupiah) ;

Demikian putusan ini dijatuhkan dalam permusyawaratan majelis yang dilangsungkan pada hari Senin tanggal 28 Desember 2020 Masehi, bertepatan dengan tanggal 13 Jumadil Awal 1442 Hijriyah, oleh Drs. Dimyati, SH, MH sebagai Ketua Majelis, dan Drs. Endang Sofwan, MH. dan Drs. Nuryadi Siswanto, MH masing-masing sebagai Hakim Anggota, putusan tersebut dibacakan dalam sidang terbuka untuk umum pada hari itu juga, oleh Ketua Majelis tersebut dengan didampingi oleh Hakim Anggota dan dibantu oleh Agus Kurnia, S. Sy sebagai Panitera Pengganti dengan dihadiri oleh Penggugat tanpa hadirnya Tergugat ;

Ketua Majelis,

Hakim Anggota,

Drs. Dimyati, SH, MH

Drs. Endang Sofwan, MH

Hakim Anggota,

Drs. Nuryadi Siswanto, MH

Panitera Pengganti

Agus Kurnia, S. Sy.

Disclaimer
Kepaniteraan Mahkamah Agung Republik Indonesia berusaha untuk selalu mencantumkan informasi paling kini dan akurat sebagai bentuk komitmen Mahkamah Agung untuk pelayanan publik, transparansi dan akuntabilitas pelaksanaan fungsi peradilan. Namun dalam hal-hal tertentu masih dimungkinkan terjadi permasalahan teknis terkait dengan akurasi dan keterkinian informasi yang kami sajikan, hal mana akan terus kami perbaiki dari waktu kewaktu.
Dalam hal Anda menemukan inakurasi informasi yang termuat pada situs ini atau informasi yang seharusnya ada, namun belum tersedia, maka harap segera hubungi Kepaniteraan Mahkamah Agung RI melalui :
Email : kepaniteraan@mahkamahagung.go.id Telp : 021-384 3348 (ext.318)

Perincian biaya :

1.	Pendaftaran	Rp. 30.000,00
2.	Proses	Rp. 50.000,00
3.	Panggilan	Rp. 360.000,00
4.	PNBP	Rp. 20.000,00
5.	Redaksi	Rp. 10.000,00
6.	Meterai	Rp. 6.000,00
	**Jumlah**	**Rp. 446.000,00**

(empat ratus empat puluh enam ribu rupiah)

Disclaimer
Kepaniteraan Mahkamah Agung Republik Indonesia berusaha untuk selalu mencantumkan informasi paling kini dan akurat sebagai bentuk komitmen Mahkamah Agung untuk pelayanan publik, transparansi dan akuntabilitas pelaksanaan fungsi peradilan. Namun dalam hal-hal tertentu masih dimungkinkan terjadi permasalahan teknis terkait dengan akurasi dan keterkinian informasi yang kami sajikan, hal mana akan terus kami perbaiki dari waktu kewaktu.
Dalam hal Anda menemukan inakurasi informasi yang termuat pada situs ini atau informasi yang seharusnya ada, namun belum tersedia, maka harap segera hubungi Kepaniteraan Mahkamah Agung RI melalui :
Email : kepaniteraan@mahkamahagung.go.id Telp : 021-384 3348 (ext.318)
