# Supplementary material for: Classification of divorce causes during the COVID-19 pandemic using convolutional neural networks
Source: PeerJ Comput Sci. 2022 Jun 30;8:e998. doi: 10.7717/peerj-cs.998 (PMC9299239; doi:10.7717/peerj-cs.998)
Supplement: Supplemental Information 2 [file peerj-cs-08-998-s002.zip › Kelalaian Dataset/Data ke-25.pdf]

P U T U S A N

Nomor 510/Pdt.G/2020/PTA.Sby

BISMILLAAHIRRAHMAANIRRAHIIM

DEMI KEADILAN BERDASARKAN KETUHANAN YANG MAHA ESA

Pengadilan Tinggi Agama Surabaya yang memeriksa dan mengadili perkara perdata pada tingkat banding dalam sidang Majelis telah menjatuhkan putusan terhadap perkara Cerai Gugat antara:

PEMBANDING, umur 45 tahun, agama Islam, pendidikan SMA, pekerjaan--, tempat kediaman di Kabupaten Sidoarjo, dalam hal ini telah memberi kuasa kepada **H. Soetomo,S.H., M.Hum., Ari Wahyu Oetomo, S.H Arif Hidayat, S.H.,M.Kn., dan Ir. H. M. Noer Hasanuddin,S.H.**, semuanya adalah para advokat, Konsultan Hukum dan Penasehat Hukum pada Kantor Hukum **"ARIE SOETOMO & ASSOCIATES"** yang beralamat di Raya Jemursari No. 17 Lt. 2, Kota Surabaya, berdasarkan Surat Kuasa Khusus tanggal 5 Oktober 2020 yang terdaftar di Kepaniteraan Pengadilan Agama Sidoarjo tanggal 14 Oktober 2020 Nomor 2120/kuasa/10/2020/PA.Sda. semula sebagai **Tergugat** sekarang sebagai **Pembanding**;

M e l a w a n

TERBANDING, umur 44 tahun, agama Islam, pendidikan ---, pekerjaan Karyawan swasta, tempat kediaman di Kabupaten Sidoarjo, dalam hal ini memberikan kuasa kepada **Indra Bayu, S.H.,** Advokat pada kantor hukum **"INDRA BAYU, SH & ASSOCIATES"** yang beralamat di Sumput No.150 RT.05 RW.02 Sidoarjo, berdasarkan surat kuasa khusus tanggal 18 November 2020 yang terdaftar di Register Kepaniteraan Pengadilan Agama Sidoarjo tanggal 19 November 2020 Nomor 2540/Kuasa/11/2020/ PA.Sda semula sebagai **Penggugat** sekarang sebagai **Terbanding**;

Disclaimer
Kepaniteraan Mahkamah Agung Republik Indonesia berusaha untuk selalu mencantumkan informasi paling kini dan akurat sebagai bentuk komitmen Mahkamah Agung untuk pelayanan publik, transparansi dan akuntabilitas pelaksanaan fungsi peradilan. Namun dalam hal-hal tertentu masih dimungkinkan terjadi permasalahan teknis terkait dengan akurasi dan keterkinian informasi yang kami sajikan, hal mana akan terus kami perbaiki dari waktu kewaktu.
Dalam hal Anda menemukan inakurasi informasi yang termuat pada situs ini atau informasi yang seharusnya ada, namun belum tersedia, maka harap segera hubungi Kepaniteraan Mahkamah Agung RI melalui :
Email : kepaniteraan@mahkamahagung.go.id   Telp : 021-384 3348 (ext.318)

Pengadilan Tinggi Agama tersebut ;

Telah mempelajari berkas perkara yang dimohonkan banding ;

DUDUK PERKARA

Memperhatikan semua uraian yang termuat dalam putusan Pengadilan Agama Sidoarjo Nomor 2867/Pdt.G/2020/PA.Sda Tanggal 23 September 2020 *Masehi*, bertepatan dengan tanggal 7 *Shafar* 1442 *Hijriah*, yang amarnya sebagai berikut:

1. Mengabulkan gugatan Penggugat ;
2. Menjatuhkan talak satu ba'in Sughro Tergugat (PEMBANDING) kepada Penggugat (TERBANDING);
3. Menetapkan anak Penggugat dan Tergugat bernama ANAK I yang lahir pada tanggal 11-07-2002 dan Achmad ANAK II yang lahir pada tanggal 30-8-2006, berada di bawah hadhonah (pemeliharaan) Penggugat dengan tetap memberikan hak kepada Tergugat (ayahnya) untuk menjenguk, mengajak serta mencurahkan kasih sayangnya kepada kedua anak tersebut sepanjang tidak mengganggu kepentingan anak ;
4. Membebankan kepada Penggugat untuk membayar biaya perkara ini sebesar Rp. 541.000,00(lima ratus empat puluh satu ribu rupiah).

Bahwa terhadap putusan tersebut Tergugat/Pembanding telah mengajukan permohonan banding pada hari Senin tanggal 5 Oktober 2020 sebagaimana tercantum dalam Akta Permohonan Banding yang dibuat oleh Panitera Pengadilan Agama Agama Sidoartjo Nomor 2867/Pdt.G/2020/PA Sda 5 Oktober 2020. Permohonan banding tersebut telah diberitahukan kepada Penggugat/Terbanding pada tanggal 12 November 2020;

Bahwa Tergugat/Pembanding telah menyerahkan Memori Banding sebagaimana Surat Tanda Terima Memori Banding yang dibuat oleh Panitera Pengadilan Agama Sidoarjo Nomor 2867/Pdt.G/2020/PA Sda tanggal 14 Oktober

Disclaimer
Kepaniteraan Mahkamah Agung Republik Indonesia berusaha untuk selalu mencantumkan informasi paling kini dan akurat sebagai bentuk komitmen Mahkamah Agung untuk pelayanan publik, transparansi dan akuntabilitas pelaksanaan fungsi peradilan. Namun dalam hal-hal tertentu masih dimungkinkan terjadi permasalahan teknis terkait dengan akurasi dan keterkinian informasi yang kami sajikan, hal mana akan terus kami perbaiki dari waktu kewaktu.
Dalam hal Anda menemukan inakurasi informasi yang termuat pada situs ini atau informasi yang seharusnya ada, namun belum tersedia, maka harap segera hubungi Kepaniteraan Mahkamah Agung RI melalui :
Email : kepaniteraan@mahkamahagung.go.id Telp : 021-384 3348 (ext.318)

2020 dan telah diberitahukan kepada Penggugat/Terbanding pada tanggal 26 November 2020;

Bahwa atas Memori Banding tersebut Penggugat/Terbanding telah mengajukan Kontra Memori Banding tertanggal 26 November 2020 sebagaimana diuraikan dalam Tanda Terima Kontra Memori Banding yang dibuat oleh Panitera Pengadilan Agama Sidoarjo Nomor 2867/Pdt.G/2020/PA Sda tanggal 26 November 2020 dan telah diberitahukan kepada Tergugat/Pembanding pada tanggal 27 November 2020;

Bahwa Tergugat/Pembanding telah diberitahu untuk memeriksa berkas perkara banding (*inzage*) pada tanggal 12 November 2020, namun Tergugat/ Pembanding tidak melaksanakan *inzage* sebagaimana Surat Keterangan Tidak Memeriksa Berkas Perkara *(inzage)* yang dibuat oleh Panitera Pengadilan Agama Sidoarjo Nomor 2867/Pdt.G/2020/PA Sda. tanggal 18 November 2020;

Bahwa Penggugat/Terbanding telah diberitahukan untuk memeriksa berkas perkara banding (*inzage*) pada tanggal 12 November 2020, dan Penggugat/Terbanding telah melaksanakan *inzage* sebagaimana Surat Keterangan Telah Memeriksa Berkas Perkara (*Inzage*) yang dibuat oleh Panitera Pengadilan Agama Sidoarjo Nomor 2867/Pdt.G/2020/PA.Sda. tanggal 19 November 2020;

Bahwa permohonan banding tersebut telah di daftar di Kepaniteraan Pengadilan Tinggi Agama Surabaya pada tanggal 17 Desember 2020 dengan Nomor 510/Pdt.G/2020/PTA.Sby dan telah diberitahukan kepada Ketua Pengadilan Agama Sidoarjo, melalui surat Nomor W13-A/5081/Hk.05/12/2020 tanggal 17 Desember 2020 yang tembusannya disampaikan kepada Tergugat/ Pembanding dan Penggugat/Terbanding;

Disclaimer
Kepaniteraan Mahkamah Agung Republik Indonesia berusaha untuk selalu mencantumkan informasi paling kini dan akurat sebagai bentuk komitmen Mahkamah Agung untuk pelayanan publik, transparansi dan akuntabilitas pelaksanaan fungsi peradilan. Namun dalam hal-hal tertentu masih dimungkinkan terjadi permasalahan teknis terkait dengan akurasi dan keterkinian informasi yang kami sajikan, hal mana akan terus kami perbaiki dari waktu kewaktu.
Dalam hal Anda menemukan inakurasi informasi yang termuat pada situs ini atau informasi yang seharusnya ada, namun belum tersedia, maka harap segera hubungi Kepaniteraan Mahkamah Agung RI melalui :
Email : kepaniteraan@mahkamahagung.go.id Telp : 021-384 3348 (ext.318)

PERTIMBANGAN HUKUM

Menimbang, bahwa dalam putusan pengadilan tingkat pertama Pembanding adalah sebagai Tergugat, karena itu berdasarkan Pasal 61 Undang-Undang Nomor 7 Tahun 1989 Tentang Peradilan Agama yang telah diubah dengan Undang-Undang Nomor 3 Tahun 2006 dan diubah untuk kedua kalinya dengan Undang-Undang Nomor 50 tahun 2009, dan pasal 26 ayat (1) Undang-Undang Nomor 48 Tahun 2009 Tentang Kekuasaan Kehakiman, maka Tergugat/Pembanding adalah sebagai *persona standi in judicio* untuk mengajukan permohonan banding dalam perkara ini. Sesuai ketentuan pasal 121 ayat (4) H.I.R. biaya perkara harus dibayar terlebih dahulu oleh pihak yang berperkara dan berdasarkan Surat Kuasa Untuk Membayar (SKUM) tanggal 5 Oktober 2020 dan Tanda Bukti Penyetoran pada BRI tanggal 5 Oktober 2020 telah lunas dibayar panjar biaya perkara banding perkara *a quo;*

Menimbang, bahwa Putusan Pengadilan Agama Sidoarjo Nomor 2867/Pdt.G/2020/PA.Sda. dijatuhkan pada tanggal 23 September 2020 *Masehi*, bertepatan dengan tanggal 7 *Shafar* 1442 *Hijriah,* dihadiri oleh Penggugat/ Terbanding diluar hadirnya Tergugat/Pembanding. Amar Putusan tersebut telah diberitahukan kepada Tergugat/Pembanding pada tanggal 5 Oktober 2020. Terhadap putusan tersebut Tergugat/Pembanding mengajukan permohonan banding pada tanggal 5 Oktober 2020, yakni sehari setelah isi putusan diberitahukan kepada Tergugat/Pembanding;

Menimbang, bahwa dengan demikian permohonan banding Tergugat/ Pembanding telah diajukan menurut cara-cara yang ditetapkan dalam undang-undang dan dalam tenggat masa banding, oleh karena itu berdasarkan Pasal 7 Undang-Undang Nomor 20 Tahun 1947 permohonan banding Tergugat/ Pembanding secara formal harus dinyatakan dapat diterima untuk diperiksa ditingkat banding;

Disclaimer
Kepaniteraan Mahkamah Agung Republik Indonesia berusaha untuk selalu mencantumkan informasi paling kini dan akurat sebagai bentuk komitmen Mahkamah Agung untuk pelayanan publik, transparansi dan akuntabilitas pelaksanaan fungsi peradilan. Namun dalam hal-hal tertentu masih dimungkinkan terjadi permasalahan teknis terkait dengan akurasi dan keterkinian informasi yang kami sajikan, hal mana akan terus kami perbaiki dari waktu kewaktu.
Dalam hal Anda menemukan inakurasi informasi yang termuat pada situs ini atau informasi yang seharusnya ada, namun belum tersedia, maka harap segera hubungi Kepaniteraan Mahkamah Agung RI melalui :
Email : kepaniteraan@mahkamahagung.go.id Telp : 021-384 3348 (ext.318)

Menimbang, bahwa Pengadilan Tinggi Agama Surabaya sebagai pengadilan ulangan pada tingkat banding, agar dapat memberikan putusan yang benar dan adil, maka perlu memeriksa ulang perkara ini untuk kemudian dipertimbangkan dan diputus ulang pada tingkat banding;

Menimbang, bahwa setelah mempelajari dan memperhatikan dengan seksama salinan resmi Putusan Pengadilan Agama Sidoarjo Nomor 2867/Pdt.G/2020/PA.Sda. tanggal 23 September 2020 *Masehi*, bertepatan dengan tanggal 7 *Shafar* 1442 *Hijriah*, berita acara sidang serta surat-surat lainnya yang berkaitan dengan perkara ini, utamanya setelah memperhatikan pertimbangan hukum Majelis Hakim Tingkat Pertama yang memutus perkara ini, memori banding dan kontra memori banding maka Majelis Hakim Tingkat Banding akan mempertimbangkan sebagai berikut:

Menimbang, bahwa Penggugat/Terbanding dalam gugatannya tanggal 13 Agustus 2020 telah mengajukan dalil-dalil gugatannya yang pada pokoknya bahwa Penggugat/Terbanding dan Tergugat/Pembanding menikah pada tanggal 14 Juni 2001, di Kantor Urusan Agama Kecamatan Sidoarjo Kabupaten Sidoarjo. Setelah akad nikah Penggugat/Terbanding dan Tergugat/Pembanding bertempat tinggal di Desa Bluru Kidul, Kecamatan Sidoarjo dan sekarang telah dikaruniai 2 (dua) orang anak. Sejak bulan Juni 2008 antara Penggugat/Terbanding dan Tergugat/Pembanading sering terjadi perselisihan dan pertengkaran yang disebabkan Tergugat/Pembanding selingkuh, sudah nikah *sirri* dan sudah dikaruniai anak, sedang Penggugat/Terbanding tidak mau dipoligami. Puncak pertengkaran terjadi pada bulan Agustus 2020, sulit dirukunkan lagi akhirnya Penggugat/Terbanding pergi meninggalkan rumah bersama berpisah dengan Tergugat/Pembanding samapai dengan gugatan ini diajukan sudah 1 (satu) bulan lamanya. Oleh karena itu Penggugat/ Terbanding mohon putusnya ikatan perkawinan dengan Tergugat/Pembanding dan mohon hak asuh/*hadhanah* 2 (dua) anak tersebut diserahkan kepada Penggugat/ Terbanding;

Menimbang, bahwa Tergugat/Pembanding dalam memori bandingnya antara lain menyatakan bahwa Majelis Hakim Tingkat Partama tidak memberi kesempatan sama

Disclaimer
Kepaniteraan Mahkamah Agung Republik Indonesia berusaha untuk selalu mencantumkan informasi paling kini dan akurat sebagai bentuk komitmen Mahkamah Agung untuk pelayanan publik, transparansi dan akuntabilitas pelaksanaan fungsi peradilan. Namun dalam hal-hal tertentu masih dimungkinkan terjadi permasalahan teknis terkait dengan akurasi dan keterkinian informasi yang kami sajikan, hal mana akan terus kami perbaiki dari waktu kewaktu.
Dalam hal Anda menemukan inakurasi informasi yang termuat pada situs ini atau informasi yang seharusnya ada, namun belum tersedia, maka harap segera hubungi Kepaniteraan Mahkamah Agung RI melalui :
Email : kepaniteraan@mahkamahagung.go.id   Telp : 021-384 3348 (ext.318)

sekali kepada Tergugat/Pembanding untuk menyampaikan pembelaan atau bantahan terhadap gugatan Penggugat/Terbanding;

Menimbang, bahwa Tergugat/Pembanding telah 4 (empat) kali dipanggil untuk menghadiri sidang tetapi hanya satu kali hadir dipersidangan yaitu sidang tanggal 9 September 2020. Sedang dalam sidang tanggal 2 September 2020, 16 September 2020 dan 23 September 2020 yang sebenarnya merupakan kesempatan Tergugat/ Pembanding untuk mengemukakan bantahannya, namun Tergugat/Pembanding tidak pernah hadir, tidak menyuruh orang lain sebagai kuasa/wakilnya, tidak memberitahukan kepada Majelis Hakim tentang alasan ketidak hadirannya tersebut, meskipun menurut berita acara sidang Tergugat/Pembanding telah dipanggil secara sah dan patut. Oleh karenanya sikap Majelis Hakim Tingkat Pertama yang telah melanjutkan pemeriksaan dan memutus perkara ini sudah tepat dan benar, sesuai dengan sabda Rasulullah SAW yang diriwayatkan oleh Al Bazzar dikuatkan oleh Ibnu Addiy dari ‘Imran bin Khushain RA dalam kitab *Majma’uz Zawa-id wa Manba’ul Fawa-id juz* 4 halaman 198 yang menyatakan :

Artinya : “*Barangsiapa dipanggil untuk menghadap Hakim namun tidak mau menghadirinya maka dia adalah dhalim dan tidak ada hak baginya”*;

Menimbang, bahwa Penggugat/Terbanding untuk meneguhkan dalil-dalil gugatannya telah menyerahkan 4 (empat) bukti surat yaitu bukti P.1 sampai dengan P.4. serta mengajukan 2 (dua) orang saksi yaitu SAKSI I dan SAKSI II (keduanya adalah kakak kandung Penggugat/Terbanding), telah memberikan keterangan dibawah sumpahnya masing-masing seperti tersebut dalam berita acara dan putusan tingkat pertama;

Menimbang, bahwa Majelis Hakim Banding berpendapat berdasarkan dalil-dalil gugatan Penggugat/Terbanding dapat disimpulkan bahwa alasan perceraian yang dikemukakan oleh Penggugat/Terbanding pada dasarnya adalah sebagaimana disebutkan dalam penjelasan pasal 39 ayat (2) Undang-Undang Nomor 1 Tahun 1974 tentang Perkawinan jis Pasal 19 huruf f Peraturan Pemerintah Nomor 9 Tahun 1975 jis pasal 116 huruf f Kompilasi Hukum Islam yakni

Disclaimer
Kepaniteraan Mahkamah Agung Republik Indonesia berusaha untuk selalu mencantumkan informasi paling kini dan akurat sebagai bentuk komitmen Mahkamah Agung untuk pelayanan publik, transparansi dan akuntabilitas pelaksanaan fungsi peradilan. Namun dalam hal-hal tertentu masih dimungkinkan terjadi permasalahan teknis terkait dengan akurasi dan keterkinian informasi yang kami sajikan, hal mana akan terus kami perbaiki dari waktu kewaktu.
Dalam hal Anda menemukan inakurasi informasi yang termuat pada situs ini atau informasi yang seharusnya ada, namun belum tersedia, maka harap segera hubungi Kepaniteraan Mahkamah Agung RI melalui :
Email : kepaniteraan@mahkamahagung.go.id Telp : 021-384 3348 (ext.318)

“antara suami isteri telah terjadi perselisihan dan pertengkaran terus menerus dan tidak ada harapan untuk hidup rukun lagi dalam satu rumah tangga”;

Menimbang, bahwa dalam pasal *a quo* terdapat 4 (empat) unsur yang harus dibuktikan oleh Penggugat/Terbanding yaitu : 1. Adanya hubungan suami isteri, 2. Adanya perselisihan dan pertengkaran, 3. Sifatnya terus menerus, 4. Tidak ada harapan untuk hidup rukun lagi dalam satu rumah tangga;

Menimbang, bahwa berdasarkan bukti P.1 dan keterangan saksi-saksi terbukti bahwa antara Penggugat/Terbanding dengan Tergugat/Pembanding terikat dalam perkawinan yang sah sebagai suami isteri, *akad nikahnya* dilaksanakan di Kantor Urusan Agama Kecamatan Sidoarjo Kabupaten Sidoarjo;

Menimbang, bahwa walaupun saksi pertama dan saksi kedua adalah saudara kandung Penggugat/Terbanding tetap dapat diterima sebagai saksi karena telah memenuhi syarat formil saksi sebagaimana ditentukan pasal 145 HIR. Kedua saksi tersebut memberikan keterangan yang sama bahwa saksi melihat pada awalnya Penggugat/Terbanding dengan Tergugat/Pembanding hidup rukun sebagaimana layaknya suami isteri, namun sebelum berpisah **saksi** mendengar penuturan Penggugat/Terbanding bahwa mereka berdua sering bertengkar. Saksi diberitahu Penggugat/Terbanding penyebab pertengkaran tersebut karena Tergugat/Pembanding sudah *nikah sirri* dan sudah dikaruniai anak, tetapi Penggugat/Terbanding tidak mau dipoligami; Oleh karena saksi mengetahui adanya pertengkaran tersebut dari penuturan Penggugat/ Terbanding, dan saksi mengetahui penyebab pertengkaran juga karena diberitahu oleh Penggugat/ Terbanding maka keterangan kedua saksi dalam 2 (dua) hal ini bersifat ***de auditu*** sehingga keterangannya tidak memenuhi syarat *materiil* saksi sebagai mana diatur dalam pasal 171 HIR. Dengan demikian dalil Penggugat/Terbanding tentang adanya perselisihan dan pertengkaran serta sebab-sebabnya tidak terbukti sebagai fakta;

Menimbang, bahwa kedua saksi menerangkan bahwa Penggugat/ Terbanding dan Tergugat/Pembanding telah berpisah tempat tinggal selama 2

Disclaimer
Kepaniteraan Mahkamah Agung Republik Indonesia berusaha untuk selalu mencantumkan informasi paling kini dan akurat sebagai bentuk komitmen Mahkamah Agung untuk pelayanan publik, transparansi dan akuntabilitas pelaksanaan fungsi peradilan. Namun dalam hal-hal tertentu masih dimungkinkan terjadi permasalahan teknis terkait dengan akurasi dan keterkinian informasi yang kami sajikan, hal mana akan terus kami perbaiki dari waktu kewaktu.
Dalam hal Anda menemukan inakurasi informasi yang termuat pada situs ini atau informasi yang seharusnya ada, namun belum tersedia, maka harap segera hubungi Kepaniteraan Mahkamah Agung RI melalui :
Email : kepaniteraan@mahkamahagung.go.id   Telp : 021-384 3348 (ext.318)

bulan, saksi-saksi sudah berupaya mendamaikan kedua pihak tetapi tidak berhasil. Berdasarkan hal-hal tersebut, ditambah dengan upaya majelis hakim dan mediator untuk mendamaikan tetapi tidak berhasil, maka timbul persangkaan bahwa antara kedua pihak telah terjadi perselisihan dan pertengkaran terus menerus dan tidak dapat dirukunkan lagi;

Menimbang, bahwa walaupun keterangan kedua saksi tentang perselisihan dan pertengkaran serta sebab-sebabnya tersebut bersifat ***de auditu,*** tetapi karena Penggugat/Terbanding dan Tergugat/Pembanding telah berpisah tempat tinggal selama 2 bulan, dan berbagai upaya untuk mendamaikan kedua pihak telah dilakukan akan tetapi tidak berhasil, kedua pihak tetap berpisah tempat tinggal dan telah mengabaikan kewajibannya masing-masing sebagai suami isteri, hal ini membuktikan bahwa antara kedua pihak sudah tidak dapat dirukunkan lagi dan sudah tidak ada harapan untuk hidup rukun sebagai suami isteri dalam satu keluarga, mempertahankan pernikahan yang seperti itu hanya akan memperpanjang penderitaan kedua belah pihak, oleh karena itu untuk menyelesaikan masalah tersebut solusi terbaik adalah menceraikan kedua orang tersebut dari ikatan suami isteri;

Menimbang, bahwa berdasarkan pertimbangan-pertimbangan tersebut terbukti bahwa alasan-alasan perceraian yang dikemukakan oleh Penggugat/ Terbanding telah memenuhi unsur-unsur yang ditentukan dalam penjelasan pasal 39 ayat (2) huruf (f) Undang-Undang Nomor 1 Tahun 1974 Jis. pasal 19 huruf (f) Peraturan Pemerintah Nomor 9 Tahun 1975 Jis. pasal 116 huruf (f) Kompilasi Hukum Islam, oleh karena itu gugatan Penggugat/Terbanding harus dikabulkan;

Menimbang, bahwa mengenai gugatan *hadhanah* terhadap 2 (dua) orang anak Tergugat/Pembanding dan Penggugat/Terbanding, Majelis Hakim Tingkat Pertama telah mempertimbangkan gugatan tersebut dengan tepat dan benar oleh karenanya pertimbangan hukum tersebut diambil alih sebagai pertimbangan

Disclaimer
Kepaniteraan Mahkamah Agung Republik Indonesia berusaha untuk selalu mencantumkan informasi paling kini dan akurat sebagai bentuk komitmen Mahkamah Agung untuk pelayanan publik, transparansi dan akuntabilitas pelaksanaan fungsi peradilan. Namun dalam hal-hal tertentu masih dimungkinkan terjadi permasalahan teknis terkait dengan akurasi dan keterkinian informasi yang kami sajikan, hal mana akan terus kami perbaiki dari waktu kewaktu.
Dalam hal Anda menemukan inakurasi informasi yang termuat pada situs ini atau informasi yang seharusnya ada, namun belum tersedia, maka harap segera hubungi Kepaniteraan Mahkamah Agung RI melalui :
Email : kepaniteraan@mahkamahagung.go.id Telp : 021-384 3348 (ext.318)

hukum Majelis Hakim Tingkat Banding dan selanjutnya gugatan *hadhanah* tersebut dapat dikabulkan;

Menimbang, bahwa mengenai keberatan-keberatan Tergugat/Pembanding yang dituangkan dalam memori bandingnya secara i*nplisit* sudah dipertimbangkan sebagaimana terurai di atas dan sesuai pula dengan *yurisprudensi* Mahkamah Agung RI tanggal 06 April 1955 Nomor : 247 K/Sip/1953, yang mengabstraksikan: *"Bahwa hakim banding tidak wajib meninjau satu persatu dalil yang termuat dalam suatu memori banding dan juga tidak wajib meninjau satu persatu segala pertimbangan hakim tingkat pertama";*

Menimbang, bahwa dengan pertimbangan-pertimbangan tersebut diatas, maka Putusan Pengadilan Agama Sidoarjo Nomor 2867/Pdt.G/2020/PA Sda. tanggal 23 September 2020 *Masehi*, bertepatan dengan tanggal 7 *Shafar* 1442 *Hijriah* harus dikuatkan;

Menimbang, bahwa oleh karena perkara *a quo* termasuk bidang perkawinan, maka berdasarkan ketentuan Pasal 89 ayat (1) Undang-Undang Nomor 7 Tahun 1989 tentang Peradilan Agama yang telah diubah dan ditambah dengan Undang-Undang Nomor 3 Tahun 2006 dan perubahan kedua dengan Undang-Undang Nomor 50 Tahun 2009, biaya perkara pada tingkat banding dibebankan kepada Tergugat/Pembanding;

Memperhatikan, Peraturan dan Perundang-undangan yang berlaku serta ketentuan-ketentuan hukum lainnya yang berkaitan dengan perkara ini;

M E N G A D I L I

- Menyatakan bahwa permohonan banding Pembanding dapat diterima untuk diperiksa ditingkat banding;
- Menguatkan Putusan Pengadilan Agama Sidoarjo Nomor 2867/Pdt.G/2020/PA.Sda tanggal 23 September 2020 *Masehi*, bertepatan dengan tanggal 7 *Shafar* 1442 *Hijriah*;
- Membebankan biaya perkara pada tingkat banding kepada Tergugat/

Disclaimer
Kepaniteraan Mahkamah Agung Republik Indonesia berusaha untuk selalu mencantumkan informasi paling kini dan akurat sebagai bentuk komitmen Mahkamah Agung untuk pelayanan publik, transparansi dan akuntabilitas pelaksanaan fungsi peradilan. Namun dalam hal-hal tertentu masih dimungkinkan terjadi permasalahan teknis terkait dengan akurasi dan keterkinian informasi yang kami sajikan, hal mana akan terus kami perbaiki dari waktu kewaktu.
Dalam hal Anda menemukan inakurasi informasi yang termuat pada situs ini atau informasi yang seharusnya ada, namun belum tersedia, maka harap segera hubungi Kepaniteraan Mahkamah Agung RI melalui :
Email : kepaniteraan@mahkamahagung.go.id Telp : 021-384 3348 (ext.318)

Pembanding sebesar Rp150.000,00 (seratus lima puluh ribu rupiah);

Demikian putusan ini dijatuhkan di Pengadilan Tinggi Agama Surabaya dalam *musyawarah* Majelis pada hari Senin tanggal 28 Desember 2020 *Masehi* bertepatan dengan tanggal 14 *Jumadil Awwal* 1442 *Hijriah* oleh kami **Drs. H. SOLIHUN, S.H., M.H.,** selaku Ketua Majelis, **Drs. H. MAS'UD** dan **SULHAN, S.H., M.Hum.** masing-masing sebagai Hakim Anggota yang ditunjuk berdasarkan Penetapan Ketua Pengadilan Tinggi Agama Surabaya Nomor 510/Pdt.G/2020/PTA.Sby tanggal 18 Desember 2020 sebagai Majelis Hakim guna memeriksa dan mengadili perkara ini dalam tingkat banding, dan pada hari Selasa tanggal 29 Desember 2020 *Masehi* bertepatan dengan tanggal 14 *Jumadil Awwal* 1442 *Hijriah* putusan tersebut dibacakan dalam sidang terbuka untuk umum oleh Hakim Ketua didampingi oleh Hakim Anggota tersebut serta dibantu oleh **Dra. SRI PRATIWININGRUM M.HES.** sebagai Panitera Pengganti, tanpa dihadiri oleh Pembanding dan Terbanding.

Ketua Majelis,

ttd

Drs. H. SOLIHUN, S.H., M.H.

Hakim Anggota,

ttd	ttd
**Drs. H. MAS'UD**	**SULHAN, S.H., M.Hum.,**

Disclaimer
Kepaniteraan Mahkamah Agung Republik Indonesia berusaha untuk selalu mencantumkan informasi paling kini dan akurat sebagai bentuk komitmen Mahkamah Agung untuk pelayanan publik, transparansi dan akuntabilitas pelaksanaan fungsi peradilan. Namun dalam hal-hal tertentu masih dimungkinkan terjadi permasalahan teknis terkait dengan akurasi dan keterkinian informasi yang kami sajikan, hal mana akan terus kami perbaiki dari waktu kewaktu.
Dalam hal Anda menemukan inakurasi informasi yang termuat pada situs ini atau informasi yang seharusnya ada, namun belum tersedia, maka harap segera hubungi Kepaniteraan Mahkamah Agung RI melalui :
Email : kepaniteraan@mahkamahagung.go.id   Telp : 021-384 3348 (ext.318)

Panitera Pengganti,

ttd

Dra. SRI PRATIWININGRUM, M.HES.

RINCIAN BIAYA PROSES:

Pemberkasan ATK	: Rp.	134.000,00
Redaksi	: Rp.	10.000,00
Meterai	: Rp.	6.000,00
Jumlah	: Rp.	150.000,00

(seratus lima puluh ribu rupiah)

UNTUK SALINAN
PENGADILAN TINGGI AGAMA SURABAYA
PANITERA,

Hj. SITI ROMIYANI,S.H., M.H.

Disclaimer
Kepaniteraan Mahkamah Agung Republik Indonesia berusaha untuk selalu mencantumkan informasi paling kini dan akurat sebagai bentuk komitmen Mahkamah Agung untuk pelayanan publik, transparansi dan akuntabilitas pelaksanaan fungsi peradilan. Namun dalam hal-hal tertentu masih dimungkinkan terjadi permasalahan teknis terkait dengan akurasi dan keterkinian informasi yang kami sajikan, hal mana akan terus kami perbaiki dari waktu kewaktu.
Dalam hal Anda menemukan inakurasi informasi yang termuat pada situs ini atau informasi yang seharusnya ada, namun belum tersedia, maka harap segera hubungi Kepaniteraan Mahkamah Agung RI melalui :
Email : kepaniteraan@mahkamahagung.go.id Telp : 021-384 3348 (ext.318)
