# Supplementary material for: Classification of divorce causes during the COVID-19 pandemic using convolutional neural networks
Source: PeerJ Comput Sci. 2022 Jun 30;8:e998. doi: 10.7717/peerj-cs.998 (PMC9299239; doi:10.7717/peerj-cs.998)
Supplement: Supplemental Information 2 [file peerj-cs-08-998-s002.zip › Kelalaian Dataset/Data ke-28.pdf]

P U T U S A N

Nomor 3190/Pdt.G/2020/PA.Lmj

بِسْمِ اللهِ الرَّحْمٰنِ الرَّحِيْمِ

DEMI KEADILAN BERDASARKAN KETUHANAN YANG MAHA ESA

Pengadilan Agama Lumajang yang memeriksa dan mengadili perkara-perkara cerai gugat dalam tingkat pertama telah menjatuhkan putusan sebagai berikut dalam perkara antara:

PENGGUGAT umur 28 tahun, agama Islam, pekerjaan Dagang, tempat tinggal di KABUPATEN LUMAJANG, selanjutnya disebut sebagai Penggugat;

Melawan

TERGUGAT umur 29 tahun, agama Islam, pekerjaan xxxxx, tempat tinggal di KABUPATEN LUMAJANG, selanjutnya disebut sebagai Tergugat;

Pengadilan Agama tersebut;

Telah mempelajari surat-surat yang berkaitan dengan perkara ini;

Telah mendengar keterangan Penggugat dan para saksi di muka sidang;

DUDUK PERKARA

Bahwa Penggugat dalam surat Gugatannya tertanggal 14 Desember 2020 yang telah terdaftar di Kepaniteraan Pengadilan Agama Lumajang tanggal 14 Desember 2020 di bawah Register Perkara Nomor 3190/Pdt.G/2020/PA.Lmj pada pokoknya mengemukakan hal-hal sebagai berikut:

1. Bahwa pada tanggal 07 Juli 2017, Penggugat dengan Tergugat melangsungkan pernikahan yang dicatat oleh Pegawai Pencatat Nikah Kantor Urusan Agama Kecamatan Tempeh Kabupaten Lumajang (Kutipan Akta Nikah Nomor : 0427/031/VII/2017 tanggal 07 Juli 2017);
2. Bahwa setelah pernikahan tersebut Penggugat dengan Tergugat bertempat tinggal di rumah Penggugat hingga Desember 2019. Selama pernikahan

Disclaimer
Kepaniteraan Mahkamah Agung Republik Indonesia berusaha untuk selalu mencantumkan informasi paling kini dan akurat sebagai bentuk komitmen Mahkamah Agung untuk pelayanan publik, transparansi dan akuntabilitas pelaksanaan fungsi peradilan. Namun dalam hal-hal tertentu masih dimungkinkan terjadi permasalahan teknis terkait dengan akurasi dan keterkinian informasi yang kami sajikan, hal mana akan terus kami perbaiki dari waktu kewaktu.
Dalam hal Anda menemukan inakurasi informasi yang termuat pada situs ini atau informasi yang seharusnya ada, namun belum tersedia, maka harap segera hubungi Kepaniteraan Mahkamah Agung RI melalui :
Email : kepaniteraan@mahkamahagung.go.id Telp : 021-384 3348 (ext.318)

tersebut Penggugat dengan Tergugat telah hidup rukun sebagaimana layaknya suami istri namun belum dikaruniai keturunan ;

3. Bahwa semula rumah tangga Penggugat dan Tergugat rukun baik akan tetapi kurang lebih sejak bulan Januari tahun 2019 ketentraman rumah tangga Penggugat dengan Tergugat mulai goyah, setelah antara Penggugat dengan Tergugat terjadi perselisihan dan pertengkaran yang penyebabnya antara lain :
   - Tergugat sering marah-marah tanpa alasan yang jelas hingga merusak barang-barang perabotan rumah tangga dan nafkah sehari-hari kurang tercukupi;
4. Bahwa puncak perselisihan dan pertengkaran antara Penggugat dan Tergugat terjadi kurang lebih pada bulan Desember tahun 2019, yang akibatnya Tergugat pergi meninggalkan Penggugat dan pulang ke rumah orangtua Tergugat sendiri dengan alamat sebagaimana tersebut di atas sehingga pisah tempat tinggal selama 1 tahun hingga sekarang dan selama itu tidak ada hubungan lagi baik lahir maupun batin;
5. Bahwa atas hal tersebut diatas kini Penggugat sudah tidak tahan lagi dengan kondisi rumah tangganya hingga kemudian mengajukan Gugatan ini;
6. Bahwa Penggugat sanggup membayar seluruh biaya yang timbul akibat perkara ini;

Berdasarkan alasan/dalil-dalil diatas, Penggugat mohon agar Ketua Pengadilan Agama Lumajang segera memeriksa dan mengadili perkara ini, selanjutnya menjatuhkan putusan yang amarnya berbunyi :

Primair :

1. Mengabulkan gugatan Penggugat;
2. Menjatuhkan talak satu ba'in sughro Tergugat terhadap Penggugat;
3. Menetapkan biaya perkara sesuai dengan peraturan yang berlaku;

Subsidair :

Atau menjatuhkan putusan lain yang seadil-adilnya;

Bahwa pada hari dan tanggal persidangan yang telah ditetapkan Penggugat menghadap ke persidangan, sedangkan Tergugat tidak menghadap

Disclaimer
Kepaniteraan Mahkamah Agung Republik Indonesia berusaha untuk selalu mencantumkan informasi paling kini dan akurat sebagai bentuk komitmen Mahkamah Agung untuk pelayanan publik, transparansi dan akuntabilitas pelaksanaan fungsi peradilan. Namun dalam hal-hal tertentu masih dimungkinkan terjadi permasalahan teknis terkait dengan akurasi dan keterkinian informasi yang kami sajikan, hal mana akan terus kami perbaiki dari waktu kewaktu.
Dalam hal Anda menemukan inakurasi informasi yang termuat pada situs ini atau informasi yang seharusnya ada, namun belum tersedia, maka harap segera hubungi Kepaniteraan Mahkamah Agung RI melalui :
Email : kepaniteraan@mahkamahagung.go.id Telp : 021-384 3348 (ext.318)

ke persidangan dan tidak menyuruh orang lain untuk menghadap sebagai kuasanya, meskipun menurut surat panggilan (Relaas) tanggal 17 Desember 2020 dan 23 Desember 2020 Nomor 3190/Pdt.G/2020/PA.Lmj yang dibacakan di persidangan, Tergugat telah dipanggil secara resmi dan patut, sedangkan tidak ternyata bahwa tidak datangnya itu disebabkan oleh sesuatu halangan yang sah, maka pemeriksaan perkara ini dilanjutkan dengan tanpa hadirnya Tergugat;

Bahwa Majelis Hakim telah berusaha menasehati dengan menyarankan kepada pihak Penggugat untuk tidak meneruskan gugatannya dan rukun kembali dengan Tergugat, akan tetapi tidak berhasil;

Bahwa oleh karena usaha perdamaian tidak berhasil, maka pemeriksaan diteruskan dengan membacakan surat gugatan Penggugat yang isinya tetap dipertahankan oleh Penggugat;

Bahwa untuk meneguhkan dalil-dalil Gugatannya di muka persidangan, Penggugat telah mengajukan bukti-bukti berupa :

A. Bukti Tertulis

1. Fotokopi Kartu Tanda Penduduk NIK 3508055202920010 tanggal 25 Januari 2018 atas nama ERNAWATI yang dikeluarkan oleh Dinas Kependudukan dan Pencatatan Sipil Kabupaten Lumajang, bermaterai cukup, telah dicocokkan sesuai dengan aslinya dan diberi tanda P.1 ;
2. Fotokopi Kutipan Akta Nikah Nomor 0427/031/VII/2017 tanggal 07 Juli 2017 yang dikeluarkan oleh Kantor Urusan Agama Kecamatan Tempeh Kabupaten Lumajang Kabupaten Lumajang, bermaterai cukup, telah dicocokkan sesuai dengan aslinya dan diberi tanda P.2;

B. Bukti Saksi

1. Nama SAKSI 1, umur 44 tahun, agama Islam, pekerjaan xxx xxxxx xxxxxx, tempat kediaman di KABUPATEN LUMAJANG, dengan di bawah sumpah telah memberikan keterangan di persidangan yang isi pokoknya sebagai berikut :
   - Bahwa saksi kenal dengan Penggugat dan Tergugat karena saksi adalah Ibu kandung Penggugat;

Disclaimer
Kepaniteraan Mahkamah Agung Republik Indonesia berusaha untuk selalu mencantumkan informasi paling kini dan akurat sebagai bentuk komitmen Mahkamah Agung untuk pelayanan publik, transparansi dan akuntabilitas pelaksanaan fungsi peradilan. Namun dalam hal-hal tertentu masih dimungkinkan terjadi permasalahan teknis terkait dengan akurasi dan keterkinian informasi yang kami sajikan, hal mana akan terus kami perbaiki dari waktu kewaktu.
Dalam hal Anda menemukan inakurasi informasi yang termuat pada situs ini atau informasi yang seharusnya ada, namun belum tersedia, maka harap segera hubungi Kepaniteraan Mahkamah Agung RI melalui :
Email : kepaniteraan@mahkamahagung.go.id Telp : 021-384 3348 (ext.318)

- Bahwa saksi mengetahui antara Penggugat dan Tergugat adalah suami istri;
- Bahwa saksi mengetahui antara Penggugat dengan Tergugat setelah menikah bertempat tinggal bersama di rumah Penggugat hingga Desember 2019;
- Bahwa saksi mengetahui antara Penggugat dengan Tergugat belum dikaruniai anak;
- Bahwa saksi mengetahui antara Penggugat dengan Tergugat telah terjadi perselisihan dan pertengkaran disebabkan nafkah sehari-hari Penggugat kurang tercukupi disamping itu Tergugat gampang marah-marah karena cemburu tanpa alasan yang jelas bahkan sampai memukul Penggugat dan merusak perabotan rumah tangga;
- Bahwa saksi mengetahui Penggugat dan Tergugat sudah pisah tempat tinggal sejak Desember 2019 sampai dengan sekarang, kurang lebih 1 tahun;
- Bahwa saksi mengetahui Tergugat pergi meninggalkan Penggugat pulang ke rumah orang tuanya sendiri hingga sekarang;
- bahwa selama pisah tersebut Penggugat dan Tergugat tidak pernah komunikasi atau kumpul layaknya suami istri;
- Bahwa saksi mengetahui keluarga sudah merukunkan Penggugat dengan Tergugat tetapi tidak berhasil
- Bahwa saksi sebagai Ibu Kandung Penggugat sudah tidak sanggup untuk merukunkan Penggugat dengan Tergugat;

2. Nama SAKSI 2, umur 52 tahun, agama Islam, pekerjaan xxxxx, tempat kediaman di KABUPATEN LUMAJANG, dengan di bawah sumpah telah memberikan keterangan di persidangan yang isi pokoknya sebagai berikut :

- Bahwa saksi kenal dengan Penggugat dan Tergugat karena saksi adalah Ayah Tiri Penggugat;
- Bahwa saksi mengetahui antara Penggugat dan Tergugat adalah suami istri;

Disclaimer
Kepaniteraan Mahkamah Agung Republik Indonesia berusaha untuk selalu mencantumkan informasi paling kini dan akurat sebagai bentuk komitmen Mahkamah Agung untuk pelayanan publik, transparansi dan akuntabilitas pelaksanaan fungsi peradilan. Namun dalam hal-hal tertentu masih dimungkinkan terjadi permasalahan teknis terkait dengan akurasi dan keterkinian informasi yang kami sajikan, hal mana akan terus kami perbaiki dari waktu kewaktu.
Dalam hal Anda menemukan inakurasi informasi yang termuat pada situs ini atau informasi yang seharusnya ada, namun belum tersedia, maka harap segera hubungi Kepaniteraan Mahkamah Agung RI melalui :
Email : kepaniteraan@mahkamahagung.go.id Telp : 021-384 3348 (ext.318)

- Bahwa saksi mengetahui antara Penggugat dengan Tergugat setelah menikah bertempat tinggal bersama di rumah Penggugat hingga Desember 2019;
- Bahwa saksi mengetahui antara Penggugat dengan Tergugat belum dikaruniai anak;
- Bahwa saksi mengetahui antara Penggugat dengan Tergugat telah terjadi perselisihan dan pertengkaran disebabkan nafkah sehari-hari Penggugat kurang tercukupi disamping itu Tergugat sering marah-marah karena cemburu tanpa alasan kepada setiap pelanggan laki-laki yang datang ke warung Penggugat;
- Bahwa saksi mengetahui Penggugat dan Tergugat sudah pisah tempat tinggal sejak Desember 2019 sampai dengan sekarang, kurang lebih 1 tahun, yakni Tergugat pergi meninggalkan Penggugat pulang ke rumah orang tuanya sendiri hingga sekarang;
- bahwa selama pisah tersebut Penggugat dan Tergugat tidak pernah komunikasi atau kumpul layaknya suami istri;
- Bahwa saksi mengetahui keluarga sudah merukunkan Penggugat dengan Tergugat tetapi tidak berhasil
- Bahwa saksi sebagai Ayah tiri Penggugat sudah tidak sanggup untuk merukunkan Penggugat dengan Tergugat;

Bahwa Penggugat telah mencukupkan dengan bukti-bukti yang telah diajukan oleh Penggugat dan mohon untuk segera diberikan putusan;

Bahwa selanjutnya untuk meringkas putusan ini, maka berita acara persidangan perkara ini ditunjuk sebagai bagian yang tak terpisahkan dari putusan ini;

PERTIMBANGAN HUKUM

Menimbang, bahwa maksud dan tujuan gugatan Penggugat pada pokoknya adalah sebagaimana terurai di atas;

Menimbang, bahwa dalam dalil gugatan Penggugat yang menyatakan Penggugat dan Tergugat beragama islam yang tidak terbantahkan, oleh karena itu berdasarkan Pasal 40 Pasal 63 Ayat (1) huruf (a) Undang-undang Nomor 1 Tahun 1974 Jo. Pasal 49 Undang-undang Nomor 7 Tahun 1989 tentang

Disclaimer
Kepaniteraan Mahkamah Agung Republik Indonesia berusaha untuk selalu mencantumkan informasi paling kini dan akurat sebagai bentuk komitmen Mahkamah Agung untuk pelayanan publik, transparansi dan akuntabilitas pelaksanaan fungsi peradilan. Namun dalam hal-hal tertentu masih dimungkinkan terjadi permasalahan teknis terkait dengan akurasi dan keterkinian informasi yang kami sajikan, hal mana akan terus kami perbaiki dari waktu kewaktu.
Dalam hal Anda menemukan inakurasi informasi yang termuat pada situs ini atau informasi yang seharusnya ada, namun belum tersedia, maka harap segera hubungi Kepaniteraan Mahkamah Agung RI melalui :
Email : kepaniteraan@mahkamahagung.go.id Telp : 021-384 3348 (ext.318)

Peradilan Agama sebagaimana diubah dengan Undang-undang Nomor 3 Tahun 2006 dan terakhir dengan Undang-undang Nomor 50 Tahun 2009 Jo. Pasal 1 huruf (b) Peraturan Pemerintah Nomor 9 Tahun 1975, Karenanya Pengadilan Agama berwenang memeriksa dan mengadili serta memutus perkara a quo;

Menimbang bahwa dalam gugatan Penggugat domisili Penggugat berada pada yuridiksi Pengadilan Agama Lumajang, sesuai dengan Pasal 73 Undang-undang Nomor 7 Tahun 1989 tentang Peradilan Agama sebagaimana diubah dengan Undang-undang Nomor 3 Tahun 2006 dan terakhir Undang-undang Nomor 50 Tahun 2009, maka perkara ini merupakan kewenangan relatif Pengadilan Agama Lumajang;

Menimbang bahwa Penggugat dalam gugatannya mendalilkan bahwa Penggugat telah melangsungkan perkawinan di hadapan Pegawai Pencatat Nikah Kantor Urusan Agama dan rumah tangga Penggugat dan Tergugat sudah tidak harmonis, oleh karena itu Penggugat memiliki *legal standing* untuk mengajukan gugatan perceraian sebagaimana diatur Pasal 49 ayat (1) huruf (a) dan Pasal 73 Undang-undang Nomor 7 Tahun 1989 tentang Peradilan Agama yang diubah dengan Undang-undang Nomor 3 Tahun 2006 dan Undang-undang Nomor 50 Tahun 2009;

Menimbang bahwa Majelis Hakim telah berusaha memberikan nasehat kepada Penggugat pada setiap persidangan secara maksimal agar Penggugat bersabar dan rukun kembali dengan Tergugat, namun tidak berhasil, karenanya ketentuan Pasal 130 HIR Jo. Pasal 83 Undang-undang Nomor 7 Tahun 1989 tentang Peradilan Agama yang diubah dengan Undang-undang Nomor 3 Tahun 2006 dan Undang-undang Nomor 50 Tahun 2009 telah terpenuhi dalam perkara ini;

Menimbang bahwa menurut Peraturan Mahkamah Agung R.I. Nomor 1 Tahun 2016 tentang Prosedur Mediasi, mediasi mengharuskan kehadiran kedua pihak yang berperkara, oleh karena Tergugat tidak pernah hadir di persidangan, maka mediasi tidak dapat dilaksanakan;

Menimbang, bahwa Tergugat telah dipanggil secara resmi dan patut tidak menghadap di persidangan dengan tanpa alasan dan Tergugat juga tidak menyuruh orang lain untuk datang menghadap sebagai wakil atau kuasanya,

Disclaimer
Kepaniteraan Mahkamah Agung Republik Indonesia berusaha untuk selalu mencantumkan informasi paling kini dan akurat sebagai bentuk komitmen Mahkamah Agung untuk pelayanan publik, transparansi dan akuntabilitas pelaksanaan fungsi peradilan. Namun dalam hal-hal tertentu masih dimungkinkan terjadi permasalahan teknis terkait dengan akurasi dan keterkinian informasi yang kami sajikan, hal mana akan terus kami perbaiki dari waktu kewaktu.
Dalam hal Anda menemukan inakurasi informasi yang termuat pada situs ini atau informasi yang seharusnya ada, namun belum tersedia, maka harap segera hubungi Kepaniteraan Mahkamah Agung RI melalui :
Email : kepaniteraan@mahkamahagung.go.id Telp : 021-384 3348 (ext.318)

maka Tergugat harus dinyatakan tidak hadir dan berdasarkan ketentuan Pasal 125 HIR, gugatan Penggugat dapat diperiksa dan diputus dengan tanpa hadirnya Tergugat (secara verstek) dan hal yang demikian ini telah sesuai pula dengan doktrin fiqih dalam Kitab Al Anwar Juz II halaman 55 yang berbunyi sebagai berikut :

فان تعزز بتعزز اوتوار او غيبة جاز اثباته بالبينة

Artinya : "Jika Tergugat membangkang atau bersembunyi atau ghoib, maka boleh memutus perkaranya dengan pembuktian"

Menimbang, bahwa Penggugat dalam surat gugatannya mendalilkan bahwa semula rumah tangga Penggugat dan Tergugat rukun baik akan tetapi kurang lebih sejak bulan Januari tahun 2019 ketentraman rumah tangga Penggugat dengan Tergugat mulai goyah, setelah antara Penggugat dengan Tergugat terus menerus terjadi perselisihan dan pertengkaran disebabkan Tergugat sering marah-marah tanpa alasan yang jelas hingga merusak barang-barang perabotan rumah tangga dan nafkah sehari-hari kurang tercukupi;

Menimbang, bahwa Penggugat telah meneguhkan dalil gugatannya dengan mengajukan bukti surat P.1 dan P.2 berupa fotokopi Akta Otentik dan fotokopi tersebut telah bermaterai cukup serta telah dicocokkan dengan aslinya dan ternyata fotokopi tersebut telah sesuai dengan aslinya, maka bukti fotokopi tersebut mempunyai nilai pembuktian yang sama dengan surat aslinya dan dengan demikian bukti surat yang diajukan oleh Penggugat tersebut mempunyai kekuatan pembuktian yang sempurna dan menentukan;

Menimbang, bahwa Penggugat selain mengajukan bukti surat juga telah mengajukan bukti dua orang saksi, yang kedua saksi tersebut dengan dibawah sumpah telah memberikan keterangan di persidangan atas pengetahuannya sendiri dan keterangannya saling bersesuaian antara satu dengan yang lainnya dan keterangan kedua saksi tersebut telah menguatkan dalil Penggugat, maka bukti kedua saksi yang diajukan oleh Penggugat telah memenuhi syarat formal dan syarat materiil saksi sehingga oleh karena itu sesuai ketentuan pasal 172 HIR keterangan kedua saksi tersebut dapat diterima sebagai bukti dalam perkara ini;

Disclaimer
Kepaniteraan Mahkamah Agung Republik Indonesia berusaha untuk selalu mencantumkan informasi paling kini dan akurat sebagai bentuk komitmen Mahkamah Agung untuk pelayanan publik, transparansi dan akuntabilitas pelaksanaan fungsi peradilan. Namun dalam hal-hal tertentu masih dimungkinkan terjadi permasalahan teknis terkait dengan akurasi dan keterkinian informasi yang kami sajikan, hal mana akan terus kami perbaiki dari waktu kewaktu.
Dalam hal Anda menemukan inakurasi informasi yang termuat pada situs ini atau informasi yang seharusnya ada, namun belum tersedia, maka harap segera hubungi Kepaniteraan Mahkamah Agung RI melalui :
Email : kepaniteraan@mahkamahagung.go.id Telp : 021-384 3348 (ext.318)

Menimbang, bahwa berdasarkan bukti P.1 telah ternyata Penggugat bertempat tinggal di wilayah hukum Pengadilan Agama Lumajang, maka gugatan Penggugat telah diajukan sesuai ketentuan pasal 73 Undang Undang Nomor 7 tahun 1989 tentang Peradilan Agama sebagaimana telah dirubah dengan Undang-undang Nomor 3 Tahun 2006 dan perubahan kedua dengan Undang-undang Nomor 50 Tahun 2009 sehingga oleh karenanya gugatan Penggugat secara formil harus diterima ;

Menimbang bahwa berdasarkan bukti P.2 tersebut di atas, maka Majelis telah menemukan fakta di persidangan bahwa pada tanggal 07 Juli 2017 Penggugat dengan Tergugat benar telah melangsungkan perkawinan di hadapan Pegawai Pencatat Nikah Kantor Urusan Agama Kecamatan Tempeh Kabupaten Lumajang, sehingga oleh karenanya harus dinyatakan terbukti bahwa Penggugat memiliki legal standing untuk mengajukan gugatan perceraian terhadap Tergugat;

Menimbang, bahwa berdasarkan keterangan kedua saksi yang diajukan oleh Penggugat tersebut di atas, Majelis telah dapat menemukan fakta di persidangan bahwa Penggugat dengan Tergugat setelah menikah benar tinggal bersama di rumah Penggugat hingga Desember 2019 dan antara Penggugat dengan Tergugat telah terjadi perselisihan dan pertengkaran disebabkan nafkah sehari-hari Penggugat kurang tercukupi disamping itu Tergugat gampang marah-marah karena cemburu dan Penggugat dengan Tergugat telah hidup berpisah rumah selama 1 tahun dan sudah tidak dapat hidup rukun lagi sebagai layaknya suami istri;

Menimbang, bahwa berdasarkan fakta tersebut di atas Majelis Hakim berkesimpulan bahwa rumah tangga Penggugat dan Tergugat tidak terdapat kesejahteraan lahir dan batin dan tidak mungkin dapat tercapai rumah tangga seperti yang disebut dalam Al-Qur'an Surat Ar-Rum ayat 21 yakni membina rumah tangga yang penuh rasa kasih sayang, serta pasal 1 Undang-Undang Nomor 1 tahun 1974 yakni membentuk rumah tangga (keluarga) yang kekal dan bahagia, karena itu Majelis Hakim berpendapat, perceraian lebih maslahat daripada mempertahankan perkawinan seperti itu karena dinilai perkawinan Penggugat dan Tergugat sudah pecah;

Disclaimer
Kepaniteraan Mahkamah Agung Republik Indonesia berusaha untuk selalu mencantumkan informasi paling kini dan akurat sebagai bentuk komitmen Mahkamah Agung untuk pelayanan publik, transparansi dan akuntabilitas pelaksanaan fungsi peradilan. Namun dalam hal-hal tertentu masih dimungkinkan terjadi permasalahan teknis terkait dengan akurasi dan keterkinian informasi yang kami sajikan, hal mana akan terus kami perbaiki dari waktu kewaktu.
Dalam hal Anda menemukan inakurasi informasi yang termuat pada situs ini atau informasi yang seharusnya ada, namun belum tersedia, maka harap segera hubungi Kepaniteraan Mahkamah Agung RI melalui :
Email : kepaniteraan@mahkamahagung.go.id Telp : 021-384 3348 (ext.318)

Menimbang, bahwa perceraian itu menimbulkan madlarat akan tetapi membiarkan rumah tangga yang diliputi oleh suasana ketegangan, kebencian dan sikap saling menyalahkan justru menimbulkan madlarat yang lebih besar karenanya harus dipilih salah satu yang madlaratnya lebih kecil sesuai dengan kaidah ushuliyah yang berbunyi:

إذا تعارض ضرران فضل أخفهما

Artinya : "Apabila ada dua hal yang sama-sama mengandung madlarat, maka harus dipilih satu di antaranya yang lebih kecil madlaratnya";

Menimbang, bahwa berdasarkan pertimbangan tersebut di atas, maka Majelis berpendapat bahwa gugatan Penggugat terbukti telah memenuhi alasan perceraian sebagaimana ditentukan dalam pasal 19 huruf (f) Peraturan Pemerintah Nomor 9 Tahun 1975 jo pasal 116 huruf (f) Kompilasi Hukum Islam dan oleh karena itu telah terdapat cukup alasan hukum untuk mengabulkan gugatan Penggugat dengan menjatuhkan talak satu ba'in sughro Tergugat terhadap Penggugat;

Menimbang, bahwa gugatan Penggugat telah beralasan hukum dan Tergugat telah dipanggil secara patut dan sah tidak hadir, maka oleh karenanya sesuai ketentuan pasal 125 HIR gugatan Penggugat dapat dikabulkan dengan tanpa hadirnya Tergugat (verstek) ;

Menimbang, bahwa berdasarkan pasal 89 ayat (1) Undang Undang Nomor 7 Tahun 1989 tentang Peradilan Agama sebagaimana telah diubah dengan Undang Undang Nomor 3 Tahun 2006 dan perubahan kedua dengan Undang-Undang Nomor 50 Tahun 2009, maka biaya perkara dibebankan kepada Penggugat;

Mengingat ketentuan hukum lain serta hukum syar'i yang berkaitan dengan perkara ini:

MENGADILI

1. Menyatakan bahwa Tergugat yang telah dipanggil dengan resmi dan patut untuk menghadap di persidangan, tidak hadir;
2. Mengabulkan gugatan Penggugat dengan verstek;

Disclaimer
Kepaniteraan Mahkamah Agung Republik Indonesia berusaha untuk selalu mencantumkan informasi paling kini dan akurat sebagai bentuk komitmen Mahkamah Agung untuk pelayanan publik, transparansi dan akuntabilitas pelaksanaan fungsi peradilan. Namun dalam hal-hal tertentu masih dimungkinkan terjadi permasalahan teknis terkait dengan akurasi dan keterkinian informasi yang kami sajikan, hal mana akan terus kami perbaiki dari waktu kewaktu.
Dalam hal Anda menemukan inakurasi informasi yang termuat pada situs ini atau informasi yang seharusnya ada, namun belum tersedia, maka harap segera hubungi Kepaniteraan Mahkamah Agung RI melalui :
Email : kepaniteraan@mahkamahagung.go.id Telp : 021-384 3348 (ext.318)

3. Menjatuhkan talak satu ba'in sughro Tergugat (TERGUGAT) terhadap Penggugat (PENGGUGAT);
4. Membebankan kepada Penggugat untuk membayar biaya perkara sebesar Rp. 591.000,- (Lima ratus sembilan puluh satu ribu rupiah);

Demikian putusan ini dijatuhkan dalam rapat permusyawaratan Majelis Hakim yang dilangsungkan pada hari Selasa tanggal 29 Desember 2020 Masehi bertepatan dengan tanggal 14 Jumadil Awwal 1442 Hijriyah oleh kami Drs. WARYONO, M.H. sebagai Hakim Ketua, dengan didampingi oleh Dra. Hj. SRIYANI, M.H. dan Drs. MUHAMMAD HILMY, M.H.E.S. masing-masing sebagai Hakim Anggota dan dibantu oleh ZUBAIDAH, S.H. sebagai Panitera Pengganti Pengadilan Agama tersebut, putusan tersebut diucapkan pada hari itu juga dalam sidang terbuka untuk umum dengan dihadiri oleh Penggugat tanpa hadirnya Tergugat;

Hakim Anggota,	Hakim Ketua,
Dra. Hj. SRIYANI, M.H.	Drs. WARYONO, M.H.
Hakim Anggota,	Panitera Pengganti,
Drs. MUHAMMAD HILMY, M.H.E.S.	ZUBAIDAH, S.H.

Disclaimer
Kepaniteraan Mahkamah Agung Republik Indonesia berusaha untuk selalu mencantumkan informasi paling kini dan akurat sebagai bentuk komitmen Mahkamah Agung untuk pelayanan publik, transparansi dan akuntabilitas pelaksanaan fungsi peradilan. Namun dalam hal-hal tertentu masih dimungkinkan terjadi permasalahan teknis terkait dengan akurasi dan keterkinian informasi yang kami sajikan, hal mana akan terus kami perbaiki dari waktu kewaktu.
Dalam hal Anda menemukan inakurasi informasi yang termuat pada situs ini atau informasi yang seharusnya ada, namun belum tersedia, maka harap segera hubungi Kepaniteraan Mahkamah Agung RI melalui :
Email : kepaniteraan@mahkamahagung.go.id Telp : 021-384 3348 (ext.318)

Rincian Biaya perkara :

1.	Biaya pendaftaran	: Rp.	30.000,-
2.	Biaya Pemberkasan/ATK	: Rp.	75.000,-
3.	Biaya Pemanggilan	: Rp.	450.000,-
4.	PNBP Relas Pertama	: Rp.	20.000,-
5.	Redaksi	: Rp.	10.000,-
6.	Meterai	: Rp.	6.000,-
	Jumlah	: Rp.	591.000,-

(Lima ratus sembilan puluh satu ribu rupiah);

Disclaimer
Kepaniteraan Mahkamah Agung Republik Indonesia berusaha untuk selalu mencantumkan informasi paling kini dan akurat sebagai bentuk komitmen Mahkamah Agung untuk pelayanan publik, transparansi dan akuntabilitas pelaksanaan fungsi peradilan. Namun dalam hal-hal tertentu masih dimungkinkan terjadi permasalahan teknis terkait dengan akurasi dan keterkinian informasi yang kami sajikan, hal mana akan terus kami perbaiki dari waktu kewaktu.
Dalam hal Anda menemukan inakurasi informasi yang termuat pada situs ini atau informasi yang seharusnya ada, namun belum tersedia, maka harap segera hubungi Kepaniteraan Mahkamah Agung RI melalui :
Email : kepaniteraan@mahkamahagung.go.id   Telp : 021-384 3348 (ext.318)
