# Supplementary material for: Classification of divorce causes during the COVID-19 pandemic using convolutional neural networks
Source: PeerJ Comput Sci. 2022 Jun 30;8:e998. doi: 10.7717/peerj-cs.998 (PMC9299239; doi:10.7717/peerj-cs.998)
Supplement: Supplemental Information 2 [file peerj-cs-08-998-s002.zip › Kelalaian Dataset/Data ke-29.pdf]

PUTUSAN

Nomor xxx/Pdt.G/2020/MS.BIR

بِسْمِ اللَّهِ الرَّحْمَنِ الرَّحِيمِ

DEMI KEADILAN BERDASARKAN KETUHANAN YANG MAHA ESA

Mahkamah Syar'iyah Bireuen, yang mengadili perkara Cerai Gugat pada tingkat pertama, dalam persidangan majelis, telah menjatuhkan putusan sebagai berikut, dalam perkara antara;

Nama Penggugat, tempat dan tanggal lahir, xxx, 10 September 1990, NIK xxx, agama Islam, pekerjaan Pelajar/Mahasiswa, pendidikan SMA, tempat kediaman di Dusun xxx, Gampong xxx, Kecamatan xxx, Kabupaten Bireuen, sebagai Penggugat;

melawan

Nama Tergugat, tempat dan tanggal lahir, xxx, 12 Juni 1982, NIK xxx, agama Islam, pekerjaan Wiraswasta, pendidikan SMA, tempat kediaman di Dusun xxx, Gampong xxx, Kecamatan xxx, Kabupaten Aceh Utara, sebagai Tergugat;

Mahkamah Syar'iyah tersebut;

Telah membaca dan mempelajari berkas perkara yang bersangkutan;

Telah mendengar keterangan Penggugat dan saksi-saksinya di depan persidangan;

DUDUK PERKARA

Bahwa Penggugat dengan surat gugatannya tanggal 06 Oktober 2020 yang terdaftar di Kepaniteraan Perkara Mahkamah Syar'iyah Bireuen di bawah Register Nomor xxx/Pdt.G/2020/MS.BIR, tertanggal 08 Oktober 2020 telah mengajukan gugatan Cerai terhadap Tergugat dengan dalil-dalil yang pada pokoknya sebagai berikut:

1. Bahwa Penggugat dan tergugat telah menikah tanggal 27 April 2015 dihadapan Pegawai Pencatat Nikah Kantor Urusan Agama Kecamatan xxx Kabupaten Bireuen Provinsi Aceh, sebagaimana terbukti buku Kutipan Akta Nikah Nomor xxx/031/IV/2015 tertanggal 27 April 2015 sehingga karenanya perkawinan tersebut adalah SAH menurut hukum agama dan sesuai

Disclaimer
Kepaniteraan Mahkamah Agung Republik Indonesia berusaha untuk selalu mencantumkan informasi paling kini dan akurat sebagai bentuk komitmen Mahkamah Agung untuk pelayanan publik, transparansi dan akuntabilitas pelaksanaan fungsi peradilan. Namun dalam hal-hal tertentu masih dimungkinkan terjadi permasalahan teknis terkait dengan akurasi dan keterkinian informasi yang kami sajikan, hal mana akan terus kami perbaiki dari waktu kewaktu.
Dalam hal Anda menemukan inakurasi informasi yang termuat pada situs ini atau informasi yang seharusnya ada, namun belum tersedia, maka harap segera hubungi Kepaniteraan Mahkamah Agung RI melalui :
Email : kepaniteraan@mahkamahagung.go.id   Telp : 021-384 3348 (ext.318)

dengan ketentuan Undang-undang Nomor 1 tahun 1974 jo. Peraturan pemerintah No. 9 Tahun 1975;

2. Bahwa setelah menikah Penggugat dan Tergugat tinggal bersama selama ± 4 tahun. Terakhir tinggal di rumah sendiri di Dusun Cot Geulumpang Gampong Jurong Kecamatan Sawang Kabupaten Aceh Utara;
3. Bahwa dalam perkawinan tersebut antara Penggugat dengan Tergugat telah hidup bersama sebagai suami istri dan telah mempunyai 1 orang anak yang bernama xxx umur 4 Tahun;
4. Bahwa oleh karena anak Penggugat dan Tergugat yang bernama xxx umur 4 tahun belum mumayiz/dewasa maka secara hukum patut ditetapkan berada dalam asuhan /hadhanah Penggugat sampai anak tersebut dewasa;
5. Bahwa dalam membina rumah tangga antara Penggugat dan Tergugat pada mulanya berada dalam keadaan aman dan damai sebagaimana dambaan setiap pasangan suami isteri, namun kebahagiaan tersebut hanya berjalan dari bulan April 2015 sampai bulan Maret 2019. Setelah itu rumah tangga Penggugat dan Tergugat tidak ada keharmonisan lagi;
6. Bahwa penyebab tidak harmonis karena :
   - Tergugat sering memaki dan mencemarkan nama baik Pengguga;
   - Tergugat tidak menakahi Penggugat lahir dan bathin selama lebih kurang 2 tahun;
   - Orang tua Tergugat sering mengancam Penggugat;
   - Tergugat tidak menghargai Penggugat;
   - Orang tua Tergugat selalu ikut campur urusan rumah tangga Penggugat dan Tergugat;
7. Bahwa Tergugat berhutang pada Penggugat senilai Rp. 6.000.000 (Enam Juta Rupiah) untuk keperluan membeli tanah sawah;
8. Bahwa terhadap permasalahan tersebut diatas, Penggugat tidak sanggup bersabar lagi dan sulit menyatukan keutuhan rumah tangga yang telah Penggugaat bina selama ini;

Disclaimer
Kepaniteraan Mahkamah Agung Republik Indonesia berusaha untuk selalu mencantumkan informasi paling kini dan akurat sebagai bentuk komitmen Mahkamah Agung untuk pelayanan publik, transparansi dan akuntabilitas pelaksanaan fungsi peradilan. Namun dalam hal-hal tertentu masih dimungkinkan terjadi permasalahan teknis terkait dengan akurasi dan keterkinian informasi yang kami sajikan, hal mana akan terus kami perbaiki dari waktu kewaktu.
Dalam hal Anda menemukan inakurasi informasi yang termuat pada situs ini atau informasi yang seharusnya ada, namun belum tersedia, maka harap segera hubungi Kepaniteraan Mahkamah Agung RI melalui :
Email : kepaniteraan@mahkamahagung.go.id Telp : 021-384 3348 (ext.318)

9. Bahwa sejak tanggal 13 Maret 2019 Penggugat dan Tergugat sudah pisah tempat tinggal sampai sekarang sudah setahun lebih kurang lamanya;
10. Bahwa terhadap permasalahan dalam rumah tangga Penggugat dan Tergugat telah dilakukan upaya musyawarah dengan keluarga/perangkat Gampong mendamaikan tapi tidak ada hasilnya;
11. Bahwa sesuai ketentuan Pasal 1 Undang-undang No. 1 tahun 1974, telah pokok-pokok Perkawinan, dinyatakan sebagai berikut bahwa : “Perkawinan adalah ikatan lahir batin antara seorang pria dengan wanita sebagai suami isteri dengan tujuan membentuk keluarga (rumah tangga) yang bahagia dan kekal berdasarkan Ketuhanan Yang Maha Esa, “Apabila Ketentuan Pasal 1 Undang-undang No.1 tahun 1974 tersebut diatas dikaitkan dengan keadaan perkawinan antara Penggugat dan Tergugat, maka jelaslah bahwa tujuan dari perkawinan tersebut sudah tidak ada lagi didalam rumah tanggaa antara penggugat dan Tergugat, dimana perkawinan Penggugat dan Tergugat terlihat telah mengandung cacat dalam pelaksanaannya, sehingga dengan demikian untuk apalah perkawinan tersebut dipertahankan lagi;
12. Berdasarkan kepada hal-hal tersebut Penggugat mohon kepada Bapak ketua Mahkamah Syari’ah Bireuen untuk dapat membuka suatu persidangan terhadap perkara ini dengan memanggil kedua belah pihak untuk di dengar keterangannya serta berkenan pula memberikan putusan sebagai berikut:
    1. Mengabulkan gugatan Penggugat;
    2. Menjatuhkan talak satu Bain Shugra Tergugat (nama Tergugat) terhadap Penggugat (nama Penggugat);
    3. Menetapkan biaya menurut hukum;
    4. Dalam persidangan yang baik mohon putusan yang memenuhi rasa keadilan;

Disclaimer
Kepaniteraan Mahkamah Agung Republik Indonesia berusaha untuk selalu mencantumkan informasi paling kini dan akurat sebagai bentuk komitmen Mahkamah Agung untuk pelayanan publik, transparansi dan akuntabilitas pelaksanaan fungsi peradilan. Namun dalam hal-hal tertentu masih dimungkinkan terjadi permasalahan teknis terkait dengan akurasi dan keterkinian informasi yang kami sajikan, hal mana akan terus kami perbaiki dari waktu kewaktu.
Dalam hal Anda menemukan inakurasi informasi yang termuat pada situs ini atau informasi yang seharusnya ada, namun belum tersedia, maka harap segera hubungi Kepaniteraan Mahkamah Agung RI melalui :
Email : kepaniteraan@mahkamahagung.go.id Telp : 021-384 3348 (ext.318)

Bahwa pada hari-hari sidang yang telah ditetapkan untuk pemeriksaan perkara ini, Penggugat dan Tergugat telah dipanggil secara resmi dan patut untuk datang menghadap di persidangan;

Bahwa terhadap panggilan tersebut Penggugat telah hadir di persidangan, sebagaimana berita acara *relaas* panggilan Nomor xxx/Pdt.G/2020/MS.Bir, sedangkan Tergugat tidak pernah hadir dan tidak pula mengutus orang lain sebagai wakil atau kuasanya yang sah, meskipun panggilan telah dilaksanakan secara resmi dan patut, sebagaimana berita acara *relaas* panggilan Nomor xxx/Pdt.G/2020/MS.Bir, sedangkan ketidakhadiran Tergugat tanpa suatu alasan yang dibenarkan oleh peraturan perundang-undangan dan perkaranya dapat diperiksa tanpa hadir Tergugat sesuai pasal 149 RBg.;

Bahwa Majelis Hakim telah berupaya mendamaikan Penggugat dengan cara menasehati Penggugat agar tidak bercerai dengan Tergugat, akan tetapi tidak berhasil;

Bahwa upaya mediasi sebagaimana yang diamanatkan oleh PERMA Nomor 1 Tahun 2016 tidak dapat dilakukan karena Tergugat tidak pernah datang ke persidangan;

Bahwa oleh karena upaya damai tidak berhasil, maka pemeriksaan perkara ini dilanjutkan dengan membacakan surat gugatan Penggugat yang pada pokoknya tetap dipertahankan oleh Penggugat ;

Bahwa atas gugatan Penggugat tersebut, jawaban Tergugat tidak dapat didengar karena Tergugat tidak pernah hadir di persidangan;

Bahwa untuk menguatkan dalil-dalil gugatannya, Penggugat telah mengajukan bukti surat berupa:

1. Fotokopi Kartu Tanda Penduduk NIK xxx tanggal 7-06-2020, atas nama Penggugat yang dikeluarkan oleh Pemerintah Kabupaten Bireuen, telah diberi meterai secukupnya dan dinazegelen, Majelis Hakim telah mencocokkan dengan aslinya di persidangan dan ternyata telah cocok, diberi kode P.1;
2. Fotokopi Buku Kutipan Akta Nikah Nomor xxx, tanggal 27-04-2015 atas nama Penggugat dan Tergugat, yang dikeluarkan oleh Kantor Urusan Agama

Disclaimer
Kepaniteraan Mahkamah Agung Republik Indonesia berusaha untuk selalu mencantumkan informasi paling kini dan akurat sebagai bentuk komitmen Mahkamah Agung untuk pelayanan publik, transparansi dan akuntabilitas pelaksanaan fungsi peradilan. Namun dalam hal-hal tertentu masih dimungkinkan terjadi permasalahan teknis terkait dengan akurasi dan keterkinian informasi yang kami sajikan, hal mana akan terus kami perbaiki dari waktu kewaktu.
Dalam hal Anda menemukan inakurasi informasi yang termuat pada situs ini atau informasi yang seharusnya ada, namun belum tersedia, maka harap segera hubungi Kepaniteraan Mahkamah Agung RI melalui :
Email : kepaniteraan@mahkamahagung.go.id Telp : 021-384 3348 (ext.318)

Kecamatan Peusangan Kabupaten Bireuen, telah diberi meterai secukupnya dan dinazegelen, Majelis Hakim telah mencocokkan dengan aslinya di persidangan dan ternyata telah cocok, diberi kode P.2;

Bahwa selain alat bukti surat tersebut Penggugat juga telah mengajukan 2 (dua) orang saksi dan telah memberikan keterangan di bawah sumpahnya sebagai berikut:

1. Nama saksi 1 , tempat/tanggal lahir xxx, 4 Juni 1965 , agama Islam, pendidikan SD, pekerjaan Petani, tempat tinggal Dusun xxx, Desa xxx, Kecamatan xxx, Kabupaten Bireuen, di bawah sumpahnya telah memberikan keterangan sebagai berikut:

- Bahwa Saksi kenal dengan Penggugat dan Tergugat sebagai adik ipar Penggugat dan mereka adalah suami istri yang menikah secara sah saksi tidak tahu kapan dqn dimana Penggugat dan Tergugat menikah karena saksi menikah dengan adik Penggugat setelah mereka menikah;
- Bahwa setelah menikah Penggugat dan Tergugat menetap di rumah orangtua Penggugat beberapa waktu setelah itubpindah kerumah bersama;
- Bahwa selama menikah Penggugat dengan Tergugat telah dikaruniai seorang anak laki-laki yang bernama xxx;
- Bahwa menurut pengetahuan saksi, pada awalnya rumah tangga antara Penggugat dan Tergugat rukun dan harmonis, kemudian mereka sering terjadi perselisihan hingga Tergugat mengusir Penggugat dan harus kembali ke rumah orangtuanya ;
- Bahwa mengenai penyebab Penggugat dan Tergugat bertengkar karena orangtua Tergugat ikut mencampuri urusan rumah tangga Penggugat dan Tergugat;
- Bahwa saksi mengetahuinya berdasarkan laporan dari Penggugat kepada saksi pada saat Penggugat diserang oleh orangtua Tergugat, Tergugat tidak membela Penggugat;
- Bahwa antara Penggugat dan Tergugat sudah pisah tempat tinggal lebih kurang 1 (satu) tahun yang lalu;

Disclaimer
Kepaniteraan Mahkamah Agung Republik Indonesia berusaha untuk selalu mencantumkan informasi paling kini dan akurat sebagai bentuk komitmen Mahkamah Agung untuk pelayanan publik, transparansi dan akuntabilitas pelaksanaan fungsi peradilan. Namun dalam hal-hal tertentu masih dimungkinkan terjadi permasalahan teknis terkait dengan akurasi dan keterkinian informasi yang kami sajikan, hal mana akan terus kami perbaiki dari waktu kewaktu.
Dalam hal Anda menemukan inakurasi informasi yang termuat pada situs ini atau informasi yang seharusnya ada, namun belum tersedia, maka harap segera hubungi Kepaniteraan Mahkamah Agung RI melalui :
Email : kepaniteraan@mahkamahagung.go.id Telp : 021-384 3348 (ext.318)

- Bahwa persoalan rumah tangga Penggugat dan Tergugat tidak pernah didamaikan oleh Perangkat Desa maupun oleh pihak keluarga;
- Bahwa saksi menyatakan tidak ada lagi hal-hal yang ingin disampaikan /cukup;

2. Nama Saksi 2, umur 52 tahun, agama Islam, pendidikan SMA, pekerjaan Pegawai kontrak, tempat tinggal Gampong xxx, Kecamatan xxx, Kabupaten Bireuen, di bawah sumpahnya telah memberikan keterangan sebagai berikut:

Bahwa Saksi kenal dengan Penggugat sebagai ayah kandung Penggugat dan Tergugat sebagai enantu saksi;

Bahwa Penggugat dan Tergugat adalah suami istri yang menikah sah secara hukum Islam sekitar 5 (lima) tahun yang lalu;

Bahwa setelah menikah Penggugat dan Tergugat menetap di rumah orangtua Penggugat (rumah saksi) , sampai sekarang selaku orangtua Penggugat di Desa xxx Kecamatan xxx Kabupaten Bireuen;

Bahwa dari perkawinan Penggugat dan Tergugat sudah dikaruniai anak/keturunan seorang sanak , saat ini anak betersebut berada dalam asuhan Penggugat;

Bahwa sepengetahuan saksi Penggugat dan Tergugat sudah pisah tempat tinggal sejak 13 Maret 2019 lebih setahun lamanya dan selama itu pula Tergugat tidak pernah memberikan nafkah kepada Penggugat;

Bahwa penyebab Penggugat dan Tergugat berpisah karena Tergugat malas bekerja sehingga kurang memberikan nafkah kepada Penggugat;

Bahwa saksi penah melihat dan mendengar antara Penggugat dan Tergugat ribut, namun Tegugat tidak melakukan KDRT terhadap Penggugat;

Bahwa antara Penggugat dan Tergugat sudah pernah didamaikan oleh Perangkat Desa sebanyak 3 kali , akan tetapi tidak berhasil;

Bahwa saksi menyatakan sudah cukup/tidak ada lagi hal-hal yang inigin disampaikan;

Menimbang, bahwa Penggugat membenarkan keterangan -keterangan saksi-saksinya tersebut dan tidak menaruh keberatan, selanjutnya atas

Disclaimer
Kepaniteraan Mahkamah Agung Republik Indonesia berusaha untuk selalu mencantumkan informasi paling kini dan akurat sebagai bentuk komitmen Mahkamah Agung untuk pelayanan publik, transparansi dan akuntabilitas pelaksanaan fungsi peradilan. Namun dalam hal-hal tertentu masih dimungkinkan terjadi permasalahan teknis terkait dengan akurasi dan keterkinian informasi yang kami sajikan, hal mana akan terus kami perbaiki dari waktu kewaktu.
Dalam hal Anda menemukan inakurasi informasi yang termuat pada situs ini atau informasi yang seharusnya ada, namun belum tersedia, maka harap segera hubungi Kepaniteraan Mahkamah Agung RI melalui :
Email : kepaniteraan@mahkamahagung.go.id Telp : 021-384 3348 (ext.318)

pertanyaan keteua Majelis menyatakan tidak akan mengajukan alat bukti lagi serta Penggugat telah menyampaikan kesimpulan secara lisan yang pokoknya mohon gugatan Penggugat dikabulkan dan mohon putusan;

Menimbang, bahwa tentang jalannya pemeriksaan lebih jauh dalam persidangan semua telah dicatat dalam berita acara persidangan perkara yang bersangkutan, maka untuk ringkasnya dalam putusan ini cukuplah menunjuk kepada berita acara persidangan tersebut, yang merupakan bagian yang tidak terpisahkan dengan putusan ini;

PERTIMBANGAN HUKUM

Menimbang, bahwa maksud dan tujuan gugatan Penggugat sebagaimana telah diuraikan di atas;

Menimbang, bahwa pada hari sidang yang telah ditetapkan Penggugat telah datang menghadap di persidangan, hal mana telah memenuhi ketentuan Pasal 26 Peraturan Pemerintah Nomor 9 Tahun 1975 Tentang Pelaksanaan Undang-Undang Nomor 1 Tahun 1974 Tentang Perkawinan jo. Pasal 145 R.Bg;

Menimbang, bahwa Tergugat telah dipanggil secara resmi dan patut untuk menghadap di persidangan, akan tetapi Tergugat tidak pernah hadir dan tidak ada mengutus orang lain sebagai wakil atau kuasanya, ketidakhadirannya tersebut bukan karena suatu alasan yang sah, dan Tergugat tidak mengajukan eksepsi, sedangkan gugatan Penggugat berdasar hukum, dengan demikian berdasarkan ketentuan Pasal 149 jo Pasal 150 R.Bg Majelis Hakim telah dapat memeriksa dan memutus perkara ini dengan tanpa hadirnya Tergugat (*verstek*);

Menimbang, bahwa Majelis Hakim telah berupaya mendamaikan Penggugat dengan cara memberikan nasihat kepada Penggugat supaya bersabar demi mempertahankan keutuhan rumah tangganya dengan Tergugat, akan tetapi Penggugat menyatakan tetap ingin bercerai dari Tergugat, dengan demikian sesuai ketentuan Pasal 82 ayat (1) Undang-Undang Nomor 7 Tahun 1989 Tentang Peradilan Agama sebagaimana yang telah diubah dan ditambah dengan Undang-Undang Nomor 3 Tahun 2006 dan terakhir diubah dengan

Disclaimer
Kepaniteraan Mahkamah Agung Republik Indonesia berusaha untuk selalu mencantumkan informasi paling kini dan akurat sebagai bentuk komitmen Mahkamah Agung untuk pelayanan publik, transparansi dan akuntabilitas pelaksanaan fungsi peradilan. Namun dalam hal-hal tertentu masih dimungkinkan terjadi permasalahan teknis terkait dengan akurasi dan keterkinian informasi yang kami sajikan, hal mana akan terus kami perbaiki dari waktu kewaktu.
Dalam hal Anda menemukan inakurasi informasi yang termuat pada situs ini atau informasi yang seharusnya ada, namun belum tersedia, maka harap segera hubungi Kepaniteraan Mahkamah Agung RI melalui :
Email : kepaniteraan@mahkamahagung.go.id Telp : 021-384 3348 (ext.318)

Undang-Undang Nomor 50 Tahun 2009 jo pasal 154 ayat (1) R.Bg dianggap telah terpenuhi;

Menimbang, bahwa kewajiban Majelis Hakim memerintahkan kepada para pihak untuk menyelesaikan sengketa rumah tangganya melalui proses mediasi sebagaimana dimaksud oleh Peraturan Mahkamah Agung Republik Indonesia Nomor 1 Tahun 2016 tidak dapat dilaksanakan karena Tergugat tidak pernah hadir menghadap di persidangan;

Menimbang, bahwa yang menjadi pokok perkara ialah Penggugat menggugat agar Penggugat diceraikan dari Tergugat dengan menjatuhkan talak satu bain sughra Tergugat terhadap Penggugat, dengan alasan rumah tangga antara Penggugat dan Tergugat sudah tidak pernah bersatu lagi, sehingga tidak ada lagi harapan hidup rukun dalam rumah tangga disebabkan dalil-dalil sebagaimana telah diuraikan dalam bagian tentang duduk perkaranya;

Menimbang, bahwa untuk menguatkan dalil-dalil gugatannya, Penggugat telah mengajukan alat bukti surat (P.1 dan P.2) dan 2 (dua) orang saksi, terhadap bukti-bukti tersebut Majelis Hakim telah mempertimbangkannya;

Menimbang, bahwa berdasarkan bukti (P.1) KTP atas nama Penggugat yang telah dinazegelen dan ternyata cocok, dengan demikian syarat formal dan syarat materil alat bukti tersebut dinilai telah terpenuhi;

Menimbang, bahwa berdasarkan bukti (P.2) fotokopi Kutipan Akta Nikah atas nama Penggugat dan Tergugat yang telah dikeluarkan oleh Pejabat yang berwenang untuk itu yaitu Pegawai Pencatat Nikah Kantor Urusan Agama Kecamatan Peusangan , Kabupaten Bireuen, pada tanggal 27 /04/2004 telah membuktikan bahwa Penggugat dan Tergugat adalah suami istri yang sah menurut hukum sesuai dengan Pasal 2 ayat (2) Undang-undang Nomor 1 tahun 1974 jo Pasal 10 ayat (3) Peraturan Pemerintah Nomor 9 tahun 1975 Tentang Pelaksanaan Undang-Undang Nomor 1 Tahun 1974 Tentang Perkawinan jo Pasal 7 ayat (1) Kompilasi Hukum Islam, dengan demikian Penggugat adalah pihak yang berkepentingan dalam mengajukan perkara ini (*Persona Standi In Judicio*);

Disclaimer
Kepaniteraan Mahkamah Agung Republik Indonesia berusaha untuk selalu mencantumkan informasi paling kini dan akurat sebagai bentuk komitmen Mahkamah Agung untuk pelayanan publik, transparansi dan akuntabilitas pelaksanaan fungsi peradilan. Namun dalam hal-hal tertentu masih dimungkinkan terjadi permasalahan teknis terkait dengan akurasi dan keterkinian informasi yang kami sajikan, hal mana akan terus kami perbaiki dari waktu kewaktu.
Dalam hal Anda menemukan inakurasi informasi yang termuat pada situs ini atau informasi yang seharusnya ada, namun belum tersedia, maka harap segera hubungi Kepaniteraan Mahkamah Agung RI melalui :
Email : kepaniteraan@mahkamahagung.go.id Telp : 021-384 3348 (ext.318)

Menimbang, bahwa Saksi I dan Saksi II yang diajukan oleh Penggugat tersebut tidak ada halangan hukum untuk diangkat menjadi saksi, dan masing-masing telah memberikan keterangan di bawah sumpah di depan persidangan, sedangkan keberadaan kedua Saksi adalah dekat dengan Penggugat, selalu melihat dan mengetahui keadaan rumah tangga Penggugat dan Tergugat, dengan demikian sesuai Pasal 175 R.Bg serta Pasal 22 ayat (2) Peraturan Pemerintah Republik Indonesia Nomor 9 Tahun 1975 Tentang Pelaksanaan Undang-Undang Republik Indonesia Nomor 1 Tahun 1974 Tentang Perkawinan, syarat formil dan syarat materilnya kedua Saksi *a quo* telah terpenuhi;

Menimbang, bahwa berdasarkan keterangan saksi-saksi Penggugat di persidangan yang telah memberikan keterangan dari penglihatan dan pengetahuan Saksi, Saksi I dan Saksi II mengetahui langsung keadaan antara Penggugat dan Tergugat, kedua Saksi *a quo* juga mengetahui bahwa Penggugat dan Tergugat tidak pernah berkumpul dan tidak pernah bersatu lagi sampai dengan sekarang. Dengan demikian keterangan kedua orang Saksi *a quo* patut dinilai telah memenuhi syarat materil sebagaimana ketentuan Pasal 308 ayat (1) dan Pasal 309 R.Bg, sehingga kesaksian tersebut dapat diterima sebagai bukti dalam perkara ini;

Menimbang, bahwa berdasarkan dalil-dalil gugatan Penggugat yang dikaitkan dengan bukti-bukti dan dihubungkan dengan sikap Penggugat di persidangan, maka ditemukan fakta-fakta sebagai berikut:

- Bahwa Penggugat dan Tergugat adalah suami istri yang sah menikah pada tanggal 27-04- 2015 di hadapan PPN KUA Kecamatan xxx, Kabupaten Bireuen;
- Bahwa Penggugat dan Tergugat sudah dikaruniai anak 1 orang, sekarang dalam asuhan Penggugat;
- Bahwa antara Penggugat dengan Tergugat telah berpisah sejak 13 Maret 2019 yang lalu sampai dengan sekarang tidak pernah bersatu lagi;
- Bahwa pihak keluarga/perangkat Gampong sudah berusaha untuk

Disclaimer
Kepaniteraan Mahkamah Agung Republik Indonesia berusaha untuk selalu mencantumkan informasi paling kini dan akurat sebagai bentuk komitmen Mahkamah Agung untuk pelayanan publik, transparansi dan akuntabilitas pelaksanaan fungsi peradilan. Namun dalam hal-hal tertentu masih dimungkinkan terjadi permasalahan teknis terkait dengan akurasi dan keterkinian informasi yang kami sajikan, hal mana akan terus kami perbaiki dari waktu kewaktu.
Dalam hal Anda menemukan inakurasi informasi yang termuat pada situs ini atau informasi yang seharusnya ada, namun belum tersedia, maka harap segera hubungi Kepaniteraan Mahkamah Agung RI melalui :
Email : kepaniteraan@mahkamahagung.go.id Telp : 021-384 3348 (ext.318)

merukunkan dan menasihati Penggugat dan Tergugat akan tetapi tidak berhasil;

Menimbang, bahwa berdasarkan fakta-fakta tersebut, Majelis Hakim berpendapat bahwa rumah tangga Penggugat dan Tergugat telah sampai pada kondisi pecah (*broken marriage*) dan tidak ada harapan akan hidup rukun damai dalam rumah tangga;

Menimbang, bahwa indikasi pecahnya rumah tangga Penggugat dan Tergugat dapat dilihat dari gugatan Penggugat dan sejak tanggal 13 Maret 2019 yang lalu Penggugat dan Tergugat telah berpisah dan tidak pernah bersatu sebagaimana layaknya pasangan suami istri;

Menimbang, bahwa oleh karena keadaan rumah tangga Penggugat dan Tergugat telah sampai pada kondisi pecah, maka tanpa mempertimbangkan lagi dari pihak mana datangnya penyebab tersebut, hal mana dalam kondisi yang demikian dipandang sudah sangat sulit untuk dapat mewujudkan tujuan perkawinan, yaitu membina rumah tangga yang kekal, bahagia, sakinah, mawadah dan rahmah, sebagaimana dimaksud oleh Pasal 1 Undang-Undang Nomor 1 Tahun 1974 Tentang Perkawinan jo. Pasal 4 (empat)Instruksi Presiden Republik Indonesia Nomor 1 Tahun 1991 Tentang Kompilasi Hukum Islam;

Menimbang, bahwa perceraian merupakan perbuatan halal yang dibenci Allah Swt. yang sedapat mungkin harus dihindari oleh setiap pasangan suami istri, akan tetapi mempertahankan perkawinan Penggugat dan Tergugat dengan kondisi tersebut di atas, Majelis Hakim berpendapat justru akan mendatangkan mafsadat yang lebih besar dari pada maslahat yang akan dicapai, diantaranya penderitaan batin yang berkepanjangan bagi Penggugat, padahal menolak mafsadat lebih diprioritaskan dari pada menarik kemaslahatan sebagaimana disebutkan dalam kaidah fikih yang telah diambil alih sebagai pendapat Majelis Hakim:

درء المفاسد مقدم على جلب المصا لح

Artinya: Menolak kemudaratan (keburukan) lebih diutamakan dari pada meraih kemaslahatan (kebaikan);

Menimbang, bahwa keadaan rumah tangga Penggugat dengan Tergugat

Disclaimer
Kepaniteraan Mahkamah Agung Republik Indonesia berusaha untuk selalu mencantumkan informasi paling kini dan akurat sebagai bentuk komitmen Mahkamah Agung untuk pelayanan publik, transparansi dan akuntabilitas pelaksanaan fungsi peradilan. Namun dalam hal-hal tertentu masih dimungkinkan terjadi permasalahan teknis terkait dengan akurasi dan keterkinian informasi yang kami sajikan, hal mana akan terus kami perbaiki dari waktu kewaktu.
Dalam hal Anda menemukan inakurasi informasi yang termuat pada situs ini atau informasi yang seharusnya ada, namun belum tersedia, maka harap segera hubungi Kepaniteraan Mahkamah Agung RI melalui :
Email : kepaniteraan@mahkamahagung.go.id Telp : 021-384 3348 (ext.318)

tersebut dinilai akan lebih mendatangkan *kemudharatan* baik bagi suami maupun isteri, oleh karena itu *kemudharatan* harus dihindari dan suatu *kemudharatan* harus dihilangkan, sebab akan menimbulkan kerusakan baik *psycologis* maupun pisik dan materil;

Menimbang, bahwa oleh karena perkara ini diajukan oleh isteri sebagai Penggugat dan *thalak* dijatuhkan oleh Pengadilan Cq. Majelis Hakim, maka perceraian (*thalak*) yang akan dijatuhkan kepada Penggugat adalah *thalak satu ba'in shugra*, sesuai dengan ketentuan Pasal 119 ayat (2) Kompilasi Hukum Islam dan sejalan dengan *qaidah fiqhiyah*, yang terdapat dalam Kitab Ghayah al-Maram (غاية المرام) halaman 162, yang sejalan dengan pendapat Majelis Hakim, sebagai berikut;

اذا اشـتـد عـدم رغبة الزوجة لـزوجـها طلق عليـه القاضـي طلقة

Artinya: *Apabila ketidaksukaan isteri terhadap suaminya itu sudah sedemikian rupa (bersangatan), maka Hakim dapat menjatuhkan talak terhadap isterinya dengan talak satu (bain shughra);*

Menimbang, bahwa berdasarkan pertimbangan-pertimbangan tersebut di atas, Majelis Hakim berkesimpulan bahwa alasan perceraian yang diajukan Penggugat telah memenuhi maksud penjelasan Pasal 39 ayat (2) Undang-Undang Nomor 1 Tahun 1974 jo. Pasal 19 huruf (f) Peraturan Pemerintah Nomor 9 Tahun 1975 dan Pasal 116 huruf (f) Kompilasi Hukum Islam Tahun 1991, dengan demikian gugatan cerai Penggugat dari Tergugat dan mohon agar Majelis Hakim menjatuhkan *talak satu ba'in suhgra* dari Tergugat terhadap Penggugat telah beralasan menurut hukum, dan gugatan Penggugat patut dikabulkan;

Menimbang, bahwa perkara ini termasuk bidang perkawinan maka sesuai dengan ketentuan pasal 89 ayat (1) Undang-undang Nomor 7 Tahun 1989 tentang Peradilan Agama , MajelisHakim mempunyai cukup alasan untuk membebankan biaya perkara ini kepada Penggugat yang jumlahnya sebagai mana akan diterakan dalam amar putusan;

Mengingat segala peraturan dan ketentuan hukum yang berlaku serta nash-nash Syara' yang berkaitan dengan perkara ini;

MENGADILI:

Disclaimer
Kepaniteraan Mahkamah Agung Republik Indonesia berusaha untuk selalu mencantumkan informasi paling kini dan akurat sebagai bentuk komitmen Mahkamah Agung untuk pelayanan publik, transparansi dan akuntabilitas pelaksanaan fungsi peradilan. Namun dalam hal-hal tertentu masih dimungkinkan terjadi permasalahan teknis terkait dengan akurasi dan keterkinian informasi yang kami sajikan, hal mana akan terus kami perbaiki dari waktu kewaktu.
Dalam hal Anda menemukan inakurasi informasi yang termuat pada situs ini atau informasi yang seharusnya ada, namun belum tersedia, maka harap segera hubungi Kepaniteraan Mahkamah Agung RI melalui :
Email : kepaniteraan@mahkamahagung.go.id Telp : 021-384 3348 (ext.318)

1. Menyatakan Tergugat yang telah dipanggil secara resmi dan patut untuk datang menghadap di persidangan, tidak hadir;
2. Mengabulkan gugatan Penggugat dengan verstek;
3. Menjatuhkan talak satu ba'in sughra Tergugat (**Nama Tergugat**) terhadap Penggugat (**Nama Penggugat**);
4. Membebankan Penggugat untuk membayar biaya perkara ini sejumlah Rp. 516.000,00 (lima ratus enam belas ribu rupiah);

Demikian diputuskan dalam rapat permusyawaratan Majelis Hakim Mahkamah Syar'iyah Bireuen pada hari Senin tanggal 28 Desember 2020 M, bertepatan dengan tanggal 13 Jumadil Awal 1442 H, oleh kami **Drs. H. Abdul Karim Usman**, Hakim yang ditunjuk oleh Ketua Mahkamah Syar'iyah Bireuen sebagai Ketua Majelis serta **Dra. Sumarni dan Drs. Ibrahim Basyah**, masing-masing sebagai Hakim Anggota dan diucapkan pada hari itu juga dalam sidang terbuka untuk umum oleh Ketua Majelis yang didampingi para Hakim Anggota yang turut bersidang dan dibantu oleh **Dra. Rosdiana** sebagai Panitera Pengganti dengan dihadiri oleh Penggugat yang didampingi kuasanya tanpa hadirnya Tergugat;

Ketua Majelis,

Drs. H. Abdul Karim Usman

Hakim Anggota,	Hakim Anggota,
**Dra. Sumarni**	**Drs. Ibrahim Basyah**

Panitera Pengganti,

Disclaimer
Kepaniteraan Mahkamah Agung Republik Indonesia berusaha untuk selalu mencantumkan informasi paling kini dan akurat sebagai bentuk komitmen Mahkamah Agung untuk pelayanan publik, transparansi dan akuntabilitas pelaksanaan fungsi peradilan. Namun dalam hal-hal tertentu masih dimungkinkan terjadi permasalahan teknis terkait dengan akurasi dan keterkinian informasi yang kami sajikan, hal mana akan terus kami perbaiki dari waktu kewaktu.
Dalam hal Anda menemukan inakurasi informasi yang termuat pada situs ini atau informasi yang seharusnya ada, namun belum tersedia, maka harap segera hubungi Kepaniteraan Mahkamah Agung RI melalui :
Email : kepaniteraan@mahkamahagung.go.id Telp : 021-384 3348 (ext.318)

Dra. Rosdiana

Rincian Biaya Perkara:

1.	Biaya Pendaftaran	Rp.	30.000,00.
2.	Biaya Proses	Rp.	50.000,00.
3.	Biaya Panggilan	Rp.	420.000,00.
4.	Redaksi	Rp.	10.000,00.
5.	Meterai	Rp.	6.000,00.
	Jumlah	Rp.	516.000,00.

(lima ratus enam belas ribu rupiah,-)

Disclaimer
Kepaniteraan Mahkamah Agung Republik Indonesia berusaha untuk selalu mencantumkan informasi paling kini dan akurat sebagai bentuk komitmen Mahkamah Agung untuk pelayanan publik, transparansi dan akuntabilitas pelaksanaan fungsi peradilan. Namun dalam hal-hal tertentu masih dimungkinkan terjadi permasalahan teknis terkait dengan akurasi dan keterkinian informasi yang kami sajikan, hal mana akan terus kami perbaiki dari waktu kewaktu.
Dalam hal Anda menemukan inakurasi informasi yang termuat pada situs ini atau informasi yang seharusnya ada, namun belum tersedia, maka harap segera hubungi Kepaniteraan Mahkamah Agung RI melalui :
Email : kepaniteraan@mahkamahagung.go.id   Telp : 021-384 3348 (ext.318)
