# Supplementary material for: Classification of divorce causes during the COVID-19 pandemic using convolutional neural networks
Source: PeerJ Comput Sci. 2022 Jun 30;8:e998. doi: 10.7717/peerj-cs.998 (PMC9299239; doi:10.7717/peerj-cs.998)
Supplement: Supplemental Information 2 [file peerj-cs-08-998-s002.zip › Kelalaian Dataset/Data ke-3.pdf]

P U T U S A N

Nomor **1945/Pdt.G/2020/PA.Bms**

بِسْمِ اللهِ الرَّحْمَنِ الرَّحِيْمِ

DEMI KEADILAN BERDASARKAN KETUHANAN YANG MAHA ESA

Pengadilan Agama Banyumas yang memeriksa dan mengadili perkara tertentu pada tingkat pertama dalam sidang majelis telah menjatuhkan putusan perkara cerai gugat antara:

PENGGUGAT, umur 40 tahun, agama Islam, pekerjaan xxxxx, pendidikan SLTA, tempat kediaman di xxxxxx xxxxx, xxxx xxxxxxxxxxx, xxxxxxxxx xxxxxxxxx, xxxxxxxxx xxxxxxxx, Sekarang beralamat di : FLAT D, xxxx,xxxxx x,xx xxx xxx xxxx xx,xxxxxxxx,xxx xxx ,xx xxxxxxxx, xxxx xxxxxxxxxxx, xxxxxxxxx, xxxx xxxxxxxx, xxxx xxxxxx, sebagai **Penggugat**;

melawan

TERGUGAT, umur 44 tahun, agama Islam, pekerjaan xxxxx, pendidikan SLTP, tempat kediaman di KABUPATEN CILACAP, KEL. DONAN, CILACAP TENGAH, KAB. CILACAP, JAWA TENGAH, sebagai **Tergugat**;

Pengadilan Agama tersebut;

Telah mempelajari berkas perkara;

Telah mendengar keterangan Penggugat serta memeriksa bukti-bukti di persidangan;

DUDUK PERKARA

Bahwa Penggugat dengan surat gugatannya tanggal 08 Desember 2020 telah mengajukan gugatan cerai gugat yang didaftar di Kepaniteraan Pengadilan Agama Banyumas dengan Nomor 1945/Pdt.G/2020/PA.Bms, tanggal 08 Desember 2020, dengan dalil-dalil yang pada pokoknya sebagai berikut:

1. Bahwa pada tanggal 22 Desember 2017, Penggugat dengan Tergugat melangsungkan pernikahan yang dicatat oleh Pegawai Pencatat Nikah Kantor Urusan Agama xxxxxxxxx xxxxxxxxx, xxxxxxxxx xxxxxxxx, sebagaimana

Disclaimer
Kepaniteraan Mahkamah Agung Republik Indonesia berusaha untuk selalu mencantumkan informasi paling kini dan akurat sebagai bentuk komitmen Mahkamah Agung untuk pelayanan publik, transparansi dan akuntabilitas pelaksanaan fungsi peradilan. Namun dalam hal-hal tertentu masih dimungkinkan terjadi permasalahan teknis terkait dengan akurasi dan keterkinian informasi yang kami sajikan, hal mana akan terus kami perbaiki dari waktu kewaktu.
Dalam hal Anda menemukan inakurasi informasi yang termuat pada situs ini atau informasi yang seharusnya ada, namun belum tersedia, maka harap segera hubungi Kepaniteraan Mahkamah Agung RI melalui :
Email : kepaniteraan@mahkamahagung.go.id Telp : 021-384 3348 (ext.318)

tersebut pada Kutipan Akta Nikah Nomor :0461 /29/XII/2017.Tanggal 22 Desember 2017.---

2. Bahwa setelah pernikahan tersebut Penggugat dengan Tergugat bertempat kediaman di rumah orang tua Penggugat di xxxxxx xxxxx, xxxx xxxxxxxxxxx, xxxxxxxxx xxxxxxxxx, xxxxxxxxx xxxxxxxx, selama 1 bulan (dari bulan Desember 2017 s/d bulan Januari 2018) ,Kemudian Penggugat dan Tergugat tinggal di rumah orang tua Tergugat di xxxxxx xxxxx,xxxxxxxxx xxxxx, Kecamatan CilacapTengah, xxxxxxxxx xxxxxxx ,selama 6 bulan (dari bulan Februari 2018 s/d bulan Juli 2018)Kemudian Penggugat dan Tergugat tinggal di rumah bersama di xxxxxx xxxxx, xxxx xxxxxxxxxxx, xxxxxxxxx xxxxxxxxx, xxxxxxxxx xxxxxxxx ,selama 5 bulan .

3. Bahwa selama pernikahan Penggugat dengan Tergugat telah melakukan hubungan suami isteri (Ba'da Dhukul), dan belum dikaruniai anak .

4. Bahwa pada semula rumah tangga Penggugat dengan Tergugat hidup rukun, namun sejak bulan juni 2018. rumah tangga antara Penggugat dengan Tergugat mulai goyah, terjadi perselisihan, dan pertengkaran dikarenakan

1) faktor ekonomi yang kurang tercukupi Tergugat kurang tanggung jawab terhadap keluarga nafkah dari Tergugat kurang mencukupi untuk kebutuhan hidup sehari - hari) .

5. Bahwa oleh karena rumah tangga Penggugat dengan Tergugat sudah tidak ada kecocokan pada akhirnya sejak bulan Januari 2019 antara Penggugat dan Tergugat terjadi pisah tempat tinggal, dimana Tergugat pulang ke rumah orang tuanya di xxxxxx xxxxx,xxxxxxxxx xxxxx, Kecamatan CilacapTengah, xxxxxxxxx xxxxxxx, sedangkan Penggugat tinggal di rumah bersama di xxxxxx xxxxx, xxxx xxxxxxxxxxx, xxxxxxxxx xxxxxxxxx, xxxxxxxxx xxxxxxxx, hingga sekarang gugatan cerai diajukan ke Pengadilan Agama Banyumas .antara Penggugat dan Tergugat sudah berpisah selama 2 tahun;

Disclaimer
Kepaniteraan Mahkamah Agung Republik Indonesia berusaha untuk selalu mencantumkan informasi paling kini dan akurat sebagai bentuk komitmen Mahkamah Agung untuk pelayanan publik, transparansi dan akuntabilitas pelaksanaan fungsi peradilan. Namun dalam hal-hal tertentu masih dimungkinkan terjadi permasalahan teknis terkait dengan akurasi dan keterkinian informasi yang kami sajikan, hal mana akan terus kami perbaiki dari waktu kewaktu.
Dalam hal Anda menemukan inakurasi informasi yang termuat pada situs ini atau informasi yang seharusnya ada, namun belum tersedia, maka harap segera hubungi Kepaniteraan Mahkamah Agung RI melalui :
Email : kepaniteraan@mahkamahagung.go.id Telp : 021-384 3348 (ext.318)

6. Bahwa Penggugat telah berusaha sabar dan berusaha menjadi istri yang baik untuk Tergugat serta kemelut rumah tangga Penggugat dengan Tergugat sudah pernah didamaikan oleh pihak keluarga, namun tidak berhasil.;

7. Atas sikap dan /atau perbuatan Tergugat tersebut, Penggugat sangat menderita lahir maupun batin dan oleh karenanya Penggugat tidak rela dan mengajukan halnya kepada Pengadilan Agama Banyumas.;

8. Bahwa dalil gugatan cerai Pengugat telah memenuhi unsur atau alasan-alasan perceraian sebagaimana di atur pada pasal 19 huruf (f) Peraturan Pemerintah No. 9 tahun 1975 Jo pasal 116 huruf (f) Kompilasi Hukum Islam.

Berdasarkan alasan/dalil-dalil diatas, maka Penggugat mohon kepada Ketua Pengadilan Agama Banyumas cq. Majelis Hakim yang memeriksa dan mengadili perkara ini, berkenan menjatuhkan putusan sebagai berikut :

1. Menerima dan mengabulkan gugatan perceraian Penggugat.

2. Menetapkan jatuhya Talak dari Tergugat (TERGUGAT) kepada Penggugat (HEVITA RONALIA Binti RONALD RICHARDO) atau menceraikan Penggugat dari Tergugat.

3. Membebankan biaya perkara menurut hukum.

Atau apabila Pengadilan Agama Banyumas berpendapat lain, mohon menjatuhkan putusan yang seadil-adilnya.

Bahwa pada hari sidang yang telah ditetapkan Penggugat telah datang menghadap ke muka sidang, sedangkan Tergugat tidak datang menghadap ke muka sidang dan tidak menyuruh orang lain untuk menghadap sebagai wakil/kuasa hukumnya meskipun telah dipanggil secara resmi dan patut menurut relaas Nomor 1945/Pdt.G/2020/PA.Bms tanggal 11 Desember 2020 dan ... yang dibacakan di dalam sidang, sedangkan tidak ternyata bahwa tidak datangnya itu disebabkan suatu halangan yang sah;

Disclaimer
Kepaniteraan Mahkamah Agung Republik Indonesia berusaha untuk selalu mencantumkan informasi paling kini dan akurat sebagai bentuk komitmen Mahkamah Agung untuk pelayanan publik, transparansi dan akuntabilitas pelaksanaan fungsi peradilan. Namun dalam hal-hal tertentu masih dimungkinkan terjadi permasalahan teknis terkait dengan akurasi dan keterkinian informasi yang kami sajikan, hal mana akan terus kami perbaiki dari waktu kewaktu.
Dalam hal Anda menemukan inakurasi informasi yang termuat pada situs ini atau informasi yang seharusnya ada, namun belum tersedia, maka harap segera hubungi Kepaniteraan Mahkamah Agung RI melalui :
Email : kepaniteraan@mahkamahagung.go.id Telp : 021-384 3348 (ext.318)

Bahwa majelis hakim telah menasehati Penggugat agar berpikir untuk tidak bercerai dengan Tergugat, tetapi Penggugat tetap pada dalil-dalil gugatannya untuk bercerai dengan Tergugat;

Bahwa perkara ini tidak dapat dimediasi karena Tergugat tidak pernah datang menghadap meskipun telah dipanggil secara resmi dan patut, selanjutnya dimulai pemeriksaan dengan membacakan surat gugatan Penggugat yang maksud dan isinya tetap dipertahankan oleh Penggugat;

Bahwa untuk menguatkan dalil-dalil gugatan Penggugat telah mengajukan alat-alat bukti berupa:

A. Surat:

1. Fotokopi Surat Keterangan atas Nama PENGGUGAT Nomor 3302/SKT/20180226/00365 Tanggal 26 Pebruari 2018 yang dikeluarkan oleh Kantor Pemerintah xxxxxxxxx xxxxxxxx, bukti surat tersebut telah diberi meterai cukup, dinazegelen pos dan telah dicocokkan dengan aslinya yang ternyata sesuai, lalu oleh Ketua Majelis diberi tanda P1;
2. Fotokopi Kutipan Akta Nikah Nomor 0461 /29/XII/201 Tanggal 22 Desember 2017, yang dikeluarkan oleh Kantor Urusan Agama xxxxxxxxx xxxxxxxxx, xxxxxxxxx xxxxxxxx, bukti surat tersebut telah diberi meterai cukup, telah dinazegelen pos dan telah dicocokkan dengan aslinya yang ternyata sesuai, lalu oleh Ketua Majelis diberi tanda P2;

B. Saksi:

1. **SAKSI 1**, umur 35 tahun, agama Islam, pekerjaan xxxxxxxxxx, bertempat tinggal di KABUPATEN BANYUMAS, di bawah sumpahnya memberikan keterangan sebagai berikut:
   - Bahwa, saksi kenal Penggugat dan Tergugat dan memiliki hubungan dengan Penggugat sebagai Adik Kandung Penggugat;
   - Bahwa, saksi mengetahui Penggugat dan Tergugat menikah pada tanggal 22 Desember 2017 di Kantor Urusan Agama xxxxxxxxx xxxxxxxxx, xxxxxxxxx xxxxxxxx;
   - Bahwa, saksi mengetahui antara Penggugat dan Tergugat belum dikaruniai anak.;

Disclaimer
Kepaniteraan Mahkamah Agung Republik Indonesia berusaha untuk selalu mencantumkan informasi paling kini dan akurat sebagai bentuk komitmen Mahkamah Agung untuk pelayanan publik, transparansi dan akuntabilitas pelaksanaan fungsi peradilan. Namun dalam hal-hal tertentu masih dimungkinkan terjadi permasalahan teknis terkait dengan akurasi dan keterkinian informasi yang kami sajikan, hal mana akan terus kami perbaiki dari waktu kewaktu.
Dalam hal Anda menemukan inakurasi informasi yang termuat pada situs ini atau informasi yang seharusnya ada, namun belum tersedia, maka harap segera hubungi Kepaniteraan Mahkamah Agung RI melalui :
Email : kepaniteraan@mahkamahagung.go.id   Telp : 021-384 3348 (ext.318)

- Bahwa, saksi mengetahui keadaan rumah tangga Penggugat dan Tergugat pada awalnya rukun dan harmonis, akan tetapi kurang lebih sejak bulan Juni 2018 sudah tidak rukun lagi sering terjadi pertengkaran;
- Bahwa, saksi sering melihat dan mendengar Penggugat dan Tergugat cekcok;
- Bahwa, saksi mengetahui yang menjadi penyebab ketidak harmonisan rumah tangga Penggugat dan Tergugat disebabkan karena masalah kurang ekonomi;
- Bahwa, saksi mengetahui antara Penggugat dan Tergugat sudah pisah rumah sejak bulan Januari 2019 sampai dengan sekarang dan sudah tidak bersatu lagi;
- Bahwa, baik saksi maupun pihak keluarga sudah berusaha merukunkan Penggugat dan Tergugat akan tetapi tidak berhasil;
- Bahwa, saksi melihat rumah tangga Penggugat dan Tergugat sudah tidak mungkin lagi dipertahankan, sulit dipertahankan dan tidak ada harapan dapat bersatu lagi sehingga lebih baik bercerai;

2. **SAKSI 2**, umur 58 tahun, agama Islam, pekerjaan xxxxxxxx xxxxx xxxxxx, bertempat tinggal di KABUPATEN BANYUMAS, di bawah sumpahnya memberikan keterangan sebagai berikut:
   - Bahwa, saksi kenal dengan Penggugat dan Tergugat dan memiliki hubungan dengan Penggugat sebagai Tetangga Penggugat;
   - Bahwa, saksi mengetahui Penggugat dan Tergugat adalah suami isteri yang sah yang menikah pada tanggal 22 Desember 2017 di Kantor Urusan Agama xxxxxxxxx xxxxxxxxx, xxxxxxxxx xxxxxxxx;
   - Bahwa, saksi mengetahui dari perkawinan Penggugat dan Tergugat belum dikaruniai anak. ;
   - Bahwa, saksi mengetahui rumah tangga Penggugat dan Tergugat sudah tidak lagi harmonis, sering terjadi perselisihan dan pertengkaran sejak bulan Juni 2018 yang mengakibatkan antara Penggugat dan Tergugat sudah pisah rumah sejak bulan Januari 2019 sampai dengan sekarang dan selama berpisah antara Penggugat dan Tergugat tidak pernah bersatu lagi;

Disclaimer
Kepaniteraan Mahkamah Agung Republik Indonesia berusaha untuk selalu mencantumkan informasi paling kini dan akurat sebagai bentuk komitmen Mahkamah Agung untuk pelayanan publik, transparansi dan akuntabilitas pelaksanaan fungsi peradilan. Namun dalam hal-hal tertentu masih dimungkinkan terjadi permasalahan teknis terkait dengan akurasi dan keterkinian informasi yang kami sajikan, hal mana akan terus kami perbaiki dari waktu kewaktu.
Dalam hal Anda menemukan inakurasi informasi yang termuat pada situs ini atau informasi yang seharusnya ada, namun belum tersedia, maka harap segera hubungi Kepaniteraan Mahkamah Agung RI melalui :
Email : kepaniteraan@mahkamahagung.go.id Telp : 021-384 3348 (ext.318)

- Bahwa, saksi pernah melihat keduanya bersitegang dan saling mendiamkan sebanyak 1 kali;
- Bahwa, saksi mengetahui penyebab perselisihan dan pertengkaran Penggugat dan Tergugat disebabkan karena masalah kurang ekonomi;
- Bahwa, pihak keluarga sudah ada upaya mendamaikan dan merukunkan Penggugat dan Tergugat akan tetapi tidak berhasil;
- Bahwa, saksi sudah tidak sanggup lagi merukunkan Penggugat dan Tergugat karena sudah sulit dirukunkan dan sudah tidak ada harapan dapat bersatu lagi dan melihat keadaan rumah tangganya lebih baik bercerai;

Bahwa Penggugat telah menyampaikan kesimpulan yang pada pokoknya tetap pada gugatan dan mohon putusan yang mengabulkan tuntutan Penggugat;

Bahwa, untuk mempersingkat uraian putusan ini maka semua hal yang termuat dalam berita acara sidang ini merupakan bagian yang tidak terpisahkan dari putusan ini;

PERTIMBANGAN HUKUM

Menimbang, bahwa maksud dan tujuan gugatan Penggugat adalah sebagaimana terurai di atas;

Menimbang, bahwa ternyata Tergugat, meskipun dipanggil secara resmi dan patut, tidak datang menghadap di muka sidang dan pula tidak ternyata bahwa tidak datangnya itu disebabkan suatu halangan yang sah;

Menimbang, bahwa Tergugat yang dipanggil secara resmi dan patut sesuai ketentuan Pasal 26 ayat (1) Peraturan Pemerintah Nomor 9 Tahun 1975, akan tetapi tidak datang menghadap harus dinyatakan tidak hadir dan gugatan tersebut harus diperiksa secara verstek;

Menimbang, bahwa oleh karena itu, maka putusan atas perkara ini dapat dijatuhkan tanpa hadirnya Tergugat (verstek);

Menimbang, bahwa sesuai dengan Pasal 39 Undang-undang Nomor 1 Tahun 1974, jo. Pasal 65 Undang-undang Nomor 7 Tahun 1989, sebagaimana telah diubah dengan Undang-undang Nomor 3 Tahun 2006 dan terakhir diubah dengan Undang-undang Nomor 50 Tahun 2009, jo. Pasal 115 Kompilasi Hukum

Disclaimer
Kepaniteraan Mahkamah Agung Republik Indonesia berusaha untuk selalu mencantumkan informasi paling kini dan akurat sebagai bentuk komitmen Mahkamah Agung untuk pelayanan publik, transparansi dan akuntabilitas pelaksanaan fungsi peradilan. Namun dalam hal-hal tertentu masih dimungkinkan terjadi permasalahan teknis terkait dengan akurasi dan keterkinian informasi yang kami sajikan, hal mana akan terus kami perbaiki dari waktu kewaktu.
Dalam hal Anda menemukan inakurasi informasi yang termuat pada situs ini atau informasi yang seharusnya ada, namun belum tersedia, maka harap segera hubungi Kepaniteraan Mahkamah Agung RI melalui :
Email : kepaniteraan@mahkamahagung.go.id   Telp : 021-384 3348 (ext.318)

Islam, Majelis Hakim pada setiap kali persidangan telah berusaha semaksimal mungkin menasehati Penggugat untuk bersabar agar bisa membina rumah tangga dengan rukun dan harmonis akan tetapi tidak berhasil, oleh karea itu perkara ini harus segera diputuskan;

Menimbang, bahwa oleh karena Tergugat tidak pernah datang menghadap meskipun telah dipanggil secara resmi dan patut maka sesuai Pasal 4 angka (2) huruf b Peraturan Mahkamah Agung Republik Indonesia Nomor 1 Tahun 2016 tentang Prosedur Mediasi di Pengadilan, perkara ini termasuk sengketa yang dikecualikan dari kewajiban mediasi;

Menimbang, bahwa berdasarkan ketentuan Pasal 125 ayat (1) HIR. yaitu putusan yang dijatuhkan tanpa hadirnya Tergugat dapat dikabulkan sepanjang berdasarkan hukum dan beralasan, oleh karena itu majelis membebani Penggugat untuk membuktikan dalil-dalil gugatannya;

Menimbang, bahwa untuk membuktikan dalil-dalil gugatannya, Penggugat telah mengajukan alat bukti surat P.1, P.2 serta dua orang saksi;

Menimbang, bahwa bukti P.1 (Fotokopi Kartu Tanda Penduduk) yang merupakan akta otentik, telah bermeterai cukup, *bernazegelen* dan cocok dengan aslinya, isi bukti tersebut menjelaskan mengenai agama dan tempat tinggal Penggugat, sehingga sejalan dengan ketentuan Pasal 165 HIR bukti tersebut telah memenuhi syarat formal dan materiil, serta mempunyai kekuatan pembuktian yang sempurna dan mengikat (*volledig en bindende bewijskracht*);

Menimbang, bahwa bukti P.2 (Fotokopi Kutipan Akta Nikah) yang merupakan akta otentik, telah bermeterai cukup, *bernazegelen* dan cocok dengan aslinya, isi bukti tersebut menjelaskan mengenai hubungan Penggugat dan Tergugat sebagai suami isteri, sehingga sejalan dengan ketentuan Pasal 165 HIR bukti tersebut telah memenuhi syarat formal dan materiil, serta mempunyai kekuatan pembuktian yang sempurna dan mengikat (*volledig en bindende bewijskracht*);

Menimbang, bahwa sesuai dengan Pasal 22 ayat (2) Peraturan Pemerintah Nomor 9 Tahun 1975, Majelis Hakim perlu mendapatkan keterangan pihak keluarga dan atau orang-orang yang dekat dengan suami isteri itu tentang adanya perselisihan dan pertengkaran antara Penggugat dan Tergugat;

Disclaimer
Kepaniteraan Mahkamah Agung Republik Indonesia berusaha untuk selalu mencantumkan informasi paling kini dan akurat sebagai bentuk komitmen Mahkamah Agung untuk pelayanan publik, transparansi dan akuntabilitas pelaksanaan fungsi peradilan. Namun dalam hal-hal tertentu masih dimungkinkan terjadi permasalahan teknis terkait dengan akurasi dan keterkinian informasi yang kami sajikan, hal mana akan terus kami perbaiki dari waktu kewaktu.
Dalam hal Anda menemukan inakurasi informasi yang termuat pada situs ini atau informasi yang seharusnya ada, namun belum tersedia, maka harap segera hubungi Kepaniteraan Mahkamah Agung RI melalui :
Email : kepaniteraan@mahkamahagung.go.id Telp : 021-384 3348 (ext.318)

Menimbang, bahwa saksi 1 Penggugat, adalah keluarga atau orang dekat Penggugat dan atau Tergugat sesuai dengan Pasal 22 ayat (2) Peraturan Pemerintah Nomor 9 Tahun 1975 jo. Pasal 134 Kompilasi Hukum Islam. Saksi 1 Penggugat sudah dewasa dan sudah disumpah, sehingga memenuhi syarat formal sebagaimana diatur dalam Pasal 145 HIR;

Menimbang, bahwa keterangan saksi 1 Penggugat mengenai hubungan Penggugat dan Tergugat serta mengenai keadaan rumah tangga Penggugat dan Tergugat, adalah fakta yang pernah dilihat dan didengar langsung dan relevan dengan dalil yang harus dibuktikan oleh Penggugat, oleh karena itu keterangan saksi tersebut telah memenuhi syarat materiil sebagaimana telah diatur dalam Pasal 171 HIR sehingga keterangan saksi tersebut memiliki kekuatan pembuktian dan dapat diterima sebagai alat bukti;

Menimbang, bahwa saksi 2 Penggugat, adalah keluarga atau orang dekat Penggugat dan atau Tergugat sesuai dengan Pasal 22 ayat (2) Peraturan Pemerintah Nomor 9 Tahun 1975 jo. Pasal 134 Kompilasi Hukum Islam. Saksi 2 Penggugat, sudah dewasa dan sudah disumpah, sehingga memenuhi syarat formal sebagaimana diatur dalam Pasal 145 HIR;

Menimbang, bahwa keterangan saksi 2 Penggugat mengenai hubungan Penggugat dan Tergugat serta mengenai keadaan rumah tangga Penggugat dan Tergugat, adalah fakta yang pernah dilihat dan didengar langsung dan relevan dengan dalil yang harus dibuktikan oleh Penggugat, oleh karena itu keterangan saksi tersebut telah memenuhi syarat materiil sebagaimana telah diatur dalam Pasal 171 HIR sehingga keterangan saksi tersebut memiliki kekuatan pembuktian dan dapat diterima sebagai alat bukti;

Menimbang, bahwa keterangan saksi 1 dan saksi 2 Penggugat bersesuaian dan cocok antara satu dengan yang lain oleh karena itu keterangan dua orang saksi tersebut memenuhi Pasal 171 dan Pasal 172 HIR;

Menimbang, bahwa berdasarkan bukti P.1 dan P. 2, Saksi 1 dan Saksi 2 Penggugat, terbukti fakta kejadian sebagai berikut:

1. Bahwa Penggugat dan Tergugat beragama Islam dan Penggugat bertempat tinggal di wilayah xxxxxxxxx xxxxxxxx;
2. Bahwa Penggugat dan Tergugat merupakan suami isteri yang menikah secara agama Islam pada tanggal 22 Desember 2017 di

Disclaimer
Kepaniteraan Mahkamah Agung Republik Indonesia berusaha untuk selalu mencantumkan informasi paling kini dan akurat sebagai bentuk komitmen Mahkamah Agung untuk pelayanan publik, transparansi dan akuntabilitas pelaksanaan fungsi peradilan. Namun dalam hal-hal tertentu masih dimungkinkan terjadi permasalahan teknis terkait dengan akurasi dan keterkinian informasi yang kami sajikan, hal mana akan terus kami perbaiki dari waktu kewaktu.
Dalam hal Anda menemukan inakurasi informasi yang termuat pada situs ini atau informasi yang seharusnya ada, namun belum tersedia, maka harap segera hubungi Kepaniteraan Mahkamah Agung RI melalui :
Email : kepaniteraan@mahkamahagung.go.id Telp : 021-384 3348 (ext.318)

Kecamatan Kantor Urusan Agama xxxxxxxxx xxxxxxxxx, xxxxxxxxx xxxxxxxx ;

3. Bahwa pada mulanya rumah tangga Penggugat dan Tergugat rukun dan belum dikaruniai anak.;

4. Bahwa rumah tangga Penggugat dengan Tergugat sejak bulan juni 2018 hingga saat ini sudah tidak rukun lagi sering berselisih dan bertengkar;

5. Bahwa perselisihan dan pertengkaran antara Penggugat dan Tergugat disebabkan karena masalah ekonomi;

6. Bahwa akibat perselisihan dan pertengkaran tersebut antara Penggugat dan Tergugat sudah pisah pisah rumah sejak kurang lebih 2 tahun yang lalu dan selama itu pula keduanya tidak pernah berkumpul lagi dan tidak melaksanakan hak dan kewajibannya masing-masing sebagaimana layaknya suami isteri;

7. Bahwa, baik majelis hakim dalam persidangan, maupun pihak keluarga Penggugat dan Tergugat sudah berusaha menasehati Penggugat dan Tergugat dan sudah ada upaya musyawarah dua keluarga untuk merukunkan Penggugat dan Tergugat, akan tetapi tidak berhasil, karena Penggugat bersikukuh ingin bercerai dengan Tergugat;

8. Bahwa kedua saksi sudah tidak sanggup lagi untuk merukunkan Penggugat dan Tergugat, karena sudah sulit merukunkan Penggugat dan Tergugat dan sudah tidak ada harapan lagi keduanya dapat rukun dalam rumah tangga;

Menimbang, bahwa berdasarkan fakta-fakta tersebut di atas dapat disimpulkan fakta hukum sebagai berikut:

1. Bahwa Penggugat dan Tergugat beragama Islam dan Penggugat bertempat tinggal di wilayah yuridiksi Pengadilan Agama Banyumas;

2. Bahwa Penggugat dengan Tergugat adalah pasangan suami isteri yang sah yang menikah secara agama Islam;

3. Bahwa dalam rumah tangga Penggugat dan Tergugat sudah tidak rukun, telah terjadi perselisihan dan pertengkaran yang terus menerus;

4. Bahwa sudah ada upaya maksimal untuk merukunkan Penggugat dan Tergugat, akan tetapi tidak berhasil;

Disclaimer
Kepaniteraan Mahkamah Agung Republik Indonesia berusaha untuk selalu mencantumkan informasi paling kini dan akurat sebagai bentuk komitmen Mahkamah Agung untuk pelayanan publik, transparansi dan akuntabilitas pelaksanaan fungsi peradilan. Namun dalam hal-hal tertentu masih dimungkinkan terjadi permasalahan teknis terkait dengan akurasi dan keterkinian informasi yang kami sajikan, hal mana akan terus kami perbaiki dari waktu kewaktu.
Dalam hal Anda menemukan inakurasi informasi yang termuat pada situs ini atau informasi yang seharusnya ada, namun belum tersedia, maka harap segera hubungi Kepaniteraan Mahkamah Agung RI melalui :
Email : kepaniteraan@mahkamahagung.go.id Telp : 021-384 3348 (ext.318)

5. Bahwa, rumah tangga Penggugat dan Tergugat telah pecah (*marriage breakdown*) sehingga rumah tangga antara Penggugat dengan Tergugat sudah tidak ada harapan untuk dipertahankan lagi (*onheel baar tweespalt*);
6. Bahwa alasan perceraian Penggugat tidak disebabkan suatu perbuatan yang melawan hukum;

Menimbang, bahwa berdasarkan fakta hukum di atas majelis dapat mengkonstituir sebagai berikut:

1. Bahwa Penggugat dan Tergugat beragama Islam dan Penggugat bertempat tinggal di wilayah yuridiksi Pengadilan Agama Banyumas, maka sesuai dengan ketentuan pasal 49 ayat (1) dan (2), jo. pasal 73 ayat (1) Undang-undang Nomor 7 Tahun 1989 sebagaimana diubah dengan Undang-undang Nomor 3 Tahun 2006 dan perubahan kedua dengan Undang-undang Nomor 50 Tahun 2009, jo. Pasal 63 ayat (1) Undang-undang Nomor 1 Tahun 1974, jo. Pasal 20 ayat (1) Peraturan Pemerintah Nomor 9 Tahun 1975, jo. Pasal 132 ayat (1) Kompilasi Hukum Islam, perkara ini merupakan kompetensi relatif Pengadilan Agama Banyumas;
2. Bahwa Penggugat dengan Tergugat adalah suami isteri yang sah dan belum pernah terjadi perceraian menurut hukum dan perundang-undangan yang berlaku sesuai dengan Pasal 2 Undang-undang Nomor 1 Tahun 1974 tentang Perkawinan, maka masing-masing pihak mempunyai hak dan kepentingan untuk mengajukan tuntutan dalam sengketa perkawinan dalam perkara ini (*persona standi in judicio),* dan perkara ini menjadi kompetensi absolut Pengadilan Agama;
3. Bahwa rumah tangga Penggugat dan Tergugat sudah tidak rukun dan telah terjadi perselisihan dan pertengkaran yang terus menerus;
4. Bahwa sudah ada upaya yang maksimal untuk merukunkan Penggugat dan Tergugat, akan tetapi Penggugat menyatakan tetap bersikukuh untuk bercerai dengan Tergugat, maka hal ini menandakan bahwa telah terdapat kebencian yang memuncak dari Penggugat terhadap Tergugat, dan oleh karenanya Majelis Hakim sependapat dan mengambil alih pendapat ahli fikih dalam kitab Al-Iqna juz II halaman 133 yang dijadikan sebagai pendapat Majelis sebagai berikut:

Disclaimer
Kepaniteraan Mahkamah Agung Republik Indonesia berusaha untuk selalu mencantumkan informasi paling kini dan akurat sebagai bentuk komitmen Mahkamah Agung untuk pelayanan publik, transparansi dan akuntabilitas pelaksanaan fungsi peradilan. Namun dalam hal-hal tertentu masih dimungkinkan terjadi permasalahan teknis terkait dengan akurasi dan keterkinian informasi yang kami sajikan, hal mana akan terus kami perbaiki dari waktu kewaktu.
Dalam hal Anda menemukan inakurasi informasi yang termuat pada situs ini atau informasi yang seharusnya ada, namun belum tersedia, maka harap segera hubungi Kepaniteraan Mahkamah Agung RI melalui :
Email : kepaniteraan@mahkamahagung.go.id Telp : 021-384 3348 (ext.318)

واذا اشتد عدم رغبة الزوجة لزوجها طلق عليه القاضى طلقة

Artinya: "Disaat istri telah memuncak rasa tidak suka terhadap suaminya, maka hakim diperkenankan menjatuhkan talak (suami) nya dengan talak satu;

5. Bahwa, rumah tangga Penggugat dan Tergugat sudah tidak ada harapan untuk dipertahankan lagi (*onheel baar tweespalt*), sehingga tujuan pernikahan untuk membina keluarga *sakinah, mawadah, warohmah* sebagaimana dimaksud Pasal 1 Undang-undang Nomor 1 Tahun 1974, jo. Pasal 3 Kompilasi Hukum Islam dan sebagaimana dimaksudkan al Qur'an Surat ar Rum [30] ayat 21, telah tidak terwujud;

6. Bahwa memaksakan untuk mempertahankan kondisi rumah tangga Penggugat dan Tergugat yang sudah pecah dan tidak ada harapan untuk dipertahankan lagi, akan menimbulkan mafsadat yang lebih besar dari pada manfaatnya, padahal menolak mafsadat diutamakan daripada mengharap maslahat, sesuai dengan Qaidah Fiqhiyah;

درء المفاسد مقدم على جلب المصالح

"*Menolak kerusakan lebih didahulukan dari pada mengambil kebaikan*"

7. Bahwa karena rumah tangga Penggugat dan Tergugat sudah tidak rukun, telah terjadi perselisihan dan pertengkaran yang terus menerus, dan tidak ada harapan akan hidup rukun lagi dalam rumah tangga, maka fakta hukum tersebut telah memenuhi alasan perceraian sebagaimana diatur dalam Pasal 19 huruf (f) Peraturan Pemerintah Nomor 9 tahun 1975 jo Pasal 116 huruf (f) Kompilasi Hukum Islam, sehingga gugatan Penggugat patut untuk dikabulkan;

8. Bahwa alasan perceraian Penggugat tidak disebabkan suatu perbuatan yang melawan hukum (*onrechtmatige daad*), maka sesuai Pasal 125 (1) HIR gugatan Penggugat dapat dikabulkan dengan verstek;

Menimbang, bahwa selama perkawinan Penggugat dan Tergugat telah bergaul sebagaimana layaknya suami isteri (ba'da dukhul), dan berdasarkan catatan perubahan NTR dalam P.2 antara Penggugat dan Tergugat belum

Disclaimer
Kepaniteraan Mahkamah Agung Republik Indonesia berusaha untuk selalu mencantumkan informasi paling kini dan akurat sebagai bentuk komitmen Mahkamah Agung untuk pelayanan publik, transparansi dan akuntabilitas pelaksanaan fungsi peradilan. Namun dalam hal-hal tertentu masih dimungkinkan terjadi permasalahan teknis terkait dengan akurasi dan keterkinian informasi yang kami sajikan, hal mana akan terus kami perbaiki dari waktu kewaktu.
Dalam hal Anda menemukan inakurasi informasi yang termuat pada situs ini atau informasi yang seharusnya ada, namun belum tersedia, maka harap segera hubungi Kepaniteraan Mahkamah Agung RI melalui :
Email : kepaniteraan@mahkamahagung.go.id Telp : 021-384 3348 (ext.318)

pernah bercerai, oleh karena itu talak Tergugat terhadap Penggugat yang akan dijatuhkan adalah talak yang kesatu, maka berdasarkan ketentuan Pasal 119 ayat (2), huruf c, Kompilasi Hukum Islam, majelis akan mengabulkan gugatan Penggugat dengan menjatuhkan talak satu ba'in sughra dari Tergugat terhadap Penggugat;

Menimbang bahwa talak satu ba'in sughra merupakan talak yang dijatuhkan oleh Hakim atas permintaan seorang isteri, yang mana akibat hukum dari talak satu ba'in sughra adalah antara suami dan isteri tidak dapat hidup bersama kembali, terkecuali dengan akad nikah yang baru;

Menimbang, bahwa karena perkara *a quo* masuk bidang perkawinan, maka berdasarkan Pasal 89 ayat (1) Undang-Undang Nomor 7 Tahun 1989 sebagaimana telah diubah dengan Undang-Undang Nomor 3 Tahun 2006 dan perubahan kedua dengan Undang-Undang Nomor 50 Tahun 2009 tentang Peradilan Agama, biaya perkara harus dibebankan kepada Penggugat;

Mengingat, semua pasal dalam peraturan perundang-undangan dan hukum Islam yang berkaitan dengan perkara ini;

M E N G A D I L I

1. Menyatakan Tergugat yang telah dipanggil secara resmi dan patut untuk menghadap sidang tidak hadir;

2. Mengabulkan gugatan Penggugat secara verstek;

3. Menjatuhkan talak satu ba'in sughra Tergugat (**TERGUGAT**) terhadap Penggugat (**PENGGUGAT**);

4. Membebankan kepada Penggugat untuk membayar biaya perkara sejumlah Rp. 381.000,00 (tiga ratus delapan puluh satu ribu rupiah);

Demikian diputuskan dalam rapat permusyawaratan Majelis Hakim yang dilangsungkan pada hari Rabu tanggal 23 Desember 2020 *Masehi*, bertepatan dengan tanggal 8 Jumadil Awwal 1442 *Hijriyah*, oleh kami Dra. Hj. Suhaimi, M.H. sebagai Ketua Majelis, Drs. Silachudin dan Rusli, S.HI.,M.H. masing-masing sebagai Hakim Anggota, putusan tersebut diucapkan dalam sidang terbuka untuk umum pada hari itu juga, oleh Ketua Majelis tersebut dengan didampingi oleh Hakim Anggota yang sama dan dibantu oleh Kusmini, SHI

Disclaimer
Kepaniteraan Mahkamah Agung Republik Indonesia berusaha untuk selalu mencantumkan informasi paling kini dan akurat sebagai bentuk komitmen Mahkamah Agung untuk pelayanan publik, transparansi dan akuntabilitas pelaksanaan fungsi peradilan. Namun dalam hal-hal tertentu masih dimungkinkan terjadi permasalahan teknis terkait dengan akurasi dan keterkinian informasi yang kami sajikan, hal mana akan terus kami perbaiki dari waktu kewaktu.
Dalam hal Anda menemukan inakurasi informasi yang termuat pada situs ini atau informasi yang seharusnya ada, namun belum tersedia, maka harap segera hubungi Kepaniteraan Mahkamah Agung RI melalui :
Email : kepaniteraan@mahkamahagung.go.id Telp : 021-384 3348 (ext.318)

sebagai Panitera Pengganti serta dihadiri oleh Penggugat tanpa hadirnya Tergugat;

Hakim Anggota	Ketua Majelis
ttd	ttd
**Drs. Silachudin**	**Dra. Hj. Suhaimi, M.H.**
Hakim Anggota	
ttd	
**Rusli, S.HI.,M.H.**	
	Panitera Pengganti
	ttd
	**Kusmini, SHI**

Perincian Biaya:

1. Pendaftaran	:	Rp.	30.000,-
2. Proses	:	Rp.	75.000,-
3. Panggilan	:	Rp.	250.000,-
4. PNBP Panggilan	:	Rp.	20.000,-
5. Redaksi	:	Rp.	10.000,-
6. Meterai	:	Rp.	6.000,-
Jumlah		Rp.	391.000,-
			(tiga ratus sembilan puluh satu ribu)

Pemberitahuan isi Putusan
Tanggal : ....

Untuk salinan putusan yang sama bunyinya oleh :
Panitera Pengadilan Agama Banyumas,

Putusan Berkekuatan

Disclaimer
Kepaniteraan Mahkamah Agung Republik Indonesia berusaha untuk selalu mencantumkan informasi paling kini dan akurat sebagai bentuk komitmen Mahkamah Agung untuk pelayanan publik, transparansi dan akuntabilitas pelaksanaan fungsi peradilan. Namun dalam hal-hal tertentu masih dimungkinkan terjadi permasalahan teknis terkait dengan akurasi dan keterkinian informasi yang kami sajikan, hal mana akan terus kami perbaiki dari waktu kewaktu.
Dalam hal Anda menemukan inakurasi informasi yang termuat pada situs ini atau informasi yang seharusnya ada, namun belum tersedia, maka harap segera hubungi Kepaniteraan Mahkamah Agung RI melalui :
Email : kepaniteraan@mahkamahagung.go.id   Telp : 021-384 3348 (ext.318)

Hukum Tetap
Tanggal : .... **Mokhamad Farid, S.Ag.,M.H.**

Disclaimer
Kepaniteraan Mahkamah Agung Republik Indonesia berusaha untuk selalu mencantumkan informasi paling kini dan akurat sebagai bentuk komitmen Mahkamah Agung untuk pelayanan publik, transparansi dan akuntabilitas pelaksanaan fungsi peradilan. Namun dalam hal-hal tertentu masih dimungkinkan terjadi permasalahan teknis terkait dengan akurasi dan keterkinian informasi yang kami sajikan, hal mana akan terus kami perbaiki dari waktu kewaktu.
Dalam hal Anda menemukan inakurasi informasi yang termuat pada situs ini atau informasi yang seharusnya ada, namun belum tersedia, maka harap segera hubungi Kepaniteraan Mahkamah Agung RI melalui :
Email : kepaniteraan@mahkamahagung.go.id Telp : 021-384 3348 (ext.318)
