# Supplementary material for: Classification of divorce causes during the COVID-19 pandemic using convolutional neural networks
Source: PeerJ Comput Sci. 2022 Jun 30;8:e998. doi: 10.7717/peerj-cs.998 (PMC9299239; doi:10.7717/peerj-cs.998)
Supplement: Supplemental Information 2 [file peerj-cs-08-998-s002.zip › Kelalaian Dataset/Data ke-30.pdf]

PENETAPAN

Nomor 1174/Pdt.G/2020/PA.Pbr

بِسْمِ اللهِ الرَّحْمٰنِ الرَّحِيْمِ

DEMI KEADILAN BERDASARKAN KETUHANAN YANG MAHA ESA

Pengadilan Agama Pekanbaru yang memeriksa dan mengadili perkara tertentu pada tingkat pertama dalam persidangan Hakim Majelis telah menjatuhkan penetapan sebagaimana tersebut di bawah ini dalam perkara **Isbath Nikah dan Cerai Gugat** antara:

PENGGUGAT, umur 39 tahun, agama Islam, pendidikan , pekerjaan Perawat, tempat tinggal di XXX Kota Pekanbaru,berdasarkan Surat Kuasa Khusus Nomor. 01/SKK-PA/OS/III/2020 tanggal 02 Maret 2020 telah memberi kuasa kepada **Aldi Sofiawan, SH** Advokat/Pengacara/Penasehat Hukum pada Kantor Otoritas Semu Law Office & Associates yang beralamat di Jalan Tegal Sari (Ujung) Gg. Mekar Sari 2 Hop 16 No.2 RT.001 RW oo5 Kel. Umbansari Kec. Rumbai Pekanbaru, selanjutnya disebut sebagai Penggugat;

Melawan

TERGUGAT, umur 46 tahun, agama Islam, pendidikan , pekerjaan Swasta, tempat tinggal di Jalan Perum Pandau Permai XXX Kabupaten Kampar, selanjutnya disebut sebagai Tergugat;

Pengadilan Agama tersebut;

Telah mempelajari semua surat dalam perkara ini;

Telah mendengar keterangan Penggugat di persidangan;

DUDUK PERKARA

Penggugat telah mengajukan gugatan secara tertulis dengan surat gugatannya bertanggal 06 Agustus 2020, yang diterima dan didaftar di Kepaniteraan Pengadilan Agama Pekanbaru Register Nomor

Disclaimer
Kepaniteraan Mahkamah Agung Republik Indonesia berusaha untuk selalu mencantumkan informasi paling kini dan akurat sebagai bentuk komitmen Mahkamah Agung untuk pelayanan publik, transparansi dan akuntabilitas pelaksanaan fungsi peradilan. Namun dalam hal-hal tertentu masih dimungkinkan terjadi permasalahan teknis terkait dengan akurasi dan keterkinian informasi yang kami sajikan, hal mana akan terus kami perbaiki dari waktu kewaktu.
Dalam hal Anda menemukan inakurasi informasi yang termuat pada situs ini atau informasi yang seharusnya ada, namun belum tersedia, maka harap segera hubungi Kepaniteraan Mahkamah Agung RI melalui :
Email : kepaniteraan@mahkamahagung.go.id Telp : 021-384 3348 (ext.318)

1174/Pdt.G/2020/PA.Pbr pada tanggal 10 Agustus 2020 dengan alasan-alasan sebagai berikut:

a. Bahwa pada tanggal 22 Februari 2016 telah dilangsungkan perkawinan antara Penggugat dengan Tergugat di wilayah hukum Kantor Urusan Agama Kecamatan Tapung sesuai dengan tuntunan ajaran agama Islam;

b. Bahwa pada saat pernikahan tersebut Penggugat berstatus janda, dan Tergugat berstatus kawin, pernikahan dilangsungkan dengan wali nikah Djamaluddin, dan dihadiri saksi nikah yang bernama Bapak Mardiansyah dengan mas kawin berupa sebentuk cincin emas yang dibayar tunai;

c. Bahwa antara Penggugat dengan Tergugat tidak ada pertalian nasab, pertalian kerabat semenda dan pertalian sesusuan serta memenuhi syarat dan tidak ada larangan untuk melangsungkan pernikahan, baik menurut ketentuan hukum Islam maupun peraturan perundang-undangan yang berlaku;

d. Bahwa perkawinan antara Penggugat dan Tergugat dilangsungkan berdasarkan kehendak kedua belah pihak dengan tujuan membentuk rumah tangga yang sakinah, mawaddah, warrahmah yang diridhoi oleh Allah SWT;

e. Bahwa benar setelah menikah, Pengguggat dan Tergugat tinggal di rumah kediaman bersama bertempat di XXX Kecamatan Siak Hulu Riau;

f. Bahwa setelah menikah Penggugat dan Tergugat hidup rukun sebagaimana layaknya suami istri dengan baik, telah berhubungan badan dan dikaruniai seorang anak bernama ANAK, Perempuan, umur 4 Tahun, lahir di Pekanbaru pada tanggal 09 September 2016;

g. Bahwa selama pernikahan tersebut tidak ada pihak ketiga yang mengganggu gugat pernikahan dan mempersoalkan pernikahan Penggugat dan Tergugat dan selama itu pula Penggugat dengan Tergugat tidak pernah terjadi perceraian;

h. Bahwa sampai sekarang Penggugat tidak mempunyai kutipan akta nikah, karena pernikahan Penggugat tidak terdaftar di Kantor Urusan Agama

Disclaimer
Kepaniteraan Mahkamah Agung Republik Indonesia berusaha untuk selalu mencantumkan informasi paling kini dan akurat sebagai bentuk komitmen Mahkamah Agung untuk pelayanan publik, transparansi dan akuntabilitas pelaksanaan fungsi peradilan. Namun dalam hal-hal tertentu masih dimungkinkan terjadi permasalahan teknis terkait dengan akurasi dan keterkinian informasi yang kami sajikan, hal mana akan terus kami perbaiki dari waktu kewaktu.
Dalam hal Anda menemukan inakurasi informasi yang termuat pada situs ini atau informasi yang seharusnya ada, namun belum tersedia, maka harap segera hubungi Kepaniteraan Mahkamah Agung RI melalui :
Email : kepaniteraan@mahkamahagung.go.id Telp : 021-384 3348 (ext.318)

Kecamatan Tapung karena keluarga tidak menyetujui sehingga pernikahan tersebut tidak tercatat di KUA setempat, sementara Penggugat sangat membutuhkan bukti keabsahan perkawinan tersebut dan karenanya Penggugat mohon agar perkawinan antara Penggugat dengan Tergugat terlebih dahulu ditetapkan keabsahannya sebagai bukti kebasahan perkawinan antara Penggugat dengan Tergugat untuk alasan hukum dalam pengurusan perceraian antara Penggugat dengan Tergugat;

i. Bahwa kehidupan rumah tangga Penggugat dan Tergugat mulai goyah dan terjadi perselisihan serta pertengkaran secara terus-menerus yang sulit diatasi sejak tahun 2017 dan pada tanggal 20 Agustus 2017 Tergugat pernah menjatuhkan/mengucapkan kata talak kepada Penggugat;

j. Bahwa Tergugat tidak memiliki tanggung jawab dan tidak menafkahi selama 1 tahun sebagaimana layaknya seorang suami yang baik serta bertanggung jawab kepada keluarganya dan juga melakukan kewajibannya sebagai seorang suami;

k. Bahwa Penggugat selalu menjadi sasaran tangan dari Tergugat di setiap pertengkaran meskipun kecil, dan bahkan Tergugat tidak segan-segan untuk melayangkan tangannya kepada Penggugat dan apabila Tergugat sedang dalam keadaan emosi selalu berkata kasar dan membentak Penggugat;

l. Bahwa pada saat Penggugat dalam keadaan hamil 8 bulan Tergugat melakukan perselingkuhan yang diketahui oleh Penggugat;

m. Bahwa sebab-sebab terjadi perselisihan dan pertengkaran antara Penggugat dan Tergugat tersebut dikarenakan:

1. Antara Penggugat dan Tergugat sudah tidak ada kecocokan lagi dan sering berbeda pendapat dalam membina rumah tangga;
2. Tergugat tidak bertanggung jawab terhadap keluarga dengan tidak cukup dalam memberikan nafkah lahir dan bathin;
3. Tergugat kerap melakukan tindak kekerasan dan bersikap kasar terhadap Penggugat;

Disclaimer
Kepaniteraan Mahkamah Agung Republik Indonesia berusaha untuk selalu mencantumkan informasi paling kini dan akurat sebagai bentuk komitmen Mahkamah Agung untuk pelayanan publik, transparansi dan akuntabilitas pelaksanaan fungsi peradilan. Namun dalam hal-hal tertentu masih dimungkinkan terjadi permasalahan teknis terkait dengan akurasi dan keterkinian informasi yang kami sajikan, hal mana akan terus kami perbaiki dari waktu kewaktu.
Dalam hal Anda menemukan inakurasi informasi yang termuat pada situs ini atau informasi yang seharusnya ada, namun belum tersedia, maka harap segera hubungi Kepaniteraan Mahkamah Agung RI melalui :
Email : kepaniteraan@mahkamahagung.go.id Telp : 021-384 3348 (ext.318)

4. Tergugat pernah melakukan perselingkuhan yang diketahui oleh Penggugat namun tidak mengakuinya;

5. Penggugat sudah tidak tahan atas perlakuan kekerasan yang dilakukan Tergugat baik secara verbal maupun fisik.

n. Bahwa atas permasalahan dan kemelut rumah tangga yang dihadapi, Penggugat telah mencoba memusyawarahkan dengan keluarga Penggugat dan Tergugat untuk mencari penyelesaian dan demi menyelamatkan perkawinan, namun usaha tersebut tidak membuahkan hasil;

o. Bahwa berdasarkan hal-hal tersebut diatas, permohonan Penggugat untuk mengajukan gugatan perceraian terhadap Tergugat atas dasar pertengkaran yang terjadi terus menerus dan tidak mungkin hidup rukun dalam suatu ikatan perkawinan juga Tergugat melakukan kekejaman atau penganiayaan berat yang membahayakan pihak lain, telah memenuhi unsur Pasal 19 huruf (f) Peraturan Pemerintah No. 9 Tahun 1975 Jo. Pasal 116 huruf (f) dan (h) Kompilasi Hukum Islam, sehingga berdasar hukum untuk menyatakan gugatan cerai ini dikabulkan;

p. Bahwa anak hasil pernikahan antara Penggugat dan Tergugat ANAK, Perempuan, umur 4 Tahun, lahir di Pekanbaru pada tanggal 09 September 2016 tersebut masih MUMAYYIZ atau dibawah umur, maka berdasarkan Pasal 105 Kompilasi Hukum Islam, dalam hal terjadinya perceraian yang berbunyi "Pemeliharaan anak yang belum mumayyiz atau belum berumur 12 tahun adalah merupakan hak ibunya", oleh karena itu Penggugat memohon kepada Ketua Pengadilan Agama Pekanbaru atau Majelis Hakim yang memeriksa dan mengadili perkara aquo menyatakan pemeliharaan anak tersebut diserahkan kepada Penggugat;

q. Bahwa Penggugat sanggup membayar biaya perkara;

Berdasarkan alasan-alasan serta dalil-dalil yang telah diuraikan oleh Penggugat sebagaimana tersebut diatas, Penggugat mohon kiranya kepada Ketua Pengadilan Agama Pekanbaru atau Majelis Hakim yang memeriksa dan mengadili perkara aquo berkenan untuk menjatuhkan Putusan dengan Amarnya yang berbunyi sebagai berikut :

Disclaimer
Kepaniteraan Mahkamah Agung Republik Indonesia berusaha untuk selalu mencantumkan informasi paling kini dan akurat sebagai bentuk komitmen Mahkamah Agung untuk pelayanan publik, transparansi dan akuntabilitas pelaksanaan fungsi peradilan. Namun dalam hal-hal tertentu masih dimungkinkan terjadi permasalahan teknis terkait dengan akurasi dan keterkinian informasi yang kami sajikan, hal mana akan terus kami perbaiki dari waktu kewaktu.
Dalam hal Anda menemukan inakurasi informasi yang termuat pada situs ini atau informasi yang seharusnya ada, namun belum tersedia, maka harap segera hubungi Kepaniteraan Mahkamah Agung RI melalui :
Email : kepaniteraan@mahkamahagung.go.id Telp : 021-384 3348 (ext.318)

PRIMAIR

1.Menerima dan mengabulkan gugatan Penggugat untuk seluruhnya;

2.Menyatakan sah pernikahan Penggugat PENGGUGAT dengan Tergugat TERGUGAT pada tanggal 22 Februari 2016 yang dilangsungkan di Kecamatan Tapung Kabupaten Kampar;

3.Menjatuhkan Talak Satu Ba'in Sughraa Tergugat atas Penggugat;

4.Menyatakan dan menetapkan secara hukum terhadap anak yang masih MUMAYYIZ atau dibawah umur bernama ANAK, Perempuan, umur 4 Tahun, lahir di Pekanbaru pada tanggal 09 September 2016, dalam pemeliharaan Penggugat;

5.Menyatakan bahwa putusan ini dapat dilaksanakan terlebih dahulu walaupun adanya upaya hukum verzet, banding dan kasasi (Uit Voerbar Bij Voorrad) dari Tergugat;

6.Menetapkan dan membebankan biaya perkara ini sesuai dengan peraturan perundang-undangan yang berlaku;

SUBSIDIER

Dan atau apabila Ketua Majelis Hakim yang memeriksa dan memutus perkara ini berpendapat lain, mohon putusan yang seadil-adilnya (ex aequo et bono).

Untuk pemeriksaan perkara ini Majelis Hakim telah memanggil Penggugat dan Tergugat untuk hadir di persidangan, panggilan-panggilan tersebut telah disampaikan secara resmi dan patut;

Pada hari sidang yang telah ditentukan Penggugat hadir di persidangan, sedangkan Tergugat tidak pernah hadir di persidangan dan tidak pula mengutus orang lain sebagai wakil atau kuasanya yang sah meskipun berdasarkan *relaas* panggilan Nomor 1174/Pdt.G/2020/PA.Pbr yang dibacakan Hakim Ketua Majelis di persidangan, Tergugat telah dipanggil secara resmi dan patut dan tidak ternyata ketidakhadiran Tergugat tersebut disebabkan alasan yang dibenarkan undang-undang;

Disclaimer
Kepaniteraan Mahkamah Agung Republik Indonesia berusaha untuk selalu mencantumkan informasi paling kini dan akurat sebagai bentuk komitmen Mahkamah Agung untuk pelayanan publik, transparansi dan akuntabilitas pelaksanaan fungsi peradilan. Namun dalam hal-hal tertentu masih dimungkinkan terjadi permasalahan teknis terkait dengan akurasi dan keterkinian informasi yang kami sajikan, hal mana akan terus kami perbaiki dari waktu kewaktu.
Dalam hal Anda menemukan inakurasi informasi yang termuat pada situs ini atau informasi yang seharusnya ada, namun belum tersedia, maka harap segera hubungi Kepaniteraan Mahkamah Agung RI melalui :
Email : kepaniteraan@mahkamahagung.go.id   Telp : 021-384 3348 (ext.318)

Oleh karena Tergugat tidak hadir di persidangan, maka mediasi terhalang dilaksanakan dan pemeriksaan terhadap perkara ini dilanjutkan;

Selanjutnya Majelis Hakim telah menasehati agar berdamai dengan Tergugat dan memberi penjelasan kepada Penggugat, dengan penjelasantersebut Penggugat menyatakan mencabut perkara secara lisan di persidangan;

Penggugat telah menyampaikan kesimpulannya secara lisan di persidangan pada tanggal 15 Desember 2020, yang pada pokoknya menyatakan tetap dengan permohonan pencabutan gugatannya dan mohon pencabutan tersebut dikabulkan;

Untuk mempersingkat uraian penetapan ini, cukuplah Majelis Hakim menunjuk berita acara sidang merupakan bagian yang tidak terpisahkan dari penetapan ini;

PERTIMBANGAN HUKUM

Menimbang, bahwa adapun maksud dan tujuan gugatan Penggugat adalah sebagaimana yang telah diuraikan di dalam bagian duduk perkara;

Menimbang, bahwa untuk pemeriksaan perkara ini Majelis Hakim telah memanggil Penggugat dan Tergugat untuk hadir di persidangan, sebagaimana yang dimaksud oleh Pasal 55 Undang-Undang Nomor 7 Tahun 1989 dan Pasal 26 ayat (1) Peraturan Pemerintah Nomor 9 Tahun 1975, panggilan kepada Penggugat dan Tergugat telah disampaikan secara resmi dan patut sebagaimana yang dimaksud Pasal 26 ayat (2), ayat (3) dan ayat (4) Peraturan Pemerintah Nomor 9 Tahun 1975;

Menimbang, bahwa pada hari sidang yang telah ditentukan Penggugat hadir di persidangan secara *in person* sedangkan Tergugat hadir di persidangan meskipun telah di panggil secara resmi dan patut;

Menimbang, bahwa Penggugat mengajukan permohonan pencabutan gugatannya secara lisan di persidangan tanggal 15 Desember 2020 yang telah diajukan Penggugat bertanggal 06 Agustus 2020;

Disclaimer
Kepaniteraan Mahkamah Agung Republik Indonesia berusaha untuk selalu mencantumkan informasi paling kini dan akurat sebagai bentuk komitmen Mahkamah Agung untuk pelayanan publik, transparansi dan akuntabilitas pelaksanaan fungsi peradilan. Namun dalam hal-hal tertentu masih dimungkinkan terjadi permasalahan teknis terkait dengan akurasi dan keterkinian informasi yang kami sajikan, hal mana akan terus kami perbaiki dari waktu kewaktu.
Dalam hal Anda menemukan inakurasi informasi yang termuat pada situs ini atau informasi yang seharusnya ada, namun belum tersedia, maka harap segera hubungi Kepaniteraan Mahkamah Agung RI melalui :
Email : kepaniteraan@mahkamahagung.go.id   Telp : 021-384 3348 (ext.318)

Menimbang, bahwa yang menjadi masalah dalam perkara ini adalah Penggugat mengajukan permohonan pencabutan gugatannya sebelum pemeriksaan terhadap pokok perkara;

Menimbang, bahwa Penggugat telah mengajukan permohonan pencabutan gugatan Penggugat sebelum pemeriksaan terhadap pokok perkara, maka Majelis Hakim berpendapat berdasarkan Pasal 271 *Rv*., yang menyatakan pencabutan gugatan sebelum jawaban tidak diperlukan izin dari Tergugat oleh karenanya permohonan pencabutan gugatan yang diajukan Penggugat patut dikabulkan;

Menimbang, bahwa oleh karena permohonan pencabutan gugatan Penggugat telah dikabulkan, maka Majelis Hakim menyatakan perkara register nomor 1174/Pdt.G/2020/PA.Pbr dicabut oleh Penggugat;

Menimbang**,** bahwa oleh karena Penggugat mencabut gugatannya, maka berdasarkan Pasal 272 Rv. semua biaya perkara yang timbul dalam perkara ini dibebankan kepada Penggugat untuk membayarnya;

Mengingat**:**

1. Pasal 55 Undang-Undang Nomor 7 Tahun 1989 Tentang Peradilan Agama;
2. Pasal 271 Rv. dan Pasal 272 Rv.;
3. Pasal 26 ayat (2), ayat (3) dan ayat (4) Peraturan Pemerintah Nomor 9 Tahun 1975 dan segala peraturan yang berkaitan dengan perkara ini;

MENETAPKAN

1. Mengabulkan permohonan pencabutan gugatan Penggugat;
2. Menyatakan perkara register Nomor 1174/Pdt.G/2020/PA.Pbr, tanggal 10 Agustus 2020 dicabut oleh Penggugat;

Disclaimer
Kepaniteraan Mahkamah Agung Republik Indonesia berusaha untuk selalu mencantumkan informasi paling kini dan akurat sebagai bentuk komitmen Mahkamah Agung untuk pelayanan publik, transparansi dan akuntabilitas pelaksanaan fungsi peradilan. Namun dalam hal-hal tertentu masih dimungkinkan terjadi permasalahan teknis terkait dengan akurasi dan keterkinian informasi yang kami sajikan, hal mana akan terus kami perbaiki dari waktu kewaktu.
Dalam hal Anda menemukan inakurasi informasi yang termuat pada situs ini atau informasi yang seharusnya ada, namun belum tersedia, maka harap segera hubungi Kepaniteraan Mahkamah Agung RI melalui :
Email : kepaniteraan@mahkamahagung.go.id   Telp : 021-384 3348 (ext.318)

3. Membebankan kepada Penggugat untuk membayar semua biaya yang timbul dalam perkara ini yang hingga saat ini dihitung sebesar Rp 738.000,00 (tujuh ratus tiga puluh delapan ribu rupiah);

Demikian Penetapan ini dijatuhkan di Pengadilan Agama Pekanbaru dalam sidang musyawarah Majelis Hakim pada hari Selasa, tanggal 29 Desember 2002 *Miladiyah*, bertepatan dengan tanggal 14 Jumadil Awal 1442 *Hijriyah*, oleh Kami **Dra. Hj. Erlis, S.H., M.H.** sebagai Hakim Ketua Majelis, **Drs. Ali Amran, S.H.** dan **Drs. Asyari, M.H.** masing-masing sebagai Hakim Anggota Majelis, penetapan tersebut diucapkan pada hari itu juga dalam sidang terbuka untuk umum, oleh Dra. Hj. Erlis, S.H., M.H. sebagai Hakim Ketua Majelis dengan dihadiri Drs. Ali Amran, S.H. dan Drs. Asyari, M.H. masing-masing sebagai Hakim Anggota Majelis, dibantu oleh **Zuriati, S.Ag.** sebagai Panitera Pengganti dengan dihadiri Penggugat tanpa hadirnya Tergugat;

Hakim Ketua Majelis

Dra. Hj. Erlis, S.H., M.H.

Hakim Anggota Majelis **Hakim Anggota Majelis**

Drs. Ali Amran, S.H. **Drs. Asyari, M.H.**

Panitera Pengganti

Disclaimer
Kepaniteraan Mahkamah Agung Republik Indonesia berusaha untuk selalu mencantumkan informasi paling kini dan akurat sebagai bentuk komitmen Mahkamah Agung untuk pelayanan publik, transparansi dan akuntabilitas pelaksanaan fungsi peradilan. Namun dalam hal-hal tertentu masih dimungkinkan terjadi permasalahan teknis terkait dengan akurasi dan keterkinian informasi yang kami sajikan, hal mana akan terus kami perbaiki dari waktu kewaktu.
Dalam hal Anda menemukan inakurasi informasi yang termuat pada situs ini atau informasi yang seharusnya ada, namun belum tersedia, maka harap segera hubungi Kepaniteraan Mahkamah Agung RI melalui :
Email : kepaniteraan@mahkamahagung.go.id Telp : 021-384 3348 (ext.318)

Zuriati, S.Ag.

Rincian Biaya Perkara:

1. Biaya pendaftaran Rp. 30.000,00
2. Biaya ATKRp. 72.000,00
3. Biaya panggilan Rp.590.000,00
4. PNBPRp. 30.000,00
5. Hak RedaksiRp. 10.000,00
6. Meterai Rp. 6.000,00

Jumlah Rp.738.000,00

(*tujuh ratus tiga puluh delapan ribu rupiah*)

Disclaimer
Kepaniteraan Mahkamah Agung Republik Indonesia berusaha untuk selalu mencantumkan informasi paling kini dan akurat sebagai bentuk komitmen Mahkamah Agung untuk pelayanan publik, transparansi dan akuntabilitas pelaksanaan fungsi peradilan. Namun dalam hal-hal tertentu masih dimungkinkan terjadi permasalahan teknis terkait dengan akurasi dan keterkinian informasi yang kami sajikan, hal mana akan terus kami perbaiki dari waktu kewaktu.
Dalam hal Anda menemukan inakurasi informasi yang termuat pada situs ini atau informasi yang seharusnya ada, namun belum tersedia, maka harap segera hubungi Kepaniteraan Mahkamah Agung RI melalui :
Email : kepaniteraan@mahkamahagung.go.id Telp : 021-384 3348 (ext.318)
