# Supplementary material for: Classification of divorce causes during the COVID-19 pandemic using convolutional neural networks
Source: PeerJ Comput Sci. 2022 Jun 30;8:e998. doi: 10.7717/peerj-cs.998 (PMC9299239; doi:10.7717/peerj-cs.998)
Supplement: Supplemental Information 2 [file peerj-cs-08-998-s002.zip › Kelalaian Dataset/Data ke-4.pdf]

P U T U S A N

Nomor 1049/Pdt.G/2020/PA.Klt

بسم الله الرحمن الرحيم

DEMI KEADILAN BERDASARKAN KETUHANAN YANG MAHA ESA

Pengadilan Agama Klaten yang memeriksa dan mengadili perkara tertentu pada tingkat pertama dalam sidang majelis telah menjatuhkan putusan perkara Gugatan Perceraian antara;

PENGGUGAT, umur 32 tahun, agama Islam, Pekerjaan xxx xxxxx xxxxxx, Pendidikan Sekolah Lanjutan Tingkat Atas, tempat kediaman di KABUPATEN KLATEN, sebagai Penggugat;

Melawan

TERGUGAT, umur 32 tahun, agama Islam, Pekerjaan xxxxx xxxxxx xxxxx, Pendidikan Sekolah Lanjutan Tingkat Pertama, tempat kediaman di xxxxx xxxxxxx, xx xxx xx xxx, xxxx xxxxxx, Kecamatan Prambanan, xxxxxxxxx xxxxxx, Sekarang tidak diketahui alamat tinggalnya dengan jelas dan pasti di Wilayah Kesatuan Republik Indonesia, sebagai Tergugat;

Pengadilan Agama tersebut;

Telah mempelajari surat-surat yang berkaitan dengan perkara ini;

Telah mendengar keterangan Penggugat dan para saksi di muka sidang;

DUDUK PERKARA

Bahwa, Penggugat dalam surat gugatannya tanggal 12 Agustus 2020 telah mengajukan gugatan perceraian yang telah didaftar di Kepaniteraan Pengadilan Agama Klaten dengan Nomor 1049/Pdt.G/2020/PA.Klt, tanggal 12 Agustus 2020, dengan dalil-dalil sebagai berikut:

1. Bahwa Penggugat dan Tergugat adalah suami-isteri sah, yang melangsungkan pernikahan pada hari Ahad tanggal 25 September 2011 yang dicatatkan oleh Pegawai Pencatat Nikah Kantor Urusan Agama (KUA)

Disclaimer
Kepaniteraan Mahkamah Agung Republik Indonesia berusaha untuk selalu mencantumkan informasi paling kini dan akurat sebagai bentuk komitmen Mahkamah Agung untuk pelayanan publik, transparansi dan akuntabilitas pelaksanaan fungsi peradilan. Namun dalam hal-hal tertentu masih dimungkinkan terjadi permasalahan teknis terkait dengan akurasi dan keterkinian informasi yang kami sajikan, hal mana akan terus kami perbaiki dari waktu kewaktu.
Dalam hal Anda menemukan inakurasi informasi yang termuat pada situs ini atau informasi yang seharusnya ada, namun belum tersedia, maka harap segera hubungi Kepaniteraan Mahkamah Agung RI melalui :
Email : kepaniteraan@mahkamahagung.go.id Telp : 021-384 3348 (ext.318)

xxxxxxxxx xxxxxxxxx, xxxxxxxxx xxxxxx sebagaimana Kutipan Akta Nikah Nomor : 0298/031/IX/2011 tertanggal 26 September 2011, Pada saat melangsungkan pernikahan Penggugat dalam kondisi Perawan dan Tergugat masih dalam kondisi Jejaka;

2. Bahwa setelah menikah Penggugat dan Tergugat bertempat tinggal di rumah orang tua Penggugat di KABUPATEN KLATEN sampai sekarang. Namun pada bulan Juni 2018 Penggugat pamit kerja dan sejak itu tidak pernah memberi kabar sampai sekarang;

3. Bahwa selama pernikahan tersebut Penggugat dengan Tergugat telah hidup rukun sebagaimana layaknya suami istri (bada dukhul) dan dikaruniai 1 (satu) orang anak yang bernama; Adil Agung Afriyanto bin Heri Ariyanto, Laki-laki, Klaten, 16 April 2012 sekarang ikut Penggugat. ;

4. Bahwa rumah tangga Penggugat dengan Tergugat semula berjalan dengan harmonis dan bahagia, namun kurang lebih sejak bulan Agustus tahun 2015, Tergugat dan Penggugat sering cekcok disebabkan;

   a. Tergugat malas bekerja dan tidak transparan masalah keuangan;
   b. Tergugat ketahuan memiliki selingkuhan;
   c. Tergugat bersifat egois dan ingin menang sendiri dalam urusan keluarga;

5. Bahwa puncaknya yakni ketika Penggugat meminta nafkah lahir sedangkan Tergugat tidak bekerja, akhirnya Tergugat pamit bekerja sejak Juni 2018 namun semenjak kepergiannya justru tidak pernah ada kabar sampai sekarang dan meniggalkan kewajibannya sebagai suami terhitung kurang lebih 2 tahun lebih 1 bulan;

6. Bahwa Penggugat dan Orang tua Penggugat telah berusaha mencari Tergugat, baik secara langsung maupun ke rumah orang tua Tergugat, akan tetapi tidak berhasil;

7. Bahwa berdasarkan hal-hal tersebut di atas, maka dalil-dalil permohonan cerai gugat telah berdasarkan atas hukum yang berlaku dan telah sesuai dengan ketentuan Pasal 39 ayat (2) Undang-undang Nomor 1 Tahun 1974 serta telah sesuai dengan alasan perceraian sebagaimana diatur dalam Pasal 19 huruf (b) PP No. 9 Tahun 1975 Jo Pasal 116 huruf (b)

Disclaimer
Kepaniteraan Mahkamah Agung Republik Indonesia berusaha untuk selalu mencantumkan informasi paling kini dan akurat sebagai bentuk komitmen Mahkamah Agung untuk pelayanan publik, transparansi dan akuntabilitas pelaksanaan fungsi peradilan. Namun dalam hal-hal tertentu masih dimungkinkan terjadi permasalahan teknis terkait dengan akurasi dan keterkinian informasi yang kami sajikan, hal mana akan terus kami perbaiki dari waktu kewaktu.
Dalam hal Anda menemukan inakurasi informasi yang termuat pada situs ini atau informasi yang seharusnya ada, namun belum tersedia, maka harap segera hubungi Kepaniteraan Mahkamah Agung RI melalui :
Email : kepaniteraan@mahkamahagung.go.id Telp : 021-384 3348 (ext.318)

Kompilasi Hukum Islam;

8. Bahwa Penggugat sanggup membayar seluruh biaya yang timbul akibat perkara ini;

PRIMAIR :;

1. Mengabulkan gugatan Penggugat;

2. Menjatuhkan talak satu bain sughro dari Tergugat (TERGUGAT) kepada Penggugat (PENGGUGAT);

3. Membebankan biaya perkara menurut hukum;

SUBSIDAIR :;

Mohon putusan yang seadil-adilnya.

Bahwa, pada hari sidang yang telah ditetapkan Penggugat telah datang menghadap ke muka sidang, sedangkan Tergugat tidak datang menghadap ke muka sidang dan tidak menyuruh orang lain untuk menghadap sebagai wakil/kuasa hukumnya meskipun telah dipanggil secara resmi dan patut yang relaas panggilannya dibacakan di dalam sidang, sedang tidak ternyata bahwa tidak datangnya itu disebabkan suatu halangan yang sah;

Bahwa, majelis hakim telah menasehati Penggugat agar berpikir untuk tidak bercerai dengan Tergugat, tetapi Penggugat tetap pada dalil-dalil gugatannya untuk bercerai dengan Tergugat;

Bahwa, perkara ini tidak dapat di mediasi karena Tergugat tidak pernah datang menghadap meskipun telah dipanggil secara resmi dan patut, selanjutnya dimulai pemeriksaan dengan membacakan surat gugatan Penggugat yang maksud dan isinya tetap dipertahankan oleh Penggugat;

Bahwa, untuk menguatkan dalil-dalil gugatannya, Penggugat telah mengajukan bukti-bukti berupa;

A. Surat;

1. Fotokopi Kartu Tanda Penduduk Nomor 3310015810870001 tanggal 13 Oktober 2012, yang bermeterai cukup dan telah sesuai dengan aslinya (Bukti P.1);

Disclaimer
Kepaniteraan Mahkamah Agung Republik Indonesia berusaha untuk selalu mencantumkan informasi paling kini dan akurat sebagai bentuk komitmen Mahkamah Agung untuk pelayanan publik, transparansi dan akuntabilitas pelaksanaan fungsi peradilan. Namun dalam hal-hal tertentu masih dimungkinkan terjadi permasalahan teknis terkait dengan akurasi dan keterkinian informasi yang kami sajikan, hal mana akan terus kami perbaiki dari waktu kewaktu.
Dalam hal Anda menemukan inakurasi informasi yang termuat pada situs ini atau informasi yang seharusnya ada, namun belum tersedia, maka harap segera hubungi Kepaniteraan Mahkamah Agung RI melalui :
Email : kepaniteraan@mahkamahagung.go.id Telp : 021-384 3348 (ext.318)

2. Fotokopi Buku Kutipan Akta Nikah dari KUA xxxxxxxxx xxxxxxxxx, xxxxxxxxx xxxxxx Nomor 0298/031/IX/2011 tanggal 26 September 2011, yang bermeterai cukup dan telah sesuai dengan aslinya (Bukti P.2);
3. Surat Keterangan Pergi nomor 474/301/01/2020, tanggal 10 Agustus 2020. yang dikeluarkan oleh Kepala xxxx xxxxxx, xxxxxxxxx xxxxxxxxx, xxxxxxxxx xxxxxx (Bukti P.3).

B. Saksi;

1. Sunardfi bin Sunar Joyo Pawiro, umur 48 tahun, Agama Islam, pekerjaan buruh, tempat tinggal di xxxxx xxxxxxx, RT 002 RW 005, xxxx xxxxxx, xxxxxxxxx xxxxxxxxx, xxxxxxxxx xxxxxx:
   - Bahwa saksi kenal Penggugat dan Tergugat, saksi tetangga Penggugat, saksi ketua RW;
   - Bahwa saksi tahu Penggugat dan Tergugat menikah, setelah menikah tinggal bersama di rumah orang tua Penggugat dan Telah dikaruniai 1 orang anak bernama Adil Agung Afriyanto bin Heri Ariyanto, sekarang ikut Penggugat;
   - Bahwa saksi tahu Tergugat pergi meninggalkan Penggugat lebih dari 2 tahun, Tergugat tidak pernah kembali kepada Penggugat,
   - Bahwa saksi tidak tahu penyebab Tergugat pergi meninggalkan Penggugat;
   - Bahwa saksi tahu selama Tergugat pergi meninggalkan Penggugat, Penggugat tetap tinggal bersama orang tua Penggugat;
2. Purwanti binti Welas Warnotiyoso, umur 29 tahun, Agama Islam, pekerjaan tidak bekerja, tempat tinggal di Dukuh Sembung, RT 003 RW 004, Desa Sembung, Kecamatan Wedi, xxxxxxxxx xxxxxx ;
   - Bahwa saksi kenal Penggugat dan Tergugat, saksi dahulu di xxxx xxxxxx tetangga Penggugat selisih 1 rumah;
   - Bahwa saksi tahu Penggugat dan Tergugat menikah, setelah menikah tinggal bersama di rumah orang tua Penggugat dan Telah dikaruniai 1 orang anak bernama Adil Agung Afriyanto bin Heri Ariyanto, sekarang ikut Penggugat;

Disclaimer
Kepaniteraan Mahkamah Agung Republik Indonesia berusaha untuk selalu mencantumkan informasi paling kini dan akurat sebagai bentuk komitmen Mahkamah Agung untuk pelayanan publik, transparansi dan akuntabilitas pelaksanaan fungsi peradilan. Namun dalam hal-hal tertentu masih dimungkinkan terjadi permasalahan teknis terkait dengan akurasi dan keterkinian informasi yang kami sajikan, hal mana akan terus kami perbaiki dari waktu kewaktu.
Dalam hal Anda menemukan inakurasi informasi yang termuat pada situs ini atau informasi yang seharusnya ada, namun belum tersedia, maka harap segera hubungi Kepaniteraan Mahkamah Agung RI melalui :
Email : kepaniteraan@mahkamahagung.go.id Telp : 021-384 3348 (ext.318)

- Bahwa saksi tahu Tergugat pergi meninggalkan Penggugat lebih dari 2 tahun, Tergugat tidak pernah kembali kepada Penggugat,
- Bahwa saksi tidak tahu penyebab Tergugat pergi meninggalkan Penggugat;
- Bahwa saksi tahu selama Tergugat pergi meninggalkan Penggugat, Penggugat tetap tinggal bersama orang tua Penggugat;

Selanjutnya untuk singkatnya uraian putusan ini, maka semua hal yang termuat dalam Berita Acara Sidang ini merupakan bagian yang tak terpisahkan dari putusan ini;

PERTIMBANGAN HUKUM

Menimbang, bahwa maksud dan tujuan gugatan Penggugat adalah sebagaimana terurai diatas;

Menimbang, bahwa berdasarkan bukti P.2, maka harus dinyatakan telah terbukti menurut hukum bahwa Penggugat dan Tergugat telah terikat dalam perkawinan yang sah sejak tanggal 26 September 2011.

Menimbang, bahwa oleh karena Penggugat dan Tergugat masih terikat sebagai suami istri yang sah, maka Penggugat sebagai *persona standi in judicio* memiliki *legal standing* sehingga Penggugat dapat dinyatakan sebagai pihak yang mempunyai hak dan berkepentingan dalam perkara ini ;

Menimbang, bahwa atas pengakuan Penggugat dalam surat gugatannya bahwa Penggugat dan Tergugat beragama Islam dan telah melakukan perkawinan secara islami, maka perkara ini merupakan kompetensi absolut Pengadilan Agama untuk memeriksa dan menyelesaikannya (vide Pasal 2 jo Pasal 49 ayat (1) Undang Undang Nomor 7 Tahun 1989 Tentang Peradilan Agama sebagai mana yang telah diubah yang kedua dengan Undang Undang Nomor 50 Tahun 2009) ;

Menimbang, bahwa berdasarkan surat gugatan, Penggugat bertempat tinggal wilayah yurisdiksi Pengadilan Agama Klaten, maka perkara ini secara relatif juga merupakan kompetensi Pengadilan Agama Klaten untuk memeriksanya (vide Pasal 73 Undang Undang Nomor 7 Tahun 1989 Tentang Peradilan Agama sebagai mana yang telah diubah yang kedua dengan Undang

Disclaimer
Kepaniteraan Mahkamah Agung Republik Indonesia berusaha untuk selalu mencantumkan informasi paling kini dan akurat sebagai bentuk komitmen Mahkamah Agung untuk pelayanan publik, transparansi dan akuntabilitas pelaksanaan fungsi peradilan. Namun dalam hal-hal tertentu masih dimungkinkan terjadi permasalahan teknis terkait dengan akurasi dan keterkinian informasi yang kami sajikan, hal mana akan terus kami perbaiki dari waktu kewaktu.
Dalam hal Anda menemukan inakurasi informasi yang termuat pada situs ini atau informasi yang seharusnya ada, namun belum tersedia, maka harap segera hubungi Kepaniteraan Mahkamah Agung RI melalui :
Email : kepaniteraan@mahkamahagung.go.id Telp : 021-384 3348 (ext.318)

Undang Nomor 50 Tahun 2009 jo Pasal 132 Kompilasi Hukum Islam) ;

Menimbang, bahwa Majelis telah berupaya mendamaikan pihak Penggugat agar rukun kembali dengan Tergugat, sebagaimana dikehendaki oleh ketentuan Pasal 130 HIR jo Pasal 82 Undang Undang Nomor 7 Tahun 1989 Tentang Peradilan Agama sebagaimana yang telah diubah terakhir dengan Undang Undang Nomor 50 Tahun 2009 jo Pasal 39 ayat (1) Undang Undang Nomor 1 Tahun 1974 Tentang Perkawinan jo Pasal 31 ayat (2) Peraturan Pemerintah Nomor 9 Tahun 1975 Tentang Pelaksanaan Undang Undang Nomor 1 Tahun 1974 Tentang Perkawinan jo Pasal 143 Kompilasi Hukum Islam, tetapi tidak berhasil ;

Menimbang, bahwa ternyata Tergugat meskipun dipanggil secara resmi dan patut, tidak datang menghadap di muka sidang dan pula tidak ternyata bahwa tidak datangnya itu disebabkan suatu halangan yang sah. Oleh karena itu, Tergugat harus dinyatakan tidak hadir dan gugatan tersebut harus diperiksa secara verstek. Sebagaimana ketentuan Pasal 125 ayat(1) HIR dan dalil syar'i :

من دعي إلى حاكم من حكام المسلمين فلم يجب فهو ظالم لا حق له

Artinya : “ Seseorang yang dipanggil Hakim (Pengadilan) tidak datang, ia dipandang sebagai dhalim dan tidak ada hak baginya. “ (Ahkamul Qur-an II : 405) ;

Menimbang, bahwa dalam perkara ini Majelis tidak menempuh prosedur mediasi karena salah satu pihak (Tergugat) tidak hadir sehingga tidak dapat dilakukan perundingan, dimana Pasal 1 ayat (1) Peraturan Mahkamah Agung R.I. Nomor 1 Tahun 2016 tentang Prosedur Mediasi di Pengadilan menyatakan, bahwa mediasi adalah cara penyelesaian sengketa melalui proses perundingan untuk memperoleh kesepakatan para pihak dengan dibantu oleh mediator. Perundingan dapat terlaksana apabila kedua belah pihak hadir ;

Menimbang, bahwa berdasarkan ketentuan Surat Edaran Mahkamah Agung RI Nomor 3 Tahun 2015 huruf C angka 3 yaitu putusan yang dijatuhkan tanpa hadirnya Tergugat dapat dikabulkan sepanjang berdasarkan hukum dan beralasan, oleh karena itu Majelis membebani Penggugat untuk membuktikan

Disclaimer
Kepaniteraan Mahkamah Agung Republik Indonesia berusaha untuk selalu mencantumkan informasi paling kini dan akurat sebagai bentuk komitmen Mahkamah Agung untuk pelayanan publik, transparansi dan akuntabilitas pelaksanaan fungsi peradilan. Namun dalam hal-hal tertentu masih dimungkinkan terjadi permasalahan teknis terkait dengan akurasi dan keterkinian informasi yang kami sajikan, hal mana akan terus kami perbaiki dari waktu kewaktu.
Dalam hal Anda menemukan inakurasi informasi yang termuat pada situs ini atau informasi yang seharusnya ada, namun belum tersedia, maka harap segera hubungi Kepaniteraan Mahkamah Agung RI melalui :
Email : kepaniteraan@mahkamahagung.go.id Telp : 021-384 3348 (ext.318)

dalil-dalil gugatannya;

Menimbang, bahwa untuk membuktikan dalil-dalil gugatannya, Penggugat telah mengajukan alat bukti surat serta 2 orang saksi;

Menimbang, bahwa saksi 1 Penggugat, sudah dewasa dan sudah bersumpah, sehingga memenuhi syarat formal sebagaimana diatur dalam Pasal 145 ayat 1 angka 3e HIR;

Menimbang, bahwa keterangan saksi 1 Penggugat mengenai dalil gugatan angka 5, adalah fakta yang dilihat/didengar/dialami sendiri dan relevan dengan dalil yang harus dibuktikan oleh Penggugat, oleh karena itu keterangan saksi tersebut telah memenuhi syarat materiil sebagaimana telah diatur dalam Pasal 171 HIR, sehingga keterangan saksi tersebut memiliki kekuatan pembuktian dan dapat diterima sebagai alat bukti;

Menimbang, bahwa saksi 2 Penggugat, sudah dewasa dan sudah bersumpah, sehingga memenuhi syarat formal sebagaimana diatur dalam Pasal 145 ayat 1 angka 3e HIR;

Menimbang, bahwa keterangan saksi 2 Penggugat mengenai dalil gugatan angka 5, adalah fakta yang dilihat/didengar/dialami sendiri dan relevan dengan dalil yang harus dibuktikan oleh Penggugat, oleh karena itu keterangan saksi tersebut telah memenuhi syarat materiil sebagaimana telah diatur dalam Pasal 171 HIR, sehingga keterangan saksi tersebut memiliki kekuatan pembuktian dan dapat diterima sebagai alat bukti;

Menimbang, bahwa keterangan saksi 1 dan saksi 2 Penggugat bersesuaian dan cocok antara satu dengan yang lain oleh karena itu keterangan dua orang saksi tersebut memenuhi Pasal 171 dan Pasal 172 HIR;

Menimbang, bahwa berdasarkan saksi 1 dan saksi 2, terbukti fakta kejadian sebagai berikut;

- Tergugat pergi meninggalkan Penggugat tanpa alasan selama lebih dari 2 tahun, Tergugat tidak pernah kembali kepada Penggugat;

Menimbang, bahwa berdasarkan fakta-fakta tersebut di atas dapat disimpulkan fakta hukum sebagai berikut;

Disclaimer
Kepaniteraan Mahkamah Agung Republik Indonesia berusaha untuk selalu mencantumkan informasi paling kini dan akurat sebagai bentuk komitmen Mahkamah Agung untuk pelayanan publik, transparansi dan akuntabilitas pelaksanaan fungsi peradilan. Namun dalam hal-hal tertentu masih dimungkinkan terjadi permasalahan teknis terkait dengan akurasi dan keterkinian informasi yang kami sajikan, hal mana akan terus kami perbaiki dari waktu kewaktu.
Dalam hal Anda menemukan inakurasi informasi yang termuat pada situs ini atau informasi yang seharusnya ada, namun belum tersedia, maka harap segera hubungi Kepaniteraan Mahkamah Agung RI melalui :
Email : kepaniteraan@mahkamahagung.go.id Telp : 021-384 3348 (ext.318)

1. Bahwa Tergugat pergi meninggalkan Penggugat tanpa alasan yang sah atau hal lain diluar kemampuannya selama lebih dari 2 tahun;
2. Bahwa antara Penggugat dan Tergugat tidak ada harapan hidup rukun kembali;

Menimbang, bahwa fakta hukum tersebut telah memenuhi norma hukum islam yang terkandung dalam Kitab Fikih Sunah Juz II halaman 251;

إذا غاب الزوج سنة فأكثر بلا عذر مقبول جاز لزوجته أن تطلب إلى القاضي تطليقها بائنا
إذا تضررت من بعده عنها ولو كان له مال تستطيع الإنفاق منه

Artinya : “*Apa bila suami meninggalkan istrinya tanpa alasan yang bisa diterima selama satu tahun atau lebih, sang istri boleh mengajukan tuntutan cerai kepada hakim dengan talak bain jika dia merasa dirugikan, walaupun sang suami memiliki harta yang dapat dijadikan nafkah untuk istrinya”;*

Menimbang, bahwa fakta hukum tersebut telah juga memenuhi Pasal 39 ayat 2 Undang-undang Nomor 1 tahun 1974 Tentang Perkawinan dan Pasal 19 huruf (b) Peraturan Pemerintah Nomor 9 Tahun 1975 tentang Pelaksanaan Undang-undang Nomor 1 tahun 1974 Tentang Perkawinan jo. Pasal 116 huruf (b) Kompilasi Hukum Islam, oleh karena itu dapat dikabulkan;

Menimbang, bahwa karena perkara *a quo* masuk bidang perkawinan, maka berdasarkan Pasal 89 ayat (1) Undang-undang Nomor 7 Tahun 1989 Tentang Peradilan Agama sebagaimana telah diubah dengan Undang-undang Nomor 3 Tahun 2006 dan perubahan kedua dengan Undang-undang Nomor 50 Tahun 2009, biaya perkara harus dibebankan kepada Penggugat;

Mengingat semua pasal dalam peraturan perundang-undangan dan hukum islam yang berkaitan dengan perkara ini;

M E N G A D I L I

1. Menyatakan Tergugat yang telah dipanggil secara resmi dan patut untuk menghadap dipersidangan tidak hadir;

Disclaimer
Kepaniteraan Mahkamah Agung Republik Indonesia berusaha untuk selalu mencantumkan informasi paling kini dan akurat sebagai bentuk komitmen Mahkamah Agung untuk pelayanan publik, transparansi dan akuntabilitas pelaksanaan fungsi peradilan. Namun dalam hal-hal tertentu masih dimungkinkan terjadi permasalahan teknis terkait dengan akurasi dan keterkinian informasi yang kami sajikan, hal mana akan terus kami perbaiki dari waktu kewaktu.
Dalam hal Anda menemukan inakurasi informasi yang termuat pada situs ini atau informasi yang seharusnya ada, namun belum tersedia, maka harap segera hubungi Kepaniteraan Mahkamah Agung RI melalui :
Email : kepaniteraan@mahkamahagung.go.id   Telp : 021-384 3348 (ext.318)

2. Mengabulkan gugata Penggugat dengan verstek;
3. Menjatuhkan talak satu bain sughro dari Tergugat (TERGUGAT) kepada Penggugat (PENGGUGAT);
4. Membebankan kepada Penggugat untuk membayar biaya perkara sebesar Rp. 381.000,00 (tiga ratus delapan puluh satu ribu rupiah);

Demikian diputuskan dalam rapat permusyawaratan Majelis yang dilangsungkan pada hari Rabu tanggal 23 Desember 2020 *Masehi*. bertepatan dengan tanggal 8 Jumadil Awwal 1442 *Hijriyah.* Oleh kami Drs. H. Tubagus Masrur, S.H. sebagai Ketua Majelis, dan Dra.Hj. Ismiyati, S.H. serta Nurman Syarif, S.HI., M.SI masing-masing sebagai Hakim Anggota, putusan tersebut diucapkan dalam sidang terbuka untuk umum pada hari Rabu tanggal 23 Desember 2020 *Masehi*. bertepatan dengan tanggal 8 Jumadil Awwal 1442 *Hijriyah.* oleh Ketua Majelis tersebut dengan didampingi oleh Hakim Anggota dan dibantu oleh Isti Wajinah,S.H. sebagai Panitera Pengganti serta dihadiri Penggugat tanpa hadirnya Tergugat;

Hakim Anggota,	Ketua Majelis,
Dra.Hj. Ismiyati, S.H.	Drs. H. Tubagus Masrur, S.H.

Hakim Anggota,

Nurman Syarif, S.HI., M.SI

Panitera Pengganti,

Isti Wajinah,S.H.

Perincian Biaya :

Biaya Pendaftaran	:	Rp	30.000,00
Biaya Proses	:	Rp	75.000,00
Biaya Pemanggilan	:	Rp	240.000,00

Disclaimer
Kepaniteraan Mahkamah Agung Republik Indonesia berusaha untuk selalu mencantumkan informasi paling kini dan akurat sebagai bentuk komitmen Mahkamah Agung untuk pelayanan publik, transparansi dan akuntabilitas pelaksanaan fungsi peradilan. Namun dalam hal-hal tertentu masih dimungkinkan terjadi permasalahan teknis terkait dengan akurasi dan keterkinian informasi yang kami sajikan, hal mana akan terus kami perbaiki dari waktu kewaktu.
Dalam hal Anda menemukan inakurasi informasi yang termuat pada situs ini atau informasi yang seharusnya ada, namun belum tersedia, maka harap segera hubungi Kepaniteraan Mahkamah Agung RI melalui :
Email : kepaniteraan@mahkamahagung.go.id Telp : 021-384 3348 (ext.318)

PNBP	:	Rp	20.000,00
Biaya Redaksi	:	Rp	10.000,00
Biaya Materai	:	Rp	6.000,00
Jumlah	:	Rp	381.000,00

Disclaimer
Kepaniteraan Mahkamah Agung Republik Indonesia berusaha untuk selalu mencantumkan informasi paling kini dan akurat sebagai bentuk komitmen Mahkamah Agung untuk pelayanan publik, transparansi dan akuntabilitas pelaksanaan fungsi peradilan. Namun dalam hal-hal tertentu masih dimungkinkan terjadi permasalahan teknis terkait dengan akurasi dan keterkinian informasi yang kami sajikan, hal mana akan terus kami perbaiki dari waktu kewaktu.
Dalam hal Anda menemukan inakurasi informasi yang termuat pada situs ini atau informasi yang seharusnya ada, namun belum tersedia, maka harap segera hubungi Kepaniteraan Mahkamah Agung RI melalui :
Email : kepaniteraan@mahkamahagung.go.id Telp : 021-384 3348 (ext.318)
