# Supplementary material for: Classification of divorce causes during the COVID-19 pandemic using convolutional neural networks
Source: PeerJ Comput Sci. 2022 Jun 30;8:e998. doi: 10.7717/peerj-cs.998 (PMC9299239; doi:10.7717/peerj-cs.998)
Supplement: Supplemental Information 2 [file peerj-cs-08-998-s002.zip › Kelalaian Dataset/Data ke-6.pdf]

***P U T U S A N***

**Nomor 592/Pdt.G/2020/PA.Sgt**

بِسْمِ اللَّهِ الرَّحْمَٰنِ الرَّحِيمِ

**DEMI KEADILAN BERDASARKAN KETUHANAN YANG MAHA ESA**

Pengadilan Agama Sengeti yang memeriksa dan mengadili perkara tertentu pada tingkat pertama dalam sidang Majelis Hakim telah menjatuhkan putusan sebagai berikut dalam perkara Cerai Gugat antara:

**PENGGUGAT**, umur 23 tahun, agama Islam, pendidikan SLTA, pekerjaan ibu rumah tangga, tempat tinggal di RT 03 Desa Baru, Kecamatan Mestong, Kabupaten Muaro Jambi, Provinsi Jambi, sebagai Penggugat;

melawan

**TERGUGAT**, umur 37 tahun, agama Islam, pendidikan SD, pekerjaan Wiraswasta, tempat tinggal di RT 01 DesaTanjungPauh, Kecamatan Mestong, Kabupaten Muaro Jambi, Provinsi Jambi., sebagai Tergugat;

Pengadilan Agama tersebut;
Setelah membaca berkas perkara yang bersangkutan;
Setelah mendengar keterangan Penggugat dan memeriksa alat bukti di persidangan;

**DUDUK PERKARA**

Bahwa Penggugat dengan surat gugatannya tertanggal 08 Desember 2020 yang telah terdaftar di Kepaniteraan Pengadilan Agama Sengeti dengan register perkara Nomor 592/Pdt.G/2020/PA.Sgt, mengemukakan dalil-dalil sebagai berikut:

1. Bahwa pada tanggal ********** telah dilangsungkan perkawinan antara Penggugat dengan Tergugat yang dilaksanakan menurut hukum dan sesuai dengan tuntunan ajaran agama Islam. Perkawinan tersebut telah dicatatkan di Kantor Urusan Agama (KUA) Kecamatan Mestong, Kabupaten Muaro



*Disclaimer*
*Kepaniteraan Mahkamah Agung Republik Indonesia berusaha untuk selalu mencantumkan informasi paling kini dan akurat sebagai bentuk komitmen Mahkamah Agung untuk pelayanan publik, transparansi dan akuntabilitas pelaksanaan fungsi peradilan. Namun dalam hal-hal tertentu masih dimungkinkan terjadi permasalahan teknis terkait dengan akurasi dan keterkinian informasi yang kami sajikan, hal mana akan terus kami perbaiki dari waktu kewaktu.*
*Dalam hal Anda menemukan inakurasi informasi yang termuat pada situs ini atau informasi yang seharusnya ada, namun belum tersedia, maka harap segera hubungi Kepaniteraan Mahkamah Agung RI melalui :*
*Email : kepaniteraan@mahkamahagung.go.id    Telp : 021-384 3348 (ext.318)*

Jambi, Provinsi Jambi,sebagaimana tercatat dalam Akta Nikah No. ********** tertanggal**********;

2. Bahwa perkawinan antara Penggugat dan Tergugat dilangsungkan berdasarkan kehendak kedua belah pihak dengan tujuan membentuk rumah tangga yang sakinah, mawaddah, warahmah yang diridhoi oleh Allah Swt;

3. Bahwa setelah menikah, Penggugat dan Tergugat tinggal di rumah kediaman bersamadi Desa Tanjung Pauh, Kecamatan Mestong, Kabupaten Muaro Jambi, Provinsi Jambi sampai terjadi pisah;

4. Bahwa selama masa perkawinan, Penggugat dan Tergugat telah berkumpul sebagaimana layaknya suami-isteri dan sudah dikaruniai sudah orang anak yang bernama ANAK PENGGUGAT DENGAN TERGUGAT, laki-laki, lahir pada tanggal 02 November 2020 di Tempino;

5. Bahwa kebahagiaan yang dirasakan Penggugat setelah berumah tangga dengan Tergugat hanya berlangsung sampai sekitar tahun 2018, ketentraman rumah tangga Penggugat dengan Tergugat mulai goyah setelah antara Penggugat dengan Tergugat terjadi perselisihan dan pertengkaran secara terus menerus sejak sekitar tahun 2018 sampai dengan saat ini, yang penyebabnya antara lain;

6. Penggugat tidak dapat menerima sikap Tergugat disaat Tergugat dan Penggugat berhubungan suami isteri, dimana Tergugat hanya mementingkan kepuasannya sendiri dan tidak mementingkan keinginan atau kepuasanIsteri;

7. Adanya pihak ketiga yaitu Tergugat menjalin hubungan dengan perempuan lain, dan hubungan tersebut telah sampai tidur berdua di suatu hotel;

8. Tergugat tidak menepati janjinya untuk berubah menjadi lebih baik, karenaTergugat telah dua kali menjalin hubungan asmara dengan perempuan lain;

9. Bahwa puncak dari percekcokan antara Penggugat dan Tergugat terjadi pada sekitar bulan November 2020. Yang menyebabkan antara Penggugat dan Tergugat telah pisah rumah, dimana Penggugat pergi dan kembali



*Disclaimer*
*Kepaniteraan Mahkamah Agung Republik Indonesia berusaha untuk selalu mencantumkan informasi paling kini dan akurat sebagai bentuk komitmen Mahkamah Agung untuk pelayanan publik, transparansi dan akuntabilitas pelaksanaan fungsi peradilan. Namun dalam hal-hal tertentu masih dimungkinkan terjadi permasalahan teknis terkait dengan akurasi dan keterkinian informasi yang kami sajikan, hal mana akan terus kami perbaiki dari waktu kewaktu.*
*Dalam hal Anda menemukan inakurasi informasi yang termuat pada situs ini atau informasi yang seharusnya ada, namun belum tersedia, maka harap segera hubungi Kepaniteraan Mahkamah Agung RI melalui :*
*Email : kepaniteraan@mahkamahagung.go.id Telp : 021-384 3348 (ext.318)*

kerumah orang tuanya. Sehingga sejak saat itu Penggugat dan Tergugat sudah tidak pernah lagi menjalin hubungan sebagaimana layaknya suami istri;

10. Bahwa atas permasalahan dan kemelut rumah tangga yang dihadapi, Penggugat telah mencoba memusyawarahkan dengan keluarga Penggugat dan Tergugat untuk mencari penyelesaian dan demi menyelamatkan perkawinan, namun usaha tersebut tidak membuahkan hasil;
11. Bahwa ikatan perkawinan antara Penggugat dan Tergugat sebagaimana yang diuraikan diatas sudah sulit dibina untuk membentuk suatu rumah tangga yang sakinah, mawaddah wa rahmah sebagaimana maksud dan tujuan dari suatu perkawinan, sehingga lebih baik diputus karena perceraian;
12. Bahwa berdasarkan hal-hal tersebut diatas, permohonan Penggugat untuk mengajukan gugatan perceraian terhadap Tergugat atas dasar pertengkaran yang terjadi terus menerus dan tidak mungkin hidup rukun dalam suatu ikatan perkawinan, telah memenuhi unsur Pasal 19 huruf (f) Peraturan Pemerintah No. 9 Tahun 1975, sehingga berdasar hukum untuk menyatakan gugatan cerai ini dikabulkan;
13. Bahwa Penggugat sanggup membayar biaya perkara;

Bahwa berdasarkan hal-hal tersebut diatas Penggugat beranggapan bahwa rumah tangga Penggugat dan Tergugat sudah tidak dapat diperbaiki lagi sehingga tujuan membina rumah tangga yang *sakinah, mawadah, warahmah* sebagaimana diamanatkan oleh syari'at Islam dan peraturan perundang-undangan yang berlaku, tidak dapat dicapai. Oleh karena itu Penggugat mohon kepada Ketua Pengadilan Agama Sengeti melalui Majelis Hakim untuk memeriksa dan mengadili perkara ini, dengan menjatuhkan putusan yang amarnya berbunyi sebagai berikut:

PRIMER:

1. Mengabulkan gugatan Penggugat untuk seluruhnya;
2. Menjatuhkan talak satu Tergugat (TERGUGAT) Terhadap Penggugat (PENGGUGAT);



*Disclaimer*
*Kepaniteraan Mahkamah Agung Republik Indonesia berusaha untuk selalu mencantumkan informasi paling kini dan akurat sebagai bentuk komitmen Mahkamah Agung untuk pelayanan publik, transparansi dan akuntabilitas pelaksanaan fungsi peradilan. Namun dalam hal-hal tertentu masih dimungkinkan terjadi permasalahan teknis terkait dengan akurasi dan keterkinian informasi yang kami sajikan, hal mana akan terus kami perbaiki dari waktu kewaktu.*
*Dalam hal Anda menemukan inakurasi informasi yang termuat pada situs ini atau informasi yang seharusnya ada, namun belum tersedia, maka harap segera hubungi Kepaniteraan Mahkamah Agung RI melalui :*
*Email : kepaniteraan@mahkamahagung.go.id Telp : 021-384 3348 (ext.318)*

3. Membebankan biaya perkara sesuai hukum;

SUBSIDER:

Atau apabila Pengadilan Agama berpendapat lain mohon putusan yang seadil-adilnya (ex aequo et bono);

Bahwa, pada hari dan tanggal sidang yang telah ditetapkan, Penggugat datang menghadap ke persidangan sedangkan Tergugat tidak pernah hadir dan tidak pula mengutus orang lain sebagai wakil atau kuasanya yang sah untuk menghadap ke persidangan meskipun telah dipanggil secara resmi dan patut serta tidak ternyata bahwa ketidakhadirannya disebabkan oleh suatu halangan yang sah;

Bahwa, Majelis Hakim dalam persidangan telah berusaha mendamaikan dengan jalan memberi nasehat, arahan dan penjelasan kepada Penggugat agar bersabar dan tetap mempertahankan keutuhan rumah tangganya dengan Tergugat akan tetapi tidak berhasil, sedangkan usaha damai melalui proses mediasi tidak dapat dilaksanakan karena Tergugat tidak pernah datang menghadap ke persidangan;

Bahwa, oleh sebab upaya damai tidak berhasil, selanjutnya pemeriksaan perkara ini dilanjutkan secara litigasi diawali dengan membacakan surat gugatan Penggugat yang pada pokoknya tetap dipertahankan oleh Penggugat;

Bahwa, terhadap gugatan Penggugat, jawaban Tergugat tidak dapat didengar dalam persidangan karena Tergugat tidak pernah hadir di persidangan;

Bahwa untuk meneguhkan dalil-dalil gugatannya, Penggugat telah mengajukan alat bukti sebagai berikut:

I. Buk
ti Surat

Fotokopi Kutipan Akta Nikah Nomor ********** Tanggal ********** atas nama Penggugat dan Tergugat yang dicatat dan dikeluarkan oleh Pegawai Pencatat Nikah Kantor Urusan Agama Kecamatan Mestong, Kabupaten Muaro Jambi, Provinsi Jambi, telah bermeterai cukup dan di-*nazegelen,* yang oleh Ketua Majelis telah dicocokkan dengan aslinya ternyata cocok, Ketua Majelis memaraf, membubuhkan tanggal dan memberi kode P;



*Disclaimer*
*Kepaniteraan Mahkamah Agung Republik Indonesia berusaha untuk selalu mencantumkan informasi paling kini dan akurat sebagai bentuk komitmen Mahkamah Agung untuk pelayanan publik, transparansi dan akuntabilitas pelaksanaan fungsi peradilan. Namun dalam hal-hal tertentu masih dimungkinkan terjadi permasalahan teknis terkait dengan akurasi dan keterkinian informasi yang kami sajikan, hal mana akan terus kami perbaiki dari waktu kewaktu.*
*Dalam hal Anda menemukan inakurasi informasi yang termuat pada situs ini atau informasi yang seharusnya ada, namun belum tersedia, maka harap segera hubungi Kepaniteraan Mahkamah Agung RI melalui :*
*Email : kepaniteraan@mahkamahagung.go.id Telp : 021-384 3348 (ext.318)*

II. Bukti Saksi

1. SAKSI 1, umur 38 tahun, agama Islam, pendidikan SLTP, pekerjaan Mengurus Rumah Tangga, tempat tinggal di RT 03, RW 02 Desa Baru, Kecamatan Mestong, Kabupaten Muaro Jambi, telah memberikan keterangan di bawah sumpah sebagai berikut:

- Bahwa saksi kenal Penggugat dan Tergugat karena saksi adalah bibi Penggugat;
- Bahwa setelah menikah Penggugat dan Tergugat tinggal di Desa Tanjung Pauh, Kecamatan Mestong, Kabupaten Muaro Jambi;
- Bahwa Penggugat dan Tergugat telah dikaruniai 1 (satu) orang anak;
- Bahwa rumah tangga Penggugat dan Tergugat awalnya rukun dan harmonis, namun sejak 2018 tidak harmonis karena sering terjadi perselisihan dan pertengkaran;
- Bahwa penyebab terjadinya perselisihan dan pertengkaran antara Penggugat dan Tergugat adalah karena Tergugat hanya mementingkan dirinya sendiri dan punya hubungan dengan perempuan lain, saksi tahu dari cerita Penggugat dan saksi melihat sendiri Tergugat memang berselingkuh dengan wanita lain;
- Bahwa saat itu saksi pergi diajak Penggugat menemaninya kesebuah kamar hotel untuk menemui Tergugat, karena Penggugat mendapat informasi bahwa Tergugat bersama perempuan lain disebuah kamar hotel, sampai disana saksi melihat langsung Tergugat bersama seorang perempuan sedang berduaan di kamar hotel, Tergugat tidak memakai baju hanya menggunakan celana pendek sedangkan perempuannya hanya menggunakan pakaian dalam saja;
- Bahwa Penggugat dan Tergugat sudah tidak satu rumah sejak kejadian di hotel itu Tergugat tidak pernah lagi menemui Penggugat;
- Bahwa sudah ada upaya untuk merukunkan Penggugat dan Tergugat, namun tidak berhasil karena keduanya sudah tidak bisa disatukan lagi dan memilih bercerai;



*Disclaimer*
*Kepaniteraan Mahkamah Agung Republik Indonesia berusaha untuk selalu mencantumkan informasi paling kini dan akurat sebagai bentuk komitmen Mahkamah Agung untuk pelayanan publik, transparansi dan akuntabilitas pelaksanaan fungsi peradilan. Namun dalam hal-hal tertentu masih dimungkinkan terjadi permasalahan teknis terkait dengan akurasi dan keterkinian informasi yang kami sajikan, hal mana akan terus kami perbaiki dari waktu kewaktu.*
*Dalam hal Anda menemukan inakurasi informasi yang termuat pada situs ini atau informasi yang seharusnya ada, namun belum tersedia, maka harap segera hubungi Kepaniteraan Mahkamah Agung RI melalui :*
*Email : kepaniteraan@mahkamahagung.go.id Telp : 021-384 3348 (ext.318)*

2. SAKSI 2, umur 40 tahun, agama Islam, pendidikan SD, pekerjaan Mengurus Rumah Tangga, bertempat tinggal di RT 03, Kecamatan Mestong, Kabupaten Muaro Jambi, di bawah sumpah memberikan keterangan yang pada pokoknya sebagai berikut :

- Bahwa saksi kenal Penggugat dan Tergugat karena saksi adalah tetangga Penggugat;
- Bahwa setelah menikah Penggugat dan Tergugat tinggal di Desa Tanjung Pauh, Kecamatan Mestong, Kabupaten Muaro Jambi;
- Bahwa Penggugat dan Tergugat telah dikaruniai 1 (satu) orang anak;
- Bahwa rumah tangga Penggugat dan Tergugat awalnya rukun dan harmonis, namun sejak 2018 tidak harmonis karena sering terjadi perselisihan dan pertengkaran;
- Bahwa perselisihan dan pertengkaran antara Penggugat dan Tergugat tersebut terjadi disebabkan Tergugat menjalin hubungan dengan perempuan lain dan hal itu juga diakui Tergugat bahkan sampai tertangkap basah dikamar hotel sedang berduaan dengan perempuan lain tersebut, saksi tahu karena saksi yang mengantarkan Penggugat ke hotel tempat Tergugat dan perempuan tersebut berada. Saksi juga melihat Tergugat bersama perempuan lain hanya memakai celana pendek sedangkan perempuan itu hanya memakai pakaian dalam;
- Bahwa sejak kejadian dihotel tersebut Penggugat dan Tergugat sudah tidak tinggal bersama;
- Bahwa pihak keluarga sudah berusaha untuk mendamaikan kembali Penggugat dan Tergugat, namun Penggugat dan Tergugat tidak mau lagi melanjutkan pernikahan mereka dan tetap ingin bercerai;

Bahwa setelah diberi kesempatan Penggugat menyatakan tidak mengajukan alat bukti apapun lagi dan menyampaikan kesimpulan secara lisan yang pada pokoknya menyatakan tetap pada gugatannya dan selanjutnya mohon putusan;



*Disclaimer*
*Kepaniteraan Mahkamah Agung Republik Indonesia berusaha untuk selalu mencantumkan informasi paling kini dan akurat sebagai bentuk komitmen Mahkamah Agung untuk pelayanan publik, transparansi dan akuntabilitas pelaksanaan fungsi peradilan. Namun dalam hal-hal tertentu masih dimungkinkan terjadi permasalahan teknis terkait dengan akurasi dan keterkinian informasi yang kami sajikan, hal mana akan terus kami perbaiki dari waktu kewaktu.*
*Dalam hal Anda menemukan inakurasi informasi yang termuat pada situs ini atau informasi yang seharusnya ada, namun belum tersedia, maka harap segera hubungi Kepaniteraan Mahkamah Agung RI melalui :*
*Email : kepaniteraan@mahkamahagung.go.id Telp : 021-384 3348 (ext.318)*

Bahwa untuk mempersingkat uraian putusan ini cukup ditunjuk segala hal yang telah termuat dalam berita acara sidang yang dianggap sebagai satu kesatuan tak terpisahkan dalam putusan ini;

**PERTIMBANGAN HUKUM**

Menimbang, bahwa maksud dan tujuan gugatan Penggugat adalah sebagaimana tersebut di atas;

Menimbang, bahwa pada hari dan tanggal persidangan yang telah ditetapkan, Penggugat datang menghadap sendiri ke persidangan, sedangkan Tergugat tidak pernah hadir dan tidak pernah mengutus orang lain sebagai kuasanya yang sah untuk menghadap ke persidangan meskipun telah dipanggil secara resmi dan patut serta tidak ternyata ketidakhadiran Tergugat disebabkan oleh suatu alasan yang sah, maka Tergugat harus dinyatakan tidak hadir dan sesuai dengan ketentuan Pasal 149 ayat (1) R.Bg perkara ini dapat diperiksa dan diputus tanpa hadirnya Tergugat (Verstek);

Menimbang, bahwa perkara ini adalah termasuk bidang perkawinan yang ternyata subjek hukum dalam perkara ini adalah beragama Islam, maka berdasarkan Pasal 49 Undang-Undang Nomor 3 Tahun 2006, tentang perubahan atas Undang-Undang Nomor 7 Tahun 1989 tentang Peradilan Agama, perkara ini termasuk kompetensi absolut Pengadilan Agama;

Menimbang, bahwa oleh sebab antara Penggugat dan Tergugat terdapat hubungan perkawinan sebagai suami istri yang sah dan belum pernah bercerai (vide bukti kode P), maka keduanya memiliki kepentingan hukum untuk bertindak sebagai pihak-pihak dalam perkara ini (persona standi in judicio);

Menimbang, bahwa berdasarkan ketentuan Pasal 65 dan Pasal 82 ayat (1) dan (4) Undang-Undang Nomor 7 Tahun 1989 tentang Peradilan Agama jo Pasal 115 Kompilasi Hukum Islam, Majelis Hakim telah berusaha mendamaikan dengan jalan memberi nasehat, arahan dan penjelasan kepada Penggugat agar bersabar dan tetap mempertahankan keutuhan rumah tangganya dengan Tergugat akan tetapi tidak berhasil, sedangkan usaha damai melalui proses mediasi sebagaimana dikehendaki Peraturan Mahkamah Agung Republik Indonesia Nomor 1 tahun 2016 tentang Prosedur Mediasi di Pengadilan tidak dapat dilaksanakan karena Tergugat tidak pernah hadir di persidangan;



*Disclaimer*
*Kepaniteraan Mahkamah Agung Republik Indonesia berusaha untuk selalu mencantumkan informasi paling kini dan akurat sebagai bentuk komitmen Mahkamah Agung untuk pelayanan publik, transparansi dan akuntabilitas pelaksanaan fungsi peradilan. Namun dalam hal-hal tertentu masih dimungkinkan terjadi permasalahan teknis terkait dengan akurasi dan keterkinian informasi yang kami sajikan, hal mana akan terus kami perbaiki dari waktu kewaktu.*
*Dalam hal Anda menemukan inakurasi informasi yang termuat pada situs ini atau informasi yang seharusnya ada, namun belum tersedia, maka harap segera hubungi Kepaniteraan Mahkamah Agung RI melalui :*
*Email : kepaniteraan@mahkamahagung.go.id    Telp : 021-384 3348 (ext.318)*

Menimbang, bahwa selanjutnya dibacakan surat gugatan Penggugat, yang isinya tetap dipertahankan tanpa ada perubahan dan penambahan;

Menimbang, bahwa berdasarkan gugatan Penggugat tersebut dalam duduk perkara, Majelis Hakim menilai bahwa posita dari gugatan Penggugat adalah antara Penggugat dan Tergugat terjadi perselisihan dan pertengkaran terus menerus dan tidak ada harapan lagi untuk rukun kembali, sebagaimana ketentuan Pasal 39 ayat (2) Undang-Undang nomor 1 tahun 1974 tentang Perkawinan, Jo. Pasal 19 huruf (f) Peraturan Pemerintah nomor 9 tahun 1975 tentang Pelaksanaan Undang-Undang nomor 1 tahun 1974 tentang Perkawinan, Jo. Pasal 116 huruf (f) Kompilasi Hukum Islam;

Menimbang, bahwa Tergugat tidak pernah hadir dalam persidangan sehingga jawaban Tergugat tidak dapat didengar dan sesuai ketentuan Pasal 149 R.Bg harus diputus tanpa melalui pembuktian, namun oleh karena perkara ini perkara perceraian sesuai hasil rumusan hukum kamar agama Mahkamah Agung R.I tahun 2015 yang dikuat dengan SEMA nomor 3 tahun 2015 terhadap Penggugat tetap dibebankan pembuktian sesuai ketentuan Pasal 22 ayat (2) Peraturan Pemerintah nomor 9 Tahun 1975 tentang Pelaksanaan Undang-Undang nomor 1 tahun 1974 tentang Perkawinan;

Menimbang, bahwa untuk dikabulkan gugatan cerai dengan alasan tersebut di atas, sebagaimana penjelasan Pasal 39 ayat (2) Undang-Undang nomor 1 tahun 1974 tentang Perkawinan, Jo. Pasal 19 huruf (f) Peraturan Pemerintah nomor 9 tahun 1975 tentang Pelaksanaan Undang-Undang nomor 1 tahun 1974 tentang Perkawinan, Jo. Pasal 116 huruf (f) Kompilasi Hukum Islam, harus terpenuhi beberapa unsur sebagai berikut: (1). Antara Penggugat dan Tergugat masih terikat dalam perkawinan yang sah, dan belum pernah bercerai, (2). Antara Penggugat dan Tergugat terjadi perselisihan dan pertengkaran secara terus menerus. (3). Kondisi perselisihan dan pertengkaran antara Penggugat dan Tergugat tersebut tidak ada harapan untuk rukun kembali;

Menimbang, bahwa untuk menguatkan dalil-dalil gugatannya, Penggugat di persidangan telah mengajukan alat bukti tertulis dan saksi-saksi;

Menimbang, bahwa terhadap alat bukti yang diajukan oleh Penggugat, Majelis Hakim memberi pertimbangan sebagai berikut;



*Disclaimer*
*Kepaniteraan Mahkamah Agung Republik Indonesia berusaha untuk selalu mencantumkan informasi paling kini dan akurat sebagai bentuk komitmen Mahkamah Agung untuk pelayanan publik, transparansi dan akuntabilitas pelaksanaan fungsi peradilan. Namun dalam hal-hal tertentu masih dimungkinkan terjadi permasalahan teknis terkait dengan akurasi dan keterkinian informasi yang kami sajikan, hal mana akan terus kami perbaiki dari waktu kewaktu.*
*Dalam hal Anda menemukan inakurasi informasi yang termuat pada situs ini atau informasi yang seharusnya ada, namun belum tersedia, maka harap segera hubungi Kepaniteraan Mahkamah Agung RI melalui :*
*Email : kepaniteraan@mahkamahagung.go.id Telp : 021-384 3348 (ext.318)*

Menimbang, bahwa untuk membuktikan antara Penggugat dan Tergugat masih terikat dalam perkawinan yang sah dan belum pernah bercerai, dalam persidangan Penggugat telah mengajukan alat bukti tertulis yang diberi kode (P);

Menimbang, bahwa terhadap alat bukti tertulis berupa fotokopi kutipan akta nikah yang diberi kode (P) yang diajukan oleh Penggugat, Majelis Hakim menilai bahwa alat bukti tersebut dikeluarkan oleh pejabat yang berwenang, telah dinazegelen dan dicocokan sesuai dengan aslinya dalam persidangan ternyata sesuai dengan akta aslinya, dengan demikian alat bukti tersebut telah memenuhi persyaratan formil sebagai alat bukti autentik yang mempunyai kekuatan pembuktian yang sempurna dan mengikat, sesuai ketentuan Pasal 1868, 1870, 1888 Kitab Undang-Undang Hukum Perdata dan Pasal 2 Undang-Undang Nomor 13 Tahun 1985 tentang Bea Meterai;

Menimbang, bahwa berdasarkan alat bukti (P) terbukti Penggugat dengan Tergugat telah melangsungkan perkawinan secara sah dan tidak ada bukti bahwa Penggugat dan Tergugat telah bercerai, maka sesuai dengan asas:

الأصل بقاء ماكان على ماكان

(hukum asal sesuatu tetap berlangsung sebagaimana yang lalu sampai ada perubahan yang pasti), oleh karenanya telah terbukti bahwa Penggugat dan Tergugat adalah suami-isteri yang sah sampai saat ini dan belum pernah bercerai, sehingga Penggugat dan Tergugat berkualitas hukum untuk bertindak sebagai pihak-pihak yang berkepentingan dalam perkara ini (persona standi in judicio);

Menimbang, bahwa untuk membuktikan antara Penggugat dan Tergugat telah terjadi perselisihan dan pertengkaran yang sedemikian rupa sehingga tidak ada harapan rukun lagi, maka berdasarkan ketentuan Pasal 22 Peraturan Pemerintah Nomor 9 Tahun 1975 tentang Pelaksanaan Undang-Undang Nomor 1 Tahun 1974 tentang Perkawinan Jo. Pasal 76 ayat (1) Undang-Undang Nomor 7 tahun 1989 tentang Peradilan Agama sebagaimana yang telah diubah dengan Undang-Undang Nomor 3 Tahun 2006 dan perubahan kedua dengan Undang-Undang Nomor 50 Tahun 2009, harus didengar saksi dari keluarga atau orang yang dekat dengan Penggugat dan Tergugat;



*Disclaimer*
*Kepaniteraan Mahkamah Agung Republik Indonesia berusaha untuk selalu mencantumkan informasi paling kini dan akurat sebagai bentuk komitmen Mahkamah Agung untuk pelayanan publik, transparansi dan akuntabilitas pelaksanaan fungsi peradilan. Namun dalam hal-hal tertentu masih dimungkinkan terjadi permasalahan teknis terkait dengan akurasi dan keterkinian informasi yang kami sajikan, hal mana akan terus kami perbaiki dari waktu kewaktu.*
*Dalam hal Anda menemukan inakurasi informasi yang termuat pada situs ini atau informasi yang seharusnya ada, namun belum tersedia, maka harap segera hubungi Kepaniteraan Mahkamah Agung RI melalui :*
*Email : kepaniteraan@mahkamahagung.go.id Telp : 021-384 3348 (ext.318)*

Menimbang, bahwa terhadap saksi-saksi yang diajukan Penggugat ke persidangan, Majelis menilai saksi-saksi tersebut adalah orang yang sudah mencapai usia di atas 15 tahun, cerdas dan boleh didengar sebagai saksi dalam perkara ini, saksi-saksi adalah keluarga atau orang yang cukup dekat dengan Penggugat maupun Tergugat, dan saksi-saksi tersebut tidak mempunyai kepentingan terhadap perkara ini dan memberikan keterangan masing-masing secara terpisah di bawah sumpahnya, oleh karena itu saksi-saksi tersebut secara formil dapat diterima sebagai saksi dalam perkara ini;

Menimbang, bahwa saksi-saksi yang dihadapkan Penggugat di bawah sumpahnya menerangkan bahwa keadaan rumah tangga Penggugat dan Tergugat sudah tidak rukun lagi sering terjadi perselisihan dan pertengkaran, serta telah diupayakan perdamaian namun tidak berhasil, dan sekarang mereka telah berpisah, keterangan tersebut saksi-saksi ketahui secara langsung dan bukan cerita dari orang lain;

Menimbang, bahwa Majelis Hakim menilai keterangan saksi-saksi tersebut saling bersesuaian, tidak bertentangan satu sama lain serta mendukung kebenaran dalil gugatan Penggugat, maka keterangan saksi-saksi tersebut telah memenuhi syarat materiil sebagai alat bukti sebagaimana dikehendaki dalam Pasal 308 dan 309 R.Bg;

Menimbang, bahwa berdasarkan keterangan saksi-saksi tersebut dapat ditemukan fakta hukum bahwa antara Penggugat dan Tergugat telah terjadi perselisihan dan pertengkaran meskipun sudah pernah diupayakan damai namun tetap tidak berhasil rukun, ditambah antara Penggugat dan Tergugat sekarang sudah sudah berpisah;

Menimbang, bahwa berdasarkan fakta antara Penggugat dan Tergugat sudah pisah, dan Majelis Hakim di setiap persidangan telah mendamaikan Penggugat agar rukun kembali dengan Tergugat tetap tidak berhasil, hal tersebut merupakan *qarinah* (petunjuk) yang dapat ditarik persangkaan hakim bahwa antara Penggugat dan Tergugat sudah tidak ada harapan untuk rukun lagi;

Menimbang, bahwa berdasarkan pertimbangan tersebut di atas, dalil gugatan Penggugat bahwa antara Penggugat dan Tergugat telah terjadi



*Disclaimer*
*Kepaniteraan Mahkamah Agung Republik Indonesia berusaha untuk selalu mencantumkan informasi paling kini dan akurat sebagai bentuk komitmen Mahkamah Agung untuk pelayanan publik, transparansi dan akuntabilitas pelaksanaan fungsi peradilan. Namun dalam hal-hal tertentu masih dimungkinkan terjadi permasalahan teknis terkait dengan akurasi dan keterkinian informasi yang kami sajikan, hal mana akan terus kami perbaiki dari waktu kewaktu.*
*Dalam hal Anda menemukan inakurasi informasi yang termuat pada situs ini atau informasi yang seharusnya ada, namun belum tersedia, maka harap segera hubungi Kepaniteraan Mahkamah Agung RI melalui :*
*Email : kepaniteraan@mahkamahagung.go.id Telp : 021-384 3348 (ext.318)*

perselisihan dan pertengkaran yang terus menerus dan tidak ada harapan untuk rukun lagi, telah terbukti;

Menimbang, bahwa secara sosiologis suatu perkawinan yang di dalamnya sering terjadi perselisihan yang terus menerus akan sulit untuk mewujudkan rumah tangga yang bahagia, yang penuh rahmah dan kasih sayang seperti yang diharapkan oleh setiap pasangan suami istri, justru sebaliknya akan menimbulkan kemudharatan yang berkepanjangan bagi salah satu atau kedua belah pihak. Hal tersebut sejalan dengan pendapat pakar Hukum lslam yang termuat dalam Kitab Madza Hurriyyatuz Zaujaeni fii ath-Thalaq yang menyatakan, "lslam memilih lembaga talak/perceraian ketika rumah tangga sudah dianggap guncang/tidak harmonis dan tidak bermanfaat lagi nasihat perdamaian dan hubungan suami isteri sudah hilang (tanpa ruh), dengan meneruskan perkawinan berarti menghukum isteri-suami dalam “penjara” yang berkepanjangan, hal tersebut merupakan sesuatu yang bertentangan dengan semangat keadilan;

Menimbang, bahwa berdasarkan pertimbangan-pertimbangan tersebut di atas, Majelis Hakim berpendapat alasan perceraian Penggugat telah memenuhi ketentuan penjelasan Pasal 39 ayat (2) Undang-Undang Nomor 1 Tahun 1974 tentang Perkawinan jo. Pasal 19 huruf (f) Peraturan Pemerintah Nomor 9 Tahun 1975 tentang Pelaksanaan Undang-Undang Nomor 1 Tahun 1974 tentang Perkawinan jo. Pasal 116 huruf (f) Kompilasi Hukum Islam, sehingga gugatan Penggugat harus dikabulkan;

Menimbang, bahwa antara Penggugat dan Tergugat belum pernah bercerai, maka sebagaimana ketentuan Pasal 119 ayat (2) huruf (c) Kompilasi hukum Islam, gugatan Penggugat dapat dikabulkan dengan menjatuhkan talak satu ba'in sughra Tergugat terhadap Penggugat;

Menimbang, bahwa hal tersebut telah relevan dengan pendapat ahli Hukum Islam Assayid Sabiq, dalam kitabnya *Fiqh As-Sunah*, Jilid II, halaman 248 yang diambil oleh Majelis Hakim sebagai pendapat sendiri yang menyatakan sebagai berikut:

[illegible]



*Disclaimer*
*Kepaniteraan Mahkamah Agung Republik Indonesia berusaha untuk selalu mencantumkan informasi paling kini dan akurat sebagai bentuk komitmen Mahkamah Agung untuk pelayanan publik, transparansi dan akuntabilitas pelaksanaan fungsi peradilan. Namun dalam hal-hal tertentu masih dimungkinkan terjadi permasalahan teknis terkait dengan akurasi dan keterkinian informasi yang kami sajikan, hal mana akan terus kami perbaiki dari waktu kewaktu.*
*Dalam hal Anda menemukan inakurasi informasi yang termuat pada situs ini atau informasi yang seharusnya ada, namun belum tersedia, maka harap segera hubungi Kepaniteraan Mahkamah Agung RI melalui :*
*Email : kepaniteraan@mahkamahagung.go.id Telp : 021-384 3348 (ext.318)*

Artinya: Jika gugatan istri menurut hakim telah kuat dengan bukti atau dengan pengakuan suami, sementara perbuatan menyakiti termasuk penyebab tidak langgengnya rumah tangga antara keduanya, di samping itu hakim juga sudah tidak bisa mendamaikan keduanya maka hakim memutuskan ikatan perkawinan keduanya dengan talak satu bain (sughra).

Menimbang, bahwa setelah putusan ini berkekuatan hukum tetap, maka bagi Penggugat terdapat waktu tunggu (masa *iddah*) selama tiga kali suci (sekurang-kurangnya sembilan puluh hari) dikarenakan perceraian tersebut *ba'da dukhul* sebagaimana maksud Pasal 153 ayat (4) dan Pasal 155 Kompilasi Hukum Islam, maka pada masa tunggu tersebut Penggugat wajib menjaga dirinya, tidak menerima pinangan dan tidak menikah dengan pria lain, hal ini sebagaimana yang telah ditentukan pada Pasal 151 Kompilasi Hukum Islam;

Menimbang, bahwa sesuai dengan ketentuan Pasal 89 ayat (1) Undang-Undang Nomor 7 Tahun 1989 Tentang Peradilan Agama sebagaimana telah diubah dengan Undang-Undang Nomor 3 Tahun 2006, terakhir dengan perubahan kedua Undang-Undang Nomor 50 Tahun 2009, biaya yang timbul dalam perkara ini harus dibebankan kepada Penggugat;

Mengingat peraturan perundang-undangan yang berlaku dan hukum syar'i yang berkaitan dengan perkara ini;

**M E N G A D I L I**

1. Menyatakan Tergugat telah dipanggil secara resmi dan patut untuk menghadap ke persidangan, tidak hadir;
2. Mengabulkan gugatan Penggugat secara verstek;
3. Menjatuhkan talak satu bain sugra Tergugat (TERGUGAT) terhadap Penggugat (PENGGUGAT);
4. Membebankan kepada Penggugat untuk membayar biaya perkara sejumlah Rp. 666.000,- ( enam ratus enam puluh enam ribu rupiah);

Demikianlah diputuskan dalam musyawarah Majelis Hakim Pengadilan Agama Sengeti pada hari Rabu tanggal 23 Desember 2020 Masehi bertepatan



*Disclaimer*
*Kepaniteraan Mahkamah Agung Republik Indonesia berusaha untuk selalu mencantumkan informasi paling kini dan akurat sebagai bentuk komitmen Mahkamah Agung untuk pelayanan publik, transparansi dan akuntabilitas pelaksanaan fungsi peradilan. Namun dalam hal-hal tertentu masih dimungkinkan terjadi permasalahan teknis terkait dengan akurasi dan keterkinian informasi yang kami sajikan, hal mana akan terus kami perbaiki dari waktu kewaktu.*
*Dalam hal Anda menemukan inakurasi informasi yang termuat pada situs ini atau informasi yang seharusnya ada, namun belum tersedia, maka harap segera hubungi Kepaniteraan Mahkamah Agung RI melalui :*
*Email : kepaniteraan@mahkamahagung.go.id Telp : 021-384 3348 (ext.318)*

dengan tanggal 8 Jumadil Awal 1442 Hijriah oleh Dra. Ma'ripah sebagai Ketua Majelis, Dra. Emaneli, M.H. dan Suwarlan, S.H., masing-masing sebagai Hakim Anggota, putusan tersebut diucapkan pada hari itu juga dalam sidang terbuka untuk umum oleh Ketua Majelis beserta para Hakim Anggota tersebut, dan didampingi oleh Roza Miftahul Jannah, S.H., M.H. sebagai Panitera Pengganti, dengan dihadiri oleh Penggugat tanpa hadirnya Tergugat.

Hakim – Hakim Anggota, Ketua Majelis,

1. **Dra. Emaneli, M. H.** **Dra. Ma'ripah**

2. **Suwarlan, S.H.**

Panitera Pengganti,

**Roza Miftahul Jannah, S.H., M.H.**

Rincian biaya:

| | | | |
|---|---|---|---|
| 1. | Biaya Pendaftaran | : | Rp. 30.000,- |
| 2. | Biaya ATK Perkara | : | Rp. 50.000,- |
| 3. | Biaya Panggilan | : | Rp. 550.000,- |
| 4. | PNBP Panggilan | : | Rp. 20.000,- |
| 5. | Biaya Redaksi | : | Rp. 10.000,- |
| 6. | Biaya Meterai | : | Rp. 6.000,- |
| | JUMLAH | **:** | Rp. 666.000,- |

(enam ratus enam puluh enam ribu rupiah).



*Disclaimer*
*Kepaniteraan Mahkamah Agung Republik Indonesia berusaha untuk selalu mencantumkan informasi paling kini dan akurat sebagai bentuk komitmen Mahkamah Agung untuk pelayanan publik, transparansi dan akuntabilitas pelaksanaan fungsi peradilan. Namun dalam hal-hal tertentu masih dimungkinkan terjadi permasalahan teknis terkait dengan akurasi dan keterkinian informasi yang kami sajikan, hal mana akan terus kami perbaiki dari waktu kewaktu.*
*Dalam hal Anda menemukan inakurasi informasi yang termuat pada situs ini atau informasi yang seharusnya ada, namun belum tersedia, maka harap segera hubungi Kepaniteraan Mahkamah Agung RI melalui :*
*Email : kepaniteraan@mahkamahagung.go.id Telp : 021-384 3348 (ext.318)*
