# Supplementary material for: Classification of divorce causes during the COVID-19 pandemic using convolutional neural networks
Source: PeerJ Comput Sci. 2022 Jun 30;8:e998. doi: 10.7717/peerj-cs.998 (PMC9299239; doi:10.7717/peerj-cs.998)
Supplement: Supplemental Information 2 [file peerj-cs-08-998-s002.zip › Kelalaian Dataset/Data ke-7.pdf]

Salinan

PUTUSAN

Nomor 7845/Pdt.G/2020/PA.Im.

بِسْمِ اللَّهِ الرَّحْمَٰنِ الرَّحِيمِ

DEMI KEADILAN BERDASARKAN KETUHANAN YANG MAHA ESA

Pengadilan Agama Indramayu yang memeriksa dan mengadili perkara tertentu dalam tingkat pertama dalam persidangan Majelis telah menjatuhkan putusan sebagai berikut dalam perkara cerai gugat antara :

PENGGUGAT, umur 22 tahun, agama Islam, pekerjaan TKW di Taiwan, tempat kediaman di Kabupaten Indramayu, dalam hal ini memberikan kuasa kepada GINANJAR YUSUF BUDIMAN, S.H., Advokat dari Kantor Hukum GINANJAR YUSUF BUDIMAN, S.H. & PARTNERS, yang beralamat di Blok Maja, Rt. 014 Rw. 003 Desa Larangan Kecamatan Lohbener Kabupaten Indramayu, berdasarkan Surat Kuasa yang terdaftar di Kepaniteraan Pengadilan Agama Indramayu Register Nomor 3595/2020 tanggal 2 Desember 2020, sebagai Penggugat;

m e l a w a n

TERGUGAT, umur 24 tahun, agama Islam, pekerjaan Wiraswasta, tempat kediaman di Kabupaten Indramayu, sebagai Tergugat;

Pengadilan Agama tersebut ;

Telah mempelajari berkas perkara ;

Telah mendengar keterangan Penggugat dan para saksi di muka sidang;

DUDUK PERKARA

Bahwa Penggugat dalam surat gugatannya tertanggal 02 Desember 2020 yang didaftarkan di Kepaniteraan Pengadilan Agama Indramayu dengan Nomor: 7845/Pdt.G/2020/PA.Im, telah mengajukan gugatan dengan dalil-dalil sebagai berikut :

Disclaimer
Kepaniteraan Mahkamah Agung Republik Indonesia berusaha untuk selalu mencantumkan informasi paling kini dan akurat sebagai bentuk komitmen Mahkamah Agung untuk pelayanan publik, transparansi dan akuntabilitas pelaksanaan fungsi peradilan. Namun dalam hal-hal tertentu masih dimungkinkan terjadi permasalahan teknis terkait dengan akurasi dan keterkinian informasi yang kami sajikan, hal mana akan terus kami perbaiki dari waktu kewaktu.
Dalam hal Anda menemukan inakurasi informasi yang termuat pada situs ini atau informasi yang seharusnya ada, namun belum tersedia, maka harap segera hubungi Kepaniteraan Mahkamah Agung RI melalui :
Email : kepaniteraan@mahkamahagung.go.id   Telp : 021-384 3348 (ext.318)

1. Bahwa Penggugat dengan Tergugat telah melangsungkan perkawinan pada tanggal 19 Desember tahun 2016 dan dicatat di Kantor Urusan Agama Kecamatan Lohbener, Kabupaten Indramayu dengan Kutipan Akta Nikah Nomor:0590/030/XII/2016 Tanggal 19 Desember 2016;
2. Bahwa setelah pernikahan tersebut Penggugat dengan Tergugat bertempat tinggal di tempat kediaman orang tua Tergugat dan telah hidup rukun sebagaimana layaknya suami isteri;
3. Bahwa selama pernikahan Penggugat dengan Tergugat belum dikaruniai anak ;
4. Bahwa Penggugat selama rumah tangga dengan Tergugat belum pernah bercerai;
5. Bahwa pada awalnya rumah tangga Penggugat dengan Tergugat berjalan dengan baik dan harmonis, namun kurang lebih sejak bulan Juni tahun 2017 sering terjadi perselisihan dan pertengkaran yang penyebabnya Tergugat tidak dapat memenuhi kebutuhan ekonomi rumah tangga dan pernah berbicara Menalak Tiga kepada Penggugat sehingga membuat perasaan Penggugat Sakit Hati;
6. Bahwa untuk memenuhi Kebutuhan Hidup, Penggugat Pergi Bekerja Keluar Negeri Di Negara Taiwan bulan November 2018 ;
7. Bahwa puncak perselisihan dan pertengkaran tersebut terjadi pada bulan April tahun 2019, Tergugat Pernah menghubungi Penggugat lewat Handphone untuk rujuk namun hal itu tidak dapat diterima Penggugat karena rasa kecewa dan sakit hati, yang akibatnya sejak itu Penggugat dan Tergugat Telah Putus Hubungan (Lost Contact) Selama Lebih Kurang selama 1 tahun 6 Bulan;
8. Bahwa atas kejadian tersebut, Penggugat merasa sudah tidak ada harapan lagi untuk hidup rukun membina rumah tangga bersama Tergugat, karena meskipun telah diupayakan perdamaian, tetapi tetap tidak berhasil;

Bahwa berdasarkan alasan atau dalil-dalil di atas, Penggugat dengan hormat mohon kiranya Majelis Hakim Pengadilan Agama Indramayu untuk memeriksa dan mengadili perkara ini, selanjutnya menjatuhkan putusan yang amarnya berbunyi :

Disclaimer
Kepaniteraan Mahkamah Agung Republik Indonesia berusaha untuk selalu mencantumkan informasi paling kini dan akurat sebagai bentuk komitmen Mahkamah Agung untuk pelayanan publik, transparansi dan akuntabilitas pelaksanaan fungsi peradilan. Namun dalam hal-hal tertentu masih dimungkinkan terjadi permasalahan teknis terkait dengan akurasi dan keterkinian informasi yang kami sajikan, hal mana akan terus kami perbaiki dari waktu kewaktu.
Dalam hal Anda menemukan inakurasi informasi yang termuat pada situs ini atau informasi yang seharusnya ada, namun belum tersedia, maka harap segera hubungi Kepaniteraan Mahkamah Agung RI melalui :
Email : kepaniteraan@mahkamahagung.go.id Telp : 021-384 3348 (ext.318)

1. Mengabulkan Gugatan Penggugat seluruhnya;
2. Menjatuhkan Talak Tergugat kepada Penggugat;
3. Membebankan biaya perkara menurut hukum;

Atau apabila Majelis Hakim berpendapat lain, mohon kiranya memberikan putusan yang seadil-adilnya (ex aequo et bono).;

Bahwa pada hari persidangan yang telah ditetapkan, Kuasa Hukum Penggugat menghadap di persidangan, sedangkan Tergugat tidak menghadap dan tidak menyuruh orang lain untuk menghadap sebagai wakilnya yang sah meskipun telah dipanggil dengan cara yang sah dan patut, sedangkan tidak ternyata bahwa ketidakhadirannya itu disebabkan oleh suatu alasan yang sah ;

Bahwa lalu pemeriksaan dimulai dengan membacakan surat gugatan Penggugat yang isinya tetap dipertahankan oleh Penggugat;

Bahwa untuk meneguhkan dalil-dalil gugatannya, Penggugat telah mengajukan bukti surat berupa Foto copy Kutipan Akta Nikah Nomor : 0590/030/XII/2016 tanggal 19 Desember 2016 yang dikeluarkan oleh Kantor Urusan Agama Kecamatan Lohbener , telah dicocokkan dengan aslinya dan telah bermaterai cukup, kemudian diberi tanda (P.1);

Bahwa disamping itu, Penggugat juga mengajukan saksi-saksi sebagai berikut :

1. **SAKSI I**, umur 40 tahun, agama Islam, pekerjaan Wiraswasta, tempat kediaman di RT.19 RW. 04 Desa Langut Kecamatan Lohbener Kabupaten Indramayu; dibawah sumpah menerangkan pada pokoknya sebagai berikut :
   - Bahwa saksi kenal Penggugat dan Tergugat karena Paman Penggugat;
   - Bahwa semula rumah tangga Penggugat dan Tergugat rukun dan harmonis, namun sejak bulan Juni tahun 2017 rumah tangganya mulai retak, karena sering terjadi perselisihan dan pertengkaran;
   - Bahwa penyebabnya Tergugat tidak dapat memenuhi kebutuhan ekonomi rumah tangga dan pernah berbicara Menalak Tiga kepada Penggugat sehingga membuat perasaan Penggugat Sakit Hati;
   - Bahwa sejak satu tahun enam bulan yang lalu Penggugat dan Tergugat telah berpisah tempat tinggal dan selama itu tidak pernah berkumpul kembali;

Disclaimer
Kepaniteraan Mahkamah Agung Republik Indonesia berusaha untuk selalu mencantumkan informasi paling kini dan akurat sebagai bentuk komitmen Mahkamah Agung untuk pelayanan publik, transparansi dan akuntabilitas pelaksanaan fungsi peradilan. Namun dalam hal-hal tertentu masih dimungkinkan terjadi permasalahan teknis terkait dengan akurasi dan keterkinian informasi yang kami sajikan, hal mana akan terus kami perbaiki dari waktu kewaktu.
Dalam hal Anda menemukan inakurasi informasi yang termuat pada situs ini atau informasi yang seharusnya ada, namun belum tersedia, maka harap segera hubungi Kepaniteraan Mahkamah Agung RI melalui :
Email : kepaniteraan@mahkamahagung.go.id Telp : 021-384 3348 (ext.318)

- Bahwa saksi pernah menasehati Penggugat dan Tergugat tetapi tidak berhasil;

2. **SAKSI II**, umur 40 tahun, agama Islam, pekerjaan Pedagang, tempat kediaman di RT.19 RW. 04 Desa Langut Kecamatan Lohbener Kabupaten Indramayu ; dibawah sumpah menerangkan pada pokoknya sebagai berikut :
   - Bahwa saksi kenal Penggugat dan Tergugat karena tetangga Penggugat;
   - Bahwa semula rumah tangga Penggugat dan Tergugat rukun dan harmonis, namun sejak bulan Juni tahun 2017 rumah tangganya mulai retak, karena sering terjadi perselisihan dan pertengkaran;
   - Bahwa penyebabnya Tergugat tidak dapat memenuhi kebutuhan ekonomi rumah tangga dan pernah berbicara Menalak Tiga kepada Penggugat sehingga membuat perasaan Penggugat Sakit Hati;
   - Bahwa sejak satu tahun enam bulan yang lalu Penggugat dan Tergugat telah berpisah tempat tinggal dan selama itu tidak pernah berkumpul kembali;
   - Bahwa saksi pernah menasehati Penggugat dan Tergugat tetapi tidak berhasil;

Bahwa Penggugat dalam kesimpulannya tetap pada gugatan semula untuk diceraikan dengan Tergugat dan selanjutnya mohon putusan;

Bahwa selanjutnya untuk mempersingkat uraian pertimbangan ini ditunjuk kepada hal-hal sebagaimana tercantum dalam berita acara persidangan perkara ini;

PERTIMBANGAN HUKUM

Menimbang, bahwa maksud dan tujuan gugatan Penggugat adalah sebagaimana terurai diatas;

Menimbang, bahwa Tergugat tidak menghadap ke muka sidang, dan tidak pula menyuruh orang lain sebagai kuasanya meskipun telah dipanggil secara resmi dan patut, dan ketidak hadirannya itu tidak disebabkan alasan yang sah menurut hukum, oleh karenanya harus dinyatakan tidak hadir ;

Menimbang, bahwa Penggugat dalam gugatannya mendalilkan telah kawin dengan Tergugat dan ikatan perkawinan tersebut tidak pernah putus,

Disclaimer
Kepaniteraan Mahkamah Agung Republik Indonesia berusaha untuk selalu mencantumkan informasi paling kini dan akurat sebagai bentuk komitmen Mahkamah Agung untuk pelayanan publik, transparansi dan akuntabilitas pelaksanaan fungsi peradilan. Namun dalam hal-hal tertentu masih dimungkinkan terjadi permasalahan teknis terkait dengan akurasi dan keterkinian informasi yang kami sajikan, hal mana akan terus kami perbaiki dari waktu kewaktu.
Dalam hal Anda menemukan inakurasi informasi yang termuat pada situs ini atau informasi yang seharusnya ada, namun belum tersedia, maka harap segera hubungi Kepaniteraan Mahkamah Agung RI melalui :
Email : kepaniteraan@mahkamahagung.go.id   Telp : 021-384 3348 (ext.318)

dengan demikian Penggugat mempunyai legal standing untuk mengajukan gugat cerai;

Menimbang, bahwa berdasarkan bukti P.1 berupa akta autentik yang memiliki kekuatan pembuktian sempurna dan mengikat, harus dinyatakan terbukti bahwa antara Penggugat dan Tergugat telah terikat dalam perkawinan yang sah sesuai dengan ketentuan Pasal 7 ayat (1) Kompilasi Hukum Islam;

Menimbang, bahwa Penggugat mendalilkan sejak Juni 2017 rumah tangga Penggugat dengan Tergugat mulai retak, sering terjadi perselisihan dan pertengkaran karena Tergugat tidak dapat memenuhi kebutuhan ekonomi rumah tangga dan pernah berbicara Menalak Tiga kepada Penggugat sehingga membuat perasaan Penggugat Sakit Hati;

Menimbang, bahwa atas bukti yang diajukan Penggugat, Majelis menilai kesaksian yang diberikan oleh kedua saksi Penggugat didasarkan pada pengetahuan, penglihatan dan pendengaran langsung saksi dan keterangannya bersesuaian antara satu dengan lainnya dan masing-masing saksi tersebut adalah keluarga dekatnya, maka berdasarkan Pasal 171 dan 172 HIR Jo Pasal 76 Undang-undang Nomor 7 Tahun 1989 yang telah diubah dengan Undang-undang Nomor 3 Tahun 2006 dan terakhir diubah dengan Undang-undang Nomor 50 Tahun 2009 Jo. Pasal 22 Peraturan Pemerintah Nomor 9 Tahun 1975, saksi-saksi tersebut dipandang telah memenuhi syarat formil dan materil sebagai saksi, maka keterangan saksi tersebut telah mempunyai nilai pembuktian;

Menimbang, bahwa berdasarkan dalil-dalil gugatan Penggugat yang dikuatkan oleh keterangan dua orang saksi sebagaimana terurai dalam duduk perkara terurai diatas maka Majelis telah dapat menemukan fakta-fakta di persidangan sebagai berikut;

- Bahwa dalam rumah tangga Penggugat dan Tergugat telah terjadi ketidak harmonisan;
- Bahwa penyebab ketidak harmonisan ialah karena Tergugat tidak dapat memenuhi kebutuhan ekonomi rumah tangga dan pernah berbicara Menalak Tiga kepada Penggugat sehingga membuat perasaan Penggugat Sakit Hati;

Disclaimer
Kepaniteraan Mahkamah Agung Republik Indonesia berusaha untuk selalu mencantumkan informasi paling kini dan akurat sebagai bentuk komitmen Mahkamah Agung untuk pelayanan publik, transparansi dan akuntabilitas pelaksanaan fungsi peradilan. Namun dalam hal-hal tertentu masih dimungkinkan terjadi permasalahan teknis terkait dengan akurasi dan keterkinian informasi yang kami sajikan, hal mana akan terus kami perbaiki dari waktu kewaktu.
Dalam hal Anda menemukan inakurasi informasi yang termuat pada situs ini atau informasi yang seharusnya ada, namun belum tersedia, maka harap segera hubungi Kepaniteraan Mahkamah Agung RI melalui :
Email : kepaniteraan@mahkamahagung.go.id Telp : 021-384 3348 (ext.318)

- Bahwa Penggugat dan Tergugat telah berpisah tempat tinggal sejak satu tahun enam bulan yang lalu;
- Bahwa saksi-saksi pernah menasehati Penggugat dan Tergugat tetapi tidak berhasil;

Menimbang, bahwa berdasarkan fakta-fakta tersebut diatas Majelis berpendapat bahwa Penggugat telah dapat membuktikan dalil-dalil gugat cerainya dan alasan-alasan gugat cerainya tersebut telah memenuhi Pasal 39 ayat (2) Undang-undang No. 1 Tahun 1974 jo Pasal 19 huruf (f) Peraturan Pemerintah No. 9 Tahun 1975 jo Pasal 116 huruf (f) Kompilasi Hukum Islam, maka gugatan Penggugat patut dikabulkan dengan verstek;

Menimbang, bahwa perkara aquo termasuk perkara bidang perkawinan, maka berdasarkan Pasal 89 ayat (1) Undang-undang Nomor 7 Tahun 1989 yang telah diubah dengan Undang-undang Nomor 3 Tahun 2006 dan terakhir diubah dengan Undang-undang Nomor 50 Tahun 2009 Tentang Peradilan Agama, seluruh biaya perkara ini dibebankan kepada Penggugat;

Mengingat Pasal 125 HIR dan segala peraturan perundang-undangan serta ketentuan hukum syara' yang berkaitan dengan perkara ini ;

M E N G A D I L I

1. Menyatakan Tergugat yang telah dipanggil dengan resmi dan patut untuk menghadap sidang, tidak hadir;
2. Mengabulkan gugatan Penggugat dengan verstek;
3. Menjatuhkan talak satu ba'in shughra Tergugat (TERGUGAT) terhadap Penggugat (PENGGUGAT);
4. Menghukum Penggugat untuk membayar biaya perkara sejumlah Rp. 491.000,- (empat ratus sembilan puluh satu ribu rupiah);

Demikian diputuskan dalam rapat permusyawaratan Majelis Hakim yang dilangsungkan pada hari Rabu tanggal 23 Desember 2020 Masehi bertepatan dengan tanggal 8 Jumadilawal 1442 Hijriyah, oleh kami **Drs. AKHMAD TOPURUDIN, M.H.** sebagai Ketua Majelis serta **Drs. HAMIDUDDIN** dan **Drs. H. AHMAD FAUZI, S.H., M.H.** masing-masing sebagai Hakim Anggota, putusan

Disclaimer
Kepaniteraan Mahkamah Agung Republik Indonesia berusaha untuk selalu mencantumkan informasi paling kini dan akurat sebagai bentuk komitmen Mahkamah Agung untuk pelayanan publik, transparansi dan akuntabilitas pelaksanaan fungsi peradilan. Namun dalam hal-hal tertentu masih dimungkinkan terjadi permasalahan teknis terkait dengan akurasi dan keterkinian informasi yang kami sajikan, hal mana akan terus kami perbaiki dari waktu kewaktu.
Dalam hal Anda menemukan inakurasi informasi yang termuat pada situs ini atau informasi yang seharusnya ada, namun belum tersedia, maka harap segera hubungi Kepaniteraan Mahkamah Agung RI melalui :
Email : kepaniteraan@mahkamahagung.go.id Telp : 021-384 3348 (ext.318)

tersebut diucapkan dalam sidang terbuka untuk umum pada hari Rabu tanggal 23 Desember 2020 Masehi bertepatan dengan tanggal 8 Jumadilawal 1442 Hijriyah oleh Ketua Majelis tersebut dengan didampingi oleh Hakim Anggota dan dibantu oleh **ERIKA ANGGRAENI YULISTIANINGSIH, S.H.** sebagai Panitera Pengganti serta dihadiri oleh Pihak Penggugat tanpa hadirnya Tergugat ;

Hakim Anggota	Ketua Majelis
ttd	ttd
Drs. HAMIDUDDIN	Drs. AKHMAD TOPURUDIN, M.H.
Hakim Anggota	
ttd	
Drs. H. AHMAD FAUZI, S.H., M.H.	
	Panitera Pengganti
	ttd
	ERIKA ANGGRAENI YULISTIANINGSIH, S.H.

Perincian Biaya Perkara :

1. Biaya Pendaftaran	Rp. 30.000,-
2. Biaya Proses	Rp. 50.000,-
3. Biaya Panggilan	Rp. 375.000,-

Disclaimer
Kepaniteraan Mahkamah Agung Republik Indonesia berusaha untuk selalu mencantumkan informasi paling kini dan akurat sebagai bentuk komitmen Mahkamah Agung untuk pelayanan publik, transparansi dan akuntabilitas pelaksanaan fungsi peradilan. Namun dalam hal-hal tertentu masih dimungkinkan terjadi permasalahan teknis terkait dengan akurasi dan keterkinian informasi yang kami sajikan, hal mana akan terus kami perbaiki dari waktu kewaktu.
Dalam hal Anda menemukan inakurasi informasi yang termuat pada situs ini atau informasi yang seharusnya ada, namun belum tersedia, maka harap segera hubungi Kepaniteraan Mahkamah Agung RI melalui :
Email : kepaniteraan@mahkamahagung.go.id Telp : 021-384 3348 (ext.318)

4. PNBP Panggilan	Rp. 20.000
5. Biaya Redaksi	Rp. 10.000,-
6. Biaya materai	Rp. 6.000,-
Jumlah	Rp. 491.000,-

Salinan Putusan ini sesuai dengan Aslinya
Pengadilan Agama Indramayu
Panitera,

Drs. H. HARUN AL RASYID

Disclaimer
Kepaniteraan Mahkamah Agung Republik Indonesia berusaha untuk selalu mencantumkan informasi paling kini dan akurat sebagai bentuk komitmen Mahkamah Agung untuk pelayanan publik, transparansi dan akuntabilitas pelaksanaan fungsi peradilan. Namun dalam hal-hal tertentu masih dimungkinkan terjadi permasalahan teknis terkait dengan akurasi dan keterkinian informasi yang kami sajikan, hal mana akan terus kami perbaiki dari waktu kewaktu.
Dalam hal Anda menemukan inakurasi informasi yang termuat pada situs ini atau informasi yang seharusnya ada, namun belum tersedia, maka harap segera hubungi Kepaniteraan Mahkamah Agung RI melalui :
Email : kepaniteraan@mahkamahagung.go.id Telp : 021-384 3348 (ext.318)
