# Supplementary material for: Classification of divorce causes during the COVID-19 pandemic using convolutional neural networks
Source: PeerJ Comput Sci. 2022 Jun 30;8:e998. doi: 10.7717/peerj-cs.998 (PMC9299239; doi:10.7717/peerj-cs.998)
Supplement: Supplemental Information 2 [file peerj-cs-08-998-s002.zip › Kelalaian Dataset/Data ke-8.pdf]

P U T U S A N

Nomor 3940/Pdt.G/2020/PA.Bks.

بسم الله الرحمن الرحيم

DEMI KEADILAN BERDASARKAN KETUHANAN YANG MAHA ESA

Pengadilan Agama Bekasi yang memeriksa dan mengadili perkara-perkara tertentu pada tingkat pertama dalam persidangan Majelis telah menjatuhkan putusan sebagai berikut dalam perkara Cerai Gugat antara :

XXX, umur 21 tahun, agama Islam, pendidikan terakhir SMK, pekerjaan Wiraswasta, tempat tinggal di XXX, selanjutnya disebut sebagai “**Penggugat**”;

Melawan

XXX, lahir di Bekasi 17 April 1996 (umur 24 tahun), agama Islam, pendidikan terakhir SMA, pekerjaan Karyawan Swasta, tempat tinggal di XXX, selanjutnya disebut sebagai **“Tergugat”;**

Pengadilan Agama tersebut;
Telah membaca surat-surat yang terkait dengan perkara ini;
Telah mendengar keterangan Penggugat dan Tergugat;
Telah memperhatikan bukti surat-surat dan mendengar keterangan saksi-saksi di persidangan;

DUDUK PERKARA

Bahwa, Penggugat dengan surat gugatannya tertanggal 24 November 2020 yang telah terdaftar di Kepaniteraan Pengadilan Agama Bekasi dengan register Nomor 3940/Pdt.G/2020/PA.Bks., tanggal yang sama telah mengemukakan hal-hal sebagai berikut :

1. Bahwa, pada tanggal 11 Juni 2019, Penggugat dengan Tergugat melangsungkan pernikahan yang dicatat oleh Pegawai Pencatat Nikah

Disclaimer
Kepaniteraan Mahkamah Agung Republik Indonesia berusaha untuk selalu mencantumkan informasi paling kini dan akurat sebagai bentuk komitmen Mahkamah Agung untuk pelayanan publik, transparansi dan akuntabilitas pelaksanaan fungsi peradilan. Namun dalam hal-hal tertentu masih dimungkinkan terjadi permasalahan teknis terkait dengan akurasi dan keterkinian informasi yang kami sajikan, hal mana akan terus kami perbaiki dari waktu kewaktu.
Dalam hal Anda menemukan inakurasi informasi yang termuat pada situs ini atau informasi yang seharusnya ada, namun belum tersedia, maka harap segera hubungi Kepaniteraan Mahkamah Agung RI melalui :
Email : kepaniteraan@mahkamahagung.go.id Telp : 021-384 3348 (ext.318)

Kantor Urusan Agama Kecamatan XXX, sesuai Kutipan Akta Nikah Nomor: XXX tertanggal 11 Juni 2019

2. Bahwa, setelah pernikahan tersebut Penggugat dengan Tergugat bertempat tinggal tinggal di XXX;

3. Bahwa, dalam perkawinan tersebut Penggugat dengan Tergugat telah behubungan sebagaimana layaknya suami dan istri (ba'da dukhul), dan belum dikaruniai anak hingga saat ini;

4. Bahwa, pada awalnya rumah tangga Penggugat dan Tergugat rukun dan harmonis, akan tetapi sejak bulan September 2020 rumah tangga Penggugat dan Tergugat sering terjadi pertengkaran, yang penyebabnya antara lain :

4.1. Bahwa Tergugat sering perhitungan kepada Penggugat seperti sesuatu yang pernah Tergugat berikan selalu diungkit-ungkit kembali;

4.2. Bahwa Tergugat kurang bertanggung bertanggung jawab sebagai suami dan Tergugat seenaknya saja dalam memberikan nafkah lahir kepada Penggugat, karena Tergugat hanya memberikan nafkah lahir sebesar Rp. 500.000,- (lima ratus ribu rupiah) sebulan;

4.3. Bahwa Tergugat setiap kali bertengkar dengan Penggugat selalu mengucapkan kata cerai kepada Penggugat;

4.4. Bahwa Tergugat sering berkata kasar hingga menyakiti hati Pengugat seperti kata-kata istri yang kurang ngajar dan lainnya;

5. Bahwa, puncaknya pada 14 November 2020 dimana antara Pengugat dan Tergugat terjadi pertengkaran, lalu Tergugat keluar dari rumah kediaman bersama dan sejak saat itu sudah tidak terjalin hubungan sebagaimana layaknya suami dan istri hingga saat ini;

6. Bahwa ikatan perkawinan antara Penggugat dan Tergugat sebagaimana diuraikan di atas sudah sulit dibina untuk membentuk suatu rumah tangga yang sakinah, mawaddah wa rahmah sebagaimana maksud dan tujuan dari suatu perkawinan, sehingga lebih baik diputus karena perceraian;

7. Bahwa, dengan fakta-fakta tersebut diatas gugatan Penggugat telah memenuhi alasan perceraian sebagaimana diatur dalam Pasal 19 PP No. 9 Tahun 1975 jo. Pasal 116 Kompilasi Hukum Islam;

Disclaimer
Kepaniteraan Mahkamah Agung Republik Indonesia berusaha untuk selalu mencantumkan informasi paling kini dan akurat sebagai bentuk komitmen Mahkamah Agung untuk pelayanan publik, transparansi dan akuntabilitas pelaksanaan fungsi peradilan. Namun dalam hal-hal tertentu masih dimungkinkan terjadi permasalahan teknis terkait dengan akurasi dan keterkinian informasi yang kami sajikan, hal mana akan terus kami perbaiki dari waktu kewaktu.
Dalam hal Anda menemukan inakurasi informasi yang termuat pada situs ini atau informasi yang seharusnya ada, namun belum tersedia, maka harap segera hubungi Kepaniteraan Mahkamah Agung RI melalui :
Email : kepaniteraan@mahkamahagung.go.id   Telp : 021-384 3348 (ext.318)

8. Bahwa, terhadap biaya yang timbul akibat perkara ini agar dibebankan menurut Peraturan Perundang-Undangan;

Berdasarkan hal-hal tersebut di atas, Pemohon memohon agar Ketua Pengadilan Agama Bekasi Bekasi C.q. Majelis Hakim yang memeriksa dan mengadili perkara ini, menjatuhkan putusan yang amarnya berbunyi sebagai berikut :

PRIMAIR:

1. Mengabulkan gugatan Penggugat;
2. Menjatuhkan talak 1 (satu) ba'in sugra Tergugat (XXX) terhadap Penggugat (XXX);
3. Membebankan biaya perkara kepada Penggugat sesuai dengan hukum yang berlaku;

SUBSIDAIR:

Dan, atau apabila Majelis Hakim berpendapat lain mohon untuk menjatuhkan putusan yang seadil-adilnya (ex aequo et bono);

Bahwa, pada hari-hari sidang yang telah ditentukan, Penggugat dan Tergugat datang sendiri menghadap ke persidangan, Majelis Hakim telah berupaya untuk mendamaikan kedua belah pihak yang berperkara, akan tetapi upaya tersebut tetap tidak berhasil;

Bahwa, Majelis Hakim telah memerintahkan kepada kedua belah pihak untuk melaksanakan mediasi sebagaimana amanat Peraturan Mahkamah Agung Republik Indonesia Nomor 1 Tahun 2016 tentang Prosedur Mediasi, di Pengadilan dengan Mediator yang telah ditunjuk dan dipilih oleh kedua belah pihak, yaitu Drs. H.M. Fadjri Rivai, S.H., M.H. (Mediator Non Hakim pada Pengadilan Agama Bekasi), akan tetapi berdasarkan laporan mediasi tanggal 07 Desember 2020 upaya mediasi tidak berhasil;

Bahwa pemeriksaan perkara dilanjutkan dengan membacakan surat gugatan tersebut yang atas pertanyaan Ketua Majelis, Penggugat menyatakan tetap bertahan dengan isi gugatannya;

Disclaimer
Kepaniteraan Mahkamah Agung Republik Indonesia berusaha untuk selalu mencantumkan informasi paling kini dan akurat sebagai bentuk komitmen Mahkamah Agung untuk pelayanan publik, transparansi dan akuntabilitas pelaksanaan fungsi peradilan. Namun dalam hal-hal tertentu masih dimungkinkan terjadi permasalahan teknis terkait dengan akurasi dan keterkinian informasi yang kami sajikan, hal mana akan terus kami perbaiki dari waktu kewaktu.
Dalam hal Anda menemukan inakurasi informasi yang termuat pada situs ini atau informasi yang seharusnya ada, namun belum tersedia, maka harap segera hubungi Kepaniteraan Mahkamah Agung RI melalui :
Email : kepaniteraan@mahkamahagung.go.id   Telp : 021-384 3348 (ext.318)

Bahwa, atas gugatan Penggugat tersebut, Tergugat tidak memberikan jawaban atau tanggapan apapun, karena Tergugat tidak pernah hadir lagi di persidangan meskipun telah dipanggil secara resmi dan patut;

Bahwa, untuk memperkuat dalil gugatannya, Penggugat telah mengajukan bukti tertulis berupa :

1. Fotokopi Kartu Tanda Penduduk No. XXX atas nama Penggugat dari Kelurahan Ciketing Udik Kecamatan Bantar Gebang (Bukti P.1);
2. Fotokopi Kutipan Akta Nikah dari Kantor Urusan Agama Kecamatan XXX Nomor : XXX Tanggal 11 Juni 2019 (Bukti P.2);

Menimbang, bahwa selain bukti-bukti tertulis sebagaimana tersebut di atas, Penggugat juga telah menghadirkan saksi-saksi sebagai berikut :

1. XXX, umur 42 tahun, agama Islam, pekerjaan Ibu rumah tangga, tempat tinggal di XXX, saksi bersumpah di muka sidang dan saksi menerangkan pada pokoknya sebagai berikut :

- Bahwa saksi adalah Ibu kandung Penggugat;
- Bahwa saksi kenal dengan Tergugat selaku suami Penggugat, mereka menikah di XXX pada tahun 2019 saksi hadir pada saat pernikahan mereka dilangsungkan;
- Bahwa setelah menikah Penggugat dan Tergugat mengambil tempat kediaman di XXX dan belum dikaruniai anak;
- Bahwa awalnya rumah tangga Penggugat dan Tergugat rukun dan harmonis, namun sejak September 2020 tidak harmonis lagi, sering terjadi perselisihan dan pertengkaran, yang menurut Penggugat disebabkan factor keuangan karena Nafkah yang diberikan Tergugat tidak mencukupi untuk kebutuhan sehari-hari;
- Bahwa saksi pernah melihat mereka bertengkar;
- Bahwa Penggugat dan Tergugat telah pisah rumah sejak bulan November 2020 yang lalu, Tergugat yang pergi meninggalkan rumah bersama;
- Bahwa pihak keluarga Penggugat, sudah berupaya mendamaikan mereka, namun tidak berhasil dan saksi tidak sanggup lagi merukunkan mereka;

Disclaimer
Kepaniteraan Mahkamah Agung Republik Indonesia berusaha untuk selalu mencantumkan informasi paling kini dan akurat sebagai bentuk komitmen Mahkamah Agung untuk pelayanan publik, transparansi dan akuntabilitas pelaksanaan fungsi peradilan. Namun dalam hal-hal tertentu masih dimungkinkan terjadi permasalahan teknis terkait dengan akurasi dan keterkinian informasi yang kami sajikan, hal mana akan terus kami perbaiki dari waktu kewaktu.
Dalam hal Anda menemukan inakurasi informasi yang termuat pada situs ini atau informasi yang seharusnya ada, namun belum tersedia, maka harap segera hubungi Kepaniteraan Mahkamah Agung RI melalui :
Email : kepaniteraan@mahkamahagung.go.id Telp : 021-384 3348 (ext.318)

2. XXX, umur 35 tahun, agama Islam, pekerjaan Karyawan Swasta, tempat tinggal di XXX, saksi bersumpah di muka sidang dan saksi menerangkan pada pokoknya sebagai berikut :

-Bahwa saksi adalah sudara sepupu Penggugat;

-Bahwa saksi kenal dengan Tergugat selaku suami Penggugat, mereka menikah di XXX tahun 2019;

-Bahwa setelah menikah Penggugat dan Tergugat mengambil tempat kediaman di XXX dan belum mempunyai anak;

-Bahwa awalnya rumah tangga Penggugat dan Tergugat rukun dan harmonis, namun sejak bulan September 2020 tidak harmonis lagi, sering terjadi perselisihan dan pertengkaran, yang menurut Penggugat disebabkan Tergugat tidak terbuka dalam masalah keuangan;

-Bahwa Penggugat dan Tergugat telah pisah rumah sejak 3 bulan yang lalu, Tergugat yang pergi dari rumah bersama;

-Bahwa pihak keluarga Penggugat, sudah berupaya mendamaikan mereka, namun tidak berhasil dan saksi tidak sanggup lagi merukunkan mereka;

Bahwa, atas keterangan saksi tersebut, Penggugat membenarkan dan menyatakan tidak keberatan;

Bahwa, Penggugat menyatakan tidak akan mengajukan bukti apapun lagi di persidangan dan telah menyampaikan kesimpulannya secara lisan yang pada pokoknya tetap dengan keinginannya semula untuk mengakhiri perkawinan dengan Tergugat dengan perceraian dan mohon putusan;

Bahwa, untuk menyingkat uraian putusan ini, maka ditunjuk Berita Acara Sidang perkara ini sebagai bagian yang tidak terpisahkan dari putusan;

PERTIMBANGAN HUKUM

Menimbang, bahwa maksud dan tujuan gugatan Penggugat adalah seperti diuraikan tersebut di atas;

Menimbang, bahwa untuk memenuhi ketentuan Pasal 130 ayat (1) HIR jo. 82 ayat (1) dan (4) Undang-Undang Republik Indonesia Nomor 7 Tahun

Disclaimer
Kepaniteraan Mahkamah Agung Republik Indonesia berusaha untuk selalu mencantumkan informasi paling kini dan akurat sebagai bentuk komitmen Mahkamah Agung untuk pelayanan publik, transparansi dan akuntabilitas pelaksanaan fungsi peradilan. Namun dalam hal-hal tertentu masih dimungkinkan terjadi permasalahan teknis terkait dengan akurasi dan keterkinian informasi yang kami sajikan, hal mana akan terus kami perbaiki dari waktu kewaktu.
Dalam hal Anda menemukan inakurasi informasi yang termuat pada situs ini atau informasi yang seharusnya ada, namun belum tersedia, maka harap segera hubungi Kepaniteraan Mahkamah Agung RI melalui :
Email : kepaniteraan@mahkamahagung.go.id Telp : 021-384 3348 (ext.318)

1989 sebagaimana telah dirubah dan ditambah dengan Undang-Undang Republik Indonesia Nomor 3 Tahun 2006 dan Undang-Undang Republik Indonesia Nomor 50 Tahun 2009 jo. Pasal 39 ayat (1) Peraturan Pemerintah Nomor 9 Tahun 1975, Majelis Hakim telah berupaya mendamaikan kedua belah pihak berperkara, namun tidak berhasil;

Menimbang, bahwa sesuai amanat PERMA RI Nomor 1 Tahun 2016, Majelis Hakim telah pula memberikan kesempatan kepada kedua belah pihak untuk melakukan mediasi dengan menunjuk Mediator yang telah ditunjuk dan dipilih oleh kedua belah pihak berperkara, yaitu Drs. H.M. Fadri Rivai, S.H., M.H. dan berdasarkan laporan dari Mediator tanggal 07 Desember 2020 mediasi tersebut tidak berhasil;

Menimbang, bahwa Penggugat telah mendalilkan bahwa rumah tangganya sudah tidak rukun dan harmonis sejak bulan September 2020, sering terjadi perselisihan dan pertengkaran yang terus menerus yang sulit untuk dirukunkan lagi, yang puncaknya terjadi pada bulan November 2020, sehingga Penggugat dan Tergugat telah pisah rumah;

Menimbang, bahwa Tergugat tidak memberikan jawaban dan atau tanggapan apapun, karena tidak hadir di persidangan, sehingga Majelis Hakim berpendapat Tergugat telah membenarkan seluruh dalil-dalil gugatan Penggugat. Oleh karenanya Majelis Hakim berpendapat Penggugat telah berhasil membuktikan dalil-dalilnya mengenai adanya perselisihan dan pertengkaran, sehingga rumah tangga mereka sudah tidak harmonis lagi;

Menimbang, bahwa meskipun dalil-dalil gugatan Penggugat bahwa rumah tangga mereka sudah tidak rukun sering terjadi perselisihan dan pertengkaran telah terbukti, namun oleh karena dasar hukum yang diajukan oleh Penggugat dapat dikualifikasikan ke dalam alasan perceraian sebagaimana disebutkan dalam Pasal 19 huruf (f) Peraturan Pemerintah Nomor 9 Tahun 1975 jo. Pasal 116 huruf (f) Kompilasi Hukum Islam, yaitu antara Penggugat dengan Tergugat terus menerus terjadi perselisihan dan pertengkaran dan tidak ada lagi harapan akan dapat hidup rukun dalam membina rumah tangga, maka selain mengajukan bukti surat-surat, berdasarkan ketentuan Pasal 22 ayat (2) Peraturan Pemerintah Nomor 9 Tahun

Disclaimer
Kepaniteraan Mahkamah Agung Republik Indonesia berusaha untuk selalu mencantumkan informasi paling kini dan akurat sebagai bentuk komitmen Mahkamah Agung untuk pelayanan publik, transparansi dan akuntabilitas pelaksanaan fungsi peradilan. Namun dalam hal-hal tertentu masih dimungkinkan terjadi permasalahan teknis terkait dengan akurasi dan keterkinian informasi yang kami sajikan, hal mana akan terus kami perbaiki dari waktu kewaktu.
Dalam hal Anda menemukan inakurasi informasi yang termuat pada situs ini atau informasi yang seharusnya ada, namun belum tersedia, maka harap segera hubungi Kepaniteraan Mahkamah Agung RI melalui :
Email : kepaniteraan@mahkamahagung.go.id Telp : 021-384 3348 (ext.318)

1975, Penggugat juga telah menghadirkan saksi dari pihak keluarga dan atau orang dekat dengan kedua belah pihak, sedangkan Tergugat tidak menghadirkan saksi dari pihak keluarga dan atau orang dekat dengan kedua belah pihak karena tidak hadir lagi di persidangan;

Menimbang, bahwa Penggugat telah mengajukan bukti surat berupa P.1, dan P.2. Bukti-bukti tersebut telah bermeterai cukup sesuai dengan ketentuan Pasal 2 Undang-Undang Nomor 13 Tahun 1985 dan cocok dengan aslinya, sehingga secara formil dapat diterima sebagai alat bukti yang sah;

Menimbang, bahwa dari posita gugatan Penggugat telah jelas menunjukan adanya sengketa perkawinan dan dengan didasarkan kepada dalil Penggugat sendiri tentang domisili Penggugat yang berada di wilayah hukum Pengadilan Agama Bekasi yang diperkuat dengan bukti P.1, maka sesuai dengan Pasal 49 ayat (1) huruf a dan Pasal 73 ayat (1) dan (2) Undang-Undang Republik Indonesia Nomor 7 Tahun 1989 yang telah diubah dan ditambah dengan Undang-Undang Republik Indonesia Nomor 3 Tahun 2006 dan Undang-Undang Nomor 50 Tahun 2009, Pengadilan Agama Bekasi berwenang memeriksa dan mengadili perkara ini;

Menimbang, bahwa berdasarkan dalil gugatan yang diperkuat dengan bukti P.2 dan keterangan saksi-saksi di persidangan yang keterangannya saling bersesuaian, harus dinyatakan terbukti dengan sangat meyakinkan bahwa Penggugat dan Tergugat terikat dalam perkawinan yang sah dan saat ini telah dikaruniai 2 orang anak, sehingga Penggugat memiliki kapasitas untuk mengajukan gugatan ini terhadap Tergugat;

Menimbang, bahwa berdasarkan keterangan saksi-saksi di bawah sumpah di muka sidang, yang keterangannya saling bersesuaian, terungkap fakta :

- Bahwa Penggugat dan Tergugat adalah pasangan suami isteri yang sah;
- Bahwa rumah tangga Penggugat dan Tergugat sudah tidak rukun dan harmonis sejak September tahun 2020;
- Bahwa Penggugat dan Tergugat sering berselisih dan bertengkar;
- Bahwa Penggugat dan Tergugat sudah pisah rumah sejak November tahun 2020;

Disclaimer
Kepaniteraan Mahkamah Agung Republik Indonesia berusaha untuk selalu mencantumkan informasi paling kini dan akurat sebagai bentuk komitmen Mahkamah Agung untuk pelayanan publik, transparansi dan akuntabilitas pelaksanaan fungsi peradilan. Namun dalam hal-hal tertentu masih dimungkinkan terjadi permasalahan teknis terkait dengan akurasi dan keterkinian informasi yang kami sajikan, hal mana akan terus kami perbaiki dari waktu kewaktu.
Dalam hal Anda menemukan inakurasi informasi yang termuat pada situs ini atau informasi yang seharusnya ada, namun belum tersedia, maka harap segera hubungi Kepaniteraan Mahkamah Agung RI melalui :
Email : kepaniteraan@mahkamahagung.go.id   Telp : 021-384 3348 (ext.318)

- Bahwa pihak keluarga telah berupaya mendamaikan kedua belah pihak, akan tetapi tidak membuahkan hasil ke arah rukunnya Penggugat dan Tergugat;

Menimbang, bahwa berdasarkan keterangan saksi-saksi keluarga tersebut di atas, cukup memberi petunjuk bahwa Penggugat dan Tergugat sudah tidak rukun sebagai suami istri, dan keyakinan Majelis Hakim akan sikap kedua belah pihak di muka sidang yang sangat sulit berkomunikasi dengan baik di antara mereka dan sangat sulit untuk didamaikan ;

Menimbang, bahwa sulitnya mendamaikan kedua belah juga ditunjukkan dengan laporan tertulis dari Mediator yang telah ditunjuk dan dipilih oleh kedua belah pihak yang menyatakan bahwa antara kedua belah pihak tidak terjadi kesepakatan ke arah rukun, sehingga mediasi dalam hal perceraian dinyatakan tidak berhasil. Sementara selama proses persidangan berlangsung, Majelis Hakim selalu berupaya memberikan nasihat kepada Penggugat pihak agar tetap mempertahankan rumah tangga dan memberikan kesempatan yang cukup kepada kedua belah pihak untuk kembali memperbaiki kondisi rumah tangga mereka, namun upaya tersebut juga tidak membuahkan hasil, karena Penggugat tetap pada pendiriannya untuk mengakhiri perkawinan dengan perceraian;

Menimbang, bahwa dalam persidangan, Majelis Hakim telah pula mendengar keterangan keluarga dan atau orang dekat dari kedua belah pihak yang dalam hal ini sekaligus bertindak sebagai saksi Penggugat yang menyatakan tidak berhasil merukunkan Penggugat dan Tergugat;

Menimbang, bahwa fakta-fakta di atas adalah fakta tetap yang sangat meyakinkan Majelis Hakim bahwa dalam rumah tangga Penggugat dengan Tergugat telah terjadi perselisihan dan pertengkaran yang terus menerus yang sulit untuk dirukunkan;

Menimbang, bahwa bagaimanapun juga pasti ada sebab musabab dari terjadinya perselisihan atau pertengkaran tersebut dan berdasarkan keterangan saksi yang dihubungkan dengan keterangan Penggugat, bahwa perselisihan dan pertengkaran antara Penggugat dengan Tergugat terutama disebabkan karena Tergugat tidak cukup dalam memberikan nafkah kepada

Disclaimer
Kepaniteraan Mahkamah Agung Republik Indonesia berusaha untuk selalu mencantumkan informasi paling kini dan akurat sebagai bentuk komitmen Mahkamah Agung untuk pelayanan publik, transparansi dan akuntabilitas pelaksanaan fungsi peradilan. Namun dalam hal-hal tertentu masih dimungkinkan terjadi permasalahan teknis terkait dengan akurasi dan keterkinian informasi yang kami sajikan, hal mana akan terus kami perbaiki dari waktu kewaktu.
Dalam hal Anda menemukan inakurasi informasi yang termuat pada situs ini atau informasi yang seharusnya ada, namun belum tersedia, maka harap segera hubungi Kepaniteraan Mahkamah Agung RI melalui :
Email : kepaniteraan@mahkamahagung.go.id Telp : 021-384 3348 (ext.318)

Penggugat. Oleh karenanya, Majelis Hakim berpendapat bahwa rumah tangga Penggugat dengan Tergugat sudah tidak layak dipertahankan lagi, karena telah pecah dan sendi-sendinya telah rapuh serta sulit untuk ditegakkan kembali, sehingga dapat dinyatakan bahwa rumah tangga Penggugat dan Tergugat telah rusak (Broken marriage);

Menimbang, bahwa terlepas dari apapun yang melatar belakangi terjadinya perselisihan dan pertengkaran tersebut, yang nampak adalah bahwa antara Penggugat dengan Tergugat sudah pisah rumah sejak bulan November 2020 lalu, sehingga antara keduanya sudah tidak lagi terjalin komunikasi, karenanya kebahagiaan dan ketenteraman rumah tangga sudah sangat tidak mungkin lagi dapat tercapai, maka sesuai dengan Yurisprudensi Mahkamah Agung RI Nomor 379 K/AG/1995 tanggal 26 Maret 1997, yang menyatakan: ”Suami isteri yang tidak berdiam serumah lagi dan tidak ada harapan untuk dapat hidup rukun kembali, maka rumah tangga tersebut telah terbukti *retak dan pecah* dan telah memenuhi alasan cerai sebagaimana disebutkan dalam Pasal 19 huruf (f) Peraturan Pemerintah Nomor 9 Tahun 1975”. Ketentuan tersebut sejalan pula dengan pendapat pakar hukum Islam yang diambil alih menjadi pendapat Majelis Hakim dalam pertimbangan hukum putusan ini, sebagaimana tersebut dalam kitab Al Mar’ah Bainal Fiqh Wal Qanun halaman 100 yang artinya :

”*Dan tidak ada manfaat yang diharapkan dalam mengumpulkan 2 (dua) orang yang saling membenci, terlepas dari masalah penyebab pertengkaran terjadi atau besar dan kecilnya pertengkaran tersebut, namun kebaikan hanya dapat diterapkan dengan mengakhiri kehidupan berumah tangga antara suami isteri tersebut*”;

Menimbang, bahwa berdasarkan ketentuan hukum Islam yang tersurat dalam Al-Qur’an surat Ar-Ruum ayat 21 dan ketentuan Pasal 1 Undang-Undang Republik Indonesia Nomor 1 Tahun 1974 yang menyebutkan bahwa “*Perkawinan ialah ikatan lahir dan batin antara seorang pria dengan seorang wanita dengan tujuan untuk membentuk keluarga (rumah tangga) yang kekal dan bahagia berdasarkan Ketuhanan Yang Maha Esa*” jo. Pasal 3 Kompilasi Hukum Islam yang menyebutkan bahwa “*Perkawinan bertujuan untuk*

Disclaimer
Kepaniteraan Mahkamah Agung Republik Indonesia berusaha untuk selalu mencantumkan informasi paling kini dan akurat sebagai bentuk komitmen Mahkamah Agung untuk pelayanan publik, transparansi dan akuntabilitas pelaksanaan fungsi peradilan. Namun dalam hal-hal tertentu masih dimungkinkan terjadi permasalahan teknis terkait dengan akurasi dan keterkinian informasi yang kami sajikan, hal mana akan terus kami perbaiki dari waktu kewaktu.
Dalam hal Anda menemukan inakurasi informasi yang termuat pada situs ini atau informasi yang seharusnya ada, namun belum tersedia, maka harap segera hubungi Kepaniteraan Mahkamah Agung RI melalui :
Email : kepaniteraan@mahkamahagung.go.id Telp : 021-384 3348 (ext.318)

membentuk rumah tangga yang sakinah, mawaddah dan rahmah”, karenanya jika antara Penggugat dengan Tergugat selaku pasangan suami isteri sudah berpisah rumah dan tidak ada lagi komunikasi yang harmonis menandakan bahwa antara keduanya sudah tidak ada lagi sikap saling mencintai, saling menyayangi, saling pengertian dan saling melindungi, apalagi Penggugat telah menyatakan sudah tidak lagi berkeinginan melanjutkan rumah tangga dengan Tergugat, sehingga tujuan perkawinan untuk membentuk keluarga yang sakinah, mawaddah dan rahmah sebagaimana dikehendaki oleh ketentuan-ketentuan tersebut di atas tidak mungkin terwujud, maka agar kedua belah pihak tidak lebih jauh melanggar norma agama dan norma hukum, perceraian merupakan satu alternatif untuk mengakhiri sengketa rumah tangga antara Penggugat dengan Tergugat;

Menimbang, bahwa Majelis Hakim tidak melihat siapa yang benar dan siapa yang salah, yang jelas bahwa hati kedua belah pihak sudah benar-benar pecah dan sangat sulit untuk dipersatukan kembali. Jika kondisi rumah tangga Penggugat dan Tergugat yang sudah demikian itu masih akan dipertahankan, dikhawatirkan akan menimbulkan mudlarat yang lebih besar bagi kedua belah pihak;

Menimbang, bahwa berdasarkan pertimbangan-pertimbangan tersebut di atas, Majelis Hakim berpendapat bahwa alasan perceraian sebagaimana tersebut dalam Pasal 39 ayat (2) Undang-Undang Republik Indonesia Nomor 1 tahun 1974 jo. Pasal 19 huruf (f) Peraturan Pemerintah Nomor 9 Tahun 1975 dan Pasal 116 huruf (f) Kompilasi Hukum Islam telah terpenuhi, karenanya gugatan Penggugat dalam petitumnya angka 2 dapat dikabulkan dengan menjatuhkan talak satu ba’in shugra Tergugat kepada Penggugat sebagaimana tertuang dalam amar putusan ini;

Menimbang, bahwa berdasarkan Pasal 89 ayat (1) Undang-Undang Republik Indonesia Nomor 7 Tahun 1989 tentang Peradilan Agama sebagaimana telah diubah dan ditambah dengan Undang-undang Republik Indonesia Nomor 3 tahun 2006 dan Undang-Undang Republik Indonesia Nomor 50 Tahun 2009, biaya yang timbul dalam perkara ini dibebankan kepada Penggugat;

Disclaimer
Kepaniteraan Mahkamah Agung Republik Indonesia berusaha untuk selalu mencantumkan informasi paling kini dan akurat sebagai bentuk komitmen Mahkamah Agung untuk pelayanan publik, transparansi dan akuntabilitas pelaksanaan fungsi peradilan. Namun dalam hal-hal tertentu masih dimungkinkan terjadi permasalahan teknis terkait dengan akurasi dan keterkinian informasi yang kami sajikan, hal mana akan terus kami perbaiki dari waktu kewaktu.
Dalam hal Anda menemukan inakurasi informasi yang termuat pada situs ini atau informasi yang seharusnya ada, namun belum tersedia, maka harap segera hubungi Kepaniteraan Mahkamah Agung RI melalui :
Email : kepaniteraan@mahkamahagung.go.id   Telp : 021-384 3348 (ext.318)

Mengingat segala ketentuan peraturan perundang-undangan yang berlaku dan hukum Islam yang berkaitan dengan perkara ini;

M E N G A D I L I

1. Mengabulkan gugatan Penggugat;
2. Menjatuhkan talak satu Ba'in Sughra Tergugat **(XXX)** terhadap Penggugat **(XXX)**;
3. Membebankan kepada Penggugat untuk membayar biaya perkara ini Sejumlah Rp 416.000,00 (empat ratus enam belas ribu rupiah) ;

Demikian putusan ini dijatuhkan dalam rapat permusyawaratan Majelis Hakim Pengadilan Agama Bekasi, pada hari Senin tanggal 28 Desember 2020 Masehi, bertepatan dengan tanggal 13 Jumadil Awwal 1442 Hijriah, oleh **Drs. Ahmad Zawawi, M.H.** sebagai Hakim Ketua Majelis, **Drs. Suyadi** dan **Dr. Ummi Azma, S.H., M.Hum**., masing-masing sebagai Hakim Anggota, putusan tersebut diucapkan dalam sidang terbuka untuk umum, oleh Hakim Ketua Majelis tersebut, dengan dihadiri oleh Hakim-hakim Anggota tersebut dan **Sulaeman Syaifudin, S.H.,** sebagai Panitera Pengganti serta dihadiri oleh Penggugat dan diluar hadirnya Tergugat.

Hakim Anggota,

Drs. Suyadi

Hakim Anggota,

Dr. Ummi Azma, S.H., M.Hum.

Ketua Majelis,

Drs. Ahmad Zawawi, M.H.

Panitera Pengganti,

Disclaimer
Kepaniteraan Mahkamah Agung Republik Indonesia berusaha untuk selalu mencantumkan informasi paling kini dan akurat sebagai bentuk komitmen Mahkamah Agung untuk pelayanan publik, transparansi dan akuntabilitas pelaksanaan fungsi peradilan. Namun dalam hal-hal tertentu masih dimungkinkan terjadi permasalahan teknis terkait dengan akurasi dan keterkinian informasi yang kami sajikan, hal mana akan terus kami perbaiki dari waktu kewaktu.
Dalam hal Anda menemukan inakurasi informasi yang termuat pada situs ini atau informasi yang seharusnya ada, namun belum tersedia, maka harap segera hubungi Kepaniteraan Mahkamah Agung RI melalui :
Email : kepaniteraan@mahkamahagung.go.id   Telp : 021-384 3348 (ext.318)

Sulaeman Syaifudin, S.H.

Rincian Biaya Perkara :

1.	Pendaftaran/PNBP	Rp 30.000,00
2.	Pemberkasan/ATK	Rp 50.000.00
3.	Panggilan Penggugat	Rp 100.000,00
4.	Panggilan Tergugat	Rp 200.000,00
5.	PNBP Relaas Pgl I PT	Rp 20.000,00
6.	Redaksi	Rp 10.000,00
7.	Materai	Rp 6.000,00
	Jumlah	Rp 416.000,00 (empat ratus enam belas ribu rupiah)

Disclaimer
Kepaniteraan Mahkamah Agung Republik Indonesia berusaha untuk selalu mencantumkan informasi paling kini dan akurat sebagai bentuk komitmen Mahkamah Agung untuk pelayanan publik, transparansi dan akuntabilitas pelaksanaan fungsi peradilan. Namun dalam hal-hal tertentu masih dimungkinkan terjadi permasalahan teknis terkait dengan akurasi dan keterkinian informasi yang kami sajikan, hal mana akan terus kami perbaiki dari waktu kewaktu.
Dalam hal Anda menemukan inakurasi informasi yang termuat pada situs ini atau informasi yang seharusnya ada, namun belum tersedia, maka harap segera hubungi Kepaniteraan Mahkamah Agung RI melalui :
Email : kepaniteraan@mahkamahagung.go.id Telp : 021-384 3348 (ext.318)
