# Supplementary material for: Classification of divorce causes during the COVID-19 pandemic using convolutional neural networks
Source: PeerJ Comput Sci. 2022 Jun 30;8:e998. doi: 10.7717/peerj-cs.998 (PMC9299239; doi:10.7717/peerj-cs.998)
Supplement: Supplemental Information 2 [file peerj-cs-08-998-s002.zip › Kelalaian Dataset/Data ke-9.pdf]

PUTUSAN

Nomor 2046/Pdt.G/2020/PA.Pt.

بسم الله الرحمن الرحيم

DEMI KEADILAN BERDASARKAN KETUHANAN YANG MAHA ESA

Pengadilan Agama Pati yang memeriksa dan mengadili perkara perdata pada tingkat pertama, yang dilangsungkan diruang sidang Pengadilan Agama tersebut, Majelis hakim telah menjatuhkan putusan dalam perkara Cerai Gugat antara:

PENGGUGAT,Tempat Tgl. Lahir, Pati, 05-07-1983, NIK X, Agama Islam, Pendidikan SD Sederajat , Pekerjaan Karyawan Swasta, beralamat di (Bu Yulaikah) Dk. Penggung RT.004 / RW.002 Desa Tlogorejo Kecamatan Tlogowungu Kabupaten Pati, dalam hal ini memberikan kuasa khusus kepada Darsono ,SH. dan Agus Aprilio,SH. adalah Advokat yang berkantor di, LBH. Perisai Jl. H Moenadi Komplek Ruko No.15 Desa Semampir, Kecamatan Pati, Kabupaten Pati, Prov.Jateng, berdasarkan Surat Kuasa Khusus tertanggal 01 September 2020 yang terdaftar di Kepaniteraan Pengadilan Agama Pati Nomor 944/Kuasa/IX/2020/PA.Pt. tanggal 02 September 2020, selanjutnya disebut sebagai " Penggugat ";

Melawan

TERGUGAT, Tempat Tgl. Lahir: Pat, 14 Mei 1979, NIK. X, Agama Islam, Pendidikan SD. Pekerjaan Petani, beralamat di Dk. Penggung RT.004 / RW.002 Desa Tlogorejo, Kecamatan. Tlogowungu, Kabupaten Pati, yang selanjutnya disebut sebagai “Tergugat”;

Pengadilan Agama tersebut;

Telah mempelajari berkas perkara;

Telah mendengar keterangan Penggugat dan Tergugat serta saksi-saksi dimuka persidangan;

TENTANG DUDUK PERKARANYA

Disclaimer
Kepaniteraan Mahkamah Agung Republik Indonesia berusaha untuk selalu mencantumkan informasi paling kini dan akurat sebagai bentuk komitmen Mahkamah Agung untuk pelayanan publik, transparansi dan akuntabilitas pelaksanaan fungsi peradilan. Namun dalam hal-hal tertentu masih dimungkinkan terjadi permasalahan teknis terkait dengan akurasi dan keterkinian informasi yang kami sajikan, hal mana akan terus kami perbaiki dari waktu kewaktu.
Dalam hal Anda menemukan inakurasi informasi yang termuat pada situs ini atau informasi yang seharusnya ada, namun belum tersedia, maka harap segera hubungi Kepaniteraan Mahkamah Agung RI melalui :
Email : kepaniteraan@mahkamahagung.go.id Telp : 021-384 3348 (ext.318)

Bahwa, Penggugat dalam surat Gugatannya tertanggal 02 September 2020 yang didaftarkan di Kepaniteraan Pengadilan Agama Pati Nomor 2046/Pdt.G/2020/PA.Pati tanggal 02 Septemebr 2020 dengan perubahannya mengajukan hal-hal yang pada pokoknya sebagai berikut:

1. Bahwa Penggugat dan Tergugat adalah suami istri yang telah menikah pada tanggal 11 April 2013, Tercatat di Kantor Urusan Agama Kecamatan Tlogowungu, Kabupaten Pati, sesuai dengan kutipan Akta Nikah nomor: 133/12/IV/2013 tanggal 11 April 2013;
2. Bahwa setelah menikah Tergugat mengucapkan Sighot taklik talak;
3. Bahwa sewaktu menikah Penggugat adalah Janda Cerai dan Tergugat adalah Duda Cerai ;
4. Bahwa setelah menikah Penggugat dan Tergugat tinggal dirumah orang tua Penggugat ;
5. Bahwa Penggugat dan Tergugat telah pernah hidup rukun dan telah berhubungan layaknya suami istri (ba'dal dhukul) telah dikaruniai seorang anak bernama Mustorifah binti TERGUGAT lahir tgl. 12 Nopember 2003 sekarang ikut Penggugat ;
6. Bahwa setelah pernikahan antara Penggugat dan Tergugat berlangsung kurang lebih 5 tahun atau terhitung sejak bulan September 2018 rumah tangga Penggugat dan tergugat tidak harmonis sering bertengkar terus-menerus yang disebabkan : Karena Tergugat tidak bertanggung jawab masalah ekonomi rumah tangga, Tergugat tidak pernah memberikan nafkah kepada Penggugat, dan Tergugat diketahui telah menjalin hubungan dengan wanita idaman lain dan sampai mempunyai seorang anak dari wanita tersebut yang ber inisial (UM), apabila dinasehati justru Tergugat malah marah-marah :
7. a. Bahwa sebagai puncak pertengkaran terjadi pada bulan Desember 2018 akhirnya antara Penggugat dan Tergugat pisah ranjang dan tidak pernah berhubungan layaknya suami istri dan selama waktu tersebut Tergugat tidak pernah memberikan nafkah wajib baik nafkah lahir maupun bathin kepada Penggugat ;
   b. Bahwa Penggugat sudah berusaha bersabar agar sikap Tergugat dapat berubah, namun tidak berhasil dan pada bulan Juli 2020

Disclaimer
Kepaniteraan Mahkamah Agung Republik Indonesia berusaha untuk selalu mencantumkan informasi paling kini dan akurat sebagai bentuk komitmen Mahkamah Agung untuk pelayanan publik, transparansi dan akuntabilitas pelaksanaan fungsi peradilan. Namun dalam hal-hal tertentu masih dimungkinkan terjadi permasalahan teknis terkait dengan akurasi dan keterkinian informasi yang kami sajikan, hal mana akan terus kami perbaiki dari waktu kewaktu.
Dalam hal Anda menemukan inakurasi informasi yang termuat pada situs ini atau informasi yang seharusnya ada, namun belum tersedia, maka harap segera hubungi Kepaniteraan Mahkamah Agung RI melalui :
Email : kepaniteraan@mahkamahagung.go.id   Telp : 021-384 3348 (ext.318)

Penggugat pulang kerumah orang tua Penggugat yang masih satu Desa dengan Tergugat;

8. Bahwa sejak bulan Desember 2018 sampai dengan sekarang atau selama kurang lebih 1 tahun 8 bulan antara Penggugat dan Tergugat telah berpisah tempat tidur dan selama pisah Tergugat tidak pernah memberi nafkah wajib kepada Penggugat;

9. Bahwa sesuai pasal 39 Undang-undang No 1 tahun 1974 jo pasal 19 huruf (f) PP. no 9 tahun 1975 jo pasal 116 huruf (f) KHI, kiranya cukup beralasan bagi Penggugat untuk mengajukan gugatan di Pengadilan Agama Pati ;

10. Bahwa Penggugat sanggup membayar biaya perkara ini ;

Berdasarkan dalil-dalil tersebut Penggugat mohon kepada Ketua Pengadilan Agama Pati Cq Majelis Hakim yang memeriksa dan mengadili perkara ini, berkenan untuk menjatuhkan putusan sebagai berikut :

1. Mengabulkan gugatan Penggugat.
2. Menjatuhkan talak satu ba'in sugro Tergugat (TERGUGAT) Terhadap Penggugat (PENGGUGAT) .
3. Menetapkan biaya perkara menurut Hukum.

Atau apabila berpendapat lain mohon putusan yang seadil-adilnya (Ex aequo et bono);

Bahwa, pada hari persidangan yang telah ditetapkan Penggugat dan Tergugat datang sendiri menghadap di persidangan;

Bahwa, Penggugat melalui Kuasanya dan Tergugat telah melaksakan mediasi, akan tetapi dalam mediasi tersebut gagal karena Penggugat dan Tergugat tidak dapat mengakhiri sengketa rumah tangganya dengan perdamaian, sebagaimana ternyata dari surat keterangan mediasi tertanggal 14 September 2020 Nomor 2046/Pdt.G/2020/PA.Pt. yang dibuat oleh Drs. H. Zaenal Arifin, MH., hakim Pengadilan Agama Pati sebagai mediator;

Bahwa, Majelis Hakim telah berusaha mendamaikan Penggugat dan Tergugat agar rukun kembali untuk membina rumah tangga akan tetapi tidak berhasil, lalu pemeriksaan dilanjutkan dengan membacakan surat Gugatan

Disclaimer
Kepaniteraan Mahkamah Agung Republik Indonesia berusaha untuk selalu mencantumkan informasi paling kini dan akurat sebagai bentuk komitmen Mahkamah Agung untuk pelayanan publik, transparansi dan akuntabilitas pelaksanaan fungsi peradilan. Namun dalam hal-hal tertentu masih dimungkinkan terjadi permasalahan teknis terkait dengan akurasi dan keterkinian informasi yang kami sajikan, hal mana akan terus kami perbaiki dari waktu kewaktu.
Dalam hal Anda menemukan inakurasi informasi yang termuat pada situs ini atau informasi yang seharusnya ada, namun belum tersedia, maka harap segera hubungi Kepaniteraan Mahkamah Agung RI melalui :
Email : kepaniteraan@mahkamahagung.go.id Telp : 021-384 3348 (ext.318)

dalam sidang tertutup untuk umum yang isinya tetap dipertahankan oleh Penggugat;

Bahwa, atas Gugatan Penggugat, Tergugat memberikan jawaban secara tertulis tanggal 12 Oktober 2020 yang pada pokonya sebagai berikut;

1. Bahwa benar antara penggugat dan tergugat adalah suami istri yang telah menikah pada tanggal 11 april 2013 yang tercatat di kantor urusan agama kec. Tlogowungu, kab. Pati, dengan kutipan akta nikah no : 133/12/IV/2013 tanggal 11 april 2013;
2. Bahwa benar setelah menikah tergugat mengacapkan sighot taklik talak;
3. Bahwa benar sewaktu menikah penggugat adalah janda cerai dan tergugat adalah duda cerai;
4. Bahwa tidak benar setelah menikah penggugat dan tergugat tinggal dirumah orang tua penggugat, yang benar adalah tinggal dirumah kedua belah pihak atau rumah sendiri;
5. Bahwa benar penggugat dan tergugat telah pernah hidup rukun dan setelah dikaruniai seorang anak yang bernama "Mustorifah binti TERGUGAT" lahir tanggal 12 november 2003 yang ikut kedua orang tuanya (tidak benar kalau ikut penggugat);
6. A. Bahwa tidak benar semenjak dimulainya pernikahan kedua kalinya antara saudari Penggugat (PENGGUGAT) dengan saudara Tergugat (TERGUGAT) tidak harmonis lagi sering bertengkar, Yang benar adalah terbukti dengan jelas kedua belah pihak mampu bertahan selama 5 tahun lamanya, dan durasi waktu itu tidaklah singkat. Dan begitu jelas juga antara saudari penggugat (PENGGUGAT) dengan saudara tergugat (TERGUGAT) telah melangsungkan/melanjutkan sesi pernikahan ulang yang kedua kalinya untuk memperbaiki kegagalannya dalam pernikahan pertamanya yang telah dikaruniai seorang anak bernama "Mustafiroh" yang sudah berumur kurang lebih 17 tahun.

B. Bahwa tidak benar jika saudara tergugat (TERGUGAT) tidak bertanggung jawab atau tidak memberikan nafkah yang telah dituduhkan / disangkakan saudari penggugat (PENGGUGAT) melalui kuasa hukumnya sejak dari bulan September 2018, Yang benar adalah terbukti sampai saat ini (berlangsungnya gugatan) kedua belah pihak masih tinggal dalam satu rumah bersama anaknya yang bernama saudari "Mustafiroh" begitu pula

Disclaimer
Kepaniteraan Mahkamah Agung Republik Indonesia berusaha untuk selalu mencantumkan informasi paling kini dan akurat sebagai bentuk komitmen Mahkamah Agung untuk pelayanan publik, transparansi dan akuntabilitas pelaksanaan fungsi peradilan. Namun dalam hal-hal tertentu masih dimungkinkan terjadi permasalahan teknis terkait dengan akurasi dan keterkinian informasi yang kami sajikan, hal mana akan terus kami perbaiki dari waktu kewaktu.
Dalam hal Anda menemukan inakurasi informasi yang termuat pada situs ini atau informasi yang seharusnya ada, namun belum tersedia, maka harap segera hubungi Kepaniteraan Mahkamah Agung RI melalui :
Email : kepaniteraan@mahkamahagung.go.id Telp : 021-384 3348 (ext.318)

keduanya masih bersama-sama saling membantu dalam bekerja/ berdagang di pasar baik dalam tenaga dan finansial/keuangan,

C. Bahwa tidak benar saudara tergugat (TERGUGAT) mempunyai wanita idaman dan sampai mempunyai seorang anak yang berinisial “UM” seperti apa yang telah dituduhkan / disangkakan saudari penggugat (PENGGUGAT) melaui kuasa hukumnya.

- Mohon harap dibuktikan dan kesaksiannya sesuai dengan alat bukti dan saksi yang sah legalitasnya dalam hukum yang berlaku. Jangan hanya sekedar tuduhan / sangkaan yang bisa membuat orang lain merasa dirugikan dan dicemarkan nama baiknya.

7. A. Bahwa tidak benar, ada sebuah pertengkaran/masalah yang terjadi pada bulan desember 2018 seperti halnya yang telah dituduhkan/ disangkakan oleh saudari penggugat (PENGGUGAT) melalui kuasa hukumnya.

- Yang benar adalah, terbukti dengan jelas sampai berlangsungnya gugatan ini, kedua belah pihak masih tinggal bersama dengan anaknya dan begitu pula dalam bekerja keduanya masih bersama-sama (sudah diperjelas dalam gugatan nomor 6 huruf B).

B. Bahwa tidak benar antara saudari penggugat (PENGGUGAT) dengan tergugat (TERGUGAT) telah pisah ranjang dan tidak pernah lagi berubungan layaknya suami istri dari bulan desember 2018 seperti halnya yang telah dituduhkan / disangkakan oleh saudari penggugat (PENGGUGAT) melalui kuasa hukumnya.

- Yang benar adalah kedua belah pihak terakhir melakukan hubungan layaknya suami istri kurang lebih 2 mingguan sebelum diajukannya gugatan perkara ini berlangsung.
- Terbukti sudah dalam point dalil gugatan nomor 7 Majelis Hakim dalam agenda persidangan kemarin sudah bertanya langsung kepada saudari penggugat (PENGGUGAT) semua jawabannya salah/tidak sesuai dengan materi dalil yang dibuat oleh kuasa hukumnya (mohon hal ini bisa dibuat Majelis Hakim untuk mempertimbangkan/menimbang ulang atas pengajuan perkara gugatan saudari penggugat (PENGGUGAT) melalui kuasa hukumnya.

Disclaimer
Kepaniteraan Mahkamah Agung Republik Indonesia berusaha untuk selalu mencantumkan informasi paling kini dan akurat sebagai bentuk komitmen Mahkamah Agung untuk pelayanan publik, transparansi dan akuntabilitas pelaksanaan fungsi peradilan. Namun dalam hal-hal tertentu masih dimungkinkan terjadi permasalahan teknis terkait dengan akurasi dan keterkinian informasi yang kami sajikan, hal mana akan terus kami perbaiki dari waktu kewaktu.
Dalam hal Anda menemukan inakurasi informasi yang termuat pada situs ini atau informasi yang seharusnya ada, namun belum tersedia, maka harap segera hubungi Kepaniteraan Mahkamah Agung RI melalui :
Email : kepaniteraan@mahkamahagung.go.id Telp : 021-384 3348 (ext.318)

8. A. Bahwa tidak benar semenjak bulan desember 2018 sampai sekarang kurang lebih 1 tahun 8 bulan antara saudari penggugat (PENGGUGAT) dengan saudara tergugat (TERGUGAT) telah pisah ranjang dan tidak memberikan nafkah;

- Yang benar adalah saudari penggugat (PENGGUGAT) dengan saudara tergugat (TERGUGAT) pisah ranjang itu terakhir dilakukan kurang lebih 2 minggu sebelum diajukannya gugatan perkara ini (sudah diperjelas dalam jawaban/tanggapan nomor 7 huruf b) “Mohon jangan diulang-ulang dalam membuat bahan materi dalil gugatan yang diajukan dalam berperkara”;
- Untuk nomor 8 ini, dalam agenda persidangan kemarin Majelis Hakim sudah bertanya langsung dengan saudari penggugat (PENGGUGAT), namun semua jawabannya salah/tidak sesuai dengan materi gugatan yang sudah dibuat oleh kuasa hukumnya (sudah jelas ini adalah rekayasa dan sandiwara yang penuh kedholiman untuk sebuah kemenangan) meskipun dengan penuh fitnah dan kebohongan yang mengabaikan dengan fakta yang ada.

B. Bahwa tidak benar pada bulan juli 2020 saudari penggugat (PENGGUGAT) telah pulang kerumah orang tuanya yang masih dalam satu desa saudari tergugat (TERGUGAT)

- Yang benar adalah saudari penggugat “PENGGUGAT” mulai pulang kerumah orang tuanya (numpang tidur) itu dilakukan /dimulai pada tanggal 22 september 2020 hal ini dilakukan setelah mendapat teguran dari Majelis Hakim pada tanggal 21 september 2020 (1 hari setelah sesi persidangan yang ke 2) dan yang benar adalah saudari penggugat kerumah orang tuanya pada jam 22.00 sampai jam 2 dini hari/ subuh untuk kembali ke rumahnya menyiapkan barang-barang dagangan untuk berjualan dipasar bersama-sama saudara tergugat “TERGUGAT” dan sepulangnya dari pasarpun juga bersama-sama kembali kerumahnya sendiri.
- Hal ini cukup jelas terbukti untuk kepulangannya saudari penggugat (PENGGUGAT) kerumah orang tauanya itu sebagai drama/skenario untuk menghadapi agenda sidang supaya mendapatkan sebuah

Disclaimer
Kepaniteraan Mahkamah Agung Republik Indonesia berusaha untuk selalu mencantumkan informasi paling kini dan akurat sebagai bentuk komitmen Mahkamah Agung untuk pelayanan publik, transparansi dan akuntabilitas pelaksanaan fungsi peradilan. Namun dalam hal-hal tertentu masih dimungkinkan terjadi permasalahan teknis terkait dengan akurasi dan keterkinian informasi yang kami sajikan, hal mana akan terus kami perbaiki dari waktu kewaktu.
Dalam hal Anda menemukan inakurasi informasi yang termuat pada situs ini atau informasi yang seharusnya ada, namun belum tersedia, maka harap segera hubungi Kepaniteraan Mahkamah Agung RI melalui :
Email : kepaniteraan@mahkamahagung.go.id Telp : 021-384 3348 (ext.318)

kemenangan yang amat dipaksakan. “mohon berhati-hati untuk membuat bahan materi dan dalil-dalil gugatan, karena ini urusannya bukan hanya sekedar duniawi saja melainkan juga di akhirat kelak.

Berdasarkan atas jawaban dari semua dalil gugatan oleh penggugat tergugat telah memberikan jawaban atau tanggapan yang sebenar-benarnya. Mohon maaf bila ada tata cara atau penulisan yang kurang benar, karena kami hanya orang bodoh yang berusaha mempertahankan hubungan rumah tangga, kami hanya memperjuangkan sebuah kebenaran bukanlah kemenangan. Mohon Majelis Hakim beserta panitera sidang yang terhormat memberikan sebuah putusan atau pertimbangan yang seadil-adilnya, karena sudah jelas dan terbukti kuasa hukum dalam membuat materi dalil gugatan semuanya tidak singkron/tidak sesuai dengan jawaban yang telah diberikan saudari penggugat (PENGGUGAT) dalam agenda sidang sebelumnya dan didukung pula kuasa hukumnya telah merevisi ulang materi dalil-dalil gugatan, itu adalah bukti yang cukup dan jelas dan kuat bahwa semua perkara ini penuh rekayasa dan sandiwara;

Bahwa, terhadap jawaban Tergugat, Penggugat menyampaikan replik secara tertulis tertanggal 19 Oktober 2020 yang pada pokoknya tetap mempertahankan dalil gugatannya, kemudian Tergugat menyampaikan duplik secara tertulis tertanggal 26 oktober 2020 yang pada pokoknya tetap mempertahankan dalil dalam jawabannya, selengkapnya telah termuat dalam berita acara sidang dan harus dianggap satu kesatuan dengan putusan ini;

Bahwa untuk memperkuat dalil gugatannya, Penggugat di depan sidang mengajukan alat bukti tertulis berupa :

1. Foto copy KTP an Penggugat Nomor X tanggal 09 Juli 2012 yang dikeluarkan oleh Kantor Kependudukan dan Pencatatan sipil Kabupaten Pati, yang oleh Ketua Majelis telah dicocokkan dengan aslinya dan telah sesuai, bermeterai cukup yang diberi tanda P.1;
2. Foto copy Kutipan Akta nikah Nomor : 133/12/IV/2013 tanggal 11 April 2013 yang dikeluarkan oleh Kantor Urusan Agama Kecamatan Tlogowungu Kabupaten Pati, yang oleh Ketua Majelis telah dicocokkan dengan aslinya dan telah sesuai, bermeterai cukup yang diberi P.2;

Disclaimer
Kepaniteraan Mahkamah Agung Republik Indonesia berusaha untuk selalu mencantumkan informasi paling kini dan akurat sebagai bentuk komitmen Mahkamah Agung untuk pelayanan publik, transparansi dan akuntabilitas pelaksanaan fungsi peradilan. Namun dalam hal-hal tertentu masih dimungkinkan terjadi permasalahan teknis terkait dengan akurasi dan keterkinian informasi yang kami sajikan, hal mana akan terus kami perbaiki dari waktu kewaktu.
Dalam hal Anda menemukan inakurasi informasi yang termuat pada situs ini atau informasi yang seharusnya ada, namun belum tersedia, maka harap segera hubungi Kepaniteraan Mahkamah Agung RI melalui :
Email : kepaniteraan@mahkamahagung.go.id Telp : 021-384 3348 (ext.318)

Bahwa, selain bukti tertulis tersebut di atas, Penggugat juga telah mengajukan 3 (tiga) orang saksi-saksi masing-masing bernama :

1. SAKSI I PENGGUGAT, umur 35 tahun, Agama Islam, Pendidikan SMP, Pekerjaan Pedagang, tempat tinggal di Desa Regolah Rt.002 Rw.002 Kecamatan Tlogowungu Kabupaten Pati, menerangkan di bawah sumpah yang pada pokoknya sebagai berikut;
   - Bahwa saksi kenal dengan Penggugat dan Tergugat karena sebagai teman dagang Penggugat dan Tergugat di pasar;
   - Bahwa saksi kenal Penggugat dan Tergugat sejak mereka jualan ayam di pasar pada tahun 2018 dan mereka sudah menjadi suami istri dan tinggal dirumah milik bersama di Desa Tlogorejo Kecamatan Tlogowungu, Kabupaten Pati dan sudah dikaruniai anak;
   - Bahwa semula rumah tangga Penggugat dan Tergugat hidup rukun dan harmonis, namun sekarang rumah tangganya tidak rukun, tidak harmonis lagi, karena antara Penggugat dan Tergugat sering terjadi perselisihan dan pertengkaran yang mengakibatkan Penggugat pulang kerumah orang tuanya sejak 2 bulan yang lalu;
   - Bahwa sebab terjadi perselisihan dan pertengkaran masalah Tergugat mencintai wanita lain bernama Umbar asal Desa Jontro Kecamatan Widarijaksa kabupaten Pati;
   - Bahwa Saksi melihat sendri terjadi pertegkaran tersebut di pasar, bahkan Tergugat sampai memukul punggung Penggugat dengan sepatu;
   - Bahwa Penggugat dan Tergugat berfrosi sama yaitu penjual ayam potong. Akan tetapi beda lapaknya (jualan sendiri-sendriri)
   - Bahwa selama berpisah antara Penggugat dan Tergugat dan sudah tidak komunikasi lagi sebagai suami istri yang baik;

   Bahwa saksi mengatakan sudah tidak ada lagi yang ingin dikemukakan, dan Penggugat dan Tergugat membenarkan serta tidak keberatan atas kesaksian tersebut;
2. SAKSI II PENGGUGAT, umur 55 tahun, Agama Islam, Pendidikan Sekolah Dasar, Pekerjaan petani, tempat tinggal di Desa Muktiharjo Rt.003 Rw.001

Disclaimer
Kepaniteraan Mahkamah Agung Republik Indonesia berusaha untuk selalu mencantumkan informasi paling kini dan akurat sebagai bentuk komitmen Mahkamah Agung untuk pelayanan publik, transparansi dan akuntabilitas pelaksanaan fungsi peradilan. Namun dalam hal-hal tertentu masih dimungkinkan terjadi permasalahan teknis terkait dengan akurasi dan keterkinian informasi yang kami sajikan, hal mana akan terus kami perbaiki dari waktu kewaktu.
Dalam hal Anda menemukan inakurasi informasi yang termuat pada situs ini atau informasi yang seharusnya ada, namun belum tersedia, maka harap segera hubungi Kepaniteraan Mahkamah Agung RI melalui :
Email : kepaniteraan@mahkamahagung.go.id   Telp : 021-384 3348 (ext.318)

Kecamatan Margorejo Kabupaten Pati, menerangkan di bawah sumpah yang pada pokoknya sebagai berikut;

- Bahwa saksi kenal dengan Penggugat dan Tergugat karena sebagai ibu kandung Penggugat;
- Bahwa Penggugat dan Tergugat menikah pada 07 tahun yang lalu dan tinggal dirumah milik bersama di Desa Tlogorejo Kecamatan Tlogowungu, Kabupaten Pati dan sudah dikaruniai anak;
- Bahwa Keadaan rumah tangga Penggugat dan Tergugat awalnya hidup rukun dan harmonis, namun sekarang rumah tangganya tidak rukun dan harmonis lagi karena antara Penggugat dan Tergugat sering berselisih dan bertengkar yang disebabkan Tergugat mencintai wanita lain bernama Umbar asal Desa Jontro Kecamatan Widarijaksa kabupaten Pati, bahkan sekarang sudah mempunyai seorang anak ;
- Bahwa saksi melihat sendiri kalau Tergugat sudah mempunyai anak dengan wanita yang bernama Umbar, karena 1 tahun yang lalu Saksi periksa ke Ibu Dokter di Tlogowungu, ketemu Tergugat dengan wanita tersebut yang memeriksakan anaknya dimana datang dan pulangnya mereka bersama-sama satu mobil;
- Bahwa akibat pertengkaran tersebut akhirnya Penggugat pergi pulang kerumah saksi dan tidak kembali lagi sampai sekarang sudah 2 bulan lamanya;
- Bahwa selama berpisah antara Penggugat dan Tergugat sudah tidak komunikasi sebagai layaknya suami istri yang baik dan saksi sudah berusaha menasehati Penggugat untuk rukun kembali dengan Tergugat, akan tetapi tidak berhasil;

Bahwa saksi mengatakan sudah tidak ada lagi yang ingin dikemukakan Penggugat dan Tergugat membenarkan, tidak keberatan kesaksian tersebut,

3.- -SAKSI III PENGGUGAT, umur 25 tahun, Agama Islam, Pendidikan SMA, Pekerjaan Scurity, tempat tinggal di Desa Jontro Rt.003 Rw.012 Kecamatan Widarijaksa Kabupaten Pati, menerangkan di bawah sumpah yang pada pokoknya sebagai berikut;

Disclaimer
Kepaniteraan Mahkamah Agung Republik Indonesia berusaha untuk selalu mencantumkan informasi paling kini dan akurat sebagai bentuk komitmen Mahkamah Agung untuk pelayanan publik, transparansi dan akuntabilitas pelaksanaan fungsi peradilan. Namun dalam hal-hal tertentu masih dimungkinkan terjadi permasalahan teknis terkait dengan akurasi dan keterkinian informasi yang kami sajikan, hal mana akan terus kami perbaiki dari waktu kewaktu.
Dalam hal Anda menemukan inakurasi informasi yang termuat pada situs ini atau informasi yang seharusnya ada, namun belum tersedia, maka harap segera hubungi Kepaniteraan Mahkamah Agung RI melalui :
Email : kepaniteraan@mahkamahagung.go.id   Telp : 021-384 3348 (ext.318)

- Bahwa saksi kenal dengan Penggugat dan Tergugat karena sebagai teman Penggugat dan Tergugat di pasar;
- Bahwa saksi kenal Penggugat pada awal jualan ayam potong di pasar pada tahun 2018 dan sebelumnya sudah kenal dengan Tergugat karena Tergugat lebih dahulu jualannya;
- Bahwa ketika saksi kenal Penggugat dan Tergugat sudah menjadi suami istri sah dan tinggal tinggal dirumah milik bersama di Desa Tlogorejo Kecamatan Tlogowungu, Kabupaten Pati dan sudah dikaruniai anak;
- Bahwa Saksi tidak mengetahui Keadaan rumah tangga Penggugat dan Tergugat, yang saksi ketahui pada tahun 2017 Tergugat menikah sirri dengan seorang wanita yang bernama Umbarwati tetangga saksi dan sekarang sudah mempunyai seorang anak umurnya 3 tahun;
- Bahwa pada waktu pernikahan yang menjadi saksi adalah saksi sendiri dengan orang tua saksi dan pada waktu itu Tergugat mengaku bersetatus duda;

Bahwa saksi mengatakan sudah tidak ada lagi yang ingin dikemukakan Penggugat dan Tergugat membenarkan, tidak keberatan kesaksian tersebut,

Bahwa, Penggugat menyatakan telah mencukupkan alat bukti tersebut diatas, dan tidak akan mengajukan suatu bukti apapun lagi;

Bahwa untuk meneguhkan dalil bantahannya, Tergugat telah mengajukan bukti-bukti tertulis dan saksi, adapun bukti-bukti surat berupa:

1. Foto Copy KTP Tergugat Nomor : 331814140579003 tanggal 11 Juli 2012 yang dikeluarkan oleh Kantor Kependudukan dan Capil Kabupaten Cilacap, yang oleh Ketua Majelis telah dicocokkan dan sesuai dengan aslinya serta bermaterai cukup selanjutnya di beri tanda T.1 ;
1. Foto copy Kutipan Akta nikah Nomor : 133/12/IV/2013 tanggal 11 April 2013 yang dikeluarkan oleh Kantor Urusan Agama Kecamatan Tlogowungu Kabupaten Pati, yang oleh Ketua Majelis telah dicocokkan dengan aslinya dan telah sesuai, bermeterai cukup selanjutnya yang diberi T.2;

Bahwa atas bukti-bukti tulisan yang diajukan oleh Tergugat tersebut, Penggugat dan Tergugat membenarkan dan tidak berkeberatan;

Disclaimer
Kepaniteraan Mahkamah Agung Republik Indonesia berusaha untuk selalu mencantumkan informasi paling kini dan akurat sebagai bentuk komitmen Mahkamah Agung untuk pelayanan publik, transparansi dan akuntabilitas pelaksanaan fungsi peradilan. Namun dalam hal-hal tertentu masih dimungkinkan terjadi permasalahan teknis terkait dengan akurasi dan keterkinian informasi yang kami sajikan, hal mana akan terus kami perbaiki dari waktu kewaktu.
Dalam hal Anda menemukan inakurasi informasi yang termuat pada situs ini atau informasi yang seharusnya ada, namun belum tersedia, maka harap segera hubungi Kepaniteraan Mahkamah Agung RI melalui :
Email : kepaniteraan@mahkamahagung.go.id Telp : 021-384 3348 (ext.318)

Bahwa, selain bukti tertulis tersebut di atas, Tergugat juga telah mengajukan 3 (tiga) orang saksi-saksi masing-masing bernama :

1. SAKSI I TERGUGAT, umur 37 tahun, Agama Islam, Pendidikan SD, Pekerjaan Penyalur ayam potong, tempat tinggal di Desa Bermi Rt.003 Rw.003 Kecamatan Gembong Kabupaten Pati, menerangkan di bawah sumpah yang pada pokoknya sebagai berikut;
   - Bahwa saksi kenal dengan Penggugat dan Tergugat karena sebagai teman kerja Tergugat di pasar;
   - Bahwa saksi kenal Penggugat dan Tergugat sejak mereka jualan ayam di pasar pada 5 tahun yang lalu dan mereka sudah menjadi suami istri dan tinggal dirumah milik bersama di Desa Tlogorejo Kecamatan Tlogowungu, Kabupaten Pati dan sudah dikaruniai seorang anak;
   - Bahwa rumah tangga Pengugat dan Tergugat baik-baik (rukun) tidak pernah ada pertengkaran akan tetapi 2 bulan yang lalu Tergugat cerita Penggugat minta cerai, karena Penggugat tidak mau ikut menanggung beban hutang Tergugat;
   - Bahwa antara Penggugat dan Tergugat masih tinggal satu rumah, karena seminggu yang lalu saksi mengantar istri kerumah Penggugat akan tetapi tidak ketemu Tergugat;
2. SAKSI II TERGUGAT, umur 23 tahun, Agama Islam, pendidikan Sekolah lanjutan Atas, Pekerjaan Mahasiswa, tempat tinggal di Rt. 004/ Rw. 002 Desa Tlogorejo Kecamatan Tlogowungu Kabupaten Pati, yang menerangkan di bawah sumpah yang pada pokoknya sebagai berikut;
   - Bahwa saksi kenal Penggugat dan Tergugat karena sebagai tetangga Penggugat dan Tergugat;
   - Bahwa Penggugat dan Tergugat menikah pada 7 tahun yang lalu dan setelah menikah Penggugat dan Tergugat hidup bersama dirumah milik bersama dan sudah dikaruniai seorang anak;
   - Bahwa rumah tangga Pengugat dan Tergugat baik-baik (rukun) tidak pernah ada pertengkaran dan masih tinggal satu rumah akan tetapi sejak 1 bulan yang lalu antara Penggugat dan Tergugat sudah tidak komunikasi sebagai layaknya suami istri yang baik, karena mereka sudah

Disclaimer
Kepaniteraan Mahkamah Agung Republik Indonesia berusaha untuk selalu mencantumkan informasi paling kini dan akurat sebagai bentuk komitmen Mahkamah Agung untuk pelayanan publik, transparansi dan akuntabilitas pelaksanaan fungsi peradilan. Namun dalam hal-hal tertentu masih dimungkinkan terjadi permasalahan teknis terkait dengan akurasi dan keterkinian informasi yang kami sajikan, hal mana akan terus kami perbaiki dari waktu kewaktu.
Dalam hal Anda menemukan inakurasi informasi yang termuat pada situs ini atau informasi yang seharusnya ada, namun belum tersedia, maka harap segera hubungi Kepaniteraan Mahkamah Agung RI melalui :
Email : kepaniteraan@mahkamahagung.go.id Telp : 021-384 3348 (ext.318)

tidak pergi bersama lagi;

- Bahwa saksi tidak mengetahui sebab Penggugat dan Tergugat sudah tidak komunikasi sebagai layaknya suami istri yang baik;

3. SAKSI III TERGUGAT, umur 19 tahun, Agama Islam, pendidikan Sekolah lanjutan Atas, Pekerjaan Mahasiswa, tempat tinggal di Rt. 004/ Rw. 002 Desa Tlogorejo Kecamatan Tlogowungu Kabupaten Pati, yang menerangkan di bawah sumpah yang pada pokoknya sebagai berikut;
   - Bahwa saksi kenal Penggugat dan Tergugat 1 tahun yang lalu karena menjadi tetangga baru Penggugat dan Tergugat;
   - Bahwa Penggugat dan Tergugat menikah pada 7 tahun yang lalu dan setelah menikah Penggugat dan Tergugat hidup bersama dirumah milik bersama dan sudah dikaruniai seorang anak;
   - Bahwa rumah tangga Pengugat dan Tergugat baik-baik (rukun) tidak pernah ada pertengkaran dan masih tinggal satu rumah akan tetapi sejak 1 bulan yang lalu antara Penggugat dan Tergugat sudah tidak komunikasi sebagai layaknya suami istri yang baik, karena mereka sudah tidak pergi bersama lagi;
   - Bahwa saksi tidak mengetahui sebab Penggugat dan Tergugat sudah tidak komunikasi sebagai layaknya suami istri yang baik;

   Bahwa saksi mengatakan sudah tidak ada lagi yang ingin dikemukakan Penggugat dan Tergugat membenarkan, tidak keberatan kesaksian tersebut,

Bahwa, Tergugat menyatakan telah mencukupkan alat bukti tersebut diatas, dan tidak akan mengajukan suatu bukti apapun lagi;

Bahwa Tergugat menyatakan tidak keberatan bercerai dengan Penggugat dan telah terjadi kesepakatan tentang pembagian harta bersama antara Penggugat dan Tergugat dan diakui bersama sebagaimana dalam berita acara sidang yang pada pokoknya sebagai berikut;

1. Bahwa Tergugat (TERGUGAT) mendapat bagian berupa tanah Kapling Nomor 0281 dengan luas tanah 11 x 10 M² terletak di Dukuh Penggung RT.004/RW.002 Desa Tlogorejo, Kecamatan. Tlogowungu, Kabupaten Pati. Dengan batas-batas sebagai berikut ;
   - Sebelah Utara : Tanah H. Suparmin;

Disclaimer
Kepaniteraan Mahkamah Agung Republik Indonesia berusaha untuk selalu mencantumkan informasi paling kini dan akurat sebagai bentuk komitmen Mahkamah Agung untuk pelayanan publik, transparansi dan akuntabilitas pelaksanaan fungsi peradilan. Namun dalam hal-hal tertentu masih dimungkinkan terjadi permasalahan teknis terkait dengan akurasi dan keterkinian informasi yang kami sajikan, hal mana akan terus kami perbaiki dari waktu kewaktu.
Dalam hal Anda menemukan inakurasi informasi yang termuat pada situs ini atau informasi yang seharusnya ada, namun belum tersedia, maka harap segera hubungi Kepaniteraan Mahkamah Agung RI melalui :
Email : kepaniteraan@mahkamahagung.go.id Telp : 021-384 3348 (ext.318)

- Sebelah Timur : Jl. Desa;
- Sebelah Selatan : Tanah Yono ;
- Sebelah Barat : Tanah H. Suparmin;

2. Bahwa Penggugat (PENGGUGAT) mendapatkan bagian Rp 10. 000.000,00 (sepuluh juta rupiah) sebagai ganti rugi tanah kapling Nomor 0281 dalam poin 1 tersebut dari Tergugat (TERGUGAT), dan uang tersebut dibayar secara tunai oleh Tergugat di depan sidang Pengadilan Agama Pati sesaat sebelum pembacaan putusan;
3. Bahwa tanah dan bangunan rumah tembok permanen ukuran 7 x 14 M² yang terletak di Dukuh Penggung RT.004/RW.002 Desa Tlogorejo, Kecamatan Tlogowungu, Kabupaten Pati. Dengan batas-batas sebagai berikut ;

- Sebelah Utara : Rumah bapak Nagsidi;
- Sebelah Timur : Jl. Pertolongan Desa;
- Sebelah Selatan : Jl. Desa ;
- Sebelah Barat : Rumah Bapak Kastam;

diberikan kepada anak Penggugat (PENGGUGAT) dan Tergugat (TERGUGAT) yang bernama Mustorifah binti TERGUGAT lahir tgl. 12 Nopember 2003;

Bahwa, bahwa meskipun telah tercapai kesepakatan perdamaian diantara pihak-pihak sebagaimana tersebut diatas, untuk memberikan keyakinan kepada Hakim mengenai letak, batas dan luas serta kwantitas serta kwalitas dari obyek sengketa berdasarkan pasal 153 HIR Jo. pasal 211 RV. jo. Peraturan Mahkamah Agung RI nomor 7 tahun 2001, Majelis Hakim memandang perlu untuk melakukan pemeriksaan setempat, dan untuk kepentingan tersebut pada hari Jumat tanggal 11 Desember 2020 telah dilakukan pemeriksaan setempat atas obyek sengketa dan berdasarkan berita acara pemeriksaan setempat di lokasi tempat barang berada/terletak telah ternyata seluruh obyek-obyek sengketa dimaksud baik luas, letak, serta batas-batas obyek sengketa telah sesuai dengan kesepakatan tersebut;

Bahwa, Pengggugat telah menyampaikan kesimpulan akhir secara lisan yang pada pokoknya rumah tangga Penggugat dan Tergugat sudah tidak harmonis lagi dan tetap pada gugatannya untuk bercerai dengan Tergugat,

Disclaimer
Kepaniteraan Mahkamah Agung Republik Indonesia berusaha untuk selalu mencantumkan informasi paling kini dan akurat sebagai bentuk komitmen Mahkamah Agung untuk pelayanan publik, transparansi dan akuntabilitas pelaksanaan fungsi peradilan. Namun dalam hal-hal tertentu masih dimungkinkan terjadi permasalahan teknis terkait dengan akurasi dan keterkinian informasi yang kami sajikan, hal mana akan terus kami perbaiki dari waktu kewaktu.
Dalam hal Anda menemukan inakurasi informasi yang termuat pada situs ini atau informasi yang seharusnya ada, namun belum tersedia, maka harap segera hubungi Kepaniteraan Mahkamah Agung RI melalui :
Email : kepaniteraan@mahkamahagung.go.id Telp : 021-384 3348 (ext.318)

sedangkan Tergugat telah menyampaikan kesimpulan akhir secara lisan yang pada pokoknya tidak keberatan bercerai dengan Penggugat dan mohon putusan;

Bahwa selanjutnya untuk mempersingkat uraian putusan ini, Majelis Hakim berpendapat cukup menunjuk Berita Acara Sidang yang merupakan bagian yang tidak terpisahkan dari putusan ini ;

TENTANG HUKUMNYA

Menimbang bahwa maksud dan tujuan gugatan Penggugat adalah sebagaimana telah diuraikan di atas

Menimbang, bahwa Majelis Hakim telah berusaha mendamaikan kedua belah pihak yang berperkara baik melalui mediasi sebagaimana laporan Hakim Mediator yang dibuat oleh Drs. H. Zaenal Arifin, MH.Hakim Pengadilan Agama Pati maupun dalam persidangan akan tetapi usaha tersebut tidak berhasil (gagal);

Menimbang, bahwa berdasarkan gugatan Penggugat perkara ini termasuk bidang perkawinan dihubungkan dengan bukti P.1, bukti mana menunjukkan identitas Penggugat sebagai seorang muslimah dan bertempat tinggal di wilayah Kabupaten Pati, dengan demikian secara relatif maupun absolut perkara ini termasuk kompetensi Pengadilan Agama Pati sesuai dengan ketentuan pasal 73 ayat (1), jo Pasal 49 huruf a Undang-Undang Republik Indonesia Nomor 7 tahun 1989 sebagaimana telah diubah pertama dengan Undang-undang Nomor 3 Tahun 2006 perubahan kedua dengan Undang-undang Nomor 50 Tahun 2009, sehingga gugatan Penggugat dapat diterima untuk dipertimbangkan lebih lanjut ;

Menimbang, bahwa berdasarkan bukti P.2 berupa fotokopi Kutipan Akta Nikah yang telah dibubuhi meterai yang cukup dan telah dicocokkan dan ternyata sesuai dengan bunyi surat aslinya, maka terbukti antara Penggugat dengan Tergugat adalah sebagai suami istri yang sah dan belum pernah bercerai sehingga Penggugat adalah pihak yang berhak dan berkepentingan mengajukan perkara ini (*persona standi in judicio*);

Menimbang, bahwa alasan gugatan Penggugat adalah sering terjadi perselisihan dan pertengkaran antara Penggugat dan Tergugat sejak

Disclaimer
Kepaniteraan Mahkamah Agung Republik Indonesia berusaha untuk selalu mencantumkan informasi paling kini dan akurat sebagai bentuk komitmen Mahkamah Agung untuk pelayanan publik, transparansi dan akuntabilitas pelaksanaan fungsi peradilan. Namun dalam hal-hal tertentu masih dimungkinkan terjadi permasalahan teknis terkait dengan akurasi dan keterkinian informasi yang kami sajikan, hal mana akan terus kami perbaiki dari waktu kewaktu.
Dalam hal Anda menemukan inakurasi informasi yang termuat pada situs ini atau informasi yang seharusnya ada, namun belum tersedia, maka harap segera hubungi Kepaniteraan Mahkamah Agung RI melalui :
Email : kepaniteraan@mahkamahagung.go.id Telp : 021-384 3348 (ext.318)

September 2018 yang disebabkan masalah Tergugat diketahui telah menjalin hubungan dengan wanita idaman lain dan sampai mempunyai seorang anak dari wanita tersebut yang ber inisial (UM), akhirnya berpisah Desember 2018 bulan Desember 2018 akhirnya antara Penggugat dan Tergugat pisah ranjang dan tidak pernah berhubungan layaknya suami istri dan selama berpisah antara Penggugat dan Tergugat sudah tidak ada komunikasi lagi sebagai layaknya suami istri yang baik, maka Penggugat menuntut agar dijatuhkan talak Tergugat kepada Penggugat;

Menimbang, bahwa atas gugatan Penggugat tersebut, Tergugat telah mengakui sebagian gugatan Penggugat dan membantah sebagian lainnya, adapun yang dibantah adalah pada pokoknya sebagai berikut ;

1. Bahwa tidak benar semenjak dimulainya pernikahan kedua kalinya antara saudari Penggugat (PENGGUGAT) dengan saudara Tergugat (TERGUGAT) tidak harmonis lagi sering bertengkar, Yang benar adalah terbukti dengan jelas kedua belah pihak mampu bertahan selama 5 tahun lamanya, dan durasi waktu itu tidaklah singkat. Dan begitu jelas juga antara saudari Penggugat (PENGGUGAT) dengan saudara tergugat (TERGUGAT) telah melangsungkan/ melanjutkan sesi pernikahan ulang yang kedua kalinya untuk memperbaiki kegagalannya dalam pernikahan pertamanya yang telah dikaruniai seorang anak bernama “Mustafiroh” yang sudah berumur kurang lebih 17 tahun.
2. Bahwa tidak benar jika saudara tergugat (TERGUGAT) tidak bertanggung jawab atau tidak memberikan nafkah sejak dari bulan September 2018, Yang benar adalah terbukti sampai saat ini kedua belah pihak masih tinggal dalam satu rumah bersama anaknya yang bernama saudari “Mustafiroh” begitu pula keduanya masih bersama-sama saling membantu dalam bekerja/ berdagang di pasar baik dalam tenaga dan finansial/keuangan,
3. Bahwa tidak benar saudara tergugat (TERGUGAT) mempunyai wanita idaman dan sampai mempunyai seorang anak yang berinisial “UM;
4. Bahwa tidak benar antara saudari penggugat (PENGGUGAT) dengan tergugat (TERGUGAT) telah pisah ranjang dan tidak pernah lagi berubungan layaknya suami istri dari bulan Desember 2018, Yang benar

Disclaimer
Kepaniteraan Mahkamah Agung Republik Indonesia berusaha untuk selalu mencantumkan informasi paling kini dan akurat sebagai bentuk komitmen Mahkamah Agung untuk pelayanan publik, transparansi dan akuntabilitas pelaksanaan fungsi peradilan. Namun dalam hal-hal tertentu masih dimungkinkan terjadi permasalahan teknis terkait dengan akurasi dan keterkinian informasi yang kami sajikan, hal mana akan terus kami perbaiki dari waktu kewaktu.
Dalam hal Anda menemukan inakurasi informasi yang termuat pada situs ini atau informasi yang seharusnya ada, namun belum tersedia, maka harap segera hubungi Kepaniteraan Mahkamah Agung RI melalui :
Email : kepaniteraan@mahkamahagung.go.id Telp : 021-384 3348 (ext.318)

adalah kedua belah pihak terakhir melakukan hubungan layaknya suami istri kurang lebih 2 mingguan sebelum diajukannya gugatan perkara ini berlangsung

Menimbang, bahwa untuk membuktikan gugatannya Penggugat mengajukan saksi-saksi Penggugat yang bernama PENGGUGAT telah memberikan keterangan di bawah sumpahnya, bahwa saksi melihat sendiri telah terjadi pertengkaran dan perselisian antara Penggugat dan Tergugat masalah Tergugat mencintai wanita lain bernama Umbar asal Desa Jontro Kecamatan Widarijaksa kabupaten Pati, bahkan Tergugat sampai memukul punggung Penggugat dengan sepatu. yang mengkibatkan Penggugat pergi pulang kerumah orang tuanya sejak 2 bulan yang lalu dan selama pisah antara Penggugat dan Tergugat sudah tidak komunikasi lagi sebagai suami istri yang baik;

Menimbang, bahwa berdasarkan keterangan saksi Penggugat yang bernama Yulaikah binti Rohmat telah memberikan keterangan di bawah sumpahnya, bahwa saksi melihat sendiri terjadinya pertengkaran antara Penggugat dan Tergugat masalah Tergugat mencintai wanita lain bernama Umbar asal Desa Jontro Kecamatan Widarijaksa kabupaten Pati, bahkan sekarang sudah mempunyai seorang anak I, yang mengakibatkan Penggugat pergi pulang kerumah saksi sejak 2 bulan yang lalu dan selama pisah antara Penggugat dan Tergugat sudah tidak komunikasi lagi sebagai suami istri yang baik;

Menimbang, bahwa berdasarkan keterangan saksi Penggugat yang bernama SAKSI PENGUGAT telah memberikan keterangan di bawah sumpahnya, bahwa saksi melihat sendiri pada tahun 2017 Tergugat menikah sirri dengan seorang wanita yang bernama Umbarwati tetangga saksi dan sekarang sudah mempunyai seorang anak umurnya 3 tahun dan Saksi menjadi saksi dalam pernikahan sirri tersebut;

Menimbang, bahwa berdasarkan keterangan saksi-saksi Penggugat yang bernama SAKSI PENGGUGAT dan SAKSI PENGGUGAT tersebut menerangkan telah terjadi perelisihan dan pertengkaran antara Penggugat dan Tergugat masalah Tergugat mencintai wanita lain bernama Umbar asal Desa Jontro Kecamatan Widarijaksa kabupaten Pati, bahkan sekarang sudah mempunyai seorang anak I, yang mengakibatkan Penggugat pergi pulang kerumah saksi sejak 2 bulan yang

Disclaimer
Kepaniteraan Mahkamah Agung Republik Indonesia berusaha untuk selalu mencantumkan informasi paling kini dan akurat sebagai bentuk komitmen Mahkamah Agung untuk pelayanan publik, transparansi dan akuntabilitas pelaksanaan fungsi peradilan. Namun dalam hal-hal tertentu masih dimungkinkan terjadi permasalahan teknis terkait dengan akurasi dan keterkinian informasi yang kami sajikan, hal mana akan terus kami perbaiki dari waktu kewaktu.
Dalam hal Anda menemukan inakurasi informasi yang termuat pada situs ini atau informasi yang seharusnya ada, namun belum tersedia, maka harap segera hubungi Kepaniteraan Mahkamah Agung RI melalui :
Email : kepaniteraan@mahkamahagung.go.id   Telp : 021-384 3348 (ext.318)

lalu dan selama pisah antara Penggugat dan Tergugat sudah tidak komunikasi lagi sebagai suami istri yang baik, maka Majelis Hakim berpendapat terbukti terjadi perselisihan dan pertengkaran antara Penggugat dan Tergugat sehinga rumah tangganya sudah tidak harmonis lagi sejak 2 bulan yang lalu;

Menimbang, bahwa berdasarkan keterangan saksi-saksi Penggugat yang bernama SAKSI PENGGUGAT tersebut menerangkan telah terjadi pernikahan sirri antara Tergugat dengan wanita lain bernama Umbar asal Desa Jontro Kecamatan Widarijaksa kabupaten Pati, bahkan sekarang sudah mempunyai seorang anak I, maka Majelis Hakim berpendapat terbukti Tergugat telah mencintai wanita lain bahkan sudah mempunyai anak 1 yang sekarang berumur 3 tahun;

Menimbang, bahwa Tergugat untuk menguatkan bantahannya mengajukan saksi-saksi yang telah memberikan keterangan di bawah sumpahnya, bahwa rumah tangga Penggugat dan Tergugat baik-baik (rukun) tidak pernah terjadi pertengkaran akan tetapi sejak 1 bulan yang lalu antara Penggugat dan Tergugat sudah tidak komunikasi sebagai layaknya suami istri yang baik, karena mereka sudah tidak pergi bersama lagi;

Menimbang, bahwa berdasarkan keterangan saksi-saksi Tergugat tersebut diatas, sudah tidak komunikasi sebagai layaknya suami istri yang baik antara Pengugat dan Tergugat sejak 1 bulan yang lalu, maka Majejelis Hakim berpendapat bahwa dengan adanya sudah tidak komunikasi lagi antara Penggugat dan Tergugat, terbukti rumah tangga Penggugat dan Tergugat sudah tidak harmonis lagi, oleh karena itu keterangan saksi-saksi Tergugat tersebut, menguatkan gugatan Penggugat dimana rumah tangganya sudah tidak harmonis lagi maka bantahan Tergugat rumah tangganya masih harmonis tidak terbukti ;

Menimbang, bahwa oleh karena dalil-dalil bantahan Tergugat tidak terbukti, maka bantahan Tergugat harus dinyatakan ditolak;

Menimbang, bahwa berdasarkan keterangan Penggugat dan pengakuan Tergugat maupun saksi-saksi Penggugat dan Tergugat ternyata bersesuaian satu dengan yang lainnya, maka Majelis Hakim menyimpulkan adanya fakta sebagai berikut:

Disclaimer
Kepaniteraan Mahkamah Agung Republik Indonesia berusaha untuk selalu mencantumkan informasi paling kini dan akurat sebagai bentuk komitmen Mahkamah Agung untuk pelayanan publik, transparansi dan akuntabilitas pelaksanaan fungsi peradilan. Namun dalam hal-hal tertentu masih dimungkinkan terjadi permasalahan teknis terkait dengan akurasi dan keterkinian informasi yang kami sajikan, hal mana akan terus kami perbaiki dari waktu kewaktu.
Dalam hal Anda menemukan inakurasi informasi yang termuat pada situs ini atau informasi yang seharusnya ada, namun belum tersedia, maka harap segera hubungi Kepaniteraan Mahkamah Agung RI melalui :
Email : kepaniteraan@mahkamahagung.go.id   Telp : 021-384 3348 (ext.318)

- Bahwa Penggugat dan Tergugat sebagai suami istri yang sah sejak April 2013, semula hidup rukun dan harmonis, telah tinggal bersama terakhir dirumah milik bersama dan sudah dikaruniai seorang anak;
- Bahwa rumah tangga Penggugat dan Tergugat tidak harmonis karena berselisih terus menerus yang disebabkan Tergugat menikah sirri dengan seorang wanita yang bernama Umbarwati akibatnya Penggugat pergi pulang kerumah orang tuanya sejak 2 bulan yang lalu dan selama pisah tersebut, antara Penggugat dan Tergugat sudah tidak ada komunikasi lagi sebagai layaknya suami istri yang baik;
- Bahwa selama berpisah tersebut keluarga Tergugat sudah berusaha mendamaikan supaya Penggugat dan Tergugat hidup rukun kembali dalam rumah tangganya akan tetapi tidak berhasil;

Menimbang, bahwa fakta tersebut diatas, Majelis Hakim berpendapat bahwa dalam konflik rumah tangga antara Penggugat dan Tergugat tersebut tidak perlu dicari siapa yang salah atau siapa yang menjadi penyebab terjadinya perselisihan dan pertengkaran sesuai Yurisprudensi (putusan Mahkamah Agung RI) Nomor 38/AG/1990 tanggal 5 Oktober 1991melainkan Majelis Hakim melihat bahwa sifat pertengkaran dan percekcokan Penggugat dan Tergugat sudah dalam katagori yang sangat kritis, yang mengakibatkan tidak menjadikan harmonisnya dalam rumah tangga antara Penggugat dan Tergugat :

Menimbang, bahwa dari apa yang telah dipertimbangkan diatas, Majelis berpendapat bahwa hubungan antara Penggugat dengan Tergugat dalam membina rumah tangganya telah pecah dan tidak ada harapan akan hidup rukun lagi sebagai suami isteri, maka dalam kondisi rumah tangga yang demikian kehidupan keluarga sakinah mawaddah dan rahmah sebagai tujuan perkawinan seperti dimaksud dalam Firman Allah swt dalam Surat Ar-Rum ayat 21 jelas tidak akan tercapai;

Menimbang, bahwa dengan demikian patut dianggap bahwa keadaan rumah tangga yang kekal berdasarkan KeTuhanan Yang Maha Esa sesuai Pasal 1 dan Pasal 33 Undang-Undang Nomor 1 Tahun 1974 sudah tidak terdapat lagi dalam rumah tangga Penggugat dan Tergugat, terbukti dalam pemeriksaan persidangan, Penggugat dan Tergugat tidak mungkin dipersatukan secara utuh,

Disclaimer
Kepaniteraan Mahkamah Agung Republik Indonesia berusaha untuk selalu mencantumkan informasi paling kini dan akurat sebagai bentuk komitmen Mahkamah Agung untuk pelayanan publik, transparansi dan akuntabilitas pelaksanaan fungsi peradilan. Namun dalam hal-hal tertentu masih dimungkinkan terjadi permasalahan teknis terkait dengan akurasi dan keterkinian informasi yang kami sajikan, hal mana akan terus kami perbaiki dari waktu kewaktu.
Dalam hal Anda menemukan inakurasi informasi yang termuat pada situs ini atau informasi yang seharusnya ada, namun belum tersedia, maka harap segera hubungi Kepaniteraan Mahkamah Agung RI melalui :
Email : kepaniteraan@mahkamahagung.go.id Telp : 021-384 3348 (ext.318)

bahkan bila dipaksakan berkumpul maka akan lebih banyak membawa kemadhorotannya daripada kemaslahatannya, kondisi semacam itu harus dihindarkan dan diakhiri, hal ini sesuai dengan qoidah fiqhiyyah yang berbunyi sebagai berikut :

د رء المفا سد مقد م على جلب المصا لح

Artinya :" Mencegah kerusakan lebih didahulukan daripada mengharap kebaikan" ;

Dan dalam kitab Fiqhus Sunnah Juz II, halaman 290, yang berbunyi sebagai berikut:

فاذا ثـبتت دعوها لدي القاضى ببينة الزوجة او اقرار الزوج- الى ان قال-

وعجز القاضى عن الاصلاح بينهما طلقها طلقة بائنة

Artinya : "*Apabila terbukti gugatan isteri di hadapan Hakim karena adanya bukti dari isteri atau pengakuan dari suami sampai pada kata-kata dan Hakim sudah tidak mampu mendamaikan keduanya, maka Hakim berwenang menjatuhkan talaknya (suami) dengan talak satu bain."*

Menimbang, bahwa dari fakta tersebut di atas telah menguatkan dalil-dalil gugatan Penggugat sehingga dalil Penggugat tersebut dinyatakan telah terbukti dan menjadi fakta hukum, maka Majelis Hakim berkesimpulan bahwa gugatan Penggugat telah memenuhi syarat dan alasan perceraian sebagaimana Pasal 39 ayat (2) Undang-Undang Nomor 1 Tahun 1974 jo Pasal 19 huruf (f) Peraturan Pemerintah Nomor 9 Tahun 1975 jo Pasal 116 huruf (f) Kompilasi Hukum Islam, oleh karenaya gugatan Penggugat tersebut dapat dikabulkan;

Menimbang, bahwa selama pernikahan Penggugat dan Tergugat belum pernah bercerai, dalam keadaan ba'da dukhul, maka sesuai maksud pasal 119 ayat (2) huruf c Kompilasi Hukum Islam di Indonersia Tahun 1991, Majelis Hakim perlu menetapkan jatuh Talak Satu Ba'in Sughro Tergugat kepada Penggugat ;

Menimbang, bahwa Penggugat dan Tergugat menyatakan setuju bercecerai dan telah terjadi kesepakatan tentang pembagian harta bersama antara Penggugat dan Tergugat yang pada pokoknya sebagai berikut;

Disclaimer
Kepaniteraan Mahkamah Agung Republik Indonesia berusaha untuk selalu mencantumkan informasi paling kini dan akurat sebagai bentuk komitmen Mahkamah Agung untuk pelayanan publik, transparansi dan akuntabilitas pelaksanaan fungsi peradilan. Namun dalam hal-hal tertentu masih dimungkinkan terjadi permasalahan teknis terkait dengan akurasi dan keterkinian informasi yang kami sajikan, hal mana akan terus kami perbaiki dari waktu kewaktu.
Dalam hal Anda menemukan inakurasi informasi yang termuat pada situs ini atau informasi yang seharusnya ada, namun belum tersedia, maka harap segera hubungi Kepaniteraan Mahkamah Agung RI melalui :
Email : kepaniteraan@mahkamahagung.go.id Telp : 021-384 3348 (ext.318)

1. Bahwa Tergugat (TERGUGAT) mendapat bagian berupa tanah Kapling Nomor 0281 dengan luas tanah 11 x 10 M² terletak di Dukuh Penggung RT.004/RW.002 Desa Tlogorejo, Kecamatan. Tlogowungu, Kabupaten Pati. Dengan batas-batas sebagai berikut ;
    - Sebelah Utara : Tanah H. Suparmin;
    - Sebelah Timur : Jl. Desa;
    - Sebelah Selatan : Tanah Yono ;
    - Sebelah Barat : Tanah H. Suparmin;
2. Bahwa Penggugat (PENGGUGAT binti Sutopo) mendapatkan bagian Rp 10. 000.000,00 (sepuluh juta rupiah) sebagai ganti rugi tanah kapling Nomor 0281 dalam poin 1 tersebut dari Tergugat (TERGUGAT), dan uang tersebut dibayar secara tunai oleh Tergugat di depan sidang Pengadilan Agama Pati sesaat sebelum pembacaan putusan;
3. Bahwa tanah dan bangunan rumah tembok permanen ukuran 7 x 14 M² yang terletak di Dukuh Penggung RT.004/RW.002 Desa Tlogorejo, Kecamatan Tlogowungu, Kabupaten Pati. Dengan batas-batas sebagai berikut ;
    - Sebelah Utara : Rumah bapak Nagsidi;
    - Sebelah Timur : Jl. Pertolongan Desa;
    - Sebelah Selatan : Jl. Desa ;
    - Sebelah Barat : Rumah Bapak Kastam;

    diberikan kepada anak Penggugat (PENGGUGAT) dan Tergugat (TERGUGAT) yang bernama ANAK PENGGUGAT DAN TERGUGAT lahir tgl. 12 Nopember 2003;

Menimbang, bahwa oleh karena Penggugat dan Tergugat telah sepakat dan terjadi perdamaian tentang pembagian harta bersaa maka kesepakatan tersebut dikabulkan dengan menghukum Penggugat dan Tergugat i untuk mentaati dan melaksanakan isi perdamaian dan kesepakatan tersebut sebagaimana ditetapkan dalam amar putusan ;

Menimbang, bahwa karena perkara ini merupakan bidang perkawinan, berdasarkan pasal 89 ayat (1) Undang-undang No. 7 tahun 1989 sebagaimana telah diubah dengan Undang-Undang No.3 tahun 2006 dan perubahan kedua

Disclaimer
Kepaniteraan Mahkamah Agung Republik Indonesia berusaha untuk selalu mencantumkan informasi paling kini dan akurat sebagai bentuk komitmen Mahkamah Agung untuk pelayanan publik, transparansi dan akuntabilitas pelaksanaan fungsi peradilan. Namun dalam hal-hal tertentu masih dimungkinkan terjadi permasalahan teknis terkait dengan akurasi dan keterkinian informasi yang kami sajikan, hal mana akan terus kami perbaiki dari waktu kewaktu.
Dalam hal Anda menemukan inakurasi informasi yang termuat pada situs ini atau informasi yang seharusnya ada, namun belum tersedia, maka harap segera hubungi Kepaniteraan Mahkamah Agung RI melalui :
Email : kepaniteraan@mahkamahagung.go.id Telp : 021-384 3348 (ext.318)

Undang-Undang Nomor 50 Tahun 2009, maka biaya perkara ini dibebankan kepada Penggugat;

Mengingat dan memperhatikan pasal-pasal peraturan perundang-undangan yang berlaku dan dalil-dalil syar'i yang berhubungan dengan perkara ini ;

M E N G A D I L I

1. Mengabulkan gugatan Penggugat;
2. Menjatuhkan talak satu bain sughro Tergugat (TERGUGAT) Terhadap Penggugat (PENGGUGAT) .
3. Menghukum kepada Penggugat dan Tergugat untuk mentaati kesepakatan tentang pembagian harta bersama yang pada pokoknya sebagai berikut;

a. Bahwa Tergugat (TERGUGAT) mendapat bagian berupa tanah Kapling Nomor 0281 dengan luas tanah 11 x 10 M² terletak di Dukuh Penggung RT.004/RW.002 Desa Tlogorejo, Kecamatan. Tlogowungu, Kabupaten Pati. Dengan batas-batas sebagai berikut ;

- Sebelah Utara : Tanah H. Suparmin;
- Sebelah Timur : Jl. Desa;
- Sebelah Selatan : Tanah Yono ;
- Sebelah Barat : Tanah H. Suparmin;

b. Bahwa Penggugat (PENGGUGAT) mendapatkan bagian Rp 10. 000.000,00 (sepuluh juta rupiah) sebagai ganti rugi tanah kapling Nomor 0281 dalam poin 1 (a) tersebut dari Tergugat (TERGUGAT), dan uang tersebut dibayar secara tunai oleh Tergugat di depan sidang Pengadilan Agama Pati sesaat sebelum pembacaan putusan;

c. Bahwa tanah dan bangunan rumah tembok permanen ukuran 7 x 14 M² yang terletak di Dukuh Penggung RT.004/RW.002 Desa Tlogorejo, Kecamatan Tlogowungu, Kabupaten Pati. Dengan batas-batas sebagai berikut ;

- Sebelah Utara : Rumah bapak Nagsidi;
- Sebelah Timur : Jl. Pertolongan Desa;
- Sebelah Selatan : Jl. Desa ;
- Sebelah Barat : Rumah Bapak Kastam;

Disclaimer
Kepaniteraan Mahkamah Agung Republik Indonesia berusaha untuk selalu mencantumkan informasi paling kini dan akurat sebagai bentuk komitmen Mahkamah Agung untuk pelayanan publik, transparansi dan akuntabilitas pelaksanaan fungsi peradilan. Namun dalam hal-hal tertentu masih dimungkinkan terjadi permasalahan teknis terkait dengan akurasi dan keterkinian informasi yang kami sajikan, hal mana akan terus kami perbaiki dari waktu kewaktu.
Dalam hal Anda menemukan inakurasi informasi yang termuat pada situs ini atau informasi yang seharusnya ada, namun belum tersedia, maka harap segera hubungi Kepaniteraan Mahkamah Agung RI melalui :
Email : kepaniteraan@mahkamahagung.go.id Telp : 021-384 3348 (ext.318)

diberikan kepada anak Penggugat (PENGGUGAT) dan Tergugat (TERGUGAT) yang bernama ANAK PENGGUGAT DAN TERGUGAT lahir tgl. 12 Nopember 2003;

3. Membebankan kepada Penggugat untuk membayar biaya perkara sejumlah Rp.1.761.000,00 (satu juta tujuh ratus enam puluh satu ribu rupiah);

Demikian diputuskan dalam rapat permusyawaratan Majelis Hakim Pengadilan Agama Pati pada hari Senin tanggal 28 Desember 2020 M, bertepatan dengan tanggal 13 Jumadil Awal 1442 H, oleh kami Drs. H. Luqman Suadi, MH**.,** selaku Hakim Ketua, Drs. Rahman Pamuji, MSI dan Drs. H. Edi Suwarsono, MH., selaku Hakim-hakim Anggota, dan pada hari itu juga diucapkan oleh Hakim Ketua dalam sidang terbuka untuk umum dengan dihadiri oleh Hakim Anggota dengan dibantu Hj. Hidayati, S.Ag. selaku Panitera Pengganti dengan dihadiri oleh Penggugat dan Kuasanya serta Tergugat;

Hakim Ketua

Drs. H. Luqman Suadi, MH

Hakim Anggota I

Drs. Rahman Pamuji, MSI,

Hakim Anggota II,

Drs. H.Edi Suwarsono, MH

Panitera Pengganti,

Hj. Hidayati, S.Ag.

<u>Perincian biaya perkara</u> :

Disclaimer
Kepaniteraan Mahkamah Agung Republik Indonesia berusaha untuk selalu mencantumkan informasi paling kini dan akurat sebagai bentuk komitmen Mahkamah Agung untuk pelayanan publik, transparansi dan akuntabilitas pelaksanaan fungsi peradilan. Namun dalam hal-hal tertentu masih dimungkinkan terjadi permasalahan teknis terkait dengan akurasi dan keterkinian informasi yang kami sajikan, hal mana akan terus kami perbaiki dari waktu kewaktu.
Dalam hal Anda menemukan inakurasi informasi yang termuat pada situs ini atau informasi yang seharusnya ada, namun belum tersedia, maka harap segera hubungi Kepaniteraan Mahkamah Agung RI melalui :
Email : kepaniteraan@mahkamahagung.go.id   Telp : 021-384 3348 (ext.318)

1. Pendaftaran	Rp.	30.000,00,-
2. BAPP	Rp.	75.000,00,-
3. Panggilan	Rp.	300.000,00,-
4. Biaya Desente	Rp.	1340.000.00,-
5. Redaksi	Rp.	10.000,00,-
6. Materai	Rp.	6.000,00,-
Jumlah	Rp.	1761.000,00,-

Disclaimer
Kepaniteraan Mahkamah Agung Republik Indonesia berusaha untuk selalu mencantumkan informasi paling kini dan akurat sebagai bentuk komitmen Mahkamah Agung untuk pelayanan publik, transparansi dan akuntabilitas pelaksanaan fungsi peradilan. Namun dalam hal-hal tertentu masih dimungkinkan terjadi permasalahan teknis terkait dengan akurasi dan keterkinian informasi yang kami sajikan, hal mana akan terus kami perbaiki dari waktu kewaktu.
Dalam hal Anda menemukan inakurasi informasi yang termuat pada situs ini atau informasi yang seharusnya ada, namun belum tersedia, maka harap segera hubungi Kepaniteraan Mahkamah Agung RI melalui :
Email : kepaniteraan@mahkamahagung.go.id Telp : 021-384 3348 (ext.318)
