# Supplementary material for: Classification of divorce causes during the COVID-19 pandemic using convolutional neural networks
Source: PeerJ Comput Sci. 2022 Jun 30;8:e998. doi: 10.7717/peerj-cs.998 (PMC9299239; doi:10.7717/peerj-cs.998)
Supplement: Supplemental Information 3 [file peerj-cs-08-998-s003.zip › KDRT Dataset/Data ke-1.pdf]

P U T U S A N

Nomor xxxx/Pdt.G/2020/PA.Dmk.

بسم الله الرحمن الرحيم

DEMI KEADILAN BERDASARKAN KETUHANAN YANG MAHA ESA

Pengadilan Agama Demak yang memeriksa dan mengadili perkara-perkara tertentu pada tingkat pertama, dalam persidangan Majelis Hakim telah menjatuhkan putusan dalam perkara cerai gugat antara :

Xxxx, umur 59 tahun, agama Islam, pendidikan SD, pekerjaan Petani, bertempat tinggal di Desa Gemulak RT02 RW02 Kecamatan Sayung, Kabupaten Demak, sebagai Penggugat;

melawan

Xxxx, umur 67 tahun, agama Islam, pendidikan SD, pekerjaan Tidak bekerja, bertempat tinggal di Desa Gemulak RT02 RW02 Kecamatan Sayung, Kabupaten Demak, sebagai Tergugat;

Pengadilan Agama tersebut ;

Telah membaca surat-surat perkara ;

Telah mendengar keterangan Penggugat dan saksi-saksinya ;

DUDUK PERKARA

Bahwa Penggugat dalam surat gugatannya tertanggal 29 Oktober 2020 terdaftar di Kepaniteraan Pengadilan Agama Demak, Nomor xxxx/Pdt.G/2020/PA.Dmk. tanggal 30 September 2020, telah mengemukakan hal-hal sebagai berikut :

1. Bahwa Penggugat adalah istri sah dari Tergugat yang telah melangsungkan pernikahan menurut agama Islam pada tanggal 9 Mei 1989 di rumah orangtua Penggugat di hadapan Pegawai Pencatat Nikah Kantor Urusan Agama kecamatan Sayung kabupaten Demak yang terdaftar dalam daftar

Disclaimer
Kepaniteraan Mahkamah Agung Republik Indonesia berusaha untuk selalu mencantumkan informasi paling kini dan akurat sebagai bentuk komitmen Mahkamah Agung untuk pelayanan publik, transparansi dan akuntabilitas pelaksanaan fungsi peradilan. Namun dalam hal-hal tertentu masih dimungkinkan terjadi permasalahan teknis terkait dengan akurasi dan keterkinian informasi yang kami sajikan, hal mana akan terus kami perbaiki dari waktu kewaktu.
Dalam hal Anda menemukan inakurasi informasi yang termuat pada situs ini atau informasi yang seharusnya ada, namun belum tersedia, maka harap segera hubungi Kepaniteraan Mahkamah Agung RI melalui :
Email : kepaniteraan@mahkamahagung.go.id   Telp : 021-384 3348 (ext.318)

yang disediakan untuk itu sebagaimana dalam Kutipan Akta Nikah nomor : 24/06/V/1989 tertanggal 9 Mei 1989 yang dikeluarkan oleh Kantor Urusan Agama Sayung, Kabupaten Demak;

2. Bahwa setelah menikah Penggugat dan Tergugat tinggal di rumah orangtua Penggugat yang ber alamat di desa Gemulak rt 02/02, kecamatan Sayung, kabupaten Demak, setelah 2 tahun kemudian tahun 1990 pindah ke rumah sendiri di di desa desa Gemulak rt 02/02 kecamatan Sayung, kabupaten Demak;
3. Bahwa antara Penggugat dan Tergugat sudah melakukan hubungan layaknya suami istri (ba'da dhukhul) selama dalam perkawinan antara Penggugat dan Tergugat dikaruniai 6 anak, masing-masing bernama :
   3.1 xxxx, umur 33 tahun, anak bawaan dari suami pertama;
   3.2 xxxx, umur : 30 tahun;
   3.3 xxxx, umur : 28 tahun;
   3.4 xxxx, umur : 25 tahun;
   3.5 xxxx, umur : 22 tahun;
   3.6 xxxx, umur : 21 tahun;

   Anak ke-3 dan ke-6 ikut bersama Penggugat;
4. Bahwa pada awalnya keadaan rumah tangga Penggugat dan Tergugat semula rukun dan harmonis, namun sejak bulan Desember tahun 2004 terjadi pertengkaran antara Penggugat dan Tergugat dengan alasan sebagai berikut :
   4.1 Tergugat pada tahun 1999 tidak bekerja sehingga tidak memberikan nafkah kepada Penggugat;
   4.2 Tergugat melakukan kekerasan rumah tangga terhadap Penggugat sejak tahun 1999 bahkan hampir saja dilaporkan ke pihak Polisi;
5. Bahwa Penggugat bersama anak-anak berusaha menyadarkan atas perilaku Tergugat namun tidak berhasil dan Penggugat terus menerus mengalami penganiayaan dari Tergugat;
6. Bahwa Penggugat tetap berusah sabar dan menerima perilaku Tergugat, namun karena Tergugat tidak bisa berhenti melakukan penganiayaan, sehingga kehidupan rumah tangga Penggugat dan Tergugat semakin jauh

Disclaimer
Kepaniteraan Mahkamah Agung Republik Indonesia berusaha untuk selalu mencantumkan informasi paling kini dan akurat sebagai bentuk komitmen Mahkamah Agung untuk pelayanan publik, transparansi dan akuntabilitas pelaksanaan fungsi peradilan. Namun dalam hal-hal tertentu masih dimungkinkan terjadi permasalahan teknis terkait dengan akurasi dan keterkinian informasi yang kami sajikan, hal mana akan terus kami perbaiki dari waktu kewaktu.
Dalam hal Anda menemukan inakurasi informasi yang termuat pada situs ini atau informasi yang seharusnya ada, namun belum tersedia, maka harap segera hubungi Kepaniteraan Mahkamah Agung RI melalui :
Email : kepaniteraan@mahkamahagung.go.id Telp : 021-384 3348 (ext.318)

dari menjadi keluarga yang Sakinah, Mawaddah, Warohmah,maka terpaksa Penggugat mengajukan Gugatan ini;

7. Bahwa atas dasar uraian diatas gugatan Penggugat telah memenuhi alasan perceraian sebagaimana diatur undang-undang no 1 tahun 1974 jo. Peraturan Pemerintah no 9 tahun 1975 Pasal 19 huruf (f) jo. Kompilsasi Hukum Islam pasal 116 huruf (f);

Bahwa berdasarkan alasan / dalil-dalil sebagaimana tersebut diatas, Penggugat mohon kepada Ketua Pengadilan Agama Demak atau Majelis Hakim yang memeriksa perkara ini agar dengan segera memeriksa dan mengadili serta memutus perkara ini yang amarnya sebagai berikut :

PRIMER

1. Mengabulkan Gugatan Penggugat untuk seluruhnya;
2. Menyatakan jatuh talak satu Ba'in Sughro Tergugat (Xxxx(alm)) atas diri Penggugat (Xxxx (alm));
3. Membebankan biaya perkara sesuai dengan ketentuan hukum yang berlaku;

SUBSIDAIR:

- Apabila Ketua Pengadilan Agama Demak berpendapat lain, mohon putusan yang seadil-adilnya.

Bahwa pada hari sidang yang telah ditentukan, Penggugat dan Tergugat telah datang menghadap di persidangan dan majelis hakim telah berusaha mendamaikan Penggugat dan Tergugat dengan mediasi, akan tetapi dalam mediasi tersebut Penggugat dan Tergugat gagal untuk mengakhiri sengketa rumah tangganya dengan perdamaian, sebagaimana ternyata dalam surat laporan mediasi tertanggal 20 Oktober 2020 nomor xxxx/Pdt.G/ 2020/PA.Dmk. yang dibuat oleh Fatkhul Mu'in, S.H., M.H., sebagai mediator;

Bahwa Majelis Hakim juga telah mendamaikan Penggugat dan Tergugat dalam persidangan agar rukun lagi dan membina rumah tangga dengan baik, akan tetapi tidak berhasil karena Penggugat tetap ingin bercerai dari Tergugat, kemudian dilanjutkan dengan pembacaan surat gugatan Penggugat yang isinya tetap dipertahankan oleh Penggugat ;

Disclaimer
Kepaniteraan Mahkamah Agung Republik Indonesia berusaha untuk selalu mencantumkan informasi paling kini dan akurat sebagai bentuk komitmen Mahkamah Agung untuk pelayanan publik, transparansi dan akuntabilitas pelaksanaan fungsi peradilan. Namun dalam hal-hal tertentu masih dimungkinkan terjadi permasalahan teknis terkait dengan akurasi dan keterkinian informasi yang kami sajikan, hal mana akan terus kami perbaiki dari waktu kewaktu.
Dalam hal Anda menemukan inakurasi informasi yang termuat pada situs ini atau informasi yang seharusnya ada, namun belum tersedia, maka harap segera hubungi Kepaniteraan Mahkamah Agung RI melalui :
Email : kepaniteraan@mahkamahagung.go.id Telp : 021-384 3348 (ext.318)

Bahwa atas gugatan Penggugat tersebut, Tergugat telah memberikan jawaban secara lisan pada persidangan tanggal 10 November 2020 sebagai berikut :

- Bahwa Tergugat menolak dengan tegas dalil-dalil gugatan Penggugat kecuali dalam hal secara tegas Tergugat mengakui kebenarannya;
- Bahwa di dalam posita nomr 1, 2, dan 3 adalah benar adanya;
- Bahwa tidak benar, antara Penggugat dengan Tergugat terjadi perselisihan dan pertengkaran sejak tahun 1999, akan tetapi yang benar, terjadi pertengkaran baru sejak 2 bulan yang lalu atau bulan September tahun 2020;
- Bahwa tidak benar, Tergugat tidak bekerja sejak tahun 1999 tetapi yang benar baru sekitar 2 tahun yang lalu disebabkan karena Tergugat mengalami sakit kaki dan sejak saat itu pula Penggugat sudah tidak mau melayani kebutuhan biologis Tergugat malah Penggugat menyuruh nglonte saja;
- Bahwa tidak benar, Tergugat melakukan kekerasan rumah tangga terhadap Penggugat dan justeru Tergugat yang dipukul oleh anak Penggugat dan Tergugat yang tinggal bersama Penggugat;
- Bahwa Penggugat pedagang buah di Pasar Sayung, berangkat jam 3 pagi, diantar jemput oleh seorang sopir, setiap mau berangkat ke Pasar, Penggugat bersolek (dandan) dan Tergugat melihat hubungan keduanya sangat dekat;
- Bahwa pada pokoknya Tergugat keberatan bercerai, namun jika Pengugat bersikeras bercerai maka Tergugat menuntut uang pengganti (pedot tersno) sejumlah Rp. 150.000.000,00,- (seratus lima puluh juta rupiah);

Bahwa terhadap jawaban tersebut, Penggugat telah mengajukan replik secara lisan yang pada pokoknya bertetap pada dalil-dalil gugatannya dan menolak memberikan uang konpensasi (pedot tresno) sebagaimana tuntutan Tergugat dan selanjutnya Tergugat pada tahap duplik juga menyatakan bertetap pada jawabannya dan tetap meminta uang konpensasi (pedot tresno) sebesara Rp. 150.000.000.00, (seratus lima puluh juta rupiah);

Disclaimer
Kepaniteraan Mahkamah Agung Republik Indonesia berusaha untuk selalu mencantumkan informasi paling kini dan akurat sebagai bentuk komitmen Mahkamah Agung untuk pelayanan publik, transparansi dan akuntabilitas pelaksanaan fungsi peradilan. Namun dalam hal-hal tertentu masih dimungkinkan terjadi permasalahan teknis terkait dengan akurasi dan keterkinian informasi yang kami sajikan, hal mana akan terus kami perbaiki dari waktu kewaktu.
Dalam hal Anda menemukan inakurasi informasi yang termuat pada situs ini atau informasi yang seharusnya ada, namun belum tersedia, maka harap segera hubungi Kepaniteraan Mahkamah Agung RI melalui :
Email : kepaniteraan@mahkamahagung.go.id Telp : 021-384 3348 (ext.318)

Bahwa untuk membuktikan dalil gugatannya, Penggugat telah mengajukan bukti-buktinya di persidangan pada tanggal 1 Desember 2020, yaitu sebagai berikut:

A. Bukti Tertulis:

1. Fotokopi kartu tanda penduduk Penggugat nomor 3321045205610005 tertanggal 03 November 2012 yang dikeluarkan Dinas Kependudukan dan Pencatatan Sipil Kabupaten Demak, (P-1);

2. Fotokopi Kutipan Akta Nikah Nomor: 24/06/V/1989 tertanggal 9 Mei 1989, yang dikeluarkan oleh Kantor Urusan Agama Kecamatan Sayung, Kabupaten Demak, (P-2).

Surat-surat bukti tersebut setelah dicocokkan dengan aslinya ternyata sesuai dan telah bermatrei cukup

A. Bukti saksi-saksi:

1. xxxx, umur 53 tahun, di bawah sumpahnya ia memberi keterangan sebagai berikut:

- Bahwa saksi kenal baik dengan Penggugat dan Tergugat karena saksi bertetangga dekat dengan Penggugat;
- Bahwa saksi kenal dengan Tergugat sejak menjadi suami Penggugat.
- Bahwa Penggugat dan Tergugat adalah suami isteri menikah sudah 30 tahun lebih dan sudah dikaruniai 5 orang anak;
- Bahwa setelah menikah Penggugat dan Tergugat tinggal di rumah sendiri di Desa Gemulak;
- Bahwa pada awalnya rumah tangga rukun dan harmonis akan tetapi sejak tahun 2004 antara Penggugat dengan Tergugat mulai terjadi perselisisihan dan pertengkaran dan saksi sering melihat sendiri pertengkaran keduanya;
- Bahwa penyebab pertengkaran tersebut karena Tergugat tidak bekerja sehingga tidak bisa memberi nafkah kepada Penggugat, menuduh Penggugat berselingkuh dengan laki-laki lain;

Disclaimer
Kepaniteraan Mahkamah Agung Republik Indonesia berusaha untuk selalu mencantumkan informasi paling kini dan akurat sebagai bentuk komitmen Mahkamah Agung untuk pelayanan publik, transparansi dan akuntabilitas pelaksanaan fungsi peradilan. Namun dalam hal-hal tertentu masih dimungkinkan terjadi permasalahan teknis terkait dengan akurasi dan keterkinian informasi yang kami sajikan, hal mana akan terus kami perbaiki dari waktu kewaktu.
Dalam hal Anda menemukan inakurasi informasi yang termuat pada situs ini atau informasi yang seharusnya ada, namun belum tersedia, maka harap segera hubungi Kepaniteraan Mahkamah Agung RI melalui :
Email : kepaniteraan@mahkamahagung.go.id Telp : 021-384 3348 (ext.318)

- Bahwa Tergugat pernah melakukan kekerasan fisik / penganiayaan kepada Penggugat dan penah pula mengancam Penggugat dengan sebilah pisau;
- Bahwa kemudian sudah sekitar 2 bulan lamanya antara Penggugat dengan Tergugat pisah rumah yaitu Pengugat pergi meninggalkan Tergugat dan kini tinggal bersama anaknya;
- Bahwa selama berpisah, antara Penggugat dan Tergugat sudah tidak ada komunikasi seperti layaknya suami istri;
- Bahwa saksi tidak tahu, apakah pihak keluarga sudah pernah mendamaikan keduanya ataukah belum;

2. **xxxx,** umur 19 tahun, di bawah sumpahnya ia memberi keterangan sebagai berikut:

- Bahwa saksi kenal baik dengan Penggugat dan Tergugat karena saksi bertetangga dekat dengan Penggugat;
- Bahwa saksi kenal dengan Tergugat sejak menjadi suami Penggugat.
- Bahwa Penggugat dan Tergugat adalah suami isteri menikah sudah 30 tahun lebih dan sudah dikaruniai 5 anak;
- Bahwa setelah menikah Penggugat dan Tergugat tinggal di rumah sendiri di Desa Gemulak;
- Bahwa pada awalnya rumah tangga rukun dan harmonis akan tetapi sejak beberapa tahun yang lalu antara Penggugat dengan Tergugat sudah terjadi perselisisihan dan pertengkaran dan saksi sering melihat sendiri pertengkaran keduanya;
- Bahwa penyebab pertengkaran tersebut karena Tergugat tidak bekerja sehingga tidak bisa memberi nafkah kepada Penggugat, menuduh Penggugat berselingkuh dengan laki-laki lain dan pernah melakukan kekerasan fisik / penganiayaan kepada Penggugat;
- Bahwa kemudian sudah sekitar 2 bulan lamanya antara Penggugat dengan Tergugat pisah rumah yaitu Pengugat pergi meninggalkan Tergugat dan kini tinggal bersama anaknya;

Disclaimer
Kepaniteraan Mahkamah Agung Republik Indonesia berusaha untuk selalu mencantumkan informasi paling kini dan akurat sebagai bentuk komitmen Mahkamah Agung untuk pelayanan publik, transparansi dan akuntabilitas pelaksanaan fungsi peradilan. Namun dalam hal-hal tertentu masih dimungkinkan terjadi permasalahan teknis terkait dengan akurasi dan keterkinian informasi yang kami sajikan, hal mana akan terus kami perbaiki dari waktu kewaktu.
Dalam hal Anda menemukan inakurasi informasi yang termuat pada situs ini atau informasi yang seharusnya ada, namun belum tersedia, maka harap segera hubungi Kepaniteraan Mahkamah Agung RI melalui :
Email : kepaniteraan@mahkamahagung.go.id   Telp : 021-384 3348 (ext.318)

- Bahwa selama berpisah, antara Penggugat dan Tergugat sudah tidak ada komunikasi seperti layaknya suami istri;
- Bahwa saksi tidak tahu, apakah pihak keluarga sudah pernah mendamaikan keduanya ataukah belum;

Bahwa terhadap ketarangan saksi-saksi tersebut Penggugat membenarkan dan menerimanya;

Bahwa atas bukti-bukti yang diajukan oleh Penggugat tersebut, Tergugat tidak mengajukan bantahannya, karena tidak hadir di persidangan;

Bahwa Penggugat telah menyampaikan kesimpulannya secara lisan di persidangan pada tanggal 01 Desember 2020, yang pada pokoknya Penggugat tetap menghendaki perceraian dan menyatakan tidak akan mengajukan suatu apapun lagi dan mohon putusan;

Bahwa untuk meringkas uraian putusan ini, maka ditunjuk hal ihwal sebagaimana tercantum dalam berita acara sidang perkara ini yang merupakan bagian yang tak terpisahkan dari putusan ini.

PERTIMBANGAN HUKUM

Menimbang, bahwa maksud dan tujuan diajukannya gugatan Penggugat adalah sebagaimana telah terurai di atas;

Menimbang, bahwa Hakim telah mempelajari berkas perkara aquo, ternyata Pengadilan Agama Demak berwenang untuk memeriksa dan mengadili perkara ini sesuai dengan maksud Pasal 49 Undang-Undang Nomor 7 tahun 1989 yang telah diubah untuk keduakalinya dengan Undang-undang Nomor 50 tahun 2009 oleh karena itu perkara ini dapat diterima untuk dipertimbangkan dan diadili;

Menimbang, bahwa berdasarkan Bukti P-1 berupa kartu tanda penduduk maka terbukti Penggugat bertempat tinggal / berdomisili di wilayah hukum Pengadilan Agama Demak, maka berdasarkan ketentuan Pasal 49 ayat (1) huruf a dan Pasal 73 ayat (1) Undang-undang Nomor 7 Tahun 1989 sebagaimana telah diubah dengan Undang-undang Nomor 3 Tahun 2006 dan perubahan kedua dengan Undang-undang Nomor 50 Tahun 2009, Pengadilan

Disclaimer
Kepaniteraan Mahkamah Agung Republik Indonesia berusaha untuk selalu mencantumkan informasi paling kini dan akurat sebagai bentuk komitmen Mahkamah Agung untuk pelayanan publik, transparansi dan akuntabilitas pelaksanaan fungsi peradilan. Namun dalam hal-hal tertentu masih dimungkinkan terjadi permasalahan teknis terkait dengan akurasi dan keterkinian informasi yang kami sajikan, hal mana akan terus kami perbaiki dari waktu kewaktu.
Dalam hal Anda menemukan inakurasi informasi yang termuat pada situs ini atau informasi yang seharusnya ada, namun belum tersedia, maka harap segera hubungi Kepaniteraan Mahkamah Agung RI melalui :
Email : kepaniteraan@mahkamahagung.go.id Telp : 021-384 3348 (ext.318)

Agama Demak berwenang untuk memeriksa, memutus, dan menyelesaikan Perkara ini;

Menimbang, bahwa berdasarkan bukti P-2, yaitu berupa Kutipan Akta Nikah yang dikeluarkan oleh Kantor Urusan Agama Kecamatan Sayung, Kabupaten Demak, maka harus dinyatakan terbukti bahwa antara Penggugat dan Tergugat telah terikat dalam suatu perkawinan yang sah sebagai pihak-pihak yang berkepentingan dalam perkara ini;

Menimbang, bahwa pada waktu yang telah ditentukan untuk persidangan perkara ini Penggugat dan Tergugat telah hadir menghadap di persidangan secara pribadi, dan sesuai dengan ketentuan Pasal 39 ayat (1) Undang-Undang Nomor 1 Tahun 1974 jo. Pasal 82 Undang-Undang Nomor 7 Tahun 1989 Majelis telah berusaha mendamaikan Penggugat dan Tergugat untuk bersatu kembali membina rumah tangga, akan tetapi tidak berhasil karena kedua belah pihak tetap pada pendiriannya masing-masing

Menimbang, bahwa sesuai dengan Peraturan Mahkamah Agung Republik Indonesia Nomor 1 Tahun 2016 terhadap pihak Penggugat dan Tergugat telah dilakukan upaya mediasi akan tetapi dalam laporannya mediator menyatakan mediasi tidak berhasil;

Menimbang bahwa gugatan Penggugat untuk bercerai dengan Tergugat pada pokoknya didasarkan kepada alasan, bahwa antara Penggugat dan Tergugat sejak bulan Desember tahun 2004 telah terjadi perselisihan dan pertengkaran terus menerus, disebabkan Tergugat tidak bekerja sehingga tidak bisa memberi nafkah kepada Penggugat dan melakukan kekerasan fisik kepada Penggugat sehingga kehidupan rumah tangga Penggugat dan Tergugat semakin jauh dari tujuan berumah tangga yaitu sakinah, mawadah warrohmah, meskipun keluarga telah berusaha mendamaikannya, akan tetapi tidak berhasil;

Menimbang bahwa atas gugatan tersebut, Tergugat telah memberikan jawaban secara lisan yang pada pokoknya menolak dalil-dalil Penggugat dan justeru pemicu ketidak rukunan antara Penggugat dengan Tergugat karena Penggugat mulai menjalin hubungan kedekatan dengan seorang sopir yang setiap hari antara jemput Pengugat ke Pasar sayung, karena itu Terguat tetap

Disclaimer
Kepaniteraan Mahkamah Agung Republik Indonesia berusaha untuk selalu mencantumkan informasi paling kini dan akurat sebagai bentuk komitmen Mahkamah Agung untuk pelayanan publik, transparansi dan akuntabilitas pelaksanaan fungsi peradilan. Namun dalam hal-hal tertentu masih dimungkinkan terjadi permasalahan teknis terkait dengan akurasi dan keterkinian informasi yang kami sajikan, hal mana akan terus kami perbaiki dari waktu kewaktu.
Dalam hal Anda menemukan inakurasi informasi yang termuat pada situs ini atau informasi yang seharusnya ada, namun belum tersedia, maka harap segera hubungi Kepaniteraan Mahkamah Agung RI melalui :
Email : kepaniteraan@mahkamahagung.go.id Telp : 021-384 3348 (ext.318)

keberatan bercerai dengan Pengugat akan tetapi jika Pengugat tetap bersikeras maka Tergugat meminta uang konpensasi (pedot tresno) sebesar Rp. 150.000.000,00 (seratus lima puluh juta rupiah);

Menimbang bahwa kemudian telah terjadi jawab jinawab (replik-duplik) antara Penggugat dengan Tergugat dan terhadap tuntutan Tergugat berupa konpensasi / tukon tresno tersebut, Penggugat menolak untuk memberikan);

Menimbang bahwa kemudian Penggugat untuk menguatkan dalil-dalil gugatannya telah mengajukan bukti surat berupa P-1 dan P-2 (sebagaimana telah dipertimbangkan sebelumnya) dan dua orang saksi yang akan dipertimbangkan sebagai berikut:

Menimbang bahwa kedua orang saksi Penggugat dibawah sumpah telah memberikan keterangan yang pada pokoknya antara Penggugat dengan Tergugat sejak tahun 2004 mulai sering terjadi perselisihan dan pertengkaran disebabkan karena Tergugat tidak bisa menafkahi Penggugat, menuduh Penggugat selingkuh, melakukan kekerasasan fisik (KDRT) kepada Penggugat dan pernah mengancam Penggugat dengan pisau yang kemudian antara Pengugat dengan Tergugat pisah rumah sudah 2 bulan lamanya yaitu Penggugat pergi meninggalkan Tergugat dan tinggal di rumah anaknya hingga sekarang ;

Menimbang, bahwa dua orang saksi Penggugat tersebut telah memenuhi syarat-syarat formil dan tidak terhalang kedudukannya sebagai saksi seperti yang diatur dalam Pasal 145 HIR, keduanya memberikan keterangan yang didasarkan pada pengetahuan dari pengalaman, penglihatan dan pendengarannya sendiri yang antara kedua saksi tersebut terdapat kesesuaian keterangan seperti yang disyaratkan dalam Pasal 172 HIR, oleh karena itu keterangan kedua saksi tersebut dapat dipertimbangkan;

Menimbang bahwa meskipun Tergugat dalam jawabannya telah membantah dalil-dalil gugatan Penggugat akan tetapi Tergugat ternyata tidak mengajukan bukti-bukti apapun sehingga bantahan Tergugat dianggap tidak terbukti;

Menimbang, bahwa dari dalil gugatan Penggugat, sikap Penggugat selama dipersidangan dihubungkan dengan bukti surat dan keterangan-

Disclaimer
Kepaniteraan Mahkamah Agung Republik Indonesia berusaha untuk selalu mencantumkan informasi paling kini dan akurat sebagai bentuk komitmen Mahkamah Agung untuk pelayanan publik, transparansi dan akuntabilitas pelaksanaan fungsi peradilan. Namun dalam hal-hal tertentu masih dimungkinkan terjadi permasalahan teknis terkait dengan akurasi dan keterkinian informasi yang kami sajikan, hal mana akan terus kami perbaiki dari waktu kewaktu.
Dalam hal Anda menemukan inakurasi informasi yang termuat pada situs ini atau informasi yang seharusnya ada, namun belum tersedia, maka harap segera hubungi Kepaniteraan Mahkamah Agung RI melalui :
Email : kepaniteraan@mahkamahagung.go.id   Telp : 021-384 3348 (ext.318)

keterangan yang disampaikan oleh para saksi di persidangan, dapat ditemukan fakta sebagai berikut :

1. Bahwa Penggugat dan Tergugat adalah suami isteri sah, menikah pada tanggal 09 Mei 1998 dan telah dikaruniai 5 orang anak ;
2. Bahwa rumah tangga Penggugat dan Tergugat yang semula rukun, namun sejak bulan Desember tahun 2004 sudah tidak harmonis lagi karena seringkali terjadi perselisihan dan pertengkaran antara Penggugat dan Tergugat yang disebabkan Tergugat tidak bisa menafkahi Penggugat, menuduh Penggugat selingkuh, melakukan kekerasasan fisik (KDRT) kepada Penggugat dan pernah mengancam Penggugat dengan pisau;
3. Bahwa kemudian sudah 2 bulan lamanya antara Penggugat dengan Tergugat pisah rumah yaitu Penggugat pergi meninggalkan Tergugat dan tinggal bersama anaknya hingga sekarang;
4. Bahwa selama berpisah, antara Penggugat dan Tergugat tidak ada komunikasi sama sekali;
5. Bahwa keluarga Penggugat sudah berusaha mendamaikannya, akan tetapi tidak berhasil dan Tergugat juga telah menyatakan tidak keberatan bercerai dengan Penggugat;

Menimbang, dari fakta-fakta tersebut diatas, pada fakta poin (1) Penggugat dan Tergugat adalah pasangan suami isteri yang sah yang tidak pernah bercerai, dengan demikian Penggugat dan Tergugat berkualitas hukum sebagai pihak-pihak dalam perkara ini, dan Penggugat berhak mengajukan gugatan terhadap Tergugat ke Pengadilan Agama sebagaimana dimaksud pasal 73 ayat (1) Undang-undang Nomor 7 Tahun 1989;

Menimbang, bahwa dari fakta point (3), (4) dan (5) sebagaimana terurai diatas telah memberikan gambaran bahwa perselisihan antara Penggugat dengan Tergugat sudah tidak mampu lagi dikendalikan dan diatasi oleh mereka berdua, bahkan sikap Penggugat yang telah pergi meninggalkan Tergugat adalah merupakan bukti bahwa perselisihan dan pertengkaran antara Penggugat dengan Tergugat sudah cukup serius dan bersifat terus menerus;

Menimbang bahwa dari pernyataan Penggugat selama dalam persidangan yang dengan tegas hendak bercerai dengan Tergugat adalah

Disclaimer
Kepaniteraan Mahkamah Agung Republik Indonesia berusaha untuk selalu mencantumkan informasi paling kini dan akurat sebagai bentuk komitmen Mahkamah Agung untuk pelayanan publik, transparansi dan akuntabilitas pelaksanaan fungsi peradilan. Namun dalam hal-hal tertentu masih dimungkinkan terjadi permasalahan teknis terkait dengan akurasi dan keterkinian informasi yang kami sajikan, hal mana akan terus kami perbaiki dari waktu kewaktu.
Dalam hal Anda menemukan inakurasi informasi yang termuat pada situs ini atau informasi yang seharusnya ada, namun belum tersedia, maka harap segera hubungi Kepaniteraan Mahkamah Agung RI melalui :
Email : kepaniteraan@mahkamahagung.go.id Telp : 021-384 3348 (ext.318)

merupakan bukti tidak adanya keinginan Penggugat untuk memperbaiki kembali keadaan rumah tangganya, karena itu merupakan fakta kongkrit yang menunjukkan bahwa hubungan Penggugat dengan Tergugat sudah sampai pada tingkat pecahnya perkawinan (*broken marriage*) dan Hakim berkesimpulan bahwa tujuan yang diharapkan dari perkawinan sebagaimana tersebut pada pasal 1 Undang-undang Nomor 1 Tahun 1974 tidak mungkin lagi terwujud;

Menimbang, bahwa meskipun Tergugat bertekat ingin rukun kembali membina rumah tangga bersama Penggugat sedanhgkan Penggugat tetap berkeras untuk bercerai, sehingga tidaklah cukup niat Tergugat tersebut untuk kembali membangun harmoni rumah tangganya bersama Penggugat, karena hal mutlak yang mesti terpenuhi adalah adanya kehendak utuh masing-masing suami dan isteri untuk hidup bersama dengan kesediaan secara bersama-sama pula menghadapi dan mengatasi semua masalah yang timbul dalam rumah tangga. Jika salah satu pihak tidak punya dorongan hati, atau sederhananya sudah tidak punya niat lagi untuk membina rumah tangga bersama, maka sangat memungkinkan rumah tangga tersebut tidak ada harapan berlangsung harmonis dan membawa kebahagiaan bagi kedua belah pihak. Jika demikian keadaannya, maka rumah tangga akan menjadi tempat yang tidak efektif lagi bagi suami dan isteri. Sebaliknya, akan timbul mudharat-mudharat lain yang merusak ketenangan hidup Penggugat dan Tergugat. Setidak-tidaknya demikianlah alasan rasional (*legal reasoning/ratio decidendi*) dari *Qaidah Fiqhiyah* yang berbunyi:

درءالمفاسد مقدم على جلب المصالح

Artinya : Menolak kerusakan itu lebih baik dari mengambil kemaslahatan.

Menimbang, bahwa Majelis Hakim juga mengambil alih pendapat ulama *fiqh* dalam kitab *Madza Hurriyah az-Zaujain fi Ath-Tholaq* untuk selanjutnya menjadi pertimbangan hukum dalam putusan ini, sebagai berikut :

قد اختارالإسلام نظام الطلاق حين تضطرب الحياة الزوجية ولم ينفع فيه نصح ولاصلح

Disclaimer
Kepaniteraan Mahkamah Agung Republik Indonesia berusaha untuk selalu mencantumkan informasi paling kini dan akurat sebagai bentuk komitmen Mahkamah Agung untuk pelayanan publik, transparansi dan akuntabilitas pelaksanaan fungsi peradilan. Namun dalam hal-hal tertentu masih dimungkinkan terjadi permasalahan teknis terkait dengan akurasi dan keterkinian informasi yang kami sajikan, hal mana akan terus kami perbaiki dari waktu kewaktu.
Dalam hal Anda menemukan inakurasi informasi yang termuat pada situs ini atau informasi yang seharusnya ada, namun belum tersedia, maka harap segera hubungi Kepaniteraan Mahkamah Agung RI melalui :
Email : kepaniteraan@mahkamahagung.go.id   Telp : 021-384 3348 (ext.318)

وحيث تصبح الربطة الزوجية صورة من غير روح لأن الاستمرار معناه أن يحكم على
أحد الزوجين بالسجن المؤبد وهذا تأباه روح العدالة

Artinya : *Islam telah memilih lembaga perceraian ketika kehidupan rumah tangga telah guncang dan nasihat serta perdamaian sudah dianggap tidak bermanfaat lagi, hubungan suami isteri telah hampa karena meneruskan perkawinan sama halnya dengan menghukum salah satu pihak dengan hukuman penjara yang berkepanjangan dan hal itu sangat bertentangan dengan rasa keadilan.*

Menimbang, bahwa tanpa harus menilai siapa penyebab pokok atau siapa yang bersalah terhadap timbulnya disharmoni rumah tangga Penggugat dan Tergugat, Majelis Hakim berkesimpulan perkawinan Penggugat dan Tergugat tidak layak lagi dipertahankan karena sudah tergolong perkawinan yang pecah (*brokendown marriage*), yaitu perkawinan yang tidak lagi menghadirkan suasana yang menenangkan bathin suami isteri melalui curahan kasih sayang, sehingga Majelis Hakim berpendapat dalam rumah tangga Penggugat dengan Tergugat sudah tidak terwujud lagi tujuan sebuah perkawinan yaitu untuk membentuk keluarga/rumah tangga yang bahagia dan kekal berdasarkan Ketuhanan Yang Maha Esa (vide Pasal 1 Undang-Undang Nomor 1 Tahun 1974) dan atau keluarga yang sakinah, penuh mawaddah dan rahmah (vide Pasal 3 Kompilasi Hukum Islam), serta keluarga yang menentramkan jiwa, tempat mencurahkan kasih dan sayang sebagaimana tertuang dalam Al quran Surat ar-Rum ayat 21, yang berbunyi :

وَمِنْ اٰيٰتِهٖٓ اَنْ خَلَقَ لَكُمْ مِّنْ اَنْفُسِكُمْ اَزْوَاجًا لِّتَسْكُنُوْٓا اِلَيْهَا وَجَعَلَ بَيْنَكُمْ مَّوَدَّةً وَّرَحْمَةً
اِنَّ فِيْ ذٰلِكَ لَاٰيٰتٍ لِّقَوْمٍ يَّتَفَكَّرُوْنَ ۝

Terjemahnya : “Dan di antara tanda-tanda kekuasaan-Nya ialah Dia menciptakan untukmu isteri-isteri dari jenismu sendiri, supaya kamu cenderung dan merasa tenteram kepadanya, dan dijadikan-Nya diantaramu rasa kasih dan sayang. Sesungguhnya pada yang demikian itu benar-benar terdapat tanda-tanda bagi kaum yang berfikir”.

Disclaimer
Kepaniteraan Mahkamah Agung Republik Indonesia berusaha untuk selalu mencantumkan informasi paling kini dan akurat sebagai bentuk komitmen Mahkamah Agung untuk pelayanan publik, transparansi dan akuntabilitas pelaksanaan fungsi peradilan. Namun dalam hal-hal tertentu masih dimungkinkan terjadi permasalahan teknis terkait dengan akurasi dan keterkinian informasi yang kami sajikan, hal mana akan terus kami perbaiki dari waktu kewaktu.
Dalam hal Anda menemukan inakurasi informasi yang termuat pada situs ini atau informasi yang seharusnya ada, namun belum tersedia, maka harap segera hubungi Kepaniteraan Mahkamah Agung RI melalui :
Email : kepaniteraan@mahkamahagung.go.id Telp : 021-384 3348 (ext.318)

Menimbang, bahwa berdasarkan pertimbangan-pertimbangan tersebut di atas, maka Majelis Hakim menyatakan gugatan Penggugat telah memenuhi unsur-unsur yang termuat dalam Pasal 19 huruf f Peraturan Pemerintah Nomor 9 Tahun 1975 jo. Pasal 116 huruf f Kompilasi Hukum Islam. Karenanya, Majelis Hakim berpendapat bahwa gugatan Penggugat beralasan hukum dan kehendaknya untuk bercerai lebih bermanfaat baginya. Karena itu, petitum angka 2 gugatan Penggugat patut untuk dikabulkan dengan menjatuhkan talak satu bain shughra Tergugat terhadap Penggugat.

Menimbang bahwa oleh karena gugatan Penggugat telah terbukti beralasan hukum maka terhadap tuntutan Tergugat mengenai tukon trisno tidak dapat dipertimbangkan dan haruslah dikesampingkan;

Menimbang, bahwa oleh karena perkara ini merupakan perkara dalam bidang perkawinan, maka sebagaimana ditetapkan Pasal 89 ayat (1) Undang-undang Nomor 7 Tahun 1989 yang telah diubah untuk kedua kalinya dengan Undang-undang Nomor 50 Tahun 2009 beralasan bagi Majelis untuk membebankan biaya perkara kepada Penggugat Konpensi / Tergugat rekonpnesi;

Mengingat, semua peraturan perundang-undangan yang berlaku serta hukum lain berhubungan dengan perkara ini;

MENGADILI :

1. Mengabulkan gugatan Penggugat;
2. Menjatuhkan talak satu bain sughra dari Tergugat (Xxxx) terhadap Penggugat (Xxxx);
3. Membebankan kepada Penggugat untuk membayar biaya perkara yang hingga kini sejumlah Rp. 741.000,- (tujuh ratus empat puluh satu ribu rupiah);

Demikianlah putusan ini dijatuhkan dalam musyawarah Majelis Hakim di Demak pada hari Selasa tanggal 22 Desember 2020 M, bertepatan dengan tanggal 07 Jumadil Awal 1442 H, oleh Kami Drs. Makali sebagai Ketua Majelis Hakim, serta Dra. Nur Immawati dan Toharudin, S.H.I., M.H. masing-masing sebagai Hakim Anggota. Putusan mana diucapkan oleh Ketua Majelis Hakim tersebut pada

Disclaimer
Kepaniteraan Mahkamah Agung Republik Indonesia berusaha untuk selalu mencantumkan informasi paling kini dan akurat sebagai bentuk komitmen Mahkamah Agung untuk pelayanan publik, transparansi dan akuntabilitas pelaksanaan fungsi peradilan. Namun dalam hal-hal tertentu masih dimungkinkan terjadi permasalahan teknis terkait dengan akurasi dan keterkinian informasi yang kami sajikan, hal mana akan terus kami perbaiki dari waktu kewaktu.
Dalam hal Anda menemukan inakurasi informasi yang termuat pada situs ini atau informasi yang seharusnya ada, namun belum tersedia, maka harap segera hubungi Kepaniteraan Mahkamah Agung RI melalui :
Email : kepaniteraan@mahkamahagung.go.id Telp : 021-384 3348 (ext.318)

hari itu juga dalam sidang terbuka untuk umum dengan dihadiri oleh para Hakim Anggota dan Moh. Rofi', S. Ag., sebagai Panitera Pengganti dan dihadiri pula oleh Penggugat diluar hadirnya Tergugat;

Ketua Majelis

Drs. Makali

Hakim Anggota	Hakim Anggota
Dra. Nur Immawati	Toharudin, S.H.I., M.H.

Panitera Pengganti

Moh. Rofi', S. Ag.

Perincian Biaya Perkara :

1.Pendaftaran	Rp	30.000,00
2. Biaya Proses	Rp	75.000,00
**3.** Panggilan	Rp	600.000,00
sidang	Rp.	20.000,00
**4.** PNBP. Panggilan		
5. Redaksi	Rp	10.000,00
6. Meterai.	Rp	6.000,00
J u m l a h	Rp	741.000,00

(tujuh ratus empat puluh satu ribu rupiah)

Disclaimer
Kepaniteraan Mahkamah Agung Republik Indonesia berusaha untuk selalu mencantumkan informasi paling kini dan akurat sebagai bentuk komitmen Mahkamah Agung untuk pelayanan publik, transparansi dan akuntabilitas pelaksanaan fungsi peradilan. Namun dalam hal-hal tertentu masih dimungkinkan terjadi permasalahan teknis terkait dengan akurasi dan keterkinian informasi yang kami sajikan, hal mana akan terus kami perbaiki dari waktu kewaktu.
Dalam hal Anda menemukan inakurasi informasi yang termuat pada situs ini atau informasi yang seharusnya ada, namun belum tersedia, maka harap segera hubungi Kepaniteraan Mahkamah Agung RI melalui :
Email : kepaniteraan@mahkamahagung.go.id Telp : 021-384 3348 (ext.318)
