# Supplementary material for: Classification of divorce causes during the COVID-19 pandemic using convolutional neural networks
Source: PeerJ Comput Sci. 2022 Jun 30;8:e998. doi: 10.7717/peerj-cs.998 (PMC9299239; doi:10.7717/peerj-cs.998)
Supplement: Supplemental Information 3 [file peerj-cs-08-998-s003.zip › KDRT Dataset/Data ke-11.pdf]

PUTUSAN

Nomor 1041/Pdt.G/2020/PA Sgm

بسم الله الرحمن الرحيم

DEMI KEADILAN BERDASARKAN KETUHANAN YANG MAHA ESA

Pengadilan Agama Sungguminasa yang memeriksa dan mengadili perkara perdata cerai gugat pada tingkat pertama dalam sidang Majelis Hakim telah menjatuhkan putusan dalam perkara antara:

#Penggugat, Umur 28 tahun, Agama Islam, Pekerjaan Tidak ada, Tempat Kabupaten Gowa, Provinsi Sulawesi-Selatan dalam hal ini memberikan kuasa kepada Muh. Safri Tunru, S.HI, dan Partner. Advokat yang berkantor di Jl. Ujung Bori Ruko Borong Bisnis Centre C-13, Kelurahan Antang Kec. Manggala Kota Makassar berdasarkan surat kuasa khusus terdaftar di kepaniteraan Pengadilan Agama Sungguminasa tanggal 4 November 2020 sebagai **Penggugat**;

melawan

#Tergugat., Umur 27 tahun, Agama Islam, Pekerjaan tidak ada, Alamat Kabupaten Maros, Provinsi Sulawesi-Selatan sebagai**Tergugat.**

Pengadilan Agama tersebut;

Telah mempelajari surat-surat yang berkaitan dengan perkara ini;

Telah mendengar keterangan Penggugat dan Tergugat serta para saksi di muka sidang;

DUDUK PERKARA

Bahwa Pemohon dalam surat permohonannya tanggal 5 November 2020 telah mengajukan permohonan Cerai Gugat, yang telah terdaftar di Kepaniteraan Pengadilan Agama, dengan Nomor 1041/Pdt.G/2020/PA Sgm, tanggal 5 November 2020, dengan dalil-dalil pada pokoknya sebagai berikut:

1. Bahwa Penggugat dan Tergugat adalah suami isteri yang sah, yang melangsungkan perkawinan pada hari Rabu, tanggal 07 November 2012, bertepatan dengan tanggal 22 DzulHijjah 1433 H, yang dicatat oleh

Disclaimer
Kepaniteraan Mahkamah Agung Republik Indonesia berusaha untuk selalu mencantumkan informasi paling kini dan akurat sebagai bentuk komitmen Mahkamah Agung untuk pelayanan publik, transparansi dan akuntabilitas pelaksanaan fungsi peradilan. Namun dalam hal-hal tertentu masih dimungkinkan terjadi permasalahan teknis terkait dengan akurasi dan keterkinian informasi yang kami sajikan, hal mana akan terus kami perbaiki dari waktu kewaktu.
Dalam hal Anda menemukan inakurasi informasi yang termuat pada situs ini atau informasi yang seharusnya ada, namun belum tersedia, maka harap segera hubungi Kepaniteraan Mahkamah Agung RI melalui :
Email : kepaniteraan@mahkamahagung.go.id Telp : 021-384 3348 (ext.318)

Pegawai Pencatat Nikah Kantor Urusan Agama Kecamatan Mappakasunggu, Kabupaten Takalar, Provinsi Sulawesi-Selatan, sesuai Kutipan Akta Nikah Nomor : 219/31/XI/2012, Tertanggal 23 Mei 2012, di Kabupaten Takalar;

2. Bahwa setelah pernikahan tersebut, Penggugat dan Tergugat tinggal bersama di rumah orangtua Tergugat di Desa Moncongloe Bulu, Kecamatan Moncongloe, Kabupaten Maros, Provinsi Sulawesi-Selatan;
3. Bahwa dari perkawinan Penggugat dan Tergugat belum dikaruniai anak/keturunan;
4. Bahwa pada awal pernikahan rumah tangga Penggugat dan Tergugat berjalan dengan rukun dan harmonis, namun **sejak awal Desember 2013 (2 Bulan Setelah Menikah)**, sudah mulai sering timbul perselisihan dan pertengkaran secara terus menerus yang disebabkan:
   - Tergugat memiliki kebiasaan suka minuma-minuman keras, serta memiliki sifat cemburu yang berlebihan;
   - Tergugat sering keluar malam tanpa alasan dan hal yang penting;
   - Penggugat dan Tergugat telah sepakat untuk bercerai
5. Bahwa **bulan Maret 2013 merupakan puncak perselisihan Penggugat dan Tergugat** dimana pada saat itu baik antara Penggugat maupun Tergugat tidak lagi saling mempedulikan lagi, dan telah pisah tempat tinggal hingga sekarang;
6. Bahwa pihak keluarga telah berupaya merukunkan Penggugat dan Tergugat namun tidak berhasil;
7. Sikap dari Tergugat tersebut diatas yang membuat Penggugat, menggugat cerai terhadap Tergugat dan tidak ingin lagi untuk melanjutkan perkawinannya dengan Tergugat;
8. Bahwa berdasarkan hal tersebut diatas, sudah cukup alasan bagi Penggugat untuk mengajukan gugatan perceraian sebagaimana dimaksud dalam pasal 19 huruf F peraturan pemerintah No. 9 tahun 1975 tentang pelaksanaan Undang-Undang No. 1 tahun 1974 tentang perkawinan menyatakan bahwa **“perceraian dapat terjadi karena suami isteri terus**

Disclaimer
Kepaniteraan Mahkamah Agung Republik Indonesia berusaha untuk selalu mencantumkan informasi paling kini dan akurat sebagai bentuk komitmen Mahkamah Agung untuk pelayanan publik, transparansi dan akuntabilitas pelaksanaan fungsi peradilan. Namun dalam hal-hal tertentu masih dimungkinkan terjadi permasalahan teknis terkait dengan akurasi dan keterkinian informasi yang kami sajikan, hal mana akan terus kami perbaiki dari waktu kewaktu.
Dalam hal Anda menemukan inakurasi informasi yang termuat pada situs ini atau informasi yang seharusnya ada, namun belum tersedia, maka harap segera hubungi Kepaniteraan Mahkamah Agung RI melalui :
Email : kepaniteraan@mahkamahagung.go.id Telp : 021-384 3348 (ext.318)

menerus terjadi perselisihan dan pertengkaran dan tidak ada harapan akan hidup rukun lagi dalam rumah tangga".

9. Bahwa tujuan perkawinan menurut UU perkawinan No. 1 Tahun 1974 adalah membentuk keluarga yang kekal dan bahagia berdasarkan Ketuhanan Yang Maha Esa tidak mungkin tercapai lagi. Dan satu-satunya jalan yang paling tepat yang dapat di tempuh untuk membebaskan Penggugat dari penderitaan dan merupakan alternatif satu-satunya yang terbaik dan juga memperjelas status hubungan, baik untuk kepentingan Penggugat maupun kepentingan Tergugat adalah Perceraian;
10. Bahwa berdasarkan pada kenyataan tersebut di atas maka berdasar hukum apabila Majelis Hakim Pengadilan Agama Sungguminasa, mengabulkan gugatan cerai Penggugat terhadap Tergugat dengan **menjatuhkan talak satu *ba'in shughraa* Tergugat #Tergugat terhadap Penggugat #Penggugat;**

Berdasarkan alasan tersebut di atas, Penggugat memohon kepada Ketua Pengadilan Agama Sungguminasa *cq* Majelis Hakim yang memeriksa dan mengadili perkara ini untuk menjatuhkan putusan sebagai berikut:

Primer:

1. Mengabulkan gugatan Penggugat;
2. Menjatuhkan talak satu *ba'in shughraa* Tergugat #Tergugat terhadap Penggugat #Penggugat;
3. Membebankan biaya perkara menurut ketentuan yang berlaku.

Subsider:

Mohon putusan yang seadil-adilnya;

Bahwa pada hari persidangan yang telah ditetapkan, Penggugat datang menghadap di persidangan, sedangkan Tergugat tidak datang menghadap serta tidak pula menyuruh orang lain sebagai wakil/kuasanya yang sah, meskipun Tergugat telah dipanggil secara resmi dan patut berdasarkan relaas panggilan Jurusita Pengganti Pengadilan Agama Sungguminasa dan ketidakhadiran Tergugat tersebut tanpa ada alasan yang dapat dibenarkan menurut hukum.

Disclaimer
Kepaniteraan Mahkamah Agung Republik Indonesia berusaha untuk selalu mencantumkan informasi paling kini dan akurat sebagai bentuk komitmen Mahkamah Agung untuk pelayanan publik, transparansi dan akuntabilitas pelaksanaan fungsi peradilan. Namun dalam hal-hal tertentu masih dimungkinkan terjadi permasalahan teknis terkait dengan akurasi dan keterkinian informasi yang kami sajikan, hal mana akan terus kami perbaiki dari waktu kewaktu.
Dalam hal Anda menemukan inakurasi informasi yang termuat pada situs ini atau informasi yang seharusnya ada, namun belum tersedia, maka harap segera hubungi Kepaniteraan Mahkamah Agung RI melalui :
Email : kepaniteraan@mahkamahagung.go.id Telp : 021-384 3348 (ext.318)

Bahwa Majelis Hakim telah berusaha menasihati dan memberikan saran kepada Penggugat agar kembali rukun dengan Tergugat, namun tidak berhasil, dan oleh karena Tergugat tidak hadir di persidangan, sehingga terhadap pihak dalam perkara ini tidak dapat dimediasi, sebagaimana ketentuan dalam Peraturan Mahkamah Agung RI Nomor 1 Tahun 2016 Tentang Prosedur Mediasi di Pengadilan, lalu persidangan pemeriksaan pokok perkara ini dilakukan dalam sidang tertutup untuk umum, selanjutnya Penggugat membacakan surat gugatannya, yang oleh Penggugat menyatakan tetap pada substansi dalil-dalil gugatannya semula;

A. Surat :
- Fotokopi Buku Kutipan Akta Nikah Nomor 219/31/XI/2012, tanggal 23 Mei 2012, yang dikeluarkan oleh Kantor Urusan Agama Kecamatan Mappakasunggu, Kabupaten Takalar, bermeterai cukup, distempel pos dan telah dicocokkan dengan aslinya ternyata cocok, oleh Ketua Majelis di beri tanda P.

B. Saksi-saksi yang memberi keterangan di persidangan secara terpisah, yaitu:
1. **#Nama saksi**umur 52 tahun, agama Islam, pendidikan terakhir S1, pekerjaan PNS, tempat kediaman Kabupaten Takalar, di bawah sumpahnya memberikan keterangan sebagai berikut :
   - Bahwa saksi kenal Penggugat dan Tergugat karena adalah ayah kandung Penggugat;
   - Bahwa setelah pernikahan tersebut Penggugat dan Tergugat tinggal bersama di rumah orang tua Tergugat di Desa Moncongloe Bulu, Kecamatan Moncongloe, Kabupaten Maros, Provinsi Sulawesi-Selatan;
   - Bahwa Penggugat dan Tergugat hingga saat ini keduanya belum dikaruniai anak;
   - Bahwa pada awal perkawinan Penggugat dan Tergugat berjalan rukun dan harmonis namun sejak awal Desember 2013 sudah mulai sering timbul perselisihan disebabkan karena Tergugat memiliki kebiasaan suka meminum-minuman keras, serta memiliki sifat cemburu yang berlebihan, Tergugat sering keluar malam tanpa

Disclaimer
Kepaniteraan Mahkamah Agung Republik Indonesia berusaha untuk selalu mencantumkan informasi paling kini dan akurat sebagai bentuk komitmen Mahkamah Agung untuk pelayanan publik, transparansi dan akuntabilitas pelaksanaan fungsi peradilan. Namun dalam hal-hal tertentu masih dimungkinkan terjadi permasalahan teknis terkait dengan akurasi dan keterkinian informasi yang kami sajikan, hal mana akan terus kami perbaiki dari waktu kewaktu.
Dalam hal Anda menemukan inakurasi informasi yang termuat pada situs ini atau informasi yang seharusnya ada, namun belum tersedia, maka harap segera hubungi Kepaniteraan Mahkamah Agung RI melalui :
Email : kepaniteraan@mahkamahagung.go.id Telp : 021-384 3348 (ext.318)

alasan dan hal yang penting, serta Penggugat dan Tergugat telah sepakat untuk bercerai;

- Bahwa puncak perselisihan Penggugat dan Tergugat pada bulan Maret 2013;
- Bahwa pada saat itu Penggugat dan Tergugat tidak lagi saling mempedulikan;
- Bahwa Penggugat dan Tergugat pisah rumah selama 7 tahun lebih lamanya;
- Bahwa saksi pernah menasihati Penggugat dan Tergugat namun tidak berhasil.

2. **#Nama saksi**, umur 52 tahun, agama Islam, pendiidkan terakhir S1, pekerjaan PNS, tempat kediaman di Kabupaten Takalar di bawah sumpahnya memberikan keterangan sebagai berikut :

- Bahwa saksi kenal Penggugat dan Tergugat karena adalah ibu kandung Penggugat;
- Bahwa setelah pernikahan tersebut Penggugat dan Tergugat tinggal bersama di rumah orang tua Tergugat di Desa Moncongloe Bulu, Kecamatan Moncongloe, Kabupaten Maros, Provinsi Sulawesi-Selatan;
- Bahwa Penggugat dan Tergugat hingga saat ini keduanya belum dikaruniai anak;
- Bahwa pada awal perkawinan Penggugat dan Tergugat berjalan rukun dan harmonis namun sejak awal Desember 2013 sudah mulai sering timbul perselisihan disebabkan karena Tergugat memiliki kebiasaan suka meminum-minuman keras, serta memiliki sifat cemburu yang berlebihan, Tergugat sering keluar malam tanpa alasan dan hal yang penting, serta Penggugat dan Tergugat telah sepakat untuk bercerai;
- Bahwa puncak perselisihan Penggugat dan Tergugat pada bulan Maret 2013;
- Bahwa pada saat itu Penggugat dan Tergugat tidak lagi saling mempedulikan;

Disclaimer
Kepaniteraan Mahkamah Agung Republik Indonesia berusaha untuk selalu mencantumkan informasi paling kini dan akurat sebagai bentuk komitmen Mahkamah Agung untuk pelayanan publik, transparansi dan akuntabilitas pelaksanaan fungsi peradilan. Namun dalam hal-hal tertentu masih dimungkinkan terjadi permasalahan teknis terkait dengan akurasi dan keterkinian informasi yang kami sajikan, hal mana akan terus kami perbaiki dari waktu kewaktu.
Dalam hal Anda menemukan inakurasi informasi yang termuat pada situs ini atau informasi yang seharusnya ada, namun belum tersedia, maka harap segera hubungi Kepaniteraan Mahkamah Agung RI melalui :
Email : kepaniteraan@mahkamahagung.go.id   Telp : 021-384 3348 (ext.318)

- Bahwa Penggugat dan Tergugat pisah rumah selama 7 tahun lebih lamanya;
- Bahwa saksi pernah menasihati Penggugat dan Tergugat namun tidak berhasil.

Bahwa, selanjutnya Penggugat menerangkan tidak akan menambah bukti-buktinya lagi, serta mengajukan kesimpulan secara lisan yang pada pokoknya tetap pada gugatannya untuk bercerai dengan Tergugat serta mohon putusan;

Bahwa, untuk mempersingkat uraian putusan ini ditunjuk hal-hal sebagaimana termuat dalam berita acara persidangan perkara ini sebagai bagian yang tidak terpisahkan dan dianggap telah termuat dalam putusan ini.

PERTIMBANGAN HUKUM

Menimbang, bahwa maksud dan tujuan surat gugatan Penggugat adalah sebagaimana terurai di atas;

Menimbang, bahwa prosedur pengajuan surat gugatan Penggugat telah sesuai dengan kompetensi Pengadilan yang berwenang berdasarkan penjelasan Pasal 49 huruf (a) angka (9) Undang-Undang RI Nomor 3 Tahun 2006 Tentang Perubahan Undang-Undang RI Nomor 7 Tahun 1989 Tentang Peradilan Agama, sebagaimana diubah kedua kalinya dengan Undang-Undang RI Nomor 50 Tahun 2009, begitu pula substansi gugatannya tidak mengandung cacat hukum, sehingga dengan demikian telah memenuhi syarat formil gugatan, sebagaimana yang diatur oleh peraturan perundang-undangan yang berlaku, oleh karenanya telah bersandar pada hukum;

Menimbang, bahwa oleh karena Tergugat telah dipanggil secara resmi dan patut berdasarkan relaas panggilan Jurusita Pengganti Pengadilan Agama Sungguminasa, dan ketidakhadiran Tergugat tersebut tanpa ada alasan yang dapat dibenarkan menurut hukum, maka pemeriksaan perkara ini dilakukan tanpa hadirnya Tergugat, sesuai ketentuan Pasal 149 *Reglement Buiteegewesten* (R.Bg.);

Menimbang, bahwa pada setiap persidangan, Majelis Hakim telah berupaya menasihati Penggugat agar kembali hidup rukun dan damai dengan Tergugat, serta tidak melanjutkan gugatan Penggugat, akan tetapi tidak

Disclaimer
Kepaniteraan Mahkamah Agung Republik Indonesia berusaha untuk selalu mencantumkan informasi paling kini dan akurat sebagai bentuk komitmen Mahkamah Agung untuk pelayanan publik, transparansi dan akuntabilitas pelaksanaan fungsi peradilan. Namun dalam hal-hal tertentu masih dimungkinkan terjadi permasalahan teknis terkait dengan akurasi dan keterkinian informasi yang kami sajikan, hal mana akan terus kami perbaiki dari waktu kewaktu.
Dalam hal Anda menemukan inakurasi informasi yang termuat pada situs ini atau informasi yang seharusnya ada, namun belum tersedia, maka harap segera hubungi Kepaniteraan Mahkamah Agung RI melalui :
Email : kepaniteraan@mahkamahagung.go.id   Telp : 021-384 3348 (ext.318)

berhasil, oleh karenanya maksud Pasal 65 Undang-Undang RI Nomor 7 Tahun 1989 Tentang Peradilan Agama, telah terpenuhi dan Penggugat pun tetap mempertahankan dalil-dalil gugatannya;

Menimbang, bahwa oleh karena Tergugat tidak hadir di persidangan, maka pihak berperkara tidak layak dimediasi sebagaimana dimaksud dalam Peraturan Mahkamah Agung RI Nomor 1 Tahun 2016 Tentang Prosedur Mediasi di Pengadilan;

Menimbang, bahwa meskipun ketidakhadiran Tergugat di dalam persidangan dapat dianggap bahwa Tergugat tidak mempergunakan hak bantahannya, namun karena perkara ini secara *lex specialis* merupakan sengketa keluarga dan untuk menghindari terjadinya kebohongan besar (*de grote leugen*) dalam perkara ini, maka Majelis Hakim tetap membebani wajib alat-alat bukti kepada Penggugat, sesuai maksud Pasal 283 *Reglement Buiteegewesten* (R.Bg.);

Menimbang, bahwa memperhatikan hadis Nabi saw. yang menyatakan bahwa adalah suatu kewajiban bagi Penggugat untuk membuktikan gugatannya, sebagaimana dalam hadis riwayat Imam Tirmizi:

أَنَّ النَّبِيَّ صَلَّى اللَّهُ عَلَيْهِ وَسَلَّمَ قَالَ: الْبَيِّنَةُ عَلَى الْمُدَّعِي وَالْيَمِينُ عَلَى الْمُدَّعَى عَلَيْه

Artinya:

Bahwasanya Nabi saw. bersabda: "Wajib alat bukti bagi Penggugat, sedangkan (jika diperlukan, juga alat bukti) sumpah bagi Tergugat";

Menimbang, bahwa untuk membuktikan dalil-dalil gugatannya, Penggugat telah mengajukan alat bukti, serta telah menghadirkan dua orang saksi;

Menimbang, bahwa alat bukti P, yang dibuat oleh pejabat yang berwenang, telah memenuhi syarat sebagai alat bukti akta otentik dan merupakan alat bukti yang sah menurut hukum sebagaimana ketentuan Pasal 285 *Reglement Buiteegewesten* (R.Bg.), dan mempunyai nilai pembuktian sempurna (*Volledig*) dan mengikat (*bindende*), maka alat bukti tersebut dapat dipertimbangkan;

Disclaimer
Kepaniteraan Mahkamah Agung Republik Indonesia berusaha untuk selalu mencantumkan informasi paling kini dan akurat sebagai bentuk komitmen Mahkamah Agung untuk pelayanan publik, transparansi dan akuntabilitas pelaksanaan fungsi peradilan. Namun dalam hal-hal tertentu masih dimungkinkan terjadi permasalahan teknis terkait dengan akurasi dan keterkinian informasi yang kami sajikan, hal mana akan terus kami perbaiki dari waktu kewaktu.
Dalam hal Anda menemukan inakurasi informasi yang termuat pada situs ini atau informasi yang seharusnya ada, namun belum tersedia, maka harap segera hubungi Kepaniteraan Mahkamah Agung RI melalui :
Email : kepaniteraan@mahkamahagung.go.id   Telp : 021-384 3348 (ext.318)

Menimbang, bahwa dua orang saksi yang diajukan Penggugat di persidangan telah memberikan keterangan berdasarkan pengetahuan langsung dan keterangan keduanya yang relevan saling bersesuaian dan saling mendukung antara satu dengan yang lain serta telah memenuhi syarat sebagai alat bukti kesaksian sebagaimana ketentuan Pasal 308 ayat (1) dan Pasal 309 *Reglement Buiteegewesten* (R.Bg.), maka kesaksian para saksi tersebut dapat dipertimbangkan;

Menimbang, bahwa pertama-tama Majelis Hakim memeriksa apakah Penggugat mempunyai kapasitas untuk mengajukan gugatan (*Legal Standing*) atas perkara ini. Berdasarkan keterangan para saksi dalam persidangan, serta alat bukti P., sehingga dengan alat-alat bukti tersebut berdasarkan Pasal 284 *Reglement Buiteegewesten* (R.Bg.), serta dengan memperhatikan ketentuan Pasal 7 ayat (1) Buku I Kompilasi Hukum Islam (Keputusan Menteri Agama RI. Nomor 154 Tahun 1991 Tentang Pelaksanaan Instruksi Presiden RI Nomor 1 Tahun 1991), maka Majelis Hakim berpendapat bahwa terhadap posita angka 1 (satu) gugatan Penggugat telah terbukti secara sah menurut hukum, sehingga antara Penggugat dan Tergugat telah terikat dalam suatu perkawinan yang sah sebagai suami-istri, telah menikah pada hari Rabu, 7 November 2012 di hadapan PPN Kantor Urusan Agama Kecamatan Mappakasunggu, Kabupaten Takalar dan oleh karenanya Penggugat telah memiliki *Legal Standing* (Kapasitas) untuk mengajukan gugatan cerai *a quo*;

Menimbang,bahwa terhadap posita angka 2 (dua) dan posita angka 3 (tiga) gugatan Penggugat, para saksi Penggugat telah memberikan keterangan yang relevan dan saling bersesuaian di persidangan, sehingga telah terbukti secara sah menurut hukum bahwa selama pernikahan tersebut, Penggugat dengan Tergugat awalnya telah hidup rukun sebagaimana layaknya suami-istri tetapi tidak dikaruniai anak dan setelah pernikahan tersebut Penggugat dan Tergugat tinggal bersama di rumah orang tua Tergugat di Desa Moncongloe Bulu, Kecamatan Moncongloe, Kabupaten Maros, Provinsi Sulawesi- Selatan;

Menimbang, bahwa terhadap substansi gugatan Penggugat pada posita angka 4 (empat) dan posita angka 5 (lima), para saksi Penggugat telah memberikan keterangan yang relevan dan saling bersesuaian di persidangan,

Disclaimer
Kepaniteraan Mahkamah Agung Republik Indonesia berusaha untuk selalu mencantumkan informasi paling kini dan akurat sebagai bentuk komitmen Mahkamah Agung untuk pelayanan publik, transparansi dan akuntabilitas pelaksanaan fungsi peradilan. Namun dalam hal-hal tertentu masih dimungkinkan terjadi permasalahan teknis terkait dengan akurasi dan keterkinian informasi yang kami sajikan, hal mana akan terus kami perbaiki dari waktu kewaktu.
Dalam hal Anda menemukan inakurasi informasi yang termuat pada situs ini atau informasi yang seharusnya ada, namun belum tersedia, maka harap segera hubungi Kepaniteraan Mahkamah Agung RI melalui :
Email : kepaniteraan@mahkamahagung.go.id Telp : 021-384 3348 (ext.318)

serta berdasarkan ketentuan Pasal 310 *Reglement Buiteegewesten* (R.Bg.), Majelis Hakim diberikan wewenang untuk mempergunakan persangkaan hakim sebagai alat bukti dalam suatu perkara, jika itu sangat penting, cermat, tertentu dan bersesuaian satu dengan yang lainnya, sehingga telah terbukti secara sah menurut hukum bahwa sejak awal Desember 2013, ketenteraman rumah tangga Penggugat dan Tergugat sudah mulai tidak harmonis lagi dengan adanya perselisihan terus menerus disebabkan karena Tergugat memiliki kebiasaan suka meminum-minuman keras, serta memiliki sifat cemburu yang berlebihan, Tergugat sering keluar malam tanpa alasan dan hal yang penting, serta Penggugat dan Tergugat telah sepakat untuk bercerai, dan antara Penggugat dan Tergugat telah pisah rumah sejak bulan Maret 2013 yang lalu dan tidak ada komunikasi lagi antara Penggugat dan Tergugat;

Menimbang, bahwa terhadap posita angka 6 (enam) gugatan Penggugat, para saksiPenggugat telah memberikan keterangan yang relevan dan saling bersesuaian di persidangan, serta berdasarkan ketentuan Pasal 310 *Reglement Buiteegewesten* (R.Bg.), Majelis Hakim diberikan wewenang untuk mempergunakan persangkaan hakim sebagai alat bukti dalam suatu perkara, jika itu sangat penting, cermat, tertentu dan bersesuaian satu dengan yang lainnya, sehingga telah terbukti secara sah menurut hukum bahwa ikatan perkawinan Penggugat dengan Tergugat sudah tidak dapat dibina lagi, dan karenanya perceraian merupakan jalan terakhir bagi Penggugat untuk menyelesaikan permasalahan antara Penggugat dengan Tergugat, dan Tergugat sudah pisah rumah sejak 7 tahun yang lalu hingga sekarang, dan Penggugat yang pergi meninggalkan kediaman bersama serta telah diupayakan untuk merukunkan Penggugat dan Tergugat, namun tidak berhasil dan di persidangan Penggugat telah memperlihatkan sikap dan tekadnya untuk bercerai dengan Tergugat;

Menimbang, bahwa berdasarkan keterangan Penggugat dan proses tahap konstatir pada pembuktian tersebut di atas, maka ditemukanlah fakta-fakta hukum sebagai berikut:

- Bahwa Penggugat dan Tergugat adalah suami istri yang sah;

Disclaimer
Kepaniteraan Mahkamah Agung Republik Indonesia berusaha untuk selalu mencantumkan informasi paling kini dan akurat sebagai bentuk komitmen Mahkamah Agung untuk pelayanan publik, transparansi dan akuntabilitas pelaksanaan fungsi peradilan. Namun dalam hal-hal tertentu masih dimungkinkan terjadi permasalahan teknis terkait dengan akurasi dan keterkinian informasi yang kami sajikan, hal mana akan terus kami perbaiki dari waktu kewaktu.
Dalam hal Anda menemukan inakurasi informasi yang termuat pada situs ini atau informasi yang seharusnya ada, namun belum tersedia, maka harap segera hubungi Kepaniteraan Mahkamah Agung RI melalui :
Email : kepaniteraan@mahkamahagung.go.id   Telp : 021-384 3348 (ext.318)

- Bahwa rumah tangga Penggugat dan Tergugat tidak harmonis lagi karena terjadi perselisihan dan pertengkaran disebabkan karena Tergugat memiliki kebiasaan suka meminum-minuman keras, serta memiliki sifat cemburu yang berlebihan, Tergugat sering keluar malam tanpa alasan dan hal yang penting, serta Penggugat dan Tergugat telah sepakat untuk bercerai;
- Bahwa kedua saksi tersebut mengetahui akibat dari perselisihan dan pertengkaran Penggugat dan Tergugat tersebut antara Penggugat dan Tergugat telah berpisah tempat tinggal sejak bulan Maret 2013 hingga sekarang telah berlangsung selama 7 tahun lebih lamanya;
- Bahwa usaha kedua saksi untuk merukunkan kembali rumah tangga Penggugat dan Tergugat ternyata tidak berhasil;

Menimbang, bahwa berdasarkan fakta-fakta hukum di atas, hal mana ketenteraman rumah tangga Penggugat dan Tergugat sudah mulai tidak harmonis dengan adanya perselisihan terus menerus disebabkan karena Tergugat memiliki kebiasaan suka meminum-minuman keras, serta memiliki sifat cemburu yang berlebihan, Tergugat sering keluar malam tanpa alasan dan hal yang penting, serta Penggugat dan Tergugat telah sepakat untuk bercerai, dan antara Penggugat dengan Tergugat telah pisah rumah sejak 7 tahun lebih lamanya dan tidak ada komunikasi lagi antara Penggugat dan Tergugat, serta telah diupayakan untuk merukunkan Penggugat dengan Tergugat, namun tidak berhasil dan di persidangan Penggugat telah memperlihatkan sikap dan tekadnya untuk bercerai dengan Tergugat, maka Majelis Hakim telah mengkualifisir, sehingga telah berkeyakinan kuat bahwa antara Penggugat dengan Tergugat telah terjadi pertengkaran dan perselisihan yang terus-menerus, dan tidak ada harapan akan hidup rukun lagi dalam membina rumah tangganya;

Menimbang, bahwa berdasarkan fakta-fakta hukum di atas, Majelis Hakim telah berkesimpulan bahwa oleh karena telah terjadi perselisihan dan pertengkaran antara Penggugat dan Tergugat yang terus-menerus, dan tidak ada harapan akan hidup rukun lagi dalam membina rumah tangganya, serta sudah tidak satu atap lagi/serumah sehingga perkawinan antara Penggugat dan

Disclaimer
Kepaniteraan Mahkamah Agung Republik Indonesia berusaha untuk selalu mencantumkan informasi paling kini dan akurat sebagai bentuk komitmen Mahkamah Agung untuk pelayanan publik, transparansi dan akuntabilitas pelaksanaan fungsi peradilan. Namun dalam hal-hal tertentu masih dimungkinkan terjadi permasalahan teknis terkait dengan akurasi dan keterkinian informasi yang kami sajikan, hal mana akan terus kami perbaiki dari waktu kewaktu.
Dalam hal Anda menemukan inakurasi informasi yang termuat pada situs ini atau informasi yang seharusnya ada, namun belum tersedia, maka harap segera hubungi Kepaniteraan Mahkamah Agung RI melalui :
Email : kepaniteraan@mahkamahagung.go.id Telp : 021-384 3348 (ext.318)

Tergugat telah pecah, dan hati kedua belah pihak pun telah pecah, berdasarkan Yurisprudensi Mahkamah Agung RI. Nomor 285 K/AG/2000, tanggal 10 November 2000, jo. Yurisprudensi Mahkamah Agung RI. Nomor 38 K/AG/1990, tanggal 5 Oktober 1991;

Menimbang, bahwa Majelis Hakim telah berupaya menasihati Penggugat agar rukun kembali dalam membina rumah tangga dengan Tergugat, akan tetapi tidak berhasil, maka Majelis Hakim berpendapat bahwa rumah tangga yang demikian, lebih baik tidak dilanjutkan dan tidak dipertahankan lagi, karena mustahil dapat mencapai tujuan perkawinan sebagaimana maksud Pasal 1 Undang-Undang RI Nomor 1 Tahun 1974 Tentang Perkawinan jo. Pasal 3 Buku I Kompilasi Hukum Islam (Keputusan Menteri Agama RI Nomor 154 Tahun 1991 Tentang Pelaksanaan Instruksi Presiden RI Nomor 1 Tahun 1991), dan putusnya perkawinan antara keduanya dalam kondisi seperti di atas, justru Allah akan memberikan limpahan karuniaNya kepada masing-masing di antara keduanya, sebagaimana firman Allah dalam al-Qur'an Surah al-Nisaa' ayat 130 sebagai berikut:

وَإِنْ يَتَفَرَّقَا يُغْنِ اللَّهُ كُلًّا مِنْ سَعَتِهِ وَكَانَ اللَّهُ وَاسِعًا حَكِيمًا

Artinya:

"Jika keduanya bercerai, maka Allah akan memberi kecukupan kepada masing-masing dari limpahan karuniaNya. Dan Dia adalah Allah Maha Luas (karuniaNya) lagi Maha Bijaksana";

Menimbang, bahwa selanjutnya Majelis Hakim melakukan tahap kostituir terhadap pertimbangan-pertimbangan hukum di atas, sehingga Majelis Hakimtelah berkeyakinan kuat bahwa dalil-dalil gugatan Penggugat tersebut telah memenuhi alasan perceraian sebagaimana yang diatur dalam Pasal 39 ayat (2) Undang-Undang RI Nomor 1 Tahun 1974 Tentang Perkawinan, berikut penjelasannya, jo. Pasal 19 huruf (f) Peraturan Pemerintah RI Nomor 9 Tahun 1975, jis. Pasal 116 huruf (f) Buku I Kompilasi Hukum Islam (Keputusan [*baca*Peraturan] Menteri Agama RI Nomor 154 Tahun 1991 Tentang Pelaksanaan Instruksi Presiden RI Nomor 1 Tahun 1991), dan secara khusus

Disclaimer
Kepaniteraan Mahkamah Agung Republik Indonesia berusaha untuk selalu mencantumkan informasi paling kini dan akurat sebagai bentuk komitmen Mahkamah Agung untuk pelayanan publik, transparansi dan akuntabilitas pelaksanaan fungsi peradilan. Namun dalam hal-hal tertentu masih dimungkinkan terjadi permasalahan teknis terkait dengan akurasi dan keterkinian informasi yang kami sajikan, hal mana akan terus kami perbaiki dari waktu kewaktu.
Dalam hal Anda menemukan inakurasi informasi yang termuat pada situs ini atau informasi yang seharusnya ada, namun belum tersedia, maka harap segera hubungi Kepaniteraan Mahkamah Agung RI melalui :
Email : kepaniteraan@mahkamahagung.go.id Telp : 021-384 3348 (ext.318)

berkaitan dengan terjadinya perselisihan terus-menerus antara suami istri berdasarkan Surat Edaran Mahkamah Agung RI. Nomor 3 Tahun 1981 perihal Perkara Perceraian, dan oleh karenanya dalil-dalil gugatan Penggugat tersebut harus dinyatakan telah terbukti secara sah menurut hukum;

Menimbang, bahwa Majelis Hakim telah berupaya menasihati Penggugat agar kembali rukun dengan Tergugat, namun tidak berhasil, dengan demikian Majelis Hakim telah memenuhi maksud Pasal 154 ayat (1) *Reglement Buiteegewesten* (R.Bg.);

Menimbang, bahwa oleh karena Tergugat telah dipanggil secara resmi dan patut berdasarkan relaas panggilan Jurusita Pengganti Pengadilan Agama Sungguminasa, dan ketidakhadiran Tergugat tersebut tanpa ada alasan yang dapat dibenarkan menurut hukum, dan pada tahap konstituir telah memenuhi maksud ketentuan Pasal 149 *Reglement Buiteegewesten* (R.Bg.), sehingga Majelis Hakimharus menyatakan Tergugat telah dipanggil secara resmi dan patut untuk menghadap di persidangan, tidak hadir;

Menimbang, bahwa oleh karena Tergugat telah dinyatakan dipanggil secara resmi dan patut untuk menghadap di persidangan namun tidak hadir, dan usaha mendamaikan tidak berhasil, serta oleh karena dalil-dalil gugatan Penggugat tersebut telah dinyatakan telah terbukti secara sah menurut hukum, dan berdasarkan ketentuan Pasal 149 ayat (1)*Reglement Buiteegewesten* (R.Bg.), sehingga terhadap angka 1 (satu) petitum primair gugatan Penggugat, oleh Majelis Hakim dalam permusyawaratannya telah sepakat untuk mengabulkan gugatan Penggugat dengan verstek;

Menimbang, bahwa berdasarkan Pasal 189 ayat (1) *Reglement Buiteegewesten* (R.Bg.), hal mana dalam rapat permusyawaratan, karena jabatannya hakim harus menambah dasar-dasar hukum yang tidak dikemukakan oleh para pihak;

Menimbang, bahwa terhadap angka 2 (dua) petitum primair gugatan Penggugat, berdasarkan sumber hukum dalam Pasal 119 ayat (2) huruf (c) Buku I Kompilasi Hukum Islam (Keputusan (*baca* Peraturan) Menteri Agama RI Nomor 154 Tahun 1991 Tentang Pelaksanaan Instruksi Presiden RI Nomor 1 Tahun 1991), menyebutkan bahwa talak yang dijatuhkan oleh Pengadilan

Disclaimer
Kepaniteraan Mahkamah Agung Republik Indonesia berusaha untuk selalu mencantumkan informasi paling kini dan akurat sebagai bentuk komitmen Mahkamah Agung untuk pelayanan publik, transparansi dan akuntabilitas pelaksanaan fungsi peradilan. Namun dalam hal-hal tertentu masih dimungkinkan terjadi permasalahan teknis terkait dengan akurasi dan keterkinian informasi yang kami sajikan, hal mana akan terus kami perbaiki dari waktu kewaktu.
Dalam hal Anda menemukan inakurasi informasi yang termuat pada situs ini atau informasi yang seharusnya ada, namun belum tersedia, maka harap segera hubungi Kepaniteraan Mahkamah Agung RI melalui :
Email : kepaniteraan@mahkamahagung.go.id   Telp : 021-384 3348 (ext.318)

Agama termasuk dalam kualifikasi Talak Ba'in Shugraa, serta berdasarkan Pasal 189 ayat (1) *Reglement Buiteegewesten* (R.Bg.) di atas, sehingga Majelis Hakim dalam rapat permusyawaratannya harus menyatakan perkawinan Penggugat dengan Tergugat putus karena Talak Ba'in Shugraa;

Menimbang, bahwa oleh karena cerai gugat yang diajukan oleh istri (Penggugat) telah dikabulkan dan berdasarkan catatan status perkawinan pada alat bukti P., yang telah menunjukkan bahwa perkara perceraian a quo antara Tergugat dengan Penggugat dijatuhkan pertama kali oleh Majelis Hakim dalam persidangan Pengadilan Agama Sungguminasa, serta berdasarkan Pasal 119 ayat (2) huruf (c) Buku I Kompilasi Hukum Islam (Keputusan (*baca* Peraturan) Menteri Agama RI Nomor 154 Tahun 1991 Tentang Pelaksanaan Instruksi Presiden RI Nomor 1 Tahun 1991), maka terkait dengan angka 2 (dua) petitum primair gugatan Penggugat, oleh Majelis Hakim dalam rapat permusyawaratannya harus menjatuhkan Talak Satu Ba'in Shugraa Tergugat (**#Tergugat**) terhadap Penggugat (**#Penggugat**);

Menimbang, bahwa oleh karena perkara ini adalah perkara perceraian dantermasuk dalam ruang lingkup perkara perkawinan, dan berdasarkan ketentuan Pasal 89 ayat (1) Undang-Undang RI Nomor 7 Tahun 1989 Tentang Peradilan Agama, yang telah diubah dengan Undang-Undang RI Nomor 3 Tahun 2006, serta Pasal 90, 91, dan 91A Undang-Undang RI Nomor 50 Tahun 2009 Tentang Perubahan Kedua Atas Undang-Undang RI Nomor 7 Tahun 1989 Tentang Peradilan Agama, maka terhadap angka 3 (tiga) petitum primer gugatan Penggugat, segala biaya yang timbul dalam perkara ini dibebankan kepada Penggugatyang jumlahnya dituangkan dalam amar putusan ini;

Memperhatikan segala ketentuan hukum syar'i dan peraturan perundang-undangan yang berlaku yang berkaitan dengan perkara ini.

MENGADILI

1. Menyatakan Tergugat telah dipanggil secara resmi dan patut untuk hadir ke persidangan, tidak hadir;
2. Mengabulkan gugatan Penggugat dengan Verstek;

Disclaimer
Kepaniteraan Mahkamah Agung Republik Indonesia berusaha untuk selalu mencantumkan informasi paling kini dan akurat sebagai bentuk komitmen Mahkamah Agung untuk pelayanan publik, transparansi dan akuntabilitas pelaksanaan fungsi peradilan. Namun dalam hal-hal tertentu masih dimungkinkan terjadi permasalahan teknis terkait dengan akurasi dan keterkinian informasi yang kami sajikan, hal mana akan terus kami perbaiki dari waktu kewaktu.
Dalam hal Anda menemukan inakurasi informasi yang termuat pada situs ini atau informasi yang seharusnya ada, namun belum tersedia, maka harap segera hubungi Kepaniteraan Mahkamah Agung RI melalui :
Email : kepaniteraan@mahkamahagung.go.id Telp : 021-384 3348 (ext.318)

3. Menjatuhkan talak satu ba'in Shugraa Tergugat
(#**Tergugat**) kepada Penggugat (#**Penggugat**);

4. Membebankan Penggugat untuk membayar biaya perkara sejumlah
Rp 686.000,00 (enam ratus delapan puluh enam ribu rupiah)

Demkian putusan ini dijatuhkan dalam rapat permusyawaratan Majelis Hakim yang dilangsungkan pada hari Rabu, tanggal 23 Desember 2020 Masehi, bertepatan dengan tanggal 8 Rabiul Awwal 1442 Hijriah, oleh kami Dra.Hj. Hadidjah, M.H sebagai Ketua Majelis, Drs. Kasang, M.H dan Ruhana Faried, S.HI.,M.HI masing-masing sebagai Hakim Anggota, penetapan tersebut diucapkan dalam sidang terbuka untuk umum, pada hari itu juga oleh Ketua Majelis tersebut dengan didampingi oleh Hakim Anggota dan dibantu oleh Rahmatiah, S.H.. sebagai Panitera Pengganti serta dihadiri oleh Penggugat tanpa hadirnya Tergugat;

Hakim Anggota,	Ketua Majelis,
Drs. Kasang, M.H	Dra.Hj. Hadidjah, M.H
Hakim Anggota,	
Ruhana Faried, S.HI.,M.HI	
	Panitera Pengganti,
	Rahmatiah, S.H.

Halaman 14 dari 15 Putusan Nomor 1041/Pdt.G/2020/PA Sgm.

Disclaimer
Kepaniteraan Mahkamah Agung Republik Indonesia berusaha untuk selalu mencantumkan informasi paling kini dan akurat sebagai bentuk komitmen Mahkamah Agung untuk pelayanan publik, transparansi dan akuntabilitas pelaksanaan fungsi peradilan. Namun dalam hal-hal tertentu masih dimungkinkan terjadi permasalahan teknis terkait dengan akurasi dan keterkinian informasi yang kami sajikan, hal mana akan terus kami perbaiki dari waktu kewaktu.
Dalam hal Anda menemukan inakurasi informasi yang termuat pada situs ini atau informasi yang seharusnya ada, namun belum tersedia, maka harap segera hubungi Kepaniteraan Mahkamah Agung RI melalui :
Email : kepaniteraan@mahkamahagung.go.id Telp : 021-384 3348 (ext.318)

Perincian Biaya Perkara :

1.	Biaya Pendaftaran	Rp	30.000,00-
2.	Biaya Administrasi	Rp	50.000,00-
3.	Biaya Panggilan	Rp	580.000,00-
4.	Biaya PNBP Panggilan	Rp	10.000,00-
5.	Biaya Redaksi	Rp	10.000,00-
6.	Biaya Materai	Rp	6.000,00-
	**J u m l a h**	**Rp**	**686.000,00-**

(enam ratus delapan puluh enam ribu rupiah)

Disclaimer
Kepaniteraan Mahkamah Agung Republik Indonesia berusaha untuk selalu mencantumkan informasi paling kini dan akurat sebagai bentuk komitmen Mahkamah Agung untuk pelayanan publik, transparansi dan akuntabilitas pelaksanaan fungsi peradilan. Namun dalam hal-hal tertentu masih dimungkinkan terjadi permasalahan teknis terkait dengan akurasi dan keterkinian informasi yang kami sajikan, hal mana akan terus kami perbaiki dari waktu kewaktu.
Dalam hal Anda menemukan inakurasi informasi yang termuat pada situs ini atau informasi yang seharusnya ada, namun belum tersedia, maka harap segera hubungi Kepaniteraan Mahkamah Agung RI melalui :
Email : kepaniteraan@mahkamahagung.go.id   Telp : 021-384 3348 (ext.318)
