# Supplementary material for: Classification of divorce causes during the COVID-19 pandemic using convolutional neural networks
Source: PeerJ Comput Sci. 2022 Jun 30;8:e998. doi: 10.7717/peerj-cs.998 (PMC9299239; doi:10.7717/peerj-cs.998)
Supplement: Supplemental Information 3 [file peerj-cs-08-998-s003.zip › KDRT Dataset/Data ke-12.pdf]

P U T U S A N

Nomor 2854/Pdt.G/2020/PA.Sda

بِسْمِ اللَّهِ الرَّحْمَنِ الرَّحِيمِ

DEMI KEADILAN BERDASARKAN KETUHANAN YANG MAHA ESA

Pengadilan Agama Sidoarjo yang memeriksa dan mengadili perkara tertentu pada tingkat pertama dalam persidangan majelis Hakim telah menjatuhkan putusan dalam perkara cerai talak antara :

PEMOHON, umur 43 tahun, agama Islam, Pendidikan SMK, pekerjaan Karyawan Swasta, tempat kediaman di Kabupaten Sidoarjo, selanjutnya disebut sebagai **Pemohon**;

m e l a w a n

TERMOHON, umur 41 tahun, agama Islam, Pendidikan SMP , pekerjaan Karyawan Swasta, tempat tinggal dahulu di Kabupaten Sidoarjo, Sekarang tidak diketahui alamat dan tempat tinggalnya dengan jelas, baik di dalam maupun di luar wilayah Republik Indonesia. selanjutnya disebut sebagai **Termohon**;

Pengadilan Agama Sidoarjo;

Telah membaca dan mempelajari berkas perkara yang bersangkutan;

Telah mendengar keterangan pihak yang berperkara dan para saksi serta memeriksa bukti lain di muka sidang;

DUDUK PERKARA

Bahwa Pemohon dalam surat permohonannya tertanggal 12 Agustus 2020 yang didaftarkan di Kepaniteraan Pengadilan Agama Sidoarjo, Nomor 2854/Pdt.G/2020/PA.Sda, telah mengajukan permohonan cerai terhadap Termohon dengan uraian/alasan sebagai berikut:

1. Bahwa Pemohon dengan Termohon telah menikah sah menurut agama Islam pada tanggal 22 Agustus 2002, dihadapan Pegawai Pencatat Nikah

Disclaimer
Kepaniteraan Mahkamah Agung Republik Indonesia berusaha untuk selalu mencantumkan informasi paling kini dan akurat sebagai bentuk komitmen Mahkamah Agung untuk pelayanan publik, transparansi dan akuntabilitas pelaksanaan fungsi peradilan. Namun dalam hal-hal tertentu masih dimungkinkan terjadi permasalahan teknis terkait dengan akurasi dan keterkinian informasi yang kami sajikan, hal mana akan terus kami perbaiki dari waktu kewaktu.
Dalam hal Anda menemukan inakurasi informasi yang termuat pada situs ini atau informasi yang seharusnya ada, namun belum tersedia, maka harap segera hubungi Kepaniteraan Mahkamah Agung RI melalui :
Email : kepaniteraan@mahkamahagung.go.id Telp : 021-384 3348 (ext.318)

Kantor Urusan Agama Kecamatan Balongbendo, Kabupaten Sidoarjo sebagaimana terbukti dalam Kutipan Akta Nikah Nomor : 357/35/VIII/2002 tanggal 22 Agustus 2002;

2. Bahwa setelah melangsungkan perkawinan tersebut, Pemohon dan Termohon telah hidup bersama sebagaimana layaknya suami istri dan hidup bersama di rumah orang tua Pemohon di Kabupaten Sidoarjo
3. Bahwa selama membina perkawinan tersebut Pemohon dan Termohon Sudah melakukan hubungan layaknya suami dan sudah dikaruniai 2 (dua) orang anak bernama;
   1. Anak I,tanggal lahir 31 Juli 2003 (17 tahun)
   2. Anak II,tanggal lahir 03 Mei 2010 (10 tahun)

   Yang saat ini tinggal dan diasuh oleh Pemohon
4. Bahwa semula kehidupan rumah tangga Pemohon dan Termohon berjalan tentram dan harmonis akan tetapi sejak bulan Januari 2016 rumah tangga Pemohon dan Termohon mulai goyah, sering terjadi perselisihan dan pertengkaran yang disebabkan :
   a. Termohon selalu merasa kurang terhadap nafkah yang diberikan oleh Pemohon;
   b. Termohon Menjalin hubungan dengan laki-laki lain
5. Bahwa perselisihan dan pertengkaran antara Pemohon dan Termohon tersebut memuncak, meskipun telah di tempuh upaya damai namun gagal, akhirnya sejak bulan Mei 2017,Termohon pergi meninggalkan Pemohon tanpa pamit dan selama pergi Termohon tidak diketahui alamat dan tempat tinggalnya, sehingga antara Pemohon dan Termohon sekarang sudah pisah rumah selama 3 tahun 3 bulan, dan selama berpisah Pemohon sudah berusaha mencari Termohon akan tetapi tidak berhasil.
6. Bahwa Pemohon bersedia membayar biaya perkara yang timbul akibat permohonan Pemohon tersebut.

Bahwa atas dasar alasan - alasan tersebut, Pemohon mohon kepada Pengadilan Agama Sidoarjo, untuk berkenan memanggil dan memeriksa Pemohon dan Termohon, selanjutnya menjatuhkan putusan sebagai berikut :

Disclaimer
Kepaniteraan Mahkamah Agung Republik Indonesia berusaha untuk selalu mencantumkan informasi paling kini dan akurat sebagai bentuk komitmen Mahkamah Agung untuk pelayanan publik, transparansi dan akuntabilitas pelaksanaan fungsi peradilan. Namun dalam hal-hal tertentu masih dimungkinkan terjadi permasalahan teknis terkait dengan akurasi dan keterkinian informasi yang kami sajikan, hal mana akan terus kami perbaiki dari waktu kewaktu.
Dalam hal Anda menemukan inakurasi informasi yang termuat pada situs ini atau informasi yang seharusnya ada, namun belum tersedia, maka harap segera hubungi Kepaniteraan Mahkamah Agung RI melalui :
Email : kepaniteraan@mahkamahagung.go.id Telp : 021-384 3348 (ext.318)

PRIMER :

1. Mengabulkan permohonan Pemohon;
2. Memberi ijin kepada Pemohon (**PEMOHON**) untuk menjatuhkan talak satu raj'i terhadap Termohon (**TERMOHON**) di depan sidang Pengadilan Agama Sidoarjo;
3. Membebankan kepada Pemohon untuk membayar biaya perkara ini sesuai dengan peraturan yang berlaku ;

SUBSIDER :

Atau apabila Pengadilan berpendapat lain, mohon putusan yang seadil - adilnya;

Bahwa pada hari sidang yang telah ditentukan Pemohon hadir di persidangan, sedangkan Termohon tidak datang ke persidangan dan tidak pula menyuruh orang lain untuk menghadap sebagai wakilnya tanpa alasan yang sah meskipun ia telah dipanggil melalui media masa sebagaimana ternyata pada relas panggilan tanggal 18 Agustus 2020 dan tanggal 18 September 2020;

Bahwa Majelis Hakim menilai bahwa oleh karena pihak Termohon tidak pernah hadir di persidangan, maka perkara ini tidak layak dilakukan Mediasi, namun selama persidangan berlangsung majelis Hakim memberikan nasehat agar Pemohon berdamai dan kembali rukun sebagai suami isteri bersama Termohon, akan tetapi upaya tersebut tidak berhasil. Selanjutnya pemeriksaan dilanjutkan dengan membacakan surat permohonan Pemohon yang isinya tetap dipertahankan oleh Pemohon;

Bahwa atas permohonan Pemohon tersebut, Termohon tidak dapat didengar tanggapan/jawabannya karena ia tidak pernah hadir di muka sidang;

Bahwa untuk meneguhkan dalil permohonannya, Pemohon telah mengajukan bukti surat berupa :

1. Fotokopi Kutipan Akta Nikah Nomor 357/35/VIII/2002 tanggal 22 Agustus 2002 yang dikeluarkan oleh PPN pada KUA Kecamatan Balongbendo, Kabupaten Sidoarjo, (P-1);

Disclaimer
Kepaniteraan Mahkamah Agung Republik Indonesia berusaha untuk selalu mencantumkan informasi paling kini dan akurat sebagai bentuk komitmen Mahkamah Agung untuk pelayanan publik, transparansi dan akuntabilitas pelaksanaan fungsi peradilan. Namun dalam hal-hal tertentu masih dimungkinkan terjadi permasalahan teknis terkait dengan akurasi dan keterkinian informasi yang kami sajikan, hal mana akan terus kami perbaiki dari waktu kewaktu.
Dalam hal Anda menemukan inakurasi informasi yang termuat pada situs ini atau informasi yang seharusnya ada, namun belum tersedia, maka harap segera hubungi Kepaniteraan Mahkamah Agung RI melalui :
Email : kepaniteraan@mahkamahagung.go.id Telp : 021-384 3348 (ext.318)

2. Fotokopi Kartu Tanda Penduduk atas nama Pemohon, Nomor [redacted] tanggal 29 April 2012, yang dikeluarkan oleh Dinas Kependudukan dan Catatan Sipil Kabupaten Sidoarjo, (P-2);
3. Fotokopi Surat Keterangan atas nama Termohon Nomor 145 / 193 / 438.7.12.5 / 2020, tanggal 23 Juli 2020, yang dikeluarkan oleh Kepala Desa Kalimati, Kecamatan Tarik Kabupaten Sidoarjo, (P-3);

Bahwa selain bukti tertulis sebagaimana terebut di atas, Pemohon juga telah menghadirkan saksi di muka sidang sebagai berikut :

Saksi I, Umur 31 tahun, Agama Islam, Pekerjaan Swasta, tempat tinggal di Kabupaten Sidoarjo, didalam sidang saksi memberikan keterangan diatas sumpah yang pokoknya adalah sebagai berikut :

--Bahwa Saksi adalah adik dari Pemohon;

--Bahwa Saksi mengetahui Pemohon dengan Termohon suami istri yang menikah pada Agustus 2002.

--Bahwa Saksi tahu selama dalam pernikahan tersebut Pemohon dengan Termohon tinggal dan membina rumah tangga di rumah orang tua Pemohon di Kabupaten Sidoarjo.

--Bahwa Saksi melihat selama dalam pernikahan Pemohon dan Termohon sudah hidup seperti layaknya suami isteri dan sudah dikaruniai 2 (dua) anak bernama Anak I, (17 tahun) dan Anak II, (10 tahun); yang saat ini tinggal dan diasuh oleh Pemohon.

--Bahwa Saksi mengetahui pada awalnya hidup rukun sebagaimana layaknya suami isteri, namun kemudian Termohon pergi meninggalkan Pemohon karena sebelumnya Saksi mengetahui bahwa Pemohon sering berselisih dan bertengkar dengan Termohon;

--Bahwa Saksi mengetahui penyebab pertengkaran Pemohon dan Termohon karena Termohon memiliki Pria Idaman Lain (PIL); Termohon selalu merasa kurang dengan uang belanja yang Pemohon berikan;

--Bahwa Saksi mengetahui Termohon pergi meninggalkan Pemohon tanpa pamit dan selama pergi Termohon tidak diketahui alamat dan tempat tinggalnya, sehingga Pemohon dengan Termohon berpisah sejak pisah rumah

Disclaimer
Kepaniteraan Mahkamah Agung Republik Indonesia berusaha untuk selalu mencantumkan informasi paling kini dan akurat sebagai bentuk komitmen Mahkamah Agung untuk pelayanan publik, transparansi dan akuntabilitas pelaksanaan fungsi peradilan. Namun dalam hal-hal tertentu masih dimungkinkan terjadi permasalahan teknis terkait dengan akurasi dan keterkinian informasi yang kami sajikan, hal mana akan terus kami perbaiki dari waktu kewaktu.
Dalam hal Anda menemukan inakurasi informasi yang termuat pada situs ini atau informasi yang seharusnya ada, namun belum tersedia, maka harap segera hubungi Kepaniteraan Mahkamah Agung RI melalui :
Email : kepaniteraan@mahkamahagung.go.id Telp : 021-384 3348 (ext.318)

sampai sekarang sudah 3 tahun; dan setelah itu mereka tidak lagi hidup dan menjalankan kewajiban sebagai layaknya suami istri.

--Bahwa Saksi tidak tahu saat ini Termohon berada dimana, namun sebelumnya Termohon tinggal di Dusun Centong, Desa Kalimati, Kecamatan Tarik, Kabupaten Sidoarjo, kemudian Termohon pergi dari rumah tersebut yang meskipun Pemohon telah mencari Termohon namun Pemohon tidak mengetahui keberadaan Termohon.

--Bahwa Saksi sudah berusaha mendamaikan mereka namun tidak berhasil.

--Bahwa Saksi telah berulang kali berusaha merukunkan mereka tapi sampai sekarang tetap tidak berhasil, sehingga Saksi tidak sanggup lagi mendamaikan mereka;

Saksi II, Umur 52 tahun, Agama Islam, Pekerjaan Swasta, tempat tinggal di Kabupaten Sidoarjo, didalam sidang saksi memberikan keterangan diatas sumpah yang pokoknya adalah sebagai berikut :

--Bahwa Saksi mengenal kedua belah pihak yang berperkara karena Saksi Paman Pemohon;

--Bahwa Saksi mengetahui selama membina rumah tangga Pemohon dengan Termohon bertempat tinggal di rumah orang tua Pemohon di Kabupaten Sidoarjo.

--Bahwa Saksi mengetahui pada awalnya Pemohon dan Termohon hidup rukun layaknya suami isteri dikaruniai 2 orang anak, namun sejak Januari 2016 sering berselisih dan bertengkar;

--Bahwa Pemohon dan Termohon sudah dikaruniai 2 (dua) anak bernama Anak I,(17 tahun) dan Anak II, yang saat ini tinggal dan diasuh oleh Pemohon.

--Bahwa Saksi mengetahui bahwa Pemohon dan Termohon) berselisih dan bertengkar.

--Bahwa Saksi mengetahui bahwa Pemohon dan Termohon berselisih dan bertengkar disebabkan karena Termohon memiliki Pria Idaman Lain (PIL); Termohon selalu merasa kurang dengan uang belanja yang Pemohon berikan;

--Bahwa Termohon pergi meninggalkan Pemohon tanpa pamit dan selama pergi Termohon tidak diketahui alamat dan tempat tinggalnya sehingga Pemohon dan Termohon berpisah sejak pisah rumah sampai sekarang sudah

Disclaimer
Kepaniteraan Mahkamah Agung Republik Indonesia berusaha untuk selalu mencantumkan informasi paling kini dan akurat sebagai bentuk komitmen Mahkamah Agung untuk pelayanan publik, transparansi dan akuntabilitas pelaksanaan fungsi peradilan. Namun dalam hal-hal tertentu masih dimungkinkan terjadi permasalahan teknis terkait dengan akurasi dan keterkinian informasi yang kami sajikan, hal mana akan terus kami perbaiki dari waktu kewaktu.
Dalam hal Anda menemukan inakurasi informasi yang termuat pada situs ini atau informasi yang seharusnya ada, namun belum tersedia, maka harap segera hubungi Kepaniteraan Mahkamah Agung RI melalui :
Email : kepaniteraan@mahkamahagung.go.id Telp : 021-384 3348 (ext.318)

3 tahun dan setelah itu Pemohon dengan Termohon tidak pernah berhubungan dan tidak saling menjalankan kewajiban sebagai suami istri.

--Bahwa Saksi tidak tahu saat ini Termohon berada dimana, namun sebelumnya Termohon tinggal di Dusun Centong, Desa Kalimati, Kecamatan Tarik, Kabupaten Sidoarjo, kemudian Termohon pergi dari rumah tersebut yang meskipun Pemohon telah mencari Termohon namun Pemohon tidak mengetahui keberadaan Termohon.

--Bahwa Pemohon dengan Termohon sudah pernah didamaikan oleh keluarga juga oleh saksi sendiri akan tetapi tidak berhasil;;

Menimbang, bahwa atas keterangan para saksi tersebut, Pemohon menyatakan tidak keberatan dan membenarkan;

Menimbang, bahwa Pemohon telah menyampaikan kesimpulannya yang pada pokoknya tetap mempertahankan permohonannya;

Menimbang, bahwa untuk mempersingkat uraian putusan ini maka hal-hal yang termuat dalam berita acara persidangan perkara ini dinyatakan sebagai bagian yang tidak terpisahkan dari Putusan ini;

PERTIMBANGAN HUKUM

Menimbang, bahwa maksud dan tujuan permohonan Pemohon adalah seperti diuraikan tersebut di atas;

Menimbang, bahwa oleh karena Termohon tidak diketahui tempat tinggalnya secara jelas maka ia dipanggil menurut ketentuan Pasal 27 Peraturan Pemerintah No.9 Tahun 1975 dan berdasarkan berita acara panggilan tanggal 18 Agustus 2020 dan 18 September 2020 Termohon telah dipanggil secara sah dan patut untuk hadir dipersidangan, tetapi Termohon tidak pernah hadir serta tidak ada orang lain yang hadir sebagai wakilnya dan ternyata ketidak hadiran Termohon tersebut bukan karena alasan yang sah menurut hukum, oleh sebab itu Termohon dinyatakan tidak hadir;

Menimbang, bahwa Majelis Hakim menilai bahwa oleh karena pihak Termohon tidak pernah hadir di persidangan, maka perkara ini tidak layak dilakukan Mediasi, namun untuk memenuhi maksud Pasal 130 HIR Jo. Pasal 82 ayat (1) dan (4) Undang-undang nomor 7 tahun 1989 tentang Peradilan Agama Jo. Pasal 31 ayat (1) dan (2) Peraturan Pemerintah nomor 9 tahun 1975

Disclaimer
Kepaniteraan Mahkamah Agung Republik Indonesia berusaha untuk selalu mencantumkan informasi paling kini dan akurat sebagai bentuk komitmen Mahkamah Agung untuk pelayanan publik, transparansi dan akuntabilitas pelaksanaan fungsi peradilan. Namun dalam hal-hal tertentu masih dimungkinkan terjadi permasalahan teknis terkait dengan akurasi dan keterkinian informasi yang kami sajikan, hal mana akan terus kami perbaiki dari waktu kewaktu.
Dalam hal Anda menemukan inakurasi informasi yang termuat pada situs ini atau informasi yang seharusnya ada, namun belum tersedia, maka harap segera hubungi Kepaniteraan Mahkamah Agung RI melalui :
Email : kepaniteraan@mahkamahagung.go.id Telp : 021-384 3348 (ext.318)

serta Pasal 18 ayat (3) PERMA Nomor 01 Tahun 2008, maka selama persidangan berlangsung majelis Hakim memberikan nasehat agar Pemohon berdamai dan kembali rukun sebagai suami isteri bersama Termohon, akan tetapi upaya tersebut tidak berhasil.

Menimbang, bahwa perkara ini termasuk dalam bidang perkawinan yang dilaksanakan berdasar Hukum Islam, maka berdasarkan Pasal I angka 37, Pasal 49 huruf (a) Undang-Undang Nomor 3 Tahun 2006 tentang perubahan pertama Undang-Undang Nomor 7 tahun 1989 tentang Peradilan Agama dan berdasarkan penjelasan pasal tersebut angka (8), perkara *a quo* menjadi kewenangan absolut Pengadilan Agama;

Menimbang, bahwa dalam permohonannya Pemohon menyatakan tempat tinggal Termohon semula berada diwilayah hukum Pengadilan Agama Sidoarjo namun sekarang tidak diketahui secara pasti di wilayah negara Repiblik Indonesia, maka berdasarkan maka berdasarkan Pasal 66 ayat (2) Undang-Undang Nomor 7 tahun 1989 tentang Peradilan Agama jo Pasal 118 HIR perkara *a quo* merupakan kewenangan relatif Pengadilan Agama Sidoarjo;

Menimbang, bahwa Pemohon mendalilkan telah menikah dengan Termohon berdasarkan Hukum Islam kemudian karena sering terjadi pertengakaran maka Pemohon menuntut agar Pengadilan Agama Sidoarjo memberi izin kepada Pemohon untuk menjatuhkan talak satu raj'i terhadap Termohon didepan sidang Pengadilan Agama Sidoarjo, oleh karenanya berdasarkan Pasal 39 Undang-undang Nomor 1 tahun 1974 tentang Perkawinan Jo Pasal 66 ayat (1) Undang-Undang Nomor 7 tahun 1989 tentang Peradilan Agama, Pemohon mempunyai *legal standing* untuk mengajukan permohonan perceraian tersebut ;

Menimbang, bahwa berdasarkan pertimbangan –pertimbangan tersebut dan karena permohonan Pemohon telah memenuhi syarat formal suatu permohonan maka terhadap petitum permohonan Pemohon angka 1 yang meminta Pengadilan menerima permohonan Pemohon secara formal dapat diterima untuk diperiksa;

Menimbang, bahwa yang menjadi pokok permohonan Pemohon adalah agar Pengadilan Agama Sidoarjo memberi izin kepada Pemohon untuk

Disclaimer
Kepaniteraan Mahkamah Agung Republik Indonesia berusaha untuk selalu mencantumkan informasi paling kini dan akurat sebagai bentuk komitmen Mahkamah Agung untuk pelayanan publik, transparansi dan akuntabilitas pelaksanaan fungsi peradilan. Namun dalam hal-hal tertentu masih dimungkinkan terjadi permasalahan teknis terkait dengan akurasi dan keterkinian informasi yang kami sajikan, hal mana akan terus kami perbaiki dari waktu kewaktu.
Dalam hal Anda menemukan inakurasi informasi yang termuat pada situs ini atau informasi yang seharusnya ada, namun belum tersedia, maka harap segera hubungi Kepaniteraan Mahkamah Agung RI melalui :
Email : kepaniteraan@mahkamahagung.go.id   Telp : 021-384 3348 (ext.318)

menjatuhkan talak satu raj'i terhadap Termohon didepan sidang Pengadilan Agama Sidoarjo dengan alasan bahwa antara Pemohon dan Termohon sering terjadi perselisihan dan pertengkaran yang disebabkan karena :

a. Termohon selalu merasa kurang terhadap nafkah yang diberikan oleh Pemohon;
b. Termohon Menjalin hubungan dengan laki-laki lain

akhirnya sejak bulan Mei tahun 2017 Termohon pergi meninggalkan Pemohon tanpa pamit dan selama pergi Termohon tidak diketahui alamat dan tempat tinggalnya, sehingga antara Pemohon dan Termohon sekarang telah pisah rumah selama 3 tahun 3 bulan yang meskipun telah di tempuh upaya damai namun tidak berhasil;

Menimbang, bahwa Termohon tidak hadir dan tidak menyampaikan bantahan terhadap permohonan Pemohon namun berdasarkan prinsip menegakkan kebenaran dan keadilan *(to enforce the truth and justice)* dan untuk memenuhi ketentuan Pasal 76 Undang-undang nomor 7 tahun 1989 Jo. Pasal 22 Peraturan Pemerintah nomor 9 tahun 1970, Majelis berpendapat bahwa Pemohon wajib membuktikan dalil permohonannya;

Menimbang bahwa untuk membuktikan dalil-dalilnya Pemohon telah mengajukan alat bukti tertulis yakni Bukti P-1, P-2 dan P-3 serta saksi I dan saksi II yang selengkapnya akan dipertimbangkan lebih lanjut;

Menimbang bahwa bukti P-1, P-2 dan P-3 adalah akta otentik yang dikeluarkan oleh pejabat yang berwenang, bermeterai cukup dan cocok dengan aslinya, oleh karena itu akta tersebut berdasarkan pasal 165 HIR / 1868 KUH Perdata, memiliki nilai pembuktian sempurna dan mengikat ;

Menimbang bahwa keterangan saksi I dan saksi II Pemohon tersebut memenuhi syarat-syarat formal sebagai saksi berdasarkan Pasal 76 Undang-undang nomor 7 tahun 1989 tentang Peradilan Agama Jo. Pasal 171 HIR Jo. Pasal 22 Peraturan Pemerintah nomor 9 tahun 1975, maka sepanjang mengenai sesuatu yang dilihat sendiri dan atau dialami sendiri, keterangan saksi tersebut bernilai sebagai alat bukti yang sah dan dapat diterima sebagai alat bukti;

Disclaimer
Kepaniteraan Mahkamah Agung Republik Indonesia berusaha untuk selalu mencantumkan informasi paling kini dan akurat sebagai bentuk komitmen Mahkamah Agung untuk pelayanan publik, transparansi dan akuntabilitas pelaksanaan fungsi peradilan. Namun dalam hal-hal tertentu masih dimungkinkan terjadi permasalahan teknis terkait dengan akurasi dan keterkinian informasi yang kami sajikan, hal mana akan terus kami perbaiki dari waktu kewaktu.
Dalam hal Anda menemukan inakurasi informasi yang termuat pada situs ini atau informasi yang seharusnya ada, namun belum tersedia, maka harap segera hubungi Kepaniteraan Mahkamah Agung RI melalui :
Email : kepaniteraan@mahkamahagung.go.id   Telp : 021-384 3348 (ext.318)

Menimbang, bahwa atas dasar tuntutan yang dikemukakan Pemohon dan berdasarkan bukti-bukti yang diajukan Pemohon, Majelis Hakim akan mempertimbangkan tuntutan Pemohon sebagaimana terurai dalam surat permohonan Pemohon petitum angka 2 sebagai berikut :

Menimbang, bahwa berdasarkan bukti P.1 serta berdasarkan keterangan saksi I dan saksi II yang saling bersesuaian, telah terbukti bahwa Pemohon dan Termohon adalah suami Istri yang menikah berdasarkan Hukum Islam, oleh karenanya maka terbukti secara sah menurut hukum bahwa Pemohon dan Termohon adalah suami istri;

Menimbang, bahwa berdasarkan keterangan saksi I dan saksi II yang saling bersesuain, telah terbukti bahwa setelah menikah Pemohon dan Termohon tinggal bersama di rumah orang tua Pemohon di Kabupaten Sidoarjo dan telah hidup layaknya suami istri dan sudah dikaruniai 2 (dua) anak bernama Anak I,tanggal lahir 31 Juli 2003 (17 tahun) dan Anak II,tanggal lahir 03 Mei 2010 (10 tahun) yang saat ini tinggal dan diasuh oleh Pemohon;

Menimbang, bahwa berdasarkan keterangan saksi I dan saksi II yang saling bersesuaian, telah terbukti bahwa dalam rumah tangga Pemohon dan Termohon sering terjadi perselisihan dan pertengkaran dan berdasarkan keterangan saksi I dan saksi II terbukti pula bahwa penyebab pertengkaran antara Pemohon dan Termohon tersebut adalah karena Termohon memiliki Pria Idaman Lain (PIL); Termohon selalu merasa kurang dengan uang belanja yang Pemohon berikan;

Menimbang, bahwa berdasarkan keterangan saksi I dan saksi II yang saling bersesuaian dapat dikonstantir sebagai fakta hukum bahwa Termohon telah meninggalkan Pemohon sehingga sampai dengan saat ini mereka berdua telah pisah rumah sampai sekarang sudah 3 tahun dan selama itu mereka berdua tidak lagi menjalankan kewajiban sebagai suami istri secara utuh;

Menimbang, bahwa atas kejadian yang menimpa rumah tangga Pemohon dan Termohon tersebut diatas saksi I dan saksi II serta Majelis hakim Pengadilan Agama Sidoarjo telah mendamaikan atau menasehati Pemohon dan Termohon agar kembali rukun namun tidak berhasil;;

Disclaimer
Kepaniteraan Mahkamah Agung Republik Indonesia berusaha untuk selalu mencantumkan informasi paling kini dan akurat sebagai bentuk komitmen Mahkamah Agung untuk pelayanan publik, transparansi dan akuntabilitas pelaksanaan fungsi peradilan. Namun dalam hal-hal tertentu masih dimungkinkan terjadi permasalahan teknis terkait dengan akurasi dan keterkinian informasi yang kami sajikan, hal mana akan terus kami perbaiki dari waktu kewaktu.
Dalam hal Anda menemukan inakurasi informasi yang termuat pada situs ini atau informasi yang seharusnya ada, namun belum tersedia, maka harap segera hubungi Kepaniteraan Mahkamah Agung RI melalui :
Email : kepaniteraan@mahkamahagung.go.id Telp : 021-384 3348 (ext.318)

Menimbang, bahwa disyariatkannya pernikahan sebagai *mitsaqan ghalidhan* mempunyai tujuan yang suci dan mulia, yakni untuk menciptakan rumah tangga yang *sakinah mawaddah dan rahmah*, sebagaimana dimaksud dalam Al-Qur'an surat Ar Rum ayat 21 dan pasal 1 Undang-undang Nomor 1 Tahun 1974 *Jo.* pasal 3 Kompilasi Hukum Islam, namun dengan keadaan rumah tangga Pemohon dan Termohon tersebut, maka tujuan pernikahan tersebut menjadi sulit untuk bisa dicapai;

Menimbang, bahwa fakta-fakta tersebut diatas telah menunjukkan bahwa perkawinan Pemohon dan Termohon benar-benar telah pecah dan telah sampai pada taraf yang sudah tidak bisa didamaikan lagi karena Pemohon telah jera dan menolak untuk melanjutkan perkawinannya dengan Termohon. Dengan demikian penyelesaian yang dipandang adil dan bermanfaat bagi kedua belah pihak adalah perceraian ;

Menimbang, bahwa pada dasarnya menurut ajaran Islam perceraian merupakan perbuatan halal yang paling dimurkai Allah SWT, namun dalam keadaan suami istri sudah tidak bisa *saling mencintai* lagi dan telah terjadi sikap *jera dan menolak* sebagaimana yang dialami oleh Pemohon tersebut, maka perceraian dibolehkan, dalam hal ini Majelis Hakim mengambil alih dan menjadikan pertimbangan sendiri, pendapat ahli Hukum Islam yang tercantum dalam Kitab Madaa Hurriyatuz Zaujaini fith Thalaaq Juz I halaman 83 ;

وقد اختار الإسلام نظام الطلاق حين تضطرب الحياة الزوجين ولم يعد ينفع فيها
نلائح ولا صلح وحيث تصبح الربطة الزوج صورة من غير روح لأن الإستمرار
معناه أن يحكم على أحد الزوجين بالسجن المؤبد وهذا تأباه روح العدالة

Artinya: Islam memilih lembaga thalaq/cerai ketika rumah tangga sudah dianggap goncang serta dianggap sudah tidak bermanfaat lagi nasehat/ perdamaian dan hubungan suami isteri menjadi tanpa ruh (hampa), sebab meneruskan perkawinan berarti menghukum salah satu suami isteri dengan penjara yang berkepanjangan. Ini adalah aniaya yang bertentangan dengan semangat keadilan.

Menimbang, bahwa dengan merujuk pada Putusan Mahkamah Agung Republik Indonesia nomor 237/K/ AG/1998, tanggal 17 Maret 1999 yang

Disclaimer
Kepaniteraan Mahkamah Agung Republik Indonesia berusaha untuk selalu mencantumkan informasi paling kini dan akurat sebagai bentuk komitmen Mahkamah Agung untuk pelayanan publik, transparansi dan akuntabilitas pelaksanaan fungsi peradilan. Namun dalam hal-hal tertentu masih dimungkinkan terjadi permasalahan teknis terkait dengan akurasi dan keterkinian informasi yang kami sajikan, hal mana akan terus kami perbaiki dari waktu kewaktu.
Dalam hal Anda menemukan inakurasi informasi yang termuat pada situs ini atau informasi yang seharusnya ada, namun belum tersedia, maka harap segera hubungi Kepaniteraan Mahkamah Agung RI melalui :
Email : kepaniteraan@mahkamahagung.go.id   Telp : 021-384 3348 (ext.318)

mengandung abstrak hukum bahwa rumah tangga yang diwarnai perselisihan, percekcekcokan, tidak bersedia tinggal dalam satu tempat kediaman bersama, salah satu pihak tidak berniat untuk meneruskan kehidupan bersama dengan pihak lain, adalah merupakan fakta hukum yang cukup untuk alasan dalam suatu perceraian sesuai dengan maksud pasal 19 huruf (f) Peraturan Pemerintah nomor 9 tahun 1975;

Menimbang, bahwa berdasarkan pertimbangan-pertimbangan tersebut di atas, maka dalil permohonan Pemohon cukup beralasan dan telah memenuhi ketentuan pasal 39 Undang-undang Nomor 1 Tahun 1974 jo. Pasal 19 huruf (f) Peraturan Pemerintah Nomor 9 Tahun 1975 jo. Pasal 116 huruf (f) Kompilasi Hukum Islam, oleh karenanya maka petitum permohonan Pemohon agar Pengadilan memberi izin kepada Pemohon untuk menjatuh talak satu raj'i terhadap Termohon dihadapan sidang Pengadilan Agama Sidoarjo dapat dikabulkan ;

Menimbang, bahwa dalam perkara ini, Majelis Hakim perlu mengetengahkan Firman Allah dalam surat Al Baqoroh ayat 227 yang berbunyi :

وإن عزموا الطلاق فإن الله سميع عليم

Artinya : "Dan jika mereka ber'azam (bertetap hati) untuk talak, sesungguhnya Allah Maha Mendengar lagi Maha Mengetahui"

Menimbang, bahwa Termohon tidak pernah hadir di muka sidang meskipun ia telah dipanggil dengan resmi dan patut sedangkan ketidak hadirannya tidak terbukti disebabkan suatu halangan yang sah maka Termohon dinyatakan tidak hadir. Kemudian oleh karena permohonan Pemohon telah memenuhi alasan hukum dan tidak melawan hak, maka berdasarkan Pasal 125 HIR permohonan Pemohon dikabulkan dengan verstek;

Menimbang, bahwa oleh karena perkara ini masuk dalam bidang perkawinan, maka sesuai Pasal 89 ayat (1) Undang-undang Nomor 7 Tahun 1989 sebagaimana telah diubah dengan Undang-undang Nomor 3 Tahun 2006 dan perubahan kedua dengan Undang-undang Nomor 50 Tahun 2009, biaya perkara dibebankan kepada Pemohon;

Disclaimer
Kepaniteraan Mahkamah Agung Republik Indonesia berusaha untuk selalu mencantumkan informasi paling kini dan akurat sebagai bentuk komitmen Mahkamah Agung untuk pelayanan publik, transparansi dan akuntabilitas pelaksanaan fungsi peradilan. Namun dalam hal-hal tertentu masih dimungkinkan terjadi permasalahan teknis terkait dengan akurasi dan keterkinian informasi yang kami sajikan, hal mana akan terus kami perbaiki dari waktu kewaktu.
Dalam hal Anda menemukan inakurasi informasi yang termuat pada situs ini atau informasi yang seharusnya ada, namun belum tersedia, maka harap segera hubungi Kepaniteraan Mahkamah Agung RI melalui :
Email : kepaniteraan@mahkamahagung.go.id Telp : 021-384 3348 (ext.318)

Mengingat, semua pasal dalam peraturan perundang-undangan dan hukum Islam yang berkaitan dengan perkara ini;

M E N G A D I L I

1. Menyatakan Termohon telah dipanggil secara resmi dan patut untuk menghadap di muka sidang, tidak hadir;
2. Mengabulkan permohonan Pemohon secara verstek;
3. Memberi izin kepada Pemohon (PEMOHON) untuk menjatuhkan talak satu raj'i terhadap Termohon (TERMOHON) di depan sidang Pengadilan Agama Sidoarjo;
4. Membebankan kepada Pemohon untuk membayar biaya perkara ini sebesar Rp. 521.000,- (lima ratus dua puluh satu ribu rupiah);

Demikian putusan ini dijatuhkan pada hari Rabu tanggal 23 Desember 2020 Masehi bertepatan dengan tanggal 8 Jumadilawal 1442 Hijriyah dalam sidang permusyawaratan Majelis Hakim Pengadilan Agama Sidoarjo oleh kami Drs. Rusli M, M.H. sebagai Ketua Majelis, Hj. Nuraeni, S. S.H., M.H. dan Drs. H. Imam Syafi'i, S.H., M.H. masing-masing sebagai Hakim Anggota; putusan mana diucapkan pada hari itu juga oleh Majelis tersebut dalam sidang terbuka untuk umum dengan dibantu oleh Aida Shofiyati, S.H., M.Kn. sebagai Panitera Pengganti, dengan dihadiri oleh Pemohon tanpa hadirnya Termohon.

Hakim Anggota,	Ketua Majelis,
**Hj. Nuraeni, S. S.H., M.H.**	**Drs. Rusli M, M.H.**
**Drs. H. Imam Syafi'i, S.H., M.H.**	

Panitera Pengganti,

Aida Shofiyati, S.H., M.Kn.

Disclaimer
Kepaniteraan Mahkamah Agung Republik Indonesia berusaha untuk selalu mencantumkan informasi paling kini dan akurat sebagai bentuk komitmen Mahkamah Agung untuk pelayanan publik, transparansi dan akuntabilitas pelaksanaan fungsi peradilan. Namun dalam hal-hal tertentu masih dimungkinkan terjadi permasalahan teknis terkait dengan akurasi dan keterkinian informasi yang kami sajikan, hal mana akan terus kami perbaiki dari waktu kewaktu.
Dalam hal Anda menemukan inakurasi informasi yang termuat pada situs ini atau informasi yang seharusnya ada, namun belum tersedia, maka harap segera hubungi Kepaniteraan Mahkamah Agung RI melalui :
Email : kepaniteraan@mahkamahagung.go.id Telp : 021-384 3348 (ext.318)

Perincian Biaya Perkara:

Pendaftaran	Rp	30.000,-
Biaya Proses	Rp	75.000,-
Panggilan	Rp	380.000,-
PNBP relaas pertama	Rp	20.000,-
Redaksi	Rp	10.000,-
Meterai	Rp	6.000,-
Jumlah	Rp	521.000,-

(empat ratus tujuh puluh satu ribu rupiah);

Disclaimer
Kepaniteraan Mahkamah Agung Republik Indonesia berusaha untuk selalu mencantumkan informasi paling kini dan akurat sebagai bentuk komitmen Mahkamah Agung untuk pelayanan publik, transparansi dan akuntabilitas pelaksanaan fungsi peradilan. Namun dalam hal-hal tertentu masih dimungkinkan terjadi permasalahan teknis terkait dengan akurasi dan keterkinian informasi yang kami sajikan, hal mana akan terus kami perbaiki dari waktu kewaktu.
Dalam hal Anda menemukan inakurasi informasi yang termuat pada situs ini atau informasi yang seharusnya ada, namun belum tersedia, maka harap segera hubungi Kepaniteraan Mahkamah Agung RI melalui :
Email : kepaniteraan@mahkamahagung.go.id Telp : 021-384 3348 (ext.318)
