# Supplementary material for: Classification of divorce causes during the COVID-19 pandemic using convolutional neural networks
Source: PeerJ Comput Sci. 2022 Jun 30;8:e998. doi: 10.7717/peerj-cs.998 (PMC9299239; doi:10.7717/peerj-cs.998)
Supplement: Supplemental Information 3 [file peerj-cs-08-998-s003.zip › KDRT Dataset/Data ke-14.pdf]

P U T U S A N

Nomor 3083/Pdt.G/2020/PA.JB

بِسْمِ اللهِ الرَّحْمٰنِ الرَّحِيْمِ

DEMI KEADILAN BERDASARKAN KETUHANAN YANG MAHA ESA

Pengadilan Agama Jakarta Barat yang memeriksa dan mengadili perkara Cerai Gugat pada tingkat pertama dalam persidangan majelis hakim telah menjatuhkan putusan sebagai berikut dalam perkara antara:

PENGGUGAT, umur 28 tahun, agama Islam, pendidikan SLTA, pekerjaan Lain-lain xxxxxxxxx xxxxxx, tempat tinggal di KOTA JAKARTA BARAT;, sebagai **Penggugat;**

melawan

TERGUGAT, umur 36 tahun, agama Islam, pendidikan SLTA, pekerjaan xxxxxxxx xxxxxx, tempat tinggal di xxxxx xxxxxxx xxxxx xxxx, xxxxx xxxxxxxxxx xxxxxx xxxxx xx xxx, xx xxx, No 53 Kelurhan Duri Kosmabi Kecamatan Cengkareng, xxxx xxxxxxx xxxxxx, sebagai **Tergugat**;

Pengadilan Agama tersebut;

Telah membaca dan mempelajari berkas perkara;

DUDUK PERKARA

Bahwa berdasarkan Gugatan tertulisnya bertanggal 30 November 2020 yang didaftarkan pada tanggal 30 November 2020 dalam Register Induk Perkara Gugatan Pengadilan Agama Jakarta Barat Nomor 3083/Pdt.G/2020/PA.JB., Penggugat mengajukan Cerai Gugat terhadap Tergugat dengan dalil-dalil sebagai berikut:

1. Bahwa, pada hari Jumat tanggal 07 November 2014, Penggugat dengan Tergugat melangsungkan pernikahan yang dicatat oleh Pegawai Pencatat Nikah Kantor Urusan Agama Kecamatan Kembangan, kota Jakarta Barat sesuai Kutipan Akta Nikah Nomor : xx tertanggal 07 November 2014;

Disclaimer
Kepaniteraan Mahkamah Agung Republik Indonesia berusaha untuk selalu mencantumkan informasi paling kini dan akurat sebagai bentuk komitmen Mahkamah Agung untuk pelayanan publik, transparansi dan akuntabilitas pelaksanaan fungsi peradilan. Namun dalam hal-hal tertentu masih dimungkinkan terjadi permasalahan teknis terkait dengan akurasi dan keterkinian informasi yang kami sajikan, hal mana akan terus kami perbaiki dari waktu kewaktu.
Dalam hal Anda menemukan inakurasi informasi yang termuat pada situs ini atau informasi yang seharusnya ada, namun belum tersedia, maka harap segera hubungi Kepaniteraan Mahkamah Agung RI melalui :
Email : kepaniteraan@mahkamahagung.go.id   Telp : 021-384 3348 (ext.318)

2. Bahwa, setelah pernikahan tersebut Penggugat dengan Tergugat bertempat tinggal di rumah orang tua Penggugat Terus pindah kerumah orang tua Tergugat,

3. Bahwa, dalam perkawinan tersebut Penggugat dengan Tergugat telah bercampur (*ba'da dukhul*) sebagaimana layaknya suami istrI, dan telah dikaruniai seorang anak yang bernama (nama anak), Laki-laki, lahir di Jakarta tanggal 19 – 02 - 2017:

4. Bahwa, semula kehidupan rumah tangga antara Penggugat dengan Tergugat berjalan dengan harmonis, namun sejak tahun 2016 keharmonisan rumah tangga Penggugat dengan Tergugat mulai goyah, disebabkan :

    4.1 Antara Penggugat dan Tergugat sering Bertengkar dalam rumah tangga dalam maslah kecil menjadi besar yang tidak ada jelan keluar

    4.2 Tergugat Melarang Penggugat kerumah orang tua Penggut;

    4.3 Dimaana Waktu Anak Penggugat dan Tergugat sakit dan Penggugat kembali kerumah orang tua untuk sementara dalam massa mengobati anak berhubung Penggugat bekerja, akan tetapi setalah satu minggu Penggugat ingin kembali kerumah orang tua Tergugat, Tergugat berkata tidak usah kembali lagi dan sejak saat ini kita cerai;

5. Bahwa, karena sebab-sebab tersebut diatas Penggugat dan Tergugat sering berselisih dan bertengkar, namun percekcokan tersebut pada awalnya masih dalam batas-batas tertentu dan dapat diatasi oleh kedua belah pihak akan tetapi akhir-akhir ini percekcokan itu semakin bertambah tajam;

6. Bahwa, puncak perselisihan dan pertengkaran antara Penggugat dengan Tergugat tersebut terjadi kurang lebih pada bulan September tahun 2018, yang akibatnya Penggugat Tidak boleh kembali keruamh orang tua Tergugat dan sejak saat itu sudah hubungan badan selayaknya suami istri;

Disclaimer
Kepaniteraan Mahkamah Agung Republik Indonesia berusaha untuk selalu mencantumkan informasi paling kini dan akurat sebagai bentuk komitmen Mahkamah Agung untuk pelayanan publik, transparansi dan akuntabilitas pelaksanaan fungsi peradilan. Namun dalam hal-hal tertentu masih dimungkinkan terjadi permasalahan teknis terkait dengan akurasi dan keterkinian informasi yang kami sajikan, hal mana akan terus kami perbaiki dari waktu kewaktu.
Dalam hal Anda menemukan inakurasi informasi yang termuat pada situs ini atau informasi yang seharusnya ada, namun belum tersedia, maka harap segera hubungi Kepaniteraan Mahkamah Agung RI melalui :
Email : kepaniteraan@mahkamahagung.go.id Telp : 021-384 3348 (ext.318)

7. Bahwa, akibat tersebut diatas Pengugat telah menderita lahir bathin dan Penggugat tidak ridho atas perlakuan Tergugat terhadap Penggugat serta Penggugat merasa tidak sanggup lagi untuk melanjutkan rumah tangga dengan Tergugat oleh karenanya Penggugat berkesimpulan satu-satunya jalan keluar yang terbaik bagi Penggugat adalah bercerai dengan Tergugat;

8. Bahwa, dengan fakta-fakta tersebut diatas gugatan Penggugat telah memenuhi alasan perceraian sebagaimana diatur dalam Pasal 19 PP No. 9 Tahun 1975 jo. Pasal 116 Kompilasi Hukum Islam;

9. Bahwa, terhadap biaya yang timbul akibat perkara ini agar dibebankan sesuai dengan peraturan perundang-undangan yang berlaku;

Berdasarkan alasan/dalil-dalil diatas, Penggugat mohon agar Ketua Pengadilan Agama Jakarta Barat segera memeriksa dan mengadili perkara ini, selanjutnya menjatuhkan putusan yang amarnya berbunyi :

PRIMER

1. Mengabulkan gugatan Penggugat;

2. Menjatuhkan thalak satu ba'in sughra Tergugat (**TERGUGAT**) terhadap Penggugat (**PENGGUGAT**);

3. Membebankan biaya perkara sesuai dengan ketentuan peraturan perundang-undangan yang berlaku;

SUBSIDER

Dan atau apabila Majelis Hakim yang menyidangkan perkara ini berpendapat lain, maka mohon untuk menjatuhkan putusan yang seadil-adilnya (*ex aequo et bono*);

Bahwa pada hari-hari persidangan yang telah ditetapkan, Penggugat menghadap ke persidangan, sedangkan Tergugat tidak menghadap dan tidak pula mengutus orang lain sebagai wakil atau kuasanya yang sah untuk menghadap ke persidangan meskipun telah dipanggil secara resmi dan patut;

Bahwa karena Tergugat tidak pernah menghadap kepersidangan, maka upaya damai dan mediasi tidak dapat dilaksanakan;

Bahwa selanjutnya Penggugat membaca Gugatannya tanpa mengajukan perubahan, dan untuk membuktikan dalil Gugatannya, Penggugat mengajukan bukti-bukti sebagai berikut;

Disclaimer
Kepaniteraan Mahkamah Agung Republik Indonesia berusaha untuk selalu mencantumkan informasi paling kini dan akurat sebagai bentuk komitmen Mahkamah Agung untuk pelayanan publik, transparansi dan akuntabilitas pelaksanaan fungsi peradilan. Namun dalam hal-hal tertentu masih dimungkinkan terjadi permasalahan teknis terkait dengan akurasi dan keterkinian informasi yang kami sajikan, hal mana akan terus kami perbaiki dari waktu kewaktu.
Dalam hal Anda menemukan inakurasi informasi yang termuat pada situs ini atau informasi yang seharusnya ada, namun belum tersedia, maka harap segera hubungi Kepaniteraan Mahkamah Agung RI melalui :
Email : kepaniteraan@mahkamahagung.go.id Telp : 021-384 3348 (ext.318)

A. Bukti Surat:

1. FotoKopi Kartu Tanda Penduduk atas nama Penggugat (bukti P.1);

2. Fotokopi Buku Kutipan Akta Nikah Nomor xxtertanggal 07 November 2014, atas nama: Penggugat dan Tergugat, (bukti P.2);

B. Bukti Saksi:

1. Saksi , umur 41 tahun, agama islam, pekerjaan ibu rumah tangga, tempat tinggal di Jl. xxxxxx di bawah sumpahnya memberikan keterangan tentang keadaan rumah tangga Penggugat dengan Tergugat sebagai berikut :

- Bahwa hubungan saksi dengan Penggugat adalah kakak kandung Penggugat;
- Bahwa Penggugat dan Tergugat menikah pada tahun 2014;
- Bahwa saksi mengetahui Penggugat dan Tergugat telah dikaruniai 1 (satu) orang anak;
- Bahwa semula rumah tangga Penggugat dan Tergugat harmonis namun sejak tahun2016 sering terjadi perselisihan dan pertengkaran;
- Bahwa penyebabnya adalah; antara Penggugat dan Tergugat sering Bertengkar, Tergugat Melarang Penggugat kerumah orang tua Penggugat, dimana waktu anak Penggugat dan Tergugat sakit Penggugat kembali kerumah orang tua untuk sementara, akan tetapi setalah satu minggu Penggugat ingin kembali kerumah orang tua Tergugat, Tergugat berkata tidak usah kembali lagi;
- Bahwa, puncak perselisihan dan pertengkaran antara Penggugat dengan Tergugat tersebut terjadi kurang lebih pada tahun 2018, yang akibatnya Penggugat tidak boleh kembali ke rumah kediaman bersama hingga sekarang;
- Bahwa keluarga dan saksi telah memberikan nasehat kepada Penggugat akan tetapi Penggugat tetap pada pendiriannya;

2. Saksi , umur 48 tahun, agama islam, pekerjaan ibu rumah tangga, tempat tinggal di Kota Jakarta Barat, di bawah sumpahnya memberikan

Disclaimer
Kepaniteraan Mahkamah Agung Republik Indonesia berusaha untuk selalu mencantumkan informasi paling kini dan akurat sebagai bentuk komitmen Mahkamah Agung untuk pelayanan publik, transparansi dan akuntabilitas pelaksanaan fungsi peradilan. Namun dalam hal-hal tertentu masih dimungkinkan terjadi permasalahan teknis terkait dengan akurasi dan keterkinian informasi yang kami sajikan, hal mana akan terus kami perbaiki dari waktu kewaktu.
Dalam hal Anda menemukan inakurasi informasi yang termuat pada situs ini atau informasi yang seharusnya ada, namun belum tersedia, maka harap segera hubungi Kepaniteraan Mahkamah Agung RI melalui :
Email : kepaniteraan@mahkamahagung.go.id Telp : 021-384 3348 (ext.318)

keterangan tentang keadaan rumah tangga Penggugat dengan Tergugat sebagai berikut :

- Bahwa hubungan saksi dengan Penggugat adalah kakak kandung Penggugat;
- Bahwa Penggugat dan Tergugat menikah pada tahun 2014;
- Bahwa saksi mengetahui Penggugat dan Tergugat telah dikaruniai 1 (satu) orang anak;
- Bahwa semula rumah tangga Penggugat dan Tergugat harmonis namun sejak tahun2016 sering terjadi perselisihan dan pertengkaran;
- Bahwa penyebabnya adalah; antara Penggugat dan Tergugat sering Bertengkar, Tergugat Melarang Penggugat kerumah orang tua Penggugat, dimana waktu anak Penggugat dan Tergugat sakit Penggugat kembali kerumah orang tua untuk sementara, akan tetapi setalah satu minggu Penggugat ingin kembali kerumah orang tua Tergugat, Tergugat berkata tidak usah kembali lagi;
- Bahwa, puncak perselisihan dan pertengkaran antara Penggugat dengan Tergugat tersebut terjadi kurang lebih pada tahun 2018, yang akibatnya Penggugat tidak boleh kembali ke rumah kediaman bersama dan sejak saat itu sudah hubungan badan selayaknya suami istri hingga sekarang;
- Bahwa keluarga dan saksi telah memberikan nasehat kepada Penggugat akan tetapi Penggugat tetap pada pendiriannya;

Bahwa selanjutnya Penggugat menyampaikan kesimpulan secara lisan yang menyatakan bahwa dalil Gugatan Penggugat telah dikuatkan oleh keterangan dua orang saksi, karena itu mohon dikabulkan;

Bahwa untuk mempersingkat uraian putusan ini ditunjuk segala hal yang tercantum dalam berita acara sidang yang merupakan bagian tak terpisahkan dari putusan ini;

PERTIMBANGAN HUKUM

Menimbang, bahwa maksud dan tujuan Gugatan Penggugat adalah sebagaimana diuraikan di atas;

Disclaimer
Kepaniteraan Mahkamah Agung Republik Indonesia berusaha untuk selalu mencantumkan informasi paling kini dan akurat sebagai bentuk komitmen Mahkamah Agung untuk pelayanan publik, transparansi dan akuntabilitas pelaksanaan fungsi peradilan. Namun dalam hal-hal tertentu masih dimungkinkan terjadi permasalahan teknis terkait dengan akurasi dan keterkinian informasi yang kami sajikan, hal mana akan terus kami perbaiki dari waktu kewaktu.
Dalam hal Anda menemukan inakurasi informasi yang termuat pada situs ini atau informasi yang seharusnya ada, namun belum tersedia, maka harap segera hubungi Kepaniteraan Mahkamah Agung RI melalui :
Email : kepaniteraan@mahkamahagung.go.id Telp : 021-384 3348 (ext.318)

Menimbang, bahwa perkara ini adalah perkara perceraian antara warga negara Indonesia yang beragama Islam, maka berdasarkan Pasal 49 ayat (1) huruf (a) Undang-Undang Nomor 7 Tahun 1989 tentang Peradilan Agama dan Pasal 49 huruf (a) Undang-Undang Nomor 3 Tahun 2006 tentang perubahan Undang-Undang Nomor 7 Tahun 1989, perkara ini menjadi wewenang absolut Pengadilan Agama;

Menimbang, bahwa berdasarkan bukti P.1 terbukti bahwa Penggugat beralamat di xxxxx xx xxxxxx xx xxx, xx xxx, xx xxx, xxxxxxxxx xxxxxxxxx xxxxx, Kecamatan Kembangan, Kota Jakarta Barat, sesuai dengan alamat yang tertera dalam Gugatan Penggugat;

Menimbang, bahwa alamat tersebut termasuk wilayah hukum (yurisdiksi) Pengadilan Agama Jakarta Barat, oleh karena itu sesuai dengan Pasal 73 ayat 1 Undang-Undang Nomor 7 tahun 1989 tentang Peradilan Agama, perkara ini termasuk wewenang relative Pengadilan Agama Jakarta Barat;

Menimbang, bahwa pada hari-hari persidangan yang telah ditetapkan, Penggugat menghadap ke persidangan, sedangkan Tergugat tidak pernah menghadap dan tidak pula menyuruh orang lain sebagai wakil atau kuasanya untuk menghadap kepersidangan;

Menimbang, bahwa panggilan terhadap Tergugat telah disampaikan secara resmi dan patut, akan tetapi Tergugat tidak hadir dan tidak pula terbukti ketidakhadiran Tergugat tersebut disebabkan oleh suatu alasan yang sah menurut hukum, oleh karena itu sesuai dengan pasal 125 ayat (1) dan pasal 126 HIR, perkara ini dapat diperiksa dan diputus tanpa hadirnya Tergugat (Verstek);

Menimbang, bahwa karena Tergugat tidak pernah menghadap ke persidangan, maka upaya damai dan mediasi sebagaimana diwajibkan pasal 130 ayat 1 HIR, dan pasal 17 ayat 1 Peraturan Mahkamah Agung Nomor 1 tahun 2016 tidak dapat dilaksanakan;

Menimbang, bahwa Penggugat mendalilkan bahwa Penggugat dan Tergugat adalah suami istri yang menikah pada tanggal 07 November 2014;

Menimbang, bahwa untuk membuktikan kebenaran dalil Gugatannya, Penggugat mengajukan bukti tertulis bertanda P.2.

Disclaimer
Kepaniteraan Mahkamah Agung Republik Indonesia berusaha untuk selalu mencantumkan informasi paling kini dan akurat sebagai bentuk komitmen Mahkamah Agung untuk pelayanan publik, transparansi dan akuntabilitas pelaksanaan fungsi peradilan. Namun dalam hal-hal tertentu masih dimungkinkan terjadi permasalahan teknis terkait dengan akurasi dan keterkinian informasi yang kami sajikan, hal mana akan terus kami perbaiki dari waktu kewaktu.
Dalam hal Anda menemukan inakurasi informasi yang termuat pada situs ini atau informasi yang seharusnya ada, namun belum tersedia, maka harap segera hubungi Kepaniteraan Mahkamah Agung RI melalui :
Email : kepaniteraan@mahkamahagung.go.id Telp : 021-384 3348 (ext.318)

Menimbang, bahwa bukti P.2 tersebut adalah fotokopi dari akta autentik berupa Buku Kutipan Akta Nikah yang dikeluarkan oleh pejabat yang berwenang, telah di-nazegelen dan telah dicocokkan dengan aslinya ternyata cocok, dan isinya menunjukkan bahwa Penggugat dan Tergugat telah menikah pada tanggal 07 November 2014;

Menimbang, bahwa berdasarkan pertimbangan di atas, harus dinyatakan bahwa bukti P.2 tersebut telah memenuhi syarat formil dan syarat materil alat bukti, dan mempunyai kekuatan bukti yang sempurna dan mengikat, oleh karena itu harus dinyatakan terbukti bahwa antara Penggugat dan Tergugat ada hubungan hukum perkawinan, dan karenanya harus dinyatakan bahwa Gugatan Penggugat mempunyai dasar hukum (*legal standing*) dan Penggugat adalah pihak yang mempunyai kepentingan hukum (*persona standi in judicio*) dalam perkara ini;

Menimbang, bahwa dalil Gugatan Penggugat untuk bercerai dengan Tergugat adalah karena;

- Semenjak tahun 2016 keharmonisan rumah tangga Penggugat dengan Tergugat mulai goyah, disebabkan: karena antara Penggugat dan Tergugat sering Bertengkar, Tergugat Melarang Penggugat kerumah orang tua Penggugat, dimana waktu anak Penggugat dan Tergugat sakit Penggugat kembali kerumah orang tua untuk sementara, akan tetapi setalah satu minggu Penggugat ingin kembali kerumah orang tua Tergugat, Tergugat berkata tidak usah kembali lagi;
- Bahwa, puncak perselisihan dan pertengkaran antara Penggugat dengan Tergugat terjadi pada tahun 2018, akibatnya Penggugat pergi meninggalkan Tergugat, dan sejak saat itu antara Penggugat dengan Tergugat sudah tidak ada lagi hubungan baik yang terjalin sampai sekarang;

Menimbang, bahwa untuk membuktikan kebenaran dalil Gugatannya, Penggugat mengajukan bukti berupa dua orang saksi sebagaimana termuat pada duduk perkara di atas;

Menimbang, bahwa saksi satu dan saksi dua Penggugat sudah dewasa, berakal sehat, dan memberikan keterangan di bawah sumpahnya tentang apa yang dilihat dan didengarnya sendiri tentang rumah tangga Penggugat dengan Tergugat dan keterangannya relevan dengan dalil Gugatan Penggugat;

Disclaimer
Kepaniteraan Mahkamah Agung Republik Indonesia berusaha untuk selalu mencantumkan informasi paling kini dan akurat sebagai bentuk komitmen Mahkamah Agung untuk pelayanan publik, transparansi dan akuntabilitas pelaksanaan fungsi peradilan. Namun dalam hal-hal tertentu masih dimungkinkan terjadi permasalahan teknis terkait dengan akurasi dan keterkinian informasi yang kami sajikan, hal mana akan terus kami perbaiki dari waktu kewaktu.
Dalam hal Anda menemukan inakurasi informasi yang termuat pada situs ini atau informasi yang seharusnya ada, namun belum tersedia, maka harap segera hubungi Kepaniteraan Mahkamah Agung RI melalui :
Email : kepaniteraan@mahkamahagung.go.id Telp : 021-384 3348 (ext.318)

Menimbang, bahwa dua saksi di atas telah memenuhi batas minimal saksi dan keterangannya saling bersesuaian satu sama lain, maka berdasarkan pasal 145 ayat 1 dan Pasal 171 HIR, Pasal 76 ayat 1 Undang-Undang Nomor 7 tahun 1989 tentang Peradilan Agama dan Pasal 22 ayat (2) Peraturan Pemerintah Nomor 9 Tahun 1975, kesaksian dua orang saksi tersebut dapat diterima sebagai bukti;

Menimbang, bahwa berdasarkan dalil Gugatan Penggugat dan keterangan dua orang saksi tersebut di atas, ditemukan fakta sebagai berikut;

- Rumah tangga Penggugat dengan Tergugat sudah tidak harmonis semenjak tahun 2016;
  - Penyebabnya adalah; antara Penggugat dan Tergugat sering Bertengkar, Tergugat Melarang Penggugat kerumah orang tua Penggugat, dimana waktu anak Penggugat dan Tergugat sakit Penggugat kembali kerumah orang tua untuk sementara, akan tetapi setalah satu minggu Penggugat ingin kembali kerumah orang tua Tergugat, Tergugat berkata tidak usah kembali lagi;
- Penggugat dan Tergugat sudah berpisah tempat tinggal semenjak tahun 2018 hingga sekarang;

Menimbang, bahwa berdasarkan fakta tersebut, majelis berkesimpulan bahwa tujuan perkawinan untuk mewujudkan rumah tangga yang bahagia, sakinah, mawaddah dan rahmah sebagaimana dimaksud dalam al-Quran surat Al-Rum ayat 21 yang telah ditransformasi menjadi undang-undang Negara yang termuat dalam pasal 1 Undang-Undang Nomor 1 Tahun 1974 tentang Perkawinan dan pasal 2 dan 3 Kompilasi Hukum Islam, tidak terwujud lagi dalam rumah tangga Penggugat dengan Tergugat;

Menimbang, bahwa secara sosiologis suatu perkawinan yang di dalamnya sering terjadi perselisihan dan pertengkaran akan sulit untuk mewujudkan rumah tangga bahagia yang penuh rahmah dan kasih sayang seperti yang diharapkan oleh setiap pasangan suami istri, justeru sebaliknya akan menimbulkan kemudharatan dan perselisihan yang berkepanjangan salah satu pihak atau kedua belah pihak. Hal ini sesuai dengan pendapat pakar Hukum Islam sebagaimana dalam kitab Madza Hurriyyatuz Zaujaeni Fii Ath-Thalaq yang diambil alih menjadi pertimbangan Majelis Hakim dalam memutus

Disclaimer
Kepaniteraan Mahkamah Agung Republik Indonesia berusaha untuk selalu mencantumkan informasi paling kini dan akurat sebagai bentuk komitmen Mahkamah Agung untuk pelayanan publik, transparansi dan akuntabilitas pelaksanaan fungsi peradilan. Namun dalam hal-hal tertentu masih dimungkinkan terjadi permasalahan teknis terkait dengan akurasi dan keterkinian informasi yang kami sajikan, hal mana akan terus kami perbaiki dari waktu kewaktu.
Dalam hal Anda menemukan inakurasi informasi yang termuat pada situs ini atau informasi yang seharusnya ada, namun belum tersedia, maka harap segera hubungi Kepaniteraan Mahkamah Agung RI melalui :
Email : kepaniteraan@mahkamahagung.go.id Telp : 021-384 3348 (ext.318)

perkara ini yang menyatakan “ Islam memilih lembaga talak/ perceraian ketika rumah tangga sudah dianggap guncang/ tidak harmonis dan tidak bermanfaat lagi nasihat perdamaian dan hubugan suami istri sudah hilang (tanpa ruh), sebab dengan meneruskan perkawinan berarti menghukum salah satu isteri atau suami dalam penjara yang berkepanjangan, hal tersebut adalah suatu bentuk penganiayaan yang bertentangan dengan semangat keadilan”;

Menimbang, bahwa berdasarkan dalil dari Kitab Fiqhus Sunah juz II halaman 89 yang berbunyi:

إن للزوجة تطلب من القاضى التفريق إذا ادعت اضرارا الزوج بها اضرارا

Artinya: “ Sungguh bagi istri dapat meminta hakim untuk diceraikan apabila terjadi keadaan yang sangat meresahkan dalam pergaulan dengan suaminya”.

Dan juga dalam Juz II yang berbunyi:

واذاثبت دعوهالدىالقاضى اضرارالزوج ببينةالزوجةاواعتراف الزوج الي ان قال.. وعجزالقاضى عن الاصلاح بينهما طلق القاضى طلقه بائنة

Artinya : Dan jika gugatan isteri terbukti dihadapan hakim tentang kemadlaratan suami dengan saksi-saksi yang diajukan oleh isteri atau pengakuan suami sampai pada kata-kata …… dan hakim tidak berhasil mendamaikan keduanya, maka hakim menjatuhkan talak ba’in;

Menimbang, bahwa terhadap perkara ini dapat diterapkan pula yurisprudensi Mahkamah Agung RI Nomor 379 K/AG/1995 tanggal 26 Maret 1997 yang abstraksi hukumnya menyatakan apabila suami istri terjadi perselisihan dan terjadi pisah tempat tinggal, maka rumah tangga mereka telah pecah dan permohonan/ gugatan cerai telah memenuhi ketentuan Pasal 19 huruf (f) Peraturan Pemerintah Nomor 9 Tahun 1975 dan gugatan tersebut harus dikabulkan;

Menimbang, bahwa berdasarkan pertimbangan-pertimbangan tersebut di atas, majelis berpendapat bahwa dalil Gugatan Penggugat telah terbukti kebenarannya, oleh karena itu sesuai dengan Pasal 19 huruf (f) Peraturan Pemerintah Nomor 9 Tahun 1975 dan Pasal 116 huruf (f) Kompilasi Hukum Islam, Gugatan Penggugat untuk bercerai dengan Tergugat dapat dikabulkan;

Menimbang, bahwa berdasarkan Pasal 89 ayat (1) Undang-Undang Nomor 7 Tahun 1989 tentang Peradilan Agama, pasal 90 ayat (1) Undang-

Disclaimer
Kepaniteraan Mahkamah Agung Republik Indonesia berusaha untuk selalu mencantumkan informasi paling kini dan akurat sebagai bentuk komitmen Mahkamah Agung untuk pelayanan publik, transparansi dan akuntabilitas pelaksanaan fungsi peradilan. Namun dalam hal-hal tertentu masih dimungkinkan terjadi permasalahan teknis terkait dengan akurasi dan keterkinian informasi yang kami sajikan, hal mana akan terus kami perbaiki dari waktu kewaktu.
Dalam hal Anda menemukan inakurasi informasi yang termuat pada situs ini atau informasi yang seharusnya ada, namun belum tersedia, maka harap segera hubungi Kepaniteraan Mahkamah Agung RI melalui :
Email : kepaniteraan@mahkamahagung.go.id Telp : 021-384 3348 (ext.318)

Undang Nomor 3 Tahun 2006 tentang perubahan Undang-Undang Nomor 7 tahun 1989, pasal 91 A Undang-Undang Nomor 50 Tahun 2009 tentang perubahan kedua Undang-Undang Nomor 7 tahun 1989, biaya perkara ini dibebankan kepada Penggugat;

Mengingat, segala peraturan perundang-undangan yang berlaku dan dalil-dalil syar'i yang berkaitan dengan perkara ini;

M E N G A D I L I

1. Menyatakan, Tergugat yang telah dipanggil secara resmi dan patut untuk menghadap di persidangan,tidak hadir;

2. Mengabulkan gugatan Penggugat dengan verstek;

3. Menjatuhkan talak satu bain shughra Tergugat (**TERGUGAT**) terhadap Penggugat (**PENGGUGAT**);

4. Membebankan kepada Penggugat untuk membayar biaya perkara yang hingga kini dihitung sejumlah Rp. 461.000 (empat ratus enam puluh satu ribu rupiah);

Demikian diputus dalam sidang permusyawaratan Majelis Hakim Pengadilan Agama Jakarta Barat pada hari Rabu tanggal 23 Desember 2020 Miladiyah bertepatan dengan tanggal 8 Jumadil Awwal 1442 Hijriyah, oleh Dr. Mustar, M.H., Ketua Majelis, Dra. Hj. Absari, M.H. dan Drs. H. Fauzi, M.H.I**,** Hakim-Hakim Anggota, yang ditunjuk oleh Ketua Pengadilan Agama Jakarta Barat untuk memeriksa dan memutus perkara ini, dan dibacakan oleh Ketua Majelis tersebut dalam sidang terbuka untuk umum pada hari itu juga dengan didampingi oleh Dra. Hj. Absari, M.H.dan Drs. H. Fauzi, M.H.I Hakim-hakim Anggota serta Hj. Nisrin, S.H., M.H., sebagai Panitera Pengganti dengan dihadiri oleh Penggugat tanpa hadirnya Tergugat;

Ketua Majelis

Dr. Mustar, M.H.

Hakim Anggota | Hakim Anggota

Disclaimer
Kepaniteraan Mahkamah Agung Republik Indonesia berusaha untuk selalu mencantumkan informasi paling kini dan akurat sebagai bentuk komitmen Mahkamah Agung untuk pelayanan publik, transparansi dan akuntabilitas pelaksanaan fungsi peradilan. Namun dalam hal-hal tertentu masih dimungkinkan terjadi permasalahan teknis terkait dengan akurasi dan keterkinian informasi yang kami sajikan, hal mana akan terus kami perbaiki dari waktu kewaktu.
Dalam hal Anda menemukan inakurasi informasi yang termuat pada situs ini atau informasi yang seharusnya ada, namun belum tersedia, maka harap segera hubungi Kepaniteraan Mahkamah Agung RI melalui :
Email : kepaniteraan@mahkamahagung.go.id Telp : 021-384 3348 (ext.318)

Dra. Hj. Absari, M.H. **Drs. H. Fauzi, M.H.I**

Panitera Pengganti

Hj. Nisrin, S.H., M.H.

Perincian Biaya :

1. 1	Pendaftaran	Rp	30.000,-
2. 2	Proses	Rp	75.000,-
3. 3	Panggilan	Rp	320.000,-
	PNBP Relaas	Rp	20.000,-
4. 4	Redaksi	Rp	10.000,-
5. 5	Meterai	Rp	6.000,-
	**J u m l a h**	**Rp**	**461.000,-**

(empat ratus enam puluh satu ribu rupiah);

Disclaimer
Kepaniteraan Mahkamah Agung Republik Indonesia berusaha untuk selalu mencantumkan informasi paling kini dan akurat sebagai bentuk komitmen Mahkamah Agung untuk pelayanan publik, transparansi dan akuntabilitas pelaksanaan fungsi peradilan. Namun dalam hal-hal tertentu masih dimungkinkan terjadi permasalahan teknis terkait dengan akurasi dan keterkinian informasi yang kami sajikan, hal mana akan terus kami perbaiki dari waktu kewaktu.
Dalam hal Anda menemukan inakurasi informasi yang termuat pada situs ini atau informasi yang seharusnya ada, namun belum tersedia, maka harap segera hubungi Kepaniteraan Mahkamah Agung RI melalui :
Email : kepaniteraan@mahkamahagung.go.id Telp : 021-384 3348 (ext.318)
