# Supplementary material for: Classification of divorce causes during the COVID-19 pandemic using convolutional neural networks
Source: PeerJ Comput Sci. 2022 Jun 30;8:e998. doi: 10.7717/peerj-cs.998 (PMC9299239; doi:10.7717/peerj-cs.998)
Supplement: Supplemental Information 3 [file peerj-cs-08-998-s003.zip › KDRT Dataset/Data ke-16.pdf]

Direktori Putusan Mahkamah Agung Republik Indonesia
putusan.mahkamahagung.go.id

SALINAN

P U T U S A N

Nomor 2249/Pdt.G/2020/PAJU

بِسْمِ اللَّهِ الرَّحْمَنِ الرَّحِيمِ

DEMI KEADILAN BERDASARKAN KETUHANAN YANG MAHA ESA

Pengadilan Agama Jakarta Utara yang memeriksa dan mengadili perkara tertentu pada tingkat pertama dalam persidangan majelis hakim telah menjatuhkan putusan perkara cerai talak yang diajukan oleh;

PEMOHON, umur 40 tahun, agama Islam, pekerjaan wiraswasta, alamat (domisili) JAKARTA UTARA dalam hal ini memberikan kuasa hukum kepada **Rohman, S.H.,** dan **Mukhlis Guntur Panahal, S.H, M.H., Para** Advokat dan Konsultan Hukum dari Kantor Hukum Rohman, S.H. & Rekan yang beralamat di Jl. Kramat Jaya Blok F 5, RT. 011, RW. 07, Kelurahan Semper Barat, Kecamatan Cilincing, Kota Administrasi Jakarta Utara 14130 **sesuai dengan** Surat Kuasa khususnya Nomor : 006 **/** SK-K **/** Pdt.GC **/** X / 2020 tertanggal 14 Oktober 2020 baik secara bersama-sama maupun sendiri-sendiri sebagai Pemohon;

Melawan

TERMOHON, umur 35 tahun, agama Islam, pekerjaan mengurus rumah tangga, alamat (domisili) Kota Medan Sumatra Utara Kode 20145 sebagai Termohon;

Pengadilan Agama tersebut;

Telah memeriksa berkas perkara yang bersangkutan;

Telah mendengar keterangan Pemohon dan Termohon serta telah memeriksa bukti-bukti dalam persidangan;

DUDUK PERKARA

Disclaimer
Kepaniteraan Mahkamah Agung Republik Indonesia berusaha untuk selalu mencantumkan informasi paling kini dan akurat sebagai bentuk komitmen Mahkamah Agung untuk pelayanan publik, transparansi dan akuntabilitas pelaksanaan fungsi peradilan. Namun dalam hal-hal tertentu masih dimungkinkan terjadi permasalahan teknis terkait dengan akurasi dan keterkinian informasi yang kami sajikan, hal mana akan terus kami perbaiki dari waktu kewaktu.
Dalam hal Anda menemukan inakurasi informasi yang termuat pada situs ini atau informasi yang seharusnya ada, namun belum tersedia, maka harap segera hubungi Kepaniteraan Mahkamah Agung RI melalui :
Email : kepaniteraan@mahkamahagung.go.id Telp : 021-384 3348 (ext.318)

Bahwa Pemohon dengan suratnya tertanggal 19 Oktober 2020 mengajukan permohonan cerai terhadap Termohon ke Pengadilan Agama Jakarta Utara yang kemudian perkaranya terdaftar dalam register sebagai perkara Nomor 2249/Pdt.G/2020/PAJU tanggal 20 Oktober 2020 dengan menerangkan atau mengajukan hal-hal sebagai berikut ;

1. Bahwa Pemohon dengan Termohon adalah pasangan suami istri yang sah, karna telah melakukan pernikahan sah pada tanggal 10 Oktober 2010 dan tercatat pada Kantor Urusan Agama dan dibukukan dalam akta nikah Nomor :;
2. Bahwa setelah menikah, Pemohon dan Termohon hidup bersama layaknya suami istri di Batam – Kepulauan Riau ;
3. Bahwa pada awal pernikahan tersebut, rumah tangga Pemohon dengan Termohon dalam keadaan harmonis dan baik-baik saja;
4. Bahwa dari hasil pernikahan Pemohon dengan Termohon sejak menikah 10 Oktober 2010 hingga sekarang dikaruniai 5 orang anak;
5. Bahwa sebenarnya sejak pernikahan berjalan 5 tahun sudah muncul percikan percikan yang membuat kondisi rumah tangga tidak harmonis, dikarnakan perbedaan prinsip diantara Pemohon dan Termohon, dan terjadi percekcokan dan perselisihan terus menerus hingga, sulit mencapai keluarga sakinah mawadah warohmah;
6. Bahwa kebiasaan Termohon yang tidak menghargai suami sebagai kepala Rumah Tangga, dan tidak mensyukuri apa yang telah diberikan suami sebagai bukti kewajiban dan sebagai bentuk tanggung jawab sebagai kepala rumah tangga, bahkan sering membandingkan kondisi keuangan suami dengan laki-laki lain;

7. Bahkan kebiasaan Termohon yang sudah di luar batas toleransi etika dalam berumah tangga, yaitu seringnya Termohon bermain judi dengan teman temannya, bahkan sampai mengorbankan waktu untuk anak dan suami, sehingga esensi Termohon sebagai seorang istri dan ibu tidak dijalankan sesuai kodratnya;

Disclaimer
Kepaniteraan Mahkamah Agung Republik Indonesia berusaha untuk selalu mencantumkan informasi paling kini dan akurat sebagai bentuk komitmen Mahkamah Agung untuk pelayanan publik, transparansi dan akuntabilitas pelaksanaan fungsi peradilan. Namun dalam hal-hal tertentu masih dimungkinkan terjadi permasalahan teknis terkait dengan akurasi dan keterkinian informasi yang kami sajikan, hal mana akan terus kami perbaiki dari waktu kewaktu.
Dalam hal Anda menemukan inakurasi informasi yang termuat pada situs ini atau informasi yang seharusnya ada, namun belum tersedia, maka harap segera hubungi Kepaniteraan Mahkamah Agung RI melalui :
Email : kepaniteraan@mahkamahagung.go.id Telp : 021-384 3348 (ext.318)

8. Bahwa Pemohon sering mengingatkan dan bahkan berkali kali memberikan maaf untuk Termohon agar bisa memperbaiki kesalahannya, dan untuk tidak mengulangi perbuatan yang dilarang oleh norma agama dan etika rumah tangga;

9. Puncak dari percekcokan tersebut adalah pada tahun 2016, yaitu Pemohon menegur untuk yang kesekian kalinya kepada Termohon untuk mengulangi kesalahan yang sama yang menurut Pemohon sudah tidak dapat lagi dimaafkan, karna sudah melampaui batas Norma dalam berumah tangga, maka untuk menghindari hal hal yang negatif diluar akal sehat manusia, maka Pemohon pergi dari rumah tinggal bersama yaitu di Batam – Kepulauan Ria, dan pindah ke Jakarta;

10. Bahwa setelah perginya Pemohon dari rumah, Pemohon menunggu itikad baik dari Termohon dengan berharap Termohon menghubungi Pemohon untuk minta maaf dan mengajak rujuk kembali, namun Temohon semakin keras untuk tidak kembali,bahkan Termohon ternyata pun meninggalkakn rumah di Batam dan pindah ke Medan, Sumata Utara untuk hidup besama anak anak nya;

11. Bahwa tahun 2016 Temohon dan Pemohon berpisah sampai dengan sekarang dengan menjalani hidup masing masing, namun Pemohon masih memberikan tanggung jawab nya sebagai seorang ayah, dengan memberikan baiya hidup dan untuk kebutuhan anak anaknya;

12. Bahwa sebagaimana diatur dalam Undang undang No. 1 th 1974 Jo Pasal 19 Peraturan Pemerintah No. 9 th 1975 tentang pelaksanaan Undang undang nomor 1 th 1974 tentang perkawinan Jo. Pasal 116 Kompilasi Hukum Islam (KHI) untuk kiranya Majelis Hakim yang Mulia di Pengadilan Agama Jakarta Utara, yang memeriksa dan mengadili perkara ini, selanjutnya menjatuhkan putusan yang amar putusannya adalah sebagai berikut :

Petitum :

1. Mengabulkan permohonan Pemohon untuk seluruhnya;

Disclaimer
Kepaniteraan Mahkamah Agung Republik Indonesia berusaha untuk selalu mencantumkan informasi paling kini dan akurat sebagai bentuk komitmen Mahkamah Agung untuk pelayanan publik, transparansi dan akuntabilitas pelaksanaan fungsi peradilan. Namun dalam hal-hal tertentu masih dimungkinkan terjadi permasalahan teknis terkait dengan akurasi dan keterkinian informasi yang kami sajikan, hal mana akan terus kami perbaiki dari waktu kewaktu.
Dalam hal Anda menemukan inakurasi informasi yang termuat pada situs ini atau informasi yang seharusnya ada, namun belum tersedia, maka harap segera hubungi Kepaniteraan Mahkamah Agung RI melalui :
Email : kepaniteraan@mahkamahagung.go.id   Telp : 021-384 3348 (ext.318)

2. Memberikan ijin kepada PEMOHON (Pemohon) sebagai Suami untuk menjatuhkan talak Raj'i kepada TERMOHON (Termohon) sebagai Istri, di hadapan Sidang Pengadilan Agama Jakarta Utara;
3. Memohon kepada Panitera Pengadilan Agama Jakarta Utara, untuk mengirimkan salinan putusan yang sudah berkuatan hukum tetap, kepada Kantor Urusan Agama;
4. Membebani biaya perkara sesuai undang undang yang berlaku;

Subsider :

Apabila putusan Pengadilan Agama Jakarta Utara Cq. Majelis Hakim berpendapat lain, maka kami memohon putusan yang seadil – adil nya;

Bahwa majelis hakim telah berusaha mendamaikan Pemohon dengan Termohon namun tidak berhasil;

Bahwa Pemohon dengan Termohon telah diupayakan mediasi dengan mediator nonhakim bernama Syakruddin, S.H., M.H., sesuai dengan laporannya berhasil sebagian yaitu sebagai berikut;

Pasal 1

Kedua belah pihak sepakat untuk bercerai

Pasal 2

Selama dalam pernikahan pihak Pemohon dan Termohon dikaruniai lima orang anak yang bernama ANAK KE I (P), ANAK KE II (P), ANAK KE III (L), ANAK KE IV (L) dan ANAK KE V (P) sampai saat ini dalam pengasuhan oleh Termohon (ibukandungnya)

Pasal 3

Kedua belah pihak sepakat bahwa Pemohon akan menanggung nafkah untuk lima orang anak minimal Rp 3.000.000 (tiga juta rupiah) setiap bulan secara terus menerus hingga anak tersebut dewasa dan atau anak tersebut bisa hidup mandiri, sepenuhnya menjadi tanggung jawab pihak Pemohon di luar biaya pendidikan dan kesehatan

Pasal 4

Disclaimer
Kepaniteraan Mahkamah Agung Republik Indonesia berusaha untuk selalu mencantumkan informasi paling kini dan akurat sebagai bentuk komitmen Mahkamah Agung untuk pelayanan publik, transparansi dan akuntabilitas pelaksanaan fungsi peradilan. Namun dalam hal-hal tertentu masih dimungkinkan terjadi permasalahan teknis terkait dengan akurasi dan keterkinian informasi yang kami sajikan, hal mana akan terus kami perbaiki dari waktu kewaktu.
Dalam hal Anda menemukan inakurasi informasi yang termuat pada situs ini atau informasi yang seharusnya ada, namun belum tersedia, maka harap segera hubungi Kepaniteraan Mahkamah Agung RI melalui :
Email : kepaniteraan@mahkamahagung.go.id Telp : 021-384 3348 (ext.318)

Kedua belah pihak sepakat bahwa biaya pendidikan dan kesehatan serta biaya yang lainnya yang berkaitan dengan kebutuhan anak sepenuhnya menjadi tanggung jawab oleh Pemohon (Ayah Kandungnya)

Pasal 5

Kedua belah pihak sepakat bahwa Pemohon akan memberikan nafkah iddah kepada Termohon sejumlah Rp 25.000.000,- (dua puluh lima juta rupiah) untuk selama masa iddah dan akan di berikan di hadapan majelis hakim

Pasal 6

Kedua belah pihak sepakat bahwa Pemohon akan memberikan Mut'ah kepada Termohon berupa uang sejumlah Rp 5.000.000 (lima juta rupiah) dan akan di berikan di hadapan majelis hakim

Pasal 7

Pernyataan Kesepakatan para pihak tentang hasil mediasi ini hanya berlaku apabila hakim pemeriksa perkara mengabulkan cerai talak yang diajukan oleh Pemohon

Pasal 8

Kedua belah pihak sepakat untuk memohon kepada hakim pemeriksa perkara untuk menguatkan Pernyataan Kesepakatan para pihak tentang hasil mediasi ini dalam putusan akhir perkara apabila surat Permohonan Cerai Talak yang diajukan oleh Pemohon dikabulkan.

Bahwa kemudian persidangan dilanjutkan kepada pemeriksaan pokok perkara dengan diawali pembacaan surat permohonan Pemohon menerangkan tetap dengan permohonannya;

Bahwa terhadap surat permohonan Pemohon tersebut Termohon memberikan jawabannya secara lisan yang pada pokoknya adalahs ebagai berikut;

- Bahwa identitas Pemohon dan Termohon sudah benar;

Disclaimer
Kepaniteraan Mahkamah Agung Republik Indonesia berusaha untuk selalu mencantumkan informasi paling kini dan akurat sebagai bentuk komitmen Mahkamah Agung untuk pelayanan publik, transparansi dan akuntabilitas pelaksanaan fungsi peradilan. Namun dalam hal-hal tertentu masih dimungkinkan terjadi permasalahan teknis terkait dengan akurasi dan keterkinian informasi yang kami sajikan, hal mana akan terus kami perbaiki dari waktu kewaktu.
Dalam hal Anda menemukan inakurasi informasi yang termuat pada situs ini atau informasi yang seharusnya ada, namun belum tersedia, maka harap segera hubungi Kepaniteraan Mahkamah Agung RI melalui :
Email : kepaniteraan@mahkamahagung.go.id   Telp : 021-384 3348 (ext.318)

- o Bahwa benar Pemohon dengan Termohon sebagai suami isteri yang menikah tanggal 10 Oktober 2010 yang tercatat pada KUA Kecamatan Batam;
- o Bahwa benar setelah menikah Pemohon dengan Termohon hidup bersama ditempat sebagaimana Pemohon sampaikan;
- o Bahwa benar awalnya kehidupan rumah tangga Pemohon dan Termohon dalam keadaan harmonis dan baik-baik saja;
- o Bahwa benar dari pernikahan Pemohon dan Termohon mempunyai anak 5 (lima) orang bernama 1. ANAK KE I (P), 2. ANAK KE II (P), 3. ANAK KE III (L), 4. ANAK KE IV (L) 5. ANAK KE V (P);
- o Bahwa benar mulainya muncul masalah dan tidak harmonis dalam rumah tangga Pemohon dan Termohon setelahnya 5 (lima) tahun hidup bersama bahkan terjadi percekcokan dan perselisihan terus menerus dikarenakan sebagaimana Pemohon sampaikan;
- o Bahwa benar puncaknya percekcokan terjadi pada tahun 2016;
- o Bahwa benar sejak tahun 2016 Pemohon dan Termohon berpisah sampai sekarang namun Pemohon masih memberikan biaya hidup untuk anak-anak;
- o Bahwa Termohon tidak keberatan atas permohonan cerai dari Pemohon dan yang lainnya juga;

Bahwa terhadap jawaban dari Termohon tersebut Pemohon menyampaikan repliknya secara lisan yaitu sama dengan permohonannya;

Bahwa terhadap replik dari Pemohon tersebut Termohon menyampaikan dupliknya secara lisan yaitu sama dengan jawabannya;

Bahwa untuk menguatkan dalil permohonannya, Pemohon telah menyerahkan alat bukti tertulis berupa fotokopi duplikat dari kutipan akta nikah atas nama Pemohon dan Termohon Nomor yang aslinya dikeluarkan oleh KUA Kecamatan yang telah bermateraikan secukupnya dan telah dicocokan dengan aslinya ternyata sesuai kemudian oleh Ketua Majelis diberi kode P1, bukti ini membuktikan bahwa Pemohon dengan Termohon adalah suami isteri yang sah;

Disclaimer
Kepaniteraan Mahkamah Agung Republik Indonesia berusaha untuk selalu mencantumkan informasi paling kini dan akurat sebagai bentuk komitmen Mahkamah Agung untuk pelayanan publik, transparansi dan akuntabilitas pelaksanaan fungsi peradilan. Namun dalam hal-hal tertentu masih dimungkinkan terjadi permasalahan teknis terkait dengan akurasi dan keterkinian informasi yang kami sajikan, hal mana akan terus kami perbaiki dari waktu kewaktu.
Dalam hal Anda menemukan inakurasi informasi yang termuat pada situs ini atau informasi yang seharusnya ada, namun belum tersedia, maka harap segera hubungi Kepaniteraan Mahkamah Agung RI melalui :
Email : kepaniteraan@mahkamahagung.go.id Telp : 021-384 3348 (ext.318)

Bahwa Pemohon juga menghadirkan saksi dari Pemohon yang bernama;

1. SAKSI I, di bawah sumpah memberikan keterangan yang pada pokoknya adalah sebagai berikut :

- Bahwa Pemohon dengan Termohon adalah suami isteri;
- Bahwa Pemohon dengan Termohon mempunyai anak 5 (lima) orang;
- Bahwa yang saksi ketahui rumah tangga Pemohon dengan Termohon awalnya baik, rukun dan harmonis kemudian tidak rukun lagi karena Pemohon dengan Termohon telah berpisah rumah sejak tahun 2016;
- Bahwa sejak berpisah rumah Pemohon dengan Termohon belum pernah hidup bersama lagi;
- Bahwa saksi mengetahui adanya pertengkaran antara Pemohon dengan Termohon sebelum berpisah dan sering;
- Bahwa saksi telah berusaha menyarankan Pemohon dan Termohon agar rukun kembali namun tidak berhasil;
- Bahwa saksi tidak akan mencoba lagi untuk menyarankan Pemohon dan Termohon agar rukun kembali;

2. SAKSI II, di bawah sumpah memberikan keterangan yang pada pokoknya adalah sebagai berikut :

- Bahwa Pemohon dengan Termohon adalah suami isteri;
- Bahwa Pemohon dengan Termohon mempunyai anak 5 (lima) orang;
- Bahwa yang saksi ketahui rumah tangga Pemohon dengan Termohon awalnya baik, rukun dan harmonis kemudian tidak rukun lagi karena Pemohon dengan Termohon telah berpisah rumah lebih kurang dari 4 (empat) tahun lamanya;
- Bahwa sejak berpisah rumah Pemohon dengan Termohon belum pernah hidup bersama lagi;

Disclaimer
Kepaniteraan Mahkamah Agung Republik Indonesia berusaha untuk selalu mencantumkan informasi paling kini dan akurat sebagai bentuk komitmen Mahkamah Agung untuk pelayanan publik, transparansi dan akuntabilitas pelaksanaan fungsi peradilan. Namun dalam hal-hal tertentu masih dimungkinkan terjadi permasalahan teknis terkait dengan akurasi dan keterkinian informasi yang kami sajikan, hal mana akan terus kami perbaiki dari waktu kewaktu.
Dalam hal Anda menemukan inakurasi informasi yang termuat pada situs ini atau informasi yang seharusnya ada, namun belum tersedia, maka harap segera hubungi Kepaniteraan Mahkamah Agung RI melalui :
Email : kepaniteraan@mahkamahagung.go.id Telp : 021-384 3348 (ext.318)

- Bahwa saksi mengetahui adanya pertengkaran antara Pemohon dengan Termohon sebelum berpisah dan sering terjadi;
- Bahwa saksi telah berusaha menyarankan Pemohon dan Termohon agar rukun kembali namun tidak berhasil;
- Bahwa saksi tidak akan mencoba lagi untuk menyarankan Pemohon dan Termohon agar rukun kembali;

Bahwa Termohon menerangkan tidak akan mengajukan bukti dan mencukupkan dengan bukti yang telah disampaikan oleh Pemohon;

Bahwa pada tahap kesimpulan Pemohon telah menyampaikan kesimpulan akhirnya yaitu sebagaimana dalam kesimpulannya;

Bahwa pada tahap kesimpulan Termohon telah menyampaikan kesimpulan akhirnya yaitu sebagaimana dalam kesimpulannya;

Bahwa mengenai jalannya pemeriksaan perkara ini selengkapnya telah dicatat dalam berita acara yang bersangkutan. Maka untuk meringkas putusan ini selanjutnya Majelis Hakim menunjuk berita acara tersebut sebagai bagian yang tak terpisahkan dari putusan ini;

PERTIMBANGAN HUKUM

Menimbang, bahwa maksud dan tujuan dari pada permohonan Pemohon adalah seperti terurai di atas;

Menimbang, bahwa majelis hakim telah berusaha mendamaikan Pemohon dengan Termohon namun tidak berhasil;

Menimbang, bahwa Pemohon dengan Termohon telah diupayakan mediasi dengan mediator nonhakim bernama Syakruddin, S.H., M.H., hal tersebut guna memenuhi PERMA Nomor 1 Tahun 2016 Tentang Prosedur Mediasi Dipengadilan;

Menimbang, bahwa perkara ini mengenai permohonan cerai talak yang diajukan oleh pihak yang beragama Islam, oleh karenanya berdasarkan Pasal 49 (a) Undang-Undang Nomor 7 Tahun 1989 Tentang Peradilan Agama sebagaimana telah diubah dengan Undang-Undang Nomor 3 Tahun 2006 dan perubahan kedua dengan Undang-Undang Nomor 50 Tahun 2009, maka

Disclaimer
Kepaniteraan Mahkamah Agung Republik Indonesia berusaha untuk selalu mencantumkan informasi paling kini dan akurat sebagai bentuk komitmen Mahkamah Agung untuk pelayanan publik, transparansi dan akuntabilitas pelaksanaan fungsi peradilan. Namun dalam hal-hal tertentu masih dimungkinkan terjadi permasalahan teknis terkait dengan akurasi dan keterkinian informasi yang kami sajikan, hal mana akan terus kami perbaiki dari waktu kewaktu.
Dalam hal Anda menemukan inakurasi informasi yang termuat pada situs ini atau informasi yang seharusnya ada, namun belum tersedia, maka harap segera hubungi Kepaniteraan Mahkamah Agung RI melalui :
Email : kepaniteraan@mahkamahagung.go.id Telp : 021-384 3348 (ext.318)

perkara a quo merupakan kewenangan absolut peradilan agama;

Menimbang, bahwa Pemohon mendalilkan telah melangsungkan perkawinan dengan Termohon yang dicatatkan di KUA Kecamatan;

Menimbang, bahwa untuk menguatkan dalil permohonannya Pemohon mengajukan bukti P 1 (fotokopi dari kutipan akta nikah) yang merupakan akta otentik dan telah bermaterai cukup dan telah cocok dengan aslinya *ternyata sesuai* oleh karena itu bukti tersebut telah memenuhi Pasal 2 ayat (3) Undang-Undang Nomor 13 Tahun 1985 Tentang Bea Materai dan Pasal 1888 KUH Perdata, sehingga bukti tersebut mempunyai kekuatan bukti yang sempurna dan mengikat;

Menimbang, bahwa berdasarkan pertimbangan tersebut di atas terbukti bahwa Pemohon adalah suami sah Termohon. Dengan demikian Pemohon dan Termohon berkualitas sebagai subjek hukum dalam perkara a quo;

Menimbang, bahwa untuk petitum angka 1 (satu) majelis hakim akan mempertimbangkannya kemudian;

Menimbang, bahwa dalam petitum angka 2 (dua) Pemohon mohon agar Memberikan ijin kepada PEMOHON (Pemohon) sebagai Suami untuk menjatuhkan talak Raj'i kepada TERMOHON (Termohon) sebagai Istri, di hadapan Sidang Pengadilan Agama Jakarta Utara maka majelis hakim akan mempertimbangkannya sebagai berikut;

Menimbang, bahwa yang menjadi dalil permohonan Pemohon untuk melakukan perceraian dengan Termohon di dasarkan kepada bahwa sejak 5 (lima) tahun setelah menikah muncul percikan-percikan yang yang membuat kondisi rumah tangga tidak harmonis dan terjadi percekcokan dan perselisihan terus menerus hingga, sulit mencapai keluarga sakinah mawadah warohmah disebabkan dengan yang Pemohon sampaikan di atas kemudian mencapai puncaknya pada tahun 2016, yaitu Pemohon menegur untuk yang kesekian kalinya kepada Termohon untuk mengulangi kesalahan yang sama yang menurut Pemohon sudah tidak dapat lagi dimaafkan, karena sudah melampaui batas norma dalam berumah tangga, maka untuk menghindari hal hal yang

Disclaimer
Kepaniteraan Mahkamah Agung Republik Indonesia berusaha untuk selalu mencantumkan informasi paling kini dan akurat sebagai bentuk komitmen Mahkamah Agung untuk pelayanan publik, transparansi dan akuntabilitas pelaksanaan fungsi peradilan. Namun dalam hal-hal tertentu masih dimungkinkan terjadi permasalahan teknis terkait dengan akurasi dan keterkinian informasi yang kami sajikan, hal mana akan terus kami perbaiki dari waktu kewaktu.
Dalam hal Anda menemukan inakurasi informasi yang termuat pada situs ini atau informasi yang seharusnya ada, namun belum tersedia, maka harap segera hubungi Kepaniteraan Mahkamah Agung RI melalui :
Email : kepaniteraan@mahkamahagung.go.id Telp : 021-384 3348 (ext.318)

negatif diluar akal sehat manusia, maka Pemohon pergi dari rumah tinggal bersama yaitu di Batam – Kepulauan Riau dan pindah ke Jakarta kemudian setelah perginya Pemohon dari rumah, Pemohon menunggu itikad baik dari Termohon dengan berharap Termohon menghubungi Pemohon untuk minta maaf dan mengajak rujuk kembali, namun Temohon semakin keras untuk tidak kembali, bahkan Termohon ternyatapun meninggalkakn rumah di Batam dan pindah ke Medan, Sumata Utara untuk hidup besama anak anak nya namun sejak berpisah Temohon dan Pemohon menjalani hidup masing masing, namun Pemohon masih memberikan tanggung jawab sebagai seorang ayah, dengan memberikan baiya hidup dan untuk kebutuhan anak anaknya;

Menimbang, bahwa terhadap dalil permohonan Pemohon tersebut Termohon menyampaikan jawabannya secara lisan yang pada pokoknya adalah mengakui dan membenarkannya dalil permohonan Pemohon tersebut dan menerangkan tidak keberatan atas permohonan cerai dari Pemohon;

Menimbang, bahwa berdasarkan ketentuan pasal 174 HIR jo. pasal 1925 KUH Perdata, kedudukan pengakuan merupakan bukti yang mengikat dan memiliki nilai pembuktian sempurna, untuk itu harus dinyatakan telah terbukti bahwa antara Pemohon dengan Termohon sejak 5 (lima) tahun setelah menikah muncul percikan-percikan yang yang membuat kondisi rumah tangga tidak harmonis dan terjadi percekcokan dan perselisihan terus menerus hingga, sulit mencapai keluarga sakinah mawadah warohmah disebabkan dengan yang Pemohon sampaikan di atas kemudian mencapai puncaknya pada tahun 2016, yaitu Pemohon menegur untuk yang kesekian kalinya kepada Termohon untuk mengulangi kesalahan yang sama yang menurut Pemohon sudah tidak dapat lagi dimaafkan, karena sudah melampaui batas norma dalam berumah tangga, maka untuk menghindari hal hal yang negatif diluar akal sehat manusia, maka Pemohon pergi dari rumah tinggal bersama yaitu di Perum. Alam Raya Blok A2 No. 2 RT. 001 RW. 015 Desa Belian Kec. Batam Kota, Kab. Kota Batam – Kepulauan Riau dan pindah ke Jakarta kemudian setelah perginya Pemohon dari rumah, Pemohon menunggu itikad baik dari Termohon dengan berharap Termohon menghubungi Pemohon untuk minta maaf dan mengajak rujuk

Disclaimer
Kepaniteraan Mahkamah Agung Republik Indonesia berusaha untuk selalu mencantumkan informasi paling kini dan akurat sebagai bentuk komitmen Mahkamah Agung untuk pelayanan publik, transparansi dan akuntabilitas pelaksanaan fungsi peradilan. Namun dalam hal-hal tertentu masih dimungkinkan terjadi permasalahan teknis terkait dengan akurasi dan keterkinian informasi yang kami sajikan, hal mana akan terus kami perbaiki dari waktu kewaktu.
Dalam hal Anda menemukan inakurasi informasi yang termuat pada situs ini atau informasi yang seharusnya ada, namun belum tersedia, maka harap segera hubungi Kepaniteraan Mahkamah Agung RI melalui :
Email : kepaniteraan@mahkamahagung.go.id Telp : 021-384 3348 (ext.318)

kembali, namun Temohon semakin keras untuk tidak kembali, bahkan Termohon ternyatapun meninggalkakn rumah di Batam dan pindah ke Medan, Sumata Utara untuk hidup besama anak anak nya namun sejak berpisah Temohon dan Pemohon menjalani hidup masing masing, namun Pemohon masih memberikan tanggung jawab sebagai seorang ayah, dengan memberikan baiya hidup dan untuk kebutuhan anak anaknya;

Menimbang, bahwa meskipun dalil permohonan Pemohon tersebut telah diakui oleh Termohon namun karena perkara perceraian menyangkut putusnya ikatan perkawinan yang bernilai sakral maka Majelis Hakim tetap akan memberikan pertimbangan-pertimbangan dengan melihat bukti lainnya untuk menentukan patut dan tidak patutnya perkawinan antara Pemohon dan Termohon diputuskan;

Menimbang, bahwa untuk bukti P1 karena telah dipertimbangkan di atas maka tidak perlu lagi untuk dipertimbangkan;

Menimbang, bahwa Pemohon juga telah mengajukan bukti saksi dari Pemohon yang telah memberikan keterangan di bawah sumpah dihadapan Majelis Hakim yang keterangannya sebagaimana dalam duduk perkara ini ternyata keterangan saksi-saksi tersebut saling bersesuaian satu dengan yang lainnya yang pada pokoknya menguatkan dalil permohonan Pemohon;

Menimbang, bahwa karena keterangan saksi-saksi berdasarkan pengetahuannya sendiri dan yang pada pokoknya menguatkan dalil-dalil permohonan Pemohon tersebut maka Majelis hakim dapat menerima keterangannya sehingga dapat dijadikan bukti dalam perkara ini;

Menimbang, bahwa Termohon menerangkan tidak akan mengajukan bukti dan mencukupkan dengan yang Pemohon ajukan maka majelis hakim akan mempertimbangkannya sebagai berikut;

Menimbang, bahwa mangajukan bukti atau tidak adalah hak para pihak namun dalam perkara a quo karena Termohon menerangkan tidak akan mengajukan bukti maka majelis hakim menilai bahwa Termohon telah

Disclaimer
Kepaniteraan Mahkamah Agung Republik Indonesia berusaha untuk selalu mencantumkan informasi paling kini dan akurat sebagai bentuk komitmen Mahkamah Agung untuk pelayanan publik, transparansi dan akuntabilitas pelaksanaan fungsi peradilan. Namun dalam hal-hal tertentu masih dimungkinkan terjadi permasalahan teknis terkait dengan akurasi dan keterkinian informasi yang kami sajikan, hal mana akan terus kami perbaiki dari waktu kewaktu.
Dalam hal Anda menemukan inakurasi informasi yang termuat pada situs ini atau informasi yang seharusnya ada, namun belum tersedia, maka harap segera hubungi Kepaniteraan Mahkamah Agung RI melalui :
Email : kepaniteraan@mahkamahagung.go.id Telp : 021-384 3348 (ext.318)

menghilangkan haknya untuk mengajukan bukti dengan demikian berarti Termohon dalam perkara a quo tidak mengajukan bukti;

Menimbang, bahwa saksi dari Pemohon telah didengar kesaksiannya dalam persidangan adalah untuk memenuhi maksud pasal 76 Ayat (1) Undang-Undang Nomor 7 Tahun 1989 Tentang Peradilan Agama sebagaimana telah diubah dengan Undang-Undang Nomor 3 Tahun 2006 dan perubahan kedua dengan Undang-Undang Nomor 50 Tahun 2009 jo pasal 22 PP Nomor 9 tahun 1975 Tentang Pelaksanaan Undang-undang Nomor 1 tahun 1974 tentang Perkawinan dan pasal 134 Kompilasi Hukum Islam;

Menimbang, bahwa berdasarkan ketentuan pasal 39 ayat (2) UU No. 1 Tahun 1974 Tentang Perkawinan “untuk melakukan perceraian harus ada cukup alasan bahwa antara suami-isteri itu tidak akan dapat hidup rukun lagi sebagai suami-isteri;

Menimbang, bahwa berdasarkan hasil pemeriksaan dalam persidangan terhadap surat permohonan Pemohon berikut keteranganya dan bukti-bukti Majelis Hakim dapat menemukan fakta sebagai berikut;

- Bahwa Pemohon dengan Termohon adalah suami isteri;
- Bahwa Pemohon dengan Termohon mempunyai anak 5 (lima) orang;
- Bahwa sejak 5 (lima) tahun setelah menikah Pemohon dan Termohon mulai muncul percikan-percikan yang membuat kondisi rumah tangga tidak harmonis dan terjadi perselisihan dan percekcokan terus-menerus hingga sulit mencapai keluarga yang sakinag mawaddah warahmah disebabkan dengan yang Pemohon sampaikan di atas yang mencapai puncaknya pada tahun 2016;
- Bahwa sejak tahun 2016 Pemohon dan Termohon sudah berpisah rumah;
- Bahwa Pemohon yang awalnya meninggalkan rumah kediaman bersama;
- Bahwa sejak tahun 2016 antara Pemohon dan Termohon pernah hidup bersama lagi;

Disclaimer
Kepaniteraan Mahkamah Agung Republik Indonesia berusaha untuk selalu mencantumkan informasi paling kini dan akurat sebagai bentuk komitmen Mahkamah Agung untuk pelayanan publik, transparansi dan akuntabilitas pelaksanaan fungsi peradilan. Namun dalam hal-hal tertentu masih dimungkinkan terjadi permasalahan teknis terkait dengan akurasi dan keterkinian informasi yang kami sajikan, hal mana akan terus kami perbaiki dari waktu kewaktu.
Dalam hal Anda menemukan inakurasi informasi yang termuat pada situs ini atau informasi yang seharusnya ada, namun belum tersedia, maka harap segera hubungi Kepaniteraan Mahkamah Agung RI melalui :
Email : kepaniteraan@mahkamahagung.go.id Telp : 021-384 3348 (ext.318)

- Bahwa Pemohon masih memberikan biaya hidup untuk anak-anaknya;

Menimbang, bahwa berdasarkan fakta tersebut di atas maka telah terbukti bahwa rumah tangga Pemohon dengan Termohon sudah tidak harmonis lagi sejak 5 (lima) tahun setelah menikah Pemohon dan Termohon mulai muncul percikan-percikan yang membuat kondisi rumah tangga tidak harmonis dan terjadi perselisihan dan percekcokan terus-menerus hingga sulit mencapai keluarga yang sakinag mawaddah warahmah disebabkan dengan yang Pemohon sampaikan di atas yang mencapai puncaknya pada tahun 2016 kemudian sejak itu Pemohon dengan Termohon berpisah rumah dan Pemohon yang awalnya meninggalkan rumah kediaman bersama bahkan sejak berpisah rumahpun belum pernah hidup bersama lagi akan tetapi Pemohon masih memberikan nafkah untuk anaknya sehingganya harapan untuk hidup rukun dalam rumah tangganya sudah sulit;

Menimbang, bahwa berdasarkan hal tersebut Majelis Hakim berpendapat bahwa hubungan antara Pemohon dan Termohon dalam membina rumah tangga sudah tidak harmonis sehingga sulit untuk mewujudkan tujuan perkawinan sebagaimana maksud dari Al Qur'an Surat Ar-Rum Ayat 21 dan Pasal 1 Undang-Undang Nomor 1 Tahun 1974 Tentang Perkawinan Jo. Pasal 3 Kompilasi Hukum Islam (INPRES Nomor 1 Tahun 1991);

Menimbang, bahwa Pasal 1 Undang-Undang Nomor 1 tahun 1974 tentang Perkawinan menyatakan bahwa perkawinan adalah ikatan lahir batin antara seorang pria dengan seorang wanita sebagai suami isteri dengan tujuan membentuk keluarga (rumah tangga) yang bahagia dan kekal berdasarkan Ketuhanan Yang Maha Esa;

Menimbang, bahwa ikatan batin menurut penjelasan pasal tersebut merupakan unsur yang penting dalam suatu perkawinan oleh karenannya Majelis Hakim berpendapat apabila ikatan batin sudah tidak ada lagi maka perkawinan tersebut sudah pecah sehingga mempertahankan perkawinan tersebut merupakan hal yang sia-sia dan tidak akan bermanfaat bagi kedua belah pihak;

Disclaimer
Kepaniteraan Mahkamah Agung Republik Indonesia berusaha untuk selalu mencantumkan informasi paling kini dan akurat sebagai bentuk komitmen Mahkamah Agung untuk pelayanan publik, transparansi dan akuntabilitas pelaksanaan fungsi peradilan. Namun dalam hal-hal tertentu masih dimungkinkan terjadi permasalahan teknis terkait dengan akurasi dan keterkinian informasi yang kami sajikan, hal mana akan terus kami perbaiki dari waktu kewaktu.
Dalam hal Anda menemukan inakurasi informasi yang termuat pada situs ini atau informasi yang seharusnya ada, namun belum tersedia, maka harap segera hubungi Kepaniteraan Mahkamah Agung RI melalui :
Email : kepaniteraan@mahkamahagung.go.id Telp : 021-384 3348 (ext.318)

Menimbang, bahwa dalam kondisi tidak harmonis tersebut Majelis Hakim berpendapat ikatan perkawinan antara Pemohon dan Termohon telah pecah yang disebabkan oleh hal-hal sebagaimana tersebut di atas bahkan adanya kehendak yang kuat dari Pemohon untuk menceraikan Termohon dengan demikian tanpa mempersoalkan siapa yang salah sehingga antara Pemohon dan Termohon sulit untuk dapat dirukunkan kembali untuk membina rumah tangga bersama sehingga permohonan Pemohon telah memenuhi maksud Pasal 39 Undang-Undang Nomor 1 Tahun 1974 Tentang Perkawinan beserta penjelasannya dan Pasal 19 huruf b dan f Peraturan Pemerintah Nomor 9 Tahun 1975 tentang Pelaksanaan Undang-undang Nomor 1 tahun 1974 tentang Perkawinan Jo. Pasal 116 huruf b dan f Kompilasi Hukum Islam;

Menimbang, bahwa Majelis Hakim perlu mengetengahkan firman Allah SWT dalam Surat Al-Baqarah ayat 227, yaitu sebagai berikut:

وَإِنْ عَزَمُوا الطَّلَاقَ فَإِنَّ اللهَ سَمِيعٌ عَلِيمٌ

Artinya: “Dan jika mereka ber’azam (bertetap hati) untuk berthalak, maka sesungguhnya Allah Maha Mendengar dan dan Maha Mengetahui”.

Menimbang, bahwa Majelis Hakim perlu mengetengahkan pendapat dalam kitab Al Iqna Juz III halaman 401 yang berbunyi :

لأن الإعتبار بالطلاق فالزوج لماروى البيهقى أن النبي صلعم
قال : الطلاق بالرجال والعدة بالنساء ولا يحرم جمع التطليقات

Artinya;Menjatuhkan talak adalah hak suami sesuai dengan riwayat Al Baihaqi bahwa nabi SAW bersabda : Cerai itu dari pihak laki-laki dan iddah dari pihak perempuan, tidak dilarang mengumpulkan beberapa talak. (Al Iqna III : 401)

Menimbang, bahwa sesuai dengan Yurisprudensi Mahkamah Agung RI Nomor 237 K/AG/1998 tanggal 17 Maret 1999 yang menetapkan bahwa cekcok, hidup berpisah tidak dalam satu tempat kediaman bersama, salah satu pihak tidak berniat meneruskan kehidupan bersama dengan pihak lain merupakan fakta yang cukup untuk dijadikan alasan perceraian;

Disclaimer
Kepaniteraan Mahkamah Agung Republik Indonesia berusaha untuk selalu mencantumkan informasi paling kini dan akurat sebagai bentuk komitmen Mahkamah Agung untuk pelayanan publik, transparansi dan akuntabilitas pelaksanaan fungsi peradilan. Namun dalam hal-hal tertentu masih dimungkinkan terjadi permasalahan teknis terkait dengan akurasi dan keterkinian informasi yang kami sajikan, hal mana akan terus kami perbaiki dari waktu kewaktu.
Dalam hal Anda menemukan inakurasi informasi yang termuat pada situs ini atau informasi yang seharusnya ada, namun belum tersedia, maka harap segera hubungi Kepaniteraan Mahkamah Agung RI melalui :
Email : kepaniteraan@mahkamahagung.go.id Telp : 021-384 3348 (ext.318)

Menimbang, bahwa sebenarnya dengan perkawinan diharapkan akan memberikan kemaslahatan bagi suami isteri (in casu Pemohon dan Termohon) akan tetapi dengan melihat kondisi rumah tangga yang sedang dialami Pemohon dan Termohon maka bukan lagi kemaslahatan yang didapat melainkan penderitaan batinlah yang dirasakan keduanya atau salah satunya, dalam keadaan demikian maka perceraian merupakan alternatif terbaik yang dapat menyelamatkan keduanya dari penderitaan batin yang terus berkepanjangan, daripada tetap mempertahankan perkawinan yang sudah rapuh, hal mana sejalan dengan maksud kaidah fiqhiyyah:

درء المفاسد مقدّم على جلب المصال

Artinya :" Menghindari kerusakan harus didahulukan daripada mendatangkan kemaslahatan ".

Menimbang, bahwa berdasarkan pertimbangan-petimbangan tersebut di atas telah cukup alasan bagi Majelis Hakim untuk mengabulkan permohonan Pemohon sebagaimana dalam petitum angka 2 (dua) yaitu dengan memberikan izin kepada Pemohon untuk ikrar menjatuhkan talak satu raj'i terhadap Termohon di depan Pengadilan Agama Jakarta Utara yang amarnya akan disebutkan dalam amar putusan ini;

Menimbang, bahwa dalam petitum angka 3 (tiga) Pemohon mohon agar memohon kepada Panitera Pengadilan Agama Jakarta Utara, untuk mengirimkan salinan putusan yang sudah berkuatan hukum tetap, kepada Kantor Urusan Agama Kota. Batam Kep. KEPRI dan terhadap hal tersebut Termohon tidak dapat didengar keterangannya karena tidak hadir dalam persidangan maka majelis hakim akan mempertimbangkannya sebagai berikut;

Menimbang, bahwa sesuai dengan Surat Edaran Ketua Mahkamah Agung Republik Indonesia Nomor 1 tahun 2017 tanggal 19 Desember 2017 tentang Pemberlakuan Rumusan Hasil Rapat Pleno Kamar Mahkamah Agung tahun 2017 sebagai Pedoman Pelaksanaan Tugas Bagi Pengadilan huruf C Rumusan Hukum Kamar Agama angka (3) menyebutkan bahwa perintah penyampaian salinan putusan/penetapan ikrar talak sesuai ketentuan pasal 84

Disclaimer
Kepaniteraan Mahkamah Agung Republik Indonesia berusaha untuk selalu mencantumkan informasi paling kini dan akurat sebagai bentuk komitmen Mahkamah Agung untuk pelayanan publik, transparansi dan akuntabilitas pelaksanaan fungsi peradilan. Namun dalam hal-hal tertentu masih dimungkinkan terjadi permasalahan teknis terkait dengan akurasi dan keterkinian informasi yang kami sajikan, hal mana akan terus kami perbaiki dari waktu kewaktu.
Dalam hal Anda menemukan inakurasi informasi yang termuat pada situs ini atau informasi yang seharusnya ada, namun belum tersedia, maka harap segera hubungi Kepaniteraan Mahkamah Agung RI melalui :
Email : kepaniteraan@mahkamahagung.go.id Telp : 021-384 3348 (ext.318)

Undang-Undang Nomor 7 tahun 1989 tidak perlu dicantumkan dalam amar putusan, dengan demikian maka terhadap petitum angka 3 (tiga) sebagai mana disebutkan di atas tidak perlu lagi dipertimbangkan lebih lanjut dan tidak perlu disebutkan dalam amar putusan ini;

Menimbang, bahwa dalam proses mediasi, antara Pemohon dan Termohon telah terjadi kesepakatan mengenai akibat perceraian yakni mengenai hak pemeliharaan/pengasuhan anak Pemohon dan Termohon, berada di bawah pengasuhan/hadhanah Termohon nomor 1 dan 3 serta nomor 2 pada Pemohon, besaran nafkah anak, besaran nafkah iddah dan mut'ah yang kedua belah pihak telah merumuskannya ke dalam kesepakatan perdamaian dan telah ditandatangani di hadapan Mediator tertanggal 18 Desember 2020;

Menimbang, bahwa atas kesepakatan yang telah dibuat oleh kedua belah pihak tersebut majelis Hakim akan mempertimbangkannya sebagai berikut;

Menimbang, bahwa apa-apa yang telah disepakati oleh kedua belah pihak tersebut adalah hal-hal yang menyangkut akibat perceraian sebagaimana yang dimaksud dalam Kompilasi Hukum Islam pasal 149 huruf (a) (b) dan (d) serta pasal 156 huruf (a), sehingga dengan demikian Majelis berpendapat bahwa kesepakatan damai tersebut adalah sesuatu yang halal/diperbolehkan untuk disepakati dan bukan sesuatu yang melanggar hukum, sebagaimana yang dimaksud dalam Pasal 1321 (KUH Perdata);

Menimbang, bahwa oleh karena Pemohon dan Termohon telah membuat kesepakatan perdamaian mengenai hak pemeliharaan/ pengasuhan anak, nafkah anak, nafkah selama masa iddah dan mut'ah, maka berdasarkan pasal 1338 Kitab Undang-Undang Hukum Perdata (KUH Perdata), perjanjian tersebut merupakan Undang-undang/peraturan sehingga harus ditaati oleh kedua belah pihak yang membuatnya, sehingga majelis hakim akan menetapkan dan menghukum hal-hal sebagaimana para pihak sepakati tersebut;

Disclaimer
Kepaniteraan Mahkamah Agung Republik Indonesia berusaha untuk selalu mencantumkan informasi paling kini dan akurat sebagai bentuk komitmen Mahkamah Agung untuk pelayanan publik, transparansi dan akuntabilitas pelaksanaan fungsi peradilan. Namun dalam hal-hal tertentu masih dimungkinkan terjadi permasalahan teknis terkait dengan akurasi dan keterkinian informasi yang kami sajikan, hal mana akan terus kami perbaiki dari waktu kewaktu.
Dalam hal Anda menemukan inakurasi informasi yang termuat pada situs ini atau informasi yang seharusnya ada, namun belum tersedia, maka harap segera hubungi Kepaniteraan Mahkamah Agung RI melalui :
Email : kepaniteraan@mahkamahagung.go.id Telp : 021-384 3348 (ext.318)

Menimbang, bahwa hal-hal yang telah disepakati antara Pemohon dengan Termohon sebagaimana laporan hasil mediasi adalah sebagaimana tersebut di atas dan Para pihak juga mohon agar hasil kesepakatan dalam mediasi tersebut dikuatkan dalam putusan akhir;

Menimbang, bahwa apa-apa yang telah disepakati antara Pemohon dengan Termohon tersebut tentang hak asuh anak, besaran nafkahnya, nafkah iddah dan mut'ah dll akan dituangkan selengkapnya dalam amar putusan ini namun untuk anak bahwa meskipun ditetapkan pada ibunya dan jika suatu saat Pemohon datang maka majelis hakim melarang Termohon untuk mempersulit Pemohon jika ingin bertemu dengan anaknya dan menyalurkan kasih sayangnya juga dapat membawanya berlibur tentunya dengan segera mengembalikannya kepada Termohon dengan terlebih dahulu dikoordinasikan dengan Termohon sebagai ibunya jika ingin bertemu dan membawa anak-anaknya tersebut;

Menimbang, bahwa untuk memberikan kepastian atas pemberian Mut'ah dan nafkah selama Iddah Pemohon kepada Termohon tersebut, sesuai Peraturan Mahkamah Agung RI Nomor 3 Tahun 2017 tentang Pedoman Mengadili Perkara Perempuan Berhadapan dengan Hukum dan Surat Edaran Mahkamah Agung RI Nomor 1 Tahun 2017 tentang Pemberlakuan Rumusan Hasil Pleno Kamar Mahkamah Agung RI Sebagai Pedoman Pelaksanaan Tugas Bagi Pengadilan, maka pembayaran tersebut harus dilakukan oleh Tergugat Rekonvensi sekaligus sesaat sebelum ikrar talak dijatuhkan;

Menimbang, bahwa dalam petitum angka 4 (empat) Pemohon juga mohon agar membebani biaya perkara sesuai undang undang yang berlaku dan terhadap hal tersebut Termohon juga menyampaikan tanggapannya secara lisan pada pokoknya menyetujui atas kehendak dari Pemohon tersebut maka majelis akan mempertimbangkannya sebagai berikut;

Menimbang, bahwa perkara ini termasuk bidang perkawinan maka sesuai dengan pasal 89 ayat 1 Undang-Undang Nomor 7 Tahun 1989 Tentang Peradilan Agama sebagaimana telah diubah dengan Undang-Undang Nomor 3 Tahun 2006 dan perubahan kedua dengan Undang-Undang Nomor 50 Tahun

Disclaimer
Kepaniteraan Mahkamah Agung Republik Indonesia berusaha untuk selalu mencantumkan informasi paling kini dan akurat sebagai bentuk komitmen Mahkamah Agung untuk pelayanan publik, transparansi dan akuntabilitas pelaksanaan fungsi peradilan. Namun dalam hal-hal tertentu masih dimungkinkan terjadi permasalahan teknis terkait dengan akurasi dan keterkinian informasi yang kami sajikan, hal mana akan terus kami perbaiki dari waktu kewaktu.
Dalam hal Anda menemukan inakurasi informasi yang termuat pada situs ini atau informasi yang seharusnya ada, namun belum tersedia, maka harap segera hubungi Kepaniteraan Mahkamah Agung RI melalui :
Email : kepaniteraan@mahkamahagung.go.id Telp : 021-384 3348 (ext.318)

2009 biaya perkara dibebankan kepada Pemohon dengan demikian cukup alasan bagi majelis hakim untuk mengabulkan permohonan Pemohon sebagaimana petitum angka 4 (empat) tersebut yaitu dengan membebankan kepada Pemohon untuk membayar biaya perkara ini yang jumlahnya akan disebutkan dalam amar putusan ini;

Menimbang, bahwa untuk petitum angka 1 (satu) majelis hakim akan mempertimbangkannya sebagai berikut;

Menimbang, bahwa karena petitum angka 2 (dua) dan 4 (empat) dikabulkan sedangkan petitum angka 3(tiga) sebagaimana dipertimbangkan di atas tidak perlu untuk dimasukan dalam amar maka majelis hakim berpendapat bahwa petitum angka 1 (satu) juga turut dikabulkan;

Memperhatikan segala peraturan dan perundang-undangan yang berlaku serta hukum syara' yang berhubungan dengan perkara ini ;

M E N G A D I L I

1. Mengabulkan permohonan Pemohon.
2. Memberikan izin kepada Pemohon (PEMOHON) untuk menjatuhkan talak satu raj'i terhadap Termohon (TERMOHON) di depan sidang Pengadilan Agama Jakarta Utara.
3. Menghukum Pemohon untuk memberikan kepada Termohon sebelum ikrar talak diucapkan berupa;
    3.1. Nafkah selama masa iddah sejumlah Rp 25.000.000,- (dua puluh lima juta rupiah);
    3.2. Mut'ah berupa uang sejumlah Rp Rp 5.000.000,- (lima juta lima ratus ribu rupiah);
4. Menetapkan anak yang bernama 1. ANAK KE I (P), 2. ANAK KE II (P), 3. ANAK KE III (L), 4. ANAK KE IV (L) 5. ANAK KE V (P) berada dalam pemeliharaan dan pengasuhan (hadhanah) Termohon;
5. Menghukum Pemohon untuk memberikan nafkah anak sebagaimana dalam diktum angka 4 (empat) tersebut di atas setiap bulannya minimal

Disclaimer
Kepaniteraan Mahkamah Agung Republik Indonesia berusaha untuk selalu mencantumkan informasi paling kini dan akurat sebagai bentuk komitmen Mahkamah Agung untuk pelayanan publik, transparansi dan akuntabilitas pelaksanaan fungsi peradilan. Namun dalam hal-hal tertentu masih dimungkinkan terjadi permasalahan teknis terkait dengan akurasi dan keterkinian informasi yang kami sajikan, hal mana akan terus kami perbaiki dari waktu kewaktu.
Dalam hal Anda menemukan inakurasi informasi yang termuat pada situs ini atau informasi yang seharusnya ada, namun belum tersedia, maka harap segera hubungi Kepaniteraan Mahkamah Agung RI melalui :
Email : kepaniteraan@mahkamahagung.go.id Telp : 021-384 3348 (ext.318)

sejumlah Rp 3.000.000,- (tiga juta rupiah) di luar biaya pendidikan dan kesehatan sampai anak tersebut dewasa atau mandiri;

6. Membebankan kepada Pemohon untuk membayar biaya perkara ini sejumlah Rp 579.000,- (lima ratus tujuh puluh sembilan ribu rupiah).

Demikian putusan ini dijatuhkan dalam rapat permusyawaratan Majelis Hakim Pengadilan Agama Jakarta Utara pada hari **Rabu** tanggal 23 Desember 2020 Masehi bertepatan dengan tanggal 8 Jumadil Awal 1442 Hijriyah, oleh Kami **Drs. Agus Abdullah, M.H.,** sebagai Ketua Majelis, **Drs. Ahd Syarwani** dan **Hj. Asmawati, S.H., M.H.,** masing-masing sebagai Hakim Anggota, putusan tersebut pada hari itu juga dibacakan oleh Ketua Majelis tersebut dalam persidangan yang terbuka untuk umum dengan didampingi Hakim-Hakim Anggota tersebut dibantu oleh **Muhammad Thahir Guhir, S.H.,** sebagai panitera Pengganti yang dihadiri oleh Pemohon dan Termohon;

Ketua Majelis

t.t.d.

Drs. Agus Abdullah, M.H.

Hakim Anggota	Hakim Anggota
t.t.d.	t.t.d.
**Drs. Ahd Syarwani**	**Hj. Asmawati, S.H., M.H.**

Panitera Pengganti

t.t.d.

Muhammad Thahir Guhir, S.H.

Perincian biaya perkara :

1. Pendaftaran Rp. 30.000,-

Disclaimer
Kepaniteraan Mahkamah Agung Republik Indonesia berusaha untuk selalu mencantumkan informasi paling kini dan akurat sebagai bentuk komitmen Mahkamah Agung untuk pelayanan publik, transparansi dan akuntabilitas pelaksanaan fungsi peradilan. Namun dalam hal-hal tertentu masih dimungkinkan terjadi permasalahan teknis terkait dengan akurasi dan keterkinian informasi yang kami sajikan, hal mana akan terus kami perbaiki dari waktu kewaktu.
Dalam hal Anda menemukan inakurasi informasi yang termuat pada situs ini atau informasi yang seharusnya ada, namun belum tersedia, maka harap segera hubungi Kepaniteraan Mahkamah Agung RI melalui :
Email : kepaniteraan@mahkamahagung.go.id Telp : 021-384 3348 (ext.318)

2. Proses	Rp.	75.000,-
3. Panggilan	Rp.	448.000,-
4. PNBP panggilan Termohon pertama	Rp	10.000,-
5. Redaksi	Rp.	10.000,-
6. Materai	Rp.	6.000,+
Jumlah	Rp.	579.000,-

Disclaimer
Kepaniteraan Mahkamah Agung Republik Indonesia berusaha untuk selalu mencantumkan informasi paling kini dan akurat sebagai bentuk komitmen Mahkamah Agung untuk pelayanan publik, transparansi dan akuntabilitas pelaksanaan fungsi peradilan. Namun dalam hal-hal tertentu masih dimungkinkan terjadi permasalahan teknis terkait dengan akurasi dan keterkinian informasi yang kami sajikan, hal mana akan terus kami perbaiki dari waktu kewaktu.
Dalam hal Anda menemukan inakurasi informasi yang termuat pada situs ini atau informasi yang seharusnya ada, namun belum tersedia, maka harap segera hubungi Kepaniteraan Mahkamah Agung RI melalui :
Email : kepaniteraan@mahkamahagung.go.id Telp : 021-384 3348 (ext.318)
