# Supplementary material for: Classification of divorce causes during the COVID-19 pandemic using convolutional neural networks
Source: PeerJ Comput Sci. 2022 Jun 30;8:e998. doi: 10.7717/peerj-cs.998 (PMC9299239; doi:10.7717/peerj-cs.998)
Supplement: Supplemental Information 3 [file peerj-cs-08-998-s003.zip › KDRT Dataset/Data ke-17.pdf]

P U T U S A N

Nomor 979/Pdt.G/2020/PA.Pal

بِسۡمِ ٱللَّهِ ٱلرَّحۡمَٰنِ ٱلرَّحِيمِ

DEMI KEADILAN BERDASARKAN KETUHANAN YANG MAHA ESA

Pengadilan Agama Palu yang memeriksa dan mengadili perkara tertentu pada tingkat pertama dalam sidang Majelis Hakim telah menjatuhkan putusan sebagai berikut dalam perkara Cerai Talak antara:

PEMOHON, umur 38 tahun, agama Islam, pendidikan SLTA, pekerjaan xxxxxxxx xxxxxx xxxxxxxx, tempat kediaman di KOTA PALU, sebagai Pemohon;

melawan

TERMOHON, umur 36 tahun, agama Islam, pendidikan S1, pekerjaan xxxx xxxxxxx xx xxx xxxx xxxxx, tempat kediaman di xxxxx xxxx sebagai Termohon;

Pengadilan Agama tersebut;
Telah membaca dan mempelajari berkas perkara;
Telah mendengar keterangan Pemohon dan Termohon;
Telah memeriksa alat-alat bukti Pemohon;

DUDUK PERKARA

Menimbang, bahwa Pemohon dengan surat permohonannya tertanggal 16 November 2020 yang terdaftar di Kepaniteraan Pengadilan Agama Palu pada tanggal 18 November 2020 dengan register perkara Nomor 979/Pdt.G/2020/PA.Pal, mengemukakan hal-hal yang pada pokoknya sebagai berikut:

1.--
Bahwa pada tanggal 20 Agustus 2006, Pemohon dan Termohon melangsungkan pernikahan sebagaimana tercatat pada Kutipan Akta Nikah Nomor : xxx xxxx xxx yang dikeluarkan oleh Pegawai Pencatat Nikah Kantor Urusan Agama Kecamatan Palu Timur, xxxx xxxx ;

Disclaimer
Kepaniteraan Mahkamah Agung Republik Indonesia berusaha untuk selalu mencantumkan informasi paling kini dan akurat sebagai bentuk komitmen Mahkamah Agung untuk pelayanan publik, transparansi dan akuntabilitas pelaksanaan fungsi peradilan. Namun dalam hal-hal tertentu masih dimungkinkan terjadi permasalahan teknis terkait dengan akurasi dan keterkinian informasi yang kami sajikan, hal mana akan terus kami perbaiki dari waktu kewaktu.
Dalam hal Anda menemukan inakurasi informasi yang termuat pada situs ini atau informasi yang seharusnya ada, namun belum tersedia, maka harap segera hubungi Kepaniteraan Mahkamah Agung RI melalui :
Email : kepaniteraan@mahkamahagung.go.id Telp : 021-384 3348 (ext.318)

2. ---
Bahwa setelah menikah Pemohon dan Termohon tinggal bersama di rumah dinas perusahaan di Luwuk. Selama pernikahan antara Pemohon dengan Termohon telah hidup sebagaimana layaknya suami istri dan telah dikaruniai 2 (dua) orang anak, masing-masing bernama :

- ANAK (perempuan), umur 13 tahun.
- ANAK (laki-laki), umur 5 tahun ;

3. Bahwa sejak awal berumah tangga antara Pemohon dan Termohon hidup rukun dan harmonis, namun sejak akhir bulan Agustus 2020 Pemohon dan Termohon sering terlibat perselisihan yang disebabkan karena :
    a. Termohon memiliki hubungan dengan pria lain.
    b. Termohon tidak mau mendengarkan perkataan dan nasehat Pemohon.
    c. Pada bulan November 2019, Pemohon pernah mengajukan gugatan perceraian di Pengadilan Agama Luwuk namun dicabut kembali karena Termohon mau berubah dan mengingat anak ;
4. Bahwa puncak dari ketidakharmonisan tersebut terjadi pada tanggal 30 Agustus 2020, dimana pada saat itu Termohon dan Pemohon yang terlibat pertengakaran disebabkan karena Termohon yang memiliki hubungan dengan pria lain lagi dan menimbulkan keributan rumah tangga, sehingga Pemohon turun dari rumah karena merasa sudah tidak di hargai lagi menjadi kepalah rumah tangga;
5. Bahwa akibat dari permasalahan tersebut sehingga mengakibatkan antara Pemohon dan Termohon telah berpisah sejak 30 Agustus 2020 sampai sekarang kurang lebih 3 bulan lamanya ;
6. Bahwa Pemohon tidak mampu lagi mempertahankan rumah tangga ini karena khawatir jika diteruskan akan terjadi hal-hal yang tidak diinginkan dikemudian hari, oleh karena itu Pemohon memutuskan untuk bercerai ;

Disclaimer
Kepaniteraan Mahkamah Agung Republik Indonesia berusaha untuk selalu mencantumkan informasi paling kini dan akurat sebagai bentuk komitmen Mahkamah Agung untuk pelayanan publik, transparansi dan akuntabilitas pelaksanaan fungsi peradilan. Namun dalam hal-hal tertentu masih dimungkinkan terjadi permasalahan teknis terkait dengan akurasi dan keterkinian informasi yang kami sajikan, hal mana akan terus kami perbaiki dari waktu kewaktu.
Dalam hal Anda menemukan inakurasi informasi yang termuat pada situs ini atau informasi yang seharusnya ada, namun belum tersedia, maka harap segera hubungi Kepaniteraan Mahkamah Agung RI melalui :
Email : kepaniteraan@mahkamahagung.go.id   Telp : 021-384 3348 (ext.318)

Berdasarkan alasan/dalil-dalil di atas, Pemohon memohon agar Ketua Peng[illegible]ilan Agama Palu segera memeriksa dan mengadili perkara ini, sel[illegible]jutnya menjatuhkan putusan yang amarnya sebagai berikut :

PRIMER

1. Mengabulkan permohonan Pemohon ;
2. Memberi izin kepada Pemohon (PEMOHON) untuk menjatuhkan talak satu raj'i kepada Termohon (TERMOHON) di hadapan sidang Pengadilan Agama Palu ;
3. Membebankan biaya perkara menurut hukum ;

SUBSIDER

Apabila Pengadilan Agama Palu berpendapat lain, mohon putusan yang seadil-adilnya ;

Bahwa pada hari-hari sidang yang telah ditetapkan Pemohon dan Termohon telah hadir sendiri menghadap di persidangan kecuali pada sidang pembuktian dan pembacaan putusan Termohon tidak hadir meskipun telah dipanggil secara sah;

Bahwa Ketua Majelis telah memerintahkan Pemohon dan Termohon untuk menempuh upaya mediasi, namun sesuai laporan Mediator (Dra. Hj. Nur Alam Baskar) tanggal 16 Desember 2020, ternyata mediasi tidak berhasil;

Bahwa meskipun mediasi tidak berhasil, Majelis Hakim tetap berusaha mendamaikan Pemohon dan Termohon agar kembali rukun membina rumah tangga, namun tidak berhasil;

Bahwa selanjutnya diperiksa pokok perkara dalam sidang tertutup untuk umum dengan terlebih dahulu dibacakan surat permohonan Pemohon yang isi dan maksudnya tetap dipertahankan oleh Pemohon;

Bahwa untuk menguatkan dalil-dalil permohonannya, Pemohon telah mengajukan alat-alat bukti sebagai berikut :

A. Surat

Disclaimer
Kepaniteraan Mahkamah Agung Republik Indonesia berusaha untuk selalu mencantumkan informasi paling kini dan akurat sebagai bentuk komitmen Mahkamah Agung untuk pelayanan publik, transparansi dan akuntabilitas pelaksanaan fungsi peradilan. Namun dalam hal-hal tertentu masih dimungkinkan terjadi permasalahan teknis terkait dengan akurasi dan keterkinian informasi yang kami sajikan, hal mana akan terus kami perbaiki dari waktu kewaktu.
Dalam hal Anda menemukan inakurasi informasi yang termuat pada situs ini atau informasi yang seharusnya ada, namun belum tersedia, maka harap segera hubungi Kepaniteraan Mahkamah Agung RI melalui :
Email : kepaniteraan@mahkamahagung.go.id Telp : 021-384 3348 (ext.318)

Fotokopi Kutipan Akta Nikah Nomor xxxx xxxx xxx yang dikeluarkan oleh Pegawai Pencatat Nikah Kantor Urusan Agama Kecamatanxxxx , xxxx xxx , teah dicocokkan aslnya bermeterai cukup, diberi kode P;

B.--

Saksi-saksi

Saksi 1 **SAKSI**, umur 30 tahun, agama Islam, pendidikan SLTA, pekerjaan xxx xxxxx xxxxxx, bertempat tinggal di jalan Ahmad Yani Nomor 13, xxxxxxxxx xxxxxx xxxxxx, xxxxxxxxx xxxx xxxxx, xxxx xxxx, saksi adalah saudara kandung Pemohon, di bawah sumpah, memberikan keterangan sebagai berikut :

- Bahwa Pemohon dan Termohon adalah suami isteri dan setelahmenikah Pemohon dan Termohon pernah tinggal di Pengawu bertempat tinggal di rumah dinas di Luwuk;
- Bahwa Pemohon dan Termohon telah dikaruniai 2 orang anak yang pertama bernama Andi Naysilla, umur 13 tahun dan Muh. Nabil, umur 5 tahun, kedua anak tersebut dalam asuhan Termohon;
- Bahwa kehidupan rumah tangga Pemohon dan Termohon pada awalnya berjalan baik dan rukun, namun sekarang ini rumah tangga mereka tidak harmonis lagi karena sering terjadi perselisihan dan pertengkaran yang terus menerus;
- Bahwa penyebab perselisihan dan pertengkaran Pemohon dan Termohon adalah karena Termohon selingkuh dengan laki-laki lain bernama Israr Bafadal, dan Termohon tidak mau mendengarkan perkataan dan nasehat Pemohon;
- Bahwa. Pada bulan November 2019, Pemohon pernah mengajukan gugatan perceraian di Pengadilan Agama Luwuk namun dicabut kembali karena Termohon mau berubah dan mengingat anak sehingga Pemohon mencabut perkaranya;
- Bahwa Sebab lainnya yang saksi ketahui adalah Termohon pernah didapat langsung dengan selingkuhannya yang bernama Isral Bafadal di rumah temannya hanya mereka berdua,

Disclaimer
Kepaniteraan Mahkamah Agung Republik Indonesia berusaha untuk selalu mencantumkan informasi paling kini dan akurat sebagai bentuk komitmen Mahkamah Agung untuk pelayanan publik, transparansi dan akuntabilitas pelaksanaan fungsi peradilan. Namun dalam hal-hal tertentu masih dimungkinkan terjadi permasalahan teknis terkait dengan akurasi dan keterkinian informasi yang kami sajikan, hal mana akan terus kami perbaiki dari waktu kewaktu.
Dalam hal Anda menemukan inakurasi informasi yang termuat pada situs ini atau informasi yang seharusnya ada, namun belum tersedia, maka harap segera hubungi Kepaniteraan Mahkamah Agung RI melalui :
Email : kepaniteraan@mahkamahagung.go.id Telp : 021-384 3348 (ext.318)

setelah kejadian tersebut Pemohon dan saksi ikut menemani Pemohon melaporkan Termohon ke Kantor Polisi dan Termohon buat perjanjian dengan selingkuhannya untuk tidak berhubungan lagi, namun pada bulan Agustus 2020 Termohon mengulang lagi dengan menjalin hubungan dengan pria yang lain lagi, sehingga Pemohon turun dari rumah karena merasa sudah tidak dihargai lagi sebagai kepala rumah tangga;

- Bahwa Pemohon dan Termohon sudah pisah tempat tinggal sejak tanggal 30 Agustus 2020 sampai sekarang sudahberjalan 3 bulan, yang meninggalkan kediaman bersama adalah Pemohon;
- Bahwa saat ini Pemohon tinggal bersama orang tuanya dan Termohon masih tetap tinggal di Luwuk ;
- Bahwa selama berpisah Pemohon dan Termohon Sudah tidak pernah lagi berkomunikasi ;
- Bahwa selama pisah Termohon tidak pernah datang, dan tidak pernah memperdulikan Pemohon lagi;
- Bahwa pernah diusahakan oleh keluarga Pemohon, namun tidak berhasil karena Pemohon tidak mau rukun lagi;

Saksi 2 SAKSI, umur 36 tahun, agama Islam, pendidikan S1, pekerjaan xxxxx xxxxxxxxx xx xxxxxx xxxxxxxxx, bertempat tinggal di KOTA PALU, saksi adalah teman Pemohon, di bawah sumpah, memberikan keterangan sebagai berikut :

- Bahwa saksi tahu antara Pemohon dan Termohon adalah suami isteri;
- Bahwa setelahmenikah Pemohon dan Termohon bertempat tinggal di rumah Dinas di Luwuk;
- Bahwa Pemohon dan Termohon telah dikaruniai 2 (dua) orang anak, yang pertama bernama Andi Naysilla, umur 13 tahun dan yang kedua Muh. Nabil, umur 5 tahun;
- Bahwa kehidupan rumah tangga Pemohon dan Termohon pada awalnya berjalan baik dan rukun, namun sekarang

Disclaimer
Kepaniteraan Mahkamah Agung Republik Indonesia berusaha untuk selalu mencantumkan informasi paling kini dan akurat sebagai bentuk komitmen Mahkamah Agung untuk pelayanan publik, transparansi dan akuntabilitas pelaksanaan fungsi peradilan. Namun dalam hal-hal tertentu masih dimungkinkan terjadi permasalahan teknis terkait dengan akurasi dan keterkinian informasi yang kami sajikan, hal mana akan terus kami perbaiki dari waktu kewaktu.
Dalam hal Anda menemukan inakurasi informasi yang termuat pada situs ini atau informasi yang seharusnya ada, namun belum tersedia, maka harap segera hubungi Kepaniteraan Mahkamah Agung RI melalui :
Email : kepaniteraan@mahkamahagung.go.id Telp : 021-384 3348 (ext.318)

inirumah tangga mereka tidak harmonis lagi karena sering terjadi perselisihan danpertengkaran yang terus menerus;

- Bahwa penyebab perselisihan dan pertengkaran Pemohon dan Termohon disebabkan Termohon memiliki hubungan dengan pria lain, dan pernah tertangkap basah kemudian Pemohon melaporkan kejadian tersebut ke kantor Polisi, dan saat itu Termon membuat perjanjian untuk tidak mengulangi perbuatanya, namun kemudian Termohon mengulangi lagi berhubungan dengan laki-laki lain, sehingga Pemohon pergi meninggalkan Termohon di Luwuk ;
- Bahwa Pemohon dan Termohon sudah pisah tempat tinggal sejak bulan Agustus 2020 sampai sekarang sudahberjalan 3 bulan;
- Bahwa selama berpisah Pemohon dan Termohon Sudah tidak pernah lagi berkomunikasi dan Termohon tidak pernah dating menemui Pemohon;
- Bahwa pernah diusahakan oleh keluarga Pemohon, namun tidak berhasil karena Pemohon tidak mau rukun lagi;

Bahwa atas keterangan 2 orang saksi tersebut, Pemohon menyatakan menerima dan membenarkan sedangkan Termohon tidak dapat didengarkan tanggapannya karena tidak hadir;

Bahwa selanjutnya Pemohon menyatakan tetap pada permohonannya dan tidak mengajukan apapun lagi selain mohon putusan;

Bahwa untuk mempersingkat uraian putusan ini, ditunjuk segala sesuatu yang tercantum dalam berita acara persidangan perkara ini sebagai bagian yang tidak terpisahkan dari isi putusan ini;

PERTIMBANGAN HUKUM

Menimbang, bahwa maksud dan tujuan permohonan Pemohon adalah sebagaimana telah diuraikan di atas;

Menimbang, bahwa antara Pemohon dan Termohon telah diupayakan untuk berdamai, baik dalam persidangan oleh Majelis Hakim maupun dalam proses mediasi oleh Mediator yang telah ditunjuk, namun upaya perdamaian

Disclaimer
Kepaniteraan Mahkamah Agung Republik Indonesia berusaha untuk selalu mencantumkan informasi paling kini dan akurat sebagai bentuk komitmen Mahkamah Agung untuk pelayanan publik, transparansi dan akuntabilitas pelaksanaan fungsi peradilan. Namun dalam hal-hal tertentu masih dimungkinkan terjadi permasalahan teknis terkait dengan akurasi dan keterkinian informasi yang kami sajikan, hal mana akan terus kami perbaiki dari waktu kewaktu.
Dalam hal Anda menemukan inakurasi informasi yang termuat pada situs ini atau informasi yang seharusnya ada, namun belum tersedia, maka harap segera hubungi Kepaniteraan Mahkamah Agung RI melalui :
Email : kepaniteraan@mahkamahagung.go.id   Telp : 021-384 3348 (ext.318)

tersebut tidak berhasil, dengan demikian telah terpenuhi ketentuan Pasal 82 ayat (2) dan (4) Undang-Undang Nomor 7 Tahun 1989 sebagaimana telah diubah dengan Undang-Undang Nomor 3 Tahun 2006 dan Undang-Undang Nomor 50 Tahun 2009 jo Pasal 154 ayat (1) Rbg dan Pasal 2 Peraturan Mahkamah Agung Republik Indonesia Nomor 01 Tahun 2008 yang telah diubah dengan Peraturan Mahkamah Agung RI Nomor 1 Tahun 2016 tentang Prosedur Mediasi di Pengadilan;

Menimbang, bahwa oleh karena upaya perdamaian tidak berhasil, maka diperiksa pokok perkara dalam sidang tertutup untuk umum, hal tersebut sesuai ketentuan Pasal 68 ayat (2) Undang-Undang Nomor 7 Tahun 1989 sebagaimana telah diubah dengan Undang-Undang Nomor 3 Tahun 2006 dan Undang-Undang Nomor 50 Tahun 2009;

Menimbang, bahwa alasan pokok Pemohon mengajukan permohonan cerai adalah bahwa sejak akhir bulan Agustus 2020 Pemohon dan Termohon sering terlibat perselisihan yang disebabkan karena Termohon memiliki hubungan dengan pria lain, Termohon tidak mau mendengarkan perkataan dan nasehat Pemohon, Pada bulan November 2019, Pemohon pernah mengajukan gugatan perceraian di Pengadilan Agama Luwuk namun dicabut kembali karena Termohon mau berubah dan mengingat anak, Pemohon dengan Termohon telah berpisah tempat tinggal selama 3 bulan karena Termohon yang pergi bersama laki-laki lain sehingga Termohon meninggalkan Pemohon dan selama berpisah tempat tinggal antara Pemohon dan Termohon sudah tidak ada komunikasi apalagi menjalankan kewajiban sebagaimana layaknya suami-istri;

Menimbang, bahwa dalam jawabannya secara lisan, Termohon mengakui adanya penyebab tersebut sampai akhirnya terjadi pisah tempat tinggal antara Pemohon dan Termohon selama kurang 3 bulan karena Termohon tidak mau lagi tinggal dengan Pemohon;

Menimbang, bahwa Pemohon telah mengajukan bukti P berupa fotokopi Kutipan Akta Nikah yang dikeluarkan oleh Pejabat yang berwenang sebagai akta autentik, bermeterai cukup, telah dicap pos (nazegelen) dan

Disclaimer
Kepaniteraan Mahkamah Agung Republik Indonesia berusaha untuk selalu mencantumkan informasi paling kini dan akurat sebagai bentuk komitmen Mahkamah Agung untuk pelayanan publik, transparansi dan akuntabilitas pelaksanaan fungsi peradilan. Namun dalam hal-hal tertentu masih dimungkinkan terjadi permasalahan teknis terkait dengan akurasi dan keterkinian informasi yang kami sajikan, hal mana akan terus kami perbaiki dari waktu kewaktu.
Dalam hal Anda menemukan inakurasi informasi yang termuat pada situs ini atau informasi yang seharusnya ada, namun belum tersedia, maka harap segera hubungi Kepaniteraan Mahkamah Agung RI melalui :
Email : kepaniteraan@mahkamahagung.go.id Telp : 021-384 3348 (ext.318)

sesuai dengan aslinya, maka bukti surat tersebut telah memenuhi syarat formil, isi bukti tersebut menerangkan bahwa Pemohon telah menikah dengan Termohon pada tanggal 20 Agustus 2006, relevan dengan dalil yang hendak dibuktikan oleh Pemohon, sehingga telah memenuhi syarat materil, maka bukti P sebagai akta otentik mempunyai kekuatan pembuktian yang sempurna dan mengikat;

Menimbang, bahwa berdasarkan bukti P terbukti bahwa Pemohon dengan Termohon adalah suami istri sah, menikah pada tanggal 20 Agustus 2006, hal tersebut sesuai ketentuan pasal 7 ayat (1) Kompilasi Hukum Islam;

Menimbang, bahwa Pemohon juga telah mengajukan saksi-saksi yaitu: **saksi 1** dan saksi 2, keduanya telah memberikan keterangan sebagaimana telah diuraikan dalam duduk perkara;

Menimbang, bahwa kedua saksi tersebut adalah orang dewasa yang memberikan keterangan secara terpisah di bawah sumpah di muka sidang, isi keterangan kedua saksi adalah fakta yang dilihat atau didengar sendiri oleh para saksi dan keterangan kedua saksi secara materil saling bersesuaian satu sama lain dan relevan dengan dalil-dalil yang hendak dibuktikan oleh Pemohon serta tidak ada halangan diterimanya kesaksian para saksi tersebut, maka kedua saksi tersebut telah memenuhi syarat formil dan materil sehingga keterangan kedua saksi tersebut memiliki kekuatan pembuktian yang dapat diterima;

Menimbang, bahwa berdasarkan keterangan Pemohon, bukti tertulis dan keterangan para saksi, Majelis Hakim telah menemukan fakta-fakta hukum yang disimpulkan sebagai berikut:

- Bahwa Pemohon dengan Termohon adalah suami istri sah dan telah dikaruniai 2 orang anak;
- Bahwa Pemohon dan Termohon pada awalnya hidup rukun namun kemudian sering terjadi pertengkaran yang disbakan Termohon seingkuh dengan laki-laki bernama Israr Bafadal, dan pada tahun 2019 ppernah mengajukan gugatan cerai namun dicabut karena Termohon berjanji tidak akan mengulangi perbuatanya;

Disclaimer
Kepaniteraan Mahkamah Agung Republik Indonesia berusaha untuk selalu mencantumkan informasi paling kini dan akurat sebagai bentuk komitmen Mahkamah Agung untuk pelayanan publik, transparansi dan akuntabilitas pelaksanaan fungsi peradilan. Namun dalam hal-hal tertentu masih dimungkinkan terjadi permasalahan teknis terkait dengan akurasi dan keterkinian informasi yang kami sajikan, hal mana akan terus kami perbaiki dari waktu kewaktu.
Dalam hal Anda menemukan inakurasi informasi yang termuat pada situs ini atau informasi yang seharusnya ada, namun belum tersedia, maka harap segera hubungi Kepaniteraan Mahkamah Agung RI melalui :
Email : kepaniteraan@mahkamahagung.go.id   Telp : 021-384 3348 (ext.318)

- Bahwa Pemohon dan Termohon telah berpisah tempat tinggal sejak bulan Agustus 2020. Dan keduanya sudah tidak saling memperdulikan lagi;
- Bahwa Pemohon telah berketetapan hati untuk bercerai dengan Termohon;

Menimbang, bahwa berdasarkan fakta-fakta tersebut di atas, Majelis Hakim berpendapat bahwa rumah tangga Pemohon dan Termohon telah mengalami keretakan, akibat perselisihan hingga terjadi pisah tempat tinggal yang sudah berlangsung sekitar 3 bulan, dan tidak ada harapan untuk kembali rukun karena keduanya sudah tidak saling mempedulikan bahkan Pemohon telah menyatakan ketetapan hati untuk bercerai dengan Termohon;

Menimbang, bahwa rumah tangga seperti tersebut di atas tentunya sudah tidak dapat diharapkan untuk merealisir tujuan perkawinan yaitu membentuk rumah tangga yang bahagia dan kekal, sesuai maksud Pasal 1 Undang-Undang Nomor 1 Tahun 1974, yang di rubah dengan Undang-Undang Nomor 16 tahun 2019, tentang Perkawinan, dan terwujudnya keluarga yang sakinah, mawaddah dan rahmah, sesuai maksud Pasal 3 Kompilasi Hukum Islam, bahkan dalam rumah tangga seperti itu, suami dan istri tentu sudah tidak dapat menjalankan hak dan kewajibannya masing-masing, yang mana untuk itulah rumah tangga diadakan;

Menimbang, bahwa mempertahankan rumah tangga Pemohon dan Termohon yang dalam kenyataannya telah pecah akibat dari perselisihan dan pertengkaran yang terus menerus, hanya akan menambah penderitaan kepada kedua belah pihak apabila dipaksakan untuk dirukunkan kembali, maka mafsadatnya lebih besar dari pada manfaatnya, sedangkan kaidah hukum Islam yang diambil sebagai pendapat Majelis Hakim sebagai berikut :

- درأالمفاسد مقدم على جلب المصالح

Artinya : ” *Menolak mafsadat harus didahulukan dengan mencari kemaslahatan*

Menimbang, bahwa Majelis Hakim perlu mengemukakan ayat Al qur’an surah al-Baqarah ayat 227 yang berbunyi:

Disclaimer
Kepaniteraan Mahkamah Agung Republik Indonesia berusaha untuk selalu mencantumkan informasi paling kini dan akurat sebagai bentuk komitmen Mahkamah Agung untuk pelayanan publik, transparansi dan akuntabilitas pelaksanaan fungsi peradilan. Namun dalam hal-hal tertentu masih dimungkinkan terjadi permasalahan teknis terkait dengan akurasi dan keterkinian informasi yang kami sajikan, hal mana akan terus kami perbaiki dari waktu kewaktu.
Dalam hal Anda menemukan inakurasi informasi yang termuat pada situs ini atau informasi yang seharusnya ada, namun belum tersedia, maka harap segera hubungi Kepaniteraan Mahkamah Agung RI melalui :
Email : kepaniteraan@mahkamahagung.go.id Telp : 021-384 3348 (ext.318)

و ان عزموا الطلاق فان الله سميع عليم -

Artinya:"*Dan jika mereka berazam (berketetapan hati) untuk talak maka sesungguhnya Allah Maha Mendengar lagi Maha Melihat*".

Menimbang, bahwa berdasarkan pertimbangan-pertimbangan tersebut di atas tanpa mempersoalkan siapa yang benar dan siapa yang salah, maka alasan perceraian dalam perkara ini dianggap telah memenuhi ketentuan Pasal 19 huruf (f) Peraturan Pemerintah Nomor 9 Tahun 1975 jo Pasal 116 huruf (f) Kompilasi Hukum Islam, atau setidak-tidaknya permohonan Pemohon untuk bercerai dengan Termohon telah memiliki cukup alasan, maka permohonan Pemohon patut dikabulkan dengan memberi izin kepada Pemohon untuk menjatuhkan talak satu raj'i terhadap Termohon;

Menimbang, bahwa oleh karena perkara ini termasuk bidang perkawinan, maka berdasarkan ketentuan pasal 89 ayat (1) Undang-Undang Nomor 7 Tahun 1989 sebagaimana telah diubah dengan Undang-Undang Nomor 3 Tahun 2006 dan Undang-Undang Nomor 50 Tahun 2009 tentang Peradilan Agama, biaya perkara dibebankan kepada Pemohon;

Memperhatikan pasal-pasal dari peraturan perundang-undangan yang berlaku dan hukum syara' yang berkenaan dengan perkara ini;

M E N G A D I L I

1. Mengabulkan permohonn Pemohon.
2. Memberi izin kepada Pemohon (PEMOHON) menjatuhkan talak satu raj'I terhadap Termohon (TERMOHON, di depan sidang Pengadilan Agama Palu setelah putusan berkekuatan hukum tetap.
3. Membebankan kepada Pemohon untuk membbayar biaya perkara sejumlah Rp.486.000,00 (empat ratus delapan puluh enam ribu rupiah)

Demikian diputuskan dalam musyawarah Majelis Hakim Pengadilan Agama Palu pada hari Rabu tanggal 23 Desember 2020 Miladiah bertepatan

Disclaimer
Kepaniteraan Mahkamah Agung Republik Indonesia berusaha untuk selalu mencantumkan informasi paling kini dan akurat sebagai bentuk komitmen Mahkamah Agung untuk pelayanan publik, transparansi dan akuntabilitas pelaksanaan fungsi peradilan. Namun dalam hal-hal tertentu masih dimungkinkan terjadi permasalahan teknis terkait dengan akurasi dan keterkinian informasi yang kami sajikan, hal mana akan terus kami perbaiki dari waktu kewaktu.
Dalam hal Anda menemukan inakurasi informasi yang termuat pada situs ini atau informasi yang seharusnya ada, namun belum tersedia, maka harap segera hubungi Kepaniteraan Mahkamah Agung RI melalui :
Email : kepaniteraan@mahkamahagung.go.id Telp : 021-384 3348 (ext.318)

dengan tanggal 8 Jumadilawal 1442 Hijriah oleh Dra. Tumisah sebagai Ketua Majelis, Hj. Muwafiqoh, S.H., M.H., dan Drs. H. Abd. Rahim T masing-masing sebagai Hakim Anggota, putusan tersebut diucapkan pada hari itu juga dalam sidang terbuka untuk umum oleh Ketua Majelis beserta para Hakim Anggota tersebut, dan didampingi oleh Khairiyah, SHI sebagai Panitera Pengganti, dengan dihadiri oleh Pemohon di luar hadirnya Termohon.

Hakim Anggota

Ketua Majelis,

Hj. Muwafiqoh, S.H., M.H.

Dra. Tumisah

Drs. H. Abd. Rahim T

Panitera Pengganti,

Khairiyah, SHI

Perincian biaya :

- Pendaftaran	: Rp	30.000,00
- ATK Perkara	: Rp	60.000,00
- Panggilan	: Rp	360.000,00
- Redaksi	: Rp	10.000,00
- Meterai	: Rp	6.000,00
J u m l a h	: Rp	486.000,00

(empat ratus delapan puluh enam ribu rupiah).

Salinan Sesuai Aslinya
Pengadilan Agama Palu

Mahkamah Agung Republik Indonesia

Disclaimer
Kepaniteraan Mahkamah Agung Republik Indonesia berusaha untuk selalu mencantumkan informasi paling kini dan akurat sebagai bentuk komitmen Mahkamah Agung untuk pelayanan publik, transparansi dan akuntabilitas pelaksanaan fungsi peradilan. Namun dalam hal-hal tertentu masih dimungkinkan terjadi permasalahan teknis terkait dengan akurasi dan keterkinian informasi yang kami sajikan, hal mana akan terus kami perbaiki dari waktu kewaktu.
Dalam hal Anda menemukan inakurasi informasi yang termuat pada situs ini atau informasi yang seharusnya ada, namun belum tersedia, maka harap segera hubungi Kepaniteraan Mahkamah Agung RI melalui :
Email : kepaniteraan@mahkamahagung.go.id Telp : 021-384 3348 (ext.318)

Panitera

Drs. H. A. Kadir, M.H.

Disclaimer
Kepaniteraan Mahkamah Agung Republik Indonesia berusaha untuk selalu mencantumkan informasi paling kini dan akurat sebagai bentuk komitmen Mahkamah Agung untuk pelayanan publik, transparansi dan akuntabilitas pelaksanaan fungsi peradilan. Namun dalam hal-hal tertentu masih dimungkinkan terjadi permasalahan teknis terkait dengan akurasi dan keterkinian informasi yang kami sajikan, hal mana akan terus kami perbaiki dari waktu kewaktu.
Dalam hal Anda menemukan inakurasi informasi yang termuat pada situs ini atau informasi yang seharusnya ada, namun belum tersedia, maka harap segera hubungi Kepaniteraan Mahkamah Agung RI melalui :
Email : kepaniteraan@mahkamahagung.go.id Telp : 021-384 3348 (ext.318)
