# Supplementary material for: Classification of divorce causes during the COVID-19 pandemic using convolutional neural networks
Source: PeerJ Comput Sci. 2022 Jun 30;8:e998. doi: 10.7717/peerj-cs.998 (PMC9299239; doi:10.7717/peerj-cs.998)
Supplement: Supplemental Information 3 [file peerj-cs-08-998-s003.zip › KDRT Dataset/Data ke-2.pdf]

S A L I N A N :

P U T U S A N

Nomor 4204/Pdt.G/2020/PA.Sbg.

بِسْمِ اللَّهِ الرَّحْمَٰنِ الرَّحِيمِ

DEMI KEADILAN BERDASARKAN KETUHANAN YANG MAHA ESA

Pengadilan Agama Subang yang memeriksa dan mengadili perkara-perkara tertentu pada tingkat pertama dalam persidangan Majelis Hakim telah menjatuhkan putusan dalam perkara **Cerai Gugat**, antara :

Penggugat, umur 46 tahun (Subang, 13 Juni 1974), Agama Islam, pendidikan terakhir SD, pekerjaan Ibu rumah tangga, tempat tinggal di Kabupaten Subang, sebagai **Penggugat**;

melawan

Tergugat, umur 60 tahun (Subang, 01 Juli 1960), Agama Islam, pendidikan terakhir SLTP, pekerjaan Wiraswasta, tempat tinggal di Kabupaten Subang, sebagai **Tergugat**;

Pengadilan Agama tersebut;
Setelah membaca berkas perkara;
Setelah mendengar keterangan Penggugat di persidangan;
Setelah memeriksa alat-alat bukti di persidangan;

DUDUK PERKARA

Bahwa Penggugat dengan surat gugatannya tanggal 26 Oktober 2020 yang telah didaftarkan di Kepaniteraan Pengadilan Agama Subang di bawah Nomor 4204/Pdt.G/2020/PA.Sbg. tanggal 26 November 2020, bermaksud mengajukan gugatan cerai terhadap Tergugat berdasarkan dalil-dalil yang pada pokoknya sebagai berikut :

1. Bahwa Penggugat saat ini bertempat tinggal di Kabupaten Subang, sebagaimana ternyata dari KTP atas nama Penggugat dengan NIK 32138760001, yang dikeluarkan oleh Pemerintah Kabupaten Subang Provinsi Jawa Barat tanggal 8 Maret 2020;

Disclaimer
Kepaniteraan Mahkamah Agung Republik Indonesia berusaha untuk selalu mencantumkan informasi paling kini dan akurat sebagai bentuk komitmen Mahkamah Agung untuk pelayanan publik, transparansi dan akuntabilitas pelaksanaan fungsi peradilan. Namun dalam hal-hal tertentu masih dimungkinkan terjadi permasalahan teknis terkait dengan akurasi dan keterkinian informasi yang kami sajikan, hal mana akan terus kami perbaiki dari waktu kewaktu.
Dalam hal Anda menemukan inakurasi informasi yang termuat pada situs ini atau informasi yang seharusnya ada, namun belum tersedia, maka harap segera hubungi Kepaniteraan Mahkamah Agung RI melalui :
Email : kepaniteraan@mahkamahagung.go.id Telp : 021-384 3348 (ext.318)

2. Bahwa pada tanggal 10 November 2000, Penggugat dengan Tergugat melangsungkan pernikahan menurut agama Islam di wilayah hukum Kantor Urusan Agama (KUA) Kecamatan Cisalak Kabupaten Subang dengan wali nikahnya adalah ayah kandung Penggugat yang bernama Bapak Parli dengan maskawin berupa uang Rp400.000,00 (empat ratus ribu rupiah) dibayar tunai dan disaksikan oleh 2 (dua) orang saksi yaitu Bapak Asep Suparno dan Bapak Engkos Koswara;
3. Bahwa pada saat pernikahan tersebut Penggugat berstatus perawan dan Tergugat berstatus jejaka, antara Penggugat dengan Tergugat pun tidak ada hubungan darah dan tidak sesusuan serta memenuhi syarat dan/atau tidak ada larangan untuk melangsungkan pernikahan, baik menurut ketentuan hukum Islam maupun peraturan perundang-undangan yang berlaku;
4. Bahwa setelah pernikahan tersebut Penggugat dan Tergugat bertempat tinggal di rumah orangtua Penggugat dan telah hidup rukun sebagaimana layaknya suami istri yang selama 20 (dua puluh) tahun namun belum dikaruniai keturunan;
5. Bahwa selama pernikahan tersebut tidak ada pihak ketiga yang mengganggu gugat pernikahan Penggugat dan Tergugat tersebut dan selama itu pula Penggugat dan Tergugat tetap beragama Islam;
6. Bahwa Penggugat dan Tergugat tidak pernah menerima Kutipan Akta Nikah dari Pegawai Pencatat Nikah Kantor Urusan Agama (KUA) Kecamatan Cisalak Kabupaten Subang, berdasarkan Surat Keterangan Nomor : B-0567/KUA.10.13.05/PW.01/11/2020 yang dikeluarkan oleh Kantor Urusan Agama (KUA) Kecamatan Cisalak Kabupaten Subang tanggal 24 November 2020, oleh karenanya Penggugat sangat membutuhkan Penetapan Pengesahan Nikah dari Pengadilan Agama Subang, guna dijadikan sebagai alasan hukum untuk perceraian di Pengadilan Agama Subang;
7. Bahwa rumah tangga Penggugat dan Tergugat awalnya bahagia dan sejahtera, namun sejak bulan Januari 2010 sering terjadi perselisihan dan pertengkaran yang disebabkan karena :

Disclaimer
Kepaniteraan Mahkamah Agung Republik Indonesia berusaha untuk selalu mencantumkan informasi paling kini dan akurat sebagai bentuk komitmen Mahkamah Agung untuk pelayanan publik, transparansi dan akuntabilitas pelaksanaan fungsi peradilan. Namun dalam hal-hal tertentu masih dimungkinkan terjadi permasalahan teknis terkait dengan akurasi dan keterkinian informasi yang kami sajikan, hal mana akan terus kami perbaiki dari waktu kewaktu.
Dalam hal Anda menemukan inakurasi informasi yang termuat pada situs ini atau informasi yang seharusnya ada, namun belum tersedia, maka harap segera hubungi Kepaniteraan Mahkamah Agung RI melalui :
Email : kepaniteraan@mahkamahagung.go.id   Telp : 021-384 3348 (ext.318)

- Tergugat kurang bertanggungjawab dalam memenuhi kebutuhan keluarga sehari-hari dimana nafkah yang diberikan Tergugat tidak menentu dan tidak bisa memenuhi kebutuhan keluarga sehari-hari;

8. Bahwa puncak pertengkaran Penggugat dan Tergugat terjadi pada bulan Desember 2013, yang akibatnya Tergugat pergi meninggalkan Penggugat dan tidak pernah hidup berumah tangga lagi sebagai suami istri, sampai saat ini sudah berpisah selama 7 (tujuh) tahun;
9. Bahwa selama berpisah tempat tinggal Penggugat tetap tinggal di rumah orangtua Penggugat, sedangkan Tergugat tinggal di rumah orangtua Tergugat;
10. Bahwa dengan adanya kejadian tersebut Penggugat tetap bersabar, dan sudah dilakukan upaya musyawarah antara Penggugat dan Tergugat beserta keluarga Penggugat dan Tergugat, namun hasilnya tetap nihil. Oleh karena rumah tangga Penggugat dengan Tergugat sudah tidak ada ketentraman dan keharmonisan maka Penggugat mengambil sikap dan keputusan untuk bercerai dengan Tergugat, karena sudah tidak sanggup lagi untuk tetap mempertahankan mahligai rumah tangga dengan Tergugat;
11. Bahwa dengan keadaan rumah tangga tersebut, Penggugat sudah tidak ada harapan untuk melanjutkan rumah tangga bersama Tergugat, sehingga tujuan perkawinan untuk membentuk rumah tangga yang sakinah, mawadah warohmah tidak dapat terwujud, dan apabila tetap dipertahankan hanya akan menimbulkan kemadorotan yang berkepanjangan;
12. Bahwa Penggugat bersedia menanggung biaya yang timbul akibat perkara ini;

Berdasarkan uraian tersebut diatas, Penggugat mohon kepada Ketua Pengadilan Agama Subang untuk memeriksa perkara ini, dan menjatuhkan putusan sebagai berikut :

1. Mengabulkan gugatan Penggugat;
2. Menyatakan sah perkawinan Penggugat (Penggugat) dan Tergugat (Tergugat) yang dilangsungkan pada tanggal 10 November 2000 di wilayah Hukum Kantor Urusan Agama Kecamatan Cisalak Kabupaten Subang

Disclaimer
Kepaniteraan Mahkamah Agung Republik Indonesia berusaha untuk selalu mencantumkan informasi paling kini dan akurat sebagai bentuk komitmen Mahkamah Agung untuk pelayanan publik, transparansi dan akuntabilitas pelaksanaan fungsi peradilan. Namun dalam hal-hal tertentu masih dimungkinkan terjadi permasalahan teknis terkait dengan akurasi dan keterkinian informasi yang kami sajikan, hal mana akan terus kami perbaiki dari waktu kewaktu.
Dalam hal Anda menemukan inakurasi informasi yang termuat pada situs ini atau informasi yang seharusnya ada, namun belum tersedia, maka harap segera hubungi Kepaniteraan Mahkamah Agung RI melalui :
Email : kepaniteraan@mahkamahagung.go.id   Telp : 021-384 3348 (ext.318)

3. Menjatuhkan talak satu ba'in shughra Tergugat (Tergugat) terhadap Penggugat (Penggugat);
4. Membebankan kepada Penggugat untuk membayar biaya perkara menurut hukum;

Apabila Majelis Hakim berpendapat lain, mohon putusan yang seadil-adilnya;

Bahwa pada hari dan tanggal yang telah ditentukan Penggugat datang menghadap persidangan, sedang Tergugat tidak datang menghadap atau menyuruh orang lain sebagai wakilnya yang sah, meskipun ia telah dipanggil dengan resmi dan patut untuk datang menghadap di sidang sebagaimana terbukti dari relaas panggilan Nomor 4204/Pdt.G/2020/PA.Sbg. tanggal 27 November 2020 dan tanggal 10 Desember 2020, sedangkan ternyata bahwa tidak hadirnya itu oleh Majelis Hakim dinilai tidak disebabkan sesuatu halangan yang sah;

Bahwa oleh karena Tergugat tidak hadir di persidangan, maka proses mediasi sebagaimana diamanatkan dalam PERMA Nomor 1 Tahun 2016 tidak dapat dilaksanakan;

Bahwa selanjutnya dibacakan surat gugatan Penggugat tertanggal 26 Oktober 2020, yang isinya tetap dipertahankan Penggugat;

Bahwa terhadap gugatan Penggugat tersebut, Tergugat tidak memberi jawaban karena tidak pernah hadir di persidangan dan pemeriksaan dilanjutkan kepada pembuktian;

Bahwa, di persidangan Penggugat telah mengajukan alat bukti (P) berupa Surat Keterangan Pernikahan Tidak Tercatat Nomor B-0567/KUA.10.13.05/PW.01/11/2020, tanggal 24 November 2020 atas nama Penggugat (Penggugat) dan Tergugat (Tergugat) yang dikeluarkan oleh Kantor Urusan Agama (KUA) Kecamatan Cisalak Kabupaten Subang;

Bahwa di samping bukti surat sebagaimana di atas, Penggugat juga mengajukan 2 (dua) orang saksi keluarga atau orang yang dekat dengan Penggugat, yang masing-masing bernama :

Disclaimer
Kepaniteraan Mahkamah Agung Republik Indonesia berusaha untuk selalu mencantumkan informasi paling kini dan akurat sebagai bentuk komitmen Mahkamah Agung untuk pelayanan publik, transparansi dan akuntabilitas pelaksanaan fungsi peradilan. Namun dalam hal-hal tertentu masih dimungkinkan terjadi permasalahan teknis terkait dengan akurasi dan keterkinian informasi yang kami sajikan, hal mana akan terus kami perbaiki dari waktu kewaktu.
Dalam hal Anda menemukan inakurasi informasi yang termuat pada situs ini atau informasi yang seharusnya ada, namun belum tersedia, maka harap segera hubungi Kepaniteraan Mahkamah Agung RI melalui :
Email : kepaniteraan@mahkamahagung.go.id   Telp : 021-384 3348 (ext.318)

1. **Saksi 1**, umur 51 tahun, agama Islam, pekerjaan Buruh, tempat tinggal di Kabupaten Subang, yang di bawah sumpah memberikan keterangan yang pada pokoknya sebagai berikut :
   - Bahwa saksi kenal kepada Penggugat yang bernama Ade Karwatin dan Tergugat yang bernama Nono Supriatna;
   - Bahwa saksi adalah sepupu Penggugat;
   - Bahwa saksi tahu dan hadir pada saat Penggugat menikah dengan Tergugat yang dilangsungkan di rumah orang tua Penggugat pada bulan November 2000 yang termasuk wilayah hukum Kantor Urusan Agama (KUA) Kecamatan Cisalak Kabupaten Subang;
   - Bahwa yang menjadi wali nikah adalah ayah kandung Penggugat yang bernama Bapak Parli, maskawinnya berupa uang Rp400.000,00 (empat ratus ribu rupiah) dibayar tunai;
   - Bahwa yang menjadi saksi nikah yaitu Bapak Asep Suparno dan Bapak Engkos Koswara, selain kedua saksi tersebut ada keluarga dan saudara yang menghadiri;
   - Bahwa yang melakukan ijab dalam pernikahan tersebut adalah ayah kandung Penggugat, dilangsungkan antara wali nikah secara langsung dengan qabulnya oleh Tergugat;
   - Bahwa antara Penggugat dengan Tergugat tidak ada hubungan darah atau hubungan sesusuan dan tidak ada hubungan perkawinan dengan pihak lain;
   - Bahwa setelah menikah Penggugat dan Tergugat tinggal bersama di rumah orangtua Penggugat, namun belum dikaruniai keturunan;
   - Bahwa selama Penggugat dan Tergugat tinggal bersama, tidak ada seorangpun diantara anggota masyarakat yang mempersoalkan dengan status hubungan suami istri antara Penggugat dan Tergugat;
   - Bahwa saksi mengetahui awalnya rumah tangga Penggugat dan Tergugat rukun dan harmonis, namun sejak awal tahun 2010 mulai tidak rukun karena sering terjadi perselisihan dan pertengkaran;
   - Bahwa penyebabnya karena nafkah yang diberikan Tergugat tidak menentu dan tidak bisa memenuhi kebutuhan keluarga sehari-hari;

Disclaimer
Kepaniteraan Mahkamah Agung Republik Indonesia berusaha untuk selalu mencantumkan informasi paling kini dan akurat sebagai bentuk komitmen Mahkamah Agung untuk pelayanan publik, transparansi dan akuntabilitas pelaksanaan fungsi peradilan. Namun dalam hal-hal tertentu masih dimungkinkan terjadi permasalahan teknis terkait dengan akurasi dan keterkinian informasi yang kami sajikan, hal mana akan terus kami perbaiki dari waktu kewaktu.
Dalam hal Anda menemukan inakurasi informasi yang termuat pada situs ini atau informasi yang seharusnya ada, namun belum tersedia, maka harap segera hubungi Kepaniteraan Mahkamah Agung RI melalui :
Email : kepaniteraan@mahkamahagung.go.id   Telp : 021-384 3348 (ext.318)

- Bahwa saksi mengetahui perselisihan dan pertengkaran tersebut karena saksi pernah 6 (enam) kali melihat pertengkaran antara Penggugat dan Tergugat;
- Bahwa Penggugat dan Tergugat sampai sekarang sudah berpisah selama 7 (tujuh) tahun, dimana Tergugat pergi meninggalkan Penggugat dan tidak pernah hidup berumah tangga lagi sebagai suami istri;
- Bahwa selama berpisah tempat tinggal, Penggugat tinggal di rumah orangtua Penggugat, sedangkan Tergugat tinggal di rumah orangtua Tergugat;
- Bahwa selama berpisah tempat tinggal Penggugat dan Tergugat sudah berusaha untuk memperbaiki rumah tangganya, namun upaya tersebut tidak berhasil;
- Bahwa saksi bersama keluarga kedua belah pihak sudah berusaha merukunkan, akan tetapi usaha tersebut tidak berhasil karena Penggugat tetap bersikeras ingin bercerai dengan Tergugat;
- Bahwa saksi tidak bersedia untuk mendamaikan kembali kedua belah pihak, karena keduanya sulit didamaikan;

2. **Saksi 1**, umur 49 tahun, agama Islam, pekerjaan Buruh, tempat tinggal di Kabupaten Subang, yang di bawah sumpah memberikan keterangan yang pada pokoknya sebagai berikut :
   - Bahwa saksi kenal kepada Penggugat dan Tergugat karena saksi adalah tetangga Penggugat;
   - Bahwa Penggugat dan Tergugat adalah suami istri yang sudah berumah tangga selama 20 (dua puluh) tahun namun belum dikaruniai keturunan;
   - Bahwa saksi tahu dan menghadiri pada saat pernikahan Penggugat dan Tergugat pada tahun 2000 yang dilangsungkan di rumah orangtua Penggugat yang termasuk wilayah hukum Kantor Urusan Agama (KUA) Kecamatan Cisalak Kabupaten Subang;
   - Bahwa yang menjadi wali nikah adalah ayah kandung Penggugat yang bernama Bapak Parli, maskawinnya berupa uang tunai namun saksi lupa berapa nominalnya;

Disclaimer
Kepaniteraan Mahkamah Agung Republik Indonesia berusaha untuk selalu mencantumkan informasi paling kini dan akurat sebagai bentuk komitmen Mahkamah Agung untuk pelayanan publik, transparansi dan akuntabilitas pelaksanaan fungsi peradilan. Namun dalam hal-hal tertentu masih dimungkinkan terjadi permasalahan teknis terkait dengan akurasi dan keterkinian informasi yang kami sajikan, hal mana akan terus kami perbaiki dari waktu kewaktu.
Dalam hal Anda menemukan inakurasi informasi yang termuat pada situs ini atau informasi yang seharusnya ada, namun belum tersedia, maka harap segera hubungi Kepaniteraan Mahkamah Agung RI melalui :
Email : kepaniteraan@mahkamahagung.go.id   Telp : 021-384 3348 (ext.318)

- Bahwa yang menjadi saksi nikah yaitu Bapak Asep Suparno dan Bapak Engkos Koswara, selain kedua saksi tersebut ada keluarga dan saudara yang menghadiri;
- Bahwa yang melakukan ijab dalam pernikahan tersebut adalah ayah kandung Penggugat, dilangsungkan antara wali nikah secara langsung dengan qabulnya oleh Tergugat
- Bahwa antara Penggugat dengan Tergugat tidak ada hubungan darah atau hubungan sesusuan dan tidak ada hubungan perkawinan dengan pihak lain;
- Bahwa setelah menikah Penggugat dan Tergugat tinggal bersama di rumah orangtua Penggugat;
- Bahwa selama Penggugat dan Tergugat tinggal bersama, tidak ada seorangpun diantara anggota masyarakat yang mempersoalkan dengan status hubungan suami istri antara Penggugat dan Tergugat;
- Bahwa Penggugat dan Tergugat sudah berpisah tempat tinggal sejak tahun 2013, dimana Tergugat pergi meninggalkan Penggugat dan tidak pernah hidup berumah tangga lagi sebagai suami istri sampai sekarang sudah berpisah sekitar 7 (tujuh) tahun;
- Bahwa selama berpisah tempat tinggal, Penggugat tinggal di rumah orangtua Penggugat, sedangkan Tergugat tinggal di rumah orangtua Tergugat;
- Bahwa sebelum berpisah, sejak bulan Januari 2010 antara Penggugat dan Tergugat sering terjadi perselisihan dan pertengkaran yang disebabkan karena Tergugat kurang bertanggungjawab dalam memenuhi kebutuhan keluarga sehari-hari;
- Bahwa saksi mengetahui perselisihan dan pertengkaran tersebut karena saksi pernah beberapa kali melihat ketika Penggugat dan Tergugat bertengkar;
- Bahwa selama berpisah tempat tinggal Penggugat dan Tergugat sudah berusaha untuk rukun, akan tetapi upaya tersebut tidak berhasil;

Disclaimer
Kepaniteraan Mahkamah Agung Republik Indonesia berusaha untuk selalu mencantumkan informasi paling kini dan akurat sebagai bentuk komitmen Mahkamah Agung untuk pelayanan publik, transparansi dan akuntabilitas pelaksanaan fungsi peradilan. Namun dalam hal-hal tertentu masih dimungkinkan terjadi permasalahan teknis terkait dengan akurasi dan keterkinian informasi yang kami sajikan, hal mana akan terus kami perbaiki dari waktu kewaktu.
Dalam hal Anda menemukan inakurasi informasi yang termuat pada situs ini atau informasi yang seharusnya ada, namun belum tersedia, maka harap segera hubungi Kepaniteraan Mahkamah Agung RI melalui :
Email : kepaniteraan@mahkamahagung.go.id Telp : 021-384 3348 (ext.318)

- Bahwa selama berpisah tempat tinggal, saksi belum pernah mendamaikan. Hanya menasehati Penggugat saja, tapi tidak berhasil karena Penggugat sudah tidak mau kembali kepada Tergugat;
- Bahwa saksi tidak bersedia mendamaikan kedua belah pihak karena sulit untuk didamaikan kembali;

Bahwa kemudian Penggugat mengajukan kesimpulan, yang pada pokoknya mengatakan bahwa berdasarkan dalil-dalil gugatan Penggugat dan bukti-bukti yang diajukan, gugatan Penggugat telah terbukti dan berdasar hukum, oleh karenanya patut dikabulkan dan selanjutnya mohon putusan;

Bahwa selanjutnya untuk mempersingkat uraian putusan ini, ditunjuklah hal-hal sebagaimana tercatat dalam berita acara sidang perkara ini sebagai bagian yang tidak terpisahkan dari putusan ini;

PERTIMBANGAN HUKUM

Menimbang, bahwa maksud dan tujuan gugatan Penggugat adalah sebagaimana diuraikan di atas;

Menimbang, bahwa berdasarkan relaas panggilan Nomor 4204/Pdt.G/2020/PA.Sbg., tanggal 27 November 2020 dan tanggal 10 Desember 2020, terbukti bahwa Tergugat telah dipanggil dengan patut untuk menghadap persidangan, tetapi Tergugat tidak menghadap persidangan dan tidak mengutus orang lain sebagai kuasanya yang sah untuk menghadap persidangan, sedang tidak terbukti bahwa tidak menghadapnya Tergugat ke persidangan tersebut berdasarkan alasan yang sah. Oleh karenanya, harus dinyatakan bahwa Tergugat tidak menghadap persidangan dan berdasarkan ketentuan Pasal 125 ayat (1) HIR Pengadilan dapat menjatuhkan putusan dengan verstek;

Menimbang, bahwa perkara ini adalah perkara perceraian. Berdasarkan azas hukum bahwa tidak ada perceraian jika tidak ada perkawinan, maka sebelum mempertimbangkan tentang perceraiannya Majelis Hakim akan mempertimbangkan terlebih dahulu mengenai ada tidaknya perkawinan antara Penggugat dan Tergugat;

Disclaimer
Kepaniteraan Mahkamah Agung Republik Indonesia berusaha untuk selalu mencantumkan informasi paling kini dan akurat sebagai bentuk komitmen Mahkamah Agung untuk pelayanan publik, transparansi dan akuntabilitas pelaksanaan fungsi peradilan. Namun dalam hal-hal tertentu masih dimungkinkan terjadi permasalahan teknis terkait dengan akurasi dan keterkinian informasi yang kami sajikan, hal mana akan terus kami perbaiki dari waktu kewaktu.
Dalam hal Anda menemukan inakurasi informasi yang termuat pada situs ini atau informasi yang seharusnya ada, namun belum tersedia, maka harap segera hubungi Kepaniteraan Mahkamah Agung RI melalui :
Email : kepaniteraan@mahkamahagung.go.id   Telp : 021-384 3348 (ext.318)

Menimbang, bahwa selain mengajukan gugatan cerai, Penggugat juga telah mengajukan permohonan penetapan nikah karena pernikahan Penggugat dengan Tergugat tidak tercatat di Kantor Urusan Agama tempat pernikahan Penggugat dengan Tergugat dilangsungkan;

Menimbang, bahwa sebelum memeriksa pokok perkara tentang perceraian Penggugat dengan Tergugat pertama-tama Majelis akan mempertimbangkan tentang pernikahan Penggugat dengan Tergugat;

Menimbang, bahwa untuk memperkuat dalil gugatannya, bahwa Penggugat telah menikah dengan Tergugat, Penggugat telah mengajukan bukti surat berupa Surat Keterangan Pernikahan Tidak Tercatat Nomor B-0567/KUA.10.13.05/PW.01/11/2020, tanggal 24 November 2020 atas nama Penggugat (Penggugat) dan Tergugat (Tergugat) yang dikeluarkan oleh Kantor Urusan Agama (KUA) Kecamatan Cisalak Kabupaten Subang (bukti P);

Menimbang, bahwa berdasarkan bukti P terbukti bahwa pernikahan Penggugat dengan Tergugat tidak tercatat di Kantor Urusan Agama (KUA) Kecamatan Cisalak Kabupaten Subang, wilayah tempat pernikahan Penggugat dengan Tergugat dilangsungkan;

Menimbang, bahwa selain bukti-bukti surat di atas, Penggugat juga telah mengajukan 2 (dua) orang saksi yang telah memberi keterangan di bawah sumpahnya masing-masing;

Menimbang, bahwa berdasarkan keterangan Penggugat dan keterangan saksi-saksi yang satu sama lain saling bersesuaian, Majelis telah mendapatkan fakta sebagai berikut :

– Bahwa Penggugat telah menikah dengan Tergugat pada tanggal 10 November 2000 di rumah orang tua Penggugat dengan wali nikah adalah ayah kandung Penggugat, saksi nikahnya Bapak Asep Suparno dan Bapak Engkos Koswara, maskawin berupa uang Rp400.000,00 (empat ratus ribu rupiah) dibayar tunai, yang melakukan ijab adalah ayah kandung Penggugat, dilangsungkan antara wali nikah secara langsung dengan qabulnya oleh Tergugat;
– Bahwa pernikahan Penggugat dengan Tergugat dilaksanakan berdasarkan tata cara agama Islam;

Halaman 9 dari 16 halaman Putusan Nomor 4204/Pdt.G/2020/PA.Sbg.

Disclaimer
Kepaniteraan Mahkamah Agung Republik Indonesia berusaha untuk selalu mencantumkan informasi paling kini dan akurat sebagai bentuk komitmen Mahkamah Agung untuk pelayanan publik, transparansi dan akuntabilitas pelaksanaan fungsi peradilan. Namun dalam hal-hal tertentu masih dimungkinkan terjadi permasalahan teknis terkait dengan akurasi dan keterkinian informasi yang kami sajikan, hal mana akan terus kami perbaiki dari waktu kewaktu.
Dalam hal Anda menemukan inakurasi informasi yang termuat pada situs ini atau informasi yang seharusnya ada, namun belum tersedia, maka harap segera hubungi Kepaniteraan Mahkamah Agung RI melalui :
Email : kepaniteraan@mahkamahagung.go.id Telp : 021-384 3348 (ext.318)

- Bahwa syarat rukun pernikahan telah terpenuhi yaitu adanya calon mempelai, adanya wali nikah, ada 2 (dua) orang saksi nikah, ada ijab kabul antara Tergugat dengan Wali Nikah dan ada mahar yang telah dibayar tunai oleh Tergugat;
- Bahwa antara Penggugat dengan Tergugat tidak ada hubungan darah, dan tidak ada hubungan susuan serta tidak ada hubungan perkawinan dengan pihak lain;
- Bahwa antara Penggugat dengan Tergugat tidak ada halangan pernikahan baik menurut syara' maupun menurut perundang-undangan yang berlaku;

Menimbang, bahwa berdasarkan fakta-fakta tersebut di atas, Majelis berkesimpulan bahwa antara Penggugat dengan Tergugat telah terjadi pernikahan secara sah berdasarkan agama Islam yang dilangsungkan pada tanggal 10 November 2000 di wilayah Kantor Urusan Agama (KUA) Kecamatan Cisalak Kabupaten Subang, dengan demikian dalil-dalil Penggugat tentang pernikahannya telah terbukti oleh karenanya Penggugat dan Tergugat dinyatakan telah terikat dalam perkawinan yang sah;

Menimbang, bahwa permohonan Penggugat untuk ditetapkan pernikahannya dengan Tergugat diajukan dalam rangka penyelesaian perceraian, dan pernikahan Penggugat dengan Tergugat dilaksanakan berdasarkan tata cara agama Islam yang dianut Penggugat dengan Tergugat serta antara Penggugat dan Tergugat tidak ada halangan perkawinan, maka berdasarkan pasal 7 ayat (3) huruf (a), (c) dan (e) Kompilasi Hukum Islam maka permohonan Penggugat untuk ditetapkan pernikahannya dengan Tergugat dapat dikabulkan;

Menimbang, bahwa Penggugat dengan Tergugat telah terikat dalam perkawinan yang sah dan selama perkawinannya belum pernah bercerai, maka Penggugat dan Tergugat mempunyai kualifikasi untuk mengajukan gugatan perceraian, oleh karenanya pemeriksaan dapat dilanjutkan pada pokok perkara tentang perceraian Penggugat dengan Tergugat;

Menimbang, bahwa sesuai dengan pasal 137 HIR dan pasal 54 Undang-undang Nomor 7 Tahun 1989 yang dirubah dengan Undang-undang Nomor: 3 Tahun 2006 jo. Pasal 22 ayat (2) Peraturan Pemerintah Nomor 9

Disclaimer
Kepaniteraan Mahkamah Agung Republik Indonesia berusaha untuk selalu mencantumkan informasi paling kini dan akurat sebagai bentuk komitmen Mahkamah Agung untuk pelayanan publik, transparansi dan akuntabilitas pelaksanaan fungsi peradilan. Namun dalam hal-hal tertentu masih dimungkinkan terjadi permasalahan teknis terkait dengan akurasi dan keterkinian informasi yang kami sajikan, hal mana akan terus kami perbaiki dari waktu kewaktu.
Dalam hal Anda menemukan inakurasi informasi yang termuat pada situs ini atau informasi yang seharusnya ada, namun belum tersedia, maka harap segera hubungi Kepaniteraan Mahkamah Agung RI melalui :
Email : kepaniteraan@mahkamahagung.go.id Telp : 021-384 3348 (ext.318)

Tahun 1975 jo. Pasal 134 Kompilasi Hukum Islam, bahwa Penggugat telah mengajukan bukti surat-surat bukti (P) serta telah mengajukan 2 (dua) orang saksi yaitu (Bapak Suhendar) dan (Bapak Surahya) yang telah memberikan keterangan masing-masing di bawah sumpahnya;

Menimbang, bahwa dalam perkara ini Penggugat mohon kepada Pengadilan agar menjatuhkan talak satu bain sughra Tergugat terhadap Penggugat berdasarkan dalil yang pada pokoknya, bahwa rumah tangga Penggugat dan Tergugat sudah tidak harmonis lagi sejak bulan Januari 2010 karena sering terjadi perselisihan dan pertengkaran yang disebabkan oleh karena Tergugat kurang bertanggungjawab dalam memenuhi kebutuhan keluarga sehari-hari dimana nafkah yang diberikan Tergugat tidak menentu dan tidak bisa memenuhi kebutuhan keluarga sehari-hari, yang mencapai puncaknya pada bulan Desember 2013 sehingga tidak ada harapan untuk hidup rukun lagi dalam rumah tangga. Sekarang antara Penggugat dan Tergugat telah berpisah tempat tidak pernah bersatu lagi selama 7 (tujuh) tahun dan selama berpisah tempat tinggal Penggugat dengan Tergugat sudah didamaikan agar rukun kembali dalam rumah tangganya namun tidak berhasil;

Menimbang, bahwa terhadap gugatan Penggugat tersebut, Tergugat tidak memberi jawaban karena tidak pernah hadir di persidangan. Namun demikian, karena perkara ini adalah perkara perceraian, maka untuk dikabulkan gugatan, Penggugat harus membuktikan kebenaran dalil gugatannya.

Menimbang, bahwa untuk membuktikan kebenaran dalil gugatannya, Penggugat telah mengajukan 2 (dua) orang saksi;

Menimbang, bahwa oleh karena kedua saksi yang diajukan Penggugat adalah orang yang dekat dengannya, maka Majelis Hakim menjadikan kedua saksi tersebut sebagai alat bukti mengenai dalil gugatannya, juga sebagai saksi untuk memenuhi ketentuan Pasal 22 ayat (2) Peraturan Pemerintah Nomor 9 tahun 1975 jo Pasal 76 ayat (1) Undang Undang Nomor 7 Tahun 1989, sebagaimana telah diubah dengan Undang-undang No. 3 tahun 2006 dan diubah untuk kedua kalinya dengan Undang-undang No. 50 tahun 2009, yang menentukan, bahwa gugatan perceraian dapat diterima apabila telah cukup jelas bagi Pengadilan mengenai sebab-sebab perselisihan dan pertengkaran itu

Disclaimer
Kepaniteraan Mahkamah Agung Republik Indonesia berusaha untuk selalu mencantumkan informasi paling kini dan akurat sebagai bentuk komitmen Mahkamah Agung untuk pelayanan publik, transparansi dan akuntabilitas pelaksanaan fungsi peradilan. Namun dalam hal-hal tertentu masih dimungkinkan terjadi permasalahan teknis terkait dengan akurasi dan keterkinian informasi yang kami sajikan, hal mana akan terus kami perbaiki dari waktu kewaktu.
Dalam hal Anda menemukan inakurasi informasi yang termuat pada situs ini atau informasi yang seharusnya ada, namun belum tersedia, maka harap segera hubungi Kepaniteraan Mahkamah Agung RI melalui :
Email : kepaniteraan@mahkamahagung.go.id Telp : 021-384 3348 (ext.318)

dan setelah mendengar keterangan pihak keluarga serta orang-orang yang dekat dengan suami istri.

Menimbang, bahwa kedua saksi atau orang dekat dengan Penggugat tersebut telah memenuhi syarat formil maupun materiil sebagai saksi, maka keterangan kedua saksi atau orang dekat tersebut dapat dipertimbangkan sebagai alat bukti dalam perkara ini.

Menimbang, bahwa ternyata isi keterangan kedua saksi tersebut sesuai dengan dalil-dalil yang dikemukakan oleh Penggugat, bahwa antara Penggugat dan Tergugat telah terjadi pisah tempat tinggal sejak bulan Desember 2013 yang disebabkan oleh pertengkaran karena nafkah yang diberikan Tergugat tidak menentu dan tidak bisa memenuhi kebutuhan sehari-hari sehingga Tergugat kurang bertanggungjawab terhadap keluarga, selama pisah tinggal sudah ada upaya untuk rukun kembali dalam membina rumah tangganya dan kedua saksi keluarga atau orang yang dekat dengan Penggugat tersebut telah menasehati Penggugat agar rukun lagi dengan Tergugat tetapi tidak berhasil.

Menimbang, bahwa berdasarkan hal-hal sebagaimana diuraikan di atas, Majelis Hakim mendapatkan fakta-fakta peristiwa sebagai berikut:

1. Bahwa Penggugat dengan Tergugat telah terikat dalam perkawinan yang sah namun belum dikaruniai keturunan;
2. Bahwa antara Penggugat dan Tergugat telah dan terjadi pisah tempat tinggal sejak bulan Desember 2013 sampai sekarang tidak pernah bersatu lagi selama 7 (tujuh) tahun;
3. Bahwa terjadinya pisah tempat tinggal tersebut disebabkan perselisihan dan pertengkaran karena nafkah yang diberikan Tergugat tidak menentu dan tidak bisa memenuhi kebutuhan sehari-hari sehingga Tergugat kurang bertanggungjawab terhadap keluarga;
4. Bahwa selama berpisah tempat tinggal Penggugat dan Tergugat sudah berusaha rukun akan tetapi upaya tersebut tidak berhasil;
5. Bahwa para saksi atau orang yang dekat dengan Penggugat telah menasehati Penggugat agar rukun kembali dengan Tergugat tetapi tidak berhasil dan dan para saksi tidak bersedia untuk merukunkan kembali karena keduanya sulit untuk didamaikan;

Disclaimer
Kepaniteraan Mahkamah Agung Republik Indonesia berusaha untuk selalu mencantumkan informasi paling kini dan akurat sebagai bentuk komitmen Mahkamah Agung untuk pelayanan publik, transparansi dan akuntabilitas pelaksanaan fungsi peradilan. Namun dalam hal-hal tertentu masih dimungkinkan terjadi permasalahan teknis terkait dengan akurasi dan keterkinian informasi yang kami sajikan, hal mana akan terus kami perbaiki dari waktu kewaktu.
Dalam hal Anda menemukan inakurasi informasi yang termuat pada situs ini atau informasi yang seharusnya ada, namun belum tersedia, maka harap segera hubungi Kepaniteraan Mahkamah Agung RI melalui :
Email : kepaniteraan@mahkamahagung.go.id   Telp : 021-384 3348 (ext.318)

Menimbang, bahwa dengan telah terbuktinya dalil Penggugat, bahwa antara Penggugat dan Tergugat telah terjadi pisah tempat tinggal sejak bulan Desember 2013 sampai sekarang tidak pernah bersatu lagi selama 7 (tujuh) tahun, di mana menurut yurisprudensi MARI No. 379 K/AG/1995 tanggal 26 Maret 1997, suami istri yang tidak berdiam serumah lagi dan tidak ada harapan untuk hidup rukun kembali, maka rumah tangga tersebut terbukti telah retak dan pecah;

Menimbang, bahwa keadaan hubungan antara Penggugat dan Tergugat yang demikian, menurut Majelis Hakim dapat dikualifikasi sebagai telah terjadi perselisihan dan pertengkaran terus menerus dan tidak ada harapan akan hidup rukun lagi dalam rumah tangga sebagaimana dimaksud oleh Pasal 19 huruf f Peraturan Pemerintah Nomor 9 Tahun 1975 jo Pasal 116 huruf f Kompilasi Hukum Islam (Instruksi Presiden Nomor 1 Tahun 1991);

Menimbang, bahwa berdasarkan fakta-fakta di atas Majelis Hakim berkesimpulan bahwa antara Penggugat dan Tergugat telah terjadi perselisihan dan pertengkaran terus menerus dan tidak ada harapan akan hidup rukun lagi dalam rumah tangga, sehingga harapan untuk mewujudkan tujuan rumah tangga yang bahagia, sakinah, mawaddah warahmah, sebagaimana yang dikehendaki oleh Pasal 1 Undang-undang Nomor 1 Tahun 1974 jo Pasal 3 Kompilasi Hukum Islam sulit terwujud. Rumah tangga Penggugat dan Tergugat yang demikian berarti telah pecah dan tidak layak untuk dipertahankan;

Menimbang, bahwa dengan tidak hadirnya Tergugat di persidangan memberi petunjuk bahwa Tergugat tidak menghendaki rukun lagi dengan Penggugat dalam rumah tangga;

Menimbang, bahwa berdasarkan pertimbangan-pertimbangan di atas Majelis Hakim berpendapat bahwa gugatan Penggugat telah memenuhi ketentuan Pasal 39 ayat (1) dan ayat (2) Undang-undang No. 1 Tahun 1974, bahwa perceraian hanya dapat dilakukan di depan sidang Pengadilan setelah Pengadilan yang bersangkutan berusaha dan tidak berhasil mendamaikan kedua belah pihak. Untuk melakukan perceraian harus ada cukup alasan bahwa antara suami-istri itu tidak akan dapat hidup rukun sebagai suami-istri. Demikian juga gugatan Penggugat telah memenuhi ketentuan Pasal 19 huruf (f)

Disclaimer
Kepaniteraan Mahkamah Agung Republik Indonesia berusaha untuk selalu mencantumkan informasi paling kini dan akurat sebagai bentuk komitmen Mahkamah Agung untuk pelayanan publik, transparansi dan akuntabilitas pelaksanaan fungsi peradilan. Namun dalam hal-hal tertentu masih dimungkinkan terjadi permasalahan teknis terkait dengan akurasi dan keterkinian informasi yang kami sajikan, hal mana akan terus kami perbaiki dari waktu kewaktu.
Dalam hal Anda menemukan inakurasi informasi yang termuat pada situs ini atau informasi yang seharusnya ada, namun belum tersedia, maka harap segera hubungi Kepaniteraan Mahkamah Agung RI melalui :
Email : kepaniteraan@mahkamahagung.go.id Telp : 021-384 3348 (ext.318)

Peraturan Pemerintah Nomor 9 Tahun 1975 jo Pasal 116 huruf (f) Kompilasi Hukum Islam, yaitu antara suami istri telah terjadi perselisihan dan pertengkaran terus menerus dan tidak ada harapan akan hidup rukun lagi dalam rumah tangga. Oleh karena itu, gugatan Penggugat patut dikabulkan;

Menimbang, bahwa menurut doktrin hukum Islam, sebagaimana ditulis dalam kitab Fiqhussunnah Jilid VIII halaman 121-122: yang artinya : *"Bahwa apabila gugatan istri telah terbukti berdasarkan alat bukti yang diajukan oleh istri atau berdasarkan pengakuan suami, sedang adanya gangguan tersebut merupakan penghalang atas kelangsungan pergaulan hidup (suami istri) sebagaimana (pergaulan hidup suami istri lain) yang sepadan dengan mereka, sementara hakim tidak berhasil mendamaikan mereka, maka hakim harus menjatuhkan gugat suaminya terhadap diri Penggugat dengan gugat bain)."* Sedang menurut ketentuan Pasal 119 ayat (2) Kompilasi Hukum Islam, salah satu talak bain sughra adalah talak yang dijatuhkan oleh Pengadilan Agama;

Menimbang, bahwa dalam perkara ini ternyata Penggugat dapat membuktikan kebenaran dalil gugatannya, dan hakim telah gagal mendamaikan keduanya, maka Majelis Hakim akan menjatuhkan talak satu bain sughra Tergugat atas Penggugat;

Menimbang, bahwa berdasarkan ketentuan Pasal 89 ayat (1) Undang-Undang Nomor 7 Tahun 1989 yang telah diubah dan ditambah dengan Undang Undang Nomor 3 Tahun 2006 terakhir dirubah dengan Undang Undang Nomor 50 Tahun 2009, maka semua biaya yang timbul dalam perkara ini dibebankan kepada Penggugat;

Memperhatikan ketentuan Pasal 125 HIR dan ketentuan hukum lainnya yang berkaitan dengan perkara ini;

M E N G A D I L I

1. Menyatakan bahwa Tergugat yang telah dipanggil secara resmi dan patut untuk menghadap persidangan, tidak hadir;
2. Mengabulkan gugatan Penggugat dengan verstek;

Disclaimer
Kepaniteraan Mahkamah Agung Republik Indonesia berusaha untuk selalu mencantumkan informasi paling kini dan akurat sebagai bentuk komitmen Mahkamah Agung untuk pelayanan publik, transparansi dan akuntabilitas pelaksanaan fungsi peradilan. Namun dalam hal-hal tertentu masih dimungkinkan terjadi permasalahan teknis terkait dengan akurasi dan keterkinian informasi yang kami sajikan, hal mana akan terus kami perbaiki dari waktu kewaktu.
Dalam hal Anda menemukan inakurasi informasi yang termuat pada situs ini atau informasi yang seharusnya ada, namun belum tersedia, maka harap segera hubungi Kepaniteraan Mahkamah Agung RI melalui :
Email : kepaniteraan@mahkamahagung.go.id   Telp : 021-384 3348 (ext.318)

3. Menyatakan sah perkawinan Penggugat (Penggugat) dengan Tergugat (Tergugat) yang dilangsungkan pada tanggal 10 November 2000 di wilayah Kantor Urusan Agama (KUA) Kecamatan Cisalak Kabupaten Subang;
4. Menjatuhkan talak satu ba'in sughra Tergugat (Tergugat) terhadap Penggugat (Penggugat);
5. Membebankan biaya perkara ini kepada Penggugat yang hingga kini dihitung sebesar Rp416.000,00 (empat ratus enam belas ribu rupiah);

Demikian dijatuhkan putusan ini dalam musyawarah Majelis Hakim yang dilangsungkan pada hari **Selasa**, tanggal **15 Desember 2020 Masehi** bertepatan dengan tanggal **29 Rabiul Akhir 1442 Hijriyah** oleh kami **Drs. Ojon Rianklana**, sebagai Ketua Majelis, **Dra. Hj. Sunaeah, M.H.** dan **Dra. Hj. Dadah Holidah, M.H.**, masing-masing sebagai Hakim Anggota. Putusan tersebut pada hari itu juga diucapkan Hakim Ketua dalam sidang terbuka untuk umum dan didampingi oleh Hakim-Hakim Anggota dengan dibantu oleh **Priyo Wicaksono, S.Kom., S.Sy.**, sebagai Panitera Pengganti serta dihadiri oleh Penggugat tanpa hadirnya Tergugat.

Hakim Anggota,

ttd.

Dra. Hj. Sunaeah, M.H.

Ketua Majelis,

ttd.

Drs. Ojon Rianklana

Hakim Anggota,

ttd.

Dra. Hj. Dadah Holidah, M.H.

Panitera Pengganti,

Disclaimer
Kepaniteraan Mahkamah Agung Republik Indonesia berusaha untuk selalu mencantumkan informasi paling kini dan akurat sebagai bentuk komitmen Mahkamah Agung untuk pelayanan publik, transparansi dan akuntabilitas pelaksanaan fungsi peradilan. Namun dalam hal-hal tertentu masih dimungkinkan terjadi permasalahan teknis terkait dengan akurasi dan keterkinian informasi yang kami sajikan, hal mana akan terus kami perbaiki dari waktu kewaktu.
Dalam hal Anda menemukan inakurasi informasi yang termuat pada situs ini atau informasi yang seharusnya ada, namun belum tersedia, maka harap segera hubungi Kepaniteraan Mahkamah Agung RI melalui :
Email : kepaniteraan@mahkamahagung.go.id Telp : 021-384 3348 (ext.318)

ttd.

Priyo Wicaksono, S.Kom., S.Sy.

Perincian Biaya :

1. Pendaftaran	:	Rp30.000,00
2. Proses	:	Rp50.000,00
3. Panggilan	:	Rp300.000,00
4. PNBP Panggilan	:	Rp20.000,00
5. Redaksi	:	Rp10.000,00
6. Meterai	:	Rp6.000,00
**Jumlah**	**:**	**Rp416.000,00**

(empat ratus enam belas ribu rupiah)

Disclaimer
Kepaniteraan Mahkamah Agung Republik Indonesia berusaha untuk selalu mencantumkan informasi paling kini dan akurat sebagai bentuk komitmen Mahkamah Agung untuk pelayanan publik, transparansi dan akuntabilitas pelaksanaan fungsi peradilan. Namun dalam hal-hal tertentu masih dimungkinkan terjadi permasalahan teknis terkait dengan akurasi dan keterkinian informasi yang kami sajikan, hal mana akan terus kami perbaiki dari waktu kewaktu.
Dalam hal Anda menemukan inakurasi informasi yang termuat pada situs ini atau informasi yang seharusnya ada, namun belum tersedia, maka harap segera hubungi Kepaniteraan Mahkamah Agung RI melalui :
Email : kepaniteraan@mahkamahagung.go.id Telp : 021-384 3348 (ext.318)
