# Supplementary material for: Classification of divorce causes during the COVID-19 pandemic using convolutional neural networks
Source: PeerJ Comput Sci. 2022 Jun 30;8:e998. doi: 10.7717/peerj-cs.998 (PMC9299239; doi:10.7717/peerj-cs.998)
Supplement: Supplemental Information 3 [file peerj-cs-08-998-s003.zip › KDRT Dataset/Data ke-20.pdf]

P U T U S A N

Nomor 989/Pdt.G/2020/PA.Pal

بِسْمِ اللهِ الرَّحْمٰنِ الرَّحِيْمِ

DEMI KEADILAN BERDASARKAN KETUHANAN YANG MAHA ESA

Pengadilan Agama Palu yang memeriksa dan mengadili perkara tertentu pada tingkat pertama dalam sidang Majelis Hakim telah menjatuhkan putusan sebagai berikut dalam perkara Cerai Talak antara:

PEMOHON, umur 32 tahun, agama Islam, pendidikan SLTA, pekerjaan xxxxxxx xx xxxxxx xxxxx xxxxxxxxxx xxxxxx xxxxxxxx xxxxxxxx xxxxxx, tempat kediaman di KOTA PALU, sebagai Pemohon;

melawan

TERMOHON, umur 25 tahun, agama Islam, pendidikan D3, pekerjaan xxxxxx xxxxx, tempat kediaman di KABUPATEN SIGI, PROVINSI SULAWESI TENGAH, sebagai Termohon;

Pengadilan Agama tersebut;

Telah membaca dan mempelajari berkas perkara;

Telah mendengar keterangan Pemohon serta para saksi di muka sidang ;

DUDUK PERKARA

Menimbang, bahwa Pemohon dengan surat permohonannya tertanggal 25 November 2020 yang terdaftar di Kepaniteraan Pengadilan Agama Palu pada tanggal 26 November 2020 dengan register perkara Nomor 989/Pdt.G/2020/PA.Pal, mengemukakan hal-hal sebagai berikut:

1. Bahwa pada tanggal 25 Agustus 2018, Pemohon dan Termohon melangsungkan pernikahan sebagaimana tercatat pada Kutipan Akta Nikah Nomor : 0175/ 007/VIII/2018 tanggal 27 Agustus 2018 yang dikeluarkan oleh Pegawai Pencatat Nikah Kantor Urusan Agama xxxxxxxxx xxxxxxxx, xxxxxxxxx xxxx, xxxxxxxx xxxxxxxx xxxxxx;

Disclaimer
Kepaniteraan Mahkamah Agung Republik Indonesia berusaha untuk selalu mencantumkan informasi paling kini dan akurat sebagai bentuk komitmen Mahkamah Agung untuk pelayanan publik, transparansi dan akuntabilitas pelaksanaan fungsi peradilan. Namun dalam hal-hal tertentu masih dimungkinkan terjadi permasalahan teknis terkait dengan akurasi dan keterkinian informasi yang kami sajikan, hal mana akan terus kami perbaiki dari waktu kewaktu.
Dalam hal Anda menemukan inakurasi informasi yang termuat pada situs ini atau informasi yang seharusnya ada, namun belum tersedia, maka harap segera hubungi Kepaniteraan Mahkamah Agung RI melalui :
Email : kepaniteraan@mahkamahagung.go.id   Telp : 021-384 3348 (ext.318)

2. Bahwa setelah menikah Pemohon dan Termohon tinggal bersama di rumah orang tua Pemohon di Palu. Selama pernikahan antara Pemohon dengan Termohon telah hidup sebagaimana layaknya suami istri namun belum di karunia anak;
3. Bahwa sejak awal berumah tangga antara Pemohon dan Termohon hidup rukun dan harmonis, namun sejak akhir bulan Agustus 2019 Pemohon dan Termohon sering terlibat perselisihan yang disebabkan karena :
   a. Termohon memiliki hubungan dengan pria idaman lain, yang di mana Teman Termohon yang melapor kepada Pemohon, dan di akui oleh Termohon;
   b. Termohon tidak mendengarkan perkataan dan nasehat Pemohon, dan tidak menghormati Pemohon sebagai Suami ;
4. Bahwa Pemohon selalu berusah sabar menghadapi tingkah lakuTermohon, namun puncak dari ketidakharmonisan tersebut terjadi pada akhir bulan September 2020, dimana pada saat itu Termohon dan Pemohon terlibat pertengkaran yang disebabkan karena Termohon ketahuan lagi memiliki hubungan dengan pria idaman lain melalui handpone milik Termohon dan Termohon akui;
5. Bahwa karena sikap Termohon yang tidak mau menghargai Pemohon lagi sebagai kepalah rumah tangga sehingga Pemohon memulangkan Termohon ke rumah orang tuanya, pihak orang tua berusaha mendamaikan Termohon dan Pemohon namun Termohon tidak mau kembali kepada Pemohon, Termohon berkeras mau berpisah dengan Pemohon;
6. Bahwa akibat dari permasalahan tersebut sehingga mengakibatkan antara Pemohon dan Termohon telah berpisah sejak akhir bulan September 2020 sampai sekarang kurang lebih 2 bulan lamanya ;
7. Bahwa Pemohon tidak mampu lagi mempertahankan rumah tangga ini karena khawatir jika diteruskan akan terjadi hal-hal yang tidak diinginkan dikemudian hari, oleh karena itu Pemohon memutuskan untuk bercerai ;

Disclaimer
Kepaniteraan Mahkamah Agung Republik Indonesia berusaha untuk selalu mencantumkan informasi paling kini dan akurat sebagai bentuk komitmen Mahkamah Agung untuk pelayanan publik, transparansi dan akuntabilitas pelaksanaan fungsi peradilan. Namun dalam hal-hal tertentu masih dimungkinkan terjadi permasalahan teknis terkait dengan akurasi dan keterkinian informasi yang kami sajikan, hal mana akan terus kami perbaiki dari waktu kewaktu.
Dalam hal Anda menemukan inakurasi informasi yang termuat pada situs ini atau informasi yang seharusnya ada, namun belum tersedia, maka harap segera hubungi Kepaniteraan Mahkamah Agung RI melalui :
Email : kepaniteraan@mahkamahagung.go.id Telp : 021-384 3348 (ext.318)

Bahwa berdasarkan alasan-alasan tersebut di atas, Pemohon mohon kepada Bapak Ketua Pengadilan Agama Palu cq. Majelis Hakim yang memeriksa perkara ini berkenan memutuskan sebagai berikut :

Primer

1. Mengabulkan permohonan Pemohon ;
2. Memberi izin kepada Pemohon (PEMOHON) untuk menjatuhkan talak satu raj'i kepada Termohon (TERMOHON) di hadapan sidang Pengadilan Agama Palu ;
3. Membebankan biaya perkara menurut hukum ;

Subsider :

Apabila Pengadilan Agama Palu berpendapat lain, mohon putusan yang seadil-adilnya ;

Bahwa pada hari-hari sidang yang telah ditetapkan, Pemohon datang menghadap sendiri di persidangan, sedangkan Termohon tidak pernah datang menghadap dan tidak pula menyuruh orang lain untuk menghadap sebagai wakil atau kuasanya yang sah, meskipun telah dipanggil secara resmi dan patut dan tidak ternyata ketidakhadirannya tersebut disebabkan oleh suatu halangan yang sah, maka perkara ini diperiksa tanpa hadirnya Termohon ;

Bahwa Majelis Hakim telah berusaha mendamaikan dengan menasehati Pemohon agar mau kembali rukun bersama Termohon akan tetapi tidak berhasil, sedangkan upaya mediasi tidak dapat dilakukan karena Termohon tidak pernah menghadiri sidang, maka selanjutnya dibacakannlah surat permohonan Pemohon didalam sidang tertutup untukj umum yang isinya tetap dipertahankan oleh Pemohon ;

Bahwa untuk menguatkan dalil-dalil permohonannya, Pemohon mengajukan alat bukti surat dan saksi-saksi sebagai berikut :

1. **Bukti Surat**

Fotokopi Kutipan Akta Nikah dari Kantor Urusan Agama xxxxxxxxx xxxxxxxx xxxxxxxxx xxxx xxxxxxxx xxxxxxxx xxxxxx, Nomor xxxxx, xxxxx, bukti surat tersebut telah diberi meterai cukup, telah dicap pos

Disclaimer
Kepaniteraan Mahkamah Agung Republik Indonesia berusaha untuk selalu mencantumkan informasi paling kini dan akurat sebagai bentuk komitmen Mahkamah Agung untuk pelayanan publik, transparansi dan akuntabilitas pelaksanaan fungsi peradilan. Namun dalam hal-hal tertentu masih dimungkinkan terjadi permasalahan teknis terkait dengan akurasi dan keterkinian informasi yang kami sajikan, hal mana akan terus kami perbaiki dari waktu kewaktu.
Dalam hal Anda menemukan inakurasi informasi yang termuat pada situs ini atau informasi yang seharusnya ada, namun belum tersedia, maka harap segera hubungi Kepaniteraan Mahkamah Agung RI melalui :
Email : kepaniteraan@mahkamahagung.go.id   Telp : 021-384 3348 (ext.318)

dan telah dicocokkan dengan aslinya yang ternyata sesuai, lalu oleh Ketua Majelis diberi tanda P...

2. **Bukti Saksi.**

Saksi 1, **SAKSI 1**, umur 30 tahun, agama Islam, pendidikan S1, pekerjaan xxxxxxx xxxxx xxxxx xxxxxxxxxx, bertempat tinggal di jalan agusalim no 29 Kelurahan baru Kecamatan palu barat kota palu, di bawah sumpah, memberikan keterangan yang pada pokoknya sebagai berikut ::

- Bahwa saksi tahu antara Pemohon dan Termohon adalah suami istri sah menikah pada tahun 2018 ;
- Bahwa setelah menikah Pemohon dan Termohon bertempat tinggal di rumah orang tua Pemohon di Palu ;
- Bahwa Pemohon dan Termohon belum dikaruniai anak,
- Bahwa kehidupan rumah tangga Pemohon dan Termohon pada awalnya berjalan baik dan rukun, namun sekarang ini rumah tangga mereka tidak harmonis lagi karena sering terjadi perselisihan dan pertengkaran yang terus menerus;
- Bahwa perselisihan dan pertengkaran Pemohon dan Termohon terjadi sejak bulan Agustus 2019, dan puncaknya pada bulan September 2020 ; ;
- Bahwa Penyebab perselisihan dan pertengkaran Pemohon dan Termohon karena Termohon memiliki hubungan dengan pria lain, yang mana Teman Termohon yang melapor kepada Pemohon dan diakui oleh Termohon, Termohon tidak mendengarkan perkataan dan nasehat Pemohon dan tidak menghormati Pemohon sebagai suami ; Bahwa saksi mengetahui karena saksi pernah melihat dan mendengar sendiri peristiwa perselisihan dan pertengkaran Pemohon dan Termohon ;
- Bahwa Pemohon dan Termohon sudah pisah tempat tinggal sejak awal bulan September 2016 dan sampai sekarang tidak pernah kembali lagi, dan sudah berlangsung selama kurang lebih 02 bulan lamanya ;

Disclaimer
Kepaniteraan Mahkamah Agung Republik Indonesia berusaha untuk selalu mencantumkan informasi paling kini dan akurat sebagai bentuk komitmen Mahkamah Agung untuk pelayanan publik, transparansi dan akuntabilitas pelaksanaan fungsi peradilan. Namun dalam hal-hal tertentu masih dimungkinkan terjadi permasalahan teknis terkait dengan akurasi dan keterkinian informasi yang kami sajikan, hal mana akan terus kami perbaiki dari waktu kewaktu.
Dalam hal Anda menemukan inakurasi informasi yang termuat pada situs ini atau informasi yang seharusnya ada, namun belum tersedia, maka harap segera hubungi Kepaniteraan Mahkamah Agung RI melalui :
Email : kepaniteraan@mahkamahagung.go.id Telp : 021-384 3348 (ext.318)

- Bahwa yang meninggalkan kediaman bersama adalah Termohon
- Bahwa selama berpisah Pemohon dan Termohon sudah tidak pernah lagi berkomunikasi.antara Pemohon dengan Termohon dan Termohon sudah tidak memperdulikan lagi Pemohon ;
- Bahwa pernah diusahakan oleh keluarga untuk rukun, namun tidak berhasil karena Pemohon tidak mau rukun lagi dengan Termohon ;
- Bahwa Pemohon dan Termohon sudah sulit untuk rukun kembali ;..

Saksi 2, **SAKSI 2**, umur 24 tahun, agama Islam, pendidikan S1, pekerjaan xxxxxxx xxxxxx xxxxxxx, bertempat tinggal di KOTA, di bawah sumpah, memberikan keterangan yang pada pokoknya sebagai berikut :

- Bahwa saksi tahu antara Pemohon dan Termohon adalah suami ister sah menikah tahun 2018;;
- Bahwa Perkawinan Pemohon dengan Termohon belum dikaruniai anak ;
- Bahwa setelah menikah Pemohon dan Termohon bertempat tinggal di rumah orang tua Pemohon di Palu ; ;
- Bahwa kehidupan rumah tangga Pemohon dan Termohon pada awalnya berjalan baik dan rukun, namun sekarang ini rumah tangga mereka tidak harmonis lagi karena sering terjadi perselisihan dan pertengkaran yang terus menerus;
- Bahwa perselisihan dan pertengkaran Pemohon dan Termohon terjadi sejak bulan Agustus 2019 dan puncaknya pada bulan September 2020 ‘
- Bahwa Penyebab perselisihan dan pertengkaran Pemohon dan Termohon karena Termohon memiliki hubungan dengan pria lain, yang mana Teman Termohon yang melapor kepada Pemohon dan diakui oleh Termohon, Termohon tidak mendengarkan perkataan dan nasehat Pemohon dan tidak menghormati Pemohon sebagai suami
- Bahwa saksi mengetahui karena saksi pernah melihat dan mendengar sendiri peristiwa perselisihan dan pertengkaran Pemohon dan Termohon ;

Disclaimer
Kepaniteraan Mahkamah Agung Republik Indonesia berusaha untuk selalu mencantumkan informasi paling kini dan akurat sebagai bentuk komitmen Mahkamah Agung untuk pelayanan publik, transparansi dan akuntabilitas pelaksanaan fungsi peradilan. Namun dalam hal-hal tertentu masih dimungkinkan terjadi permasalahan teknis terkait dengan akurasi dan keterkinian informasi yang kami sajikan, hal mana akan terus kami perbaiki dari waktu kewaktu.
Dalam hal Anda menemukan inakurasi informasi yang termuat pada situs ini atau informasi yang seharusnya ada, namun belum tersedia, maka harap segera hubungi Kepaniteraan Mahkamah Agung RI melalui :
Email : kepaniteraan@mahkamahagung.go.id Telp : 021-384 3348 (ext.318)

- Bahwa Pemohon dan Termohon sudah pisah tempat tinggal sejak awal bulan September 2020 dan sampai sekarang tidak pernah kembali lagi, dan sudah berlangsung selama kurang lebih 02 bulan lamanya
- Bahwa yang meninggalkan kediaman bersama adalah Termohon ;
- Bahwa selama berpisah Pemohon dan Termohon sudah tidak pernah lagi berkomunikasi dan Termohon tidak memperdulikan lagi Pemohon;
- Bahwa Pemohon dan Termohon pernah diusahakan oleh keluarga untuk rukun, namun tidak berhasil karena Permohon tidak mau rukun lagi dengan Termohoin ;
- Bahwa Pemohon dan Termohon sudah sulit untuk rukun kembali ;

Bahwa atas keterangan para saksi tersebut, Pemohon menyatakan menerima dan membenarkan, sedangkan Termohon tidak dapat didengarkan tanggapannya;

Bahwa selanjutnya Pemohon menyatakan tidak akan mengajukan apapun lagi dan mohon putusan;

Bahwa untuk mempersingkat uraian putusan ini, ditunjuk segala sesuatu yang tercantum dalam berita acara persidangan perkara ini sebagai bagian yang tidak terpisahkan dari isi putusan ini;

PERTIMBANGAN HUKUM

Menimbang, bahwa maksud dan tujuan permohonan Pemohon adalah sebagaimana telah diuraikan di atas;

Menimbang, bahwa selama persidangan hanya Pemohon yang selalu hadir sedangkan Termohon tidak pernah hadir, maka upaya mediasi sebagaimana ketentuan Peraturan Mahkamah Agung RI Nomor 1 Tahun 2008 yang telah diubah dengan Peraturan Mahkamah Agung RI Nomor 1 Tahun 2016 tentang Prosedur Mediasi di Pengadilan, tidak dapat dilaksanakan;

Menimbang, bahwa oleh karena Termohon tidak pernah hadir di persidangan meskipun telah dipanggil secara resmi dan patut dan tidak

Disclaimer
Kepaniteraan Mahkamah Agung Republik Indonesia berusaha untuk selalu mencantumkan informasi paling kini dan akurat sebagai bentuk komitmen Mahkamah Agung untuk pelayanan publik, transparansi dan akuntabilitas pelaksanaan fungsi peradilan. Namun dalam hal-hal tertentu masih dimungkinkan terjadi permasalahan teknis terkait dengan akurasi dan keterkinian informasi yang kami sajikan, hal mana akan terus kami perbaiki dari waktu kewaktu.
Dalam hal Anda menemukan inakurasi informasi yang termuat pada situs ini atau informasi yang seharusnya ada, namun belum tersedia, maka harap segera hubungi Kepaniteraan Mahkamah Agung RI melalui :
Email : kepaniteraan@mahkamahagung.go.id Telp : 021-384 3348 (ext.318)

ternyata tidak hadirnya tersebut disebabkan oleh suatu halangan yang sah, maka Termohon harus dinyatakan tidak hadir, dan perkara ini dilanjutkan tanpa hadirnya Termohon;

Menimbang, bahwa selanjutnya dibacakanlah surat permohonan Pemohon dalam sidang yang tertutup untuk umum, dan atas pertanyaan Majelis Hakim Pemohon menyatakan tetap pada dalil-dalil permohonannya .

Menimbang, bahwa Termohon yang telah dipanggil secara resmi dan patut akan tetapi tidak datang menghadap, harus dinyatakan tidak bersungguh-sungguh dan telah melalaikan haknya untuk menjawab dalil permohonan Pemohon, sehingga karenanya Termohon dapat dipandang telah mengakui semua dalil permohonan Pemohon tersebut ;

Menimbang, bahwa meskipun Termohon tidak hadir akan tetapi demi untuk memenuhi maksud Pasal 149 Ayat (1) R.Bg. yang maksudnya bahwa Putusan yang dijatuhkan tanpa hadirnya Termohon dapat dikabulkan sepanjang permohonannya cukup beralasan dan berdasarkan hukum, begitu juga Pasal 283 R.Bg. yang maksudnya bahwa barang siapa yang beranggapan mempunyai suatu hak atau suatu keadaan, maka untuk menguatkan haknya tersebut ia harus membuktikannya terlebih dahulu, untuk itulah Pemohon di depan persidangan telah mengajukan alat bukti (P) dan dua orang saksi seperti tersebut di muka;

Menimbang, bahwa tentang bukti (P) yaitu fotokopi Kutipan Akta Nikah yang diajukan Pemohon, oleh Majelis Hakim dinilai telah memenuhi syarat formal dan syarat materil sebagai alat bukti karena surat tersebut merupakan akta autentik yang sengaja dibuat untuk dijadikan alat bukti dan ditanda tangani oleh Pejabat yang berwenang serta berisikan pernyataan yang menerangkan bahwa pada tanggal 25 Agustus 2018 telah terjadi ikatan perkawinan antara Pemohon dan Termohon,alat bukti tersebut telah dicocokkan dan sesuai dengan aslinya, telah bermeterai cukup, sehingga alat bukti tersebut mempunyai kekuatan pembuktian yang sempurna dan mengikat;

Disclaimer
Kepaniteraan Mahkamah Agung Republik Indonesia berusaha untuk selalu mencantumkan informasi paling kini dan akurat sebagai bentuk komitmen Mahkamah Agung untuk pelayanan publik, transparansi dan akuntabilitas pelaksanaan fungsi peradilan. Namun dalam hal-hal tertentu masih dimungkinkan terjadi permasalahan teknis terkait dengan akurasi dan keterkinian informasi yang kami sajikan, hal mana akan terus kami perbaiki dari waktu kewaktu.
Dalam hal Anda menemukan inakurasi informasi yang termuat pada situs ini atau informasi yang seharusnya ada, namun belum tersedia, maka harap segera hubungi Kepaniteraan Mahkamah Agung RI melalui :
Email : kepaniteraan@mahkamahagung.go.id   Telp : 021-384 3348 (ext.318)

Menimbang, bahwa dari alat bukti tersebut diperoleh fakta hukum bahwa Pemohon dengan Termohoin adalah suami istri yang sah, menikah tanggal 09 November 1997 dan karenanya Pemohon dapat dinyatakan telah mempunyai legal standing dalam perkara ini;

Menimbang, bahwa selain bukti surat yang diajukan oleh Pemohon, Pemohon juga telah mengajukan dua orang saksi bernama , **SAKSI 1** dan **SAKSI 2** kedua orang saksi tersebut dibawah sumpahnya telah memberikan keterangan yang satu sama lain saling mendukung dalil-dalil permohonan Pemohon ;

Menimbang, bahwa demikian pula kedua saksi yang diajukan Pemohon, oleh Majelis Hakim juga dinilai telah memenuhi syarat formal dan syarat materil sesuai Pasal 308 dan 309 R.Bg. karena saksi tersebut telah dewasa, telah datang menghadap di persidangan dan saksi itu bukanlah orang yang tidak boleh didengar keterangannya sebagai saksi, saksi tersebut telah memberikan keterangan setelah bersumpah bahwa ia melihat langsung Pemohon dengan Termohon bertengkar dan saksi mengetahui Pemohon dan Termohon sudah tidak sama-sama lagi karena mereka telah berpisah tempat tinggal selama kurang lebih 02 bulan lamanya, setelah mereka cekcok terus disebabkan karena Termohon memiliki hubungan dengan pria lain, yang mana Teman Termohon yang melapor kepada Pemohon dan diakui oleh Termohon, Termohon tidak mendengarkan perkataan dan nasehat Pemohon dan tidak menghormati Pemohon sebagai suami, semua keterangan saksi tersebut telah sesuai dengan dalil-dalil permohonan Pemohon ;

Menimbang, bahwa jika dihubungkan antara bukti surat dan bukti saksi maka diantara keduanya terlihat kesamaan dan saling bersesuaian antara satu dan yang lainnya dalam mendukung kebenaran dalil permohonan Pemohon dan kedua alat bukti tersebut juga sangat relevan dengan pokok perkara ini;

Menimbang, bahwa berdasarkan keterangan Pemohon, bukti tertulis dan keterangan para saksi, Majelis Hakim telah menemukan fakta-fakta hukum yang disimpulkan sebagai berikut:

Disclaimer
Kepaniteraan Mahkamah Agung Republik Indonesia berusaha untuk selalu mencantumkan informasi paling kini dan akurat sebagai bentuk komitmen Mahkamah Agung untuk pelayanan publik, transparansi dan akuntabilitas pelaksanaan fungsi peradilan. Namun dalam hal-hal tertentu masih dimungkinkan terjadi permasalahan teknis terkait dengan akurasi dan keterkinian informasi yang kami sajikan, hal mana akan terus kami perbaiki dari waktu kewaktu.
Dalam hal Anda menemukan inakurasi informasi yang termuat pada situs ini atau informasi yang seharusnya ada, namun belum tersedia, maka harap segera hubungi Kepaniteraan Mahkamah Agung RI melalui :
Email : kepaniteraan@mahkamahagung.go.id Telp : 021-384 3348 (ext.318)

- Bahwa Pemohon dengan Termohon adalah suami istri sah menikah pada tanggal 25 Agustus 2018 ;
- Bahwa Pemohon dengan Termohoin pernah hidup rukun dan belum dikaruniai anak ;
- Bahwa setelah Pemohon dan Termohon menikah mereka tinggal di rumah orang tua Pemohon di Palu ;
- Bahwa Pemohon dan Termohon sudah tidak rukun dan harmonis lagi, karena sering terjadi perselisihan dan pertengkaran ;
- Bahwa penyebab terjadinya perselisihan dan pertengkaran karena karena Termohon memiliki hubungan dengan pria lain, yang mana Teman Termohon yang melapor kepada Pemohon dan diakui oleh Termohon, Termohon tidak mendengarkan perkataan dan nasehat Pemohon dan tidak menghormati Pemohon sebagai suami
- Bahwa Pemohon dan Termohon telah pisah tempat tinggal sejak bulan September 2020, dan sampai sekarang tidak pernah kembali lag dan sudah berjalan selama kurang lebih 02 bulan lamanya ;
- Bahwa selama berpisah Pemohon dan Termohon sudah tidak ada komunikasi antara Pemohon dan Termohon, serta Termohon sudah tidak memperdulikan lagi Pemohon ;
- Bahwa ada usaha keluarga untuk merukunkan Pemohon dan Termohon nmun tidak berhasil,
- Bahwa Pemohon dan Termohon sudah sulit untuk rukun kembali ;

Menimbang, bahwa percekcokan terus-menerus seperti yang terjadi di dalam rumah tangga antara Pemohon dan Termohon adalah merupakan petunjuk nyata yang menandakan tidak adanya lagi tali kasih sayang diantara mereka, lagi pula pihak keluarga telah berusaha mendamaikan Pemohon dengan Termohon agar bisa mempertahankan rumah tangganya ternyata tidak berhasil;

Menimbang, bahwa idealnya sebuah perkawinan adalah jika suami dengan isteri dalam menjalani kehidupan rumah tangganya selalu hidup berdampingan secara damai dan bahagia, saling kasih-mengasihi, saling

Disclaimer
Kepaniteraan Mahkamah Agung Republik Indonesia berusaha untuk selalu mencantumkan informasi paling kini dan akurat sebagai bentuk komitmen Mahkamah Agung untuk pelayanan publik, transparansi dan akuntabilitas pelaksanaan fungsi peradilan. Namun dalam hal-hal tertentu masih dimungkinkan terjadi permasalahan teknis terkait dengan akurasi dan keterkinian informasi yang kami sajikan, hal mana akan terus kami perbaiki dari waktu kewaktu.
Dalam hal Anda menemukan inakurasi informasi yang termuat pada situs ini atau informasi yang seharusnya ada, namun belum tersedia, maka harap segera hubungi Kepaniteraan Mahkamah Agung RI melalui :
Email : kepaniteraan@mahkamahagung.go.id   Telp : 021-384 3348 (ext.318)

cinta-mencintai serta saling melayani kebutuhan masing-masing, baik kebutuhan lahir terutama kebutuhan bathin diantara keduanya;

Menimbang, bahwa akan tetapi ternyata pada kehidupan rumah tangga antara Pemohon dan Termohon, yang terjadi adalah percekcokan terus menerus sampai berlanjut dengan perpisahan tempat tinggal, sehingga kesempatan untuk saling menunaikan kewajiban masing-masing sebagai sarana untuk mencapai tujuan perkawinan yakni kehidupan yang sakinah mawaddah warahmah sebagaimana yang dikehendaki oleh Al-Quran Surat Ar-Ruum Ayat 21 dan Pasal 1 Undang-Undang Nomor 1 Tahun 1974 tentang perkawinan, tidak mungkin dicapai lagi;

Menimbang, bahwa begitupun sikap Pemohon yang benar-benar sudah tidak mau lagi mempertahankan ikatan perkawinannya bersama dengan Termohon hal tersebut disaksikan oleh kedua saksi Pemohon yang melihat langsung Pemohon dengan Termohon selalu cekcok terus sehingga Pemohon dan Termohon sudah pisah tempat tinggal selama kurang lebih 07 bulan lamanya dan sampai sekarang tidak pernah kembali lagi, saksi juga telah berusaha dengan maksimal untuk mendamaikan kedua belah pihak akan tetapi tidak berhasil, sehingga berdasarkan hal-hal tersebut, maka Majelis Hakim berpendapat bahwa perkawinan Pemohon dengan Termohon sudah benar-benar berantakan dan sudah selayaknya untuk diputuskan, karena jika tetap dipertahankan maka dapat dipastikan akan menimbulkan fitnah dan kemudaratan bagi kedua belah pihak ;

Menimbang, bahwa jika salah satu pihak atau keduanya telah tidak ingin mempertahankan keutuhan rumah tangganya lagi maka telah terbukti bahwa ikatan batin di antara keduanya telah putus dan tidak ada harapan untuk rukun sebagai suami istri ;

Menimbang, bahwa mempertahankan rumah tangga Pemohon dan Termohon yang dalam kenyataannya telah pecah karena tidak saling mencintai karena sering terjadi perselisihan dan pertengkaran, sebagai kenyataan bahwa rumah tangga Pemohon dan Termohon yang tidak bisa dirukunkan lagi dan bila diteruskan, hanya akan menambah penderitaan

Disclaimer
Kepaniteraan Mahkamah Agung Republik Indonesia berusaha untuk selalu mencantumkan informasi paling kini dan akurat sebagai bentuk komitmen Mahkamah Agung untuk pelayanan publik, transparansi dan akuntabilitas pelaksanaan fungsi peradilan. Namun dalam hal-hal tertentu masih dimungkinkan terjadi permasalahan teknis terkait dengan akurasi dan keterkinian informasi yang kami sajikan, hal mana akan terus kami perbaiki dari waktu kewaktu.
Dalam hal Anda menemukan inakurasi informasi yang termuat pada situs ini atau informasi yang seharusnya ada, namun belum tersedia, maka harap segera hubungi Kepaniteraan Mahkamah Agung RI melalui :
Email : kepaniteraan@mahkamahagung.go.id   Telp : 021-384 3348 (ext.318)

kepada kedua belah pihak dan mafsadatnya lebih besar dari pada manfaatnya, oleh karenanya sesuai dengan kaidah hukum Islam dalam kitab D[illegible]usu Lisysyaekh Su'udi Sysyariim halaman 7 yang sekaligus diambil sebagai pendapat Majelis Hakim sebagai berikut ;

درأالمفاسد مقدم على جلب المصالح -

Artinya : " *Menolak mafsadat harus didahulukan dengan mencari kemaslahatan*

Oleh karena itu perkawinan Pemohon dan Termohon harus diakhiri dengan perceraian ;

Menimbang, bahwa Majelis Hakim perlu mengemukakan ayat Al qur'an surah al-Baqarah ayat 227 yang berbunyi:

و ان عزموا الطلاق فان الله سميع عليم -

Artinya:*"Dan jika mereka berazam (berketetapan hati) untuk talak maka sesungguhnya Allah Maha Mendengar lagi Maha Melihat"*.

Menimbang, bahwa berdasarkan pertimbangan-pertimbangan tersebut di atas tanpa mempersoalkan siapa yang benar dan siapa yang salah, maka alasan-alasan perceraian dalam perkara ini dianggap telah memenuhi ketentuan Pasal 19 huruf f Peraturan Pemerintah Nomor 9 Tahun 1975 jo Pasal 116 huruf f Kompilasi Hukum Islam, atau setidak-tidaknya permohonan Pemohon untuk bercerai dengan Termohon telah memiliki cukup alasan, maka permohonan Pemohon patut dikabulkan dengan memberi izin kepada Pemohon untuk menjatuhkan talak satu raj'i terhadap Termohon;

Menimbang, bahwa permohonan Pemohon dipandang cukup alasan dan tidak melawan hukum dapat diperiksa dan diputus dengan verstek tanpa hadirnya Termohon (vide Pasal 149 ayat (1) R.Bg),

Menimbang, bahwa oleh karena perkara ini termasuk bidang perkawinan, maka berdasarkan ketentuan pasal 89 ayat (1) Undang-Undang Nomor 7 Tahun 1989 sebagaimana telah diubah dengan Undang-Undang Nomor 3 Tahun 2006 dan Undang-Undang Nomor 50 Tahun 2009 tentang Peradilan Agama, biaya perkara dibebankan kepada Pemohon ;

Disclaimer
Kepaniteraan Mahkamah Agung Republik Indonesia berusaha untuk selalu mencantumkan informasi paling kini dan akurat sebagai bentuk komitmen Mahkamah Agung untuk pelayanan publik, transparansi dan akuntabilitas pelaksanaan fungsi peradilan. Namun dalam hal-hal tertentu masih dimungkinkan terjadi permasalahan teknis terkait dengan akurasi dan keterkinian informasi yang kami sajikan, hal mana akan terus kami perbaiki dari waktu kewaktu.
Dalam hal Anda menemukan inakurasi informasi yang termuat pada situs ini atau informasi yang seharusnya ada, namun belum tersedia, maka harap segera hubungi Kepaniteraan Mahkamah Agung RI melalui :
Email : kepaniteraan@mahkamahagung.go.id Telp : 021-384 3348 (ext.318)

Memperhatikan pasal-pasal dari peraturan perundang-undangan yang berlaku dan hukum syara' yang berkenaan dengan perkara ini;

M E N G A D I L I

1. Menyatakan Termohon telah dipanggil dengan resmi dan patut untuk menghadap di persidangan, tidak hadir;
2. Mengabulkan permohonan Pemohon dengan verstek;
3. Memberi izin kepada Pemohon (PEMOHON) untuk menjatuhkan talak satu raj'i terhadap Termohon (TERMOHON) di depan sidang Pengadilan Agama Palu ;
4. Membebankan kepada Pemohon untuk membayar biaya perkara sejumlah Rp.616.000,- (enam ratus enam belas ribu rupiah...

Demikian diputuskan dalam musyawarah Majelis Hakim Pengadilan Agama Palu pada hari Rabu tanggal .23 Desember 2020 Miladiyah bertepatan dengan tanggal 08 Jumadil Awal 1442 Hijriah oleh Drs. H. M. Natsir sebagai Ketua Majelis, Drs. M. Taman dan Dra. Hj. St. Sabiha, M.H., masing-masing sebagai Hakim Anggota, putusan tersebut diucapkan pada hari itu juga dalam sidang terbuka untuk umum oleh Ketua Majelis beserta para Hakim Anggota tersebut, dan didampingi oleh Nuniek Widriyani, SH. sebagai Panitera Pengganti, dengan dihadiri oleh Pemohon tanpa hadirnya Termohon.

Hakim Anggota

Drs. M. Taman

Dra. Hj. St. Sabiha, M.H.

Ketua Majelis,

Drs. H. M. Natsir

Panitera Pengganti,

Disclaimer
Kepaniteraan Mahkamah Agung Republik Indonesia berusaha untuk selalu mencantumkan informasi paling kini dan akurat sebagai bentuk komitmen Mahkamah Agung untuk pelayanan publik, transparansi dan akuntabilitas pelaksanaan fungsi peradilan. Namun dalam hal-hal tertentu masih dimungkinkan terjadi permasalahan teknis terkait dengan akurasi dan keterkinian informasi yang kami sajikan, hal mana akan terus kami perbaiki dari waktu kewaktu.
Dalam hal Anda menemukan inakurasi informasi yang termuat pada situs ini atau informasi yang seharusnya ada, namun belum tersedia, maka harap segera hubungi Kepaniteraan Mahkamah Agung RI melalui :
Email : kepaniteraan@mahkamahagung.go.id Telp : 021-384 3348 (ext.318)

Nuniek Widriyani, SH.

Perincian biaya :

- Pendaftaran	: Rp	30.000,00
- ATK Perkara	: Rp	60.000,00
- Panggilan	: Rp	490.000,00
- PNBP Panggilan	: Rp	20.000,00
- Redaksi	: Rp	10.000,00
- Meterai	: Rp	6.000,00
J u m l a h	: Rp	616..000,00

(enam ratus enam belas ribu rupiah).

Salinan Sesuai Aslinya
Pengadilan Agama Palu
Panitera

Drs. H. A. Kadir, M.H.

Disclaimer
Kepaniteraan Mahkamah Agung Republik Indonesia berusaha untuk selalu mencantumkan informasi paling kini dan akurat sebagai bentuk komitmen Mahkamah Agung untuk pelayanan publik, transparansi dan akuntabilitas pelaksanaan fungsi peradilan. Namun dalam hal-hal tertentu masih dimungkinkan terjadi permasalahan teknis terkait dengan akurasi dan keterkinian informasi yang kami sajikan, hal mana akan terus kami perbaiki dari waktu kewaktu.
Dalam hal Anda menemukan inakurasi informasi yang termuat pada situs ini atau informasi yang seharusnya ada, namun belum tersedia, maka harap segera hubungi Kepaniteraan Mahkamah Agung RI melalui :
Email : kepaniteraan@mahkamahagung.go.id Telp : 021-384 3348 (ext.318)
