# Supplementary material for: Classification of divorce causes during the COVID-19 pandemic using convolutional neural networks
Source: PeerJ Comput Sci. 2022 Jun 30;8:e998. doi: 10.7717/peerj-cs.998 (PMC9299239; doi:10.7717/peerj-cs.998)
Supplement: Supplemental Information 3 [file peerj-cs-08-998-s003.zip › KDRT Dataset/Data ke-21.pdf]

PUTUSAN

Nomor 615/Pdt.G/2020/PA.LK

بِسْمِ اللهِ الرَّحْمٰنِ الرَّحِيْمِ

DEMI KEADILAN BERDASARKAN KETUHANAN YANG MAHA ESA

Pengadilan Agama Tanjung Pati yang memeriksa dan mengadili perkara tertentu pada tingkat pertama dalam sidang Majelis telah menjatuhkan putusan sebagai berikut dalam perkara Cerai Talak antara:

Pemohon, NIK 3173071509620011, tempat dan tanggal lahir: Koto Tinggi/ 15 September 1962, agama Islam, pendidikan SLTP, pekerjaan Petani, tempat kediaman di Kabupaten Lima Puluh Kota, sebagai **Pemohon**;

melawan

Termohon, tempat dan tanggal lahir: Padang/ 18 Juli 1969, agama Islam, pendidikan SD, pekerjaan Ibu Rumah Tangga, tempat kediaman di Kabupaten Lima Puluh Kota, sebagai **Termohon**;

Pengadilan Agama tersebut;

Telah membaca dan mempelajari berkas perkara;

Telah mendengar keterangan Pemohon;

Telah memeriksa alat-alat bukti di persidangan;

DUDUK PERKARA

Bahwa Pemohon dengan surat permohonannya tertanggal 13 Oktober 2020 yang telah terdaftar di Kepaniteraan Pengadilan Agama Tanjung Pati dengan register perkara Nomor 615/Pdt.G/2020/PA.LK, mengemukakan dalil-dalil sebagai berikut:

1. Bahwa Pemohon dan Termohon adalah suami istri yang sah menikah pada tanggal 17 Desember 2007 di Kantor Urusan Agama Kecamatan Rumpin, Kabupaten Bogor, Provinsi Jawa Barat, sesuai dengan Penetapan Nomor 857/53/XII/2007 tanggal 18 Desember 2007 yang dikeluarkan oleh Kantor Urusan Agama Kecamatan Rumpin, Kab. Bogor, Prov. Jawa Barat;

2. Bahwa setelah pernikahan Pemohon dan Termohon membina rumah tangga di rumah kontrakan di DKI Jakarta selama 1 tahun, setelah itu pindah ke rumah kontrakan di F9 Kuansi, Taluak Kuantan, Provisi Riau

Disclaimer
Kepaniteraan Mahkamah Agung Republik Indonesia berusaha untuk selalu mencantumkan informasi paling kini dan akurat sebagai bentuk komitmen Mahkamah Agung untuk pelayanan publik, transparansi dan akuntabilitas pelaksanaan fungsi peradilan. Namun dalam hal-hal tertentu masih dimungkinkan terjadi permasalahan teknis terkait dengan akurasi dan keterkinian informasi yang kami sajikan, hal mana akan terus kami perbaiki dari waktu kewaktu.
Dalam hal Anda menemukan inakurasi informasi yang termuat pada situs ini atau informasi yang seharusnya ada, namun belum tersedia, maka harap segera hubungi Kepaniteraan Mahkamah Agung RI melalui :
Email : kepaniteraan@mahkamahagung.go.id   Telp : 021-384 3348 (ext.318)

selama 8 tahun, dan setelah itu pindah ke rumah sendiri di Kabupaten Lima Puluh Kota, dan selama pernikahan tersebut Pemohon dengan Termohon belum dikaruniai anak, namun ba'da dukhul;

3. Bahwa pada awalnya rumah tangga Pemohon dan Termohon berjalan harmonis, namun sejak bulan April 2020 rumah tangga Pemohon dan Termohon sudah tidak berjalan harmonis, antara Pemohon dan Termohon sering terjadi perselisihan dan pertengkaran yang disebabkan:

3.1. Termohon tidak menghargai Pemohon sebagai suami Termohon, yakni Termohon seringkali membantah perkataan Pemohon dalam rangka membina rumah tangga yang harmonis, bahkan Termohon sering berkata-kata kasar kepada Pemohon di saat terjadi permasalahan antara Pemohon dan Termohon;

3.2. Termohon kurang memperhatikan Pemohon, yakni Termohon lebih mementingkan diri sendiri dari pada Termohon, seperti memasak makanan dan membuatkan minum untuk Pemohon, maka tak jarang Pemohon sendiri yang melakukannya meskipun hal tersebut merupakan kewajiban Termohon selaku istri sah Pemohon;

3.3. Termohon sering menolak untuk melakukan hubungan suami istri tanpa alasan yang jelas;

4. Bahwa Pemohon sudah menasehati Termohon untuk merubah sikap Termohon, namun usaha Pemohon tidak berhasil;

5. Bahwa perselisihan dan pertengkaran tersebut terjadi terus-menerus, sehingga sejak bulan September 2020 hingga sekarang kurang lebih 1 bulan, antara Pemohon dan Termohon telah berpisah tempat tinggal, karena Pemohon sudah merasa tidak tahan lagi dengan sikap Termohon tersebut, akhirnya Pemohon pergi meninggalkan tempat kediaman bersama, dan pergi ke rumah saudara Pemohon di alamat yang tertera pada identitas Pemohon di atas, serta selama itu tidak saling memperdulikan lagi;

6. Bahwa akibat perselisihan dan pertengkaran yang terjadi tersebut mengakibatkan rumah tangga Pemohon dan Termohon tidak ada kebahagian lahir dan batin dan tidak ada harapan untuk kembali membina rumah tangga, serta selama itu sudah ada usaha damai, namun tidak berhasil;

Disclaimer
Kepaniteraan Mahkamah Agung Republik Indonesia berusaha untuk selalu mencantumkan informasi paling kini dan akurat sebagai bentuk komitmen Mahkamah Agung untuk pelayanan publik, transparansi dan akuntabilitas pelaksanaan fungsi peradilan. Namun dalam hal-hal tertentu masih dimungkinkan terjadi permasalahan teknis terkait dengan akurasi dan keterkinian informasi yang kami sajikan, hal mana akan terus kami perbaiki dari waktu kewaktu.
Dalam hal Anda menemukan inakurasi informasi yang termuat pada situs ini atau informasi yang seharusnya ada, namun belum tersedia, maka harap segera hubungi Kepaniteraan Mahkamah Agung RI melalui :
Email : kepaniteraan@mahkamahagung.go.id Telp : 021-384 3348 (ext.318)

7. Bahwa Pemohon menyimpulkan sudah tidak ada kecocokan lagi dalam membina rumah tangga bersama Termohon karena sering terjadi perselisihan dan pertengkaran dan tidak mungkin disatukan lagi, maka cukup alasan bagi permohonan Pemohon;

Berdasarkan hal-hal tersebut di atas, Pemohon memohon kepada Ketua Pengadilan Agama Tanjung Pati c.q. Majelis Hakim kiranya berkenan menerima, memeriksa, mengadili permohonan Pemohon ini dengan menjatuhkan putusan sebagai berikut:

Primer

1. Mengabulkan permohonan Pemohon;

2. Memberi izin kepada Pemohon (**Pemohon**) untuk menjatuhkan talak satu raj'i terhadap Termohon (**Termohon**) di depan sidang Pengadilan Agama Tanjung Pati;

3. Menetapkan biaya perkara menurut hukum yang berlaku;

Subsider

Jika majelis hakim berpendapat lain mohon putusan yang seadil-adilnya (*ex aequo et bono*);

Bahwa pada hari sidang yang telah ditetapkan Pemohon telah menghadap sendiri ke muka sidang, sedangkan Termohon tidak menghadap dan tidak menyuruh orang lain untuk menghadap sebagai Wakil/Kuasa hukumnya meskipun telah dipanggil secara resmi dan patut yang relaas panggilannya dibacakan di dalam sidang, sedangkan tidak ternyata bahwa tidak datangnya itu disebabkan suatu halangan yang sah;

Bahwa, Majelis dalam persidangan telah berusaha mendamaikan dengan jalan memberi saran dan nasehat kepada Pemohon agar mengurungkan keinginannya untuk bercerai dan mencoba kembali membina rumah tangga yang *sakinah, mawaddah wa rahmah* namun tidak berhasil, sedangkan usaha damai melalui proses mediasi tidak dapat dilaksanakan karena Termohon tidak pernah datang menghadap ke persidangan;

Bahwa, oleh sebab upaya damai tidak berhasil, selanjutnya pemeriksaan perkara ini dilanjutkan secara litigasi diawali dengan membacakan surat permohonan Pemohon yang pada pokoknya tetap dipertahankan oleh Pemohon;

Disclaimer
Kepaniteraan Mahkamah Agung Republik Indonesia berusaha untuk selalu mencantumkan informasi paling kini dan akurat sebagai bentuk komitmen Mahkamah Agung untuk pelayanan publik, transparansi dan akuntabilitas pelaksanaan fungsi peradilan. Namun dalam hal-hal tertentu masih dimungkinkan terjadi permasalahan teknis terkait dengan akurasi dan keterkinian informasi yang kami sajikan, hal mana akan terus kami perbaiki dari waktu kewaktu.
Dalam hal Anda menemukan inakurasi informasi yang termuat pada situs ini atau informasi yang seharusnya ada, namun belum tersedia, maka harap segera hubungi Kepaniteraan Mahkamah Agung RI melalui :
Email : kepaniteraan@mahkamahagung.go.id Telp : 021-384 3348 (ext.318)

Bahwa, terhadap permohonan Pemohon, Termohon tidak dapat didengar jawabannya dalam persidangan karena Termohon tidak pernah hadir di persidangan;

Bahwa untuk meneguhkan dalil-dalil permohonannya, Pemohon telah mengajukan bukti-bukti sebagai berikut:

I. Bukti Surat

Fotokopi Kutipan Akta Nikah Nomor 857/53/XII/2007 tanggal 18 Desember 2007 atas nama Pemohon dan Termohon yang dicatat dan dikeluarkan oleh Pegawai Pencatat Nikah Kantor Urusan Agama (KUA) Kecamatan Rumpin, Kabupaten Bogor, telah bermeterai cukup dan di-*nazegeling,* yang oleh Hakim Ketua telah dicocokkan dengan aslinya ternyata cocok, diberi tanggal, diberi tanda P dan diparaf;

II. Bukti Saksi

1.

Saksi I, umur 39 tahun, agama Islam, pendidikan SD, pekerjaan Petani, tempat tinggal di Kabupaten Limapuluh Kota, Provinsi Sumatera Barat, telah memberikan keterangan di bawah sumpah sebagai berikut:

- Bahwa saksi kenal dengan Pemohon dan Termohon karena saksi adalah tetangga Pemohon sejak lebih kurang 2 (dua) tahun yang lalu;
- Bahwa Pemohon dan Termohon adalah suami isteri sah;
- Bahwa Pemohon dan Termohon bertempat tinggal terakhir di rumah bersama Pemohon dengan Termohon di Kabupaten Lima Puluh Kota;
- Bahwa dari pernikahan tersebut Pemohon dan Termohon belum dikaruniai anak;
- Bahwa awalnya rumah tangga Pemohon dengan Termohon berjalan rukun dan harmonis, namun sejak lebih kurang 4 (empat) bulan yang lalu rumah tangga Pemohon dengan Termohon sudah tidak rukun dan tidak harmonis karena adanya perselisihan dan pertengkaran antara Pemohon dengan Termohon;
- Bahwa saksi tidak mengetahui penyebab perselisihan dan pertengkaran antara Pemohon dengan Termohon namun saksi

Disclaimer
Kepaniteraan Mahkamah Agung Republik Indonesia berusaha untuk selalu mencantumkan informasi paling kini dan akurat sebagai bentuk komitmen Mahkamah Agung untuk pelayanan publik, transparansi dan akuntabilitas pelaksanaan fungsi peradilan. Namun dalam hal-hal tertentu masih dimungkinkan terjadi permasalahan teknis terkait dengan akurasi dan keterkinian informasi yang kami sajikan, hal mana akan terus kami perbaiki dari waktu kewaktu.
Dalam hal Anda menemukan inakurasi informasi yang termuat pada situs ini atau informasi yang seharusnya ada, namun belum tersedia, maka harap segera hubungi Kepaniteraan Mahkamah Agung RI melalui :
Email : kepaniteraan@mahkamahagung.go.id   Telp : 021-384 3348 (ext.318)

mengetahui bahwa Pemohon dengan Termohon pernah didamaikan oleh keluarga kedua belah pihak namun tidak berhasil;

-

Bahwa Pemohon dan Termohon telah berpisah tempat tinggal sejak 2 (dua) bulan yang lalu, Pemohon pergi dari kediaman bersama;

- Bahwa sejak pisah rumah antara Pemohon dan Termohon sudah tidak saling berkominukasi dan tidak saling memperdulikan lagi;

2. Saksi II, umur 36 tahun, agama Islam, pendidikan SMK, pekerjaan Petani, bertempat tinggal di Kabupaten Lima Puluh Kota, Provinsi Sumatera Barat, di bawah sumpah memberikan keterangan yang pada pokoknya sebagai berikut:

- Bahwa saksi kenal dengan Pemohon dan Termohon karena saksi adalah keponakan Pemohon;
- Bahwa Pemohon dan Termohon adalah suami isteri sah;
- Bahwa Pemohon dan Termohon bertempat tinggal terakhir di rumah bersama Pemohon dengan Termohon di Kabupaten Lima Puluh Kota;
- Bahwa dari pernikahan tersebut Pemohon dan Termohon belum dikaruniai anak;
- Bahwa awalnya rumah tangga Pemohon dengan Termohon berjalan rukun dan harmonis, namun sejak lebih kurang 4 (empat) bulan yang lalu rumah tangga Pemohon dengan Termohon sudah tidak rukun dan tidak harmonis karena adanya perselisihan dan pertengkaran antara Pemohon dengan Termohon;
- Bahwa penyebab perselisihan dan pertengkaran antara Pemohon dengan Termohon adalah karena Termohon kurang melayani Pemohon dalam hal menyiapkan makanan dan minuman Pemohon serta Termohon yang kurang memperhatikan anak-anak bawaan Pemohon;
- Bahwa saksi mengetahui perselisihan dan pertengkaran antara Pemohon dengan Termohon dari cerita Pemohon kepada saksi;
- Bahwa Pemohon dengan Termohon pernah didamaikan oleh keluarga kedua belah pihak namun tidak berhasil;

Disclaimer
Kepaniteraan Mahkamah Agung Republik Indonesia berusaha untuk selalu mencantumkan informasi paling kini dan akurat sebagai bentuk komitmen Mahkamah Agung untuk pelayanan publik, transparansi dan akuntabilitas pelaksanaan fungsi peradilan. Namun dalam hal-hal tertentu masih dimungkinkan terjadi permasalahan teknis terkait dengan akurasi dan keterkinian informasi yang kami sajikan, hal mana akan terus kami perbaiki dari waktu kewaktu.
Dalam hal Anda menemukan inakurasi informasi yang termuat pada situs ini atau informasi yang seharusnya ada, namun belum tersedia, maka harap segera hubungi Kepaniteraan Mahkamah Agung RI melalui :
Email : kepaniteraan@mahkamahagung.go.id   Telp : 021-384 3348 (ext.318)

-
Bahwa Pemohon dan Termohon telah berpisah tempat tinggal sejak 2 (dua) bulan yang lalu, Pemohon pergi dari kediaman bersama;

- Bahwa sejak pisah rumah antara Pemohon dan Termohon sudah tidak saling berkominukasi dan tidak saling memperdulikan lagi;

Bahwa setelah diberi kesempatan Pemohon menyatakan tidak mengajukan alat bukti apapun lagi dan menyampaikan kesimpulan secara lisan yang pada pokoknya menyatakan tetap pada permohonannya dan selanjutnya mohon putusan;

Bahwa untuk mempersingkat uraian putusan ini cukup ditunjuk segala hal yang telah termuat dalam berita acara sidang yang dianggap sebagai satu kesatuan tak terpisahkan dalam putusan ini;

PERTIMBANGAN HUKUM

Menimbang, bahwa maksud dan tujuan permohonan Pemohon adalah sebagaimana tersebut di atas;

Menimbang, bahwa Pemohon telah datang menghadap *(in person)* di persidangan, sedangkan Termohon tidak pernah hadir di persidangan dan tidak pula mengirimkan orang lain selaku wakil atau kuasanya yang sah untuk hadir di persidangan meskipun telah dipanggil secara resmi dan patut sesuai Pasal 26 Peraturan Pemerintah Nomor 9 Tahun 1975, dan tidak ternyata ketidakhadiran Termohon itu disebabkan suatu alasan yang sah menurut hukum (*default without reason*) sesuai Pasal 149 ayat (1) R.bg, maka harus dinyatakan Termohon tidak hadir dan perkara *a quo* dapat diperiksa tanpa hadirnya Termohon;

Menimbang, bahwa berdasarkan ketentuan Pasal 39 ayat (1) Undang-Undang Nomor 1 Tahun 1974 jo. Pasal 65 dan Pasal 82 Undang-Undang Nomor 7 Tahun 1989 sebagaimana telah diubah dengan Undang-Undang Nomor 3 Tahun 2006, terakhir dengan perubahan kedua Undang-Undang Nomor 50 Tahun 2009 jo. Pasal 31 Peraturan Pemerintah Nomor 9 Tahun 1975 serta Pasal 143 ayat (1) Kompilasi Hukum Islam, maka Majelis telah berusaha mendamaikan dengan menasehati Pemohon agar bersabar dan rukun kembali dengan Termohon akan tetapi tidak berhasil;

Disclaimer
Kepaniteraan Mahkamah Agung Republik Indonesia berusaha untuk selalu mencantumkan informasi paling kini dan akurat sebagai bentuk komitmen Mahkamah Agung untuk pelayanan publik, transparansi dan akuntabilitas pelaksanaan fungsi peradilan. Namun dalam hal-hal tertentu masih dimungkinkan terjadi permasalahan teknis terkait dengan akurasi dan keterkinian informasi yang kami sajikan, hal mana akan terus kami perbaiki dari waktu kewaktu.
Dalam hal Anda menemukan inakurasi informasi yang termuat pada situs ini atau informasi yang seharusnya ada, namun belum tersedia, maka harap segera hubungi Kepaniteraan Mahkamah Agung RI melalui :
Email : kepaniteraan@mahkamahagung.go.id Telp : 021-384 3348 (ext.318)

Menimbang, bahwa berdasarkan Peraturan Mahkamah Agung Republik Indonesia Nomor 1 Tahun 2016 Tentang Prosedur Mediasi di Pengadilan, semua perkara yang masuk ke pengadilan terlebih dahulu harus dilaksanakan mediasi, akan tetapi dalam perkara yang bersangkutan karena pihak Termohon tidak pernah hadir, maka mediasi tidak dapat dilaksanakan;

Menimbang, bahwa oleh sebab antara Pemohon dan Termohon terdapat hubungan perkawinan sebagai suami istri yang belum pernah putus (bercerai), maka keduanya memiliki kepentingan hukum untuk bertindak sebagai pihak-pihak dalam perkara ini (*persona standi in judicio*);

Menimbang, bahwa setelah meneliti surat permohonan Pemohon dan mendengar keterangan Pemohon maka yang menjadi masalah pokok dalam perkara ini adalah Pemohon bermohon untuk menceraikan Termohon dengan alasan bahwa Pemohon dan Termohon terus menerus terjadi perselisihan dan pertengkaran serta tidak ada harapan akan rukun lagi dalam rumah tangga;

Menimbang, bahwa Termohon tidak dapat didengar jawabannya di persidangan karena Termohon tidak pernah hadir, maka secara yuridis formal Termohon dapat dianggap tidak bermaksud untuk mempertahankan hak-hak keperdataannya dan atau membela kepentingannya di persidangan, mengakui dan membenarkan semua Posita dan Petitum dalam surat permohonan Pemohon, namun tidak serta merta hal tersebut menjadikan permohonan Pemohon dapat dikabulkan, karena mengingat perkara ini merupakan perkara yang dikhawatirkan dapat menimbulkan suatu kebohongan besar (*de groten langen*);

Menimbang, bahwa Majelis sependapat dan mengambil alih pendapat ahli fiqih dalam *Kitab Ahkamul Qur'an* Juz II hal 405 yang berbunyi:

مَنْ دُعِيَ اِلَى حَاكِمٍ مِنْ حُكَّامِ الْمُسْلِمِيْنَ وَلَمْ يُجِبْ فَهُوَ ظَالِمٌ لَا حق لَهُ

Artinya: *"Barang siapa yang dipanggil untuk menghadap Hakim Islam, kemudian tidak menghadap maka ia termasuk orang yang zalim, dan gugurlah haknya";*

Menimbang, bahwa berdasarkan alasan-alasan tersebut di atas, maka Majelis menilai alasan perceraian yang didalilkan oleh Pemohon adalah termasuk dalam alasan-alasan perceraian sebagaimana diatur dalam ketentuan

Disclaimer
Kepaniteraan Mahkamah Agung Republik Indonesia berusaha untuk selalu mencantumkan informasi paling kini dan akurat sebagai bentuk komitmen Mahkamah Agung untuk pelayanan publik, transparansi dan akuntabilitas pelaksanaan fungsi peradilan. Namun dalam hal-hal tertentu masih dimungkinkan terjadi permasalahan teknis terkait dengan akurasi dan keterkinian informasi yang kami sajikan, hal mana akan terus kami perbaiki dari waktu kewaktu.
Dalam hal Anda menemukan inakurasi informasi yang termuat pada situs ini atau informasi yang seharusnya ada, namun belum tersedia, maka harap segera hubungi Kepaniteraan Mahkamah Agung RI melalui :
Email : kepaniteraan@mahkamahagung.go.id Telp : 021-384 3348 (ext.318)

Pasal 19 huruf (f) Peraturan Pemerintah Nomor 9 Tahun 1975 jo. Pasal 116 huruf (f) Kompilasi Hukum Islam;

Menimbang, bahwa untuk dikabulkannya sebuah perceraian dengan alasan dasar tersebut di atas, maka sebagaimana Pasal 39 ayat (2) Undang-Undang Nomor 1 Tahun 1974 Tentang Perkawinan jo. Pasal 19 huruf (f) Peraturan Pemerintah Nomor 9 Tahun 1975 Tentang Pelaksanaan Undang-Undang Nomor 1 Tahun 1974 Tentang Perkawinan jo. Pasal 116 huruf (f) dan Pasal 134 Kompilasi Hukum Islam, harus terpenuhi beberapa unsur sebagai berikut, (1). Rumah tangga sudah tidak rukun dan harmonis, sering terjadi perselisihan dan pertengkaran secara terus menerus. (2). Perselisihan dan pertengkaran tersebut menyebabkan suami istri tidak ada harapan untuk rukun kembali. (3). Pengadilan sudah mendamaikan, namun tidak berhasil;

Menimbang, bahwa karena perkara ini adalah mengenai bidang perceraian yang dinilai penting untuk ditemukan kebenaran materiilnya, dan untuk lebih meyakinkan Majelis atas dalil-dalil permohonan Pemohon, maka sesuai dengan Keputusan Mahkamah Agung RI Nomor KMA/032/SK/IV/2006 tanggal 4 April 2006 tentang Pemberlakuan Buku II Pedoman Teknis Administrasi dan Teknis Peradilan Agama, Edisi Revisi 2013, Majelis berpendapat Pemohon diwajibkan untuk mengajukan bukti-bukti yang dapat mendukung dalil-dalil posita dan petitum permohonannya;

Menimbang, bahwa Majelis sependapat dan mengambil alih *hujjah syari'ah* dalam Kitab Al-Anwar Juz II halaman 55 yang untuk selanjutnya diambil alih sebagai pendapat Majelis yang berbunyi sebagai berikut:

فان تعزز بتعزز أو توار أو غيبة جازا ثباته بالبينة

Artinya: *"Apabila dia (Termohon) enggan hadir, atau bersembunyi atau tidak diketahui alamatnya, perkara ini dapat diputus berdasarkan bukti";*

Menimbang, bahwa untuk menguatkan dalil-dalil permohonannya Pemohon telah mengajukan alat bukti berupa bukti surat dan bukti saksi sebagaimana dalam duduk perkaranya di atas;

Menimbang, bahwa terhadap bukti surat yang diajukan Pemohon tersebut, Majelis berpendapat bukti tersebut merupakan fotokopi sah dari suatu akta autentik, khusus dibuat sebagai alat bukti, telah diberi meterai cukup

Disclaimer
Kepaniteraan Mahkamah Agung Republik Indonesia berusaha untuk selalu mencantumkan informasi paling kini dan akurat sebagai bentuk komitmen Mahkamah Agung untuk pelayanan publik, transparansi dan akuntabilitas pelaksanaan fungsi peradilan. Namun dalam hal-hal tertentu masih dimungkinkan terjadi permasalahan teknis terkait dengan akurasi dan keterkinian informasi yang kami sajikan, hal mana akan terus kami perbaiki dari waktu kewaktu.
Dalam hal Anda menemukan inakurasi informasi yang termuat pada situs ini atau informasi yang seharusnya ada, namun belum tersedia, maka harap segera hubungi Kepaniteraan Mahkamah Agung RI melalui :
Email : kepaniteraan@mahkamahagung.go.id Telp : 021-384 3348 (ext.318)

sesuai ketentuan Pasal 2 ayat (1) Undang-Undang Nomor 13 Tahun 1985 dan telah di-nazegeling sehingga alat bukti tersebut telah memenuhi persyaratan formil dan harus dinyatakan dapat diterima, dan secara materiil dapat dipertimbangkan karena alat bukti tersebut memuat keterangan yang menguatkan dan relevan dengan dalil permohonan Pemohon;

Menimbang, bahwa bukti P (Fotokopi Kutipan Akta Nikah Nomor 857/53/XII/2007 tanggal 18 Desember 2007) merupakan akta otentik yang berdaya bukti sempurna dan mengikat yang memberi bukti Pemohon dengan Termohon telah dan masih terikat dalam perkawinan sah yang tercatat Kantor Urusan Agama (KUA) Kecamatan Rumpin, Kabupaten Bogor, dan telah sesuai dengan ketentuan Pasal 285 R.Bg jo. Pasal 2 ayat (1) dan (2) Undang-Undang Nomor 1 Tahun 1974 jo. Pasal 7 ayat (1) Kompilasi Hukum Islam, dengan demikian Pemohon dengan Termohon telah mempunyai hubungan dan kapasitas hukum untuk menjadi pihak dalam perkara ini (*persona standi in judicio*), karenanya Pemohon mempunyai kualitas untuk mengajukan tuntutan dalam sengketa bidang perkawinan;

Menimbang, bahwa oleh karena alasan perceraian yang diajukan Pemohon berkenaan dengan ketentuan Pasal 19 huruf (f) Peraturan Pemerintah Nomor 9 Tahun 1975 jo. Pasal 116 huruf (f) Kompilasi Hukum Islam di Indonesia, sehingga berdasarkan ketentuan Pasal 22 ayat (2) Peraturan Pemerintah Nomor 9 Tahun 1975 dan Pasal 138 Kompilasi Hukum Islam, maka Majelis perlu mendengarkan keterangan saksi-saksi yang berasal dari keluarga dan atau orang-orang yang dekat dengan suami istri tersebut;

Menimbang, bahwa Majelis telah mendengarkan keterangan 2 (dua) orang saksi yang berasal dari pihak keluarga dan orang yang dekat dengan Pemohon yang dalam penilaian Majelis kedua orang saksi tersebut telah memenuhi persyaratan formil sebagai saksi sesuai dengan ketentuanPasal 171-172 R.Bg jo. Pasal 22 ayat (2) Peraturan Pemerintah Nomor 9 Tahun 1975, dan kesaksiannya telah diberikan di bawah sumpah sesuai dengan ketentuan Pasal 175 R.Bg sehingga dengan demikian dapat diterima sebagai alat bukti yang sah di persidangan;

Disclaimer
Kepaniteraan Mahkamah Agung Republik Indonesia berusaha untuk selalu mencantumkan informasi paling kini dan akurat sebagai bentuk komitmen Mahkamah Agung untuk pelayanan publik, transparansi dan akuntabilitas pelaksanaan fungsi peradilan. Namun dalam hal-hal tertentu masih dimungkinkan terjadi permasalahan teknis terkait dengan akurasi dan keterkinian informasi yang kami sajikan, hal mana akan terus kami perbaiki dari waktu kewaktu.
Dalam hal Anda menemukan inakurasi informasi yang termuat pada situs ini atau informasi yang seharusnya ada, namun belum tersedia, maka harap segera hubungi Kepaniteraan Mahkamah Agung RI melalui :
Email : kepaniteraan@mahkamahagung.go.id Telp : 021-384 3348 (ext.318)

Menimbang, bahwa keterangan saksi-saksi yang dihadirkan oleh Pemohon mengenai pertengkaran, adalah fakta yang tidak dilihat sendiri/ tidak didengar sendiri/tidak dialami sendiri sehingga keterangan saksi tersebut tidak memenuhi syarat materiil sebagaimana telah diatur dalam Pasal 308 R.Bg. oleh karena itu keterangan saksi tersebut harus dikesampingkan;

Menimbang, bahwa keterangan saksi-saksi yang dihadirkan oleh Pemohon mengenai pisah rumah dan tidak saling memperdulikan sebagai suami istri antara Pemohon dengan Termohon sejak lebih kurang 2 (dua) bulan yang lalu dan telah diupayakan damai oleh keluarga kedua belah pihak namun tidak berhasil adalah fakta yang dilihat sendiri dan relevan dengan dalil yang harus dibuktikan oleh Pemohon, oleh karena itu keterangan saksi-saksi tersebut telah memenuhi syarat materiil sebagaimana telah diatur dalam Pasal 308 R.Bg. sehingga keterangan saksi tersebut memiliki kekuatan pembuktian dan dapat diterima sebagai alat bukti;

Menimbang, bahwa saksi-saksi yang dihadirkan oleh Pemohon tidak mengetahui langsung pertengkaran antara Pemohon dengan Termohon dan hanya berdasarkan cerita Pemohon, sehingga kesaksiannya bersifat kesaksian *De Auditu (testimonium De Auditu)*, namun saksi-saksi tersebut menyatakan mengetahui bahwa antara Pemohon dan Termohon telah berpisah rumah dan tidak saling memperdulikan sebagai suami istri sejak 2 (dua) bulan yang lalu;

Menimbang, bahwa keterangan saksi-saksi yang mengetahui antara Pemohon dan Termohon telah berpisah (hanya menerangkan suatu akibat hukum *"Rechts Gevotg"*) tanpa terlebih dahulu adanya sebab-sebab/alasan hukum *(Vreem de Oozaak)* timbulnya perpisahan tersebut yang menurut Majelis Hakim dengan berpisahnya tempat tinggal tersebut tanpa saling memperdulikan lagi sebagai suami istri menunjukkan bahwa antara Pemohon dan Termohon telah terjadi perselisihan yang tidak ada harapan untuk rukun lagi sebagai suami istri, dengan demikian persaksian saksi dimaksud mempunyai kekuatan hukum;

Menimbang, bahwa terhadap keterangan 2 (dua) orang saksi Pemohon tersebut, maka secara materiil dalam penilaian Majelis kedua orang saksi tersebut telah memberikan keterangan berdasarkan pengetahuan saksi-saksi

Disclaimer
Kepaniteraan Mahkamah Agung Republik Indonesia berusaha untuk selalu mencantumkan informasi paling kini dan akurat sebagai bentuk komitmen Mahkamah Agung untuk pelayanan publik, transparansi dan akuntabilitas pelaksanaan fungsi peradilan. Namun dalam hal-hal tertentu masih dimungkinkan terjadi permasalahan teknis terkait dengan akurasi dan keterkinian informasi yang kami sajikan, hal mana akan terus kami perbaiki dari waktu kewaktu.
Dalam hal Anda menemukan inakurasi informasi yang termuat pada situs ini atau informasi yang seharusnya ada, namun belum tersedia, maka harap segera hubungi Kepaniteraan Mahkamah Agung RI melalui :
Email : kepaniteraan@mahkamahagung.go.id Telp : 021-384 3348 (ext.318)

sendiri, mempunyai keterkaitan dan hubungan, serta saling bersesuaian dan atau saling menguatkan antara satu dengan lainnya yang dapat digunakan untuk menguatkan suatu perbuatan sesuai ketentuan Pasal 307-309 R.Bg., yang pada pokoknya menyatakan sebagai berikut:

- Bahwa Pemohon dan Termohon adalah suami istri namun belum dikaruniai anak;
- Bahwa Pemohon telah berpisah rumah dengan Termohon sejak 2 (dua) bulan yang lalu, Pemohon pergi dari kediaman bersama;
- Bahwa keluarga kedua belah pihak sudah pernah berupaya untuk mendamaikan Pemohon dengan Termohon namun tidak berhasil;
- Bahwa sejak pisah rumah antara Pemohon dan Termohon sudah tidak saling berkominukasi dan tidak saling memperdulikan lagi;

Menimbang, bahwa apabila bukti surat dan kesaksian dua orang saksi serta anggapan hukum sebagaimana tersebut di atas dihubungkan dengan dalil-dalil permohonan Pemohon, Majelis menilai dalil-dalil permohonan Pemohon telah terbukti, dan dari pembuktian tersebut diketemukan fakta-fakta yuridis sebagai berikut:

- Bahwa Pemohon dan Termohon adalah suami istri dengan Kutipan Akta Nikah Nomor Nomor 857/53/XII/2007 tanggal 18 Desember 2007dikeluarkan oleh Pegawai Pencatat Nikah Kantor Urusan Agama (KUA) Kecamatan Rumpin, Kabupaten Bogor dan dari pernikahan tersebut Pemohon dan Termohon belum dikaruniai anak;
- Bahwa Pemohon telah berpisah rumah dengan Termohon sejak 2 (dua) bulan yang lalu, Pemohon pergi dari kediaman bersama;
- Bahwa sejak pisah rumah antara Pemohon dan Termohon sudah tidak saling berkominukasi dan tidak saling memperdulikan lagi;
- Bahwa Majelis berpendapat pisah rumah tanpa saling memperdulikan sebagai suami istri merupakan faktor yang sangat prinsipal dan sangat berpengaruh terhadap keutuhan rumah tangga;

Disclaimer
Kepaniteraan Mahkamah Agung Republik Indonesia berusaha untuk selalu mencantumkan informasi paling kini dan akurat sebagai bentuk komitmen Mahkamah Agung untuk pelayanan publik, transparansi dan akuntabilitas pelaksanaan fungsi peradilan. Namun dalam hal-hal tertentu masih dimungkinkan terjadi permasalahan teknis terkait dengan akurasi dan keterkinian informasi yang kami sajikan, hal mana akan terus kami perbaiki dari waktu kewaktu.
Dalam hal Anda menemukan inakurasi informasi yang termuat pada situs ini atau informasi yang seharusnya ada, namun belum tersedia, maka harap segera hubungi Kepaniteraan Mahkamah Agung RI melalui :
Email : kepaniteraan@mahkamahagung.go.id   Telp : 021-384 3348 (ext.318)

- Bahwa upaya yang dilakukan untuk mendamaikan pihak berperkara, melalui Majelis di persidangan ternyata tidak berhasil karenanya harapan untuk bisa menyatukan mereka kembali sangat sulit untuk bisa diwujudkan;
- Bahwa Pemohon sampai pada tahap kesimpulan masih tetap bersikeras ingin bercerai dengan Termohon;
- Bahwa dengan keadaan ini disimpulkan antara Pemohon dan Termohon sudah tidak ada lagi harapan akan hidup rukun kembali dalam meneruskan rumah tangganya;

Menimbang, bahwa berdasarkan fakta tersebut, Majelis berpendapat bahwa terhadap fakta tersebut dapat diterapkan ketentuan Pasal 19 huruf (f) Peraturan Pemerintah Nomor 9 Tahun 1975 jo. Pasal 116 huruf (f) Kompilasi Hukum Islam tahun 1991, yaitu rumah tangga Pemohon dan Termohon tidak ada harapan akan hidup rukun lagi dalam rumah tangga (*onheelbaare tweespalt*, atas dasar tersebut Majelis Hakim berpendapat bahwa rumah tangga Pemohon dan Termohon telah berada dalam kondisi pecah (*broken marriage*) dan sudah sulit untuk dipertahankan lagi;

Menimbang, bahwa mengenai siapa yang penyebabkan perselisihan dan pertengkaran dalam rumah tangga Pemohon dan Termohon, maka dalam hal ini Majelis juga sependapat dengan Yurisprudensi Mahkamah Agung RI Nomor 38 K/AG/1990 tanggal 5 Oktober 1991 yang mengandung abstraksi hukum bahwa tidak perlu lagi mempertimbangkan siapa yang menyebabkan timbulnya perselisihan tersebut, melainkan ditekankan pada keadaan itu sendiri, apakah telah pecah/retak dan sulit dipertahankan dan jika Hakim telah yakin pecahnya hati kedua belah pihak yang berperkara yang menyebabkan pecahnya rumah tangga mereka, maka berarti telah terpenuhi maksud Pasal 19 huruf (f) Peraturan Pemerintah Nomor 9 tahun 1975;

Menimbang, bahwa Majelis berpendapat, dengan meneruskan rumah tangga yang sudah tidak terjalin harmonis hanya akan membuat salah satu pihak atau bahkan kedua belah pihak dalam keadaan teraniaya *(dzulm),* maka hal tersebut merupakan bukti adanya kemudhorotan dalam rumah tangga Pemohon dan Termohon, maka sudah sepatutnya kemadhorotan tersebut dihilangkan. Terkait hal tersebut, Majelis sependapat dengan pendapat ahli

Disclaimer
Kepaniteraan Mahkamah Agung Republik Indonesia berusaha untuk selalu mencantumkan informasi paling kini dan akurat sebagai bentuk komitmen Mahkamah Agung untuk pelayanan publik, transparansi dan akuntabilitas pelaksanaan fungsi peradilan. Namun dalam hal-hal tertentu masih dimungkinkan terjadi permasalahan teknis terkait dengan akurasi dan keterkinian informasi yang kami sajikan, hal mana akan terus kami perbaiki dari waktu kewaktu.
Dalam hal Anda menemukan inakurasi informasi yang termuat pada situs ini atau informasi yang seharusnya ada, namun belum tersedia, maka harap segera hubungi Kepaniteraan Mahkamah Agung RI melalui :
Email : kepaniteraan@mahkamahagung.go.id Telp : 021-384 3348 (ext.318)

hukum Islam dalam kitab *Madza* Hurriyat *Azzauzaini Fii Athalaq*, Juz 1, halaman 83 yang diambil alih oleh Majelis sebagai pendapat sendiri, yang berbunyi berbunyi:

وقد اختار الاسلام نظام للطلاق حين تضطرب الحياة الزوجين ولم يعد ينفع فيها نصائح ولا صلح وحيث تصبح الربطة الزواج صورة من غير روح لان الاستمرار معناه ان يحكم على احد الزوجين بالسجن للمؤبد وهذا ظلم تأباه روح العداله

Artinya: *"Islam memilih lembaga talak/perceraian ketika rumah tangga sudah dianggap goncang serta dianggap sudah tidak bermanfaat lagi nasehat perdamaian dan hubungan suami isteri menjadi tanpa ruh (hampa) sebab dengan meneruskan perkawinan berarti menghukum salah satu suami isteri dengan penjara yang berkepanjangan. Hal ini adalah aniaya yang bertentangan dengan semangat keadilan";*

Menimbang, bahwa Majelis perlu mengambil alih pendapat Dr. Musthafa As Siba'i dalam Kitab Al Mar'ah bainal Fiqh wal Qanun halaman 100 sebagai berikut *"Sesungguhnya kehidupan suami isteri tidak akan tegak dengan adanya perpecahan dan pertentangan, selain itu justru akan menimbulkan bahaya yang serius terhadap pendidikan anak-anak dan perkembangan mereka, dan tidak ada kebaikannya mengumpulkan dua orang yang saling membenci. Dan kadang-kadang apapun sebab-sebab timbulnya perselisihan ini, baik yang membahayakan atau patut dapat diduga membahayakan, sesungguhnya yang lebih baik adalah mengakhiri hubungan perkawinan antara dua orang suami istri ini. Mudah-mudahan (sesudah itu) Allah menyediakan bagi mereka pasangan lain dalam hidupnya, barangkali dengan pasangan baru itu diperoleh ketenangan dan kedamaian";*

Menimbang, bahwa hukum perceraian menurut Islam berkisar pada hukum haram, wajib, sunat, mubah dan makruh, dan dalam perkara ini perceraian menjadi diperbolehkan, dan oleh karena *Imsak bil Ma'ruf* tidak berhasil maka perceraian dianggap sebagai *Tasrih bi Ihsan*;

Menimbang, bahwa secara sosiologis suatu perkawinan yang didalamnya sering terjadi perselisihan dan pertengkaran akan sulit untuk mewujudkan rumah tangga bahagia yang penuh rahmah dan kasih sayang seperti yang diharapkan setiap pasangan suami istri, justru sebaliknya mempertahankan

Disclaimer
Kepaniteraan Mahkamah Agung Republik Indonesia berusaha untuk selalu mencantumkan informasi paling kini dan akurat sebagai bentuk komitmen Mahkamah Agung untuk pelayanan publik, transparansi dan akuntabilitas pelaksanaan fungsi peradilan. Namun dalam hal-hal tertentu masih dimungkinkan terjadi permasalahan teknis terkait dengan akurasi dan keterkinian informasi yang kami sajikan, hal mana akan terus kami perbaiki dari waktu kewaktu.
Dalam hal Anda menemukan inakurasi informasi yang termuat pada situs ini atau informasi yang seharusnya ada, namun belum tersedia, maka harap segera hubungi Kepaniteraan Mahkamah Agung RI melalui :
Email : kepaniteraan@mahkamahagung.go.id   Telp : 021-384 3348 (ext.318)

perkawinan seperti itu (rumah tangga yang sudah pecah/retak) bisa menimbulkan dan mengakibatkan akibat negatif bagi semua pihak dan kesemuanya itu bisa mendatangkan mudharat, oleh karena itu harus dicari kemaslahatannya (yang terbaik), hal ini sesuai pula dengan kaidah fiqh yang berbunyi sebagai berikut:

درء المفاسد مقدم على جلب المصالح

Artinya: *“Menolak kesusahan (madlarat) itu harus didahulukan (diutamakan) daripada mengambil kemaslahatan”;*

Menimbang, bahwa berdasarkan pertimbangan-pertimbangan tersebut di atas, Majelis berkesimpulan, harus dinyatakan Termohon tidak hadir sesuai dengan Pasal 149 ayat (1) R.bg dan alasan perceraian yang didalilkan oleh Pemohon telah terbukti dan telah memenuhi maksud ketentuan Pasal 39 ayat (2) huruf (f) Penjelasan Undang-Undang Nomor 1 Tahun 1974 *jo* Pasal 19 huruf (f) Peraturan Pemerintah Nomor 9 Tahun 1975 *jo* Pasal 116 huruf (f) Kompilasi Hukum Islam, oleh karena itu dengan memperhatikan ketentuan Pasal 65 dan Pasal 70 ayat (1) Undang-undang Nomor 7 tahun 1989 sebagaimana telah diubah dengan Undang-undang Nomor 3 Tahun 2006 dan Undang-undang Nomor 50 Tahun 2009 *jo* Pasal 131 ayat (2) Kompilasi Hukum Islam, maka Majelis berkesimpulan permohonan Pemohon pada petitum angka 1 (satu) dan 2 (dua) dalam surat permohonannya, dapat dikabulkan dengan memberi izin kepada Pemohon untuk menjatuhkan talak satu raj’i terhadap Termohon di hadapan sidang Pengadilan Agama Tanjung Pati;

Menimbang, bahwa dengan dikabulkannya petitum angka 1 (satu) dan 2 (dua) dalam surat permohonan Pemohon tersebut maka Pengadilan telah memberikan izin kepada Pemohon untuk menjatuhkan talak terhadap Termohon, dan oleh karena perceraian ini adalah perceraian pertama antara Pemohon dan Termohon, maka berdasarkan Pasal 118 Kompilasi Hukum Islam talak yang dijatuhkan terhadap Termohon adalah talak raj’i;

Menimbang, bahwa untuk pelaksanaan ikrar talak dimaksud, maka berdasarkan ketentuan Pasal 70 ayat (3) Undang-Undang Peradilan Agama yang telah diubah dengan Undang-Undang Nomor 3 Tahun 2006 dan

Disclaimer
Kepaniteraan Mahkamah Agung Republik Indonesia berusaha untuk selalu mencantumkan informasi paling kini dan akurat sebagai bentuk komitmen Mahkamah Agung untuk pelayanan publik, transparansi dan akuntabilitas pelaksanaan fungsi peradilan. Namun dalam hal-hal tertentu masih dimungkinkan terjadi permasalahan teknis terkait dengan akurasi dan keterkinian informasi yang kami sajikan, hal mana akan terus kami perbaiki dari waktu kewaktu.
Dalam hal Anda menemukan inakurasi informasi yang termuat pada situs ini atau informasi yang seharusnya ada, namun belum tersedia, maka harap segera hubungi Kepaniteraan Mahkamah Agung RI melalui :
Email : kepaniteraan@mahkamahagung.go.id   Telp : 021-384 3348 (ext.318)

perubahan kedua dengan Undang-Undang Nomor 50 Tahun 2009 dan Pasal 123 Kompilasi Hukum Islam, akan ditetapkan setelah putusan izin cerai ini berkekuatan hukum tetap, dengan memanggil masing-masing pihak;

Menimbang, bahwa perkara ini termasuk dalam bidang perkawinan, maka sesuai ketentuan Pasal 89 ayat (1) Undang-Undang Nomor 7 Tahun 1989 Tentang Peradilan Agama sebagaimana yang telah di ubah dengan Undang-Undang Nomor 3 Tahun 2006, terakhir dengan perubahan kedua Undang-Undang Nomor 50 Tahun 2009, biaya perkara dibebankan kepada Pemohon;

Mengingat pasal-pasal peraturan perundang-undangan yang berlaku dan hukum syara yang berkaitan dengan perkara ini;

MENGADILI

1. Menyatakan Termohon telah dipanggil secara resmi dan patut untuk menghadap di persidangan, tidak hadir;

2. Mengabulkan permohonan Pemohon secara verstek;

3. Memberi izin kepada Pemohon **(Pemohon)** untuk menjatuhkan talak satu raj'i terhadap Termohon **(Termohon)** di depan sidang Pengadilan Agama Tanjung Pati;

4. Membebankan kepada Pemohon untuk membayar biaya perkara sejumlah Rp1.616.000,00 (satu juta enam ratus enam belas ribu rupiah);

Demikianlah diputuskan dalam musyawarah Majelis Hakim Pengadilan Agama Tanjung Pati pada hari Rabu tanggal 23 Desember 2020 Masehi bertepatan dengan tanggal 08 Jumadilawal 1442 Hijriah oleh Rahmiwati Andreas, S.H.I. sebagai Hakim Ketua, Dina Hayati, S.H.I., dan Fauziah Rahmah, S.H., masing-masing sebagai Hakim Anggota, putusan tersebut diucapkan pada hari itu juga dalam sidang terbuka untuk umum oleh Hakim Ketua beserta para Hakim Anggota tersebut, dan didampingi oleh Yeni Marliza, S.Sy., sebagai Panitera Pengganti, dengan dihadiri oleh Pemohon tanpa hadirnya Termohon.

Hakim Ketua,

Ttd

Disclaimer
Kepaniteraan Mahkamah Agung Republik Indonesia berusaha untuk selalu mencantumkan informasi paling kini dan akurat sebagai bentuk komitmen Mahkamah Agung untuk pelayanan publik, transparansi dan akuntabilitas pelaksanaan fungsi peradilan. Namun dalam hal-hal tertentu masih dimungkinkan terjadi permasalahan teknis terkait dengan akurasi dan keterkinian informasi yang kami sajikan, hal mana akan terus kami perbaiki dari waktu kewaktu.
Dalam hal Anda menemukan inakurasi informasi yang termuat pada situs ini atau informasi yang seharusnya ada, namun belum tersedia, maka harap segera hubungi Kepaniteraan Mahkamah Agung RI melalui :
Email : kepaniteraan@mahkamahagung.go.id   Telp : 021-384 3348 (ext.318)

Rahmiwati Andreas, S.H.I..

Hakim Anggota,

Ttd

Dina Hayati, S.H.I.

Hakim Anggota,

Ttd

Fauziah Rahmah, S.H.

Panitera Pengganti,

Ttd

Yeni Marliza, S.Sy.

Rincian biaya:

1. Pendaftaran	:	Rp	30.000,00
2. Proses	:	Rp	50.000,00
3. Panggilan	:	Rp	1.500.000,00
4. PNBP Panggilan	:	Rp	20.000,00
5. Redaksi	:	Rp	10.000,00
6. Meterai	:	Rp	6.000,00
Jumlah	:	Rp	1.616.000,00

(satu juta enam ratus enam belas ribu rupiah);

Disclaimer
Kepaniteraan Mahkamah Agung Republik Indonesia berusaha untuk selalu mencantumkan informasi paling kini dan akurat sebagai bentuk komitmen Mahkamah Agung untuk pelayanan publik, transparansi dan akuntabilitas pelaksanaan fungsi peradilan. Namun dalam hal-hal tertentu masih dimungkinkan terjadi permasalahan teknis terkait dengan akurasi dan keterkinian informasi yang kami sajikan, hal mana akan terus kami perbaiki dari waktu kewaktu.
Dalam hal Anda menemukan inakurasi informasi yang termuat pada situs ini atau informasi yang seharusnya ada, namun belum tersedia, maka harap segera hubungi Kepaniteraan Mahkamah Agung RI melalui :
Email : kepaniteraan@mahkamahagung.go.id Telp : 021-384 3348 (ext.318)
