# Supplementary material for: Classification of divorce causes during the COVID-19 pandemic using convolutional neural networks
Source: PeerJ Comput Sci. 2022 Jun 30;8:e998. doi: 10.7717/peerj-cs.998 (PMC9299239; doi:10.7717/peerj-cs.998)
Supplement: Supplemental Information 3 [file peerj-cs-08-998-s003.zip › KDRT Dataset/Data ke-22.pdf]

P U T U S A N

Nomor 3094/Pdt.G/2020/PA.JB

بِسْمِ اللهِ الرَّحْمٰنِ الرَّحِيْمِ

DEMI KEADILAN BERDASARKAN KETUHANAN YANG MAHA ESA

Pengadilan Agama Jakarta Barat yang memeriksa dan mengadili perkara Cerai Talak pada tingkat pertama dalam persidangan majelis hakim telah menjatuhkan putusan sebagai berikut dalam perkara antara:

PEMOHON, umur 32 tahun, lahir tanggal 22 September 1988 agama Islam, pendidikan SD, pekerjaan xxxx xxxxxx, tempat tinggal di KOTA JAKARTA BARAT;, sebagai **Pemohon**;

melawan

TERMOHON, umur 23 tahun, agama Islam, pendidikan SLTP, pekerjaan xxx xxxxx xxxxxx, tempat tinggal di KOTA JAKARTA BARAT;, sebagai **Termohon**;

Pengadilan Agama tersebut;

Telah membaca dan mempelajari berkas perkara;

Telah mendengar keterangan Pemohon dan memeriksa bukti-bukti di persidangan;

DUDUK PERKARA

Bahwa berdasarkan Permohonan Talak tertulisnya bertanggal 01 Desember 2020 yang didaftarkan pada tanggal 01 Desember 2020 dalam Register Induk Perkara Permohonan Talak Pengadilan Agama Jakarta Barat Nomor 3094/Pdt.G/2020/PA.JB., Pemohon mengajukan Cerai Talak terhadap Termohon dengan dalil-dalil sebagai berikut:

1. Bahwa, pada hari Selasa tanggal 26 Januari 2016, Pemohon dengan Termohon melangsungkan pernikahan yang dicatat oleh Pegawai Pencatat Nikah Kantor Urusan Agama Kecamatan Taman Sari Kota Jakarta Barat sesuai Kutipan Akta Nikah Nomor : xx tertanggal 26 Januari 2016;
2. Bahwa, setelah pernikahan tersebut Pemohon dengan Termohon bertempat tinggal di rumah orang tua Pemohon Kampung Duri Dalam RT.

Disclaimer
Kepaniteraan Mahkamah Agung Republik Indonesia berusaha untuk selalu mencantumkan informasi paling kini dan akurat sebagai bentuk komitmen Mahkamah Agung untuk pelayanan publik, transparansi dan akuntabilitas pelaksanaan fungsi peradilan. Namun dalam hal-hal tertentu masih dimungkinkan terjadi permasalahan teknis terkait dengan akurasi dan keterkinian informasi yang kami sajikan, hal mana akan terus kami perbaiki dari waktu kewaktu.
Dalam hal Anda menemukan inakurasi informasi yang termuat pada situs ini atau informasi yang seharusnya ada, namun belum tersedia, maka harap segera hubungi Kepaniteraan Mahkamah Agung RI melalui :
Email : kepaniteraan@mahkamahagung.go.id   Telp : 021-384 3348 (ext.318)

003 RW. 003 No. 19 Kelurahan Duri Selatan Kecamatan Tambora Kota Jakarta Barat dan setelah itu Pemohon dan Termohon pindah ke rumah orangtua Termohon di KOTA JAKARTA BARAT;

3. Bahwa, dalam perkawinan tersebut Pemohon dengan Termohon telah bercampur (*ba'da dukhul*) sebagaimana layaknya suami istri dan telah dikaruniai 2 (dua) orang anak yang masing-masing bernama :

3.1 (nama anak),, laki-laki, lahir di Jakarta tanggal 28 Juni 2016;

3.2 (nama anak),, laki-laki, lahir di Jakarta tanggal 07 Juni 2018;

4. Bahwa, pada mulanya kehidupan rumah tangga Pemohon dan Termohon dalam keadaan harmonis, namun kurang lebih sejak pertengahan tahun 2017 ketentraman rumah tangga Pemohon dengan Termohon mulai goyah, yang disebabkan oleh :

4.1 Termohon telah memiliki hubungan dengan pria idaman lain yang telah diketahui oleh Pemohon melalui foto di sosial media Facebook;

4.2 Termohon sulit untuk dinasehati dan diarahkan sehingga Pemohon merasa keberatan;

4.3 Termohon sering berkata-kata kasar kepada Pemohon seperti berkata nama binatang;

4.4 Termohon sering pergi meninggalkan tempat kediaman bersama tampa izin dari Pemohon;

4.5 Orangtua Termohon sering mencampuri urusan rumah tangga Pemohon dan Termohon;

4.6 Termohon sering mencerikan aib Pemohon kepada orang lain;

5. Bahwa, karena sebab-sebab tersebut diatas Pemohon dan Termohon sering berselisih dan bertengkar, namun percekcokan tersebut pada awalnya masih dalam batas-batas tertentu dan dapat diatasi oleh kedua belah pihak akan tetapi akhir-akhir ini percekcokan itu semakin bertambah tajam;

Disclaimer
Kepaniteraan Mahkamah Agung Republik Indonesia berusaha untuk selalu mencantumkan informasi paling kini dan akurat sebagai bentuk komitmen Mahkamah Agung untuk pelayanan publik, transparansi dan akuntabilitas pelaksanaan fungsi peradilan. Namun dalam hal-hal tertentu masih dimungkinkan terjadi permasalahan teknis terkait dengan akurasi dan keterkinian informasi yang kami sajikan, hal mana akan terus kami perbaiki dari waktu kewaktu.
Dalam hal Anda menemukan inakurasi informasi yang termuat pada situs ini atau informasi yang seharusnya ada, namun belum tersedia, maka harap segera hubungi Kepaniteraan Mahkamah Agung RI melalui :
Email : kepaniteraan@mahkamahagung.go.id Telp : 021-384 3348 (ext.318)

6. Bahwa, puncak keretakan hubungan antara Pemohon dengan Termohon tersebut terjadi kurang lebih pada bulan September tahun 2020, yang akibatnya Pemohon pergi meninggalkan rumah kediaman bersama;

7. Bahwa, akibat tersebut di atas, Pemohon sudah tidak sanggup lagi memberikan nasehat dan bimbingan kepada Termohon dan Pemohon sudah tidak sanggup lagi untuk melanjutkan rumah tangga dengan Termohon, maka jalan keluar yang terbaik bagi Pemohon menceraikan Termohon dihadapan sidang Pengadilan Agama Jakarta Barat;

8. Bahwa, dengan fakta-fakta tersebut diatas permohonan Pemohon telah memenuhi alasan perceraian sebagaimana diatur dalam Pasal 19 PP No. 9 Tahun 1975 jo. Pasal 116 Kompilasi Hukum Islam;

9. Bahwa, terhadap biaya yang timbul akibat perkara ini agar dibebankan sesuai dengan peraturan perundang-undangan yang berlaku;

Berdasarkan alasan/dalil-dalil diatas, Pemohon mohon agar Ketua Pengadilan Agama Jakarta Barat segera memeriksa dan mengadili perkara ini, selanjutnya menjatuhkan putusan yang amarnya berbunyi :

PRIMER

1. Mengabulkan permohonan Pemohon;

2. Memberikan izin kepada Pemohon (**PEMOHON**) untuk menjatuhkan talak satu Raj'i kepada Termohon (**TERMOHON**) dihadapan sidang Pengadilan Agama Jakarta Barat;

3. Menetapkan anak bernama :

3.1 (nama anak), laki-laki, lahir di Jakarta tanggal 28 Juni 2016;

3.2 (nama anak),, laki-laki, lahir di Jakarta tanggal 07 Juni 2018; berada dibawah hadhonah Pemohon (**PEMOHON**);

4. Menghukum Termohon untuk menyerahkan anak yang bernama : Arlinno Habib Ramadhan, laki-laki, lahir di Jakarta tanggal 07 Juni 2018 kepada Pemohon (**PEMOHON**);

5. Membebankan biaya perkara sesuai dengan ketentuan peraturan perundang-undangan yang berlaku;

SUBSIDER

Disclaimer
Kepaniteraan Mahkamah Agung Republik Indonesia berusaha untuk selalu mencantumkan informasi paling kini dan akurat sebagai bentuk komitmen Mahkamah Agung untuk pelayanan publik, transparansi dan akuntabilitas pelaksanaan fungsi peradilan. Namun dalam hal-hal tertentu masih dimungkinkan terjadi permasalahan teknis terkait dengan akurasi dan keterkinian informasi yang kami sajikan, hal mana akan terus kami perbaiki dari waktu kewaktu.
Dalam hal Anda menemukan inakurasi informasi yang termuat pada situs ini atau informasi yang seharusnya ada, namun belum tersedia, maka harap segera hubungi Kepaniteraan Mahkamah Agung RI melalui :
Email : kepaniteraan@mahkamahagung.go.id Telp : 021-384 3348 (ext.318)

Dan atau apabila Majelis Hakim yang menyidangkan perkara ini berpendapat lain, maka mohon untuk menjatuhkan putusan yang seadil-adilnya (*ex aequo et bono*);

Bahwa pada hari-hari persidangan yang telah ditetapkan, Pemohon menghadap ke persidangan, sedangkan Termohon tidak menghadap dan tidak pula mengutus orang lain sebagai wakil atau kuasanya yang sah untuk menghadap ke persidangan meskipun telah dipanggil secara resmi dan patut;

Bahwa karena Termohon tidak pernah menghadap kepersidangan, maka upaya damai dan mediasi tidak dapat dilaksanakan;

Bahwa selanjutnya Pemohon membaca Permohonan Talaknya tanpa mengajukan perubahan dan menacabut petitum No.3, 3.1, dan 3.2, dan untuk membuktikan dalil Permohonan Talaknya, Pemohon mengajukan bukti-bukti sebagai berikut;

A. Bukti Surat

1. Fotokopi Kartu Tanda Penduduk atas nama Pemohon (P.1);
2. Fotokopi Kutipan Akta Nikah Nomor xx tertanggal 26 Januari 2016, atas nama: Pemohon dan Termohon, (P.2);

B. Bukti Saksi:

1. **SAKSI 1**, umur 39 tahun, agama Islam, pekerjaan xxx xxxxx xxxxxx, tempat tinggal di KOTA JAKARTA BARAT, di bawah sumpahnya memberikan keterangan tentang keadaan rumah tangga Pemohon dengan Termohon sebagai berikut :

- Bahwa, hubungan saya dengan Pemohon adalah kakak kandung Pemohon;
- Bahwa, Pemohon adalah suami sah Termohon yang menikah pada tahun 2016;
- Bahwa, dari pernikahan tersebut sudah dikaruniai 2 (dua) orang anak;
- Bahwa, Pemohon dan Termohon terakhir tinggal bersama di rumah orang tua Pemohon di daerah Kampung Duri Kecamatan Tambora Kota Jakarta Barat;

Disclaimer
Kepaniteraan Mahkamah Agung Republik Indonesia berusaha untuk selalu mencantumkan informasi paling kini dan akurat sebagai bentuk komitmen Mahkamah Agung untuk pelayanan publik, transparansi dan akuntabilitas pelaksanaan fungsi peradilan. Namun dalam hal-hal tertentu masih dimungkinkan terjadi permasalahan teknis terkait dengan akurasi dan keterkinian informasi yang kami sajikan, hal mana akan terus kami perbaiki dari waktu kewaktu.
Dalam hal Anda menemukan inakurasi informasi yang termuat pada situs ini atau informasi yang seharusnya ada, namun belum tersedia, maka harap segera hubungi Kepaniteraan Mahkamah Agung RI melalui :
Email : kepaniteraan@mahkamahagung.go.id Telp : 021-384 3348 (ext.318)

- Bahwa, saya mengetahui keadaan rumah tangga Pemohon dan Termohon, sudah tidak harmonis lagi, sering berselisih dan bertengkar dari sejak tahun 2017;
- Bahwa, penyebabnya adalah; Termohon telah memiliki hubungan dengan pria idaman lain, Termohon sulit untuk dinasehati dan diarahkan, Termohon sering berkata-kata kasar kepada Pemohon, Termohon sering pergi meninggalkan tempat kediaman bersama tanpa izin dari Pemohon, orangtua Termohon sering mencampuri urusan rumah tangga Pemohon dan Termohon, dan Termohon sering mencerikan aib Pemohon kepada orang lain;
- Bahwa, puncak keretakan hubungan antara Pemohon dengan Termohon tersebut terjadi kurang lebih pada tahun 2020, yang akibatnya Pemohon pergi meninggalkan rumah kediaman bersama dan sejak saat itu antara Pemohon dan Termohon sudah brtidak lagi menjalin hubungan sebagaimana layaknya suami istri hingga sekarang;
- Bahwa, saksi dan keluaarga sudah menasehati Pemohon dan Termohon akan tetapi tidak berhasil ;

2. **SAKSI 2**, umur 36 tahun, agama Islam, pekerjaan xxx xxxxx xxxxxx, tempat tinggal di KOTA JAKARTA BARAT di bawah sumpahnya memberikan keterangan tentang keadaan rumah tangga Pemohon dengan Termohon sebagai berikut :

- Bahwa, hubungan saya dengan Pemohon adalah kakak kandung Pemohon;
- Bahwa, Pemohon adalah suami sah Termohon yang menikah pada tahun 2016;
- Bahwa, dari pernikahan tersebut sudah dikaruniai 2 (dua) orang anak;
- Bahwa, Pemohon dan Termohon terakhir tinggal bersama di rumah orang tua Pemohon di daerah Kampung Duri Kecamatan Tambora Kota Jakarta Barat;

Disclaimer
Kepaniteraan Mahkamah Agung Republik Indonesia berusaha untuk selalu mencantumkan informasi paling kini dan akurat sebagai bentuk komitmen Mahkamah Agung untuk pelayanan publik, transparansi dan akuntabilitas pelaksanaan fungsi peradilan. Namun dalam hal-hal tertentu masih dimungkinkan terjadi permasalahan teknis terkait dengan akurasi dan keterkinian informasi yang kami sajikan, hal mana akan terus kami perbaiki dari waktu kewaktu.
Dalam hal Anda menemukan inakurasi informasi yang termuat pada situs ini atau informasi yang seharusnya ada, namun belum tersedia, maka harap segera hubungi Kepaniteraan Mahkamah Agung RI melalui :
Email : kepaniteraan@mahkamahagung.go.id Telp : 021-384 3348 (ext.318)

- Bahwa, saya mengetahui keadaan rumah tangga Pemohon dan Termohon, sudah tidak harmonis lagi, sering berselisih dan bertengkar dari sejak tahun 2017;
- Bahwa, penyebabnya adalah; Termohon telah memiliki hubungan dengan pria idaman lain, Termohon sulit untuk dinasehati dan diarahkan, Termohon sering berkata-kata kasar kepada Pemohon, Termohon sering pergi meninggalkan tempat kediaman bersama tanpa izin dari Pemohon, orangtua Termohon sering mencampuri urusan rumah tangga Pemohon dan Termohon, dan Termohon sering mencerikan aib Pemohon kepada orang lain;
- Bahwa, puncak keretakan hubungan antara Pemohon dengan Termohon tersebut terjadi kurang lebih pada tahun 2020, yang akibatnya Pemohon pergi meninggalkan rumah kediaman bersama hingga sekarang;
- Bahwa, saksi dan keluaarga sudah menasehati Pemohon dan Termohon akan tetapi tidak berhasil ;

Bahwa selanjutnya Pemohon menyampaikan kesimpulan secara lisan yang menyatakan bahwa dalil Permohonan Talak Pemohon telah dikuatkan oleh keterangan dua orang saksi, karena itu mohon dikabulkan;

Bahwa untuk mempersingkat uraian putusan ini ditunjuk segala hal yang tercantum dalam berita acara sidang yang merupakan bagian tak terpisahkan dari putusan ini;

PERTIMBANGAN HUKUM

Menimbang, bahwa maksud dan tujuan Permohonan Talak Pemohon adalah sebagaimana diuraikan di atas;

Menimbang, bahwa perkara ini adalah perkara perceraian antara warga negara Indonesia yang beragama Islam, maka berdasarkan Pasal 49 ayat (1) huruf (a) Undang-Undang Nomor 7 Tahun 1989 tentang Peradilan Agama dan Pasal 49 huruf (a) Undang-Undang Nomor 3 Tahun 2006 tentang perubahan Undang-Undang Nomor 7 Tahun 1989, perkara ini menjadi wewenang absolut Pengadilan Agama;

Menimbang, bahwa Termohon beralamat di KOTA JAKARTA BARAT;; sesuai dengan permohonan Pemohon;

Disclaimer
Kepaniteraan Mahkamah Agung Republik Indonesia berusaha untuk selalu mencantumkan informasi paling kini dan akurat sebagai bentuk komitmen Mahkamah Agung untuk pelayanan publik, transparansi dan akuntabilitas pelaksanaan fungsi peradilan. Namun dalam hal-hal tertentu masih dimungkinkan terjadi permasalahan teknis terkait dengan akurasi dan keterkinian informasi yang kami sajikan, hal mana akan terus kami perbaiki dari waktu kewaktu.
Dalam hal Anda menemukan inakurasi informasi yang termuat pada situs ini atau informasi yang seharusnya ada, namun belum tersedia, maka harap segera hubungi Kepaniteraan Mahkamah Agung RI melalui :
Email : kepaniteraan@mahkamahagung.go.id Telp : 021-384 3348 (ext.318)

Menimbang, bahwa alamat Termohon tersebut, termasuk wilayah hukum (yurisdiksi) Pengadilan Agama Jakarta Barat, oleh karena itu sesuai dengan pasal 66 ayat (1) Undang-Undang Nomor 7 tahun 1989 tentang Peradilan Agama, perkara ini termasuk wewenang relative Pengadilan Agama Jakarta Barat;

Menimbang, bahwa pada hari-hari persidangan yang telah ditetapkan, Pemohon menghadap ke persidangan, sedangkan Termohon tidak pernah menghadap dan tidak pula menyuruh orang lain sebagai wakil atau kuasanya untuk menghadap kepersidangan;

Menimbang, bahwa panggilan terhadap Termohon telah disampaikan secara resmi dan patut, akan tetapi Termohon tidak hadir dan tidak pula terbukti ketidakhadiran Termohon tersebut disebabkan oleh suatu alasan yang sah menurut hukum, oleh karena itu sesuai dengan pasal 125 ayat (1) dan pasal 126 HIR, perkara ini dapat diperiksa dan diputus tanpa hadirnya Termohon (Verstek);

Menimbang, bahwa karena Termohon tidak pernah menghadap ke persidangan, maka upaya damai dan mediasi sebagaimana diwajibkan pasal 130 ayat 1 HIR, dan pasal 17 ayat 1 Peraturan Mahkamah Agung Nomor 1 tahun 2016 tidak dapat dilaksanakan;

Menimbang, bahwa Pemohon mendalilkan bahwa Pemohon dan Termohon adalah suami istri yang menikah pada tanggal 26 Januari 2016;

Menimbang, bahwa untuk membuktikan kebenaran dalil Permohonan Talaknya, Pemohon mengajukan bukti tertulis bertanda P.2.

Menimbang, bahwa bukti P.2 tersebut adalah fotokopi dari akta autentik berupa Buku Kutipan Akta Nikah yang dikeluarkan oleh pejabat yang berwenang, telah di-nazegelen dan telah dicocokkan dengan aslinya ternyata cocok, dan isinya menunjukkan bahwa Pemohon dan Termohon telah menikah pada tanggal 26 Januari 2016;

Menimbang, bahwa berdasarkan pertimbangan di atas, harus dinyatakan bahwa bukti P.1 tersebut telah memenuhi syarat formil dan syarat materil alat bukti, dan mempunyai kekuatan bukti yang sempurna dan mengikat, oleh karena itu harus dinyatakan terbukti bahwa antara Pemohon dan Termohon ada hubungan hukum perkawinan, dan karenanya harus dinyatakan

Disclaimer
Kepaniteraan Mahkamah Agung Republik Indonesia berusaha untuk selalu mencantumkan informasi paling kini dan akurat sebagai bentuk komitmen Mahkamah Agung untuk pelayanan publik, transparansi dan akuntabilitas pelaksanaan fungsi peradilan. Namun dalam hal-hal tertentu masih dimungkinkan terjadi permasalahan teknis terkait dengan akurasi dan keterkinian informasi yang kami sajikan, hal mana akan terus kami perbaiki dari waktu kewaktu.
Dalam hal Anda menemukan inakurasi informasi yang termuat pada situs ini atau informasi yang seharusnya ada, namun belum tersedia, maka harap segera hubungi Kepaniteraan Mahkamah Agung RI melalui :
Email : kepaniteraan@mahkamahagung.go.id Telp : 021-384 3348 (ext.318)

bahwa Permohonan Talak Pemohon mempunyai dasar hukum (*legal standing*) dan Pemohon adalah pihak yang mempunyai kepentingan hukum (*persona standi in judicio*) dalam perkara ini;

Menimbang, bahwa dalil Permohonan Talak Pemohon untuk bercerai dengan Termohon adalah karena;

- Sejak tahun 2017 keharmonisan rumah tangga Pemohon dengan Termohon mulai goyah, disebabkan:
  - Karena; Termohon telah memiliki hubungan dengan pria idaman lain, Termohon sulit untuk dinasehati dan diarahkan, Termohon sering berkata-kata kasar kepada Pemohon, Termohon sering pergi meninggalkan tempat kediaman bersama tanpa izin dari Pemohon, orangtua Termohon sering mencampuri urusan rumah tangga Pemohon dan Termohon, dan Termohon sering mencerikan aib Pemohon kepada orang lain;
  - Bahwa, puncak perselisihan dan pertengkaran antara Pemohon dengan Termohon terjadi pada tahun 2020, Pemohon pergi meninggalkan rumah kediaman bersama hingga sekarang;

Menimbang, bahwa untuk membuktikan kebenaran dalil Permohonan Talaknya, Pemohon mengajukan bukti berupa dua orang saksi sebagaimana termuat pada duduk perkara di atas;

Menimbang, bahwa saksi satu dan saksi dua Pemohon sudah dewasa, berakal sehat, dan memberikan keterangan di bawah sumpahnya tentang apa yang dilihat dan didengarnya sendiri tentang rumah tangga Pemohon dengan Termohon dan keterangannya relevan dengan dalil Permohonan Talak Pemohon;

Menimbang, bahwa dua saksi di atas telah memenuhi batas minimal saksi dan keterangannya saling bersesuaian satu sama lain, maka berdasarkan pasal 145 ayat 1 dan Pasal 171 HIR, Pasal 76 ayat 1 Undang-Undang Nomor 7 tahun 1989 tentang Peradilan Agama dan Pasal 22 ayat (2) Peraturan Pemerintah Nomor 9 Tahun 1975, kesaksian dua orang saksi tersebut dapat diterima sebagai bukti;

Menimbang, bahwa berdasarkan dalil Permohonan Talak Pemohon dan keterangan dua orang saksi tersebut di atas, ditemukan fakta sebagai berikut;

Disclaimer
Kepaniteraan Mahkamah Agung Republik Indonesia berusaha untuk selalu mencantumkan informasi paling kini dan akurat sebagai bentuk komitmen Mahkamah Agung untuk pelayanan publik, transparansi dan akuntabilitas pelaksanaan fungsi peradilan. Namun dalam hal-hal tertentu masih dimungkinkan terjadi permasalahan teknis terkait dengan akurasi dan keterkinian informasi yang kami sajikan, hal mana akan terus kami perbaiki dari waktu kewaktu.
Dalam hal Anda menemukan inakurasi informasi yang termuat pada situs ini atau informasi yang seharusnya ada, namun belum tersedia, maka harap segera hubungi Kepaniteraan Mahkamah Agung RI melalui :
Email : kepaniteraan@mahkamahagung.go.id Telp : 021-384 3348 (ext.318)

- Rumah tangga Pemohon dengan Termohon sudah tidak harmonis sejak tahun 2017 sebabnya adalah; Termohon telah memiliki hubungan dengan pria idaman lain, Termohon sulit untuk dinasehati dan diarahkan, Termohon sering berkata-kata kasar kepada Pemohon, Termohon sering pergi meninggalkan tempat kediaman bersama tanpa izin dari Pemohon, orangtua Termohon sering mencampuri urusan rumah tangga Pemohon dan Termohon, dan Termohon sering mencerikan aib Pemohon kepada orang lain;

Menimbang, bahwa berdasarkan fakta tersebut, majelis berkesimpulan bahwa tujuan perkawinan untuk mewujudkan rumah tangga yang bahagia, sakinah, mawaddah dan rahmah sebagaimana dimaksud dalam al-Quran surat Al-Rum ayat 21 yang telah ditransformasi menjadi undang-undang Negara yang termuat dalam pasal 1 Undang-Undang Nomor 1 Tahun 1974 tentang Perkawinan dan pasal 2 dan 3 Kompilasi Hukum Islam, tidak terwujud lagi dalam rumah tangga Pemohon dengan Termohon;

Menimbang, bahwa Pemohon dalam petitum angka 2 (dua) menuntut agar Pemohon diberi izin untuk menjatuhkan talak terhadap Termohon, dan permohonan tersebut akan dipertimbangkan sebagai berikut;

Menimbang, bahwa berdasarkan Pasal 39 Undang Undang Nomor 1 Tahun 1974 tentang perkawinan menentukan bahwa untuk melakukan suatu perceraian harus ada cukup alasan dimana suami istri tidak akan dapat hidup rukun sebagai suami istri sebagaimana dimaksud dalam Pasal 1 Undang-Undang Nomor 1 Tahun 1974 jo. Pasal 3 Kompilasi Hukum Islam dan juga al-Qur’an surat ar-Rum ayat 21;

Menimbang, bahwa sebagaimana terbukti Pemohon dan Termohon telah berpisah dan sudah tidak ada komunikasi lagi, dapat diambil suatu kesimpulan bahwa sudah menjadi pecah *(broken marriage)* dipandang telah memenuhi unsur-unsur terjadinya perceraian sebagaimana ditetapkan dalam Pasal 39 ayat (2) Undang Undang Nomor 1 Tahun 1974 jo. Pasal 19 huruf (f) Peraturan Pemerintah Nomor 9 Tahun 1975 Jo. Pasal 116 huruf (f) Kompilasi Hukum Islam;

Menimbang, bahwa terhadap perkara ini dapat diterapkan pula yurisprudensi Mahkamah Agung RI Nomor 379 K/AG/1995 tanggal 26 Maret

Disclaimer
Kepaniteraan Mahkamah Agung Republik Indonesia berusaha untuk selalu mencantumkan informasi paling kini dan akurat sebagai bentuk komitmen Mahkamah Agung untuk pelayanan publik, transparansi dan akuntabilitas pelaksanaan fungsi peradilan. Namun dalam hal-hal tertentu masih dimungkinkan terjadi permasalahan teknis terkait dengan akurasi dan keterkinian informasi yang kami sajikan, hal mana akan terus kami perbaiki dari waktu kewaktu.
Dalam hal Anda menemukan inakurasi informasi yang termuat pada situs ini atau informasi yang seharusnya ada, namun belum tersedia, maka harap segera hubungi Kepaniteraan Mahkamah Agung RI melalui :
Email : kepaniteraan@mahkamahagung.go.id Telp : 021-384 3348 (ext.318)

1997 yang abstraksi hukumnya menyatakan apabila suami istri terjadi perselisihan dan terjadi pisah tempat tinggal, maka rumah tangga mereka telah pecah dan permohonan/ gugatan cerai telah memenuhi ketentuan Pasal 19 huruf (f) Peraturan Pemerintah Nomor 9 Tahun 1975 dan permohonan tersebut harus dikabulkan;

Menimbang, bahwa dalam hal ini Majelis Hakim perlu mengetengahkan Firman Allah SWT dalam Al Qur'an Surat Al Baqarah ayat 229 yang berbunyi artinya : "Talak (yang dapat dirujuk) itu dua kali, setelah itu boleh dirujuk lagi dengan cara yang ma'ruf atau menceraikan dengan cara yang baik;

Menimbang, bahwa berdasarkan pertimbangan-pertimbangan tersebut diatas dan Pemohon belum pernah menjatuhkan talak/ baru menjatuhkan talak satu raj'i satu kali, maka petitum permohonan Pemohon mengenai izin talak raj'i tersebut telah memenuhi ketentuan Pasal 118 Kompilasi Hukum Islam, oleh karena permohonan Pemohon dapat dikabulkan;

Menimbang, bahwa Pemohon menacabut petitum No.3, 3.1, dan 3.2, maka tidak perlu ditetapkan;

Menimbang, bahwa berdasarkan Pasal 89 ayat (1) Undang-Undang Nomor 7 Tahun 1989 tentang Peradilan Agama, pasal 90 ayat (1) Undang-Undang Nomor 3 Tahun 2006 tentang perubahan Undang-Undang Nomor 7 tahun 1989, pasal 91 A Undang-Undang Nomor 50 Tahun 2009 tentang perubahan kedua Undang-Undang Nomor 7 tahun 1989, dan Peraturan Pemerintah Nomor 5 tahun 2019 tentang Penerimaan Negara Bukan Pajak (PNBP), biaya perkara ini dibebankan kepada Pemohon;

Mengingat, segala peraturan perundang-undangan yang berlaku dan dalil-dalil syar'i yang berkaitan dengan perkara ini;

M E N G A D I L I

1. Menyatakan, Termohon yang telah dipanggil secara resmi dan patut untuk menghadap di persidangan,tidak hadir;

2. Mengabulkan permohonan Pemohon dengan verstek;

3. Memberikan izin kepada Pemohon (**PEMOHON**) untuk menjatuhkan talak satu Raj'i kepada Termohon (**TERMOHON**) dihadapan sidang Pengadilan Agama Jakarta Barat;

Disclaimer
Kepaniteraan Mahkamah Agung Republik Indonesia berusaha untuk selalu mencantumkan informasi paling kini dan akurat sebagai bentuk komitmen Mahkamah Agung untuk pelayanan publik, transparansi dan akuntabilitas pelaksanaan fungsi peradilan. Namun dalam hal-hal tertentu masih dimungkinkan terjadi permasalahan teknis terkait dengan akurasi dan keterkinian informasi yang kami sajikan, hal mana akan terus kami perbaiki dari waktu kewaktu.
Dalam hal Anda menemukan inakurasi informasi yang termuat pada situs ini atau informasi yang seharusnya ada, namun belum tersedia, maka harap segera hubungi Kepaniteraan Mahkamah Agung RI melalui :
Email : kepaniteraan@mahkamahagung.go.id Telp : 021-384 3348 (ext.318)

4. Membebankan kepada Pemohon untuk membayar biaya perkara yang hingga kini dihitung sejumlah Rp. 531.000 (lima ratus tiga puluh satu ribu rupiah);

Demikian diputus dalam sidang permusyawaratan Majelis Hakim Pengadilan Agama Jakarta Barat pada Rabu tanggal 23 Desember 2020 Miladiyah bertepatan dengan 8 Jumadil Awwal 1442 Hijriah, oleh Dr. Mustar, M.H.., Ketua Majelis, Dra. Hj. Absari, M.H. dan Drs. H. Fauzi, M.H.I**,** Hakim-Hakim Anggota, yang ditunjuk oleh Ketua Pengadilan Agama Jakarta Barat untuk memeriksa dan memutus perkara ini, dan dibacakan oleh Ketua Majelis tersebut dalam sidang terbuka untuk umum pada hari itu juga dengan didampingi oleh Dra. Hj. Absari, M.H. dan Drs. H. Fauzi, M.H.I. Hakim-hakim Anggota serta Ahlan, S.H., sebagai Panitera Pengganti dengan dihadiri oleh Pemohon tanpa hadirnya Termohon;

Ketua Majelis

Dr. Mustar, M.H.

Hakim Anggota	Hakim Anggota
**Dra. Hj. Absari, M.H.**	**Drs. H. Fauzi, M.H.I**

Panitera Pengganti

Ahlan, S.H.

Perincian Biaya :

1. 1	Pendaftaran	Rp	30.000,-
2 . 2	Proses	Rp	75.000,-
3	Panggilan	Rp	390.000,-

Disclaimer
Kepaniteraan Mahkamah Agung Republik Indonesia berusaha untuk selalu mencantumkan informasi paling kini dan akurat sebagai bentuk komitmen Mahkamah Agung untuk pelayanan publik, transparansi dan akuntabilitas pelaksanaan fungsi peradilan. Namun dalam hal-hal tertentu masih dimungkinkan terjadi permasalahan teknis terkait dengan akurasi dan keterkinian informasi yang kami sajikan, hal mana akan terus kami perbaiki dari waktu kewaktu.
Dalam hal Anda menemukan inakurasi informasi yang termuat pada situs ini atau informasi yang seharusnya ada, namun belum tersedia, maka harap segera hubungi Kepaniteraan Mahkamah Agung RI melalui :
Email : kepaniteraan@mahkamahagung.go.id Telp : 021-384 3348 (ext.318)

3	PNBP Relaas	Rp	20.000,-
4.4	Redaksi	Rp	10.000,-
5.5	Meterai	Rp	6.000,-
	**J u m l a h**	**Rp**	**531.000,-**

(lima ratus tiga puluh satu ribu rupiah);)

Disclaimer
Kepaniteraan Mahkamah Agung Republik Indonesia berusaha untuk selalu mencantumkan informasi paling kini dan akurat sebagai bentuk komitmen Mahkamah Agung untuk pelayanan publik, transparansi dan akuntabilitas pelaksanaan fungsi peradilan. Namun dalam hal-hal tertentu masih dimungkinkan terjadi permasalahan teknis terkait dengan akurasi dan keterkinian informasi yang kami sajikan, hal mana akan terus kami perbaiki dari waktu kewaktu.
Dalam hal Anda menemukan inakurasi informasi yang termuat pada situs ini atau informasi yang seharusnya ada, namun belum tersedia, maka harap segera hubungi Kepaniteraan Mahkamah Agung RI melalui :
Email : kepaniteraan@mahkamahagung.go.id Telp : 021-384 3348 (ext.318)
