# Supplementary material for: Classification of divorce causes during the COVID-19 pandemic using convolutional neural networks
Source: PeerJ Comput Sci. 2022 Jun 30;8:e998. doi: 10.7717/peerj-cs.998 (PMC9299239; doi:10.7717/peerj-cs.998)
Supplement: Supplemental Information 3 [file peerj-cs-08-998-s003.zip › KDRT Dataset/Data ke-23.pdf]

PUTUSAN

Nomor 4620/Pdt.G/2020/PA.Sda

بِسْمِ اللهِ الرَّحْمَنِ الرَّحِيْمِ

DEMI KEADILAN BERDASARKAN KETUHANAN YANG MAHA ESA

Pengadilan Agama Sidoarjo yang memeriksa dan mengadili perkara perdata pada tingkat pertama dalam sidang majelis hakim telah menjatuhkan putusan dalam perkara Cerai Talak antara:

PEMOHON, tempat tanggal lahir, Lamongan 13 Juli 1978/ umur 42 tahun, agama Islam, pekerjaan Wiraswasta, Pendidikan SMA, tempat kediaman di Kabupaten Sidoarjo, dalam hal ini telah memberi kuasa kepada **Mansur, S.H.** Advokat & Konsultan Hukum, berkedudukan dan berkantor pada kantor Advokat **“MANSUR, S.H. dan REKAN”** di Perumahan Florencia Regency CF-10 Kelurahan Gebang Kecamatan Sidoarjo Kabupaten Sidoarjo, berdasarkan Surat Kuasa Khusus tanggal 15 Desember 2020, sebagai Pemohon**;**

melawan

TERMOHON, tempat tanggal lahir, Lamongan 10 Mei 1980/ umur 40 tahun, agama Islam, pekerjaan Karyawan Toko, Pendidikan SMP, tempat kediaman di Kabupaten Sidoarjo, sebagai Termohon;

Pengadilan Agama tersebut;

Telah mempelajari surat-surat yang berkaitan dengan perkara ini;

Telah mendengar keterangan Pemohon/kuasanya, dan para saksi di muka sidang;

DUDUK PERKARA

Bahwa Pemohon dalam surat pemohonannya tanggal 15 Desember 2020 telah mengajukan permohonan Cerai Talak, yang telah terdaftar di Kepaniteraan Pengadilan Agama Sidoarjo, dengan Nomor 4620/Pdt.G/2020/PA.Sda, tanggal 15 Desember 2020 dengan dalil-dalil pada pokoknya sebagai berikut:

Disclaimer
Kepaniteraan Mahkamah Agung Republik Indonesia berusaha untuk selalu mencantumkan informasi paling kini dan akurat sebagai bentuk komitmen Mahkamah Agung untuk pelayanan publik, transparansi dan akuntabilitas pelaksanaan fungsi peradilan. Namun dalam hal-hal tertentu masih dimungkinkan terjadi permasalahan teknis terkait dengan akurasi dan keterkinian informasi yang kami sajikan, hal mana akan terus kami perbaiki dari waktu kewaktu.
Dalam hal Anda menemukan inakurasi informasi yang termuat pada situs ini atau informasi yang seharusnya ada, namun belum tersedia, maka harap segera hubungi Kepaniteraan Mahkamah Agung RI melalui :
Email : kepaniteraan@mahkamahagung.go.id Telp : 021-384 3348 (ext.318)

1. Bahwa Pemohon dan Termohon telah menikah pada tanggal 18 Januari 2006 dihadapan Pegawai Pencatat Nikah pada Kantor Urusan Agama Kecamatan Karanggeneng Kabupaten Lamongan dengan Kutipan Akta Nikah Nomor 37/37/I/2006;

2. Bahwa setelah melangsungkan perkawinan Pemohon dan Termohon telah hidup bersama sebagaimana layaknya suami istri dan hidup bersama di Kabupaten Sidoarjo;

3. Bahwa selama perkawinan tersebut Pemohon dan Termohon dikaruniai 1 (satu) orang anak:

- **Anak I**, laki-laki, umur 11 tahun (ikut Termohon).

4. Bahwa semula kehidupan rumah tangga Pemohon dan Termohon berjalan tentram, bahagia dan harmonis. Namun sejak 2,5 tahun yang lalu, sering terjadi perselisihan dan pertengkaran yang terjadi disebabkan karena:

- Termohon selalu menolak hubungan suami istri.

5. Bahwa sejak 2 tahun yang lalu, perselisihan dan pertengkaran antara Pemohon dan Termohon tersebut kerap kali terjadi, sulit dirukunkan kembali, hingga akhirnya 2 tahun yang lalu, Termohon keluar rumah dan memutuskan untuk bertempat tinggal di Kabupaten Sidoarjo. Dengan demikian Pemohon dan Termohon sudah pisah rumah ± 2 tahun.

6. Bahwa dengan kejadian tersebut rumah tangga antara Pemohon dan Termohon sudah tidak lagi dapat dibina dengan baik sehingga tujuan perkawinan untuk membentuk rumah tangga yang sakinah, mawaddah dan warahmah sudah sulit dipertahankan lagi, dan karenanya agar masing-masing pihak lebih jauh melanggar norma hukum dan norma agama maka perceraian merupakan alternative terakhir untuk menyelesaikan permasalahan rumah tangga antara Pemohon dengan Termohon.

7. Bahwa Pemohon bersedia membayar biaya perkara yang timbul akibat permohonan tersebut.

Bahwa atas dasar alasan-alasan tersebut, Pemohon mohon kepada Pengadilan Agama Sidoarjo, untuk berkenan memanggil dan memeriksa Pemohon dan Termohon, selanjutnya menjatuhkan putusan sebagai berikut:

Disclaimer
Kepaniteraan Mahkamah Agung Republik Indonesia berusaha untuk selalu mencantumkan informasi paling kini dan akurat sebagai bentuk komitmen Mahkamah Agung untuk pelayanan publik, transparansi dan akuntabilitas pelaksanaan fungsi peradilan. Namun dalam hal-hal tertentu masih dimungkinkan terjadi permasalahan teknis terkait dengan akurasi dan keterkinian informasi yang kami sajikan, hal mana akan terus kami perbaiki dari waktu kewaktu.
Dalam hal Anda menemukan inakurasi informasi yang termuat pada situs ini atau informasi yang seharusnya ada, namun belum tersedia, maka harap segera hubungi Kepaniteraan Mahkamah Agung RI melalui :
Email : kepaniteraan@mahkamahagung.go.id   Telp : 021-384 3348 (ext.318)

PRIMER:

1. Mengabulkan permohonan Pemohon;

2. Memberi ijin kepada Pemohon (**PEMOHON**) untuk menjatuhkan talak satu raj'i terhadap Termohon (**TERMOHON**) di depan sidang Pengadilan Agama Sidoarjo.

3. Membebankan kepada Pemohon untuk membayar biaya perkara ini sesuai dengan peraturan yang berlaku.

SUBSIDER:

Atau apabila Majelis Hakim Pengadilan Agama Sidoarjo berpendapat lain, mohon putusan yang seadil-adilnya.

Bahwa Pemohon telah menguasakan kepada **Mansur, S.H.,** Advokat, berdasarkan surat kuasa khusus tertanggal 15 Desember 2020, telah dilampiri fotokopi Kartu Tanda Pengenal Advokat dan Berita Acara Sumpah serta telah terdaftar di Kepaniteraan Pengadilan Agama Sidoarjo dengan Nomor 2813/kuasa/12/2020/PA.Sda tanggal 15 Desember 2020;

Bahwa pada persidangan yang telah ditetapkan Pemohon telah hadir menghadap di persidangan, sedangkan Termohon tidak hadir menghadap di persidangan dan tidak menyuruh orang lain untuk menghadap sebagai wakil/kuasanya yang sah, meskipun Termohon telah dipanggil secara resmi dan patut, sedangkan tidak ternyata bahwa tidak datangnya Termohon tersebut disebabkan oleh suatu alasan yang sah;

Bahwa majelis hakim telah menasehati Pemohon agar berpikir untuk tidak bercerai dengan Termohon, tetapi Pemohon tetap pada dalil-dalil pemohonannya untuk bercerai dengan Termohon;

Bahwa perkara ini tidak dapat dimediasi karena Termohon tidak pernah datang menghadap meskipun telah dipanggil secara resmi dan patut, selanjutnya dimulai pemeriksaan dengan membacakan surat pemohonan Pemohon yang maksud dan tujuannya tetap dipertahankan oleh Pemohon;

Bahwa untuk menguatkan dalil-dalil pemohonannya, Pemohon telah mengajukan bukti surat berupa:

Disclaimer
Kepaniteraan Mahkamah Agung Republik Indonesia berusaha untuk selalu mencantumkan informasi paling kini dan akurat sebagai bentuk komitmen Mahkamah Agung untuk pelayanan publik, transparansi dan akuntabilitas pelaksanaan fungsi peradilan. Namun dalam hal-hal tertentu masih dimungkinkan terjadi permasalahan teknis terkait dengan akurasi dan keterkinian informasi yang kami sajikan, hal mana akan terus kami perbaiki dari waktu kewaktu.
Dalam hal Anda menemukan inakurasi informasi yang termuat pada situs ini atau informasi yang seharusnya ada, namun belum tersedia, maka harap segera hubungi Kepaniteraan Mahkamah Agung RI melalui :
Email : kepaniteraan@mahkamahagung.go.id Telp : 021-384 3348 (ext.318)

1. Fotokopi Fotokopi Kartu Tanda Penduduk atas nama **Pemohon** yang dikeluarkan oleh Kepala Dinas Kependudukan dan Catatan Sipil Kabupaten Sidoarjo, bermeterai cukup dan telah sesuai dengan aslinya, (P-1);

2. Fotokopi Kutipan Akta Nikah Nomor 37/37/I/2006, yang dikeluarkan oleh PPN pada Kantor Urusan Agama Kecamatan Karanggeneng Kabupaten Lamongan, bermeterai cukup dan telah sesuai dengan aslinya, (P-2);

Saksi I, umur 48 tahun, agama Islam, pekerjaan ibu rumah tangga, tempat tinggal di Kabupaten Sidoarjo, didalam sidang saksi memberikan keterangan di atas sumpah yang pokoknya adalah sebagai berikut :

- Bahwa saksi kenal dengan Pemohon dan Termohon, saksi sebagai bibi Pemohon;
- Bahwa Saksi mengetahui Pemohon dengan Termohon sebagai suami istri yang menikah pada bulan Januari 2006;
- Bahwa Saksi mengetahui selama dalam pernikahan Pemohon dengan Termohon tinggal dan membina rumah tangga di Kabupaten Sidoarjo;
- Bahwa Saksi mengetahui selama dalam pernikahan Pemohon dan Termohon sudah hidup seperti layaknya suami isteri, dan telah dikaruniai 1 orang anak bernama **Anak I,** umur 11 tahun;
- Bahwa Saksi mengtahui bahwa Pemohon berselisih dan bertengkar dengan Termohon sejak 2,5 tahun yang lalu;
- Bahwa Saksi mengetahui penyebab pertengkaran tersebut karena Termohon suka menolak diajak hubungan suami istri oleh Pemohon;
- Bahwa saksi tahu Pemohon dan Termohon sudah pisah rumah sekitar 1,5 tahun yang lalu, Termohon pergi dari rumah meninggalkan Pemohon, dan sejak saat itu mereka tidak lagi menjalankan kewajiban sebagai layaknya suami istri;
- Bahwa Saksi telah berulang kali berusaha merukunkan mereka tapi sampai sekarang tetap tidak berhasil;

Saksi II, umur 37 tahun, agama Islam, pekerjaan Wiraswasta, tempat tinggal di Kabupaten Sidoarjo, didalam sidang saksi memberikan keterangan di atas sumpahnya yang pokoknya adalah sebagai berikut :

Disclaimer
Kepaniteraan Mahkamah Agung Republik Indonesia berusaha untuk selalu mencantumkan informasi paling kini dan akurat sebagai bentuk komitmen Mahkamah Agung untuk pelayanan publik, transparansi dan akuntabilitas pelaksanaan fungsi peradilan. Namun dalam hal-hal tertentu masih dimungkinkan terjadi permasalahan teknis terkait dengan akurasi dan keterkinian informasi yang kami sajikan, hal mana akan terus kami perbaiki dari waktu kewaktu.
Dalam hal Anda menemukan inakurasi informasi yang termuat pada situs ini atau informasi yang seharusnya ada, namun belum tersedia, maka harap segera hubungi Kepaniteraan Mahkamah Agung RI melalui :
Email : kepaniteraan@mahkamahagung.go.id   Telp : 021-384 3348 (ext.318)

- Bahwa saksi kenal dengan Pemohon dan Termohon, saksi sebagai adik sepupu Pemohon;
- Bahwa Saksi mengetahui Pemohon dengan Termohon sebagai suami istri yang menikah pada bulan Januari 2006;
- Bahwa Saksi mengetahui selama dalam pernikahan Pemohon dengan Termohon tinggal dan membina rumah tangga di Kabupaten Sidoarjo;
- Bahwa Saksi mengetahui selama dalam pernikahan Pemohon dan Termohon sudah hidup seperti layaknya suami isteri, dan telah dikaruniai 1 orang anak bernama **Anak I,** umur 11 tahun;
- Bahwa Saksi mengtahui bahwa Pemohon berselisih dan bertengkar dengan Termohon sejak 2,5 tahun yang lalu;
- Bahwa menurut cerita Pemohon penyebab pertengkaran tersebut karena Termohon sering menolak diajak hubungan suami istri oleh Pemohon;
- Bahwa saksi tahu Pemohon dan Termohon sudah pisah rumah sekitar 1,5 tahun yang lalu, Termohon pergi dari rumah meninggalkan Pemohon, dan sejak saat itu mereka tidak lagi menjalankan kewajiban sebagai layaknya suami istri;
- Bahwa Saksi telah berulang kali berusaha merukunkan mereka tapi sampai sekarang tetap tidak berhasil;

Bahwa Pemohon menyampaikan kesimpulan yang isinya sebagaimana tertuang dalam berita acara sidang;

Selanjutnya untuk singkatnya uraian putusan ini, maka semua hal yang termuat dalam berita acara sidang merupakan bagian yang tidak terpisahkan dari putusan ini;

PERTIMBANGAN HUKUM

Menimbang, bahwa maksud dan tujuan pemohonan Pemohon adalah sebagaimana terurai di atas;

Menimbang, bahwa Pemohon telah menguasakan kepada kuasa hukum **Mansur, S.H.,** Advokat, berdasarkan surat kuasa khusus tertanggal 15 Desember 2020, dan telah terdaftar di Kepaniteraan Pengadilan Agama

Disclaimer
Kepaniteraan Mahkamah Agung Republik Indonesia berusaha untuk selalu mencantumkan informasi paling kini dan akurat sebagai bentuk komitmen Mahkamah Agung untuk pelayanan publik, transparansi dan akuntabilitas pelaksanaan fungsi peradilan. Namun dalam hal-hal tertentu masih dimungkinkan terjadi permasalahan teknis terkait dengan akurasi dan keterkinian informasi yang kami sajikan, hal mana akan terus kami perbaiki dari waktu kewaktu.
Dalam hal Anda menemukan inakurasi informasi yang termuat pada situs ini atau informasi yang seharusnya ada, namun belum tersedia, maka harap segera hubungi Kepaniteraan Mahkamah Agung RI melalui :
Email : kepaniteraan@mahkamahagung.go.id   Telp : 021-384 3348 (ext.318)

Sidoarjo, sehingga karenanya kuasa hukum tersebut secara formal dapat diterima untuk beracara dalam perkara ini;

Menimbang, bahwa ternyata Termohon tidak datang menghadap di persidangan dan tidak pula menyuruh orang lain untuk menghadap sebagai wakil/kuasanya yang sah, meskipun Termohon telah dipanggil secara resmi dan patut, sedangkan tidak ternyata bahwa tidak datangnya Termohon tersebut disebabkan oleh suatu alasan yang sah;

Menimbang, bahwa karena Termohon tidak menghadiri persidangan, maka sikap Termohon tersebut dinilai sebagai sikap yang mengabaikan hak-haknya di muka persidangan, oleh karena itu sesuai dengan Pasal 125 dan 126 HIR permohonan Pemohon dapat diputus dengan verstek sekiranya permohonan Pemohon tersebut tidak melawan hak dan beralasan pada hukum;

Menimbang, bahwa yang menjadi pokok permohonan Pemohon adalah agar Pengadilan Agama Sidoarjo memberi izin kepada Pemohon untuk menjatuhkan talak satu raj'i terhadap Termohon didepan sidang Pengadilan Agama Sidoarjo dengan alasan bahwa antara Pemohon dan Termohon sering terjadi perselisihan dan pertengkaran yang disebabkan karena Termohon sering menolak diajak hubungan suami istri oleh Pemohon, akhirnya sejak 2 tahun yang lalu Pemohon dan Termohon pisah rumah, Termohon pergi meninggalkan Pemohon, sehingga antara Pemohon dan Termohon sudah tidak melaksanakan kewajiban sebagai suami istri, yang meskipun telah di tempuh upaya damai namun tidak berhasil;

Menimbang, bahwa walaupun Termohon dinilai telah mengabaikan hak-haknya di muka persidangan karena tidak hadir dan tidak mewakilkan kepada orang lain sebagai kuasa hukumnya padahal telah dipanggil secara sah dan patut, akan tetapi karena dalam posita surat permohonan terurai kejadian perselisihan dan pertengkaran dan untuk memenuhi Pasal 76 ayat (1) Undang-Undang Nomor 7 Tahun 1989 jo. Pasal 22 ayat (2) Peraturan Pemerintah Nomor 9 Tahun 1975, maka Majelis akan tetap membebankan pembuktian kepada Pemohon untuk menghadirkan saksi dari keluarga atau teman dekat dengan Pemohon atau Termohon sebelum memutuskan perkawinannya;

Disclaimer
Kepaniteraan Mahkamah Agung Republik Indonesia berusaha untuk selalu mencantumkan informasi paling kini dan akurat sebagai bentuk komitmen Mahkamah Agung untuk pelayanan publik, transparansi dan akuntabilitas pelaksanaan fungsi peradilan. Namun dalam hal-hal tertentu masih dimungkinkan terjadi permasalahan teknis terkait dengan akurasi dan keterkinian informasi yang kami sajikan, hal mana akan terus kami perbaiki dari waktu kewaktu.
Dalam hal Anda menemukan inakurasi informasi yang termuat pada situs ini atau informasi yang seharusnya ada, namun belum tersedia, maka harap segera hubungi Kepaniteraan Mahkamah Agung RI melalui :
Email : kepaniteraan@mahkamahagung.go.id Telp : 021-384 3348 (ext.318)

Menimbang, bahwa untuk membuktikan dalil-dalil permohonannya, Pemohon telah mengajukan bukti surat P.1, dan P.2 serta dua orang saksi;

Menimbang bahwa bukti P-1 dan P-2 adalah akta otentik yang dikeluarkan oleh pejabat yang berwenang, bermeterai cukup dan cocok dengan aslinya, oleh karena itu akta tersebut berdasarkan pasal 165 HIR / 1868 KUH Perdata, memiliki nilai pembuktian sempurna dan mengikat ;

Menimbang bahwa keterangan saksi 1 dan saksi 2 Pemohon tersebut memenuhi syarat-syarat formal sebagai saksi berdasarkan Pasal 76 Undang-undang nomor 7 tahun 1989 tentang Peradilan Agama Jo. Pasal 171 HIR Jo. Pasal 22 Peraturan Pemerintah nomor 9 tahun 1975, maka sepanjang mengenai sesuatu yang dilihat sendiri dan atau dialami sendiri, keterangan saksi tersebut bernilai sebagai alat bukti yang sah dan dapat diterima sebagai alat bukti;

Menimbang, bahwa berdasarkan bukti P.2 serta berdasarkan keterangan saksi I dan saksi II yang saling bersesuaian, telah terbukti bahwa Pemohon dan Termohon adalah suami Istri yang menikah berdasarkan Hukum Islam, oleh karenanya maka terbukti secara sah menurut hukum bahwa Pemohon dan Termohon adalah suami istri;

Menimbang, bahwa berdasarkan bukti P.1 dan P- 2, Saksi 1 dan Saksi 2 Pemohon telah terbukti fakta sebagai berikut:

- Bahwa para saksi mengetahui Pemohon dengan Termohon pasangan suami-istri yang membina rumah tangga di Kabupaten Sidoarjo serta telah dikaruniai 1 orang anak;
- Bahwa para saksi mengetahui semula rumah tangga Pemohon dan Termohon rukun, kemudian sekitar 2,5 tahun yang lalu sering terjadi pertengkaran dan atau percekcokan;
- Bahwa pengetahuan para saksi tentang percekcokan dan pertengkaran Pemohon dengan Termohon merupakan pengetahuan langsung, penyebabnya karena Termohon sering menolak diajak hubungan suami istri oleh Pemohon;

Disclaimer
Kepaniteraan Mahkamah Agung Republik Indonesia berusaha untuk selalu mencantumkan informasi paling kini dan akurat sebagai bentuk komitmen Mahkamah Agung untuk pelayanan publik, transparansi dan akuntabilitas pelaksanaan fungsi peradilan. Namun dalam hal-hal tertentu masih dimungkinkan terjadi permasalahan teknis terkait dengan akurasi dan keterkinian informasi yang kami sajikan, hal mana akan terus kami perbaiki dari waktu kewaktu.
Dalam hal Anda menemukan inakurasi informasi yang termuat pada situs ini atau informasi yang seharusnya ada, namun belum tersedia, maka harap segera hubungi Kepaniteraan Mahkamah Agung RI melalui :
Email : kepaniteraan@mahkamahagung.go.id Telp : 021-384 3348 (ext.318)

- Bahwa para saksi mengetahui sejak 1,5 tahun yang lalu Pemohon dengan Termohon pisah rumah, Termohon pergi meninggalkan Pemohon, dan tidak menjalankan kewajiban sebagai pasangan suami-istri;
- Bahwa Pemohon dengan Termohon telah dirukunkan atau didamaikan, tetapi tidak berhasil;

Menimbang, bahwa sebagaimana telah dipertimbangkan, Termohon dinilai telah mengabaikan hak-haknya untuk berperkara di muka sidang sehingga dalam perkara a quo maupun kesimpulan kesaksian para saksi di atas dinilai benar, dan bernilai fakta yang merupakan pengetahuan langsung para saksi;

Menimbang, bahwa fakta sejak 1,5 tahun yang lalu hingga sekarang Pemohon dan Termohon berpisah rumah, hal ini telah memberi petunjuk sejak 1,5 tahun yang lalu atau sejak sekitar waktu itu hingga sekarang antara Pemohon dengan Termohon sudah pisah rumah, Pemohon dan Termohon sudah tidak dapat menjalankan kewajibannya sebagai suami atau istri, *personal care* dari Pemohon sebagain suami terhadap Termohon sebagai istrinya atau sebaliknya sebagaimana umumnya terjadi dalam rumah tangga, sudah tidak ada lagi dalam rumah tangga Pemohon dan Termohon;

Menimbang, bahwa disyariatkannya pernikahan sebagai *mitsaqan ghalidhan* mempunyai tujuan yang suci dan mulia, yakni untuk menciptakan rumah tangga yang *sakinah mawaddah dan rahmah*, sebagaimana dimaksud dalam Al-Qur'an surat Ar Rum ayat 21 dan Pasal 1 Undang-undang Nomor 1 Tahun 1974 Jo. pasal 3 Kompilasi Hukum Islam, namun dengan keadaan rumah tangga Pemohon dan Termohon tersebut, maka tujuan pernikahan tersebut menjadi sulit untuk bisa dicapai;

Menimbang, bahwa fakta-fakta tersebut di atas telah menunjukkan bahwa perkawinan Pemohon dan Termohon benar-benar telah pecah dan telah sampai pada taraf yang sudah tidak bisa didamaikan lagi karena Pemohon telah jera dan menolak untuk melanjutkan perkawinannya dengan Termohon. Dengan demikian penyelesaian yang dipandang adil dan bermanfaat bagi kedua belah pihak adalah perceraian ;

Disclaimer
Kepaniteraan Mahkamah Agung Republik Indonesia berusaha untuk selalu mencantumkan informasi paling kini dan akurat sebagai bentuk komitmen Mahkamah Agung untuk pelayanan publik, transparansi dan akuntabilitas pelaksanaan fungsi peradilan. Namun dalam hal-hal tertentu masih dimungkinkan terjadi permasalahan teknis terkait dengan akurasi dan keterkinian informasi yang kami sajikan, hal mana akan terus kami perbaiki dari waktu kewaktu.
Dalam hal Anda menemukan inakurasi informasi yang termuat pada situs ini atau informasi yang seharusnya ada, namun belum tersedia, maka harap segera hubungi Kepaniteraan Mahkamah Agung RI melalui :
Email : kepaniteraan@mahkamahagung.go.id Telp : 021-384 3348 (ext.318)

Menimbang, bahwa dengan merujuk pada Putusan Mahkamah Agung Republik Indonesia nomor 237/K/AG/1998, tanggal 17 Maret 1999 yang mengandung abstrak hukum bahwa rumah tangga yang diwarnai perselisihan, percekcekcokan, tidak bersedia tinggal dalam satu tempat kediaman bersama, salah satu pihak tidak berniat untuk meneruskan kehidupan bersama dengan pihak lain, adalah merupakan fakta hukum yang cukup untuk alasan dalam suatu perceraian sesuai dengan maksud pasal 19 huruf (f) Peraturan Pemerintah nomor 9 tahun 1975;

Menimbang, bahwa sengketa perkawinan berbeda dengan sengketa kebendaan umumnya, ikatan perkawinan tidak hanya berhubungan dengan ikatan *lahiriah* (pisik) melainkan juga ikatan *bathiniah* antara suami dengan isteri, bahkan unsur bathin mempunyai peran yang penting dalam suatu perkawinan, sehingga jika unsur tersebut sudah tidak ada lagi, maka perkawinan tersebut dinilai sudah rapuh;

Menimbang, bahwa dengan melihat kondisi rumah tangga Pemohon dan Termohon tersebut, jelas ikatan bathin antara antara Pemohon dengan Termohon yang merupakan kunci kokohnya keutuhan rumah tangga sudah tidak ada lagi, sehingga antara Pemohon dengan Termohon tidak mungkin akan terwujud kehidupan rumah tangga yang menjadi tujuan perkawinan dalam Pasal 1 Undang-Undang Nomor 1 Tahun 1974 jo. Pasal 3 KHI, yaitu mewujudkan rumah tangga yang bahagia, *sakinah, mawaddah, rahmah,* kekal dan berdasarkan Ketuhanan Yang Maha Esa dan membiarkan suasana rumah tangga yang demikian, justru akan menimbulkan mudlarat bagi kedua belah pihak, oleh karena itu menceraikan Pemohon dan Termohon dipandang jalan yang terbaik dan lebih maslahat bagi keduanya;

Menimbang, bahwa dengan demikian dari fakta hukum tersebut telah cukup beralasan bagi Majelis untuk menerapkan Pasal 19 huruf f Peraturan Pemerintah Nomor 9 Tahun 1975 jo. Pasal 116 huruf f Kompilasi Hukum Islam untuk memutuskan perkara ini;

Menimbang, bahwa dalam perkara ini, Majelis Hakim perlu mengetengahkan Firman Allah dalam surat Al Baqoroh ayat 227 yang berbunyi:

Disclaimer
Kepaniteraan Mahkamah Agung Republik Indonesia berusaha untuk selalu mencantumkan informasi paling kini dan akurat sebagai bentuk komitmen Mahkamah Agung untuk pelayanan publik, transparansi dan akuntabilitas pelaksanaan fungsi peradilan. Namun dalam hal-hal tertentu masih dimungkinkan terjadi permasalahan teknis terkait dengan akurasi dan keterkinian informasi yang kami sajikan, hal mana akan terus kami perbaiki dari waktu kewaktu.
Dalam hal Anda menemukan inakurasi informasi yang termuat pada situs ini atau informasi yang seharusnya ada, namun belum tersedia, maka harap segera hubungi Kepaniteraan Mahkamah Agung RI melalui :
Email : kepaniteraan@mahkamahagung.go.id Telp : 021-384 3348 (ext.318)

وإن عزموا الطلاق فإن الله سميع عليم

Artinya : “Dan jika mereka ber'azam (bertetap hati) untuk talak, sesungguhnya Allah Maha Mendengar lagi Maha Mengetahui”;

Menimbang, bahwa berdasarkan pertimbangan-pertimbangan sesuai dengan maksud Pasal 39 Undang-Undang Nomor 1 Tahun 1974 jo Pasal 70 ayat (1) Undang-undang Nomor 7 Tahun 1989 jo. Pasal 131 Kompilasi Hukum Islam beralasan bagi Majelis untuk mengabulkan permohonan Pemohon dengan memberi izin kepada Pemohon untuk menjatuhkan talak satu raj’i terhadap Termohon di depan sidang Pengadilan Agama Sidoarjo setelah putusan ini berkekuatan hukum tetap;

Menimbang, bahwa Termohon tidak pernah hadir di muka sidang meskipun ia telah dipanggil dengan resmi dan patut sedangkan ketidak hadirannya tidak terbukti disebabkan suatu halangan yang sah maka Termohon dinyatakan tidak hadir. Kemudian oleh karena permohonan Pemohon telah memenuhi alasan hukum dan tidak melawan hak, maka berdasarkan Pasal 125 HIR permohonan Pemohon dikabulkan dengan verstek;

Menimbang, bahwa sebagaimana ditetapkan Pasal 89 ayat (1) Undang-Undang Nomor 7 Tahun 1989 jo. Pasal 90 Undang-Undang Nomor 3 Tahun 2006 sebagaimana diubah oleh Undang-Undang Nomor 50 Tahun 2009 beralasan bagi Majelis untuk membebankan biaya perkara kepada Pemohon;

Mengingat, semua pasal dalam peraturan perundang-undangan dan hukum Islam yang berkaitan dengan perkara ini;

MENGADILI:

1. Menyatakan Termohon telah dipanggil secara resmi dan patut untuk menghadap di muka sidang, tidak hadir;
2. Mengabulkan permohonan Pemohon secara verstek;
3. Memberi izin kepada Pemohon (**PEMOHON**) untuk menjatuhkan talak satu raj'i terhadap Termohon **(TERMOHON**) di depan sidang Pengadilan Agama Sidoarjo;

Disclaimer
Kepaniteraan Mahkamah Agung Republik Indonesia berusaha untuk selalu mencantumkan informasi paling kini dan akurat sebagai bentuk komitmen Mahkamah Agung untuk pelayanan publik, transparansi dan akuntabilitas pelaksanaan fungsi peradilan. Namun dalam hal-hal tertentu masih dimungkinkan terjadi permasalahan teknis terkait dengan akurasi dan keterkinian informasi yang kami sajikan, hal mana akan terus kami perbaiki dari waktu kewaktu.
Dalam hal Anda menemukan inakurasi informasi yang termuat pada situs ini atau informasi yang seharusnya ada, namun belum tersedia, maka harap segera hubungi Kepaniteraan Mahkamah Agung RI melalui :
Email : kepaniteraan@mahkamahagung.go.id Telp : 021-384 3348 (ext.318)

4. Membebankan kepada Pemohon untuk membayar biaya perkara ini sebesar Rp. 441.000,- (empat ratus empat puluh satu ribu rupiah);

Demikian putusan ini dijatuhkan dalam rapat permusyawaratan Majelis Hakim yang dilangsungkan pada hari Rabu tanggal 23 Desember 2020 Masehi, bertepatan dengan tanggal 08 Jumadil Awal 1442 Hijriah, oleh kami Dra. Hj. Imas Salamah, M.H. sebagai Ketua Majelis, Dra. Siti Muarofah Sa'adah, S.H., dan Drs. Akhmad Khoiron, masing-masing sebagai Hakim Anggota, putusan tersebut diucapkan dalam sidang terbuka untuk umum pada hari itu juga, oleh Ketua Majelis tersebut dengan didampingi oleh Hakim Anggota dan dibantu oleh Andri Dwi Perwitasari, S.H. sebagai Panitera Pengganti serta dihadiri oleh Pemohon dan kuasanya tanpa hadirnya Termohon;

Hakim Anggota,	Ketua Majelis,
**Dra. Siti Muarofah Sa'adah, S.H.**	**Dra. Hj. Imas Salamah, M.H.**

Hakim Anggota,

Drs. Akhmad Khoiron

Panitera Pengganti

Andri Dwi Perwitasari, S.H.

<u>Perincian Biaya Perkara:</u>

Disclaimer
Kepaniteraan Mahkamah Agung Republik Indonesia berusaha untuk selalu mencantumkan informasi paling kini dan akurat sebagai bentuk komitmen Mahkamah Agung untuk pelayanan publik, transparansi dan akuntabilitas pelaksanaan fungsi peradilan. Namun dalam hal-hal tertentu masih dimungkinkan terjadi permasalahan teknis terkait dengan akurasi dan keterkinian informasi yang kami sajikan, hal mana akan terus kami perbaiki dari waktu kewaktu.
Dalam hal Anda menemukan inakurasi informasi yang termuat pada situs ini atau informasi yang seharusnya ada, namun belum tersedia, maka harap segera hubungi Kepaniteraan Mahkamah Agung RI melalui :
Email : kepaniteraan@mahkamahagung.go.id Telp : 021-384 3348 (ext.318)

Pendaftaran	Rp	30.000,-
Biaya Proses	Rp	75.000,-
Panggilan	Rp	300.000,-
PNBP Panggilan	Rp	20.000,-
Redaksi	Rp	10.000,-
Meterai	Rp	6.000,-
Jumlah	Rp	441.000,-

(empat ratus empat puluh satu ribu rupiah)

Disclaimer
Kepaniteraan Mahkamah Agung Republik Indonesia berusaha untuk selalu mencantumkan informasi paling kini dan akurat sebagai bentuk komitmen Mahkamah Agung untuk pelayanan publik, transparansi dan akuntabilitas pelaksanaan fungsi peradilan. Namun dalam hal-hal tertentu masih dimungkinkan terjadi permasalahan teknis terkait dengan akurasi dan keterkinian informasi yang kami sajikan, hal mana akan terus kami perbaiki dari waktu kewaktu.
Dalam hal Anda menemukan inakurasi informasi yang termuat pada situs ini atau informasi yang seharusnya ada, namun belum tersedia, maka harap segera hubungi Kepaniteraan Mahkamah Agung RI melalui :
Email : kepaniteraan@mahkamahagung.go.id   Telp : 021-384 3348 (ext.318)
