# Supplementary material for: Classification of divorce causes during the COVID-19 pandemic using convolutional neural networks
Source: PeerJ Comput Sci. 2022 Jun 30;8:e998. doi: 10.7717/peerj-cs.998 (PMC9299239; doi:10.7717/peerj-cs.998)
Supplement: Supplemental Information 3 [file peerj-cs-08-998-s003.zip › KDRT Dataset/Data ke-24.pdf]

PUTUSAN

Nomor 757/Pdt.G/2020/PA.Mpr

بسم الله الرحمن الرحيم

DEMI KEADILAN BERDASARKAN KETUHANAN YANG MAHA ESA

Pengadilan Agama Martapura yang memeriksa dan mengadili perkara tertentu pada tingkat pertama dalam sidang majelis hakim telah menjatuhkan putusan dalam perkara Cerai Talak antara:

xxxxxx, umur 37 tahun, agama Islam, pekerjaan Swasta, pendidikan Sekolah Lanjutan Tingkat Atas, tempat kediaman di RT.06 RW.02 Desa XXXXXX Kecamatan Belitang Madang Raya Kabupaten Ogan Komering Ulu Timur, sebagai Pemohon Konvensi/Tergugat Rekonvensi;

melawan

XXXXXX, umur 29 tahun, agama Islam, pekerjaan mengurus rumah tangga, pendidikan Sekolah Lanjutan Tingkat Pertama, tempat kediaman di Desa XXXXXX I Kecamatan Belitang Madang Raya Kabupaten Ogan Komering Ulu Timur, sebagai Termohon Konvensi/ Penggugat Rekonvensi**;**

Pengadilan Agama tersebut;

Telah mempelajari surat-surat yang berkaitan dengan perkara ini;

Telah mendengar keterangan Pemohon dan Termohon, serta memeriksa bukti surat dan saksi-saksi di persidangan;

DUDUK PERKARA

Bahwa Pemohon dalam surat permohonannya tertanggal 23 Oktober 2020 telah mengajukan permohonan Cerai Talak, yang telah terdaftar di Kepaniteraan Pengadilan Agama Martapura dengan Nomor

Disclaimer
Kepaniteraan Mahkamah Agung Republik Indonesia berusaha untuk selalu mencantumkan informasi paling kini dan akurat sebagai bentuk komitmen Mahkamah Agung untuk pelayanan publik, transparansi dan akuntabilitas pelaksanaan fungsi peradilan. Namun dalam hal-hal tertentu masih dimungkinkan terjadi permasalahan teknis terkait dengan akurasi dan keterkinian informasi yang kami sajikan, hal mana akan terus kami perbaiki dari waktu kewaktu.
Dalam hal Anda menemukan inakurasi informasi yang termuat pada situs ini atau informasi yang seharusnya ada, namun belum tersedia, maka harap segera hubungi Kepaniteraan Mahkamah Agung RI melalui :
Email : kepaniteraan@mahkamahagung.go.id Telp : 021-384 3348 (ext.318)

757/Pdt.G/2020/PA.Mpr, tertanggal 23 Oktober 2020, mengemukakan hal-hal sebagai berikut:

1. Bahwa, Pemohon adalah suami sah dari Termohon yang akad nikah dilaksanakan pada tanggal 21 April 2020 di Desa XXXXXX I Kecamatan Belitang Madang Raya Kabupaten Ogan Komering Ulu Timur wali nikah(bapak kandung Termohon), dengan mas kawin berupa Uang Rp.200.000 tunai, sebagaimana dapat dibuktikan dengan Kutipan Akta Nikah Nomor XXXXXX yang dikeluarkan oleh Kantor Urusan Agama Kecamatan Belitang Madang Raya Kabupaten Ogan Komering Ulu Timur, tertanggal 21 April 2020;
2. Bahwa, Pemohon dan Termohon dahulu suami istri yang sah dan pernah bercerai pada tahun 2006, kemudian Pemohon dan Termohon rujuk kembali pada bulan April 2020;
3. Bahwa, setelah menikah Pemohon dengan Termohon tinggal bersama dirumah kontrakan di Desa XXXXXX I Kecamatan Belitang Madang Raya Kabupaten Ogan Komering Ulu Timur, sampai dengan berpisah;
4. Bahwa, selama pernikahan tersebut antara Pemohon dan Termohon telah berhubungan sebagaimana layaknya suami istri dan telah dikaruniai 2 orang anak yang bernama XXXXXX (22-11-2008) dan XXXXXX (10-07-2015), yang saat ini anak nomor pertama bernama XXXXXX bertempat tinggal bersama Pemohon, sedangkan anak nomor dua bernama XXXXXX bertempat tinggal bersama Termohon;
5. Bahwa, pada mulanya kehidupan rumah tangga Pemohon dan Termohon telah hidup rukun dan harmonis selama kurang lebih 1 bulan, namun sejak Mei 2020 rumah tangga Pemohon dengan Termohon tidak harmonis lagi dan sering terjadi perselisihan dan pertengkaran yang disebabkan :
   5.1 Orang Tua Termohon selalu ikut campur dalam rumah tangga Pemohon dan Termohon;

Disclaimer
Kepaniteraan Mahkamah Agung Republik Indonesia berusaha untuk selalu mencantumkan informasi paling kini dan akurat sebagai bentuk komitmen Mahkamah Agung untuk pelayanan publik, transparansi dan akuntabilitas pelaksanaan fungsi peradilan. Namun dalam hal-hal tertentu masih dimungkinkan terjadi permasalahan teknis terkait dengan akurasi dan keterkinian informasi yang kami sajikan, hal mana akan terus kami perbaiki dari waktu kewaktu.
Dalam hal Anda menemukan inakurasi informasi yang termuat pada situs ini atau informasi yang seharusnya ada, namun belum tersedia, maka harap segera hubungi Kepaniteraan Mahkamah Agung RI melalui :
Email : kepaniteraan@mahkamahagung.go.id   Telp : 021-384 3348 (ext.318)

5.2 Termohon tidak mau mengurus anak nomor 1 karena anak tersebut berkebutuhan khusus;

5.3 Selain itu, Termohon sering menuntut lebih diluar batas kemampuan Pemohon;

6. Bahwa, perselisihan dan pertengkaran antara Pemohon dengan Termohon semakin memuncak yang terjadi pada bulan September 2020 yang disebabkan pada saat itu Orang tua Termohon ingin meminjam uang kepada Pemohon, akan tetapi tidak dipinjami oleh Pemohon karena orang tua Termohon sering meminjam uang namun tidak pernah dipulangkan, namun Termohon marah sehingga terjadilah perselisihan dan pertengkaran antara Pemohon dengan Termohon. Setelah kejadian itu, Termohon pergi dari rumah meninggalkan Pemohon;

7. Bahwa, persoalan rumah tangga Pemohon dan Termohon sudah pernah didamaikan oleh pihak keluarga Pemohon dan Termohon, akan tetapi tidak berhasil;

8. Bahwa, oleh karena rumah tangga Pemohon dengan Termohon sudah sulit dipertahankan untuk membina rumah tangga yang bahagia rukun dan damai sebagaimana yang diamanatkan dalam Undang-undang Nomor 1 tahun 1974 tentang perkawinan tidak akan terwujud dan telah berpisah selama kurang lebih , dan tidak saling memperdulikan lagi antara Pemohon dan Termohon, maka Pemohon telah berketetapan hati untuk mengajukan permohonan carai talak;

9. Bahwa, berdasarkan alasan-alasan tersebut maka Pemohon mohon Kepada Ketua Pengadilan Agama Martapura melalui Yth. Majelis Hakim kiranya dapat menyidangkan perkara ini serta berkenan memberikan putusan yang amarnya sebagai berikut :

Primer:

1. Menerima dan Mengabulkan Permohonan Pemohon;

Disclaimer
Kepaniteraan Mahkamah Agung Republik Indonesia berusaha untuk selalu mencantumkan informasi paling kini dan akurat sebagai bentuk komitmen Mahkamah Agung untuk pelayanan publik, transparansi dan akuntabilitas pelaksanaan fungsi peradilan. Namun dalam hal-hal tertentu masih dimungkinkan terjadi permasalahan teknis terkait dengan akurasi dan keterkinian informasi yang kami sajikan, hal mana akan terus kami perbaiki dari waktu kewaktu.
Dalam hal Anda menemukan inakurasi informasi yang termuat pada situs ini atau informasi yang seharusnya ada, namun belum tersedia, maka harap segera hubungi Kepaniteraan Mahkamah Agung RI melalui :
Email : kepaniteraan@mahkamahagung.go.id Telp : 021-384 3348 (ext.318)

2. Memberi izin kepada Pemohon (XXXXXX) untuk menjatuhkan talak satu raj'i terhadap Termohon (XXXXXX) di depan sidang Pengadilan Agama Martapura;

3. Membebankan biaya perkara sesuai dengan hukum yang berlaku;

Subsider:

Apabila majelis hakim berpendapat lain, maka Pemohon mohon Putusan yang seadil-adilnya;

Bahwa, pada hari dan tanggal sidang yang telah ditetapkan Pemohon dan Termohon hadir di depan persidangan. majelis hakim telah berusaha untuk mendamaikan Pemohon dan Termohon sebagaimana diatur dalam Pasal 154 RBg namun tidak berhasil. Bahkan Pemohon dan Termohon telah melalui proses mediasi oleh mediator bernama Ari Ferdinansyah, S.H., sesuai Peraturan Mahkamah Agung Republik Indonesia Nomor 1 tahun 2016, dan mediator tersebut telah melaporkan secara tertulis tertanggal 19 November 2020 yang pada pokoknya menyatakan bahwa mediasi tidak berhasil;

Bahwa, selanjutnya majelis hakim melanjutkan persidangan dengan membacakan surat permohonan Pemohon yang maksud dan isinya tetap dipertahankan oleh Pemohon dengan menyampaikan perbaikan secara lisan terhadap poin nomor 8, bahwa yang benar hingga sekarang Pemohon dan Termohon telah berpisah tempat tinggal selama kurang lebih 2 (dua) bulan, Pemohon bertempat tinggal di rumah orang tua Pemohon, dan sedangkan Termohon bertempat tinggal di rumah orang tua Termohon;

Bahwa, atas surat permohonan tersebut, Termohon telah memberikan jawaban konvensi dan gugatan rekonvensi secara lisan, yang pada pokoknya sebagai berikut:

1. Bahwa, poin 1 posita permohonan Pemohon adalah benar;
2. Bahwa, poin 2 posita permohonan Pemohon adalah benar;
3. Bahwa, poin 3 posita permohonan Pemohon adalah benar;

Disclaimer
Kepaniteraan Mahkamah Agung Republik Indonesia berusaha untuk selalu mencantumkan informasi paling kini dan akurat sebagai bentuk komitmen Mahkamah Agung untuk pelayanan publik, transparansi dan akuntabilitas pelaksanaan fungsi peradilan. Namun dalam hal-hal tertentu masih dimungkinkan terjadi permasalahan teknis terkait dengan akurasi dan keterkinian informasi yang kami sajikan, hal mana akan terus kami perbaiki dari waktu kewaktu.
Dalam hal Anda menemukan inakurasi informasi yang termuat pada situs ini atau informasi yang seharusnya ada, namun belum tersedia, maka harap segera hubungi Kepaniteraan Mahkamah Agung RI melalui :
Email : kepaniteraan@mahkamahagung.go.id Telp : 021-384 3348 (ext.318)

4. Bahwa, poin 4 posita permohonan Pemohon adalah benar

5. Bahwa, poin 5 posita permohonan Pemohon tidak benar. Karena rumah tangga Pemohon dan Termohon harmonis selama kurang lebih 3 (tiga) bulan. Baru sejak bulan Juli 2020 mulai terjadi pertengkaran dan perselisihan:

5.1. Bahwa, benar;

5.2. Bahwa, tidak benar Termohon tidak mau mengurus anak nomor pertama;

5.3 Bahwa, tidak benar Termohon menuntut nafkah di luar batas kemampuan Pemohon. Termohon hanya menuntut nafkah yang layak;

6. Bahwa, benar pada bulan September 2020 terjadi pertengkaran antara Pemohon dan Termohon karena orang tua Termohon mau meminjam uang dari Pemohon. Namun setelah pertengkaran tersebut Termohon tidak meninggalkan rumah, melainkan tetap berada di rumah;

7. Bahwa, posita nomor 7 permohonan Pemohon adalah benar;

8. Bahwa, posita nomor 7 permohonan Pemohon adalah benar;

9. Bahwa, atas permohonan cerai Pemohon, Termohon tidak keberatan dicerai oleh Pemohon;

Bahwa, selain jawaban konvensi Termohon juga mengajukan gugatan rekonvensi secara lisan jika permohonan Pemohon dikabulkan oleh majelis hakim, yang pada pokoknya sebagai berikut:

1. Bahwa, Termohon menuntut nafkah *‘iddah* sebesar Rp.100.000,-(seratus ribu rupiah) per bulan selama 3 (tiga) bulan. Jadi total uang nafkah *‘iddah* yang dituntut oleh Termohon adalah sebesar Rp.300.000,- (tiga ratus ribu rupiah);

2. Bahwa, untuk *mut’ah,* Termohon menuntut Pemohon membayar uang sebesar Rp.300.000,- (tiga ratus ribu rupiah);

Disclaimer
Kepaniteraan Mahkamah Agung Republik Indonesia berusaha untuk selalu mencantumkan informasi paling kini dan akurat sebagai bentuk komitmen Mahkamah Agung untuk pelayanan publik, transparansi dan akuntabilitas pelaksanaan fungsi peradilan. Namun dalam hal-hal tertentu masih dimungkinkan terjadi permasalahan teknis terkait dengan akurasi dan keterkinian informasi yang kami sajikan, hal mana akan terus kami perbaiki dari waktu kewaktu.
Dalam hal Anda menemukan inakurasi informasi yang termuat pada situs ini atau informasi yang seharusnya ada, namun belum tersedia, maka harap segera hubungi Kepaniteraan Mahkamah Agung RI melalui :
Email : kepaniteraan@mahkamahagung.go.id Telp : 021-384 3348 (ext.318)

3. Bahwa, Termohon menuntut nafkah anak bernama XXXXXX sebesar Rp.100.000,-(seratus ribu rupiah) tiap bulan hingga anak tersebut berusia dewasa;
4. Bahwa, Termohon menuntut nafkah *madhiyah* (lampau) sebesar Rp.600.000,-(enam ratus ribu rupiah) per bulan sebagaimana yang disepakati saat mediasi;
5. Bahwa, tuntutan Termohon tersebut didasari penghasilan Pemohon dari usaha pembibitan ikan lele sebesar Rp.1.500.000,- (satu juta lima ratus ribu rupiah) per bulan, dan upah harian buruh ikan sebesar Rp.50.000,- (lima puluh ribu rupiah) per hari;

Bahwa, terhadap jawaban Termohon dalam konvensi dan gugatan dalam rekonvensi tersebut, Pemohon Konvensi/Tergugat Rekonvensi menyampaikan replik dalam konvensi dan serta jawaban rekonvensi secara lisan, yang pada pokoknya sebagai berikut:

1. Bahwa, jawaban Termohon Konvensi/ Penggugat Rekonvensi pada angka 1 dalam konvensi adalah benar;
2. Bahwa, jawaban Termohon Konvensi/ Penggugat Rekonvensi pada angka 2 dalam konvensi adalah benar;
3. Bahwa, jawaban Termohon Konvensi/ Penggugat Rekonvensi pada angka 3 dalam konvensi adalah benar;;
4. Bahwa, jawaban Termohon Konvensi/ Penggugat Rekonvensi pada angka 4 dalam konvensi adalah benar;
5. Bahwa, tidak benar Pemohon dan Termohon rukun selama 3 (tiga) bulan, melainkan hanya 2 (dua) bulan, karena sejak bulan Juni 2020 mulai terjadi pertengkaran:

`5.1 Bahwa, adalah benar;

5.2 Bahwa, tidak benar, karena Termohon selalu mengabaikan anak nomor pertama;

Disclaimer
Kepaniteraan Mahkamah Agung Republik Indonesia berusaha untuk selalu mencantumkan informasi paling kini dan akurat sebagai bentuk komitmen Mahkamah Agung untuk pelayanan publik, transparansi dan akuntabilitas pelaksanaan fungsi peradilan. Namun dalam hal-hal tertentu masih dimungkinkan terjadi permasalahan teknis terkait dengan akurasi dan keterkinian informasi yang kami sajikan, hal mana akan terus kami perbaiki dari waktu kewaktu.
Dalam hal Anda menemukan inakurasi informasi yang termuat pada situs ini atau informasi yang seharusnya ada, namun belum tersedia, maka harap segera hubungi Kepaniteraan Mahkamah Agung RI melalui :
Email : kepaniteraan@mahkamahagung.go.id Telp : 021-384 3348 (ext.318)

5.3 Bahwa, tidak benar, karena Termohon menuntut nafkah di luar batas kemampuan Pemohon;

6. Bahwa, tidak benar Pemohon yang lebih dulu meninggalkan rumah kontrakan, justru Termohon yang sempat pergi dari rumah kontrakan selam dua hari, kemudian kembali ke kontrakan lagi. Setelah itu Termohon pulang ke rumah orang tuanya di desa XXXXXX;

7. Bahwa, benar jawaban Termohon pada angka 7;

8. Bahwa, benar jawaban Termohon pada angka 8;

Bahwa, Pemohon Konvensi/Tergugat Rekonvensi juga menyampaikan jawaban atas gugatan rekonvensi Termohon secara lisan, yang pada pokoknya sebagai berikut:

1. Bahwa, Pemohon Konvensi/Tergugat Rekonvensi menyanggupi membayar nafkah *‘iddah* sebesar Rp.100.000,- (seratus ribu rupiah) per bulan selama 3 (tiga) bulan. Sehingga total nafkah *‘iddah* yang disanggupi oleh Pemohon sebesar Rp.300.000,- (tiga ratus ribu rupiah);

2. Bahwa, Pemohon Konvensi/Tergugat Rekonvensi hanya menyanggupi membayar *mut’ah* sebesar Rp.200.000,- (dua ratus ribu rupiah);

3. Bahwa, Pemohon Konvensi/Tergugat Rekonvensi menyanggupi membayar nafkah anak bernama XXXXXX sebesar Rp.100.000,- (seratus ribu rupiah) per bulan hingga anak tersebut dewasa;

4. Bahwa, Pemohon Konvensi/Tergugat Rekonvensi menyanggupi membayar nafkah *madhiyah*/lampau sebesar Rp.600.000,- (enam ratus ribu rupiah);

5. Bahwa, usaha pembibitan ikan lele yang dilakukan Pemohon baru satu kali menghasilkan yaitu pada bulan Agustus, sebesar kurang lebih Rp.3.000.000,- (tiga juta rupiah), dan sedangkan upah dari

Disclaimer
Kepaniteraan Mahkamah Agung Republik Indonesia berusaha untuk selalu mencantumkan informasi paling kini dan akurat sebagai bentuk komitmen Mahkamah Agung untuk pelayanan publik, transparansi dan akuntabilitas pelaksanaan fungsi peradilan. Namun dalam hal-hal tertentu masih dimungkinkan terjadi permasalahan teknis terkait dengan akurasi dan keterkinian informasi yang kami sajikan, hal mana akan terus kami perbaiki dari waktu kewaktu.
Dalam hal Anda menemukan inakurasi informasi yang termuat pada situs ini atau informasi yang seharusnya ada, namun belum tersedia, maka harap segera hubungi Kepaniteraan Mahkamah Agung RI melalui :
Email : kepaniteraan@mahkamahagung.go.id Telp : 021-384 3348 (ext.318)

buruh jaring ikan Pemohon baru 3 (tiga) kali kali mendapat upah, yaitu Rp.50.000,-(lima puluh ribu rupiah) per hari, sehingga totalnya sebesar Rp.150.000,- (seratus lima puluh ribu rupiah);

Bahwa, terhadap replik Pemohon dalam konvensi dan jawaban Tergugat dalam Rekonvensi tersebut, Penggugat Rekonvensi/ Termohon Konvensi telah menyampaikan duplik dalam konvensi dan replik rekonvensi secara lisan, yang pada pokoknya sebagai berikut:

1. Bahwa, terhadap terhadap replik Pemohon Konvensi/Tergugat Rekonvensi, Penggugat Rekonvensi/ Termohon Konvensi tetap bertahan pada jawaban dalam jawaban konvensi;
2. Bahwa, terhadap jawaban rekonvensi Tergugat Rekonvensi, Penggugat Rekonvensi menyampaikan replik rekonvensi sebagai berikut;
   2.1 Bahwa, benar usaha pembibitan ikan lele Pemohon Konvensi/Tergugat Rekonvensi baru menghasilkan satu kali, namun hasilnya adalah sebesar Rp.5.000.000,- (lima juta rupiah);
   2.1 Bahwa, hasil upah harian dari jaring ikan yang didapatkan oleh Pemohon Konvensi/Tergugat Rekonvensi adalah Rp.210.000,- (dua ratus sepuluh ribu rupiah)

Bahwa, terhadap selain menyampaikan duplik, Termohon Konvensi/Penggugat Rekonvensi juga menyampaikan replik rekonvensi secara lisan yang pada pokoknya sebagai berikut:

1. Bahwa, terhadap *mut'ah* Termohon Konvensi/Penggugat Rekonvensi menyapakati berdasarkan kesanggupan Pemohon Konvensi/Tergugat Rekonvensi uang sebesar Rp.200.000,-(dua ratus ribu rupiah);

Bahwa, setelah tahapan jawab jinawab, majelis hakim memberikan kesempatan secara berimbang kepada Pemohon Konvensi/Tergugat

Disclaimer
Kepaniteraan Mahkamah Agung Republik Indonesia berusaha untuk selalu mencantumkan informasi paling kini dan akurat sebagai bentuk komitmen Mahkamah Agung untuk pelayanan publik, transparansi dan akuntabilitas pelaksanaan fungsi peradilan. Namun dalam hal-hal tertentu masih dimungkinkan terjadi permasalahan teknis terkait dengan akurasi dan keterkinian informasi yang kami sajikan, hal mana akan terus kami perbaiki dari waktu kewaktu.
Dalam hal Anda menemukan inakurasi informasi yang termuat pada situs ini atau informasi yang seharusnya ada, namun belum tersedia, maka harap segera hubungi Kepaniteraan Mahkamah Agung RI melalui :
Email : kepaniteraan@mahkamahagung.go.id   Telp : 021-384 3348 (ext.318)

Rekonvensi dan Termohon Konvensi/Penggugat Rekonvensi untuk mengajukan alat bukti masing-masing;

Bahwa, untuk menguatkan dalil-dalil permohonannya Pemohon Konvensi/Tergugat Rekonvensi telah mengajukan bukti surat, berupa Kutipan Akta Nikah Nomor XXXXXX, tertanggal 21 April 2020 yang dikeluarkan oleh Kantor Urusan Agama Kecamatan Martapura Kabupaten Ogan Komering Ullu Timur, yang telah bermeterai cukup, dinazegelen Kantor Pos, dan telah dicocokkan dengan aslinya yang ternyata sesuai, lalu oleh Ketua majelis bukti tersebut diparaf dan dan diberi tanda bukti (P);

Bahwa, selain bukti tertulis Pemohon Konvensi/Tergugat Rekonvensi juga mengajukan 2 (dua) orang saksi di muka persidangan, yaitu:

1. XXXXXX, umur 34 tahun, agama Islam, aparatur desa, tempat kediaman di RT.01 RW.04 Desa XXXXXX Kecamatan Belitang II Kabupaten Ogan Komering Ulu Timur, yang di bawah sumpah telah menerangkan pada pokoknya sebagai berikut:

- Bahwa, saksi adalah adik kandung Pemohon, dan mengenal Termohon yang merupakan istri Pemohon dan ayah kandung saksi;
- Bahwa, hubungan Pemohon dengan Termohon dahulu merupakan suami isteri, namun bercerai pada tahun 2006, kemudian menikah kembali pada bulan Apri 2020;
- Bahwa, dari pernikahan sebelumnya Pemohon dan Termohon telah dikaruniai 2 (dua) orang anak bernama XXXXXX dan XXXXXXn;
- Bahwa, setelah akad nikah Pemohon dan Termohon bertempat tingggal di rumah kontrakan XXXXXX I sampai dengan berpisah;
- Bahwa, saat ini anak pertama bernama XXXXXX diasuh oleh Pemohon, dan sedangkan anak kedua bernama XXXXXXn diasuh oleh Termohon;

Disclaimer
Kepaniteraan Mahkamah Agung Republik Indonesia berusaha untuk selalu mencantumkan informasi paling kini dan akurat sebagai bentuk komitmen Mahkamah Agung untuk pelayanan publik, transparansi dan akuntabilitas pelaksanaan fungsi peradilan. Namun dalam hal-hal tertentu masih dimungkinkan terjadi permasalahan teknis terkait dengan akurasi dan keterkinian informasi yang kami sajikan, hal mana akan terus kami perbaiki dari waktu kewaktu.
Dalam hal Anda menemukan inakurasi informasi yang termuat pada situs ini atau informasi yang seharusnya ada, namun belum tersedia, maka harap segera hubungi Kepaniteraan Mahkamah Agung RI melalui :
Email : kepaniteraan@mahkamahagung.go.id   Telp : 021-384 3348 (ext.318)

- Bahwa, rumah tangga Pemohon dan Termohon pada awalnya rukun selama kurang lebih 1 (satu) bulan. Namun sejak bulan Mei 2020 terakhir mulai terjadi pertengkaran;
- Bahwa, saksi melihat Pemohon dan Termohon cekcok mulut di rumah orang tua Pemohon, yang disebabkan Termohon tidak terima penghasilan Pemohon yang sedikit, dan Termohon tidak menghargai usaha orang tua Pemohon sebagai pembibit ikan lele;
- Bahwa, saksi melihat Termohon tidak memberlakukan orang tua Pemohon dengan baik selayaknya mertua;
- Bahwa, sepengetahuan saksi Pemohon dan Termohon saat ini sudah tidak bersama selama kurang lebih 3 (tiga) bulan;
- Bahwa, saksi sudah dua kali menasehati Pemohon untuk kembali hidup rukun, namun tidak berhasil;
- Bahwa, pihak keluarga Pemohon telah berupaya merukunkan Pemohon dan Termohon namun tidak berhasil;
- Bahwa, pekerjaan Pemohon adalah pembibitan ikan lele dan buruh jaring ikan;
- Bahwa, dari usaha pembibitan ikan lele tersebut baru satu kali menghasilkan atau panen, dengan nilai sebesar Rp.2.000.000,- (dua juta rupiah);
- Bahwa, Pemohon tidak setiap waktu menjadi buruh jaring ikan, hanya ketika ada permintaan saja. Adapun upah harian buruh jaring ikan di bawah delapan puluh ribu rupiah;

Bahwa, terhadap keterangan saksi pertama Pemohon Konvensi/Tergugat Rekonvensi, Pemohon Konvensi/Tergugat Rekonvensi tidak membantah. Sedangkan Termohon Konvensi/Penggugat Rekonvensi

Disclaimer
Kepaniteraan Mahkamah Agung Republik Indonesia berusaha untuk selalu mencantumkan informasi paling kini dan akurat sebagai bentuk komitmen Mahkamah Agung untuk pelayanan publik, transparansi dan akuntabilitas pelaksanaan fungsi peradilan. Namun dalam hal-hal tertentu masih dimungkinkan terjadi permasalahan teknis terkait dengan akurasi dan keterkinian informasi yang kami sajikan, hal mana akan terus kami perbaiki dari waktu kewaktu.
Dalam hal Anda menemukan inakurasi informasi yang termuat pada situs ini atau informasi yang seharusnya ada, namun belum tersedia, maka harap segera hubungi Kepaniteraan Mahkamah Agung RI melalui :
Email : kepaniteraan@mahkamahagung.go.id Telp : 021-384 3348 (ext.318)

membantah keterangan saksi pertama Pemohon Konvensi/Tergugat Rekonvensi tersebut sebagai berikut:

- Bahwa, saksi pertama Pemohon tidak melihat langsung pertengkaran antara Pemohon dan Termohon di rumah orang tua Pemohon;

2. XXXXXX (Alm), umur 33 tahun, agama Islam, pekerjaan wiraswasta, tempat kediaman di Desa XXXXXX Kecamatan Belitang Madang Raya Kabupaten Ogan Komering Ulu Timur, yang di bawah sumpah telah menerangkan pada pokoknya sebagai berikut;

- Bahwa, saksi adalah tetangga Pemohon dan Termohon;
- Bahwa, Pemohon dan Termohon merupakan pasangan suami istri;
- Bahwa, setelah menikah Pemohon dan Termohon bertempat tinggal di rumah kontrakan di desa XXXXXX;
- Bahwa, setelah menikah Pemohon dan Termohon telah dikaruniai 2 (dua) orang anak, anak pertama bernama XXXXXX diasuh oleh Pemohon, dan anak kedua bernama XXXXXXn diasuh oleh Termohon;
- Bahwa, anak pertama Pemohon dan Termohon berkebutuhan khusus;
- Bahwa, saksi mengetahui Pemohon dan Termohon bertengkar mulut, disebabkan faktor ekonomi keluarga yang kurang dan orang tua Termohon yang sering ikut campur urusan rumah tangga Pemohon dan Termohon;
- Bahwa, saat ini Pemohon sudah berpisah tempat tinggal selama kurang lebih 3 bulan, Pemohon bertempat tinggal di rumah orang tua Pemohon, dan sedangkan Termohon bertempat tinggal di rumah orang tua Termohon;
- Bahwa, kadang-kadang Pemohon dipanggil untuk menjadi buruh jaring ikan dengan upah kurang lebih sebesar Rp.40.000,- (empat puluh ribu) per hari;

Disclaimer
Kepaniteraan Mahkamah Agung Republik Indonesia berusaha untuk selalu mencantumkan informasi paling kini dan akurat sebagai bentuk komitmen Mahkamah Agung untuk pelayanan publik, transparansi dan akuntabilitas pelaksanaan fungsi peradilan. Namun dalam hal-hal tertentu masih dimungkinkan terjadi permasalahan teknis terkait dengan akurasi dan keterkinian informasi yang kami sajikan, hal mana akan terus kami perbaiki dari waktu kewaktu.
Dalam hal Anda menemukan inakurasi informasi yang termuat pada situs ini atau informasi yang seharusnya ada, namun belum tersedia, maka harap segera hubungi Kepaniteraan Mahkamah Agung RI melalui :
Email : kepaniteraan@mahkamahagung.go.id Telp : 021-384 3348 (ext.318)

- Bahwa, Pemohon pernah usaha pembibitan ikan lele, namun hanya satu kali berhasil;
- Bahwa, saksi sudah menasehati Pemohon untuk kembali rukun dengan Termohon, namun tidak berhasil;
- Bahwa, saksi tidak mengetahui apakah ada musyawarah damai yang dilakukan keluarga Pemohon dan Termohon atau kah tidak;

Bahwa, terhadap keterangan saksi kedua Pemohon Konvensi/Tergugat Rekonvensi menyatakan benar. Begitupula Termohon Konvensi/Penggugat Rekonvensi, menyatakan bahwa apa yang disampaikan oleh saksi kedua Pemohon Konvensi/Tergugat Rekonvensi adalah benar. Hanya saja Termohon Konvensi/Penggugat Rekonvensi tidak mengetahui kalau saat ini Pemohon Konvensi/Tergugat Rekonvensi sedang tidak memiliki pekerjaan;

Bahwa, setelah mengajukan alat bukti tertulis dan 2 (dua) orang saksi, Pemohon Konvensi/Tergugat Rekonvensi menyatakan cukup dan tidak mengajukan bukti lain;

Bahwa, selanjutnya majelis hakim memberikan kesempatan kepada Termohon Konvensi/Penggugat Rekonvensi untuk mengajukan bukti di persidangan. Kemudian Termohon Konvensi/Penggugat Rekonvensi mengajukan dua orang saksi untuk menguatkan dalil-dalil jawaban dalam konvensi dan gugatan rekonvesinya, sebagai berikut:

1. XXXXXX, umur 52 tahun, agama Islam, pekerjaan Tani, tempat kediaman di RT.07 RW.04 Desa XXXXXX I Kecamatan Belitang Madang Raya Kabupaten Ogan Komering Ulu Timur, yang di bawah sumpah telah menerangkan pada pokoknya sebagai berikut;
   - Bahwa, saksi merupakan ayah kandung Termohon, sekaligus mertua Pemohon;
   - Bahwa, Pemohon dan Termohon adalah pasangan suami istri, serta telah dikaruniai dua orang anak laki-laki yang saat ini anak pertama bernama XXXXXX diasuh oleh Pemohon, dan

Disclaimer
Kepaniteraan Mahkamah Agung Republik Indonesia berusaha untuk selalu mencantumkan informasi paling kini dan akurat sebagai bentuk komitmen Mahkamah Agung untuk pelayanan publik, transparansi dan akuntabilitas pelaksanaan fungsi peradilan. Namun dalam hal-hal tertentu masih dimungkinkan terjadi permasalahan teknis terkait dengan akurasi dan keterkinian informasi yang kami sajikan, hal mana akan terus kami perbaiki dari waktu kewaktu.
Dalam hal Anda menemukan inakurasi informasi yang termuat pada situs ini atau informasi yang seharusnya ada, namun belum tersedia, maka harap segera hubungi Kepaniteraan Mahkamah Agung RI melalui :
Email : kepaniteraan@mahkamahagung.go.id Telp : 021-384 3348 (ext.318)

sedangkan anak kedua bernama XXXXXXn dalam asuhan Termohon;

- Bahwa, Pemohon dan Termohon dahulu sudah pernah menikah dan bercerai pada tahun 2006, kemudian keduanya menikah kembali pada bulan April 2020;
- Bahwa, setelah menikah Pemohon dan Termohon sempat bertempat tinggal di rumah saksi, kemudian pindah ke rumah kontrakan di desa XXXXXX;
- Bahwa, rumah tangga Pemohon dan Termohon sempat rukun selama 2 (dua) bulan, kemudian setelah itu mulai terjadi perselisihan dan pertengkaran;
- Bahwa, saksi pernah melihat Pemohon dan Termohon bertengkar mulut, yang disebabkan ekonomi keluarga yang tidak cukup, dan Pemohon yang tidak terbuka dalam masalah penghasilan kepada Termohon;
- Bahwa, Termohon tidak pilih kasih dalam mengasuh anak;
- Bahwa, saat ini Pemohon dan Termohon telah berpisah tempat tinggal selama kurang lebih 4 (empat) bulan, Pemohon yang terlebih dahulu pergi dari rumah kontrakan dan meninggalkan Termohon;
- Bahwa, Pemohon bekerja usaha pembibitan ikan lele, yang telah tiga kali panen, dan ketika panen hasilnya kurang lebih sebesar tiga juta rupiah;
- Bahwa, Pemohon juga bekerja sampingan, yaitu jual beli ikan;
- Bahwa, saksi sudah menasehati Pemohon dan Termohon, namun tidak berhasil;
- Bahwa, pihak keluarga telah berupaya mendamaikan Pemohon dan Termohon, namun tidak berhasil;

Bahwa, terhadap keterangan saksi pertama yang diajukan tersebut Termohon Konvensi/Penggugat Rekonvensi membenarkan dan tidak

Disclaimer
Kepaniteraan Mahkamah Agung Republik Indonesia berusaha untuk selalu mencantumkan informasi paling kini dan akurat sebagai bentuk komitmen Mahkamah Agung untuk pelayanan publik, transparansi dan akuntabilitas pelaksanaan fungsi peradilan. Namun dalam hal-hal tertentu masih dimungkinkan terjadi permasalahan teknis terkait dengan akurasi dan keterkinian informasi yang kami sajikan, hal mana akan terus kami perbaiki dari waktu kewaktu.
Dalam hal Anda menemukan inakurasi informasi yang termuat pada situs ini atau informasi yang seharusnya ada, namun belum tersedia, maka harap segera hubungi Kepaniteraan Mahkamah Agung RI melalui :
Email : kepaniteraan@mahkamahagung.go.id Telp : 021-384 3348 (ext.318)

membantah. Adapun Pemohon Konvensi/Tergugat Rekonvensi memberikan bantahan sebagai berikut:

- Bahwa, usaha pembibitan ikan lele Pemohon baru panen satu kali hingga sekarang;
- Bahwa, Termohon pilih kasih dalam mengasuh kedua anak mereka;
- Bahwa, Pemohon mengakui yang lebih dahulu meninggalkan rumah kontrakan dan meninggalkan Termohon;

2. XXXXXX, umur 20 tahun, agama Islam, pekerjaan wiraswasta, tempat kediaman di RT.04 RW.02 Desa XXXXXX I Kecamatan Belitang Madang Raya Kabupaten Ogan Komering Ulu Timur, yang di bawah sumpah telah menerangkan pada pokoknya sebagai berikut;
   - Bahwa, saksi merupakan tetangga rumah kontrakan Termohon dan Pemohon di desa XXXXXX I;
   - Bahwa, saksi mendengar dan melihat Pemohon dan Termohon bertengkar mulut pada bulan Juni 2020, yang disebabkan Pemohon tidak memberi Termohon nafkah, dan Pemohon pilih kasih dalam mengasuh anak, karena Pemohon hanya mau mengasuh anak kandung Pemohon dan tidak mau mengasuh anak tiri Pemohon yang merupakan anak kandung Termohon dari suami sebelumnya;
   - Bahwa, sepengetahuan saksi saat ini Pemohon dan Termohon telah berpisah tempat tinggal selama kurang lebih 3 (tiga) bulan, dan Pemohon yang lebih dahulu pergi dari rumah kontrakan dan meninggalkan Termohon;
   - Bahwa, sepengetahuan saksi, Pemohon bekerja hanya sebagai pembibit ikan lele, dan tidak ada pekerjaan lain selain pembibitan ikan lele;
   - Bahwa, sepengetahuan saksi, usaha pembibitan ikan lele yang dilakukan Pemohon baru satu kali panen, dengan penghasilan kurang lebih lima juta rupiah;

Disclaimer
Kepaniteraan Mahkamah Agung Republik Indonesia berusaha untuk selalu mencantumkan informasi paling kini dan akurat sebagai bentuk komitmen Mahkamah Agung untuk pelayanan publik, transparansi dan akuntabilitas pelaksanaan fungsi peradilan. Namun dalam hal-hal tertentu masih dimungkinkan terjadi permasalahan teknis terkait dengan akurasi dan keterkinian informasi yang kami sajikan, hal mana akan terus kami perbaiki dari waktu kewaktu.
Dalam hal Anda menemukan inakurasi informasi yang termuat pada situs ini atau informasi yang seharusnya ada, namun belum tersedia, maka harap segera hubungi Kepaniteraan Mahkamah Agung RI melalui :
Email : kepaniteraan@mahkamahagung.go.id   Telp : 021-384 3348 (ext.318)

- Bahwa, saksi sudah pernah menasehati Termohon untuk kembali rukun dengan Pemohon, namun tidak berhasil;
- Bahwa, pihak keluarga Termohon telah berupaya mendamaikan Pemohon dan Termohon, namun tidak berhasil;

Bahwa, atas keterangan saksi kedua yang telah diajukan Termohon Konvensi/Penggugat Rekonvensi membenarkan. Sedangkan Pemohon Konvensi/Tergugat Rekonvensi membantah dengan bantahan sebagai berikut:

1. Bahwa, Pemohon tidak pilih kasih dalam mengasuh anak, termasuk terhadap anak kandung Termohon. Namun memang Pemohon lebih memperhatikan anak pertama Pemohon dan Termohon, karena anak tersebut berkebutuhan khusus;

Bahwa, atas keterangan kedua orang saksi tersebut, Termohon Konvensi/Penggugat Rekonvensi menyatakan cukup dan tidak akan mengajukan alat bukti lain;

Bahwa, oleh karena Pemohon Konvensi/Tergugat Rekonvensi dan Termohon Konvensi/Penggugat Rekonvensi telah mencukupkan buktinya, maka majelis hakim memberikan kesempatan bagi keduanya untuk mengajukan kesimpulan;

Bahwa, Pemohon Konvensi/Tergugat Rekonvensi mengajukan kesimpulan secara lisan di muka persidangan yang pada pokoknya tetap bertahan dengan dalil-dalil permohonan konvensi/jawaban rekonvensi dan menyatakan tetap ingin bercerai dari Termohon Konvensi/Penggugat Rekonvensi. Begitu juga Termohon Konvensi/Penggugat Rekonvensi mengajukan kesimpulan secara lisan yang pada pokoknya tetap bertahan dengan dalil-dalil jawaban konvensi dan gugatan rekonvensi, dan menyatakan juga ingin bercerai dari Pemohon Konvensi/Tergugat Rekonvensi;

Bahwa, Pemohon dan Termohon menyatakan tidak lagi mengajukan sesuatu apapun dan memohon untuk dijatuhkan putusan ;

Disclaimer
Kepaniteraan Mahkamah Agung Republik Indonesia berusaha untuk selalu mencantumkan informasi paling kini dan akurat sebagai bentuk komitmen Mahkamah Agung untuk pelayanan publik, transparansi dan akuntabilitas pelaksanaan fungsi peradilan. Namun dalam hal-hal tertentu masih dimungkinkan terjadi permasalahan teknis terkait dengan akurasi dan keterkinian informasi yang kami sajikan, hal mana akan terus kami perbaiki dari waktu kewaktu.
Dalam hal Anda menemukan inakurasi informasi yang termuat pada situs ini atau informasi yang seharusnya ada, namun belum tersedia, maka harap segera hubungi Kepaniteraan Mahkamah Agung RI melalui :
Email : kepaniteraan@mahkamahagung.go.id Telp : 021-384 3348 (ext.318)

Bahwa, untuk mempersingkat uraian putusan ini, maka segala sesuatu yang tercantum dalam Berita Acara Sidang merupakan satu kesatuan yang tak terpisahkan dengan putusan ini;

PERTIMBANGAN HUKUM

A. Dalam Konvensi

Menimbang, bahwa maksud dan tujuan permohonan Pemohon sebagaimana diuraikan dalam duduk perkara di atas;

Menimbang, bahwa antara Pemohon dan Termohon telah diupayakan perdamaian oleh majelis hakim dalam persidangan dan melalui proses mediasi oleh mediator dalam proses mediasi, akan tetapi tidak berhasil;

Menimbang, bahwa berdasarkan posita permohonan Pemohon pokok perkara ini adalah mengenai sengketa perkawinan yaitu Cerai Talak antara orang-orang yang beragama Islam, maka berdasarkan Pasal 49 ayat (1) huruf a Undang-Undang Nomor 7 Tahun 1989 tentang Peradilan Agama, sebagaimana telah diubah dengan Undang-Undang Nomor 3 Tahun 2006 dan Undang-Undang Nomor 50 Tahun 2009, perkara ini termasuk kompetensi absolut Pengadilan Agama;

Menimbang, bahwa berdasarkan surat gugatan Pemohon, Termohon bertempat tinggal di Desa XXXXXX I Kecamatan Belitang Madang Raya Kabupaten Ogan Komering Ulu Timur yang merupakan wilayah hukum Pengadilan Agama Martapura, maka berdasarkan Pasal 73 ayat (1) Undang-Undang Nomor 7 Tahun 1989 tentang Peradilan Agama sebagaimana telah diubah dengan Undang-Undang Nomor 3 Tahun 2006 dan perubahan keduanya dengan Undang-Undang Nomor 50 Tahun 2009, Pengadilan Agama Martapura berwenang secara relatif memeriksa dan mengadili gugatan Pemohon;

Menimbang, bahwa sebelum mempertimbangkan pokok perkara, majelis hakim akan mempertimbangkan sebagai berikut:

Disclaimer
Kepaniteraan Mahkamah Agung Republik Indonesia berusaha untuk selalu mencantumkan informasi paling kini dan akurat sebagai bentuk komitmen Mahkamah Agung untuk pelayanan publik, transparansi dan akuntabilitas pelaksanaan fungsi peradilan. Namun dalam hal-hal tertentu masih dimungkinkan terjadi permasalahan teknis terkait dengan akurasi dan keterkinian informasi yang kami sajikan, hal mana akan terus kami perbaiki dari waktu kewaktu.
Dalam hal Anda menemukan inakurasi informasi yang termuat pada situs ini atau informasi yang seharusnya ada, namun belum tersedia, maka harap segera hubungi Kepaniteraan Mahkamah Agung RI melalui :
Email : kepaniteraan@mahkamahagung.go.id Telp : 021-384 3348 (ext.318)

Menimbang, bahwa Pemohon dan Termohon adalah suami isteri sah yang telah melangsungkan perkawinan secara hukum Islam di hadapan Pegawai Pencatat Nikah Kantor Urusan Agama Kecamatan Belitang Madang Raya Kabupaten Ogan Komering Ulu Timur dengan Kutipan Akta Nikah Nomor XXXXXX tertanggal 21 April 2020, maka telah terpenuhi maksud Pasal 2 ayat (1) dan (2) Undang-Undang Nomor 1 Tahun 1974 tentang Perkawinan;

Menimbang, bahwa oleh karena antara Pemohon dan Termohon memiliki hubungan hukum yaitu suami isteri, dan ikatan perkawinan antara keduanya belum pernah putus, kemudian Pemohon mengajukan permohonan Cerai Talak dengan alasan yang dibenarkan oleh Undang-undang, maka Pemohon memiliki kewenangan dan kepentingan hukum (*legitima persona standi in judicio*) untuk mengajukan permohonan cerai terhadap Termohon;

Menimbang, bahwa berdasarkan berdasarkan Pasal 82 ayat (1) dan (4) Undang-undang Nomor 7 Tahun 1989 tentang Peradilan Agama, sebagaimana telah diubah dengan Undang-Undang Nomor 3 Tahun 2006 dan perubahan kedua dengan Undang-Undang Nomor 50 Tahun 2009 majelis hakim telah berusaha mendamaikan pihak yang berperkara dengan cara mendamaikan Pemohon dan Termohon untuk kembali hidup rukun seperti semula sebagai suami isteri, dan bahkan Pemohon dan Termohon telah menempuh mediasi sebagaimana yang diamanatkan Peraturan Mahkamah Agung RI. Nomor 1 Tahun 2016 tentang Proses Mediasi di Pengadilan dengan mediator Ari Ferdinansyah, S.H., akan tetapi upaya mediasi tersebut tidak berhasil;

Menimbang, bahwa yang menjadi dalil pokok Pemohon mengajukan permohonanya adalah:

1. Bahwa semula rumah tangga Pemohon dengan Termohon dalam keadaan rukun dan bahagia selama kurang lebih 1 (satu) bulamn. Namun sejak Mei 2020 rumah tangga Pemohon dengan Termohon mulai tidak harmonis karena telah terjadi perselesihan dan

Disclaimer
Kepaniteraan Mahkamah Agung Republik Indonesia berusaha untuk selalu mencantumkan informasi paling kini dan akurat sebagai bentuk komitmen Mahkamah Agung untuk pelayanan publik, transparansi dan akuntabilitas pelaksanaan fungsi peradilan. Namun dalam hal-hal tertentu masih dimungkinkan terjadi permasalahan teknis terkait dengan akurasi dan keterkinian informasi yang kami sajikan, hal mana akan terus kami perbaiki dari waktu kewaktu.
Dalam hal Anda menemukan inakurasi informasi yang termuat pada situs ini atau informasi yang seharusnya ada, namun belum tersedia, maka harap segera hubungi Kepaniteraan Mahkamah Agung RI melalui :
Email : kepaniteraan@mahkamahagung.go.id   Telp : 021-384 3348 (ext.318)

pertengkaran diantara Pemohon dan Termohon disebabkan hal-hal berikut:

- Orang tua Termohon selalu ikut campur dalam rumah tangga Pemohon dan Termohon;
- Termohon tidak mau mengurus anak pertama Pemohon dan Termohon, karena anak tersebut berkebutuhan khusus;
- Termohon sering menuntut nafkah lebih di luar batas kemampuan Pemohon;

2. Bahwa, pertengkaran antara Pemohon dan Termohon terus menerus terjadi hingga bulan September 2020 yang disebabkan orang tua Termohon ingin meminjam uang kepada Pemohon, namun Pemohon tidak mau meminjamkan uang, karena orang tua Termohon sering meminjam uang, namun tidak pernah mengembalikan pinjaman. Kemudian terjadilah pertengkaran antara Pemohon dan Termohon. Akibat dari pertengkaran tersebut Pemohon dan Termohon telah berpisah tempat tinggal hingga sekarang selama ± 2 (dua) bulan. Termohon bertempat tinggal di rumah orang tua Termohon, dan sedangkan Pemohon bertempat tinggal di rumah orang tua Pemohon;

3. Bahwa, meski pihak keluarga sudah mendamaikan Pemohon dan Termohon, namun Pemohon tidak sanggup lagi membina rumah tangga dengan Termohon dan telah berketetapan hati ingin bercerai dari Termohon di hadapan sidang Pengadilan Agama Martapura;

Menimbang, bahwa terhadap dalil-dalil permohonan Pemohon tersebut, Termohon dalam jawabannya telah mengakui sebagian, baik dengan pengakuan murni (*aveu pur et simple*) maupun pengakuan dengan kualifikasi (*gequaliceerde bekentenis, aveu qualifie*), dan membantah selebihnya dari permohonan Permohon;

Menimbang, bahwa dengan demikian Pemohon mengajukan permohonan cerai ini adalah dengan alasan sebagaimana tercantum dalam Pasal 19 huruf (f) Peraturan Pemerintah Nomor 9 Tahun 1975 jo.

Disclaimer
Kepaniteraan Mahkamah Agung Republik Indonesia berusaha untuk selalu mencantumkan informasi paling kini dan akurat sebagai bentuk komitmen Mahkamah Agung untuk pelayanan publik, transparansi dan akuntabilitas pelaksanaan fungsi peradilan. Namun dalam hal-hal tertentu masih dimungkinkan terjadi permasalahan teknis terkait dengan akurasi dan keterkinian informasi yang kami sajikan, hal mana akan terus kami perbaiki dari waktu kewaktu.
Dalam hal Anda menemukan inakurasi informasi yang termuat pada situs ini atau informasi yang seharusnya ada, namun belum tersedia, maka harap segera hubungi Kepaniteraan Mahkamah Agung RI melalui :
Email : kepaniteraan@mahkamahagung.go.id Telp : 021-384 3348 (ext.318)

Pasal 116 huruf (f) Kompilasi Hukum Islam, oleh karena itu maka yang menjadi pokok masalah dalam perkara a quo adalah benarkah kondisi rumah tangga Pemohon dengan Termohon telah terjadi perselisihan dan pertengkaran yang terus menerus yang termasuk dalam kategori telah pecah (*broken marriage)* dan tidak ada harapan akan hidup rukun lagi dalam rumah tangga sehingga terpenuhi syarat atau alasan untuk terjadinya perceraian;

Menimbang, bahwa oleh karena Pemohon dan Termohon saling membantah sebagaimana yang tergambar dari tahapan jawab jinawab mulai dari permohonan Pemohon, jawaban Termohon, replik dan duplik, maka sesuai ketentuan Pasal 283 R.Bg:"*Barangsiapa beranggapan mempunyai suatu hak atau suatu keadaan untuk menguatkan haknya atau menyangkal hak seseorang lain, harus membuktikan hak atau keadaan itu*", karenanya kedua belah pihak akan dibebani wajib pembuktian secara berimbang;

Menimbang, bahwa untuk membuktikan kebenaran dalil-dalil permohonannya Pemohon telah mengajukan alat bukti surat P, dan 2 (dua) orang saksi;

Menimbang, bahwa Pemohon telah mengajukan alat bukti tertulis yaitu bukti P, yang telah memenuhi syarat formil dan materil sebagaimana ketentuan dalam Pasal 285 RBg sehingga dapat diterima sebagai bukti dalam perkara ini dan dapat dipertimbangkan;

Menimbang bahwa bukti surat (P) berupa fotokopi fotokopi Kutipan Akta Nikah, bermeterai cukup, telah dicocokkan sesuai dengan aslinya sehingga majelis hakim menilai alat bukti tertulis tersebut sah sebagai alat bukti berdasarkan Pasal 1888 Kitab Undang-Undang Hukum Perdata dan Pasal 2 ayat (3) Undang-Undang Nomor 13 tahun 1985 tentang Bea Meterai serta pasal 2 ayat (1) Peraturan Pemerintah Nomor 24 Tahun 2000 tentang Perubahan Tarif Bea Meterai dan Besarnya Batas Pengenaan Harga Nominal Yang Dikenakan Bea Meterai;

Disclaimer
Kepaniteraan Mahkamah Agung Republik Indonesia berusaha untuk selalu mencantumkan informasi paling kini dan akurat sebagai bentuk komitmen Mahkamah Agung untuk pelayanan publik, transparansi dan akuntabilitas pelaksanaan fungsi peradilan. Namun dalam hal-hal tertentu masih dimungkinkan terjadi permasalahan teknis terkait dengan akurasi dan keterkinian informasi yang kami sajikan, hal mana akan terus kami perbaiki dari waktu kewaktu.
Dalam hal Anda menemukan inakurasi informasi yang termuat pada situs ini atau informasi yang seharusnya ada, namun belum tersedia, maka harap segera hubungi Kepaniteraan Mahkamah Agung RI melalui :
Email : kepaniteraan@mahkamahagung.go.id Telp : 021-384 3348 (ext.318)

Menimbang bahwa alat bukti P tersebut merupakan akta autentik yang dibuat oleh pejabat yang berwenang, dan isinya tersebut tidak dibantah oleh pihak Termohon, maka nilai kekuatan pembuktianya adalah bersifat sempurna dan mengikat berdasarkan Pasal 1870 KUH Perdata dan Pasal 285 RBg;

Menimbang, bahwa bukti P yaitu berupa Kutipan Akta Nikah atas nama Pemohon dan Termohon yang dikeluarkan oleh Kantor Urusan Agama Kecamatan Belitang Madang Raya Kabupaten Ogan Komering Ulu Timur Nomor XXXXXX, tanggal 21 April 2020, isi bukti tersebut menjelaskan bahwa Pemohon dengan Termohon adalah suami istri sah yang telah menikah tercatat di Kantor Urusan Agama Kecamatan Martapura Kabupaten Ogan Komering Ulu Timur. Oleh karena bukti P merupakan akta otentik yang mempunyai nilai pembuktian yang sempurna dan mengikat maka alat bukti tersebut diterima kebenarannya sepanjang tidak dibuktikan sebaliknya, membuktikan bahwa antara Pemohon dan Termohon terikat dalam perkawinan yang sah belum pernah bercerai. Dengan demikian ketentuan Pasal 7 ayat (1) Kompilasi Hukum Islam telah terpenuhi, dan kedua belah pihak telah berkualitas hukum untuk bertindak sebagai pihak-pihak dalam perkara ini *(persona standi in judicio)*;

Menimbang, bahwa selain mengajukan bukti tertulis, Pemohon juga menghadirkan dua orang saksi yaitu XXXXXX dan XXXXXX (Alm), yang masing-masing saksi tersebut berakal, sudah dewasa, dan telah disumpah sesuai agamanya, karenanya telah terpenuhi syarat formil untuk menjadi saksi sebagaimana ketentuan dalam Pasal 171, 172 dan 175 RBg, sehingga patut didengar keterangannya;

Menimbang bahwa majelis hakim telah mendengar keterangan dua orang saksi Pemohon yang telah memberikan keterangan di bawah sumpahnya sebagaimana telah terurai selengkapnya dalam duduk perkara;

Menimbang, bahwa saksi pertama adalah keluarga Pemohon yaitu adik kandung Pemohon, sedangkan saksi kedua merupakan orang dekat

Disclaimer
Kepaniteraan Mahkamah Agung Republik Indonesia berusaha untuk selalu mencantumkan informasi paling kini dan akurat sebagai bentuk komitmen Mahkamah Agung untuk pelayanan publik, transparansi dan akuntabilitas pelaksanaan fungsi peradilan. Namun dalam hal-hal tertentu masih dimungkinkan terjadi permasalahan teknis terkait dengan akurasi dan keterkinian informasi yang kami sajikan, hal mana akan terus kami perbaiki dari waktu kewaktu.
Dalam hal Anda menemukan inakurasi informasi yang termuat pada situs ini atau informasi yang seharusnya ada, namun belum tersedia, maka harap segera hubungi Kepaniteraan Mahkamah Agung RI melalui :
Email : kepaniteraan@mahkamahagung.go.id Telp : 021-384 3348 (ext.318)

Pemohon yaitu tetangga Pemohon, dengan demikian telah terpenuhi maksud Pasal 22 Peraturan Pemerintah Nomor 9 Tahun 1975 jo. Pasal 134 Kompilasi Hukum Islam;

Menimbang, bahwa kedua orang saksi yang diajukan oleh Pemohon yang bernama XXXXXX dan XXXXXX (Alm) memberi keterangan di muka sidang sebagai berikut:

1. Bahwa, hubungan Pemohon dengan Termohon adalah suami isteri yang menikah pada bulan April 2020;

2. Bahwa, hubungan Pemohon dengan Termohon dahulu merupakan suami isteri, namun bercerai pada tahun 2006, kemudian menikah kembali pada bulan Apri 2020;;

3. Bahwa, dari pernikahan sebelumnya Pemohon dan Termohon telah dikaruniai 2 (dua) orang anak bernama XXXXXX dan XXXXXXn;

4. Bahwa, setelah akad nikah Pemohon dan Termohon bertempat tingggal di rumah kontrakan XXXXXX I sampai dengan berpisah;

5. Bahwa, saat ini anak pertama bernama XXXXXX diasuh oleh Pemohon, dan sedangkan anak kedua bernama XXXXXXn diasuh oleh Termohon;

6. Bahwa, anak pertama Pemohon dan Termohon berkebutuhan khusus;

7. Bahwa, , rumah tangga Pemohon dan Termohon pada awalnya rukun selama kurang lebih 1 (satu) bulan. Namun sejak bulan Mei 2020 terakhir mulai terjadi pertengkaran;

8. Bahwa, saksi melihat Pemohon dan Termohon cekcok mulut di rumah orang tua Pemohon, yang disebabkan faktor ekonomi keluarga yang kurang, orang tua Termohon yang sering ikut campur

Disclaimer
Kepaniteraan Mahkamah Agung Republik Indonesia berusaha untuk selalu mencantumkan informasi paling kini dan akurat sebagai bentuk komitmen Mahkamah Agung untuk pelayanan publik, transparansi dan akuntabilitas pelaksanaan fungsi peradilan. Namun dalam hal-hal tertentu masih dimungkinkan terjadi permasalahan teknis terkait dengan akurasi dan keterkinian informasi yang kami sajikan, hal mana akan terus kami perbaiki dari waktu kewaktu.
Dalam hal Anda menemukan inakurasi informasi yang termuat pada situs ini atau informasi yang seharusnya ada, namun belum tersedia, maka harap segera hubungi Kepaniteraan Mahkamah Agung RI melalui :
Email : kepaniteraan@mahkamahagung.go.id Telp : 021-384 3348 (ext.318)

urusan rumah tangga Pemohon dan Termohon, Termohon tidak terima penghasilan Pemohon yang sedikit, dan Termohon tidak menghargai usaha orang tua Pemohon sebagai pembibit ikan lele;

9. Bahwa, saksi melihat Termohon tidak memberlakukan orang tua Pemohon dengan baik selayaknya mertua;

10. Bahwa, saat ini Pemohon sudah berpisah tempat tinggal selama kurang lebih 3 bulan, Pemohon bertempat tinggal di rumah orang tua Pemohon, dan sedangkan Termohon bertempat tinggal di rumah orang tua Termohon;

11. Bahwa, saksi sudah menasehati Pemohon untuk hidup rukun dengan Termohon, namun tidak berhasil;

12. Bahwa, pihak keluarga Pemohon telah berupaya mendamaikan Pemohon dan Termohon, namun tidak berhasil;

13. Bahwa, pekerjaan Pemohon adalah pembibitan ikan lele dan buruh jaring ikan;

14. Bahwa, dari usaha pembibitan ikan lele tersebut baru satu kali menghasilkan atau panen, dengan nilai sebesar Rp.2.000.000,- (dua juta rupiah);

15. Bahwa, Pemohon tidak setiap waktu menjadi buruh jaring ikan, hanya ketika ada permintaan saja. Adapun upah harian buruh jaring ikan di bawah delapan puluh ribu rupiah;

Menimbang, bahwa kedua orang saksi Pemohon memberikan keterangan berdasarkan fakta kejadian yang dilihat, didengar dan dialami sendiri, bukan cerita, pendapat atau perasaan pribadi saksi serta keterangan saksi-saksi tersebut relevan dengan dalil-dalil gugatan Pemohon serta saling bersesuaian sehingga telah memenuhi syarat materil sebagaimana ketentuan dalam Pasal 307, 308 dan 309 RBg jo. Pasal 76 ayat (1) Undang-Undang Nomor 7 tahun 1989 jo. Pasal 22 ayat

Disclaimer
Kepaniteraan Mahkamah Agung Republik Indonesia berusaha untuk selalu mencantumkan informasi paling kini dan akurat sebagai bentuk komitmen Mahkamah Agung untuk pelayanan publik, transparansi dan akuntabilitas pelaksanaan fungsi peradilan. Namun dalam hal-hal tertentu masih dimungkinkan terjadi permasalahan teknis terkait dengan akurasi dan keterkinian informasi yang kami sajikan, hal mana akan terus kami perbaiki dari waktu kewaktu.
Dalam hal Anda menemukan inakurasi informasi yang termuat pada situs ini atau informasi yang seharusnya ada, namun belum tersedia, maka harap segera hubungi Kepaniteraan Mahkamah Agung RI melalui :
Email : kepaniteraan@mahkamahagung.go.id Telp : 021-384 3348 (ext.318)

(2) Peraturan Pemerintah Nomor 9 Tahun 1975 jo. Pasal 134 Kompilasi Hukum Islam, karenanya keterangan saksi-saksi tersebut mempunyai nilai pembuktian ;

Menimbang, bahwa majelis hakim telah mendengar keterangan dua orang saksi Termohon yang telah memberikan keterangan di bawah sumpahnya sebagaimana telah terurai selengkapnya dalam duduk perkara;

Menimbang, bahwa saksi pertama merupakan keluarga Termohon yaitu ayah kandung Termohon, sedangkan saksi kedua Termohon adalah orang dekat Termohon yaitu teman Termohon, dengan demikian telah terpenuhi maksud Pasal 22 Peraturan Pemerintah Nomor 9 Tahun 1975 jo. Pasal 134 Kompilasi Hukum Islam;

Menimbang, bahwa kedua orang saksi yang diajukan oleh Termohon yang bernama XXXXXX, dan XXXXXX menerangkan sebagai berikut:

1. Bahwa, Pemohon dan Termohon dahulu sudah pernah menikah dan bercerai pada tahun 2006, kemudian keduanya menikah kembali pada bulan April 2020;
2. Bahwa, Pemohon dan Termohon adalah pasangan suami istri, serta telah dikaruniai dua orang anak laki-laki yang saat ini anak pertama bernama XXXXXX diasuh oleh Pemohon, dan sedangkan anak kedua bernama XXXXXXn dalam asuhan Termohon;
3. Bahwa, setelah menikah Pemohon dan Termohon sempat bertempat tinggal di rumah saksi, kemudian pindah ke rumah kontrakan di desa XXXXXX;
4. Bahwa, rumah tangga Pemohon dan Termohon sempat rukun selama 2 (dua) bulan, kemudian setelah itu mulai terjadi perselisihan dan pertengkaran, yang disebabkan ekonomi keluarga yang tidak cukup, Pemohon yang tidak terbuka dalam masalah

Disclaimer
Kepaniteraan Mahkamah Agung Republik Indonesia berusaha untuk selalu mencantumkan informasi paling kini dan akurat sebagai bentuk komitmen Mahkamah Agung untuk pelayanan publik, transparansi dan akuntabilitas pelaksanaan fungsi peradilan. Namun dalam hal-hal tertentu masih dimungkinkan terjadi permasalahan teknis terkait dengan akurasi dan keterkinian informasi yang kami sajikan, hal mana akan terus kami perbaiki dari waktu kewaktu.
Dalam hal Anda menemukan inakurasi informasi yang termuat pada situs ini atau informasi yang seharusnya ada, namun belum tersedia, maka harap segera hubungi Kepaniteraan Mahkamah Agung RI melalui :
Email : kepaniteraan@mahkamahagung.go.id Telp : 021-384 3348 (ext.318)

penghasilan kepada Termohon, dan Pemohon tidak memberi Termohon nafkah;

5. Bahwa, saat ini Pemohon dan Termohon telah berpisah tempat tinggal selama kurang lebih 3 (tiga) bulan, Pemohon yang terlebih dahulu pergi dari rumah kontrakan dan meninggalkan Termohon;

6. Bahwa, Pemohon bekerja usaha pembibitan ikan lele, yang telah tiga kali panen, dan ketika panen hasilnya kurang lebih sebesar tiga juta rupiah;

7. Bahwa, Pemohon juga bekerja sampingan, yaitu jual beli ikan;

8. Bahwa, saksi sudah menasehati Pemohon dan Termohon, namun tidak berhasil;

9. Bahwa, pihak keluarga Termohon sudah berupaya mendamaikan Pemohon dan Termohon, namun tidak berhasil;

Menimbang, bahwa kedua orang saksi Termohon memberikan keterangan berdasarkan fakta kejadian yang dilihat, didengar dan dialami sendiri, bukan cerita, pendapat atau perasaan pribadi saksi serta keterangan saksi-saksi tersebut relevan dengan dalil-dalil gugatan Pemohon serta saling bersesuaian sehingga telah memenuhi syarat materil sebagaimana ketentuan dalam Pasal 307, 308 dan 309 RBg jo. Pasal 76 ayat (1) Undang-Undang Nomor 7 tahun 1989 jo. Pasal 22 ayat (2) Peraturan Pemerintah Nomor 9 Tahun 1975 jo. Pasal 134 Kompilasi Hukum Islam, karenanya keterangan saksi-saksi tersebut mempunyai nilai pembuktian;

Menimbang, bahwa berdasarkan keterangan saksi baik Pemohon dan Termohon, majelis hakim mempertimbangkan bahwa keterangan saksi dalam perkara perceraian yang hanya menerangkan tentang suatu akibat hukum (*rechts gevolg*) mempunyai kekuatan hukum dalam pembuktian;

Disclaimer
Kepaniteraan Mahkamah Agung Republik Indonesia berusaha untuk selalu mencantumkan informasi paling kini dan akurat sebagai bentuk komitmen Mahkamah Agung untuk pelayanan publik, transparansi dan akuntabilitas pelaksanaan fungsi peradilan. Namun dalam hal-hal tertentu masih dimungkinkan terjadi permasalahan teknis terkait dengan akurasi dan keterkinian informasi yang kami sajikan, hal mana akan terus kami perbaiki dari waktu kewaktu.
Dalam hal Anda menemukan inakurasi informasi yang termuat pada situs ini atau informasi yang seharusnya ada, namun belum tersedia, maka harap segera hubungi Kepaniteraan Mahkamah Agung RI melalui :
Email : kepaniteraan@mahkamahagung.go.id Telp : 021-384 3348 (ext.318)

Menimbang, bahwa berdasarkan hal-hal yang diakui atau setidak-tidaknya tidak dibantah oleh Termohon dan bukti-bukti yang diajukan oleh Pemohon dan Termohon, maka majelis hakim telah menemukan fakta hukum sebagai berikut;

1. Bahwa, Pemohon dan Termohon adalah pasangan suami istri yang sah yang pernah bercerai pada tahun 2006 dan menikah kembali pada tanggal 21 April 2020;
2. Bahwa, dari setelah menikah Pemohon dan Termohon telah dikaruniai 2 (dua) orang anak laki-laki bernama XXXXXX dan XXXXXX, yang saat ini anak bernama Khusesn diasuh oleh Pemohon, dan sedangkan anak bernama XXXXXX berada dalam asuhan Termohon;
3. Bahwa, bahwa setelah menikah Pemohon dan Termohon bertempat tingggal di rumah kontrakan di desa XXXXXX I sampai dengan berpisah;
4. Bahwa, keadaan rumah tangga Pemohon dan Termohon awalnya rukun dan harmonis selama kurang lebih 2 (dua) bulan. Namun sejak bulan Juni 2020 mulai terjadi perselisihan dan pertengkaran yang disebabkan Termohon menuntut nafkah di luar batas kemampuan Pemohon;
5. Bahwa, pada bulan September 2020 pertengkaran antara Pemohon dan Termohon semakin memuncak disebabkan orang tua Termohon ingin meminjam uang kepada Pemohon, namun Pemohon menolak untuk meminjamkan uang, karena orang tua Termohon sering meminjam uang, namun tidak pernah mengembalikan. Sehingga terjadilah pertengkaran antara Pemohon dan Termohon;
6. Bahwa, akibat pertengkaran pada bulan September 2020 tersebut Pemohon pergi dari rumah kontrakan dan meninggalkan Termohon. Sehingga antara Pemohon dan Termohon telah berpisah tempat tinggal hingga sekarang selama kurang lebih 3 (tiga) bulan. Pemohon

Disclaimer
Kepaniteraan Mahkamah Agung Republik Indonesia berusaha untuk selalu mencantumkan informasi paling kini dan akurat sebagai bentuk komitmen Mahkamah Agung untuk pelayanan publik, transparansi dan akuntabilitas pelaksanaan fungsi peradilan. Namun dalam hal-hal tertentu masih dimungkinkan terjadi permasalahan teknis terkait dengan akurasi dan keterkinian informasi yang kami sajikan, hal mana akan terus kami perbaiki dari waktu kewaktu.
Dalam hal Anda menemukan inakurasi informasi yang termuat pada situs ini atau informasi yang seharusnya ada, namun belum tersedia, maka harap segera hubungi Kepaniteraan Mahkamah Agung RI melalui :
Email : kepaniteraan@mahkamahagung.go.id Telp : 021-384 3348 (ext.318)

bertempat tinggal di rumah orang tua Pemohon, dan sedangkan Termohon bertempat tinggal di rumah orang tua Termohon, dan antara keduanya sudah tidak saling memperdulikan lagi layaknya suami istri ;

6. Bahwa, para saksi dan pihak keluarga Pemohon dan Termohon telah berupaya menasehati dan mendamaikan Pemohon dan Temohon, namun tidak berhasil;
7. Bahwa, Pemohon tidak sanggup lagi membina rumah tangga dengan Termohon, dan Pemohon tetap ingin bercerai dari Termohon;

Menimbang bahwa fakta hukum yang telah dirumuskan di atas, perlu dianalisis dan dipertimbangkan lebih lanjut;

Menimbang, bahwa berdasarkan fakta hukum pertama, kedua, dan ketiga, Pemohon dan Termohon merupakan suami istri yang sah dan telah dikaruniai dua orang anak laki-laki bernama XXXXXX dan XXXXXX, membuktikan rumah tangga Pemohon dan Termohon pada awalnya rukun harmonis;

Menimbang, bahwa berdasarkan fakta hukum keempat, kelima, dan keenam, membuktikan telah terjadi pertengkaran antara Pemohon dan Termohon yang berujung pada perpisahan tempat tinggal selama 3 (tiga) bulan, dapat dikategorikan sebagai perselisihan dan pertengkaran terus menerus yang merupakan gejala hilangnya rasa cinta dan kasih sayang diantara suami isteri sekaligus sebagai pertanda kehidupan rumah tangga telah hancur, sehingga dalam kondisi yang sedemikian berat sulit membangun kembali rumah tangga ideal;

Menimbang, bahwa Pemohon dan Termohon sudah berpisah tempat tinggal selama 3 (tiga) bulan dan sudah tidak saling memperdulikan layaknya suami isteri, sehingga fakta tersebut membuktikan bahwa diantara Pemohon dan Termohon sudah tidak mampu membangun rasa kasih sayang;

Menimbang, bahwa berdasarkan fakta hukum keenam dan ketujuh, para saksi dan pihak keluarga Pemohon telah berupaya menasehati dan

Disclaimer
Kepaniteraan Mahkamah Agung Republik Indonesia berusaha untuk selalu mencantumkan informasi paling kini dan akurat sebagai bentuk komitmen Mahkamah Agung untuk pelayanan publik, transparansi dan akuntabilitas pelaksanaan fungsi peradilan. Namun dalam hal-hal tertentu masih dimungkinkan terjadi permasalahan teknis terkait dengan akurasi dan keterkinian informasi yang kami sajikan, hal mana akan terus kami perbaiki dari waktu kewaktu.
Dalam hal Anda menemukan inakurasi informasi yang termuat pada situs ini atau informasi yang seharusnya ada, namun belum tersedia, maka harap segera hubungi Kepaniteraan Mahkamah Agung RI melalui :
Email : kepaniteraan@mahkamahagung.go.id   Telp : 021-384 3348 (ext.318)

mendamaikan Pemohon dan Termohon, akan tetapi tidak berhasil. Bahkan, Pemohon dan Termohon telah menyampaikan secara lisan di persidangan kesimpulan masing-masing di muka persidangan yang pada pokonya sama-sama ingin bercerai. Hal demikian secara nyata membuktikan rumah tangga Pemohon dan Termohon telah pecah sedemikian rupa dan tidak ada harapan akan hidup rukun lagi sebagaimana tujuan adanya pernikahan;

Menimbang bahwa berdasarkan fakta hukum dan analisis atas fakta hukum diatas, maka petitum permohonan Pemohon nomor 1 dan 2 dapat dipertimbangkan sebagai berikut :

Menimbang bahwa berdasarkan ketentuan Pasal 39 Undang-Undang Nomor 1 Tahun 1974 tentang Perkawinan yaitu untuk melakukan suatu perceraian harus ada cukup alasan dimana suami isteri tidak akan dapat hidup rukun sebagai suami isteri dan pengadilan telah berusaha dan tidak berhasil mendamaikan kedua belah pihak. Selanjutnya dalam Pasal 19 huruf (f) Peraturan Pemerintah Nomor 9 Tahun 1975 jo. Pasal 116 huruf (f) Kompilasi Hukum Islam menegaskan salah satu alasan perceraian yaitu adanya perselisihan dan pertengkaran terus menerus antara suami istri dan tidak ada harapan lagi untuk kembali rukun;

Menimbang bahwa dari ketentuan pasal-pasal tersebut terdapat beberapa unsur yang harus dipenuhi terjadinya perceraian yaitu :

1. Adanya alasan terjadinya perselisihan dan pertengkaran terus menerus;
2. Perselisihan dan pertengkaran menyebabkan suami istri sudah tidak ada harapan untuk kembali rukun;
3. Pengadilan telah berupaya mendamaikan suami istri tapi tidak berhasil;

Menimbang berdasarkan fakta hukum tersebut di atas telah terbukti terjadinya perselisihan dan pertengkaran terus menerus antara Pemohon dan Termohon sejak bulan Juni hingga September 2020, disebabkan

Disclaimer
Kepaniteraan Mahkamah Agung Republik Indonesia berusaha untuk selalu mencantumkan informasi paling kini dan akurat sebagai bentuk komitmen Mahkamah Agung untuk pelayanan publik, transparansi dan akuntabilitas pelaksanaan fungsi peradilan. Namun dalam hal-hal tertentu masih dimungkinkan terjadi permasalahan teknis terkait dengan akurasi dan keterkinian informasi yang kami sajikan, hal mana akan terus kami perbaiki dari waktu kewaktu.
Dalam hal Anda menemukan inakurasi informasi yang termuat pada situs ini atau informasi yang seharusnya ada, namun belum tersedia, maka harap segera hubungi Kepaniteraan Mahkamah Agung RI melalui :
Email : kepaniteraan@mahkamahagung.go.id Telp : 021-384 3348 (ext.318)

Termohon menuntut nafkah di luar batas kemampuan Pemohon dan orang tua Termohon yang sering meminjam uang kepada Pemohon namun tidak pernah mengembalikan, karenanya majelis hakim berpendapat unsur pertama telah terpenuhi dalam perkara ini;

Menimbang bahwa akibat dari perselisihan dan pertengkaran yang terjadi antara Pemohon dengan Termohon adalah telah terjadi pisah tempat tinggal selama 3 (tiga) bulan, dan selama berpisah tempat tinggal antara Pemohon dan Termohon sudah tidak saling memperdulikan layaknya suami istri;

Menimbang, bahwa majelis hakim menilai tindakan Pemohon dan Termohon yang sudah tidak saling memperdulikan dan menghiraukan antara satu dengan lainnya dalam kurun waktu 3 (tiga) bulan menunjukkan rumah tangga Pemohon dan Termohon sudah tidak ada harapan untuk rukun kembali, karenanya majelis hakim berpendapat unsur kedua telah terpenuhi dalam perkara ini;

Menimbang, bahwa majelis hakim telah berupaya untuk memberikan nasehat pada setiap persidangan, serta para saksi dan pihak keluarga Pemohon dan Termohon juga telah berusaha menasehati dan mendamaikan Pemohon dan Termohon agar rukun kembali, sebagaimana ketentuan dalam Pasal 82 ayat (1) dan (4) Undang-Undang Nomor 7 Tahun 1989 Tentang Peradilan Agama sebagaimana telah diubah dengan Undang-Undang Nomor 3 Tahun 2006 dan Undang-Undang Nomor 50 Tahun 2009 jis. Pasal 31 Peraturan Pemerintah Nomor 9 Tahun 1975 jo. Pasal 143 ayat (1) dan (2) Kompilasi Hukum Islam, namun upaya tersebut tidak berhasil. Bahkan upaya untuk mendamaikan Pemohon dan Termohon juga telah ditempuh melalui mediasi oleh mediator, akan tetapi Pemohon tetap ingin bercerai dari Termohon, karenanya majelis hakim berpendapat unsur ketiga telah terpenuhi dalam perkara ini;

Menimbang, bahwa berdasarkan Pasal 1 Undang-undang Nomor 1 Tahun 1974 Tentang Perkawinan:"*Perkawinan ialah ikatan lahir bathin*

Disclaimer
Kepaniteraan Mahkamah Agung Republik Indonesia berusaha untuk selalu mencantumkan informasi paling kini dan akurat sebagai bentuk komitmen Mahkamah Agung untuk pelayanan publik, transparansi dan akuntabilitas pelaksanaan fungsi peradilan. Namun dalam hal-hal tertentu masih dimungkinkan terjadi permasalahan teknis terkait dengan akurasi dan keterkinian informasi yang kami sajikan, hal mana akan terus kami perbaiki dari waktu kewaktu.
Dalam hal Anda menemukan inakurasi informasi yang termuat pada situs ini atau informasi yang seharusnya ada, namun belum tersedia, maka harap segera hubungi Kepaniteraan Mahkamah Agung RI melalui :
Email : kepaniteraan@mahkamahagung.go.id Telp : 021-384 3348 (ext.318)

antara seorang pria dengan seorang wanita sebagai suami isteri dengan tujuan membentuk keluarga (rumah tangga) yang bahagia dan kekal berdasarkan Ketuhanan Yang Maha esa", dan Pasal 3 Kompilasi Hukum Islam:"*Perkawinan bertujuan untuk mewujudkan kehidupan rumah tangga yang sakinah, mawaddah, dan rahmah*";

Menimbang, bahwa untuk mewujudkan tujuan perkawinan sebagaimana ketentuan dalam Pasal 1 Undang-undang Nomor 1 Tahun 1974 Tentang Perkawinan, dan Pasal 3 Kompilasi Hukum Islam sudah seharusnya pasangan suami istri dapat hidup bersama dalam satu atap membina rumah tangga, kecuali terdapat alasan tertentu yang sejalan dan tidak bertentangan dengan nilai-nilai agama dan hukum perundang-undangan, misalnya karena bekerja untuk memenuhi kebutuhan keluarga.

Menimbang, bahwa Pemohon dan Termohon telah berpisah tempat tinggal, tidak lagi saling memperdulikan layaknya suami istri, dan telah menjalani kehidupan masing-masing selama 3 (tiga) bulan tanpa alasan yang sejalan dengan nilai-nilai agama dan hukum perundang-undangan, maka majelis hakim berpendapat Pemohon dan Termohon tidak dapat mewujudkan kehidupan rumah tangga yang sakinah, mawaddah, dan rahmah, bahagia, dan kekal sebagaimana ketentuan dalam Pasal 1 Undang-undang Nomor 1 Tahun 1974 Tentang Perkawinan, dan Pasal 3 Kompilasi Hukum Islam;

Menimbang, bahwa "*Suami isteri wajib saling cinta-mencintai hormat-menghormati, setia dan memberi bantuan lahir bathin yang satu kepada yang lain*" sebagaimana dalam ketentuan Pasal 33 Undang-Undang Nomor 1 Tahun 1974 tentang Perkawinan jo. Pasal 77 huruf (a) dan (b) Kompilasi Hukum Islam;

Menimbang, sikap Pemohon yang tidak transparan dalam penghasilan, dan Termohon yang menuntut nafkah di luar batas kemampuan Pemohon, menunjukkan Pemohon dan Termohon tidak saling mencintai, menghormati dan memberi bantuan lahir bathin dalam rumah tangga. Sebab jika Pemohon mencintai dan menghormati Termohon

Disclaimer
Kepaniteraan Mahkamah Agung Republik Indonesia berusaha untuk selalu mencantumkan informasi paling kini dan akurat sebagai bentuk komitmen Mahkamah Agung untuk pelayanan publik, transparansi dan akuntabilitas pelaksanaan fungsi peradilan. Namun dalam hal-hal tertentu masih dimungkinkan terjadi permasalahan teknis terkait dengan akurasi dan keterkinian informasi yang kami sajikan, hal mana akan terus kami perbaiki dari waktu kewaktu.
Dalam hal Anda menemukan inakurasi informasi yang termuat pada situs ini atau informasi yang seharusnya ada, namun belum tersedia, maka harap segera hubungi Kepaniteraan Mahkamah Agung RI melalui :
Email : kepaniteraan@mahkamahagung.go.id   Telp : 021-384 3348 (ext.318)

sebagai istri, maka tentu Pemohon akan bersikap terbuka masalah penghasilan yang telah didapat kepada Termohon sebagai istri. Begitupula jika Termohon menghormati dan memberikan bantuan lahir bathin kepada Pemohon, maka tentu Termohon tidak akan menuntut nafkah di luar batas kemampuan Pemohon;

Menimbang, bahwa dengan sikap Pemohon yang tidak terbuka masalah keuangan, Termohon yang tidak dapat memahami batas kemampuan keuangan Pemohon, serta antara keduanya sudah tidak saling memperdulikan layaknya suami istri selama kurang lebih 3 (tiga) bulan, maka majelis hakim berpendapat Pemohon dan Termohon sudah tidak dapat mewujudkan maksud Pasal 33 Undang-Undang Nomor 1 Tahun 1974 tentang Perkawinan jo. Pasal 77 huruf (a) dan (b) Kompilasi Hukum Islam untuk saling mencintai, menghormati, dan memberi bantuan lahir bathin;

Menimbang, bahwa setiap orang berhak mendapatkan hak-hak kemanusiannya sebagaimana yang disebutkan dalam ketentuan Undang-undang Nomor 39 Tahun 1999 tentang Hak Asasi Manusia Pasal 9 ayat (2) menyebutkan: "*Setiap orang berhak tenteram, aman, damai, bahagia, sejahtera lahir dan batin*";

Menimbang, bahwa berdasarkan fakta hukum dalam rumah tangga Pemohon dan Termohon, majelis hakim berpendapat tidak ada lagi kententraman, kedamaian, kebahagiaan lahir batin dalam rumah tangga Pemohon dan Termohon. Padahal semestinya melalui pernikahan setiap orang dapat merasakan kehidupan yang tenteram, aman, damai, bahagia, sejahtera lahir dan batin, karenanya majelis hakim berpendapat rumah tangga Pemohon dan Termohon tidak dapat dipertahankan lagi, yang apabila tetap dipaksakan hidup bersama dalam ikatan pernikahan hanya akan menyengsarakan kedua belah pihak dan bertentangan dengan maksud Pasal 9 ayat (2) Undang-undang Nomor 39 Tahun 1999 tentang Hak Asasi Manusia;

Disclaimer
Kepaniteraan Mahkamah Agung Republik Indonesia berusaha untuk selalu mencantumkan informasi paling kini dan akurat sebagai bentuk komitmen Mahkamah Agung untuk pelayanan publik, transparansi dan akuntabilitas pelaksanaan fungsi peradilan. Namun dalam hal-hal tertentu masih dimungkinkan terjadi permasalahan teknis terkait dengan akurasi dan keterkinian informasi yang kami sajikan, hal mana akan terus kami perbaiki dari waktu kewaktu.
Dalam hal Anda menemukan inakurasi informasi yang termuat pada situs ini atau informasi yang seharusnya ada, namun belum tersedia, maka harap segera hubungi Kepaniteraan Mahkamah Agung RI melalui :
Email : kepaniteraan@mahkamahagung.go.id Telp : 021-384 3348 (ext.318)

Menimbang, bahwa berdasarkan ketentuan Pasal 5 Undang-undang Republik Indonesia Nomor 23 Tahun 24 Tentang Penghapusan Kekerasan Dalam Rumah Tangga: "*Setiap orang dilarang melakukan kekerasan dalam rumah tangga terhadap orang dalam lingkup rumah tangganya, dengan cara : a. kekerasan fisik; b. kekerasan psikis; c. kekerasan seksual; atau d. penelantaran rumah tangga*", dan Pasal 9: "*Setiap orang dilarang menelantarkan orang dalam lingkup rumah tangganya, padahal menurut hukum yang berlaku baginya atau karena persetujuan atau perjanjian ia wajib memberikan kehidupan, perawatan, atau pemeliharaan kepada orang tersebut*":

Menimbang, bahwa sikap Pemohon yang tidak terbuka masalah keuangan, pergi dari rumah kontrakan meninggalkan Termohon, dan tidak lagi memperdulikan Termohon layaknya suami istri adalah merupakan kekerasan dalam rumah tangga dalam bentuk psikis dan penelantaran, sebagaimana yang dimaksud dalam ketentuan Pasal 5 huruf (a) dan (d) dan Pasal 9 Undang-undang Republik Indonesia Nomor 23 Tahun 24 Tentang Penghapusan Kekerasan Dalam Rumah Tangga;

Menimbang, bahwa kekerasan psikis dan penelantaran dalam rumah tangga yang telah dilakukan Pemohon kepada Termohon dapat berpotensi menimbulkan dampak psikis bagi Termohon, dan juga bahkan bagi kedua anak Pemohon dan Termohon, berupa hilangnya rasa percaya diri, hilangnya kemampuan untuk bertindak, rasa tidak berdaya, dan/atau penderitaan, sebagaimana ketentuan dalam Pasal 7 Undang-undang Republik Indonesia Nomor 23 Tahun 24 Tentang Penghapusan Kekerasan Dalam Rumah Tangga: *"Kekerasan psikis sebagaimana dimaksud dalam Pasal 5 huruf b adalah perbuatan yang mengakibatkan ketakutan, hilangnya rasa percaya diri, hilangnya kemampuan untuk bertindak, rasa tidak berdaya, dan/atau penderitaan"*;

Menimbang, bahwa majelis hakim perlu mempertimbangkan perceraian sebagai bentuk upaya untuk mengambalikan rasa percaya diri,

Disclaimer
Kepaniteraan Mahkamah Agung Republik Indonesia berusaha untuk selalu mencantumkan informasi paling kini dan akurat sebagai bentuk komitmen Mahkamah Agung untuk pelayanan publik, transparansi dan akuntabilitas pelaksanaan fungsi peradilan. Namun dalam hal-hal tertentu masih dimungkinkan terjadi permasalahan teknis terkait dengan akurasi dan keterkinian informasi yang kami sajikan, hal mana akan terus kami perbaiki dari waktu kewaktu.
Dalam hal Anda menemukan inakurasi informasi yang termuat pada situs ini atau informasi yang seharusnya ada, namun belum tersedia, maka harap segera hubungi Kepaniteraan Mahkamah Agung RI melalui :
Email : kepaniteraan@mahkamahagung.go.id Telp : 021-384 3348 (ext.318)

kemampuan bertindak, rasa berdaya, dan menghindari penderitaan bagi Termohon dan kedua anak Pemohon dan Termohon;

Menimbang, bahwa tujuan agama (*maqashid al-syari'ah*) adalah mewujudkan kebaikan bagi semua orang (*al-mashlahah al-'ammah*) dengan memberikan perlindungan keyakinan (*hifzhu al-dien*), keselamatan jiwa (*hifzhu al-nafs*), kesehatan akal (*hifzhu al-'aql*), keamanan harta (*hifzhu al-maal*), dan keberlangsungan keturunan (*hifzhu al-nasl*);

Menimbang, bahwa mempertimbangkan perceraian sebagai upaya untuk memberikan perlindungan bagi keselamatan jiwa, mengembalikan rasa percaya diri, kemampuan bertindak/berpikir, rasa berdaya, dan menghindari penderitaan bagi Termohon dan kedua anak Pemohon dan Termohon telah sejalan dengan tujuan agama (*maqashid al-syari'ah*), yaitu keselamatan jiwa (*hifzhu al-nafs*) dan kesehatan akal (*hifzhu al-'aql*);

Menimbang, bahwa majelis hakim perlu mempertimbangkan perceraian sebagai bentuk perlindungan dari lembaga Pengadilan bagi Termohon dan anak Pemohon dan Termohon akibat dampak pertengkaran terus menerus dalam rumah tangga agar Termohon dan kedua anaknya tidak terus menerus mengalami kekerasan psikis, sebagaimana ketentuan Pasal 10 huruf (a) Undang-undang Republik Indonesia Nomor 23 Tahun 24 Tentang Penghapusan Kekerasan Dalam Rumah Tangga:"*Korban berhak mendapatkan: a. perlindungan dari pihak keluarga, kepolisian, kejaksaan, pengadilan, advokat, lembaga sosial, atau pihak lainnya baik sementara maupun berdasarkan penetapan perintah perlindungan dari pengadilan*";

Menimbang, bahwa syeikh Muhammad al-Thahir ibnu 'Asyur dalam kitab *Maqashid al-Syari'ah al-islamiyah* yang diambil alih sebagai pendapat dan bahan pertimbangan majelis hakim menyatakan salah tujuan agama (*maqashid al-syariah*) adalah melindungi kehormatan manusia (*hifzhu al-'irdh*);

Disclaimer
Kepaniteraan Mahkamah Agung Republik Indonesia berusaha untuk selalu mencantumkan informasi paling kini dan akurat sebagai bentuk komitmen Mahkamah Agung untuk pelayanan publik, transparansi dan akuntabilitas pelaksanaan fungsi peradilan. Namun dalam hal-hal tertentu masih dimungkinkan terjadi permasalahan teknis terkait dengan akurasi dan keterkinian informasi yang kami sajikan, hal mana akan terus kami perbaiki dari waktu kewaktu.
Dalam hal Anda menemukan inakurasi informasi yang termuat pada situs ini atau informasi yang seharusnya ada, namun belum tersedia, maka harap segera hubungi Kepaniteraan Mahkamah Agung RI melalui :
Email : kepaniteraan@mahkamahagung.go.id   Telp : 021-384 3348 (ext.318)

Menimbang, bahwa majelis hakim juga perlu mempertimbangkan perceraian sebagai upaya memberi kepastian hukum bagi status Pemohon dan Termohon dalam kehidupan bermasyarakat, serta dalam rangka menjaga kehormatan kedua belah pihak. Sehingga dengan adanya kepastian status perkawinan tersebut Pemohon dan Termohon dapat memilih jalan yang terbaik untuk keberlanjutan kehidupan rumah tangga masing-masing;

Menimbang, bahwa karena perceraian merupakan upaya untuk memberikan kepastian hukum bagi status Pemohon dan Termohon di tengah masyarakat, serta menjaga kehormatan kedua belah, maka majelis hakim berpendapat mempertimbangkan perceraian bagi Pemohon dan Termohon sudah sesuai dengan tujuan agama (*maqashid al-syariah*) yaitu melindungi kehormatan manusia (*hifzhu al-'irdh*);

Menimbang, bahwa segala hal yang dapat menimbulkan kemudharatan dalam kehidupan seseorang semaksimal mungkin harus dihindari atau dihilangkan, begitu pula kesengsaraan fisik maupun psikis yang dirasakan Pemohon dan Termohon akibat pertengkaran dan pecahnya rumah tangga hendaknya pula dihilangkan, sebagaimana kaidah fikih (*al-Qawaid al-Fiqhiyah*) yang diambil alih sebagai pendapat dan bahan pertimbangan majelis hakim berbunyi:

اَلضَّرَرُ يُزَالُ

Artinya: "*sesuatu yang dapat menimbulkan penderitaan (mudharat) harus segera dihilangkan*";

Menimbang, bahwa suami istri berkewajiban menjaga keutuhan dan berupaya menghindari perceraian, namun dalam kondisi kehidupan rumah tangga yang sudah pecah dimana suami istri tidak dapat hidup bersama lagi, serta tidak saling memperdulikan lagi yang menyebabkan ruh ketenangan, rasa cinta, dan kasih sayang dalam pernikahan menjadi hilang, sehingga mempertahankan ikatan perkawinan hanya akan

Disclaimer
Kepaniteraan Mahkamah Agung Republik Indonesia berusaha untuk selalu mencantumkan informasi paling kini dan akurat sebagai bentuk komitmen Mahkamah Agung untuk pelayanan publik, transparansi dan akuntabilitas pelaksanaan fungsi peradilan. Namun dalam hal-hal tertentu masih dimungkinkan terjadi permasalahan teknis terkait dengan akurasi dan keterkinian informasi yang kami sajikan, hal mana akan terus kami perbaiki dari waktu kewaktu.
Dalam hal Anda menemukan inakurasi informasi yang termuat pada situs ini atau informasi yang seharusnya ada, namun belum tersedia, maka harap segera hubungi Kepaniteraan Mahkamah Agung RI melalui :
Email : kepaniteraan@mahkamahagung.go.id Telp : 021-384 3348 (ext.318)

mendatangkan *mafsadah* bagi kedua pasangan suami istri, maka perceraian menjadi jalan terakhir yang dapat ditempuh, sebagaimana doktrin hukum Islam dalam kitab *Madaa Hurriyatu al-Zaujaini fi al-Thalaq,* juz I halaman 83 yang diambil alih sebagai pendapat dan bahan pertimbangan majelis hakim menyatakan:

وقد اختار الإسلام نظام الطلاق حين تضطرب الحياة الزوجين ولم يعد ينفع فيها نصائح ولا صلح وحيث تصبح الربطة الزواج صورة من غير روح لأن الإستمرار معناه أن يحكم على أحد الزوجين بالسجن المؤبد وهذا ظلم تأباه روح العدالة

Artinya :*"Islam memilih peraturan perceraian ketika rumah tangga sudah dianggap goncang serta nasehat/perdamaian sudah tidak bermanfaat lagi, dan hubungan suami isteri menjadi tanpa ruh (hampa), sebab meneruskan perkawinan berarti menghukum salah satu suami isteri dengan penjara yang berkepanjangan. Ini adalah aniaya yang bertentangan dengan semangat keadilan";*

Menimbang, bahwa secara sosiologis pula pemaksaan terhadap suami isteri untuk hidup rukun dalam kondisi rumah tangga yang sudah pecah hanya akan semakin memperburuk keadaan, sebagaimana pendapat Ibnu Sina yang dikutip oleh Sayyid Sabiq dalam kitab *Fiqhus Sunnah juz II* halaman 208 dan diambil alih sebagai pendapat dan bahan pertimbangan majelis hakim yang berbunyi sebagai berikut:

فكلما اجتهد فى الجمع بينهما زاد الشر والنبو(أي لخلاف) وتنغصت المعا يش

Artinya : "Maka jika kedua belah pihak dipaksakan untuk tetap rukun sebagai suami isteri, niscaya keadaan akan bertambah buruk".

Menimbang, bahwa perceraian menjadi jalan keluar darurat ketika pertengkaran dalam rumah tangga semakin tajam dan menimbulkan *mudharat*, sebagaiman pendapat Prof. Dr. Wahbah Zuhaili dalam kitab *Al-Fiqhiyatul Islamiyah wa Adillatuhu* Juz VII halaman 527, yang diambil alih

Disclaimer
Kepaniteraan Mahkamah Agung Republik Indonesia berusaha untuk selalu mencantumkan informasi paling kini dan akurat sebagai bentuk komitmen Mahkamah Agung untuk pelayanan publik, transparansi dan akuntabilitas pelaksanaan fungsi peradilan. Namun dalam hal-hal tertentu masih dimungkinkan terjadi permasalahan teknis terkait dengan akurasi dan keterkinian informasi yang kami sajikan, hal mana akan terus kami perbaiki dari waktu kewaktu.
Dalam hal Anda menemukan inakurasi informasi yang termuat pada situs ini atau informasi yang seharusnya ada, namun belum tersedia, maka harap segera hubungi Kepaniteraan Mahkamah Agung RI melalui :
Email : kepaniteraan@mahkamahagung.go.id Telp : 021-384 3348 (ext.318)

sebagai pendapat dan bahan pertimbangan majelis hakim yang berbunyi sebagai berikut yang berbunyi :

التفريق للشقاق أو للضرر منعا للنزاع وحتى لا تصبح الحياة الزوجية جحيما و بلاء

Artinya :"Perceraian didasarkan atas adanya pertengkaran yang tajam atau adanya madharat sebagai pemecahan atas terjadinya persengketaan, sehingga dengan perceraian tersebut kehidupan perkawinan tidak akan merupakan neraka dan bencana";

Menimbang, bahwa perceraian memang menimbulkan mudharat bagi kehidupan pasangan suami istri, namun mempertahankan rumah tangga dalam suasana kebencian dan pertengkaran terus-menerus juga mendatangkan *mudharat* yang jauh lebih besar, baik secara psikis, fisik, maupun mental, karenanya dalam dua kondisi yang sama-sama mendatangkan kemudharatan yang tidak dapat dihindari tersebut hendaklah dipilih *mudharat* yang paling ringan, sesuai dengan kaidah fikih yang diambil alih sebagai pendapat dan bahan pertimbangan majelis hakim yang berbunyi:

إِذَا تَعَارَضَ مَفْسَدَتَانِ رُوْعِيَ أَعْظَمُهُمَا ضَرَرًا بِارْتِكَابِ أَخَفِّهِمَا

Artinya: "*Jika ada dua mafsadat yang bertemu (yang harus dikerjakan salah satunya), maka yang dihindari adalah mudharat yang lebih besar, dengan memilih mudharat yang lebih ringan*".

Menimbang, bahwa dengan terjadinya perselisihan dan pertengkaran terus menerus antara Pemohon dan, serta kondisi kehidupan rumah tangga yang tidak dapat dirukunkan lagi, akan sulit bagi Pemohon dan Termohon untuk mewujudkan tujuan perkawinan yaitu membina rumah tangga yang bahagia dan kekal, sebagaimana maksud Pasal 1 Undang-Undang Nomor 1 Tahun 1974 jo Pasal 3 Kompilasi Hukum Islam (KHI), atau mewujudkan kehidupan pernikahan yang memberikan ketenangan (*sakinah*), saling mencintai (*mawaddah*), dan bertabur kasih sayang (*wa rahmah*) sebagaimana dikehendaki Allah SWT

Disclaimer
Kepaniteraan Mahkamah Agung Republik Indonesia berusaha untuk selalu mencantumkan informasi paling kini dan akurat sebagai bentuk komitmen Mahkamah Agung untuk pelayanan publik, transparansi dan akuntabilitas pelaksanaan fungsi peradilan. Namun dalam hal-hal tertentu masih dimungkinkan terjadi permasalahan teknis terkait dengan akurasi dan keterkinian informasi yang kami sajikan, hal mana akan terus kami perbaiki dari waktu kewaktu.
Dalam hal Anda menemukan inakurasi informasi yang termuat pada situs ini atau informasi yang seharusnya ada, namun belum tersedia, maka harap segera hubungi Kepaniteraan Mahkamah Agung RI melalui :
Email : kepaniteraan@mahkamahagung.go.id Telp : 021-384 3348 (ext.318)

dalam surah Ar-Ruum ayat 21:"*Dan diantara tanda-tanda kebesaranNya ialah Dia menciptakan untuk isteri-isteri dari jenismu sendiri, supaya kamu cenderung dan merasa tenteram kepadanya, dan menjadikan diantaramu rasa kasih dan sayang. Sesungguhnya pada yang demikian itu benar-benar terdapat tanda-tanda bagi kaum yang berfikir"*;

Menimbang, bahwa terhadap perkara ini majelis hakim merujuk kepada Yurisprudensi Mahkamah Agung RI Nomor 273 K/AG/1998 tanggal 17 Maret 1999 yang diambil alih menjadi pertimbangan majlis hakim yang pada pokoknya bahwa, cekcok, hidup berpisah tidak dalam satu tempat kediaman bersama dengan pihak lain, merupakan fakta yang cukup untuk perceraian berdasar pasal 19 huruf (f) PP. Nomor 9 tahun 1975 tentang Pelaksanaan Undang-Undang Nomor 1 tahun 1974 tentang Perkawinan:

Menimbang, bahwa berdasarkan Yurisprudensi Mahkamah Agung RI Nomor 379/K/AG/1995 tangal 26 Maret 1997 yang abstraksi hukumnya menyatakan apabila suami isteri terjadi perselisihan dan terjadi pisah tempat tinggal, maka rumah tangga mereka telah pecah dan dalil-dalil permohonan perceraian telah memenuhi ketentuan pasal 19 huruf (f) Peraturan Pemerintah Nomor 9 Tahun 1975 jo pasal 116 huruf (f) Kompilasi Hukum Islam;

Menimbang, bahwa berdasarkan Yurisprudensi Mahkamah Agung Republik Indonesia Nomor 38/K/AG/1990 tanggal 22 Agustus 1991, bahwa apabila terbukti suatu rumah tangga sudah pecah dan tidak dapat diperbaiki dan mempertahankan rumah tangga membawa akibat negatif (*mafsadat* yang lebih besar) bagi kedua belah pihak, maka perceraian dapat dipertimbangkan untuk dikabulkan;

Menimbang, bahwa dengan demikian gugatan Pemohon telah beralasan hukum dan memenuhi unsur-unsur yang terkandung dalam Pasal 39 ayat (2) Undang-Undang Nomor 1 Tahun 1974 tentang Perkawinan jis. Pasal 19 huruf (f) Peraturan Pemerintah Nomor 9 Tahun 1975, juncto Pasal 116 huruf (f) Kompilasi Hukum Islam, telah cukup

Disclaimer
Kepaniteraan Mahkamah Agung Republik Indonesia berusaha untuk selalu mencantumkan informasi paling kini dan akurat sebagai bentuk komitmen Mahkamah Agung untuk pelayanan publik, transparansi dan akuntabilitas pelaksanaan fungsi peradilan. Namun dalam hal-hal tertentu masih dimungkinkan terjadi permasalahan teknis terkait dengan akurasi dan keterkinian informasi yang kami sajikan, hal mana akan terus kami perbaiki dari waktu kewaktu.
Dalam hal Anda menemukan inakurasi informasi yang termuat pada situs ini atau informasi yang seharusnya ada, namun belum tersedia, maka harap segera hubungi Kepaniteraan Mahkamah Agung RI melalui :
Email : kepaniteraan@mahkamahagung.go.id Telp : 021-384 3348 (ext.318)

alasan bagi majelis hakim untuk mengabulkan petitum angka 1 permohonan Pemohon;

Menimbang, bahwa antara Pemohon dengan Termohon telah bergaul sebagaimana layaknya suami isteri dan belum pernah bercerai, sehingga majelis hakim mempunyai alasan yang cukup untuk menetapkan bahwa talak yang diizinkan oleh pengadilan untuk diikrarkan oleh Pemohon terhadap Termohon adalah talak satu raj'i;

B. Dalam Rekonvensi

Menimbang, bahwa maksud dan tujuan gugatan Penggugat Rekonvensi adalah sebagaimana telah diuraikan diatas ;

Menimbang, bahwa gugatan Penggugat Rekonvensi tersebut dilakukan dengan cara yang telah sesuai dengan ketentuan Undang-undang, dan dalam tahapan yang benar dengan demikian gugatan Penggugat secara formil dapat diperiksa dan dipertimbangkan ;

Menimbang, bahwa dengan demikian maka kedudukan Termohon Konvensi menjadi Penggugat Rekonvensi dan Pemohon Konvensi menjadi Tergugat Rekonvensi:

Menimbang bahwa oleh karena gugatan rekonvensi ini berkaitan langsung dengan permohonan Cerai Talak dalam konvensi, maka pertimbangan dalam konvensi dinyatakan sebagai satu kesatuan yang tidak terpisahkan dengan pertimbangan dalam rekonvensi ini, untuk itu apa yang telah dipertimbangkan dalam konvensi sepanjang ada relevansinya dinyatakan sebagai pertimbangan dalam rekonvensi;

Menimbang, bahwa gugatan Penggugat Rekonvensi pada pokoknya menuntut agar Tergugat Rekonvensi membayar uang nafkah *iddah*, nafkah *madliyah*, *mut'ah* dan nafkah anak dengan rincian sebagai berikut:

1. Bahwa, Penggugat Rekonvensi menuntut nafkah *'iddah* sebesar Rp.100.000,-(seratus ribu rupiah) per bulan selama 3 (tiga)

Disclaimer
Kepaniteraan Mahkamah Agung Republik Indonesia berusaha untuk selalu mencantumkan informasi paling kini dan akurat sebagai bentuk komitmen Mahkamah Agung untuk pelayanan publik, transparansi dan akuntabilitas pelaksanaan fungsi peradilan. Namun dalam hal-hal tertentu masih dimungkinkan terjadi permasalahan teknis terkait dengan akurasi dan keterkinian informasi yang kami sajikan, hal mana akan terus kami perbaiki dari waktu kewaktu.
Dalam hal Anda menemukan inakurasi informasi yang termuat pada situs ini atau informasi yang seharusnya ada, namun belum tersedia, maka harap segera hubungi Kepaniteraan Mahkamah Agung RI melalui :
Email : kepaniteraan@mahkamahagung.go.id   Telp : 021-384 3348 (ext.318)

bulan. Jadi total uang nafkah *‘iddah* yang dituntut sebesar Rp.300.000,- (tiga ratus ribu rupiah);

2. Bahwa, Penggugat Rekonvensi menuntut nafkah *madhiyah* (lampau) sebesar Rp.600.000,-(enam ratus ribu rupiah);

3. Bahwa, Penggugat Rekonvensi menuntut *mut’ah* uang sebesar Rp.300.000,-(tiga ratus ribu rupiah) Tergugat Rekonvensi;

4. Bahwa, Penggugat Rekonvensi menuntut nafkah anak bernama XXXXXX sebesar Rp.100.000,-(seratus ribu rupiah) per bulan hingga anak tersebut berusia dewasa;

Menimbang, bahwa terhadap gugatan rekonvensi, Tergugat Rekonvensi mengajukan jawaban rekonvensi yang pada pokoknya menyanggupi membayar nafkah *’iddah*, nafkah *madhiyah*/lampau, nafkah anak sebagaimana yang dituntut oleh Penggugat Rekonvensi. Adapun untuk *mut’ah*, Tergugat Rekonvensi hanya menyanggupi membayar uang sebesar Rp.200.000,-(dua ratus ribu rupiah);

Menimbang, bahwa terhadap kesanggupan Tergugat Rekonvensi yang menyanggupi membayar *mut’ah* uang sebesar Rp.200.000,-(dua ratus ribu rupiah) tersebut, Penggugat Rekonvensi menyatakan bersedia menerima;

Menimbang, bahwa bila perkawinan putus karena talak maka berdasarkan ketentuan Pasal 149 huruf (b) Kompilasi Hukum Islam:”b. *memberi nafkah, maskan dan kiswah kepada bekas isteri selama dalam iddah, kecuali bekas isteri telahdi jatuhi talak bain atau nusyuz dan dalam keadaan tidak hamil*”;

Menimbang, bahwa terhadap kewajiban Tergugat Rekonvensi memberikan nafkah *iddah* kepada Penggugat Rekonvensi, juga didasarkan doktrin hukum Islam pada kitab *Al Muhadzdzab* juz II halaman 176, yang diambil alih sebagai pendapat dan bahan pertimbangan majelis hakim:

Disclaimer
Kepaniteraan Mahkamah Agung Republik Indonesia berusaha untuk selalu mencantumkan informasi paling kini dan akurat sebagai bentuk komitmen Mahkamah Agung untuk pelayanan publik, transparansi dan akuntabilitas pelaksanaan fungsi peradilan. Namun dalam hal-hal tertentu masih dimungkinkan terjadi permasalahan teknis terkait dengan akurasi dan keterkinian informasi yang kami sajikan, hal mana akan terus kami perbaiki dari waktu kewaktu.
Dalam hal Anda menemukan inakurasi informasi yang termuat pada situs ini atau informasi yang seharusnya ada, namun belum tersedia, maka harap segera hubungi Kepaniteraan Mahkamah Agung RI melalui :
Email : kepaniteraan@mahkamahagung.go.id Telp : 021-384 3348 (ext.318)

إذا طلق إمرأته بعد الدخول طلاقا رجعيا وجب لها ال
سكنى والنفقة في العدة

Artinya:"*Apabila suami mencerai isteri sesudah dukhul dengan talak raj'i, maka isteri berhak mendapat tempat tinggal dan nafkah semasa iddah*";

Menimbang, bahwa nafkah *iddah* merupakan nafkah untuk mencukup kebutuhan hidup istri yang meliputi nafkah *maskan* (tempat tinggal), dan *kiswah* (pakaian) selama masa *iddah*;

Menimbang, bahwa dalam gugatan rekonvensi Penggugat Rekonvensi menuntut nafkah *iddah* dari Tergugat Rekonvensi untuk membayar uang sebesar Rp.100.000,- (seratus ribu rupiah) per bulan selama 3 (tiga) bulan, sehingga total uang nafkah yang dituntut oleh Penggugat Rekonvensi adalah sebesar Rp.300.000 (tiga ratus ribu rupiah);

Menimbang, bahwa Tergugat Rekonvensi menyatakan menyanggupi membayar nafkah *'iddah* sebesar Rp.100.000,- (seratus ribu rupiah) per bulan selama 3 (tiga) bulan sebagaimana yang dituntut oleh Penggugat Rekonvensi dalam gugatan rekonvensi, karenanya majelis hakim menetapkan uang *iddah* yang harus dibayar oleh Tergugat Rekonvensi kepada Penggugat Rekonvensi adalah sebesar Rp.100.000,- (seratus ribu rupiah) per bulan selama 3 (tiga) bulan, sehingga total uang nafkah *'iddah* tersebut adalah sebesar Rp.300.000 (tiga ratus ribu rupiah);

Menimbang, bahwa dengan uraian pertimbangan tersebut majelis hakim berpendapat gugatan Penggugat Rekonvensi tentang nafkah *iddah* dapat dikabulkan, dan karenanya majelis hakim mewajibkan Tergugat Rekonvensi untuk membayar nafkah *iddah* kepada Penggugat Rekonvensi sebesar Rp.300.000 (tiga ratus ribu rupiah);

Menimbang, bahwa tentang nafkah *madliyah*/lampau majelis hakim mempertimbangkan kewajiban syar'i seorang suami yang akan

Disclaimer
Kepaniteraan Mahkamah Agung Republik Indonesia berusaha untuk selalu mencantumkan informasi paling kini dan akurat sebagai bentuk komitmen Mahkamah Agung untuk pelayanan publik, transparansi dan akuntabilitas pelaksanaan fungsi peradilan. Namun dalam hal-hal tertentu masih dimungkinkan terjadi permasalahan teknis terkait dengan akurasi dan keterkinian informasi yang kami sajikan, hal mana akan terus kami perbaiki dari waktu kewaktu.
Dalam hal Anda menemukan inakurasi informasi yang termuat pada situs ini atau informasi yang seharusnya ada, namun belum tersedia, maka harap segera hubungi Kepaniteraan Mahkamah Agung RI melalui :
Email : kepaniteraan@mahkamahagung.go.id Telp : 021-384 3348 (ext.318)

menjatuhkan talak terhadap istrinya, dan kewajiban suami memberikan nafkah *madliah* kepada istri, karenanya majelis hakim mendasarkan kepada kitab *I'anatut Thalibin* juz IV halaman 85 :

فالنفقة أو الكسوة لجميع ما مضى من تلك المدة دين لها عليه لأنها استحق ذلك فى ذمته

Artinya: "Nafkah dan pakaian yang telah lewat dari batas waktunya menjadi hutang suami kepada isterinya yang harus ditanggung/dilunasi";

Menimbang, bahwa dalam gugatan rekonvensi Penggugat Rekonvensi menuntut nafkah *madliyah*/lampau kepada Tergugat Rekonvensi sebagaimana yang telah mereka berdua sepakati dalam proses mediasi, yaitu uang sebesar Rp.600.000,- (enam ratus ribu rupiah);

Menimbang, bahwa terhadap gugatan mut'ah tersebut Tergugat Rekonvensi menyatakan kesanggupannya untuk untuk membayar uang sebesar Rp.600.000,- (enam ratus ribu rupiah) sebagaimana yang telah disepakati oleh Penggugat Rekonvensi dan Tergugat Rekonvensi dalam proses mediasi, karenanya majelis hakim menetapkan nafkah *madliyah*/lampau yang harus dibayar oleh Tergugat Rekonvensi kepada Penggugat Rekonvensi adalah uang sebesar Rp.600.000,- (enam ratus ribu rupiah);

Menimbang, bahwa dengan uraian pertimbangan tersebut majelis hakim berpendapat gugatan Penggugat Rekonvensi tentang nafkah nafkah *madliyah*/lampau dapat dikabulkan, dan karenanya majelis hakim mewajibkan Tergugat Rekonvensi untuk membayar nafkah nafkah *madliyah*/lampau kepada Penggugat Rekonvensi sebesar Rp.600.000,- (enam ratus ribu rupiah);

Menimbang, bahwa bila perkawinan putus karena talak maka berdasarkan ketentuan Pasal 149 huruf (a) Kompilasi Hukum Islam:" a.*memberikan mut`ah yang layak kepada bekas isterinya, baik berupa uang atau benda, kecuali bekas isteri tersebut qobla al dukhul*";

Disclaimer
Kepaniteraan Mahkamah Agung Republik Indonesia berusaha untuk selalu mencantumkan informasi paling kini dan akurat sebagai bentuk komitmen Mahkamah Agung untuk pelayanan publik, transparansi dan akuntabilitas pelaksanaan fungsi peradilan. Namun dalam hal-hal tertentu masih dimungkinkan terjadi permasalahan teknis terkait dengan akurasi dan keterkinian informasi yang kami sajikan, hal mana akan terus kami perbaiki dari waktu kewaktu.
Dalam hal Anda menemukan inakurasi informasi yang termuat pada situs ini atau informasi yang seharusnya ada, namun belum tersedia, maka harap segera hubungi Kepaniteraan Mahkamah Agung RI melalui :
Email : kepaniteraan@mahkamahagung.go.id Telp : 021-384 3348 (ext.318)

Menimbang, bahwa berdasarkan Pasal 158 Kompilasi Hukum Islam: “*Mut`ah wajib diberikan oleh bekas suami dengan syarat : a. belum ditetapkan mahar bagi isteriba`da al dukhul; b. perceraian itu atas kehendak suami*”, dan Pasal 159: ”*Mut`ah sunnat diberikan oleh bekas suami tanpa syarat tersebut pada pasal 158*”;

Menimbang, bahwa hakikat pemberian *mut’ah* adalah sebagai wujud menceraikan isteri secara baik (*ma’ruf*), berdasarkan firman Allah Swt Surat Al Baqarah ayat 241 :

وللمطلقات متاع بالمعروف

Artinya : “Kepada wanita-wanita yang diceraikan (hendaklah diberikan oleh suaminya) mut’ah (pemberian) menurut yang ma’ruf”;

Menimbang, bahwa pemberian *mut’ah* merupakan upaya untuk mengurangi kepedihan dan kesedian istri, sebagaimana pendapat Dr. Wahbah Zuhaili dalam kitabnya *Fiqh Al-Islami wa adillatuhu* juz VII halaman 320, yang diambil alih sebagai pendapat dan bahan pertimbangan majelis hakim :

لتطييب خاطر المرأة وتخفيف ألم الفراق ولإيجاد باعث على العودة إلى الزوجية إن لم تكن البينونة كبرى

Artinya : “Pemberian mut’ah itu agar isteri terhibur hatinya, dapat mengurangi kepedihan akibat cerai talak, dan untuk menumbuhkan keinginan rukun kembali sebagai suami isteri, jika talak itu bukan ba’in kubra”;

Menimbang, bahwa dalam gugatan rekonvensi Penggugat Rekonvensi menuntut Tergugat Rekonvensi untuk memberikan mut’ah uang sebesar Rp.300.000,- (tiga ratus ribu rupiah), dan terhadap gugatan *mut’ah* tersebut Tergugat Rekonvensi menyatakan berdasarkan kemampuannya hanya menyanggupi membayar uang sebesar Rp.200.000,- (dua ratus ribu rupiah);

Disclaimer
Kepaniteraan Mahkamah Agung Republik Indonesia berusaha untuk selalu mencantumkan informasi paling kini dan akurat sebagai bentuk komitmen Mahkamah Agung untuk pelayanan publik, transparansi dan akuntabilitas pelaksanaan fungsi peradilan. Namun dalam hal-hal tertentu masih dimungkinkan terjadi permasalahan teknis terkait dengan akurasi dan keterkinian informasi yang kami sajikan, hal mana akan terus kami perbaiki dari waktu kewaktu.
Dalam hal Anda menemukan inakurasi informasi yang termuat pada situs ini atau informasi yang seharusnya ada, namun belum tersedia, maka harap segera hubungi Kepaniteraan Mahkamah Agung RI melalui :
Email : kepaniteraan@mahkamahagung.go.id   Telp : 021-384 3348 (ext.318)

Menimbang, bahwa atas kesanggupan Tergugat Rekonvensi membayar *mut'ah* uang sebesar Rp.200.000,- (dua ratus ribu rupiah), Penggugat Rekonvensi menyatakan bersedia menerima, karenanya majelis hakim menetapkan *mut'ah* yang harus dibayar oleh Tergugat Rekonvensi kepada Penggugat Rekonvensi adalah uang sebesar Rp.200.000,- (dua ratus ribu rupiah);

Menimbang, bahwa dengan uraian pertimbangan tersebut majelis hakim berpendapat gugatan Penggugat Rekonvensi tentang *mut'ah* dapat dikabulkan, dan karenanya majelis hakim mewajibkan Tergugat Rekonvensi untuk membayar *mut'ah* kepada Penggugat Rekonvensi sebesar Rp.200.000,- (dua ratus ribu rupiah);

Menimbang, bahwa bila perkawinan putus karena talak maka berdasarkan ketentuan Pasal 149 huruf (d) Kompilasi Hukum Islam:"*d. memberikan biaya hadhanan untuk anak-anaknya yang belum mencapai umur 21 tahun*";

Menimbang, bahwa Penggugat Rekonvensi dan Tergugat Rekonvensi terbukti telah dikaruniai dua orang anak laki-laki bernama XXXXXX dan XXXXXX. Kemudian setelah Penggugat Rekonvensi dan Tergugat Rekonvensi berpisah tempat tinggal, anak pertama bernama Khusesn berada dalam asuhan Tergugat Rekonvensi, dan anak kedua bernama XXXXXX diasuh oleh Penggugat Rekonvensi;

Menimbang, bahwa anak kedua Penggugat Rekonvensi dan Tergugat Rekonvensi bernama XXXXXX saat ini berada dalam pengasuhan Penggugat Rekonvensi, karenanya Tergugat Rekonvensi tetap berkewajiban untuk menanggung biaya pengasuhan dan pemeliharaan dengan mendasarkan pada dalil syar'I dalam kitab *I'anatut Thalibin* juz IV halaman 99 :

من له أب وأم فنفقته على أب

Disclaimer
Kepaniteraan Mahkamah Agung Republik Indonesia berusaha untuk selalu mencantumkan informasi paling kini dan akurat sebagai bentuk komitmen Mahkamah Agung untuk pelayanan publik, transparansi dan akuntabilitas pelaksanaan fungsi peradilan. Namun dalam hal-hal tertentu masih dimungkinkan terjadi permasalahan teknis terkait dengan akurasi dan keterkinian informasi yang kami sajikan, hal mana akan terus kami perbaiki dari waktu kewaktu.
Dalam hal Anda menemukan inakurasi informasi yang termuat pada situs ini atau informasi yang seharusnya ada, namun belum tersedia, maka harap segera hubungi Kepaniteraan Mahkamah Agung RI melalui :
Email : kepaniteraan@mahkamahagung.go.id Telp : 021-384 3348 (ext.318)

Artinya : "Anak yang masih punya bapak dan ibu, maka bapaknyalah yang wajib menafkahinya;

Menimbang, bahwa nafkah anak yang dimaksud di atas harus diberikan oleh Tergugat Rekonvensi setiap bulan hingga XXXXXX berusia 21 (dua puluh satu) tahun;

Menimbang, bahwa dalam gugatan rekonvensi Penggugat Rekonvensi menuntut nafkah anak bernama XXXXXX kepada Tergugat Rekonvensi uang sebesar sebesar Rp.100.000,- (seratus ribu rupiah) per bulan hingga anak tersebut berusia dewasa;

Menimbang, bahwa Tergugat Rekonvensi menyatakan menyanggupi membayar nafkah anak bernama XXXXXX sebesar Rp.100.000,- (seratus ribu rupiah) per bulan hingga anak tersebut dewasa sebagaimana yang dituntut oleh Penggugat Rekonvensi, karenanya majelis hakim menetapkan nafkah anak yang harus dibayar oleh Tergugat Rekonvensi kepada Penggugat Rekonvensi untuk XXXXXX adalah sebesar Rp.100.000,- (seratus ribu rupiah) per bulan hingga anak tersebut berusia 21 (dua puluh satu);

Menimbang, bahwa kebutuhan hidup anak Penggugat Rekonvensi dan Tergugat Rekonvensi akan terus meningkat seiring bertambah usianya, begitu pula terjadinya kenaikan harga kebutuhan pokok sebagai dampak adanya inflasi di Indonesia yang mencapai rata-rata 3,5% - 5% pertahun, maka majelis hakim perlu menetapkan kenaikan nominal nafkah anak yang rasional tiap tahunnya, sebagaimana poin 14 Rumusan Hukum Kamar Agama Surat Edaran Mahkamah Agung RI No 3 Tahun 2015 Tentang Pemberlakuan Rumusan Hasil Rapat Pleno Kamar Mahkamah Agung Tahun 2015:"*Amar mengenai pembebanan nafkah anak hendaknya diikuti dengan penambahan 10% sampai dengan 20% per tahun dari jumlah yang ditetapkan, di luar biaya pendidikan dan kesehatan*", karenanya majelis hakim menetapkan penambahan 10% tiap

Disclaimer
Kepaniteraan Mahkamah Agung Republik Indonesia berusaha untuk selalu mencantumkan informasi paling kini dan akurat sebagai bentuk komitmen Mahkamah Agung untuk pelayanan publik, transparansi dan akuntabilitas pelaksanaan fungsi peradilan. Namun dalam hal-hal tertentu masih dimungkinkan terjadi permasalahan teknis terkait dengan akurasi dan keterkinian informasi yang kami sajikan, hal mana akan terus kami perbaiki dari waktu kewaktu.
Dalam hal Anda menemukan inakurasi informasi yang termuat pada situs ini atau informasi yang seharusnya ada, namun belum tersedia, maka harap segera hubungi Kepaniteraan Mahkamah Agung RI melalui :
Email : kepaniteraan@mahkamahagung.go.id Telp : 021-384 3348 (ext.318)

tahun atas uang nafkah anak yang harus dibayarkan Tergugat Rekonvensi;

Menimbang, bahwa berdasarkan pertimbangan di atas majelis hakim berpendapat gugatan Penggugat Rekonvensi tentang nafkah anak patut dikabulkan, dan karenanya Tergugat Rekonvensi diwajibkan membayar nafkah anak Penggugat Rekonvensi dan Tergugat yang bernama XXXXXX sebesar Rp.100.000,- (seratus ribu rupiah), per bulan hingga berusia 21 (dua puluh satu) tahun dengan kenaikan sejumlah 10% setiap tahunnya diluar biaya pendidikan dan kesehatan, karena biaya pendidikan dan kesehatan sifatnya berkembang dan tidak bisa ditentukan pada waktu dan keadaan putusan ini dijatuhkan;

Menimbang, bahwa dalam rangka pelaksanaan Peraturan Mahkamah Agung RI No 3 Tahun 2017 Tentang Pedoman Mengadili Perkara Perempuan Berhadapan dengan Hukum untuk memberi perlindungan hukum bagi hak-hak perempuan pasca perceraian, maka berdasarkan poin angka 1 Surat Edaran Mahkamah Agung RI No 1 Tahun 2017 Tentang pemberlakukan Rumusan Hasil Rapat Pleno Kamar Mahkamah Agung Tahun 2017, pembayaran kewajiban nafkah *iddah*, *mut'ah*, dan nafkah *madliyah*, dibayar sebelum pengucapan ikrar talak.

C. Dalam Konvensi dan Rekonvensi

Menimbang, bahwa perkara ini termasuk dalam bidang perkawinan, maka sesuai ketentuan Pasal 89 ayat (1) Undang-Undang Nomor 7 Tahun 1989 Tentang Peradilan Agama sebagaimana telah diubah dengan Undang-Undang Nomor 3 Tahun 2006 dan Undang-Undang Nomor 50 Tahun 2009, maka biaya perkara dibebankan kepada Pemohon;

Mengingat pasal-pasal peraturan perundang-undangan yang berlaku dan hukum *syara'* yang berkaitan dengan perkara ini;

MENGADILI

A. Dalam Konvensi

Disclaimer
Kepaniteraan Mahkamah Agung Republik Indonesia berusaha untuk selalu mencantumkan informasi paling kini dan akurat sebagai bentuk komitmen Mahkamah Agung untuk pelayanan publik, transparansi dan akuntabilitas pelaksanaan fungsi peradilan. Namun dalam hal-hal tertentu masih dimungkinkan terjadi permasalahan teknis terkait dengan akurasi dan keterkinian informasi yang kami sajikan, hal mana akan terus kami perbaiki dari waktu kewaktu.
Dalam hal Anda menemukan inakurasi informasi yang termuat pada situs ini atau informasi yang seharusnya ada, namun belum tersedia, maka harap segera hubungi Kepaniteraan Mahkamah Agung RI melalui :
Email : kepaniteraan@mahkamahagung.go.id   Telp : 021-384 3348 (ext.318)

1. Mengabulkan permohonan Pemohon;

2. Memberikan izin kepada Pemohon (XXXXXX) untuk menjatuhkan talak satu raj'i terhadap Termohon (XXXXXX) di depan persidangan Pengadilan Agama Martapura;

B. Dalam Rekonvensi:

1. Mengabulkan gugatan Penggugat Rekonvensi;
2. Menghukum Tergugat Rekonvensi untuk membayar kepada Penggugat Rekonvensi, berupa;
   a. Nafkah *iddah* selama 3 (tiga) bulan sebesar Rp.300.000,- (tiga ratus ribu rupiah);
   b. *Mut`ah* sebesar Rp.200.000,- (dua ratus ribu rupiah);
   c. Nafkah *madhiyah*/lampau sebesar Rp.600.000,- (enam ratus ribu rupiah);

   yang wajib dibayarkan dan diserahkan oleh Tergugat Rekonvensi kepada Penggugat Rekonvensi sebelum pengucapan ikrar talak;
3. Menghukum Tergugat Rekonvensi untuk membayar nafkah anak Penggugat Rekonvensi dan Tergugat Rekonvensi yang bernama XXXXXX sebesar Rp.100.000,- (seratus ribu rupiah) setiap bulan dengan kenaikan 10% setiap tahun sampai dengan anak tersebut berumur 21 (dua puluh satu) tahun;

C. Dalam Konvensi dan Rekonvensi:

Membebankan kepada Pemohon membayar biaya perkara sejumlah Rp.716.000,-(tujuh ratus enam belas ribu rupiah);

Demikian putusan ini dijatuhkan dalam rapat permusyawaratan majelis hakim yang dilangsungkan pada hari Rabu tanggal 23 Desember 2020 Masehi, bertepatan dengan tanggal 8 Jumadil Ula 1442 Hijriah, oleh kami Wildi Raihanda, Lc, sebagai Ketua Majelis, Muhammad Zhamir Islami, S.H.I., dan M. Ja'far Shiddiq Sunariya, S.H., masing-masing sebagai Hakim Anggota, putusan tersebut diucapkan dalam sidang

Disclaimer
Kepaniteraan Mahkamah Agung Republik Indonesia berusaha untuk selalu mencantumkan informasi paling kini dan akurat sebagai bentuk komitmen Mahkamah Agung untuk pelayanan publik, transparansi dan akuntabilitas pelaksanaan fungsi peradilan. Namun dalam hal-hal tertentu masih dimungkinkan terjadi permasalahan teknis terkait dengan akurasi dan keterkinian informasi yang kami sajikan, hal mana akan terus kami perbaiki dari waktu kewaktu.
Dalam hal Anda menemukan inakurasi informasi yang termuat pada situs ini atau informasi yang seharusnya ada, namun belum tersedia, maka harap segera hubungi Kepaniteraan Mahkamah Agung RI melalui :
Email : kepaniteraan@mahkamahagung.go.id   Telp : 021-384 3348 (ext.318)

terbuka untuk umum pada hari itu juga oleh Ketua Majelis tersebut dengan didampingi oleh Hakim Anggota dan dibantu oleh Fahrizal, S.H.I., sebagai Panitera Pengganti serta dihadiri oleh Pemohon dan Termohon;

Hakim Anggota,	Ketua Majelis,
ttd	ttd
Muhammad Zhamir Islami, S.H.I	Wildi Raihanda, Lc

Hakim Anggota,

ttd

M. Ja'far Shiddiq Sunariya, S.H.,

Panitera Pengganti,

ttd

Fahrizal, S.H.I.

Rincian Biaya

1. Biaya Pendaftaran	: Rp.	30.000,-
2. Biaya Proses	: Rp.	50.000,-
3. Biaya Panggilan	: Rp.	600.000,-
4. PNBP	: Rp.	20.000,-
5. Redaksi	: Rp.	10.000,-
6. Meterai	: Rp.	6.000,-
**Jumlah**	**:Rp.**	**716.000,-**

(tujuh ratus enam belas ribu rupiah)

Disclaimer
Kepaniteraan Mahkamah Agung Republik Indonesia berusaha untuk selalu mencantumkan informasi paling kini dan akurat sebagai bentuk komitmen Mahkamah Agung untuk pelayanan publik, transparansi dan akuntabilitas pelaksanaan fungsi peradilan. Namun dalam hal-hal tertentu masih dimungkinkan terjadi permasalahan teknis terkait dengan akurasi dan keterkinian informasi yang kami sajikan, hal mana akan terus kami perbaiki dari waktu kewaktu.
Dalam hal Anda menemukan inakurasi informasi yang termuat pada situs ini atau informasi yang seharusnya ada, namun belum tersedia, maka harap segera hubungi Kepaniteraan Mahkamah Agung RI melalui :
Email : kepaniteraan@mahkamahagung.go.id Telp : 021-384 3348 (ext.318)
