# Supplementary material for: Classification of divorce causes during the COVID-19 pandemic using convolutional neural networks
Source: PeerJ Comput Sci. 2022 Jun 30;8:e998. doi: 10.7717/peerj-cs.998 (PMC9299239; doi:10.7717/peerj-cs.998)
Supplement: Supplemental Information 3 [file peerj-cs-08-998-s003.zip › KDRT Dataset/Data ke-25.pdf]

Mahkamah Agung Republik Indonesia

PUTUSAN

Nomor xxxx/Pdt.G/2020/PAJT

بِسْمِ اللهِ الرَّحْمَنِ الرَّحِيْمِ

DEMI KEADILAN BERDASARKAN KETUHANAN YANG MAHA ESA

Pengadilan Agama Jakarta Timur yang memeriksa dan mengadili perkara tertentu pada tingkat pertama, dalam sidang Majelis telah menjatuhkan putusan atas perkara **Cerai Gugat** antara :

Xxxx bt xxxx, umur 25 tahun, agama Islam, pekerjaan Ibu rumah tangga, tempat kediaman di xxxxxxxxxx Kota Jakarta Timur, selanjutnya disebut Penggugat. ---

melawan

xxxx bin xxxx , umur 26 tahun, agama Islam, pekerjaan Ojek online, tempat kediaman di xxxxxx Kota Jakarta Timur, selanjutnya disebut Tergugat. ---

Pengadilan Agama tersebut.--

Telah mempelajari surat-surat yang berkaitan dengan perkara ini.-------------------

Telah mendengar Penggugat dan para saksi di muka sidang.-------------------------

DUDUK PERKARA

Bahwa Penggugat dengan surat gugatannya tertanggal 10 Desember 2020 yang terdaftar di Kepaniteraan Pengadilan Agama Jakarta Timur dalam register perkara Nomor 4899/Pdt.G/2020/PAJT tanggal 10 Desember 2020, mengajukan perkara Cerai Gugat dengan dalil-dalil sebagai berikut :

1. Bahwa, pada tanggal 13 Juli 2020 Penggugat dengan Tergugat melangsungkan pernikahan yang dicatat oleh Pegawai Pencatat Nikah Kantor Urusan Agama Kecamatan CIracas, Kota Jakarta Timur, sesuai Kutipan Akta Nikah Nomor: 703/49/VII/2020 tertanggal 13 Juli 2020;

Disclaimer
Kepaniteraan Mahkamah Agung Republik Indonesia berusaha untuk selalu mencantumkan informasi paling kini dan akurat sebagai bentuk komitmen Mahkamah Agung untuk pelayanan publik, transparansi dan akuntabilitas pelaksanaan fungsi peradilan. Namun dalam hal-hal tertentu masih dimungkinkan terjadi permasalahan teknis terkait dengan akurasi dan keterkinian informasi yang kami sajikan, hal mana akan terus kami perbaiki dari waktu kewaktu.
Dalam hal Anda menemukan inakurasi informasi yang termuat pada situs ini atau informasi yang seharusnya ada, namun belum tersedia, maka harap segera hubungi Kepaniteraan Mahkamah Agung RI melalui :
Email : kepaniteraan@mahkamahagung.go.id Telp : 021-384 3348 (ext.318)

2. Bahwa, selama pernikahan Penggugat dan Tergugat bertempat tinggal di Jalan Tanah Merdeka No. 10 Rt. 9 Rw. 2 Kelurahan Rambutan Kecamatan Ciracas Kota Jakarta Timur;
3. Bahwa, selama pernikahan Penggugat dan Tergugat sudah bercampur (ba'da dukhul) sebagaimana layaknya suami istri dan belum memiliki keturunan;
4. Bahwa, semula rumah tangga Penggugat dan Tergugat sudah berjalan rukun dan harmonis, namun sejak Agustus 2020 sering terjadi perselisihan dan pertengkaran, yang disebabkan:
   4.1. Bahwa Tergugat merupakan seorang pemabuk;
   4.2. Bahwa tergugat jarang tinggal di rumah dan lebih sering berada di rumah orang tua Tergugat;
   4.3. Bahwa Tergugat tidak menghormati orang tua Penggugat dengan tidak mau mendengarkan perkataan orang tua Penggugat;
5. Bahwa, puncak perselisihan dan pertengkaran antara Penggugat dan Tergugat terjadi pada awal Bulan September 2020 dimana saat itu Tergugat meninggalkan rumah kediaman bersama. Dan sejak itu antara Penggugat dan Tergugat sudah tidak berhubungan badan layaknya suami istri;
6. Bahwa atas rangkaian kejadian sebagaimana telah disebutkan di atas sudah tidak memungkinkan untuk kembali merajut tali pernikahan yang sakinah, mawaddah, dan rahmah. Sehingga atas hal tersebut, Penggugat mengajukan gugatan perceraian;
7. Bahwa, terhadap biaya yang timbul akibat perkara ini agar dibebankan sesuai dengan peraturan perundang-undangan yang berlaku;

Berdasarkan alasan/dalil-dalil diatas, Penggugat mohon agar Ketua Pengadilan Agama Jakarta Timur C.q Majelis Hakim segera memeriksa dan mengadili perkara ini, selanjutnya menjatuhkan putusan yang amarnya berbunyi:

PRIMER:

1. Mengabulkan gugatan Penggugat;
2. Menjatuhkan talak 1 (satu) ba'in sughra Tergugat (xxxxxxx) terhadap Penggugat (xxxxxxxx);

Disclaimer
Kepaniteraan Mahkamah Agung Republik Indonesia berusaha untuk selalu mencantumkan informasi paling kini dan akurat sebagai bentuk komitmen Mahkamah Agung untuk pelayanan publik, transparansi dan akuntabilitas pelaksanaan fungsi peradilan. Namun dalam hal-hal tertentu masih dimungkinkan terjadi permasalahan teknis terkait dengan akurasi dan keterkinian informasi yang kami sajikan, hal mana akan terus kami perbaiki dari waktu kewaktu.
Dalam hal Anda menemukan inakurasi informasi yang termuat pada situs ini atau informasi yang seharusnya ada, namun belum tersedia, maka harap segera hubungi Kepaniteraan Mahkamah Agung RI melalui :
Email : kepaniteraan@mahkamahagung.go.id Telp : 021-384 3348 (ext.318)

3. Membebankan biaya perkara sesuai dengan ketentuan hukum yang berlaku;

SUBSIDER:

Dan atau apabila Majelis Hakim yang menyidangkan perkara ini berpendapat lain, maka mohon untuk menjatuhkan putusan yang seadil-adilnya(ex aequo et bono;

Bahwa pada hari sidang yang telah ditetapkan, Penggugat datang menghadap dalam sidang, sedangkan Tergugat tidak hadir menghadap dalam sidang dan tidak menyuruh orang lain menghadap sebagai wakilnya/kuasanya, meskipun telah dipanggil secara resmi dan patut sesuai dengan relaas panggilan yang dibacakan di dalam sidang, sedangkan tidak ternyata bahwa tidak datangnya itu disebabkan oleh suatu halangan yang sah.-----------------------

Bahwa perkara ini tidak dapat dilakukan Mediasi karena Tergugat tidak pernah hadir dalam sidang.---

Bahwa Majelis Hakim telah berusaha mendamaikan pihak berperkara dengan cara menasihati Penggugat agar tetap mempertahankan rumah tangganya dan tidak bercerai dengan Tergugat, namun Penggugat tetap pada dalil-dalil gugatannya untuk bercerai dengan Tergugat; lalu dibacakan surat gugatan Penggugat tersebut yang maksud dan isinya tetap dipertahankan oleh Penggugat.--

Bahwa untuk meneguhkan dalil gugatannya, Penggugat telah mengajukan bukti surat berupa : Fotocopy Kutipan Akta Nikah Nomor : 703/49/VII/2020 tanggal 13 Juli 2020 (tertanda P.).--------------------------------------

Bahwa selain bukti surat tersebut di atas, Penggugat juga mengajukan saksi-saksi yang telah memberikan keterangan di bawah sumpah pada pokoknya sebagai berikut :

1.---

Saksi Pertama : **xxxxxxxxxxxx**.

- -

Bahwa saksi kenal dengan Penggugat dan Tergugat karena saksi adalah xxxxxxxxxxxxxxx Penggugat .

Disclaimer
Kepaniteraan Mahkamah Agung Republik Indonesia berusaha untuk selalu mencantumkan informasi paling kini dan akurat sebagai bentuk komitmen Mahkamah Agung untuk pelayanan publik, transparansi dan akuntabilitas pelaksanaan fungsi peradilan. Namun dalam hal-hal tertentu masih dimungkinkan terjadi permasalahan teknis terkait dengan akurasi dan keterkinian informasi yang kami sajikan, hal mana akan terus kami perbaiki dari waktu kewaktu.
Dalam hal Anda menemukan inakurasi informasi yang termuat pada situs ini atau informasi yang seharusnya ada, namun belum tersedia, maka harap segera hubungi Kepaniteraan Mahkamah Agung RI melalui :
Email : kepaniteraan@mahkamahagung.go.id Telp : 021-384 3348 (ext.318)

- -

Bahwa Penggugat dan Tergugat adalah suami isteri namun belum dikaruniai anak. --

- -

Bahwa sejak bulan Agustus 2020 antara Penggugat dan Tergugat terjadi pertengkaran terus-menerus disebabkan Tergugat suka mabuk dan jarang tinggal di rumah malahan lebih banyak tinggal di rumah orang tua Tergugat. --

- -

Bahwa sejak bulan September 2020 terjadi pisah tempat kediaman disebabkan Tergugat pergi meninggalkan tempat kediaman bersama sampai sekarang selama 4 bulan. --

- -

Bahwa selama pisah, antara Penggugat dan Tergugat tidak ada yang berusaha untuk rukun kembali. --

- -

Bahwa selama pisah, Tergugat tidak pernah datang mengunjungi Penggugat dan tidak pernah memberi nafkah kepada Penggugat. --

- -

Bahwa saksi selaku orang yang dekat dengan pihak berperkara sudah berusaha mendamaikan Penggugat dan Tergugat, tetapi tidak berhasil, karena itu saksi sudah tidak sanggup untuk mendamaikan lagi. --

2. Saksi Kedua : **xxxxxxxxxxx.**--

- -

Bahwa saksi kenal dengan Penggugat dan Tergugat karena saksi adalah xxxxxxxxxxxx Penggugat. --

Disclaimer
Kepaniteraan Mahkamah Agung Republik Indonesia berusaha untuk selalu mencantumkan informasi paling kini dan akurat sebagai bentuk komitmen Mahkamah Agung untuk pelayanan publik, transparansi dan akuntabilitas pelaksanaan fungsi peradilan. Namun dalam hal-hal tertentu masih dimungkinkan terjadi permasalahan teknis terkait dengan akurasi dan keterkinian informasi yang kami sajikan, hal mana akan terus kami perbaiki dari waktu kewaktu.
Dalam hal Anda menemukan inakurasi informasi yang termuat pada situs ini atau informasi yang seharusnya ada, namun belum tersedia, maka harap segera hubungi Kepaniteraan Mahkamah Agung RI melalui :
Email : kepaniteraan@mahkamahagung.go.id Telp : 021-384 3348 (ext.318)

Bahwa Penggugat dan Tergugat adalah suami isteri namun belum dikaruniai anak.

Bahwa sejak bulan Agustus 2020 antara Penggugat dan Tergugat terjadi pertengkaran terus-menerus disebabkan Tergugat suka minuman keras sampai mabuk, Tergugat jarang tinggal bersama Penggugat dan sering tinggal di rumah orang tua Tergugat. .

Bahwa sejak bulan September 2020 terjadi pisah tempat kediaman disebabkan Tergugat pergi meninggalkan tempat kediaman bersama sampai sekarang selama 4 bulan.

Bahwa selama pisah, antara Penggugat dan Tergugat tidak ada yang berusaha untuk rukun kembali.

Bahwa selama pisah, Tergugat tidak pernah datang mengunjungi Penggugat dan tidak pernah memberi nafkah kepada Penggugat.

Bahwa saksi selaku orang yang dekat dengan pihak berperkara sudah berusaha mendamaikan Penggugat dan Tergugat, tetapi tidak berhasil, karena itu saksi sudah tidak sanggup untuk mendamaikan lagi.

Bahwa selanjutnya Penggugat menyatakan tidak akan mengajukan tanggapan apapun dan akhirnya mohon putusan.---------------------------------------

Disclaimer
Kepaniteraan Mahkamah Agung Republik Indonesia berusaha untuk selalu mencantumkan informasi paling kini dan akurat sebagai bentuk komitmen Mahkamah Agung untuk pelayanan publik, transparansi dan akuntabilitas pelaksanaan fungsi peradilan. Namun dalam hal-hal tertentu masih dimungkinkan terjadi permasalahan teknis terkait dengan akurasi dan keterkinian informasi yang kami sajikan, hal mana akan terus kami perbaiki dari waktu kewaktu.
Dalam hal Anda menemukan inakurasi informasi yang termuat pada situs ini atau informasi yang seharusnya ada, namun belum tersedia, maka harap segera hubungi Kepaniteraan Mahkamah Agung RI melalui :
Email : kepaniteraan@mahkamahagung.go.id Telp : 021-384 3348 (ext.318)

Bahwa untuk mempersingkat uraian putusan ini, maka ditunjuk hal-hal sebagaimana tercantum dalam Berita Acara Sidang dan harus dianggap telah termuat dalam pertimbangan Majelis secara keseluruhan.----------------------------

PERTIMBANGAN HUKUM

Menimbang, bahwa maksud dan tujuan gugatan Penggugat adalah sebagaimana telah diuraikan di atas.--

Menimbang, bahwa dari posita gugatan menunjukkan bahwa perkara ini termasuk sengketa di bidang perkawinan, dan berdasarkan bukti P.1 berupa fotocopy Kartu Tanda Penduduk a.n. Penggugat Nomor : tanggal .... yang dikeluarkan oleh pejabat yang berwenang dan telah bermeterai cukup serta cocok sesuai dengan aslinya. Bukti ini merupakan akta otentik, telah memenuhi syarat formil dan materiil pembuktian sehingga alat bukti tersebut dapat diterima dan dipertimbangkan. Dan berdasarkan alat bukti tersebut Penggugat berdomisili di wilayah hukum Pengadilan Agama Jakarta Timur, karena itu permohonan Pemohon secara formal dapat diterima Pengadilan Agama Jakarta Timur, maka berdasarkan ketentuan Pasal 49 ayat (1) huruf a dan Pasal 73 ayat (1) UU No. 7 Tahun 1989 tentang Peradilan Agama sebagaimana telah diubah dengan UU No. 3 Tahun 2006 dan UU No. 50 Tahun 2009, Pengadilan Agama Jakarta Timur berwenang secara absolut maupun relatif untuk memeriksa, memutus, dan menyelesaikan gugatan Penggugat.---------------------

Menimbang, bahwa Penggugat mendalilkan, sejak Agustus 2020 sering terjadi perselisihan dan pertengkaran, yang disebabkan: Tergugat merupakan seorang pemabuk. Tergugat jarang tinggal di rumah dan lebih sering berada di rumah orang tua Tergugat. Tergugat tidak menghormati orang tua Penggugat dengan tidak mau mendengarkan perkataan orang tua Penggugat. , kemudian sejak bulan September 2020 terjadi pisah tempat kediaman disebabkan Tergugat pergi meninggalkan tempat kediaman bersama sampai sekarang selama 4 bulan dan selama itu tidak ada yang berusaha untuk rukun kembali; bahkan Tergugat tidak pernah datang mengunjungi Penggugat dan tidak pernah memberi nafkah kepada Penggugat.---

Disclaimer
Kepaniteraan Mahkamah Agung Republik Indonesia berusaha untuk selalu mencantumkan informasi paling kini dan akurat sebagai bentuk komitmen Mahkamah Agung untuk pelayanan publik, transparansi dan akuntabilitas pelaksanaan fungsi peradilan. Namun dalam hal-hal tertentu masih dimungkinkan terjadi permasalahan teknis terkait dengan akurasi dan keterkinian informasi yang kami sajikan, hal mana akan terus kami perbaiki dari waktu kewaktu.
Dalam hal Anda menemukan inakurasi informasi yang termuat pada situs ini atau informasi yang seharusnya ada, namun belum tersedia, maka harap segera hubungi Kepaniteraan Mahkamah Agung RI melalui :
Email : kepaniteraan@mahkamahagung.go.id Telp : 021-384 3348 (ext.318)

Menimbang, bahwa Tergugat tidak pernah hadir dalam sidang dan tidak pula menyuruh orang lain menghadap sebagai wakilnya, meskipun ia telah dipanggil secara resmi dan patut, maka Tergugat harus dinyatakan dalam keadaan tidak hadir, karenanya putusan atas perkara ini dapat dijatuhkan secara verstek.--

Menimbang, bahwa berdasarkan ketentuan Pasal 125 ayat (1) HIR, bahwa gugatan Penggugat dapat dikabulkan dengan verstek sepanjang gugatan tersebut beralasan dan tidak melawan hak, oleh karena itu Majelis membebani Penggugat untuk membuktikan kebenaran dalil-dalil gugatannya.---

Menimbang, bahwa untuk meneguhkan kebenaran dalil-dalil gugatannya, Penggugat telah mengajukan bukti surat tertanda P serta saksi-saksi.---

Menimbang, bahwa berdasarkan bukti surat tertanda P. berupa fotocopy Surat Nikah atas nama Penggugat dan Tergugat, yang telah bermeterai serta telah dicocokkan dengan aslinya ternyata cocok, maka bukti ini merupakan akta otentik, telah memenuhi syarat formil dan materiil pembuktian serta dikuatkan oleh keterangan para saksi telah terbukti bahwa Penggugat dan Tergugat telah dan masih terikat dalam perkawinan yang sah sejak tanggal 13 Juli 2020, dengan demikian Penggugat dan Tergugat adalah orang yang berkepentingan, memiliki legal standing, dan patut menjadi pihak-pihak dalam perkara ini.--------

Menimbang, bahwa Penggugat pada pokoknya mendalilkan alasan gugatan cerainya dikarenakan telah terjadi perselisihan dan pertengkaran antara Penggugat dengan Tergugat secara terus menerus, maka untuk memenuhi maksud pasal 22 ayat (2) Peraturan Pemerintah Nomor 9 Tahun 1975 jo. pasal 76 ayat (1) Undang-Undang Nomor 7 Tahun 1989 jo. Undang-Undang Nomor 3 Tahun 2006, Majelis Hakim telah mendengar dua orang saksi keluarga atau orang yang dekat dengan kedua belah pihak, menerangkan dibawah sumpahnya.---

Menimbang bahwa Penggugat telah mengajukan dua orang saksi masing-masing bernama **xxxxxxxxxxxx** dan **xxxxxxxxxxx** adalah orang-orang yang dekat dengan Penggugat, di bawah sumpah telah memberikan keterangan berdasarkan pengalaman dan atau penglihatan mereka sendiri, kesaksian para saksi telah saling bersesuaian, karenanya kesaksian para saksi

Disclaimer
Kepaniteraan Mahkamah Agung Republik Indonesia berusaha untuk selalu mencantumkan informasi paling kini dan akurat sebagai bentuk komitmen Mahkamah Agung untuk pelayanan publik, transparansi dan akuntabilitas pelaksanaan fungsi peradilan. Namun dalam hal-hal tertentu masih dimungkinkan terjadi permasalahan teknis terkait dengan akurasi dan keterkinian informasi yang kami sajikan, hal mana akan terus kami perbaiki dari waktu kewaktu.
Dalam hal Anda menemukan inakurasi informasi yang termuat pada situs ini atau informasi yang seharusnya ada, namun belum tersedia, maka harap segera hubungi Kepaniteraan Mahkamah Agung RI melalui :
Email : kepaniteraan@mahkamahagung.go.id   Telp : 021-384 3348 (ext.318)

a quo telah memenuhi syarat formil maupun materiil sebagai saksi, sebagaimana makud pasal 172 HIR, oleh karenanya keterangan para saksi dapat diterima yang pada pokoknya menerangkan bahwa Penggugat dan Tergugat adalah suami isteri yang sah, rumah tangga Penggugat dengan Tergugat sudah tidak harmonis sejak bulan Agustus 2020, telah terjadi pertengkaran dan pertengkaran secara terus-menerus yang disebabkan karena Tergugat tidak memberi nafkah kepada Penggugat. Dan sejak bulan September 2020 terjadi pisah tempat kediaman disebabkan Tergugat pergi meninggalkan tempat kediaman bersama sampai sekarang selama 4 bulan. Semenjak berpisah tersebut menyebabkan Penggugat dan Tergugat sudah saling tidak melaksanakan kewajiban sebagaimana layaknya kewajiban suami-isteri.---

Menimbang bahwa berdasarkan alat-alat bukti yang diajukan ke persidangan oleh Penggugat Majelis Hakim telah menemukan fakta hukum sebagai berikut:

- bahwa antara Penggugat dan Tergugat telah terikat dalam perkawinan yang sah sejak tanggal 13 Juli 2020.
- bahwa sejak Agustus 2020 sering terjadi perselisihan dan pertengkaran, yang disebabkan: Tergugat merupakan seorang pemabuk. Tergugat jarang tinggal di rumah dan lebih sering berada di rumah orang tua Tergugat. Tergugat tidak menghormati orang tua Penggugat dengan tidak mau mendengarkan perkataan orang tua Penggugat. .
- bahwa sejak bulan September 2020 terjadi pisah tempat kediaman disebabkan Tergugat pergi meninggalkan tempat kediaman bersama sampai sekarang selama 4 bulan dan selama itu sudah tidak ada yang berusaha untuk rukun kembali dalam rumah tangga.

Menimbang, bahwa walaupun pada dasarnya perceraian adalah suatu yang tidak disenangi oleh Allah SWT, namun membiarkan rumah tangga Penggugat dan Tergugat dalam kondisi yang demikian dimana sudah tidak ada lagi keadaan yang sakinah mawaddah dan rahmah sebagaimana maksud dan tujuan perkawinan, maka perceraian dapat dimungkinkan sebagai pintu darurat atas kemelut rumah tangga yang terjadi antara Penggugat dengan Tergugat.----

Disclaimer
Kepaniteraan Mahkamah Agung Republik Indonesia berusaha untuk selalu mencantumkan informasi paling kini dan akurat sebagai bentuk komitmen Mahkamah Agung untuk pelayanan publik, transparansi dan akuntabilitas pelaksanaan fungsi peradilan. Namun dalam hal-hal tertentu masih dimungkinkan terjadi permasalahan teknis terkait dengan akurasi dan keterkinian informasi yang kami sajikan, hal mana akan terus kami perbaiki dari waktu kewaktu.
Dalam hal Anda menemukan inakurasi informasi yang termuat pada situs ini atau informasi yang seharusnya ada, namun belum tersedia, maka harap segera hubungi Kepaniteraan Mahkamah Agung RI melalui :
Email : kepaniteraan@mahkamahagung.go.id Telp : 021-384 3348 (ext.318)

Menimbang, bahwa dengan tidak melihat dari pihak mana yang menjadi penyebab timbulnya perselisihan dan pertengkaran antara Penggugat dengan Tergugat namun telah jelas bahwa akibat perselisihan yang terjadi antara Penggugat dan Tergugat dimana Penggugat dan Tergugat sudah 1 tahun tidak lagi melaksankan kewajiban sebagai mana layaknya kewajiban suai-isteri. Dan selama itu pula Penggugat dengan Tergugat tidak pernah bersatu kembali sebagaimana rumah tangga yang baik. Hal ini semakin menegaskan bahwa rumah tangga Penggugat dan Tergugat sudah benar-benar tidak dapat dipertahankan lagi.---

Menimbang, bahwa terlepas dari apapun yang melatarbelakanginya, dan ternyata bahwa keduanya sudah tidak lagi melaksanakan kewajiban sebagaimana layaknya kewajiban suami-isteri, tidak pernah berhubungan sebagaimana layaknya hubungan suami-isteri, maka sesuai dengan Yurisprudensi Mahkamah Agung RI. Nomor : 379 K/AG/1995 tanggal 26 Maret 1997, menyatakan : “Suami istri yang sudah tidak lagi saling melaksanakan kewajiban dan sudah saling tidak memperdulikan bahkan sudah pisah tempat tinggal tidak ada harapan untuk dapat hidup rukun kembali, maka rumah tangga tersebut telah terbukti retak dan pecah dan telah memenuhi alasan cerai Pasal 19 huruf (f) Peraturan Pemerintah Nomor 9 Tahun 1975, dan telah sejalan pula dengan pendapat pakar hukum Islam yang diambil alih menjadi pendapat dalam pertimbangan hukum putusan ini, sebagaimana tersebut dalam kitab *Al Mar’ah Bainal Fiqh Wal Qanun*, halaman 100 yang artinya : “Dan tidak ada manfaatnya yang dapat diharapkan dalam mengumpulkan dua manusia yang saling membenci terlepas dari masalah apakah, sebab terjadinya pertengkaran ini besar atau kecil namun kebaikan hanya dapat diterapkan dengan mengakhiri kehidupan berumah tangga antara suami istri ini”.---------------------------------------

Menimbang, bahwa dengan demikian alasan perceraian yang diajukan oleh Penggugat bahwa telah terjadi perselisihan dan pertengkaran terus menerus dengan sebab-sebab sebagaimana diuraikan di atas, halmana upaya perdamaian telah ditempuh dan tidak berhasil sehingga tidak ada harapan lagi rumah tangga Pengugat dan Tergugat dapat rukun seperti sediakala, oleh karenanya telah terbukti dan telah memenuhi ketentuan pasal 39 ayat (2) Undang-Undang Nomor 1 Tahun 1974 jo. pasal 19 huruf (f) Peraturan

Disclaimer
Kepaniteraan Mahkamah Agung Republik Indonesia berusaha untuk selalu mencantumkan informasi paling kini dan akurat sebagai bentuk komitmen Mahkamah Agung untuk pelayanan publik, transparansi dan akuntabilitas pelaksanaan fungsi peradilan. Namun dalam hal-hal tertentu masih dimungkinkan terjadi permasalahan teknis terkait dengan akurasi dan keterkinian informasi yang kami sajikan, hal mana akan terus kami perbaiki dari waktu kewaktu.
Dalam hal Anda menemukan inakurasi informasi yang termuat pada situs ini atau informasi yang seharusnya ada, namun belum tersedia, maka harap segera hubungi Kepaniteraan Mahkamah Agung RI melalui :
Email : kepaniteraan@mahkamahagung.go.id   Telp : 021-384 3348 (ext.318)

Pemerintah Nomor 9 Tahun 1975 jo. pasal 116 huruf (f) Kompilasi Hukum Islam.---

Menimbang, bahwa disamping itu alasan tersebut telah sesuai dengan maksud dalil syar'i yang terdapat dalam Al-Qur'an Surat Al Baqarah ayat 229 yang menjadi salah satu pertimbangan Majelis Hakim yang berbunyi :

الطَّلاقُ مَرَّتَانِ فَإِمْسَاكٌ بِمَعْـرُوفٍ أَوْ تَسْـرِيحٌ بِإِحْسَانٍ

Artinya : " Talak (yang dapat dirujuk) itu dua kali, setelah itu boleh rujuk lagi dengan cara yang ma'ruf, atau menceraikan dengan cara yang baik".

Menimbang, bahwa dapat dianggap sebagai penyalahgunaan dan berdosa jika suami istri tanpa sebab yang pasti mereka harus bercerai dan juga termasuk perkosaan terhadap hukum dan moral, jika memaksakan suami istri harus tetap hidup dalam rumah tangga yang kehidupan interpersonal tidak lagi terkoordinasi dan hilangnya tujuan bersama dalam rumah tangga sebagaimana diamanatkan dalam Al Qur'an surat Ar Rum ayat 21 dan dalam Pasal 1 Undang-Undang Nomor 1 Tahun 1974. Dalam hal ini rumah tangga seperti tersebut di atas dan upaya perdamaian dari berbagai pihak sudah dilakukan, termasuk sebagaimana dimaksud oleh Pasal 31 Peraturan Pemerintah Nomor 9 Tahun 1975, dan Pasal 82 Undang-Undang Nomor 7 Tahun 1989, namun tidak berhasil, maka perceraian dipandang lebih baik untuk menentukan kehidupan berikutnya atau dianggap sebagai “*tasrih bi ihsan*“ (melepaskan ikatan perkawinan dengan cara yang baik) sebagaimana tersurat dalam ayat al-Qur'an di atas.---

Menimbang, bahwa dengan demikian dalil-dalil gugatan Penggugat yang dijadikan alasan untuk bercerai sebagaimana yang dimaksud Pasal 19 huruf " a, f " PP No. 9 Tahun 1975 Jo. Pasal 116 huruf " a, f " Kompilasi Hukum Islam, telah dapat dibuktikan oleh Penggugat.--

Menimbang, bahwa Majelis sependapat dengan Ahli Fiqih yang selanjutnya diambil alih menjadi pendapat Majelis seperti tercantum dalam Kitab Fiqhus Sunnah Juz II halaman 248 berbunyi :

Disclaimer
Kepaniteraan Mahkamah Agung Republik Indonesia berusaha untuk selalu mencantumkan informasi paling kini dan akurat sebagai bentuk komitmen Mahkamah Agung untuk pelayanan publik, transparansi dan akuntabilitas pelaksanaan fungsi peradilan. Namun dalam hal-hal tertentu masih dimungkinkan terjadi permasalahan teknis terkait dengan akurasi dan keterkinian informasi yang kami sajikan, hal mana akan terus kami perbaiki dari waktu kewaktu.
Dalam hal Anda menemukan inakurasi informasi yang termuat pada situs ini atau informasi yang seharusnya ada, namun belum tersedia, maka harap segera hubungi Kepaniteraan Mahkamah Agung RI melalui :
Email : kepaniteraan@mahkamahagung.go.id Telp : 021-384 3348 (ext.318)

فـاذا ثبتت دعواها لـدى القاضى ببينة الزوجة او اعـتراف الــزوج وكــان الإيــذاء مما يطــاق معه دوام العشــرة بين امثالهما وعجز القاضى عن الإصـــلاح بينهما طلقها طلقة بائنة

Artinya: "Apabila gugatan isteri itu diterima oleh hakim berdasarkan pada bukti-bukti yang diajukan oleh isteri atau adanya pengakuan suami, dan isteri merasa menderita jika tetap bertahan hidup bersama suaminya, sedangkan Hakim tidak berhasil mendamaikan mereka, maka Hakim menceraikan isteri itu dengan talak satu ba'in". --

Menimbang, bahwa berdasarkan pertimbangan-pertimbangan tersebut diatas, maka gugatan Penggugat *a quo* telah beralasan dan harus dikabulkan dengan menjatuhkan talak satu ba'in sughra dari Tergugat kepada Penggugat.--

Menimbang, bahwa Cerai Gugat termasuk perkara bidang perkawinan, maka sesuai ketentuan Pasal 89 ayat (1) UU Nomor 7 Tahun 1989 tentang Peradilan Agama sebagaimana telah diubah dengan UU Nomor 3 Tahun 2006 dan UU Nomor 50 Tahun 2009, biaya perkara dibebankan kepada Penggugat.- -

Mengingat, segala peraturan perundang-undangan yang berlaku dan Hukum Islam yang berkaitan dengan perkara ini.--

M E N G A D I L I

1. Menyatakan, Tergugat yang telah dipanggil secara resmi dan patut untuk menghadap di persidangan, tidak hadir.
2. Mengabulkan gugatan Penggugat secara verstek.
3. Menjatuhkan talak satu ba'in shughra Tergugat (xxxxxxxx) terhadap Penggugat (xxxxxxxx).
4. Membebankan kepada Penggugat untuk membayar biaya perkara ini sejumlah Rp 391000 (tiga ratus Sembilan puluh satu ribu rupiah).

Demikian diputuskan dalam rapat permusyawaratan Majelis Hakim Pengadilan Agama Jakarta Timur pada hari **Rabu** tanggal **06 Januari 2021** Masehi bertepatan dengan tanggal 22 Jumadilawal 1442 Hijriyah, oleh kami **Drs. M. Nasir, M.H.** sebagai Ketua Majelis, **Drs. H. Moh. Faizin, S.H., M.H.** dan

Disclaimer
Kepaniteraan Mahkamah Agung Republik Indonesia berusaha untuk selalu mencantumkan informasi paling kini dan akurat sebagai bentuk komitmen Mahkamah Agung untuk pelayanan publik, transparansi dan akuntabilitas pelaksanaan fungsi peradilan. Namun dalam hal-hal tertentu masih dimungkinkan terjadi permasalahan teknis terkait dengan akurasi dan keterkinian informasi yang kami sajikan, hal mana akan terus kami perbaiki dari waktu kewaktu.
Dalam hal Anda menemukan inakurasi informasi yang termuat pada situs ini atau informasi yang seharusnya ada, namun belum tersedia, maka harap segera hubungi Kepaniteraan Mahkamah Agung RI melalui :
Email : kepaniteraan@mahkamahagung.go.id   Telp : 021-384 3348 (ext.318)

Drs. H. Muhammad Anwar Saleh, S.H., M.H. masing-masing sebagai Hakim Anggota, yang pada hari itu juga diucapkan dalam sidang terbuka untuk umum oleh Ketua Majelis tersebut dengan didampingi Hakim-hakim Anggota dan dibantu oleh Hamdani, S.H.I. sebagai Panitera Pengganti serta dihadiri oleh Penggugat tanpa hadirnya Tergugat.---

Ketua Majelis

Drs. M. Nasir, M.H.

Hakim Anggota	Hakim Anggota
Drs. H. Moh. Faizin, S.H., M.H.	Drs. H. Muhammad Anwar Saleh, S.H., M.H.

Panitera Pengganti,

Hamdani, S.H.I.

Rincian Biaya Perkara :

1. Biaya pendaftaran	:	Rp 30.000,00
2. Biaya Proses	:	Rp 75.000,00
3. Biaya panggilan	:	Rp 250.000,00
4. Biaya PNBP panggilan	:	Rp 20.000,00
5. Redaksi putusan	:	Rp 10.000,00
6. Meterai	:	Rp 6.000,00
Jumlah	:	Rp 391000 (tiga ratus Sembilan puluh satu ribu rupiah).

Disclaimer
Kepaniteraan Mahkamah Agung Republik Indonesia berusaha untuk selalu mencantumkan informasi paling kini dan akurat sebagai bentuk komitmen Mahkamah Agung untuk pelayanan publik, transparansi dan akuntabilitas pelaksanaan fungsi peradilan. Namun dalam hal-hal tertentu masih dimungkinkan terjadi permasalahan teknis terkait dengan akurasi dan keterkinian informasi yang kami sajikan, hal mana akan terus kami perbaiki dari waktu kewaktu.
Dalam hal Anda menemukan inakurasi informasi yang termuat pada situs ini atau informasi yang seharusnya ada, namun belum tersedia, maka harap segera hubungi Kepaniteraan Mahkamah Agung RI melalui :
Email : kepaniteraan@mahkamahagung.go.id Telp : 021-384 3348 (ext.318)
