# Supplementary material for: Classification of divorce causes during the COVID-19 pandemic using convolutional neural networks
Source: PeerJ Comput Sci. 2022 Jun 30;8:e998. doi: 10.7717/peerj-cs.998 (PMC9299239; doi:10.7717/peerj-cs.998)
Supplement: Supplemental Information 3 [file peerj-cs-08-998-s003.zip › KDRT Dataset/Data ke-26.pdf]

PUTUSAN

Nomor 4290/Pdt.G/2020/PA.JT

بِسْمِ اللَّهِ الرَّحْمَنِ الرَّحِيمِ

DEMI KEADILAN BERDASARKAN KETUHANAN YANG MAHA ESA

Pengadilan Agama Jakarta Timur yang memeriksa dan mengadili perkara tertentu pada tingkat pertama dalam sidang majelis hakim telah menjatuhkan putusan dalam perkara Cerai Gugat antara:

Penggugat, tempat dan tanggal lahir cimahi, 07 September 1974, agama Islam, pekerjaan karyawan swasta, pendidikan Sekolah Lanjutan Tingkat Atas, tempat kediaman di Kota Jakarta Timur, dalam hal ini memberikan kuasa kepada H. Dody Haryanto, S.H., Aris Munandar, S.H., dan Dasriel, S.H., Advokat yang berkantor di Jalan Raya PKP. Nomor 19 Kelapa Dua Wetan, Jakarta Timur, berdasarkan Surat Kuasa Khusus yang terdaftar di Kepanitaraan Pengadilan Agama Jakarta Timur tanggal 3 November 2020, sebagai Penggugat;

melawan

Tergugat, tempat dan tanggal lahir Jakarta, 21 Maret 1980, agama Islam, pekerjaan karyawan swasta, pendidikan Strata I, tempat kediaman di Propinsi Jawa Barat, sebagai Tergugat.

Pengadilan Agama tersebut.

Telah mempelajari surat-surat yang berkaitan dengan perkara ini.

Disclaimer
Kepaniteraan Mahkamah Agung Republik Indonesia berusaha untuk selalu mencantumkan informasi paling kini dan akurat sebagai bentuk komitmen Mahkamah Agung untuk pelayanan publik, transparansi dan akuntabilitas pelaksanaan fungsi peradilan. Namun dalam hal-hal tertentu masih dimungkinkan terjadi permasalahan teknis terkait dengan akurasi dan keterkinian informasi yang kami sajikan, hal mana akan terus kami perbaiki dari waktu kewaktu.
Dalam hal Anda menemukan inakurasi informasi yang termuat pada situs ini atau informasi yang seharusnya ada, namun belum tersedia, maka harap segera hubungi Kepaniteraan Mahkamah Agung RI melalui :
Email : kepaniteraan@mahkamahagung.go.id   Telp : 021-384 3348 (ext.318)

DUDUK PERKARA

Bahwa Penggugat dalam surat gugatannya tanggal 03 November 2020 telah mengajukan Cerai Gugat, yang telah terdaftar di Kepaniteraan Pengadilan Agama Jakarta Timur, dengan Nomor 0000/Pdt.G/2020/PA.JT, tanggal 04 November 2020, dengan dalil-dalil sebagai berikut:

1. Bahwa Penggugat adalah sebagai isteri sah Tergugat yang telah menikah pada hari Sabtu pada tanggal 17 Maret 2018 di tercatat pada Kantor Urusan Agama Kecamatan Cimahi Tengah, Kota Cimahi, Propinsi Jawa Barat berdasarkan dengan Kutipan Akta Nikah Nomor : xxx yang dikeluarkan pada tanggal 19 Maret 2018.
2. Bahwa antara Penggugat dan Tergugat selama menjalankan kehidupan berumah tangga berkediaman terakhir di Kota Jakarta Timur. Selama masa dalam pernikahan belum dikaruniai keturunan.
3. Bahwa kehidupan rumah tangga Penggugat dan Tergugat awal terjadi keributan sejak pernikahan tahun 2018 dan sudah tidak ada lagi komunikasi yang sehat antara Penggugat dan Tergugat. Keadaan ini memicu timbul keributan yang sulit untuk mewujudkan tujuan perkawinan, adapun hal-hal yang menimbulkan ketidakharmonisan dalam rumah tangga sebagai berikut:
   - Bahwa Tergugat sejak awal pernikahan tanggl 17 Maret 2018 sampai dengan sekarang Tergugat tidak memberikan nafkah lahir kepada Penggugat dan tidak bertanggung jawab sebagai kepala keluarga;
   - Bahwa Tergugat sering melakukan kekerasan dalam rumah tangga baik fisik maupun physikis kepada Penggugat;
   - Bahwa Tergugat sering meminjam uang kepada saudara, kerabat atau kenalan Penggugat untuk kepentingan pribadi Tergugat untuk membeli obat penenang dan judi online, sehingga hutang-hutang Tergugat yang menanggung Penggugat;
   - Bahwa Tergugat menuntut Penggugat untuk memenuhi kebutuhan Tergugat setiap hari sebesar Rp 150.000,- Penggugat merasa sangat

Disclaimer
Kepaniteraan Mahkamah Agung Republik Indonesia berusaha untuk selalu mencantumkan informasi paling kini dan akurat sebagai bentuk komitmen Mahkamah Agung untuk pelayanan publik, transparansi dan akuntabilitas pelaksanaan fungsi peradilan. Namun dalam hal-hal tertentu masih dimungkinkan terjadi permasalahan teknis terkait dengan akurasi dan keterkinian informasi yang kami sajikan, hal mana akan terus kami perbaiki dari waktu kewaktu.
Dalam hal Anda menemukan inakurasi informasi yang termuat pada situs ini atau informasi yang seharusnya ada, namun belum tersedia, maka harap segera hubungi Kepaniteraan Mahkamah Agung RI melalui :
Email : kepaniteraan@mahkamahagung.go.id Telp : 021-384 3348 (ext.318)

keberatan atas tuntutan Tergugat apabila tidak dipenuhi oleh Penggugat dan Tergugat selalu marah-marah;

- Bahwa pada tanggal 13 September 2020, Tergugat menggunakan obat-obatan penenang dari uang yang diberikan oleh Penggugat;
- Bahwa Penggugat dan Tergugat sudah tidak terjadi komunikasi yang sehat dan sudah masing-masing dalam hidup berumahtangga.
- Bahwa, antara Penggugat dan Tergugat sering sekali terjadi berselisih terus menerus yang mengakibatkan pertengkaran dan perselisihan yang pada akhirnya Penggugat tidak nyaman hidup berumahtangga dengan Tergugat;
- Bahwa sejak tanggal 30 September 2020 sampai dengan sekarang Penggugat dan Tergugat sudah pisah ranjang dan pisah rumah yang mana Penggugat bertempat tinggal di Kota Jakarta Timur dan Tergugat bertempat tinggal di Propinsi Jawa Barat.

5. Bahwa Penggugat sudah berusaha membicarakan masalah kehidupan rumah tangga yang sudah tidak harmonis dengan Tergugat, namun tidak berhasil Penggugat dan Tergugat sudah sulit untuk mengatasi masalah rumah tangga Penggugat dengan Tergugat.
6. Bahwa Penggugat dengan Tergugat berdasarkan fakta tersebut, hubungan perkawinan Pengugat dengan Tergugat sudah tidak dapat lagi dipertahankan, untuk membina rumah tangga yang kekal berdasarkan Ketuhanan Yang Maha Esa dan sesuai dengan maksud dalam Pasal 3 Kompilasi Hukum Islam jo. Pasal 1 Undang-Undang No. 1 Tahun 1974 tentang Perkawinan.
7. Bahwa dengan demikian terbukti dengan jelas, cerai gugat yang diajukan oleh Penggugat tersebut adalah telah memenuhi ketentuan sebagaimana yang diatur dalam Undang-undang No. 1 Tahun 1974 dalam Pasal 39 ayat 2 dan Pasal 19 huruf (f) Peraturan Pemerintah No. 9 Tahun 1975 Jo. Pasal 116 huruf (f) Kompilasi Hukum Islam. Oleh karena itu maka sangatlah beralasan hukum apabila Majelis Hakim Yang Mulia menetapkan dan mengabulkan permohonan Penggugat dan jatuh talak satu dari Tergugat kepada Penggugat.

Disclaimer
Kepaniteraan Mahkamah Agung Republik Indonesia berusaha untuk selalu mencantumkan informasi paling kini dan akurat sebagai bentuk komitmen Mahkamah Agung untuk pelayanan publik, transparansi dan akuntabilitas pelaksanaan fungsi peradilan. Namun dalam hal-hal tertentu masih dimungkinkan terjadi permasalahan teknis terkait dengan akurasi dan keterkinian informasi yang kami sajikan, hal mana akan terus kami perbaiki dari waktu kewaktu.
Dalam hal Anda menemukan inakurasi informasi yang termuat pada situs ini atau informasi yang seharusnya ada, namun belum tersedia, maka harap segera hubungi Kepaniteraan Mahkamah Agung RI melalui :
Email : kepaniteraan@mahkamahagung.go.id Telp : 021-384 3348 (ext.318)

8. Bahwa Penggugat mohon biaya perkara yang timbul dalam perkara ini, Majelis Hakim Yang Mulia dapat menetapkan sesuai dengan ketentuan hukum yang berlaku.

Maka berdasakan hal-hal tersebut di atas dapat kiranya Majelis Hakim Pengadilan Agama Jakarta Timur yang menyidangkan perkara aquo menjatuhkan putusan sebagai berikut :

1. Menerima dan mengabulkan gugatan Penggugat ;
2. Menjatuhkantalak satu bain shughra Tergugat terhadap Penggugat.
3. Menetapkan biaya perkara sesuai dengan ketentuan hukum yang berlaku.

Bahwa pada hari sidang yang telah ditetapkan Penggugat telah datang menghadap di muka sidang, sedangkan Tergugat tidak datang menghadap di muka sidang dan tidak menghadirkan orang lain untuk menghadap sebagai kuasa hukumnya yang sah meskipun Tergugat telah dipanggil secara resmi dan patut sebagaimana relaas panggilan yang dibacakan di dalam sidang, sedangkan tidak ternyata ketidakhadiran Tergugat tersebut disebabkan oleh halangan yang sah.

Bahwa perkara ini tidak dapat dimediasi karena Tergugat tidak pernah datang menghadap meskipun telah dipanggil secara resmi dan patut, selanjutnya dimulai pemeriksaan dengan membacakan surat gugatan Penggugat.

Bahwa ketidakhadiran Tergugat dalam persidangan perkara ini dan tidak menggunakan hak jawab atas dalil-dalil gugatan Penggugat, akan tetapi karena perkara ini adalah perkara perceraian yang didasarkan atas perselisihan dan pertengkaran antara Penggugat dengan Tergugat yang berlaku aturan khusus, maka Pengadilan membebankan Penggugat untuk membuktikan dalil-dalil gugatannya.

Bahwa untuk membuktikan dalil gugatannya, Penggugat telah mengajukan bukti, sebagai berikut :

A. Surat :

Fotokopi Kutipan Akta Nikah Nomor xxx yang dikeluarkan Kantor Urusan Agama Kecamatan Cimahi Tengah, Kota Cimahi, Provinsi Jawa Barat

Disclaimer
Kepaniteraan Mahkamah Agung Republik Indonesia berusaha untuk selalu mencantumkan informasi paling kini dan akurat sebagai bentuk komitmen Mahkamah Agung untuk pelayanan publik, transparansi dan akuntabilitas pelaksanaan fungsi peradilan. Namun dalam hal-hal tertentu masih dimungkinkan terjadi permasalahan teknis terkait dengan akurasi dan keterkinian informasi yang kami sajikan, hal mana akan terus kami perbaiki dari waktu kewaktu.
Dalam hal Anda menemukan inakurasi informasi yang termuat pada situs ini atau informasi yang seharusnya ada, namun belum tersedia, maka harap segera hubungi Kepaniteraan Mahkamah Agung RI melalui :
Email : kepaniteraan@mahkamahagung.go.id Telp : 021-384 3348 (ext.318)

tanggal 19 Maret 2018. Bukti surat tersebut telah diberi materai cukup dan telah dicocokkan dengan aslinya yang ternyata sesuai, bukti P.

B. Saksi:

1. xxx (kakak kandung Penggugat), di bawah sumpahnya memberikan keterangan sebagai berikut:
   - Bahwa saksi kenal Tergugat sebagai suami Penggugat.
   - Bahwa sejak satu bulan setelah Penggugat dan Tergugat menikah, saksi lebih dari dua kali melihat Penggugat dan Tergugat bertengkar disebabkan Tergugat tidak memberikan nafkah kepada Penggugat.
   - Bahwa Penggugat dan Tergugat telah pisah tempat tinggal sejak bulan September 2020 sampai sekarang, Tergugat meninggalkan kediaman bersama.
   - Bahwa selama pisah tempat tinggal tersebut, Tergugat tidak pernah datang ke tempat tinggal Penggugat.
   - Bahwa Penggugat dan Tergugat telah didamaikan, namun tidak berhasil.

2. xxx (kakak kandung Penggugat), di bawah sumpahnya memberikan keterangan sebagai berikut :
   - Bahwa saksi kenal Tergugat sebagai suami Penggugat.
   - Bahwa sejak satu bulan setelah Penggugat dan Tergugat menikah, saksi dua kali melihat Penggugat dan Tergugat bertengkar disebabkan Tergugat tidak memberikan nafkah kepada Penggugat.
   - Bahwa Penggugat dan Tergugat telah pisah tempat tinggal sejak bulan September 2020 sampai sekarang, Tergugat meninggalkan kediaman bersama.
   - Bahwa selama pisah tempat tinggal tersebut, Tergugat tidak pernah datang ke tempat tinggal Penggugat.
   - Bahwa Penggugat dan Tergugat telah didamaikan, namun tidak berhasil.

Disclaimer
Kepaniteraan Mahkamah Agung Republik Indonesia berusaha untuk selalu mencantumkan informasi paling kini dan akurat sebagai bentuk komitmen Mahkamah Agung untuk pelayanan publik, transparansi dan akuntabilitas pelaksanaan fungsi peradilan. Namun dalam hal-hal tertentu masih dimungkinkan terjadi permasalahan teknis terkait dengan akurasi dan keterkinian informasi yang kami sajikan, hal mana akan terus kami perbaiki dari waktu kewaktu.
Dalam hal Anda menemukan inakurasi informasi yang termuat pada situs ini atau informasi yang seharusnya ada, namun belum tersedia, maka harap segera hubungi Kepaniteraan Mahkamah Agung RI melalui :
Email : kepaniteraan@mahkamahagung.go.id   Telp : 021-384 3348 (ext.318)

Bahwa Penggugat telah mengajukan kesimpulan tetap pada gugatannya untuk bercerai dengan Tergugat dan mohon putusan.

Selanjutnya untuk singkatnya uraian putusan, semua hal yang termuat dalam berita acara sidang ini merupakan bagian yang tidak terpisahkan dari putusan ini.

PERTIMBANGAN HUKUM

Menimbang, bahwa maksud dan tujuan gugatan Penggugat adalah sebagaimana terurai di atas.

Menimbang, bahwa Tergugat yang dipanggil secara resmi dan patut akan tetapi tidak datang menghadap harus dinyatakan tidak hadir dan gugatan tersebut harus diperiksa secara verstek.

Menimbang, bahwa oleh karena itu, maka putusan atas perkara ini dapat dijatuhkan tanpa hadirnya Tergugat (verstek).

Menimbang, bahwa karena Tergugat tidak pernah hadir, upaya damai dan prosedur mediasi sebagaimana diatur dalam Perma Nomor 01 Tahun 2016 tidak dapat dilaksanakan.

Menimbang, bahwa perkara ini mengenai gugatan perceraian antara Penggugat dengan Tergugat yang beragama Islam dan perkawinannya dilangsungkan secara hukum Islam di hadapan Pegawai Pencatat Nikah pada Kantor Urusan Agama, maka berdasarkan ketentuan Pasal 49 Undang-Undang Nomor 7 Tahun 1989 sebagaimana telah diubah dengan Undang-Undang Nomor 3 Tahun 2006 dan perubahan kedua dengan Undang-Undang Nomor 50 Tahun 2009, maka Pengadilan Agama berwenang memeriksa, mengadili dan memutus perkara a quo.

Menimbang, bahwa perkara ini adalah cerai gugat, Penggugat bertempat tinggal di wilayah Jakarta Timur, maka berdasarkan Pasal 73 ayat (1) Undang-Undang Nomor 7 Tahun 1989 sebagaimana telah diubah dengan Undang-Undang Nomor 3 Tahun 2006 dan Undang-Undang Nomor 50 Tahun 2009, perkara a quo merupakan kompetensi relatif Pengadilan Agama Jakarta Timur.

Menimbang, bahwa Pengugat mendalilkan bahwa ia telah melangsungkan perkawinan dengan Tergugat yang tercatat pada Kantor

Disclaimer
Kepaniteraan Mahkamah Agung Republik Indonesia berusaha untuk selalu mencantumkan informasi paling kini dan akurat sebagai bentuk komitmen Mahkamah Agung untuk pelayanan publik, transparansi dan akuntabilitas pelaksanaan fungsi peradilan. Namun dalam hal-hal tertentu masih dimungkinkan terjadi permasalahan teknis terkait dengan akurasi dan keterkinian informasi yang kami sajikan, hal mana akan terus kami perbaiki dari waktu kewaktu.
Dalam hal Anda menemukan inakurasi informasi yang termuat pada situs ini atau informasi yang seharusnya ada, namun belum tersedia, maka harap segera hubungi Kepaniteraan Mahkamah Agung RI melalui :
Email : kepaniteraan@mahkamahagung.go.id Telp : 021-384 3348 (ext.318)

Urusan Agama Kecamatan Cimahi Tengah, Kota Cimahi, Provinsi Jawa Barat dan antara Penggugat dan Tergugat telah terjadi perselisihan dan pertengkaran. Untuk membuktikan adanya hubungan hukum sahnya pernikahan antara Penggugat dengan Tergugat, bukti yang diajukan Penggugat adalah bukti P yang merupakan fotokopi dari akta otentik, telah bermaterai cukup dan telah cocok dengan aslinya, oleh karena itu bukti tersebut telah memenuhi Pasal 2 ayat (3) Undang-Undang Nomor 13 Tahun 1985 dan Pasal 165 HIR, sehingga bukti tersebut mempunyai kekuatan bukti yang sempurna dan mengikat sepanjang berkenaan dengan isi akta tersebut. Berdasarkan bukti P tersebut harus dinyatakan terbukti bahwa Penggugat dengan Tergugat telah menikah secara sah pada hari Sabtu tgl 17 Maret 2018 yang dicatatkan oleh Pegawai Pencatat Nikah Kantor Urusan Agama Kecamatan Cimahi Tengah, Kota Cimahi, Provinsi Jawa Barat. Dengan terbuktinya sahnya pernikahan Penggugat dan Tergugat tersebut, maka berdasarkan ketentuan Pasal 73 ayat (1) Undang-Undang Nomor 7 Tahun 1989 sebagaimana telah diubah dengan Undang-Undang Nomor 3 Tahun 2006 dan perubahan kedua dengan Undang-Undang Nomor 50 Tahun 2009, maka Penggugat memiliki *legal standing* mengajukan gugatan cerai kepada Tergugat.

Menimbang, bahwa pokok perkara ini sebagaimana petitum angka 2 gugatan Penggugat adalah gugatan cerai dengan alasan bahwa sejak awal pernikahan terjadi perselisihan dan pertengkaran antara Penggugat dan Tergugat dengan sebab sebagaimana tersebut di atas. Akibat dari perselisihan dan pertengkaran tersebut Penggugat dan Tergugat telah pisah tempat tinggal sejak bulan September 2020 sampai sekarang, Tergugat meninggalkan kediaman bersama. Penggugat mendasarkan gugatannya tersebut pada ketentuan Pasal 19 huruf f Peraturan Pemerintah Nomor 9 Tahun 1975 jo. Pasal 116 huruf f Kompilasi Hukum Islam.

Menimbang, bahwa oleh karena Penggugat mendasarkan alasan cerainya antara lain pada ketentuan Pasal 19 huruf f Peraturan Pemerintah Nomor 9 Tahun 1975, maka guna memenuhi ketentuan Pasal 22 ayat (2) Peraturan Pemerintah Nomor 9 Tahun 1975 telah didengar keterangan saksi keluarga dan orang dekat dengan Penggugat, masing-masing telah memberi

Disclaimer
Kepaniteraan Mahkamah Agung Republik Indonesia berusaha untuk selalu mencantumkan informasi paling kini dan akurat sebagai bentuk komitmen Mahkamah Agung untuk pelayanan publik, transparansi dan akuntabilitas pelaksanaan fungsi peradilan. Namun dalam hal-hal tertentu masih dimungkinkan terjadi permasalahan teknis terkait dengan akurasi dan keterkinian informasi yang kami sajikan, hal mana akan terus kami perbaiki dari waktu kewaktu.
Dalam hal Anda menemukan inakurasi informasi yang termuat pada situs ini atau informasi yang seharusnya ada, namun belum tersedia, maka harap segera hubungi Kepaniteraan Mahkamah Agung RI melalui :
Email : kepaniteraan@mahkamahagung.go.id Telp : 021-384 3348 (ext.318)

keterangan di bawah sumpah dan materi keterangan saksi-saksi tersebut saling bersesuaian dan relevan dengan pokok perkara, maka keterangan saksi-saksi tersebut memenuhi syarat pembuktian saksi sebagaimana diatur dalam Pasal 171 dan 172 HIR.

Menimbang, bahwa dari keterangan saksi-saksi tersebut dapat ditemukan fakta kejadian yang telah terbukti kebenarannya, sebagai berikut :

- Telah terjadi pertengkaran antara Penggugat dan Tergugat sejak sebulan setelah Penggugat dan Tergugat menikah disebabkan Tergugat tidak memberikan nafkah kepada Penggugat.
- Penggugat dan Tergugat telah pisah tempat tinggal sejak bulan September 2020 sampai sekarang, Tergugat meninggalkan kediaman bersama.
- Selama pisah tempat tinggal tersebut, Tergugat tidak pernah datang ke tempat tinggal Penggugat.

Penggugat dan Tergugat sudah didamaikan, namun tidak berhasil.

Menimbang, bahwa perselisihan dan pertengkaran antara suami istri adalah keadaan tidak rukunnya suami istri tersebut, terbukti dari fakta-fakta yang menunjukkan tidak rukunnya suami istri tersebut. Sebagaimana fakta-fakta kejadian tentang pertengkaran antara Penggugat dengan Tergugat kemudian Penggugat dan Tergugat telah pisah tempat tinggal sejak bulan September 2020 sampai sekarang dan selama pisah tersebut Tergugat tidak pernah datang ke tempat tinggal Penggugat, merupakan rangkaian fakta tidak rukunnya Penggugat dan Tergugat yang patut diduga rangkaian fakta itu terjadi karena adanya perselisihan dan pertengkaran antara Penggugat dan Tergugat. Dengan demikian, terbukti telah terjadi perselisihan dan pertengkaran antara Penggugat dan Tergugat, sehingga antara keduanya tidak ada harapan akan rukun lagi dalam rumah tangga.

Menimbang, bahwa hal tidak ada harapan akan rukun lagi tersebut selain didasarkan atas fakta Penggugat dan Tergugat sudah didamaikan namun tidak berhasil, juga selama pisah tempat tinggal tersebut tidak ada tanda membaiknya keadaan rumah tangga Penggugat dan Tergugat. Jika Penggugat

Disclaimer
Kepaniteraan Mahkamah Agung Republik Indonesia berusaha untuk selalu mencantumkan informasi paling kini dan akurat sebagai bentuk komitmen Mahkamah Agung untuk pelayanan publik, transparansi dan akuntabilitas pelaksanaan fungsi peradilan. Namun dalam hal-hal tertentu masih dimungkinkan terjadi permasalahan teknis terkait dengan akurasi dan keterkinian informasi yang kami sajikan, hal mana akan terus kami perbaiki dari waktu kewaktu.
Dalam hal Anda menemukan inakurasi informasi yang termuat pada situs ini atau informasi yang seharusnya ada, namun belum tersedia, maka harap segera hubungi Kepaniteraan Mahkamah Agung RI melalui :
Email : kepaniteraan@mahkamahagung.go.id   Telp : 021-384 3348 (ext.318)

tetap ingin bercerai dengan Tergugat karena upaya damai sudah tidak berhasil karena keadaan rumah tangga Penggugat dan Tergugat sebagaimana tersebut di atas, maka sudah ada bukti antara keduanya tidak ada harapan akan rukun lagi dalam rumah tangga.

Menimbang, bahwa dengan demikian, dalam perkara a quo Penggugat telah dapat membuktikan dalil gugatan cerainya, ditemukan fakta hukum telah terjadi perselisihan dan pertengkaran antara Penggugat dengan Tergugat dan antara keduanya tidak ada harapan akan rukun lagi dalam rumah tangga.

Menimbang, bahwa dari fakta hukum tersebut, maka perkawinan antara Penggugat dengan Tergugat tersebut telah pecah, tidak ada lagi ikatan batin antara keduanya, sehingga substansi perkawinan sebagaimana Pasal 1 Undang-Undang Nomor 1 Tahun 1974 yaitu ikatan lahir batin dengan tujuan membentuk keluarga yang bahagia dan kekal serta tujuan perkawinan sebagaimana dikehendaki Pasal 3 Kompilasi Hukum Islam yaitu untuk mewujudkan kehidupan rumah tangga yang sakinah mawaddah warahmah (bahagia dan sejahtera) tidak dapat diwujudkan lagi. Jika kehidupan bahagia atau sakinah mawaddah dan rahmah tidak terwujud dalam suatu rumah tangga, maka hanya akan menimbulkan *mafsadat* (kerusakan) yang lebih besar, terutama bagi para pihak berperkara. Hukum Islam menentukan bahwa menolak *mafsadat* harus didahulukan dari mengharap kemaslahatan, sesuai dengan kaidah *fiqhiyah*, sebagai berikut:

درء المفاسد مقدم على جلب المصالح

artinya: “Menghindari *mafsadat* (kerusakan) harus didahulukan dari pada menarik kemaslahatan”.

Menimbang, bahwa berdasarkan kaedah *fiqhiyah* tersebut, untuk mengakhiri *mafsadat* yang lebih besar di antara Penggugat dan Tergugat, Majelis menilai bahwa perceraian sebagai solusi terbaik bagi Penggugat dan Tergugat.

Menimbang bahwa berdasarkan kesimpulan Penggugat di depan persidangan, yakni Penggugat berketetapan hati ingin bercerai dengan

Disclaimer
Kepaniteraan Mahkamah Agung Republik Indonesia berusaha untuk selalu mencantumkan informasi paling kini dan akurat sebagai bentuk komitmen Mahkamah Agung untuk pelayanan publik, transparansi dan akuntabilitas pelaksanaan fungsi peradilan. Namun dalam hal-hal tertentu masih dimungkinkan terjadi permasalahan teknis terkait dengan akurasi dan keterkinian informasi yang kami sajikan, hal mana akan terus kami perbaiki dari waktu kewaktu.
Dalam hal Anda menemukan inakurasi informasi yang termuat pada situs ini atau informasi yang seharusnya ada, namun belum tersedia, maka harap segera hubungi Kepaniteraan Mahkamah Agung RI melalui :
Email : kepaniteraan@mahkamahagung.go.id Telp : 021-384 3348 (ext.318)

Tergugat dan Penggugat dalam keadaan sehat jasmani dan rohani, maka dalam hal ini Majelis Hakim sependapat sekaligus mengambil alih pendapat pakar hukum Islam sebagaimana yang termuat dalam kitab *Al Iqna* Juz II halaman 133 sebagai berikut :

وان اشتد عدم رغبة الزوجة لزوجها طلق عليه القاضى طلقة

Artinya:

Apabila ketidaksenangan seorang isteri kepada suaminya telah mencapai puncaknya, maka pada saat itu hakim diperbolehkan menjatuhkan talak seorang suami kepada istrinya dengan talak satu".

Menimbang, bahwa meskipun perselisihan dan pertengkaran antara Penggugat dan Tergugat tidak terbukti terjadi secara terus menerus, akan tetapi telah mengakibatkan antara keduanya sudah tidak ada harapan akan rukun lagi dalam rumah tangga, maka telah sesuai dengan ketantuan Pasal 39 ayat (1) dan (2) Undang-Undang Nomor 1 Tahun1974 jo. Pasal 19 huruf f Peraturan Pemerintah Nomor 9 Tahun 1975 dan Pasal 116 huruf f Kompilasi Hukum Islam. Hal ini sesuai dengan kaedah hukum Yurisprudensi sebagaimana Putusan Mahkamah Agung Republik Indonesia Nomor 237 K/AG/1998 bahwa perceraian dapat dikabulkan apabila telah memenuhi ketentuan Pasal 19 huruf f Peraturan Pemerintah Nomor 9 Tahun 1975.

Menimbang, bahwa berdasarkan pertimbangan-pertimbangan tersebut di atas, karena ternyata Tergugat meskipun telah dipanggil dengan patut akan tetapi tidak pernah datang menghadap di persidangan dan tidak pula ternyata bahwa tidak datangnya itu disebabkan sesuatu halangan yang sah, serta gugatan Penggugat tidak melawan hukum dan cukup beralasan, maka sesuai dengan ketentuan Pasal 126 H.I.R. Tergugat yang dipanggil dengan patut akan tetapi tidak datang menghadap harus dinyatakan tidak hadir dan gugatan pada petitum angka 2 tersebut dapat dikabulkan dengan verstek. Oleh karena Penggugat dan Tergugat sebelumnya belum pernah bercerai dan talak yang dijatuhkan dalam perkara ini adalah talak yang dijatuhkan Pengadilan Agama, sesuai ketentuan Pasal 119 ayat 2 huruf c Kompilasi Hukum Islam, maka

Disclaimer
Kepaniteraan Mahkamah Agung Republik Indonesia berusaha untuk selalu mencantumkan informasi paling kini dan akurat sebagai bentuk komitmen Mahkamah Agung untuk pelayanan publik, transparansi dan akuntabilitas pelaksanaan fungsi peradilan. Namun dalam hal-hal tertentu masih dimungkinkan terjadi permasalahan teknis terkait dengan akurasi dan keterkinian informasi yang kami sajikan, hal mana akan terus kami perbaiki dari waktu kewaktu.
Dalam hal Anda menemukan inakurasi informasi yang termuat pada situs ini atau informasi yang seharusnya ada, namun belum tersedia, maka harap segera hubungi Kepaniteraan Mahkamah Agung RI melalui :
Email : kepaniteraan@mahkamahagung.go.id Telp : 021-384 3348 (ext.318)

Pengadilan menjatuhkan talak satu *bain shughra* dari Tergugat kepada Penggugat.

Menimbang, bahwa karena perkara a quo masuk bidang perkawinan, maka berdasarkan Pasal 89 ayat (1) Undang-Undang Nomor 7 Tahun 1989 sebagaimana telah diubah dengan Undang-Undang Nomor 3 Tahun 2006 dan perubahan kedua dengan Undang-Undang Nomor 50 Tahun 2009, biaya perkara harus dibebankan kepada Penggugat.

Mengingat, semua pasal dalam peraturan perundang-undangan dan hukum Islam yang berkaitan dengan perkara ini.

M E N G A D I L I

1. Menyatakan, Tergugat yang telah dipanggil secara resmi dan patut untuk menghadap di persidangan, tidak hadir.
2. Mengabulkan gugatan Penggugat dengan verstek.
3. Menjatuhkan talak satu *bain shughra* Tergugat terhadap Penggugat.
4. Membebankan kepada Penggugat untuk membayar biaya perkara ini sejumlah Rp 51.000,00 (empat ratus delapan puluh satu ribu rupiah).

Demikian diputuskan dalam rapat permusyawaratan Majelis Hakim Pengadilan Agama Jakarta Timur pada hari Rabu tanggal 23 Desember 2020 Masehi bertepatan dengan tanggal 8 Jumadil Awwal 1442 Hijriyah, oleh kami Drs. Azhar Mayang, M.H.I. sebagai Ketua Majelis, Drs. Salwi, S.H. dan Hj. Ira Puspita Sari, S.H., M.H. dan masing-masing sebagai Hakim Anggota, putusan tersebut diucapkan dalam sidang terbuka untuk umum pada hari Rabu tanggal 23 Desember 2020 Masehi bertepatan dengan tanggal 8 Jumadil Awwal 1442 Hijriyah oleh Ketua Majelis tersebut dengan didampingi Hakim-Hakim Anggota dan dibantu oleh Nur Holia, S.H., M.H. sebagai Panitera Pengganti serta dihadiri oleh Penggugat tanpa hadirnya Tergugat.

Ketua Majelis

Drs. Azhar Mayang, M.H.I.

Disclaimer
Kepaniteraan Mahkamah Agung Republik Indonesia berusaha untuk selalu mencantumkan informasi paling kini dan akurat sebagai bentuk komitmen Mahkamah Agung untuk pelayanan publik, transparansi dan akuntabilitas pelaksanaan fungsi peradilan. Namun dalam hal-hal tertentu masih dimungkinkan terjadi permasalahan teknis terkait dengan akurasi dan keterkinian informasi yang kami sajikan, hal mana akan terus kami perbaiki dari waktu kewaktu.
Dalam hal Anda menemukan inakurasi informasi yang termuat pada situs ini atau informasi yang seharusnya ada, namun belum tersedia, maka harap segera hubungi Kepaniteraan Mahkamah Agung RI melalui :
Email : kepaniteraan@mahkamahagung.go.id   Telp : 021-384 3348 (ext.318)

Hakim Anggota	Hakim Anggota
Drs. Salwi, S.H.	Hj. Ira Puspita Sari, S.H., M.H.

Panitera Pengganti,

Nur Holia, S.H., M.H.

Perincian biaya :

1. Pendaftaran	:	Rp	30.000,00
2. Proses	:	Rp	75.000,00
3. Panggilan	:	Rp	350.000,00
4. PNBP Panggilan	:	Rp	10.000,00
5. Redaksi	:	Rp	10.000,00
6. Meterai	:	Rp	6.000,00
Jumlah	:	Rp	481.000,00

(empat ratus delapan puluh satu ribu rupiah)

Disclaimer
Kepaniteraan Mahkamah Agung Republik Indonesia berusaha untuk selalu mencantumkan informasi paling kini dan akurat sebagai bentuk komitmen Mahkamah Agung untuk pelayanan publik, transparansi dan akuntabilitas pelaksanaan fungsi peradilan. Namun dalam hal-hal tertentu masih dimungkinkan terjadi permasalahan teknis terkait dengan akurasi dan keterkinian informasi yang kami sajikan, hal mana akan terus kami perbaiki dari waktu kewaktu.
Dalam hal Anda menemukan inakurasi informasi yang termuat pada situs ini atau informasi yang seharusnya ada, namun belum tersedia, maka harap segera hubungi Kepaniteraan Mahkamah Agung RI melalui :
Email : kepaniteraan@mahkamahagung.go.id   Telp : 021-384 3348 (ext.318)
