# Supplementary material for: Classification of divorce causes during the COVID-19 pandemic using convolutional neural networks
Source: PeerJ Comput Sci. 2022 Jun 30;8:e998. doi: 10.7717/peerj-cs.998 (PMC9299239; doi:10.7717/peerj-cs.998)
Supplement: Supplemental Information 3 [file peerj-cs-08-998-s003.zip › KDRT Dataset/Data ke-27.pdf]

P U T U S A N

Nomor 832/Pdt.G/2020/PA.Blk

بِسْمِ اللَّهِ الرَّحْمَنِ الرَّحِيمِ

DEMI KEADILAN BERDASARKAN KETUHANAN YANG MAHA ESA

Pengadilan Agama Bulukumba yang memeriksa dan mengadili perkara tertentu pada tingkat pertama dalam sidang, Majelis Hakim telah menjatuhkan putusan sebagai berikut dalam perkara Cerai Gugat antara:

PENGGUGAT, NIK 7302045502750004, umur 45 tahun, agama Islam, pendidikan SLTA, pekerjaan xxxxxxxx xxxxxxx, tempat kediaman di KABUPATEN BULUKUMBA,, sebagai Penggugat;

melawan

TERGUGAT, umur 47 tahun, agama Islam, pendidikan SD, pekerjaan xxxxxx, tempat kediaman di KABUPATEN BULUKUMB, sebagai Tergugat;

Pengadilan Agama tersebut;
Telah membaca dan mempelajari berkas perkara;
Telah mendengar keterangan Penggugat;
Telah memeriksa alat-alat bukti Penggugat;

DUDUK PERKARA

Menimbang, bahwa Penggugat dengan surat gugatannya tertanggal 04 Desember 2020 yang terdaftar di Kepaniteraan Pengadilan Agama Bulukumba pada hari itu juga dengan register perkara Nomor 832/Pdt.G/2020/PA.Blk, mengemukakan hal-hal sebagai berikut:

1. Bahwa Penggugat dan Tergugat adalah suami isteri sah yang menikah pada hari Senin, 4 September 1995, di KABUPATEN BULUKUMBA, berdasarkan Kutipan Akta Nikah Nomor: 107/18/IX/1995 tanggal 29 September 1995 yang dikeluarkan oleh Pegawai Pencatat Nikah Kantor Urusan Agama xxxxxxxxx xxxxx xxxx, xxxxxxxxx xxxxxxxxx;
2. Bahwa setelah menikah Penggugat dan Tergugat tinggal di rumah orang tua Tergugat di KABUPATEN BULUKUMBa selama kurang lebih 2 tahun, Kemudian tinggal dirumah perusahaan di Malaysia selama kurang lebih 15 tahun, kemudian pindah dirumah bersama di KABUPATEN BULUKUMBa selama kurang lebih 7 tahun dan telah dikaruniai 3 orang anak yang bernama,

Disclaimer
Kepaniteraan Mahkamah Agung Republik Indonesia berusaha untuk selalu mencantumkan informasi paling kini dan akurat sebagai bentuk komitmen Mahkamah Agung untuk pelayanan publik, transparansi dan akuntabilitas pelaksanaan fungsi peradilan. Namun dalam hal-hal tertentu masih dimungkinkan terjadi permasalahan teknis terkait dengan akurasi dan keterkinian informasi yang kami sajikan, hal mana akan terus kami perbaiki dari waktu kewaktu.
Dalam hal Anda menemukan inakurasi informasi yang termuat pada situs ini atau informasi yang seharusnya ada, namun belum tersedia, maka harap segera hubungi Kepaniteraan Mahkamah Agung RI melalui :
Email : kepaniteraan@mahkamahagung.go.id   Telp : 021-384 3348 (ext.318)

xxxxxxxxxxx, perempuan 24 tahun, xxxxxxxxxxxxxx, perempuan, 19 tahun, xxxxxxxxxxxxxxxxx laki-laki, 15 tahun, dan anak tersebut berada dalam asuhan Penggugat;

3. Bahwa keadaan rumah tangga Penggugat dan Tergugat semula berjalan rukun dan baik, tetapi sejak bulan Oktober 1996 mulai muncul perselisihan dan pertengkaran yang mengakibatkan hubungan Penggugat dan Tergugat pada akhirnya menjadi tidak harmonis lagi;

4. Bahwa perselisihan antara Penggugat dan Tergugat pada intinya disebabkan karena :

a. Tergugat sering marah-marah tidak jelas dan kurang memberikan nafkah kepada Penggugat;

b. Tergugat sering melakukan KDRT kepada Penggugat;

5. Bahwa, puncak perselisihan dan pertengkaran terjadi sejak bulan Juni 2020 di mana pada saat itu Penggugat dan Tergugat sedang nonton bersama dengan anaknya, kemudian tanpa alasan yang jelas Tergugat tiba-tiba marah tidak jelas, sehingga Penggugat dan Tergugat bertengkar, karena merasa tidak tahan dengan sikap Tergugat yang sering cemburuan tidak jelas, sehingga sejak kejadian itu Penggugat pergi meninggalkan Tergugat dan tidak pernah lagi kembali sampai sekarang;

6. Bahwa sejak kejadian itu Penggugat dan Tergugat berpisah tempat tinggal dan tidak pernah lagi hidup bersama sebagai suami istri sampai sekarang yang sudah berlangsung selama kurang lebih 6 bulan;

7. Bahwa kondisi rumah tangga Penggugat dan Tergugat sudah sedemikian parahnya, sehingga dengan cara apapun, Penggugat dan Tergugat tidak mungkin lagi hidup bersama sebagai suami istri dan solusi yang terbaik bagi Penggugat adalah bercerai dengan Tergugat;

Berdasarkan alasan-alasan tersebut, Penggugat mohon kepada Ketua Pengadilan Agama Bulukumba Cq. Majelis Hakim yang memeriksa dan mengadili perkara ini, berkenan menjatuhkan putusan sebagai berikut :

Primer

1. Mengabulkan gugatan Penggugat;
2. Menjatuhkan talak satu ba'in shughra Tergugat **TERGUGAT**, terhadap Penggugat **PENGGUGAT;**
3. Membebankan biaya perkara sesuai dengan ketentuan hukum yang berlaku;

Disclaimer
Kepaniteraan Mahkamah Agung Republik Indonesia berusaha untuk selalu mencantumkan informasi paling kini dan akurat sebagai bentuk komitmen Mahkamah Agung untuk pelayanan publik, transparansi dan akuntabilitas pelaksanaan fungsi peradilan. Namun dalam hal-hal tertentu masih dimungkinkan terjadi permasalahan teknis terkait dengan akurasi dan keterkinian informasi yang kami sajikan, hal mana akan terus kami perbaiki dari waktu kewaktu.
Dalam hal Anda menemukan inakurasi informasi yang termuat pada situs ini atau informasi yang seharusnya ada, namun belum tersedia, maka harap segera hubungi Kepaniteraan Mahkamah Agung RI melalui :
Email : kepaniteraan@mahkamahagung.go.id Telp : 021-384 3348 (ext.318)

Subsider

Jika Majelis Hakim berpendapat lain mohon putusan yang seadil-adilnya;

Bahwa pada hari-hari sidang yang telah ditetapkan, Penggugat datang menghadap sendiri di persidangan, sedangkan Tergugat tidak pernah datang menghadap dan tidak pula menyuruh orang lain untuk menghadap sebagai wakil atau kuasanya yang sah, meskipun telah dipanggil secara resmi dan patut dan tidak ternyata ketidakhadirannya tersebut disebabkan oleh suatu halangan yang sah, maka perkara ini diperiksa tanpa hadirnya Tergugat;

Bahwa upaya perdamaian dan mediasi tidak dapat dilaksanakan karena Tergugat tidak pernah hadir di persidangan;

Bahwa selanjutnya pemeriksaan perkara dilakukan dalam sidang tertutup untuk umum dengan terlebih dahulu dibacakan surat gugatan Penggugat yang isi dan maksudnya tetap dipertahankan oleh Penggugat;

Bahwa oleh karena Tergugat atau kuasanya yang sah tidak pernah hadir di persidangan, maka jawaban Tergugat atas gugatan Penggugat tidak dapat didengarkan;

Bahwa untuk menguatkan dalil-dalil gugatannya, Penggugat mengajukan alat bukti surat dan saksi-saksi sebagai berikut :

1. **Bukti Surat.**

   Fotokopi Kutipan Akta Nikah dari Kantor Urusan Agama xxxxxxxxx xxxxx xxxx, xxxxxxxxx xxxxxxxxx, Nomor 107/18/IX/1995 Tanggal 29 September 1995, bukti surat tersebut telah diberi meterai cukup, telah dicap pos dan telah dicocokkan dengan aslinya yang ternyata sesuai, lalu oleh Ketua Majelis diberi tanda P1;

2. **Bukti Saksi.**

   Saksi 1, **SAKSI 1**, umur 43 tahun, agama Islam, pendidikan SLTP, pekerjaan xxx xxxxx xxxxxx, bertempat tinggal di KABUPATEN BULUKUMBA, di bawah sumpah memberikan keterangan yang pada pokoknya sebagai berikut :

   - Bahwa saksi mengenal Penggugat dan Tergugat karena saksi adalah sepupu Penggugat;
   - Bahwa saksi megetahui Penggugat dan Tergugat adalah suami istri sah, setelah menikah Penggugat dan Tergugat tinggal di rumah orang tua Tergugat selama kurang lebih 2 tahun, Kemudian tinggal dirumah

Disclaimer
Kepaniteraan Mahkamah Agung Republik Indonesia berusaha untuk selalu mencantumkan informasi paling kini dan akurat sebagai bentuk komitmen Mahkamah Agung untuk pelayanan publik, transparansi dan akuntabilitas pelaksanaan fungsi peradilan. Namun dalam hal-hal tertentu masih dimungkinkan terjadi permasalahan teknis terkait dengan akurasi dan keterkinian informasi yang kami sajikan, hal mana akan terus kami perbaiki dari waktu kewaktu.
Dalam hal Anda menemukan inakurasi informasi yang termuat pada situs ini atau informasi yang seharusnya ada, namun belum tersedia, maka harap segera hubungi Kepaniteraan Mahkamah Agung RI melalui :
Email : kepaniteraan@mahkamahagung.go.id   Telp : 021-384 3348 (ext.318)

perusahaan di Malaysia selama kurang lebih 15 tahun, kemudian pindah dirumah bersama selama kurang lebih 7 tahun telah dikaruniai 3 orang anak ;

- Bahwa saksi mengetahui rumah tangga Penggugat dan Tergugat pada awalnya rukun dan harmonis, namun sekarang sudah tidak harmonis lagi karena antara Penggugat dan Tergugat sering terjadi perselisihan dan pertengkaran ;
- Bahwa saksi mengetahui yang menjadi penyebab perselisihan dan pertengkaran Penggugat dan Tergugat adalah karena Tergugat sering marah-marah tidak jelas dan kurang memberikan nafkah kepada Penggugat dan Tergugat sering melakukan KDRT kepada Penggugat; ;
- Bahwa saksi mengetahui Penggugat dan Tergugat telah terjadi pisah selama 8 bulan selama itu sudah tidak ada komunikasi dan sudah tidak saling memperdulikan lagi ;
- Bahwa antara Penggugat dan Tergugat telah ada usaha untuk merukunkan, namun tidak berhasil ;

Saksi 2, **SAKSI 2**, umur 55 tahun, agama Islam, pendidikan SLTP, pekerjaan xxxxxxxx, bertempat tinggal di KABUPATEN BULUKUMBA, di bawah sumpah memberikan keterangan yang pada pokoknya sebagai berikut:

- Bahwa saksi mengenal Penggugat dan Tergugat karena saksi adalah Sepupu Penggugat ;
- Bahwa saksi megetahui Penggugat dan Tergugat adalah suami istri sah, setelah menikah Penggugat dan Tergugat tinggal di rumah orang tua Tergugat selama kurang lebih 2 tahun, Kemudian tinggal dirumah perusahaan di Malaysia selama kurang lebih 15 tahun, kemudian pindah dirumah bersama selama kurang lebih 7 tahun telah dikaruniai 3 orang anak ;
- Bahwa saksi mengetahui rumah tangga Penggugat dan Tergugat pada awalnya rukun dan harmonis, namun sekarang sudah tidak harmonis lagi karena antara Penggugat dan Tergugat sering terjadi perselisihan dan pertengkaran ;
- Bahwa saksi mengetahui yang menjadi penyebab perselisihan dan pertengkaran Penggugat dan Tergugat adalah karena Tergugat sering

Disclaimer
Kepaniteraan Mahkamah Agung Republik Indonesia berusaha untuk selalu mencantumkan informasi paling kini dan akurat sebagai bentuk komitmen Mahkamah Agung untuk pelayanan publik, transparansi dan akuntabilitas pelaksanaan fungsi peradilan. Namun dalam hal-hal tertentu masih dimungkinkan terjadi permasalahan teknis terkait dengan akurasi dan keterkinian informasi yang kami sajikan, hal mana akan terus kami perbaiki dari waktu kewaktu.
Dalam hal Anda menemukan inakurasi informasi yang termuat pada situs ini atau informasi yang seharusnya ada, namun belum tersedia, maka harap segera hubungi Kepaniteraan Mahkamah Agung RI melalui :
Email : kepaniteraan@mahkamahagung.go.id Telp : 021-384 3348 (ext.318)

marah-marah tidak jelas dan kurang memberikan nafkah kepada Penggugat dan Tergugat sering melakukan KDRT kepada Penggugat; ;

- Bahwa saksi mengetahui Penggugat dan Tergugat telah terjadi pisah selama 8 bulan selama itu sudah tidak ada komunikasi dan sudah tidak saling memperdulikan lagi ;
- Bahwa antara Penggugat dan Tergugat telah ada usaha untuk merukunkan, namun tidak berhasil ;

Bahwa atas keterangan para saksi tersebut, Penggugat menyatakan menerima dan membenarkan, sedangkan Tergugat tidak dapat didengarkan tanggapannya;

Bahwa selanjutnya Penggugat atas pertanyaan Majelis Hakim menyatakan dalam keadaan Suci dan Penggugat menyampaikan kesimpulan secara lisan yang pada pokoknya tetap pada gugatan dan mohon putusan;

Bahwa untuk mempersingkat uraian putusan ini, ditunjuk segala sesuatu yang tercantum dalam berita acara persidangan perkara ini sebagai bagian yang tidak terpisahkan dari isi putusan ini;

PERTIMBANGAN HUKUM

Menimbang, bahwa maksud dan tujuan gugatan Penggugat adalah sebagaimana telah diuraikan di atas;

Menimbang, bahwa selama persidangan hanya Penggugat yang selalu hadir sedangkan Tergugat tidak pernah hadir, maka upaya mediasi sebagaimana ketentuan Peraturan Mahkamah Agung RI Nomor 1 Tahun 2008 yang telah diubah dengan Peraturan Mahkamah Agung RI Nomor 1 Tahun 2016 tentang Prosedur Mediasi di Pengadilan, tidak dapat dilaksanakan;

Menimbang, bahwa oleh karena Tergugat tidak pernah hadir di persidangan meskipun telah dipanggil secara resmi dan patut dan tidak ternyata tidak hadirnya tersebut disebabkan oleh suatu halangan yang sah, maka Tergugat harus dinyatakan tidak hadir, dan perkara ini dilanjutkan tanpa hadirnya Tergugat;

Menimbang, bahwa oleh karena Tergugat telah dinyatakan tidak hadir, dan gugatan Penggugat mempunyai alasan serta tidak bertentangan dengan hukum, maka berdasarkan ketentuan Pasal 149 Ayat (1) R.Bg., gugatan Penggugat dapat diperiksa dan diputus secara verstek;

Menimbang, bahwa alasan pokok Penggugat mengajukan gugatan cerai adalah bahwa Tergugat sering marah-marah tidak jelas dan kurang memberikan

Disclaimer
Kepaniteraan Mahkamah Agung Republik Indonesia berusaha untuk selalu mencantumkan informasi paling kini dan akurat sebagai bentuk komitmen Mahkamah Agung untuk pelayanan publik, transparansi dan akuntabilitas pelaksanaan fungsi peradilan. Namun dalam hal-hal tertentu masih dimungkinkan terjadi permasalahan teknis terkait dengan akurasi dan keterkinian informasi yang kami sajikan, hal mana akan terus kami perbaiki dari waktu kewaktu.
Dalam hal Anda menemukan inakurasi informasi yang termuat pada situs ini atau informasi yang seharusnya ada, namun belum tersedia, maka harap segera hubungi Kepaniteraan Mahkamah Agung RI melalui :
Email : kepaniteraan@mahkamahagung.go.id Telp : 021-384 3348 (ext.318)

nafkah kepada Penggugat dan Tergugat sering melakukan KDRT kepada Penggugat; yang akibatnya antara Penggugat dan Tergugat terjadi pisah tempat tinggal selama 8 bulan dengan tanpa ada komunikasi da sudah tidak saling memperdulikan lagi, sehingga Penggugat tidak sanggup berumah tangga dengan Tergugat dan ingin mengakhirinya dengan jalan perceraian;

Menimbang, bahwa meskipun Tergugat dianggap mengakui atau setidak-tidaknya tidak membantah dalil-dalil gugatan Penggugat karena Tergugat tidak pernah hadir di persidangan, namun karena perkara ini perkara perceraian, maka Penggugat tetap diwajibkan untuk membuktikan alasan-alasan perceraiannya dengan mengajukan alat-alat bukti yang cukup;

Menimbang, bahwa Penggugat telah mengajukan bukti P.1 berupa fotokopi Kutipan Akta Nikah yang dikeluarkan oleh Pejabat yang berwenang sebagai akta autentik, bermeterai cukup, telah dicap pos (nazegelen) dan sesuai dengan aslinya, maka alat bukti tersebut telah memenuhi syarat formil, isi bukti tersebut menerangkan bahwa Penggugat telah menikah dengan Tergugat pada tanggal 04 September 1995, alat bukti tersebut adalah akta otentik mempunyai kekuatan pembuktian yang sempurna dan mengikat;

Menimbang, bahwa berdasarkan bukti P.1 tersebut harus dinyatakan terbukti bahwa Penggugat dengan Tergugat adalah suami istri sah, hal tersebut sesuai ketentuan pasal 7 ayat (1) Kompilasi Hukum Islam;

Menimbang, bahwa Penggugat juga telah mengajukan saksi-saksi yaitu: SAKSI 1 dan SAKSI 2, keduanya telah memberikan keterangan sebagaimana telah diuraikan dalam duduk perkara;

Menimbang, bahwa kedua saksi tersebut adalah orang dewasa yang memberikan keterangan secara terpisah di bawah sumpah di muka sidang, keterangan kedua saksi secara materil saling bersesuaian satu sama lain dan relevan dengan dalil-dalil yang hendak dibuktikan oleh Penggugat serta tidak ada halangan diterimanya kesaksian para saksi tersebut, maka kedua saksi tersebut telah memenuhi syarat formil dan materil sehingga keterangan kedua saksi tersebut memiliki kekuatan pembuktian yang dapat diterima;

Menimbang, bahwa berdasarkan keterangan Penggugat, bukti tertulis dan keterangan para saksi, Majelis Hakim telah menemukan fakta-fakta hukum yang disimpulkan sebagai berikut:

- Bahwa Penggugat dengan Tergugat adalah suami istri sah;

Disclaimer
Kepaniteraan Mahkamah Agung Republik Indonesia berusaha untuk selalu mencantumkan informasi paling kini dan akurat sebagai bentuk komitmen Mahkamah Agung untuk pelayanan publik, transparansi dan akuntabilitas pelaksanaan fungsi peradilan. Namun dalam hal-hal tertentu masih dimungkinkan terjadi permasalahan teknis terkait dengan akurasi dan keterkinian informasi yang kami sajikan, hal mana akan terus kami perbaiki dari waktu kewaktu.
Dalam hal Anda menemukan inakurasi informasi yang termuat pada situs ini atau informasi yang seharusnya ada, namun belum tersedia, maka harap segera hubungi Kepaniteraan Mahkamah Agung RI melalui :
Email : kepaniteraan@mahkamahagung.go.id Telp : 021-384 3348 (ext.318)

- Bahwa Penggugat dengan Tergugat pernah hidup rukun dan telah dikaruniai 3 orang anak;
- Bahwa rumah tangga Penggugat dan Tergugat yang semula rukun dan harmonis, namun kemudian sering terjadi perselisihan dan pertengkaran yang disebabkan Tergugat sering marah-marah tidak jelas dan kurang memberikan nafkah kepada Penggugat dan Tergugat sering melakukan KDRT kepada Penggugat; ;
- Bahwa akibat pertengkaran tersebut antara Penggugat dan Tergugat telah terjadi pisah tempat tinggal selama 8 bulan selama itu tidak ada komunikasi dan sudah tidak saling memperdulikan lagi ;
- Bahwa antara Penggugat dan Tergugat telah didamaikan namun tidak berhasil ;

Menimbang, bahwa berdasarkan fakta-fakta tersebut di atas, Majelis Hakim berpendapat bahwa antara Penggugat dan Tergugat telah terjadi keretakan ikatan batin sebagai suami istri, akibat perselisihan hingga terjadi pisah tempat tinggal yang sudah berlangsung selama 8 bulan, dan tidak ada harapan untuk kembali rukun karena keduanya sudah tidak saling mempedulikan bahkan Penggugat telah berketetapan hati untuk bercerai dengan Tergugat, yang tentunya rumah tangga seperti itu sudah tidak dapat diharapkan untuk merealisir tujuan perkawinan yaitu membentuk rumah tangga yang bahagia dan kekal, sesuai maksud Pasal 1 Undang-Undang Nomor 1 Tahun 1974 tentang Perkawinan, dan terwujudnya keluarga yang sakinah, mawaddah dan rahmah, sesuai maksud Pasal 3 Kompilasi Hukum Islam, bahkan dalam rumah tangga seperti itu, suami dan istri sudah tidak dapat menjalankan hak dan kewajibannya masing-masing, yang mana untuk itulah rumah tangga diadakan;

Menimbang, bahwa suatu ikatan pernikahan adalah dimaksudkan untuk memberikan kemaslahatan bagi suami maupun istri, tetapi dengan melihat kondisi rumah tangga Penggugat dengan Tergugat sebagaimana tersebut di atas, Majelis Hakim berpendapat bahwa rumah tangga seperti itu bukan saja tidak lagi mendatangkan kemaslahatan, tapi justru hanya akan memberikan penderitaan batin baik bagi Penggugat maupun Tergugat, karena itu perceraian dapat menjadi jalan keluar dari kemelut rumah tangga tersebut;

Menimbang, bahwa berdasarkan pertimbangan-pertimbangan tersebut di atas tanpa mempersoalkan siapa yang benar dan siapa yang salah, maka alasan-

Disclaimer
Kepaniteraan Mahkamah Agung Republik Indonesia berusaha untuk selalu mencantumkan informasi paling kini dan akurat sebagai bentuk komitmen Mahkamah Agung untuk pelayanan publik, transparansi dan akuntabilitas pelaksanaan fungsi peradilan. Namun dalam hal-hal tertentu masih dimungkinkan terjadi permasalahan teknis terkait dengan akurasi dan keterkinian informasi yang kami sajikan, hal mana akan terus kami perbaiki dari waktu kewaktu.
Dalam hal Anda menemukan inakurasi informasi yang termuat pada situs ini atau informasi yang seharusnya ada, namun belum tersedia, maka harap segera hubungi Kepaniteraan Mahkamah Agung RI melalui :
Email : kepaniteraan@mahkamahagung.go.id Telp : 021-384 3348 (ext.318)

alasan perceraian dalam perkara ini dianggap telah memenuhi ketentuan Pasal 19 huruf (f) Peraturan Pemerintah Nomor 9 Tahun 1975 jo Pasal 116 huruf (f) Kompilasi Hukum Islam, atau setidak-tidaknya gugatan Penggugat untuk bercerai dengan Tergugat telah memiliki cukup alasan, maka gugatan Penggugat patut dikabulkan dengan menjatuhkan talak satu bain sugra Tergugat terhadap Penggugat;

Menimbang, bahwa perceraian yang dijatuhkan oleh Pengadilan Agama Bulukumba adalah talak satu bain sugra, Penggugat dalam keadaan Suci dan sebagaimana maksud Pasal 119 ayat (1) Kompilasi Hukum Islam, bekas istri (Penggugat) meskipun dalam masa iddah tidak boleh rujuk dengan bekas suaminya (Tergugat), tetapi keduanya boleh melakukan akad nikah baru ;

Menimbang, bahwa oleh karena perkara ini termasuk bidang perkawinan, maka berdasarkan ketentuan pasal 89 ayat (1) Undang-Undang Nomor 7 Tahun 1989 sebagaimana telah diubah dengan Undang-Undang Nomor 3 Tahun 2006 dan Undang-Undang Nomor 50 Tahun 2009 tentang Peradilan Agama, biaya perkara dibebankan kepada Penggugat;

Memperhatikan pasal-pasal dari peraturan perundang-undangan yang berlaku dan hukum syara' yang berkenaan dengan perkara ini;

M E N G A D I L I

1. Menyatakan Tergugat telah dipanggil secara resmi dan patut untuk datang menghadap di persidangan, tidak hadir ;

2. Mengabulkan gugatan Penggugat, dengan verstek ;

3. Menjatuhkan talak satu ba'in sughra Tergugat (**TERGUGAT**) terhadap Penggugat (**PENGGUGAT**)

4. Membebankan biaya perkara kepada Penggugat sejumlah Rp566.000,00 (lima ratus enam puluh enam ribu rupiah);

Demikian diputuskan dalam musyawarah Majelis Hakim Pengadilan Agama Bulukumba pada hari Rabu tanggal 23 Desember 2020 Masehi bertepatan dengan tanggal 8 Jumadil Awwal 1442 Hijriah oleh M. Safi'i, S.Ag sebagai Ketua Majelis, Indriyani Nasir, SH dan Fadhliyatun Mahmudah, S.H.I, masing-masing sebagai Hakim Anggota, putusan tersebut diucapkan pada hari itu juga dalam sidang terbuka untuk umum oleh Ketua Majelis beserta para Hakim Anggota tersebut, dan didampingi oleh Nurwahidah, S.Ag sebagai Panitera Pengganti, dengan dihadiri oleh Penggugat tanpa hadirnya Tergugat.

Disclaimer
Kepaniteraan Mahkamah Agung Republik Indonesia berusaha untuk selalu mencantumkan informasi paling kini dan akurat sebagai bentuk komitmen Mahkamah Agung untuk pelayanan publik, transparansi dan akuntabilitas pelaksanaan fungsi peradilan. Namun dalam hal-hal tertentu masih dimungkinkan terjadi permasalahan teknis terkait dengan akurasi dan keterkinian informasi yang kami sajikan, hal mana akan terus kami perbaiki dari waktu kewaktu.
Dalam hal Anda menemukan inakurasi informasi yang termuat pada situs ini atau informasi yang seharusnya ada, namun belum tersedia, maka harap segera hubungi Kepaniteraan Mahkamah Agung RI melalui :
Email : kepaniteraan@mahkamahagung.go.id   Telp : 021-384 3348 (ext.318)

Hakim Anggota	Ketua Majelis,
**Indriyani Nasir, SH**	**M. Safi'i, S.Ag**
**Fadhliyatun Mahmudah, S.H.I**	
	Panitera Pengganti,
	**Nurwahidah, S.Ag**

Rincian Biaya Perkara :

No.	Uraian		Jumlah
1.	Biaya Pencatatan	Rp.	30.000,-
2.	Biaya Proses	Rp.	50.000,-
3.	Biaya Panggilan	Rp.	450.000,-
4.	Biaya PNBP Panggilan	Rp.	20.000,-
5.	Biaya Redaksi	Rp.	10.000,-
6.	Biaya Materai	Rp.	6.000,-
	Jumlah	Rp.	566.000,-

(lima ratus enam puluh enam ribu rupiah)

Disclaimer
Kepaniteraan Mahkamah Agung Republik Indonesia berusaha untuk selalu mencantumkan informasi paling kini dan akurat sebagai bentuk komitmen Mahkamah Agung untuk pelayanan publik, transparansi dan akuntabilitas pelaksanaan fungsi peradilan. Namun dalam hal-hal tertentu masih dimungkinkan terjadi permasalahan teknis terkait dengan akurasi dan keterkinian informasi yang kami sajikan, hal mana akan terus kami perbaiki dari waktu kewaktu.
Dalam hal Anda menemukan inakurasi informasi yang termuat pada situs ini atau informasi yang seharusnya ada, namun belum tersedia, maka harap segera hubungi Kepaniteraan Mahkamah Agung RI melalui :
Email : kepaniteraan@mahkamahagung.go.id   Telp : 021-384 3348 (ext.318)
