# Supplementary material for: Classification of divorce causes during the COVID-19 pandemic using convolutional neural networks
Source: PeerJ Comput Sci. 2022 Jun 30;8:e998. doi: 10.7717/peerj-cs.998 (PMC9299239; doi:10.7717/peerj-cs.998)
Supplement: Supplemental Information 3 [file peerj-cs-08-998-s003.zip › KDRT Dataset/Data ke-28.pdf]

P E N E T A P A N
Nomor 3043/Pdt.G/2020/PA.TA

بِسْمِ اللَّهِ الرَّحْمَنِ الرَّحِيمِ

DEMI KEADILAN BERDASARKAN KETUHANAN YANG MAHA ESA

Pengadilan Agama Tulungagung yang memeriksa dan mengadili perkara perdata pada tingkat pertama telah menjatuhkan putusan dalam perkara gugatan cerai antara :

Penggugat umur 39 tahun, agama Islam, pekerjaan Swasta, tempat tinggal di Kabupaten Tulungagung, sekarang di Taiwan , yang dalam hal ini dikuasakan kepada PURHADI, SH, Advokat yang beralamat di Desa Mergayu Kecamatan Bandung Kabupaten Tulungagung sebagai **Penggugat;**

MELAWAN

Tergugatumur 52 tahun, agama Islam, pekerjaan Swasta, tempat tinggal di Kabupaten Tulungagung , sebagai **Tergugat;**

Pengadilan Agama tersebut ;
Setelah membaca dan mempelajari berkas perkara;

TENTANG DUDUK PERKARA

Menimbang, bahwa Penggugat dengan surat gugatannya tertanggal 11 Desember 2020 yang telah terdaftar di kepaniteraan Pengadilan Agama Tulungagung Nomor 3043/Pdt.G/2020/PA.TA mengemukakan hal-hal sebagai berikut :

1. Bahwa pada tanggal 09 Februari 1998, Penggugat dengan Tergugat melangsungkan pernikahan yang dicatat oleh Pegawai Pencatat Nikah Kantor Urusan Agama kecamatan Kabupaten Tulungagung, sebagaimana Kutipan Akta Nikah Nomor 4xx/12/II/1998 tanggal 09 Februari 1998 ;
2. Bahwa setelah akad nikah antara Penggugat dan Tergugat hidup bersama di rumah orang tua tergugat selama kurang lebih 2 tahun, karena

Disclaimer
Kepaniteraan Mahkamah Agung Republik Indonesia berusaha untuk selalu mencantumkan informasi paling kini dan akurat sebagai bentuk komitmen Mahkamah Agung untuk pelayanan publik, transparansi dan akuntabilitas pelaksanaan fungsi peradilan. Namun dalam hal-hal tertentu masih dimungkinkan terjadi permasalahan teknis terkait dengan akurasi dan keterkinian informasi yang kami sajikan, hal mana akan terus kami perbaiki dari waktu kewaktu.
Dalam hal Anda menemukan inakurasi informasi yang termuat pada situs ini atau informasi yang seharusnya ada, namun belum tersedia, maka harap segera hubungi Kepaniteraan Mahkamah Agung RI melalui :
Email : kepaniteraan@mahkamahagung.go.id Telp : 021-384 3348 (ext.318)

faktor ekonomi kemudian penggugat menjadi TKW tahun 2001 s.d tahun 2014 (Jadi TKW 3 kali). Pernah cuti pada tahun 2004 dan 2008.

3. Bahwa dalam perkawinan mereka mempunyai mempunyai anak 2 bernama ANAK I PENGGUGAT DAN TERGUGAT umur kurang lebih 21 tahun dan ANAK II PENGGUGAT DAN TERGUGAT umur kurang lebih 11 tahun yang sekarang ikut orang tua penggugat.

4. Bahwa semula rumah tangga antara penggugat dan tergugat dijalani dalam keadaan rukun dan bahagia seperti keluarga pada umumnya, akan tetapi sejak TAHUN 2010 antara penggugat dan tergugat selalu terjadi pertengkaran terus menerus yang tidak dapat dirukunkan kembali, disebabkan karena: Faktor ekonomi, Tergugat kurang perhatian terhadap istri dan anak, lebih mementingkan keluarganya dan Tergugat bekerja semaunya.

5. Bahwa puncaknya terjadi pada tahun 2016 antara penggugat dan tergugat sudah tidak ada komunikasi layaknya suami istri pada umumnya sampai dengan sekarang kurang lebih 4 tahun lamanya.

6. Bahwa dengan demikian Penggugat menganggap bahwa Tergugat adalah seorang suami yang tidak baik, tidak setia, dan tidak bertanggung jawab.

7. Bahwa pihak keluarga sudah mencoba untuk merukunkan kembali, tetapi tidak berhasil.

8. Bahwa dengan demikian usaha penggugat untuk membangun rumah tangga yang bahagia dan sejahtera dengan tergugat tidak berhasil, oleh karena itu penggugat bermaksud mengakhiri perkawinan ini dengan perceraian.;

9. Bahwa Penggugat sanggup membayar seluruh biaya yang timbul akibat perkara ini;

Berdasarkan alasan/dalil-dalil diatas, Penggugat merasa sudah tidak sanggup lagi meneruskan hidup berumah tangga bersama Tergugat, oleh karena itu Penggugat mohon agar Ketua Pengadilan Agama Tulungagung

Disclaimer
Kepaniteraan Mahkamah Agung Republik Indonesia berusaha untuk selalu mencantumkan informasi paling kini dan akurat sebagai bentuk komitmen Mahkamah Agung untuk pelayanan publik, transparansi dan akuntabilitas pelaksanaan fungsi peradilan. Namun dalam hal-hal tertentu masih dimungkinkan terjadi permasalahan teknis terkait dengan akurasi dan keterkinian informasi yang kami sajikan, hal mana akan terus kami perbaiki dari waktu kewaktu.
Dalam hal Anda menemukan inakurasi informasi yang termuat pada situs ini atau informasi yang seharusnya ada, namun belum tersedia, maka harap segera hubungi Kepaniteraan Mahkamah Agung RI melalui :
Email : kepaniteraan@mahkamahagung.go.id Telp : 021-384 3348 (ext.318)

memeriksa dan mengadili perkara ini, selanjutnya menjatuhkan putusan yang amarnya berbunyi:

PRIMER :

1. Mengabulkan gugatan Penggugat ;
2. Menjatuhkan talak satu ba'in sughro Tergugat (Tergugat) terhadap Penggugat (Penggugat) ;
3. Membebankan biaya perkara kepada Penggugat menurut hukum yang berlaku ;

SUBSIDER:

Mohon putusan yang seadil-adilnya;

Bahwa pada hari sidang yang telah ditentukan, kuasa Penggugat datang menghadap dipersidangan, dan Tergugat tidak hadir dipersidangan dan tidak pula mengutus orang lain sebagai wakilnya karena Tergugat belum dipanggil secara sah dan patut;

Bahwa dalam persidangan kuasa Penggugat menyatakan mengurungkan niatnya untuk meneruskan perkaranya karena akan menentukan alamat Tergugat secara jelas dan pasti terlebih dahulu;

Bahwa selanjutnya kuasa Penggugat mengajukan pencabutan perkaranya di depan sidang Pengadilan Agama Tulungagung tertanggal 23 Desember 2020;

Bahwa untuk mempersingkat uraian dalam penetapan ini, Majelis Hakim cukup menunjuk kepada segala hal ihwal sebagaimana tercantum dalam Berita Acara Persidangan perkara ini yang harus dianggap ikut dipertimbangkan karena merupakan bagian tidak terpisahkan dari penetapan ini;

TENTANG PERTIMBANGAN HUKUM

Menimbang, bahwa gugatan Penggugat diajukan sesuai dengan peraturan perundang-undangan yang berlaku, karenanya formil dapat diterima;

Menimbang, bahwa maksud dan tujuan pencabutan gugatan Penggugat adalah sebagaimana diuraikan diatas;

Disclaimer
Kepaniteraan Mahkamah Agung Republik Indonesia berusaha untuk selalu mencantumkan informasi paling kini dan akurat sebagai bentuk komitmen Mahkamah Agung untuk pelayanan publik, transparansi dan akuntabilitas pelaksanaan fungsi peradilan. Namun dalam hal-hal tertentu masih dimungkinkan terjadi permasalahan teknis terkait dengan akurasi dan keterkinian informasi yang kami sajikan, hal mana akan terus kami perbaiki dari waktu kewaktu.
Dalam hal Anda menemukan inakurasi informasi yang termuat pada situs ini atau informasi yang seharusnya ada, namun belum tersedia, maka harap segera hubungi Kepaniteraan Mahkamah Agung RI melalui :
Email : kepaniteraan@mahkamahagung.go.id Telp : 021-384 3348 (ext.318)

Menimbang, bahwa Penggugat telah mengajukan pencabutan perkara pada Ketua Majelis pada tanggal 23 Desember 2020;

Menimbang, bahwa permohonan pencabutan perkara yang dilakukan oleh Penggugat dilaksanakan sebelum pemeriksaan pokok perkara, oleh karena itu tidak memerlukan persetujuan Tergugat maka pencabutan tersebut patut dikabulkan;

Menimbang, bahwa tentang biaya sebagaimana dalam petitum angka tiga (3), Majelis Hakim mempertimbangkan, bahwa perkara ini termasuk dalam lingkup bidang perkawinan, maka sesuai ketentuan Pasal 89 ayat (1) Undang-undang Nomor 7 Tahun 1989 tentang Peradilan Agama yang telah dirubah dengan Undang-Undang Nomor 3 Tahun 2006 dan perubahan kedua dengan Undang-Undang Nomor 50 Tahun 2009, maka biaya perkara dibebankan kepada Penggugat;

Mengingat, ketentuan-ketentuan peraturan perundang-undangan yang berlaku dan hukum syar'i yang berkaitan dengan perkara ini**;**

M E N E T A P K A N

1. Mengabulkan permohonan Penggugat untuk mencabut perkaranya;
2. Menyatakan perkara Nomor 3043/Pdt.G/2020/PA.TA tanggal 11 Desember 2020 telah selesai karena dicabut;
3. Membebankan kepada Penggugat untuk membayar biaya perkara sebesar Rp 231.000,00 (dua ratus tiga puluh satu ribu rupiah)

Demikian ditetapkan dalam rapat permusyawaratan Majelis Hakim yang dilangsungkan pada hari Rabu tanggal 23 Desember 2020 masehi bertepatan dengan tanggal 8 Jumadilawal 1442 hijriyah, oleh kami **Drs. H. Nuril Huda, M.H.** sebagai Ketua Majelis serta **Drs. H. Muqoddar, S.H.** dan **Drs. Jureimi Arief** masing-masing sebagai Hakim Anggota. Penetapan tersebut diucapkan pada hari itu juga dalam sidang terbuka untuk umum oleh Ketua Majelis tersebut dengan di dampingi oleh Hakim Anggota dan dibantu dibantu **Abdul**

Disclaimer
Kepaniteraan Mahkamah Agung Republik Indonesia berusaha untuk selalu mencantumkan informasi paling kini dan akurat sebagai bentuk komitmen Mahkamah Agung untuk pelayanan publik, transparansi dan akuntabilitas pelaksanaan fungsi peradilan. Namun dalam hal-hal tertentu masih dimungkinkan terjadi permasalahan teknis terkait dengan akurasi dan keterkinian informasi yang kami sajikan, hal mana akan terus kami perbaiki dari waktu kewaktu.
Dalam hal Anda menemukan inakurasi informasi yang termuat pada situs ini atau informasi yang seharusnya ada, namun belum tersedia, maka harap segera hubungi Kepaniteraan Mahkamah Agung RI melalui :
Email : kepaniteraan@mahkamahagung.go.id Telp : 021-384 3348 (ext.318)

Rachman, S.H. sebagai panitera pengganti dengan dihadiri oleh Penggugat dan Tergugat;

Ketua Majelis

Ttd.

Drs. H. Nuril Huda, M.H.

Hakim Anggota I	Hakim Anggota II
Ttd.	*Ttd.*
**Drs. H. Muqoddar, S.H.**	**Drs. Jureimi Arief**

Panitera Pengganti

Ttd.

Abdul Rachman, S.H.

Perincian Biaya Perkara :

1. Pendaftaran	*Rp.*	30.000,-
2. Biaya Panggilan	*Rp.*	0,-
3. Biaya Proses	*Rp.*	50.000,-
4. HHKL	*Rp.*	0.000,-
5. Materai Putusan	*Rp.*	6.000,-
6. Redaksi	*Rp.*	10.000,-
Jumlah	*Rp.*	231.000,-

(dua ratus tiga puluh satu ribu rupiah)

Disclaimer
Kepaniteraan Mahkamah Agung Republik Indonesia berusaha untuk selalu mencantumkan informasi paling kini dan akurat sebagai bentuk komitmen Mahkamah Agung untuk pelayanan publik, transparansi dan akuntabilitas pelaksanaan fungsi peradilan. Namun dalam hal-hal tertentu masih dimungkinkan terjadi permasalahan teknis terkait dengan akurasi dan keterkinian informasi yang kami sajikan, hal mana akan terus kami perbaiki dari waktu kewaktu.
Dalam hal Anda menemukan inakurasi informasi yang termuat pada situs ini atau informasi yang seharusnya ada, namun belum tersedia, maka harap segera hubungi Kepaniteraan Mahkamah Agung RI melalui :
Email : kepaniteraan@mahkamahagung.go.id Telp : 021-384 3348 (ext.318)
