# Supplementary material for: Classification of divorce causes during the COVID-19 pandemic using convolutional neural networks
Source: PeerJ Comput Sci. 2022 Jun 30;8:e998. doi: 10.7717/peerj-cs.998 (PMC9299239; doi:10.7717/peerj-cs.998)
Supplement: Supplemental Information 3 [file peerj-cs-08-998-s003.zip › KDRT Dataset/Data ke-29.pdf]

{\rtf1\ansi\ansicpg1256\uc1\deff1{\fonttbl
{\f0\fswiss\fcharset178\fprq2 Arial;}
{\f1\froman\fcharset0\fprq2 Times New Roman;}
{\f2\fswiss\fcharset0\fprq2 Calibri;}
{\f3\fswiss\fcharset0\fprq2 Segoe UI;}
{\f4\fscript\fcharset0\fprq2 Lucida Calligraphy;}
{\f5\fswiss\fcharset0\fprq2 Arial;}
{\f6\froman\fcharset2\fprq2 Symbol;}}
{\colortbl;\red0\green0\blue0;}
{\stylesheet{\s0\itap0\widctlpar\f1\fs24 Normal;}{*\cs10\additive Default Paragraph Font;}{\s16\itap0\nowidctlpar\f0\fs24 [Normal];}
{\s17\itap0\widctlpar\tqc\tx4153\tqr\tx8306\f1\fs24\sbasedon0 footer;}
{\s18\itap0\widctlpar\qc\f2\fs24\b\sbasedon0 Title;}
{\s19\itap0\widctlpar\li360\sa120\sl480\slmult1\f2\fs24\sbasedon0 Body Text Indent 2;}
{\s20\itap0\widctlpar\qc\li720\sa200\sl276\slmult1\f2\fs22\sbasedon0 List Paragraph;}{\s21\itap0\widctlpar\tqc\tx4153\tqr\tx8306\f1\fs24\sbasedon0 header;}{\s22\itap0\widctlpar\f3\fs18\sbasedon0 Balloon Text;}
{\s23\itap0\widctlpar\f2\fs22\sbasedon16 No Spacing;}
{*\cs24\additive\sbasedon10 Footer Char;}{*\cs25\additive\sbasedon10 Header Char;}{*\cs26\f3\fs18\additive\sbasedon10 Balloon Text Char;}
{*\cs27\fs24\additive\sbasedon10 Body Text Indent 2 Char;}
{*\cs28\fs24\b\additive\sbasedon10 Title Char;}}
{*\listtable{\list\listtemplateid1{\listlevel\levelnfc0\leveljc0\levelfollow0\levelstartat1\levelspace0\levelindent0{\leveltext\'02\'00.;}
{\levelnumbers\'01;}\f5\fs24\b0\i0\strike0\ul0\cf0\cb0\fi-360\li360}
{\listlevel\levelnfc0\leveljc0\levelfollow0\levelstartat1\levelspace0\levelindent0{\leveltext\'02\'01.;}
{\levelnumbers\'01;}\f5\fs24\b0\i0\strike0\ul0\cf0\cb0\fi-360\li720}
{\listlevel\levelnfc0\leveljc0\levelfollow0\levelstartat1\levelspace0\levelindent0{\leveltext\'02\'02.;}
{\levelnumbers\'01;}\f5\fs24\b0\i0\strike0\ul0\cf0\cb0\fi-360\li1080}
{\listlevel\levelnfc0\leveljc0\levelfollow0\levelstartat1\levelspace0\levelindent0{\leveltext\'02\'03.;}
{\levelnumbers\'01;}\f5\fs24\b0\i0\strike0\ul0\cf0\cb0\fi-284\li284}
{\listlevel\levelnfc0\leveljc0\levelfollow0\levelstartat1\levelspace0\levelindent0{\leveltext\'02\'04.;}
{\levelnumbers\'01;}\f5\fs24\b0\i0\strike0\ul0\cf0\cb0\fi-284\li644}
{\listlevel\levelnfc0\leveljc0\levelfollow0\levelstartat1\levelspace0\levelindent0{\leveltext\'02\'05.;}
{\levelnumbers\'01;}\f5\fs24\b0\i0\strike0\ul0\cf0\cb0\fi-284\li1004}
{\listlevel\levelnfc0\leveljc0\levelfollow0\levelstartat1\levelspace0\levelindent0{\leveltext\'02\'06.;}
{\levelnumbers\'01;}\f5\fs24\b0\i0\strike0\ul0\cf0\cb0\fi-284\li1364}
{\listlevel\levelnfc0\leveljc0\levelfollow0\levelstartat1\levelspace0\levelindent0{\leveltext\'02\'07.;}

Disclaimer
Kepaniteraan Mahkamah Agung Republik Indonesia berusaha untuk selalu mencantumkan informasi paling kini dan akurat sebagai bentuk komitmen Mahkamah Agung untuk pelayanan publik, transparansi dan akuntabilitas pelaksanaan fungsi peradilan. Namun dalam hal-hal tertentu masih dimungkinkan terjadi permasalahan teknis terkait dengan akurasi dan keterkinian informasi yang kami sajikan, hal mana akan terus kami perbaiki dari waktu kewaktu.
Dalam hal Anda menemukan inakurasi informasi yang termuat pada situs ini atau informasi yang seharusnya ada, namun belum tersedia, maka harap segera hubungi Kepaniteraan Mahkamah Agung RI melalui :
Email : kepaniteraan@mahkamahagung.go.id   Telp : 021-384 3348 (ext.318)

{\levelnumbers\'01;}\f5\fs24\b0\i0\strike0\ul0\cf0\cb0\fi-284\li1724}
{\listlevel\levelnfc0\leveljc0\levelfollow0\levelstartat1\levelspace0\levelinden
t0{\leveltext\'02\'08.;}
{\levelnumbers\'01;}\f5\fs24\b0\i0\strike0\ul0\cf0\cb0\fi-284\li2084}
{\listname ;}\listid1}
{\list\listtemplateid2{\listlevel\levelnfc23\leveljc0\levelfollow0\levelstartat1\l
evelspace0\levelindent0{\leveltext\'01\u-4051 ?;}
{\levelnumbers;}\f6\fs24\fbias0\b0\i0\strike0\ul0\cf0\cb0\fi-284\li284}
{\listlevel\levelnfc23\leveljc0\levelfollow0\levelstartat1\levelspace0\levelinde
nt0{\leveltext\'01\u-4051 ?;}
{\levelnumbers;}\f6\fs24\fbias0\b0\i0\strike0\ul0\cf0\cb0\fi-284\li644}
{\listlevel\levelnfc23\leveljc0\levelfollow0\levelstartat1\levelspace0\levelinde
nt0{\leveltext\'01\u-4051 ?;}
{\levelnumbers;}\f6\fs24\fbias0\b0\i0\strike0\ul0\cf0\cb0\fi-284\li1004}
{\listlevel\levelnfc23\leveljc0\levelfollow0\levelstartat1\levelspace0\levelinde
nt0{\leveltext\'01\u-4051 ?;}
{\levelnumbers;}\f6\fs24\fbias0\b0\i0\strike0\ul0\cf0\cb0\fi-284\li1364}
{\listlevel\levelnfc23\leveljc0\levelfollow0\levelstartat1\levelspace0\levelinde
nt0{\leveltext\'01\u-4051 ?;}
{\levelnumbers;}\f6\fs24\fbias0\b0\i0\strike0\ul0\cf0\cb0\fi-284\li1724}
{\listlevel\levelnfc23\leveljc0\levelfollow0\levelstartat1\levelspace0\levelinde
nt0{\leveltext\'01\u-4051 ?;}
{\levelnumbers;}\f6\fs24\fbias0\b0\i0\strike0\ul0\cf0\cb0\fi-284\li2084}
{\listlevel\levelnfc23\leveljc0\levelfollow0\levelstartat1\levelspace0\levelinde
nt0{\leveltext\'01\u-4051 ?;}
{\levelnumbers;}\f6\fs24\fbias0\b0\i0\strike0\ul0\cf0\cb0\fi-284\li2444}
{\listlevel\levelnfc23\leveljc0\levelfollow0\levelstartat1\levelspace0\levelinde
nt0{\leveltext\'01\u-4051 ?;}
{\levelnumbers;}\f6\fs24\fbias0\b0\i0\strike0\ul0\cf0\cb0\fi-284\li2804}
{\listlevel\levelnfc23\leveljc0\levelfollow0\levelstartat1\levelspace0\levelinde
nt0{\leveltext\'01\u-4051 ?;}
{\levelnumbers;}\f6\fs24\fbias0\b0\i0\strike0\ul0\cf0\cb0\fi-284\li3164}
{\listname ;}\listid2}}
{*\listoverridetable{\listoverride\listid1\listoverridecount0\ls1}
{\listoverride\listid2\listoverridecount0\ls2}}
{*\generator TX_RTF32 19.0.542.500;}{\info{*\hlinkbase D:\'5cMASTER
PUTUSAN OK\'5c}}
\
paperw11906\paperh16838\margl1440\margt1440\margr1440\margb1440\d
eftab1134\widowctrl\lytexcttp\formshade\sectd\pgbrdrhead\pgbrdrfoot
\
headery720\footery720\pgwsxn11907\pghsxn16840\marglsxn2268\margtsxn
1701\margrsxn1134\margbsxn1134\pgbrdropt0\pgbrdrhead\pgbrdrfoot
{\footer\pard\itap0\widctlpar\qr\plain\f4\fs24\tab\plain\f5\fs12 Hal.
\plain\f5\fs12{\txfielddef{*\txfieldstart\txfieldtype3\txfieldflags0}
{*\txfielddatadef\txfielddatatype3\txfielddata 00030001}{*\txfieldtext

Disclaimer
Kepaniteraan Mahkamah Agung Republik Indonesia berusaha untuk selalu mencantumkan informasi paling kini dan akurat sebagai bentuk komitmen Mahkamah Agung untuk pelayanan publik, transparansi dan akuntabilitas pelaksanaan fungsi peradilan. Namun dalam hal-hal tertentu masih dimungkinkan terjadi permasalahan teknis terkait dengan akurasi dan keterkinian informasi yang kami sajikan, hal mana akan terus kami perbaiki dari waktu kewaktu.
Dalam hal Anda menemukan inakurasi informasi yang termuat pada situs ini atau informasi yang seharusnya ada, namun belum tersedia, maka harap segera hubungi Kepaniteraan Mahkamah Agung RI melalui :
Email : kepaniteraan@mahkamahagung.go.id   Telp : 021-384 3348 (ext.318)

2{*\txfieldend}}{\field{*\fldinst{ PAGE * Arabic }}
{\fldrslt{1}}}}\plain\f5\fs12 dari
\plain\f5\fs12{\txfielddef{*\txfieldstart\txfieldtype9\txfieldflags0}
{*\txfielddatadef\txfielddatatype9\txfielddata 00030000}{*\txfieldtext
6{*\txfieldend}}{\field{*\fldinst{ NUMPAGES * Arabic }}
{\fldrslt{1}}}}\plain\f5\fs12 hal.
\par\pard\s17\itap0\widctlpar\qr\ri357\tqr\tx8306\tx10208\tx12760\tx15312\t
x17864\tx20416\tx22968\tx25520\tx28072\tx30624\plain\f4\fs24
\plain\f5\fs22\par\pard\s17\itap0\widctlpar\tqc\tx4153\tqr\tx8306\tx10208\tx
12760\tx15312\tx17864\tx20416\tx22968\tx25520\tx28072\tx30624\plain\f1
\fs24\par\pard\s17\itap0\widctlpar\tx3325\tqc\tx4153\tqr\tx8306\tqr\tx8505\p
ar}\pard\s18\itap0\widctlpar\qc\plain\f5\fs32\b P E N E T A P A
N\par\pard\itap0\widctlpar\qc\plain\f5\fs24 Nomor
6592/Pdt.G/2020/PA.Sbr\par{\pict{*\picprop{\sp{\sn shapeType}{\sv 75}}
{\sp{\sn fFlipH}{\sv 0}}{\sp{\sn fFlipV}{\sv 0}}{\sp{\sn wzName}{\sv
Picture 1}}{\sp{\sn fLayoutInCell}{\sv
1}}}\wmetafile8\picw23813\pich4445\picwgoal13500\pichgoal2520\picscale
x25\picscaley25\blipupi96
0100090000036a260000000000511000000000161000026060f002220574d4
6430100000000000100d1fc000000000002000000
00200000080a0000082a000001000006c0000000000000000000003f020
00077000000000000000000000000634f00008910
000020454d4600000100082a00000c0000000100000000000000000000000
000000056050000000030000e20100000f010000
000000000000000000000000665a070055220400460000002c00000020000
000454d462b014001001c00000010000000210
c0db01000000600000000600000004600000064060000058060000454d462b2
24004000c00000000000001e4009000c000000
0000000024400100c0000000000000030400200100000000400000000008
03f214007000c000000000000000008400005b005
0000a40500000210c0db01000000000000000000000000000000000000000
100000089504e470d0a1a0a0000000d49484452
00000240000000780103000000020e2df9c0000000017352474200aece1ce900
000006504c5445000000ffffffa5d99fdd0000
052f4944415468deedd83f6fe336140070aa1a34aa638702ec472850a0e870a
85af4a3f40374bda1382948812c87f39a0245
fd4d6a051e7c4be1f53a85410aa45b64780883d07c7def91fa2f5b17c543815a
401c27667e7a7c241fa90838d2254ed0093a
41ff4548a747828a63412a7931b4e2d7fce550442fbb772fee9a0d39d797d3a0
c7faad71909836fcaa01fdc883164c8372ff
7d07f08193fd77f83208bb9851b21fee9269d06ff4b206d860bb18dfde4d5c22
4f9fd1eb0ce00aaca0582e26422eb5184a08
f63b7e6ba6418a526b2598082c8fdc5428a7046b0945ec2654212742efa55ba
64a6abfd02642d7f492a59041416387dff524
881d1bd0b8a974cdcb6d7f5d3b00a5335e5d912b4308193909b20e72954c25
f803f6cb4c804cca63750b055245c2ea620a54

Disclaimer
Kepaniteraan Mahkamah Agung Republik Indonesia berusaha untuk selalu mencantumkan informasi paling kini dan akurat sebagai bentuk komitmen Mahkamah Agung untuk pelayanan publik, transparansi dan akuntabilitas pelaksanaan fungsi peradilan. Namun dalam hal-hal tertentu masih dimungkinkan terjadi permasalahan teknis terkait dengan akurasi dan keterkinian informasi yang kami sajikan, hal mana akan terus kami perbaiki dari waktu kewaktu.
Dalam hal Anda menemukan inakurasi informasi yang termuat pada situs ini atau informasi yang seharusnya ada, namun belum tersedia, maka harap segera hubungi Kepaniteraan Mahkamah Agung RI melalui :
Email : kepaniteraan@mahkamahagung.go.id   Telp : 021-384 3348 (ext.318)

bc89fc58c554aa199af7201b8c42b90d1d44cb55d3428127d9838c484620bc9
587dec6b0aba0eeead7a3900e4b681ec39f9a
6bff577de8311b835404170ec2517fefa02f13db85b6d584d807e19d660e2a9
2dd0707893e54ef98fb205cf83e2205bb4b93
32945ad9bbe1d8a82de09a21c3897050007d281c5f22d72b82f82b30e992d2
de87eabeee8576b3152592bf10ba75bf95034b
e9e3209a823a32c97e281981fe9aad08a19963520feda8803f17fad5419687d
c41d60c417204ba8c6f115a5ba963e208327a
0032f1089449822e40e21c47e8c641b30950728bf93907990714ff661f64a33
128bdc5ec0430cf455a43fdbdd68623d00a3c
94094c2741f7e6f5d0a67d3e36b3193a2728aaa074d56f76350add20b482f98
2fa46d07a026468726c78ae2d201721c882a0
9f87207508a2b98310710b2b108a3543c900541c820a1a8a8d5b5985c02451
918b26404ad450ee2183901c80f46128a15b49
37a17061205444e68d1c38fcf9aa3d0ce57464d40ec231bb26288fccb7f15daf
b4dd9b43d09510af3ce44e8f3303f9ca2264
9e0761df84831eec171e3a5fd924bed3adf5ff04d7eb2ed43e439912da9ad704
adb114adac8cef8b16b485f9b20bb51ee9e6
5a0408cd71e8f42b84d618915cd97806dbd66a5bd20aea40cd473a2b550961
1f133a9118fb79642384ceda9b91cc4467f8f3
46167524e2651712910d66b0c91a91ebd0443de85ddc1c7d010f9b124a1932
e20783934209d96a875707fa3d6c663ae4733f
42c5270c4984bed70cb5da09f14dd08166f544c8033ea505b048703e3394ec8
cc84516fbae36024aee3b5054c55c889086d0
415a66ae54983017984725aa90b4102aa852564142f818b16c70ba10fa23f12
be986c73528181251d92ed2610e7dc835d0c2
05bfc6331beeebeea181f75915e2b6f20be7f727807fa89d8d1f87a0f2e287a2
1837ac0a5a728874a328cfab76ad07dc0aca
6a8892a5e9046ba009f1961b73c8753b1fed2014d2e3a74a9a50b52ef0a1ab6
ae903ca3bd0a7611933ef55fc245441d4297e
478f01654849b993b62023cfadfb1c7fbf3dc38db8006f71877c6de6bf3265bb2
1886e05e5845701a65931a4cb0e7117e889
6141adbfae174ad6ee9a6a56113a37d0c6879388e328e8cf0297efde09f9a60
d65ad924dfb39d60b9d50b30d1dbae90999e3
365de869db8428e66d5db2efb9d65097ce78634b7d54db4ee8f5dc28219cc6f
e176e6ad89422c05368e8ca6e95d376e8beba
35205aee51e3431babb8ac27c033c64f25d3fb378b7b582da1cceff7f58ca779
6bdcfa538d4fdac5bf515a050cd55abfd796
85a0196ad7b1dde26f452b8b9a7f6c54b17d97e99e21bb9d5fba1a08063eee1
27b63f689d4cf85f65dea58507624c8064782
74782448c54782b2f44850044782e0049da01374824ed009fa3f42ff02c21e32
85687fb84f0000000049454e44ae42608200
08400108240000000180000000210c0db010000000300000000000000000000
000000000001b4000004000000003400000000100
0000020000000000000bf000000bf000010440000f0420300000000000000b300
0000b3ffff0f44000000b3000000b3ffffef42

Disclaimer
Kepaniteraan Mahkamah Agung Republik Indonesia berusaha untuk selalu mencantumkan informasi paling kini dan akurat sebagai bentuk komitmen Mahkamah Agung untuk pelayanan publik, transparansi dan akuntabilitas pelaksanaan fungsi peradilan. Namun dalam hal-hal tertentu masih dimungkinkan terjadi permasalahan teknis terkait dengan akurasi dan keterkinian informasi yang kami sajikan, hal mana akan terus kami perbaiki dari waktu kewaktu.
Dalam hal Anda menemukan inakurasi informasi yang termuat pada situs ini atau informasi yang seharusnya ada, namun belum tersedia, maka harap segera hubungi Kepaniteraan Mahkamah Agung RI melalui :
Email : kepaniteraan@mahkamahagung.go.id   Telp : 021-384 3348 (ext.318)

2100000008000000620000000c0000000100000015000000 0c00000004000
000150000000c0000000400000051000000 4022
000000000000000000003f020000770000000000000000000000000000000
000000040020000780000005000000030000000
80000000c0210000000000002000cc0040020000780000002800000040020
000780000000100010000000000000000000000
0000000000002000000000000000000000000ffffff00fffffffffffffffffffffffffffffffffffff
ffffffffffffffffffff
ffffffffffffffffffffffffffffffffffffffffffffffffffffffffffffffffffffffffffffffffffffffffffffffffffffff
ffffffffffffffffffffffffffffffffffffffffffffffffffffffffffffffffffffffffffffffffffffffffffffffffffffff
ffffffffffffffffffffffffffffffffffffffffffffffffffffffffffffffffffffffffffffffffffffffffffffffffffffff
ffffffffffffffffffffffffffffffffffffffffffffffffffffffffffffffffffffffffffffffffffffffffffffffffffffff
ffffffffffffffffffffffffffffffffffffffffffffffffffffffffffffffffffffffffffffffffffffffffffffffffffffff
ffffffffffffffffffffffffffffffffffffffffffffffffffffffffffffffffffffffffffffffffffffffffffffffffffffff
ffffffffffffffffffffffffffffffffffffffffffffffffffffffffffffffffffffffffffffffffffffffffffffffffffffff
ffffffffffffffffffffffffffffffffffffffffffffffffffffffffffffffffffffffffffffffffffffffffffffffffffffff
ffffffffffffffffffffffffffffffffffffffffffffffffffffffffffffffffffffffffffffffffffffffffffffffffffffff
ffffffffffffffffffffffffffffffffffffffffffffffffffffffffffffffffffffffffffffffffffffffffffffffffffffff
ffffffffffffffffffffffffffffffffffffffffffffffffffffffffffffffffffffffffffffffffffffffffffffffffffffff
ffffffffffffffffffffffffffffffffffffffffffffffffffffffffffffffffffffffffffffffffffffffffffffffffffffff
ffffffffffffffffffffffffffffffffffffffffffffffffffffffffffffffffffffffffffffffffffffffffffffffffffffff
ffffffffffffffffffffffffffffffffffffffffffffffffffffffffffffffffffffffffffffffffffffffffffffffffffffff
ffffffffffffffffffffffffffffffffffffffffffffffffffffffffffffffffffffffffffffffffffffffffffffffffffffff
ffffffffffffffffffffffffffffffffffffffffffffffffffffffffffffffffffffffffffffffffffffffffffffffffffffff
ffffffffffffffffffffffffffffffffffffffffffffffffffffffffffffffffffffffffffffffffffffffffffffffffffffff
ffffffffffffffffffffffffffffffffffffffffffffffffffffffffffffffffffffffffffffffffffffffffffffffffffffff
ffffffffffffffffffffffffffffffffffffffffffffffffffffffffffffffffffffffffffffffffffffffffffffffffffffff
ffffffffffffffffffffffffffffffffffffffffffffffffffffffffffffffffffffffffffffffffffffffffffffffffffffff
ffffffffffffffffffffffffffffffffffffffffffffffffffffffffffffffffffffffffffffffffffffffffffffffffffffff
ffffffffffffffffffffffffffffffffffffffffffffffffffffffffffffffffffffffffffffffffffffffffffffffffffffff
ffffffffffffffffffffffffffffffffffffffffffffffffffffffffffffffffffffffffffffffffffffffffffffffffffffff
ffffffffffffffffffffffffffffffffffffffffffffffffffffffffffffffffffffffffffffffffffffffffffffffffffffff
ffffffffffffffffffffffffffffffffffffffffffffffffffffffffffffffffffffffffffffffffffffffffffffffffffffff
ffffffffffffffffffffffffffffffffffffffffffffffffffffffffffffffffffffffffffffffffffffffffffffffffffffff
ffffffffffffffffffffffffffffffffffffffffffffffffffffffffffffffffffffffffffffffffffffffffffffffffffffff
ffffffffffffffffffffffffffffffffffffffffffffffffffffffffffffffffffffffffffffffffffffffffffffffffffffff
ffffffffffffffffffffffffffffffffffffffffffffffffffffffffffffffffffffffffffffffffffffffffffffffffffffff
ffffffffffffffffffffffffffffffffffffffffffffffffffffffffffffffffffffffffffffffffffffffffffffffffffffff
ffffffffffffffffffffffffffffffffffffffffffffffffffffffffffffffffffffffffffffffffffffffffffffffffffffff
ffffffffffffffffffffffffffffffffffffffffffffffffffffffffffffffffffffffffffffffffffffffffffffffffffffff
ffffffffffffffffffffffffffffffffffffffffffffffffffffffffffffffffffffffffffffffffffffffffffffffffffffff
ffffffffffffffffffffffffffffffffffffffffffffffffffffffffffffffffffffffffffffffffffffffffffffffffffffff
ffffffffffffffffffffffffffffffffffffffffffffffffffffffffffffffffffffffffffffffffffffffffffffffffffffff
ffffff07ffffffffffffffffffffffffffffffffffffffffffffffffffffffffffffffffffffffffffffffffffffffffffff
fffffffffffffffffffffffffffffffffffffffffffffffffff807ffffffffffffffffffffffffffffffffffffffffffffffff

Disclaimer
Kepaniteraan Mahkamah Agung Republik Indonesia berusaha untuk selalu mencantumkan informasi paling kini dan akurat sebagai bentuk komitmen Mahkamah Agung untuk pelayanan publik, transparansi dan akuntabilitas pelaksanaan fungsi peradilan. Namun dalam hal-hal tertentu masih dimungkinkan terjadi permasalahan teknis terkait dengan akurasi dan keterkinian informasi yang kami sajikan, hal mana akan terus kami perbaiki dari waktu kewaktu.
Dalam hal Anda menemukan inakurasi informasi yang termuat pada situs ini atau informasi yang seharusnya ada, namun belum tersedia, maka harap segera hubungi Kepaniteraan Mahkamah Agung RI melalui :
Email : kepaniteraan@mahkamahagung.go.id   Telp : 021-384 3348 (ext.318)

ffffffffffffffffffffffffffffffffffffffffffffffffffffffffffffffffffffffffffffffffffffffffffffffe00fff
fffffffffffffffffffffffffffffffffffffffffffffffffffffffffffffffffffffffffffffffffffffffffffffffffffff
ffffffffffffffffffffffffffffffffffffffff803fffffffffffffffffffffffffffffffffffffffffffffffffffffffffff
ffffffffffffffffffffffffffffffffffffffffffffffffffffffffffffffffffffffffffffffffffffffe01ffffffffffff
fffffffffffffffffffffffffffffffffffffffffffffffffffffffffffffffffffffffffffffffffffffffffffffffffffff
ffffffffffffffffffffffffffff80fffffffffffffffffffffffffffffffffffffffffffffffffffffffffffffffffffffff
fffffffffffffffffffffffffffffffffffffffffffffffffffffffffffffffffffffffe0ffffffffffffffffffffffffff
ffffffffffffffffffffffffffffffffffffffffffffffffffffffffffffffffffffffeffffffffffffffffffffffffffffff
fffffffffff807fff8fffffffffffffffffffffffffffffffffffffffffffffffffffffffffffffffffffffffffffffffffff
ffffffffffffc03ffffffffffffffffffffffffffffc3fffffffc000fffcfffffffffffffffffffffffffffffffffffffff
ffffffffffffffffffffffffffffffffffffffffffffffffffffffffffe007ffffffffffffffffffffffffff8007ffffff00
003fffffffffffffffffffffffffffffffffffffffffffffffffffffffffffffffffffc07fffffffffffffffffffffffffffff
ffc01fffffffffffffffffffffffffe0003ffffffe00000ffffffffffffffffffffffffffffffeffffffffffffffffffffff
ffffffffffffe007fffffffffffffffffffffff80fffff80ffffffffffffffffff00001ffffffc000007ffff
ffe0
0fffffffffffffffffffff00fffffffffffffffffffffffffffffffffc01ffffffffffffffffffffc00fffff07ffffff
ffffffffffffe0fe00ffffffc000003fffff0001fffff87ffffffffffc03ffffffffffffffffffffffff
ffffff
fff807fffffffffffffffffffffff803ffffc3ffffffffffffffffffffffffe3fffe7ffc07fffff807fc03fffffc0000ff
ffff807f
fffffffffff80fffffffffffffffffffffffffffffffffffffffffff03ffffffffffffffffffffffffffffe01fffff3ffffff
c1efffffff03fffff81fff81fffff000007ffffff01ffffffff3ffffe07ffffffffffffffffffffffffffffffffffffc3
ffffff
fffffffffffffffff80ffffffffffffffffffffffffc083ffffff81fffff83fffe1fffff1ff803ffffffc07ffffff0
3fff
fc3fffffffffffffffffffffffffffffffffbf3fffffffffffffffffffffffffe07ffffffffffffffffffffffc001ffffffc1
fffff87ffff0fffffffff01fffffff01fffffff80ffffe3ffffffffffffffffffffffffffffffffe1ffffffffffffffffff
fffffff03fffffff00000000fffffffe001fe7fffe0fffff8fffff8ffffffffff81fffffffc0ffffffe01ffff
bffffffff0
361fffffffffffffffffff80ffffffffffffffffffffffffffffc1f83fe0000000000007ffffff381fe07fff0
fffff8fffffc
fffffffffe0fffffffff0fffffffff807fffffffc00000000007fffffffffffffff807fffffffffffffffffffffffffff
f1003
c0000000000000000ffffff83ff80fff8fffffcfffffcffffffffff07fffffffcffffffffff03ffffff80000
00000000003fff
ffffffffff807fffffffffffffffffffffffffffff80000000000000000000003ffffcfffe03ffc7ffffcffff
fcffffffffff87
ffffffffffffffff81fffff8000000000000000001ffffffffffffc0fffffffffffffffffffffffffffff80000
00000000000
0000001ffffffff81ffc7ffffcfffffcffffffffffc3fffffffff80ffffffe0ffff8000000000000000
0001fffffffffffe3
fffffffffffffffffffffffffff000000000000000000000003fffffffe0ffc0ffffe7ffffcffffffffff
c3ffffffffff007
ffffff07c0c000000000000000000001ffffffffffffffffffffffffffffffffffffffffffffff00000007ffff
fffff80000003fff
ffffcff803fffe7ffffcffffffffffe3ffffffffe003fc3fff8400000000006fffffe80000003ffe0
Disclaimer
Kepaniteraan Mahkamah Agung Republik Indonesia berusaha untuk selalu mencantumkan informasi paling kini dan akurat sebagai bentuk komitmen Mahkamah Agung untuk pelayanan publik, transparansi dan akuntabilitas pelaksanaan fungsi peradilan. Namun dalam hal-hal tertentu masih dimungkinkan terjadi permasalahan teknis terkait dengan akurasi dan keterkinian informasi yang kami sajikan, hal mana akan terus kami perbaiki dari waktu kewaktu.
Dalam hal Anda menemukan inakurasi informasi yang termuat pada situs ini atau informasi yang seharusnya ada, namun belum tersedia, maka harap segera hubungi Kepaniteraan Mahkamah Agung RI melalui :
Email : kepaniteraan@mahkamahagung.go.id   Telp : 021-384 3348 (ext.318)

ff9ffffffffffffff
ffffffffffffff000001ffffffffffffe00000ffffffffff001ffbe3ffff87fffffffffe1ffffffffe001f0
1fffe00000
0007ffffffffffe000007f803c0ffffffffffffffffffffffffffffffff07001ffffffffffffff00003f07
ffffff001ff
bf3ffff81f80ffffffe0fffffc03c000c01fffe0000003ffffffffffffff80000e001807ffffffff
ffffffffffffffffff
ffff8f00fffffffffffffffffc001c00ffffff001ffbf3ffff007007fffffc03ffdfc00c000800fffe
000003ffffffffff
ffffffc000000003fefffffffffffffffffffffffffff9e03fffffffffffffffffc000003fffff001ff9
ffffff00200
7fffffc01ffcfc0041f8000fffe00003ffffffffffffffffffc000000003801fffffffffffffffffff
ffffff8e0fffff
ffffffffffffffffc00000fff7f1f8ff9fffffe000007fffffc00ffcfc0043fc000fffe0001ffffffff
ffffffffffffc000
00000000ffffffffffffffffffffffffffffc01ffffffffffffffffffffff807000fc3fff8ff9fffffe000003
fffffc00ffc
fe0007fe018ffff1807ffffffffffffffffffffffff803fc078000fffffffffffffffffffffffffffe03ffffffff
fffffffff
fffffe1fe00001fff8ff9fffffe000003fffffc00ffcfe1c07fe078ffff381ffffffffffffffffffffff
03ff1f8000ffff
fffffffffffffffffffffffff07fffffffffffffffffffffff9ff80001fff8ff9ffffff1f83c3fffffcf8ffcff1fc7f
e0f8f
fff387ffffffffffffffffffffffffc3ff9f80007fffffffffffffffffffffffffffff8fffffffffffffffffffffffffffc00
00fff8ff1ffffff3fc783fffffffcffcff0fc7fe1f0feff00fffffffffffffffffffffffffff3ff9fc3fc7ffffff
fffffffff
ffffffffffffffffffffffffffffffffffffffffffe00000fff8ff1ffffff7fe203fffffff8ffcff83c7fe3f0fe7f8
0fffffff
ffffffffffffffffffffbffdfcffc7fffffffffffffffffffffffffffffffffffffffffffffffffffffffffc100003cfff8ff1fff
fffffe003fffffff8ffcffc0c7fe3e0fe7fc1ffffffffffffffffffffffffffffffeffc7ffffffffffffffffffffff
fffff
ffffffffffffffffffffffffffffc0000ffffff8ff1fffffffff007fffdfff8ff8ffc007fc3e0fe7fe1ffffffffff
fffffffff
ffffffffffffeffc7ffffffffffffffffffffffffffffffffffffffffffffffffffffe0003ffffff0ff1fffffffff807fff
0fff8ff8fff007fc7e1fe7ffffffffffffffffffffffffffffffffffffff87fffffffffffffffffffffffffffffffffffffff
ffffffffffffffe000fffffff1ff1fffffffffc0000007ff8ff8fffc07fc7c1fc7fffffffffffffffffffffffffffff
ffffffff
fff8ffffffffffffffffffffffffffffffffffffffffffffffffffffffff003fffffff1ff1fffffffff0000007ff8ff8ffff
07fc7c3fc7fffffffffffffffffffffffffffffffffffffffff0fffffffffffffffffffffffffffffffffffffffffffffffffffffffff
fff80ffffffff1fe1fffffffbffe000003ff0ff8ffffcffc7c3fc7ffffffffffffffffffffffffffffffffffffffffff0ff
ffffff
fffffffffffffffffffffffffffffffffffffffffffffffc1ffffffff1fe3fffffffbfffc00003ff1ff0ffffcffc7c7fc
7ff
ffffffffffffffffffffffffffffffffffffe0fffffffffffffffffffffffffffffffffffffffffffffffffffffffffffffffffffffff
e1fe3fffc7ff9fbfc0007fff1ff1ffffcffc787fc7fffffffffffffffffffffffffffffffffffffffc1fffffffffffff
ffffff

Disclaimer
Kepaniteraan Mahkamah Agung Republik Indonesia berusaha untuk selalu mencantumkan informasi paling kini dan akurat sebagai bentuk komitmen Mahkamah Agung untuk pelayanan publik, transparansi dan akuntabilitas pelaksanaan fungsi peradilan. Namun dalam hal-hal tertentu masih dimungkinkan terjadi permasalahan teknis terkait dengan akurasi dan keterkinian informasi yang kami sajikan, hal mana akan terus kami perbaiki dari waktu kewaktu.
Dalam hal Anda menemukan inakurasi informasi yang termuat pada situs ini atau informasi yang seharusnya ada, namun belum tersedia, maka harap segera hubungi Kepaniteraan Mahkamah Agung RI melalui :
Email : kepaniteraan@mahkamahagung.go.id   Telp : 021-384 3348 (ext.318)

fffffffffffffffffffffffffffffffffffffffffffffe3fe3fff83ff9f800007ffff1ff1fffeffc787fc7fffffffff
ffffff
ffffffffffffffffffffffffc1ffffffffffffffffffffffffffffffffffffffffffffffffffffffffffffffc7ffe3fe3fff01ff
9f80001fffff1ff1ffffeffc78ff87ffffffffffffffffffffffffffffffffffffff83ffffffffffffffffffffffffffffff
ff
ffffffffffffffffffffffffffff807fe3fe3fff01ff9fc0007ffffe1ff1ffffffcf8ff87fffffffffffffffffffff
ffffff
ffffffffe03fffffffffffffffffffffffffffffffffffffffffffffffffffffffffffff803fe3fc3fff83ff1fc001fffff
e
1ff1ffffffcf8ff8fffffffffffffffffffffffffffffffffffffe07fffffffffffffffffffffffffffffffffffffffffffff
ffffffffffffffff801fe3fc7fff87ff1fe007fffffe3fe1ffffffcf8ff8fffffffffffffffffffffffffffffffffff
ffc0f
fffffffffffffffffffffffffffffffffffffffffffffffffffffffffffff980fe3fc7fffcffe1ff80fffffe3fe3fffffff
c
f8ff0fffffffffffffffffffffffffffffffffffffffc0fffffffffffffffffffffffffffffffffffffffffffffffffffffffff
ffff998fc7fc7ffffffe3ffe3ffffffc3fe3ffffffefcff0ffffffffffffffffffffffffffffffffffffffffffffffffe1fffffffff
fff
fffffffffffffffffffffffffffffffffffffffffffffffffd98fc7fc7ffffffc3ffffffc7ffc3fe3ffffffffdff0ffffff
fffffffffffffffffffffffffffffffe3fffffffffffffffffffffffffffffffffffffffffffffffffffffffffffffd8fc7f8
7ffffffc7ffffff807fc3fe3ffffffffff1ffffffffffffffffffffffffffffffffffffffe3ffffffffffffffffffffffffff
fffffffffffffffffffffffffffffffffffffd8fc7f87ffffff87ffffff801fc7fe3ffffffffff1fffffffffffffffffff
ffffffffffffffffffffe7fffffffffffffffffffffffffffffffffffffffffffffffffffffffffffffffdfc7f8ffffff8ffff
fff801fc7fc3ffffff9ffffe1ffffffffffffffffffffffffffffffffe3ffffffffffffffffffffffffffffffffffffffffffff
ffffffffffffffffffffffbfffdf87f8ffffffcffffff980fc7fc7ffffff01fffe1fffffffffffffffffffffffffffffff
f
ff03ffffffffffffffffffffffffffffffffffffffffffffffffffffffffffffffff03ffff87f8ffffffffffe0fffd98fc7fc7
ffffff007ffe1ffffffffffffffffffffffffffffffffff80fffffffffffffffffffffffffffffffffffffffffffffffffffff
fffffffff803fff8ff8ffffffffffff03ffdd8f8ffc7ffffff003ffe3fffffffffffffffffffffffffffffffffff07ff
fffff
fffffffffffffffffffffffffffffffffffffffffffffffffffffffe007ff87f8ffffffffffc1fffc8f8ffc7ffffff303ff
e
3fffffffffffffffffffffffffffffffff3f87ffffffffffffffffffffffffffffffffffffffffffffffffffffffffffffffff8
03ff03f8fffffffff9f0fffcdf8ffc7ffffff331ffc3fffffffffffffffffffffffffffffffffff9803ffffffffffff
fffff
ffffffffffffffffffffffffffffffffffffffffffffe01ff03f8fffffffff8c0ffffdf8ff8ffffffffb1ffc3ffffffffff
fffffffffffffffffffff8003ffffffffffffffffffffffffffffffffffffffffffffffffffffffffffffffc0fe03f8ffff
ffffff8007fffff8ff8ffffffffb1ffc7fffffffffffffffffffffffffffffffffc003ffffffffffffffffffffffffffffffff
fffffffffffffffffffffffffffffffff07e07f8ffffffffffc00ffffff07f8fffffffff9ffc7fffffffffffffffffffffff
fffffffffe01ffffffffffffffffffffffffffffffffffffffffffffffffffffffffffffffffffffffc7f0ff9ffffffe3fff03fff
fff03f8f1a05000026060f002a0a574d464301000000000000010000000000000
02000000080a000000000000000082a0000ffff
ffffbffc7fffffffffffffffffffffffffffffffffffffffffffffffffffffffffffffffffffffffffffffffffffffffffff
fffffffffe7f8ffffffffffe03fffffffffe03f8ffffff8fffffc7ffffffffffffffffffffffffffffffffffffffffffffffffffffffff
fffffffffffffffffffffffffffffffffffffffffffffffffffffff7f8ffffffffff807ffffffffe03fcffffffc1ffff87fff
ffffffffffffffffffffffffffffffffffffffffffffffffffffffffffffffffffffffffffffffffffffffffffffffffffffff
dffffffffffe01ffff8fffe07fcffffffe03fff87ffffffffffffffffffffffffffffffffffffffffffffffffffffffffffff

Disclaimer
Kepaniteraan Mahkamah Agung Republik Indonesia berusaha untuk selalu mencantumkan informasi paling kini dan akurat sebagai bentuk komitmen Mahkamah Agung untuk pelayanan publik, transparansi dan akuntabilitas pelaksanaan fungsi peradilan. Namun dalam hal-hal tertentu masih dimungkinkan terjadi permasalahan teknis terkait dengan akurasi dan keterkinian informasi yang kami sajikan, hal mana akan terus kami perbaiki dari waktu kewaktu.
Dalam hal Anda menemukan inakurasi informasi yang termuat pada situs ini atau informasi yang seharusnya ada, namun belum tersedia, maka harap segera hubungi Kepaniteraan Mahkamah Agung RI melalui :
Email : kepaniteraan@mahkamahagung.go.id   Telp : 021-384 3348 (ext.318)

ffffffffffffffffffffffffffffffffffffffffffffffffffffffffffdfffffffff807fff80fff0ffcffffff80fff8ffffffffffffffffffffff
ffffffffffffffffffffffffffffffffffffffffffffffffffffffffffffffffffffffffffffffffffffffffffffffffffffffffffffffffffffc81ffffff
e03fffe01ff8fffffffffc03ffcffffffffffffffffffffffffffffffffffffffffffffffffffffffffffffffffffffffffffffffffffffffffffffffff
fffffffffffffffffffffffffffffffffffffffffffc007ffffff81ffff00ff9fffffffff01ffcfffffffffffffffffffffffffffffffffffffffffffffffffff
ffffffffffffffffffffffffffffffffffffffffffffffffffffffffffffffffffffffffffffffffffffffffffffffffffffe003ffffffe1ffffc07ff
feffffffffc0ffcffffffffffffffffffffffffffffffffffffffffffffffffffffffffffffffffffffffffffffffffffffffffffffffffffffffffffffff
ffffffffffffffffffffffffffffff001fffffff0fffff03fffe40fffffff0ffcffffffffffffffffffffffffffffffffffffffffffffffffffffffffffffffffff
ffffffffffffffffffffffffffffffffffffffffffffffffffffffffffffffffffffffffffffffffffffffb18fffffff8fffffc1fffe003ffffff
87fffffffffffffffffffffffffffffffffffffffffffffffffffffffffffffffffffffffffffffffffffffffffffffffffffffffffffffffffffffffffffff
fffffffffffffff9cfffffffcffffff1ffff000ffffffe7fffffffffffffffffffffffffffffffffffffffffffffffffffffffffffffffffffff
ffffffffffffffffffffffffffffffffffffffffffffffffffffffffffffffffffffffffc0ffffffffffffff9ffff8007ffffff7e73ffffff
ffffffffffffffffffffffffffffffffffffffffffffffffffffffffffffffffffffffffffffffffffffffffffffffffffffffffffffffffffffffffffffffff
c0fffffffffffffffdffffd8c7fffffffe403ffffffffffffffffffffffffffffffffffffffffffffffffffffffffffffffffff
ffffffffffffffffffffffffffffffffffffffffffffffffffffffffe0fffffffffffffffffffffffffffffc63fffffffff001ffffffffffffffff
fffffffffffffffffffffffffffffffffffffffffffffffffffffffffffffffffffffffffffffffffffffffffffffffffffffffffffffffff9fffffffff
ffffffffffffffc03ffffffff800fffffffffffffffffffffffffffffffffffffffffffffffffffffffffffffffffffffffffffffffffff
fffffffffffffffffffffffffffffffffffffffffffffffffffffffffffffffffffffffe03ffffffff8987fffffffffffffffffffffffffffffffffffff
ffffffffffffffffffffffffffffffffffffffffffffffffffffffffffffffffffffffffffffffffffffffffffffffffffffffffffffffffffffffffffffffff
07ffffffffd8e7fffffffffffffffffffffffffffffffffffffffffffffffffffffffffffffffffffffffffffffffffffffffffffffffffffffffff
ffffffffffffffffffffffffffffffffffffffffffffffffffffffffc7fffffffffc03fffffffffffffffffffffffffffffffffffffffffffffffffffff
ffffffffffffffffffffffffffffffffffffffffffffffffffffffffffffffffffffffffffffffffffffffffffffffffffffffffffffffffffffffffffffffff
e03fffffffffffffffffffffffffffffffffffffffffffffffffffffffffffffffffffffffffffffffffffffffffffffffffffffffffffffffffffffffffffff
ffffffffffffffffffffffffffffffffffffffffffffffffffffffffff07ffffffffffffffffffffffffffffffffffffffffffffffffffffffffffffffffffff
ffffffffffffffffffffffffffffffffffffffffffffffffffffffffffffffffffffffffffffffffffffffffffffffffffffffffffffffffff87ffffffff
ffffffffffffffffffffffffffffffffffffffffffffffffffffffffffffffffffffffffffffffffffffffffffffffffffffffffffffffffffffffffffffffff
ffffffffffffffffffffffffffffffffffffffffffffffffffffffffffffffffffffffffffffffffffffffffffffffffffffffffffffffffffffffffffffffff
ffffffffffffffffffffffffffffffffffffffffffffffffffffffffffffffffffffffffffffffffffffffffffffffffffffffffffffffffffffffffffffffff
ffffffffffffffffffffffffffffffffffffffffffffffffffffffffffffffffffffffffffffffffffffffffffffffffffffffffffffffffffffffffffffffff
ffffffffffffffffffffffffffffffffffffffffffffffffffffffffffffffffffffffffffffffffffffffffffffffffffffffffffffffffffffffffffffffff
ffffffffffffffffffffffffffffffffffffffffffffffffffffffffffffffffffffffffffffffffffffffffffffffffffffffffffffffffffffffffffffffff
ffffffffffffffffffffffffffffffffffffffffffffffffffffffffffffffffffffffffffffffffffffffffffffffffffffffffffffffffffffffffffffffff
ffffffffffffffffffffffffffffffffffffffffffffffffffffffffffffffffffffffffffffffffffffffffffffffffffffffffffffffffffffffffffffffff
ffffffffffffffffffffffffffffffffffffffffffffffffffffffffffffffffffffffffffffffffffffffffffffffffffffffffffffffffffffffffffffffff
ffffffffffffffffffffffffffffffffffffffffffffffffffffffffffffffffffffffffffffffffffffffffffffffffffffffffffffffffffffffffffffffff
ffffffffffffffffffffffffffffffffffffffffffffffffffffffffffffffffffffffffffffffffffffffffffffffffffffffffffffffffffffffffffffffff
ffffffffffffffffffffffffffffffffffffffffffffffffffffffffffffffffffffffffffffffffffffffffffffffffffffffffffffffffffffffffffffffff
ffffffffffffffffffffffffffffffffffffffffffffffffffffffffffffffffffffffffffffffffffffffffffffffffffffffffffffffffffffffffffffffff
ffffffffffffffffffffffffffffffffffffffffffffffffffffffffffffffffffffffffffffffffffffffffffffffffffffffffffffffffffffffffffffffff
ffffffffffffffffffffffffffffffffffffffffffffffffffffffffffffffffffffffffffffffffffffffffffffffffffffffffffffffffffffffffffffffff
ffffffffffffffffffffffffffffffffffffffffffffffffffffffffffffffffffffffffffffffffffffffffffffffffffffffffffffffffffffffffffffffff
ffffffffffffffffffffffffffffffffffffffffffffffffffffffffffffffffffffffffffffffffffffffffffffffffffffffffffffffffffffffffffffffff
ffffffffffffffffffffffffffffffffffffffffffffffffffffffffffffffffffffffffffffffffffffffffffffffffffffffffffffffffffffffffffffffff
ffffffffffffffffffffffffffffffffffffffffffffffffffffffffffffffffffffffffffffffffffffffffffffffffffffffffffffffffffffffffffffffff
fffffffffff4c000000640000000000000000000000003f02000077000000000000000
000000000400200007800000002900aa00
0000000000000000000803f0000000000000000000803f0000000000000

Disclaimer
Kepaniteraan Mahkamah Agung Republik Indonesia berusaha untuk selalu mencantumkan informasi paling kini dan akurat sebagai bentuk komitmen Mahkamah Agung untuk pelayanan publik, transparansi dan akuntabilitas pelaksanaan fungsi peradilan. Namun dalam hal-hal tertentu masih dimungkinkan terjadi permasalahan teknis terkait dengan akurasi dan keterkinian informasi yang kami sajikan, hal mana akan terus kami perbaiki dari waktu kewaktu.
Dalam hal Anda menemukan inakurasi informasi yang termuat pada situs ini atau informasi yang seharusnya ada, namun belum tersedia, maka harap segera hubungi Kepaniteraan Mahkamah Agung RI melalui :
Email : kepaniteraan@mahkamahagung.go.id   Telp : 021-384 3348 (ext.318)

0000000000000000000000000000000000000
000000000000220000000c000000ffffffff460000001c000000100000000454d4
62b024000000c000000000000000e000000
14000000000000000100000001400000004000000030108000500000000b020
0000000050000000c02780040020300000001e00
040000000701040004000000070104000511000041 0b2000cc00780040020
0000000780040020000000002800000040020000
780000000100010000000000000000000000000000000000200000000000
00000000000ffffff00ffffffffffffffffffff
ffffffffffffffffffffffffffffffffffffffffffffffffffffffffffffffffffffffffffffffffffffffffffffffffffff
ffffffffffffffffffffffffffffffffffffffffffffffffffffffffffffffffffffffffffffffffffffffffffffffffffff
ffffffffffffffffffffffffffffffffffffffffffffffffffffffffffffffffffffffffffffffffffffffffffffffffffff
ffffffffffffffffffffffffffffffffffffffffffffffffffffffffffffffffffffffffffffffffffffffffffffffffffff
ffffffffffffffffffffffffffffffffffffffffffffffffffffffffffffffffffffffffffffffffffffffffffffffffffff
ffffffffffffffffffffffffffffffffffffffffffffffffffffffffffffffffffffffffffffffffffffffffffffffffffff
ffffffffffffffffffffffffffffffffffffffffffffffffffffffffffffffffffffffffffffffffffffffffffffffffffff
ffffffffffffffffffffffffffffffffffffffffffffffffffffffffffffffffffffffffffffffffffffffffffffffffffff
ffffffffffffffffffffffffffffffffffffffffffffffffffffffffffffffffffffffffffffffffffffffffffffffffffff
ffffffffffffffffffffffffffffffffffffffffffffffffffffffffffffffffffffffffffffffffffffffffffffffffffff
ffffffffffffffffffffffffffffffffffffffffffffffffffffffffffffffffffffffffffffffffffffffffffffffffffff
ffffffffffffffffffffffffffffffffffffffffffffffffffffffffffffffffffffffffffffffffffffffffffffffffffff
ffffffffffffffffffffffffffffffffffffffffffffffffffffffffffffffffffffffffffffffffffffffffffffffffffff
ffffffffffffffffffffffffffffffffffffffffffffffffffffffffffffffffffffffffffffffffffffffffffffffffffff
ffffffffffffffffffffffffffffffffffffffffffffffffffffffffffffffffffffffffffffffffffffffffffffffffffff
ffffffffffffffffffffffffffffffffffffffffffffffffffffffffffffffffffffffffffffffffffffffffffffffffffff
ffffffffffffffffffffffffffffffffffffffffffffffffffffffffffffffffffffffffffffffffffffffffffffffffffff
ffffffffffffffffffffffffffffffffffffffffffffffffffffffffffffffffffffffffffffffffffffffffffffffffffff
ffffffffffffffffffffffffffffffffffffffffffffffffffffffffffffffffffffffffffffffffffffffffffffffffffff
ffffffffffffffffffffffffffffffffffffffffffffffffffffffffffffffffffffffffffffffffffffffffffffffffffff
ffffffffffffffffffffffffffffffffffffffffffffffffffffffffffffffffffffffffffffffffffffffffffffffffffff
ffffffffffffffffffffffffffffffffffffffffffffffffffffffffffffffffffffffffffffffffffffffffffffffffffff
ffffffffffffffffffffffffffffffffffffffffffffffffffffffffffffffffffffffffffffffffffffffffffffffffffff
ffffffffffffffffffffffffffffffffffffffffffffffffffffffffffffffffffffffffffffffffffffffffffffffffffff
ffffffffffffffffffffffffffffffffffffffffffffffffffffffffffffffffffffffffffffffffffffffffffffffffffff
ffffffffffffffffffffffffffffffffffffffffffffffffffffffffffffffffffffffffffffffffffffffffffffffffffff
ffffffffffffffffffffffffffffffffffffffffffffffffffffffffffffffffffffffffffffffffffffffffffffffffffff
ffffffffffffffffffffffffffffffffffffffffffffffffffffffffffffffffffffffffffffffffffffffffffffffffffff
ffffffffffffffffffffffffffffffffffffffffffffffffffffffffffffffffffffffffffffffffffffffffffffffffffff
ffffffffffffffffffffffffffffffffffffffffffffffffffffffffffffffffffffffffffffffffffffffffffffffffffff
ffffffffffffffffffffffffffffffffffffffffffffffffffffffffffffffffffffffffffffffffffffffffffffffffffff
ffffffffffffffffffffffffffffffffffffffffffffffffffffffffffffffffffffffffffffffffffffffffffffffffffff
ffffffffffffffffffffffffffffffffffffffffffffffffffffffffffffffffffffffffffffffffffffffffffffffffffff
ffffffffffffffffffffffffffffffffffffffffffffffffffffffffffffffffffffffffffffffffffffffffffffffffffff
ffffffffffffffffffffffffffffffffffffffffffffffffffffffffffffffffffffffffffffffffffffffffffffffffffff
ffffffffffffffffffffffffffffffffffffffffff07ffffffffffffffffffffffffffffffffffffffffffffffffffffffff

Disclaimer
Kepaniteraan Mahkamah Agung Republik Indonesia berusaha untuk selalu mencantumkan informasi paling kini dan akurat sebagai bentuk komitmen Mahkamah Agung untuk pelayanan publik, transparansi dan akuntabilitas pelaksanaan fungsi peradilan. Namun dalam hal-hal tertentu masih dimungkinkan terjadi permasalahan teknis terkait dengan akurasi dan keterkinian informasi yang kami sajikan, hal mana akan terus kami perbaiki dari waktu kewaktu.
Dalam hal Anda menemukan inakurasi informasi yang termuat pada situs ini atau informasi yang seharusnya ada, namun belum tersedia, maka harap segera hubungi Kepaniteraan Mahkamah Agung RI melalui :
Email : kepaniteraan@mahkamahagung.go.id   Telp : 021-384 3348 (ext.318)

ffffffffffffffffffffffffffffffffffffffffffffffffffffffffffffffffffffffffffffffffffffff807fffffffffff
ffffffffffffffffffffffffffffffffffffffffffffffffffffffffffffffffffffffffffffffffffffffffffffffffffff
ffffffffffffffffffffffffffffffe00fffffffffffffffffffffffffffffffffffffffffffffffffffffffffffffffffff
fffffffffffffffffffffffffffffffffffffffffffffffffffffffffffffffffffffffffff803ffffffffffffffffffffff
ffffffffffffffffffffffffffffffffffffffffffffffffffffffffffffffffffffffffffffffffffffffffffffffffffff
fffffffffffffffffffe01ffffffffffffffffffffffffffffffffffffffffffffffffffffffffffffffffffffffffffffff
ffffffffffffffffffffffffffffffffffffffffffffffffffffffffffffffff80ffffffffffffffffffffffffffffffffff
ffffffffffffffffffffffffffffffffffffffffffffffffffffffffffffffffffffffffffffffffffffffffffffffffffff
ffffffffe0ffffffffffffffffffffffffffffffffffffffffffffffffffffffffffffffffffffffffffffffffffffffffff
fffffefffffffffffffffffffffffffffffffffff807fff8ffffffffffffffffffffffffffffffffffffffffffffff
fffffffffffffffffffffffffffffffffffffffffffffffffc03fffffffffffffffffffffffffffffc3fffffffc000fffcff
fffffffffffffffffffffffffffffffffffffffffffffffffffffffffffffffffffffffffffffffffffffffffffffe007fff
fffffffffffffffffffffff8007fffffff00003fffffffffffffffffffffffffffffffffffffffffffffffffffffffffffff
fffffc07fffffffffffffffffffffffffffffffffffc01ffffffffffffffffffffe0003ffffffe00000ffffffffffffffff
f
fffffffffffffffeffffffffffffffffffffffffffffffffffe007fffffffffffffffffffffff80fffff80fffffffffffffff
fffffffff00001ffffc000007fffffffe00ffffffffffffffffffffffffffff00fffffffffffffffffffffffffffffc0
1fff
ffffffffffffffffffc00ffff07ffffffffffffffffffffe0fe00fffffc000003fffff0001ffffff87ffff
ffffff
fffc03ffffffffffffffffffffffffffffff807ffffffffffffffffffff803ffffc3ffffffffffffffffe3fffe7f
fc07fffff807fc03fffffc0000ffffff807ffffffffffff80ffffffffffffffffffffffffffffffffff03fffffffff
ffff
ffffffffe01fffff3fffffffffffffffffc1effffff03fffff81fff81fffff000007ffffff01ffffffff3ffffe
07fffff
ffffffffffffffffffffffffffc3ffffffffffffffffffffff80ffffffffffffffffffffffc083ffffff81fffff83f
ffe1fffff1ff803ffffffc07ffffff03ffffc3ffffffffffffffffffffffffffffffffbf3ffffffffffffffffffffffe
0
7fffffffffffffffffffffffc001ffffffc1fffff87ffff0ffffffffff01ffffffff01ffffffff80ffffe3fffffffffffff
fff
ffffffffffffe1fffffffffffffffffffffffffff03fffffff00000000ffffffffe001fe7fffe0fffff8fffff8ff
ffffff
f81ffffffffc0ffffffffe01ffffbffffffff0361fffffffffffffffffff80ffffffffffffffffffffffffffffffc1f83fe0
00000
0000007ffffff381fe07fff0ffffff8fffffcffffffffffe0ffffffff0ffffffff807ffffffffc000000000
07fffffffffffff
ff807ffffffffffffffffffffffffffffff1003c0000000000000000ffffff83ff80fff8fffffcfffffcffffffff
ff07ffffffffc
ffffffff03ffffff8000000000000003ffffffffffff807ffffffffffffffffffffffffffff8000000000
000000000003ff
ffcfffe03ffc7ffffcfffffcffffffffff87ffffffffffffffff81fffff8000000000000000001ffffffffff
ffc0ffffffff
ffffffffffffffffff800000000000000000000001ffffffff81ffc7ffffcfffffcffffffffffc3ffffff
fff80ffffffe0f
fff800000000000000000001ffffffffffe3fffffffffffffffffffffffffff0000000000000000
00000003ffffffe0ffc

Disclaimer
Kepaniteraan Mahkamah Agung Republik Indonesia berusaha untuk selalu mencantumkan informasi paling kini dan akurat sebagai bentuk komitmen Mahkamah Agung untuk pelayanan publik, transparansi dan akuntabilitas pelaksanaan fungsi peradilan. Namun dalam hal-hal tertentu masih dimungkinkan terjadi permasalahan teknis terkait dengan akurasi dan keterkinian informasi yang kami sajikan, hal mana akan terus kami perbaiki dari waktu kewaktu.
Dalam hal Anda menemukan inakurasi informasi yang termuat pada situs ini atau informasi yang seharusnya ada, namun belum tersedia, maka harap segera hubungi Kepaniteraan Mahkamah Agung RI melalui :
Email : kepaniteraan@mahkamahagung.go.id   Telp : 021-384 3348 (ext.318)

0fffe7ffffcfffffffffffc3ffffffff007ffffff07c0c0000000000000000000001ffffffffffffff
ffffffffffffff
fffffff00000007ffffffff80000003fffffffcff803fffe7ffffcffffffffffe3fffffffe003fc3fff
8400000000006f
ffffe80000003ffe0ff9ffffffffffffffffffffffffffffffffffff000001fffffffffffe00000ffffffffff001
ffbe3ffff8
7fffffffffe1ffffffffe001f01fffe000000007ffffffffffe000007f803c0fffffffffffffffffffffff
ffffffffff0700
1ffffffffffffff00003f07ffffff001ffbf3ffff81f80ffffffe0fffffc03c000c01fffe0000003
ffffffffffffff800
00e001807ffffffffffffffffffffffffffffffff8f00fffffffffffffffffc001c00ffffff001ffbf3ffff007
007fffffc0
3ffdfc00c000800fffe000003fffffffffffffffffc0000000003feffffffffffffffffffffffffffffff9e
03ffffffffffff
fffffffc000003fffff001ff9ffffff002007fffffc01ffcfc0041f8000fffe00003ffffffffffffff
fffffc000000003801
ffffffffffffffffffffffffffff8e0fffffffffffffffffffffc00000fff7f1f8ff9fffffe000007fffffc00ffcf
c0043fc
000fffe0001ffffffffffffffffffffffc00000000000ffffffffffffffffffffffffffffc01ffffffffffffffffff
ffff807
000fc3fff8ff9fffffe000003fffffc00ffcfe0007fe018ffff1807ffffffffffffffffffffff803fc0
78000ffffffffffff
ffffffffffffffe03fffffffffffffffffffffffe1fe00001fff8ff9fffffe000003fffffc00ffcfe1c07fe
078ffff381ff
ffffffffffffffffffffff03ff1f8000ffffffffffffffffffffffffffffffff07ffffffffffffffffffffff9ff80001fff8
ff
9ffffff1f83c3fffffcf8ffcff1fc7fe0f8ffff387ffffffffffffffffffffffffc3ff9f80007ffffffffffffff
fffffffff
fffff8ffffffffffffffffffffffffffffffc0000fff8ff1ffffff3fc783fffffffcffcff0fc7fe1f0feff00ffffffff
fffffff
ffffffffff3ff9fc3fc7fffffffffffffffffffffffffffffffffffffffffffffffffffffffffe00000fff8ff1ffffff7fe2
0
3fffffff8ffcff83c7fe3f0fe7f80ffffffffffffffffffffffffffffbffdfcffc7ffffffffffffffffffffffffffffffff
fff
ffffffffffffffffffc100003cfff8ff1ffffffffe003fffffff8ffcffc0c7fe3e0fe7fc1ffffffffffffffffff
fffffffff
ffffeffc7fffffffffffffffffffffffffffffffffffffffffffffffffffffffffc0000ffffff8ff1ffffffffff007fffdfff8ff8
ffc007fc3e0fe7fe1fffffffffffffffffffffffffffffffffffeffc7ffffffffffffffffffffffffffffffffffffffffffffffff
ffffffe0003ffffff0ff1ffffffffff807fff0fff8ff8fff007fc7e1fe7fffffffffffffffffffffffffffffffffffffff
f87fff
ffffffffffffffffffffffffffffffffffffffffffffffffffffffe000fffffff1ff1ffffffffffc0000007ff8ff8fffc07fc
7c1f
c7fffffffffffffffffffffffffffffffffffffffff8ffffffffffffffffffffffffffffffffffffffffffffffffffffffffffff003ff
fffff1ff1fffffffffff0000007ff8ff8ffff07fc7c3fc7ffffffffffffffffffffffffffffffffffffffffff0ffffffffffff
ffff
ffffffffffffffffffffffffffffffffffffffffff80ffffffff1fe1fffffffbffe000003ff0ff8ffffcffc7c3fc7ffff
Disclaimer
Kepaniteraan Mahkamah Agung Republik Indonesia berusaha untuk selalu mencantumkan informasi paling kini dan akurat sebagai bentuk komitmen Mahkamah Agung untuk pelayanan publik, transparansi dan akuntabilitas pelaksanaan fungsi peradilan. Namun dalam hal-hal tertentu masih dimungkinkan terjadi permasalahan teknis terkait dengan akurasi dan keterkinian informasi yang kami sajikan, hal mana akan terus kami perbaiki dari waktu kewaktu.
Dalam hal Anda menemukan inakurasi informasi yang termuat pada situs ini atau informasi yang seharusnya ada, namun belum tersedia, maka harap segera hubungi Kepaniteraan Mahkamah Agung RI melalui :
Email : kepaniteraan@mahkamahagung.go.id Telp : 021-384 3348 (ext.318)

ffffff
fffffffffffffffffffffffff0ffffffffffffffffffffffffffffffffffffffffffffffffffffffc1fffffff1fe3fff
ffffbfffc00003ff1ff0ffffcffc7c7fc7fffffffffffffffffffffffffffffffffffffe0fffffffffffffffffffffffff
ff
ffffffffffffffffffffffffffffffffffffe1fe3fffc7ff9fbfc0007fff1ff1ffffcffc787fc7ffffffffffffffff
ffffff
fffffffffffffc1fffffffffffffffffffffffffffffffffffffffffffffffffffffffffffffffe3fe3fff83ff9f800007
ffff1ff1ffffeffc787fc7ffffffffffffffffffffffffffffffffffffffffc1ffffffffffffffffffffffffffffffffffffff
fffffffffffffffffffffc7ffe3fe3fff01ff9f80001fffff1ff1ffffeffc78ff87ffffffffffffffffffffffffffffff
ffffff
ff83ffffffffffffffffffffffffffffffffffffffffffffffffffffffffffffffff807fe3fe3fff01ff9fc0007ffffe1ff1
ffff
fffcf8ff87ffffffffffffffffffffffffffffffffffffffffffe03fffffffffffffffffffffffffffffffffffffffffffffffffffff
ffffffff803fe3fc3fff83ff1fc001fffffe1ff1ffffffffcf8ff8ffffffffffffffffffffffffffffffffffffffffffffffffe07ff
ffffff
ffffffffffffffffffffffffffffffffffffffffffffffffffff801fe3fc7fff87ff1fe007fffffe3fe1ffffffffcf8ff
8fff
ffffffffffffffffffffffffffffffffffffc0ffffffffffffffffffffffffffffffffffffffffffffffffffffffffffffffffffff980f
e3fc7fffcffe1ff80ffffffe3fe3ffffffffcf8ff0fffffffffffffffffffffffffffffffffffffffffc0ffffffffffffffffffff
f
fffffffffffffffffffffffffffffffffffffffffffff998fc7fc7ffffffe3ffe3ffffffc3fe3ffffffffefcff0ffffffffffffff
f
ffffffffffffffffffffe1ffffffffffffffffffffffffffffffffffffffffffffffffffffffffffffffffffd98fc7fc7ffffffc
3ffffffc7ffc3fe3ffffffffffdff0ffffffffffffffffffffffffffffffffffffffffe3ffffffffffffffffffffffffffffffffff
ffffffffffffffffffffffffffffffffffd8fc7f87ffffffc7ffffff807fc3fe3ffffffffffff1fffffffffffffffffffffffffffff
ffffffffffe3fffffffffffffffffffffffffffffffffffffffffffffffffffffffffffffffffffffffffd8fc7f87ffffff87ffffff801fc
7fe3ffffffffffff1fffffffffffffffffffffffffffffffffffffffffffe7ffffffffffffffffffffffffffffffffffffffffffffffff
fffffffffffffffffffffdfc7f8ffffffff8ffffffff801fc7fc3ffffff9ffffe1ffffffffffffffffffffffffffffffffffffffe3ffff
f
fffffffffffffffffffffffffffffffffffffffffffffffffffffffffffffffffbfffdf87f8ffffffffcffffffff980fc7fc7ffffff01
fffe1fffffffffffffffffffffffffffffffffffff03fffffffffffffffffffffffffffffffffffffffffffffffffffffffffffffffff
ff03ffff87f8ffffffffffe0fffd98fc7fc7ffffff007ffe1fffffffffffffffffffffffffffffffffffffff80fffffffffff
ffffff
fffffffffffffffffffffffffffffffffffffffffffffffffffffff803fff8ff8fffffffffff03ffdd8f8ffc7ffffff003ffe3ffff
fff
ffffffffffffffffffffffffffffffff07ffffffffffffffffffffffffffffffffffffffffffffffffffffffffffffffffffffffe007ff87f8
fffffffffffffc1fffc8f8ffc7ffffff303ffe3ffffffffffffffffffffffffffffffffffffff3f87ffffffffffffffffffffffffffffff
ffffffffffffffffffffffffffffffffffffffffff803ff03f8fffffffffff9f0fffcdf8ffc7ffffff331ffc3ffffffffffffffffff
fff
fffffffffffff9803ffffffffffffffffffffffffffffffffffffffffffffffffffffffffffffffffffffffffe01ff03f8ffffffffffff8c
0ffffdf8ff8ffffffffffb1ffc3ffffffffffffffffffffffffffffffffffffffff8003ffffffffffffffffffffffffffffffffffffffffffff
fffffffffffffffffffffffffffffc0fe03f8fffffffffffff8007fffff8ff8ffffffffffb1ffc7ffffffffffffffffffffffffffffffffffff
fc003fffffffffffffffffffffffffffffffffffffffffffffffffffffffffffffffffffffffffff07e07f8fffffffffffc00ffffff07f8f
ffffffffff9ffc7fffffffffffffffffffffffffffffffffffffffe01ffffffffffffffffffffffffffffffffffffffffffffffffffffffff
ffffffffffffffc7f0ff9ffffffe3fff03ffffff03f8fffffffffffbffc7ffffffffffffffffffffffffffffffffffffffffffffffffff
ffffffffffffffffffffffffffffffffffffffffffffffffffffffffffffffffffe7f8ffffffffffe03ffffffffffe03f8ffffff8fffffc

Disclaimer
Kepaniteraan Mahkamah Agung Republik Indonesia berusaha untuk selalu mencantumkan informasi paling kini dan akurat sebagai bentuk komitmen Mahkamah Agung untuk pelayanan publik, transparansi dan akuntabilitas pelaksanaan fungsi peradilan. Namun dalam hal-hal tertentu masih dimungkinkan terjadi permasalahan teknis terkait dengan akurasi dan keterkinian informasi yang kami sajikan, hal mana akan terus kami perbaiki dari waktu kewaktu.
Dalam hal Anda menemukan inakurasi informasi yang termuat pada situs ini atau informasi yang seharusnya ada, namun belum tersedia, maka harap segera hubungi Kepaniteraan Mahkamah Agung RI melalui :
Email : kepaniteraan@mahkamahagung.go.id   Telp : 021-384 3348 (ext.318)

7ffffffffffffffffffffffffffffffffffffffffffffffffffffffffffffffffffffffffffffffffffffffffffffffffffffffffff
ff7f8ffffffffff807ffffffffe03fcffffffc1ffff87fffffffffffffffffffffffffffffffffffffffffffffffffffffffffffff
fffffffffffffffffffffffffffffffffffffffffffffffffffdffffffffffe01ffff8fffe07fcffffffe03fff87ffffffffffff
fffffffffffffffffffffffffffffffffffffffffffffffffffffffffffffffffffffffffffffffffffffffffffffffffffffdfffff
ffff807fff80fff0ffcffffff80fff8fffffffffffffffffffffffffffffffffffffffffffffffffffffffffffffffffffffffff
fffffffffffffffffffffffffffffffffffffffffc81ffffffe03fffe01ff8fffffffffc03ffcfffffffffffffffffffffffffff
ffffffffffffffffffffffffffffffffffffffffffffffffffffffffffffffffffffffffffffffffffffffffc007fffffff81ffff0
0ff9ffffffffffff01ffcffffffffffffffffffffffffffffffffffffffffffffffffffffffffffffffffffffffffffffffffff
ffffffffffffffffffffffffffffe003ffffffe1ffffc07fffeffffffffc0ffcfffffffffffffffffffffffffffffffffff
ffffffffffffffffffffffffffffffffffffffffffffffffffffffffffffffffffffffffffff001fffffff0fffff03fffe40ffff
ffff0ffcfffffffffffffffffffffffffffffffffffffffffffffffffffffffffffffffffffffffffffffffffffffffffffffffff
ffffffffffffffffb18fffffff8fffffc1fffe003ffffff87ffffffffffffffffffffffffffffffffffffffffffffffffffffff
ffffffffffffffffffffffffffffffffffffffffffffffffffffffffffffffff9cffffffcffffff1ffff000ffffffe7ffffff
fffffffffffffffffffffffffffffffffffffffffffffffffffffffffffffffffffffffffffffffffffffffffffffffffffffffffff
ffffc0ffffffffffffffff9ffff8007ffffff7e73fffffffffffffffffffffffffffffffffffffffffffffffffffffffffffffff
fffffffffffffffffffffffffffffffffffffffffffffffffffc0ffffffffffffffffdffffd8c7fffffffe403ffffffffffffff
fffffffffffffffffffffffffffffffffffffffffffffffffffffffffffffffffffffffffffffffffffffffffffffffffffe0ffffff
ffffffffffffffffc63ffffffff001fffffffffffffffffffffffffffffffffffffffffffffffffffffffffffffffffffffffffff
ffffffffffffffffffffffffffffffffffffffff9ffffffffffffffffffffffc03ffffffff800ffffffffffffffffffffffffffff
fffffffffffffffffffffffffffffffffffffffffffffffffffffffffffffffffffffffffffffffffffffffffffffffffffffffffff
fffe03ffffffff8987fffffffffffffffffffffffffffffffffffffffffffffffffffffffffffffffffffffffffffffffffffffff
fffffffffffffffffffffffffffffffffffffffffffffffffff07ffffffffd8e7ffffffffffffffffffffffffffffffffffffffff
ffffffffffffffffffffffffffffffffffffffffffffffffffffffffffffffffffffffffffffffffffffffffffffffffffc7ffffff
ffffc03ffffffffffffffffffffffffffffffffffffffffffffffffffffffffffffffffffffffffffffffffffffffffffffffffffff
fffffffffffffffffffffffffffffffffffffffffffffffffffe03fffffffffffffffffffffffffffffffffffffffffffffffffffff
ffffffffffffffffffffffffffffffffffffffffffffffffffffffffffffffffffffffffffffffffffffffffffffffffffffff07fffff
fffffffffffffffffffffffffffffffffffffffffffffffffffffffffffffffffffffffffffffffffffffffffffffffffffffffffff
ffffffffffffffffffffffffffffffffffffffff87ffffffffffffffffffffffffffffffffffffffffffffffffffffffffffffffffff
fffffffffffffffffffffffffffffffffffffffffffffffffffffffffffffffffffffffffffffffffffffffffffffffffffffffffff
fffffffffffffffffffffffffffffffffffffffffffffffffffffffffffffffffffffffffffffffffffffffffffffffffffffffffff
fffffffffffffffffffffffffffffffffffffffffffffffffffffffffffffffffffffffffffffffffffffffffffffffffffffffffff
fffffffffffffffffffffffffffffffffffffffffffffffffffffffffffffffffffffffffffffffffffffffffffffffffffffffffff
fffffffffffffffffffffffffffffffffffffffffffffffffffffffffffffffffffffffffffffffffffffffffffffffffffffffffff
fffffffffffffffffffffffffffffffffffffffffffffffffffffffffffffffffffffffffffffffffffffffffffffffffffffffffff
fffffffffffffffffffffffffffffffffffffffffffffffffffffffffffffffffffffffffffffffffffffffffffffffffffffffffff
fffffffffffffffffffffffffffffffffffffffffffffffffffffffffffffffffffffffffffffffffffffffffffffffffffffffffff
fffffffffffffffffffffffffffffffffffffffffffffffffffffffffffffffffffffffffffffffffffffffffffffffffffffffffff
fffffffffffffffffffffffffffffffffffffffffffffffffffffffffffffffffffffffffffffffffffffffffffffffffffffffffff
fffffffffffffffffffffffffffffffffffffffffffffffffffffffffffffffffffffffffffffffffffffffffffffffffffffffffff
fffffffffffffffffffffffffffffffffffffffffffffffffffffffffffffffffffffffffffffffffffffffffffffffffffffffffff
fffffffffffffffffffffffffffffffffffffffffffffffffffffffffffffffffffffffffffffffffffffffffffffffffffffffffff
fffffffffffffffffffffffffffffffffffffffffffffffffffffffffffffffffffffffffffffffffffffffffffffffffffffffffff
fffffffffffffffffffffffffffffffffffffffffffffffffffffffffffffffffffffffffffffffffffffffffffffffffffffffffff
fffffffffffffffffffffffffffffffffffffffffffffffffffffffffffffffffffffffffffffffffffffffffffffffffffffffffff
fffffffffffffffffffffffffffffffffffffffffffffffffffffffffffffffffffffffffffffffffffffffffffffffffffffffffff
fffffffffffffffffffffffffffffffffffffffffffffffffffffffffffffffffffffffffffffffffffffffffffffffffffffffffff

Disclaimer
Kepaniteraan Mahkamah Agung Republik Indonesia berusaha untuk selalu mencantumkan informasi paling kini dan akurat sebagai bentuk komitmen Mahkamah Agung untuk pelayanan publik, transparansi dan akuntabilitas pelaksanaan fungsi peradilan. Namun dalam hal-hal tertentu masih dimungkinkan terjadi permasalahan teknis terkait dengan akurasi dan keterkinian informasi yang kami sajikan, hal mana akan terus kami perbaiki dari waktu kewaktu.
Dalam hal Anda menemukan inakurasi informasi yang termuat pada situs ini atau informasi yang seharusnya ada, namun belum tersedia, maka harap segera hubungi Kepaniteraan Mahkamah Agung RI melalui :
Email : kepaniteraan@mahkamahagung.go.id   Telp : 021-384 3348 (ext.318)

ffffffffffffffffffffffffffffffffffffffffffffffffffffffffffffff0c00000040092900aa00000000000
00078004002
000000000040000002701ffff030000000000}\par\plain\f5\fs24\b DEMI KEADILAN BERDASARKAN KETUHANAN YANG MAHA ESA\par\pard\s23\itap0\widctlpar\qj\fi850\sl360\slmult1\plain\f5\fs24\par\pard\s23\itap0\widctlpar\qj\fi709\sl360\slmult1 Pengadilan Agama Sumber yang memeriksa dan mengadili perkara perdata pada tingkat pertama dalam persidangan Majelis telah menjatuhkan putusan sebagai berikut dalam perkara antara :\par\pard\itap0\widctlpar\qj\fi-1800\li1800\sb120\sl360\slmult1\tx1800\plain\f1\fs24{\txfielddef{*\txfieldstart\txfieldtype4\txfieldflags0}{*\txfielddatadef\txfielddatatype4\txfielddata 540065007800740031000000}{*\txfieldtext {*\txfieldend}}{*\bkmkstart Text1}{*\bkmkend Text1}}\plain\f5\fs24\b\cf1 SATIAH BINTI MISTA\plain\f5\fs24\cf1 , umur 33 tahun, agama Islam, pendidikan SLTP, pekerjaan Mengurus Rumah Tangga / Tenaga Kerja Indonesia, tempat kediaman di \plain\f5\fs24\cf1{\txfielddef{*\txfieldstart\txfieldtype4\txfieldflags0}{*\txfielddatadef\txfielddatatype4\txfielddata 540065007800740033000000}{*\txfieldtext {*\txfieldend}}{*\bkmkstart Text3}{*\bkmkend Text3}}\plain\f5\fs24\cf1 Dusun 02, RT 006 RW 002, Desa Bayalangu Lor, Kecamatan Gegesik, Kabupaten Cirebon Alamat di Taiwan : No.12, Lane 35, Section 2, Ziyou Road, Central District, Taichung City, Taiwan R.O.C, sebagai \plain\f5\fs24\cf1{\txfielddef{*\txfieldstart\txfieldtype4\txfieldflags0}{*\txfielddatadef\txfielddatatype4\txfielddata 540065007800740050000000}{*\txfieldtext {*\txfieldend}}{*\bkmkstart TextP}{*\bkmkend TextP}}\plain\f5\fs24\cf1 Penggugat\plain\f5\fs24 ;\par\pard\itap0\widctlpar\qc\sl360\slmult1 melawan\par\pard\itap0\widctlpar\qj\fi-1800\li1800\sl360\slmult1\tx1800\plain\f1\fs24{\txfielddef{*\txfieldstart\txfieldtype4\txfieldflags0}{*\txfielddatadef\txfielddatatype4\txfielddata 540065007800740032000000}{*\txfieldtext {*\txfieldend}}{*\bkmkstart Text2}{*\bkmkend Text2}}\plain\f5\fs24\b\cf1 SARIKA BIN DABAS\plain\f5\fs24\cf1 ,\plain\f5\fs24\b\cf1 \plain\f5\fs24\cf1 umur 37 tahun, agama Islam, pendidikan SLTP, pekerjaan Wiraswast, tempat kediaman di \plain\f5\fs24\cf1{\txfielddef{*\txfieldstart\txfieldtype4\txfieldflags0}{*\txfielddatadef\txfielddatatype4\txfielddata 540065007800740034000000}{*\txfieldtext {*\txfieldend}}{*\bkmkstart Text4}{*\bkmkend Text4}}\plain\f5\fs24\cf1 Dusun 02, RT 006 RW 002, Desa Bayalangu Lor, Kecamatan Gegesik, Kabupaten Cirebon, sebagai \plain\f5\fs24\cf1{\txfielddef{*\txfieldstart\txfieldtype4\txfieldflags0}{*\txfielddatadef\txfielddatatype4\txfielddata 540065007800740054000000}{*\txfieldtext {*\txfieldend}}{*\bkmkstart TextT}{*\bkmkend TextT}}\plain\f5\fs24\cf1 Tergugat;\plain\f5\fs24\par\pard\s23\itap0\widctlpar\qj\sl360\slmult1

Disclaimer
Kepaniteraan Mahkamah Agung Republik Indonesia berusaha untuk selalu mencantumkan informasi paling kini dan akurat sebagai bentuk komitmen Mahkamah Agung untuk pelayanan publik, transparansi dan akuntabilitas pelaksanaan fungsi peradilan. Namun dalam hal-hal tertentu masih dimungkinkan terjadi permasalahan teknis terkait dengan akurasi dan keterkinian informasi yang kami sajikan, hal mana akan terus kami perbaiki dari waktu kewaktu.
Dalam hal Anda menemukan inakurasi informasi yang termuat pada situs ini atau informasi yang seharusnya ada, namun belum tersedia, maka harap segera hubungi Kepaniteraan Mahkamah Agung RI melalui :
Email : kepaniteraan@mahkamahagung.go.id   Telp : 021-384 3348 (ext.318)

Pengadilan Agama tersebut;\par Setelah mempelajari berkas perkara yang bersangkutan;\par Setelah mendengar keterangan Penggugat di muka persidangan;\par\pard\itap0\widctlpar\qc\sb120\sa120\sl360\slmult1 DUDUK PERKARA\par\pard\itap0\widctlpar\qj\fi709\sl360\slmult1 Bahwa, Penggugat dengan surat gugatannya tertanggal 16 November 2020 yang telah terdaftar di Kepaniteraan Pengadilan Agama Sumber dibawah Nomor 6592/Pdt.G/2020/PA.Sbr tanggal 16 November 2020, telah mengajukan hal-hal sebagai berikut :\par{\listtext\pard\plain\f5\fs24 1.\tab}\pard\itap0\widctlpar\qj\fi-360\li360\sl360\slmult1\tx360\ls1\ilvl0 Bahwa Penggugat telah melangsungkan pernikahan dengan Tergugat pada tanggal 11 Juli 2005, dicatat di \plain\f5\fs24{\txfielddef{*\txfieldstart\txfieldtype4\txfieldflags0}{*\txfielddatadef\txfielddatatype4\txfielddata 740065007800740035000000}{*\txfieldtext {*\txfieldend}}{*\bkmkstart text5}{*\bkmkend text5}}\plain\f5\fs24 Kantor Urusan Agama Kecamatan Gegesik Kabupaten Cirebon, dengan Akta Nikah Nomor 390/45/III/2005 tanggal 11 Juli 2005;\par{\listtext\pard\plain\f5\fs24 2.\tab}\ls1\ilvl0 Bahwa setelah akad nikah Penggugat dan Tergugat membina rumah tangga dengan bertempat tinggal terakhir di rumah ...., telah hidup rukun sebagai mana layaknya suami isteri \plain\f5\fs24{\txfielddef{*\txfieldstart\txfieldtype4\txfieldflags0}{*\txfielddatadef\txfielddatatype4\txfielddata 540065007800740036000000}{*\txfieldtext {*\txfieldend}}{*\bkmkstart Text6}{*\bkmkend Text6}}\plain\f5\fs24 belum dikaruniai anak ..../ .... sudah dikaruniai anak ...... orang bernama :;\par{\listtext\pard\plain\f5\fs24 3.\tab}\ls1\ilvl0 Bahwa selama berumah tangga Penggugat dengan Tergugat belum pernah bercerai ;\par{\listtext\pard\plain\f5\fs24 4.\tab}\ls1\ilvl0 Bahwa awalnya keadaan rumah tangga Penggugat dengan Tergugat berjalan dengan rukun dan harmonis, akan tetapi sekitar bulan 1 keharmonisan tersebut mulai pudar, karena antara Penggugat dan Tergugat sering terjadi perseilsihan dan pertengkaran yang mencapai puncaknya pada bulan 1;\par{\listtext\pard\plain\f5\fs24 5.\tab}\ls1\ilvl0 Bahwa perselisihan dan pertengkaran Penggugat dan Tergugat tersebut disebabkan \plain\f5\fs24{\txfielddef{*\txfieldstart\txfieldtype4\txfieldflags0}{*\txfielddatadef\txfielddatatype4\txfielddata 5400650078007400310030000000}{*\txfieldtext {*\txfieldend}}{*\bkmkstart Text10}{*\bkmkend Text10}}\plain\f5\fs24 1;\par{\listtext\pard\plain\f5\fs24 6.\tab}\ls1\ilvl0 Bahwa akibat rumah tangga Penggugat dan Tergugat yang sering terjadi perselisihan dan pertengkaran tersebut, maka Penggugat dan #0047 berpisah tempat tinggal selama .....bulan/tahun;\par{\listtext\pard\plain\f5\fs24 7.\tab}\pard\itap0\widctlpar\qj\fi-357\li357\sl360\slmult1\tx357\ls1\ilvl0 Bahwa Penggugat telah berusaha bersabar dan berupaya mempertahankan keutuhan rumah tangga, akan tetapi tidak berhasil, sehingga untuk mencapai tujuan perkawinan yang sakinah, mawaddah dan rahmah tidak mungkin bisa terwujud, oleh karenanya cukup alasan bagi Penggugat untuk

Disclaimer
Kepaniteraan Mahkamah Agung Republik Indonesia berusaha untuk selalu mencantumkan informasi paling kini dan akurat sebagai bentuk komitmen Mahkamah Agung untuk pelayanan publik, transparansi dan akuntabilitas pelaksanaan fungsi peradilan. Namun dalam hal-hal tertentu masih dimungkinkan terjadi permasalahan teknis terkait dengan akurasi dan keterkinian informasi yang kami sajikan, hal mana akan terus kami perbaiki dari waktu kewaktu.
Dalam hal Anda menemukan inakurasi informasi yang termuat pada situs ini atau informasi yang seharusnya ada, namun belum tersedia, maka harap segera hubungi Kepaniteraan Mahkamah Agung RI melalui :
Email : kepaniteraan@mahkamahagung.go.id   Telp : 021-384 3348 (ext.318)

mengajukan perceraian ini;\par\pard\itap0\widctlpar\qj\fi709\sl360\slmult1 Berdasarkan dalil-dalil/alasan tersebut diatas, Penggugat mohon dengan hormat, kiranya bapak Ketua Pengadilan Agama Sumber cq. Majelis Hakim yang memeriksa dan mengadili perkara ini, berkenan untuk menjatuhkan putusan yang amarnya berbunyi sebagai berikut :\par{\listtext\pard\plain\f5\fs24 1.\tab}\pard\itap0\widctlpar\qj\fi-284\li284\sl360\slmult1\tx284\ls1\ilvl3 Mengabulkan gugatan Penggugat;\par{\listtext\pard\plain\f5\fs24 2.\tab}\ls1\ilvl3 Menjatuhkan talak satu bain sughra Tergugat (\plain\f5\fs24\cf1 SARIKA BIN DABAS\plain\f5\fs24) terhadap Penggugat (SATIAH BINTI MISTA);\par{\listtext\pard\plain\f5\fs24 3.\tab}\ls1\ilvl3 Membebankan biaya perkara menurut hukum;\par\pard\itap0\widctlpar\qj\sl360\slmult1 Atau apabila Pengadilan berpendapat lain, mohon putusan yang seadil-adilnya;\par\pard\itap0\widctlpar\qj\fi709\sl360\slmult1 Bahwa, pada hari sidang yang telah ditetapkan, Penggugat datang menghadap di muka persidangan, sedangkan \plain\f5\fs24\cf1 Tergugat \plain\f5\fs24 tidak datang dan tidak pula menyuruh orang lain sebagai wakil atau kuasanya yang sah, padahal kepadanya telah dipanggil dengan resmi dan patut berdasarkan berita acara relaas panggilan tanggal 17 Desember 2020 dan 17 Desember 2020, sedangkan tidak ternyata ketidakhadirannya itu disebabkan oleh suatu alasan yang sah menurut hukum;\par Bahwa, Majelis Hakim telah berusaha menasehati Penggugat agar Penggugat dapat membina kembali rumah tangganya dengan \plain\f5\fs24\cf1 Tergugat\plain\f5\fs24 ;\par Bahwa, atas nasehat tersebut Penggugat mengatakan akan mencabut perkaranya dengan alasan Penggugat akan kembali rukun membina rumah tangga dengan Tergugat;\par Bahwa, selanjutnya Majelis Hakim bermusyawarah;\par\pard\s23\itap0\widctlpar\qj\fi720\sl360\slmult1\plain\f5\fs22 Bahwa, \plain\f5\fs24 untuk mempersingkat uraian pertimbangan ini ditunjuk hal-hal sebagaimana tercantum dalam berita acara persidangan perkara ini;\par\pard\itap0\widctlpar\qc\fi720\sb120\sa120\sl360\slmult1 PERTIMBANGAN HUKUM\par\pard\s23\itap0\widctlpar\qj\fi720\sl360\slmult1 Menimbang, bahwa maksud dan tujuan gugatan Penggugat adalah sebagaimana terurai diatas;\par Menimbang, bahwa sesuai dengan maksud dan ketentuan Pasal \plain\f5\fs24\cf1 82 Undang-Undang Nomor 7 Tahun 1989 \plain\f5\fs24 tentang Peradilan Agama sebagaimana telah diubah dengan Undang-Undang Nomor 3 Tahun 2006 dan perubahan kedua dengan Undang-Undang Nomor 50 Tahun 2009, Majelis Hakim telah berusaha maksimal menasehati Penggugat agar Penggugat dapat rukun kembali membina rumah dengan Tergugat dan ternyata usaha tersebut berhasil;\par\pard\s19\itap0\widctlpar\qj\fi709\sl360\slmult1 Menimbang, bahwa Penggugat di muka sidang mengatakan akan mencabut perkaranya dengan alasan akan kembali rukun dengan Tergugat;\par\pard\s23\itap0\widctlpar\qj\fi720\sl360\slmult1 Menimbang, bahwa oleh karena Penggugat mengatakan akan mencabut perkaranya dengan alasan sebagai mana tersebut di atas, maka Majelis Hakim

Disclaimer
Kepaniteraan Mahkamah Agung Republik Indonesia berusaha untuk selalu mencantumkan informasi paling kini dan akurat sebagai bentuk komitmen Mahkamah Agung untuk pelayanan publik, transparansi dan akuntabilitas pelaksanaan fungsi peradilan. Namun dalam hal-hal tertentu masih dimungkinkan terjadi permasalahan teknis terkait dengan akurasi dan keterkinian informasi yang kami sajikan, hal mana akan terus kami perbaiki dari waktu kewaktu.
Dalam hal Anda menemukan inakurasi informasi yang termuat pada situs ini atau informasi yang seharusnya ada, namun belum tersedia, maka harap segera hubungi Kepaniteraan Mahkamah Agung RI melalui :
Email : kepaniteraan@mahkamahagung.go.id Telp : 021-384 3348 (ext.318)

berpendapat bahwa perkara Nomor 6592/Pdt.G/2020/PA.Sbr dinyatakan dicabut;\par Menimbang, bahwa oleh karena perkara tersebut telah dinyatakan dicabut, maka proses penyelesaian perkara ini harus dinyatakan selesai;\par\pard\s19\itap0\widctlpar\qj\fi720\sl360\slmult1 Menimbang, bahwa perkara ini termasuk bidang perkawinan, oleh karenanya berdasarkan Pasal 89 ayat (1) Undang-Undang Nomor 7 Tahun 1989 tentang Peradilan Agama sebagaimana telah diubah dengan Undang-Undang Nomor 3 Tahun 2006 dan perubahan kedua dengan Undang-Undang Nomor 50 Tahun 2009, maka seluruh biaya perkara ini dibebankan kepada Penggugat;\par Mengingat segala peraturan perundang-undangan yang berlaku serta ketentuan hukum syara\loch\f5\hich\f5\'92 yang berkaitan dengan perkara ini;\par\pard\itap0\widctlpar\qc\sl360\slmult1\plain\f5\fs24\b M E N E T A P K A N\par\pard\itap0\widctlpar\qj\fi-284\li284\sl360\slmult1\tx284\plain\f5\fs24
1. Menerima pencabutan perkara Nomor 6592/Pdt.G/2020/PA.Sbr dari Penggugat; \par
2.MemerintakanPanitera Pengadilan Agama Sumberuntuk mencatat pencabutan perkara tersebut dalam register perkara; \par
3.Membebankan kepada Penggugat untuk membayar biaya perkara sejumlah Rp. 501000,00 (lima ratus satu ribu rupiah); \par

\par\par\pard\s23\itap0\widctlpar\qj\fi782\sl360\slmult1 Demikian Penetapan ini dijatuhkan dalam permusyawaratan Majelis Hakim Pengadilan Agama Sumber\plain\f5\fs22 \plain\f5\fs24 pada hari Rabu tanggal 23 Desember 2020 Masehi, bertepatan dengan tanggal 8 Jumadil Awwal 1442 Hijriyah, oleh kami Drs. H. MUHAMMAD MAULUDIN sebagai Ketua Majelis, Drs. H. TAUFIQURROKHMAN, M.H. dan H. ABDUL HANAN, SH.,MH masing-masing sebagai Hakim Anggota, yang dibacakan dalam sidang terbuka untuk umum oleh Ketua Majelis tersebut dengan dihadiri para Hakim Anggota dan FERI NURJAMAN S.H.I. sebagai Panitera Pengganti serta dihadiri oleh Penggugat tanpa hadirnya
Tergugat;\par\pard\itap0\widctlpar\qj\fi720\sl360\slmult1\par\trowd\irow0\irowband0\lastrow\trgaph108\trleft0\trftsWidth1\trftsWidthB3\trftsWidthA3\trpaddl108\trpaddr108\trpaddfl3\trpaddft3\trpaddfr3\trpaddfb3\clvertalt\clftsWidth3\clwWidth8789\cellx8789\pard\intbl\widctlpar\qc\sl276\slmult1\plain\f5\fs24 Ketua
Majelis\cell\trowd\irow0\irowband0\lastrow\trgaph108\trleft0\trftsWidth1\trftsWidthB3\trftsWidthA3\trpaddl108\trpaddr108\trpaddfl3\trpaddft3\trpaddfr3\trpaddfb3\clvertalt\clftsWidth3\clwWidth8789\cellx8789\row\pard\itap0\widctlpar\qj\par\par\par\par\trowd\irow0\irowband0\lastrow\trgaph108\trleft0\trftsWidth1\trftsWidthB3\trftsWidthA3\trpaddl108\trpaddr108\trpaddfl3\trpaddft3\trpaddfr3\trpaddfb3\clvertalt\clftsWidth3\clwWidth8789\cellx8789\pard\intbl\widctlpar\qc\sl276\slmult1\plain\f5\fs24\b Drs. H. MUHAMMAD MAULUDIN \cell\trowd\irow0\irowband0\lastrow\trgaph108\trleft0\trftsWidth1\trftsWidthB3\trftsWidthA3\trpaddl108\trpaddr108\trpaddfl3\trpaddft3\trpaddfr3\trpaddfb3\clvertalt\clftsWidth3\clwWidth8789\cellx8789\row\pard\itap0\widctlpar\qj\plain\f5\fs24\par\trowd\irow0\irowband0\lastrow\trgaph108\trleft0\trftsWidth1\t

Disclaimer
Kepaniteraan Mahkamah Agung Republik Indonesia berusaha untuk selalu mencantumkan informasi paling kini dan akurat sebagai bentuk komitmen Mahkamah Agung untuk pelayanan publik, transparansi dan akuntabilitas pelaksanaan fungsi peradilan. Namun dalam hal-hal tertentu masih dimungkinkan terjadi permasalahan teknis terkait dengan akurasi dan keterkinian informasi yang kami sajikan, hal mana akan terus kami perbaiki dari waktu kewaktu.
Dalam hal Anda menemukan inakurasi informasi yang termuat pada situs ini atau informasi yang seharusnya ada, namun belum tersedia, maka harap segera hubungi Kepaniteraan Mahkamah Agung RI melalui :
Email : kepaniteraan@mahkamahagung.go.id   Telp : 021-384 3348 (ext.318)

rftsWidthB3\trftsWidthA3\trpaddl108\trpaddr108\trpaddfl3\trpaddft3\trpaddfr3\trpaddfb3\clvertalt\clftsWidth3\clwWidth4395\cellx4395\clvertalt\clftsWidth3\clwWidth4394\cellx8789\pard\intbl\widctlpar\qc\sl276\slmult1 Hakim Anggota\cell Hakim Anggota\cell\trowd\irow0\irowband0\lastrow\trgaph108\trleft0\trftsWidth1\trftsWidthB3\trftsWidthA3\trpaddl108\trpaddr108\trpaddfl3\trpaddft3\trpaddfr3\trpaddfb3\clvertalt\clftsWidth3\clwWidth4395\cellx4395\clvertalt\clftsWidth3\clwWidth4394\cellx8789\row\pard\itap0\widctlpar\qj\par\par\par\pard\itap0\widctlpar
\par\trowd\irow0\irowband0\lastrow\trgaph108\trleft0\trftsWidth1\trftsWidthB3\trftsWidthA3\trpaddl108\trpaddr108\trpaddfl3\trpaddft3\trpaddfr3\trpaddfb3\clvertalt\clftsWidth3\clwWidth4395\cellx4395\clvertalt\clftsWidth3\clwWidth4394\cellx8789\pard\intbl\widctlpar\qc\sl276\slmult1\plain\f5\fs24\b Drs. H. TAUFIQURROKHMAN, M.H. \cell H. ABDUL HANAN, SH.,MH
\cell\trowd\irow0\irowband0\lastrow\trgaph108\trleft0\trftsWidth1\trftsWidthB3\trftsWidthA3\trpaddl108\trpaddr108\trpaddfl3\trpaddft3\trpaddfr3\trpaddfb3\clvertalt\clftsWidth3\clwWidth4395\cellx4395\clvertalt\clftsWidth3\clwWidth4394\cellx8789\row\pard\itap0\widctlpar\qj\li4111\tx4111\plain\f5\fs24\par\trowd\irow0\irowband0\lastrow\trgaph108\trleft0\trftsWidth1\trftsWidthB3\trftsWidthA3\trpaddl108\trpaddr108\trpaddfl3\trpaddft3\trpaddfr3\trpaddfb3\clvertalt\clftsWidth3\clwWidth8789\cellx8789\pard\intbl\widctlpar\qc\sl276\slmult1 Panitera Pengganti\cell\trowd\irow0\irowband0\lastrow\trgaph108\trleft0\trftsWidth1\trftsWidthB3\trftsWidthA3\trpaddl108\trpaddr108\trpaddfl3\trpaddft3\trpaddfr3\trpaddfb3\clvertalt\clftsWidth3\clwWidth8789\cellx8789\row\pard\itap0\widctlpar\qj\par\par\par\par\trowd\irow0\irowband0\lastrow\trgaph108\trleft0\trftsWidth1\trftsWidthB3\trftsWidthA3\trpaddl108\trpaddr108\trpaddfl3\trpaddft3\trpaddfr3\trpaddfb3\clvertalt\clftsWidth3\clwWidth8789\cellx8789\pard\intbl\widctlpar\qc\sl276\slmult1\plain\f5\fs24\b FERI NURJAMAN S.H.I.
\cell\trowd\irow0\irowband0\lastrow\trgaph108\trleft0\trftsWidth1\trftsWidthB3\trftsWidthA3\trpaddl108\trpaddr108\trpaddfl3\trpaddft3\trpaddfr3\trpaddfb3\clvertalt\clftsWidth3\clwWidth8789\cellx8789\row\pard\itap0\widctlpar\qj\sl360\slmult1\plain\f5\fs24\par\pard\itap0\widctlpar\qj\sl360\slmult1\tx720\tx1440\tx2160\tx2880\tx3600\tx4320\tx5040\tx5760\tx6480\tx7200\tx7920\tx8640\tx9360\tx10080 Perincian Biaya Perkara :\par\pard\itap0\widctlpar\qj\sl360\slmult1\tx3686\tx3969\tqr\tx6237\tx6480\tx7200\tx7920\tx8640\tx9360\tx10080\tx10800\tx11520\tx12240\tx12960\tx13680 1. Biaya Pendaftaran\tab : \tab Rp.\tab 30.000,00\par 2. Biaya Proses\tab :\tab Rp.\tab 50.000,00\par 3. Biaya Panggilan \tab :\tab Rp. \tab 385.000,00\par 4. Biaya PNBP Panggilan\tab : \tab Rp.\tab 20.000,00\par 5. Biaya Redaksi\tab :\tab Rp.\tab 10.000,00\par 6. Biaya materai\tab :\plain\f5\fs24\ul\tab Rp.\tab 6.000,00\plain\f5\fs24\par   Jumlah\tab\tab Rp.\tab 501.000,00
\par\pard\itap0\widctlpar\qj\sl360\slmult1\tqr\tx6237\tx6480\tx7200\tx7920\tx8640\tx9360\tx10080\tx10800\tx11520\tx12240\tx12960\tx13680\tx14400\tx15120\tab (lima ratus satu ribu

Disclaimer
Kepaniteraan Mahkamah Agung Republik Indonesia berusaha untuk selalu mencantumkan informasi paling kini dan akurat sebagai bentuk komitmen Mahkamah Agung untuk pelayanan publik, transparansi dan akuntabilitas pelaksanaan fungsi peradilan. Namun dalam hal-hal tertentu masih dimungkinkan terjadi permasalahan teknis terkait dengan akurasi dan keterkinian informasi yang kami sajikan, hal mana akan terus kami perbaiki dari waktu kewaktu.
Dalam hal Anda menemukan inakurasi informasi yang termuat pada situs ini atau informasi yang seharusnya ada, namun belum tersedia, maka harap segera hubungi Kepaniteraan Mahkamah Agung RI melalui :
Email : kepaniteraan@mahkamahagung.go.id   Telp : 021-384 3348 (ext.318)

rupiah)\par\pard\itap0\widctlpar\qj\sl360\slmult1\tqr\tx6237\par\pard\itap0\wi
dctlpar\qj\sl360\slmult1 Dicatat disini
:\par{\pntext\pard\plain\f6\fs24\'2d\tab}\pard\s20\itap0\widctlpar\qj\fi-
284\li284\sl360\slmult1\tx284\ls2\ilvl0{*\pn\pnlvlblt\ilvl0\ls2\pnstart1\pnf6\p
nfs24\pnb0\pni0\pnstrike0\pnulnone\pncf0\pncb0{\pntxtb \'2d}}Amar
penetapan tersebut telah diberitahukan kepada Tergugat pada tanggal
\par\pard\itap0\widctlpar\qj\sl360\slmult1\tqr\tx6237\par\pard\itap0\widctlpar
....\par\par\trowd\irow0\irowband0\lastrow\trga
ph108\trleft0\trftsWidth1\trftsWidthB3\trftsWidthA3\trpaddl108\trpaddr108\tr
paddfl3\trpaddft3\trpaddfr3\trpaddfb3\clvertalt\clftsWidth3\clwWidth8789\cel
lx8789\pard\intbl\widctlpar\qc\sl276\slmult1\par Ketua
Majelis\cell\trowd\irow0\irowband0\lastrow\trgaph108\trleft0\trftsWidth1\trfts
WidthB3\trftsWidthA3\trpaddl108\trpaddr108\trpaddfl3\trpaddft3\trpaddfr3\tr
paddfb3\clvertalt\clftsWidth3\clwWidth8789\cellx8789\row\pard\itap0\widctlp
ar\qj\par\pard\itap0\widctlpar\qj\li3600\tx3600
ttd\par\pard\itap0\widctlpar\qj\par\par\trowd\irow0\irowband0\lastrow\trgaph
108\trleft0\trftsWidth1\trftsWidthB3\trftsWidthA3\trpaddl108\trpaddr108\trpa
ddfl3\trpaddft3\trpaddfr3\trpaddfb3\clvertalt\clftsWidth3\clwWidth8789\cellx
8789\pard\intbl\widctlpar\qc\sl276\slmult1\plain\f5\fs24\b Drs. H.
MUHAMMAD MAULUDIN
\cell\trowd\irow0\irowband0\lastrow\trgaph108\trleft0\trftsWidth1\trftsWidthB
3\trftsWidthA3\trpaddl108\trpaddr108\trpaddfl3\trpaddft3\trpaddfr3\trpaddfb
3\clvertalt\clftsWidth3\clwWidth8789\cellx8789\row\trowd\irow0\irowband0\la
strow\trgaph108\trleft0\trftsWidth1\trftsWidthB3\trftsWidthA3\trpaddl108\trp
addr108\trpaddfl3\trpaddft3\trpaddfr3\trpaddfb3\clvertalt\clftsWidth3\clwWid
th4395\cellx4395\clvertalt\clftsWidth3\clwWidth4394\cellx8789\pard\intbl\wi
dctlpar\qc\sl276\slmult1\plain\f5\fs24\par Hakim Anggota\cell\par Hakim
Anggota\cell\trowd\irow0\irowband0\lastrow\trgaph108\trleft0\trftsWidth1\trft
sWidthB3\trftsWidthA3\trpaddl108\trpaddr108\trpaddfl3\trpaddft3\trpaddfr3\t
rpaddfb3\clvertalt\clftsWidth3\clwWidth4395\cellx4395\clvertalt\clftsWidth3\c
lwWidth4394\cellx8789\row\pard\itap0\widctlpar\qj\par\pard\itap0\widctlpar\
qj\li1440\tx1440 ttd \tab\tab\tab
ttd\par\pard\itap0\widctlpar\qj\par\pard\itap0\widctlpar
\par\trowd\irow0\irowband0\lastrow\trgaph108\trleft0\trftsWidth1\trftsWidthB
3\trftsWidthA3\trpaddl108\trpaddr108\trpaddfl3\trpaddft3\trpaddfr3\trpaddfb
3\clvertalt\clftsWidth3\clwWidth4395\cellx4395\clvertalt\clftsWidth3\clwWidth
4394\cellx8789\pard\intbl\widctlpar\qc\sl276\slmult1\plain\f5\fs24\b Drs. H.
TAUFIQURROKHMAN, M.H. \cell H. ABDUL HANAN, SH.,MH
\cell\trowd\irow0\irowband0\lastrow\trgaph108\trleft0\trftsWidth1\trftsWidthB
3\trftsWidthA3\trpaddl108\trpaddr108\trpaddfl3\trpaddft3\trpaddfr3\trpaddfb
3\clvertalt\clftsWidth3\clwWidth4395\cellx4395\clvertalt\clftsWidth3\clwWidth
4394\cellx8789\row\trowd\irow0\irowband0\lastrow\trgaph108\trleft0\trftsWid
th1\trftsWidthB3\trftsWidthA3\trpaddl108\trpaddr108\trpaddfl3\trpaddft3\trp
addfr3\trpaddfb3\clvertalt\clftsWidth3\clwWidth8789\cellx8789\pard\intbl\wid
ctlpar\qc\sl276\slmult1\plain\f5\fs24\par Panitera
Pengganti\cell\trowd\irow0\irowband0\lastrow\trgaph108\trleft0\trftsWidth1\tr

Disclaimer
Kepaniteraan Mahkamah Agung Republik Indonesia berusaha untuk selalu mencantumkan informasi paling kini dan akurat sebagai bentuk komitmen Mahkamah Agung untuk pelayanan publik, transparansi dan akuntabilitas pelaksanaan fungsi peradilan. Namun dalam hal-hal tertentu masih dimungkinkan terjadi permasalahan teknis terkait dengan akurasi dan keterkinian informasi yang kami sajikan, hal mana akan terus kami perbaiki dari waktu kewaktu.
Dalam hal Anda menemukan inakurasi informasi yang termuat pada situs ini atau informasi yang seharusnya ada, namun belum tersedia, maka harap segera hubungi Kepaniteraan Mahkamah Agung RI melalui :
Email : kepaniteraan@mahkamahagung.go.id   Telp : 021-384 3348 (ext.318)

ftsWidthB3\trftsWidthA3\trpaddl108\trpaddr108\trpaddfl3\trpaddft3\trpaddfr3
\trpaddfb3\clvertalt\clftsWidth3\clwWidth8789\cellx8789\row\pard\itap0\widct
lpar\qj\par\pard\itap0\widctlpar\qj\li3600\tx3600
ttd\par\pard\itap0\widctlpar\qj\par\par\trowd\irow0\irowband0\lastrow\trgaph
108\trleft0\trftsWidth1\trftsWidthB3\trftsWidthA3\trpaddl108\trpaddr108\trpa
ddfl3\trpaddft3\trpaddfr3\trpaddfb3\clvertalt\clftsWidth3\clwWidth8789\cellx
8789\pard\intbl\widctlpar\qc\sl276\slmult1\plain\f5\fs24\b FERI NURJAMAN
S.H.I.\cell\trowd\irow0\irowband0\lastrow\trgaph108\trleft0\trftsWidth1\trftsWi
dthB3\trftsWidthA3\trpaddl108\trpaddr108\trpaddfl3\trpaddft3\trpaddfr3\trpa
ddfb3\clvertalt\clftsWidth3\clwWidth8789\cellx8789\row\pard\itap0\widctlpar\
qj\sl360\slmult1\plain\f5\fs24\par\pard\itap0\widctlpar\qj\sl360\slmult1\tx720\
tx1440\tx2160\tx2880\tx3600\tx4320\tx5040\tx5760\tx6480\tx7200\tx7920\
tx8640\tx9360\tx10080 Perincian Biaya Perkara
:\par\pard\itap0\widctlpar\qj\sl360\slmult1\tx3686\tx3969\tqr\tx6237\tx6480\
tx7200\tx7920\tx8640\tx9360\tx10080\tx10800\tx11520\tx12240\tx12960\tx
13680 1. Biaya Pendaftaran\tab : \tab Rp.\tab 30.000,00\par 2. Biaya
Proses\tab :\tab Rp.\tab 50.000,00\par 3. Biaya Panggilan \tab :\tab Rp. \tab
385.000,00\par 4. Biaya PNBP Panggilan\tab : \tab Rp.\tab 20.000,00\par 5.
Biaya Redaksi\tab :\tab Rp.\tab 10.000,00\par 6. Biaya materai\tab
:\plain\f5\fs24\ul\tab Rp.\tab 6.000,00\plain\f5\fs24\par   Jumlah\tab\tab
Rp.\tab 501.000,00
\par\pard\itap0\widctlpar\qj\sl360\slmult1\tqr\tx6237\tx6480\tx7200\tx7920\t
x8640\tx9360\tx10080\tx10800\tx11520\tx12240\tx12960\tx13680\tx14400\
tx15120\tab (lima ratus satu ribu
rupiah)\par\pard\itap0\widctlpar\qj\sl360\slmult1\par Dicatat disini
:\par{\pntext\pard\plain\f6\fs24\'2d\tab}\pard\s20\itap0\widctlpar\qj\fi-
284\li284\sl360\slmult1\tx284\ls2\ilvl0{*\pn\pnlvlblt\ilvl0\ls2\pnstart1\pnf6\p
nfs24\pnb0\pni0\pnstrike0\pnulnone\pncf0\pncb0{\pntxtb \'2d}}Amar
penetapan tersebut telah diberitahukan kepada Tergugat pada tanggal
\par\pard\itap0\widctlpar\qj\sl360\slmult1\tqr\tx6237\par\pard\itap0\widctlpar
\li4536\sb120\tx4536 Untuk salinan yang sama bunyinya\par
Panitera,\par\pard\itap0\widctlpar\qc\li4536\sb120\tx4536\par\par\pard\itap0\
widctlpar\qj\li4536\sb120\sl360\slmult1\tx4536\plain\f5\fs24\b Drs. H. Jaenal,
MH.\plain\f5\fs24\par\pard\itap0\widctlpar\plain\f1\fs24\par\par }

Mahkamah Agung Republik Indonesia

Disclaimer
Kepaniteraan Mahkamah Agung Republik Indonesia berusaha untuk selalu mencantumkan informasi paling kini dan akurat sebagai bentuk komitmen Mahkamah Agung untuk pelayanan publik, transparansi dan akuntabilitas pelaksanaan fungsi peradilan. Namun dalam hal-hal tertentu masih dimungkinkan terjadi permasalahan teknis terkait dengan akurasi dan keterkinian informasi yang kami sajikan, hal mana akan terus kami perbaiki dari waktu kewaktu.
Dalam hal Anda menemukan inakurasi informasi yang termuat pada situs ini atau informasi yang seharusnya ada, namun belum tersedia, maka harap segera hubungi Kepaniteraan Mahkamah Agung RI melalui :
Email : kepaniteraan@mahkamahagung.go.id   Telp : 021-384 3348 (ext.318)
