# Supplementary material for: Classification of divorce causes during the COVID-19 pandemic using convolutional neural networks
Source: PeerJ Comput Sci. 2022 Jun 30;8:e998. doi: 10.7717/peerj-cs.998 (PMC9299239; doi:10.7717/peerj-cs.998)
Supplement: Supplemental Information 3 [file peerj-cs-08-998-s003.zip › KDRT Dataset/Data ke-3.pdf]

S A L I N A N :

P U T U S A N

Nomor 4229/Pdt.G/2020/PA.Sbg.

بِسۡمِ ٱللَّهِ ٱلرَّحۡمَٰنِ ٱلرَّحِيمِ

DEMI KEADILAN BERDASARKAN KETUHANAN YANG MAHA ESA

Pengadilan Agama Subang yang memeriksa dan mengadili perkara-perkara tertentu pada tingkat pertama dalam persidangan Majelis Hakim telah menjatuhkan putusan dalam perkara **Cerai Gugat**, antara :

Penggugat, umur 21 tahun (Subang, 10 Oktober 1999), Agama Islam, pendidikan terakhir SLTA, pekerjaan Mengurus Rumah Tangga, tempat tinggal di Kabupaten Subang, dalam hal ini memberikan kuasa kepada **Bambang Subarkah, S.H..** Advokat-Penasihat Hukum pada Kantor Advokat & Konsultan Hukum Masri Bambang & Partners yang beralamat kantor di Jalan Mayjen Sutoyo Gang Rambutan No.22, Kelurahan Karanganyar Kecamatan Subang, Kabupaten Subang, berdasarkan Surat Kuasa Khusus Nomor tanggal 23 Agustus 2020 yang telah didaftar di Kepaniteraan Pengadilan Agama Subang dengan Register Nomor 1029/Adv/2020 tertanggal 30 November 2020, sebagai **Penggugat**;

melawan

Tergugat, umur 26 tahun (Subang, 30 November 1994), Agama Islam, pendidikan terakhir SD, pekerjaan Wiraswasta, tempat tinggal di Kabupaten Subang, sebagai **Tergugat**;

Pengadilan Agama tersebut;
Setelah membaca berkas perkara;
Setelah mendengar keterangan Penggugat di persidangan;

Disclaimer
Kepaniteraan Mahkamah Agung Republik Indonesia berusaha untuk selalu mencantumkan informasi paling kini dan akurat sebagai bentuk komitmen Mahkamah Agung untuk pelayanan publik, transparansi dan akuntabilitas pelaksanaan fungsi peradilan. Namun dalam hal-hal tertentu masih dimungkinkan terjadi permasalahan teknis terkait dengan akurasi dan keterkinian informasi yang kami sajikan, hal mana akan terus kami perbaiki dari waktu kewaktu.
Dalam hal Anda menemukan inakurasi informasi yang termuat pada situs ini atau informasi yang seharusnya ada, namun belum tersedia, maka harap segera hubungi Kepaniteraan Mahkamah Agung RI melalui :
Email : kepaniteraan@mahkamahagung.go.id Telp : 021-384 3348 (ext.318)

Setelah memeriksa alat-alat bukti di persidangan;

DUDUK PERKARA

Bahwa Penggugat dengan surat gugatannya tanggal 30 November 2020 yang telah didaftarkan di Kepaniteraan Pengadilan Agama Subang di bawah Nomor 4229/Pdt.G/2020/PA.Sbg. tanggal 30 November 2020, bermaksud mengajukan gugatan cerai terhadap Tergugat berdasarkan dalil-dalil yang pada pokoknya sebagai berikut :

1. Bahwa Penggugat dengan Tergugat adalah suami isteri yang sah. Menikah padatanggal 19 Oktober 2014, sebagaimana terbukti dari buku Register Akta Nikah Nomor :2014, tanggal 19 Oktober 2014, yang diterbitkan oleh Kantor Urusan Agama Kecamatan Cibogo, Kabupaten Subang;
2. Bahwa setelah akad nikah Penggugat dan Tergugat hidup bersama sebagai suami isteri di rumah Orang Tua Penggugat.
3. Bahwa perkawinan antara Penggugat dengan Tergugat sampai saat ini telah berjalan Kurang Lebih selama 6 Tahun Dan Telah Bergaul Sebagaimana Layaknya Suami Istri Yang Baik Dan Dikaruniai 1 Orang Anak Yang Bernama
    1. HAIDAR ARSYAD Berusia 5 Tahun
4. Bahwa Semula Rumah Tangga penggugat dan tergugat cukup harmonis dan bahagia , Namun Awal sejak Bulan Oktober 2018 Sudah mulai tidak harmonis karena sering terjadi perselisihan dan pertengkaran.
5. Bahwa Akibat perselisihan dan pertengkaran terakhir terjadi pada Tanggal November 2018 yang disebabkan…
    - Bahwa tergugat telah melalaikan kewajibanya dengan tidak member nafkah wajib kepada penggugat Kadang tidak Menentu dan sampai sekarang dan untuk memenuhi kebutuhan sehari-hari mengandalkan dari penghasilan sehari-hari penggugat sendiri Dengan Mengandalkan penggugat bekerja.
    - Bahwa Tegugat Selalu Bersikap kasar dan Bersifat emosional dan sering berkata-kata kasar kepada penggugat apabila tergugat sedang

Disclaimer
Kepaniteraan Mahkamah Agung Republik Indonesia berusaha untuk selalu mencantumkan informasi paling kini dan akurat sebagai bentuk komitmen Mahkamah Agung untuk pelayanan publik, transparansi dan akuntabilitas pelaksanaan fungsi peradilan. Namun dalam hal-hal tertentu masih dimungkinkan terjadi permasalahan teknis terkait dengan akurasi dan keterkinian informasi yang kami sajikan, hal mana akan terus kami perbaiki dari waktu kewaktu.
Dalam hal Anda menemukan inakurasi informasi yang termuat pada situs ini atau informasi yang seharusnya ada, namun belum tersedia, maka harap segera hubungi Kepaniteraan Mahkamah Agung RI melalui :
Email : kepaniteraan@mahkamahagung.go.id Telp : 021-384 3348 (ext.318)

cemburu kepada penggugat dan pernah seketika tergugat menuduh penggugat berselingkuh.

Bahwa Tergugat dan penggugat sudah tidak ada komunikasi sebagaimana layaknya suami istri dan antara penggugat dan tergugat lebih memilih hidup terpisah dimana penggugat memilih hidup bersama orang tuanya dan tergugat bersama orang tua tergugat..

7. Bahwa akibat perselisihan dan pertengkaran tersebut, sehingga pada Januari 2019 antara Penggugat dengan Tergugat terjadi pisah tempat tinggal Kurang Lebih Selama 10 Bulan, dan selama itu sulit dilakukan komunikasi yang wajar sebagaimana layaknya suami istri, sehingga penggugat merasa berat untuk melanjutkan untuk melanjutkan perkawinan dengan tergugat**;**

8. Bahwa pihak keluarga kedua belah pihak telah berusaha untuk mendamaikan kedua belah pihak, akan tetapi tidak berhasil;
9. Bahwa Berdasarkan Alasan-Alasan Tersebut Diatas , Penggugat Mengajukan Gugatan Terhadap Tergugat Dengan Alasan , Antara Penggugat Dan Tergugat terus Menerus Terus Terjadi Perselisihan Dan Pertengkaran dan tidak ada harapan untuk hidup rukun lagi dalam perkawinan dengan tergugat.
10. Bahwa berdasarkanfakta di atas, maka Gugatan Penggugat telah memenuhi ketentuan Pasal 19 huruf f Peraturan Pemerintah No.9 Tahun 1975 jo Pasal 116 huruf f Kompilasi Hukum Islam;
11. Bahwa perkawinan (rumah tangga) yang demikian menurut Penggugat sulit dipertahankan karena yang menjadi tujuan perkawinan (berumah tangga) sulit tercapai. Maka untuk mengakhiri perkawinan tersebut Penggugat bermaksud menceraikanTergugat di depan siding Pengadilan Agama Subang,.

Bahwa berdasarkan dalil-dalil tersebut di atas, Penggugat mohon kepada Ketua Pengadilan Agama Subang untuk memanggil pihak-pihak, memeriksa, mengadili dan memutuskan perkara ini dengan amarnya yang berbunyi sebagai berikut:

Disclaimer
Kepaniteraan Mahkamah Agung Republik Indonesia berusaha untuk selalu mencantumkan informasi paling kini dan akurat sebagai bentuk komitmen Mahkamah Agung untuk pelayanan publik, transparansi dan akuntabilitas pelaksanaan fungsi peradilan. Namun dalam hal-hal tertentu masih dimungkinkan terjadi permasalahan teknis terkait dengan akurasi dan keterkinian informasi yang kami sajikan, hal mana akan terus kami perbaiki dari waktu kewaktu.
Dalam hal Anda menemukan inakurasi informasi yang termuat pada situs ini atau informasi yang seharusnya ada, namun belum tersedia, maka harap segera hubungi Kepaniteraan Mahkamah Agung RI melalui :
Email : kepaniteraan@mahkamahagung.go.id   Telp : 021-384 3348 (ext.318)

1. Mengabulkan gugatan Penggugat;
2. Menjatuhkan talak satu ba'in shugro Tergugat (**Tergugat**) terhadap Penggugat (**Penggugat**) ;
3. Membebankan kepada Penggugat untuk membayar biaya perkara menurut hukum;

Apabila Majelis Hakim berpendapat lain, mohon putusan yang seadil adilnya;

Bahwa pada hari dan tanggal yang telah ditentukan Penggugat datang menghadap didampingi Kuasanya persidangan, sedang Tergugat tidak datang menghadap atau menyuruh orang lain sebagai wakilnya yang sah, meskipun ia telah dipanggil dengan resmi dan patut untuk datang menghadap di sidang sebagaimana terbukti dari relaas panggilan Nomor 4229/Pdt.G/2020/PA.Sbg. tanggal 01 Desember 2020 dan 08 Desember 2020, sedangkan ternyata bahwa tidak hadirnya itu oleh Majelis Hakim dinilai tidak disebabkan sesuatu halangan yang sah;

Bahwa oleh karena Tergugat tidak hadir di persidangan, maka proses mediasi sebagaimana diamanatkan dalam PERMA Nomor 1 Tahun 2016 tidak dapat dilaksanakan;

Bahwa selanjutnya dibacakan surat gugatan Penggugat tertanggal 30 November 2020, yang isinya tetap dipertahankan Kuasa Penggugat;

Bahwa terhadap gugatan Penggugat tersebut, Tergugat tidak memberi jawaban karena tidak pernah hadir di persidangan;

Bahwa untuk memperkuat dalil gugatannya, bahwa Penggugat telah menikah dengan Tergugat, Kuasa Penggugat telah mengajukan alat bukti (P) berupa fotokopi Kutipan Akta Nikah Nomor 0484/063/X/2014 yang dikeluarkan oleh Kantor Urusan Agama Kecamatan Cibogo, Kabupaten Subang tanggal 19 Oktober 2014, fotokopi mana telah dinachtzegelen/bermeterai cukup;

Bahwa di samping bukti surat sebagaimana di atas, Kuasa Penggugat juga mengajukan dua orang saksi keluarga atau orang yang dekat dengan Penggugat, yang masing-masing bernama :

Disclaimer
Kepaniteraan Mahkamah Agung Republik Indonesia berusaha untuk selalu mencantumkan informasi paling kini dan akurat sebagai bentuk komitmen Mahkamah Agung untuk pelayanan publik, transparansi dan akuntabilitas pelaksanaan fungsi peradilan. Namun dalam hal-hal tertentu masih dimungkinkan terjadi permasalahan teknis terkait dengan akurasi dan keterkinian informasi yang kami sajikan, hal mana akan terus kami perbaiki dari waktu kewaktu.
Dalam hal Anda menemukan inakurasi informasi yang termuat pada situs ini atau informasi yang seharusnya ada, namun belum tersedia, maka harap segera hubungi Kepaniteraan Mahkamah Agung RI melalui :
Email : kepaniteraan@mahkamahagung.go.id Telp : 021-384 3348 (ext.318)

1. Saksi 1, umur 29 tahun, agama Islam, pekerjaan Dagang, tempat tinggal di Kabupaten Subang, yang di bawah sumpah memberikan keterangan yang pada pokoknya sebagai berikut :
   - Bahwa saksi kenal kepada Penggugat yang bernama Cucu Yulia dan Tergugat yang bernama Gugun Gunawan;
   - Bahwa saksi adalah Sepupu Penggugat;
   - Bahwa Penggugat dan Tergugat adalah suami istri yang menikah pada bulan Oktober 2014 di hadapan Pegawai Pencatat Nikah Kantor Urusan Agama Kecamatan Cibogo, Kabupaten Subang;
   - Bahwa setelah menikah Penggugat dan Tergugat tinggal bersama di rumah orangtua Penggugat, dan telah dikaruniai keturunan seorang anak bernama Haidar Arsyad;
   - Bahwa saksi mengetahui awalnya rumah tangga Penggugat dan Tergugat rukun dan harmonis, namun sejak bulan Oktober 2018 mulai tidak rukun karena sering terjadi perselisihan dan pertengkaran;
   - Bahwa penyebabnya karena Tergugat telah melalaikan kewajibannya dalam hal nafkah, dimana nafkah yang diberikan Tergugat tidak dapat memenuhi kebutuhan sehari-hari keluarga dan untuk menutupinya dari hasil Penggugat bekerja selain itu bersifat emosional juga bersikap dan berkata-kata kasar kepada Penggugat;
   - Bahwa saksi mengetahui perselisihan dan pertengkaran tersebut karena saksi pernah melihat antara Penggugat dengan Tergugat bertengkar;
   - Bahwa Penggugat dan Tergugat sampai sekarang sudah berpisah selama 10 bulan, dimana Tergugat pergi meninggalkan Penggugat;
   - Bahwa selama berpisah tempat tinggal, Penggugat tinggal di rumah orangtua Penggugat, sedangkan Tergugat tinggal di rumah orangtua Tergugat;
   - Bahwa selama berpisah tempat tinggal Penggugat dan Tergugat sudah berusaha untuk memperbaiki rumah tangganya, namun upaya tersebut tidak berhasil;

Disclaimer
Kepaniteraan Mahkamah Agung Republik Indonesia berusaha untuk selalu mencantumkan informasi paling kini dan akurat sebagai bentuk komitmen Mahkamah Agung untuk pelayanan publik, transparansi dan akuntabilitas pelaksanaan fungsi peradilan. Namun dalam hal-hal tertentu masih dimungkinkan terjadi permasalahan teknis terkait dengan akurasi dan keterkinian informasi yang kami sajikan, hal mana akan terus kami perbaiki dari waktu kewaktu.
Dalam hal Anda menemukan inakurasi informasi yang termuat pada situs ini atau informasi yang seharusnya ada, namun belum tersedia, maka harap segera hubungi Kepaniteraan Mahkamah Agung RI melalui :
Email : kepaniteraan@mahkamahagung.go.id Telp : 021-384 3348 (ext.318)

- Bahwa saksi bersama keluarga kedua belah pihak sudah berusaha merukunkan, akan tetapi usaha tersebut tidak berhasil karena Penggugat tetap bersikeras ingin bercerai dengan Tergugat;
- Bahwa saksi tidak bersedia untuk mendamaikan kembali kedua belah pihak, karena keduanya sulit didamaikan;

2. Saksi 2, umur 28 tahun, agama Islam, pekerjaan Karyawan Pabrik, tempat tinggal di Kabupaten Subang, yang di bawah sumpah memberikan keterangan yang pada pokoknya sebagai berikut :
- Bahwa saksi kenal kepada Penggugat dan Tergugat karena saksi adalah tetangga Penggugat;
- Bahwa Penggugat dan Tergugat adalah suami istri yang sudah berumah tangga selama 6 tahun dan telah dikaruniai keturunan seorang anak;
- Bahwa setelah menikah Penggugat dan Tergugat tinggal bersama di rumah orangtua Penggugat;
- Bahwa Penggugat dan Tergugat sudah berpisah tempat tinggal sejak Januari 2020, dimana Tergugat pergi meninggalkan Penggugat sampai sekarang sudah berpisah sekitar 10 bulan;
- Bahwa selama berpisah tempat tinggal, Penggugat tinggal di rumah orangtua Penggugat, sedangkan Tergugat tinggal di rumah orangtua Tergugat;
- Bahwa sebelum berpisah, sejak bulan Oktober 2018 antara Penggugat dan Tergugat sering terjadi perselisihan dan pertengkaran yang disebabkan karena Tergugat telah melalaikan kewajibanya dengan tidak memberi nafkah wajib kepada penggugat kadang tidak Menentu dan sampai sekarang dan untuk memenuhi kebutuhan sehari-hari mengandalkan dari penghasilan sehari-hari penggugat sendiri Dengan Mengandalkan penggugat bekerja disamping itu Tegugat Selalu Bersikap kasar dan Bersifat emosional dan sering berkata-kata kasar kepada penggugat apabila tergugat sedang cemburu kepada penggugat dan pernah seketika tergugat menuduh penggugat berselingkuh.;

Disclaimer
Kepaniteraan Mahkamah Agung Republik Indonesia berusaha untuk selalu mencantumkan informasi paling kini dan akurat sebagai bentuk komitmen Mahkamah Agung untuk pelayanan publik, transparansi dan akuntabilitas pelaksanaan fungsi peradilan. Namun dalam hal-hal tertentu masih dimungkinkan terjadi permasalahan teknis terkait dengan akurasi dan keterkinian informasi yang kami sajikan, hal mana akan terus kami perbaiki dari waktu kewaktu.
Dalam hal Anda menemukan inakurasi informasi yang termuat pada situs ini atau informasi yang seharusnya ada, namun belum tersedia, maka harap segera hubungi Kepaniteraan Mahkamah Agung RI melalui :
Email : kepaniteraan@mahkamahagung.go.id   Telp : 021-384 3348 (ext.318)

- Bahwa saksi mengetahui perselisihan dan pertengkaran tersebut karena saksi pernah melihat antara Penggugat dengan Tergugat bertengkar;
- Bahwa selama berpisah tempat tinggal Penggugat dan Tergugat sudah berusaha untuk rukun, akan tetapi upaya tersebut tidak berhasil;
- Bahwa selama berpisah tempat tinggal, saksi belum pernah mendamaikan. Hanya menasehati Penggugat saja, tapi tidak berhasil karena Penggugat sudah tidak mau kembali kepada Tergugat;
- Bahwa saksi tidak bersedia mendamaikan kedua belah pihak karena sulit untuk didamaikan kembali;

Bahwa kemudian Kuasa Penggugat mengajukan kesimpulan, yang pada pokoknya mengatakan bahwa berdasarkan dalil-dalil gugatan Penggugat dan bukti-bukti yang diajukan, gugatan Penggugat telah terbukti dan berdasar hukum, oleh karenanya patut dikabulkan dan selanjutnya mohon putusan;

Bahwa selanjutnya untuk mempersingkat uraian putusan ini, ditunjuklah hal-hal sebagaimana tercatat dalam berita acara sidang perkara ini sebagai bagian yang tidak terpisahkan dari putusan ini;

PERTIMBANGAN HUKUM

Menimbang, bahwa maksud dan tujuan gugatan Penggugat adalah sebagaimana diuraikan di atas;

Menimbang, bahwa berdasarkan relaas panggilan Nomor 4229/Pdt.G/2020/PA.Sbg. tanggal 01 Desember 2020 dan 08 Desember 2020, terbukti bahwa Tergugat telah dipanggil dengan resmi dan patut untuk datang menghadap di persidangan, tetapi Tergugat tidak datang menghadap atau menyuruh orang lain sebagai wakilnya yang sah, sedangkan ternyata bahwa tidak hadirnya itu oleh Majelis Hakim dinilai tidak disebabkan sesuatu halangan yang sah. Oleh karenanya, harus dinyatakan bahwa Tergugat tidak menghadap persidangan dan berdasarkan ketentuan Pasal 125 ayat (1) HIR Pengadilan dapat menjatuhkan putusan dengan verstek;

Disclaimer
Kepaniteraan Mahkamah Agung Republik Indonesia berusaha untuk selalu mencantumkan informasi paling kini dan akurat sebagai bentuk komitmen Mahkamah Agung untuk pelayanan publik, transparansi dan akuntabilitas pelaksanaan fungsi peradilan. Namun dalam hal-hal tertentu masih dimungkinkan terjadi permasalahan teknis terkait dengan akurasi dan keterkinian informasi yang kami sajikan, hal mana akan terus kami perbaiki dari waktu kewaktu.
Dalam hal Anda menemukan inakurasi informasi yang termuat pada situs ini atau informasi yang seharusnya ada, namun belum tersedia, maka harap segera hubungi Kepaniteraan Mahkamah Agung RI melalui :
Email : kepaniteraan@mahkamahagung.go.id   Telp : 021-384 3348 (ext.318)

Menimbang, bahwa perkara ini adalah perkara perceraian. Berdasarkan azas hukum bahwa tidak ada perceraian jika tidak ada perkawinan, maka sebelum mempertimbangkan tentang perceraiannya Majelis Hakim akan mempertimbangkan terlebih dahulu mengenai ada tidaknya perkawinan antara Penggugat dan Tergugat;

Menimbang, bahwa untuk memperkuat dalil gugatannya, bahwa Penggugat telah menikah dengan Tergugat, Kuasa Penggugat telah mengajukan bukti surat yang berupa fotokopi Kutipan Akta Nikah Nomor 0484/063/X/2014 yang dikeluarkan oleh Kantor Urusan Agama Kecamatan Cibogo, Kabupaten Subang tanggal 19 Oktober 2014, bermeterai cukup (bukti P), sehingga sah untuk dipertimbangkan sebagai alat bukti dalam perkara ini;

Menimbang, bahwa bukti P sebagaimana tersebut di atas merupakan akta otentik yang bernilai pembuktian mengikat dan sempurna. Maka berdasarkan alat bukti tersebut Majelis Hakim berkesimpulan, telah terbukti benar, dalil Penggugat yang menyatakan bahwa antara Penggugat dan Tergugat terikat dalam perkawinan yang sah;

Menimbang, bahwa dalam perkara ini Penggugat melalui Kuasanya mohon kepada Pengadilan agar menjatuhkan talak satu bain sughra Tergugat terhadap Penggugat berdasarkan dalil yang pada pokoknya, bahwa rumah tangga Penggugat dan Tergugat sudah tidak harmonis lagi karena sering terjadi perselisihan dan pertengkaran yang disebabkan oleh karena Tergugat telah melalaikan kewajibannya dalam hal nafkah, dimana nafkah yang diberikan Tergugat tidak dapat memenuhi kebutuhan sehari-hari keluarga dan untuk menutupinya dari hasil Penggugat bekerja selain itu bersifat emosional juga bersikap dan berkata-kata kasar kepada Penggugat yang mencapai puncaknya pada Januari 2020 sehingga sekarang antara Penggugat dan Tergugat telah berpisah tempat tinggal selama 10 bulan dan selama berpisah tempat tinggal tersebut Penggugat dan Tergugat sudah berusaha rukun akan tetapi upaya tersebut tidak berhasil;

Menimbang, bahwa terhadap gugatan Penggugat tersebut, Tergugat tidak memberi jawaban karena tidak pernah hadir di persidangan. Namun

Disclaimer
Kepaniteraan Mahkamah Agung Republik Indonesia berusaha untuk selalu mencantumkan informasi paling kini dan akurat sebagai bentuk komitmen Mahkamah Agung untuk pelayanan publik, transparansi dan akuntabilitas pelaksanaan fungsi peradilan. Namun dalam hal-hal tertentu masih dimungkinkan terjadi permasalahan teknis terkait dengan akurasi dan keterkinian informasi yang kami sajikan, hal mana akan terus kami perbaiki dari waktu kewaktu.
Dalam hal Anda menemukan inakurasi informasi yang termuat pada situs ini atau informasi yang seharusnya ada, namun belum tersedia, maka harap segera hubungi Kepaniteraan Mahkamah Agung RI melalui :
Email : kepaniteraan@mahkamahagung.go.id Telp : 021-384 3348 (ext.318)

demikian, karena perkara ini adalah perkara perceraian, maka untuk dikabulkan gugatan, Penggugat harus membuktikan kebenaran dalil gugatannya;

Menimbang, bahwa untuk membuktikan kebenaran dalil gugatannya, Penggugat telah mengajukan dua orang saksi;

Menimbang, bahwa oleh karena kedua saksi yang diajukan Penggugat adalah saksi keluarga dan orang yang dekat dengannya, maka Majelis Hakim menjadikan kedua saksi tersebut sebagai alat bukti mengenai dalil gugatannya, juga sebagai saksi keluarga untuk memenuhi ketentuan Pasal 22 ayat (2) Peraturan Pemerintah Nomor 9 tahun 1975 jo Pasal 76 ayat (1) Undang Undang Nomor 7 Tahun 1989, sebagaimana telah diubah dengan Undang-undang No. 3 tahun 2006 dan diubah untuk kedua kalinya dengan Undang-undang No. 50 tahun 2009, yang menentukan, bahwa gugatan perceraian dapat diterima apabila telah cukup jelas bagi Pengadilan mengenai sebab-sebab perselisihan dan pertengkaran itu dan setelah mendengar keterangan pihak keluarga serta orang-orang yang dekat dengan suami istri;

Menimbang, bahwa kedua saksi keluarga atau orang dekat dengan Penggugat tersebut telah memenuhi syarat formil maupun materiil sebagai saksi, maka keterangan kedua saksi atau orang dekat tersebut dapat dipertimbangkan sebagai alat bukti dalam perkara ini;

Menimbang, bahwa ternyata isi keterangan kedua saksi tersebut sesuai dengan dalil-dalil yang dikemukakan oleh Penggugat, bahwa antara Penggugat dan Tergugat telah terjadi pisah tempat tinggal selama 10 bulan yang disebabkan oleh pertengkaran karena Tergugat telah melalaikan kewajibannya dalam hal nafkah, dimana nafkah yang diberikan Tergugat tidak dapat memenuhi kebutuhan sehari-hari keluarga dan untuk menutupinya dari hasil Penggugat bekerja selain itu bersifat emosional juga bersikap dan berkata-kata kasar kepada Penggugat dan selama berpisah tempat tinggal Penggugat dan Tergugat sudah berusaha rukun akan tetapi upaya tersebut tidak berhasil. Demikian juga kedua saksi keluarga atau orang yang dekat dengan Penggugat tersebut telah menasehati Penggugat agar rukun lagi dengan Tergugat tetapi tidak berhasil;

Disclaimer
Kepaniteraan Mahkamah Agung Republik Indonesia berusaha untuk selalu mencantumkan informasi paling kini dan akurat sebagai bentuk komitmen Mahkamah Agung untuk pelayanan publik, transparansi dan akuntabilitas pelaksanaan fungsi peradilan. Namun dalam hal-hal tertentu masih dimungkinkan terjadi permasalahan teknis terkait dengan akurasi dan keterkinian informasi yang kami sajikan, hal mana akan terus kami perbaiki dari waktu kewaktu.
Dalam hal Anda menemukan inakurasi informasi yang termuat pada situs ini atau informasi yang seharusnya ada, namun belum tersedia, maka harap segera hubungi Kepaniteraan Mahkamah Agung RI melalui :
Email : kepaniteraan@mahkamahagung.go.id   Telp : 021-384 3348 (ext.318)

Menimbang, bahwa berdasarkan hal-hal sebagaimana diuraikan di atas, Majelis Hakim mendapatkan fakta-fakta peristiwa sebagai berikut:

1. Bahwa Penggugat dengan Tergugat telah terikat dalam pernikahan yang sah dan telah dikaruniai keturunan seorang anak bernama Haidar Arsyad;
2. Bahwa antara Penggugat dan Tergugat telah terjadi pisah tempat tinggal selama 10 bulan;
3. Bahwa terjadinya perpisahan tempat tinggal tersebut disebabkan oleh pertengkaran karena Tergugat telah melalaikan kewajibannya dalam hal nafkah, dimana nafkah yang diberikan Tergugat tidak dapat memenuhi kebutuhan sehari-hari keluarga dan untuk menutupinya dari hasil Penggugat bekerja selain itu bersifat emosional juga bersikap dan berkata-kata kasar kepada Penggugat;
4. Bahwa selama berpisah tempat tinggal Penggugat dan Tergugat sudah berusaha rukun akan tetapi upaya tersebut tidak berhasil;
5. Bahwa para saksi keluarga atau orang yang dekat dengan Penggugat telah menasehati Penggugat agar rukun kembali dengan Tergugat tetapi tidak berhasil dan para saksi tidak bersedia untuk merukunkan kembali karena keduanya sulit untuk didamaikan;

Menimbang, bahwa dengan telah terbuktinya dalil Penggugat, bahwa antara Penggugat dan Tergugat telah terjadi pisah tempat tinggal selama 10 bulan, di mana menurut yurisprudensi MARI No. 379 K/AG/1995 tanggal 26 Maret 1997, suami istri yang tidak berdiam serumah lagi dan tidak ada harapan untuk hidup rukun kembali, maka rumah tangga tersebut terbukti telah retak dan pecah;

Menimbang, bahwa keadaan hubungan antara Penggugat dan Tergugat yang demikian, menurut Majelis Hakim dapat dikualifikasi sebagai telah terjadi perselisihan dan pertengkaran terus menerus dan tidak ada harapan akan hidup rukun lagi dalam rumah tangga sebagaimana yang dimaksud oleh Pasal 19 huruf f Peraturan Pemerintah Nomor 9 Tahun 1975 jo Pasal 116 huruf f Kompilasi Hukum Islam (Instruksi Presiden Nomor 1 Tahun 1991);

Disclaimer
Kepaniteraan Mahkamah Agung Republik Indonesia berusaha untuk selalu mencantumkan informasi paling kini dan akurat sebagai bentuk komitmen Mahkamah Agung untuk pelayanan publik, transparansi dan akuntabilitas pelaksanaan fungsi peradilan. Namun dalam hal-hal tertentu masih dimungkinkan terjadi permasalahan teknis terkait dengan akurasi dan keterkinian informasi yang kami sajikan, hal mana akan terus kami perbaiki dari waktu kewaktu.
Dalam hal Anda menemukan inakurasi informasi yang termuat pada situs ini atau informasi yang seharusnya ada, namun belum tersedia, maka harap segera hubungi Kepaniteraan Mahkamah Agung RI melalui :
Email : kepaniteraan@mahkamahagung.go.id Telp : 021-384 3348 (ext.318)

Menimbang, bahwa berdasarkan fakta-fakta di atas Majelis Hakim berkesimpulan bahwa antara Penggugat dan Tergugat telah terjadi perselisihan dan pertengkaran terus menerus dan tidak ada harapan akan hidup rukun lagi dalam rumah tangga, sehingga harapan untuk mewujudkan tujuan rumah tangga yang bahagia, sakinah, mawaddah warahmah, sebagaimana yang dikehendaki oleh Pasal 1 Undang-undang Nomor 1 Tahun 1974 jo Pasal 3 Kompilasi Hukum Islam sulit terwujud. Rumah tangga Penggugat dan Tergugat yang demikian berarti telah pecah dan tidak layak untuk dipertahankan;

Menimbang, bahwa dengan tidak hadirnya Tergugat di persidangan memberi petunjuk bahwa Tergugat tidak menghendaki rukun lagi dengan Penggugat dalam rumah tangga;

Menimbang, bahwa berdasarkan pertimbangan-pertimbangan di atas Majelis Hakim berpendapat bahwa gugatan Penggugat telah memenuhi ketentuan Pasal 39 ayat (1) dan ayat (2) Undang-undang No. 1 Tahun 1974, bahwa perceraian hanya dapat dilakukan di depan sidang Pengadilan setelah Pengadilan yang bersangkutan berusaha dan tidak berhasil mendamaikan kedua belah pihak. Untuk melakukan perceraian harus ada cukup alasan bahwa antara suami-istri itu tidak akan dapat hidup rukun sebagai suami-istri. Demikian juga gugatan Penggugat telah memenuhi ketentuan Pasal 19 huruf (f) Peraturan Pemerintah Nomor 9 Tahun 1975 jo Pasal 116 huruf (f) Kompilasi Hukum Islam, yaitu antara suami istri telah terjadi perselisihan dan pertengkaran terus menerus dan tidak ada harapan akan hidup rukun lagi dalam rumah tangga. Oleh karena itu, gugatan Penggugat dikabulkan;

Menimbang, bahwa menurut doktrin hukum Islam, sebagaimana ditulis dalam kitab Fiqhussunnah Jilid VIII halaman 121-122, yang artinya : *”Bahwa apabila gugatan istri telah terbukti berdasarkan alat bukti yang diajukan oleh istri atau berdasarkan pengakuan suami, sedang adanya gangguan tersebut merupakan penghalang atas kelangsungan pergaulan hidup (suami istri) sebagaimana (pergaulan hidup suami istri lain) yang sepadan dengan mereka, sementara hakim tidak berhasil mendamaikan mereka, maka hakim harus menjatuhkan gugat suaminya terhadap diri penggugat dengan gugat bain”.*

Disclaimer
Kepaniteraan Mahkamah Agung Republik Indonesia berusaha untuk selalu mencantumkan informasi paling kini dan akurat sebagai bentuk komitmen Mahkamah Agung untuk pelayanan publik, transparansi dan akuntabilitas pelaksanaan fungsi peradilan. Namun dalam hal-hal tertentu masih dimungkinkan terjadi permasalahan teknis terkait dengan akurasi dan keterkinian informasi yang kami sajikan, hal mana akan terus kami perbaiki dari waktu kewaktu.
Dalam hal Anda menemukan inakurasi informasi yang termuat pada situs ini atau informasi yang seharusnya ada, namun belum tersedia, maka harap segera hubungi Kepaniteraan Mahkamah Agung RI melalui :
Email : kepaniteraan@mahkamahagung.go.id Telp : 021-384 3348 (ext.318)

Sedang menurut ketentuan Pasal 119 ayat (2) Kompilasi Hukum Islam, salah satu talak bain sughra adalah talak yang dijatuhkan oleh Pengadilan Agama;

Menimbang, bahwa dalam perkara ini ternyata Penggugat dapat membuktikan kebenaran dalil gugatannya, dan hakim telah gagal mendamaikan keduanya, maka Majelis Hakim akan menjatuhkan talak satu bain sughra Tergugat atas Penggugat;

Menimbang, bahwa berdasarkan Pasal 89 ayat (1) Undang-undang Nomor 7 Tahun 1989 sebagaimana telah diubah dengan Undang-undang No. 3 tahun 2006 dan diubah untuk kedua kalinya dengan Undang-undang No. 50 tahun 2009, biaya perkara ini dibebankan kepada Penggugat;

Memperhatikan ketentuan Pasal 125 HIR dan ketentuan hukum lainnya yang berkaitan dengan perkara ini;

M E N G A D I L I

1. Menyatakan bahwa Tergugat yang telah dipanggil secara resmi dan patut untuk menghadap persidangan, tidak hadir;
2. Mengabulkan gugatan Penggugat dengan verstek;
3. Menjatuhkan talak satu ba'in sughra Tergugat (Tergugat) terhadap Penggugat (Penggugat);
4. Membebankan biaya perkara ini kepada Penggugat yang hingga kini dihitung sebesar Rp371.000,00 (tiga ratus tujuh puluh satu ribu rupiah);

Demikian dijatuhkan putusan ini dalam musyawarah Majelis Hakim yang dilangsungkan pada hari **Selasa**, tanggal **15 Desember 2020 Masehi** bertepatan dengan tanggal **29 Rabiul Akhir 1442 Hijriyah** oleh **Drs. Arifin** sebagai Ketua Majelis serta **Drs. H. Humaidi** dan **Dede Rika Nurhasanah, S.Ag., M.H.** masing-masing sebagai Hakim Anggota, dibantu oleh **Dra. Hj. Popon Susilawaty**, sebagai Panitera Pengganti. Putusan tersebut pada hari itu juga diucapkan Hakim Ketua dalam sidang terbuka untuk umum dengan dihadiri Kuasa Penggugat tanpa hadirnya Tergugat.

Hakim Anggota, Ketua Majelis,

Disclaimer
Kepaniteraan Mahkamah Agung Republik Indonesia berusaha untuk selalu mencantumkan informasi paling kini dan akurat sebagai bentuk komitmen Mahkamah Agung untuk pelayanan publik, transparansi dan akuntabilitas pelaksanaan fungsi peradilan. Namun dalam hal-hal tertentu masih dimungkinkan terjadi permasalahan teknis terkait dengan akurasi dan keterkinian informasi yang kami sajikan, hal mana akan terus kami perbaiki dari waktu kewaktu.
Dalam hal Anda menemukan inakurasi informasi yang termuat pada situs ini atau informasi yang seharusnya ada, namun belum tersedia, maka harap segera hubungi Kepaniteraan Mahkamah Agung RI melalui :
Email : kepaniteraan@mahkamahagung.go.id Telp : 021-384 3348 (ext.318)

ttd. ttd.

Drs. H. Humaidi **Drs. Arifin**

Hakim Anggota,

ttd.

Dede Rika Nurhasanah, S.Ag., M.H.

Panitera Pengganti,

ttd.

Dra. Hj. Popon Susilawaty

Perincian Biaya :

1. Pendaftaran	:	Rp 30.000,00
2. Proses	:	Rp 50.000,00
3. Panggilan	:	Rp 255.000,00
4. PNBP Panggilan	:	Rp 20.000,00
5. Redaksi	:	Rp 10.000,00
6. Meterai	:	Rp 6.000,00
**Jumlah**	**:**	**Rp371.000,00**

(tiga ratus tujuh puluh satu ribu rupiah)

Untuk **Salinan** yang sama bunyinya oleh :
PANITERA PENGADILAN AGAMA SUBANG

Drs. H. Dadang Zaenal, M.M.

Disclaimer
Kepaniteraan Mahkamah Agung Republik Indonesia berusaha untuk selalu mencantumkan informasi paling kini dan akurat sebagai bentuk komitmen Mahkamah Agung untuk pelayanan publik, transparansi dan akuntabilitas pelaksanaan fungsi peradilan. Namun dalam hal-hal tertentu masih dimungkinkan terjadi permasalahan teknis terkait dengan akurasi dan keterkinian informasi yang kami sajikan, hal mana akan terus kami perbaiki dari waktu kewaktu.
Dalam hal Anda menemukan inakurasi informasi yang termuat pada situs ini atau informasi yang seharusnya ada, namun belum tersedia, maka harap segera hubungi Kepaniteraan Mahkamah Agung RI melalui :
Email : kepaniteraan@mahkamahagung.go.id Telp : 021-384 3348 (ext.318)
