# Supplementary material for: Classification of divorce causes during the COVID-19 pandemic using convolutional neural networks
Source: PeerJ Comput Sci. 2022 Jun 30;8:e998. doi: 10.7717/peerj-cs.998 (PMC9299239; doi:10.7717/peerj-cs.998)
Supplement: Supplemental Information 3 [file peerj-cs-08-998-s003.zip › KDRT Dataset/Data ke-30.pdf]

PUTUSAN

Nomor 1499/Pdt.G/2020/PA.Pmk.

بسم الله الرحمن الرحيم

DEMI KEADILAN BERDASARKAN KETUHANAN YANG MAHA ESA

Pengadilan Agama Pamekasan yang memeriksa dan mengadili perkara tertentu pada tingkat pertama telah menjatuhkan putusan sebagai berikut dalam perkara cerai talak yang diajukan oleh :

Pemohon, NIK : 3528131104640001, tempat dan tanggal lahir Pamekasan,11 April 1964, umur 56 tahun, agama Islam, pendidikan Tidak Sekolah, pekerjaan Petani, bertempat tinggal di **Kabupaten Pamekasan,** selanjutnya disebut **Pemohon** ;

M E L A W A N

Termohon, NIK : 3528134411720001, tempat dan tanggal lahir, Pamekasan,04 Nopember 1972 umur 48 tahun, agama Islam, pendidikan SD, pekerjaan Petani, bertempat tinggal di **Kabupaten Pamekasan,** selanjutnya disebut **Termohon** ;

Pengadilan Agama tersebut;

Telah membaca dan mempelajari berkas perkara;

Telah mendengar keterangan Pemohon dan telah memeriksa bukti-bukti di persidangan;

DUDUK PERKARA

Bahwa Pemohon telah mengajukan Permohonan cerai dengan suratnya tertanggal 10 Desember 2020 dan telah terdaftar di Kepaniteraan Perkara Pengadilan Agama Pamekasan pada Register Nomor 1499/Pdt.G/2020/PA.Pmk, tanggal 10 Desember 2020 telah mengemukakan hal-hal sebagai berikut:

1. Bahwa Pemohon dan Termohon adalah suami isteri menikah dilaksanakan pada tanggal 30 Mei 1989, yang dicatat oleh Pegawai pencatat Nikah Kantor Urusan Agama Kecamatan Waru Kabupaten Pamekasan sebagaimana bukti Duplikat Kutipan Akta Nikah Nomor 53/06/VI/1989 tanggal 03 Desember 2020 ;
2. Bahwa setelah menikah Pemohon dan Termohon hidup rukun sebagaimana layaknya suami isteri di rumah Termohon selama 25 tahun 6 bulan 10 hari dan selama tersebut telah melakukan hubungan sebagaimana layaknya suami isteri (ba'da dukhul) dan telah dikaruniai 3 orang anak bernama : **Anak ke 1 Pemohon dengan Termohon,** Umur 29 Tahun saat ini sudah berkeluarga,

Disclaimer
Kepaniteraan Mahkamah Agung Republik Indonesia berusaha untuk selalu mencantumkan informasi paling kini dan akurat sebagai bentuk komitmen Mahkamah Agung untuk pelayanan publik, transparansi dan akuntabilitas pelaksanaan fungsi peradilan. Namun dalam hal-hal tertentu masih dimungkinkan terjadi permasalahan teknis terkait dengan akurasi dan keterkinian informasi yang kami sajikan, hal mana akan terus kami perbaiki dari waktu kewaktu.
Dalam hal Anda menemukan inakurasi informasi yang termuat pada situs ini atau informasi yang seharusnya ada, namun belum tersedia, maka harap segera hubungi Kepaniteraan Mahkamah Agung RI melalui :
Email : kepaniteraan@mahkamahagung.go.id Telp : 021-384 3348 (ext.318)

Anak ke 2 Pemohon dengan Termohon, Umur 18 tahun **Anak ke 3 Pemohon dengan Termohon,** Umur 14 tahun saat ini kedua anak tersebut dalam asuhan dan pemeliharaan Pemohon ;

3. Bahwa semula rumah tangga Pemohon dan Termohon hidup rukun dan harmonis, akan tetapi sejak 7 tahun yang lalu, rumah tangga Pemohon dan Termohon mulai goyah dan mengalami keretakan karena terjadi perselisihan dan pertengkaran ;
4. Bahwa penyebab perselisihan dan pertengkaran karena Termohon berangkat ke Malaysia pada tahun 2013 tanpa pamit kepada Pemohon, Termohon pulang dari Malaysia pada tahun 2017 dan tinggal bersama dengan Pemohon serta keluarga sekitar 4 bulan namun dalam keadaan pisah ranjang, setelah kedatangan Termohon selama 4 bulan tersebut Termohon berangkat tanpa pamit lagi kepada Pemohon ke Surabaya dan sekarang Termohon datang kembali, Termohon meminta untuk diceraikan kepada Pemohon ;
5. Bahwa akibat perselisihan dan pertengkaran tersebut, kini Pemohon dan Termohon telah pisah rumah sejak 7 Tahun yang lalu, Pemohon dan Termohon tetap dalam satu rumah namun pisah ranjang ;
6. Bahwa atas sikap Termohon yang demikian itu, menyebabkan Pemohon merasa tidak sanggup lagi meneruskan hubungan rumah tangga bersama Termohon, dan bermaksud mengakhirinya dengan perceraian ;
7. Bahwa, Pemohon sanggup membayar seluruh biaya yang timbul akibat perkara ini ;

Berdasarkan alasan/dalil-dalil diatas, Pemohon mohon Kepada Bapak Ketua melalui Majelis Hakim Pengadilan Agama Pamekasan agar memeriksa dan mengadili perkara ini, selanjutnya menjatuhkan putusan yang amarnya :

Primair :

1. Mengabulkan permohonan Pemohon;
2. Memberikan ijin kepada Pemohon (**Pemohon**) untuk menjatuhkan talak satu kepada Termohon (**Termohon**) di hadapan sidang Pengadilan Agama Pamekasan ;
3. Membebankan biaya perkara kepada Pemohon;

Subsidair :

- Atau menjatuhkan putusan lain yang seadil-adilnya;

Bahwa pada hari dan tanggal persidangan yang telah ditetapkan Pemohon hadir menghadap sendiri, sedangkan Termohon tidak hadir dan tidak pula mengutus

Disclaimer
Kepaniteraan Mahkamah Agung Republik Indonesia berusaha untuk selalu mencantumkan informasi paling kini dan akurat sebagai bentuk komitmen Mahkamah Agung untuk pelayanan publik, transparansi dan akuntabilitas pelaksanaan fungsi peradilan. Namun dalam hal-hal tertentu masih dimungkinkan terjadi permasalahan teknis terkait dengan akurasi dan keterkinian informasi yang kami sajikan, hal mana akan terus kami perbaiki dari waktu kewaktu.
Dalam hal Anda menemukan inakurasi informasi yang termuat pada situs ini atau informasi yang seharusnya ada, namun belum tersedia, maka harap segera hubungi Kepaniteraan Mahkamah Agung RI melalui :
Email : kepaniteraan@mahkamahagung.go.id Telp : 021-384 3348 (ext.318)

kuasanya yang sah, meskipun Termohon telah dipanggil dengan resmi dan patut, sedangkan tidak ternyata bahwa tidak datangnya itu disebabkan oleh suatu halangan yang sah, maka pemeriksaan perkara ini dilanjutkan tanpa hadirnya Termohon ;

Bahwa Majelis Hakim telah berusaha mendamaikan dengan menyarankan kepada pihak Pemohon agar tidak meneruskan Permohonannya dan rukun kembali dengan Termohon, akan tetapi tidak berhasil ;

Bahwa oleh karena usaha perdamaian tidak berhasil, maka pemeriksaan diteruskan dengan membacakan surat Permohonan Pemohon yang isinya tetap dipertahankan oleh Pemohon ;

Bahwa Pemohon telah meneguhkan dalil-dalil Permohonannya dengan menyerahkan alat bukti di persidangan berupa surat sebagai berikut :

a. Fotokopi Kartu Tanda Penduduk an. **Pemohon**, NIK : 3528131104640001 tanggal 06 Juni 2012 yang dikeluarkan oleh Kantor Dinas Kependudukan dan Pencatatan Sipil Kabupaten Pamekasan. Bukti tersebut telah bermeterai cukup dan dicap pos. Setelah dicocokkan dengan aslinya, ternyata sesuai, lalu oleh Ketua Majelis diberi tanda (P.1).
b. Fotokopi Duplikat Kutipan Akta Nikah yang dikeluarkan oleh Kantor Urusan Agama Kecamatan Waru Kabupaten Pamekasan Nomor 53/06/VI/1989 Tanggal 03 Desember 2020. Bukti tersebut bermeterai cukup dan dicap pos. Setelah dicocokkan dengan aslinya, ternyata sesuai, lalu oleh Ketua Majelis diberi tanda (P.2);

Bahwa selain bukti surat Pemohon juga mengajukan bukti saksi-saksi/ keluarganya, yaitu :

1. **Saksi ke 1 Pemohon,** umur 50 tahun, agama Islam, pekerjaan wiraswasta, bertempat tinggal di **Kabupaten Pamekasan,** sebagai saksi I;

Bahwa saksi I di hadapan sidang telah memberikan keterangan dibawah sumpah yang pada pokoknya sebagai berikut :

- Bahwa saksi kenal dengan Pemohon dan Termohon sebab saksi adalah tetangga dari Pemohon ;
- Bahwa saksi mengetahui antara Pemohon dan Termohon adalah suami isteri , dan telah dikaruniai 3 orang anak ;
- Bahwa saksi mengetahui rumah tangga Pemohon dan Termohon semula rukun dan harmonis, namun kemudian sering terjadi perselisihan dan pertengkaran, bahkan sekarang antara Pemohon dan Termohon telah terjadi pisah ranjang ;

Disclaimer
Kepaniteraan Mahkamah Agung Republik Indonesia berusaha untuk selalu mencantumkan informasi paling kini dan akurat sebagai bentuk komitmen Mahkamah Agung untuk pelayanan publik, transparansi dan akuntabilitas pelaksanaan fungsi peradilan. Namun dalam hal-hal tertentu masih dimungkinkan terjadi permasalahan teknis terkait dengan akurasi dan keterkinian informasi yang kami sajikan, hal mana akan terus kami perbaiki dari waktu kewaktu.
Dalam hal Anda menemukan inakurasi informasi yang termuat pada situs ini atau informasi yang seharusnya ada, namun belum tersedia, maka harap segera hubungi Kepaniteraan Mahkamah Agung RI melalui :
Email : kepaniteraan@mahkamahagung.go.id Telp : 021-384 3348 (ext.318)

- Bahwa saksi mengetahui penyebab terjadi perselisihan dan pertengkaran antara Pemohon dan Termohon karena Termohon berangkat ke Malaysia tanpa pamit kepada Pemohon dan setelah dinasehati oleh Pemohon, Termohon meminta untuk diceraikan kepada Pemohon ;
- Bahwa saksi mengetahui Pemohon dan Termohon sudah pisah ranjang sejak lebih dari 7 Tahun yang lalu sampai sekarang;
- Bahwa saksi sudah mendamaikan Pemohon dan Termohon, namun tidak berhasil ;

2. **Saksi ke 2 Pemohon,** umur 24 tahun, agama Islam, pekerjaan swasta, bertempat tinggal di **Kabupaten Pamekasan,** sebagai saksi II ;

Bahwa saksi II di hadapan sidang telah memberikan keterangan dibawah sumpah yang pada pokoknya sebagai berikut :

- Bahwa saksi kenal dengan Pemohon dan Termohon sebab saksi adalah tetangga dari Pemohon ;
- Bahwa saksi mengetahui antara Pemohon dan Termohon adalah suami isteri, dan telah dikaruniai 3 orang anak ;
- Bahwa saksi mengetahui rumah tangga Pemohon dan Termohon semula rukun dan harmonis, namun kemudian sering terjadi perselisihan dan pertengkaran, bahkan sekarang antara Pemohon dan Termohon telah terjadi pisah ranjang ;
- Bahwa saksi mengetahui penyebab antara Pemohon dan Termohon terjadi perselisihan dan pertengkaran karena Termohon berangkat ke Malaysia tanpa pamit kepada Pemohon ;
- Bahwa saksi mengetahui Pemohon dan Termohon sudah pisah ranjang sejak lebih dari 7 Tahun yang lalu sampai sekarang;
- Bahwa saksi sudah mendamaikan Pemohon dan Termohon, namun tidak berhasil;

Bahwa Pemohon tidak mengajukan tambahan keterangan dan alat bukti lagi, kemudian menyampaikan kesimpulan yang pada pokoknya tetap dengan permohonannya dan mohon agar perkaranya segera diputus;

Bahwa untuk mempersingkat uraian putusan ini cukup ditunjuk segala hal ikhwal yang telah termuat dalam Berita Acara Sidang perkara ini dianggap sebagai bagian dalam putusan ini;

PERTIMBANGAN HUKUM

Menimbang, bahwa maksud dan tujuan Permohonan Pemohon adalah sebagaimana telah diuraikan di atas;

Disclaimer
Kepaniteraan Mahkamah Agung Republik Indonesia berusaha untuk selalu mencantumkan informasi paling kini dan akurat sebagai bentuk komitmen Mahkamah Agung untuk pelayanan publik, transparansi dan akuntabilitas pelaksanaan fungsi peradilan. Namun dalam hal-hal tertentu masih dimungkinkan terjadi permasalahan teknis terkait dengan akurasi dan keterkinian informasi yang kami sajikan, hal mana akan terus kami perbaiki dari waktu kewaktu.
Dalam hal Anda menemukan inakurasi informasi yang termuat pada situs ini atau informasi yang seharusnya ada, namun belum tersedia, maka harap segera hubungi Kepaniteraan Mahkamah Agung RI melalui :
Email : kepaniteraan@mahkamahagung.go.id Telp : 021-384 3348 (ext.318)

Menimbang, bahwa untuk menegakkan ketentuan pasal 130 HIR. jo. pasal 31 ayat (1) dan (2) Peraturan Pemerintah Nomor 9 Tahun 1975 jo. pasal 82 ayat (4) Undang-Undang Nomor 7 Tahun 1989 sebagaimana telah diubah dengan Undang-Undang Nomor 3 Tahun 2006 dan perubahan kedua dengan Undang-Undang Nomor 50 Tahun 2009 jo. pasal 143 ayat (2) Kompilasi Hukum Islam Tahun 1991, Majelis Hakim telah berusaha mendamaikan dengan cara menasehati Pemohon agar hidup rukun kembali dengan Termohon sebagaimana layaknya suami isteri, akan tetapi tidak berhasil karena Pemohon tetap ingin bercerai dengan Termohon;

Menimbang, bahwa oleh karena Termohon tidak pernah datang menghadap di muka persidangan meskipun telah dipanggil secara resmi dan patut, maka proses mediasi sebagaimana diamanahkan oleh Peraturan Mahkamah Agung Nomor 03 Tahun 2016 tidak dapat dilaksanakan;

Menimbang, bahwa Termohon tidak hadir di persidangan meskipun telah dipanggil secara resmi dan patut, lagi pula ketidakhadirannya tersebut tidak disebabkan oleh suatu halangan yang sah, maka Termohon yang telah dipanggil secara resmi dan patut tidak datang menghadap harus dinyatakan tidak hadir dan putusan atas perkara ini dijatuhkan dengan Verstek (Pasal 125 ayat (1) HIR);

Menimbang, bahwa Pemohon dalam surat Permohonannya mendalilkan bahwa Pemohon dan Termohon adalah suami isteri. Pada mulanya rumah tangga Pemohon dan Termohon rukun dan harmonis, namun sejak 7 tahun yang lalu, rumah tangga Pemohon dan Termohon mulai goyah dan tidak harmonis, penyebabnya karena Termohon berangkat ke Malaysia pada tahun 2013 tanpa pamit kepada Pemohon, Termohon pulang dari Malaysia pada tahun 2017 dan tinggal bersama dengan Pemohon serta keluarga sekitar 4 bulan namun dalam keadaan pisah ranjang, setelah kedatangan Termohon selama 4 bulan tersebut Termohon berangkat tanpa pamit lagi kepada Pemohon ke Surabaya dan sekarang Termohon datang kembali, Termohon meminta untuk diceraikan kepada Pemohon . Akibat dari perselisihan dan pertengkaran tersebut, Pemohon dan Termohon berpisah ranjang sejak 7 Tahun yang lalu sampai sekarang, dan atas dasar tersebut Pemohon ingin menceraikan Termohon;

Menimbang, bahwa oleh karena Termohon tidak hadir di persidangan, maka Termohon telah tidak menggunakan haknya untuk melawan Permohonan Pemohon;

Menimbang, bahwa berdasarkan ketentuan pasal 125 ayat (1) HIR, yaitu putusan yang dijatuhkan tanpa hadirnya Termohon dapat dikabulkan sepanjang

Disclaimer
Kepaniteraan Mahkamah Agung Republik Indonesia berusaha untuk selalu mencantumkan informasi paling kini dan akurat sebagai bentuk komitmen Mahkamah Agung untuk pelayanan publik, transparansi dan akuntabilitas pelaksanaan fungsi peradilan. Namun dalam hal-hal tertentu masih dimungkinkan terjadi permasalahan teknis terkait dengan akurasi dan keterkinian informasi yang kami sajikan, hal mana akan terus kami perbaiki dari waktu kewaktu.
Dalam hal Anda menemukan inakurasi informasi yang termuat pada situs ini atau informasi yang seharusnya ada, namun belum tersedia, maka harap segera hubungi Kepaniteraan Mahkamah Agung RI melalui :
Email : kepaniteraan@mahkamahagung.go.id Telp : 021-384 3348 (ext.318)

berdasarkan hukum dan beralasan, oleh karena itu Majelis Hakim berpendapat bahwa Pemohon dibebani untuk membuktikan dalil-dalil Permohonannya ;

Menimbang, bahwa untuk meneguhkan dalil-dalilnya tersebut Pemohon mengajukan bukti surat yang diberi tanda P.1 dan P.2 serta menghadirkan 2 orang saksi di persidangan;

Menimbang, bahwa bukti P.1 (fotokopi Kartu Tanda Penduduk) yang aslinya merupakan akta otentik dan telah bermeterai cukup serta cocok dengan aslinya, isi bukti tersebut menjelaskan mengenai identitas Pemohon yang meliputi nama, tempat tanggal lahir, jenis kelamin, alamat, agama, status perkawinan, pekerjaan, dan kewarganegaraan, sehingga bukti tersebut telah memenuhi syarat formal dan materiil, serta mempunyai kekuatan pembuktian yang sempurna dan mengikat;

Menimbang, bahwa berdasarkan bukti P.1, telah terbukti Pemohon tercatat sebagai penduduk Kecamatan Pasean Kabupaten Pamekasan dan bertempat tinggal sebagaimana tersebut dalam surat permohonannya, dan Termohon bertempat tinggal di wilayah hukum Pengadilan Agama Pamekasan, yang berdasarkan ketentuan Pasal 4 Undang Undang Nomor 7 Tahun 1989 tentang Peradilan Agama yang telah diubah dengan Undang-Undang Nomor 3 Tahun 2006 serta perubahan kedua dengan Undang-Undang Nomor 50 Tahun 2009 merupakan daerah yurisdiksi Pengadilan Agama Pamekasan, maka dengan berdasarkan pada Pasal 49 ayat (1) dan Pasal 73 ayat (1) Undang-Undang tersebut maka Pengadilan Agama Pamekasan berwenang untuk mengadili perkara ini;

Menimbang, bahwa bukti P.2 (fotokopi Duplikat Kutipan Akta Nikah) yang aslinya merupakan akta otentik, telah bermeterai cukup dan telah dicocokkan dengan aslinya, isi bukti tersebut menjelaskan mengenai Pemohon dan Termohon telah melangsungkan perkawinan pada tanggal 30 Mei 1989, yang dicatat oleh Pegawai Pencatat Nikah Kantor Urusan Agama Kecamatan Waru Kabupaten Pamekasan, sehingga bukti tersebut telah memenuhi syarat formal dan materiil, serta mempunyai kekuatan pembuktian yang sempurna dan mengikat;

Menimbang, bahwa berdasarkan bukti P.2 maka terbukti Pemohon dan Termohon telah mempunyai hubungan hukum sebagai suami istri sah, sehingga Pemohon telah mempunyai *legal standing* dalam mengajukan Permohonan ini, serta masing-masing berkualitas untuk bertindak sebagai pihak-pihak dalam perkara ini;

Disclaimer
Kepaniteraan Mahkamah Agung Republik Indonesia berusaha untuk selalu mencantumkan informasi paling kini dan akurat sebagai bentuk komitmen Mahkamah Agung untuk pelayanan publik, transparansi dan akuntabilitas pelaksanaan fungsi peradilan. Namun dalam hal-hal tertentu masih dimungkinkan terjadi permasalahan teknis terkait dengan akurasi dan keterkinian informasi yang kami sajikan, hal mana akan terus kami perbaiki dari waktu kewaktu.
Dalam hal Anda menemukan inakurasi informasi yang termuat pada situs ini atau informasi yang seharusnya ada, namun belum tersedia, maka harap segera hubungi Kepaniteraan Mahkamah Agung RI melalui :
Email : kepaniteraan@mahkamahagung.go.id Telp : 021-384 3348 (ext.318)

Menimbang, bahwa saksi 1 dan saksi 2 Pemohon, sudah dewasa dan sudah disumpah, sehingga memenuhi syarat formal sebagaimana diatur dalam Pasal 145 ayat 1 angka 3e HIR dan Pasal 147 HIR;

Menimbang, bahwa keterangan saksi 1 dan saksi 2 Pemohon tentang keadaan rumah tangga, perselisihan dan penyebabnya serta pisah rumah, adalah fakta yang dilihat sendiri dan didengar sendiri serta relevan dengan dalil yang harus dibuktikan oleh Pemohon, oleh karena itu keterangan saksi-saksi tersebut telah memenuhi syarat materiil sebagaimana telah diatur dalam Pasal 171 HIR. sehingga keterangan saksi-saksi tersebut memiliki kekuatan pembuktian dan dapat diterima sebagai alat bukti;

Menimbang, bahwa keterangan saksi 1 dan saksi 2 Pemohon bersesuaian dan cocok antara satu dengan yang lain oleh karena itu keterangan dua orang saksi tersebut memenuhi Pasal 171 dan Pasal 172 HIR;

Menimbang, bahwa dari saksi-saksi tersebut diperoleh keterangan mengenai keadaan rumah tangga Pemohon dengan Termohon yang pada pokoknya sebagai berikut:

- Bahwa Pemohon dan Termohon adalah pasangan suami-istri yang sah dan telah dikaruniai anak;
- Bahwa semula rumah tangga Pemohon dan Termohon berjalan rukun, tetapi kemudian rumah tangga mereka bermasalah dan terjadi perselisihan dan pertengkaran yang penyebabnya karena Termohon berangkat ke Malaysia tanpa pamit kepada Pemohon dan setelah dinasehati oleh Pemohon, Termohon meminta untuk diceraikan kepada Pemohon ;
- Bahwa Pemohon dan Termohon telah berpisah ranjang tinggal sejak lebih dari 7 Tahun yang lalu sampai sekarang;
- Bahwa saksi-saksi sudah menasihati Pemohon dan Termohon, namun tidak berhasil;

Menimbang, bahwa berdasarkan dalil Pemohon, bukti P.1 dan P.2 serta keterangan saksi-saksi tersebut di atas maka Majelis Hakim telah dapat menemukan fakta hukum dalam persidangan yang pada pokoknya sebagai berikut:

- Bahwa rumah tangga Pemohon dan Termohon yang telah dibina sejak 30 Mei 1989, kini sudah tidak rukun dan harmonis lagi, sering berselisih dan bertengkar yang disebabkan karena Termohon berangkat ke Malaysia tanpa pamit kepada

Disclaimer
Kepaniteraan Mahkamah Agung Republik Indonesia berusaha untuk selalu mencantumkan informasi paling kini dan akurat sebagai bentuk komitmen Mahkamah Agung untuk pelayanan publik, transparansi dan akuntabilitas pelaksanaan fungsi peradilan. Namun dalam hal-hal tertentu masih dimungkinkan terjadi permasalahan teknis terkait dengan akurasi dan keterkinian informasi yang kami sajikan, hal mana akan terus kami perbaiki dari waktu kewaktu.
Dalam hal Anda menemukan inakurasi informasi yang termuat pada situs ini atau informasi yang seharusnya ada, namun belum tersedia, maka harap segera hubungi Kepaniteraan Mahkamah Agung RI melalui :
Email : kepaniteraan@mahkamahagung.go.id Telp : 021-384 3348 (ext.318)

Pemohon dan setelah dinasehati oleh Pemohon, Termohon meminta untuk diceraikan kepada Pemohon ;

- Bahwa akibat perselisihan Pemohon dan Termohon yang terus-menerus, Pemohon dan Termohon telah pisah ranjang sejak lebih dari 7 Tahun yang lalu sampai sekarang;
- Bahwa para saksi sudah menasihati Pemohon dan Termohon, tetapi tidak berhasil;

Menimbang, bahwa berdasarkan fakta-fakta tersebut di atas Majelis Hakim berpendapat bahwa perselisihan dan pertengkaran antara Pemohon dan Termohon telah memuncak dan mengakibatkan suami istri hidup berpisah dan komunikasi tidak terjalin baik atau tidak berjalan sebagaimana mestinya, dengan demikian akibat yang ditimbulkan dari perselisihan dan pertengkaran sudah sedemikian rupa dan berpengaruh pada keutuhan kehidupan rumah tangganya, karena itu Majelis Hakim menilai bagi rumah tangga Pemohon dan Termohon penyebab perselisihan dan pertengkaran tersebut merupakan sesuatu yang bersifat prinsipil;

Menimbang, bahwa dalam hal tidak adanya harapan untuk hidup rukun antara Pemohon dan Termohon, ini dapat diketahui dari keterlibatan keluarga yang telah secara maksimal berupaya merukunkan keduanya tetapi tidak berhasil, juga fakta di persidangan di mana Pemohon tidak dapat menerima saran serta nasehat Majelis Hakim untuk hidup rukun lagi dengan Termohon;

Menimbang, berdasarkan fakta-fakta tersebut di atas Majelis Hakim berpendapat bahwa rumah tangga Pemohon dan Termohon sudah pecah sedemikian rupa dan tidak mungkin dapat dirukunkan lagi, sehingga tujuan perkawinan untuk membentuk keluarga bahagia dan kekal berdasarkan Ketuhanan Yang Maha Esa (Pasal 1 Undang-Undang Nomor 1 Tahun 1974) dan atau keluarga sakinah, mawadah dan rahmah (Pasal 3 Kompilasi Hukum Islam) tidak tercapai, begitu pula firman Allah SWT. dalam al-Qur'an surat ar-Rum ayat 21 yang artinya: “Dan diantara tanda-tanda kebesaran-Nya ialah Dia menciptakan untuk istri-istri dari jenismu sendiri, supaya kamu cenderung dan merasa tentram kepadanya, dan menjadikan diantaramu rasa kasih dan sayang. Sesungguhnya pada yang demikian itu benar-benar terdapat tanda-tanda bagi kaum yang berfikir”;

Menimbang, bahwa pada setiap persidangan Majelis Hakim telah berusaha untuk menasehati Pemohon, demikian pula keluarga dan/atau orang dekat kedua belah pihak telah berusaha merukunkan kembali namun ternyata tidak berhasil

Disclaimer
Kepaniteraan Mahkamah Agung Republik Indonesia berusaha untuk selalu mencantumkan informasi paling kini dan akurat sebagai bentuk komitmen Mahkamah Agung untuk pelayanan publik, transparansi dan akuntabilitas pelaksanaan fungsi peradilan. Namun dalam hal-hal tertentu masih dimungkinkan terjadi permasalahan teknis terkait dengan akurasi dan keterkinian informasi yang kami sajikan, hal mana akan terus kami perbaiki dari waktu kewaktu.
Dalam hal Anda menemukan inakurasi informasi yang termuat pada situs ini atau informasi yang seharusnya ada, namun belum tersedia, maka harap segera hubungi Kepaniteraan Mahkamah Agung RI melalui :
Email : kepaniteraan@mahkamahagung.go.id Telp : 021-384 3348 (ext.318)

karena Pemohon tetap bersikeras untuk bercerai, sehingga Majelis Hakim berkesimpulan bahwa antara Pemohon dan Termohon telah tidak ada harapan untuk dapat rukun kembali dalam sebuah rumah tangga;

Menimbang, bahwa mempertahankan rumah tangga yang telah pecah sedemikian rupa adalah sia-sia belaka, bahkan apabila keadaannya seperti sekarang ini dipaksakan atau dibiarkan maka justru akan menimbulkan madharat dan penderitaan lahir batin yang berkepanjangan bagi Pemohon, dan Termohon, sehingga oleh karenanya Majelis Hakim berpandapat bahwa rumah tangga Pemohon dengan Termohon telah tidak dapat dipertahankan lagi;

Menimbang, bahwa menurut ajaran Islam perceraian adalah merupakan perbuatan yang tidak terpuji, namun demikian dalam hal suatu perkawinan yang sudah tidak dapat dipertahankan lagi keutuhannya karena telah pecah sehingga menimbulkan mafsadat yang lebih besar dari pada maslahatnya, maka perceraian dibolehkan. Hal ini sesuai dengan kaidah fiqhiyah yang berbunyi:

[illegible]

Artinya: *”Menolak kerusakan harus lebih didahulukan dari pada menarik kemaslahatan”.*

Menimbang, bahwa Majelis perlu mengemukakan dalil dalam surat Al-Baqarah ayat 227 yang berbunyi sebagai berikut:

وإن عزموا الطلاق فإن الله سميع عليم

Artinya : *”Dan jika mereka berketetapan hati untuk talak, maka sesungguhnya Allah Maha Mendengar lagi Maha Mengetahui”;*

Menimbang, bahwa dengan demikian Permohonan Pemohon telah beralasan hukum sesuai dengan Pasal 39 ayat (1) Undang-Undang Nomor 1 Tahun 1974 jo. Pasal 19 huruf f Peraturan Pemerintah Nomor 9 Tahun 1975 jo. Pasal 116 huruf f Kompilasi Hukum Islam;

Menimbang, bahwa berdasarkan pertimbangan-pertimbangan tersebut di atas dan Pemohon belum pernah menjatuhkan talak, maka petitum permohonan Pemohon untuk diberi izin mengucapkan ikrar talak terhadap Termohon, sesuai ketentuan Pasal 118 Kompilasi Hukum Islam dapat dikabulkan dengan memberi izin

Disclaimer
Kepaniteraan Mahkamah Agung Republik Indonesia berusaha untuk selalu mencantumkan informasi paling kini dan akurat sebagai bentuk komitmen Mahkamah Agung untuk pelayanan publik, transparansi dan akuntabilitas pelaksanaan fungsi peradilan. Namun dalam hal-hal tertentu masih dimungkinkan terjadi permasalahan teknis terkait dengan akurasi dan keterkinian informasi yang kami sajikan, hal mana akan terus kami perbaiki dari waktu kewaktu.
Dalam hal Anda menemukan inakurasi informasi yang termuat pada situs ini atau informasi yang seharusnya ada, namun belum tersedia, maka harap segera hubungi Kepaniteraan Mahkamah Agung RI melalui :
Email : kepaniteraan@mahkamahagung.go.id Telp : 021-384 3348 (ext.318)

kepada Pemohon untuk menjatuhkan talak satu raj'i terhadap Termohon di depan sidang Pengadilan Agama Pamekasan;

Menimbang, bahwa karena perkara tersebut termasuk bidang perkawinan, dan sesuai dengan Pasal 89 ayat (1) Undang-Undang Nomor 7 Tahun 1989 sebagaimana telah diubah dengan Undang-Undang Nomor 3 Tahun 2006 dan perubahan kedua dengan Undang-Undang Nomor 50 Tahun 2009, maka biaya perkara ini dibebankan kepada Pemohon;

Mengingat Pasal 49 Undang-Undang Nomor 7 Tahun 1989 sebagaimana telah diubah dengan Undang-Undang Nomor 3 Tahun 2006 dan perubahan kedua dengan Undang-Undang Nomor 50 Tahun 2009 dan ketentuan hukum lain serta hukum syar'i yang berkaitan dengan perkara ini;

M E N G A D I L I

1. Menyatakan bahwa Termohon yang telah dipanggil secara resmi dan patut untuk menghadap persidangan, tidak hadir ;
2. Mengabulkan permohonan Pemohon dengan verstek;
3. Memberi izin kepada Pemohon (**Pemohon**) untuk menjatuhkan talak satu raj'i terhadap Termohon (**Termohon**) di depan sidang Pengadilan Agama Pamekasan;
4. Membebankan kepada Pemohon untuk membayar biaya perkara sebesar Rp.466.000 ,- (empat ratus enam puluh enam ribu rupiah);

Demikian putusan ini dijatuhkan dalam sidang permusyawaratan Majelis Hakim Pengadilan Agama Pamekasan yang dilaksanakan pada hari Rabu tanggal 23 Desember 2020 Masehi bertepatan dengan tanggal 08 Jumadil Awal 1442 Hijriyah, oleh kami **Dra. Hj. Farhanah, M.H.** selaku Ketua Majelis, **Drs. Ainurrofiq ZA.** dan **Nurjumaatun Agustinah, S.Ag.** masing-masing selaku Hakim Anggota dan pada hari itu juga diucapkan pada sidang yang terbuka untuk umum oleh Ketua Majelis dengan didampingi Hakim-Hakim Anggota serta dibantu oleh **R. Ayu Fitrotin Nuzuliyah, S.Psi., S.H.** sebagai Panitera Pengganti, dengan dihadiri oleh Pemohon tanpa hadirnya Termohon;

Hakim Anggota	Ketua Majelis
ttd	ttd
**Drs. Ainurrofiq ZA.**	**Dra. Hj. Farhanah, M.H.**

Disclaimer
Kepaniteraan Mahkamah Agung Republik Indonesia berusaha untuk selalu mencantumkan informasi paling kini dan akurat sebagai bentuk komitmen Mahkamah Agung untuk pelayanan publik, transparansi dan akuntabilitas pelaksanaan fungsi peradilan. Namun dalam hal-hal tertentu masih dimungkinkan terjadi permasalahan teknis terkait dengan akurasi dan keterkinian informasi yang kami sajikan, hal mana akan terus kami perbaiki dari waktu kewaktu.
Dalam hal Anda menemukan inakurasi informasi yang termuat pada situs ini atau informasi yang seharusnya ada, namun belum tersedia, maka harap segera hubungi Kepaniteraan Mahkamah Agung RI melalui :
Email : kepaniteraan@mahkamahagung.go.id Telp : 021-384 3348 (ext.318)

Hakim Anggota	Panitera Pengganti
ttd	ttd
**Nurjumaatun Agustinah, S.Ag.**	**R. Ayu Fitrotin Nuzuliyah, S.Psi., S.H.**

Perincian Biaya Perkara :

1.	Biaya pendaftaran	Rp	30.000,00
2.	Biaya Proses	Rp	50.000,00
3.	Biaya Panggilan	Rp	350.000,00
4.	PNBP Panggilan Pemohon	Rp	10.000,00
5.	PNBP Panggilan Termohon	Rp	10.000,00
6.	Redaksi	Rp	10.000,00
7.	Materai	Rp	6.000,00
	**J U M L A H**	Rp	466.000,00

(empat ratus enam puluh enam ribu rupiah)

Disclaimer
Kepaniteraan Mahkamah Agung Republik Indonesia berusaha untuk selalu mencantumkan informasi paling kini dan akurat sebagai bentuk komitmen Mahkamah Agung untuk pelayanan publik, transparansi dan akuntabilitas pelaksanaan fungsi peradilan. Namun dalam hal-hal tertentu masih dimungkinkan terjadi permasalahan teknis terkait dengan akurasi dan keterkinian informasi yang kami sajikan, hal mana akan terus kami perbaiki dari waktu kewaktu.
Dalam hal Anda menemukan inakurasi informasi yang termuat pada situs ini atau informasi yang seharusnya ada, namun belum tersedia, maka harap segera hubungi Kepaniteraan Mahkamah Agung RI melalui :
Email : kepaniteraan@mahkamahagung.go.id Telp : 021-384 3348 (ext.318)
