# Supplementary material for: Classification of divorce causes during the COVID-19 pandemic using convolutional neural networks
Source: PeerJ Comput Sci. 2022 Jun 30;8:e998. doi: 10.7717/peerj-cs.998 (PMC9299239; doi:10.7717/peerj-cs.998)
Supplement: Supplemental Information 3 [file peerj-cs-08-998-s003.zip › KDRT Dataset/Data ke-4.pdf]

P U T U S A N

Nomor 5206/Pdt.G/2020/PA.Badg

بِسْمِ اللهِ الرَّحْمٰنِ الرَّحِيْمِ

DEMI KEADILAN BERDASARKAN KETUHANAN YANG MAHA ESA

Pengadilan Agama Bandung yang memeriksa dan mengadili perkara tertentu pada tingkat pertama dalam sidang majelis telah menjatuhkan putusan perkara Cerai Gugat antara :

Nama Penggugat, NIK XXXXXXXXXXXXXXXXX, tempat dan tanggal lahir Kebumen, 25 Juli 1968 (umur 52), agama Islam, pekerjaan Wirausaha, pendidikan SLTA, bertempat tinggal di XXXXXXXXXXXXXXXXX, Kota Bandung, sebagai **Penggugat**;

melawan

Nama Tergugat, NIK , tempat dan tanggal lahir Mojokerto, 19 April 1970 (umur 50), agama Islam, pekerjaan Tidak Diketahui, pendidikan SLTP, bertempat tinggal di XXXXXXXXXXXXXXXXX, sebagai **Tergugat**;

Pengadilan Agama tersebut;

Telah mempelajari surat-surat yang berkaitan dengan perkara ini;

Telah mendengar keterangan Penggugat serta para saksi di muka sidang;

DUDUK PERKARA

Menimbang, bahwa Penggugat dalam surat gugatannya tanggal 23 Oktober 2020 telah mengajukan gugatan cerai yang telah terdaftar di Kepaniteraan Pengadilan Agama Bandung dengan Nomor 5206/Pdt.G/2020/PA.Badg tanggal 26 Oktober 2020 dengan dalil-dalil sebagai berikut :

1. Bahwa antara Penggugat dan Tergugat adalah sepasang suami istri sah yang menikah pada tanggal 23 Oktober 1992 dan telah dicatatkan di Kantor Urusan Agama (KUA) Kecamatan Kebumen, Kabupaten

Disclaimer
Kepaniteraan Mahkamah Agung Republik Indonesia berusaha untuk selalu mencantumkan informasi paling kini dan akurat sebagai bentuk komitmen Mahkamah Agung untuk pelayanan publik, transparansi dan akuntabilitas pelaksanaan fungsi peradilan. Namun dalam hal-hal tertentu masih dimungkinkan terjadi permasalahan teknis terkait dengan akurasi dan keterkinian informasi yang kami sajikan, hal mana akan terus kami perbaiki dari waktu kewaktu.
Dalam hal Anda menemukan inakurasi informasi yang termuat pada situs ini atau informasi yang seharusnya ada, namun belum tersedia, maka harap segera hubungi Kepaniteraan Mahkamah Agung RI melalui :
Email : kepaniteraan@mahkamahagung.go.id Telp : 021-384 3348 (ext.318)

Kebumen dengan bukti Kutipan Akta Nikah Nomor: 763/101/X/651/1992 tanggal 22 Oktober 1992.

2. Bahwa setelah menikah antara Penggugat dengan Tergugat tinggal dan hidup bersama terakhir di rumah bersama di XXXXXXXXXXXXXXXXX, Kota Bandung.
3. Bahwa semula keadaan rumah tangga antara Penggugat dengan Tergugat hidup rukun dan harmonis kurang lebih selama 26 tahun. Dan dari hasil perkawinan antara Penggugat dengan Tergugat telah dikaruniai anak sebanyak 3 (tiga) orang yang bernama:
   1. Nama Anak I (laki-laki), tanggal lahir 17 Agustus 1993;
   2. Nama Anak II (laki-laki), tanggal lahir 12 November 199;
   3. Nama Anak III (perempuan), tanggal lahir 07 Januari 2002;
4. Bahwa selama berumah tangga dengan Tergugat, keadaan tidak selamanya rukun dan harmonis, rumah tangga antara Penggugat dan Tergugat sejak bulan **Oktober 2018** mulai terjadi perselisihan dan pertengkaran terus menerus, sehingga Penggugat mengajukan gugatan cerai ke Pengadilan Agama Bandung. Adapun sebab-sebab perceraian ini diajukan adalah sebagai berikut:

   4.1. Bahwa Tergugat kurang bertanggungjawab mengenai nafkah keluarga, Tergugat tidak terbuka mengenai penghasilan yang diperolehnya, nafkah yang diberikan pun tidak menentu, bahkan sejak tahun 2018 Tergugat sudah tidak pernah memberikan nafkah kepada Penggugat, sehingga untuk mencukupi kebutuhan sehari-hari dan biaya anak mengandalkan hasil bekerja Penggugat.
5. Bahwa Penggugat telah berusaha sabar untuk mempertahankan rumah tangga, tetapi Tergugat tidak berubah dan merubah sikapnya. Puncaknya pada bulan **Oktober 2019**, antara Penggugat dan Tergugat terlibat pertengkaran, Tergugat pergi meninggalkan kediaman bersama, sehingga antara Penggugat dengan Tergugat sudah pisah tempat tinggal, dan Penggugat dengan Tergugat sudah tidak melaksanakan kewajiban masing-masing layaknya suami istri lagi;

Disclaimer
Kepaniteraan Mahkamah Agung Republik Indonesia berusaha untuk selalu mencantumkan informasi paling kini dan akurat sebagai bentuk komitmen Mahkamah Agung untuk pelayanan publik, transparansi dan akuntabilitas pelaksanaan fungsi peradilan. Namun dalam hal-hal tertentu masih dimungkinkan terjadi permasalahan teknis terkait dengan akurasi dan keterkinian informasi yang kami sajikan, hal mana akan terus kami perbaiki dari waktu kewaktu.
Dalam hal Anda menemukan inakurasi informasi yang termuat pada situs ini atau informasi yang seharusnya ada, namun belum tersedia, maka harap segera hubungi Kepaniteraan Mahkamah Agung RI melalui :
Email : kepaniteraan@mahkamahagung.go.id   Telp : 021-384 3348 (ext.318)

6. Bahwa setelah pisah tempat tinggal, Penggugat tinggal di rumah Penggugat, sedangkan Tergugat tinggal di rumah Tergugat;
7. Bahwa setelah pisah tempat tinggal, antara Penggugat dengan Tergugat tidak berkomunikasi, dan Tergugat tidak memberikan nafkah kepada Penggugat;
8. Bahwa atas permasalahan tersebut di atas Penggugat sudah tidak sanggup lagi untuk mempertahankan perkawinan ini, oleh karena itu Penggugat telah berketetapan hati untuk bercerai dengan Tergugat;
9. Bahwa pengajuan gugatan cerai Penggugat telah memenuhi alasan-alasan perceraian sebagaimana diatur dalam Pasal 19 huruf f Peraturan Pemerintah Nomor 9 tahun 1975 jo Pasal 116 huruf f Instruksi Presiden Nomor 1 tahun 1991 tentang Kompilasi Hukum Islam.

Berdasarkan alasan/dalil-dalil gugatan diatas, Penggugat memohon kepada Bapak Ketua Pengadilan Agama Bandung berkenan, untuk segera memeriksa dan mengadili perkara ini, selanjutnya menjatuhkan putusan yang amarnya sebagai berikut:

1. Mengabulkan gugatan Penggugat;
2. Menjatuhkan talak satu ba'in sughra dari Tergugat (Nama Tergugat) terhadap Penggugat (Nama Penggugat);
3. Membebankan biaya perkara menurut hukum;

Atau apabila Majelis Hakim Pengadilan Agama Bandung berpendapat lain, mohon putusan yang seadil adilnya (ex aequo et bono).

Menimbang, bahwa pada hari sidang yang telah ditetapkan Penggugat telah datang menghadap ke muka sidang, sedangkan Tergugat tidak datang menghadap dan tidak menyuruh orang lain untuk menghadap sebagai wakil/kuasa hukumnya meskipun telah dipanggil secara resmi dan patut yang relaas panggilannya dibacakan di dalam sidang, sedangkan tidak ternyata bahwa tidak datangnya itu disebabkan suatu halangan yang sah;

Menimbang, bahwa bahwa majelis hakim telah menasehati Penggugat agar berpikir untuk tidak bercerai dengan Tergugat, tetapi Penggugat tetap pada dalil-dalil gugatannya untuk bercerai dengan Tergugat;

Disclaimer
Kepaniteraan Mahkamah Agung Republik Indonesia berusaha untuk selalu mencantumkan informasi paling kini dan akurat sebagai bentuk komitmen Mahkamah Agung untuk pelayanan publik, transparansi dan akuntabilitas pelaksanaan fungsi peradilan. Namun dalam hal-hal tertentu masih dimungkinkan terjadi permasalahan teknis terkait dengan akurasi dan keterkinian informasi yang kami sajikan, hal mana akan terus kami perbaiki dari waktu kewaktu.
Dalam hal Anda menemukan inakurasi informasi yang termuat pada situs ini atau informasi yang seharusnya ada, namun belum tersedia, maka harap segera hubungi Kepaniteraan Mahkamah Agung RI melalui :
Email : kepaniteraan@mahkamahagung.go.id Telp : 021-384 3348 (ext.318)

Menimbang, bahwa perkara ini tidak dapat dimediasi karena Tergugat tidak pernah datang menghadap meskipun telah dipanggil secara resmi dan patut, selanjutnya dimulai pemeriksaan dengan membacakan surat gugatan Penggugat yang maksud dan isinya tetap dipertahankan oleh Penggugat;

Menimbang, bahwa untuk menguatkan dalil-dalil gugatannya, Penggugat telah mengajukan alat-alat bukti berupa:

A. Surat:

Fotokopi Buku Kutipan Akta Nikah Nomor: 763/101/X/651/1992, tertanggal 23 Oktober 1992, yang dikeluarkan oleh Kecamatan Kebumen, Kabupaten Kebumen, fotokopi tersebut telah dinazegelen dan oleh Ketua Majelis telah dicocokkan dengan aslinya ternyata telah sesuai selanjutnya diberi tanda bukti (P.);

B. Saksi:

1. Saksi I, umur 66 tahun, agama Islam, pekerjaan Pensiunan, tempat tinggal di XXXXXXXXXXXXXXXXX Kota Bandung, di bawah sumpahnya memberikan keterangan sebagai berikut:
   - Bahwa saksi kenal dengan Penggugat dan Tergugat karena saksi adalah paman Penggugat;
   - Bahwa Penggugat dan Tergugat adalah suami istri dan telah dikaruniai 3 (tiga) orang anak;
   - Bahwa semula keadaan rumah tangga rumah tangga Penggugat dan Tergugat rukun dan harmonis, namun sejak akhit tahun 2018 sudah tidak harmonis lagi, karena sering terjadi perselisihan dan pertengkaran, disebabkan Tergugat kurang bertanggungjawab terhadap nafkah keluarga bahkan untuk keperluan sehari-hari dan biaya sekolah anaknya lebih mengandalkan hasil kerja Penggugat;
   - Bahwa saksi sering melihat dan menyaksikan pertengkaran penggugat dan Tergugat;
   - Bahwa Penggugat sudah cukup bersabar tapi akhirnya berpisah sekitar bulan Oktober 2020;
   - Bahwa saksi sudah berusaha untuk merukunkan Penggugat dengan Tergugat, namun tidak berhasil;

Disclaimer
Kepaniteraan Mahkamah Agung Republik Indonesia berusaha untuk selalu mencantumkan informasi paling kini dan akurat sebagai bentuk komitmen Mahkamah Agung untuk pelayanan publik, transparansi dan akuntabilitas pelaksanaan fungsi peradilan. Namun dalam hal-hal tertentu masih dimungkinkan terjadi permasalahan teknis terkait dengan akurasi dan keterkinian informasi yang kami sajikan, hal mana akan terus kami perbaiki dari waktu kewaktu.
Dalam hal Anda menemukan inakurasi informasi yang termuat pada situs ini atau informasi yang seharusnya ada, namun belum tersedia, maka harap segera hubungi Kepaniteraan Mahkamah Agung RI melalui :
Email : kepaniteraan@mahkamahagung.go.id Telp : 021-384 3348 (ext.318)

2. **Saksi II,** umur 65 tahun, agama Islam, pekerjaan Karyawan Swasta, tempat tinggal di XXXXXXXXXXXXXXXXX di bawah sumpahnya memberikan keterangan sebagai berikut:
   - Bahwa saksi kenal dengan Penggugat dan Tergugat karena saksi adalah tetangga Penggugat;
   - Bahwa Penggugat dan Tergugat adalah suami istri dan dan telah dikaruniai 3 (tiga) orang anak;
   - Bahwa rumah tangga Penggugat dan Tergugat sejak akhir tahun 2018 sudah tidak harmonis lagi, sering terjadi perselisihan dan pertengkaran, disebabkan masalah ekonomi karena Tergugat sudah tidak memperhatikan lagi nafkah Penggugat dan ketiga anaknya sehingga untuk memenuhi kebutuhan rumah tangga dan biaya sekolah anak lebih mengandalkan hasil kerja Penggugat;
   - Bahwa saksi tidak melihat pertengkarang Penggugat dan Tergugat hanya sering mendapat laporan perselisihan Penggugat dan Tergugat karena sebagai ketua RW dan telah menasehi serta mendamaikan mereka;
   - Bahwa Penggugat dan Tergugat saat ini telah pisah tempat sejak akhir tahin 2019;
   - Bahwa saksi sudah berusaha untuk merukunkan Penggugat dengan Tergugat namun tidak berhasil;

Menimbang, bahwa selanjutnya untuk singkatnya uraian putusan ini, maka semua hal yang termuat dalam berita acara sidang ini merupakan bagian yang tidak terpisahkan dari putusan ini;

PERTIMBANGAN HUKUM

Menimbang, bahwa maksud dan tujuan gugatan Penggugat adalah sebagaimana terurai di atas;

Disclaimer
Kepaniteraan Mahkamah Agung Republik Indonesia berusaha untuk selalu mencantumkan informasi paling kini dan akurat sebagai bentuk komitmen Mahkamah Agung untuk pelayanan publik, transparansi dan akuntabilitas pelaksanaan fungsi peradilan. Namun dalam hal-hal tertentu masih dimungkinkan terjadi permasalahan teknis terkait dengan akurasi dan keterkinian informasi yang kami sajikan, hal mana akan terus kami perbaiki dari waktu kewaktu.
Dalam hal Anda menemukan inakurasi informasi yang termuat pada situs ini atau informasi yang seharusnya ada, namun belum tersedia, maka harap segera hubungi Kepaniteraan Mahkamah Agung RI melalui :
Email : kepaniteraan@mahkamahagung.go.id Telp : 021-384 3348 (ext.318)

Menimbang, bahwa ternyata Tergugat, meskipun telah dipanggil secara resmi dan patut, tidak datang menghadap di muka sidang dan pula tidak ternyata bahwa tidak datangnya itu disebabkan suatu halangan yang sah;

Menimbang, bahwa Tergugat yang dipanggil secara resmi dan patut akan tetapi tidak datang menghadap harus dinyatakan tidak hadir dan gugatan tersebut harus diperiksa secara verstek;

Menimbang, bahwa oleh karena itu, maka putusan atas perkara ini dapat dijatuhkan tanpa hadirnya Tergugat (verstek);

Menimbang, bahwa berdasarkan ketentuan Pasal 125 ayat (1) HIR, yaitu putusan yang dijatuhkan tanpa hadirnya Tergugat dapat dikabulkan sepanjang berdasarkan hukum dan beralasan, oleh karena itu majelis membebani Penggugat untuk membuktikan dalil-dalil gugatannya;

Menimbang, bahwa gugatan Penggugat didasarkan pada dalil yang pada pokoknya adalah rumah tangga Penggugat dan Tergugat tidak harmonis lagi sering terjadi perselisihan dan pertengkaran dan tidak ada harapan akan hidup rukun lagi dalam satu rumah tangga, sehingga Penggugat memohon agar Pengadilan Agama menjatuhkan talak satu bain sughra Tergugat terhadap Penggugat;

Menimbang, bahwa untuk membuktikan dalil-dalilnya, Penggugat telah mengajukan alat bukti surat P. dan dua orang saksi;

Menimbang, bahwa bukti P. (fotokopi Duplikat Kutipan Akta Nikah) yang merupakan akta otentik dan telah bermeterai cukup dan cocok dengan aslinya, isi bukti tersebut menjelaskan mengenai pernikahan Penggugat dan Tergugat, sehingga bukti tersebut telah memenuhi syarat formil dan materil, serta mempunyai kekuatan pembuktian yang sempurna dan mengikat;

Menimbang, bahwa berdasarkan bukti (P.) maka dapat dinyatakan terbukti bahwa Penggugat dan Tergugat adalah suami istri yang sah, sehingga mempunyai landasan hukum untuk mengajukan cerai gugat terhadap Tergugat;

Menimbang, bahwa oleh karena alasan Cerai Gugat Penggugat didasarkan pada pasal 19 huruf (f) Peraturan Pemerintah Nomor 9 Tahun 1975 jo. Pasal 116 huruf (f) Kompilasi Hukum Islam, maka Majelis telah mendengar keterangan saksi dari pihak keluarga Penggugat, sehingga telah terpenuhi

Disclaimer
Kepaniteraan Mahkamah Agung Republik Indonesia berusaha untuk selalu mencantumkan informasi paling kini dan akurat sebagai bentuk komitmen Mahkamah Agung untuk pelayanan publik, transparansi dan akuntabilitas pelaksanaan fungsi peradilan. Namun dalam hal-hal tertentu masih dimungkinkan terjadi permasalahan teknis terkait dengan akurasi dan keterkinian informasi yang kami sajikan, hal mana akan terus kami perbaiki dari waktu kewaktu.
Dalam hal Anda menemukan inakurasi informasi yang termuat pada situs ini atau informasi yang seharusnya ada, namun belum tersedia, maka harap segera hubungi Kepaniteraan Mahkamah Agung RI melalui :
Email : kepaniteraan@mahkamahagung.go.id Telp : 021-384 3348 (ext.318)

maksud pasal 76 ayat (1) Undang Undang Nomor 7 Tahun 1989, yang telah mengalami 2 kali perubahan dengan Undang-undang Nomor 3 tahun 2006 dan Undang-Undang Nomor 50 tahun 2009;

Menimbang, bahwa dua orang saksi yang diajukan oleh Penggugat secara formil dan materil telah memenuhi syarat sebagai saksi, oleh karena itu dapat dipertimbangkan lebih lanjut;

Menimbang, bahwa berdasarkan keterangan saksi-saksi tersebut di atas yang keterangannya saling bersesuaian, maka Majelis telah dapat menemukan fakta yang pada pokoknya sebagai berikut:

1. Suami atau isteri yang mengabaikan norma perkawinan maka ikatan perkawinan akan sangat rentan terhadap adanya perceraian. Kenyataan inilah yang terjadi dalam rumah tangga Penggugat dan Tergugat;
2. Berpisahnya tempat tinggal dan keengganan Penggugat untuk berdamai (rukun kembali) dengan Tergugat selama dalam proses persidangan, merupakan fakta kongkrit yang menunjukkan bahwa hubungan antara Penggugat dengan Tergugat sudah sampai pada tingkat pecahnya perkawinan (broken marriage);
3. Rumah tangga Penggugat dan Tergugat sudah sulit untuk dirukunkan kembali, perkawinan yang telah pecah seperti itu tidak akan membawa maslahat, bahkan akan menyebabkan mudlarat yang lebih besar lagi bagi kedua belah pihak, serta sulit untuk mewujudkan kehidupan rumah tangga yang sakinah, mawaddah dan rahmah, sebagaimana yang diamanatkan al-Qur'an dalam surat ar-Ruum ayat 21;

Menimbang, bahwa berdasarkan fakta tersebut di atas, Majelis berpendapat rumah tangga Penggugat dengan Tergugat tidak harmonis lagi prilaku Tergugat tersebut menjadi pemicu terjadinya percekcokan sehingga hubungan Penggugat dan Tergugat semakin tidak baik, dengan demikian Majelis berkesimpulan keadaan rumah tangga Penggugat dengan Tergugat telah pecah sedemikian rupa sehingga tujuan perkawinan untuk membentuk keluarga/rumah tangga yang bahagia dan kekal berdasarkan Ketuhanan Yang Maha Esa (*vide* pasal 1 Undang-Undang Nomor 1 Tahun 1974) dan atau keluarga yang sakinah, penuh mawaddah dan rahmah (*vide* pasal 3

Disclaimer
Kepaniteraan Mahkamah Agung Republik Indonesia berusaha untuk selalu mencantumkan informasi paling kini dan akurat sebagai bentuk komitmen Mahkamah Agung untuk pelayanan publik, transparansi dan akuntabilitas pelaksanaan fungsi peradilan. Namun dalam hal-hal tertentu masih dimungkinkan terjadi permasalahan teknis terkait dengan akurasi dan keterkinian informasi yang kami sajikan, hal mana akan terus kami perbaiki dari waktu kewaktu.
Dalam hal Anda menemukan inakurasi informasi yang termuat pada situs ini atau informasi yang seharusnya ada, namun belum tersedia, maka harap segera hubungi Kepaniteraan Mahkamah Agung RI melalui :
Email : kepaniteraan@mahkamahagung.go.id Telp : 021-384 3348 (ext.318)

Kompilasi Hukum Islam) telah tidak terwujud dalam rumah tangga Penggugat dengan Tergugat;

Menimbang, bahwa unsur pokok tegaknya suatu bangunan rumah tangga adalah adanya ikatan lahir batin yang kokoh antara suami dan isteri. Apabila terjadi perselisihan dan pertengkaran antara suami-isteri yang berakibat kepada tidak lagi saling peduli dan telah diupayakan untuk rukun kembali tetapi tidak berhasil maka hal tersebut mengindikasikan bahwa ikatan lahir-batin diantara suami-isteri tersebut telah sedemikian rapuh atau bahkan telah lepas sama sekali, sehingga telah tidak ada lagi kecocokan dan kesamaan kehendak diantara keduanya;

Menimbang, bahwa mempertahankan rumah tangga yang telah pecah sedemikian rupa adalah sia-sia belaka, bahkan apabila keadaannya seperti sekarang ini jika dipaksakan atau dibiarkan maka justru akan menimbulkan mudharat dan penderitaan lahir batin yang berkepanjangan bagi Penggugat, sehingga oleh karenanya Majelis berpendapat bahwa rumah tangga Penggugat dengan Tergugat telah tidak dapat dipertahankan lagi;

Menimbang, bahwa Majelis perlu mengemukakan doktrin ulama yang kemudian diambil alih sebagai pendapat Majelis dalam Kitab Madaa Hurriyatuz Zaujaini fith Thalaaq Juz I halaman 83:

وقد اختار الإسلام نظام الطلاق حين تضطرب الحياة الزوجين ولم يعد ينفع فيها نصائح ولا صلح وحيث تصبح الربطة الزواج صورة من غير روح لأن الإستمرار معناه أن يحكم على أحد الزوجين بالسجن المؤبد وهذا ظلم تأباه روح العدالة

"*Islam memilih lembaga talak/cerai ketika rumah tangga sudah dianggap goncang serta dianggap sudah tidak bermanfaat lagi nasehat/perdamaian dan hubungan suami isteri menjadi tanpa ruh (hampa), sebab meneruskan perkawinan berarti menghukum salah satu suami isteri dengan penjara yang berkepanjangan. Ini adalah aniaya yang bertentangan dengan semangat keadilan*"

Menimbang, bahwa berdasarkan hal-hal tersebut di atas maka gugatan Penggugat telah terbukti beralasan hukum sesuai ketentuan pasal 39 ayat (2) Undang-Undang Nomor 1 Tahun 1974 yang telah diubah dengan Undang-undang Nomor 16 Tahun 2019 jis Pasal 19 huruf (f) Peraturan Pemerintah

Disclaimer
Kepaniteraan Mahkamah Agung Republik Indonesia berusaha untuk selalu mencantumkan informasi paling kini dan akurat sebagai bentuk komitmen Mahkamah Agung untuk pelayanan publik, transparansi dan akuntabilitas pelaksanaan fungsi peradilan. Namun dalam hal-hal tertentu masih dimungkinkan terjadi permasalahan teknis terkait dengan akurasi dan keterkinian informasi yang kami sajikan, hal mana akan terus kami perbaiki dari waktu kewaktu.
Dalam hal Anda menemukan inakurasi informasi yang termuat pada situs ini atau informasi yang seharusnya ada, namun belum tersedia, maka harap segera hubungi Kepaniteraan Mahkamah Agung RI melalui :
Email : kepaniteraan@mahkamahagung.go.id   Telp : 021-384 3348 (ext.318)

Nomor 9 Tahun 1975 dan Pasal 116 huruf (f) Kompilasi Hukum Islam, oleh karena itu gugatan Penggugat dapat dikabulkan dengan menjatuhkan talak satu bain sughra dari Tergugat terhadap Penggugat;

Menimbang, bahwa oleh karena perkara ini dalam bidang perkawinan, maka sesuai Pasal 89 ayat (1) Undang-Undang Nomor 7 Tahun 1989 sebagaimana telah diubah dengan Undang-Undang Nomor 3 Tahun 2006 dan perubahan kedua dengan Undang-Undang Nomor 50 Tahun 2009, biaya perkara dibebankan kepada Penggugat;

Mengingat, semua pasal dalam peraturan perundang-undangan dan hukum Islam yang berkaitan dengan perkara ini;

MENGADILI

1. Menyatakan Tergugat yang telah dipanggil dengan resmi dan patut untuk menghadap di persidangan, tidak hadir;
2. Mengabulkan gugatan Penggugat dengan verstek;
3. Menjatuhkan talak satu bain shughra Tergugat (**Nama Tergugat**) terhadap Penggugat (**Nama Penggugat**);
4. Membebankan biaya perkara kepada Penggugat sejumlah Rp. 476.000 (empat ratus tujuh puluh enam ribu rupiah);

Demikianlah diputus dalam sidang permusyawaratan majelis Pengadilan Agama Bandung pada hari Senin tanggal 21 Desember 2020 Masehi bertepatan dengan tanggal 06 Jumadil Awal 1442 Hijriyah oleh kami Dra. Hj. Inne Noor Faidah sebagai Ketua Majelis, Drs. H. Badruddin, M.H. dan Drs. Asep Gupron, S.H., M.H., masing-masing sebagai Hakim Anggota, dan diucapkan pada hari itu juga dalam sidang terbuka untuk umum oleh Ketua Majelis tersebut didampingi para Hakim Anggota, dibantu oleh Astria Lestari Ningsih, S.HI. sebagai Panitera Pengganti dengan dihadiri oleh Penggugat tanpa hadirnya Tergugat;

Ketua Majelis

Disclaimer
Kepaniteraan Mahkamah Agung Republik Indonesia berusaha untuk selalu mencantumkan informasi paling kini dan akurat sebagai bentuk komitmen Mahkamah Agung untuk pelayanan publik, transparansi dan akuntabilitas pelaksanaan fungsi peradilan. Namun dalam hal-hal tertentu masih dimungkinkan terjadi permasalahan teknis terkait dengan akurasi dan keterkinian informasi yang kami sajikan, hal mana akan terus kami perbaiki dari waktu kewaktu.
Dalam hal Anda menemukan inakurasi informasi yang termuat pada situs ini atau informasi yang seharusnya ada, namun belum tersedia, maka harap segera hubungi Kepaniteraan Mahkamah Agung RI melalui :
Email : kepaniteraan@mahkamahagung.go.id Telp : 021-384 3348 (ext.318)

Dra. Hj. Inne Noor Faidah

Hakim Anggota | Hakim Anggota

Drs. H. Badruddin, M.H. | Drs. Asep Gupron, S.H., M.H.,

Panitera Pengganti

Astria Lestari Ningsih, S.HI.

Perincian Biaya Perkara :

1. Pendaftaran	:	Rp.	30.000,-
2. Proses	:	Rp.	40.000,-
3. Panggilan Penggugat	:	Rp.	50.000,-
4. Panggilan Tergugat	:	Rp.	320.000,-
5. PNBP Panggilan	:	Rp.	20.000,-
6. Redaksi	:	Rp.	10.000,-
7. Materai	:	Rp.	6.000,-
Jumlah	:	Rp.	476.000,-

empat ratus tujuh puluh enam ribu rupiah

Disclaimer
Kepaniteraan Mahkamah Agung Republik Indonesia berusaha untuk selalu mencantumkan informasi paling kini dan akurat sebagai bentuk komitmen Mahkamah Agung untuk pelayanan publik, transparansi dan akuntabilitas pelaksanaan fungsi peradilan. Namun dalam hal-hal tertentu masih dimungkinkan terjadi permasalahan teknis terkait dengan akurasi dan keterkinian informasi yang kami sajikan, hal mana akan terus kami perbaiki dari waktu kewaktu.
Dalam hal Anda menemukan inakurasi informasi yang termuat pada situs ini atau informasi yang seharusnya ada, namun belum tersedia, maka harap segera hubungi Kepaniteraan Mahkamah Agung RI melalui :
Email : kepaniteraan@mahkamahagung.go.id Telp : 021-384 3348 (ext.318)
