# Supplementary material for: Classification of divorce causes during the COVID-19 pandemic using convolutional neural networks
Source: PeerJ Comput Sci. 2022 Jun 30;8:e998. doi: 10.7717/peerj-cs.998 (PMC9299239; doi:10.7717/peerj-cs.998)
Supplement: Supplemental Information 3 [file peerj-cs-08-998-s003.zip › KDRT Dataset/Data ke-5.pdf]

P U T U S A N
NOMOR 283/Pdt.G/e-court/2020/PN Bdg

DEMI KEADILAN BERDASARKAN KETUHANAN YANG MAHA ESA

Pengadilan Negeri Bandung, yang memeriksa dan memutus perkara-perkara perdata pada peradilan tingkat pertama telah menjatuhkan putusan sebagai berikut dalam perkara antara :

JESSICA DESIANA, bertempat tinggal di Jl. Sharon Garden 1 No. 15 RT. 004 RW. 011 Kelurahan Cipamokolan Kecamatan Rancasari Kota Bandung, dalam hal ini diwakili oleh 1. HAMZAH AHMAD, S.H., dan MEYHANA SIHOMBING, S.H, para advokat/pengacara pada kantor HA.AH LAW FIRM yang beralamat di Perumahan Kiara Asri Jl. Kiarasari V No. 3 Kelurahan Margasari Kecamatan Buahbatu Kota Bandung, berdasarkan surat Kuasa Khusus tanggal 7 Juli 2020, Selanjutnya disebut sebagai **PENGGUGAT** ;

L A W A N

HENRY SETIADY, bertempat tinggal di Toko Setia Kawan Jl. Dulatip No. 1B Kelurahan Kebon Jeruk, Kecamatan Andir, Kota Bandung, selanjutnya disebut sebagai **TERGUGAT**;

Pengadilan Negeri tersebut ;

Setelah membaca surat-surat dalam berkas perkara ini;

Setelah memperhatikan surat- surat bukti.

Setelah mendengar keterangan para saksi.

Setelah mendengar kedua belah pihak yang berperkara.

TENTANG DUDUK PERKARA

Menimbang, bahwa Penggugat telah mengajukan surat gugatannya secara tertulis tanggal 27 Juli 2020 dan telah didaftarkan di Kepaniteraan Pengadilan Negeri Bandung tertanggal 28 Juli 2020 dibawah nomor : 283/Pdt.G/e-court/2020/PNBdg yang pada pokoknya berbunyi sebagai berikut :

1. Bahwa PENGGUGAT pada tanggal 12 Mei 2018 telah melangsungkan perkawinan di Paroki St. Gabriel – Bandung. Sesuai dengan *Testimonium Matrimonii* Nomor 05 Anno 2018 tertanggal 12 Mei 2018 yang dikeluarkan oleh Parochiae Sancti Gabrielis Bandung – Indonesia.

Disclaimer
Kepaniteraan Mahkamah Agung Republik Indonesia berusaha untuk selalu mencantumkan informasi paling kini dan akurat sebagai bentuk komitmen Mahkamah Agung untuk pelayanan publik, transparansi dan akuntabilitas pelaksanaan fungsi peradilan. Namun dalam hal-hal tertentu masih dimungkinkan terjadi permasalahan teknis terkait dengan akurasi dan keterkinian informasi yang kami sajikan, hal mana akan terus kami perbaiki dari waktu kewaktu.
Dalam hal Anda menemukan inakurasi informasi yang termuat pada situs ini atau informasi yang seharusnya ada, namun belum tersedia, maka harap segera hubungi Kepaniteraan Mahkamah Agung RI melalui :
Email : kepaniteraan@mahkamahagung.go.id Telp : 021-384 3348 (ext.318)

2. Bahwa PENGGUGAT mencatatkan perkawinannya dengan TERGUGAT di kantor Pencatatan Sipil Kota Bandung. Sesuai dengan Kutipan Akta Perkawinan Nomor 3273-KW-02082018-0002 tertanggal 3 Agustus 2018 yang dikeluarkan oleh Dinas Kependudukan dan Pencatatan Sipil Kota Bandung.
3. Bahwa PENGGUGAT selama perkawinannya dengan TERGUGAT belum dikaruniai anak.
4. Bahwa PENGGUGAT sudah 2 (dua) tahun 2 (dua) bulan berumah tangga dengan TERGUGAT.
5. Bahwa PENGGUGAT dan TERGUGAT dari awal perkawinannya sampai Januari 2020 bertempat tinggal di Jalan Sharon Garden 1 No. 15 dan perkawinannya berjalan harmonis. Pada bulan Februari 2020 TERGUGAT mengajak kepada PENGGUGAT untuk pindah tempat tinggal ke Jalan Kalipah Apo Gg. Siti No. 57 Kelurahan Karanganyar Kecamatan Astanaanyar Kota Bandung dan mulailah sering terjadi keributan dalam rumah tangganya.
6. Bahwa PENGGUGAT mengalami perlakuan Kekerasan Dalam Rumah Tangga (KDRT) yang dilakukan oleh TERGUGAT pada hari Minggu tanggal 3 Mei 2020 dan berulang kembali pada hari Jumat tanggal 3 Juli 2020.
7. Bahwa antara PENGGUGAT dengan TERGUGAT sudah tidak terjalin komunikasi yang baik dan telah melaksanakan pisah ranjang.
8. Bahwa antara PENGGUGAT dengan TERGUGAT sudah beberapa kali coba untuk didamaikan oleh kedua belah pihak dari keluarga besar namun tidak pernah berhasil.
9. Bahwa antara PENGGUGAT terhadap TERGUGAT sudah tidak ada perasaan cinta lagi, sehingga apabila tetap berlangsung hubungan pernikahan dikhawatirkan akan kembali terjadi pertengkaran yang terus berulang-ulang dan terjadi kembali kekerasan fisik yang lebih fatal lagi.
10. Bahwa Sesuai dengan bunyi Pasal 19 huruf (d) Peraturan Pemerintah Nomor 9 Tahun 1975 yang berbunyi sebagai berikut:
    Perceraian dapat terjadi karena alasan atau alasan-alasan:
    (d) Salah satu pihak melakukan kekejaman atau penganiayaan berat yang membahayakan pihak yang lain;
11. Bahwa dikarenakan domisili TERGUGAT tersebut berada di Kota Bandung maka gugatan inipun diajukan kepada Pengadilan Negeri Bandung kelas 1A Khusus.
12. Bahwa untuk mewujudkan maksud PENGGUGAT tersebut haruslah terlebih dahulu adanya putusan perceraian dari Pengadilan dalam hal ini Pengadilan

Disclaimer
Kepaniteraan Mahkamah Agung Republik Indonesia berusaha untuk selalu mencantumkan informasi paling kini dan akurat sebagai bentuk komitmen Mahkamah Agung untuk pelayanan publik, transparansi dan akuntabilitas pelaksanaan fungsi peradilan. Namun dalam hal-hal tertentu masih dimungkinkan terjadi permasalahan teknis terkait dengan akurasi dan keterkinian informasi yang kami sajikan, hal mana akan terus kami perbaiki dari waktu kewaktu.
Dalam hal Anda menemukan inakurasi informasi yang termuat pada situs ini atau informasi yang seharusnya ada, namun belum tersedia, maka harap segera hubungi Kepaniteraan Mahkamah Agung RI melalui :
Email : kepaniteraan@mahkamahagung.go.id   Telp : 021-384 3348 (ext.318)

Negeri Bandung Kelas 1A Khusus, sesuai dengan bunyi Pasal 39 Ayat (1) Undang-undang Nomor 1 Tahun 1974 sebagai berikut:

(1) Perceraian hanya dapat dilakukan di depan Sidang Pengadilan setelah Pengadilan yang bersangkutan berusaha dan tidak berhasil mendamaikan kedua belah pihak.

Bahwa berdasarkan dalil-dalil tersebut diatas, PENGGUGAT mohon kepada Ketua Pengadilan Negeri Bandung Kelas 1A Khusus melalui Majelis Hakim yang memeriksa dan mengadili perkara ini untuk berkenan memberikan putusan yang mana amar putusannya sebagai berikut:

1. Mengabulkan Gugatan Cerai dari PENGGUGAT untuk seluruhnya.
2. Menyatakan perkawinan antara PENGGUGAT dengan TERGUGAT telah putus karena perceraian.
3. Memerintahkan kepada Panitera Pengadilan Negeri Bandung untuk mengirimkan salinan putusan perceraian ini yang telah berkekuatan hukum tetap kepada Dinas Kependudukan dan Pencatatan Sipil Kota Bandung.
4. Membebankan Biaya Perkara Sesuai Peraturan Yang Berlaku.

Apabila Majelis Hakim yang memeriksa dan mengadili perkara ini berpendapat lain, mohon putusan yang seadil-adilnya (ex aquo et bono) ;

Menimbang, bahwa pada hari sidang yang telah ditentukan Penggugat hadir dan kuasanya bernama 1. HAMZAH AHMAD, S.H., dan MEYHANA SIHOMBING, S.H, para advokat/pengacara pada kantor HA.AH LAW FIRM yang beralamat di Perumahan Kiara Asri Jl. Kiarasari V No. 3 Kelurahan Margasari Kecamatan Buahbatu Kota Bandung, berdasarkan surat Kuasa Khusus tanggal 7 Juli 2020 dan Tergugat hadir kuasanya bernama IWAN AGUSTIAN, S.H. dan INDERA KESUMA, S.H., Advokat pada Kantor Hukum IWAN AGUSTIAN, S.H. & ASSOCIATES, berkantor di Jl. H. Samsudin No.79, Kota Bandung, berdasarkan Surat Kuasa, tertanggal 04 Agustus 2020 ;

Menimbang bahwa pada kesempatan pertama, majelis Hakim telah berusaha untuk menyelesaikan perkara ini dengan jalan damai melalui lembaga mediasi dengan mediator yaitu I Dewa Gede Suarditha, SH, MH., akan tetapi berdasarkan surat laporan dari mediator tertanggal 1 September 2020 menyatakan bahwa mediasi gagal;

Menimbang, bahwa oleh karena upaya perdamaian melalui lembaga

Disclaimer
Kepaniteraan Mahkamah Agung Republik Indonesia berusaha untuk selalu mencantumkan informasi paling kini dan akurat sebagai bentuk komitmen Mahkamah Agung untuk pelayanan publik, transparansi dan akuntabilitas pelaksanaan fungsi peradilan. Namun dalam hal-hal tertentu masih dimungkinkan terjadi permasalahan teknis terkait dengan akurasi dan keterkinian informasi yang kami sajikan, hal mana akan terus kami perbaiki dari waktu kewaktu.
Dalam hal Anda menemukan inakurasi informasi yang termuat pada situs ini atau informasi yang seharusnya ada, namun belum tersedia, maka harap segera hubungi Kepaniteraan Mahkamah Agung RI melalui :
Email : kepaniteraan@mahkamahagung.go.id Telp : 021-384 3348 (ext.318)

mediasi tidak berhasil, maka pemeriksaan perkara ini dilanjutkan dengan pembacaan surat gugatan.

Menimbang bahwa atas surat gugatan tersebut, Penggugat menyatakan tetap pada gugatannya.

Menimbang bahwa atas gugatan Penggugat tersebut, Tergugat telah menyampaikan jawabannya secara tertulis tertanggal 21 September 2020, yang pada pokoknya berbunyi sebagai berikut:

1. Bahwa Tergugat menolak seluruh dalil pada posita Gugatan Penggugat kecuali diakui secara tegas ;
2. Bahwa benar antara Tergugat dan Penggugat telah melangsungkan pernikahan di Paroki St. Gabriel Bandung pada tanggal 12 Mei 2018 sesuai dengan *Testimonium Matrimonii* No. 05 Anno 2018 tertanggal 12 Mei 2018 yang dikeluarkan Parochiae Sancti Gabrielis Bandung – Indonesia;
3. Bahwa benar perkawinan Tergugat dengan Penggugat dicatat di Kantor Pencatatan Sipil Kota Bandung, sesuai dengan kutipan Akta Perkawinan Nomor 3273-KW-02082018-0002 tertanggal 3 Agustus 2018 yang dikeluarkan oleh Dinas Kependudukan dan Pencatatan Sipil Kota Bandung;
4. Bahwa benar selama perkawinan Tergugat dengan Penggugat belum dikaruniai anak;
5. Bahwa benar sejak perkawinan sampai dengan Bulan Januari 2020 Tergugat dan Penggugat bertempat tinggal di Jalan Sharon Garden 1 No. 15 dan perkawinannya berjalan rukun dan harmonis, dan sejak bulan Februari 2020, Tergugat mengajak Penggugat untuk pindah tempat tinggal ke Jalan Kalipah Apo Gang Siti No. 57 Kelurahan Karang Anyar Kecamatan Astana Anyar Kota Bandung dan sejak itu sering terjadi keributan dalam rumah tangga;
6. Bahwa tidak benar “Bahwa PENGGUGAT mengalami perlakuan Kekerasan Dalam Rumah Tangga (KDRT) yang dilakukan TERGUGAT pada hari Minggu, tanggal 3 Mei 2020 dan pada hari Jum’at, 3 Juli 2020”

   Yang benar pada hari jumat tanggal 3 juli 2020 “Bahwa saat itu Tergugat membeli makanan khas Surabaya (ote-ote) dan meminta dimasakan oleh Penggugat akan tetapi Penggugat malah ngomel dan marah-marah. Tergugat yang saat itu sedang istirahat di tempat tidur, diinjak kakinya oleh Penggugat, secara reflex tangan Tergugat menampar Penggugat, lalu Penggugat memukul ke wajah Tergugat menggunakan gagang sapu besi memukul dan di tangkis oleh tangannya sampai luka memar sambil teriak-teriak memaki

Disclaimer
Kepaniteraan Mahkamah Agung Republik Indonesia berusaha untuk selalu mencantumkan informasi paling kini dan akurat sebagai bentuk komitmen Mahkamah Agung untuk pelayanan publik, transparansi dan akuntabilitas pelaksanaan fungsi peradilan. Namun dalam hal-hal tertentu masih dimungkinkan terjadi permasalahan teknis terkait dengan akurasi dan keterkinian informasi yang kami sajikan, hal mana akan terus kami perbaiki dari waktu kewaktu.
Dalam hal Anda menemukan inakurasi informasi yang termuat pada situs ini atau informasi yang seharusnya ada, namun belum tersedia, maka harap segera hubungi Kepaniteraan Mahkamah Agung RI melalui :
Email : kepaniteraan@mahkamahagung.go.id Telp : 021-384 3348 (ext.318)

Tergugat, namun ditepis oleh Tergugat, lalu Tergugat pergi untuk menghindar tetapi Penggugat terus ngoceh dan teriak-teriak memaki Tergugat walau telah ditenangkan oleh orang tua Tergugat"

7. Bahwa pada posita perkara gugatan Penggugat pada point 8 yang intinya "bahwa antara Penggugat dan Tergugat sudah beberapa kali coba untuk didamaikan oleh kedua belah pihak dari keluarga besar namun tidak pernah berhasil.

   Terhadap posita Gugatan Penggugat tersebut, Tergugat tanggapi sebagai berikut :

   "Bahwa memang keluarga Penggugat pernah datang kerumah Tergugat, rencananya mau mendamaikan Penggugat dan Tergugat, akan tetapi malah membuat onar dan teriak-teriak memaki Tergugat, mengatakan "banci" kepada Tergugat dan menyuruh mengembalikan barang-barang Penggugat".

8. Bahwa Penggugat sering melakukan kebohongan dan Tergugat telah menegur berulang kali tetapi dilakukan lagi oleh Penggugat di tambah lagi Penggugat jarang berlaku sopan sama orang tua Tergugat.

9. Bahwa benar antara Penggugat dan Tergugat sudah tidak terjalin komunikasi yang baik dan telah melaksanakan pisah ranjang.

10. Bahwa dari rangkaian kronologis tersebut lebih tepatnya pasal yang digunakannya adalah Peraturan Pemerintah No. 9 Tahun 1975, pasal 19 huruf F : "Antara suami dan istri terus menerus terjadi perselisihan dan pertengkaran dan tidak ada harapan akan hidup rukun lagi dalam rumah tangga".

11. Bahwa apabila alasannya memakai Peraturan Pemerintah No. 9 Tahun 1975, pasal 19 huruf D : "Salah satu pihak melakukan kekejaman atau penganiayaan berat yang membahayakan pihak yang lain", maka Tergugatlah sebetulnya yang diperlakukan oleh penggugat terjadi kekejaman atau penganiayaan tersebut karena yang memulai adalah Penggugat

12. Bahwa berdasarkan hal-hal tersebut diatas, Tergugat dapat mengabulkan keinginan Penggugat untuk bercerai dengan alasan Peraturan Pemerintah No. 9 Tahun 1975 Pasal 19 huruf F : "Antara suami dan istri terus menerus terjadi perselisihan dan pertengkaran dan tidak ada harapan akan hidup rukun lagi dalam rumah tangga".

Berdasarkan hal-hal yang telah diuraikan tersebut diatas, maka Tergugat mohon kiranya Yth. Ketua dan hakim anggota yang mengadili dan memeriksa perkara ini berkenan untuk memberikan putusan sebagai berikut :

Disclaimer
Kepaniteraan Mahkamah Agung Republik Indonesia berusaha untuk selalu mencantumkan informasi paling kini dan akurat sebagai bentuk komitmen Mahkamah Agung untuk pelayanan publik, transparansi dan akuntabilitas pelaksanaan fungsi peradilan. Namun dalam hal-hal tertentu masih dimungkinkan terjadi permasalahan teknis terkait dengan akurasi dan keterkinian informasi yang kami sajikan, hal mana akan terus kami perbaiki dari waktu kewaktu.
Dalam hal Anda menemukan inakurasi informasi yang termuat pada situs ini atau informasi yang seharusnya ada, namun belum tersedia, maka harap segera hubungi Kepaniteraan Mahkamah Agung RI melalui :
Email : kepaniteraan@mahkamahagung.go.id Telp : 021-384 3348 (ext.318)

PRIMAIR :

1. Menyatakan bahwa perkawinan TERGUGAT dan PENGGUGAT Putus karena Perceraian;
2. Menyatakan perkawinan antara PENGGUGAT dengan TERGUGAT telah putus karena perceraian berdasarkan ketentuan Peraturan Pemerintah No. 9 Tahun 1975 Pasal 19 huruf F yaitu : Antara suami dan istri terus menerus terjadi perselisihan dan pertengkaran dan tidak ada harapan akan hidup rukun lagi dalam rumah tangga
3. Memerintahkan kepada Panitera Pengadilan Negeri Bandung untuk mengirim salinan putusan perceraian ini yang telah berkekuatan hukum yang tetap kepada Dinas Kependudukan dan pencatatan Sipil Kota Bandung;
4. Menghukum Penggugat untuk membayar biaya perkara yang timbul dalam perkara ini;

SUBSIDAIR :

Atau apabila Yth. Ketua Majelis dan hakim anggota yang mengadili dan memeriksa perkara ini berpendapat lain, mohon kiranya memberikan putusan yang seadil-adilnya *(ex aequo et bono).*

Menimbang, bahwa atas jawaban Tergugat tersebut diatas, Penggugat telah menyampaikan Replik secara tertulis sebagaimana selengkapnya dalam Repliknya tertanggal 29 September 2020, sedangkan atas Replik dari Penggugat tersebut Tergugat mengajukan Dupliknya tertanggal 6 Oktober 2020 ;

Menimbang bahwa untuk memperkuat dalil gugatannya Penggugat telah mengajukan bukti – bukti tertulis berupa photo copy surat – surat yang telah dibubuhi materai secukupnya dan telah dicocokkan dengan aslinya masing – masing berupa:

1. Photo copy sesuai dengan aslinyaKartu Tanda Penduduk atas nama Jessica Desiana, di beritanda P-1;
2. Photo copy sesuai dengan aslinya Kartu Keluarga atas nama Kepala Keluarga Jessica Desiana, diberitanda P-2 ;
3. Photo copy sesuai dengan aslinya Testimonium Matrimonii (surat kawin dari gereja), diberitanda P-3 ;
4. Photo copy sesuai dengan aslinya Kutipan Akta Perkawinan antara Hendri Setiadi dengan Jessica Desiana, diberitanda P-4 ;
5. Photo Penggugat sesuai dengan print out, di beritanda P-5;

Disclaimer
Kepaniteraan Mahkamah Agung Republik Indonesia berusaha untuk selalu mencantumkan informasi paling kini dan akurat sebagai bentuk komitmen Mahkamah Agung untuk pelayanan publik, transparansi dan akuntabilitas pelaksanaan fungsi peradilan. Namun dalam hal-hal tertentu masih dimungkinkan terjadi permasalahan teknis terkait dengan akurasi dan keterkinian informasi yang kami sajikan, hal mana akan terus kami perbaiki dari waktu kewaktu.
Dalam hal Anda menemukan inakurasi informasi yang termuat pada situs ini atau informasi yang seharusnya ada, namun belum tersedia, maka harap segera hubungi Kepaniteraan Mahkamah Agung RI melalui :
Email : kepaniteraan@mahkamahagung.go.id Telp : 021-384 3348 (ext.318)

Menimbang bahwa selain mengajukan bukti tertulis sebagaimana tersebut diatas Penggugat juga telah mengajukan saksi yang telah memberikan keterangan dibawah sumpah dimuka persidangan menurut tata cara agamanya masing – masing yang pada pokoknya keterangannya sebagai berikut:

1. Saksi ENDANG NURJANTI, menerangkan :
   - Bahwa Saksi merupakan Ibu kandung dari Penggugat;
   - Bahwa saksi tahu Penggugat dan Tergugat telah melangsungkan Perkawinan pada tanggal 12 Mei 2018;
   - Bahwa setelah Penggugat dan Tergugat menikah sempat tinggal di rumah Saksi selama 2 tahun kurang 2 bulan yang beralamat di Jl. Sharon Garden I No. 15 Kelurahan Cipamokolan Kecamatan Rancasari Kota Bandung;
   - Bahwa sepengetahuan saksi selama tinggal di Jl. Sharon Garden I No. 15 rumah tangga antara Penggugat dan Tergugat berjalan harmonis tidak ada masalah;
   - Bahwa setahu saksi pada awal tahun 2020 Penggugat mengajak Tergugat pindah ke ruko dan tinggal bersama dengan orangtua Tergugat yang beralamat di Jl. Kalipah Apo gg. Siti No. 57 Kota Bandung ;
   - Bahwa setelah pindah dan tinggal bersama di rumah orangtua Tergugat, rumah tangga antara Penggugat dan Tergugat mulai tidak harmonis dan sering bertengkar;
   - Bahwa saksi mengetahui Penggugat dan Tergugat sempat melakukan program kehamilan dan Penggugat sempat hamil selama 2 bulan namun janin dalam kandungan Penggugat meninggal (keguguran) akibat Penggugat terlalu kecapean;
   - Bahwa saksitahu dari Penggugat Pada bulan Mei 2020 bahwa sempat terjadi pertengkaran Tergugat telah melakukan kekerasan pada pada Penggugat ;
   - Bahwa saksi diperlihatkan bukti P-5, dan setelah dilihat saksi tahu dan membenarkan pernah melihat poto di hp ;
   - Bahwa saksi tidak pernah melihat langsung kejadian keributan antara Penggugat dan Tergugat ;
   - Bahwa sekitar tanggal 5 Juli 2020 saksi beserta suami dan menantu laki-laki (Kakak Ipar Penggugat/saksi Wendi), sempat datang ke rumah Tergugat berniat untuk mendamaikan Penggugat dan Tergugat namun sesampainya didepan rumah Tergugat, yang ada Tergugat langsung memaki-maki dan mengusir saksi dan suami beserta menantu laki-laki (Kakak Ipar Penggugat)

Disclaimer
Kepaniteraan Mahkamah Agung Republik Indonesia berusaha untuk selalu mencantumkan informasi paling kini dan akurat sebagai bentuk komitmen Mahkamah Agung untuk pelayanan publik, transparansi dan akuntabilitas pelaksanaan fungsi peradilan. Namun dalam hal-hal tertentu masih dimungkinkan terjadi permasalahan teknis terkait dengan akurasi dan keterkinian informasi yang kami sajikan, hal mana akan terus kami perbaiki dari waktu kewaktu.
Dalam hal Anda menemukan inakurasi informasi yang termuat pada situs ini atau informasi yang seharusnya ada, namun belum tersedia, maka harap segera hubungi Kepaniteraan Mahkamah Agung RI melalui :
Email : kepaniteraan@mahkamahagung.go.id Telp : 021-384 3348 (ext.318)

dengan mengeluarkan barang-barang milik saksi yang diangkut ketika Penggugat dan Tergugat pindah rumah dengan cara di lempar keluar ;

- Bahwa pada saat itu ada mengatakan banci kepada Penggugat , karena saat itu kami emosi ;

2. Saksi MULYANA, menerangkan :
   - Bahwa saksi merupakan Ayah kandung dari Penggugat ;
   - Bahwa sepengetahuan Saksi pada awal bulan Pebuari 2020 terjadi percekcokan/ pertengkaran semenjak Penggugat dan Tergugat tinggal bersama dengan orangtua Tergugat;
   - Bahwa pada tanggal 5 Juli 2020 saksi sempat datang kerumah Tergugat untuk berencana mendamaikan Penggugat dan Tergugat namun yang ada Tergugat malah mengusir dan memaki –maki Saksi dan isteri beserta menantu laki-laki, bahkan Tergugat juga sempat meludahi Saksi;
   - Bahwa Saksi Mengetahui dari Penggugat pada bulan Mei 2020 bahwa sempat terjadi pertengkaran Tergugat telah melakukan kekerasan pada pada Penggugat dengan memukul kepala Penggugat ;
   - Bahwa saksi diperlihatkan bukti P-5, dan setelah dilihat saksi tahu dan membenarkan pernah melihat poto di hp ;
   - Bahwa pada saat itu ada mengatakan banci kepada Penggugat , karena saat itu kami emosi ;

3. Saksi WENDI, menerangkan :
   - Bahwa Saksi merupakan Kakak Ipar dari Penggugat;
   - Bahwa sepengetahuan saksi Penggugat dan Tergugat awal bertemu di Gereja, kemudian pada bulan Mei 2018 menikah;
   - Bahwa pada awalnya rumah tangga antara Penggugat dan Tergugat berjalan harmonis, namun sejak Penggugat diajak pindah dan tinggal bersama orangtua Tergugat barulah sering terjadi pertengkaran;
   - Bahwa Saksi mengetahui dari group keluarga via aplikasi Whatsapp Tergugat sering melakukan pemukulan terhadap Penggugat saat Penggugat dan Tergugat sedang bertengkar;
   - Bahwa saksi tidak pernah melihat langsung kejadian keributan antara Penggugat dan Tergugat ;
   - Bahwa pada tanggal 5 Juli 2020 saksi diajak oleh mertua untuk ikut ke rumah Tergugat untuk mendamaikan Penguggat dan Tergugat namun yang ada sesampainya di depan rumah Tergugat langsung mengusir saksi

Disclaimer
Kepaniteraan Mahkamah Agung Republik Indonesia berusaha untuk selalu mencantumkan informasi paling kini dan akurat sebagai bentuk komitmen Mahkamah Agung untuk pelayanan publik, transparansi dan akuntabilitas pelaksanaan fungsi peradilan. Namun dalam hal-hal tertentu masih dimungkinkan terjadi permasalahan teknis terkait dengan akurasi dan keterkinian informasi yang kami sajikan, hal mana akan terus kami perbaiki dari waktu kewaktu.
Dalam hal Anda menemukan inakurasi informasi yang termuat pada situs ini atau informasi yang seharusnya ada, namun belum tersedia, maka harap segera hubungi Kepaniteraan Mahkamah Agung RI melalui :
Email : kepaniteraan@mahkamahagung.go.id Telp : 021-384 3348 (ext.318)

beserta mertua sambil mengeluarkan barang-barang milik mertua ke luar dengan cara di lempar;

- Bahwa saksi diperlihatkan bukti P-5, dan setelah dilihat saksi tahu dan membenarkan pernah melihat poto di hp ;
- Bahwa pada saat itu ada mengatakan banci kepada Penggugat , karena saat itu kami emosi ;

Menimbang bahwa untuk mendukung bantahannya Tergugat telah mengajukan bukti – bukti tertulis berupa photo copy surat – surat yang telah dibubuhi materai secukupnya sesuai dengan Foto Copy masing – masing berupa :

1. Photo copy sesuai dengan aslinya Kartu Tanda Penduduk atas nama Hendri Setiadi, di beritanda T-1;
2. Photo copy sesuai dengan aslinya Kartu Keluarga atas nama Kepala Keluarga Hendri Setiadi, diberitanda T-2 ;
3. Photo copy sesuai dengan aslinya Testimonium Matrimonii (surat kawin dari gereja), diberitanda T-3 ;
4. Photo copy sesuai dengan aslinya percakapan What App antara Penggugat dan Tergugat, diberitanda T-4 ;
5. Photo Tergugat sesuai dengan print out, diberitanda T-5 ;

Menimbang bahwa selain mengajukan bukti tertulis sebagaimana tersebut diatas Tergugat juga telah mengajukan saksi yang telah memberikan keterangan dibawah sumpah dimuka persidangan menurut tata cara agamanya masing – masing yang pada pokoknya keterangannya sebagai berikut :

Saksi HENDI SETIADI, menerangkan :

- Bahwa Saksi merupakan Kakak kandung Tergugat;
- Bahwa Saksi mengetahui saksi dari cerita Ibu saksi dan Tergugat pada bulan Juni 2020 telah terjadi percekcokan antara Penggugat dan Tergugat ;
- Bahwa setahu saksi masalahnya ketika dirumah banyak sampah dan kotor, selanjutnya Tergugat meminta untuk di bersihkannya akan tetapi Penggugat menolaknya sehingga terjadi percekcokan, dan hal tersebut terjadi berulang-ulang ;
- Bahwa saksi tidak pernah melihat Penggugat dan Tergugat berkelahi dan tidak melihat Tergugat memukul Penggugat ;

Disclaimer
Kepaniteraan Mahkamah Agung Republik Indonesia berusaha untuk selalu mencantumkan informasi paling kini dan akurat sebagai bentuk komitmen Mahkamah Agung untuk pelayanan publik, transparansi dan akuntabilitas pelaksanaan fungsi peradilan. Namun dalam hal-hal tertentu masih dimungkinkan terjadi permasalahan teknis terkait dengan akurasi dan keterkinian informasi yang kami sajikan, hal mana akan terus kami perbaiki dari waktu kewaktu.
Dalam hal Anda menemukan inakurasi informasi yang termuat pada situs ini atau informasi yang seharusnya ada, namun belum tersedia, maka harap segera hubungi Kepaniteraan Mahkamah Agung RI melalui :
Email : kepaniteraan@mahkamahagung.go.id   Telp : 021-384 3348 (ext.318)

- Bahwa Saksi tidak pernah melihat secara langsung Penggugat dan Tergugat bertengkar;
- Bahwa Saksi pernah mengetahui pada awal tahun 2020 Penggugat dan Tergugat sempat melakukan program kehamilan;
- Bahwa Saksi mengetahui bahwa Penggugat pada bulan Pebuari sempat hamil selama 2 bulan namun tidak lama kemudian mengalami keguguran;
- Bahwa setahu saksi dahulu antara Penggugat dan Tergugat sebelum menikah ada pacaran terlebih dahulu ;

Menimbang, bahwa selanjutnya kuasa Penggugat dan Kuasa Tergugat telah menyerahkan kesimpulannya masing-masing tertanggal 24 November 2020 ;

Menimbang, bahwa untuk mempersingkat uraian putusan ini maka ditunjuk segala sesuatu sebagaimana tercantum dalam berita acara perkara ini dianggap seluruhnya masuk dalam putusan ini;

Menimbang, bahwa akhirnya Para Pihak menyatakan tidak mengajukan sesuatu lagi dan mohon putusan;

TENTANG HUKUMNYA

Menimbang, bahwa maksud dan tujuan gugatan Penggugat adalah sebagaimana tersebut diatas;

Menimbang, bahwa Penggugat di dalam gugatannya pada pokoknya mendalilkan :

1. Bahwa Penggugat telah melangsungkan perkawinan dengan Tergugat di Paroki St. Gabriel – Bandung pada tanggal 12 Mei 2018, sesuai dengan *Testimonium Matrimonii* Nomor 05 Anno 2018 tertanggal 12 Mei 2018 yang dikeluarkan oleh Parochiae Sancti Gabrielis Bandung – Indonesia
2. Bahwa Penggugat dan Tergugat telah mencatatkan perkawinannya di kantor Pencatatan Sipil Kota Bandung. Sesuai dengan Kutipan Akta Perkawinan Nomor 3273-KW-02082018-0002 tertanggal 3 Agustus 2018 yang dikeluarkan oleh Dinas Kependudukan dan Pencatatan Sipil Kota Bandung;
3. Bahwa Penggugat selama perkawinannya dengan Tergugat belum dikaruniai anak ;

Disclaimer
Kepaniteraan Mahkamah Agung Republik Indonesia berusaha untuk selalu mencantumkan informasi paling kini dan akurat sebagai bentuk komitmen Mahkamah Agung untuk pelayanan publik, transparansi dan akuntabilitas pelaksanaan fungsi peradilan. Namun dalam hal-hal tertentu masih dimungkinkan terjadi permasalahan teknis terkait dengan akurasi dan keterkinian informasi yang kami sajikan, hal mana akan terus kami perbaiki dari waktu kewaktu.
Dalam hal Anda menemukan inakurasi informasi yang termuat pada situs ini atau informasi yang seharusnya ada, namun belum tersedia, maka harap segera hubungi Kepaniteraan Mahkamah Agung RI melalui :
Email : kepaniteraan@mahkamahagung.go.id Telp : 021-384 3348 (ext.318)

4. Bahwa setelah melangsungkan perkawinannya sampai Januari 2020 Penggugat dan Tergugat bertempat tinggal di Jalan Sharon Garden 1 No. 15 dan perkawinannya berjalan harmonis.selanjutnya pada bulan Februari 2020 Tergugat mengajak kepada Penggugat untuk pindah tempat tinggal ke Jalan Kalipah Apo Gg. Siti No. 57 Kelurahan Karanganyar Kecamatan Astanaanyar Kota Bandung dan mulailah sering terjadi keributan dalam rumah tangganya.
5. Bahwa Penggugat mengalami perlakuan Kekerasan Dalam Rumah Tangga (KDRT) yang dilakukan oleh Tergugat pada hari Minggu tanggal 3 Mei 2020 dan berulang kembali pada hari Jumat tanggal 3 Juli 2020.
6. Bahwa antara Penggugat dengan Tergugat sudah tidak terjalin komunikasi yang baik dan telah melaksanakan pisah ranjang.
7. Bahwa antara Penggugat dengan Tergugat sudah beberapa kali coba untuk didamaikan oleh kedua belah pihak dari keluarga besar namun tidak pernah berhasil.
8. Bahwa antara Penggugat dengan Tergugat sudah tidak ada perasaan cinta lagi, sehingga apabila tetap berlangsung hubungan pernikahan dikhawatirkan akan kembali terjadi pertengkaran yang terus berulang-ulang dan terjadi kembali kekerasan fisik yang lebih fatal lagi
9. Bahwa Sesuai dengan bunyi Pasal 19 huruf (d) Peraturan Pemerintah Nomor 9 Tahun 1975 yang berbunyi sebagai berikut:

   Perceraian dapat terjadi karena alasan atau alasan-alasan:

   (d) Salah satu pihak melakukan kekejaman atau penganiayaan berat yang membahayakan pihak yang lain;

Menimbang, bahwa Tergugat di dalam jawabannya membenarkan beberpa dalil gugatan Penggugat sehingga dinyatakan sudah terbukti atau tidak perlu dibuktikan lagi diantaranya:

1. Bahwa Penggugat telah melangsungkan perkawinan dengan Tergugat di Paroki St. Gabriel – Bandung pada tanggal 12 Mei 2018, sesuai dengan *Testimonium Matrimonii* Nomor 05 Anno 2018 tertanggal 12 Mei 2018 yang dikeluarkan oleh Parochiae Sancti Gabrielis Bandung – Indonesia (bukti P.3/T.3)
2. Bahwa Penggugat dan Tergugat telah mencatatkan perkawinannya di kantor Pencatatan Sipil Kota Bandung. Sesuai dengan Kutipan Akta Perkawinan Nomor 3273-KW-02082018-0002 tertanggal 3 Agustus 2018

Disclaimer
Kepaniteraan Mahkamah Agung Republik Indonesia berusaha untuk selalu mencantumkan informasi paling kini dan akurat sebagai bentuk komitmen Mahkamah Agung untuk pelayanan publik, transparansi dan akuntabilitas pelaksanaan fungsi peradilan. Namun dalam hal-hal tertentu masih dimungkinkan terjadi permasalahan teknis terkait dengan akurasi dan keterkinian informasi yang kami sajikan, hal mana akan terus kami perbaiki dari waktu kewaktu.
Dalam hal Anda menemukan inakurasi informasi yang termuat pada situs ini atau informasi yang seharusnya ada, namun belum tersedia, maka harap segera hubungi Kepaniteraan Mahkamah Agung RI melalui :
Email : kepaniteraan@mahkamahagung.go.id Telp : 021-384 3348 (ext.318)

yang dikeluarkan oleh Dinas Kependudukan dan Pencatatan Sipil Kota Bandung (P.4);

3. Bahwa Penggugat selama perkawinannya dengan Tergugat belum dikaruniai anak ;
4. Bahwa setelah melangsungkan perkawinannya sampai Januari 2020 Penggugat dan Tergugat bertempat tinggal di Jalan Sharon Garden 1 No. 15 dan perkawinannya berjalan harmonis.selanjutnya pada bulan Februari 2020 Tergugat mengajak kepada Penggugat untuk pindah tempat tinggal ke Jalan Kalipah Apo Gg. Siti No. 57 Kelurahan Karanganyar Kecamatan Astanaanyar Kota Bandung dan mulailah sering terjadi keributan dalam rumah tangganya

Menimbang, bahwa karena dalil gugatan Pihak Penggugat dibantah oleh Pihak Tergugat pada pokoknya yaitu tidak benar “Bahwa Penggugat mengalami perlakuan Kekerasan Dalam Rumah Tangga (KDRT) yang dilakukan Tergugat pada hari Minggu, tanggal 3 Mei 2020 dan pada hari Jum’at, 3 Juli 2020”, yang benar pada hari jumat tanggal 3 Juli 2020 bahwa saat itu Tergugat membeli makanan khas Surabaya (ote-ote) dan meminta dimasakan oleh Penggugat akan tetapi Penggugat malah ngomel dan marah-marah. Tergugat yang saat itu sedang istirahat di tempat tidur, diinjak kakinya oleh Penggugat, secara reflex tangan Tergugat menampar Penggugat, lalu Penggugat memukul ke wajah Tergugat menggunakan gagang sapu besi memukul dan di tangkis oleh tangannya sampai luka memar sambil teriak-teriak memaki Tergugat, namun ditepis oleh Tergugat, lalu Tergugat pergi untuk menghindar tetapi Penggugat terus ngoceh dan teriak-teriak memaki Tergugat walau telah ditenangkan oleh orang tua Tergugat”,

Menimbang, bahwa hal yang dibantah Tergugat juga pada posita perkara gugatan Penggugat pada point 8 yang intinya “bahwa antara Penggugat dan Tergugat sudah beberapa kali coba untuk didamaikan oleh kedua belah pihak dari keluarga besar namun tidak pernah berhasil, yang sebenarnya terjadi adalah bahwa memang keluarga Penggugat pernah datang kerumah Tergugat, rencananya mau mendamaikan Penggugat dan Tergugat, akan tetapi malah membuat onar dan teriak-teriak memaki Tergugat, mengatakan “banci” kepada Tergugat dan menyuruh mengembalikan barang-barang Penggugat”., maka menjadi kewajiban Pihak Penggugat untuk membuktikan dalil gugatannya;

Disclaimer
Kepaniteraan Mahkamah Agung Republik Indonesia berusaha untuk selalu mencantumkan informasi paling kini dan akurat sebagai bentuk komitmen Mahkamah Agung untuk pelayanan publik, transparansi dan akuntabilitas pelaksanaan fungsi peradilan. Namun dalam hal-hal tertentu masih dimungkinkan terjadi permasalahan teknis terkait dengan akurasi dan keterkinian informasi yang kami sajikan, hal mana akan terus kami perbaiki dari waktu kewaktu.
Dalam hal Anda menemukan inakurasi informasi yang termuat pada situs ini atau informasi yang seharusnya ada, namun belum tersedia, maka harap segera hubungi Kepaniteraan Mahkamah Agung RI melalui :
Email : kepaniteraan@mahkamahagung.go.id Telp : 021-384 3348 (ext.318)

Menimbang, bahwa sebelum membuktikan dalil gugatan Pihak Penggugat akan dipertimbangkan terlebih dahulu hal hal sebagai berikut, bahwa hakim yang memeriksa perkara perdata berwenang membagi beban pembuktian di antara para pihak yang bersengketa, pembagian beban pembuktian tersebut dilaksanakan dengan mengingat asas *fair trial* dalam persidangan sehingga harus dilakukan dengan adil dan tidak berat sebelah;

Menimbang, bahwa hukum pembuktian mengajarkan bahwa pembagian beban pembuktian dilaksanakan berdasarkan atas prinsip setiap orang yang mendalilkan bahwa ia mempunyai suatu hak, atau guna meneguhkan haknya sendiri maupun membantah hak orang lain, menunjukkan pada suatu peristiwa, diwajibkan membuktikan adanya hak atau peristiwa tersebut (Pasal 163 HIR Jo Pasal 1865 KUHPerdata);

Menimbang, bahwa Penggugat mendalilkan bahwa Penggugat mengalami perlakuan Kekerasan Dalam Rumah Tangga (KDRT) yang dilakukan Tergugat pada hari Minggu, tanggal 3 Mei 2020 dan pada hari Jum'at, 3 Juli 2020", yang dibantah oleh pihak Tergugat bahwa kejadiannya yaitu yang benar pada hari jumat tanggal 3 Juli 2020 bahwa saat itu Tergugat membeli makanan khas Surabaya (ote-ote) dan meminta dimasakan oleh Penggugat akan tetapi Penggugat malah ngomel dan marah-marah. Tergugat yang saat itu sedang istirahat di tempat tidur, diinjak kakinya oleh Penggugat, secara reflex tangan Tergugat menampar Penggugat, lalu Penggugat memukul ke wajah Tergugat menggunakan gagang sapu besi memukul dan di tangkis oleh tangannya sampai luka memar sambil teriak-teriak memaki Tergugat, namun ditepis oleh Tergugat, lalu Tergugat pergi untuk menghindar tetapi Penggugat terus ngoceh dan teriak-teriak memaki Tergugat walau telah ditenangkan oleh orang tua Tergugat, maka Majelis Hakim akan melihat fakta hukum berdasarkan keterangan Saksi Endang Nurjanti, Mulyana dan Wendi yang pada pokoknya menerangkan para saksi tidak mengetahui dan tidak melihat terjadinya perlakuan Kekerasan Dalam Rumah Tangga (KDRT) yang dilakukan Tergugat dan hanya mengetahui adanya lebam-lebam dari gambar Whast App group dan dari ceritera Penggugat ;

Menimbang bahwa sanggahan pihak Tergugat yang mendalilkan bahwa pada hari Jumat tanggal 3 Juli 2020 bahwa saat itu Tergugat membeli makanan khas Surabaya (ote-ote) dan meminta dimasakan oleh Penggugat akan tetapi Penggugat malah ngomel dan marah-marah. Tergugat yang saat itu sedang

Disclaimer
Kepaniteraan Mahkamah Agung Republik Indonesia berusaha untuk selalu mencantumkan informasi paling kini dan akurat sebagai bentuk komitmen Mahkamah Agung untuk pelayanan publik, transparansi dan akuntabilitas pelaksanaan fungsi peradilan. Namun dalam hal-hal tertentu masih dimungkinkan terjadi permasalahan teknis terkait dengan akurasi dan keterkinian informasi yang kami sajikan, hal mana akan terus kami perbaiki dari waktu kewaktu.
Dalam hal Anda menemukan inakurasi informasi yang termuat pada situs ini atau informasi yang seharusnya ada, namun belum tersedia, maka harap segera hubungi Kepaniteraan Mahkamah Agung RI melalui :
Email : kepaniteraan@mahkamahagung.go.id Telp : 021-384 3348 (ext.318)

istirahat di tempat tidur, diinjak kakinya oleh Penggugat, secara reflex tangan Tergugat menampar Penggugat, lalu Penggugat memukul ke wajah Tergugat menggunakan gagang sapu besi memukul dan di tangkis oleh tangannya sampai luka memar sambil teriak-teriak memaki Tergugat, namun ditepis oleh Tergugat, lalu Tergugat pergi untuk menghindar tetapi Penggugat terus ngoceh dan teriak-teriak memaki Tergugat walau telah ditenangkan oleh orang tua Tergugat ;

Menimbang bahwa berdasarkan bukti photo Penggugat (P5) yang dibenarkan oleh saksi Endang Nurjanti, Mulyana dan Wendi dan bukti photo Tergugat (T5) yang dibenarkan juga oleh saksi Hendi Setiadi, menurut majelis Hakim kejadian tersebut bukanlah KDRT yang dilakukan oleh salah satu pihak, akan tetapi antara Penggugat dan Tergugat terjadi pertengkaran dan percekcokan sehingga baik Penggugat maupun Tergugat masing-masing saling menyerang;

Menimbang bahwa Penggugat mendalilkan bahwa antara Penggugat dan Tergugat sudah beberapa kali coba untuk didamaikan oleh kedua belah pihak dari keluarga besar namun tidak pernah berhasil, hal tersebut dibantah oleh Tergugat yang mendalilkan bahwa memang keluarga Penggugat pernah datang kerumah Tergugat, rencananya mau mendamaikan Penggugat dan Tergugat, akan tetapi malah membuat onar dan teriak-teriak memaki Tergugat, mengatakan “banci” kepada Tergugat dan menyuruh mengembalikan barang-barang Penggugat”, maka Majelis Hakim akan melihat fakta hukum berdasarkan keterangan Saksi Endang Nurjanti, Mulyana dan Wendi yang pada pokoknya menerangkan para saksi pernah datang ke rumah Tergugat dengan maksud akan mendamaikan, akan tetapi belum sempat terjadi malah terjadi keributan dan percekcokan antara pihak Penggugat dan Tergugat ;

Menimbang, bahwa berdasarkan pertimbangan tersebut diatas, maka Majelis berpendapat benar bahwa hubungan antara Penggugat dan Tergugat sudah tidak harmonis lagi dengan sering terjadinya pertengkaran dan percekcokan yang terus menerus, bahkan terjadinya adu pisik antara Penggugat dan Tergugat sehingga dengan keadaan tersebut diatas maka rumah tangga Penggugat Penggugat dan Tergugat sudah tidak sehat, sejahtera, tentram dan bahagia sebagaimana harapan bagi setiap orang yang berumah tangga yaitu keluarga yang harmonis;

Disclaimer
Kepaniteraan Mahkamah Agung Republik Indonesia berusaha untuk selalu mencantumkan informasi paling kini dan akurat sebagai bentuk komitmen Mahkamah Agung untuk pelayanan publik, transparansi dan akuntabilitas pelaksanaan fungsi peradilan. Namun dalam hal-hal tertentu masih dimungkinkan terjadi permasalahan teknis terkait dengan akurasi dan keterkinian informasi yang kami sajikan, hal mana akan terus kami perbaiki dari waktu kewaktu.
Dalam hal Anda menemukan inakurasi informasi yang termuat pada situs ini atau informasi yang seharusnya ada, namun belum tersedia, maka harap segera hubungi Kepaniteraan Mahkamah Agung RI melalui :
Email : kepaniteraan@mahkamahagung.go.id Telp : 021-384 3348 (ext.318)

Menimbang, bahwa berdasarkan keadaan sebagaimana tersebut diatas maka Majelis mengkhawatirkan apabila mereka dipaksa berkumpul, sebagai suami isteri maka bisa terjadi sesuatu yang lebih parah dari pada sekedar bertengkar, maka apa yang menjadi tujuan dari hidup berumah tangga yaitu membentuk satu keluarga yang harmonis tidak akan pernah tercapai lagi ;

Menimbang, bahwa oleh karena demikian keadaan rumah tangga Penggugat dan Tergugat tersebut maka Majelis Hakim berpendapat bahwa perkawinan Penggugat dan Tergugat tidaklah dapat dipertahankan lagi dan karenanya sudah sepatutnya diputuskan dengan perceraian sebagaimana yang dimaksud oleh pasal 19 huruf f PP No.9 tahun 1975 ;

Menimbang, bahwa berdasarkan pertimbangan sebagaimana tersebut diatas Majelis Hakim berkesimpulan bahwa Gugatan Penggugat cukup bukti dan beralasan serta menurut hukum dan oleh karenanya Gugatan Penggugat pada petitum ke-2 dapat dikabulkan ;

Menimbang, bahwa oleh karena gugatan cerai dikabulkan maka sesuai dengan ketentuan PP No.9 Tahun 1975 Pasal 35 Ayat (1) diperintahkan kepada panitera atau pejabat yang ditunjuk untuk itu supaya mengirimkan salinan putusan ini kepada Kantor Dinas Kependudukan dan Pencatatan Sipil Pemerintah Kota Bandung, agar dapat didaftarkan dan dicatat pada register untuk itu dan oleh karenanya terhadap petitum ke-3 dapatlah dikabulkan;

Menimbang,bahwa Penggugat dapat membuktikan Dalil dalam Gugatannya maka Tergugat dihukum membayar biaya Perkara yang timbul dalam perkara ini yang mana total biaya yang timbul akan dimuat dalam amar putusan ini,oleh karenanya terhadap Petitum ke-4 dapatlah dikabulkan;

Menimbang Petitum ke-2 sampai dengan Petitum ke-4 dikabulkan maka oleh karenanya Petitum ke1 dapat dikabulkan;

Menimbang, bahwa dengan berlakunya Undang-undang RI Nomor 23 Tahun 2006 tentang Administrasi Kependudukan,Jo Undang-undang Nomor 24 tahun 2013 Tentang Perubahan atas Undang-undang RI Nomor 23 Tahun 2006 tentang Administrasi Kependudukan didalam pasal 40 undang-undang tersebut ditentukan bahwa pihak yang bercerai diwajibkan juga melaporkan perceraiannya

Disclaimer
Kepaniteraan Mahkamah Agung Republik Indonesia berusaha untuk selalu mencantumkan informasi paling kini dan akurat sebagai bentuk komitmen Mahkamah Agung untuk pelayanan publik, transparansi dan akuntabilitas pelaksanaan fungsi peradilan. Namun dalam hal-hal tertentu masih dimungkinkan terjadi permasalahan teknis terkait dengan akurasi dan keterkinian informasi yang kami sajikan, hal mana akan terus kami perbaiki dari waktu kewaktu.
Dalam hal Anda menemukan inakurasi informasi yang termuat pada situs ini atau informasi yang seharusnya ada, namun belum tersedia, maka harap segera hubungi Kepaniteraan Mahkamah Agung RI melalui :
Email : kepaniteraan@mahkamahagung.go.id Telp : 021-384 3348 (ext.318)

kepada Instansi Pelaksana Pencatatan Sipil setempat paling lama 60 (enam puluh) hari sejak putusan perkara perceraian berkekuatan hukum tetap sehingga Majelis

Hakim menambah petitum karena Perintah Undang-Undang sebagaimana sudah diatur dalam Undang-Undang RI nomor Undang-undang RI Nomor 23 Tahun 2006 tentang Administrasi Kependudukan,Jo Undang-undang Nomor 24 tahun 2013 Tentang Perubahan atas Undang-undang RI Nomor 23 Tahun 2006 tentang Administrasi Kependudukan ;

Menimbang, bahwa berdasarkan hal tersebut diatas dan permohonan Ex Aequo et Bono maka Majelis akan menambahkan dalam amar putusan ;

Menimbang, bahwa dengan demikian, maka gugatan pihak Penggugat dapat dikabulkan untuk seluruhnya;

Menimbang, bahwa untuk bukti surat dan bukti saksi baik dari pihak Penggugat maupun Tergugat yang tidak relevan dengan perkara ini tidak dipertimbangkan oleh Majelis;

Mengingat akan pasal-pasal dari peraturan yang bersangkutan yang berkenaan dengan perkara ini terutama pasal 19 huruf f PP No.9 tahun 1975 :

M E N G A D I L I

1. Menerima dan mengabulkan gugatan Penggugat untuk seluruhnya;
2. Menyatakan perkawinan Penggugat dengan Tergugat yang telah dilangsungkan di Paroki St. Gabriel-Bandung pada tanggal 12 Mei 2018 dengan Nomor 05 Anno 2018, yang mana perkawinan tersebut telah dicatatkan/didaftarkan di Dinas Kependudukan Kota Bandung dan Catatan Sipil dengan nomor 3273-KW-02082018-0002 tertanggal 3 Agustus 2018 putus karena perceraian, dengan segala akibat hukumnya ;
3. Memerintahkan kepada Panitera Pengadilan Negeri Kelas IA Bandung untuk mengirimkan salinan turunan putusan ini yang telah mempunyai kekuatan hukum tetap dan mencoret dari daftar register perkawinan pada Dinas Kependudukan dan Catatan Sipil Kota Bandung untuk dicatat dalam daftar/register yang disediakan untuk itu dan menerbitkan Akta Perceraiannya;

Disclaimer
Kepaniteraan Mahkamah Agung Republik Indonesia berusaha untuk selalu mencantumkan informasi paling kini dan akurat sebagai bentuk komitmen Mahkamah Agung untuk pelayanan publik, transparansi dan akuntabilitas pelaksanaan fungsi peradilan. Namun dalam hal-hal tertentu masih dimungkinkan terjadi permasalahan teknis terkait dengan akurasi dan keterkinian informasi yang kami sajikan, hal mana akan terus kami perbaiki dari waktu kewaktu.
Dalam hal Anda menemukan inakurasi informasi yang termuat pada situs ini atau informasi yang seharusnya ada, namun belum tersedia, maka harap segera hubungi Kepaniteraan Mahkamah Agung RI melalui :
Email : kepaniteraan@mahkamahagung.go.id Telp : 021-384 3348 (ext.318)

4. Memerintahkan kepada Para Pihak yang bercerai diwajibkan juga melaporkan perceraiannya kepada Instansi Pelaksana Pencatatan Sipil setempat paling lama 60 (enam puluh) hari sejak putusan perkara perceraian berkekuatan hukum tetap;
5. Menghukum Tergugat untuk membayar biaya yang timbul dalam perkara ini sejumlah Rp.326.000,- (tiga ratus dua puluh enam ribu rupiah)

Demikian diputuskan dalam permusyawaratan Majelis Hakim Pengadilan Negeri Bandung pada hari : Selasa, tanggal **1 Desember 2020**, oleh kami : **SULISTIYONO, SH.** .sebagai Hakim Ketua, dengan **SRI KUNCORO, SH.MH** dan **DENNIE ARSAN FATRIKA, SH.MH** .masing–masing sebagai Hakim Anggota, putusan mana diucapkan pada hari **SELASA** tanggal **15 Desember 2020** di muka sidang yang terbuka untuk umum oleh Hakim Ketua tersebut dengan didampingi Hakim – Hakim Anggota tersebut, dibantu **ENGKUS KUSMANA, SH.MH** Panitera Pengganti pada Pengadilan Negeri Bandung dan dihadiri Kuasa Penggugat dan Kuasa Tergugat;

Hakim – Hakim Anggota | Hakim Ketua Majelis

Sri kuncoro, SH.MH | **Sulistiyono, SH.**

Panitera Pengganti

Dennie Arsan Fatrika, SH.MH

ENGKUS KUSMANA, SH.MH

Rincian biaya Perkara :

-	Biaya pendaftaran	:	Rp	30.000,-
-	Proses	:	Rp	50.000,-
-	Panggilan	:	Rp	220.000,-
-	Materai	:	Rp	6.000,-
-	Redaksi	:	Rp	20.000,-
	T o t a l	:	Rp	326.000,- (tiga ratus duapuluh enam ribu rupiah)

Disclaimer
Kepaniteraan Mahkamah Agung Republik Indonesia berusaha untuk selalu mencantumkan informasi paling kini dan akurat sebagai bentuk komitmen Mahkamah Agung untuk pelayanan publik, transparansi dan akuntabilitas pelaksanaan fungsi peradilan. Namun dalam hal-hal tertentu masih dimungkinkan terjadi permasalahan teknis terkait dengan akurasi dan keterkinian informasi yang kami sajikan, hal mana akan terus kami perbaiki dari waktu kewaktu.
Dalam hal Anda menemukan inakurasi informasi yang termuat pada situs ini atau informasi yang seharusnya ada, namun belum tersedia, maka harap segera hubungi Kepaniteraan Mahkamah Agung RI melalui :
Email : kepaniteraan@mahkamahagung.go.id Telp : 021-384 3348 (ext.318)
