# Supplementary material for: Classification of divorce causes during the COVID-19 pandemic using convolutional neural networks
Source: PeerJ Comput Sci. 2022 Jun 30;8:e998. doi: 10.7717/peerj-cs.998 (PMC9299239; doi:10.7717/peerj-cs.998)
Supplement: Supplemental Information 3 [file peerj-cs-08-998-s003.zip › KDRT Dataset/Data ke-6.pdf]

P U T U S A N
Nomor --/Pdt.G/----/PN Sda.

DEMI KEADILAN BERDASARKAN KETUHANAN YANG MAHA ESA

Pengadilan Negeri Sidoarjo Kelas 1A Khusus yang memeriksa dan memutus perkara perdata pada tingkat pertama, telah menjatuhkan putusan sebagai berikut dalam perkara gugatan antara:

PENGGUGAT, bertempat tinggal di Kabupaten Sidoarjo dalam hal ini memberikan kuasa kepada Johanes Dipa Widjaja, S.H., S.Psi., C.L.A.; Satrya Ardyrespati Wicaksana, S.H.; Beryl Cholif Arrachman, S.H.; Wachid Aditya Ansory, S.H., para Advokat dan para advokat magang, beralamat di Kantor Hukum **“Johanes Dipa Widjaja & Partners”** Taman Rivera Regency E-6, Surabaya berdasarkan Surat Kuasa Khusus tanggal 27 Februari 2020, selanjutnya disebut sebagai Penggugat;

Lawan

TERGUGAT, bertempat tinggal di Kabupaten Sidoarjo, selanjutnya disebut sebagai Tergugat;

Pengadilan Negeri tersebut;

Setelah membaca berkas perkara;

Setelah mendengar Penggugat;

TENTANG DUDUK PERKARA

Menimbang, bahwa Penggugat dengan surat gugatan tanggal 3 Maret 2020 yang diterima dan didaftarkan di Kepaniteraan Pengadilan Negeri Sidoarjo pada tanggal 5 Maret 2020 dalam Register Nomor 75/Pdt.G/2020/PN Sda., telah mengajukan gugatan sebagai berikut:

1. Bahwa pada tanggal 16 Juni 2012 Penggugat dan Tergugat telah melangsungkan perkawinan yang sah di Surabaya, dan kemudian mencatatkan perkawinan yang sah di Kantor Catatan Sipil Surabaya sebagaimana tertuang dalam Akta Perkawinan Nomor: 3578-KW-19062012-0017 tanggal 20 Juni 2012 (*vide* **bukti P.1**);

2. Bahwa pada mulanya perkawinan antara Penggugat dan Tergugat hidup rukun, harmonis layaknya suami istri yang bahagia ;

3. Bahwa sekitar Oktober 2019 mulai sering terjadi pertengkaran antara Penggugat dan Tergugat, namun Penggugat tetap bertahan dan masih ingin mempertahankan perkawinannya, namun pertengkaran antara Penggugat

Disclaimer
Kepaniteraan Mahkamah Agung Republik Indonesia berusaha untuk selalu mencantumkan informasi paling kini dan akurat sebagai bentuk komitmen Mahkamah Agung untuk pelayanan publik, transparansi dan akuntabilitas pelaksanaan fungsi peradilan. Namun dalam hal-hal tertentu masih dimungkinkan terjadi permasalahan teknis terkait dengan akurasi dan keterkinian informasi yang kami sajikan, hal mana akan terus kami perbaiki dari waktu kewaktu.
Dalam hal Anda menemukan inakurasi informasi yang termuat pada situs ini atau informasi yang seharusnya ada, namun belum tersedia, maka harap segera hubungi Kepaniteraan Mahkamah Agung RI melalui :
Email : kepaniteraan@mahkamahagung.go.id Telp : 021-384 3348 (ext.318)

dan Tergugat kian hari kian memuncak, sehingga tidak ada lagi kemungkinan untuk hidup rukun dalam rumah tangga sebagaimana umumnya suami istri yang bahagia ;

4. Bahwa yang menjadi penyebab pertengkaran antara Penggugat dan Tergugat adalah dikarenakan Tergugat tidak menghargai Penggugat sebagai suami ;

5. Bahwa karena tidak tahan dengan sikap Tergugat sebagaimana tersebut di atas, Penggugat sejak akhir Oktober 2019 sudah tidak tinggal satu rumah lagi dengan Tergugat, dan saat ini Penggugat tinggal di rumah orang tua Penggugat ;

6. Bahwa dari uraian tersebut di atas, maka nampak jelas alasan serta fakta hukum bahwa perkawinan antara Penggugat dengan Tergugat tidak dapat disatukan lagi sebagaimana layaknya dalam suatu rumah tangga. Oleh karenanya berdasarkan Undang-Undang Republik Indonesia Nomor 1 Tahun 1974 Pasal 39 ayat(1) dan (2) Jo. Peraturan Pemerintah Nomor 9 Tahun 1975 Pasal 19f, perkawinan antara Penggugat dan Tergugat yang telah dicatatkan pada tanggal 20 Juni 2012 di Kantor Catatan Sipil Surabaya Nomor :3578-KW-19062012-0017tanggal 16 Juni 2012 sudah sepatutnya diputus karena perceraian dengan segala akibat hukumnya ;

Berdasarkan alasan-alasan tersebut di atas, maka Penggugat mohon dengan segala kerendahan hati kepada Ketua Pengadilan Negeri Sidoarjo cq Majelis Hakim yang memeriksa dan mengadili perkara ini berkenan menjatuhkan putusan sebagai berikut :

- Mengabulkan gugatan Penggugat seluruhnya;
- Menyatakan perkawinan antara Penggugat dan Tergugat yang telah dicatatkan di Kantor Catatan Sipil Surabaya pada tanggal 20 Juni 2012, berdasarkan akta perkawinan Nomor : 3578-KW-19062012-0017 tanggal 16 Juni 2012, putus karena perceraian dengan segala akibat hukumnya;
- Memerintahkan kepada Panitera Pengadilan Negeri Sidoarjo atau Pejabat yang ditunjuk untuk itu mengirimkan Salinan putusan dalam perkara ini yang telah mempunyai kekuatan hukum tetap kepada:

  I. Dinas Kependudukan dan Pencatatan Sipil Kabupaten Sidoarjo, untuk dicatat dalam daftar perceraian dalam tahun yang sedang berjalan untuk diterbitkan Akta Perceraian;

Disclaimer
Kepaniteraan Mahkamah Agung Republik Indonesia berusaha untuk selalu mencantumkan informasi paling kini dan akurat sebagai bentuk komitmen Mahkamah Agung untuk pelayanan publik, transparansi dan akuntabilitas pelaksanaan fungsi peradilan. Namun dalam hal-hal tertentu masih dimungkinkan terjadi permasalahan teknis terkait dengan akurasi dan keterkinian informasi yang kami sajikan, hal mana akan terus kami perbaiki dari waktu kewaktu.
Dalam hal Anda menemukan inakurasi informasi yang termuat pada situs ini atau informasi yang seharusnya ada, namun belum tersedia, maka harap segera hubungi Kepaniteraan Mahkamah Agung RI melalui :
Email : kepaniteraan@mahkamahagung.go.id Telp : 021-384 3348 (ext.318)

II. Dinas Kependudukan dan Pencatatan Sipil Kota Surabaya, untuk dicatat pada bagian pinggir dari daftar catatan perkawinan.

- Menghukum Tergugat untuk membayar biaya perkara;

Atau :

Jika Majelis Hakim berpendapat lain mohon putusan yang seadil-adilnya (*Ex Aequo Et Bono*).

Menimbang, bahwa pada hari sidang yang telah ditentukan Penggugat telah datang menghadap di persidangan, akan tetapi Tergugat tidak datang menghadap ataupun menyuruh orang lain menghadap untuk mewakilinya, meskipun berdasarkan risalah panggilan sidang tanggal 9 Maret 2020, tanggal 13 Maret 2020, tanggal 26 Maret 2020, dan 6 April 2020 telah dipanggil dengan patut, sedangkan tidak ternyata bahwa tidak datangnya itu disebabkan oleh sesuatu halangan yang sah;

Menimbang, bahwa untuk membuktikan dalil-dalil gugatannya Kuasa Penggugat telah menyerahkan alat bukti tertulis berupa fotokopi surat-surat sebagai berikut:

1. Fotokopi Kutipan Akta Perkawinan tertanggal 20 Juni 2012 antara Penggugat dengan Tergugat, Bukti P-1;
2. Fotokopi Surat Keterangan pengganti KTP-el atas nama Penggugat, Bukti P-2;
3. Fotokopi Kartu Keluarga No. 3603192407180024 atas nama Penggugat, Bukti P-3;

Menimbang, bahwa Penggugat menghadirkan 2 (dua) orang saksi bernama SAKSI 1 dan SAKSI 2 yang masing-masing memberikan keterangan di bawah janji;

Menimbang, bahwa untuk menyingkat putusan, maka segala sesuatu yang termuat dalam berita acara persidangan, dianggap telah termuat dan menjadi bagian yang tak terpisahkan dari putusan ini;

Menimbang, bahwa akhirnya Penggugat menyatakan tidak ada hal-hal yang diajukan lagi dan mohon putusan;

TENTANG PERTIMBANGAN HUKUM

Menimbang, bahwa maksud dan tujuan gugatan Penggugat pada pokoknya adalah perkawinan antara Penggugat dan Tergugat putus karena perceraian dengan segala akibat hukumnya;

Menimbang, bahwa menurut Pasal 1 Undang-Undang Nomor 1 Tahun 1974 tentang Perkawinan, yang dimaksud perkawinan ialah ikatan lahir batin antara seorang pria dengan seorang wanita sebagai suami isteri dengan tujuan

Disclaimer
Kepaniteraan Mahkamah Agung Republik Indonesia berusaha untuk selalu mencantumkan informasi paling kini dan akurat sebagai bentuk komitmen Mahkamah Agung untuk pelayanan publik, transparansi dan akuntabilitas pelaksanaan fungsi peradilan. Namun dalam hal-hal tertentu masih dimungkinkan terjadi permasalahan teknis terkait dengan akurasi dan keterkinian informasi yang kami sajikan, hal mana akan terus kami perbaiki dari waktu kewaktu.
Dalam hal Anda menemukan inakurasi informasi yang termuat pada situs ini atau informasi yang seharusnya ada, namun belum tersedia, maka harap segera hubungi Kepaniteraan Mahkamah Agung RI melalui :
Email : kepaniteraan@mahkamahagung.go.id Telp : 021-384 3348 (ext.318)

membentuk keluarga (rumah tangga) yang bahagia dan kekal berdasarkan Ketuhanan Yang Maha Esa;

Menimbang, bahwa dari alat bukti yang diajukan oleh Penggugat yaitu alat bukti surat P-1 tentang Kutipan Akta Perkawinan tertanggal 20 Juni 2012 antara Penggugat dengan Tergugat terbukti para pihak terikat dalam perkawinan yang sah;

Menimbang, bahwa Pasal 39 ayat (2) Undang-Undang Nomor 1 Tahun 1974 tentang Perkawinan mensyaratkan bahwa untuk melakukan perceraian harus terdapat cukup alasan, bahwa antara suami istri itu *tidak akan hidup rukun sebagai suami istri*. Adapun alasan-alasan yang dapat dipergunakan untuk menuntut perceraian terurai dalam Penjelasan pasal tersebut dan Pasal 19 Peraturan Pemerintah Nomor 9 Tahun 1975 tentang Pelaksanaan Undang-Undang Nomor 1 Tahun 1974 tentang Perkawinan. Perceraian dapat terjadi karena alasan atau alasan-alasan:

a. Salah satu pihak berbuat zina atau menjadi pemabok, pemadat, penjudi, dan lain sebagainya yang sukar disembuhkan;
b. Salah satu pihak meninggalkan pihak lain selama 2 (dua) tahun berturut-turut tanpa izin pihak lain dan tanpa alasan yang sah atau karena hal lain diluar kemampuannya;
c. Salah satu pihak mendapat hukuman penjara 5 (lima) tahun atau hukuman yang lebih berat setelah perkawinan berlangsung;
d. Salah satu pihak melakukan kekejaman atau penganiayaan berat yang membahayakan pihak yang lain;
e. Salah satu pihak mendapat cacat badan atau penyakit dengan akibat tidak dapat menjalankan kewajibannya sebagai suami/isteri;
f. Antara suami dan isteri terus-menerus terjadi perselisihan dan pertengkaran dan tidak ada harapan akan hidup rukun lagi dalam rumah tangga.

Menimbang, bahwa berdasarkan keterangan para saksi Penggugat yang pada pokoknya menerangkan antara Penggugat sebagai suami dan Tergugat sebagai istri terus-menerus terjadi perselisihan dan pertengkaran dan tidak ada harapan akan hidup rukun lagi dalam rumah tangga;

Menimbang, bahwa oleh karena jangka waktu dan formalitas panggilan menurut hukum telah diindahkan dengan sepatutnya serta gugatan tersebut tidak melawan hukum dan beralasan, maka Tergugat yang telah dipanggil dengan patut akan tetapi tidak datang menghadap di persidangan dan tidak menyuruh orang lain menghadap sebagai wakilnya, harus dinyatakan tidak hadir dan gugatan tersebut dikabulkan dengan verstek seluruhnya;

Disclaimer
Kepaniteraan Mahkamah Agung Republik Indonesia berusaha untuk selalu mencantumkan informasi paling kini dan akurat sebagai bentuk komitmen Mahkamah Agung untuk pelayanan publik, transparansi dan akuntabilitas pelaksanaan fungsi peradilan. Namun dalam hal-hal tertentu masih dimungkinkan terjadi permasalahan teknis terkait dengan akurasi dan keterkinian informasi yang kami sajikan, hal mana akan terus kami perbaiki dari waktu kewaktu.
Dalam hal Anda menemukan inakurasi informasi yang termuat pada situs ini atau informasi yang seharusnya ada, namun belum tersedia, maka harap segera hubungi Kepaniteraan Mahkamah Agung RI melalui :
Email : kepaniteraan@mahkamahagung.go.id   Telp : 021-384 3348 (ext.318)

Menimbang, bahwa Perceraian wajib dilaporkan oleh yang bersangkutan kepada Instansi Pelaksana paling lambat 60 (enam puluh) hari sejak putusan pengadilan tentang perceraian yang telah memperoleh kekuatan hukum tetap. Berdasarkan laporan tersebut, Pejabat Pencatatan Sipil mencatat pada Register Akta Perceraian dan menerbitkan Kutipan Akta Perceraian (vide Pasal 40 ayat (1) dan (2) Undang-Undang Nomor 23 Tahun 2006 tentang Administrasi Kependudukan);

Menimbang, bahwa Panitera Pengadilan atau Pejabat Pengadilan yang ditunjuk berkewajiban mengirimkan satu helai salinan putusan Pengadilan yang telah mempunyai kekuatan hukum yang tetap/yang telah dikukuhkan, tanpa bermeterai kepada Pegawai Pencatat ditempat perceraian itu terjadi, dan Pegawai Pencatat mendaftar putusan perceraian dalam sebuah daftar yang diperuntukkan untuk itu (vide Pasal 35 ayat (1) Peraturan Pemerintah Nomor 9 Tahun 1975 tentang Pelaksanaan Undang-Undang Nomor 1 Tahun 1974 tentang Perkawinan);

Menimbang, bahwa oleh karena gugatan Penggugat dikabulkan dengan *verstek* dan Tergugat ada di pihak yang kalah maka Tergugat dihukum membayar biaya perkara ini;

Memperhatikan Pasal 125 HIR dan peraturan-peraturan lain yang bersangkutan;

MENGADILI:

1. Menyatakan Tergugat telah dipanggil dengan patut tetapi tidak hadir;
2. Mengabulkan gugatan Penggugat seluruhnya dengan *verstek*;
3. Menyatakan perkawinan antara Penggugat dan Tergugat yang telah dicatatkan di Kantor Catatan Sipil Surabaya pada tanggal 20 Juni 2012, berdasarkan akta perkawinan Nomor: 3578-KW-19062012-0017 tanggal 16 Juni 2012, putus karena perceraian dengan segala akibat hukumnya;
4. Memerintahkan kepada Panitera Pengadilan Negeri Sidoarjo atau Pejabat yang ditunjuk untuk itu mengirimkan Salinan putusan dalam perkara ini yang telah mempunyai kekuatan hukum tetap kepada:
   I. **Dinas Kependudukan dan Pencatatan Sipil Kabupaten Sidoarjo,** untuk dicatat dalam daftar perceraian dalam tahun yang sedang berjalan untuk diterbitkan Akta Perceraian;
   II. **Dinas Kependudukan dan Pencatatan Sipil Kota Surabaya,** untuk dicatat pada bagian pinggir dari daftar catatan perkawinan;
5. Menghukum Tergugat untuk membayar biaya perkara sejumlah Rp596.000,00 (lima ratus sembilan puluh enam ribu rupiah);

Demikian diputuskan dalam sidang pemusyawaratan Majelis Hakim Pengadilan Negeri Sidoarjo, pada hari Kamis, tanggal 30 April 2020, oleh kami,

Disclaimer
Kepaniteraan Mahkamah Agung Republik Indonesia berusaha untuk selalu mencantumkan informasi paling kini dan akurat sebagai bentuk komitmen Mahkamah Agung untuk pelayanan publik, transparansi dan akuntabilitas pelaksanaan fungsi peradilan. Namun dalam hal-hal tertentu masih dimungkinkan terjadi permasalahan teknis terkait dengan akurasi dan keterkinian informasi yang kami sajikan, hal mana akan terus kami perbaiki dari waktu kewaktu.
Dalam hal Anda menemukan inakurasi informasi yang termuat pada situs ini atau informasi yang seharusnya ada, namun belum tersedia, maka harap segera hubungi Kepaniteraan Mahkamah Agung RI melalui :
Email : kepaniteraan@mahkamahagung.go.id Telp : 021-384 3348 (ext.318)

Dameria Frisella Simanjuntak, S.H., M.Hum., sebagai Hakim Ketua, Achmad Peten Sili, S.H., M.H. dan Joedi Prajitno, S.H., M.H. masing-masing sebagai Hakim Anggota, yang ditunjuk berdasarkan Surat Penetapan Ketua Pengadilan Negeri Sidoarjo Nomor 75/Pdt.G/2020/PN Sda. tanggal 5 Maret 2020, putusan tersebut pada hari itu juga diucapkan dalam persidangan terbuka untuk umum oleh Hakim Ketua dengan dihadiri oleh para Hakim Anggota tersebut, Nunik Apriani Isminarningtyas, S.H., Panitera Pengganti dan Penggugat tanpa dihadiri oleh Tergugat.

Hakim Anggota, Hakim Ketua,

Achmad Peten Sili, S.H., M.H. Dameria Frisella Simanjuntak, S.H., M.Hum.

Joedi Prajitno, S.H., M.H.

Panitera Pengganti,

Nunik Apriani Isminarningtyas, S.H.

Perincian biaya :

1. Biaya Pendaftaran..................	:	Rp 30.000,00;
2. Biaya Proses/ATK...................	:	Rp 100.000,00;
3...B iaya Panggilan	:	Rp 400.000,00;
4..P NBP	:	Rp 20.000,00;
5..B iaya Sumpah	:	Rp 30.000,00;
6..M eterai	:	Rp 6.000,00;
7. Redaksi	:	Rp 10.000,00;
Jumlah	:	Rp596.000,00;

(lima ratus sembilan puluh enam ribu rupiah)

Disclaimer
Kepaniteraan Mahkamah Agung Republik Indonesia berusaha untuk selalu mencantumkan informasi paling kini dan akurat sebagai bentuk komitmen Mahkamah Agung untuk pelayanan publik, transparansi dan akuntabilitas pelaksanaan fungsi peradilan. Namun dalam hal-hal tertentu masih dimungkinkan terjadi permasalahan teknis terkait dengan akurasi dan keterkinian informasi yang kami sajikan, hal mana akan terus kami perbaiki dari waktu kewaktu.
Dalam hal Anda menemukan inakurasi informasi yang termuat pada situs ini atau informasi yang seharusnya ada, namun belum tersedia, maka harap segera hubungi Kepaniteraan Mahkamah Agung RI melalui :
Email : kepaniteraan@mahkamahagung.go.id Telp : 021-384 3348 (ext.318)
