# Supplementary material for: Classification of divorce causes during the COVID-19 pandemic using convolutional neural networks
Source: PeerJ Comput Sci. 2022 Jun 30;8:e998. doi: 10.7717/peerj-cs.998 (PMC9299239; doi:10.7717/peerj-cs.998)
Supplement: Supplemental Information 3 [file peerj-cs-08-998-s003.zip › KDRT Dataset/Data ke-7.pdf]

Pdt.I.C.3

P U T U S A N
Nomor: 83/Pdt.G/2020/PN.SDA.

DEMI KEADILAN BERDASARKAN KETUHANAN YANG MAHA ESA

Pengadilan Negeri Sidoarjo yang memeriksa dan memutus perkara perdata pada tingkat pertama, telah menjatuhkan putusan sebagai berikut dalam perkara gugatan antara:

Penggugat, Perempuan, 31 tahun, Lahir di Surabaya pada tanggal 02 September 1988, Warga Negara Indonesia, Agama Kristen, Tempat tinggal di Wonoayu Nomor 193, Rungkut, Surabaya, Pekerjaan Karyawan Swasta, selanjutnya disebut sebagai **Penggugat;**

Lawan:

Tergugat, Laki-laki, 34 tahun, Lahir di Surabaya pada tanggal 09 Mei 1988, Warga Negara Indonesia, Agama Kristen, Tempat tinggal di Jalan Bimbing II Nomor 64, Pondok Candra, Waru, Sidoarjo, Pekerjaan: -, selanjutnya disebut sebagai **Tergugat;**

Pengadilan Negeri tersebut;

Setelah membaca berkas perkara;

Setelah mendengar Penggugat;

TENTANG DUDUK PERKARA

Menimbang, bahwa Penggugat dengan surat gugatan tertanggal Surabaya, 10 Maret 2020, yang diterima dan didaftarkan di Kepaniteraan Pengadilan Negeri Sidoarjo pada tanggal 10 Maret 2020 dalam Register Nomor: 83/Pdt.G/2020/PN.SDA., telah mengajukan gugatan beserta perubahannya sebagai berikut:-

1. Bahwa, PENGGUGAT dan TERGUGAT adalah Suami-Istri yang sah di kota Surabaya pada tanggal 2 November 2016 yang tercatat sesuai dengan Kutipan Akta Perkawinan No. 3578-KW-03112016-0004 yang dikeluarkan oleh Kantor Dinas Kependudukan dan Catatan Sipil Kota Surabaya pada tanggal 3 November 2016;

2. Bahwa, sejak awal pernikahan PENGGUGAT dan TERGUGAT hidup bersama sebagai Suami-Istri di rumah di Jl. Kutisari Indah Utara IV No. 42 Surabaya, terakhir TERGUGAT tinggal di Jl. Kutisari Indah Utara IV No.42 Surabaya;

3. Bahwa, dalam perkawinan antara PENGGUGAT dan TERGUGAT diperoleh keturunan:

3.1. Anak, yang berumur 3 tahun (07 Februari 2017);

4. Bahwa, sejak semula kehidupan perkawinan antara PENGGUGAT dan TERGUGAT rukun-rukun saja, namun sejak tahun 2018 sering terjadi percekcokan dan pertengkaran yang tidak dapat didamaikan dikarenakan :

4.1. Masalah Ekonomi (Tidak Menafkahi);

Disclaimer
Kepaniteraan Mahkamah Agung Republik Indonesia berusaha untuk selalu mencantumkan informasi paling kini dan akurat sebagai bentuk komitmen Mahkamah Agung untuk pelayanan publik, transparansi dan akuntabilitas pelaksanaan fungsi peradilan. Namun dalam hal-hal tertentu masih dimungkinkan terjadi permasalahan teknis terkait dengan akurasi dan keterkinian informasi yang kami sajikan, hal mana akan terus kami perbaiki dari waktu kewaktu.
Dalam hal Anda menemukan inakurasi informasi yang termuat pada situs ini atau informasi yang seharusnya ada, namun belum tersedia, maka harap segera hubungi Kepaniteraan Mahkamah Agung RI melalui :
Email : kepaniteraan@mahkamahagung.go.id Telp : 021-384 3348 (ext.318)

4.2. Sifat Kasar;

4.3. Emosional;

4.4. Sering Terjadi Pertengkaran;

5. Bahwa, dalam kondisi rumah tangga yang demikian, PENGGUGAT merasa tersiksa lahir dan batin dan tidak sanggup untuk melanjutkan kehidupan perkawinan dengan TERGUGAT, sehingga lebih baik perkawinan antara PENGGUGAT dan TERGUGAT diakhiri dan diputus dengan sebab perceraian.

6. Bahwa, oleh karena anak-anak yang lahir Anak, yang berumur 3 tahun masih dibawah umur, demi perkembangan jiwa anak tersebut yang masih membutuhkan perhatian dan kasih sayang orang tuanya, maka PENGGUGAT mohon anak tersebut tetap diasuh oleh PENGGUGAT.

7. Bahwa, oleh karena anak-anak PENGGUGAT dan TERGUGAT masih memerlukan biaya untuk penghidupan dan pendidikannya, maka PENGGUGAT mohon agar TERGUGAT memberikan biaya/ nafkah bagi anak-anak tersebut setiap sebesar Rp 5.000.000,- (Lima Juta Rupiah) hingga anak-anak tersebut dewasa dan dapat berdiri sendiri.

Berdasarkan hal-hal yang tersebut di atas, PENGGUGAT memohon kepada Ketua Pengadilan Negeri Surabaya berkenan untuk memanggil kedua belah pihak dan memeriksa perkara ini serta untuk selanjutnya menjatuhkan putusan sebagai berikut:

1. Mengabulkan Gugatan Cerai PENGGUGAT untuk seluruhnya;

2. Menyatakan perkawinan antara PENGGUGAT dan TERGUGAT sebagaimana tertuang dalam Kutipan Akta Perkawinan No. 3578-KW-03112016-0004 yang dikeluarkan oleh Kantor Dinas Kependudukan dan Catatan Sipil Kota Surabaya pada tanggal 3 November 2016 putus karena sebab perceraian;

3. Memerintahkan PENGGUGAT dan TERGUGAT untuk mengirimkan salinan putusan perkara yang telah berkekuatan hukum tetap kepada Kantor Dinas Kependudukan dan Catatan Sipil Kota Surabaya, untuk melakukan pendaftaran terhadap putusan ini, pada daftar yang dipergunakan untuk itu dan selanjutnya menerrbitkan masing-masing 1 (satu) helai kutipan akta perceraian bagi Penggugat dan Tergugat;

4. Menetapkan hak asuh anak yang bernama:

4.1 Anak, yang berumur 3 tahun (07 Februari 2017)

Kepada PENGGUGAT;

5. Memerintahkan kepada TERGUGAT untuk memberikan biaya/nafkah bagi anak-anak dan Pendidikan tersebut setiap bulannya selambat-lambat nya tanggal 5 kalender sebesar Rp.5.000.000,- (lima juta rupiah) hingga anak-anak tersebut dewasa dan dapat beridiri sendiri;

6. Membebankan biaya perkara ini menurut hukum;

Atau apabila Majelis berpendapat lain mohon putusan yang seadil-adilnya.

Disclaimer
Kepaniteraan Mahkamah Agung Republik Indonesia berusaha untuk selalu mencantumkan informasi paling kini dan akurat sebagai bentuk komitmen Mahkamah Agung untuk pelayanan publik, transparansi dan akuntabilitas pelaksanaan fungsi peradilan. Namun dalam hal-hal tertentu masih dimungkinkan terjadi permasalahan teknis terkait dengan akurasi dan keterkinian informasi yang kami sajikan, hal mana akan terus kami perbaiki dari waktu kewaktu.
Dalam hal Anda menemukan inakurasi informasi yang termuat pada situs ini atau informasi yang seharusnya ada, namun belum tersedia, maka harap segera hubungi Kepaniteraan Mahkamah Agung RI melalui :
Email : kepaniteraan@mahkamahagung.go.id   Telp : 021-384 3348 (ext.318)

Menimbang, bahwa pada hari sidang yang telah di tentukan Penggugat telah datang menghadap di persidangan, akan tetapi para Tergugat tidak datang menghadap ataupun menyuruh orang lain menghadap untuk mewakilinya, meskipun berdasarkan risalah panggilan sidang hari Kamis, tanggal 12 Maret 2020 untuk sidang pada hari Rabu, tanggal 18 Maret 2020 dan risalah panggilan sidang pada hari Kamis, tanggal 19 Maret 2020 untuk sidang pada hari Kamis, tanggal 26 Maret 2020, telah dipanggil dengan patut, sedangkan tidak ternyata bahwa tidak datangnya itu disebabkan oleh sesuatu halangan yang sah;

Menimbang, bahwa selanjutnya diajukan bukti-bukti surat oleh Penggugat sebagai berikut:

1. Photocopy Kutipan Akta Perkawinan Nomor: 3578-KW-03112016-0004 tertanggal Kota Surabaya, 03 November 2016 atas nama Penggugat, Penggugat dan Tergugat, Tergugat, diberi tanda bukti P-1;
2. Photocopy Kutipan Akta Kelahiran Nomor: 3578-LU-24032017-0126, tertanggal Kota Surabaya, 27 Maret 2017 atas nama Anak, diberi tanda bukti P-2;
3. Photocopy Kartu Keluarga Nomor: 3578240110180001, tanggal 01-10-2018 dengan Kepala Keluarga atas nama Tergugat, Tergugat, diberi tanda bukti P-3;
4. Photocopy Kartu Tanda Penduduk Republik Indonesia, Provinsi Jawa Timur, Kota Surabaya Nomor: 3578284209880002 atas nama Penggugat, diberi tanda bukti P-4;
5. Print Out Screen Shoot Chat Whatsapp dan SMS dari Tergugat kepada Penggugat, diberi tanda bukti P-5;
6. Print Out Photo HP Penggugat yang pecah dan rusak dibanting oleh Tergugat, diberi tanda bukti P-6;

Menimbang, bahwa bukti-buti surat P-1, P-2, dan P-4 telah dicocokkan dengan aslinya dan ternyata cocok sehingga dapat dipergunakan untuk pembuktian surat lebih lanjut dalam perkara ini;

Menimbang, bahwa bukti surat P-3 adalah photocopy surat tanpa ditunjukkan aslinya namun bersesuaian dengan alat bukti lainnya yakni keterangan para saksi dalam perkara ini sehingga dapat menjadi prasangka bagi majelis hakim untuk kebenaran tentang isi dari bukti surat tersebut;

Menimbang, bahwa sedangkan bukti surat P-5 dan P-6 adalah print out dari handphone yang juga menjadi prasangka bagi majelis hakim untuk kebenaran isinya akan pertengkaran-pertengkaran yang terjadi diantara Penggugat dan Tergugat;

Menimbang, bahwa diajukan bukti saksi-saksi oleh Penggugat sebagai berikut:

1. Saksi Henrijata Wardojo;

Disclaimer
Kepaniteraan Mahkamah Agung Republik Indonesia berusaha untuk selalu mencantumkan informasi paling kini dan akurat sebagai bentuk komitmen Mahkamah Agung untuk pelayanan publik, transparansi dan akuntabilitas pelaksanaan fungsi peradilan. Namun dalam hal-hal tertentu masih dimungkinkan terjadi permasalahan teknis terkait dengan akurasi dan keterkinian informasi yang kami sajikan, hal mana akan terus kami perbaiki dari waktu kewaktu.
Dalam hal Anda menemukan inakurasi informasi yang termuat pada situs ini atau informasi yang seharusnya ada, namun belum tersedia, maka harap segera hubungi Kepaniteraan Mahkamah Agung RI melalui :
Email : kepaniteraan@mahkamahagung.go.id Telp : 021-384 3348 (ext.318)

- Bahwa saksi adalah ayah/ orang tua laki-laki dari Penggugat, Penggugat;
- Bahwa antara saksi dan ibu/ orang tua Perempuan Penggugat yakni saksi Rita Oliy sudah bercerai dan saksi sudah dengan keluarga barunya;
- Bahwa anak perempuan saksi, Penggugat, Penggugat telah melangsungkan perkawinannya dengan Tergugat pada tanggal 29 Oktober 2016 menurut tatacara agama Kristen di Gereja Kasih Karunia Indonesia Jemaat Anggur Baru Surabaya;
- Bahwa perkawinan antara Penggugat, Penggugat dengan Tergugat dilangsungkan menurut tatacara agama Kristen di hadapan Pdt. Hendrick Lusikooy, M.Th., dari Gereja Kasih Karunia Indonesia Jemaat Anggur Baru Surabaya;
- Bahwa dalam perkawinan antara Penggugat, Penggugat dan Tergugat, Tergugat telah dilahirkan 1 (satu) orang anak yang bernama Anak, laki-laki, lahir di Surabaya pada tanggal 07 Februari 2017;
- Bahwa sebelum melangsungkan perkawinan dengan Tergugat maka Penggugat, Penggugat berstatus janda dari seorang laki-laki bernama Ramadha Sabri dengan seorang anak yang bernama Ananda Ramsi, laki-laki, lahir di Surabaya pada tanggal 11-01-2010;

- Bahwa dalam perkawinannya dengan Penggugat maka Tergugat tidak mempunyai pekerjaan yang tetap bahkan saat ini tidak lagi bekerja dan akibatnya tidak bisa memberikan nafkah kepada anak dan isterinya;
- Bahwa Tergugat juga mempunyai sifat perangai yang jahat dan selalu mengusir Penggugat dari tempat kediaman bersama serta Tergugat selalu berkata cerai saat bertengkar;
- Bahwa Penggugat bekerja sebagai karyawan swasta;
- Bahwa menurut saksi perkawinan antara Penggugat dan Tergugat diputuskan saja perkawinannya supaya masing-masing pihak bisa berinterospeksi serta memperbaiki diri dan meningkatkannya kemampuan ekonominya terlebih dahulu;

2. Saksi Rita Oliy;

- Bahwa saksi adalah ibu/ orang tua perempuan dari Penggugat, Penggugat;
- Bahwa antara saksi dan ayah/ orang tua laki-laki Penggugat, Penggugat sudah bercerai dan saat ini saksi hidup tinggal bersama dengan Penggugat, Penggugat;
- Bahwa anak perempuan saksi, Penggugat, Penggugat telah melangsungkan perkawinannya dengan Tergugat pada tanggal 29 Oktober 2016;
- Bahwa perkawinan antara Penggugat, Penggugat dengan Tergugat dilangsungkan menurut tatacara agama Kristen dihadapan Pdt. Hendrick Lusikooy, M.Th., di Gereja Kasih Karunia Indonesia Jemaat Anggur Baru Surabaya;

Disclaimer
Kepaniteraan Mahkamah Agung Republik Indonesia berusaha untuk selalu mencantumkan informasi paling kini dan akurat sebagai bentuk komitmen Mahkamah Agung untuk pelayanan publik, transparansi dan akuntabilitas pelaksanaan fungsi peradilan. Namun dalam hal-hal tertentu masih dimungkinkan terjadi permasalahan teknis terkait dengan akurasi dan keterkinian informasi yang kami sajikan, hal mana akan terus kami perbaiki dari waktu kewaktu.
Dalam hal Anda menemukan inakurasi informasi yang termuat pada situs ini atau informasi yang seharusnya ada, namun belum tersedia, maka harap segera hubungi Kepaniteraan Mahkamah Agung RI melalui :
Email : kepaniteraan@mahkamahagung.go.id   Telp : 021-384 3348 (ext.318)

- Bahwa dalam perkawinan antara Penggugat, Penggugat dan Tergugat, Tergugat telah dilahirkan 1 (satu) orang anak laki-laki yang bernama Anak, laki-laki, lahir di Surabaya pada tanggal 07 Februari 2017;
- Bahwa sebelum melangsungkan perkawinan dengan Tergugat maka Penggugat, Penggugat berstatus janda dari seorang laki-laki bernama Ramadha Sabri dengan seorang anak yang bernama Ananda Ramsi, laki-laki, lahir di Surabaya pada tanggal 11-01-2010;
- Bahwa dalam perkawinannya dengan Penggugat maka Tergugat tidak mempunyai pekerjaan yang tetap bahkan saat ini tidak lagi bekerja dan akibatnya tidak bisa memberikan nafkah kepada anak dan isterinya;
- Bahwa Tergugat juga mempunyai sifat perangai yang jahat dan selalu mengusir Penggugat dari tempat kediaman bersama serta Tergugat selalu berkata cerai saat bertengkar;
- Bahwa Penggugat bekerja sebagai karyawan swasta;
- Bahwa menurut saksi perkawinan antara Penggugat dan Tergugat diputuskan saja perkawinannya supaya masing-masing pihak bisa berinterospeksi serta memperbaiki diri dan meningkatkannya kemampuan ekonominya terlebih dahulu;

Menimbang, bahwa diajukan kesimpulan oleh Penggugat tertanggal Surabaya, 16 April 2020 terlampir dalam berkas perkara yang dianggap termuat dan menjadi satu kesatuan dengan putusan perkara ini;

Menimbang, bahwa untuk menyingkat putusan, maka segala sesuatu yang termuat dalam berita acara persidangan, dianggap telah termuat dan menjadi bagian yang tak terpisahkan dari putusan ini;

Menimbang, bahwa akhirnya Penggugat menyatakan tidak ada hal-hal yang diajukan lagi dan mohon putusan;

TENTANG PERTIMBANGAN HUKUM

Menimbang, bahwa maksud dan tujuan gugatan Penggugat pada pokoknya adalah mengenai putusnya perkawinan antara Penggugat dan Tergugat serta nafkah alimentasi anak yang dilahirkan dalam perkawinan antara Penggugat dan Tergugat;

Menimbang, bahwa pada pokoknya surat gugatan Penggugat memuat dalil-dalil gugatan sebagai berikut yang hal tersebut dikuatkan dengan bukti-bukti surat serta keterangan kedua orang tuanya sebagai saksi-saksi sebagaimana tersebut diatas:-

- Bahwa di Kota Surabaya pada tanggal 29 Oktober 2016 telah dilangsungkan perkawinan antara Penggugat, Penggugat dengan Tergugat, Tergugat menurut tata cara agama Kristen di Gereja Kasih Karunia Indonesia Jemaat Anggur Baru Surabaya sesuai dengan Kutipan Akta Perkawinan No. 3578-KW-

Disclaimer
Kepaniteraan Mahkamah Agung Republik Indonesia berusaha untuk selalu mencantumkan informasi paling kini dan akurat sebagai bentuk komitmen Mahkamah Agung untuk pelayanan publik, transparansi dan akuntabilitas pelaksanaan fungsi peradilan. Namun dalam hal-hal tertentu masih dimungkinkan terjadi permasalahan teknis terkait dengan akurasi dan keterkinian informasi yang kami sajikan, hal mana akan terus kami perbaiki dari waktu kewaktu.
Dalam hal Anda menemukan inakurasi informasi yang termuat pada situs ini atau informasi yang seharusnya ada, namun belum tersedia, maka harap segera hubungi Kepaniteraan Mahkamah Agung RI melalui :
Email : kepaniteraan@mahkamahagung.go.id Telp : 021-384 3348 (ext.318)

03112016-0004 yang diterbitkan oleh Kepala Dinas Kependudukan dan Pencatatan Sipil Kota Surabaya tertanggal Kota Surabaya, 03 November 2016;

- Bahwa dari pernikahan antara Penggugat, Penggugat dengan Tergugat, Tergugat, telah mempunyai 1 (satu) orang anak kandung yaitu:

- Anak, laki-laki, lahir di Surabaya, pada tanggal 07 Februari 2017, yang diterbitkan oleh Kepala Dinas Kependudukan dan Pencatatan Sipil Kota Surabaya Nomor: 3578-LU-24032017-0126, tertanggal Kota Surabaya, 27 Maret 2017;

- Bahwa antara Penggugat dan Tergugat sudah pisah ranjang dan tempat kediaman bersama, karena sudah diusir Tergugat;

- Bahwa Tergugat tidak bekerja dan tidak memberi nafkah lahir kepada Penggugat dan anak-anak;

Menimbang, bahwa tujuan perkawinan menurut UU Perkawinan No.1 Tahun 1974 menyatakan bahwa perkawinan adalah ikatan lahir bathin antara seorang perempuan dan seorang pria sebagai suami isteri dengan tujuan membentuk keluarga (rumah tangga) yang bahagia dan kekal berdasarkan Ketuhanan Yang Maha Esa, Pasal 33: menyebutkan bahwa suami isteri wajib saling mencintai, hormat menghormati, setia dan memberi bantuan lahir dan bathin antara yang satu dengan lainnya. Tujuan maupun harapan terhadap perkawinan tersebut tidak pernah tercapai karena tidak ada keharmonisan lagi antara Penggugat dan Tergugat;-

Menimbang, bahwa dengan pertimbangan hukum sebagaimana tersebut diatas maka telah memenuhi ketentuan Pasal 19 huruf (f) Peraturan Pemerintah No.09 Tahun 1975 yang menentukan, bahwa alasan untuk dapat mengabulkan perceraian, adalah apabila diantara suami-isteri terus menerus terjadi perselisihan dan pertengkaran dan dalam perkara ini antara Penggugat dan Tergugat terus menerus berselisih tentang keadaan keluarga pada umumnya yang sumber utamanya adalah masalah ekonomi dan diantara Penggugat dan Tergugat sudah tidak ada harapan akan hidup rukun lagi dalam rumah tangga, sehingga Majelis Hakim berkesimpulan, bahwa dalil gugatan Penggugat pada petitum No.2 yang menyatakan agar perkawinan Penggugat dan Tergugat putus karena perceraian dapat dikabulkan;-

Menimbang, bahwa terhadap anak yang dilahirkan dalam perkawinan Penggugat dan Tergugat majelis hakim mempertimbangkan untuk tetap berada dalam asuhan Penggugat tanpa mengurangi hak Tergugat untuk turut serta merawat dan membiayai anak-anaknya tersebut hingga anak-anak tersebut dewasa dan mandiri.

Menimbang, bahwa sedangkan petitum untuk diberikannya nafkah alimentasi atas anak yang dilahirkan dalam perkawinan antara Penggugat dn Tergugat maka oleh karena Tergugat belum ternyata mempunyai pekerjaan dan

Disclaimer
Kepaniteraan Mahkamah Agung Republik Indonesia berusaha untuk selalu mencantumkan informasi paling kini dan akurat sebagai bentuk komitmen Mahkamah Agung untuk pelayanan publik, transparansi dan akuntabilitas pelaksanaan fungsi peradilan. Namun dalam hal-hal tertentu masih dimungkinkan terjadi permasalahan teknis terkait dengan akurasi dan keterkinian informasi yang kami sajikan, hal mana akan terus kami perbaiki dari waktu kewaktu.
Dalam hal Anda menemukan inakurasi informasi yang termuat pada situs ini atau informasi yang seharusnya ada, namun belum tersedia, maka harap segera hubungi Kepaniteraan Mahkamah Agung RI melalui :
Email : kepaniteraan@mahkamahagung.go.id Telp : 021-384 3348 (ext.318)

tidak pula ternyata mempunyai penghasilan yang sah untuk menghidupi baik bagi dirinya sendiri maupun bagi keluarganya maka petitum yang demikian harus dinyatakan tidak dapat diterima;

Menimbang, bahwa oleh karena jangka waktu dan formalitas panggilan menurut hukum telah diindahkan dengan sepatutnya serta gugatan tersebut tidak melawan hukum dan beralasan, maka Tergugat yang telah dipanggil dengan patut akan tetapi tidak datang menghadap di persidangan dan tidak menyuruh orang lain menghadap sebagai wakilnya, harus dinyatakan tidak hadir dan gugatan tersebut dikabulkan sebagian dengan verstek;

Menimbang, bahwa oleh karena Tergugat berada di pihak yang kalah, maka segala biaya yang timbul di dalam perkara ini, sudah sepatutnya dibebankan kepada Tergugat yang besarnya akan ditentukan di dalam amar putusan ini;-

Mengingat Pasal 19 huruf (f) Peraturan Pemerintah No. 09 Tahun 1975 dan Pasal 125 HIR/ 149 RBg serta ketentuan perundang-undangan lainnya yang bersangkutan dengan perkara ini;-

MENGADILI:

- Menyatakan sebagai hukum bahwa Tergugat yang telah dipanggil secara sah tidak hadir dipersidangan.
- Mengabulkan gugatan Penggugat untuk sebagian dengan verstek.
- Menyatakan perkawinan antara Penggugat, Penggugat dengan Tergugat, Tergugat, yang terjadi di Kota Surabaya pada tanggal 29 Oktober 2016, menurut tata cara agama Kristen di Gereja Kasih Karunia Indonesia Jemaat Anggur Baru Surabaya sesuai dengan Kutipan Akta Perkawinan Nomor: 3578-KW-03112016-0004 yang diterbitkan oleh Kepala Dinas Kependudukan dan Pencatatan Sipil Kota Surabaya tertanggal Kota Surabaya, 03 November 2016, PUTUS KARENA PERCERAIAN dengan segala akibat hukumnya;-
- Menyatakan secara hukum anak hasil perkawinan antara Penggugat dan Tergugat yaitu:
    - Anak, laki-laki, lahir di Kota Surabaya, tanggal 07 Februari 2017 sebagaimana tercatat pada Akta Kelahiran Nomor: 3578-LU-24032017-0126, tertanggal Kota Surabaya, 27 Maret 2017, berada dalam asuhan Penggugat;
- Memerintahkan para pihak, Penggugat dan Tergugat untuk mengirim salinan putusan yang telah mempunyai kekuatan hukum tetap ke Kantor Dinas Kependudukan dan Pencatatan Sipil Kabupaten Sidoarjo dan Kantor Dinas Kependudukan dan Pencatatan Sipil Kota Surabaya dalam waktu paling lambat 60 (enam puluh) hari sejak putusan dalam perkara ini mempunyai kekuatan hukum tetap untuk mencoret perkawinan dalam register yang dipergunakan untuk itu dan mencatat perceraian tersebut dalam daftar yang sedang berjalan serta menerbitkan akta cerainya.

Disclaimer
Kepaniteraan Mahkamah Agung Republik Indonesia berusaha untuk selalu mencantumkan informasi paling kini dan akurat sebagai bentuk komitmen Mahkamah Agung untuk pelayanan publik, transparansi dan akuntabilitas pelaksanaan fungsi peradilan. Namun dalam hal-hal tertentu masih dimungkinkan terjadi permasalahan teknis terkait dengan akurasi dan keterkinian informasi yang kami sajikan, hal mana akan terus kami perbaiki dari waktu kewaktu.
Dalam hal Anda menemukan inakurasi informasi yang termuat pada situs ini atau informasi yang seharusnya ada, namun belum tersedia, maka harap segera hubungi Kepaniteraan Mahkamah Agung RI melalui :
Email : kepaniteraan@mahkamahagung.go.id Telp : 021-384 3348 (ext.318)

- Menghukum Tergugat untuk membayar biaya perkara yang timbul dalam perkara ini yang hingga kini ditaksir sebesar Rp.436.000,- (empat ratus tiga puluh enam ribu rupiah);
- Menyatakan tidak dapat diterima gugatan Penggugat untuk selain dan selebihnya;

Demikian diputuskan dalam sidang pemusyawaratan Majelis Hakim Pengadilan Negeri Sidoarjo, pada hari Kamis, tanggal 30 April 2020, oleh kami, Joedi Prajitno, S.H., M.H., sebagai Hakim Ketua, Dameria Frisella Simanjuntak, S.H., M.Hum., dan Achmad Petensili, S.H., M.H., masing-masing sebagai Hakim Anggota, yang ditunjuk berdasarkan Surat Penetapan Ketua Pengadilan Negeri Sidoarjo Nomor: 83/Pdt.G/2020/PN.SDA., tertanggal Sidoarjo, 10 Maret 2020, putusan tersebut pada hari itu juga diucapkan dalam persidangan terbuka untuk umum oleh Hakim Ketua dengan dihadiri oleh para Hakim Anggota tersebut, Endang Kusrini, S.H, Panitera Pengganti dan Penggugat tanpa dihadiri oleh Tergugat ataupun kuasa hukumnya.

Hakim-hakim Anggota, Hakim Ketua,

Dameria Frisella Simanjuntak, S.H., M.Hum., Joedi Prajitno, S.H., M.H.,

Achmad Petensili, S.H., M.H.,

Panitera Pengganti,

Endang Kusrini, S.H.

Perincian biaya:

1. Biaya pendaftaran ...	: Rp. 30.000,00
2. Biaya proses/ atk ...	: Rp.100.000,00
3. Biaya panggilan ...	: Rp.240.000,00
4. PNBP ...	: Rp. 20.000,00
5. Biaya sumpah ...	: Rp. 30.000,00
6. Materai ...	: Rp. 6.000,00
7. Redaksi ...	: Rp. 10.000,00
Jumlah	: Rp.436.000,00

Disclaimer
Kepaniteraan Mahkamah Agung Republik Indonesia berusaha untuk selalu mencantumkan informasi paling kini dan akurat sebagai bentuk komitmen Mahkamah Agung untuk pelayanan publik, transparansi dan akuntabilitas pelaksanaan fungsi peradilan. Namun dalam hal-hal tertentu masih dimungkinkan terjadi permasalahan teknis terkait dengan akurasi dan keterkinian informasi yang kami sajikan, hal mana akan terus kami perbaiki dari waktu kewaktu.
Dalam hal Anda menemukan inakurasi informasi yang termuat pada situs ini atau informasi yang seharusnya ada, namun belum tersedia, maka harap segera hubungi Kepaniteraan Mahkamah Agung RI melalui :
Email : kepaniteraan@mahkamahagung.go.id Telp : 021-384 3348 (ext.318)

(empat ratus tiga puluh enam ribu rupiah);

Disclaimer
Kepaniteraan Mahkamah Agung Republik Indonesia berusaha untuk selalu mencantumkan informasi paling kini dan akurat sebagai bentuk komitmen Mahkamah Agung untuk pelayanan publik, transparansi dan akuntabilitas pelaksanaan fungsi peradilan. Namun dalam hal-hal tertentu masih dimungkinkan terjadi permasalahan teknis terkait dengan akurasi dan keterkinian informasi yang kami sajikan, hal mana akan terus kami perbaiki dari waktu kewaktu.
Dalam hal Anda menemukan inakurasi informasi yang termuat pada situs ini atau informasi yang seharusnya ada, namun belum tersedia, maka harap segera hubungi Kepaniteraan Mahkamah Agung RI melalui :
Email : kepaniteraan@mahkamahagung.go.id Telp : 021-384 3348 (ext.318)
