# Supplementary material for: Classification of divorce causes during the COVID-19 pandemic using convolutional neural networks
Source: PeerJ Comput Sci. 2022 Jun 30;8:e998. doi: 10.7717/peerj-cs.998 (PMC9299239; doi:10.7717/peerj-cs.998)
Supplement: Supplemental Information 3 [file peerj-cs-08-998-s003.zip › KDRT Dataset/Data ke-8.pdf]

P U T U S A N

Nomor 4171/Pdt.G/2020/PA.Smdg

بِسْــــــــــمِ ٱللَّهِ ٱلرَّحْمَٰنِ ٱلرَّحِيمِ

DEMI KEADILAN BERDASARKAN KETUHANAN YANG MAHA ESA

Pengadilan Agama Sumedang yang memeriksa dan mengadili perkara tertentu pada tingkat pertama telah menjatuhkan putusan perkara Cerai Gugat yang diajukan oleh:

Rika Yuningsih Binti Nahrowi, umur 20 tahun, agama Islam, pekerjaan Ibu Rumah Tangga, pendidikan SD, tempat kediaman di Dusun Cijerah RT. 05 RW. 01, Desa Jayamandiri, Cibugel, Kab. Sumedang, Jawa Barat selanjutnya telah memberikan kuasa kepada H.M. Yusuf T. Insyaf, S.H. M.M., Slamet Riyadi, SH., Dahliah Sobarna, SH., R. Ateng Ruchana, SH., Siti Citra Erliana, SH., Citra Erica Arba Yusuf, SH., yang berkantor di Lembaga Bantuan Hukum dan Perlindungan Masyarakat (LBH PERMAS) yang beralamat di Jalan Pangaduan Hebeul, Rt. 03 / Rw. 11, Kelurahan Situ, Kecamatan Sumedang Utara, Kabupaten Sumedang, berdasarkan Surat Kuasa Khusus tertanggal 02 Desember 2020; sebagai **Penggugat**;

melawan

Rahmat Cahyana Bin Dayat umur 25 tahun, agama Islam, pendidikan SD, pekerjaan Karyawan Swasta, tempat kediaman di Dusun Cijerah RT. 05 RW. 01, Desa Jayamandiri, Cibugel, Kab. Sumedang, Jawa Barat; sebagai **Tergugat**;

Pengadilan Agama tersebut;

Telah mempelajari surat-surat yang berkaitan dengan perkara ini;

Telah mendengar keterangan Penggugat dan para saksi di depan sidang;

DUDUK PERKARA

Menimbang, bahwa Penggugat dalam surat gugatannya tanggal 02

Disclaimer
Kepaniteraan Mahkamah Agung Republik Indonesia berusaha untuk selalu mencantumkan informasi paling kini dan akurat sebagai bentuk komitmen Mahkamah Agung untuk pelayanan publik, transparansi dan akuntabilitas pelaksanaan fungsi peradilan. Namun dalam hal-hal tertentu masih dimungkinkan terjadi permasalahan teknis terkait dengan akurasi dan keterkinian informasi yang kami sajikan, hal mana akan terus kami perbaiki dari waktu kewaktu.
Dalam hal Anda menemukan inakurasi informasi yang termuat pada situs ini atau informasi yang seharusnya ada, namun belum tersedia, maka harap segera hubungi Kepaniteraan Mahkamah Agung RI melalui :
Email : kepaniteraan@mahkamahagung.go.id   Telp : 021-384 3348 (ext.318)

Desember 2020 telah mengajukan gugatan cerai, yang telah didaftar di Kepaniteraan Pengadilan Agama Sumedang, dengan Nomor 4171/Pdt.G/2020/PA.Smdg, tanggal 02 Desember 2020, dengan dalil-dalil sebagai berikut:

1. Bahwa Penggugat dan Tergugat adalah suami isteri yang sah sebagaimana terbukti dalam Kutipan Akta Nikah No. 0140 / 017 / VIII / 2018, yang menikah di hadapan Pejabat Kantor Urusan Agama (KUA), Kecamatan Cibugel, Kabupaten Sumedang, pada tanggal 20 Agustus 2018;
2. Bahwa setelah menikah baik Penggugat maupun Tergugat membina rumah tangga di Dusun Cijerah, RT. 005 / RW. 001, Desa Jayamandiri, Kecamatan Cibugel, Kabupaten Sumedang, di rumah kediaman milik bersama ;
3. Bahwa semula kehidupan rumah tangga antara Penggugat dan Tergugat berjalan rukun dan harmonis diliputi kebahagiaan sebagaimana layaknya suami istri yang hidup dalam rumah tangga yang harmonis, sehingga dari hasil pernikahan antara Penggugat dan Tergugat sudah dikaruniai satu orang anak yang bernama Zhafiratul Hanna (berusia 1 tahun) ;
4. Bahwa kehidupan rumah tangga antara Penggugat dan Tergugat mulai goyah dan sering terjadi perselisihan dan pertengkaran kurang lebih terjadi pada bulan Juni tahun 2019 hal tersebut terjadi disebabkan karena faktor kekurangan ekonomi dimana nafkah yang diberikan oleh Tergugat tidak mencukupi untuk menutupi kebutuhan hidup sehari-hari karena nafkah yang diberikan oleh Tergugat rata-rata hanya sebesar satu juta lima ratus ribu rupiah per bulan itupun kadang tidak menentu sementara kebutuhan hidup Penggugat jauh lebih besar dari nafkah yang selama ini diberikan oleh Tergugat apalagi ditambah biaya hidup anak yang memerlukan biaya tidak sedikit per bulannya;
5. Bahwa permasalahan tersebut tidak jarang akhirnya memicu terjadinya perselisihan dan pertengkaran antara Penggugat dan Tergugat sehingga puncak dari perselisihan dan pertengkaran antara Penggugat dengan Tergugat kurang lebih terjadi pada bulan Nopember tahun

Disclaimer
Kepaniteraan Mahkamah Agung Republik Indonesia berusaha untuk selalu mencantumkan informasi paling kini dan akurat sebagai bentuk komitmen Mahkamah Agung untuk pelayanan publik, transparansi dan akuntabilitas pelaksanaan fungsi peradilan. Namun dalam hal-hal tertentu masih dimungkinkan terjadi permasalahan teknis terkait dengan akurasi dan keterkinian informasi yang kami sajikan, hal mana akan terus kami perbaiki dari waktu kewaktu.
Dalam hal Anda menemukan inakurasi informasi yang termuat pada situs ini atau informasi yang seharusnya ada, namun belum tersedia, maka harap segera hubungi Kepaniteraan Mahkamah Agung RI melalui :
Email : kepaniteraan@mahkamahagung.go.id Telp : 021-384 3348 (ext.318)

2019 dimana antara Penggugat dan Tergugat sudah tidak serumah lagi sebagaimana layaknya pasangan suami istri dan yang pergi meninggalkan rumah adalah Tergugat ;

6. Bahwa walaupun telah dilakukan beberapa kali upaya untuk rukun kembali bahkan dilakukan dengan bantuan dari keluarga Penggugat akan tetapi perselisihan dan pertengkaran antara Penggugat dan Tergugat tetap saja berlangsung terus menerus hal tersebut telah memenuhi salah satu alasan perceraian sebagaimana yang tercantum di dalam PP No. 9 Tahun 1975 Pasal 19 Huruf F Jo Kompilasi Hukum Islam Pasal 116 Huruf f ;
7. Bahwa dengan demikian hubungan pernikahan antara Penggugat dengan Tergugat termasuk kriteria pernikahan yang telah pecah sulit untuk dipertahankan, apabila tetap dipertahankan akan menimbulkan kemudhorotan yang berkepanjangan bagi kedua belah pihak ;
8. Bahwa berdasarkan hal tersebut diatas keadaan rumah tangga Penggugat dan Tergugat sudah tidak dapat dibina dengan baik sehingga sulit untuk membentuk rumah tangga yang sakinah, mawaddah, dan rahmah, Penggugat menderita lahir batin dan tidak sanggup lagi meneruskan rumah tangga dengan Tergugat serta tidak ada jalan lagi yang terbaik kecuali perceraian ;

bahwa berdasarkan alasan - alasan dan peristiwa - peristiwa tersebut di atas Penggugat mohon kepada Bapak Ketua Pengadilan Agama Klas I A Sumedang melalui Majelis Hakim Pengadilan Agama Klas I A Sumedang yang mengadili dan memeriksa perkara ini berkenan menjatuhkan putusan sebagai berikut ;

Primair :

1. Mengabulkan Gugatan Penggugat
2. Menjatuhkan talak satu Ba`in Sughra dari Tergugat (Rahmat Cahyana Bin Dayat) terhadap Penggugat (Rika Yuningsih Binti Nahrowi)
3. Menetapkan biaya perkara menurut Hukum.

Disclaimer
Kepaniteraan Mahkamah Agung Republik Indonesia berusaha untuk selalu mencantumkan informasi paling kini dan akurat sebagai bentuk komitmen Mahkamah Agung untuk pelayanan publik, transparansi dan akuntabilitas pelaksanaan fungsi peradilan. Namun dalam hal-hal tertentu masih dimungkinkan terjadi permasalahan teknis terkait dengan akurasi dan keterkinian informasi yang kami sajikan, hal mana akan terus kami perbaiki dari waktu kewaktu.
Dalam hal Anda menemukan inakurasi informasi yang termuat pada situs ini atau informasi yang seharusnya ada, namun belum tersedia, maka harap segera hubungi Kepaniteraan Mahkamah Agung RI melalui :
Email : kepaniteraan@mahkamahagung.go.id Telp : 021-384 3348 (ext.318)

Bahwa pada hari sidang yang telah ditetapkan Penggugat telah datang menghadap ke persidangan, sedangkan Tergugat tidak datang menghadap ke persidangan dan tidak menyuruh orang lain untuk menghadap sebagai wakil/kuasa hukumnya, meskipun telah dipanggil secara resmi dan patut yang relaas panggilannya dibacakan di dalam sidang, sedangkan tidak ternyata bahwa tidak datangnya itu disebabkan suatu alasan yang sah;

Bahwa majelis hakim telah menasehati Penggugat agar berpikir untuk tidak bercerai dengan Tergugat, tetapi Penggugat tetap pada dalil-dalil gugatannya untuk bercerai dengan Tergugat;

Bahwa perkara ini tidak dapat dimediasi karena Tergugat tidak pernah datang menghadap meskipun telah dipanggil secara resmi dan patut, selanjutnya dimulai pemeriksaan dengan membacakan surat gugatan Penggugat yang maksud dan tujuannya tetap dipertahankan oleh Penggugat;

Bahwa terhadap gugatan Penggugat tersebut, Tergugat tidak memberikan jawaban karena tidak hadir di persidangan;

Bahwa untuk menguatkan dalil-dalil gugatannya, Penggugat telah mengajukan bukti berupa:

A. Surat :

Buku Kutipan Akta Nikah atas nama Penggugat dan Penggugat yang menikah pada tanggal 20 Agustus 2018, dengan Nomor 0140 / 017 / VIII / 2018 tertanggal 20 Agustus 2018, yan g dikeluarkan oleh Kantor Urusan Agama KUA Kecamatan Cibugel, Kabupaten Sumedang. Fotocopi tersebut dibubuhi materai cukup dan setelah dicocokan dengan aslinya ternyata sesuai, kemudian oleh Ketua Majelis diparaf dan diberi kode P.1;

B. Saksi :

1. Nanang bin Momo, umur 50 tahun, agama Islam, pekerjaan Buruh, tempat tinggal di Dusun Cijerah RT. 05 RW. 01, Desa Jayamandiri, Cibugel, Kab. Sumedang, Jawa Barat;di bawah sumpahnya

Disclaimer
Kepaniteraan Mahkamah Agung Republik Indonesia berusaha untuk selalu mencantumkan informasi paling kini dan akurat sebagai bentuk komitmen Mahkamah Agung untuk pelayanan publik, transparansi dan akuntabilitas pelaksanaan fungsi peradilan. Namun dalam hal-hal tertentu masih dimungkinkan terjadi permasalahan teknis terkait dengan akurasi dan keterkinian informasi yang kami sajikan, hal mana akan terus kami perbaiki dari waktu kewaktu.
Dalam hal Anda menemukan inakurasi informasi yang termuat pada situs ini atau informasi yang seharusnya ada, namun belum tersedia, maka harap segera hubungi Kepaniteraan Mahkamah Agung RI melalui :
Email : kepaniteraan@mahkamahagung.go.id Telp : 021-384 3348 (ext.318)

memberikan keterangan sebagai berikut:

- Bahwa saksi kenal kepada Penggugat dalam hubungan selaku Tetangga Penggugat;
- Bahwa antara Penggugat dengan Tergugat telah hidup rukun sebagaimana layaknya suami istri sudah dikaruniai satu orang anak yang bernama Zhafiratul Hanna (berusia 1 tahun) ;
- Bahwa sepengetahuan saksi hubungan rumah tangga Penggugat dengan Tergugat mulai tidak lagi harmonis sejak bulan Juni tahun 2019 disebabkan karena faktor kekurangan ekonomi dimana nafkah yang diberikan oleh Tergugat tidak mencukupi untuk menutupi kebutuhan hidup sehari-hari,
- Bahwa akibatnya bulan Nopember tahun 2019 dimana antara Penggugat dan Tergugat sudah tidak serumah lagi;
- Bahwa, saksi sudah menasehati Penggugat agar tetap sabar dan rukun kembali dengan Tergugat, tetapi tidak berhasil;
- Bahwa, saksi sudah tidak sanggup merukunkan Penggugat dan Tergugat;

2. Cepi Somantri bin Nahrowi umur 25 tahun, agama Islam, pekerjaan Buruh, tempat tinggal di Dusun Cijerah RT. 05 RW. 01, Desa Jayamandiri, Cibugel, Kab. Sumedang, Jawa Barat di bawah sumpahnya memberikan keterangan sebagai berikut:

- Bahwa saksi kenal kepada Penggugat dalam hubungan selaku kakak Penggugat;
- Bahwa antara Penggugat dengan Tergugat telah hidup rukun sebagaimana layaknya suami istri sudah dikaruniai satu orang anak yang bernama Zhafiratul Hanna (berusia 1 tahun) ;
- Bahwa sepengetahuan saksi hubungan rumah tangga Penggugat dengan Tergugat mulai tidak lagi harmonis sejak bulan Juni tahun 2019 disebabkan karena faktor kekurangan ekonomi dimana nafkah yang diberikan oleh Tergugat tidak mencukupi untuk menutupi

Disclaimer
Kepaniteraan Mahkamah Agung Republik Indonesia berusaha untuk selalu mencantumkan informasi paling kini dan akurat sebagai bentuk komitmen Mahkamah Agung untuk pelayanan publik, transparansi dan akuntabilitas pelaksanaan fungsi peradilan. Namun dalam hal-hal tertentu masih dimungkinkan terjadi permasalahan teknis terkait dengan akurasi dan keterkinian informasi yang kami sajikan, hal mana akan terus kami perbaiki dari waktu kewaktu.
Dalam hal Anda menemukan inakurasi informasi yang termuat pada situs ini atau informasi yang seharusnya ada, namun belum tersedia, maka harap segera hubungi Kepaniteraan Mahkamah Agung RI melalui :
Email : kepaniteraan@mahkamahagung.go.id Telp : 021-384 3348 (ext.318)

kebutuhan hidup sehari-hari,

- Bahwa akibatnya bulan Nopember tahun 2019 dimana antara Penggugat dan Tergugat sudah tidak serumah lagi;
- Bahwa, saksi sudah menasehati Penggugat agar tetap sabar dan rukun kembali dengan Tergugat, tetapi tidak berhasil;
- Bahwa, saksi sudah tidak sanggup merukunkan Penggugat dan Tergugat;

Bahwa selanjutnya Penggugat menyampaikan kesimpulan yang pada pokoknya Penggugat tetap dengan gugatannya ingin bercerai dengan Tergugat dan mohon agar Majelis Hakim segera menjatuhkan putusan;

Bahwa selanjutnya untuk singkatnya uraian putusan ini, maka semua hal yang termuat dalam berita acara sidang ini, merupakan bagian yang tidak terpisahkan dari putusan ini;

PERTIMBANGAN HUKUM

Menimbang, bahwa maksud dan tujuan gugatan Penggugat adalah sebagaimana terurai di atas;

Menimbang, bahwa gugatan Penggugat telah diajukan sesuai dengan peraturan perundang-undangan yang berlaku, oleh karena itu gugatan Penggugat tersebut secara formil dapat diterima dan dapat dipertimbangkan lebih lanjut;

Menimbang bahwa, Tergugat yang dipanggil secara resmi dan patut akan tetapi tidak datang menghadap, harus dinyatakan tidak hadir, dan gugatan tersebut harus diperiksa secara verstek;

Menimbang, bahwa berdasarkan ketentuan Pasal 125 ayat (1) HIR, yaitu putusan yang dijatuhkan tanpa hadirnya Tergugat dapat dikabulkan sepanjang berdasarkan hukum dan beralasan, oleh karena perkara perceraian memiliki aspek-aspek yang khusus (lex specialis) serta dalam rangka menghindari adanya penyelundupan hukum, mak kepada Penggugat dibebani bukti-bukti , oleh karena itu Majelis Hakim membebani Penggugat untuk membuktikan dlil-dalil gugatanya;

Disclaimer
Kepaniteraan Mahkamah Agung Republik Indonesia berusaha untuk selalu mencantumkan informasi paling kini dan akurat sebagai bentuk komitmen Mahkamah Agung untuk pelayanan publik, transparansi dan akuntabilitas pelaksanaan fungsi peradilan. Namun dalam hal-hal tertentu masih dimungkinkan terjadi permasalahan teknis terkait dengan akurasi dan keterkinian informasi yang kami sajikan, hal mana akan terus kami perbaiki dari waktu kewaktu.
Dalam hal Anda menemukan inakurasi informasi yang termuat pada situs ini atau informasi yang seharusnya ada, namun belum tersedia, maka harap segera hubungi Kepaniteraan Mahkamah Agung RI melalui :
Email : kepaniteraan@mahkamahagung.go.id Telp : 021-384 3348 (ext.318)

Menimbang, bahwa sebelum memeriksa pokok gugatanya Penggugat Pengadilan akan mempertimbangkan terlebih dhulu ikatan perkawinan Penggugat dan Tergugat;

Menimbang, untuk membuktikan dalil angka 1, Penggugat telah mengajukan alat bukti P.1;

Menimbang, bahwa berdasarkan keterangan Penggugat yang diperkuat oleh bukti tertulis P.1 berupa Fotokopi Kutipan Akta Nikah yang merupakan akta otentik, dan telah bermeterai cukup serta cocok dengan aslinya, isi bukti tersebut menjelaskan mengenai pernikahan antara Penggugat dengan Tergugat, sehingga bukti tersebut telah memenuhi syarat formal dan materiil, serta mempunyai kekuatan pembuktian yang sempurna dan mengikat, maka harus dinyatakan terbukti bahwa Penggugat dengan Tergugat telah terikat dalam perkawinan yang sah sebagaimana diatur dalam Pasal 2 Undang-Undang Nomor 1 Tahun 1974 yang telah di ubah dengan Undang-Undang Nomor 16 tahun 2019;

Menimbang, bahwa saksi pertama Penggugat, sudah dewasa dan sudah disumpah, sehingga memenuhi syarat formal sebagaimana diatur dalam Pasal 145 ayat (1) angka 3e HIR;

Menimbang, bahwa keterangan saksi pertama Penggugat adalah fakta yang relevan dengan dalil yang harus dibuktikan oleh Penggugat, oleh karena itu keterangan saksi tersebut telah memenuhi syarat materiil sebagaimana telah diatur dalam Pasal 171 HIR, sehingga keterangan saksi tersebut memiliki kekuatan pembuktian, dan dapat diterima sebagai alat bukti;

Menimbang, bahwa saksi kedua Penggugat, sudah dewasa dan sudah disumpah, sehingga memenuhi syarat formal sebagaimana diatur dalam Pasal 145 ayat (1) angka 3e HIR;

Menimbang, bahwa keterangan saksi kedua Penggugat adalah fakta yang relevan dengan dalil yang harus dibuktikan oleh Penggugat, oleh karena itu keterangan saksi tersebut telah memenuhi syarat materiil sebagaimana telah diatur dalam Pasal 171 HIR, sehingga keterangan saksi tersebut memiliki kekuatan pembuktian, dan dapat diterima sebagai alat bukti;

Disclaimer
Kepaniteraan Mahkamah Agung Republik Indonesia berusaha untuk selalu mencantumkan informasi paling kini dan akurat sebagai bentuk komitmen Mahkamah Agung untuk pelayanan publik, transparansi dan akuntabilitas pelaksanaan fungsi peradilan. Namun dalam hal-hal tertentu masih dimungkinkan terjadi permasalahan teknis terkait dengan akurasi dan keterkinian informasi yang kami sajikan, hal mana akan terus kami perbaiki dari waktu kewaktu.
Dalam hal Anda menemukan inakurasi informasi yang termuat pada situs ini atau informasi yang seharusnya ada, namun belum tersedia, maka harap segera hubungi Kepaniteraan Mahkamah Agung RI melalui :
Email : kepaniteraan@mahkamahagung.go.id   Telp : 021-384 3348 (ext.318)

Menimbang, bahwa keterangan saksi kesatu dan saksi kedua Penggugat bersesuaian dan cocok antara satu dengan yang lain, oleh karena itu keterangan dua orang saksi tersebut memenuhi Pasal 171 dan Pasal 172 HIR;

Menimbang, bahwa berdasarkan keterangan kedua orang saksi Penggugat, terbukti fakta kejadian sebagai berikut:

- Bahwa, Penggugat adalah isteri sah Tergugat, yang pernikahannya dilaksanakan pada tanggal 20 Agustus 2018, sebagaimana tercatat di Kantor Urusan Agama KUA Kecamatan Cibugel, Kabupaten Sumedang;
- Bahwa, selama berumah tangga sampai sekarang Penggugat dengan Tergugat sudah dikaruniai satu orang anak yang bernama Zhafiratul Hanna (berusia 1 tahun) ;;
- Bahwa, sejak bulan Juni tahun 2019 disebabkan karena faktor kekurangan ekonomi dimana nafkah yang diberikan oleh Tergugat tidak mencukupi untuk menutupi kebutuhan hidup sehari-hari
- Bahwa sekarang Penggugat dan tergugat nerpisah tempat tinggal
- Bahwa, saksi telah berusaha menasehati Penggugat agar tetap mempertahankan rumah tangganya dengan Tergugat tetapi tidak berhasil;

Menimbang, bahwa berdasarkan fakta-fakta tersebut di atas, dapat disimpulkan fakta hukum sebagai berikut:

- Bahwa sejak bulan Juni tahun 2019 Penggugat dan tergugat berselih dan bertengkar disebabkan karena faktor kekurangan ekonomi dimana nafkah yang diberikan oleh Tergugat tidak mencukupi untuk menutupi kebutuhan hidup sehari-hari.
- Bahwa, sejak bulan bulan November 2019 disebabkan karena faktor kekurangan ekonomi Penggugat dengan Tergugat telah pisah rumah;
- Bahwa, sifat dari perselisihan dan pertengkaran tersebut sudah sulit untuk dirukunkan lagi, karena Penggugat tetap pada pendiriannya untuk bercerai dengan Tergugat;

Disclaimer
Kepaniteraan Mahkamah Agung Republik Indonesia berusaha untuk selalu mencantumkan informasi paling kini dan akurat sebagai bentuk komitmen Mahkamah Agung untuk pelayanan publik, transparansi dan akuntabilitas pelaksanaan fungsi peradilan. Namun dalam hal-hal tertentu masih dimungkinkan terjadi permasalahan teknis terkait dengan akurasi dan keterkinian informasi yang kami sajikan, hal mana akan terus kami perbaiki dari waktu kewaktu.
Dalam hal Anda menemukan inakurasi informasi yang termuat pada situs ini atau informasi yang seharusnya ada, namun belum tersedia, maka harap segera hubungi Kepaniteraan Mahkamah Agung RI melalui :
Email : kepaniteraan@mahkamahagung.go.id Telp : 021-384 3348 (ext.318)

Menimbang, bahwa fakta hukum tersebut telah memenuhi norma hukum Islam yang terkandung dalam kaidah fiqhiyyah yang diambil alih menjadi pendapat Majelis yang berbunyi:

من دعي إلى حاكم من حكام المسلمين فلم يجب فهو ظالم لا حق له

Artinya: "Barang siapa yang dipanggil hakim muslim untuk menghadap dipersidangan, kemudian ia tidak menghadap, maka ia termasuk orang yang dholim, dan gugurlah haknya" (Ahkamul Qur'an Jilid II hal.405);

فإن تعزز بتعزز أو توار أو غيبة جاز إثباته بالبينة

Artinya: "Apabila Tergugat enggan, bersembunyi, atau dia ghoib, maka perkara itu boleh diputus berdasarkan bukti-bukti (persaksian)" (Al-Anwar jilid II hal. 55);

Menimbang, bahwa berdasarkan fakta-fakta tersebut di atas, Majelis Hakim berpendapat gugatan Penggugat telah memenuhi alasan perceraian sebagaimana diatur dalam Pasal39 Undang Undang Nomor 1 Tahun 1974 jis Pasal 19 haruf (f) Peraturan Pemerintah Nomor 9 tahun 1975 jo. Pasal 116 huruf (f) Kompilasi Hukum Islam;

Menimbang, bahwa berdasarkan pertimbangan-pertimbangan tersebut di atas, maka gugatan Penggugat yang meminta agar dijatuhkan talak satu ba'in shughra terhadap Penggugat beralasan untuk dikabulkan;

Menimbang, bahwa Karena perkara *aquo* termasuk bidang perkawinan, maka berdasarkan Pasal 89 ayat (1) Undang-Undang Nomor 7 Tahun 1989 tentang Peradilan Agama, sebagaimana telah diubah dengan Pasal 90 Undang-Undang Nomor 3 Tahun 2006, dan perubahan kedua dengan Pasal 91A Undang-Undang Nomor 50 Tahun 2009, biaya perkara harus dibebankan kepada Penggugat;

Disclaimer
Kepaniteraan Mahkamah Agung Republik Indonesia berusaha untuk selalu mencantumkan informasi paling kini dan akurat sebagai bentuk komitmen Mahkamah Agung untuk pelayanan publik, transparansi dan akuntabilitas pelaksanaan fungsi peradilan. Namun dalam hal-hal tertentu masih dimungkinkan terjadi permasalahan teknis terkait dengan akurasi dan keterkinian informasi yang kami sajikan, hal mana akan terus kami perbaiki dari waktu kewaktu.
Dalam hal Anda menemukan inakurasi informasi yang termuat pada situs ini atau informasi yang seharusnya ada, namun belum tersedia, maka harap segera hubungi Kepaniteraan Mahkamah Agung RI melalui :
Email : kepaniteraan@mahkamahagung.go.id Telp : 021-384 3348 (ext.318)

Mengingat, semua Pasal dalam peraturan perundang-undangan, dan hukum Islam yang berkaitan dengan perkara ini;

MENGADILI

1. Menyatakan Tergugat yang telah dipanggil secara resmi dan patut untuk menghadap di persidangan, tidak hadir;
2. Mengabulkan gugatan Penggugat dengan verstek;
3. Menjatuhkan talak satu Bain Sugro Tergugat (Rahmat Cahyana Bin Dayat) terhadap Penggugat (Rika Yuningsih Binti Nahrowi);
4. Membebankan kepada Penggugat untuk membayar biaya perkara ini sejumlah Rp421.000,00 (empat ratus dua puluh satu ribu rupiah);

Demikian diputuskan dalam rapat permusyawaratan Majelis Hakim yang dilangsungkan pada hari Selasa tanggal .22 Desember 2020 Masehi, bertepatan dengan tanggal 7 Jumadil awal 1442 Hijriyah, oleh kami Drs. H. Syamsul Falah, M.H. sebagai Ketua Majelis, Drs. Dimyati, SH, MH dan Drs. Solihudin, S.H, masing-masing sebagai Hakim Anggota, putusan tersebut diucapkan dalam sidang terbuka untuk umum pada hari itu juga, oleh Ketua Majelis tersebut dengan didampingi oleh Hakim Anggota dan dibantu oleh Drs. Harun Nur Rasyid sebagai Panitera Pengganti serta dihadiri oleh Penggugat tanpa kehadiran Tergugat;

Ketua Majelis

Drs. H. Syamsul Falah, M.H.

Disclaimer
Kepaniteraan Mahkamah Agung Republik Indonesia berusaha untuk selalu mencantumkan informasi paling kini dan akurat sebagai bentuk komitmen Mahkamah Agung untuk pelayanan publik, transparansi dan akuntabilitas pelaksanaan fungsi peradilan. Namun dalam hal-hal tertentu masih dimungkinkan terjadi permasalahan teknis terkait dengan akurasi dan keterkinian informasi yang kami sajikan, hal mana akan terus kami perbaiki dari waktu kewaktu.
Dalam hal Anda menemukan inakurasi informasi yang termuat pada situs ini atau informasi yang seharusnya ada, namun belum tersedia, maka harap segera hubungi Kepaniteraan Mahkamah Agung RI melalui :
Email : kepaniteraan@mahkamahagung.go.id Telp : 021-384 3348 (ext.318)

Hakim Anggota	Hakim Anggota
Drs. Dimyati, SH, MH	Drs. Solihudin, S.H

Panitera Pengganti

Drs. Harun Nur Rasyid

Perincian Biaya Perkara :

1.	Pendaftaran	: Rp. 30.000,-
2.	Proses	: Rp. 75.000,-
3.	Panggilan Tergugat	: Rp. 280.000,-
4.	PNBP	: Rp 20.000,-
5.	Redaksi	: Rp. 10.000,-
6.	Materai	: Rp. 6.000,-

Jumlah : Rp. 421.000,-

Disclaimer
Kepaniteraan Mahkamah Agung Republik Indonesia berusaha untuk selalu mencantumkan informasi paling kini dan akurat sebagai bentuk komitmen Mahkamah Agung untuk pelayanan publik, transparansi dan akuntabilitas pelaksanaan fungsi peradilan. Namun dalam hal-hal tertentu masih dimungkinkan terjadi permasalahan teknis terkait dengan akurasi dan keterkinian informasi yang kami sajikan, hal mana akan terus kami perbaiki dari waktu kewaktu.
Dalam hal Anda menemukan inakurasi informasi yang termuat pada situs ini atau informasi yang seharusnya ada, namun belum tersedia, maka harap segera hubungi Kepaniteraan Mahkamah Agung RI melalui :
Email : kepaniteraan@mahkamahagung.go.id Telp : 021-384 3348 (ext.318)
