# Supplementary material for: Classification of divorce causes during the COVID-19 pandemic using convolutional neural networks
Source: PeerJ Comput Sci. 2022 Jun 30;8:e998. doi: 10.7717/peerj-cs.998 (PMC9299239; doi:10.7717/peerj-cs.998)
Supplement: Supplemental Information 3 [file peerj-cs-08-998-s003.zip › KDRT Dataset/Data ke-9.pdf]

Direktori Putusan Mahkamah Agung Republik Indonesia
putusan.mahkamahagung.go.id

SALINAN

PUTUSAN

Nomor 2649/Pdt.G/2020/PA.JS

بِسْمِ اللهِ الرَّحْمَنِ الرَّحِيْمِ

DEMI KEADILAN BERDASARKAN KETUHANAN YANG MAHA ESA

Pengadilan Agama Jakarta Selatan yang memeriksa dan mengadili perkara tertentu pada tingkat pertama dalam sidang Majelis Hakim telah menjatuhkan putusan sebagai berikut dalam perkara Cerai Gugat antara:

PENGGUGAT, umur 35 tahun, agama Islam, pendidikan SLTA, pekerjaan Mengurus Rumah Tangga, tempat kediaman di --- Kota Jakarta Selatan, sebagai **Penggugat;**

melawan

TERGUGAT, umur 44 tahun, agama Islam, pendidikan SLTA, pekerjaan Dahulu Karyawan Swasta, tempat kediaman di ---Kota Jakarta Pusat Dan sekarang tidak diketahui dengan jelas alamat dan keberadaanya, baik didalam maupun di luar Wilayah Republik Indonesia (GHOIB), sebagai **Tergugat;**

Pengadilan Agama tersebut;
Telah membaca dan mempelajari berkas perkara;
Telah mendengar keterangan Penggugat;
Telah memeriksa alat-alat bukti Penggugat;

DUDUK PERKARA

Menimbang, bahwa Penggugat dengan surat gugatannya tertanggal 05 Agustus 2020 yang terdaftar di Kepaniteraan Pengadilan Agama Jakarta Selatan pada hari itu juga dengan register perkara Nomor XXXX/Pdt.G/2020/PA.JS, mengemukakan hal-hal sebagai berikut:

Disclaimer
Kepaniteraan Mahkamah Agung Republik Indonesia berusaha untuk selalu mencantumkan informasi paling kini dan akurat sebagai bentuk komitmen Mahkamah Agung untuk pelayanan publik, transparansi dan akuntabilitas pelaksanaan fungsi peradilan. Namun dalam hal-hal tertentu masih dimungkinkan terjadi permasalahan teknis terkait dengan akurasi dan keterkinian informasi yang kami sajikan, hal mana akan terus kami perbaiki dari waktu kewaktu.
Dalam hal Anda menemukan inakurasi informasi yang termuat pada situs ini atau informasi yang seharusnya ada, namun belum tersedia, maka harap segera hubungi Kepaniteraan Mahkamah Agung RI melalui :
Email : kepaniteraan@mahkamahagung.go.id Telp : 021-384 3348 (ext.318)

1. Bahwa, pada tanggal 21 Mei 2006 telah dilangsungkan perkawinan antara Penggugat dengan Tergugat yang dilaksanakan menurut hukum dan sesuai dengan tuntunan ajaran agama Islam. Perkawinan tersebut telah dicatatkan di Kantor Urusan Agama (KUA) Kecamatan Setia Budi Kota Jakarta Selatan, sebagaimana tercatat dalam kutipan Akta Nikah dengan Nomor : XXXX/88/V/2006 Tertanggal 22 Mei 2006;

2. Bahwa perkawinan antara Penggugat dan Tergugat dilangsungkan berdasarkan kehendak kedua belah pihak dengan tujuan membentuk rumah tangga yang sakinah, mawaddah, warahmah yang diridhoi oleh Allah Swt;

3. Bahwa setelah menikah, Penggugat dan Tergugat tinggal terakhir bersama di --Kota Jakarta Pusat dan selama Pernikahan Penggugat dan Tergugat telah dikaruniai 2 (dua) orang anak yang bernama:

3.1. ANAK I PENGGUGAT DAN TERGUGAT, Laki laki , Jakarta, 15 Oktober 2006;

3.2. ANAK II PENGGUGAT DAN TERGUGAT , Perempuan , Jakarta, 02 Juli 2010

4. Bahwa kehidupan rumah tangga Penggugat dan Tergugat mulai goyah dan terjadi pertengkaran dan perselisihan kurang lebih sejak tahun 2014 yang penyebabnya antara lain:

4.1. Tergugat tidak bertanggungjawab dalam memberikan nafkah lahir dan batin kepada Penggugat;

4.2. Tergugat tidak jujur dalam masalah keuangan kepada Penggugat;

4.3. Tergugat tidak perhatian dan kasih sayang kepada Penggugat dan anak;

4.4. Tergugat jarang pulang dalam jangka waktu Berhari- hari tanpa alasan yang jelas;

4.5. Tergugat diketahui memiliki wanita idaman lain yang dimana sering berkomunikasi secara terus menerus;

Disclaimer
Kepaniteraan Mahkamah Agung Republik Indonesia berusaha untuk selalu mencantumkan informasi paling kini dan akurat sebagai bentuk komitmen Mahkamah Agung untuk pelayanan publik, transparansi dan akuntabilitas pelaksanaan fungsi peradilan. Namun dalam hal-hal tertentu masih dimungkinkan terjadi permasalahan teknis terkait dengan akurasi dan keterkinian informasi yang kami sajikan, hal mana akan terus kami perbaiki dari waktu kewaktu.
Dalam hal Anda menemukan inakurasi informasi yang termuat pada situs ini atau informasi yang seharusnya ada, namun belum tersedia, maka harap segera hubungi Kepaniteraan Mahkamah Agung RI melalui :
Email : kepaniteraan@mahkamahagung.go.id Telp : 021-384 3348 (ext.318)

5. Bahwa, puncak kekecewaan Penggugat terjadi pada Bulan Juli 2015 Tergugat pergi meninggalkan rumah kediaman bersama tanpa sebab dan Tergugat sudah tidak kembali dan Tergugat sudah tidak diketahui keberadaannya diluar maupun diseluruh wilayah Indonesia (GHOIB),

6. Bahwa Penggugat dan keluarga telah berusaha semaksimal mungkin untuk mencari dimana keberadaan Tergugat, namun sampai saat ini tidak diketahui keberadaanya, baik didalam maupun diluar wilayah republik Indonesia (GHOIB) sesuai dengan surat keterangan GHOIB yang dikeluarkan oleh Kelurahan Menteng Kecamatan Menteng Kota Jakarta Pusat dengan Nomor: XXXX/1.755.6/2020 yang di keluarkan pada tanggal 04 Agustus 2020;

7. Bahwa dengan bukti-bukti diatas Penggugat dengan Tergugat sudah tidak dapat melanjutkan hubungan rumah tangga yang sakinah mawaddah warrahmah, sehingga menempuh jalur perceraian;

8. Bahwa Penggugat sanggup membayar seluruh biaya yang timbul akibat perkara ini;

Berdasarkan dalil dan alasan-alasan tersebut diatas, maka dengan ini Penggugat memohon kepada Ketua Pengadilan Agama Jakarta selatan cq. Majelis Hakim yang memeriksa dan mengadili perkara ini untuk dapat menentukan hari persidangan, kemudian memanggil Penggugat dan Tergugat untuk diperiksa dan diadili, selanjutnya memberikan putusan yang amarnya sebagai berikut:

PRIMER:

1. Menerima dan Mengabulkan Gugatan Penggugat;
2. Menjatuhkan thalaq satu bain syughro dari Tergugat (TERGUGAT) kepada Penggugat (PENGGUGAT);
3. Menetapkan Biaya Perkara ini sesuai dengan peraturan perundang – undangan yang berlaku;

SUBSIDER:

Atau apabila Pengadilan Agama berpendapat lain mohon putusan yang seadil-adilnya (*ex aequo et bono*);

Disclaimer
Kepaniteraan Mahkamah Agung Republik Indonesia berusaha untuk selalu mencantumkan informasi paling kini dan akurat sebagai bentuk komitmen Mahkamah Agung untuk pelayanan publik, transparansi dan akuntabilitas pelaksanaan fungsi peradilan. Namun dalam hal-hal tertentu masih dimungkinkan terjadi permasalahan teknis terkait dengan akurasi dan keterkinian informasi yang kami sajikan, hal mana akan terus kami perbaiki dari waktu kewaktu.
Dalam hal Anda menemukan inakurasi informasi yang termuat pada situs ini atau informasi yang seharusnya ada, namun belum tersedia, maka harap segera hubungi Kepaniteraan Mahkamah Agung RI melalui :
Email : kepaniteraan@mahkamahagung.go.id Telp : 021-384 3348 (ext.318)

Bahwa pada hari persidangan yang telah ditetapkan, Penggugat telah hadir di persidangan, sedangkan Tergugat tidak hadir dan tidak menyuruh orang lain untuk hadir sebagai wakilnya, meskipun menurut Surat Panggilan (Relaas) yang dibacakan di persidangan, Tergugat telah dipanggil secara resmi dan patut, sedang tidak ternyata bahwa tidak hadirnya itu disebabkan oleh suatu halangan yang sah;

Bahwa Majelis hakim telah berusaha menasehati Penggugat untuk rukun kembali membina rumah tangga bersama dengan Tergugat, namun Penggugat tetap pada pendiriannya untuk bercerai dengan Tergugat;

Bahwa perkara ini tidak dilaksanakan upaya mediasi karena Tergugat tidak pernah datang menghadap meskipun telah dipanggil secara resmi dan patut, selanjutnya dibacakan gugatan Penggugat tanpa ada perubahan dan isinya tetap dipertahankan oleh Penggugat;

Bahwa meskipun Tergugat tidak pernah hadir di persidangan dan tidak menggunakan hak jawabnya, akan tetapi karena perkara ini adalah perkara perceraian yang berlaku aturan khusus maka Penggugat dibebani wajib bukti;

Bahwa untuk membuktikan dalil gugatannya, Penggugat mengajukan bukti sebagai berikut :

A. Bukti Surat

1. Fotokopi Kartu Tanda Penduduk Penggugat. Bukti surat tersebut telah diberi materai cukup dan telah dicocokkan dengan aslinya yang ternyata sesuai. (Bukti P.1.);
2. Fotokopi Kutipan Akta Nikah Nomor XXX/88/V/2006 yang dikeluarkan KUA Kecamatan Setia Budi Kota Jakarta Selatan tanggal 22 Mei 2006. Bukti surat tersebut telah diberi materai cukup dan telah dicocokkan dengan aslinya yang ternyata sesuai. (Bukti P.2.);

B. Saksi-saksi :

Saksi 1, SAKSI I, umur 49 tahun, pekerjaan Ibu Rumah Tangga, tempat kediaman di -- Jakarta Selatan, di bawah sumpah memberikan keterangan yang pada pokoknya sebagai berikut :

Disclaimer
Kepaniteraan Mahkamah Agung Republik Indonesia berusaha untuk selalu mencantumkan informasi paling kini dan akurat sebagai bentuk komitmen Mahkamah Agung untuk pelayanan publik, transparansi dan akuntabilitas pelaksanaan fungsi peradilan. Namun dalam hal-hal tertentu masih dimungkinkan terjadi permasalahan teknis terkait dengan akurasi dan keterkinian informasi yang kami sajikan, hal mana akan terus kami perbaiki dari waktu kewaktu.
Dalam hal Anda menemukan inakurasi informasi yang termuat pada situs ini atau informasi yang seharusnya ada, namun belum tersedia, maka harap segera hubungi Kepaniteraan Mahkamah Agung RI melalui :
Email : kepaniteraan@mahkamahagung.go.id   Telp : 021-384 3348 (ext.318)

- Bahwa saksi kenal dengan Penggugat dan Tergugat karena saksi adalah Kakak Penggugat;
- Bahwa Penggugat dan Tergugat adalah pasangan suami isteri yang sah;
- Bahwa selama pernikahan Penggugat dan Tergugat telah dikaruniai 2 (dua) orang anak;
- Bahwa sepengetahuan saksi sejak tahun 2014 kehidupan rumah tangga Penggugat dan Tergugat sering terjadi perselisihan dan pertengkaran yang terus menerus;
- Bahwa penyebab perselisihan tersebut karena Tergugat tidak bertanggung jawab dalam memberikan nafkah lahir kepada Penggugat;
- Bahwa Penggugat dan Tergugat sejak bulan Juli 2015 dan sejak saat itu sudah tidak menjalankan kewajiban masing-masing sebagai suami istri;
- Bahwa keluarga sudah berusaha mendamaikan, namun tidak berhasil;

Saksi 2, SAKSI II, umur 37 tahun, pekerjaan Ibu Rumah Tangga, tempat kediaman di -- Jakarta Selatan, di bawah sumpah memberikan keterangan yang pada pokoknya sebagai berikut :

- Bahwa saksi kenal dengan Penggugat dan Tergugat karena saksi adalah Sepupu Penggugat;
- Bahwa Penggugat dan Tergugat adalah pasangan suami isteri yang sah;
- Bahwa selama pernikahan Penggugat dan Tergugat telah dikaruniai 2 (dua) orang anak;
- Bahwa sepengetahuan saksi sejak tahun 2014 kehidupan rumah tangga Penggugat dan Tergugat sering terjadi perselisihan dan pertengkaran yang terus menerus;
- Bahwa penyebab perselisihan tersebut karena Tergugat telah memiliki wanita idaman lain (WIL);

Disclaimer
Kepaniteraan Mahkamah Agung Republik Indonesia berusaha untuk selalu mencantumkan informasi paling kini dan akurat sebagai bentuk komitmen Mahkamah Agung untuk pelayanan publik, transparansi dan akuntabilitas pelaksanaan fungsi peradilan. Namun dalam hal-hal tertentu masih dimungkinkan terjadi permasalahan teknis terkait dengan akurasi dan keterkinian informasi yang kami sajikan, hal mana akan terus kami perbaiki dari waktu kewaktu.
Dalam hal Anda menemukan inakurasi informasi yang termuat pada situs ini atau informasi yang seharusnya ada, namun belum tersedia, maka harap segera hubungi Kepaniteraan Mahkamah Agung RI melalui :
Email : kepaniteraan@mahkamahagung.go.id Telp : 021-384 3348 (ext.318)

- Bahwa Penggugat dan Tergugat sejak bulan Juli 2015 dan sejak saat itu sudah tidak menjalankan kewajiban masing-masing sebagai suami istri;
- Bahwa keluarga sudah berusaha mendamaikan, namun tidak berhasil;

Bahwa Penggugat telah mengajukan kesimpulan yang pada pokoknya tetap dengan dalil-dalil gugatannya dan akhirnya mohon putusan;

Bahwa untuk mempersingkat uraian putusan ini, segala sesuatu yang berlangsung dalam persidangan dapat dilihat dalam Berita Acara Sidang (BAS) yang dianggap telah termasuk dan merupakan bagian yang tak terpisahkan dari putusan ini;

PERTIMBANGAN HUKUM

Menimbang, bahwa maksud dan tujuan gugatan Penggugat adalah sebagaimana telah diuraikan di atas;

Menimbang, bahwa majelis hakim telah berupaya menasehati Penggugat agar rukun kembali membina rumah tangga dengan Tergugat, namun upaya tersebut tidak berhasil;

Menimbang, bahwa ternyata Tergugat tidak datang menghadap di persidangan dan tidak pula menyuruh orang lain untuk menghadap sebagai wakil/kuasanya yang sah, meskipun berdasarkan Surat Panggilan (relaas) yang dibacakan di persidangan, Tergugat telah dipanggil secara resmi dan patut, sedangkan tidak ternyata bahwa tidak datangnya Tergugat tersebut disebabkan oleh suatu alasan yang sah;

Menimbang, bahwa oleh karena selama persidangan hanya Penggugat yang hadir sedangkan Tergugat tidak pernah hadir, maka upaya mediasi sebagaimana ketentuan Peraturan Mahkamah Agung RI Nomor 1 Tahun 2016 tentang Prosedur Mediasi di Pengadilan, tidak dapat dilaksanakan;

Menimbang, bahwa yang menjadi alasan pokok diajukannya gugatan perceraian ini adalah bahwa rumah tangga Penggugat dan Tergugat awalnya rukun dan harmonis namun sejak tahun 2014 sering terjadi perselisihan dan percekcokan yang terus menerus yang disebabkan hal-hal sebagaimana dalam surat gugatannya, sehingga antara Penggugat dengan Tergugat sejak bulan

Disclaimer
Kepaniteraan Mahkamah Agung Republik Indonesia berusaha untuk selalu mencantumkan informasi paling kini dan akurat sebagai bentuk komitmen Mahkamah Agung untuk pelayanan publik, transparansi dan akuntabilitas pelaksanaan fungsi peradilan. Namun dalam hal-hal tertentu masih dimungkinkan terjadi permasalahan teknis terkait dengan akurasi dan keterkinian informasi yang kami sajikan, hal mana akan terus kami perbaiki dari waktu kewaktu.
Dalam hal Anda menemukan inakurasi informasi yang termuat pada situs ini atau informasi yang seharusnya ada, namun belum tersedia, maka harap segera hubungi Kepaniteraan Mahkamah Agung RI melalui :
Email : kepaniteraan@mahkamahagung.go.id   Telp : 021-384 3348 (ext.318)

Juli 2015 hingga sekarang dan Penggugat merasakan hidup berumah tangga dengan Tergugat sudah tidak dapat dipertahankan lagi;

Menimbang, bahwa terhadap dalil-dalil gugatan Penggugat tersebut, Tergugat tidak membantah atau membenarkannya, karena Tergugat tidak pernah hadir atau menyuruh orang lain hadir sebagai wakilnya meskipun telah dipanggil secara resmi dan patut. Oleh karenanya majelis hakim berpendapat Tergugat telah mengakui kebenaran dalil-dalil gugatan Penggugat, sehingga dengan demikian putusan ini dapat dijatuhkan dengan tanpa hadirnya Tergugat;

Menimbang, bahwa meskipun dengan ketidakhadiran Tergugat dianggap telah mengakui dalil-dalil gugatan Penggugat, namun karena perkara ini khusus mengenai perkawinan dalam hal perceraian, maka Penggugat tetap dibebankan untuk mengajukan bukti-buktinya di depan persidangan;

Menimbang, bahwa untuk membuktikan dalil-dalil gugatannya Penggugat mengajukan bukti P.1 dan P.2 serta 2 (dua) orang saksi;

Menimbang, bahwa berdasarkan bukti P.1, terbukti domisili tempat kediaman Penggugat, sehingga bukti tersebut telah memenuhi syarat formil dan materiil, serta mempunyai kekuatan pembuktian yang sempurna dan mengikat;

Menimbang, bahwa berdasarkan bukti P.2 (Kutipan Akta Nikah), surat yang dikeluarkan oleh pejabat yang berwenang berdasarkan Undang-Undang, bermaterai cukup dan cocok dengan aslinya, oleh karena itu bukti tersebut merupakan bukti autentik yang memenuhi syarat formil dan materil, sehingga bukti tersebut dapat diterima dan mengikat;

Menimbang, bahwa berdasarkan bukti P.2, maka Penggugat dan Tergugat harus dinyatakan terbukti sebagai suami istri yang sah. Dengan demikian Penggugat memiliki *legal standing* untuk mengajukan gugatan cerai sebagaimana diatur dalam Pasal 14 Peraturan Pemerintah Nomor 9 Tahun 1975 Tentang Peraturan Pelaksanaan Undang-undang Nomor 1 Tahun 1974 Tentang Perkawinan jo. Pasal 73 Undang-Undang Nomor 7 Tahun 1989 Tentang Peradilan Agama yang telah diubah dan ditambah dengan Undang-Undang Nomor 3 Tahun 2006 dan Undang-Undang No. 50 Tahun 2009;

Menimbang, bahwa Penggugat juga telah mengajukan saksi-saksi yang keduanya telah memberikan keterangan sebagaimana telah diuraikan dalam

Disclaimer
Kepaniteraan Mahkamah Agung Republik Indonesia berusaha untuk selalu mencantumkan informasi paling kini dan akurat sebagai bentuk komitmen Mahkamah Agung untuk pelayanan publik, transparansi dan akuntabilitas pelaksanaan fungsi peradilan. Namun dalam hal-hal tertentu masih dimungkinkan terjadi permasalahan teknis terkait dengan akurasi dan keterkinian informasi yang kami sajikan, hal mana akan terus kami perbaiki dari waktu kewaktu.
Dalam hal Anda menemukan inakurasi informasi yang termuat pada situs ini atau informasi yang seharusnya ada, namun belum tersedia, maka harap segera hubungi Kepaniteraan Mahkamah Agung RI melalui :
Email : kepaniteraan@mahkamahagung.go.id   Telp : 021-384 3348 (ext.318)

duduk perkara dan kedua saksi tersebut adalah orang dewasa yang memberikan keterangan secara terpisah di bawah sumpah di muka sidang, keterangan kedua saksi secara materil saling bersesuaian satu sama lain dan relevan dengan dalil-dalil yang hendak dibuktikan oleh Penggugat serta tidak ada halangan diterimanya kesaksian para saksi tersebut, maka kedua saksi tersebut telah memenuhi syarat formil dan materil sehingga keterangan kedua saksi tersebut memiliki kekuatan pembuktian yang dapat diterima;

Menimbang, bahwa berdasarkan keterangan Penggugat, bukti tertulis dan keterangan para saksi, Majelis Hakim telah menemukan fakta-fakta hukum yang disimpulkan sebagai berikut:

- Bahwa Penggugat dan Tergugat adalah pasangan suami isteri yang menikah pada tanggal 21 Mei 2006;
- Bahwa sejak tahun 2014 antara Penggugat dengan Tergugat sering terjadi perselisihan dan pertengkaran yang disebabkan oleh karena Tergugat tidak bertanggung jawab dalam memberikan nafkah lahir kepada Penggugat dan Tergugat telah memiliki wanita idaman lain (WIL);
- Bahwa antara Penggugat dan Tergugat sudah pisah sejak bulan Juli 2015 dan sudah tidak menjalankan kewajiban masing-masing sebagai suami istri;
- Bahwa pihak keluarga sudah berusaha mendamaikan namun tidak berhasil.

Menimbang, bahwa dari fakta-fakta yang diuraikan di atas majelis hakim berkeyakinan bahwa rumah tangga Penggugat dengan Tergugat sudah dapat dikatakan pecah dan sangat sulit untuk dirukunkan kembali;

Menimbang, bahwa berdasarkan fakta hukum diatas, telah terbukti bahwa rumah tangga Penggugat dan Tergugat telah terjadi perselisihan dan pertengkaran dan sudah tidak menjalankan kewajiban sebagaimana layaknya suami istri sejak tahun 2014 hingga sekarang, hal ini telah menunjukkan bahwa rumah tangga yang seperti itu tidak lagi dapat dikatakan rumah tangga yang bahagia yang dilandasi oleh adanya mawaddah dan rahmah, sehingga majelis hakim berkesimpulan bahwa Penggugat dan Tergugat telah gagal untuk

Disclaimer
Kepaniteraan Mahkamah Agung Republik Indonesia berusaha untuk selalu mencantumkan informasi paling kini dan akurat sebagai bentuk komitmen Mahkamah Agung untuk pelayanan publik, transparansi dan akuntabilitas pelaksanaan fungsi peradilan. Namun dalam hal-hal tertentu masih dimungkinkan terjadi permasalahan teknis terkait dengan akurasi dan keterkinian informasi yang kami sajikan, hal mana akan terus kami perbaiki dari waktu kewaktu.
Dalam hal Anda menemukan inakurasi informasi yang termuat pada situs ini atau informasi yang seharusnya ada, namun belum tersedia, maka harap segera hubungi Kepaniteraan Mahkamah Agung RI melalui :
Email : kepaniteraan@mahkamahagung.go.id   Telp : 021-384 3348 (ext.318)

membentuk rumah tangga yang bahagia dan kekal dan tidak ada harapan lagi untuk rukun kembali;

Menimbang, bahwa hal lain yang menjadikan majelis hakim yakin bahwa rumah tangga Penggugat dan Tergugat tidak ada harapan untuk rukun kembali adalah sikap pihak Penggugat yang tetap bertekad hendak bercerai dengan Tergugat sekalipun majelis telah berupaya menasehati Penggugat agar rukun kembali dengan Tergugat, begitu juga sikap saksi yang telah berupaya untuk menasehati Penggugat namun tidak berhasil;

Menimbang, bahwa perkawinan itu adalah ikatan lahir-bathin antara suami istri dengan tujuan untuk membentuk rumah tangga yang sakinah (vide Pasal 1 Undang-undang Nomor 1 Tahun 1974 Tentang Perkawinan), sehingga apabila salah satu pihak (*ic.* Penggugat) sudah menyatakan tekadnya untuk tidak mempertahankan perkawinannya dan sudah meminta cerai, maka disini sudah ada petunjuk bahwa antara suami isteri (*ic*. Penggugat dan Tergugat) tersebut sudah tidak ada ikatan lahir dan bathin lagi, sehingga perkawinan yang seperti itu sudah dapat dikatakan telah rapuh dan tidak akan dapat lagi mewujudkan rumah tangga yang sakinah sebagaimana tujuan dari perkawinan itu sendiri;

Menimbang, bahwa perceraian itu sedapat mungkin untuk dapat dihindari, namun apabila kondisi sebuah keluarga sudah sebagaimana yang diuraikan di atas, maka mempertahankannya adalah suatu usaha yang sia-sia saja dan bahkan akan membawa mafsadat bagi kedua belah pihak;

Menimbang, bahwa menghindari terjadinya mafsadat dalam rumah tangga harus lebih diutamakan dari pada mendatangkan kemaslahatan sesuai qaidah Fiqhiyyah yang berbunyi :

درء المفاسد مقدم على جلب المصالح

Artinya : *Menolak mafsadat lebih diutamakan untuk menjaga kemaslahatan*;

Menimbang, bahwa dari apa yang diuraikan di atas, maka majelis berpendapat pintu perceraian dapat dibuka guna menghindarkan para pihak dari kemelut rumah tangga yang berkepanjangan;

Disclaimer
Kepaniteraan Mahkamah Agung Republik Indonesia berusaha untuk selalu mencantumkan informasi paling kini dan akurat sebagai bentuk komitmen Mahkamah Agung untuk pelayanan publik, transparansi dan akuntabilitas pelaksanaan fungsi peradilan. Namun dalam hal-hal tertentu masih dimungkinkan terjadi permasalahan teknis terkait dengan akurasi dan keterkinian informasi yang kami sajikan, hal mana akan terus kami perbaiki dari waktu kewaktu.
Dalam hal Anda menemukan inakurasi informasi yang termuat pada situs ini atau informasi yang seharusnya ada, namun belum tersedia, maka harap segera hubungi Kepaniteraan Mahkamah Agung RI melalui :
Email : kepaniteraan@mahkamahagung.go.id   Telp : 021-384 3348 (ext.318)

Menimbang, bahwa berdasarkan pertimbangan-pertimbangan hukum tersebut di atas, maka majelis hakim berkesimpulan bahwa alasan perceraian yang diajukan oleh Penggugat telah terbukti dan telah memenuhi unsur Pasal 39 Undang-Undang Nomor 1 Tahun 1974 Tentang Perkawinan Jo. Pasal 19 huruf (f), Peraturan Pemerintah Nomor 9 Tahun 1975 Tentang Peraturan Pelaksanaan Undang-undang Nomor 1 Tahun 1974 Tentang Perkawinan Jo. Pasal 116 huruf (f) Kompilasi Hukum Islam, maka gugatan Penggugat tersebut beralasan dan tidak melawan hukum;

Menimbang, bahwa oleh karena Tergugat sekalipun telah dipanggil secara resmi dan patut untuk menghadap dipersidangan tidak hadir, dan ketidak hadirannya tersebut bukan disebabkan oleh suatu halangan yang sah, maka berdasarkan ketentuan Pasal 125 HIR, gugatan Penggugat untuk dapat diceraikan dari Tergugat tersebut patut untuk dikabulkan dengan verstek dengan menjatuhkan talak satu ba'in sughra dari Tergugat terhadap Penggugat;

Menimbang, bahwa oleh karena perkara tersebut dalam bidang perkawinan, maka berdasarkan ketentuan Pasal 89 ayat (1) Undang-Undang Nomor 7 Tahun 1989 Tentang Peradilan Agama sebagaimana yang telah diubah dan ditambah dengan Undang-Undang Nomor 3 Tahun 2006, dan perubahan kedua dengan Undang-Undang Nomor 50 Tahun 2009, maka Penggugat dibebankan untuk membayar biaya perkara;

Memperhatikan, pasal-pasal dari peraturan Perundang-undangan yang berlaku serta dalil-dalil yang berkaitan dengan perkara ini;

MENGADILI

1. Menyatakan Tergugat yang telah dipanggil secara resmi dan patut untuk menghadap di persidangan, tidak hadir;

2. Mengabulkan gugatan Penggugat dengan verstek;

3. Menjatuhkan talak satu ba'in shughra Tergugat (**TERGUGAT**) terhadap Penggugat (**PENGGUGAT**);

4. Membebankan kepada Penggugat untuk membayar biaya perkara hingga putusan ini diucapkan sejumlah **Rp. 541.000,00** (lima ratus empat puluh satu ribu rupiah).

Disclaimer
Kepaniteraan Mahkamah Agung Republik Indonesia berusaha untuk selalu mencantumkan informasi paling kini dan akurat sebagai bentuk komitmen Mahkamah Agung untuk pelayanan publik, transparansi dan akuntabilitas pelaksanaan fungsi peradilan. Namun dalam hal-hal tertentu masih dimungkinkan terjadi permasalahan teknis terkait dengan akurasi dan keterkinian informasi yang kami sajikan, hal mana akan terus kami perbaiki dari waktu kewaktu.
Dalam hal Anda menemukan inakurasi informasi yang termuat pada situs ini atau informasi yang seharusnya ada, namun belum tersedia, maka harap segera hubungi Kepaniteraan Mahkamah Agung RI melalui :
Email : kepaniteraan@mahkamahagung.go.id   Telp : 021-384 3348 (ext.318)

Demikian diputuskan dalam musyawarah Majelis Hakim Pengadilan Agama Jakarta Selatan pada hari Rabu tanggal 23 Desember 2020 Masehi bertepatan dengan tanggal 8 Jumadil Awwal 1442 Hijriah oleh **Drs. H. Haryadi Hasan, M.H.** sebagai Ketua Majelis, **Drs. Eko Budiono, S.H., M.H.** dan **H. S. Shalahuddin, S.H., M.H.**, masing-masing sebagai Hakim Anggota, putusan tersebut diucapkan pada hari itu juga dalam sidang terbuka untuk umum oleh Ketua Majelis beserta para Hakim Anggota tersebut dan dibantu oleh **Rohimah, S.H., M.H.** sebagai Panitera Pengganti dengan dihadiri oleh Penggugat tanpa hadirnya Tergugat.

Hakim Anggota,	Ketua Majelis,
Ttd	Ttd
**Drs. Eko Budiono, S.H., M.H.**	**Drs. H. Haryadi Hasan, M.H.**
**Hakim Anggota,**	
**Ttd**	
**H. S. Shalahuddin, S.H., M.H.**	
	**Panitera Pengganti,**
	**Ttd**
	**Rohimah, S.H., M.H.**

Perincian biaya :

1.	PNBP	Rp	50.000,00
2.	Biaya Proses	Rp	75.000,00
3.	Panggilan	Rp	400.000,00
4.	Redaksi	Rp	10.000,00
5.	Meterai	Rp	6.000,00
	**Jumlah**	Rp	**541.000,00** (lima ratus empat puluh satu ribu rupiah)

Untuk Salinan Sesuai Dengan Aslinya,
Panitera Pengadilan Agama Jakarta Selatan,

Saiful Bahry, S.H., M.H.

Disclaimer
Kepaniteraan Mahkamah Agung Republik Indonesia berusaha untuk selalu mencantumkan informasi paling kini dan akurat sebagai bentuk komitmen Mahkamah Agung untuk pelayanan publik, transparansi dan akuntabilitas pelaksanaan fungsi peradilan. Namun dalam hal-hal tertentu masih dimungkinkan terjadi permasalahan teknis terkait dengan akurasi dan keterkinian informasi yang kami sajikan, hal mana akan terus kami perbaiki dari waktu kewaktu.
Dalam hal Anda menemukan inakurasi informasi yang termuat pada situs ini atau informasi yang seharusnya ada, namun belum tersedia, maka harap segera hubungi Kepaniteraan Mahkamah Agung RI melalui :
Email : kepaniteraan@mahkamahagung.go.id Telp : 021-384 3348 (ext.318)
