# Supplementary material for: Classification of divorce causes during the COVID-19 pandemic using convolutional neural networks
Source: PeerJ Comput Sci. 2022 Jun 30;8:e998. doi: 10.7717/peerj-cs.998 (PMC9299239; doi:10.7717/peerj-cs.998)
Supplement: Supplemental Information 4 [file peerj-cs-08-998-s004.zip › Konflik Dataset/Data ke-1.pdf]

P U T U S A N

Nomor xxxx/Pdt.G/2020/PA.Dmk.

بسم الله الرحمن الرحيم

DEMI KEADILAN BERDASARKAN KETUHANAN YANG MAHA ESA

Pengadilan Agama Demak yang memeriksa dan mengadili perkara-perkara tertentu dalam tingkat pertama telah menjatuhkan putusan sebagai berikut dalam perkara Cerai Talak antara :

Xxxx, umur 53 tahun, agama Islam, pendidikan SD, pekerjaan Swasta, bertempat tinggal di Dusun Tanubayan, RT.06, RW.09, Kelurahan Bintoro, Kecamatan Demak, Kabupaten Demak, sebagai Pemohon;

melawan

Xxxx, umur 44 tahun, agama Islam, pendidikan SD, pekerjaan Mengurus Rumah Tangga, bertempat tinggal di Dusun Klawean, RT.01, RW.02, Desa Botorejo, Kecamatan Wonosalam, Kabupaten Demak, sebagai Termohon;

Pengadilan Agama tersebut;

Telah membaca surat-surat perkara ;

Telah mendengar Pemohon dan saksi-saksi;

DUDUK PERKARA

Bahwa Pemohon dalam surat permohonannya tertanggal 08 Desember 2020, terdaftar di Kepaniteraan Pengadilan Agama Demak pada tanggal 08 Desember 2020 Nomor xxxx/Pdt.G/2020/PA.Dmk. telah mengemukakan hal-hal sebagai berikut :

1. Bahwa Pemoho (berstatus duda mati) dengan Termohon (berstatus janda cerai) telah melangsungkan pernikahan pada tanggal 24 Juli 2016, yang dicatat oleh Pegawai Pencatat Nikah Kantor Urusan Agama Kecamatan

Disclaimer
Kepaniteraan Mahkamah Agung Republik Indonesia berusaha untuk selalu mencantumkan informasi paling kini dan akurat sebagai bentuk komitmen Mahkamah Agung untuk pelayanan publik, transparansi dan akuntabilitas pelaksanaan fungsi peradilan. Namun dalam hal-hal tertentu masih dimungkinkan terjadi permasalahan teknis terkait dengan akurasi dan keterkinian informasi yang kami sajikan, hal mana akan terus kami perbaiki dari waktu kewaktu.
Dalam hal Anda menemukan inakurasi informasi yang termuat pada situs ini atau informasi yang seharusnya ada, namun belum tersedia, maka harap segera hubungi Kepaniteraan Mahkamah Agung RI melalui :
Email : kepaniteraan@mahkamahagung.go.id Telp : 021-384 3348 (ext.318)

Wonoslam, Kabupaten Demak sebagaimana Kutipan Akta Nikah Nomor: 0378/115/VII/2016, tanggal 24 Juli 2016;

2. Bahwa setelah menikah, Pemohon dengan Termohon bertempat tinggal di rumah Termohon selama 1 hari, lalu tinggal di rumh Pemohon selama 4 tahun, sudah melakukan hubungan layaknya suami istri (ba'da dukhul), namun belum dikaruniia keturúrnan;
3. Bahwa sejak bulan Desember 2019, antara Pemohon dan Termohon sering terjadi perselisihan dan pertengkaran yang disebabkan oleh masalah:
   a. anak bawaan Termohon tidak suka dan tidak menghormati Pemohon sebagai ayah tirinya, serta sering berkata-kata yang tidak sepantasnya kepada Pemohon. Bila antara Pemohon dan anak bawaan Termohon bertengkar, Termohon selalu membela anak bawaan Termohon;
   b. Termohon sering keluar rumah tanpa izin Pemohon, kemudian pulang ke rumah Termohon;

   Akhirnya menjadikan perselisihan dan pertengkaran antara Pemohon dan Termohon semakin memuncak;
4. Bahwa akibatnya, sejak tanggal 24 Juli 2020, tanpa pamit Pemohon, Termohon pulang ke rumah Termohon hingga sekarang sudah ± 5 bulan lamanya. Selama pisah rumah tersebut, antara Pemohon dan Termohon sudah tidak pernah melakukan hubungan layaknya suami istri dan tidak ada komunikasi. Adapun Pemohon pada tanggal 26 Juli 2020 pernah datang sekali ke rumah Termohon untuk bertemu dengan anak bawaan Termohon serta mengajak Termohon agar mau kembali lagi kepada Pemohon dan rukun lagi dalam rumah tangga dengan Pemohon, tetapi Termohon menolak;
5. Bahwa berdasarkan hal-hal tersebut diatas, permohonan Pemohon untuk mengajukan permohonan cerai talak terhadap Termohon atas dasar perselisihan dan pertengkaran yang terjadi terus-menerus dan tidak mungkin hidup rukun dalam suatu ikatan perkawinan, telah memenuhi unsur pasal 19 huruf (f) Peraturan Pemerintah No. 9 Tahun 1975 Jo. Pasal

Disclaimer
Kepaniteraan Mahkamah Agung Republik Indonesia berusaha untuk selalu mencantumkan informasi paling kini dan akurat sebagai bentuk komitmen Mahkamah Agung untuk pelayanan publik, transparansi dan akuntabilitas pelaksanaan fungsi peradilan. Namun dalam hal-hal tertentu masih dimungkinkan terjadi permasalahan teknis terkait dengan akurasi dan keterkinian informasi yang kami sajikan, hal mana akan terus kami perbaiki dari waktu kewaktu.
Dalam hal Anda menemukan inakurasi informasi yang termuat pada situs ini atau informasi yang seharusnya ada, namun belum tersedia, maka harap segera hubungi Kepaniteraan Mahkamah Agung RI melalui :
Email : kepaniteraan@mahkamahagung.go.id   Telp : 021-384 3348 (ext.318)

116 huruf (f) Kompilasi Hukum Islam, sehingga berdasar hukum untuk menyatakan permohonan cerai talak ini dikabulkan;

6. Bahwa Pemohon sanggup membayar biaya perkara;

Berdasarkan alasan-alasan/dalil-dalil tersebut diatas, Pemohon mohon kepada Ketua Pengadilan Agama Demak Cq Majelis Hakim yang memeriksa dan mengadili perkara ini, berkenan menjatuhkan putusan sebagai berikut:

PRIMER:

1. Mengabulkan permohonan Pemohon;
2. Memberi izin kepada Pemohon (Xxxx) untuk menjatuhkan talak satu raj'i terhadap Termohon (Xxxx) di depan sidang Pengadilan Agama Demak;
3. Membebankan biaya perkara ini menurut hukum;

SUBSIDER:

Atau mohon putusan lain yang seadil-adilnya;

Bahwa pada sidang yang telah ditentukan Pemohon telah datang menghadap sendiri, akan tetapi Termohon tidak datang menghadap atau menyuruh orang lain menghadap sebagai wakilnya yang sah, meskipun menurut berita acara panggilan sidang yang dibuat oleh Jurusita Pengganti Pengadilan Agama Demak yang dibacakan di sidang, Termohon telah dipanggil secara resmi dan patut dan tidak ternyata, bahwa tidak datangnya itu disebabkan oleh sesuatu halangan yang sah;

Bahwa Ketua Majelis telah berusaha menasehati Pemohon supaya bersabar untuk damai hidup rukun lagi dengan Termohon sebagai suami istri yang baik, akan tetapi tidak berhasil, lalu pemeriksaan perkara ini dimulai dengan membacakan surat permohonan tersebut yang isinya dipertahankan oleh Pemohon;

Bahwa atas surat permohonan Pemohon tersebut, Termohon tidak mengajukan jawabannya, karena tidak hadir di persidangan;

Bahwa meskipun Termohon tidak hadir, karena perkara ini termasuk ruang lingkup sengketa perkawinan, maka kepada Pemohon tetap dibebani wajib bukti;

Disclaimer
Kepaniteraan Mahkamah Agung Republik Indonesia berusaha untuk selalu mencantumkan informasi paling kini dan akurat sebagai bentuk komitmen Mahkamah Agung untuk pelayanan publik, transparansi dan akuntabilitas pelaksanaan fungsi peradilan. Namun dalam hal-hal tertentu masih dimungkinkan terjadi permasalahan teknis terkait dengan akurasi dan keterkinian informasi yang kami sajikan, hal mana akan terus kami perbaiki dari waktu kewaktu.
Dalam hal Anda menemukan inakurasi informasi yang termuat pada situs ini atau informasi yang seharusnya ada, namun belum tersedia, maka harap segera hubungi Kepaniteraan Mahkamah Agung RI melalui :
Email : kepaniteraan@mahkamahagung.go.id Telp : 021-384 3348 (ext.318)

Bahwa Pemohon telah mengajukan bukti-buktinya di persidangan, pada tanggal 15 Desember 2020, yaitu sebagai berikut:

A. Bukti tertulis:

1. Fotokopi kartu tanda penduduk, Nomor xxxx tertanggal 07 Nopember 2017, yang dikeluarkan oleh Kepala Dinas Kependudukan dan Pencatatan Sipil Kabupaten Demak, (P-1);
2. Fotokopi Kutipan Akta Nikah Nomor: 0378/115/VII/2016, tanggal 24 Juli 2016 yang dikeluarkan oleh Kantor Urusan Agama Kecamatan Wonosalam, Kabupaten Demak, (P-2).

   Surat-surat bukti yang berupa fotokopi tersebut setelah dicocokkan dengan aslinya ternyata sesuai dan telah bermaterai cukup;

B. Saksi-saksi:

1. xxxx, umur 59 tahun di bawah sumpahnya ia memberi keterangan sebagai berikut:

- Bahwa saksi Kakak kandung Pemohon dan kenal Termohon yaitu sebagai isteri Pemohon;
- Bahwa Pemohon dan Termohon menikah pada tahun 2016 dan belum dikaruniai anak;
- Bahwa setelah menikah Pemohon dan Termohon bertempat tinggal di rumah Pemohon;
- Bahwa semula rumah tangga Pemohon dan Termohon tampak rukun harmonis, akan tetapi sejak 1 tahun yang lalu antara Pemohon dengan Termohon mulai terjadi perselisihan dan pertengkaran;
- Bahwa penyebab pertengkaran tersebut karena Termohon sering pergi meninggalkan rumah tanpa seijin Pemohon dan anak bawaan Termohon tidak suka kepada Pemohon;
- Bahwa kemudian sejak bulan Juli tahun 2020 Termohon pergi meninggalkan Pemohon dan pulang ke rumah orang tuanya hingga sekarang;
- Bahwa keluarga Pemohon sudah berusaha merukunkan Pemohon dengan Termohon, akan tetapi tidak berhasil;

Disclaimer
Kepaniteraan Mahkamah Agung Republik Indonesia berusaha untuk selalu mencantumkan informasi paling kini dan akurat sebagai bentuk komitmen Mahkamah Agung untuk pelayanan publik, transparansi dan akuntabilitas pelaksanaan fungsi peradilan. Namun dalam hal-hal tertentu masih dimungkinkan terjadi permasalahan teknis terkait dengan akurasi dan keterkinian informasi yang kami sajikan, hal mana akan terus kami perbaiki dari waktu kewaktu.
Dalam hal Anda menemukan inakurasi informasi yang termuat pada situs ini atau informasi yang seharusnya ada, namun belum tersedia, maka harap segera hubungi Kepaniteraan Mahkamah Agung RI melalui :
Email : kepaniteraan@mahkamahagung.go.id Telp : 021-384 3348 (ext.318)

2. xxxx, umur 66 tahun di bawah sumpahnya ia memberi keterangan sebagai berikut:

- Bahwa saksi Kakak kandung Pemohon dan kenal Termohon yaitu sebagai isteri Pemohon;
- Bahwa Pemohon dan Termohon menikah pada tahun 2016 dan belum dikaruniai anak;
- Bahwa setelah menikah Pemohon dan Termohon bertempat tinggal di rumah Pemohon;
- Bahwa semula rumah tangga Pemohon dan Termohon tampak rukun harmonis, akan tetapi sejak 1 tahun yang lalu antara Pemohon dengan Termohon mulai terjadi perselisihan dan pertengkaran;
- Bahwa penyebab pertengkaran tersebut karena Termohon sering pergi meninggalkan rumah tanpa seijin Pemohon dan anak bawaan Termohon tidak suka kepada Pemohon;
- Bahwa kemudian sejak bulan Juli tahun 2020 Termohon pergi meninggalkan Pemohon dan pulang ke rumah orang tuanya hingga sekarang;
- Bahwa keluarga Pemohon sudah berusaha merukunkan Pemohon dengan Termohon, akan tetapi tidak berhasil;

Bahwa terhadap keterangan saksi-saksi tersebut Pemohon membenarkan dan menerimanya dan telah mencukupkan bukti-buktinya di persidangan;

Bahwa atas bukti-bukti yang diajukan oleh Pemohon tersebut, Termohon tidak mengajukan bantahannya karena tidak hadir di persidangan;

Bahwa Pemohon telah menyampaikan kesimpulannya secara lisan di persidangan pada tanggal 22 Desember 2020, yang pada pokoknya tetap pada permohonannya (bercerai) dan terakhir mohon putusan;

Bahwa untuk meringkas uraian putusan ini, maka ditunjuk hal ihwal sebagaimana tercantum dalam berita acara sidang perkara ini yang merupakan bagian yang tak terpisahkan dari putusan ini.

Disclaimer
Kepaniteraan Mahkamah Agung Republik Indonesia berusaha untuk selalu mencantumkan informasi paling kini dan akurat sebagai bentuk komitmen Mahkamah Agung untuk pelayanan publik, transparansi dan akuntabilitas pelaksanaan fungsi peradilan. Namun dalam hal-hal tertentu masih dimungkinkan terjadi permasalahan teknis terkait dengan akurasi dan keterkinian informasi yang kami sajikan, hal mana akan terus kami perbaiki dari waktu kewaktu.
Dalam hal Anda menemukan inakurasi informasi yang termuat pada situs ini atau informasi yang seharusnya ada, namun belum tersedia, maka harap segera hubungi Kepaniteraan Mahkamah Agung RI melalui :
Email : kepaniteraan@mahkamahagung.go.id   Telp : 021-384 3348 (ext.318)

PERTIMBANGAN HUKUM

Menimbang, bahwa maksud dan tujuan permohonan Pemohon adalah sebagai mana telah terurai di atas ;

Menimbang, bahwa Pemohon dan Termohon beragama Islam dan telah melakukan perkawinan secara Islami, maka perkara ini merupakan kompetensi absolut Pengadilan Agama untuk memeriksa dan menyelesaikannya. (vide Pasal 2 jo Pasal 49 ayat (1) Undang-Undang Nomor 7 Tahun 1989 yang telah diubah kedua Undang-Undang Nomor 50 Tahun 2009;

Menimbang, bahwa berdasarkan Bukti P-1 yang berupa kartu tanda penduduk maka terbukti Pemohon bertempat tinggal wilayah Kabupaten Demak dan jika dihubungkan dengan alamat Termohon yang juga bertempat tinggal di wilayah Kabupaten Demak, maka perkara ini secara relatif juga merupakan kompetensi Pengadilan Agama Demak untuk memeriksanya, vide Pasal 66 ayat (2) Undang-undang Nomor 7 Tahun 1989 sebagaimana telah diubah dengan Undang-undang Nomor 3 Tahun 2006 dan perubahan kedua dengan Undang-undang Nomor 50 Tahun 2009;

Menimbang, bahwa Termohon tidak pernah hadir di persidangan dan tidak pula mengirimkan orang lain sebagai wakilnya yang sah untuk menghadap di persidangan dan tidak ternyata bahwa ketidakhadirannya itu atas dasar / alasan yang dibenarkan oleh hukum maka patutlah dianggap bahwa Termohon dalam keadaan tidak hadir, karenanya putusan ini dijatuhkan tanpa hadirnya Termohon (verstek) hal mana sesuai dengan kehendak pasal 125 HIR ;

Menimbang bahwa oleh karena Termohon tidak pernah hadir dipersidangan sehingga proses mediasi sebagaimana ketentuan PERMA Nomor 01 Tahun 2016 tidak dapat dilaksanakan;

Menimbang, bahwa berdasarkan bukti surat P-2 yang berupa foto kopi Kutipan Akta Nikah maka patut dinyatakan terbukti bahwa benar, antara Pemohon dengan Termohon telah terikat dalam perkawinan yang sah, karena itu permohon Pemohon untuk menceraikan Termohon memiliki *dasar hukum yang sah*;

Disclaimer
Kepaniteraan Mahkamah Agung Republik Indonesia berusaha untuk selalu mencantumkan informasi paling kini dan akurat sebagai bentuk komitmen Mahkamah Agung untuk pelayanan publik, transparansi dan akuntabilitas pelaksanaan fungsi peradilan. Namun dalam hal-hal tertentu masih dimungkinkan terjadi permasalahan teknis terkait dengan akurasi dan keterkinian informasi yang kami sajikan, hal mana akan terus kami perbaiki dari waktu kewaktu.
Dalam hal Anda menemukan inakurasi informasi yang termuat pada situs ini atau informasi yang seharusnya ada, namun belum tersedia, maka harap segera hubungi Kepaniteraan Mahkamah Agung RI melalui :
Email : kepaniteraan@mahkamahagung.go.id Telp : 021-384 3348 (ext.318)

Menimbang, bahwa Pemohon mendalilkan yang pada pokoknya semula rumah tangganya rukun dan harmonis akan tetapi sejak bulan Desember tahun 2019 antara Pemohon dengan Termohon mulai terjadi perselisihan dan pertengkaran yang penyebabnya karena Termohon sering pergi meninggalkan rumah tanpa seijin Pemohon dan anak bawaan Termohon tidak suka kepada Pemohon yang kemudian sejak bulan Juni tahuan 2020 Termohon pergi meninggalkan Pemohon dan kembali ke rumah orang tuanya hingga sekarang dan sejak itu pula antara Pemohon dengan Termohon sudah tidak ada komonkasi lagi layaknya suami isteri;

Menimbang bahwa secara yuridis Pemohon telah menyandarkan alasan permohonannya pada ketentuan pasal 19 huruf f Peraturan Pemerintah Nomor 9 tahun 1975 jo pasal 116 huruf f Kompilasi Hukum Islam yaitu " *Antara suami dan isteri terus menerus terjadi perselisihan dan pertengkaran dan tidak ada harapan akan hidup rukun lagi dalam rumah tangga*",;

Menimbang bahwa ketidak hadiran Termohon dalam persidangan tanpa alasan yang sah dianggap telah mengabaikan hak bantah Termohon atas dalil-dalil Pemohon yang karenanya dinilai sebagai pengakuan Termohon secara-diam-diam (iqrar bi as-sukut);

Menimbang, bahwa dua orang saksi tersebut telah memenuhi syarat-syarat formil dan tidak terhalang kedudukannya sebagai saksi seperti yang diatur dalam Pasal 145 HIR, keduanya memberikan keterangan yang didasarkan pada pengetahuan dari pengalaman, penglihatan dan pendengarannya sendiri yang antara kedua saksi tersebut terdapat kesesuaian keterangan seperti yang disyaratkan dalam Pasal 172 HIR, oleh karena itu keterangan kedua saksi tersebut dapat dipertimbangkan;

Menimbang, bahwa dari dalil gugatan Pemohon, sikap Pemohon selama dipersidangan dihubungkan dengan bukti surat dan keterangan-keterangan yang disampaikan oleh para saksi di persidangan, dapat ditemukan fakta sebagai berikut :

1. Bahwa Pemohon dan Termohon adalah suami isteri sah, menikah pada tanggal 24 Juli 2016 dan belum dikaruniai anak;

Disclaimer
Kepaniteraan Mahkamah Agung Republik Indonesia berusaha untuk selalu mencantumkan informasi paling kini dan akurat sebagai bentuk komitmen Mahkamah Agung untuk pelayanan publik, transparansi dan akuntabilitas pelaksanaan fungsi peradilan. Namun dalam hal-hal tertentu masih dimungkinkan terjadi permasalahan teknis terkait dengan akurasi dan keterkinian informasi yang kami sajikan, hal mana akan terus kami perbaiki dari waktu kewaktu.
Dalam hal Anda menemukan inakurasi informasi yang termuat pada situs ini atau informasi yang seharusnya ada, namun belum tersedia, maka harap segera hubungi Kepaniteraan Mahkamah Agung RI melalui :
Email : kepaniteraan@mahkamahagung.go.id Telp : 021-384 3348 (ext.318)

2. Bahwa kemudian sejak bulan Desember tahun 2019 antara Pemohon dengan Termohon mulai sering terjadi perselisihan dan pertengkaran disebabkan Termohon sering pergi meninggalkan rumah tanpa seijin Pemohon dan anak bawaan Termohon tidak suka kepada;
3. Bahwa kemudian sejak bulan Juli tahun 2020Termohon pergi meninggalkan Pemohon dan pulang ke rumah orang tuanya hingga sekarang;
4. Bahwa selama berpisah, antara Pemohon dan Termohon tidak ada komunikasi sama sekali;
5. Bahwa keluarga Pemohon dan Termohon sudah berusaha mendamaikannya, akan tetapi tidak berhasil.

Menimbang, bahwa berdasarkan fakta poin (1) menunjukkan bahwa Pemohon dan Termohon berkualitas hukum sebagai pihak-pihak dalam perkara ini, dan Pemohon berhak mengajukan permohonan cerai talak terhadap Termohon ke Pengadilan Agama sebagaimana dimaksud pasal 66 ayat (1) Undang-undang Nomor 7 Tahun 1989;

Menimbang, bahwa dari fakta point 2, 3, 4 dan 5 sebagaimana terurai diatas telah memberikan gambaran bahwa perselisihan antara Pemohon dan Termohon sudah tidak mampu lagi dikendalikan dan diatasi oleh mereka berdua, bahkan sikap Termohon yang telah memilih pergi meninggalkan Pemohon adalah merupakan bukti bahwa perselisihan antara Pemohon dan Termohon sudah cukup serius dan bersifat terus menerus;

Menimbang bahwa dari pernyataan Pemohon selama dalam persidangan yang dengan tegas hendak menceraikan Termohon, begitu pula sikap Termohon yang tidak pernah menghadiri persidangan adalah merupakan indikasi tidak adanya kehendak dari kedua belah pihak untuk memperbaiki keadaan rumah tangganya ;

Menimbang bahwa sebagaimana tersebut dalam kitab Hurriatuz Zaujani fith Thalaq Juz I halaman 83 yang diambil alih menjadi pendapat Majelis Hakim sebagai berikut :

وقد اختارالإسلام نظام الطلاق حين تضطر ب الحياة الزوجين ولم يعد ينفع فيها نصا ئح ولا صلح وحيث تصبح الربطة الزواج صورة

Disclaimer
Kepaniteraan Mahkamah Agung Republik Indonesia berusaha untuk selalu mencantumkan informasi paling kini dan akurat sebagai bentuk komitmen Mahkamah Agung untuk pelayanan publik, transparansi dan akuntabilitas pelaksanaan fungsi peradilan. Namun dalam hal-hal tertentu masih dimungkinkan terjadi permasalahan teknis terkait dengan akurasi dan keterkinian informasi yang kami sajikan, hal mana akan terus kami perbaiki dari waktu kewaktu.
Dalam hal Anda menemukan inakurasi informasi yang termuat pada situs ini atau informasi yang seharusnya ada, namun belum tersedia, maka harap segera hubungi Kepaniteraan Mahkamah Agung RI melalui :
Email : kepaniteraan@mahkamahagung.go.id Telp : 021-384 3348 (ext.318)

من غير روح لأن استمرار معناه ان يحكم على احد الزوجين
بالسجن الموء بد وهذا ظلم تأ باه روح العدالة

Artinya : "*Islam memilih lembaga thalaq (perceraian) ketika rumah tangga sudah dianggap goncang serta dianggap sudah tidak bermanfaat lagi nasehat/ perdamaian, dan hubungan suami isteri menjadi tanpa ruh (hampa), sebab meneruskan perkawinan berarti menghukum salah satu suami isteri dengan penjara yang berkepanjangan. Ini adalah aniaya (kedhaliman) yang bertentangan dengan semangat keadilan*".

Menimbang bahwa sehubungan dengan hal tersebut Yurisprudensi Mahkamah Agung RI. Nomor 379 K / AG / 1995 tanggal 26 Maret 1997 menyatakan "*Suami isteri yang tidak berdiam serumah lagi dan tidak ada harapan untuk hidup rukun kembali maka rumah tanga tersebut telah terbukti retak dan pecah dan telah memenuhi alasan cerai Pasal 19 huruf f Peraturan Pemerintah Nomor 9 tahun 1975*";

Menimbang, bahwa berdasarkan pertimbangan-pertimbangan tersebut di atas, maka permohonan Pemohon dinilai oleh majelis hakim telah memenuhi ketentuan Pasal 19 huruf f Peraturan Pemerintah Nomor 9 tahun 1975 jo pasal 116 huruf f Kompilasi Hukum Islam dan dengan mengingat ketentuan Pasal 125 HIR maka permohonan Pemohon sebagaimana tersebut dalam petitum 2 permohonan Pemohon dapat dikabulkan dengan verstek;

Menimbang, bahwa berdasarkan pasal 118 Kompilasi Hukum Islam maka permohonan Pemohon dikabulkan dengan memberi ijin kepada Pemohon untuk menjatuhkan talak satu raj'i atas Termohon dihadapan sidang Pengadilan Agama Demak setelah putusan ini berkekuatan hukum tetap;

Menimbang, bahwa oleh karena perkara ini termasuk dalam bidang perkawinan, berdasarkan Pasal 89 ayat (1) Undang-Undang 7 Tahun 1989 yang telah diubah dengan Undang-Undang Nomor 3 Tahun 2006 dan perubahan kedua dengan Undang-Undang Nomor 50 Tahun 2009, maka biaya perkara ini dibebankan kepada Pemohon ;

Memperhatikan Pasal 125 HIR serta ketentuan Hukum Syara' yang bersangkutan;

Disclaimer
Kepaniteraan Mahkamah Agung Republik Indonesia berusaha untuk selalu mencantumkan informasi paling kini dan akurat sebagai bentuk komitmen Mahkamah Agung untuk pelayanan publik, transparansi dan akuntabilitas pelaksanaan fungsi peradilan. Namun dalam hal-hal tertentu masih dimungkinkan terjadi permasalahan teknis terkait dengan akurasi dan keterkinian informasi yang kami sajikan, hal mana akan terus kami perbaiki dari waktu kewaktu.
Dalam hal Anda menemukan inakurasi informasi yang termuat pada situs ini atau informasi yang seharusnya ada, namun belum tersedia, maka harap segera hubungi Kepaniteraan Mahkamah Agung RI melalui :
Email : kepaniteraan@mahkamahagung.go.id Telp : 021-384 3348 (ext.318)

M E N G A D I L I :

1. Menyatakan Termohon telah dipanggil secara resmi dan patut untuk datang menghadap di persidangan, tidak hadir;
2. Mengabulkan permohonan Pemohon dengan verstek;
3. Memberi izin kepada Pemohon (Xxxx) untuk menjatuhkan talak satu raj'i terhadap Termohon (Xxxx) di depan sidang Pengadilan Agama Demak setelah putusan berkekuatan hukum tetap.
4. Membebankan kepada Pemohon untuk membayar biaya perkara yang hingga kini sejumlah Rp 481.000,- (empat ratus delapan puluh satu ribu rupiah);

Demikian putusan ini dijatuhkan dalam permusyawaratan Majelis Hakim di Demak pada hari Selasa tanggal 22 Desember 2020 M, bertepatan dengan tanggal 07 Jumadil Akhir 1442 H, oleh kami Drs. MAKALI sebagai Hakim Ketua Majelis, Dra. Nur Immawati dan Toharudin, S.H.I., M.H. masing-masing sebagai Hakim Anggota. Putusan ini dibacakan oleh Ketua Majelis Hakim tersebut pada hari itu juga dalam sidang terbuka untuk umum dengan dihadiri oleh para Hakim Anggota dan Hj. Siti Hajar Zulaikha, SH. sebagai Panitera Pengganti dan dihadiri pula oleh Pemohon tanpa hadirnya Termohon;

Ketua Majelis

Drs. Makali

Hakim Anggota | Hakim Anggota

Disclaimer
Kepaniteraan Mahkamah Agung Republik Indonesia berusaha untuk selalu mencantumkan informasi paling kini dan akurat sebagai bentuk komitmen Mahkamah Agung untuk pelayanan publik, transparansi dan akuntabilitas pelaksanaan fungsi peradilan. Namun dalam hal-hal tertentu masih dimungkinkan terjadi permasalahan teknis terkait dengan akurasi dan keterkinian informasi yang kami sajikan, hal mana akan terus kami perbaiki dari waktu kewaktu.
Dalam hal Anda menemukan inakurasi informasi yang termuat pada situs ini atau informasi yang seharusnya ada, namun belum tersedia, maka harap segera hubungi Kepaniteraan Mahkamah Agung RI melalui :
Email : kepaniteraan@mahkamahagung.go.id Telp : 021-384 3348 (ext.318)

Dra. Nur Immawati **Toharudin, S.H.I., M.H.**

Panitera Pengganti

Hj. Siti Hajar Zulaikha, SH.

Perincian Biaya Perkara :

1.Pendaftaran	Rp	30.000,00
2. Biaya Proses	Rp	75.000,00
2. Panggilan sidang	Rp	340.000,00
3. PNBP Panggilan	Rp	20.000,00
5. Redaksi	Rp	10.000,00
6. Meterai.	Rp	6.000,00
J u m l a h	Rp	481.000,00

(empat ratus delapan puluh satu ribu rupiah)

Disclaimer
Kepaniteraan Mahkamah Agung Republik Indonesia berusaha untuk selalu mencantumkan informasi paling kini dan akurat sebagai bentuk komitmen Mahkamah Agung untuk pelayanan publik, transparansi dan akuntabilitas pelaksanaan fungsi peradilan. Namun dalam hal-hal tertentu masih dimungkinkan terjadi permasalahan teknis terkait dengan akurasi dan keterkinian informasi yang kami sajikan, hal mana akan terus kami perbaiki dari waktu kewaktu.
Dalam hal Anda menemukan inakurasi informasi yang termuat pada situs ini atau informasi yang seharusnya ada, namun belum tersedia, maka harap segera hubungi Kepaniteraan Mahkamah Agung RI melalui :
Email : kepaniteraan@mahkamahagung.go.id Telp : 021-384 3348 (ext.318)
