# Supplementary material for: Classification of divorce causes during the COVID-19 pandemic using convolutional neural networks
Source: PeerJ Comput Sci. 2022 Jun 30;8:e998. doi: 10.7717/peerj-cs.998 (PMC9299239; doi:10.7717/peerj-cs.998)
Supplement: Supplemental Information 4 [file peerj-cs-08-998-s004.zip › Konflik Dataset/Data ke-10.pdf]

Direktori Putusan Mahkamah Agung Republik Indonesia
putusan.mahkamahagung.go.id

P U T U S A N

Nomor 4223/Pdt.G/2020/PA.Mjl

بِسْمِ اللَّهِ الرَّحْمَٰنِ الرَّحِيمِ

DEMI KEADILAN BERDASARKAN KETUHANAN YANG MAHA ESA

Pengadilan Agama Majalengka yang memeriksa dan mengadili perkara perdata pada tingkat pertama dalam persidangan Majelis telah menjatuhkan putusan sebagai berikut dalam perkara antara :

Penggugat, umur 51 tahun, agama Islam, pendidikan S1, pekerjaan Pegawai Negeri Sipil, tempat kediaman di Kabupaten Majalengka, dalam hal ini memberikan kuasa khusus kepada Cucu Samsul Falah S.H.I., Advokat pada Firma Hukum IKTAN LAW FIRM & PARTNERS, yang berkantor di Jl.Siliwangi No. 3 Depan Kantor Pengadilan Agama Majalengka, Desa Karyamukti Kecamatan Panyingkiran Kabupaten Majalengka berdasarkan surat kuasa khusus Nomor 0978/ADVXII/2020/PA.Mjl tanggal 10 Desember 2020, sebagai Penggugat;

melawan

Tergugat, umur 57 tahun, agama Islam, pendidikan SLTA, pekerjaan Pegawai Negeri Sipil, tempat kediaman di Kabupaten Majalengka, sebagai Tergugat;

Pengadilan Agama tersebut;

Setelah mempelajari berkas perkara yang bersangkutan;

Setelah mendengar keterangan Penggugat/kuasa hukumnya dan saksi-saksi di muka persidangan;

DUDUK PERKARA

Bahwa Penggugat dalam surat gugatannya tanggal 10 Desember 2020 telah mengajukan gugatan yang telah didaftar di Kepaniteraan Pengadilan Agama Majalengka, dengan Nomor 4223/Pdt.G/2020/PA.Mjl, tanggal 10 Desember 2020, mengajukan gugatan Cerai dengan dalil-dalil sebagai berikut:

1. Bahwa Penggugat dan Tergugat telah melangsungkan pernikahan pada Hari Sabtu tanggal 16 Agustus 2014 Masehi, bertepatan dengan tanggal 20

Disclaimer
Kepaniteraan Mahkamah Agung Republik Indonesia berusaha untuk selalu mencantumkan informasi paling kini dan akurat sebagai bentuk komitmen Mahkamah Agung untuk pelayanan publik, transparansi dan akuntabilitas pelaksanaan fungsi peradilan. Namun dalam hal-hal tertentu masih dimungkinkan terjadi permasalahan teknis terkait dengan akurasi dan keterkinian informasi yang kami sajikan, hal mana akan terus kami perbaiki dari waktu kewaktu.
Dalam hal Anda menemukan inakurasi informasi yang termuat pada situs ini atau informasi yang seharusnya ada, namun belum tersedia, maka harap segera hubungi Kepaniteraan Mahkamah Agung RI melalui :
Email : kepaniteraan@mahkamahagung.go.id Telp : 021-384 3348 (ext.318)

Syawwal 1435 Hijriyah dihadapan Pegawai Pencatat Nikah pada Kantor Urusan Agama Kecamatan Sindang, Kabupaten Majalengka, sebagaimana tercantum dalam Kutipan Akta Nikah Nomor. 116/31/VIII/2014;

2. Bahwa setelah pernikahan tersebut Penggugat dan Tergugat telah melakukan hubungan badan (Ba'da dukhul), namun belum dikaruniai anak;
3. Bahwa setelah menikah tersebut, Penggugat dan Tergugat bertempat tinggal di rumah bersama di Kabupaten Majalengka - Jawa Barat;
4. Bahwa pada awalnya rumah tangga Penggugat dan Tergugat tentram, harmonis dan bahagia namun sejak Bulan Januari tahun 2018 rumah tangga Penggugat dan Tergugat mulai goyah dan tidak harmonis lagi, sering terjadi perselisihan dan pertengkaran, hal tersebut terjadi karena :

- Tergugat kurang bertanggung jawab dalam memberikan nafkah rumah tanggga, sehingga untuk kebutuhan sehari - hari tidak tercukupi;
- Tergugat jarang komunikasi dengan Pengggugat dan sering mendiamkan Penggugat;

5. Bahwa puncak keretakan rumah tangga Penggugat dan Tergugat terjadi pada Bulan Juli tahun 2020 dimana Penggugat pergi dari rumah bersama dan pulang ke rumah Penggugat dengan alamat di Kabupaten Majalengka dan sampai dengan sekarang tidak pernah berkumpul bersama lagi;
6. Bahwa Penggugat sudah berusaha mempertahankan keutuhan rumah tangga ini dengan cara bersabar dan menunggu kesadaran Tergugat untuk melaksanakan tanggung jawabnya, akan tetapi tidak berhasil;
7. Bahwa pihak keluarga / orang tua kedua belah pihak juga sudah berupaya merukunkan, akan tetapi tidak berhasil;
8. Bahwa tujuan pernikahan Penggugat dan Tergugat yaitu keluarga yang Sakinah Mawaddah Warahmah sudah tidak dapat diharapkan lagi, sebaliknya yang terjadi adalah penderitaan / kesengsaraan bagi Penggugat;
9. Bahwa Gugatan Penggugat telah memenuhi alasan perceraian sebagamana diatur dalam pasal 38 Undang - Undang Nomor 1 tahun 1974 tentang perkawinan Jo. Peraturan Pemerintah Nomor 9 Tahun 1975 Pasal 19 (huruf f) Jo. Kompilasi Hukum Islam pasal 116;
10. Penggugat sanggup membayar biaya yang timbul akibat perkara ini;

Disclaimer
Kepaniteraan Mahkamah Agung Republik Indonesia berusaha untuk selalu mencantumkan informasi paling kini dan akurat sebagai bentuk komitmen Mahkamah Agung untuk pelayanan publik, transparansi dan akuntabilitas pelaksanaan fungsi peradilan. Namun dalam hal-hal tertentu masih dimungkinkan terjadi permasalahan teknis terkait dengan akurasi dan keterkinian informasi yang kami sajikan, hal mana akan terus kami perbaiki dari waktu kewaktu.
Dalam hal Anda menemukan inakurasi informasi yang termuat pada situs ini atau informasi yang seharusnya ada, namun belum tersedia, maka harap segera hubungi Kepaniteraan Mahkamah Agung RI melalui :
Email : kepaniteraan@mahkamahagung.go.id Telp : 021-384 3348 (ext.318)

Berdasarkan uraian tersebut diatas, Penggugat mohon kepada Ketua Pengadilan Agama Majalengka c.q majelis hakim yang memeriksa dan mengadili perkara ini dapat menjatuhkan putusan sebagai berikut:

I. PRIMAIR :

1. Mengabulkan gugatan Penggugat;
2. Menjatuhkan talak satu Ba'in Sughro Tergugat (Tergugat) Terhadap Penggugat (Penggugat);
3. Membebankan biaya perkara menurut hukum;

II. SUBSIDAIR :

- Mohon putusan yang seadil-adilnya;

Bahwa, Penggugat telah menguasakan kepada kuasa hukumnya Cucu Samsul Falah S.H.I./Advokat berdasarkan Surat Kuasa Khusus tertanggal 10 Desember 2020 dan telah terdaftar di Kepaniteraan Pengadilan Agama Majalengka dengan Nomor 0978/ADVXII/2020/PA.Mjl;

Bahwa, pada hari sidang yang telah ditetapkan, Penggugat dengan didampingi kuasa hukumnya datang menghadap di muka persidangan, sedangkan Tergugat tidak datang dan tidak pula menyuruh orang lain sebagai wakil atau kuasanya yang sah, padahal kepadanya telah dipanggil dengan resmi dan patut berdasarkan berita acara relaas panggilan tanggal 14 Desember 2020, sedangkan tidak ternyata ketidakhadirannya itu disebabkan oleh suatu alasan yang sah menurut hukum;

Bahwa, Majelis Hakim telah berusaha menasehati Penggugat baik secara prinsipal maupun melalui kuasa hukumnya agar Penggugat dapat membina kembali rumah tangganya dengan Tergugat, akan tetapi tidak berhasil dan Majelis Hakim memandang perkara a quo tidak mungkin untuk dilaksanakan mediasi karena Tergugat tidak pernah hadir di persidangan;

Bahwa, selanjutnya pemeriksaan perkara ini dimulai dengan membacakan surat gugatan Penggugat yang maksud dan isinya tetap dipertahankan oleh Penggugat;

Disclaimer
Kepaniteraan Mahkamah Agung Republik Indonesia berusaha untuk selalu mencantumkan informasi paling kini dan akurat sebagai bentuk komitmen Mahkamah Agung untuk pelayanan publik, transparansi dan akuntabilitas pelaksanaan fungsi peradilan. Namun dalam hal-hal tertentu masih dimungkinkan terjadi permasalahan teknis terkait dengan akurasi dan keterkinian informasi yang kami sajikan, hal mana akan terus kami perbaiki dari waktu kewaktu.
Dalam hal Anda menemukan inakurasi informasi yang termuat pada situs ini atau informasi yang seharusnya ada, namun belum tersedia, maka harap segera hubungi Kepaniteraan Mahkamah Agung RI melalui :
Email : kepaniteraan@mahkamahagung.go.id Telp : 021-384 3348 (ext.318)

Bahwa, untuk membuktikan dalil gugatannya, Penggugat telah mengajukan alat bukti surat berupa :

1.Fotokopi Kutipan Akta Nikah dari KUA Sindang Kabupaten Majalengka Propinsi Jawa Barat, Nomor 116/31/VIII/2014 Tanggal 16 Agustus 2014, bukti surat tersebut telah diberi meterai cukup, telah dicap pos dan telah dicocokkan dengan aslinya yang ternyata sesuai, lalu oleh Ketua Majelis diberi tanda P1;

2.Surat Keputusan Tentang Izin Perceraian Nomor 873.4/Kep.496-BKPSDM/2020 tanggal 04 Desember 2020 yang dikeluarkan oleh Badan Kepegawaian dan Pengembangan Daya Manusia, lalu oleh Ketua Majelis diberi tanda P2;

Bahwa, di samping itu, Penggugat juga telah mengajukan alat bukti saksi yaitu :

1. Sukimi bin Ehon, umur 67 tahun, agama Islam, pekerjaan wiraswasta, tempat tinggal di Blok Jumat RT 001 RW 001 Desa Gunung Kuning Kecamatan Sindang Kabupaten Majalengka. Saksi sebagai kakak sepupu Penggugat, dibawah sumpah telah memberikan keterangan yang pada pokoknya sebagai berikut:

- Bahwa Saksi kenal dengan Penggugat bernama Penggugat dan Tergugat bernama Tergugat;
- Bahwa Saksi tahu Penggugat dengan Tergugat sudah menikah, mereka menikah pada tanggal 16 Agustus 2014;
- Bahwa setelah menikah Penggugat dengan Tergugat membina rumah tangga tinggal bersama di rumah bersama;
- Bahwa selama pernikahan Penggugat dengan Tergugat belum dikaruniai anak;
- Bahwa semula rumah tangga Penggugat dengan Tergugat rukun dan harmonis tinggal bersama di rumah bersama akan tetapi sejak bulan Januari 2018 rumah tangganya sudah tidak harmonis lagi, sering terjadi perselisihan dan pertengkaran;

Disclaimer
Kepaniteraan Mahkamah Agung Republik Indonesia berusaha untuk selalu mencantumkan informasi paling kini dan akurat sebagai bentuk komitmen Mahkamah Agung untuk pelayanan publik, transparansi dan akuntabilitas pelaksanaan fungsi peradilan. Namun dalam hal-hal tertentu masih dimungkinkan terjadi permasalahan teknis terkait dengan akurasi dan keterkinian informasi yang kami sajikan, hal mana akan terus kami perbaiki dari waktu kewaktu.
Dalam hal Anda menemukan inakurasi informasi yang termuat pada situs ini atau informasi yang seharusnya ada, namun belum tersedia, maka harap segera hubungi Kepaniteraan Mahkamah Agung RI melalui :
Email : kepaniteraan@mahkamahagung.go.id   Telp : 021-384 3348 (ext.318)

- Bahwa penyebabnya karena Tergugat kurang bertanggung jawab dalam memberikan nafkah rumah tangga, sehingga kebutuhan sehari-hari tidak terpenuhi;;
- Bahwa sejak bulan Juli 2020 yang lalu antara Penggugat dan Tergugat telah berpisah tempat tinggal dan tidak pernah berkumpul kembali;
- Bahwa saksi pernah berusaha menasehati Penggugat, akan tetapi tidak berhasil;
- Bahwa saksi sudah tidak sanggup lagi untuk merukunkan kembali;

Bahwa, terhadap keterangan saksi tersebut Penggugat membenarkannya;

2. Didi Sutardi bin Iyat Sutisna, umur 50 tahun, agama Islam, pekerjaan wiraswasta, tempat tinggal di Blok Selasa RT 005 RW 002 Desa Gunung Kuning Kecamatan Sindang Kabupaten Majalengka. Saksi sebagai adik sepupu Penggugat, dibawah sumpahnya telah memberikan keterangan yang pada pokoknya sebagai berikut:

- Bahwa Saksi kenal dengan Penggugat bernama Penggugat dan Tergugat bernama Tergugat;
- Bahwa awalnya rumah tangga Penggugat dan Tergugat rukun dan harmonis akan tetapi sejak bulan Januari 2018 rumah tangganya sudah tidak harmonis lagi sering terjadi perselisihan dan pertengkaran;
- Bahwa penyebab perselisihan dan pertengkaran tersebut karena Tergugat kurang bertanggung jawab dalam memberikan nafkah rumah tangga, sehingga kebutuhan sehari-hari tidak terpenuhi;;
- Bahwa sejak bulan Juli 2020 yang lalu Penggugat dan Tergugat telah berpisah tempat tinggal dan tidak pernah berkumpul kembali;
- Bahwa saksi sudah tidak sanggup lagi untuk merukunkannya;

Bahwa, terhadap keterangan saksi tersebut Penggugat membenarkannya;

Bahwa, Penggugat dalam kesimpulannya mengatakan tidak akan mengajukan suatu apapun dan memohon kepada Majelis Hakim agar menjatuhkan putusannya;

Disclaimer
Kepaniteraan Mahkamah Agung Republik Indonesia berusaha untuk selalu mencantumkan informasi paling kini dan akurat sebagai bentuk komitmen Mahkamah Agung untuk pelayanan publik, transparansi dan akuntabilitas pelaksanaan fungsi peradilan. Namun dalam hal-hal tertentu masih dimungkinkan terjadi permasalahan teknis terkait dengan akurasi dan keterkinian informasi yang kami sajikan, hal mana akan terus kami perbaiki dari waktu kewaktu.
Dalam hal Anda menemukan inakurasi informasi yang termuat pada situs ini atau informasi yang seharusnya ada, namun belum tersedia, maka harap segera hubungi Kepaniteraan Mahkamah Agung RI melalui :
Email : kepaniteraan@mahkamahagung.go.id Telp : 021-384 3348 (ext.318)

Bahwa, untuk mempersingkat uraian pertimbangan ini ditunjuk hal-hal sebagaimana tercantum dalam berita acara persidangan perkara ini;

PERTIMBANGAN HUKUM

Menimbang, bahwa maksud dan tujuan gugatan Penggugat adalah sebagaimana terurai diatas;

Menimbang, bahwa Penggugat telah menguasakan kepada kuasa hukumnya Cucu Samsul Falah S.H.I./Advokat berdasarkan Surat Kuasa Khusus tertanggal 10 Desember 2020, dan telah terdaftar di Kepaniteraan Pengadilan Agama Majalengka, sehingga karenanya kuasa hukum tersebut secara formal dapat diterima untuk beracara dalam perkara ini;

Menimbang, bahwa Tergugat telah dipanggil dengan resmi dan patut, akan tetapi tidak datang dan tidak pula menyuruh orang lain sebagai wakilnya/kuasanya dan tidak ternyata tidak datangnya itu disebabkan suatu alasan yang sah, sedangkan gugatan Penggugat telah cukup beralasan dan tidak melawan hukum, maka Tergugat yang telah dipanggil tersebut harus dinyatakan tidak hadir dan sesuai dengan Pasal 125 ayat (1) HIR perkaranya dapat diputuskan dengan verstek;

Menimbang, bahwa sesuai dengan maksud dan ketentuan Pasal 82 Undang-Undang Nomor 7 Tahun 1989 tentang Peradilan Agama sebagaimana telah diubah dengan Undang-Undang Nomor 3 Tahun 2006 dan perubahan kedua dengan Undang-Undang Nomor 50 Tahun 2009, Majelis Hakim telah berusaha maksimal menasehati Penggugat baik secara prinsipal maupun melalui kuasa hukumnya agar Penggugat dapat rukun kembali membina rumah tangga dengan Tergugat akan tetapi usaha tersebut tidak berhasil;

Menimbang, bahwa di samping itu oleh karena Tergugat tidak pernah hadir di persidangan, maka proses mediasi tidak dapat dilaksanakan (vide PERMA Nomor 1 Tahun 2016 Pasal 4 ayat 2 huruf b);

Menimbang, bahwa selanjutnya berdasarkan alat bukti (P.1) yang telah memenuhi syarat formil dan materil, harus dinyatakan terbukti bahwa hubungan hukum antara Penggugat dan Tergugat yaitu sebagai suami isteri sah yang menikah pada tanggal 16 Agustus 2014, dan tercatat pada Kutipan Akta Nikah

Disclaimer
Kepaniteraan Mahkamah Agung Republik Indonesia berusaha untuk selalu mencantumkan informasi paling kini dan akurat sebagai bentuk komitmen Mahkamah Agung untuk pelayanan publik, transparansi dan akuntabilitas pelaksanaan fungsi peradilan. Namun dalam hal-hal tertentu masih dimungkinkan terjadi permasalahan teknis terkait dengan akurasi dan keterkinian informasi yang kami sajikan, hal mana akan terus kami perbaiki dari waktu kewaktu.
Dalam hal Anda menemukan inakurasi informasi yang termuat pada situs ini atau informasi yang seharusnya ada, namun belum tersedia, maka harap segera hubungi Kepaniteraan Mahkamah Agung RI melalui :
Email : kepaniteraan@mahkamahagung.go.id Telp : 021-384 3348 (ext.318)

dari KUA Sindang Kabupaten Majalengka Propinsi Jawa Barat, Nomor 116/31/VIII/2014 dan telah terikat dalam perkawinan yang sah sebagaimana diatur dalam Pasal 2 Undang-Undang Nomor 1 Tahun 1974 sekaligus memiliki kedudukan hukum sebagai pihak-pihak yang berkepentingan dalam perkara ini (*persona standi in yudicio*);

Menimbang, bahwa pada pokoknya Penggugat telah mendasarkan gugatan cerainya terhadap Tergugat dengan alasan sejak bulan Januari 2018 rumah tangga Penggugat dengan Tergugat sudah tidak harmonis, sering terjadi perselisihan dan pertengkaran yang mencapai puncaknya pada bulan Juli 2020 sehingga tidak ada harapan akan hidup rukun lagi dalam membina rumah tangga, dimana penyebabnya karena Tergugat kurang bertanggung jawab dalam memberikan nafkah rumah tangga, sehingga kebutuhan sehari-hari tidak terpenuhi, sehingga sejak bulan Juli 2020 Penggugat dan Tergugat telah pisah tempat tinggal;

Menimbang, bahwa di muka persidangan Penggugat telah mengajukan dua orang saksi yang dari keterangannya saling bersesuaian, Majelis Hakim telah dapat menemukan fakta hukum sebagai berikut :

- Bahwa Penggugat dan Tergugat adalah suami isteri yang sah;
- Bahwa semula rumah tangga Penggugat dan Tergugat rukun dan harmonis;
- Bahwa sejak bulan Januari 2018 keadaan rumah tangganya tersebut sudah tidak harmonis lagi, sering terjadi perselisihan dan pertengkaran;
- Bahwa penyebabnya karena Tergugat kurang bertanggung jawab dalam memberikan nafkah rumah tangga, sehingga kebutuhan sehari-hari tidak terpenuhi;;
- Bahwa sejak bulan Juli 2020 yang lalu Penggugat dan Tergugat telah berpisah tempat tinggal dan tidak pernah berkumpul kembali;

Menimbang, bahwa berdasarkan fakta hukum tersebut di atas, harus dinyatakan terbukti bahwa dalam kehidupan rumah tangga Penggugat dan Tergugat sudah tidak harmonis lagi, telah terjadi perselisihan dan pertengkaran yang mengakibatkan antara keduanya telah berpisah tempat tinggal dan tidak pernah berkumpul kembali dalam satu rumah tangga;

Disclaimer
Kepaniteraan Mahkamah Agung Republik Indonesia berusaha untuk selalu mencantumkan informasi paling kini dan akurat sebagai bentuk komitmen Mahkamah Agung untuk pelayanan publik, transparansi dan akuntabilitas pelaksanaan fungsi peradilan. Namun dalam hal-hal tertentu masih dimungkinkan terjadi permasalahan teknis terkait dengan akurasi dan keterkinian informasi yang kami sajikan, hal mana akan terus kami perbaiki dari waktu kewaktu.
Dalam hal Anda menemukan inakurasi informasi yang termuat pada situs ini atau informasi yang seharusnya ada, namun belum tersedia, maka harap segera hubungi Kepaniteraan Mahkamah Agung RI melalui :
Email : kepaniteraan@mahkamahagung.go.id   Telp : 021-384 3348 (ext.318)

Menimbang, bahwa disamping itu para saksi sudah pernah berusaha menasehati Penggugat dan mengatakan tidak ada kesanggupan untuk dapat merukunkannya kembali, sehingga Majelis Hakim berpendapat bahwa antara Penggugat dan Tergugat sudah sulit dan tidak ada harapan untuk dapat hidup rukun kembali di dalam membina rumah tangganya;

Menimbang, bahwa menurut ketentuan Pasal 19 huruf (f) PP Nomor 9 tahun 1975 jo. Pasal 116 huruf (f) Kompilasi Hukum Islam, perceraian dapat terjadi apabila "antara suami dan isteri terus menerus terjadi perselisihan dan pertengkaran dan tidak ada harapan akan hidup rukun kembali dalam membina rumah tangga";

Menimbang, bahwa demikian juga menurut Mahkamah Agung sebagaimana tertuang dalam putusannya No. 38/K/AG/1990 tanggal 5 Oktober 1990 yang kemudian diambil alih menjadi pendapat Majelis Hakim bahwa apabila ternyata adanya perselisihan sebagaimana dimaksud Pasal 19 huruf (f) PP No. 9 Tahun 1975, maka hal itu semata-mata ditujukan kepada perkawinannya itu sendiri tanpa mempersoalkan siapa yang salah dalam hal terjadinya perselisihan yang mengakibatkan tidak ada harapan akan hidup rukun lagi, karena pernikahan adalah suatu perjanjian yang suci (mitsaqan gholidzon/Pasal 2 KHI) yang untuk memutuskannya tidak boleh diukur dengan kesalahan salah satu pihak, sehingga apabila perkawinan itu telah pecah berarti hati kedua belah pihak telah pecah pula;

Menimbang, bahwa terlepas dari peristiwa atau keadaan yang menjadi penyebab terjadinya perselisihan yang terjadi antara Penggugat dan Tergugat, yang jelas Majelis Hakim telah dapat menemukan fakta hukum bahwa perselisihan antara Penggugat dan Tergugat tersebut telah sedemikian rupa, rumah tangga Penggugat dan Tergugat sudah hancur dan tidak mungkin akan dapat hidup rukun kembali dalam membina rumah tangga;

Menimbang, bahwa dengan melihat kondisi rumah tangga Penggugat dan Tergugat tersebut, jelas tidak mungkin akan terwujud kehidupan rumah tangga yang bahagia dan kekal lahir bathin sebagaimana yang menjadi tujuan perkawinan dalam Pasal 1 Undang-Undang Nomor 1 Tahun 1974 jo. Pasal 3 KHI, dan membiarkan suasana rumah tangga yang demikian, justru akan

Disclaimer
Kepaniteraan Mahkamah Agung Republik Indonesia berusaha untuk selalu mencantumkan informasi paling kini dan akurat sebagai bentuk komitmen Mahkamah Agung untuk pelayanan publik, transparansi dan akuntabilitas pelaksanaan fungsi peradilan. Namun dalam hal-hal tertentu masih dimungkinkan terjadi permasalahan teknis terkait dengan akurasi dan keterkinian informasi yang kami sajikan, hal mana akan terus kami perbaiki dari waktu kewaktu.
Dalam hal Anda menemukan inakurasi informasi yang termuat pada situs ini atau informasi yang seharusnya ada, namun belum tersedia, maka harap segera hubungi Kepaniteraan Mahkamah Agung RI melalui :
Email : kepaniteraan@mahkamahagung.go.id Telp : 021-384 3348 (ext.318)

menimbulkan mudlarat bagi kedua belah pihak, oleh karena itu perceraian dipandang jalan yang terbaik dan lebih maslahat bagi keduanya;

Menimbang, bahwa Majelis Hakim perlu mengetengahkan dalil dalam kitab Al-Muhadzab juz II halaman 82 yang kemudian diambil alih menjadi pendapat Majelis Hakim sebagai berikut :

Ejnb æÈ°jÛ¯ tvnÌ ØnÊ°ã¸äSÛ E¸äSÛ¯ ECĐÀ Þ¾Ì
¾FÄ¯¯¿ ªä

Artinya: "Disaat istri telah memuncak ketidaksukaan terhadap suaminya, maka Hakim diperkenankan menjatuhkan talak (suami) nya dengan talak satu ;

Menimbang, bahwa berdasarkan pertimbangan tersebut diatas, maka gugatan Penggugat telah memenuhi alasan perceraian sebagaimana diatur dalam Pasal 39 ayat (2) Undang-Undang Nomor 1 Tahun 1974, jo. Pasal 19 huruf (f) Peraturan Pemerintah Nomor 9 Tahun 1975, jo. Pasal 116 huruf (f) Kompilasi Hukum Islam dan karenanya gugatan Penggugat dapat dikabulkan dengan menjatuhkan talak satu ba'in sughro Tergugat terhadap Penggugat;

Menimbang, bahwa perkara ini termasuk bidang perkawinan, oleh karenanya berdasarkan Pasal 89 ayat (1) Undang-Undang Nomor 7 Tahun 1989 tentang Peradilan Agama sebagaimana telah diubah dengan Undang-Undang Nomor 3 Tahun 2006 dan perubahan kedua dengan Undang-Undang Nomor 50 Tahun 2009, maka seluruh biaya perkara ini dibebankan kepada Penggugat;

Mengingat segala peraturan perundang-undangan yang berlaku serta ketentuan hukum syara' yang berkaitan dengan perkara ini;

M E N G A D I L I

1. Menyatakan Tergugat yang telah dipanggil secara resmi dan patut untuk menghadap di persidangan, tidak hadir;
2. Mengabulkan gugatan Penggugat dengan verstek;
3. Menjatuhkan talak satu bain sughra Tergugat (Tergugat) terhadap Penggugat (Penggugat);
4. Membebankan kepada Penggugat untuk membayar biaya perkara sejumlah Rp. 376000,00 (tiga ratus tujuh puluh enam ribu rupiah).

Disclaimer
Kepaniteraan Mahkamah Agung Republik Indonesia berusaha untuk selalu mencantumkan informasi paling kini dan akurat sebagai bentuk komitmen Mahkamah Agung untuk pelayanan publik, transparansi dan akuntabilitas pelaksanaan fungsi peradilan. Namun dalam hal-hal tertentu masih dimungkinkan terjadi permasalahan teknis terkait dengan akurasi dan keterkinian informasi yang kami sajikan, hal mana akan terus kami perbaiki dari waktu kewaktu.
Dalam hal Anda menemukan inakurasi informasi yang termuat pada situs ini atau informasi yang seharusnya ada, namun belum tersedia, maka harap segera hubungi Kepaniteraan Mahkamah Agung RI melalui :
Email : kepaniteraan@mahkamahagung.go.id   Telp : 021-384 3348 (ext.318)

Demikian Putusan ini dijatuhkan dalam permusyawaratan Majelis Hakim Pengadilan Agama Majalengka pada hari Selasa tanggal 22 Desember 2020 Masehi, bertepatan dengan tanggal 7 Jumadil Awwal 1442 Hijriyah, oleh kami Drs. H. Epoy Rosmana, S.H. sebagai Ketua Majelis, Drs. H. Mambaul Izzi, S.H., M.H. dan Drs. Abdul Malik, M.Si masing-masing sebagai Hakim Anggota, yang dibacakan dalam sidang terbuka untuk umum oleh Ketua Majelis tersebut dengan dihadiri para Hakim Anggota dan Drs. Udin Bahrudin sebagai Panitera Pengganti serta dihadiri oleh Penggugat/kuasa hukumnya tanpa hadirnya Tergugat;

Ketua Majelis

Drs. H. Epoy Rosmana, S.H.

Hakim Anggota	Hakim Anggota
Drs. H. Mambaul Izzi, S.H., M.H.	Drs. Abdul Malik, M.Si

Panitera Pengganti

Drs. Udin Bahrudin

Rincian biaya perkara:

1. Biaya pendaftaran	:Rp 30.000,00
2. Biaya proses	:Rp 50.000,00
3. Biaya panggilan Penggugat	:Rp 100.000,00
4. Biaya PNBP panggilan Penggugat	:Rp 10.000,00
5. Biaya panggilan Tergugat	:Rp 150.000,00
6. Biaya PNBP panggilan Tergugat	:Rp 10.000,00
7. Biaya redaksi	:Rp 10.000,00
8. Biaya meterai	:Rp 6.000,00
J u m l a h	:Rp 366.000,00

(tiga ratus enam puluh enam ribu rupiah)

Disclaimer
Kepaniteraan Mahkamah Agung Republik Indonesia berusaha untuk selalu mencantumkan informasi paling kini dan akurat sebagai bentuk komitmen Mahkamah Agung untuk pelayanan publik, transparansi dan akuntabilitas pelaksanaan fungsi peradilan. Namun dalam hal-hal tertentu masih dimungkinkan terjadi permasalahan teknis terkait dengan akurasi dan keterkinian informasi yang kami sajikan, hal mana akan terus kami perbaiki dari waktu kewaktu.
Dalam hal Anda menemukan inakurasi informasi yang termuat pada situs ini atau informasi yang seharusnya ada, namun belum tersedia, maka harap segera hubungi Kepaniteraan Mahkamah Agung RI melalui :
Email : kepaniteraan@mahkamahagung.go.id   Telp : 021-384 3348 (ext.318)

Hal. 11 dari 12 hal.

Disclaimer
Kepaniteraan Mahkamah Agung Republik Indonesia berusaha untuk selalu mencantumkan informasi paling kini dan akurat sebagai bentuk komitmen Mahkamah Agung untuk pelayanan publik, transparansi dan akuntabilitas pelaksanaan fungsi peradilan. Namun dalam hal-hal tertentu masih dimungkinkan terjadi permasalahan teknis terkait dengan akurasi dan keterkinian informasi yang kami sajikan, hal mana akan terus kami perbaiki dari waktu kewaktu.
Dalam hal Anda menemukan inakurasi informasi yang termuat pada situs ini atau informasi yang seharusnya ada, namun belum tersedia, maka harap segera hubungi Kepaniteraan Mahkamah Agung RI melalui :
Email : kepaniteraan@mahkamahagung.go.id   Telp : 021-384 3348 (ext.318)

Dicatat disini :

- Amar putusan tersebut telah diberitahukan kepada Tergugat pada tanggal
- Putusan tersebut telah mempunyai kekuatan hukum tetap sejak tanggal

Untuk salinan yang sama bunyinya

Oleh :

Panitera

Drs. H. Jaenal, M.H.

Disclaimer
Kepaniteraan Mahkamah Agung Republik Indonesia berusaha untuk selalu mencantumkan informasi paling kini dan akurat sebagai bentuk komitmen Mahkamah Agung untuk pelayanan publik, transparansi dan akuntabilitas pelaksanaan fungsi peradilan. Namun dalam hal-hal tertentu masih dimungkinkan terjadi permasalahan teknis terkait dengan akurasi dan keterkinian informasi yang kami sajikan, hal mana akan terus kami perbaiki dari waktu kewaktu.
Dalam hal Anda menemukan inakurasi informasi yang termuat pada situs ini atau informasi yang seharusnya ada, namun belum tersedia, maka harap segera hubungi Kepaniteraan Mahkamah Agung RI melalui :
Email : kepaniteraan@mahkamahagung.go.id Telp : 021-384 3348 (ext.318)
