# Supplementary material for: Classification of divorce causes during the COVID-19 pandemic using convolutional neural networks
Source: PeerJ Comput Sci. 2022 Jun 30;8:e998. doi: 10.7717/peerj-cs.998 (PMC9299239; doi:10.7717/peerj-cs.998)
Supplement: Supplemental Information 4 [file peerj-cs-08-998-s004.zip › Konflik Dataset/Data ke-12.pdf]

P U T U S A N

Nomor 4202/Pdt.G/2020/PA.Sbg

بِسْمِ اللهِ الرَّحْمٰنِ الرَّحِيْمِ

DEMI KEADILAN BERDASARKAN KETUHANAN YANG MAHA ESA

Pengadilan Agama Subang yang memeriksa dan mengadili perkara tertentu pada tingkat pertama dalam persidangan Majelis Hakim telah menjatuhkan putusan dalam perkara Cerai Gugat, antara:

Penggugat, umur 27 tahun, agama Islam, pendidikan terakhir SLTA, pekerjaan Ibu Rumah Tangga, tempat tinggal di Kabupaten Subang, disebut sebagai Penggugat;

m e l a w a n

Tergugat, umur 34 tahun, agama Islam, pendidikan SLTP, pekerjaan Buruh, tempat tinggal di Kabupaten Subang, disebut sebagai Tergugat;

Pengadilan Agama tersebut;

Setelah membaca berkas perkara;

Setelah mendengar keterangan Penggugat;

Setelah memeriksa bukti-bukti dalam persidangan;

DUDUK PERKARA

Bahwa, Penggugat dengan surat gugatannya tertanggal 26 November 2020 yang terdaftar di Kepaniteraan Pengadilan Agama Subang pada tanggal 26 November 2020 dengan register perkara Nomor 4202/Pdt.G/2020/PA.Sbg, telah mengemukakan hal-hal dan alasan-alasan sebagai berikut:

1. Bahwa Penggugat saat ini bertempat tinggal di Kabupaten Subang sebagaimana ternyata dari KTP atas nama Penggugat nomor 321001, yang diterbitkan oleh Dinas Kependudukan dan Catatan Sipil Kabupaten Subang;
2. Bahwa pada tanggal 14 November 2012, Penggugat dengan Tergugat melangsungkan pernikahan di Wilayah Hukum Kantor Urusan Agama

Disclaimer
Kepaniteraan Mahkamah Agung Republik Indonesia berusaha untuk selalu mencantumkan informasi paling kini dan akurat sebagai bentuk komitmen Mahkamah Agung untuk pelayanan publik, transparansi dan akuntabilitas pelaksanaan fungsi peradilan. Namun dalam hal-hal tertentu masih dimungkinkan terjadi permasalahan teknis terkait dengan akurasi dan keterkinian informasi yang kami sajikan, hal mana akan terus kami perbaiki dari waktu kewaktu.
Dalam hal Anda menemukan inakurasi informasi yang termuat pada situs ini atau informasi yang seharusnya ada, namun belum tersedia, maka harap segera hubungi Kepaniteraan Mahkamah Agung RI melalui :
Email : kepaniteraan@mahkamahagung.go.id Telp : 021-384 3348 (ext.318)

Kecamatan Kasomalang Kabupaten Subang sebagaimana ternyata dari Buku Kutipan Akta Nikah yang dikeluarkan oleh Pegawai Pencatat Nikah Kantor Urusan Agama Kecamatan Kasomalang Kabupaten Subang Nomor 2012, tertanggal 14 November 2012;

3. Bahwa setelah akad nikah Penggugat dan Tergugat hidup bersama sebagai suami istri di rumah orangtua Penggugat;
4. Bahwa perkawinan antara Penggugat dan Tergugat sampai saat ini telah berjalan selama 8 tahun, dan telah dikaruniai anak 1 (satu) yang bernama, Zahra Hoerunnisa, berusia 7 tahun;
5. Bahwa semula rumah tangga Penggugat dan Tergugat harmonis dan bahagia, namun sejak bulan Juli 2020 sudah tidak harmonis lagi karena sering terjadi perselisihan dan pertengkaran yang disebabkan karena:
   5.1. Tergugat kurang tanggungjawab terhadap nafkah keluarga, Tergugat tidak mempunyai pekerjaan tetap, nafkah yang diberikan Tergugat tidak pernah menentu, sehingga Tergugat tidak mampu mencukupi kebutuhan rumah tangga sehari-hari, untuk mencukupi kebutuhan rumah tangga mengandalkan dari Penggugat dan orangtua Penggugat;
   5.2. Tergugat sering cemburu berlebihan terhadap Penggugat tanpa alasan, Penggugat telah berusaha bersabar, terbuka dan menjelaskan kepada Tergugat namun Tergugat tidak merubah sikapnya, sehinga Penggugat merasa tidak sanggup lagi menjalani rumah tangga bersama Tergugat;
6. Bahwa puncak perselisihan dan pertengkaran terjadi pada bulan Agustus 2020, yang berakibat Tergugat pergi meninggalkan Penggugat, sehingga antara Penggugat dan Tergugat telah pisah tempat tinggal dan sejak saat itu tidak lagi berhubungan layaknya suami istri, terhitung selama lebih dari 3 bulan;
7. Bahwa selama berpisah tempat tinggal Penggugat tinggal di rumah orangtua Penggugat, sedangkan Tergugat tinggal di orangtua Tergugat;
8. Bahwa dengan adanya kejadian tersebut Penggugat tetap bersabar, dan sudah dilakukan upaya musyawarah antara Penggugat dan Tergugat beserta keluarga Penggugat dan Tergugat, namun upaya tersebut tidak

Disclaimer
Kepaniteraan Mahkamah Agung Republik Indonesia berusaha untuk selalu mencantumkan informasi paling kini dan akurat sebagai bentuk komitmen Mahkamah Agung untuk pelayanan publik, transparansi dan akuntabilitas pelaksanaan fungsi peradilan. Namun dalam hal-hal tertentu masih dimungkinkan terjadi permasalahan teknis terkait dengan akurasi dan keterkinian informasi yang kami sajikan, hal mana akan terus kami perbaiki dari waktu kewaktu.
Dalam hal Anda menemukan inakurasi informasi yang termuat pada situs ini atau informasi yang seharusnya ada, namun belum tersedia, maka harap segera hubungi Kepaniteraan Mahkamah Agung RI melalui :
Email : kepaniteraan@mahkamahagung.go.id Telp : 021-384 3348 (ext.318)

berhasil. Oleh karena rumah tangga Penggugat dengan Tergugat sudah tidak ada ketentraman dan keharmonisan maka Penggugat mengambil sikap dan keputusan untuk bercerai dengan Tergugat, karena sudah tidak sanggup lagi untuk tetap mempertahankan mahligai rumah tangga dengan Tergugat;

9. Bahwa dengan keadaan rumah tangga tersebut, Penggugat sudah tidak ada harapan untuk melanjutkan rumah tangga bersama Tergugat, sehingga tujuan perkawinan untuk membentuk rumah tangga yang sakinah, mawadah warohmah tidak dapat terwujud, dan apabila tetap dipertahankan hanya akan menimbulkan kemudaratan yang berkepanjangan. Maka untuk mengakhiri perkawinan tersebut Penggugat bermaksud menggugat cerai Tergugat di depan sidang Pengadilan Agama Subang;
10. Bahwa Penggugat sanggup membayar biaya perkara;

Bahwa berdasarkan dalil-dalil tersebut di atas, Penggugat mohon kepada Ketua Pengadilan Agama Subang Cq. Majelis Hakim yang menyidangkan perkara ini kiranya berkenan untuk memanggil Penggugat dan Tergugat, memeriksa, mengadili dan memutuskan perkara ini dengan amarnya yang berbunyi sebagai berikut:

1. Mengabulkan gugatan Penggugat;
2. Menjatuhkan talaq satu ba'in shughra Tergugat (Tergugat) terhadap Penggugat (Penggugat);
3. Membebankan kepada Penggugat biaya perkara menurut hukum;

Apabila Majelis Hakim berpendapat lain, mohon putusan yang seadil-adilnya;

Bahwa pada hari dan tanggal yang telah ditentukan Penggugat menghadap persidangan, sedangkan Tergugat tidak pernah datang menghadap dan tidak pula mengirimkan orang lain sebagai wakil atau kuasanya yang sah untuk menghadap, meskipun telah dipanggil secara resmi dan patut pada tanggal 27 November 2020 dan tanggal 07 Desember 2020 dengan masing-masing relaas panggilan Nomor 4202/Pdt.G/2020/PA.Sbg, dan ketidakhadiran Tergugat tersebut tanpa berita dan alasan yang sah;

Bahwa karena Tergugat tidak hadir maka mediasi tidak dapat dilaksanakan, Majelis Hakim disetiap persidangan telah menasehati Penggugat,

Disclaimer
Kepaniteraan Mahkamah Agung Republik Indonesia berusaha untuk selalu mencantumkan informasi paling kini dan akurat sebagai bentuk komitmen Mahkamah Agung untuk pelayanan publik, transparansi dan akuntabilitas pelaksanaan fungsi peradilan. Namun dalam hal-hal tertentu masih dimungkinkan terjadi permasalahan teknis terkait dengan akurasi dan keterkinian informasi yang kami sajikan, hal mana akan terus kami perbaiki dari waktu kewaktu.
Dalam hal Anda menemukan inakurasi informasi yang termuat pada situs ini atau informasi yang seharusnya ada, namun belum tersedia, maka harap segera hubungi Kepaniteraan Mahkamah Agung RI melalui :
Email : kepaniteraan@mahkamahagung.go.id Telp : 021-384 3348 (ext.318)

namun tidak berhasil. Bahwa telah pula dibacakan surat gugatan Penggugat yang isinya tetap dipertahankan oleh Penggugat;

Bahwa, oleh karena Tergugat tidak hadir dipersidangan, pemeriksaan dilanjutkan kepada pembuktian;

Bahwa, di persidangan Penggugat telah mengajukan alat–alat bukti surat berupa:

1. Fotokopi Kartu Tanda Penduduk atas nama Penggugat (Siti Maryam), NIK: 321326930001 tanggal 22 Oktober 2018, yang dikeluarkan oleh Pemerintah Kabupaten Subang Provinsi Jawa Barat, bukti surat tersebut telah diberi meterai cukup dan telah dicocokkan dengan aslinya yang ternyata sesuai, lalu oleh Ketua Majelis diberi tanda P.1;
2. Fotokopi Buku Kutipan Akta Nikah atas nama Penggugat (Penggugat) dengan Tergugat (Tergugat), Nomor 2012, yang dikeluarkan oleh Kantor Urusan Agama Kecamatan Kasomalang Kabupaten Subang, tanggal 14 November 2012, bukti surat tersebut telah diberi meterai cukup dan telah dicocokkan dengan aslinya yang ternyata sesuai, oleh Ketua Majelis diberi tanda P.2;

Bahwa selain alat bukti surat tersebut Penggugat juga telah mengajukan saksi-saksi, yaitu:

1. Saksi 1, umur 39 tahun, agama Islam, pekerjaan dagang, tempat tinggal di Kabupaten Subang;

   Dibawah sumpahnya saksi tersebut memberikan keterangan yang pada pokoknya sebagai berikut:

   – Bahwa saksi kenal dengan Penggugat karena saksi adalah Kakak kandung Penggugat dan kenal dengan Tergugat semenjak menikah dengan Penggugat;
   – Bahwa Penggugat dan Tergugat adalah suami isteri yang menikah pada bulan November 2012 di Kantor Urusan Agama Kecamatan Kasomalang Kabupaten Subang;
   – Bahwa Penggugat dan Tergugat setelah menikah hidup bersama membina rumah tangga di rumah orangtua Penggugat dan telah dikaruniai seorang anak bernama Zahra Hoerunnisa, berusia 7 tahun;

Disclaimer
Kepaniteraan Mahkamah Agung Republik Indonesia berusaha untuk selalu mencantumkan informasi paling kini dan akurat sebagai bentuk komitmen Mahkamah Agung untuk pelayanan publik, transparansi dan akuntabilitas pelaksanaan fungsi peradilan. Namun dalam hal-hal tertentu masih dimungkinkan terjadi permasalahan teknis terkait dengan akurasi dan keterkinian informasi yang kami sajikan, hal mana akan terus kami perbaiki dari waktu kewaktu.
Dalam hal Anda menemukan inakurasi informasi yang termuat pada situs ini atau informasi yang seharusnya ada, namun belum tersedia, maka harap segera hubungi Kepaniteraan Mahkamah Agung RI melalui :
Email : kepaniteraan@mahkamahagung.go.id Telp : 021-384 3348 (ext.318)

- Bahwa antara Penggugat dan Tergugat dalam membina rumah tangga sering terjadi perselisihan dan pertengkaran semenjak bulan Juli 2020 disebabkan Tergugat kurang tanggungjawab terhadap nafkah keluarga, Tergugat tidak mempunyai pekerjaan tetap dan Tergugat sering cemburu berlebihan terhadap Penggugat tanpa alasan;
- Bahwa antara Penggugat dan Tergugat telah berpisah tempat tinggal semenjak bulan Agustus tahun 2020, karena Tergugat pergi meninggalkan Penggugat dan pulang kerumah orang tuanya sampai sekarang tidak pernah bersatu lagi selama 3 (tiga) bulan;
- Bahwa pihak keluarga telah berusaha untuk menasehati Penggugat dan Tergugat untuk rukun kembali namun tidak berhasil;

2. Saksi 2, umur 43 tahun, agama Islam, pekerjaan Tani, tempat tinggal di Kabupaten Subang;

   Dibawah sumpahnya saksi tersebut memberikan keterangan yang pada pokoknya sebagai berikut;

- Bahwa saksi kenal dengan Penggugat karena saksi adalah tetangga Penggugat dan kenal dengan Tergugat semenjak menikah dengan Penggugat;
- Bahwa Penggugat dan Tergugat adalah suami isteri yang menikah pada tahun 2012 di Kantor Urusan Agama Kecamatan Kasomalang Kabupaten Subang;
- Bahwa Penggugat dan Tergugat setelah menikah hidup bersama membina rumah tangga di rumah orangtua Penggugat dan telah dikaruniai seorang anak bernama Zahra Hoerunnisa, berusia 7 tahun;
- Bahwa antara Penggugat dan Tergugat dalam membina rumah tangga sering terjadi perselisihan dan pertengkaran semenjak bulan Juli 2020 disebabkan Tergugat kurang bertanggung jawab terhadap nafkah keluarga dan Tergugat sering cemburu tanpa alasan terhadap Penggugat;
- Bahwa antara Penggugat dan Tergugat telah berpisah tempat tinggal semenjak bulan Agustus tahun 2020, karena Tergugat pergi meninggalkan Penggugat dan pulang kerumah orang tuanya sampai sekarang tidak pernah bersatu lagi selama 3 (tiga) bulan;

Disclaimer
Kepaniteraan Mahkamah Agung Republik Indonesia berusaha untuk selalu mencantumkan informasi paling kini dan akurat sebagai bentuk komitmen Mahkamah Agung untuk pelayanan publik, transparansi dan akuntabilitas pelaksanaan fungsi peradilan. Namun dalam hal-hal tertentu masih dimungkinkan terjadi permasalahan teknis terkait dengan akurasi dan keterkinian informasi yang kami sajikan, hal mana akan terus kami perbaiki dari waktu kewaktu.
Dalam hal Anda menemukan inakurasi informasi yang termuat pada situs ini atau informasi yang seharusnya ada, namun belum tersedia, maka harap segera hubungi Kepaniteraan Mahkamah Agung RI melalui :
Email : kepaniteraan@mahkamahagung.go.id   Telp : 021-384 3348 (ext.318)

– Bahwa pihak keluarga telah berusaha untuk menasehati Penggugat dan Tergugat untuk rukun kembali namun tidak berhasil;

Bahwa, selanjutnya Penggugat menerangkan sudah tidak akan mengajukan alat bukti maupun keterangan lagi dan menyampaikan kesimpulan secara lisan yang pada pokoknya tetap pada gugatannya dan mohon putusan;

Bahwa untuk mempersingkat uraian dalam putusan ini, maka hal hal selengkapnya dapat dilihat dalam berita acara pemeriksaan perkara ini dan dianggap telah termuat dan menjadi bagian yang tidak terpisahkan dari putusan ini.

PERTIMBANGAN HUKUM

Menimbang, bahwa maksud dan tujuan gugatan Penggugat adalah sebagai mana tersebut di atas;

Menimbang, bahwa ternyata Tergugat tidak datang menghadap di persidangan dan tidak pula menyuruh orang lain untuk menghadap sebagai wakil/kuasanya yang sah, meskipun telah dipanggil secara resmi dan patut, sedangkan tidak ternyata bahwa tidak datangnya Tergugat tersebut disebabkan oleh suatu alasan yang sah;

Menimbang, bahwa Tergugat yang dipanggil secara resmi dan patut akan tetapi tidak menghadap harus dinyatakan tidak hadir dan gugatan tersebut harus diperiksa secara verstek;

Menimbang, bahwa berdasarkan ketentuan Pasal 125 ayat (1) HIR, yaitu putusan yang dijatuhkan tanpa hadirnya Tergugat dapat dikabulkan sepanjang berdasarkan hukum dan beralasan, oleh karena itu majelis hakim membebani Penggugat untuk membuktikan dalil-dalil gugatannya;

Menimbang, bahwa yang menjadi pokok gugatan Penggugat dalam perkara ini adalah Penggugat menggugat cerai terhadap Tergugat agar dijatuhkan talak Tergugat kepada Penggugat dengan alasan bahwa antara Penggugat dan Tergugat tidak harmonis semenjak bulan Juli 2020 karena sering terjadi perselisihan dan pertengkaran yang disebabkan Tergugat kurang tanggungjawab terhadap nafkah keluarga, Tergugat tidak mempunyai pekerjaan tetap, nafkah yang diberikan Tergugat tidak pernah menentu, sehingga Tergugat tidak mampu mencukupi kebutuhan rumah tangga sehari-hari, untuk mencukupi kebutuhan rumah tangga mengandalkan dari

Disclaimer
Kepaniteraan Mahkamah Agung Republik Indonesia berusaha untuk selalu mencantumkan informasi paling kini dan akurat sebagai bentuk komitmen Mahkamah Agung untuk pelayanan publik, transparansi dan akuntabilitas pelaksanaan fungsi peradilan. Namun dalam hal-hal tertentu masih dimungkinkan terjadi permasalahan teknis terkait dengan akurasi dan keterkinian informasi yang kami sajikan, hal mana akan terus kami perbaiki dari waktu kewaktu.
Dalam hal Anda menemukan inakurasi informasi yang termuat pada situs ini atau informasi yang seharusnya ada, namun belum tersedia, maka harap segera hubungi Kepaniteraan Mahkamah Agung RI melalui :
Email : kepaniteraan@mahkamahagung.go.id Telp : 021-384 3348 (ext.318)

Penggugat dan orangtua Penggugat, Tergugat sering cemburu berlebihan terhadap Penggugat tanpa alasan, Penggugat telah berusaha bersabar, terbuka dan menjelaskan kepada Tergugat namun Tergugat tidak merubah sikapnya, sehinga Penggugat merasa tidak sanggup lagi menjalani rumah tangga bersama Tergugat; akibat dari hal tersebut sejak bulan Agustus tahun 2020 Tergugat pergi meninggalkan Penggugat dan tidak pernah hidup berumah tangga lagi sebagai suami istri;

Menimbang, bahwa untuk membuktikan dalil gugatan Penggugat telah mengajukan alat bukti surat P.1, P.2 serta 2 orang saksi;

Menimbang, bahwa bukti P.1 (Fotokopi Kartu Tanda Penduduk Penggugat) yang telah bermeterai cukup dan cocok dengan aslinya, isi bukti tersebut menjelaskan mengenai tempat tinggal Penggugat, sehingga bukti tersebut telah memenuhi syarat formal dan materiil, serta mempunyai kekuatan pembuktian yang sempurna dan mengikat, oleh karenanya Pengadilan Agama Subang berwenang memeriksa dan mengadili perkara ini sesuai dengan ketentuan Pasal 49 huruf (a) Undang-undang Nomor 3 Tahun 2006 tentang perubahan pertama Undang-undang Nomor 7 Tahun 1989 jo. Pasal 73 ayat (1) Undang-undang Nomor 7 Tahun 1989 jo. Pasal 132 Kompilasi Hukum Islam di Indonesia Tahun 1991;

Menimbang, bahwa bukti P.2 (Fotokopi Buku Kutipan Akta Nikah) yang merupakan akta otentik dan telah bermeterai cukup dan cocok dengan aslinya, isi bukti tersebut menjelaskan mengenai data perkawinan Penggugat dan Tergugat, sehingga bukti tersebut telah memenuhi syarat formal dan materiil, sehingga bukti tersebut mempunyai kekuatan yang sempurna dan mengikat, oleh karenanya Penggugat dan Tergugat adalah pihak yang berkepentingan dalam perkara ini (*persona standi in judicio*) sesuai dengan ketentuan Pasal 2 ayat 2 Undang-undang Nomor 1 Tahun 1974 jo. Pasal 7 ayat 1 Kompilasi Hukum Islam di Indonesia Tahun 1991;

Menimbang, bahwa saksi 1 dan saksi 2 Penggugat, sudah dewasa, berakal sehat, dan sebelum memberikan keterangannya telah disumpah terlebih dahulu, sehingga memenuhi syarat formil sebagaimana diatur dalam Pasal 145 ayat (1) HIR dan merupakan keluarga dan orang dekat Penggugat,

Disclaimer
Kepaniteraan Mahkamah Agung Republik Indonesia berusaha untuk selalu mencantumkan informasi paling kini dan akurat sebagai bentuk komitmen Mahkamah Agung untuk pelayanan publik, transparansi dan akuntabilitas pelaksanaan fungsi peradilan. Namun dalam hal-hal tertentu masih dimungkinkan terjadi permasalahan teknis terkait dengan akurasi dan keterkinian informasi yang kami sajikan, hal mana akan terus kami perbaiki dari waktu kewaktu.
Dalam hal Anda menemukan inakurasi informasi yang termuat pada situs ini atau informasi yang seharusnya ada, namun belum tersedia, maka harap segera hubungi Kepaniteraan Mahkamah Agung RI melalui :
Email : kepaniteraan@mahkamahagung.go.id Telp : 021-384 3348 (ext.318)

sesuai ketentuan Pasal 22 ayat (2) Peraturan Pemerintah Nomor 9 Tahun 1975 jo. pasal 134 Kompilasi Hukum Islam;

Menimbang, bahwa keterangan 2 (dua) orang saksi Penggugat adalah fakta yang dilihat sendiri, didengar sendiri, dialami sendiri dan relevan dengan dalil yang harus dibuktikan oleh Penggugat, oleh karena itu keterangan saksi tersebut telah memenuhi syarat materiil sebagaimana telah diatur dalam Pasal 172 HIR. sehingga keterangan saksi tersebut memiliki kekuatan pembuktian dan dapat diterima sebagai alat bukti;

Menimbang, bahwa keterangan saksi 1 dan saksi 2 Penggugat bersesuaian dan cocok antara satu dengan yang lain oleh karena itu keterangan dua orang saksi tersebut memenuhi Pasal 172 dan Pasal 173 HIR;

Menimbang, bahwa berdasarkan keterangan Penggugat dan 2 orang saksi terbukti fakta kejadian sebagai berikut:

- Bahwa sejak bulan Juli 2020 antara Penggugat dengan Tergugat telah terjadi pertengkaran terus menerus dan sulit untuk rukun kembali, disebabkan Tergugat kurang bertanggungjawab terhadap nafkah keluarga dan sering cemburu berlebihan terhadap Penggugat tanpa alasan;
- Bahwa sejak bulan Agustus tahun 2020 Penggugat dan Tergugat sudah berpisah tempat tinggal sampai dengan sekarang selama 3 (tiga) bulan dan tidak saling memperdulikan lagi;
- Bahwa antara Penggugat dengan Tergugat telah diupayakan untuk rukun, namun tidak berhasil dan kini Penggugat tetap bersikeras untuk bercerai dengan Tergugat;

Menimbang, bahwa berdasarkan fakta-fakta tersebut di atas dapat disimpulkan fakta hukum sebagai berikut:

1. Penggugat dan Tergugat sudah tidak mempunyai rasa saling mencintai, hormat menghormati, setia dan memberi bantuan lahir batin, dimana rasa cinta, hormat menghormati, setia dan saling memberi bantuan lahir batin adalah merupakan sendi dasar dan menjadi kewajiban suami istri dalam hidup berumah tangga (vide: pasal 33 Undang Undang Nomor 1 Tahun 1974 Tentang Perkawinan jo. pasal 77 ayat (2) Kompilasi Hukum Islam);
2. Rumah tangga Penggugat dan Tergugat benar benar telah pecah (*broken marriage*) serta telah terjadi perselisihan dan pertengkaran secara terus

Disclaimer
Kepaniteraan Mahkamah Agung Republik Indonesia berusaha untuk selalu mencantumkan informasi paling kini dan akurat sebagai bentuk komitmen Mahkamah Agung untuk pelayanan publik, transparansi dan akuntabilitas pelaksanaan fungsi peradilan. Namun dalam hal-hal tertentu masih dimungkinkan terjadi permasalahan teknis terkait dengan akurasi dan keterkinian informasi yang kami sajikan, hal mana akan terus kami perbaiki dari waktu kewaktu.
Dalam hal Anda menemukan inakurasi informasi yang termuat pada situs ini atau informasi yang seharusnya ada, namun belum tersedia, maka harap segera hubungi Kepaniteraan Mahkamah Agung RI melalui :
Email : kepaniteraan@mahkamahagung.go.id Telp : 021-384 3348 (ext.318)

menerus yang tidak ada harapan akan hidup rukun lagi dalam rumah tangga, sehingga tujuan perkawinan untuk membentuk rumah tangga yang bahagia dan kekal sebagaimana dimaksud pasal 1 Undang Undang Nomor 1 Tahun 1974 jo. pasal 3 Kompilasi Hukum Islam (KHI) tidak dapat diwujudkan dalam rumah tangga Penggugat dan Tergugat;

3. Penggugat dengan Tergugat telah terjadi pisah tempat tinggal sejak bulan Agustus tahun 2020 dan sejak saat itu tidak hidup bersama lagi sebagai suami istri, di mana menurut yurisprudensi MARI Nomor 379 K/AG/1995 tanggal 26 Maret 1997, suami isteri yang tidak berdiam serumah lagi dan tidak ada harapan untuk hidup rukun kembali, maka rumah tangga tersebut terbukti telah retak dan pecah;

Menimbang, bahwa fakta hukum tersebut telah memenuhi norma hukum Islam yang terkandung dalam *Al-Qur'an* surat *Ar-Rum* ayat (21) :

وَمِنْ آيَاتِهِ أَنْ خَلَقَ لَكُمْ مِنْ أَنْفُسِكُمْ أَزْوَاجًا لِتَسْكُنُوا إِلَيْهَا وَجَعَلَ بَيْنَكُمْ مَوَدَّةً وَرَحْمَةً ۚ إِنَّ فِي ذَٰلِكَ لَآيَاتٍ لِقَوْمٍ يَتَفَكَّرُونَ

Artinya : "Dan di antara tanda-tanda kekuasaan-Nya ialah Dia menciptakan untukmu isteri-isteri dari jenismu sendiri, supaya kamu cenderung dan merasa tenteram kepadanya, dan dijadikan-Nya diantaramu rasa kasih dan sayang. Sesungguhnya pada yang demikian itu benar-benar terdapat tanda-tanda bagi kaum yang berfikir."

Menimbang, bahwa berdasarkan pertimbangan pertimbangan tersebut di atas, maka Majelis Hakim dalam permusyawaratannya menilai bahwa gugatan Penggugat telah terbukti dan memenuhi alasan perceraian sebagaimana dimaksud dalam penjelasan pasal 39 ayat (2) huruf (f) Undang Undang Nomor 1 Tahun 1974 jo. pasal 19 huruf (f) Peraturan Pemerintah Nomor 9 Tahun 1975 jo. pasal 116 huruf (f) Kompilasi Hukum Islam, oleh karenanya gugatan Penggugat patut dikabulkan dengan diktum menjatuhkan talak satu ba'in sughra Tergugat terhadap Penggugat sesuai dengan ketentuan pasal 119 ayat 2 huruf (c) Kompilasi Hukum Islam;

Menimbang, bahwa berdasarkan ketentuan Pasal 89 ayat (1) Undang-Undang Nomor 7 Tahun 1989 yang telah diubah dan ditambah dengan Undang Undang Nomor 3 Tahun 2006 terakhir dirubah dengan Undang Undang Nomor

Disclaimer
Kepaniteraan Mahkamah Agung Republik Indonesia berusaha untuk selalu mencantumkan informasi paling kini dan akurat sebagai bentuk komitmen Mahkamah Agung untuk pelayanan publik, transparansi dan akuntabilitas pelaksanaan fungsi peradilan. Namun dalam hal-hal tertentu masih dimungkinkan terjadi permasalahan teknis terkait dengan akurasi dan keterkinian informasi yang kami sajikan, hal mana akan terus kami perbaiki dari waktu kewaktu.
Dalam hal Anda menemukan inakurasi informasi yang termuat pada situs ini atau informasi yang seharusnya ada, namun belum tersedia, maka harap segera hubungi Kepaniteraan Mahkamah Agung RI melalui :
Email : kepaniteraan@mahkamahagung.go.id Telp : 021-384 3348 (ext.318)

50 Tahun 2009, maka semua biaya yang timbul dalam perkara ini dibebankan kepada Penggugat;

Mengingat, ketentuan dalam Peraturan Perundang undangan yang berlaku dan dalil syar'i yang berkaitan dengan perkara ini;

M E N G A D I L I

1. Menyatakan Tergugat telah dipanggil secara resmi dan patut untuk menghadap persidangan, tidak hadir;
2. Mengabulkan gugatan Penggugat dengan verstek;
3. Menjatuhkan talak satu ba'in sughra Tergugat (Tergugat) terhadap Penggugat (Penggugat);
4. Membebankan biaya perkara ini kepada Penggugat yang hingga kini dihitung sebesar Rp516.000,00 (lima ratus enam belas ribu rupiah);

Demikian diputuskan dalam musyawarah Majelis Hakim pada hari Senin, tanggal 14 Desember 2020 Masehi bertepatan dengan tanggal 28 Rabiul Akhir 1442 Hijriyyah. oleh Hakim Pengadilan Agama Subang yang terdiri dari Dede Rika Nurhasanah, S.Ag., M.H., sebagai Ketua Majelis dan Drs. Esib Jaelani, M.H. serta Drs. H. Humaidi masing-masing sebagai Hakim Anggota, dibantu oleh Drs. Budiana, S.H.I. sebagai Panitera Pengganti. Putusan tersebut pada hari itu juga diucapkan dalam persidangan terbuka untuk umum oleh Ketua Majelis tersebut dengan dihadiri oleh Penggugat tanpa hadirnya Tergugat.

Ketua Majelis,

ttd.

Dede Rika Nurhasanah, S.Ag., M.H.

Hakim Anggota, Hakim Anggota,

T

Disclaimer
Kepaniteraan Mahkamah Agung Republik Indonesia berusaha untuk selalu mencantumkan informasi paling kini dan akurat sebagai bentuk komitmen Mahkamah Agung untuk pelayanan publik, transparansi dan akuntabilitas pelaksanaan fungsi peradilan. Namun dalam hal-hal tertentu masih dimungkinkan terjadi permasalahan teknis terkait dengan akurasi dan keterkinian informasi yang kami sajikan, hal mana akan terus kami perbaiki dari waktu kewaktu.
Dalam hal Anda menemukan inakurasi informasi yang termuat pada situs ini atau informasi yang seharusnya ada, namun belum tersedia, maka harap segera hubungi Kepaniteraan Mahkamah Agung RI melalui :
Email : kepaniteraan@mahkamahagung.go.id Telp : 021-384 3348 (ext.318)

t

Drs. Esib Jaelani, M.H. Drs. H. Humaidi

Panitera Pengganti,

tt

Drs. Budiana, S.H.I.

Perincian Biaya :

1. Pendaftaran	:	Rp30.000,00
2. Proses	:	Rp50.000,00
3. Panggilan	:	Rp400.000,00
4. PNBP Panggilan	:	Rp20.000,00
5. Redaksi	:	Rp10.000,00
6. Meterai	:	Rp6.000,00
**Jumlah**	**:**	**Rp516.000,00**

(lima ratus enam belas ribu rupiah)

Untuk **Salinan** yang sama bunyinya oleh :
PANITERA PENGADILAN AGAMA SUBANG

Drs. H. Dadang Zaenal, M.M.

Disclaimer
Kepaniteraan Mahkamah Agung Republik Indonesia berusaha untuk selalu mencantumkan informasi paling kini dan akurat sebagai bentuk komitmen Mahkamah Agung untuk pelayanan publik, transparansi dan akuntabilitas pelaksanaan fungsi peradilan. Namun dalam hal-hal tertentu masih dimungkinkan terjadi permasalahan teknis terkait dengan akurasi dan keterkinian informasi yang kami sajikan, hal mana akan terus kami perbaiki dari waktu kewaktu.
Dalam hal Anda menemukan inakurasi informasi yang termuat pada situs ini atau informasi yang seharusnya ada, namun belum tersedia, maka harap segera hubungi Kepaniteraan Mahkamah Agung RI melalui :
Email : kepaniteraan@mahkamahagung.go.id Telp : 021-384 3348 (ext.318)
