# Supplementary material for: Classification of divorce causes during the COVID-19 pandemic using convolutional neural networks
Source: PeerJ Comput Sci. 2022 Jun 30;8:e998. doi: 10.7717/peerj-cs.998 (PMC9299239; doi:10.7717/peerj-cs.998)
Supplement: Supplemental Information 4 [file peerj-cs-08-998-s004.zip › Konflik Dataset/Data ke-13.pdf]

P U T U S A N

Nomor **4203/Pdt.G/2020/PA.Sbg**

بسم الله الرحمن الرحيم

DEMI KEADILAN BERDASARKAN KETUHANAN YANG MAHA ESA

Pengadilan Agama Subang yang memeriksa dan mengadili perkara tertentu pada tingkat pertama dalam persidangan Majelis Hakim telah menjatuhkan putusan dalam perkara **Cerai Talak**, antara:

Pemohon, umur 36 tahun, agama Islam, pekerjaan Wiraswasta, bertempat tinggal di Subang, untuk selanjutnya disebut sebagai **Pemohon**;

Melawan

Termohon, umur 40 tahun, agama Islam, pekerjaan Guru Honorer, bertempat tinggal di rumah Kontrakan an. Ibu Rosi Indahsari di kabupaten Subang, untuk selanjutnya disebut sebagai **Termohon**;

Pengadilan Agama tersebut;

Telah membaca berkas perkara;

Telah mendengar keterangan Pemohon;

Telah memeriksa bukti-bukti dalam persidangan;

DUDUK PERKARA

Bahwa, Pemohon dengan Surat permohonannya tertanggal 26 November 2020 yang terdaftar di Kepaniteraan Pengadilan Agama Subang pada tanggal 26 November 2020 dengan register perkara Nomor 4203/Pdt.G/2020/PA.Sbg, telah mengemukakan hal-hal dan alasan-alasan sebagai berikut:

Disclaimer
Kepaniteraan Mahkamah Agung Republik Indonesia berusaha untuk selalu mencantumkan informasi paling kini dan akurat sebagai bentuk komitmen Mahkamah Agung untuk pelayanan publik, transparansi dan akuntabilitas pelaksanaan fungsi peradilan. Namun dalam hal-hal tertentu masih dimungkinkan terjadi permasalahan teknis terkait dengan akurasi dan keterkinian informasi yang kami sajikan, hal mana akan terus kami perbaiki dari waktu kewaktu.
Dalam hal Anda menemukan inakurasi informasi yang termuat pada situs ini atau informasi yang seharusnya ada, namun belum tersedia, maka harap segera hubungi Kepaniteraan Mahkamah Agung RI melalui :
Email : kepaniteraan@mahkamahagung.go.id Telp : 021-384 3348 (ext.318)

1. Bahwa Pemohon saat ini bertempat tinggal di kabupaten Subang sebagaimana ternyata dari KTP atas nama Pemohon dengan NIK 32130640003, tanggal 13 April 2019 yang diterbitkan oleh Kepala Dinas Kependudukan dan Pencatatan Sipil Kabupaten Subang;
2. Bahwa pada tanggal 31 Agustus 2007, Pemohon dengan Termohon melangsungkan pernikahan di Wilayah Hukum Kantor Urusan Agama Kecamatan Purwadadi Kabupaten Subang sebagaimana ternyata dari Buku Kutipan Akta Nikah yang dikeluarkan oleh Pegawai Pencatat Nikah Kantor Urusan Agama Kecamatan Purwadadi Kabupaten Subang Nomor 2007 tertanggal 31 Agustus 2007;
3. Bahwa setelah akad nikah Pemohon dan Termohon hidup bersama sebagai suami istri di rumah kontrakan;
4. Bahwa perkawinan antara Pemohon dan Termohon sampai saat ini telah berjalan selama 13 tahun 3 bulan dan telah dikaruniai keturunan 2 orang anak yang masing-masing bernama : Ayesha Agrichaila berusia 12 tahun dan Qilauna Argeulis berusia 7 tahun;
5. Bahwa semula rumah tangga Pemohon dan Termohon harmonis dan bahagia, namun sejak bulan Desember 2019 sudah tidak harmonis lagi karena sering terjadi perselisihan dan pertengkaran yang disebabkan karena :
   a. Termohon diketahui memiliki hubungan dengan seorang pria lain yang bernama Adi yang berasal dari Bekasi, saat ditanyakan Termohon mengakuinya ;
   b. Selain itu, Termohon tidak mau melayani kebutuhan Pemohon, dan apabila terjadi peselisihan Termohon sering meminta untuk di ceraikan bahkan Pemohon pernah di usir oleh Termohon;
6. Bahwa puncak perselisihan dan pertengkaran antara Pemohon dan Termohon terjadi pada tanggal 03 Agustus 2020, yang berakibat Pemohon di usir oleh Termohon dan tidak pernah hidup berumah tangga lagi sebagai suami istri dan sampai sekarang sudah berpisah selama 3 bulan;
7. Bahwa selama berpisah tempat tinggal Pemohon tinggal di rumah

Disclaimer
Kepaniteraan Mahkamah Agung Republik Indonesia berusaha untuk selalu mencantumkan informasi paling kini dan akurat sebagai bentuk komitmen Mahkamah Agung untuk pelayanan publik, transparansi dan akuntabilitas pelaksanaan fungsi peradilan. Namun dalam hal-hal tertentu masih dimungkinkan terjadi permasalahan teknis terkait dengan akurasi dan keterkinian informasi yang kami sajikan, hal mana akan terus kami perbaiki dari waktu kewaktu.
Dalam hal Anda menemukan inakurasi informasi yang termuat pada situs ini atau informasi yang seharusnya ada, namun belum tersedia, maka harap segera hubungi Kepaniteraan Mahkamah Agung RI melalui :
Email : kepaniteraan@mahkamahagung.go.id   Telp : 021-384 3348 (ext.318)

orangtua Pemohon, sedangkan Termohon tinggal di rumah kontrakan Termohon;

8. Bahwa dengan adanya kejadian tersebut Pemohon tetap bersabar, dan sudah dilakukan upaya musyawarah antara Pemohon dan Termohon beserta keluarga Pemohon dan Termohon, namun upaya tersebut tidak berhasil;
9. Bahwa dengan keadaan rumah tangga tersebut, Pemohon sudah tidak ada harapan untuk melanjutkan rumah tangga bersama Termohon, sehingga tujuan perkawinan untuk membentuk rumah tangga yang sakinah, mawadah warohmah tidak dapat terwujud, dan apabila tetap dipertahankan hanya akan menimbulkan kemudaratan yang berkepanjangan. Maka untuk mengakhiri perkawinan tersebut Pemohon bermaksud menceraikan Termohon di depan sidang Pengadilan Agama Subang;
10. Bahwa Pemohon sanggup membayar biaya perkara;

Bahwa berdasarkan dalil-dalil tersebut di atas, Pemohon mohon kepada Ketua Pengadilan Agama Subang Cq. Majelis Hakim yang menyidangkan perkara ini kiranya berkenan untuk memanggil Pemohon dan Termohon, memeriksa, mengadili dan memutuskan perkara ini dengan amarnya yang berbunyi sebagai berikut:

1. Mengabulkan permohonan Pemohon;
2. Memberi izin kepada Pemohon (Pemohon) untuk menjatuhkan talak satu raj'i terhadap Termohon (Termohon) didepan sidang Pengadilan Agama Subang;
3. Membebankan biaya perkara ini menurut hukum;

Apabila majelis hakim berpendapat lain, mohon putusan yang seadil-adilnya;

Bahwa, pada hari sidang yang telah ditetapkan Pemohon datang menghadap sendiri di muka persidangan, sedangkan Termohon tidak pernah datang menghadap dan tidak pula mengirimkan orang lain sebagai wakil atau kuasanya yang sah untuk menghadap, meskipun telah dipanggil secara resmi dan patut pada tanggal 02 Desember 2020 dan tanggal 10 Desember 2020

Disclaimer
Kepaniteraan Mahkamah Agung Republik Indonesia berusaha untuk selalu mencantumkan informasi paling kini dan akurat sebagai bentuk komitmen Mahkamah Agung untuk pelayanan publik, transparansi dan akuntabilitas pelaksanaan fungsi peradilan. Namun dalam hal-hal tertentu masih dimungkinkan terjadi permasalahan teknis terkait dengan akurasi dan keterkinian informasi yang kami sajikan, hal mana akan terus kami perbaiki dari waktu kewaktu.
Dalam hal Anda menemukan inakurasi informasi yang termuat pada situs ini atau informasi yang seharusnya ada, namun belum tersedia, maka harap segera hubungi Kepaniteraan Mahkamah Agung RI melalui :
Email : kepaniteraan@mahkamahagung.go.id Telp : 021-384 3348 (ext.318)

dengan masing masing relaas panggilan Nomor 4203/Pdt.G/2020/PA.Sbg, dan ketidakhadiran Termohon tersebut tanpa berita dan alasan yang sah.

Bahwa, oleh karena Termohon tidak hadir dipersidangan walaupun telah dipanggil secara resmi dan patut maka upaya mediasi tidak dapat dilaksanakan. Majelis dipersidangan telah pula menasehati Pemohon, namun tidak berhasil. Selanjutnya pemeriksaan terhadap perkara dimulai dengan membacakan Surat permohonan Pemohon yang isinya tetap dipertahankan oleh Pemohon;

Bahwa, oleh karena Termohon tidak hadir, pemeriksaan dilanjutkan kepada pembuktian:

Bahwa, untuk meneguhkan dalil dalil permohonan cerai talaknya di muka persidangan Pemohon mengajukan alat bukti surat berupa:

1. Fotokopi Kartu Tanda Penduduk atas nama Pemohon (Pemohon), NIK: 321306840003, yang dikeluarkan tanggal 13 April 2019 oleh Pemerintah Daerah Kabupaten Subang Provinsi Jawa Barat, bukti surat tersebut telah diberi materai cukup dan telah dicocokkan dengan aslinya yang ternyata sesuai, oleh Ketua Majelis diberi tanda **P.1**;
2. Fotokopi Buku Kutipan Akta Nikah Akta Nikah atas nama Pemohon (Pemohon) dengan Termohon (Termohon), Nomor 2007, yang dikeluarkan oleh Kantor Urusan Agama Kecamatan Purwadadi Kabupaten Subang, tanggal 31 Agustus 2007, bukti surat tersebut telah diberi materai cukup dan telah dicocokkan dengan aslinya yang ternyata sesuai, oleh Ketua Majelis diberi tanda **P.2**;

Bahwa, selain mengajukan alat bukti surat, Pemohon juga telah mengajukan saksi-saksi, yaitu:

1. Saksi 1 , umur 47 tahun, agama Islam, pekerjaan Tani , tempat tinggal di kabupaten Subang;

   Dibawah sumpahnya saksi tersebut memberikan keterangan sebagai berikut:

   – Bahwa saksi kenal dengan Pemohon karena saksi Kakak Kandung Pemohon dan kenal dengan Termohon semenjak menikah dengan Pemohon ;

Disclaimer
Kepaniteraan Mahkamah Agung Republik Indonesia berusaha untuk selalu mencantumkan informasi paling kini dan akurat sebagai bentuk komitmen Mahkamah Agung untuk pelayanan publik, transparansi dan akuntabilitas pelaksanaan fungsi peradilan. Namun dalam hal-hal tertentu masih dimungkinkan terjadi permasalahan teknis terkait dengan akurasi dan keterkinian informasi yang kami sajikan, hal mana akan terus kami perbaiki dari waktu kewaktu.
Dalam hal Anda menemukan inakurasi informasi yang termuat pada situs ini atau informasi yang seharusnya ada, namun belum tersedia, maka harap segera hubungi Kepaniteraan Mahkamah Agung RI melalui :
Email : kepaniteraan@mahkamahagung.go.id Telp : 021-384 3348 (ext.318)

- Bahwa Pemohon dan Termohon adalah suami isteri yang menikah pada tahun 2007 di wilayah Kantor Urusan Agama Kecamatan Purwadadi Kabupaten Subang;
- Bahwa selama dalam perkawinan Pemohon dan Termohon dan telah dikaruniai keturunan 2 orang anak yang masing-masing bernama Ayesha Agrichaila, umur 12 tahun dan Qilauna Argeulis, umur 7 tahun ;
- Bahwa Pemohon dan Termohon setelah menikah bertempat tinggal di rumah kontrakan;
- Bahwa Pemohon dan Termohon dalam membina rumah tangga semenjak bulan Desember 2019 sering terjadi perselisihan dan pertengkaran dikarenakan Termohon menjalin hubungan dengan laki-laki lain yang bernama Adi yang berasal dari Bekasi, dan setiap terjadi pertengkaran Termohon suka meminta untuk diceraikan oleh Pemohon selain itu Termohon suka mengusir Pemohon;
- Bahwa setiap terjadi pertengkaran Termohon suka berkata kasar kepada Pemohon;
- Bahwa antara Pemohon dan Termohon telah berpisah tempat tinggal semenjak awal bulan Agustus 2020 karena Termohon pulang kerumah orang tuanya sampai sekarang tidak pernah bersatu lagi selama 4 (empat) bulan;
- Bahwa pihak keluarga telah berusaha menasehati kedua belah pihak untuk kembali rukun namun tidak berhasil ;

2. Saksi 2, umur 62 tahun, agama Islam, pekerjaan dagang , tempat tinggal di kabupaten Subang;

Dibawah sumpahnya saksi tersebut memberikan keterangan sebagai berikut:

- Bahwa saksi kenal dengan Pemohon karena saksi adalah Saudara Sepupu Pemohon dan kenal dengan Termohon semenjak menikah dengan Pemohon ;
- Bahwa Pemohon dan Termohon adalah suami isteri yang menikah pada tahun 2007 di wilayah Kantor Urusan Agama Kecamatan Purwadadi Kabupaten Subang;

Disclaimer
Kepaniteraan Mahkamah Agung Republik Indonesia berusaha untuk selalu mencantumkan informasi paling kini dan akurat sebagai bentuk komitmen Mahkamah Agung untuk pelayanan publik, transparansi dan akuntabilitas pelaksanaan fungsi peradilan. Namun dalam hal-hal tertentu masih dimungkinkan terjadi permasalahan teknis terkait dengan akurasi dan keterkinian informasi yang kami sajikan, hal mana akan terus kami perbaiki dari waktu kewaktu.
Dalam hal Anda menemukan inakurasi informasi yang termuat pada situs ini atau informasi yang seharusnya ada, namun belum tersedia, maka harap segera hubungi Kepaniteraan Mahkamah Agung RI melalui :
Email : kepaniteraan@mahkamahagung.go.id Telp : 021-384 3348 (ext.318)

- Bahwa selama dalam perkawinan Pemohon dan Termohon dan telah dikaruniai keturunan 2 orang anak yang masing-masing bernama Ayesha Agrichaila, umur 12 tahun dan Qilauna Argeulis, umur 7 tahun;
- Bahwa Pemohon dan Termohon setelah menikah bertempat tinggal di rumah kontrakan;
- Bahwa Pemohon dan Termohon dalam membina rumah tangga semenjak bulan Desember 2019 sering terjadi perselisihan dan pertengkaran dikarenakan Termohon menjalin hubungan dengan laki-laki lain yang bernama Adi yang berasal dari Bekasi, dan setiap terjadi pertengkaran Termohon suka meminta untuk diceraikan oleh Pemohon selain itu Termohon suka mengusir Pemohon;
- Bahwa setiap terjadi pertengkaran Termohon suka meminta berkata kasar kepada Pemohon ;
- Bahwa antara Pemohon dan Termohon telah berpisah tempat tinggal semenjak awal bulan Agustus 2020 karena Termohon pulang kerumah orang tuanya sampai sekarang tidak pernah bersatu lagi selama 4 (empat) bulan;
- Bahwa pihak keluarga telah berusaha menasehati kedua belah pihak untuk kembali rukun namun tidak berhasil ;

Bahwa, selanjutnya Pemohon menerangkan sudah tidak akan mengajukan alat bukti maupun keterangan lagi dan menyampaikan kesimpulan secara lisan yang pada pokoknya tetap pada permohonannya dan mohon putusan;

Bahwa, untuk mempersingkat uraian putusan ini, maka hal-hal selengkapnya dianggap telah termuat dalam Berita Acara Sidang dan menjadi bagian yang tidak terpisahkan dari putusan ini;

PERTIMBANGAN HUKUM

Menimbang, bahwa maksud dan tujuan permohonan Pemohon adalah sebagaimana tersebut di atas;

Disclaimer
Kepaniteraan Mahkamah Agung Republik Indonesia berusaha untuk selalu mencantumkan informasi paling kini dan akurat sebagai bentuk komitmen Mahkamah Agung untuk pelayanan publik, transparansi dan akuntabilitas pelaksanaan fungsi peradilan. Namun dalam hal-hal tertentu masih dimungkinkan terjadi permasalahan teknis terkait dengan akurasi dan keterkinian informasi yang kami sajikan, hal mana akan terus kami perbaiki dari waktu kewaktu.
Dalam hal Anda menemukan inakurasi informasi yang termuat pada situs ini atau informasi yang seharusnya ada, namun belum tersedia, maka harap segera hubungi Kepaniteraan Mahkamah Agung RI melalui :
Email : kepaniteraan@mahkamahagung.go.id Telp : 021-384 3348 (ext.318)

Menimbang, bahwa oleh karena Termohon tidak hadir dipersidangan maka upaya mediasi sebagaimana diatur dalam PERMA Nomor 1 Tahun 2016 tidak dapat dilaksanakan;

Menimbang, bahwa Termohon tidak pernah hadir menghadap dan tidak mengirimkan orang lain sebagai wakil atau kuasanya yang sah untuk datang menghadap, sedangkan tidak ternyata bahwa ketidakhadiran Termohon tersebut berdasarkan atas alasan yang sah menurut hukum. Oleh karena itu, maka perkara ini dapat diperiksa dengan tanpa hadirnya Termohon. Hal ini sesuai dengan ketentuan Pasal 126 HIR dan sejalan pula dengan sebuah pendapat Ulama di dalam Kitab al-Anwar juz: II halaman 149, yang artinya sebagai berikut :

"Apabila Termohon tidak hadir karena bersembunyi atau enggan, maka Hakim dapat memeriksa permohonan tersebut dan alat alat bukti yang diajukan dan memberikan keputusannya";

Menimbang, bahwa yang menjadi pokok permasalahan dalam perkara ini adalah Pemohon mengajukan permohonan mohon izin untuk menjatuhkan thalak kepada Termohon dengan alasan bahwa antara Pemohon dan Termohon sering terjadi perselisihan dan pertengkaran semenjak bulan Desember 2019 disebabkan Termohon diketahui memiliki hubungan dengan seorang pria lain yang bernama Adi yang berasal dari Bekasi, saat ditanyakan Termohon mengakuinya, selain itu Termohon tidak mau melayani kebutuhan Pemohon, dan apabila terjadi peselisihan Termohon sering meminta untuk di ceraikan bahkan Pemohon pernah di usir oleh Termohon, yang mengakibatkan antara Pemohon dan Termohon telah berpisah tempat tinggal semenjak tanggal 03 Agustus 2020 sampai sekarang tidak pernah bersatu lagi selama 4 (empat) bulan;

Menimbang, oleh karena Termohon tidak pernah hadir, maka tidak diketahui jawaban atau bantahan Termohon, namun karena perkara ini menyangkut Undang-undang Perkawinan yang memerlukan acara khusus, maka Pemohon dibebani wajib bukti ;

Disclaimer
Kepaniteraan Mahkamah Agung Republik Indonesia berusaha untuk selalu mencantumkan informasi paling kini dan akurat sebagai bentuk komitmen Mahkamah Agung untuk pelayanan publik, transparansi dan akuntabilitas pelaksanaan fungsi peradilan. Namun dalam hal-hal tertentu masih dimungkinkan terjadi permasalahan teknis terkait dengan akurasi dan keterkinian informasi yang kami sajikan, hal mana akan terus kami perbaiki dari waktu kewaktu.
Dalam hal Anda menemukan inakurasi informasi yang termuat pada situs ini atau informasi yang seharusnya ada, namun belum tersedia, maka harap segera hubungi Kepaniteraan Mahkamah Agung RI melalui :
Email : kepaniteraan@mahkamahagung.go.id Telp : 021-384 3348 (ext.318)

Menimbang, bahwa untuk menguatkan dalil-dalil permohonannya, Pemohon telah mengajukan bukti surat dan saksi yang telah memenuhi syarat formil dan materil, sehingga dapat diterima sebagai alat bukti;

Menimbang, bahwa berdasarkan Bukti **P.1** dan relas panggilan Termohon, telah terbukti menurut hukum bahwa tempat kediaman Pemohon dan Termohon berada di wilayah Kabupaten Subang, maka pengajuan permohonan Pemohon aquo secara formil dapat diterima, hal ini sesuai dengan ketentuan Pasal 66 ayat (2) Undang-Undang Nomor 7 Tahun 1989, seperti yang telah diubah dan ditambah dengan Undang-Undang Nomor 3 Tahun 2006, dan terakhir dirubah dengan Undang-Undang Nomor 50 Tahun 2009;

Menimbang, berdasarkan Bukti **P.2**, terbukti menurut hukum bahwa antara Pemohon (Pemohon) dengan Termohon (Termohon) sejak tanggal 31 Agustus 2007 telah terikat ikatan tali perkawinan yang sah, sesuai dengan ketentuan Pasal 2 Undang-Undang Nomor 1 Tahun 1974, Jo. Pasal 4 Kompilasi Hukum Islam;

Menimbang, bahwa untuk menguatkan dalil permohonan Pemohon, maka Pemohon telah mengajukan dua orang saksi dalam persidangan dibawah sumpahnya menerangkan bahwa antara Pemohon dan Termohon dalam membina rumah tangga semenjak bulan Desember 2019 sering terjadi perselisihan dan pertengkaran disebabkan Termohon menjalin hubungan dengan laki-laki lain yang bernama Adi yang berasal dari Bekasi, dan setiap terjadi pertengkaran Termohon suka meminta untuk diceraikan oleh Pemohon selain itu Termohon suka mengusir Pemohon, yang mengakibat dari hal tersebut antara Pemohon dan Termohon telah berpisah tempat tinggal semenjak awal bulan Agustus 2020 sampai sekarang tidak pernah bersatu lagi selama 4 (empat) bulan;

Menimbang, bahwa dari keterangan saksi diatas ditemukan fakta kejadian bahwa antara Pemohon dan Termohon semenjak bulan Desember 2019 sering terjadi perselisihan dan pertengkaran yang mendalam, sehingga antara kedua belah pihak telah berpisah tempat tinggal semenjak tanggal 03 Agustus 2020 sampai sekarang tidak pernah bersatu lagi selama 4 (empat)

Disclaimer
Kepaniteraan Mahkamah Agung Republik Indonesia berusaha untuk selalu mencantumkan informasi paling kini dan akurat sebagai bentuk komitmen Mahkamah Agung untuk pelayanan publik, transparansi dan akuntabilitas pelaksanaan fungsi peradilan. Namun dalam hal-hal tertentu masih dimungkinkan terjadi permasalahan teknis terkait dengan akurasi dan keterkinian informasi yang kami sajikan, hal mana akan terus kami perbaiki dari waktu kewaktu.
Dalam hal Anda menemukan inakurasi informasi yang termuat pada situs ini atau informasi yang seharusnya ada, namun belum tersedia, maka harap segera hubungi Kepaniteraan Mahkamah Agung RI melalui :
Email : kepaniteraan@mahkamahagung.go.id Telp : 021-384 3348 (ext.318)

bulan, hal ini telah menunjukkan bahwa rumah tangga Pemohon dan Termohon telah pecah;

Menimbang, bahwa keterangan saksi-saksi tersebut merupakan keterangan yang dilihat dan didengar sendiri, dan keterangan saksi-saksi tersebut relevan dengan dalil permohonan Pemohon, dan sebagaimana diatur dalam Pasal 171 HIR, keterangan saksi tersebut memiliki kekuatan pembuktian dan dapat diterima sebagai alat bukti;

Menimbang, bahwa dengan sikap Termohon tidak pernah datang menghadap ke persidangan serta permohonan Pemohon tidak melawan hukum dan beralasan maka perkara ini dapat diputus dengan verstek dan berdasarkan penilaian terhadap keterangan kedua orang saksi tersebut diatas, Majelis Hakim menemukan fakta hukum bahwa antara Pemohon dan Termohon dalam membina rumah tangga semenjak bulan Desember 2019 telah terjadi perserlisihan dan pertengkaran yang berkepanjangan atau terus menerus, sehingga antara kedua belah pihak telah pisah tempat tinggal semenjak tanggal 03 Agustus 2020 sampai dengan saat ini tidak pernah bersatu lagi selama 4 (empat) bulan, hal itu menunjukkan rumah tangga antara Pemohon dan Termohon telah pecah sehingga tidak mungkin untuk rukun kembali karena lebih besar mudharat dari pada maslahatnya ;

Menimbang, bahwa berdasarkan fakta-fakta hukum tersebut diatas, Majelis Hakim berpendapat, rumah tangga Pemohon dengan Termohon telah pecah sedemikian rupa (broken mariage) disebabkan karena perselisihan dan pertengkaran yang terus menerus yang terjadi antara Pemohon dan Termohon dan apalagi antara kedua belah pihak telah berpisah tempat tinggal semenjak tanggal 03 Agustus 2020 sampai dengan saat ini selama 4 (empat) bulan, yang akibatnya kewajiban dan tanggung jawab suami ataupun isteri tidak dapat dilaksanakan;

Menimbang, bahwa Pemohon di persidangan tetap bersikeras untuk bercerai dengan Termohon walaupun telah dinasehati oleh Majelis Hakim, keadaan tersebut menunjukkan bahwa rumah tangga Pemohon dan Termohon saat ini tidak lagi mencerminkan keadaan yang rukun dan harmonis, sehingga keadaan tersebut sulit diharapkan untuk bertahan

Disclaimer
Kepaniteraan Mahkamah Agung Republik Indonesia berusaha untuk selalu mencantumkan informasi paling kini dan akurat sebagai bentuk komitmen Mahkamah Agung untuk pelayanan publik, transparansi dan akuntabilitas pelaksanaan fungsi peradilan. Namun dalam hal-hal tertentu masih dimungkinkan terjadi permasalahan teknis terkait dengan akurasi dan keterkinian informasi yang kami sajikan, hal mana akan terus kami perbaiki dari waktu kewaktu.
Dalam hal Anda menemukan inakurasi informasi yang termuat pada situs ini atau informasi yang seharusnya ada, namun belum tersedia, maka harap segera hubungi Kepaniteraan Mahkamah Agung RI melalui :
Email : kepaniteraan@mahkamahagung.go.id Telp : 021-384 3348 (ext.318)

dalam suatu keluarga yang sakinah mawaddah warohmah sebagaimana yang diharapkan oleh Pasal 1 Undang-undang Nomor 1 Tahun 1974, dengan adanya terjadinya perselisihan dan pertengkaran yang terus menerus semenjak bulan Desember 2019 apalagi antara Pemohon dan Termohon telah berpisah tempat tinggal semenjak tanggal 03 Agustus 2020 sampai sekarang selama 4 (empat) bulan. Dengan adanya fakta tersebut telah mengindikasikan bahwa hak dan kewajiban yang seharusnya dilaksanakan oleh suami atau istri tidak terlaksana disebabkan perselisihan dan pertengkaran terus menerus tersebut, oleh karenanya syarat dan alasan permohonan Pemohon sudah terpenuhi ;

Menimbang, bahwa dengan telah terbuktinya dalil Pemohon, bahwa antara Pemohon dan Termohon telah terjadi pisah tempat tinggal semenjak tanggal 03 Agustus 2020 sampai sekarang selama 4 (empat) bulan, di mana menurut yurisprudensi MARI No. 379 K/AG/1995 tanggal 26 Maret 1997, yang abstraksi hukumnya menyatakan apabila suami istri terjadi perselisihan dan pertengkaran yang kemudian terjadi pisah tempat, dan suami istri yang tidak berdiam serumah lagi dan tidak ada harapan untuk hidup rukun kembali, maka rumah tangga tersebut terbukti telah retak dan pecah.

Menimbang, bahwa berdasarkan fakta-fakta hukum di atas Majelis berpendapat bahwa rumah tangga Pemohon dengan Termohon telah pecah sehingga sulit diharapkan untuk bertahan dalam suatu keluarga yang sakinah, mawaddah, warohmah sesuai Pasal 1 Undang-undang Nomor:1 Tahun 1974 dan petunjuk al-Qur'an surat al-Rum ayat 21;

Menimbang, bahwa berdasarkan pertimbangan-pertimbangan di atas, maka dalil-dalil permohonan Pemohon telah terbukti yaitu terjadinya pertengkaran dan perselisihan yang terus menerus semenjak bulan Desember 2019 dan antara kedua belah pihak telah berpisah tempat tinggal semenjak tanggal 03 Agustus 2020 sampai sekarang selama 4 (empat) bulan, dengan demikian alasan tersebut telah memenuhi ketentuan Pasal 39 ayat (2) Undang-undang Nomor 1 Tahun 1974, jo Pasal 19 huruf (f) Peraturan Pemerintah Nomor 9 Tahun 1975, jo Pasal 116 huruf (f) Kompilasi Hukukm Islam, oleh karenanya permohonan Pemohon pada petitum angka 2

Disclaimer
Kepaniteraan Mahkamah Agung Republik Indonesia berusaha untuk selalu mencantumkan informasi paling kini dan akurat sebagai bentuk komitmen Mahkamah Agung untuk pelayanan publik, transparansi dan akuntabilitas pelaksanaan fungsi peradilan. Namun dalam hal-hal tertentu masih dimungkinkan terjadi permasalahan teknis terkait dengan akurasi dan keterkinian informasi yang kami sajikan, hal mana akan terus kami perbaiki dari waktu kewaktu.
Dalam hal Anda menemukan inakurasi informasi yang termuat pada situs ini atau informasi yang seharusnya ada, namun belum tersedia, maka harap segera hubungi Kepaniteraan Mahkamah Agung RI melalui :
Email : kepaniteraan@mahkamahagung.go.id Telp : 021-384 3348 (ext.318)

aquo dapat dikabulkan dengan memberi izin kepada Pemohon untuk menjatuhkan talak satu roj’i terhadap Termohon dihadapan sidang Pengadilan Agama Subang ;

Menimbang, bahwa berdasarkan ketentuan Pasal 89 ayat (1) Undang-Undang Nomor 7 Tahun 1989 tentang Peradilan Agama, seperti yang telah diubah dengan Undang-Undang Nomor 3 Tahun 2006, dan perubahan kedua dengan Undang-Undang Nomor 50 Tahun 2009, maka biaya yang timbul dalam perkara ini harus dibebankan kepada Pemohon;

Mengingat akan ketentuan di dalam Peraturan Perundang-undangan yang berlaku dan dalil Syara yang berkaitan dengan perkara ini;

M E N G A D I L I

1. Menyatakan bahwa Termohon yang telah dipanggil secara resmi dan patut untuk menghadap persidangan, tidak hadir;
2. Mengabulkan permohonan Pemohon dengan verstek;
3. Memberi izin kepada Pemohon (Pemohon) untuk menjatuhkan talak satu raj'i terhadap Termohon (Termohon) di depan sidang Pengadilan Agama Subang;
4. Membebankan biaya perkara ini kepada Pemohon yang hingga kini dihitung sebesar Rp416.000,00 (empat ratus enam belas ribu rupiah);

Demikian diputuskan dalam musyawarah Majelis Hakim pada hari **Selasa**, tanggal **15 Desember 2020 Masehi,** yang bertepatan dengan tanggal **29 Rabiul Akhir 1442 Hijriyyah**., oleh Hakim Pengadilan Agama Subang yang terdiri dari **Dra. Hj. Suherni, M.H.** sebagai Ketua Majelis dan **Drs. Esib Jaelani, M.H.** serta **Drs. H. Abdul Hamid Mayeli, S.H., M.H.** masing-masing sebagai Hakim Anggota, dibantu oleh **Drs. Budiana, S.H.I.** sebagai Panitera Pengganti. Putusan tersebut pada hari itu juga diucapkan dalam persidangan terbuka untuk umum oleh Ketua Majelis dihadiri Pemohon tanpa hadirnya Termohon.

Hakim Anggota, Ketua Majelis Hakim,

Disclaimer
Kepaniteraan Mahkamah Agung Republik Indonesia berusaha untuk selalu mencantumkan informasi paling kini dan akurat sebagai bentuk komitmen Mahkamah Agung untuk pelayanan publik, transparansi dan akuntabilitas pelaksanaan fungsi peradilan. Namun dalam hal-hal tertentu masih dimungkinkan terjadi permasalahan teknis terkait dengan akurasi dan keterkinian informasi yang kami sajikan, hal mana akan terus kami perbaiki dari waktu kewaktu.
Dalam hal Anda menemukan inakurasi informasi yang termuat pada situs ini atau informasi yang seharusnya ada, namun belum tersedia, maka harap segera hubungi Kepaniteraan Mahkamah Agung RI melalui :
Email : kepaniteraan@mahkamahagung.go.id Telp : 021-384 3348 (ext.318)

Drs. Esib Jaelani, M.H.

Dra. Hj. Suherni, M.H.

Hakim Anggota,

Drs. H. Abdul Hamid Mayeli, S.H., M.H.

Panitera Pengganti,

Drs. Budiana, S.H.I.

Perincian Biaya :

1. Pendaftaran	:	Rp30.000,00
2. Proses	:	Rp50.000,00
3. Panggilan	:	Rp300.000,00
4. PNBP Panggilan	:	Rp20.000,00
5. Redaksi	:	Rp10.000,00
6. Meterai	:	Rp6.000,00
**Jumlah**	**:**	**Rp416.000,00**

(empat ratus enam belas ribu rupiah)

Disclaimer
Kepaniteraan Mahkamah Agung Republik Indonesia berusaha untuk selalu mencantumkan informasi paling kini dan akurat sebagai bentuk komitmen Mahkamah Agung untuk pelayanan publik, transparansi dan akuntabilitas pelaksanaan fungsi peradilan. Namun dalam hal-hal tertentu masih dimungkinkan terjadi permasalahan teknis terkait dengan akurasi dan keterkinian informasi yang kami sajikan, hal mana akan terus kami perbaiki dari waktu kewaktu.
Dalam hal Anda menemukan inakurasi informasi yang termuat pada situs ini atau informasi yang seharusnya ada, namun belum tersedia, maka harap segera hubungi Kepaniteraan Mahkamah Agung RI melalui :
Email : kepaniteraan@mahkamahagung.go.id Telp : 021-384 3348 (ext.318)
