# Supplementary material for: Classification of divorce causes during the COVID-19 pandemic using convolutional neural networks
Source: PeerJ Comput Sci. 2022 Jun 30;8:e998. doi: 10.7717/peerj-cs.998 (PMC9299239; doi:10.7717/peerj-cs.998)
Supplement: Supplemental Information 4 [file peerj-cs-08-998-s004.zip › Konflik Dataset/Data ke-16.pdf]

S A L I N A N :

P U T U S A N

Nomor 4209/Pdt.G/2020/PA.Sbg.

بِسْمِ اللهِ الرَّحْمٰنِ الرَّحِيْمِ

DEMI KEADILAN BERDASARKAN KETUHANAN YANG MAHA ESA

Pengadilan Agama Subang yang memeriksa dan mengadili perkara-perkara tertentu pada tingkat pertama dalam persidangan Majelis Hakim telah menjatuhkan putusan dalam perkara **Cerai Gugat**, antara :

Penggugat, umur 28 tahun (Subang, 04 Februari 1992), Agama Islam, pendidikan terakhir D3, pekerjaan Wiraswasta, tempat tinggal di Kabupaten Subang, sebagai **Penggugat**;

melawan

Tergugat, umur 32 tahun (Garut, 18 Juli 1988), Agama Islam, pendidikan terakhir SLTA, pekerjaan Wiraswasta, tempat tinggal di Kabupaten Subang, sebagai **Tergugat**;

Pengadilan Agama tersebut;
Setelah membaca berkas perkara;
Setelah mendengar keterangan Penggugat di persidangan;
Setelah memeriksa alat-alat bukti di persidangan;

DUDUK PERKARA

Bahwa Penggugat dengan surat gugatannya tanggal 27 November 2020 yang telah didaftarkan di Kepaniteraan Pengadilan Agama Subang di bawah Nomor 4209/Pdt.G/2020/PA.Sbg. tanggal 27 November 2020, bermaksud mengajukan gugatan cerai terhadap Tergugat berdasarkan dalil-dalil yang pada pokoknya sebagai berikut :

1. Bahwa Penggugat saat ini bertempat tinggal di Kabupaten Subang sebagaimana ternyata dari KTP atas nama Penggugat dengan NIK 3213074930001, tanggal 15 Oktober 2012 yang diterbitkan oleh Kepala Dinas Kependudukan dan Pencatatan Sipil Kabupaten Subang;

Disclaimer
Kepaniteraan Mahkamah Agung Republik Indonesia berusaha untuk selalu mencantumkan informasi paling kini dan akurat sebagai bentuk komitmen Mahkamah Agung untuk pelayanan publik, transparansi dan akuntabilitas pelaksanaan fungsi peradilan. Namun dalam hal-hal tertentu masih dimungkinkan terjadi permasalahan teknis terkait dengan akurasi dan keterkinian informasi yang kami sajikan, hal mana akan terus kami perbaiki dari waktu kewaktu.
Dalam hal Anda menemukan inakurasi informasi yang termuat pada situs ini atau informasi yang seharusnya ada, namun belum tersedia, maka harap segera hubungi Kepaniteraan Mahkamah Agung RI melalui :
Email : kepaniteraan@mahkamahagung.go.id Telp : 021-384 3348 (ext.318)

2. Bahwa pada tanggal 04 Desember 2010, Penggugat dengan Tergugat melangsungkan pernikahan di Wilayah Hukum Kantor Urusan Agama Kecamatan Pagaden Kabupaten Subang sebagaimana ternyata dari Buku Kutipan Akta Nikah yang dikeluarkan oleh Pegawai Pencatat Nikah Kantor Urusan Agama Kecamatan Pagaden Kabupaten Subang Nomor 2010 tertanggal 06 Desember 2010;
3. Bahwa setelah akad nikah Penggugat dan Tergugat hidup bersama sebagai suami istri di rumah milik Penggugat;
4. Bahwa perkawinan antara Penggugat dan Tergugat sampai saat ini telah berjalan selama 9 tahun 11 bulan dan telah dikaruniai keturunan 2 (dua) orang anak yang masing-masing bernama : M. Rifki Taufik (berusia 9 tahun) dan Zidan Misbahul Anwar Taufik (berusia 6 tahun);
5. Bahwa semula rumah tangga Penggugat dan Tergugat harmonis dan bahagia, namun sejak bulan Februari tahun 2019 sudah tidak harmonis lagi karena sering terjadi perselisihan dan pertengkaran yang disebabkan karena :
   – Tergugat diketahui mempunyai wanita idaman lain, awalnya kabar tersebut diketahui Penggugat melaui handphone Tergugat yang berisi chattan mersa dan foto bersama dengan wanita tersebut. bahkan ketika ditanyakan langsung Tergugat dengan wanita tersebut mengakuinya, sehingga Penggugat merasa kecewa dan terlibat pertengkaran;
6. Bahwa puncak perselisihan dan pertengkaran antara Penggugat dan Tergugat terjadi pada bulan September tahun 2020, yang berakibat Tergugat pergi meninggalkan Penggugat dan tidak pernah hidup berumah tangga lagi sebagai suami istri dan sampai sekarang sudah berpisah selama 2 bulan;
7. Bahwa selama berpisah tempat tinggal Penggugat tinggal di rumah milik Penggugat, sedangkan Tergugat tinggal di rumah kontrakan a.n Bapak Ujang;
8. Bahwa dengan adanya kejadian tersebut Penggugat tetap bersabar, dan

Disclaimer
Kepaniteraan Mahkamah Agung Republik Indonesia berusaha untuk selalu mencantumkan informasi paling kini dan akurat sebagai bentuk komitmen Mahkamah Agung untuk pelayanan publik, transparansi dan akuntabilitas pelaksanaan fungsi peradilan. Namun dalam hal-hal tertentu masih dimungkinkan terjadi permasalahan teknis terkait dengan akurasi dan keterkinian informasi yang kami sajikan, hal mana akan terus kami perbaiki dari waktu kewaktu.
Dalam hal Anda menemukan inakurasi informasi yang termuat pada situs ini atau informasi yang seharusnya ada, namun belum tersedia, maka harap segera hubungi Kepaniteraan Mahkamah Agung RI melalui :
Email : kepaniteraan@mahkamahagung.go.id Telp : 021-384 3348 (ext.318)

sudah dilakukan upaya musyawarah antara Penggugat dan Tergugat beserta keluarga Penggugat dan Tergugat, namun upaya tersebut tidak berhasil;

9. Bahwa dengan keadaan rumah tangga tersebut, Penggugat sudah tidak ada harapan untuk melanjutkan rumah tangga bersama Tergugat, sehingga tujuan perkawinan untuk membentuk rumah tangga yang sakinah, mawadah warohmah tidak dapat terwujud, dan apabila tetap dipertahankan hanya akan menimbulkan kemudaratan yang berkepanjangan. Maka untuk mengakhiri perkawinan tersebut Penggugat bermaksud menggugat cerai Tergugat di depan sidang Pengadilan Agama Subang;
10. Bahwa Penggugat sanggup membayar biaya perkara;

Bahwa berdasarkan dalil-dalil tersebut di atas, Penggugat mohon kepada Ketua Pengadilan Agama Subang Cq. Majelis Hakim yang menyidangkan perkara ini kiranya berkenan untuk memanggil Penggugat dan Tergugat, memeriksa, mengadili dan memutuskan perkara ini dengan amarnya yang berbunyi sebagai berikut :

1. Mengabulkan gugatan Penggugat;
2. Menjatuhkan talak satu bain shugra Tergugat (**Tergugat**) terhadap Penggugat (**Penggugat**);
3. Membebankan biaya perkara ini menurut hukum;

Apabila majelis hakim berpendapat lain, mohon putusan yang seadil-adilnya;

Bahwa pada hari dan tanggal yang telah ditentukan Penggugat menghadap persidangan, sedang Tergugat tidak datang menghadap atau menyuruh orang lain sebagai wakilnya yang sah, meskipun ia telah dipanggil dengan resmi dan patut untuk datang menghadap di sidang sebagaimana terbukti dari relaas panggilan Nomor 4209/Pdt.G/2020/PA.Sbg. tanggal 01 Desember 2020 dan 08 Desember 2020, sedangkan ternyata bahwa tidak

Disclaimer
Kepaniteraan Mahkamah Agung Republik Indonesia berusaha untuk selalu mencantumkan informasi paling kini dan akurat sebagai bentuk komitmen Mahkamah Agung untuk pelayanan publik, transparansi dan akuntabilitas pelaksanaan fungsi peradilan. Namun dalam hal-hal tertentu masih dimungkinkan terjadi permasalahan teknis terkait dengan akurasi dan keterkinian informasi yang kami sajikan, hal mana akan terus kami perbaiki dari waktu kewaktu.
Dalam hal Anda menemukan inakurasi informasi yang termuat pada situs ini atau informasi yang seharusnya ada, namun belum tersedia, maka harap segera hubungi Kepaniteraan Mahkamah Agung RI melalui :
Email : kepaniteraan@mahkamahagung.go.id Telp : 021-384 3348 (ext.318)

hadirnya itu oleh Majelis Hakim dinilai tidak disebabkan sesuatu halangan yang sah;

Bahwa oleh karena Tergugat tidak hadir di persidangan, maka proses mediasi sebagaimana diamanatkan dalam PERMA Nomor 1 Tahun 2016 tidak dapat dilaksanakan;

Bahwa selanjutnya dibacakan surat gugatan Penggugat tertanggal 27 November 2020, yang isinya tetap dipertahankan Penggugat;

Bahwa terhadap gugatan Penggugat tersebut, Tergugat tidak memberi jawaban karena tidak pernah hadir di persidangan;

Bahwa untuk memperkuat dalil-dalil gugatannya, bahwa Penggugat telah menikah dengan Tergugat, Penggugat telah mengajukan alat bukti (P) berupa fotokopi Kutipan Akta Nikah Nomor 2010 yang dikeluarkan oleh Kantor Urusan Agama Kecamatan Pagaden Kabupaten Subang tanggal 06 Desember 2010, fotokopi mana telah dinachtzegelen/bermeterai cukup;

Bahwa di samping bukti surat sebagaimana di atas, Penggugat juga mengajukan 2 (dua) orang saksi keluarga atau orang yang dekat dengan Penggugat, yang masing-masing bernama :

1. Saksi 1, umur 40 tahun, agama Islam, pekerjaan Dagang, tempat tinggal di Kabupaten Subang, yang di bawah sumpah memberikan keterangan yang pada pokoknya sebagai berikut :
   - Bahwa saksi kenal kepada Penggugat yang bernama dan Tergugat yang bernama;
   - Bahwa saksi adalah Kakak ipar Penggugat;
   - Bahwa Penggugat dan Tergugat adalah suami istri yang menikah pada bulan Desember 2010 di hadapan Pegawai Pencatat Nikah Kantor Urusan Agama Kecamatan Pagaden Kabupaten Subang;
   - Bahwa setelah menikah Penggugat dan Tergugat tinggal bersama di rumah milik Penggugat, dan telah dikaruniai keturunan 2 (dua) orang anak yang masing-masing bernama M. Rifki Taufik dan Zidan Misbahul Anwar Taufik;

Disclaimer
Kepaniteraan Mahkamah Agung Republik Indonesia berusaha untuk selalu mencantumkan informasi paling kini dan akurat sebagai bentuk komitmen Mahkamah Agung untuk pelayanan publik, transparansi dan akuntabilitas pelaksanaan fungsi peradilan. Namun dalam hal-hal tertentu masih dimungkinkan terjadi permasalahan teknis terkait dengan akurasi dan keterkinian informasi yang kami sajikan, hal mana akan terus kami perbaiki dari waktu kewaktu.
Dalam hal Anda menemukan inakurasi informasi yang termuat pada situs ini atau informasi yang seharusnya ada, namun belum tersedia, maka harap segera hubungi Kepaniteraan Mahkamah Agung RI melalui :
Email : kepaniteraan@mahkamahagung.go.id Telp : 021-384 3348 (ext.318)

- Bahwa saksi mengetahui awalnya rumah tangga Penggugat dan Tergugat rukun dan harmonis, namun sejak bulan Februari tahun 2019 mulai tidak rukun karena sering terjadi perselisihan dan pertengkaran;
- Bahwa penyebabnya karena Tergugat diketahui mempunyai wanita idaman lain, awalnya kabar tersebut diketahui Penggugat melaui handphone Tergugat yang berisi chattan mersa dan foto bersama dengan wanita tersebut. bahkan ketika ditanyakan langsung Tergugat dengan wanita tersebut mengakuinya;
- Bahwa saksi mengetahui perselisihan dan pertengkaran tersebut karena saksi pernah melihat antara Penggugat dengan Tergugat bertengkar;
- Bahwa Penggugat dan Tergugat sampai sekarang sudah berpisah selama 2 bulan, dimana Tergugat pergi meninggalkan Penggugat dan tidak pernah hidup berumah tangga lagi sebagai suami istri;
- Bahwa selama berpisah tempat tinggal, Penggugat tinggal di rumah milik Penggugat, sedangkan Tergugat tinggal di rumah kontrakan a.n Bapak Ujang;
- Bahwa selama berpisah tempat tinggal Penggugat dan Tergugat sudah berusaha untuk memperbaiki rumah tangganya, namun upaya tersebut tidak berhasil;
- Bahwa saksi bersama keluarga kedua belah pihak sudah berusaha merukunkan, akan tetapi usaha tersebut tidak berhasil karena Penggugat tetap bersikeras ingin bercerai dengan Tergugat;
- Bahwa saksi tidak bersedia untuk mendamaikan kembali kedua belah pihak, karena keduanya sulit didamaikan;

2. Saksi 2, umur 36 tahun, agama Islam, pekerjaan Ibu rumah tangga, tempat tinggal di Kabupaten Subang, yang di bawah sumpah memberikan keterangan yang pada pokoknya sebagai berikut :
   - Bahwa saksi kenal kepada Penggugat dan Tergugat karena saksi adalah teman Penggugat;

Disclaimer
Kepaniteraan Mahkamah Agung Republik Indonesia berusaha untuk selalu mencantumkan informasi paling kini dan akurat sebagai bentuk komitmen Mahkamah Agung untuk pelayanan publik, transparansi dan akuntabilitas pelaksanaan fungsi peradilan. Namun dalam hal-hal tertentu masih dimungkinkan terjadi permasalahan teknis terkait dengan akurasi dan keterkinian informasi yang kami sajikan, hal mana akan terus kami perbaiki dari waktu kewaktu.
Dalam hal Anda menemukan inakurasi informasi yang termuat pada situs ini atau informasi yang seharusnya ada, namun belum tersedia, maka harap segera hubungi Kepaniteraan Mahkamah Agung RI melalui :
Email : kepaniteraan@mahkamahagung.go.id Telp : 021-384 3348 (ext.318)

- Bahwa Penggugat dan Tergugat adalah suami istri yang sudah berumah tangga selama 9 tahun 11 bulan dan telah dikaruniai keturunan 2 (dua) orang anak;
- Bahwa setelah menikah Penggugat dan Tergugat tinggal bersama di rumah milik Penggugat;
- Bahwa Penggugat dan Tergugat sudah berpisah tempat tinggal sejak bulan September tahun 2020, dimana Tergugat pergi meninggalkan Penggugat dan tidak pernah hidup berumah tangga lagi sebagai suami istri sampai sekarang sudah berpisah sekitar 2 bulan;
- Bahwa selama berpisah tempat tinggal, Penggugat tinggal di rumah milik Penggugat, sedangkan Tergugat tinggal di rumah kontrakan a.n Bapak Ujang;
- Bahwa sebelum berpisah, antara Penggugat dan Tergugat sering terjadi perselisihan dan pertengkaran yang disebabkan karena Tergugat diketahui mempunyai hubungan istimewa dengan wanita idaman lain, hal tersebut diketahui oleh Penggugat selain melalui fhoto dan Chat juga atas dasar pengakuan Tergugat;
- Bahwa saksi mengetahui perselisihan dan pertengkaran tersebut karena saksi pernah melihat antara Penggugat dengan Tergugat bertengkar;
- Bahwa selama berpisah tempat tinggal Penggugat dan Tergugat sudah berusaha untuk rukun, akan tetapi upaya tersebut tidak berhasil;
- Bahwa selama berpisah tempat tinggal, saksi belum pernah mendamaikan. Hanya menasehati Penggugat saja, tapi tidak berhasil karena Penggugat sudah tidak mau kembali kepada Tergugat;
- Bahwa saksi tidak bersedia mendamaikan kedua belah pihak karena sulit untuk didamaikan kembali;

Bahwa kemudian Penggugat mengajukan kesimpulan, yang pada pokoknya mengatakan bahwa berdasarkan dalil-dalil gugatan Penggugat dan bukti-bukti yang diajukan, gugatan Penggugat telah terbukti dan berdasar hukum, oleh karenanya patut dikabulkan dan selanjutnya mohon putusan;

Disclaimer
Kepaniteraan Mahkamah Agung Republik Indonesia berusaha untuk selalu mencantumkan informasi paling kini dan akurat sebagai bentuk komitmen Mahkamah Agung untuk pelayanan publik, transparansi dan akuntabilitas pelaksanaan fungsi peradilan. Namun dalam hal-hal tertentu masih dimungkinkan terjadi permasalahan teknis terkait dengan akurasi dan keterkinian informasi yang kami sajikan, hal mana akan terus kami perbaiki dari waktu kewaktu.
Dalam hal Anda menemukan inakurasi informasi yang termuat pada situs ini atau informasi yang seharusnya ada, namun belum tersedia, maka harap segera hubungi Kepaniteraan Mahkamah Agung RI melalui :
Email : kepaniteraan@mahkamahagung.go.id Telp : 021-384 3348 (ext.318)

Bahwa selanjutnya untuk mempersingkat uraian putusan ini, ditunjuklah hal-hal sebagaimana tercatat dalam berita acara sidang perkara ini sebagai bagian yang tidak terpisahkan dari putusan ini;

PERTIMBANGAN HUKUM

Menimbang, bahwa maksud dan tujuan gugatan Penggugat adalah sebagaimana diuraikan di atas;

Menimbang, bahwa berdasarkan relaas panggilan Nomor 4209/Pdt.G/2020/PA.Sbg. tanggal 01 Desember 2020 dan 08 Desember 2020, terbukti bahwa Tergugat telah dipanggil dengan resmi dan patut untuk datang menghadap di persidangan, tetapi Tergugat tidak datang menghadap atau menyuruh orang lain sebagai wakilnya yang sah, sedangkan ternyata bahwa tidak hadirnya itu oleh Majelis Hakim dinilai tidak disebabkan sesuatu halangan yang sah. Oleh karenanya, harus dinyatakan bahwa Tergugat tidak menghadap persidangan dan berdasarkan ketentuan Pasal 125 ayat (1) HIR Pengadilan dapat menjatuhkan putusan dengan verstek;

Menimbang, bahwa perkara ini adalah perkara perceraian. Berdasarkan azas hukum bahwa tidak ada perceraian jika tidak ada perkawinan, maka sebelum mempertimbangkan tentang perceraiannya Majelis Hakim akan mempertimbangkan terlebih dahulu mengenai ada tidaknya perkawinan antara Penggugat dan Tergugat;

Menimbang, bahwa untuk memperkuat dalil gugatannya, bahwa Penggugat telah menikah dengan Tergugat, Penggugat telah mengajukan bukti surat yang berupa fotokopi Kutipan Akta Nikah Nomor 676/06/XII/2010 yang dikeluarkan oleh Kantor Urusan Agama Kecamatan Pagaden Kabupaten Subang tanggal 06 Desember 2010, bermeterai cukup (bukti P), sehingga sah untuk dipertimbangkan sebagai alat bukti dalam perkara ini;

Menimbang, bahwa bukti P sebagaimana tersebut di atas merupakan akta otentik yang bernilai pembuktian mengikat dan sempurna. Maka berdasarkan alat bukti tersebut Majelis Hakim berkesimpulan, telah terbukti

Disclaimer
Kepaniteraan Mahkamah Agung Republik Indonesia berusaha untuk selalu mencantumkan informasi paling kini dan akurat sebagai bentuk komitmen Mahkamah Agung untuk pelayanan publik, transparansi dan akuntabilitas pelaksanaan fungsi peradilan. Namun dalam hal-hal tertentu masih dimungkinkan terjadi permasalahan teknis terkait dengan akurasi dan keterkinian informasi yang kami sajikan, hal mana akan terus kami perbaiki dari waktu kewaktu.
Dalam hal Anda menemukan inakurasi informasi yang termuat pada situs ini atau informasi yang seharusnya ada, namun belum tersedia, maka harap segera hubungi Kepaniteraan Mahkamah Agung RI melalui :
Email : kepaniteraan@mahkamahagung.go.id Telp : 021-384 3348 (ext.318)

benar, dalil Penggugat yang menyatakan bahwa antara Penggugat dan Tergugat terikat dalam perkawinan yang sah;

Menimbang, bahwa dalam perkara ini Penggugat mohon kepada Pengadilan agar menjatuhkan talak satu bain sughra Tergugat terhadap Penggugat berdasarkan dalil yang pada pokoknya, bahwa rumah tangga Penggugat dan Tergugat sudah tidak harmonis lagi karena sering terjadi perselisihan dan pertengkaran yang disebabkan oleh karena Tergugat diketahui mempunyai hubungan istimewa dengan wanita idaman lain yang mencapai puncaknya pada bulan September tahun 2020 sehingga sekarang antara Penggugat dan Tergugat telah berpisah tempat tinggal selama 2 bulan dan selama berpisah tempat tinggal tersebut Penggugat dan Tergugat sudah berusaha rukun akan tetapi upaya tersebut tidak berhasil;

Menimbang, bahwa terhadap gugatan Penggugat tersebut, Tergugat tidak memberi jawaban karena tidak pernah hadir di persidangan. Namun demikian, karena perkara ini adalah perkara perceraian, maka untuk dikabulkan gugatan, Penggugat harus membuktikan kebenaran dalil gugatannya;

Menimbang, bahwa untuk membuktikan kebenaran dalil gugatannya, Penggugat telah mengajukan dua orang saksi;

Menimbang, bahwa oleh karena kedua saksi yang diajukan Penggugat adalah saksi keluarga dan orang yang dekat dengannya, maka Majelis Hakim menjadikan kedua saksi tersebut sebagai alat bukti mengenai dalil gugatannya, juga sebagai saksi keluarga untuk memenuhi ketentuan Pasal 22 ayat (2) Peraturan Pemerintah Nomor 9 tahun 1975 jo Pasal 76 ayat (1) Undang Undang Nomor 7 Tahun 1989, sebagaimana telah diubah dengan Undang-undang No. 3 tahun 2006 dan diubah untuk kedua kalinya dengan Undang-undang No. 50 tahun 2009, yang menentukan, bahwa gugatan perceraian dapat diterima apabila telah cukup jelas bagi Pengadilan mengenai sebab-sebab perselisihan dan pertengkaran itu dan setelah mendengar keterangan pihak keluarga serta orang-orang yang dekat dengan suami istri;

Menimbang, bahwa kedua saksi keluarga atau orang dekat dengan Penggugat tersebut telah memenuhi syarat formil maupun materiil sebagai

Disclaimer
Kepaniteraan Mahkamah Agung Republik Indonesia berusaha untuk selalu mencantumkan informasi paling kini dan akurat sebagai bentuk komitmen Mahkamah Agung untuk pelayanan publik, transparansi dan akuntabilitas pelaksanaan fungsi peradilan. Namun dalam hal-hal tertentu masih dimungkinkan terjadi permasalahan teknis terkait dengan akurasi dan keterkinian informasi yang kami sajikan, hal mana akan terus kami perbaiki dari waktu kewaktu.
Dalam hal Anda menemukan inakurasi informasi yang termuat pada situs ini atau informasi yang seharusnya ada, namun belum tersedia, maka harap segera hubungi Kepaniteraan Mahkamah Agung RI melalui :
Email : kepaniteraan@mahkamahagung.go.id   Telp : 021-384 3348 (ext.318)

saksi, maka keterangan kedua saksi atau orang dekat tersebut dapat dipertimbangkan sebagai alat bukti dalam perkara ini;

Menimbang, bahwa ternyata isi keterangan kedua saksi tersebut sesuai dengan dalil-dalil yang dikemukakan oleh Penggugat, bahwa antara Penggugat dan Tergugat telah terjadi pisah tempat tinggal selama 2 bulan yang disebabkan oleh pertengkaran karena Tergugat diketahui mempunyai hubungan istimewa dengan wanita idaman lain dan selama berpisah tempat tinggal Penggugat dan Tergugat sudah berusaha rukun akan tetapi upaya tersebut tidak berhasil. Demikian juga kedua saksi keluarga atau orang yang dekat dengan Penggugat tersebut telah menasehati Penggugat agar rukun lagi dengan Tergugat tetapi tidak berhasil;

Menimbang, bahwa berdasarkan hal-hal sebagaimana diuraikan di atas, Majelis Hakim mendapatkan fakta-fakta peristiwa sebagai berikut :

1. Bahwa Penggugat dengan Tergugat telah terikat dalam pernikahan yang sah dan telah dikaruniai keturunan 2 (dua) orang anak yang masing-masing bernama : M. Rifki Taufik (berusia 9 tahun) dan Zidan Misbahul Anwar Taufik (berusia 6 tahun);
2. Bahwa antara Penggugat dan Tergugat telah terjadi pisah tempat tinggal selama 2 bulan;
3. Bahwa terjadinya perpisahan tempat tinggal tersebut disebabkan oleh pertengkaran karena Tergugat diketahui mempunyai hubungan istimewa dengan wanita idaman lain;
4. Bahwa selama berpisah tempat tinggal Penggugat dan Tergugat sudah berusaha rukun akan tetapi upaya tersebut tidak berhasil;
5. Bahwa para saksi keluarga atau orang yang dekat dengan Penggugat telah menasehati Penggugat agar rukun kembali dengan Tergugat tetapi tidak berhasil dan para saksi tidak bersedia untuk merukunkan kembali karena keduanya sulit untuk didamaikan;

Menimbang, bahwa dengan telah terbuktinya dalil Penggugat, bahwa antara Penggugat dan Tergugat telah terjadi pisah tempat tinggal selama 2 bulan, di mana menurut yurisprudensi MARI No. 379 K/AG/1995 tanggal 26

Disclaimer
Kepaniteraan Mahkamah Agung Republik Indonesia berusaha untuk selalu mencantumkan informasi paling kini dan akurat sebagai bentuk komitmen Mahkamah Agung untuk pelayanan publik, transparansi dan akuntabilitas pelaksanaan fungsi peradilan. Namun dalam hal-hal tertentu masih dimungkinkan terjadi permasalahan teknis terkait dengan akurasi dan keterkinian informasi yang kami sajikan, hal mana akan terus kami perbaiki dari waktu kewaktu.
Dalam hal Anda menemukan inakurasi informasi yang termuat pada situs ini atau informasi yang seharusnya ada, namun belum tersedia, maka harap segera hubungi Kepaniteraan Mahkamah Agung RI melalui :
Email : kepaniteraan@mahkamahagung.go.id Telp : 021-384 3348 (ext.318)

Maret 1997, suami istri yang tidak berdiam serumah lagi dan tidak ada harapan untuk hidup rukun kembali, maka rumah tangga tersebut terbukti telah retak dan pecah;

Menimbang, bahwa keadaan hubungan antara Penggugat dan Tergugat yang demikian, menurut Majelis Hakim dapat dikualifikasi sebagai telah terjadi perselisihan dan pertengkaran terus menerus dan tidak ada harapan akan hidup rukun lagi dalam rumah tangga sebagaimana yang dimaksud oleh Pasal 19 huruf f Peraturan Pemerintah Nomor 9 Tahun 1975 jo Pasal 116 huruf f Kompilasi Hukum Islam (Instruksi Presiden Nomor 1 Tahun 1991);

Menimbang, bahwa berdasarkan fakta-fakta di atas Majelis Hakim berkesimpulan bahwa antara Penggugat dan Tergugat telah terjadi perselisihan dan pertengkaran terus menerus dan tidak ada harapan akan hidup rukun lagi dalam rumah tangga, sehingga harapan untuk mewujudkan tujuan rumah tangga yang bahagia, sakinah, mawaddah warahmah, sebagaimana yang dikehendaki oleh Pasal 1 Undang-undang Nomor 1 Tahun 1974 jo Pasal 3 Kompilasi Hukum Islam sulit terwujud. Rumah tangga Penggugat dan Tergugat yang demikian berarti telah pecah dan tidak layak untuk dipertahankan;

Menimbang, bahwa dengan tidak hadirnya Tergugat di persidangan memberi petunjuk bahwa Tergugat tidak menghendaki rukun lagi dengan Penggugat dalam rumah tangga;

Menimbang, bahwa berdasarkan pertimbangan-pertimbangan di atas Majelis Hakim berpendapat bahwa gugatan Penggugat telah memenuhi ketentuan Pasal 39 ayat (1) dan ayat (2) Undang-undang No. 1 Tahun 1974, bahwa perceraian hanya dapat dilakukan di depan sidang Pengadilan setelah Pengadilan yang bersangkutan berusaha dan tidak berhasil mendamaikan kedua belah pihak. Untuk melakukan perceraian harus ada cukup alasan bahwa antara suami-istri itu tidak akan dapat hidup rukun sebagai suami-istri. Demikian juga gugatan Penggugat telah memenuhi ketentuan Pasal 19 huruf (f) Peraturan Pemerintah Nomor 9 Tahun 1975 jo Pasal 116 huruf (f) Kompilasi Hukum Islam, yaitu antara suami istri telah terjadi perselisihan dan

Disclaimer
Kepaniteraan Mahkamah Agung Republik Indonesia berusaha untuk selalu mencantumkan informasi paling kini dan akurat sebagai bentuk komitmen Mahkamah Agung untuk pelayanan publik, transparansi dan akuntabilitas pelaksanaan fungsi peradilan. Namun dalam hal-hal tertentu masih dimungkinkan terjadi permasalahan teknis terkait dengan akurasi dan keterkinian informasi yang kami sajikan, hal mana akan terus kami perbaiki dari waktu kewaktu.
Dalam hal Anda menemukan inakurasi informasi yang termuat pada situs ini atau informasi yang seharusnya ada, namun belum tersedia, maka harap segera hubungi Kepaniteraan Mahkamah Agung RI melalui :
Email : kepaniteraan@mahkamahagung.go.id Telp : 021-384 3348 (ext.318)

pertengkaran terus menerus dan tidak ada harapan akan hidup rukun lagi dalam rumah tangga. Oleh karena itu, gugatan Penggugat dikabulkan;

Menimbang, bahwa menurut doktrin hukum Islam, sebagaimana ditulis dalam kitab Fiqhussunnah Jilid VIII halaman 121-122, yang artinya : *"Bahwa apabila gugatan istri telah terbukti berdasarkan alat bukti yang diajukan oleh istri atau berdasarkan pengakuan suami, sedang adanya gangguan tersebut merupakan penghalang atas kelangsungan pergaulan hidup (suami istri) sebagaimana (pergaulan hidup suami istri lain) yang sepadan dengan mereka, sementara hakim tidak berhasil mendamaikan mereka, maka hakim harus menjatuhkan gugat suaminya terhadap diri penggugat dengan gugat bain".* Sedang menurut ketentuan Pasal 119 ayat (2) Kompilasi Hukum Islam, salah satu talak bain sughra adalah talak yang dijatuhkan oleh Pengadilan Agama;

Menimbang, bahwa dalam perkara ini ternyata Penggugat dapat membuktikan kebenaran dalil gugatannya, dan hakim telah gagal mendamaikan keduanya, maka Majelis Hakim akan menjatuhkan talak satu bain sughra Tergugat atas Penggugat;

Menimbang, bahwa berdasarkan Pasal 89 ayat (1) Undang-undang Nomor 7 Tahun 1989 sebagaimana telah diubah dengan Undang-undang No. 3 tahun 2006 dan diubah untuk kedua kalinya dengan Undang-undang No. 50 tahun 2009, biaya perkara ini dibebankan kepada Penggugat;

Memperhatikan ketentuan Pasal 125 HIR dan ketentuan hukum lainnya yang berkaitan dengan perkara ini;

M E N G A D I L I

1. Menyatakan bahwa Tergugat yang telah dipanggil secara resmi dan patut untuk menghadap persidangan, tidak hadir;

Disclaimer
Kepaniteraan Mahkamah Agung Republik Indonesia berusaha untuk selalu mencantumkan informasi paling kini dan akurat sebagai bentuk komitmen Mahkamah Agung untuk pelayanan publik, transparansi dan akuntabilitas pelaksanaan fungsi peradilan. Namun dalam hal-hal tertentu masih dimungkinkan terjadi permasalahan teknis terkait dengan akurasi dan keterkinian informasi yang kami sajikan, hal mana akan terus kami perbaiki dari waktu kewaktu.
Dalam hal Anda menemukan inakurasi informasi yang termuat pada situs ini atau informasi yang seharusnya ada, namun belum tersedia, maka harap segera hubungi Kepaniteraan Mahkamah Agung RI melalui :
Email : kepaniteraan@mahkamahagung.go.id Telp : 021-384 3348 (ext.318)

2. Mengabulkan gugatan Penggugat dengan verstek;
3. Menjatuhkan talak satu ba'in sughra Tergugat (Tergugat) terhadap Penggugat (Penggugat);
4. Membebankan biaya perkara ini kepada Penggugat yang hingga kini dihitung sebesar Rp386.000,00 (tiga ratus delapan puluh enam ribu rupiah);

Demikian dijatuhkan putusan ini dalam musyawarah Majelis Hakim yang dilangsungkan pada hari **Selasa**, tanggal **15 Desember 2020 Masehi** bertepatan dengan tanggal **29 Rabiul Akhir 1442 Hijriyah** oleh **Drs. Arifin** sebagai Ketua Majelis serta **Drs. H. Humaidi** dan **Dede Rika Nurhasanah, S.Ag., M.H.** masing-masing sebagai Hakim Anggota, dibantu oleh **Dra. Hj. Popon Susilawaty**, sebagai Panitera Pengganti. Putusan tersebut pada hari itu juga diucapkan Hakim Ketua dalam sidang terbuka untuk umum dengan dihadiri Penggugat tanpa hadirnya Tergugat.

Hakim Anggota,	Ketua Majelis,
ttd.	ttd.
**Drs. H. Humaidi**	**Drs. Arifin**
Hakim Anggota,	
ttd.	
**Dede Rika Nurhasanah, S.Ag., M.H.**	
	Panitera Pengganti,
	ttd.
	**Dra. Hj. Popon Susilawaty**

Disclaimer
Kepaniteraan Mahkamah Agung Republik Indonesia berusaha untuk selalu mencantumkan informasi paling kini dan akurat sebagai bentuk komitmen Mahkamah Agung untuk pelayanan publik, transparansi dan akuntabilitas pelaksanaan fungsi peradilan. Namun dalam hal-hal tertentu masih dimungkinkan terjadi permasalahan teknis terkait dengan akurasi dan keterkinian informasi yang kami sajikan, hal mana akan terus kami perbaiki dari waktu kewaktu.
Dalam hal Anda menemukan inakurasi informasi yang termuat pada situs ini atau informasi yang seharusnya ada, namun belum tersedia, maka harap segera hubungi Kepaniteraan Mahkamah Agung RI melalui :
Email : kepaniteraan@mahkamahagung.go.id Telp : 021-384 3348 (ext.318)

Perincian Biaya :

1. Pendaftaran	:	Rp 30.000,00
2. Proses	:	Rp 50.000,00
3. Panggilan	:	Rp 270.000,00
4. PNBP Panggilan	:	Rp 20.000,00
5. Redaksi	:	Rp 10.000,00
6. Meterai	:	Rp 6.000,00
**Jumlah**	**:**	**Rp386.000,00**
		(tiga ratus delapan puluh enam ribu rupiah)

Untuk **Salinan** yang sama bunyinya oleh :
PANITERA PENGADILAN AGAMA SUBANG

Drs. H. Dadang Zaenal, M.M.

Disclaimer
Kepaniteraan Mahkamah Agung Republik Indonesia berusaha untuk selalu mencantumkan informasi paling kini dan akurat sebagai bentuk komitmen Mahkamah Agung untuk pelayanan publik, transparansi dan akuntabilitas pelaksanaan fungsi peradilan. Namun dalam hal-hal tertentu masih dimungkinkan terjadi permasalahan teknis terkait dengan akurasi dan keterkinian informasi yang kami sajikan, hal mana akan terus kami perbaiki dari waktu kewaktu.
Dalam hal Anda menemukan inakurasi informasi yang termuat pada situs ini atau informasi yang seharusnya ada, namun belum tersedia, maka harap segera hubungi Kepaniteraan Mahkamah Agung RI melalui :
Email : kepaniteraan@mahkamahagung.go.id Telp : 021-384 3348 (ext.318)
