# Supplementary material for: Classification of divorce causes during the COVID-19 pandemic using convolutional neural networks
Source: PeerJ Comput Sci. 2022 Jun 30;8:e998. doi: 10.7717/peerj-cs.998 (PMC9299239; doi:10.7717/peerj-cs.998)
Supplement: Supplemental Information 4 [file peerj-cs-08-998-s004.zip › Konflik Dataset/Data ke-17.pdf]

PUTUSAN

Nomor 5947/Pdt.G/2020/PA.Jr

بِسْمِ اللهِ الرَّحْمٰنِ الرَّحِيْمِ

DEMI KEADILAN BERDASARKAN KETUHANAN YANG MAHA ESA

Pengadilan Agama Jember yang memeriksa dan mengadili perkara perkara tertentu dalam tingkat pertama telah menjatuhkan putusan dalam perkara cerai gugat antara:

PENGGUGAT, umur 50 tahun, agama Islam, pendidikan MTs, pekerjaan tidak bekerja, tempat kediaman di Kabupaten Jember, sebagai Penggugat;

melawan

TERGUGAT, umur 50 tahun, agama Islam, pendidikan MTs, pekerjaan petani, tempat kediaman di Kabupaten Jember, sebagai Tergugat;

Pengadilan Agama tersebut;

Telah mempelajari surat-surat yang berkaitan dengan perkara ini;

Telah mendengar keterangan Penggugat serta para saksi dimuka sidang;

DUDUK PERKARA

Bahwa Penggugat dalam surat gugatannya tanggal 02 Desember 2020 yang telah didaftar di Kepaniteraan Pengadilan Agama Jember Nomor 5947/Pdt.G/2020/PA.Jr tanggal 02 Desember 2020, dengan dalil-dalil sebagai berikut:

1. Bahwa Penggugat dan Tergugat telah menikah pada tanggal 05 Nopember 1992, yang dicatatkan pada Kantor Urusan Agama Kecamatan Patrang, Kabupaten Jember dengan Kutipan Akta Nikah Nomor 498/08/XI/1992 tanggal 05 Nopember 1992 dengan status Penggugat perawan sedang Tergugat jejaka;
2. Bahwa setelah pernikahan itu antara Penggugat dan Tergugat hidup bersama sebagai suami istri selama 27 tahun 8 bulan telah berhubungan layaknya suami istri (ba'dad dukhul) dan terakhir mengambil tempat kediaman di rumah Penggugat di Jalan Srikoyo Gang Delima RT.003 RW. 009 Kelurahan Patrang

Disclaimer
Kepaniteraan Mahkamah Agung Republik Indonesia berusaha untuk selalu mencantumkan informasi paling kini dan akurat sebagai bentuk komitmen Mahkamah Agung untuk pelayanan publik, transparansi dan akuntabilitas pelaksanaan fungsi peradilan. Namun dalam hal-hal tertentu masih dimungkinkan terjadi permasalahan teknis terkait dengan akurasi dan keterkinian informasi yang kami sajikan, hal mana akan terus kami perbaiki dari waktu kewaktu.
Dalam hal Anda menemukan inakurasi informasi yang termuat pada situs ini atau informasi yang seharusnya ada, namun belum tersedia, maka harap segera hubungi Kepaniteraan Mahkamah Agung RI melalui :
Email : kepaniteraan@mahkamahagung.go.id Telp : 021-384 3348 (ext.318)

Mahkamah Agung Republik Indonesia

Kecamatan Patrang Kabupaten Jember sudah mempunyai anak 3 masing-masing bernama 1) ANAK I, perempuan, umur 26 tahun (sudah menikah) ANAK II, laki-laki, umur 20 tahun 3) ANAK III, laki-laki, umur 10 tahun, sekarang keduanya dalam asuhan Penggugat;

3. Bahwa pada mulanya rumah tangga Penggugat dan Tergugat berjalan dengan baik, akan tetapi sejak bulan April 2020 yang lalu, rumah tangga Penggugat dan Tergugat mulai goyah, sering terjadi perselisihan dan pertengkaran yang disebabkan karena Tergugat tidak menghargai Penggugat sebagai seorang istri dimana Tergugat tidak pernah musyawaroh dengan Penggugat setiap kali ada kepentingan dalam rumah tangga Penggugat dan Tergugat, misalnya menggadaikan sawah tanpa sepengetahuan Penggugat.;
4. Bahwa perselisihan dan pertengkaran antara Penggugat dan Tergugat tersebut makin lama makin memuncak, akhirnya Tergugat pergi meninggalkan tempat kediaman bersama pamit kepada Penggugat sejak bulan September 2020 dan sekarang berada dirumah saudara Tergugat di Jalan Sriti Lingkungan Kebon Kidul RT.001 RW. 005 Kelurahan Banjarsengon Kecamatan Patrang Kabupaten Jember;
5. Bahwa sejak itu antara Penggugat dan Tergugat telah hidup berpisah yang hingga sekarang sudah 3 bulan dan selama hidup berpisah tersebut sudah tidak ada hubungan lagi layaknya suami istri;
6. Bahwa atas sikap dan perbuatan Tergugat yang demikian itu, Penggugat mengalami penderitaan lahir dan bathin yang berkepanjangan;
7. Bahwa Penggugat sudah berusaha memulihkan ketentraman rumah tangga dengan Tergugat akan tetapi tidak berhasil, akhirnya Penggugat sudah tidak sanggup lagi membina rumah tangga dengan Tergugat;
8. Bahwa Penggugat sanggup membayar biaya perkara;
9. Bahwa atas dasar alasan-alasan sebagaimana tersebut diatas, Penggugat mohon kepada Pengadilan Agama Jember agar berkenan memeriksa perkara ini dan menjatuhkan putusan sebagai berikut :

   Primair :

Disclaimer
Kepaniteraan Mahkamah Agung Republik Indonesia berusaha untuk selalu mencantumkan informasi paling kini dan akurat sebagai bentuk komitmen Mahkamah Agung untuk pelayanan publik, transparansi dan akuntabilitas pelaksanaan fungsi peradilan. Namun dalam hal-hal tertentu masih dimungkinkan terjadi permasalahan teknis terkait dengan akurasi dan keterkinian informasi yang kami sajikan, hal mana akan terus kami perbaiki dari waktu kewaktu.
Dalam hal Anda menemukan inakurasi informasi yang termuat pada situs ini atau informasi yang seharusnya ada, namun belum tersedia, maka harap segera hubungi Kepaniteraan Mahkamah Agung RI melalui :
Email : kepaniteraan@mahkamahagung.go.id Telp : 021-384 3348 (ext.318)

1. Mengabulkan gugatan Penggugat;
2. Menjatuhkan talak satu ba'in dari Tergugat terhadap Penggugat;
3. Membebankan biaya perkara ini sesuai ketentuan hukum yang berlaku;

Subsidair :

Atau mohon putusan yang seadil-adilnya :

Bahwa pada hari dan tanggal persidangan yang telah ditetapkan Penggugat telah hadir sendiri, sedangkan Tergugat telah tidak datang menghadap dan tidak menyuruh orang lain untuk menghadap sebagai wakil/kuasanya, meskipun telah dipanggil secara patut;

Bahwa Majelis Hakim telah berusaha menasehati Penggugat agar mau rukun kembali dengan Tergugat, akan tetapi tidak berhasil, kemudian dibacakan gugatan Penggugat, yang ternyata isinya tetap dipertahankan oleh Penggugat;

Bahwa untuk menguatkan dalil gugatannya, Penggugat telah dapat mengajukan alat bukti tertulis berupa :

1. Fotokopi Kartu Tanda Penduduk an. Penggugat yang dikeluarkan oleh Kantor Dinas Kependudukan dan Pencatatan Sipil Kabupaten Jember tanggal 11 Agustus 2012 (P.1);
2. Fotokopi Kutipan Akta Nikah Nomor 498/08/XI/1992 yang dikeluarkan oleh Kepala Kantor Urusan Agama Kecamatan Patrang, Kabupaten Jember tanggal 05 Nopember 1992 (P.2);

Bahwa Penggugat telah mengajukan saksi-saksi dipersidangan yaitu :

1. SAKSI I, umur 33 tahun, agama Islam, pekerjaan Ibu rumah tangga, bertempat tinggal di Kabupaten Jember

Saksi memberikan keterangan di bawah sumpah pada pokoknya sebagai berikut:

- Saksi tahu dan kenal dengan para pihak karena Saksi tetangga Penggugat;
- Penggugat dan Tergugat sebagai suami istri setelah menikah mereka tinggal di rumah Penggugat di Jalan Srikoyo Gang Delima RT.003 RW. 009 Kelurahan Patrang Kecamatan Patrang Kabupaten Jember sudah mempunyai anak yang masing-masing bernama 1) ANAK I, perempuan, umur 26 tahun (sudah menikah) ANAK II, laki-laki, umur 20 tahun 3) ANAK III, laki-laki, umur 10 tahun, sekarang keduanya dalam asuhan Penggugat;

Disclaimer
Kepaniteraan Mahkamah Agung Republik Indonesia berusaha untuk selalu mencantumkan informasi paling kini dan akurat sebagai bentuk komitmen Mahkamah Agung untuk pelayanan publik, transparansi dan akuntabilitas pelaksanaan fungsi peradilan. Namun dalam hal-hal tertentu masih dimungkinkan terjadi permasalahan teknis terkait dengan akurasi dan keterkinian informasi yang kami sajikan, hal mana akan terus kami perbaiki dari waktu kewaktu.
Dalam hal Anda menemukan inakurasi informasi yang termuat pada situs ini atau informasi yang seharusnya ada, namun belum tersedia, maka harap segera hubungi Kepaniteraan Mahkamah Agung RI melalui :
Email : kepaniteraan@mahkamahagung.go.id   Telp : 021-384 3348 (ext.318)

- Bahwa Saksi tahu kini rumah tangga Penggugat dan Tergugat sudah tidak harmonis lagi dan penyebab percekcokan mereka itu karena Tergugat tidak menghargai Penggugat sebagai seorang istri dimana Tergugat tidak pernah musyawaroh dengan Penggugat setiap kali ada kepentingan dalam rumah tangga Penggugat dan Tergugat, misalnya menggadaikan sawah tanpa sepengetahuan Penggugat.;
- Bahwa sekarang Penggugat dan Tergugat telah berpisah selama 3 bulan lamanya dan selama pisah itu saksi tidak pernah tahu keduanya rukun lagi layaknya suami istri;
- Saksi sudah berusaha menasehati kedua belah pihak untuk rukun lagi membina rumah tangga akan tetapi tidak berhasil, Penggugat tetap pada pendiriannya untuk bercerai dengan Tergugat;

2. SAKSI II, umur 43 tahun, agama Islam, pekerjaan Ibu rumah tangga, bertempat tinggal di Kabupaten Jember;

Saksi memberikan keterangan dibawah sumpah pada pokoknya sebagai berikut :

- Saksi tahu dan kenal dengan para pihak karena saksi tetangga Penggugat;
- Penggugat dan Tergugat sebagai suami istri setelah menikah mereka tinggal di rumah Penggugat di Jalan Srikoyo Gang Delima RT.003 RW. 009 Kelurahan Patrang Kecamatan Patrang Kabupaten Jember sudah mempunyai anak yang masing-masing bernama 1) ANAK I, perempuan, umur 26 tahun (sudah menikah) ANAK II, laki-laki, umur 20 tahun 3) ANAK III, laki-laki, umur 10 tahun, sekarang keduanya dalam asuhan Penggugat;
- Bahwa Saksi tahu kini rumah tangga Penggugat dan Tergugat sudah tidak harmonis lagi dan penyebab percekcokan mereka itu karena Tergugat tidak menghargai Penggugat sebagai seorang istri dimana Tergugat tidak pernah musyawaroh dengan Penggugat setiap kali ada kepentingan dalam rumah tangga Penggugat dan Tergugat, misalnya menggadaikan sawah tanpa sepengetahuan Penggugat.;
- Bahwa sekarang Penggugat dan Tergugat telah berpisah selama 3 bulan lamanya dan selama pisah itu saksi tidak pernah tahu keduanya rukun lagi layaknya suami istri;

Disclaimer
Kepaniteraan Mahkamah Agung Republik Indonesia berusaha untuk selalu mencantumkan informasi paling kini dan akurat sebagai bentuk komitmen Mahkamah Agung untuk pelayanan publik, transparansi dan akuntabilitas pelaksanaan fungsi peradilan. Namun dalam hal-hal tertentu masih dimungkinkan terjadi permasalahan teknis terkait dengan akurasi dan keterkinian informasi yang kami sajikan, hal mana akan terus kami perbaiki dari waktu kewaktu.
Dalam hal Anda menemukan inakurasi informasi yang termuat pada situs ini atau informasi yang seharusnya ada, namun belum tersedia, maka harap segera hubungi Kepaniteraan Mahkamah Agung RI melalui :
Email : kepaniteraan@mahkamahagung.go.id   Telp : 021-384 3348 (ext.318)

– Saksi sudah berusaha menasehati kedua belah pihak untuk rukun lagi membina rumah tangga akan tetapi tidak berhasil, Penggugat tetap pada pendiriannya untuk bercerai dengan Tergugat;

Bahwa atas bukti bukti tersebut telah dibenarkan oleh Penggugat;

Bahwa selanjutnya Penggugat tidak mengajukan tanggapan apapun dan mohon putusan;

Selanjutnya untuk mempersingkat uraian putusan ini ditunjuk hal hal sebagaimana tercantum dalam Berita Acara Sidang perkara ini yang merupakan bagian tak terpisahkan dari putusan ini;

PERTIMBANGAN HUKUM

Menimbang bahwa maksud dan tujuan gugatan Penggugat adalah seperti diuraikan tersebut di atas;

Menimbang bahwa berdasarkan bukti P.1 telah ternyata Penggugat bertempat kediaman di wilayah Kabupaten Jember, dengan demikian berdasarkan Pasal 73 ayat (1) Undang undang Nomor 7 Tahun 1989 tentang Peradilan Agama yang telah diubah dengan Undang Undang Nomor 3 Tahun 2006, terakhir diubah dengan Undang Undang Nomor 50 Tahun 2009 Pengadilan Agama Jember berwenang untuk memeriksa dan mengadili perkara aquo;

Menimbang bahwa karena ternyata Tergugat meskipun telah dipanggil secara patut, tidak menghadap, pula tidak ternyata bahwa ketidakhadirannya itu disebabkan oleh suatu halangan yang sah, maka Tergugat harus dinyatakan tidak hadir;

Menimbang, bahwa Majelis Hakim telah berusaha menasehati Penggugat agar mau rukun kembali dengan Tergugat akan tetapi tidak berhasil;

Menimbang, bahwa berdasarkan bukti P.2, telah terbukti bahwa Penggugat dan Tergugat adalah suami isteri yang sah;

Menimbang, bahwa gugatan Penggugat didasarkan pada dalil yang pada pokoknya adalah bahwa setelah 27 tahun 8 bulan hidup bersama rumah tangga di antara Penggugat dan Tergugat telah terjadi perselisihan dan pertengkaran disebabkan karena Tergugat tidak menghargai Penggugat sebagai seorang istri dimana Tergugat tidak pernah musyawaroh dengan Penggugat setiap kali ada

Disclaimer
Kepaniteraan Mahkamah Agung Republik Indonesia berusaha untuk selalu mencantumkan informasi paling kini dan akurat sebagai bentuk komitmen Mahkamah Agung untuk pelayanan publik, transparansi dan akuntabilitas pelaksanaan fungsi peradilan. Namun dalam hal-hal tertentu masih dimungkinkan terjadi permasalahan teknis terkait dengan akurasi dan keterkinian informasi yang kami sajikan, hal mana akan terus kami perbaiki dari waktu kewaktu.
Dalam hal Anda menemukan inakurasi informasi yang termuat pada situs ini atau informasi yang seharusnya ada, namun belum tersedia, maka harap segera hubungi Kepaniteraan Mahkamah Agung RI melalui :
Email : kepaniteraan@mahkamahagung.go.id Telp : 021-384 3348 (ext.318)

kepentingan dalam rumah tangga Penggugat dan Tergugat, misalnya menggadaikan sawah tanpa sepengetahuan Penggugat.;

Menimbang bahwa sikap Tergugat yang telah tidak hadir dipersidangan dapat dipandang bahwa ia tidak hendak membantah dalil-dalil gugatan Penggugat, dengan demikian dalil gugatan Penggugat telah menjadi fakta yang tetap;

Menimbang bahwa Penggugat telah mengajukan dua orang saksi, dan dari keterangan para saksi tersebut yang bersesuaian satu sama lain telah terungkap fakta yang pada pokoknya menguatkan dalil gugatan Penggugat;

Menimbang bahwa sejak terjadinya perselisihan dan pertengkaran tersebut sampai dengan tahap akhir persidangan, selama 3 bulan, telah ternyata Penggugat dan Tergugat sudah tidak berhubungan lagi sebagai suami isteri/hidup berpisah, halmana membuktikan bahwa perselisihan dan pertengkaran Penggugat dan Tergugat telah berlangsung terus menerus;

Menimbang bahwa Majelis Hakim dan pihak keluarga telah tidak berhasil menasehati Penggugat dengan Tergugat, pula telah ternyata Penggugat menyatakan tetap pada gugatannya, dengan demikian hal tersebut telah menunjukkan bahwa di antara Penggugat dan Tergugat sudah tidak ada harapan akan hidup rukun dalam rumah tangga;

Menimbang bahwa disyariatkannya pernikahan sebagai *mitsaqon gholidhon* mempunyai tujuan yang suci dan mulia, yakni untuk menciptakan rumah tangga yang *sakinah, mawaddah dan rahmah*, sebagaimana dimaksud dalam al Qur'an surat Ar Rum ayat 21 dan pasal 1 Undang-Undang Nomor 1 tahun 1974 Jo. pasal 3 Kompilasi Hukum Islam, namun dengan keadaan rumah tangga Penggugat dan Tergugat tersebut, maka tujuan pernikahan menjadi tidak bisa dicapai;

Menimbang bahwa fakta-fakta tersebut diatas telah menunjukkan bahwa perkawinan Penggugat dan Tergugat benar-benar telah pecah dan telah sampai pada taraf yang sudah tidak bisa didamaikan lagi, karena sudah tidak adanya kehendak dari para pihak untuk melanjutkan perkawinannya, dengan demikian penyelesaian yang dipandang adil dan bermanfaat bagi kedua belah pihak adalah perceraian;

Disclaimer
Kepaniteraan Mahkamah Agung Republik Indonesia berusaha untuk selalu mencantumkan informasi paling kini dan akurat sebagai bentuk komitmen Mahkamah Agung untuk pelayanan publik, transparansi dan akuntabilitas pelaksanaan fungsi peradilan. Namun dalam hal-hal tertentu masih dimungkinkan terjadi permasalahan teknis terkait dengan akurasi dan keterkinian informasi yang kami sajikan, hal mana akan terus kami perbaiki dari waktu kewaktu.
Dalam hal Anda menemukan inakurasi informasi yang termuat pada situs ini atau informasi yang seharusnya ada, namun belum tersedia, maka harap segera hubungi Kepaniteraan Mahkamah Agung RI melalui :
Email : kepaniteraan@mahkamahagung.go.id Telp : 021-384 3348 (ext.318)

Menimbang bahwa pada dasarnya menurut ajaran Islam perceraian merupakan perbuatan tercela, namun begitu dalam keadaan suami isteri sudah tidak saling mencintai lagi dan yang terjadi hanya sikap permusuhan dan saling membenci sebagaimana yang dialami oleh Penggugat dan Tergugat tersebut, maka perceraian dibolehkan, sesuai dengan doktrin hukum Islam yang tercantum dalam Kitab Fiqih Sunnah Juz II halaman 248 :

ذهب الإمام مالك : أن الزوجة أن تطلب من القاضى التفريق إذا ادعت إضرار الزوج بها إضرارا لا يستطاع معه دوام العشرة بين إمثالهما, مثل : ضربها, او سبها, أو إيذائها بأي نوع من أنواع الإيذاء الذى لا يطاق, او إكراهها على المنكر من القول أوالفعل. فإذا ثبتت دعواها لدى القاضى ببينة الزوجة, أو إعتراف الزوج, و كان الإيذاء مما لايطاق معه دوام العشرة بين أمثالها و عجز القاضى عن الإصلاح بينهما طلقها طلقة بائنة

Artinya: *“Menurut Imam Malik, bahwa isteri berhak mengajukan gugatan cerai kepada hakim bila terdapat alasan bahwa suaminya telah membuatnya menderita sehingga ia tidak sanggup lagi melanjutkan bergaul dengan suaminya, misalnya karena suaminya suka memukul, memaki atau menyakiti dengan cara lain yang tidak tertahankan lagi atau memaksanya berbuat mungkar, baik tindakannya itu berupa ucapan atau perbuatan; bila dakwaan tersebut telah terbukti dengan dasar bukti atau pengakuan suami dan isteri telah tidak sanggup lagi mempertahankan rumah tangganya, serta hakim tidak mampu menasehatinya, maka hakim berhak menjatuhkan talak satu bain suami”.*

dan dalam Kitab Al-Anwar Juz II halaman 55 :

فاءن تعزز بتعزز او توار او غيبة جاز اثباته بالبينة

Artinya: *“Apabila ia (Tergugat) enggan untuk hadir atau bersembunyi atau tidak diketahui tempat kediamannya, maka perkaranya boleh diputuskan dengan berdasarkan pada pembuktian”.*

Menimbang, bahwa berdasarkan pertimbangan tersebut di atas, gugatan Penggugat telah mempunyai cukup alasan dan telah terbukti serta memenuhi pasal 39 ayat (2) Undang-Undang Nomor 1 tahun 1974 jo. Pasal 19 huruf (f) Peraturan Pemerintah Nomor 9 Tahun 1975 Jo. Pasal 116 huruf (f) Kompilasi

Disclaimer
Kepaniteraan Mahkamah Agung Republik Indonesia berusaha untuk selalu mencantumkan informasi paling kini dan akurat sebagai bentuk komitmen Mahkamah Agung untuk pelayanan publik, transparansi dan akuntabilitas pelaksanaan fungsi peradilan. Namun dalam hal-hal tertentu masih dimungkinkan terjadi permasalahan teknis terkait dengan akurasi dan keterkinian informasi yang kami sajikan, hal mana akan terus kami perbaiki dari waktu kewaktu.
Dalam hal Anda menemukan inakurasi informasi yang termuat pada situs ini atau informasi yang seharusnya ada, namun belum tersedia, maka harap segera hubungi Kepaniteraan Mahkamah Agung RI melalui :
Email : kepaniteraan@mahkamahagung.go.id   Telp : 021-384 3348 (ext.318)

Hukum Islam, oleh karena itu berdasarkan Pasal 125 HIR dapat dikabulkan dengan verstek;

Menimbang, bahwa berdasarkan Pasal 89 ayat (1) Undang-Undang Nomor 7 Tahun 1989 yang telah diubah dengan Undang-Undang Nomor 3 Tahun 2006 dan perubahan kedua dengan Undang-Undang Nomor 50 tahun 2009, maka biaya perkara ini dibebankan kepada Penggugat;

Memperhatikan, segala ketentuan peraturan perundang-undangan yang berlaku dan hukum syara' yang berkaitan dengan perkara ini;

MENGADILI

1. Menyatakan Tergugat telah dipanggil secara resmi dan patut untuk menghadap ke persidangan, tidak hadir;
2. Mengabulkan gugatan Penggugat dengan verstek;
3. Menjatuhkan talak satu ba'in sughra Tergugat (TERGUGAT) terhadap Penggugat (PENGGUGAT)
4. Membebankan kepada Penggugat membayar biaya perkara sejumlah Rp. 416000.- (empat ratus enam belas ribu rupiah);

Demikian diputuskan dalam permusyawaratan Majelis yang dilangsungkan pada hari Rabu tanggal 23 Desember 2020 M bertepatan dengan tanggal 9 Jumadilawal 1442 H, oleh kami Drs. H. Umar Jaya, S.H., M.H. sebagai Ketua Majelis, Drs. H. Nur Chozin, S.H., M.Hum. dan Drs. H. M. Ijmak, S.H., M.H. masing-masing sebagai Hakim Anggota, putusan dibacakan dalam sidang terbuka untuk umum dengan didampingi oleh Abdur Rohman, S.H. sebagai Panitera Pengganti Pengadilan Agama tersebut dan dihadiri oleh Penggugat tanpa hadirnya Tergugat;

Hakim Anggota I	Ketua Majelis
ttd	ttd
Drs. H. Nur Chozin, S.H., M.Hum.	Drs. H. Umar Jaya, S.H., M.H.

Disclaimer
Kepaniteraan Mahkamah Agung Republik Indonesia berusaha untuk selalu mencantumkan informasi paling kini dan akurat sebagai bentuk komitmen Mahkamah Agung untuk pelayanan publik, transparansi dan akuntabilitas pelaksanaan fungsi peradilan. Namun dalam hal-hal tertentu masih dimungkinkan terjadi permasalahan teknis terkait dengan akurasi dan keterkinian informasi yang kami sajikan, hal mana akan terus kami perbaiki dari waktu kewaktu.
Dalam hal Anda menemukan inakurasi informasi yang termuat pada situs ini atau informasi yang seharusnya ada, namun belum tersedia, maka harap segera hubungi Kepaniteraan Mahkamah Agung RI melalui :
Email : kepaniteraan@mahkamahagung.go.id   Telp : 021-384 3348 (ext.318)

Hakim Anggota II

ttd

Drs. H. M. Ijmak, S.H., M.H.

Panitera Pengganti,

ttd

Abdur Rohman, S.H.

Perincian Biaya Perkara :

1.	Pendaftaran	: Rp.	30.000,00
2.	Biaya Proses	: Rp.	50.000,00
3.	Biaya Panggilan	: Rp.	300.000,00
4.	Biaya PNBP	: Rp.	20.000,00
5.	Redaksi	: Rp.	10.000,00
6.	Materai	: Rp.	6.000,00
	Jumlah	Rp.	416.000,00

(empat ratus enam belas ribu rupiah)

Untuk salinan yang sama bunyinya,
Oleh,
Pengadilan Agama Jember
Panitera

Akhmad Muzaeri, S.H.

Disclaimer
Kepaniteraan Mahkamah Agung Republik Indonesia berusaha untuk selalu mencantumkan informasi paling kini dan akurat sebagai bentuk komitmen Mahkamah Agung untuk pelayanan publik, transparansi dan akuntabilitas pelaksanaan fungsi peradilan. Namun dalam hal-hal tertentu masih dimungkinkan terjadi permasalahan teknis terkait dengan akurasi dan keterkinian informasi yang kami sajikan, hal mana akan terus kami perbaiki dari waktu kewaktu.
Dalam hal Anda menemukan inakurasi informasi yang termuat pada situs ini atau informasi yang seharusnya ada, namun belum tersedia, maka harap segera hubungi Kepaniteraan Mahkamah Agung RI melalui :
Email : kepaniteraan@mahkamahagung.go.id   Telp : 021-384 3348 (ext.318)
