# Supplementary material for: Classification of divorce causes during the COVID-19 pandemic using convolutional neural networks
Source: PeerJ Comput Sci. 2022 Jun 30;8:e998. doi: 10.7717/peerj-cs.998 (PMC9299239; doi:10.7717/peerj-cs.998)
Supplement: Supplemental Information 4 [file peerj-cs-08-998-s004.zip › Konflik Dataset/Data ke-19.pdf]

P E N E T A P A N

Nomor **4211/Pdt.G/2020/PA.Sbg**

بِسْمِ اللَّهِ الرَّحْمَٰنِ الرَّحِيمِ

DEMI KEADILAN BERDASARKAN KETUHANAN YANG MAHA ESA

Pengadilan Agama Subang yang memeriksa dan mengadili perkara tertentu pada tingkat pertama dalam sidang Majelis Hakim telah menjatuhkan putusan perkara **Cerai Talak**, antara :

Cepi Supriadi bin Aceng Kushaeri, umur 40 tahun (Subang, 21 Agustus 1980), agama Islam, Pendidikan SLTP, pekerjaan Karyawan Swasta, tempat tinggal di Blok Dangdeur RT 001 RW 001, Kelurahan Dangdeur, Kecamatan Subang, Kabupaten Subang., sebagai **Pemohon**;

melawan

Yuli Yulyana binti Udin, umur 34 tahun (Purwakarta, 12 Februari 1986), agama Islam, Pendidikan SLTP, pekerjaan Karyawan Swasta, tempat tinggal di Blok Dangdeur RT 001 RW 001, Kelurahan Dangdeur, Kecamatan Subang, Kabupaten Subang., sebagai **Termohon**;

Pengadilan Agama tersebut;

Telah mempelajari surat-surat yang berkaitan dengan perkara ini;

Telah mendengar keterangan Pemohon dipersidangan;

DUDUK PERKARA

Bahwa Pemohon dengan surat permohonannya tertanggal 27 November 2020 yang telah didaftarkan di Kepaniteraan Pengadilan Agama Subang Nomor 4211/Pdt.G/2020/PA.Sbg tanggal 27 November 2020 bermaksud mengajukan permohonan cerai terhadap Termohon berdasarkan dalil-dalil sebagaimana dalam surat permohonan sebagai berikut :

Disclaimer
Kepaniteraan Mahkamah Agung Republik Indonesia berusaha untuk selalu mencantumkan informasi paling kini dan akurat sebagai bentuk komitmen Mahkamah Agung untuk pelayanan publik, transparansi dan akuntabilitas pelaksanaan fungsi peradilan. Namun dalam hal-hal tertentu masih dimungkinkan terjadi permasalahan teknis terkait dengan akurasi dan keterkinian informasi yang kami sajikan, hal mana akan terus kami perbaiki dari waktu kewaktu.
Dalam hal Anda menemukan inakurasi informasi yang termuat pada situs ini atau informasi yang seharusnya ada, namun belum tersedia, maka harap segera hubungi Kepaniteraan Mahkamah Agung RI melalui :
Email : kepaniteraan@mahkamahagung.go.id Telp : 021-384 3348 (ext.318)

1. Bahwa Pemohon saat ini bertempat tinggal di Blok Dangdeur RT.001 RW.001 Kelurahan Dangdeur Kecamatan Subang Kabupaten Subang sebagaimana ternyata dari KTP atas nama Pemohon dengan NIK 3213032108800001, tanggal 01 Oktober 2018 yang diterbitkan oleh Kepala Dinas Kependudukan dan Pencatatan Sipil Kabupaten Subang;

2. Bahwa pada tanggal 11 Agustus 2012, Pemohon dengan Termohon melangsungkan pernikahan di Wilayah Hukum Kantor Urusan Agama Kecamatan Wanayasa Kabupaten Purwakarta sebagaimana ternyata dari Register Akta Nikah yang dikeluarkan oleh Pegawai Pencatat Nikah Kantor Urusan Agama Kecamatan Wanayasa Kabupaten Purwakarta Nomor 283/02/VIII/2012 tertanggal 11 Agustus 2012;

3. Bahwa Buku Kutipan Akta Nikah yang asli saat ini berada dalam penguasaan Termohon;

4. Bahwa setelah akad nikah Pemohon dan Termohon hidup bersama sebagai suami istri di rumah orangtua Pemohon;

5. Bahwa perkawinan antara Pemohon dan Termohon sampai saat ini telah berjalan selama 8 bulan 3 bulan dan telah dikaruniai seorang keturunan bernama: Ananda Faizal Nugraha (berusia 8 tahun);

6. Bahwa semula rumah tangga Pemohon dan Termohon harmonis dan bahagia, namun sejak bulan Januari tahun 2020 sudah tidak harmonis lagi karena sering terjadi perselisihan dan pertengkaran yang disebabkan karena :

a. Termohon sering mengeluh dan merasa kekurangan terhadap nafkah yang Pemohon berikan, padahal Pemohon telah berusaha terbuka dan menasihati Termohon untuk bersabar, namun Termohon selalu melawan dan tidak merubah sikapnya;

b. Termohon bersifat cemburu berlebihan terhadap Pemohon, sehingga Pemohon merasa tidak nyaman dalam berumah tangga;

7. Bahwa puncak perselisihan dan pertengkaran antara Pemohon dan Termohon terjadi pada bulan Maret tahun 2020, yang berakibat Pemohon dan Termohon pisah ranjang dan sudah tidak menjalankan kewajibannya sebagai suami istri dan sampai sekarang sudah selama 8 bulan;

Disclaimer
Kepaniteraan Mahkamah Agung Republik Indonesia berusaha untuk selalu mencantumkan informasi paling kini dan akurat sebagai bentuk komitmen Mahkamah Agung untuk pelayanan publik, transparansi dan akuntabilitas pelaksanaan fungsi peradilan. Namun dalam hal-hal tertentu masih dimungkinkan terjadi permasalahan teknis terkait dengan akurasi dan keterkinian informasi yang kami sajikan, hal mana akan terus kami perbaiki dari waktu kewaktu.
Dalam hal Anda menemukan inakurasi informasi yang termuat pada situs ini atau informasi yang seharusnya ada, namun belum tersedia, maka harap segera hubungi Kepaniteraan Mahkamah Agung RI melalui :
Email : kepaniteraan@mahkamahagung.go.id Telp : 021-384 3348 (ext.318)

8. Bahwa selama berpisah ranjang Pemohon tinggal di rumah orangtua Pemohon, sedangkan Termohon tinggal di rumah orangtua Pemohon;

9. Bahwa dengan adanya kejadian tersebut Pemohon tetap bersabar, dan sudah dilakukan upaya musyawarah antara Pemohon dan Termohon beserta keluarga Pemohon dan Termohon, namun upaya tersebut tidak berhasil;

10. Bahwa dengan keadaan rumah tangga tersebut, Pemohon sudah tidak ada harapan untuk melanjutkan rumah tangga bersama Termohon, sehingga tujuan perkawinan untuk membentuk rumah tangga yang sakinah, mawadah warohmah tidak dapat terwujud, dan apabila tetap dipertahankan hanya akan menimbulkan kemudaratan yang berkepanjangan. Maka untuk mengakhiri perkawinan tersebut Pemohon bermaksud menceraikan Termohon di depan sidang Pengadilan Agama Subang;

11. Bahwa Pemohon sanggup membayar biaya perkara;

Berdasarkan dalil-dalil tersebut di atas, Pemohon mohon kepada Ketua Pengadilan Agama Subang Cq. Majelis Hakim yang menyidangkan perkara ini kirana berkenan untuk memanggil Pemohon dan Termohon, memeriksa mengadili dan memutuskan perkara ini dengan amarnya yang berbunyi sebagai berikut:

1. Mengabulkan permohonan Pemohon;
2. Memberi izin kepada Pemohon (Cepi Supriadi bin Aceng Kushaeri) untuk menjatuhkan talak satu raj'i terhadap Termohon (Yuli Yulyana binti Udin) didepan sidang Pengadilan Agama Subang;
3. Membebankan biaya perkara ini menurut hukum;

Apabila majelis hakim berpendapat lain, mohon putusan yang seadil-adilnya;

Bahwa pada hari dan tanggal persidangan yang telah ditetapkan, Pemohon telah datang menghadap di persidangan, sedangkan Termohon tidak datang menghadap di persidangan dan tidak menyuruh orang lain sebagai wakil atau kuasanya yang sah;

Disclaimer
Kepaniteraan Mahkamah Agung Republik Indonesia berusaha untuk selalu mencantumkan informasi paling kini dan akurat sebagai bentuk komitmen Mahkamah Agung untuk pelayanan publik, transparansi dan akuntabilitas pelaksanaan fungsi peradilan. Namun dalam hal-hal tertentu masih dimungkinkan terjadi permasalahan teknis terkait dengan akurasi dan keterkinian informasi yang kami sajikan, hal mana akan terus kami perbaiki dari waktu kewaktu.
Dalam hal Anda menemukan inakurasi informasi yang termuat pada situs ini atau informasi yang seharusnya ada, namun belum tersedia, maka harap segera hubungi Kepaniteraan Mahkamah Agung RI melalui :
Email : kepaniteraan@mahkamahagung.go.id Telp : 021-384 3348 (ext.318)

Bahwa Majelis Hakim telah mengupayakan perdamaian, atas upaya tersebut Pemohon menyatakan akan berdamai dan rukun kembali dengan Termohon serta mencabut permohonannya, oleh karenanya Majelis Hakim telah mencukupkan pemeriksaannya;

Bahwa selanjutnya untuk mempersingkat uraian penetapan ini, maka semua hal yang termuat dalam berita acara sidang perkara ini sebagai bagian yang tidak terpisahkan dari penetapan ini;

PERTIMBANGAN HUKUM

Menimbang, bahwa maksud dan tujuan permohonan Pemohon seperti telah diuraikan diatas;

Menimbang, bahwa berdasarkan Pasal 130 HIR Jo. Pasal 82 Undang Undang N0. 7 Tahun 1989 yang telah dirubah dengan Undang-undang nomor 3 Tahun 2006 dan perubahan kedua dengan Undang-undang nomor 50 Tahun 2009 Majelis Hakim telah mengupayakan perdamaian, dan atas upaya perdamaian tersebut Pemohon menyatakan mencabut permohonannya karena telah berdamai dan rukun kembali dengan Termohon;

Menimbang, bahwa dalam hal ini Majelis berpendapat, oleh karena pencabutan tersebut dilakukan Pemohon sebelum ada jawaban dari pihak Termohon serta tidak merugikan pihak Termohon, dengan demikian pencabutan tersebut harus dikabulkan dengan menyatakan bahwa perkara tersebut telah selesai karena dicabut;

Menimbang, bahwa dalam perkara ini telah timbul biaya, maka sesuai ketentuan pasal 89 ayat (1) Undang-Undang Nomor 7 Tahun 1989 yang telah dirubah dengan Undang-undang nomor 3 Tahun 2006 dan perubahan kedua dengan Undang-undang nomor 50 Tahun 2009 biaya perkara harus dibebankan kepada Pemohon;

Mengingat semua pasal dalam peraturan perundang-undangan yang berlaku dan ketentuan hukum lainnya yang berkaitan dengan perkara ini;

Disclaimer
Kepaniteraan Mahkamah Agung Republik Indonesia berusaha untuk selalu mencantumkan informasi paling kini dan akurat sebagai bentuk komitmen Mahkamah Agung untuk pelayanan publik, transparansi dan akuntabilitas pelaksanaan fungsi peradilan. Namun dalam hal-hal tertentu masih dimungkinkan terjadi permasalahan teknis terkait dengan akurasi dan keterkinian informasi yang kami sajikan, hal mana akan terus kami perbaiki dari waktu kewaktu.
Dalam hal Anda menemukan inakurasi informasi yang termuat pada situs ini atau informasi yang seharusnya ada, namun belum tersedia, maka harap segera hubungi Kepaniteraan Mahkamah Agung RI melalui :
Email : kepaniteraan@mahkamahagung.go.id Telp : 021-384 3348 (ext.318)

M E N E T A P K A N

1. Mengabulkan permohonan untuk mencabut permohonannya;
2. Menyatakan perkara ini telah selesai karena dicabut;
3. Membebankan biaya perkara ini kepada Pemohon yang hingga kini dihitung sebesar Rp. 351.000,00 (Tiga ratus lima puluh satu ribu rupiah);

Demikian penetapan ini dijatuhkan pada hari Senin tanggal **14 Desember 2020 Masehi**, bertepatan dengan tanggal 28 Robiul Akhir 1442 **Hijriyyah**., oleh kami **Dra. Hj. Sunaeah, M.H.,** sebagai Ketua Majelis, **Drs. Ojon Rianklana** dan **Dra. Hj. Suherni, M.H.,** masing-masing sebagai Hakim Anggota. Penetapan tersebut diucapkan dalam sidang terbuka untuk umum pada hari itu juga oleh Ketua Majelis tersebut dengan didampingi oleh Hakim Anggota dan dibantu oleh **Dra. N. Euis Siti Palahiah** sebagai Panitera Pengganti serta dihadiri oleh Pemohon tanpa dihadiri Termohon;

Ketua Majelis,

ttd.

Dra. Hj. Sunaeah, M.H.

Hakim Anggota I,

ttd.

Drs. Ojon Rianklana

Hakim Anggota II,

ttd.

Dra. Hj. Suherni, M.H.

Panitera Pengganti,

Tt
d.

Dra. N. Euis Siti Palahiah

Halaman 5 dari 6 halaman Penetapan Nomor 4211/Pdt.G/2020/PA.Sbg.

Disclaimer
Kepaniteraan Mahkamah Agung Republik Indonesia berusaha untuk selalu mencantumkan informasi paling kini dan akurat sebagai bentuk komitmen Mahkamah Agung untuk pelayanan publik, transparansi dan akuntabilitas pelaksanaan fungsi peradilan. Namun dalam hal-hal tertentu masih dimungkinkan terjadi permasalahan teknis terkait dengan akurasi dan keterkinian informasi yang kami sajikan, hal mana akan terus kami perbaiki dari waktu kewaktu.
Dalam hal Anda menemukan inakurasi informasi yang termuat pada situs ini atau informasi yang seharusnya ada, namun belum tersedia, maka harap segera hubungi Kepaniteraan Mahkamah Agung RI melalui :
Email : kepaniteraan@mahkamahagung.go.id Telp : 021-384 3348 (ext.318)

Perincian Biaya :

1.	Pendaftaran	:	…………………………………….	Rp.	30.000,00
2.	Proses	:	……………………………….........	Rp.	50.000,00
3.	Panggilan	:	……………………………….........	Rp.	225.000,00
4.	PNBP Panggilan	:	……………………………….........	Rp.	20.000,00
5.	PNBP cabut	:	…………………………………….	Rp.	10.000,00
6.	Redaksi	:	……………………………….........	Rp.	10.000,00
7.	Meterai	:	……………………………….........	Rp.	6.000,00
	**Jumlah**	**:**	**……………………………….........**	**Rp.**	**351.000,00**

(tiga ratus empat puluh satu ribu rupiah)

Halaman 6 dari 6 halaman Penetapan Nomor 4211/Pdt.G/2020/PA.Sbg.

Disclaimer
Kepaniteraan Mahkamah Agung Republik Indonesia berusaha untuk selalu mencantumkan informasi paling kini dan akurat sebagai bentuk komitmen Mahkamah Agung untuk pelayanan publik, transparansi dan akuntabilitas pelaksanaan fungsi peradilan. Namun dalam hal-hal tertentu masih dimungkinkan terjadi permasalahan teknis terkait dengan akurasi dan keterkinian informasi yang kami sajikan, hal mana akan terus kami perbaiki dari waktu kewaktu.
Dalam hal Anda menemukan inakurasi informasi yang termuat pada situs ini atau informasi yang seharusnya ada, namun belum tersedia, maka harap segera hubungi Kepaniteraan Mahkamah Agung RI melalui :
Email : kepaniteraan@mahkamahagung.go.id Telp : 021-384 3348 (ext.318)
