# Supplementary material for: Classification of divorce causes during the COVID-19 pandemic using convolutional neural networks
Source: PeerJ Comput Sci. 2022 Jun 30;8:e998. doi: 10.7717/peerj-cs.998 (PMC9299239; doi:10.7717/peerj-cs.998)
Supplement: Supplemental Information 4 [file peerj-cs-08-998-s004.zip › Konflik Dataset/Data ke-2.pdf]

P U T U S A N

Nomor XXXX/Pdt.G/2020/PA.Dmk

بسم الله الرحمن الرحيم

DEMI KEADILAN BERDASARKAN KETUHANAN YANG MAHA ESA

Pengadilan Agama Demak yang memeriksa dan mengadili perkara-perkara tertentu pada tingkat pertama, dalam persidangan Majelis Hakim telah menjatuhkan putusan dalam perkara Cerai Gugat dan Hadhonah antara :

Penggugat, umur 23 tahun, agama Islam, Pekerjaan Karyawati Pabrik, Pendidikan Sekolah Lanjutan Tingkat Atas, tempat kediaman di XXXXKabupaten Demak, sebagai **Penggugat**;

Melawan

Tergugat, umur 25 tahun, agama Islam, Pekerjaan Swasta, Pendidikan Sekolah Dasar, tempat kediaman di XXXX, Kabupaten Demak dan sekarang tingggal di kos Mas Agus di XXXXKabupaten Demak, sebagai **Tergugat**;

Pengadilan Agama tersebut ;
Telah membaca surat-surat perkara;
Telah mendengar keterangan Penggugat dan saksi-saksinya ;

DUDUK PERKARA

Bahwa Penggugat dalam surat gugatan tertanggal 08 Desember 2020, terdaftar di Kepaniteraan Pengadilan Agama Demak, Nomor XXXX/Pdt.G/2020/PA.Dmk. Tanggal 08 Desember 2020 , telah mengemukakan hal-hal yang pada pokoknya sebagai berikut :

1. Bahwa Penggugat dengan Tergugat telah melangsungkan pernikahan pada tanggal 11 Februari 2015, yang dicatat oleh Pegawai Pencatat Nikah Kantor Urusan Agama Kecamatan Mranggen, Kabupaten Demak, sebagaimana Kutipan Akta Nikah Nomor : 0173/54/II/2015, tanggal 11 Februari 2015;
2. Bahwa setelah menikah, Penggugat dengan Tergugat bertempat tinggal di rumah orang tua Penggugat selama Â± 5 bulan, kemudian tinggal di rumah orang tua Penggugat Â± 3 tahun, sudah melakukan hubungan layaknya suami istri (ba'da dukhul), dan telah dikaruniai 2 orang anak yang masing-masing bernama: ;

Disclaimer
Kepaniteraan Mahkamah Agung Republik Indonesia berusaha untuk selalu mencantumkan informasi paling kini dan akurat sebagai bentuk komitmen Mahkamah Agung untuk pelayanan publik, transparansi dan akuntabilitas pelaksanaan fungsi peradilan. Namun dalam hal-hal tertentu masih dimungkinkan terjadi permasalahan teknis terkait dengan akurasi dan keterkinian informasi yang kami sajikan, hal mana akan terus kami perbaiki dari waktu kewaktu.
Dalam hal Anda menemukan inakurasi informasi yang termuat pada situs ini atau informasi yang seharusnya ada, namun belum tersedia, maka harap segera hubungi Kepaniteraan Mahkamah Agung RI melalui :
Email : kepaniteraan@mahkamahagung.go.id Telp : 021-384 3348 (ext.318)

2.1. XXXX, laki-laki, lahir pada tanggal 18 Maret 2016/ umur ± 4.5 tahun, sekarang dalam asuhan Penggugat;

2.2. XXXX, laki-laki, lahir pada tanggal 23 Mei 2018/ umur ± 2.5 tahun, sekarang dalam asuhan Penggugat;

3. Bahwa sejak Oktober 2017, antara Penggugat dan Tergugat sering terjadi perselisihan dan pertengkaran yang disebabkan oleh masalah Tergugat tidak memberi nafkah kepada Penggugat, karena Tergugat kalau bekerja, seluruh penghasilannya digunakan oleh Tergugat sendiri. Bila Penggugat meminta nafkah kepada Tergugat, Tergugat tidak pernah memberi. Sehingga untuk memenuhi kebutuhan rumah tangga Penggugat dan Tergugat, Penggugat harus bekerja sendiri dan dibantu oleh orang tua Penggugat. Penggugat juga sudah berusaha untuk menasehati Tergugat, namun Tergugat hanya diam saja;

   Akhirnya menjadikan perselisihan dan pertengkaran antara Penggugat dan Tergugat semakin memuncak;

4. Bahwa akibatnya, sejak juli 2018, Penggugat seijin Tergugat pulang kembali kerumah orang tua Penggugat sampai sekarang Â± 2 tahun 5 bulan lamanya, Selama pisah ranjang tersebut, antara Penggugat dan Tergugat tidak pernah melakukan hubungan layaknya suami istri dan tidak ada komunikasi;

5. Bahwa pihak keluarga dari Penggugat dan keluarga dari Tergugat sudah pernah mendamaikan Penggugat dengan Tergugat agar rukun lagi dan membina rumah tangga secara baik, tetapi tidak berhasil, karena Penggugat tetap berkeinginan bercerai dari Tergugat;

6. Bahwa ikatan perkawinan antara Penggugat dan Tergugat sebagaimana yang diuraikan diatas sudah sulit dibina untuk membentuk suatu rumah tangga yang sakinah, mawaddah wa rahmah sebagaimana maksud dan tujuan dari suatu perkawinan, sehingga lebih baik diputus karena perceraian;

7. Bahwa berdasarkan hal-hal tersebut diatas, alasan Penggugat untuk mengajukan gugatan perceraian terhadap Tergugat atas dasar perselisihan dan pertengkaran yang terjadi terus menerus dan tidak mungkin hidup

Disclaimer
Kepaniteraan Mahkamah Agung Republik Indonesia berusaha untuk selalu mencantumkan informasi paling kini dan akurat sebagai bentuk komitmen Mahkamah Agung untuk pelayanan publik, transparansi dan akuntabilitas pelaksanaan fungsi peradilan. Namun dalam hal-hal tertentu masih dimungkinkan terjadi permasalahan teknis terkait dengan akurasi dan keterkinian informasi yang kami sajikan, hal mana akan terus kami perbaiki dari waktu kewaktu.
Dalam hal Anda menemukan inakurasi informasi yang termuat pada situs ini atau informasi yang seharusnya ada, namun belum tersedia, maka harap segera hubungi Kepaniteraan Mahkamah Agung RI melalui :
Email : kepaniteraan@mahkamahagung.go.id   Telp : 021-384 3348 (ext.318)

rukun dalam suatu ikatan perkawinan, telah memenuhi unsur Pasal 19 huruf (f) Peraturan Pemerintah No. 9 Tahun 1975 Jo. Pasal 116 huruf (f) Kompilasi Hukum Islam, sehingga berdasar hukum untuk menyatakan gugatan cerai ini dikabulkan;

8. Bahwa anak hasil perkawinan antara Penggugat dengan Tergugat yang diberi nama XXXX dan XXXX, belum mumayyiz atau belum berumur 12 tahun, maka untuk kepentingan anak itu dan rasa kasih sayang Penggugat maka sepatutnya keberadaan anak tersebut tetap di bawah pengasuhan dan pemeliharaan Penggugat;

9. Bahwa Penggugat sanggup membayar biaya perkara;

Berdasarkan alasan-alasan/dalil-dalil tersebut diatas, Penggugat mohon kepada Ketua Pengadilan Agama Demak Cq Majelis Hakim yang memeriksa dan mengadili perkara ini, berkenan menjatuhkan putusan sebagai berikut: ;

PRIMER:;

1. Mengabulkan gugatan Penggugat;
2. Menjatuhkan talak satu ba'in sughra Tergugat (Tergugat) terhadap Penggugat (Penggugat);
3. Menetapkan anak yang bernama: XXXX, laki-laki, lahir pada tanggal 18 Maret 2016/ umur Â± 4.5 tahun dan XXXX, laki-laki, lahir pada tanggal 23 Mei 2018/ umur Â± 2.5 tahun berada dibawah pemeliharaan (hadhonah) Penggugat;
4. Membebankan biaya perkara ini menurut hukum;

SUBSIDER:;

Atau mohon putusan lain yang seadil-adilnya;

Bahwa Penggugat telah datang menghadap di persidangan yang telah ditentukan, tetapi Tergugat telah tidak datang menghadap atau menyuruh orang lain untuk datang menghadap sebagai kuasanya, meskipun menurut Berita Acara Panggilan dari Jurusita Pengganti Pengadilan Agama Demak Nomor XXXX/Pdt.G/2020/PA.Dmk yang dibacakan di persidangan telah dipanggil secara resmi dan patut, dan tidak ternyata, bahwa tidak datangnya itu disebabkan oleh suatu halangan yang sah;

Bahwa Majelis Hakim telah menasehati Penggugat dalam rangka untuk mendamaikan agar rukun lagi dan membina rumah tangga dengan Tergugat, akan tetapi tidak berhasil karena Penggugat tetap ingin bercerai dari

Disclaimer
Kepaniteraan Mahkamah Agung Republik Indonesia berusaha untuk selalu mencantumkan informasi paling kini dan akurat sebagai bentuk komitmen Mahkamah Agung untuk pelayanan publik, transparansi dan akuntabilitas pelaksanaan fungsi peradilan. Namun dalam hal-hal tertentu masih dimungkinkan terjadi permasalahan teknis terkait dengan akurasi dan keterkinian informasi yang kami sajikan, hal mana akan terus kami perbaiki dari waktu kewaktu.
Dalam hal Anda menemukan inakurasi informasi yang termuat pada situs ini atau informasi yang seharusnya ada, namun belum tersedia, maka harap segera hubungi Kepaniteraan Mahkamah Agung RI melalui :
Email : kepaniteraan@mahkamahagung.go.id Telp : 021-384 3348 (ext.318)

Penggugat, kemudian dilanjutkan dengan pembacaan surat gugatan Penggugat yang isinya tetap dipertahankan oleh Penggugat;

Bahwa atas surat gugatan Penggugat tersebut, Tergugat tidak mengajukan jawabannya, karena tidak hadir, karena itu pemeriksaan dilakukan tanpa hadirnya Tergugat;

Bahwa karena Tergugat tidak hadir, dan perkara ini termasuk lingkup sengketa perceraian, maka pemeriksaan dilanjutkan dengan pembuktian;

Bahwa untuk membuktikan dalil gugatannya, Penggugat telah mengajukan bukti-buktinya di persidangan pada tanggal 10 November 2020, yaitu sebagai berikut:

A. Bukti Tertulis:

1. Fotokopi Kartu Tanda Penduduk Nomor 3321014509970003 tanggal 07 Januari 2016, (P.1);
2. Fotokopi Buku Kutipan Akta Nikah dari KUA Kecamatan Mranggen, Kabupaten Demak Propinsi Jawa Tengah Nomor 0173/54/II/2015 tanggal 11 Februari 2015, (P.2);
3. Fotokopi Kutipan Akta Kelahiran an. XXXX, nomor 3321-LT-30092016-0002, tertanggal 04 Oktober 2016, dikeluarkan oleh Dinas Kependudukan dan Pencatatan Sipil Demak, (P.3);
4. Fotokopi Kutipan Akta Kelahiran an.XXXX, nomor 3321-LT-28082018-0004, tertanggal 28 Agustus 2018, dikeluarkan oleh Dinas Kependudukan dan Pencatatan Sipil Demak, (P.4);

Surat –surat bukti yang berupa fotokopi tersebut setelah dicocokkan dengan aslinya ternyata sesuai dan telah bermaterai cukup;

B. Bukti saksi-saksi:

1. **Saksi I**, umur 51 tahun**,** di bawah sumpahnya ia memberi keterangan sebagai berikut:

- Bahwa saksi kenal baik dengan Penggugat dan Tergugat karena saksi adalah ayah Penggugat;
- Bahwa saksi kenal dengan Tergugat sejak menjadi suami Penggugat;
- Bahwa Penggugat dan Tergugat adalah suami isteri, menikah sudah 5 tahun lebih dan telah dikaruniai 2 orang anak;

Disclaimer
Kepaniteraan Mahkamah Agung Republik Indonesia berusaha untuk selalu mencantumkan informasi paling kini dan akurat sebagai bentuk komitmen Mahkamah Agung untuk pelayanan publik, transparansi dan akuntabilitas pelaksanaan fungsi peradilan. Namun dalam hal-hal tertentu masih dimungkinkan terjadi permasalahan teknis terkait dengan akurasi dan keterkinian informasi yang kami sajikan, hal mana akan terus kami perbaiki dari waktu kewaktu.
Dalam hal Anda menemukan inakurasi informasi yang termuat pada situs ini atau informasi yang seharusnya ada, namun belum tersedia, maka harap segera hubungi Kepaniteraan Mahkamah Agung RI melalui :
Email : kepaniteraan@mahkamahagung.go.id Telp : 021-384 3348 (ext.318)

- Bahwa anaknya tersebut sekarang dalam asuhan Penggugat dan setahu saksi Penggugat sangat perhatian kepada anaknya tersebut ;
- Bahwa Penggugat dan Tergugat semula tinggal bersama di rumah orang tua Penggugat , tidak lama kemudian pindah dan tinggal bersama di rumah orang tua Tergugat;
- Bahwa pada awalnya rumah tangga Penguggat dan Tergugat tampak rukun dan harmonis akan tetapi sejak 3 tahun yang lalu mulai tidak rukun, karena sering terjadi perselisihan dan pertengkaran;
- Bahwa penyebab pertengkaran tersebut karena Tergugat tidak memberikan nafkah yang layak kepada Penggugat;
- Bahwa kemudian pada bulan Juli tahun 2018 Penggugat pergi meninggalkan Tergugat dan pulang ke rumah orang tuanya di Desa Kalitengah, hingga sekarang;
- Bahwa selama berpisah, antara Penggugat dan Tergugat tidak ada komonikasi sama sekali;
- Bahwa keluarga Penggugat dan Tergugat sudah berusaha menasehati Penggugat agar rukun kembali akan tetapi tidak berhasil.

2. **Saksi II**, umur 25 tahun**,** di bawah sumpahnya ia memberi keterangan sebagai berikut:

- Bahwa saksi kenal baik dengan Penggugat dan Tergugat karena saksi adalah tetangga Penggugat;
- Bahwa saksi kenal dengan Tergugat sejak menjadi suami Penggugat;
- Bahwa Penggugat dan Tergugat adalah suami isteri, menikah sudah 5 tahun lebih dan telah dikaruniai 2 orang anak;
- Bahwa anaknya tersebut sekarang dalam asuhan Penggugat dan setahu saksi Penggugat sangat perhatian kepada anaknya tersebut ;
- Bahwa Penggugat dan Tergugat semula tinggal bersama di rumah orang tua Penggugat , tidak lama kemudian pindah dan tinggal bersama di rumah orang tua Tergugat;

Disclaimer
Kepaniteraan Mahkamah Agung Republik Indonesia berusaha untuk selalu mencantumkan informasi paling kini dan akurat sebagai bentuk komitmen Mahkamah Agung untuk pelayanan publik, transparansi dan akuntabilitas pelaksanaan fungsi peradilan. Namun dalam hal-hal tertentu masih dimungkinkan terjadi permasalahan teknis terkait dengan akurasi dan keterkinian informasi yang kami sajikan, hal mana akan terus kami perbaiki dari waktu kewaktu.
Dalam hal Anda menemukan inakurasi informasi yang termuat pada situs ini atau informasi yang seharusnya ada, namun belum tersedia, maka harap segera hubungi Kepaniteraan Mahkamah Agung RI melalui :
Email : kepaniteraan@mahkamahagung.go.id Telp : 021-384 3348 (ext.318)

- Bahwa pada awalnya rumah tangga Penguggat dan Tergugat tampak rukun dan harmonis akan tetapi sejak 3 tahun yang lalu mulai tidak rukun, karena sering terjadi perselisihan dan pertengkaran;
- Bahwa penyebab pertengkaran tersebut karena Tergugat tidak memberikan nafkah yang layak kepada Penggugat;
- Bahwa kemudian pada bulan Juli tahun 2018 Penggugat pergi meninggalkan Tergugat dan pulang ke rumah orang tuanya di Desa Kalitengah, hingga sekarang;
- Bahwa selama berpisah, antara Penggugat dan Tergugat tidak ada komonikasi sama sekali;
- Bahwa keluarga Penggugat dan Tergugat sudah berusaha menasehati Penggugat agar rukun kembali akan tetapi tidak berhasil.

Bahwa atas bukti-bukti yang diajukan oleh Penggugat tersebut, Tergugat tidak mengajukan bantahannya, karena tidak hadir di persidangan;

Bahwa Penggugat telah menyampaikan kesimpulannya secara lisan di persidangan pada tanggal 22 Desember 2020, yang pada pokoknya Penggugat tetap menghendaki perceraian, tetap memohon hak asuh terhadap kedua orang anaknya dan menyatakan tidak akan mengajukan suatu apapun lagi dan mohon putusan;

Bahwa untuk meringkas uraian putusan ini, maka ditunjuk hal ihwal sebagaimana tercantum dalam berita acara sidang perkara ini yang merupakan bagian yang tak terpisahkan dari putusan ini.

PERTIMBANGAN HUKUM

Menimbang bahwa maksud dan tujuan gugatan Penggugat adalah sebagaimana telah terurai diatas;

Menimbang, bahwa Penggugat dan Tergugat beragama Islam dan telah melakukan perkawinan secara Islami, maka perkara ini merupakan kompetensi absolut Pengadilan Agama untuk memeriksa dan menyelesaikannya. (vide Pasal 40 dan Pasal 63 ayat (1) Undang Undang No. 1 Tahun 1974 Jo. Pasal 14 dan Pasal 1 huruf b. PP. N0. 9 Tahun 1975, Jo Pasal 2 dan Pasal 49 ayat (1) Undang-Undang Nomor 7 Tahun 1989 yang telah diubah dengan Undang Undang No 3 Tahun 2006 dan Undang-Undang Nomor 50 Tahun 2009;

Disclaimer
Kepaniteraan Mahkamah Agung Republik Indonesia berusaha untuk selalu mencantumkan informasi paling kini dan akurat sebagai bentuk komitmen Mahkamah Agung untuk pelayanan publik, transparansi dan akuntabilitas pelaksanaan fungsi peradilan. Namun dalam hal-hal tertentu masih dimungkinkan terjadi permasalahan teknis terkait dengan akurasi dan keterkinian informasi yang kami sajikan, hal mana akan terus kami perbaiki dari waktu kewaktu.
Dalam hal Anda menemukan inakurasi informasi yang termuat pada situs ini atau informasi yang seharusnya ada, namun belum tersedia, maka harap segera hubungi Kepaniteraan Mahkamah Agung RI melalui :
Email : kepaniteraan@mahkamahagung.go.id Telp : 021-384 3348 (ext.318)

Menimbang, bahwa dari bukti P-1 yang berupa kartu tanda Penduduk maka telah terbukti Penggugat bertempat tinggal di wilayah yurisdiksi Pengadilan Agama Demak, maka perkara ini secara relatif merupakan kompetensi Pengadilan Agama Demak, (vide Pasal 73 ayat (1) Undang-Undang Nomor 7 Tahun 1989 yang telah diubah dengan Undang-Undang Nomor No 3 Tahun 2006 dan Undang Undang No. 50 Tahun 2009 jo Pasal 132 ayat (1) Kompilasi Hukum Islam);

Menimbang, bahwa berdasarkan bukti P-2 yang berupa Kutipan Akta Nikah maka telah terbukti bahwa benar, antara Penggugat dengan Tergugat telah terikat dalam perkawinan yang sah oleh karena itu gugatan Penggugat terhadap Tergugat *memiliki dasar hukum yang sah*;-

Menimbang, bahwa karena ternyata Tergugat meskipun dipanggil dengan patut tidak pernah datang menghadap dan tidak pula menyuruh orang lain untuk menghadap sebagai wakilnya serta ketidak datangannya itu tanpa disebabkan oleh suatu halangan yang sah, maka patutlah dianggap bahwa Tergugat dalam keadaan tidak hadir dan sesuai dengan kehendak pasal 125 HIR putusan perkara ini dijatuhkan dengan verstek;

Menimbang, bahwa oleh karena Tergugat tidak pernah hadir dipersidangan sehingga proses mediasi sebagaimana ketentuan PERMA Nomor 01 Tahun 2016 tidak dapat dilaksanakan;

Menimbang, bahwa Penggugat pada pokoknya menuntut perceraian dengan alasan karena sejak bulan Oktober tahun 2017 rumah tangganya mulai tidak harmonis yaitu antara Penggugat dengan Tergugat sering terjadi perselisihan dan pertengkaran disebabkan karena Tergugat tidak memberi nafkah kepada Pengguat kareana penghasilan Tergugat hanya dihabiskan untuk memenuhi kebutuhannya sendiri yang kemudian pada bulan Juli tahun 2018 Penggugat pergi meninggalkan Tergugat dan pulang ke rumah orang tuanya di Kelurahan Kalitengah, hingga sekarang dan sejak itu pula antara Penggugat dengan Tergugat tidak ada lagi komonikasi layaknya suami isteri;

Menimbang bahwa secara yuridis Pemohon telah menyandarkan alasan permohonannya pada ketentuan pasal 19 huruf f Peraturan Pemerintah Nomor 9 tahun 1975 jo pasal 116 huruf f Kompilasi Hukum Islam yaitu “ *Antara suami*

Disclaimer
Kepaniteraan Mahkamah Agung Republik Indonesia berusaha untuk selalu mencantumkan informasi paling kini dan akurat sebagai bentuk komitmen Mahkamah Agung untuk pelayanan publik, transparansi dan akuntabilitas pelaksanaan fungsi peradilan. Namun dalam hal-hal tertentu masih dimungkinkan terjadi permasalahan teknis terkait dengan akurasi dan keterkinian informasi yang kami sajikan, hal mana akan terus kami perbaiki dari waktu kewaktu.
Dalam hal Anda menemukan inakurasi informasi yang termuat pada situs ini atau informasi yang seharusnya ada, namun belum tersedia, maka harap segera hubungi Kepaniteraan Mahkamah Agung RI melalui :
Email : kepaniteraan@mahkamahagung.go.id Telp : 021-384 3348 (ext.318)

dan isteri terus menerus terjadi perselisihan dan pertengkaran dan tidak ada harapan akan hidup rukun lagi dalam rumah tangga",;

Menimbang bahwa ketidak hadiran Termohon dalam persidangan tanpa alasan yang sah dianggap telah mengabaikan hak bantah Termohon atas dalil-dalil Pemohon yang karenanya dinilai sebagai pengakuan Termohon secara-diam-diam (iqrar bi as-sukut)

Menimbang, bahwa dua orang saksi Penggugat tersebut telah memenuhi syarat-syarat formil dan tidak terhalang kedudukannya sebagai saksi seperti yang diatur dalam Pasal 145 HIR, keduanya memberikan keterangan yang didasarkan pada pengetahuan dari pengalaman, penglihatan dan pendengarannya sendiri yang antara kedua saksi tersebut terdapat kesesuaian keterangan seperti yang disyaratkan dalam Pasal 172 HIR, oleh karena itu keterangan kedua saksi tersebut dapat dipertimbangkan;

Menimbang, bahwa dari dalil gugatan Penggugat, sikap Penggugat selama dipersidangan dihubungkan dengan bukti surat dan keterangan-keterangan yang disampaikan oleh para saksi di persidangan, dapat ditemukan fakta sebagai berikut :

1. Bahwa Penggugat dan Tergugat adalah suami isteri sah, menikah pada tanggal 11 Februari 2011 dan telah dikaruniai 2 orang anak dalam asuhan Penggugat;
2. Bahwa kemudian sejak Oktober tahun 2017, antara Penggugat dengan Tergugat mulai sering terjadi perselisihan dan pertengkaran disebabkan karena Tergugat Tergugat tidak memberi nafkah kepada Pengguat kareana penghasilan Tergugat hanya dihabiskan untuk memenuhi kebutuhannya sendiri ;
3. Bahwa kemudian puncaknya terjadi pada bulan Juli tahun 2018 menyebabkan antara Penggugat dengan Tergugat pisah rumah yaitu Penggugat pergi meninggalkan Tergugat hingga sekarang;
4. Bahwa selama berpisah rumah tersebut antara Penggugat dan Tergugat tidak ada komunikasi sama sekali;
5. Bahwa keluarga Penggugat sudah berusaha mendamaikannya, akan tetapi tidak berhasil.

Disclaimer
Kepaniteraan Mahkamah Agung Republik Indonesia berusaha untuk selalu mencantumkan informasi paling kini dan akurat sebagai bentuk komitmen Mahkamah Agung untuk pelayanan publik, transparansi dan akuntabilitas pelaksanaan fungsi peradilan. Namun dalam hal-hal tertentu masih dimungkinkan terjadi permasalahan teknis terkait dengan akurasi dan keterkinian informasi yang kami sajikan, hal mana akan terus kami perbaiki dari waktu kewaktu.
Dalam hal Anda menemukan inakurasi informasi yang termuat pada situs ini atau informasi yang seharusnya ada, namun belum tersedia, maka harap segera hubungi Kepaniteraan Mahkamah Agung RI melalui :
Email : kepaniteraan@mahkamahagung.go.id Telp : 021-384 3348 (ext.318)

Menimbang, bahwa berdasarkan fakta poin (1) menunjukkan bahwa Penggugat dan Tergugat berkualitas hukum sebagai pihak-pihak dalam perkara ini, dan Penggugat berhak mengajukan permohonan cerai gugat terhadap Tergugat ke Pengadilan Agama sebagaimana dimaksud pasal 66 ayat (1) Undang-undang Nomor 7 Tahun 1989;

Menimbang, bahwa dari fakta point 2, 3, 4 dan 5 sebagaimana terurai diatas telah memberikan gambaran bahwa perselisihan antara Penggugat dengan Tergugat sudah tidak mampu lagi dikendalikan dan diatasi oleh mereka berdua, bahkan sikap Penggugat yang telah pergi meninggalkan Tergugat adalah merupakan bukti bahwa perselisihan antara Penggugat dengan Tergugat sudah cukup serius dan bersifat terus menerus;

Menimbang bahwa dari pernyataan Penggugat selama dalam persidangan yang dengan tegas hendak bercerai dengan Tergugat dan juga sikap Tergugat yang tidak pernah menghadiri persidangan adalah merupakan indikasi tidak adanya kehendak dari kedua belah pihak untuk memperbaiki kembali keadaan rumah tangganya, karena itu merupakan fakta kongkrit yang menunjukkan bahwa hubungan Penggugat dengan Tergugat sudah sampai pada tingkat pecahnya perkawinan (*broken marriage*) dan Hakim berkesimpulan bahwa tujuan yang diharapkan dari perkawinan sebagaimana tersebut pada pasal 1 Undang-undang Nomor 1 Tahun 1974 tidak mungkin lagi terwujud;

Menimbang bahwa para saksi saksi telah menerangkan adanya usaha pihak keluarga merukunkan Penggugat dengan Tergugat akan tetapi tidak berhasil, kerena itu dianggap telah memenui ketentuan Pasal 22 ayat (2) Peraturan Pemerintah Nomor 9 Tahun 1974;

Menimbang bahwa mengutip referensi fiqh yang terdapat dalam kitab Hurriatuz Zaujani fith Thalaq Juz I halaman 83 yang diambil alih menjadi pendapat Hakim sebagai berikut :

æÞÏ ÇÎÊÇÑÇáÅÓáÇã äÙÇã ÇáØáÇÞ Ííä ÊÖØÑ È ÇáÍíÇÉ ÇáÒæÌíä æáã íÚÏ

íäÝÚ ÝíåÇ äÕÇ ÆÍ æáÇ Õáí æÍíË ÊÕÈÍ ÇáÑÈØÉ ÇáÒæÇÌ ÕæÑÉ ãä ÛíÑ Ñæí

Putusan Nomor 2213/Pdt.G/2020/PA.Dmk
Halaman 9 dari 14 Halaman

Disclaimer
Kepaniteraan Mahkamah Agung Republik Indonesia berusaha untuk selalu mencantumkan informasi paling kini dan akurat sebagai bentuk komitmen Mahkamah Agung untuk pelayanan publik, transparansi dan akuntabilitas pelaksanaan fungsi peradilan. Namun dalam hal-hal tertentu masih dimungkinkan terjadi permasalahan teknis terkait dengan akurasi dan keterkinian informasi yang kami sajikan, hal mana akan terus kami perbaiki dari waktu kewaktu.
Dalam hal Anda menemukan inakurasi informasi yang termuat pada situs ini atau informasi yang seharusnya ada, namun belum tersedia, maka harap segera hubungi Kepaniteraan Mahkamah Agung RI melalui :
Email : kepaniteraan@mahkamahagung.go.id   Telp : 021-384 3348 (ext.318)

áÃä ÇÓÊãÑÇÑ ãÚäÇå Çä íßã Úáì ÇÎÏ ÇáÒæÌíä ÈÇáÓÌä ÇáãæÁ ÈÏ æåÐÇ Ùáã ÊÃ ÈÇå ÑæÍ ÇáÚÏÇáÉ

Artinya : "*Islam memilih lembaga thalaq (perceraian) ketika rumah tangga sudah dianggap goncang serta dianggap sudah tidak bermanfaat lagi nasehat/ perdamaian, dan hubungan suami isteri menjadi tanpa ruh (hampa), sebab meneruskan perkawinan berarti menghukum salah satu suami isteri dengan penjara yang berkepanjangan. Ini adalah aniaya (kedhaliman) yang bertentangan dengan semangat keadilan*".

Menimbang bahwa sehubungan dengan hal tersebut Yurisprudensi Mahkamah Agung RI. Nomor 379 K / AG / 1995 tanggal 26 Maret 1997 menyatakan "*Suami isteri yang tidak berdiam serumah lagi dan tidak ada harapan untuk hidup rukun kembali maka rumah tanga tersebut telah terbukti retak dan pecah dan telah memenuhi alasan cerai Pasal 19 huruf f Peraturan Pemerintah Nomor 9 tahun 1975*";

Menimbang, bahwa aspek penting dari bunyi pasal 19 huruf f Peraturan Pemerintah Nomor 9 tahun 1975 jo pasal 116 huruf KHI diatas adalah tidak adanya harapan untuk menata dan mempertahankan rumah tangga, dengan demikian apabila maksud pasal tersebut dikomparasikan dengan keadaan rumah tangga Penggugat dan Tergugat maka telah terdapat cukup alasan hukum untuk mengabulkan gugatan Penggugat dan menjatuhkan talak satu ba'in sughro dari Tergugat terhadap Penggugat;

Menimbang bahwa selaian perceraian tersebut, Penggugat juga menuntut hadlonah/ pemeliharaan terhadap kedua orang anaknya masing-masing bernama XXXX, lahir tanggal 17 Juli 2011, dan XXXX, lahir tanggal 16 Februari 2018), yang keduanya saat ini ikut Penggugat;

Menimbang bahwa, berdasarkan bukti P-3 yang berupa kutipan akta lahir atas nama XXXX, lahir tanggal 18 Maret 2016 dan P-4 berupa kutipan akta kelahiran atas nama XXXX, lahir tanggal 23 Mei 2018, yang berarti kedua anak tersebut belum mumayyiz atau belum berumur 12 tahun dan berdasarkan keterangan para saksi bahwa kedua anak tersebut dalam asuhan Penggugat

Disclaimer
Kepaniteraan Mahkamah Agung Republik Indonesia berusaha untuk selalu mencantumkan informasi paling kini dan akurat sebagai bentuk komitmen Mahkamah Agung untuk pelayanan publik, transparansi dan akuntabilitas pelaksanaan fungsi peradilan. Namun dalam hal-hal tertentu masih dimungkinkan terjadi permasalahan teknis terkait dengan akurasi dan keterkinian informasi yang kami sajikan, hal mana akan terus kami perbaiki dari waktu kewaktu.
Dalam hal Anda menemukan inakurasi informasi yang termuat pada situs ini atau informasi yang seharusnya ada, namun belum tersedia, maka harap segera hubungi Kepaniteraan Mahkamah Agung RI melalui :
Email : kepaniteraan@mahkamahagung.go.id Telp : 021-384 3348 (ext.318)

dan Penggugat selaku Ibu penuh perhatian dan sayang kepada kedua anaknya tersebut;

Menimbang bahwa karena kedua anak tersebut belum mumayyiz dan masih sangat membutuhkan pelayanan, perhatian, kesabaran dan sentuhan kasih sayang dari seorang Ibu dan secara nyata sejak lahir hingga sekarang anak tersebut dalam asuhan Penggugat, sehingga hukum memandang Ibunya (Penggugat) lebih berhak mengasuh, mendidik serta pantas ditunjuk sebagai pemegang hak hadlonah atas kedua anak tersebut, hal ini sesuai ketentuan Pasal 105 ayat (1) Kompilasi Hukum Islam dan hadis Nabi yang berbunyi:

أنت أحق به مالم تنكحى

Artinya "*Engkau (Ibu) lebih berhak terhadap anakmu selama engkau belum menikah*"

Menimbang bahwa berdasarkan pertimbangan tersebut diatas maka Pengadilan patut menetapkan kedua anak Penggugat dan Tergugat bernama XXXX dan XXXX tetap berada dalam hadlonah/ asuhan Penggugat;

Menimbang bahwa bapak atau Ibu yang tidak mengasuh anak, tetap mempunyai hak untuk mencurahkan kasih sayangnya seperti melihat dan mengajak bepergian anak tersebut kapan saja waktunya dengan tujuan untuk mendatangkan kemaslahatan bagi anak dan orang tua yang tidak mengasuh anak tersebut, dengan demikian anak tersebut akan merasa tetap dekat dengan kedua orang tuanya, sebagaimana pendapat DR. Wahbah Az-Zuhaily dalam kitab al-Fiqhul Islamy wa Adillatuhu, jilid VII halaman 70 yang diambil alih sebagain pendapat Majelis Hakim sebagai berikut:

حق الرؤية أو الزيارة لاحد الابوين غير الحاضن مقرر شرعا باتفاق الفقهاء لصلة ولكنهم ذكروا اراء مختلفة نسيبا بحسب تقدير المصلحة, لكل من الولد والولد الذي ولده فى حضانه غيره

Artinya: " Ulama' ahli fiqh sepakat, hak untuk melihat atau berkunjung bagi salah satu dari orang tua yang tidak mengasuh anak, ditetapkan berdasarkan syara' untuk menyambung hubungan antara anak dengan salah

Disclaimer
Kepaniteraan Mahkamah Agung Republik Indonesia berusaha untuk selalu mencantumkan informasi paling kini dan akurat sebagai bentuk komitmen Mahkamah Agung untuk pelayanan publik, transparansi dan akuntabilitas pelaksanaan fungsi peradilan. Namun dalam hal-hal tertentu masih dimungkinkan terjadi permasalahan teknis terkait dengan akurasi dan keterkinian informasi yang kami sajikan, hal mana akan terus kami perbaiki dari waktu kewaktu.
Dalam hal Anda menemukan inakurasi informasi yang termuat pada situs ini atau informasi yang seharusnya ada, namun belum tersedia, maka harap segera hubungi Kepaniteraan Mahkamah Agung RI melalui :
Email : kepaniteraan@mahkamahagung.go.id Telp : 021-384 3348 (ext.318)

satu orang tuanya yang tidka mengasuh anak, sekira bisa mendatangkankan kemaslahatan bagi anak dan orang tua yang melahirkan anak tersebut, yang berada dibawah asuhan salah satu borang tuanya"

Menimbang bahwa karena itu orang yang mengasuh anak serta seluruh keluarganya tidak boleh menghalang-halangi anak untuk bertemu dengan orang tuanya yang tidak mengasuh anak atau menyembunyikan anak tersebut, sehingga tidak ada kesempatan bagi anak untuk bertemu dengan orang tuanya yang tidak mengasuhnya;

Menimbang bahwa berdasarkan pertimbangan tersebut diatas, maka kepada Tergugat selaku ayah dari kedua anak bernama XXXX dan XXXX tersebut berhak untuk menengok/ mengunjungi dan mengajak anak tersebut bepergian dengan sepengetahuan /izin Penggugat;

Menimbang, bahwa oleh karena perkara ini termasuk dalam bidang perkawinan, berdasarkan Pasal 89 ayat (1) Undang-Undang 7 Tahun 1989 yang telah diubah dengan Undang-Undang Nomor 3 Tahun 2006 dan perubahan kedua dengan Undang-Undang Nomor 50 Tahun 2009, maka biaya perkara ini dibebankan kepada Penggugat ;

Memperhatikan Pasal 125 dan 126 HIR serta ketentuan dalil syar'i yang berkaitan dengan perkara ini ;

M E N G A D I L I

1. *Menyatakan Tergugat yang telah dipanggil secara resmi dan patut untuk menghadap ke persidangan, tidak hadir;*
2. *Mengabulkan gugatan Penggugat dengan verstek;*
3. *Menjatuhkan talak satu ba'in sughra Tergugat (**Tergugat**) kepada Penggugat (**Penggugat**);*
4. *Menetapkan kedua orang anak Penggugat dan Tergugat yang bernama* XXXX, lahir 18 Maret 2016 dan XXXX, lahir tanggal 23 Mei 2018 berada *dibawah pemeliharaan (hadhonah) Penggugat;*
5. *Membebankan kepada Penggugat untuk membayar biaya perkara yang hingga kini sejumlah Rp. 501.000,- (lima ratus satu ribu rupiah);*

Disclaimer
Kepaniteraan Mahkamah Agung Republik Indonesia berusaha untuk selalu mencantumkan informasi paling kini dan akurat sebagai bentuk komitmen Mahkamah Agung untuk pelayanan publik, transparansi dan akuntabilitas pelaksanaan fungsi peradilan. Namun dalam hal-hal tertentu masih dimungkinkan terjadi permasalahan teknis terkait dengan akurasi dan keterkinian informasi yang kami sajikan, hal mana akan terus kami perbaiki dari waktu kewaktu.
Dalam hal Anda menemukan inakurasi informasi yang termuat pada situs ini atau informasi yang seharusnya ada, namun belum tersedia, maka harap segera hubungi Kepaniteraan Mahkamah Agung RI melalui :
Email : kepaniteraan@mahkamahagung.go.id Telp : 021-384 3348 (ext.318)

Demikianlah putusan ini dijatuhkan dalam musyawarah Majelis Hakim di Demak pada hari Selasa tanggal 22 Desember 2020 Masehi, bertepatan dengan tanggal 07 Jumadil Awal 1442 Hijriyah, oleh Kami ***Drs. Makali*** *sebagai Ketua Majelis Hakim, dan* ***Dra. Nur Immawati*** *serta* ***Toharudin, S.H.I., M.H.*** *masing-masing sebagai Hakim Anggota. Putusan mana diucapkan oleh Ketua Majelis Hakim tersebut pada hari itu juga dalam sidang terbuka untuk umum dengan dihadiri oleh para Hakim Anggota dan* ***Hj. Siti Hajar Zulaikha, S.H.*** *sebagai Panitera Pengganti dan dihadiri pula oleh Penggugat tanpa hadirnya Tergugat;*

Ketua Majelis

Drs. Makali

Hakim Anggota	Hakim Anggota
**Dra. Nur Immawati**	**Toharudin, S.H.I., M.H.**

Panitera Pengganti

Hj. Siti Hajar Zulaikha, S.H.

Perincian Biaya :

1. Biaya Pendaftaran	:	Rp	30.000,00
2. Biaya Proses	:	Rp	75.000,00
3. Biaya Pemanggilan	:	Rp	360.000,00
4. PNBP Panggilan	:	Rp	20.000,00
5. Biaya Redaksi	:	Rp	10000,00
6. Biaya	:	Rp	6000,00

Disclaimer
Kepaniteraan Mahkamah Agung Republik Indonesia berusaha untuk selalu mencantumkan informasi paling kini dan akurat sebagai bentuk komitmen Mahkamah Agung untuk pelayanan publik, transparansi dan akuntabilitas pelaksanaan fungsi peradilan. Namun dalam hal-hal tertentu masih dimungkinkan terjadi permasalahan teknis terkait dengan akurasi dan keterkinian informasi yang kami sajikan, hal mana akan terus kami perbaiki dari waktu kewaktu.
Dalam hal Anda menemukan inakurasi informasi yang termuat pada situs ini atau informasi yang seharusnya ada, namun belum tersedia, maka harap segera hubungi Kepaniteraan Mahkamah Agung RI melalui :
Email : kepaniteraan@mahkamahagung.go.id   Telp : 021-384 3348 (ext.318)

Meterai			
Jumlah	:	Rp	501.000,00

(lima ratus satu ribu rupiah)

Disclaimer
Kepaniteraan Mahkamah Agung Republik Indonesia berusaha untuk selalu mencantumkan informasi paling kini dan akurat sebagai bentuk komitmen Mahkamah Agung untuk pelayanan publik, transparansi dan akuntabilitas pelaksanaan fungsi peradilan. Namun dalam hal-hal tertentu masih dimungkinkan terjadi permasalahan teknis terkait dengan akurasi dan keterkinian informasi yang kami sajikan, hal mana akan terus kami perbaiki dari waktu kewaktu.
Dalam hal Anda menemukan inakurasi informasi yang termuat pada situs ini atau informasi yang seharusnya ada, namun belum tersedia, maka harap segera hubungi Kepaniteraan Mahkamah Agung RI melalui :
Email : kepaniteraan@mahkamahagung.go.id   Telp : 021-384 3348 (ext.318)
