# Supplementary material for: Classification of divorce causes during the COVID-19 pandemic using convolutional neural networks
Source: PeerJ Comput Sci. 2022 Jun 30;8:e998. doi: 10.7717/peerj-cs.998 (PMC9299239; doi:10.7717/peerj-cs.998)
Supplement: Supplemental Information 4 [file peerj-cs-08-998-s004.zip › Konflik Dataset/Data ke-20.pdf]

P U T U S A N

Nomor 4198/Pdt.G/2020/PA.Smdg

بسم الله الرحمن الرحيم

DEMI KEADILAN BERDASARKAN KETUHANAN YANG MAHA ESA

Pengadilan Agama Sumedang yang memeriksa dan mengadili perkara tertentu dalam tingkat pertama telah menjatuhkan putusan perkara Cerai Talak yang diajukan oleh:

Teten Juanda bin Endang, umur 24 tahun, agama Islam, pekerjaan Buruh, pendidikan SLTA, tempat kediaman di Dusun Sindulang 03 RW 01 Desa Sindulang Kecamatan Cimanggung Kabupaten Sumedang ...; sebagai **Pemohon**;

melawan

Siti Halimah Sa'adah binti Dindin Zaenudin umur 23 tahun, agama Islam, pendidikan SLTP, pekerjaan Ibu Rumah Tangga, tempat kediaman di Bertempat Tinggal Di Kediaman Oarngtuanya a,n Dusun Binong RT 01 RW 12 Desa Girimukti Kecamatan Sumedang Utara Kabupaten Sumedang; sebagai **Termohon**;

Pengadilan Agama tersebut;

Telah mempelajari surat-surat yang berkaitan dengan perkara ini;

Telah mendengar keterangan Pemohon dan para saksi di depan sidang;

DUDUK PERKARA

Menimbang, bahwa Pemohon dalam surat permohonannya tanggal 04 Desember 2020 telah mengajukan permohonan cerai, yang telah didaftar di Kepaniteraan Pengadilan Agama Sumedang, dengan Nomor 4198/Pdt.G/2020/PA.Smdg, tanggal 04 Desember 2020, dengan dalil-dalil sebagai berikut:

1. Bahwa Pemohon dan Termohon adalah suami isteri sah menikah pada tanggal 11 Juli 2019 berdasarkan Kutipan Akta Nikah dari KUA KUA Kecamatan Sumedang Utara Kabupaten Sumedang, No: 0442/033/VII/2019. Tertanggal 11 Juli 2019;

Disclaimer
Kepaniteraan Mahkamah Agung Republik Indonesia berusaha untuk selalu mencantumkan informasi paling kini dan akurat sebagai bentuk komitmen Mahkamah Agung untuk pelayanan publik, transparansi dan akuntabilitas pelaksanaan fungsi peradilan. Namun dalam hal-hal tertentu masih dimungkinkan terjadi permasalahan teknis terkait dengan akurasi dan keterkinian informasi yang kami sajikan, hal mana akan terus kami perbaiki dari waktu kewaktu.
Dalam hal Anda menemukan inakurasi informasi yang termuat pada situs ini atau informasi yang seharusnya ada, namun belum tersedia, maka harap segera hubungi Kepaniteraan Mahkamah Agung RI melalui :
Email : kepaniteraan@mahkamahagung.go.id Telp : 021-384 3348 (ext.318)

2. Bahwa sebelum membina rumahtangga, Pemohon berstatus Jejaka begitupun Termohon berstatus Perawan;
3. Bahwa antara Pemohon dan Termohon **terakhir berumah tangga tinggal di rumah kediaman orangtua Pemohon** di Dusun Sindulang 03 RW 01 Desa Sindulang Kecamatan Cimanggung Kabupaten Sumedang dan telah bergaul baik sebagaimana layaknya suami isteri, dan telah dikaruniai seorang anak bernama: Muhammad Abidzar Al-Ghifari (berusia 6 bulan);
4. Bahwa antara Pemohon dan Termohon sejak **bulan Oktober 2019** sudah tidak harmonis lagi dalam menjalani hidup berumah tangga dikarenakan antara Pemohon dan Termohon sering terjadi perselisihan dan pertengkaran terus-menerus yang disebabkan:
5. Bahwa antara Pemohon dan Termohon sering berselisih paham terkait tempat tinggal dimana Termohon tidak mau tinggal bersama di rumah kediaman milik Pemohon dengan alasan yang tidak pasti, sedangkan Pemohon merasa tidak betah tinggal di rumah kediaman milik orangtua Termohon dengan alasan jauh dari tempat kerja, sehingga tidak ada yang mau mengalah yang mengakibatkan rumah tangga sering terlibat pertengkaran.
6. Bahwa Termohon ketika ada permasalahan rumah tangga, sering pulang ke rumah orangtuanya, selain itu Termohon lebih mendengarkan perkataan orangtuanya dibandingkan Pemohon sebagai seorang suami, selain itu juga Termohon sering membantah kata-kata Pemohon dalam hal membina rumah tangga yang lebih baik lagi, yang mana apabila Termohon dinasehati oleh Pemohon, Termohon sering tidak menerimanya, sehingga hal itu menjadi pemicu keretakan dalam rumah tangga.
7. Bahwa keretakan rumah tangga antara Pemohon dan Termohon telah berlangsung lama. Pemohon berusaha sabar, tetapi Termohon tidak berusaha berubah dan merubah sifatnya. Oleh karena itu Pemohon merasa tidak nyaman berumah tangga dengan Termohon. Puncaknya **pada bulan November 2019** antara Pemohon dan Termohon terjadi

Disclaimer
Kepaniteraan Mahkamah Agung Republik Indonesia berusaha untuk selalu mencantumkan informasi paling kini dan akurat sebagai bentuk komitmen Mahkamah Agung untuk pelayanan publik, transparansi dan akuntabilitas pelaksanaan fungsi peradilan. Namun dalam hal-hal tertentu masih dimungkinkan terjadi permasalahan teknis terkait dengan akurasi dan keterkinian informasi yang kami sajikan, hal mana akan terus kami perbaiki dari waktu kewaktu.
Dalam hal Anda menemukan inakurasi informasi yang termuat pada situs ini atau informasi yang seharusnya ada, namun belum tersedia, maka harap segera hubungi Kepaniteraan Mahkamah Agung RI melalui :
Email : kepaniteraan@mahkamahagung.go.id   Telp : 021-384 3348 (ext.318)

pertengkaran besar dan **Termohon pergi meninggalkan rumah kediaman orangtua Pemohon**, sejak itu Pemohon dengan Termohon pisah tempat tinggal dan tidak lagi bergaul layaknya suami isteri. **Dimana sekarang ini Pemohon tinggal di kediaman orangtua Pemohon** dan **Termohon tinggal di rumah kediaman orangtua Termohon;**

8. Bahwa keretakan rumah tangga Pemohon dan Termohon sudah pernah didamaikan oleh keluarga, tetapi tidak berhasil.
9. Bahwa Permohonan Pemohon tersebut telah memenuhi syarat sesuai ketentuan Pasal 19 huruf (f) Peraturan Pemerintah Nomor 9 Tahun 1975 jo. Pasal 116 huruf (f) Kompilasi Hukum Islam.
10. Bahwa atas permasalahan tersebut di atas Pemohon sudah tidak sanggup lagi untuk mempertahankan perkawinan ini, oleh karena itu Pemohon telah berketetapan hati untuk bercerai dengan Termohon.

Bahwa berdasarkan alsan-alasan tersebut diatas, Pemohon mohon kepada Majelis Hakim Pengadilan Agama Sumedang yang memeriksa dan mengadili perkara ini, berkenan untuk memutus dengan amar sebagai berikut :

1. Menerima dan mengabulkan Permohonan Ikrar Talak Pemohon.
2. Memberikan izin kepada Pemohon (Teten Juanda bin Endang) untuk mengikrarkan Talak Satu Raj'i kepada Termohon (Siti Halimah Sa'adah binti Dindin Zaenudin) di depan sidang Pengadilan Agama Klas IA Sumedang.
3. Menetapkan biaya perkara menurut hukum.

Bahwa pada hari sidang yang telah ditetapkan Pemohon telah datang menghadap ke persidangan, sedangkan Termohon tidak datang menghadap ke persidangan dan tidak menyuruh orang lain untuk menghadap sebagai wakil/kuasa hukumnya, meskipun telah dipanggil secara resmi dan patut yang relaas panggilannya dibacakan di dalam sidang, sedangkan tidak ternyata bahwa tidak datangnya itu disebabkan suatu alasan yang sah;

Disclaimer
Kepaniteraan Mahkamah Agung Republik Indonesia berusaha untuk selalu mencantumkan informasi paling kini dan akurat sebagai bentuk komitmen Mahkamah Agung untuk pelayanan publik, transparansi dan akuntabilitas pelaksanaan fungsi peradilan. Namun dalam hal-hal tertentu masih dimungkinkan terjadi permasalahan teknis terkait dengan akurasi dan keterkinian informasi yang kami sajikan, hal mana akan terus kami perbaiki dari waktu kewaktu.
Dalam hal Anda menemukan inakurasi informasi yang termuat pada situs ini atau informasi yang seharusnya ada, namun belum tersedia, maka harap segera hubungi Kepaniteraan Mahkamah Agung RI melalui :
Email : kepaniteraan@mahkamahagung.go.id   Telp : 021-384 3348 (ext.318)

Bahwa majelis hakim telah menasehati Pemohon agar berpikir untuk tidak bercerai dengan Termohon, tetapi Pemohon tetap pada dalil-dalil permohonannya untuk bercerai dengan Termohon ;

Bahwa perkara ini tidak dapat dimediasi karena Termohon tidak pernah datang menghadap meskipun telah dipanggil secara resmi dan patut, selanjutnya dimulai pemeriksaan dengan membacakan surat permohonan Pemohon yang maksud dan tujuannya tetap dipertahankan oleh Pemohon ;

Bahwa terhadap permohonan Pemohon tersebut, Termohon tidak memberikan jawaban karena tidak hadir di persidangan;

Bahwa untuk menguatkan dalil-dalil permohonannya, Pemohon telah mengajukan bukti berupa:

A. Surat :

Buku Kutipan Akta Nikah atas nama Pemohon dan Pemohon yang menikah pada tanggal 11 Juli 2019, dengan Nomor 0442/033/VII/2019 tertanggal 11 Juli 2019, yang dikeluarkan oleh Kantor Urusan Agama KUA Kecamatan Sumedangn Utara Kabupaten Sumedang. Fotocopi tersebut dibubuhi materai cukup dan setelah dicocokan dengan aslinya ternyata sesuai, kemudian oleh Ketua Majelis diparaf dan diberi kode P.1;

B. Saksi :

1. Lili bin Endang umur 37 tahun, agama Islam, pekerjaan Buruh, tempat tinggal di Dusun Sindulang RT 03 RW 01 Desa Sindulang Kecamatan Sumedang Utara Kabupaten Sumedang;di bawah sumpahnya memberikan keterangan sebagai berikut:
   - Bahwa, saksi adalah kakak Kandung Pemohon
   - Bahwa, selama rumah tangga Pemohon dengan Termohon telah dikaruniai seorang anak bernama: Muhammad Abidzar Al-Ghifari (berusia 6 bulan);
   - Bahwa, sepengetahuan saksi rumah tangga antara Pemohon dan Termohon semula rukun dan harmonis tetapi sejak bulan Oktober 2019 sudah tidak harmonis lagi dalam menjalani hidup berumah

Disclaimer
Kepaniteraan Mahkamah Agung Republik Indonesia berusaha untuk selalu mencantumkan informasi paling kini dan akurat sebagai bentuk komitmen Mahkamah Agung untuk pelayanan publik, transparansi dan akuntabilitas pelaksanaan fungsi peradilan. Namun dalam hal-hal tertentu masih dimungkinkan terjadi permasalahan teknis terkait dengan akurasi dan keterkinian informasi yang kami sajikan, hal mana akan terus kami perbaiki dari waktu kewaktu.
Dalam hal Anda menemukan inakurasi informasi yang termuat pada situs ini atau informasi yang seharusnya ada, namun belum tersedia, maka harap segera hubungi Kepaniteraan Mahkamah Agung RI melalui :
Email : kepaniteraan@mahkamahagung.go.id Telp : 021-384 3348 (ext.318)

tangga dikarenakan antara Pemohon dan Termohon sering terjadi perselisihan dan pertengkaran terus-menerus yang disebabkan antara Pemohon dan Termohon sering berselisih paham terkait tempat tinggal dimana Termohon tidak mau tinggal bersama di rumah kediaman milik Pemohon dengan alasan yang tidak pasti, sedangkan Pemohon merasa tidak betah tinggal di rumah kediaman milik orangtua Termohon dengan alasan jauh dari tempat kerja

- Bahwa, sejak bulan November 2019 antara Pemohon dan Termohon pisah tempat tinggal.
- Bahwa, saksi sudah menasehatiPemohon agar tetap sabar dan rukun kembali dengan Termohon, tetapi tidak berhasil;
- Bahwa, saksi sudah tidak sanggup lagi untuk merukunkanPemohon dan Termohon;

2. Didin bin Iyan ., umur 20 tahun, agama Islam, pekerjaan Buruh, tempat tinggal di Dusun Sindulang RT 03 RW 01 Desa Sindulang Kecamatan Sumedang Utara Kabupaten Sumedang di bawah sumpahnya memberikan keterangan sebagai berikut:

- Bahwa, saksi adalah Sepupu Pemohon
- Bahwa, selama rumah tangga Pemohon dengan Termohon telah dikaruniai seorang anak bernama: Muhammad Abidzar Al-Ghifari (berusia 6 bulan);
- Bahwa, sepengetahuan saksi rumah tangga antara Pemohon dan Termohon semula rukun dan harmonis tetapi sejak bulan Oktober 2019 sudah tidak harmonis lagi dalam menjalani hidup berumah tangga dikarenakan antara Pemohon dan Termohon sering terjadi perselisihan dan pertengkaran terus-menerus yang disebabkan antara Pemohon dan Termohon sering berselisih paham terkait tempat tinggal
- Bahwa, sejak bulan November 2019 antara Pemohon dan Termohon pisah tempat tinggal yang lalu Pemohon dengan Termohon sampai sekarang telah pisah rumah;

Disclaimer
Kepaniteraan Mahkamah Agung Republik Indonesia berusaha untuk selalu mencantumkan informasi paling kini dan akurat sebagai bentuk komitmen Mahkamah Agung untuk pelayanan publik, transparansi dan akuntabilitas pelaksanaan fungsi peradilan. Namun dalam hal-hal tertentu masih dimungkinkan terjadi permasalahan teknis terkait dengan akurasi dan keterkinian informasi yang kami sajikan, hal mana akan terus kami perbaiki dari waktu kewaktu.
Dalam hal Anda menemukan inakurasi informasi yang termuat pada situs ini atau informasi yang seharusnya ada, namun belum tersedia, maka harap segera hubungi Kepaniteraan Mahkamah Agung RI melalui :
Email : kepaniteraan@mahkamahagung.go.id Telp : 021-384 3348 (ext.318)

- Bahwa, saksi sudah menasehatiPemohon agar tetap sabar dan rukun kembali dengan Termohon, tetapi tidak berhasil;
- Bahwa, saksi sudah tidak sanggup lagi untuk merukunkanPemohon dan Termohon;

Bahwa oleh karena Termohon tidak pernah hadir di persidangan, maka Majelis Hakim tidak dapat mendengar keterangan saksi keluarga dari pihak Termohon di persidangan dan juga Termohon tidak dapat dimintai tanggapannya;

Bahwa selanjutnya Pemohon menyampaikan kesimpulan yang pada pokoknya Pemohon tetap dengan permohonannya ingin bercerai dengan Termohon dan mohon agar Majelis Hakim segera menjatuhkan putusan;

Bahwa selanjutnya untuk singkatnya uraian putusan ini, maka semua hal yang termuat dalam berita acara sidang ini, merupakan bagian yang tidak terpisahkan dari putusan ini;

PERTIMBANGAN HUKUM

Menimbang, bahwa maksud dan tujuan permohonan Pemohon adalah sebagaimana terurai di atas;

Menimbang, bahwa permohonan Pemohon telah diajukan sesuai dengan peraturan perundang-undangan yang berlaku, oleh karena itu permohonan Pemohon tersebut secara formil dapat diterima dan dapat dipertimbangkan lebih lanjut;

Menimbang, bahwa ternyata Termohon meskipun dipanggil secara resmi dan patut, tidak datang menghadap di depan sidang, dan juga tidak ternyata bahwa tidak datangnya itu disebabkan sesuatu halangan yang sah;

Menimbang bahwa, Termohon yang dipanggil secara resmi dan patut akan tetapi tidak datang menghadap, harus dinyatakan tidak hadir, dan permohonan tersebut harus diperiksa secara verstek;

Menimbang, bahwa guna memenuhi Pasal 82 ayat (1) dan (4) Majelis Hakim telah berusaha mendamaikan para pihak yang berperkara dengan

Disclaimer
Kepaniteraan Mahkamah Agung Republik Indonesia berusaha untuk selalu mencantumkan informasi paling kini dan akurat sebagai bentuk komitmen Mahkamah Agung untuk pelayanan publik, transparansi dan akuntabilitas pelaksanaan fungsi peradilan. Namun dalam hal-hal tertentu masih dimungkinkan terjadi permasalahan teknis terkait dengan akurasi dan keterkinian informasi yang kami sajikan, hal mana akan terus kami perbaiki dari waktu kewaktu.
Dalam hal Anda menemukan inakurasi informasi yang termuat pada situs ini atau informasi yang seharusnya ada, namun belum tersedia, maka harap segera hubungi Kepaniteraan Mahkamah Agung RI melalui :
Email : kepaniteraan@mahkamahagung.go.id Telp : 021-384 3348 (ext.318)

menasehati Pemohon agar bersabar dan dapat rukun kembali dalam membinna rumah tangga, namun tidak berhasil;

Menimbang, bahwa berdasarkan ketentuan Pasal 125 ayat (1) HIR, yaitu putusan yang dijatuhkan tanpa hadirnya Termohon dapat dikabulkan sepanjang berdasarkan hukum dan beralasan, oleh karena perkara perceraian memiliki aspek-aspek yang khusus (lex specialis) serta dalam rangka menghindari adanya penyelundupan hukum, mak kepada Pemohon dibebani bukti-bukti , oleh karena itu Majelis Hakim membebani Pemohon untuk membuktikan dlil-dalil permohonanya;

Menimbang, bahwa sebelum memeriksa pokok permohonanya Pemohon Pengadilan akan mempertimbangkan terlebih dahulu ikatan perkawinan Pemohon dan Termohon;

Menimbang, untuk membuktikan dalil angka 1, Pemohon telah mengajukan alat bukti (P.1);

Menimbang, bahwa berdasarkan keterangan Pemohon yang diperkuat oleh bukti tertulis (P.1) berupa Fotokopi Kutipan Akta Nikah yang merupakan akta otentik, dan telah bermeterai cukup serta cocok dengan aslinya, isi bukti tersebut menjelaskan mengenai pernikahan antara Pemohon dengan Termohon, sehingga bukti tersebut telah memenuhi syarat formal dan materiil, serta mempunyai kekuatan pembuktian yang sempurna dan mengikat, maka harus dinyatakan terbukti bahwa Pemohon dengan Termohon telah terikat dalam perkawinan yang sah sebagaimana diatur dalam Pasal 2 Undang-Undang Nomor 1 Tahun 1974 yang telah di ubah dengan Undang undang nomor : 16 tahun 2019.

Menimbang, bahwa saksi pertama Pemohon, sudah dewasa dan sudah disumpah, sehingga memenuhi syarat formal sebagaimana diatur dalam Pasal 145 ayat (1) angka 3e HIR;

Menimbang, bahwa keterangan saksi pertama Pemohon adalah fakta yang relevan dengan dalil yang harus dibuktikan oleh Pemohon, oleh karena itu keterangan saksi tersebut telah memenuhi syarat materiil sebagaimana telah diatur dalam Pasal 171 HIR, sehingga keterangan saksi tersebut memiliki kekuatan pembuktian, dan dapat diterima sebagai alat bukti;

Disclaimer
Kepaniteraan Mahkamah Agung Republik Indonesia berusaha untuk selalu mencantumkan informasi paling kini dan akurat sebagai bentuk komitmen Mahkamah Agung untuk pelayanan publik, transparansi dan akuntabilitas pelaksanaan fungsi peradilan. Namun dalam hal-hal tertentu masih dimungkinkan terjadi permasalahan teknis terkait dengan akurasi dan keterkinian informasi yang kami sajikan, hal mana akan terus kami perbaiki dari waktu kewaktu.
Dalam hal Anda menemukan inakurasi informasi yang termuat pada situs ini atau informasi yang seharusnya ada, namun belum tersedia, maka harap segera hubungi Kepaniteraan Mahkamah Agung RI melalui :
Email : kepaniteraan@mahkamahagung.go.id Telp : 021-384 3348 (ext.318)

Menimbang, bahwa saksi kedua Pemohon, sudah dewasa dan sudah disumpah, sehingga memenuhi syarat formal sebagaimana diatur dalam Pasal 145 ayat (1) angka 3e HIR;

Menimbang, bahwa keterangan saksi kedua Pemohon adalah fakta yang relevan dengan dalil yang harus dibuktikan oleh Pemohon, oleh karena itu keterangan saksi tersebut telah memenuhi syarat materiil sebagaimana telah diatur dalam Pasal 171 HIR, sehingga keterangan saksi tersebut memiliki kekuatan pembuktian, dan dapat diterima sebagai alat bukti;

Menimbang, bahwa keterangan saksi kesatu dan saksi kedua Pemohon bersesuaian dan cocok antara satu dengan yang lain, oleh karena itu keterangan dua orang saksi tersebut memenuhi Pasal 171 dan Pasal 172 HIR;

Menimbang, bahwa berdasarkan keterangan kedua orang saksi Pemohon, terbukti fakta kejadian sebagai berikut:

- Bahwa, Pemohon adalah isteri sah Termohon, yang pernikahannya dilaksanakan pada tanggal 11 Juli 2019, sebagaimana tercatat di Kantor Urusan Agama KUA Kecamatan Sumedangn Utara Kabupaten Sumedang;
- Bahwa, selama berumah tangga sampai sekarang Pemohon dengan Termohon telah dikaruniai seorang anak bernama: Muhammad Abidzar Al-Ghifari (berusia 6 bulan);;
- Bahwa, rumah tangga Pemohon dan Termohon semula rukun dan harmonis tetapi sejak bulan bulan Oktober 2019 sudah tidak harmonis lagi dalam menjalani hidup berumah tangga dikarenakan antara Pemohon dan Termohon sering terjadi perselisihan dan pertengkaran terus-menerus yang disebabkan antara Pemohon dan Termohon sering berselisih paham terkait tempat tinggal dimana Termohon tidak mau tinggal bersama di rumah kediaman milik Pemohon dengan alasan yang tidak pasti, sedangkan Pemohon merasa tidak betah tinggal di rumah kediaman milik orangtua Termohon dengan alasan jauh dari tempat kerja
- Bahwa, sejak bulan bulan November 2019 antara Pemohon dan Termohon pisah tempat tinggal

Disclaimer
Kepaniteraan Mahkamah Agung Republik Indonesia berusaha untuk selalu mencantumkan informasi paling kini dan akurat sebagai bentuk komitmen Mahkamah Agung untuk pelayanan publik, transparansi dan akuntabilitas pelaksanaan fungsi peradilan. Namun dalam hal-hal tertentu masih dimungkinkan terjadi permasalahan teknis terkait dengan akurasi dan keterkinian informasi yang kami sajikan, hal mana akan terus kami perbaiki dari waktu kewaktu.
Dalam hal Anda menemukan inakurasi informasi yang termuat pada situs ini atau informasi yang seharusnya ada, namun belum tersedia, maka harap segera hubungi Kepaniteraan Mahkamah Agung RI melalui :
Email : kepaniteraan@mahkamahagung.go.id Telp : 021-384 3348 (ext.318)

- Bahwa, pihak keluarga telah berusaha menasehati Pemohon agar tetap mempertahankan rumah tangganya dengan Termohon tetapi tidak berhasil;

Menimbang, bahwa berdasarkan fakta-fakta tersebut di atas, dapat disimpulkan fakta hukum sebagai berikut:

- Bahwa, rumah tangga antara Pemohon dan Termohon semula rukun dan harmonis tetapi sejak bulan bulan Oktober 2019 sudah tidak harmonis lagi dalam menjalani hidup berumah tangga dikarenakan antara Pemohon dan Termohon sering terjadi perselisihan dan pertengkaran terus-menerus yang disebabkan antara Pemohon dan Termohon sering berselisih paham terkait tempat tinggal dimana Termohon tidak mau tinggal bersama di rumah kediaman milik Pemohon dengan alasan yang tidak pasti, sedangkan Pemohon merasa tidak betah tinggal di rumah kediaman milik orangtua Termohon dengan alasan jauh dari tempat kerja
- Bahwa, sejak bulan bulan November 2019 antara Pemohon dan Termohon pisah tempat tinggal
- Bahwa, sifat dari perselisihan dan pertengkaran tersebut sudah sulit untuk dirukunkan lagi, karena Pemohon tetap pada pendiriannya untuk bercerai dengan Termohon;

Menimbang, bahwa fakta hukum tersebut telah memenuhi norma hukum Islam yang terkandung dalam Al-Qur'an Surat Al-Baqarah ayat 227 yang berbunyi:

وا ن عزموا الطلا ق فا ن ا لله سميع عليم

Artinya : "Dan jika mereka ber'azam (bertetap hati untuk) talak, maka sesungguhnya Allah Maha Mendengar lagi Maha Mengetahui";

Menimbang, bahwa fakta hukum tersebut telah juga memenuhi Pasal 39 ayat (2) Undang-undang Nomor 1 Tahun 1974, jo. Pasal 19 huruf (f) Peraturan Pemerintah Nomor 9 tahun 1975 jo. Pasal 116 huruf (f) Kompilasi Hukum Islam;

Menimbang, bahwa dalam ketentuan Pasal 19 huruf (f) Peraturan Pemerintah Nomor 9 tahun 1975 jo. Pasal 116 huruf (f) Kompilasi Hukum

Disclaimer
Kepaniteraan Mahkamah Agung Republik Indonesia berusaha untuk selalu mencantumkan informasi paling kini dan akurat sebagai bentuk komitmen Mahkamah Agung untuk pelayanan publik, transparansi dan akuntabilitas pelaksanaan fungsi peradilan. Namun dalam hal-hal tertentu masih dimungkinkan terjadi permasalahan teknis terkait dengan akurasi dan keterkinian informasi yang kami sajikan, hal mana akan terus kami perbaiki dari waktu kewaktu.
Dalam hal Anda menemukan inakurasi informasi yang termuat pada situs ini atau informasi yang seharusnya ada, namun belum tersedia, maka harap segera hubungi Kepaniteraan Mahkamah Agung RI melalui :
Email : kepaniteraan@mahkamahagung.go.id Telp : 021-384 3348 (ext.318)

Islam, menyatakan bahwa alasan yang memungkinkan dapat terjadinya perceraian yaitu “Antara suami isteri terus menerus terjadi perselisihan dan pertengkaran dan tidak ada harapan akan hidup rukun lagi dalam rumah tangga”;

Menimbang, bahwa aspek penting dari maksud pasal tersebut yaitu tidak adanya harapan akan hidup rukun lagi dalam rumah tangga, antara Pemohon dan Termohon terus menerus telah terjadi perselisihan dan pertengkaran yang sulit didamaikan, dengan demikian apabinla maksud pasal tersebut dihubungkan dengan keadaan rumah tangga Pemohon dan Termohon, maka telah terdapat cukup alasan perceraian untuk mengabulkan permohonan Pemohon;

Menimbang, bahwa berdasarkan pertimbangan-pertimbangan tersebut di atas dan Pemohon belum pernah menjatuhkan talak, maka petitum permohonan Pemohon mengenai izin talak raj’i tersebut memenuhi pasal 118 Kompilasi Hukum Islam, oleh karena itu dapat dikabulkan;

Menimbang, bahwa karena perkara a quo termasuk bindang perkawinan, maka berdasarkan Pasal 89 ayat (1) Undang-Undang Nomor 7 Tahun 1989 tentang Peradilan Agama, sebagaimana telah diubah dengan Pasal 90 Undang-Undang Nomor 3 Tahun 2006, dan perubahan kedua dengan Pasal 91A Undang-Undang Nomor 50 Tahun 2009, binaya perkara harus dibebankan kepada Pemohon;

Mengingat, semua Pasal dalam peraturan perundang-undangan, dan hukum Islam yang berkaitan dengan perkara ini;

MENGADILI

1. Menyatakan Termohon yang telah dipanggil secara resmi dan patut untuk menghadap di persidangan, tidak hadir;
2. Mengabulkan Permohonan Pemohon dengan verstek;
3. Memberi izin kepada Pemohon (Teten Juanda bin Endang) untuk menjatuhkan talak satu raj'i terhadap Termohon (Siti Halimah Sa'adah binti Dindin Zaenudin) di depan sidang Pengadilan Agama Sumedang;

Disclaimer
Kepaniteraan Mahkamah Agung Republik Indonesia berusaha untuk selalu mencantumkan informasi paling kini dan akurat sebagai bentuk komitmen Mahkamah Agung untuk pelayanan publik, transparansi dan akuntabilitas pelaksanaan fungsi peradilan. Namun dalam hal-hal tertentu masih dimungkinkan terjadi permasalahan teknis terkait dengan akurasi dan keterkinian informasi yang kami sajikan, hal mana akan terus kami perbaiki dari waktu kewaktu.
Dalam hal Anda menemukan inakurasi informasi yang termuat pada situs ini atau informasi yang seharusnya ada, namun belum tersedia, maka harap segera hubungi Kepaniteraan Mahkamah Agung RI melalui :
Email : kepaniteraan@mahkamahagung.go.id Telp : 021-384 3348 (ext.318)

4. Membebankan kepada Pemohon untuk membayar biaya perkara ini sejumlah Rp436000,00 (empat ratus tiga puluh enam ribu rupiah).

Demikian diputuskan dalam rapat permusyawaratan Majelis Hakim yang dilangsungkan pada hari Selasa tanggal 22 Desember 2020 Masehi, bertepatan dengan tanggal 7 Jumadil Awwal 1442 Hijriyah, oleh kami Drs. H. Syamsul Falah, M.H. sebagai Ketua Majelis, Drs. Solihudin, S.H dan Drs. Dimyati, SH, MH, masing-masing sebagai Hakim Anggota, putusan tersebut diucapkan dalam sidang terbuka untuk umum pada hari itu juga, oleh Ketua Majelis tersebut dengan didampingi oleh Hakim Anggota dan dibantu oleh Drs. Harun Nur Rasyid sebagai Panitera Pengganti serta dihadiri oleh Pemohon tanpa kehadiran Termohon;

Ketua Majelis

Drs. H. Syamsul Falah, M.H.

Hakim Anggota	Hakim Anggota
Drs. Solihudin, S.H	Drs. Dimyati, SH, MH

Panitera Pengganti

Drs. Harun Nur Rasyid

Perincian Biaya Perkara :

Disclaimer
Kepaniteraan Mahkamah Agung Republik Indonesia berusaha untuk selalu mencantumkan informasi paling kini dan akurat sebagai bentuk komitmen Mahkamah Agung untuk pelayanan publik, transparansi dan akuntabilitas pelaksanaan fungsi peradilan. Namun dalam hal-hal tertentu masih dimungkinkan terjadi permasalahan teknis terkait dengan akurasi dan keterkinian informasi yang kami sajikan, hal mana akan terus kami perbaiki dari waktu kewaktu.
Dalam hal Anda menemukan inakurasi informasi yang termuat pada situs ini atau informasi yang seharusnya ada, namun belum tersedia, maka harap segera hubungi Kepaniteraan Mahkamah Agung RI melalui :
Email : kepaniteraan@mahkamahagung.go.id Telp : 021-384 3348 (ext.318)

1.	Pendaftaran	: Rp. 30.000,-
2.	Proses	: Rp. 50.000,-
3.	Panggilan Pemohon	: Rp. 140.000,-
4.	Panggilan Termohon	: Rp. 180.000,-
5.	PNBP	: Rp 20.000,-
6.	Redaksi	: Rp. 10.000,-
7.	Materai	: Rp. 6.000,-
	Jumlah	: Rp. 436.000,-

Disclaimer
Kepaniteraan Mahkamah Agung Republik Indonesia berusaha untuk selalu mencantumkan informasi paling kini dan akurat sebagai bentuk komitmen Mahkamah Agung untuk pelayanan publik, transparansi dan akuntabilitas pelaksanaan fungsi peradilan. Namun dalam hal-hal tertentu masih dimungkinkan terjadi permasalahan teknis terkait dengan akurasi dan keterkinian informasi yang kami sajikan, hal mana akan terus kami perbaiki dari waktu kewaktu.
Dalam hal Anda menemukan inakurasi informasi yang termuat pada situs ini atau informasi yang seharusnya ada, namun belum tersedia, maka harap segera hubungi Kepaniteraan Mahkamah Agung RI melalui :
Email : kepaniteraan@mahkamahagung.go.id Telp : 021-384 3348 (ext.318)
