# Supplementary material for: Classification of divorce causes during the COVID-19 pandemic using convolutional neural networks
Source: PeerJ Comput Sci. 2022 Jun 30;8:e998. doi: 10.7717/peerj-cs.998 (PMC9299239; doi:10.7717/peerj-cs.998)
Supplement: Supplemental Information 4 [file peerj-cs-08-998-s004.zip › Konflik Dataset/Data ke-21.pdf]

PUTUSAN
Nomor 537/Pdt.G/2020/MS.Sgi

بِسْمِ اللَّهِ الرَّحْمَنِ الرَّحِيمِ

DEMI KEADILAN BERDASARKAN KETUHANAN YANG MAHA ESA

Mahkamah Syar'iyah Sigli yang memeriksa dan mengadili perkara pada tingkat pertama dalam sidang Majelis telah menjatuhkan putusan perkara cerai gugat antara:

Penggugat, NIK 1107084107710169, tempat/tanggal lahir Aron Asan Kumbang, 01 Juli 1971, agama Islam, pendidikan SMA, pekerjaan Mengurus rumah tangga, tempat tinggal di Gampong Aron Asan Kumbang, Kecamatan Kembang Tanjong, Kabupaten Pidie, selanjutnya sebagai **Penggugat**;

melawan

Tergugat, NIK -, tempat/tgl lahir Aron Asan Kumbang, 01 Juli 1964, agama Islam, pendidikan SD, pekerjaan Buruh Harian Lepas, tempat tinggal Gampong Aron Asan Kumbang, Kecamatan Kembang Tanjong, Kabupaten Pidie, selanjutnya sebagai **Tergugat**;

Mahkamah Syar'iyah tersebut;
Telah mempelajari surat-surat yang berkaitan dengan perkara ini;
Telah mendengar keterangan Penggugat serta para saksi di muka sidang;

DUDUK PERKARA

Bahwa Penggugat dalam surat gugatannya tanggal 13 Juli 2020 telah mengajukan gugatan cerai gugat secara prodeo yang telah didaftar di Kepaniteraan Mahkamah Syar'iyah Sigli dengan nomor 537/Pdt.G/2020/MS. Sgi, tanggal 07 Desember 2020, dengan dalil-dalil sebagai berikut:

1. Bahwa pada tanggal 20 Maret 2005, Penggugat dengan Tergugat melangsungkan pernikahan yang dicatat oleh Pegawai Pencatat Nikah Kantor Urusan Agama (KUA) Kembang Tanjong Kabupaten Pidie, Kutipan Akta Nikah Nomor 22/08/III/2005, tanggal 22 Maret 2005;

Disclaimer
Kepaniteraan Mahkamah Agung Republik Indonesia berusaha untuk selalu mencantumkan informasi paling kini dan akurat sebagai bentuk komitmen Mahkamah Agung untuk pelayanan publik, transparansi dan akuntabilitas pelaksanaan fungsi peradilan. Namun dalam hal-hal tertentu masih dimungkinkan terjadi permasalahan teknis terkait dengan akurasi dan keterkinian informasi yang kami sajikan, hal mana akan terus kami perbaiki dari waktu kewaktu.
Dalam hal Anda menemukan inakurasi informasi yang termuat pada situs ini atau informasi yang seharusnya ada, namun belum tersedia, maka harap segera hubungi Kepaniteraan Mahkamah Agung RI melalui :
Email : kepaniteraan@mahkamahagung.go.id   Telp : 021-384 3348 (ext.318)

2. Bahwa setelah menikah Penggugat dan Tergugat tinggal bersama diTakengon selama 3 tahun, kemudian sekira akhir tahun 2008 pulang ke Gamong Aron Asan Kumbang, Kecamatan Kembang tanjong Kabupaten Pidie sampai dengan sekarang, sedangkan Tergugat masih di Takengon karena bekerja;
3. Bahwa dari perkawinan Penggugat dengan Tergugat telah dikaruniai 2 (dua) orang anak yang bernama:
   3.1. Nurhidayah umur 13 tahun;
   3.2. Muhammad Danis telahmeninggal dunia tahun 2008;
4. Bahwa kehidupan rumah tangga Penggugat dengan Tergugat pada mulanya hidup rukun dan harmonis selama lebih kurang 2 tahun, lalu selanjutnya kondisi rumah tangga antara Penggugat dengan tergugat tidak harmonis lagi karena terjadi perselisihan dan percekcokan disebabkan:
   4.1. Tergugat tidak pernah memberikan nafkah yang layak kepada Penggugat;
   4.2. Tergugat kasar dan sering memukul Penggugat;
   4.3. Tergugat telah menjatuhkan talak pada Penggugat;
   4.4. Tergugat tidak memberikan nafkah lahir batin kepada Penggugat lebih kurang sejak tahun 2008 sampai dengan sekarang;
5. Bahwa antara Penggugat dengan Tergugat telah pernah didamaikan oleh pihak perngkat gampong akan tetapi tidak berhasil;
6. Bahwa untuk menghindari penderitaan batin yang berkepanjangan bagi Penggugat dan dapat mengganggu mental psikis Penggugat, maka Penggugat terpaksa melakukan upaya terakhir dengan mengajukan gugatan perceraian ini;
7. Bahwa Penggugat adalah seorang yang tidak mampu sesuai dengan Surat Keterangan Kurang Mampu, Nomor 200/AK//2020 yang dikeluarkan oleh Keuchik Gampong Aron Asan Kumbang Kecmatan Kembang Tanjong Kabupaten Pidie tanggal 13 Juli 2020;

Berdasarkan alasan/dalil-dalil tersebut diatas, Penggugat mohon agar Ketua Mahkamah Syar’iyah Sigli segera memeriksa dan mengdili perkara ini

Disclaimer
Kepaniteraan Mahkamah Agung Republik Indonesia berusaha untuk selalu mencantumkan informasi paling kini dan akurat sebagai bentuk komitmen Mahkamah Agung untuk pelayanan publik, transparansi dan akuntabilitas pelaksanaan fungsi peradilan. Namun dalam hal-hal tertentu masih dimungkinkan terjadi permasalahan teknis terkait dengan akurasi dan keterkinian informasi yang kami sajikan, hal mana akan terus kami perbaiki dari waktu kewaktu.
Dalam hal Anda menemukan inakurasi informasi yang termuat pada situs ini atau informasi yang seharusnya ada, namun belum tersedia, maka harap segera hubungi Kepaniteraan Mahkamah Agung RI melalui :
Email : kepaniteraan@mahkamahagung.go.id Telp : 021-384 3348 (ext.318)

dengan memanggil Penggugat dan Tergugat dan selanjutnya menjatuhkan putusan sebagai berikut:

PRIMER:

1. Mengabulkan gugatan Penggugat;
1. Menetapkan member izin kepada Penggugat untuk berperkara secara Cuma-Cuma;
2. Menjatuhkan talak satu ba'in sughra Tergugat (Tergugat**)** terhadap Penggugat (Penggugat**);**
3. Membebaskan Penggugat dari biaya perkara;

SUBSIDER:

- Apabila Majelis Hakim berpendapat lain mohon putusan yang seadil-adilnya;

Bahwa pada hari dan tanggal sidang yang telah ditetapkan Penggugat datang menghadap sendiri di persidangan, sedangkan Tergugat tidak datang menghadapi persidangan dan tidak menyuruh orang lain untuk menghadap sebagai wakil/kuasanya yang sah meskipun berdasarkan surat panggilan (relaas) Nomor 537/Pdt.G/2020/MS.Sgi yang dibacakan di persidangan, Tergugat telah dipanggil secara resmi dan patut, sedangkan tidak ternyata bahwa tidak datangnya Tergugat tersebut disebabkan oleh suatu alasan yang sah;

Bahwa Majelis Hakim telah menasehati Penggugat agar berfikir untuk tidak bercerai dengan Tergugat, akan tetapi Penggugat tetap pada dalil-dalil gugatannya untuk bercerai dengan Tergugat;

Bahwa selanjutnya Majelis Hakim membacakan surat gugatan Penggugat dimana Penggugat tetap dengan gugatannya;

Bahwa untuk menguatkan dalil-dalil gugatannya Penggugat dalam persidangan telah mengajukan alat bukti berupa:

- Fotocopy Kutipan Akta Nikah Nomor: 22/08/III/2005 tanggal 22 Maret 2005, yang diterbitkan oleh Kantor urusan Agama Kecamatan Kembang Tanjong Kabupaten Pidie, telah dinazagelen, lalu dicocokkan oleh Ketua Majelis dengan aslinya dan diberi tanda P;

Bahwa disamping alat bukti tersebut Penggugat juga telah

Disclaimer
Kepaniteraan Mahkamah Agung Republik Indonesia berusaha untuk selalu mencantumkan informasi paling kini dan akurat sebagai bentuk komitmen Mahkamah Agung untuk pelayanan publik, transparansi dan akuntabilitas pelaksanaan fungsi peradilan. Namun dalam hal-hal tertentu masih dimungkinkan terjadi permasalahan teknis terkait dengan akurasi dan keterkinian informasi yang kami sajikan, hal mana akan terus kami perbaiki dari waktu kewaktu.
Dalam hal Anda menemukan inakurasi informasi yang termuat pada situs ini atau informasi yang seharusnya ada, namun belum tersedia, maka harap segera hubungi Kepaniteraan Mahkamah Agung RI melalui :
Email : kepaniteraan@mahkamahagung.go.id   Telp : 021-384 3348 (ext.318)

menghadirkan saksi-saksi sebagai berikut:

1. **Saksi pertama,** Umur 63 tahun, agama Islam, pendidikan SMA, pekerjaan Tani, tempat tinggal Gampong Aron Asan Kumbang, Kecamatan Kembang Tanjong, Kabupaten Pidie, di bawah sumpahnya memberikan keterangan sebagai berikut:
    - Bahwa saksi kenal dengan Penggugat karena Penggugat adalah warga saksi, kenal pula dengan Tergugat sebagai suami Penggugat yang bernama Bustamam;
    - Bahwa Penggugat dengan Tergugat adalah suami isteri yang menikah tahun 2005 dan telah mempnyai anak 2 orang sekarang dalam asuhan Penggugat;
    - Bahwa Penggugat dan Tergugat setelah menikah tinggal di rumah orang tua Penggugat, kemudian pindah ke Takengon;
    - Bahwa antara Penggugat dengan Tergugat telah berpisah sejak tahun 2008;
    - Bahwa penyebab Penggugat berpisah dengan Tergugat karena Tergugat karena tidak ada lagi kecocokan dalam rumah tangga karena Tegugat kurang memberi nafkah pada Penggugat dan anak-anaknya, Terguggat selalu berlaku kasar dan main pukul pada Penggugat, Tergugat telah mentalak Penggugat dengan talak tiga;
    - Bahwa antara Penggugat pernah didamaikan oleh aparat gampong termasuk saksi sendiri pada tahun 2008 akan tetapi tidak berhasil;
    - Bahwa slama berpisah Tergugat tidak ada lagi memberi nakfah pada Penggugat kecuali sekedarnya saja untuk anak Penggugat dengan Tergugat dan tidak ada pula ada harta yang ditinggalkan oleh Tergugat;
    - Bahwa Penggugat di gampong adalah tergolong miskin;
    - Bahwa antara Penggugat dengan Tergugat tidak mungkin lagi disatukan dalam satu rumah tangga, karena Penggugat tidak mau lagi sama Tergugat;
2. **Saksi kedua**, umur 49 tahun, agama Islam, pendidikan SMA, pekerjaan Mengurus rumah tangga, tempat tinggal Gampong Keuniree, Kecamatan

Disclaimer
Kepaniteraan Mahkamah Agung Republik Indonesia berusaha untuk selalu mencantumkan informasi paling kini dan akurat sebagai bentuk komitmen Mahkamah Agung untuk pelayanan publik, transparansi dan akuntabilitas pelaksanaan fungsi peradilan. Namun dalam hal-hal tertentu masih dimungkinkan terjadi permasalahan teknis terkait dengan akurasi dan keterkinian informasi yang kami sajikan, hal mana akan terus kami perbaiki dari waktu kewaktu.
Dalam hal Anda menemukan inakurasi informasi yang termuat pada situs ini atau informasi yang seharusnya ada, namun belum tersedia, maka harap segera hubungi Kepaniteraan Mahkamah Agung RI melalui :
Email : kepaniteraan@mahkamahagung.go.id   Telp : 021-384 3348 (ext.318)

Pidie, Kabupaten Pidie dibawah sumpahnya memberikan keterangan sebagai berikut:

- Bahwa saksi kenal dengan Penggugat karena saksi adalah saudara sepupu Penggugat dan kenal pula dengan Tergugat sebagai suami Penggugat yang bernama Bustaman:
- Bahwa Penggugat dengan Tergugat menikah pada tahun 2005 yang lalu dan telah dikaruniai dua orang anak;
- Bahwa setelah menikah Penggugat dan Tergugat tinggal du rumah orang tua Penggugat kemudian pindah ke Takengon sampai dengan berpisah;
- Bahwa pada awalnya rumah tangga Penggugat dengan Tergugat pada awalnya rukun dan damai saja, setelah dua tahun kemudian Tergugat mulai terjadi pertengkara dan percekcokan dalam rumah tangga;
- Bahwa penyebab terjadinya pertengkaran dan percekcokan antara Penggugat dengan Tergugat adalah karena Tergugat kurang member nafkah pada Penggugat dan anak-anaknya, Tergugat berlaku kasar dan suka main pukul,Tergugat telah meceraikan Penggugat dengan talak tiga;
- Bahwa antara Penggugat dengan Tergugat telah berpisah rumah sejak tahun 2008 sampai dengan sekarang;
- Bahwa setahu saksi keluarga kedua belah pihak pernah mendamaikan akan tetapi tidak berhasil karena Penggugat tidak mau lagi sama Tergugat;
- Bahwa semenjak Tergugat pergi tidak ada lagi memberikan nafkah pada Penggugat, sedangkan untuk anaknya cuma sekedarnya saja;
- Bahwa menurut saksi tidak mungkin lagi ditunggu kepulangan Tergugat lebih baik pisah saja antara Penggugat dengan Tergugat;
- Bahwa Penggugat adalah tergolong orang miskin di gampong;

Bahwa Penggugat telah menyampaikan kesimpulan secara lisan yang isinya sebagaimana telah dicatat dalam berita acara sidang;

Bahwa untuk mempersingkat uraian putusan ini, segala yang tercatat dalam berita acara sidang merupakan bagian yang tidak terpisahkan dari

Disclaimer
Kepaniteraan Mahkamah Agung Republik Indonesia berusaha untuk selalu mencantumkan informasi paling kini dan akurat sebagai bentuk komitmen Mahkamah Agung untuk pelayanan publik, transparansi dan akuntabilitas pelaksanaan fungsi peradilan. Namun dalam hal-hal tertentu masih dimungkinkan terjadi permasalahan teknis terkait dengan akurasi dan keterkinian informasi yang kami sajikan, hal mana akan terus kami perbaiki dari waktu kewaktu.
Dalam hal Anda menemukan inakurasi informasi yang termuat pada situs ini atau informasi yang seharusnya ada, namun belum tersedia, maka harap segera hubungi Kepaniteraan Mahkamah Agung RI melalui :
Email : kepaniteraan@mahkamahagung.go.id Telp : 021-384 3348 (ext.318)

putusan ini;

PERTIMBANGAN HUKUM

Menimbang, bahwa maksud dan tujuan gugatan Penggugat adalah sebagaimana telah diuraikan di atas;

Menimbang, bahwa Penggugat telah hadir sendiri menghadap ke persidangan, sedangkan Tergugat telah dipanggil secara resmi dan patut, akan tetapi tidak pernah hadir dalam persidangan, ketidakhadiran Tergugat tersebut juga tanpa alasan yang sah menurut hukum, maka sesuai dengan pasal 149 ayat (1) R Bg, maka gugatan Penggugat dapat diputus secara verstek;

Menimbang, bahwa Majeis Hakim telah berusaha secara sungguh-sungguh untuk menasehati Penggugat agar bersabar dan tetap berusaha menanti Tergugat, sebagaimana dimaksud pasal 39 ayat (1) Undang-Undang Nomor 1 Tahun 1974 jo Pasal 65 dan 70 ayat (1) Undang-Undang Nomor 7 Tahun 1989 jo pasal 31 ayat (1) Peraturan Pemerintah Nomor 9 Tahun 1975 dan Pasal 131 ayat (2) Kompilasi Hukum Islam di Indonesia, namun tidak memperoleh hasil pasitif dari Penggugat dan tetap ingin bercerai dengan Tergugat, sedangkan proses mediasi sesuai dengan PERMA Nomor 1 Tahun 2016 tidak dapat dilaksanakan karena Tergugat tidak pernah hadir dipersidangan;

Menimbang, bahwa sekalipun Tergugat tidak pernah datang kepersidangan dan tidak pula mengirim jawaban secara tertulis, namun karena perkara ini termasuk bidang perkawinan, maka kepada Penggugat tetap dibebani wajib membuktikan gugatannya sesuai dengan ketentuan Pasal 54 Undang-Undang Nomor 7 Tahun 1989, oleh karena itu diperlukan pembuktian terhadap dalil-dalil gugatan yang diajukan Penggugat, sebab yang diputus secara verstek itu bukan hanya ketidakhadiran Tergugat akan tetapi juga pokok perkara yaitu perceraian itu sendiri;

Menimbang, bahwa Majelis Hakim berpendapat beban pembuktian terhadap dalil-dalil gugatannya Penggugat wajib membuktikan gugatannya;

Menimbang, bahwa untuk mebuktikan dalil-dalil gugatannya Penggugat telah mengajukan alat bukti tertulis berupa P dan dua orang saksi, yang dinilai oleh Majelis sebagai berikut:

Disclaimer
Kepaniteraan Mahkamah Agung Republik Indonesia berusaha untuk selalu mencantumkan informasi paling kini dan akurat sebagai bentuk komitmen Mahkamah Agung untuk pelayanan publik, transparansi dan akuntabilitas pelaksanaan fungsi peradilan. Namun dalam hal-hal tertentu masih dimungkinkan terjadi permasalahan teknis terkait dengan akurasi dan keterkinian informasi yang kami sajikan, hal mana akan terus kami perbaiki dari waktu kewaktu.
Dalam hal Anda menemukan inakurasi informasi yang termuat pada situs ini atau informasi yang seharusnya ada, namun belum tersedia, maka harap segera hubungi Kepaniteraan Mahkamah Agung RI melalui :
Email : kepaniteraan@mahkamahagung.go.id Telp : 021-384 3348 (ext.318)

Menimbang, bahwa Penggugat telah mengajukan alat bukti tertulis P, berupa fotocopy Kutipan Akta Nikah Nomor; 22/08/III/2005 tanggal 22 Maret 2005 an Penggugat dan Tergugat, telah dinazegelen di kantor Pos, aslinya dikeuarkan oleh Kantor Urusan Agama Kecamatan Kembang Tanjong Kabupaten Pidie, telah dicocokkan dengan yang aslinya oleh Ketua Majelis, maka bukti tersebut dapat dipertimbangkan sebagai alat bukti yang sah yang mempunyai nilai pembuktian sebagaimana ditentukan dalam pasal 7 ayat (1) Kompilasi Hukum Islam, bahwa perkawinan hanya dapat dibuktikan dengan Akta Nikah yang dibuat oleh Pegawai Pencatat Nikah;

Menimbang, bahwa untuk membuktikan dalil-dalil gugatan Penggugat telah mengajukan alat bukti berupa 2 (dua) orang saksi;

Menimbang, bahwa saksi I Penggugat, sudah dewasa dan sudah disumpah, sehingga memenuhi syarat formal sebagaimana diatur dalam Pasal 172 ayat 1 angka 4 R.Bg.;

Menimbang, bahwa keterangan saksi I Penggugat mengenai dalil dalil gugatan Penggugat adalah fakta yang dilihat sendiri dan relevan dengan dalil yang harus dibuktikan oleh Penggugat, oleh karena itu keterangan saksi tersebut telah memenuhi syarat materiil sebagaimana telah diatur dalam Pasal 308 R.Bg., sehingga keterangan saksi tersebut memiliki kekuatan pembuktian dan dapat diterima sebagai alat bukti;

Menimbang, bahwa saksi II Penggugat, sudah dewasa dan sudah disumpah, sehingga memenuhi syarat formal sebagaimana diatur dalam Pasal 172 ayat 1 angka 4 R.Bg.;

Menimbang, bahwa keterangan saksi II Penggugat mengenai dalil dalil-dalil dan mengenai ketidak harmonisan rumah tangga antara Tergugat dengan Penggugat sejak lebih kurang 2 tahun setelah menikah yang disebabkan oleh Tergugat tidak memberi nafkah kepada Penggugat, berlaku kasar, Tergugat telah menjatuhkan talak pada Penggugat, adalah fakta yang dilihat sendiri dan relevan dengan dalil yang harus dibuktikan oleh Penggugat, oleh karena itu keterangan saksi tersebut telah memenuhi syarat materiil sebagaimana telah diatur dalam Pasal 308 R.Bg. sehingga keterangan saksi tersebut memiliki kekuatan pembuktian dan dapat diterima sebagai alat bukti;

Disclaimer
Kepaniteraan Mahkamah Agung Republik Indonesia berusaha untuk selalu mencantumkan informasi paling kini dan akurat sebagai bentuk komitmen Mahkamah Agung untuk pelayanan publik, transparansi dan akuntabilitas pelaksanaan fungsi peradilan. Namun dalam hal-hal tertentu masih dimungkinkan terjadi permasalahan teknis terkait dengan akurasi dan keterkinian informasi yang kami sajikan, hal mana akan terus kami perbaiki dari waktu kewaktu.
Dalam hal Anda menemukan inakurasi informasi yang termuat pada situs ini atau informasi yang seharusnya ada, namun belum tersedia, maka harap segera hubungi Kepaniteraan Mahkamah Agung RI melalui :
Email : kepaniteraan@mahkamahagung.go.id Telp : 021-384 3348 (ext.318)

Menimbang, bahwa keterangan saksi I dan II Penggugat bersesuaian dan cocok antara satu dengan yang lain, sehubungan dengan keabsahan pernikahan antara Penggugat dengan Tergugat, oleh karena itu keterangan saksi-saksi tersebut memenuhi Pasal 308 dan Pasal 309 R.Bg.;

Menimbang, bahwa berdasarkan bukti saksi I, saksi II Penggugat, terbukti fakta-fakta kejadian sebagai berikut:

1. Bahwa Penggugat dengan Tergugat telah melangsungkan perkawinan pada tahun 20 Maret 2005 yang terdaftar di Kantor Urusan Agama Kecamatan Kembang Tanjong Kabupaten Pidie;
2. Bahwa antara Penggugat dengan Tergugat telah terjadi perselisihan dan pertengkaran yang disebabkan Tergugat tidak pernah memberikan nafkah yang layak pada Penggugat, berlaku kasar dan telah menjatuhkan talak pada Penggugat, sehingga Tergugat telah berpisah rumah dengan Penggugat sejak tahun 2008 yang lalu sampai dengan sekarang tidak pernah pulang lagi pada Penggugat;
3. Bahwa selama Tergugat berpisah dengan Penggugat Tergugat tidak pernah ada lagi komunikasi dan tidak pernah lagi memberikan nafkah pada Penggugat;

Menimbang, bahwa berdasarkan fakta-fakta tersebut di atas dapat disimpulkan fakta hukum sebagai berikut:

1.. Bahwa antara Penggugat dengan Tergugat telah menikah pada tanggal 22 Maret 2005;

2. Rumah tangga Penggugat dengan Tergugat tidak harmonis lagi sejak dua tahun setelah menikah, sehingga antara Penggugat dengan Tergugat telah berpisah rumah sejak tahun 2008 yang lalu;

3. Antara Penggugat dengan Tergugat telah pernah didamaikan oleh aparat gampong akan tetapi tidak berhasil;

Menimbang, bahwa fakta hukum tersebut telah memenuhi norma hukum Islam yang terkandung dalam:

1. Al Qur’an Surat An Nisa ayat 130:

وإن يتفرقا يغن الله كلا من سعته وكان الله واسعا حكيما

“Jika keduanya bercerai maka Allah akan memberi kecukupan kepada

Disclaimer
Kepaniteraan Mahkamah Agung Republik Indonesia berusaha untuk selalu mencantumkan informasi paling kini dan akurat sebagai bentuk komitmen Mahkamah Agung untuk pelayanan publik, transparansi dan akuntabilitas pelaksanaan fungsi peradilan. Namun dalam hal-hal tertentu masih dimungkinkan terjadi permasalahan teknis terkait dengan akurasi dan keterkinian informasi yang kami sajikan, hal mana akan terus kami perbaiki dari waktu kewaktu.
Dalam hal Anda menemukan inakurasi informasi yang termuat pada situs ini atau informasi yang seharusnya ada, namun belum tersedia, maka harap segera hubungi Kepaniteraan Mahkamah Agung RI melalui :
Email : kepaniteraan@mahkamahagung.go.id Telp : 021-384 3348 (ext.318)

masing-masingnya dari limpahan karunianya. Dan adalah Allah Maha Luas (karunia Nya) lagi Maha Bijaksana";

2. Qaidah Fiqh yang terdapat dalam kitab Al-Asbah wa al-Nazair halaman 59 yang telah diambilalih menjadi pendapat majelis hakim yang berbunyi:

الضرار
يزال

"Kemudharatan itu harus dihilangkan"

3. Doktrin Ulama Fiqh yang terdapat di dalam Kitab Ghayah Al Maram, halaman 162 yang telah diambil alih menjadi pendapat Majelis Hakim yang berbunyi:

وإذا اشتد عدم رغبة الزوجة لزوجها طلق عليه القاضى طلقة

"Dan ketika isteri sudah sangat tidak senang terhadap suaminya maka Hakim dibolehkan menjatuhkan talak satu suami".

Menimbang, bahwa fakta hukum tersebut telah juga memenuhi pasal 2 ayat (1) Undang-undang Nomor 1 Tahun 1974, pasal 116 huruf (f) Kompilasi Hukum Islam di Indonesia Tahun dan pasal 19 huruf (f) Peraturan Pemerintah Nomor 9 Tahun 1975;

Menimbang, bahwa berdasarkan pertimbangan-pertimbangan tersebut di atas, maka dalil gugatan Penggugat pada angka 4 (sehubungan dengan tidak harmonisnya rumah tangga Penggugat dengan Tergugat telah berpisah rumah dengan Penggugat sejak tahun 2008 yang lalu telah terbukti kebenarannya dan telah sesuai dengan maksud Pasal 19 huruf (f) Peraturan Pemerintah Nomor 9 Tahun 1975 *jo.* pasal 116 huruf (f) Kompilasi Hukum Islam di Indonesia Tahun 1991, oleh karenanya, Majelis Hakim berpendapat dalil gugatan Penggugat tersebut telah patut untuk dikabulkan;

Menimbang, bahwa karena perkara *a quo* termasuk bidang perkawinan, maka berdasarkan Penetapan Ketua Mahkamah Syar'iyah Sigli Nomor 537/Pdt.G/2020/MS.Sgi tanggal 7 Desember 2020, ternyata Penggugat miskin dan dibebaskan dari kewajiban membayar biaya perkara dan selanjutnya biaya perkara yang timbul akibat pengajuan perkara ini dibebankan

Disclaimer
Kepaniteraan Mahkamah Agung Republik Indonesia berusaha untuk selalu mencantumkan informasi paling kini dan akurat sebagai bentuk komitmen Mahkamah Agung untuk pelayanan publik, transparansi dan akuntabilitas pelaksanaan fungsi peradilan. Namun dalam hal-hal tertentu masih dimungkinkan terjadi permasalahan teknis terkait dengan akurasi dan keterkinian informasi yang kami sajikan, hal mana akan terus kami perbaiki dari waktu kewaktu.
Dalam hal Anda menemukan inakurasi informasi yang termuat pada situs ini atau informasi yang seharusnya ada, namun belum tersedia, maka harap segera hubungi Kepaniteraan Mahkamah Agung RI melalui :
Email : kepaniteraan@mahkamahagung.go.id Telp : 021-384 3348 (ext.318)

kepada Negara sepenuhnya melalui DIPA Mahkamah Syar'iyah Sigli tahun 2020;

Mengingat, semua pasal dalam peraturan perundang-undangan dan hukum Islam yang berkaitan dengan perkara ini;

MENGADILI

1. Menyatakan Tergugat yang telah dipanggil secara resmi dan patut untuk menghadap ke persidangan tidak hadir;
2. Mengabulkan gugatan Penggugat dengan verstek;
3. Menjatuhkan talak satu bain sughra Tergugat (**Tergugat**) terhadap Penggugat (**Penggugat**);
4. Membebankan biaya perkara ini kepada Negara melalui DIPA Mahkamah Syar'iyah Sigli tahun 2020 sejumlah Rp. 311.000,00 (tiga ratus sebelas ribu rupiah);

Demikian diputuskan dalam rapat permusyawaratan Majelis pada hari Selasa tanggal 22 Desember 2020 Miladiyah bertepatan dengan tanggal 07 Jumadil Awwal 1442 *Hijriyah*, oleh kami **Dr. Indra Suhardi, M. Ag**, sebagai Ketua Majelis, **Drs. Ramli, MH** dan **Drs. Zukri, SH,** masing-masing sebagai Hakim Anggota, putusan tersebut diucapkan dalam sidang terbuka untuk umum pada hari itu juga, oleh Ketua Majelis tersebut dengan didampingi oleh Hakim-Hakim Anggota dan dibantu oleh **Dedy Afrizal, SHI** sebagai Panitera Pengganti serta dihadiri oleh Pengugat tanpa hadir Tergugat;

Ketua Majelis,

Dr. Indra Suhardi, M. Ag

Hakim Anggota,

Drs. R a m l I, M H

Hakim Anggota,

Disclaimer
Kepaniteraan Mahkamah Agung Republik Indonesia berusaha untuk selalu mencantumkan informasi paling kini dan akurat sebagai bentuk komitmen Mahkamah Agung untuk pelayanan publik, transparansi dan akuntabilitas pelaksanaan fungsi peradilan. Namun dalam hal-hal tertentu masih dimungkinkan terjadi permasalahan teknis terkait dengan akurasi dan keterkinian informasi yang kami sajikan, hal mana akan terus kami perbaiki dari waktu kewaktu.
Dalam hal Anda menemukan inakurasi informasi yang termuat pada situs ini atau informasi yang seharusnya ada, namun belum tersedia, maka harap segera hubungi Kepaniteraan Mahkamah Agung RI melalui :
Email : kepaniteraan@mahkamahagung.go.id   Telp : 021-384 3348 (ext.318)

Drs. Z u k r i, S H

Panitera Pengganti,

Dedy Afrizal, SH

Rincian Biaya Perkara:

1. Biaya Proses:	Rp.	50.000,00
2. Biaya Panggilan:	Rp.	255.000,00
3. Biaya Meterai	Rp.	6.000,00
J u m l a h	Rp.	311,000,00

(tiga ratus sebelas ribu rupiah)

1.
11
1

3.
3

Disclaimer
Kepaniteraan Mahkamah Agung Republik Indonesia berusaha untuk selalu mencantumkan informasi paling kini dan akurat sebagai bentuk komitmen Mahkamah Agung untuk pelayanan publik, transparansi dan akuntabilitas pelaksanaan fungsi peradilan. Namun dalam hal-hal tertentu masih dimungkinkan terjadi permasalahan teknis terkait dengan akurasi dan keterkinian informasi yang kami sajikan, hal mana akan terus kami perbaiki dari waktu kewaktu.
Dalam hal Anda menemukan inakurasi informasi yang termuat pada situs ini atau informasi yang seharusnya ada, namun belum tersedia, maka harap segera hubungi Kepaniteraan Mahkamah Agung RI melalui :
Email : kepaniteraan@mahkamahagung.go.id Telp : 021-384 3348 (ext.318)
