# Supplementary material for: Classification of divorce causes during the COVID-19 pandemic using convolutional neural networks
Source: PeerJ Comput Sci. 2022 Jun 30;8:e998. doi: 10.7717/peerj-cs.998 (PMC9299239; doi:10.7717/peerj-cs.998)
Supplement: Supplemental Information 4 [file peerj-cs-08-998-s004.zip › Konflik Dataset/Data ke-22.pdf]

P U T U S A N

Nomor 3562/Pdt.G/2020/PA.Badg

بِسْمِ اللَّهِ الرَّحْمَنِ الرَّحِيمِ

DEMI KEADILAN BERDASARKAN KETUHANAN YANG MAHA ESA

Pengadilan Agama Bandung kelas I.A yang memeriksa dan mengadili perkara perdata tertentu pada tingkat pertama dalam persidangan Majelis telah menjatuhkan putusan sebagai berikut dalam perkara cerai gugat yang diajukan oleh:

Nama Penggugat, NIK.[redacted], tempat dan tanggal lahir : Bandung, 20 Nopember 1987, umur 33 tahun, agama Islam, pendidikan SMK, pekerjaan Buruh, bertempat tinggal di Jalan Mekarsari, RT 001 RW 017, Kelurahan Babakansari, Kecamatan Kiaracondong, Kota Bandung, selanjutnya disebut Penggugat;

m e l a w a n

Nama Tergugat, tempat dan tanggal lahir : Banyumas, 12 Juni 1992, umur 28 tahun, agama Islam, pendidikan STM, pekerjaan Buruh Harian Lepas, bertempat tinggal dahulu di Jalan Mekarsari, RT 001 RW 017, Kelurahan Babakansari, Kecamatan Kiaracondong, Kota Bandung, adapun sekarang tidak diketahui tempat tinggalnya, selanjutnya disebut Tergugat;

Pengadilan Agama tersebut;

Telah mempelajari berkas perkara;

Telah mendengar keterangan Penggugat;

Telah memperhatikan alat-alat bukti di persidangan;

TENTANG DUDUK PERKARA

Menimbang, bahwa Penggugat dengan surat gugatannya tanggal 12 Agustus 2020 yang didaftar di Kepaniteraan Pengadilan Agama Bandung pada tanggal 12 Agustus 2020 di bawah register Nomor 3562/Pdt.G/2020/PA.Badg, mengajukan hal-hal sebagai berikut :

Disclaimer
Kepaniteraan Mahkamah Agung Republik Indonesia berusaha untuk selalu mencantumkan informasi paling kini dan akurat sebagai bentuk komitmen Mahkamah Agung untuk pelayanan publik, transparansi dan akuntabilitas pelaksanaan fungsi peradilan. Namun dalam hal-hal tertentu masih dimungkinkan terjadi permasalahan teknis terkait dengan akurasi dan keterkinian informasi yang kami sajikan, hal mana akan terus kami perbaiki dari waktu kewaktu.
Dalam hal Anda menemukan inakurasi informasi yang termuat pada situs ini atau informasi yang seharusnya ada, namun belum tersedia, maka harap segera hubungi Kepaniteraan Mahkamah Agung RI melalui :
Email : kepaniteraan@mahkamahagung.go.id   Telp : 021-384 3348 (ext.318)

1. Bahwa antara Penggugat dan Tergugat adalah sepasang suami isteri sah yang menikah pada tanggal 11 Januari 2013 dan telah dicatatkan di Kantor Urusan Agama (KUA) Kecamatan Kiaracondong, Kota Bandung dengan bukti Kutipan Akta Nikah Nomor: xxx/14/**I**/2013 tanggal 11 Januari 2013;

2. Bahwa setelah menikah antara Penggugat dengan Tergugat tinggal dan hidup bersama terakhir di **rumah orangtua Penggugat** beralamat di Jalan Mekarsari, RT 001, RW 017, Kelurahan Babakan Sari, Kecamatan Kiaracondong, Kota Bandung;

3. Bahwa semula keadaan rumah tangga antara Penggugat dengan Tergugat hidup rukun dan harmonis. Dan dari perkawinan tersebut dikaruniai 2 (dua) orang anak yang bernama :

a. Anak 1, tanggal lahir 10 Oktober 2013;

b. Anak 2, tanggal lahir 29 Oktober 2014;

4. Bahwa seiring berjalannya waktu ternyata keadaan rumah tangga antara Penggugat dengan Tergugat tidak selamanya rukun dan harmonis, rumah tangga antara Penggugat dan Tergugat sejak tanggal **03 Mei 2014** mulai terjadi perselisihan dan pertengkaran terus menerus, sehingga Penggugat merasakan apa yang menjadi tujuan dari perkawinan seperti yang tercantum dalam undang-undang perkawinan tidak berhasil dicapai Penggugat dan Tergugat. Sebagai sepasang suami isteri Penggugat dan Tergugat telah beruapaya sedemikian rupa untuk dapat mempertahankan perkawinan ini tetapi upaya tersebut sepertinya semakin sulit terwujud, sehingga Penggugat mengajukan gugatan cerai ke Pengadilan Agama Bandung. Adapun sebab-sebab perceraian ini diajukan adalah sebagai berikut:

4.1. Tergugat diketahui memiliki wanita idaman lain, Penggugat mengetahui hal tersebut karena Tergugat membawa wanita tersebut ke rumah dan bertemu langsung dengan Penggugat;

4.2. Sejak kejadian tersebut Tergugat pergi meninggalkan Penggugat tanpa ada alasan yang jelas, dengan membawa sepedah motor,

Disclaimer
Kepaniteraan Mahkamah Agung Republik Indonesia berusaha untuk selalu mencantumkan informasi paling kini dan akurat sebagai bentuk komitmen Mahkamah Agung untuk pelayanan publik, transparansi dan akuntabilitas pelaksanaan fungsi peradilan. Namun dalam hal-hal tertentu masih dimungkinkan terjadi permasalahan teknis terkait dengan akurasi dan keterkinian informasi yang kami sajikan, hal mana akan terus kami perbaiki dari waktu kewaktu.
Dalam hal Anda menemukan inakurasi informasi yang termuat pada situs ini atau informasi yang seharusnya ada, namun belum tersedia, maka harap segera hubungi Kepaniteraan Mahkamah Agung RI melalui :
Email : kepaniteraan@mahkamahagung.go.id Telp : 021-384 3348 (ext.318)

membawa perhiasan Penggugat dan meninggalkan hutang yang menjadi tanggungan Penggugat;

5. Bahwa Penggugat telah berusaha sabar untuk mempertahankan rumah tangga, tetapi Tergugat tidak berubah dan merubah sikapnya. Puncaknya pada **tanggal 20 mei 2014** antara Penggugat dan Tergugat bertengkar hebat, **Tergugat pergi meninggalkan Penggugat** sehingga antara Penggugat dengan Tergugat sudah pisah tempat tinggal dan sudah tidak melaksanakan kewajiban masing-masing layaknya suami istri lagi terhitung **selama 6 (enam) tahun**;

6. Bahwa selama pisah tempat tinggal Penggugat tinggal di rumah orangtua Penggugat, sedangkan Tergugat tidak diketahui keberadaannya berdasarkan surat keterangan no. 967/Ket/VIII/2020, tertanggal 12 Agustus 2020 dari Kelurahan Babakan Sari, Kecamatan Kiaracondong, Kota Bandung;

7. Bahwa keluarga pihak Penggugat dan keluarga Tergugat telah berusaha untuk menasehati serta mendamaikan Penggugat dan Tergugat, akan tetapi tidak berhasil;

8. Bahwa berdasarkan uraian tersebut di atas, jelas bahwasanya antara Penggugat dengan Tergugat sudah tidak ada lagi rasa saling menyanyangi, cinta mencintai sehingga apa yang menjadi tujuan dan sendi-sendi dasar sebuah perkawinan sebagaimana yang tersebut dalam Pasal 1 Undang-Undang Nomor 1 Tahun 1974 Tentang Perkawinan yaitu bertujuan untuk membentuk keluarga (rumah tangga) yang bahagia dan kekal tidak dapat tercapai dan terwujud, sehingga Penggugat berketetapan hati untuk bercerai dengan Tergugat;

9. Bahwa pengajuan gugatan cerai Penggugat telah memenuhi alasan-alasan perceraian sebagaimana diatur dalam Pasal 19 huruf f Peraturan Pemerintah Nomor 9 tahun 1975 jo Pasal 116 huruf f Instruksi Presiden Nomor 1 tahun 1991 tentang Kompilasi Hukum Islam;

10. Bahwa Penggugat tidak mampu membayar panjar biaya perkara cerai gugat di Pengadilan Agama Bandung, berdasarkan Surat Keterangan

Disclaimer
Kepaniteraan Mahkamah Agung Republik Indonesia berusaha untuk selalu mencantumkan informasi paling kini dan akurat sebagai bentuk komitmen Mahkamah Agung untuk pelayanan publik, transparansi dan akuntabilitas pelaksanaan fungsi peradilan. Namun dalam hal-hal tertentu masih dimungkinkan terjadi permasalahan teknis terkait dengan akurasi dan keterkinian informasi yang kami sajikan, hal mana akan terus kami perbaiki dari waktu kewaktu.
Dalam hal Anda menemukan inakurasi informasi yang termuat pada situs ini atau informasi yang seharusnya ada, namun belum tersedia, maka harap segera hubungi Kepaniteraan Mahkamah Agung RI melalui :
Email : kepaniteraan@mahkamahagung.go.id   Telp : 021-384 3348 (ext.318)

No. 1208/KET/VIII/2020, tertanggal 11 Agustus 2020 dari Kelurahan Babakansari, Kecamatan Kiaracondong, Kota Bandung;

Bahwa berdasarkan hal-hal dan alasan tersebut di atas, maka gugatan cerai Penggugat ini telah sesuai dan memenuhi ketentuan hukum dan mempunyai alasan-alasan yang cukup. Maka oleh karenanya Penggugat memohon kepada Ketua Pengadilan Agama Bandung Cq. Majelis Hakim berkenan membuka persidangan untuk memeriksa dan mengadili perkara ini dan berkenan menjatuhkan putusan yang amarnya sebagai berikut :

1. Mengabulkan gugatan Penggugat;

2. Menjatuhkan talak satu ba'in sughra dari Tergugat (**Nama Tergugat**) terhadap Penggugat (**Nama Penggugat**);

3. Membebaskan biaya perkara menurut hukum;

- Atau apabila Majelis Hakim Pengadilan Agama Bandung berpendapat lain, mohon putusan yang seadil adilnya *(ex aequo et bono);*

Bahwa pada hari persidangan yang telah ditetapkan Penggugat datang menghadap sendiri ke persidangan, sedangkan Tergugat tidak pernah datang dan tidak menyuruh orang lain sebagai wakil/kuasa hukumnya yang sah meskipun telah dipanggil secara resmi dan patut oleh Jurusita Pengganti Pengadilan Agama Bandung melalui RRI Bandung;

Bahwa Majelis Hakim telah mendamaikan dengan mengingatkan Penggugat agar mempertahankan rumah tangga, tetapi tidak berhasil;

Bahwa sebelum memeriksa pokok perkara, Majelis Hakim terlebih dahulu telah memeriksa permohonan Penggugat untuk mendapatkan keringanan biaya perkara dan Majelis telah menjatuhkan Putusan Sela Nomor 3562/Pdt.G/2020/PA.Badg tanggal 21 Desember 2020 yang amarnya berbunyi sebagai berikut:

M E N G A D I L I

Sebelum menjatuhkan putusan akhir:

1. Mengabulkan permohonan Penggugat untuk mendapat keringanan biaya perkara;
2. Memerintahkan kepada Penggugat untuk melanjutkan perkaranya;

Disclaimer
Kepaniteraan Mahkamah Agung Republik Indonesia berusaha untuk selalu mencantumkan informasi paling kini dan akurat sebagai bentuk komitmen Mahkamah Agung untuk pelayanan publik, transparansi dan akuntabilitas pelaksanaan fungsi peradilan. Namun dalam hal-hal tertentu masih dimungkinkan terjadi permasalahan teknis terkait dengan akurasi dan keterkinian informasi yang kami sajikan, hal mana akan terus kami perbaiki dari waktu kewaktu.
Dalam hal Anda menemukan inakurasi informasi yang termuat pada situs ini atau informasi yang seharusnya ada, namun belum tersedia, maka harap segera hubungi Kepaniteraan Mahkamah Agung RI melalui :
Email : kepaniteraan@mahkamahagung.go.id   Telp : 021-384 3348 (ext.318)

Bahwa terhadap perkara ini tidak dapat dilakukan upaya perdamaian melalui mediasi karena pihak Tergugat tidak pernah hadir di persidangan, maka pemeriksaan dilanjutkan dengan pembacaan surat gugatan Penggugat tersebut yang isinya tetap dipertahankan oleh Penggugat;

Bahwa terhadap dalil-dalil gugatan yang diajukan oleh Penggugat tersebut, Tergugat tidak memberikan jawaban karena tidak pernah hadir ke persidangan;

Bahwa untuk menguatkan dalil gugatannya, Penggugat telah mengajukan alat-alat bukti berupa :

A. Bukti Surat

- Fotokopi Surat Keterangan Nomor :967/Ket/VIII/2020, tertanggal 12 Agustus 2020 yang dikeluarkan oleh Kelurahan Babakansari, Kecamatan Kiaracondong, Kota Bandung (bukti P-2);
- Fotokopi Kutipan Akta Nikah Nomor : xxx/14/I/2013 tanggal 11 Januari 2013 atas nama Penggugat dan Tergugat yang dikeluarkan oleh Kantor Urusan Agama Kecamatan Kiaracondong, Kota Bandung (bukti P-3);

B. Bukti Saksi

1. Saksi 1, umur 55 tahun, menerangkan di bawah sumpah yang pada pokoknya sebagai berikut :

- Bahwa saksi kenal dengan Penggugat dan Tergugat, saksi adalah ibu kandung Penggugat;
- Bahwa Penggugat dan Tergugat adalah suami istri dan sudah dikaruniai 2 (dua) orang anak bernama Muhammad Rizki dan Riska Dwi Nurhayati;
- Bahwa Penggugat dan Tergugat terakhir tinggal bersama di rumah orang tua Penggugat di Jl. Mekarsari, Kelurahan Babakansari, Kecamatan Kiaracondong, Kota Bandung;
- Bahwa saksi tahu rumah tangga Penggugat dengan Tergugat awalnya berjalan rukun, namun sejak bulan Mei 2014 sudah tidak rukun karena sering terjadi perselisihan dan pertengkaran;

Disclaimer
Kepaniteraan Mahkamah Agung Republik Indonesia berusaha untuk selalu mencantumkan informasi paling kini dan akurat sebagai bentuk komitmen Mahkamah Agung untuk pelayanan publik, transparansi dan akuntabilitas pelaksanaan fungsi peradilan. Namun dalam hal-hal tertentu masih dimungkinkan terjadi permasalahan teknis terkait dengan akurasi dan keterkinian informasi yang kami sajikan, hal mana akan terus kami perbaiki dari waktu kewaktu.
Dalam hal Anda menemukan inakurasi informasi yang termuat pada situs ini atau informasi yang seharusnya ada, namun belum tersedia, maka harap segera hubungi Kepaniteraan Mahkamah Agung RI melalui :
Email : kepaniteraan@mahkamahagung.go.id Telp : 021-384 3348 (ext.318)

- Bahwa perselisihan dan pertengkaran Penggugat dan Tergugat tersebut disebabkan Tergugat selingkuh dengan wanita lain;
- Bahwa saksi tahu Penggugat dan Tergugat sudah pisah rumah sejak bulan Mei 2014, Tergugat yang pergi meninggalkan tempat kediaman bersama;
- Bahwa Tergugat sudah tidak pernah memberi nafkah lahir dan batin kepada Penggugat bahkan Tergugat tidak diketahui lagi keberadaannya;
- Bahwa Penggugat sudah berusaha mencari keberadaan Tergugat melalui keluarganya, tetapi tidak berhasil;

2. Saksi 2, umur 60 tahun, menerangkan di bawah sumpah yang pada pokoknya sebagai berikut :

- Bahwa saksi kenal dengan Penggugat dan Tergugat, saksi adalah ayah kandung Penggugat;
- Bahwa Penggugat dan Tergugat adalah suami istri dan sudah dikaruniai 2 (dua) orang anak bernama Anak 1 dan Anak 2;
- Bahwa Penggugat dan Tergugat terakhir tinggal bersama di rumah orang tua Penggugat di Jl. Mekarsari, Kelurahan Babakansari, Kecamatan Kiaracondong, Kota Bandung;
- Bahwa saksi tahu rumah tangga Penggugat dengan Tergugat awalnya berjalan rukun, namun mulai bulan Mei 2014 menjadi sering terjadi perselisihan dan pertengkaran disebabkan Tergugat memiliki wanita idaman lain;
- Bahwa saksi tahu Penggugat dan Tergugat sudah pisah rumah selama 6 (enam) tahun, dan sudah tidak ada komunikasi lagi bahkan Tergugat tidak diketahui keberadaannya;
- Bahwa saksi sudah berusaha mencari keberadaan Tergugat melalui keluarganya, tetapi tidak berhasil;

Bahwa Penggugat telah menyampaikan kesimpulannya secara lisan yang pada pokoknya Penggugat tetap ingin bercerai dari Tergugat, serta mohon putusan;

Disclaimer
Kepaniteraan Mahkamah Agung Republik Indonesia berusaha untuk selalu mencantumkan informasi paling kini dan akurat sebagai bentuk komitmen Mahkamah Agung untuk pelayanan publik, transparansi dan akuntabilitas pelaksanaan fungsi peradilan. Namun dalam hal-hal tertentu masih dimungkinkan terjadi permasalahan teknis terkait dengan akurasi dan keterkinian informasi yang kami sajikan, hal mana akan terus kami perbaiki dari waktu kewaktu.
Dalam hal Anda menemukan inakurasi informasi yang termuat pada situs ini atau informasi yang seharusnya ada, namun belum tersedia, maka harap segera hubungi Kepaniteraan Mahkamah Agung RI melalui :
Email : kepaniteraan@mahkamahagung.go.id Telp : 021-384 3348 (ext.318)

Bahwa untuk meringkas uraian putusan ini ditunjuk kepada hal-hal sebagaimana tercantum di dalam berita acara sidang perkara ini, yang merupakan bagian tidak terpisahkan dari putusan ini;

TENTANG PERTIMBANGAN HUKUM

Menimbang, bahwa maksud gugatan Penggugat adalah sebagaimana diuraikan di atas;

Menimbang bahwa Pengadilan Agama Bandung telah melakukan pemanggilan kepada Tergugat secara resmi dan patut, namun demikian Tergugat tidak pernah hadir ke persidangan, sedangkan tidak ternyata ketidak hadirannya tersebut disebabkan suatu alasan yang sah, oleh karena itu Tergugat harus dinyatakan tidak hadir di persidangan;

Menimbang, bahwa Majelis Hakim guna memenuhi Pasal 82 ayat (1) dan (4) Undang-undang Nomor 7 Tahun 1989 sebagaimana diubah dengan Undang-undang Nomor 3 Tahun 2006 dan perubahan kedua dengan Undang-undang Nomor 50 Tahun 2009, telah berusaha mendamaikan dengan mengingatkan Penggugat agar dapat mempertahankan rumah tangga, tetapi tidak berhasil;

Menimbang, bahwa oleh karena Tergugat tidak pernah hadir di persidangan, maka upaya perdamaian melalui mediasi sebagaimana kehendak PERMA Nomor 1 tahun 2016 tidak dapat dilaksanakan, dan merujuk kepada ketentuan Pasal 4 ayat 2 huruf (b) PERMA Nomor 1 tahun 2016 tersebut maka perkara ini dapat diproses sebagaimana mestinya;

Menimbang, bahwa oleh karena Tergugat telah dinyatakan tidak hadir di persidangan, maka perkara ini diperiksa dan diputus tanpa hadirnya Tergugat (verstek) sesuai dengan maksud Pasal 125 HIR;

Menimbang, bahwa yang menjadi permasalahan dari dalil-dalil gugatan Penggugat pada pokoknya adalah bahwa Penggugat mohon diceraikan dari Tergugat dengan alasan bahwa rumah tangga Penggugat dan Tergugat sejak bulan Mei 2014 sudah tidak rukun karena sering terjadi perselisihan dan pertengkaran yang disebabkan Tergugat memiliki wanita idaman lain, akhirnya sejak bulan Mei 2014 tersebut Tergugat pergi meninggalkan Penggugat bahkan hingga sekarang Tergugat tidak diketahui keberadaannya;

Disclaimer
Kepaniteraan Mahkamah Agung Republik Indonesia berusaha untuk selalu mencantumkan informasi paling kini dan akurat sebagai bentuk komitmen Mahkamah Agung untuk pelayanan publik, transparansi dan akuntabilitas pelaksanaan fungsi peradilan. Namun dalam hal-hal tertentu masih dimungkinkan terjadi permasalahan teknis terkait dengan akurasi dan keterkinian informasi yang kami sajikan, hal mana akan terus kami perbaiki dari waktu kewaktu.
Dalam hal Anda menemukan inakurasi informasi yang termuat pada situs ini atau informasi yang seharusnya ada, namun belum tersedia, maka harap segera hubungi Kepaniteraan Mahkamah Agung RI melalui :
Email : kepaniteraan@mahkamahagung.go.id   Telp : 021-384 3348 (ext.318)

Menimbang, bahwa terhadap dalil-dalil gugatan Penggugat tersebut Tergugat tidak memberikan jawabannya karena Tergugat tidak pernah hadir ke persidangan meskipun telah dipanggil secara resmi dan patut;

Menimbang, bahwa oleh karena Tergugat yang telah dipanggil secara resmi dan patut supaya menghadap di depan sidang tidak pernah hadir, sedangkan dalil-dalil gugatan Penggugat sepanjang yang dijadikan alasan perceraian tersebut tidak melawan hukum, maka dengan demikian dalil-dalil gugatan Penggugat tersebut menjadi tetap adanya;

Menimbang, bahwa meskipun dalil-dalil gugatan Penggugat tersebut telah menjadi tetap, namun karena perkara ini masalah perceraian maka kepada Penggugat masih dibebani bukti atas dalil-dalil gugatannya tersebut;

Menimbang, bahwa untuk memenuhi pembuktian tersebut Penggugat telah mengajukan bukti surat yaitu P-2 dan P-3 serta 2 (dua) orang saksi sebagaimana diuraikan dalam duduk perkara;

Menimbang, bahwa berdasarkan bukti P-2 ternyata Tergugat tidak diketahui tempat tinggalnya maka dari itu Pengadilan Agama Bandung melakukan pemanggilan kepada Tergugat dengan perpedoman kepada Pasal 27 Peraturan Pemerintah Nomor 9 Tahun 1975;

Menimbang, bahwa berdasarkan bukti P.3, yaitu fotokopi Kutipan Akta Nikah yang telah dinazegelend dan sesuai dengan aslinya sehingga bukti surat tersebut formal dapat diterima sebagai alat bukti, hal mana sesuai dengan maksud Pasal 165 HIR, dan sesuai pula dengan maksud Pasal 7 ayat (1) Kompilasi Hukum Islam, harus dinyatakan terbukti bahwa antara Penggugat dengan Tergugat telah terikat dalam perkawinan yang sah sebagaimana diatur dalam Pasal 2 Undang-undang Nomor 1 Tahun 1974, oleh karenanya Penggugat dipandang berkepentingan untuk mengajukan perkara ini;

Menimbang, bahwa dari 2 (dua) orang saksi Penggugat yang memberikan keterangan di bawah sumpah yang pada pokoknya saksi-saksi tersebut mengetahui dan membenarkan bahwa rumah tangga Penggugat dan Tergugat sudah tidak harmonis karena sering terjadi perselisihan dan pertengkaran yang disebabkan oleh Tergugat selingkuh dengan wanita lain,

Disclaimer
Kepaniteraan Mahkamah Agung Republik Indonesia berusaha untuk selalu mencantumkan informasi paling kini dan akurat sebagai bentuk komitmen Mahkamah Agung untuk pelayanan publik, transparansi dan akuntabilitas pelaksanaan fungsi peradilan. Namun dalam hal-hal tertentu masih dimungkinkan terjadi permasalahan teknis terkait dengan akurasi dan keterkinian informasi yang kami sajikan, hal mana akan terus kami perbaiki dari waktu kewaktu.
Dalam hal Anda menemukan inakurasi informasi yang termuat pada situs ini atau informasi yang seharusnya ada, namun belum tersedia, maka harap segera hubungi Kepaniteraan Mahkamah Agung RI melalui :
Email : kepaniteraan@mahkamahagung.go.id Telp : 021-384 3348 (ext.318)

sekarang Penggugat dan Tergugat sudah pisah rumah sejak bulan Mei 2014 dan Tergugat tidak diketahui lagi keberadaannya;

Menimbang, bahwa saksi-saksi yang diajukan oleh Penggugat adalah saksi yang berasal dari keluarga dan/atau orang yang dekat dengan Penggugat, hal ini sejalan dengan ketentuan Pasal 22 ayat (2) Peraturan Pemerintah Nomor 9 Tahun 1975 jo. Pasal 76 ayat (1) Undang-undang Nomor 7 Tahun 1989 sebagaimana diubah dan ditambah dengan Undang-undang Nomor 3 Tahun 2006 dan perubahan kedua dengan Undang-undang Nomor 50 tahun 2009 tentang Peradilan Agama serta Pasal 134 Kompilasi Hukum Islam, maka keterangan saksi tersebut dapat dipakai sebagai bukti dalam perkara ini;

Menimbang, bahwa berdasarkan dalil-dalil gugatan Penggugat yang telah menjadi tetap dihubungkan dengan keterangan di bawah sumpah saksi-saksi yang diajukan oleh Penggugat, Majelis Hakim menemukan sejumlah fakta hukum sebagai berikut:

Bahwa Penggugat dan Tergugat adalah pasangan suami istri yang menikah pada tanggal 11 Januari 2013;

Bahwa Penggugat dan Tergugat sudah dikaruniai 2 (dua) orang anak;

Bahwa rumah tangga Penggugat dengan Tergugat sudah tidak harmonis sejak bulan Mei 2014, karena sering terjadi perselisihan dan pertengkaran yang disebabkan Tergugat mempunyai wanita idaman lain;

Bahwa Penggugat dengan Tergugat sudah berpisah rumah sejak bulan Mei 2014 bahkan Tergugat sudah tidak diketahui tempat tinggalnya;

Bahwa telah diupayakan agar Penggugat mempertahankan rumah tangganya, namun tidak berhasil;

Menimbang, bahwa berdasarkan fakta-fakta tersebut di atas, maka dalil-dalil gugatan Penggugat tentang telah tidak harmonisnya lagi rumah tangga

Disclaimer
Kepaniteraan Mahkamah Agung Republik Indonesia berusaha untuk selalu mencantumkan informasi paling kini dan akurat sebagai bentuk komitmen Mahkamah Agung untuk pelayanan publik, transparansi dan akuntabilitas pelaksanaan fungsi peradilan. Namun dalam hal-hal tertentu masih dimungkinkan terjadi permasalahan teknis terkait dengan akurasi dan keterkinian informasi yang kami sajikan, hal mana akan terus kami perbaiki dari waktu kewaktu.
Dalam hal Anda menemukan inakurasi informasi yang termuat pada situs ini atau informasi yang seharusnya ada, namun belum tersedia, maka harap segera hubungi Kepaniteraan Mahkamah Agung RI melalui :
Email : kepaniteraan@mahkamahagung.go.id Telp : 021-384 3348 (ext.318)

sebagaimana dikemukakan dalam surat gugatan Penggugat tanggal 12 Agustus 2020 dapat dinyatakan terbukti;

Menimbang, bahwa dengan telah terbuktinya dalil-dalil gugatan Penggugat tersebut, Majelis Hakim berpendapat bahwa perselisihan dan pertengkaran yang terjadi antara Penggugat dan Tergugat sudah sampai kepada tahap yang sulit untuk dapat dirukunkan lagi, hal demikian merupakan indikasi bahwa jiwa rumah tangga Penggugat dengan Tergugat telah pecah, sehingga Majelis Hakim berkesimpulan bahwa mempertahankan perkawinan Penggugat dengan Tergugat adalah sia-sia karena sudah tidak lagi memberi manfaat, serta tidak akan dapat mewujudkan tujuan perkawinan sebagaimana dimaksud Pasal 1 Undang-Undang Nomor 1 Tahun 1974 yaitu membentuk keluarga (rumah tangga) yang bahagia dan kekal berdasarkan Ketuhanan Yang Mahaesa, oleh karena itu perceraian merupakan alternatif terbaik untuk Penggugat dan Tergugat;

Menimbang, bahwa berdasarkan pertimbangan-pertimbangan di atas, maka gugatan Penggugat untuk dapat bercerai dari Tergugat dipandang telah memenuhi maksud Pasal 39 ayat (2) Undang-undang Nomor 1 Tahun 1974 Jo. Pasal 19 huruf (b) dan (f) Peraturan pemerintah Nomor 9 Tahun 1975 Jo. Pasal 116 huruf (b) dan (f) Kompilasi Hukum Islam, karenanya gugatan Penggugat tersebut dapat dikabulkan;

Menimbang, bahwa oleh karena perkara ini bidang perkawinan, maka sesuai Pasal 89 ayat (1) Undang-Undang Nomor 7 Tahun 1989 sebagaimana telah diubah dengan Undang-Undang Nomor 3 Tahun 2006 dan perubahan kedua dengan Undang-Undang Nomor 50 Tahun 2009, biaya perkara dibebankan kepada Penggugat, namun berdasarkan Putusan Sela Nomor 3562/Pdt.G/2020/PA.Badg tanggal 21 Desember 2020 Penggugat akan diberi keringanan dalam membayar biaya perkara;

Mengingat, segala peraturan perundang-undangan yang berlaku dan hukum syara' yang berkaitan dengan perkara ini;

M E N G A D I L I

1. Menyatakan, Tergugat yang telah dipanggil secara resmi dan patut untuk datang menghadap di persidangan, tidak hadir;

Disclaimer
Kepaniteraan Mahkamah Agung Republik Indonesia berusaha untuk selalu mencantumkan informasi paling kini dan akurat sebagai bentuk komitmen Mahkamah Agung untuk pelayanan publik, transparansi dan akuntabilitas pelaksanaan fungsi peradilan. Namun dalam hal-hal tertentu masih dimungkinkan terjadi permasalahan teknis terkait dengan akurasi dan keterkinian informasi yang kami sajikan, hal mana akan terus kami perbaiki dari waktu kewaktu.
Dalam hal Anda menemukan inakurasi informasi yang termuat pada situs ini atau informasi yang seharusnya ada, namun belum tersedia, maka harap segera hubungi Kepaniteraan Mahkamah Agung RI melalui :
Email : kepaniteraan@mahkamahagung.go.id Telp : 021-384 3348 (ext.318)

2. Mengabulkan gugatan Penggugat dengan verstek;
3. Menjatuhkan talak satu ba'in sughra Tergugat (Nama Tergugat) terhadap Penggugat (Nama Penggugat);
4. Membebankan biaya perkara kepada Penggugat sejumlah Rp 46.000,- (empat puluh enam ribu rupiah);

Demikian putusan ini dijatuhkan dalam musyawarah Majelis Hakim Pengadilan Agama Bandung pada hari Senin tanggal 21 Desember 2020 Masehi, bertepatan dengan tanggal 06 Jumadilawwal 1442 Hijriah oleh Drs. H. Nana Supriatna, Ketua Majelis, Drs. H. W. Setiawan, S.H. dan Dra. Hj. Dedeh Saidah, M.H., masing-masing sebagai Hakim Anggota, putusan tersebut pada hari itu juga dibacakan oleh Ketua Majelis dalam persidangan terbuka untuk umum dengan didampingi hakim-hakim anggota, serta dibantu oleh Astria Lestari Ningsih, S.HI. sebagai Panitera Pengganti, dengan dihadiri oleh Penggugat tanpa hadirnya Tergugat;

Ketua Majelis,

Ttd.

Drs. H. Nana Supriatna

Hakim Anggota,	Hakim Anggota,
Ttd.	Ttd.
Drs. H. Ahmad, M.Hum	Drs. H. Endang Tamami, M.H.

Panitera Pengganti,

Ttd.

Astria Lestari Ningsih, S.HI.

Disclaimer
Kepaniteraan Mahkamah Agung Republik Indonesia berusaha untuk selalu mencantumkan informasi paling kini dan akurat sebagai bentuk komitmen Mahkamah Agung untuk pelayanan publik, transparansi dan akuntabilitas pelaksanaan fungsi peradilan. Namun dalam hal-hal tertentu masih dimungkinkan terjadi permasalahan teknis terkait dengan akurasi dan keterkinian informasi yang kami sajikan, hal mana akan terus kami perbaiki dari waktu kewaktu.
Dalam hal Anda menemukan inakurasi informasi yang termuat pada situs ini atau informasi yang seharusnya ada, namun belum tersedia, maka harap segera hubungi Kepaniteraan Mahkamah Agung RI melalui :
Email : kepaniteraan@mahkamahagung.go.id Telp : 021-384 3348 (ext.318)

Perincian biaya perkara :

1.	Pendaftaran	Rp	0,-
2.	Proses	Rp	40.000,-
3.	Panggilan Penggugat	Rp	0,-
4.	Panggilan Tergugat	Rp	0,-
5.	PNBP	Rp	0,-
6.	Redaksi	Rp	0,-
7.	Biaya Meterai	Rp	6.000,-
	Jumlah	Rp.	46.000,-

(empat puluh enam ribu rupiah);

Disclaimer
Kepaniteraan Mahkamah Agung Republik Indonesia berusaha untuk selalu mencantumkan informasi paling kini dan akurat sebagai bentuk komitmen Mahkamah Agung untuk pelayanan publik, transparansi dan akuntabilitas pelaksanaan fungsi peradilan. Namun dalam hal-hal tertentu masih dimungkinkan terjadi permasalahan teknis terkait dengan akurasi dan keterkinian informasi yang kami sajikan, hal mana akan terus kami perbaiki dari waktu kewaktu.
Dalam hal Anda menemukan inakurasi informasi yang termuat pada situs ini atau informasi yang seharusnya ada, namun belum tersedia, maka harap segera hubungi Kepaniteraan Mahkamah Agung RI melalui :
Email : kepaniteraan@mahkamahagung.go.id Telp : 021-384 3348 (ext.318)
