# Supplementary material for: Classification of divorce causes during the COVID-19 pandemic using convolutional neural networks
Source: PeerJ Comput Sci. 2022 Jun 30;8:e998. doi: 10.7717/peerj-cs.998 (PMC9299239; doi:10.7717/peerj-cs.998)
Supplement: Supplemental Information 4 [file peerj-cs-08-998-s004.zip › Konflik Dataset/Data ke-23.pdf]

**P U T U S A N**

**Nomor 5898/Pdt.G/2020/PA.Badg**

بسم الله الرحمن الرحيم

**DEMI KEADILAN BERDASARKAN KETUHANAN YANG MAHA ESA**

Pengadilan Agama Bandung yang memeriksa dan mengadili perkara tertentu pada tingkat pertama dalam sidang majelis telah menjatuhkan putusan perkara Cerai Gugat antara :

**PENGGUGAT**, tempat dan tanggal lahir Bandung, 09 Maret 1972, agama Islam, pekerjaan Mengurus Rumah Tangga, pendidikan S1, tempat kediaman di Jalan Sadang Serang, Gang Intan VIII, No. 3, RT 005, RW 014, Kelurahan Sekeloa, Kecamatan Coblong, Kota Bandung., sebagai **Penggugat**;

melawan

**TERGUGAT**, tempat dan tanggal lahir Sukabumi, 03 April 1969, agama Islam, pekerjaan Wiraswasta, pendidikan S1, tempat kediaman di Jalan Babakan Sirna, No. 12, RT 003, RW 003, Desa Benteng, Kecamatan Warungdoyong, Kota Sukabumi, sebagai **Tergugat**;

Pengadilan Agama tersebut;

Telah mempelajari surat-surat yang berkaitan dengan perkara ini;

Telah mendengar keterangan Penggugat serta para saksi di muka sidang;

**DUDUK PERKARA**

Menimbang, bahwa Penggugat dalam surat gugatannya tanggal 30 November 2020 telah mengajukan gugatan cerai yang telah terdaftar di Kepaniteraan Pengadilan Agama Bandung dengan Nomor 5898/Pdt.G/2020/PA.Badg tanggal 30 November 2020 dengan dalil-dalil sebagai berikut:

**1.** Bahwa antara Penggugat dan Tergugat adalah sepasang suami istri sah yang menikah pada tanggal 22 Mei 2016 dan telah dicatatkan di Kantor



Mahkamah Agung Republik Indonesia

*Disclaimer*
*Kepaniteraan Mahkamah Agung Republik Indonesia berusaha untuk selalu mencantumkan informasi paling kini dan akurat sebagai bentuk komitmen Mahkamah Agung untuk pelayanan publik, transparansi dan akuntabilitas pelaksanaan fungsi peradilan. Namun dalam hal-hal tertentu masih dimungkinkan terjadi permasalahan teknis terkait dengan akurasi dan keterkinian informasi yang kami sajikan, hal mana akan terus kami perbaiki dari waktu kewaktu.*
*Dalam hal Anda menemukan inakurasi informasi yang termuat pada situs ini atau informasi yang seharusnya ada, namun belum tersedia, maka harap segera hubungi Kepaniteraan Mahkamah Agung RI melalui :*
*Email : kepaniteraan@mahkamahagung.go.id    Telp : 021-384 3348 (ext.318)*

Urusan Agama (KUA) Kecamatan Coblong, Kota Bandung dengan bukti Kutipan Akta Nikah Nomor : 329/75/V/2016 tanggal 23 Mei 2016.

**2.** Bahwa setelah menikah antara Penggugat dengan Tergugat tinggal dan hidup bersama terakhir di Jalan Babakan Sirna, No. 12, RT 003, RW 003, Desa Benteng, Kecamatan Warungdoyong, Kota Sukabumi.

**3.** Bahwa semula keadaan rumah tangga antara Penggugat dengan Tergugat hidup rukun dan harmonis. Namun dari perkawinan tersebut tidak dikaruniai anak**;**

**4.** Bahwa selama berumah tangga dengan Tergugat, keadaan tidak selamanya rukun dan harmonis, rumah tangga antara Penggugat dan Tergugat sejak bulan **Juli 2016** mulai terjadi perselisihan dan pertengkaran terus menerus, sehingga Penggugat mengajukan gugatan cerai ke Pengadilan Agama Bandung. Adapun sebab-sebab perceraian ini diajukan adalah sebagai berikut:

4.1. Bahwa Tergugat sering marah tidak jelas kepada Penggugat bahkan jika dalam keadaan tersebut Tergugat tidak pernah berkomunikasi dan memberi kabar kepada Penggugat, sehingga hal tersebut membuat tidak nyaman Penggugat;

4.2. Disamping itu Tergugat kurang bertanggung jawab dalam memberikan nafkah kepada Penggugat seperti jika memberikan nafkah rata-rata hanya Rp. 100.000,00 (seratus ribu rupiah) namun itupun tidak menentu, yang pemberian tersebut dilakukan hanya untuk mengugurkan kewajiban Tergugat saja.

**5.** Bahwa Penggugat telah berusaha sabar untuk mempertahankan rumah tangga, tetapi Tergugat tidak berubah dan merubah sikapnya. Puncaknya pada bulan **Oktober 2020**, antara Penggugat dan Tergugat terlibat pertengkaran, sehingga antara Penggugat dengan Tergugat sudah pisah tempat tinggal dan sudah tidak melaksanakan kewajiban masing-masing layaknya suami istri lagi, kurang lebih selama 2 (dua) bulan.

**6.** Bahwa selama pisah tempat tinggal Penggugat tinggal di tempat kediaman Penggugat dan Tergugat tinggal di tempat kediaman Tergugat



*Disclaimer*
*Kepaniteraan Mahkamah Agung Republik Indonesia berusaha untuk selalu mencantumkan informasi paling kini dan akurat sebagai bentuk komitmen Mahkamah Agung untuk pelayanan publik, transparansi dan akuntabilitas pelaksanaan fungsi peradilan. Namun dalam hal-hal tertentu masih dimungkinkan terjadi permasalahan teknis terkait dengan akurasi dan keterkinian informasi yang kami sajikan, hal mana akan terus kami perbaiki dari waktu kewaktu.*
*Dalam hal Anda menemukan inakurasi informasi yang termuat pada situs ini atau informasi yang seharusnya ada, namun belum tersedia, maka harap segera hubungi Kepaniteraan Mahkamah Agung RI melalui :*
*Email : kepaniteraan@mahkamahagung.go.id Telp : 021-384 3348 (ext.318)*

**7.** Bahwa setelah pisah tempat tinggal, antara Penggugat dengan Tergugat sudah tidak berkomunikasi.

**8.** Bahwa pihak keluarga Pemohon dan Termohon telah berupaya menasehati dan mendamaikan Penggugat dan Tergugat, akan tetapi tidak berhasil.

**9.** Bahwa atas permasalahan tersebut di atas Penggugat sudah tidak sanggup lagi untuk mempertahankan perkawinan ini, oleh karena itu Penggugat telah berketetapan hati untuk bercerai dengan Tergugat.

**10.** Bahwa pengajuan gugatan cerai Penggugat telah memenuhi alasan-alasan perceraian sebagaimana diatur dalam Pasal 19 huruf f Peraturan Pemerintah Nomor 9 tahun 1975 jo Pasal 116 huruf f Instruksi Presiden Nomor 1 tahun 1991 tentang Kompilasi Hukum Islam.

Bahwa berdasarkan hal-hal dan alasan tersebut di atas, maka gugatan cerai Penggugat ini telah sesuai dan memenuhi ketentuan hukum dan mempunyai alasan-alasan yang cukup. Maka oleh karenanya Penggugat memohon kepada Ketua Pengadilan Agama Bandung Cq. Majelis Hakim berkenan membuka persidangan untuk memeriksa dan mengadili perkara ini dan berkenan menjatuhkan putusan yang amarnya sebagai berikut:

**1.** Mengabulkan gugatan Penggugat;

**2.** Menjatuhkan talak satu ba'in sughra dari Tergugat (**TERGUGAT**) terhadap Penggugat (**PENGGUGAT**.);

**3.** Membebankan biaya perkara menurut hukum;

Atau apabila Majelis Hakim Pengadilan Agama Bandung berpendapat lain, mohon putusan yang seadil adilnya *(ex aequo et bono).*

Menimbang, bahwa pada hari sidang yang telah ditetapkan Penggugat telah datang menghadap ke muka sidang, sedangkan Tergugat tidak datang menghadap dan tidak menyuruh orang lain untuk menghadap sebagai wakil/kuasa hukumnya meskipun telah dipanggil secara resmi dan patut yang relaas panggilannya dibacakan di dalam sidang, sedangkan tidak ternyata bahwa tidak datangnya itu disebabkan suatu halangan yang sah;



*Disclaimer*
*Kepaniteraan Mahkamah Agung Republik Indonesia berusaha untuk selalu mencantumkan informasi paling kini dan akurat sebagai bentuk komitmen Mahkamah Agung untuk pelayanan publik, transparansi dan akuntabilitas pelaksanaan fungsi peradilan. Namun dalam hal-hal tertentu masih dimungkinkan terjadi permasalahan teknis terkait dengan akurasi dan keterkinian informasi yang kami sajikan, hal mana akan terus kami perbaiki dari waktu kewaktu.*
*Dalam hal Anda menemukan inakurasi informasi yang termuat pada situs ini atau informasi yang seharusnya ada, namun belum tersedia, maka harap segera hubungi Kepaniteraan Mahkamah Agung RI melalui :*
*Email : kepaniteraan@mahkamahagung.go.id Telp : 021-384 3348 (ext.318)*

Menimbang, bahwa bahwa majelis hakim telah menasehati Penggugat agar berpikir untuk tidak bercerai dengan Tergugat, tetapi Penggugat tetap pada dalil-dalil gugatannya untuk bercerai dengan Tergugat;

Menimbang, bahwa perkara ini tidak dapat dimediasi karena Tergugat tidak pernah datang menghadap meskipun telah dipanggil secara resmi dan patut, selanjutnya dimulai pemeriksaan dengan membacakan surat gugatan Penggugat yang maksud dan isinya tetap dipertahankan oleh Penggugat;

Menimbang, bahwa untuk menguatkan dalil-dalil gugatannya, Penggugat telah mengajukan alat-alat bukti berupa:

A. Bukti Surat:

Fotokopi Buku Kutipan Akta Nikah Nomor: 329/75/V/2016, tertanggal 23 Mei 2016, yang dikeluarkan oleh Kantor Urusan Agama (KUA) Kecamatan Coblong, Kota Bandung, fotokopi tersebut telah dinazegelen dan oleh Ketua Majelis telah dicocokkan dengan aslinya ternyata telah sesuai selanjutnya diberi tanda bukti (P.1);

B. Bukti Saksi:

1. **SAKSI I, umur 21 tahun, agama Islam, pekerjaan Freelen, tempat tinggal di Perumnas Sadang Serang Gang Intan 8 No. 03 RT. 05 RW. 14 Kelurahan Sekeloa Kecamatan Coblong Kota Bandung,** di bawah sumpahnya memberikan keterangan sebagai berikut:
   - Bahwa saksi kenal dengan Penggugat dan Tergugat karena saksi adalah Anak Tiri Tergugat;
   - Bahwa, Penggugat dan Tergugat adalah suami isteri yang menikah sekitar tahun 2016;
   - Bahwa, perkawinan Penggugat dan Tergugat belum dikaruniai anak;
   - Bahwa semula keadaan rumah tangga Penggugat dan Tergugat rukun dan harmonis, namun sejak Juli 2015 sudah tidak harmonis lagi, karena sering terjadi perselisihan dan pertengkaran;
   - Bahwa, penyebab perselisihan dan pertengkaran dikarenakan Tergugat sering marah-marah yang tidak jelas kepada Penggugat dan Tergugat tidak memberi nafkah kepada Penggugat;



Disclaimer
Kepaniteraan Mahkamah Agung Republik Indonesia berusaha untuk selalu mencantumkan informasi paling kini dan akurat sebagai bentuk komitmen Mahkamah Agung untuk pelayanan publik, transparansi dan akuntabilitas pelaksanaan fungsi peradilan. Namun dalam hal-hal tertentu masih dimungkinkan terjadi permasalahan teknis terkait dengan akurasi dan keterkinian informasi yang kami sajikan, hal mana akan terus kami perbaiki dari waktu kewaktu.
Dalam hal Anda menemukan inakurasi informasi yang termuat pada situs ini atau informasi yang seharusnya ada, namun belum tersedia, maka harap segera hubungi Kepaniteraan Mahkamah Agung RI melalui :
Email : kepaniteraan@mahkamahagung.go.id Telp : 021-384 3348 (ext.318)

- Bahwa, antara Penggugat dan Tergugat sudah pisah rumah sejak bulan Oktober 2020;
- Bahwa, keluarga kedua belah pihak dan saksi sudah berusaha untuk merukunkan Penggugat dengan Tergugat, namun tidak berhasil;

2. **SAKSI II, umur 44 tahun, agama Islam, pekerjaan Wiraswasta, tempat kediaman di di Perumnas Sadang Serang Gang Intan 8 No. 03 RT. 05 RW. 14 Kelurahan Sekeloa Kecamatan Coblong Kota Bandung,** di bawah sumpahnya memberikan keterangan sebagai berikut:
   - Bahwa saksi kenal dengan Penggugat dan Tergugat karena saksi adalah adik kandung Penggugat;
   - Bahwa, Penggugat dan Tergugat adalah suami isteri yang menikah sekitar tahun 2016;
   - Bahwa, perkawinan Penggugat dan Tergugat belum dikaruniai anak;
   - Bahwa semula keadaan rumah tangga Penggugat dan Tergugat rukun dan harmonis, namun sejak Juli 2015 sudah tidak harmonis lagi, karena sering terjadi perselisihan dan pertengkaran;
   - Bahwa, penyebab perselisihan dan pertengkaran dikarenakan Tergugat sering marah-marah yang tidak jelas kepada Penggugat dan Tergugat tidak memberi nafkah kepada Penggugat;
   - **Bahwa, antara Penggugat dan Tergugat sudah pisah rumah sejak bulan Oktober 2020;**
   - Bahwa, keluarga kedua belah pihak dan saksi sudah berusaha untuk merukunkan Penggugat dengan Tergugat, namun tidak berhasil;

Menimbang, bahwa selanjutnya untuk singkatnya uraian putusan ini, maka semua hal yang termuat dalam berita acara sidang ini merupakan bagian yang tidak terpisahkan dari putusan ini;

**PERTIMBANGAN HUKUM**



*Disclaimer*
*Kepaniteraan Mahkamah Agung Republik Indonesia berusaha untuk selalu mencantumkan informasi paling kini dan akurat sebagai bentuk komitmen Mahkamah Agung untuk pelayanan publik, transparansi dan akuntabilitas pelaksanaan fungsi peradilan. Namun dalam hal-hal tertentu masih dimungkinkan terjadi permasalahan teknis terkait dengan akurasi dan keterkinian informasi yang kami sajikan, hal mana akan terus kami perbaiki dari waktu kewaktu.*
*Dalam hal Anda menemukan inakurasi informasi yang termuat pada situs ini atau informasi yang seharusnya ada, namun belum tersedia, maka harap segera hubungi Kepaniteraan Mahkamah Agung RI melalui :*
*Email : kepaniteraan@mahkamahagung.go.id Telp : 021-384 3348 (ext.318)*

Menimbang, bahwa maksud dan tujuan gugatan Penggugat adalah sebagaimana terurai di atas;

Menimbang, bahwa ternyata Tergugat, meskipun telah dipanggil secara resmi dan patut, tidak datang menghadap di muka sidang dan pula tidak ternyata bahwa tidak datangnya itu disebabkan suatu halangan yang sah;

Menimbang, bahwa Tergugat yang dipanggil secara resmi dan patut akan tetapi tidak datang menghadap harus dinyatakan tidak hadir dan gugatan tersebut harus diperiksa secara verstek;

Menimbang, bahwa oleh karena itu, maka putusan atas perkara ini dapat dijatuhkan tanpa hadirnya Tergugat (verstek);

Menimbang, bahwa berdasarkan ketentuan Pasal 125 ayat ( 1) HIR, yaitu putusan yang dijatuhkan tanpa hadirnya Tergugat dapat dikabulkan sepanjang berdasarkan hukum dan beralasan, oleh karena itu majelis membebani Penggugat untuk membuktikan dalil-dalil gugatannya;

Menimbang, bahwa gugatan Penggugat didasarkan pada dalil yang pada pokoknya adalah rumah tangga Penggugat dan Tergugat tidak harmonis lagi sering terjadi perselisihan dan pertengkaran dan tidak ada harapan akan hidup rukun lagi dalam satu rumah tangga, sehingga Penggugat memohon agar Pengadilan Agama menjatuhkan talak satu bain sughra Tergugat terhadap Penggugat;

Menimbang, bahwa untuk membuktikan dalil-dalilnya, Penggugat telah mengajukan alat bukti surat P.1 dan dua orang saksi;

Menimbang, bahwa bukti P.1 (fotokopi Buku Kutipan Akta Nikah) yang merupakan akta otentik dan telah bermeterai cukup dan cocok dengan aslinya, isi bukti tersebut menjelaskan mengenai pernikahan Penggugat dan Tergugat, sehingga bukti tersebut telah memenuhi syarat formil dan materil, serta mempunyai kekuatan pembuktian yang sempurna dan mengikat;

Menimbang, bahwa berdasarkan bukti (P.1) maka dapat dinyatakan terbukti bahwa Penggugat dan Tergugat adalah suami istri yang sah, sehingga mempunyai landasan hukum untuk mengajukan cerai gugat terhadap Tergugat;

Menimbang, bahwa oleh karena alasan Cerai Gugat Penggugat didasarkan pada pasal 19 huruf (f) Peraturan Pemerintah Nomor 9 Tahun 1975



*Disclaimer*
*Kepaniteraan Mahkamah Agung Republik Indonesia berusaha untuk selalu mencantumkan informasi paling kini dan akurat sebagai bentuk komitmen Mahkamah Agung untuk pelayanan publik, transparansi dan akuntabilitas pelaksanaan fungsi peradilan. Namun dalam hal-hal tertentu masih dimungkinkan terjadi permasalahan teknis terkait dengan akurasi dan keterkinian informasi yang kami sajikan, hal mana akan terus kami perbaiki dari waktu kewaktu.*
*Dalam hal Anda menemukan inakurasi informasi yang termuat pada situs ini atau informasi yang seharusnya ada, namun belum tersedia, maka harap segera hubungi Kepaniteraan Mahkamah Agung RI melalui :*
*Email : kepaniteraan@mahkamahagung.go.id Telp : 021-384 3348 (ext.318)*

jo. Pasal 116 huruf (f) Kompilasi Hukum Islam, maka Majelis telah mendengar keterangan saksi dari pihak keluarga Penggugat, sehingga telah terpenuhi maksud pasal 76 ayat (1) Undang Undang Nomor 7 Tahun 1989, yang telah mengalami 2 kali perubahan dengan Undang-undang Nomor 3 tahun 2006 dan Undang-Undang Nomor 50 tahun 2009;

Menimbang, bahwa dua orang saksi yang diajukan oleh Penggugat secara formil dan materil telah memenuhi syarat sebagai saksi, oleh karena itu dapat dipertimbangkan lebih lanjut;

Menimbang, bahwa berdasarkan keterangan saksi-saksi tersebut di atas yang keterangannya saling bersesuaian, maka Majelis telah dapat menemukan fakta yang pada pokoknya sebagai berikut:

- Bahwa, Penggugat dan Tergugat adalah suami isteri yang menikah sekitar tahun 2016;
- Bahwa, perkawinan Penggugat dan Tergugat belum dikaruniai anak;
- Bahwa semula keadaan rumah tangga Penggugat dan Tergugat rukun dan harmonis, namun sejak Juli 2015 sudah tidak harmonis lagi, karena sering terjadi perselisihan dan pertengkaran;
- Bahwa, penyebab perselisihan dan pertengkaran dikarenakan Tergugat sering marah-marah yang tidak jelas kepada Penggugat dan Tergugat tidak memberi nafkah kepada Penggugat;
- Bahwa, antara Penggugat dan Tergugat sudah pisah rumah sejak bulan Oktober 2020;
- Bahwa, keluarga kedua belah pihak dan saksi sudah berusaha untuk merukunkan Penggugat dengan Tergugat, namun tidak berhasil;

Menimbang, bahwa berdasarkan fakta tersebut di atas, Majelis berpendapat rumah tangga Penggugat dengan Tergugat tidak harmonis lagi prilaku Tergugat tersebut menjadi pemicu terjadinya percekcokan sehingga hubungan Penggugat dan Tergugat semakin tidak baik, dengan demikian Majelis berkesimpulan keadaan rumah tangga Penggugat dengan Tergugat telah pecah sedemikian rupa sehingga tujuan perkawinan untuk membentuk keluarga/rumah tangga yang bahagia dan kekal berdasarkan Ketuhanan Yang Maha Esa (*vide* pasal 1 Undang-Undang Nomor 1 Tahun 1974) dan atau



Disclaimer
Kepaniteraan Mahkamah Agung Republik Indonesia berusaha untuk selalu mencantumkan informasi paling kini dan akurat sebagai bentuk komitmen Mahkamah Agung untuk pelayanan publik, transparansi dan akuntabilitas pelaksanaan fungsi peradilan. Namun dalam hal-hal tertentu masih dimungkinkan terjadi permasalahan teknis terkait dengan akurasi dan keterkinian informasi yang kami sajikan, hal mana akan terus kami perbaiki dari waktu kewaktu.
Dalam hal Anda menemukan inakurasi informasi yang termuat pada situs ini atau informasi yang seharusnya ada, namun belum tersedia, maka harap segera hubungi Kepaniteraan Mahkamah Agung RI melalui :
Email : kepaniteraan@mahkamahagung.go.id Telp : 021-384 3348 (ext.318)

keluarga yang sakinah, penuh mawaddah dan rahmah (*vide* pasal 3 Kompilasi Hukum Islam) telah tidak terwujud dalam rumah tangga Penggugat dengan Tergugat;

Menimbang, bahwa unsur pokok tegaknya suatu bangunan rumah tangga adalah adanya ikatan lahir batin yang kokoh antara suami dan isteri. Apabila terjadi perselisihan dan pertengkaran antara suami-isteri yang berakibat kepada tidak lagi saling peduli dan telah diupayakan untuk rukun kembali tetapi tidak berhasil maka hal tersebut mengindikasikan bahwa ikatan lahir-batin diantara suami-isteri tersebut telah sedemikian rapuh atau bahkan telah lepas sama sekali, sehingga telah tidak ada lagi kecocokan dan kesamaan kehendak diantara keduanya;

Menimbang, bahwa mempertahankan rumah tangga yang telah pecah sedemikian rupa adalah sia-sia belaka, bahkan apabila keadaannya seperti sekarang ini jika dipaksakan atau dibiarkan maka justru akan menimbulkan mudharat dan penderitaan lahir batin yang berkepanjangan bagi Penggugat, sehingga oleh karenanya Majelis berpendapat bahwa rumah tangga Penggugat dengan Tergugat telah tidak dapat dipertahankan lagi;

Menimbang, bahwa Majelis perlu mengemukakan doktrin ulama yang kemudian diambil alih sebagai pendapat Majelis dalam Kitab Madaa Hurriyatuz Zaujaini fith Thalaaq Juz I halaman 83:

وقد اختار الإسلام نظام الطلاق حين تضطرب الحياة الزوجين ولم يعد ينفع فيها نصائح ولا صلح وحيث تصبح الربطة الزواج صورة من غير روح لأن الإستمرار معناه أن يحكم على أحد الزوجين بالسجن المؤبد وهذا ظلم تأباه روح العدالة

"*Islam memilih lembaga talak/cerai ketika rumah tangga sudah dianggap goncang serta dianggap sudah tidak bermanfaat lagi nasehat/perdamaian dan hubungan suami isteri menjadi tanpa ruh (hampa), sebab meneruskan perkawinan berarti menghukum salah satu suami isteri dengan penjara yang berkepanjangan. Ini adalah aniaya yang bertentangan dengan semangat keadilan*"

Menimbang, bahwa berdasarkan hal-hal tersebut di atas maka gugatan Penggugat telah terbukti beralasan hukum sesuai ketentuan pasal 39 ayat (2) Undang-Undang Nomor 1 Tahun 1974 jis Pasal 19 huruf (f) Peraturan



Disclaimer
Kepaniteraan Mahkamah Agung Republik Indonesia berusaha untuk selalu mencantumkan informasi paling kini dan akurat sebagai bentuk komitmen Mahkamah Agung untuk pelayanan publik, transparansi dan akuntabilitas pelaksanaan fungsi peradilan. Namun dalam hal-hal tertentu masih dimungkinkan terjadi permasalahan teknis terkait dengan akurasi dan keterkinian informasi yang kami sajikan, hal mana akan terus kami perbaiki dari waktu kewaktu.
Dalam hal Anda menemukan inakurasi informasi yang termuat pada situs ini atau informasi yang seharusnya ada, namun belum tersedia, maka harap segera hubungi Kepaniteraan Mahkamah Agung RI melalui :
Email : kepaniteraan@mahkamahagung.go.id Telp : 021-384 3348 (ext.318)

Pemerintah Nomor 9 Tahun 1975 dan Pasal 116 huruf (f) Kompilasi Hukum Islam, oleh karena itu gugatan Penggugat dapat dikabulkan dengan menjatuhkan talak satu bain sughra dari Tergugat terhadap Penggugat;

Menimbang, bahwa oleh karena perkara ini dalam bidang perkawinan, maka sesuai Pasal 89 ayat (1) Undang-Undang Nomor 7 Tahun 1989 sebagaimana telah diubah dengan Undang-Undang Nomor 3 Tahun 2006 dan perubahan kedua dengan Undang-Undang Nomor 50 Tahun 2009, biaya perkara dibebankan kepada Penggugat;

Mengingat, semua pasal dalam peraturan perundang-undangan dan hukum Islam yang berkaitan dengan perkara ini;

**MENGADILI**

**1.** Menyatakan Tergugat yang telah dipanggil dengan resmi dan patut untuk datang menghadap di persidangan, tidak hadir;

**2.** Mengabulkan gugatan Penggugat dengan Verstek;

**3.** Menjatuhkan talak satu ba'in sughra dari Tergugat (**TERGUGAT)** terhadap Penggugat (**PENGGUGAT**);

**4.** Membebankan kepada Penggugat untuk membayar biaya perkara sejumlah Rp. 366.000.- (tiga ratus enam puluh enam ribu rupiah);

Demikianlah diputus dalam sidang permusyawaratan majelis Pengadilan Agama Bandung pada hari Senin tanggal 21 Desember 2020 Masehi bertepatan dengan tanggal 6 Jumadil Awal 1442 Hijriyah oleh kami Drs. H. Cece Rukmana Ibrahim, S.H., M.H. sebagai Ketua Majelis, Drs. H. Endang Tamami, M.H. dan Drs. Mustopa, SH. masing-masing sebagai Hakim Anggota, dan diucapkan pada hari itu juga dalam sidang terbuka untuk umum oleh Ketua Majelis tersebut didampingi para Hakim Anggota, dibantu oleh Achmad Sadikin S.H. sebagai Panitera Pengganti dengan dihadiri oleh Penggugat tanpa hadirnya Tergugat;

Ketua Majelis



*Disclaimer*
*Kepaniteraan Mahkamah Agung Republik Indonesia berusaha untuk selalu mencantumkan informasi paling kini dan akurat sebagai bentuk komitmen Mahkamah Agung untuk pelayanan publik, transparansi dan akuntabilitas pelaksanaan fungsi peradilan. Namun dalam hal-hal tertentu masih dimungkinkan terjadi permasalahan teknis terkait dengan akurasi dan keterkinian informasi yang kami sajikan, hal mana akan terus kami perbaiki dari waktu kewaktu.*
*Dalam hal Anda menemukan inakurasi informasi yang termuat pada situs ini atau informasi yang seharusnya ada, namun belum tersedia, maka harap segera hubungi Kepaniteraan Mahkamah Agung RI melalui :*
*Email : kepaniteraan@mahkamahagung.go.id Telp : 021-384 3348 (ext.318)*

Drs. H. Cece Rukmana Ibrahim, S.H., M.H.

Hakim Anggota Hakim Anggota

Drs. H. Endang Tamami, M.H. Drs. Mustopa, SH.

Panitera Pengganti

Achmad Sadikin S.H.

Perincian Biaya Perkara :

| | | | |
|---|---|---|---|
| 1. Pendaftaran | : | Rp. | 30.000,- |
| 2. Proses | : | Rp. | 50.000,- |
| 3. Panggilan Penggugat | : | Rp. | 100.000,- |
| 4. Panggilan Tergugat | : | Rp. | 150.000,- |
| 5. Biaya PNBP | ; | Rp. | 20.000.- |
| 5. Redaksi | : | Rp. | 10.000,- |
| 6. Materai | : | Rp. | 6.000,- |

-----------------------------------------------------------------------------------------

| | | | |
|---|---|---|---|
| Jumlah | : | Rp. | 366.000,- |

tiga ratus enam puluh enam ribu rupiah



*Disclaimer*
*Kepaniteraan Mahkamah Agung Republik Indonesia berusaha untuk selalu mencantumkan informasi paling kini dan akurat sebagai bentuk komitmen Mahkamah Agung untuk pelayanan publik, transparansi dan akuntabilitas pelaksanaan fungsi peradilan. Namun dalam hal-hal tertentu masih dimungkinkan terjadi permasalahan teknis terkait dengan akurasi dan keterkinian informasi yang kami sajikan, hal mana akan terus kami perbaiki dari waktu kewaktu.*
*Dalam hal Anda menemukan inakurasi informasi yang termuat pada situs ini atau informasi yang seharusnya ada, namun belum tersedia, maka harap segera hubungi Kepaniteraan Mahkamah Agung RI melalui :*
*Email : kepaniteraan@mahkamahagung.go.id Telp : 021-384 3348 (ext.318)*
