# Supplementary material for: Classification of divorce causes during the COVID-19 pandemic using convolutional neural networks
Source: PeerJ Comput Sci. 2022 Jun 30;8:e998. doi: 10.7717/peerj-cs.998 (PMC9299239; doi:10.7717/peerj-cs.998)
Supplement: Supplemental Information 4 [file peerj-cs-08-998-s004.zip › Konflik Dataset/Data ke-24.pdf]

PUTUSAN

Nomor 325/Pdt. G/2020/PA. Pspk.

بسم الله الرحمن الرحيم

DEMI KEADILAN BERDASARKAN KETUHANAN YANG MAHA ESA

Pengadilan Agama Kota Padang Sidempuan yang memeriksa dan mengadili perkara perdata tertentu pada tingkat pertama dalam sidang Majelis Hakim telah menjatuhkan putusan perkara cerai gugat antara:

Nora Mayang Sari binti Poniran, Tempat/Tgl Lahir, T. Manunggang, 25-05-1991, NIK. 2171116505910004, agama Islam, pendidikan Sekolah Lanjutan Tingkat Pertama, Pekerjaan Karyawan Swasta, tempat kediaman di Dusun III, Desa Labuhan Rasoki, Kecamatan Padangsidimpuan Tengara, Kota Padangsidimpuan, sebagai Penggugat;

Melawan

Haryono bin Koming, Tempat/Tgl Lahir, Goti/20-10-1984, NIK. 2171112010840001 agama Islam, pendidikan Sekolah Lanjutan Tingkat Atas, pekerjaan Karyawan Swasta, tempat kediaman di KP. Warung Ceuri RT/RW 011/005 Nomor 29, Kelurahan Pondok Kaso Landeuh, Kecamatan Parung Kuda, Kota Sukabumi, Propinsi Jawa Barat, sebagai Tergugat;

Pengadilan Agama tersebut ;

Telah mempelajari surat-surat yang berkaitan dengan perkara ini ;

Telah mendengar keterangan Penggugat serta para saksi di muka sidang ;

DUDUK PERKARA

Bahwa Penggugat dalam surat gugatannya tanggal 23 Nopember 2020 telah mengajukan gugatan cerai gugat yang telah didaftar di Kepaniteraan

Disclaimer
Kepaniteraan Mahkamah Agung Republik Indonesia berusaha untuk selalu mencantumkan informasi paling kini dan akurat sebagai bentuk komitmen Mahkamah Agung untuk pelayanan publik, transparansi dan akuntabilitas pelaksanaan fungsi peradilan. Namun dalam hal-hal tertentu masih dimungkinkan terjadi permasalahan teknis terkait dengan akurasi dan keterkinian informasi yang kami sajikan, hal mana akan terus kami perbaiki dari waktu kewaktu.
Dalam hal Anda menemukan inakurasi informasi yang termuat pada situs ini atau informasi yang seharusnya ada, namun belum tersedia, maka harap segera hubungi Kepaniteraan Mahkamah Agung RI melalui :
Email : kepaniteraan@mahkamahagung.go.id Telp : 021-384 3348 (ext.318)

Pengadilan Agama Kota Padangsidimpuan dengan Nomor 325/Pdt.G/2020/PA. Pspk, tanggal 23 Nopember 2020, dengan dalil-dalil sebagai berikut :

1. Bahwa Penggugat dan Tergugat adalah suami isteri yang sah, menikah secara Islam pada tanggal 15 September 2010, tercatat dan terdaftar di KUA Kecamatan Padangsidimpuan Tenggara, Kota Padangsidimpuan Nomor : 207/01/X/2010, tanggal 23-09-2010;

1. Bahwa atas dasar pernikahan tersebut Penggugat dengan Tergugat telah bergaul dalam rumah tangga dan telah dikaruniai anak 2 (dua) orang yang bernama : Khansa Nuraulia, Perempuan, lahir tanggal 23-06-2011, saat ini bersama Tergugat dan Naufal Afkar, Laki-laki, lahir tangga 14-02-2015 saat ini bersama Tergugat;

3. Bahwa setelah menikah Penggugat dengan Tergugat tinggal bersama di rumah orang tua Tergugat di Desa Hutakoje, Kecamatan Padangsidimpuan Tenggara, Kota Padangsidimpuan dan kemudian pindah-pindah rumah dan terakhir tinggal bersama di Dusun III, Desa Labuhan Rasoki, Kecamatan Padangsidimpuan Tengara, Kota Padangsidimpuan;

4. Bahwa keadaan rumah tangga yang telah di bina oleh Penggugat dan Tergugat dalam keadaan rukun dan damai selama lebih kurang 6 (enam) tahun, kemudian sejak tahun 2016 Tergugat pergi meninggalkan Penggugat dari rumah yang selama ini menjadi tempat tinggal bersama Penggugat dengan Tergugat tanpa ada alasan yang sah dan kepergian Tergugat tersebut juga tanpa sepengetahuan Penggugat, dan tanpa izin Penggugat, sejak kepergian Tergugat tersebut sampai dengan saat sekarang ini Tergugat tidak pernah kembali lagi untuk menemui atau pun menjenguk Penggugat untuk hidup bersama lagi dalam rumah tangga, bahkan Tergugat tidak pernah ada lagi memberikan nafkah lahir dan bathin kepada Penggugat dan Tergugat tidak lagi memperdulikan Penggugat sudah lebih 4 (empat) tahun lamanya;

5. Bahwa atas perbuatan Tergugat tersebut, yang meninggalkan Penggugat lebih 4 tahun berturut-turut tanpa izin Penggugat dan tanpa alasan yang sah maka sudah cukup alasan hukum untuk bercerai sesuai dengan

Disclaimer
Kepaniteraan Mahkamah Agung Republik Indonesia berusaha untuk selalu mencantumkan informasi paling kini dan akurat sebagai bentuk komitmen Mahkamah Agung untuk pelayanan publik, transparansi dan akuntabilitas pelaksanaan fungsi peradilan. Namun dalam hal-hal tertentu masih dimungkinkan terjadi permasalahan teknis terkait dengan akurasi dan keterkinian informasi yang kami sajikan, hal mana akan terus kami perbaiki dari waktu kewaktu.
Dalam hal Anda menemukan inakurasi informasi yang termuat pada situs ini atau informasi yang seharusnya ada, namun belum tersedia, maka harap segera hubungi Kepaniteraan Mahkamah Agung RI melalui :
Email : kepaniteraan@mahkamahagung.go.id   Telp : 021-384 3348 (ext.318)

Peraturan Pemerintah No.9 tahun 1975 pasal (19) huruf (b) dan lebih baik berpisah untuk mendapatkan jalan keluar dari penderitaan lahir dan bathin Penggugat dan demi masa depan yang mungkin lebih baik bagi Penggugat dan Tergugat;

6. Bahwa berdasarkan uraian-uraian tersebut, Penggugat bermohon kepada Bapak Ketua Pengadilan Agama Kota Padangsidimpuan Cq. Majelis Hakim yang memeriksa dan mengadili perkara ini kiranya dapat menentukan suatu hari persidangan, memanggil Penggugat dan Tergugat ke persidangan guna memeriksa dan mengadili perkara ini dan selanjutnya Penggugat mohon putusan yang amarnya berbunyi sebagai berikut:

    1. Mengabulkan gugatan Penggugat.
    2. Menjatuhkan talak satu ba'in sughra Tergugat (**Haryono bin Koming**) atas diri Penggugat (**Nora Mayang Sari binti Poniran**).
    3. Membebankan kepada Penggugat untuk membayar biaya perkara ini sesuai dengan ketentuan peraturan perundang-undangan yang berlaku.

    Atau : Apabila pengadilan berpendapat lain mohon putusan yang seadil-adilnya.

Bahwa pada hari sidang yang telah ditetapkan Penggugat telah datang menghadap ke muka sidang, sedangkan Tergugat tidak datang menghadap ke muka sidang dan tidak menyuruh orang lain untuk menghadap sebagai wakil/kuasa hukumnya meskipun telah dipanggil secara resmi dan patut yang relaas panggilannya dibacakan di dalam sidang, sedangkan tidak ternyata bahwa tidak datangnya itu disebabkan suatu halangan yang sah ;

Bahwa majelis hakim telah menasehati Penggugat agar berpikir untuk tidak bercerai dengan Tergugat, tetapi Penggugat tetap pada dalil-dalil gugatannya untuk bercerai dengan Tergugat ;

Bahwa perkara ini tidak dapat dimediasi karena Tergugat tidak pernah datang menghadap meskipun telah dipanggil secara resmi dan patut, selanjutnya dimulai pemeriksaan dengan membacakan surat gugatan Penggugat dimana Penggugat tidak menyampaikan perubahan apapun ;

Disclaimer
Kepaniteraan Mahkamah Agung Republik Indonesia berusaha untuk selalu mencantumkan informasi paling kini dan akurat sebagai bentuk komitmen Mahkamah Agung untuk pelayanan publik, transparansi dan akuntabilitas pelaksanaan fungsi peradilan. Namun dalam hal-hal tertentu masih dimungkinkan terjadi permasalahan teknis terkait dengan akurasi dan keterkinian informasi yang kami sajikan, hal mana akan terus kami perbaiki dari waktu kewaktu.
Dalam hal Anda menemukan inakurasi informasi yang termuat pada situs ini atau informasi yang seharusnya ada, namun belum tersedia, maka harap segera hubungi Kepaniteraan Mahkamah Agung RI melalui :
Email : kepaniteraan@mahkamahagung.go.id Telp : 021-384 3348 (ext.318)

Bahwa untuk menguatkan dalil-dalil gugatan, Penggugat telah mengajukan alat-alat bukti berupa :

A. Surat :

- Fotokopi Kutipan Akta Nikah Nomor : 207/01/X/2010, tanggal 23-09-2010, telah dinazagelen (P) ;

B. Saksi :

1. Sukirto Bin Saring, umur 41 tahun, Agama Islam, pekerjaan Wiraswasta, tempat tinggal di Jl. Mayor Bejo, Desa Labuhan Rasoki, Kecamatan Padangsidimpuan Tenggara, Kota Padangsidimpuan, di bawah sumpahnya memberikan keterangan sebagai berikut :
   - Saksi kenal dengan Penggugat dan Tergugat karena saksi sebagai adik Penggugat;
   - Hubungan Penggugat dengan Tergugat adalah suami isteri, menikah pada tahun 2010;
   - Setelah menikah Penggugat dan Tergugat hidup bersama terakhir di Dusun III, Desa Labuhan Rasoki, Kecamatan Padangsidimpuan Tengara, Kota Padangsidimpuan;
   - Selama berumah tangga Penggugat dan Tergugat sudah dikaruniai anak sebanyak 2 (dua) orang;
   - Yang saksi ketahui keadaan rumah tangga Penggugat dengan Tergugat, semula rumah tangga Penggugat dan Tergugat rukun dan harmonis, akan tetapi sejak tahun 2016 Tergugat pergi meninggalkan Penggugat dari rumah yang selama ini menjadi tempat tinggal bersama, tanpa izin dan tanpa pamit kepada Penggugat;
   - Sejak kepergian Tergugat tahun 2016 yang lalu, antara Penggugat dengan Tergugat tidak pernah lagi tinggal satu rumah atau berjumpa dengan Tergugat;
   - Tergugat tidak meninggalkan sesuatu apapun yang dapat dijadikan

Disclaimer
Kepaniteraan Mahkamah Agung Republik Indonesia berusaha untuk selalu mencantumkan informasi paling kini dan akurat sebagai bentuk komitmen Mahkamah Agung untuk pelayanan publik, transparansi dan akuntabilitas pelaksanaan fungsi peradilan. Namun dalam hal-hal tertentu masih dimungkinkan terjadi permasalahan teknis terkait dengan akurasi dan keterkinian informasi yang kami sajikan, hal mana akan terus kami perbaiki dari waktu kewaktu.
Dalam hal Anda menemukan inakurasi informasi yang termuat pada situs ini atau informasi yang seharusnya ada, namun belum tersedia, maka harap segera hubungi Kepaniteraan Mahkamah Agung RI melalui :
Email : kepaniteraan@mahkamahagung.go.id Telp : 021-384 3348 (ext.318)

nafkah atau belanja Penggugat;

- Saksi tidak sanggup lagi untuk merukunkan Penggugat dengan Tergugat;

2. Yetti irma suryani Binti Salamuddin, umur 31 tahun, Agama Islam, pekerjaan berjualan, tempat tinggal di Jl. Imam Bonjol. Kelurahan Aek Tampang, Kecamatan Padangsidimpuan Selatan, Kota Padangsidimpuan;
   - Saksi kenal dengan Penggugat yang bernama Nora Mayang Sari karena saksi adalah Tetangga Penggugat, dan juga kenal dengan Tergugat bernama Haryono;
   - Saksi kenal dengan Tergugat hanya pada saat pernikahan Penggugat dengan Tergugat;
   - Saksi tidak mengetahui apapun tentang keluarga Penggugat dengan Tergugat;

Bahwa, terhadap keterangan saksi kedua tersebut Penggugat tidak membantahnya, akan tetapi saksi kedua yang dihadirkan Pengguat tersebut tidak mengetahui tentang permasalahan keluarga Pengugat dengan Tergugat. Lalu Penggugat menyatakan tidak sanggup menghadirkan saksi lain namun Penggugat menyatakan ianya sanggup untuk disumpah sebagai tambahan untuk melengkapi kesaksian kemudian pada tahap kesimpulan Penggugat menyatakan tetap pada keinginannya untuk bercerai dari Tergugat ;

Bahwa terhadap keterangan 1 orang saksi Penggugat tersebut dari keterangannya baru dapat diambil sebagai bukti awal dan belum dapat diambil sebagai bukti yang kuat terkecuali dikuatkan dengan bukti lainnya ;

Bahwa oleh karena Penggugat telah mencukupkan 1 orang Saksi yang telah diajukan dipersidangan dan menyatakan tidak dapat menghadirkan Saksi lainnya untuk didengar keterangan di persidangan ;

Bahwa oleh karena Penggugat hanya dapat mengajukan 1 orang Saksi tersebut, kesaksian mana baru dapat dikatakan sebagai bukti permulaan, maka berdasarkan ketentuan pasal 182 R.Bg, Majelis Hakim berwenang untuk memerintahkan Penggugat untuk mengucapkan sumpah Suplatoir di depan persidangan ;

Disclaimer
Kepaniteraan Mahkamah Agung Republik Indonesia berusaha untuk selalu mencantumkan informasi paling kini dan akurat sebagai bentuk komitmen Mahkamah Agung untuk pelayanan publik, transparansi dan akuntabilitas pelaksanaan fungsi peradilan. Namun dalam hal-hal tertentu masih dimungkinkan terjadi permasalahan teknis terkait dengan akurasi dan keterkinian informasi yang kami sajikan, hal mana akan terus kami perbaiki dari waktu kewaktu.
Dalam hal Anda menemukan inakurasi informasi yang termuat pada situs ini atau informasi yang seharusnya ada, namun belum tersedia, maka harap segera hubungi Kepaniteraan Mahkamah Agung RI melalui :
Email : kepaniteraan@mahkamahagung.go.id Telp : 021-384 3348 (ext.318)

Bahwa Saksi yang diajukan oleh Penggugat di persidangan 1 orang, yaitu Sukirto Bin Saring adalah Saksi orang dekat Pemohon, telah memberikan keterangan di bawah sumpah, yang pada pokoknya adalah sesuai dengan dalil-dalil gugatan Pemohon, dengan demikian kesaksiannya dapat dipertimbangkan sebagai bukti permulaan. Oleh karena pembuktian Pemohon belum mencapai batas minimal pembuktian, maka sesuai dengan ketentuan pasal 182 R.Bg. Majelis Hakim beralasan untuk membebankan Pemohon mengucapkan sumpah suplatoir sebagaimana tersebut yang berbunyi *“ Bismillahirrahmanirrahin, Wallahi Demi saya bersumpah bahwa seluruh dalil gugatan saya dan seluruh keterangan yang saya sampaikan dipersidangan ini adalah benar, tidak ada lain melainkan yang sebenarnya dan saya tahu akibat sumpah saya ini”* ;

Bahwa selanjutnya untuk singkatnya uraian putusan ini, maka semua hal yang termuat dalam berita acara sidang ini merupakan bagian yang tidak terpisahkan dari putusan ini;

PERTIMBANGAN HUKUM

Menimbang, bahwa maksud dan tujuan gugatan Penggugat adalah sebagaimana terurai di atas;

Menimbang, bahwa ternyata Tergugat, meskipun dipanggil secara resmi dan patut, tidak datang menghadap di muka sidang dan pula tidak ternyata bahwa tidak datangnya itu disebabkan suatu halangan yang sah;

Menimbang, bahwa Tergugat yang dipanggil secara resmi dan patut akan tetapi tidak datang menghadap harus dinyatakan tidak hadir dan gugatan tersebut harus diperiksa secara verstek;

Menimbang, bahwa oleh karena itu, maka putusan atas perkara ini dapat dijatuhkan tanpa hadirnya Tergugat (verstek) ;

Menimbang, bahwa berdasarkan ketentuan Pasal 149 ayat (1) R.Bg. yaitu putusan yang dijatuhkan tanpa hadirnya Tergugat dapat dikabulkan sepanjang berdasarkan hukum dan beralasan, oleh karena itu majelis membebani Penggugat untuk membuktikan dalil-dalil gugatannya ;

Menimbang, bahwa bukti P (Fotokopi Kutipan Akta Nikah) yang merupakan akta otentik dan telah bermeterai cukup dan cocok dengan aslinya,

Disclaimer
Kepaniteraan Mahkamah Agung Republik Indonesia berusaha untuk selalu mencantumkan informasi paling kini dan akurat sebagai bentuk komitmen Mahkamah Agung untuk pelayanan publik, transparansi dan akuntabilitas pelaksanaan fungsi peradilan. Namun dalam hal-hal tertentu masih dimungkinkan terjadi permasalahan teknis terkait dengan akurasi dan keterkinian informasi yang kami sajikan, hal mana akan terus kami perbaiki dari waktu kewaktu.
Dalam hal Anda menemukan inakurasi informasi yang termuat pada situs ini atau informasi yang seharusnya ada, namun belum tersedia, maka harap segera hubungi Kepaniteraan Mahkamah Agung RI melalui :
Email : kepaniteraan@mahkamahagung.go.id   Telp : 021-384 3348 (ext.318)

isi bukti tersebut menjelaskan mengenai status perkawinan antara Penggugat dengan Tergugat, sehingga bukti tersebut telah memenuhi syarat formal dan materiil, serta mempunyai kekuatan pembuktian yang sempurna dan mengikat ;

Menimbang, bahwa saksi I Penggugat, sudah dewasa dan sudah disumpah, sehingga memenuhi syarat formal sebagaimana diatur dalam Pasal 172 ayat 1 angka 4 R.Bg.;

Menimbang, bahwa keterangan saksi I Penggugat mengenai angka 3, 4 dan 5 posita gugatan Penggugat adalah fakta yang dilihat sendiri dan relevan dengan dalil yang harus dibuktikan oleh Penggugat ditambah dengan sumpah suplatoir, oleh karena itu keterangan saksi tersebut telah memenuhi syarat materiil sebagaimana telah diatur dalam 308 R.Bg. sehingga keterangan saksi tersebut memiliki kekuatan pembuktian dan dapat diterima sebagai alat bukti ;

Menimbang, bahwa keterangan saksi I dan ditambah dengan sumpah suplatoir Penggugat bersesuaian dan cocok antara satu dengan yang lain oleh karena itu keterangan dua orang saksi tersebut memenuhi Pasal 308 dan Pasal 309 R.Bg.;

Menimbang, bahwa berdasarkan bukti P saksi I dan sumpah suplatoir terbukti fakta kejadian sebagai berikut:

1. Bahwa Penggugat dengan Tergugat telah menikah pada tanggal 23-09-2010 di Kecamatan Padangsidimpuan Tenggara ;
2. Bahwa dalam menjalani kehidupan berumah tangga, antara Penggugat dengan Tergugat tidak harmonis lagi;
3. Bahwa Tergugat telah pergi meninggalkan Penggugat sejak tahun 2016 yang lalu ;

Menimbang, bahwa berdasarkan fakta-fakta tersebut di atas dapat disimpulkan fakta hukum sebagai berikut :

1. Bahwa Penggugat dengan Tergugat adalah suami istri ;
2. Bahwa antara Penggugat dengan Tergugat telah berpisah selama kurang lebih 4 (empat) tahun;

Disclaimer
Kepaniteraan Mahkamah Agung Republik Indonesia berusaha untuk selalu mencantumkan informasi paling kini dan akurat sebagai bentuk komitmen Mahkamah Agung untuk pelayanan publik, transparansi dan akuntabilitas pelaksanaan fungsi peradilan. Namun dalam hal-hal tertentu masih dimungkinkan terjadi permasalahan teknis terkait dengan akurasi dan keterkinian informasi yang kami sajikan, hal mana akan terus kami perbaiki dari waktu kewaktu.
Dalam hal Anda menemukan inakurasi informasi yang termuat pada situs ini atau informasi yang seharusnya ada, namun belum tersedia, maka harap segera hubungi Kepaniteraan Mahkamah Agung RI melalui :
Email : kepaniteraan@mahkamahagung.go.id Telp : 021-384 3348 (ext.318)

3. Bahwa Keadaan tersebut telah menimbulkan kemuderatan (kesulitan) bagi Penggugat dan Tergugat dan jika tidak diakhiri makan dikhawatirkan akan menimbulkan kesulitan-kesulitan yang lain ;

Menimbang, bahwa fakta hukum tersebut telah memenuhi norma hukum Islam yang terkandung dalam:

1. Al Qur’an Surat An Nisa ayat 130:

وإن يتفرقا يغن الله كلا من سعته وكان الله واسعا حكيما

Jika keduanya bercerai maka Allah akan memberi kecukupan kepada masing-masingnya dari limpahan karunianya. Dan adalah Allah Maha Luas (karunia Nya) lagi Maha Bijaksana.

2. Qaidah Fiqh yang terdapat dalam kitab Al-Asbah wa al-Nazair halaman 59

لازيررضلا

“Kemudharatan itu harus dihilangkan”

3. Kitab Ghoyatul Marom :

إذا اشتد عدم رغبة الزوجة لزوجها طلق عليه القاضى طلقة

Dan ketika isteri sudah sangat tidak senang terhadap suaminya maka Hakim dibolehkan menjatuhkan talak satu suami.

Menimbang, bahwa fakta hukum tersebut telah juga memenuhi pasal 19 huruf b Peraturan Pemerintah Nomor 9 Tahun 1975 *jo.* pasal 116 huruf b Kompilasi Hukum Islam di Indonesia Tahun 1991;

Menimbang, bahwa berdasarkan pertimbangan-pertimbangan tersebut di atas, maka petitum gugatan Penggugat mengenai keinginannya untuk bercerai dari Tergugat tersebut memenuhi Pasal 19 huruf b Peraturan Pemerintah Nomor 9 Tahun 1975 *jo.* pasal 116 huruf b Kompilasi Hukum Islam di Indonesia Tahun 1991, oleh karena itu dapat dikabulkan;

Disclaimer
Kepaniteraan Mahkamah Agung Republik Indonesia berusaha untuk selalu mencantumkan informasi paling kini dan akurat sebagai bentuk komitmen Mahkamah Agung untuk pelayanan publik, transparansi dan akuntabilitas pelaksanaan fungsi peradilan. Namun dalam hal-hal tertentu masih dimungkinkan terjadi permasalahan teknis terkait dengan akurasi dan keterkinian informasi yang kami sajikan, hal mana akan terus kami perbaiki dari waktu kewaktu.
Dalam hal Anda menemukan inakurasi informasi yang termuat pada situs ini atau informasi yang seharusnya ada, namun belum tersedia, maka harap segera hubungi Kepaniteraan Mahkamah Agung RI melalui :
Email : kepaniteraan@mahkamahagung.go.id Telp : 021-384 3348 (ext.318)

Menimbang, bahwa karena perkara *a quo* termasuk bidang perkawinan, maka berdasarkan Pasal 89 ayat (1) Undang-undang Nomor 7 Tahun 1989 sebagaimana telah diubah dengan Undang-undang Nomor 3 Tahun 2006 dan perubahan kedua dengan Undang-undang Nomor 50 Tahun 2009, biaya perkara harus dibebankan kepada Penggugat ;

Mengingat, semua pasal dalam peraturan perundang-undangan dan hukum Islam yang berkaitan dengan perkara ini ;

MENGADILI

1. Menyatakan Tergugat yang telah dipanggil secara resmi dan patut untuk menghadap ke persidangan tidak hadir.
2. Mengabulkan gugatan Penggugat secara verstek.
3. Menjatuhkan talak satu bain sughra Tergugat (**Haryono bin Koming**) terhadap Penggugat (**Nora Mayang Sari binti Poniran**(.
4. Membebankan kepada Penggugat untuk membayar biaya perkara ini sejumlah Rp646.000,00 (enam ratus empat puluh enam ribu rupiah) ;

Demikian diputuskan di Pengadilan Agama Kota Padangsidimpuan pada hari Senin tanggal 21 Desember 2020 Masehi bertepatan dengan tanggal 6 Jumadil Awwal 1442 Hijriah, oleh kami Fadlah Mardiyah Pulungan, S.HI, M.A, selaku Ketua Majelis dan Muzhirul Haq, S. Ag. serta Hasybi Hassadiqi, SHI, masing – masing sebagai Hakim Anggota putusan tersebut diucapkan dalam sidang terbuka untuk umum pada hari itu juga oleh Ketua Majelis dan dibantu oleh Yulita Fifprawati, S.H, sebagai Panitera Pengganti serta dihadiri oleh Penggugat dan tanpa hadirnya Tergugat;

Ketua Majelis

Fadlah Mardiyah Pulungan, S.HI, M.A

Disclaimer
Kepaniteraan Mahkamah Agung Republik Indonesia berusaha untuk selalu mencantumkan informasi paling kini dan akurat sebagai bentuk komitmen Mahkamah Agung untuk pelayanan publik, transparansi dan akuntabilitas pelaksanaan fungsi peradilan. Namun dalam hal-hal tertentu masih dimungkinkan terjadi permasalahan teknis terkait dengan akurasi dan keterkinian informasi yang kami sajikan, hal mana akan terus kami perbaiki dari waktu kewaktu.
Dalam hal Anda menemukan inakurasi informasi yang termuat pada situs ini atau informasi yang seharusnya ada, namun belum tersedia, maka harap segera hubungi Kepaniteraan Mahkamah Agung RI melalui :
Email : kepaniteraan@mahkamahagung.go.id Telp : 021-384 3348 (ext.318)

Hakim Anggota,	Hakim Anggota,
Muzhirul Haq, S. Ag	Hasybi Hassadiqi, S. HI

Panitera pengganti,

Yulita Fifprawati, S.H.

Perincian biaya :

1.	Pendaftaran	Rp. 30.000,00
2.	Proses	Rp. 50.000,00
3.	Panggilan	Rp. 500.000,00
4.	Redaksi	Rp. 5.000,00
5.	Meterai	Rp. 6.000,00
	**J u m l a h**	**Rp. 646.000,00**

Disclaimer
Kepaniteraan Mahkamah Agung Republik Indonesia berusaha untuk selalu mencantumkan informasi paling kini dan akurat sebagai bentuk komitmen Mahkamah Agung untuk pelayanan publik, transparansi dan akuntabilitas pelaksanaan fungsi peradilan. Namun dalam hal-hal tertentu masih dimungkinkan terjadi permasalahan teknis terkait dengan akurasi dan keterkinian informasi yang kami sajikan, hal mana akan terus kami perbaiki dari waktu kewaktu.
Dalam hal Anda menemukan inakurasi informasi yang termuat pada situs ini atau informasi yang seharusnya ada, namun belum tersedia, maka harap segera hubungi Kepaniteraan Mahkamah Agung RI melalui :
Email : kepaniteraan@mahkamahagung.go.id   Telp : 021-384 3348 (ext.318)
