# Supplementary material for: Classification of divorce causes during the COVID-19 pandemic using convolutional neural networks
Source: PeerJ Comput Sci. 2022 Jun 30;8:e998. doi: 10.7717/peerj-cs.998 (PMC9299239; doi:10.7717/peerj-cs.998)
Supplement: Supplemental Information 4 [file peerj-cs-08-998-s004.zip › Konflik Dataset/Data ke-25.pdf]

P U T U S A N

Nomor 2742/Pdt.G/2020/PA.Kng

بِسْمِ اللهِ الرَّحْمَنِ الرَّحِيمِ

DEMI KEADILAN BERDASARKAN KETUHANAN YANG MAHA ESA

Pengadilan Agama Kuningan yang memeriksa dan mengadili perkara perdata pada tingkat pertama dalam persidangan Majelis telah menjatuhkan putusan sebagai berikut dalam perkara antara :

PEMOHON, umur xxx tahun, agama Islam, pendidikan xxx, pekerjaan xxx, tempat kediaman di xxx, sebagai Pemohon;

melawan

TERMOHON, umur xxx tahun, agama Islam, pendidikan xxx, pekerjaan xxx, tempat kediaman di xxx, sebagai Termohon;

Pengadilan Agama tersebut;

Setelah mempelajari berkas perkara yang bersangkutan;

Setelah mendengar keterangan Pemohon dan saksi-saksi di muka persidangan;

DUDUK PERKARA

Bahwa, Pemohon dengan surat permohonannya tertanggal 10 Desember 2020 yang telah terdaftar di Kepaniteraan Pengadilan Agama Kuningan dibawah Nomor 2742/Pdt.G/2020/PA.Kng tanggal 10 Desember 2020, telah mengajukan hal-hal sebagai berikut :

1. Bahwa Pemohon dan #0047# telah melangsungkan akad nikah di hadapan Pejabat Kantor Urusan Agama Kecamatan xxx pada tanggal xxx tercatat dalam Register Akta Nikah Nomor : 0783/052/X/2016 tanggal 28 Oktober 2016, yang dikeluarkan oleh Kantor Urusan Agama Kecamatan xxx;
2. Bahwa sesudah akad nikah Pemohon dengan Termohon telah hidup berumah tangga selama xxx dan harmonisnya selama xxx dengan memilih tempat tinggal di xxx serta telah bergaul sebagaimana layaknya suami isteri dan xxx dikaruniai xxx;

Disclaimer
Kepaniteraan Mahkamah Agung Republik Indonesia berusaha untuk selalu mencantumkan informasi paling kini dan akurat sebagai bentuk komitmen Mahkamah Agung untuk pelayanan publik, transparansi dan akuntabilitas pelaksanaan fungsi peradilan. Namun dalam hal-hal tertentu masih dimungkinkan terjadi permasalahan teknis terkait dengan akurasi dan keterkinian informasi yang kami sajikan, hal mana akan terus kami perbaiki dari waktu kewaktu.
Dalam hal Anda menemukan inakurasi informasi yang termuat pada situs ini atau informasi yang seharusnya ada, namun belum tersedia, maka harap segera hubungi Kepaniteraan Mahkamah Agung RI melalui :
Email : kepaniteraan@mahkamahagung.go.id Telp : 021-384 3348 (ext.318)

3. Bahwa selama berumah tangga antara Pemohon dan Termohon xxx pernah bercerai ;

4. Bahwa pada awal bulan xxx rumah tangga Pemohon dan Termohon mulai goyah, Pemohon dan Termohon sering terjadi perselisihan dan pertengkaran yang terus menerus disebabkan karena xxx;

5. Bahwa puncak pertengkaran dan perselisihan antara Pemohon dengan Termohon terjadi pada bulan xxx yang mengakibatkan Pemohon dan Termohon berpisah tempat tinggal hingga sekarang telah lebih kurang xxx lamanya;

6. Bahwa selama berpisah pihak keluarga Pemohon dan Termohon telah berupaya merukunkan rumah tangga Pemohon dan Termohon agar bisa damai dalam rumah tangga, akan tetapi Pemohon sudah tidak mau lagi melanjutkan ikatan perkawinan dengan Termohon ;

7. Berdasarkan hal-hal tersebut di atas, Pemohon memohon kepada Bapak Ketua Pengadilan Agama Kuningan cq. Majelis Hakim yang memeriksa perkara ini agar berkenan membuka persidangan dan menjatuhkan putusan yang amarnya sebagai berikut :

PRIMER

1. Mengabulkan permohonan Pemohon.
2. Memberi ijin kepada Pemohon untuk menjatuhkan talak satu raj'i kepada Termohon dihadapan sidang Pengadilan Agama Kuningan.
3. Membebankan biaya perkara kepada Pemohon;

SUBSIDER

Apabila Majelis Hakim berpendapat lain mohon putusan yang seadil-adilnya.

Bahwa, pada hari sidang yang telah ditetapkan, Pemohon datang menghadap sendiri di muka persidangan, sedangkan Termohon tidak datang dan tidak pula menyuruh orang lain sebagai wakil atau kuasanya yang sah, padahal kepadanya telah dipanggil dengan resmi dan patut berdasarkan berita acara relaas panggilan tanggal 11 Desember 2020 dan #0120#, sedangkan tidak ternyata ketidakhadirannya itu disebabkan oleh suatu alasan yang sah menurut hukum;

Disclaimer
Kepaniteraan Mahkamah Agung Republik Indonesia berusaha untuk selalu mencantumkan informasi paling kini dan akurat sebagai bentuk komitmen Mahkamah Agung untuk pelayanan publik, transparansi dan akuntabilitas pelaksanaan fungsi peradilan. Namun dalam hal-hal tertentu masih dimungkinkan terjadi permasalahan teknis terkait dengan akurasi dan keterkinian informasi yang kami sajikan, hal mana akan terus kami perbaiki dari waktu kewaktu.
Dalam hal Anda menemukan inakurasi informasi yang termuat pada situs ini atau informasi yang seharusnya ada, namun belum tersedia, maka harap segera hubungi Kepaniteraan Mahkamah Agung RI melalui :
Email : kepaniteraan@mahkamahagung.go.id Telp : 021-384 3348 (ext.318)

Bahwa, Majelis Hakim telah berusaha menasehati Pemohon agar dapat membina kembali rumah tangganya dengan Termohon, akan tetapi tidak berhasil dan Majelis Hakim memandang perkara a quo tidak mungkin untuk dilaksanakan mediasi karena Termohon tidak pernah hadir di persidangan;

Bahwa, selanjutnya pemeriksaan perkara ini dimulai dengan membacakan surat permohonan Pemohon yang maksud dan isinya tetap dipertahankan oleh #0046#;

Bahwa, untuk membuktikan dalil permohonannya, Pemohon telah mengajukan alat bukti surat berupa :

1. Fotokopi Kartu Tanda Penduduk/Surat Keterangan Domisili an. Pemohon, bukti tersebut telah bermaterai cukup dan telah dicocokkan dengan aslinya yang ternyata sesuai, lalu oleh Ketua Majelis diparaf dan diberi tanda (P.1);
2. Fotokopi Buku Kutipan Akta Nikah Nomor 0062/015/II/2018 tanggal 26 Februari 2018 yang dikeluarkan oleh Kepala Kantor Urusan Agama Kecamatan Cibingbin Kabupaten Kuningan, bukti tersebut telah bermeterai cukup dan telah dicocokkan dengan aslinya yang ternyata sesuai, lalu oleh Ketua Majelis diberi tanda (P.2);

Bahwa, di samping itu, Pemohon juga telah mengajukan alat bukti saksi yaitu :

1. Saksi I, dibawah sumpah menerangkan pada pokoknya sebagai berikut :
   - Bahwa saksi adalah ...;
   - Bahwa semula rumah tangga Pemohon dengan Termohon rukun dan harmonis di di rumah Pemohon akan tetapi sejak bulan bulan Februari 2019 rumah tangganya sudah tidak harmonis lagi, sering terjadi perselisihan dan pertengkaran;
   - Bahwa penyebabnya karena Termohon tidak ridha dengan nafkah yang diberikan Pemohon sesuai kemampuan;
   - Bahwa sejak satu tahun yang lalu antara Pemohon dan Termohon telah berpisah tempat tinggal dan tidak pernah berkumpul kembali;
   - Bahwa saksi pernah berusaha menasehati Pemohon, akan tetapi tidak berhasil;

Disclaimer
Kepaniteraan Mahkamah Agung Republik Indonesia berusaha untuk selalu mencantumkan informasi paling kini dan akurat sebagai bentuk komitmen Mahkamah Agung untuk pelayanan publik, transparansi dan akuntabilitas pelaksanaan fungsi peradilan. Namun dalam hal-hal tertentu masih dimungkinkan terjadi permasalahan teknis terkait dengan akurasi dan keterkinian informasi yang kami sajikan, hal mana akan terus kami perbaiki dari waktu kewaktu.
Dalam hal Anda menemukan inakurasi informasi yang termuat pada situs ini atau informasi yang seharusnya ada, namun belum tersedia, maka harap segera hubungi Kepaniteraan Mahkamah Agung RI melalui :
Email : kepaniteraan@mahkamahagung.go.id   Telp : 021-384 3348 (ext.318)

- Bahwa saksi sudah tidak sanggup lagi untuk merukunkan kembali;

Bahwa, terhadap keterangan saksi tersebut Pemohon membenarkannya;

1. Saksi II, dibawah sumpah menerangkan pada pokoknya sebagai berikut :

- Bahwa saksi adalah ...;
- Bahwa awalnya rumah tangga Pemohon dan Termohon rukun dan harmonis akan tetapi sejak bulan bulan Februari 2019 rumah tangganya sudah tidak harmonis lagi sering terjadi perselisihan dan pertengkaran;
- Bahwa penyebab perselisihan dan pertengkaran tersebut karena Termohon tidak ridha dengan nafkah yang diberikan Pemohon;
- Bahwa sejak satu tahun yang lalu Pemohon dan Termohon telah berpisah tempat tinggal dan tidak pernah berkumpul kembali;
- Bahwa saksi sudah tidak sanggup lagi untuk merukunkannya;

Bahwa, terhadap keterangan saksi tersebut Pemohon membenarkannya;

Bahwa, Pemohon dalam kesimpulannya mengatakan tidak akan mengajukan suatu apapun dan memohon kepada Majelis Hakim agar menjatuhkan putusannya;

Bahwa, untuk mempersingkat uraian pertimbangan ini ditunjuk hal-hal sebagaimana tercantum dalam berita acara persidangan perkara ini;

PERTIMBANGAN HUKUM

Menimbang, bahwa maksud dan tujuan permohonan Pemohon adalah sebagaimana terurai diatas;

Menimbang, bahwa Termohon telah dipanggil dengan resmi dan patut, akan tetapi tidak datang dan tidak pula menyuruh orang lain sebagai wakilnya/kuasanya dan tidak ternyata tidak datangnya itu disebabkan suatu alasan yang sah, sedangkan permohonan Pemohon telah cukup beralasan dan tidak melawan hukum, maka Termohon yang telah dipanggil tersebut harus dinyatakan tidak hadir dan sesuai dengan Pasal 125 ayat (1) HIR perkaranya dapat diputuskan dengan verstek;

Menimbang, bahwa sesuai dengan maksud dan ketentuan Pasal 82 Undang-Undang Nomor 7 Tahun 1989 tentang Peradilan Agama sebagaimana

Disclaimer
Kepaniteraan Mahkamah Agung Republik Indonesia berusaha untuk selalu mencantumkan informasi paling kini dan akurat sebagai bentuk komitmen Mahkamah Agung untuk pelayanan publik, transparansi dan akuntabilitas pelaksanaan fungsi peradilan. Namun dalam hal-hal tertentu masih dimungkinkan terjadi permasalahan teknis terkait dengan akurasi dan keterkinian informasi yang kami sajikan, hal mana akan terus kami perbaiki dari waktu kewaktu.
Dalam hal Anda menemukan inakurasi informasi yang termuat pada situs ini atau informasi yang seharusnya ada, namun belum tersedia, maka harap segera hubungi Kepaniteraan Mahkamah Agung RI melalui :
Email : kepaniteraan@mahkamahagung.go.id Telp : 021-384 3348 (ext.318)

telah diubah dengan Undang-Undang Nomor 3 Tahun 2006 dan perubahan kedua dengan Undang-Undang Nomor 50 Tahun 2009, Majelis Hakim telah berusaha maksimal menasehati Pemohon agar Pemohon dapat rukun kembali membina rumah tangga dengan Termohon akan tetapi usaha tersebut tidak berhasil;

Menimbang, bahwa di samping itu oleh karena Termohon tidak pernah hadir di persidangan, maka proses mediasi tidak dapat dilaksanakan (vide PERMA Nomor 1 Tahun 2016 Pasal 4 ayat 2 huruf b);

Menimbang, bahwa terlebih dahulu Majelis Hakim harus menyatakan bahwa perkara ini adalah menjadi yurisdiksi Pengadilan Agama Kuningan, dan Pemohon berkapasitas dalam perkara ini (vide Pasal 66 ayat (2) Undang-Undang Nomor 7 Tahun 1989 tentang Peradilan Agama sebagaimana telah diubah dengan Undang-Undang Nomor 3 Tahun 2006 dan perubahan kedua dengan Undang-Undang Nomor 50 Tahun 2009 jo. Pasal 14 Peraturan Pemerintah Nomor 9 Tahun 1975 jo. Pasal 129 Kompilasi Hukum Islam dan berdasarkan alat bukti (P.1) Pemohon bertempat tinggal di wilayah Kabupaten Kuningan;

Menimbang, bahwa selanjutnya berdasarkan alat bukti (P.2) yang telah memenuhi syarat formil dan materil, harus dinyatakan terbukti bahwa hubungan hukum antara Pemohon dan Termohon telah terikat dalam perkawinan yang sah sebagaimana diatur dalam Pasal 2 Undang-Undang Nomor 1 Tahun 1974;

Menimbang, bahwa pada pokoknya Pemohon telah mendasarkan permohonannya agar diberi ijin untuk menjatuhkan talak terhadap Termohon dengan alasan sejak bulan bulan Februari 2019 rumah tangga Pemohon dengan Termohon mulai tidak harmonis, sering terjadi perselisihan dan pertengkaran yang mencapai puncaknya pada bulan #9031# sehingga tidak ada harapan akan hidup rukun lagi dalam membina rumah tangga, dimana penyebabnya karena Termohon tidak ridlo dengan nafkah yang kurang dari Pemohon, padahal Pemohon telah memenuhinya sesuai kemampuan dan penghasilan yang ada sehingga sejak satu tahun Pemohon dan Termohon telah pisah tempat tinggal;

Disclaimer
Kepaniteraan Mahkamah Agung Republik Indonesia berusaha untuk selalu mencantumkan informasi paling kini dan akurat sebagai bentuk komitmen Mahkamah Agung untuk pelayanan publik, transparansi dan akuntabilitas pelaksanaan fungsi peradilan. Namun dalam hal-hal tertentu masih dimungkinkan terjadi permasalahan teknis terkait dengan akurasi dan keterkinian informasi yang kami sajikan, hal mana akan terus kami perbaiki dari waktu kewaktu.
Dalam hal Anda menemukan inakurasi informasi yang termuat pada situs ini atau informasi yang seharusnya ada, namun belum tersedia, maka harap segera hubungi Kepaniteraan Mahkamah Agung RI melalui :
Email : kepaniteraan@mahkamahagung.go.id   Telp : 021-384 3348 (ext.318)

Menimbang, bahwa di muka persidangan Pemohon telah mengajukan dua orang saksi yang dari keterangannya saling bersesuaian, Majelis Hakim telah dapat menemukan fakta hukum sebagai berikut :

- Bahwa Pemohon dan Termohon adalah suami isteri yang sah;
- Bahwa semula rumah tangga Pemohon dan Termohon rukun dan harmonis;
- Bahwa sejak bulan bulan Februari 2019 keadaan rumah tangganya tersebut sudah tidak harmonis lagi, sering terjadi perselisihan dan pertengkaran;
- Bahwa penyebabnya karena Termohon tidak ridha dengan nafkah yang diberikan Pemohon sesuai kemampuan;
- Bahwa sejak satu tahun yang lalu Pemohon dan Termohon telah berpisah tempat tinggal dan tidak pernah berkumpul kembali;

Menimbang, bahwa berdasarkan fakta hukum tersebut di atas, harus dinyatakan terbukti bahwa dalam kehidupan rumah tangga Pemohon dan Termohon sudah tidak harmonis lagi, telah terjadi perselisihan dan pertengkaran yang mengakibatkan antara keduanya telah berpisah tempat tinggal dan tidak pernah berkumpul kembali dalam satu rumah tangga;

Menimbang, bahwa disamping itu para saksi sudah pernah berusaha menasehati Pemohon dan mengatakan tidak ada kesanggupan untuk dapat merukunkannya kembali, sehingga Majelis Hakim berpendapat bahwa antara Pemohon dan Termohon sudah sulit dan tidak ada harapan untuk dapat hidup rukun kembali di dalam membina rumah tangganya;

Menimbang, bahwa menurut ketentuan Pasal 19 huruf (f) PP Nomor 9 tahun 1975 jo. Pasal 116 huruf (f) Kompilasi Hukum Islam, perceraian dapat terjadi apabila “antara suami dan isteri terus menerus terjadi perselisihan dan pertengkaran dan tidak ada harapan akan hidup rukun kembali dalam membina rumah tangga”;

Menimbang, bahwa demikian juga menurut Mahkamah Agung sebagaimana tertuang dalam putusannya No. 38/K/AG/1990 tanggal 5 Oktober 1990 yang kemudian diambil alih menjadi pendapat Majelis Hakim bahwa apabila ternyata adanya perselisihan sebagaimana dimaksud Pasal 19 huruf (f)

Disclaimer
Kepaniteraan Mahkamah Agung Republik Indonesia berusaha untuk selalu mencantumkan informasi paling kini dan akurat sebagai bentuk komitmen Mahkamah Agung untuk pelayanan publik, transparansi dan akuntabilitas pelaksanaan fungsi peradilan. Namun dalam hal-hal tertentu masih dimungkinkan terjadi permasalahan teknis terkait dengan akurasi dan keterkinian informasi yang kami sajikan, hal mana akan terus kami perbaiki dari waktu kewaktu.
Dalam hal Anda menemukan inakurasi informasi yang termuat pada situs ini atau informasi yang seharusnya ada, namun belum tersedia, maka harap segera hubungi Kepaniteraan Mahkamah Agung RI melalui :
Email : kepaniteraan@mahkamahagung.go.id Telp : 021-384 3348 (ext.318)

PP No. 9 Tahun 1975, maka hal itu semata-mata ditujukan kepada perkawinannya itu sendiri tanpa mempersoalkan siapa yang salah dalam hal terjadinya perselisihan yang mengakibatkan tidak ada harapan akan hidup rukun lagi, karena pernikahan adalah suatu perjanjian yang suci (mitsaqan gholidzon/Pasal 2 KHI) yang untuk memutuskannya tidak boleh diukur dengan kesalahan salah satu pihak, sehingga apabila perkawinan itu telah pecah berarti hati kedua belah pihak telah pecah pula;

Menimbang, bahwa terlepas dari peristiwa atau keadaan yang menjadi penyebab terjadinya perselisihan yang terjadi antara Pemohon dan Termohon, yang jelas Majelis Hakim telah dapat menemukan fakta hukum bahwa perselisihan antara Pemohon dan Termohon tersebut telah sedemikian rupa, rumah tangga Pemohon dan Termohon sudah hancur dan tidak mungkin akan dapat hidup rukun kembali dalam membina rumah tangga;

Menimbang, bahwa dengan melihat kondisi rumah tangga Pemohon dan Termohon tersebut, jelas tidak mungkin akan terwujud kehidupan rumah tangga yang bahagia dan kekal lahir bathin sebagaimana yang menjadi tujuan perkawinan dalam Pasal 1 Undang-Undang Nomor 1 Tahun 1974 jo. Pasal 3 KHI, dan membiarkan suasana rumah tangga yang demikian, justru akan menimbulkan mudlarat bagi kedua belah pihak, oleh karena itu perceraian dipandang jalan yang terbaik dan lebih maslahat bagi keduanya;

Menimbang, bahwa Majelis Hakim perlu mengetengahkan firman Allah dalam Al-Quran surat Al-Baqarah ayat 227, sebagai berikut;

qvnÌ ÏvpÂ tnÛ¯ ÿ°Õ ØzcÛ¯ ¯uÝSÌ ÿªä

Artinya : "Dan jika mereka berazam (berketetapan hati) untuk menjatuhkan talak, maka sesungguhnya Allah Maha Mendengar lagi Maha Mengetahui";

Menimbang, bahwa berdasarkan pertimbangan tersebut diatas, maka permohonan Pemohon telah memenuhi alasan perceraian sebagaimana diatur dalam Pasal 39 ayat (2) Undang-Undang Nomor 1 Tahun 1974, jo. Pasal 19 huruf (f) Peraturan Pemerintah Nomor 9 Tahun 1975, jo. Pasal 116 huruf (f)

Disclaimer
Kepaniteraan Mahkamah Agung Republik Indonesia berusaha untuk selalu mencantumkan informasi paling kini dan akurat sebagai bentuk komitmen Mahkamah Agung untuk pelayanan publik, transparansi dan akuntabilitas pelaksanaan fungsi peradilan. Namun dalam hal-hal tertentu masih dimungkinkan terjadi permasalahan teknis terkait dengan akurasi dan keterkinian informasi yang kami sajikan, hal mana akan terus kami perbaiki dari waktu kewaktu.
Dalam hal Anda menemukan inakurasi informasi yang termuat pada situs ini atau informasi yang seharusnya ada, namun belum tersedia, maka harap segera hubungi Kepaniteraan Mahkamah Agung RI melalui :
Email : kepaniteraan@mahkamahagung.go.id Telp : 021-384 3348 (ext.318)

Kompilasi Hukum Islam dan karenanya permohonan Pemohon agar diberi ijin untuk berikrar menjatuhkan talaknya terhadap Termohon dapat dikabulkan;

Menimbang, bahwa perkara ini termasuk bidang perkawinan, oleh karenanya berdasarkan Pasal 89 ayat (1) Undang-Undang Nomor 7 Tahun 1989 tentang Peradilan Agama sebagaimana telah diubah dengan Undang-Undang Nomor 3 Tahun 2006 dan perubahan kedua dengan Undang-Undang Nomor 50 Tahun 2009, maka seluruh biaya perkara ini dibebankan kepada Pemohon;

Mengingat segala peraturan perundang-undangan yang berlaku serta ketentuan hukum syara' yang berkaitan dengan perkara ini;

M E N G A D I L I

1. Menyatakan Termohon yang telah dipanggil dengan resmi dan patut untuk menghadap sidang, tidak hadir;
2. Mengabulkan permohonan Pemohon dengan verstek;
3. Memberi izin kepada Pemohon (XXX) untuk menjatuhkan talak satu raji terhadap Termohon (XXX) di depan sidang Pengadilan Agama Kuningan;
4. Membebankan Pemohon untuk membayar biaya perkara sejumlah Rp. 416.000,00 (empat ratus enam belas ribu rupiah)

Demikian Putusan ini dijatuhkan dalam permusyawaratan Majelis Hakim Pengadilan Agama Kuningan pada hari Selasa tanggal 22 Desember 2020 Masehi, bertepatan dengan tanggal 07 Jumadil Awal 1442 Hijriyah, oleh kami Drs. Sartino, S.H.,M.H. sebagai Ketua Majelis, Drs. M.G. Zulzamar, S.H., M.H.I. dan Dra. Arnetis masing-masing sebagai Hakim Anggota, yang dibacakan dalam sidang terbuka untuk umum oleh Ketua Majelis tersebut dengan dihadiri para Hakim Anggota dan Hj. N. Juriah, S.H sebagai Panitera Pengganti serta dihadiri oleh Pemohon tanpa hadirnya Termohon;

Ketua Majelis

Drs. Sartino, S.H.,M.H.

Disclaimer
Kepaniteraan Mahkamah Agung Republik Indonesia berusaha untuk selalu mencantumkan informasi paling kini dan akurat sebagai bentuk komitmen Mahkamah Agung untuk pelayanan publik, transparansi dan akuntabilitas pelaksanaan fungsi peradilan. Namun dalam hal-hal tertentu masih dimungkinkan terjadi permasalahan teknis terkait dengan akurasi dan keterkinian informasi yang kami sajikan, hal mana akan terus kami perbaiki dari waktu kewaktu.
Dalam hal Anda menemukan inakurasi informasi yang termuat pada situs ini atau informasi yang seharusnya ada, namun belum tersedia, maka harap segera hubungi Kepaniteraan Mahkamah Agung RI melalui :
Email : kepaniteraan@mahkamahagung.go.id Telp : 021-384 3348 (ext.318)

Hakim Anggota	Hakim Anggota
**Drs. M.G. Zulzamar, S.H., M.H.I.**	**Dra. Arnetis**

Panitera Pengganti

Hj. N. Juriah, S.H

Perincian Biaya Perkara :

1. Biaya Pendaftaran	:	Rp.	30.000,00
2. Biaya Proses	:	Rp.	50.000,00
3. Biaya Panggilan	:	Rp.	300.000,00
4. Biaya PNBP Panggilan	:	Rp.	20.000,00
5. Biaya Redaksi	:	Rp.	10.000,00
6. Biaya materai	:	Rp.	6.000,00
Jumlah		Rp.	416.000,00

(empat ratus enam belas ribu rupiah)

Dicatat disini :

- Amar putusan tersebut telah diberitahukan kepada Termohon pada tanggal
- Putusan tersebut telah mempunyai kekuatan hukum tetap sejak tanggal

Disclaimer
Kepaniteraan Mahkamah Agung Republik Indonesia berusaha untuk selalu mencantumkan informasi paling kini dan akurat sebagai bentuk komitmen Mahkamah Agung untuk pelayanan publik, transparansi dan akuntabilitas pelaksanaan fungsi peradilan. Namun dalam hal-hal tertentu masih dimungkinkan terjadi permasalahan teknis terkait dengan akurasi dan keterkinian informasi yang kami sajikan, hal mana akan terus kami perbaiki dari waktu kewaktu.
Dalam hal Anda menemukan inakurasi informasi yang termuat pada situs ini atau informasi yang seharusnya ada, namun belum tersedia, maka harap segera hubungi Kepaniteraan Mahkamah Agung RI melalui :
Email : kepaniteraan@mahkamahagung.go.id   Telp : 021-384 3348 (ext.318)
