# Supplementary material for: Classification of divorce causes during the COVID-19 pandemic using convolutional neural networks
Source: PeerJ Comput Sci. 2022 Jun 30;8:e998. doi: 10.7717/peerj-cs.998 (PMC9299239; doi:10.7717/peerj-cs.998)
Supplement: Supplemental Information 4 [file peerj-cs-08-998-s004.zip › Konflik Dataset/Data ke-26.pdf]

PUTUSAN

Nomor 1823/Pdt.G/2020/PA.Kng

بِسْــــــمِ اللهِ الرَّحْمٰنِ الرَّحِيْــــــمِ

DEMI KEADILAN BERDASARKAN KETUHANAN YANG MAHA ESA

Pengadilan Agama Kuningan yang memeriksa dan mengadili perkara perdata pada tingkat pertama dalam persidangan Majelis telah menjatuhkan putusan sebagai berikut dalam perkara antara :

Xxxx, umur 48 tahun, agama Islam, pendidikan S1, pekerjaan Guru SD, tempat kediaman di xxxx Lebakwangi Kabupaten Kuningan. Berdasarkan Surat Kuasa Khusus tertanggal 19 Agustus 2020 Penggugat memberikan kuasa kepada : **xxx** Advokat / Pengacara & Konsultan Hukum yang berkantor pada **Kantor Hukum "GMM & PARTNERS"** yang beralamat di Jln. Raya Perjuangan No. 38 Ancaran Kuningan Jawa Barat, yang terdaftar di Kepaniteraan Pengadilan Agama Kuningan nomor 897/Adv/VIII/2020 tanggal 24 Agustus 2020, sebagai Penggugat;

melawan

Xxxx, umur 49 tahun, agama Islam, pendidikan SLTA, pekerjaan Buruh, tempat kediaman di xxx Garawangi Kabupten Kuningan. Dalam hal ini Tergugat telah menguasakan kepada kuasa hukumnya **xxx**, Adalah **Advokat**, yang beralamat Kantor di **Kantor Hukum** "**RPE & Rekan** '' di Jalan Nusa Indah Raya No. 234 Ciporang Kabupaten Kuningan 45514 berdasarkan Surat Kuasa Khusus yang terdaftar di Kepaniteraan Pengadilan Agama Kuningan tertanggal 8 September 2020 nomor 973/Adv/IX/2020, sebagai Tergugat;

Pengadilan Agama tersebut;

Setelah mempelajari berkas perkara yang bersangkutan;

Disclaimer
Kepaniteraan Mahkamah Agung Republik Indonesia berusaha untuk selalu mencantumkan informasi paling kini dan akurat sebagai bentuk komitmen Mahkamah Agung untuk pelayanan publik, transparansi dan akuntabilitas pelaksanaan fungsi peradilan. Namun dalam hal-hal tertentu masih dimungkinkan terjadi permasalahan teknis terkait dengan akurasi dan keterkinian informasi yang kami sajikan, hal mana akan terus kami perbaiki dari waktu kewaktu.
Dalam hal Anda menemukan inakurasi informasi yang termuat pada situs ini atau informasi yang seharusnya ada, namun belum tersedia, maka harap segera hubungi Kepaniteraan Mahkamah Agung RI melalui :
Email : kepaniteraan@mahkamahagung.go.id   Telp : 021-384 3348 (ext.318)

Setelah mendengar keterangan Penggugat, Tergugat dan saksi-saksi di muka persidangan;

DUDUK PERKARA

Bahwa, Penggugat dengan surat gugatannya tertanggal 19 Agustus 2020 yang telah terdaftar di Kepaniteraan Pengadilan Agama Kuningan dibawah Nomor 1823/Pdt.G/2020/PA.Kng tanggal 24 Agustus 2020, telah mengajukan hal-hal sebagai berikut :

1. Bahwa pada hari Kamis tanggal 12 Agustus 2004 telah dilangsungkan perkawinan antara Penggugat dan Tergugat yang dilaksanakan menurut hukum dan sesuai dengan tuntunan ajaran Islam. Perkawinan tersebut telah dicatatkan di Kantor Urusan Agama (KUA) Kecamatan Lebakwangi Kabupaten Kuningan sebagaimana tercatat dalam Kutipan Akta Nikah Nomor. 560/45/VIII/2004, tertanggal 12 Agustus 2004;

2. Bahwa sesudah akad nikah Penggugat dan Tergugat telah hidup berumah tangga selama 16 tahun dan harmonisnya selama 11 tahun dengan memilih tempat tinggal di rumah kediaman milik bersama serta telah bergaul sebagaimana layaknya suami isteri dan sudah di karuniai 2 anak yang bernama :
   - xxx

3. Bahwa pada mulanya rumah tangga Penggugat dengan Tergugat dalam keadaan rukun dan harmonis, namun sejak awal bulan April 2014 rumah tangga Penggugat dengan Tergugat mulai goyah, sering terjadi perselisihan dan pertengkaran disebabkan karena Tergugat suka melakukan kekerasan dalam rumah tangga (KDRT) terhadap Penggugat, dan Tergugat juga tidak bertanggung jawab dalam hal memberikan nafkah, yaitu tidak memenuhi kebutuhan sehari-hari Penggugat;

4. Bahwa perselisihan dan pertengkaran tersebut berkelanjutan terus-menerus sehingga puncaknya sejak awal bulan Desember 2015 mengakibatkan Tergugat pergi meninggalkan rumah kediaman milik bersama

Disclaimer
Kepaniteraan Mahkamah Agung Republik Indonesia berusaha untuk selalu mencantumkan informasi paling kini dan akurat sebagai bentuk komitmen Mahkamah Agung untuk pelayanan publik, transparansi dan akuntabilitas pelaksanaan fungsi peradilan. Namun dalam hal-hal tertentu masih dimungkinkan terjadi permasalahan teknis terkait dengan akurasi dan keterkinian informasi yang kami sajikan, hal mana akan terus kami perbaiki dari waktu kewaktu.
Dalam hal Anda menemukan inakurasi informasi yang termuat pada situs ini atau informasi yang seharusnya ada, namun belum tersedia, maka harap segera hubungi Kepaniteraan Mahkamah Agung RI melalui :
Email : kepaniteraan@mahkamahagung.go.id Telp : 021-384 3348 (ext.318)

ke rumah orang tuanya di alamat tersebut di atas dan telah berpisah tempat tinggal sampai dengan sekarang selama 4 tahun 8 bulan ;

5. Bahwa adanya perselisihan dan pertengkaran tersebut mengakibatkan rumah tangga Penggugat dan Tergugat tidak ada kebahagiaan lahir batin dan tidak ada harapan untuk kembali membina rumah tangga;

6. Bahwa pihak keluarga sudah berupaya untuk mendamaikan antara Penggugat dengan Tergugat, namun tidak berhasil karena Penggugat tidak mau melanjutkan ikatan perkawinan dengan Tergugat;

Berdasarkan dalil dan alasan di atas, maka dengan ini Penggugat memohon kepada Ketua Pengadilan Agama Kuningan cq. Majelis Hakim yang memeriksa perkara ini untuk berkenan memberikan putusan yang amarnya sebagai berikut:

PRIMAIR :

1. Mengabulkan Gugatan Penggugat untuk seluruhnya ;

2. Menjatuhkan talak satu ba'in sughro Tergugat **(Xxxx)** terhadap Penggugat **(Xxxx)** ;

3. Membebankan biaya perkara kepada Penggugat;

SUBSIDAIR :

Atau apabila Majelis Hakim Pengadilan Agama Kuningan berpendapat lain mohon putusan yang seadil-adilnya (ex aequo et bono)

Bahwa, pada hari sidang yang telah ditetapkan, Penggugat dan Tergugat datang menghadap di muka persidangan;

Bahwa, Majelis Hakim telah berusaha menasehati dan mendamaikan Penggugat dan Tergugat agar dapat membina kembali rumah tangganya secara rukun dan bahkan Majelis Hakim telah memerintahkan kepada Penggugat dan Tergugat agar melaksanakan mediasi dalam rangka perdamaian sebagaimana surat penetapan mediasi bertanggal 06 Oktober 2020, akan tetapi tidak berhasil;

Disclaimer
Kepaniteraan Mahkamah Agung Republik Indonesia berusaha untuk selalu mencantumkan informasi paling kini dan akurat sebagai bentuk komitmen Mahkamah Agung untuk pelayanan publik, transparansi dan akuntabilitas pelaksanaan fungsi peradilan. Namun dalam hal-hal tertentu masih dimungkinkan terjadi permasalahan teknis terkait dengan akurasi dan keterkinian informasi yang kami sajikan, hal mana akan terus kami perbaiki dari waktu kewaktu.
Dalam hal Anda menemukan inakurasi informasi yang termuat pada situs ini atau informasi yang seharusnya ada, namun belum tersedia, maka harap segera hubungi Kepaniteraan Mahkamah Agung RI melalui :
Email : kepaniteraan@mahkamahagung.go.id Telp : 021-384 3348 (ext.318)

Bahwa, selanjutnya pemeriksaan perkara ini dimulai dengan membacakan surat gugatan Penggugat yang maksud dan isinya tetap dipertahankan oleh Penggugat;

Bahwa, terhadap gugatan Penggugat tersebut, Tergugat telah memberikan jawaban secara lisan yang pada pokoknya Tergugat menerima dan membenarkan apa yang didalilkan oleh Penggugat tersebut dan bersedia bercerai;

Bahwa, Penggugat dalam repliknya mengatakan tetap pada gugatannya, demikian juga Tergugat dalam dupliknya mengatakan tetap berpegang pada jawabannya;

Bahwa, untuk membuktikan dalil gugatannya, Penggugat telah mengajukan alat bukti surat berupa :

1. Fotokopi Kartu Tanda Penduduk an. Penggugat, bukti tersebut telah bermaterai cukup dan telah dicocokkan dengan aslinya yang ternyata sesuai, lalu oleh Ketua Majelis diparaf dan diberi tanda (P.1);
2. Fotokopi Buku Kutipan Akta Nikah Nomor 560/45/VIII/2004 tanggal 12 Agustus 2004, yang dikeluarkan oleh Kepala Kantor Urusan Agama (KUA) Kecamatan Lebakwangi Kabupaten Kuningan, bukti tersebut telah bermeterai cukup dan telah dicocokkan dengan aslinya yang ternyata sesuai, lalu oleh Ketua Majelis diberi tanda (P.2);
3. Asli Surat Keputusan Bupati Kuningan nomor 474.12/KPTS.674 - BKPSDM/2020 Tentang Pemberian Izin Perceraian Kepada Sdr. Titin Sumartini, S.Pd. dengan Sdr. Lukman Mulyadi tanggal 8 Desember 2020, diberi tanda P-3;

Bahwa, di samping itu, Penggugat juga telah mengajukan alat bukti saksi yaitu :

1. Saksi 1 :
   - Bahwa saksi kenal dengan Penggugat dan Tergugat;
   - Bahwa semula rumah tangga Penggugat dengan Tergugat rukun dan harmonis di di rumah milik bersama akan tetapi sejak sejak tahun 2014 rumah tangganya sudah tidak harmonis lagi, sering terjadi perselisihan dan pertengkaran;

Disclaimer
Kepaniteraan Mahkamah Agung Republik Indonesia berusaha untuk selalu mencantumkan informasi paling kini dan akurat sebagai bentuk komitmen Mahkamah Agung untuk pelayanan publik, transparansi dan akuntabilitas pelaksanaan fungsi peradilan. Namun dalam hal-hal tertentu masih dimungkinkan terjadi permasalahan teknis terkait dengan akurasi dan keterkinian informasi yang kami sajikan, hal mana akan terus kami perbaiki dari waktu kewaktu.
Dalam hal Anda menemukan inakurasi informasi yang termuat pada situs ini atau informasi yang seharusnya ada, namun belum tersedia, maka harap segera hubungi Kepaniteraan Mahkamah Agung RI melalui :
Email : kepaniteraan@mahkamahagung.go.id Telp : 021-384 3348 (ext.318)

- Bahwa penyebabnya karena Tergugat tidak bertanggung jawab masalah nafkah;
- Bahwa sejak 4 tahun 8 bulan yang lalu antara Penggugat dan Tergugat telah berpisah tempat tinggal dan tidak pernah berkumpul kembali;
- Bahwa saksi pernah berusaha menasehati Penggugat, akan tetapi tidak berhasil;
- Bahwa saksi sudah tidak sanggup lagi untuk merukunkan kembali;

Bahwa, terhadap keterangan saksi tersebut Tergugat membenarkannya;

2. Saksi 2 :

- Bahwa saksi kenal dengan Penggugat dan Tergugat;
- Bahwa awalnya rumah tangga Penggugat dan Tergugat rukun dan harmonis akan tetapi sejak bulan sejak tahun 2014 rumah tangganya sudah tidak harmonis lagi sering terjadi perselisihan dan pertengkaran;
- Bahwa penyebab perselisihan dan pertengkaran tersebut karena karena Tergugat tidak bertanggung jawab masalah nafkah;
- Bahwa sejak 4 tahun 8 bulan Penggugat dan Tergugat telah berpisah tempat tinggal dan tidak pernah berkumpul kembali;
- Bahwa saksi sudah tidak sanggup lagi untuk merukunkannya;

Bahwa, terhadap keterangan saksi tersebut Tergugat membenarkannya;

Bahwa, Tergugat mengatakan tidak akan mengajukan alat-alat buktinya termasuk alat bukti saksi dan telah mencukupkan alat-alat bukti tersebut sebagaimana telah diajukan oleh Penggugat;

Bahwa Penggugat dan Tergugat dalam kesimpulannya mengatakan tidak akan mengajukan suatu apapun dan memohon kepada Majelis Hakim agar menjatuhkan putusannya;

Bahwa, untuk mempersingkat uraian pertimbangan ini ditunjuk hal-hal sebagaimana tercantum dalam berita acara persidangan perkara ini;

PERTIMBANGAN HUKUM

Menimbang, bahwa maksud dan tujuan gugatan Penggugat adalah sebagaimana terurai diatas;

Menimbang, bahwa sesuai dengan maksud dan ketentuan Pasal 82 Undang-Undang Nomor 7 Tahun 1989 tentang Peradilan Agama sebagaimana telah diubah dengan Undang-Undang Nomor 3 Tahun 2006 dan perubahan

Disclaimer
Kepaniteraan Mahkamah Agung Republik Indonesia berusaha untuk selalu mencantumkan informasi paling kini dan akurat sebagai bentuk komitmen Mahkamah Agung untuk pelayanan publik, transparansi dan akuntabilitas pelaksanaan fungsi peradilan. Namun dalam hal-hal tertentu masih dimungkinkan terjadi permasalahan teknis terkait dengan akurasi dan keterkinian informasi yang kami sajikan, hal mana akan terus kami perbaiki dari waktu kewaktu.
Dalam hal Anda menemukan inakurasi informasi yang termuat pada situs ini atau informasi yang seharusnya ada, namun belum tersedia, maka harap segera hubungi Kepaniteraan Mahkamah Agung RI melalui :
Email : kepaniteraan@mahkamahagung.go.id   Telp : 021-384 3348 (ext.318)

kedua dengan Undang-Undang Nomor 50 Tahun 2009, Majelis Hakim telah berusaha maksimal menasehati dan mendamaikan Penggugat dan Tergugat, agar Penggugat dan Tergugat dapat membina kembali rumah tangganya secara rukun, akan tetapi usaha tersebut tidak berhasil;

Menimbang, bahwa disamping itu untuk memenuhi Pasal 7 ayat (1) PERMA Nomor 1 Tahun 2016, Majelis Hakim telah memerintahkan kepada Penggugat dan Tergugat agar melaksanakan mediasi dalam rangka perdamaian akan tetapi tidak berhasil, sebagaimana laporan mediator Drs. Akhmad Jalaludin tanggal 06 Oktober 2020;

Menimbang, bahwa terlebih dahulu berdasarkan alat bukti (P.1) Majelis Hakim harus menyatakan bahwa oleh karena Penggugat berdomisili di wilayah hukum Pengadilan Agama Kuningan, maka gugatan Penggugat dapat diterima untuk diperiksa dan diadili;

Menimbang, bahwa selanjutnya berdasarkan alat bukti (P.2) yang telah memenuhi syarat formil dan materil, harus dinyatakan terbukti bahwa hubungan hukum antara Penggugat dan Tergugat telah terikat dalam perkawinan yang sah sebagaimana diatur dalam Pasal 2 Undang-Undang Nomor 1 Tahun 1974;

Menimbang, bahwa Penggugat sebagai Pegawai Negeri Sipil telah memperoleh izin untuk melakukan perceraian dari pejabat yang berwenang, sesuai bukti P.3, yaitu Surat Keputusan Bupati Kuningan nomor 474.12/KPTS.674 - BKPSDM/2020 Tentang Pemberian Izin Perceraian Kepada Sdr. Titin Sumartini, S.Pd. dengan Sdr. Lukman Mulyadi tanggal 8 Desember 2020. Hal sesuai Pasal 3 Peraturan Pemerintah nomor 10 tahun 1983 tentang Izin Perkawinan dan Perceraian bagi Pegawai Negeri Sipil, sebagaimana telah diubah dengan Peraturan Pemerintah nomor 45 tahun 1990

Menimbang, bahwa pada pokoknya Penggugat telah mendasarkan gugatan cerainya terhadap Tergugat dengan alasan sejak bulan sejak tahun 2014 rumah tangga Penggugat dengan Tergugat sudah tidak harmonis, sering terjadi perselisihan dan pertengkaran yang mencapai puncaknya pada bulan Bulan Desember 2015 sehingga tidak ada harapan akan hidup rukun lagi dalam membina rumah tangga, dimana penyebabnya karena Tergugat tidak bertanggung jawab dalam hal memberi nafkah kepada Penggugat sehingga sejak 4 tahun 8 bulan Penggugat dan Tergugat telah pisah tempat tinggal;

Disclaimer
Kepaniteraan Mahkamah Agung Republik Indonesia berusaha untuk selalu mencantumkan informasi paling kini dan akurat sebagai bentuk komitmen Mahkamah Agung untuk pelayanan publik, transparansi dan akuntabilitas pelaksanaan fungsi peradilan. Namun dalam hal-hal tertentu masih dimungkinkan terjadi permasalahan teknis terkait dengan akurasi dan keterkinian informasi yang kami sajikan, hal mana akan terus kami perbaiki dari waktu kewaktu.
Dalam hal Anda menemukan inakurasi informasi yang termuat pada situs ini atau informasi yang seharusnya ada, namun belum tersedia, maka harap segera hubungi Kepaniteraan Mahkamah Agung RI melalui :
Email : kepaniteraan@mahkamahagung.go.id Telp : 021-384 3348 (ext.318)

Menimbang, bahwa Tergugat dalam jawabannya telah mengakui dan membenarkan apa yang didalilkan oleh Penggugat tersebut;

Menimbang, bahwa terhadap jawaban Tergugat sebagai mana tersebut di atas, Majelis Hakim akan menjadikannya sebagai bukti yang mengikat dalam perkara ini (Vide Pasal 174 HIR);

Menimbang, bahwa di muka persidangan Penggugat telah mengajukan dua orang saksi yang dari keterangannya saling bersesuaian, sehingga keterangan para saksi tersebut dapat diterima untuk dipertimbangkan;

Menimbang, bahwa dengan didasarkan pada jawaban Tergugat yang kemudian dihubungkan dengan keterangan para saksi, Majelis Hakim telah dapat menemukan fakta hukum sebagai berikut :

- Bahwa Penggugat dan Tergugat adalah suami isteri yang sah;
- Bahwa semula rumah tangga Penggugat dan Tergugat rukun dan harmonis;
- Bahwa sejak bulan sejak tahun 2014 keadaan rumah tangganya tersebut sudah tidak harmonis lagi, sering terjadi perselisihan dan pertengkaran;
- Bahwa penyebabnya karena Tergugat tidak bertanggung jawab masalah nafkah;
- Bahwa sejak 4 tahun 8 bulan yang lalu Penggugat dan Tergugat telah berpisah tempat tinggal dan tidak pernah berkumpul kembali;

Menimbang, bahwa berdasarkan fakta hukum tersebut di atas, harus dinyatakan terbukti bahwa dalam kehidupan rumah tangga Penggugat dan Tergugat sudah tidak harmonis lagi, telah terjadi perselisihan dan pertengkaran yang mengakibatkan antara keduanya telah berpisah tempat tinggal dan tidak pernah berkumpul kembali dalam satu rumah tangga;

Menimbang, bahwa disamping itu para saksi sudah pernah berusaha menasehati Penggugat dan mengatakan tidak ada kesanggupan untuk dapat merukunkannya kembali, sehingga Majelis Hakim berpendapat bahwa antara Penggugat dan Tergugat sudah sulit dan tidak ada harapan untuk dapat hidup rukun kembali di dalam membina rumah tangganya;

Menimbang, bahwa menurut ketentuan Pasal 19 huruf (f) PP Nomor 9 tahun 1975 jo. Pasal 116 huruf (f) Kompilasi Hukum Islam, perceraian dapat terjadi apabila “antara suami dan isteri terus menerus terjadi perselisihan dan

Disclaimer
Kepaniteraan Mahkamah Agung Republik Indonesia berusaha untuk selalu mencantumkan informasi paling kini dan akurat sebagai bentuk komitmen Mahkamah Agung untuk pelayanan publik, transparansi dan akuntabilitas pelaksanaan fungsi peradilan. Namun dalam hal-hal tertentu masih dimungkinkan terjadi permasalahan teknis terkait dengan akurasi dan keterkinian informasi yang kami sajikan, hal mana akan terus kami perbaiki dari waktu kewaktu.
Dalam hal Anda menemukan inakurasi informasi yang termuat pada situs ini atau informasi yang seharusnya ada, namun belum tersedia, maka harap segera hubungi Kepaniteraan Mahkamah Agung RI melalui :
Email : kepaniteraan@mahkamahagung.go.id   Telp : 021-384 3348 (ext.318)

pertengkaran dan tidak ada harapan akan hidup rukun kembali dalam membina rumah tangga";

Menimbang, bahwa demikian juga menurut Mahkamah Agung sebagaimana tertuang dalam putusannya No. 38/K/AG/1990 tanggal 5 Oktober 1990 yang kemudian diambil alih menjadi pendapat Majelis Hakim bahwa apabila ternyata adanya perselisihan sebagaimana dimaksud Pasal 19 huruf (f) PP No. 9 Tahun 1975, maka hal itu semata-mata ditujukan kepada perkawinannya itu sendiri tanpa mempersoalkan siapa yang salah dalam hal terjadinya perselisihan yang mengakibatkan tidak ada harapan akan hidup rukun lagi, karena pernikahan adalah suatu perjanjian yang suci (mitsaqan gholidzon/Pasal 2 KHI) yang untuk memutuskannya tidak boleh diukur dengan kesalahan salah satu pihak, sehingga apabila perkawinan itu telah pecah berarti hati kedua belah pihak telah pecah pula;

Menimbang, bahwa terlepas dari peristiwa atau keadaan yang menjadi penyebab terjadinya perselisihan yang terjadi antara Penggugat dan Tergugat, yang jelas Majelis Hakim telah dapat menemukan fakta hukum bahwa perselisihan antara Penggugat dan Tergugat tersebut telah sedemikian rupa, rumah tangga Penggugat dan Tergugat sudah hancur dan tidak mungkin akan dapat hidup rukun kembali dalam membina rumah tangga;

Menimbang, bahwa dengan melihat kondisi rumah tangga Penggugat dan Tergugat tersebut, jelas tidak mungkin akan terwujud kehidupan rumah tangga yang bahagia dan kekal lahir bathin sebagaimana yang menjadi tujuan perkawinan dalam Pasal 1 Undang-Undang Nomor 1 Tahun 1974 jo. Pasal 3 KHI, dan membiarkan suasana rumah tangga yang demikian, justru akan menimbulkan mudlarat bagi kedua belah pihak, oleh karena itu perceraian dipandang jalan yang terbaik dan lebih maslahat bagi keduanya;

Menimbang, bahwa Majelis Hakim perlu mengetengahkan dalil dalam kitab Al-Muhadzab juz II halaman 82 yang kemudian diambil alih menjadi pendapat Majelis Hakim *Artinya: "Disaat istri telah memuncak ketidaksukaan terhadap suaminya, maka Hakim diperkenankan menjatuhkan talak (suami) nya dengan talak satu ;*

Menimbang, bahwa berdasarkan pertimbangan tersebut diatas, maka gugatan Penggugat telah memenuhi alasan perceraian sebagaimana diatur

Disclaimer
Kepaniteraan Mahkamah Agung Republik Indonesia berusaha untuk selalu mencantumkan informasi paling kini dan akurat sebagai bentuk komitmen Mahkamah Agung untuk pelayanan publik, transparansi dan akuntabilitas pelaksanaan fungsi peradilan. Namun dalam hal-hal tertentu masih dimungkinkan terjadi permasalahan teknis terkait dengan akurasi dan keterkinian informasi yang kami sajikan, hal mana akan terus kami perbaiki dari waktu kewaktu.
Dalam hal Anda menemukan inakurasi informasi yang termuat pada situs ini atau informasi yang seharusnya ada, namun belum tersedia, maka harap segera hubungi Kepaniteraan Mahkamah Agung RI melalui :
Email : kepaniteraan@mahkamahagung.go.id Telp : 021-384 3348 (ext.318)

dalam Pasal 39 ayat (2) Undang-Undang Nomor 1 Tahun 1974, jo. Pasal 19 huruf (f) Peraturan Pemerintah Nomor 9 Tahun 1975, jo. Pasal 116 huruf (f) Kompilasi Hukum Islam dan karenanya gugatan Penggugat dapat dikabulkan dengan menjatuhkan talak satu ba'in sughro Tergugat terhadap Penggugat;

Menimbang, bahwa perkara ini termasuk bidang perkawinan, oleh karenanya berdasarkan Pasal 89 ayat (1) Undang-Undang Nomor 7 Tahun 1989 tentang Peradilan Agama sebagaimana telah diubah dengan Undang-Undang Nomor 3 Tahun 2006 dan perubahan kedua dengan Undang-Undang Nomor 50 Tahun 2009, maka seluruh biaya perkara ini dibebankan kepada Penggugat;

Mengingat segala peraturan perundang-undangan yang berlaku serta ketentuan hukum syara' yang berkaitan dengan perkara ini;

M E N G A D I L I

1. Mengabulkan gugatan Penggugat;

2. Menjatuhkan talak satu ba'in shughra Tergugat (**Xxxx**) terhadap Penggugat (**Xxxx**);

3. Membebankan Penggugat untuk membayar biaya perkara sejumlah Rp306.000,00 (tiga ratus enam ribu rupiah)

Demikian Putusan ini dijatuhkan dalam permusyawaratan Majelis Hakim Pengadilan Agama Kuningan pada hari Selasa tanggal 22 Desember 2020 Masehi, bertepatan dengan tanggal 2 Jumadil Ula 1442 Hijriyah, oleh kami Drs. Sartino, S.H.,M.H. sebagai Ketua Majelis, Drs. M.G. Zulzamar, S.H., M.H.I. dan Dra. Arnetis masing-masing sebagai Hakim Anggota, yang dibacakan dalam sidang terbuka untuk umum oleh Ketua Majelis tersebut dengan dihadiri para Hakim Anggota dan Hj. N. Juriah, S.H sebagai Panitera Pengganti serta dihadiri oleh Kuasa Penggugat dan Kuasa Tergugat;

Ketua Majelis

Drs. Sartino, S.H.,M.H.

Disclaimer
Kepaniteraan Mahkamah Agung Republik Indonesia berusaha untuk selalu mencantumkan informasi paling kini dan akurat sebagai bentuk komitmen Mahkamah Agung untuk pelayanan publik, transparansi dan akuntabilitas pelaksanaan fungsi peradilan. Namun dalam hal-hal tertentu masih dimungkinkan terjadi permasalahan teknis terkait dengan akurasi dan keterkinian informasi yang kami sajikan, hal mana akan terus kami perbaiki dari waktu kewaktu.
Dalam hal Anda menemukan inakurasi informasi yang termuat pada situs ini atau informasi yang seharusnya ada, namun belum tersedia, maka harap segera hubungi Kepaniteraan Mahkamah Agung RI melalui :
Email : kepaniteraan@mahkamahagung.go.id   Telp : 021-384 3348 (ext.318)

Hakim Anggota Hakim Anggota

Drs. M.G. Zulzamar, S.H., M.H.I. **Dra. Arnetis**

Panitera Pengganti

Hj. N. Juriah, S.H

Perincian Biaya Perkara :

1. Biaya Pendaftaran	:	Rp.	30.000,00
2. Biaya Proses	:	Rp.	50.000,00
3. Biaya Panggilan	:	Rp.	190.000,00
4. Biaya PNBP Panggilan	:	Rp.	20.000,00
5. Biaya Redaksi	:	Rp.	10.000,00
6. Biaya materai	:	Rp.	6.000,00
Jumlah		Rp.	306.000,00

(tiga ratus enam ribu rupiah)

Disclaimer
Kepaniteraan Mahkamah Agung Republik Indonesia berusaha untuk selalu mencantumkan informasi paling kini dan akurat sebagai bentuk komitmen Mahkamah Agung untuk pelayanan publik, transparansi dan akuntabilitas pelaksanaan fungsi peradilan. Namun dalam hal-hal tertentu masih dimungkinkan terjadi permasalahan teknis terkait dengan akurasi dan keterkinian informasi yang kami sajikan, hal mana akan terus kami perbaiki dari waktu kewaktu.
Dalam hal Anda menemukan inakurasi informasi yang termuat pada situs ini atau informasi yang seharusnya ada, namun belum tersedia, maka harap segera hubungi Kepaniteraan Mahkamah Agung RI melalui :
Email : kepaniteraan@mahkamahagung.go.id Telp : 021-384 3348 (ext.318)
