# Supplementary material for: Classification of divorce causes during the COVID-19 pandemic using convolutional neural networks
Source: PeerJ Comput Sci. 2022 Jun 30;8:e998. doi: 10.7717/peerj-cs.998 (PMC9299239; doi:10.7717/peerj-cs.998)
Supplement: Supplemental Information 4 [file peerj-cs-08-998-s004.zip › Konflik Dataset/Data ke-27.pdf]

Pdt.I.C.1

P U T U S A N

Nomor 87/Pdt.G/2020/PN SDA

DEMI KEADILAN BERDASARKAN KETUHANAN YANG MAHA ESA

Pengadilan Negeri Sidoarjo yang memeriksa dan memutus perkara perdata pada tingkat pertama, telah menjatuhkan putusan sebagai berikut dalam perkara gugatan antara:

Penggugat, bertempat tinggal di Griyo Wage Asri II Blok AG / 04 - Sidoarjo , sebagai **Penggugat I**;

Lawan:

Tergugat, bertempat tinggal di Griyo Wage Asri II Blok AG / 04 - Sidoarjo , sebagai **Tergugat I**;

Pengadilan Negeri tersebut;

Setelah membaca berkas perkara beserta surat-surat yang bersangkutan;

Setelah mendengar kedua belah pihak yang berperkara;

TENTANG DUDUK PERKARA

Menimbang, bahwa Penggugat dengan surat gugatan tanggal 17 Maret 2020 yang diterima dan didaftarkan di Kepaniteraan Pengadilan Negeri Sidoarjo pada tanggal 18 Maret 2020 dalam Register Nomor 87/Pdt.G/2020/PN SDA, telah mengajukan gugatan sebagai berikut:

1. Bahwa PENGGUGAT dan TERGUGAT adalah suami istri sah telah melangsungkan Perkawinan p ada tanggal 08 - 08 - 2008 di Kota Surabaya (sesuai kutipan Akta Perkawinan nomor : 1460/WNI/2008 Tanggal 11 - 08 - 2008 yang telah diterbitkan oleh kantor Dinas Kependudukan Dan Pencatatan Sipil Kota Surabaya
2. Bahwa setelah melaksanakan perkawinan antara PENGGUGAT dan TERGUGAT tinggal bersama di Ploso IX B / 4 kemudian, Penggugat dan anaknya tinggal di Ploso IX B / 4 Sedangkan Tergugat bertempat tinggal di Ploso IX B / 4 hingga gugatan cerai ini diajukan di Pengadilan Negeri

Disclaimer
Kepaniteraan Mahkamah Agung Republik Indonesia berusaha untuk selalu mencantumkan informasi paling kini dan akurat sebagai bentuk komitmen Mahkamah Agung untuk pelayanan publik, transparansi dan akuntabilitas pelaksanaan fungsi peradilan. Namun dalam hal-hal tertentu masih dimungkinkan terjadi permasalahan teknis terkait dengan akurasi dan keterkinian informasi yang kami sajikan, hal mana akan terus kami perbaiki dari waktu kewaktu.
Dalam hal Anda menemukan inakurasi informasi yang termuat pada situs ini atau informasi yang seharusnya ada, namun belum tersedia, maka harap segera hubungi Kepaniteraan Mahkamah Agung RI melalui :
Email : kepaniteraan@mahkamahagung.go.id   Telp : 021-384 3348 (ext.318)

Sidoarjo:

3. Bahwa dari perkawinan antara Penggugat dan Tergugat tersebut telah dikaruniai anak Perempuan yang belum dewasa nama : Gitarja Maheswari Emmylea Andhiny lahir di Surabaya tanggal : 07 - 11 - 2011.
4. Bahwa pada awal mula Perkawinan antara Penggugat dan Tergugat baik-baik saja akan tetapi sejak tahun 2017 antara Penggugat dan Tergugat sudah mulai sering timbul perselisihan-perselisihan dan pertengkaran-pertengkaran yang penyebabnya:
   - Financial
   - Tidak di nafkahi
5. Bahwa tujuan perkawinan adalah untuk membentuk keluarga yang bahagia dan kekal berdasarkan pada Ketuhanan Yang Maha Esa tidak dapat dipertahankan lagi antara PENGGUGAT dan TERGUGAT dikarenakan sudah tidak ada lagi kecocokan dan keharmonisan rumah tangga dan Tergugat sering minta cerai kepada Penggugat.
6. Tergugat tersebut pada puncaknya antara Penggugat dan Tergugat telah pisah tempat tinggal yakni Penggugat dan anaknya bertempat tinggal di Jl.Ploso IX B / 4 sedangkan Tergugat bertempat tinggal di Jl Pondok Wisata Estate G/11 Malang, hingga gugatan cerai ini diajukan di Pengadilan Negeri Sidoarjo;
7. Bahwa antara Penggugat dan Tergugat sejak sekitar bulan Januari Sudah tidak pernah melakukan hubungan suami istri hingga gugatan cerai dan hak asuh ini diajukan di Pengadilan Negeri Sidoarjo;
8. Bahwa akibat dari itu semua PENGGUGAT sangat menderita baik lahir maupun batin dan PENGGUGAT sudah tidak kuat lagi berumah tangga dengan Tergugat dan PENGGUGAT sudah tidak ingin lagi untuk mempertahankan mahligai perkawinan dengan TERGUGAT:
9. Bahwa PENGGUGAT selaku Ibu kandung dari : Gitarja Maheswari Emmylea Andhiny perempuan lahir ditanggal 07 - 08 - 2011 tidak keberatan anak PENGGUGATdan TERGUGAT tersebut hak asuh pada PENGGUGAT akan tetapi TERGUGAT selaku ayah kandungnya sewaktu-waktu tetap diperbolehkan untuk menjenguk dan mengajak anak tersebut jalan-jalan dari

Disclaimer
Kepaniteraan Mahkamah Agung Republik Indonesia berusaha untuk selalu mencantumkan informasi paling kini dan akurat sebagai bentuk komitmen Mahkamah Agung untuk pelayanan publik, transparansi dan akuntabilitas pelaksanaan fungsi peradilan. Namun dalam hal-hal tertentu masih dimungkinkan terjadi permasalahan teknis terkait dengan akurasi dan keterkinian informasi yang kami sajikan, hal mana akan terus kami perbaiki dari waktu kewaktu.
Dalam hal Anda menemukan inakurasi informasi yang termuat pada situs ini atau informasi yang seharusnya ada, namun belum tersedia, maka harap segera hubungi Kepaniteraan Mahkamah Agung RI melalui :
Email : kepaniteraan@mahkamahagung.go.id   Telp : 021-384 3348 (ext.318)

rumah PENGGUGAT untuk beberapa waktu.

10. Bahwa PENGGUGAT pada tanggal 10-03-2020 membuat surat pernyataan tidak keberatan untuk bercerai dengan Penggugat:

Maka berdasarkan hal-hal tersebut di atas, PENGGUGAT dengan hormat mohon kehadapan Ketua Pengadilan Negeri Sidoarjo berkenan untuk memanggil para pihak guna pemeriksaan perkara Gugatan Perceraian dan hak asuh anak tersebut dan selanjutnya memberi putusan yang amarnya sebagai berikut:

1. Mengabulkan gugatan PENGGUGAT seluruhnya:
2. Menyatakan bahwa Perkawinan antara PENGGUGAT dan TERGUGAT yang telah dilaksanakan pada tanggal 08 - 08 - 2008 di Kota Surabaya (sesuai kutipan Akta Perkawinan nomor : 1460/WNI/2008 tanggal 11 - 08 - 2008yangtelah diterbitkan oleh kantor Dinas Kependudukan Dan Pencatatan Sipil Kota Surabaya putus karena perceraian dengan segala akibat hukumnya;
3. Menyatakan PENGGUGAT sebagai pemegang Hak asuh atas anak TERGUGAT yang belum dewasa bernama Gitarja Maheswari Emmylea Andhiny perempuan lahir di Surabaya tanggal 07 - 08 - 2008 sejak perkara ini mempunyai kekuatan hukum tetap hingga anak tersebut dewasa dengan mengijinkan dan tidak menghalangi TERGUGAT sewaktu-waktu untuk menjenguk dan mengajak anak tersebut untuk pergi jala-jalan dan dari rumah Penggugat.
4. Memerintahkan para pihak untuk mengirimkan/menyerahkan salinan Putusan perkara ini yang telah berkekuatan hukum tetap kepada pegawai pencatat pada Kantor Dinas Pendaftaran Penduduk dan Pencatatan Sipil kota Surabaya untuk didaftarkan/dicatatkan adanya perceraian ini ke dalam register yang sedang berjalan dengan mengeluarkan kutipan/turunannya.
5. Menetapkan beaya perkara yang timbul dalam perkara ini menurut hukum;

Atau : Apabila Pengadilan Negeri Sidoarjo berpendapat lain

Disclaimer
Kepaniteraan Mahkamah Agung Republik Indonesia berusaha untuk selalu mencantumkan informasi paling kini dan akurat sebagai bentuk komitmen Mahkamah Agung untuk pelayanan publik, transparansi dan akuntabilitas pelaksanaan fungsi peradilan. Namun dalam hal-hal tertentu masih dimungkinkan terjadi permasalahan teknis terkait dengan akurasi dan keterkinian informasi yang kami sajikan, hal mana akan terus kami perbaiki dari waktu kewaktu.
Dalam hal Anda menemukan inakurasi informasi yang termuat pada situs ini atau informasi yang seharusnya ada, namun belum tersedia, maka harap segera hubungi Kepaniteraan Mahkamah Agung RI melalui :
Email : kepaniteraan@mahkamahagung.go.id   Telp : 021-384 3348 (ext.318)

Maka : Mohon putusan yang adil dan bijaksana.

Menimbang, bahwa pada hari persidangan yang telah ditentukan, untuk Penggugat hadir sendiri, sedangkan Tergugat tidak pernah hadir dipersidangan walaupun telah dipanggil secara patut seta tidak menunjuk wakilnya yang sah;

Menimbang, bahwa oleh karena itu pemeriksaan perkara dilanjutkan dengan pembacaan surat gugatan yang isinya tetap dipertahankan oleh Penggugat;

Menimbang, bahwa terhadap gugatan Penggugat , Tergugat tidak mengajukan jawaban;

Menimbang, bahwa untuk membukikan Dalil-dalil Gugagatannya, Penggugat telah mengajukan Surat-Surat sebagai berikut :

1. Fotocopy KTP an. Penggugat HENY MERRYYANTI WENNY, diberi tanda P-1;
2. Fotocopy KK an YUDI SETIAWAN, dibeti tanda P-2;
3. Fotocopy Kutipan Akte Perkawinan an. Yudhi Setiawan dan HENY MERRYYANTI WENNY, diberi tanda P-3;
4. Fotocopy Kutipan Akta Kelahiran an. Gitarja Maheswari Emmylea Andhiny, diberi tanda P-4;

Menimbang, bahwa selain Surat-surat juga telah diajukan dua orang saksi yang masing-masing nenerangkan sebagai berikut :

Disclaimer
Kepaniteraan Mahkamah Agung Republik Indonesia berusaha untuk selalu mencantumkan informasi paling kini dan akurat sebagai bentuk komitmen Mahkamah Agung untuk pelayanan publik, transparansi dan akuntabilitas pelaksanaan fungsi peradilan. Namun dalam hal-hal tertentu masih dimungkinkan terjadi permasalahan teknis terkait dengan akurasi dan keterkinian informasi yang kami sajikan, hal mana akan terus kami perbaiki dari waktu kewaktu.
Dalam hal Anda menemukan inakurasi informasi yang termuat pada situs ini atau informasi yang seharusnya ada, namun belum tersedia, maka harap segera hubungi Kepaniteraan Mahkamah Agung RI melalui :
Email : kepaniteraan@mahkamahagung.go.id Telp : 021-384 3348 (ext.318)

1. **ETIK**

- Bahwa saksi kenal dengan Penggugat dan Tergugat karena masih ada hubungan famili dengan Penggugat yaitu sepupu;
- Bahwa saksi bersama Penggugat sering saling curhat masalah kehidupan rumah tangganya;
- Bahwa Penggugat dan Tergugat adalah suami istri sejak tahun 2008, yang waktu perkawinannya saksi ikut menghadiri di Gereja Kristen Residen Sudirman Surabaya;
- Bahwa perkawinan Penggugat dengan Tergugat telah dikaruniai seorang anak perempuan bernama Maheswari Emmylea Andhiny ;
- Bahwa anak tersebut masih belum dewasa dan selama ini selalu ikut ibunya;
- Bahwa pada awalnya hubungan suami istri tersebut baik-baik saja, tetapi belakangan ini sudah pisah rumah sudah cukup lama sedangkan anaknya ikut dengan ibunya;
- Bahwa saksi pernah melihat mereka bertengkar hebat, tetapi saksi tidak mengerti sebab apa mereka bertengkar;
- Bahwa Tergugat sampai sekarang tidak diketahui lagi dimana berada;

2. **SOFIA:**

- Bahwa saksi kenal dengan Penggugat tetapi tidak kenal dengan Tergugat;
- Bahwa Penggugat dan Tergugat adalah Suami Istri yang melangsungkan perkawinan secara agama Kristen ;
- Bahwa Penggugat dan Tergugat melakuka perkawinan di Gereja Kristen Residen Sudirman Surabaya;
- Bahwa perkawinan Penggugat dengan Tergugat telah dikaruniai seorang anak perempuan bernama Maheswari Emmylea Andhiny ;
- Bahwa anak tersebut masih belum dewasa dan selama ini selalu ikut ibunya;
- Bahwa pada awalnya hubungan suami istri tersebut baik-baik saja, tetapi belakangan ini sudah pisah rumah sudah cukup lama sedangkan anaknya ikut dengan ibunya;
- Bahwa saksi belum pernah melihat mereka bertengkar, tetapi tahu mereka sudah pisah rumah sudah lama sekali;
- Bahwa saksi tidak tahu sekarang Tergugat tinggal dimana;

Disclaimer
Kepaniteraan Mahkamah Agung Republik Indonesia berusaha untuk selalu mencantumkan informasi paling kini dan akurat sebagai bentuk komitmen Mahkamah Agung untuk pelayanan publik, transparansi dan akuntabilitas pelaksanaan fungsi peradilan. Namun dalam hal-hal tertentu masih dimungkinkan terjadi permasalahan teknis terkait dengan akurasi dan keterkinian informasi yang kami sajikan, hal mana akan terus kami perbaiki dari waktu kewaktu.
Dalam hal Anda menemukan inakurasi informasi yang termuat pada situs ini atau informasi yang seharusnya ada, namun belum tersedia, maka harap segera hubungi Kepaniteraan Mahkamah Agung RI melalui :
Email : kepaniteraan@mahkamahagung.go.id Telp : 021-384 3348 (ext.318)

Menimbang, bahwa selanjutnya segala sesuatu yang termuat dalam berita acara persidangan perkara ini, untuk menyingkat putusan ini dianggap telah termuat dan menjadi bagian yang tak terpisahkan dengan putusan ini;

Menimbang, bahwa akhirnya para pihak menyatakan tidak ada hal-hal yang diajukan lagi dan mohon putusan;

TENTANG PERTIMBANGAN HUKUM

Menimbang, bahwa Tergugat walaupun telah dipanggil secara patut sebagaimana Relas tanggal 31 Maret 2020 dan 21 April 2020, tetapi Tergugat tidak datang menghadap ke persidangan sedangkan ketidak hadirannya tersebt tanpa alasan yang sah dan tidak menunjuk wakilnya untuk menghadap dipersidangan;

Menimbang, bahwa oleh karena tergugat tidak pernah datang walaupun telah dipanggil secara patut dan sah, berdasarkan pasal pasa 125 HIR pemeriksaan perkara dilanjutkan dengan verstek, selanjutnya surat gugatan dibacakan didepan persidanagan dan isinya tetap dipertahankan oleh Penggugat;

Menimbang, bahwa maksud dan tujuan gugatan Penggugat yang pada pokoknya adalah mengenai :

Menimbang, bahwa Bahwa PENGGUGAT dan TERGUGAT adalah suami istri sah telah melangsungkan Perkawinan pada tanggal 08 - 08 - 2008 di Kota Surabaya (sesuai kutipan Akta Perkawinan nomor : 1460/WNI/2008 Tanggal 11 - 08 - 2008 yang telah diterbitkan oleh kantor Dinas Kependudukan Dan Pencatatan Sipil Kota Surabaya, dari perkawinannya telah dikaruniai anak Perempuan yang belum dewasa nama : Gitarja Maheswari Emmylea Andhiny lahir di Surabaya tanggal : 07 - 11 – 2011;

Menimbang, bahwa pada awal mula Perkawinan antara Penggugat dan Tergugat baik-baik saja akan tetapi sejak tahun 2017 antara Penggugat dan Tergugat sudah mulai sering timbul perselisihan-perselisihan dan pertengkaran-pertengkaran yang penyebabnya:

- Financial;

Disclaimer
Kepaniteraan Mahkamah Agung Republik Indonesia berusaha untuk selalu mencantumkan informasi paling kini dan akurat sebagai bentuk komitmen Mahkamah Agung untuk pelayanan publik, transparansi dan akuntabilitas pelaksanaan fungsi peradilan. Namun dalam hal-hal tertentu masih dimungkinkan terjadi permasalahan teknis terkait dengan akurasi dan keterkinian informasi yang kami sajikan, hal mana akan terus kami perbaiki dari waktu kewaktu.
Dalam hal Anda menemukan inakurasi informasi yang termuat pada situs ini atau informasi yang seharusnya ada, namun belum tersedia, maka harap segera hubungi Kepaniteraan Mahkamah Agung RI melalui :
Email : kepaniteraan@mahkamahagung.go.id   Telp : 021-384 3348 (ext.318)

- Tidak di nafkahi;

Menimbang, bahwa pertengkaran Tergugat tersebut pada puncaknya antara Penggugat dan Tergugat telah pisah tempat tinggal yakni Penggugat dan anaknya bertempat tinggal di Jl.Ploso IX B / 4 sedangkan Tergugat bertempat tinggal di Jl Pondok Wisata Estate G/11 Malang, hingga gugatan cerai ini diajukan di Pengadilan Negeri Sidoarjo;

Menimbang, bahwa PENGGUGAT selaku Ibu kandung dari : Gitarja Maheswari Emmylea Andhiny perempuan lahir di Surabaya, pada tanggal 07 - 08 – 2011;

Menimbang, bahwa hak asuh anak tersebut karena masih dibawah umur akan diberikan kepada PENGGUGAT akan tetapi TERGUGAT selaku ayah kandungnya sewaktu-waktu tetap diperbolehkan untuk menjenguk dan mengajak anak tersebut jalan-jalan dari rumah PENGGUGAT untuk beberapa waktu;

Menimbang, bahwa PENGGUGAT pada tanggal 10 - 03 - 2020 membuat surat pernyataan tidak keberatan untuk bercerai dengan Penggugat;

Menimbang, bahwa meskipun Tergugat tidak hadir, agar mendapatkan Putusan yang fair, Pengadilan berpendapat sesuai Pasal 163 HIR Penggugat diberi beban untuk membuktikan dalil-dalil gugatannya;

Menimbang, bahwa untuk membuktikan dalil-dalil gugatannya tersebut, Penggugat telah mengajukan surat-surat bukti yang diberi tanda bukti P.1 dan P.4 serta 2 (dua) orang saksi yang masing-masing telah memberikan keterangan di bawah sumpah / janji di depan persidangan ;

Menimbang, bahwa berdasarkan surat bukti P.3 benar antara Penggugat dengan Tergugat telah melangsungkan Perkawinan menurut Hukum Agama Kristen dilaksanakan di Gereja Kristen Residen Sudirman Surabaya pada tanggal 08 Agustus 2008;

Menimbang, bahwa berdasarkan surat bukti P.4, selama dalam perkawinan antara Penggugat dengan Tergugat telah dikaruniai seorang anak

Disclaimer
Kepaniteraan Mahkamah Agung Republik Indonesia berusaha untuk selalu mencantumkan informasi paling kini dan akurat sebagai bentuk komitmen Mahkamah Agung untuk pelayanan publik, transparansi dan akuntabilitas pelaksanaan fungsi peradilan. Namun dalam hal-hal tertentu masih dimungkinkan terjadi permasalahan teknis terkait dengan akurasi dan keterkinian informasi yang kami sajikan, hal mana akan terus kami perbaiki dari waktu kewaktu.
Dalam hal Anda menemukan inakurasi informasi yang termuat pada situs ini atau informasi yang seharusnya ada, namun belum tersedia, maka harap segera hubungi Kepaniteraan Mahkamah Agung RI melalui :
Email : kepaniteraan@mahkamahagung.go.id Telp : 021-384 3348 (ext.318)

yaitu : Gitarja Maheswari Emmylea Andhiny perempuan lahir di Surabaya, pada tanggal 07-08-2011;

Menimbang, bahwa berdasarkan keterangan saksi-saksi yang diajukan oleh Penggugat bahwa benar saat ini hubungan perkawinan antara Penggugat dengan Tergugat sudah tidak harmonis lagi dan selalu terjadi percekcokan antara Penggugat dengan Tergugat ;

Menimbang, bahwa benar sebagai puncak percekcokan antara Penggugat dengan Tergugat tersebut, Tergugat telah pisah rumah dengan Penggugat;

Menimbang, bahwa dari suasana kehidupan rumah tangga antara Penggugat dengan Tergugat sebagaimana terungkap di atas, menurut hemat Majelis Hakim jelas sudah tidak dapat lagi dipertahankan keutuhannya, dan sudah bertentangan dengan tujuan perkawinan itu sendiri yakni membentuk keluarga yang bahagia dan kekal berdasarkan Ketuhanan Yang Maha Esa ;

Menimbang, bahwa berdasarkan fakta dan pertimbangan sebagaimana tersebut di atas, menurut Majelis Hakim Penggugat telah berhasil membuktikan dalil pokok gugatannya maka berdasarkan ketentuan dalam pasal 38, jo. pasal 39, jo. pasal 40 Undang-Undang Nomor 1 Tahun 1974 tentang Perkawinan jo. Pasal 19 huruf f Peraturan Pemerintah Republik Indonesia Nomor 9 Tahun 1975 tentang Pelaksanaan Undang-Undang Nomor 1 Tahun 1974 tentang Perkawinan maka petitum gugatan Penggugat pada angka 2 (dua) tersebut beralasan dan patut untuk dikabulkan sehingga perkawinan antara Penggugat dengan Tergugat haruslah dinyatakan putus karena perceraian dengan segala akibat hukumnya ;

Menimbang, bahwa sejak diajukannya gugatan perceraian ini, antara Penggugat dengan Tergugat sudah tidak tinggal satu rumah lagi dan anak tersebut di rawat dan diasuh oleh Penggugat ;

Disclaimer
Kepaniteraan Mahkamah Agung Republik Indonesia berusaha untuk selalu mencantumkan informasi paling kini dan akurat sebagai bentuk komitmen Mahkamah Agung untuk pelayanan publik, transparansi dan akuntabilitas pelaksanaan fungsi peradilan. Namun dalam hal-hal tertentu masih dimungkinkan terjadi permasalahan teknis terkait dengan akurasi dan keterkinian informasi yang kami sajikan, hal mana akan terus kami perbaiki dari waktu kewaktu.
Dalam hal Anda menemukan inakurasi informasi yang termuat pada situs ini atau informasi yang seharusnya ada, namun belum tersedia, maka harap segera hubungi Kepaniteraan Mahkamah Agung RI melalui :
Email : kepaniteraan@mahkamahagung.go.id   Telp : 021-384 3348 (ext.318)

Menimbang, bahwa berdasarkan Yurisprudensi Mahkamah Agung Republik Indonesia Nomor : 102 K/Sip/1973 tanggal 24 April 1975 ditegaskan mengenai perwalian anak semata-mata untuk kepentingan anak yang menjadi kriteria khususnya anak yang belum dewasa perwalian anak diserahkan kepada ibu kandungnya, kecuali terbukti bahwa ibu tersebut tidak wajar dalam memelihara anaknya ;

Menimbang, bahwa oleh karena perkawinan antara Penggugat dengan Tergugat telah dinyatakan putus karena perceraian dengan segala akibat hukumnya, dan kenyataannya selama dalam perkawinan antara Penggugat dengan Tergugat telah dikaruniai 1 (satu) orang anak yang belum dewasa dan faktanya menurut keterangan para saksi yang diajukan oleh Penggugat bahwa Tergugat sebagai ibu kandungnya telah merawat, mengasuh, serta mendidik anak tersebut dengan baik serta anak-anak tersebut lebih dekat dengan Pengugat, maka demi perkembangan mental dan psychologis yang baik untuk anak-anak tersebut ke depannya, Majelis Hakim memandang adil apabila hak pengasuhan dan perawatan anak-anak tersebut sebagaimana tuntutan Penggugat dalam petitum angka 3 (tiga) diberikan kepada Penggugat sebagai ibu kandungnya, namun Tergugat sebagai ayah kandungnya tetap diperbolehkan untuk berkomunikasi, bertemu, dan berkunjung setiap saat dengan anak-anaknya tersebut sehingga tidak memutuskan hubungan antara anak dengan ayah kandungnya, sehingga petitum angka 3 (tiga) cukup beralasan hukum dan patut untuk dikabulkan ;

Menimbang, bahwa sebagai konsekwensi logis dikabulkannya Petitum no.2 dan 4, maka petitum No. 4 juga dikabulkan;

Menimbang, bahwa oleh karena gugatan Penggugat dikabulkan dan Tergugat ada di pihak yang kalah maka Tergugat dihukum membayar biaya yang timbul dalam perkara ini;

Disclaimer
Kepaniteraan Mahkamah Agung Republik Indonesia berusaha untuk selalu mencantumkan informasi paling kini dan akurat sebagai bentuk komitmen Mahkamah Agung untuk pelayanan publik, transparansi dan akuntabilitas pelaksanaan fungsi peradilan. Namun dalam hal-hal tertentu masih dimungkinkan terjadi permasalahan teknis terkait dengan akurasi dan keterkinian informasi yang kami sajikan, hal mana akan terus kami perbaiki dari waktu kewaktu.
Dalam hal Anda menemukan inakurasi informasi yang termuat pada situs ini atau informasi yang seharusnya ada, namun belum tersedia, maka harap segera hubungi Kepaniteraan Mahkamah Agung RI melalui :
Email : kepaniteraan@mahkamahagung.go.id   Telp : 021-384 3348 (ext.318)

Memperhatikan pasal 125 HIR, Undang-Undang Nomor 1 Tahun 1974 tentang Perkawinan jo. Peraturan Pemerintah Republik Indonesia Nomor 9 Tahun 1975 tentang Pelaksanaan Undang-Undang Nomor 1 Tahun 1974 tentang Perkawinan serta peraturan perundang-undangan lainnya yang bersangkutan dengan perkara ini ;

MENGADILI:

1. Menyatakan Tergugat tidak hadir;
2. Mengabulkan gugatan PENGGUGAT seluruhnya dengan verstek:
3. Menyatakan bahwa Perkawinan antara PENGGUGAT dan TERGUGAT yang telah dilaksanakan pada tanggal 08 - 08 - 2008 di Kota Surabaya (sesuai kutipan Akta Perkawinan nomor : 1460/WNI/2008 tanggal 11 - 08 - 2008yangtelah diterbitkan oleh kantor Dinas Kependudukan Dan Pencatatan Sipil Kota Surabaya putus karena perceraian dengan segala akibat hukumnya;
4. Menyatakan PENGGUGAT sebagai pemegang Hak asuh atas anak TERGUGAT yang belum dewasa bernama Gitarja Maheswari Emmylea Andhiny perempuan lahir di Surabaya tanggal 07 - 08 - 2008 sejak perkara ini mempunyai kekuatan hukum tetap hingga anak tersebut dewasa dengan mengijinkan dan tidak menghalangi TERGUGAT sewaktu-waktu untuk menjenguk dan mengajak anak tersebut untuk pergi jala-jalan dan dari rumah Penggugat.
5. Memerintahkan para pihak untuk mengirimkan/menyerahkan salinan Putusan perkara ini yang telah berkekuatan hukum tetap kepada pegawai pencatat pada Kantor Dinas Pendaftaran Penduduk dan Pencatatan Sipil kota Surabaya untuk didaftarkan/dicatatkan adanya perceraian ini ke dalam register yang sedang berjalan dengan mengeluarkan kutipan/turunannya.
6. Menghukum Tergugat membayar beaya perkara yang timbul dalam perkara ini yang hingga saat ini diperhitungkan sejumlah Rp.556.000,- (lima ratus lima puluh enam ribu rupiah);

Disclaimer
Kepaniteraan Mahkamah Agung Republik Indonesia berusaha untuk selalu mencantumkan informasi paling kini dan akurat sebagai bentuk komitmen Mahkamah Agung untuk pelayanan publik, transparansi dan akuntabilitas pelaksanaan fungsi peradilan. Namun dalam hal-hal tertentu masih dimungkinkan terjadi permasalahan teknis terkait dengan akurasi dan keterkinian informasi yang kami sajikan, hal mana akan terus kami perbaiki dari waktu kewaktu.
Dalam hal Anda menemukan inakurasi informasi yang termuat pada situs ini atau informasi yang seharusnya ada, namun belum tersedia, maka harap segera hubungi Kepaniteraan Mahkamah Agung RI melalui :
Email : kepaniteraan@mahkamahagung.go.id Telp : 021-384 3348 (ext.318)

Demikian diputuskan dalam sidang permusyawaratan Majelis Hakim Pengadilan Negeri Sidoarjo, pada hari Rabu, tanggal 29 April 2020, oleh kami, Afandi Widarijanto, S.H., sebagai Hakim Ketua, Lie Sonny, S.H.dan Eni Sri Rahayu, S.H., M.H. masing-masing sebagai Hakim Anggota,, yang ditunjuk berdasarkan Surat Penetapan Ketua Pengadilan Negeri Sidoarjo Nomor 87/Pdt.G/2020/PN SDA tanggal 18 Maret 2020, putusan tersebut pada hari Rabu, tanggal 6 Mei 2020 diucapkan dalam persidangan terbuka untuk umum oleh Hakim Ketua dengan dihadiri oleh para Hakim Anggota tersebut, Ika Yunia Ratnawati, S.H.,M.H., Panitera Pengganti dan Penggugat, akan tetapi tidak dihadiri oleh pihak Tergugat maupun Kuasanya;

Hakim Anggota, Hakim Ketua,

Lie Sonny, S.H.. Afandi Widarijanto, S.H.

Eni Sri Rahayu, S.H., M.H. Panitera Pengganti,

Ika Yunia Ratnawati, S.H.,M.H.

Perincian biaya :

1.	Pendaftaran	: Rp. 30.000,-
2.	Proses ATK	: Rp.100.000,-
3.	PNBP	: Rp. 20.000,-
4.	Panggilan	: Rp.360.000,-
5.	Sumpah	: Rp. 30.000,-
6.	Materai	: Rp. 6.000,-
7.	Redaksi	: Rp. 10.000,-

Disclaimer
Kepaniteraan Mahkamah Agung Republik Indonesia berusaha untuk selalu mencantumkan informasi paling kini dan akurat sebagai bentuk komitmen Mahkamah Agung untuk pelayanan publik, transparansi dan akuntabilitas pelaksanaan fungsi peradilan. Namun dalam hal-hal tertentu masih dimungkinkan terjadi permasalahan teknis terkait dengan akurasi dan keterkinian informasi yang kami sajikan, hal mana akan terus kami perbaiki dari waktu kewaktu.
Dalam hal Anda menemukan inakurasi informasi yang termuat pada situs ini atau informasi yang seharusnya ada, namun belum tersedia, maka harap segera hubungi Kepaniteraan Mahkamah Agung RI melalui :
Email : kepaniteraan@mahkamahagung.go.id Telp : 021-384 3348 (ext.318)

Jumlah : Rp. 556.000,- (lima ratus lima puluh enam ribu rupiah)

Disclaimer
Kepaniteraan Mahkamah Agung Republik Indonesia berusaha untuk selalu mencantumkan informasi paling kini dan akurat sebagai bentuk komitmen Mahkamah Agung untuk pelayanan publik, transparansi dan akuntabilitas pelaksanaan fungsi peradilan. Namun dalam hal-hal tertentu masih dimungkinkan terjadi permasalahan teknis terkait dengan akurasi dan keterkinian informasi yang kami sajikan, hal mana akan terus kami perbaiki dari waktu kewaktu.
Dalam hal Anda menemukan inakurasi informasi yang termuat pada situs ini atau informasi yang seharusnya ada, namun belum tersedia, maka harap segera hubungi Kepaniteraan Mahkamah Agung RI melalui :
Email : kepaniteraan@mahkamahagung.go.id Telp : 021-384 3348 (ext.318)
