# Supplementary material for: Classification of divorce causes during the COVID-19 pandemic using convolutional neural networks
Source: PeerJ Comput Sci. 2022 Jun 30;8:e998. doi: 10.7717/peerj-cs.998 (PMC9299239; doi:10.7717/peerj-cs.998)
Supplement: Supplemental Information 4 [file peerj-cs-08-998-s004.zip › Konflik Dataset/Data ke-28.pdf]

PUTUSAN

Nomor 865/Pdt.G/2020/PA.Ppg

بِسْمِ اللَّهِ الرَّحْمَنِ الرَّحِيمِ

DEMI KEADILAN BERDASARKAN KETUHANAN YANG MAHA ESA

Pengadilan Agama Pasir Pengaraian yang memeriksa dan mengadili perkara-perkara tertentu pada peradilan tingkat pertama dalam sidang Majelis telah menjatuhkan putusan sebagai berikut dalam perkara Cerai Talak antara:

Pemohon, tempat dan tanggal lahir Tingkok 02 April 1981, umur 39 tahun, agama Islam, pendidikan SLTA, pekerjaan Dagang, alamat kediaman di Dusun I, RT.002 RW.003, Desa Tingkok, Kecamatan Tambusai, Kabupaten Rokan Hulu, selanjutnya disebut sebagai **Pemohon**;

Melawan

Termohon, tempat dan tanggal lahir Binjai 27 November 1981, umur 39 tahun, agama Islam, pendidikan SLTA, pekerjaan Mengurus rumah tangga, alamat kediaman di Dusun I, RT.002 RW.003, Desa Tingkok, Kecamatan Tambusai, Kabupaten Rokan Hulu, selanjutnya disebut sebagai **Termohon**;

Pengadilan Agama tersebut;

Setelah membaca berkas perkara, mendengar keterangan Pemohon, dan memeriksa bukti-bukti di persidangan;

DUDUK PERKARA

Bahwa, Pemohon yang dalam surat permohonannya tertanggal 17 Desember 2020 yang telah terdaftar di Kepaniteraan Pengadilan Agama Pasir Pengaraian pada tanggal 17 Desember 2020 dengan Nomor 865/Pdt.G/2020/PA.Ppg telah mengemukakan hal-hal sebagai berikut:

1. Bahwa Pemohon dan Termohon telah melaksanakan pernikahan pada tanggal 24 Juli 2004 di Kecamatan Batang Angkola, Kabupaten

Disclaimer
Kepaniteraan Mahkamah Agung Republik Indonesia berusaha untuk selalu mencantumkan informasi paling kini dan akurat sebagai bentuk komitmen Mahkamah Agung untuk pelayanan publik, transparansi dan akuntabilitas pelaksanaan fungsi peradilan. Namun dalam hal-hal tertentu masih dimungkinkan terjadi permasalahan teknis terkait dengan akurasi dan keterkinian informasi yang kami sajikan, hal mana akan terus kami perbaiki dari waktu kewaktu.
Dalam hal Anda menemukan inakurasi informasi yang termuat pada situs ini atau informasi yang seharusnya ada, namun belum tersedia, maka harap segera hubungi Kepaniteraan Mahkamah Agung RI melalui :
Email : kepaniteraan@mahkamahagung.go.id   Telp : 021-384 3348 (ext.318)

Tapanuli Selatan, Propinsi Sumatera Utara, yang dicatat oleh Pegawai Pencatat Nikah Kantor Urusan Agama Kecamatan Batang Angkola, Kabupaten Tapanuli Selatan, Propinsi Sumatera Utara, sebagaimana ternyata dari Kutipan Akta Nikah Nomor: 230/16/IX/2004 tanggal 24 Juli 2004;

2. Bahwa setelah menikah Pemohon dan Termohon kumpul baik dan tinggal di rumah orangtua Pemohon di Desa Tingkok selama 2 tahun, terakhir pindah di rumah kediaman bersama di Desa Tingkok;

3. Bahwa selama pernikahan antara Pemohon dan Termohon telah hidup rukun sebagaiman layaknya suami istri (ba'da dukhul) dan telah dan dikaruniai 3 orang anak bernama yaitu :

   a. Rona Bila Lubis (perempuan) umur 14 tahun;
   b. Daffa Yusri Mahendra Lubis (laki-laki) umur 9 tahun;
   c. Abi (laki-laki) umur 5 tahun, anak tersebut sekarang berada dibawah asuhan Termohon;

4. Bahwa rumah tangga Pemohon dengan Termohon pada awalnya berjalan baik-baik dan rukun saja selama 2 tahun namun setelah itu mulai tidak harmonis karena adanya perselisihan dan pertengkaran yang terus menerus dan sulit untuk dirukunkan lagi yang disebabkan yaitu :

   a. Termohon apabila di nasehati Pemohon, Termohon selalu memabntah ucapan Pemohon;
   b. Termohon tidak pernah jujur kepada Pemohon;

5. Bahwa puncak dari perselisihan dan pertengkaran tersebut terjadi pada tanggal 25 Agustus 2015, yang akhirnya menyebabkan antara Pemohon dengan Termohon telah pisah rumah dan yang pergi meninggalkan kediaman bersama adalah Termohon, karena ada perselisihan dan pertengkaran antara Pemohon dan Termohon, dan Pemohon telah menajtuhkan talak kepada Termohon;

6. Bahwa antara Pemohon dan Termohon sudah pernah didamaikan oleh pihak keluarga namun tidak berhasil;

Bahwa, berdasarkan alasan-alasan tersebut diatas, maka Pemohon memohon kepada Bapak Ketua Pengadilan Agama Pasir Pengaraian Cq.

Disclaimer
Kepaniteraan Mahkamah Agung Republik Indonesia berusaha untuk selalu mencantumkan informasi paling kini dan akurat sebagai bentuk komitmen Mahkamah Agung untuk pelayanan publik, transparansi dan akuntabilitas pelaksanaan fungsi peradilan. Namun dalam hal-hal tertentu masih dimungkinkan terjadi permasalahan teknis terkait dengan akurasi dan keterkinian informasi yang kami sajikan, hal mana akan terus kami perbaiki dari waktu kewaktu.
Dalam hal Anda menemukan inakurasi informasi yang termuat pada situs ini atau informasi yang seharusnya ada, namun belum tersedia, maka harap segera hubungi Kepaniteraan Mahkamah Agung RI melalui :
Email : kepaniteraan@mahkamahagung.go.id Telp : 021-384 3348 (ext.318)

Majelis Hakim yang memeriksa perkara ini, kiranya berkenan menerima, memeriksa, mengadili serta memutuskan sebagai berikut:

1. Mengabulkan permohonan Pemohon seluruhnya;

2. Memberi izin kepada Pemohon **(Pemohon)** untuk menjatuhkan talak satu raj'i terhadap diri Termohon **(Termohon)** di depan sidang Pengadilan Agama Pasir Pengaraian;

3. Membebankan kepada Pemohon untuk membayar semua biaya yang timbul dalam perkara ini;

Dan Atau,

Apabila Majelis Hakim berpendapat lain mohon putusan yang seadil-adilnya:

Menimbang, bahwa pada hari dan tanggal yang telah ditetapkan untuk pemeriksaan perkara ini, Pemohon hadir dan menghadap sendiri di persidangan, sedangkan Termohon tidak pernah hadir di persidangan dan tidak pula menyuruh orang lain untuk hadir sebagai wakil atau kuasanya yang sah meskipun Termohon telah dipanggil secara sah dan patut, dan tidak ternyata bahwa ketidakhadiran Termohon tersebut didasarkan oleh Undang-Undang;

Bahwa, Majelis hakim telah menasehati Pemohon agar berpikir untuk tidak bercerai dengan Termohon, tetapi Pemohon tetap pada dalil-dalil permohonannya untuk bercerai dengan Termohon;

Bahwa, perkara ini tidak dapat di mediasi karena Termohon tidak pernah datang menghadap meskipun telah dipanggil secara resmi dan patut, selanjutnya dimulai pemeriksaan dengan membacakan surat Permohonan Pemohon yang maksud dan isinya tetap dipertahankan oleh Pemohon;

Bahwa, terhadap permohonan Pemohon tersebut, Termohon tidak dapat didengar jawabannya karena tidak pernah hadir di persidangan;

Bahwa, untuk menguatkan dalil-dalil permohonannya, Pemohon telah mengajukan bukti berupa fotokopi Kutipan Akta Nikah Nomor 230/16/IX/2004 an. Termohon dan Pemohon, yang aslinya dikeluarkan oleh Kantor Urusan Agama Kecamatan Batang Angkola, Kabupaten Tapanuli Selatan, Propinsi Sumatera Utara, pada tanggal 24 Juli 2004, bukti surat tersebut telah diberi meterai cukup dan telah dicocokkan dengan aslinya yang ternyata sesuai (P.1);

Disclaimer
Kepaniteraan Mahkamah Agung Republik Indonesia berusaha untuk selalu mencantumkan informasi paling kini dan akurat sebagai bentuk komitmen Mahkamah Agung untuk pelayanan publik, transparansi dan akuntabilitas pelaksanaan fungsi peradilan. Namun dalam hal-hal tertentu masih dimungkinkan terjadi permasalahan teknis terkait dengan akurasi dan keterkinian informasi yang kami sajikan, hal mana akan terus kami perbaiki dari waktu kewaktu.
Dalam hal Anda menemukan inakurasi informasi yang termuat pada situs ini atau informasi yang seharusnya ada, namun belum tersedia, maka harap segera hubungi Kepaniteraan Mahkamah Agung RI melalui :
Email : kepaniteraan@mahkamahagung.go.id   Telp : 021-384 3348 (ext.318)

Bahwa, selain bukti tertulis, Pemohon telah mengajukan saksi-saksi sebagai berikut:

1. Saksi I, umur 37 tahun, agama Islam, pendidikan SLTA, pekerjaan Ibu Rumah Tangga, tempat kediaman di RT 02 RW 03, Desa Tingkok, Kecamatan Tambusai, Kabupaten Rokan hulu, Saksi adalah tetangga Pemohon, telah memberikan keterangan di bawah sumpah yang pada pokoknya sebagai berikut :

- Bahwa Pemohon dengan Termohon adalah suami isteri;
- Bahwa Pemohon dan Termohon tinggal di rumah orangtua Pemohon di Desa Tingkok selama 2 tahun, terakhir pindah di rumah kediaman bersama di Desa Tingkok;
- Bahwa Pemohon dan Termohon telah dikaruniai 3 orang anak bernama yaitu :
  a. Rona Bila Lubis (perempuan) umur 14 tahun;
  b. Daffa Yusri Mahendra Lubis (laki-laki) umur 9 tahun;
  c. Abi (laki-laki) umur 5 tahun, anak tersebut sekarang berada dibawah asuhan Termohon;
- Bahwa setahu saksi awalnya rumah tangga antara Pemohon dengan Termohon baik-baik saja;
- Bahwa Saksi tidak mengetahui apa yang terjadi dalam rumah tangga antara Pemohon dengan Termohon;
- Bahwa Pemohon dan Termohon telah pisah rumah sejak Agustus 2015 yang lalu, Termohon telah pergi dari rumah kediaman bersama;
- Bahwa Pemohon telah berusaha untuk mencari Termohon kepada keluarga dan teman-teman Termohon, namun tidak berhasil;
- Bahwa Pemohon dan Termohon sudah didamaikan oleh keluarga, tetapi tidak berhasil;
- Bahwa Saksi tidak mampu mendamaikan Pemohon dan Termohon;

2. Saksi II, umur 37 tahun, agama Islam, pendidikan SLTP, pekerjaan Petani, tempat kediaman di RT 11 RW 06, Desa Tingkok, Kecamatan Tambusai, Kabupaten Rokan hulu, Saksi adalah kawan Pemohon, telah

Disclaimer
Kepaniteraan Mahkamah Agung Republik Indonesia berusaha untuk selalu mencantumkan informasi paling kini dan akurat sebagai bentuk komitmen Mahkamah Agung untuk pelayanan publik, transparansi dan akuntabilitas pelaksanaan fungsi peradilan. Namun dalam hal-hal tertentu masih dimungkinkan terjadi permasalahan teknis terkait dengan akurasi dan keterkinian informasi yang kami sajikan, hal mana akan terus kami perbaiki dari waktu kewaktu.
Dalam hal Anda menemukan inakurasi informasi yang termuat pada situs ini atau informasi yang seharusnya ada, namun belum tersedia, maka harap segera hubungi Kepaniteraan Mahkamah Agung RI melalui :
Email : kepaniteraan@mahkamahagung.go.id   Telp : 021-384 3348 (ext.318)

memberikan keterangan di bawah sumpah yang pada pokoknya sebagai berikut :

- Bahwa Pemohon dengan Termohon adalah suami isteri;
- Bahwa Pemohon dan Termohon tinggal di rumah orangtua Pemohon di Desa Tingkok selama 2 tahun, terakhir pindah di rumah kediaman bersama di Desa Tingkok;
- Bahwa Pemohon dan Termohon telah dikaruniai 3 orang anak bernama yaitu :
  a. Rona Bila Lubis (perempuan) umur 14 tahun;
  b. Daffa Yusri Mahendra Lubis (laki-laki) umur 9 tahun;
  c. Abi (laki-laki) umur 5 tahun, anak tersebut sekarang berada dibawah asuhan Termohon;
- Bahwa setahu saksi awalnya rumah tangga antara Pemohon dengan Termohon baik-baik saja;
- Bahwa Saksi tidak mengetahui apa yang terjadi dalam rumah tangga antara Pemohon dengan Termohon;
- Bahwa Pemohon dan Termohon telah pisah rumah sejak Agustus 2015 yang lalu, Termohon telah pergi dari rumah kediaman bersama;
- Bahwa Pemohon telah berusaha untuk mencari Termohon kepada keluarga dan teman-teman Termohon, namun tidak berhasil;
- Bahwa Pemohon dan Termohon sudah didamaikan oleh keluarga, tetapi tidak berhasil;
- Bahwa Saksi tidak mampu mendamaikan Pemohon dan Termohon;

Bahwa, terhadap keterangan saksi-saksi tersebut Pemohon tidak mengajukan pertanyaan tambahan;

Bahwa, Pemohon telah menyampaikan kesimpulannya secara lisan yang pada pokoknya tetap pada permohonannya dan memohon Putusan;

Bahwa, untuk mempersingkat uraian putusan ini, maka segala sesuatu yang tercantum dalam berita acara sidang harus dipandang termuat dalam putusan ini;

Disclaimer
Kepaniteraan Mahkamah Agung Republik Indonesia berusaha untuk selalu mencantumkan informasi paling kini dan akurat sebagai bentuk komitmen Mahkamah Agung untuk pelayanan publik, transparansi dan akuntabilitas pelaksanaan fungsi peradilan. Namun dalam hal-hal tertentu masih dimungkinkan terjadi permasalahan teknis terkait dengan akurasi dan keterkinian informasi yang kami sajikan, hal mana akan terus kami perbaiki dari waktu kewaktu.
Dalam hal Anda menemukan inakurasi informasi yang termuat pada situs ini atau informasi yang seharusnya ada, namun belum tersedia, maka harap segera hubungi Kepaniteraan Mahkamah Agung RI melalui :
Email : kepaniteraan@mahkamahagung.go.id Telp : 021-384 3348 (ext.318)

PERTIMBANGAN HUKUM

Menimbang, bahwa pokok permohonan Pemohon adalah tentang permohonan cerai talak sebagaimana tersebut di atas;

Menimbang, bahwa perkara ini mengenai perceraian antara pihak-pihak yang beragama Islam dan perkawinannya dilangsungkan secara Islam, maka berdasarkan pasal 49 ayat (1) Undang-undang nomor 7 tahun 1989 Tentang Peradilan Agama yang diubah dengan Undang-undang nomor 3 tahun 2006 dan Undang-undang nomor 50 tahun 2009, maka Pengadilan Agama berwenang memeriksa, mengadili dan menyelesaikan perkara ini secara Absolut;

Menimbang, Pemohon telah hadir di persidangan sementara Termohon tidak pernah hadir untuk menghadap di persidangan meskipun terhadapnya telah dilakukan pemanggilan secara resmi dan patut, dan telah ternyata bahwa ketidakhadiran Termohon tersebut tidak disebabkan oleh suatu alasan yang sah, oleh karena itu menurut hukum pembuktian Termohon dianggap telah mengakui atau setidak-tidaknya tidak membantah dalil-dalil Pemohon;

Menimbang, bahwa Termohon meskipun telah dipanggil secara resmi dan patut ternyata tidak datang menghadap di depan persidangan dan tidak menyuruh orang lain sebagai kuasanya, sedangkan ketidak hadirannya itu tidak disebabkan sesuatu halangan yang sah serta Permohonan Pemohon beralasan dan tidak melawan hukum, maka Termohon yang dipanggil secara resmi dan patut tetapi tidak datang menghadap di persidangan harus dinyatakan tidak hadir, dan sesuai dengan Pasal 149 ayat (1) RBg *jo* Surat Edaran Mahkamah Agung RI Nomor 9 Tahun 1964, maka Majelis Hakim dapat memeriksa dan memutus perkara ini tanpa hadirnya Termohon (*verstek*);

Menimbang bahwa sedianya akan diupayakan mediasi antara Pemohon dengan Termohon sebagaimana ketentuan Peraturan Mahkamah Agung Nomor 1 Tahun 2016, akan tetapi karena Termohon tidak pernah hadir memenuhi panggilan sidang, maka upaya mediasi tersebut tidak dapat dilaksanakan;

Menimbang, bahwa di dalam persidangan, Majelis Hakim telah berusaha menasihati Pemohon agar bersabar dan mengurungkan niatnya yang hendak bercerai dengan Termohon, serta menasihati agar Pemohon mempertahankan

Disclaimer
Kepaniteraan Mahkamah Agung Republik Indonesia berusaha untuk selalu mencantumkan informasi paling kini dan akurat sebagai bentuk komitmen Mahkamah Agung untuk pelayanan publik, transparansi dan akuntabilitas pelaksanaan fungsi peradilan. Namun dalam hal-hal tertentu masih dimungkinkan terjadi permasalahan teknis terkait dengan akurasi dan keterkinian informasi yang kami sajikan, hal mana akan terus kami perbaiki dari waktu kewaktu.
Dalam hal Anda menemukan inakurasi informasi yang termuat pada situs ini atau informasi yang seharusnya ada, namun belum tersedia, maka harap segera hubungi Kepaniteraan Mahkamah Agung RI melalui :
Email : kepaniteraan@mahkamahagung.go.id Telp : 021-384 3348 (ext.318)

rumah tangganya dengan baik sesuai ketentuan Pasal 82 ayat 1 dan 4 Undang-undang 7 Tahun 1989 tentang Peradilan Agama, dan Pasal 131 ayat 2 Kompilasi Hukum Islam, akan tetapi tidak berhasil;

Menimbang, bahwa Pemohon bermohon agar diberi izin untuk menjatuhkan talak satu raj'i terhadap Termohon, dengan dalil-dalil bahwa Pemohon dan Termohon adalah suami isteri sah yang menikah pada tanggal 24 Juli 2004 dan pernah hidup rukun dan harmonis dan sudah dikaruniai 3 (tiga) orang anak, tetapi sejak 2 tahun setelah menikah rumah tangga Pemohon dan Termohon mulai tidak harmonis lagi karena sering terjadi perselisihan dan pertengkaran yang disebabkan karena Termohon apabila dinasehati Pemohon Termohon selalu membantah ucapan Pemohon, dan puncaknya pertengkaran Pemohon dan Termohon terjadi pada tanggal 25 Agustus 2015 yang akhirnya menyebabkan Pemohon dan Termohon pisah rumah dan yang pergi meninggalkan kediaman bersama adalah Termohon; bahwa pihak keluarga sudah mendamaikan Pemohon dan Termohon namun tidak berhasil;

Menimbang bahwa sekalipun Termohon tidak pernah hadir dalam persidangan, Pemohon tetap dibebankan untuk membuktikan dalil-dalilnya Permohonannya untuk menghindari adanya kesepakatan untuk bercerai sebagaimana Yurisprudensi Mahkamah Agung Republik Indonesia Nomor : 863 K/Pdt/1990, tanggal 28 Nopember 1991 dalam perkara perceraian tidak dibenarkan semata-mata didasarkan pada adanya pengakuan dan atau adanya kesepakatan saja karena dikawatirkan timbulnya kebohongan besar (de grote langen) pasal 208 BW;

Menimbang, bahwa untuk menguatkan dalil permohonannya, Pemohon telah mengajukan alat bukti berupa (P) serta 2 (dua) orang saksi di persidangan;

Menimbang, bahwa alat bukti surat (P) yang diajukan Pemohon merupakan fotokopi sah dari suatu akta otentik yang telah dinazagelen dan setelah diteliti oleh Majelis Hakim ternyata sesuai dengan aslinya, dan alat bukti tersebut memuat keterangan yang menguatkan dan relevan dengan Permohonan Pemohon, dengan demikian Majelis Hakim berpendapat bahwa alat bukti tersebut telah memenuhi syarat formil dan materil sebagai alat bukti;

Disclaimer
Kepaniteraan Mahkamah Agung Republik Indonesia berusaha untuk selalu mencantumkan informasi paling kini dan akurat sebagai bentuk komitmen Mahkamah Agung untuk pelayanan publik, transparansi dan akuntabilitas pelaksanaan fungsi peradilan. Namun dalam hal-hal tertentu masih dimungkinkan terjadi permasalahan teknis terkait dengan akurasi dan keterkinian informasi yang kami sajikan, hal mana akan terus kami perbaiki dari waktu kewaktu.
Dalam hal Anda menemukan inakurasi informasi yang termuat pada situs ini atau informasi yang seharusnya ada, namun belum tersedia, maka harap segera hubungi Kepaniteraan Mahkamah Agung RI melalui :
Email : kepaniteraan@mahkamahagung.go.id   Telp : 021-384 3348 (ext.318)

Menimbang, bahwa berdasarkan bukti P tersebut harus dinyatakan terbukti bahwa Pemohon dan Termohon adalah sebagai suami istri yang sah, oleh karena itu Pemohon dan Termohon adalah pihak yang berkepentingan dalam perkara ini (*persona standi in judicio*) sesuai dengan ketentuan Pasal 2 ayat 2 Undang-undang 1 Tahun 1974 tentang Perkawinan *jo.* Pasal 7 ayat 1 Kompilasi Hukum Islam di Indonesia Tahun 1991;

Menimbang, bahwa terhadap dua orang saksi yang diajukan oleh Pemohon, Majelis Hakim berpendapat bahwa dua orang saksi tersebut telah memenuhi persyaratan formil pembuktian, saksi mana telah disumpah menurut tata cara agama Islam dan di samping itu tidak terhalang untuk didengar keterangannya sebagai saksi, dengan demikian dapat diterima dan sesuai dengan ketentuan Pasal 22 ayat (2) Peraturan Pemerintah Nomor 9 Tahun 1975;

Menimbang, bahwa dengan mempertimbangkan keadaan dan kedudukan para saksi, yaitu saksi yang bernama **Saksi I** sebagai tetangga Pemohon dan **Saksi II** kawan Pemohon, maka cukup beralasan apabila saksi tersebut mengetahui keadaan rumah tangga Pemohon dan Termohon dari apa yang didengarnya secara langsung dan keterangan saksi tersebut sebagaimana telah diuraikan dalam bagian duduk perkara. Atas dasar tersebut Majelis Hakim berpendapat bahwa keterangan saksi-saksi tersebut adalah sebenarnya dan sejujurnya serta mempunyai relevansi dengan dalil permohonan Pemohon. Dengan demikian keterangan saksi-saksi tersebut secara materil dapat diterima dan dijadikan sebagai bukti yang sah untuk mendukung kebenaran dalil-dalil permohonan Pemohon sesuai dengan ketentuan Pasal 308 dan 309 R.Bg;

Menimbang, bahwa dari bukti-bukti yang diajukan oleh Pemohon, baik bukti surat P, maupun saksi-saksi, maka terhadap permohonan Pemohon telah mempunyai cukup pembuktian;

Menimbang, bahwa dari pembuktian tersebut ditemukan fakta-fakta yang dapat dikonstatir sebagai berikut:

1. Pemohon dan Termohon merupakan pasangan suami istri yang terikat perkawinan yang sah menikah 24 Juli 2004;

Disclaimer
Kepaniteraan Mahkamah Agung Republik Indonesia berusaha untuk selalu mencantumkan informasi paling kini dan akurat sebagai bentuk komitmen Mahkamah Agung untuk pelayanan publik, transparansi dan akuntabilitas pelaksanaan fungsi peradilan. Namun dalam hal-hal tertentu masih dimungkinkan terjadi permasalahan teknis terkait dengan akurasi dan keterkinian informasi yang kami sajikan, hal mana akan terus kami perbaiki dari waktu kewaktu.
Dalam hal Anda menemukan inakurasi informasi yang termuat pada situs ini atau informasi yang seharusnya ada, namun belum tersedia, maka harap segera hubungi Kepaniteraan Mahkamah Agung RI melalui :
Email : kepaniteraan@mahkamahagung.go.id Telp : 021-384 3348 (ext.318)

2. Semula rumah tangga Penggugat dan Tergugat hidup rukun dan harmonis, namun selanjutnya kerukunan dan keharmonisan tersebut tidak dapat dipertahankan lagi, karena sejak Agustus 2015 Termohon pergi meninggalkan Pemohon tanpa izin dan alasan yang jelas dan hingga saat ini tidak pernah kembali;
3. Bahwa antara Pemohon dan Termohon telah berpisah tempat tinggal dan antara Pemohon dan Termohon sudah tidak ada komunikasi dan keduanya sudah tidak melaksanakan hak dan kewajibannya lagi sebagai suami isteri;
4. Bahwa pihak keluarga sudah berusaha mendamaikan Pemohon dan Termohon namun tidak berhasil;
5. Bahwa Pemohon sudah tidak sanggup lagi melanjutkan hubungan rumah tangga dengan Termohon dan bertekad untuk bercerai dengan Termohon;

Menimbang, bahwa berdasarkan fakta-fakta tersebut di atas, maka Majelis Hakim berpendapat (*feetelijk vermoeden*) bahwa ikatan perkawinan antara Pemohon dengan Termohon pada hakikatnya sudah sangat rapuh dan ikatan batin yang menjadi unsur terpenting di dalam membina rumah tangga sudah tidak terwujud lagi, sehingga mempertahankan kehidupan rumah tangga yang seperti itu, bukanlah kedamaian dan kebahagiaan yang akan tercapai, tetapi sebaliknya akan dapat menyebabkan tekanan jiwa, penderitaan batin serta kerusakan mental (*mental illness*), baik bagi salah satu pihak maupun bagi kedua belah pihak suami dan istri;

Menimbang, bahwa dengan kenyataan di atas, maka harapan untuk dapat membina kehidupan rumah tangga yang *sakinah*, *mawaddah*, *wa rahmah* yang menjadi tujuan dari sebuah perkawinan sulit untuk dapat diwujudkan, padahal tujuan tersebut merupakan asas-asas mendasar yang melandasi hubungan pasangan suami istri dalam membangun suatu rumah tangga dalam Islam, sebagaimana yang dimaksudkan dalam firman Allah SWT dalam surah Ar-Rum ayat 21 yang berbunyi sebagai berikut:

وَمِنْ آيَاتِهِ أَنْ خَلَقَ لَكُمْ مِنْ أَنْفُسِكُمْ أَزْوَاجًا لِتَسْكُنُوا إِلَيْهَا وَجَعَلَ بَيْنَكُمْ مَوَدَّةً وَرَحْمَةً إِنَّ فِي ذَلِكَ لَآيَاتٍلِقَوْمٍ يَتَفَكَّرُونَ

Disclaimer
Kepaniteraan Mahkamah Agung Republik Indonesia berusaha untuk selalu mencantumkan informasi paling kini dan akurat sebagai bentuk komitmen Mahkamah Agung untuk pelayanan publik, transparansi dan akuntabilitas pelaksanaan fungsi peradilan. Namun dalam hal-hal tertentu masih dimungkinkan terjadi permasalahan teknis terkait dengan akurasi dan keterkinian informasi yang kami sajikan, hal mana akan terus kami perbaiki dari waktu kewaktu.
Dalam hal Anda menemukan inakurasi informasi yang termuat pada situs ini atau informasi yang seharusnya ada, namun belum tersedia, maka harap segera hubungi Kepaniteraan Mahkamah Agung RI melalui :
Email : kepaniteraan@mahkamahagung.go.id Telp : 021-384 3348 (ext.318)

Artinya:“*Dan di antara tanda-tanda kekuasaan-Nya ialah Dia menciptakan untukmu isteri-isteri dari jenismu sendiri, supaya kamu cenderung dan merasa tenteram kepadanya, dan dijadikan-Nya di antara kalian rasa kasih dan sayang. Sesungguhnya pada yang demikian itu benar-benar terdapat tanda-tanda bagi kaum yang berfikir*”.

Menimbang, bahwa perceraian merupakan perbuatan halal yang sangat dibenci oleh Allah SWT yang sedapat mungkin dihindari oleh setiap pasangan suami istri, akan tetapi mempertahankan suatu ikatan perkawinan yang rusak, yang mana dalam ikatan perkawinan tersebut hak-hak seorang suami maupun istri terabaikan, serta masing-masing suami dan istri hidup dalam kesendirian tanpa adanya ikatan batin layaknya rumah tangga yang normal, maka mempertahankan perkawinan yang demikian itu diduga kuat dapat mendatangkan *mudharat* atau *mafsadat* yang lebih besar dari pada maslahatnya juga bertentangan kaidah-kaidah hukum Islam (*maqâshid al-syari’ah*), sebab perkawinan semacam itu akan menyebabkan timbulnya penderitaan batin yang berkepanjangan yang tentu memudharatkan kepada salah satu pihak atau kedua belah pihak suami maupun istri;

Menimbang, bahwa dengan keadaan rumah tangga Pemohon dan Termohon seperti terungkap di atas apabila tetap dipertahankan, maka tentu akan berpotensi menimbulkan kemudharatan dan *mafsadah* yang lebih besar dari pada manfaat yang didapat, padahal menghindarkan bahaya lebih diutamakan dari mengambil manfaat, hal mana sesuai dengan *qaidah fiqhiyyah* yang berbunyi:

دَرْءُ المَفَاسِدِ مُقَدَّمٌ عَلَى جَلْبِ المَصَالِحِ

Artinya : “*Menghindarkan kerusakan / bahaya harus lebih diutamakan daripada mengharapkan kemaslahatan*”.

Menimbang, bahwa menurut Pasal 19 huruf (b) Peraturan Pemerintah Nomor 9 Tahun 1975 *juncto* Pasal 116 huruf (b) Kompilasi Hukum Islam ditegaskan salah satu alasan perceraian salah satu pihak meninggalkan pihak lain selama 2 (dua) tahun berturut-turut tanpa izin pihak lain dan tanpa alasan yang sah atau karena hal lain di luar kemampuannya, sedangkan Pasal 39 Undang-Undang Nomor 1 Tahun 1974 tentang Perkawinan

Disclaimer
Kepaniteraan Mahkamah Agung Republik Indonesia berusaha untuk selalu mencantumkan informasi paling kini dan akurat sebagai bentuk komitmen Mahkamah Agung untuk pelayanan publik, transparansi dan akuntabilitas pelaksanaan fungsi peradilan. Namun dalam hal-hal tertentu masih dimungkinkan terjadi permasalahan teknis terkait dengan akurasi dan keterkinian informasi yang kami sajikan, hal mana akan terus kami perbaiki dari waktu kewaktu.
Dalam hal Anda menemukan inakurasi informasi yang termuat pada situs ini atau informasi yang seharusnya ada, namun belum tersedia, maka harap segera hubungi Kepaniteraan Mahkamah Agung RI melalui :
Email : kepaniteraan@mahkamahagung.go.id   Telp : 021-384 3348 (ext.318)

menentukan bahwa untuk melakukan suatu perceraian harus ada cukup alasan, yakni ketika suami-isteri tidak akan dapat hidup rukun sebagai suami isteri dan Pengadilan telah berusaha dan tidak berhasil mendamaikan kedua belah pihak;

Menimbang, bahwa dari ketentuan pasal-pasal tersebut, terdapat beberapa unsur yang harus dipenuhi untuk terjadinya perceraian:

1. Salah satu pihak meninggalkan pihak lain selama 2 (dua) tahun berturut-turut tanpa izin pihak lain dan tanpa alasan yang sah atau karena hal lain di luar kemampuannya;
2. Suami isteri sudah tidak ada harapan untuk kembali rukun;
3. Pengadilan telah berupaya mendamaikan kedua belah pihak, namun tidak berhasil;

Menimbang, bahwa unsur-unsur tersebut akan dipertimbangkan satu persatu dengan mengaitkan fakta-fakta yang terjadi dalam rumah tangga Pemohon dan Termohon, sehingga antara Pemohon dan Termohon dipandang telah memenuhi unsur-unsur terjadinya suatu perceraian;

Menimbang, bahwa berdasarkan fakta yang telah dipertimbangkan di atas terbukti bahwa Tergugat telah lebih dari 2 (dua) tahun berturut-turut pergi meninggalkan Pemohon dengan tanpa izin dan alasan yang jelas, sehingga telah memenuhi alasan cerai Pasal 19 huruf b Peraturan Pemerintah Nomor 9 Tahun 1975 *juncto* Pasal 116 huruf b Kompilasi Hukum Islam. Berdasarkan pertimbangan-pertimbangan tersebut, maka unsur perceraian yang pertama harus dinyatakan telah terpenuhi;

Menimbang, bahwa selanjutnya terbukti pula bahwa sejak berpisah rumah tersebut, antara Pemohon dan Termohon tidak pernah saling pedulikan lagi, hal ini menunjukkan rumah tangga antara Pemohon dan Termohon sudah tidak ada harapan untuk kembali rukun, dengan demikian unsur kedua juga telah terpenuhi;

Menimbang, bahwa Majelis Hakim telah berupaya menasihati Pemohon pada setiap persidangan sesuai ketentuan Pasal 31 Peraturan Pemerintah Nomor 9 Tahun 1975, namun upaya tersebut tidak berhasil, dengan demikian maka unsur ketiga juga telah terpenuhi;

Disclaimer
Kepaniteraan Mahkamah Agung Republik Indonesia berusaha untuk selalu mencantumkan informasi paling kini dan akurat sebagai bentuk komitmen Mahkamah Agung untuk pelayanan publik, transparansi dan akuntabilitas pelaksanaan fungsi peradilan. Namun dalam hal-hal tertentu masih dimungkinkan terjadi permasalahan teknis terkait dengan akurasi dan keterkinian informasi yang kami sajikan, hal mana akan terus kami perbaiki dari waktu kewaktu.
Dalam hal Anda menemukan inakurasi informasi yang termuat pada situs ini atau informasi yang seharusnya ada, namun belum tersedia, maka harap segera hubungi Kepaniteraan Mahkamah Agung RI melalui :
Email : kepaniteraan@mahkamahagung.go.id   Telp : 021-384 3348 (ext.318)

Menimbang, bahwa oleh karena Permohonan Pemohon beralasan dan tidak melawan hukum, sedangkan Termohon tidak pernah hadir dalam persidangan meskipun telah dipanggil secara resmi dan patut, maka berdasarkan Pasal 149 R.Bg Permohonan Pemohon tersebut dikabulkan dengan tanpa hadirnya Termohon (verstek);

Menimbang, bahwa oleh karena dalil-dalil permohonan Pemohon cukup beralasan dan Pemohon dan Termohon belum pernah bercerai sebelumnya. Maka berdasarkan Pasal 131 ayat (2) jo. Pasal 118 Kompilasi Hukum Islam Pengadilan Agama Pasir Pengaraian akan menjatuhkan putusan terhadap perkara a quo dengan memberi izin kepada Pemohon menjatuhkan talak 1 (satu) raj'i terhadap Termohon;

Menimbang, bahwa oleh karena perkara ini termasuk ke dalam bidang perkawinan, maka berdasarkan ketentuan pasal 89 ayat (1) Undang-Undang Nomor 7 Tahun 1989 sebagaimana telah diubah dengan Undang-Undang Nomor 3 Tahun 2006, dan perubahan kedua dengan Undang-Undang Nomor 50 tahun 2009, maka seluruh biaya perkara dibebankan kepada Pemohon;;

Mengingat semua peraturan perundang-undangan yang berlaku serta dalil-dalil hukum Islam yang berhubungan dengan perkara ini;

M E N G A D I L I

1. Menyatakan Termohon yang telah dipanggil secara resmi dan patut untuk menghadap sidang tidak hadir;
2. Mengabulkan permohonan Pemohon secara Verstek;
3. Memberi izin kepada Pemohon **(Pemohon)** untuk menjatuhkan talak satu raj'i terhadap Termohon **(Termohon)** di depan sidang Pengadilan Agama Pasir Pengaraian;
4. Membebankan kepada Pemohon untuk membayar biaya perkara ini sejumlah Rp476.000,00 (empat ratus tujuh puluh enam ribu rupiah);

Demikian putusan ini dijatuhkan dalam musyawarah Majelis Hakim pada hari Rabu, tanggal 23 Desember 2020 Masehi/ 08 Jumadil awal 1442 Hijriah oleh Ahmad Zainul Anam, S.H.I., M.S.I., selaku Ketua Majelis, Liza, S.Sy dan Gustomo Try Budiharjo, S.H.I selaku Hakim-hakim Anggota dan putusan

Disclaimer
Kepaniteraan Mahkamah Agung Republik Indonesia berusaha untuk selalu mencantumkan informasi paling kini dan akurat sebagai bentuk komitmen Mahkamah Agung untuk pelayanan publik, transparansi dan akuntabilitas pelaksanaan fungsi peradilan. Namun dalam hal-hal tertentu masih dimungkinkan terjadi permasalahan teknis terkait dengan akurasi dan keterkinian informasi yang kami sajikan, hal mana akan terus kami perbaiki dari waktu kewaktu.
Dalam hal Anda menemukan inakurasi informasi yang termuat pada situs ini atau informasi yang seharusnya ada, namun belum tersedia, maka harap segera hubungi Kepaniteraan Mahkamah Agung RI melalui :
Email : kepaniteraan@mahkamahagung.go.id Telp : 021-384 3348 (ext.318)

tersebut diucapkan pada hari itu juga dalam sidang yang terbuka untuk umum oleh Ketua Majelis, didampingi oleh Hakim-hakim Anggota, dan dibantu oleh Syurya Gusmardi, S.H., selaku Panitera Pengganti, yang dihadiri oleh Pemohon tanpa kehadiran Termohon;

Hakim Anggota,

Ketua Majelis,

Liza, S.Sy

Ahmad Zainul Anam, S.H.I., M.S.I.

Hakim Anggota,

Gustomo Try Budiharjo, S.H.I

Panitera Pengganti,

Syurya Gusmardi, S.H.

Perincian biaya perkara :

1. Pendaftaran	Rp30.000,00
2. Proses	Rp50.000,00
3. Panggilan	Rp360.000,00
4. PNBP Panggilan	Rp20.000,00
4. Redaksi	Rp10.000,00
5. Meterai	Rp6.000,00
Jumlah	Rp476.000,00

(empat ratus tujuh puluh enam ribu rupiah);

Disclaimer
Kepaniteraan Mahkamah Agung Republik Indonesia berusaha untuk selalu mencantumkan informasi paling kini dan akurat sebagai bentuk komitmen Mahkamah Agung untuk pelayanan publik, transparansi dan akuntabilitas pelaksanaan fungsi peradilan. Namun dalam hal-hal tertentu masih dimungkinkan terjadi permasalahan teknis terkait dengan akurasi dan keterkinian informasi yang kami sajikan, hal mana akan terus kami perbaiki dari waktu kewaktu.
Dalam hal Anda menemukan inakurasi informasi yang termuat pada situs ini atau informasi yang seharusnya ada, namun belum tersedia, maka harap segera hubungi Kepaniteraan Mahkamah Agung RI melalui :
Email : kepaniteraan@mahkamahagung.go.id Telp : 021-384 3348 (ext.318)
