# Supplementary material for: Classification of divorce causes during the COVID-19 pandemic using convolutional neural networks
Source: PeerJ Comput Sci. 2022 Jun 30;8:e998. doi: 10.7717/peerj-cs.998 (PMC9299239; doi:10.7717/peerj-cs.998)
Supplement: Supplemental Information 4 [file peerj-cs-08-998-s004.zip › Konflik Dataset/Data ke-29.pdf]

PUTUSAN

Nomor 3050/Pdt.G/2020/PA.JB

بِسْمِ اللهِ الرَّحْمٰنِ الرَّحِيْمِ

DEMI KEADILAN BERDASARKAN KETUHANAN YANG MAHA ESA

Pengadilan Agama Jakarta Barat yang memeriksa dan mengadili perkara tertentu pada tingkat pertama dalam sidang majelis hakim telah menjatuhkan putusan dalam perkara Cerai Gugat antara:

Penggugat, NIK: XXX, Lahir di Jakarta, tanggal XXX, Nomor Telp: XXX, Agama Islam, Pendidikan SMK, Pekerjaan Mengurus Rumah Tangga, Tempat tinggal di Kota Jakarta Barat, selanjutnya disebut sebagai **Penggugat;**

melawan

Tergugat, NIK:-, Lahir di Jakarta, tanggal XXX, Agama Islam, Pendidikan SMK, Pekerjaan Karyawan Swasta, Tempat tinggal di Kota Jakarta Barat, selanjutnya disebut sebagai **Tergugat**;

Pengadilan Agama tersebut;
Telah mempelajari surat-surat yang berkaitan dengan perkara ini;
Telah mendengar keterangan Penggugat dan para saksi di muka sidang;

DUDUK PERKARA

Bahwa Penggugat dalam surat gugatannnya tanggal 25 November 2020, yang telah terdaftar di Kepaniteraan Pengadilan Agama Jakarta Barat, dengan Nomor 3050/Pdt.G/2020/PA.JB, tanggal 25 November 2020, dengan dalil-dalil pada pokoknya sebagai berikut:

1. Bahwa, pada hari Minggu tanggal 30 November 2014, Penggugat dengan Tergugat melangsungkan pernikahan yang dicatat oleh Pegawai Pencatat Nikah Kantor Urusan Agama Kecamatan Kalideres Kota Jakarta Barat sesuai Duplikat Kutipan Akta Nikah Nomor : XXX tertanggal 02 Desember 2011;

Disclaimer
Kepaniteraan Mahkamah Agung Republik Indonesia berusaha untuk selalu mencantumkan informasi paling kini dan akurat sebagai bentuk komitmen Mahkamah Agung untuk pelayanan publik, transparansi dan akuntabilitas pelaksanaan fungsi peradilan. Namun dalam hal-hal tertentu masih dimungkinkan terjadi permasalahan teknis terkait dengan akurasi dan keterkinian informasi yang kami sajikan, hal mana akan terus kami perbaiki dari waktu kewaktu.
Dalam hal Anda menemukan inakurasi informasi yang termuat pada situs ini atau informasi yang seharusnya ada, namun belum tersedia, maka harap segera hubungi Kepaniteraan Mahkamah Agung RI melalui :
Email : kepaniteraan@mahkamahagung.go.id Telp : 021-384 3348 (ext.318)

2. Bahwa, setelah pernikahan tersebut Penggugat dengan Tergugat bertempat tinggal di rumah kontrakan di Kota Jakarta Barat;

3. Bahwa, dalam perkawinan tersebut Penggugat dengan Tergugat telah bercampur (*ba'da dukhul*) sebagaimana layaknya suami istrI, dan telah dikaruniai seorang anak yang bernama :

3.1 XXX, laki-laki, lahir di Jakarta, tanggal XXX;

4. Bahwa, semula kehidupan rumah tangga antara Penggugat dengan Tergugat berjalan dengan harmonis, namun sejak pertengahan awal tahun 2015 keharmonisan rumah tangga Penggugat dengan Tergugat mulai goyah, disebabkan :

4.1 Tergugat kurang bertanggungjawab dalam menafkahi keluarga yang mana nafkah dari Tergugat tidak mencukupi untuk memenuhi kebutuhan sehari-hari;

4.2 Tergugat sering mengucapakan perkataan kasar terhadap Penggugat seperti kata-kata hinaan dan binatang;

4.3 Tergugat kurang dalam memperhatikan dan memberikan kasih sayang terhadap Penggugat;

4.4 Tergugat sering mabuk-mabukkan;

4.5 Tergugat sering keluar rumah tanpa alasan yang jelas;

4.6 Tergugat pernah mengucapkan perkataan cerai terhadap Penggugat;

5. Bahwa, karena sebab-sebab tersebut diatas Penggugat dan Tergugat sering berselisih dan bertengkar, namun percekcokan tersebut pada awalnya masih dalam batas-batas tertentu dan dapat diatasi oleh kedua belah pihak akan tetapi akhir-akhir ini percekcokan itu semakin bertambah tajam;

6. Bahwa, puncak perselisihan dan pertengkaran antara Penggugat dengan Tergugat tersebut terjadi kurang lebih pada bulan Mei tahun 2017, yang akibatnya Tergugat pergi meninggalkan Penggugat dan sejak saat itu antara Penggugat dan Tergugat sudah tidak menjalin hubungan selayaknya hubungan suami isteri;

Disclaimer
Kepaniteraan Mahkamah Agung Republik Indonesia berusaha untuk selalu mencantumkan informasi paling kini dan akurat sebagai bentuk komitmen Mahkamah Agung untuk pelayanan publik, transparansi dan akuntabilitas pelaksanaan fungsi peradilan. Namun dalam hal-hal tertentu masih dimungkinkan terjadi permasalahan teknis terkait dengan akurasi dan keterkinian informasi yang kami sajikan, hal mana akan terus kami perbaiki dari waktu kewaktu.
Dalam hal Anda menemukan inakurasi informasi yang termuat pada situs ini atau informasi yang seharusnya ada, namun belum tersedia, maka harap segera hubungi Kepaniteraan Mahkamah Agung RI melalui :
Email : kepaniteraan@mahkamahagung.go.id Telp : 021-384 3348 (ext.318)

7. Bahwa, akibat tersebut diatas Pengugat telah menderita lahir bathin dan Penggugat tidak ridho atas perlakuan Tergugat terhadap Penggugat serta Penggugat merasa tidak sanggup lagi untuk melanjutkan rumah tangga dengan Tergugat oleh karenanya Penggugat berkesimpulan satu-satunya jalan keluar yang terbaik bagi Penggugat adalah bercerai dengan Tergugat;

8. Bahwa, dengan fakta-fakta tersebut diatas gugatan Penggugat telah memenuhi alasan perceraian sebagaimana diatur dalam Pasal 19 PP No. 9 Tahun 1975 jo. Pasal 116 Kompilasi Hukum Islam;

9. Bahwa, terhadap biaya yang timbul akibat perkara ini agar dibebankan sesuai dengan peraturan perundang-undangan yang berlaku;

Berdasarkan alasan/dalil-dalil diatas, Penggugat mohon agar Ketua Pengadilan Agama Jakarta Barat segera memeriksa dan mengadili perkara ini, selanjutnya menjatuhkan putusan yang amarnya berbunyi :

PRIMER

1. Mengabulkan gugatan Penggugat;

2. Menjatuhkan thalak satu ba'in sughra Tergugat (**Tergugat**) terhadap Penggugat (**Penggugat**);

3. Membebankan biaya perkara sesuai dengan ketentuan peraturan perundang-undangan yang berlaku;

SUBSIDER

Dan atau apabila Majelis Hakim yang menyidangkan perkara ini berpendapat lain, maka mohon untuk menjatuhkan putusan yang seadil-adilnya (*ex aequo et bono*);

Bahwa pada hari dan sidang yang telah ditetapkan, Penggugat datang menghadap sendiri di persidangan, sedangkan Tergugat tidak pernah datang menghadap dan tidak pula menyuruh orang lain untuk menghadap sebagai wakil atau kuasanya yang sah, meskipun berdasarkan surat panggilan Nomor 3050/Pdt.G/2020/PA.JB yang dibacakan di persidangan, Tergugat telah

Disclaimer
Kepaniteraan Mahkamah Agung Republik Indonesia berusaha untuk selalu mencantumkan informasi paling kini dan akurat sebagai bentuk komitmen Mahkamah Agung untuk pelayanan publik, transparansi dan akuntabilitas pelaksanaan fungsi peradilan. Namun dalam hal-hal tertentu masih dimungkinkan terjadi permasalahan teknis terkait dengan akurasi dan keterkinian informasi yang kami sajikan, hal mana akan terus kami perbaiki dari waktu kewaktu.
Dalam hal Anda menemukan inakurasi informasi yang termuat pada situs ini atau informasi yang seharusnya ada, namun belum tersedia, maka harap segera hubungi Kepaniteraan Mahkamah Agung RI melalui :
Email : kepaniteraan@mahkamahagung.go.id Telp : 021-384 3348 (ext.318)

dipanggil secara resmi dan patut, sedangkan tidak ternyata, bahwa tidak datangnya Tergugat tersebut disebabkan oleh suatu alasan yang sah;

Bahwa karena Tergugat tidak pernah datang kepersidangan, maka perintah PERMA nomor 1 tahun 2016 tentang mediasi tidak dapat dilaksanakan;

Bahwa Majlis hakim telah berusaha menasehati Penggugat agar rukun kembali membina rumah tangganya dengan Tergugat, apalagi perkawinan Penggugat dengan Tergugat sudah lama dan sudah dikaruniai seorang anak, kasihan kepada anak jika terjadi, sebab yang akan menjadi korban dari perceraian adalah anak itu sendiri. Oleh karena itu Penggugat harus berfikir ulang untuk bercerai dengan Tergugat, karena dalam berumah tangga harus saling mengalah dan saling menghormati, sehingga Penggugat harus berfikir ulang untuk bercerai. Akan tetapi Penggugat tetap pada pendiriannya untuk bercerai dengan Tergugat;

Bahwa perkara ini tidak dapat dimediasi karena Tergugat tidak pernah datang menghadap meskipun telah dipanggil secara resmi dan patut, selanjutnya pemeriksaan perkara dilakukan dalam sidang tertutup untuk umum dengan terlebih dahulu dibacakan surat gugatan Penggugat yang isi dan maksudnya tetap dipertahankan oleh Penggugat;

Bahwa untuk menguatkan dalil-dalil gugatannya, Penggugat mengajukan alat bukti sebagai berikut :

A. Bukti Surat

1. Fotokopi Kartu Tanda Penduduk NIK XXX, atas nama (Penggugat) yang dikeluarkan oleh Pemerintah Kota Jakarta Barat, telah di-*nazegelen* dan telah dicocokkan dengan aslinya ternyata cocok, lalu diberi tanda “P1” dan diparaf oleh Ketua Majelis;
2. Fotokopi Kutipan Akta Nikah Nomor : XXX tanggal 02 Desember 2011 yang dikeluarkan oleh Kepala Kantor Urusan Agama Kecamatan Kalideres Jakarta Barat, telah di-*nazegelen* dan telah dicocokkan dengan aslinya ternyata cocok, lalu diberi tanda “P2” dan diparaf oleh Ketua Majelis;

B. Bukti Saksi.

Disclaimer
Kepaniteraan Mahkamah Agung Republik Indonesia berusaha untuk selalu mencantumkan informasi paling kini dan akurat sebagai bentuk komitmen Mahkamah Agung untuk pelayanan publik, transparansi dan akuntabilitas pelaksanaan fungsi peradilan. Namun dalam hal-hal tertentu masih dimungkinkan terjadi permasalahan teknis terkait dengan akurasi dan keterkinian informasi yang kami sajikan, hal mana akan terus kami perbaiki dari waktu kewaktu.
Dalam hal Anda menemukan inakurasi informasi yang termuat pada situs ini atau informasi yang seharusnya ada, namun belum tersedia, maka harap segera hubungi Kepaniteraan Mahkamah Agung RI melalui :
Email : kepaniteraan@mahkamahagung.go.id   Telp : 021-384 3348 (ext.318)

Saksi 1, XXX, umur 42 tahun, agama Islam, pekerjaan Buruh, tempat kediaman di Jakarta Barat, saksi di bawah sumpah memberikan keterangan yang pada pokoknya sebagai berikut :

- Bahwa sepengetahuan saksi perkawinan Penggugat dengan Tergugat berstatus gadis dan pejaka dan mereka bertempat tinggal di rumah kontrakan di XXX;
- Bahwa sepengetahuan saksi perkawinan Penggugat dengan Tergugat telah berhubungan sebagaimana layaknya suami istri dan telah dikaruniai seorang anak, masing-masing bernama XXX;
- Bahwa sepengetahuan saksi pada awalnya rumah tengga Penggugat dengan Tergugat rukun dan harmonis, akan tetapi sejak pertengahan awal tahun 2015 yang lalu rumah tangga Penggugat dan Tergugat mulai tidak harmonis lagi sering terjadi perselisihan dan pertengkaran yang terus menerus;
- Bahwa setahu saksi penyebab terjadinya perselisihan dan pertengkaran tersebut karena Tergugat kurang bertanggungjawab dalam menafkahi keluarga, Tergugat sering mengucapkan perkataan kasar, Tergugat sering mabuk-mabukan, Tergugat sering keluar rumah tanpa alasan yang jelas;
- Bahwa puncak perselisihan dan pertengkaran antara Penggugat dan Tergugat terjadi pada bulan Mei tahun 2017 yang lalu, yang akhirnya Tergugat pergi meninggalkan kediaman bersama;
- Bahwa sejak pisah rumah pada bulan Mei tahun 2017 sampai sekarang, antara Penggugat dan Tergugat sudah tidak ada hubungan layaknya suami istri;
- Bahwa setahu saksi ke dua orang tua Penggugat dan orang tua Tergugat pernah merukunkan rumah tangga mereka mereka, tetapi tidak berhasil dan saksi juga pernah menasehati Penggugat dengan Tergugat, tetapi tidak berhasil, saksi sudah tidak sanggup lagi menasehati mereka;

Saksi 2, XXX, umur 56 tahun, agama Islam, pekerjaan Ibu Rumah Tangga, tempat kediaman di Jakarta Barat, saksi di bawah sumpah memberikan keterangan yang pada pokoknya sebagai berikut:

Disclaimer
Kepaniteraan Mahkamah Agung Republik Indonesia berusaha untuk selalu mencantumkan informasi paling kini dan akurat sebagai bentuk komitmen Mahkamah Agung untuk pelayanan publik, transparansi dan akuntabilitas pelaksanaan fungsi peradilan. Namun dalam hal-hal tertentu masih dimungkinkan terjadi permasalahan teknis terkait dengan akurasi dan keterkinian informasi yang kami sajikan, hal mana akan terus kami perbaiki dari waktu kewaktu.
Dalam hal Anda menemukan inakurasi informasi yang termuat pada situs ini atau informasi yang seharusnya ada, namun belum tersedia, maka harap segera hubungi Kepaniteraan Mahkamah Agung RI melalui :
Email : kepaniteraan@mahkamahagung.go.id Telp : 021-384 3348 (ext.318)

- Bahwa sepengetahuan saksi perkawinan Penggugat dengan Tergugat berstatus gadis dan pejaka dan mereka bertempat tinggal di rumah kontrakan di XXX;
- Bahwa sepengetahuan saksi perkawinan Penggugat dengan Tergugat telah berhubungan sebagaimana layaknya suami istri dan telah dikaruniai seorang anak, masing-masing bernama XXX;
- Bahwa sepengetahuan saksi pada awalnya rumah tengga Penggugat dengan Tergugat rukun dan harmonis, akan tetapi sejak pertengahan awal tahun 2015 yang lalu rumah tangga Penggugat dan Tergugat mulai tidak harmonis lagi sering terjadi perselisihan dan pertengkaran yang terus menerus;
- Bahwa setahu saksi penyebab terjadinya perselisihan dan pertengkaran tersebut karena Tergugat kurang bertanggungjawab dalam menafkahi keluarga, Tergugat sering mengucapkan perkataan kasar, Tergugat sering mabuk-mabukan, Tergugat sering keluar rumah tanpa alasan yang jelas;
- Bahwa puncak perselisihan dan pertengkaran antara Penggugat dan Tergugat terjadi pada bulan Mei tahun 2017 yang lalu, yang akhirnya Tergugat pergi meninggalkan kediaman bersama;
- Bahwa sejak pisah rumah pada bulan Mei tahun 2017 sampai sekarang, antara Penggugat dan Tergugat sudah tidak ada hubungan layaknya suami istri;
- Bahwa setahu saksi ke dua orang tua Penggugat dan orang tua Tergugat pernah merukunkan rumah tangga mereka mereka, tetapi tidak berhasil dan saksi juga pernah menasehati Penggugat dengan Tergugat, tetapi tidak berhasil, saksi sudah tidak sanggup lagi menasehati mereka;

Bahwa, Penggugat telah mengajukan kesimpulan yang pada pokoknya tetap dengan dalil-dalil gugatannya dan mohon putusan;

Bahwa untuk mempersingkat uraian putusan ini, segala sesuatu yang tercatat dalam Berita Acara Sidang (BAS) dianggap telah termasuk dan merupakan bagian yang tak terpisahkan dari putusan ini;

Disclaimer
Kepaniteraan Mahkamah Agung Republik Indonesia berusaha untuk selalu mencantumkan informasi paling kini dan akurat sebagai bentuk komitmen Mahkamah Agung untuk pelayanan publik, transparansi dan akuntabilitas pelaksanaan fungsi peradilan. Namun dalam hal-hal tertentu masih dimungkinkan terjadi permasalahan teknis terkait dengan akurasi dan keterkinian informasi yang kami sajikan, hal mana akan terus kami perbaiki dari waktu kewaktu.
Dalam hal Anda menemukan inakurasi informasi yang termuat pada situs ini atau informasi yang seharusnya ada, namun belum tersedia, maka harap segera hubungi Kepaniteraan Mahkamah Agung RI melalui :
Email : kepaniteraan@mahkamahagung.go.id Telp : 021-384 3348 (ext.318)

PERTIMBANGAN HUKUM

Menimbang, bahwa maksud dan tujuan gugatan Penggugat adalah sebagaimana telah diuraikan di atas;

Menimbang, bahwa ternyata Tergugat tidak datang menghadap di persidangan dan tidak pula menyuruh orang lain untuk menghadap sebagai wakil/kuasanya yang sah, meskipun berdasarkan Surat Panggilan (relaas) Nomor 3050/Pdt.G/2020/PA.JB yang dibacakan di persidangan, Tergugat telah dipanggil secara resmi dan patut, sedangkan tidak ternyata bahwa tidak datangnya Tergugat tersebut disebabkan oleh suatu alasan yang sah;

Menimbang, bahwa oleh karena selama persidangan hanya Penggugat yang hadir sedangkan Tergugat tidak pernah hadir, maka upaya mediasi sebagaimana ketentuan Peraturan Mahkamah Agung RI Nomor 1 Tahun 2016 tentang Prosedur Mediasi di Pengadilan, tidak dapat dilaksanakan;

Menimbang bahwa Majlis hakim telah berusaha menasehati Penggugat agar rukun kembali membinan rumah tangganya dengan Tergugat, apalagi perkawinan Penggugat dengan Tergugat sudah lama dan sudah dikaruniai seorang anak, kasihan kepada anak-anak jika terjadi, sebab yang akan menjadi korban dari perceraian adalah anak itu sendiri. Oleh karena itu Penggugat harus berfikir ulang untuk bercerai dengan Tergugat, karena dalam berumah tangga harus saling mengalah dan saling menghormati, sehingga Penggugat harus berfikir ulang untuk bercerai. Akan tetapi Penggugat tetap pada pendiriannya untuk bercerai dengan Tergugat;

Menimbang, bahwa yang dijadikan dasar bagi Penggugat mengajukan gugatannya kepada Tergugat dengan alasan bahwa rumah tangga Penggugat dengan Tergugat sejak pertengahan awal tahun 2015 sudah tidak rukun, sering terjadi pertengkaran, yang disebabkan hal-hal sebagaimana dalam surat gugatannya, yaitu : “Tergugat kurang bertanggungjawab dalam menafkahi keluarga, Tergugat sering mengucapkan perkataan kasar, Tergugat sering mabuk-mabukan, Tergugat sering keluar rumah tanpa alasan yang jelas”. Sehingga antara Penggugat dengan Tergugat pisah tempat tinggal pada bulan Mei tahun 2017 hingga sekarang dan Penggugat merasakan hidup berumah tangga dengan Tergugat sudah tidak dapat dipertahankan lagi;

Disclaimer
Kepaniteraan Mahkamah Agung Republik Indonesia berusaha untuk selalu mencantumkan informasi paling kini dan akurat sebagai bentuk komitmen Mahkamah Agung untuk pelayanan publik, transparansi dan akuntabilitas pelaksanaan fungsi peradilan. Namun dalam hal-hal tertentu masih dimungkinkan terjadi permasalahan teknis terkait dengan akurasi dan keterkinian informasi yang kami sajikan, hal mana akan terus kami perbaiki dari waktu kewaktu.
Dalam hal Anda menemukan inakurasi informasi yang termuat pada situs ini atau informasi yang seharusnya ada, namun belum tersedia, maka harap segera hubungi Kepaniteraan Mahkamah Agung RI melalui :
Email : kepaniteraan@mahkamahagung.go.id Telp : 021-384 3348 (ext.318)

Menimbang, bahwa untuk menguatkan dalil gugatannya Penggugat telah membawa bukti tertulis berupa P.1 dan P.2 serta 2 (dua) orang saksi;

Menimbang, bahwa bukti P.1, menjelaskan mengenai Kartu Tanda Penduduk dan tempat kediaman Penggugat, sehingga bukti tersebut telah memenuhi syarat formal dan materiil, serta mempunyai kekuatan pembuktian yang sempurna dan mengikat, sehingga Pengadilan Agama Jakarta Barat berwengan untuk memeriksa dan mengadili perkara ini;

Menimbang, bahwa bukti P.2 merupakan fotokopi dari Akta Nikah telah bermeterai cukup dan telah cocok dengan aslinya, oleh karena itu bukti tersebut telah memenuhi Pasal 2 ayat (3) Undang-Undang Nomor 13 Tahun 1985 dan Pasal 165 HIR, sehingga bukti tersebut mempunyai kekuatan bukti yang sempurna dan mengikat, maka Penggugat dan Tergugat harus dinyatakan terbukti sebagai suami istri yang sah, dengan demikian Penggugat memiliki *legal standing* untuk mengajukan gugatan cerai sebagaimana diatur dalam Pasal 14 Peraturan Pemerintah No. 9 Tahun 1975 jo. Pasal 73 Undang-Undang No. 7 Tahun 1989 yang sudah diubah dengan Undang-Undang No. 3 Tahun 2006 dan Undang-Undang No. 50 Tahun 2009;

Menimbang, bahwa telah didengar keterangan saksi-saksi yang diajukan oleh Penggugat, diatas sumpahnya dan keterangan saksi-saksi tersebut bersesuaian satu dengan yang lainnya, oleh karena itu memenuhi syarat keterangan saksi sebagaimana diatur dalam Pasal 171 dan 172 HIR, oleh karena itu keterangan saksi tersebut dapat diterima;

Menimbang, bahwa atas dalil-dalil gugatan Penggugat dihubungkan dengan alat bukti yang diajukan serta hasil pemeriksaan dalam sidang, Majelis hakim telah menemukan fakta-fakta sebagai berikut :

- Bahwa sepengetahuan saksi-saksi perkawinan Penggugat dengan Tergugat berstatus gadis dan pejaka dan mereka bertempat tinggal di rumah kontrakan di XXX;
- Bahwa sepengetahuan saksi-saksi perkawinan Penggugat dengan Tergugat telah berhubungan sebagaimana layaknya suami istri dan telah dikaruniai seorang anak, masing-masing bernama : XXX;

Disclaimer
Kepaniteraan Mahkamah Agung Republik Indonesia berusaha untuk selalu mencantumkan informasi paling kini dan akurat sebagai bentuk komitmen Mahkamah Agung untuk pelayanan publik, transparansi dan akuntabilitas pelaksanaan fungsi peradilan. Namun dalam hal-hal tertentu masih dimungkinkan terjadi permasalahan teknis terkait dengan akurasi dan keterkinian informasi yang kami sajikan, hal mana akan terus kami perbaiki dari waktu kewaktu.
Dalam hal Anda menemukan inakurasi informasi yang termuat pada situs ini atau informasi yang seharusnya ada, namun belum tersedia, maka harap segera hubungi Kepaniteraan Mahkamah Agung RI melalui :
Email : kepaniteraan@mahkamahagung.go.id Telp : 021-384 3348 (ext.318)

- Bahwa sepengetahuan saksi-saksi pada awalnya rumah tengga Penggugat dengan Tergugat rukun dan harmonis, akan tetapi sejak pertengahan awal tahun 2015 yang lalu rumah tangga Penggugat dan Tergugat mulai tidak harmonis lagi sering terjadi perselisihan dan pertengkaran yang terus menerus;
- Bahwa setahu saksi-saksi penyebab terjadinya perselisihan dan pertengkaran tersebut karena Tergugat kurang bertanggungjawab dalam menafkahi keluarga, Tergugat sering mengucapkan perkataan kasar, Tergugat sering mabuk-mabukan, Tergugat sering keluar rumah tanpa alasan yang jelas;
- Bahwa puncak perselisihan dan pertengkaran antara Penggugat dan Tergugat terjadi pada bulan Mei tahun 2017 yang lalu, yang akhirnya Tergugat pergi meninggalkan kediaman bersama;
- Bahwa sejak pisah rumah pada bulan Mei tahun 2017 sampai sekarang, antara Penggugat dan Tergugat sudah tidak ada hubungan layaknya suami istri;
- Bahwa setahu saksi-saksi ke dua orang tua Penggugat dan orang tua Tergugat pernah merukunkan rumah tangga mereka mereka, tetapi tidak berhasil dan saksi-saksi juga pernah menasehati Penggugat dengan Tergugat, tetapi tidak berhasil, saksi-saksi sudah tidak sanggup lagi menasehati mereka;

Menimbang, bahwa berdasarkan fakta hukum diatas, ternyatalah bahwa rumah tangga Penggugat dan Tergugat telah terjadi perselisihan dan pertengkaran dan telah hidup berpisah ranjang serta sudah tidak menjalankan kewajiban sebagaimana layaknya suami istri sejak pada bulan Mei tahun 2017 yang lalu hingga sekarang sudah berjalan 2 tahun lebih tidak pernah bersatu lagi, hal ini telah menunjukkan bahwa rumah tangga yang seperti itu tidak lagi dapat dikatakan rumah tangga yang bahagia yang dilandasi oleh adanya mawaddah dan rahmah, sehingga oleh karenanya dari fakta tersebut telah menunjukkan pula bahwa Penggugat dan Tergugat telah gagal untuk membentuk rumah tangga yang bahagia ;

Disclaimer
Kepaniteraan Mahkamah Agung Republik Indonesia berusaha untuk selalu mencantumkan informasi paling kini dan akurat sebagai bentuk komitmen Mahkamah Agung untuk pelayanan publik, transparansi dan akuntabilitas pelaksanaan fungsi peradilan. Namun dalam hal-hal tertentu masih dimungkinkan terjadi permasalahan teknis terkait dengan akurasi dan keterkinian informasi yang kami sajikan, hal mana akan terus kami perbaiki dari waktu kewaktu.
Dalam hal Anda menemukan inakurasi informasi yang termuat pada situs ini atau informasi yang seharusnya ada, namun belum tersedia, maka harap segera hubungi Kepaniteraan Mahkamah Agung RI melalui :
Email : kepaniteraan@mahkamahagung.go.id Telp : 021-384 3348 (ext.318)

Menimbang, bahwa sejalan dengan firman Allah dalam surat Ar-Rumm ayat 21 yang berbunyi :

وَمِنْ ءَايَاتِهِ أَنْ خَلَقَ لَكُمْ مِنْ أَنْفُسِكُمْ أَزْوَاجًا لِتَسْكُنُوا إِلَيْهَا وَجَعَلَ بَيْنَكُمْ مَوَدَّةً

وَرَحْمَةً إِنَّ فِي ذَلِكَ لَآيَاتٍ لِقَوْمٍ يَتَفَكَّرُونَ

Artinya: Dan di antara tanda-tanda kekuasaan-Nya ialah Dia menciptakan untukmu isteri-isteri dari jenismu sendiri, supaya kamu cenderung dan merasa tenteram kepadanya, dan dijadikan-Nya di antaramu rasa kasih dan sayang. Sesungguhnya pada yang demikian itu benar-benar terdapat tanda-tanda bagi kaum yang berfikir;

Menimbang, bahwa menghindari terjadinya mafsadat dalam rumah tangga harus lebih diutamakan dari pada mendatangkan kemaslahatan sesuai qaidah Fiqhiyyah yang berbunyi :

درء المفاسد مقدم على جلب المصالح

Artinya : *Menolak mafsadat lebih diutamakan untuk menjaga kemaslahatan*;

Menimbang, bahwa berdasarkan pertimbangan-pertimbangan tersebut di atas, majelis hakim berpendapat bahwa terdapat fakta hukum yang cukup untuk menyatakan bahwa gugatan cerai yang diajukan oleh Penggugat telah terbukti beralasan dan tidak melawan hukum serta telah memenuhi unsur yang tercantum pada Pasal 19 huruf (f) Peraturan Pemerintah Nomor 9 Tahun 1975 Jo. Pasal 116 huruf (f) Kompilasi Hukum Islam, oleh karena itu gugatan Penggugat sudah sepatutnya dikabulkan dengan menjatuhkan talak satu bain shugra Tergugat terhadap Penggugat secara verstek, sebagaimana ketentuan Pasal 125 ayat (1) HIR;

Menimbang, bahwa oleh karena perkara tersebut dalam bidang perkawinan, maka berdasarkan ketentuan Pasal 89 ayat (1) Undang-Undang Nomor 7 Tahun 1989 sebagaimana yang telah diubah dengan Undang-Undang Nomor 3 Tahun 2006, dan perubahan kedua dengan Undang-Undang Nomor 50 Tahun 2009, maka biaya perkara di bebankan kepada Penggugat;

Disclaimer
Kepaniteraan Mahkamah Agung Republik Indonesia berusaha untuk selalu mencantumkan informasi paling kini dan akurat sebagai bentuk komitmen Mahkamah Agung untuk pelayanan publik, transparansi dan akuntabilitas pelaksanaan fungsi peradilan. Namun dalam hal-hal tertentu masih dimungkinkan terjadi permasalahan teknis terkait dengan akurasi dan keterkinian informasi yang kami sajikan, hal mana akan terus kami perbaiki dari waktu kewaktu.
Dalam hal Anda menemukan inakurasi informasi yang termuat pada situs ini atau informasi yang seharusnya ada, namun belum tersedia, maka harap segera hubungi Kepaniteraan Mahkamah Agung RI melalui :
Email : kepaniteraan@mahkamahagung.go.id Telp : 021-384 3348 (ext.318)

Mengingat, bunyi dari pasal-pasal dari peraturan Perundang-undangan yang berlaku serta dalil-dalil yang berkaitan dengan perkara ini;

MENGADILI

1. Menyatakan Tergugat yang telah dipanggil secara resmi dan patut untuk menghadap di muka sidang, tidak hadir;

2. Mengabulkan gugatan Penggugat dengan verstek;

3. Menjatuhkan talak satu ba'in shughra Tergugat (**Tergugat**) terhadap Penggugat (**Penggugat**)

4. Membebankan kepada Penggugat untuk membayar biaya perkara sejumlah Rp. 401.000,- (empat ratus satu ribu rupiah);

Demikian putusan ini dijatuhkan dalam rapat permusyawaratan Majelis Hakim Pengadilan Agama Jakarta Barat yang dilangsungkan pada hari Rabu tanggal 23 Desember 2020 Masehi, bertepatan dengan tanggal 08 Jumadil Awwal 1442 Hijriyah, oleh kami Drs. H. Soleman., M.H. sebagai Ketua Majelis, Dr. Agus Yunih., S.H., M.H.I. dan Dra. Hj. Fauziah masing-masing sebagai Hakim Anggota, putusan tersebut diucapkan dalam sidang terbuka untuk umum pada hari itu juga, oleh Ketua Majelis tersebut dengan didampingi oleh Hakim Anggota dan dibantu oleh H. Waluyo, S.H. sebagai Panitera Pengganti serta dihadiri oleh Penggugat tanpa kehadiran Tergugat;

Ketua Majelis,

Drs. H. Soleman., M.H.

Hakim Anggota, **Hakim Anggota,**

Disclaimer
Kepaniteraan Mahkamah Agung Republik Indonesia berusaha untuk selalu mencantumkan informasi paling kini dan akurat sebagai bentuk komitmen Mahkamah Agung untuk pelayanan publik, transparansi dan akuntabilitas pelaksanaan fungsi peradilan. Namun dalam hal-hal tertentu masih dimungkinkan terjadi permasalahan teknis terkait dengan akurasi dan keterkinian informasi yang kami sajikan, hal mana akan terus kami perbaiki dari waktu kewaktu.
Dalam hal Anda menemukan inakurasi informasi yang termuat pada situs ini atau informasi yang seharusnya ada, namun belum tersedia, maka harap segera hubungi Kepaniteraan Mahkamah Agung RI melalui :
Email : kepaniteraan@mahkamahagung.go.id Telp : 021-384 3348 (ext.318)

Dr. Agus Yunih., S.H., M.H.I. Dra. Hj. Fauziah

Panitera Pengganti,

H. Waluyo, S.H.

Perincian biaya :

1.	Pendaftaran	Rp. 30.000,-
2.	BAPP	Rp. 75.000,-
3.	Panggilan	Rp. 260.000,-
4.	PNBP	Rp. 20.000,-
5.	Redaksi	Rp. 10.000,-
6.	Materai	Rp. 6.000,-
Jumlah	Rp. 401.000,- (empat ratus satu ribu rupiah);	

Disclaimer
Kepaniteraan Mahkamah Agung Republik Indonesia berusaha untuk selalu mencantumkan informasi paling kini dan akurat sebagai bentuk komitmen Mahkamah Agung untuk pelayanan publik, transparansi dan akuntabilitas pelaksanaan fungsi peradilan. Namun dalam hal-hal tertentu masih dimungkinkan terjadi permasalahan teknis terkait dengan akurasi dan keterkinian informasi yang kami sajikan, hal mana akan terus kami perbaiki dari waktu kewaktu.
Dalam hal Anda menemukan inakurasi informasi yang termuat pada situs ini atau informasi yang seharusnya ada, namun belum tersedia, maka harap segera hubungi Kepaniteraan Mahkamah Agung RI melalui :
Email : kepaniteraan@mahkamahagung.go.id Telp : 021-384 3348 (ext.318)
