# Supplementary material for: Classification of divorce causes during the COVID-19 pandemic using convolutional neural networks
Source: PeerJ Comput Sci. 2022 Jun 30;8:e998. doi: 10.7717/peerj-cs.998 (PMC9299239; doi:10.7717/peerj-cs.998)
Supplement: Supplemental Information 4 [file peerj-cs-08-998-s004.zip › Konflik Dataset/Data ke-3.pdf]

P U T U S A N

Nomor XXXX/Pdt.G/2020/PA.Dmk

بِسْمِ اللَّهِ الرَّحْمَٰنِ الرَّحِيمِ

DEMI KEADILAN BERDASARKAN KETUHANAN YANG MAHA ESA

Pengadilan Agama Demak yang memeriksa dan mengadili perkara tertentu pada tingkat pertama dalam sidang Majelis telah menjatuhkan putusan perkara Gugatan Perceraian antara;

Penggugat, umur 34 tahun, agama Islam, Pekerjaan online Shop, Pendidikan Sekolah Lanjutan Tingkat Atas, tempat kediaman di XXXXKabupaten Demak, sebagai **Penggugat**;

Melawan

Tergugat, umur 38 tahun, agama Islam, Pekerjaan Penjahit, Pendidikan Sekolah Lanjutan Tingkat Atas, tempat kediaman di XXXXKabupaten Demak, tetapi sekarang tidak diketahui tempat kediamannya, baik di dalam maupun di luar wilayah Republik Indonesia, sebagai **Tergugat**;

Pengadilan Agama tersebut;

Telah mempelajari surat-surat yang berkaitan dengan perkara ini;

Telah mendengar keterangan Penggugat dan para saksi di muka sidang;

DUDUK PERKARA

Bahwa, Penggugat dalam surat gugatannya tanggal 14 Agustus 2020 telah mengajukan gugatan perceraian yang telah didaftar di Kepaniteraan Pengadilan Agama Demak dengan Nomor XXXX/Pdt.G/2020/PA.Dmk, tanggal 18 Agustus 2020, dengan dalil-dalil sebagai berikut:

1. Bahwa Penggugat dengan Tergugat telah melangsungkan pernikahan pada tanggal 29 Juni 2006, dicatat oleh Pegawai Pencatat Nikah Kantor Urusan Agama Kecamatan Wedung, Kabupaten Demak, sebagaimana Kutipan Akta Nikah Nomor: 385/60/VI/2006, tanggal 29 Juni 2006;

Disclaimer
Kepaniteraan Mahkamah Agung Republik Indonesia berusaha untuk selalu mencantumkan informasi paling kini dan akurat sebagai bentuk komitmen Mahkamah Agung untuk pelayanan publik, transparansi dan akuntabilitas pelaksanaan fungsi peradilan. Namun dalam hal-hal tertentu masih dimungkinkan terjadi permasalahan teknis terkait dengan akurasi dan keterkinian informasi yang kami sajikan, hal mana akan terus kami perbaiki dari waktu kewaktu.
Dalam hal Anda menemukan inakurasi informasi yang termuat pada situs ini atau informasi yang seharusnya ada, namun belum tersedia, maka harap segera hubungi Kepaniteraan Mahkamah Agung RI melalui :
Email : kepaniteraan@mahkamahagung.go.id Telp : 021-384 3348 (ext.318)

2. Bahwa setelah menikah, Penggugat dengan Tergugat bertempat tinggal di rumah orang tua Penggugat selama 13 tahun 2 bulan, sudah melakukan hubungan layaknya suami istri (ba'da dukhul), dan telah dikaruniai 2 (dua) orang anak yang masing-masing bernama:;

- XXXX, laki-laki, lahir pada tanggal 18 Agustus 2008, sekarang dalam asuhan Penggugat;
- XXXX, perempuan, lahir pada tanggal 13 Juli 2017, sekarang dalam asuhan Penggugat;

3. Bahwa sejak bulan Juni 2018, antara Penggugat dan Tergugat sering terjadi perselisihan dan pertengkaran yang disebabkan Tergugat kurang, dalam memberi nafkah kepada Penggugat, karena Tergugat orangnya malas bekerja dan lebih sering menganggurnya. Tergugat hanya memberi nafkah kepada Penggugat setiap minggunya rata-rata sebesar Rp200.000,00 (dua ratus ribu rupiah). Padahal, untuk memenuhi kebutuhan rumah tangga Penggugat dan Tergugat, setiap minggunya Penggugat membutuhkan paling tidak sebesar Rp500.000,00 (lima ratus ribu rupiah). Bila dinasehati oleh Penggugat, Tergugat justru marah-marah. Sehingga untuk menutup kekurangannya, Penggugat harus bekerja sendiri dan dibantu oleh orang tua Penggugat. Akhirnya menjadikan perselisihan dan pertengkaran antara Penggugat dan Tergugat semakin memuncak;

4. Bahwa akibatnya, sejak bulan Agustus 2019, tanpa pamit Penggugat, Tergugat pergi meninggalkan Penggugat hingga sekarang sudah 1 tahun lamanya, dan selama itu pula, Tergugat tidak pernah kembali lagi pada Penggugat, serta Tergugat tidak memberi kabar (berita) pada Penggugat, bahkan sekarang Tergugat sudah tidak diketahui tempat kediamannya, baik di dalam maupun di luar wilayah Negara RI;

5. Bahwa Penggugat sudah berusaha mencari keberadaan Tergugat, tetapi tidak berhasil;

6. Bahwa berdasarkan hal-hal tersebut diatas, alasan Penggugat untuk mengajukan gugatan perceraian terhadap Tergugat atas dasar perselisihan dan pertengkaran yang terjadi terus menerus dan tidak

Disclaimer
Kepaniteraan Mahkamah Agung Republik Indonesia berusaha untuk selalu mencantumkan informasi paling kini dan akurat sebagai bentuk komitmen Mahkamah Agung untuk pelayanan publik, transparansi dan akuntabilitas pelaksanaan fungsi peradilan. Namun dalam hal-hal tertentu masih dimungkinkan terjadi permasalahan teknis terkait dengan akurasi dan keterkinian informasi yang kami sajikan, hal mana akan terus kami perbaiki dari waktu kewaktu.
Dalam hal Anda menemukan inakurasi informasi yang termuat pada situs ini atau informasi yang seharusnya ada, namun belum tersedia, maka harap segera hubungi Kepaniteraan Mahkamah Agung RI melalui :
Email : kepaniteraan@mahkamahagung.go.id Telp : 021-384 3348 (ext.318)

mungkin hidup rukun dalam suatu ikatan perkawinan, kemudian Tergugat telah pergi meninggalkan Penggugat hingga sekarang sudah 1 tahun lamanya, dan selama itu pula, Tergugat tidak pernah kembali lagi pada Penggugat, serta Tergugat tidak memberi kabar (berita) pada Penggugat, telah memenuhi unsur Pasal 19 huruf (f) Peraturan Pemerintah No. 9 Tahun 1975 Jo. Pasal 116 huruf (f) Kompilasi Hukum Islam, sehingga berdasar hukum untuk menyatakan gugatan cerai ini dikabulkan;

7. Bahwa kedua anak Penggugat dan Tergugat tersebut masih di bawah umur dan masih sangat membutuhkan kasih sayang seorang ibu, oleh karena itu Penggugat mohon agar ditetapkan sebagai pemegang hak hadhanah (pemeliharaan) atas kedua anak tersebut di atas;

8. Bahwa Penggugat sanggup membayar biaya perkara;

PRIMER:;

1. Mengabulkan gugatan Penggugat;
2. Menjatuhkan talak satu ba'in sughra Tergugat (Tergugat) terhadap Penggugat (Penggugat);
3. Menetapkan 2 (dua) orang anak dari perkawinan Penggugat dan Tergugat yang bernama: ;
   - XXXX, laki-laki, lahir pada tanggal 18 Agustus 2008;
   - XXXX, perempuan, lahir pada tanggal 13 Juli 2017;

   berada di bawah pemeliharaan (hadhanah) Penggugat;
4. Membebankan biaya perkara ini menurut hukum;

SUBSIDER:;

- Atau mohon putusan lain yang seadil-adilnya;

Bahwa, pada hari sidang yang telah ditetapkan Penggugat telah datang menghadap ke muka sidang, sedangkan Tergugat tidak datang menghadap ke muka sidang dan tidak menyuruh orang lain untuk menghadap sebagai wakil/kuasa hukumnya meskipun telah dipanggil secara resmi dan patut yang relaas panggilannya telah dibacakan di dalam sidang, sedangkan tidak ternyata bahwa tidak datangnya itu disebabkan suatu halangan yang sah;

Bahwa, Majelis hakim telah menasehati Penggugat agar berpikir untuk tidak bercerai dengan Tergugat, tetapi Penggugat tetap pada dalil-dalil

Disclaimer
Kepaniteraan Mahkamah Agung Republik Indonesia berusaha untuk selalu mencantumkan informasi paling kini dan akurat sebagai bentuk komitmen Mahkamah Agung untuk pelayanan publik, transparansi dan akuntabilitas pelaksanaan fungsi peradilan. Namun dalam hal-hal tertentu masih dimungkinkan terjadi permasalahan teknis terkait dengan akurasi dan keterkinian informasi yang kami sajikan, hal mana akan terus kami perbaiki dari waktu kewaktu.
Dalam hal Anda menemukan inakurasi informasi yang termuat pada situs ini atau informasi yang seharusnya ada, namun belum tersedia, maka harap segera hubungi Kepaniteraan Mahkamah Agung RI melalui :
Email : kepaniteraan@mahkamahagung.go.id Telp : 021-384 3348 (ext.318)

gugatannya untuk bercerai dengan Tergugat;

Bahwa, perkara ini tidak dapat di mediasi karena Tergugat tidak pernah datang menghadap meskipun telah dipanggil secara resmi dan patut, selanjutnya dimulai pemeriksaan dengan membacakan surat gugatan Penggugat yang maksud dan isinya tetap dipertahankan oleh Penggugat;

Bahwa, untuk menguatkan dalil-dalil gugatannya, Penggugat telah mengajukan bukti-bukti berupa;

A. Surat;

1. Fotokopi Kartu Tanda Penduduk Nomor 3321135508860006 tanggal 06 Januari 2016, bermeterai cukup dan telah sesuai dengan aslinya (Bukti P.1);
2. Fotokopi Buku Kutipan Akta Nikah dari KUA Kecamatan Wedung, Kabupaten Demak Propinsi Jawa Tengah Nomor 385/60/VI/2006 tanggal 29 Juni 2006, bermeterai cukup dan telah sesuai dengan aslinya (Bukti P.2);
3. Fotokopi Surat Keterangan Pergi nomor 472.2/1433, tanggal 13 Agustus 2020. yang dikeluarkan oleh XXXXKabupaten Demak (Bukti P.3).
4. Fotokopi kutipan akta kelahiran No. AL. 6590067664 tertanggal 15 Mei 2009, dikeluarkan oleh Dinad Kependudukan dan Pencatatan Sipil Kabupaten Demak, (bukti P-3);
5. Fotokopi kutipan akta kelahiran No. AL. 6590485116 tertanggal 13 Oktober 2017, dikeluarkan oleh Dinad Kependudukan dan Pencatatan Sipil Kabupaten Demak, (bukti P-4);

B. Saksi;

1. **Saksi I**, umur 52 tahun, di bawah sumpahnya menerangkan sebagai berikut:
   - Bahwa saksi kenal baik dengan Penggugat dan Tergugat karena saksi tetangga Penggugat;
   - Bahwa saksi kenal dengan Tergugat sejak menjadi suami Penggugat.

Disclaimer
Kepaniteraan Mahkamah Agung Republik Indonesia berusaha untuk selalu mencantumkan informasi paling kini dan akurat sebagai bentuk komitmen Mahkamah Agung untuk pelayanan publik, transparansi dan akuntabilitas pelaksanaan fungsi peradilan. Namun dalam hal-hal tertentu masih dimungkinkan terjadi permasalahan teknis terkait dengan akurasi dan keterkinian informasi yang kami sajikan, hal mana akan terus kami perbaiki dari waktu kewaktu.
Dalam hal Anda menemukan inakurasi informasi yang termuat pada situs ini atau informasi yang seharusnya ada, namun belum tersedia, maka harap segera hubungi Kepaniteraan Mahkamah Agung RI melalui :
Email : kepaniteraan@mahkamahagung.go.id Telp : 021-384 3348 (ext.318)

- Bahwa Penggugat dan Tergugat adalah suami isteri menikah tahun 2006 dan telah dikaruniai 2 orang anak;
- Bahwa kedua anak tersebut sekarang dalam asuhan Penggugat dan setahu saksi Penggugat sangat perhatian dan sayang kepada kedua anaknya tersebut;
- Bahwa setelah menikah Penggugat dan Tergugat semula tinggal di rumah orang tua Penggugat di Desa Wedung;
- Bahwa pada awalnya rumah tangga rukun dan harmonis akan tetapi sejak pertengahan tahun 2018 antara Penggugat dengan Tergugat mulai sering terjadi perselisisihan dan pertengkaran;
- Bahwa penyebab pertengkaran tersebut karena Tergugat malas bekerja sehingga tidak dapat memberikan nafkah yang layak kepada Pengugat;
- Bahwa kemudian sejak bulan Agustus tahun 2019 Tergugat pergi meninggalkan Penggugat yang hingga sekarang tidak diketahui alamatnya yang jelas;
- Bahwa keluarga Penggugat sudah berusaha menasehati Penggugat akan tetapi tidak berhasil.

2 **Saksi II,** umur 46 tahun, di bawah sumpahnya ia memberi keterangan sebagai berikut:

- Bahwa saksi kenal baik dengan Penggugat dan Tergugat karena saksi tetangga Penggugat;
- Bahwa saksi kenal dengan Tergugat sejak menjadi suami Penggugat.
- Bahwa Penggugat dan Tergugat adalah suami isteri menikah tahun 2006 dan telah dikaruniai 2 orang anak;
- Bahwa kedua anak tersebut sekarang dalam asuhan Penggugat dan setahu saksi Penggugat sangat perhatian dan sayang kepada kedua anaknya tersebut;
- Bahwa setelah menikah Penggugat dan Tergugat semula tinggal di rumah orang tua Penggugat di Desa Wedung;

Disclaimer
Kepaniteraan Mahkamah Agung Republik Indonesia berusaha untuk selalu mencantumkan informasi paling kini dan akurat sebagai bentuk komitmen Mahkamah Agung untuk pelayanan publik, transparansi dan akuntabilitas pelaksanaan fungsi peradilan. Namun dalam hal-hal tertentu masih dimungkinkan terjadi permasalahan teknis terkait dengan akurasi dan keterkinian informasi yang kami sajikan, hal mana akan terus kami perbaiki dari waktu kewaktu.
Dalam hal Anda menemukan inakurasi informasi yang termuat pada situs ini atau informasi yang seharusnya ada, namun belum tersedia, maka harap segera hubungi Kepaniteraan Mahkamah Agung RI melalui :
Email : kepaniteraan@mahkamahagung.go.id Telp : 021-384 3348 (ext.318)

- Bahwa pada awalnya rumah tangga rukun dan harmonis akan tetapi sejak pertengahan tahun 2018 antara Penggugat dengan Tergugat mulai sering terjadi perselisisihan dan pertengkaran;
- Bahwa penyebab pertengkaran tersebut karena Tergugat malas bekerja sehingga tidak dapat memberikan nafkah yang layak kepada Penggugat;
- Bahwa kemudian sejak bulan Agustus tahun 2019 Tergugat pergi meninggalkan Penggugat yang hingga sekarang tidak diketahui alamatnya yang jelas;
- Bahwa keluarga Penggugat sudah berusaha menasehati Penggugat akan tetapi tidak berhasil.

Bahwa atas bukti-bukti yang diajukan oleh Penggugat tersebut, Tergugat tidak mengajukan bantahannya, karena tidak hadir di persidangan;

Bahwa Penggugat telah menyampaikan kesimpulannya secara lisan di persidangan pada tanggal 22 Desember 2020, yang pada pokoknya Penggugat tetap menghendaki perceraian dan mohon asuh anak lalu menyatakan tidak akan mengajukan suatu apapun lagi dan mohon putusan;

Bahwa untuk meringkas uraian putusan ini, maka ditunjuk hal ihwal sebagaimana tercantum dalam berita acara sidang perkara ini yang merupakan bagian yang tak terpisahkan dari putusan ini.

PERTIMBANGAN HUKUM

Menimbang bahwa maksud dan tujuan gugatan Penggugat adalah sebagaimana telah terurai diatas;

Menimbang, bahwa Penggugat dan Tergugat beragama Islam dan telah melakukan perkawinan secara Islami, maka perkara ini merupakan kompetensi absolut Pengadilan Agama untuk memeriksa dan menyelesaikannya. (vide Pasal 40 dan Pasal 63 ayat (1) Undang Undang No. 1 Tahun 1974 Jo. Pasal 14 dan Pasal 1 huruf b. PP. N0. 9 Tahun 1975, Jo Pasal 2 dan Pasal 49 ayat (1) Undang-Undang Nomor 7 Tahun 1989 yang telah diubah dengan Undang Undang No 3 Tahun 2006 dan Undang-Undang Nomor 50 Tahun 2009;

Menimbang, bahwa dari bukti P-1 yang berupa kartu tanda Penduduk maka telah terbukti Penggugat bertempat tinggal di wilayah yurisdiksi Pengadilan Agama

Disclaimer
Kepaniteraan Mahkamah Agung Republik Indonesia berusaha untuk selalu mencantumkan informasi paling kini dan akurat sebagai bentuk komitmen Mahkamah Agung untuk pelayanan publik, transparansi dan akuntabilitas pelaksanaan fungsi peradilan. Namun dalam hal-hal tertentu masih dimungkinkan terjadi permasalahan teknis terkait dengan akurasi dan keterkinian informasi yang kami sajikan, hal mana akan terus kami perbaiki dari waktu kewaktu.
Dalam hal Anda menemukan inakurasi informasi yang termuat pada situs ini atau informasi yang seharusnya ada, namun belum tersedia, maka harap segera hubungi Kepaniteraan Mahkamah Agung RI melalui :
Email : kepaniteraan@mahkamahagung.go.id Telp : 021-384 3348 (ext.318)

Demak, maka perkara ini secara relatif merupakan kompetensi Pengadilan Agama Demak, (vide Pasal 73 ayat (1) Undang-Undang Nomor 7 Tahun 1989 yang telah diubah dengan Undang-Undang Nomor No 3 Tahun 2006 dan Undang Undang No. 50 Tahun 2009 jo Pasal 132 ayat (1) Kompilasi Hukum Islam);

Menimbang, bahwa berdasarkan bukti P-2 yang berupa Kutipan Akta Nikah maka telah terbukti bahwa benar, antara Penggugat dengan Tergugat telah terikat dalam perkawinan yang sah oleh karena itu gugatan Penggugat terhadap Tergugat *memiliki dasar hukum yang sah*;-

Menimbang, bahwa karena ternyata Tergugat meskipun dipanggil dengan patut tidak pernah datang menghadap dan tidak pula menyuruh orang lain untuk menghadap sebagai wakilnya serta ketidak datangannya itu tanpa disebabkan oleh suatu halangan yang sah, maka patutlah dianggap bahwa Tergugat dalam keadaan tidak hadir dan sesuai dengan kehendak pasal 125 HIR putusan perkara ini dijatuhkan dengan verstek;

Menimbang, bahwa oleh karena Tergugat tidak pernah hadir dipersidangan sehingga proses mediasi sebagaimana ketentuan PERMA Nomor 01 Tahun 2016 tidak dapat dilaksanakan;

Menimbang, bahwa Penggugat pada pokoknya menuntut perceraian dengan alasan karena sejak bulan Juni tahun 2018, antara Penggugat dengan Tergugat sering terjadi perselisihan dan pertengkaran disebabkan Tergugat malas bekerja sehingga tidak dapat memebrikan nafkah yang layak kepada Penggugat, kemudian pada bulan Agustus tahun 2019 Tergugat pergi meninggalkan Penggugat danhingga sedkarang gtidak diketahui alamatnya yang jelas dan sejak itu pula antara Penggugat dengan Tergugat tidak ada komunikasi lagi layaknya suami isteri;

Menimbang bahwa Penggugat telah menyandarkan alasan tersebut pada Pasal 19 huruf f Peraturan Pemerintah Nomor 9 tahun 1975 jo pasal 116 huruf f KHI. "*Antara suami dan isteri terus menerus terjadi perselisihan dan pertengkaran dan tidak ada harapan akan hidup rukun lagi dalam rumah tangga*";

Menimbang bahwa ketidak hadiran Tergugat dalam persidangan ini dianggap telah mengabaikan hak bantah Tergugat atas dalil-dalil gugatan Penggugat, namun demikian Penggugat tetap mengajukan saksi-saksi yang akan dipertimbangkan sebagai berikut:

Menimbang, bahwa alat bukti P.3 yang berupa Surat Keterangan pergi atas nama Tergugat yang dikeluarkan oleh Kepala Desa Wedung, majelis hakim berpendapat bahwa alat bukti P.3 tersebut telah memenuhi syarat formil sebagai bukti

Disclaimer
Kepaniteraan Mahkamah Agung Republik Indonesia berusaha untuk selalu mencantumkan informasi paling kini dan akurat sebagai bentuk komitmen Mahkamah Agung untuk pelayanan publik, transparansi dan akuntabilitas pelaksanaan fungsi peradilan. Namun dalam hal-hal tertentu masih dimungkinkan terjadi permasalahan teknis terkait dengan akurasi dan keterkinian informasi yang kami sajikan, hal mana akan terus kami perbaiki dari waktu kewaktu.
Dalam hal Anda menemukan inakurasi informasi yang termuat pada situs ini atau informasi yang seharusnya ada, namun belum tersedia, maka harap segera hubungi Kepaniteraan Mahkamah Agung RI melalui :
Email : kepaniteraan@mahkamahagung.go.id Telp : 021-384 3348 (ext.318)

karena telah dikeluarkan oleh pejabat yang berwenang untuk itu dan secara materil bukti tersebut telah mendukung dan membuktikan dalil gugatan Penggugat bahwa sejak bulan Juni tahun 2018 Tergugat sudah pergi meninggalkan kediaman bersama dan tidak pernah kembali serta tidak diketahui lagi alamatnya dengan jelas;

Menimbang, bahwa dua orang saksi Penggugat tersebut telah memenuhi syarat-syarat formil dan tidak terhalang kedudukannya sebagai saksi seperti yang diatur dalam Pasal 145 HIR, keduanya memberikan keterangan yang didasarkan pada pengetahuan dari pengalaman, penglihatan dan pendengarannya sendiri yang antara kedua saksi tersebut terdapat kesesuaian keterangan seperti yang disyaratkan dalam Pasal 172 HIR, oleh karena itu keterangan kedua saksi tersebut dapat dipertimbangkan;

Menimbang, bahwa dari dalil gugatan Penggugat, sikap Penggugat selama dipersidangan dihubungkan dengan bukti surat dan keterangan-keterangan yang disampaikan oleh para saksi di persidangan, dapat ditemukan fakta sebagai berikut :

1. Bahwa Penggugat dan Tergugat adalah suami isteri sah, menikah pada tanggal 29 Juni 2006 dan sudah dikaruniai 2 orang anak sekarang dalam asuhan Penggugat;
2. Bahwa kemudian sejak bulan Juni tahun 2018, antara Penggugat dengan Tergugat mulai sering terjadi perselisihan dan pertengkaran disebabkan Tergugat malas bekerja sehingga tidak dapat memebrikan nafkah yang layak kepada Penggugat;
3. Bahwa kemudian sejak bulan Agsutust tahun 2019 Tergugat pergi meninggalkan Penggugat hingga sekarang dan tidak diketahui alamatnya yang jelas;
4. Bahwa selama berpisah tersebut antara Penggugat dan Tergugat tidak ada komunikasi sama sekali;
5. Bahwa keluarga Penggugat sudah berusaha mendamaikannya, akan tetapi tidak berhasil.

Menimbang, bahwa berdasarkan fakta poin (1) menunjukkan bahwa Penggugat dan Tergugat berkualitas hukum sebagai pihak-pihak dalam perkara ini, dan Penggugat berhak mengajukan permohonan cerai gugat terhadap Tergugat ke Pengadilan Agama sebagaimana dimaksud pasal 66 ayat (1) Undang-undang Nomor 7 Tahun 1989;

Menimbang, bahwa dari fakta point 2, 3, 4 dan 5 sebagaimana terurai diatas telah memberikan gambaran bahwa perselisihan antara Penggugat dengan Tergugat

Disclaimer
Kepaniteraan Mahkamah Agung Republik Indonesia berusaha untuk selalu mencantumkan informasi paling kini dan akurat sebagai bentuk komitmen Mahkamah Agung untuk pelayanan publik, transparansi dan akuntabilitas pelaksanaan fungsi peradilan. Namun dalam hal-hal tertentu masih dimungkinkan terjadi permasalahan teknis terkait dengan akurasi dan keterkinian informasi yang kami sajikan, hal mana akan terus kami perbaiki dari waktu kewaktu.
Dalam hal Anda menemukan inakurasi informasi yang termuat pada situs ini atau informasi yang seharusnya ada, namun belum tersedia, maka harap segera hubungi Kepaniteraan Mahkamah Agung RI melalui :
Email : kepaniteraan@mahkamahagung.go.id Telp : 021-384 3348 (ext.318)

sudah tidak mampu lagi dikendalikan dan diatasi oleh mereka berdua, bahkan sikap Tergugat yang telah pergi meninggalkan Penggugat adalah merupakan bukti bahwa perselisihan antara Penggugat dengan Tergugat sudah cukup serius dan bersifat terus menerus;

Menimbang bahwa dari pernyataan Penggugat selama dalam persidangan yang dengan tegas hendak bercerai dengan Tergugat dan juga sikap Tergugat yang tidak pernah menghadiri persidangan adalah merupakan indikasi tidak adanya kehendak dari kedua belah pihak untuk memperbaiki kembali keadaan rumah tangganya, karena itu merupakan fakta kongkrit yang menunjukkan bahwa hubungan Penggugat dengan Tergugat sudah sampai pada tingkat pecahnya perkawinan (*broken marriage*) dan Hakim berkesimpulan bahwa tujuan yang diharapkan dari perkawinan sebagaimana tersebut pada pasal 1 Undang-undang Nomor 1 Tahun 1974 tidak mungkin lagi terwujud;

Menimbang bahwa para saksi saksi telah menerangkan adanya usaha pihak keluarga menasehati Penggugat untuk memepertahankan rumah tanggannya akan tetapi tidak berhasil, kerena itu dianggap telah memenui ketentuan Pasal 22 ayat (2) Peraturan Pemerintah Nomor 9 Tahun 1974;

Menimbang bahwa mengutip referensi fiqh yang terdapat dalam kitab Hurriatuz Zaujani fith Thalaq Juz I halaman 83 yang diambil alih menjadi pendapat Hakim sebagai berikut :

æÞÏ ÇÎÊÇÑÇáÅÓáÇã äÙÇã ÇáØáÇÞ Ííä ÊÖØÑ È ÇáÍíÇÉ ÇáÒæÌíä æáã íÚÏ íäÝÚ ÝíåÇ äÕÇ ÆÍ æáÇ Õáí æíÍË ÊÕÈÍ ÇáÑÈØÉ ÇáÒæÇÌ ÕæÑÉ ãä ÛíÑ Ñæí áÃä ÇÓÊãÑÇÑ ãÚäÇå Çä íÍßã Úáì ÇÍÏ ÇáÒæÌíä ÈÇáÓÌä ÇáãæÁ ÈÏ æåÐÇ Ùáã ÊÃ ÈÇå Ñæí ÇáÚÏÇáÉ

Artinya : "*Islam memilih lembaga thalaq (perceraian) ketika rumah tangga sudah dianggap goncang serta dianggap sudah tidak bermanfaat lagi nasehat/ perdamaian, dan hubungan suami isteri menjadi tanpa ruh (hampa), sebab meneruskan perkawinan berarti menghukum salah satu suami isteri dengan penjara yang berkepanjangan. Ini adalah aniaya (kedhaliman) yang bertentangan dengan semangat keadilan*".

Menimbang bahwa sehubungan dengan hal tersebut Yurisprudensi Mahkamah Agung RI. Nomor 379 K / AG / 1995 tanggal 26 Maret 1997 menyatakan "*Suami isteri yang tidak berdiam serumah lagi dan tidak ada harapan untuk hidup rukun kembali*

Disclaimer
Kepaniteraan Mahkamah Agung Republik Indonesia berusaha untuk selalu mencantumkan informasi paling kini dan akurat sebagai bentuk komitmen Mahkamah Agung untuk pelayanan publik, transparansi dan akuntabilitas pelaksanaan fungsi peradilan. Namun dalam hal-hal tertentu masih dimungkinkan terjadi permasalahan teknis terkait dengan akurasi dan keterkinian informasi yang kami sajikan, hal mana akan terus kami perbaiki dari waktu kewaktu.
Dalam hal Anda menemukan inakurasi informasi yang termuat pada situs ini atau informasi yang seharusnya ada, namun belum tersedia, maka harap segera hubungi Kepaniteraan Mahkamah Agung RI melalui :
Email : kepaniteraan@mahkamahagung.go.id Telp : 021-384 3348 (ext.318)

maka rumah tanga tersebut telah terbukti retak dan pecah dan telah memenuhi alasan cerai Pasal 19 huruf f Peraturan Peraturan Pemerintah Nomor 9 tahun 1975";

Menimbang, bahwa aspek penting dari bunyi pasal 19 huruf f Peraturan Pemerintah Nomor 9 tahun 1975 jo pasal 116 huruf KHI diatas adalah tidak adanya harapan untuk menata dan mempertahankan rumah tangga, dengan demikian apabila maksud pasal tersebut dikomparasikan dengan keadaan rumah tangga Penggugat dan Tergugat maka telah terdapat cukup alasan hukum untuk mengabulkan gugatan Penggugat dan menjatuhkan talak satu ba'in sughro dari Tergugat terhadap Penggugat;

Menimbang bahwa selaian perceraian tersebut, Penggugat juga menuntut hadlonah/ pemeliharaan terhadap dua orang anaknya masing-masing Agus Devin Fadihila dan Putri Najwa Salsa Azzahra;

Menimbang bahwa berdasarkan bukti surat yang bertanda P-3 dan P-4 masing – masing berupa kutipan akta lahir dan keterangan para saksi maka telah terbukti bahwa Penggugat dan Tergugat telah dikaruniai dua orang anak masing-masing bernama Agus Devin Fadihila, perempuan, lahir tanggal 18 Agustus 2008 dan Putri Najwa Salsa Azzahra, perempuan lahir tanggal 13 Juli 2017 yang berarti kedua anak tersebut belum mumayyiz dan dari keterangan para saksi terbukti selama ini kedua anak tersebut dalam asuhan Penggugat dan Penggugat selaku Ibu penuh perhatian dan sayang kepada anaknya tersebut;

Menimbang bahwa karena anak tersebut belum mumayyiz dan masih sangat membutuhkan pelayanan, perhatian, kesabaran dan sentuhan kasih sayang dari seorang Ibu, sehingga hukum memandang Ibunya (Penggugat) lebih berhak mengasuh, mendidik serta pantas ditunjuk sebagai pemegang hak hadlonah atas anak tersebut, hal ini sesuai ketentuan Pasal 105 ayat (1) Kompilasi Hukum Islam dan hadis Nabi yang berbunyi:

أنت أحق به مالم تنكحى

Artinya "*Engkau (Ibu) lebih berhak terhadap anakmu selama engkau belum menikah"*

Menimbang bahwa berdasarkan pertimbangan tersebut diatas maka Pengadilan patut menetapkan dua orang anak Penggugat dan Tergugat bernama Agus Devin Fadihila dan Putri Najwa Salsa Azzahra berada dalam hadlonah/ asuhan Penggugat;

Menimbang bahwa bapak atau Ibu yang tidak mengasuh anak, tetap mempunyai hak untuk mencurahkan kasih sayangnya seperti melihat dan mengajak bepergian anak tersebut kapan saja waktunya dengan tujuan untuk mendatangkan

Disclaimer
Kepaniteraan Mahkamah Agung Republik Indonesia berusaha untuk selalu mencantumkan informasi paling kini dan akurat sebagai bentuk komitmen Mahkamah Agung untuk pelayanan publik, transparansi dan akuntabilitas pelaksanaan fungsi peradilan. Namun dalam hal-hal tertentu masih dimungkinkan terjadi permasalahan teknis terkait dengan akurasi dan keterkinian informasi yang kami sajikan, hal mana akan terus kami perbaiki dari waktu kewaktu.
Dalam hal Anda menemukan inakurasi informasi yang termuat pada situs ini atau informasi yang seharusnya ada, namun belum tersedia, maka harap segera hubungi Kepaniteraan Mahkamah Agung RI melalui :
Email : kepaniteraan@mahkamahagung.go.id Telp : 021-384 3348 (ext.318)

kemaslahatan bagi anak dan orang tua yang tidak mengasuh anak tersebut, dengan demikian anak tersebut akan merasa tetap dekat dengan kedua orang tuanya, sebagaimana pendapat DR. Wahbah Az-Zuhaily dalam kitab al-Fiqhul Islamy wa Adillatuhu, jilid VII halaman 70 yang diambil alih sebagain pendapat Majelis Hakim sebagai berikut:

حق الرؤية أو الزيارة لاحد الابوين غير الحاضن مقرر شرعا باتفاق الفقهاء لصلة ولكنهم ذكروا اراء مختلفة نسيبا بحسب تقدير المصلحة, لكل من الولد والولد الذي ولده فى حضانه غيره

Artinya: " Ulama' ahli fiqh sepakat, hak untuk melihat atau berkunjung bagi salah satu dari orang tua yang tidak mengasuh anak, ditetapkan berdasarkan syara' untuk menyambung hubungan antara anak dengan salah satu orang tuanya yang tidka mengasuh anak, sekira bisa mendatangkankan kemaslahatan bagi anak dan orang tua yang melahirkan anak tersebut, yang berada dibawah asuhan salah satu borang tuanya"

Menimbang bahwa karena itu orang yang mengasuh anak serta seluruh keluarganya tidak boleh menghalang-halangi anak untuk bertemu dengan orang tuanya yang tidak mengasuh anak atau menyembunyikan anak tersebut, sehingga tidak ada kesempatan bagi anak untuk bertemu dengan orang tuanya yang tidak mengasuhnya;

Menimbang bahwa berdasarkan pertimbangan tersebut diatas, maka kepada Tergugat selaku ayah dari kedua orang anak bernama Agus Devin Fadihila dan Putri Najwa Salsa Azzahra tersebut berhak untuk menengok/ mengunjungi dan mengajak anak tersebut bepergian dengan sepengetahuan /izin Penggugat;

Menimbang, bahwa oleh karena perkara ini termasuk dalam bidang perkawinan, berdasarkan Pasal 89 ayat (1) Undang-Undang 7 Tahun 1989 yang telah diubah dengan Undang-Undang Nomor 3 Tahun 2006 dan perubahan kedua dengan Undang-Undang Nomor 50 Tahun 2009, maka biaya perkara ini dibebankan kepada Penggugat;

Memperhatikan Pasal 125 dan 126 HIR serta ketentuan dalil syar'i yang berkaitan dengan perkara ini;

M E N G A D I L I

1. Menyatakan Tergugat telah dipanggil secara resmi dan patut untuk datang menghadap di persidangan, tidak hadir;
2. Mengabulkan gugatan Penggugat dengan verstek;

Disclaimer
Kepaniteraan Mahkamah Agung Republik Indonesia berusaha untuk selalu mencantumkan informasi paling kini dan akurat sebagai bentuk komitmen Mahkamah Agung untuk pelayanan publik, transparansi dan akuntabilitas pelaksanaan fungsi peradilan. Namun dalam hal-hal tertentu masih dimungkinkan terjadi permasalahan teknis terkait dengan akurasi dan keterkinian informasi yang kami sajikan, hal mana akan terus kami perbaiki dari waktu kewaktu.
Dalam hal Anda menemukan inakurasi informasi yang termuat pada situs ini atau informasi yang seharusnya ada, namun belum tersedia, maka harap segera hubungi Kepaniteraan Mahkamah Agung RI melalui :
Email : kepaniteraan@mahkamahagung.go.id   Telp : 021-384 3348 (ext.318)

3. Menjatuhkan talak satu bain sughra dari Tergugat (Tergugat) terhadap Penggugat (Penggugat);
4. Menetapkan kedua orang anak Penggugat dan Tergugat yang bernama XXXX, laki-laki, lahir tanggal 18 Agustus 2008 dan XXXX, perempuan, lahir tanggal 13 Juli 2017 berada dibawah pemeliharaan (hadhonah) Penggugat;
5. Membebankan kepada Penggugat untuk membayar biaya perkara yang hingga kini sejumlah Rp. 461.000,00- (empat ratus enam puluh satu ribu rupiah);

Demikianlah putusan ini dijatuhkan dalam musyawarah Majelis Hakim di Demak pada hari Selasa tanggal 22 Desember 2020 M, bertepatan dengan tanggal 07 Jumadil Awal 1442 H, oleh Kami **Drs. Makali** sebagai Ketua Majelis Hakim, serta **Dra. Nur Immawati** dan **Toharudin, S.H.I., M.H.** masing-masing sebagai Hakim Anggota. Putusan mana diucapkan oleh Ketua Majelis Hakim tersebut pada hari itu juga dalam sidang terbuka untuk umum dengan dihadiri oleh para Hakim Anggota dan **Moh. Rofi', S. Ag.,** sebagai Panitera Pengganti dan dihadiri pula oleh Penggugat tanpa hadir nya Tergugat;

Ketua Majelis

Drs. Makali

Hakim Anggota	Hakim Anggota
**Dra. Nur Immawati**	**Toharudin, S.H.I., M.H.**

Panitera Pengganti

Disclaimer
Kepaniteraan Mahkamah Agung Republik Indonesia berusaha untuk selalu mencantumkan informasi paling kini dan akurat sebagai bentuk komitmen Mahkamah Agung untuk pelayanan publik, transparansi dan akuntabilitas pelaksanaan fungsi peradilan. Namun dalam hal-hal tertentu masih dimungkinkan terjadi permasalahan teknis terkait dengan akurasi dan keterkinian informasi yang kami sajikan, hal mana akan terus kami perbaiki dari waktu kewaktu.
Dalam hal Anda menemukan inakurasi informasi yang termuat pada situs ini atau informasi yang seharusnya ada, namun belum tersedia, maka harap segera hubungi Kepaniteraan Mahkamah Agung RI melalui :
Email : kepaniteraan@mahkamahagung.go.id Telp : 021-384 3348 (ext.318)

Moh. Rofi', S. Ag.

Perincian Biaya :

Biaya Pendaftaran	:	Rp	30.000,00
Biaya Proses	:	Rp	75.000,00
Biaya Pemanggilan	:	Rp	320.000,00
PNBP panggilan		Rp	20.000,00
Biaya Redaksi	:	Rp	10000,00
Biaya Materai	:	Rp	6.000,00
Jumlah	:	Rp	461.000,00

(empat ratus enam puluh satu ribu rupiah)

Disclaimer
Kepaniteraan Mahkamah Agung Republik Indonesia berusaha untuk selalu mencantumkan informasi paling kini dan akurat sebagai bentuk komitmen Mahkamah Agung untuk pelayanan publik, transparansi dan akuntabilitas pelaksanaan fungsi peradilan. Namun dalam hal-hal tertentu masih dimungkinkan terjadi permasalahan teknis terkait dengan akurasi dan keterkinian informasi yang kami sajikan, hal mana akan terus kami perbaiki dari waktu kewaktu.
Dalam hal Anda menemukan inakurasi informasi yang termuat pada situs ini atau informasi yang seharusnya ada, namun belum tersedia, maka harap segera hubungi Kepaniteraan Mahkamah Agung RI melalui :
Email : kepaniteraan@mahkamahagung.go.id Telp : 021-384 3348 (ext.318)
