# Supplementary material for: Classification of divorce causes during the COVID-19 pandemic using convolutional neural networks
Source: PeerJ Comput Sci. 2022 Jun 30;8:e998. doi: 10.7717/peerj-cs.998 (PMC9299239; doi:10.7717/peerj-cs.998)
Supplement: Supplemental Information 4 [file peerj-cs-08-998-s004.zip › Konflik Dataset/Data ke-30.pdf]

PUTUSAN

Nomor 498/Pdt.G/2020/PA.Pyb

بِسْمِ اللَّهِ الرَّحْمَٰنِ الرَّحِيمِ

DEMI KEADILAN BERDASARKAN KETUHANAN YANG MAHA ESA

Pengadilan Agama Panyabungan yang memeriksa dan mengadili perkara tertentu pada tingkat pertama dalam sidang Majelis telah menjatuhkan putusan perkara Cerai Talak antara:

Xxxx, lahir di Panyabungan tanggal 06 Desember 1981, agama Islam, NIK 1213010512810001, pekerjaan buruh bangunan, pendidikan SD, tempat kediaman di Aek Galoga Desa Pidoli Lombang, Kecamatan Panyabungan Kabupaten Mandailing Natal, sebagai **Pemohon**;

MELAWAN

Xxxx, lahir di Jambur Pd Matinggi tanggal 24 April 1989, agama Islam, pekerjaan ibu rumah tangga, pendidikan SLTA, tempat kediaman di Desa Jambur padang Matinggi Kecamatan Panyabungan Utara Kabupaten Mandailing Natal, sebagai **Termohon;**

Pengadilan Agama tersebut;

Setelah mempelajari berkas perkara yang bersangkutan;

Setelah mendengar keterangan Pemohon di persidangan;

Setelah memeriksa alat-alat bukti di persidangan;

Setelah memperhatikan hal ihwal yang terjadi di persidangan;

DUDUK PERKARA

Bahwa Pemohon dengan surat permohonannya tanggal 11 Desember 2020 telah mengajukan permohonan cerai talak yang telah didaftar di Kepaniteraan Pengadilan Agama Panyabungan dengan nomor 498/Pdt.G/2020/PA.Pyb tanggal 11 Desember 2020 dengan dalil-dalil sebagai berikut:

1. Bahwa pada tanggal 19 Februari 2010, Pemohon dengan Termohon melangsungkan pernikahan yang dicatat oleh Pegawai Pencatat Nikah

Disclaimer
Kepaniteraan Mahkamah Agung Republik Indonesia berusaha untuk selalu mencantumkan informasi paling kini dan akurat sebagai bentuk komitmen Mahkamah Agung untuk pelayanan publik, transparansi dan akuntabilitas pelaksanaan fungsi peradilan. Namun dalam hal-hal tertentu masih dimungkinkan terjadi permasalahan teknis terkait dengan akurasi dan keterkinian informasi yang kami sajikan, hal mana akan terus kami perbaiki dari waktu kewaktu.
Dalam hal Anda menemukan inakurasi informasi yang termuat pada situs ini atau informasi yang seharusnya ada, namun belum tersedia, maka harap segera hubungi Kepaniteraan Mahkamah Agung RI melalui :
Email : kepaniteraan@mahkamahagung.go.id   Telp : 021-384 3348 (ext.318)

Kantor Urusan Agama Kecamatan Panyabungan Utara Kabupaten Mandailing Natal sesuai Kutipan Akta Nikah Nomor: 32/03/III/2010, tertanggal 01 Maret 2010;

2. Bahwa setelah menikah Pemohon dan Termohon tinggal bersama di rumah kontrakan di kelurahan Panyabungan II Kecamatan Panyabungan Kabupaten Mandailing Natal selama 3 tahun, kemudian pindah kerumah orangtua Termohon di Kelurahan Sipolu-polu Kecamatan Panyabungan Kabupaten Mandailing Natal selama 1 tahun, kemudian pindah kerumah kontrakan Kelurahan Panyabungan II Kecamatan Panyabungan Kabupaten Mandailing Natal,sampai pisah;
3. Bahwa selama pernikahan antara Pemohon dan Termohon sudah berhubungan layaknya suami isteri (*ba'da ad-dukhul*), dan telah dikaruniai seorang anak bernama Muhammad Ilyas Ali (laki-laki), umur 9 tahun saat ini tinggal dengan Termohon;
4. Bahwa keharmonisan dan kerukunan rumah tangga Pemohon dan Termohon, tidak dapat dipertahankan lagi dikarenakan sejak tahun 2011 mulai terjadi perselisihan yang secara terus menerus antara Pemohon dan Termohon yang pada pokoknya disebabkan oleh:
   a) Termohon memiliki penyakit kejiwaan;
   b) Pemohon merasa Termohon tidak bisa diajak musyawarah sabagaimana orang normal biasanya;
5. Bahwa puncak perselisihan antara Pemohon dengan Termohon terjadi pada tahun 2014, penyebabnya karena saat Pemohon ingin berhubungan suami isteri dengan Termohon, namun Termohon tidak mau, sehingga Pemohon keluar rumah agar tidak ada pertengkaran, pagi harinya sewaktu Pemohon pergi bekerja, Termohon menulis surat bahwa Termohon pergi kerumah orangtua Termohon, Termohon pun menghubungi Pemohon dan meminta untuk diceraikan, seminggu kemudian Pemohon pun menjatuhkan talak Termohon;
6. Bahwa sejak itu Pemohon dengan Termohon tidak lagi tinggal satu rumah dan tidak pernah bersatu kembali;

Disclaimer
Kepaniteraan Mahkamah Agung Republik Indonesia berusaha untuk selalu mencantumkan informasi paling kini dan akurat sebagai bentuk komitmen Mahkamah Agung untuk pelayanan publik, transparansi dan akuntabilitas pelaksanaan fungsi peradilan. Namun dalam hal-hal tertentu masih dimungkinkan terjadi permasalahan teknis terkait dengan akurasi dan keterkinian informasi yang kami sajikan, hal mana akan terus kami perbaiki dari waktu kewaktu.
Dalam hal Anda menemukan inakurasi informasi yang termuat pada situs ini atau informasi yang seharusnya ada, namun belum tersedia, maka harap segera hubungi Kepaniteraan Mahkamah Agung RI melalui :
Email : kepaniteraan@mahkamahagung.go.id Telp : 021-384 3348 (ext.318)

7. Bahwa pihak keluarga Pemohon dan Termohon tidak pernah mendamaikan Pemohon dan Termohon;
8. Bahwa saat ini Pemohon merasa tidak sanggup lagi untuk meneruskan ikatan perkawinan dengan Termohon, maka antara Pemohon dengan Termohon tidak mungkin dirukunkan sehingga rumah tangga yang *sakinah, mawaddah, warohmah* sangat sulit untuk diwujudkan;

   Berdasarkan alasan/dalil-dalil di atas, Pemohon mohon agar Ketua Pengadilan Agama Panyabungan cq. Majelis Hakim memeriksa dan mengadili perkara ini, selanjutnya menjatuhkan putusan yang amarnya berbunyi:

PRIMAIR:

1. Mengabulkan permohonan Pemohon;
2. Memberi izin kepada Pemohon (Xxxx) untuk menjatuhkan talak satu raj'i terhadap Termohon (Xxxx) dihadapan sidang Pengadilan Agama Panyabungan;
3. Membebankan biaya perkara menurut peraturan dan hukum yang berlaku;

SUBSIDAIR;

Jika Majelis Hakim berpendapat lain, mohon putusan yang seadil-adilnya;

Bahwa pada hari sidang yang telah ditetapkan Pemohon telah datang menghadap sendiri di persidangan, sedangkan Termohon tidak pernah datang menghadap di persidangan, meskipun berdasarkan berita acara panggilan, Termohon telah dipanggil secara resmi dan patut sebanyak 2 (dua) kali pada tanggal 11 dan 17 Desember 2020 yang diterima oleh sekretaris desa Jambur Padang Matinggi dan selanjutnya untuk diteruskan kepada Termohon, sedangkan tidak ternyata bahwa tidak datangnya itu disebabkan suatu halangan yang sah;

Bahwa, perkara ini tidak dapat dimediasi karena Termohon tidak pernah datang menghadap meskipun telah dipanggil secara resmi dan patut;

Menimbang, bahwa pemeriksaan perkara ini dimulai dengan dibacakan surat permohonan Pemohon tanggal 11 Desember 2020 yang telah terdaftar pada kepaniteraan Pangadilan Agama Panyabungan Nomor

Disclaimer
Kepaniteraan Mahkamah Agung Republik Indonesia berusaha untuk selalu mencantumkan informasi paling kini dan akurat sebagai bentuk komitmen Mahkamah Agung untuk pelayanan publik, transparansi dan akuntabilitas pelaksanaan fungsi peradilan. Namun dalam hal-hal tertentu masih dimungkinkan terjadi permasalahan teknis terkait dengan akurasi dan keterkinian informasi yang kami sajikan, hal mana akan terus kami perbaiki dari waktu kewaktu.
Dalam hal Anda menemukan inakurasi informasi yang termuat pada situs ini atau informasi yang seharusnya ada, namun belum tersedia, maka harap segera hubungi Kepaniteraan Mahkamah Agung RI melalui :
Email : kepaniteraan@mahkamahagung.go.id   Telp : 021-384 3348 (ext.318)

498/Pdt.G/2020/PA.Pyb tanggal 11 Desember 2020 yang pada pokoknya tetap dipertahankan oleh Pemohon;

Bahwa untuk menguatkan dalil-dalil permohonannya, Pemohon telah mengajukan alat-alat bukti berupa:

A. Bukti tertulis berupa Fotokopi Buku Kutipan Akta Nikah dari Pegawai Pencatat Nikah Kantor Urusan Agama Kecamatan Panyabungan Utara Kabupaten Mandailing Natal, Nomor 32/03/III/2010, tertanggal 01 Maret 2010, yang bermeterai cukup dan telah sesuai dengan aslinya (Bukti P);

B. Saksi

1. saksi, mengaku hubungannya dengan Pemohon adalah sebagai saudara kandung, saksi menerangkan di bawah sumpah pada pokoknya sebagai berikut,
   - Bahwa, saksi kenal dengan Pemohon dan Termohon;
   - Bahwa, hubungan Pemohon dengan Termohon adalah suami isteri;
   - Bahwa, selama pernikahan antara Pemohon dan Termohon sudah berhubungan layaknya suami isteri (*ba'da ad-dukhul*), dan sudah dikaruniai satu orang anak yang saat ini tinggal bersama Termohon;
   - Bahwa, setelah menikah Pemohon dengan Termohon terakhir bertempat kediaman di Kelurahan Panyabungan II Kecamatan Panyabungan Kabupaten Mandailing Natal, sampai pisah;
   - Bahwa, saksi mengetahui semula keadaan rumah tangga Pemohon dengan Termohon rukun dan harmonis, namun sejak tahun 2011 antara Pemohon dan Termohon sering terjadi perselisihan;
   - Bahwa, yang menjadi penyebab perselisihan Pemohon dengan Termohon adalah karena Termohon selalu bersikap kasar terhadap Pemohon;
   - Bahwa, saksi mengetahui perselisihan Pemohon dan Termohon tidak satu rumah dalam jangka waktu yang lama;
   - Bahwa, Pemohon dan Termohon sudah pisah tempat kediaman, sehingga sampai sekarang sudah lebih kurang 6 tahun;
   - Bahwa, Pemohon pergi meninggalkan tempat kediaman bersama,

Disclaimer
Kepaniteraan Mahkamah Agung Republik Indonesia berusaha untuk selalu mencantumkan informasi paling kini dan akurat sebagai bentuk komitmen Mahkamah Agung untuk pelayanan publik, transparansi dan akuntabilitas pelaksanaan fungsi peradilan. Namun dalam hal-hal tertentu masih dimungkinkan terjadi permasalahan teknis terkait dengan akurasi dan keterkinian informasi yang kami sajikan, hal mana akan terus kami perbaiki dari waktu kewaktu.
Dalam hal Anda menemukan inakurasi informasi yang termuat pada situs ini atau informasi yang seharusnya ada, namun belum tersedia, maka harap segera hubungi Kepaniteraan Mahkamah Agung RI melalui :
Email : kepaniteraan@mahkamahagung.go.id Telp : 021-384 3348 (ext.318)

dimana saat ini Pemohon tinggal di Aek Galoga Desa Pidoli Lombang Kecamatan Panyabungan Kabupaten Mandailing Natal dan Termohon tinggal di Desa Jambur Padang Matinggi Kecamatan Panyabungan Utara Kabupaten Mandailing;Natal

- Bahwa, selama berpisah antara Pemohon dengan Termohon tidak pernah kumpul bersama lagi dan tidak saling mengunjungi lagi;
- Bahwa, antara Pemohon dan Termohon sudah pernah di upayakan agar rukun kembali, akan tetapi tidak berhasil;
- Bahwa, saksi sudah pernah merukunkan Pemohon dan Termohon, tetapi tidak berhasil;

2. saksi, mengaku hubungannya dengan Pemohon adalah sebagai tetangga, saksi menerangkan di bawah sumpah pada pokoknya sebagai berikut;
   - Bahwa, saksi kenal dengan Pemohon dan Termohon;
   - Bahwa, hubungan Pemohon dengan Termohon adalah suami isteri;
   - Bahwa, selama pernikahan antara Pemohon dan Termohon sudah berhubungan layaknya suami isteri (*ba'da ad-dukhul*), dan sudah dikaruniai satu orang anak yang saat ini tinggal bersama Termohon;
   - Bahwa, setelah menikah Pemohon dengan Termohon terakhir bertempat kediaman di Kelurahan Panyabungan II Kecamatan Panyabungan Kabupaten Mandailing Natal, sampai pisah;
   - Bahwa, saksi mengetahui semula keadaan rumah tangga Pemohon dengan Termohon rukun dan harmonis, namun sejak tahun 2011 antara Pemohon dan Termohon sering terjadi pertengkaran;
   - Bahwa, yang menjadi penyebab pertengkaran Pemohon dengan Termohon adalah karena Termohon selalu bersikap kasar terhadap Pemohon;
   - Bahwa, saksi sering melihat dan mendengar pertengkaran Pemohon dan Termohon karena pertengkaran tersebut sampai ke rumah saksi;
   - Bahwa, Pemohon dan Termohon sudah pisah tempat kediaman,

Disclaimer
Kepaniteraan Mahkamah Agung Republik Indonesia berusaha untuk selalu mencantumkan informasi paling kini dan akurat sebagai bentuk komitmen Mahkamah Agung untuk pelayanan publik, transparansi dan akuntabilitas pelaksanaan fungsi peradilan. Namun dalam hal-hal tertentu masih dimungkinkan terjadi permasalahan teknis terkait dengan akurasi dan keterkinian informasi yang kami sajikan, hal mana akan terus kami perbaiki dari waktu kewaktu.
Dalam hal Anda menemukan inakurasi informasi yang termuat pada situs ini atau informasi yang seharusnya ada, namun belum tersedia, maka harap segera hubungi Kepaniteraan Mahkamah Agung RI melalui :
Email : kepaniteraan@mahkamahagung.go.id Telp : 021-384 3348 (ext.318)

sehingga sampai sekarang sudah lebih kurang 6 tahun;

- Bahwa, Pemohon pergi meninggalkan tempat kediaman bersama, dimana saat ini Pemohon tinggal di Aek Galoga Desa Pidoli Lombang Kecamatan Panyabungan Kabupaten Mandailing Natal dan Termohon tinggal di Desa Jambur Padang Matinggi Kecamatan Panyabungan Utara Kabupaten Mandailing;Natal
- Bahwa, selama berpisah antara Pemohon dengan Termohon tidak pernah kumpul bersama lagi dan tidak saling mengunjungi lagi;
- Bahwa, antara Pemohon dan Termohon sudah pernah di upayakan agar rukun kembali, akan tetapi tidak berhasil;
- Bahwa, saksi sudah pernah merukunkan Pemohon dan Termohon, tetapi tidak berhasil;

Bahwa, selanjutnya Pemohon menyatakan mencukupkan bukti-buktinya;

Bahwa, dikarenakan Termohon tidak hadir meskipun telah dipanggil secara resmi dan patut tanpa alasan yang sah, maka Majelis Hakim menyatakan bahwa tahapan pembuktian dinyatakan selesai;

Kemudian Pemohon menyampaikan kesimpulan akhirnya secara lisan yang pada pokoknya tetap dengan permohonannya dan mohon segera diputus dengan mengabulkan permohonan Pemohon;

Bahwa mengenai segala kejadian yang terjadi di persidangan telah tercantum di dalam berita acara sidang yang bersangkutan, maka untuk mempersingkat uraian dalam keputusan ini, maka segala sesuatunya dianggap telah termuat dalam putusan ini;

PERTIMBANGAN HUKUM

Menimbang, bahwa maksud dan tujuan permohonan Pemohon adalah sebagaimana yang telah diuraikan di atas;

Menimbang, bahwa berdasarkan Pasal 49 ayat (1) dan (2) serta penjelasan Pasal 49 ayat (2) Undang-Undang Nomor 3 Tahun 2006 tentang perubahan Undang-Undang Nomor 7 Tahun 1989 Tentang Peradilan Agama

Disclaimer
Kepaniteraan Mahkamah Agung Republik Indonesia berusaha untuk selalu mencantumkan informasi paling kini dan akurat sebagai bentuk komitmen Mahkamah Agung untuk pelayanan publik, transparansi dan akuntabilitas pelaksanaan fungsi peradilan. Namun dalam hal-hal tertentu masih dimungkinkan terjadi permasalahan teknis terkait dengan akurasi dan keterkinian informasi yang kami sajikan, hal mana akan terus kami perbaiki dari waktu kewaktu.
Dalam hal Anda menemukan inakurasi informasi yang termuat pada situs ini atau informasi yang seharusnya ada, namun belum tersedia, maka harap segera hubungi Kepaniteraan Mahkamah Agung RI melalui :
Email : kepaniteraan@mahkamahagung.go.id Telp : 021-384 3348 (ext.318)

dan perubahan keduanya dengan Undang-Undang Nomor 50 Tahun 2009, maka perkara ini termasuk kompetensi absolute Pengadilan Agama;

Menimbang, bahwa pada hari dan tanggal persidangan yang telah ditentukan Pemohon hadir di persidangan, sedangkan Termohon tidak hadir menghadap di persidangan dan tidak pula mengutus orang lain untuk datang sebagai wakil atau kuasanya yang sah, meskipun telah dipanggil secara resmi dan patut, karena itu Majelis Hakim berkesimpulan bahwa Termohon telah dengan sengaja tidak mengindahkan panggilan tersebut dan telah melepaskan hak jawabnya;

Menimbang, bahwa Majelis Hakim perlu mengetengahkan dalil syar'i dari Kitab Ahkamul Qur'an, Juz 2, halaman 45 :

نم يعد ىلامكاح نماكح نيملسملا ملف بجي وهف ملاظ لا قح هل

Artinya: *Barang siapa yang dipanggil oleh Hakim di dalam persidangan sedangkan orang tersebut tidak memenuhi panggilan tersebut, maka dia termasuk orang zalim, maka gugurlah haknya;*

Menimbang, bahwa Termohon yang dipanggil secara resmi dan patut akan tetapi tidak datang menghadap harus dinyatakan tidak hadir dan permohonan tersebut harus diperiksa secara verstek;

Menimbang, bahwa oleh karena itu, maka putusan atas perkara ini dapat dijatuhkan tanpa hadirnya Termohon (verstek);

Menimbang, bahwa sesuai dengan ketentuan Pasal 154 ayat 1 R.Bg Jis. Pasal 39 ayat (1) Undang-Undang Nomor 1 Tahun 1974 tentang Perkawinan, Pasal 82 Undang-Undang Nomor 7 Tahun 1989 tentang Peradilan Agama sebagaimana telah diubah dengan Undang-Undang Nomor 3 Tahun 2006 dan perubahan kedua Undang-Undang Nomor 50 tahun 2009, Majelis Hakim telah berusaha memberikan nasehat kepada Pemohon agar kembali rukun sebagai suami isteri guna membina rumah tangga dengan baik, akan tetapi tidak berhasil;

Menimbang, bahwa oleh karena Termohon tidak pernah hadir di persidangan, maka proses mediasi sebagaimana diamanatkan oleh

Disclaimer
Kepaniteraan Mahkamah Agung Republik Indonesia berusaha untuk selalu mencantumkan informasi paling kini dan akurat sebagai bentuk komitmen Mahkamah Agung untuk pelayanan publik, transparansi dan akuntabilitas pelaksanaan fungsi peradilan. Namun dalam hal-hal tertentu masih dimungkinkan terjadi permasalahan teknis terkait dengan akurasi dan keterkinian informasi yang kami sajikan, hal mana akan terus kami perbaiki dari waktu kewaktu.
Dalam hal Anda menemukan inakurasi informasi yang termuat pada situs ini atau informasi yang seharusnya ada, namun belum tersedia, maka harap segera hubungi Kepaniteraan Mahkamah Agung RI melalui :
Email : kepaniteraan@mahkamahagung.go.id Telp : 021-384 3348 (ext.318)

Peraturan Mahkamah Agung RI (PERMA) Nomor 01 Tahun 2016 tentang Prosedur Mediasi di Pengadilan, tidak dapat dilaksanakan dalam perkara ini;

Menimbang, bahwa berdasarkan ketentuan Surat Edaran Mahkamah Agung RI Nomor 3 Tahun 2015 huruf C angka 3 yaitu putusan yang dijatuhkan tanpa hadirnya Tergugat dapat dikabulkan sepanjang berdasarkan hukum dan beralasan;

Menimbang, bahwa dari posita permohonan Pemohon, yang menjadi dalil pokok dari permohonan Pemohon adalah bahwa Pemohon mohon agar diberi izin untuk menjatuhkan talak satu raj'i terhadap Termohon dengan alasan bahwa:

1. Bahwa keharmonisan dan kerukunan rumah tangga Pemohon dan Termohon, tidak dapat dipertahankan lagi dikarenakan sejak tahun 2011, mulai terjadi perselisihan dan pertengkaran yang secara terus menerus antara Pemohon dan Termohon yang pada pokoknya disebabkan karena Termohon selalu bersikap kasar terhadap Pemohon;
2. Bahwa puncak perselisihan dan pertengkaran antara Pemohon dengan Termohon terjadi sejak tahun 2014, Pemohon dengan Termohon tidak lagi tinggal satu rumah dan hingga sekarang tidak pernah bersatu kembali;
4. Bahwa pihak keluarga Pemohon dan Termohon sudah pernah mendamaikan Pemohon dan Termohon, namun tidak berhasil;
5. Bahwa saat ini Pemohon merasa tidak sanggup lagi untuk meneruskan ikatan perkawinan dengan Termohon, maka antara Pemohon dengan Termohon tidak mungkin dirukunkan sehingga rumah tangga yang *sakinah, mawaddah, warohmah* sangat sulit untuk diwujudkan;

Menimbang, bahwa Pemohon dalam petitum permohonannya angka 2 memohon kepada Majelis Hakim untuk memberikan izin kepada Pemohon untuk menjatuhkan talak satu raj'i terhadap Termohon di depan sidang Pengadilan Agama Panyabungan;

Menimbang, bahwa berdasarkan ketentuan Pasal 149 ayat (1) R.Bg. yaitu putusan yang dijatuhkan tanpa hadirnya Termohon dapat dikabulkan, oleh karena perkara ini termasuk perkara perdata khusus yang tunduk kepada

Disclaimer
Kepaniteraan Mahkamah Agung Republik Indonesia berusaha untuk selalu mencantumkan informasi paling kini dan akurat sebagai bentuk komitmen Mahkamah Agung untuk pelayanan publik, transparansi dan akuntabilitas pelaksanaan fungsi peradilan. Namun dalam hal-hal tertentu masih dimungkinkan terjadi permasalahan teknis terkait dengan akurasi dan keterkinian informasi yang kami sajikan, hal mana akan terus kami perbaiki dari waktu kewaktu.
Dalam hal Anda menemukan inakurasi informasi yang termuat pada situs ini atau informasi yang seharusnya ada, namun belum tersedia, maka harap segera hubungi Kepaniteraan Mahkamah Agung RI melalui :
Email : kepaniteraan@mahkamahagung.go.id   Telp : 021-384 3348 (ext.318)

hukum acara yang bersifat khusus pula, maka berdasarkan *azas lex specialis derogat legi generalis* ketidakhadiran Termohon dalam perkara ini tidaklah dapat dianggap sebagai pengakuannya yang memiliki kekuatan pembuktian sempurna dan mengikat, melainkan hanyalah menggugurkan hak jawabnya terhadap permohonan Pemohon, karena menurut Pasal 311 R.Bg, pengakuan yang mempunyai nilai pembuktian yang lengkap hanyalah pengakuan yang dilakukan di depan hakim, namun berdasarkan Pasal 208 Kitab Undang-Undang Hukum Perdata bahwa perceraian tidak boleh terjadi karena kesepakatan, oleh karenanya sesuai dengan Pasal 283 R.Bg jo Pasal 1865 Kitab Undang-Undang Hukum Perdata, barang siapa mendalilkan sesuatu maka harus membuktikan kebenarannya, oleh karena itu beban pembuktian dibebankan kepada Pemohon;

Menimbang, bahwa namun demikian dalam praktek peradilan Agama, khusus perkara perceraian sebagaimana yang telah diatur dalam Pasal 39 ayat (2) Undang-Undang Nomor 1 Tahun 1974 tentang Perkawinan bahwa perceraian dibolehkan jika telah cukup alasan untuk bercerai, alasan-alasan yang dimaksud mengacu kepada alasan-alasan yang telah diatur dalam peraturan perundangan (vide Pasal 19 Peraturan Pemerintah Nomor 9 Tahun 1975 tentang Pelaksanaan Undang-Undang Nomor 1 Tahun 1974 tentang Perkawinan Jo. Pasal 116 Kompilasi Hukum Islam), maka dengan demikian Pemohon tetap dibebani pembuktian untuk membuktikan alasan perceraiannya tersebut;

Menimbang, bahwa oleh karena alasan permohonan Pemohon dalam pokok sengketa perkara ini adalah adanya perselisihan dan pertengkaran yang terus menerus, dan telah sulit didamaikan untuk hidup rukun kembali dalam rumah tangga maka sesuai ketentuan berdasarkan Pasal 22 ayat (2) Peraturan Pemerintah Nomor 9 tahun 1975 tentang Pelaksanaan Undang-Undang Nomor 1 Tahun 1974 Tentang Perkawinan, maka terlebih dahulu mendengar pihak keluarga serta orang-orang yang dekat dengan suami isteri;

Menimbang, bahwa untuk membuktikan dalil-dalilnya Pemohon telah mengajukan alat bukti tertulis fotokopi Kutipan Akta Nikah (bukti P);

Disclaimer
Kepaniteraan Mahkamah Agung Republik Indonesia berusaha untuk selalu mencantumkan informasi paling kini dan akurat sebagai bentuk komitmen Mahkamah Agung untuk pelayanan publik, transparansi dan akuntabilitas pelaksanaan fungsi peradilan. Namun dalam hal-hal tertentu masih dimungkinkan terjadi permasalahan teknis terkait dengan akurasi dan keterkinian informasi yang kami sajikan, hal mana akan terus kami perbaiki dari waktu kewaktu.
Dalam hal Anda menemukan inakurasi informasi yang termuat pada situs ini atau informasi yang seharusnya ada, namun belum tersedia, maka harap segera hubungi Kepaniteraan Mahkamah Agung RI melalui :
Email : kepaniteraan@mahkamahagung.go.id Telp : 021-384 3348 (ext.318)

Menimbang, bahwa alat bukti berupa surat yaitu bukti P yang diajukan Pemohon, yang telah memenuhi syarat formil dan materil sebagaimana ketentuan dalam Pasal 285 RBg dan Pasal 1868 Kitab Undang-Undang Hukum Perdata sehingga dapat diterima sebagai bukti dalam perkara ini dan dapat dipertimbangkan;

Menimbang, bahwa bukti P adalah fotokopi Kutipan Akta Nikah yang dikeluarkan oleh pejabat yang berwenang, telah bermeterai cukup, telah dinazegelen, telah dilegalisir dan telah dicocokkan dengan aslinya dan cocok, sehingga bukti P telah memenuhi syarat formil dan materil oleh karenanya alat bukti P tersebut mempunyai nilai pembuktian yang sempurna dan mengikat sebagaimana maksud Pasal 7 ayat (1) Kompilasi Hukum Islam, dengan demikian Pemohon dan Termohon telah terbukti sebagai suami istri yang sah sampai saat ini, sehingga Pemohon dalam perkara ini merupakan pihak yang memiliki kepentingan hukum secara langsung dari akibat putusan ini. Sehingga permohonan Pemohon tidak melawan hak dan Pemohon dapat dinyatakan sebagai orang yang berhak untuk mengajukan permohonan dalam perkara ini *(persona standi in judicio)*;

Menimbang, bahwa bukti P berupa fotokopi Kutipan Akta Nikah yang merupakan Akta otentik dan telah bermeterai cukup dan cocok dengan aslinya, isi bukti tersebut menjelaskan mengenai ikatan perkawinan antara Pemohon dengan Termohon, sehingga bukti tersebut telah memenuhi syarat formal dan materiil, serta mempunyai kekuatan pembuktian yang sempurna dan mengikat;

Menimbang, bahwa Pemohon telah pula mengajukan saksi-saksi di mana Majelis Hakim telah mendengar keterangan saksi-saksi yang telah dipanggil satu persatu dalam persidangan dan telah pula ditanya identitas dan hubungannya dengan Pemohon dan Termohon serta di bawah sumpahnya yang mana para saksi tersebut telah memenuhi syarat formil sebagaimana maksud Pasal 171, 172 dan 175 RBg, sehingga patut didengar keterangannya;

Menimbang, bahwa saksi Pemohon terdiri dari 2 orang berdasarkan atas pengetahuan sendiri, bukan pendapat, kesan maupun perasaan pribadi saksi serta keterangan tersebut saling bersesuaian sebagaimana ketentuan

Disclaimer
Kepaniteraan Mahkamah Agung Republik Indonesia berusaha untuk selalu mencantumkan informasi paling kini dan akurat sebagai bentuk komitmen Mahkamah Agung untuk pelayanan publik, transparansi dan akuntabilitas pelaksanaan fungsi peradilan. Namun dalam hal-hal tertentu masih dimungkinkan terjadi permasalahan teknis terkait dengan akurasi dan keterkinian informasi yang kami sajikan, hal mana akan terus kami perbaiki dari waktu kewaktu.
Dalam hal Anda menemukan inakurasi informasi yang termuat pada situs ini atau informasi yang seharusnya ada, namun belum tersedia, maka harap segera hubungi Kepaniteraan Mahkamah Agung RI melalui :
Email : kepaniteraan@mahkamahagung.go.id Telp : 021-384 3348 (ext.318)

dalam Pasal 307, 308 dan 309 RBg sehingga telah memenuhi syarat materil karenanya patut didengar keterangannya;

Menimbang, bahwa untuk membuktikan dalil tentang alasan perceraiannya Pemohon telah mengajukan alat bukti 2 orang saksi;

Menimbang, bahwa saksi-saksi Pemohon, sudah dewasa dan sudah disumpah, sehingga memenuhi syarat formal sebagaimana diatur dalam pasal 172 ayat 1 angka 4 R.Bg;

Menimbang, bahwa keterangan saksi-saksi Pemohon mengenai dalil alasan perceraian Pemohon dan Termohon adalah fakta yang dilihat sendiri/didengar sendiri/dialami sendiri dan relevan dengan dalil yang harus dibuktikan oleh Pemohon, oleh karena itu keterangan saksi tersebut telah memenuhi syarat materiil sebagaimana telah diatur dalam Pasal 308 R.Bg. sehingga keterangan saksi tersebut memiliki kekuatan pembuktian dan dapat diterima sebagai alat bukti;

Menimbang, bahwa berdasarkan bukti tertulis dan keterangan para saksi Pemohon bahwa antara Pemohon dan Termohon masih terikat perkawinan yang sah dan belum pernah bercerai;

Menimbang, bahwa para saksi Pemohon di persidangan menerangkan bahwa pada awalnya kehidupan rumah tangga harmonis;

Menimbang, bahwa saksi pertama Pemohon di persidangan menerangkan bahwa antara Pemohon dan Termohon telah terjadi perselisihan;

Menimbang, bahwa saksi kedua Pemohon di persidangan menerangkan bahwa terjadi pertengkaran dan penyebab pertengkaran adalah karena Termohon selalu bersikap kasar terhadap Pemohon;

Menimbang, bahwa menurut para saksi Pemohon memberikan keterangan bahwa antara Pemohon dan Termohon sudah tidak tinggal bersama selama lebih kurang 6 tahun, dimana Pemohon dan Termohon tidak pernah hidup bersama kembali bahkan antara Pemohon dan Termohon sudah tidak saling mempedulikan lagi dan tidak ada komunikasi antara Pemohon dan Termohon dan tidak pula menjalankan kewajiban sebagai suami isteri;

Disclaimer
Kepaniteraan Mahkamah Agung Republik Indonesia berusaha untuk selalu mencantumkan informasi paling kini dan akurat sebagai bentuk komitmen Mahkamah Agung untuk pelayanan publik, transparansi dan akuntabilitas pelaksanaan fungsi peradilan. Namun dalam hal-hal tertentu masih dimungkinkan terjadi permasalahan teknis terkait dengan akurasi dan keterkinian informasi yang kami sajikan, hal mana akan terus kami perbaiki dari waktu kewaktu.
Dalam hal Anda menemukan inakurasi informasi yang termuat pada situs ini atau informasi yang seharusnya ada, namun belum tersedia, maka harap segera hubungi Kepaniteraan Mahkamah Agung RI melalui :
Email : kepaniteraan@mahkamahagung.go.id   Telp : 021-384 3348 (ext.318)

Menimbang, bahwa para saksi Pemohon di persidangan menerangkan bahwa sudah ada diupayakan perdamaian dari pihak keluarga tetapi tidak berhasil bahkan para saksi telah memberikan nasehat tetapi tidak berhasil;

Menimbang, bahwa berdasarkan fakta-fakta tersebut di atas dapat disimpulkan fakta hukum sebagai berikut:

1. Bahwa, Pemohon dan Termohon telah terikat perkawinan yang sah dan belum pernah bercerai;
2. Bahwa, antara Pemohon dan Termohon sering terjadi perselisihan dan pertengkaran;
3. Bahwa, Pemohon dan Termohon telah berpisah tempat tinggal yang hingga saat ini sudah berlangsung selama lebih kurang 6 tahun;
4. Bahwa, selama selama berpisah, Pemohon dan Termohon tidak pernah hidup bersama kembali bahkan antara Pemohon dan Termohon sudah tidak saling mempedulikan lagi dan tidak ada komunikasi dan tidak pula menjalankan kewajiban sebagai suami isteri
5. Bahwa, Pemohon dan Termohon telah diupayakan untuk didamaikan namun tidak berhasil;

Menimbang, bahwa menurut Pasal 39 Undang-undang Nomor 1 Tahun 1974 tentang perkawinan menentukan bahwa untuk melakukan suatu perceraian harus ada cukup alasan dimana suami istri tidak akan dapat hidup rukun sebagai suami istri dan pengadilan telah berusaha dan tidak berhasil mendamaikan kedua belah pihak sebagaimana maksud pasal 143 ayat (1) dan (2) Kompilasi Hukum Islam. Selanjutnya dalam Pasal 19 huruf (f) Peraturan Pemerintah Nomor 9 Tahun 1975 jo Pasal 116 huruf (f) Kompilasi Hukum Islam (KHI) menegaskan salah satu alasan perceraian yaitu adanya perselisihan dan pertengkaran yang terus menerus antara suami istri dan tidak ada harapan lagi untuk kembali rukun.

Menimbang, bahwa dari ketentuan pasal – pasal tersebut terdapat unsur – unsur yang harus dipenuhi untuk terjadinya perceraian baik cerai talak maupun cerai gugat yaitu :

Disclaimer
Kepaniteraan Mahkamah Agung Republik Indonesia berusaha untuk selalu mencantumkan informasi paling kini dan akurat sebagai bentuk komitmen Mahkamah Agung untuk pelayanan publik, transparansi dan akuntabilitas pelaksanaan fungsi peradilan. Namun dalam hal-hal tertentu masih dimungkinkan terjadi permasalahan teknis terkait dengan akurasi dan keterkinian informasi yang kami sajikan, hal mana akan terus kami perbaiki dari waktu kewaktu.
Dalam hal Anda menemukan inakurasi informasi yang termuat pada situs ini atau informasi yang seharusnya ada, namun belum tersedia, maka harap segera hubungi Kepaniteraan Mahkamah Agung RI melalui :
Email : kepaniteraan@mahkamahagung.go.id Telp : 021-384 3348 (ext.318)

- Adanya alasan telah terjadinya perselisihan dan pertengkaran yang terus menerus.
- Perselisihan dan pertengkaran yang menyebabkan suami istri sudah tidak ada harapan untuk kembali rukun.
- Pengadilan telah berupaya mendamaikan suami istri tapi tidak berhasil.

Menimbang, bahwa unsur-unsur tersebut akan dipertimbangkan satu persatu dengan menghubungkan fakta – fakta yang terjadi dalam rumah tangga Pemohon dengan Termohon sehingga antara Pemohon dan Termohon dipandang telah memenuhi unsur – unsur terjadinya suatu perceraian;

Menimbang, yurisprudensi Mahkamah Agung Nomor 1354 K/Pdt/2000, tanggal 8 September 2003 "Suami isteri yang telah pisah tempat tinggal selama 4 tahun dan tidak saling mempedulikan sudah merupakan fakta adanya perselisihan dan pertengkaran sehingga tidak ada harapan untuk hidup rukun dalam rumah tangga dapat dijadikan alasan untuk mengabulkan gugatan perceraian".

Menimbang, bahwa antara Pemohon dan Termohon telah pisah rumah selama kurang lebih 6 tahun, dimana Termohon tidak pernah kembali untuk hidup bersama, tidak pernah memberikan nafkah kepada Penggugat, tidak ada komunikasi serta sudah tidak saling mempedulikan dan sudah tidak lagi menjalankan kewajibannya sebagai suami isteri, sehingga Majelis Hakim berpendapat bahwa antara Pemohon dan Termohon telah terjadi perselisihan yang nyata yang dapat dijadikan sebagai alasan perceraian antara Pemohon dan Tergugat, dengan demikian berdasarkan pertimbangan di atas bahwa unsur pertama dan kedua juga telah terpenuhi;

Menimbang, bahwa Mejelis Hakim telah berupaya mendamaikan Pemohon dan Termohon dari awal persidangan dan pada setiap persidangan sesuai ketentuan Pasal 82 ayat (1) dan (4) Undang-Undang Nomor 4 tahun 1989 tentang Peradilan Agama sebagaimana telah diubah dengan Undang-Undang Nomor 3 Tahun 2006 dan perubahan kedua Undang-Undang Nomor 50 tahun 2009 Jis Pasal 31 Peraturan Pemerintah Nomor 9 Tahun 1975, Pasal 115 dan Pasal 143 ayat (1) dan (2) Kompilasi Hukum Islam dan para saksi juga

Disclaimer
Kepaniteraan Mahkamah Agung Republik Indonesia berusaha untuk selalu mencantumkan informasi paling kini dan akurat sebagai bentuk komitmen Mahkamah Agung untuk pelayanan publik, transparansi dan akuntabilitas pelaksanaan fungsi peradilan. Namun dalam hal-hal tertentu masih dimungkinkan terjadi permasalahan teknis terkait dengan akurasi dan keterkinian informasi yang kami sajikan, hal mana akan terus kami perbaiki dari waktu kewaktu.
Dalam hal Anda menemukan inakurasi informasi yang termuat pada situs ini atau informasi yang seharusnya ada, namun belum tersedia, maka harap segera hubungi Kepaniteraan Mahkamah Agung RI melalui :
Email : kepaniteraan@mahkamahagung.go.id Telp : 021-384 3348 (ext.318)

telah berupaya menasehati Pemohon agar dapat mempertahankan rumah tangganya namun tidak berhasil serta Pemohon tetap bersikukuh untuk tetap bercerai dengan Termohon sebagaimana dalam kesimpulannya, Majelis Hakim berpendapat unsur ketiga juga telah terpenuhi;

Menimbang, bahwa berdasarkan fakta-fakta tersebut di atas, Majelis Hakim telah menemukan fakta hukum bahwa antara Pemohon dan Termohon telah terjadi perselisihan terus menerus yang nyata sulit diperbaiki, serta tidak ada harapan untuk hidup rukun lagi dalam rumah tangga, secara syar'i bahwa rumah tangga seperti itu tidak dapat dipertahankan lagi, apabila mereka tetap dipaksa hidup di bawah satu atap, bukan keharmonisan yang akan dicapai, melainkan besar kemungkinan akan menimbulkan kemudhoratan sedangkan kemadaratan itu harus dihindarkan , sebagaimana Qaidah Ushul Fiqih yang berbunyi:

درء المفاسد مقدم على جلب المصالح

Artinya : *Menolak kemudharatan lebih utama daripada menarik (mempertahankan) kebaikan*;

إذا تعارض مفسدتان روعي أعظمهما ضررا بارتكاب أخفهما

Artinya : "Apabila dua mafsadah bertentangan, maha perhatikan mana yang lebih besar mudaratnya dengan mengerjakan yang lebih ringan mudaratnya";

Menimbang, bahwa secara sosiologis, dengan tetap mempertahankan pernikahan Pemohon dan Termohon akan menyebabkan penderitaan bagi kedua belah pihak yang akan membahayakan kehidupan rumah tangga keduanya dan juga membahayakan bagi diri dan perkembangan anak-anak Pemohon dan Termohon sebagaimana amanat yang ditegaskan oleh Rasulallah SAW dalam Sunan Ibnu Majah, *Kitab al-Ahkam*, Hadits nomor 2331 yang berbunyi:

لا ضرر ولا ضرار

Disclaimer
Kepaniteraan Mahkamah Agung Republik Indonesia berusaha untuk selalu mencantumkan informasi paling kini dan akurat sebagai bentuk komitmen Mahkamah Agung untuk pelayanan publik, transparansi dan akuntabilitas pelaksanaan fungsi peradilan. Namun dalam hal-hal tertentu masih dimungkinkan terjadi permasalahan teknis terkait dengan akurasi dan keterkinian informasi yang kami sajikan, hal mana akan terus kami perbaiki dari waktu kewaktu.
Dalam hal Anda menemukan inakurasi informasi yang termuat pada situs ini atau informasi yang seharusnya ada, namun belum tersedia, maka harap segera hubungi Kepaniteraan Mahkamah Agung RI melalui :
Email : kepaniteraan@mahkamahagung.go.id Telp : 021-384 3348 (ext.318)

Artinya: *Tidak boleh memudharatkan diri sendiri dan tidak boleh pula membahayakan orang lain;*

Menimbang, bahwa tujuan pernikahan adalah mencapai keluarga sakinah dan mawaddah, dihubungkan dengan fakta-fakta hukum tersebut di atas, maka tujuan pernikahan tersebut sudah tidak dapat diwujudkan, karena Pemohon tetap ingin berpisah dengan Termohon;

Menimbang, bahwa "Suami isteri wajib saling cinta-mencintai hormat-menghormati, setia dan memberi bantuan lahir bathin yang satu kepada yang lain" sebagaimana pasal 33 Undang-Undang Nomor 1 Tahun 1974 tentang Perkawinan jo Pasal 77 huruf (a) dan (b) Kompilasi Hukum Islam, dihubungkan dengan fakta hukum di atas, maka Majelis Hakim berpendapat, secara psikologis, Pemohon dan Termohon sudah tidak dapat mewujudkan maksud dari pasal tersebut dan tidak mampu mewujudkan tujuan perkawinan;

Menimbang, bahwa "Suami wajib melindungi istrinya dan memberikan segala sesuatu keperluan hidup berumah tangga sesuai dengan kemampuannya" sebagaimana Pasal 80 ayat (2) Kompilasi Hukum Islam, dihubungkan dengan fakta hukum di atas, maka Majelis Hakim berpendapat Pemohon sudah tidak dapat mewujudkan maksud dari pasal tersebut dan mencapai tujuan perkawinan;

Menimbang, bahwa "Kewajiban utama bagi seorang istri ialah berbakti lahir dan batin kepada suami di dalam batas-batas yang dibenarkan hukum Islam" sebagaimana Pasal 83 ayat (1) Kompilasi Hukum Islam, dihubungkan dengan fakta hukum di atas, maka Majelis Hakim berpendapat Termohon sudah tidak dapat melaksanakan maksud dari pasal tersebut;

Menimbang, bahwa berdasarkan Yurisprudensi Mahkamah Agung Republik Indonesia Nomor 38/K/AG/1990 tanggal 22 Agustus 1991, bahwa apabila terbukti suatu rumah tangga sudah pecah dan tidak dapat diperbaiki dan mempertahankan rumah tangga membawa akibat negatif (mafsadat yang lebih besar) bagi kedua belah pihak, maka perceraian dapat dipertimbangkan untuk dikabulkan;

Disclaimer
Kepaniteraan Mahkamah Agung Republik Indonesia berusaha untuk selalu mencantumkan informasi paling kini dan akurat sebagai bentuk komitmen Mahkamah Agung untuk pelayanan publik, transparansi dan akuntabilitas pelaksanaan fungsi peradilan. Namun dalam hal-hal tertentu masih dimungkinkan terjadi permasalahan teknis terkait dengan akurasi dan keterkinian informasi yang kami sajikan, hal mana akan terus kami perbaiki dari waktu kewaktu.
Dalam hal Anda menemukan inakurasi informasi yang termuat pada situs ini atau informasi yang seharusnya ada, namun belum tersedia, maka harap segera hubungi Kepaniteraan Mahkamah Agung RI melalui :
Email : kepaniteraan@mahkamahagung.go.id Telp : 021-384 3348 (ext.318)

Menimbang, bahwa yurisprudensi Mahkamah Agung Nomor 534 K/Pdt/1996, tanggal 18 Juni 1996: “Bahwa dalam hal perceraian tidak perlu dilihat dari siapa penyebab percekcokan atau salah satu pihak telah meninggalkan pihak lain, tetapi yang perlu dilihat adalah perkawinan itu sendiri apakah perkawinan itu masih dapat dipertahankan lagi atau tidak”.

Menimbang, bahwa berdasarkan yurisprudensi tersebut, maka perceraian antara Pemohon dengan Termohon tidak perlu dilihat dari siapa penyebab percekcokan yang terjadi antara Pemohon dan Termohon;

Menimbang, bahwa firman Allah dalam surat Al Baqarah ayat 227

وَإِنْ عَزَمُوا الطَّلَاقَ فَإِنَّ اللَّهَ سَمِيعٌ عَلِيمٌ

Artinya: “dan jika mereka ber'azam (bertetap hati untuk) talak, Maka Sesungguhnya Allah Maha mendengar lagi Maha mengetahui”;

Menimbang, bahwa firman Allah dalam surat An Nisa ayat 130

وَإِن يَتَفَرَّقَا يُغْنِ اللَّهُ كُلًّا مِّن سَعَتِهِ ۚ وَكَانَ اللَّهُ وَاسِعًا حَكِيمًا

Artinya: “Jika keduanya bercerai, Maka Allah akan memberi kecukupan kepada masing-masingnya dari limpahan karunia-Nya. dan adalah Allah Maha luas (karunia-Nya) lagi Maha Bijaksana”;

Menimbang, bahwa oleh karena permohonan Pemohon telah memenuhi ketentuan Pasal 39 ayat (2) dan Pasal 70 ayat (1) Undang – Undang Nomor 1 Tahun 1974 tentang Perkawinan, maka Majelis Hakim berkesimpulan permohonan Pemohon tersebut telah beralasan hukum dan terbukti sebagaimana diatur dalam ketentuan Pasal 19 huruf (b) dan (f) Peraturan Pemerintah Nomor 9 Tahun 1975 jo Pasal 116 huruf (b) dan (f) Kompilasi Hukum Islam di Indonesia, sehingga permohonan Pemohon pada petitum angka 2 dapat dikabulkan;

Menimbang, bahwa perceraian antara Pemohon dan Termohon merupakan perceraian yang terjadi karena talak sebagaimana maksud pasal 114 Kompilasi Hukum Islam dan dijatuhkan oleh Pengadilan Agama

Disclaimer
Kepaniteraan Mahkamah Agung Republik Indonesia berusaha untuk selalu mencantumkan informasi paling kini dan akurat sebagai bentuk komitmen Mahkamah Agung untuk pelayanan publik, transparansi dan akuntabilitas pelaksanaan fungsi peradilan. Namun dalam hal-hal tertentu masih dimungkinkan terjadi permasalahan teknis terkait dengan akurasi dan keterkinian informasi yang kami sajikan, hal mana akan terus kami perbaiki dari waktu kewaktu.
Dalam hal Anda menemukan inakurasi informasi yang termuat pada situs ini atau informasi yang seharusnya ada, namun belum tersedia, maka harap segera hubungi Kepaniteraan Mahkamah Agung RI melalui :
Email : kepaniteraan@mahkamahagung.go.id Telp : 021-384 3348 (ext.318)

sebagaimana maksud Pasal 131 ayat (2), maka talak antara Pemohon dan Termohon adalah talak raj'i sebagaimana yang tercantum dalam amar putusan ini;

Menimbang, bahwa selama dalam perkawinan antara Pemohon dengan Termohon belum pernah terjadi perceraian, maka talak yang terjadi adalah talak satu;

Menimbang, bahwa perkara ini adalah perkara cerai talak dimana Pemohon baru diberi izin untuk menjatuhkan talaknya maka berdasarkan Pasal 71 ayat (2) Undang-Undang nomor 7 Tahun 1989 tentang Peradilan Agama sebagaimana yang telah diubah menjadi Undang - Undang Nomor 3 Tahun 2006 dan perubahan kedua dengan Undang – Undang Nomor 50 Tahun 2009 bahwa Hakim membuat penetapan yang isinya menyatakan bahwa perkawinan putus sejak ikrar talak diucapkan, dimana penetapan tersebut dikirim kepada Pegawai Pencatat Nikah yang mewilayahi tempat tinggal suami untuk diadakan pencatatan sebagaimana maksud Pasal 131 ayat (5) Kompilasi Hukum Islam;

Menimbang, bahwa berdasarkan Pasal 85 Undang-undang Nomor 7 Tahun 1989 tentang Peradilan Agama sebagaimana yang telah diubah menjadi Undang-undang Nomor 3 Tahun 2006 dan perubahan kedua dengan Undang – Undang Nomor 50 Tahun 2009, bahwa kelalaian pengiriman tersebut, menjadi tanggung jawab Panitera yang bersangkutan atau pejabat Pengadilan yang ditunjuk, apabila yang demikian itu mengakibatkan kerugian bagi bekas suami atau istri atau keduanya, maka Majelis Hakim memerintahkan Penitera Pengadilan Agama Panyabungan untuk mengirimkan salinan ikrar talak tersebut;

Menimbang, bahwa berdasarkan Pasal 89 ayat (1) Undang-undang Nomor 4 tahun 1989 tentang Peradilan Agama yang telah diubah dengan Undang-undang Nomor 3 tahun 2006 dan perubahan kedua dengan Undang-undang Nomor 50 tahun 2009, maka kepada Pemohon dibebani untuk membayar biaya perkara ini;

Disclaimer
Kepaniteraan Mahkamah Agung Republik Indonesia berusaha untuk selalu mencantumkan informasi paling kini dan akurat sebagai bentuk komitmen Mahkamah Agung untuk pelayanan publik, transparansi dan akuntabilitas pelaksanaan fungsi peradilan. Namun dalam hal-hal tertentu masih dimungkinkan terjadi permasalahan teknis terkait dengan akurasi dan keterkinian informasi yang kami sajikan, hal mana akan terus kami perbaiki dari waktu kewaktu.
Dalam hal Anda menemukan inakurasi informasi yang termuat pada situs ini atau informasi yang seharusnya ada, namun belum tersedia, maka harap segera hubungi Kepaniteraan Mahkamah Agung RI melalui :
Email : kepaniteraan@mahkamahagung.go.id Telp : 021-384 3348 (ext.318)

Mengingat ketentuan hukum *syara'* serta peraturan perundangan berlaku yang berhubungan dengan perkara ini;

MENGADILI

1. Menyatakan Termohon yang telah dipanggil secara resmi dan patut untuk menghadap di persidangan tidak hadir;
2. Mengabulkan permohonan Pemohon dengan verstek;
3. Memberi izin kepada Pemohon (Xxxx) untuk menjatuhkan talak satu raj'i terhadap Termohon (Xxxx) di depan sidang Pengadilan Agama Panyabungan;
4. Membebankan kepada Pemohon untuk membayar biaya perkara sejumlah Rp401.000,00 (empat ratus satu ribu rupiah);

Demikian perkara ini diputus di Panyabungan berdasarkan musyawarah Majelis Hakim Pengadilan Agama Panyabungan pada hari Rabu tanggal 23 Desember 2020 Masehi bertepatan dengan tanggal 08 Jumadil Awal 1442 H, oleh Hasanuddin, S.Ag., yang ditunjuk oleh Ketua Pengadilan Agama Panyabungan sebagai Ketua Majelis, Nurlaini M. Siregar, S.H.I., dan Abdul Aziz Alhamid, S.H.I., sebagai Hakim-Hakim Anggota, putusan mana diucapkan dalam sidang terbuka untuk umum pada hari itu juga oleh Ketua Majelis tersebut, didampingi oleh Hakim-Hakim Anggota dan dibantu oleh Fatimah, S.H., sebagai Panitera Pengganti dengan dihadiri Pemohon tanpa hadirnya Termohon;

Ketua Majelis,

Hasanuddin, S.Ag.

Hakim Anggota,

Ketua Majelis,

Abdul Aziz Alhamid, S.H.I.

Nurlaini M. Siregar, S.H.I.

Panitera Pengganti,

Disclaimer
Kepaniteraan Mahkamah Agung Republik Indonesia berusaha untuk selalu mencantumkan informasi paling kini dan akurat sebagai bentuk komitmen Mahkamah Agung untuk pelayanan publik, transparansi dan akuntabilitas pelaksanaan fungsi peradilan. Namun dalam hal-hal tertentu masih dimungkinkan terjadi permasalahan teknis terkait dengan akurasi dan keterkinian informasi yang kami sajikan, hal mana akan terus kami perbaiki dari waktu kewaktu.
Dalam hal Anda menemukan inakurasi informasi yang termuat pada situs ini atau informasi yang seharusnya ada, namun belum tersedia, maka harap segera hubungi Kepaniteraan Mahkamah Agung RI melalui :
Email : kepaniteraan@mahkamahagung.go.id Telp : 021-384 3348 (ext.318)

Fatimah, S.H.

<u>Perincian biaya perkara</u> :

1.	Biaya Pendaftaran	Rp	30.000,00
2.	Biaya proses	Rp	50.000,00
3.	Biaya Panggilan	Rp	285.000,00
4.	Biaya PNBP panggilan Pemohon	Rp	10.000,00
5.	Biaya PNBP panggilan Termohon	Rp	10.000,00
6.	Meterai	Rp	6.000,00
7.	Redaksi	Rp	10.000,00
	Jumlah	Rp	401.000,00

Terbilang : empat ratus satu ribu rupiah.

Disclaimer
Kepaniteraan Mahkamah Agung Republik Indonesia berusaha untuk selalu mencantumkan informasi paling kini dan akurat sebagai bentuk komitmen Mahkamah Agung untuk pelayanan publik, transparansi dan akuntabilitas pelaksanaan fungsi peradilan. Namun dalam hal-hal tertentu masih dimungkinkan terjadi permasalahan teknis terkait dengan akurasi dan keterkinian informasi yang kami sajikan, hal mana akan terus kami perbaiki dari waktu kewaktu.
Dalam hal Anda menemukan inakurasi informasi yang termuat pada situs ini atau informasi yang seharusnya ada, namun belum tersedia, maka harap segera hubungi Kepaniteraan Mahkamah Agung RI melalui :
Email : kepaniteraan@mahkamahagung.go.id Telp : 021-384 3348 (ext.318)
