# Supplementary material for: Classification of divorce causes during the COVID-19 pandemic using convolutional neural networks
Source: PeerJ Comput Sci. 2022 Jun 30;8:e998. doi: 10.7717/peerj-cs.998 (PMC9299239; doi:10.7717/peerj-cs.998)
Supplement: Supplemental Information 4 [file peerj-cs-08-998-s004.zip › Konflik Dataset/Data ke-4.pdf]

P U T U S A N
Nomor xxxx/Pdt.G/2020/PA.Dmk
بسم الله الرحمن الرحيم
DEMI KEADILAN BERDASARKAN KETUHANAN YANG MAHA ESA

Pengadilan Agama Demak yang memeriksa dan mengadili perkara-perkara tertentu pada tingkat pertama, dalam persidangan Majelis Hakim telah menjatuhkan putusan dalam perkara Cerai Gugat antara :

Xxxx, umur 34 tahun, agama Islam, Pekerjaan karyawan Toko, Pendidikan Sekolah Lanjutan Tingkat Pertama, tempat kediaman di Desa Trengguli 02 Rw 08 Kecamatan Wonosalam Kabupaten Demak, dalam hal ini memberikan kuasa kepada **xxxx.,** Advokat yang berkantor di Advokat/Penasihat Hukum pada Kantor Hukum dan Mediator *"Toro Masiran, SH.MH dan Rekan"*, berkantor di Perum Nusa Indah Permai Blok A No. 1 Demak, berdasarkan surat kuasa khusus tanggal 27 November 2020, sebagai Penggugat

Melawan

Xxxx, umur 37 tahun, agama Islam, Pekerjaan Tukang Rosok, Pendidikan Sekolah Lanjutan Tingkat Pertama, tempat kediaman di Desa Trengguli 02 Rw 08 Kecamatan Wonosalam Kabupaten Demak, sebagai Tergugat

Pengadilan Agama tersebut ;
Telah membaca surat-surat perkara;
Telah mendengar keterangan Penggugat dan saksi-saksinya ;

DUDUK PERKARA

Bahwa Penggugat dalam surat gugatan tertanggal 27 November 2020, terdaftar di Kepaniteraan Pengadilan Agama Demak, Nomor xxxx/Pdt.G/2020/PA.Dmk. Tanggal 08 Desember 2020 , telah mengemukakan hal-hal sebagai berikut :

1. Bahwa Penggugat adalah istri sah dari Tergugat yang telah melangsungkan pernikahan menurut Agama Islam pada tanggal 4 Februari 2005 di Rumah Penggugat hadapan Pegawai Pencatatan Nikah Kantor Urusan Agama kecamatan Wonosalam yang terdaftar dalam daftar yang disediakan untuk itu sebagaimana dalam Kutipan Akta Nikah nomor :

Disclaimer
Kepaniteraan Mahkamah Agung Republik Indonesia berusaha untuk selalu mencantumkan informasi paling kini dan akurat sebagai bentuk komitmen Mahkamah Agung untuk pelayanan publik, transparansi dan akuntabilitas pelaksanaan fungsi peradilan. Namun dalam hal-hal tertentu masih dimungkinkan terjadi permasalahan teknis terkait dengan akurasi dan keterkinian informasi yang kami sajikan, hal mana akan terus kami perbaiki dari waktu kewaktu.
Dalam hal Anda menemukan inakurasi informasi yang termuat pada situs ini atau informasi yang seharusnya ada, namun belum tersedia, maka harap segera hubungi Kepaniteraan Mahkamah Agung RI melalui :
Email : kepaniteraan@mahkamahagung.go.id Telp : 021-384 3348 (ext.318)

89/44/II/2005 tertanggal 4 Februari 2005 yang dikeluarkan oleh Kantor Urusan Agama kecamatan Wonosalam Kabupaten Demak;

2. Bahwa ketika menikah Penggugat berstatus Perawan dan Tergugat Ketika menikah berstatus Jejaka;

3. Bahwa akad nikah dilakukan di rumah orangtua Penggugat di Desa Trengguli 02 Rw 08 Kecamatan Wonosalam Kabupaten Demak;

4. Bahwa setelah akad nikah, Penggugat dan Tergugat bertempat tinggal dirumah orangtua Penggugat yang beralamat di Desa Trengguli 02 Rw 08 Kecamatan Wonosalam Kabupaten Demak;

5. Bahwa semula rumah tangga antara Penggugat dan Tergugat harmonis dan Penggugat belum pernah bercerai dengan Tergugat, dan sudah pernah melakukan hubungan layaknya suami isteri dengan Tergugat (bada dukhul) dan telah dikaruniai 2 (dua) orang anak yang bernama;

   a. xxxx, Jenis kelamin laki-laki, umur kurang lebih 14 tahun, Anak tersebut ikut Penggugat;
   b. Xxxx, Jenis kelamin Laki-laki, Umur 9 tahun 1 bulan, Anak tersebut ikut Penggugat;

6. Bahwa awal mula Pernikahan antara Penggugat dan Tergugat harmonis, Akan tetapi sejak bulan Desember 2019 perselisihan dan pertengkaran antara Penggugat dan Tergugat mulai terjadi dikarenakan :;

   a. Tergugat sering minum-minuman keras, selain itu Termohon juga sering berjudi;
   b. Tergugat tidak memberikan nafkah kepada Penggugat;
   c. Tergugat mempunyai banyak hutang, Akan tetapi Tergugat tidak mau tanggung jawab dengan Hutangnya tersebut;

7. Bahwa puncak pertengkaran terjadi pada bulan Juli 2020, Penggugat dan Tergugat akhirnya memutuskan untuk pisah ranjang, Kemudian Penggugat dan Tergugat sudah pisah ranjang selama 5 (lima) bulan;

8. Bahwa anak Penggugat dan Tergugat yang bernama Xxxx, jenis kelamin Perempuan, berumur 9 (Sembilan) tahun 1 (satu) bulan, Masih dibawah umur (belum Mumayiz) dan Tergugat juga tidak pernah mengurusi dan memberikan nafkah kepada anaknya tersebut maka sudah sepatutnya hak asuk anak jatuh kepada Penggugat, Apalagi Tergugat

Disclaimer
Kepaniteraan Mahkamah Agung Republik Indonesia berusaha untuk selalu mencantumkan informasi paling kini dan akurat sebagai bentuk komitmen Mahkamah Agung untuk pelayanan publik, transparansi dan akuntabilitas pelaksanaan fungsi peradilan. Namun dalam hal-hal tertentu masih dimungkinkan terjadi permasalahan teknis terkait dengan akurasi dan keterkinian informasi yang kami sajikan, hal mana akan terus kami perbaiki dari waktu kewaktu.
Dalam hal Anda menemukan inakurasi informasi yang termuat pada situs ini atau informasi yang seharusnya ada, namun belum tersedia, maka harap segera hubungi Kepaniteraan Mahkamah Agung RI melalui :
Email : kepaniteraan@mahkamahagung.go.id Telp : 021-384 3348 (ext.318)

sering meminum-minuman keras dan berjudi, Selain itu Tergugat tidak memberikan nafkah kepada Penggugat dan anak-anaknya;

9. Bahwa semenjak Penggugat dan Tergugat bermasalah pihak keluarga sudah berusaha untuk mendamaikannya, Akan tetapi Penggugat dan Tergugat tetap tidak mau melanjutkan perkawinan ini sehingga tidak ada hasilnya oleh karena itu, Penggugat menyerahkan sepenuhnya penyelesaian lewat Pengadilan Agama Demak;

10. Bahwa berdasarkan hal-hal tersebut, kiranya telah memenuhi alasan hukum Penggugat mengajukan cerai gugat terhadap Tergugat sebagaimana diatur dalam Pasal 19 huruf (f) Peraturan Pemerintah No. 9 Tahun 1975 jo. Pasal 116 huruf (f) Kompilasi Hukum Islam, sehingga berdasar hukum untuk menyatakan gugatan cerai gugat ini dikabulkan;

11. Bahwa oleh karena kehidupan rumah tangga antara Penggugat dan Tergugat sudah tidak ada kecocokan lagi dan sudah tidak ada lagi kebahagiaan dan ketentraman dalam hidup bersama sebagaimana layaknya suami isteri dan sudah tidak mungkin untuk dipersatukan kembali, maka tidak ada jalan lagi kecuali untuk diputus perkawinan ini dengan perceraian;

12. Bahwa Penggugat sanggup membayar biaya perkara;

PRIMER;

1. Mengabulkan gugatan Penggugat;
2. Menjatuhkan Talak satu dari Tergugat (Xxxx) atas diri Penggugat (Xxxx) dengan jatuhnya Talak satu Bain Shugro;
3. Menetapkan hak asuh anak yang bernama Xxxx, jenis kelamin Perempuan, berumur 9 tahun 1 bulan Jatuh Kepada Penggugat;
4. Menetapkan besarnya biaya perkara ini beserta pembebanannya menurut ketentuan hukum yang berlaku;

SUBSIDER ;

- Apabila Pengadilan Agama Demak berpendapat lain mohon putusan yang seadil-adilnya.

Bahwa Penggugat telah datang menghadap di persidangan yang telah ditentukan, tetapi Tergugat telah tidak datang menghadap atau menyuruh orang lain untuk datang menghadap sebagai kuasanya, meskipun menurut Berita Acara Panggilan dari Jurusita Pengganti Pengadilan Agama Demak Nomor

Disclaimer
Kepaniteraan Mahkamah Agung Republik Indonesia berusaha untuk selalu mencantumkan informasi paling kini dan akurat sebagai bentuk komitmen Mahkamah Agung untuk pelayanan publik, transparansi dan akuntabilitas pelaksanaan fungsi peradilan. Namun dalam hal-hal tertentu masih dimungkinkan terjadi permasalahan teknis terkait dengan akurasi dan keterkinian informasi yang kami sajikan, hal mana akan terus kami perbaiki dari waktu kewaktu.
Dalam hal Anda menemukan inakurasi informasi yang termuat pada situs ini atau informasi yang seharusnya ada, namun belum tersedia, maka harap segera hubungi Kepaniteraan Mahkamah Agung RI melalui :
Email : kepaniteraan@mahkamahagung.go.id Telp : 021-384 3348 (ext.318)

xxxx/Pdt.G/2020/PA.Dmk yang dibacakan di persidangan telah dipanggil secara resmi dan patut, dan tidak ternyata, bahwa tidak datangnya itu disebabkan oleh suatu halangan yang sah;

Bahwa Majelis Hakim telah menasehati Penggugat dalam rangka untuk mendamaikan agar rukun lagi dan membina rumah tangga dengan Tergugat, akan tetapi tidak berhasil karena Penggugat tetap ingin bercerai dari Penggugat, kemudian dilanjutkan dengan pembacaan surat gugatan Penggugat yang isinya tetap dipertahankan oleh Penggugat;

Bahwa atas surat gugatan Penggugat tersebut, Tergugat tidak mengajukan jawabannya, karena tidak hadir, karena itu pemeriksaan dilakukan tanpa hadirnya Tergugat;

Bahwa untuk membuktikan dalil gugatannya, Penggugat telah mengajukan bukti-buktinya di persidangan pada tanggal 01 Desember 2020, yaitu sebagai berikut:

A. Bukti Surat:

1. Fotokopi Kartu Tanda Penduduk Penggugat, nomor 3321066005860001 tertanggal 16 Nopember 2012, dikeluarkan oleh Dinas Kependudukan dan Pencatatan Sipil Kabupaten Demak,(P-1);

2. Fotokopi Kutipan Akta Nikah, nomor 89/44/II/2005 tertanggal 25 Februari 2005, yang dikeluarkan oleh Kantor Urusan Agama Kecamatan Wonosalam, Kabupaten Demak, (P-3).

3. Fotokopi Kutipan Akta Kelahiran nomor AL. 659.0270476 tertanggal 19 Oktober 2011, dikeluarkan oleh Dinas Kependudukan dan Pencatatan Sipil Demak, (P-4);

Surat-surat bukti tersebut setelah dicocokkan dengan aslinya ternyata sesuai dan telah bermatrei cukup

B. Bukti saksi-saksi:

1. xxxx, umur 46 tahun, di bawah sumpahnya ia memberi keterangan sebagai berikut:

- Bahwa saksi kenal baik dengan Penggugat dan Tergugat karena saksi saudara sepupu dengan Penggugat;
- Bahwa saksi kenal dengan Tergugat sejak menjadi suami Penggugat.

Disclaimer
Kepaniteraan Mahkamah Agung Republik Indonesia berusaha untuk selalu mencantumkan informasi paling kini dan akurat sebagai bentuk komitmen Mahkamah Agung untuk pelayanan publik, transparansi dan akuntabilitas pelaksanaan fungsi peradilan. Namun dalam hal-hal tertentu masih dimungkinkan terjadi permasalahan teknis terkait dengan akurasi dan keterkinian informasi yang kami sajikan, hal mana akan terus kami perbaiki dari waktu kewaktu.
Dalam hal Anda menemukan inakurasi informasi yang termuat pada situs ini atau informasi yang seharusnya ada, namun belum tersedia, maka harap segera hubungi Kepaniteraan Mahkamah Agung RI melalui :
Email : kepaniteraan@mahkamahagung.go.id Telp : 021-384 3348 (ext.318)

- Bahwa Penggugat dan Tergugat adalah suami isteri menikah tahun 2005 dan telah dikaruniai 2 orang anak;
- Bahwa anak tersebut sekarang dalam asuhan Penggugat dan setahu saksi Penggugat sangat perhatian dan sayang kepada anaknya tersebut;
- Bahwa setelah menikah Penggugat dan Tergugat semula tinggal di rumah orang tua Penggugat di Desa Trengguli;
- Bahwa pada awalnya rumah tangga rukun dan harmonis akan tetapi sejak bulan Desember tahun 2019 antara Penggugat dengan Tergugat mulai sering terjadi perselisisihan dan pertengkaran;
- Bahwa penyebab pertengkaran tersebut karena Tergugat jarang-jarang memberikan nafkah kepada Penggugat, banyak pinjaman uang kepada orang lain, suka meminum minuman keras dan suka bermain judi ;
- Bahwa kemudian sejak sudah 5 bulan lamanya antara Pengugat dengan Tergugat pisah ranjang hingga sekarang;
- Bahwa saksi mengetahui Penggugat dan Tergugat pisah ranjang karena antara Penggugat dengan Tergugat sudah tidak pernah kelihatan bersama-sama dan saling mendiamkan;
- Bahwa keluarga Penggugat sudah berusaha menasehati Penggugat akan tetapi tidak berhasil;

2. xxxx, umur 46 tahun, di bawah sumpahnya ia memberi keterangan sebagai berikut:

- Bahwa saksi kenal baik dengan Penggugat dan Tergugat karena saksi bertetangga dekat dengan Penggugat;
- Bahwa saksi kenal dengan Tergugat sejak menjadi suami Penggugat.
- Bahwa Penggugat dan Tergugat adalah suami isteri menikah tahun 2005 dan telah dikaruniai 2 orang anak;
- Bahwa anak tersebut sekarang dalam asuhan Penggugat dan setahu saksi Penggugat sangat perhatian dan sayang kepada anaknya tersebut;

Disclaimer
Kepaniteraan Mahkamah Agung Republik Indonesia berusaha untuk selalu mencantumkan informasi paling kini dan akurat sebagai bentuk komitmen Mahkamah Agung untuk pelayanan publik, transparansi dan akuntabilitas pelaksanaan fungsi peradilan. Namun dalam hal-hal tertentu masih dimungkinkan terjadi permasalahan teknis terkait dengan akurasi dan keterkinian informasi yang kami sajikan, hal mana akan terus kami perbaiki dari waktu kewaktu.
Dalam hal Anda menemukan inakurasi informasi yang termuat pada situs ini atau informasi yang seharusnya ada, namun belum tersedia, maka harap segera hubungi Kepaniteraan Mahkamah Agung RI melalui :
Email : kepaniteraan@mahkamahagung.go.id Telp : 021-384 3348 (ext.318)

- Bahwa setelah menikah Penggugat dan Tergugat semula tinggal di rumah orang tua Penggugat di Desa Trengguli;
- Bahwa pada awalnya rumah tangga rukun dan harmonis akan tetapi sejak bulan Desember tahun 2019 antara Penggugat dengan Tergugat mulai sering terjadi perselisisihan dan pertengkaran;
- Bahwa penyebab pertengkaran tersebut karena Tergugat tidak pernah memberikan nafkah kepada Penggugat, banyak pinjaman uang kepada orang lain, dan juga Tergugat suka meminum minuman keras dan bermain judi ;
- Bahwa kemudian sejak sudah 5 bulan lamanya antara Pengugat dengan Tergugat pisah ranjang hingga sekarang;
- Bahwa saksi mengetahui Penggugat dan Tergugat pisah ranjang karena antara Penggugat dengan Tergugat sudah tidak pernah kelihatan bersama-sama dan saling mendiamkan;
- Bahwa keluarga Penggugat sudah berusaha menasehati Penggugat akan tetapi tidak berhasil;

Bahwa atas bukti-bukti yang diajukan oleh Penggugat tersebut, Tergugat tidak mengajukan bantahannya, karena tidak hadir di persidangan;

Bahwa Penggugat telah menyampaikan kesimpulannya secara lisan di persidangan pada tanggal 22 Desember 2020, yang pada pokoknya Penggugat tetap menghendaki perceraian dan mohon asuh anak, lalu menyatakan tidak akan mengajukan suatu apapun lagi dan mohon putusan;

Bahwa untuk meringkas uraian putusan ini, maka ditunjuk hal ihwal sebagaimana tercantum dalam berita acara sidang perkara ini yang merupakan bagian yang tak terpisahkan dari putusan ini.

PERTIMBANGAN HUKUM

Menimbang bahwa maksud dan tujuan gugatan Penggugat adalah sebagaimana telah terurai diatas;

Menimbang, bahwa Penggugat dan Tergugat beragama Islam dan telah melakukan perkawinan secara Islami, maka perkara ini merupakan kompetensi absolut Pengadilan Agama untuk memeriksa dan menyelesaikannya. (vide

Disclaimer
Kepaniteraan Mahkamah Agung Republik Indonesia berusaha untuk selalu mencantumkan informasi paling kini dan akurat sebagai bentuk komitmen Mahkamah Agung untuk pelayanan publik, transparansi dan akuntabilitas pelaksanaan fungsi peradilan. Namun dalam hal-hal tertentu masih dimungkinkan terjadi permasalahan teknis terkait dengan akurasi dan keterkinian informasi yang kami sajikan, hal mana akan terus kami perbaiki dari waktu kewaktu.
Dalam hal Anda menemukan inakurasi informasi yang termuat pada situs ini atau informasi yang seharusnya ada, namun belum tersedia, maka harap segera hubungi Kepaniteraan Mahkamah Agung RI melalui :
Email : kepaniteraan@mahkamahagung.go.id Telp : 021-384 3348 (ext.318)

Pasal 40 dan Pasal 63 ayat (1) Undang Undang No. 1 Tahun 1974 Jo. Pasal 14 dan Pasal 1 huruf b. PP. N0. 9 Tahun 1975, Jo Pasal 2 dan Pasal 49 ayat (1) Undang-Undang Nomor 7 Tahun 1989 yang telah diubah dengan Undang Undang No 3 Tahun 2006 dan Undang-Undang Nomor 50 Tahun 2009;

Menimbang, bahwa dari bukti P-1 yang berupa kartu tanda Penduduk maka telah terbukti Penggugat bertempat tinggal di wilayah yurisdiksi Pengadilan Agama Demak, maka perkara ini secara relatif merupakan kompetensi Pengadilan Agama Demak, (vide Pasal 73 ayat (1) Undang-Undang Nomor 7 Tahun 1989 yang telah diubah dengan Undang-Undang Nomor No 3 Tahun 2006 dan Undang Undang No. 50 Tahun 2009 jo Pasal 132 ayat (1) Kompilasi Hukum Islam);

Menimbang, bahwa berdasarkan bukti P-2 yang berupa Kutipan Akta Nikah maka telah terbukti bahwa benar, antara Penggugat dengan Tergugat telah terikat dalam perkawinan yang sah oleh karena itu gugatan Penggugat terhadap Tergugat *memiliki dasar hukum yang sah*;-

Menimbang, bahwa karena ternyata Tergugat meskipun dipanggil dengan patut tidak pernah datang menghadap dan tidak pula menyuruh orang lain untuk menghadap sebagai wakilnya serta ketidak datangannya itu tanpa disebabkan oleh suatu halangan yang sah, maka patutlah dianggap bahwa Tergugat dalam keadaan tidak hadir dan sesuai dengan kehendak pasal 125 HIR putusan perkara ini dijatuhkan dengan verstek;

Menimbang, bahwa oleh karena Tergugat tidak pernah hadir dipersidangan sehingga proses mediasi sebagaimana ketentuan PERMA Nomor 01 Tahun 2016 tidak dapat dilaksanakan;

Menimbang, bahwa Penggugat pada pokoknya menuntut perceraian dengan alasan karena sejak bulan Desember tahun 2019, antara Penggugat dengan Tergugat mulai sering terjadi perselisihan dan pertengkaran disebabkan Tergugat jarang-jarang memberikan nafkah kepada Penggugat, banyak pinjaman uang kepada orang lain, suka meminum minuman keras dan suka bermain judi yang kemudian sejak bulan Juli 2020 antara Pengugat dengan Tergugat pisah ranjang hingga sekarang dan sejak itu pula antara Penggugat dengan Tergugat tidak ada komunikasi lagi layaknya suami isteri;

Disclaimer
Kepaniteraan Mahkamah Agung Republik Indonesia berusaha untuk selalu mencantumkan informasi paling kini dan akurat sebagai bentuk komitmen Mahkamah Agung untuk pelayanan publik, transparansi dan akuntabilitas pelaksanaan fungsi peradilan. Namun dalam hal-hal tertentu masih dimungkinkan terjadi permasalahan teknis terkait dengan akurasi dan keterkinian informasi yang kami sajikan, hal mana akan terus kami perbaiki dari waktu kewaktu.
Dalam hal Anda menemukan inakurasi informasi yang termuat pada situs ini atau informasi yang seharusnya ada, namun belum tersedia, maka harap segera hubungi Kepaniteraan Mahkamah Agung RI melalui :
Email : kepaniteraan@mahkamahagung.go.id Telp : 021-384 3348 (ext.318)

Menimbang bahwa Penggugat telah menyandarkan alasan tersebut pada Pasal 19 huruf f Peraturan Pemerintah Nomor 9 tahun 1975 jo pasal 116 huruf f KHI. "*Antara suami dan isteri terus menerus terjadi perselisihan dan pertengkaran dan tidak ada harapan akan hidup rukun lagi dalam rumah tangga*";

Menimbang bahwa ketidak hadiran Tergugat dalam persidangan ini dianggap telah mengabaikan hak bantah Tergugat atas dalil-dalil gugatan Penggugat, namun demikian Penggugat tetap mengajukan saksi-saksi yang akan dipertimbangkan sebagai berikut:

Menimbang, bahwa dua orang saksi Penggugat tersebut telah memenuhi syarat-syarat formil dan tidak terhalang kedudukannya sebagai saksi seperti yang diatur dalam Pasal 145 HIR, keduanya memberikan keterangan yang didasarkan pada pengetahuan dari pengalaman, penglihatan dan pendengarannya sendiri yang antara kedua saksi tersebut terdapat kesesuaian keterangan seperti yang disyaratkan dalam Pasal 172 HIR, oleh karena itu keterangan kedua saksi tersebut dapat dipertimbangkan;

Menimbang, bahwa dari dalil gugatan Penggugat, sikap Penggugat selama dipersidangan dihubungkan dengan bukti surat dan keterangan-keterangan yang disampaikan oleh para saksi di persidangan, dapat ditemukan fakta sebagai berikut :

1. Bahwa Penggugat dan Tergugat adalah suami isteri sah, menikah pada tanggal 04 Februari 2005 dan sudah dikaruniai 2 orang anak sekarang dalam asuhan Penggugat;
2. Bahwa kemudian sejak bulan Desember tahun 2019, antara Penggugat dengan Tergugat mulai sering terjadi perselisihan dan pertengkaran disebabkan Tergugat jarang-jarang memberikan nafkah kepada Penggugat, banyak pinjaman uang kepada orang lain, suka meminum minuman keras dan suka bermain judi;
3. Bahwa kemudian puncaknya terjadi pada bulan Juli tahun 2020 hingga antara Penggugat dengan Tergugat pisah ranjang hinga sekarang dan sejak itu pula antara Penggugat dan Tergugat tidak ada komunikasi sama sekali;

Disclaimer
Kepaniteraan Mahkamah Agung Republik Indonesia berusaha untuk selalu mencantumkan informasi paling kini dan akurat sebagai bentuk komitmen Mahkamah Agung untuk pelayanan publik, transparansi dan akuntabilitas pelaksanaan fungsi peradilan. Namun dalam hal-hal tertentu masih dimungkinkan terjadi permasalahan teknis terkait dengan akurasi dan keterkinian informasi yang kami sajikan, hal mana akan terus kami perbaiki dari waktu kewaktu.
Dalam hal Anda menemukan inakurasi informasi yang termuat pada situs ini atau informasi yang seharusnya ada, namun belum tersedia, maka harap segera hubungi Kepaniteraan Mahkamah Agung RI melalui :
Email : kepaniteraan@mahkamahagung.go.id   Telp : 021-384 3348 (ext.318)

4. Bahwa keluarga Penggugat sudah berusaha mendamaikannya, akan tetapi tidak berhasil.

Menimbang, bahwa berdasarkan fakta poin (1) menunjukkan bahwa Penggugat dan Tergugat berkualitas hukum sebagai pihak-pihak dalam perkara ini, dan Penggugat berhak mengajukan permohonan cerai gugat terhadap Tergugat ke Pengadilan Agama sebagaimana dimaksud pasal 66 ayat (1) Undang-undang Nomor 7 Tahun 1989;

Menimbang, bahwa dari fakta point 2, 3, 4 dan 5 sebagaimana terurai diatas telah memberikan gambaran bahwa perselisihan antara Penggugat dengan Tergugat sudah tidak mampu lagi dikendalikan dan diatasi oleh mereka berdua, bahkan sikap Pengguat dan Tergugat yang kini telah pisah ranjang adalah merupakan bukti bahwa perselisihan antara Penggugat dengan Tergugat sudah cukup serius dan bersifat terus menerus;

Menimbang bahwa dari pernyataan Penggugat selama dalam persidangan yang dengan tegas hendak bercerai dengan Tergugat dan juga sikap Tergugat yang tidak pernah menghadiri persidangan adalah merupakan indikasi tidak adanya kehendak dari kedua belah pihak untuk memperbaiki kembali keadaan rumah tangganya, karena itu merupakan fakta kongkrit yang menunjukkan bahwa hubungan Penggugat dengan Tergugat sudah sampai pada tingkat pecahnya perkawinan (*broken marriage*) dan Hakim berkesimpulan bahwa tujuan yang diharapkan dari perkawinan sebagaimana tersebut pada pasal 1 Undang-undang Nomor 1 Tahun 1974 tidak mungkin lagi terwujud;

Menimbang bahwa para saksi saksi telah menerangkan adanya usaha pihak keluarga merukunkan Penggugat dengan Tergugat akan tetapi tidak berhasil, kerena itu dianggap telah memenui ketentuan Pasal 22 ayat (2) Peraturan Pemerintah Nomor 9 Tahun 1974;

Menimbang bahwa mengutip referensi fiqh yang terdapat dalam kitab Hurriatuz Zaujani fith Thalaq Juz I halaman 83 yang diambil alih menjadi pendapat Hakim sebagai berikut :

æÞÏ ÇÎÊÇÑÇáÅÓáÇã äÙÇã ÇáØáÇÞ Ííä ÊÖØÑ È ÇáÍíÇÉ ÇáÒæÌíä æáã íÚÏ íäÝÚ ÝíåÇ äÕÇ ÆÍ æáÇ Õáí æíÍË ÊÕÈÍ ÇáÑÈØÉ ÇáÒæÇÌ ÕæÑÉ ãä ÛíÑ Ñæí

Disclaimer
Kepaniteraan Mahkamah Agung Republik Indonesia berusaha untuk selalu mencantumkan informasi paling kini dan akurat sebagai bentuk komitmen Mahkamah Agung untuk pelayanan publik, transparansi dan akuntabilitas pelaksanaan fungsi peradilan. Namun dalam hal-hal tertentu masih dimungkinkan terjadi permasalahan teknis terkait dengan akurasi dan keterkinian informasi yang kami sajikan, hal mana akan terus kami perbaiki dari waktu kewaktu.
Dalam hal Anda menemukan inakurasi informasi yang termuat pada situs ini atau informasi yang seharusnya ada, namun belum tersedia, maka harap segera hubungi Kepaniteraan Mahkamah Agung RI melalui :
Email : kepaniteraan@mahkamahagung.go.id Telp : 021-384 3348 (ext.318)

áÃä ÇÓÊãÑÇÑ ãÚäÇå Çä íÍßã Úáì ÇÎÏ ÇáÒæÌíä ÈÇáÓÌä ÇáãæÄ ÈÏ æåÐÇ Ùáã ÊÃ ÈÇå Ñæí ÇáÚÏÇáÉ

Artinya : "*Islam memilih lembaga thalaq (perceraian) ketika rumah tangga sudah dianggap goncang serta dianggap sudah tidak bermanfaat lagi nasehat/ perdamaian, dan hubungan suami isteri menjadi tanpa ruh (hampa), sebab meneruskan perkawinan berarti menghukum salah satu suami isteri dengan penjara yang berkepanjangan. Ini adalah aniaya (kedhaliman) yang bertentangan dengan semangat keadilan*".

Menimbang, bahwa aspek penting dari bunyi pasal 19 huruf f Peraturan Pemerintah Nomor 9 tahun 1975 jo pasal 116 huruf KHI diatas adalah tidak adanya harapan untuk menata dan mempertahankan rumah tangga, dengan demikian apabila maksud pasal tersebut dikomparasikan dengan keadaan rumah tangga Penggugat dan Tergugat maka telah terdapat cukup alasan hukum untuk mengabulkan gugatan Penggugat dan menjatuhkan talak satu ba'in sughro dari Tergugat terhadap Penggugat;

Menimbang bahwa selaian perceraian tersebut, Penggugat juga menuntut hadlonah/ pemeliharaan terhadap anaknya yang nomor 2 bernama Xxxx ;

Menimbang bahwa berdasarkan bukti surat yang bertanda P-3 yang berupa kutipan akta lahir dan keterangan para saksi maka telah terbukti bahwa Penggugat dan Tergugat telah dikaruniai seorang anak bernama Xxxx, umur 9 tahun yang berarti anak tersebut belum mumayyiz dan dari keterangan para saksi terbukti anak tersejut sekarang dalam asuhan Penggugat dan Penggugat selaku Ibu penuh perhatian dan sayang kepada anaknya tersebut;

Menimbang bahwa karena anak tersebut belum mumayyiz dan masih sangat membutuhkan pelayanan, perhatian, kesabaran dan sentuhan kasih sayang dari seorang Ibu dan anak tersebut hingga sekarang dalam asuhan Penggugat, sehingga hukum memandang Ibunya (Penggugat) lebih berhak mengasuh, mendidik serta pantas ditunjuk sebagai pemegang hak hadlonah

Disclaimer
Kepaniteraan Mahkamah Agung Republik Indonesia berusaha untuk selalu mencantumkan informasi paling kini dan akurat sebagai bentuk komitmen Mahkamah Agung untuk pelayanan publik, transparansi dan akuntabilitas pelaksanaan fungsi peradilan. Namun dalam hal-hal tertentu masih dimungkinkan terjadi permasalahan teknis terkait dengan akurasi dan keterkinian informasi yang kami sajikan, hal mana akan terus kami perbaiki dari waktu kewaktu.
Dalam hal Anda menemukan inakurasi informasi yang termuat pada situs ini atau informasi yang seharusnya ada, namun belum tersedia, maka harap segera hubungi Kepaniteraan Mahkamah Agung RI melalui :
Email : kepaniteraan@mahkamahagung.go.id Telp : 021-384 3348 (ext.318)

atas anak tersebut, hal ini sesuai ketentuan Pasal 105 ayat (1) Kompilasi Hukum Islam dan hadis Nabi yang berbunyi:

أنت أحق به مالم تنكحى

Artinya "*Engkau (Ibu) lebih berhak terhadap anakmu selama engkau belum menikah"*

Menimbang bahwa berdasarkan pertimbangan tersebut diatas maka Pengadilan patut menetapkan anak Penggugat dan Tergugat nomor 2 bernama Xxxx berada dalam hadlonah/ asuhan Penggugat;

Menimbang bahwa bapak atau Ibu yang tidak mengasuh anak, tetap mempunyai hak untuk mencurahkan kasih sayangnya seperti melihat dan mengajak bepergian anak tersebut kapan saja waktunya dengan tujuan untuk mendatangkan kemaslahatan bagi anak dan orang tua yang tidak mengasuh anak tersebut, dengan demikian anak tersebut akan merasa tetap dekat dengan kedua orang tuanya, sebagaimana pendapat DR. Wahbah Az-Zuhaily dalam kitab al-Fiqhul Islamy wa Adillatuhu, jilid VII halaman 70 yang diambil alih sebagain pendapat Majelis Hakim sebagai berikut:

حق الرؤية أو الزيارة لاحد الابوين غير الحاضن مقرر شرعا باتفاق الفقهاء لصلة ولكنهم ذكروا اراء مختلفة نسيبا بحسب تقدير المصلحة, لكل من الولد والولد الذي ولده فى حضانه غيره

Artinya: " Ulama' ahli fiqh sepakat, hak untuk melihat atau berkunjung bagi salah satu dari orang tua yang tidak mengasuh anak, ditetapkan berdasarkan syara' untuk menyambung hubungan antara anak dengan salah satu orang tuanya yang tidka mengasuh anak, sekira bisa mendatangkankan kemaslahatan bagi anak dan orang tua yang melahirkan anak tersebut, yang berada dibawah asuhan salah satu borang tuanya"

Menimbang bahwa karena itu orang yang mengasuh anak serta seluruh keluarganya tidak boleh menghalang-halangi anak untuk bertemu dengan orang tuanya yang tidak mengasuh anak atau menyembunyikan anak tersebut, sehingga tidak ada kesempatan bagi anak untuk bertemu dengan orang tuanya yang tidak mengasuhnya;

Disclaimer
Kepaniteraan Mahkamah Agung Republik Indonesia berusaha untuk selalu mencantumkan informasi paling kini dan akurat sebagai bentuk komitmen Mahkamah Agung untuk pelayanan publik, transparansi dan akuntabilitas pelaksanaan fungsi peradilan. Namun dalam hal-hal tertentu masih dimungkinkan terjadi permasalahan teknis terkait dengan akurasi dan keterkinian informasi yang kami sajikan, hal mana akan terus kami perbaiki dari waktu kewaktu.
Dalam hal Anda menemukan inakurasi informasi yang termuat pada situs ini atau informasi yang seharusnya ada, namun belum tersedia, maka harap segera hubungi Kepaniteraan Mahkamah Agung RI melalui :
Email : kepaniteraan@mahkamahagung.go.id Telp : 021-384 3348 (ext.318)

Menimbang bahwa berdasarkan pertimbangan tersebut diatas, maka kepada Tergugat selaku ayah dari anak bernama Xxxx tersebut berhak untuk menengok/ mengunjungi dan mengajak anak tersebut bepergian dengan sepengetahuan /izin Penggugat;

Menimbang, bahwa oleh karena perkara ini termasuk dalam bidang perkawinan, berdasarkan Pasal 89 ayat (1) Undang-Undang 7 Tahun 1989 yang telah diubah dengan Undang-Undang Nomor 3 Tahun 2006 dan perubahan kedua dengan Undang-Undang Nomor 50 Tahun 2009, maka biaya perkara ini dibebankan kepada Penggugat;

Memperhatikan Pasal 125 dan 126 HIR serta ketentuan dalil syar'i yang berkaitan dengan perkara ini;

M E N G A D I L I :

1. *Menyatakan Tergugat yang telah dipanggil secara resmi dan patut untuk menghadap ke persidangan, tidak hadir;*
2. *Mengabulkan gugatan Penggugat dengan verstek;*
3. *Menjatuhkan talak satu ba'in sughra Tergugat (Xxxx) kepada Penggugat (Xxxx);*
4. *Menetapkan anak Pengugat dan Tergugat nomor 2 yang bernama Xxxx, berada dibawah pemeliharaan (hadhonah) Penggugat;.*
5. *Membebankan kepada Penggugat untuk membayar biaya perkara yang hingga kini sejumlah Rp. 406.000,- (empat ratus enam ribu rupiah).*

Demikianlah putusan ini dijatuhkan dalam musyawarah Majelis Hakim di Demak pada hari Selasa tanggal 22 Desember 2020 Masehi, bertepatan dengan tanggal 07 Jumadil awal 1442 H.oleh Kami Drs. Makali sebagai Ketua Majelis Hakim, dan Dra. Nur Immawati serta Toharudin, S.H.I., M.H. masing-masing sebagai Hakim Anggota. Putusan mana diucapkan oleh Ketua Majelis Hakim tersebut pada hari itu juga dalam sidang terbuka untuk umum dengan dihadiri oleh para Hakim Anggota dan Hj. Siti Hajar Zulaikha, S.H. sebagai Panitera Pengganti dan dihadiri pula oleh Penggugat tanpa hadirnya Tergugat;

Ketua Majelis

Drs. Makali

Disclaimer
Kepaniteraan Mahkamah Agung Republik Indonesia berusaha untuk selalu mencantumkan informasi paling kini dan akurat sebagai bentuk komitmen Mahkamah Agung untuk pelayanan publik, transparansi dan akuntabilitas pelaksanaan fungsi peradilan. Namun dalam hal-hal tertentu masih dimungkinkan terjadi permasalahan teknis terkait dengan akurasi dan keterkinian informasi yang kami sajikan, hal mana akan terus kami perbaiki dari waktu kewaktu.
Dalam hal Anda menemukan inakurasi informasi yang termuat pada situs ini atau informasi yang seharusnya ada, namun belum tersedia, maka harap segera hubungi Kepaniteraan Mahkamah Agung RI melalui :
Email : kepaniteraan@mahkamahagung.go.id Telp : 021-384 3348 (ext.318)

Hakim Anggota	Hakim Anggota
**Dra. Nur Immawati**	**Toharudin, S.H.I., M.H.**

Panitera Pengganti

Hj. Siti Hajar Zulaikha, S.H.

Perincian Biaya :

1. Biaya Pendaftaran	:	Rp	30.000,00
2. Biaya Proses	:	Rp	100.000,00
3. Biaya Pemanggilan	:	Rp	240.000,00
4. PNBP Panggilan	:	Rp	20.000,00
5. Biaya Redaksi	:	Rp	10000,00
6. Biaya Meterai	:	Rp	6000,00
Jumlah	:	Rp	406.000,00

(empat ratus enam ribu rupiah)

Disclaimer
Kepaniteraan Mahkamah Agung Republik Indonesia berusaha untuk selalu mencantumkan informasi paling kini dan akurat sebagai bentuk komitmen Mahkamah Agung untuk pelayanan publik, transparansi dan akuntabilitas pelaksanaan fungsi peradilan. Namun dalam hal-hal tertentu masih dimungkinkan terjadi permasalahan teknis terkait dengan akurasi dan keterkinian informasi yang kami sajikan, hal mana akan terus kami perbaiki dari waktu kewaktu.
Dalam hal Anda menemukan inakurasi informasi yang termuat pada situs ini atau informasi yang seharusnya ada, namun belum tersedia, maka harap segera hubungi Kepaniteraan Mahkamah Agung RI melalui :
Email : kepaniteraan@mahkamahagung.go.id   Telp : 021-384 3348 (ext.318)
