# Supplementary material for: Classification of divorce causes during the COVID-19 pandemic using convolutional neural networks
Source: PeerJ Comput Sci. 2022 Jun 30;8:e998. doi: 10.7717/peerj-cs.998 (PMC9299239; doi:10.7717/peerj-cs.998)
Supplement: Supplemental Information 4 [file peerj-cs-08-998-s004.zip › Konflik Dataset/Data ke-5.pdf]

P U T U S A N

Nomor 1305/Pdt.G/2020/PA.Kds

بسم الله الرحمن الرحيم

DEMI KEADILAN BERDASARKAN KETUHANAN YANG MAHA ESA

Pengadilan Agama Kudus yang memeriksa dan mengadili perkara tertentu pada tingkat pertama dalam sidang majelis telah menjatuhkan putusan dalam perkara Cerai Talak antara:

Rija Revano bin Muchtar, NIK. 3319081706880007, tempat tanggal lahir Kudus, 17 Juni 1988, umur 33 tahun, agama Islam, pekerjaan guru swasta, pendidikan Sarjana, bertempat tinggal di RT.003 RW.003, Desa Getassrabi, Kecamatan Gebog, Kabupaten Kudus, sebagai **Pemohon**;

m e l a w a n

Nina Sulistiyani binti H.Mustain, tempat tanggal lahir Jepara, 11 Juli 1981 umur 30 tahun, agama Islam, pekerjaan karyawan swasta, pendidikan SMA, tempat kediaman di RT.003 RW003, Desa Getassrabi, Kecamatan Gebog, Kabupaten Kudus, sebagai **Termohon;**

Pengadilan Agama tersebut;

Telah mempelajari surat-surat yang berkaitan dengan perkara ini;

Telah mendengar keterangan Pemohon dan para saksi di muka sidang;

DUDUK PERKARA

Bahwa Pemohon dalam surat permohonannya tanggal 21 November 2020 telah mengajukan Cerai Talak, yang telah didaftar di Kepaniteraan Pengadilan Agama Kudus dengan Nomor 1305/Pdt.G/2020/PA.Kds, tanggal 21 November 2020 dengan dalil-dalil permohonannya sebagai berikut:

1. Bahwa pada tanggal 30 November 2013, No. 0844/017/XI/2013 Pemohon telah melangsungkan pernikahan dengan Termohon yang dicatat oleh Pegawai Pencatat Nikah Kantor Urusan Agama Kecamatan Welahan Kabupaten Jepara;
2. Bahwa sebelum menikah Pemohon berstatus jejaka dan Termohon perawan;

Disclaimer
Kepaniteraan Mahkamah Agung Republik Indonesia berusaha untuk selalu mencantumkan informasi paling kini dan akurat sebagai bentuk komitmen Mahkamah Agung untuk pelayanan publik, transparansi dan akuntabilitas pelaksanaan fungsi peradilan. Namun dalam hal-hal tertentu masih dimungkinkan terjadi permasalahan teknis terkait dengan akurasi dan keterkinian informasi yang kami sajikan, hal mana akan terus kami perbaiki dari waktu kewaktu.
Dalam hal Anda menemukan inakurasi informasi yang termuat pada situs ini atau informasi yang seharusnya ada, namun belum tersedia, maka harap segera hubungi Kepaniteraan Mahkamah Agung RI melalui :
Email : kepaniteraan@mahkamahagung.go.id Telp : 021-384 3348 (ext.318)

3. Bahwa setelah pernikahan tersebut, Pemohon dengan Termohon bertempat tinggal di rumah orang tua Pemohon di RT.003/RW003, Desa Getassrabi, Kecamatan Gebog, Kabupaten Kudus, selama 3 Tahun kemudian bertempat tinggal di Perumahan Pondok Pesantren selama 2 tahun, kemudian membangun rumah di RT.003/RW003, Desa Getassrabi, Kecamatan Gebog, Kabupaten Kudus, dan dalam perkawinan telah hidup rukun sebagaimana layaknya suami istri (ba'da dukhul) dan dikaruniai 2 orang anak bernama:
   a. SYAHRIVAR REVANO umur 6 tahun;
   b. MAHREZ REVANO umur 8 bulan;
4. Bahwa selama dalam pernikahan Pemohon dan Termohon belum pernah bercerai;
5. Bahwa semula rumah tangga Pemohon dan Termohon berjalan harmonis, namun sejak bulan April tahun 2020 Pemohon dan Termohon mulai tidak harmonis lagi sebab:
   a. Termohon semaunya sendiri dan tidak mau dinasehati;
   b. Termohon merasa kurang menerima nafkah yang diberikan oleh Pemohon;
   c. Antara Pemohon dengan Termohon sering terjadi perbedaan pendapat yang mengakibatkan pertengkaran;
6. Bahwa puncak perselisihan dan pertengkaran tersebut pada bulan Juni 2020 pisah rumah sampai sekarang. Bahwa atas sikap dan perbuatan Termohon tersebut, Pemohon merasa sangat menderita lahir batin dan oleh karenanya Pemohon tidak rela dan berkesimpulan bahwa Termohon adalah istri yang tidak sholihah, sehingga tujuan perkawinan untuk membentuk rumah tangga yang sakinah, mawaddah dan rahmah sudah sulit dipertahankan lagi;
7. Bahwa berdasarkan uraian di atas, permohonan Pemohon telah memenuhi alasan perceraian sebagaimana diatur dalam Undang-Undang Nomor 1 Tahun 1974 dan Peraturan Pemerintah Nomor 9 Tahun 1975 dan Kompilasi Hukum Islam;
8. Bahwa Pemohon sanggup membayar seluruh biaya yang timbul dalam penyelesaian perkara ini.

Disclaimer
Kepaniteraan Mahkamah Agung Republik Indonesia berusaha untuk selalu mencantumkan informasi paling kini dan akurat sebagai bentuk komitmen Mahkamah Agung untuk pelayanan publik, transparansi dan akuntabilitas pelaksanaan fungsi peradilan. Namun dalam hal-hal tertentu masih dimungkinkan terjadi permasalahan teknis terkait dengan akurasi dan keterkinian informasi yang kami sajikan, hal mana akan terus kami perbaiki dari waktu kewaktu.
Dalam hal Anda menemukan inakurasi informasi yang termuat pada situs ini atau informasi yang seharusnya ada, namun belum tersedia, maka harap segera hubungi Kepaniteraan Mahkamah Agung RI melalui :
Email : kepaniteraan@mahkamahagung.go.id   Telp : 021-384 3348 (ext.318)

Berdasarkan alasan/dalil-dalil di atas, Pemohon merasa sudah tidak tahan lagi untuk meneruskan kehidupan berumah tangganya dengan Termohon, oleh karenanya Pemohon mohon agar Ketua Pengadilan Agama Kudus c.q. Majelis Hakim segera memeriksa dan mengadili perkara ini, selanjutnya menjatuhkan putusan yang amarnya berbunyi :

PRIMER :

1. Mengabulkan permohonan Permohon;
2. Memberi izin kepada Pemohon (**Rija Revano bin Muchtar**) untuk menjatuhkan talak satu raj'i terhadap Termohon (**Nina Sulistiyani Binti H.Mustain**) didepan sidang Pengadilan Agama Kudus;
3. Membebankan biaya perkara menurut hukum;

SUBSIDIER :

Apabila Pengadilan Agama berpendapat lain, mohon putusan yang seadil-adilnya;

Bahwa pada hari sidang yang telah ditetapkan Pemohon telah datang menghadap ke muka sidang, sedangkan Termohon tidak datang menghadap ke muka sidang dan tidak menyuruh orang lain untuk menghadap sebagai wakil/ kuasa hukumnya meskipun telah dipanggil secara resmi dan patut yang relaas panggilannya dibacakan di dalam sidang, sedangkan tidak ternyata bahwa tidak datangnya itu disebabkan suatu halangan yang sah;

Bahwa majelis hakim telah menasehati Pemohon agar rukun lagi dan tidak bercerai dengan Termohon, tetapi Pemohon tetap pada dalil-dalil permohonannya untuk bercerai dengan Termohon;

Bahwa perkara ini tidak dapat dimediasi karena Temohon tidak pernah datang menghadap meskipun telah dipanggil secara resmi dan patut, selanjutnya dimulai pemeriksaan dengan membacakan surat permohonan Pemohon yang maksud dan isinya tetap dipertahankan oleh Pemohon;

Bahwa untuk menguatkan dalil-dalil permohonannya, Pemohon telah mengajukan alat-alat bukti di persidangan, yaitu sebagai berikut:

I. **Alat bukti Surat**

Disclaimer
Kepaniteraan Mahkamah Agung Republik Indonesia berusaha untuk selalu mencantumkan informasi paling kini dan akurat sebagai bentuk komitmen Mahkamah Agung untuk pelayanan publik, transparansi dan akuntabilitas pelaksanaan fungsi peradilan. Namun dalam hal-hal tertentu masih dimungkinkan terjadi permasalahan teknis terkait dengan akurasi dan keterkinian informasi yang kami sajikan, hal mana akan terus kami perbaiki dari waktu kewaktu.
Dalam hal Anda menemukan inakurasi informasi yang termuat pada situs ini atau informasi yang seharusnya ada, namun belum tersedia, maka harap segera hubungi Kepaniteraan Mahkamah Agung RI melalui :
Email : kepaniteraan@mahkamahagung.go.id Telp : 021-384 3348 (ext.318)

1. Fotokopi Kartu Tanda Penduduk Nomor 3319081706880007 tanggal 20 September 2020, telah bermeterai cukup, telah dicocokan sesuai dengan aslinya (bukti P.1);
2. Fotokopi Kutipan Akta Nikah dari Kantor Urusan Agama Kecamatan Welahan, Kabupaten Jepara Nomor 372/Kua.11.20.11/PW.01/ 10/2020 tanggal 30 November 2013, telah bermeterai cukup, telah dicocokan sesuai dengan aslinya (bukti P.2);

II. Alat bukti Saksi:

1. Hamim Prawoto bin Jambari, umur 28 tahun, agama Islam, pekerjaan karyawan swasta, tempat tinggal di RT.003 RW.003, Desa Getassrabi, Kecamatan Gebog, Kabupaten Kudus, di bawah sumpahnya telah memberikan keterangan yang pada pokoknya sebagai berikut :

- Bahwa saksi kenal dengan Pemohon dan Termohon karena saksi adalah saudara sepupu Pemohon;
- Bahwa setelah menikah Pemohon dan Termohon tinggal di rumah orang tua Pemohon, kemudian di rumah kediaman bersama, sudah mempunyai 2 (dua) orang anak;
- Bahwa saksi sering main ke tempat tempat tinggal Pemohon dan Termohon serta mengetahui Pemohon dan Termohon telah pisah hingga sekarang lebih kurang 5 (lima) bulan karena Termohon pergi meningggalkan Pemohon dan pulang ke rumah orang tuanya;
- Bahwa sebelum berpisah Pemohon dan Termohon sering terjadi perselisihan dan pertengkaran masalah ekomoni, Termohon merasa kurang nafkah, saksi ada 4 (empat) kali melihat dan mendengar sendiri pertengkaran tersebut;
- Bahwa saksi sudah menasehatai Pemohon agar rukun lagi dengan Termohon, tetapi tidak berhasil;

2. Zaenatun binti Yamin, umur 59 tahun, agama Islam, pekerjaan mengurus rumah tangga, tempat tinggal di RT.003 RW.003, Desa Getassrabi, Kecamatan Gebog, Kabupaten Kudus, telah memberikan keterangan di bawah sumpah yang pada pokoknya sebagai berikut :

Disclaimer
Kepaniteraan Mahkamah Agung Republik Indonesia berusaha untuk selalu mencantumkan informasi paling kini dan akurat sebagai bentuk komitmen Mahkamah Agung untuk pelayanan publik, transparansi dan akuntabilitas pelaksanaan fungsi peradilan. Namun dalam hal-hal tertentu masih dimungkinkan terjadi permasalahan teknis terkait dengan akurasi dan keterkinian informasi yang kami sajikan, hal mana akan terus kami perbaiki dari waktu kewaktu.
Dalam hal Anda menemukan inakurasi informasi yang termuat pada situs ini atau informasi yang seharusnya ada, namun belum tersedia, maka harap segera hubungi Kepaniteraan Mahkamah Agung RI melalui :
Email : kepaniteraan@mahkamahagung.go.id Telp : 021-384 3348 (ext.318)

- Bahwa saksi kenal dengan Pemohon dan Termohon karena saksi adalah
ibu kandung Pemohon;
- Bahwa setelah menikah Pemohon dan Termohon tinggal di rumah orang tua Pemohon, kemudian di rumah kediaman bersama, sudah mempunyai 2 (dua) orang anak;
- Bahwa saksi sering ke tempat tinggal Pemohon dan Termohon sehingga mengetahui Pemohon dan Termohon telah pisah hingga sekarang lebih kurang 5 (lima) bulan karena Termohon pergi meninggalkan Pemohon dan pulang ke rumah orang tuanya;
- Bahwa sebelum berpisah, Pemohon dan Termohon sering terjadi perseli-sihan dan pertengkaran karena Termohon merasa kurang atas pemberian nafkah dari Pemohon, saksi ada 5 (lima) kali melihat dan mendengar sendiri pertengkaran tersebut;
- Bahwa saksi sudah menasehati Pemohon agar rukun lagi dengan Termohon, tetapi tidak berhasil;

Bahwa atas keterangan saksi-saksi tersebut, Pemohon menyatakan cukup dan tidak akan mengajukan alat bukti lainnya lagi;

Bahwa Pemohon telah menyampaikan kesimpulan secara lisan dengan menyatakan tetap pada permohonannya dan mohon putusan;

Bahwa untuk singkatnya uraian putusan ini, maka semua hal yang termuat dalam berita acara sidang perkara ini merupakan bagian yang tidak terpisahkan dari putusan ini;

PERTIMBANGAN HUKUM

Menimbang, bahwa maksud dan tujuan permohonan Pemohon adalah sebagaimana telah diuraikan di atas;

Menimbang, bahwa oleh karena perkara ini merupakan bidang perkawinan, maka sesuai dengan Pasal 49 huruf a Undang-Undang Nomor 7 Tahun 1989 tentang Peradilan Agama sebagaimana telah diubah dengan Undang-Undang Nomor 3 Tahun 2006 dan perubahan kedua dengan Undang-Undang Nomor 50 Tahun 2009, perkara ini merupakan kewenangan Pengadilan Agama (*absolute competentie*);

Disclaimer
Kepaniteraan Mahkamah Agung Republik Indonesia berusaha untuk selalu mencantumkan informasi paling kini dan akurat sebagai bentuk komitmen Mahkamah Agung untuk pelayanan publik, transparansi dan akuntabilitas pelaksanaan fungsi peradilan. Namun dalam hal-hal tertentu masih dimungkinkan terjadi permasalahan teknis terkait dengan akurasi dan keterkinian informasi yang kami sajikan, hal mana akan terus kami perbaiki dari waktu kewaktu.
Dalam hal Anda menemukan inakurasi informasi yang termuat pada situs ini atau informasi yang seharusnya ada, namun belum tersedia, maka harap segera hubungi Kepaniteraan Mahkamah Agung RI melalui :
Email : kepaniteraan@mahkamahagung.go.id Telp : 021-384 3348 (ext.318)

Menimbang, bahwa berdasarkan identitas para pihak, Pemohon dan Termohon bertempat tinggal di wilayah Kabupaten Kudus yang merupakan yurisdiksi Pengadilan Agama Kudus, maka sesuai dengan Pasal 66 ayat (2) Undang-Undang Nomor 7 Tahun 1989 tentang Peradilan Agama, sebagaimana telah diubah dengan Undang-Undang Nomor 3 Tahun 2006 dan perubahan kedua dengan Undang-Undang Nomor 50 Tahun 2009, Pengadilan Agama Kudus berwenang untuk memeriksa perkara ini (relative competentie);

Menimbang, bahwa ternyata Termohon meskipun telah dipanggil secara resmi dan patut, tidak datang menghadap di muka sidang dan pula tidak ternyata bahwa tidak datangnya itu disebabkan oleh suatu halangan yang sah menurut hukum;

Menimbang, bahwa Termohon yang dipanggil secara resmi dan patut akan tetapi tidak datang menghadap harus dinyatakan tidak hadir dan surat permohonan tersebut harus diperiksa secara verstek;

Menimbang, bahwa oleh karena itu, maka putusan atas perkara ini dapat dijatuhkan tanpa hadirnya Termohon (verstek);

Menimbang, bahwa berdasarkan ketentuan Pasal 125 ayat (1) HIR yaitu putusan yang dijatuhkan tanpa hadirnya Termohon dapat dikabulkan sepanjang berdasarkan hukum dan beralasan, oleh karena itu majelis membebani Pemohon untuk membuktikan dalil-dalil permohonannya;

Menimbang, bahwa untuk membuktikan dalil-dalil permohonannya, Pemohon telah mengajukan alat bukti surat P.1, P.2 dan 2 orang saksi;

Menimbang, bahwa alat bukti P.1 bermeterai cukup, telah dicocokkan dengan aslinya, terbukti Pemohon bertempat tinggal di RT.003 RW.003, Desa Getassrabi, Kecamatan Gebog, Kabupaten Kudus, ternyata sesuai dengan identitas Pemohon pada surat permohonannya, oleh karena itu dapat diterima sebagai alat bukti;

Menimbang, bahwa bukti P.2 berupa Fotokopi Kutipan Akta Nikah yang aslinya merupakan akta otentik dan telah bermeterai cukup dan cocok dengan aslinya, isi bukti tersebut menjelaskan mengenai Pemohon dan Termohon telah melangsungkan perkawinan pada tanggal 30 November 2013, tercatat di Kantor Urusan Agama Kecamatan Welahan, Kabupaten Jepara, sehingga bukti tersebut

Disclaimer
Kepaniteraan Mahkamah Agung Republik Indonesia berusaha untuk selalu mencantumkan informasi paling kini dan akurat sebagai bentuk komitmen Mahkamah Agung untuk pelayanan publik, transparansi dan akuntabilitas pelaksanaan fungsi peradilan. Namun dalam hal-hal tertentu masih dimungkinkan terjadi permasalahan teknis terkait dengan akurasi dan keterkinian informasi yang kami sajikan, hal mana akan terus kami perbaiki dari waktu kewaktu.
Dalam hal Anda menemukan inakurasi informasi yang termuat pada situs ini atau informasi yang seharusnya ada, namun belum tersedia, maka harap segera hubungi Kepaniteraan Mahkamah Agung RI melalui :
Email : kepaniteraan@mahkamahagung.go.id Telp : 021-384 3348 (ext.318)

telah memenuhi syarat formal dan materiil, serta mempunyai kekuatan yang sempurna dan mengikat;

Menimbang, bahwa saksi 1 dan saksi 2 Pemohon, sudah dewasa dan sudah disumpah, sehingga memenuhi syarat formal sebagaimana diatur dalam Pasal 145 HIR;

Menimbang, bahwa saksi 1 dan saksi 2 Pemohon telah memberikan kete-rangan yang pada pokoknya Pemohon dan Termohon sering terjadi perselisihan dan pertengkaran masalah kurang nafkah, Pemohon dan Termohon sudah berpisah tempat tinggal lebih kurang 1 (satu) tahun 7 (tujuh) bulan karena Termohon pergi mening-galkan Pemohon, saksi 1 dan saksi 2 sudah menasehati Pemohon agar rukun lagi dengan Termohon, tetapi tidak berhasil;

Menimbang, bahwa keterangan saksi 1 dan saksi 2 diberikan atas dasar pengetahuannya sendiri dan keterangannya saling bersesuaian dan bersesuaian pula serta relevan dengan dalil permohonan Pemohon, oleh karena itu keterangan saksi tersebut telah memenuhi syarat materiil sebagaimana telah diatur dalam Pasal 171 dan Pasal 172 HIR, sehingga keterangan saksi tersebut memiliki kekuatan pembuktian dan dapat diterima sebagai alat bukti;

Menimbang, bahwa berdasarkan permohonan Pemohon, bukti P.1, P.2 serta saksi 1 dan saksi 2 terbukti fakta-fakta sebagai berikut:

1. Bahwa benar Pemohon dan Termohon adalah suami istri yang menikah pada tanggal 30 November 2013, sudah mempunyai 2 (dua) orang anak;
2. Bahwa benar rumah tangga Pemohon dan Temohon tidak harmonis lagi karena
   sering terjadi perselisihan dan pertengkaran dan tidak ada harapan akan dapat hidup rukun lagi dalam rumah tangga;
3. Bahwa benar Pemohon dan Termohon telah pisah 5 (lima) bulan;

Menimbang, bahwa berdasarkan fakta-fakta tersebut di atas, tujuan perkawinan sebagaimana dikehendaki oleh Pasal 1 Undang-Undang Nomor 1 Tahun 1974 jo. Pasal 3 Kompilasi Hukum Islam yaitu, membentuk keluarga (rumah tangga) yang bahagia dan kekal berdasarkan Ketuhanan Yang Maha Esa serta membina kehidupan rumah tangga yang sakinah, mawaddah dan rahmah seperti

Disclaimer
Kepaniteraan Mahkamah Agung Republik Indonesia berusaha untuk selalu mencantumkan informasi paling kini dan akurat sebagai bentuk komitmen Mahkamah Agung untuk pelayanan publik, transparansi dan akuntabilitas pelaksanaan fungsi peradilan. Namun dalam hal-hal tertentu masih dimungkinkan terjadi permasalahan teknis terkait dengan akurasi dan keterkinian informasi yang kami sajikan, hal mana akan terus kami perbaiki dari waktu kewaktu.
Dalam hal Anda menemukan inakurasi informasi yang termuat pada situs ini atau informasi yang seharusnya ada, namun belum tersedia, maka harap segera hubungi Kepaniteraan Mahkamah Agung RI melalui :
Email : kepaniteraan@mahkamahagung.go.id Telp : 021-384 3348 (ext.318)

yang dimaksud dalam Al-Qur'an surat Ar-Rum ayat 21, tidak dapat terwujud karena Pemohon dan Termohon sudah berpisah;

Menimbang, bahwa dengan keadaan demikian, ikatan perkawinan Pemohon dan Termohon sudah tidak bisa dipertahankan lagi, hal ini terbukti dengan kuatnya tekad Pemohon untuk bercerai dengan Termohon, hal ini dapat diterapkan surat al-Baqarah ayat 227 yang berbunyi:

وإن عزموا الطلاق فإن الله سميع عليم

Artinya: "Jika mereka (para suami) telah berketetapan hati untuk menjatuhkan talak, maka sesungguhnya Allah Maha Mendengar lagi Maha Mengetahui";

Menimbang, bahwa melihat fakta tersebut perlu dicarikan jalan keluarnya, maka majelis berpendapat bahwa perceraian adalah solusi yang terbaik bagi Pemohon dan Termohon, agar keduanya terlepas dari beban penderitaan lahir dan batin yang berkepanjangan, kalau dipaksakan juga untuk mempertahankan rumah tangganya, patut diduga bahwa hal itu akan menimbulkan mafsadah yang lebih besar dari maslahahnya sebagaimana kaidah Fiqih yang berbunyi:

درؤ المفاسد مقدم على جلب المصالح

Artinya : "Menolak kemudharatan lebih utama daripada menarik (mempertahankan) kebaikan";

Menimbang, bahwa dengan fakta dan pertimbangan tersebut, maka mejelis menilai permohonan Pemohon telah memenuhi Pasal 19 huruf f Peraturan Pemerintah Nomor 9 Tahun 1975 jo. Pasal 116 huruf f Kompilasi Hukum Islam;

Menimbang, bahwa berdasarkan pertimbangan-pertimbangan tersebut di atas, dan berdasarkan Pasal 125 HIR, maka Termohon harus dinyatakan tidak hadir dan permohonan Pemohon dapat dikabulkan secara verstek dengan memberi izin kepada Pemohon untuk menjatuhkan talak satu raj'i terhadap Termohon;

Menimbang, bahwa oleh karena perkara a quo masuk bidang perkawinan, maka berdasarkan Pasal 89 ayat (1) Undang-Undang Nomor 7 Tahun 1989 sebagaimana telah diubah dengan Undang-Undang Nomor 3 Tahun 2006 dan perubahan kedua dengan Undang-Undang Nomor 50 Tahun 2009, biaya perkara dibebankan kepada Pemohon;

Mengingat, semua pasal dalam peraturan perundang-undangan dan hukum Islam yang berkaitan dengan perkara ini;

Disclaimer
Kepaniteraan Mahkamah Agung Republik Indonesia berusaha untuk selalu mencantumkan informasi paling kini dan akurat sebagai bentuk komitmen Mahkamah Agung untuk pelayanan publik, transparansi dan akuntabilitas pelaksanaan fungsi peradilan. Namun dalam hal-hal tertentu masih dimungkinkan terjadi permasalahan teknis terkait dengan akurasi dan keterkinian informasi yang kami sajikan, hal mana akan terus kami perbaiki dari waktu kewaktu.
Dalam hal Anda menemukan inakurasi informasi yang termuat pada situs ini atau informasi yang seharusnya ada, namun belum tersedia, maka harap segera hubungi Kepaniteraan Mahkamah Agung RI melalui :
Email : kepaniteraan@mahkamahagung.go.id Telp : 021-384 3348 (ext.318)

M E N G A D I L I

1. Menyatakan Termohon yang telah dipanggil secara resmi dan patut untuk menghadap sidang, tidak hadir;
2. Mengabulkan permohonan Pemohon dengan verstek;
3. Memberi izin kepada Pemohon (**Rija Revano bin Muchtar)** untuk menjatuhkan talak satu raj'i terhadap Termohon (**Nina Sulistiyani binti H.Mustain)** di depan sidang Pengadilan Agama Kudus;
4. Membebankan kepada Pemohon untuk membayar biaya perkara sejumlah Rp 411.000,00 (empat ratus sebelas ribu rupiah);

Demikian diputuskan dalam rapat permusyawaratan Majelis yang dilangsungkan pada hari Senin tanggal 21 Desember 2020 Masehi bertepatan dengan tanggal 6 Jumadilawal 1442 Hijriah, oleh **Sulomo, S.Ag** sebagai Ketua Majelis, **H. Ah. Sholih, S.H.** dan **Dra. Ulfah** masing-masing sebagai Hakim Anggota, putusan tersebut diucapkan dalam sidang terbuka untuk umum pada hari itu juga oleh Ketua Majelis tersebut dengan didampingi oleh Hakim-Hakim Anggota dan dibantu oleh **Nur Cholifah, S.H.** sebagai Panitera Pengganti serta dihadiri oleh Pemohon tanpa hadirnya Termohon.

Hakim Anggota,

H. Ah. Sholih, S.H.

Hakim Anggota,

Dra. Ulfah

Ketua Majelis,

Sulomo, S.Ag.

Panitera Pengganti,

Nur Cholifah, S.H.

<u>PERINCIAN BIAYA :</u>

1.	Pendaftaran	: Rp	30.000,00
2.	ATK	: Rp	75.000,00

Disclaimer
Kepaniteraan Mahkamah Agung Republik Indonesia berusaha untuk selalu mencantumkan informasi paling kini dan akurat sebagai bentuk komitmen Mahkamah Agung untuk pelayanan publik, transparansi dan akuntabilitas pelaksanaan fungsi peradilan. Namun dalam hal-hal tertentu masih dimungkinkan terjadi permasalahan teknis terkait dengan akurasi dan keterkinian informasi yang kami sajikan, hal mana akan terus kami perbaiki dari waktu kewaktu.
Dalam hal Anda menemukan inakurasi informasi yang termuat pada situs ini atau informasi yang seharusnya ada, namun belum tersedia, maka harap segera hubungi Kepaniteraan Mahkamah Agung RI melalui :
Email : kepaniteraan@mahkamahagung.go.id   Telp : 021-384 3348 (ext.318)

3.	Panggilan	: Rp 270.000,00
4.	PNBP pgl	: Rp 20.000,00
5.	Redaksi	: Rp 10.000,00
6.	Meterai	: Rp 6.000,00
Jumlah	: Rp 411.000,00	

Putusan ini telah diberitahukan kepada Termohon tanggal . . . .

Putusan ini telah memperoeh kekuatan hukum tetap tanggal . . . .

Disclaimer
Kepaniteraan Mahkamah Agung Republik Indonesia berusaha untuk selalu mencantumkan informasi paling kini dan akurat sebagai bentuk komitmen Mahkamah Agung untuk pelayanan publik, transparansi dan akuntabilitas pelaksanaan fungsi peradilan. Namun dalam hal-hal tertentu masih dimungkinkan terjadi permasalahan teknis terkait dengan akurasi dan keterkinian informasi yang kami sajikan, hal mana akan terus kami perbaiki dari waktu kewaktu.
Dalam hal Anda menemukan inakurasi informasi yang termuat pada situs ini atau informasi yang seharusnya ada, namun belum tersedia, maka harap segera hubungi Kepaniteraan Mahkamah Agung RI melalui :
Email : kepaniteraan@mahkamahagung.go.id Telp : 021-384 3348 (ext.318)
