# Supplementary material for: Classification of divorce causes during the COVID-19 pandemic using convolutional neural networks
Source: PeerJ Comput Sci. 2022 Jun 30;8:e998. doi: 10.7717/peerj-cs.998 (PMC9299239; doi:10.7717/peerj-cs.998)
Supplement: Supplemental Information 4 [file peerj-cs-08-998-s004.zip › Konflik Dataset/Data ke-6.pdf]

P U T U S A N

Nomor 1324/Pdt.G/2020/PA.Kds

بسم الله الرحمن الرحيم

DEMI KEADILAN BERDASARKAN KETUHANAN YANG MAHA ESA

Pengadilan Agama Kudus yang memeriksa dan mengadili perkara tertentu pada tingkat pertama dalam sidang majelis telah menjatuhkan putusan dalam perkara Cerai Gugat antara:

Munti'ah binti Karjo, NIK. 3319034502950003, tempat tanggal lahir Kudus, 05 Februari 1995, agama Islam, pekerjaan buruh harian lepas, pendi-dikan SMPkewarga-negaraan WNI, alamat Desa Loram Kulon RT 004 RW 005, Keca-matan Jati, Kabupaten Kudus, dalam hal ini telah memberikan kuasa kepada ANDRIAS WIBOWO, SH, Advokat/ Konsultan Hukum pada Kantor Advokat/Pengacara ANDRIAS WIBOWO SH & Rekan yang beralamat di Desa Loram Kulon, RT.004 RW.002 Karang Watu, Kecamatan Jati, Kabupaten Kudus, berda-sarkan Surat Kuasa Khusus dengan meterai cukup tertanggal 24 November 2020, telah terdaftar pada Register Surat Kuasa Khusus di Kepaniteraan Pengadilan Agama Kudus dengan nomor 622/BH/ 2020/PA.Kds, tertanggal 2 Desember 2020, sebagai **Penggugat**;

m e l a w a n

Moch Handayani bin Yasak, NIK. 3319073011850002, tempat tanggal lahir Kudus, 30 November 1985, agama Islam, pekerjaan sopir, pendidikan SMP, kewarganegaraan WNI, alamat Desa Loram Kulon RT 004 RW 005, Kecamatan Jati, Kabupaten Kudus, sebagai **Tergugat;**

Pengadilan Agama tersebut;
Telah mempelajari surat-surat yang berkaitan dengan perkara ini;
Telah mendengar keterangan Penggugat dan para saksi di muka sidang;

DUDUK PERKARA

Bahwa Penggugat dalam surat gugatannya tanggal 30 November 2020 telah mengajukan gugatan Cerai Gugat yang telah didaftar di Kepaniteraan

Disclaimer
Kepaniteraan Mahkamah Agung Republik Indonesia berusaha untuk selalu mencantumkan informasi paling kini dan akurat sebagai bentuk komitmen Mahkamah Agung untuk pelayanan publik, transparansi dan akuntabilitas pelaksanaan fungsi peradilan. Namun dalam hal-hal tertentu masih dimungkinkan terjadi permasalahan teknis terkait dengan akurasi dan keterkinian informasi yang kami sajikan, hal mana akan terus kami perbaiki dari waktu kewaktu.
Dalam hal Anda menemukan inakurasi informasi yang termuat pada situs ini atau informasi yang seharusnya ada, namun belum tersedia, maka harap segera hubungi Kepaniteraan Mahkamah Agung RI melalui :
Email : kepaniteraan@mahkamahagung.go.id   Telp : 021-384 3348 (ext.318)

Pengadilan Agama Kudus dengan Nomor 1324/Pdt.G/2020/PA.Kds., tanggal 2 Desember 2020 dengan dalil-dalil gugatan sebagai berikut:

1. Bahwa pada tanggal 18 November 2011, Penggugat dan Tergugat telah menikah secara sah diKantor Urusan Agama (KUA) Kecamatan Jati Kabupaten Kudus sebagaimana Kutipan Akta Nikah Nomor :918/212/XI/2011;

2. Bahwa sesudah Akad nikah antara Penggugat dan Tergugat membina rumah tangga dirumah orang tua Penggugat, dan telah melakukan hubungan intim seperti layaknya suami istri (ba'da dukhul) serta telah dikaruniai keturunan atau anak 2 (dua), yang pertama perempuan bernama ***Yunanda Mufhalina Alsa***, TTL: Kudus, 17 Juli 2012 (8 Tahun), yang kedua laki-laki bernama: ***Muhammad Akbar Maulana***, *TTL: Kudus, 15 Maret 2016 (4 tahun);*

3. Bahwa semula rumah tangga antara Penggugat dan Tergugat hidup rukun, damai dan tenteram, akan tetapi setelah melahirkan anak ke dua atau mulai pertengahan tahun 2016 kehidupan rumah tangga antara Penggugat dan Tergugat ada gejala perpecahan ikatan perkawinan yaitu dengan sering kali terjadi kesalahpahaman, perbedaan prinsip, pertengkaran dan percekcokan yang tidak ada harapan untuk rukun kembali sebagaimana diatur dalam Pasal 19 huruf (f) Peraturan Pemerintah Nomor 9 Tahun 1975;

4. Bahwa faktor penyebab terjadinya perselisihan, pertengkaran dan percekcokan antara Penggugat dan Tergugat adalah :

a. Antara Penggugat dan Tergugat tidak ada kecocokan dan sering berselisih pendapat bertengkar terus menerus;

b. Tergugat saat cekcok tidak memberi nafkah kepada Penggugat dan anaknya sehingga kebutuhan sehari-hari lebih dari hasil jerih payah bekerja Penggugat sebagai buruh;

c. Tergugat jika ada masalah sering mengancam jiwa penggugat dan anak sehingga membuat Penggugat takut dan tidak nyaman;

d. Tergugat sering mengucapkan kata-kata yang tidak pantas saat terjadi cekcok atau pertengakaran dan selalu memojokkan Penggugat tentang masa lalu sehingga membuat Penggugat menjadi tekanan batin;

5. Bahwa Penggugat sering mengingatkan Tergugat agar bersikap yang

Disclaimer
Kepaniteraan Mahkamah Agung Republik Indonesia berusaha untuk selalu mencantumkan informasi paling kini dan akurat sebagai bentuk komitmen Mahkamah Agung untuk pelayanan publik, transparansi dan akuntabilitas pelaksanaan fungsi peradilan. Namun dalam hal-hal tertentu masih dimungkinkan terjadi permasalahan teknis terkait dengan akurasi dan keterkinian informasi yang kami sajikan, hal mana akan terus kami perbaiki dari waktu kewaktu.
Dalam hal Anda menemukan inakurasi informasi yang termuat pada situs ini atau informasi yang seharusnya ada, namun belum tersedia, maka harap segera hubungi Kepaniteraan Mahkamah Agung RI melalui :
Email : kepaniteraan@mahkamahagung.go.id Telp : 021-384 3348 (ext.318)

baik, menghargai Penggugat sebagai seorang istri, tetapi Tergugat mengabaikan saran dari Penggugat dan jika diajak berkomunikasi selalu saja ngajak bertengkar;

6. Bahwa untuk memenuhi kebutuhan hidup, Penggugat akhirnya memutuskan untuk bekerja sebagai buruh, namun selama Penggugat bekerja justru Tergugat semakin tidak peduli dengan Penggugat dan jika berkomunikasi berujung percekcokan;

7. Bahwa puncaknya Penggugat dan Tergugat sering cekcok dan bertengkar maka sejak pada bulan Agustus 2020 Penggugat dan tergugat pisah ranjang, sebagaimana hingga sekarang, sehingga antara Penggugat dan Tergugat sudah pisah ranjang selama kurang lebih 4 (empat) bulan;

8. Bahwa dengan demikian Penggugat merasa sudah tidak kuat lagi hidup berumah tangga bersama Tergugat dalam suasana yang tidak harmonis, tidak bahagia, maka Penggugat dengan rela hati ingin dicerai Tergugat di depan pengadilan;

Bahwa berdasarkan alasan-alasan tersebut diatas kiranya cukup alasan gugatan Penggugat minta cerai dari Tergugat dapat diproses dan dikabulkan sesuai dengan peraturan perundang-undangan yang berlaku oleh karena itu Penggugat merasa tidak rela dan mohon kepada Ketua Pengadilan Agama Kudus berkenan untuk memanggil pihak-pihak untuk memeriksa, mengadili dan menjatuhkan putusan sebagai berikut:

PRIMER:

1. Mengabulkan gugatan Penggugat;
2. Menjatuhkan talak ba'in sughro dari Tergugat (*Moch Handayani bin Yasak)* kepada Penggugat (*Munti'ah Binti Karjo);*.
3. Menetapkan biaya perkara menurut hukum;

SUBSIDER:

Atau apabila Pengadilan Agama Kudus berpendapat lain mohon putusan yang seadil-adilnya.

Bahwa pada hari sidang yang telah ditetapkan Penggugat telah datang menghadap ke muka sidang, sedangkan Tergugat tidak datang menghadap ke muka sidang dan tidak menyuruh orang lain untuk menghadap sebagai

Disclaimer
Kepaniteraan Mahkamah Agung Republik Indonesia berusaha untuk selalu mencantumkan informasi paling kini dan akurat sebagai bentuk komitmen Mahkamah Agung untuk pelayanan publik, transparansi dan akuntabilitas pelaksanaan fungsi peradilan. Namun dalam hal-hal tertentu masih dimungkinkan terjadi permasalahan teknis terkait dengan akurasi dan keterkinian informasi yang kami sajikan, hal mana akan terus kami perbaiki dari waktu kewaktu.
Dalam hal Anda menemukan inakurasi informasi yang termuat pada situs ini atau informasi yang seharusnya ada, namun belum tersedia, maka harap segera hubungi Kepaniteraan Mahkamah Agung RI melalui :
Email : kepaniteraan@mahkamahagung.go.id Telp : 021-384 3348 (ext.318)

wakil/kuasa hukumnya meskipun telah dipanggil secara resmi dan patut yang relaas panggilannya dibacakan di dalam sidang, sedangkan tidak ternyata bahwa tidak datangnya itu disebabkan suatu halangan yang sah;

Bahwa majelis hakim telah menasehati Penggugat agar rukun lagi dan tidak bercerai dengan Tergugat, tetapi Penggugat tetap pada dalil-dalil gugatannya untuk bercerai dengan Tergugat;

Bahwa perkara ini tidak dapat dimediasi karena Tergugat tidak pernah datang menghadap meskipun telah dipanggil secara resmi dan patut, selanjutnya dimulai pemeriksaan dengan membacakan surat gugatan Penggugat yang maksud dan isinya tetap dipertahankan oleh Penggugat;

Bahwa untuk menguatkan dalil-dalil gugatannya, Penggugat telah mengajukan alat-alat bukti di persidangan, yaitu sebagai berikut:

I. Alat bukti Surat:

1. Fotokopi Kartu Tanda Penduduk Nomor 3319034502950003 tanggal 9 Oktober 2012, bermeterai cukup, telah dicocokkan sesuai dengan aslinya (bukti P.1);
2. Fotokopi Kutipan Akta Nikah dari Kantor Urusan Agama Kecamatan Jati, Kabupaten Kudus Nomor 918/212/XI/2011 tanggal 18 November 2011, telah bermeterai cukup, telah dicocokkan sesuai dengan aslinya (bukti P.2);

II. Alat bukti Saksi:

1. **Edi Pornomo bin Ngadiyono**, 40 tahun, agama Islam, pekerjaan buruh harian lepas, tempat tinggal di Desa Loram Kulon RT 004 RW 005, Kecamatan Jati, Kabupaten Kudus, telah memberikan keterangan di bawah sumpah yang pada pokoknya sebagai berikut :
   - Bahwa saksi kenal dengan Penggugat dan Tergugat karena saksi adalah kakak kandung Penggugat;
   - Bahwa setelah menikah Penggugat dan Tergugat bertempat tinggal di rumah orang tua Penggugat, sudah mempunyai 2 (dua) orang anak;
   - Bahwa saksi sering main ke tempat tinggal Penggugat dan Tergugat, saksi mengetahui Penggugat dan Tergugat sering terjadi perselisihan dan pertengkaran masalah kurang nafkah, saksi ada 3 (tiga) kali melihat dan mendengar sendiri pertengkaran tersebut;

Disclaimer
Kepaniteraan Mahkamah Agung Republik Indonesia berusaha untuk selalu mencantumkan informasi paling kini dan akurat sebagai bentuk komitmen Mahkamah Agung untuk pelayanan publik, transparansi dan akuntabilitas pelaksanaan fungsi peradilan. Namun dalam hal-hal tertentu masih dimungkinkan terjadi permasalahan teknis terkait dengan akurasi dan keterkinian informasi yang kami sajikan, hal mana akan terus kami perbaiki dari waktu kewaktu.
Dalam hal Anda menemukan inakurasi informasi yang termuat pada situs ini atau informasi yang seharusnya ada, namun belum tersedia, maka harap segera hubungi Kepaniteraan Mahkamah Agung RI melalui :
Email : kepaniteraan@mahkamahagung.go.id Telp : 021-384 3348 (ext.318)

- Bahwa saksi sudah menasehati Penggugat agar rukun lagi dengan Tergugat, tetapi tidak berhasil;

2. **Sholikin Bakdawi bin Sutarwi**, umur 51 tahun, agama Islam, pekerjaan buruh harian lepas, tempat tinggal di Desa Loram Kulon RT 004 RW 005, Keca-matan Jati, Kabupaten Kudus, telah memberikan keterangan di bawah sumpah yang pada pokoknya sebagai berikut :

- Bahwa saksi kenal dengan Penggugat dan Tergugat karena saksi adalah tetangga Penggugat;
- Bahwa setelah menikah Penggugat dan Tergugat tinggal di rumah orang tua Penggugat, sudah mempunyai 2 (dua) orang anak;
- Bahwa saksi sering main ke tempat tinggal Penggugat dan Tergugat, saksi
mengetahui Penggugat dan Tergugat sering terjadi pertengkaran dan perselisihan masalah Tergugat tidak tanggung jawab nafkah, saksi ada 4 (empat) kali melihat dan mendengar sendiri pertengkaran tersebut;
- Bahwa saksi sudah menasehati Penggugat agar rukun lagi dengan Tergugat, tetapi tidak berhasil;

Bahwa terhadap keterangan saksi-saksi tersebut, Penggugat menyatakan cukup dan tidak akan mengajukan saksi lainnya lagi;

Bahwa Penggugat telah menyampaikan kesimpulan secara lisan dengan menyatakan tetap pada gugatannya dan mohon putusan;

---Bahwa untuk singkatnya uraian putusan ini, maka semua hal yang termuat dalam berita acara sidang perkara ini merupakan bagian yang tidak terpisahkan dari putusan ini;

PERTIMBANGAN HUKUM

Menimbang, bahwa maksud dan tujuan gugatan Penggugat adalah sebagaimana telah diuraikan di atas;

Menimbang, bahwa oleh karena perkara ini merupakan bidang perkawinan, maka sesuai dengan Pasal 49 huruf a Undang-Undang Nomor 7 Tahun 1989 tentang Peradilan Agama, sebagaimana telah diubah dengan Undang-Undang Nomor 3 Tahun 2006 dan perubahan kedua dengan Undang-Undang Nomor 50 Tahun 2009, perkara ini merupakan kewenangan Pengadilan Agama (*absolute competentie*);

Disclaimer
Kepaniteraan Mahkamah Agung Republik Indonesia berusaha untuk selalu mencantumkan informasi paling kini dan akurat sebagai bentuk komitmen Mahkamah Agung untuk pelayanan publik, transparansi dan akuntabilitas pelaksanaan fungsi peradilan. Namun dalam hal-hal tertentu masih dimungkinkan terjadi permasalahan teknis terkait dengan akurasi dan keterkinian informasi yang kami sajikan, hal mana akan terus kami perbaiki dari waktu kewaktu.
Dalam hal Anda menemukan inakurasi informasi yang termuat pada situs ini atau informasi yang seharusnya ada, namun belum tersedia, maka harap segera hubungi Kepaniteraan Mahkamah Agung RI melalui :
Email : kepaniteraan@mahkamahagung.go.id Telp : 021-384 3348 (ext.318)

Menimbang, bahwa berdasarkan identitas para pihak, Penggugat dan Tergugat bertempat tinggal di wilayah Kabupaten Kudus yang merupakan yurisdiksi Pengadilan Agama Kudus, maka sesuai dengan Pasal 73 ayat (1) Undang-Undang Nomor 7 Tahun 1989 tentang Peradilan Agama, sebagaimana telah diubah dengan Undang-Undang Nomor 3 Tahun 2006 dan perubahan kedua dengan Undang-Undang Nomor 50 Tahun 2009, Pengadilan Agama Kudus berwenang untuk memeriksa perkara ini (*relative competentie*);

Menimbang, bahwa ternyata Tergugat meskipun dipanggil secara resmi dan patut, tidak datang menghadap di muka sidang dan pula tidak ternyata bahwa tidak datangnya itu disebabkan suatu halangan yang sah;

Menimbang, bahwa Tergugat yang dipanggil secara resmi dan patut akan tetapi tidak datang menghadap harus dinyatakan tidak hadir dan gugatan tersebut harus diperiksa secara verstek;

Menimbang, bahwa oleh karena itu, maka putusan atas perkara ini dapat dijatuhkan tanpa hadirnya Tergugat (verstek);

Menimbang, bahwa berdasarkan ketentuan Pasal 125 HIR yaitu, putusan yang dijatuhkan tanpa hadirnya Tergugat dapat dikabulkan, kecuali kalau nyata gugatan itu melawan hak atau tidak beralasan, oleh karena itu majelis membebani Penggugat untuk membuktikan dalil-dalil gugatannya;

Menimbang, bahwa untuk membuktikan dalil-dalil gugatannya, Penggugat telah mengajukan alat bukti surat P.1, P.2 dan 2 orang saksi;

Menimbang, bahwa bukti P.1 terbukti Penggugat bertempat kediaman di Desa Loram Kulon RT 004 RW 005, Keca-matan Jati, Kabupaten Kudus, ternyata sesuai dengan identitas Penggugat pada surat gugatannya, oleh karena itu dapat diterima sebagai alat bukti;

Menimbang, bahwa bukti P.2 berupa Fotokopi Kutipan Akta Nikah yang aslinya merupakan akta otentik dan fotokopinya telah bermeterai cukup dan cocok dengan aslinya, isi bukti tersebut menjelaskan mengenai Penggugat dan Tergugat telah melangsungkan perkawinan pada tanggal 18 November 2011 tercatat di Kantor Urusan Agama Kecamatan Jati, Kabupaten Kudus, sehingga bukti tersebut telah memenuhi syarat formal dan materiil, serta mempunyai kekuatan yang sempurna dan mengikat;

Disclaimer
Kepaniteraan Mahkamah Agung Republik Indonesia berusaha untuk selalu mencantumkan informasi paling kini dan akurat sebagai bentuk komitmen Mahkamah Agung untuk pelayanan publik, transparansi dan akuntabilitas pelaksanaan fungsi peradilan. Namun dalam hal-hal tertentu masih dimungkinkan terjadi permasalahan teknis terkait dengan akurasi dan keterkinian informasi yang kami sajikan, hal mana akan terus kami perbaiki dari waktu kewaktu.
Dalam hal Anda menemukan inakurasi informasi yang termuat pada situs ini atau informasi yang seharusnya ada, namun belum tersedia, maka harap segera hubungi Kepaniteraan Mahkamah Agung RI melalui :
Email : kepaniteraan@mahkamahagung.go.id Telp : 021-384 3348 (ext.318)

Menimbang, bahwa saksi 1 dan saksi 2 Penggugat, sudah dewasa dan sudah disumpah, sehingga memenuhi syarat formal sebagaimana diatur dalam Pasal 145 HIR;

Menimbang, bahwa saksi 1 dan saksi 2 Penggugat telah memberikan keterangan yang pada pokoknya Penggugat dan Tergugat sering terjadi perselisihan dan pertengkaran masalah kurang nafkah, saksi 1 dan saksi 2 sudah menasehati Penggugat dan Tergugat agar rukun lagi, tetapi tidak berhasil;

Menimbang, bahwa keterangan saksi 1 dan saksi 2 diberikan atas dasar pengetahuannya sendiri dan keterangannya saling bersesuaian satu sama lain dan bersesuaian pula serta relevan dengan gugatan Penggugat, maka keterangan saksi-saksi tersebut telah memenuhi syarat materiil sebagaimana telah diatur dalam Pasal 171 dan Pasal 172 HIR, sehingga keterangan saksi-saksi tersebut memiliki kekuatan pembuktian dan dapat diterima sebagai alat bukti;

Menimbang, bahwa berdasarkan gugatan Penggugat, bukti P.1, P.2 serta saksi 1 dan saksi 2 terbukti fakta-fakta sebagai berikut:

1. Bahwa benar Penggugat dan Tergugat adalah suami istri yang menikah pada
tanggal 18 November 2011, sudah mempunyai 2 (dua) orang anak;
2. Bahwa benar rumah tangga Penggugat dan Tergugat tidak harmonis lagi karena
telah sering terjadi perselisihan dan pertengkaran dan tidak ada harapan untuk hidup rukun lagi dalam rumah tangga;
3. Bahwa saksi 1 dan saksi 2 sudah menasehati Penggugat dan Tergugat agar rukun lagi, tetapi tidak berhasil;

Menimbang, bahwa berdasarkan fakta-fakta tersebut di atas tujuan perkawinan sebagaimana dikehendaki oleh Pasal 1 Undang-Undang Nomor 1 Tahun 1974 jo. Pasal 3 Kompilasi Hukum Islam yaitu, membentuk keluarga (rumah tangga) yang bahagia dan kekal berdasarkan Ketuhanan Yang Maha Esa serta membina kehidupan rumah tangga yang sakinah, mawaddah dan rahmah seperti yang dimaksud dalam Al-Qur'an surat Ar-Rum ayat 21 tidak dapat terwujud karena masing-masing telah hidup berpisah;

Menimbang, bahwa majelis telah menasehati Penggugat di setiap kali sidang agar rukun lagi dengan Tergugat, tetapi Penggugat tetap menunjukkan

Disclaimer
Kepaniteraan Mahkamah Agung Republik Indonesia berusaha untuk selalu mencantumkan informasi paling kini dan akurat sebagai bentuk komitmen Mahkamah Agung untuk pelayanan publik, transparansi dan akuntabilitas pelaksanaan fungsi peradilan. Namun dalam hal-hal tertentu masih dimungkinkan terjadi permasalahan teknis terkait dengan akurasi dan keterkinian informasi yang kami sajikan, hal mana akan terus kami perbaiki dari waktu kewaktu.
Dalam hal Anda menemukan inakurasi informasi yang termuat pada situs ini atau informasi yang seharusnya ada, namun belum tersedia, maka harap segera hubungi Kepaniteraan Mahkamah Agung RI melalui :
Email : kepaniteraan@mahkamahagung.go.id Telp : 021-384 3348 (ext.318)

tidak senangnya kepada Tergugat dan bersikeras untuk bercerai dengan Tergugat, sehingga ikatan perkawinan Penggugat dengan Tergugat sudah tidak bisa dipertahankan lagi, hal ini dapat diterapkan pendapat ulama dalam Kitab Ghoyatul Marom halaman 162 yang selanjutnya diambilalih menjadi pendapat majelis, yaitu yang berbunyi:

واذا اشتد عدم رغبة الزوجة لزوجها طلق عليه القاضى طلقة

Artinya : *Diwaktu istri sudah sangat tidak senang pada suaminya, maka Hakim diperkenankan menjatuhkan talak si suami*;

Menimbang, bahwa dengan melihat fakta-fakta seperti tersebut di atas perlu dicarikan jalan keluarnya, maka majelis berpendapat bahwa perceraian adalah solusi yang terbaik bagi Penggugat dan Tergugat, kalau dipaksakan juga untuk mempertahankan rumah tangganya patut diduga bahwa hal itu akan menimbulkan mafsadah yang lebih besar dari maslahahnya, padahal menolak mafsadah lebih diutamakan dari mencapai kemaslahatan, hal ini sesuai dengan kaidah Fiqih yang berbunyi:

درؤ المفاسد مقدم على جلب المصالح

Artinya : *Menolak kemudharatan lebih utama daripada menarik (memper-tahankan) kebaikan;*

Menimbang, bahwa dengan pertimbangan tersebut majelis menilai gu-gatan Penggugat telah berdasar hukum dan telah memenuhi Pasal 19 huruf f Peraturan Pemerintah Nomor 9 Tahun 1975 jo. Pasal 116 huruf f Kompilasi Hukum Islam;

Menimbang, bahwa berdasarkan Pasal 125 HIR harus dinyatakan Tergugat tidak hadir menghadap sidang dan gugatan Penggugat dikabulkan dengan menjatuhkan talak satu bain sughra Tergugat terhadap Penggugat;

Menimbang, bahwa oleh karena perkara ini termasuk dalam bidang perkawinan, maka sesuai dengan ketentuan Pasal 89 ayat (1) Undang-Undang Nomor 7 Tahun 1989 Tentang Peradilan Agama sebagaimana telah diubah dengan Undang-Undang Nomor 3 Tahun 2006 dan perubahan kedua dengan Undang-Undang Nomor 50 Tahun 2009, biaya perkara ini dibebankan kepada Penggugat;

Mengingat, semua pasal dalam peraturan perundang-undangan dan hukum Islam yang berkaitan dengan perkara ini;

M E N G A D I L I

Disclaimer
Kepaniteraan Mahkamah Agung Republik Indonesia berusaha untuk selalu mencantumkan informasi paling kini dan akurat sebagai bentuk komitmen Mahkamah Agung untuk pelayanan publik, transparansi dan akuntabilitas pelaksanaan fungsi peradilan. Namun dalam hal-hal tertentu masih dimungkinkan terjadi permasalahan teknis terkait dengan akurasi dan keterkinian informasi yang kami sajikan, hal mana akan terus kami perbaiki dari waktu kewaktu.
Dalam hal Anda menemukan inakurasi informasi yang termuat pada situs ini atau informasi yang seharusnya ada, namun belum tersedia, maka harap segera hubungi Kepaniteraan Mahkamah Agung RI melalui :
Email : kepaniteraan@mahkamahagung.go.id Telp : 021-384 3348 (ext.318)

1. Menyatakan Tergugat yang telah dipanggil secara resmi dan patut untuk menghadap sidang, tidak hadir;
2. Mengabulkan gugatan Penggugat dengan verstek;
3. Menjatuhkan talak satu ba'in sughra Tergugat (**Moch Handayani bin Yasak**) terhadap Penggugat (**Munti'ah binti Karjo**);
4. Membebankan kepada Penggugat untuk membayar biaya perkara sejumlah Rp 291.000,00 (dua ratus sembilan puluh satu ribu rupiah);

Demikian diputuskan dalam rapat permusyawaratan Majelis Hakim yang dilangsungkan pada hari Senin tanggal 21 Desember 2020 Masehi bertepatan dengan tanggal 6 Jumadilawal 1442 Hijriah, oleh **Sulomo, S.Ag** sebagai Ketua Majelis, **H. Ah. Sholih, S.H.** dan **Dra. Ulfah** masing-masing sebagai Hakim Anggota, putusan tersebut diucapkan dalam sidang terbuka untuk umum pada hari itu juga oleh Ketua Majelis tersebut dengan didampingi oleh Hakim-Hakim Anggota dan dibantu oleh **Nur Cholifah, S.H.** sebagai Panitera Pengganti serta dihadiri oleh Penggugat tanpa hadirnya Tergugat.

Hakim Anggota,	Ketua Majelis,
**H. Ah. Sholih, S.H.**	**Sulomo, S.Ag**.
Hakim Anggota,	
**Dra. Ulfah**	Panitera Pengganti,
	**Nur Cholifah, S.H.**

PERINCIAN BIAYA :

1.	Pendaftaran	:	Rp 30.000,00
2.	ATK	:	Rp 75.000,00
3.	Panggilan	:	Rp 150.000,00
4.	PNBP pgl	:	Rp 20.000,00
5.	Redaksi	:	Rp 10.000,00

Disclaimer
Kepaniteraan Mahkamah Agung Republik Indonesia berusaha untuk selalu mencantumkan informasi paling kini dan akurat sebagai bentuk komitmen Mahkamah Agung untuk pelayanan publik, transparansi dan akuntabilitas pelaksanaan fungsi peradilan. Namun dalam hal-hal tertentu masih dimungkinkan terjadi permasalahan teknis terkait dengan akurasi dan keterkinian informasi yang kami sajikan, hal mana akan terus kami perbaiki dari waktu kewaktu.
Dalam hal Anda menemukan inakurasi informasi yang termuat pada situs ini atau informasi yang seharusnya ada, namun belum tersedia, maka harap segera hubungi Kepaniteraan Mahkamah Agung RI melalui :
Email : kepaniteraan@mahkamahagung.go.id   Telp : 021-384 3348 (ext.318)

6. Meterai : Rp 6.000,00
Jumlah : Rp 291.000,00

Putusan ini telah diberitahukan kepada Tergugat tanggal . . . .

Putusan ini telah memperoleh kekuatan hukum tetap tanggal . . . .

Disclaimer
Kepaniteraan Mahkamah Agung Republik Indonesia berusaha untuk selalu mencantumkan informasi paling kini dan akurat sebagai bentuk komitmen Mahkamah Agung untuk pelayanan publik, transparansi dan akuntabilitas pelaksanaan fungsi peradilan. Namun dalam hal-hal tertentu masih dimungkinkan terjadi permasalahan teknis terkait dengan akurasi dan keterkinian informasi yang kami sajikan, hal mana akan terus kami perbaiki dari waktu kewaktu.
Dalam hal Anda menemukan inakurasi informasi yang termuat pada situs ini atau informasi yang seharusnya ada, namun belum tersedia, maka harap segera hubungi Kepaniteraan Mahkamah Agung RI melalui :
Email : kepaniteraan@mahkamahagung.go.id Telp : 021-384 3348 (ext.318)
