# Supplementary material for: Classification of divorce causes during the COVID-19 pandemic using convolutional neural networks
Source: PeerJ Comput Sci. 2022 Jun 30;8:e998. doi: 10.7717/peerj-cs.998 (PMC9299239; doi:10.7717/peerj-cs.998)
Supplement: Supplemental Information 4 [file peerj-cs-08-998-s004.zip › Konflik Dataset/Data ke-7.pdf]

P U T U S A N

Nomor 1334/Pdt.G/2020/PA.Kds

بسم الله الرحمن الرحيم

DEMI KEADILAN BERDASARKAN KETUHANAN YANG MAHA ESA

Pengadilan Agama Kudus yang memeriksa dan mengadili perkara tertentu pada tingkat pertama dalam sidang majelis telah menjatuhkan putusan dalam perkara Cerai Gugat antara:

Jumisih binti Sukerno (alm), NIK. 3319035504810003, umur 39 tahun, agama Islam, pekerjaan mengurus rumah tangga, pendidikan SLTP, tempat kediaman, Desa Getas Pejaten RT. 08 RW. 03, Kecamatan Jati, Kabupaten Kudus, sebagai **Penggugat**;

m e l a w a n

Sardi bin Pariden (alm), NIK. 3319033112620175, umur 58 tahun, agama Islam, pekerjaan pedagang, pendidikan SD, tempat kediaman di Desa Ngloran Kulon RT. 04 RW. 02, Kecamatan Jati Kabupaten Kudus sebagai **Tergugat**;

Pengadilan Agama tersebut;
Telah mempelajari surat-surat yang berkaitan dengan perkara ini;
Telah mendengar keterangan Penggugat dan para saksi di muka sidang;

DUDUK PERKARA

Bahwa Penggugat dalam surat gugatannya tanggal 3 Desember 2020 telah mengajukan gugatan Cerai Gugat yang telah didaftar di Kepaniteraan Pengadilan Agama Kudus dengan Nomor 1334/Pdt.G/2020/PA.Kds., tanggal 3 Desember 2020 dengan dalil-dalil gugatan sebagai berikut:

1. Bahwa antara Penggugat dengan Tergugat adalah suami istri yang sah yang telah melangsungkan pernikahan di hadapan Pegawai Pencatat Nikah Kantor Urusan Agama (KUA) Kecamatan Jati, Kabupaten Kudus, pada tanggal 8-06-2014 sebagaimana dalam Kutipan Akta Nikah Nomor : 0369/027/VI/2014 yang dikeluarkan oleh Kantor Urusan Agama Kecamatan Jati, Kabupaten Kudus;

Disclaimer
Kepaniteraan Mahkamah Agung Republik Indonesia berusaha untuk selalu mencantumkan informasi paling kini dan akurat sebagai bentuk komitmen Mahkamah Agung untuk pelayanan publik, transparansi dan akuntabilitas pelaksanaan fungsi peradilan. Namun dalam hal-hal tertentu masih dimungkinkan terjadi permasalahan teknis terkait dengan akurasi dan keterkinian informasi yang kami sajikan, hal mana akan terus kami perbaiki dari waktu kewaktu.
Dalam hal Anda menemukan inakurasi informasi yang termuat pada situs ini atau informasi yang seharusnya ada, namun belum tersedia, maka harap segera hubungi Kepaniteraan Mahkamah Agung RI melalui :
Email : kepaniteraan@mahkamahagung.go.id   Telp : 021-384 3348 (ext.318)

2. Bahwa sebelum menikah Penggugat berstatus janda dan Tergugat berstatus masih beristri;

3. Bahwa setelah menikah antara Penggugat dengan Tergugat bertempat tinggal di warung Bakso Donoklliwir yang beralamat di Desa Getas Pejaten RT.08 RW.03, Kecamatan Jati, Kabupaten Kudus selama kurang lebih 5 tahun, kemudian Tergugat meninggalkan Penggugat pulang ke rumahnya sendiri di Desa Ngloram Kulon RT.04 RW.02, Kecamatan Jati, Kabupaten Kudus dan Penggugat tetap tinggal di warung Bakso Donoklliwir yang beralamat di Desa Getas Pejaten RT.08 RW.03 Kecamatan Jati, Kabupaten Kudus;

4. Bahwa antara Penggugat dengan Tergugat telah melakukan hubungan sebagai-mana selayaknya suami istri (ba'dadukhul) namun belum dikaruniai anak;

5. Bahwa semula kehidupan rumah tangga antara Penggugat dengan Tergugat berjalan harmonis dan hidup rukun sebagai suami istri, namun sejak bulan Juni tahun 2019 keadaan rumah tangga Penggugat dengan Tergugat mulai goyah dan retak karena adanya perselisihan dan pertengkaran terus-menerus yang tidak ada harapan untuk rukun kembali yang disebabkan Tergugat berperilaku tidak baik sering kali menggoda perempuan lain, bahkan adik kandung Penggugat pernah hampir diperkosa selain itu juga keponakan Penggugat juga diperlakukan sama;

6. Bahwa puncak perselisihan dan pertengkaran antara Penggugat dengan Tergugat terjadi pada bulan Juli 2019, antara Penggugat dan Tergugat terjadi perselisihan dengan permasalahan point 5 di atas, kemudian Tergugat pergi meninggalkan Penggugat ke rumahnya sendiri di Desa Ngloran Kulon RT.04 RW.02, Kecamatan Jati, Kabupaten Kudus sampai dengan sekarang, akibat-nya Penggugat dengan Tergugat sudah berpisah selama 1 tahun 5 bulan;

7. Bahwa atas sikap dan perbuatan Tergugat tersebut, Penggugat merasa sangat menderita lahir batin dan oleh karenanya Penggugat tidak rela dan berkesimpulan bahwa Tergugat adalah suami yang tidak baik;

8. Bahwa berdasarkan uraian di atas permohonan Gugat Cerai Penggugat telah memenuhi alasan perceraian sebagaimana diatur dalam Pasal 19 huruf

Disclaimer
Kepaniteraan Mahkamah Agung Republik Indonesia berusaha untuk selalu mencantumkan informasi paling kini dan akurat sebagai bentuk komitmen Mahkamah Agung untuk pelayanan publik, transparansi dan akuntabilitas pelaksanaan fungsi peradilan. Namun dalam hal-hal tertentu masih dimungkinkan terjadi permasalahan teknis terkait dengan akurasi dan keterkinian informasi yang kami sajikan, hal mana akan terus kami perbaiki dari waktu kewaktu.
Dalam hal Anda menemukan inakurasi informasi yang termuat pada situs ini atau informasi yang seharusnya ada, namun belum tersedia, maka harap segera hubungi Kepaniteraan Mahkamah Agung RI melalui :
Email : kepaniteraan@mahkamahagung.go.id Telp : 021-384 3348 (ext.318)

(f) PP No.9 Tahun 1975 jo Pasal 116 huruf (f) Kompilasi Hukum Islam, oleh karena itu patut dikabulkan;

Bahwa berdasarkan alasan/dalil-dalil tersebut di atas, Penggugat merasa sudah tidak tahan lagi untuk meneruskan kehidupan rumah tangganya bersama dengan Tergugat, maka Penggugat mohon kepada Ketua Pengadilan Agama Kudus melalui Majelis Hakim yang memeriksa dan mengadili perkara ini agar berkenan memanggil para pihak untuk memeriksa dan selanjutnya menjatuhkan putusan sebagai berikut :

PRIMER :

1. Mengabulkan gugatan cerai Pengugat;
2. Menjatuhkan talak ba'in dari Tergugat (Sardi bin Pariden (Alm) kepada Penggugat (Jumisih binti Sukerno (alm);
3. Menetapkan biaya perkara menurut peraturan hukum yang berlaku;

SUBSIDER :

Atau apabila Pengadilan Agama Kudus berpendapat lain mohon putusan yang seadil-adilnya (*Ex Aequo Et Bono);*

Bahwa pada hari sidang yang telah ditetapkan Penggugat telah datang menghadap ke muka sidang, sedangkan Tergugat tidak datang menghadap ke muka sidang dan tidak menyuruh orang lain untuk menghadap sebagai wakil/kuasa hukumnya meskipun telah dipanggil secara resmi dan patut yang relaas panggilannya dibacakan di dalam sidang, sedangkan tidak ternyata bahwa tidak datangnya itu disebabkan suatu halangan yang sah;

Bahwa majelis hakim telah menasehati Penggugat agar berpikir untuk tidak bercerai dengan Tergugat, tetapi Penggugat tetap pada dalil-dalil gugatannya untuk bercerai dengan Tergugat;

Bahwa perkara ini tidak dapat dimediasi karena Tergugat tidak pernah datang menghadap meskipun telah dipanggil secara resmi dan patut, selanjutnya dimulai pemeriksaan dengan membacakan surat gugatan Penggugat yang maksud dan isinya tetap dipertahankan oleh Penggugat;

Bahwa untuk menguatkan dalil-dalil gugatannya, Penggugat telah mengajukan alat-alat bukti di persidangan, yaitu sebagai berikut:

I. Alat bukti Surat:

Disclaimer
Kepaniteraan Mahkamah Agung Republik Indonesia berusaha untuk selalu mencantumkan informasi paling kini dan akurat sebagai bentuk komitmen Mahkamah Agung untuk pelayanan publik, transparansi dan akuntabilitas pelaksanaan fungsi peradilan. Namun dalam hal-hal tertentu masih dimungkinkan terjadi permasalahan teknis terkait dengan akurasi dan keterkinian informasi yang kami sajikan, hal mana akan terus kami perbaiki dari waktu kewaktu.
Dalam hal Anda menemukan inakurasi informasi yang termuat pada situs ini atau informasi yang seharusnya ada, namun belum tersedia, maka harap segera hubungi Kepaniteraan Mahkamah Agung RI melalui :
Email : kepaniteraan@mahkamahagung.go.id Telp : 021-384 3348 (ext.318)

1. Fotokopi Kartu Tanda Penduduk Nomor 3319035504810003 tanggal 12 September 2012, telah bermeterai cukup, telah dicocokkan sesuai dengan aslinya (bukti P.1);

2. Fotokopi Kutipan Akta Nikah dari Kantor Urusan Agama Kecamatan Jati,
Kabupaten Kudus Nomor 0369/027/VI/2014 tanggal 8 Juni 2014, telah bermeterai cukup, telah dicocokkan sesuai dengan aslinya (bukti P.2);

II. Alat bukti Saksi:

1. **Khoirul Sholeh bin Noor Rhoim**, umur 52 tahun, agama Islam, pekerjaan buruh, tempat tinggal di Desa Getas Pejaten RT.08 RW.03, Kecamatan Jati, Kabupaten Kudus, telah memberikan keterangan di bawah sumpah yang pada pokoknya sebagai berikut :
    - Bahwa saksi kenal dengan Penggugat dan Tergugat karena saksi adalah tetangga Penggugat;
    - Bahwa setelah menikah Penggugat dan Tergugat bertempat tinggal di Warung Bakso Donokliwir;
    - Bahwa saksi sering main ke tempat tinggal Penggugat dan mengetahui
    Penggugat dengan Tergugat telah berpisah hingga sekarang lebih kurang 1 (satu) tahun 5 (lima) bulan karena Tergugat pergi meninggalkan Penggugat dan pulang ke rumahnya sendiri;
    - Bahwa sebelum pisah rumah, Penggugat dan Tergugat sering bertengkar masalah Tergugat suka menghgoda wanita lain, saksi ada 2 (dua) kali melihat dan mendengar sendiri pertengkaran tersebut;
    - Bahwa saksi sudah menasehati Penggugat agar rukun lagi dengan Tergugat, tetapi tidak berhasil;

2. **M. Arifin bin Sunardi**, umur 38 tahun, agama Islam, pekerjaan buruh lepas, tempat tinggal di Desa Getas Pejaten RT.08 RW.03, Kecamatan Jati, Kabupaten Kudus, telah memberikan keterangan di bawah sumpah yang pada pokoknya sebagai berikut :
    - Bahwa saksi kenal dengan Penggugat dan Tergugat karena saksi adalah tetangga Penggugat;

Disclaimer
Kepaniteraan Mahkamah Agung Republik Indonesia berusaha untuk selalu mencantumkan informasi paling kini dan akurat sebagai bentuk komitmen Mahkamah Agung untuk pelayanan publik, transparansi dan akuntabilitas pelaksanaan fungsi peradilan. Namun dalam hal-hal tertentu masih dimungkinkan terjadi permasalahan teknis terkait dengan akurasi dan keterkinian informasi yang kami sajikan, hal mana akan terus kami perbaiki dari waktu kewaktu.
Dalam hal Anda menemukan inakurasi informasi yang termuat pada situs ini atau informasi yang seharusnya ada, namun belum tersedia, maka harap segera hubungi Kepaniteraan Mahkamah Agung RI melalui :
Email : kepaniteraan@mahkamahagung.go.id Telp : 021-384 3348 (ext.318)

- Bahwa setelah menikah Penggugat dan Tergugat bertempat tinggal di Warung Bakso Donokliwir;
- Bahwa saksi sering main ke tempat tinggal Penggugat dan mengetahui
Penggugat dengan Tergugat telah berpisah hingga sekarang lebih kurang 1 (satu) tahun 5 (lima) bulan karena Tergugat telah pergi meninggalkan Penggugat dan pulang ke rumahnya sendiri;
- Bahwa sebelum pisah, Penggugat dan Tergugat sering bertengkar masalah kurang nafkah, saksi pernah satu kali melihat dan mendengar sendiri pertengkaran tersebut;
- Bahwa saksi sudah menasehati Penggugat agar rukun lagi dengan Tergugat, tetapi tidak berhasil;

Bahwa terhadap keterangan saksi-saksi tersebut, Penggugat menyatakan cukup dan tidak akan mengajukan saksi lainnya lagi;

Bahwa Penggugat telah menyampaikan kesimpulan secara lisan dengan menyatakan tetap pada gugatannya dan mohon putusan;

---Bahwa untuk singkatnya uraian putusan ini, maka semua hal yang termuat dalam berita acara sidang perkara ini merupakan bagian yang tidak terpisahkan dari putusan ini;

PERTIMBANGAN HUKUM

Menimbang, bahwa maksud dan tujuan gugatan Penggugat adalah sebagaimana telah diuraikan di atas;

Menimbang, bahwa oleh karena perkara ini merupakan bidang perkawinan, maka sesuai dengan Pasal 49 huruf a Undang-Undang Nomor 7 Tahun 1989 tentang Peradilan Agama, sebagaimana telah diubah dengan Undang-Undang Nomor 3 Tahun 2006 dan perubahan kedua dengan Undang-Undang Nomor 50 Tahun 2009, perkara ini merupakan kewenangan Pengadilan Agama;

Menimbang, bahwa berdasarkan identitas para pihak, Penggugat dan Tergugat bertempat tinggal di wilayah Kabupaten Kudus yang merupakan yurisdiksi Pengadilan Agama Kudus, maka sesuai dengan Pasal 73 ayat (1) Undang-Undang Nomor 7 Tahun 1989 tentang Peradilan Agama, sebagaimana telah diubah dengan Undang-Undang Nomor 3 Tahun 2006 dan perubahan kedua dengan Undang-

Disclaimer
Kepaniteraan Mahkamah Agung Republik Indonesia berusaha untuk selalu mencantumkan informasi paling kini dan akurat sebagai bentuk komitmen Mahkamah Agung untuk pelayanan publik, transparansi dan akuntabilitas pelaksanaan fungsi peradilan. Namun dalam hal-hal tertentu masih dimungkinkan terjadi permasalahan teknis terkait dengan akurasi dan keterkinian informasi yang kami sajikan, hal mana akan terus kami perbaiki dari waktu kewaktu.
Dalam hal Anda menemukan inakurasi informasi yang termuat pada situs ini atau informasi yang seharusnya ada, namun belum tersedia, maka harap segera hubungi Kepaniteraan Mahkamah Agung RI melalui :
Email : kepaniteraan@mahkamahagung.go.id Telp : 021-384 3348 (ext.318)

Undang Nomor 50 Tahun 2009, Pengadilan Agama Kudus berwenang untuk memeriksa perkara ini (*relative competentie*);

Menimbang, bahwa ternyata Tergugat meskipun dipanggil secara resmi dan patut, tidak datang menghadap di muka sidang dan pula tidak ternyata bahwa tidak datangnya itu disebabkan suatu halangan yang sah;

Menimbang, bahwa Tergugat yang dipanggil secara resmi dan patut akan tetapi tidak datang menghadap harus dinyatakan tidak hadir dan gugatan tersebut harus diperiksa secara verstek;

Menimbang, bahwa oleh karena itu, maka putusan atas perkara ini dapat dijatuhkan tanpa hadirnya Tergugat (verstek);

Menimbang, bahwa berdasarkan ketentuan Pasal 125 HIR yaitu, putusan yang dijatuhkan tanpa hadirnya Tergugat dapat dikabulkan, kecuali kalau nyata gugatan itu melawan hak atau tidak beralasan, oleh karena itu majelis membebani Penggugat untuk membuktikan dalil-dalil gugatannya;

Menimbang, bahwa untuk membuktikan dalil-dalil gugatannya, Penggugat telah mengajukan alat bukti surat P.1, P.2 dan 2 orang saksi;

Menimbang, bahwa bukti P.1 terbukti Penggugat bertempat kediaman di Desa Getas Pejaten RT.08 RW.03, Kecamatan Jati, Kabupaten Kudus, ternyata sesuai dengan identitas Penggugat pada surat gugatannya, oleh karena itu dapat diterima sebagai alat bukti;

Menimbang, bahwa bukti P.2 berupa Fotokopi Kutipan Akta Nikah yang aslinya merupakan akta otentik dan fotokopinya telah bermeterai cukup dan cocok dengan aslinya, isi bukti tersebut menjelaskan mengenai Penggugat dan Tergugat telah melangsungkan perkawinan pada tanggal 8 Juni 2014 tercatat di Kantor Urusan Agama Kecamatan Jati, Kabupaten Kudus, sehingga bukti tersebut telah memenuhi syarat formal dan materiil, serta mempunyai kekuatan yang sempurna dan mengikat;

Menimbang, bahwa saksi 1 dan saksi 2 Penggugat, sudah dewasa dan sudah disumpah, sehingga memenuhi syarat formal sebagaimana diatur dalam Pasal 145 HIR;

Menimbang, bahwa saksi 1 dan saksi 2 Penggugat telah memberikan keterangan yang pada pokoknya Penggugat dan Tergugat sering terjadi perselisihan

Disclaimer
Kepaniteraan Mahkamah Agung Republik Indonesia berusaha untuk selalu mencantumkan informasi paling kini dan akurat sebagai bentuk komitmen Mahkamah Agung untuk pelayanan publik, transparansi dan akuntabilitas pelaksanaan fungsi peradilan. Namun dalam hal-hal tertentu masih dimungkinkan terjadi permasalahan teknis terkait dengan akurasi dan keterkinian informasi yang kami sajikan, hal mana akan terus kami perbaiki dari waktu kewaktu.
Dalam hal Anda menemukan inakurasi informasi yang termuat pada situs ini atau informasi yang seharusnya ada, namun belum tersedia, maka harap segera hubungi Kepaniteraan Mahkamah Agung RI melalui :
Email : kepaniteraan@mahkamahagung.go.id Telp : 021-384 3348 (ext.318)

dan pertengkaran masalah Tergugat suka menggoda wanita lain, Penggugat dan Tergugat telah pisah rumah lebih kurang 1 (satu) tahun 5 (lima) bulan lamanya, saksi 1 dan saksi 2 sudah menasehati Penggugat agar rukun lagi dengan Tergugat, tetapi tidak berhasil;

Menimbang, bahwa keterangan saksi 1 dan saksi 2 diberikan atas dasar pengetahuannya sendiri dan keterangannya saling bersesuaian satu sama lain dan bersesuaian pula serta relevan dengan gugatan Penggugat, maka keterangan saksi-saksi tersebut telah memenuhi syarat materiil sebagaimana telah diatur dalam Pasal 171 dan Pasal 172 HIR, sehingga keterangan saksi-saksi tersebut memiliki kekuatan pembuktian dan dapat dipertimbangkan;

Menimbang, bahwa berdasarkan gugatan Penggugat, bukti P.1, P.2 serta saksi 1 dan saksi 2 terbukti fakta-fakta sebagai berikut:

1. Bahwa benar Penggugat dan Tergugat adalah suami istri yang menikah pada
tanggal 8 Juni 2014;
2. Bahwa benar rumah tangga Penggugat dan Tergugat tidak harmonis lagi karena
telah sering terjadi perselisihan dan pertengkaran dan tidak ada harapan untuk hidup rukun lagi dalam rumah tangga;
3. Bahwa benar terbukti antara Penggugat dengan Tergugat telah pisah rumah 1 (satu) tahun 5 (lima) bulan;

Menimbang, bahwa berdasarkan fakta-fakta tersebut di atas tujuan perkawinan sebagaimana dikehendaki oleh Pasal 1 Undang-Undang Nomor 1 Tahun 1974 jo. Pasal 3 Kompilasi Hukum Islam yaitu, membentuk keluarga (rumah tangga) yang bahagia dan kekal berdasarkan Ketuhanan Yang Maha Esa serta membina kehidupan rumah tangga yang sakinah, mawaddah dan rahmah seperti yang dimaksud dalam Al-Qur'an surat Ar-Rum ayat 21 tidak dapat terwujud karena masing-masing telah hidup berpisah;

Menimbang, bahwa majelis telah menasehati Penggugat di setiap kali sidang agar rukun lagi dengan Tergugat, tetapi Penggugat tetap menunjukkan tidak senangnya kepada Tergugat dan bersikeras untuk bercerai dengan Tergugat,

Disclaimer
Kepaniteraan Mahkamah Agung Republik Indonesia berusaha untuk selalu mencantumkan informasi paling kini dan akurat sebagai bentuk komitmen Mahkamah Agung untuk pelayanan publik, transparansi dan akuntabilitas pelaksanaan fungsi peradilan. Namun dalam hal-hal tertentu masih dimungkinkan terjadi permasalahan teknis terkait dengan akurasi dan keterkinian informasi yang kami sajikan, hal mana akan terus kami perbaiki dari waktu kewaktu.
Dalam hal Anda menemukan inakurasi informasi yang termuat pada situs ini atau informasi yang seharusnya ada, namun belum tersedia, maka harap segera hubungi Kepaniteraan Mahkamah Agung RI melalui :
Email : kepaniteraan@mahkamahagung.go.id   Telp : 021-384 3348 (ext.318)

sehingga ikatan perkawinan Penggugat dengan Tergugat sudah tidak bisa dipertahankan lagi, hal ini dapat diterapkan pendapat ulama dalam Kitab Ghoyatul Marom halaman 162 yang selanjutnya diambilalih menjadi pendapat majelis, yaitu yang berbunyi:

واذا اشتد عدم رغبة الزوجة لزوجها طلق عليه القاضى طلقة

Artinya : *Diwaktu istri sudah sangat tidak senang pada suaminya, maka Hakim diperkenankan menjatuhkan talak si suami*;

Menimbang, bahwa dengan melihat fakta-fakta seperti tersebut di atas perlu dicarikan jalan keluarnya, maka majelis berpendapat bahwa perceraian adalah solusi yang terbaik bagi Penggugat dan Tergugat, kalau dipaksakan juga untuk mempertahankan rumah tangganya patut diduga bahwa hal itu akan menimbul- mafsadah yang lebih besar dari maslahahnya, padahal menolak mafsadah lebih diutamakan dari mencapai kemaslahatan, hal ini sesuai dengan kaidah Fiqih yang berbunyi:

درؤ المفاسد مقدم على جلب المصالح

Artinya : *Menolak kemudharatan lebih utama daripada menarik (memper-tahankan) kebaikan;*

Menimbang, bahwa dengan pertimbangan tersebut majelis menilai gugatan Penggugat telah berdasar hukum dan telah memenuhi Pasal 19 huruf f Peraturan Pemerintah Nomor 9 Tahun 1975 jo. Pasal 116 huruf f Kompilasi Hukum Islam;

Menimbang, bahwa berdasarkan Pasal 125 HIR harus dinyatakan Tergugat tidak hadir menghadap sidang dan gugatan Penggugat dikabulkan dengan menjatuhkan talak satu bain sughra Tergugat terhadap Penggugat;

Menimbang, bahwa oleh karena perkara ini termasuk dalam bidang perkawinan, maka sesuai dengan ketentuan Pasal 89 ayat (1) Undang-Undang Nomor 7 Tahun 1989 Tentang Peradilan Agama sebagaimana telah diubah dengan Undang-Undang Nomor 3 Tahun 2006 dan perubahan kedua dengan Undang-Undang Nomor 50 Tahun 2009, biaya perkara ini dibebankan kepada Penggugat;

Mengingat, semua pasal dalam peraturan perundang-undangan dan hukum Islam yang berkaitan dengan perkara ini;

M E N G A D I L I

Disclaimer
Kepaniteraan Mahkamah Agung Republik Indonesia berusaha untuk selalu mencantumkan informasi paling kini dan akurat sebagai bentuk komitmen Mahkamah Agung untuk pelayanan publik, transparansi dan akuntabilitas pelaksanaan fungsi peradilan. Namun dalam hal-hal tertentu masih dimungkinkan terjadi permasalahan teknis terkait dengan akurasi dan keterkinian informasi yang kami sajikan, hal mana akan terus kami perbaiki dari waktu kewaktu.
Dalam hal Anda menemukan inakurasi informasi yang termuat pada situs ini atau informasi yang seharusnya ada, namun belum tersedia, maka harap segera hubungi Kepaniteraan Mahkamah Agung RI melalui :
Email : kepaniteraan@mahkamahagung.go.id   Telp : 021-384 3348 (ext.318)

1. Menyatakan Tergugat yang telah dipanggil secara resmi dan patut untuk menghadap sidang, tidak hadir;
2. Mengabulkan gugatan Penggugat dengan verstek;
3. Menjatuhkan talak satu ba'in sughra Tergugat (**Sardi bin Pariden (alm)** terhadap Penggugat (**Jumisih binti Sukerno (alm)**;
4. Membebankan kepada Penggugat untuk membayar biaya perkara sejumlah Rp 381.000,00 (tiga ratus delapan puluh satu ribu rupiah);

Demikian diputuskan dalam rapat permusyawaratan Majelis Hakim yang dilangsungkan pada hari Senin tanggal 21 Desember 2020 Masehi bertepatan dengan tanggal 6 Jumadilawal 1442 Hijriah, oleh **Sulomo, S.Ag** sebagai Ketua Majelis, **H. Ah. Sholih, S.H.** dan **Dra. Ulfah** masing-masing sebagai Hakim Anggota, putusan tersebut diucapkan dalam sidang terbuka untuk umum pada hari itu juga oleh Ketua Majelis tersebut dengan didampingi oleh Hakim-Hakim Anggota dan dibantu oleh **Nur Cholifah, S.H.** sebagai Panitera Pengganti serta dihadiri oleh Penggugat tanpa hadirnya Tergugat.

Hakim Anggota,

H. Ah. Sholih, S.H.

Ketua Majelis,

Sulomo, S.Ag.

Hakim Anggota,

Dra. Ulfah

Panitera Pengganti,

Nur Cholifah, S.H.

PERINCIAN BIAYA :

1.	Pendaftaran	:	Rp 30.000,00
2.	ATK	:	Rp 75.000,00
3.	Panggilan	:	Rp 240.000,00
4.	PNBP pgl	:	Rp 20.000,00

Disclaimer
Kepaniteraan Mahkamah Agung Republik Indonesia berusaha untuk selalu mencantumkan informasi paling kini dan akurat sebagai bentuk komitmen Mahkamah Agung untuk pelayanan publik, transparansi dan akuntabilitas pelaksanaan fungsi peradilan. Namun dalam hal-hal tertentu masih dimungkinkan terjadi permasalahan teknis terkait dengan akurasi dan keterkinian informasi yang kami sajikan, hal mana akan terus kami perbaiki dari waktu kewaktu.
Dalam hal Anda menemukan inakurasi informasi yang termuat pada situs ini atau informasi yang seharusnya ada, namun belum tersedia, maka harap segera hubungi Kepaniteraan Mahkamah Agung RI melalui :
Email : kepaniteraan@mahkamahagung.go.id   Telp : 021-384 3348 (ext.318)

5. Redaksi : Rp 10.000,00
6. Meterai : Rp 6.000,00
Jumlah : Rp 381.000,00

Putusan ini telah diberitahukan kepada Tergugat tanggal . . . .

Putusan ini telah memperoleh kekuatan hukum tetap tanggal . . . .

Disclaimer
Kepaniteraan Mahkamah Agung Republik Indonesia berusaha untuk selalu mencantumkan informasi paling kini dan akurat sebagai bentuk komitmen Mahkamah Agung untuk pelayanan publik, transparansi dan akuntabilitas pelaksanaan fungsi peradilan. Namun dalam hal-hal tertentu masih dimungkinkan terjadi permasalahan teknis terkait dengan akurasi dan keterkinian informasi yang kami sajikan, hal mana akan terus kami perbaiki dari waktu kewaktu.
Dalam hal Anda menemukan inakurasi informasi yang termuat pada situs ini atau informasi yang seharusnya ada, namun belum tersedia, maka harap segera hubungi Kepaniteraan Mahkamah Agung RI melalui :
Email : kepaniteraan@mahkamahagung.go.id Telp : 021-384 3348 (ext.318)
