# Supplementary material for: Classification of divorce causes during the COVID-19 pandemic using convolutional neural networks
Source: PeerJ Comput Sci. 2022 Jun 30;8:e998. doi: 10.7717/peerj-cs.998 (PMC9299239; doi:10.7717/peerj-cs.998)
Supplement: Supplemental Information 5 [file peerj-cs-08-998-s005.zip › Masalah Ekonomi Dataset/Data ke-1.pdf]

P U T U S A N

Nomor XXXX/Pdt.G/2020/PA.Dmk

بِسْمِ اللهِ الرَّحْمَنِ الرَّحِيْمِ

DEMI KEADILAN BERDASARKAN KETUHANAN YANG MAHA ESA

Pengadilan Agama Demak yang memeriksa dan mengadili perkara tertentu pada tingkat pertama dalam sidang Majelis telah menjatuhkan putusan perkara Gugatan Perceraian antara;

Penggugat, umur 33 tahun, agama Islam, Pekerjaan Karyawan Swasta (Guru), Pendidikan Strata I, tempat kediaman di XXXXKabupaten Demak, dalam hal ini memberikan kuasa kepada Agus Muhammad Ali Magfur, S.HI., MH., Advokat yang berkantor di adalah Para Advokat pada Kantor Advokat dan Konsultan Hukum Lembaga Bantuan Hukum (LBH) Loka Jaya, yang beralamat di Desa Tambakroto Rt. 001 Rw. 00I. Kecamatan Sayung, Kabupaten Demak, Provinsi Jawa Tengah, No. Hp., 081 225 144 991 berdasarkan surat kuasa khusus tanggal 30 November 2020, sebagai **Penggugat**

Melawan

Tergugat, umur 40 tahun, agama Islam, Pekerjaan Wiraswasta, Pendidikan Sekolah Lanjutan Tingkat Atas, tempat kediaman di XXXXKabupaten Demak, sebagai **Tergugat;**

Pengadilan Agama tersebut;

Telah mempelajari surat-surat yang berkaitan dengan perkara ini;

Telah mendengar keterangan Penggugat dan para saksi di muka sidang;

DUDUK PERKARA

Bahwa, Penggugat dalam surat gugatannya tanggal 08 Desember 2020 telah mengajukan gugatan perceraian yang telah didaftar di Kepaniteraan

Disclaimer
Kepaniteraan Mahkamah Agung Republik Indonesia berusaha untuk selalu mencantumkan informasi paling kini dan akurat sebagai bentuk komitmen Mahkamah Agung untuk pelayanan publik, transparansi dan akuntabilitas pelaksanaan fungsi peradilan. Namun dalam hal-hal tertentu masih dimungkinkan terjadi permasalahan teknis terkait dengan akurasi dan keterkinian informasi yang kami sajikan, hal mana akan terus kami perbaiki dari waktu kewaktu.
Dalam hal Anda menemukan inakurasi informasi yang termuat pada situs ini atau informasi yang seharusnya ada, namun belum tersedia, maka harap segera hubungi Kepaniteraan Mahkamah Agung RI melalui :
Email : kepaniteraan@mahkamahagung.go.id   Telp : 021-384 3348 (ext.318)

Pengadilan Agama Demak dengan Nomor XXXX/Pdt.G/2020/PA.Dmk, tanggal 08 Desember 2020, dengan dalil-dalil sebagai berikut:

1. Bahwa Penggugat dan Tergugat adalah pasangan suami istri sah, yang telah melangsungkan pernikahan sesuai dengan hukum Islam dan telah dicatatkan di Kantor Urusan Agama Kecamatan Guntur Kabupaten Demak Jawa tengah pada hari Kamis 31 Juli 2014, sebagaimana tercantum dalam Kutipan Akta Nikah Nomor : 0460/18/VII/2014;
2. Bahwa Penggugat dan Tergugat dalam membina rumah tangga bertempat tinggal XXXXKabupaten Demak, selama 2 (dua) tahun, kemudian pindah kerumah orang tua Penggugat sampai sekarang;
3. Bahwa pada mulanya kehidupan rumah tangga Penggugat dan Tergugat pernah hidup rukun serta telah melakukan hubungan layaknya suami istri (bada dukhul) selama 6 (Enam) Tahun dan belum dikaruniai seorang anak, serta selama dalam perkawinan antara Penggugat dengan Tergugat tidak pernah bercerai;
4. Bahwa tanda-tanda ketidak harmonisan rumah tangga Penggugat dan Tergugat mulai terjadi sejak tahun 2016, sehingga sering terjadi percekcokan dan pertengkaran yang terusmenerus, yang disebabkan karena:;
   - Tergugat yang seharusnya menjadi Pemimpin dan bertanggung jawab dalam rumah tangga namun selama membina rumah tangga dengan Penggugat, Tergugat tidak mencerminkan sebagai kepala keluarga yang baik atau memberikan kenyamanan dengan Penggugat;
   - Tergugat dalam memenuhi kebutuhan (Nafkah) terhadap Penggugat, setiap penggugat membutuhkan keuangan yang lebih untuk keperluan penggugat, Tergugat tidak mauberupaya mencari solusi yang terbaik, dan terkesan tergugat tidak menghiraukan akan kebutuha ntersebut;
   - Bahwa Tergugat setiap kali Penggugat membicarakan permasalahan keluarga Tergugat setiap memberikan jawaban menyakitkan hati Penggugat dan itu terjadi berulang - ulang kali;
   - Bahwa Penggugat setiap berkeluh kesah dengan Tergugat tidak bisa memberikan ketenangan, kenyamanan kepada Penggugat bahkan

Disclaimer
Kepaniteraan Mahkamah Agung Republik Indonesia berusaha untuk selalu mencantumkan informasi paling kini dan akurat sebagai bentuk komitmen Mahkamah Agung untuk pelayanan publik, transparansi dan akuntabilitas pelaksanaan fungsi peradilan. Namun dalam hal-hal tertentu masih dimungkinkan terjadi permasalahan teknis terkait dengan akurasi dan keterkinian informasi yang kami sajikan, hal mana akan terus kami perbaiki dari waktu kewaktu.
Dalam hal Anda menemukan inakurasi informasi yang termuat pada situs ini atau informasi yang seharusnya ada, namun belum tersedia, maka harap segera hubungi Kepaniteraan Mahkamah Agung RI melalui :
Email : kepaniteraan@mahkamahagung.go.id Telp : 021-384 3348 (ext.318)

Tergugat membuat bertambahnya masalah;

- Bahwa sikap Tergugat terlalu cemburu / Protektif dengan Penggugat, Setiap kali Penggugat mengikuti kegiatan - kegiatan di sekolah, Tergugat tidak memperbolehkan, seperti kegiatan Pramuka dan Extra sekolah;
- Dan antara Penggugat dengan Tergugat apabila terjadi Permasalahan di keluarga Tergugat, Penggugat merasakan di tekan dan disalahkan, Tergugat tidak bisa melindungi / membela Penggugat agar permasalahan bisa selesai dengan baik;
- Bahwa Pertengkaran dan Percekcokan Penggugat dan Tergugat terjadi terus menerus sejak tahun 2016 sampai dengan September 2020, sehingga Penggugat sudah pisah ranjang dengan Tergugat;
- Penggugat sudah sangat bersabar untuk mempertahankan keutuhan rumah tangga dengan Tergugat Namun karena permasalahan tidak ada penyelesaianya sejak tahun 2016 sampai dengan bulan September 2020, dan sampai sekarang Tergugat tidak ada niat untuk merubah sikapnya sehingga Penggugat memutuskan mengajukan Permohonan cerai Gugat di Pengadilan Agama Demak;
- Pengugat sudah beberapa kali berbagai usaha berbicara baik-baik dengan Tergugat, namun tidak ada perubahan sikap terhadap Tergugat,
- Bahwa puncak pertengkaran terjadi pada bulan September 2020 yang menyebabkan pisah ranjang hingga sekarang;

5. Bahwa berbagai usaha telah dilakukan oleh Penggugat dan keluarga untuk mencari jalan keluar permasalahan tersebut di atas, agar rumah tangga Penggugat dan Tergugat dapat utuh kembali, namun hingga sekarang belum berhasil;
6. Bahwa Penggugat sanggup membayar biaya Perkara:

Bahwa berdasarkan hal-hal tersebut di atas, telah cukup alasan bagi Penggugat untuk mengajukan Gugatan ini sebagaimana dimaksud dalam Pasal 19 huruf f Peraturan Pemerintah No 9 tahun 1975, jo Paasal 116 huruf (f) Kompilasi Hukum Islam, dan untuk itu Penggugat mohon Kepada Bapak Kepala Pengadilan Agama Demak kiranya berkenan menerima dan memeriksa perkara ini. Selanjutnya memberikan putusan sebagai berikut :

Disclaimer
Kepaniteraan Mahkamah Agung Republik Indonesia berusaha untuk selalu mencantumkan informasi paling kini dan akurat sebagai bentuk komitmen Mahkamah Agung untuk pelayanan publik, transparansi dan akuntabilitas pelaksanaan fungsi peradilan. Namun dalam hal-hal tertentu masih dimungkinkan terjadi permasalahan teknis terkait dengan akurasi dan keterkinian informasi yang kami sajikan, hal mana akan terus kami perbaiki dari waktu kewaktu.
Dalam hal Anda menemukan inakurasi informasi yang termuat pada situs ini atau informasi yang seharusnya ada, namun belum tersedia, maka harap segera hubungi Kepaniteraan Mahkamah Agung RI melalui :
Email : kepaniteraan@mahkamahagung.go.id Telp : 021-384 3348 (ext.318)

PRIMER

1. Menerima dan mengabulkan gugatan Penggugat:
2. Menyatakan perkawinan Penggugat (**Penggugat (Alm)**) Melawan dengan Tergugat (**Tergugat (Alm)**,Putus karena talak *bain sughra;*
3. Membebankan biaya perkara menurut Hukum;

Subsider

Atau jika Majlis Hakim berpendapat lain, mohon putusan yang seadil-adilnya (*Ex equo et Bono)*:

Bahwa, pada hari sidang yang telah ditetapkan Penggugat telah datang menghadap ke muka sidang, sedangkan Tergugat tidak datang menghadap ke muka sidang dan tidak menyuruh orang lain untuk menghadap sebagai wakil/kuasa hukumnya meskipun telah dipanggil secara resmi dan patut yang relaas panggilannya telah dibacakan di dalam sidang, sedangkan tidak ternyata bahwa tidak datangnya itu disebabkan suatu halangan yang sah;

Bahwa, Majelis hakim telah menasehati Penggugat agar berpikir untuk tidak bercerai dengan Tergugat, tetapi Penggugat tetap pada dalil-dalil gugatannya untuk bercerai dengan Tergugat;

Bahwa, perkara ini tidak dapat di mediasi karena Tergugat tidak pernah datang menghadap meskipun telah dipanggil secara resmi dan patut, selanjutnya dimulai pemeriksaan dengan membacakan surat gugatan Penggugat yang maksud dan isinya tetap dipertahankan oleh Penggugat;

Bahwa, untuk menguatkan dalil-dalil gugatannya, Penggugat telah mengajukan bukti-bukti berupa;

A. Surat;
   1. Fotokopi Kartu Tanda Penduduk Nomor 3321035306870001 tanggal 01 Maret 2013, yang bermeterai cukup dan telah sesuai dengan aslinya (Bukti P.1);
   2. Fotokopi Buku Kutipan Akta Nikah dari KUA Kecamatan Guntur Kabupaten Demak Jawa tengah propinsi Jawa Tengah Nomor 0460/18/VII/2014 tanggal 31 Juli 2014, yang bermeterai cukup dan telah sesuai dengan aslinya (Bukti P.2);

B. Saksi;

Disclaimer
Kepaniteraan Mahkamah Agung Republik Indonesia berusaha untuk selalu mencantumkan informasi paling kini dan akurat sebagai bentuk komitmen Mahkamah Agung untuk pelayanan publik, transparansi dan akuntabilitas pelaksanaan fungsi peradilan. Namun dalam hal-hal tertentu masih dimungkinkan terjadi permasalahan teknis terkait dengan akurasi dan keterkinian informasi yang kami sajikan, hal mana akan terus kami perbaiki dari waktu kewaktu.
Dalam hal Anda menemukan inakurasi informasi yang termuat pada situs ini atau informasi yang seharusnya ada, namun belum tersedia, maka harap segera hubungi Kepaniteraan Mahkamah Agung RI melalui :
Email : kepaniteraan@mahkamahagung.go.id Telp : 021-384 3348 (ext.318)

1. **Saksi I,** umur 32 tahun, Agama Islam, pekerjaan Swasta tempat tinggal di XXXXKabupaten Demak, di bawah sumpahnya menerangkan sebagai berikut:
   - Bahwa saksi kenal dengan Penggugat dan Tergugat karena saksi adalah Saudara Sepupu Penggugat;
   - Bahwa Penggugat dan Tergugat adalah suami isteri, menikah pada bulan Juli 2014, danselama menikah belum dikaruniai anak;
   - Bahwa setelah menikah Penggugat dan Tergugat tinggal bersama di XXXXKabupaten Demak, selama 2 (dua) tahun, kemudian pindah kerumah orang tua Penggugat;
   - Bahwa pada awalnya rumah tangga Penggugat dan Tergugat rukun dan harmonis, akan tetapi sejak tahun 2016, rumah tangganya mulai tidak rukun, karena sering terjadi perselisihan dan pertengkaran;
   - Bahwa saksi tahu dan melihat perselisihan dan pertengkaran antara Penggugat dan Tergugat;
   - Bahwa penyebab pertengkaran Penggugat dan Tergugat karena ekonomi kurang karena Tergugat malas bekerja dan Tergugat orangnya cemberu buta kepada Penggugat tanpa alasan yang jelas;
   - Bahwa akibat pertengkaran Penggugat dan Tergugat telah pisah ranjang, sejak akhir tahun tahun 2016 Penggugat dan Tergugat tidak pernah memperdulikan satu sama lain selama kurang lebih 4 tahun;
   - Bahwa keluarga Penggugat sudah berusaha menasihati Penggugat, akan tetapi tidak berhasil;
2. **Saksi II,** umur 32 tahun, Agama Islam, pekerjaan Swasta tempat tinggal di XXXXKabupaten Demak, di bawah sumpahnya menerangkan sebagai berikut:
   - Bahwa saksi kenal dengan Penggugat dan Tergugat karena saksi adalah teman Penggugat;
   - Bahwa Penggugat dan Tergugat adalah suami isteri, menikah pada bulan Juli 2014, danselama menikah belum dikaruniai anak;
   - Bahwa setelah menikah Penggugat dan Tergugat tinggal bersama di XXXXKabupaten Demak, selama 2 (dua) tahun, kemudian pindah kerumah orang tua Penggugat;
   - Bahwa semula rumah tangga Penggugat dan Tergugat tidak ada masalah, akan tetapi sejak tahun 2016, rumah tangganya mulai tidak rukun, karena sering terjadi perselisihan dan pertengkaran;

Disclaimer
Kepaniteraan Mahkamah Agung Republik Indonesia berusaha untuk selalu mencantumkan informasi paling kini dan akurat sebagai bentuk komitmen Mahkamah Agung untuk pelayanan publik, transparansi dan akuntabilitas pelaksanaan fungsi peradilan. Namun dalam hal-hal tertentu masih dimungkinkan terjadi permasalahan teknis terkait dengan akurasi dan keterkinian informasi yang kami sajikan, hal mana akan terus kami perbaiki dari waktu kewaktu.
Dalam hal Anda menemukan inakurasi informasi yang termuat pada situs ini atau informasi yang seharusnya ada, namun belum tersedia, maka harap segera hubungi Kepaniteraan Mahkamah Agung RI melalui :
Email : kepaniteraan@mahkamahagung.go.id   Telp : 021-384 3348 (ext.318)

- Bahwa saksi tahu dan melihat perselisihan dan pertengkaran antara Penggugat dan Tergugat;
- Bahwa penyebab pertengkaran Penggugat dan Tergugat disebabkan oleh nafkah kurang karena Tergugat malas bekerja dan lebih sering menganggurnya dan Tergugat orangnya cemberu buta kepada Penggugat tanpa alasan yang jelas;
- Bahwa akibat pertengkaran Penggugat dan Tergugat telah pisah ranjang, sejak akhir tahun tahun 2016 Penggugat dan Tergugat tidak pernah memperdulikan satu sama lain;
- Bahwa keluarga Penggugat sudah berusaha menasihati Penggugat, akan tetapi tidak berhasil;

Selanjutnya untuk singkatnya uraian putusan ini, maka semua hal yang termuat dalam Berita Acara Sidang ini merupakan bagian yang tak terpisahkan dari putusan ini;

PERTIMBANGAN HUKUM

Menimbang, bahwa maksud dan tujuan gugatan Penggugat adalah sebagaimana telah diuraikan di atas;

Menimbang, bahwa pada hari dan tanggal sidang yang telah ditetapkan Penggugat telah datang menghadap sendiri di muka sidang, sedangkan Tergugat tidak pernah datang menghadap di muka sidang dan tidak pula menyuruh orang lain sebagai wakil/kuasanya yang sah untuk datang menghadap, meskipun Tergugat telah dipanggil secara resmi dan patut, sedangkan tidak ternyata tidak datangnya itu disebabkan suatu halangan yang sah, maka Tergugat harus dinyatakan tidak hadir dan gugatan tersebut diperiksa tanpa hadirnya Tergugat;

Menimbang, bahwa Majelis Hakim dalam setiap persidangan telah berusaha mendamaikan dengan cara menasihati Penggugat agar kembali rukun membina rumah tangganya dengan Tergugat sebagaimana ketentuan Pasal 130 HIR Jo. Pasal 31 ayat (1) dan (2) Peraturan Pemerintah Nomor 9 Tahun 1975 Jo. Pasal 82 ayat (1) dan (4) Undang-Undang Nomor 7 Tahun 1989 tentang Peradilan Agama sebagaimana yang telah diubah dengan Undang-Undang Nomor 3 Tahun 2006 dan perubahan kedua dengan Undang-Undang

Disclaimer
Kepaniteraan Mahkamah Agung Republik Indonesia berusaha untuk selalu mencantumkan informasi paling kini dan akurat sebagai bentuk komitmen Mahkamah Agung untuk pelayanan publik, transparansi dan akuntabilitas pelaksanaan fungsi peradilan. Namun dalam hal-hal tertentu masih dimungkinkan terjadi permasalahan teknis terkait dengan akurasi dan keterkinian informasi yang kami sajikan, hal mana akan terus kami perbaiki dari waktu kewaktu.
Dalam hal Anda menemukan inakurasi informasi yang termuat pada situs ini atau informasi yang seharusnya ada, namun belum tersedia, maka harap segera hubungi Kepaniteraan Mahkamah Agung RI melalui :
Email : kepaniteraan@mahkamahagung.go.id   Telp : 021-384 3348 (ext.318)

Nomor 50 Tahun 2009 Jo. Pasal 143 ayat (2) Kompilasi Hukum Islam Tahun 1991, namun usaha tersebut tidak berhasil;

Menimbang, bahwa Tergugat tidak pernah datang menghadap di muka sidang, maka upaya damai melalui mediasi sebagaimana dimaksud oleh Peraturan Mahkamah Agung Republik Indonesia Nomor 01 Tahun 2016 tidak dapat dilaksanakan;

Menimbang, bahwa oleh karena usaha penasihatan tidak berhasil, maka pemeriksaan perkara ini dilanjutkan dengan pembacaan surat gugatan Penggugat dalam sidang yang tertutup untuk umum, sesuai ketentuan Pasal 80 ayat (2) Undang-Undang Nomor 7 Tahun 1989 sebagaimana telah diubah dengan Undang-Undang Nomor 3 Tahun 2006 dan perubahan kedua dengan Undang-Undang Nomor 50 Tahun 2009;

Menimbang, bahwa dasar hukum yang diajukan oleh Penggugat sebagai dasar alasan cerai gugat ini adalah sebagaimana dalam Pasal 19 huruf (f) Peraturan Pemerintah Nomor 9 Tahun 1975 juncto Pasal 116 huruf (f) Instruksi Presiden Nomor 1 Tahun 1991 tentang Kompilasi Hukum Islam, yang berbunyi bahwa antara suami istri terus menerus terjadi perselisihan dan pertengkaran dan tidak ada harapan akan hidup rukun lagi dalam rumah tangga;

Menimbang, bahwa untuk meneguhkan dalil-dalil gugatannya, Penggugat telah mengajukan bukti-bukti surat yang diberi tanda P.1 dan P.2, yang seluruhnya dikeluarkan oleh pejabat yang berwenang, telah bermeterai cukup, dan bukti surat yang berupa fotokopi telah cocok dengan aslinya, sehingga bukti-bukti surat tersebut telah memenuhi syarat formil dan materiil sebagai alat bukti.

Menimbang, bahwa berdasarkan bukti P.1 maka terbukti, Penggugat berdomisili di Desa Blerong, Rt,02, Rw,02, Kecamatan Guntur, Kabupaten Demak, wilayah yurisdiksi Pengadilan Agama Demak, dengan demikian berdasarkan ketentuan Pasal 73 ayat (1) Undang-undang Nomor 7 Tahun 1989 Tentang Peradilan Agama sebagaimana telah diubah dengan Undang-undang Nomor 3 Tahun 2006 dan perubahan kedua dengan Undang-undang Nomor 50 Tahun 2009, maka Pengadilan Agama Demak berwenang secara relatif untuk memeriksa, memutus dan menyelesaikan perkara ini;

Disclaimer
Kepaniteraan Mahkamah Agung Republik Indonesia berusaha untuk selalu mencantumkan informasi paling kini dan akurat sebagai bentuk komitmen Mahkamah Agung untuk pelayanan publik, transparansi dan akuntabilitas pelaksanaan fungsi peradilan. Namun dalam hal-hal tertentu masih dimungkinkan terjadi permasalahan teknis terkait dengan akurasi dan keterkinian informasi yang kami sajikan, hal mana akan terus kami perbaiki dari waktu kewaktu.
Dalam hal Anda menemukan inakurasi informasi yang termuat pada situs ini atau informasi yang seharusnya ada, namun belum tersedia, maka harap segera hubungi Kepaniteraan Mahkamah Agung RI melalui :
Email : kepaniteraan@mahkamahagung.go.id   Telp : 021-384 3348 (ext.318)

Menimbang, bahwa berdasarkan bukti P.2, maka terbukti, bahwa antara Penggugat dengan Tergugat adalah suami-istri yang sah menurut hukum Islam, oleh karena itu Penggugat telah memenuhi syarat kedudukan hukum (*legal standing*) untuk mengajukan gugatan cerai ini sehingga berdasarkan Pasal 49 ayat (1) huruf (a) Undang-Undang Nomor 7 Tahun 1989 Tentang Peradilan Agama sebagaimana telah diubah dengan Undang-Undang Nomor 3 Tahun 2006 dan perubahan kedua dengan Undang-Undang Nomor 50 Tahun 2009 tersebut, Pengadilan Agama Demak berwenang untuk memeriksa, mengadili dan menyelesaikan perkara a quo;

Menimbang, bahwa selain mengajukan bukti surat Penggugat juga menghadirkan saksi-saksi, Majelis Hakim telah mendengarkan keterangan saksi keluarga dan atau orang dekat dengan kedua belah pihak, guna memenuhi ketentuan Pasal 76 Undang-Undang Nomor 7 tahun 1989 yang telah diubah oleh Undang-Undang Nomor 3 Tahun 2006 jo. Pasal 22 Ayat (2) Peraturan Pemerintah Nomor 9 tahun 1975 dan Pasal 134 Kompilasi Hukum Islam;

Menimbang, bahwa dari keterangan saksi-saksi keterangannya saling mendukung dan memperkuat dalil-dalil gugatan Penggugat, kesaksian mana telah memenuhi ketentuan Pasal 171 Ayat (1) dan 172 HIR. setelah dihubungkan dengan keterangan pihak-pihak serta bukti lain.

Menimbang, bahwa berdasarkan fakta-fakta tersebut di atas dapat disimpulkan fakta hukum sebagai berikut;

- Bahwa Penggugat dengan Tergugat adalah suami istri sah.
- Bahwa setelah menikah Penggugat dengan Tergugat tinggal di XXXXKabupaten Demak, selama 2 (dua) tahun, kemudian pindah kerumah orang tua Penggugat dan selama berumah tangga belum dikaruniai anak;
- Bahwa sejak tahun 2016 antara Penggugat dan Tergugat sering terjadi perselisihan dan pertengkaran yang disebabkan karena nafkah kurang karena Tergugat malas bekerja dan lebih sering menganggurnya dan Tergugat orangnya cemberu buta kepada Penggugat tanpa alasan yang jelas;
- Bahwa akibat perselisihan dan pertengkaran Penggugat dan Tergugat sejak akhir tahun 2016, Penggugat dan Tergugat telah pisah ranjang, Penggugat

Disclaimer
Kepaniteraan Mahkamah Agung Republik Indonesia berusaha untuk selalu mencantumkan informasi paling kini dan akurat sebagai bentuk komitmen Mahkamah Agung untuk pelayanan publik, transparansi dan akuntabilitas pelaksanaan fungsi peradilan. Namun dalam hal-hal tertentu masih dimungkinkan terjadi permasalahan teknis terkait dengan akurasi dan keterkinian informasi yang kami sajikan, hal mana akan terus kami perbaiki dari waktu kewaktu.
Dalam hal Anda menemukan inakurasi informasi yang termuat pada situs ini atau informasi yang seharusnya ada, namun belum tersedia, maka harap segera hubungi Kepaniteraan Mahkamah Agung RI melalui :
Email : kepaniteraan@mahkamahagung.go.id Telp : 021-384 3348 (ext.318)

dan Tergugat tidak pernah memperdulikan satu sama lain selama kurang lebih 4 tahun;

- Bahwa pihak keluarga sudah berusaha mendamaikan kedua belah pihak, akan tetapi tidak berhasil;

Menimbang, bahwa dari fakta sebagaimana terurai di atas telah memberikan gambaran bahwa perselisihan antara Penggugat dengan Tergugat sudah tidak mampu lagi dikendalikan dan diatasi oleh mereka berdua, bahkan perpisahan antara Penggugat dan Tergugat merupakan bukti bahwa perselisihan antara Penggugat dengan Tergugat sudah cukup serius dan bersifat terus menerus;

Menimbang bahwa dari pernyataan Penggugat selama dalam persidangan yang dengan tegas hendak bercerai dengan Tergugat dan juga sikap Tergugat yang tidak pernah menghadiri persidangan adalah merupakan indikasi tidak adanya kehendak dari kedua belah pihak untuk memperbaiki kembali keadaan rumah tangganya, karena itu merupakan fakta kongkrit yang menunjukkan bahwa hubungan Penggugat dengan Tergugat sudah sampai pada tingkat pecahnya perkawinan (*broken marriage*) dan Hakim berkesimpulan bahwa tujuan yang diharapkan dari perkawinan sebagaimana tersebut pada pasal 1 Undang-undang Nomor 1 Tahun 1974 tidak mungkin lagi terwujud;

Menimbang bahwa para saksi saksi telah menerangkan adanya usaha pihak keluarga merukunkan Penggugat dengan Tergugat akan tetapi tidak berhasil, kerena itu dianggap telah memenui ketentuan Pasal 22 ayat (2) Peraturan Pemerintah Nomor 9 Tahun 1974;

Menimbang bahwa mengutip referensi fiqh yang terdapat dalam kitab Hurriatuz Zaujani fith Thalaq Juz I halaman 83 yang diambil alih menjadi pendapat Hakim sebagai berikut :

وقد اختارالإسلام نظام الطلاق حين تضطر ب الحياة الزوجين ولم يعد ينفع فيها نصا ئح ولا صلح وحيث تصبح الربطة الزواج صورة من غير روح لأن استمرار معناه ان يحكم على احد الزوجين بالسجن الموء بد وهذا ظلم تأ باه روح العدالة

Artinya : “*Islam memilih lembaga thalaq (perceraian) ketika rumah tangga sudah dianggap goncang serta dianggap sudah tidak bermanfaat lagi nasehat/ perdamaian, dan hubungan suami isteri menjadi tanpa ruh (hampa), sebab meneruskan perkawinan berarti menghukum*

Disclaimer
Kepaniteraan Mahkamah Agung Republik Indonesia berusaha untuk selalu mencantumkan informasi paling kini dan akurat sebagai bentuk komitmen Mahkamah Agung untuk pelayanan publik, transparansi dan akuntabilitas pelaksanaan fungsi peradilan. Namun dalam hal-hal tertentu masih dimungkinkan terjadi permasalahan teknis terkait dengan akurasi dan keterkinian informasi yang kami sajikan, hal mana akan terus kami perbaiki dari waktu kewaktu.
Dalam hal Anda menemukan inakurasi informasi yang termuat pada situs ini atau informasi yang seharusnya ada, namun belum tersedia, maka harap segera hubungi Kepaniteraan Mahkamah Agung RI melalui :
Email : kepaniteraan@mahkamahagung.go.id   Telp : 021-384 3348 (ext.318)

salah satu suami isteri dengan penjara yang berkepanjangan. Ini adalah aniaya (kedhaliman) yang bertentangan dengan semangat keadilan".

Menimbang bahwa sehubungan dengan hal tersebut Yurisprudensi Mahkamah Agung RI. Nomor 379 K / AG / 1995 tanggal 26 Maret 1997 menyatakan "*Suami isteri yang tidak berdiam serumah lagi dan tidak ada harapan untuk hidup rukun kembali maka rumah tanga tersebut telah terbukti retak dan pecah dan telah memenuhi alasan cerai Pasal 19 huruf f Peraturan Pemerintah Nomor 9 tahun 1975*";

Menimbang, bahwa aspek penting dari bunyi Pasal 19 huruf f Peraturan Pemerintah Nomor 9 tahun 1975 jo. Pasal 116 huruf f Kompilasi Hukum Islam di atas adalah tidak adanya harapan untuk menata dan mempertahankan rumah tangga, dengan demikian apabila maksud pasal tersebut dikomparasikan dengan keadaan rumah tangga Penggugat dan Tergugat maka telah terdapat cukup alasan hukum untuk mengabulkan gugatan Penggugat dan menjatuhkan talak satu ba'in sughro dari Tergugat terhadap Penggugat;

Menimbang, bahwa karena perkara a quo masuk bidang perkawinan, maka berdasarkan Pasal 89 Ayat (1) Undang-Undang Nomor 7 Tahun 1989 tentang Peradilan Agama yang telah diubah pertama dengan Undang-Undang Nomor 3 Tahun 2006 dan perubahan kedua dengan Undang-Undang Nomor 50 tahun 2009, biaya perkara harus dibebankan kepada Penggugat;

Mengingat segala peraturan perundang-undangan yang berlaku serta hukum syara' yang berkaitan dengan perkara ini;

M E N G A D I L I

1. Menyatakan Tergugat yang telah dipanggil secara resmi dan patut untuk menghadap ke persidangan, tidak hadir;
2. Mengabulkan gugatan Penggugat dengan verstek;
3. Menjatuhkan talak satu ba'in sughra Tergugat (**Tergugat**) kepada Penggugat (**Penggugat**);
4. Membebankan kepada Penggugat untuk membayar biaya perkara yang hingga kini sejumlah Rp386.000,00 (tiga ratus delapan puluh enam ribu rupiah);

Disclaimer
Kepaniteraan Mahkamah Agung Republik Indonesia berusaha untuk selalu mencantumkan informasi paling kini dan akurat sebagai bentuk komitmen Mahkamah Agung untuk pelayanan publik, transparansi dan akuntabilitas pelaksanaan fungsi peradilan. Namun dalam hal-hal tertentu masih dimungkinkan terjadi permasalahan teknis terkait dengan akurasi dan keterkinian informasi yang kami sajikan, hal mana akan terus kami perbaiki dari waktu kewaktu.
Dalam hal Anda menemukan inakurasi informasi yang termuat pada situs ini atau informasi yang seharusnya ada, namun belum tersedia, maka harap segera hubungi Kepaniteraan Mahkamah Agung RI melalui :
Email : kepaniteraan@mahkamahagung.go.id Telp : 021-384 3348 (ext.318)

Demikian diputuskan dalam rapat permusyawaratan Majelis yang dilangsungkan pada hari Selasa tanggal 22 Desember 2020 *Masehi*. bertepatan dengan tanggal 7 Jumadil Awwal 1442 *Hijriyah.* Oleh kami **Drs. Makali** sebagai Ketua Majelis, dan **Dra. Nur Immawati** serta **Toharudin, S.H.I., M.H.** masing-masing sebagai Hakim Anggota. Putusan mana diucapkan oleh Ketua Majelis Hakim tersebut pada hari itu juga dalam sidang terbuka untuk umum dengan dihadiri oleh para Hakim Anggota **dan Hj. Siti Hajar Zulaikha, S.H.** sebagai Panitera Pengganti dan dihadiri pula oleh Kuasa Hukum Penggugat tanpa hadirnya Tergugat;

Ketua Majelis,

Drs. Makali

Hakim Anggota,	Hakim Anggota,
**Dra. Nur Immawati**	**Toharudin, S.H.I., M.H.**

Panitera Pengganti,

Hj. Siti Hajar Zulaikha, S.H.

Perincian Biaya :

1. Biaya Pendaftaran	:	Rp	30.000,00
2. Biaya Proses	:	Rp	100.000,00

Disclaimer
Kepaniteraan Mahkamah Agung Republik Indonesia berusaha untuk selalu mencantumkan informasi paling kini dan akurat sebagai bentuk komitmen Mahkamah Agung untuk pelayanan publik, transparansi dan akuntabilitas pelaksanaan fungsi peradilan. Namun dalam hal-hal tertentu masih dimungkinkan terjadi permasalahan teknis terkait dengan akurasi dan keterkinian informasi yang kami sajikan, hal mana akan terus kami perbaiki dari waktu kewaktu.
Dalam hal Anda menemukan inakurasi informasi yang termuat pada situs ini atau informasi yang seharusnya ada, namun belum tersedia, maka harap segera hubungi Kepaniteraan Mahkamah Agung RI melalui :
Email : kepaniteraan@mahkamahagung.go.id   Telp : 021-384 3348 (ext.318)

3. Biaya Pemanggilan	:	Rp	220.000,00
4. PNBP Panggilan	:	Rp	20.000,00
5. Biaya Redaksi	:	Rp	10.000,00
6. Biaya Meterai	:	Rp	6.000,00
Jumlah	:	Rp	386.000,00

Disclaimer
Kepaniteraan Mahkamah Agung Republik Indonesia berusaha untuk selalu mencantumkan informasi paling kini dan akurat sebagai bentuk komitmen Mahkamah Agung untuk pelayanan publik, transparansi dan akuntabilitas pelaksanaan fungsi peradilan. Namun dalam hal-hal tertentu masih dimungkinkan terjadi permasalahan teknis terkait dengan akurasi dan keterkinian informasi yang kami sajikan, hal mana akan terus kami perbaiki dari waktu kewaktu.
Dalam hal Anda menemukan inakurasi informasi yang termuat pada situs ini atau informasi yang seharusnya ada, namun belum tersedia, maka harap segera hubungi Kepaniteraan Mahkamah Agung RI melalui :
Email : kepaniteraan@mahkamahagung.go.id   Telp : 021-384 3348 (ext.318)
