# Supplementary material for: Classification of divorce causes during the COVID-19 pandemic using convolutional neural networks
Source: PeerJ Comput Sci. 2022 Jun 30;8:e998. doi: 10.7717/peerj-cs.998 (PMC9299239; doi:10.7717/peerj-cs.998)
Supplement: Supplemental Information 5 [file peerj-cs-08-998-s005.zip › Masalah Ekonomi Dataset/Data ke-10.pdf]

PUTUSAN

Nomor 528/Pdt.G/2020/PA.Ppg

بِسْمِ اللهِ الرَّحْمَنِ الرَّحِيْمِ

DEMI KEADILAN BERDASARKAN KETUHANAN YANG MAHA ESA

Pengadilan Agama Pasir Pengaraian yang memeriksa dan mengadili perkara-perkara tertentu pada peradilan tingkat pertama dalam sidang Majelis telah menjatuhkan putusan sebagai berikut dalam perkara Cerai Gugat antara:

Penggugat, tempat dan tanggal lahir Kuala Piasa 15 Juni 1992 umur 28 tahun, agama Islam, pendidikan SLTA, pekerjaan Mengurus Rumah Tangga, tempat tinggal di Kabupaten Rokan Hulu, selanjutnya disebut sebagai **Penggugat;**

melawan

Tergugat, tempat dan tanggal lahir Aek Nabara 12 Desember 1991 umur 29 tahun, agama Islam, pendidikan SLTA, pekerjaan Buruh Harian Lepas, dahulu bertempat tinggal di Kabupaten Rokan Hulu, sekarang tidak diketahui alamatnya dengan jelas dan pasti di wilayah Republik Indonesia (ghaib), yang untuk selanjutnya disebut sebagai **Tergugat**;

Pengadilan Agama tersebut;

Setelah membaca semua surat dalam perkara ini;

Setelah mendengar keterangan Penggugat dan memeriksa bukti-bukti di persidangan;

DUDUK PERKARA

Bahwa, Penggugat dalam surat gugatan tertanggal 11 Agustus 2020 yang telah terdaftar di Kepaniteraan Pengadilan Agama Pasir Pengaraian pada tanggal 11 Agustus 2020 dengan Nomor Register 528/Pdt.G/2020/PA.Ppg. telah mengemukakan hal-hal sebagai berikut:

1. Bahwa pada tanggal 16 Maret 2014, Penggugat dan Tergugat melangsungkan pernikahan yang dicatat oleh Pegawai Pencatat Nikah

Disclaimer
Kepaniteraan Mahkamah Agung Republik Indonesia berusaha untuk selalu mencantumkan informasi paling kini dan akurat sebagai bentuk komitmen Mahkamah Agung untuk pelayanan publik, transparansi dan akuntabilitas pelaksanaan fungsi peradilan. Namun dalam hal-hal tertentu masih dimungkinkan terjadi permasalahan teknis terkait dengan akurasi dan keterkinian informasi yang kami sajikan, hal mana akan terus kami perbaiki dari waktu kewaktu.
Dalam hal Anda menemukan inakurasi informasi yang termuat pada situs ini atau informasi yang seharusnya ada, namun belum tersedia, maka harap segera hubungi Kepaniteraan Mahkamah Agung RI melalui :
Email : kepaniteraan@mahkamahagung.go.id Telp : 021-384 3348 (ext.318)

Kantor Urusan Agama Kecamatan Tambusai Utara, Kabupaten Rokan Hulu, sebagaimana tertera dari Kutipan Akta Nikah Nomor : 126/36/III/2014 tanggal 17 Maret 2014;

2. Bahwa setelah menikah Penggugat dan Tergugat kumpul baik dan tinggal di rumah orangtua Penggugat di Desa Mahato selama 1 tahun, terakhir pindah di rumah kediaman bersama di Desa Mahato;
3. Bahwa selama pernikahan antara Penggugat dan Tergugat telah hidup rukun sebagaimana layaknya suami istri (ba'da dukhul) dan telah dikaruniai seorang anak, bernama Aqila Pranaja (laki-laki) umur 5 tahun, anak tersebut sekarang berada dibawah asuhan Penggugat;
4. Bahwa kurang lebih sejak 1 tahun sesudah menikah antara Penggugat dan Tergugat telah terjadi perselisihan dan pertengkaran dalam rumah tangga yang disebabkan antara lain
   a. Tergugat kurang memberi nafkah kepada Penggugat;
   b. Tergugat suka berkata kasar kepada Penggugat;
5. Bahwa lebih kurang sejak tanggal 15 Oktober 2019 berturut-turut hingga sekarang, Tergugat pergi meninggalkan Penggugat tanpa izin Penggugat dan tanpa alasan yang sah. sejak itu Tergugat tidak pulang dan tidak mengirim kabar, tidak memberi nafkah serta tidak diketahui alamatnya yang jelas dan pasti di dalam maupun di luar wilayah Republik Indonesia sesuai dengan keterangan Gaib yang dikeluarkan oleh kepala Desa Mahato, Kecamatan Tambusai Utara, Kabupaten Rokan Hulu, Nomor: 470/1203/Pem-Mt/VII/2020;
6. Bahwa Penggugat telah berusaha mencari Tergugat, antara lain menanyakan keberadaan Tergugat kepada keluarga Tergugat juga kepada teman-teman dekat Tergugat, mereka tidak mengetahui secara persis keberadaan Tergugat namun tidak bertemu juga;
7. Bahwa dengan kejadian tersebut rumah tangga antara Penggugat dan Tergugat sudah tidak lagi dapat dibina dengan baik sehingga tujuan perkawinan untuk membentuk rumah tangga yang sakinah, mawaddah dan rahmah sudah sulit dipertahankan lagi dan karenanya agar masing-masing pihak tidak lebih jauh melanggar norma hukum dan norma agama maka

Disclaimer
Kepaniteraan Mahkamah Agung Republik Indonesia berusaha untuk selalu mencantumkan informasi paling kini dan akurat sebagai bentuk komitmen Mahkamah Agung untuk pelayanan publik, transparansi dan akuntabilitas pelaksanaan fungsi peradilan. Namun dalam hal-hal tertentu masih dimungkinkan terjadi permasalahan teknis terkait dengan akurasi dan keterkinian informasi yang kami sajikan, hal mana akan terus kami perbaiki dari waktu kewaktu.
Dalam hal Anda menemukan inakurasi informasi yang termuat pada situs ini atau informasi yang seharusnya ada, namun belum tersedia, maka harap segera hubungi Kepaniteraan Mahkamah Agung RI melalui :
Email : kepaniteraan@mahkamahagung.go.id   Telp : 021-384 3348 (ext.318)

perceraian merupakan alternative terakhir bagi Penggugat untuk menyelesaikan permasalahan antara Penggugat dan Tergugat;

Berdasarkan alasan/dalil-dalil diatas, Penggugat mohon agar Ketua Pengadilan Agama Pasir Pengaraian segera memeriksa dan mengadili perkara ini, selanjutnya menjatuhkan putusan yang amarnya berbunyi:

1. Mengabulkan gugatan Penggugat;

2. Menjatuhkan talak satu Ba'in Shughra Tergugat **(Tergugat)** dengan Penggugat **(Penggugat)**;

3. Membebankan seluruh biaya perkara ini sesuai dengan peraturan dan perundang-undangan yang berlaku;

Dan apabila Majelis Hakim berpendapat lain, mohon putusan yang seadil-adilnya;

Bahwa, Penggugat mendalilkan bahwa Tergugat telah pergi meninggalkan tempat kediaman bersama dan hingga saat ini tidak diketahui keberadaannya baik di dalam maupun di luar wilayah Republik Indonesia, oleh karena itu Penggugat mengajukan surat keterangan Nomor 470/1203/Pem-Mt/VII/2010, yang dikeluarkan oleh Kepala Desa Mahato, tanggal 24 Julir 2020;

Bahwa, pada hari-hari persidangan yang telah ditetapkan, Penggugat telah datang mengahadap sendiri di persidangan, sedangkan Tergugat tidak pernah datang menghadap di persidangan dan tidak pula mengutus orang lain sebagai wakil atau kuasanya yang sah, meskipun telah dipanggil secara resmi dan patut;

Bahwa, Majelis Hakim telah berusaha mendamaikan dengan cara menasihati Penggugat agar rukun kembali dalam membina rumah tangga bersama Tergugat, namun tidak berhasil. Adapun upaya mediasi sebagaimana yang diamanatkan Perma No. 1 tahun 2016 tidak dapat dilaksanakan karena Tergugat tidak pernah datang menghadap pada hari-hari persidangan yang telah ditetapkan;

Bahwa, pemeriksaan terhadap perkara ini dilanjutkan dengan membacakan gugatan Penggugat yang dalil-dalilnya tetap dipertahankan oleh Penggugat;

Disclaimer
Kepaniteraan Mahkamah Agung Republik Indonesia berusaha untuk selalu mencantumkan informasi paling kini dan akurat sebagai bentuk komitmen Mahkamah Agung untuk pelayanan publik, transparansi dan akuntabilitas pelaksanaan fungsi peradilan. Namun dalam hal-hal tertentu masih dimungkinkan terjadi permasalahan teknis terkait dengan akurasi dan keterkinian informasi yang kami sajikan, hal mana akan terus kami perbaiki dari waktu kewaktu.
Dalam hal Anda menemukan inakurasi informasi yang termuat pada situs ini atau informasi yang seharusnya ada, namun belum tersedia, maka harap segera hubungi Kepaniteraan Mahkamah Agung RI melalui :
Email : kepaniteraan@mahkamahagung.go.id Telp : 021-384 3348 (ext.318)

Bahwa, untuk menguatkan dalil-dalil gugatan Penggugat, Penggugat menyerahkan bukti tertulis berupa: Fotokopi Kutipan Akta Nikah Nomor 126/36/III/2014, an. Tergugat dan Penggugat yang aslinya dikeluarkan oleh Kantor Urusan Agama Kecamatan Tambusai Utara, Kabupaten Rokan Hulu, tanggal 17 Maret 2014, bermeterai cukup, telah *dinazegelen* dan telah dicocokkan dengan aslinya (P.1);

Bahwa, selain bukti tertulis tersebut, Penggugat telah menghadirkan saksi-saksi di persidangan sebagai berikut:

1. Saksi I, umur 64 tahun, agama Islam, pendidikan SD, pekerjaan Petani, bertempat tinggal di Kabupaten Rokan hulu, Saksi adalah ayah kandung Penggugat, telah memberikan keterangan di bawah sumpah yang pada pokoknya sebagai berikut:
   - Bahwa Saksi kenal dengan Penggugat dan Tergugat;
   - Bahwa Saksi tahu antara Penggugat dan Tergugat adalah suami isteri yang sah;
   - Bahwa setelah menikah Penggugat dan Tergugat bertempat tinggal di rumah orangtua Penggugat di Desa Mahato selama 1 tahun, terakhir pindah di rumah kediaman bersama di Desa Mahato;
   - Bahwa Penggugat dan Tergugat sudah dikaruniai 1 (satu) orang anak;
   - Bahwa anak Penggugat dan Tergugat diasuh oleh Penggugat;
   - Bahwa kehidupan rumah tangga Penggugat dan Tergugat pada awalnya berjalan baik dan rukun, namun sekarang ini rumah tangga mereka tidak harmonis lagi karena sering terjadi perselisihan dan pertengkaran;
   - Bahwa perselisihan dan pertengkaran Penggugat dan Tergugat terjadi sejak 1 tahun menikah;
   - Bahwa penyebab perselisihan dan pertengkaran Penggugat dan Tergugat karena Tergugat kurang memberi nafkah kepada Penggugat, Tergugat suka berkata kasar kepada Penggugat;
   - Bahwa Saksi mengetahui perselisihan dan pertengkaran tersebut karena saksi pernah melihat sendiri tentang peristiwa perselisihan dan

Disclaimer
Kepaniteraan Mahkamah Agung Republik Indonesia berusaha untuk selalu mencantumkan informasi paling kini dan akurat sebagai bentuk komitmen Mahkamah Agung untuk pelayanan publik, transparansi dan akuntabilitas pelaksanaan fungsi peradilan. Namun dalam hal-hal tertentu masih dimungkinkan terjadi permasalahan teknis terkait dengan akurasi dan keterkinian informasi yang kami sajikan, hal mana akan terus kami perbaiki dari waktu kewaktu.
Dalam hal Anda menemukan inakurasi informasi yang termuat pada situs ini atau informasi yang seharusnya ada, namun belum tersedia, maka harap segera hubungi Kepaniteraan Mahkamah Agung RI melalui :
Email : kepaniteraan@mahkamahagung.go.id Telp : 021-384 3348 (ext.318)

pertengkaran Penggugat dan Tergugat tersebut ketika Penggugat dan Tergugat masih tinggal serumah;

- Bahwa Penggugat dan Tergugat sudah pisah tempat tinggal sejak bulan Oktober tahun 2019 sampai sekarang;
- Bahwa yang meninggalkan kediaman bersama adalah Tergugat;
- Bahwa selama pisah Tergugat tidak pernah datang, dan tidak pernah lagi memberi nafkah untuk Penggugat, dan Tergugat sudah tidak mempedulikan Penggugat lagi;
- Bahwa Penggugat dan Tergugat pernah diusahakan damai oleh keluarga Penggugat, namun tidak berhasil karena Penggugat tidak mau rukun lagi;

2. **Saksi II**, umur 41 tahun, agama Islam, pendidikan SLTA, pekerjaan Ibu Rumah Tangga, bertempat tinggal di Kabupaten Rokan hulu, Saksi adalah tetangga Penggugat, telah memberikan keterangan di bawah sumpah yang pada pokoknya sebagai berikut:
   - Bahwa Saksi kenal dengan Penggugat dan Tergugat;
   - Bahwa Saksi tahu antara Penggugat dan Tergugat adalah suami isteri yang sah;
   - Bahwa setelah menikah Penggugat dan Tergugat bertempat tinggal di rumah orangtua Penggugat di Desa Mahato selama 1 tahun, terakhir pindah di rumah kediaman bersama di Desa Mahato;
   - Bahwa Penggugat dan Tergugat sudah dikaruniai 1 (satu) orang anak;
   - Bahwa anak Penggugat dan Tergugat diasuh oleh Penggugat;
   - Bahwa kehidupan rumah tangga Penggugat dan Tergugat pada awalnya berjalan baik dan rukun, namun sekarang ini rumah tangga mereka tidak harmonis lagi karena sering terjadi perselisihan dan pertengkaran;
   - Bahwa perselisihan dan pertengkaran Penggugat dan Tergugat terjadi sejak 1 tahun menikah;

Disclaimer
Kepaniteraan Mahkamah Agung Republik Indonesia berusaha untuk selalu mencantumkan informasi paling kini dan akurat sebagai bentuk komitmen Mahkamah Agung untuk pelayanan publik, transparansi dan akuntabilitas pelaksanaan fungsi peradilan. Namun dalam hal-hal tertentu masih dimungkinkan terjadi permasalahan teknis terkait dengan akurasi dan keterkinian informasi yang kami sajikan, hal mana akan terus kami perbaiki dari waktu kewaktu.
Dalam hal Anda menemukan inakurasi informasi yang termuat pada situs ini atau informasi yang seharusnya ada, namun belum tersedia, maka harap segera hubungi Kepaniteraan Mahkamah Agung RI melalui :
Email : kepaniteraan@mahkamahagung.go.id Telp : 021-384 3348 (ext.318)

- Bahwa penyebab perselisihan dan pertengkaran Penggugat dan Tergugat karena Tergugat kurang memberi nafkah kepada Penggugat, Tergugat suka berkata kasar kepada Penggugat;
- Bahwa Saksi mengetahui perselisihan dan pertengkaran tersebut karena saksi pernah melihat sendiri tentang peristiwa perselisihan dan pertengkaran Penggugat dan Tergugat tersebut ketika Penggugat dan Tergugat masih tinggal serumah;
- Bahwa Penggugat dan Tergugat sudah pisah tempat tinggal sejak bulan Oktober 2019 sampai sekarang;
- Bahwa yang meninggalkan kediaman bersama adalah Tergugat;
- Bahwa selama pisah Tergugat tidak pernah datang, dan tidak pernah lagi memberi nafkah untuk Penggugat, dan Tergugat sudah tidak mempedulikan Penggugat lagi;
- Bahwa Penggugat dan Tergugat pernah diusahakan damai oleh keluarga Penggugat, namun tidak berhasil karena Penggugat tidak mau rukun lagi;

Bahwa, atas keterangan kedua orang saksi tersebut Penggugat tidak mengajukan pertanyaan tambahan;

Bahwa, Penggugat telah menyampaikan kesimpulannya secara lisan di persidangan, yang pada pokoknya menyatakan tetap dengan gugatan Penggugat dan mohon kepada Majelis Hakim mengabulkan gugatan Penggugat;

Bahwa, untuk mempersingkat uraian dalam putusan ini, Majelis Hakim cukup menunjuk kepada berita acara sidang yang merupakan bagian yang tidak terpisahkan dari putusan ini;

PERTIMBANGAN HUKUM

Menimbang, bahwa maksud dan tujuan gugatan Penggugat adalah sebagaimana telah diuraikan dalam duduk perkara;

Menimbang, bahwa Penggugat dalam gugatannya mendalilkan bahwa Penggugat dengan Tergugat telah melangsungkan perkawinan dan telah tercatat secara resmi sebagaimana termatub dalam Kutipan Akta Nikah Nomor

Disclaimer
Kepaniteraan Mahkamah Agung Republik Indonesia berusaha untuk selalu mencantumkan informasi paling kini dan akurat sebagai bentuk komitmen Mahkamah Agung untuk pelayanan publik, transparansi dan akuntabilitas pelaksanaan fungsi peradilan. Namun dalam hal-hal tertentu masih dimungkinkan terjadi permasalahan teknis terkait dengan akurasi dan keterkinian informasi yang kami sajikan, hal mana akan terus kami perbaiki dari waktu kewaktu.
Dalam hal Anda menemukan inakurasi informasi yang termuat pada situs ini atau informasi yang seharusnya ada, namun belum tersedia, maka harap segera hubungi Kepaniteraan Mahkamah Agung RI melalui :
Email : kepaniteraan@mahkamahagung.go.id   Telp : 021-384 3348 (ext.318)

126/36/III/2014, tanggal 17 Maret 2014, oleh karena itu berdasarkan Pasal 73 Ayat (1) Undang-Undang Nomor 7 Tahun 1989 tentang Peradilan Agama sebagaimana telah diubah dengan Undang-Undang Nomor 3 Tahun 2006 dan perubahan kedua dengan Undang-Undang Nomor 50 Tahun 2009, Penggugat memiliki *legal standing* untuk mengajukan perkara *a quo*;

Menimbang, bahwa perkawinan antara Penggugat dan Tergugat dilaksanakan menurut syari'at Islam, maka berdasarkan ketentuan Pasal 40 dan Pasal 63 Ayat (1) Huruf (a) Undang-Undang Nomor 1 Tahun 1974 tentang Perkawinan *juncto* Pasal 49 (ayat 1 huruf a) Undang-Undang Nomor 7 tahun 1989 tentang Peradilan Agama yang telah diubah dengan Undang-Undang Nomor 3 Tahun 2006 dan perubahan kedua dengan Undang-Undang Nomor 50 Tahun 2009, *juncto* Pasal 14 dan Pasal 1 huruf (b) Peraturan Pemerintah No. 9 tahun 1975 tentang Pelaksanaan Undang-Undang No. 1 tahun 1974 tentang Perkawinan, maka perkara ini menjadi kewenangan absolut Pengadilan Agama;

Menimbang, bahwa berdasarkan gugatan Penggugat, ternyata Penggugat bertempat tinggal di wilayah hukum Pengadilan Agama Pasir Pengaraian, maka berdasarkan Pasal 73 Ayat (1) Undang-Undang Nomor 7 Tahun 1989 tentang Peradilan Agama sebagaimana telah diubah dengan Undang-Undang Nomor 3 Tahun 2006 dan perubahan kedua dengan Undang-Undang Nomor 50 Tahun 2009 *juncto* Pasal 129 Kompilasi Hukum Islam (KHI), pemeriksaan perkara *a quo* menjadi kewenangan relatif Pengadilan Agama Pasir Pengaraian;

Menimbang, bahwa oleh karena Penggugat mendalilkan bahwa Tergugat telah pergi meninggalkan tempat kediaman bersama dan hingga saat ini tidak diketahui keberadaannya baik di dalam maupun di luar wilayah Republik Indonesia, maka Penggugat mengajukan surat keterangan sebagaimana telah disebutkan dalam duduk perkara, sehingga gugatan Penggugat telah sesuai dengan ketentuan Pasal 142 R.Bg. *juncto* Pasal 20 Peraturan Pemerintah No 9 tahun 1975 tentang Pelaksanaan Undang-undang Nomor 1 tahun 1974;

Menimbang, bahwa untuk memenuhi perintah Pasal 82 ayat (1) Undang-undang Nomor 7 tahun 1989 tentang Peradilan Agama yang telah diubah dengan Undang-undang Nomor 3 Tahun 2006 dan Undang-undang Nomor 50

Disclaimer
Kepaniteraan Mahkamah Agung Republik Indonesia berusaha untuk selalu mencantumkan informasi paling kini dan akurat sebagai bentuk komitmen Mahkamah Agung untuk pelayanan publik, transparansi dan akuntabilitas pelaksanaan fungsi peradilan. Namun dalam hal-hal tertentu masih dimungkinkan terjadi permasalahan teknis terkait dengan akurasi dan keterkinian informasi yang kami sajikan, hal mana akan terus kami perbaiki dari waktu kewaktu.
Dalam hal Anda menemukan inakurasi informasi yang termuat pada situs ini atau informasi yang seharusnya ada, namun belum tersedia, maka harap segera hubungi Kepaniteraan Mahkamah Agung RI melalui :
Email : kepaniteraan@mahkamahagung.go.id Telp : 021-384 3348 (ext.318)

Tahun 2009, Majelis Hakim telah berusaha mendamaikan agar Penggugat dan Tergugat rukun kembali dengan cara menasihati Penggugat, namun tidak berhasil. Selanjutnya oleh karena Tergugat tidak pernah datang dalam persidangan maka proses mediasi sesuai Perma nomor 1 tahun 2016 tidak dapat dilaksanakan;

Menimbang, bahwa dalam gugatannya, Penggugat pada pokoknya mohon kepada Pengadilan Agama untuk menjatuhkan talak satu ba'in sughra Tergugat terhadap Penggugat, dengan dalil-dalil sebagaimana termaktub dalam gugatan Penggugat;

Menimbang, bahwa berdasarkan gugatan Penggugat dan keterangan saksi-saksi dalam persidangan, dapat disimpulkan bahwa hal yang menjadi pokok sengketa gugatan Penggugat adalah adanya perselisihan dan pertengkaran yang terus-menerus antara Penggugat dan Tergugat yang disebabkan karena:

a. Tergugat kurang memberi nafkah kepada Penggugat;
b. Tergugat suka berkata kasar kepada Penggugat;

Sehingga akibatnya antara Penggugat dan Tergugat berpisah tempat tinggal tanpa saling peduli sejak bulan Oktober 2019 hingga saat ini dan tidak ada harapan untuk rukun lagi dalam rumah tangga;

Menimbang, bahwa terhadap gugatan Penggugat tersebut, Tergugat tidak pernah hadir, tidak pula menyuruh orang lain untuk menghadap di persidangan sebagai wakil atau kuasanya yang sah, sehingga Tergugat tidak dapat didengar keteranganya, meskipun Tergugat telah dipanggil secara resmi dan patut sebagaimana ketentuan Pasal 26 Peraturan Pemerintah Nomor 9 Tahun 1975, sedang ketidakhadirannya tersebut bukan disebabkan oleh suatu halangan yang sah. Oleh karena itu, berdasarkan Pasal 149 R.Bg., perkara ini dapat diputus secara verstek;

Menimbang, bahwa untuk meneguhkan dalil-dalil gugatannya, sesuai dengan ketentuan Pasal 283 R.Bg., Penggugat mengajukan bukti tertulis berupa P.1;

Menimbang, bahwa bukti P.1 merupakan akta autentik dengan nilai kekuatan pembuktian sempurna dan mengikat *(volledig en bindende*

Disclaimer
Kepaniteraan Mahkamah Agung Republik Indonesia berusaha untuk selalu mencantumkan informasi paling kini dan akurat sebagai bentuk komitmen Mahkamah Agung untuk pelayanan publik, transparansi dan akuntabilitas pelaksanaan fungsi peradilan. Namun dalam hal-hal tertentu masih dimungkinkan terjadi permasalahan teknis terkait dengan akurasi dan keterkinian informasi yang kami sajikan, hal mana akan terus kami perbaiki dari waktu kewaktu.
Dalam hal Anda menemukan inakurasi informasi yang termuat pada situs ini atau informasi yang seharusnya ada, namun belum tersedia, maka harap segera hubungi Kepaniteraan Mahkamah Agung RI melalui :
Email : kepaniteraan@mahkamahagung.go.id Telp : 021-384 3348 (ext.318)

bewijskracht), sesuai dengan aslinya, bermeterai cukup, dan telah *dinazegelen*, maka berdasarkan ketentuan Pasal 284 dan 285 R.Bg. *juncto* Pasal 2 Ayat (1) Undang-Undang No. 13 Tahun 1985 tentang Bea Meterai *juncto* Pasal 2 Ayat (1) Peraturan Pemerintah Nomor 24 Tahun 2000 tentang Perubahan Tarif Bea Meterai dan Besarnya Batas Pengenaan Harga Nominal yang Dikenakan Bea Materai, bukti-bukti tersebut secara formil dapat diterima sebagai alat bukti;

Menimbang, bahwa berdasarkan bukti P.1, berupa fotokopi Kutipan Akta Nikah, berdasarkan ketentuan Pasal 2 Undang-Undang No. 1 Tahun 1974, *juncto* Pasal 4, 5 dan 6 ayat (1) Kompilasi Hukum Islam (KHI), terbukti bahwa Penggugat dan Tergugat telah terikat dalam perkawinan yang sah;

Menimbang, bahwa karena alasan gugatan Penggugat didasarkan pada terjadinya perselisihan dan pertengkaran sebagaimana dimaksud dalam Pasal 19 huruf (f) Peraturan Pemerintah Nomor 9 Tahun 1975 *juncto* Pasal 116 Huruf (f) Kompilasi Hukum Islam, maka untuk memenuhi maksud Pasal 22 ayat (2) Peraturan Pemerintah Nomor 9 Tahun 1975 *juncto* Pasal 134 Kompilasi Hukum Islam, Majelis Hakim wajib terlebih dahulu mendengar keterangan dari keluarga Penggugat dan Tergugat atau orang terdekat kedua belah pihak;

Menimbang, bahwa Penggugat telah menghadirkan 2 (dua) orang dekat sebagai saksi dalam persidangan yang bernama **Saksi I** dan **Saksi II**. Kedua saksi tersebut di bawah sumpahnya memberikan keterangan sebagaimana yang termaktub dalam duduk perkara, keterangan lengkap saksi-saksi tersebut *mutatis-mutandis* dianggap terulang dalam pertimbangan ini, yang pada pokoknya secara jelas mendukung dalil-dalil gugatan Penggugat;

Menimbang, bahwa kesaksian yang diberikan saksi-saksi Penggugat disampaikan di bawah sumpah dengan secara bergilir dan terpisah, didasarkan atas pengetahuannya apa yang dilihat, didengar, dan dialami sendiri, saling bersesuaian, serta tidak termasuk orang yang dilarang menjadi saksi, sehingga saksi tersebut memenuhi syarat formil maupun materiil suatu kesaksian sebagaimana yang ditentukan Pasal 171, 172, 175, 307, dan 308 R.Bg. Oleh sebab itu, keterangan saksi-saksi tersebut dapat diterima sebagai alat bukti yang mempunyai nilai kekuatan pembuktian;

Disclaimer
Kepaniteraan Mahkamah Agung Republik Indonesia berusaha untuk selalu mencantumkan informasi paling kini dan akurat sebagai bentuk komitmen Mahkamah Agung untuk pelayanan publik, transparansi dan akuntabilitas pelaksanaan fungsi peradilan. Namun dalam hal-hal tertentu masih dimungkinkan terjadi permasalahan teknis terkait dengan akurasi dan keterkinian informasi yang kami sajikan, hal mana akan terus kami perbaiki dari waktu kewaktu.
Dalam hal Anda menemukan inakurasi informasi yang termuat pada situs ini atau informasi yang seharusnya ada, namun belum tersedia, maka harap segera hubungi Kepaniteraan Mahkamah Agung RI melalui :
Email : kepaniteraan@mahkamahagung.go.id Telp : 021-384 3348 (ext.318)

Menimbang, bahwa berdasarkan gugatan Penggugat, bukti surat, serta keterangan saksi-saksi yang telah dipertimbangkan tersebut di atas, Majelis Hakim dapat menemukan dan menyimpulkan fakta-fakta sebagai berikut:

1. Bahwa Penggugat dan Tergugat adalah suami-isteri sah, semula hidup rukun dan harmonis;
2. Bahwa sejak 1 tahun menikah keharmonisan rumah tangga Penggugat dan Tergugat tidak dapat dipertahankan, karena terjadi perselisihan dan pertengkaran yang terus-menerus;
3. Penyebab perselisihan dan pertengkaran tersebut adalah karena Tergugat kurang memberi nafkah kepada Penggugat, Tergugat suka berkata kasar kepada Penggugat;
4. Bahwa Penggugat dan Tergugat telah berpisah rumah dan tidak saling memedulikan lagi sejak bulan Oktober 2019, Tergugat pergi meninggalkan tempat kediaman bersama;
5. Pihak keluarga telah berusaha mendamaikan Penggugat dan Tergugat, namun tidak berhasil;

Menimbang, bahwa menurut Pasal 19 huruf (f) Peraturan Pemerintah Nomor 9 Tahun 1975 *juncto* Pasal 116 huruf (f) Kompilasi Hukum Islam ditegaskan bahwa salah satu alasan perceraian yaitu adanya perselisihan dan pertengkaran yang terus-menerus, selanjutnya Pasal 39 Undang-Undang Nomor 1 Tahun 1974 tentang Perkawinan menentukan bahwa untuk melakukan suatu perceraian harus cukup alasan, yakni ketika suami-isteri tidak akan dapat hidup rukun sebagai suami isteri dan Pengadilan telah berusaha dan tidak berhasil mendamaikan kedua belah pihak;

Menimbang, bahwa dari ketentuan pasal-pasal tersebut, terdapat beberapa unsur yang harus dipenuhi untuk terjadinya sebuah perceraian:

1. Adanya alasan telah terjadinya perselisihan dan pertengkaran yang terus-menerus;
2. Perselisihan dan pertengkaran menyebabkan suami-isteri sudah tidak ada harapan untuk rukun kembali;
3. Pengadilan telah berupaya mendamaikan kedua belah pihak, namun tidak berhasil;

Disclaimer
Kepaniteraan Mahkamah Agung Republik Indonesia berusaha untuk selalu mencantumkan informasi paling kini dan akurat sebagai bentuk komitmen Mahkamah Agung untuk pelayanan publik, transparansi dan akuntabilitas pelaksanaan fungsi peradilan. Namun dalam hal-hal tertentu masih dimungkinkan terjadi permasalahan teknis terkait dengan akurasi dan keterkinian informasi yang kami sajikan, hal mana akan terus kami perbaiki dari waktu kewaktu.
Dalam hal Anda menemukan inakurasi informasi yang termuat pada situs ini atau informasi yang seharusnya ada, namun belum tersedia, maka harap segera hubungi Kepaniteraan Mahkamah Agung RI melalui :
Email : kepaniteraan@mahkamahagung.go.id Telp : 021-384 3348 (ext.318)

Menimbang, bahwa unsur-unsur tersebut akan dipertimbangkan satu persatu dengan mengaitkan fakta-fakta yang terjadi dalam rumah tangga Penggugat dan Tergugat, sehingga antara Penggugat dan Tergugat dipandang telah memenuhi unsur-unsur terjadinya suatu perceraian;

Menimbang, bahwa berdasarkan fakta hukum yang telah dipertimbangkan di atas, terbukti antara Penggugat dan Tergugat terus-menerus terjadi perselisihan dan pertengkaran yang disebabkan karena Tergugat kurang memberi nafkah kepada Penggugat, Tergugat suka berkata kasar kepada Penggugat;

Menimbang, bahwa akibat dari perselisihan dan pertengkaran tersebut, antara Penggugat dan Tergugat berpisah tempat tinggal. Fakta pisah tempat tinggal antara Penggugat dengan Tergugat bukan merupakan upaya sementara untuk meredam konflik yang mereka hadapi, melainkan sudah merupakan akibat dari konflik yang berkelanjutan dan menunjukkan peningkatan kualitas perselisihan dan pertengkaran di antara Penggugat dan Tergugat;

Menimbang, bahwa sesuai dengan Yurisprudensi Mahkamah Agung Nomor 379/ K/AG/1995 tanggal 26 Maret 1997, yang diambil-alih menjadi pendapat Majelis Hakim, suami isteri yang tidak bertempat tinggal serumah lagi dan tidak ada harapan untuk hidup rukun kembali, maka rumah tangga tersebut telah terbukti retak dan pecah dan telah memenuhi alasan cerai Pasal 19 huruf f Peraturan Pemerintah Nomor 9 Tahun 1975 *juncto* Pasal 116 huruf f Kompilasi Hukum Islam. Berdasarkan pertimbangan-pertimbangan tersebut, maka unsur perceraian yang pertama harus dinyatakan telah terpenuhi;

Menimbang, bahwa selanjutnya terbukti pula bahwa antara Penggugat dan Tergugat telah diupayakan damai oleh pihak keluarga agar Penggugat dan Tergugat dapat rukun kembali, tapi upaya tersebut tidak berhasil. Begitu juga selama persidangan berlangsung, Penggugat telah menunjukkan sikap dan tekadnya untuk bercerai, yang berarti tidak mau lagi mempertahankan perkawinannya. Selain itu, fakta pisah tempat tinggal antara Penggugat dan Tergugat merupakan bukti bahwa Penggugat dan Tergugat sudah tidak peduli terhadap masa depan rumah tangganya, hal ini menunjukkan rumah tangga antara Penggugat dan Tergugat sudah tidak ada harapan untuk kembali rukun.

Disclaimer
Kepaniteraan Mahkamah Agung Republik Indonesia berusaha untuk selalu mencantumkan informasi paling kini dan akurat sebagai bentuk komitmen Mahkamah Agung untuk pelayanan publik, transparansi dan akuntabilitas pelaksanaan fungsi peradilan. Namun dalam hal-hal tertentu masih dimungkinkan terjadi permasalahan teknis terkait dengan akurasi dan keterkinian informasi yang kami sajikan, hal mana akan terus kami perbaiki dari waktu kewaktu.
Dalam hal Anda menemukan inakurasi informasi yang termuat pada situs ini atau informasi yang seharusnya ada, namun belum tersedia, maka harap segera hubungi Kepaniteraan Mahkamah Agung RI melalui :
Email : kepaniteraan@mahkamahagung.go.id Telp : 021-384 3348 (ext.318)

Berdasarkan pertimbangan tersebut, maka unsur kedua juga harus dinyatakan telah terpenuhi;

Menimbang, bahwa Majelis Hakim telah berupaya mendamaikan dengan cara menasihati Penggugat pada setiap persidangan sesuai ketentuan Pasal 31 Peraturan Pemerintah Nomor 9 Tahun 1975, namun upaya tersebut tidak berhasil, dengan demikian maka unsur ketiga juga telah terpenuhi;

Menimbang, bahwa menurut pasal 1 Undang-Undang Nomor 1 tahun 1974 tujuan perkawinan adalah untuk membina rumah tangga yang bahagia dan kekal. Apabila dalam suatu rumah tangga ternyata kebahagiaan dan kerukunan sudah tidak ada lagi antara suami isteri, kemudian salah satu pihak telah bertekad untuk bercerai, maka mempertahankan rumah tangga yang demikian mafsadatnya akan lebih besar daripada manfaat dan mashlahatnya, untuk hal yang demikian Majelis hakim dapat menunjuk kepada kaidah fiqh yang berbunyi:

درأ المفاسد مقدم على جلب المصالح

Artinya: *Bahwa menghindarkan mafsadat harus lebih diprioritaskan daripada mendambakan kemaslahatan ;*

Menimbang, bahwa Majelis perlu mendeskripsikan pendapat ahli fiqh, sebagaimana tercantum dalam Kitab *Fiqh as-Sunnah,* Jilid II, halaman 291 yang diambil-alih sebagai pendapat Majelis yang berbunyi:

يجوز لها ان تطلب من القاضى التفريق وحينئذ يطلقها القاضى
طلقة بائنة اذا ثبت الضرر و عجز عن الا صلاح بينهما

Artinya :"*Dan bagi seorang isteri boleh mengajukan perceraian terhadap suaminya. Dan Hakim boleh menjatuhkan talak satu ba'in, apabila terbukti adanya kemadharatan dalam pernikahan dan keduanya sulit didamaikan*";

Menimbang, bahwa berdasarkan kajian filosofis, yang diambil-alih menjadi pendapat Majelis, menyatakan bahwa secara ontologis perkawinan merupakan ikatan lahir-bathin antara seorang laki-laki dan perempuan sebagai suami-isteri. Sedangkan secara aksiologis, perkawinan bertujuan membentuk keluarga sakinah, mawaddah, dan rahmah. Adapun fakta hukum yang terbukti

Disclaimer
Kepaniteraan Mahkamah Agung Republik Indonesia berusaha untuk selalu mencantumkan informasi paling kini dan akurat sebagai bentuk komitmen Mahkamah Agung untuk pelayanan publik, transparansi dan akuntabilitas pelaksanaan fungsi peradilan. Namun dalam hal-hal tertentu masih dimungkinkan terjadi permasalahan teknis terkait dengan akurasi dan keterkinian informasi yang kami sajikan, hal mana akan terus kami perbaiki dari waktu kewaktu.
Dalam hal Anda menemukan inakurasi informasi yang termuat pada situs ini atau informasi yang seharusnya ada, namun belum tersedia, maka harap segera hubungi Kepaniteraan Mahkamah Agung RI melalui :
Email : kepaniteraan@mahkamahagung.go.id   Telp : 021-384 3348 (ext.318)

dalam persidangan perkara *a quo* menunjukkan bahwa rumah tangga antara Penggugat dan Tergugat sudah pecah dan jauh menyimpang dari kondisi ideal ontologi dan aksiologi perkawinan itu sendiri;

Menimbang, bahwa berdasarkan kajian sosiologis, dalam teori peran (*role theory*) yang dicetuskan oleh Robert Linton, yang diambil-alih menjadi pendapat Majelis, menyatakan bahwa dalam sebuah interaksi sosial, hak dan kewajiban masing-masing subjek (suami dan isteri) haruslah dipenuhi secara berimbang. Jika salah satu pihak, dalam konteks ini suami atau isteri sudah tidak mempedulikan hak dan kewajibannya, tentulah kondisi sosial keluarga antara Penggugat dan Tergugat tidak berimbang dan mengalami guncangan (*turbulence*);

Menimbang, bahwa berdasarkan kajian antropologis, dalam penelitian Hedi Sri Ahimsyah Putra yang dituangkan dalam buku berjudul Strukturalisme Levi Straus, Mitos dan Karya Sastra, yang diambil-alih menjadi pendapat Majelis, menyatakan bahwa terdapat keteraturan “struktur luar” (realitas yang tampak secara empiris) dan “struktur dalam” (realitas yang tidak selalu tampak dan dapat mempengaruhi struktur luar). Kontekstualisasi dalam perkara *a quo*, bahwa fakta hukum dalam sidang menunjukkan telah terjadi perpecahan pada “struktur luar” rumah tangga Penggugat dan Tergugat, dan realitas tersebut tentu dipengaruhi oleh “struktur dalam”, yakni perpecahan bathin antara Penggugat dan Tergugat itu sendiri. Sehingga tujuan perkawinan untuk membentuk keluarga sakinah, mawaddah, dan rahmah sulit tercapai;

Menimbang, bahwa berdasarkan pertimbangan tersebut diatas Majelis berpendapat bahwa perkawinan Penggugat dan Tergugat sudah pecah *(broken marriage),* sulit untuk disatukan dalam sebuah rumah tangga yang harmonis, sedangkan upaya Majelis Hakim untuk merukunkan kembali mereka sudah tidak berhasil. Dengan demikian, Majelis berpendapat gugatan Penggugat patut untuk dikabulkan;

Menimbang, bahwa oleh karena gugatan Penggugat telah beralaskan hukum, maka Majelis akan menjatuhkan putusan yang amarnya antara lain menjatuhkan talak satu ba’in shughra dari Tergugat terhadap Penggugat;

Disclaimer
Kepaniteraan Mahkamah Agung Republik Indonesia berusaha untuk selalu mencantumkan informasi paling kini dan akurat sebagai bentuk komitmen Mahkamah Agung untuk pelayanan publik, transparansi dan akuntabilitas pelaksanaan fungsi peradilan. Namun dalam hal-hal tertentu masih dimungkinkan terjadi permasalahan teknis terkait dengan akurasi dan keterkinian informasi yang kami sajikan, hal mana akan terus kami perbaiki dari waktu kewaktu.
Dalam hal Anda menemukan inakurasi informasi yang termuat pada situs ini atau informasi yang seharusnya ada, namun belum tersedia, maka harap segera hubungi Kepaniteraan Mahkamah Agung RI melalui :
Email : kepaniteraan@mahkamahagung.go.id Telp : 021-384 3348 (ext.318)

Menimbang, bahwa oleh karena perkara *a quo* mengenai sengketa di bidang perkawinan, maka sesuai dengan Pasal 89 ayat (1) Undang–undang Nomor 7 Tahun 1989 tentang Peradilan Agama sebagaimana telah diubah dengan Undang-undang Nomor 3 tahun 2006 dan Undang-undang Nomor 50 tahun 2009 maka segala biaya yang timbul dalam perkara ini dibebankan kepada Penggugat;

Menginggat segala ketentuan peraturan perundang-undangan yang berlaku dan hukum syara' yang berkaitan dengan perkara ini;

MENGADILI

1. Menyatakan Tergugat yang telah dipanggil secara resmi dan patut untuk menghadap sidang tidak hadir;
2. Mengabulkan gugatan Penggugat secara Verstek;
3. Menjatuhkan talak satu Ba'in Shughra Tergugat **(**Tergugat**)** terhadap Penggugat **(**Penggugat**)**;
4. Membebankan kepada Penggugat untuk membayar biaya perkara ini sejumlah Rp676.000,00 (enam ratus tujuh puluh enam ribu rupiah);

Demikian putusan ini dijatuhkan dalam musyawarah Majelis Hakim pada hari Rabu, tanggal 23 Desember 2020 Masehi/ 08 Jumadil Awal 1442 Hijriah oleh Ahmad Zainul Anam, S.H.I., M.S.I. selaku Ketua Majelis, Liza, S.Sy. dan Gustomo Try Budiharjo, S.H.I selaku Hakim-hakim Anggota dan putusan tersebut pada hari itu juga diucapkan dalam sidang yang terbuka untuk umum oleh Ketua Majelis, didampingi oleh Hakim-hakim Anggota, dan dibantu oleh Syurya Gusmardi, S.H., selaku Panitera Pengganti, yang dihadiri oleh Penggugat tanpa kehadiran Tergugat;

Hakim Anggota,

Ketua Majelis,

Liza, S.Sy

Ahmad Zainul Anam, S.H.I., M.S.I.

Hakim Anggota,

Disclaimer
Kepaniteraan Mahkamah Agung Republik Indonesia berusaha untuk selalu mencantumkan informasi paling kini dan akurat sebagai bentuk komitmen Mahkamah Agung untuk pelayanan publik, transparansi dan akuntabilitas pelaksanaan fungsi peradilan. Namun dalam hal-hal tertentu masih dimungkinkan terjadi permasalahan teknis terkait dengan akurasi dan keterkinian informasi yang kami sajikan, hal mana akan terus kami perbaiki dari waktu kewaktu.
Dalam hal Anda menemukan inakurasi informasi yang termuat pada situs ini atau informasi yang seharusnya ada, namun belum tersedia, maka harap segera hubungi Kepaniteraan Mahkamah Agung RI melalui :
Email : kepaniteraan@mahkamahagung.go.id Telp : 021-384 3348 (ext.318)

Gustomo Try Budiharjo, S.H.I

Panitera Pengganti,

Syurya Gusmardi, S.H.

Perincian biaya perkara :

1. Biaya Pendaftaran	: Rp30.000,00
2. Biaya Proses	: Rp50.000,00
3. Biaya Panggilan	: Rp560.000,00
4. PNBP Panggilan	: Rp20.000,00
5. Biaya Redaksi	: Rp10.000,00
6. Biaya Meterai	: Rp6.000,00
Jumlah	: Rp676.000

(enam ratus tujuh puluh enam ribu rupiah)

Disclaimer
Kepaniteraan Mahkamah Agung Republik Indonesia berusaha untuk selalu mencantumkan informasi paling kini dan akurat sebagai bentuk komitmen Mahkamah Agung untuk pelayanan publik, transparansi dan akuntabilitas pelaksanaan fungsi peradilan. Namun dalam hal-hal tertentu masih dimungkinkan terjadi permasalahan teknis terkait dengan akurasi dan keterkinian informasi yang kami sajikan, hal mana akan terus kami perbaiki dari waktu kewaktu.
Dalam hal Anda menemukan inakurasi informasi yang termuat pada situs ini atau informasi yang seharusnya ada, namun belum tersedia, maka harap segera hubungi Kepaniteraan Mahkamah Agung RI melalui :
Email : kepaniteraan@mahkamahagung.go.id   Telp : 021-384 3348 (ext.318)
