# Supplementary material for: Classification of divorce causes during the COVID-19 pandemic using convolutional neural networks
Source: PeerJ Comput Sci. 2022 Jun 30;8:e998. doi: 10.7717/peerj-cs.998 (PMC9299239; doi:10.7717/peerj-cs.998)
Supplement: Supplemental Information 5 [file peerj-cs-08-998-s005.zip › Masalah Ekonomi Dataset/Data ke-12.pdf]

P E N E T A P A N

Nomor **2354/Pdt.G/2020/PA.Tmk**

بســـم الله الرحمن الرحــيم

DEMI KEADILAN BERDASARKAN KETUHANAN YANG MAHA ESA

Pengadilan Agama Kota Tasikmalaya yang memeriksa dan mengadili perkara tertentu pada tingkat pertama dalam sidang majelis telah menjatuhkan penetapan dalam perkara cerai gugat antara:

xxxxxx, umur 41 tahun, agama Islam, pekerjaan Mengurus Rumah Tangga, pendidikan D3, tempat kediaman di Kota Tasikmalaya namun sekarang beralamat di Kota Tasikmalaya. Jawa Barat, dalam hal ini memberikan kuasa khusus kepada Asep Iwan Ristiawan, S.H dan Jajang Nurhidayat, SH. dan Asep Supriatna, SH., Advokat/Pengacara yang berkantor di Perum Baitul Marhamah 2 blok E.6 Kota Tasikmalaya berdasarkan surat Kuasa khusus tanggal 09 Desember 2020 yang telah didaftar dalam Register Kuasa Nomor 2648/Reg.K/2020/PA.Tmk tanggal 10 Desember 2020,sebagai **Penggugat**;

melawan

xxxxx, umur 29 tahun, agama Islam, pekerjaan Guru Honorer, tempat kediaman di Kota Tasikmalaya Jawa Barat, sebagai **Tergugat**;

Pengadilan Agama tersebut;

Telah mempelajari berkas perkara;

Telah mendengar keterangan Penggugat serta memeriksa bukti-bukti di persidangan;

DUDUK PERKARA

Bahwa Penggugat dengan surat gugatannya tanggal 10 Desember 2020 telah mengajukan gugatan cerai gugat yang didaftar di Kepaniteraan Pengadilan Agama Kota Tasikmalaya dengan Nomor 2354/Pdt.G/2020/PA.Tmk,

Disclaimer
Kepaniteraan Mahkamah Agung Republik Indonesia berusaha untuk selalu mencantumkan informasi paling kini dan akurat sebagai bentuk komitmen Mahkamah Agung untuk pelayanan publik, transparansi dan akuntabilitas pelaksanaan fungsi peradilan. Namun dalam hal-hal tertentu masih dimungkinkan terjadi permasalahan teknis terkait dengan akurasi dan keterkinian informasi yang kami sajikan, hal mana akan terus kami perbaiki dari waktu kewaktu.
Dalam hal Anda menemukan inakurasi informasi yang termuat pada situs ini atau informasi yang seharusnya ada, namun belum tersedia, maka harap segera hubungi Kepaniteraan Mahkamah Agung RI melalui :
Email : kepaniteraan@mahkamahagung.go.id   Telp : 021-384 3348 (ext.318)

tanggal 10 Desember 2020, dengan dalil-dalil yang pada pokoknya sebagai berikut:

1. Bahwa penggugat dan tergugat telah menikah pada tanggal 14 Juli 2013 di hadapan Petugas Pencatat Nikah Kantor Urusan Agama (KUA) Kecamatan Tamansari Kota Tasikmalaya, sebagaimana tercatat dalam buku kutipan akta nikah nomor: 347/14/VII/2013, tertanggal 14 Juli 2013;
2. Bahwa setelah menikah Penggugat dengan Tergugat pernah merasakan kehidupan berumah tangga yang rukun bahagia dan bergaul layaknya suami istri dan sudah dikaruniai keturunan 1 (satu) orang anak:
3. Bahwa setelah menikah Penggugat dengan tergugat tinggal bersama di Kota Tasikmalaya.
4. Bahwa keharmonisan rumah tangga Penggugat dengan Tergugat bertahan selama kurang lebih 7 (tujuh) tahun lamanya, karena sejak sekitar September 2020 rumah tangga penggugat dan tergugat mulai retak, karena sering terjadi perselisihan dan pertengkaran yang terus menerus yang sulit untuk di damaikan dan keadaan tersebut memuncak pada sekitar November 2020 sehingga menyebabkan pisah selama kurang lebih 1 (satu) bulan lamanya;
5. Bahwa sebab terjadinya perselisihan dan pertengkaran tersebut dikarenakan tergugat tidak ada kesepahaman dalam menjalankan rumah tangga, sehingga penggugat tidak menerima keadaan tersebut;
6. Bahwa usaha untuk mempertahankan kebutuhan rumah tangga antara Penggugat dengan Tergugat telah ditempuh, namun usaha tersebut tidak berhasil;
7. Bahwa selama pisah Penggugat dan sudah Tergugat tidak lagi saling melaksanakan kewajibannya masing-masing sebagai suami-istri;
8. Bahwa keutuhan rumah tangga antara Penggugat dengan Tergugat sudah tidak dapat dipertahankan lagi dan sudah tidak ada harapan berumah tangga dengan tentram dan damai maka Penggugat berketetapan hati untuk mengakhirinya dengan perceraian;

Disclaimer
Kepaniteraan Mahkamah Agung Republik Indonesia berusaha untuk selalu mencantumkan informasi paling kini dan akurat sebagai bentuk komitmen Mahkamah Agung untuk pelayanan publik, transparansi dan akuntabilitas pelaksanaan fungsi peradilan. Namun dalam hal-hal tertentu masih dimungkinkan terjadi permasalahan teknis terkait dengan akurasi dan keterkinian informasi yang kami sajikan, hal mana akan terus kami perbaiki dari waktu kewaktu.
Dalam hal Anda menemukan inakurasi informasi yang termuat pada situs ini atau informasi yang seharusnya ada, namun belum tersedia, maka harap segera hubungi Kepaniteraan Mahkamah Agung RI melalui :
Email : kepaniteraan@mahkamahagung.go.id Telp : 021-384 3348 (ext.318)

9. Bahwa tujuan perkawinan untuk membentuk keluarga yang bahagia sebagaimana yang dimaksud dalam UU. No. 1 tahun 1974 sudah tidak dapat tercapai lagi oleh penggugat dengan tergugat;

10. Bahwa oleh karenanya, Gugatan Penggugat telah memenuhi alasan perceraian sebagaimana diatur dalam pasal 19 hurup (f) PP. No. 9 tahun 1975 jo pasal 116 hurup (f) Kompilasi Hukum Islam, maka sudah selayaknya menurut hukum apabila pengadilan Agama Kota Tasikmalaya mengabulkan gugatan penggugat aquo;

Berdasarkan alasan/dalil-dalil diatas, maka Penggugat mohon kepada Ketua Pengadilan Agama Kota Tasikmalaya cq. Majelis Hakim yang memeriksa dan mengadili perkara ini, berkenan menjatuhkan putusan sebagai berikut :

1. Menerima dan Mengabulkan Gugatan Penggugat;
2. Menjatuhkan Thalaq Satu Bain Sughro Tergugat Terhadap Penggugat;
3. Menetapkan Biaya Perkara Menurut Hukum;

Bahwa dalam perkara aquo, Penggugat telah menguasakan kepada kuasa hukum Asep Iwan Ristiawan, S.H dan Jajang Nurhidayat, SH. dan Asep Supriatna, SH., Advokat dan Penasehat Hukum yang mengambil domisili di Perum Baitul Marhamah 2 blok E.6 Kota Tasikmalaya berdasarkan Surat Kuasa Khusus tertanggal 09 Desember 2020, dan telah terdaftar di Kepaniteraan Pengadilan Agama Kota Tasikmalaya dengan Nomor 2648/Reg.K/2020/PA.Tmk tanggal 10 Desember 2020;

Bahwa, Majelis Hakim telah memeriksa surat Kuasa Penggugat tersebut diatas, dan memeriksa pula Kartu Tanda Pengenal Advokat (KTPA) yang bersangkutan;

Bahwa pada hari sidang yang telah ditetapkan Penggugat diwakili Kuasanya telah datang menghadap ke muka sidang, sedangkan Tergugat tidak datang menghadap ke muka sidang dan tidak menyuruh orang lain untuk menghadap sebagai wakil/kuasa hukumnya meskipun telah dipanggil secara resmi dan patut menurut relaas Nomor 2354/Pdt.G/2020/PA.Tmk tanggal 11 Desember 2020 dan 17 Desember 2020 yang dibacakan di dalam sidang,

Disclaimer
Kepaniteraan Mahkamah Agung Republik Indonesia berusaha untuk selalu mencantumkan informasi paling kini dan akurat sebagai bentuk komitmen Mahkamah Agung untuk pelayanan publik, transparansi dan akuntabilitas pelaksanaan fungsi peradilan. Namun dalam hal-hal tertentu masih dimungkinkan terjadi permasalahan teknis terkait dengan akurasi dan keterkinian informasi yang kami sajikan, hal mana akan terus kami perbaiki dari waktu kewaktu.
Dalam hal Anda menemukan inakurasi informasi yang termuat pada situs ini atau informasi yang seharusnya ada, namun belum tersedia, maka harap segera hubungi Kepaniteraan Mahkamah Agung RI melalui :
Email : kepaniteraan@mahkamahagung.go.id Telp : 021-384 3348 (ext.318)

sedangkan tidak ternyata bahwa tidak datangnya itu disebabkan suatu halangan yang sah;

Bahwa majelis hakim telah menasehati Penggugat melalui Kuasanya agar berpikir untuk tidak bercerai dengan Tergugat, dan atas nasehat majelis hakim Kuasa Penggugat menyatakan memohon kepada majelis hakim mencabut perkaranya;

Selanjutnya untuk singkatnya uraian penetapan ini, maka semua hal yang termuat dalam berita acara sidang ini merupakan bagian yang tidak terpisahkan dari penetapan ini;

PERTIMBANGAN HUKUM

Menimbang, bahwa maksud dan tujuan gugatan Penggugat adalah sebagaimana terurai di atas;

Menimbang, bahwa Majelis Hakim telah memeriksa Surat Kuasa Khusus Penggugat tertanggal 09 Desember 2020, yang telah terdaftar di Kepaniteraan Pengadilan Agama Kota Tasikmalaya dengan Nomor 2648/Reg.K/2020/PA.Tmk tanggal 10 Desember 2020, ternyata telah sesuai dengan ketentuan Pasal 123 HIR jo. Surat Edaran Mahkamah Agung RI Nomor 6 Tahun 1994 tentang Surat Kuasa Khusus, dan Majelis Hakim telah pula memeriksa Kartu Tanda Pengenal Advokat (KTPA) Kuasa Penggugat, dan ternyata telah sesuai dengan Ketentuan Pasal 30 Undang-undang Nomor 18 Tahun 2003 tentang Advokat, oleh karena itu Majelis Hakim harus menyatakan bahwa Kuasa Penggugat merupakan subjek pemberi bantuan hukun yang sah sehingga Majelis Hakim dapat menerima dan memberi izin kepada Kuasa Penggugat tersebut untuk beracara dalam perkara *aquo* sebagai advokat profesional;

Menimbang, bahwa ternyata Tergugat, meskipun dipanggil secara resmi dan patut, tidak datang menghadap di muka sidang dan pula tidak ternyata bahwa tidak datangnya itu disebabkan suatu halangan yang sah;

Menimbang, bahwa sesuai dengan Pasal 39 Undang-undang Nomor 1 Tahun 1974, jo. Pasal 65 Undang-undang Nomor 7 Tahun 1989, sebagaimana telah diubah dengan Undang-undang Nomor 3 Tahun 2006 dan terakhir diubah dengan Undang-undang Nomor 50 Tahun 2009, jo. Pasal 115 Kompilasi Hukum Islam, majelis hakim pada persidangan pertama telah berusaha semaksimal

Disclaimer
Kepaniteraan Mahkamah Agung Republik Indonesia berusaha untuk selalu mencantumkan informasi paling kini dan akurat sebagai bentuk komitmen Mahkamah Agung untuk pelayanan publik, transparansi dan akuntabilitas pelaksanaan fungsi peradilan. Namun dalam hal-hal tertentu masih dimungkinkan terjadi permasalahan teknis terkait dengan akurasi dan keterkinian informasi yang kami sajikan, hal mana akan terus kami perbaiki dari waktu kewaktu.
Dalam hal Anda menemukan inakurasi informasi yang termuat pada situs ini atau informasi yang seharusnya ada, namun belum tersedia, maka harap segera hubungi Kepaniteraan Mahkamah Agung RI melalui :
Email : kepaniteraan@mahkamahagung.go.id   Telp : 021-384 3348 (ext.318)

mungkin menasehati Penggugat melalui Kuasanya untuk bersabar agar bisa membina rumah tangga dengan rukun dan harmonis dan ternyata berhasil;

Menimbang, bahwa Kuasa Penggugat di muka sidang menyatakan memohon mencabut perkaranya dengan alasan Penggugat akan kembali rukun dengan Tergugat;

Menimbang, bahwa pencabutan perkara oleh Penggugat tersebut dilakukan sebelum pemeriksaan pokok perkara, maka sesuai dengan Pasal 271-272 Rv permohonan Penggugat untuk mencabut perkara tersebut dapat dibenarkan secara hukum, karenanya permohonan tersebut harus dikabulkan;

Menimbang, bahwa oleh karena permohonan dicabut dan perkara ini telah didaftar dalam register perkara, maka Majelis Hakim memandang perlu memerintahkan Panitera untuk mencatat pencabutan perkara tersebut dalam register perkara dalam kolom keterangan;

Menimbang, bahwa meskipun gugatan Penggugat dicabut, oleh karena perkara ini telah didaftar dalam register perkara dan termasuk dalam bidang perkawinan, maka sesuai Pasal 89 Undang-Undang Nomor 7 Tahun 1989 tentang Peradilan Agama, sebagaimana diubah dengan Undang-Undang Nomor 3 Tahun 2006 dan perubahan kedua dengan Undang-undang Nomor 50 Tahun 2009, maka biaya perkara dibebankan kepada kepada Penggugat;

Mengingat, semua pasal dalam peraturan perundang-undangan dan hukum Islam yang berkaitan dengan perkara ini;

M E N G A D I L I

1. Menyatakan perkara Nomor 2354/Pdt.G/2019/PA.Tmk, gugur.
2. Membebankan biaya perkara kepada Penggugat sejumlah Rp 351000,00 (tiga ratus lima puluh satu ribu rupiah);

Demikian ditetapkan dalam rapat permusyawaratan Majelis Hakim yang dilangsungkan pada hari Rabu tanggal 23 Desember 2020 *Masehi*, bertepatan dengan tanggal 8 Jumadil Awwal 1442 *Hijriyah*, oleh kami Marwan Ibrahim Piinga, S.Ag. sebagai Ketua Majelis, Ahmad Mudlofar, S.H.I. dan Ahmad Mufid Bisri, S.H.I. masing-masing sebagai Hakim Anggota, penetapan tersebut diucapkan dalam sidang terbuka untuk umum pada hari itu juga, oleh Ketua Majelis tersebut dengan didampingi oleh Hakim Anggota yang sama dan

Disclaimer
Kepaniteraan Mahkamah Agung Republik Indonesia berusaha untuk selalu mencantumkan informasi paling kini dan akurat sebagai bentuk komitmen Mahkamah Agung untuk pelayanan publik, transparansi dan akuntabilitas pelaksanaan fungsi peradilan. Namun dalam hal-hal tertentu masih dimungkinkan terjadi permasalahan teknis terkait dengan akurasi dan keterkinian informasi yang kami sajikan, hal mana akan terus kami perbaiki dari waktu kewaktu.
Dalam hal Anda menemukan inakurasi informasi yang termuat pada situs ini atau informasi yang seharusnya ada, namun belum tersedia, maka harap segera hubungi Kepaniteraan Mahkamah Agung RI melalui :
Email : kepaniteraan@mahkamahagung.go.id Telp : 021-384 3348 (ext.318)

dibantu oleh Mamat Rakhmat, S.H.I. sebagai Panitera Pengganti serta dihadiri oleh Kuasa Penggugat tanpa hadirnya Tergugat;

Hakim Anggota	Ketua Majelis
**Ahmad Mudlofar, S.H.I.**	**Marwan Ibrahim Piinga, S.Ag.**
Hakim Anggota	
**Ahmad Mufid Bisri, S.H.I.**	
	Panitera Pengganti
	**Mamat Rakhmat, S.H.I.**

Perincian Biaya:

1. Pendaftaran	:	Rp.	30.000,-
2. Proses	:	Rp.	60.000,-
3. Panggilan	:	Rp.	225.000,-
4. PNBP	:	Rp.	20.000,-
5. Redaksi	:	Rp.	10.000,-
6. Meterai	:	Rp.	6.000,-
Jumlah		Rp.	351.000,-

(tiga ratus lima puluh satu ribu rupiah)

Disclaimer
Kepaniteraan Mahkamah Agung Republik Indonesia berusaha untuk selalu mencantumkan informasi paling kini dan akurat sebagai bentuk komitmen Mahkamah Agung untuk pelayanan publik, transparansi dan akuntabilitas pelaksanaan fungsi peradilan. Namun dalam hal-hal tertentu masih dimungkinkan terjadi permasalahan teknis terkait dengan akurasi dan keterkinian informasi yang kami sajikan, hal mana akan terus kami perbaiki dari waktu kewaktu.
Dalam hal Anda menemukan inakurasi informasi yang termuat pada situs ini atau informasi yang seharusnya ada, namun belum tersedia, maka harap segera hubungi Kepaniteraan Mahkamah Agung RI melalui :
Email : kepaniteraan@mahkamahagung.go.id Telp : 021-384 3348 (ext.318)
