# Supplementary material for: Classification of divorce causes during the COVID-19 pandemic using convolutional neural networks
Source: PeerJ Comput Sci. 2022 Jun 30;8:e998. doi: 10.7717/peerj-cs.998 (PMC9299239; doi:10.7717/peerj-cs.998)
Supplement: Supplemental Information 5 [file peerj-cs-08-998-s005.zip › Masalah Ekonomi Dataset/Data ke-13.pdf]

P U T U S A N

Nomor 869/Pdt.G/2020/PA.Ppg

بسم الله الرحمن الرحيم

DEMI KEADILAN BERDASARKAN KETUHANAN YANG MAHA ESA

Pengadilan Agama Pasir Pengaraian yang memeriksa dan mengadili perkara-perkara tertentu pada peradilan tingkat pertama dalam sidang Majelis telah menjatuhkan putusan sebagai berikut dalam perkara Cerai Gugat antara:

Penggugat, umur 30 tahun, agama Islam, pendidikan SLTP, pekerjaan mengurus rumah tangga, Kabupaten Rokan Hulu, sebagai **Penggugat**;

melawan

Tergugat, umur 42 tahun, agama Islam, pendidikan SLTA, pekerjaan Dagang, tempat tinggal di Kabupaten Rokan Hulu, sebagai **Tergugat**;

Pengadilan Agama tersebut;

Setelah membaca semua surat dalam perkara ini;

Setelah mendengar keterangan Penggugat dan memeriksa bukti-bukti di persidangan;

DUDUK PERKARA

Bahwa, Penggugat dalam surat gugatan tertanggal 14 Desember 2020 yang telah terdaftar di Kepaniteraan Pengadilan Agama Pasir Pengaraian pada tanggal 17 Desember 2020 dengan Nomor Register 869/Pdt.G/2020/PA.Ppg telah mengemukakan hal-hal sebagai berikut:

1. Bahwa pada tanggal 19 Juni 2010 Penggugat dan Tergugat melangsungkan pernikahan yang dicatat oleh Pegawai Pencatat Nikah Kantor Urusan Agama Kecamatan Tanjung Pura, Kabupaten Langkat, Propinsi Sumatera Utara, sebagaimana tertera dari Kutipan Akta Nikah Nomor : 388/47/VI/2020 tanggal 21 Juni 2010;

Disclaimer
Kepaniteraan Mahkamah Agung Republik Indonesia berusaha untuk selalu mencantumkan informasi paling kini dan akurat sebagai bentuk komitmen Mahkamah Agung untuk pelayanan publik, transparansi dan akuntabilitas pelaksanaan fungsi peradilan. Namun dalam hal-hal tertentu masih dimungkinkan terjadi permasalahan teknis terkait dengan akurasi dan keterkinian informasi yang kami sajikan, hal mana akan terus kami perbaiki dari waktu kewaktu.
Dalam hal Anda menemukan inakurasi informasi yang termuat pada situs ini atau informasi yang seharusnya ada, namun belum tersedia, maka harap segera hubungi Kepaniteraan Mahkamah Agung RI melalui :
Email : kepaniteraan@mahkamahagung.go.id   Telp : 021-384 3348 (ext.318)

2. Bahwa setelah menikah Penggugat dan Tergugat kumpul baik dan tinggal di rumah orangtua Tergugat di Langkat selama 1 minggu, terakahir pindah di rumah kediaman bersama di Desa Suka Damai;
3. Bahwa selama pernikahan antara Penggugat dan Tergugat telah hidup rukun sebagaimana layaknya suami istri (ba'da dukhul) dan telah dikaruniai 2 orang anak, bernama yaitu :
   1. anak (perempuan) umur 9 tahun;
   2. anak (laki-laki) umur 7 tahun, anak tersebut sekarang berada dibawah asuhan keluarga Tergugat;
4. Bahwa kehidupan rumah tangga Penggugat dan Tergugat pada awalnya berlangsung harmonis, akan tetapi sejak 2 tahun sesudah menikah antara Penggugat dan Tergugat mulai terjadi perselisihan dan pertengkaran dalam rumah tangga yang disebabkan yaitu :
   a. Tergugat kurang memberi nafkah kepada Penggugat;
   b. Tergugat memakai narkoba;
5. Bahwa Penggugat telah berupaya mengingatkan Tergugat agar Tergugat mengubah sikapnya untuk tidak melakukan perbuatannya pada poin di atas, akan tetapi Tergugat tidak terima, sehingga pertengkaran terus terjadi;
6. Bahwa puncak dari perselisihan dan pertengkaran tersebut terjadi pada tanggal 27 Desember 2014 yang akhirnya menyebabkan antara Penggugat dengan Tergugat telah pisah rumah dan yang pergi meninggalkan kediaman bersama adalah Tergugat, karenan ada perselisihan dan pertengkaran antara Penggugat dan Tergugat, dan Tergugat telah menajtuhkan talak kepada Penggugat;
7. Bahwa atas permasalahan rumah tangga Penggugat dan Tergugat pihak keluarga telah berupaya mendamaikan, namun tidak berhasil;

Bahwa berdasarkan alasan-alasan tersebut di atas, maka Penggugat mohon kepada Bapak Ketua Pengadilan Agama Pasir Pengaraian melalui Majelis Hakim yang memeriksa perkara ini, kiranya berkenan menerima, memeriksa dan mengadili serta memutuskan sebagai berikut:

1. Mengabulkan gugatan Penggugat;

Disclaimer
Kepaniteraan Mahkamah Agung Republik Indonesia berusaha untuk selalu mencantumkan informasi paling kini dan akurat sebagai bentuk komitmen Mahkamah Agung untuk pelayanan publik, transparansi dan akuntabilitas pelaksanaan fungsi peradilan. Namun dalam hal-hal tertentu masih dimungkinkan terjadi permasalahan teknis terkait dengan akurasi dan keterkinian informasi yang kami sajikan, hal mana akan terus kami perbaiki dari waktu kewaktu.
Dalam hal Anda menemukan inakurasi informasi yang termuat pada situs ini atau informasi yang seharusnya ada, namun belum tersedia, maka harap segera hubungi Kepaniteraan Mahkamah Agung RI melalui :
Email : kepaniteraan@mahkamahagung.go.id Telp : 021-384 3348 (ext.318)

2. Menjatuhkan talak satu ba'in shughra Tergugat **(Tergugat)** terhadap Penggugat **(Penggugat);**
3. Membebankan seluruh biaya perkara ini sesuai dengan peraturan dan perundang-undangan yang berlaku.

Dan Atau, Apabila Majelis Hakim bependapat lain, mohon putusan yang seadil-adilnya;

Bahwa, pada hari-hari persidangan yang telah ditetapkan, Penggugat telah datang menghadap sendiri di persidangan, sedangkan Tergugat tidak pernah datang menghadap di persidangan dan tidak pula mengutus orang lain sebagai wakil atau kuasanya yang sah, meskipun telah dipanggil secara resmi dan patut;

Bahwa, Majelis Hakim telah berusaha mendamaikan dengan cara menasihati Penggugat agar rukun kembali dalam membina rumah tangga bersama Tergugat, namun tidak berhasil. Adapun upaya mediasi sebagaimana yang diamanatkan Perma No. 1 tahun 2016 tidak dapat dilaksanakan karena Tergugat tidak pernah datang menghadap pada hari-hari persidangan yang telah ditetapkan;

Bahwa, pemeriksaan terhadap perkara ini dilanjutkan dengan membacakan gugatan Penggugat yang dalil-dalilnya tetap dipertahankan oleh Penggugat;

Bahwa, untuk menguatkan dalil-dalil gugatan Penggugat, Penggugat menyerahkan bukti tertulis berupa:

1. Fotokopi Kutipan Akta Nikah Nomor 388/47/VI/2020, an. Tergugat dan Penggugat yang aslinya dikeluarkan oleh Kantor Urusan Agama Kecamatan Tanjung Pura, Kabupaten Langkat, Propinsi Sumatera Utara,, tanggal 21 Juni 2010, bermeterai cukup, telah *dinazegelen* dan telah dicocokkan dengan aslinya (P.1)

2. Asli Salinan Putusan atas nama Nomor 246/Pid.Sus/2020/PN Prp tanggal 14 September 2020 yang dikeluarkan oleh Pengadilan Negeri Pasir Pengaraian, bukti surat tersebut telah diberi meterai cukup, namun tidak dicap pos, lalu oleh Ketua Majelis diberi tanda P2;

Disclaimer
Kepaniteraan Mahkamah Agung Republik Indonesia berusaha untuk selalu mencantumkan informasi paling kini dan akurat sebagai bentuk komitmen Mahkamah Agung untuk pelayanan publik, transparansi dan akuntabilitas pelaksanaan fungsi peradilan. Namun dalam hal-hal tertentu masih dimungkinkan terjadi permasalahan teknis terkait dengan akurasi dan keterkinian informasi yang kami sajikan, hal mana akan terus kami perbaiki dari waktu kewaktu.
Dalam hal Anda menemukan inakurasi informasi yang termuat pada situs ini atau informasi yang seharusnya ada, namun belum tersedia, maka harap segera hubungi Kepaniteraan Mahkamah Agung RI melalui :
Email : kepaniteraan@mahkamahagung.go.id Telp : 021-384 3348 (ext.318)

Bahwa, selain bukti tertulis tersebut, Penggugat telah menghadirkan saksi-saksi di persidangan sebagai berikut:

1. Saksi I, umur 28 tahun, agama Islam, pendidikan SLTP, pekerjaan Wiraswasta, tempat tinggal di Kabupaten Rokan Hulu, Saksi adalah Keponakan Penggugat, telah memberikan keterangan di bawah sumpah yang pada pokoknya sebagai berikut :

- Bahwa hubungan Penggugat dengan Tergugat adalah suami isteri;
- Bahwa Penggugat dan Tergugat menikah sekitar tahun 2010;
- Bahwa setelah menikah Penggugat dan Tergugat tinggal di rumah orangtua Tergugat di Langkat selama 1 minggu, terakahir pindah di rumah kediaman bersama di Desa Suka Damai;
- Bahwa Penggugat dan Tergugat telah dikaruniai dua orang anak, anak tersebut sekarang berada dibawah asuhan Penggugat;
- Bahwa pada awalnya rumah tangga Penggugat dan Tergugat baik dan harmonis, akan tetapi sejak 2 tahun sesudah menikah, tidak harmonis lagi. Penggugat dan Tergugat telah pisah rumah;
- Bahwa Saksi pernah melihat dan mendengar Penggugat dan Tergugat bertengkar;
- Bahwa Penggugat dan Tergugat bertengkar karena Tergugat kurang memberi nafkah kepada Penggugat dan Tergugat memakai narkoba;
- Bahwa Penggugat dan Tergugat telah pisah rumah sejak Desember 2014 yang lalu, Penggugat telah pergi dari kediaman bersama;
- Bahwa Penggugat dan Tergugat sudah didamaikan oleh keluarga, tetapi tidak berhasil;
- Bahwa Saksi tidak mampu mendamaikan Penggugat dan Tergugat;

2. Saksi II, umur 47 tahun, agama Islam, pendidikan SD, pekerjaan Mengurus Rumah Tangga, tempat kediaman di Kabupaten Rokan Hulu,

Disclaimer
Kepaniteraan Mahkamah Agung Republik Indonesia berusaha untuk selalu mencantumkan informasi paling kini dan akurat sebagai bentuk komitmen Mahkamah Agung untuk pelayanan publik, transparansi dan akuntabilitas pelaksanaan fungsi peradilan. Namun dalam hal-hal tertentu masih dimungkinkan terjadi permasalahan teknis terkait dengan akurasi dan keterkinian informasi yang kami sajikan, hal mana akan terus kami perbaiki dari waktu kewaktu.
Dalam hal Anda menemukan inakurasi informasi yang termuat pada situs ini atau informasi yang seharusnya ada, namun belum tersedia, maka harap segera hubungi Kepaniteraan Mahkamah Agung RI melalui :
Email : kepaniteraan@mahkamahagung.go.id Telp : 021-384 3348 (ext.318)

Saksi adalah tetangga Penggugat, telah memberikan keterangan di bawah sumpah yang pada pokoknya sebagai berikut:

- Bahwa hubungan Penggugat dengan Tergugat adalah suami isteri;
- Bahwa Penggugat dan Tergugat menikah sekitar tahun 2010;
- Bahwa setelah menikah Penggugat dan Tergugat tinggal di rumah orangtua Tergugat di Langkat selama 1 minggu, terakahir pindah di rumah kediaman bersama di Desa Suka Damai;
- Bahwa Penggugat dan Tergugat telah dikaruniai dua orang anak, anak tersebut sekarang berada dibawah asuhan Penggugat;
- Bahwa pada awalnya rumah tangga Penggugat dan Tergugat baik dan harmonis, akan tetapi sejak 2 tahun sesudah menikah, tidak harmonis lagi. Penggugat dan Tergugat telah pisah rumah;
- Bahwa Saksi pernah melihat dan mendengar Penggugat dan Tergugat bertengkar;
- Bahwa Penggugat dan Tergugat bertengkar karena Tergugat kurang memberi nafkah kepada Penggugat dan Tergugat memakai narkoba;
- Bahwa Penggugat dan Tergugat telah pisah rumah sejak Desember 2014 yang lalu, Tergugat telah pergi dari kediaman bersama;
- Bahwa Penggugat dan Tergugat sudah didamaikan oleh keluarga, tetapi tidak berhasil;
- Bahwa Saksi tidak mampu mendamaikan Penggugat dan Tergugat;

Bahwa, atas keterangan kedua orang saksi tersebut Penggugat tidak mengajukan pertanyaan tambahan;

Bahwa, Penggugat telah menyampaikan kesimpulannya secara lisan di persidangan, yang pada pokoknya menyatakan tetap dengan gugatan Penggugat dan mohon kepada Majelis Hakim mengabulkan gugatan Penggugat;

Disclaimer
Kepaniteraan Mahkamah Agung Republik Indonesia berusaha untuk selalu mencantumkan informasi paling kini dan akurat sebagai bentuk komitmen Mahkamah Agung untuk pelayanan publik, transparansi dan akuntabilitas pelaksanaan fungsi peradilan. Namun dalam hal-hal tertentu masih dimungkinkan terjadi permasalahan teknis terkait dengan akurasi dan keterkinian informasi yang kami sajikan, hal mana akan terus kami perbaiki dari waktu kewaktu.
Dalam hal Anda menemukan inakurasi informasi yang termuat pada situs ini atau informasi yang seharusnya ada, namun belum tersedia, maka harap segera hubungi Kepaniteraan Mahkamah Agung RI melalui :
Email : kepaniteraan@mahkamahagung.go.id Telp : 021-384 3348 (ext.318)

Bahwa, untuk mempersingkat uraian dalam putusan ini, Majelis Hakim cukup menunjuk kepada berita acara sidang yang merupakan bagian yang tidak terpisahkan dari putusan ini;

PERTIMBANGAN HUKUM

Menimbang, bahwa maksud dan tujuan gugatan Penggugat adalah sebagaimana telah diuraikan dalam duduk perkara;

Menimbang, bahwa Penggugat dalam gugatannya mendalilkan bahwa Penggugat dengan Tergugat telah melangsungkan perkawinan dan telah tercatat secara resmi sebagaimana termaktub dalam Kutipan Akta Nikah Nomor 388/47/VI/2020, tanggal 21 Juni 2010, oleh karena itu berdasarkan Pasal 73 Ayat (1) Undang-Undang Nomor 7 Tahun 1989 tentang Peradilan Agama sebagaimana telah diubah dengan Undang-Undang Nomor 3 Tahun 2006 dan perubahan kedua dengan Undang-Undang Nomor 50 Tahun 2009, Penggugat memiliki *legal standing* untuk mengajukan perkara *a quo*;

Menimbang, bahwa perkawinan antara Penggugat dan Tergugat dilaksanakan menurut syari'at Islam, maka berdasarkan ketentuan Pasal 40 dan Pasal 63 Ayat (1) Huruf (a) Undang-Undang Nomor 1 Tahun 1974 tentang Perkawinan *juncto* Pasal 49 (ayat 1 huruf a) Undang-Undang Nomor 7 tahun 1989 tentang Peradilan Agama yang telah diubah dengan Undang-Undang Nomor 3 Tahun 2006 dan perubahan kedua dengan Undang-Undang Nomor 50 Tahun 2009, *juncto* Pasal 14 dan Pasal 1 huruf (b) Peraturan Pemerintah No. 9 tahun 1975 tentang Pelaksanaan Undang-Undang No. 1 tahun 1974 tentang Perkawinan, maka perkara ini menjadi kewenangan absolut Pengadilan Agama;

Menimbang, bahwa berdasarkan gugatan Penggugat, ternyata Penggugat bertempat tinggal di wilayah hukum Pengadilan Agama Pasir Pengaraian, maka berdasarkan Pasal 73 Ayat (1) Undang-Undang Nomor 7 Tahun 1989 tentang Peradilan Agama sebagaimana telah diubah dengan Undang-Undang Nomor 3 Tahun 2006 dan perubahan kedua dengan Undang-Undang Nomor 50 Tahun 2009 *juncto* Pasal 129 Kompilasi Hukum Islam (KHI),

Disclaimer
Kepaniteraan Mahkamah Agung Republik Indonesia berusaha untuk selalu mencantumkan informasi paling kini dan akurat sebagai bentuk komitmen Mahkamah Agung untuk pelayanan publik, transparansi dan akuntabilitas pelaksanaan fungsi peradilan. Namun dalam hal-hal tertentu masih dimungkinkan terjadi permasalahan teknis terkait dengan akurasi dan keterkinian informasi yang kami sajikan, hal mana akan terus kami perbaiki dari waktu kewaktu.
Dalam hal Anda menemukan inakurasi informasi yang termuat pada situs ini atau informasi yang seharusnya ada, namun belum tersedia, maka harap segera hubungi Kepaniteraan Mahkamah Agung RI melalui :
Email : kepaniteraan@mahkamahagung.go.id Telp : 021-384 3348 (ext.318)

pemeriksaan perkara *a quo* menjadi kewenangan relatif Pengadilan Agama Pasir Pengaraian;

Menimbang, bahwa untuk memenuhi perintah Pasal 82 ayat (1) Undang-undang Nomor 7 tahun 1989 tentang Peradilan Agama yang telah diubah dengan Undang-undang Nomor 3 Tahun 2006 dan Undang-undang Nomor 50 Tahun 2009, Majelis Hakim telah berusaha mendamaikan agar Penggugat dan Tergugat rukun kembali dengan cara menasihati Penggugat, namun tidak berhasil. Selanjutnya oleh karena Tergugat tidak pernah datang dalam persidangan maka proses mediasi sesuai Perma nomor 1 tahun 2016 tidak dapat dilaksanakan;

Menimbang, bahwa dalam gugatannya, Penggugat pada pokoknya mohon kepada Pengadilan Agama untuk menjatuhkan talak satu ba'in sughra Tergugat terhadap Penggugat, dengan dalil-dalil sebagaimana termaktub dalam gugatan Penggugat;

Menimbang, bahwa berdasarkan gugatan Penggugat dan keterangan saksi-saksi dalam persidangan, dapat disimpulkan bahwa hal yang menjadi pokok sengketa gugatan Penggugat adalah adanya perselisihan dan pertengkaran yang terus-menerus antara Penggugat dan Tergugat yang disebabkan karena:

a. Tergugat kurang memberi nafkah kepada Penggugat;
b. Tergugat memakai narkoba;

Sehingga akibatnya antara Penggugat dan Tergugat berpisah tempat tinggal tanpa saling peduli sejak bulan tahun 2014 hingga saat ini dan tidak ada harapan untuk rukun lagi dalam rumah tangga;

Menimbang, bahwa terhadap gugatan Penggugat tersebut, Tergugat tidak pernah hadir, tidak pula menyuruh orang lain untuk menghadap di persidangan sebagai wakil atau kuasanya yang sah, sehingga Tergugat tidak dapat didengar keterangannya, meskipun Tergugat telah dipanggil secara resmi dan patut sebagaimana ketentuan Pasal 26 Peraturan Pemerintah Nomor 9 Tahun 1975, sedang ketidakhadirannya tersebut bukan disebabkan oleh suatu halangan yang sah. Oleh karena itu, berdasarkan Pasal 149 R.Bg., perkara ini dapat diputus secara verstek;

Disclaimer
Kepaniteraan Mahkamah Agung Republik Indonesia berusaha untuk selalu mencantumkan informasi paling kini dan akurat sebagai bentuk komitmen Mahkamah Agung untuk pelayanan publik, transparansi dan akuntabilitas pelaksanaan fungsi peradilan. Namun dalam hal-hal tertentu masih dimungkinkan terjadi permasalahan teknis terkait dengan akurasi dan keterkinian informasi yang kami sajikan, hal mana akan terus kami perbaiki dari waktu kewaktu.
Dalam hal Anda menemukan inakurasi informasi yang termuat pada situs ini atau informasi yang seharusnya ada, namun belum tersedia, maka harap segera hubungi Kepaniteraan Mahkamah Agung RI melalui :
Email : kepaniteraan@mahkamahagung.go.id Telp : 021-384 3348 (ext.318)

Menimbang, bahwa untuk meneguhkan dalil-dalil gugatannya, sesuai dengan ketentuan Pasal 283 R.Bg., Penggugat mengajukan bukti tertulis berupa P.1

Menimbang, bahwa bukti P.1 merupakan akta autentik dengan nilai kekuatan pembuktian sempurna dan mengikat *(volledig en bindende bewijskracht)*, sesuai dengan aslinya, bermeterai cukup, dan telah *dinazegelen*, maka berdasarkan ketentuan Pasal 284 dan 285 R.Bg. *juncto* Pasal 2 Ayat (1) Undang-Undang No. 13 Tahun 1985 tentang Bea Meterai *juncto* Pasal 2 Ayat (1) Peraturan Pemerintah Nomor 24 Tahun 2000 tentang Perubahan Tarif Bea Meterai dan Besarnya Batas Pengenaan Harga Nominal yang Dikenakan Bea Materai, bukti-bukti tersebut secara formil dapat diterima sebagai alat bukti;

Menimbang, bahwa bukti P.2 merupakan akta autentik, bermeterai cukup, namun tidak dinazegelen. Meskipun bukti tersebut tidak dinazegelen, oleh karena bukti tersebut bersesuaian dengan keterangan saksi, sehingga Majelis Hakim berpendapat bukti tersebut dapat dipertimbangkan lebih lanjut;

Menimbang, bahwa berdasarkan bukti P.1, berupa fotokopi Kutipan Akta Nikah, berdasarkan ketentuan Pasal 2 Undang-Undang No. 1 Tahun 1974, *juncto* Pasal 4, 5 dan 6 ayat (1) Kompilasi Hukum Islam (KHI), terbukti bahwa Penggugat dan Tergugat telah terikat dalam perkawinan yang sah;

Menimbang, bahwa berdasarkan bukti P.1 yang dikuatkan oleh keterangan saksi-saksi, dapat dinyatakan terbukti bahwa Termohon terlibat kasus pidana narkotika;

Menimbang, bahwa karena alasan gugatan Penggugat didasarkan pada terjadinya perselisihan dan pertengkaran sebagaimana dimaksud dalam Pasal 19 huruf (f) Peraturan Pemerintah Nomor 9 Tahun 1975 *juncto* Pasal 116 Huruf (f) Kompilasi Hukum Islam, maka untuk memenuhi maksud Pasal 22 ayat (2) Peraturan Pemerintah Nomor 9 Tahun 1975 *juncto* Pasal 134 Kompilasi Hukum Islam, Majelis Hakim wajib terlebih dahulu mendengar keterangan dari keluarga Penggugat dan Tergugat atau orang terdekat kedua belah pihak;

Menimbang, bahwa Penggugat telah menghadirkan 2 (dua) orang dekat sebagai saksi dalam persidangan yang bernama Saksi I dan Saksi II. Kedua saksi tersebut di bawah sumpahnya memberikan keterangan sebagaimana

Disclaimer
Kepaniteraan Mahkamah Agung Republik Indonesia berusaha untuk selalu mencantumkan informasi paling kini dan akurat sebagai bentuk komitmen Mahkamah Agung untuk pelayanan publik, transparansi dan akuntabilitas pelaksanaan fungsi peradilan. Namun dalam hal-hal tertentu masih dimungkinkan terjadi permasalahan teknis terkait dengan akurasi dan keterkinian informasi yang kami sajikan, hal mana akan terus kami perbaiki dari waktu kewaktu.
Dalam hal Anda menemukan inakurasi informasi yang termuat pada situs ini atau informasi yang seharusnya ada, namun belum tersedia, maka harap segera hubungi Kepaniteraan Mahkamah Agung RI melalui :
Email : kepaniteraan@mahkamahagung.go.id Telp : 021-384 3348 (ext.318)

yang termaktub dalam duduk perkara, keterangan lengkap saksi-saksi tersebut *mutatis-mutandis* dianggap terulang dalam pertimbangan ini, yang pada pokoknya secara jelas mendukung dalil-dalil gugatan Penggugat;

Menimbang, bahwa kesaksian yang diberikan saksi-saksi Penggugat disampaikan di bawah sumpah dengan secara bergilir dan terpisah, didasarkan atas pengetahuannya apa yang dilihat, didengar, dan dialami sendiri, saling bersesuaian, serta tidak termasuk orang yang dilarang menjadi saksi, sehingga saksi tersebut memenuhi syarat formil maupun materiil suatu kesaksian sebagaimana yang ditentukan Pasal 171, 172, 175, 307, dan 308 R.Bg. Oleh sebab itu, keterangan saksi-saksi tersebut dapat diterima sebagai alat bukti yang mempunyai nilai kekuatan pembuktian;

Menimbang, bahwa berdasarkan gugatan Penggugat, bukti surat, serta keterangan saksi-saksi yang telah dipertimbangkan tersebut di atas, Majelis Hakim dapat menemukan dan menyimpulkan fakta-fakta sebagai berikut:

1. Penggugat dan Tergugat adalah suami-isteri sah, semula hidup rukun dan harmonis;
2. Sejak bulan Juni 2012 keharmonisan rumah tangga Penggugat dan Tergugat tidak dapat dipertahankan, karena terjadi perselisihan dan pertengkaran yang terus-menerus;
3. Penyebab perselisihan dan pertengkaran tersebut adalah karena Tergugat kurang memberi nafkah kepada Penggugat dan Tergugat memakai narkoba;
4. Bahwa Penggugat dan Tergugat telah berpisah rumah dan tidak saling memedulikan lagi sejak bulan Desember 2014, Tergugat pergi meninggalkan tempat kediaman bersama;
5. Pihak keluarga telah berusaha mendamaikan Penggugat dan Tergugat, namun tidak berhasil;

Menimbang, bahwa menurut Pasal 19 huruf (f) Peraturan Pemerintah Nomor 9 Tahun 1975 *juncto* Pasal 116 huruf (f) Kompilasi Hukum Islam ditegaskan bahwa salah satu alasan perceraian yaitu adanya perselisihan dan pertengkaran yang terus-menerus, selanjutnya Pasal 39 Undang-Undang Nomor 1 Tahun 1974 tentang Perkawinan menentukan bahwa untuk melakukan

Disclaimer
Kepaniteraan Mahkamah Agung Republik Indonesia berusaha untuk selalu mencantumkan informasi paling kini dan akurat sebagai bentuk komitmen Mahkamah Agung untuk pelayanan publik, transparansi dan akuntabilitas pelaksanaan fungsi peradilan. Namun dalam hal-hal tertentu masih dimungkinkan terjadi permasalahan teknis terkait dengan akurasi dan keterkinian informasi yang kami sajikan, hal mana akan terus kami perbaiki dari waktu kewaktu.
Dalam hal Anda menemukan inakurasi informasi yang termuat pada situs ini atau informasi yang seharusnya ada, namun belum tersedia, maka harap segera hubungi Kepaniteraan Mahkamah Agung RI melalui :
Email : kepaniteraan@mahkamahagung.go.id   Telp : 021-384 3348 (ext.318)

suatu perceraian harus cukup alasan, yakni ketika suami-isteri tidak akan dapat hidup rukun sebagai suami isteri dan Pengadilan telah berusaha dan tidak berhasil mendamaikan kedua belah pihak;

Menimbang, bahwa dari ketentuan pasal-pasal tersebut, terdapat beberapa unsur yang harus dipenuhi untuk terjadinya sebuah perceraian:

1. Adanya alasan telah terjadinya perselisihan dan pertengkaran yang terus-menerus;
2. Perselisihan dan pertengkaran menyebabkan suami-isteri sudah tidak ada harapan untuk rukun kembali;
3. Pengadilan telah berupaya mendamaikan kedua belah pihak, namun tidak berhasil;

Menimbang, bahwa unsur-unsur tersebut akan dipertimbangkan satu persatu dengan mengaitkan fakta-fakta yang terjadi dalam rumah tangga Penggugat dan Tergugat, sehingga antara Penggugat dan Tergugat dipandang telah memenuhi unsur-unsur terjadinya suatu perceraian;

Menimbang, bahwa berdasarkan fakta hukum yang telah dipertimbangkan di atas, terbukti antara Penggugat dan Tergugat terus-menerus terjadi perselisihan dan pertengkaran yang disebabkan karena Tergugat kurang memberi nafkah kepada Penggugat dan Tergugat memakai narkoba;

Menimbang, bahwa akibat dari perselisihan dan pertengkaran tersebut, antara Penggugat dan Tergugat berpisah tempat tinggal. Fakta pisah tempat tinggal antara Penggugat dengan Tergugat bukan merupakan upaya sementara untuk meredam konflik yang mereka hadapi, melainkan sudah merupakan akibat dari konflik yang berkelanjutan dan menunjukkan peningkatan kualitas perselisihan dan pertengkaran di antara Penggugat dan Tergugat;

Menimbang, bahwa sesuai dengan Yurisprudensi Mahkamah Agung Nomor 379/ K/AG/1995 tanggal 26 Maret 1997, yang diambil-alih menjadi pendapat Majelis Hakim, suami isteri yang tidak bertempat tinggal serumah lagi dan tidak ada harapan untuk hidup rukun kembali, maka rumah tangga tersebut telah terbukti retak dan pecah dan telah memenuhi alasan cerai Pasal 19 huruf f Peraturan Pemerintah Nomor 9 Tahun 1975 *juncto* Pasal 116 huruf f

Disclaimer
Kepaniteraan Mahkamah Agung Republik Indonesia berusaha untuk selalu mencantumkan informasi paling kini dan akurat sebagai bentuk komitmen Mahkamah Agung untuk pelayanan publik, transparansi dan akuntabilitas pelaksanaan fungsi peradilan. Namun dalam hal-hal tertentu masih dimungkinkan terjadi permasalahan teknis terkait dengan akurasi dan keterkinian informasi yang kami sajikan, hal mana akan terus kami perbaiki dari waktu kewaktu.
Dalam hal Anda menemukan inakurasi informasi yang termuat pada situs ini atau informasi yang seharusnya ada, namun belum tersedia, maka harap segera hubungi Kepaniteraan Mahkamah Agung RI melalui :
Email : kepaniteraan@mahkamahagung.go.id Telp : 021-384 3348 (ext.318)

Kompilasi Hukum Islam. Berdasarkan pertimbangan-pertimbangan tersebut, maka unsur perceraian yang pertama harus dinyatakan telah terpenuhi;

Menimbang, bahwa selanjutnya terbukti pula bahwa antara Penggugat dan Tergugat telah diupayakan damai oleh pihak keluarga agar Penggugat dan Tergugat dapat rukun kembali, tapi upaya tersebut tidak berhasil. Begitu juga selama persidangan berlangsung, Penggugat telah menunjukkan sikap dan tekadnya untuk bercerai, yang berarti tidak mau lagi mempertahankan perkawinannya. Selain itu, fakta pisah tempat tinggal antara Penggugat dan Tergugat merupakan bukti bahwa Penggugat dan Tergugat sudah tidak peduli terhadap masa depan rumah tangganya, hal ini menunjukkan rumah tangga antara Penggugat dan Penggugat sudah tidak ada harapan untuk kembali rukun. Berdasarkan pertimbangan tersebut, maka unsur kedua juga harus dinyatakan telah terpenuhi;

Menimbang, bahwa Majelis Hakim telah berupaya mendamaikan dengan cara menasihati Penggugat pada setiap persidangan sesuai ketentuan Pasal 31 Peraturan Pemerintah Nomor 9 Tahun 1975, namun upaya tersebut tidak berhasil, dengan demikian maka unsur ketiga juga telah terpenuhi;

Menimbang, bahwa menurut pasal 1 Undang-Undang Nomor 1 tahun 1974 tujuan perkawinan adalah untuk membina rumah tangga yang bahagia dan kekal. Apabila dalam suatu rumah tangga ternyata kebahagiaan dan kerukunan sudah tidak ada lagi antara suami isteri, kemudian salah satu pihak telah bertekad untuk bercerai, maka mempertahankan rumah tangga yang demikian mafsadatnya akan lebih besar daripada manfaat dan mashlahatnya, untuk hal yang demikian Majelis hakim dapat menunjuk kepada kaidah fiqh yang berbunyi:

درأ المفاسد مقدم على جلب المصالح

Artinya: *Bahwa menghindarkan mafsadat harus lebih diprioritaskan daripada mendambakan kemaslahatan ;*

Menimbang, bahwa Majelis perlu mendeskripsikan pendapat ahli fiqh, sebagaimana tercantum dalam Kitab *Fiqh as-Sunnah,* Jilid II, halaman 291 yang diambil-alih sebagai pendapat Majelis yang berbunyi:

Disclaimer
Kepaniteraan Mahkamah Agung Republik Indonesia berusaha untuk selalu mencantumkan informasi paling kini dan akurat sebagai bentuk komitmen Mahkamah Agung untuk pelayanan publik, transparansi dan akuntabilitas pelaksanaan fungsi peradilan. Namun dalam hal-hal tertentu masih dimungkinkan terjadi permasalahan teknis terkait dengan akurasi dan keterkinian informasi yang kami sajikan, hal mana akan terus kami perbaiki dari waktu kewaktu.
Dalam hal Anda menemukan inakurasi informasi yang termuat pada situs ini atau informasi yang seharusnya ada, namun belum tersedia, maka harap segera hubungi Kepaniteraan Mahkamah Agung RI melalui :
Email : kepaniteraan@mahkamahagung.go.id Telp : 021-384 3348 (ext.318)

يجوز لها ان تطلب من القاضى التفريق وحينئذ يطلقها القاضى طلقة بائنة اذا ثبت الضرر و عجز عن الا صلاح بينهما

Artinya :"*Dan bagi seorang isteri boleh mengajukan perceraian terhadap suaminya. Dan Hakim boleh menjatuhkan talak satu ba'in, apabila terbukti adanya kemadharatan dalam pernikahan dan keduanya sulit didamaikan*";

Menimbang, bahwa berdasarkan kajian filosofis, yang diambil-alih menjadi pendapat Majelis, menyatakan bahwa secara ontologis perkawinan merupakan ikatan lahir-bathin antara seorang laki-laki dan perempuan sebagai suami-isteri. Sedangkan secara aksiologis, perkawinan bertujuan membentuk keluarga sakinah, mawaddah, dan rahmah. Adapun fakta hukum yang terbukti dalam persidangan perkara *a quo* menunjukkan bahwa rumah tangga antara Penggugat dan Tergugat sudah pecah dan jauh menyimpang dari kondisi ideal ontologi dan aksiologi perkawinan itu sendiri;

Menimbang, bahwa berdasarkan kajian sosiologis, dalam teori peran (*role theory*) yang dicetuskan oleh Robert Linton, yang diambil-alih menjadi pendapat Majelis, menyatakan bahwa dalam sebuah interaksi sosial, hak dan kewajiban masing-masing subjek (suami dan isteri) haruslah dipenuhi secara berimbang. Jika salah satu pihak, dalam konteks ini suami atau isteri sudah tidak mempedulikan hak dan kewajibannya, tentulah kondisi sosial keluarga antara Penggugat dan Tergugat tidak berimbang dan mengalami guncangan (*turbulence*);

Menimbang, bahwa berdasarkan kajian antropologis, dalam penelitian Hedi Sri Ahimsyah Putra yang dituangkan dalam buku berjudul Strukturalisme Levi Straus, Mitos dan Karya Sastra, yang diambil-alih menjadi pendapat Majelis, menyatakan bahwa terdapat keteraturan "struktur luar" (realitas yang tampak secara empiris) dan "struktur dalam" (realitas yang tidak selalu tampak dan dapat mempengaruhi struktur luar). Kontekstualisasi dalam perkara *a quo*, bahwa fakta hukum dalam sidang menunjukkan telah terjadi perpecahan pada "struktur luar" rumah tangga Penggugat dan Tergugat, dan realitas tersebut tentu dipengaruhi oleh "struktur dalam", yakni perpecahan bathin antara

Disclaimer
Kepaniteraan Mahkamah Agung Republik Indonesia berusaha untuk selalu mencantumkan informasi paling kini dan akurat sebagai bentuk komitmen Mahkamah Agung untuk pelayanan publik, transparansi dan akuntabilitas pelaksanaan fungsi peradilan. Namun dalam hal-hal tertentu masih dimungkinkan terjadi permasalahan teknis terkait dengan akurasi dan keterkinian informasi yang kami sajikan, hal mana akan terus kami perbaiki dari waktu kewaktu.
Dalam hal Anda menemukan inakurasi informasi yang termuat pada situs ini atau informasi yang seharusnya ada, namun belum tersedia, maka harap segera hubungi Kepaniteraan Mahkamah Agung RI melalui :
Email : kepaniteraan@mahkamahagung.go.id Telp : 021-384 3348 (ext.318)

Penggugat dan Tergugat itu sendiri. Sehingga tujuan perkawinan untuk membentuk keluarga sakinah, mawaddah, dan rahmah sulit tercapai;

Menimbang, bahwa berdasarkan pertimbangan tersebut diatas Majelis berpendapat bahwa perkawinan Penggugat dan Tergugat sudah pecah *(broken marriage),* sulit untuk disatukan dalam sebuah rumah tangga yang harmonis, sedangkan upaya Majelis Hakim untuk merukunkan kembali mereka sudah tidak berhasil. Dengan demikian, Majelis berpendapat gugatan Penggugat patut untuk dikabulkan;

Menimbang, bahwa oleh karena gugatan Penggugat telah beralaskan hukum, maka Majelis akan menjatuhkan putusan yang amarnya antara lain menjatuhkan talak satu ba'in shughra dari Tergugat terhadap Penggugat;

Menimbang, bahwa oleh karena perkara *a quo* mengenai sengketa di bidang perkawinan, maka sesuai dengan Pasal 89 ayat (1) Undang–undang Nomor 7 Tahun 1989 tentang Peradilan Agama sebagaimana telah diubah dengan Undang-undang Nomor 3 tahun 2006 dan Undang-undang Nomor 50 tahun 2009 maka segala biaya yang timbul dalam perkara ini dibebankan kepada Penggugat;

Menginggat segala ketentuan peraturan perundang-undangan yang berlaku dan hukum syara' yang berkaitan dengan perkara ini;

M E N G A D I L I

1. Menyatakan Tergugat telah dipanggil secara resmi dan patut untuk menghadap sidang, tidak hadir;
2. Mengabulkan Gugatan Penggugat secara verstek;
3. Menjatuhkan talak satu ba'in shugra Tergugat (Tergugat) terhadap Penggugat (Penggugat);
4. Membebankan kepada Penggugat untuk membayar biaya perkara sejumlah Rp406.000,00 (empat ratus enam ribu rupiah);

Demikian putusan ini dijatuhkan dalam musyawarah Majelis Hakim pada hari Rabu, tanggal 23 Desember 2020 Masehi/ 8 Jumadil Awwal 1442 Hijriah oleh Ahmad Zainul Anam, S.H.I., M.S.I., selaku Ketua Majelis, Liza, S.Sy dan

Disclaimer
Kepaniteraan Mahkamah Agung Republik Indonesia berusaha untuk selalu mencantumkan informasi paling kini dan akurat sebagai bentuk komitmen Mahkamah Agung untuk pelayanan publik, transparansi dan akuntabilitas pelaksanaan fungsi peradilan. Namun dalam hal-hal tertentu masih dimungkinkan terjadi permasalahan teknis terkait dengan akurasi dan keterkinian informasi yang kami sajikan, hal mana akan terus kami perbaiki dari waktu kewaktu.
Dalam hal Anda menemukan inakurasi informasi yang termuat pada situs ini atau informasi yang seharusnya ada, namun belum tersedia, maka harap segera hubungi Kepaniteraan Mahkamah Agung RI melalui :
Email : kepaniteraan@mahkamahagung.go.id Telp : 021-384 3348 (ext.318)

Gustomo Try Budiharjo, S.H.I selaku Hakim-hakim Anggota dan putusan tersebut diucapkan pada hari itu juga dalam sidang yang terbuka untuk umum oleh Ketua Majelis, didampingi oleh Hakim-hakim Anggota, dan dibantu oleh Syurya Gusmardi, S.H. selaku Panitera Pengganti, yang dihadiri oleh Penggugat, dengan tanpa kehadiran Tergugat;

Hakim Anggota, Ketua Majelis,

Liza, S.Sy Ahmad Zainul Anam, S.H.I., M.S.I.

Hakim Anggota,

Gustomo Try Budiharjo, S.H.I

Panitera Pengganti,

Syurya Gusmardi, S.H..

Perincian biaya perkara :

1. Pendaftaran	Rp30.000,00
2. Proses	Rp50.000,00
3. Panggilan	Rp290.000,00
4. PNBP Panggilan	Rp20.000,00
4. Redaksi	Rp10.000,00
5. Meterai	Rp6.000,00
Jumlah	Rp406.000,00 (empat ratus enam ribu rupiah);

Disclaimer
Kepaniteraan Mahkamah Agung Republik Indonesia berusaha untuk selalu mencantumkan informasi paling kini dan akurat sebagai bentuk komitmen Mahkamah Agung untuk pelayanan publik, transparansi dan akuntabilitas pelaksanaan fungsi peradilan. Namun dalam hal-hal tertentu masih dimungkinkan terjadi permasalahan teknis terkait dengan akurasi dan keterkinian informasi yang kami sajikan, hal mana akan terus kami perbaiki dari waktu kewaktu.
Dalam hal Anda menemukan inakurasi informasi yang termuat pada situs ini atau informasi yang seharusnya ada, namun belum tersedia, maka harap segera hubungi Kepaniteraan Mahkamah Agung RI melalui :
Email : kepaniteraan@mahkamahagung.go.id Telp : 021-384 3348 (ext.318)
