# Supplementary material for: Classification of divorce causes during the COVID-19 pandemic using convolutional neural networks
Source: PeerJ Comput Sci. 2022 Jun 30;8:e998. doi: 10.7717/peerj-cs.998 (PMC9299239; doi:10.7717/peerj-cs.998)
Supplement: Supplemental Information 5 [file peerj-cs-08-998-s005.zip › Masalah Ekonomi Dataset/Data ke-15.pdf]

Direktori Putusan Mahkamah Agung Republik Indonesia
putusan.mahkamahagung.go.id

SALINAN

P U T U S A N

Nomor : 2653/Pdt.G/2020/PAJU

بسم الله الرحمن الرحيم

DEMI KEADILAN BERDASARKAN KETUHANAN YANG MAHA ESA

Pengadilan Agama Jakarta Utara yang memeriksa dan mengadili perkara tertentu pada tingkat pertama dalam persidangan majelis hakim telah menjatuhkan putusan perkara cerai talak yang diajukan oleh;

PEMOHON, NIK., tempat tanggal lahir Jakarta, 31 Agustus 1987, agama Islam, pendidikan terakhir SLTP, pekerjaan wiraswasta, tempat kediaman di JAKARTA UTARA (No.Hp: 0812XXXXXXX) selanjutnya di sebut sebagai Pemohon,

melawan

TERMOHON, tempat tanggal lahir Jakarta, 4 November 1990, agama Islam, pendidikan SLTA, pekerjaan ibu rumah tangga, tempat kediaman di JAKARTA UTARA selanjutnya di sebut sebagai Termohon;

Pengadilan Agama tersebut;

Telah memeriksa berkas perkara yang bersangkutan;

Telah mendengar keterangan Pemohon dan telah memeriksa bukti-bukti dalam persidangan;

DUDUK PERKARA

Bahwa Pemohon dengan suratnya tertanggal 4 Desember 2020 mengajukan permohonan cerai terhadap Termohon ke Pengadilan Agama Jakarta Utara yang kemudian perkaranya terdaftar dalam register sebagai perkara Nomor 2653/Pdt.G/2020/PAJU tanggal 4 Desember 2020 dengan menerangkan atau mengajukan hal-hal sebagai berikut ;

1. Bahwa pemohon dengan termohon adalah suami isteri sah, menikah pada tanggal 9 Oktober 2014 dihadapan Pejabat Kantor Urusan Agama

Disclaimer
Kepaniteraan Mahkamah Agung Republik Indonesia berusaha untuk selalu mencantumkan informasi paling kini dan akurat sebagai bentuk komitmen Mahkamah Agung untuk pelayanan publik, transparansi dan akuntabilitas pelaksanaan fungsi peradilan. Namun dalam hal-hal tertentu masih dimungkinkan terjadi permasalahan teknis terkait dengan akurasi dan keterkinian informasi yang kami sajikan, hal mana akan terus kami perbaiki dari waktu kewaktu.
Dalam hal Anda menemukan inakurasi informasi yang termuat pada situs ini atau informasi yang seharusnya ada, namun belum tersedia, maka harap segera hubungi Kepaniteraan Mahkamah Agung RI melalui :
Email : kepaniteraan@mahkamahagung.go.id Telp : 021-384 3348 (ext.318)

Kecamatan, sebagaimana tercantum dalam Kutipan nikah Nomor: tertanggal 9 Oktober 2014).

2. Bahwa setelah pernikahan tersebut Pemohon dengan Termohon bertempat tinggal di rumah orangtua Pemohon Kota Jakarta Utara. Selama pernikahan tersebut Pemohon dengan Termohon telah hidup rukun sebagaimana layaknya suami istri namun tidak dikaruniai anak keturunan;

3. Bahwa pada awalnya kehidupan rumah tangga Pemohon dengan Termohon berjalan dengan baik, harmonis sebagaimana layaknya suami-isteri, akan tetapi sejak Oktober tahun 2018, Pemohon dengan Termohon mulai terjadi perselisihan dan percecokan yang terus-menerus dan sulit untuk didamaikan yang disebabkan antara lain:

   a. Termohon hendak berpisah dengan Pemohon karena selama pernikahan belum juga dikaruniai anak keturunan;

   b. Termohon dengan Pemohon sudah mediasi dan melakukan hakamain dengan orangtua Pemohon maupun Termohon dengan kesimpulan berpisah secara baik-baik ;

4. Bahwa Pemohon sudah berusaha mempertahankan rumah tangga dengan memberi nasehat serta saran kepada Termohon agar ia dapat merubah sikapnya namun Termohon tetap tidak dapat berubah. Pihak keluarga pun sudah berusaha mendamaikan namun tidak dapat dirukunkan. Oleh karena itu Pemohon telah berketetapan hati untuk bercerai dengan Termohon;

5. Bahwa puncak keretakan hubungan antara Pemohon dengan Termohon tersebut terjadi kurang lebih pada bulan Juli tahun 2019, yang akibatnya Termohon pergi meninggalkan Pemohon dan tinggal sebagaimana dengan alamat tersebut di atas, selama itu sudah tidak ada lagi hubungan baik lahir maupun batin;

6. Bahwa Pemohon telah menepis harapan terciptanya suasana hidup rukun dan tentram dalam mahligai rumah tangga, dengan keadaan yang sudah sedemikian itu Pemohon sudah tidak ada kecocokan lagi dalam

Disclaimer
Kepaniteraan Mahkamah Agung Republik Indonesia berusaha untuk selalu mencantumkan informasi paling kini dan akurat sebagai bentuk komitmen Mahkamah Agung untuk pelayanan publik, transparansi dan akuntabilitas pelaksanaan fungsi peradilan. Namun dalam hal-hal tertentu masih dimungkinkan terjadi permasalahan teknis terkait dengan akurasi dan keterkinian informasi yang kami sajikan, hal mana akan terus kami perbaiki dari waktu kewaktu.
Dalam hal Anda menemukan inakurasi informasi yang termuat pada situs ini atau informasi yang seharusnya ada, namun belum tersedia, maka harap segera hubungi Kepaniteraan Mahkamah Agung RI melalui :
Email : kepaniteraan@mahkamahagung.go.id   Telp : 021-384 3348 (ext.318)

membina rumah tangga dan sudah tidak ada harapan serta sudah tidak sanggup lagi untuk melanjutkan hidup berumah tangga dengan Termohon;

7. Bahwa pemohon sanggup membayar biaya yang timbul dalam perkara ini;

Berdasarkan dalil-dalil di atas, Pemohon mohon agar Ketua Pengadilan Agama Jakarta Utara segera memeriksa dan mengadili perkara ini, selanjutnya menjatuhkan putusan yang amarnya berbunyi:

Primer

1. Mengabulkan permohonan Pemohon;
2. Memberikan ijin kepada Pemohon PEMOHON untuk ikrar menjatuhkan talak 1 (satu) raj'i terhadap Termohon TERMOHON di depan sidang Pengadilan Agama Jakarta Utara;
3. Biaya perkara menurut hukum;

Subsider

Dan atau menjatuhkan putusan yang seadil-adilnya;

Bahwa pada hari persidangan yang telah ditentukan Pemohon hadir dalam persidangan sedangkan Termohon tidak pernah hadir dan tidak pula menyuruh orang lain sebagai wakil atau kuasanya meskipun telah dipanggil secara resmi dan patut;

Bahwa kemudian persidangan dilanjutkan kepada pemeriksaan pokok perkara dengan diawali pembacaan surat permohonan Pemohon menerangkan tetap dengan permohonannya;

Bahwa terhadap surat permohonan Pemohon tersebut Termohon tidak dapat didengar keterangan atau jawabannya karena tidak hadir dalam persidangan dan tidak pula menyuruh orang lain sebagai wakil atau kuasanya meskipun telah dipanggil secara resmi dan patut sehingga tidak dapat dikonfirmasikan permohonan Pemohon tersebut kepada Termohon;

Bahwa untuk menguatkan dalil permohonannya, Pemohon telah menyerahkan alat bukti tertulis berupa fotokopi dari kutipan akta nikah atas nama Pemohon dan Termohon Nomor yang aslinya dikeluarkan oleh KUA Kecamatan yang telah bermateraikan secukupnya dan telah dicocokan dengan

Disclaimer
Kepaniteraan Mahkamah Agung Republik Indonesia berusaha untuk selalu mencantumkan informasi paling kini dan akurat sebagai bentuk komitmen Mahkamah Agung untuk pelayanan publik, transparansi dan akuntabilitas pelaksanaan fungsi peradilan. Namun dalam hal-hal tertentu masih dimungkinkan terjadi permasalahan teknis terkait dengan akurasi dan keterkinian informasi yang kami sajikan, hal mana akan terus kami perbaiki dari waktu kewaktu.
Dalam hal Anda menemukan inakurasi informasi yang termuat pada situs ini atau informasi yang seharusnya ada, namun belum tersedia, maka harap segera hubungi Kepaniteraan Mahkamah Agung RI melalui :
Email : kepaniteraan@mahkamahagung.go.id Telp : 021-384 3348 (ext.318)

aslinya ternyata sesuai kemudian oleh Ketua Majelis diberi kode P1, bukti ini membuktikan bahwa Pemohon dengan Termohon adalah suami isteri yang sah;

Bahwa Pemohon juga menghadirkan saksi dari Pemohon yang bernama;

1. SAKSI I, di bawah sumpah memberikan keterangan yang pada pokoknya adalah sebagai berikut :

- Bahwa Pemohon dengan Termohon adalah suami isteri;
- Bahwa Pemohon dengan Termohon belum mempunyai anak;
- Bahwa yang saksi ketahui rumah tangga Pemohon dengan Termohon awalnya baik, rukun dan harmonis kemudian tidak rukun lagi karena Pemohon dengan Termohon telah berpisah rumah sejak bulan Juli tahun 2019;
- Bahwa Termohon yang meninggalkan rumah kediaman bersama;
- Bahwa sejak berpisah rumah Pemohon dengan Termohon belum pernah hidup bersama lagi;
- Bahwa saksi tidak mengetahui adanya pertengkaran antara Pemohon dengan Termohon sebelum berpisah ;
- Bahwa saksi telah berusaha menyarankan Pemohon agar rukun kembali dengan Termohon namun tidak berhasil;
- Bahwa saksi tidak akan mencoba lagi untuk menyarankan Pemohon agar rukun kembali dengan Termohon;

2. SAKSI II, di bawah sumpah memberikan keterangan yang pada pokoknya adalah sebagai berikut :

- Bahwa Pemohon dengan Termohon adalah suami isteri;
- Bahwa Pemohon dengan Termohon belum mempunyai anak;
- Bahwa yang saksi ketahui rumah tangga Pemohon dengan Termohon awalnya baik, rukun dan harmonis kemudian tidak rukun lagi karena Pemohon dengan Termohon telah berpisah rumah lebih kurang dari 1 (satu) tahun 5 (lima) bulan lamanya;
- Bahwa Termohon yang meninggalkan rumah kediaman bersama;

Disclaimer
Kepaniteraan Mahkamah Agung Republik Indonesia berusaha untuk selalu mencantumkan informasi paling kini dan akurat sebagai bentuk komitmen Mahkamah Agung untuk pelayanan publik, transparansi dan akuntabilitas pelaksanaan fungsi peradilan. Namun dalam hal-hal tertentu masih dimungkinkan terjadi permasalahan teknis terkait dengan akurasi dan keterkinian informasi yang kami sajikan, hal mana akan terus kami perbaiki dari waktu kewaktu.
Dalam hal Anda menemukan inakurasi informasi yang termuat pada situs ini atau informasi yang seharusnya ada, namun belum tersedia, maka harap segera hubungi Kepaniteraan Mahkamah Agung RI melalui :
Email : kepaniteraan@mahkamahagung.go.id Telp : 021-384 3348 (ext.318)

- Bahwa sejak berpisah rumah Pemohon dengan Termohon belum pernah hidup bersama lagi;
- Bahwa saksi tidak mengetahui adanya pertengkaran antara Pemohon dengan Termohon sebelum berpisah;
- Bahwa saksi telah berusaha menyarankan Pemohon agar rukun kembali dengan Termohon namun tidak berhasil;
- Bahwa saksi tidak akan mencoba lagi untuk menyarankan Pemohon agar rukun kembali dengan Termohon;

Bahwa terhadap keterangan saksi tersebut Pemohon membenarkannya;

Bahwa pada tahap kesimpulan Pemohon telah menyampaikan kesimpulan akhirnya yaitu sebagaimana dalam kesimpulannya;

Bahwa mengenai jalannya pemeriksaan perkara ini selengkapnya telah dicatat dalam berita acara yang bersangkutan. Maka untuk meringkas putusan ini selanjutnya Majelis Hakim menunjuk berita acara tersebut sebagai bagian yang tak terpisahkan dari putusan ini;

PERTIMBANGAN HUKUM

Menimbang, bahwa maksud dan tujuan dari pada permohonan Pemohon adalah seperti terurai di atas;

Menimbang, bahwa perkara ini mengenai permohonan cerai talak yang diajukan oleh pihak yang beragama Islam, oleh karenanya berdasarkan Pasal 49 (a) Undang-Undang Nomor 7 Tahun 1989 Tentang Peradilan Agama sebagaimana telah diubah dengan Undang-Undang Nomor 3 Tahun 2006 dan perubahan kedua dengan Undang-Undang Nomor 50 Tahun 2009, maka perkara a quo merupakan kewenangan absolut peradilan agama;

Menimbang, bahwa Pemohon mendalilkan telah melangsungkan perkawinan dengan Termohon yang dicatatkan di KUA Kecamatan Koja;

Menimbang, bahwa untuk menguatkan dalil permohonannya Pemohon mengajukan bukti P 1 (fotokopi dari kutipan akta nikah) yang merupakan akta otentik dan telah bermaterai cukup dan telah cocok dengan aslinya *ternyata*

Disclaimer
Kepaniteraan Mahkamah Agung Republik Indonesia berusaha untuk selalu mencantumkan informasi paling kini dan akurat sebagai bentuk komitmen Mahkamah Agung untuk pelayanan publik, transparansi dan akuntabilitas pelaksanaan fungsi peradilan. Namun dalam hal-hal tertentu masih dimungkinkan terjadi permasalahan teknis terkait dengan akurasi dan keterkinian informasi yang kami sajikan, hal mana akan terus kami perbaiki dari waktu kewaktu.
Dalam hal Anda menemukan inakurasi informasi yang termuat pada situs ini atau informasi yang seharusnya ada, namun belum tersedia, maka harap segera hubungi Kepaniteraan Mahkamah Agung RI melalui :
Email : kepaniteraan@mahkamahagung.go.id Telp : 021-384 3348 (ext.318)

sesuai oleh karena itu bukti tersebut telah memenuhi Pasal 2 ayat (3) Undang-Undang Nomor 13 Tahun 1985 Tentang Bea Materai dan Pasal 1888 KUH Perdata, sehingga bukti tersebut mempunyai kekuatan bukti yang sempurna dan mengikat;

Menimbang, bahwa berdasarkan pertimbangan tersebut di atas terbukti bahwa Pemohon adalah suami sah Termohon. Dengan demikian Pemohon berkualitas sebagai subjek hukum dalam perkara a quo;

Menimbang, bahwa untuk petitum angka 1 (satu) majelis hakim akan mempertimbangkannya kemudian;

Menimbang, bahwa dalam petitum angka 2 (dua) Pemohon mohon agar memberikan ijin kepada Pemohon PEMOHON untuk ikrar menjatuhkan talak 1 (satu) raj'i terhadap Termohon TERMOHON di depan sidang Pengadilan Agama Jakarta Utara maka majelis hakim akan mempertimbangkannya sebagai berikut;

Menimbang, bahwa yang menjadi dalil permohonan Pemohon untuk melakukan perceraian dengan Termohon di dasarkan kepada bahwa sejak bulan Oktober tahun 2018 Pemohon dengan Termohon mulai terjadi perselisihan dan percekcokan terus-menerus yang sulit untuk didamaikan disebabkan dengan yang Pemohon sampaikan di atas yang mencapai puncaknya pada bulan Juli tahun 2019 yang akibatnya Termohon pergi meninggalkan Pemohon dan sejak saat itu sudah tidak ada hubungan baik lahir maupun batin;

Menimbang, bahwa terhadap dalil permohonan Pemohon tersebut Termohon tidak dapat didengar keterangannya karena tidak hadir dalam persidangan dan tidak pula menyuruh orang lain sebagai wakil atau kuasanya meskipun telah dipanggil secara resmi dan patut sehingga perkara aquo diperiksa dengan tanpa adanya Termohon dan Termohon yang tidak hadir tersebut dinyatakan tidak hadir;

Menimbang, bahwa dengan tidak hadirnya Termohon tersebut maka perkara ini diputus dengan verstek sesuai dengan pasal 125 HIR;

Disclaimer
Kepaniteraan Mahkamah Agung Republik Indonesia berusaha untuk selalu mencantumkan informasi paling kini dan akurat sebagai bentuk komitmen Mahkamah Agung untuk pelayanan publik, transparansi dan akuntabilitas pelaksanaan fungsi peradilan. Namun dalam hal-hal tertentu masih dimungkinkan terjadi permasalahan teknis terkait dengan akurasi dan keterkinian informasi yang kami sajikan, hal mana akan terus kami perbaiki dari waktu kewaktu.
Dalam hal Anda menemukan inakurasi informasi yang termuat pada situs ini atau informasi yang seharusnya ada, namun belum tersedia, maka harap segera hubungi Kepaniteraan Mahkamah Agung RI melalui :
Email : kepaniteraan@mahkamahagung.go.id Telp : 021-384 3348 (ext.318)

Menimbang, bahwa dengan tidak hadirnya Termohon tersebut berarti Termohon menghilangkan haknya untuk menjawab dalil permohonan Pemohon tersebut berarti dalil permohonan Pemohon tersebut dinilai oleh majelis hakim tidak terbantahkan;

Menimbang, bahwa meskipun dalil permohonan Pemohon tersebut tidak dijawab oleh Termohon sehingganya tidak terbantahkan oleh Termohon namun karena perkara perceraian menyangkut putusnya ikatan perkawinan yang bernilai sakral maka Majelis Hakim tetap akan memberikan pertimbangan-pertimbangan dengan melihat bukti lainnya untuk menentukan patut dan tidak patutnya perkawinan antara Pemohon dan Termohon diputuskan;

Menimbang, bahwa untuk bukti P1 karena telah dipertimbangkan di atas maka tidak perlu lagi untuk dipertimbangkan;

Menimbang, bahwa Pemohon juga telah mengajukan bukti saksi dari Pemohon yang telah memberikan keterangan di bawah sumpah dihadapan Majelis Hakim yang keterangannya sebagaimana dalam duduk perkara ini ternyata keterangan saksi-saksi tersebut saling bersesuaian satu dengan yang lainnya yang pada pokoknya menguatkan dalil permohonan Pemohon;

Menimbang, bahwa karena keterangan saksi-saksi berdasarkan pengetahuannya sendiri dan yang pada pokoknya menguatkan dalil-dalil permohonan Pemohon tersebut maka Majelis hakim dapat menerima keterangannya sehingga dapat dijadikan bukti dalam perkara ini;

Menimbang, bahwa saksi dari Pemohon telah didengar kesaksiannya dalam persidangan adalah untuk memenuhi maksud pasal 76 Ayat (1) Undang-Undang Nomor 7 Tahun 1989 Tentang Peradilan Agama sebagaimana telah diubah dengan Undang-Undang Nomor 3 Tahun 2006 dan perubahan kedua dengan Undang-Undang Nomor 50 Tahun 2009 jo pasal 22 PP Nomor 9 tahun 1975 Tentang Pelaksanaan Undang-undang Nomor 1 tahun 1974 tentang Perkawinan dan pasal 134 Kompilasi Hukum Islam;

Menimbang, bahwa berdasarkan ketentuan pasal 39 ayat (2) UU No. 1 Tahun 1974 Tentang Perkawinan “untuk melakukan perceraian harus ada

Disclaimer
Kepaniteraan Mahkamah Agung Republik Indonesia berusaha untuk selalu mencantumkan informasi paling kini dan akurat sebagai bentuk komitmen Mahkamah Agung untuk pelayanan publik, transparansi dan akuntabilitas pelaksanaan fungsi peradilan. Namun dalam hal-hal tertentu masih dimungkinkan terjadi permasalahan teknis terkait dengan akurasi dan keterkinian informasi yang kami sajikan, hal mana akan terus kami perbaiki dari waktu kewaktu.
Dalam hal Anda menemukan inakurasi informasi yang termuat pada situs ini atau informasi yang seharusnya ada, namun belum tersedia, maka harap segera hubungi Kepaniteraan Mahkamah Agung RI melalui :
Email : kepaniteraan@mahkamahagung.go.id   Telp : 021-384 3348 (ext.318)

cukup alasan bahwa antara suami-isteri itu tidak akan dapat hidup rukun lagi sebagai suami-isteri;

Menimbang, bahwa berdasarkan hasil pemeriksaan dalam persidangan terhadap surat permohonan Pemohon berikut keteranganya dan bukti-bukti Majelis Hakim dapat menemukan fakta sebagai berikut;

- Bahwa Pemohon dengan Termohon adalah suami isteri;
- Bahwa Pemohon dengan Termohon belum mempunyai anak;
- Bahwa sejak bulan Oktober tahun 2018 Pemohon dengan Termohon mulai terjadi perselisihan dan percekcokan terus-menerus yang sulit untuk didamaikan disebabkan dengan yang Pemohon sampaikan di atas yang mencapai puncaknya pada bulan Juli tahun 2019;
- Bahwa sejak bulan Juli tahun 2019 Pemohon dan Termohon sudah berpisah rumah;
- Bahwa Termohon yang meninggalkan rumah kediaman bersama
- Bahwa sejak bulan Juli tahun 2019 antara Pemohon dan Termohon sudah tidak ada lagi hubungan baik lahir maupun batin juga belum pernah hidup bersama lagi;

Menimbang, bahwa berdasarkan fakta tersebut di atas maka telah terbukti bahwa rumah tangga Pemohon dengan Termohon sudah tidak harmonis lagi sejak bulan Oktober tahun 2018 karena Pemohon dengan Termohon mulai terjadi perselisihan dan percekcokan terus-menerus yang sulit untuk didamaikan disebabkan dengan sebagaimana Pemohon sampaikan di atas yang mencapai puncaknya pada bulan Juli tahun 2019 kemudian sejak itu Pemohon dengan Termohon berpisah rumah dan Termohon yang meninggalkan rumah kediaman bersama bahkan sejak berpisah rumahpun sudah tidak ada lagi hubungan baik lahir maupun batin juga belum pernah hidup bersama lagi sehingganya harapan untuk hidup rukun dalam rumah tangganya sudah sulit;

Menimbang, bahwa berdasarkan hal tersebut Majelis Hakim berpendapat bahwa hubungan antara Pemohon dan Termohon dalam

Disclaimer
Kepaniteraan Mahkamah Agung Republik Indonesia berusaha untuk selalu mencantumkan informasi paling kini dan akurat sebagai bentuk komitmen Mahkamah Agung untuk pelayanan publik, transparansi dan akuntabilitas pelaksanaan fungsi peradilan. Namun dalam hal-hal tertentu masih dimungkinkan terjadi permasalahan teknis terkait dengan akurasi dan keterkinian informasi yang kami sajikan, hal mana akan terus kami perbaiki dari waktu kewaktu.
Dalam hal Anda menemukan inakurasi informasi yang termuat pada situs ini atau informasi yang seharusnya ada, namun belum tersedia, maka harap segera hubungi Kepaniteraan Mahkamah Agung RI melalui :
Email : kepaniteraan@mahkamahagung.go.id Telp : 021-384 3348 (ext.318)

membina rumah tangga sudah tidak harmonis sehingga sulit untuk mewujudkan tujuan perkawinan sebagaimana maksud dari Al Qur'an Surat Ar-Rum Ayat 21 dan Pasal 1 Undang-Undang Nomor 1 Tahun 1974 Tentang Perkawinan Jo. Pasal 3 Kompilasi Hukum Islam (INPRES Nomor 1 Tahun 1991);

Menimbang, bahwa Pasal 1 Undang-Undang Nomor 1 tahun 1974 tentang Perkawinan menyatakan bahwa perkawinan adalah ikatan lahir batin antara seorang pria dengan seorang wanita sebagai suami isteri dengan tujuan membentuk keluarga (rumah tangga) yang bahagia dan kekal berdasarkan Ketuhanan Yang Maha Esa;

Menimbang, bahwa ikatan batin menurut penjelasan pasal tersebut merupakan unsur yang penting dalam suatu perkawinan oleh karenannya Majelis Hakim berpendapat apabila ikatan batin sudah tidak ada lagi maka perkawinan tersebut sudah pecah sehingga mempertahankan perkawinan tersebut merupakan hal yang sia-sia dan tidak akan bermanfaat bagi kedua belah pihak;

Menimbang, bahwa dalam kondisi tidak harmonis tersebut Majelis Hakim berpendapat ikatan perkawinan antara Pemohon dan Termohon telah pecah yang disebabkan oleh hal-hal sebagaimana tersebut di atas bahkan adanya kehendak yang kuat dari Pemohon untuk menceraikan Termohon dengan demikian tanpa mempersoalkan siapa yang salah sehingga antara Pemohon dan Termohon sulit untuk dapat dirukunkan kembali untuk membina rumah tangga bersama sehingga permohonan Pemohon telah memenuhi maksud Pasal 39 Undang-Undang Nomor 1 Tahun 1974 Tentang Perkawinan beserta penjelasannya dan Pasal 19 huruf f Peraturan Pemerintah Nomor 9 Tahun 1975 tentang Pelaksanaan Undang-undang Nomor 1 tahun 1974 tentang Perkawinan Jo. Pasal 116 huruf f Kompilasi Hukum Islam;

Menimbang, bahwa Majelis Hakim perlu mengetengahkan firman Allah SWT dalam Surat Al-Baqarah ayat 227, yaitu sebagai berikut:

وَإِنْ عَزَمُوا الطَّلَاقَ فَإِنَّ الله سَمِيعٌ عَلِيمُ

Disclaimer
Kepaniteraan Mahkamah Agung Republik Indonesia berusaha untuk selalu mencantumkan informasi paling kini dan akurat sebagai bentuk komitmen Mahkamah Agung untuk pelayanan publik, transparansi dan akuntabilitas pelaksanaan fungsi peradilan. Namun dalam hal-hal tertentu masih dimungkinkan terjadi permasalahan teknis terkait dengan akurasi dan keterkinian informasi yang kami sajikan, hal mana akan terus kami perbaiki dari waktu kewaktu.
Dalam hal Anda menemukan inakurasi informasi yang termuat pada situs ini atau informasi yang seharusnya ada, namun belum tersedia, maka harap segera hubungi Kepaniteraan Mahkamah Agung RI melalui :
Email : kepaniteraan@mahkamahagung.go.id Telp : 021-384 3348 (ext.318)

Artinya: “Dan jika mereka ber’azam (bertetap hati) untuk berthalak, maka sesungguhnya Allah Maha Mendengar dan dan Maha Mengetahui”.

Menimbang, bahwa Majelis Hakim perlu mengetengahkan pendapat dalam kitab Al Iqna Juz III halaman 401 yang berbunyi :

لأن الإعتبار بالطلاق فالزوج لماروى البيهقى أن النبي صلعم قال : الطلاق بالرجال والعدة بالنساء ولا يحرم جمع التطليقات

Artinya;Menjatuhkan talak adalah hak suami sesuai dengan riwayat Al Baihaqi bahwa nabi SAW bersabda : Cerai itu dari pihak laki-laki dan iddah dari pihak perempuan, tidak dilarang mengumpulkan beberapa talak. (Al Iqna III : 401)

Menimbang, bahwa sesuai dengan Yurisprudensi Mahkamah Agung RI Nomor 237 K/AG/1998 tanggal 17 Maret 1999 yang menetapkan bahwa cekcok, hidup berpisah tidak dalam satu tempat kediaman bersama, salah satu pihak tidak berniat meneruskan kehidupan bersama dengan pihak lain merupakan fakta yang cukup untuk dijadikan alasan perceraian;

Menimbang, bahwa berdasarkan pertimbangan-petimbangan tersebut di atas telah cukup alasan bagi Majelis Hakim untuk mengabulkan permohonan Pemohon sebagaimana dalam petitum angka 2 (dua) yaitu dengan memberikan izin kepada Pemohon untuk ikrar menjatuhkan talak satu raj'i terhadap Termohon di depan Pengadilan Agama Jakarta Utara yang amarnya akan disebutkan dalam amar putusan ini;

Menimbang, bahwa dalam petitum angka 3 (tiga) Pemohon juga mohon agar biaya perkara menurut hukum dengan alasan sebagaimana Pemohon sampaikan dan terhadap hal tersebut Termohon tidak dapat didengar keterangannya karena tidak hadir dalam persidangan juga tidak mengirimkan wakilnya yang sah maka majelis akan mempertimbangkannya sebagai berikut;

Menimbang, bahwa perkara ini termasuk bidang perkawinan maka sesuai dengan pasal 89 ayat 1 Undang-Undang Nomor 7 Tahun 1989 Tentang Peradilan Agama sebagaimana telah diubah dengan Undang-Undang Nomor 3 Tahun 2006 dan perubahan kedua dengan Undang-Undang Nomor 50 Tahun

Disclaimer
Kepaniteraan Mahkamah Agung Republik Indonesia berusaha untuk selalu mencantumkan informasi paling kini dan akurat sebagai bentuk komitmen Mahkamah Agung untuk pelayanan publik, transparansi dan akuntabilitas pelaksanaan fungsi peradilan. Namun dalam hal-hal tertentu masih dimungkinkan terjadi permasalahan teknis terkait dengan akurasi dan keterkinian informasi yang kami sajikan, hal mana akan terus kami perbaiki dari waktu kewaktu.
Dalam hal Anda menemukan inakurasi informasi yang termuat pada situs ini atau informasi yang seharusnya ada, namun belum tersedia, maka harap segera hubungi Kepaniteraan Mahkamah Agung RI melalui :
Email : kepaniteraan@mahkamahagung.go.id Telp : 021-384 3348 (ext.318)

2009 biaya perkara dibebankan kepada Pemohon dengan demikian cukup alasan bagi majelis hakim untuk mengabulkan permohonan Pemohon sebagaimana petitum angka 3 (tiga) tersebut yaitu dengan membebankan kepada Pemohon untuk membayar biaya perkara ini yang jumlahnya akan disebutkan dalam amar putusan ini;

Menimbang, bahwa untuk petitum angka 1 (satu) majelis hakim akan mempertimbangkannya sebagai berikut;

Menimbang, bahwa karena petitum angka 2 (dua) dan 3 (tiga) dikabulkan maka majelis hakim berpendapat bahwa petitum angka 1 (satu) juga turut dikabulkan;

Memperhatikan segala peraturan dan perundang-undangan yang berlaku serta hukum syara' yang berhubungan dengan perkara ini ;

M E N G A D I L I

1. Menyatakan Termohon yang telah dipanggil secara resmi dan patut untuk menghadap kepersidangan tidak hadir;
2. Mengabulkan permohonan Pemohon dengan verstek.
3. Memberikan izin kepada Pemohon (PEMOHON) untuk menjatuhkan talak satu raj'i terhadap Termohon (TERMOHON) di depan sidang Pengadilan Agama Jakarta Utara.
4. Membebankan biaya perkara kepada Pemohon sejumlah Rp 516.000,- (lima ratus enam belas ribu rupiah).

Demikian putusan ini dijatuhkan dalam rapat permusyawaratan Majelis Hakim Pengadilan Agama Jakarta Utara pada hari **Rabu** tanggal 23 Desember 2020 Masehi bertepatan dengan tanggal 8 Jumadil Awal 1442 Hijriyah, oleh Kami **Drs. Agus Abdullah, M.H.,** sebagai Ketua Majelis, **Drs. Ahd Syarwani** dan **Hj. Asmawati, S.H., M.H.,** masing-masing sebagai Hakim Anggota, putusan tersebut pada hari itu juga dibacakan oleh Ketua Majelis tersebut dalam persidangan yang terbuka untuk umum dengan didampingi Hakim-Hakim Anggota tersebut dibantu oleh **Muhammad Thahir Guhir, S.H.,** sebagai panitera Pengganti yang dihadiri oleh Pemohon tanpa hadirnya Termohon;

Disclaimer
Kepaniteraan Mahkamah Agung Republik Indonesia berusaha untuk selalu mencantumkan informasi paling kini dan akurat sebagai bentuk komitmen Mahkamah Agung untuk pelayanan publik, transparansi dan akuntabilitas pelaksanaan fungsi peradilan. Namun dalam hal-hal tertentu masih dimungkinkan terjadi permasalahan teknis terkait dengan akurasi dan keterkinian informasi yang kami sajikan, hal mana akan terus kami perbaiki dari waktu kewaktu.
Dalam hal Anda menemukan inakurasi informasi yang termuat pada situs ini atau informasi yang seharusnya ada, namun belum tersedia, maka harap segera hubungi Kepaniteraan Mahkamah Agung RI melalui :
Email : kepaniteraan@mahkamahagung.go.id Telp : 021-384 3348 (ext.318)

Ketua Majelis

t.t.d.

Drs. Agus Abdullah, M.H.

Hakim Anggota Hakim Anggota

t.t.d. t.t.d.

Drs. Ahd Syarwani **Hj. Asmawati, S.H., M.H.**

Panitera Pengganti

t.t.d.

Muhammad Thahir Guhir, S.H.

Perincian biaya perkara :

1. Pendaftaran	Rp.	30.000,-
2. Proses	Rp.	75.000,-
3. Panggilan	Rp.	375.000,-
4. PNBP panggilan Pemohon pertama	Rp	10.000,-
5. PNBP panggilan Termohon pertama	Rp	10.000,-
6. Redaksi	Rp.	10.000,-
7. Materai	Rp.	6.000,+
Jumlah	Rp.	516.000,-

Disclaimer
Kepaniteraan Mahkamah Agung Republik Indonesia berusaha untuk selalu mencantumkan informasi paling kini dan akurat sebagai bentuk komitmen Mahkamah Agung untuk pelayanan publik, transparansi dan akuntabilitas pelaksanaan fungsi peradilan. Namun dalam hal-hal tertentu masih dimungkinkan terjadi permasalahan teknis terkait dengan akurasi dan keterkinian informasi yang kami sajikan, hal mana akan terus kami perbaiki dari waktu kewaktu.
Dalam hal Anda menemukan inakurasi informasi yang termuat pada situs ini atau informasi yang seharusnya ada, namun belum tersedia, maka harap segera hubungi Kepaniteraan Mahkamah Agung RI melalui :
Email : kepaniteraan@mahkamahagung.go.id Telp : 021-384 3348 (ext.318)
