# Supplementary material for: Classification of divorce causes during the COVID-19 pandemic using convolutional neural networks
Source: PeerJ Comput Sci. 2022 Jun 30;8:e998. doi: 10.7717/peerj-cs.998 (PMC9299239; doi:10.7717/peerj-cs.998)
Supplement: Supplemental Information 5 [file peerj-cs-08-998-s005.zip › Masalah Ekonomi Dataset/Data ke-16.pdf]

P U T U S A N

Nomor 3013/Pdt.G/2020/PA.Ckr

بِسْمِ اللهِ الرَّحْمٰنِ الرَّحِيْمِ

DEMI KEADILAN BERDASARKAN KETUHANAN YANG MAHA ESA

Pengadilan Agama Cikarang yang memeriksa dan mengadili perkara tertentu pada tingkat pertama dalam sidang Majelis Hakim telah menjatuhkan putusan sebagai berikut, dalam perkara Cerai Gugat antara:

Penggugat, tempat dan tanggal lahir Jakarta 28 Maret 1987, umur 33 tahun, agama Islam, pendidikan SLTA, pekerjaan Mengurus Rumah Tangga, tempat tinggal di Kabupaten Bekasi, Provinsi Jawa Barat, selanjutnya disebut sebagai Penggugat;

lawan

Tergugat, tempat dan tanggal lahir Jakarta 19 Oktober 1987, umur 33 tahun, agama Islam, pendidikan SLTA, pekerjaan Karyawan Swasta, tempat tinggal di Kota Jakarta Pusat, Provinsi DKI Jakarta, selanjutnya disebut sebagai Tergugat;

Pengadilan Agama tersebut;

Telah membaca dan mempelajari berkas perkara dan semua surat yang berhubungan dengan perkara ini;

Telah mendengar Penggugat serta saksi-saksinya;

DUDUK PERKARA

Menimbang, bahwa Penggugat dengan surat gugatannya tertanggal 03 November 2020 yang telah terdaftar di Kepaniteraan Pengadilan Agama Cikarang dengan register perkara Nomor 3013/Pdt.G/2020/PA.Ckr tanggal 03 November 2020, mengemukakan dalil-dalil sebagai berikut:

1. Bahwa pada tanggal 16 Juni 2015, Penggugat dengan Tergugat mencatatkan pernikahan di wilayah hukum Kantor Urusan Agama Kecamatan Cempaka Putih Kota Jakarta Pusat Provinsi DKI Jakarta sebagaimana ternyata dalam Kutipan Akta Nikah yang dikeluarkan oleh

Disclaimer
Kepaniteraan Mahkamah Agung Republik Indonesia berusaha untuk selalu mencantumkan informasi paling kini dan akurat sebagai bentuk komitmen Mahkamah Agung untuk pelayanan publik, transparansi dan akuntabilitas pelaksanaan fungsi peradilan. Namun dalam hal-hal tertentu masih dimungkinkan terjadi permasalahan teknis terkait dengan akurasi dan keterkinian informasi yang kami sajikan, hal mana akan terus kami perbaiki dari waktu kewaktu.
Dalam hal Anda menemukan inakurasi informasi yang termuat pada situs ini atau informasi yang seharusnya ada, namun belum tersedia, maka harap segera hubungi Kepaniteraan Mahkamah Agung RI melalui :
Email : kepaniteraan@mahkamahagung.go.id   Telp : 021-384 3348 (ext.318)

Pegawai Pencatat Nikah Kantor Urusan Agama Kecamatan Cempaka Putih Kota Jakarta Pusat Provinsi DKI Jakarta Nomor 214/39/VI/2015 tertanggal 16 Juni 2015;

2. Bahwa saat pernikahan, Penggugat berstatus Janda Cerai dan Tergugat berstatus Perjaka;
3. Bahwa setelah menikah, Penggugat dan Tergugat terakhir tinggal bersama di Kabupaten Bekasi;
4. Bahwa setelah menikah Penggugat dengan Tergugat telah bergaul baik selayaknya suami istri;
5. Bahwa Penggugat dengan Tergugat sudah dikaruniai 3 (tiga) orang anak yang masing-masing bernama:
   1) Anak 1, Laki-laki, Umur 6 tahun
   2) Anak 2 Laki-laki, Umur 5 tahun
   3) Anak 3, Laki-laki, Umur 2 tahun
6. Bahwa, pada awalnya rumah tangga Penggugat dan Tergugat berjalan rukun dan harmonis, namun sejak bulan Juni 2020 sering terjadi perselisihan dan pertengkaran yang di sebabkan antara lain:
   1) Tergugat tidak terbuka soal keuangan kepada Penggugat;
   2) Tergugat melakukan Kekerasan Dalam Rumah Tangga (KDRT) kepada Penggugat;
7. Bahwa pada bulan Agustus 2020 terjadi puncak perselisihan dan pertengkaran yang mengakibatkan Tergugat pergi dari tempat kediaman bersama sehingga antara Penggugat dengan Tergugat pisah rumah, sampai saat ini sudah pisah rumah 3 bulan lamanya dan sampai saat ini sudah tidak ada hubungan baik dan sudah tidak memberi nafkah lahir maupun batin lagi kepada Penggugat;
8. Bahwa dengan adanya kejadian tersebut Penggugat tetap bersabar, dan sudah dilakukan upaya musyawarah antara Penggugat dan Tergugat beserta keluarga Penggugat dan Tergugat, namun hasilnya tetap nihil. Oleh karena rumah tangga Penggugat dengan Tergugat sudah tidak ada ketentraman dan keharmonisan maka Penggugat mengambil sikap dan

Disclaimer
Kepaniteraan Mahkamah Agung Republik Indonesia berusaha untuk selalu mencantumkan informasi paling kini dan akurat sebagai bentuk komitmen Mahkamah Agung untuk pelayanan publik, transparansi dan akuntabilitas pelaksanaan fungsi peradilan. Namun dalam hal-hal tertentu masih dimungkinkan terjadi permasalahan teknis terkait dengan akurasi dan keterkinian informasi yang kami sajikan, hal mana akan terus kami perbaiki dari waktu kewaktu.
Dalam hal Anda menemukan inakurasi informasi yang termuat pada situs ini atau informasi yang seharusnya ada, namun belum tersedia, maka harap segera hubungi Kepaniteraan Mahkamah Agung RI melalui :
Email : kepaniteraan@mahkamahagung.go.id Telp : 021-384 3348 (ext.318)

keputusan untuk bercerai dengan Tergugat, karena sudah tidak sanggup lagi untuk tetap mempertahankan mahligai rumah tangga dengan Tergugat;

9. Bahwa dengan keadaan rumah tangga tersebut, Penggugat sudah tidak ada harapan untuk melanjutkan rumah tangga bersama Tergugat, sehingga tujuan perkawinan untuk membentuk rumah tangga yang sakinah, mawadah warohmah tidak dapat terwujud, dan apabila tetap dipertahankan hanya akan menimbulkan kemadorotan yang berkepanjangan;
10. Bahwa Penggugat bersedia menanggung biaya yang timbul akibat perkara ini;

Bahwa berdasarkan dalil-dalil tersebut di atas, maka Penggugat mohon kepada Ketua Pengadilan Agama Cikarang cq. Majelis Hakim untuk memeriksa dan mengadili perkara ini, dan selanjutnya dapat memutuskan sebagai berikut:

1. Mengabulkan gugatan Penggugat;
2. Menjatuhkan talak 1 (satu) Ba'in Sughra Tergugat (Tergugat) terhadap Penggugat (Penggugat);
3. Membebankan biaya perkara sesuai dengan peraturan yang berlaku;

Atau apabila Ketua Pengadilan Agama cq. Majelis Hakim yang memeriksa perkara berpendapat lain mohon putusan yang seadil-adilnya;

Menimbang, bahwa Penggugat telah datang menghadap di persidangan, sedangkan Tergugat tidak datang menghadap dan tidak menyuruh orang lain untuk datang menghadap sebagai kuasanya, meskipun Tergugat telah dipanggil dengan resmi dan patut, sedangkan ketidakhadirannya tidak disebabkan oleh sesuatu alasan yang sah;

Menimbang, bahwa terhadap perkara ini tidak layak dilaksanakan mediasi karena Tergugat tidak pernah hadir di persidangan;

Menimbang, bahwa kemudian dibacakan surat gugatan Penggugat yang isi pokoknya tetap dipertahankan oleh Penggugat;

Menimbang, bahwa, untuk menguatkan dalil-dalil gugatannya, Penggugat telah mengajukan alat bukti surat berupa:

1. Fotokopi Kutipan Akta Nikah Nomor 214/39/VI/2015 tanggal 16 Juni 2015 atas nama Penggugat dan Tergugat yang dicatat dan dikeluarkan oleh

Disclaimer
Kepaniteraan Mahkamah Agung Republik Indonesia berusaha untuk selalu mencantumkan informasi paling kini dan akurat sebagai bentuk komitmen Mahkamah Agung untuk pelayanan publik, transparansi dan akuntabilitas pelaksanaan fungsi peradilan. Namun dalam hal-hal tertentu masih dimungkinkan terjadi permasalahan teknis terkait dengan akurasi dan keterkinian informasi yang kami sajikan, hal mana akan terus kami perbaiki dari waktu kewaktu.
Dalam hal Anda menemukan inakurasi informasi yang termuat pada situs ini atau informasi yang seharusnya ada, namun belum tersedia, maka harap segera hubungi Kepaniteraan Mahkamah Agung RI melalui :
Email : kepaniteraan@mahkamahagung.go.id Telp : 021-384 3348 (ext.318)

Pegawai Pencatat Nikah Kantor Urusan Agama Kecamatan Cempaka Putih Kota Jakarta Pusat Provinsi DKI Jakarta, telah bermeterai cukup dan di-*nazegeling*, yang oleh Ketua Majelis telah dicocokkan dengan aslinya ternyata cocok, diberi tanggal, diberi tanda P dan diparaf;

Menimbang, bahwa selain alat bukti tersebut di atas, Penggugat menghadirkan dua orang saksi, yaitu:

1. Saksi I, umur 27 tahun, agama Islam, pekerjaan Wiraswasta, tempat tinggal di Kabupaten Bekasi;

   Saksi tersebut telah memberikan keterangannya di bawah sumpah di muka sidang yang secara rinci sebagaimana tertuang dalam berita acara perkara ini yang untuk mempersingkat putus pada pokoknya adalah sebagai berikut:

   - Bahwa saksi kenal dengan Penggugat dan Tergugat karena saksi adalah teman Penggugat;
   - Bahwa benar Penggugat dan Tergugat adalah suami istri sah dan telah menikah pada tanggal 16 Juni 2015 dan telah dikaruniai keturunan tiga orang anak;
   - Bahwa rumah tangga Penggugat dan Tergugat awalnya rukun dan harmonis namun sejak Juni 2020 sudah tidak rukun lagi sering terjadi pertengkaran dan perselisihan;
   - Bahwa penyebab pertengkaran karena Tergugat tidak terbuka soal keuangan kepada Penggugat, Tergugat melakukan Kekerasan Dalam Rumah Tangga (KDRT) kepada Penggugat;
   - Bahwa Penggugat dan Tergugat telah pisah rumah tiga bulan lamanya;
   - Bahwa sudah diadakan musyawarah keluarga, namun tidak berhasil;
   - Bahwa saksi tidak sanggup merukunkan Penggugat dan Tergugat, karena Penggugat tetap menginginkan bercerai dengan Tergugat;

2. Saksi II, umur 48 tahun, agama Islam, pekerjaan Wiraswasta, bertempat tinggal di Kabupaten Bekasi;

   Saksi tersebut telah memberikan keterangan di bawah sumpah menurut agama Islam di muka sidang, yang pada pokoknya adalah sebagai berikut:

Disclaimer
Kepaniteraan Mahkamah Agung Republik Indonesia berusaha untuk selalu mencantumkan informasi paling kini dan akurat sebagai bentuk komitmen Mahkamah Agung untuk pelayanan publik, transparansi dan akuntabilitas pelaksanaan fungsi peradilan. Namun dalam hal-hal tertentu masih dimungkinkan terjadi permasalahan teknis terkait dengan akurasi dan keterkinian informasi yang kami sajikan, hal mana akan terus kami perbaiki dari waktu kewaktu.
Dalam hal Anda menemukan inakurasi informasi yang termuat pada situs ini atau informasi yang seharusnya ada, namun belum tersedia, maka harap segera hubungi Kepaniteraan Mahkamah Agung RI melalui :
Email : kepaniteraan@mahkamahagung.go.id   Telp : 021-384 3348 (ext.318)

- Bahwa saksi kenal dengan Penggugat dan Tergugat karena saksi adalah sepupu Penggugat;
- Bahwa benar Penggugat dan Tergugat adalah suami isteri sah dan telah menikah pada tanggal 16 Juni 2015 dan telah dikaruniai keturunan tiga orang anak;
- Bahwa rumah tangga Penggugat dan Tergugat awalnya rukun dan harmonis namun sejak Juni 2020 sudah tidak rukun lagi sering terjadi pertengkaran dan perselisihan;
- Bahwa penyebab pertengkaran karena Tergugat tidak terbuka soal keuangan kepada Penggugat, Tergugat melakukan Kekerasan Dalam Rumah Tangga (KDRT) kepada Penggugat;
- Bahwa Penggugat dan Tergugat telah pisah rumah tiga bulan lamanya;
- Bahwa sudah diadakan musyawarah keluarga, namun tidak berhasil;
- Bahwa saksi tidak sanggup merukunkan Penggugat dan Tergugat, karena Penggugat tetap menginginkan bercerai dengan Tergugat;

Menimbang, bahwa atas keterangan para saksi tersebut, Penggugat menyatakan tidak keberatan;

Menimbang, bahwa selanjutnya Penggugat telah menyampaikan kesimpulan secara lisan yang pada pokoknya Penggugat tetap ingin bercerai dari Tergugat, selanjutnya Penggugat tidak akan mengajukan sesuatu apapun lagi dan mohon putusan;

Menimbang, bahwa tentang jalannya pemeriksaan perkara ini selengkapnya telah dicatat dalam berita acara persidangan yang bersangkutan dan untuk mempersingkat cukuplah Majelis Hakim menunjuk kepada berita acara tersebut, yang merupakan bagian dan rangkaian tidak terpisahkan dari putusan ini;

PERTIMBANGAN HUKUM

Menimbang, bahwa maksud dan tujuan gugatan Penggugat adalah sebagaimana tersebut di atas;

Menimbang bahwa Penggugat dalam gugatannya mendalilkan bahwa Penggugat telah melangsungkan perkawinan di hadapan Pegawai Pencatat Nikah Kantor Urusan Agama Kecamatan Cempaka Putih Kota Jakarta Pusat

Disclaimer
Kepaniteraan Mahkamah Agung Republik Indonesia berusaha untuk selalu mencantumkan informasi paling kini dan akurat sebagai bentuk komitmen Mahkamah Agung untuk pelayanan publik, transparansi dan akuntabilitas pelaksanaan fungsi peradilan. Namun dalam hal-hal tertentu masih dimungkinkan terjadi permasalahan teknis terkait dengan akurasi dan keterkinian informasi yang kami sajikan, hal mana akan terus kami perbaiki dari waktu kewaktu.
Dalam hal Anda menemukan inakurasi informasi yang termuat pada situs ini atau informasi yang seharusnya ada, namun belum tersedia, maka harap segera hubungi Kepaniteraan Mahkamah Agung RI melalui :
Email : kepaniteraan@mahkamahagung.go.id   Telp : 021-384 3348 (ext.318)

Provinsi DKI Jakarta, sebagaimana ternyata dalam Kutipan Akta Nikah Nomor 214/39/VI/2015 tertanggal 16 Juni 2015; dan rumah tangga Penggugat dan Tergugat tidak harmonis, oleh karena itu Penggugat memiliki *legal standing* untuk mengajukan gugatan perceraian sebagaimana diatur dalam Pasal 73 Undang-Undang Nomor 7 Tahun 1989 yang telah diubah dengan Undang-Undang Nomor 3 Tahun 2006 dan terakhir diubah dengan Undang-Undang Nomor 50 Tahun 2009;

Menimbang, bahwa Penggugat dan Tergugat beragama Islam dan perkawinan mereka dilangsungkan berdasarkan Hukum Islam oleh karena itu berdasarkan Pasal 40 dan Pasal 63 ayat (1) huruf (a) Undang-Undang Nomor 1 Tahun 1974 jis. Pasal 14 dan Pasal 1 huruf (b) Peraturan Pemerintah Nomor 9 Tahun 1975, Pasal 49 ayat (1) huruf (a) Undang-Undang Nomor 7 Tahun 1989 yang telah diubah dengan Undang-Undang Nomor 3 Tahun 2006 dan terakhir diubah dengan Undang-Undang Nomor 50 Tahun 2009, Pengadilan Agama berwewenang memeriksa dan mengadili dan memutus perkara *a quo*;

Menimbang, bahwa berdasarkan ketentuan Pasal 82 Undang-Undang Nomor 7 Tahun 1989 yang telah diubah dengan Undang-Undang Nomor 3 Tahun 2006 dan terakhir diubah dengan Undang-Undang Nomor 50 Tahun 2009, Majelis Hakim telah berusaha mendamaikan dengan memberi nasehat kepada Penggugat, tetapi tidak berhasil;

Menimbang, bahwa berdasarkan ketentuan Pasal 4 ayat (1) Peraturan Mahkamah Agung Nomor 1 Tahun 2016, dimana setiap perkara sengketa perdata yang diajukan ke Pengadilan Tingkat Pertama diwajibkan terlebih dahulu diupayakan perdamaian melalui bantuan mediator, akan tetapi dalam perkara ini Tergugat tidak pernah datang menghadap persidangan dan tidak menyuruh orang lain untuk datang menghadap sebagai kuasanya sehingga upaya mediasi tidak dapat dilaksanakan;

Menimbang, bahwa Tergugat tidak pernah hadir di persidangan dan tidak pula mengutus orang lain sebagai kuasanya, meskipun dipanggil dengan resmi dan patut, sedangkan ketidakhadiran tersebut tidak disebabkan oleh suatu halangan yang sah, Tergugat yang tidak hadir itu dinyatakan tidak hadir, berdasarkan Pasal 125 ayat (1) HIR, perkara ini dapat diputus dengan *verstek*;

Disclaimer
Kepaniteraan Mahkamah Agung Republik Indonesia berusaha untuk selalu mencantumkan informasi paling kini dan akurat sebagai bentuk komitmen Mahkamah Agung untuk pelayanan publik, transparansi dan akuntabilitas pelaksanaan fungsi peradilan. Namun dalam hal-hal tertentu masih dimungkinkan terjadi permasalahan teknis terkait dengan akurasi dan keterkinian informasi yang kami sajikan, hal mana akan terus kami perbaiki dari waktu kewaktu.
Dalam hal Anda menemukan inakurasi informasi yang termuat pada situs ini atau informasi yang seharusnya ada, namun belum tersedia, maka harap segera hubungi Kepaniteraan Mahkamah Agung RI melalui :
Email : kepaniteraan@mahkamahagung.go.id   Telp : 021-384 3348 (ext.318)

Menimbang, bahwa berdasarkan bukti surat bertanda P berupa fotokopi sah Kutipan Akta Nikah adalah suatu akta autentik, karena dibuat oleh pejabat yang berwenang dan berdasarkan peraturan perundang-undangan yang berlaku, sehingga mempunyai kekuatan pembuktian sempurna (Pasal 165 HIR) selama tidak dibuktikan kepalsuannya, oleh karena itu harus dinyatakan terbukti bahwa hubungan hukum antara Penggugat dengan Tergugat adalah terikat dalam pernikahan yang sah, oleh karena itu harus dipandang Penggugat sebagai pihak yang berkepentingan langsung dalam perkara ini;

Menimbang, bahwa gugatan Penggugat pada pokoknya memohon kepada Majelis Hakim agar menceraikan Penggugat dengan Tergugat, dengan mengemukakan alasan yang secara lengkapnya telah termuat dalam surat gugatan dan telah dicantumkan dalam tentang duduk perkaranya di atas adalah telah memenuhi syarat formal sebagaimana sebuah surat gugatan, sehingga perkaranya dapat diterima untuk dipertimbangkan;

Menimbang, bahwa untuk menguatkan dalil-dalil gugatannya, Penggugat telah menghadirkan saksi di persidangan sebagaimana yang dikehendaki ketentuan Pasal 22 ayat (2) Peraturan Pemerintah Nomor 9 Tahun 1975;

Menimbang, bahwa berdasarkan keterangan saksi-saksi Penggugat di muka persidangan telah ditemukan fakta bahwasanya antara Penggugat dan Tergugat telah terjadi pertengkaran dan perselisihan yang terus menerus sehingga mengakibatkan Penggugat dan Tergugat telah pisah rumah dan telah ada usaha untuk merukunkan Penggugat dan Tergugat namun tidak berhasil;

Menimbang, bahwa kesaksian para saksi ini satu dengan lainnya saling berkesesuaian dan mendukung dalil gugatan Penggugat sehingga kesaksian tersebut dapat diterima, sesuai dengan ketentuan Pasal 172 HIR, maka Majelis berpendapat bahwa rumah tangga Penggugat dan Tergugat tidak harmonis lagi dan harapan terciptanya rumah tangga yang bahagia sebagaimana dikehendaki Pasal 1 Undang-Undang Nomor 1 Tahun 1974 tidak tercapai;

Menimbang, bahwa berdasarkan keterangan Penggugat dan saksi-saksi di persidangan yang menyatakan telah terjadi perselisihan dan pertengkaran antara Penggugat dan Tergugat. Dan keluarga Penggugat tersebut menyatakan bahwa telah diupayakan mendamaikan Penggugat dengan Tergugat tetapi tidak

Disclaimer
Kepaniteraan Mahkamah Agung Republik Indonesia berusaha untuk selalu mencantumkan informasi paling kini dan akurat sebagai bentuk komitmen Mahkamah Agung untuk pelayanan publik, transparansi dan akuntabilitas pelaksanaan fungsi peradilan. Namun dalam hal-hal tertentu masih dimungkinkan terjadi permasalahan teknis terkait dengan akurasi dan keterkinian informasi yang kami sajikan, hal mana akan terus kami perbaiki dari waktu kewaktu.
Dalam hal Anda menemukan inakurasi informasi yang termuat pada situs ini atau informasi yang seharusnya ada, namun belum tersedia, maka harap segera hubungi Kepaniteraan Mahkamah Agung RI melalui :
Email : kepaniteraan@mahkamahagung.go.id   Telp : 021-384 3348 (ext.318)

berhasil, serta tidak sanggup lagi untuk mengusahakan perdamaian, maka Majelis berpendapat bahwasanya Pasal 22 ayat (2) Peraturan Pemerintah Nomor 9 Tahun 1975 telah terpenuhi karenanya gugatan Penggugat dapat dipertimbangkan;

Menimbang, bahwa Majelis sependapat dan mengambil alih pendapat ahli fiqih dalam Kitab *Ahkamul Qur'an* Juz II hal. 405 yang berbunyi:

من دعى الى حاكم من حكام المسلمين ولم يجب فهو ظالم لا حق له

"Barang siapa yang dipanggil untuk menghadap Hakim Islam, kemudian tidak menghadap maka ia termasuk orang yang dlalim, dan gugurlah haknya”

Menimbang, bahwa berdasarkan fakta-fakta tersebut di atas Majelis berkesimpulan bahwasanya telah terbukti antara Penggugat dengan Tergugat telah terjadi perselisihan dan pertengkaran terus-menerus dan tidak mungkin lagi untuk dapat didamaikan maka dengan demikian gugatan Penggugat untuk melakukan perceraian telah cukup beralasan dan tidak melawan hukum, sebagaimana maksud Pasal 39 ayat (2) Undang-Undang Nomor 1 Tahun 1974 *jo* Pasal 19 huruf (f) Peraturan Pemerintah Nomor 9 Tahun 1975 *jo* Pasal 116 huruf (f) Kompilasi Hukum Islam, oleh karenanya Majelis hakim mengabulkan gugatan Penggugat;

Menimbang, bahwa berdasarkan ketentuan Pasal 89 ayat (1) Undang-Undang Nomor 7 Tahun 1989 yang telah diubah dengan Undang-Undang Nomor 3 Tahun 2006 dan terakhir diubah dengan Undang-Undang Nomor 50 Tahun 2009, maka kepada Penggugat dibebani untuk membayar biaya perkara yang timbul dari perkara ini;

Mengingat segala peraturan perundang-undangan yang berlaku dan ketentuan Hukum *Syar'i* yang berkaitan dengan perkara ini;

M E N G A D I L I

1. Menyatakan Tergugat yang telah dipanggil secara resmi dan patut untuk menghadap di persidangan, tidak hadir;
2. Mengabulkan Gugatan Penggugat dengan verstek;
3. Menjatuhkan talak satu ba'in sughra Tergugat (Tergugat) terhadap Penggugat (Penggugat);

Disclaimer
Kepaniteraan Mahkamah Agung Republik Indonesia berusaha untuk selalu mencantumkan informasi paling kini dan akurat sebagai bentuk komitmen Mahkamah Agung untuk pelayanan publik, transparansi dan akuntabilitas pelaksanaan fungsi peradilan. Namun dalam hal-hal tertentu masih dimungkinkan terjadi permasalahan teknis terkait dengan akurasi dan keterkinian informasi yang kami sajikan, hal mana akan terus kami perbaiki dari waktu kewaktu.
Dalam hal Anda menemukan inakurasi informasi yang termuat pada situs ini atau informasi yang seharusnya ada, namun belum tersedia, maka harap segera hubungi Kepaniteraan Mahkamah Agung RI melalui :
Email : kepaniteraan@mahkamahagung.go.id   Telp : 021-384 3348 (ext.318)

4. Membebankan kepada Penggugat untuk membayar biaya perkara sejumlah Rp 741.000,- (tujuh ratus empat puluh satu ribu rupiah);

Demikianlah diputuskan dalam musyawarah Majelis Hakim Pengadilan Agama Cikarang pada hari Rabu tanggal 23 Desember 2020 Masehi bertepatan dengan tanggal 8 Jumadil Awwal 1442 Hijriah oleh Alvi Syafiatin, S.Ag. sebagai Ketua Majelis, Drs. H. A. Jazuli, M.Ag. dan Drs. H. Sayuti, masing-masing sebagai Hakim Anggota, putusan tersebut diucapkan pada hari itu juga dalam sidang terbuka untuk umum oleh Ketua Majelis beserta para Hakim Anggota tersebut, dan didampingi oleh Nove Ratnawati, S.H. sebagai Panitera Pengganti, dengan dihadiri oleh Penggugat tanpa hadirnya Tergugat.

Hakim Anggota, Ketua Majelis,

Drs. H. A. Jazuli, M.Ag. **Alvi Syafiatin, S.Ag.**

Hakim Anggota,

Drs. H. Sayuti

Panitera Pengganti,

Nove Ratnawati, S.H.

Rincian biaya:

1. Biaya Pendaftaran	:	Rp.	30.000,00
2. Biaya Proses	:	Rp.	50.000,00
3. PNBP Panggilan	:	Rp.	20.000,00
4. Biaya Panggilan	:	Rp.	625.000,00

Disclaimer
Kepaniteraan Mahkamah Agung Republik Indonesia berusaha untuk selalu mencantumkan informasi paling kini dan akurat sebagai bentuk komitmen Mahkamah Agung untuk pelayanan publik, transparansi dan akuntabilitas pelaksanaan fungsi peradilan. Namun dalam hal-hal tertentu masih dimungkinkan terjadi permasalahan teknis terkait dengan akurasi dan keterkinian informasi yang kami sajikan, hal mana akan terus kami perbaiki dari waktu kewaktu.
Dalam hal Anda menemukan inakurasi informasi yang termuat pada situs ini atau informasi yang seharusnya ada, namun belum tersedia, maka harap segera hubungi Kepaniteraan Mahkamah Agung RI melalui :
Email : kepaniteraan@mahkamahagung.go.id Telp : 021-384 3348 (ext.318)

5. Biaya Redaksi	:	Rp.	10.000,00
6. Biaya Meterai	:	Rp.	6.000,00
**JUMLAH**	**:**	Rp.	741.000,00

(tujuh ratus empat puluh satu ribu rupiah).

Disclaimer
Kepaniteraan Mahkamah Agung Republik Indonesia berusaha untuk selalu mencantumkan informasi paling kini dan akurat sebagai bentuk komitmen Mahkamah Agung untuk pelayanan publik, transparansi dan akuntabilitas pelaksanaan fungsi peradilan. Namun dalam hal-hal tertentu masih dimungkinkan terjadi permasalahan teknis terkait dengan akurasi dan keterkinian informasi yang kami sajikan, hal mana akan terus kami perbaiki dari waktu kewaktu.
Dalam hal Anda menemukan inakurasi informasi yang termuat pada situs ini atau informasi yang seharusnya ada, namun belum tersedia, maka harap segera hubungi Kepaniteraan Mahkamah Agung RI melalui :
Email : kepaniteraan@mahkamahagung.go.id Telp : 021-384 3348 (ext.318)
