# Supplementary material for: Classification of divorce causes during the COVID-19 pandemic using convolutional neural networks
Source: PeerJ Comput Sci. 2022 Jun 30;8:e998. doi: 10.7717/peerj-cs.998 (PMC9299239; doi:10.7717/peerj-cs.998)
Supplement: Supplemental Information 5 [file peerj-cs-08-998-s005.zip › Masalah Ekonomi Dataset/Data ke-17.pdf]

P U T U S A N

Nomor 1650/Pdt.G/2020/PA.Bgr

بِسْمِ اللَّهِ الرَّحْمَنِ الرَّحِيمِ

DEMI KEADILAN BERDASARKAN KETUHANAN YANG MAHA ESA

Pengadilan Agama Bogor yang memeriksa dan mengadili perkara tertentu pada tingkat pertama dalam sidang Majelis Hakim telah menjatuhkan putusan sebagai berikut dalam perkara cerai gugat antara:

Penggugat, tempat/tangal lahir Bogor/17 Oktober 1962, umur 58 tahun, agama Islam, pendidikan SLTA, pekerjaan Pensiunan, tempat kediaman di Jalan xxxxxxxxxxxxxxxxx, Kota Bogor Provinsi, Jawa Barat, sebagai Penggugat;

Melawan

Tergugat, tempat/tangal lahir Bogor/07 Juli 1965, umur 55 tahun, agama Islam, pendidikan SLTA, pekerjaan Pensiunan, tempat kediaman di xxxxxxxxxxxxxxxx, Kota Bogor Provinsi, Jawa Barat, sebagai Tergugat;

Pengadilan Agama tersebut;

Telah membaca dan mempelajari berkas perkara;

Telah mendengar keterangan Penggugat dan memeriksa alat-alat bukti Penggugat di persidangan;

DUDUK PERKARA

Bahwa Penggugat dengan surat gugatannya tertanggal 14 Desember 2020 yang terdaftar di Kepaniteraan Pengadilan Agama Bogor pada hari itu juga dengan register perkara Nomor 1650/Pdt.G/2020/PA.Bgr, mengemukakan hal-hal sebagai berikut:

1. Bahwa Penggugat adalah istri sah dari Tergugat yang telah melangsungkan pernikahan pada hari Sabtu tanggal 17 Oktober 1992 di hadapan Pejabat Pencatat Nikah Kantor Urusan Agama Kecamatan Bogor Barat, Kota Bogor, Provinsi Jawa Barat, sebagaimana terbukti dalam Buku Kutipan Akta Nikah Nomor xxxxxxxxxxx tertanggal 17 Oktober 1992;

Disclaimer
Kepaniteraan Mahkamah Agung Republik Indonesia berusaha untuk selalu mencantumkan informasi paling kini dan akurat sebagai bentuk komitmen Mahkamah Agung untuk pelayanan publik, transparansi dan akuntabilitas pelaksanaan fungsi peradilan. Namun dalam hal-hal tertentu masih dimungkinkan terjadi permasalahan teknis terkait dengan akurasi dan keterkinian informasi yang kami sajikan, hal mana akan terus kami perbaiki dari waktu kewaktu.
Dalam hal Anda menemukan inakurasi informasi yang termuat pada situs ini atau informasi yang seharusnya ada, namun belum tersedia, maka harap segera hubungi Kepaniteraan Mahkamah Agung RI melalui :
Email : kepaniteraan@mahkamahagung.go.id Telp : 021-384 3348 (ext.318)

2. Bahwa pada saat menikah Penggugat berstatus perawan dan Tergugat berstatus perjaka;

3. Bahwa setelah menikah Penggugat dengan Tergugat tinggal bersama terakhir di Jalan xxxxxxxxxxxxxxxx, Kota Bogor Provinsi, Jawa Barat;

4. Bahwa selama hidup berumah tangga antara Penggugat dengan Tergugat sudah berhubungan sebagaimana layaknya suami isteri dan sudah dikaruniai 3 orang anak yang bernama:

4.1. Anak 1, perempuan, lahir di Bogor, 17 Juni 1993;

4.2. Anak 2, perempuan, lahir di Bogor, 25 Agustus 1994;

4.3. Anak 3, laki-laki, lahir di Bogor, 03 November 2001;

5. Bahwa sejak awal Tahun 2012 Tergugat pergi meninggalkan Penggugat dan anak-anak, sejak itu Tergugat tidak pernah memperdulikan nasib Penggugat dan anak-anak, dan tidak pernah lagi memberikan nafkah lahir dan bathin. Pada Bulan Maret Tahun 2019, Tergugat sempat kembali kerumah hanya untuk menikahkan anak Penggugat dan Tergugat, setelah itu Tergugat pergi lagi, dan sampai sekarang tidak pernah kembali;

6. Bahwa puncak perselisihan antara Penggugat dengan Tergugat terjadi sekitar Bulan Maret tahun 2019 yang mana Tergugat pergi Penggugat dengan Tergugat sudah pisah kamar dan sejak itu tidak ada hubungan seperti layaknya suami istri lagi;

7. Bahwa Penggugat yakin tidak ada lagi harapan untuk meneruskan rumah tangga dengan Tergugat karena tujuan perkawinan untuk membina rumah tangga yang Sakinah, Mawaddah wa Rahmah sudah tidak terwujud lagi sebagaimana yang diamanatkan oleh Undang-undang Nomor 1 Tahun 1974 tentang Perkawinan dan Instruksi Presiden Nomor 1 Tahun 1991 tentang Kompilasi Hukum Islam, sehingga sangat beralasan apabila gugatan ini dikabulkan;

8. Bahwa Penggugat sanggup membayar biaya yang timbul dari perkara ini;

Berdasarkan alasan-alasan tersebut, Penggugat mohon kepada Ketua Pengadilan Agama Bogor Cq. Majelis Hakim yang memeriksa dan mengadili perkara ini untuk menjatuhkan putusan sebagai berikut:

Disclaimer
Kepaniteraan Mahkamah Agung Republik Indonesia berusaha untuk selalu mencantumkan informasi paling kini dan akurat sebagai bentuk komitmen Mahkamah Agung untuk pelayanan publik, transparansi dan akuntabilitas pelaksanaan fungsi peradilan. Namun dalam hal-hal tertentu masih dimungkinkan terjadi permasalahan teknis terkait dengan akurasi dan keterkinian informasi yang kami sajikan, hal mana akan terus kami perbaiki dari waktu kewaktu.
Dalam hal Anda menemukan inakurasi informasi yang termuat pada situs ini atau informasi yang seharusnya ada, namun belum tersedia, maka harap segera hubungi Kepaniteraan Mahkamah Agung RI melalui :
Email : kepaniteraan@mahkamahagung.go.id Telp : 021-384 3348 (ext.318)

Primer:

1. Mengabulkan gugatan Penggugat;

2. Menjatuhkan talak satu ba'in shugro Tergugat (**Tergugat**) kepada Penggugat (**Penggugat**);

3. Menetapkan biaya perkara menurut hukum;

Subsider:

- Apabila Majelis Hakim berpendapat lain mohon keputusan yang seadil-adilnya.

Bahwa pada hari-hari sidang yang telah ditetapkan, Penggugat datang menghadap sendiri di persidangan, sedangkan Tergugat tidak pernah datang menghadap dan tidak pula menyuruh orang lain untuk menghadap sebagai wakil atau kuasanya yang sah, meskipun telah dipanggil secara resmi dan patut dan tidak ternyata ketidakhadirannya tersebut disebabkan oleh suatu halangan yang sah, maka perkara ini diperiksa tanpa hadirnya Tergugat;

Bahwa upaya perdamaian dan mediasi tidak dapat dilaksanakan karena Tergugat tidak hadir di persidangan;

Bahwa Majelis Hakim dalam persidangan telah berusaha mendamaikan dengan jalan memberikan nasihat kepada Penggugat agar bersabar dan tetap mempertahankan keutuhan rumah tangganya dengan Tergugat, akan tetapi tidak berhasil. Sedangkan usaha damai melalui proses mediasi tidak dapat dilaksanakan karena Tergugat tidak pernah datang menghadap ke persidangan;

Bahwa selanjutnya pemeriksaan perkara dilakukan dalam sidang tertutup untuk umum dengan terlebih dahulu dibacakan surat gugatan Penggugat yang isi dan maksudnya tetap dipertahankan oleh Penggugat;

Bahwa, oleh karena Tergugat tidak datang di persidangan meskipun telah dipanggil secara resmi dan patut, maka jawaban Tergugat tidak dapat didengar dalam persidangan ;

Bahwa untuk meneguhkan dalil-dalil gugatannya, Penggugat telah mengajukan bukti surat berupa :

a. Fotokopi Kutipan Akta Nikah dari Kantor Urusan Agama Kecamatan Bogor Barat, Kota bogor, Nomor : xxxxxxxxxxxxx tertanggal 17 Oktober

Disclaimer
Kepaniteraan Mahkamah Agung Republik Indonesia berusaha untuk selalu mencantumkan informasi paling kini dan akurat sebagai bentuk komitmen Mahkamah Agung untuk pelayanan publik, transparansi dan akuntabilitas pelaksanaan fungsi peradilan. Namun dalam hal-hal tertentu masih dimungkinkan terjadi permasalahan teknis terkait dengan akurasi dan keterkinian informasi yang kami sajikan, hal mana akan terus kami perbaiki dari waktu kewaktu.
Dalam hal Anda menemukan inakurasi informasi yang termuat pada situs ini atau informasi yang seharusnya ada, namun belum tersedia, maka harap segera hubungi Kepaniteraan Mahkamah Agung RI melalui :
Email : kepaniteraan@mahkamahagung.go.id Telp : 021-384 3348 (ext.318)

1992, dan setelah dicocokan dengan aslinya ternyata sama serta bermaterai cukup, selanjutnya di beri tanda bukti P.1;

b. Fotokopi Kartu Tanda penduduk atas nama **Penggugat**, yang dikeluarkan oleh pemerintah Kota Bogor, dengan NIK : xxxxxxxxxxxx tanggal 24 Juni 2017, dan setelah dicocokan dengan aslinya ternyata sama serta bermeterai cukup, lalu diberi kode P.2;

Bahwa selain bukti surat tersebut, Penggugat juga menghadirkan 2 orang saksi sebagai berikut:

1. **Saksi 1**, umur 64 tahun, agama Islam, pekerjaan Ibu Rumah Tangga, bertempat tinggal di xxxxxxxxxxxxxxxxx, Kota Bogor;

   Saksi adalah kakak kandung Penggugat dan dibawah sumpahnya menerangkan hal-hal yang pada pokoknya sebagai berikut :

   - Bahwa Penggugat dan Tergugat adalah suami isteri yang menikah pada 17 Oktober 1992;
   - Bahwa setelah menikah antara Penggugat dan Tergugat betempat tinggal terakhir di daerah Kelurahan kedung Halang, Kecamatan Bogor Utara, Kota Bogor ;
   - Bahwa Penggugat dan Tergugat dikaruniai 3 orang anak bernama **Anak 1, anak 2 dan Anak 3**, dan ketiganya tinggal bersama Penggugat ;
   - Bahwa saksi mengerti keadaan rumah tangga mereka yaitu semula mereka rukun dan damai dalam membina rumah tangganya, namun sejak awal tahun 2012 Penggugat dan Tergugat tidak rukun lagi ;
   - Bahwa Penyebabnya adalah Tergugat kurang memperhatikan dan tidak peduli terhadap keadaan rumah tangganya, dan anak-anak selalu dititip pada saksi jika Penggugat pergi kerja, dan pada tahun 2012 tersebut Tergugat meninggalkan Penggugat, dan Tergugat datang pada Maret 2019 pada saat anak Penggugat dan Tergugat menikah, dan kedatangan Tergugat hanya untuk menjadi wali terhadap anaknya ;
   - Bahwa selama kepergian Tergugat maka Tergugat tidak pernah memberi nafkah kepada Penggugat dan anak-anaknya ;
   - Bahwa saksi mendengar dari anak Penggugat dan Tergugat bahwa Tergugat telah menikah dan tinggal dengan isteri yang lainnya ;

Disclaimer
Kepaniteraan Mahkamah Agung Republik Indonesia berusaha untuk selalu mencantumkan informasi paling kini dan akurat sebagai bentuk komitmen Mahkamah Agung untuk pelayanan publik, transparansi dan akuntabilitas pelaksanaan fungsi peradilan. Namun dalam hal-hal tertentu masih dimungkinkan terjadi permasalahan teknis terkait dengan akurasi dan keterkinian informasi yang kami sajikan, hal mana akan terus kami perbaiki dari waktu kewaktu.
Dalam hal Anda menemukan inakurasi informasi yang termuat pada situs ini atau informasi yang seharusnya ada, namun belum tersedia, maka harap segera hubungi Kepaniteraan Mahkamah Agung RI melalui :
Email : kepaniteraan@mahkamahagung.go.id Telp : 021-384 3348 (ext.318)

- Bahwa dengan sikap Tergugat tersebut maka pihak keluarga sudah memediasi Penggugat dan Tergugat, namun tetap saja tidak berubah ;
- Bahwa saksi sudah berusaha menasehati dan mendamaikan Penggugat dan Tergugat agar rukun kembali dalam membina rumah tangganya, namun usaha tersebut tidak berhasil ;
- Bahwa saksi sudah tidak sanggup lagi dan menyerahkan keputusannya kepada Pengadilan ;

2. **Saksi 2,**, umur 35 tahun, agama Islam, pekerjaan Karyawan, bertempat tinggal di Kompleks xxxxxxxxxxxxxxxx, Kota Bogor,

Saksi adalah keponakan Penggugat dan di persidangan saksi tersebut telah memberikan keterangan di bawah sumpah yang pada pokoknya sebagai berikut:

- Bahwa Penggugat dan Tergugat adalah suami isteri yang menikah pada tahun 1992;
- Bahwa setelah Setelah menikah antara Penggugat dan Tergugat betempat tinggal terakhir di daerah Kelurahan kedung Halang, Kecamatan Bogor Utara, Kota Bogor ;
- Bahwa Penggugat dan Tergugat dikaruniai 3 orang anak bernama **Anak 1, Anak 2 dan Anak 3**, dan ketiganya tinggal bersama Penggugat ;
- Bahwa saksi mengerti keadaan rumah tangga mereka yaitu semula mereka rukun dan damai dalam membina rumah tangganya, namun sejak awal tahun 2012 Penggugat dan Tergugat tidak rukun lagi ;
- Bahwa Penyebabnya adalah Tergugat kurang memperhatikan dan tidak peduli terhadap keadaan rumah tangganya, dan anak-anak selalu dititip pada saksi I jika Penggugat pergi kerja, dan pada tahun 2012 tersebut Tergugat meninggalkan Penggugat, dan Tergugat datang pada saat menikah anaknya pada Maret 2019 dan datang hanya untuk menjadi wali terhadap anaknya ;
- Bahwa setelah kepergian Tergugat maka Tergugat tidak pernah memberi nafkah kepada Penggugat dan anak-anaknya ;

Disclaimer
Kepaniteraan Mahkamah Agung Republik Indonesia berusaha untuk selalu mencantumkan informasi paling kini dan akurat sebagai bentuk komitmen Mahkamah Agung untuk pelayanan publik, transparansi dan akuntabilitas pelaksanaan fungsi peradilan. Namun dalam hal-hal tertentu masih dimungkinkan terjadi permasalahan teknis terkait dengan akurasi dan keterkinian informasi yang kami sajikan, hal mana akan terus kami perbaiki dari waktu kewaktu.
Dalam hal Anda menemukan inakurasi informasi yang termuat pada situs ini atau informasi yang seharusnya ada, namun belum tersedia, maka harap segera hubungi Kepaniteraan Mahkamah Agung RI melalui :
Email : kepaniteraan@mahkamahagung.go.id Telp : 021-384 3348 (ext.318)

- Bahwa saksi ketahui dari anak Penggugat dan Tergugat bahwa Tergugat telah menikah dan tinggal dengan isteri yang barunya ;
- Bahwa dengan sikap Tergugat tersebut maka pihak keluarga sudah memediasi Penggugat dan Tergugat dan Tergugat berjanji akan berubah dan akan memperbaiki sikapnya, namun tetap saja tidak berubah ;
- Bahwa saksi sudah berusaha menasehati dan mendamaikan Penggugat dan Tergugat agar rukun kembali dalam membina rumah tangganya, namun usaha tersebut tidak berhasil ;
- Bahwa saksi sudah tidak sanggup lagi dan menyerahkan keputusannya kepada Pengadilan ;

Bahwa selanjutnya Penggugat mencukupkan alat buktinya dan mengajukan kesimpulan secara lisan yang pada pokoknya tetap pada pendiriannya untuk bercerai dengan Tergugat dan mohon putusan;

Bahwa untuk mempersingkat uraian putusan ini, ditunjuk segala sesuatu yang tercantum dalam Berita Acara Sidang perkara ini sebagai bagian yang tidak terpisahkan dari isi putusan ini;

PERTIMBANGAN HUKUM

Menimbang, bahwa maksud dan tujuan gugatan Penggugat sebagaimana telah diuraikan di atas;

Menimbang, bahwa Penggugat dan Tergugat dalam kedudukannya sebagai suami isteri sebagaimana yang didasarkan pada posita Penggugat dan dikuatkan dengan bukti P1 (akta autentik), maka Penggugat memiliki *persona standi in yudisio* atau memiliki kedudukan hukum untuk mengajukan perkara ini;

Menimbang, bahwa atas dasar identitas diri bukti P1 yang juga sebagai bukti autentik serta Pengakuan Penggugat bahwa Penggugat sebagai orang-orang yang beragama Islam dan beralamat diwilayah hukum Pengadilan Agama Bogor, serta perkara yang diajukan oleh Penggugat termasuk dalam bidang perkawinan, maka menurut Pasal 49 ayat (1) dan ayat (2) berikut Undang-Undang Nomor 7 Tahun 1989, sebagaimana telah diubah dua kali yaitu

Disclaimer
Kepaniteraan Mahkamah Agung Republik Indonesia berusaha untuk selalu mencantumkan informasi paling kini dan akurat sebagai bentuk komitmen Mahkamah Agung untuk pelayanan publik, transparansi dan akuntabilitas pelaksanaan fungsi peradilan. Namun dalam hal-hal tertentu masih dimungkinkan terjadi permasalahan teknis terkait dengan akurasi dan keterkinian informasi yang kami sajikan, hal mana akan terus kami perbaiki dari waktu kewaktu.
Dalam hal Anda menemukan inakurasi informasi yang termuat pada situs ini atau informasi yang seharusnya ada, namun belum tersedia, maka harap segera hubungi Kepaniteraan Mahkamah Agung RI melalui :
Email : kepaniteraan@mahkamahagung.go.id Telp : 021-384 3348 (ext.318)

oleh Undang-Undang Nomor 3 Tahun 2006 dan Undang-Undang Nomor: 50 Tahun 2009 berikut penjelasannya, perkara ini menjadi kewenangan Pengadilan Agama Bogor;

Menimbang, bahwa Penggugat telah datang menghadap di persidangan sedangkan Tergugat meskipun telah dipanggil secara resmi dan patut tidak datang menghadap dipersidangan dan tidak pula menyuruh orang lain sebagai wakil atau kuasanya yang sah dan tidak ternyata bahwa ketidakhadiran Tergugat itu disebabkan oleh suatu halangan yang sah, maka berdasarkan Pasal 125 dan 126 HIR perkara ini dapat diputus tanpa hadirnya Tergugat *(verstek);*

Menimbang, bahwa oleh karena Tergugat tidak pernah hadir di persidangan maka upaya mediasi tidak dapat dilaksanakan, sebagaimana ketentuan PERMA Nomor 01 Tahun 2008 tentang Prosedur Mediasi di Pengadilan, meskipun demikian Pengadilan telah bersungguh-sungguh mengupayakan perdamaian akan tetapi tidak berhasil karena Penggugat tetap pada gugatannya untuk bercerai dengan Tergugat ;

Menimbang, bahwa alasan pokok gugatan Penggugat untuk menceraikan Tergugat adalah karena antara Penggugat dan Tergugat sebagai suami isteri awalnya rukun 3 orang anak bernma **Anak 1, Anak 2dan Anak 3**, dan sejak tahun 2012 antara Penggugat dengan tidak rukun lagi Tergugat meninggalkan Penggugat dan tidak memperdulikan Penggugat dan anak-anaknya serta tidak pernah memberi nafkah kepada Penggugat dan anak-anaknya, dan pada Maret 2019 Tergugat sempat kembali hanya untuk menikahkan anaknya dan setelah itu Tergugat pergi lagi dan tidak kembali lagi hingga saat ini, dan sejak saat tidak ada lagi hubungan antara Penggugat dengan Tergugat sampai saat ini ;

Menimbang, bahwa meskipun Tergugat tidak pernah hadir dipersidangan dapat dinilai bahwa Tergugat tidak hendak membantah dalil-dalil gugatan Penggugat, akan tetapi oleh karena alasan yang diajukan Penggugat dalam kasus ini didasarkan pada adanya perselisihan dan pertengkaran yang terus-menerus serta perkara ini menganut aturan khusus *(lex specialis)*, maka Penggugat dibebani wajib bukti ;

Disclaimer
Kepaniteraan Mahkamah Agung Republik Indonesia berusaha untuk selalu mencantumkan informasi paling kini dan akurat sebagai bentuk komitmen Mahkamah Agung untuk pelayanan publik, transparansi dan akuntabilitas pelaksanaan fungsi peradilan. Namun dalam hal-hal tertentu masih dimungkinkan terjadi permasalahan teknis terkait dengan akurasi dan keterkinian informasi yang kami sajikan, hal mana akan terus kami perbaiki dari waktu kewaktu.
Dalam hal Anda menemukan inakurasi informasi yang termuat pada situs ini atau informasi yang seharusnya ada, namun belum tersedia, maka harap segera hubungi Kepaniteraan Mahkamah Agung RI melalui :
Email : kepaniteraan@mahkamahagung.go.id   Telp : 021-384 3348 (ext.318)

Menimbang, bahwa sebelum mempertimbangkan mengenai bukti tentang alasan perceraian, terlebih dahulu dipertimbangkan tentang sahnya perkawinan Penggugat dan Tergugat sebagai dasar Penggugat mengajukan gugatan cerai ini ;

Menimbang, bahwa berdasarkan bukti P1 yang diajukan Penggugat yang dinilai oleh Pengadilan sebagai akta otentik dan nilai pembuktiannya sempurna dan mengikat, maka harus dinyatakan terbukti sah perkawinan Penggugat dan Tergugat yang dilaksanakan pada tanggal 17 oktober 1992 dan tercatat pada Kantor urusan agama kecamatan Bogor Barat, Kota Bogor ;

Menimbang, bahwa Penggugat dalam meneguhkan dalil-dalil pokok tentang perceraiannya, maka Penggugat mengajukan 2 (dua) orang saksi untuk didengar keterangannya di persidangan, dan kedua saksi tersebut meskipun keduanya adalah orang yang terdekat dengan Penggugat yakni kakak kandung dan keponakan Penggugat, namun alasan Penggugat untuk menceraikan Tergugat adalah didasarkan pada adanya perselisihan terus, maka kedua saksi tersebut dapat diterima sebagai pembuktian dalam perkara ini (Vide pasal 22 ayat (2) Peraturan Pemerintah nomor 9 tahun 1975), serta keduanya telah memberikan keterangan dipersidangan dibawah sumpahnya, dengan demikian saksi-saksi tersebut memenuhi syarat formil saksi ;

Menimbang, bahwa kedua saksi tersebut telah memberikan keterangan yang saling bersesuaian dan mendukung dalil gugatan Penggugat, dan keterangan-keterangan saksi tersebut yakni kedua saksi mengetahui secara langsung bahwa antara Penggugat dengan Tergugat awalnya rukun 3 orang anak bernama **Anak 1, Anak 2**dan **Anak 3** dan ketiganya tinggal bersama Penggugat, dan sejak tahun 2012 antara Penggugat dengan Tergugat tidak rukun lagi disebabkan Tergugat telah meninggalkan Penggugat dan Tergugat tidak memperhatikan dan tidak peduli lagi terhadap keadaan rumah tangganya, dan selama berpisah Tergugat tidak memberi nafkah kepada Penggugat dan anak-anaknya, Tergugat pernah datang pada Maret 2019 hanya untuk menjadi wali pada pernikahan anaknya ;

Disclaimer
Kepaniteraan Mahkamah Agung Republik Indonesia berusaha untuk selalu mencantumkan informasi paling kini dan akurat sebagai bentuk komitmen Mahkamah Agung untuk pelayanan publik, transparansi dan akuntabilitas pelaksanaan fungsi peradilan. Namun dalam hal-hal tertentu masih dimungkinkan terjadi permasalahan teknis terkait dengan akurasi dan keterkinian informasi yang kami sajikan, hal mana akan terus kami perbaiki dari waktu kewaktu.
Dalam hal Anda menemukan inakurasi informasi yang termuat pada situs ini atau informasi yang seharusnya ada, namun belum tersedia, maka harap segera hubungi Kepaniteraan Mahkamah Agung RI melalui :
Email : kepaniteraan@mahkamahagung.go.id   Telp : 021-384 3348 (ext.318)

Menimbang, bahwa berdasarkan pertimbangan pembuktian tersebut diatas maka pengadilan menemukan fakta-fakta yang pada pokoknya sebagai berikut:

- Bahwa antara Penggugat dengan Tergugat adalah sebagai suami isteri sah yang menikah pada tanggal 17 oktober 1992 dan tercatat pada Kantor urusan agama kecamatan Bogor Barat, Kota Bogor ;
- Bahwa Penggugat dan Tergugat 3 orang anak bernma **Anak 1, Anak 2**dan **Anak 3**, ketiga tinggal bersama Penggugat;
- Bahwa kehidupan rumah tangga Penggugat dengan Tergugat awalnya rukun, namun kemudian sejak tahun 2012 sudah tidak rukun lagi karena Tergugat meninggalkan Penggugat dan tidak peduli kepada Penggugat dan anak-anaknya ;
- Bahwa penyebab tidak rukunnya Penggugat dengan Tergugat tersebut karena Tergugat tidak memperhatikan dan tidak peduli terhadap keadaan Penggugat dan anaknya-anaknya, dan selama kepergian Tergugat tersebut tidak memberi nafkah kepada Penggugat dan anak-anaknya ;
- Bahwa Tergugat pernah datang pada Maret 2019 hanya untuk menjadi wali dalam pernikahan anaknya ;
- Bahwa antara Penggugat dengan Tergugat telah pisahsejak tahun 2012 tersebut karena Tergugat meninggalkan kediaman bersamanya dan sampai saat ini tidak bersama lagi dan tidak lagi memberi nafkah kepada Penggugat ;
- Bahwa Penggugat dengan Tergugat telah diupayakan rukun oleh pihak keluarga, namun tidak berhasil ;

Menimbang, bahwa berdasarkan fakta tersebut diatas maka pengadilan telah dapat menilai bahwa di dalam rumah tangga antara Penggugat dengan Tergugat sudah tidak harmonis lagi apalagi Tergugat telah nyata meninggalkan Penggugat sejak tahun 2012 atau dihitung 8 (delapan) tahun lalu sampai saat ini tanpa nafkah ;

Menimbang, bahwa prilaku Tergugat yang kurang memperhatikan dan tidak peduli terhadap keadaan rumah tangganya, dan sikap Tergugat yang

Disclaimer
Kepaniteraan Mahkamah Agung Republik Indonesia berusaha untuk selalu mencantumkan informasi paling kini dan akurat sebagai bentuk komitmen Mahkamah Agung untuk pelayanan publik, transparansi dan akuntabilitas pelaksanaan fungsi peradilan. Namun dalam hal-hal tertentu masih dimungkinkan terjadi permasalahan teknis terkait dengan akurasi dan keterkinian informasi yang kami sajikan, hal mana akan terus kami perbaiki dari waktu kewaktu.
Dalam hal Anda menemukan inakurasi informasi yang termuat pada situs ini atau informasi yang seharusnya ada, namun belum tersedia, maka harap segera hubungi Kepaniteraan Mahkamah Agung RI melalui :
Email : kepaniteraan@mahkamahagung.go.id Telp : 021-384 3348 (ext.318)

tidak peduli dan tidak memebri nafkah kepada Penggugat dan anak-anaknya, hal ini menandakan Tergugat selaku suami tidak dapat melaksanakan kewajibannya kepada Penggugat sehingga Penggugat tidak merasakan kesejahteraan dan kebahagian bersuamikan Tergugat, dengan keadaan itu maka timbullah perselisihan terus menerus apalagi keduan telah berpisah karena Tergugat meninggalkan Penggugat sampai saat ini sudah 8 (delapan) tahun lamanya ;

Menimbang, bahwa sikap dan prilaku Tergugat yang meninggalkan Penggugat tersebut pengadilan menilainya pula Tergugat selaku suami telah tidak memberikan tanggungjawabnya untuk memberikan kasih sayangnya, serta telah tidak peduli akan keutuhan rumah tangganya, dan hal dapat dikatakan Tergugat telah menelantarkan Penggugat dan anaknya, dengan demikian menandakan Tergugat selaku suami tidak dapat melaksanakan kewajibannya kepada Penggugat sehingga Penggugat tidak merasakan kebahagian bersuamikan Tergugat

Menimbang, bahwa dengan sikap-sikap Tergugat tersebut maka Penggugat sudah sangat tidak senang dan telah benci atas prilaku Tergugat tersebut, hal ini dapat dilihat dari keinginan Penggugat yang mengajukan perkara dan tetap bersikukuh atas keinginannya tersebut, maka dalam hal itu Majelis Hakim juga perlu mengemukakan dalil fiqih sebagaimana termaktub dalam Kitab Al-Muhazzab jilid II halaman 81 dan mengambil alih menjadi pertimbangan dalam putusan ini, sebagai berikut:

واذا اشتد عدم الرغبة الزوجة لزوجها طلق عليها القاضي طلقة

Artinya: Apabila isteri sudah memuncak kebenciannya kepada suami, Hakim boleh menjatuhkan talak satu suami ;

Menimbang, bahwa keterpisahan hidup antara Penggugat dengan Tergugat yang dihitung sudah 8 (delapan) tahun sampai saat ini, dan selama kurun waktu tersebut keduanya tidak terjalin lagi komunikasi yang baik sebagaimana yang dibutuhkan dalam suatu rumah tangga, sehingga dengan kondisi tersebut maka pengadilan menyatakan rumah tangga antara Penggugat dan Tergugat tidak mencerminkan lagi suatu rumah tangga yang dicita-citakan, dan keterpisahan hidup tersebut diartikan pula sebagai bentuk perselisihan

Disclaimer
Kepaniteraan Mahkamah Agung Republik Indonesia berusaha untuk selalu mencantumkan informasi paling kini dan akurat sebagai bentuk komitmen Mahkamah Agung untuk pelayanan publik, transparansi dan akuntabilitas pelaksanaan fungsi peradilan. Namun dalam hal-hal tertentu masih dimungkinkan terjadi permasalahan teknis terkait dengan akurasi dan keterkinian informasi yang kami sajikan, hal mana akan terus kami perbaiki dari waktu kewaktu.
Dalam hal Anda menemukan inakurasi informasi yang termuat pada situs ini atau informasi yang seharusnya ada, namun belum tersedia, maka harap segera hubungi Kepaniteraan Mahkamah Agung RI melalui :
Email : kepaniteraan@mahkamahagung.go.id Telp : 021-384 3348 (ext.318)

terus menerus secara batiniyah karena pada dasarnya perkawinan itu dilaksanakan adalah untuk menyatukan seorang pria (suami) dengan seorang perempuan (isteri) dalam satu atap (satu rumah), namun hal itu tidak terwujud lagi;

Menimbang, bahwa dengan keadaan rumah tangga Penggugat dengan Tergugat tersebut diatas, maka pengadilan menyatakan rumah tangga Penggugat dengan Tergugat telah pecah karena tujuan utama dilaksanakannya perkawinan sebagaimana disebut dalam Pasal 1 Undang-Undang Nomor 1 Tahun 1974 jo. Pasal 3 Kompilasi Hukum Islam yaitu untuk membentuk rumah tangga yang kekal, bahagia dan sejahtera tidak terwujud lagi ;

Menimbang, bahwa berdasarkan pertimbangan-pertimbangan tersebut diatas, maka gugatan Penggugat telah beralasan hukum sebagaimana disebutkan dalam Pasal 19 huruf (f) Peraturan Pemerintah Nomor 9 Tahun 1975 jo. Pasal 116 huruf (f) Kompilasi Hukum Islam ;

Menimbang, bahwa berdasarkan apa yang telah dipertimbangkan oleh pengadilan diatas maka terdapat cukup alasan hukum untuk mengabulkan gugatan Penggugat sehingga pengadilan menyatakan menjatuhkan talak satu bain shughra dari Tergugat kepada Penggugat ;

Menimbang, bahwa berdasarkan ketentuan Pasal 89 ayat (1) Undang-Undang Nomor 7 Tahun 1989 sebagaimana telah diubah dengan Undang-Undang Nomor 3 Tahun 2006 dan telah diubah dengan Undang-Undang Nomor 50 tahun 2009 Tentang perubahan kedua atas Undang-Undang Nomor 7 tahun 1989 Tentang Peradilan Agama, maka biaya perkara dibebankan kepada Penggugat;

Mengingat dan memperhatikan segala ketentuan peraturan perundang-undangan yang berlaku serta hukum syara' yang berkaitan dengan perkara ini;

MENGADILI

1. Menyatakan bahwa Tergugat yang telah dipanggil secara resmi dan patut untuk menghadap di persidangan, tidak hadir.
2. Mengabulkan gugatan Penggugat dengan verstek.
3. Menjatuhkan talak 1 (satu) bain sughra dari Tergugat (**Tergugat**) terhadap Penggugat (**Penggugat**).

Disclaimer
Kepaniteraan Mahkamah Agung Republik Indonesia berusaha untuk selalu mencantumkan informasi paling kini dan akurat sebagai bentuk komitmen Mahkamah Agung untuk pelayanan publik, transparansi dan akuntabilitas pelaksanaan fungsi peradilan. Namun dalam hal-hal tertentu masih dimungkinkan terjadi permasalahan teknis terkait dengan akurasi dan keterkinian informasi yang kami sajikan, hal mana akan terus kami perbaiki dari waktu kewaktu.
Dalam hal Anda menemukan inakurasi informasi yang termuat pada situs ini atau informasi yang seharusnya ada, namun belum tersedia, maka harap segera hubungi Kepaniteraan Mahkamah Agung RI melalui :
Email : kepaniteraan@mahkamahagung.go.id Telp : 021-384 3348 (ext.318)

4. Membebankan Penggugat untuk membayar biaya perkara sejumlah Rp. 316.000,00 (tiga ratus enam belas ribu rupiah).

Demikian diputuskan dalam permusyawaratan Majelis Hakim Pengadilan Agama Bogor pada hari Rabu tanggal 23 Desember 2020 Masehi bertepatan dengan tanggal 8 Jumadil awal 1442 Hijriah, oleh kami Dra. Hj. Mukasipa, M.H. sebagai Ketua Majelis, Dra. Hj. Nuroniah, S.H., M.H. dan Dra. Sulfita Netti, S.H., M.H. masing-masing sebagai Hakim Anggota, putusan tersebut pada hari itu juga diucapkan dalam sidang terbuka untuk umum oleh Ketua Majelis beserta Hakim Anggota tersebut dan dibantu oleh Wardah Hamzah, S.H.I. sebagai Panitera Pengganti, dihadiri oleh Penggugat tanpa hadirnya Tergugat;

Hakim Anggota,	Ketua Majelis,
**Dra. Hj. Nuroniah, S.H., M.H.**	**Dra. Hj. Mukasipa, M.H.**
Hakim Anggota,	
**Dra. Sulfita Netti, S.H., M.H.**	
	Panitera Pengganti,
	**Wardah Hamzah, S.H.I.**

Perincian biaya :

- Pendaftaran	: Rp	30.000,-
- Biaya Proses	: Rp	50.000,-
- Panggilan	: Rp	200.000,-
- PNBP Panggilan	: Rp	20.000,-

Disclaimer
Kepaniteraan Mahkamah Agung Republik Indonesia berusaha untuk selalu mencantumkan informasi paling kini dan akurat sebagai bentuk komitmen Mahkamah Agung untuk pelayanan publik, transparansi dan akuntabilitas pelaksanaan fungsi peradilan. Namun dalam hal-hal tertentu masih dimungkinkan terjadi permasalahan teknis terkait dengan akurasi dan keterkinian informasi yang kami sajikan, hal mana akan terus kami perbaiki dari waktu kewaktu.
Dalam hal Anda menemukan inakurasi informasi yang termuat pada situs ini atau informasi yang seharusnya ada, namun belum tersedia, maka harap segera hubungi Kepaniteraan Mahkamah Agung RI melalui :
Email : kepaniteraan@mahkamahagung.go.id   Telp : 021-384 3348 (ext.318)

- Redaksi	: Rp	10.000,-
- Meterai	: Rp	6.000,-
J u m l a h	: Rp	316.000,-

(tiga ratus enam belas ribu rupiah);

Disclaimer
Kepaniteraan Mahkamah Agung Republik Indonesia berusaha untuk selalu mencantumkan informasi paling kini dan akurat sebagai bentuk komitmen Mahkamah Agung untuk pelayanan publik, transparansi dan akuntabilitas pelaksanaan fungsi peradilan. Namun dalam hal-hal tertentu masih dimungkinkan terjadi permasalahan teknis terkait dengan akurasi dan keterkinian informasi yang kami sajikan, hal mana akan terus kami perbaiki dari waktu kewaktu.
Dalam hal Anda menemukan inakurasi informasi yang termuat pada situs ini atau informasi yang seharusnya ada, namun belum tersedia, maka harap segera hubungi Kepaniteraan Mahkamah Agung RI melalui :
Email : kepaniteraan@mahkamahagung.go.id Telp : 021-384 3348 (ext.318)
