# Supplementary material for: Classification of divorce causes during the COVID-19 pandemic using convolutional neural networks
Source: PeerJ Comput Sci. 2022 Jun 30;8:e998. doi: 10.7717/peerj-cs.998 (PMC9299239; doi:10.7717/peerj-cs.998)
Supplement: Supplemental Information 5 [file peerj-cs-08-998-s005.zip › Masalah Ekonomi Dataset/Data ke-18.pdf]

PUTUSAN

Nomor 1147/Pdt.G/2020/PA.Sim

بِسْمِ اللَّهِ الرَّحْمَٰنِ الرَّحِيمِ

DEMI KEADILAN BERDASARKAN KETUHANAN YANG MAHA ESA

Pengadilan Agama Simalungun yang memeriksa dan mengadili perkara tertentu pada tingkat pertama dalam sidang majelis telah menjatuhkan putusan dalam perkara cerai gugat antara :

Penggugat, NIK------------, tempat tanggal lahir 20 Juni 1995, agama Islam, pendidikan S.1, pekerjaan Buruh, tempat kediaman di Kabupaten Simalungun, sebagai **Penggugat**;

melawan

Tergugat, NIK ------------, tempat tanggal lahir 19 Juli 1995, agama Islam, pendidikan SMA, pekerjaan Petani, tempat kediaman di Kabupaten Simalungun, sebagai **Tergugat**;

Pengadilan Agama tersebut ;

Telah mempelajari surat-surat yang berkaitan dengan perkara ini;

Telah mendengar keterangan Penggugat dan para saksi di muka sidang ;

DUDUK PERKARA

Menimbang, bahwa Penggugat dalam surat gugatannya tanggal 07 Desember 2020 telah mengajukan cerai gugat yang telah didaftar di Kepaniteraan Pengadilan Agama Simalungun Nomor 1147/Pdt.G/2020/PA.Sim., tanggal 07 Desember 2020, dengan dalil-dalil sebagai berikut:

1. Bahwa pada tanggal 28 Oktober 2018, Penggugat dengan Tergugat melangsungkan pernikahan yang dicatat oleh Pegawai Pencatat Nikah Kantor Urusan Agama Kecamatan Bandar, Kabupaten Simalungun sesuai dengan Kutipan Akta Nikah Nomor-----------, tanggal 29 Oktober 2018, sesaat setelah akad nikah Tergugat mengucapkan sighat taklik talak sebagaimana tertera dalam Akta Nikah tersebut;

Disclaimer
Kepaniteraan Mahkamah Agung Republik Indonesia berusaha untuk selalu mencantumkan informasi paling kini dan akurat sebagai bentuk komitmen Mahkamah Agung untuk pelayanan publik, transparansi dan akuntabilitas pelaksanaan fungsi peradilan. Namun dalam hal-hal tertentu masih dimungkinkan terjadi permasalahan teknis terkait dengan akurasi dan keterkinian informasi yang kami sajikan, hal mana akan terus kami perbaiki dari waktu kewaktu.
Dalam hal Anda menemukan inakurasi informasi yang termuat pada situs ini atau informasi yang seharusnya ada, namun belum tersedia, maka harap segera hubungi Kepaniteraan Mahkamah Agung RI melalui :
Email : kepaniteraan@mahkamahagung.go.id Telp : 021-384 3348 (ext.318)

2. Bahwa setelah akad nikah tersebut Penggugat dengan Tergugat bertempat tinggal di rumah orang tua Penggugat di Kelurahan Perdagangan I selama 1 hari, kemudian Penggugat dan Tergugat berpindah-pindah tempat tinggal dan terakhir Penggugat dan Tergugat tinggal di rumah orang tua Penggugat di Kelurahan Perdagangan I selama 9 bulan;
3. Bahwa dalam perkawinan tersebut Penggugat dengan Tergugat telah dikaruniai 1 orang anak yang bernama Anak ke I, perempuan, tanggal lahir 14 Mei 2019, anak Penggugat dan Tergugat saat ini tinggal dengan Penggugat;
4. Bahwa pada awalnya rumah tangga Penggugat dengan Tergugat rukun dan harmonis, namun pada bulan Juni 2019, Tergugat pergi meninggalkan Penggugat dengan alasan ingin berkunjung ke rumah orang tua Tergugat namun sejak saat itu, Tergugat tidak pernah kembali lagi sampai sekarang;
5. Bahwa beberapa minggu setelah Tergugat pergi, Penggugat meminta Tergugat pulang namun Tergugat tidak mau;
6. Bahwa sejak pergi, Tergugat tidak pernah memberikan atau mengirimkan nafkah kepada Penggugat sampai sekarang, Tergugat juga tidak ada meninggalkan harta maupun barang-barang yang dapat dijual untuk memenuhi kebutuhan sehari-hari Penggugat dan anak Penggugat dan Tergugat;
7. Bahwa akibat sikap dan perilaku Tergugat tersebut, Penggugat merasa telah dibiarkan atau tidak diperdulikan oleh Tergugat sehingga penggugat menderita lahir maupun bathin sehingga tidak rela serta tidak ingin lagi melanjutkan ikatan perkawinan dengan Tergugat;
8. Bahwa berdasarkan hal tersebut, Tergugat telah melanggar sighat taklik talak yang pernah diucapkannya sesaat setelah akad nikah pada poin (2) dan (4);
9. Bahwa Penggugat sanggup membayar seluruh biaya yang timbul akibat perkara ini;

Disclaimer
Kepaniteraan Mahkamah Agung Republik Indonesia berusaha untuk selalu mencantumkan informasi paling kini dan akurat sebagai bentuk komitmen Mahkamah Agung untuk pelayanan publik, transparansi dan akuntabilitas pelaksanaan fungsi peradilan. Namun dalam hal-hal tertentu masih dimungkinkan terjadi permasalahan teknis terkait dengan akurasi dan keterkinian informasi yang kami sajikan, hal mana akan terus kami perbaiki dari waktu kewaktu.
Dalam hal Anda menemukan inakurasi informasi yang termuat pada situs ini atau informasi yang seharusnya ada, namun belum tersedia, maka harap segera hubungi Kepaniteraan Mahkamah Agung RI melalui :
Email : kepaniteraan@mahkamahagung.go.id Telp : 021-384 3348 (ext.318)

Mahkamah Agung Republik Indonesia

Berdasarkan alasan/dalil-dalil di atas, Penggugat mohon agar Bapak Ketua Pengadilan Agama Simalungun memeriksa dan mengadili perkara ini, selanjutnya menjatuhkan putusan yang amarnya:

1. Mengabulkan gugatan Penggugat;
2. Menjatuhkan talak satu khul'i Tergugat (Tergugat) terhadap Penggugat (Penggugat) dengan membayar iwadl sebesar Rp.10.000 (*sepuluh ribu rupiah*);
3. Membebankan Penggugat untuk membayar biaya perkara ini sesuai ketentuan yang berlaku;

Jika Majelis Hakim berpendapat lain, mohon putusan yang seadil-adilnya;

Bahwa pada hari sidang yang telah ditetapkan untuk pemeriksaan perkara ini, Penggugat dan Tergugat telah dipanggil secara resmi dan patut untuk datang menghadap di persidangan, dan terhadap panggilan tersebut Penggugat telah hadir secara *in person*, sedangkan Tergugat tidak hadir dan tidak mengutus orang lain sebagai wakil atau kuasanya yang sah meskipun panggilan telah dilaksanakan secara resmi dan patut;

Bahwa Majelis Hakim telah berusaha secara maksimal melakukan upaya damai dengan cara menasihati Penggugat agar bersabar menunggu kepulangan Tergugat demi mempertahankan keutuhan rumah tangganya dengan Tergugat, akan tetapi Penggugat menyatakan tetap ingin bercerai dari Tergugat;

Bahwa upaya perundingan melalui jalur mediasi tidak dapat dilaksanakan karena Tergugat tidak hadir di persidangan;

Bahwa pada persidangan tanggal 23 Desember 2020 dibacakanlah surat gugatan Penggugat yang isi dan dalil-dalilnya tetap dipertahankan oleh Penggugat, selengkapnya sebagaimana telah dicatat dan diuraikan dalam Berita Acara Sidang yang bersangkutan;

Bahwa Tergugat tidak dapat didengar jawabannya karena Tergugat sudah tidak pernah menghadiri persidangan;

Disclaimer
Kepaniteraan Mahkamah Agung Republik Indonesia berusaha untuk selalu mencantumkan informasi paling kini dan akurat sebagai bentuk komitmen Mahkamah Agung untuk pelayanan publik, transparansi dan akuntabilitas pelaksanaan fungsi peradilan. Namun dalam hal-hal tertentu masih dimungkinkan terjadi permasalahan teknis terkait dengan akurasi dan keterkinian informasi yang kami sajikan, hal mana akan terus kami perbaiki dari waktu kewaktu.
Dalam hal Anda menemukan inakurasi informasi yang termuat pada situs ini atau informasi yang seharusnya ada, namun belum tersedia, maka harap segera hubungi Kepaniteraan Mahkamah Agung RI melalui :
Email : kepaniteraan@mahkamahagung.go.id Telp : 021-384 3348 (ext.318)

Bahwa untuk menguatkan dalil-dalil gugatannya, Penggugat telah mengajukan bukti surat berupa:

1. Fotokopi Kutipan Akta Nikah----------, tanggal 29 Oktober 2018, yang dikeluarkan oleh Pegawai Pencatat Nikah Kantor Urusan Agama Kecamatan Bandar, Kabupaten Simalungun, Provinsi Sumatera Utara, telah diberi meterai secukupnya dan dinazegelen di kantor pos, Majelis Hakim telah mencocokkan fotokopi *surat* dengan aslinya di persidangan, dan ternyata telah cocok, selanjutnya Ketua Majelis telah memberi paraf serta tanda bukti P;

Bahwa selain bukti surat, Penggugat juga mengajukan bukti lain dengan menghadirkan 2 (dua) orang saksi sebagai berikut:

1. **Saksi dari Penggugat ke I**, umur 22 tahun, agama Islam, pendidikan SMA, pekerjaan Buruh, tempat tinggal di Kabupaten Simalungun, di bawah sumpahnya memberikan keterangan sebagai berikut:
   - Bahwa Saksi kenal dengan Penggugat karena Saksi adalah keponakan Penggugat dan kenal Tergugat bernama Tergugat sebagai suami Penggugat;
   - Bahwa Penggugat dan Tergugat adalah suami istri yang menikah pada bulan Oktober 2018, dan Saksi hadir dalam pernikahan tersebut;
   - Bahwa sesaat setelah akad nikah, Tergugat mengucapkan *sighat* taklik talak;
   - Bahwa setelah menikah, Penggugat dan Tergugat tinggal bersama sebagai suami istri terakhir di rumah orang tua Penggugat di Keluraha Perdagangan I;
   - Bahwa selama perkawinan Penggugat dan Tergugat telah dikaruniai 1 orang anak;
   - Bahwa pada awalnya rumah tangga Penggugat dan Tergugat rukun dan harmonis, namun sejak bulan Juni 2019 Tergugat pergi meninggalkan Penggugat dan sampai sekarang Tergugat tidak pernah pulang lagi;

Disclaimer
Kepaniteraan Mahkamah Agung Republik Indonesia berusaha untuk selalu mencantumkan informasi paling kini dan akurat sebagai bentuk komitmen Mahkamah Agung untuk pelayanan publik, transparansi dan akuntabilitas pelaksanaan fungsi peradilan. Namun dalam hal-hal tertentu masih dimungkinkan terjadi permasalahan teknis terkait dengan akurasi dan keterkinian informasi yang kami sajikan, hal mana akan terus kami perbaiki dari waktu kewaktu.
Dalam hal Anda menemukan inakurasi informasi yang termuat pada situs ini atau informasi yang seharusnya ada, namun belum tersedia, maka harap segera hubungi Kepaniteraan Mahkamah Agung RI melalui :
Email : kepaniteraan@mahkamahagung.go.id Telp : 021-384 3348 (ext.318)

- Bahwa selama Tergugat pergi sampai dengan sekarang, Tergugat tidak pernah memberi kabar, tidak pernah mengirimkan nafkah belanja, dan tidak pula meninggalkan harta atau usaha kepada Penggugat;
- Bahwa Saksi sudah pernah menasehati Penggugat agar bersabar menunggu kepulangan Tergugat, akan tetapi Penggugat menyatakan sudah tidak sabar lagi;

2. Saksi dari Penggugat ke II, umur 37 tahun, agama Islam, pendidikan SMA, pekerjaan Buruh, tempat kediaman di Kabupaten Simalungun, di bawah sumpahnya memberikan keterangan sebagai berikut:

- Bahwa Saksi kenal dengan Penggugat karena Saksi adalah Kakak Penggugat dan kenal Tergugat bernama Tergugat sebagai suami Penggugat;
- Bahwa Penggugat dan Tergugat adalah suami istri yang menikah pada bulan Oktober 2018, dan Saksi hadir dalam pernikahan tersebut;
- Bahwa sesaat setelah akad nikah, Tergugat mengucapkan *sighat* taklik talak;
- Bahwa setelah menikah, Penggugat dan Tergugat tinggal bersama terakhir di rumah orang tua Penggugat di Kelurahan Perdagangan I;
- Bahwa Penggugat dan Tergugat telah dikaruniai 1 orang anak;
- Bahwa pada awalnya rumah tangga Penggugat dan Tergugat rukun dan harmonis, namun sejak bulan Juni 2019 Tergugat pergi meninggalkan Penggugat dan sampai sekarang Tergugat tidak pernah pulang lagi;
- Bahwa selama Tergugat pergi sampai dengan sekarang, Tergugat tidak pernah memberi kabar, tidak pernah mengirimkan nafkah belanja, dan tidak pula meninggalkan harta atau usaha kepada Penggugat;
- Bahwa Saksi sudah pernah menasehati Penggugat agar bersabar menunggu kepulangan Tergugat, akan tetapi Penggugat menyatakan sudah tidak sabar lagi;

Bahwa Penggugat menyatakan tidak mengajukan sesuatu apapun lagi sebagai alat bukti;

Disclaimer
Kepaniteraan Mahkamah Agung Republik Indonesia berusaha untuk selalu mencantumkan informasi paling kini dan akurat sebagai bentuk komitmen Mahkamah Agung untuk pelayanan publik, transparansi dan akuntabilitas pelaksanaan fungsi peradilan. Namun dalam hal-hal tertentu masih dimungkinkan terjadi permasalahan teknis terkait dengan akurasi dan keterkinian informasi yang kami sajikan, hal mana akan terus kami perbaiki dari waktu kewaktu.
Dalam hal Anda menemukan inakurasi informasi yang termuat pada situs ini atau informasi yang seharusnya ada, namun belum tersedia, maka harap segera hubungi Kepaniteraan Mahkamah Agung RI melalui :
Email : kepaniteraan@mahkamahagung.go.id Telp : 021-384 3348 (ext.318)

Bahwa Penggugat telah menyampaikan kesimpulan secara lisan yang pada pokoknya tetap dengan gugatannya dan Penggugat telah menyerahkan iwad berupa uang sejumlah Rp10.000,00 (sepuluh ribu rupiah) dan memohon putusan dengan mengabulkan gugatan Penggugat;

Bahwa tentang jalannya pemeriksaan di persidangan, selengkapnya telah dicatat dalam Berita Acara Sidang, dan untuk menyempurnakan uraian putusan ini cukuplah Pengadilan menunjuk kepada Berita Acara Sidang tersebut;

PERTIMBANGAN HUKUM

Menimbang, bahwa maksud dan tujuan gugatan Penggugat sebagaimana telah diuraikan di atas;

Menimbang, bahwa berdasarkan *relaas-relaas* yang telah disampaikan kepada Penggugat dan Tergugat, ternyata bahwa panggilan telah dilakukan secara resmi dan patut, dengan demikian ketentuan Pasal 145 ayat (1) dan (2) dan 718 ayat (1) R.Bg. jo. Pasal 27 ayat (1) dan (2) Peraturan Pemerintah Republik Indonesia Nomor 9 Tahun 1975 Tentang Pelaksanaan Undang-Undang Republik Indonesia Nomor 1 Tahun 1974 Tentang Perkawinan telah terpenuhi;

Menimbang, bahwa pada hari sidang yang telah ditetapkan Penggugat *in person* telah datang menghadap sendiri di muka persidangan, hal mana telah memenuhi ketentuan Pasal 30 Peraturan Pemerintah Republik Indonesia Nomor 9 Tahun 1975 Tentang Pelaksanaan Undang-Undang Republik Indonesia Nomor 1 Tahun 1974 Tentang Perkawinan jo. Pasal 142 ayat (1) Instruksi Presiden Republik Indonesia Nomor 1 Tahun 1991 Tentang Kompilasi Hukum Islam;

Menimbang, bahwa dengan melaksanakan ketentuan Pasal 27 ayat (1), (2) dan (3) Peraturan Pemerintah Nomor 9 Tahun 1975 Tentang Pelaksanaan Undang-Undang Nomor 1 Tahun 1974 Tentang Perkawinan, Tergugat telah dipanggil secara resmi dan patut untuk menghadap di persidangan, sesuai relaas panggilan Nomor 1147/Pdt.G/2020/PA.Sim., akan tetapi Tergugat tidak hadir dan tidak ada mengutus serta menunjuk orang lain sebagai wakil atau

Disclaimer
Kepaniteraan Mahkamah Agung Republik Indonesia berusaha untuk selalu mencantumkan informasi paling kini dan akurat sebagai bentuk komitmen Mahkamah Agung untuk pelayanan publik, transparansi dan akuntabilitas pelaksanaan fungsi peradilan. Namun dalam hal-hal tertentu masih dimungkinkan terjadi permasalahan teknis terkait dengan akurasi dan keterkinian informasi yang kami sajikan, hal mana akan terus kami perbaiki dari waktu kewaktu.
Dalam hal Anda menemukan inakurasi informasi yang termuat pada situs ini atau informasi yang seharusnya ada, namun belum tersedia, maka harap segera hubungi Kepaniteraan Mahkamah Agung RI melalui :
Email : kepaniteraan@mahkamahagung.go.id Telp : 021-384 3348 (ext.318)

kuasanya, sedangkan ketidakhadirannya tersebut bukan karena suatu alasan yang sah, dengan demikian berdasarkan ketentuan Pasal 27 ayat (4) Peraturan Pemerintah Nomor 9 Tahun 1975 Tentang Pelaksanaan Undang-Undang Nomor 1 Tahun 1974 Tentang Perkawinan Majelis Hakim telah dapat memeriksa dan memutus perkara ini dengan tanpa hadirnya Tergugat (*verstek*);

Menimbang, bahwa Majelis Hakim telah melakukan upaya damai secara maksimal dengan cara memberi nasihat kepada Penggugat agar mau bersabar menanti kepulangan Tergugat dan kembali rukun dalam membina rumah tangganya, sebagaimana dimaksud pada ketentuan Pasal 82 ayat (1) dan (4) Undang-Undang Republik Indonesia Nomor 7 Tahun 1989 Tentang Peradilan Agama sebagaimana telah diubah dengan Undang-Undang Republik Indonesia Nomor 3 Tahun 2006 dan Perubahan Kedua dengan Undang-Undang Republik Indonesia Nomor 50 Tahun 2009 jo. Pasal 31 ayat (1) dan (2) Peraturan Pemerintah Republik Indonesia Nomor 9 Tahun 1975 Tentang Pelaksanaan Undang-Undang Republik Indonesia Nomor 1 Tahun 1974 Tentang Perkawinan, akan tetapi tidak berhasil damai;

Menimbang, bahwa berdasarkan ketentuan Pasal 4 ayat (2) huruf (b) Peraturan Mahkamah Agung Republik Indonesia Nomor 1 Tahun 2016 Tentang Prosedur Mediasi di Pengadilan, maka proses mediasi dalam perkara ini tidak dilaksanakan karena Tergugat tidak hadir menghadap di persidangan;

Menimbang, bahwa yang menjadi pokok perkara ialah Penggugat menggugat agar Penggugat diceraikan dari Tergugat dengan alasan karena Tergugat telah melanggar sighat taklik talak pada poin (2) dan (4);

Menimbang, bahwa terhadap petitum gugatan Penggugat poin 1 dan 2, Majelis Hakim memberikan pertimbangan hukum sebagai berikut;

Menimbang, bahwa alasan perceraian yang dikemukakan oleh Penggugat dinilai oleh Majelis Hakim mengarah kepada ketentuan yang termuat dalam Pasal 116 huruf (g) Instruksi Presiden Republik Indonesia Nomor 1 Tahun 1991 Tentang Kompilasi Hukum Islam, yang isinya menentukan bahwa perceraian dapat terjadi karena suami melanggar taklik talak;

Disclaimer
Kepaniteraan Mahkamah Agung Republik Indonesia berusaha untuk selalu mencantumkan informasi paling kini dan akurat sebagai bentuk komitmen Mahkamah Agung untuk pelayanan publik, transparansi dan akuntabilitas pelaksanaan fungsi peradilan. Namun dalam hal-hal tertentu masih dimungkinkan terjadi permasalahan teknis terkait dengan akurasi dan keterkinian informasi yang kami sajikan, hal mana akan terus kami perbaiki dari waktu kewaktu.
Dalam hal Anda menemukan inakurasi informasi yang termuat pada situs ini atau informasi yang seharusnya ada, namun belum tersedia, maka harap segera hubungi Kepaniteraan Mahkamah Agung RI melalui :
Email : kepaniteraan@mahkamahagung.go.id Telp : 021-384 3348 (ext.318)

Menimbang, bahwa berdasarkan ketentuan Pasal 27 ayat (4) Peraturan Pemerintah Nomor 9 Tahun 1975 Tentang Pelaksanaan Undang-Undang Nomor 1 Tahun 1974 Tentang Perkawinan, maka Majelis Hakim telah dapat memeriksa dan memutus perkara ini dengan mengabulkan gugatan Penggugat meskipun tanpa hadirnya Tergugat, namun oleh karena perkara ini merupakan perkara perceraian, maka berdasarkan ketentuan Pasal 283 R.Bg., Majelis Hakim membebankan pembuktian kepada Penggugat dengan memeriksa bukti-bukti, dan oleh karena perkara ini adalah perkara perceraian dengan alasan pelanggaran *sighat* taklik talak, maka Penggugat diwajibkan menghadirkan Saksi dari orang-orang terdekat dengan Penggugat dan Tergugat untuk didengar keterangannya di persidangan;

Menimbang, bahwa untuk menguatkan dalil-dalil gugatannya, Penggugat telah mengajukan alat bukti surat (P), serta menghadirkan 2 (dua) orang saksi, terhadap bukti-bukti *a quo* Majelis Hakim mempertimbangkannya;

Menimbang, bahwa bukti P merupakan akta otentik dan dinilai telah memenuhi syarat formil pembuktian karena dibuat dan ditandatangani oleh pejabat yang berwenang untuk itu, serta telah dinazegelen di kantor pos untuk kepentingan pembuktian, dan telah dicocokkan dengan aslinya di muka persidangan, maka sesuai Pasal 301 R.Bg. bukti *a quo* dapat dijadikan sebagai alat bukti, sedangkan substansinya akan dipertimbangkan selanjutnya;

Menimbang, bahwa bukti P yang diajukan Penggugat di persidangan merupakan *conditio sine qua non* dan telah diteliti kebenaran isinya bahwa Penggugat dan Tergugat adalah suami istri yang sah menikah di Kecamatan Bandar, Kabupaten Simalungun, Provinsi Sumatera Utara pada tanggal 29 Oktober 2018 dan belum pernah bercerai, dan pernikahan tersebut telah dilakukan secara sah sesuai dengan ketentuan Pasal 2 ayat (1) dan (2) Undang-Undang Nomor 1 Tahun 1974 Tentang Perkawinan, dengan demikian syarat materil pembuktian telah terpenuhi, dan harus dinyatakan terbukti Penggugat dan Tergugat adalah pihak yang berkepentingan dalam perkara ini (*persona standi in judicio*) dan sepatutnya dinyatakan tidak melawan hak;

Disclaimer
Kepaniteraan Mahkamah Agung Republik Indonesia berusaha untuk selalu mencantumkan informasi paling kini dan akurat sebagai bentuk komitmen Mahkamah Agung untuk pelayanan publik, transparansi dan akuntabilitas pelaksanaan fungsi peradilan. Namun dalam hal-hal tertentu masih dimungkinkan terjadi permasalahan teknis terkait dengan akurasi dan keterkinian informasi yang kami sajikan, hal mana akan terus kami perbaiki dari waktu kewaktu.
Dalam hal Anda menemukan inakurasi informasi yang termuat pada situs ini atau informasi yang seharusnya ada, namun belum tersedia, maka harap segera hubungi Kepaniteraan Mahkamah Agung RI melalui :
Email : kepaniteraan@mahkamahagung.go.id Telp : 021-384 3348 (ext.318)

Menimbang, bahwa kedua orang Saksi yang dihadirkan oleh Penggugat di persidangan tersebut tidak ada halangan hukum untuk diangkat menjadi saksi (*vide* Pasal 172 R.Bg.), telah menyatakan bersedia diangkat menjadi saksi (*vide* Pasal 174 R.Bg.), dan masing-masing secara seorang demi seorang telah memberikan keterangan di bawah sumpah di depan persidangan (*vide* Pasal 175 R.Bg.), sedangkan keberadaan kedua Saksi adalah sebagai orang-orang terdekat yang selalu berkomunikasi dengan Penggugat dan Tergugat, selalu melihat keadaan rumah tangga Penggugat dan Tergugat, dan pernah mendengar cerita dan keluhan Penggugat dan Tergugat tentang keadaan rumah tangga Penggugat dan Tergugat, dan jumlah 2 (dua) orang Saksi telah memenuhi batas minimal bukti Saksi (*vide* Pasal 306 R.Bg.), dengan demikian syarat formil kedua Saksi *a quo* telah terpenuhi, sedangkan syarat materilnya akan dipertimbangkan selanjutnya;

Menimbang, bahwa kesaksian 2 (dua) orang Saksi Penggugat sepanjang mengenai Tergugat yang telah pergi meninggalkan Penggugat sejak bulan Juni 2019 yang lalu dan hingga sekarang Tergugat tidak pernah pulang lagi, tidak memberi kabar serta nafkah wajib lahir dan batin, tidak saling bertentangan satu sama lain dan sejalan dengan dalil-dalil gugatan Penggugat, dengan demikian keterangan kedua orang Saksi *a quo* patut dinilai telah memenuhi syarat materil sebagaimana ketentuan Pasal 309 R.Bg., sehingga kesaksian tersebut dapat diterima sebagai bukti dalam perkara ini;

Menimbang, bahwa pada petitum poin 2 gugatan, Penggugat memohon agar Majelis Hakim Pengadilan Agama Simalungun menjatuhkan talak satu khul'i Tergugat (Tergugat) terhadap Penggugat (Penggugat) dengan iwad berupa uang sejumlah Rp10.000,00 (sepuluh ribu rupiah), sedangkan di persidangan ditemukan fakta-fakta bahwa Tergugat mengucapkan dan membaca *sighat* taklik, hal mana menjadi syarat mutlak kumulatif tentang ada atau tidaknya pelanggaran *sighat* taklik talak, dengan demikian harus dinyatakan telah terbukti bahwa Tergugat telah memenuhi syarat mutlak kumulasi tentang adanya pengucapan *sighat* taklik talak;

Disclaimer
Kepaniteraan Mahkamah Agung Republik Indonesia berusaha untuk selalu mencantumkan informasi paling kini dan akurat sebagai bentuk komitmen Mahkamah Agung untuk pelayanan publik, transparansi dan akuntabilitas pelaksanaan fungsi peradilan. Namun dalam hal-hal tertentu masih dimungkinkan terjadi permasalahan teknis terkait dengan akurasi dan keterkinian informasi yang kami sajikan, hal mana akan terus kami perbaiki dari waktu kewaktu.
Dalam hal Anda menemukan inakurasi informasi yang termuat pada situs ini atau informasi yang seharusnya ada, namun belum tersedia, maka harap segera hubungi Kepaniteraan Mahkamah Agung RI melalui :
Email : kepaniteraan@mahkamahagung.go.id   Telp : 021-384 3348 (ext.318)

Menimbang, bahwa berdasarkan dalil-dalil gugatan Penggugat yang dikaitkan dengan bukti-bukti, maka ditemukan fakta-fakta sebagai berikut:

- Bahwa Penggugat dan Tergugat adalah suami istri yang sah menikah di Kecamatan Bandar, Kabupaten Simalungun, Provinsi Sumatera Utara, pada tanggal 28 Oktober 2018;
- Bahwa sesaat setelah akad nikah Tergugat ada mengucapkan sighat taklik talak;
- Bahwa selama masa perkawinan Penggugat dan Tergugat telah dikaruniai 1 orang anak;
- Bahwa Pada bulan Juni 2019 Tergugat telah pamit pergi meninggalkan Penggugat dan selama itu pula Tergugat tidak pernah pulang lagi;
- Bahwa Tergugat juga tidak pernah lagi memberikan nafkah wajib lahir dan batin kepada Penggugat, dan tidak juga mempedulikan atau telah membiarkan Penggugat tanpa memberi kabar kepada Penggugat;
- Bahwa Penggugat sudah tidak sanggup dan tidak sabar lagi menunggu kepulangan Tergugat;

Menimbang, bahwa dalam suatu rumah tangga manakala suami telah tidak memberikan nafkah wajib kepada istrinya selama 3 (tiga) bulan lamanya, dan bahkan suami telah membiarkan (tidak mempedulikan) istrinya selama 6 (enam) bulan lamanya, sedangkan istrinya tidak ridha dan mengadukan halnya kepada Pengadilan Agama, maka perceraian dapat terjadi karena alasan suami melanggar taklik talak sebagaimana bunyi Pasal 116 huruf (g) Instruksi Presiden Republik Indonesia Nomor 1 Tahun 1991 Tentang Kompilasi Hukum Islam;

Menimbang, bahwa Tergugat telah pergi meninggalkan Penggugat sekurang-kurangnya sejak bulan Juni 2019, dan selama itu pula Tergugat tidak pernah pulang lagi, bahkan Tergugat telah membiarkan (tidak mempedulikan) Penggugat dengan tidak lagi memberikan nafkah wajib lahir dan batin kepada Penggugat;

Menimbang, bahwa pada petitum poin 2 gugatan, Penggugat memohon agar Majelis Hakim Pengadilan Agama Simalungun menjatuhkan talak satu

Disclaimer
Kepaniteraan Mahkamah Agung Republik Indonesia berusaha untuk selalu mencantumkan informasi paling kini dan akurat sebagai bentuk komitmen Mahkamah Agung untuk pelayanan publik, transparansi dan akuntabilitas pelaksanaan fungsi peradilan. Namun dalam hal-hal tertentu masih dimungkinkan terjadi permasalahan teknis terkait dengan akurasi dan keterkinian informasi yang kami sajikan, hal mana akan terus kami perbaiki dari waktu kewaktu.
Dalam hal Anda menemukan inakurasi informasi yang termuat pada situs ini atau informasi yang seharusnya ada, namun belum tersedia, maka harap segera hubungi Kepaniteraan Mahkamah Agung RI melalui :
Email : kepaniteraan@mahkamahagung.go.id Telp : 021-384 3348 (ext.318)

khul’i Tergugat (Tergugat) terhadap Penggugat (Penggugat) dengan iwad berupa uang sejumlah Rp10.000,00 (sepuluh ribu rupiah), sedangkan di persidangan ditemukan fakta-fakta yang membuktikan bahwa Tergugat telah membaca dan mengucapkan *sighat* taklik talak sesaat setelah akad nikah, dan sejak bulan Juni 2019 yang lalu hingga sekarang, Tergugat telah pergi meninggalkan Penggugat, dan selama itu pula Tergugat tidak pernah pulang lagi, tidak memberikan nafkah wajib lahir dan batin kepada Penggugat, dan tidak juga mempedulikan atau telah membiarkan Penggugat tanpa memberi kabar kepada Penggugat, maka Majelis Hakim menilai bahwa Tergugat telah terbukti melanggar *sighat* taklik talak angka (2) dan (4) yang telah diucapkannya setelah akad nikah;

Menimbang, bahwa Penggugat menyatakan tidak rela dan tidak sabar lagi terhadap sikap Tergugat, dan telah bersedia menyerahkan iwad berupa uang sejumlah Rp10.000,00 (sepuluh ribu rupiah) kepada Pengadilan Agama Simalungun agar diserahkan kepada Direktorat Jenderal Bimas Islam dan Penyelenggaraan Haji cq. Direktorat Urusan Agama Islam untuk keperluan ibadah sosial, demi jatuhnya talak satu khul’i Tergugat terhadap Penggugat;

Menimbang, bahwa berdasarkan ketentuan dalil fikih yang telah diambil sebagai alas hukum pertimbangan pendapat Majelis Hakim sebagaimana berikut:

من علق طلاقا بصفة وقع بوجودها عملا بمقتضى اللفظ

Artinya: Siapa yang menggantungkan talaknya dengan satu sifat (janji), maka jatuhlah talak tersebut dengan terjadinya sifat (janji) itu sesuai dengan bunyi ucapannya;

Menimbang, bahwa berdasarkan pertimbangan-pertimbangan di atas, Majelis Hakim berpendapat bahwa gugatan Penggugat dengan alasan pelanggaran *sighat* taklik talak angka (2) dan (4) telah terpenuhi, telah cukup alasan hukum dan terbukti memenuhi alasan perceraian sebagaimana ketentuan Pasal 116 huruf (g) Instruksi Presiden Republik Indonesia Tahun 1991 Tentang Kompilasi Hukum Islam, dan oleh karena itu poin 1 dan 2 petitum gugatan Penggugat sudah sepatutnya dikabulkan dengan menjatuhkan talak

Disclaimer
Kepaniteraan Mahkamah Agung Republik Indonesia berusaha untuk selalu mencantumkan informasi paling kini dan akurat sebagai bentuk komitmen Mahkamah Agung untuk pelayanan publik, transparansi dan akuntabilitas pelaksanaan fungsi peradilan. Namun dalam hal-hal tertentu masih dimungkinkan terjadi permasalahan teknis terkait dengan akurasi dan keterkinian informasi yang kami sajikan, hal mana akan terus kami perbaiki dari waktu kewaktu.
Dalam hal Anda menemukan inakurasi informasi yang termuat pada situs ini atau informasi yang seharusnya ada, namun belum tersedia, maka harap segera hubungi Kepaniteraan Mahkamah Agung RI melalui :
Email : kepaniteraan@mahkamahagung.go.id Telp : 021-384 3348 (ext.318)

satu khul'i Tergugat terhadap Penggugat dengan iwad berupa uang sejumlah Rp10.000,00 (sepuluh ribu rupiah);

Menimbang, bahwa sesuai dengan ketentuan Pasal 89 ayat (1) Undang-Undang Nomor 7 Tahun 1989 tentang Peradilan Agama sebagaimana diubah dengan Undang-Undang Nomor 3 Tahun 2006 dan Perubahan Kedua dengan Undang-Undang Nomor 50 Tahun 2009, maka terhadap petitum gugatan Penggugat poin angka 3, Majelis Hakim membebankan kepada Penggugat untuk membayar biaya dalam perkara ini;

Mengingat, bunyi peraturan perundang-undangan yang berlaku dan dalil-dalil *syar'i* yang berkenaan dengan perkara ini;

MENGADILI

1. Menyatakan Tergugat yang telah dipanggil secara resmi dan patut untuk menghadap di persidangan tidak hadir;
2. Mengabulkan gugatan Penggugat dengan verstek;
3. Menjatuhkan talak satu khul'i Tergugat (Tergugat) terhadap Penggugat (Penggugat) dengan membayar iwadl sebesar Rp.10.000 (sepuluh ribu rupiah);
4. Membebankan Penggugat untuk membayar biaya perkara sejumlah Rp566.000,00 (lima ratus enam puluh enam ribu rupiah);

Demikian diputuskan di Pengadilan Agama Simalungun, pada hari Rabu, 23 Desember 2020 Masehi bertepatan dengan tanggal 08 Jumadil Awwal 1442 Hijriyah, dalam permusyawaratan Majelis Hakim Pengadilan Agama Simalungun yang terdiri dari Muhammad Irsyad, S.Sy. Hakim yang ditetapkan sebagai Ketua Majelis, Muhammad Ali Imron Nst, S.H.I. dan Muhammad Tsabbit Abdullah, S.H. masing-masing sebagai Hakim Anggota, dan diucapkan pada hari itu juga dalam sidang terbuka untuk umum oleh Ketua Majelis tersebut didampingi para Hakim Anggota yang turut bersidang, dibantu oleh Umi Ulfah Tarigan, S.H., M.H sebagai Panitera Pengganti dan dihadiri oleh Penggugat tanpa dihadiri oleh Tergugat.

Disclaimer
Kepaniteraan Mahkamah Agung Republik Indonesia berusaha untuk selalu mencantumkan informasi paling kini dan akurat sebagai bentuk komitmen Mahkamah Agung untuk pelayanan publik, transparansi dan akuntabilitas pelaksanaan fungsi peradilan. Namun dalam hal-hal tertentu masih dimungkinkan terjadi permasalahan teknis terkait dengan akurasi dan keterkinian informasi yang kami sajikan, hal mana akan terus kami perbaiki dari waktu kewaktu.
Dalam hal Anda menemukan inakurasi informasi yang termuat pada situs ini atau informasi yang seharusnya ada, namun belum tersedia, maka harap segera hubungi Kepaniteraan Mahkamah Agung RI melalui :
Email : kepaniteraan@mahkamahagung.go.id Telp : 021-384 3348 (ext.318)

Ketua Majelis

Muhammad Irsyad, S.Sy.

Hakim Anggota **Hakim Anggota**

Muhammad Ali Imron Nst, S.H.I. **Muhammad Tsabbit Abdullah, S.H.**

Panitera Pengganti

Umi Ulfah Tarigan, S.H., M.H

Perincian Biaya Perkara :

1.	Pendaftaran	Rp 30.000,00
2.	Proses	Rp 50.000,00
3.	PNBP Panggilan	Rp 20.000,00
4.	Panggilan	Rp450.000,00
5.	Redaksi	Rp 10.000,00
6.	Meterai	Rp. 6.000,00
	Jumlah	Rp566.000,00

(lima ratus enam puluh enam ribu rupiah)

Disclaimer
Kepaniteraan Mahkamah Agung Republik Indonesia berusaha untuk selalu mencantumkan informasi paling kini dan akurat sebagai bentuk komitmen Mahkamah Agung untuk pelayanan publik, transparansi dan akuntabilitas pelaksanaan fungsi peradilan. Namun dalam hal-hal tertentu masih dimungkinkan terjadi permasalahan teknis terkait dengan akurasi dan keterkinian informasi yang kami sajikan, hal mana akan terus kami perbaiki dari waktu kewaktu.
Dalam hal Anda menemukan inakurasi informasi yang termuat pada situs ini atau informasi yang seharusnya ada, namun belum tersedia, maka harap segera hubungi Kepaniteraan Mahkamah Agung RI melalui :
Email : kepaniteraan@mahkamahagung.go.id Telp : 021-384 3348 (ext.318)
