# Supplementary material for: Classification of divorce causes during the COVID-19 pandemic using convolutional neural networks
Source: PeerJ Comput Sci. 2022 Jun 30;8:e998. doi: 10.7717/peerj-cs.998 (PMC9299239; doi:10.7717/peerj-cs.998)
Supplement: Supplemental Information 5 [file peerj-cs-08-998-s005.zip › Masalah Ekonomi Dataset/Data ke-19.pdf]

P U T U S A N

Nomor 1061/Pdt.G/2020/PA.Tnk

بِسْمِ اللَّهِ الرَّحْمَنِ الرَّحِيمِ

DEMI KEADILAN BERDASARKAN KETUHANAN YANG MAHA ESA

Pengadilan Agama Tanjungkarang yang memeriksa dan mengadili perkara tertentu pada tingkat pertama dalam persidangan Majelis Hakim telah menjatuhkan putusan sebagai berikut dalam perkara Cerai Gugat yang diajukan oleh :

PENGGUGAT, umur 34 tahun, agama Islam, pendidikan SLTA, pekerjaan Mengurus Rumah Tangga, tempat tinggal di Kelurahan Way Halim Permai Kecamatan Way Halim Kota Bandar Lampung, disebut Penggugat;

melawan

TERGUGAT, umur 47 tahun, agama Islam, pendidikan S1, pekerjaan Pemborong Bangunan, tempat tinggal di Kelurahan Way Halim Permai Kecamatan Way Halim Kota Bandar Lampung, disebut Tergugat;

Pengadilan Agama tersebut ;
Telah membaca dan mempelajari berkas perkara;

DUDUK PERKARA

Menimbang, bahwa Penggugat berdasarkan surat gugatannya tertanggal 06 Juli 2020 yang didaftar di Kepaniteraan Pengadilan Agama Tanjungkarang Register Nomor 1061/Pdt.G/2020/PA.Tnk tanggal 14 Agustus 2020 pada pokoknya mengajukan hal-hal sebagai berikut :

1. Bahwa Penggugat adalah istri sah Tergugat yang akad nikahnya berlangsung di KUA Kecamatan Way Halim Kota Bandar Lampung, pada tanggal XXXX berdasarkan Akta Nikah yang dikeluarkan oleh Pegawai

Disclaimer
Kepaniteraan Mahkamah Agung Republik Indonesia berusaha untuk selalu mencantumkan informasi paling kini dan akurat sebagai bentuk komitmen Mahkamah Agung untuk pelayanan publik, transparansi dan akuntabilitas pelaksanaan fungsi peradilan. Namun dalam hal-hal tertentu masih dimungkinkan terjadi permasalahan teknis terkait dengan akurasi dan keterkinian informasi yang kami sajikan, hal mana akan terus kami perbaiki dari waktu kewaktu.
Dalam hal Anda menemukan inakurasi informasi yang termuat pada situs ini atau informasi yang seharusnya ada, namun belum tersedia, maka harap segera hubungi Kepaniteraan Mahkamah Agung RI melalui :
Email : kepaniteraan@mahkamahagung.go.id Telp : 021-384 3348 (ext.318)

Pencatat Nikah Kantor Urusan Agama Kecamatan Way Halim, Nomor : XXXX. tertanggal 18 Mei 2015

2. Bahwa Perkawinan antara Penggugat dan Tergugat dilangsungkan berdasarkan kehendak kedua belah pihak dengan tujuan membentuk rumah tangga yang Sakinah, Mawadah, dan Warahmah yang diridhoi oleh Allah SWT sewaktu menikah Penggugat berstatus Perawan dan Tergugat berstatus Perjaka dan dilaksanakan atas dasar Suka sama suka;

3. Bahwa Penggugat dan Tergugat setelah menikah tinggal bersama orang tua Penggugat di Way Halim Permai sampai dengan berpisah

4. Bahwa antara Penggugat dan Tergugat telah melakukan hubungan sebagaimana layaknya suami istri dan di karuniai 1 orang anak yang bernama;

Anak Umur 4 tahun saat ini dalam asuhan Penggugat

5. Bahwa pada awalnya rumah tangga Penggugat dan Tergugat berjalan rukun dan harmonis, namun sejak tahun 2018 rumah tangga Penggugat dan Tergugat mulai goyah:

a. Tergugat tidak pernah pernah jujur terhadap Penggugat

b. Tergugat terlalu egois dan keras kepala

c. Tergugat pergi meninggal kan rumah dari tahun 2019 dengan alasan mencari kerja dan hingga kini tidak kembali

6. Bahwa puncaknya Sejak November tahun 2019 Tergugat berprilaku tidak baik, Tergugat tidak pernah jujur terhadap Pengugat mengenai masalah ekonomi jika di tanya oleh Penggugat tidak pernah menjawab, Tergugat terlalu egois dan keras kepala, Tergugat tidak bertanggung jawab secara lahir dan batin kepada Penggugat dan tidak memberikan nafkah secara layak kepada anak dan setelah itu Tergugat meninggalkan penggugat dan anak tanpa izin yang jelas, sejak bulan November 2019 itu Tergugat tidak pernah pulang dan tidak pernah kirim kabar berita kepada penggugat serta tidak di ketahui alamat yang jelas berdasarkan Surat Pernyataan Ghoib dari Kelurahan Way Halim Permai, Kecamatan Way Halim, Kota Bandar Lampung dan pasti di wilayah Republik Indonesia serta selama itu pula

Disclaimer
Kepaniteraan Mahkamah Agung Republik Indonesia berusaha untuk selalu mencantumkan informasi paling kini dan akurat sebagai bentuk komitmen Mahkamah Agung untuk pelayanan publik, transparansi dan akuntabilitas pelaksanaan fungsi peradilan. Namun dalam hal-hal tertentu masih dimungkinkan terjadi permasalahan teknis terkait dengan akurasi dan keterkinian informasi yang kami sajikan, hal mana akan terus kami perbaiki dari waktu kewaktu.
Dalam hal Anda menemukan inakurasi informasi yang termuat pada situs ini atau informasi yang seharusnya ada, namun belum tersedia, maka harap segera hubungi Kepaniteraan Mahkamah Agung RI melalui :
Email : kepaniteraan@mahkamahagung.go.id   Telp : 021-384 3348 (ext.318)

antara Penggugat dan Tergugat tidak ada lagi saling berkomunikasi dan Tergugat tidak meninggalkan suatu apapun sebagai nafkah wajibnya.

7. Bahwa Penggugat telah berusaha keras mencari Tergugat, antara lain kepada keluarga serta orang tua Tergugat namun tidak berhasil

8. Bahwa dengan demikin Tergugat telah dengan sengaja pergi meninggalkan Penggugat selama kurang lebih 8 bulan tanpa ada rasa tanggung jawab;

9. Bahwa berdasarkan hal-hal tersebut diatas, Gugatan Penggugat untuk mengajukan gugatan percerai hadap Tergugat atas dasar telah meninggalkan Penggugat secara berturut-turut dan tanpa alasan yang sah atau karena hallain diluar kemampuannya dan sudah tidak ada lagi yang namamyna kelurga yang Sakinah Mawaddah dan Warohmah, sebagaiman diatur dalam Undang-undang No. 1 tahun 1974 Jo. Peraturan Pemerintah No. 9 tahun 1975 Pasal 19 ayat (2) Jo. Kompilasi Hukum Islam Pasal 116 huruf (b), sehingga berdasarkan hukum untuk menyatakan gugatan cerai (Ghoib) ini dikabulkan :

10. Bahwa Penggugat adalah keluarga tidak mampu, dengan bukti surat keterangan tidak mampu nomor : XXXX yang di keluarkan oleh kelurahan Way Halim Permai Kecamatan Way Halim Kota Bandar Lampung pada tanggal 06 Juli 2020.

Berdasarkan dalil-dalil diatas, Penggugat mohon kepada Ketua Pengadilan Agama Kelas I A Tanjung Karang dalam hal ini Majelis Hakim yang memeriksa dan mengadili perkara ini, kiranya berkenan memberikan putusan yang amarnya berbunyi, sebagai berikut :

PRIMAIR:

1. Mengabulkan gugatan Penggugat untuk seluruhnya;
2. Menjatuhkan talak satu ba’in sughra Tergugat (Feriyansyah .SE .SH Bin H. Roesli Darwin) terhadap Penggugat (PENGGUGAT) ;
3. Membebankan biaya perkara kepada Negara melalui DIPA PA Tnk. th 2020;;

SUBSIDAIR:

Apa bila Majelis Hakim berpendapat lain, mohon putusan yang seadil-adilnya;

Disclaimer
Kepaniteraan Mahkamah Agung Republik Indonesia berusaha untuk selalu mencantumkan informasi paling kini dan akurat sebagai bentuk komitmen Mahkamah Agung untuk pelayanan publik, transparansi dan akuntabilitas pelaksanaan fungsi peradilan. Namun dalam hal-hal tertentu masih dimungkinkan terjadi permasalahan teknis terkait dengan akurasi dan keterkinian informasi yang kami sajikan, hal mana akan terus kami perbaiki dari waktu kewaktu.
Dalam hal Anda menemukan inakurasi informasi yang termuat pada situs ini atau informasi yang seharusnya ada, namun belum tersedia, maka harap segera hubungi Kepaniteraan Mahkamah Agung RI melalui :
Email : kepaniteraan@mahkamahagung.go.id   Telp : 021-384 3348 (ext.318)

Menimbang, bahwa pada hari persidangan yang telah ditetapkan yaitu tanggal 23 Desember 2020, Penggugat dan Tergugat tidak datang menghadap di persidangan dan tidak menyuruh orang lain sebagai wakil atau kuasanya yang sah meskipun telah dipanggil secara resmi dan patut, dan ternyata ketidakhadirannya tidak disebabkan suatu alasan yang sah menurut hukum;

Menimbang, bahwa untuk meringkas uraian putusan ini Majelis Hakim menunjuk berita acara persidangan perkara ini yang dinyatakan sebagai hal yang tidak terlepas kaitannya dengan putusan ini;

PERTIMBANGAN HUKUM

Menimbang, bahwa maksud dan tujuan gugatan Penggugat adalah sebagaimana tersebut diatas;

Menimbang, bahwa perkara ini berkenaan dengan perceraian bagi orang yang beragama Islam, maka sesuai dengan Pasal 49 Undang-Undang Nomor 7 Tahun 1989 tentang Peradilan Agama yang telah diubah dengan Undang-Undang Nomor 3 Tahun 2006 dan Undang-Undang Nomor 50 Tahun 2009 tentang Perubahan kedua atas Undang-Undang Nomor 7 Tahun 1989 maka perkara ini termasuk Absolute kompetensi Pengadilan Agama;

Menimbang, bahwa sesuai dengan alamat Penggugat sebagaimana dalam surat gugatan, Penggugat berdomisili di Kelurahan Way Halim Permai Kecamatan Way Halim Kota Bandar Lampung, maka sesuai dengan Pasal 73 Undang-Undang Nomor 7 tahun 1989 tentang Peradilan Agama yang telah diubah dengan Undang-Undang Nomor 3 Tahun 2006 dan Undang-Undang Nomor 50 Tahun 2009 tentang Perubahan kedua atas Undang-Undang Nomor 7 Tahun 1989 perkara ini merupakan relatif kompetensi Pengadilan Agama Tanjungkarang;

Menimbang, bahwa Penggugat yang telah dipanggil secara resmi dan patut tidak pernah hadir menghadap ke persidangan dan tidak pula menyuruh orang lain sebagai wakil atau kuasanya yang sah untuk hadir, dan ketidakhadiran Penggugat tersebut tidak disebabkan suatu alasan yang sah

Disclaimer
Kepaniteraan Mahkamah Agung Republik Indonesia berusaha untuk selalu mencantumkan informasi paling kini dan akurat sebagai bentuk komitmen Mahkamah Agung untuk pelayanan publik, transparansi dan akuntabilitas pelaksanaan fungsi peradilan. Namun dalam hal-hal tertentu masih dimungkinkan terjadi permasalahan teknis terkait dengan akurasi dan keterkinian informasi yang kami sajikan, hal mana akan terus kami perbaiki dari waktu kewaktu.
Dalam hal Anda menemukan inakurasi informasi yang termuat pada situs ini atau informasi yang seharusnya ada, namun belum tersedia, maka harap segera hubungi Kepaniteraan Mahkamah Agung RI melalui :
Email : kepaniteraan@mahkamahagung.go.id   Telp : 021-384 3348 (ext.318)

menurut hukum, maka berdasarkan ketentuan Pasal 148 R.Bg, Majelis Hakim berpendapat gugatan Penggugat harus dinyatakan gugur;

Menimbang, bahwa Penggugat dalam posita dan petitum gugatannya memohon dibebaskan dari biaya perkara dan berdasarkan Penetapan Ketua Pengadilan Agama Tanjungkarang Nomor 1061/Pdt.G/2020/PA.Tnk yang memberi izin kepada Penggugat untuk berperkara secara prodeo dan biaya perkara dibebankan kepada Negara, maka oleh karenanya dalam perkara a quo biaya perkara dibebankan kepada Negara melalui DIPA Pengadilan Agama Tanjungkarang;

Memperhatikan segala peraturan perundang-undangan yang berlaku dan Hukum Islam yang berkaitan dalam perkara ini;

M E N E T A P K A N

1. Menyatakan gugatan Penggugat gugur;
2. Membebankan biaya perkara sejumlah Rp 351.000,- (tiga ratus lima puluh satu ribu rupiah), kepada Negara melalui DIPA Pengadilan Agama Tanjungkarang tahun anggaran 2020;

Demikianlah diputuskan dalam musyawarah Majelis Hakim Pengadilan Agama Tanjungkarang pada hari Rabu tanggal 23 Desember 2020 M bertepatan dengan tanggal 8 Jumadilawal 1442 H, oleh kami Drs. H. Nurkholish, M.H. sebagai Ketua Majelis dan Drs. H.K.M. Junaidi, S.H., M.H. serta Dra. Hj. Rabiah Adawiyah Nasution, S.H., M.H. masing-masing sebagai Hakim Anggota, putusan mana diucapkan dalam sidang terbuka untuk umum pada hari itu juga oleh Ketua Majelis dengan didampingi oleh Para Hakim Anggota tersebut dan dibantu Rahmatiah Oktafiana, SHI, M.H. sebagai Panitera Pengganti dengan tanpa dihadiri Penggugat dan Tergugat;

Ketua Majelis

Drs. H. Nurkholish, M.H.

Disclaimer
Kepaniteraan Mahkamah Agung Republik Indonesia berusaha untuk selalu mencantumkan informasi paling kini dan akurat sebagai bentuk komitmen Mahkamah Agung untuk pelayanan publik, transparansi dan akuntabilitas pelaksanaan fungsi peradilan. Namun dalam hal-hal tertentu masih dimungkinkan terjadi permasalahan teknis terkait dengan akurasi dan keterkinian informasi yang kami sajikan, hal mana akan terus kami perbaiki dari waktu kewaktu.
Dalam hal Anda menemukan inakurasi informasi yang termuat pada situs ini atau informasi yang seharusnya ada, namun belum tersedia, maka harap segera hubungi Kepaniteraan Mahkamah Agung RI melalui :
Email : kepaniteraan@mahkamahagung.go.id   Telp : 021-384 3348 (ext.318)

Hakim Anggota, Hakim Anggota,

Drs. H.K.M. Junaidi, S.H., M.H. Dra. Hj. Rabiah Adawiyah Nasution, S.H., M.H.

Panitera Pengganti,

Rahmatiah Oktafiana, SHI, M.H.

Rincian Biaya Perkara :

1.	Biaya Pendaftaran	:	Rp	30.000,-
1.	Biaya Proses	:	Rp	50.000,-
2.	Biaya Panggilan	:	Rp	255.000,-
3.	Biaya Redaksi	:	Rp	10.000,-
4.	Biaya Materai	:	Rp	6.000,-
	Jumlah	:	Rp	351.000,-

(tiga ratus lima puluh satu ribu rupiah);

Disclaimer
Kepaniteraan Mahkamah Agung Republik Indonesia berusaha untuk selalu mencantumkan informasi paling kini dan akurat sebagai bentuk komitmen Mahkamah Agung untuk pelayanan publik, transparansi dan akuntabilitas pelaksanaan fungsi peradilan. Namun dalam hal-hal tertentu masih dimungkinkan terjadi permasalahan teknis terkait dengan akurasi dan keterkinian informasi yang kami sajikan, hal mana akan terus kami perbaiki dari waktu kewaktu.
Dalam hal Anda menemukan inakurasi informasi yang termuat pada situs ini atau informasi yang seharusnya ada, namun belum tersedia, maka harap segera hubungi Kepaniteraan Mahkamah Agung RI melalui :
Email : kepaniteraan@mahkamahagung.go.id Telp : 021-384 3348 (ext.318)
