# Supplementary material for: Classification of divorce causes during the COVID-19 pandemic using convolutional neural networks
Source: PeerJ Comput Sci. 2022 Jun 30;8:e998. doi: 10.7717/peerj-cs.998 (PMC9299239; doi:10.7717/peerj-cs.998)
Supplement: Supplemental Information 5 [file peerj-cs-08-998-s005.zip › Masalah Ekonomi Dataset/Data ke-2.pdf]

PUTUSAN

Nomor 1219/Pdt.G/2020/PA.Kds

بسم الله الرحمن الرحيم

DEMI KEADILAN BERDASARKAN KETUHANAN YANG MAHA ESA

Pengadilan Agama Kudus yang memeriksa dan mengadili perkara tertentu pada tingkat pertama dalam sidang majelis telah menjatuhkan putusan dalam perkara Cerai Gugat antara:

Siwi Purwo Cahyani binti Moch. Mahmud, NIK. 3319065707800002, tempat tanggal lahir Kudus, 17 Juli 1980, umur 40 tahun, agama Islam, pekerjaan Karyawan Djarum, pendidikan SLTP, alamat berdasarkan KTP di RT. 03 RW. 08, Desa Tanjungrejo, Kecamatan Jekulo, Kabupaten Kudus, sekarang berdomisili di Desa Jekulo Rt. 03 Rw. 01, Kecamatan Jekulo, Kabupaten Kudus, sebagai **Penggugat**;

m e l a w a n

Buchori bin Said, NIK. 3319061411730002, tempat tanggal lahir Kudus, 14 November 1973, umur 47 tahun, agama Islam, pekerjaan buruh, pendidikan SLTP, tempat kediaman di RT.03 RW.08, Desa Tanjungrejo, Kecamatan Jekulo Kabupaten Kudus, sebagai **Tergugat**;

Pengadilan Agama tersebut;

Telah mempelajari surat-surat yang berkaitan dengan perkara ini;

Telah mendengar keterangan Penggugat dan para saksi di muka sidang;

DUDUK PERKARA

Bahwa Penggugat dalam surat gugatannya tanggal 9 November 2020 telah mengajukan gugatan Cerai Gugat yang telah didaftar di Kepaniteraan Pengadilan Agama Kudus dengan Nomor 1219/Pdt.G/2020/PA.Kds., tanggal 9 November 2020 dengan dalil-dalil gugatan sebagai berikut:

1. Bahwa pada tanggal 21 Juli 2017, Penggugat telah melangsungkan perni-kahan dengan Tergugat yang dicatat oleh Pegawai Pencatat Nikah Kantor Urusan

Disclaimer
Kepaniteraan Mahkamah Agung Republik Indonesia berusaha untuk selalu mencantumkan informasi paling kini dan akurat sebagai bentuk komitmen Mahkamah Agung untuk pelayanan publik, transparansi dan akuntabilitas pelaksanaan fungsi peradilan. Namun dalam hal-hal tertentu masih dimungkinkan terjadi permasalahan teknis terkait dengan akurasi dan keterkinian informasi yang kami sajikan, hal mana akan terus kami perbaiki dari waktu kewaktu.
Dalam hal Anda menemukan inakurasi informasi yang termuat pada situs ini atau informasi yang seharusnya ada, namun belum tersedia, maka harap segera hubungi Kepaniteraan Mahkamah Agung RI melalui :
Email : kepaniteraan@mahkamahagung.go.id Telp : 021-384 3348 (ext.318)

Agama Kecamatan Jekulo Kabupaten Kudus sebagaimana dalam Kutipan Akta Nikah nomor : 0245/053/VII/2017 tanggal 21 Juli 2017 dan setelah akad nikah Tergugat mengucapkan sighat taklik talak terhadap Penggugat;

2. Bahwa pada saat menikah Penggugat berstatus janda cerai dan Tergugat berstatus duda mati;
3. Bahwa setelah pernikahan tersebut, Penggugat dengan Tergugat bertempat tinggal di rumah Tergugat di Desa Tanjungrejo Kecamatan Jekulo Kabupaten Kudus selama 3 tahun 1 bulan, telah hidup rukun sebagaimana layaknya suami istri namun belum dikaruniai keturunan ;
4. Bahwa selama dalam pernikahan Penggugat dan Tergugat belum pernah bercerai;
5. Bahwa semula rumah tangga Penggugat dan Tergugat berjalan harmonis, namun sejak 1 bulan menikah rumah tangga Penggugat dengan Tergugat mulai goyah dan sering terjadi perselisihan dan pertengkaran yang disebabkan :
   a. Tergugat tidak terbuka masalah keuangan;
   b. setelah menikah Tergugat baru jujur dan mengaku mempunyai hutang;
6. Bahwa puncak perselisihan dan pertengkaran tersebut terjadi pada bulan Agustus tahun 2020, yang akibatnya Penggugat pulang kerumah orangtua Penggugat sendiri dengan alamat sebagaimana tersebut diatas selama 3 bulan hingga sekarang. Selama itu sudah tidak ada komunikasi baik;
7. Bahwa atas sikap dan perbuatan Tergugat tersebut, Penggugat merasa sangat menderita lahir batin dan oleh karenanya Penggugat tidak rela dan berkesimpulan bahwa Tergugat adalah suami yang tidak bertanggung jawab, sehingga tujuan perkawinan untuk membentuk rumah tangga yang sakinah, mawaddah dan rahmah sudah sulit dipertahankan lagi, oleh karena itu perceraian merupakan alternative terakhir bagi Penggugat untuk menyelesai-kan permasalahan Penggugat dengan Tergugat;
8. Bahwa berdasarkan uraian di atas, gugatan Penggugat telah memenuhi alasan perceraian sebagaimana diatur dalam Undang-Undang Nomor 1 Tahun 1974 jo. Peraturan Pemerintah Nomor 9 Tahun 1975 dan Kompilasi Hukum Islam;
9. Bahwa Penggugat sanggup membayar seluruh biaya yang timbul dalam penyelesaian perkara ini.

Disclaimer
Kepaniteraan Mahkamah Agung Republik Indonesia berusaha untuk selalu mencantumkan informasi paling kini dan akurat sebagai bentuk komitmen Mahkamah Agung untuk pelayanan publik, transparansi dan akuntabilitas pelaksanaan fungsi peradilan. Namun dalam hal-hal tertentu masih dimungkinkan terjadi permasalahan teknis terkait dengan akurasi dan keterkinian informasi yang kami sajikan, hal mana akan terus kami perbaiki dari waktu kewaktu.
Dalam hal Anda menemukan inakurasi informasi yang termuat pada situs ini atau informasi yang seharusnya ada, namun belum tersedia, maka harap segera hubungi Kepaniteraan Mahkamah Agung RI melalui :
Email : kepaniteraan@mahkamahagung.go.id   Telp : 021-384 3348 (ext.318)

Berdasarkan alasan/dalil-dalil di atas, Penggugat merasa sudah tidak tahan lagi untuk meneruskan kehidupan rumah tangganya dengan Tergugat, oleh karenanya Penggugat mohon agar Ketua Pengadilan Agama Kudus c.q. Majelis Hakim segera memeriksa dan mengadili perkara ini, selanjutnya menjatuhkan putusan yang amarnya berbunyi :

PRIMER:

1. Mengabulkan gugatan Penggugat;
2. Menjatuhkan talak satu ba'in sughra Tergugat (Buchori bin Said) terhadap Penggugat (Siwi Purwo Cahyani binti Moch. Mahmud);
3. Membebankan biaya perkara menurut hukum;

SUBSIDER :

Apabila Pengadilan Agama berpendapat lain, mohon putusan yang seadil-adilnya;

Bahwa pada hari persidangan yang telah ditetapkan, Penggugat dan Tergugat datang sendiri menghadap ke persidangan, Majelis Hakim telah berusaha mendamaikan Penggugat dan Tergugat, akan tetapi tidak berhasil;

Bahwa untuk memenuhi kehendak Perma Nomor 1 Tahun 2016 Majelis Hakim telah memerintahkan kepada Penggugat dan Tergugat agar terlebih dahulu menjalani proses mediasi. Mediasi tersebut telah dilaksanakan oleh Hakim Mediator dan ternyata mediasi yang telah dilaksanakan tanggal 23 November 2020 juga tidak berhasil mendamaikan Penggugat dan Tergugat;

Bahwa pada hari-hari dan tanggal sidang selanjutnya Tergugat tidak hadir di persidangan meskipun telah diberitahu serta dipanggil secara resmi dan patut dan tidak menyuruh orang lain atau kuasanya untuk hadir menghadap sidang;

Bahwa selanjutnya dimulai pemeriksaan perkara dengan dibacakan surat gugatan Penggugat yang maksud dan isinya tetap dipertahankan oleh Penggugat;

Bahwa atas gugatan Penggugat tersebut, Tergugat tidak memberikan jawaban karena setelah proses mediasi, Tergugat tidak pernah datang lagi menghadap sidang;

Bahwa untuk menguatkan dalil-dalil gugatannya, Penggugat telah mengajukan alat-alat bukti di persidangan, yaitu sebagai berikut:

I. Alat bukti Surat:

Disclaimer
Kepaniteraan Mahkamah Agung Republik Indonesia berusaha untuk selalu mencantumkan informasi paling kini dan akurat sebagai bentuk komitmen Mahkamah Agung untuk pelayanan publik, transparansi dan akuntabilitas pelaksanaan fungsi peradilan. Namun dalam hal-hal tertentu masih dimungkinkan terjadi permasalahan teknis terkait dengan akurasi dan keterkinian informasi yang kami sajikan, hal mana akan terus kami perbaiki dari waktu kewaktu.
Dalam hal Anda menemukan inakurasi informasi yang termuat pada situs ini atau informasi yang seharusnya ada, namun belum tersedia, maka harap segera hubungi Kepaniteraan Mahkamah Agung RI melalui :
Email : kepaniteraan@mahkamahagung.go.id   Telp : 021-384 3348 (ext.318)

1. Fotokopi Kartu Tanda Penduduk Nomor 3319065707800002 tanggal 21 September 2012, telah bermeterai cukup, telah dicocokan sesuai dengan aslinya (bukti P.1);

2. Fotokopi Kutipan Akta Nikah dari Kantor Urusan Agama Kecamatan Jekulo, Kabupaten Kudus Nomor 0245/053/VII/2017 tanggal 21 Juli 2017, telah bermeterai cukup, telah dicocokkan sesuai dengan aslinya (bukti P.2);

II. Alat bukti Saksi:

1. **Masrukin bin Sumardi**, umur 37 tahun, agama Islam, pekerjaan sopir, tempat tinggal di Desa Jekulo Rt. 03 Rw. 01, Kecamatan Jekulo, Kabupaten Kudus, telah memberikan keterangan di bawah sumpah yang pada pokoknya sebagai berikut :

   - Bahwa saksi kenal dengan Penggugat dan Tergugat karena saksi adalah ayah kandung Penggugat dan tinggal serumah dengan Penggugat;
   - Bahwa setelah menikah Penggugat dan Tergugat bertempat tinggal di rumah orang tua Tergugat;
   - Bahwa saksi mengetahui Penggugat dengan Tergugat telah berpisah hingga sekarang lebih kurang 3 (tiga) bulan karena Penggugat telah pergi meninggalkan Tergugat dan pulang ke rumah orang tuanya;
   - Bahwa sebelum pisah rumah, Penggugat dan Tergugat sering bertengkar dalam bentuk saling diam (serik-serikan) masalah ekonomi dan Tergugat mempunyai utang, saksi melihat ada 2 (dua) kali melihat dan mendengar sendiri pertengkaran tersebut;
   - Bahwa saksi sudah menasehati Penggugat agar rukun lagi dengan Tergugat, tetapi tidak berhasil;

2. **Zumrotun binti Ngadiran**, umur 47 tahun, agama Islam, pekerjaan dagang, tempat tinggal di Desa Jekulo Rt. 03 Rw. 01, Kecamatan Jekulo, Kabupaten Kudus, telah memberikan keterangan di bawah sumpah yang pada pokoknya sebagai berikut :

   - Bahwa saksi kenal dengan Penggugat dan Tergugat karena saksi adalah tetangga dekat Penggugat;
   - Bahwa setelah menikah Penggugat dan Tergugat bertempat tinggal di rumah orang tua Tergugat;

Disclaimer
Kepaniteraan Mahkamah Agung Republik Indonesia berusaha untuk selalu mencantumkan informasi paling kini dan akurat sebagai bentuk komitmen Mahkamah Agung untuk pelayanan publik, transparansi dan akuntabilitas pelaksanaan fungsi peradilan. Namun dalam hal-hal tertentu masih dimungkinkan terjadi permasalahan teknis terkait dengan akurasi dan keterkinian informasi yang kami sajikan, hal mana akan terus kami perbaiki dari waktu kewaktu.
Dalam hal Anda menemukan inakurasi informasi yang termuat pada situs ini atau informasi yang seharusnya ada, namun belum tersedia, maka harap segera hubungi Kepaniteraan Mahkamah Agung RI melalui :
Email : kepaniteraan@mahkamahagung.go.id Telp : 021-384 3348 (ext.318)

- Bahwa karena sering main ke tempat tinggal Penggugat, saksi mengetahui Penggugat dengan Tergugat telah berpisah hingga sekarang lebih kurang 3 (tiga) bulan karena Penggugat telah pergi meninggalkan Tergugat dan pulang ke rumah orang tuanya;
- Bahwa sebelum pisah rumah, Penggugat dan Tergugat sering bertengkar

masalah Tergugat mempunyai utang dan masalah ekonomi, tetapi saksi tidak melihat maupun mendengar sendiri pertengkaran tersebut dan hanya mendapat cerita dari Penggugat;
- Bahwa saksi sudah menasehati Penggugat agar rukun lagi dengan Tergugat, tetapi tidak berhasil;

Bahwa terhadap keterangan saksi-saksi tersebut, Penggugat menyatakan cukup dan tidak akan mengajukan saksi lainnya lagi;

Bahwa Penggugat telah menyampaikan kesimpulan secara lisan dengan menyatakan tetap pada gugatannya dan mohon putusan;

---Bahwa untuk singkatnya uraian putusan ini, maka semua hal yang termuat dalam berita acara sidang perkara ini merupakan bagian yang tidak terpisahkan dari putusan ini;

PERTIMBANGAN HUKUM

Menimbang, bahwa maksud dan tujuan gugatan Penggugat adalah sebagaimana telah diuraikan di atas;

Menimbang, bahwa oleh karena perkara ini termasuk dalam bidang perkawinan, maka sesuai dengan Pasal 49 huruf a Undang-Undang Nomor 7 Tahun 1989 tentang Peradilan Agama, sebagaimana telah diubah dengan Undang-Undang Nomor 3 Tahun 2006 dan perubahan kedua dengan Undang-Undang Nomor 50 Tahun 2009, perkara ini merupakan kewenangan Pengadilan Agama;

Menimbang, bahwa berdasarkan identitas para pihak, Penggugat dan Tergugat bertempat tinggal di wilayah Kabupaten Kudus yang merupakan yurisdiksi Pengadilan Agama Kudus, maka sesuai dengan Pasal 73 ayat (1) Undang-Undang Nomor 7 Tahun 1989 tentang Peradilan Agama, sebagaimana telah diubah dengan Undang-Undang Nomor 3 Tahun 2006 dan perubahan kedua dengan Undang-Undang Nomor 50

Disclaimer
Kepaniteraan Mahkamah Agung Republik Indonesia berusaha untuk selalu mencantumkan informasi paling kini dan akurat sebagai bentuk komitmen Mahkamah Agung untuk pelayanan publik, transparansi dan akuntabilitas pelaksanaan fungsi peradilan. Namun dalam hal-hal tertentu masih dimungkinkan terjadi permasalahan teknis terkait dengan akurasi dan keterkinian informasi yang kami sajikan, hal mana akan terus kami perbaiki dari waktu kewaktu.
Dalam hal Anda menemukan inakurasi informasi yang termuat pada situs ini atau informasi yang seharusnya ada, namun belum tersedia, maka harap segera hubungi Kepaniteraan Mahkamah Agung RI melalui :
Email : kepaniteraan@mahkamahagung.go.id Telp : 021-384 3348 (ext.318)

Tahun 2009, Pengadilan Agama Kudus berwenang untuk memeriksa perkara ini (*relative competentie*);

Menimbang, bahwa Majelis Hakim telah berusaha mendamaikan Penggugat dan Tergugat, akan tetapi tidak berhasil, dan untuk memenuhi kehendak Perma Nomor 1 Tahun 2016 Majelis Hakim telah memerintahkan kepada Penggugat dan Tergugat agar terlebih dahulu menjalani proses mediasi, dan mediasi tersebut telah dilaksanakan oleh Mediator **Sunarto, S.H., M.H.** ternyata juga tidak berhasil mendamaikan Penggugat dan Tergugat;

Menimbang, bahwa alasan Penggugat mengajukan Cerai Gugat pada pokoknya adalah pada awalnya rumah tangga Penggugat dengan Tergugat harmonis, namun namun sejak 1 bulan menikah antara Penggugat dengan Tergugat sering terjadi perselisihan dan pertengkaran yang disebabkan Tergugat tidak terbuka masalah keuangan dan setelah menikah Tergugat baru jujur mengaku mempunyai hutang, dan puncaknya terjadi pada bulan Agustus tahun 2020, yang akibatnya Penggugat pulang ke rumah orang tua Penggugat sendiri dengan alamat sebagaimana tersebut diatas selama 3 bulan hingga sekarang, selama itu sudah tidak ada komunikasi baik;

Menimbang, bahwa terhadap gugatan tersebut, Tergugat tidak memberikan jawaban karena setelah proses mediasi, Tergugat tidak pernah datang lagi meng-hadap sidang, meskipun telah diberi tahu dan dipanggil secara resmi dan patut dan tidak pula menyuruh orang lain sebagai kuasanya untuk menghadap sidang;

Menimbang, bahwa meskipun Tergugat tidak memberikan jawabannya, namun sesuai prinsip dan asas yang terdapat dalam penjelasan Undang-Undang Nomor 1 Tahun 1974 angka (4) huruf (e), jo Pasal 163 HIR, maka Majelis Hakim membani Penggugat untuk membuktikan dalil-dalil gugatannya;

Menimbang, bahwa untuk membuktikan dalil-dalil gugatannya, Penggugat telah mengajukan alat bukti surat P.1, P.2, P.3 dan 2 orang saksi;

Menimbang, bahwa bukti P.1 terbukti Penggugat berdasarkan KTP beralamat di RT. 03 RW. 08, Desa Tanjungrejo, Kecamatan Jekulo, Kabupaten Kudus, sekarang berdomisili di Desa Jekulo Rt. 03 Rw. 01, Kecamatan Jekulo, Kabupaten Kudus, ternyata sesuai dengan identitas Penggugat pada surat gugatannya, oleh karena itu dapat diterima sebagai alat bukti;

Disclaimer
Kepaniteraan Mahkamah Agung Republik Indonesia berusaha untuk selalu mencantumkan informasi paling kini dan akurat sebagai bentuk komitmen Mahkamah Agung untuk pelayanan publik, transparansi dan akuntabilitas pelaksanaan fungsi peradilan. Namun dalam hal-hal tertentu masih dimungkinkan terjadi permasalahan teknis terkait dengan akurasi dan keterkinian informasi yang kami sajikan, hal mana akan terus kami perbaiki dari waktu kewaktu.
Dalam hal Anda menemukan inakurasi informasi yang termuat pada situs ini atau informasi yang seharusnya ada, namun belum tersedia, maka harap segera hubungi Kepaniteraan Mahkamah Agung RI melalui :
Email : kepaniteraan@mahkamahagung.go.id Telp : 021-384 3348 (ext.318)

Menimbang, bahwa bukti P.2 berupa Fotokopi Kutipan Akta Nikah yang aslinya merupakan akta otentik dan fotokopinya telah bermeterai cukup dan cocok dengan aslinya, isi bukti tersebut menjelaskan mengenai Penggugat dan Tergugat telah melangsungkan perkawinan pada tanggal 21 Juli 2017 tercatat di Kantor Urusan Agama Kecamatan Jekulo, Kabupaten Kudus, sehingga bukti tersebut telah memenuhi syarat formal dan materiil, serta mempunyai kekuatan yang sempurna dan mengikat;

Menimbang, bahwa saksi 1 dan saksi 2 Penggugat, sudah dewasa dan sudah disumpah, sehingga memenuhi syarat formal sebagaimana diatur dalam Pasal 145 HIR;

Menimbang, bahwa saksi 1 dan saksi 2 Penggugat telah memberikan keterangan yang pada pokoknya Penggugat dan Tergugat sering terjadi perselisihan dan pertengkaran masalah ekonomi dan Tergugat mempunyai utang, saksi 1 melihat dan mendengar sendiri pertengkaran tersebut, sedangkan saksi 2 tidak melihat maupun mendengar sendiri pertengkaran tersebut dan hanya mendapat cerita dari Pengugat, namun saksi 1 dan saksi 2 sama-sama mengetahui Penggugat dan Tergugat telah pisah rumah lebih kurang 3 (tiga) bulan lamanya, saksi 1 dan saksi 2 sudah menasehati Penggugat agar rukun lagi dengan Tergugat, tetapi tidak berhasil;

Menimbang, bahwa saksi 2 mendapat cerita dari Penggugat mengenai telah sering terjadi perselisihan dan pertengkaran antara Penggugat dan Tergugat, keterangan saksi hanya bersifat testimonium de auditu, majelis sependapat dengan Yurisprudensi Mahkamah Agung RI Nomor 308K/Sip/1959 tanggal 11 November 1959 menyatakan, bahwa meskipun testimonium de auditu tidak dapat digunakan sebagai alat bukti langsung, tetapi penggunaan kesaksian yang demikian sebagai persangkaan yang dari persangkaan itu dibuktikan sesuatu, tidak dilarang;

Menimbang, bahwa saksi 1 dan saksi 2 sama-sama mengetahui Penggugat dan Tergugat telah pisah rumah lebih kurang 3 (tiga) bulan, majelis menilai pisah rumah merupakan indikasi telah terjadinya perselisihan dan pertengkarn antara Penggugat dan Tergugat, hal ini sesuai dengan Yurisprudensi Mahkamah Agung RI Nomor 136/K/AG/1997 tanggal 26 Februari 1998 yang menyatakan bahwa pisah rumah merupakan indikasi terjadinya perselisihan dan pertengkaran yang terus menerus, maka majelis berpendapat Penggugat dan Tergugat yang pisah

Disclaimer
Kepaniteraan Mahkamah Agung Republik Indonesia berusaha untuk selalu mencantumkan informasi paling kini dan akurat sebagai bentuk komitmen Mahkamah Agung untuk pelayanan publik, transparansi dan akuntabilitas pelaksanaan fungsi peradilan. Namun dalam hal-hal tertentu masih dimungkinkan terjadi permasalahan teknis terkait dengan akurasi dan keterkinian informasi yang kami sajikan, hal mana akan terus kami perbaiki dari waktu kewaktu.
Dalam hal Anda menemukan inakurasi informasi yang termuat pada situs ini atau informasi yang seharusnya ada, namun belum tersedia, maka harap segera hubungi Kepaniteraan Mahkamah Agung RI melalui :
Email : kepaniteraan@mahkamahagung.go.id Telp : 021-384 3348 (ext.318)

rumah lebih kurang 3 (tiga) bulan telah sesuai dengan maksud yurisprudensi tersebut;

Menimbang, bahwa keterangan saksi 1 dan saksi 2 mengenai Penggugat dan Tergugat telah pisah rumah lebih kurang 3 (tiga) bulan diberikan atas dasar pengetahuannya sendiri dan keterangannya saling bersesuaian satu sama lain dan bersesuaian pula serta relevan dengan gugatan Penggugat, maka keterangan saksi-saksi tersebut telah memenuhi syarat materiil sebagaimana telah diatur dalam Pasal 171 dan Pasal 172 HIR, sehingga keterangan saksi-saksi tersebut memiliki kekuatan pembuktian dan dapat dipertimbangkan;

Menimbang, bahwa berdasarkan gugatan Penggugat, bukti P.1, P.2, P.3 serta saksi 1 dan saksi 2 terbukti fakta-fakta sebagai berikut:

1. Bahwa benar Penggugat dan Tergugat adalah suami istri yang menikah pada
tanggal 21 Juli 2017;
2. Bahwa benar rumah tangga Penggugat dan Tergugat tidak harmonis lagi karena
telah sering terjadi perselisihan dan pertengkaran dan tidak ada harapan untuk hidup rukun lagi dalam rumah tangga;
3. Bahwa benar terbukti antara Penggugat dengan Tergugat telah pisah rumah 3 (tiga) bulan;

Menimbang, bahwa berdasarkan fakta-fakta tersebut di atas tujuan perkawinan sebagaimana dikehendaki oleh Pasal 1 Undang-Undang Nomor 1 Tahun 1974 jo. Pasal 3 Kompilasi Hukum Islam yaitu, membentuk keluarga (rumah tangga) yang bahagia dan kekal berdasarkan Ketuhanan Yang Maha Esa serta membina kehidupan rumah tangga yang sakinah, mawaddah dan rahmah seperti yang dimaksud dalam Al-Qur’an surat Ar-Rum ayat 21 tidak dapat terwujud karena masing-masing telah hidup berpisah;

Menimbang, bahwa majelis telah menasehati Penggugat di setiap kali sidang agar rukun lagi dengan Tergugat, tetapi Penggugat tetap menunjukkan tidak senangnya kepada Tergugat dan bersikeras untuk bercerai dengan Tergugat, sehingga ikatan perkawinan Penggugat dengan Tergugat sudah tidak bisa dipertahankan lagi, hal ini dapat diterapkan pendapat ulama dalam Kitab

Disclaimer
Kepaniteraan Mahkamah Agung Republik Indonesia berusaha untuk selalu mencantumkan informasi paling kini dan akurat sebagai bentuk komitmen Mahkamah Agung untuk pelayanan publik, transparansi dan akuntabilitas pelaksanaan fungsi peradilan. Namun dalam hal-hal tertentu masih dimungkinkan terjadi permasalahan teknis terkait dengan akurasi dan keterkinian informasi yang kami sajikan, hal mana akan terus kami perbaiki dari waktu kewaktu.
Dalam hal Anda menemukan inakurasi informasi yang termuat pada situs ini atau informasi yang seharusnya ada, namun belum tersedia, maka harap segera hubungi Kepaniteraan Mahkamah Agung RI melalui :
Email : kepaniteraan@mahkamahagung.go.id   Telp : 021-384 3348 (ext.318)

Ghoyatul Marom halaman 162 yang selanjutnya diambilalih menjadi pendapat majelis, yaitu:

واذا اشتد عدم رغبة الزوجة لزوجها طلق عليه القاضى طلقة

Artinya : *Diwaktu istri sudah sangat tidak senang pada suaminya, maka Hakim diperkenankan menjatuhkan talak si suami*;

Menimbang, bahwa dengan melihat fakta-fakta seperti tersebut di atas perlu dicarikan jalan keluarnya, maka majelis berpendapat bahwa perceraian adalah solusi yang terbaik bagi Penggugat dan Tergugat, kalau dipaksakan juga untuk mempertahankan rumah tangganya patut diduga bahwa hal itu akan menimbulkan mafsadah yang lebih besar dari maslahahnya, padahal menolak mafsadah lebih diutamakan dari mencapai kemaslahatan, hal ini sesuai dengan kaidah Fiqih yang berbunyi:

درؤ المفاسد مقدم على جلب المصالح

Artinya : *Menolak kemudharatan lebih utama daripada menarik (memper-tahankan) kebaikan;*

Menimbang, bahwa dengan pertimbangan tersebut majelis menilai gugatan Penggugat telah berdasar hukum dan telah memenuhi Pasal 19 huruf f Peraturan Pemerintah Nomor 9 Tahun 1975 jo. Pasal 116 huruf f Kompilasi Hukum Islam;

Menimbang, bahwa berdasarkan pertimbangan tersebut, maka sudah sepatutnya gugatan Penggugat dikabulkan dengan menjatuhkan talak satu bain sughra Tergugat terhadap Penggugat;

Menimbang, bahwa oleh karena perkara ini termasuk dalam bidang perka-winan, maka sesuai dengan Pasal 89 ayat (1) Undang-Undang Nomor 7 Tahun 1989 Tentang Peradilan Agama sebagaimana telah diubah dengan Undang-Undang Nomor 3 Tahun 2006 dan perubahan kedua dengan Undang-Undang Nomor 50 Tahun 2009, biaya perkara ini dibebankan kepada Penggugat;

Mengingat, semua pasal dalam peraturan perundang-undangan dan hukum Islam yang berkaitan dengan perkara ini;

M E N G A D I L I

1. Mengabulkan gugatan Penggugat;

Disclaimer
Kepaniteraan Mahkamah Agung Republik Indonesia berusaha untuk selalu mencantumkan informasi paling kini dan akurat sebagai bentuk komitmen Mahkamah Agung untuk pelayanan publik, transparansi dan akuntabilitas pelaksanaan fungsi peradilan. Namun dalam hal-hal tertentu masih dimungkinkan terjadi permasalahan teknis terkait dengan akurasi dan keterkinian informasi yang kami sajikan, hal mana akan terus kami perbaiki dari waktu kewaktu.
Dalam hal Anda menemukan inakurasi informasi yang termuat pada situs ini atau informasi yang seharusnya ada, namun belum tersedia, maka harap segera hubungi Kepaniteraan Mahkamah Agung RI melalui :
Email : kepaniteraan@mahkamahagung.go.id   Telp : 021-384 3348 (ext.318)

2. Menjatuhkan talak satu ba'in sughra Tergugat (**Buchori bin Said)** terhadap Penggugat (**Siwi Purwo Cahyani binti Moch. Mahmud**);
3. Membebankan kepada Penggugat untuk membayar biaya perkara sejumlah Rp 461.000,00 (empat ratus enam puluh satu ribu rupiah);

Demikian diputuskan dalam rapat permusyawaratan Majelis Hakim yang dilangsungkan pada hari Senin tanggal 21 Desember 2020 Masehi bertepatan dengan tanggal 6 Jumadilawal 1442 Hijriah, oleh **Sulomo, S.Ag** sebagai Ketua Majelis, **H. Ah. Sholih, S.H**. dan **Dra. Ulfah** masing-masing sebagai Hakim Anggota, putusan tersebut diucapkan dalam sidang terbuka untuk umum pada hari itu juga oleh Ketua Majelis tersebut dengan didampingi oleh Hakim-Hakim Anggota dan dibantu oleh **Nur Cholifah, S.H.** sebagai Panitera Pengganti serta dihadiri oleh Penggugat di luar hadirnya Tergugat.

Hakim Anggota,	Ketua Majelis,
**H. Ah. Sholih, S.H**.	**Sulomo, S.Ag**.
Hakim Anggota,	
**Dra. Ulfah**	Panitera Pengganti,
	**Nur Cholifah, S.H.**

PERINCIAN BIAYA :

1. Pendaftaran--: Rp 30.000,00
2. ATK--: Rp 75.000,00
3. Panggilan ---: Rp 320.000,00
4. PNBP pgl---: Rp 20.000,00
5. Redaksi : Rp 10.000,00
6. Meterai--: Rp 6.000,00

Jumlah : Rp 461.000,00

Disclaimer
Kepaniteraan Mahkamah Agung Republik Indonesia berusaha untuk selalu mencantumkan informasi paling kini dan akurat sebagai bentuk komitmen Mahkamah Agung untuk pelayanan publik, transparansi dan akuntabilitas pelaksanaan fungsi peradilan. Namun dalam hal-hal tertentu masih dimungkinkan terjadi permasalahan teknis terkait dengan akurasi dan keterkinian informasi yang kami sajikan, hal mana akan terus kami perbaiki dari waktu kewaktu.
Dalam hal Anda menemukan inakurasi informasi yang termuat pada situs ini atau informasi yang seharusnya ada, namun belum tersedia, maka harap segera hubungi Kepaniteraan Mahkamah Agung RI melalui :
Email : kepaniteraan@mahkamahagung.go.id Telp : 021-384 3348 (ext.318)

Putusan ini telah diberitahukan kepada Tergugat tanggal . . . .

Putusan ini telah memperoleh kekuatan hukum tetap tanggal . . . .

Disclaimer
Kepaniteraan Mahkamah Agung Republik Indonesia berusaha untuk selalu mencantumkan informasi paling kini dan akurat sebagai bentuk komitmen Mahkamah Agung untuk pelayanan publik, transparansi dan akuntabilitas pelaksanaan fungsi peradilan. Namun dalam hal-hal tertentu masih dimungkinkan terjadi permasalahan teknis terkait dengan akurasi dan keterkinian informasi yang kami sajikan, hal mana akan terus kami perbaiki dari waktu kewaktu.
Dalam hal Anda menemukan inakurasi informasi yang termuat pada situs ini atau informasi yang seharusnya ada, namun belum tersedia, maka harap segera hubungi Kepaniteraan Mahkamah Agung RI melalui :
Email : kepaniteraan@mahkamahagung.go.id   Telp : 021-384 3348 (ext.318)
