# Supplementary material for: Classification of divorce causes during the COVID-19 pandemic using convolutional neural networks
Source: PeerJ Comput Sci. 2022 Jun 30;8:e998. doi: 10.7717/peerj-cs.998 (PMC9299239; doi:10.7717/peerj-cs.998)
Supplement: Supplemental Information 5 [file peerj-cs-08-998-s005.zip › Masalah Ekonomi Dataset/Data ke-20.pdf]

P U T U S A N

Nomor 2385/Pdt.G/2020/PA.Cbd

بِسْمِ اللهِ الرَّحْمٰنِ الرَّحِيْمِ

DEMI KEADILAN BERDASARKAN KETUHANAN YANG MAHA ESA

Pengadilan Agama Cibadak yang memeriksa dan mengadili perkara tertentu pada tingkat pertama dalam persidangan majelis telah menjatuhkan Putusan sebagai berikut dalam perkara Cerai Gugat yang diajukan oleh:

Citra binti Dedi, umur 25 tahun, agama Islam, pendidikan SLTP, pekerjaan Karyawan Swasta, tempat kediaman di Kampung Babakan Jampang RT 002 RW 009 Desa Jayabakti Kecamatan Cidahu Kabupaten Sukabumi, sebagai Penggugat;

melawan

Sandi Qodir bin Supardi, umur 24 tahun, agama Islam, pendidikan SD, pekerjaan Buruh, tempat kediaman di Kampung Papisangan RT 001 RW 004 Desa Caringin Kecamatan Cicurug Kabupaten Sukabumi, sebagai Tergugat;**;**

Pengadilan Agama tersebut;

Telah membaca dan mempelajari berkas perkara;

Telah mendengar keterangan Penggugat dan memeriksa alat-alat bukti di persidangan;

DUDUK PERKARA

Bahwa Penggugat dalam surat gugatannya tertanggal 02 Desember 2020, yang terdaftar di Kepaniteraan Pengadilan Agama Cibadak pada tanggal hari 02 Desember 2020 dalam register perkara Nomor 2385/Pdt.G/2020/PA.Cbd, telah mengajukan dalil-dalil sebagai berikut:

Bahwa Penggugat dengan Tergugat pada tanggal 05 September 2013 telah melangsungkan pernikahan di wilayah hukum Kantor Urusan Agama Kecamatan Cidahu Kabupaten Sukabumi dengan Kutipan Akta Nikah Nomor: 562/18/IX/2013 tertanggal 05 September 2013;

Bahwa setelah pernikahan tersebut Penggugat dengan Tergugat membina rumah tangga di rumah orang tua Penggugat yang beralamat di Kampung

Disclaimer
Kepaniteraan Mahkamah Agung Republik Indonesia berusaha untuk selalu mencantumkan informasi paling kini dan akurat sebagai bentuk komitmen Mahkamah Agung untuk pelayanan publik, transparansi dan akuntabilitas pelaksanaan fungsi peradilan. Namun dalam hal-hal tertentu masih dimungkinkan terjadi permasalahan teknis terkait dengan akurasi dan keterkinian informasi yang kami sajikan, hal mana akan terus kami perbaiki dari waktu kewaktu.
Dalam hal Anda menemukan inakurasi informasi yang termuat pada situs ini atau informasi yang seharusnya ada, namun belum tersedia, maka harap segera hubungi Kepaniteraan Mahkamah Agung RI melalui :
Email : kepaniteraan@mahkamahagung.go.id   Telp : 021-384 3348 (ext.318)

Babakan Jampang RT 002 RW 009 Desa Jayabakti Kecamatan Cidahu Kabupaten Sukabumi, sebagai tempat kediaman bersama;

Bahwa selama berumah tangga antara Penggugat dan Tergugat telah hidup rukun sebagaimana layaknya suami isteri dan telah dikaruniai 1 (Satu) orang anak/keturunan yang bernama;

Najwa Maulida, Sukabumi 31 Maret 2014 (umur 6 tahun);

Bahwa pada awalnya rumah tangga antara Penggugat dengan Tergugat berjalan baik, rukun dan harmonis sebagaimana layaknya rumah tangga yang baik, akan tetapi sejak sekitar bulan Desember 2013 kehidupan dan ketentraman rumah tangga antara Penggugat dengan Tergugat mulai tidak harmonis sering terjadi perselisihan dan pertengkaran, hal itu disebabkan antara lain:

Bahwa Tergugat sudah tidak bertanggungjawab dalam hal nafkah keluarga, sehingga Penggugat yang harus menanggung beban dalam rumah tangga dengan cara bekerja;

Bahwa Tergugat tidak peduli terhadap Penggugat dan anaknya sehingga Penggugat dan anaknya merasa terabaikan;

Bahwa puncak permasalahan antara Penggugat dengan Tergugat terjadi pada bulan April 2014, dimana Tergugat menjatuhkan talak secara dibawah tangan kepada Penggugat diluar Pengadilan Agama, kemudian Tergugat pergi meninggalkan tempat kediaman bersama, sehingga sejak itu antara Penggugat dan Tergugat pisah rumah, tidak pernah lagi saling peduli dan menjalin hubungan sebagaimana layaknya suami istri dan selama itu pula Tergugat tidak memberikan nafkah kepada Penggugat sampai dengan sekarang;

Bahwa menghadapi keadaan rumah tangga Penggugat yang sudah tidak rukun lagi tersebut Penggugat sudah berusaha bersabar, akan tetapi tidak berhasil ;

Bahwa keadaan rumah tangga Penggugat dengan Tergugat makin hari semakin memburuk sehingga dengan kondisi seperti itu Penggugat merasa tidak sanggup lagi untuk melanjutkan rumah tangga dengan Tergugat

Disclaimer
Kepaniteraan Mahkamah Agung Republik Indonesia berusaha untuk selalu mencantumkan informasi paling kini dan akurat sebagai bentuk komitmen Mahkamah Agung untuk pelayanan publik, transparansi dan akuntabilitas pelaksanaan fungsi peradilan. Namun dalam hal-hal tertentu masih dimungkinkan terjadi permasalahan teknis terkait dengan akurasi dan keterkinian informasi yang kami sajikan, hal mana akan terus kami perbaiki dari waktu kewaktu.
Dalam hal Anda menemukan inakurasi informasi yang termuat pada situs ini atau informasi yang seharusnya ada, namun belum tersedia, maka harap segera hubungi Kepaniteraan Mahkamah Agung RI melalui :
Email : kepaniteraan@mahkamahagung.go.id   Telp : 021-384 3348 (ext.318)

karena tujuan perkawinan untuk membentuk rumah tangga yang sakinah, mawaddah, dan warohmah tidak mungkin terwujud;

Bahwa Penggugat sanggup membayar biaya perkara;

;

Bahwa berdasarkan alasan-alasan tersebut di atas, Penggugat mohon kepada Bapak Ketua Pengadilan Agama Cibadak cq. Majelis Hakim yang memeriksa perkara ini berkenan memutuskan sebagai berikut :

Mengabulkan gugatan Penggugat;

Menjatuhkan talak satu bain sughra Tergugat **(Sandi Qodir bin Supardi)** kepada Penggugat **(Citra binti Dedi)**

Menetapkan biaya perkara menurut hukum

ATAU : Apabila Pengadilan Agama Cibadak Cq Majelis Hakim berpendapat lain, dapat menjatuhkan putusan yang sesuai dengan rasa keadilan;

Bahwa pada hari-hari dan tanggal sidang yang telah ditetapkan Penggugat telah hadir menghadap di persidangan, sedangkan Tergugat tidak pernah datang menghadap dan tidak pula menyuruh orang lain sebagai kuasa atau wakilnya yang sah dan tidak ternyata ketidakhadirannya tersebut disebabkan oleh suatu alasan yang sah, meskipun telah dipanggil secara resmi dan patut menurut hukum, maka perkara ini diperiksa tanpa hadirnya Tergugat;;

Bahwa majelis hakim dalam persidangan telah berusaha mendamaikan dengan jalan memberikan nasihat kepada Penggugat agar bersabar dan tetap mempertahankan keutuhan rumah tangganya denganTergugat, akan tetapi tidak berhasil. Sedangkan usaha damai melalui proses mediasi tidak dapat dilaksanakan karena Tergugat tidak pernah datang menghadap ke persidangan;

Bahwa selanjutnya Ketua Majelis membacakan surat gugatan Penggugat dalam sidang tertutup untuk umum. Kemudian Penggugat menyatakan tetap mempertahankan semua dalil gugatannya untuk bercerai dengan Penggugat tanpa perubahan ataupun keterangan tambahan;

Bahwa, terhadap gugatan Penggugat tersebut, jawaban Tergugat tidak dapat didengar dalam persidangan karena Tergugat tidak pernah hadir di persidangan meskipun telah dipanggil secara resmi dan patut;

Disclaimer
Kepaniteraan Mahkamah Agung Republik Indonesia berusaha untuk selalu mencantumkan informasi paling kini dan akurat sebagai bentuk komitmen Mahkamah Agung untuk pelayanan publik, transparansi dan akuntabilitas pelaksanaan fungsi peradilan. Namun dalam hal-hal tertentu masih dimungkinkan terjadi permasalahan teknis terkait dengan akurasi dan keterkinian informasi yang kami sajikan, hal mana akan terus kami perbaiki dari waktu kewaktu.
Dalam hal Anda menemukan inakurasi informasi yang termuat pada situs ini atau informasi yang seharusnya ada, namun belum tersedia, maka harap segera hubungi Kepaniteraan Mahkamah Agung RI melalui :
Email : kepaniteraan@mahkamahagung.go.id Telp : 021-384 3348 (ext.318)

Bahwa untuk meneguhkan dalil-dalil gugatannya, Penggugat telah mengajukan bukti surat berupa ...;

Bahwa selain bukti surat tersebut, Penggugat juga menghadirkan 2 orang saksi sebagai berikut:

1. **...**, umur ... tahun, agama ..., pekerjaan ..., bertempat tinggal di ... di persidangan saksi tersebut telah memberikan keterangan di bawah sumpah yang pada pokoknya sebagai berikut:
   ...
2. **...**, umur ... tahun, agama ..., pekerjaan ..., bertempat tinggal di ..., di persidangan saksi tersebut telah memberikan keterangan di bawah sumpah yang pada pokoknya sebagai berikut:
   ...

Bahwa selanjutnya Penggugat mencukupkan alat buktinya dan mengajukan kesimpulan secara lisan yang pada pokoknya tetap pada pendiriannya untuk bercerai dengan Tergugat dan mohon putusan;

Bahwa untuk meringkas uraian putusan ini, cukup ditunjuk segala hal sebagaimana tercantum dalam berita acara sidang perkara ini, yang merupakan bagian tidak terpisahkan dari putusan ini;

PERTIMBANGAN HUKUM

Menimbang, bahwa maksud dan tujuan gugatan Penggugat sebagaimana telah diuraikan di atas;

Menimbang, bahwa pada hari dan tanggal persidangan yang telah ditetapkan, Penggugat telah datang menghadap sendiri ke persidangan sedangkan Tergugat yang telah dipanggil secara resmi dan patut serta ternyata tidak pernah hadir ke persidangan atau menyuruh orang lain sebagai kuasa atau wakilnya yang sah dan ketidakhadirannya tanpa alasan yang sah, maka Tergugat harus dinyatakan tidak hadir dan sesuai ketentuan Pasal 125 ayat (1), 126 HIR, gugatan Penggugat diperiksa dan diputus dengan tanpa hadirnya Tergugat (verstek);

Disclaimer
Kepaniteraan Mahkamah Agung Republik Indonesia berusaha untuk selalu mencantumkan informasi paling kini dan akurat sebagai bentuk komitmen Mahkamah Agung untuk pelayanan publik, transparansi dan akuntabilitas pelaksanaan fungsi peradilan. Namun dalam hal-hal tertentu masih dimungkinkan terjadi permasalahan teknis terkait dengan akurasi dan keterkinian informasi yang kami sajikan, hal mana akan terus kami perbaiki dari waktu kewaktu.
Dalam hal Anda menemukan inakurasi informasi yang termuat pada situs ini atau informasi yang seharusnya ada, namun belum tersedia, maka harap segera hubungi Kepaniteraan Mahkamah Agung RI melalui :
Email : kepaniteraan@mahkamahagung.go.id Telp : 021-384 3348 (ext.318)

Menimbang, bahwa sesuai ketentuan Pasal 65 dan Pasal 82 ayat (1) Undang-Undang Nomor 7 Tahun 1989 Tentang Peradilan Agama sebagaimana yang telah di ubah dengan Undang-Undang Nomor 3 Tahun 2006, terakhir dengan perubahan kedua Undang-Undang Nomor 50 Tahun 2009, Jo. Pasal 31 ayat (2) PP Nomor 9 Tahun 1975, majelis hakim telah berusaha mendamaikan rumah tangga Penggugat dan Tergugat pada setiap persidangan dengan jalan memberikan nasihat kepada Penggugat agar bersabar dan tetap mempertahankan keutuhan rumah tangganya dengan Tergugat akan tetapi tidak berhasil, sedangkan usaha damai melalui proses mediasi sebagaimana yang dikehendaki dalam Peraturan Mahkamah Agung RI Nomor 01 Tahun 2016 Tentang Prosedur Mediasi di Pengadilan tidak dapat dilaksanakan karena Tergugat tidak pernah hadir ke persidangan atau menyuruh orang lain sebagai kuasa atau wakilnya yang sah dan ketidakhadirannya tanpa alasan yang sah meskipun Tergugat telah dipanggil secara resmi dan patut;

Menimbang, bahwa yang menjadi dalil gugatan Penggugat untuk melakukan perceraian adalah adanya perselisihan dan pertengkaran yang terus menerus yang sulit untuk didamaikan sejak _____ yang disebabkan _______;

Menimbang, bahwa atas gugatan Penggugat tersebut, jawaban Tergugat tidak dapat didengar di persidangan karena Tergugat tidak pernah hadir dan tidak pula mengutus orang lain sebagai wakil atau kuasanya yang sah untuk menghadap ke persidangan meskipun telah dipanggil secara resmi dan patut serta tidak ternyata bahwa ketidakhadiran Tergugat disebabkan oleh suatu halangan yang sah, maka secara yuridis formal dalil atau alasan gugatan Penggugat tersebut dapat dianggap sebagai fakta yang benar atau Tergugat dapat dianggap mengakui semua dalil gugatan Penggugat, namun tidak serta merta gugatan Penggugat dikabulkan, mengingat perkara ini merupakan perkara perceraian dengan pertimbangan dikhawatirkan akan timbul suatu kebohongan besar (*de groten langen*), maka perlu didukung dengan alat-alat bukti, yang kemudian Penggugat mengajukan alat bukti tertulis berkode (P) dan 2 orang saksinya, yang keterangannya sebagaimana terurai dalam duduk perkara di atas;

Disclaimer
Kepaniteraan Mahkamah Agung Republik Indonesia berusaha untuk selalu mencantumkan informasi paling kini dan akurat sebagai bentuk komitmen Mahkamah Agung untuk pelayanan publik, transparansi dan akuntabilitas pelaksanaan fungsi peradilan. Namun dalam hal-hal tertentu masih dimungkinkan terjadi permasalahan teknis terkait dengan akurasi dan keterkinian informasi yang kami sajikan, hal mana akan terus kami perbaiki dari waktu kewaktu.
Dalam hal Anda menemukan inakurasi informasi yang termuat pada situs ini atau informasi yang seharusnya ada, namun belum tersedia, maka harap segera hubungi Kepaniteraan Mahkamah Agung RI melalui :
Email : kepaniteraan@mahkamahagung.go.id   Telp : 021-384 3348 (ext.318)

Menimbang, bahwa terhadap alat bukti tertulis (kode P), majelis hakim menilai bahwa karena alat bukti tersebut merupakan fotokopi akta autentik yang telah sesuai dengan aslinya, maka Majelis Hakim dapat menerimanya sebagai alat bukti yang sempurna dan mengikat dan telah memenuhi syarat formil dan materil alat bukti, oleh karenanya telah dapat diterima sebagai bukti yang sah berdasarkan Pasal 165 HIR Jo. Pasal 1870 KUHPerdata dan terbukti Penggugat dengan Tergugat, terikat dalam satu perkawinan yang sah dan belum pernah bercerai, oleh karenanya Penggugat dan Tergugat, mempunyai hak dan berkepentingan dalam perkara ini (*persona standi in judicio*);

Menimbang, bahwa untuk memenuhi Pasal 22 Peraturan Pemerintah Nomor 9 Tahun 1975, Penggugat telah menghadirkan dua orang saksi, yakni keluarga dan orang-orang yang dekat dan telah memberikan keterangan di persidangan, keterangan mana bersumber dari pengetahuan saksi sendiri karena saksi mengetahui langsung peristiwa perselisihan dan pertengkarannya dan ternyata pula keterangan keduanya saling bersesuaian dan mendukung gugatan Penggugat, sehingga dinilai oleh Majelis Hakim telah memenuhi syarat formil dan materiil sebuah kesaksian, oleh karena itu keterangan saksi tersebut dapat dijadikan sebagai dasar pertimbangan;

Menimbang, bahwa berdasarkan keterangan Penggugat, bukti surat dan dua orang saksi, Majelis Hakim telah dapat menemukan fakta-fakta yang dapat disimpulkan sebagai berikut:

1. Bahwa antara Penggugat dengan Tergugat telah terikat dalam perkawinan yang sah;
2. Bahwa antara Penggugat dengan Tergugat sering terjadi perselisihan dan pertengkaran ________ ;
3. Bahwa __________ telah pisah rumah;
4. Bahwa pihak keluarga telah berusaha merukunkan Penggugat dan Tergugat namun tidak berhasil dan Penggugat sampai pada tahap kesimpulan masih tetap bersikeras ingin bercerai dengan Tergugat;

Menimbang, bahwa berdasarkan fakta-fakta tersebut di atas, Majelis Hakim berkesimpulan bahwa rumah tangga Penggugat dan Tergugat benar-benar sudah tidak rukun, yang sudah tidak ada harapan untuk dapat

Disclaimer
Kepaniteraan Mahkamah Agung Republik Indonesia berusaha untuk selalu mencantumkan informasi paling kini dan akurat sebagai bentuk komitmen Mahkamah Agung untuk pelayanan publik, transparansi dan akuntabilitas pelaksanaan fungsi peradilan. Namun dalam hal-hal tertentu masih dimungkinkan terjadi permasalahan teknis terkait dengan akurasi dan keterkinian informasi yang kami sajikan, hal mana akan terus kami perbaiki dari waktu kewaktu.
Dalam hal Anda menemukan inakurasi informasi yang termuat pada situs ini atau informasi yang seharusnya ada, namun belum tersedia, maka harap segera hubungi Kepaniteraan Mahkamah Agung RI melalui :
Email : kepaniteraan@mahkamahagung.go.id   Telp : 021-384 3348 (ext.318)

dipertahankan lagi (*Onheel baar tweespalt*), sehingga rumah tangga Penggugat dan Tergugat sudah pecah sedemikian rupa (*broken/down marriage*);

Menimbang, bahwa berdasarkan Yurisprudensi Mahkamah Agung RI Nomor 38/K/AG/1990 tanggal 22 Agustus 1991, bahwa apabila terbukti suatu rumah tangga sudah pecah dan tidak dapat diperbaiki lagi serta mempertahankan rumah tangga membawa dampak negatif (*mafsadat* yang lebih besar) bagi kedua belah pihak, maka tanpa mempersoalkan siapa yang salah dan mencari kesalahan salah satu pihak, perceraian dapat dipertimbangkan untuk dikabulkan;

Menimbang, bahwa berdasarkan pertimbangan-pertimbangan dan fakta-fakta hukum yang terurai di atas, Majelis Hakim berpendapat bahwa rumah tangga Penggugat dan Tergugat sudah tidak harmonis lagi, sering terjadi perselisihan dan pertengkaran terus menerus yang sulit didamaikan, sehingga rumah tangga Penggugat dan Tergugat sudah tidak sesuai dengan tujuan perkawinan sebagaimana dimaksud dalam Pasal 1 Undang-Undang Nomor 1 Tahun 1974 yaitu untuk membentuk keluarga (rumah tangga) yang bahagia dan kekal berdasarkan Ketuhanan Yang Maha Esa atau pasal 3 Kompilasi Hukum Islam yaitu mewujudkan rumah tangga/keluarga yang sakinah, mawaddah dan rahmah. Dengan demikian Majelis Hakim berkesimpulan bahwa dalil gugatan Penggugat telah terbukti beralasan dan telah memenuhi maksud Pasal 19 huruf (f) Peraturan Pemerintah Nomor 9 Tahun 1975 jo Pasal 116 huruf (f) Kompilasi Hukum Islam, sehingga gugatan Penggugat patut untuk dikabulkan dengan menjatuhkan talak satu bain sughro Tergugat terhadap Penggugat;

Menimbang, bahwa oleh karena perkara ini termasuk dalam bidang perkawinan, maka berdasarkan Pasal 89 Ayat (1) Undang-Undang Nomor 7 Tahun 1989 tentang Peradilan Agama sebagaimana telah diubah dengan Undang-Undang Nomor 3 Tahun 2006 dan perubahan kedua dengan Undang-Undang Nomor 50 tahun 2009, semua biaya yang timbul dalam perkara ini dibebankan kepada Penggugat;

Disclaimer
Kepaniteraan Mahkamah Agung Republik Indonesia berusaha untuk selalu mencantumkan informasi paling kini dan akurat sebagai bentuk komitmen Mahkamah Agung untuk pelayanan publik, transparansi dan akuntabilitas pelaksanaan fungsi peradilan. Namun dalam hal-hal tertentu masih dimungkinkan terjadi permasalahan teknis terkait dengan akurasi dan keterkinian informasi yang kami sajikan, hal mana akan terus kami perbaiki dari waktu kewaktu.
Dalam hal Anda menemukan inakurasi informasi yang termuat pada situs ini atau informasi yang seharusnya ada, namun belum tersedia, maka harap segera hubungi Kepaniteraan Mahkamah Agung RI melalui :
Email : kepaniteraan@mahkamahagung.go.id Telp : 021-384 3348 (ext.318)

Mengingat pasal-pasal peraturan perundang-undangan yang berlaku dan hukum syar'i yang berkaitan dengan perkara ini;

M E N G A D I L I

Menyatakan Tergugat yang telah dipanggil secara resmi dan patut untuk menghadap ke persidangan, tidak hadir;

Mengabulkan gugatan Penggugat secara verstek;

Menjatuhkan talak satu bain sughra Tergugat (Sandi Qodir bin Supardi) terhadap Penggugat (Citra binti Dedi);

Membebankan kepada Penggugat untuk membayar biaya perkara sejumlah Rp566000,00 (lima ratus enam puluh enam ribu);

Demikian diputuskan dalam permusyawaratan Majelis Hakim Pengadilan Agama Cibadak pada hari Rabu tanggal 23 Desember 2020 Masehi bertepatan dengan tanggal 8 Jumadil Awwal 1442 Hijriah, oleh kami Drs. Hendi Rustandi, S.H., M.Si. sebagai Ketua Majelis, A. Mahfudin, S.Ag., M.H. dan Drs. Usman Ali, S.H masing-masing sebagai Hakim Anggota, putusan tersebut pada hari itu juga diucapkan dalam sidang terbuka untuk umum oleh Ketua Majelis beserta Hakim Anggota tersebut dan dibantu oleh Pupu Saripuddin, S.Ag sebagai Panitera Pengganti, dihadiri oleh Penggugat tanpa hadirnya Tergugat.

Hakim Anggota

t.t.d.

A. Mahfudin, S.Ag., M.H.

t.t.d.

Drs. Usman Ali, S.H

Ketua Majelis,

t.t.d.

Drs. Hendi Rustandi, S.H., M.Si.

Panitera Pengganti,

t.t.d.

Pupu Saripuddin, S.Ag

Disclaimer
Kepaniteraan Mahkamah Agung Republik Indonesia berusaha untuk selalu mencantumkan informasi paling kini dan akurat sebagai bentuk komitmen Mahkamah Agung untuk pelayanan publik, transparansi dan akuntabilitas pelaksanaan fungsi peradilan. Namun dalam hal-hal tertentu masih dimungkinkan terjadi permasalahan teknis terkait dengan akurasi dan keterkinian informasi yang kami sajikan, hal mana akan terus kami perbaiki dari waktu kewaktu.
Dalam hal Anda menemukan inakurasi informasi yang termuat pada situs ini atau informasi yang seharusnya ada, namun belum tersedia, maka harap segera hubungi Kepaniteraan Mahkamah Agung RI melalui :
Email : kepaniteraan@mahkamahagung.go.id Telp : 021-384 3348 (ext.318)

Perincian biaya :

-	Pendaftaran	: Rp. 30.000,00
-	ATK Perkara	: Rp. 50.000,00
-	Panggilan	: Rp. 450.000,00
-	PNBP Panggilan Pertama Penggugat	: Rp. 10.000,00
-	PNBP Panggilan Pertama Tergugat	: Rp. 10.000,00
-	Redaksi	: Rp. 10.000,00
-	Meterai	: Rp. 6.000,00
**J u m l a h**		**: Rp. 566.000,00**

Catatan:

Putusan ini belum berkekuatan hukum tetap dan akan berkekuatan hukum tetap sejak tanggal ....

Salinan putusan ini sesuai dengan Aslinya
Panitera Pengadilan Agama Cibadak

Pupu Saripuddin, S.Ag

Disclaimer
Kepaniteraan Mahkamah Agung Republik Indonesia berusaha untuk selalu mencantumkan informasi paling kini dan akurat sebagai bentuk komitmen Mahkamah Agung untuk pelayanan publik, transparansi dan akuntabilitas pelaksanaan fungsi peradilan. Namun dalam hal-hal tertentu masih dimungkinkan terjadi permasalahan teknis terkait dengan akurasi dan keterkinian informasi yang kami sajikan, hal mana akan terus kami perbaiki dari waktu kewaktu.
Dalam hal Anda menemukan inakurasi informasi yang termuat pada situs ini atau informasi yang seharusnya ada, namun belum tersedia, maka harap segera hubungi Kepaniteraan Mahkamah Agung RI melalui :
Email : kepaniteraan@mahkamahagung.go.id Telp : 021-384 3348 (ext.318)

Disclaimer
Kepaniteraan Mahkamah Agung Republik Indonesia berusaha untuk selalu mencantumkan informasi paling kini dan akurat sebagai bentuk komitmen Mahkamah Agung untuk pelayanan publik, transparansi dan akuntabilitas pelaksanaan fungsi peradilan. Namun dalam hal-hal tertentu masih dimungkinkan terjadi permasalahan teknis terkait dengan akurasi dan keterkinian informasi yang kami sajikan, hal mana akan terus kami perbaiki dari waktu kewaktu.
Dalam hal Anda menemukan inakurasi informasi yang termuat pada situs ini atau informasi yang seharusnya ada, namun belum tersedia, maka harap segera hubungi Kepaniteraan Mahkamah Agung RI melalui :
Email : kepaniteraan@mahkamahagung.go.id   Telp : 021-384 3348 (ext.318)
