# Supplementary material for: Classification of divorce causes during the COVID-19 pandemic using convolutional neural networks
Source: PeerJ Comput Sci. 2022 Jun 30;8:e998. doi: 10.7717/peerj-cs.998 (PMC9299239; doi:10.7717/peerj-cs.998)
Supplement: Supplemental Information 5 [file peerj-cs-08-998-s005.zip › Masalah Ekonomi Dataset/Data ke-21.pdf]

PUTUSAN

Nomor 2274/Pdt.G/2020/PA.Tbn

بِسْمِ اللهِ الرَّحْمٰنِ الرَّحِيْمِ

DEMI KEADILAN BERDASARKAN KETUHANAN YANG MAHA ESA

Pengadilan Agama Tuban yang memeriksa dan mengadili perkara tertentu pada tingkat pertama dalam Majelis telah menjatuhkan putusan perkara permohonan cerai talak antara :

PEMOHON, umur 47 tahun, agama Islam, pendidikan SLTA, pekerjaan sopir, tempat tinggal di xxxxxxxxxxxxxx Kelurahan Xxxxxxxxx xxxxxxxxx xxxxx xxxxxxxxx xxxxx dalam hal ini dikuasakan kepada HERI TRI WIDODO, SH., MH. & VEVI YULISTIAN, SH., MH. Para Advokat dan Konsultan Hukum Pada Kantor Advokat "HERI TRI WIDODO, SH., MH. & Partners" yang beralamat di Perum Karang Indah Gg. Sejahtera I Barat No. 78 RT. 11 RW. 05 Kel. Karang, Kec. Semanding, Kab. Tuban berdasarakn surat kuasa tertanggal 30 Oktober 2020, yang terdaftar pada register kuasa nomor 736/ADV/XI/2020/PA Tbn, tanggal 09-11-2020 sebagai "Pemohon".,

melawan

TERMOHON, umur 49 tahun, agama Islam, pendidikan SLTA, pekerjaan xxxxxxxxxx, tempat tinggal di Perum Xxxxxxxxx Xxxxxxxxx RT.01 RW. 06 No. CC 18 Kelurahan Xxxxxxxxx xxxxxxxxx xxxxx xxxxxxxxx xxxxx, dalam hal ini memberi kuasa kepada SYAHBIAN ALAM SAPUTRO, S.H. dan TRINAH ASI ISLAMI, S.H., M.H., Para Advokat yang berkantor di Jl. Samratulangi nomor 18, Jombang, berdasarkan surat kuasa tanggal 19 Nopember 2020 yang terdaftar pada register kuasa nomor 785/ADV/XI/2020/PA Tbn, tanggal 25-11-2020, sebagai "Termohon".;

Pengadilan Agama tersebut.;

Telah mempelajari surat-surat yang berkaitan dalam perkara ini.;

Disclaimer
Kepaniteraan Mahkamah Agung Republik Indonesia berusaha untuk selalu mencantumkan informasi paling kini dan akurat sebagai bentuk komitmen Mahkamah Agung untuk pelayanan publik, transparansi dan akuntabilitas pelaksanaan fungsi peradilan. Namun dalam hal-hal tertentu masih dimungkinkan terjadi permasalahan teknis terkait dengan akurasi dan keterkinian informasi yang kami sajikan, hal mana akan terus kami perbaiki dari waktu kewaktu.
Dalam hal Anda menemukan inakurasi informasi yang termuat pada situs ini atau informasi yang seharusnya ada, namun belum tersedia, maka harap segera hubungi Kepaniteraan Mahkamah Agung RI melalui :
Email : kepaniteraan@mahkamahagung.go.id Telp : 021-384 3348 (ext.318)

Telah mendengar keterangan pihak yang berperkara dan para saksi dimuka sidang.;

DUDUK PERKARA

Bahwa Pemohon dalam surat permohonannya tertanggal 09 Nopember 2020 telah mengajukan *permohonan cerai talak* terhadap Termohon yang didaftarkan di Kepaniteraan Pengadilan Agama Tuban, Nomor 2274/Pdt.G/2020/PA.Tbn., tanggal 09 Nopember 2020 dengan dalil-dalil sebagai berikut :

1. Bahwa Pemohon dan Termohon berkedudukan sebagai suami istri, menikah menurut syariat Agama Islam pada hari Jumat tanggal 25-10-1996, yang dicatat Pegawai Pencatat Nikah (PPN) KUA Kec. Krembangan, Kota Surabaya, Propinsi Jawa Timur sesuai bukti Kutipan Akta Nikah Nomor: 422/36/X/1996 tanggal 19 Agustus 2010.

2. Bahwa pada saat menikah, Pemohon berstatus jejaka dan Termohon berstatus perawan.

3. Bahwa pada awal pernikahan, Pemohon dan Termohon hidup rukun dengan baik sebagaimana layaknya suami istri dan pada awalnya bertempat tinggal di rumah kediaman bersama kumpul dengan orang tua (ibu) Termohon di Sidotopo Wetan, Kec. Kenjeran, Kota Surabaya selama ± 2 (dua) tahun, kemudian pada tahun 1998 pindah ke Tuban dan bertempat tinggal di rumah kontrakan tempat kediaman bersama di Perumahan Xxxxxxxxx Xxxxxxxxx Blok KK, xxxx xxxxxxxx, xxxx xxx xxxx xxxxx, dan pada tahun 2000 Pemohon dan Termohon telah menempati rumah kediaman bersama yang dibeli selama dalam perkawinan terletak di Xxxxxxxxx Xxxxxxxxx CC 18 RT. 01 RW. 06, xxxx xxxxxxxx, xxxx xxx xxxx xxxxx.

4. Bahwa selama dalam perkawinan, Pemohon dan Termohon telah melakukan hubungan sebagaimana layaknya suami istri (ba'da dhukul), dan telah dikaruniai 3 (tiga) orang anak bernama:

1. Nama anak, lahir tanggal 03-05-1998, dan pada tanggal 25-11-2015 meninggal dunia;

Disclaimer
Kepaniteraan Mahkamah Agung Republik Indonesia berusaha untuk selalu mencantumkan informasi paling kini dan akurat sebagai bentuk komitmen Mahkamah Agung untuk pelayanan publik, transparansi dan akuntabilitas pelaksanaan fungsi peradilan. Namun dalam hal-hal tertentu masih dimungkinkan terjadi permasalahan teknis terkait dengan akurasi dan keterkinian informasi yang kami sajikan, hal mana akan terus kami perbaiki dari waktu kewaktu.
Dalam hal Anda menemukan inakurasi informasi yang termuat pada situs ini atau informasi yang seharusnya ada, namun belum tersedia, maka harap segera hubungi Kepaniteraan Mahkamah Agung RI melalui :
Email : kepaniteraan@mahkamahagung.go.id Telp : 021-384 3348 (ext.318)

2. Nama anak, lahir tanggal 08-10-2001, dan sekarang berusia 19 (sembilan belas) tahun;

3. Nama anak, lahir tanggal 23-06-2005, dan sekarang berusia 15 (lima belas) tahun;

5. Bahwa sejak tahun 2010 (dua ribu sepuluh) sampai sekarang, antara Pemohon dan Termohon telah berpisah rumah disebabkan Pemohon telah pergi dari rumah kediaman bersama karena sudah tidak sanggup lagi untuk mempertahankan keutuhan rumah tangganya dengan Termohon, dimana keserasian dan kedamaian dalam rumah tangga seperti yang diharapkan dalam tujuan perkawinan guna mewujudkan rumah tangga yang sakinah, mawaddah dan rahmah tidak mungkin diwujudkan kembali;

6. Bahwa berdasarkan hal-hal tersebut di atas, Pemohon merasa rumah tangganya dengan Termohon sudah tidak bisa dipertahankan lagi, dan berkesimpulan lebih baik bercerai dengan Termohon, karena apabila perkawinan tetap dipertahankan dikhawatirkan akan menimbulkan mudharat yang lebih besar;

7. Bahwa dengan demikian gugatan Pemohon telah sesuai ketentuan pasal 19 huruf b PP No. 9 Tahun 1975 tentang pelaksanaan UU No. 1 Tahun 1974 tentang Perkawinan jo. pasal 39 ayat 2 UU No. 1 tahun 1974 tentang Perkawinan jo. pasal 116 huruf (b) tentang Kompilasi Hukum Islam;

8. Bahwa, terhadap biaya yang timbul akibat perkara ini agar dibebankan menurut peraturan perundang-undangan;

Berdasar dalil-dalil sebagaimana tersebut diatas, mohon kepada Ketua Pengadilan Agama Tuban untuk segera memeriksa dan mengadili perkara ini, selanjutnya menjatuhkan putusan yang amarnya berbunyi sebagai berikut:

PRIMER :

1. Mengabulkan permohonan Pemohon.

Disclaimer
Kepaniteraan Mahkamah Agung Republik Indonesia berusaha untuk selalu mencantumkan informasi paling kini dan akurat sebagai bentuk komitmen Mahkamah Agung untuk pelayanan publik, transparansi dan akuntabilitas pelaksanaan fungsi peradilan. Namun dalam hal-hal tertentu masih dimungkinkan terjadi permasalahan teknis terkait dengan akurasi dan keterkinian informasi yang kami sajikan, hal mana akan terus kami perbaiki dari waktu kewaktu.
Dalam hal Anda menemukan inakurasi informasi yang termuat pada situs ini atau informasi yang seharusnya ada, namun belum tersedia, maka harap segera hubungi Kepaniteraan Mahkamah Agung RI melalui :
Email : kepaniteraan@mahkamahagung.go.id Telp : 021-384 3348 (ext.318)

2. Menetapkan, memberi ijin kepada Pemohon (PEMOHON) untuk menjatuhkan talak satu raj'i kepada Termohon (Tri Wahyuningsih Binti H. Syafi'i) dihadapan sidang Pengadilan Agama Tuban.

3. Membebankan biaya perkara kepada Pemohon.

SUBSIDER :

Mohon Putusan yang seadil-adilnya.

Bahwa pada hari persidangan yang telah ditetapkan, Pemohon dan Termohon telah hadir dengan didampingi Kuasa masing-masing dipersidangan, kemudian Majelis Hakim berusaha mendamaikan kedua belah pihak dan telah diupayakan melalui proses mediasi oleh seorang Mediator bersertifikat di Pengadilan Agama Tuban bernama FARUQ ABDIL HAQ, S.HI.,M.HI., yang ditunjuk oleh Ketua Majelis dengan penetapan tanggal 25 Nopember 2020 untuk menjalankan fungsi sebagai mediator, namun usaha dan upaya mediasi tersebut tidak berhasil, namun Pemohon dan Termohon mencapai kesepakatan tentang nafkah anak dan harta bersama, selanjutnya dibacakan Permohonan Pemohon, yang isinya tetap dipertahankan oleh Pemohon.;

Bahwa, Pemohon menjelaskan, sikap Termohon yang terlalu berani kepada Pemohon, sering memicu pertengkaran, sampai akhirnya Pemohon meninggalkan Termohon dan sekarang Pemohon sudah menikah sirri dengan wanita lain.

Bahwa, ketika Pemohon dan termohon melaksanakan mediasi, telah terjadi kesepakatan bahwa Pemohon akan memberikan :

- Nafkah untuk anak-anak Pemohon dan Termohon, setiap bulan minimal sebesar Rp1.000.000,00 (Satu juta rupiah), di luar biaya pendidikan dan kesehatan dengan penambahan 10 % untuk setiap tahun;
- Sebuah rumah yang terletak di Perum Xxxxxxxxx Xxxxxxxxx RT.01 RW. 06 No. CC 18 Kelurahan Xxxxxxxxx xxxxxxxxx xxxxx xxxxxxxxx xxxxx, dengan luas 105m2 (seratus lima meter persegi) atas nama Didik Subiyantoro (Pemohon) beserta sertifikat HGB. No 449 kepada Termohon.

Disclaimer
Kepaniteraan Mahkamah Agung Republik Indonesia berusaha untuk selalu mencantumkan informasi paling kini dan akurat sebagai bentuk komitmen Mahkamah Agung untuk pelayanan publik, transparansi dan akuntabilitas pelaksanaan fungsi peradilan. Namun dalam hal-hal tertentu masih dimungkinkan terjadi permasalahan teknis terkait dengan akurasi dan keterkinian informasi yang kami sajikan, hal mana akan terus kami perbaiki dari waktu kewaktu.
Dalam hal Anda menemukan inakurasi informasi yang termuat pada situs ini atau informasi yang seharusnya ada, namun belum tersedia, maka harap segera hubungi Kepaniteraan Mahkamah Agung RI melalui :
Email : kepaniteraan@mahkamahagung.go.id Telp : 021-384 3348 (ext.318)

Bahwa atas permohonan Pemohon tersebut, Termohon telah memberikan jawaban yang pada pokoknya sebagai berikut :

- Bahwa, benar, Pemohon dan Termohon adalah suami istri yang sudah hidup rukun dan sudah dikarunai 3 orang anak, dan anak yang nomor satu sudah meninggal dunia.
- Bahwa benar Pemohon dan Termohon sering berselisih dan bertengkar, disebabkan sering salah faham lalu Pemohon meninggalkan Termohon dan sekarang Pemohon sudah menikah lagi dengan wanita lain. ;
- Bahwa benar antara Pemohon dan Termohon telah pisah tempat tinggal selama 10 (sepuluh) tahun lebih;
- Bahwa, Termohon tidak keberatan bercerai dengan Pemohon;
- Bahwa, benar, Pemohon dan Termohon pada saat melaksanakan mediasi, telah sepakat tentang nafkah anak dan harta bersama berupa rumah.

Bahwa untuk meneguhkan dalil permohonannya, Pemohon telah mengajukan alat-alat bukti berupa :

A. Surat :

1. Fotokopi Kutipan Akta Nikah, yang dikeluarkan oleh Kepala Kantor Urusan Agama (KUA) Kecamatan Krembangan Kota Surabaya, Nomor 422/36/X/1996, tanggal 19 Agustus 2010. Bukti tersebut bermeterai cukup. Setelah dicocokkan dengan aslinya, ternyata sesuai, lalu oleh Ketua Majelis diberi tanda P.1.dan paraf;
2. Fotokopi Kartu tanda Penduduk Pemohon, Nomor 352316211073002, yang dikeluarkan oleh Kepala Dinas Kependudukan dan Catatan Sipil xxxxxxxxx xxxxx, tanggal 20-07-2013. Bukti tersebut bermeterai cukup. Setelah dicocokkan dengan aslinya, ternyata sesuai, lalu oleh Ketua Majelis diberi tanda P.2. dan paraf;

Bahwa atas bukti surat Pemohon tersebut, Termohon tidak keberatan dan membenarkannya.;

Disclaimer
Kepaniteraan Mahkamah Agung Republik Indonesia berusaha untuk selalu mencantumkan informasi paling kini dan akurat sebagai bentuk komitmen Mahkamah Agung untuk pelayanan publik, transparansi dan akuntabilitas pelaksanaan fungsi peradilan. Namun dalam hal-hal tertentu masih dimungkinkan terjadi permasalahan teknis terkait dengan akurasi dan keterkinian informasi yang kami sajikan, hal mana akan terus kami perbaiki dari waktu kewaktu.
Dalam hal Anda menemukan inakurasi informasi yang termuat pada situs ini atau informasi yang seharusnya ada, namun belum tersedia, maka harap segera hubungi Kepaniteraan Mahkamah Agung RI melalui :
Email : kepaniteraan@mahkamahagung.go.id Telp : 021-384 3348 (ext.318)

B. Saksi :

Bahwa Pemohon juga mengajukan saksi-saksi keluarga/orang dekat yaitu:

Saksi I : SAKSI 1, umur 52 tahun, agama Islam, pekerjaan tani, tempat kediaman di KABUPATEN TUBAN, dihadapan persidangan memberikan keterangan dibawah sumpah yang pada pokoknya sebagai berikut :

- Bahwa saksi kenal dengan Pemohon dan Termohon, karena saksi adalah Teman kerja Pemohon di Pabrik Semen.;
- Bahwa saksi mengetahui, Pemohon dan Termohon suami istri, setelah menikah Pemohon dan Termohon tinggal di rumah orang tua Termohon kemudian pindah ke tuban dan terakhir tinggal di Perum Xxxxxxxxx Xxxxxxxxx, sudah hidup rukun dan sudah dikaruniai 3 (tiga) orang anak.;
- Bahwa saksi mengetahui, keadaan rumah tangga Pemohon dan Termohon sekarang ini sudah tidak rukun dan tidak harmonis;
- Bahwa saksi mengetahui Pemohon dan Termohon sering berselisih dan bertengkar berdasarkan cerita dari Pemohon, bahwa Termohon mempunyai karakter yang keras;
- Bahwa saksi mengetahui, Pemohon dan Termohon berpisah tempat tinggal selama 10 tahun sejak Pemohon pergi pulang kerumah orang tuanya sendiri dan selama itu mereka sudah tidak pernah saling mengunjungi.;
- Bahwa saksi selaku keluarga/orang dekat, telah berusaha mendamaikan Pemohon dan Termohon, akan tetapi tidak berhasil.;

Saksi II : SAKSI 2, umur 40 tahun, agama Islam, pekerjaan Karyawan sawasta, tempat kediaman di Perum Parengan asri Blok F.5-2, xx xx, xx xx, xxxx xxxxxxxx xxxxx , xxxxxxxxx xxxxxxxx, xxxxxxxxx xxxxxxxx, dihadapan persidangan memberikan keterangan dibawah sumpah yang pada pokoknya sebagai berikut :

- Bahwa saksi kenal dengan Pemohon dan Termohon, karena saksi adalah adik kandung Pemohon.;
- Bahwa saksi mengetahui, Pemohon dan Termohon suami istri, setelah menikah Pemohon dan Termohon tinggal di rumah orang tua Termohon dan

Disclaimer
Kepaniteraan Mahkamah Agung Republik Indonesia berusaha untuk selalu mencantumkan informasi paling kini dan akurat sebagai bentuk komitmen Mahkamah Agung untuk pelayanan publik, transparansi dan akuntabilitas pelaksanaan fungsi peradilan. Namun dalam hal-hal tertentu masih dimungkinkan terjadi permasalahan teknis terkait dengan akurasi dan keterkinian informasi yang kami sajikan, hal mana akan terus kami perbaiki dari waktu kewaktu.
Dalam hal Anda menemukan inakurasi informasi yang termuat pada situs ini atau informasi yang seharusnya ada, namun belum tersedia, maka harap segera hubungi Kepaniteraan Mahkamah Agung RI melalui :
Email : kepaniteraan@mahkamahagung.go.id Telp : 021-384 3348 (ext.318)

terakhir tinggal di Perum Xxxxxxxxx Xxxxxxxxx, sudah hidup rukun dan sudah dikaruniai 3 (tiga) orang anak.

- Bahwa saksi mengetahui, keadaan rumah tangga Pemohon dan Termohon sekarang ini sudah tidak rukun dan tidak harmonis;
- Bahwa saksi tidak mengetahui dengan pasti apa yang menjadi penyebab pertengkaran mereka, saksi hanya mengetahui sikap Pemohon dan Termohon yang saling mendiamkan dan tidak berbicara ketika pulang kerumah orang tua saksi dan berkumpul bersama keluarga;
- Bahwa saksi mengetahui, Pemohon dan Termohon berpisah tempat tinggal selama 10 tahun lebih sejak Pemohon meninggalkan Termohon, dan selama itu mereka sudah tidak pernah saling mengunjungi.;
- Bahwa saksi selaku keluarga/orang dekat, telah berusaha mendamaikan Pemohon dan Termohon, akan tetapi tidak berhasil.;

Bahwa selanjutnya Pemohon dan Termohon memberikan kesimpulan dan mohon putusan.;

Bahwa selanjutnya untuk mempersingkat uraian putusan ini, maka semua hal yang termuat dalam berita acara sidang ini merupakan bagian yang tidak terpisahkan dari putusan ini.;

PERTIMBANGAN HUKUM

Menimbang, bahwa maksud dan tujuan Permohonann Pemohon pada pokoknya adalah sebagaimana telah diuraikan diatas.;

Menimbang, pada hari sidang yang sudah ditetapkan, Pemohon dan Termohon hadir sendiri di persidangan.

Menimbang, bahwa Majelis Hakim dalam setiap persidangan telah berupaya mendamaikan Pemohon dan Termohon agar mempertahankan rumah tangganya, sebagaimana ketentuan Pasal 82 Undang-Undang Nomor 7 Tahun 1989, tentang Peradilan Agama, sebagaimana telah diubah dengan Undang-Undang Nomor 3 Tahun 2006, dan perubahan kedua dengan Undang-Undang Nomor 50 Tahun 2009, juga telah ditempuh melalui mediasi sebagaimana ketentuan Peraturan Mahkamah Agung RI, Nomor 1 Tahun 2016, tentang Proses Mediasi di Pengadilan, namun upaya tersebut tidak berhasil;

Disclaimer
Kepaniteraan Mahkamah Agung Republik Indonesia berusaha untuk selalu mencantumkan informasi paling kini dan akurat sebagai bentuk komitmen Mahkamah Agung untuk pelayanan publik, transparansi dan akuntabilitas pelaksanaan fungsi peradilan. Namun dalam hal-hal tertentu masih dimungkinkan terjadi permasalahan teknis terkait dengan akurasi dan keterkinian informasi yang kami sajikan, hal mana akan terus kami perbaiki dari waktu kewaktu.
Dalam hal Anda menemukan inakurasi informasi yang termuat pada situs ini atau informasi yang seharusnya ada, namun belum tersedia, maka harap segera hubungi Kepaniteraan Mahkamah Agung RI melalui :
Email : kepaniteraan@mahkamahagung.go.id   Telp : 021-384 3348 (ext.318)

Menimbang, bahwa atas permohonan Pemohon, Termohon telah memberikan jawaban yang pada pokoknya sebagaimana terurai diatas;

Menimbang, bahwa untuk melakukan perceraian, antara Pemohon dan Termohon harus ada hubungan hukum sebagai suami istri;

Menimbang, bahwa untuk membuktikan dalil permohonannya, Pemohon di persidangan telah mengajukan bukti surat, P.1, bermeterai cukup dan telah dinazegelen. Setelah dicocokkan dengan aslinya, ternyata sesuai. Oleh karena itu, berdasarkan ketentuan Pasal 165 HIR. juncto Pasal 1888 KUH Perdata juncto Peraturan Pemerintah Republik Indonesia, Nomor 24 Tahun 2000, Tentang Perubahan Tarif Bea Meterai, bukti tersebut telah memenuhi syarat formil, serta mempunyai kekuatan pembuktian sempurna dan mengikat;

Menimbang, bahwa bukti P.1 tersebut adalah fotokopi Kutipan Akta Nikah yang menerangkan tentang perkawinan Pemohon dengan Termohon, merupakan akta otentik dan telah diakui Termohon, sehingga telah terbukti Pemohon dan Termohon adalah suami isteri sah, sehingga Pemohon memiliki legal standing untuk mengajukan cerai talak terhadap Termohon. Berdasarkan Pasal 49 ayat (1) Undang-Undang Nomor 7 Tahun 1989, tentang Peradilan Agama, sebagaimana telah diubah dengan Undang-Undang Nomor 3 Tahun 2006, dan perubahan kedua dengan Undang-Undang Nomor 50 Tahun 2009, maka perkara a quo menjadi *kompetensi absolut* Pengadilan Agama Tuban;

Menimbang, bahwa Pemohon telah mendalilkan sejak Januari 2019, rumah tangga Pemohon dan Termohon sudah tidak rukun dan harmonis, sering berselisih dan bertengkar, disebabkan Termohon sikap Termohon yang terlalu berani kepada Pemohon, sering memicu pertengkaran, sampai akhirnya Pemohon meninggalkan Termohon dan sekarang Pemohon sudah menikah sirri dengan wanita lain, yang kemudian mengakibatkan Pemohon meninggalkan Termohon pada tahun 2010 sampai sekarang, dan selama itu Pemohon dan Termohon, sudah tidak pernah saling mengunjungi hingga sekarang;

Menimbang bahwa, Termohon pada pokoknya mengakui tentang adanya perselisihan dan pertengkaran antara Pemohon dan Termohon, sudah berpisah

Disclaimer
Kepaniteraan Mahkamah Agung Republik Indonesia berusaha untuk selalu mencantumkan informasi paling kini dan akurat sebagai bentuk komitmen Mahkamah Agung untuk pelayanan publik, transparansi dan akuntabilitas pelaksanaan fungsi peradilan. Namun dalam hal-hal tertentu masih dimungkinkan terjadi permasalahan teknis terkait dengan akurasi dan keterkinian informasi yang kami sajikan, hal mana akan terus kami perbaiki dari waktu kewaktu.
Dalam hal Anda menemukan inakurasi informasi yang termuat pada situs ini atau informasi yang seharusnya ada, namun belum tersedia, maka harap segera hubungi Kepaniteraan Mahkamah Agung RI melalui :
Email : kepaniteraan@mahkamahagung.go.id   Telp : 021-384 3348 (ext.318)

tempat tinggal selama 10 tahun dan Termohon tidak keberatan bercerai dengan Pemohon;

Menimbang, meskipun dalil permohonan Pemohon telah diakui kebenarannya oleh Termohon, tetapi untuk memenuhi ketentuan Pasal 22 (2) Peraturan Pemerintah Nomor 9 Tahun 1975 jo. Pasal 70 ayat (1) Undang-Undang Nomor 7 Tahun 1989, Majelis Hakim telah mendengar keterangan 2 (dua) orang saksi keluarga/orang dekat dengan Pemohon dan Termohon;

Menimbang, bahwa berdasarkan keterangan 2 (dua) orang saksi, masing-masing bernama dan , yang mengetahui sendiri bahwa Pemohon dan Termohon sering berselisih dan bertengkar, dan sudah berpisah selama sekitar 10 (sepuluh) tahun sejak Pemohon meninggalkan Termohon, dan selama itu, Pemohon dan Termohon sudah tidak pernah saling mengunjungi dan bergaul sebagaimana layaknya suami istri hingga sekarang;

Menimbang, bahwa dua orang saksi tersebut telah menerangkan berdasarkan pengetahuan, penglihatan dan pendengaran secara langsung sesuai ketentuan Pasal 171 HIR, dan keterangannya saling bersesuaian sesuai ketentuan Pasal 170 HIR, sehingga secara materiil, keterangan dua orang saksi tersebut mempunyai nilai pembuktian;

Menimbang, bahwa berdasarkan pertimbangan tersebut, telah terbukti rumah tangga Pemohon dan Termohon sudah tidak rukun dan harmonis, sering berselisih dan bertengkar dan sudah sulit dipertahankan lagi. Hal ini dapat diketahui dari beberapa indikasi sebagai berikut :

- Bahwa Pengadilan telah berusaha mendamaikan dan merukunkan Pemohon dan Termohon, baik di dalam maupun di luar sidang, namun tidak berhasil;
- Bahwa Pemohon tetap bertekad untuk menceraikan Termohon;
- Bahwa Pemohon dan Termohon telah berpisah tempat tinggal sekitar 10 (sepuluh) tahun sejak Pemohon meninggalkan Termohon, dan selama itu, sudah tidak mau hidup bersama hingga sekarang;

Menimbang, bahwa fakta tersebut adalah merupakan indikasi kuat bahwa sendi bangunan rumah tangga Pemohon dan Termohon telah runtuh, sudah sulit untuk bisa hidup harmonis, apalagi setelah diketahui Pemohon

Disclaimer
Kepaniteraan Mahkamah Agung Republik Indonesia berusaha untuk selalu mencantumkan informasi paling kini dan akurat sebagai bentuk komitmen Mahkamah Agung untuk pelayanan publik, transparansi dan akuntabilitas pelaksanaan fungsi peradilan. Namun dalam hal-hal tertentu masih dimungkinkan terjadi permasalahan teknis terkait dengan akurasi dan keterkinian informasi yang kami sajikan, hal mana akan terus kami perbaiki dari waktu kewaktu.
Dalam hal Anda menemukan inakurasi informasi yang termuat pada situs ini atau informasi yang seharusnya ada, namun belum tersedia, maka harap segera hubungi Kepaniteraan Mahkamah Agung RI melalui :
Email : kepaniteraan@mahkamahagung.go.id Telp : 021-384 3348 (ext.318)

sudah tidak mau untuk hidup bersama sebagai suami istri dengan Termohon, maka sesuai dengan Yurisprudensi Mahkamah Agung RI. Nomor 379 K/AG/1995, tanggal 26 Maret 1997, yang menyatakan bahwa *"Suami istri yang tidak berdiam serumah lagi dan tidak ada harapan untuk dapat hidup rukun kembali, maka rumah tangga tersebut telah terbukti retak dan pecah, sehingga* telah memenuhi alasan cerai Pasal 19 huruf (f) Peraturan Pemerintah Nomor 9 Tahun 1975, juncto Pasal 116 huruf (f) Kompilasi Hukum Islam;

Menimbang, bahwa atas dasar pertimbangan tersebut di atas, karena Pemohon telah dapat membuktikan kebenaran dalil permohonannya, sedangkan permohonan Pemohon tidak melawan hukum, oleh sebab itu permohonan Pemohon a quo telah beralasan dan patut dikabulkan;

Menimbang bahwa oleh karena perkara ini adalah cerai talak, maka Majelis Hakim perlu merujuk Firman Allah dalam Al Qur'an, surat Al Baqarah ayat 229 :

à°Tº°± MçRT´ä¯ ÖäRÎp± Ú°TÝ°Õ à °´RÝ Ø zcÛ¯

"Talak (yang dapat dirujuk) adalah dua kali, (apabila masih dapat diperbaiki) tahanlah dengan cara yang baik, (bila tidak bisa diperbaiki), pisahlah dengan cara yang baik (pula)";

Menimbang, bahwa sebagai akibat dari permohonan cerai talak ini, Pemohon sanggup memberikan kepada Termohon berupa :

Bahwa, ketika Pemohon dan termohon melaksanakan mediasi, telah terjadi kesepakatan bahwa Pemohon akan memberikan :

- Nafkah untuk anak-anak Pemohon dan Termohon, setiap bulan minimal sebesar Rp1.000.000,00 (Satu juta rupiah), di luar biaya pendidikan dan kesehatan dengan penambahan 10 % untuk setiap tahun;
- Sebuah rumah yang terletak di Perum Xxxxxxxxx Xxxxxxxxx RT.01 RW. 06 No. CC 18 Kelurahan Xxxxxxxxx xxxxxxxxx xxxxx xxxxxxxxx xxxxx, dengan luas 105m2 (seratus lima meter persegi) atas nama Didik Subiyantoro (Pemohon) beserta sertifikat HGB. No 449 kepada Termohon,

Disclaimer
Kepaniteraan Mahkamah Agung Republik Indonesia berusaha untuk selalu mencantumkan informasi paling kini dan akurat sebagai bentuk komitmen Mahkamah Agung untuk pelayanan publik, transparansi dan akuntabilitas pelaksanaan fungsi peradilan. Namun dalam hal-hal tertentu masih dimungkinkan terjadi permasalahan teknis terkait dengan akurasi dan keterkinian informasi yang kami sajikan, hal mana akan terus kami perbaiki dari waktu kewaktu.
Dalam hal Anda menemukan inakurasi informasi yang termuat pada situs ini atau informasi yang seharusnya ada, namun belum tersedia, maka harap segera hubungi Kepaniteraan Mahkamah Agung RI melalui :
Email : kepaniteraan@mahkamahagung.go.id Telp : 021-384 3348 (ext.318)

dan Termohon menyetujuinya, karenanya Majelis harus mempertimbangkannya dalam putusan ini;

Menimbang, bahwa karena Pemohon telah menyanggupi memberikan biaya tersebut yang telah sesuai dengan maksud pasal 34 ayat (1) dan pasal 41 huruf (c) Undang-undang Nomor 1 tahun 1974 tentang Perkawinan jo 149 huruf (d) jo pasal 156 huruf (d) Kompilasi Hukum Islam maka Majelis Hakim menghukum Pemohon untuk menyerahkan kepada Termohon : Sebuah rumah yang terletak di Perum Xxxxxxxxx Xxxxxxxxx RT.01 RW. 06 No. CC 18 Kelurahan Xxxxxxxxx xxxxxxxxx xxxxx xxxxxxxxx xxxxx, dengan luas 105m2 (seratus lima meter persegi) atas nama Didik Subiyantoro (Pemohon) beserta sertifikat HGB. No 449, dan sertifikatnya diserahkan pada saat sidang ikrar talak

Menimbang, bahwa Pemohon juga dihukum Pemohon untuk memberikan nafkah untuk anak-anak Pemohon dan Termohon yang bernama Nama anak, lahir tanggal 08-10-2001, dan Nama anak, lahir tanggal 23-06-2005, setiap bulan minimal sebesar Rp1.000.000,00 (Satu juta rupiah), sejak bulan Desember 2020 sampai dengan anak-anak tersebut dewasa, di luar biaya pendidikan dan kesehatan;

Menimbang, bahwa dengan bertambahnya umur anak-anak Pemohon dan Termohon nanti, yang berarti bertambah juga biaya dan kebutuhan hidup mereka, maka Pemohon juga harus menyesuaikan biaya yang diberikan dengan biaya kebutuhan hidup anak tersebut dengan penambahan minimal sebesar Rp. 10 persen pertahun;

Menimbang, bahwa hak menjatuhkan talak yang diberikan oleh Pengadilan kepada Pemohon harus seimbang dengan kewajibannya itu sendiri dan justru menunaikan kewajiban harus didahulukan dari pada menerima haknya, lagi pula dengan mempertimbangkan kemampuan Pemohon maka layak dan adil jika kewajiban Pemohon dilaksanakan bersamaan dengan haknya untuk menjatuhkan talak. Dalam hal dibolehkan membayar nafkah tersebut pada waktu sidang ikrar talak, Majelis Hakim sependapat dengan pendapat Mahkamah Agung yang termuat dalam putusannya Nomor 84

Disclaimer
Kepaniteraan Mahkamah Agung Republik Indonesia berusaha untuk selalu mencantumkan informasi paling kini dan akurat sebagai bentuk komitmen Mahkamah Agung untuk pelayanan publik, transparansi dan akuntabilitas pelaksanaan fungsi peradilan. Namun dalam hal-hal tertentu masih dimungkinkan terjadi permasalahan teknis terkait dengan akurasi dan keterkinian informasi yang kami sajikan, hal mana akan terus kami perbaiki dari waktu kewaktu.
Dalam hal Anda menemukan inakurasi informasi yang termuat pada situs ini atau informasi yang seharusnya ada, namun belum tersedia, maka harap segera hubungi Kepaniteraan Mahkamah Agung RI melalui :
Email : kepaniteraan@mahkamahagung.go.id Telp : 021-384 3348 (ext.318)

K/AG/2009 tanggal 17 April 2009 yang selanjutnya diambil alih sebagai keputusan Majelis;

Menimbang, bahwa menjatuhkan talak dan menyerahkan sebuah rumah sebagaimana kesanggupan Pemohon tersebut adalah menjadi satu kesatuan yang tidak dapat dipisahkan, oleh karena itu Pemohon harus menyerahkan Sebuah rumah yang terletak di Perum Xxxxxxxxx Xxxxxxxxx RT.01 RW. 06 No. CC 18 Kelurahan Xxxxxxxxx xxxxxxxxx xxxxx xxxxxxxxx xxxxx, dengan luas 105m2 (seratus lima meter persegi) atas nama Didik Subiyantoro (Pemohon) beserta sertifikat HGB. No 449 untuk Termohon tersebut pada saat sidang ikrar talak. Apabila Pemohon tidak secara suka rela membayarnya pada saat sidang ikrar talak, maka sidang ikar talak dapat ditunda guna memberi kesempatan kepada Pemohon dan diberikan tenggang waktu paling lama 6 bulan sejak ditetapkannya sidang ikrar talak tersebut;

Menimbang, bahwa berdasarkan pasal 89 ayat (1) Undang-undang Nomor 7 tahun 1989 yang telah diubah dengan Undang-undang Nomor 3 Tahun 2006 yang kemudian diubah untuk kedua kali dengan Undang-undang Nomor 50 tahun 2009 biaya perkara dibebankan kepada Pemohon. ;

Mengingat, pasal 49 Undang-undang Nomor 7 tahun 1989 yang telah diubah untuk kedua kali menjadi Undang-undang Nomor 3 Tahun 2006 yang kemudian diubah dengan Undang-undang Nomor 50 tahun 2009 tentang Peradilan Agama serta segala ketentuan perundang-undangan yang berlaku, dan dalil syar'i yang bersangkutan dengan perkara ini ;

M E N G A D I L I

1. Mengabulkan permohonan Pemohon;

2. Memberi izin kepada Pemohon (PEMOHON) untuk menjatuhkan talak satu raj'i terhadap Termohon (TERMOHON) di depan sidang Pengadilan Agama Tuban;

3. Menghukum Pemohon untuk menyerahkan kepada Termohon : Sebuah rumah yang terletak di Perum Xxxxxxxxx Xxxxxxxxx RT.01 RW. 06 No. CC 18 Kelurahan Xxxxxxxxx xxxxxxxxx xxxxx xxxxxxxxx xxxxx, dengan luas 105m2 (seratus lima meter persegi) atas nama Didik

Disclaimer
Kepaniteraan Mahkamah Agung Republik Indonesia berusaha untuk selalu mencantumkan informasi paling kini dan akurat sebagai bentuk komitmen Mahkamah Agung untuk pelayanan publik, transparansi dan akuntabilitas pelaksanaan fungsi peradilan. Namun dalam hal-hal tertentu masih dimungkinkan terjadi permasalahan teknis terkait dengan akurasi dan keterkinian informasi yang kami sajikan, hal mana akan terus kami perbaiki dari waktu kewaktu.
Dalam hal Anda menemukan inakurasi informasi yang termuat pada situs ini atau informasi yang seharusnya ada, namun belum tersedia, maka harap segera hubungi Kepaniteraan Mahkamah Agung RI melalui :
Email : kepaniteraan@mahkamahagung.go.id Telp : 021-384 3348 (ext.318)

Subiyantoro (Pemohon) beserta sertifikat HGB. No 449, dan sertifikatnya diserahkan pada saat sidang ikrar talak.

4. Menghukun Pemohon untuk memberikan nafkah untuk anak-anak Pemohon dan Termohon yang bernama Nama anak, lahir tanggal 08-10-2001, dan Nama anak, lahir tanggal 23-06-2005, setiap bulan minimal sebesar Rp1.000.000,00 (Satu juta rupiah), sejak bulan Desember 2020 sampai dengan anak-anak tersebut dewasa, di luar biaya pendidikan dan kesehatan dengan penambahan 10 % untuk setiap tahun;

5. Membebankan kepada Pemohon untuk membayar biaya perkara sejumlah Rp 442.500,00 (empat ratus empat puluh dua ribu lima ratus rupiah);

Demikian diputuskan pada hari Rabu tanggal 23 Desember 2020 M, bertepatan dengan tanggal 8 Jumadil awwal 1442 Hijriyah, oleh Majelis Hakim Pengadilan Agama Tuban terdiri dari Dra. HJ. UMMU LAILA,MHI sebagai Hakim Ketua, Drs. H.ABU AMAR dan Drs. MUNTASIR, M.H.P masing masing sebagai Hakim Anggota. Putusan tersebut diucapkan dalam sidang terbuka untuk umum pada hari Rabu, tanggal 23 Desember 2020 Masehi bertepatan dengan tanggal 8 Jumadil awwal 1442 Hijriyah oleh Ketua Majelis, didampingi Hakim Anggota tersebut, dibantu FAKHRUR ROZI, SH sebagai Panitera Pengganti dengan dihadiri oleh Kuasa Pemohon dan Kuasa Termohon.;

Hakim Anggota I	Ketua Majelis
Drs. H.ABU AMAR	Dra. HJ. UMMU LAILA,MHI
Hakim Anggota II	
Drs. MUNTASIR, M.H.P	

Disclaimer
Kepaniteraan Mahkamah Agung Republik Indonesia berusaha untuk selalu mencantumkan informasi paling kini dan akurat sebagai bentuk komitmen Mahkamah Agung untuk pelayanan publik, transparansi dan akuntabilitas pelaksanaan fungsi peradilan. Namun dalam hal-hal tertentu masih dimungkinkan terjadi permasalahan teknis terkait dengan akurasi dan keterkinian informasi yang kami sajikan, hal mana akan terus kami perbaiki dari waktu kewaktu.
Dalam hal Anda menemukan inakurasi informasi yang termuat pada situs ini atau informasi yang seharusnya ada, namun belum tersedia, maka harap segera hubungi Kepaniteraan Mahkamah Agung RI melalui :
Email : kepaniteraan@mahkamahagung.go.id Telp : 021-384 3348 (ext.318)

Panitera Pengganti

FAKHRUR ROZI, SH

Rincian Biaya Perkara :

1. Biaya Pendaftaran	: Rp 30.000,00
2. Biaya proses	: Rp 51.500,00
3. Biaya Panggilan	: Rp325.000,00
4. Biaya PNBP Panggilan I	: Rp 20.000,00
5. Biaya Redaksi	: Rp 10.000,00
6. Biaya Meterai	: Rp 6.000,00
Jumlah	Rp442.500,00

(empat ratus empat puluh dua ribu lima ratus rupiah)

Disclaimer
Kepaniteraan Mahkamah Agung Republik Indonesia berusaha untuk selalu mencantumkan informasi paling kini dan akurat sebagai bentuk komitmen Mahkamah Agung untuk pelayanan publik, transparansi dan akuntabilitas pelaksanaan fungsi peradilan. Namun dalam hal-hal tertentu masih dimungkinkan terjadi permasalahan teknis terkait dengan akurasi dan keterkinian informasi yang kami sajikan, hal mana akan terus kami perbaiki dari waktu kewaktu.
Dalam hal Anda menemukan inakurasi informasi yang termuat pada situs ini atau informasi yang seharusnya ada, namun belum tersedia, maka harap segera hubungi Kepaniteraan Mahkamah Agung RI melalui :
Email : kepaniteraan@mahkamahagung.go.id Telp : 021-384 3348 (ext.318)
