# Supplementary material for: Classification of divorce causes during the COVID-19 pandemic using convolutional neural networks
Source: PeerJ Comput Sci. 2022 Jun 30;8:e998. doi: 10.7717/peerj-cs.998 (PMC9299239; doi:10.7717/peerj-cs.998)
Supplement: Supplemental Information 5 [file peerj-cs-08-998-s005.zip › Masalah Ekonomi Dataset/Data ke-23.pdf]

P U T U S A N

Nomor 4193/Pdt.G/2020/PA.Smdg

بِسْمِ اللَّهِ الرَّحْمَنِ الرَّحِيمِ

DEMI KEADILAN BERDASARKAN KETUHANAN YANG MAHA ESA

Pengadilan Agama Sumedang yang memeriksa dan mengadili perkara perdata pada tingkat pertama dalam persidangan Majelis telah menjatuhkan putusan sebagai berikut dalam perkara antara :

Awa bin Suta, umur 63 tahun, agama Islam, pendidikan SD, pekerjaan Wiraswasta, tempat kediaman di Dusun Ciaseum RT 03 RW 01 Desa Karanglayung Kecamatan Conggeang Kabupaten Sumedang, sebagai Pemohon;

melawan

Eros binti Surya, umur 54 tahun, agama Islam, pendidikan SD, pekerjaan Mengurus Rumah Tangga, tempat kediaman di Dusun Ciaseum RT 03 RW 01 Desa Karanglayung Kecamtaan Conggeang Kabupaten Sumedang, sebagai Termohon;

Pengadilan Agama tersebut;

Setelah mempelajari berkas perkara yang bersangkutan;

Setelah mendengar keterangan Pemohon dan saksi-saksi di muka persidangan;

DUDUK PERKARA

Bahwa, Pemohon dengan surat permohonannya tertanggal 04 Desember 2020 yang telah terdaftar di Kepaniteraan Pengadilan Agama Sumedang dibawah Nomor 4193/Pdt.G/2020/PA.Smdg tanggal 04 Desember 2020, telah mengajukan hal-hal sebagai berikut :

1. Bahwa Pemohon dan Termohon adalah suami isteri sah menikah pada tanggal 24 September 2018 berdasarkan Kutipan Akta Nikah dari KUA Kecamatan Conggeang Kabupaten Sumedang, No: 0226/027/IX/2018. Tertanggal 24 September 2018;

Disclaimer
Kepaniteraan Mahkamah Agung Republik Indonesia berusaha untuk selalu mencantumkan informasi paling kini dan akurat sebagai bentuk komitmen Mahkamah Agung untuk pelayanan publik, transparansi dan akuntabilitas pelaksanaan fungsi peradilan. Namun dalam hal-hal tertentu masih dimungkinkan terjadi permasalahan teknis terkait dengan akurasi dan keterkinian informasi yang kami sajikan, hal mana akan terus kami perbaiki dari waktu kewaktu.
Dalam hal Anda menemukan inakurasi informasi yang termuat pada situs ini atau informasi yang seharusnya ada, namun belum tersedia, maka harap segera hubungi Kepaniteraan Mahkamah Agung RI melalui :
Email : kepaniteraan@mahkamahagung.go.id Telp : 021-384 3348 (ext.318)

2. Bahwa sebelum membina rumahtangga, Pemohon berstatus Duda mati begitupun Termohon berstatus janda mati;
3. Bahwa antara Pemohon dan Termohon terakhir berumah tangga tinggal di rumah milik Termohon di Dusun Ciasem RT 03 RW 01 Desa Karanglayung Kecamatan Conggeang Kabupaten Sumedang dan telah bergaul baik sebagaimana layaknya suami isteri, namun belum dikaruniai anak;
4. Bahwa antara Pemohon dan Termohon sejak bulan **Oktober 2020** sudah tidak harmonis lagi dalam menjalani hidup berumah tangga dikarenakan antara Pemohon dengan Termohon sering terjadi perselisihan dan pertengkaran terus-menerus yang disebabkan Termohon kurang menerima dengan kondisi Pemohon saat ini yang sudah lanjut usia yang berakibat terhadap Pemohon dalam mencari penghasilan tidak seperti dahulu sehingga dalam memberikan nafkah terhdap Termohon sesuai kemampuan Pemohon rata-rata setiap minggunya Rp. 50.000 (lima puluh ribu rupiah), namun Termohon sering mempermasalahkannya dan meminta/menuntut lebih dari kemampuan Pemohon bahkan sering mempermasalahkan dengan sikap marah terhadap Pemohon sehingga dirasa rumah tangga dengan Termohon sudah tidak nyaman dan harmonis;
5. Bahwa keretakan rumah tangga antara Pemohon dan Termohon telah berlangsung lama. Pemohon berusaha sabar, tetapi Termohon tidak berusaha berubah dan merubah sifatnya. Oleh karena itu Pemohon merasa tidak nyaman berumah tangga dengan Termohon. Puncaknya bulan **Desember 2020**, **antara Pemohon dengan Termohon pisah ranjanga** dan tidak lagi bergaul layaknya suami isteri meskipun masih tinggal satu rumah;
6. Bahwa keretakan rumah tangga Pemohon dan Termohon sudah pernah didamaikan oleh keluarga, tetapi tidak berhasil;
7. Bahwa permohonan Pemohon tersebut telah memenuhi syarat sesuai ketentuan Pasal 19 huruf (f) Peraturan Pemerintah Nomor 9 Tahun 1975 jo. Pasal 116 huruf (f) Kompilasi Hukum Islam.

Disclaimer
Kepaniteraan Mahkamah Agung Republik Indonesia berusaha untuk selalu mencantumkan informasi paling kini dan akurat sebagai bentuk komitmen Mahkamah Agung untuk pelayanan publik, transparansi dan akuntabilitas pelaksanaan fungsi peradilan. Namun dalam hal-hal tertentu masih dimungkinkan terjadi permasalahan teknis terkait dengan akurasi dan keterkinian informasi yang kami sajikan, hal mana akan terus kami perbaiki dari waktu kewaktu.
Dalam hal Anda menemukan inakurasi informasi yang termuat pada situs ini atau informasi yang seharusnya ada, namun belum tersedia, maka harap segera hubungi Kepaniteraan Mahkamah Agung RI melalui :
Email : kepaniteraan@mahkamahagung.go.id Telp : 021-384 3348 (ext.318)

8. Bahwa atas permasalahan tersebut di atas Pemohon sudah tidak sanggup lagi untuk mempertahankan perkawinan ini, oleh karena itu Pemohon telah berketetapan hati untuk bercerai dengan Termohon;

Berdasarkan dalil-dalil/alasan tersebut diatas, Pemohon mohon dengan hormat, kiranya bapak Ketua Pengadilan Agama Sumedang cq. Majelis Hakim yang memeriksa dan mengadili perkara ini, berkenan untuk menjatuhkan putusan yang amarnya berbunyi sebagai berikut :

1. Menerima dan mengabulkan Permohonan Ikrar Talak Pemohon.
2. Memberikan izin kepada Pemohon (Awa bin Suta) untuk mengikrarkan Talak Satu Raj'i kepada Termohon (Eros binti Surya) di depan sidang Pengadilan Agama Kelas IA Sumedang.
3. Menetapkan biaya perkara menurut hukum.

Atau apabila Pengadilan berpendapat lain, mohon putusan yang seadil-adilnya;

Bahwa, pada hari sidang yang telah ditetapkan, Pemohon datang menghadap sendiri di muka persidangan, sedangkan Termohon tidak datang dan tidak pula menyuruh orang lain sebagai wakil atau kuasanya yang sah, padahal kepadanya telah dipanggil dengan resmi dan patut, sedangkan tidak ternyata ketidakhadirannya itu disebabkan oleh suatu alasan yang sah menurut hukum;

Bahwa, Majelis Hakim telah berusaha menasehati Pemohon agar dapat membina kembali rumah tangganya dengan Termohon, akan tetapi tidak berhasil dan Majelis Hakim memandang perkara a quo tidak mungkin untuk dilaksanakan mediasi karena Termohon tidak pernah hadir di persidangan;

Bahwa, selanjutnya pemeriksaan perkara ini dimulai dengan membacakan surat permohonan Pemohon yang maksud dan isinya tetap dipertahankan oleh Pemohon;

Bahwa, untuk membuktikan dalil permohonannya, Pemohon telah mengajukan alat bukti surat berupa Fotocopi Kutipan Akta Nikah, Nomor 0226/027/IX/2018, tertanggal 24 September 2018 yang diterbitkan oleh Kantor Urusan Agama KUA Kecamatan Conggeang Kabupaten Sumedang (Bukti P);

Disclaimer
Kepaniteraan Mahkamah Agung Republik Indonesia berusaha untuk selalu mencantumkan informasi paling kini dan akurat sebagai bentuk komitmen Mahkamah Agung untuk pelayanan publik, transparansi dan akuntabilitas pelaksanaan fungsi peradilan. Namun dalam hal-hal tertentu masih dimungkinkan terjadi permasalahan teknis terkait dengan akurasi dan keterkinian informasi yang kami sajikan, hal mana akan terus kami perbaiki dari waktu kewaktu.
Dalam hal Anda menemukan inakurasi informasi yang termuat pada situs ini atau informasi yang seharusnya ada, namun belum tersedia, maka harap segera hubungi Kepaniteraan Mahkamah Agung RI melalui :
Email : kepaniteraan@mahkamahagung.go.id Telp : 021-384 3348 (ext.318)

Bahwa, di samping itu, Pemohon juga telah mengajukan alat bukti saksi yaitu :

1. Dadan S bin Tata, dibawah sumpah menerangkan pada pokoknya sebagai berikut :
   - Bahwa saksi adalah Keponakan Termohon;
   - Bahwa semula rumah tangga Pemohon dengan Termohon rukun dan harmonis akan tetapi sejak bulan bulan Oktober 2020 rumah tangganya sudah tidak harmonis lagi, sering terjadi perselisihan dan pertengkaran;
   - Bahwa penyebabnya karena Termohon menuntut nafkah diluar kemampuan Pemohon;
   - Bahwa sejak bulan Desember 2020 yang lalu antara Pemohon dan Termohon telah berpisah tempat tinggal dan tidak pernah berkumpul kembali;
   - Bahwa saksi pernah berusaha menasehati Pemohon, akan tetapi tidak berhasil;
   - Bahwa saksi sudah tidak sanggup lagi untuk merukunkan kembali;

   Bahwa, terhadap keterangan saksi tersebut Pemohon membenarkannya;
2. Akum bin Sarradi, dibawah sumpah menerangkan pada pokoknya sebagai berikut :
   - Bahwa saksi adalah tetangga Pemohon;
   - Bahwa awalnya rumah tangga Pemohon dan Termohon rukun dan harmonis akan tetapi sejak bulan bulan Oktober 2020 rumah tangganya sudah tidak harmonis lagi sering terjadi perselisihan dan pertengkaran;
   - Bahwa penyebab perselisihan dan pertengkaran tersebut karena Termohon menuntut nafkah diluar kemampuan Pemohon;
   - Bahwa sejak bulan Desember 2020 yang lalu Pemohon dan Termohon telah berpisah tempat tinggal dan tidak pernah berkumpul kembali;
   - Bahwa saksi sudah tidak sanggup lagi untuk merukunkannya;

   Bahwa, terhadap keterangan saksi tersebut Pemohon membenarkannya;

Bahwa, Pemohon dalam kesimpulannya mengatakan tidak akan mengajukan suatu apapun dan memohon kepada Majelis Hakim agar menjatuhkan putusannya;

Disclaimer
Kepaniteraan Mahkamah Agung Republik Indonesia berusaha untuk selalu mencantumkan informasi paling kini dan akurat sebagai bentuk komitmen Mahkamah Agung untuk pelayanan publik, transparansi dan akuntabilitas pelaksanaan fungsi peradilan. Namun dalam hal-hal tertentu masih dimungkinkan terjadi permasalahan teknis terkait dengan akurasi dan keterkinian informasi yang kami sajikan, hal mana akan terus kami perbaiki dari waktu kewaktu.
Dalam hal Anda menemukan inakurasi informasi yang termuat pada situs ini atau informasi yang seharusnya ada, namun belum tersedia, maka harap segera hubungi Kepaniteraan Mahkamah Agung RI melalui :
Email : kepaniteraan@mahkamahagung.go.id   Telp : 021-384 3348 (ext.318)

Bahwa, untuk mempersingkat uraian pertimbangan ini ditunjuk hal-hal sebagaimana tercantum dalam berita acara persidangan perkara ini;

PERTIMBANGAN HUKUM

Menimbang, bahwa maksud dan tujuan permohonan Pemohon adalah sebagaimana terurai diatas;

Menimbang, bahwa Termohon telah dipanggil dengan resmi dan patut, akan tetapi tidak datang dan tidak pula menyuruh orang lain sebagai wakilnya/kuasanya dan tidak ternyata tidak datangnya itu disebabkan suatu alasan yang sah, sedangkan permohonan Pemohon telah cukup beralasan dan tidak melawan hukum, maka Termohon yang telah dipanggil tersebut harus dinyatakan tidak hadir dan sesuai dengan Pasal 125 ayat (1) HIR perkaranya dapat diputuskan dengan verstek;

Menimbang, bahwa sesuai dengan maksud dan ketentuan Pasal 82 Undang-Undang Nomor 7 Tahun 1989 tentang Peradilan Agama sebagaimana telah diubah dengan Undang-Undang Nomor 3 Tahun 2006 dan perubahan kedua dengan Undang-Undang Nomor 50 Tahun 2009, Majelis Hakim telah berusaha maksimal menasehati Pemohon agar Pemohon dapat rukun kembali membina rumah tangga dengan Termohon akan tetapi usaha tersebut tidak berhasil;

Menimbang, bahwa di samping itu oleh karena Termohon tidak pernah hadir di persidangan, maka proses mediasi tidak dapat dilaksanakan (vide PERMA Nomor 1 Tahun 2016 Pasal 4 ayat 2 huruf b);

Menimbang, bahwa terlebih dahulu Majelis Hakim harus menyatakan bahwa perkara ini adalah menjadi yurisdiksi Pengadilan Agama Sumedang, dan Pemohon yang bertempat tinggal di wilayah Kabupaten Sumedang berkapasitas dalam perkara ini (vide Pasal 66 ayat (2) Undang-Undang Nomor 7 Tahun 1989 tentang Peradilan Agama sebagaimana telah diubah dengan Undang-Undang Nomor 3 Tahun 2006 dan perubahan kedua dengan Undang-Undang Nomor 50 Tahun 2009 jo. Pasal 14 Peraturan Pemerintah Nomor 9 Tahun 1975 jo. Pasal 129 Kompilasi Hukum Islam;

Menimbang, bahwa selanjutnya berdasarkan alat bukti (P) yang telah memenuhi syarat formil dan materil, harus dinyatakan terbukti bahwa hubungan

Disclaimer
Kepaniteraan Mahkamah Agung Republik Indonesia berusaha untuk selalu mencantumkan informasi paling kini dan akurat sebagai bentuk komitmen Mahkamah Agung untuk pelayanan publik, transparansi dan akuntabilitas pelaksanaan fungsi peradilan. Namun dalam hal-hal tertentu masih dimungkinkan terjadi permasalahan teknis terkait dengan akurasi dan keterkinian informasi yang kami sajikan, hal mana akan terus kami perbaiki dari waktu kewaktu.
Dalam hal Anda menemukan inakurasi informasi yang termuat pada situs ini atau informasi yang seharusnya ada, namun belum tersedia, maka harap segera hubungi Kepaniteraan Mahkamah Agung RI melalui :
Email : kepaniteraan@mahkamahagung.go.id Telp : 021-384 3348 (ext.318)

hukum antara Pemohon dan Termohon telah terikat dalam perkawinan yang sah sebagaimana diatur dalam Pasal 2 Undang-Undang Nomor 1 Tahun 1974;

Menimbang, bahwa pada pokoknya Pemohon telah mendasarkan permohonannya agar diberi ijin untuk menjatuhkan talak terhadap Termohon dengan alasan sejak bulan bulan Oktober 2020 rumah tangga Pemohon dengan Termohon mulai tidak harmonis, sering terjadi perselisihan dan pertengkaran yang mencapai puncaknya pada bulan bulan Desember 2020 sehingga tidak ada harapan akan hidup rukun lagi dalam membina rumah tangga, dimana penyebabnya karena Termohon menuntut nafkah diluar kemampuan Pemohon sehingga sejak bulan Desember 2020 Pemohon dan Termohon telah pisah tempat tinggal;

Menimbang, bahwa di muka persidangan Pemohon telah mengajukan dua orang saksi yang dari keterangannya saling bersesuaian, Majelis Hakim telah dapat menemukan fakta hukum sebagai berikut:

– Bahwa Pemohon dan Termohon adalah suami isteri yang sah;
– Bahwa semula rumah tangga Pemohon dan Termohon rukun dan harmonis;
– Bahwa sejak bulan bulan Oktober 2020 keadaan rumah tangganya tersebut sudah tidak harmonis lagi, sering terjadi perselisihan dan pertengkaran;
– Bahwa penyebabnya karena Termohon menuntut nafkah diluar kemampuan Pemohon;
– Bahwa sejak bulan Desember 2020 yang lalu Pemohon dan Termohon telah berpisah tempat tinggal dan tidak pernah berkumpul kembali;

Menimbang, bahwa berdasarkan fakta hukum tersebut di atas, harus dinyatakan terbukti bahwa dalam kehidupan rumah tangga Pemohon dan Termohon sudah tidak harmonis lagi, telah terjadi perselisihan dan pertengkaran yang mengakibatkan antara keduanya telah berpisah tempat tinggal dan tidak pernah berkumpul kembali dalam satu rumah tangga;

Menimbang, bahwa disamping itu para saksi sudah pernah berusaha menasehati Pemohon dan mengatakan tidak ada kesanggupan untuk dapat merukunkannya kembali, sehingga Majelis Hakim berpendapat bahwa antara Pemohon dan Termohon sudah sulit dan tidak ada harapan untuk dapat hidup rukun kembali di dalam membina rumah tangganya;

Disclaimer
Kepaniteraan Mahkamah Agung Republik Indonesia berusaha untuk selalu mencantumkan informasi paling kini dan akurat sebagai bentuk komitmen Mahkamah Agung untuk pelayanan publik, transparansi dan akuntabilitas pelaksanaan fungsi peradilan. Namun dalam hal-hal tertentu masih dimungkinkan terjadi permasalahan teknis terkait dengan akurasi dan keterkinian informasi yang kami sajikan, hal mana akan terus kami perbaiki dari waktu kewaktu.
Dalam hal Anda menemukan inakurasi informasi yang termuat pada situs ini atau informasi yang seharusnya ada, namun belum tersedia, maka harap segera hubungi Kepaniteraan Mahkamah Agung RI melalui :
Email : kepaniteraan@mahkamahagung.go.id Telp : 021-384 3348 (ext.318)

Menimbang, bahwa menurut ketentuan Pasal 19 huruf (f) PP Nomor 9 tahun 1975 jo. Pasal 116 huruf (f) Kompilasi Hukum Islam, perceraian dapat terjadi apabila “antara suami dan isteri terus menerus terjadi perselisihan dan pertengkaran dan tidak ada harapan akan hidup rukun kembali dalam membina rumah tangga”;

Menimbang, bahwa demikian juga menurut Mahkamah Agung sebagaimana tertuang dalam putusannya No. 38/K/AG/1990 tanggal 5 Oktober 1990 yang kemudian diambil alih menjadi pendapat Majelis Hakim bahwa apabila ternyata adanya perselisihan sebagaimana dimaksud Pasal 19 huruf (f) PP No. 9 Tahun 1975, maka hal itu semata-mata ditujukan kepada perkawinannya itu sendiri tanpa mempersoalkan siapa yang salah dalam hal terjadinya perselisihan yang mengakibatkan tidak ada harapan akan hidup rukun lagi, karena pernikahan adalah suatu perjanjian yang suci (mitsaqan gholidzon/Pasal 2 KHI) yang untuk memutuskannya tidak boleh diukur dengan kesalahan salah satu pihak, sehingga apabila perkawinan itu telah pecah berarti hati kedua belah pihak telah pecah pula;

Menimbang, bahwa terlepas dari peristiwa atau keadaan yang menjadi penyebab terjadinya perselisihan yang terjadi antara Pemohon dan Termohon, yang jelas Majelis Hakim telah dapat menemukan fakta hukum bahwa perselisihan antara Pemohon dan Termohon tersebut telah sedemikian rupa, rumah tangga Pemohon dan Termohon sudah hancur dan tidak mungkin akan dapat hidup rukun kembali dalam membina rumah tangga;

Menimbang, bahwa dengan melihat kondisi rumah tangga Pemohon dan Termohon tersebut, jelas tidak mungkin akan terwujud kehidupan rumah tangga yang bahagia dan kekal lahir bathin sebagaimana yang menjadi tujuan perkawinan dalam Pasal 1 Undang-Undang Nomor 1 Tahun 1974 jo. Pasal 3 KHI, dan membiarkan suasana rumah tangga yang demikian, justru akan menimbulkan mudlarat bagi kedua belah pihak, oleh karena itu perceraian dipandang jalan yang terbaik dan lebih maslahat bagi keduanya;

Menimbang, bahwa Majelis Hakim perlu mengetengahkan firman Allah dalam Al-Quran surat Al-Baqarah ayat 227, sebagai berikut;

qvnÌ ÏvpÂ tnÛ¯ ÿ°Õ ØzcÛ¯ ¯uÝSÌ ÿªä

Disclaimer
Kepaniteraan Mahkamah Agung Republik Indonesia berusaha untuk selalu mencantumkan informasi paling kini dan akurat sebagai bentuk komitmen Mahkamah Agung untuk pelayanan publik, transparansi dan akuntabilitas pelaksanaan fungsi peradilan. Namun dalam hal-hal tertentu masih dimungkinkan terjadi permasalahan teknis terkait dengan akurasi dan keterkinian informasi yang kami sajikan, hal mana akan terus kami perbaiki dari waktu kewaktu.
Dalam hal Anda menemukan inakurasi informasi yang termuat pada situs ini atau informasi yang seharusnya ada, namun belum tersedia, maka harap segera hubungi Kepaniteraan Mahkamah Agung RI melalui :
Email : kepaniteraan@mahkamahagung.go.id   Telp : 021-384 3348 (ext.318)

Artinya : "Dan jika mereka berazam (berketetapan hati) untuk menjatuhkan talak, maka sesungguhnya Allah Maha Mendengar lagi Maha Mengetahui";

Menimbang, bahwa berdasarkan pertimbangan tersebut diatas, maka permohonan Pemohon telah memenuhi alasan perceraian sebagaimana diatur dalam Pasal 39 ayat (2) Undang-Undang Nomor 1 Tahun 1974, jo. Pasal 19 huruf (f) Peraturan Pemerintah Nomor 9 Tahun 1975, jo. Pasal 116 huruf (f) Kompilasi Hukum Islam dan karenanya permohonan Pemohon agar diberi ijin untuk berikrar menjatuhkan talaknya terhadap Termohon dapat dikabulkan;

Menimbang, bahwa perkara ini termasuk bidang perkawinan, oleh karenanya berdasarkan Pasal 89 ayat (1) Undang-Undang Nomor 7 Tahun 1989 tentang Peradilan Agama sebagaimana telah diubah dengan Undang-Undang Nomor 3 Tahun 2006 dan perubahan kedua dengan Undang-Undang Nomor 50 Tahun 2009, maka seluruh biaya perkara ini dibebankan kepada Pemohon;

Mengingat segala peraturan perundang-undangan yang berlaku serta ketentuan hukum syara' yang berkaitan dengan perkara ini;

M E N G A D I L I

1. Menyatakan Termohon yang telah dipanggil secara resmi dan patut untuk menghadap di persidangan, tidak hadir;
2. Mengabulkan Permohonan Pemohon dengan verstek;
3. Memberi izin kepada Pemohon (Awa bin Suta) untuk menjatuhkan talak satu raj'i terhadap Termohon (Eros binti Surya) di depan sidang Pengadilan Agama Sumedang;
4. Membebankan kepada Pemohon untuk membayar biaya perkara ini sejumlah Rp. 960.00,00 (sembilan puluh enam ribu rupiah).

Demikian Putusan ini dijatuhkan dalam permusyawaratan Majelis Hakim Pengadilan Agama Sumedang pada hari Selasa tanggal 22 Desember 2020 Masehi, bertepatan dengan tanggal 07 Jumadil Awwal 1442 Hijriyah, oleh kami Drs. Endang Sofwan, M.H sebagai Ketua Majelis, Drs. Erik Sumarna, S.H., M.A. dan Dra. Hj. Budi Purwantini, MH masing-masing sebagai Hakim

Disclaimer
Kepaniteraan Mahkamah Agung Republik Indonesia berusaha untuk selalu mencantumkan informasi paling kini dan akurat sebagai bentuk komitmen Mahkamah Agung untuk pelayanan publik, transparansi dan akuntabilitas pelaksanaan fungsi peradilan. Namun dalam hal-hal tertentu masih dimungkinkan terjadi permasalahan teknis terkait dengan akurasi dan keterkinian informasi yang kami sajikan, hal mana akan terus kami perbaiki dari waktu kewaktu.
Dalam hal Anda menemukan inakurasi informasi yang termuat pada situs ini atau informasi yang seharusnya ada, namun belum tersedia, maka harap segera hubungi Kepaniteraan Mahkamah Agung RI melalui :
Email : kepaniteraan@mahkamahagung.go.id Telp : 021-384 3348 (ext.318)

Anggota, yang dibacakan dalam sidang terbuka untuk umum oleh Ketua Majelis tersebut dengan dihadiri para Hakim Anggota dan N. Popon Nurhayati, S. Ag, M. M sebagai Panitera Pengganti serta dihadiri oleh Pemohon tanpa hadirnya Termohon;

Ketua Majelis

Drs. Endang Sofwan, M.H

Hakim Anggota	Hakim Anggota
**Drs. Erik Sumarna, S.H., M.A.**	**Dra. Hj. Budi Purwantini, MH**

Panitera Pengganti

N. Popon Nurhayati, S. Ag, M. M

Perincian Biaya Perkara :

1. Biaya Pendaftaran	:	Rp.	30.000,00
2. Biaya Proses	:	Rp.	50.000,00
3. Biaya Panggilan	:	Rp.	0,00
4. Biaya PNBP Panggilan	:	Rp.	...,00
5. Biaya Redaksi	:	Rp.	10.000,00
6. Biaya materai	:	Rp.	6.000,00
Jumlah		Rp.	96.000,00

(sembilan puluh enam ribu rupiah)

Dicatat disini :

Disclaimer
Kepaniteraan Mahkamah Agung Republik Indonesia berusaha untuk selalu mencantumkan informasi paling kini dan akurat sebagai bentuk komitmen Mahkamah Agung untuk pelayanan publik, transparansi dan akuntabilitas pelaksanaan fungsi peradilan. Namun dalam hal-hal tertentu masih dimungkinkan terjadi permasalahan teknis terkait dengan akurasi dan keterkinian informasi yang kami sajikan, hal mana akan terus kami perbaiki dari waktu kewaktu.
Dalam hal Anda menemukan inakurasi informasi yang termuat pada situs ini atau informasi yang seharusnya ada, namun belum tersedia, maka harap segera hubungi Kepaniteraan Mahkamah Agung RI melalui :
Email : kepaniteraan@mahkamahagung.go.id Telp : 021-384 3348 (ext.318)

- Amar putusan tersebut telah diberitahukan kepada Termohon pada tanggal ....
- Putusan tersebut telah mempunyai kekuatan hukum tetap sejak tanggal ....

Disclaimer
Kepaniteraan Mahkamah Agung Republik Indonesia berusaha untuk selalu mencantumkan informasi paling kini dan akurat sebagai bentuk komitmen Mahkamah Agung untuk pelayanan publik, transparansi dan akuntabilitas pelaksanaan fungsi peradilan. Namun dalam hal-hal tertentu masih dimungkinkan terjadi permasalahan teknis terkait dengan akurasi dan keterkinian informasi yang kami sajikan, hal mana akan terus kami perbaiki dari waktu kewaktu.
Dalam hal Anda menemukan inakurasi informasi yang termuat pada situs ini atau informasi yang seharusnya ada, namun belum tersedia, maka harap segera hubungi Kepaniteraan Mahkamah Agung RI melalui :
Email : kepaniteraan@mahkamahagung.go.id Telp : 021-384 3348 (ext.318)
