# Supplementary material for: Classification of divorce causes during the COVID-19 pandemic using convolutional neural networks
Source: PeerJ Comput Sci. 2022 Jun 30;8:e998. doi: 10.7717/peerj-cs.998 (PMC9299239; doi:10.7717/peerj-cs.998)
Supplement: Supplemental Information 5 [file peerj-cs-08-998-s005.zip › Masalah Ekonomi Dataset/Data ke-24.pdf]

P U T U S A N

Nomor 4278/Pdt.G/2020/PA.Sbg.

بِسْمِ اللَّهِ الرَّحْمَنِ الرَّحِيمِ

DEMI KEADILAN BERDASARKAN KETUHANAN YANG MAHA ESA

Pengadilan Agama Subang yang memeriksa dan mengadili perkara-perkara tertentu pada tingkat pertama dalam persidangan Majelis Hakim telah menjatuhkan putusan dalam perkara **Cerai Talak**, antara :

Pemohon, umur 39 tahun (Subang, 17 Juni 1981), Agama Islam, pendidikan terakhir SD, pekerjaan Buruh Harian Lepas, tempat tinggal di Kabupaten Subang, sebagai **Pemohon**;

melawan

Termohon, umur 31 tahun (Subang, 29 November 1989), Agama Islam, pendidikan terakhir SD, pekerjaan Ibu Rumah Tangga, tempat tinggal di Kabupaten Subang, sebagai **Termohon**;

Pengadilan Agama tersebut;
Setelah membaca berkas perkara;
Setelah mendengar keterangan Pemohon di persidangan;
Setelah memeriksa alat-alat bukti di persidangan;

DUDUK PERKARA

Bahwa Pemohon dengan surat permohonannya tanggal 02 Desember 2020 yang telah didaftarkan di Kepaniteraan Pengadilan Agama Subang di bawah Nomor 4278/Pdt.G/2020/PA.Sbg. tanggal 02 Desember 2020, bermaksud mengajukan permohonan cerai terhadap Termohon berdasarkan dalil-dalil yang pada pokoknya sebagai berikut :

1. Bahwa Pemohon saat ini bertempat tinggal di Kampung Cibarubus RT.022 RW.011 Desa Cijengkol Kecamatan Serang Panjang Kabupaten Subang sebagaimana ternyata dari KTP atas nama Pemohon dengan NIK

Disclaimer
Kepaniteraan Mahkamah Agung Republik Indonesia berusaha untuk selalu mencantumkan informasi paling kini dan akurat sebagai bentuk komitmen Mahkamah Agung untuk pelayanan publik, transparansi dan akuntabilitas pelaksanaan fungsi peradilan. Namun dalam hal-hal tertentu masih dimungkinkan terjadi permasalahan teknis terkait dengan akurasi dan keterkinian informasi yang kami sajikan, hal mana akan terus kami perbaiki dari waktu kewaktu.
Dalam hal Anda menemukan inakurasi informasi yang termuat pada situs ini atau informasi yang seharusnya ada, namun belum tersedia, maka harap segera hubungi Kepaniteraan Mahkamah Agung RI melalui :
Email : kepaniteraan@mahkamahagung.go.id Telp : 021-384 3348 (ext.318)

32706810009 tertanggal 25 Oktober 2012 yang di terbitkan oleh Kepala Dinas Kependudukan dan Catatan Sipil Kabupaten Subang;

2. Bahwa pada tanggal 23 Juli 2007, Pemohon dengan Termohon melangsungkan pernikahan di Wilayah Hukum Kantor Urusan Agama Kecamatan Sagalaherang Kabupaten Subang sebagaimana ternyata dari Buku Kutipan Akta Nikah yang dikeluarkan oleh Pegawai Pencatat Nikah Kantor Urusan Agama Kecamatan Sagalaherang Kabupaten Subang Nomor 2007 tertanggal 23 Juli 2007;
3. Bahwa setelah akad nikah Pemohon dan Termohon hidup bersama sebagai suami istri terakhir di rumah orangtua Termohon selama 3 tahun setelah itu pindah ke rumah kediaman bersama;
4. Bahwa perkawinan antara Pemohon dan Termohon sampai saat ini telah berjalan selama 13 tahun 4 bulan dan telah dikaruniai 1(satu) orang keturunan yang bernama: Risnawati (Perempuan) berusia 12 tahun;
5. Bahwa semula rumah tangga Pemohon dan Termohon harmonis dan bahagia, namun sejak bulan Maret 2013 sudah tidak harmonis lagi karena sering terjadi perselisihan dan pertengkaran yang disebabkan karena :
   a. Termohon sering keluar malam tanpa pamit dan izin kepada Pemohon, sehingga Pemohon merasa tidak dihargai oleh Termohon ketika dinasehati Termohon selalu melawan dan tidak menerima;
6. Bahwa puncak perselisihan dan pertengkaran antara Pemohon dan Termohon terjadi pada bulan Desember tahun 2013, yang berakibat Termohon pergi meninggalkan Pemohon dan tidak pernah hidup berumah tangga lagi sebagai suami istri dan sampai sekarang sudah berpisah selama 6 tahun 11 bulan;
7. Bahwa selama berpisah tempat tinggal Pemohon tinggal di rumah kediaman bersama, sedangkan Termohon tinggal di rumah orangtua Termohon;
8. Bahwa dengan adanya kejadian tersebut Pemohon tetap bersabar, dan sudah dilakukan upaya musyawarah antara Pemohon dan Termohon beserta keluarga Pemohon dan Termohon, namun upaya tersebut tidak berhasil. Oleh karena rumah tangga Pemohon dengan Termohon sudah

Disclaimer
Kepaniteraan Mahkamah Agung Republik Indonesia berusaha untuk selalu mencantumkan informasi paling kini dan akurat sebagai bentuk komitmen Mahkamah Agung untuk pelayanan publik, transparansi dan akuntabilitas pelaksanaan fungsi peradilan. Namun dalam hal-hal tertentu masih dimungkinkan terjadi permasalahan teknis terkait dengan akurasi dan keterkinian informasi yang kami sajikan, hal mana akan terus kami perbaiki dari waktu kewaktu.
Dalam hal Anda menemukan inakurasi informasi yang termuat pada situs ini atau informasi yang seharusnya ada, namun belum tersedia, maka harap segera hubungi Kepaniteraan Mahkamah Agung RI melalui :
Email : kepaniteraan@mahkamahagung.go.id Telp : 021-384 3348 (ext.318)

tidak ada ketentraman dan keharmonisan maka Pemohon mengambil sikap dan keputusan untuk bercerai dengan Termohon, karena sudah tidak sanggup lagi untuk tetap mempertahankan mahligai rumah tangga dengan Termohon;

9. Bahwa dengan keadaan rumah tangga tersebut, Pemohon sudah tidak ada harapan untuk melanjutkan rumah tangga bersama Termohon, sehingga tujuan perkawinan untuk membentuk rumah tangga yang sakinah, mawadah warohmah tidak dapat terwujud, dan apabila tetap dipertahankan hanya akan menimbulkan kemudaratan yang berkepanjangan. Maka untuk mengakhiri perkawinan tersebut Pemohon bermaksud menceraikan Termohon di depan sidang Pengadilan Agama Subang;
10. Bahwa Pemohon sanggup membayar biaya perkara;

Bahwa berdasarkan alasan-alasan tersebut di atas, Pemohon mohon kepada Ketua Pengadilan Agama Subang Cq. Majelis Hakim, untuk memanggil Pemohon dan Termohon, selanjutnya memeriksa dan mengadili perkara ini dengan menjatuhkan putusan yang amarnya sebagai berikut :

1. Mengabulkan permohonan Pemohon;
2. Memberi izin kepada Pemohon (Pemohon) untuk menjatuhkan talak satu raj'i terhadap Termohon (Termohon) di depan sidang Pengadilan Agama Subang;
3. Membebankan kepada Pemohon untuk membayar biaya perkara menurut hukum;

Apabila Majelis Hakim berpendapat lain, mohon putusan yang seadil-adilnya;

Bahwa pada hari dan tanggal yang telah ditentukan Pemohon menghadap persidangan, sedang Termohon tidak datang menghadap atau menyuruh orang lain sebagai wakilnya yang sah, meskipun ia telah dipanggil dengan resmi dan patut untuk datang menghadap di sidang sebagaimana terbukti dari relaas panggilan Nomor 4278/Pdt.G/2020/PA.Sbg. tanggal 03 Desember 2020, sedangkan ternyata bahwa tidak hadirnya itu oleh Majelis Hakim dinilai tidak disebabkan sesuatu halangan yang sah;

Disclaimer
Kepaniteraan Mahkamah Agung Republik Indonesia berusaha untuk selalu mencantumkan informasi paling kini dan akurat sebagai bentuk komitmen Mahkamah Agung untuk pelayanan publik, transparansi dan akuntabilitas pelaksanaan fungsi peradilan. Namun dalam hal-hal tertentu masih dimungkinkan terjadi permasalahan teknis terkait dengan akurasi dan keterkinian informasi yang kami sajikan, hal mana akan terus kami perbaiki dari waktu kewaktu.
Dalam hal Anda menemukan inakurasi informasi yang termuat pada situs ini atau informasi yang seharusnya ada, namun belum tersedia, maka harap segera hubungi Kepaniteraan Mahkamah Agung RI melalui :
Email : kepaniteraan@mahkamahagung.go.id   Telp : 021-384 3348 (ext.318)

Bahwa oleh karena Termohon tidak hadir di persidangan, maka proses mediasi sebagaimana diamanatkan dalam PERMA Nomor 1 Tahun 2016 tidak dapat dilaksanakan;

Bahwa selanjutnya dibacakan surat permohonan Pemohon tertanggal 02 Desember 2020, yang isinya tetap dipertahankan Pemohon;

Bahwa terhadap permohonan Pemohon tersebut, Termohon tidak memberi jawaban karena tidak pernah hadir di persidangan;

Bahwa untuk memperkuat dalil permohonannya, bahwa Pemohon telah menikah dengan Termohon, Pemohon telah mengajukan alat bukti (P) berupa fotokopi Kutipan Akta Nikah Nomor 2007 yang dikeluarkan oleh Kantor Urusan Agama Kecamatan Sagalaherang Kabupaten Subang tanggal 23 Juli 2007, fotokopi mana telah dinachtzegelen/bermeterai cukup;

Bahwa di samping bukti surat sebagaimana di atas, Pemohon juga mengajukan 2 (dua) orang saksi keluarga atau orang yang dekat dengan Pemohon, yang masing-masing bernama :

1. **Saksi 1**, umur 35 tahun, agama Islam, pekerjaan Cleaning Service, tempat tinggal di Kabupaten Subang, yang di bawah sumpah memberikan keterangan yang pada pokoknya sebagai berikut :
   - Bahwa saksi kenal kepada Pemohon yang bernama Didin Saripudin dan Termohon yang bernama Lena Mardalena;
   - Bahwa saksi adalah adik kandung Pemohon;
   - Bahwa Pemohon dan Termohon adalah suami istri yang menikah pada pertengahan tahun 2007 di hadapan Pegawai Pencatat Nikah Kantor Urusan Agama Kecamatan Sagalaherang Kabupaten Subang;
   - Bahwa setelah menikah Pemohon dan Termohon tinggal bersama di rumah kediaman bersama dan telah dikaruniai keturunan seorang anak bernama Risnawati;
   - Bahwa saksi mengetahui awalnya rumah tangga Pemohon dan Termohon rukun dan harmonis, namun sejak awal tahun 2013 mulai tidak rukun karena sering terjadi perselisihan dan pertengkaran;

Disclaimer
Kepaniteraan Mahkamah Agung Republik Indonesia berusaha untuk selalu mencantumkan informasi paling kini dan akurat sebagai bentuk komitmen Mahkamah Agung untuk pelayanan publik, transparansi dan akuntabilitas pelaksanaan fungsi peradilan. Namun dalam hal-hal tertentu masih dimungkinkan terjadi permasalahan teknis terkait dengan akurasi dan keterkinian informasi yang kami sajikan, hal mana akan terus kami perbaiki dari waktu kewaktu.
Dalam hal Anda menemukan inakurasi informasi yang termuat pada situs ini atau informasi yang seharusnya ada, namun belum tersedia, maka harap segera hubungi Kepaniteraan Mahkamah Agung RI melalui :
Email : kepaniteraan@mahkamahagung.go.id   Telp : 021-384 3348 (ext.318)

- Bahwa penyebabnya karena Termohon sering keluar malam tanpa pamit dan izin kepada Pemohon, sehingga Pemohon merasa tidak dihargai oleh Termohon;
- Bahwa saksi mengetahui perselisihan dan pertengkaran tersebut karena saksi sering melihat pertengkaran antara Pemohon dan Termohon;
- Bahwa Pemohon dan Termohon sampai sekarang sudah berpisah selama 7 (tujuh) tahun, dimana Termohon pergi meninggalkan Pemohon dan tidak pernah hidup berumah tangga lagi sebagai suami istri;
- Bahwa selama berpisah tempat tinggal, Pemohon tinggal di rumah kediaman bersama, sedangkan Termohon tinggal di rumah orangtua Termohon;
- Bahwa selama berpisah tempat tinggal Pemohon dan Termohon sudah berusaha untuk memperbaiki rumah tangganya, namun upaya tersebut tidak berhasil;
- Bahwa saksi bersama keluarga kedua belah pihak sudah berusaha merukunkan, akan tetapi usaha tersebut tidak berhasil karena Pemohon tetap bersikeras ingin bercerai dengan Termohon;
- Bahwa saksi tidak bersedia untuk mendamaikan kembali kedua belah pihak, karena keduanya sulit didamaikan;

2. **Saksi 2**, umur 53 tahun, agama Islam, pekerjaan Buruh, tempat tinggal di Kabupaten Subang, yang di bawah sumpah memberikan keterangan yang pada pokoknya sebagai berikut :
   - Bahwa saksi kenal kepada Pemohon dan Termohon karena saksi adalah tetangga Pemohon;
   - Bahwa Pemohon dan Termohon adalah suami istri yang sudah lama berumah tangga dan telah dikaruniai keturunan seorang anak;
   - Bahwa setelah menikah Pemohon dan Termohon tinggal bersama di rumah kediaman bersama;
   - Bahwa Pemohon dan Termohon sudah berpisah tempat tinggal sejak akhir tahun 2013, dimana Termohon pergi meninggalkan Pemohon dan

Disclaimer
Kepaniteraan Mahkamah Agung Republik Indonesia berusaha untuk selalu mencantumkan informasi paling kini dan akurat sebagai bentuk komitmen Mahkamah Agung untuk pelayanan publik, transparansi dan akuntabilitas pelaksanaan fungsi peradilan. Namun dalam hal-hal tertentu masih dimungkinkan terjadi permasalahan teknis terkait dengan akurasi dan keterkinian informasi yang kami sajikan, hal mana akan terus kami perbaiki dari waktu kewaktu.
Dalam hal Anda menemukan inakurasi informasi yang termuat pada situs ini atau informasi yang seharusnya ada, namun belum tersedia, maka harap segera hubungi Kepaniteraan Mahkamah Agung RI melalui :
Email : kepaniteraan@mahkamahagung.go.id Telp : 021-384 3348 (ext.318)

tidak pernah hidup berumah tangga lagi sebagai suami istri sampai sekarang sudah berpisah sekitar 7 (tujuh) tahun;

- Bahwa selama berpisah tempat tinggal, Pemohon tinggal di rumah kediaman bersama, sedangkan Termohon tinggal di rumah orangtua Termohon;
- Bahwa sebelum berpisah, antara Pemohon dan Termohon sering terjadi perselisihan dan pertengkaran yang disebabkan karena Termohon sering melawan kepada Pemohon;
- Bahwa saksi mengetahui perselisihan dan pertengkaran tersebut karena saksi pernah beberapa kali mendengar ketika Pemohon dan Termohon bertengkar;
- Bahwa selama berpisah tempat tinggal Pemohon dan Termohon sudah berusaha untuk rukun, akan tetapi upaya tersebut tidak berhasil;
- Bahwa selama berpisah tempat tinggal, saksi belum pernah mendamaikan. Hanya menasehati Pemohon saja, tapi tidak berhasil karena Pemohon sudah tidak mau kembali kepada Termohon;
- Bahwa saksi tidak bersedia mendamaikan kedua belah pihak karena sulit untuk didamaikan kembali;

Bahwa kemudian Pemohon mengajukan kesimpulan, yang pada pokoknya mengatakan bahwa berdasarkan dalil-dalil permohonan Pemohon dan bukti-bukti yang diajukan, permohonan Pemohon telah terbukti dan berdasar hukum, oleh karenanya patut dikabulkan dan selanjutnya mohon putusan;

Bahwa selanjutnya untuk mempersingkat uraian putusan ini, ditunjuklah hal-hal sebagaimana tercatat dalam berita acara sidang perkara ini sebagai bagian yang tidak terpisahkan dari putusan ini;

PERTIMBANGAN HUKUM

Menimbang, bahwa maksud dan tujuan permohonan Pemohon adalah sebagaimana diuraikan di atas;

Menimbang, bahwa berdasarkan relaas panggilan Nomor 4278/Pdt.G/2020/PA.Sbg. tanggal 03 Desember 2020, terbukti bahwa

Disclaimer
Kepaniteraan Mahkamah Agung Republik Indonesia berusaha untuk selalu mencantumkan informasi paling kini dan akurat sebagai bentuk komitmen Mahkamah Agung untuk pelayanan publik, transparansi dan akuntabilitas pelaksanaan fungsi peradilan. Namun dalam hal-hal tertentu masih dimungkinkan terjadi permasalahan teknis terkait dengan akurasi dan keterkinian informasi yang kami sajikan, hal mana akan terus kami perbaiki dari waktu kewaktu.
Dalam hal Anda menemukan inakurasi informasi yang termuat pada situs ini atau informasi yang seharusnya ada, namun belum tersedia, maka harap segera hubungi Kepaniteraan Mahkamah Agung RI melalui :
Email : kepaniteraan@mahkamahagung.go.id Telp : 021-384 3348 (ext.318)

Termohon telah dipanggil dengan resmi dan patut untuk datang menghadap di persidangan, tetapi Termohon tidak datang menghadap atau menyuruh orang lain sebagai wakilnya yang sah, sedangkan ternyata bahwa tidak hadirnya itu oleh Majelis Hakim dinilai tidak disebabkan sesuatu halangan yang sah. Oleh karenanya, harus dinyatakan bahwa Termohon tidak menghadap persidangan dan berdasarkan ketentuan Pasal 125 ayat (1) HIR Pengadilan dapat menjatuhkan putusan dengan verstek;

Menimbang, bahwa perkara ini adalah perkara perceraian. Berdasarkan azas hukum bahwa tidak ada perceraian jika tidak ada perkawinan, maka sebelum mempertimbangkan tentang perceraiannya Majelis Hakim akan mempertimbangkan terlebih dahulu mengenai ada tidaknya perkawinan antara Pemohon dan Termohon;

Menimbang, bahwa untuk memperkuat dalil permohonannya, bahwa Pemohon telah menikah dengan Termohon, Pemohon telah mengajukan bukti surat yang berupa fotokopi Kutipan Akta Nikah Nomor 348/43/VII/2007 yang dikeluarkan oleh Kantor Urusan Agama Kecamatan Sagalaherang Kabupaten Subang tanggal 23 Juli 2007, bermeterai cukup (bukti P), sehingga sah untuk dipertimbangkan sebagai alat bukti dalam perkara ini;

Menimbang, bahwa bukti P sebagaimana tersebut di atas merupakan akta otentik yang bernilai pembuktian mengikat dan sempurna. Maka berdasarkan alat bukti tersebut Majelis Hakim berkesimpulan, telah terbukti benar, dalil Pemohon yang menyatakan bahwa antara Pemohon dan Termohon terikat dalam perkawinan yang sah;

Menimbang, bahwa dalam perkara ini Pemohon mendalilkan, bahwa rumah tangga Pemohon dan Termohon sudah tidak harmonis lagi karena sering terjadi pertengkaran yang disebabkan oleh karena Termohon sering keluar malam tanpa pamit dan izin kepada Pemohon, sehingga Pemohon merasa tidak dihargai oleh Termohon ketika dinasehati Termohon selalu melawan dan tidak menerima yang mencapai puncaknya pada bulan Desember 2013, sehingga antara Pemohon dan Termohon telah berpisah tempat tinggal selama 7 (tujuh) tahun dan selama berpisah tempat tinggal tersebut Pemohon

Disclaimer
Kepaniteraan Mahkamah Agung Republik Indonesia berusaha untuk selalu mencantumkan informasi paling kini dan akurat sebagai bentuk komitmen Mahkamah Agung untuk pelayanan publik, transparansi dan akuntabilitas pelaksanaan fungsi peradilan. Namun dalam hal-hal tertentu masih dimungkinkan terjadi permasalahan teknis terkait dengan akurasi dan keterkinian informasi yang kami sajikan, hal mana akan terus kami perbaiki dari waktu kewaktu.
Dalam hal Anda menemukan inakurasi informasi yang termuat pada situs ini atau informasi yang seharusnya ada, namun belum tersedia, maka harap segera hubungi Kepaniteraan Mahkamah Agung RI melalui :
Email : kepaniteraan@mahkamahagung.go.id Telp : 021-384 3348 (ext.318)

dan Termohon sudah berusaha rukun akan tetapi upaya tersebut tidak berhasil. Oleh karena itu Pemohon mohon agar diizinkan untuk menjatuhkan talak satu terhadap Termohon di depan sidang Pengadilan Agama Subang;

Menimbang, bahwa terhadap permohonan Pemohon tersebut, Termohon tidak memberi jawaban karena tidak pernah hadir di persidangan;

Menimbang, bahwa untuk memenuhi ketentuan Pasal 22 ayat (2) Peraturan Pemerintah Nomor 9 tahun 1975, bahwa permohonan perceraian dapat diterima apabila telah cukup jelas bagi Pengadilan mengenai sebab-sebab perselisihan dan pertengkaran itu dan setelah mendengar keterangan pihak keluarga serta orang-orang yang dekat dengan suami istri, Majelis Hakim telah mendengar keterangan keluarga atau orang-orang yang dekat dengan kedua belah pihak;

Menimbang, bahwa kedua saksi keluarga atau orang dekat dengan Pemohon tersebut telah memenuhi syarat formil maupun materiil sebagai saksi, maka keterangan kedua saksi atau orang dekat tersebut dapat dipertimbangkan sebagai alat bukti dalam perkara ini;

Menimbang, bahwa ternyata isi keterangan kedua saksi tersebut sesuai dengan dalil-dalil yang dikemukakan oleh Pemohon, bahwa antara Pemohon dan Termohon telah terjadi pisah tempat tinggal selama 7 (tujuh) tahun yang disebabkan oleh pertengkaran karena Termohon sering keluar malam tanpa pamit dan izin kepada Pemohon, sehingga Pemohon merasa tidak dihargai oleh Termohon bahkan Termohon sering melawan kepada Pemohon dan selama berpisah tempat tinggal Pemohon dan Termohon sudah berusaha rukun akan tetapi upaya tersebut tidak berhasil. Demikian juga kedua saksi keluarga atau orang yang dekat dengan Pemohon tersebut telah menasehati Pemohon agar rukun lagi dengan Termohon tetapi tidak berhasil;

Menimbang, bahwa berdasarkan hal-hal sebagaimana diuraikan di atas, Majelis Hakim mendapatkan fakta-fakta peristiwa sebagai berikut :

1. Bahwa Pemohon dengan Termohon telah terikat dalam pernikahan yang sah dan telah dikaruniai keturunan seorang anak bernama Risnawati;

Disclaimer
Kepaniteraan Mahkamah Agung Republik Indonesia berusaha untuk selalu mencantumkan informasi paling kini dan akurat sebagai bentuk komitmen Mahkamah Agung untuk pelayanan publik, transparansi dan akuntabilitas pelaksanaan fungsi peradilan. Namun dalam hal-hal tertentu masih dimungkinkan terjadi permasalahan teknis terkait dengan akurasi dan keterkinian informasi yang kami sajikan, hal mana akan terus kami perbaiki dari waktu kewaktu.
Dalam hal Anda menemukan inakurasi informasi yang termuat pada situs ini atau informasi yang seharusnya ada, namun belum tersedia, maka harap segera hubungi Kepaniteraan Mahkamah Agung RI melalui :
Email : kepaniteraan@mahkamahagung.go.id Telp : 021-384 3348 (ext.318)

2. Bahwa antara Pemohon dan Termohon telah terjadi pisah tempat tinggal selama 7 (tujuh) tahun;
3. Bahwa terjadinya perpisahan tempat tinggal tersebut disebabkan oleh pertengkaran karena Termohon sering keluar malam tanpa pamit dan izin kepada Pemohon, sehingga Pemohon merasa tidak dihargai oleh Termohon bahkan Termohon sering melawan kepada Pemohon;
4. Bahwa selama berpisah tempat tinggal Pemohon dan Termohon sudah berusaha rukun akan tetapi upaya tersebut tidak berhasil;
5. Bahwa para saksi keluarga atau orang yang dekat dengan Pemohon telah menasehati Pemohon agar rukun kembali dengan Termohon tetapi tidak berhasil dan para saksi tidak bersedia untuk merukunkan kembali karena keduanya sulit untuk didamaikan;

Menimbang, bahwa dengan telah terbuktinya dalil Pemohon, bahwa antara Pemohon dan Termohon telah terjadi pisah tempat tinggal selama 7 (tujuh) tahun, di mana menurut yurisprudensi MARI No. 379 K/AG/1995 tanggal 26 Maret 1997, suami istri yang tidak berdiam serumah lagi dan tidak ada harapan untuk hidup rukun kembali, maka rumah tangga tersebut terbukti telah retak dan pecah;

Menimbang, bahwa keadaan hubungan antara Pemohon dan Termohon yang demikian, menurut Majelis Hakim dapat dikualifikasi sebagai telah terjadi perselisihan dan pertengkaran terus menerus dan tidak ada harapan akan hidup rukun lagi dalam rumah tangga sebagaimana yang dimaksud oleh Pasal 19 huruf f Peraturan Pemerintah Nomor 9 Tahun 1975 jo Pasal 116 huruf f Kompilasi Hukum Islam (Instruksi Presiden Nomor 1 Tahun 1991);

Menimbang, bahwa berdasarkan fakta-fakta di atas Majelis Hakim berkesimpulan bahwa antara Pemohon dan Termohon telah terjadi perselisihan dan pertengkaran terus menerus dan tidak ada harapan akan hidup rukun lagi dalam rumah tangga, sehingga harapan untuk mewujudkan tujuan rumah tangga yang bahagia, sakinah, mawaddah warahmah, sebagaimana yang dikehendaki oleh Pasal 1 Undang-undang Nomor 1 Tahun 1974 jo Pasal 3 Kompilasi Hukum Islam sulit terwujud. Rumah tangga Pemohon dan Termohon yang demikian berarti telah pecah dan tidak layak untuk dipertahankan;

Disclaimer
Kepaniteraan Mahkamah Agung Republik Indonesia berusaha untuk selalu mencantumkan informasi paling kini dan akurat sebagai bentuk komitmen Mahkamah Agung untuk pelayanan publik, transparansi dan akuntabilitas pelaksanaan fungsi peradilan. Namun dalam hal-hal tertentu masih dimungkinkan terjadi permasalahan teknis terkait dengan akurasi dan keterkinian informasi yang kami sajikan, hal mana akan terus kami perbaiki dari waktu kewaktu.
Dalam hal Anda menemukan inakurasi informasi yang termuat pada situs ini atau informasi yang seharusnya ada, namun belum tersedia, maka harap segera hubungi Kepaniteraan Mahkamah Agung RI melalui :
Email : kepaniteraan@mahkamahagung.go.id Telp : 021-384 3348 (ext.318)

Menimbang, bahwa dengan tidak hadirnya Termohon di persidangan memberi petunjuk bahwa Termohon tidak menghendaki rukun lagi dengan Pemohon dalam rumah tangga;

Menimbang, bahwa berdasarkan pertimbangan-pertimbangan di atas Majelis Hakim berpendapat bahwa permohonan Pemohon telah memenuhi ketentuan Pasal 39 ayat (1) dan ayat (2) Undang-undang No. 1 Tahun 1974, bahwa perceraian hanya dapat dilakukan di depan sidang Pengadilan setelah Pengadilan yang bersangkutan berusaha dan tidak berhasil mendamaikan kedua belah pihak. Untuk melakukan perceraian harus ada cukup alasan bahwa antara suami-istri itu tidak akan dapat hidup rukun sebagai suami-istri. Demikian juga permohonan Pemohon telah memenuhi ketentuan Pasal 19 huruf (f) Peraturan Pemerintah Nomor 9 Tahun 1975 jo Pasal 116 huruf (f) Kompilasi Hukum Islam, yaitu antara suami istri telah terjadi perselisihan dan pertengkaran terus menerus dan tidak ada harapan akan hidup rukun lagi dalam rumah tangga. Oleh karena itu, permohonan Pemohon dikabulkan;

Menimbang, bahwa menurut doktrin hukum Islam, bahwa talak yang dijatuhkan oleh suami adalah satu persatu, tidak boleh dijatuhkan talak tiga sekaligus, sebagaimana ditegaskan dalam kitab *Ath-Thalaq Fi Syariatil Islamiyyah wal Qanun* halaman 242, yang artinya : *“...Allah telah mensyariatkan talak satu persatu, tidak mensyariatkan talak dijatuhkan sekaligus. Maka barang siapa yang menjatuhkan talak tiga menjadi satu, berarti dia melanggar ketentuan Allah, berbuat dzalim terhadap dirinya sendiri dan mempermainkan kitab Allah. Oleh sebab itu, dia berhak untuk disiksa karena tidak bertakwa kepada Allah dan tidak mentalak sebagiamana yang diperintah-Nya ...* , sedang talak satu yang dijatuhkan oleh suami adalah termasuk talak raj’i, karena suami berhak melakukan rujuk isteri yang telah ditalak satu tersebut, sebagaimana ditentukan dalam Al-Quran Surat Al-Baqarah : 228, yang artinya : “*bahwa para suami dari isteri yang telah ditalak adalah lebih berhak untuk merujuknya*;

Disclaimer
Kepaniteraan Mahkamah Agung Republik Indonesia berusaha untuk selalu mencantumkan informasi paling kini dan akurat sebagai bentuk komitmen Mahkamah Agung untuk pelayanan publik, transparansi dan akuntabilitas pelaksanaan fungsi peradilan. Namun dalam hal-hal tertentu masih dimungkinkan terjadi permasalahan teknis terkait dengan akurasi dan keterkinian informasi yang kami sajikan, hal mana akan terus kami perbaiki dari waktu kewaktu.
Dalam hal Anda menemukan inakurasi informasi yang termuat pada situs ini atau informasi yang seharusnya ada, namun belum tersedia, maka harap segera hubungi Kepaniteraan Mahkamah Agung RI melalui :
Email : kepaniteraan@mahkamahagung.go.id   Telp : 021-384 3348 (ext.318)

Menimbang, bahwa berdasarkan pertimbangan di atas, Majlis Hakim dalam perkara ini memberi izin Pemohon untuk menjatuhkan talak satu raj'i terhadap Termohon;

Menimbang, bahwa berdasarkan Pasal 89 ayat (1) Undang-undang Nomor 7 Tahun 1989 sebagaimana telah diubah dengan Undang-undang No. 3 tahun 2006 dan diubah untuk kedua kalinya dengan Undang-undang No. 50 tahun 2009, biaya perkara ini dibebankan kepada Pemohon;

Memperhatikan ketentuan Pasal 125 HIR dan ketentuan hukum lainnya yang berkaitan dengan perkara ini;

M E N G A D I L I

1. Menyatakan bahwa Termohon yang telah dipanggil secara resmi dan patut untuk menghadap persidangan, tidak hadir;
2. Mengabulkan permohonan Pemohon dengan verstek;
3. Memberi izin kepada Pemohon (Pemohon) untuk menjatuhkan talak satu raj'i terhadap Termohon (Termohon) di depan sidang Pengadilan Agama Subang;
4. Membebankan biaya perkara ini kepada Pemohon yang hingga kini dihitung sebesar Rp316.000,00 (tiga ratus enam belas ribu rupiah);

Demikian dijatuhkan putusan ini dalam musyawarah Majelis Hakim yang dilangsungkan pada hari **Selasa**, tanggal **15 Desember 2020 Masehi** bertepatan dengan tanggal **29 Rabiul Akhir 1442 Hijriyah** oleh **Drs. Ojon Rianklana** sebagai Ketua Majelis serta **Dra. Hj. Sunaeah, M.H.** dan **Dra. Hj. Dadah Holidah, M.H.** masing-masing sebagai Hakim Anggota, dibantu oleh **Priyo Wicaksono, S.Kom., S.Sy.**, sebagai Panitera Pengganti. Putusan tersebut pada hari itu juga diucapkan Hakim Ketua dalam sidang terbuka untuk umum dengan dihadiri Pemohon tanpa hadirnya Termohon.

Hakim Anggota,	Ketua Majelis,
ttd.	ttd.

Disclaimer
Kepaniteraan Mahkamah Agung Republik Indonesia berusaha untuk selalu mencantumkan informasi paling kini dan akurat sebagai bentuk komitmen Mahkamah Agung untuk pelayanan publik, transparansi dan akuntabilitas pelaksanaan fungsi peradilan. Namun dalam hal-hal tertentu masih dimungkinkan terjadi permasalahan teknis terkait dengan akurasi dan keterkinian informasi yang kami sajikan, hal mana akan terus kami perbaiki dari waktu kewaktu.
Dalam hal Anda menemukan inakurasi informasi yang termuat pada situs ini atau informasi yang seharusnya ada, namun belum tersedia, maka harap segera hubungi Kepaniteraan Mahkamah Agung RI melalui :
Email : kepaniteraan@mahkamahagung.go.id Telp : 021-384 3348 (ext.318)

Dra. Hj. Sunaeah, M.H. **Drs. Ojon Rianklana**

Hakim Anggota,

ttd.

Dra. Hj. Dadah Holidah, M.H.

Panitera Pengganti,

ttd.

Priyo Wicaksono, S.Kom., S.Sy.

Perincian Biaya :

1. Pendaftaran	:	Rp30.000,00
2. Proses	:	Rp50.000,00
3. Panggilan	:	Rp200.000,00
4. PNBP Panggilan	:	Rp20.000,00
5. Redaksi	:	Rp10.000,00
6. Meterai	:	Rp6.000,00
**Jumlah**	**:**	**Rp316.000,00**

(tiga ratus enam belas ribu rupiah)

Disclaimer
Kepaniteraan Mahkamah Agung Republik Indonesia berusaha untuk selalu mencantumkan informasi paling kini dan akurat sebagai bentuk komitmen Mahkamah Agung untuk pelayanan publik, transparansi dan akuntabilitas pelaksanaan fungsi peradilan. Namun dalam hal-hal tertentu masih dimungkinkan terjadi permasalahan teknis terkait dengan akurasi dan keterkinian informasi yang kami sajikan, hal mana akan terus kami perbaiki dari waktu kewaktu.
Dalam hal Anda menemukan inakurasi informasi yang termuat pada situs ini atau informasi yang seharusnya ada, namun belum tersedia, maka harap segera hubungi Kepaniteraan Mahkamah Agung RI melalui :
Email : kepaniteraan@mahkamahagung.go.id Telp : 021-384 3348 (ext.318)
