# Supplementary material for: Classification of divorce causes during the COVID-19 pandemic using convolutional neural networks
Source: PeerJ Comput Sci. 2022 Jun 30;8:e998. doi: 10.7717/peerj-cs.998 (PMC9299239; doi:10.7717/peerj-cs.998)
Supplement: Supplemental Information 5 [file peerj-cs-08-998-s005.zip › Masalah Ekonomi Dataset/Data ke-25.pdf]

P U T U S A N
Nomor 27/Pdt.G/2020/PN Mre

DEMI KEADILAN BERDASARKAN KETUHANAN YANG MAHA ESA

Pengadilan Negeri Muara Enim yang memeriksa dan memutus perkara perdata pada tingkat pertama, telah menjatuhkan putusan sebagai berikut dalam perkara gugatan antara:

Maryoto, Tempat dan tanggal lahir: Lampung Tengah, 25 Januari 1983, Jenis Kelamin: Laki-laki, Kebangsaan: Indonesia, Agama: Islam Pekerjaan: Petani, Alamat: Dusun IV Desa Gemawang Kecamatan Rambang Niru Kabupaten Muara Enim, selanjutnya disebut sebagai --**Penggugat**;

Lawan:

Ni Komang Sukreni, Tempat dan tanggal lahir: Lampung Tengah, 05 Februari 1986, Jenis Kelamin: Perempuan, Kebangsaan: Indonesia, Agama: Hindu Pekerjaan: Wiraswasta, Alamat: Dusun II Desa Air Enau Kecamatan Rambang Niru Kabupaten Muara Enim, selanjutnya disebut sebagai ---------------------------------**Tergugat**;

Pengadilan Negeri tersebut;

Membaca berkas perkara yang bersangkutan;
Mendengar pihak Penggugat yang berperkara;
Memperhatikan bukti-bukti yang diajukan oleh Penggugat;

TENTANG DUDUK PERKARA

Menimbang, bahwa Penggugat dengan surat gugatan tanggal 16 Oktober 2020 yang diterima dan didaftarkan di Kepaniteraan Pengadilan Negeri Muara Enim pada tanggal 19 Oktober 2020 dalam Nomor Register 27/Pdt.G/2020/PN Mre, telah mengajukan gugatan sebagai berikut:

1. Bahwa Penggugat adalah suami sah dari Tergugat yang telah menikah di hadapan pemuka agama Hindu yang bernama Nengah Rembawa pada tanggal 10 September 2009 di Muara Enim, sesuai dengan Kutipan Akta Perkawinan No. 1603CPK10092009005674, dari daftar perkawinan Stbld yang dikeluarkan oleh Kantor Pencatatan Sipil Dinas Kependudukan dan Capil Muara Enim;

Disclaimer
Kepaniteraan Mahkamah Agung Republik Indonesia berusaha untuk selalu mencantumkan informasi paling kini dan akurat sebagai bentuk komitmen Mahkamah Agung untuk pelayanan publik, transparansi dan akuntabilitas pelaksanaan fungsi peradilan. Namun dalam hal-hal tertentu masih dimungkinkan terjadi permasalahan teknis terkait dengan akurasi dan keterkinian informasi yang kami sajikan, hal mana akan terus kami perbaiki dari waktu kewaktu.
Dalam hal Anda menemukan inakurasi informasi yang termuat pada situs ini atau informasi yang seharusnya ada, namun belum tersedia, maka harap segera hubungi Kepaniteraan Mahkamah Agung RI melalui :
Email : kepaniteraan@mahkamahagung.go.id   Telp : 021-384 3348 (ext.318)

2. Bahwa dari pernikahan tersebut Penggugat dan Tergugat telah dikarunia 2 orang anak yang bernama Niluh Putriyani yang saat ini berusia 19 Tahun berdasarkan Kutipan Akta Kelahiran No.1603CLT0709200913682, dan Made Wardani yang saat ini berusia 10 Tahun berdasarkan Kutipan Akta Kelahiran No. 1603-LT-30122013-0113;
3. Bahwa setelah pernikahan Penggugat dan Tergugat bertempat tinggal di rumah Penggugat yang terletak di Desa Air Enau Kecamatan Rambang Niru Kabupaten Muara Enim;
4. Bahwa kehidupan rumah tangga antara Penggugat dengan Tergugat telah berjalan rukun dan damai dan jika ada perselisihan dan pertengkaran itu di anggap sebagai ujian dalam membina keluarga (rumah tangga);
5. Bahwa pada tahun 2017 dikarenakan perbedaan prinsip dan tidak ada kecocokan lagi antara Penggugat dan Tergugat, sehingga Penggugat pergi meninggalkan kediaman bersama di Desa Air Enau menuju kediaman orang tua Penggugat;
6. Bahwa pada tanggal 18 Juni 2017 Penggugat menyatakan memeluk agama Islam sebagaimana surat pernyataan memeluk agama islam tanggal 18 Juni 2017;
7. Bahwa Penggugat dan Tergugat telah pisah rumah selama kurang lebih 3 tahun;
8. Bahwa dengan telah beralihnya keyakinan antara Penggugat dan Tergugat, sehingga menimbulkan perselisihan dan pertengkaran antara Penggugat dan Tergugat secara terus menerus dan berlarut -larut, sehingga antara Penggugat dengan Tergugat tidak ada harapan akan hidup rukun lagi dalam rumah tangga, karena itu terpenuhilah Pasal 19 huruf (b) Peraturan Pemerintah RI No. 9 tahun 1975 tentang pelaksanaan Undang-undang No. 1 tahun 1974 tentang perkawinan, yang berbunyi sebagai berikut: “salah satu pihak meninggalkan pihak lain selama 2 tahun berturut-turut tanpa izin pihak lain dan tanpa alasan yang sah atau karena hal lain diluar kemampuannya ”;
9. Bahwa berdasarkan alasan-alasan tersebut di atas maka cukup alasan bagi Penggugat untuk menuntut perceraian berdasarkan putusan Pengadilan;

Disclaimer
Kepaniteraan Mahkamah Agung Republik Indonesia berusaha untuk selalu mencantumkan informasi paling kini dan akurat sebagai bentuk komitmen Mahkamah Agung untuk pelayanan publik, transparansi dan akuntabilitas pelaksanaan fungsi peradilan. Namun dalam hal-hal tertentu masih dimungkinkan terjadi permasalahan teknis terkait dengan akurasi dan keterkinian informasi yang kami sajikan, hal mana akan terus kami perbaiki dari waktu kewaktu.
Dalam hal Anda menemukan inakurasi informasi yang termuat pada situs ini atau informasi yang seharusnya ada, namun belum tersedia, maka harap segera hubungi Kepaniteraan Mahkamah Agung RI melalui :
Email : kepaniteraan@mahkamahagung.go.id   Telp : 021-384 3348 (ext.318)

Bahwa Berdasarkan hal–hal tersebut di atas dengan ini Penggugat mohon kehadapan Bapak Ketua Pengadilan Negeri Muara Enim cq. Majelis Hakim, agar berkenan kiranya:

1. Mengabulkan gugatan Penggugat untuk seluruhnya;
2. Menyatakan bahwa perkawinan antara Penggugat dan Tergugat yang telah menikah di hadapan pemuka agama hindu yang bernama Nengah Rembawa pada tanggal 10 September 2009 di Muara Enim, sesuai dengan Kutipan Akta Perkawinan No. 1603CPK10092009005674, dari daftar perkawinan Stbld yang dikeluarkan oleh Kantor Pencatatan Sipil Dinas Kependudukan dan Capil Muara Enim, putus karena perceraian dengan segala akibat hukumnya;
3. Memerintahkan kepada Panitera Pengadilan Negeri Muara Enim untuk mengirim salinan resmi putusan yang telah mempunyai kekuatan hukum tetap kepada Kantor Catatan Sipil Kabupaten Muara Enim agar dapat didaftarkan perceraian ini dalam suatu daftar perceraian;
4. Menghukum Tergugat untuk membayar seluruh biaya yang timbul karena perkara ini

Atau apabila Pengadilan Negeri Muara Enim berpendapat lain, mohon putusan yang seadil-adilnya (ex aequo et bono);

Menimbang, bahwa pada hari persidangan yang telah ditetapkan, Penggugat datang sendiri, akan tetapi Tergugat tidak datang ataupun menyuruh orang lain sebagai kuasanya, meskipun berdasarkan risalah panggilan sidang tanggal 26 Oktober 2020 dan tanggal 16 November 2020 telah dipanggil secara sah dan patut, sedangkan tidak ternyata, bahwa tidak datangnya disebabkan sesuatu halangan yang sah, oleh karena itu pemeriksaan perkara ini tetap dilanjutkan tanpa hadirnya Tergugat;

Menimbang, bahwa telah dibacakan surat gugatan Penggugat yang isinya tetap dipertahankan;

Menimbang, bahwa untuk membuktikan dalil gugatannya, Penggugat telah mengajukan bukti surat berupa fotocopy yang telah disesuaikan dengan aslinya dan telah dibubuhi biaya meterai secukupnya, kecuali bukti surat P-1 berupa fotocopy dari hasil print out:

Disclaimer
Kepaniteraan Mahkamah Agung Republik Indonesia berusaha untuk selalu mencantumkan informasi paling kini dan akurat sebagai bentuk komitmen Mahkamah Agung untuk pelayanan publik, transparansi dan akuntabilitas pelaksanaan fungsi peradilan. Namun dalam hal-hal tertentu masih dimungkinkan terjadi permasalahan teknis terkait dengan akurasi dan keterkinian informasi yang kami sajikan, hal mana akan terus kami perbaiki dari waktu kewaktu.
Dalam hal Anda menemukan inakurasi informasi yang termuat pada situs ini atau informasi yang seharusnya ada, namun belum tersedia, maka harap segera hubungi Kepaniteraan Mahkamah Agung RI melalui :
Email : kepaniteraan@mahkamahagung.go.id Telp : 021-384 3348 (ext.318)

Bukti P-1 : Fotocopy Kartu Keluarga No. 1603032506200002 atas nama Kepala Keluarga Maryoto, tertanggal 03-07-2020;

Bukti P-2 : Fotocopy Kutipan Akta Perkawinan Nomor 1603CPK1009200900567 antara Maryoto dengan Ni Komang Sukreni, tertanggal 11 September 2009;

Bukti P-3 : Fotocopy Surat Keterangan Perceraian Agama Hindu, Nomor 01/SDCH/LNIDN/AE/VII/2017, tanggal Air Enau 08 Juli 2017;

Bukti P-4 : Fotocopy Surat Pernyataan Memeluk Agama Islam, atasnama Maryoto, tanggal 18 Juni 2017;

Bukti P-5 : Fotocopy Kutipan Akta Kelahiran Nomor 1603CLT0709200913683 atas nama Maryoto, tertanggal 15 September 2009;

Menimbang, bahwa untuk menguatkan dalil gugatannya, Penggugat telah pula mengajukan 2 (dua) orang Saksi ke persidangan, yang setelah bersumpah menurut cara agamanya masing-masing, memberikan keterangan yang pada pokoknya sebagai berikut:

1. Saksi I Wayan Sugindra;
   - Bahwa antara Penggugat dengan Tergugat adalah suami istri;
   - Bahwa Saksi tidak tahu kapan antara Penggugat dengan Tergugat menikah;
   - Bahwa Penggugat dengan Tergugat menikah secara adat berdasarkan agama Hindu dihadapan ketua adat Nenga Rimbawa;
   - Bahwa dari perkawinan antara Penggugat dengan Tergugat sudah dikaruni 2 (dua) orang dan kedua anaknya tersebut sekarang ini ikut dengan Tergugat;
   - Bahwa sebelum menikah Penggugat beragama Islam, sekarang ini Penggugat memeluk agama Islam kembali;
   - Bahwa Saksi pernah mendengar cekcok antara Penggugat dengan Tergugat sekira tiga tahun yang lalu dan mereka langsung pisah rumah;
   - Bahwa antara Penggugat dengan Tergugat sekarang sudah tidak satu rumah lagi;
   - Bahwa Penggugat dan Tergugat sudah pernah di damaikan oleh keluarganya tapi tidak berhasil;
   - Bahwa sekarang Penggugat sudah menikah, sedangkan Tergugat belum menikah;

Disclaimer
Kepaniteraan Mahkamah Agung Republik Indonesia berusaha untuk selalu mencantumkan informasi paling kini dan akurat sebagai bentuk komitmen Mahkamah Agung untuk pelayanan publik, transparansi dan akuntabilitas pelaksanaan fungsi peradilan. Namun dalam hal-hal tertentu masih dimungkinkan terjadi permasalahan teknis terkait dengan akurasi dan keterkinian informasi yang kami sajikan, hal mana akan terus kami perbaiki dari waktu kewaktu.
Dalam hal Anda menemukan inakurasi informasi yang termuat pada situs ini atau informasi yang seharusnya ada, namun belum tersedia, maka harap segera hubungi Kepaniteraan Mahkamah Agung RI melalui :
Email : kepaniteraan@mahkamahagung.go.id Telp : 021-384 3348 (ext.318)

- Bahwa Saksi tidak hadir pada pernikahan Penggugat dengan istrinya yang baru;

2. Saksi Made Indra;
   - Bahwa, antara Penggugat dengan Tergugat merupakan suami istri;
   - Bahwa Penggugat dengan Tergugat menikah pada tanggal 10 September 2009;
   - Bahwa Penggugat dengan Tergugat menikah secara adat berdasarkan agama Hindu dihadapan ketua adat Nenga Rimbawa;
   - Bahwa dari perkawinan Penggugat dengan Tergugat sudah dikaruni 2 (dua) orang dan kedua anaknya tersebut sekarang ini ikut dengan Tergugat;
   - Bahwa sebelum menikah Penggugat beragama Islam, sekarang ini Penggugat memeluk agama Islam kembali;
   - Bahwa Saksi pernah mendengar cekcok antara Penggugat dengan Tergugat sekira tiga tahun yang lalu dan mereka langsung pisah rumah;
   - Bahwa antara Penggugat dengan Tergugat sekarang sudah tidak satu rumah lagi;
   - Bahwa Penggugat dan Tergugat sudah pernah di damaikan oleh keluarganya tapi tidak berhasil;
   - Bahwa sekarang Penggugat sudah menikah, sedangkan Tergugat belum menikah;
   - Bahwa Saksi tidak hadir pada pernikahan Penggugat dengan istrinya yang baru;

Menimbang, bahwa Penggugat menyatakan tidak ada lagi hal-hal yang akan diajukan dan mohon putusan;

Menimbang, bahwa untuk menyingkat putusan, segala sesuatu yang termuat dalam berita acara persidangan dianggap telah termuat dan menjadi bagian yang tidak terpisahkan dengan putusan ini;

TENTANG PERTIMBANGAN HUKUM

Menimbang, bahwa maksud dan tujuan gugatan Penggugat pada pokoknya adalah mengenai adanya gugatan perceraian yang diajukan oleh Penggugat dikarenakan sejak tahun 2017 terjadi perbedaan prinsip keyakinan antara penggugat dengan Tergugat dan tidak ada kecocokan lagi serta antara Penggugat dan Tergugat sejak tahun 2017 tidak satu rumah lagi, oleh karena

Disclaimer
Kepaniteraan Mahkamah Agung Republik Indonesia berusaha untuk selalu mencantumkan informasi paling kini dan akurat sebagai bentuk komitmen Mahkamah Agung untuk pelayanan publik, transparansi dan akuntabilitas pelaksanaan fungsi peradilan. Namun dalam hal-hal tertentu masih dimungkinkan terjadi permasalahan teknis terkait dengan akurasi dan keterkinian informasi yang kami sajikan, hal mana akan terus kami perbaiki dari waktu kewaktu.
Dalam hal Anda menemukan inakurasi informasi yang termuat pada situs ini atau informasi yang seharusnya ada, namun belum tersedia, maka harap segera hubungi Kepaniteraan Mahkamah Agung RI melalui :
Email : kepaniteraan@mahkamahagung.go.id   Telp : 021-384 3348 (ext.318)

itulah Penggugat dalam petitumnya memohon agar perkawinannya putus karena perceraian dengan segala akibat hukumnya dan memerintahkan kepada Panitera Pengadilan Negeri Muara Enim untuk mengirim salinan resmi putusan yang telah mempunyai kekuatan hukum tetap kepada Kantor Catatan Sipil Kabupaten Muara Enim agar dapat didaftarkan perceraian ini dalam suatu daftar perceraian;

Menimbang, bahwa oleh karena jangka waktu dan formalitas panggilan menurut hukum telah dilaksanakan secara sah dan patut, maka Tergugat yang tidak datang menghadap dipersidangan dan tidak menyuruh orang lain menghadap sebagai Kuasanya, harus dinyatakan tidak hadir;

Menimbang, bahwa setelah Majelis Hakim membaca, mempelajari dan mencermati, ternyata gugatan Penggugat cukup beralasan dan tidak bertentangan dengan hukum, oleh karena itu dapat menjadi dasar pemeriksaan dalam perkara ini;

Menimbang, bahwa untuk menguatkan dalil-dalil gugatannya, Penggugat telah mengajukan bukti surat tertanda P-1 sampai dengan P-5, serta keterangan 2(dua) orang Saksi;

Menimbang, bahwa oleh karena gugatan ini adalah mengenai gugatan perceraian, maka akan dipertimbangkan dan diputus dengan mengingat ketentuan-ketentuan dalam Undang-undang Republik Indonesia Nomor 1 Tahun 1974 tentang Perkawinan dan peraturan perundangan lain yang berkaitan;

Menimbang, bahwa berdasarkan ketentuan Pasal 1 Undang-undang Republik Indonesia Nomor 1 Tahun 1974 Tentang Perkawinan, yang dimaksud dengan perkawinan adalah ikatan lahir batin antara seorang pria dengan seorang wanita sebagai suami istri dengan tujuan membentuk keluarga (rumah tangga) yang bahagia dan kekal berdasarkan Ketuhanan Yang Maha Esa, namun berdasarkan Pasal 19 Peraturan Pemerintah Republik Indonesia Nomor 9 Tahun 1975 Tentang Pelaksanaan Undang-Undang Nomor 1 Tahun 1974 Tentang Perkawinan bahwa perceraian dapat terjadi karena alasan atau alasan-alasan:

a. Salah satu pihak berbuat zina atau menjadi pemabuk, pemadat, pejudi dan lain sebagainya yang sukar disembuhkan;

Disclaimer
Kepaniteraan Mahkamah Agung Republik Indonesia berusaha untuk selalu mencantumkan informasi paling kini dan akurat sebagai bentuk komitmen Mahkamah Agung untuk pelayanan publik, transparansi dan akuntabilitas pelaksanaan fungsi peradilan. Namun dalam hal-hal tertentu masih dimungkinkan terjadi permasalahan teknis terkait dengan akurasi dan keterkinian informasi yang kami sajikan, hal mana akan terus kami perbaiki dari waktu kewaktu.
Dalam hal Anda menemukan inakurasi informasi yang termuat pada situs ini atau informasi yang seharusnya ada, namun belum tersedia, maka harap segera hubungi Kepaniteraan Mahkamah Agung RI melalui :
Email : kepaniteraan@mahkamahagung.go.id Telp : 021-384 3348 (ext.318)

b. Salah satu pihak meninggalkan pihak lain selama 2 (dua) tahun berturut-turut tanpa izin pihak lain dan tanpa alasan yang sah atau karena hal lain diluar kemampuannya;
c. Salah satu pihak mendapat hukuman penjara 5 (lima) tahun atau hukuman yang lebih berat setelah perkawinan berlangsung;
d. Salah satu pihak melakukan kekejaman atau penganiayaan berat yang membahayakan pihak yang lain;
e. Salah satu pihak mendapat cacat badan atau penyakit dengan akibat tidak dapat menjalankan kewajibannya sebagai suami/istri;
f. Antara suami dan istri terus menerus terjadi perselisihan dan pertengkaran dan tidak ada harapan akan hidup rukun lagi dalam rumah tangga;

Menimbang, bahwa selanjutnya mengenai gugatan Penggugat yang mendalilkan bahwa sejak tahun 2017 terjadi perbedaan prinsip keyakinan antara penggugat dengan Tergugat dan tidak ada kecocokan lagi serta antara Penggugat dan Tergugat sejak tahun 2017 tidak satu rumah lagi, akan Majelis Hakim pertimbangkan apakah alasan gugatan Penggugat tersebut memenuhi alasan sebagaimana yang ditentukan dalam Pasal 19 Peraturan Pemerintah Republik Indonesia Nomor 9 Tahun 1975 Tentang Pelaksanaan Undang-Undang Nomor 1 Tahun 1974 Tentang Perkawinan tersebut di atas;

Menimbang, bahwa berdasarkan bukti surat bertanda P-1 sampai dengan P-5 dihubungkan dengan keterangan Saksi-saksi di persidangan yang sesuai satu dengan yang lainnya dan dengan memperhatikan pula fakta persidangan, diperoleh fakta-fakta sebagai berikut:

- Bahwa Penggugat dan Tergugat adalah pasangan suami isteri yang telah menikah di hadapan pemuka agama Hindu yang bernama Nengah Rembawa pada tanggal 10 September 2009 di Muara Enim, sesuai dengan Kutipan Akta Perkawinan No. 1603CPK10092009005674, dari daftar perkawinan Stbld yang dikeluarkan oleh Kantor Pencatatan Sipil Dinas Kependudukan dan Capil Muara Enim (Vide bukti surat P-2);
- Bahwa berdasarkan Surat Keterangan Perceraian Agama Hindu, Nomor 01/SDCH/LNIDN/AE/VII/2017, tanggal Air Enau 08 Juli 2017, antara Penggugat dengan Tergugat sudah bercerai menurut hukum adat dan agama Hindu, (Vide bukti surat P-3);
- Bahwa dalam perkawinan tersebut Penggugat dan Tergugat telah dikaruniai 2(dua) orang anak;

Disclaimer
Kepaniteraan Mahkamah Agung Republik Indonesia berusaha untuk selalu mencantumkan informasi paling kini dan akurat sebagai bentuk komitmen Mahkamah Agung untuk pelayanan publik, transparansi dan akuntabilitas pelaksanaan fungsi peradilan. Namun dalam hal-hal tertentu masih dimungkinkan terjadi permasalahan teknis terkait dengan akurasi dan keterkinian informasi yang kami sajikan, hal mana akan terus kami perbaiki dari waktu kewaktu.
Dalam hal Anda menemukan inakurasi informasi yang termuat pada situs ini atau informasi yang seharusnya ada, namun belum tersedia, maka harap segera hubungi Kepaniteraan Mahkamah Agung RI melalui :
Email : kepaniteraan@mahkamahagung.go.id   Telp : 021-384 3348 (ext.318)

- Bahwa pada tanggal 18 Juni 2017 Penggugat menyatakan memeluk agama Islam sebagaimana surat pernyataan memeluk agama islam tanggal 18 Juni 2017 (vide bukti surat P-4);
- Bahwa dengan telah beralihnya keyakinan antara Penggugat dan Tergugat, sehingga menimbulkan perselisihan dan pertengkaran antara Penggugat dan Tergugat secara terus menerus dan berlarut-larut, sehingga antara Penggugat dengan Tergugat tidak ada harapan akan hidup rukun lagi dalam rumah tangga;
- Bahwa Penggugat dan Tergugat telah pisah rumah selama kurang lebih 3 (tiga) tahun dan Penggugat sekarang sudah menikah lagi;

Menimbang, bahwa berdasarkan fakta-fakta tersebut di atas Majelis Hakim berkesimpulan bahwa antara Penggugat dan Tergugat memang sudah tidak mungkin dipersatukan lagi karena dengan adanya fakta sering terjadi perselisihan dan pertengkaran karena perbedaan keyakinan, sehingga tidak ada harapan untuk hidup rukun dalam rumah tangga dan dengan memperhatikan pula fakta sejak tahun 2017 Penggugat dan Tergugat tidak lagi hidup dalam satu kediaman serta Penggugat sekarang sudah menikah lagi, oleh karena itu Majelis Hakim berpendapat bahwa tujuan perkawinan untuk membentuk rumah tangga yang bahagia berdasarkan Ketuhanan Yang Maha Esa tidak mungkin lagi dicapai dalam perkawinan Penggugat dan Tergugat;

Menimbang, bahwa berdasarkan pertimbangan hukum tersebut di atas, maka Penggugat dipandang telah dapat membuktikan kebenaran dalil-dalil gugatannya. Demikian pula Tergugat dengan ketidak hadirannya, Majelis Hakim berkesimpulan bahwa Tergugat tidak mau mengindahkan hak-haknya dan ataupun mempertahankan keutuhan perkawinannya dengan Penggugat;

Menimbang, bahwa dengan demikian tuntutan Penggugat dalam petitum gugatannya angka 2 (dua), yaitu agar menyatakan perkawinan antara Penggugat dan Tergugat yang telah menikah di hadapan pemuka agama hindu yang bernama Nengah Rembawa pada tanggal 10 September 2009 di Muara Enim, sesuai dengan Kutipan Akta Perkawinan No. 1603CPK10092009005674, dari daftar perkawinan Stbld yang dikeluarkan oleh Kantor Pencatatan Sipil Dinas Kependudukan dan Capil Muara Enim, putus karena perceraian dengan segala akibat hukumnya adalah cukup beralasan dan tidak melawan hukum karena telah memenuhi alasan yang ditentukan dalam Pasal 19 Peraturan Pemerintah Republik Indonesia Nomor 9 Tahun 1975 Tentang Pelaksanaan

Disclaimer
Kepaniteraan Mahkamah Agung Republik Indonesia berusaha untuk selalu mencantumkan informasi paling kini dan akurat sebagai bentuk komitmen Mahkamah Agung untuk pelayanan publik, transparansi dan akuntabilitas pelaksanaan fungsi peradilan. Namun dalam hal-hal tertentu masih dimungkinkan terjadi permasalahan teknis terkait dengan akurasi dan keterkinian informasi yang kami sajikan, hal mana akan terus kami perbaiki dari waktu kewaktu.
Dalam hal Anda menemukan inakurasi informasi yang termuat pada situs ini atau informasi yang seharusnya ada, namun belum tersedia, maka harap segera hubungi Kepaniteraan Mahkamah Agung RI melalui :
Email : kepaniteraan@mahkamahagung.go.id   Telp : 021-384 3348 (ext.318)

Undang-Undang Nomor 1 Tahun 1974 Tentang Perkawinan tersebut di atas, karenanya patut untuk dikabulkan dengan *verstek*;

Menimbang, bahwa terhadap tuntutan Penggugat pada petitum angka 3 (tiga) yaitu memerintahkan kepada Panitera Pengadilan Negeri Muara Enim untuk mengirim salinan resmi putusan yang telah mempunyai kekuatan hukum tetap kepada Kantor Catatan Sipil Kabupaten Muara Enim agar dapat didaftarkan perceraian ini dalam suatu daftar perceraian, menurut Majelis Hakim dengan memperhatikan ketentuan Pasal 40 Undang-undang Nomor 23 Tahun 2006 tentang Administrasi Kependudukan yang pada pokoknya mewajibkan pada yang bersangkutan untuk melaporkan perceraian pada instansi pelaksana paling lambat 60(enam puluh) hari sejak putusan perceraian telah berkekuatan hukum tetap dan berdasarkan laporan tersebut Pejabat Pencatatan Sipil mencatat pada Register Akta Perceraian dan menerbitkan Kutipan Akta Percaraian, maka terhadap tuntutan Penggugat pada angka tiga patut dikabulkan dengan redaksi sebagaimana ditentukan dalam amar putusan ini;

Menimbang, bahwa terhadap tuntutan Penggugat pada petitum angka 4 (empat), oleh karena gugatan Penggugat dikabulkan dengan *verstek* dan dengan mengingat ketentuan Pasal 192 Rbg, maka sudah sepatutnya Tergugat dihukum untuk membayar biaya yang timbul dalam perkara ini, yang besarnya sebagaimana ditentukan dalam amar putusan ini;

Mengingat dan memperhatikan Undang-undang Nomor 1 Tahun 1974 Tentang Perkawinan *juncto* Undang-undang No.23 tahun 2006 tentang Administrasi Kependudukan *junctis* Pemerintah Republik Indonesia Nomor 9 Tahun 1975 Tentang Pelaksanaan Undang-Undang Nomor 1 Tahun 1974 Tentang Perkawinan, Pasal 149 Rbg dan peraturan perundangan yang berhubungan dengan perkara ini;

MENGADILI:

1. Menyatakan Tergugat dipanggil secara sah dan patut tetapi tidak hadir;
2. Mengabulkan gugatan Penggugat seluruhnya dengan *verstek*;
3. Menyatakan perkawinan antara Penggugat dan Tergugat yang telah menikah di hadapan pemuka agama Hindu yang bernama Nengah Rembawa pada tanggal 10 September 2009 di Muara Enim, sesuai dengan Kutipan Akta Perkawinan No. 1603CPK10092009005674, dari daftar perkawinan Stbld

Disclaimer
Kepaniteraan Mahkamah Agung Republik Indonesia berusaha untuk selalu mencantumkan informasi paling kini dan akurat sebagai bentuk komitmen Mahkamah Agung untuk pelayanan publik, transparansi dan akuntabilitas pelaksanaan fungsi peradilan. Namun dalam hal-hal tertentu masih dimungkinkan terjadi permasalahan teknis terkait dengan akurasi dan keterkinian informasi yang kami sajikan, hal mana akan terus kami perbaiki dari waktu kewaktu.
Dalam hal Anda menemukan inakurasi informasi yang termuat pada situs ini atau informasi yang seharusnya ada, namun belum tersedia, maka harap segera hubungi Kepaniteraan Mahkamah Agung RI melalui :
Email : kepaniteraan@mahkamahagung.go.id Telp : 021-384 3348 (ext.318)

yang dikeluarkan oleh Kantor Pencatatan Sipil Dinas Kependudukan dan Capil Muara Enim, putus karena perceraian dengan segala akibat hukumnya;

4. Memerintahkan kepada Penggugat dan/atau Tergugat untuk melaporkan perceraian ini kepada instansi pelaksana pada Kantor Dinas Kependudukan dan Catatan Sipil Kabupaten Muara Enim paling lambat 60(enam puluh) hari sejak putusan perceraian ini telah berkekuatan hukum tetap dan berdasarkan laporan tersebut Pejabat Pencatatan Sipil mencatat pada Register Akta Perceraian dan menerbitkan Kutipan Akta Percaraian;
5. Menghukum Tergugat untuk membayar biaya perkara sejumlah Rp566.000,00 (lima ratus enam puluh enam ribu rupiah);

Demikian diputuskan dalam sidang permusyawaratan Majelis Hakim Pengadilan Negeri Muara Enim, pada hari Selasa, tanggal 1 Desember 2020, oleh kami, Hartati, S.H., sebagai Hakim Ketua, Sera Ricky Swanri S, S.H., dan Titis Ayu Wulandari, S.H., masing-masing sebagai Hakim Anggota, putusan tersebut telah diucapkan dalam persidangan terbuka untuk umum pada hari Kamis tanggal 3 Desember 2020, oleh Hakim Ketua dengan didampingi oleh para Hakim Anggota tersebut, dibantu oleh Arman, S.H., Panitera Pengganti serta dihadiri Penggugat dan tanpa dihadiri Tergugat.

Hakim Anggota,

Sera Ricky Swanri S, S.H.

Titis Ayu Wulandari, S.H.

Hakim Ketua,

Hartati, S.H.

Panitera Pengganti,

Arman, S.H.

Disclaimer
Kepaniteraan Mahkamah Agung Republik Indonesia berusaha untuk selalu mencantumkan informasi paling kini dan akurat sebagai bentuk komitmen Mahkamah Agung untuk pelayanan publik, transparansi dan akuntabilitas pelaksanaan fungsi peradilan. Namun dalam hal-hal tertentu masih dimungkinkan terjadi permasalahan teknis terkait dengan akurasi dan keterkinian informasi yang kami sajikan, hal mana akan terus kami perbaiki dari waktu kewaktu.
Dalam hal Anda menemukan inakurasi informasi yang termuat pada situs ini atau informasi yang seharusnya ada, namun belum tersedia, maka harap segera hubungi Kepaniteraan Mahkamah Agung RI melalui :
Email : kepaniteraan@mahkamahagung.go.id Telp : 021-384 3348 (ext.318)

Perincian biaya :

1. Pendaftaran	Rp	30.000,00
2. ATK	Rp	50.000,00
3. PNBP Panggilan Pengguat	Rp.	10.000,00
4. Biaya Panggilan Tergugat	Rp.	450.000,00
5. PNBP Panggilan Tergugat	Rp.	10.000,00
6. Redaksi	Rp	10.000,00
7. Meterai	Rp	6.000,00
Jumlah	Rp	566.000,00

(lima ratus enam puluh enam ribu rupiah)

Disclaimer
Kepaniteraan Mahkamah Agung Republik Indonesia berusaha untuk selalu mencantumkan informasi paling kini dan akurat sebagai bentuk komitmen Mahkamah Agung untuk pelayanan publik, transparansi dan akuntabilitas pelaksanaan fungsi peradilan. Namun dalam hal-hal tertentu masih dimungkinkan terjadi permasalahan teknis terkait dengan akurasi dan keterkinian informasi yang kami sajikan, hal mana akan terus kami perbaiki dari waktu kewaktu.
Dalam hal Anda menemukan inakurasi informasi yang termuat pada situs ini atau informasi yang seharusnya ada, namun belum tersedia, maka harap segera hubungi Kepaniteraan Mahkamah Agung RI melalui :
Email : kepaniteraan@mahkamahagung.go.id Telp : 021-384 3348 (ext.318)
