# Supplementary material for: Classification of divorce causes during the COVID-19 pandemic using convolutional neural networks
Source: PeerJ Comput Sci. 2022 Jun 30;8:e998. doi: 10.7717/peerj-cs.998 (PMC9299239; doi:10.7717/peerj-cs.998)
Supplement: Supplemental Information 5 [file peerj-cs-08-998-s005.zip › Masalah Ekonomi Dataset/Data ke-26.pdf]

P U T U S A N

Nomor 3694/Pdt.G/2020/PA.Smdg

بِسْمِ اللَّهِ الرَّحْمَنِ الرَّحِيمِ

DEMI KEADILAN BERDASARKAN KETUHANAN YANG MAHA ESA

Pengadilan Agama Sumedang yang memeriksa dan mengadili perkara perdata pada tingkat pertama dalam persidangan Majelis telah menjatuhkan putusan sebagai berikut dalam perkara antara :

Ayu Yuhanah Rahyu binti Kana, umur 29 tahun, agama Islam, pendidikan SD, pekerjaan Ibu Rumah Tangga, tempat kediaman di Dusun Lebaktulang RT 03 RW 04 Desa Nagarawangi Kecamatan Rancakalong Kabupaten Sumedang, sebagai Penggugat;

melawan

Gina Ginanjar bin Deden Ruhyat, umur 32 tahun, agama Islam, pendidikan SLTP, pekerjaan Wiraswasta, tempat kediaman di Dusun Lebaktulang RT 02 RW 04 Desa Nagarawangi Kecamatan Rancakalong Kabupaten Sumedang, sebagai Tergugat;

Pengadilan Agama tersebut;

Setelah mempelajari berkas perkara yang bersangkutan;

Setelah mendengar keterangan Penggugat dan saksi-saksi di muka persidangan;

DUDUK PERKARA

Bahwa, Penggugat dengan surat gugatannya tertanggal 23 Oktober 2020 yang telah terdaftar di Kepaniteraan Pengadilan Agama Sumedang dibawah Nomor 3694/Pdt.G/2020/PA.Smdg tanggal 23 Oktober 2020, telah mengajukan hal-hal sebagai berikut :

1. Bahwa Penggugat dan Tergugat adalah suami isteri sah menikah pada tanggal 17 Desember 2008 berdasarkan Kutipan Akta Nikah dari KUA KUA Kecamatan Rancakalong Kabupaten Sumedang, No: 423/36/XII/2008. Tertanggal 17 Desember 2008;

Disclaimer
Kepaniteraan Mahkamah Agung Republik Indonesia berusaha untuk selalu mencantumkan informasi paling kini dan akurat sebagai bentuk komitmen Mahkamah Agung untuk pelayanan publik, transparansi dan akuntabilitas pelaksanaan fungsi peradilan. Namun dalam hal-hal tertentu masih dimungkinkan terjadi permasalahan teknis terkait dengan akurasi dan keterkinian informasi yang kami sajikan, hal mana akan terus kami perbaiki dari waktu kewaktu.
Dalam hal Anda menemukan inakurasi informasi yang termuat pada situs ini atau informasi yang seharusnya ada, namun belum tersedia, maka harap segera hubungi Kepaniteraan Mahkamah Agung RI melalui :
Email : kepaniteraan@mahkamahagung.go.id Telp : 021-384 3348 (ext.318)

2. Bahwa sebelum membina rumahtangga, Penggugat berstatus Perawan begitupun Tergugat berstatus Jejaka;
3. Bahwa antara Penggugat dan Tergugat terakhir berumah tangga tinggal di rumah kediaman bersama Dusun Lebaktulang RT 03 RW 04 Desa Nagarawangi Kecamatan Rancakalong Kabupaten Sumedang dan telah bergaul baik sebagaimana layaknya suami isteri, dan telah dikaruniai seorang anak bernama: Ulwan Khautal Ginanjar (berusia 10 tahun);
4. Bahwa antara Penggugat dan Tergugat sejak bulan Juni 2017 sudah tidak harmonis lagi dalam menjalani hidup berumah tangga dikarenakan antara Penggugat dengan Tergugat sering terjadi perselisihan dan pertengkaran terus-menerus yang disebabkan Tergugat kurang bertanggung jawab terkait nafkah keluarga, yakni Tergugat jarang bekerja dan Tergugat hanya dapat memberikan penghasilan setiap minggunya rata-rata sebesar Rp. 300.000,-(satu juta rupiah), itupun pemberiannya tidak menentu, sehingga tidak dapat mencukupi kebutuhan rumah tangga bersama, dan untuk mencukupi kebutuhan rumah tanggga bersama Penggugat bekerja, disamping itu Tergugat mudah marah, yakni apabila terjadi perselisihan Tergugat suka mengeluarkan kata-kata kasar kepada Penggugat, bahkan Tergugat berani melakukan kekerasan fisik terhadap Penggugat, seperti Tergugat pernah memukul pada bagian muka Penggugat, di sehingga keadaan rumah tangga dirasa Penggugat sudah tidak nyaman/tentram
5. Bahwa keretakan rumah tangga antara Penggugat dan Tergugat telah berlangsung lama. Penggugat berusaha sabar, tetapi Tergugat tidak berusaha berubah dan merubah sifatnya. Oleh karena itu Penggugat merasa tidak nyaman berumah tangga dengan Tergugat. Puncaknya pada **bulan Oktober 2019** antara Penggugat dan Tergugat terjadi pertengkaran besar **hingga Penggugat pergi meningalkan rumah kediaman milik bersama**, dan sejak itu antara Penggugat dan Tergugat sudah pisah tempat tinggal dan tidak lagi bergaul layaknya suami isteri. Dimana sekarang ini **Penggugat tinggal di rumah kediaman orangtua Penggugat** sedangkan **Tergugat tinggal di tempat rumah kediaman milik bersama**;

Disclaimer
Kepaniteraan Mahkamah Agung Republik Indonesia berusaha untuk selalu mencantumkan informasi paling kini dan akurat sebagai bentuk komitmen Mahkamah Agung untuk pelayanan publik, transparansi dan akuntabilitas pelaksanaan fungsi peradilan. Namun dalam hal-hal tertentu masih dimungkinkan terjadi permasalahan teknis terkait dengan akurasi dan keterkinian informasi yang kami sajikan, hal mana akan terus kami perbaiki dari waktu kewaktu.
Dalam hal Anda menemukan inakurasi informasi yang termuat pada situs ini atau informasi yang seharusnya ada, namun belum tersedia, maka harap segera hubungi Kepaniteraan Mahkamah Agung RI melalui :
Email : kepaniteraan@mahkamahagung.go.id   Telp : 021-384 3348 (ext.318)

6. Bahwa keretakan rumah tangga Penggugat dan Tergugat sudah pernah didamaikan oleh keluarga, tetapi tidak berhasil;
7. Bahwa gugatan Penggugat tersebut telah memenuhi syarat sesuai ketentuan Pasal 19 huruf (f) Peraturan Pemerintah Nomor 9 Tahun 1975 jo. Pasal 116 huruf (f) Kompilasi Hukum Islam.;
8. Bahwa atas permasalahan tersebut di atas Penggugat sudah tidak sanggup lagi untuk mempertahankan perkawinan ini, oleh karena itu Penggugat telah berketetapan hati untuk menggugat cerai dari Tergugat;

Berdasarkan dalil-dalil/alasan tersebut diatas, Penggugat mohon dengan hormat, kiranya bapak Ketua Pengadilan Agama Sumedang cq. Majelis Hakim yang memeriksa dan mengadili perkara ini, berkenan untuk menjatuhkan putusan yang amarnya berbunyi sebagai berikut :

1. Menerima dan mengabulkan gugatan Penggugat.
2. Menjatuhkan talak satu ba'in sughro dari Tergugat (Gina Ginanjar bin Deden Ruhyat) kepada Penggugat (Ayu Yuhanah Rahyu binti Kana).
3. Menetapkan biaya perkara menurut hukum.

Atau apabila Pengadilan berpendapat lain, mohon putusan yang seadil-adilnya;

Bahwa, pada hari sidang yang telah ditetapkan, Penggugat dan Tergugat datang menghadap di muka persidangan;

Bahwa, Majelis Hakim telah berusaha menasehati dan mendamaikan Penggugat dan Tergugat agar dapat membina kembali rumah tangganya secara rukun dan bahkan Majelis Hakim telah memerintahkan kepada Penggugat dan Tergugat agar melaksanakan mediasi dalam rangka perdamaian sebagaimana surat penetapan mediasi bertanggal 01 Desember 2020, akan tetapi tidak berhasil;

Bahwa, selanjutnya pemeriksaan perkara ini dimulai dengan membacakan surat gugatan Penggugat yang maksud dan isinya tetap dipertahankan oleh Penggugat;

Bahwa, pada persidangan selanjutnya dengan agenda pemeriksaan, Tergugat tidak pernah hadir lagi dipersidangan meskipun telah diperintahkan dan telah dipanggil dengan resmi dan patut agar hadir lagi di persidangan,

Disclaimer
Kepaniteraan Mahkamah Agung Republik Indonesia berusaha untuk selalu mencantumkan informasi paling kini dan akurat sebagai bentuk komitmen Mahkamah Agung untuk pelayanan publik, transparansi dan akuntabilitas pelaksanaan fungsi peradilan. Namun dalam hal-hal tertentu masih dimungkinkan terjadi permasalahan teknis terkait dengan akurasi dan keterkinian informasi yang kami sajikan, hal mana akan terus kami perbaiki dari waktu kewaktu.
Dalam hal Anda menemukan inakurasi informasi yang termuat pada situs ini atau informasi yang seharusnya ada, namun belum tersedia, maka harap segera hubungi Kepaniteraan Mahkamah Agung RI melalui :
Email : kepaniteraan@mahkamahagung.go.id Telp : 021-384 3348 (ext.318)

sedangkan tidak pula ternyata bahwa ketidakhadirannya tersebut disebabkan oleh suatu halangan yang sah, oleh karenanya tanggapan atau jawaban Tergugat terhadap gugatan Penggugat tidak dapat di dengar;

Bahwa, untuk membuktikan dalil gugatannya, Penggugat telah mengajukan alat bukti surat berupa Fotocopi Kutipan Akta Nikah Nomor 423/36/XII/2008, tertanggal 17 Desember 2008 yang diterbitkan oleh Kantor Urusan Agama KUA Kecamatan Rancakalong Kabupaten Sumedang (Bukti P);

Bahwa, di samping itu, Penggugat juga telah mengajukan alat bukti saksi yaitu :

1. **Yayu Sukaedah binti Abun**, dibawah sumpah menerangkan pada pokoknya sebagai berikut :
   - Bahwa saksi adalah sepupu Penggugat;
   - Bahwa semula rumah tangga Penggugat dengan Tergugat rukun dan harmonis akan tetapi sejak bulan bulan Juni 2017 rumah tangganya sudah tidak harmonis lagi, sering terjadi perselisihan dan pertengkaran;
   - Bahwa penyebabnya karena Bahwa Tergugat kurang bertanggung jawab terkait nafkah keluarga, yakni Tergugat jarang bekerja dan Tergugat hanya dapat memberikan penghasilan setiap minggunya rata-rata sebesar Rp. 300.000,-(satu juta rupiah), itupun pemberiannya tidak menentu, sehingga tidak dapat mencukupi kebutuhan rumah tangga bersama, dan untuk mencukupi kebutuhan rumah tanggga bersama Penggugat bekerja,
   - Bahwa Tergugat mudah marah, apabila terjadi perselisihan suka mengeluarkan kata-kata kasar, bahkan Tergugat berani melakukan kekerasan fisik terhadap Penggugat, seperti Tergugat pernah memukul pada bagian muka Penggugat, di sehingga keadaan rumah tangga dirasa Penggugat sudah tidak nyaman/tentram;
   - Bahwa sejak bulan Oktober 2019 yang lalu antara Penggugat dan Tergugat telah berpisah tempat tinggal dan tidak pernah berkumpul kembali;
   - Bahwa saksi pernah berusaha menasehati Penggugat, akan tetapi tidak berhasil;
   - Bahwa saksi sudah tidak sanggup lagi untuk merukunkan kembali;

Disclaimer
Kepaniteraan Mahkamah Agung Republik Indonesia berusaha untuk selalu mencantumkan informasi paling kini dan akurat sebagai bentuk komitmen Mahkamah Agung untuk pelayanan publik, transparansi dan akuntabilitas pelaksanaan fungsi peradilan. Namun dalam hal-hal tertentu masih dimungkinkan terjadi permasalahan teknis terkait dengan akurasi dan keterkinian informasi yang kami sajikan, hal mana akan terus kami perbaiki dari waktu kewaktu.
Dalam hal Anda menemukan inakurasi informasi yang termuat pada situs ini atau informasi yang seharusnya ada, namun belum tersedia, maka harap segera hubungi Kepaniteraan Mahkamah Agung RI melalui :
Email : kepaniteraan@mahkamahagung.go.id Telp : 021-384 3348 (ext.318)

Bahwa, terhadap keterangan saksi tersebut Penggugat membenarkannya;

2. **Taryat bin Lili**, dibawah sumpah menerangkan pada pokoknya sebagai berikut :
   - Bahwa saksi adalah kakak sepupu Penggugat;
   - Bahwa awalnya rumah tangga Penggugat dan Tergugat rukun dan harmonis akan tetapi sejak bulan bulan Juni 2017 rumah tangganya sudah tidak harmonis lagi sering terjadi perselisihan dan pertengkaran;
   - Bahwa penyebab perselisihan dan pertengkaran tersebut karena Bahwa Tergugat kurang bertanggung jawab terkait nafkah keluarga, yakni Tergugat jarang bekerja dan Tergugat hanya dapat memberikan penghasilan setiap minggunya rata-rata sebesar Rp. 300.000,-(satu juta rupiah), itupun pemberiannya tidak menentu, sehingga tidak dapat mencukupi kebutuhan rumah tangga bersama, dan untuk mencukupi kebutuhan rumah tanggga bersama Penggugat bekerja,
   - Bahwa Tergugat mudah marah, apabila terjadi perselisihan suka mengeluarkan kata-kata kasar, bahkan Tergugat berani melakukan kekerasan fisik terhadap Penggugat, seperti Tergugat pernah memukul pada bagian muka Penggugat, di sehingga keadaan rumah tangga dirasa Penggugat sudah tidak nyaman/tentram;
   - Bahwa sejak bulan Oktober 2019 yang lalu Penggugat dan Tergugat telah berpisah tempat tinggal dan tidak pernah berkumpul kembali;
   - Bahwa saksi sudah tidak sanggup lagi untuk merukunkannya;

Bahwa, terhadap keterangan saksi tersebut Penggugat membenarkannya;

Bahwa, Penggugat dalam kesimpulannya mengatakan tidak akan mengajukan suatu apapun dan memohon kepada Majelis Hakim agar menjatuhkan putusannya;

Bahwa, untuk mempersingkat uraian pertimbangan ini ditunjuk hal-hal sebagaimana tercantum dalam berita acara persidangan perkara ini;

Disclaimer
Kepaniteraan Mahkamah Agung Republik Indonesia berusaha untuk selalu mencantumkan informasi paling kini dan akurat sebagai bentuk komitmen Mahkamah Agung untuk pelayanan publik, transparansi dan akuntabilitas pelaksanaan fungsi peradilan. Namun dalam hal-hal tertentu masih dimungkinkan terjadi permasalahan teknis terkait dengan akurasi dan keterkinian informasi yang kami sajikan, hal mana akan terus kami perbaiki dari waktu kewaktu.
Dalam hal Anda menemukan inakurasi informasi yang termuat pada situs ini atau informasi yang seharusnya ada, namun belum tersedia, maka harap segera hubungi Kepaniteraan Mahkamah Agung RI melalui :
Email : kepaniteraan@mahkamahagung.go.id Telp : 021-384 3348 (ext.318)

PERTIMBANGAN HUKUM

Menimbang, bahwa maksud dan tujuan gugatan Penggugat adalah sebagaimana terurai diatas;

Menimbang, bahwa sesuai dengan maksud dan ketentuan Pasal 82 Undang-Undang Nomor 7 Tahun 1989 tentang Peradilan Agama sebagaimana telah diubah dengan Undang-Undang Nomor 3 Tahun 2006 dan perubahan kedua dengan Undang-Undang Nomor 50 Tahun 2009, Majelis Hakim telah berusaha maksimal menasehati Penggugat dan Tergugat agar kembali membina rumah tangga dengan rukun dan harmonis, akan tetapi usaha tersebut tidak berhasil;

Menimbang, bahwa disamping itu untuk memenuhi Pasal 7 ayat (1) PERMA Nomor 1 Tahun 2016, Majelis Hakim telah memerintahkan kepada Penggugat dan Terggat agar melaksanakan mediasi dalam rangka perdamaian akan tetapi tidak berhasil, sebagaimana laporan mediator Drs. Eman Sulaeman, SH. tanggal 01 Desember 2020;

Menimbang, bahwa selanjutnya berdasarkan alat bukti (P) yang telah memenuhi syarat formil dan materil, harus dinyatakan terbukti bahwa hubungan hukum antara Penggugat dan Tergugat telah terikat dalam perkawinan yang sah sebagaimana diatur dalam Pasal 2 Undang-Undang Nomor 1 Tahun 1974;

Menimbang, bahwa pada pokoknya Penggugat telah mendasarkan gugatan cerainya terhadap Tergugat dengan alasan sejak bulan bulan Juni 2017 rumah tangga Penggugat dengan Tergugat sudah tidak harmonis, sering terjadi perselisihan dan pertengkaran yang mencapai puncaknya pada bulan Oktober 2019 sehingga tidak ada harapan akan hidup rukun lagi dalam membina rumah tangga, dimana penyebabnya karena Tergugat kurang bertanggung jawab terkait nafkah keluarga, yakni Tergugat jarang bekerja dan Tergugat hanya dapat memberikan penghasilan setiap minggunya rata-rata sebesar Rp. 300.000,-(tiga ratus ribu rupiah), itupun pemberiannya tidak menentu, sehingga tidak dapat mencukupi kebutuhan rumah tangga bersama, dan untuk mencukupi kebutuhan rumah tanggga bersama Penggugat bekerja, disamping itu Tergugat mudah marah, apabila terjadi perselisihan suka mengeluarkan kata-kata kasar, bahkan Tergugat berani melakukan kekerasan

Disclaimer
Kepaniteraan Mahkamah Agung Republik Indonesia berusaha untuk selalu mencantumkan informasi paling kini dan akurat sebagai bentuk komitmen Mahkamah Agung untuk pelayanan publik, transparansi dan akuntabilitas pelaksanaan fungsi peradilan. Namun dalam hal-hal tertentu masih dimungkinkan terjadi permasalahan teknis terkait dengan akurasi dan keterkinian informasi yang kami sajikan, hal mana akan terus kami perbaiki dari waktu kewaktu.
Dalam hal Anda menemukan inakurasi informasi yang termuat pada situs ini atau informasi yang seharusnya ada, namun belum tersedia, maka harap segera hubungi Kepaniteraan Mahkamah Agung RI melalui :
Email : kepaniteraan@mahkamahagung.go.id Telp : 021-384 3348 (ext.318)

fisik terhadap Penggugat, seperti Tergugat pernah memukul pada bagian muka Penggugat, di sehingga keadaan rumah tangga dirasa Penggugat sudah tidak nyaman/tentram sehingga sejak bulan Oktober 2019 Penggugat dan Tergugat telah pisah tempat tinggal;

Menimbang, bahwa di muka persidangan Penggugat telah mengajukan dua orang saksi yang dari keterangannya saling bersesuaian, Majelis Hakim telah dapat menemukan fakta hukum sebagai berikut :

- Bahwa Penggugat dan Tergugat adalah suami isteri yang sah;
- Bahwa semula rumah tangga Penggugat dan Tergugat rukun dan harmonis;
- Bahwa sejak bulan bulan Juni 2017 keadaan rumah tangganya tersebut sudah tidak harmonis lagi, sering terjadi perselisihan dan pertengkaran;
- Bahwa penyebabnya karena Bahwa Tergugat kurang bertanggung jawab terkait nafkah keluarga, yakni Tergugat jarang bekerja dan Tergugat hanya dapat memberikan penghasilan setiap minggunya rata-rata sebesar Rp. 300.000,-(satu juta rupiah), itupun pemberiannya tidak menentu, sehingga tidak dapat mencukupi kebutuhan rumah tangga bersama, dan untuk mencukupi kebutuhan rumah tanggga bersama Penggugat bekerja,
- Bahwa Tergugat mudah marah, apabila terjadi perselisihan suka mengeluarkan kata-kata kasar, bahkan Tergugat berani melakukan kekerasan fisik terhadap Penggugat, seperti Tergugat pernah memukul pada bagian muka Penggugat, di sehingga keadaan rumah tangga dirasa Penggugat sudah tidak nyaman/tentram;
- Bahwa sejak bulan Oktober 2019 yang lalu Penggugat dan Tergugat telah berpisah tempat tinggal dan tidak pernah berkumpul kembali;

Menimbang, bahwa berdasarkan fakta hukum tersebut di atas, harus dinyatakan terbukti bahwa dalam kehidupan rumah tangga Penggugat dan Tergugat sudah tidak harmonis lagi, telah terjadi perselisihan dan pertengkaran yang mengakibatkan antara keduanya telah berpisah tempat tinggal dan tidak pernah berkumpul kembali dalam satu rumah tangga;

Menimbang, bahwa disamping itu para saksi sudah pernah berusaha menasehati Penggugat dan mengatakan tidak ada kesanggupan untuk dapat merukunkannya kembali, sehingga Majelis Hakim berpendapat bahwa antara

Disclaimer
Kepaniteraan Mahkamah Agung Republik Indonesia berusaha untuk selalu mencantumkan informasi paling kini dan akurat sebagai bentuk komitmen Mahkamah Agung untuk pelayanan publik, transparansi dan akuntabilitas pelaksanaan fungsi peradilan. Namun dalam hal-hal tertentu masih dimungkinkan terjadi permasalahan teknis terkait dengan akurasi dan keterkinian informasi yang kami sajikan, hal mana akan terus kami perbaiki dari waktu kewaktu.
Dalam hal Anda menemukan inakurasi informasi yang termuat pada situs ini atau informasi yang seharusnya ada, namun belum tersedia, maka harap segera hubungi Kepaniteraan Mahkamah Agung RI melalui :
Email : kepaniteraan@mahkamahagung.go.id Telp : 021-384 3348 (ext.318)

Penggugat dan Tergugat sudah sulit dan tidak ada harapan untuk dapat hidup rukun kembali di dalam membina rumah tangganya;

Menimbang, bahwa menurut ketentuan Pasal 19 huruf (f) PP Nomor 9 tahun 1975 jo. Pasal 116 huruf (f) Kompilasi Hukum Islam, perceraian dapat terjadi apabila "antara suami dan isteri terus menerus terjadi perselisihan dan pertengkaran dan tidak ada harapan akan hidup rukun kembali dalam membina rumah tangga";

Menimbang, bahwa demikian juga menurut Mahkamah Agung sebagaimana tertuang dalam putusannya No. 38/K/AG/1990 tanggal 5 Oktober 1990 yang kemudian diambil alih menjadi pendapat Majelis Hakim bahwa apabila ternyata adanya perselisihan sebagaimana dimaksud Pasal 19 huruf (f) PP No. 9 Tahun 1975, maka hal itu semata-mata ditujukan kepada perkawinannya itu sendiri tanpa mempersoalkan siapa yang salah dalam hal terjadinya perselisihan yang mengakibatkan tidak ada harapan akan hidup rukun lagi, karena pernikahan adalah suatu perjanjian yang suci (mitsaqan gholidzon/Pasal 2 KHI) yang untuk memutuskannya tidak boleh diukur dengan kesalahan salah satu pihak, sehingga apabila perkawinan itu telah pecah berarti hati kedua belah pihak telah pecah pula;

Menimbang, bahwa terlepas dari peristiwa atau keadaan yang menjadi penyebab terjadinya perselisihan yang terjadi antara Penggugat dan Tergugat, yang jelas Majelis Hakim telah dapat menemukan fakta hukum bahwa perselisihan antara Penggugat dan Tergugat tersebut telah sedemikian rupa, rumah tangga Penggugat dan Tergugat sudah hancur dan tidak mungkin akan dapat hidup rukun kembali dalam membina rumah tangga;

Menimbang, bahwa dengan melihat kondisi rumah tangga Penggugat dan Tergugat tersebut, jelas tidak mungkin akan terwujud kehidupan rumah tangga yang bahagia dan kekal lahir bathin sebagaimana yang menjadi tujuan perkawinan dalam Pasal 1 Undang-Undang Nomor 1 Tahun 1974 jo. Pasal 3 KHI, dan membiarkan suasana rumah tangga yang demikian, justru akan menimbulkan mudlarat bagi kedua belah pihak, oleh karena itu perceraian dipandang jalan yang terbaik dan lebih maslahat bagi keduanya;

Disclaimer
Kepaniteraan Mahkamah Agung Republik Indonesia berusaha untuk selalu mencantumkan informasi paling kini dan akurat sebagai bentuk komitmen Mahkamah Agung untuk pelayanan publik, transparansi dan akuntabilitas pelaksanaan fungsi peradilan. Namun dalam hal-hal tertentu masih dimungkinkan terjadi permasalahan teknis terkait dengan akurasi dan keterkinian informasi yang kami sajikan, hal mana akan terus kami perbaiki dari waktu kewaktu.
Dalam hal Anda menemukan inakurasi informasi yang termuat pada situs ini atau informasi yang seharusnya ada, namun belum tersedia, maka harap segera hubungi Kepaniteraan Mahkamah Agung RI melalui :
Email : kepaniteraan@mahkamahagung.go.id Telp : 021-384 3348 (ext.318)

Menimbang, bahwa Majelis Hakim perlu mengetengahkan dalil dalam kitab Al-Muhadzab juz II halaman 82 yang kemudian diambil alih menjadi pendapat Majelis Hakim sebagai berikut :

Ejnb æÈ°jÛ¯ tvnÌ ØnÊ°ã¸äSÛ E¸äSÛ¯ ECĐÀ Þ¾Ì
¾FÄ¯¯¿ ªä

Artinya: "Disaat istri telah memuncak ketidaksukaan terhadap suaminya, maka Hakim diperkenankan menjatuhkan talak (suami) nya dengan talak satu ;

Menimbang, bahwa berdasarkan pertimbangan tersebut diatas, maka gugatan Penggugat telah memenuhi alasan perceraian sebagaimana diatur dalam Pasal 39 ayat (2) Undang-Undang Nomor 1 Tahun 1974, jo. Pasal 19 huruf (f) Peraturan Pemerintah Nomor 9 Tahun 1975, jo. Pasal 116 huruf (f) Kompilasi Hukum Islam dan karenanya gugatan Penggugat dapat dikabulkan dengan menjatuhkan talak satu ba'in sughro Tergugat terhadap Penggugat;

Menimbang, bahwa perkara ini termasuk bidang perkawinan, oleh karenanya berdasarkan Pasal 89 ayat (1) Undang-Undang Nomor 7 Tahun 1989 tentang Peradilan Agama sebagaimana telah diubah dengan Undang-Undang Nomor 3 Tahun 2006 dan perubahan kedua dengan Undang-Undang Nomor 50 Tahun 2009, maka seluruh biaya perkara ini dibebankan kepada Penggugat;

Mengingat segala peraturan perundang-undangan yang berlaku serta ketentuan hukum syara' yang berkaitan dengan perkara ini;

M E N G A D I L I

1. Mengabulkan gugatan Penggugat ;
2. Menjatuhkan talak satu bain sughra Tergugat (Gina Ginanjar bin Deden Ruhyat) terhadap Penggugat (Ayu Yuhanah Rahyu binti Kana);
3. Membebankan kepada Penggugat untuk membayar biaya perkara ini sejumlah Rp. 736.000,00 (tujuh ratus tiga puluh enam ribu rupiah).

Demikian Putusan ini dijatuhkan dalam permusyawaratan Majelis Hakim Pengadilan Agama Sumedang pada hari Selasa tanggal 22 Desember 2020

Disclaimer
Kepaniteraan Mahkamah Agung Republik Indonesia berusaha untuk selalu mencantumkan informasi paling kini dan akurat sebagai bentuk komitmen Mahkamah Agung untuk pelayanan publik, transparansi dan akuntabilitas pelaksanaan fungsi peradilan. Namun dalam hal-hal tertentu masih dimungkinkan terjadi permasalahan teknis terkait dengan akurasi dan keterkinian informasi yang kami sajikan, hal mana akan terus kami perbaiki dari waktu kewaktu.
Dalam hal Anda menemukan inakurasi informasi yang termuat pada situs ini atau informasi yang seharusnya ada, namun belum tersedia, maka harap segera hubungi Kepaniteraan Mahkamah Agung RI melalui :
Email : kepaniteraan@mahkamahagung.go.id Telp : 021-384 3348 (ext.318)

Masehi, bertepatan dengan tanggal 07 Jumadil Awwal 1442 Hijriyah, oleh kami Drs. Endang Sofwan, M.H sebagai Ketua Majelis, Drs. Erik Sumarna, S.H., M.A. dan Dra. Hj. Budi Purwantini, MH masing-masing sebagai Hakim Anggota, yang dibacakan dalam sidang terbuka untuk umum oleh Ketua Majelis tersebut dengan dihadiri para Hakim Anggota dan N. Popon Nurhayati, S. Ag, M. M sebagai Panitera Pengganti serta dihadiri oleh Penggugat diluar hadirnya Tergugat;

Ketua Majelis

Drs. Endang Sofwan, M.H

Hakim Anggota | Hakim Anggota

Drs. Erik Sumarna, S.H., M.A. | **Dra. Hj. Budi Purwantini, MH**

Panitera Pengganti

N. Popon Nurhayati, S. Ag, M. M

Perincian Biaya Perkara :

1. Biaya Pendaftaran	:	Rp.	30.000,00
2. Biaya Proses	:	Rp.	50.000,00
3. Biaya Panggilan	:	Rp.	620.000,00
4. Biaya PNBP Panggilan	:	Rp.	20.000,00
5. Biaya Redaksi	:	Rp.	10.000,00
6. Biaya materai	:	Rp.	6.000,00
Jumlah		Rp.	736.000,00

(tujuh ratus tiga puluh enam ribu rupiah)

Dicatat disini :

– Amar putusan telah diberitahukan pada Tergugat tanggal ....

Hal. 10 Putusan Nomor 3694/Pdt.G/2020/PA.Smdg.

Disclaimer
Kepaniteraan Mahkamah Agung Republik Indonesia berusaha untuk selalu mencantumkan informasi paling kini dan akurat sebagai bentuk komitmen Mahkamah Agung untuk pelayanan publik, transparansi dan akuntabilitas pelaksanaan fungsi peradilan. Namun dalam hal-hal tertentu masih dimungkinkan terjadi permasalahan teknis terkait dengan akurasi dan keterkinian informasi yang kami sajikan, hal mana akan terus kami perbaiki dari waktu kewaktu.
Dalam hal Anda menemukan inakurasi informasi yang termuat pada situs ini atau informasi yang seharusnya ada, namun belum tersedia, maka harap segera hubungi Kepaniteraan Mahkamah Agung RI melalui :
Email : kepaniteraan@mahkamahagung.go.id Telp : 021-384 3348 (ext.318)

- Putusan telah berkekuatan hukum tetap sejak tanggal ……………………………….

Ketua Majelis

ttd

Drs. Endang Sofwan, M.H

Hakim Anggota   Hakim Anggota

Disclaimer
Kepaniteraan Mahkamah Agung Republik Indonesia berusaha untuk selalu mencantumkan informasi paling kini dan akurat sebagai bentuk komitmen Mahkamah Agung untuk pelayanan publik, transparansi dan akuntabilitas pelaksanaan fungsi peradilan. Namun dalam hal-hal tertentu masih dimungkinkan terjadi permasalahan teknis terkait dengan akurasi dan keterkinian informasi yang kami sajikan, hal mana akan terus kami perbaiki dari waktu kewaktu.
Dalam hal Anda menemukan inakurasi informasi yang termuat pada situs ini atau informasi yang seharusnya ada, namun belum tersedia, maka harap segera hubungi Kepaniteraan Mahkamah Agung RI melalui :
Email : kepaniteraan@mahkamahagung.go.id   Telp : 021-384 3348 (ext.318)

ttd	ttd
**Drs. Erik Sumarna, S.H., M.A.**	**Dra. Hj. Budi Purwantini, MH**

Panitera Pengganti

ttd

N. Popon Nurhayati, S. Ag, M. M

Perincian Biaya Perkara :

1. Biaya Pendaftaran	:	Rp.	30.000,00
2. Biaya Proses	:	Rp.	50.000,00
3. Biaya Panggilan	:	Rp.	360.000,00
4. Biaya PNBP Panggilan	:	Rp.	...,00
5. Biaya Redaksi	:	Rp.	10.000,00
6. Biaya materai	:	Rp.	6.000,00
Jumlah		Rp.	456.000,00

(empat ratus lima puluh enam ribu rupiah)

Dicatat disini :

- Amar putusan tersebut telah diberitahukan kepada Tergugat pada tanggal
- Putusan tersebut telah mempunyai kekuatan hukum tetap sejak tanggal

Untuk salinan yang sama bunyinya
Panitera,

Rohili, S.H.

Disclaimer
Kepaniteraan Mahkamah Agung Republik Indonesia berusaha untuk selalu mencantumkan informasi paling kini dan akurat sebagai bentuk komitmen Mahkamah Agung untuk pelayanan publik, transparansi dan akuntabilitas pelaksanaan fungsi peradilan. Namun dalam hal-hal tertentu masih dimungkinkan terjadi permasalahan teknis terkait dengan akurasi dan keterkinian informasi yang kami sajikan, hal mana akan terus kami perbaiki dari waktu kewaktu.
Dalam hal Anda menemukan inakurasi informasi yang termuat pada situs ini atau informasi yang seharusnya ada, namun belum tersedia, maka harap segera hubungi Kepaniteraan Mahkamah Agung RI melalui :
Email : kepaniteraan@mahkamahagung.go.id   Telp : 021-384 3348 (ext.318)
