# Supplementary material for: Classification of divorce causes during the COVID-19 pandemic using convolutional neural networks
Source: PeerJ Comput Sci. 2022 Jun 30;8:e998. doi: 10.7717/peerj-cs.998 (PMC9299239; doi:10.7717/peerj-cs.998)
Supplement: Supplemental Information 5 [file peerj-cs-08-998-s005.zip › Masalah Ekonomi Dataset/Data ke-27.pdf]

P U T U S A N

Nomor 2115/Pdt.G/ 2020/PA Sr

بسم الله الرحمن الرحيم

DEMI KEADILAN BERDASARKAN KETUHANAN YANG MAHA ESA

Pengadilan Agama Sragen yang memeriksa dan mengadili perkara-perkara tertentu dalam tingkat pertama telah menjatuhkan putusan dalam perkara yang diajukan oleh :

Xxxxxxxxxxxxxx, Tempat tanggal lahir: Sragen, 09 Agustus 1979 (Umur: ± 41 tahun), Agama Islam, Pekerjaan Karyawan Swasta, Pendidikan S1, NIK : 3314064908790007, Beralamat di Dukuh Gondang Tani RT. 018, Desa Gondang, Kecamatan Gondang, Kabupaten Sragen, dalam hal ini berdasarkan surat kuasa khusus tanggal 5 November 2020 telah memberikan kuasa kepada **H. Slamet Widodo, SH.,** KTA No: 92.10061, NIK : 3314102903650002 **dan Ridhi Yantoro, SH.,** KTA No: 13.01675, NIK : 3314072709840003 Advokat dan Konsultan Hukum yang berkantor di Jl. Dr. Sutomo No: 24 Bangunsari, Sragen, Jawa Tengah, selanjutnya disebut sebagai Penggugat;

melawan

Xxxxxxxxxxxxxx, Tempat tanggal lahir: Surabaya, 16 Januari 1976 (Umur: ± 44 tahun), Agama Islam, Pekerjaan Wiraswasta (Ekspedisi), Pendidikan SMA, Beralamat di Dukuh Gondang Tani RT. 018, Desa Gondang, Kecamatan Gondang, Kabupaten Sragen, selanjutnya disebut sebagai Tergugat;

Pengadilan Agama tersebut ;

Telah membaca dan mempelajari berkas perkara yang bersangkutan ;

Disclaimer
Kepaniteraan Mahkamah Agung Republik Indonesia berusaha untuk selalu mencantumkan informasi paling kini dan akurat sebagai bentuk komitmen Mahkamah Agung untuk pelayanan publik, transparansi dan akuntabilitas pelaksanaan fungsi peradilan. Namun dalam hal-hal tertentu masih dimungkinkan terjadi permasalahan teknis terkait dengan akurasi dan keterkinian informasi yang kami sajikan, hal mana akan terus kami perbaiki dari waktu kewaktu.
Dalam hal Anda menemukan inakurasi informasi yang termuat pada situs ini atau informasi yang seharusnya ada, namun belum tersedia, maka harap segera hubungi Kepaniteraan Mahkamah Agung RI melalui :
Email : kepaniteraan@mahkamahagung.go.id Telp : 021-384 3348 (ext.318)

Telah mendengar keterangan Penggugat dan saksi-saksi Penggugat di persidangan ;

DUDUK PERKARANYA

Menimbang, bahwa Penggugat telah mengajukan gugatan cerai tanggal 10 Nopember 2020, dan telah terdaftar di Kepaniteraan Pengadilan Agama tersebut dengan register perkara Nomor 2115/Pdt.G/2020/PA Sr. tanggal 17 Nopember 2020 yang isinya sebagai berikut :

1. Bahwa Penggugat telah menikah secara sah dengan Tergugat pada tanggal 21 Oktober 2003 dihadapan Pegawai Pencatat Nikah Kantor Urusan Agama Kecamatan Gondang, Kabupaten Sragen sesuai dengan Kutipan Akta Nikah Nomor: 393/25/X/2003 pada tanggal 21 Oktober 2003 dalam status Perawan dan Jejaka yang sampai sekarang belum pernah bercerai.
2. Bahwa pada waktu akad nikah Tergugat mengucapkan sighot taklik talak yang bunyinya sesuai dengan buku nikah.
3. Bahwa Penggugat dengan Tergugat setelah akad nikah hidup berumah tangga dengan rukun baik selayaknya suami istri (ba'da dukhul) di rumah orang tua Penggugat di Dukuh Gondang Tani RT. 018, Desa Gondang, Kecamatan Gondang, Kabupaten Sragen selama 6 bulan, setelah itu di rumah orang tua Tergugat di Jalan Banyurip Kidul No: 4b. 14 Surabaya selama kurang lebih 2 tahun, lalu Penggugat dan Tergugat merantau ke Mataram NTB selama 2 tahun, setelah itu kembali ke rumah orang tua Tergugat di Surabaya lagi selama 6 bulan, kemudian ke rumah orang tua Penggugat selama 8 tahun, adapun sejak tahun 2016 Tergugat sering pergi pulang ke rumah orang tua Tergugat hingga sekarang yang sudah pisah rumah kurang lebih 4 tahun lamanya.
4. Bahwa Penggugat dengan Tergugat dalam kehidupan berumah tangga hingga sekarang sudah dikaruniai keturunan satu orang anak yaitu yang bernama: **Nasywa Fajriah Noviana,** yang lahir pada tanggal 08 November 2004, sekarang semua ikut Penggugat.
5. Bahwa Penggugat dengan Tergugat dalam kehidupan berumah tangga semula rukun baik akan tetapi sejak sekitar bulan Januari 2010 rumah tangga mulai tidak harmonis dan terjadi pertengkaran terus-menerus yang

Disclaimer
Kepaniteraan Mahkamah Agung Republik Indonesia berusaha untuk selalu mencantumkan informasi paling kini dan akurat sebagai bentuk komitmen Mahkamah Agung untuk pelayanan publik, transparansi dan akuntabilitas pelaksanaan fungsi peradilan. Namun dalam hal-hal tertentu masih dimungkinkan terjadi permasalahan teknis terkait dengan akurasi dan keterkinian informasi yang kami sajikan, hal mana akan terus kami perbaiki dari waktu kewaktu.
Dalam hal Anda menemukan inakurasi informasi yang termuat pada situs ini atau informasi yang seharusnya ada, namun belum tersedia, maka harap segera hubungi Kepaniteraan Mahkamah Agung RI melalui :
Email : kepaniteraan@mahkamahagung.go.id Telp : 021-384 3348 (ext.318)

disebabkan masalah ekonomi karena Tergugat dalam memberi nafkah kepada Penggugat kurang, kemudian Tergugat egois dan sering marah tanpa sebab yang jelas kepada Penggugat, lalu Tergugat tidak mau di ikuti oleh Penggugat dan anaknya serta antara Penggugat dan Tergugat sudah bermusyawarah tetapi tidak ada titik temunya.

6. Bahwa setelah Penggugat dan Tergugat pisah tersebut, Tergugat sudah tidak pernah memberi nafkah wajib dan juga telah membiarkan dan tidak memperdulikan lagi terhadap Penggugat hingga sekarang yang sudah kurang lebih 4 tahun lamanya.
7. Bahwa karena keadaan rumah tangga Penggugat dengan Tergugat seperti tersebut diatas maka Penggugat sudah merasa tidak mau lagi untuk hidup berumah tangga dengan Tergugat dan juga sudah tidak mungkin lagi dirukunkan kembali seperti selayaknya keluarga yang sakinah mawaddah warohmah.
8. Bahwa berdasarkan hal-hal tersebut diatas maka dalil-dalil gugatan cerai penggugat telah berdasar atas hukum yang berlaku dan telah memenuhi yang dimaksud pasal 39 ayat (2) Undang-Undang nomor 1 tahun 1974 serta telah sesuai dengan alasan perceraian sebagaimana diatur dalam pasal 19 huruf (f) Peraturan Pemerintah Nomor 9 tahun 1975 dan pasal 116 huruf (f), Kompilasi Hukum Islam.

Berdasarkan hal-hal tersebut diatas penggugat mohon kepada Bapak Ketua Pengadilan Agama Sragen agar berkenan memanggil dan memeriksa orang-orang yang perlu didengar keterangannya dan menjatuhkan putusan sebagai berikut:

PRIMAIR :

1. Mengabulkan cerai gugat Penggugat untuk seluruhnya.
2. Menjatuhkan talak satu ba'in shughro dari Tergugat **(Xxxxxxxxxxxxxxx)** terhadap Penggugat **(Xxxxxxxxxxxxxx)**.
3. Membebankan biaya perkara ini menurut peraturan hukum yang berlaku.

SUBSIDAIR :

Mohon perkara ini untuk diputus dengan seadil-adilnya

Menimbang, bahwa pada hari sidang yang telah ditetapkan Penggugat hadir sendiri di persidangan, sedang Tergugat tidak pernah hadir

Disclaimer
Kepaniteraan Mahkamah Agung Republik Indonesia berusaha untuk selalu mencantumkan informasi paling kini dan akurat sebagai bentuk komitmen Mahkamah Agung untuk pelayanan publik, transparansi dan akuntabilitas pelaksanaan fungsi peradilan. Namun dalam hal-hal tertentu masih dimungkinkan terjadi permasalahan teknis terkait dengan akurasi dan keterkinian informasi yang kami sajikan, hal mana akan terus kami perbaiki dari waktu kewaktu.
Dalam hal Anda menemukan inakurasi informasi yang termuat pada situs ini atau informasi yang seharusnya ada, namun belum tersedia, maka harap segera hubungi Kepaniteraan Mahkamah Agung RI melalui :
Email : kepaniteraan@mahkamahagung.go.id   Telp : 021-384 3348 (ext.318)

meskipun telah dipanggil secara sah dan patut dan tidak ternyata bahwa ketidakhadiran Tergugat disebabkan suatu halangan yang sah ;

Menimbang, bahwa selanjutnya pemeriksaan dimulai dengan membacakan gugatan Penggugat dan atas pertanyaan Majelis Hakim Penggugat tetap pada gugatan Penggugat tanpa ada perubahan;

Menimbang, bahwa untuk menguatkan dalil-dalil gugatannya Penggugat telah mengajukan bukti-bukti sebagai berikut :

A. Bukti Tertulis :

1. Fotokopi Kartu Tanda Penduduk an. Penggugat Nomor 3314064908790007 yang dikeluarkan oleh Kepala Denas kependudukan dan Catatan Sipil Kabupaten Sragen, alat bukti tersebut bermeterai cukup dan telah dicocokkan ternyata sesuai dengan aslinya, diberi kode P1
2. Fotokopi Kutipan Akta Nikah Nomor 393/25/X/2003 pada tanggal 21 Oktober 2003 yang dikeluarkan oleh Kantor Urusan Agama Kecamatan Gondang, Kabupaten Sragen, alat bukti tersebut bermeterai cukup dan telah dicocokkan ternyata sesuai dengan aslinya, diberi kode P2;

B. Bukti Saksi :

1. Sukimin bin Kartodikromo, umur 54 tahun, agama Islam, pekerjaan Guru, tempat kediaman di Dukuh Damping Rt.19 Desa Jambeyan, Kecamatan Sambirejo, setelah berjanji saksi telah memberikan keterangan yang pada pokoknya sebagai berikut :
   - Bahwa saksi kenal dengan Penggugat dan Tergugat karena saksi sebagai paman Penggugat;
   - Bahwa Penggugat dan Tergugat adalah suami isteri;
   - Bahwa setelah menikah Penggugat dan Tergugat hidup bersama di rumah orang tua Penggugat, dikaruniai anak 1 orang;

Disclaimer
Kepaniteraan Mahkamah Agung Republik Indonesia berusaha untuk selalu mencantumkan informasi paling kini dan akurat sebagai bentuk komitmen Mahkamah Agung untuk pelayanan publik, transparansi dan akuntabilitas pelaksanaan fungsi peradilan. Namun dalam hal-hal tertentu masih dimungkinkan terjadi permasalahan teknis terkait dengan akurasi dan keterkinian informasi yang kami sajikan, hal mana akan terus kami perbaiki dari waktu kewaktu.
Dalam hal Anda menemukan inakurasi informasi yang termuat pada situs ini atau informasi yang seharusnya ada, namun belum tersedia, maka harap segera hubungi Kepaniteraan Mahkamah Agung RI melalui :
Email : kepaniteraan@mahkamahagung.go.id Telp : 021-384 3348 (ext.318)

- Bahwa sejak tahun 2010 Penggugat dan Tergugat telah terjadi perselisihan dan pertengkaran yang disebabkan masalah ekonomi karena Tergugat dalam memberi nafkah kepada Penggugat kurang;
- Bahwa sejak tahun 2016 Penggugat dan Tergugat telah berpisah tempat tinggal Tergugat yang pergi meninggalkan Penggugat ke rumah orang tua Tergugat sampai sekarang selama 4 tahun lebih tidak pernah bertemu lagi dan tidak ada komunikasi;
- Bahwa Penggugat dan tergugat sudah pernah dirukunkan tetapi tidak berhasil;

2. Fatah Yasin bin Iksan Purnomo, umur 55 tahun, agama Islam, pekerjaan Perangkat Desa, tempat kediaman di Dukuh Gondangtani Rt.25, Desa Gondang, Kecamatan Gondang, Kabupaten Sragen, Provinsi Jawa Tengah, setelah berjanji, setelah bersumpah saksi telah memberikan keterangan yang pada pokoknya sebagai berikut :
   - Bahwa saksi kenal dengan Penggugat dan Tergugat karena saksi sebagai tetangga Penggugat;
   - Bahwa Penggugat dan Tergugat adalah suami isteri;
   - Bahwa setelah menikah Penggugat dan Tergugat hidup bersama di rumah orang tua ~~Penggugat~~, dikaruniai anak 1 orang;
   - Bahwa sejak tahun 2010 Penggugat dan Tergugat telah terjadi perselisihan dan pertengkaran yang disebabkan masalah ekonomi karena Tergugat dalam memberi nafkah kepada Penggugat kurang;
   - Bahwa sejak tahun 2016 Penggugat dan Tergugat telah berpisah tempat tinggal Tergugat yang pergi meninggalkan Penggugat ke rumah orang tua Tergugat sampai sekarang selama 4 tahun lebih tidak pernah bertemu lagi dan tidak ada komunikasi;

Disclaimer
Kepaniteraan Mahkamah Agung Republik Indonesia berusaha untuk selalu mencantumkan informasi paling kini dan akurat sebagai bentuk komitmen Mahkamah Agung untuk pelayanan publik, transparansi dan akuntabilitas pelaksanaan fungsi peradilan. Namun dalam hal-hal tertentu masih dimungkinkan terjadi permasalahan teknis terkait dengan akurasi dan keterkinian informasi yang kami sajikan, hal mana akan terus kami perbaiki dari waktu kewaktu.
Dalam hal Anda menemukan inakurasi informasi yang termuat pada situs ini atau informasi yang seharusnya ada, namun belum tersedia, maka harap segera hubungi Kepaniteraan Mahkamah Agung RI melalui :
Email : kepaniteraan@mahkamahagung.go.id Telp : 021-384 3348 (ext.318)

- Bahwa Penggugat dan tergugat sudah pernah dirukunkan tetapi tidak berhasil;

Menimbang, bahwa selanjutnya Penggugat telah memberikan kesimpulan tetap pada gugatan Penggugat dan mohon putusan ;

Menimbang, bahwa untuk menyingkat uraian dalam putusan ini, maka segala sesuatu yang tercantum dalam Berita Acara Persidangan perkara ini dianggap telah masuk dan merupakan bagian yang tidak terpisahkan dari putusan ini ;

PERTIMBANGAN HUKUM

Menimbang, bahwa maksud dan tujuan gugatan Penggugat adalah seperti tersebut di atas ;

Menimbang, bahwa dari bukti kode P1 yang merupakan bukti otentik, dan dapat diterima sebagai bukti, maka terbukti bahwa Penggugat bertempat tinggal di wilayah Kabupaten Sragen, sehingga sesuai Pasal 73 Ayat 1 Undang-Undang Nomor 7 Tahun 1989 tentang Peradilan Agama, Pengadilan Agama Sragen berwenang mengadili perkara tersebut dan gugatan Penggugat aquo formil dapat diterima;

Menimbang, bahwa dari bukti kode P2 yang merupakan bukti otentik dan dapat diterima sebagai bukti, maka terbukti bahwa Penggugat dan Tergugat adalah suami isteri yang sah, oleh karena itu gugatan Penggugat terhadap Tergugat telah memiliki dasar hukum yang sah;

Menimbang, bahwa Penggugat dalam gugatannya telah mendalilkan hal-hal yang pada pokoknya sebagai berikut :

- Bahwa Penggugat dengan Tergugat dalam kehidupan berumah tangga semula rukun baik akan tetapi sejak sekitar bulan Januari 2010 rumah tangga mulai tidak harmonis dan terjadi pertengkaran terus-menerus yang disebabkan masalah ekonomi karena Tergugat dalam memberi nafkah kepada Penggugat kurang, kemudian Tergugat egois dan sering marah tanpa sebab yang jelas kepada Penggugat, lalu Tergugat tidak mau di ikuti oleh Penggugat dan anaknya serta antara Penggugat dan Tergugat sudah bermusyawarah tetapi tidak ada titik temunya.

Disclaimer
Kepaniteraan Mahkamah Agung Republik Indonesia berusaha untuk selalu mencantumkan informasi paling kini dan akurat sebagai bentuk komitmen Mahkamah Agung untuk pelayanan publik, transparansi dan akuntabilitas pelaksanaan fungsi peradilan. Namun dalam hal-hal tertentu masih dimungkinkan terjadi permasalahan teknis terkait dengan akurasi dan keterkinian informasi yang kami sajikan, hal mana akan terus kami perbaiki dari waktu kewaktu.
Dalam hal Anda menemukan inakurasi informasi yang termuat pada situs ini atau informasi yang seharusnya ada, namun belum tersedia, maka harap segera hubungi Kepaniteraan Mahkamah Agung RI melalui :
Email : kepaniteraan@mahkamahagung.go.id Telp : 021-384 3348 (ext.318)

- Bahwa sejak tahun 2016 Tergugat sering pergi pulang ke rumah orang tua Tergugat hingga sekarang yang sudah pisah rumah kurang lebih 4 tahun lamanya, tidak pernah memberi nafkah wajib dan juga telah membiarkan dan tidak memperdulikan lagi terhadap Penggugat;

Menimbang, bahwa untuk mendukung dalil-dalil gugatannya, Penggugat telah mengajukan bukti 2 (dua) orang saksi yang telah memberikan keterangan di bawah sumpah masing-masing saksi;

Menimbang, bahwa oleh karena kesaksian para saksi didasarkan pada apa yang dilihat, didengar dan dialami oleh para saksi, maka kesaksian tersebut telah memenuhi syarat formil dan materiil dan oleh karenanya kesaksian tersebut dapat diterima sebagai bukti;

Menimbang, bahwa dari kesaksian saksi-saksi Penggugat para saksi telah menyaksikan bahwa sejak tahun 2010 Penggugat dan Tergugat telah terjadi perselisihan dan pertengkaran yang disebabkan masalah ekonomi karena Tergugat dalam memberi nafkah kepada Penggugat kurang, dan sejak tahun 2016 Penggugat dan Tergugat telah berpisah tempat tinggal Tergugat yang pergi meninggalkan Penggugat ke rumah orang tua Tergugat sampai sekarang selama 4 tahun lebih tidak pernah bertemu lagi dan tidak ada komunikasi, sudah pernah dirukunkan tetapi tidak berhasil, sehingga gugatan Penggugat telah didukung bukti;

Menimbang, bahwa dari kesaksian dua orang saksi tersebut Majelis Hakim telah memperoleh fakta sebagai berikut :

- Bahwa sejak tahun 2010 Penggugat dan Tergugat secara terus menerus telah terjadi perselisihan dan pertengkaran yang disebabkan masalah ekonomi karena Tergugat dalam memberi nafkah kepada Penggugat kurang ;
- Bahwa sejak tahun 2016 Penggugat dan Tergugat telah berpisah tempat tinggal Tergugat yang pergi meninggalkan Penggugat ke rumah orang tua Tergugat sampai sekarang selama 4 tahun lebih tidak pernah bertemu lagi, sudah pernah dirukunkan tetapi tidak berhasil

Menimbang, bahwa berdasarkan fakta tersebut, maka gugatan Penggugat telah memenuhi alasan perceraian Penjelasan Pasal 39 ayat (2)

Disclaimer
Kepaniteraan Mahkamah Agung Republik Indonesia berusaha untuk selalu mencantumkan informasi paling kini dan akurat sebagai bentuk komitmen Mahkamah Agung untuk pelayanan publik, transparansi dan akuntabilitas pelaksanaan fungsi peradilan. Namun dalam hal-hal tertentu masih dimungkinkan terjadi permasalahan teknis terkait dengan akurasi dan keterkinian informasi yang kami sajikan, hal mana akan terus kami perbaiki dari waktu kewaktu.
Dalam hal Anda menemukan inakurasi informasi yang termuat pada situs ini atau informasi yang seharusnya ada, namun belum tersedia, maka harap segera hubungi Kepaniteraan Mahkamah Agung RI melalui :
Email : kepaniteraan@mahkamahagung.go.id Telp : 021-384 3348 (ext.318)

huruf f, Undang-Undang nomor 1 Tahun 1974 tentang Perkawinan, jo. Pasal 19 huruf f, Peraturan Pemerintah Nomor 9 Tahun 1975 dan pasal 116 huruf f, Kompilasi Hukum Islam;

Menimbang, bahwa berdasarkan pertimbangan-pertimbangan tersebut diatas Majelis hakim berpendapat bahwa rumah tangga Penggugat dan Tergugat telah pecah dan tidak ada harapan untuk dapat mewujudkan tujuan perkawinan sebagaimana pasal 1 Undang-Undang Nomor 1 Tahun 1974 jo. Pasal 3 Kompilasi Hukum Islam, dan karenanya hak dan kewajiban Penggugat dan Tergugat sebagai suami isteri sebagaimana Pasal 33 dan 34 Ayat (1) dan Ayat (2) Undang-Undang Nomor 1 Tahun 1974 jo. Pasal 77 Kompilasi Hukum Islam juga tidak dapat ditegakkan, sehingga gugatan Penggugat agar dijatuhkan talak satu ba’in sughra Tergugat terhadap Penggugat dapat dikabulkan;

Menimbang pula, bahwa walaupun perceraian merupakan perbuatan halal yang sangat dimurkai Allah, akan tetapi karena keadaan rumah tangga Penggugat dan Tergugat sudah terbukti tidak harmonis, telah pecah, dan telah memenuhi alasan perceraian, maka perceraian merupakan jalan keluar dari keadaan tersebut dan jalan untuk menghilangkan mafsadat yang lebih besar bagi Penggugat dan Tergugat;

Menimbang, bahwa oleh karena Tergugat tidak pernah hadir dan tidak menyuruh orang lain sebagai wakil/kuasanya meskipun telah dipanggil secara sah dan patut sedang gugatan Penggugat beralasan dan tidak melawan hukum, maka sesuai pasal 125 HIR gugatan Penggugat diputus tanpa hadirnya Tergugat. (verstek) ;

Menimbang, bahwa oleh karena perkara ini menyangkut bidang perkawinan, maka sesuai dengan Pasal 89 Ayat (1) Undang-Undang Nomor 7 Tahun 1989 Tentang Peradilan Agama yang telah diubah dengan Undang-Undang Nomor 3 Tahun 2006 dan perubahan ke dua dengan Undang Undang Nomor 50 Tahun 2009, maka biaya perkara dibebankan kepada Penggugat ;

Mengingat ketentuan peraturan perundang-undangan yang berlaku dan hukum syara’ yang berkaitan dengan perkara ini ;

Disclaimer
Kepaniteraan Mahkamah Agung Republik Indonesia berusaha untuk selalu mencantumkan informasi paling kini dan akurat sebagai bentuk komitmen Mahkamah Agung untuk pelayanan publik, transparansi dan akuntabilitas pelaksanaan fungsi peradilan. Namun dalam hal-hal tertentu masih dimungkinkan terjadi permasalahan teknis terkait dengan akurasi dan keterkinian informasi yang kami sajikan, hal mana akan terus kami perbaiki dari waktu kewaktu.
Dalam hal Anda menemukan inakurasi informasi yang termuat pada situs ini atau informasi yang seharusnya ada, namun belum tersedia, maka harap segera hubungi Kepaniteraan Mahkamah Agung RI melalui :
Email : kepaniteraan@mahkamahagung.go.id Telp : 021-384 3348 (ext.318)

MENGADILI

1. Menyatakan Tergugat yang telah dipanggil secara resmi dan patut untuk menghadap di persidangan tidak hadir;
2. Mengabulkan gugatan Penggugat dengan verstek;
3. Menjatuhkan talak satu ba'in sughro Tergugat **(Xxxxxxxx)** terhadap Penggugat **(Xxxxxxxx)**.
4. Membebankan kepada Penggugat untuk membayar biaya perkara sejumlah Rp 371.000,00 (tiga ratus tujuh puluh satu ribu rupiah);

Demikian diputuskan dalam rapat permusyawaratan majelis hakim yang dilangsungkan pada hari Selasa tanggal 1 Desember 2020 Masehi, bertepatan dengan tanggal 15 Rabi'ul Akhir 1442 Hijriyah, oleh kami Drs. Amiruddin, S.H. sebagai Ketua Majelis, Drs. H. Muhammad Fatchan, M.A. dan Drs Muh. Mahfudz, masing-masing sebagai Hakim Anggota, dan pada hari itu juga putusan diucapkan dalam sidang terbuka untuk umum oleh Ketua Majelis tersebut dengan didampingi Hakim-Hakim Anggota tersebut dan dibantu oleh Muhammad Abdus Shobur, S.H., sebagai panitera pengganti serta dihadiri oleh Penggugat tanpa hadirnya Tergugat;

Hakim Anggota,	Ketua Majelis,
Drs. H. Muhammad Fatchan, M.A.	Drs. Amiruddin, S.H.
Hakim Anggota,	
Drs. Muh. Mahfudz	
	Panitera Pengganti,
	Muhammad Abdus Shobur, S.H.

Perincian Biaya Perkara :

1. Pendaftaran: Rp 30.000,00
2. Proses : Rp 75.000,00

Disclaimer
Kepaniteraan Mahkamah Agung Republik Indonesia berusaha untuk selalu mencantumkan informasi paling kini dan akurat sebagai bentuk komitmen Mahkamah Agung untuk pelayanan publik, transparansi dan akuntabilitas pelaksanaan fungsi peradilan. Namun dalam hal-hal tertentu masih dimungkinkan terjadi permasalahan teknis terkait dengan akurasi dan keterkinian informasi yang kami sajikan, hal mana akan terus kami perbaiki dari waktu kewaktu.
Dalam hal Anda menemukan inakurasi informasi yang termuat pada situs ini atau informasi yang seharusnya ada, namun belum tersedia, maka harap segera hubungi Kepaniteraan Mahkamah Agung RI melalui :
Email : kepaniteraan@mahkamahagung.go.id Telp : 021-384 3348 (ext.318)

3. PNBP : Rp 20.000,00
4. Panggilan : Rp 230.000,00
5. Redaksi : Rp 10.000,00
6. Meterai : Rp 6.000,00

J u m l a h : Rp 371.000,00

(tiga ratus tujuh puluh satu ribu rupiah);

Disclaimer
Kepaniteraan Mahkamah Agung Republik Indonesia berusaha untuk selalu mencantumkan informasi paling kini dan akurat sebagai bentuk komitmen Mahkamah Agung untuk pelayanan publik, transparansi dan akuntabilitas pelaksanaan fungsi peradilan. Namun dalam hal-hal tertentu masih dimungkinkan terjadi permasalahan teknis terkait dengan akurasi dan keterkinian informasi yang kami sajikan, hal mana akan terus kami perbaiki dari waktu kewaktu.
Dalam hal Anda menemukan inakurasi informasi yang termuat pada situs ini atau informasi yang seharusnya ada, namun belum tersedia, maka harap segera hubungi Kepaniteraan Mahkamah Agung RI melalui :
Email : kepaniteraan@mahkamahagung.go.id Telp : 021-384 3348 (ext.318)
