# Supplementary material for: Classification of divorce causes during the COVID-19 pandemic using convolutional neural networks
Source: PeerJ Comput Sci. 2022 Jun 30;8:e998. doi: 10.7717/peerj-cs.998 (PMC9299239; doi:10.7717/peerj-cs.998)
Supplement: Supplemental Information 5 [file peerj-cs-08-998-s005.zip › Masalah Ekonomi Dataset/Data ke-28.pdf]

P U T U S A N

Nomor 2048/Pdt.G/2020/PA.Sr.

بِسْمِ اللَّهِ الرَّحْمَنِ الرَّحِيمِ

DEMI KEADILAN BERDASARKAN KETUHANAN YANG MAHA ESA

Pengadilan Agama Sragen yang memeriksa dan mengadili perkara tertentu pada tingkat pertama dalam sidang Majelis Hakimtelah menjatuhkan putusan dalam perkara Cerai Gugat antara:

Penggugat, umur 56 tahun, NIK 3314117112690119, (Tempat Lahir Sragen, Tanggal Lahir 04 Juli 1964), agama Islam, pendidikan SD, pekerjaan Swasta, tempat kediaman di Dukuh Taraman Rt.01/Rw.01, Desa Taraman, Kecamatan Sidoharjo, Kabupaten Sragen, Propinsi Jawa Tengah, Kewarganegaraan Indonesia, dalam hal ini berdasarkan surat kuasa khusus tanggal 07 Nopember 2020 memberikan kuasa kepada Totok Sumanto, S.H. Advokat yang berkantor di Jl. Raya Sukowati Barat Km.3/30 Dukuh Ngepos, Desa Jetak, Kecamatan Sidoharjo, Kabupaten Sragen-Jawa Tengah sebagai **Penggugat;**

lawan

Tergugat, umur 56 tahun, agama Islam, pendidikan SLTP, pekerjaan Swasta, tempat kediaman di Dukuh Taraman Rt.01/Rw.01, Desa Taraman, Kecamatan Sidoharjo, Kabupaten Sragen, Propinsi Jawa Tengah, Kewarganegaraan Indonesia, sebagai **Tergugat**;

Pengadilan Agama tersebut;

Telah mempelajari surat-surat yang berkaitan dengan perkara ini;

Telah mendengar keterangan Penggugat dan para saksi di muka sidang;

Disclaimer
Kepaniteraan Mahkamah Agung Republik Indonesia berusaha untuk selalu mencantumkan informasi paling kini dan akurat sebagai bentuk komitmen Mahkamah Agung untuk pelayanan publik, transparansi dan akuntabilitas pelaksanaan fungsi peradilan. Namun dalam hal-hal tertentu masih dimungkinkan terjadi permasalahan teknis terkait dengan akurasi dan keterkinian informasi yang kami sajikan, hal mana akan terus kami perbaiki dari waktu kewaktu.
Dalam hal Anda menemukan inakurasi informasi yang termuat pada situs ini atau informasi yang seharusnya ada, namun belum tersedia, maka harap segera hubungi Kepaniteraan Mahkamah Agung RI melalui :
Email : kepaniteraan@mahkamahagung.go.id Telp : 021-384 3348 (ext.318)

DUDUK PERKARA

Bahwa Penggugat dalam surat gugatannnya tanggal 11 November 2020 telah mengajukan Cerai Gugat, yang telah terdaftar di Kepaniteraan Pengadilan Agama Sragen, dengan Nomor 2048/Pdt.G/2020/PA.Sr., tanggal 11 November 2020, dengan dalil-dalil pada pokoknya sebagai berikut :

1. Bahwa antara Penggugat dan Tergugat pada hari Kemis Wage, tanggal 24 Oktober 1985 telah melangsungkan pernikahan, seperti tersebut dari Kutipan Kutipan Akta Nikah Nomor: 304/13/X/1985, yang dikeluarkan oleh Kantor Urusan Agama (KUA) Kecamatan Sidoharjo, Kabupaten Sragen, Jawa Tengah. Dan sesaat setelah menikah Tergugat telah mengucapkan Sighot Ta'lik Talak yang bunyinya seperti di dalam Buku Nikah.
2. Bahwa setelah menikah Penggugat dan Tergugat hidup bersama berumah tangga di rumah Penggugat di Dukuh Taraman Rt.01/Rw.01, Desa Taraman, Kecamatan Sidoharjo, Kabupaten Sragen, Jawa Tengah sampai ± bulan Februari tahun 2017, setelah itu Penggugat dan Tergugat berpisah.
3. Bahwa dalam perkawinannya Penggugat berstatus perawan dan Tergugat berstatus jejaka dan dan telah melakukan hubungan suami istri (ba'da dukhul) dan telah di karuniai seorang anak nama: Winarno, lahir 28 April 1987, ikut Penggugat.
4. Bahwa rumah tangga Penggugat dan Tergugat semula baik dan harmonis akan tetapi sejak ± tahun 2000 terjadi permasalahan atau terjadi perselisihan dan pertengkaran terus menerus yang dikarenakan masalah yang antara lain yaitu:
   - Bahwa Tergugat jarang memberi nafkah kepada Penggugat
   - Bahwa ekonomi kurang
   - Bahwa Tergugat malas bekerja
5. Bahwa Penggugat sudah berusaha untuk bersabar, memperbaiki rumah tangganya agar menjadi keluarga yang bahagia

Disclaimer
Kepaniteraan Mahkamah Agung Republik Indonesia berusaha untuk selalu mencantumkan informasi paling kini dan akurat sebagai bentuk komitmen Mahkamah Agung untuk pelayanan publik, transparansi dan akuntabilitas pelaksanaan fungsi peradilan. Namun dalam hal-hal tertentu masih dimungkinkan terjadi permasalahan teknis terkait dengan akurasi dan keterkinian informasi yang kami sajikan, hal mana akan terus kami perbaiki dari waktu kewaktu.
Dalam hal Anda menemukan inakurasi informasi yang termuat pada situs ini atau informasi yang seharusnya ada, namun belum tersedia, maka harap segera hubungi Kepaniteraan Mahkamah Agung RI melalui :
Email : kepaniteraan@mahkamahagung.go.id Telp : 021-384 3348 (ext.318)

dan kekal juga sakinah, mawaddah, warohmah sebagaimana tujuan perkawinan akan tetapi tidak berhasil.

6. Bahwa Puncak permasalahan atau perselisihan dan pertengkaran Penggugat dan Tergugat tersebut pada ± bulan Februari tahun 2017, dimana Tergugat pergi meninggalkan Penggugat, sampai sekarang sudah ± 3 (tiga) tahun 9 (sembilan) bulan lamanya dan selama itu Tergugat tidak memperdulikan (tidak mengurusi) Penggugat dan juga tidak memberi nafkah kepada Penggugat. Tergugat pergi dari alamat tempat tinggal Tergugat terakhir yaitu di Dukuh Taraman Rt.01/Rw.01, Desa Taraman, Kecamatan Sidoharjo, Kabupaten Sragen, Jawa Tengah, sedangkan Penggugat di rumah Penggugat.

7. Bahwa mohon untuk Tergugat di panggil di alamat tempat tinggal terakhir Tergugat pergi meninggalkan Penggugat tersebut, seperti pada alamat tempat tinggal terakhir Tergugat tersebut di atas.

8. Bahwa Penggugat sudah berusaha untuk bersabar, menunggu dan mencari Tergugat akan tetapi belum berhasil, dan bahwa antara Penggugat dan Tergugat sudah tidak akan mungkin lagi dapat hidup rukun kembali sebagai suami istri.

9. Bahwa atas perbuatan Tergugat tersebut maka Penggugat tidak rela, sehingga Penggugat sangat menderita dan sudah tidak sanggup lagi menjadi istri Tergugat, maka demi kemaslakhatan kedua belah pihak lebih baik Penggugat mengajukan Cerai Gugat.

10. Bahwa berdasarkan hal-hal tersebut diatas, maka dalil-dalil permohonan Cerai Gugat Penggugat telah berdasarkan hukum dan telah beralasan hukum karena telah sesuai dengan pasal 39 ayat (2) Undang-undang Nomor 1 tahun 1974. jo. Pasal 116 huruf (b), (f) Kompilasi Hukum Islam Indonesia.

Atas dasar hal-hal tersebut diatas, Penggugat mohon kepada Bapak Ketua Pengadilan Agama Sragen berkenaan untuk memeriksa, memanggil orang-orang yang perlu dimintai keterangannya selanjutnya menjatuhkan putusan sebagai berikut:

Disclaimer
Kepaniteraan Mahkamah Agung Republik Indonesia berusaha untuk selalu mencantumkan informasi paling kini dan akurat sebagai bentuk komitmen Mahkamah Agung untuk pelayanan publik, transparansi dan akuntabilitas pelaksanaan fungsi peradilan. Namun dalam hal-hal tertentu masih dimungkinkan terjadi permasalahan teknis terkait dengan akurasi dan keterkinian informasi yang kami sajikan, hal mana akan terus kami perbaiki dari waktu kewaktu.
Dalam hal Anda menemukan inakurasi informasi yang termuat pada situs ini atau informasi yang seharusnya ada, namun belum tersedia, maka harap segera hubungi Kepaniteraan Mahkamah Agung RI melalui :
Email : kepaniteraan@mahkamahagung.go.id Telp : 021-384 3348 (ext.318)

PRIMER :

1. Mengabulkan gugatan Penggugat;
2. Menjatuhkan talak satu ba'in sughro dari Tergugat (Tergugat) terhadap Penggugat (Penggugat);
3. Membebankan biaya perkara menurut hukum;

SUBSIDER :

Mohon putusan yang seadil-adilnya;

Bahwa pada persidangan yang telah ditetapkan Penggugat telah datang menghadap di persidangan, sedangkan Tergugat tidak datang menghadap di persidangan dan tidak menyuruh orang lain untuk menghadap sebagai wakil/kuasanya yang sah, meskipun kepada Tergugat telah dipanggil secara resmi dan patut, sedangkan tidak ternyata bahwa tidak datangnya Tergugat tersebut disebabkan oleh suatu alasan yang sah;

Bahwa Majelis Hakim telah menasehati Penggugat agar berpikir untuk tidak bercerai dengan Tergugat, tetapi Penggugat tetap pada dalil-dalil gugatannya untuk bercerai dengan Tergugat;

Bahwa perkara ini tidak dapat dimediasi karena Tergugat tidak pernah datang menghadap meskipun telah dipanggil secara resmi dan patut, selanjutnya dimulai pemeriksaan dengan membacakan surat gugatan Penggugat yang maksud dan tujuannya tetap dipertahankan oleh Penggugat;

Bahwa untuk menguatkan dalil-dalil gugatannya, Penggugat telah mengajukan bukti berupa:

1.--- Surat:

a. Fotokopi Kartu Tanda Penduduk atas nama Penggugat NIK : 3314117112690119 tanggal 24-05-2016 yang dikeluarkan oleh Pemerintah Kabupaten Sragen, Provinsi Jawa Tengah. Bukti surat tersebut telah diberi meterai cukup dan telah dicocokkan dengan

Disclaimer
Kepaniteraan Mahkamah Agung Republik Indonesia berusaha untuk selalu mencantumkan informasi paling kini dan akurat sebagai bentuk komitmen Mahkamah Agung untuk pelayanan publik, transparansi dan akuntabilitas pelaksanaan fungsi peradilan. Namun dalam hal-hal tertentu masih dimungkinkan terjadi permasalahan teknis terkait dengan akurasi dan keterkinian informasi yang kami sajikan, hal mana akan terus kami perbaiki dari waktu kewaktu.
Dalam hal Anda menemukan inakurasi informasi yang termuat pada situs ini atau informasi yang seharusnya ada, namun belum tersedia, maka harap segera hubungi Kepaniteraan Mahkamah Agung RI melalui :
Email : kepaniteraan@mahkamahagung.go.id Telp : 021-384 3348 (ext.318)

aslinya yang ternyata sesuai, lalu oleh Majelis Hakimdiberi tanda P.1;

b. Fotokopi Kutipan Akta Nikah Nomor 304/13/X/1985 tanggal 24 Oktober 1985, yang dikeluarkan oleh Kantor Urusan Agama Kantor Urusan Agama (KUA) Kecamatan Sidoharjo, Kabupaten Sragen, Jawa Tengah. Bukti surat tersebut telah diberi meterai cukup dan telah dicocokkan dengan aslinya yang ternyata ternyata sesuai, lalu oleh Majelis Hakimdiberi tanda P.2;

2.-- Saksi:

1. Saksi pertama, umur 50 tahun, Agama Islam, Pekerjaan Swasta, tempat tinggal di Dukuh Taraman Rt.03/Rw.01, Desa Taraman, Kecamatan Sidoharjo, Kabupaten Sragen, Propinsi Jawa Tengah, Kewarganegaraan Indonesia, dibawah sumpahnya ia memberikan keterangan sebagai berikut :

- Bahwa saksi kenal dengan Penggugat dan Tergugat karena saksi sebagai adik ipar Penggugat;
- Bahwa Penggugat dengan Tergugat menikah pada 24 Oktober 1985, telah dikaruniai seorang anak;
- Bahwa setelah menikah Penggugat dengan Tergugat tinggal bersama di rumah Penggugat di Dukuh Taraman Rt.01/Rw.01, Desa Taraman, Kecamatan Sidoharjo, Kabupaten Sragen;
- Bahwa setahu saksi rumah tangga antara Penggugat dan Tergugat pada awalnya harmonis akan tetapi sejak tahun 2000 mulai tidak rukun lagi sering berselisih dan bertengkar masalah Tergugat jarang memberi nafkah kepada Penggugat, karena Tergugat malas bekerja, puncaknya pada Februari tahun 2017 Tergugat pergi meninggalkan Penggugat sehingga pisah rumah sampai sekarang sudah lebih dari 3 tahun lamanya;
- Bahwa sejak kejadian tersebut Tergugat sudah tidak pernah ada komunikasi layaknya suami isteri hingga sekarang;

Disclaimer
Kepaniteraan Mahkamah Agung Republik Indonesia berusaha untuk selalu mencantumkan informasi paling kini dan akurat sebagai bentuk komitmen Mahkamah Agung untuk pelayanan publik, transparansi dan akuntabilitas pelaksanaan fungsi peradilan. Namun dalam hal-hal tertentu masih dimungkinkan terjadi permasalahan teknis terkait dengan akurasi dan keterkinian informasi yang kami sajikan, hal mana akan terus kami perbaiki dari waktu kewaktu.
Dalam hal Anda menemukan inakurasi informasi yang termuat pada situs ini atau informasi yang seharusnya ada, namun belum tersedia, maka harap segera hubungi Kepaniteraan Mahkamah Agung RI melalui :
Email : kepaniteraan@mahkamahagung.go.id Telp : 021-384 3348 (ext.318)

- Bahwa saksi sebagai keluarga sudah berusaha mendamaikan mereka akan tetapi tidak berhasil;

2. Saksi Kedua, umur 41 tahun, Agama Islam, Pekerjaan Swasta, tempat tinggal di Dukuh Bugel RT 02 RW 10, Desa Tangkil, Kecamatan Sragen, Kabupaten Sragen, dibawah sumpahnya ia memberikan keterangan sebagai berikut :
   - Bahwa saksi kenal dengan Penggugat dan Tergugat karena saksi sebagai keponakanPenggugat;
   - Bahwa Penggugat dengan Tergugat menikah pada 24 Oktober 1985, telah dikaruniai seorang anak;
   - Bahwa setelah menikah Penggugat dengan Tergugat tinggal bersama di rumah Penggugat di Dukuh Taraman Rt.01/Rw.01, Desa Taraman, Kecamatan Sidoharjo, Kabupaten Sragen;
   - Bahwa setahu saksi rumah tangga antara Penggugat dan Tergugat pada awalnya harmonis akan tetapi sejak tahun 2000 mulai tidak rukun lagi sering berselisih dan bertengkar masalah Tergugat jarang memberi nafkah kepada Penggugat, karena Tergugat malas bekerja, puncaknya pada Februari tahun 2017 Tergugat pergi meninggalkan Penggugat sehingga pisah rumah sampai sekarang sudah lebih dari 3 tahun lamanya;
   - Bahwa sejak kejadian tersebut Tergugat sudah tidak pernah ada komunikasi layaknya suami isteri hingga sekarang;
   - Bahwa saksi sebagai keluarga sudah berusaha mendamaikan mereka akan tetapi tidak berhasil;

Menimbang, bahwa Penggugat telah mencukupkan bukti-buktinya di persidangan ;

Menimbang, bahwa Penggugat telah menyampaikan kesimpulannya secara lisan di persidangan yang pada pokoknya Penggugat tetap menghendaki perceraian serta Penggugat mohon agar Hakim segera menjatuhkan putusan yang seadil-adilnya ;

Disclaimer
Kepaniteraan Mahkamah Agung Republik Indonesia berusaha untuk selalu mencantumkan informasi paling kini dan akurat sebagai bentuk komitmen Mahkamah Agung untuk pelayanan publik, transparansi dan akuntabilitas pelaksanaan fungsi peradilan. Namun dalam hal-hal tertentu masih dimungkinkan terjadi permasalahan teknis terkait dengan akurasi dan keterkinian informasi yang kami sajikan, hal mana akan terus kami perbaiki dari waktu kewaktu.
Dalam hal Anda menemukan inakurasi informasi yang termuat pada situs ini atau informasi yang seharusnya ada, namun belum tersedia, maka harap segera hubungi Kepaniteraan Mahkamah Agung RI melalui :
Email : kepaniteraan@mahkamahagung.go.id Telp : 021-384 3348 (ext.318)

Selanjutnya untuk singkatnya uraian putusan ini, maka semua hal yang termuat dalam berita acara sidang merupakan bagian yang tidak terpisahkan dari putusan ini;

PERTIMBANGAN HUKUM

Menimbang, bahwa maksud dan tujuan gugatan Penggugat adalah sebagaimana terurai di atas;

Menimbang, bahwa pada hari-hari persidangan yang telah ditentukan, Penggugat telah datang dan menghadap sendiri di persidangan, sedangkan Tergugat tidak pernah datang dan menghadap di persidangan, dan tidak juga mengirimkan orang lain sebagai kuasanya yang sah, meskipun menurut berita acara relaas panggilan yang dibacakan di persidangan, ia telah dipanggil dengan sah dan patut, sesuai pasal 27 PP No. 9 tahun 1975, dan ketidak hadiran Tergugat tersebut oleh Majelis Hakim dinyatakan tidak disebabkan sesuatu alasan yang sah menurut hukum, oleh sebab itu pemeriksaan perkara ini dilangsungkan di luar hadirnya Tergugat, sesuai pasal 125 HIR, yakni diputus secara verstek;

Menimbang, bahwa Majelis Hakim berdasarkan pasal 82 Undang-undang Nomor 7 tahun 1989, telah berusaha menasihati Penggugat, supaya bersabar dan rukun kembali untuk membina rumah tangganya dengan Tergugat, akan tetapi tidak berhasil;

Menimbang, bahwa berdasarkan pasal 7 ayat (2) Peraturan Mahkamah Agung RI (PERMA), No. : 1 Tahun 2016 tentang Mediasi, mejelis hakim tidak dapat memberikan kesempatan untuk melakukan mediasi, karena Tergugat tidak pernah hadir, karena itu usaha perdamaian dinyatakan gagal ;

Menimbang bahwa gugatan Penggugat untuk bercerai dengan Tergugat yang pada pokoknya didasarkan kepada alasan, bahwa rumah tangga Penggugat dan Tergugat awalnya baik dan rukun namun sejak tahun 2000 mulai tidak rukun sering berselisih dan bertengkar masalah Tergugat jarang memberi nafkah kepada Penggugat, karena Tergugat malas bekerja, puncaknya pada Februari tahun 2017 PTergugat pergi

Disclaimer
Kepaniteraan Mahkamah Agung Republik Indonesia berusaha untuk selalu mencantumkan informasi paling kini dan akurat sebagai bentuk komitmen Mahkamah Agung untuk pelayanan publik, transparansi dan akuntabilitas pelaksanaan fungsi peradilan. Namun dalam hal-hal tertentu masih dimungkinkan terjadi permasalahan teknis terkait dengan akurasi dan keterkinian informasi yang kami sajikan, hal mana akan terus kami perbaiki dari waktu kewaktu.
Dalam hal Anda menemukan inakurasi informasi yang termuat pada situs ini atau informasi yang seharusnya ada, namun belum tersedia, maka harap segera hubungi Kepaniteraan Mahkamah Agung RI melalui :
Email : kepaniteraan@mahkamahagung.go.id Telp : 021-384 3348 (ext.318)

meninggalkan Penggugat sehingga pisah rumah tanpa saling berkomunikasi lagi sampai sekarang sudah selama lebih dari 3 tahun lamanya;

Menimbang, bahwa atas surat gugatan Penggugat tersebut, Tergugat tidak mengajukan jawabannya, karena tidak hadir di persidangan.

Menimbang, bahwa meskipun Tergugat tidak hadir, karena perkara ini termasuk ruang lingkup sengketa perkawinan, maka kepada Penggugat tetap dibebani wajib bukti.

Menimbang, bahwa dalam perkara ini Penggugat telah mengajukan bukti tertulis (P-1), (P-2) dan (P-3) yang telah memenuhi formil materiil sebagai alat bukti dan dua orang saksi dan kedua orang saksi tersebut telah disumpah sesuai dengan agamanya, oleh sebab itu keterangan saksi tersebut adalah formal dan dapat dijadikan bukti dalam perkara ini;

Menimbang, bahwa berdasarkan bukti (P-1) terbukti Penggugat bertempat tinggal di wilayah hukum Pengadilan Agama Sragen maka adalah tepat dan beralasan Penggugat mengajukan gugatannya ke Pengadilan Agama Sragen (pasal 73 Undang-undang Nomor 7 tahun 1989 yang telah diubah dan ditambah dengan Undang-undang Nomor 3 tahun 2006 dan Undang-undang Nomor 50 tahun 2009 tentang Peradilan Agama);

Menimbang, bahwa surat bukti (P.2) berupa Fotocopy sah Kutipan Akta Nikah adalah merupaka alat bukti autentik karena dibuat berdasarkan Peraturan Perundang-undangan oleh Pejabat yang berwenang, sehingga mempunyai kekuatan pembuktian sempurna selama tidak dibuktikan kepalsuannya (pasal 165 HIR);

Menimbang, bahwa berdasarkan pasal 7 ayat 1 Kompilasi Hukum Islam yang menyatakan bahwa perkawinan hanya dapat dibuktikan dengan Akta Nikah yang dibuat oleh Pegawai Pencatat Nikah, maka harus dinyatakan terbukti bahwa antara Penggugat dan Tergugat telah terikat dengan perkawinan yang sah, oleh karena itu Penggugat mempunyai alasan hukum untuk mengajukan gugatan ini;

Disclaimer
Kepaniteraan Mahkamah Agung Republik Indonesia berusaha untuk selalu mencantumkan informasi paling kini dan akurat sebagai bentuk komitmen Mahkamah Agung untuk pelayanan publik, transparansi dan akuntabilitas pelaksanaan fungsi peradilan. Namun dalam hal-hal tertentu masih dimungkinkan terjadi permasalahan teknis terkait dengan akurasi dan keterkinian informasi yang kami sajikan, hal mana akan terus kami perbaiki dari waktu kewaktu.
Dalam hal Anda menemukan inakurasi informasi yang termuat pada situs ini atau informasi yang seharusnya ada, namun belum tersedia, maka harap segera hubungi Kepaniteraan Mahkamah Agung RI melalui :
Email : kepaniteraan@mahkamahagung.go.id Telp : 021-384 3348 (ext.318)

Menimbang, bahwa berdasarkan bukti (P-3) terbukti Tergugat benar warga setempat namun sekarang sudah tidak diketahui tempat tinggalnya di seluruh wilayah RI selama lebih dari lebih dari 3 tahun lamanya;

Menimbang, bahwa saksi-saksi adalah orang-orang yang dekat dengan kedua pihak yang diajukan telah memberikan keterangan lisan di persidangan dibawah sumpahnya, keterangan mana berdasarkan peristiwa yang dialamai sendiri, saling berhubungan satu dengan yang lain dan mendukung dalil-dalil gugatan Penggugat yang pada pokoknya benar rumah tangga Penggugat dan Tergugat sekurang-kurangnya sejak tahun 2000 mulai tidak rukun sering berselisih dan bertengkar masalah Tergugat jarang memberi nafkah kepada Penggugat, karena Tergugat malas bekerja, puncaknya pada Februari tahun 2017 PTergugat pergi meninggalkan Penggugat sehingga pisah rumah tanpa saling berkomunikasi lagi sampai sekarang sudah selama lebih dari 3 tahun lamanya;

Menimbang, bahwa berdasarkan bukti (P-1), (P-2) dan (P-3) serta keterangan dua orang saksi yang saling bersesuaian, terungkap fakta sebagai berikut :

- Bahwa Penggugat dan Tergugat adalah suami isteri sah, menikah pada tanggal 24 Oktober 1985, dalam keadaan bakda dukhul, telah dikaruniai seorang anak;
- Bahwa setelah menikah Penggugat dan Tergugat tinggal bersama di rumah Penggugat di Dukuh Taraman Rt.01/Rw.01, Desa Taraman, Kecamatan Sidoharjo, Kabupaten Sragen;
- Bahwa rumah tangga Penggugat dan Tergugat awalnya baik dan rukun akan tetapi sejak tahun 2000 mulai tidak rukun sering berselisih dan bertengkar masalah Tergugat jarang memberi nafkah kepada Penggugat, karena Tergugat malas bekerja, puncaknya pada Februari tahun 2017 Tergugat pergi meninggalkan Penggugat sehingga pisah rumah tanpa saling berkomunikasi lagi sampai sekarang sudah selama lebih dari 3 tahun lamanya;
- Bahwa selama berpisah kedua pihak sudah tidak saling berkomunikasi layaknya suami isteri;

Disclaimer
Kepaniteraan Mahkamah Agung Republik Indonesia berusaha untuk selalu mencantumkan informasi paling kini dan akurat sebagai bentuk komitmen Mahkamah Agung untuk pelayanan publik, transparansi dan akuntabilitas pelaksanaan fungsi peradilan. Namun dalam hal-hal tertentu masih dimungkinkan terjadi permasalahan teknis terkait dengan akurasi dan keterkinian informasi yang kami sajikan, hal mana akan terus kami perbaiki dari waktu kewaktu.
Dalam hal Anda menemukan inakurasi informasi yang termuat pada situs ini atau informasi yang seharusnya ada, namun belum tersedia, maka harap segera hubungi Kepaniteraan Mahkamah Agung RI melalui :
Email : kepaniteraan@mahkamahagung.go.id Telp : 021-384 3348 (ext.318)

- Bahwa keluarga sudah berusaha mendamaikan kedua pihak tetapi tidak berhasil;

Menimbang, bahwa berdasarkan fakta tersebut diatas terbukti rumah tangga antara Penggugat dan Tergugat telah pecah dan sulit dirukunkan kembali karena selalu diwarnai perselisihan dan pertengkaran terus menerus yang sulit didamaikan disebabkan masalah Tergugat jarang memberi nafkah kepada Penggugat, karena Tergugat malas bekerja, sehingga pisah rumah tanpa komunikasi lagi layaknya suami isteri sampai sekarang selama lebih dari 3 tahun lamanya

Menimbang, bahwa oleh karena rumah tangga Penggugat dan Tergugat telah nyata pecah, maka apabila perkawinan antara Penggugat dan Tergugat tersebut tetap dipertahankan niscaya akan menimbulkan madlarat yang lebih besar bagi kedua belah pihak atau salah satu pihak ;

Menimbang, bahwa Majelis sependapat dengan pendapat Fuqaha dalam kitab Fiqhus Sunnah Juz II hal. 291 yang berbunyi :

اذا ادعت الزوجة اضرار الزوج بها بما لا يستطاع معه دوام العشرة بين امثا لهما يجوز لها ان تطلب من القاضى التفريق وحينئذ يطلقها القاضى طلقة بائنة لوثبت الضرر وعجز عن الاصلاح بينهما

artinya ; “Apabila isteri menggugat kemadlorotan suami karena tidak dapat melangsungkan kehidupan berkeluarga diantara keduanya, isteri boleh meminta kepada Hakim untuk dipisahkan/ diceraikan seketika itu juga, maka Hakim dapat menjatuhkan thalaknya dengan Thalak Bain, apabila terbukti kemadlorotan tersebut dan tidak tercapainya perdamaian diantara keduanya”;

Menimbang, bahwa berdasarkan pertimbangan tersebut diatas, maka gugatan Penggugat cukup beralasan, karena telah memenuhi pasal 39 ayat (2) Undang-undang Nomor 1 tahun 1974 jo. Pasal 19 huruf (f) Peraturan Pemerintah No. 9 tahun 1975 jo. Pasal 116 huruf (f) Kompilasi Hukum Islam, maka sesuai dengan pasal 19 angka (2) huruf (c) Kompilasi Hukum Islam gugatan Penggugat tersebut patut dikabulkan dengan

Disclaimer
Kepaniteraan Mahkamah Agung Republik Indonesia berusaha untuk selalu mencantumkan informasi paling kini dan akurat sebagai bentuk komitmen Mahkamah Agung untuk pelayanan publik, transparansi dan akuntabilitas pelaksanaan fungsi peradilan. Namun dalam hal-hal tertentu masih dimungkinkan terjadi permasalahan teknis terkait dengan akurasi dan keterkinian informasi yang kami sajikan, hal mana akan terus kami perbaiki dari waktu kewaktu.
Dalam hal Anda menemukan inakurasi informasi yang termuat pada situs ini atau informasi yang seharusnya ada, namun belum tersedia, maka harap segera hubungi Kepaniteraan Mahkamah Agung RI melalui :
Email : kepaniteraan@mahkamahagung.go.id Telp : 021-384 3348 (ext.318)

dijatuhkannya talak ba'in sughro Tergugat kepada Penggugat, dengan verstek (pasal 125 HIR);

Menimbang, bahwa perkara ini termasuk dalam bidang perkawinan maka berdasarkan pasal 89 ayat 1 Undang-undang Nomor 7 tahun 1989 yang telah diubah dengan Undang-undang nomor: 3 tahun 2006 dan diubah dengan Undang-undang nomor: 50 tahun 2009 tentang Peradilan Agama, maka kepada Penggugat dihukum membayar biaya perkara;

Memperhatikan ketentuan Hukum Islam dan peraturan perundang-undangan yang berkaitan dengan perkara ini;

MENGADILI

1. Menyatakan Tergugat yang telah dipanggil secara resmi dan patut untuk menghadap di persidangan tidak hadir;

2. Mengabulkan gugatan Penggugat secara verstek;

3. Menjatuhkan talak satu ba'in sughro Tergugat (Tergugat) terhadap Penggugat (Penggugat);

4. Membebankan kepada Penggugat untuk membayar biaya perkara sejumlah Rp 271.000,00 (dua ratus tujuh puluh satu ribu rupiah);

Demikian diputuskan dalam rapat musyawarah Majelis Hakim Pengadilan Agama Sragen pada hari Selasa tanggal 01 Desember 2020 Masehi, bertepatan dengan tanggal 15 Rabiul Akhir 1442 Hijriah, oleh kami Drs. H. Muhd. Jazuli sebagai Hakim Ketua, Drs.Jayin.S.H dan Hadi Suyoto, S. Ag., M. Hum sebagai Hakim Anggota, putusan tersebut diucapkan pada hari itu juga oleh Hakim Ketua tersebut dalam sidang terbuka untuk umum, dihadiri para Hakim Anggota dan Suminah, S.H., M.H. sebagai Panitera Pengganti serta dihadiri oleh Penggugat tanpa hadirnya Tergugat;

Ketua Majelis,

Disclaimer
Kepaniteraan Mahkamah Agung Republik Indonesia berusaha untuk selalu mencantumkan informasi paling kini dan akurat sebagai bentuk komitmen Mahkamah Agung untuk pelayanan publik, transparansi dan akuntabilitas pelaksanaan fungsi peradilan. Namun dalam hal-hal tertentu masih dimungkinkan terjadi permasalahan teknis terkait dengan akurasi dan keterkinian informasi yang kami sajikan, hal mana akan terus kami perbaiki dari waktu kewaktu.
Dalam hal Anda menemukan inakurasi informasi yang termuat pada situs ini atau informasi yang seharusnya ada, namun belum tersedia, maka harap segera hubungi Kepaniteraan Mahkamah Agung RI melalui :
Email : kepaniteraan@mahkamahagung.go.id Telp : 021-384 3348 (ext.318)

Drs. H. Muhd. Jazuli

Hakim Anggota Hakim Anggota

Drs. Jayin, S.H. **Hadi Suyoto, S. Ag., M.Hum.**

Panitera Pengganti,

Suminah, S.H., M.H.

Rincian Biaya Perkara ;

1.	Pendaftaran	: Rp 30.000,00
2.	ATK Perkara: Rp	75.000,00
3.	Panggilan	: Rp 140.000,00
4.	PNBP Panggilan	: Rp 20.000,00
5.	Redaksi	: Rp 10.000,00
6.	Meterai	: Rp 6.000,00

J u m l a h : Rp 271.000,00

(dua ratus tujuh puluh satu ribu rupiah).

Disclaimer
Kepaniteraan Mahkamah Agung Republik Indonesia berusaha untuk selalu mencantumkan informasi paling kini dan akurat sebagai bentuk komitmen Mahkamah Agung untuk pelayanan publik, transparansi dan akuntabilitas pelaksanaan fungsi peradilan. Namun dalam hal-hal tertentu masih dimungkinkan terjadi permasalahan teknis terkait dengan akurasi dan keterkinian informasi yang kami sajikan, hal mana akan terus kami perbaiki dari waktu kewaktu.
Dalam hal Anda menemukan inakurasi informasi yang termuat pada situs ini atau informasi yang seharusnya ada, namun belum tersedia, maka harap segera hubungi Kepaniteraan Mahkamah Agung RI melalui :
Email : kepaniteraan@mahkamahagung.go.id Telp : 021-384 3348 (ext.318)
