# Supplementary material for: Classification of divorce causes during the COVID-19 pandemic using convolutional neural networks
Source: PeerJ Comput Sci. 2022 Jun 30;8:e998. doi: 10.7717/peerj-cs.998 (PMC9299239; doi:10.7717/peerj-cs.998)
Supplement: Supplemental Information 5 [file peerj-cs-08-998-s005.zip › Masalah Ekonomi Dataset/Data ke-29.pdf]

P U T U S A N

Nomor 2091/Pdt.G/ 2020/PA Sr

بسم الله الرحمن الرحيم

DEMI KEADILAN BERDASARKAN KETUHANAN YANG MAHA ESA

Pengadilan Agama Sragen yang memeriksa dan mengadili perkara-perkara tertentu dalam tingkat pertama telah menjatuhkan putusan dalam perkara yang diajukan oleh :

Xxxxxxxxxxx, NIK 3312035604880003, tempat lahir di Wonogiri, tanggal 06 April 1988 (umur 32 tahun), No HP: 081228790994, agama Islam, pendidikan SLTP/Sederajat, Pekerjaan Asisten Rumah Tangga, tempat tinggal di Dukuh Tegalrejo RT 023, Desa Kadipiro, Kecamatan Sambirejo, Kabupaten Sragen, selanjutnya disebut sebagai Penggugat;

melawan

Xxxxxxxxxxx, NIK -, tempat lahir di Karanganyar, tanggal 11 Juli 1987 (umur 33 tahun), No HP -, agama Islam, pendidikan SLTA/Sederajat, Pekerjaan Sopir Truck, tempat tinggal di Dukuh Tegalrejo RT 023, Desa Kadipiro, Kecamatan Sambirejo, Kabupaten Sragen, selanjutnya disebut sebagai Tergugat;

Pengadilan Agama tersebut ;

Telah membaca dan mempelajari berkas perkara yang bersangkutan ;

Telah mendengar keterangan Penggugat dan saksi-saksi Penggugat di persidangan ;

DUDUK PERKARANYA

Menimbang, bahwa Penggugat telah mengajukan gugatan cerai tanggal 16 Nopember 2020, dan telah terdaftar di Kepaniteraan Pengadilan

Disclaimer
Kepaniteraan Mahkamah Agung Republik Indonesia berusaha untuk selalu mencantumkan informasi paling kini dan akurat sebagai bentuk komitmen Mahkamah Agung untuk pelayanan publik, transparansi dan akuntabilitas pelaksanaan fungsi peradilan. Namun dalam hal-hal tertentu masih dimungkinkan terjadi permasalahan teknis terkait dengan akurasi dan keterkinian informasi yang kami sajikan, hal mana akan terus kami perbaiki dari waktu kewaktu.
Dalam hal Anda menemukan inakurasi informasi yang termuat pada situs ini atau informasi yang seharusnya ada, namun belum tersedia, maka harap segera hubungi Kepaniteraan Mahkamah Agung RI melalui :
Email : kepaniteraan@mahkamahagung.go.id Telp : 021-384 3348 (ext.318)

Agama tersebut dengan register perkara Nomor 2091/Pdt.G/2020/PA Sr. tanggal 16 Nopember 2020 yang isinya sebagai berikut :

1. Bahwa antara Penggugat dan Tergugat adalah suami istri yang telah melangsungkan perkawinan secara sah pada tanggal 24 April 2008, dihadapan Pegawai Pencatat Nikah Kantor Urusan Agama (KUA) Kecamatan Giriwoyo Kabupaten Wonogiri, sebagaimana tercantum dalam Buku Kutipan Akta Nikah Nomor : 108/33/IV/2008 yang dikeluarkan oleh Kantor Urusan Agama Kecamatan Giriwoyo Kabupaten Wonogiri tertanggal 24 April 2008 dalam status Jejaka dan Perawan;
2. Bahwa setelah menikah Penggugat dan Tergugat pernah merasakan kehidupan berumah tangga yang bahagia dan dikaruniai 2 (dua) orang anak yang bernama:
   - **Niken Adelia Putri**, jenis kelamin perempuan umur 9 tahun;
   - **Octaviana Jasmine Marfiza,** jenis kelamin perempuan umur 5 tahun;
3. Bahwa setelah menikah Penggugat dan Tergugat kumpul membina rumah tangga di rumah orangtua Penggugat di Tegalrejo RT 024, Desa Kadipiro, Kecamatan Sambirejo, Kabupaten Sragen selama kurang lebih 5 tahun kemudian di rumah bersama Tegalrejo RT 023, Desa Kadipiro, Kecamatan Sambirejo, Kabupaten Sragen selama kurang lebih 6 tahun ;
4. Bahwa sejak bulan Januari tahun 2019 rumah tangga Penggugat dan Tergugat mulai goyah,dan sering terjadi perselisihan dan pertengkaran terus-menerus yang sulit untuk didamaikan, hal ini disebabkan:
   - Bahwa Tergugat sering menyakiti badan/jasmani Penggugat;
   - Bahwa Tergugat telah lalai memberikan nafkah wajibnya (ekonomi) pada Penggugat kurang lebih 1 tahun ;
   - Bahwa Penggugat pernah di usir oleh Tergugat dari rumah kediaman bersama bila tidak pergi Penggugat diancam di bunuh;

Disclaimer
Kepaniteraan Mahkamah Agung Republik Indonesia berusaha untuk selalu mencantumkan informasi paling kini dan akurat sebagai bentuk komitmen Mahkamah Agung untuk pelayanan publik, transparansi dan akuntabilitas pelaksanaan fungsi peradilan. Namun dalam hal-hal tertentu masih dimungkinkan terjadi permasalahan teknis terkait dengan akurasi dan keterkinian informasi yang kami sajikan, hal mana akan terus kami perbaiki dari waktu kewaktu.
Dalam hal Anda menemukan inakurasi informasi yang termuat pada situs ini atau informasi yang seharusnya ada, namun belum tersedia, maka harap segera hubungi Kepaniteraan Mahkamah Agung RI melalui :
Email : kepaniteraan@mahkamahagung.go.id Telp : 021-384 3348 (ext.318)

5. Bahwa puncak perselisihan dan pertengkaran tersebut mengakibatkan Penggugat dengan Tergugat berpisah tempat kediaman bersama selama kurang lebih 1 tahun 1 bulan terhitung sejak bulan Oktober tahun 2019, Penggugat saat ini tinggal di rumah bulik nya bernama Atun Bebek yang beralamat di Tegalrejo RT 002 RW 002, Kelurahan Jebres, Kecamatan Jebres, Kota Surakarta dan Tergugat di Dukuh Tegalrejo RT 023, Desa Kadipiro, Kecamatan Sambirejo, Kabupaten Sragen;
6. Bahwa dengan keadaan rumah tangga tersebut di atas Penggugat sudah tidak sanggup lagi meneruskan perkawinan dengan Tergugat, karena dikhawatirkan akan menimbulkan permasalahan yang berkepanjangan;
7. Bahwa Penguggat telah berusaha mempertahankan keutuhan rumah tangga dengan meminta nasihat baik kepada keluarga Penggugat dan Tergugat maupun kepada pemuka agama, akan tetapi usaha tersebut tidak berhasil, maka Penggugat ingin bercerai saja;

Bahwa berdasarkan hal-hal tersebut diatas, gugatan Penggugat telah memenuhi alasan perceraian, maka Penggugat mohon kepada Yth Bapak Ketua Pengadilan Agama Sragen agar menerima, memanggil, memeriksa dan mengadili dan menjatuhkan putusan sebagai berikut :

1. Mengabulkan gugatan Penggugat;
2. Menyatakan jatuh talak satu bain shugro Tergugat (**Xxxxxxxxxxx**) terhadap Penggugat (**Xxxxxxxxxxx**);
3. Membebankan seluruh biaya perkara menurut hukum;

Apabila majelis hakim berpendapat lain, mohon putusan yang seadil-adilnya;

Menimbang, bahwa pada hari sidang yang telah ditetapkan Penggugat hadir sendiri di persidangan, sedang Tergugat tidak pernah hadir meskipun telah dipanggil secara sah dan patut dan tidak ternyata bahwa ketidakhadiran Tergugat disebabkan suatu halangan yang sah ;

Disclaimer
Kepaniteraan Mahkamah Agung Republik Indonesia berusaha untuk selalu mencantumkan informasi paling kini dan akurat sebagai bentuk komitmen Mahkamah Agung untuk pelayanan publik, transparansi dan akuntabilitas pelaksanaan fungsi peradilan. Namun dalam hal-hal tertentu masih dimungkinkan terjadi permasalahan teknis terkait dengan akurasi dan keterkinian informasi yang kami sajikan, hal mana akan terus kami perbaiki dari waktu kewaktu.
Dalam hal Anda menemukan inakurasi informasi yang termuat pada situs ini atau informasi yang seharusnya ada, namun belum tersedia, maka harap segera hubungi Kepaniteraan Mahkamah Agung RI melalui :
Email : kepaniteraan@mahkamahagung.go.id Telp : 021-384 3348 (ext.318)

Menimbang, bahwa selanjutnya pemeriksaan dimulai dengan membacakan gugatan Penggugat dan atas pertanyaan Majelis Hakim Penggugat tetap pada gugatan Penggugat tanpa ada perubahan;

Menimbang, bahwa untuk menguatkan dalil-dalil gugatannya Penggugat telah mengajukan bukti-bukti sebagai berikut :

A. Bukti Tertulis :

1. Fotokopi Kartu Tanda Penduduk an. Penggugat Nomor 3314086501830001, yang dikeluarkan oleh Kepala Dinas Kependudukan dan Pencatatan Sipil Kabupaten Sragen, alat bukti tersebut bermeterai cukup dan telah dicocokkan ternyata sesuai dengan aslinya, diberi kode P1
2. Fotokopi Kutipan Akta Nikah Nomor 344/08/X/2010 tertanggal 3 Oktober 2010 yang dikeluarkan oleh Kantor Urusan Agama Kecamatan Ngrampal, Kabupaten Sragen, alat bukti tersebut bermeterai cukup dan telah dicocokkan ternyata sesuai dengan aslinya, diberi kode P2;

B. Bukti Saksi :

1. Supriyatun binti Wagino, umur 47 tahun, agama Islam, pekerjaan pedagang, tempat kediaman di Tegalrejo Rt.002 Rw.002, Kelurahan Tegalrejo, Kecamatan Jebres, Kota Surakarta, setelah bersumpah saksi telah memberikan keterangan yang pada pokoknya sebagai berikut :
   - Bahwa saksi kenal dengan Penggugat dan Tergugat karena saksi sebagai bibi Penggugat;
   - Bahwa Penggugat dan Tergugat adalah suami isteri;
   - Bahwa setelah menikah Penggugat dan Tergugat tinggal di rumah bersama, dikaruniai anak 2 orang;
   - Bahwa sejak tahun 2019 Penggugat dan Tergugat telah terjadi perselisihan dan pertengkaran yang disebabkan

Disclaimer
Kepaniteraan Mahkamah Agung Republik Indonesia berusaha untuk selalu mencantumkan informasi paling kini dan akurat sebagai bentuk komitmen Mahkamah Agung untuk pelayanan publik, transparansi dan akuntabilitas pelaksanaan fungsi peradilan. Namun dalam hal-hal tertentu masih dimungkinkan terjadi permasalahan teknis terkait dengan akurasi dan keterkinian informasi yang kami sajikan, hal mana akan terus kami perbaiki dari waktu kewaktu.
Dalam hal Anda menemukan inakurasi informasi yang termuat pada situs ini atau informasi yang seharusnya ada, namun belum tersedia, maka harap segera hubungi Kepaniteraan Mahkamah Agung RI melalui :
Email : kepaniteraan@mahkamahagung.go.id Telp : 021-384 3348 (ext.318)

Tergugat sering menyakiti badan Penggugat, tidak memberi nafkah kepada Penggugat, dan pernah mengancam mau membunuh Penggugat;

- Bahwa sejak tahun 2019 Penggugat dan Tergugat telah berpisah tempat tinggal Penggugat yang pergi meninggalkan Tergugat ke rumah bibi Penggugat sampai sekarang selama 1 tahun lebih tidak pernah bertemu lagi dan tidak ada komunikasi;
- Bahwa Penggugat dan tergugat sudah pernah dirukunkan tetapi tidak berhasil;

2. Tri Wandoko bin Suradi, umur 29 tahun, agama Islam, pekerjaan swasta , tempat kediaman di Kampung Tawangsari Rt.01 Rw.34, Kelurahan Mojosongo, Kecamatan Jebres, Kota Surakarta, setelah bersumpah saksi telah memberikan keterangan yang pada pokoknya sebagai berikut :
    - Bahwa saksi kenal dengan Penggugat dan Tergugat karena saksi sebagai tetangga Penggugat;
    - Bahwa Penggugat dan Tergugat adalah suami isteri;
    - Bahwa setelah menikah Penggugat dan Tergugat tinggal di rumah bersama, dikaruniai anak 2 orang;
    - Bahwa sejak tahun 2019 Penggugat dan Tergugat telah terjadi perselisihan dan pertengkaran yang disebabkan Tergugat sering menyakiti badan Penggugat, tidak memberi nafkah kepada Penggugat, dan pernah mengancam mau membunuh Penggugat;
    - Bahwa sejak tahun 2019 Penggugat dan Tergugat telah berpisah tempat tinggal Penggugat yang pergi meninggalkan Tergugat ke rumah bibi Penggugat sampai sekarang selama 1 tahun lebih tidak pernah bertemu lagi dan tidak ada komunikasi;
    - Bahwa Penggugat dan tergugat sudah pernah dirukunkan tetapi tidak berhasil;

Disclaimer
Kepaniteraan Mahkamah Agung Republik Indonesia berusaha untuk selalu mencantumkan informasi paling kini dan akurat sebagai bentuk komitmen Mahkamah Agung untuk pelayanan publik, transparansi dan akuntabilitas pelaksanaan fungsi peradilan. Namun dalam hal-hal tertentu masih dimungkinkan terjadi permasalahan teknis terkait dengan akurasi dan keterkinian informasi yang kami sajikan, hal mana akan terus kami perbaiki dari waktu kewaktu.
Dalam hal Anda menemukan inakurasi informasi yang termuat pada situs ini atau informasi yang seharusnya ada, namun belum tersedia, maka harap segera hubungi Kepaniteraan Mahkamah Agung RI melalui :
Email : kepaniteraan@mahkamahagung.go.id Telp : 021-384 3348 (ext.318)

Menimbang, bahwa selanjutnya Penggugat telah memberikan kesimpulan tetap pada gugatan Penggugat dan mohon putusan ;

Menimbang, bahwa untuk menyingkat uraian dalam putusan ini, maka segala sesuatu yang tercantum dalam Berita Acara Persidangan perkara ini dianggap telah masuk dan merupakan bagian yang tidak terpisahkan dari putusan ini ;

PERTIMBANGAN HUKUM

Menimbang, bahwa maksud dan tujuan gugatan Penggugat adalah seperti tersebut di atas ;

Menimbang, bahwa dari bukti kode P1 yang merupakan bukti otentik, dan dapat diterima sebagai bukti, maka terbukti bahwa Penggugat bertempat tinggal di wilayah Kabupaten Sragen, sehingga sesuai Pasal 73 Ayat 1 Undang-Undang Nomor 7 Tahun 1989 tentang Peradilan Agama, Pengadilan Agama Sragen berwenang mengadili perkara tersebut dan gugatan Penggugat aquo formil dapat diterima;

Menimbang, bahwa dari bukti kode P2 yang merupakan bukti otentik dan dapat diterima sebagai bukti, maka terbukti bahwa Penggugat dan Tergugat adalah suami isteri yang sah, oleh karena itu gugatan Penggugat terhadap Tergugat telah memiliki dasar hukum yang sah;

Menimbang, bahwa Penggugat dalam gugatannya telah mendalilkan hal-hal yang pada pokoknya sebagai berikut :

- Bahwa sejak bulan Januari tahun 2019 rumah tangga Penggugat dan Tergugat mulai goyah,dan sering terjadi perselisihan dan pertengkaran terus-menerus yang sulit untuk didamaikan, hal ini disebabkan:
  - Bahwa Tergugat sering menyakiti badan/jasmani Penggugat;
  - Bahwa Tergugat telah lalai memberikan nafkah wajibnya (ekonomi) pada Penggugat kurang lebih 1 tahun ;
  - Bahwa Penggugat pernah di usir oleh Tergugat dari rumah kediaman bersama bila tidak pergi Penggugat diancam di bunuh;
- Bahwa puncak perselisihan dan pertengkaran tersebut mengakibatkan Penggugat dengan Tergugat berpisah tempat kediaman bersama

Disclaimer
Kepaniteraan Mahkamah Agung Republik Indonesia berusaha untuk selalu mencantumkan informasi paling kini dan akurat sebagai bentuk komitmen Mahkamah Agung untuk pelayanan publik, transparansi dan akuntabilitas pelaksanaan fungsi peradilan. Namun dalam hal-hal tertentu masih dimungkinkan terjadi permasalahan teknis terkait dengan akurasi dan keterkinian informasi yang kami sajikan, hal mana akan terus kami perbaiki dari waktu kewaktu.
Dalam hal Anda menemukan inakurasi informasi yang termuat pada situs ini atau informasi yang seharusnya ada, namun belum tersedia, maka harap segera hubungi Kepaniteraan Mahkamah Agung RI melalui :
Email : kepaniteraan@mahkamahagung.go.id Telp : 021-384 3348 (ext.318)

selama kurang lebih 1 tahun 1 bulan terhitung sejak bulan Oktober tahun 2019, Penggugat saat ini tinggal di rumah bulik nya bernama Atun Bebek yang beralamat di Tegalrejo RT 002 RW 002, Kelurahan Jebres, Kecamatan Jebres, Kota Surakarta dan Tergugat di Dukuh Tegalrejo RT 023, Desa Kadipiro, Kecamatan Sambirejo, Kabupaten Sragen;

Menimbang, bahwa untuk mendukung dalil-dalil gugatannya, Penggugat telah mengajukan bukti 2 (dua) orang saksi yang telah memberikan keterangan di bawah sumpah masing-masing saksi;

Menimbang, bahwa oleh karena kesaksian para saksi didasarkan pada apa yang dilihat, didengar dan dialami oleh para saksi, maka kesaksian tersebut telah memenuhi syarat formil dan materiil dan oleh karenanya kesaksian tersebut dapat diterima sebagai bukti;

Menimbang, bahwa dari kesaksian saksi-saksi Penggugat para saksi telah menyaksikan bahwa sejak tahun 2019 Penggugat dan Tergugat telah terjadi perselisihan dan pertengkaran yang disebabkan Tergugat sering menyakiti badan Penggugat, tidak memberi nafkah kepada Penggugat, dan pernah mengancam mau membunuh Penggugat;, dan sejak tahun 2019 Penggugat dan Tergugat telah berpisah tempat tinggal Penggugat yang pergi meninggalkan Tergugat ke rumah bibi Penggugat sampai sekarang selama 1 tahun lebih tidak pernah bertemu lagi dan tidak ada komunikasi, sudah pernah dirukunkan tetapi tidak berhasil, sehingga gugatan Penggugat telah didukung bukti;

Menimbang, bahwa dari kesaksian dua orang saksi tersebut Majelis Hakim telah memperoleh fakta sebagai berikut :

- Bahwa sejak tahun 2019 Penggugat dan Tergugat secara terus menerus telah terjadi perselisihan dan pertengkaran yang disebabkan Tergugat sering menyakiti badan Penggugat, tidak memberi nafkah kepada Penggugat, dan pernah mengancam mau membunuh Penggugat;;
- Bahwa sejak tahun 2019 Penggugat dan Tergugat telah berpisah tempat tinggal Penggugat yang pergi meninggalkan Tergugat ke rumah bibi Penggugat sampai sekarang selama 1 tahun lebih tidak pernah

Disclaimer
Kepaniteraan Mahkamah Agung Republik Indonesia berusaha untuk selalu mencantumkan informasi paling kini dan akurat sebagai bentuk komitmen Mahkamah Agung untuk pelayanan publik, transparansi dan akuntabilitas pelaksanaan fungsi peradilan. Namun dalam hal-hal tertentu masih dimungkinkan terjadi permasalahan teknis terkait dengan akurasi dan keterkinian informasi yang kami sajikan, hal mana akan terus kami perbaiki dari waktu kewaktu.
Dalam hal Anda menemukan inakurasi informasi yang termuat pada situs ini atau informasi yang seharusnya ada, namun belum tersedia, maka harap segera hubungi Kepaniteraan Mahkamah Agung RI melalui :
Email : kepaniteraan@mahkamahagung.go.id   Telp : 021-384 3348 (ext.318)

bertemu lagi dan tidak ada komunikasi, sudah pernah dirukunkan tetapi tidak berhasil

Menimbang, bahwa berdasarkan fakta tersebut, maka gugatan Penggugat telah memenuhi alasan perceraian Penjelasan Pasal 39 ayat (2) huruf f, Undang-Undang nomor 1 Tahun 1974 tentang Perkawinan, jo. Pasal 19 huruf f, Peraturan Pemerintah Nomor 9 Tahun 1975 dan pasal 116 huruf f, Kompilasi Hukum Islam;

Menimbang, bahwa berdasarkan pertimbangan-pertimbangan tersebut diatas Majelis hakim berpendapat bahwa rumah tangga Penggugat dan Tergugat telah pecah dan tidak ada harapan untuk dapat mewujudkan tujuan perkawinan sebagaimana pasal 1 Undang-Undang Nomor 1 Tahun 1974 jo. Pasal 3 Kompilasi Hukum Islam, dan karenanya hak dan kewajiban Penggugat dan Tergugat sebagai suami isteri sebagaimana Pasal 33 dan 34 Ayat (1) dan Ayat (2) Undang-Undang Nomor 1 Tahun 1974 jo. Pasal 77 Kompilasi Hukum Islam juga tidak dapat ditegakkan, sehingga gugatan Penggugat agar dijatuhkan talak satu ba'in sughra Tergugat terhadap Penggugat dapat dikabulkan;

Menimbang pula, bahwa walaupun perceraian merupakan perbuatan halal yang sangat dimurkai Allah, akan tetapi karena keadaan rumah tangga Penggugat dan Tergugat sudah terbukti tidak harmonis, telah pecah, dan telah memenuhi alasan perceraian, maka perceraian merupakan jalan keluar dari keadaan tersebut dan jalan untuk menghilangkan mafsadat yang lebih besar bagi Penggugat dan Tergugat;

Menimbang, bahwa oleh karena Tergugat tidak pernah hadir dan tidak menyuruh orang lain sebagai wakil/kuasanya meskipun telah dipanggil secara sah dan patut sedang gugatan Penggugat beralasan dan tidak melawan hukum, maka sesuai pasal 125 HIR gugatan Penggugat diputus tanpa hadirnya Tergugat. (verstek) ;

Menimbang, bahwa oleh karena perkara ini menyangkut bidang perkawinan, maka sesuai dengan Pasal 89 Ayat (1) Undang-Undang Nomor 7 Tahun 1989 Tentang Peradilan Agama yang telah diubah dengan Undang-Undang Nomor 3 Tahun 2006 dan perubahan ke dua dengan Undang

Disclaimer
Kepaniteraan Mahkamah Agung Republik Indonesia berusaha untuk selalu mencantumkan informasi paling kini dan akurat sebagai bentuk komitmen Mahkamah Agung untuk pelayanan publik, transparansi dan akuntabilitas pelaksanaan fungsi peradilan. Namun dalam hal-hal tertentu masih dimungkinkan terjadi permasalahan teknis terkait dengan akurasi dan keterkinian informasi yang kami sajikan, hal mana akan terus kami perbaiki dari waktu kewaktu.
Dalam hal Anda menemukan inakurasi informasi yang termuat pada situs ini atau informasi yang seharusnya ada, namun belum tersedia, maka harap segera hubungi Kepaniteraan Mahkamah Agung RI melalui :
Email : kepaniteraan@mahkamahagung.go.id Telp : 021-384 3348 (ext.318)

Undang Nomor 50 Tahun 2009, maka biaya perkara dibebankan kepada Penggugat ;

Mengingat ketentuan peraturan perundang-undangan yang berlaku dan hukum syara' yang berkaitan dengan perkara ini ;

MENGADILI

1. Menyatakan Tergugat yang telah dipanggil secara resmi dan patut untuk menghadap di persidangan tidak hadir;
2. Mengabulkan gugatan Penggugat dengan verstek;
3. Menjatuhkan talak satu ba'in sughro (**Xxxxxxxxxxx**) terhadap Penggugat (**Xxxxxxxxxxx**);
4. Membebankan kepada Penggugat untuk membayar biaya perkara sejumlah Rp 396.000,00 (tiga ratus sembilan puluh enam ribu rupiah);

Demikian diputuskan dalam rapat permusyawaratan majelis hakim yang dilangsungkan pada hari Selasa tanggal 1 Desember 2020 Masehi, bertepatan dengan tanggal 15 Rabi'ul Akhir 1442 Hijriyah, oleh kami Drs. Amiruddin, S.H. sebagai Ketua Majelis, Drs. H. Muhammad Fatchan, M.A. dan Drs. H. Muh. Mahfudz, masing-masing sebagai Hakim Anggota, dan pada hari itu juga putusan diucapkan dalam sidang terbuka untuk umum oleh Ketua Majelis tersebut dengan didampingi Hakim-Hakim Anggota tersebut dan dibantu oleh Muhammad Abdus Shobur, S.H., sebagai panitera pengganti serta dihadiri oleh Penggugat tanpa hadirnya Tergugat;

Hakim Anggota,

Drs. H. Muhammad Fatchan, M.A.

Ketua Majelis,

Drs. Amiruddin, S.H.

Hakim Anggota,

Drs. H. Muh. Mahfudz

Panitera Pengganti,

Disclaimer
Kepaniteraan Mahkamah Agung Republik Indonesia berusaha untuk selalu mencantumkan informasi paling kini dan akurat sebagai bentuk komitmen Mahkamah Agung untuk pelayanan publik, transparansi dan akuntabilitas pelaksanaan fungsi peradilan. Namun dalam hal-hal tertentu masih dimungkinkan terjadi permasalahan teknis terkait dengan akurasi dan keterkinian informasi yang kami sajikan, hal mana akan terus kami perbaiki dari waktu kewaktu.
Dalam hal Anda menemukan inakurasi informasi yang termuat pada situs ini atau informasi yang seharusnya ada, namun belum tersedia, maka harap segera hubungi Kepaniteraan Mahkamah Agung RI melalui :
Email : kepaniteraan@mahkamahagung.go.id Telp : 021-384 3348 (ext.318)

Muhammad Abdus Shobur, S.H.

Perincian Biaya Perkara :

1. Pendaftaran: Rp 30.000,00
2. Proses : Rp 75.000,00
3. PNBP : Rp 20.000,00
4. Panggilan : Rp 255.000,00
5. Redaksi : Rp 10.000,00
6. Meterai : Rp 6.000,00

J u m l a h : Rp 396.000,00

(tiga ratus sembilan puluh enam ribu rupiah);

Disclaimer
Kepaniteraan Mahkamah Agung Republik Indonesia berusaha untuk selalu mencantumkan informasi paling kini dan akurat sebagai bentuk komitmen Mahkamah Agung untuk pelayanan publik, transparansi dan akuntabilitas pelaksanaan fungsi peradilan. Namun dalam hal-hal tertentu masih dimungkinkan terjadi permasalahan teknis terkait dengan akurasi dan keterkinian informasi yang kami sajikan, hal mana akan terus kami perbaiki dari waktu kewaktu.
Dalam hal Anda menemukan inakurasi informasi yang termuat pada situs ini atau informasi yang seharusnya ada, namun belum tersedia, maka harap segera hubungi Kepaniteraan Mahkamah Agung RI melalui :
Email : kepaniteraan@mahkamahagung.go.id Telp : 021-384 3348 (ext.318)
