# Supplementary material for: Classification of divorce causes during the COVID-19 pandemic using convolutional neural networks
Source: PeerJ Comput Sci. 2022 Jun 30;8:e998. doi: 10.7717/peerj-cs.998 (PMC9299239; doi:10.7717/peerj-cs.998)
Supplement: Supplemental Information 5 [file peerj-cs-08-998-s005.zip › Masalah Ekonomi Dataset/Data ke-3.pdf]

P U T U S A N

Nomor 5889/Pdt.G/2020/PA.Badg

بِسْمِ اللهِ الرَّحْمَنِ الرَّحِيْمِ

DEMI KEADILAN BERDASARKAN KETUHANAN YANG MAHA ESA

Pengadilan Agama Bandung yang memeriksa dan mengadili perkara tertentu pada tingkat pertama dalam sidang majelis telah menjatuhkan putusan perkara Cerai Gugat antara :

PENGGUGAT, tempat dan tanggal lahir Bandung, 03 Februari 1974, agama Islam, pekerjaan Wiraswasta, pendidikan SLTA, tempat kediaman Jalan Sadireja No. 369/41, RT. 006 RW. 003, Kelurahan Sukamaju, Kecamatan Cibeunying Kidul, Kota Bandung, sebagai **Penggugat**;

melawan

TERGUGAT, tempat dan tanggal lahir Bandung, 10 Maret 1972, agama Islam, pekerjaan Tidak diketahui, pendidikan D3, tempat kediaman di Jalan Sadireja No. 369/41, RT. 006 RW. 003, Kelurahan Sukamaju, Kecamatan Cibeunying Kidul, Kota Bandung, sebagai **Tergugat**;

Pengadilan Agama tersebut;

Telah mempelajari surat-surat yang berkaitan dengan perkara ini;

Telah mendengar keterangan Penggugat serta para saksi di muka sidang;

DUDUK PERKARA

Menimbang, bahwa Penggugat dalam surat gugatannya tanggal 30 November 2020 telah mengajukan gugatan cerai yang telah terdaftar di Kepaniteraan Pengadilan Agama Bandung dengan Nomor 5889/Pdt.G/2020/PA.Badg tanggal 30 November 2020 dengan dalil-dalil sebagai berikut:

1. Bahwa antara Penggugat dan Tergugat adalah sepasang suami istri sah yang menikah pada tanggal 15 Maret 1998 dan telah dicatatkan di Kantor Urusan Agama (KUA) Kecamatan Cibeunying Kidul, Kota Bandung

Disclaimer
Kepaniteraan Mahkamah Agung Republik Indonesia berusaha untuk selalu mencantumkan informasi paling kini dan akurat sebagai bentuk komitmen Mahkamah Agung untuk pelayanan publik, transparansi dan akuntabilitas pelaksanaan fungsi peradilan. Namun dalam hal-hal tertentu masih dimungkinkan terjadi permasalahan teknis terkait dengan akurasi dan keterkinian informasi yang kami sajikan, hal mana akan terus kami perbaiki dari waktu kewaktu.
Dalam hal Anda menemukan inakurasi informasi yang termuat pada situs ini atau informasi yang seharusnya ada, namun belum tersedia, maka harap segera hubungi Kepaniteraan Mahkamah Agung RI melalui :
Email : kepaniteraan@mahkamahagung.go.id Telp : 021-384 3348 (ext.318)

dengan bukti Kutipan Akta Nikah Nomor: 880/50/III/1998 tanggal 16 Maret 1998.

2. Bahwa setelah menikah antara Penggugat dengan Tergugat tinggal dan hidup bersama terakhir di rumah Bersama di Jalan Sadireja No. 369/41, RT. 006 RW. 003, Kelurahan Sukamaju, Kecamatan Cibeunying Kidul, Kota Bandung.

3. Bahwa semula keadaan rumah tangga antara Penggugat dengan Tergugat hidup rukun dan harmonis kurang lebih selama 12 tahun. Dan dari hasil perkawinan antara Penggugat dengan Tergugat telah dikaruniai anak sebanyak 2 (dua) orang yang bernama:

3.1. ANAK I (perempuan), tanggal lahir 24 Agustus 1998.

3.2. ANAK II (perempuan), tanggal lahir 06 Mei 2003.

4. Bahwa selama berumah tangga dengan Tergugat, keadaan tidak selamanya rukun dan harmonis, rumah tangga antara Penggugat dan Tergugat sejak bulan **Juni 2010** mulai terjadi perselisihan dan pertengkaran terus menerus, sehingga Penggugat mengajukan gugatan cerai ke Pengadilan Agama Bandung. Adapun sebab-sebab perceraian ini diajukan adalah sebagai berikut:

4.1. Bahwa antara Penggugat dan Tergugat ketika sedang berselisih, Tergugat sering mengucapkan talak kepada Penggugat, meskipun Penggugat telah berupaya untuk sabar, namun Tergugat tetap bersikap seperti itu ketika sedang berselisih, bahkan ketika Tergugat sedang sakit pun Tergugat tetap tidak merubah sikapnya.

4.2. Bahwa selain alasan diatas, Tergugat pun sudah tidak pernah memberikan nafkah kepada Penggugat, dan sejak tahun 2010 sampai dengan sekarang untuk mencukupi kebutuhan sehari-hari dan biaya anak mengandalkan hasil usaha Penggugat.

5. Bahwa Penggugat telah berusaha sabar untuk mempertahankan rumah tangga, tetapi Tergugat tidak berubah dan merubah sikapnya. Puncaknya pada bulan **Juni 2010**, antara Penggugat dan Tergugat terlibat pertengkaran, Penggugat dan Tergugat masih tinggal dikediaman bersama,

Disclaimer
Kepaniteraan Mahkamah Agung Republik Indonesia berusaha untuk selalu mencantumkan informasi paling kini dan akurat sebagai bentuk komitmen Mahkamah Agung untuk pelayanan publik, transparansi dan akuntabilitas pelaksanaan fungsi peradilan. Namun dalam hal-hal tertentu masih dimungkinkan terjadi permasalahan teknis terkait dengan akurasi dan keterkinian informasi yang kami sajikan, hal mana akan terus kami perbaiki dari waktu kewaktu.
Dalam hal Anda menemukan inakurasi informasi yang termuat pada situs ini atau informasi yang seharusnya ada, namun belum tersedia, maka harap segera hubungi Kepaniteraan Mahkamah Agung RI melalui :
Email : kepaniteraan@mahkamahagung.go.id Telp : 021-384 3348 (ext.318)

namun antara Penggugat dengan Tergugat sudah pisah ranjang atau pisah kamar, dan Penggugat dengan Tergugat sudah tidak melaksanakan kewajiban masing-masing layaknya suami istri lagi.

6. Bahwa sampai saat ini, antara Penggugta dan Tergugat masih tinggal dikediaman Bersama, akan tetapi sudah pisah ranjang atau pisah kamar.

7. Bahwa sampai saat ini, antara Penggugat dengan Tergugat jarang berkomunikasi, dan Tergugat tidak memberikan nafkah kepada Penggugat.

8. Bahwa atas permasalahan tersebut di atas Penggugat sudah tidak sanggup lagi untuk mempertahankan perkawinan ini, oleh karena itu Penggugat telah berketetapan hati untuk bercerai dengan Tergugat.

9. Bahwa pengajuan gugatan cerai Penggugat telah memenuhi alasan-alasan perceraian sebagaimana diatur dalam Pasal 19 huruf f Peraturan Pemerintah Nomor 9 tahun 1975 jo Pasal 116 huruf f Instruksi Presiden Nomor 1 tahun 1991 tentang Kompilasi Hukum Islam.

Bahwa berdasarkan hal-hal dan alasan tersebut di atas, maka gugatan cerai Penggugat ini telah sesuai dan memenuhi ketentuan hukum dan mempunyai alasan-alasan yang cukup. Maka oleh karenanya Penggugat memohon kepada Ketua Pengadilan Agama Bandung Cq. Majelis Hakim berkenan membuka persidangan untuk memeriksa dan mengadili perkara ini dan berkenan menjatuhkan putusan yang amarnya sebagai berikut:

1. Mengabulkan gugatan Penggugat;
2. Menjatuhkan talak satu ba'in sughra dari Tergugat (TERGUGAT) terhadap Penggugat (PENGGUGAT);
3. Membebankan biaya perkara menurut hukum;

Atau apabila Majelis Hakim Pengadilan Agama Bandung berpendapat lain, mohon putusan yang seadil adilnya *(ex aequo et bono).*

Menimbang, bahwa pada hari sidang yang telah ditetapkan Penggugat telah datang menghadap ke muka sidang, sedangkan Tergugat tidak datang menghadap dan tidak menyuruh orang lain untuk menghadap sebagai wakil/kuasa hukumnya meskipun telah dipanggil secara resmi dan patut yang

Disclaimer
Kepaniteraan Mahkamah Agung Republik Indonesia berusaha untuk selalu mencantumkan informasi paling kini dan akurat sebagai bentuk komitmen Mahkamah Agung untuk pelayanan publik, transparansi dan akuntabilitas pelaksanaan fungsi peradilan. Namun dalam hal-hal tertentu masih dimungkinkan terjadi permasalahan teknis terkait dengan akurasi dan keterkinian informasi yang kami sajikan, hal mana akan terus kami perbaiki dari waktu kewaktu.
Dalam hal Anda menemukan inakurasi informasi yang termuat pada situs ini atau informasi yang seharusnya ada, namun belum tersedia, maka harap segera hubungi Kepaniteraan Mahkamah Agung RI melalui :
Email : kepaniteraan@mahkamahagung.go.id Telp : 021-384 3348 (ext.318)

relaas panggilannya dibacakan di dalam sidang, sedangkan tidak ternyata bahwa tidak datangnya itu disebabkan suatu halangan yang sah;

Menimbang, bahwa bahwa majelis hakim telah menasehati Penggugat agar berpikir untuk tidak bercerai dengan Tergugat, tetapi Penggugat tetap pada dalil-dalil gugatannya untuk bercerai dengan Tergugat;

Menimbang, bahwa perkara ini tidak dapat dimediasi karena Tergugat tidak pernah datang menghadap meskipun telah dipanggil secara resmi dan patut, selanjutnya dimulai pemeriksaan dengan membacakan surat gugatan Penggugat yang maksud dan isinya tetap dipertahankan oleh Penggugat;

Menimbang, bahwa untuk menguatkan dalil-dalil gugatannya, Penggugat telah mengajukan alat-alat bukti berupa:

A. Bukti Surat:

Fotokopi Buku Kutipan Akta Nikah Nomor: 880/50/III/1998, tertanggal 16 Maret 1998, yang dikeluarkan oleh (KUA) Kecamatan Cibeunying Kidul, Kota Bandung, fotokopi tersebut telah dinazegelen dan oleh Ketua Majelis telah dicocokkan dengan aslinya ternyata telah sesuai selanjutnya diberi tanda bukti (P.1);

B. Bukti Saksi:

1. **SAKSI I**, umur 65 tahun, agama Islam, pekerjaan Ibu Rumah Tangga, tempat tinggal di Jalan Sadireja No. 369 RT. 06 RW. 03 Kelurahan Sukamaju Kecamatan Cibeunying Kidul Kota Bandung, di bawah sumpahnya memberikan keterangan sebagai berikut:
   - Bahwa saksi kenal dengan Penggugat dan Tergugat karena saksi adalah ibu kandung Penggugat;
   - Bahwa, Penggugat dan Tergugat adalah pasangan suami isteri yang menikah sekitar tahun 1998;
   - Bahwa, perkawinan Penggugat dan Tergugat telah dikaruniai 2 (dua) orang anak;
   - Bahwa semula keadaan rumah tangga rumah tangga Penggugat dan Tergugat rukun dan harmonis, namun sejak bulan Juni tahun 2020 sudah tidak harmonis lagi, karena sering terjadi perselisihan dan pertengkaran;

Disclaimer
Kepaniteraan Mahkamah Agung Republik Indonesia berusaha untuk selalu mencantumkan informasi paling kini dan akurat sebagai bentuk komitmen Mahkamah Agung untuk pelayanan publik, transparansi dan akuntabilitas pelaksanaan fungsi peradilan. Namun dalam hal-hal tertentu masih dimungkinkan terjadi permasalahan teknis terkait dengan akurasi dan keterkinian informasi yang kami sajikan, hal mana akan terus kami perbaiki dari waktu kewaktu.
Dalam hal Anda menemukan inakurasi informasi yang termuat pada situs ini atau informasi yang seharusnya ada, namun belum tersedia, maka harap segera hubungi Kepaniteraan Mahkamah Agung RI melalui :
Email : kepaniteraan@mahkamahagung.go.id Telp : 021-384 3348 (ext.318)

- Bahwa, penyebab perselisihan dan pertengkaran dikarenakan Tergugat sering mengucapkan kata talak dan Tergugat tidak memberi nafkah kepada Penggugat;
- Bahwa, antara Penggugat dan Tergugat telah berpisah rumah selama 6 (enam) bulan;
- Bahwa, keluarga kedua belah pihak dan saksi sudah berusaha untuk merukunkan Penggugat dengan Tergugat, namun tidak berhasil;

2. **SAKSI II,** umur 30 tahun, agama Islam, pekerjaan Ibu Rumah Tangga, tempat tinggal di Jalan Sadireja No. 369 RT. 06 RW. 03 Kelurahan Sukamaju Kecamatan Cibeunying Kidul Kota Bandung, di bawah sumpahnya memberikan keterangan sebagai berikut:
   - Bahwa saksi kenal dengan Penggugat dan Tergugat karena saksi adalah adik kandung Penggugat;
   - Bahwa, Penggugat dan Tergugat adalah pasangan suami isteri yang menikah sekitar tahun 1998;
   - Bahwa, perkawinan Penggugat dan Tergugat telah dikaruniai 2 (dua) orang anak;
   - Bahwa semula keadaan rumah tangga rumah tangga Penggugat dan Tergugat rukun dan harmonis, namun sejak bulan Juni tahun 2020 sudah tidak harmonis lagi, karena sering terjadi perselisihan dan pertengkaran;
   - Bahwa, penyebab perselisihan dan pertengkaran dikarenakan Tergugat sering mengucapkan kata talak dan Tergugat tidak memberi nafkah kepada Penggugat;
   - Bahwa, antara Penggugat dan Tergugat telah berpisah rumah selama 6 (enam) bulan;
   - Bahwa, keluarga kedua belah pihak dan saksi sudah berusaha untuk merukunkan Penggugat dengan Tergugat, namun tidak berhasil;

Disclaimer
Kepaniteraan Mahkamah Agung Republik Indonesia berusaha untuk selalu mencantumkan informasi paling kini dan akurat sebagai bentuk komitmen Mahkamah Agung untuk pelayanan publik, transparansi dan akuntabilitas pelaksanaan fungsi peradilan. Namun dalam hal-hal tertentu masih dimungkinkan terjadi permasalahan teknis terkait dengan akurasi dan keterkinian informasi yang kami sajikan, hal mana akan terus kami perbaiki dari waktu kewaktu.
Dalam hal Anda menemukan inakurasi informasi yang termuat pada situs ini atau informasi yang seharusnya ada, namun belum tersedia, maka harap segera hubungi Kepaniteraan Mahkamah Agung RI melalui :
Email : kepaniteraan@mahkamahagung.go.id   Telp : 021-384 3348 (ext.318)

Menimbang, bahwa selanjutnya untuk singkatnya uraian putusan ini, maka semua hal yang termuat dalam berita acara sidang ini merupakan bagian yang tidak terpisahkan dari putusan ini;

PERTIMBANGAN HUKUM

Menimbang, bahwa maksud dan tujuan gugatan Penggugat adalah sebagaimana terurai di atas;

Menimbang, bahwa ternyata Tergugat, meskipun telah dipanggil secara resmi dan patut, tidak datang menghadap di muka sidang dan pula tidak ternyata bahwa tidak datangnya itu disebabkan suatu halangan yang sah;

Menimbang, bahwa Tergugat yang dipanggil secara resmi dan patut akan tetapi tidak datang menghadap harus dinyatakan tidak hadir dan gugatan tersebut harus diperiksa secara verstek;

Menimbang, bahwa oleh karena itu, maka putusan atas perkara ini dapat dijatuhkan tanpa hadirnya Tergugat (verstek);

Menimbang, bahwa berdasarkan ketentuan Pasal 125 ayat (1) HIR, yaitu putusan yang dijatuhkan tanpa hadirnya Tergugat dapat dikabulkan sepanjang berdasarkan hukum dan beralasan, oleh karena itu majelis membebani Penggugat untuk membuktikan dalil-dalil gugatannya;

Menimbang, bahwa gugatan Penggugat didasarkan pada dalil yang pada pokoknya adalah rumah tangga Penggugat dan Tergugat tidak harmonis lagi sering terjadi perselisihan dan pertengkaran dan tidak ada harapan akan hidup rukun lagi dalam satu rumah tangga, sehingga Penggugat memohon agar Pengadilan Agama menjatuhkan talak satu bain sughra Tergugat terhadap Penggugat;

Menimbang, bahwa untuk membuktikan dalil-dalilnya, Penggugat telah mengajukan alat bukti surat P.1 dan dua orang saksi;

Menimbang, bahwa bukti P.1 (fotokopi Buku Kutipan Akta Nikah) yang merupakan akta otentik dan telah bermeterai cukup dan cocok dengan aslinya, isi bukti tersebut menjelaskan mengenai pernikahan Penggugat dan Tergugat, sehingga bukti tersebut telah memenuhi syarat formil dan materil, serta mempunyai kekuatan pembuktian yang sempurna dan mengikat;

Disclaimer
Kepaniteraan Mahkamah Agung Republik Indonesia berusaha untuk selalu mencantumkan informasi paling kini dan akurat sebagai bentuk komitmen Mahkamah Agung untuk pelayanan publik, transparansi dan akuntabilitas pelaksanaan fungsi peradilan. Namun dalam hal-hal tertentu masih dimungkinkan terjadi permasalahan teknis terkait dengan akurasi dan keterkinian informasi yang kami sajikan, hal mana akan terus kami perbaiki dari waktu kewaktu.
Dalam hal Anda menemukan inakurasi informasi yang termuat pada situs ini atau informasi yang seharusnya ada, namun belum tersedia, maka harap segera hubungi Kepaniteraan Mahkamah Agung RI melalui :
Email : kepaniteraan@mahkamahagung.go.id Telp : 021-384 3348 (ext.318)

Menimbang, bahwa berdasarkan bukti (P.1) maka dapat dinyatakan terbukti bahwa Penggugat dan Tergugat adalah suami istri yang sah, sehingga mempunyai landasan hukum untuk mengajukan cerai gugat terhadap Tergugat;

Menimbang, bahwa oleh karena alasan Cerai Gugat Penggugat didasarkan pada pasal 19 huruf (f) Peraturan Pemerintah Nomor 9 Tahun 1975 jo. Pasal 116 huruf (f) Kompilasi Hukum Islam, maka Majelis telah mendengar keterangan saksi dari pihak keluarga Penggugat, sehingga telah terpenuhi maksud pasal 76 ayat (1) Undang Undang Nomor 7 Tahun 1989, yang telah mengalami 2 kali perubahan dengan Undang-undang Nomor 3 tahun 2006 dan Undang-Undang Nomor 50 tahun 2009;

Menimbang, bahwa dua orang saksi yang diajukan oleh Penggugat secara formil dan materil telah memenuhi syarat sebagai saksi, oleh karena itu dapat dipertimbangkan lebih lanjut;

Menimbang, bahwa berdasarkan keterangan saksi-saksi tersebut di atas yang keterangannya saling bersesuaian, maka Majelis telah dapat menemukan fakta yang pada pokoknya sebagai berikut:

- Bahwa, Penggugat dan Tergugat adalah pasangan suami isteri yang menikah sekitar tahun 1998;
- Bahwa, perkawinan Penggugat dan Tergugat telah dikaruniai 2 (dua) orang anak;
- Bahwa semula keadaan rumah tangga rumah tangga Penggugat dan Tergugat rukun dan harmonis, namun sejak bulan Juni tahun 2020 sudah tidak harmonis lagi, karena sering terjadi perselisihan dan pertengkaran;
- Bahwa, penyebab perselisihan dan pertengkaran dikarenakan Tergugat sering mengucapkan kata talak dan Tergugat tidak memberi nafkah kepada Penggugat;
- Bahwa, antara Penggugat dan Tergugat telah berpisah rumah selama 6 (enam) bulan;

Menimbang, bahwa berdasarkan fakta tersebut di atas, Majelis berpendapat rumah tangga Penggugat dengan Tergugat tidak harmonis lagi prilaku Tergugat tersebut menjadi pemicu terjadinya percekcokan sehingga

Disclaimer
Kepaniteraan Mahkamah Agung Republik Indonesia berusaha untuk selalu mencantumkan informasi paling kini dan akurat sebagai bentuk komitmen Mahkamah Agung untuk pelayanan publik, transparansi dan akuntabilitas pelaksanaan fungsi peradilan. Namun dalam hal-hal tertentu masih dimungkinkan terjadi permasalahan teknis terkait dengan akurasi dan keterkinian informasi yang kami sajikan, hal mana akan terus kami perbaiki dari waktu kewaktu.
Dalam hal Anda menemukan inakurasi informasi yang termuat pada situs ini atau informasi yang seharusnya ada, namun belum tersedia, maka harap segera hubungi Kepaniteraan Mahkamah Agung RI melalui :
Email : kepaniteraan@mahkamahagung.go.id Telp : 021-384 3348 (ext.318)

hubungan Penggugat dan Tergugat semakin tidak baik, dengan demikian Majelis berkesimpulan keadaan rumah tangga Penggugat dengan Tergugat telah pecah sedemikian rupa sehingga tujuan perkawinan untuk membentuk keluarga/rumah tangga yang bahagia dan kekal berdasarkan Ketuhanan Yang Maha Esa (*vide* pasal 1 Undang-Undang Nomor 1 Tahun 1974) dan atau keluarga yang sakinah, penuh mawaddah dan rahmah (*vide* pasal 3 Kompilasi Hukum Islam) telah tidak terwujud dalam rumah tangga Penggugat dengan Tergugat;

Menimbang, bahwa unsur pokok tegaknya suatu bangunan rumah tangga adalah adanya ikatan lahir batin yang kokoh antara suami dan isteri. Apabila terjadi perselisihan dan pertengkaran antara suami-isteri yang berakibat kepada tidak lagi saling peduli dan telah diupayakan untuk rukun kembali tetapi tidak berhasil maka hal tersebut mengindikasikan bahwa ikatan lahir-batin diantara suami-isteri tersebut telah sedemikian rapuh atau bahkan telah lepas sama sekali, sehingga telah tidak ada lagi kecocokan dan kesamaan kehendak diantara keduanya;

Menimbang, bahwa mempertahankan rumah tangga yang telah pecah sedemikian rupa adalah sia-sia belaka, bahkan apabila keadaannya seperti sekarang ini jika dipaksakan atau dibiarkan maka justru akan menimbulkan mudharat dan penderitaan lahir batin yang berkepanjangan bagi Penggugat, sehingga oleh karenanya Majelis berpendapat bahwa rumah tangga Penggugat dengan Tergugat telah tidak dapat dipertahankan lagi;

Menimbang, bahwa Majelis perlu mengemukakan doktrin ulama yang kemudian diambil alih sebagai pendapat Majelis dalam Kitab Madaa Hurriyatuz Zaujaini fith Thalaaq Juz I halaman 83:

وقد اختار الإسلام نظام الطلاق حين تضطرب الحياة الزوجين ولم يعد ينفع فيها نصائح ولا صلح وحيث تصبح الربطة الزواج صورة من غير روح لأن الإستمرار معناه أن يحكم على أحد الزوجين بالسجن المؤبد وهذا ظلم تأباه روح العدالة

"Islam memilih lembaga talak/cerai ketika rumah tangga sudah dianggap goncang serta dianggap sudah tidak bermanfaat lagi nasehat/perdamaian dan hubungan suami isteri menjadi tanpa ruh (hampa), sebab meneruskan

Disclaimer
Kepaniteraan Mahkamah Agung Republik Indonesia berusaha untuk selalu mencantumkan informasi paling kini dan akurat sebagai bentuk komitmen Mahkamah Agung untuk pelayanan publik, transparansi dan akuntabilitas pelaksanaan fungsi peradilan. Namun dalam hal-hal tertentu masih dimungkinkan terjadi permasalahan teknis terkait dengan akurasi dan keterkinian informasi yang kami sajikan, hal mana akan terus kami perbaiki dari waktu kewaktu.
Dalam hal Anda menemukan inakurasi informasi yang termuat pada situs ini atau informasi yang seharusnya ada, namun belum tersedia, maka harap segera hubungi Kepaniteraan Mahkamah Agung RI melalui :
Email : kepaniteraan@mahkamahagung.go.id Telp : 021-384 3348 (ext.318)

perkawinan berarti menghukum salah satu suami isteri dengan penjara yang berkepanjangan. Ini adalah aniaya yang bertentangan dengan semangat keadilan"

Menimbang, bahwa berdasarkan hal-hal tersebut di atas maka gugatan Penggugat telah terbukti beralasan hukum sesuai ketentuan pasal 39 ayat (2) Undang-Undang Nomor 1 Tahun 1974 jis Pasal 19 huruf (f) Peraturan Pemerintah Nomor 9 Tahun 1975 dan Pasal 116 huruf (f) Kompilasi Hukum Islam, oleh karena itu gugatan Penggugat dapat dikabulkan dengan menjatuhkan talak satu bain sughra dari Tergugat terhadap Penggugat;

Menimbang, bahwa oleh karena perkara ini dalam bidang perkawinan, maka sesuai Pasal 89 ayat (1) Undang-Undang Nomor 7 Tahun 1989 sebagaimana telah diubah dengan Undang-Undang Nomor 3 Tahun 2006 dan perubahan kedua dengan Undang-Undang Nomor 50 Tahun 2009, biaya perkara dibebankan kepada Penggugat;

Mengingat, semua pasal dalam peraturan perundang-undangan dan hukum Islam yang berkaitan dengan perkara ini;

MENGADILI

1. Menyatakan Tergugat yang telah dipanggil dengan resmi dan patut untuk datang menghadap di persidangan, tidak hadir;

2. Mengabulkan gugatan Penggugat dengan Verstek;

3. Menjatuhkan talak satu ba'in sughra dari Tergugat (**TERGUGAT**) terhadap Penggugat (**PENGGUGAT);**

4. Membebankan kepada Penggugat untuk membayar biaya perkara sejumlah Rp. 341.000.- (tiga ratus empat puluh satu ribu rupiah);

Demikianlah diputus dalam sidang permusyawaratan majelis Pengadilan Agama Bandung pada hari Senin tanggal 21 Desember 2020 Masehi bertepatan dengan tanggal 6 Jumadil Awal 1442 Hijriyah oleh kami Drs. H. Cece Rukmana Ibrahim, S.H., M.H. sebagai Ketua Majelis, Drs. H. Endang Tamami, M.H. dan Drs. Mustopa, SH. masing-masing sebagai Hakim Anggota, dan diucapkan pada hari itu juga dalam sidang terbuka untuk umum oleh Ketua Majelis tersebut didampingi para Hakim Anggota, dibantu oleh Achmad Sadikin S.H. sebagai Panitera Pengganti dengan dihadiri oleh Penggugat tanpa hadirnya Tergugat;

Disclaimer
Kepaniteraan Mahkamah Agung Republik Indonesia berusaha untuk selalu mencantumkan informasi paling kini dan akurat sebagai bentuk komitmen Mahkamah Agung untuk pelayanan publik, transparansi dan akuntabilitas pelaksanaan fungsi peradilan. Namun dalam hal-hal tertentu masih dimungkinkan terjadi permasalahan teknis terkait dengan akurasi dan keterkinian informasi yang kami sajikan, hal mana akan terus kami perbaiki dari waktu kewaktu.
Dalam hal Anda menemukan inakurasi informasi yang termuat pada situs ini atau informasi yang seharusnya ada, namun belum tersedia, maka harap segera hubungi Kepaniteraan Mahkamah Agung RI melalui :
Email : kepaniteraan@mahkamahagung.go.id Telp : 021-384 3348 (ext.318)

Ketua Majelis

Drs. H. Cece Rukmana Ibrahim, S.H., M.H.

Hakim Anggota   Hakim Anggota

Drs. H. Endang Tamami, M.H.   Drs. Mustopa, SH.

Panitera Pengganti

Achmad Sadikin S.H.

Perincian Biaya Perkara :

1. Pendaftaran	:	Rp.	30.000,-
2. Proses	:	Rp.	50.000,-
3. Panggilan Penggugat	:	Rp.	75.000,-
4. Panggilan Tergugat	:	Rp.	150.000,-
5. Biaya PNBP	;	P.	20.000.-
6. Redaksi	:	Rp.	10.000,-
7. Materai	:	Rp.	6.000,-

Jumlah	:	Rp.	341.000,-

tiga ratus empat puluh satu ribu rupiah

Disclaimer
Kepaniteraan Mahkamah Agung Republik Indonesia berusaha untuk selalu mencantumkan informasi paling kini dan akurat sebagai bentuk komitmen Mahkamah Agung untuk pelayanan publik, transparansi dan akuntabilitas pelaksanaan fungsi peradilan. Namun dalam hal-hal tertentu masih dimungkinkan terjadi permasalahan teknis terkait dengan akurasi dan keterkinian informasi yang kami sajikan, hal mana akan terus kami perbaiki dari waktu kewaktu.
Dalam hal Anda menemukan inakurasi informasi yang termuat pada situs ini atau informasi yang seharusnya ada, namun belum tersedia, maka harap segera hubungi Kepaniteraan Mahkamah Agung RI melalui :
Email : kepaniteraan@mahkamahagung.go.id   Telp : 021-384 3348 (ext.318)
