# Supplementary material for: Classification of divorce causes during the COVID-19 pandemic using convolutional neural networks
Source: PeerJ Comput Sci. 2022 Jun 30;8:e998. doi: 10.7717/peerj-cs.998 (PMC9299239; doi:10.7717/peerj-cs.998)
Supplement: Supplemental Information 5 [file peerj-cs-08-998-s005.zip › Masalah Ekonomi Dataset/Data ke-30.pdf]

PUTUSAN

Nomor 2080/Pdt.G/2020/PA.Sr

بِسْمِ اللَّهِ الرَّحْمَنِ الرَّحِيمِ

DEMI KEADILAN BERDASARKAN KETUHANAN YANG MAHA ESA

Pengadilan Agama Sragen yang memeriksa dan mengadili perkara pada tingkat pertama dalam sidang majelis hakim telah menjatuhkan putusan dalam perkara Cerai Gugat antara:

yani Binti warno tempat dan tanggal lahir Sragen, 12 November 1983, agama Islam, pekerjaan Mengurus rumah tangga, Pendidikan Sekolah Lanjutan Tingkat Atas, tempat kediaman di L. H. Tahir Gg Remaja No 30, Rt.002 Rw.011, Kelurahan Kranji, Kecamatan Bekasi Barat, Kota Bekasi, Provinsi Jawa Barat, Sekarang Berdomisili (sebagai Relaas Panggilan) Di Tawengan, Rt.22, Desa Pilangsari, Kecamatan Ngrampal, Kabupaten Sragen dalam hal ini memberikan kuasa kepada Moegiyono, S.H., Budi Rohmat Anggoro, S.H Dan Faqih Arsyad Herlambang, S.H, Advokat yang berkantor di Jalan Veteran Taman Asri Gg. Ii / 36 Sragen berdasarkan surat kuasa khusus tanggal 16 November 2020 sebagai Penggugat;

melawan

lana Bin ohim, tempat dan tanggal lahir Bekasi, 08 April 1981, agama Islam, pekerjaan Swasta, Pendidikan Sekolah Lanjutan Tingkat Atas, tempat kediaman di Jl. H. Tahir Gg Remaja No 37, Rt.002 Rw.011, Kelurahan Kranji, Kecamatan Bekasi Barat, Kota Bekasi, Provinsi Jawa Barat. sebagai Tergugat;

Pengadilan Agama tersebut;

Telah mempelajari surat-surat yang berkaitan dengan perkara ini;

Telah mendengar keterangan Penggugat dan para saksi di muka sidang;

DUDUK PERKARA

Bahwa Penggugat dalam surat gugatannya tanggal 16 November 2020 telah mengajukan Cerai Gugat, yang telah terdaftar di Kepaniteraan Pengadilan Agama Sragen, dengan Nomor 2080/Pdt.G/2020/PA.Sr, tanggal 16 November 2020, dengan dalil-dalil pada pokoknya sebagai berikut:

1. Bahwa Penggugat menikah dengan Tergugat secara sah, pada tanggal 30 Desember 2006, dihadapan Pegawai Pencatat Nikah Kantor Urusan Agama

Disclaimer
Kepaniteraan Mahkamah Agung Republik Indonesia berusaha untuk selalu mencantumkan informasi paling kini dan akurat sebagai bentuk komitmen Mahkamah Agung untuk pelayanan publik, transparansi dan akuntabilitas pelaksanaan fungsi peradilan. Namun dalam hal-hal tertentu masih dimungkinkan terjadi permasalahan teknis terkait dengan akurasi dan keterkinian informasi yang kami sajikan, hal mana akan terus kami perbaiki dari waktu kewaktu.
Dalam hal Anda menemukan inakurasi informasi yang termuat pada situs ini atau informasi yang seharusnya ada, namun belum tersedia, maka harap segera hubungi Kepaniteraan Mahkamah Agung RI melalui :
Email : kepaniteraan@mahkamahagung.go.id Telp : 021-384 3348 (ext.318)

Kecamatan Ngrampal, Kabupaten Sragen sesuai dengan Kutipan Akta Nikah Nomor: 19/19/I/2007, tertanggal 2 Januari 2007, dalam status Perawan dan Jejaka, sesaat akad nikah Tergugat mengucapkan Sighot Taklik Talak yang bunyinya sebagai mana tersebut dalam buku nikah;

2. Bahwa setelah Penggugat menikah dengan Tergugat, pada awalnya membentuk rumah tangga dan menetap dirumah orang tua Tergugat selama + 12 tahun, pada awalnya rukun dan harmonis;
3. Bahwa selama dalam perkawinan antara Penggugat dengan Tergugat, telah melakukan hubungan layaknya sebagai suami isteri (Ba’dha Dhukul) dan dikaruniai keturunan seorang anak laki-laki bernama Ahmad Odie Prasetyo, Lahir di Bekasi, 17 Desember 2007;
4. Bahwa sekitar bulan Oktober tahun 2019, rumah tangga Penggugat dan Tergugat mulai goyah sering terjadi pertengkaran dan perselisihan, dikarenakan Tergugat jarang memberi nafkah kepada Penggugat, dan kalau Penggugat meminta uang tidak diberi serta Tergugat marah-marah berkata kasar, lalu Penggugat di pulangkan kerumah orang tuanya;
5. Bahwa setelah Penggugat di pulangkan kerumah orang tuanya, Tergugat sudah tidak bisa dihubungi dan tidak memperdulikan Penggugat lagi sampai sekarang serta tidak memberi nafkah wajib, sehingga antara Penggugat dengan Tergugat sudah pisah rumah + 1 tahun;
6. Bahwa atas perbuatan Tergugat tersebut, Penggugat tidak rela dan karenanya Tergugat telah melalaikan kewajibannya sebagai seorang suami, maka Penggugat tidak sanggup lagi menjadi istri Tergugat.
7. Bahwa atas hal-hal tersebut diatas, maka dalil cerai gugat Penggugat sudah sesuai dengan ketentuan hukum yang berlaku yaitu : pasal 39 ayat (2) UU No.1 Tahun 1974 jo pasal 19 huruf (f) PP No. 9 Tahun 1975 jo pasal 116 huruf (f) Kompilasi Hukum Islam.

Bahwa berdasarkan dalil-dalil tersebut, Pemohon memohon kepada Pengadilan Agama Sragen, agar menjatuhkan putusan yang amarnya sebagai berikut:

P R I M A I R

1. Mengabulkan Cerai Gugat Penggugat.
2. Menjatuhkan talak satu bain sughro Tergugat (Ahmad Maulana bin ABD. Rohim) terhadap Penggugat (Anik Handayani binti Hadi Suwarno Padi).

Disclaimer
Kepaniteraan Mahkamah Agung Republik Indonesia berusaha untuk selalu mencantumkan informasi paling kini dan akurat sebagai bentuk komitmen Mahkamah Agung untuk pelayanan publik, transparansi dan akuntabilitas pelaksanaan fungsi peradilan. Namun dalam hal-hal tertentu masih dimungkinkan terjadi permasalahan teknis terkait dengan akurasi dan keterkinian informasi yang kami sajikan, hal mana akan terus kami perbaiki dari waktu kewaktu.
Dalam hal Anda menemukan inakurasi informasi yang termuat pada situs ini atau informasi yang seharusnya ada, namun belum tersedia, maka harap segera hubungi Kepaniteraan Mahkamah Agung RI melalui :
Email : kepaniteraan@mahkamahagung.go.id Telp : 021-384 3348 (ext.318)

3. Menetapkan biaya perkara menurut hukum.

SUBSIDAIR

- Memohon putusan yang seadil-adilnya.

Bahwa pada persidangan yang telah ditetapkan Penggugat telah datang menghadap di persidangan, sedangkan Tergugat tidak datang menghadap di persidangan dan tidak menyuruh orang lain untuk menghadap sebagai wakil/kuasanya yang sah, meskipun berdasarkan surat panggilan (relaas) yang dibacakan di persidangan, Tergugat telah dipanggil secara resmi dan patut, sedangkan tidak ternyata bahwa tidak datangnya Tergugat tersebut disebabkan oleh suatu alasan yang sah;

Bahwa majelis hakim telah menasehati Penggugat agar berpikir untuk tidak bercerai dengan Tergugat, tetapi Penggugat tetap pada dalil-dalil pemohonannya untuk bercerai dengan Tergugat;

Bahwa perkara ini tidak dapat dimediasi karena Tergugat tidak pernah datang menghadap meskipun telah dipanggil secara resmi dan patut, selanjutnya dimulai pemeriksaan dengan membacakan surat gugatan Penggugat yang maksud dan tujuannya tetap dipertahankan oleh Penggugat;

Bahwa untuk menguatkan dalil-dalil gugatannya, Penggugat telah mengajukan bukti berupa:

A. Surat:

1. Foto copy Kartu Tanda Penduduk a.n. Penggugat, dikeluarkan oleh pejabat yang berwenang, sesuai dengan aslinya dan bermaterai cukup, P.1;
2. Foto kopi kutipant Akta Nikah nomor 19/19/I/2007, tertanggal 2 Januari 2007, tercatat di Kantor Urusan Agama Kecamatan Ngrampal Kabupaten Sragen, meterai cukup sesuai aslinya sebagai bukti P.2

B. Saksi:

- Bahwa saksi kenal Penggugat dan Tergugat karena saksi adalah ayah kandung Penggugat;
- Bahwa Penggugat menikah dengan Tergugat secara sah, pada tanggal 30 Desember 2006, di Ngrampal, Kabupaten Sragen dalam status Perawan dan Jejaka, sesaat akad nikah Tergugat mengucapkan Sighot Taklik Talak;

Disclaimer
Kepaniteraan Mahkamah Agung Republik Indonesia berusaha untuk selalu mencantumkan informasi paling kini dan akurat sebagai bentuk komitmen Mahkamah Agung untuk pelayanan publik, transparansi dan akuntabilitas pelaksanaan fungsi peradilan. Namun dalam hal-hal tertentu masih dimungkinkan terjadi permasalahan teknis terkait dengan akurasi dan keterkinian informasi yang kami sajikan, hal mana akan terus kami perbaiki dari waktu kewaktu.
Dalam hal Anda menemukan inakurasi informasi yang termuat pada situs ini atau informasi yang seharusnya ada, namun belum tersedia, maka harap segera hubungi Kepaniteraan Mahkamah Agung RI melalui :
Email : kepaniteraan@mahkamahagung.go.id Telp : 021-384 3348 (ext.318)

- Bahwa setelah Penggugat menikah dengan Tergugat, pada awalnya membentuk rumah tangga dan menetap dirumah orang tua Tergugat selama + 12 tahun, pada awalnya rukun dan harmonis;
- Bahwa selama dalam perkawinan antara Penggugat dengan Tergugat, telah dikaruniai keturunan seorang anak;
- Bahwa sekitar bulan Oktober tahun 2019, rumah tangga Penggugat dan Tergugat mulai goyah sering terjadi pertengkaran dan perselisihan, dikarenakan Tergugat jarang memberi nafkah kepada Penggugat, dan kalau Penggugat meminta uang tidak diberi serta Tergugat marah-marah berkata kasar, lalu Penggugat di pulangkan kerumah orang tuanya;
- Bahwa setelah Penggugat di pulangkan kerumah orang tuanya, Tergugat sudah tidak bisa dihubungi dan tidak memperdulikan Penggugat lagi sampai sekarang serta tidak memberi nafkah wajib, sehingga antara Penggugat dengan Tergugat sudah pisah rumah sekitar 1 tahun;

- Bahwa saksi kenal Penggugat dan Tergugat karena saksi adalah ayah kandung Penggugat;
- Bahwa Penggugat menikah dengan Tergugat secara sah, pada tanggal 30 Desember 2006, di Ngrampal, Kabupaten Sragen dalam status Perawan dan Jejaka, sesaat akad nikah Tergugat mengucapkan Sighot Taklik Talak;
- Bahwa setelah Penggugat menikah dengan Tergugat, pada awalnya membentuk rumah tangga dan menetap dirumah orang tua Tergugat selama sekitar 12 tahun, pada awalnya rukun dan harmonis;
- Bahwa selama dalam perkawinan antara Penggugat dengan Tergugat, telah dikaruniai keturunan seorang anak;
- Bahwa sekitar bulan Oktober tahun 2019, rumah tangga Penggugat dan Tergugat mulai goyah sering terjadi pertengkaran dan perselisihan, dikarenakan Tergugat jarang memberi nafkah kepada Penggugat, dan kalau Penggugat meminta uang tidak diberi serta Tergugat marah-marah berkata kasar, lalu Penggugat di pulangkan kerumah orang tuanya;
- Bahwa setelah Penggugat di pulangkan kerumah orang tuanya, Tergugat sudah tidak bisa dihubungi dan tidak memperdulikan Penggugat lagi sampai sekarang serta tidak memberi nafkah wajib, sehingga antara Penggugat dengan Tergugat sudah pisah rumah sekitar 1 tahun;

Disclaimer
Kepaniteraan Mahkamah Agung Republik Indonesia berusaha untuk selalu mencantumkan informasi paling kini dan akurat sebagai bentuk komitmen Mahkamah Agung untuk pelayanan publik, transparansi dan akuntabilitas pelaksanaan fungsi peradilan. Namun dalam hal-hal tertentu masih dimungkinkan terjadi permasalahan teknis terkait dengan akurasi dan keterkinian informasi yang kami sajikan, hal mana akan terus kami perbaiki dari waktu kewaktu.
Dalam hal Anda menemukan inakurasi informasi yang termuat pada situs ini atau informasi yang seharusnya ada, namun belum tersedia, maka harap segera hubungi Kepaniteraan Mahkamah Agung RI melalui :
Email : kepaniteraan@mahkamahagung.go.id Telp : 021-384 3348 (ext.318)

Bahwa Penggugat menyampaikan kesimpulan yang isinya sebagaimana tertuang dalam berita acara sidang;

Selanjutnya untuk singkatnya uraian putusan ini, maka semua hal yang termuat dalam berita acara sidang merupakan bagian yang tidak terpisahkan dari putusan ini;

PERTIMBANGAN HUKUM

Menimbang bahwa maksud dan tujuan gugatan Penggugat adalah seperti diuraikan tersebut di muka;

Menimbang, bahwa berdasarkan alat bukti P.1 dikaitkan pasal 4, pasal 49 dan pasal 73 Undang–Undang Nomor 7 Tahun 1989 tentang Peradilan Agama yang telah diubah dan di tambah dengan Undang-Undang Nomor 3 Tahun 2006 dan telah diubah yang kedua dengan Undang-Undang Nomor 50 Tahun 2009, dinyatakan terbukti secara absolute dan relatif bahwa perkara ini adalah wewenang Pengadilan Agama Sragen;

Menimbang bahwa Majelis Hakim telah berusaha secara maksimal mendamaikan dan menasehati Penggugat dan Tergugat di depan persidangan agar Penggugat dan Tergugat tetap membina rumah tangga dengan baik, namun tidak berhasil.

Menimbang bahwa berdasarkan PERMA Nomor 1 Tahun 2016 tentang mediasi, bahwa setiap perkara perdata harus dimediasi, namun karena Tergugat tidak pernah hadir di muka sidang dan tidak pula mengutus orang lain sebagai wakil atau kuasanya, sehingga Majelis Hakim menganggap perkara ini tidak dimediasi.

Menimbang, bahwa dari posita gugatan Penggugat, majelis menilai bahwa yang dijadikan alasan gugatan Penggugat adalah sering terjadinya perselisihan dan pertengkaran dikarenakan Tergugat jarang memberi nafkah kepada Penggugat, dan kalau Penggugat meminta uang tidak diberi serta Tergugat marah-marah berkata kasar, lalu Penggugat di pulangkan kerumah orang tuanya dan setelah Penggugat di pulangkan kerumah orang tuanya, Tergugat sudah tidak bisa dihubungi dan tidak memperdulikan Penggugat lagi sampai sekarang serta tidak memberi nafkah wajib, sehingga antara Penggugat dengan Tergugat sudah pisah rumah sekitar 1 tahun;

Menimbang bahwa alasan tersebut telah sesuai dengan ketentuan Pasal 34 ayat (3) Undang-Undang Nomor 1 Tahun 1974 jo Pasal 19 huruf (f) Peraturan

Disclaimer
Kepaniteraan Mahkamah Agung Republik Indonesia berusaha untuk selalu mencantumkan informasi paling kini dan akurat sebagai bentuk komitmen Mahkamah Agung untuk pelayanan publik, transparansi dan akuntabilitas pelaksanaan fungsi peradilan. Namun dalam hal-hal tertentu masih dimungkinkan terjadi permasalahan teknis terkait dengan akurasi dan keterkinian informasi yang kami sajikan, hal mana akan terus kami perbaiki dari waktu kewaktu.
Dalam hal Anda menemukan inakurasi informasi yang termuat pada situs ini atau informasi yang seharusnya ada, namun belum tersedia, maka harap segera hubungi Kepaniteraan Mahkamah Agung RI melalui :
Email : kepaniteraan@mahkamahagung.go.id Telp : 021-384 3348 (ext.318)

Pemerintah Nomor 9 Tahun 1975 jo. Pasal 116 huruf (f) Kompilasi Hukum Islam, oleh karenanya akan dipertimbangkan untuk diperiksa lebih lanjut.

Menimbang bahwa untuk membuktikan dalil-dalil gugatannya sebagaimana angka 1 sampai 7, Penggugat telah mengajukan alat bukti surat P dan 2 (dua) orang saksi yang akan dipertimabngkan sebagai berikut:

Menimbang bahwa bukti P. (Fotokopi Kutipan Akta Nikah) yang merupakan akta otentik dan telah bermeterai cukup dan cocok dengan aslinya, isi bukti tersebut menjelaskan mengenai perkawinan Penggugat dengan Tergugat, lagi pula dibuat oleh pejabat yang berwenang dan berkaitan langsung dengan apa yang dipersengketakan di Pengadilan sehingga bukti tersebut telah memenuhi syarat formil dan materiil, serta mempunyai kekuatan pembuktian yang sempurna dan mengikat sebagaimana ketentuan Pasal 1868 dan Pasal 1871 KUH Perdata, dengan demikian harus dinyatakan terbukti bahwa antara Penggugat dan Tergugat adalah suami-isteri yang sah, dan merupakan legal standing perkara ini.

Menimbang bahwa saksi pertama Penggugat sudah dewasa dan sudah disumpah, sehingga memenuhi syarat formil sebagaimana yang diatur dalam Pasal 145 ayat 1 angka 3e HIR.;

Menimbang bahwa keterangan saksi pertama Penggugat mengenai angka 1, 2, 3, 4, 5, 6 adalah fakta yang didengar sendiri/dialami sendiri dan relevan dengan dalil yang harus dibuktikan oleh Penggugat, oleh karena itu keterangan saksi tersebut telah memenuhi syarat materiil sebagaimana telah diatur dalam Pasal 171 HIR sehingga keterangan saksi tersebut memiliki kekuatan pembuktian dan dapat diterima sebagai alat bukti;

Menimbang bahwa saksi kedua Penggugat, sudah dewasa dan sudah disumpah, sehingga memenuhi syarat formal sebagaimana diatur dalam Pasal 145 ayat 1 angka 3e HIR.;

Menimbang bahwa keterangan saksi kedua Penggugat mengenai angka 2, 3, 4, 5, 6 adalah fakta yang dilihat sendiri/didengar sendiri dan relevan dengan dalil yang harus dibuktikan oleh Penggugat, oleh karena itu keterangan saksi tersebut telah memenuhi syarat materiil sebagaimana telah diatur dalam Pasal 145 ayat 1 angka 3e HIR. sehingga keterangan saksi tersebut memiliki kekuatan pembuktian dan dapat diterima sebagai alat bukti;

Disclaimer
Kepaniteraan Mahkamah Agung Republik Indonesia berusaha untuk selalu mencantumkan informasi paling kini dan akurat sebagai bentuk komitmen Mahkamah Agung untuk pelayanan publik, transparansi dan akuntabilitas pelaksanaan fungsi peradilan. Namun dalam hal-hal tertentu masih dimungkinkan terjadi permasalahan teknis terkait dengan akurasi dan keterkinian informasi yang kami sajikan, hal mana akan terus kami perbaiki dari waktu kewaktu.
Dalam hal Anda menemukan inakurasi informasi yang termuat pada situs ini atau informasi yang seharusnya ada, namun belum tersedia, maka harap segera hubungi Kepaniteraan Mahkamah Agung RI melalui :
Email : kepaniteraan@mahkamahagung.go.id Telp : 021-384 3348 (ext.318)

Menimbang, bahwa keterangan saksi 1 dan saksi 2 tersebut bersesuaian dan cocok antara satu dengan yang lain oleh karena itu keterangan dua orang saksi tersebut memenuhi Pasal 171 dan Pasal 172 HIR;

Menimbang bahwa berdasarkan pengakuan Penggugat, diperkuat dengan alat bukti P dan 2 (dua) orang saksi Penggugat. Maka Majelis Hakim telah memperoleh fakta yang pada pokoknya sebagai berikut:

- Bahwa Penggugat menikah dengan Tergugat secara sah, pada tanggal 30 Desember 2006, dihadapan Pegawai Pencatat Nikah Kantor Urusan Agama Kecamatan Ngrampal, Kabupaten Sragen;
- Bahwa Penggugat dan Tergugat telah hidup bersama dalam rumah tangga sebagai suami-isteri pada awalnya rukun dan harmonis dan dikaruniai 1 anak, namun keharmonisan tersebut sudah tidak terwujud lagi karena sering terjadi perselisihan dan pertengkaran;
- Bahwa perselisihan dan pertengkaran dikarenakan Tergugat jarang memberi nafkah kepada Penggugat, dan kalau Penggugat meminta uang tidak diberi serta Tergugat marah-marah berkata kasar, lalu Penggugat di pulangkan kerumah orang tuanya dan setelah Penggugat di pulangkan kerumah orang tuanya, Tergugat sudah tidak bisa dihubungi dan tidak memperdulikan Penggugat lagi sampai sekarang serta tidak memberi nafkah wajib, sehingga antara Penggugat dengan Tergugat sudah pisah rumah sekitar 1 tahun;

Menimbang bahwa pecahnya perkawinan tersebut dapat dilihat dari keadaan Penggugat dan Tergugat yang sering terjadi perselisihan dan pertengkaran dan akibatnya antara Penggugat dan Tergugat telah berpisah tempat tinggal bersama sampai saat ini dan ditunjukkan Penggugat dalam sidang ketidakmaunnya lagi hidup bersama dengan Tergugat, hal mana mengindikasikan bahwa perselisihan dan pertengkaran antara Penggugat dan Tergugat adalah perselisihan dan pertengkaran yang terus menerus.

Menimbang bahwa yang dimaksud perselisihan dalam rumah tangga tidaklah identik dengan pertengkaran mulut, rumah tangga dapat dinyatakan terjadi perselisihan jika hubungan suami isteri sudah tidak selaras, tidak saling percaya dan saling melindungi, maka dengan ditemukannya fakta antara Penggugat dengan Tergugat telah berpisah tempat kediaman bersama, menunjukkan bahwa antara Penggugat dengan Tergugat sudah tidak lagi saling percaya dan saling pengertian

Disclaimer
Kepaniteraan Mahkamah Agung Republik Indonesia berusaha untuk selalu mencantumkan informasi paling kini dan akurat sebagai bentuk komitmen Mahkamah Agung untuk pelayanan publik, transparansi dan akuntabilitas pelaksanaan fungsi peradilan. Namun dalam hal-hal tertentu masih dimungkinkan terjadi permasalahan teknis terkait dengan akurasi dan keterkinian informasi yang kami sajikan, hal mana akan terus kami perbaiki dari waktu kewaktu.
Dalam hal Anda menemukan inakurasi informasi yang termuat pada situs ini atau informasi yang seharusnya ada, namun belum tersedia, maka harap segera hubungi Kepaniteraan Mahkamah Agung RI melalui :
Email : kepaniteraan@mahkamahagung.go.id Telp : 021-384 3348 (ext.318)

dan sudah tidak ada lagi komunikasi suami isteri yang harmonis yang merupakan bagian dari gejala perselisihan dalam rumah tangga.

Menimbang bahwa dengan merujuk pada Putusan Mahkamah Agung Republik Indonesia tanggal 17 Maret 1999 Nomor 237/K/AG/1998 yang mengandung abstrak hukum, bahwa berselisih, cekcok, hidup berpisah, tidak dalam satu tempat kediaman bersama, salah satu pihak tidak berniat untuk meneruskan kehidupan bersama dengan pihak lain, hal itu adalah merupakan fakta hukum yang cukup untuk menjadi alasan dalam suatu perceraian sesuai dengan maksud Pasal 19 huruf (f) Peraturan Pemerintah Nomor 9 Tahun 1975.

Menimbang bahwa berdasarkan fakta tersebut di atas, maka Penggugat telah membuktikan dalil-dalil gugatannya tentang adanya perselisihan dan pertengkaran terus-menerus tanpa adanya penyelesaian yang baik. Sehingga Penggugat telah cukup bukti adanya alasan perceraian, menurut ketentuan Pasal 39 ayat (2) Undang-Undang Nomor 1 Tahun 1974 dan Pasal 116 huruf (f) Kompilasi Hukum Islam.

Menimbang bahwa maksud dan tujuan perkawinan untuk mencapai keluarga yang sakinah, mawaddah, wa rohmah sebagaimana yang diamanatkan pasal 3 Kompilasi Hukum Islam (KHI) jo. pasal 1 Undang-undang nomor 1 tahun 1974. Hal ini senafas dengan Al-Qur’an, surah Arrum ayat (21) sebagai berikut:

وَمِنْ آيَاتِهِ أَنْ خَلَقَ لَكُمْ مِنْ أَنْفُسِكُمْ أَزْوَاجًا لِتَسْكُنُوا إِلَيْهَا وَجَعَلَ بَيْنَكُمْ مَوَدَّةً وَرَحْمَةً إِنَّ فِي ذَلِكَ لَآيَاتٍ لِقَوْمٍ يَتَفَكَّرُونَ

Artinya:
"Dan di antara tanda-tanda kekuasaan-Nya ialah dia menciptakan untukmu isteri-isteri dari jenismu sendiri, supaya kamu cenderung dan merasa tenteram kepadanya, dan dijadikan-Nya diantaramu rasa kasih dan sayang. Sesungguhnya pada yang demikian itu benar-benar terdapat tanda-tanda bagi kaum yang berfikir".

ternyata telah tidak terwujud lagi dalam kehidupan rumah tangga antara Penggugat dan Tergugat.

Menimbang bahwa berdasarkan fakta yang telah terungkap serta bukti-bukti yang sah dalam sidang, maka Majelis Hakim berkeyakinan bahwa keutuhan rumah tangga antara Penggugat dengan Tergugat tidak dapat dipertahankan lagi, sehingga yang dipandang adil untuk menyelesaikan kemaslahatan kedua belah pihak adalah dengan perceraian.

Disclaimer
Kepaniteraan Mahkamah Agung Republik Indonesia berusaha untuk selalu mencantumkan informasi paling kini dan akurat sebagai bentuk komitmen Mahkamah Agung untuk pelayanan publik, transparansi dan akuntabilitas pelaksanaan fungsi peradilan. Namun dalam hal-hal tertentu masih dimungkinkan terjadi permasalahan teknis terkait dengan akurasi dan keterkinian informasi yang kami sajikan, hal mana akan terus kami perbaiki dari waktu kewaktu.
Dalam hal Anda menemukan inakurasi informasi yang termuat pada situs ini atau informasi yang seharusnya ada, namun belum tersedia, maka harap segera hubungi Kepaniteraan Mahkamah Agung RI melalui :
Email : kepaniteraan@mahkamahagung.go.id Telp : 021-384 3348 (ext.318)

Menimbang bahwa dengan kondisi rumah tangga yang demikian apabila dipaksakan untuk diteruskan, maka akan membawa mafsadat lebih besar daripada maslahatnya yaitu Penggugat dan Tergugat akan terus menerus dalam penderitaan lahir batin, hal ini perlu dihindari sesuai dengan kaidah fiqhiyah yang berbunyi :

د ر ء المفاسد مقد م على جلب المصالح

Artinya:
"Menolak banyak mafsadah lebih didahulukan dari pada menarik banyak maslahah".

Menimbang bahwa berdasarkan kesimpulan Penggugat di depan persidangan, yakni Penggugat berketetapan hati ingin bercerai dengan Tergugat dan Penggugat dalam keadaan sehat jasmani dan rohani, maka dalam hal ini majelis hakim perlu mempertimbangkan pendapat pakar hukum Islam sebagaimana yang termuat dalam kitab Al Iqna Juz II halaman 133 sebagai berikut:

وان اشتد عدم رغبة الزوجة لزوجها طلق عليه القاضى طلقة

Artinya:
"Apabila ketidaksenangan seorang isteri kepada suaminya telah mencapai puncaknya, maka pada saat itu hakim diperbolehkan menjatuhkan talak seorang suami kepada istrinya dengan talak satu".

Majelis hakim sependapat sekaligus mengambil alih pendapat pakar hukum Islam tersebut karena berkaitan erat dengan perkara ini.

Menimbang bahwa berdasarkan pertimbangan-pertimbangan tersebut di atas, dan alasan-alasan perceraian telah terpenuhi sebagaimana petunjuk Pasal 70 ayat (1) Undang-Undang Nomor 7 Tahun 1989 jo. Pasal 39 ayat (1) dan (2) Undang-Undang Nomor 1 Tahun 1974. Maka Majelis Hakim berkesimpulan bahwa gugatan Penggugat sebagaimana petitum angka 2 (dua) patut untuk dikabulkan dengan menjatuhkan talak ba'in.

Menimbang bahwa oleh karena talak yang akan dijatuhkan oleh Pengadilan adalah talak yang pertama, maka dengan demikian talak yang dikabulkan dan yang akan dijatuhkan oleh Pengadilan adalah talak satu bain shughro Tergugat kepada Penggugat sebagaimana yang diatur dalam pasal 119 (2) Kompilasi Hukum Islam;

Menimbang bahwa Tergugat tidak datang menghadap di sidang dan tidak pula menyuruh orang lain sebagai kuasanya meskipun telah dipanggil secara resmi

Disclaimer
Kepaniteraan Mahkamah Agung Republik Indonesia berusaha untuk selalu mencantumkan informasi paling kini dan akurat sebagai bentuk komitmen Mahkamah Agung untuk pelayanan publik, transparansi dan akuntabilitas pelaksanaan fungsi peradilan. Namun dalam hal-hal tertentu masih dimungkinkan terjadi permasalahan teknis terkait dengan akurasi dan keterkinian informasi yang kami sajikan, hal mana akan terus kami perbaiki dari waktu kewaktu.
Dalam hal Anda menemukan inakurasi informasi yang termuat pada situs ini atau informasi yang seharusnya ada, namun belum tersedia, maka harap segera hubungi Kepaniteraan Mahkamah Agung RI melalui :
Email : kepaniteraan@mahkamahagung.go.id Telp : 021-384 3348 (ext.318)

dan patut, dan tidak ternyata atas ketidakhadirnya disebabkan suatu halangan yang sah, dan lagi pula gugatan Penggugat dinilai cukup beralasan dan tidak melawan hukum, oleh karenanya Tergugat harus dinyatakan tidak hadir dan putusan ini harus dijatuhkan dengan Verstek atau tanpa hadirnya Tergugat, sesuai petunjuk pasal 125 HIR;

Menimbang bahwa perkara ini termasuk dalam bidang perkawinan, maka berdasarkan ketentuan Pasal 89 ayat (1) Undang-Undang Nomor 7 Tahun 1989 yang telah diubah dengan Undang-Undang Republik Indonesia Nomor 3 Tahun 2006 dan perubahan kedua Undang-Undang Republik Indonesia Nomor 50 Tahun 2009 Tentang Peradilan Agama, maka semua biaya perkara yang timbul dalam perkara ini dibebankan kepada Penggugat.

Mengingat segala ketentuan Peraturan Perundang-undangan yang berlaku dan hukum Islam yang berkaitan dengan perkara ini.

MENGADILI:

1. Menyatakan Tergugat yang telah dipanggil secara resmi dan patut untuk menghadap di persidangan tidak hadir;
2. Mengabulkan gugatan Penggugat dengan verstek;
3. Menjatuhkan talak satu bain sughro Tergugat (lana bin ohim) terhadap Penggugat (yani binti warno);
4. Membebankan kepada Penggugat untuk membayar biaya perkara ini sebesar Rp489.000,- (empat ratus delapan puluh sembilan ribu rupiah);

Demikian putusan ini dijatuhkan dalam rapat permusyawaratan Majelis Hakim yang dilangsungkan pada hari Senin tanggal 30 November 2020 Masehi, bertepatan dengan tanggal 15 R.Akhir 1442 Hijriah, oleh kami Drs. H. Yasin Irfan, M.H. sebagai Ketua Majelis, Drs. Ikhsan SH.MA dan Drs. H.Muhammad Fatchan MA masing-masing sebagai Hakim Anggota, putusan tersebut diucapkan dalam sidang terbuka untuk umum pada hari itu juga pada hari Senin tanggal 30 November 2020 Masehi, bertepatan dengan tanggal 15 R. Akhir 1442 Hijriah, oleh Ketua Majelis tersebut dengan didampingi oleh Hakim Anggota dan dibantu Drs.Amir. sebagai Panitera Pengganti serta dihadiri oleh Penggugat tanpa hadirnya Tergugat;

Hakim Anggota, Ketua Majelis,

Disclaimer
Kepaniteraan Mahkamah Agung Republik Indonesia berusaha untuk selalu mencantumkan informasi paling kini dan akurat sebagai bentuk komitmen Mahkamah Agung untuk pelayanan publik, transparansi dan akuntabilitas pelaksanaan fungsi peradilan. Namun dalam hal-hal tertentu masih dimungkinkan terjadi permasalahan teknis terkait dengan akurasi dan keterkinian informasi yang kami sajikan, hal mana akan terus kami perbaiki dari waktu kewaktu.
Dalam hal Anda menemukan inakurasi informasi yang termuat pada situs ini atau informasi yang seharusnya ada, namun belum tersedia, maka harap segera hubungi Kepaniteraan Mahkamah Agung RI melalui :
Email : kepaniteraan@mahkamahagung.go.id Telp : 021-384 3348 (ext.318)

Drs. Ikhsan SH.MA Drs. H. Yasin Irfan, M.H.
Hakim Anggota

Drs. H.Muhammad Fatchan MA
Panitera Pengganti

Drs.Amir.

Perincian biaya :

1.	Pendaftaran	Rp	30.000,00
2.	Proses	Rp	75.000,00
3.	Panggilan	Rp	368.000,00
4.	Redaksi	Rp	10.000,00
5.	Meterai	Rp	6.000,00
	Jumlah	Rp	489.000,00

(empat ratus delapan puluh sembilan ribu rupiah);

Disclaimer
Kepaniteraan Mahkamah Agung Republik Indonesia berusaha untuk selalu mencantumkan informasi paling kini dan akurat sebagai bentuk komitmen Mahkamah Agung untuk pelayanan publik, transparansi dan akuntabilitas pelaksanaan fungsi peradilan. Namun dalam hal-hal tertentu masih dimungkinkan terjadi permasalahan teknis terkait dengan akurasi dan keterkinian informasi yang kami sajikan, hal mana akan terus kami perbaiki dari waktu kewaktu.
Dalam hal Anda menemukan inakurasi informasi yang termuat pada situs ini atau informasi yang seharusnya ada, namun belum tersedia, maka harap segera hubungi Kepaniteraan Mahkamah Agung RI melalui :
Email : kepaniteraan@mahkamahagung.go.id   Telp : 021-384 3348 (ext.318)
