# Supplementary material for: Classification of divorce causes during the COVID-19 pandemic using convolutional neural networks
Source: PeerJ Comput Sci. 2022 Jun 30;8:e998. doi: 10.7717/peerj-cs.998 (PMC9299239; doi:10.7717/peerj-cs.998)
Supplement: Supplemental Information 5 [file peerj-cs-08-998-s005.zip › Masalah Ekonomi Dataset/Data ke-4.pdf]

P U T U S A N

Nomor 4192/Pdt.G/2020/PA.Smdg

بِسْمِ اللهِ الرَّحْمٰنِ الرَّحِيْمِ

DEMI KEADILAN BERDASARKAN KETUHANAN YANG MAHA ESA

Pengadilan Agama Sumedang yang memeriksa dan mengadili perkara tertentu pada tingkat pertama dalam sidang majelis hakim telah menjatuhkan putusan dalam perkara Cerai Gugat antara:

Yani Suryani Binti Suryana, tempat dan tanggal lahir Sumedang, 19 Juni 1985, agama Islam, pekerjaan Ibu Rumah Tangga, Pendidikan Sekolah Lanjutan Tingkat Pertama, tempat kediaman di Dusun Silegok Rt 21 Rw 06 Desa Paseh Kidul Kecamatan Paseh Kaupaten Sumedang sebagai Penggugat**;**

melawan

Acep Suherman Bin Aja, tempat dan tanggal lahir Sumedang, 16 Juni 1980, agama Islam, pekerjaan Wiraswasta, Pendidikan Sekolah Dasar, tempat kediaman di Dusun Cileuksa Rt 03 Rw 05 Desa Lego Kaler Kecamatan Paseh Kabupaten Sumedang sebagai Tergugat;

Pengadilan Agama tersebut;

Telah mempelajari surat-surat yang berkaitan dengan perkara ini;

Telah mendengar keterangan Penggugat dan para saksi di muka sidang;

DUDUK PERKARA

Bahwa Penggugat dalam surat gugatan tanggal 04 Desember 2020 telah mengajukan permohonan Cerai Gugat, yang telah terdaftar di Kepaniteraan Pengadilan Agama Sumedang, dengan Nomor 4192/Pdt.G/2020/PA.Smdg, tanggal 04 Desember 2020, dengan dalil-dalil pada pokoknya sebagai berikut:

Disclaimer
Kepaniteraan Mahkamah Agung Republik Indonesia berusaha untuk selalu mencantumkan informasi paling kini dan akurat sebagai bentuk komitmen Mahkamah Agung untuk pelayanan publik, transparansi dan akuntabilitas pelaksanaan fungsi peradilan. Namun dalam hal-hal tertentu masih dimungkinkan terjadi permasalahan teknis terkait dengan akurasi dan keterkinian informasi yang kami sajikan, hal mana akan terus kami perbaiki dari waktu kewaktu.
Dalam hal Anda menemukan inakurasi informasi yang termuat pada situs ini atau informasi yang seharusnya ada, namun belum tersedia, maka harap segera hubungi Kepaniteraan Mahkamah Agung RI melalui :
Email : kepaniteraan@mahkamahagung.go.id Telp : 021-384 3348 (ext.318)

1. Bahwa Penggugat dan Tergugat adalah suami isteri sah menikah pada tanggal 12 November 2013 berdasarkan Kutipan Akta Nikah KUA Kecamatan Paseh Kabupaten Sumedang, No: 0399/013/XI/2013, tertanggal 12 November 2013;
2. Bahwa sebelum membina rumahtangga, Penggugat berstatus Janda Cerai begitupun Tergugat berstatus Duda Cerai;
3. Bahwa antara Penggugat dan Tergugat terakhir berumah tangga tinggal di rumah kediaman milik Tergugat Dusun Cileuksa RT 03 RW 05 Desa Legok Kaler Kecamatan Paseh Kabupaten Sumedang dan telah bergaul baik sebagaimana layaknya suami isteri, namun belum dikaruniai anak;
4. Bahwa antara Penggugat dan Tergugat sejak **bulan Juni 2019** sudah tidak harmonis lagi dalam menjalani hidup berumah tangga dikarenakan antara Penggugat dengan Tergugat sering terjadi perselisihan dan pertengkaran terus-menerus yang disebabkan karena Tergugat sering bersikap acuh dan kurang peduli terhadap Penggugat, yakni Tergugat kurang menujukan perhatian dan kasih sayangnya terhadap Penggugat. Selain itu Tergugat sering berikap acuh dan mengabaikan Penggugat, apabila Penggugat meminta ijin kepada Tergugat untuk menjenguk dan merawat orangtuanya yang sedang sakit, sehingga hal itu menyebabkan rumah tangga di rasa tidak nyaman dan harmonis.
5. Bahwa keretakan rumah tangga antara Penggugat dan Tergugat telah berlangsung lama. Penggugat berusaha sabar, tetapi Tergugat tidak berusaha berubah dan merubah sifatnya. Oleh karena itu Penggugat merasa tidak nyaman berumah tangga dengan Tergugat. Puncaknya pada **bulan Agustus 2019 Penggugat pergi meningalkan rumah kediaman milik Tergugat** dan sejak itu antara Penggugat dan Tergugat sudah pisah tempat tinggal dan tidak lagi bergaul layaknya suami isteri. Dimana sekarang ini **Penggugat tinggal di rumah kediaman orangtua Penggugat** dan **Tergugat tinggal di rumah kediaman milik Tergugat**;
6. Bahwa keretakan rumah tangga Penggugat dan Termohon sudah pernah didamaikan oleh keluarga, tetapi tidak berhasil;

Disclaimer
Kepaniteraan Mahkamah Agung Republik Indonesia berusaha untuk selalu mencantumkan informasi paling kini dan akurat sebagai bentuk komitmen Mahkamah Agung untuk pelayanan publik, transparansi dan akuntabilitas pelaksanaan fungsi peradilan. Namun dalam hal-hal tertentu masih dimungkinkan terjadi permasalahan teknis terkait dengan akurasi dan keterkinian informasi yang kami sajikan, hal mana akan terus kami perbaiki dari waktu kewaktu.
Dalam hal Anda menemukan inakurasi informasi yang termuat pada situs ini atau informasi yang seharusnya ada, namun belum tersedia, maka harap segera hubungi Kepaniteraan Mahkamah Agung RI melalui :
Email : kepaniteraan@mahkamahagung.go.id Telp : 021-384 3348 (ext.318)

7. Bahwa Penggugat tidak mampu untuk membayar biaya perkara atas pengajuan Cerai Gugat di Pengadilan Agama Sumedang. Ketidakmampuan Penggugat tersebut dilampirkan juga Surat Keterangan Tidak Mampu Nomor: 470/806/Ds./2020 yang dikeluarkan oleh Desa Paseh Kidul Kecamatan Paseh Kabupaten Sumedang., tertanggal 03 Desember 2020, untuk itu Penggugat mohon dibebaskan dari biaya berperkara (cuma-cuma);
8. Bahwa gugatan Penggugat tersebut telah memenuhi syarat sesuai ketentuan Pasal 19 huruf (f) Peraturan Pemerintah Nomor 9 Tahun 1975 jo. Pasal 116 huruf (f) Kompilasi Hukum Islam;
9. Bahwa atas permasalahan tersebut di atas Penggugat sudah tidak sanggup lagi untuk mempertahankan perkawinan ini, oleh karena itu Penggugat telah berketetapan hati untuk menggugat cerai dari Tergugat;

Bahwa berdasarkan dalil-dalil tersebut, Penggugat memohon kepada Pengadilan Agama Sumedang, agar menjatuhkan putusan yang amarnya sebagai berikut:

1. Menerima dan mengabulkan gugatan Penggugat.
2. Menjatuhkan talak satu ba'in sughro dari Tergugat (Acep Suherman bin Aja) kepada Penggugat (Yani Suryani binti Suryana).
3. Membebaskan biaya perkara menurut hukum.

Bahwa pada persidangan yang telah ditetapkan Penggugat telah datang menghadap di persidangan, sedangkan Tergugat tidak datang menghadap di persidangan dan tidak menyuruh orang lain untuk menghadap sebagai wakil/kuasanya yang sah, meskipun berdasarkan surat panggilan (relaas) yang dibacakan di persidangan, Tergugat telah dipanggil secara resmi dan patut, sedangkan tidak ternyata bahwa tidak datangnya Tergugat tersebut disebabkan oleh suatu alasan yang sah;

Bahwa majelis hakim telah menasehati Penggugat agar berpikir untuk tidak bercerai dengan Tergugat, tetapi Penggugat tetap pada dalil-dalil Penggugatannya untuk bercerai dengan Tergugat;

Disclaimer
Kepaniteraan Mahkamah Agung Republik Indonesia berusaha untuk selalu mencantumkan informasi paling kini dan akurat sebagai bentuk komitmen Mahkamah Agung untuk pelayanan publik, transparansi dan akuntabilitas pelaksanaan fungsi peradilan. Namun dalam hal-hal tertentu masih dimungkinkan terjadi permasalahan teknis terkait dengan akurasi dan keterkinian informasi yang kami sajikan, hal mana akan terus kami perbaiki dari waktu kewaktu.
Dalam hal Anda menemukan inakurasi informasi yang termuat pada situs ini atau informasi yang seharusnya ada, namun belum tersedia, maka harap segera hubungi Kepaniteraan Mahkamah Agung RI melalui :
Email : kepaniteraan@mahkamahagung.go.id   Telp : 021-384 3348 (ext.318)

Bahwa perkara ini tidak dapat dimediasi karena Tergugat tidak pernah datang menghadap meskipun telah dipanggil secara resmi dan patut, selanjutnya dimulai pemeriksaan dengan membacakan surat Penggugatan Penggugat yang maksud dan tujuannya tetap dipertahankan oleh Penggugat;

Bahwa untuk menguatkan dalil-dalil Penggugatannya, Penggugat telah mengajukan bukti berupa :

A. Surat :

Fotocopy Kutipan Akta Nikah Nomor : 0399/013/XI/2013, tertanggal 12 November 2013 yang dikeluarkan oleh Kantor Urusan Agama Kecamatan Paseh, Kabupaten Sumedang, telah bermeterai cukup dan dicocokan dengan aslinya, kemudian diberi tanda alat bukti (P) ;

B. Saksi :

1. Emar binti Daim, umur 62 tahun, agama Islam, pekerjaan ibu rumah tangga, tempat kediaman di Dusun Silegok Rt 21 Rw 06 Desa Paseh Kidul Kecamatan Paseh Kaupaten Sumedang, di bawah sumpahnya memberikan keterangan sebagai berikut :
   - Bahwa saksi kenal kepada Penggugat dan Tergugat karena saksi sebagai Bibi Penggugat;
   - Bahwa saksi mengetahui Penggugat dan Tergugat setelah menikah bertempat tinggal bersama terakhir tinggal bersama di rumah milik Penggugat di bersama di rumah kediaman milik Tergugat Dusun Cileuksa RT 03 RW 05 Desa Legok Kaler Kecamatan Paseh Kabupaten Sumedang ;
   - Bahwa saksi mengetahui rumah tangga Penggugat dan Tergugat semula rukun dan harmonis meskipun tidak dikaruniai anak, akan tetapi sejak bulan Juni 2019 rumah tangga Penggugat dan Tergugat mulai terjadi perselisihan dan pertengkaran;
   - Bahwa saksi melihat antara Penggugat dan Tergugat telah berselisih dan bertengkar karena Tergugat sering bersikap acuh dan kurang peduli terhadap Penggugat ;

Disclaimer
Kepaniteraan Mahkamah Agung Republik Indonesia berusaha untuk selalu mencantumkan informasi paling kini dan akurat sebagai bentuk komitmen Mahkamah Agung untuk pelayanan publik, transparansi dan akuntabilitas pelaksanaan fungsi peradilan. Namun dalam hal-hal tertentu masih dimungkinkan terjadi permasalahan teknis terkait dengan akurasi dan keterkinian informasi yang kami sajikan, hal mana akan terus kami perbaiki dari waktu kewaktu.
Dalam hal Anda menemukan inakurasi informasi yang termuat pada situs ini atau informasi yang seharusnya ada, namun belum tersedia, maka harap segera hubungi Kepaniteraan Mahkamah Agung RI melalui :
Email : kepaniteraan@mahkamahagung.go.id Telp : 021-384 3348 (ext.318)

- Bahwa pada bulan Agustus 2019 Penggugat pergi meninggalkan rumah tempat kediaman bersama sampai sekarang, sehingga sejak saat itu antara Penggugat dan Tergugat telah pisah rumah hingga sekarang;
- Bahwa pihak keluarga Penggugat dan Tergugat telah mendamaikan dan menasehati Penggugat dan Tergugat akan tetapi tidak berhasil ;
- Bahwa saksi sudah tidak ada kesanggupan merukunkan Penggugat dan Tergugat ;

2. Titing binti Nana, umur 49 tahun, agama Islam, pekerjaan ibu rumah tangga, tempat kediaman di Dusun Silegok Rt 21 Rw 06 Desa Paseh Kidul Kecamatan Paseh Kaupaten Sumedang, di bawah sumpahnya memberikan keterangan sebagai berikut :
   - Bahwa saksi kenal kepada Penggugat dan Tergugat karena saksi sebagai Bibi Penggugat;
   - Bahwa saksi mengetahui semula rumah tangga Penggugat dan Tergugat rukun dan harmonis meskipun tidak dikaruniai anak, namun sejak bulan Juni 2019 rumah tangga Penggugat dan Tergugat mulai berselisih dan bertengkar ;
   - Bahwa saksi pernah mendengar antara Penggugat dan Tergugat berselisih dan bertengkar, yaitu pada bulan Juni 2019 disebabkan karena Tergugat sering bersikap acuh dan kurang peduli terhadap Penggugat ;
   - Bahwa puncak pertengkaran terjadi pada bulan Agustus 2019 dimana Tergugat pergi meninggalkan rumah tempat kediaman bersama sampai sekarang, maka sejak saat itu antara Penggugat dan Tergugat telah pisah rumah hingga sekarang ;
   - Bahwa saksi dan keluarga Penggugat dan keluarga Tergugat telah mendamaikan dan menasehati Penggugat dan Tergugat supaya rukun kembali membina rumah tangga akan tetapi tidak berhasil ;
   - Bahwa saksi sudah tidak kesanggupan lagi merukunkan Penggugat dan Tergugat;

Disclaimer
Kepaniteraan Mahkamah Agung Republik Indonesia berusaha untuk selalu mencantumkan informasi paling kini dan akurat sebagai bentuk komitmen Mahkamah Agung untuk pelayanan publik, transparansi dan akuntabilitas pelaksanaan fungsi peradilan. Namun dalam hal-hal tertentu masih dimungkinkan terjadi permasalahan teknis terkait dengan akurasi dan keterkinian informasi yang kami sajikan, hal mana akan terus kami perbaiki dari waktu kewaktu.
Dalam hal Anda menemukan inakurasi informasi yang termuat pada situs ini atau informasi yang seharusnya ada, namun belum tersedia, maka harap segera hubungi Kepaniteraan Mahkamah Agung RI melalui :
Email : kepaniteraan@mahkamahagung.go.id   Telp : 021-384 3348 (ext.318)

Bahwa Penggugat menyampaikan kesimpulan yang isinya sebagaimana tertuang dalam berita acara sidang;

Selanjutnya untuk singkatnya uraian putusan ini, maka semua hal yang termuat dalam berita acara sidang merupakan bagian yang tidak terpisahkan dari putusan ini ;

PERTIMBANGAN HUKUM

Menimbang, bahwa maksud dan tujuan Penggugatan Penggugat adalah sebagaimana terurai di atas;

Menimbang, bahwa ternyata Tergugat tidak datang menghadap di persidangan dan tidak pula menyuruh orang lain untuk menghadap sebagai wakil/kuasanya yang sah, meskipun berdasarkan Surat Panggilan (relaas) yang dibacakan di persidangan, Tergugat telah dipanggil secara resmi dan patut, sedangkan tidak ternyata bahwa tidak datangnya Tergugat tersebut disebabkan oleh suatu alasan yang sah;

Menimbang, bahwa Tergugat yang dipanggil secara resmi dan patut akan tetapi tidak menghadap harus dinyatakan tidak hadir dan Penggugatan tersebut harus diperiksa secara verstek;

Menimbang, bahwa berdasarkan ketentuan Pasal 125 ayat (1) HIR/ Pasal 149 ayat (1) R.Bg, yaitu putusan yang dijatuhkan tanpa hadirnya Tergugat dapat dikabulkan sepanjang berdasarkan hukum dan beralasan, oleh karena itu majelis hakim membebani Penggugat untuk membuktikan dalil-dalil Penggugatannya;

Menimbang, bahwa untuk menguatkan perkara tersebut berdasarkan hokum dan beralasan, maka majelis membebani Penggugat untuk membuktikan dalil-dalil gugatannya ;

Menimbang, bahwa untuk membuktikan dalil permohonannya, Penggugat telah mengajukan alat bukti surat dan 2 orang saksi;

Disclaimer
Kepaniteraan Mahkamah Agung Republik Indonesia berusaha untuk selalu mencantumkan informasi paling kini dan akurat sebagai bentuk komitmen Mahkamah Agung untuk pelayanan publik, transparansi dan akuntabilitas pelaksanaan fungsi peradilan. Namun dalam hal-hal tertentu masih dimungkinkan terjadi permasalahan teknis terkait dengan akurasi dan keterkinian informasi yang kami sajikan, hal mana akan terus kami perbaiki dari waktu kewaktu.
Dalam hal Anda menemukan inakurasi informasi yang termuat pada situs ini atau informasi yang seharusnya ada, namun belum tersedia, maka harap segera hubungi Kepaniteraan Mahkamah Agung RI melalui :
Email : kepaniteraan@mahkamahagung.go.id Telp : 021-384 3348 (ext.318)

Menimbang, bahwa bukti P (Fotokopi Kutipan Akta Nikah) yang merupakan akta otentik dan telah bermeterai cukup dan cocok dengan aslinya, isi bukti tersebut menjelaskan mengenai hubungan suami isteri antara Pemohon dengan Termohon, sehingga bukti tersebut telah memenuhi syarat formal dan materiil, serta mempunyai kekuatan pembuktian yang sempurna dan mengikat;

Menimbang, bahwa berdasarkan bukti P.1 tentang kompetensi absolut dalam perkara ini, Majelis Hakim menyatakan bahwa perkara ini adalah wewenang Pengadilan Agama Sumedang ;

Menimbang, bahwa saksi 1 Penggugat, sudah dewasa dan sudah bersumpah, sehingga memenuhi syarat formal sebagaimana diatur dalam Pasal 145 ayat 1 angka 3e HIR;

Menimbang, bahwa keterangan saksi 1 Penggugat mengenai pernikahan Penggugat dengan Tergugat, adalah fakta yang dilihat sendiri dan relevan dengan dalil yang harus dibuktikan oleh Penggugat, oleh karena itu keterangan saksi tersebut telah memenuhi syarat materiil sebagaimana telah diatur dalam Pasal 171 HIR. sehingga keterangan saksi tersebut memiliki kekuatan pembuktian dan dapat diterima sebagai alat bukti;

Menimbang, bahwa saksi 2 Penggugat, sudah dewasa dan sudah bersumpah, sehingga memenuhi syarat formal sebagaimana diatur dalam Pasal 145 ayat 1 angka 3e HIR;

Menimbang, bahwa keterangan saksi 2 Penggugat mengenai terjadinya pernikahan Penggugat dengan Tergugat, adalah fakta yang didengar sendiri dan relevan dengan dalil yang harus dibuktikan oleh Penggugat, oleh karena itu keterangan saksi tersebut telah memenuhi syarat materiil sebagaimana telah diatur dalam Pasal 171 HIR. sehingga keterangan saksi tersebut memiliki kekuatan pembuktian dan dapat diterima sebagai alat bukti;

Disclaimer
Kepaniteraan Mahkamah Agung Republik Indonesia berusaha untuk selalu mencantumkan informasi paling kini dan akurat sebagai bentuk komitmen Mahkamah Agung untuk pelayanan publik, transparansi dan akuntabilitas pelaksanaan fungsi peradilan. Namun dalam hal-hal tertentu masih dimungkinkan terjadi permasalahan teknis terkait dengan akurasi dan keterkinian informasi yang kami sajikan, hal mana akan terus kami perbaiki dari waktu kewaktu.
Dalam hal Anda menemukan inakurasi informasi yang termuat pada situs ini atau informasi yang seharusnya ada, namun belum tersedia, maka harap segera hubungi Kepaniteraan Mahkamah Agung RI melalui :
Email : kepaniteraan@mahkamahagung.go.id   Telp : 021-384 3348 (ext.318)

Menimbang, bahwa keterangan saksi 1 dan saksi 2 Penggugat telah terbukti bahwa Penggugat dengan Tergugat telah menikah secara sah berdasarkan syariat Islam ;

Menimbang, bahwa keterangan saksi 1 Penggugat mengenai perselisihan dan pertengkaran, adalah fakta yang dilihat sendiri dan relevan dengan dalil yang harus dibuktikan oleh Penggugat, oleh karena itu keterangan saksi tersebut telah memenuhi syarat materiil sebagaimana telah diatur dalam Pasal 171 HIR sehingga keterangan saksi tersebut memiliki kekuatan pembuktian dan dapat diterima sebagai alat bukti;

Menimbang, bahwa keterangan saksi 2 Penggugat mengenai perselisihan dan pertengkaran, adalah fakta yang didengar sendiri dan relevan dengan dalil yang harus dibuktikan oleh Penggugat, oleh karena itu keterangan saksi tersebut telah memenuhi syarat materiil sebagaimana telah diatur dalam Pasal 171 HIR sehingga keterangan saksi tersebut memiliki kekuatan pembuktian dan dapat diterima sebagai alat bukti;

Menimbang, bahwa keterangan saksi 1 dan saksi 2 Penggugat bersesuaian dan cocok antara satu dengan yang lain oleh karena itu keterangan dua orang saksi tersebut memenuhi Pasal 171 dan Pasal 172 HIR ;

Menimbang, bahwa berdasarkan bukti P, Saksi 1 dan Saksi 2 terbukti fakta kejadian sebagai berikut:

1. Bahwa Penggugat telah menikah dengan Tergugat dan belum bercerai sampai dengan sekarang ;
2. Bahwa Penggugat dan Tergugat sering terjadi perselisihan dan pertengkaran ;
3. Bahwa perselisihan dan pertengkaran antara Penggugat dan Tergugat telah didamaikan oleh pihak keluarga dan tokoh masyarakat setempat ;
4. Bahwa antara Penggugat dan Tergugat sudah pisah rumah tempat tinggal ;

Disclaimer
Kepaniteraan Mahkamah Agung Republik Indonesia berusaha untuk selalu mencantumkan informasi paling kini dan akurat sebagai bentuk komitmen Mahkamah Agung untuk pelayanan publik, transparansi dan akuntabilitas pelaksanaan fungsi peradilan. Namun dalam hal-hal tertentu masih dimungkinkan terjadi permasalahan teknis terkait dengan akurasi dan keterkinian informasi yang kami sajikan, hal mana akan terus kami perbaiki dari waktu kewaktu.
Dalam hal Anda menemukan inakurasi informasi yang termuat pada situs ini atau informasi yang seharusnya ada, namun belum tersedia, maka harap segera hubungi Kepaniteraan Mahkamah Agung RI melalui :
Email : kepaniteraan@mahkamahagung.go.id Telp : 021-384 3348 (ext.318)

Menimbang, bahwa berdasarkan fakta-fakta tersebut di atas dapat disimpulkan fakta hukum sebagai berikut:

1. Bahwa perkara dimaksud adalah kewenangan absolut Pengadilan Agama Sumedang ;
2. Bahwa Penggugat dan Tergugat adalah suami isteri yang sah dan tidak pernah berceri ;
3. Bahwa antara Penggugat dan Tergugat terus-menerus terjadi perselisihan dan pertengkaran dan tidak ada harapan akan hidup rukun lagi dalam rumah tangga ;
4. Bahwa antara Penggugat dan Tergugat telah pisah rumah;

Menimbang, bahwa fakta hukum tersebut telah pula memenuhi doktrin hukum Islam sebagai berikut :

إذا اشتدّ رغبة الزوجة لزوجها طلق عليها القاضى طلقة

Artinya: "Disaat istri telah memuncak kebencian terhadap suaminya, maka hakim diperkenankan menjatuhkan talak (suami) nya dengan talak satu";

Menimbang, bahwa fakta hukum tersebut telah juga memenuhi Pasal 22 jo Pasal 19 huruf (f) Peraturan Pemerintah Nomor 9 Tahun 1975 jo. Pasal 19 huruf (f) Kompilasi Hukum Islam ;

Menimbang, bahwa berdasarkan pertimbangan-pertimbangan tersebut di atas dan Penggugat belum pernah cerai dengan Tergugat, maka petitum permohonan Penggugat mengenai cerai gugat tersebut memenuhi Pasal 119 Kompilasi Hukum Islam, oleh karena itu dapat dikabulkan;

Menimbang, bahwa oleh karena Penggugat telah diizinkan berperkara secara prodeo maka Penggugat dibebaskan untuk membayar biaya perkara ini;

Mengingat, semua pasal dalam peraturan perundang-undangan dan hukum Islam yang berkaitan dengan perkara ini;

Disclaimer
Kepaniteraan Mahkamah Agung Republik Indonesia berusaha untuk selalu mencantumkan informasi paling kini dan akurat sebagai bentuk komitmen Mahkamah Agung untuk pelayanan publik, transparansi dan akuntabilitas pelaksanaan fungsi peradilan. Namun dalam hal-hal tertentu masih dimungkinkan terjadi permasalahan teknis terkait dengan akurasi dan keterkinian informasi yang kami sajikan, hal mana akan terus kami perbaiki dari waktu kewaktu.
Dalam hal Anda menemukan inakurasi informasi yang termuat pada situs ini atau informasi yang seharusnya ada, namun belum tersedia, maka harap segera hubungi Kepaniteraan Mahkamah Agung RI melalui :
Email : kepaniteraan@mahkamahagung.go.id Telp : 021-384 3348 (ext.318)

MENGADILI:

1. Menyatakan Tergugat yang telah dipanggil secara resmi dan patut untuk menghadap di persidangan tidak hadir ;
2. Mengabulkan gugatan Penggugat dengan verstek ;
3. Menjatuhkan talak satu ba'in sughra dari Tergugat **(Acep Suherman bin Aja**) terhadap Penggugat (**Yani Suryani binti Suryana)** ;
4. Membebaskan Penggugat membayar biaya perkara ini ;

Demikian putusan ini dijatuhkan dalam permusyawaratan majelis yang dilangsungkan pada hari Senin tanggal 21 Desember 2020 Masehi, bertepatan dengan tanggal 06 Jumadil Awal 1442 Hijriyah, oleh Drs. Dimyati, SH, MH sebagai Ketua Majelis, dan Drs. Endang Sofwan, MH. dan Drs. Nuryadi Siswanto, MH masing-masing sebagai Hakim Anggota, putusan tersebut dibacakan dalam sidang terbuka untuk umum pada hari itu juga, oleh Ketua Majelis tersebut dengan didampingi oleh Hakim Anggota dan dibantu oleh Agus Kurnia, S. Sy sebagai Panitera Pengganti dengan dihadiri oleh Penggugat tanpa hadirnya Tergugat ;

Ketua Majelis,

Hakim Anggota,

Drs. Dimyati, SH, MH

Drs. Endang Sofwan, MH

Hakim Anggota,

Drs. Nuryadi Siswanto, MH

*Halaman **10** dari **11** putusan Nomor 4192/Pdt.G/2020/PA.Smdg*

Disclaimer
Kepaniteraan Mahkamah Agung Republik Indonesia berusaha untuk selalu mencantumkan informasi paling kini dan akurat sebagai bentuk komitmen Mahkamah Agung untuk pelayanan publik, transparansi dan akuntabilitas pelaksanaan fungsi peradilan. Namun dalam hal-hal tertentu masih dimungkinkan terjadi permasalahan teknis terkait dengan akurasi dan keterkinian informasi yang kami sajikan, hal mana akan terus kami perbaiki dari waktu kewaktu.
Dalam hal Anda menemukan inakurasi informasi yang termuat pada situs ini atau informasi yang seharusnya ada, namun belum tersedia, maka harap segera hubungi Kepaniteraan Mahkamah Agung RI melalui :
Email : kepaniteraan@mahkamahagung.go.id Telp : 021-384 3348 (ext.318)

Panitera Pengganti

Agus Kurnia, S. Sy.

Perincian biaya :

1.	Pendaftaran	Rp0,00
2.	Proses	Rp0,00
3.	Panggilan	Rp0,00
4.	Redaksi	Rp0,00
5.	Meterai	Rp0,00
	**Jumlah**	**Rp0,00**
	(rupiah)	

Disclaimer
Kepaniteraan Mahkamah Agung Republik Indonesia berusaha untuk selalu mencantumkan informasi paling kini dan akurat sebagai bentuk komitmen Mahkamah Agung untuk pelayanan publik, transparansi dan akuntabilitas pelaksanaan fungsi peradilan. Namun dalam hal-hal tertentu masih dimungkinkan terjadi permasalahan teknis terkait dengan akurasi dan keterkinian informasi yang kami sajikan, hal mana akan terus kami perbaiki dari waktu kewaktu.
Dalam hal Anda menemukan inakurasi informasi yang termuat pada situs ini atau informasi yang seharusnya ada, namun belum tersedia, maka harap segera hubungi Kepaniteraan Mahkamah Agung RI melalui :
Email : kepaniteraan@mahkamahagung.go.id Telp : 021-384 3348 (ext.318)
