# Supplementary material for: Classification of divorce causes during the COVID-19 pandemic using convolutional neural networks
Source: PeerJ Comput Sci. 2022 Jun 30;8:e998. doi: 10.7717/peerj-cs.998 (PMC9299239; doi:10.7717/peerj-cs.998)
Supplement: Supplemental Information 5 [file peerj-cs-08-998-s005.zip › Masalah Ekonomi Dataset/Data ke-5.pdf]

Direktori Putusan Mahkamah Agung Republik Indonesia
putusan.mahkamahagung.go.id

P E N E T A P A N

Nomor 5585/Pdt.G/2020/PA.Badg

بسم الله الرحمن الرحيم

DEMI KEADILAN BERDASARKAN KETUHANAN YANG MAHA ESA

Pengadilan Agama Bandung yang memeriksa dan mengadili perkara-perkara tertentu pada tingkat pertama telah memberikan penetapan atas perkara Cerai Talak yang diajukan oleh:

PEMOHON, umur 34, agama Islam, pekerjaan Karyawan Honorer, tempat tinggal di di rumah orangtua di Jalan Cibogo Tengah, RT. 005 RW. 003, Kelurahan Sukawarna, Kecamatan Sukajadi, Kota Bandung., Selanjutnya disebut sebagai “Pemohon”;

melawan

TERMOHON umur 26 tahun, agama Islam, pendidikan S1, pekerjaan Pegawai BUMN/BUMD, tempat kediaman di di rumah orangtua di Jalan Cijawura Hilir Gg. Neglasari IV No. 21, RT. 004 RW. 008, Kelurahan Neglasari, Kecamatan Buah Batu, Kota Bandung; sebagai **Termohon**;

Pengadilan Agama tersebut;

Telah membaca dan mempelajari berkas perkara;

Telah mendengar penjelasan Pemohon dan Termohon di persidangan;

DUDUK PERKARA

Bahwa, Pemohon dengan surat permohonannya tanggal 16 November 2020 yang terdaftar di Kepaniteraan Pengadilan Agama Bandung dalam register Nomor 5585/Pdt.G/2020/PA.Badg, tanggal16 November 2020 telah mengajukan perkara Cerai Talak yang secara rinci sebagaimana tertuang dalam Berita Acara perkara ini;

Bahwa pada hari persidangan yang telah ditentukan untuk pemeriksaan perkara ini Pemohon dan Termohon mohon menghadap secara *inperson* ke persidangan;

Bahwa, selanjutnya sebelum Termohon mengajukan jawaban Pemohon

Disclaimer
Kepaniteraan Mahkamah Agung Republik Indonesia berusaha untuk selalu mencantumkan informasi paling kini dan akurat sebagai bentuk komitmen Mahkamah Agung untuk pelayanan publik, transparansi dan akuntabilitas pelaksanaan fungsi peradilan. Namun dalam hal-hal tertentu masih dimungkinkan terjadi permasalahan teknis terkait dengan akurasi dan keterkinian informasi yang kami sajikan, hal mana akan terus kami perbaiki dari waktu kewaktu.
Dalam hal Anda menemukan inakurasi informasi yang termuat pada situs ini atau informasi yang seharusnya ada, namun belum tersedia, maka harap segera hubungi Kepaniteraan Mahkamah Agung RI melalui :
Email : kepaniteraan@mahkamahagung.go.id Telp : 021-384 3348 (ext.318)

menyatakan akan mencabut permohonannya dengan alasan persoalan yang dihadapinya akan di selesaikan diluar lembaga pengadilan dengan musyawarah kekeluargaan;

Menimbang, bahwa untuk mempersingkat penetapan ini, ditunjuklah hal-ihwal sebagaimana telah tercantum dalam berita acara sidang yang merupakan bagian tak terpisahkan dari penetapan ini;

PERTIMBANGAN HUKUM

Menimbang, bahwa maksud dan tujuan permohonan Pemohon adalah sebagaimana diuraikan di atas.

Menimbang, bahwa oleh karena Pemohon telah menyatakan mencabut permohonannya, maka majelis tidak perlu lagi memeriksa dan menyelesaikan permohonan Pemohon tersebut yang selanjutnya dengan didasarkan kepada ketentuan pasal 271 dan 272 RV majelis berpendapat permohonan pencabutan Pemohon beralaskan hukum dan karenanya patut diterima dan dikabulkan;

Menimbang, bahwa berdasarkan maksud Pasal. 89 Undang-undang Nomor 50 Tahun 2009 tentang perubahan keduan atas Undang Nomor 7 Tahun 1989 tentang Peradilan Agama kepada Pemohon dibebankan untuk membayar seluruh biaya perkara yang ditimbulkan dalam perkara ini.

Mengingat, segala Undang-undang dan peraturan yang berlaku serta ketentuan Hukum Syar'i yang berkaitan dengan perkara ini.

M E N E T A P K A N

1. Mengabulkan permohonan pencabutan perkara Nomor 5585/Pdt.G/2020/PA.Badg dari Pemohon;
2. Menyatakan perkara Nomor 5585/Pdt.G/2020/PA.Badg selesai karena dicabut;
3. Memerintahkan Panitera Pengadilan Agama Bandung untuk mencatat pencabutan perkara tersebut dalam register perkara;
4. Membebankan biaya perkara kepada Pemohon sejumlah Rp. 416.000.- (empat ratus enam belas ribu rupiah).

Demikian ditetapkan dalam rapat permusyawaratan Majelis yang

Disclaimer
Kepaniteraan Mahkamah Agung Republik Indonesia berusaha untuk selalu mencantumkan informasi paling kini dan akurat sebagai bentuk komitmen Mahkamah Agung untuk pelayanan publik, transparansi dan akuntabilitas pelaksanaan fungsi peradilan. Namun dalam hal-hal tertentu masih dimungkinkan terjadi permasalahan teknis terkait dengan akurasi dan keterkinian informasi yang kami sajikan, hal mana akan terus kami perbaiki dari waktu kewaktu.
Dalam hal Anda menemukan inakurasi informasi yang termuat pada situs ini atau informasi yang seharusnya ada, namun belum tersedia, maka harap segera hubungi Kepaniteraan Mahkamah Agung RI melalui :
Email : kepaniteraan@mahkamahagung.go.id   Telp : 021-384 3348 (ext.318)

dilangsungkan pada hari Senin tanggal 21 Desember 2020 Masehi, bertepatan dengan tanggal 6 Jumadil Awwal 1442 Hijriyah, oleh kami Drs. H. Cece Rukmana Ibrahim, S.H., M.H. sebagai Ketua Majelis, Drs. H. Endang Tamami, M.H. dan Drs. Mustopa, SH. masing-masing sebagai Hakim Anggota, penetapan tersebut diucapkan dalam sidang terbuka untuk umum pada hari Senin tanggal 21 Desember 2020 Masehi, bertepatan dengan tanggal 6 Jumadil Awwal 1442 Hijriyah, oleh Ketua Majelis tersebut dengan didampingi oleh Hakim Anggota dan dibantu oleh Achmad Sadikin S.H. sebagai Panitera Pengganti serta dihadiri oleh Pemohon tanpa kehadiran Termohon;

Ketua Majelis

Drs. H. Cece Rukmana Ibrahim, S.H., M.H.

Hakim Anggota	Hakim Anggota
Drs. H. Endang Tamami, M.H.	Drs. Mustopa, SH.

Panitera Pengganti

Achmad Sadikin S.H.

Perincian Biaya Perkara :

1. Pendaftaran : Rp. 30.000,-
2. Proses : Rp. 50.000,-

Disclaimer
Kepaniteraan Mahkamah Agung Republik Indonesia berusaha untuk selalu mencantumkan informasi paling kini dan akurat sebagai bentuk komitmen Mahkamah Agung untuk pelayanan publik, transparansi dan akuntabilitas pelaksanaan fungsi peradilan. Namun dalam hal-hal tertentu masih dimungkinkan terjadi permasalahan teknis terkait dengan akurasi dan keterkinian informasi yang kami sajikan, hal mana akan terus kami perbaiki dari waktu kewaktu.
Dalam hal Anda menemukan inakurasi informasi yang termuat pada situs ini atau informasi yang seharusnya ada, namun belum tersedia, maka harap segera hubungi Kepaniteraan Mahkamah Agung RI melalui :
Email : kepaniteraan@mahkamahagung.go.id Telp : 021-384 3348 (ext.318)

Direktori Putusan Mahkamah Agung Republik Indonesia
putusan.mahkamahagung.go.id

3. Panggilan Penggugat : Rp. 100.000,-
4. Panggilan Tergugat : Rp. 200.000,-
5. Redaksi : Rp. 10.000,-
6. Materai : Rp. 6.000,-

Jumlah : Rp. 416.000,-

Empat ratus enam belas ribu rupiah

Disclaimer
Kepaniteraan Mahkamah Agung Republik Indonesia berusaha untuk selalu mencantumkan informasi paling kini dan akurat sebagai bentuk komitmen Mahkamah Agung untuk pelayanan publik, transparansi dan akuntabilitas pelaksanaan fungsi peradilan. Namun dalam hal-hal tertentu masih dimungkinkan terjadi permasalahan teknis terkait dengan akurasi dan keterkinian informasi yang kami sajikan, hal mana akan terus kami perbaiki dari waktu kewaktu.
Dalam hal Anda menemukan inakurasi informasi yang termuat pada situs ini atau informasi yang seharusnya ada, namun belum tersedia, maka harap segera hubungi Kepaniteraan Mahkamah Agung RI melalui :
Email : kepaniteraan@mahkamahagung.go.id Telp : 021-384 3348 (ext.318)
