# Supplementary material for: Classification of divorce causes during the COVID-19 pandemic using convolutional neural networks
Source: PeerJ Comput Sci. 2022 Jun 30;8:e998. doi: 10.7717/peerj-cs.998 (PMC9299239; doi:10.7717/peerj-cs.998)
Supplement: Supplemental Information 5 [file peerj-cs-08-998-s005.zip › Masalah Ekonomi Dataset/Data ke-6.pdf]

SALINAN ***P U T U S A N***

Nomor 387/Pdt.G/2020/PA.Mmj

بسم الله الرحمن الرحيم

DEMI KEADILAN BERDASARKAN KETUHANAN YANG MAHA ESA

Pengadilan Agama Mamuju yang memeriksa dan mengadili perkara tertentu pada tingkat pertama telah menjatuhkan putusan sebagai berikut dalam perkara cerai gugat antara:

PENGGUGAT;

melawan

TERGUGAT;

Pengadilan Agama tersebut;

Telah membaca dan mempelajari berkas perkara;

Telah mendengar keterangan Penggugat dan Tergugat;

DUDUK PERKARA

Menimbang, bahwa Penggugat dengan surat gugatannya tertanggal 02 November 2020 yang terdaftar di Kepaniteraan Pengadilan Agama Mamuju pada tanggal 02 November 2020 dengan register perkara Nomor 387/Pdt.G/2020/PA. Mmj, mengemukakan hal-hal sebagai berikut:

1. Bahwa antara Penggugat dan Tergugat adalah pasangan suami istri yang telah menikah secara Islam pada hari Minggu 10 April 1994 atau bertepatan dengan 28 Syawal 1414 H di Kecamatan Patampanua, Kabupaten Pinrang Provinsi Sulawesi Selatan, berdasarkan Kutipan Akta Nikah Nomor 102/12/94 tertanggal 14 April 1994;
2. Bahwa sebelum menikah, Penggugat berstatus Perawan dan Tergugat berstatus Perjaka;
3. Bahwa antara Penggugat dan Tergugat telah berhubungan suami istri (ba'da dukhul) dan telah dikarunai 4 orang anak yang masing-masing bernama:
   1) ANAK PENGGUGAT DAN TERGUGAT Umur 25 Tahun;
   2) ANAK PENGGUGAT DAN TERGUGAT Umur 20 Tahun;
   3) ANAK PENGGUGAT DAN TERGUGAT umur 19 Tahun;

Disclaimer
Kepaniteraan Mahkamah Agung Republik Indonesia berusaha untuk selalu mencantumkan informasi paling kini dan akurat sebagai bentuk komitmen Mahkamah Agung untuk pelayanan publik, transparansi dan akuntabilitas pelaksanaan fungsi peradilan. Namun dalam hal-hal tertentu masih dimungkinkan terjadi permasalahan teknis terkait dengan akurasi dan keterkinian informasi yang kami sajikan, hal mana akan terus kami perbaiki dari waktu kewaktu.
Dalam hal Anda menemukan inakurasi informasi yang termuat pada situs ini atau informasi yang seharusnya ada, namun belum tersedia, maka harap segera hubungi Kepaniteraan Mahkamah Agung RI melalui :
Email : kepaniteraan@mahkamahagung.go.id Telp : 021-384 3348 (ext.318)

4) ANAK PENGGUGAT DAN TERGUGAT umur 14 tahun;

4. Bahwa antara Penggugat dan Tergugat pada awal mengarungi bahtera rumah tangga berjalan baik-baik saja tanpa ada perselisihan dan pertengkaran hingga kemudian tahun 1994 antara Penggugat dan Tergugat mulai terjadi perselisihan dan pertengakaran;

5. Bahwa alasan perselisihan dan pertengkaran antara Penggugat dan Tergugat diakibatkan karena sifat Tergugat yang sangat emosional seperti pada saat Penggugat menyajikan makanan yang tidak sesuai dengan keinginan Tergugat atau Pengggguat menjawab pertanyaan yang tidak diharapakan Tergugat maka Tergugat akan marah dan terkadang melampiaskannya dengan memukul Penggugat;

6. Bahwa Tergugat juga sudah menikah lagi dengan perempuan lain tanpa sepengetahuan Penggugat pada tahun 2018, yang diketahui oleh Penggugat dari isteri selingkuhan Tergugat yang pada saat itu sementara berbulan madu, dengan bukti sms serta foto yang ada di Hp Penggugat;

7. Bahwa pada 21 Juli 2019 Penggugat sudah mendaftarkan perceraiannya dipengadilan agama mamuju dengan Nomor Perkara 210/Pdt/6/2019/PA,Mmj, namun akhirnya mengurungkan niatnya disaat menjelang terselenggaranya sidang ke-3 dengan alasan bahwa Tergugat memberi sinyal kepada Penggugat untuk membatalkan perkara dengan harapan bahwa Tergugat akan merubah sikapnya namun selang beberapa hari setelah kejadian itu Tergugat Kembali lagi mengulangi perbuatanhya dan tidak memberikan tanda untuk bersungguh-sungguh berubah;

8. Bahwa pada tanggal 6 Juni 2020 Penggugat mengetahui bahwa Tergugat telah berselingkuh lagi dengan beberapa perempuan dan salah satu diantaranya telah dinikahi diawal Juni 2020 tanpa seizin Penggugat, dengan bukti sms dan foto yang ada di HP Penggugat dan kemudian pada tanggal 12 Agustus 2020 Tergugat mengakui dengan sendirinya dan mengatakan kepada Penggugat bahwa benar Tergugat telah menikah lagi tanpa seizin Penggugat;

9. Bahwa adapun perilaku Tergugat kepada Penggugat yang semakin buruk setelah Tergugat menikah dengan wanita lain yakni Tergugat sering menutupi kesalahannya dengan cara marah, melempar dan membanting barang-barang yang ada di rumah Penggugat dan Tergugat, bahkan sampai memukul

Disclaimer
Kepaniteraan Mahkamah Agung Republik Indonesia berusaha untuk selalu mencantumkan informasi paling kini dan akurat sebagai bentuk komitmen Mahkamah Agung untuk pelayanan publik, transparansi dan akuntabilitas pelaksanaan fungsi peradilan. Namun dalam hal-hal tertentu masih dimungkinkan terjadi permasalahan teknis terkait dengan akurasi dan keterkinian informasi yang kami sajikan, hal mana akan terus kami perbaiki dari waktu kewaktu.
Dalam hal Anda menemukan inakurasi informasi yang termuat pada situs ini atau informasi yang seharusnya ada, namun belum tersedia, maka harap segera hubungi Kepaniteraan Mahkamah Agung RI melalui :
Email : kepaniteraan@mahkamahagung.go.id Telp : 021-384 3348 (ext.318)

Penggugat yang membuat Penggugat selalu merasa terancam saat berada di rumah hingga Penggugat seringkali tidak tidur semalaman;

10. Bahwa pada tanggal 26 Juli 2020 Tergugat marah dan melukai Penggugat yakni dengan cara mencakar lengan dan leher Penggugat karena Penggugat menegur Tergugat untuk tidak memanggil Penggugat dengan sebutan mami dikarenakan panggilan tersebut adalah panggilan kepada isteri baru Tergugat, yang diketahui oleh anak serta karyawan;

11. Bahwa pada 28 Juli 2020 Tergugat marah lagi sehingga menimbulkan keributan antara Penggugat dan Tergugat dengan alasan Penggugat menolak berhubungan badan karena luka Penggugat belum sembuh dan Tergugat menginginkan berhubungan dengan cara kasar yakni dengan menendang Penggugat, meskipun Penggugat sudah meminta Tergugat untuk tidak kasar karena Penggugat merasa belum sehat dan kemudian Tergugat meninggalkan rumah tanpa pamit kepada Penggugat;

12. Bahwa pada 12 Agustus 2020 antara Penggugat dan Tergugat terjadi keributan dikarenakan Tergugat menuduh Penggugat telah memeriksa HP Tergugat yang kemudian disangkal oleh Penggugat sehingga menyebabkan Tergugat marah dan disaat itu pula Tergugat mengakui bahwa dia telah menikah lagi dengan wanita lain;

13. Bahwa dengan adanya perselisihan dan pertengkaran yang terjadi antara Penggugat dan Tergugat secara terus menerus yang tidak memungkinkan lagi untuk hidup rukun dan damai kembali, maka terdapat alasan hukum yang cukup bagi Penggugat untuk mengajukan gugatan cerai ini;

14. Bahwa oleh karena gugatan ini dikenakan biaya, mohon untuk ditetapkan biaya perkara sesuai dengan peraturan perundang-undangan yang berlaku.

Berdasarkan uraian-uraian tersebut diatas, maka Penggugat mohon kepada Ketua Pengadilan Agama Mamuju cq. Majelis Hakim yang memeriksa dan mengadili perkara ini, untuk berkenan mengabulkan gugatan Penggugat dengan menjatuhkan putusan sebagai berikut :

1. Menerima dan mengabulkan gugatan Penggugat;

Disclaimer
Kepaniteraan Mahkamah Agung Republik Indonesia berusaha untuk selalu mencantumkan informasi paling kini dan akurat sebagai bentuk komitmen Mahkamah Agung untuk pelayanan publik, transparansi dan akuntabilitas pelaksanaan fungsi peradilan. Namun dalam hal-hal tertentu masih dimungkinkan terjadi permasalahan teknis terkait dengan akurasi dan keterkinian informasi yang kami sajikan, hal mana akan terus kami perbaiki dari waktu kewaktu.
Dalam hal Anda menemukan inakurasi informasi yang termuat pada situs ini atau informasi yang seharusnya ada, namun belum tersedia, maka harap segera hubungi Kepaniteraan Mahkamah Agung RI melalui :
Email : kepaniteraan@mahkamahagung.go.id Telp : 021-384 3348 (ext.318)

2. Menyatakan perkawinan antara Penggugat (**PENGGUGAT**) dan Tergugat (**TERGUGAT**) putus disebabkan perceraian;

3. Membebankan biaya perkara sesuai dengan peraturan perundang-undangan yang berlaku;

ATAU

Apabila Majelis Hakim berpendapat lain, mohon putusan seadil-adilnya (*ex aquo et bono*).

Bahwa pada hari dan tanggal sidang yang telah ditetapkan Penggugat dan Tergugat, masing-masing telah datang menghadap sendiri ke persidangan;

Bahwa Majelis Hakim telah berusaha mendamaikan Penggugat dan Tergugat agar rukun kembali dalam membina rumah tangga, namun tidak berhasil;

Bahwa Penggugat dan Tergugat telah melaksanakan proses mediasi dengan mediator bernama **DR. H. Muh. Arasy Latif, Lc., M.A.**, Hakim Pengadilan Agama Mamuju, namun berdasarkan Laporan Hasil Mediasi dari mediator tersebut pada tanggal 24 November 2020, mediasi dinyatakan tidak berhasil;

Bahwa selanjutnya dibacakan surat gugatan Penggugat dalam sidang tertutup untuk umum yang isi dan maksudnya tetap dipertahankan oleh Penggugat;

Bahwa atas gugatan Penggugat tersebut, Tergugat telah menyampaikan jawaban secara lisan yang pada pokoknya membantah sepanjang adanya persesihan dan pertengkaran antara Penggugat dengan Tergugat, karena selama ini antara Penggugat dengan Tergugat masih rukun dan tidak ada masalah, selain itu Tergugat menyatakan keberatan bercerai dengan Penggugat;

Bahwa atas jawaban Tergugat tersebut, Penggugat telah mengajukan replik secara lisan yang pada pokoknya tetap pada dalil-dalil gugatannya semula, kemudian terhadap replik Penggugat tersebut, Tergugat telah mengajukan duplik secara lisan yang pada pokoknya tetap pada jawabannya semula;

Bahwa untuk menguatkan dalil-dalil gugatannya, Penggugat telah mengajukan alat bukti surat berupa fotokopi Kutipan Akta Nikah Nomor 102/12/94, atas nama Penggugat dengan Tergugat, yang dikeluarkan oleh Kantor Urusan Agama Kecamatan Patampanua, Kabupaten Pinrang, Provinsi Sulawesi Selatan, pada tanggal 14 April 1994, bukti (P);

Bahwa, terhadap bukti surat Penggugat tersebut, Tergugat membenarkan dan tidak keberatan;

Disclaimer
Kepaniteraan Mahkamah Agung Republik Indonesia berusaha untuk selalu mencantumkan informasi paling kini dan akurat sebagai bentuk komitmen Mahkamah Agung untuk pelayanan publik, transparansi dan akuntabilitas pelaksanaan fungsi peradilan. Namun dalam hal-hal tertentu masih dimungkinkan terjadi permasalahan teknis terkait dengan akurasi dan keterkinian informasi yang kami sajikan, hal mana akan terus kami perbaiki dari waktu kewaktu.
Dalam hal Anda menemukan inakurasi informasi yang termuat pada situs ini atau informasi yang seharusnya ada, namun belum tersedia, maka harap segera hubungi Kepaniteraan Mahkamah Agung RI melalui :
Email : kepaniteraan@mahkamahagung.go.id Telp : 021-384 3348 (ext.318)

Bahwa, Majelis Hakim telah memberi kesempatan kepada Penggugat dan Tergugat untuk mengajukan alat bukti lain (pembuktian lanjutan);

Bahwa, untuk mempersingkat uraian putusan ini, ditunjuk segala sesuatu yang tercantum dalam berita acara sidang perkara ini sebagai bagian yang tidak terpisahkan dari isi putusan ini;

PERTIMBANGAN HUKUM

Menimbang, bahwa maksud dan tujuan gugatan Penggugat adalah sebagaimana tersebut di atas;

Menimbang, bahwa terlebih dahulu Majelis Hakim akan mempertimbangkan status perkawinan Penggugat dan Tergugat untuk mengetahui apakah Penggugat mempunyai kedudukan hukum (*legal standing*) atau tidak dalam mengajukan perkara *a quo*;

Menimbang, bahwa berdasarkan bukti (P) telah nyata terbukti bahwa antara Penggugat dan Tergugat telah terikat dalam perkawinan yang sah, sehingga harus dinyatakan Penggugat mempunyai kedudukan hukum (*legal standing*) untuk mengajukan gugatan cerai terhadap Tergugat;

Menimbang, bahwa pada hari persidangan yang telah ditetapkan Penggugat dan Tergugat datang menghadap ke persidangan;

Menimbang, bahwa Majelis Hakim telah berusaha mendamaikan Penggugat dan Tergugat agar rukun kembali dan mengurungkan niatnya untuk bercerai, sesuai dengan ketentuan Pasal 65, Pasal 70 ayat (1), Pasal 82 ayat (1) dan (4) Undang-Undang Nomor 7 Tahun 1989 tentang Peradilan Agama sebagaimana telah diubah pertama dengan Undang-Undang Nomor 3 Tahun 2006 dan perubahan kedua dengan Undang-Undang Nomor 50 Tahun 2009 jo. Pasal 16 Peraturan Pemerintah Nomor 9 Tahun 1975 tentang Pelaksanaan Undang-Undang Nomor 1 Tahun 1974 tentang Perkawinan jo. Pasal 115 dan Pasal 131 ayat (2) Instruksi Presiden Nomor 1 Tahun 1991 tentang Kompilasi Hukum Islam, namun tidak berhasil;

Menimbang, bahwa untuk memenuhi ketentuan Pasal 154 R.Bg. jo. Peraturan Mahkamah Agung Republik Indonesia Nomor 1 Tahun 2016 tentang Prosedur Mediasi di Pengadilan telah dilakukan proses mediasi terhadap Penggugat dan Tergugat dengan seorang mediator bernama **DR. H. Muh. Arasy**

Disclaimer
Kepaniteraan Mahkamah Agung Republik Indonesia berusaha untuk selalu mencantumkan informasi paling kini dan akurat sebagai bentuk komitmen Mahkamah Agung untuk pelayanan publik, transparansi dan akuntabilitas pelaksanaan fungsi peradilan. Namun dalam hal-hal tertentu masih dimungkinkan terjadi permasalahan teknis terkait dengan akurasi dan keterkinian informasi yang kami sajikan, hal mana akan terus kami perbaiki dari waktu kewaktu.
Dalam hal Anda menemukan inakurasi informasi yang termuat pada situs ini atau informasi yang seharusnya ada, namun belum tersedia, maka harap segera hubungi Kepaniteraan Mahkamah Agung RI melalui :
Email : kepaniteraan@mahkamahagung.go.id Telp : 021-384 3348 (ext.318)

Latif, Lc., M.A., Hakim Pengadilan Agama Mamuju, namun berdasarkan Laporan Hasil Mediasi dari mediator tersebut pada tanggal 24 November 2020, mediasi dinyatakan tidak berhasil;

Menimbang, bahwa selanjutnya dibacakan surat gugatan Penggugat yang isi dan maksudnya tetap dipertahankan oleh Penggugat;

Menimbang, bahwa terhadap dalil-dalil gugatan Penggugat tersebut, Tergugat telah membantah dalil gugatan Penggugat sepanjang adanya perselisihan dan pertengkaran antara Penggugat dengan Tergugat, oleh karena itu Penggugat wajib membuktikan dalil-dalil gugatannya yang dibantah oleh Tergugat tersebut;

Menimbang, bahwa Majelis Hakim telah memberi kesempatan 3 (tiga) kali kepada Penggugat untuk membuktikan dalil-dalil gugatannya, namun pada kesempatan kali ketiga Penggugat justru tidak datang menghadap ke persidangan tanpa alasan yang sah, sehingga Majelis Hakim berpendapat Penggugat tidak dapat membuktikan dalil-dalil gugatannya;

Menimbang, bahwa oleh karena Penggugat tidak dapat membuktikan dalil-dalil gugatannya, maka gugatan Penggugat tersebut harus dinyatakan tidak terbukti dan harus ditolak;

Menimbang, bahwa perkara ini masuk bidang perkawinan dalam hal perceraian, maka sesuai dengan ketentuan Pasal 89 ayat (1) Undang-Undang Nomor 7 Tahun 1989 tentang Peradilan Agama sebagaimana telah diubah pertama dengan Undang-Undang Nomor 3 Tahun 2006 dan perubahan kedua dengan Undang-Undang Nomor 50 Tahun 2009, biaya perkara ini dibebankan kepada Penggugat;

Mengingat Pasal 49 Undang-Undang Nomor 7 Tahun 1989 tentang Peradilan Agama sebagaimana telah diubah pertama dengan Undang-Undang Nomor 3 Tahun 2006 dan perubahan kedua dengan Undang-Undang Nomor 50 Tahun 2009 dan ketentuan hukum lain serta hukum syar'i yang berkaitan dengan perkara ini:

M E N G A D I L I

1. Menolak gugatan Penggugat;

Disclaimer
Kepaniteraan Mahkamah Agung Republik Indonesia berusaha untuk selalu mencantumkan informasi paling kini dan akurat sebagai bentuk komitmen Mahkamah Agung untuk pelayanan publik, transparansi dan akuntabilitas pelaksanaan fungsi peradilan. Namun dalam hal-hal tertentu masih dimungkinkan terjadi permasalahan teknis terkait dengan akurasi dan keterkinian informasi yang kami sajikan, hal mana akan terus kami perbaiki dari waktu kewaktu.
Dalam hal Anda menemukan inakurasi informasi yang termuat pada situs ini atau informasi yang seharusnya ada, namun belum tersedia, maka harap segera hubungi Kepaniteraan Mahkamah Agung RI melalui :
Email : kepaniteraan@mahkamahagung.go.id   Telp : 021-384 3348 (ext.318)

2. Membebankan biaya perkara kepada Penggugat sejumlah Rp.466.000,- (empat ratus enam puluh enam ribu rupiah).

Demikian diputusan dalam musyawarah majelis pada hari Selasa tanggal 22 Desember 2020 Miladiah bertepatan dengan tanggal 07 Jumadil Awwal 1442 Hijriah oleh kami **M. Syaefuddin, S.HI., M.Sy.** sebagai Ketua Majelis, **Abdul Hizam Monoarfa, S.H.** dan **Tri Hasan Bashori, S.HI.** masing-masing sebagai Hakim Anggota, putusan mana diucapkan pada hari itu juga oleh Ketua Majelis dalam sidang terbuka untuk umum dengan didampingi oleh Hakim-Hakim Anggota tersebut serta dibantu oleh **Muh. Fauzan, S.Ag., M.H.** sebagai Panitera Pengganti di luar hadirnya Penggugat dan Tergugat;

Ketua Majelis,

ttd

M. Syaefuddin, S.HI., M.Sy.

Hakim Anggota,

ttd

Abdul Hizam Monoarfa, S.H.

Hakim Anggota,

ttd

Tri Hasan Bashori, S.HI.

Panitera Pengganti,

ttd

Muh. Fauzan, S.Ag., M.H.

Perincian Biaya Perkara :

1. Biaya Pendaftaran	Rp	30.000,-
2. Biaya ATK Perkara	Rp	50.000,-
3. Biaya Panggilan	Rp	350.000,-
4. Biaya PNBP Relaas Panggilan	Rp	20.000,-
5. Biaya Redaksi	Rp	10.000,-
6. Biaya Meterai	Rp	6.000,-
**Jumlah**	**Rp**	**466.000,-**

(empat ratus enam puluh enam ribu rupiah)

Mamuju, 22 Desember 2020
Salinan sesuai aslinya
PANITERA,

Drs. H. Sudarno, M.H.

Disclaimer
Kepaniteraan Mahkamah Agung Republik Indonesia berusaha untuk selalu mencantumkan informasi paling kini dan akurat sebagai bentuk komitmen Mahkamah Agung untuk pelayanan publik, transparansi dan akuntabilitas pelaksanaan fungsi peradilan. Namun dalam hal-hal tertentu masih dimungkinkan terjadi permasalahan teknis terkait dengan akurasi dan keterkinian informasi yang kami sajikan, hal mana akan terus kami perbaiki dari waktu kewaktu.
Dalam hal Anda menemukan inakurasi informasi yang termuat pada situs ini atau informasi yang seharusnya ada, namun belum tersedia, maka harap segera hubungi Kepaniteraan Mahkamah Agung RI melalui :
Email : kepaniteraan@mahkamahagung.go.id   Telp : 021-384 3348 (ext.318)
