# Supplementary material for: Classification of divorce causes during the COVID-19 pandemic using convolutional neural networks
Source: PeerJ Comput Sci. 2022 Jun 30;8:e998. doi: 10.7717/peerj-cs.998 (PMC9299239; doi:10.7717/peerj-cs.998)
Supplement: Supplemental Information 5 [file peerj-cs-08-998-s005.zip › Masalah Ekonomi Dataset/Data ke-7.pdf]

P U T U S A N

Nomor **4226/Pdt.G/2020/PA.Sbg**

بِسْمِ اللَّهِ الرَّحْمَنِ الرَّحِيمِ

DEMI KEADILAN BERDASARKAN KETUHANAN YANG MAHA ESA

Pengadilan Agama Subang yang memeriksa dan mengadili perkara tertentu pada tingkat pertama dalam persidangan Majelis Hakim telah menjatuhkan putusan perkara Cerai Gugat, antara :

Penggugat, umur 18 tahun, agama Islam, pekerjaan Ibu Rumah Tangga, bertempat tinggal di Kabupaten Subang, untuk selanjutnya disebut sebagai **Penggugat**;

Melawan

Tergugat, umur 23 tahun, agama Islam, pekerjaan Wiraswasta, bertempat tinggal di Kabupaten Subang, untuk selanjutnya disebut sebagai **Tergugat**;

Pengadilan Agama tersebut;
Setelah membaca berkas perkara;
Setelah mendengar keterangan Penggugat;
Setelah memeriksa bukti-bukti dalam persidangan;

DUDUK PERKARA

Bahwa, Penggugat dengan surat gugatannya tertanggal 30 November 2020 yang terdaftar di Kepaniteraan Pengadilan Agama Subang pada tanggal 30 November 2020 dengan register perkara Nomor 4226/Pdt.G/2020/PA.Sbg., telah mengemukakan hal-hal dan alasan-alasan sebagai berikut :

1. Bahwa Penggugat saat ini bertempat tinggal di Kabupaten Subang

Disclaimer
Kepaniteraan Mahkamah Agung Republik Indonesia berusaha untuk selalu mencantumkan informasi paling kini dan akurat sebagai bentuk komitmen Mahkamah Agung untuk pelayanan publik, transparansi dan akuntabilitas pelaksanaan fungsi peradilan. Namun dalam hal-hal tertentu masih dimungkinkan terjadi permasalahan teknis terkait dengan akurasi dan keterkinian informasi yang kami sajikan, hal mana akan terus kami perbaiki dari waktu kewaktu.
Dalam hal Anda menemukan inakurasi informasi yang termuat pada situs ini atau informasi yang seharusnya ada, namun belum tersedia, maka harap segera hubungi Kepaniteraan Mahkamah Agung RI melalui :
Email : kepaniteraan@mahkamahagung.go.id Telp : 021-384 3348 (ext.318)

Mahkamah Agung Republik Indonesia

sebagaimana ternyata dari Surat Keterangan Domisili Nomor 474/305/Pem atas nama Penggugat dengan NIK 32130001 yang diterbitkan oleh Kelurahan Cigadung, Kecamatan Subang, Kabupaten Subang tertanggal 30 November 2020;

2. Bahwa pada tanggal 10 Mei 2018, Penggugat dengan Tergugat melangsungkan pernikahan di Wilayah Hukum Kantor Urusan Agama Kecamatan Pagaden Kabupaten Subang sebagaimana ternyata dari Buku Kutipan Akta Nikah yang dikeluarkan oleh Pegawai Pencatat Nikah Kantor Urusan Agama Kecamatan Pagaden Kabupaten Subang Nomor 2018 tertanggal 10 Mei 2018;
3. Bahwa setelah akad nikah Penggugat dan Tergugat hidup bersama sebagai suami istri di rumah orangtua Penggugat;
4. Bahwa perkawinan antara Penggugat dan Tergugat sampai saat ini telah berjalan selama 2 tahun 6 bulan namun belum dikaruniai keturunan;
5. Bahwa semula rumah tangga Penggugat dan Tergugat harmonis dan bahagia, namun sejak awal bulan Juli 2018 sudah tidak harmonis lagi karena sering terjadi perselisihan dan pertengkaran yang disebabkan karena :
   - Masalah ekonomi dimana Tergugat kurang mampu memberikan nafkah lahir kepada Penggugat yang disebabkan karena Tergugat tidak memiliki pekerjaan yang tetap dan penghasilan yang tetap, sehingga untuk mencukupi kebutuhan sehari-hari harus di bantu oleh orangtua Penggugat;
6. Bahwa puncak perselisihan dan pertengkaran antara Penggugat dan Tergugat terjadi pada akhir bulan Juli 2018, yang berakibat Tergugat pergi meninggalkan Penggugat dan tidak pernah hidup berumah tangga lagi sebagai suami istri dan sampai sekarang sudah berpisah selama 2 tahun 4 bulan;
7. Bahwa selama berpisah tempat tinggal Penggugat tinggal di rumah orangtua Penggugat, sedangkan Tergugat tinggal di rumah orangtua Tergugat;
8. Bahwa dengan adanya kejadian tersebut Penggugat tetap bersabar, dan sudah dilakukan upaya musyawarah antara Penggugat dan Tergugat

Disclaimer
Kepaniteraan Mahkamah Agung Republik Indonesia berusaha untuk selalu mencantumkan informasi paling kini dan akurat sebagai bentuk komitmen Mahkamah Agung untuk pelayanan publik, transparansi dan akuntabilitas pelaksanaan fungsi peradilan. Namun dalam hal-hal tertentu masih dimungkinkan terjadi permasalahan teknis terkait dengan akurasi dan keterkinian informasi yang kami sajikan, hal mana akan terus kami perbaiki dari waktu kewaktu.
Dalam hal Anda menemukan inakurasi informasi yang termuat pada situs ini atau informasi yang seharusnya ada, namun belum tersedia, maka harap segera hubungi Kepaniteraan Mahkamah Agung RI melalui :
Email : kepaniteraan@mahkamahagung.go.id   Telp : 021-384 3348 (ext.318)

beserta keluarga Penggugat dan Tergugat, namun upaya tersebut tidak berhasil;

9. Bahwa dengan keadaan rumah tangga tersebut, Penggugat sudah tidak ada harapan untuk melanjutkan rumah tangga bersama Tergugat, sehingga tujuan perkawinan untuk membentuk rumah tangga yang sakinah, mawadah warohmah tidak dapat terwujud, dan apabila tetap dipertahankan hanya akan menimbulkan kemudaratan yang berkepanjangan. Maka untuk mengakhiri perkawinan tersebut Penggugat bermaksud menggugat cerai Tergugat di depan sidang Pengadilan Agama Subang;
10. Bahwa Penggugat sanggup membayar biaya perkara;

Bahwa berdasarkan dalil-dalil tersebut di atas, Penggugat mohon kepada Ketua Pengadilan Agama Subang Cq. Majelis Hakim yang menyidangkan perkara ini kiranya berkenan untuk memanggil Penggugat dan Tergugat, memeriksa, mengadili dan memutuskan perkara ini dengan amarnya yang berbunyi sebagai berikut :

1. Mengabulkan gugatan Penggugat;
2. Menjatuhkan talak satu bain shugra Terguggat (Saepul Basri bin Kholik) terhadap Penggugat (Penggugat);
3. Membebankan biaya perkara ini menurut hukum;

Apabila majelis hakim berpendapat lain, mohon putusan yang seadil-adilnya;

Bahwa pada hari dan tanggal yang telah ditentukan Penggugat menghadap persidangan, sedang Tergugat tidak datang menghadap atau menyuruh orang lain sebagai wakilnya yang sah, meskipun ia telah dipanggil dengan resmi dan patut untuk datang menghadap di sidang sebagaimana terbukti dari relaas panggilan Nomor 4226/Pdt.G/2020/PA.Sbg., tanggal 01 Desember 2020 dan tanggal 08 Desember 2020, sedangkan ternyata bahwa tidak hadirnya itu oleh Majelis Hakim dinilai tidak disebabkan sesuatu halangan yang sah.

Bahwa karena Tergugat tidak hadir maka mediasi tidak dapat dilaksanakan, Majelis Hakim disetiap persidangan telah menasehati Penggugat,

Disclaimer
Kepaniteraan Mahkamah Agung Republik Indonesia berusaha untuk selalu mencantumkan informasi paling kini dan akurat sebagai bentuk komitmen Mahkamah Agung untuk pelayanan publik, transparansi dan akuntabilitas pelaksanaan fungsi peradilan. Namun dalam hal-hal tertentu masih dimungkinkan terjadi permasalahan teknis terkait dengan akurasi dan keterkinian informasi yang kami sajikan, hal mana akan terus kami perbaiki dari waktu kewaktu.
Dalam hal Anda menemukan inakurasi informasi yang termuat pada situs ini atau informasi yang seharusnya ada, namun belum tersedia, maka harap segera hubungi Kepaniteraan Mahkamah Agung RI melalui :
Email : kepaniteraan@mahkamahagung.go.id   Telp : 021-384 3348 (ext.318)

namun tidak berhasil. Bahwa telah pula dibacakan surat gugatan Penggugat yang yang isinya tetap dipertahankan oleh Penggugat ;

Bahwa, oleh karena Tergugat tidak hadir dipersidangan, pemeriksaan dilanjutkan kepada pembuktian;

Bahwa untuk meneguhkan dalil-dalil gugatannya, Penggugat telah mengajukan alat bukti surat sebagai berikut :

1. Fotokopi Kartu Tanda Penduduk atas nama Penggugat (Penggugat), NIK: 321303520001,03 November 2020 yang dikeluarkan oleh Pemerintah Daerah Kabupaten Subang Provinsi Jawa Barat, bukti surat tersebut telah diberi materai cukup dan telah dicocokkan dengan aslinya yang ternyata sesuai, oleh Ketua Majelis diberi tanda **P.1**;
2. Fotokopi Buku Kutipan Akta Nikah Akta Nikah atas nama Penggugat (Penggugat) dengan Tergugat (Saepul Basri bin Kholik), Nomor 2018, yang dikeluarkan oleh Kantor Urusan Agama Kecamatan Pagaden Kabupaten Subang, tanggal 10 Mei 2018, bukti surat tersebut telah diberi materai cukup dan telah dicocokkan dengan aslinya yang ternyata sesuai, oleh Ketua Majelis diberi tanda **P.2**;

Bahwa selain alat bukti surat tersebut Penggugat juga telah mengajukan bukti saksi-saksi, yaitu :

1. Saksi 1 umur 23 tahun, agama Islam, pekerjaan Mahasiswa, tempat tinggal di Kabupaten Subang;

   Dibawah sumpahnya saksi tersebut memberikan keterangan yang pada pokoknya sebagai berikut :

   – Bahwa saksi kenal dengan Penggugat karena saksi adalah saudara sepupu Penggugat dan kenal dengan Tergugat semenjak menikah dengan Penggugat ;
   – Bahwa Penggugat dan Tergugat adalah suami isteri yang menikah pada bulan Mei 2018 di wilayah Kantor Urusan Agama Kecamatan Pagaden Kabupaten Subang;
   – Bahwa Penggugat dan Tergugat setelah menikah telah hidup bersama sebagai suami isteri membina rumah tangga terakhir bertempat tinggal di rumah orangtua Penggugat;

Disclaimer
Kepaniteraan Mahkamah Agung Republik Indonesia berusaha untuk selalu mencantumkan informasi paling kini dan akurat sebagai bentuk komitmen Mahkamah Agung untuk pelayanan publik, transparansi dan akuntabilitas pelaksanaan fungsi peradilan. Namun dalam hal-hal tertentu masih dimungkinkan terjadi permasalahan teknis terkait dengan akurasi dan keterkinian informasi yang kami sajikan, hal mana akan terus kami perbaiki dari waktu kewaktu.
Dalam hal Anda menemukan inakurasi informasi yang termuat pada situs ini atau informasi yang seharusnya ada, namun belum tersedia, maka harap segera hubungi Kepaniteraan Mahkamah Agung RI melalui :
Email : kepaniteraan@mahkamahagung.go.id Telp : 021-384 3348 (ext.318)

- Bahwa Penggugat dan Tergugat selama membina rumah tangga namun belum dikaruniai keturunan;
- Bahwa antara Penggugat dan Tergugat dalam membina rumah sudah tidak rukun dan harmonis semenjak awal bulan Juli 2018 sering terjadi perselisihan dan pertengkaran disebabkan masalah ekonomi yang selalu kekurangan dikarenakan Tergugat tidak memberikan nafkah untuk memenuhi kebutuhan sehari-hari dalam rumah tangga karena Tergugat tidak mempunyai pekerjaan untuk memenuhi kebutuhan rumah tangga dibantu orang tua Penggugat.:
- Bahwa semenjak akhir bulan Juli 2018, Tergugat pergi meninggalkan Penggugat dan pulang kerumah orang tuanya sampai sekarang tidak pernah bersatu lagi sudah 2 (dua) tahun 5 (lima) bulan;
- Bahwa semenjak kepergiannya Tergugat tidak pernah memberikan nafkah kepada Penggugat serta tidak ada meninggalkan harta yang dapat dijadikan sebagai nafkah untuk Penggugat;
- Bahwa pihak keluarga telah berusaha untuk menasehati Penggugat dan Tergugat untuk rukun kembali namun tidak berhasil karena Penggugat tetap dengan pendiriannya ingin bercerai dengan Tergugat;

2. Saksi 2 , umur 43 tahun, agama Islam, pekerjaan dagang , tempat tinggal di Kabupaten Subang;

Dibawah sumpahnya saksi tersebut memberikan keterangan yang pada pokoknya sebagai berikut:

- Bahwa saksi kenal dengan Penggugat semenjak kecil karena saksi adalah Paman Penggugat dan kenal dengan Tergugat semenjak menikah dengan Penggugat;
- Bahwa Penggugat dan Tergugat adalah suami isteri yang menikah pada bulan Mei 2018 di wilayah Kantor Urusan Agama Kecamatan Pagaden Kabupaten Subang;
- Bahwa Penggugat dan Tergugat setelah menikah telah hidup bersama sebagai suami isteri membina rumah tangga di rumah orangtua Penggugat;
- Bahwa Penggugat dan Tergugat selama membina rumah tangga namun belum dikaruniai keturunan;

Disclaimer
Kepaniteraan Mahkamah Agung Republik Indonesia berusaha untuk selalu mencantumkan informasi paling kini dan akurat sebagai bentuk komitmen Mahkamah Agung untuk pelayanan publik, transparansi dan akuntabilitas pelaksanaan fungsi peradilan. Namun dalam hal-hal tertentu masih dimungkinkan terjadi permasalahan teknis terkait dengan akurasi dan keterkinian informasi yang kami sajikan, hal mana akan terus kami perbaiki dari waktu kewaktu.
Dalam hal Anda menemukan inakurasi informasi yang termuat pada situs ini atau informasi yang seharusnya ada, namun belum tersedia, maka harap segera hubungi Kepaniteraan Mahkamah Agung RI melalui :
Email : kepaniteraan@mahkamahagung.go.id Telp : 021-384 3348 (ext.318)

- Bahwa antara Penggugat dan Tergugat dalam membina rumah sudah tidak rukun dan harmonis semenjak awal bulan Juli 2018 sering terjadi perselisihan dan pertengkaran disebabkan msalah ekonomi yang selalu kekurangan dikarenakan Tergugat tidak memberikan nafkah untuk memenuhi kebutuhan sehari-hari dalam rumah tangga karena Tergugat tidak mempunyai pekerjaan untuk memenauhi kebutuhan rumah tangga dibantu orang tua Penggugat.:
- Bahwa semenjak akhir bulan Juli 2018, Tergugat pergi meninggalkan Penggugat dan pulang kerumah orang tuanya sampai sekarang tidak pernah bersatu lagi selama 2 (dua) tahun 5 (lima) bulan;
- Bahwa semenjak kepergiannya Tergugat tidak pernah memberikan nafkah kepada Penggugat serta tidak ada meninggalkan harta yang dapat dijadikan sebagai nafkah untuk Penggugat ;
- Bahwa pihak keluarga telah berusaha untuk menasehati Penggugat dan Tergugat untuk rukun kembali namun tidak berhasil karena Penggugat tetap dengan pendiriannya ingin bercerai dengan Tergugat ;

Bahwa, Penggugat telah menyampaikan kesimpulan secara lisan yang pada pokoknya tetap pada gugatannya dan mohon putusan;

Bahwa untuk mempersingkat uraian dalam putusan ini, maka hal hal selengkapnya dianggap telah termuat dalam berita acara sidang perkara ini yang merupakan bagian dan dijadikan dasar pertimbangan dalam putusan ini;

PERTIMBANGAN HUKUM

Menimbang, bahwa maksud dan tujuan gugatan Penggugat adalah sebagai mana tersebut di atas;

Menimbang, bahwa berdasarkan gugatan tersebut Tergugat dipanggil untuk menghadap kepersidangan, namun tidak hadir dan tidak pula mengirimkan orang lain sebagai wakil atau kuasanya, sedang ketidak hadiran Tergugat tersebut bukan disebabkan suatu alasan yang sah menurut hukum. Oleh karenanya kepada Tergugat terlebih dahulu harus dinyatakan tidak pernah

Disclaimer
Kepaniteraan Mahkamah Agung Republik Indonesia berusaha untuk selalu mencantumkan informasi paling kini dan akurat sebagai bentuk komitmen Mahkamah Agung untuk pelayanan publik, transparansi dan akuntabilitas pelaksanaan fungsi peradilan. Namun dalam hal-hal tertentu masih dimungkinkan terjadi permasalahan teknis terkait dengan akurasi dan keterkinian informasi yang kami sajikan, hal mana akan terus kami perbaiki dari waktu kewaktu.
Dalam hal Anda menemukan inakurasi informasi yang termuat pada situs ini atau informasi yang seharusnya ada, namun belum tersedia, maka harap segera hubungi Kepaniteraan Mahkamah Agung RI melalui :
Email : kepaniteraan@mahkamahagung.go.id Telp : 021-384 3348 (ext.318)

hadir dipersidangan, sehingga perkara ini berdasarkan Pasal 126 HIR dapat diperiksa dan diputus dengan tanpa hadirnya Tergugat (Verstek) ;

Menimbang, bahwa oleh karena Tergugat tidak hadir maka mediasi sebagaimana diatur dalam PERMA Nomor 1 Tahun 2016 tidak dapat dilaksanakan, berdasarkan Pasal 82 Undang-undang Nomor 7 Tahun 1989 jo. Pasal 31 Peraturan Pemerintah Nomor 9 Tahun 1975 dan Pasal 143 ayat 1 dan 2 Kompilasi Hukum Islam, Majelis Hakim telah berupaya menasehati Penggugat untuk rukun dan tetap membina rumah tangga dengan Tergugat , akan tetapi tidak berhasil;

Menimbang, bahwa yang menjadi pokok permasalahan dalam perkara ini adalah Penggugat menggugat cerai terhadap Tergugat agar dijatuhkan talak Tergugat kepada Penggugat dengan alasan bahwa antara Penggugat dan Tergugat dalam membina rumah tangga semenjak awal bulan Juli 2018 telah terjadi persilisihan dan pertengkaran disebabkan masalah ekonomi dimana Tergugat kurang mampu memberikan nafkah lahir kepada Penggugat yang disebabkan karena Tergugat tidak memiliki pekerjaan yang tetap dan penghasilan yang tetap, sehingga untuk mencukupi kebutuhan sehari-hari harus di bantu oleh orangtua Penggugat, akibat dari hal tersebut Tergugat pergi meninggalkan Penggugat semenjak bulan Juli 2018 dan pulang kerumah orang tuanya sampai sekarang tidak pernah bersatu lagi selama 2 (dua) tahun 5 (lima) bulan;

Menimbang, oleh karena Tergugat tidak pernah hadir, maka tidak diketahui jawaban atau bantahan Tergugat, namun karena perkara ini menyangkut Undang-undang Perkawinan yang memerlukan acara khusus, maka Penggugat dibebani wajib bukti, hal dan sejalan pula dengan sebuah pendapat Ulama dalam Kitab al-Anwar juz: II halaman 149, yang artinya sebagai berikut:

“Apabila Tergugat berhalangan hadir karena bersembunyi atau enggan, maka Hakim boleh memeriksa gugatan tersebut dan alat alat bukti yang diajukan dan memberikan keputusannya“;

Menimbang, bahwa dipersidangan Penggugat telah mengajukan bukti surat-surat berupa **P.1** dan **P.2** serta (2) dua orang saksi yang telah memenuhi

Disclaimer
Kepaniteraan Mahkamah Agung Republik Indonesia berusaha untuk selalu mencantumkan informasi paling kini dan akurat sebagai bentuk komitmen Mahkamah Agung untuk pelayanan publik, transparansi dan akuntabilitas pelaksanaan fungsi peradilan. Namun dalam hal-hal tertentu masih dimungkinkan terjadi permasalahan teknis terkait dengan akurasi dan keterkinian informasi yang kami sajikan, hal mana akan terus kami perbaiki dari waktu kewaktu.
Dalam hal Anda menemukan inakurasi informasi yang termuat pada situs ini atau informasi yang seharusnya ada, namun belum tersedia, maka harap segera hubungi Kepaniteraan Mahkamah Agung RI melalui :
Email : kepaniteraan@mahkamahagung.go.id Telp : 021-384 3348 (ext.318)

syarat formil dan materil, dan Majelis Hakim mempertimbangkannya sebagai berikut;

Menimbang, bahwa berdasarkan Bukti **P.1**, telah terbukti menurut hukum bahwa tempat kediaman Penggugat berada di Wilayah Kabupaten Subang, bukti tersebut telah memenuhi syarat formil dan materil serta mempunyai kekuatan yang sempurna dan mengikat, oleh karenanya bukti tersebut secara aquo dapat diterima, hal ini sesuai dengan pasal 73 ayat (1) Undang Undang Nomor 7 Tahun 1989 yang telah diubah dan ditambah dengan Undang Undang Nomor 3 Tahun 2006 terakhir dirubah dengan Undang Undang Nomor 50 Tahun 2009;

Menimbang, bahwa berdasarkan Bukti **P.2**, maka telah terbukti menurut hukum bahwa antara Penggugat (Penggugat) dengan Tergugat (Saepul Basri bin Kholik) sejak tanggal 10 Mei 2018 telah terikat dalam ikatan tali perkawinan yang sah, sesuai dengan ketetuan Pasal 2 Undang-undang Nomor 1 Tahun 1974 Tentang Perkawinan jo. Pasal 4 Kompilasi Hukum Islam;

Menimbang, bahwa untuk menguat dalil gugatan Penggugat, maka Penggugat telah mengajukan dua orang saksi dalam persidangan dibawah sumpahnya menerangkan bahwa antara Penggugat dan Tergugat dalam membina rumah tangga semenjak awal bulan Juli 2018 sering terjadi perselisihan dan Pertengkaran dikarenakan masalah ekonomi yang selalu kekurangan dikarenakan Tergugat tidak memberikan nafkah untuk memenuhi kebutuhan sehari-hari dalam rumah tangga karena Tergugat tidak mempunyai pekerjaan untuk memenuhi kebutuhan rumah tangga dibantu orang tua Penggugat., akibat dari hal tersebut antara Penggugat dan Tergugat telah berpisah tempat tinggal semenjak buan Juli 2018 disebabkan Tergugat pergi meninggalkan Penggugat dan tidak pernah bersatu lagi sampai sekarang selama 2 (dua) tahun 5 (lima) bulan ;

Menimbang, bahwa dari keterangan saksi diatas ditemukan fakta kejadian bahwa antara Penggugat dan Tergugat semenjak akhir bulan Juli 2018 telah berpisah tempat tinggal sampai saat ini karena Tergugat pergi meninggalkan Penggugat sampai sekarang tidak pernah bersatu lagi;

Disclaimer
Kepaniteraan Mahkamah Agung Republik Indonesia berusaha untuk selalu mencantumkan informasi paling kini dan akurat sebagai bentuk komitmen Mahkamah Agung untuk pelayanan publik, transparansi dan akuntabilitas pelaksanaan fungsi peradilan. Namun dalam hal-hal tertentu masih dimungkinkan terjadi permasalahan teknis terkait dengan akurasi dan keterkinian informasi yang kami sajikan, hal mana akan terus kami perbaiki dari waktu kewaktu.
Dalam hal Anda menemukan inakurasi informasi yang termuat pada situs ini atau informasi yang seharusnya ada, namun belum tersedia, maka harap segera hubungi Kepaniteraan Mahkamah Agung RI melalui :
Email : kepaniteraan@mahkamahagung.go.id Telp : 021-384 3348 (ext.318)

Menimbang, bahwa kesaksian yang diberikan oleh dua orang saksi Penggugat didasarkan pengetahuan, penglihatan dan pendengaran langsung saksi dan keterangannya saling terkait satu dengan yang lain, maka berdasarkan Pasal 172 HIR jo. Pasal 76 Undang-Undang Nomor 7 Tahun 1989 dengan demikian dua orang saksi Penggugat dipandang telah memenuhi syarat formal dan materil kesaksiannya, maka keterangan saksi tersebut merupakan alat bukti yang mempunyai nilai pembuktian ;

Menimbang, bahwa dengan sikap Tergugat yang tidak pernah datang menghadap di muka persidangan serta gugatan Penggugat tidak melawan hukum dan beralasan maka perkara ini dapat diputus dengan verstek dan berdasarkan penilaian terhadap keterangan kedua orang saksi tersebut diatas, Majelis Hakim menemukan fakta hukum bahwa antara Penggugat dan Tergugat telah pisah tempat tinggal semenjak akhir bulan Juli 2018 sampai dengan saat ini tidak pernah bersatu lagi, dengan tidak bersatunya Penggugat dan Tergugat sampai sekarang, hal tersebut telah menunjukkan bahwa rumah tangga Penggugat dan Tergugat telah pecah dan tidak mungkin untuk bersatu lagi karena lebih besar mudarat dari pada maslahatnya;

Menimbang, bahwa berdasarkan fakta-fakta hukum di atas Majelis berpendapat bahwa rumah tangga Penggugat dengan Tergugat telah pecah sehingga sulit diharapkan dan sudah tidak mungkin akan sakinah, mawaddah, warohmah karena antara kedua belah pihak sudah tidak melaksanakan kewajiban dan tanggung jawab antara satu dengan lainnya semenjak 2 tahun 4 bulan, maka alasan gugatan Penggugat ini telah sesuai Pasal 1 Undang-undang Nomor 1 Tahun 1974 dan petunjuk al-Qur'an surat al-Rum ayat 21;

Menimbang, bahwa berdasarkan fakta-fakta hukum tersebut diatas, Majelis Hakim berpendapat, rumah tangga Penggugat dengan Tergugat telah pecah sedemikian rupa (broken mariage) disebabkan antara kedua belah pihak telah berpisah tempat tinggal semenjak akhir bulan Juli 2018 sampai dengan saat ini sudah tidak bersatu lagi;

Menimbang, bahwa berdasarkan pertimbangan tersebut diatas, maka perceraian merupakan alternatif yang terbaik yang dapat menyelamatkan

Disclaimer
Kepaniteraan Mahkamah Agung Republik Indonesia berusaha untuk selalu mencantumkan informasi paling kini dan akurat sebagai bentuk komitmen Mahkamah Agung untuk pelayanan publik, transparansi dan akuntabilitas pelaksanaan fungsi peradilan. Namun dalam hal-hal tertentu masih dimungkinkan terjadi permasalahan teknis terkait dengan akurasi dan keterkinian informasi yang kami sajikan, hal mana akan terus kami perbaiki dari waktu kewaktu.
Dalam hal Anda menemukan inakurasi informasi yang termuat pada situs ini atau informasi yang seharusnya ada, namun belum tersedia, maka harap segera hubungi Kepaniteraan Mahkamah Agung RI melalui :
Email : kepaniteraan@mahkamahagung.go.id Telp : 021-384 3348 (ext.318)

keduanya dari penderitaan yang berkepanjangan, dari pada tetap mempertahankan perkawinan yang sudah rapuh, hal mana sejalan pula dengan maksud kaidah fiqhiyah yang artinya "menghindari kerusakan harus didahulukan daripada mendatangkan kemaslahatan". Sejalan pula dengan sebuah pendapat Ulama dalam Kitab Fiqh al-Sunnah juz II : 248, yang artinya sebagai berikut:

"Bahwa sesungguhnya boleh bagi seorang isteri minta kepada Hakim untuk diceraikan dari suaminya dengan dasar tuntutan bahwa apabila telah ternyata di dalam perkawinan terdapat kemadlaratan dimana suami isteri tersebut sudah tidak mampu lagi untuk mempertahankan kelangsungan rumah tangga itu. … dan Hakim sudah tidak dapat mendamaikan suami isteri tersebut, maka Hakim menceraikannya dengan talak satu bain " ;

Menimbang, bahwa berdasarkan pertimbangan-pertimbangan sebagaimana tersebut diatas, maka dalil-dalil gugatan Penggugat bahwa antara Penggugat dan Tergugat telah berpisah tempat tinggal semenjak akhir bulan Juli 2018 sampai dengan saat ini tidak bersatu lagi, maka dalil-dalil gugatan Penggugat telah beralasan hukum dan terbukti serta telah memenuhi ketentuan Pasal 39 ayat (2) Undang-undang Nomor 1 Tahun 1974, Jo. Pasal 19 huruf (b) Peraturan Pemerintah Nomor 9 Tahun 1975, Jo Pasal 116 huruf (b) Kompilasi Hukum Islam yang berbunyi "Salah satu pihak meninggalkan pihak lain 2 (dua) tahun berturut-turuttanpa izin pihak lain dan tanpa alasan yang sah atau karena hal lain diluar kemampuannya", oleh karenanya gugatan Penggugat patut dikabulkan dengan menjatuhkan talak satu bain sughra Tergugat terhadap Penggugat;

Menimbang, bahwa Tergugat tidak pernah hadir dipersidangan sedangkan gugatan Penggugat telah beralasan hukum oleh karenanya berdasarkan Pasal 126 HIR maka gugatan Penggugat patut dikabulkan dengan Verstek dengan menjatuhkan thalak satu bain shugra Tergugat terhadap Penggugat;

Menimbang, bahwa berdasarkan ketentuan Pasal 89 ayat (1) Undang-undang Nomor 7 Tahun 1989 yang telah diubah dan ditambah dengan

Disclaimer
Kepaniteraan Mahkamah Agung Republik Indonesia berusaha untuk selalu mencantumkan informasi paling kini dan akurat sebagai bentuk komitmen Mahkamah Agung untuk pelayanan publik, transparansi dan akuntabilitas pelaksanaan fungsi peradilan. Namun dalam hal-hal tertentu masih dimungkinkan terjadi permasalahan teknis terkait dengan akurasi dan keterkinian informasi yang kami sajikan, hal mana akan terus kami perbaiki dari waktu kewaktu.
Dalam hal Anda menemukan inakurasi informasi yang termuat pada situs ini atau informasi yang seharusnya ada, namun belum tersedia, maka harap segera hubungi Kepaniteraan Mahkamah Agung RI melalui :
Email : kepaniteraan@mahkamahagung.go.id Telp : 021-384 3348 (ext.318)

Undang-undang Nomor 3 Tahun 2006 terakhir dirubah dengan Undang-undang Nomor 50 Tahun 2009, maka semua biaya yang timbul dalam perkara ini dibebankan kepada Penggugat;

Mengingat, ketentuan dalam Peraturan Per Undang-undangan yang berlaku dan dalil syar'i yang berkaitan dengan perkara ini;

M E N G A D I L I

1. Menyatakan bahwa Tergugat yang telah dipanggil secara resmi dan patut untuk menghadap persidangan, tidak hadir;
2. Mengabulkan gugatan Penggugat dengan verstek;
3. Menjatuhkan talak satu ba'in sughra Tergugat (Saepul Basri bin Kholik) terhadap Penggugat (Penggugat);
4. Membebankan biaya perkara ini kepada Penggugat yang hingga kini dihitung sebesar Rp391.000,00 (tiga ratus sembilan puluh satu ribu rupiah);

Demikian diputuskan dalam musyawarah Majelis Hakim pada hari **Selasa**, tanggal **15 Desember 2020 Masehi,** yang bertepatan dengan tanggal **29 Rabiul Akhir 1442 Hijriyyah**, oleh Hakim Pengadilan Agama Subang yang terdiri dari **Dra. Hj. Suherni, M.H.** sebagai Ketua Majelis dan **Drs. Esib Jaelani, M.H.** serta **Drs. H. Abdul Hamid Mayeli, S.H., M.H.** masing-masing sebagai Hakim Anggota, dibantu oleh **Drs. Budiana, S.H.I.** sebagai Panitera Pengganti. Putusan tersebut pada hari itu juga diucapkan dalam persidangan terbuka untuk umum oleh Ketua Majelis dihadiri Penggugat tanpa hadirnya Tergugat.

Hakim Anggota,	Ketua Majelis Hakim,
**Drs. Esib Jaelani, M.H.**	**Dra. Hj. Suherni, M.H.**

Disclaimer
Kepaniteraan Mahkamah Agung Republik Indonesia berusaha untuk selalu mencantumkan informasi paling kini dan akurat sebagai bentuk komitmen Mahkamah Agung untuk pelayanan publik, transparansi dan akuntabilitas pelaksanaan fungsi peradilan. Namun dalam hal-hal tertentu masih dimungkinkan terjadi permasalahan teknis terkait dengan akurasi dan keterkinian informasi yang kami sajikan, hal mana akan terus kami perbaiki dari waktu kewaktu.
Dalam hal Anda menemukan inakurasi informasi yang termuat pada situs ini atau informasi yang seharusnya ada, namun belum tersedia, maka harap segera hubungi Kepaniteraan Mahkamah Agung RI melalui :
Email : kepaniteraan@mahkamahagung.go.id Telp : 021-384 3348 (ext.318)

Hakim Anggota,

Drs. H. Abdul Hamid Mayeli, S.H., M.H.

Panitera Pengganti,

Drs. Budiana, S.H.I.

Perincian Biaya :

1. Pendaftaran	:	Rp30.000,00
2. Proses	:	Rp50.000,00
3. Panggilan	:	Rp275.000,00
4. PNBP Panggilan	:	Rp20.000,00
5. Redaksi	:	Rp10.000,00
6. Meterai	:	Rp6.000,00
**Jumlah**	**:**	**Rp391.000,00**

(tiga ratus sembilan puluh satu ribu rupiah)

Disclaimer
Kepaniteraan Mahkamah Agung Republik Indonesia berusaha untuk selalu mencantumkan informasi paling kini dan akurat sebagai bentuk komitmen Mahkamah Agung untuk pelayanan publik, transparansi dan akuntabilitas pelaksanaan fungsi peradilan. Namun dalam hal-hal tertentu masih dimungkinkan terjadi permasalahan teknis terkait dengan akurasi dan keterkinian informasi yang kami sajikan, hal mana akan terus kami perbaiki dari waktu kewaktu.
Dalam hal Anda menemukan inakurasi informasi yang termuat pada situs ini atau informasi yang seharusnya ada, namun belum tersedia, maka harap segera hubungi Kepaniteraan Mahkamah Agung RI melalui :
Email : kepaniteraan@mahkamahagung.go.id   Telp : 021-384 3348 (ext.318)
