# Supplementary material for: Classification of divorce causes during the COVID-19 pandemic using convolutional neural networks
Source: PeerJ Comput Sci. 2022 Jun 30;8:e998. doi: 10.7717/peerj-cs.998 (PMC9299239; doi:10.7717/peerj-cs.998)
Supplement: Supplemental Information 5 [file peerj-cs-08-998-s005.zip › Masalah Ekonomi Dataset/Data ke-8.pdf]

P U T U S A N

Nomor 4202/Pdt.G/2020/PA.Smdg

بِسْمِ اللهِ الرَّحْمٰنِ الرَّحِيْمِ

DEMI KEADILAN BERDASARKAN KETUHANAN YANG MAHA ESA

Pengadilan Agama Sumedang yang memeriksa dan mengadili perkara tertentu pada tingkat pertama dalam sidang majelis telah menjatuhkan putusan perkara Cerai Gugat antara:

Rina Nurmalasari, S.Pd binti Nana Mulyana, umur 37 tahun, agama Islam, pekerjaan PNS, pendidikan S2, tempat kediaman di Dusun Selaawi, Rt. 002, Rw. 006, Desa Sukahayu, Kecamatan Rancakalong, Kabupaten Sumedang., Desa Sukahayu, Rancakalong, Kab. Sumedang, Jawa Barat selanjutnya telah memberikan kuasa kepada Humaedi Abdurohman, S.sy, yang berkantor di Biro Konsultasi dan Bantuan Hukum Mitra Keluarga (BKBH MK) yang beralamat di Jalan Statistik No. 76, Kelurahan Situ, Rt. 001/ Rw. 011 Kecamatan Sumedang Utara, Kabupaten Sumedang. 45325, berdasarkan Surat Kuasa Khusus tertanggal 04 Desember 2020; sebagai **Penggugat**;

melawan

Muhamad Fahrudianto bin Sugiri, umur 44 tahun, agama Islam, pendidikan D3, pekerjaan Karyawan Swasta, tempat kediaman di Dusun Selaawi, Rt.002, Rw. 006, Desa Sukahayu, Kecamatan Rancakalong, Kabupaten Sumedang., Desa Sukahayu, Rancakalong, Kab. Sumedang, Jawa Barat; sebagai **Tergugat**;

Pengadilan Agama tersebut;

Telah mempelajari surat-surat yang berkaitan dengan perkara ini;

Telah mendengar keterangan Penggugat serta para saksi di muka sidang;

DUDUK PERKARA

Disclaimer
Kepaniteraan Mahkamah Agung Republik Indonesia berusaha untuk selalu mencantumkan informasi paling kini dan akurat sebagai bentuk komitmen Mahkamah Agung untuk pelayanan publik, transparansi dan akuntabilitas pelaksanaan fungsi peradilan. Namun dalam hal-hal tertentu masih dimungkinkan terjadi permasalahan teknis terkait dengan akurasi dan keterkinian informasi yang kami sajikan, hal mana akan terus kami perbaiki dari waktu kewaktu.
Dalam hal Anda menemukan inakurasi informasi yang termuat pada situs ini atau informasi yang seharusnya ada, namun belum tersedia, maka harap segera hubungi Kepaniteraan Mahkamah Agung RI melalui :
Email : kepaniteraan@mahkamahagung.go.id Telp : 021-384 3348 (ext.318)

Bahwa Penggugat dalam surat gugatannya tanggal 04 Desember 2020 telah mengajukan gugatan, yang telah didaftar di Kepaniteraan Pengadilan Agama Sumedang, dengan Nomor 4202/Pdt.G/2020/PA.Smdg, tanggal 04 Desember 2020, dengan dalil-dalil sebagai berikut:

1. Bahwa Penggugat dan Tergugat adalah suami isteri sah, menikah pada tanggal 07 Agustus 2003 M/08 Jumadil Akhir 1424 H, berdasarkan Kutipan Akta Nikah dari KUA Kecamatan Rancakalong, Kabupaten Sumedang, dengan memenuhi syarat rukun nikah, sebagaimana tertera dalam Kutipan Akta Nikah Nomor: 116/05/VIII/2003, tertanggal 07Agustus 2003.
2. Bahwa setelah pernikahan tersebut, Penggugat dengan Tergugat terakhir tinggal bersama di rumah kediaman milik Penggugatdi Dusun Selaawi, RT. 002/ RW. 006, Desa Sukahayu, Kecamatan Rancakalong, Kabupaten Sumedang. Serta telah melakukan hubungan layaknya suami istri (ba'da dukhul), sehingga rumah tangga antara Penggugat dengan tergugat hingga sekarang telah dikaruniai seorang anak yang bernama **Faza Indira Pratama Putri**(berusia 15 tahun).
3. Bahwa seiring perjalanan rumah tangga, keadaan tidak selamanya rukun dan harmonis, rumah tangga antara Penggugat dan Tergugat sejak bulan **Juli 2018** diwarnai dengan perselisihan dan pertengkaran terus menerus yang disebabkandiantaranya karena hubungan antara Tergugat dengan orangtua Penggugat kurang harmonis yang mana Tergugat seringkali menjelek-jelekan orangtua Penggugat, sehingga masalah tersebut tidak jarang menjadi pemicu terjadinya perselisihan bahkan berujung pada pertengkaraen terus-menerus;
4. Bahwa Penggugat telah berusaha sabar untuk mempertahankan rumah tangga, tetapi kondisi rumah tangga semakin tidak harmonis. Puncaknya terjadi pada bulan **Oktober 2019**Tergugat pergi meninggalkan rumah kediaman orangtua Penggugat dan sejak itu Penggugat dan Tergugat pisah tempat tinggal, bahkan berakibat tidak lagi berhubungan layaknya suami isteri (hingga sekarang sudah sampai 1 tahun 2 bulan lamanya).
5. Bahwa Penggugat telah berusaha untuk mempertahankan rumah

Disclaimer
Kepaniteraan Mahkamah Agung Republik Indonesia berusaha untuk selalu mencantumkan informasi paling kini dan akurat sebagai bentuk komitmen Mahkamah Agung untuk pelayanan publik, transparansi dan akuntabilitas pelaksanaan fungsi peradilan. Namun dalam hal-hal tertentu masih dimungkinkan terjadi permasalahan teknis terkait dengan akurasi dan keterkinian informasi yang kami sajikan, hal mana akan terus kami perbaiki dari waktu kewaktu.
Dalam hal Anda menemukan inakurasi informasi yang termuat pada situs ini atau informasi yang seharusnya ada, namun belum tersedia, maka harap segera hubungi Kepaniteraan Mahkamah Agung RI melalui :
Email : kepaniteraan@mahkamahagung.go.id Telp : 021-384 3348 (ext.318)

tangga bersama Tergugat bahkan Penggugat telah meminta bantuan kepada keluarga akan tetapi tidak berhasil.

6. Bahwa gugatan Penggugat tersebut telah memenuhi syarat sesuai ketentuan **Pasal 19 huruf (f) Peraturan Pemerintah Nomor 9 Tahun 1975 jo. Pasal 116 huruf (f) Kompilasi Hukum Islam**.
7. Bahwa atas permasalahan tersebut di atas Penggugat sudah tidak sanggup lagi untuk mempertahankan perkawinan ini, oleh karena itu Penggugat telah berketetapan hati untuk bercerai dengan Tergugat.
8. Bahwa sekarang ini Penggugat bekerja sebagai PNS dan telah memiliki izin dari BKD untuk mengajukan Gugatan Cerai ke Pengadilan Agama Sumedang berdasarkan Surat Keputusan Bupati Sumedang Nomor: 873.4/Kep.112/BKPSDM/2002, tertanggal 26 November 2020;

Berdasarkan uraian tersebut diatas, Penggugat mohon kepada Ketua Pengadilan Agama Sumedang untuk memeriksa perkara ini, dan menjatuhkan putusan sebagai berikut :

1. Mengabulkan gugatan Penggugat;
2. Menjatuhkan talak satu ba'in sughra Tergugat (**Muhamad Fahrudianto bin Sugiri**) terhadap Penggugat (**Rina Nurmalasari, S.Pd binti Nana Mulyana**);
3. Membebankan biaya perkara menurut hukum;

Atau apabila Ketua Pengadilan Agama Sumedang Cq. Majelis Hakim berpendapat lain mohon putusan yang seadil-adilnya (ex aequo et bono) ;

Bahwa pada persidangan yang telah ditetapkan Penggugat didampingi kuasanya telah datang menghadap ke muka sidang, sedangkan Tergugat tidak datang menghadap di persidangan dan tidak menyuruh orang lain untuk menghadap sebagai wakil/kuasa yang sah, meskipun berdasarkan surat panggilan (relass) Nomor 4202/Pdt.G/2020/PA.Smdg, tanggal 08 Desember 2020 dan tanggal 15 Desember 2020, yang dibacakan di persidangan, Tergugat telah dipanggil secara resmi dan patut, sedangkan tidak ternyata bahwa tidak datangnya itu disebabkan suatu alasan yang sah;

Bahwa majelis hakim telah menasehati Penggugat agar berpikir untuk tidak bercerai dengan Tergugat, tetapi Penggugat tetap pada dalil-dalil

Disclaimer
Kepaniteraan Mahkamah Agung Republik Indonesia berusaha untuk selalu mencantumkan informasi paling kini dan akurat sebagai bentuk komitmen Mahkamah Agung untuk pelayanan publik, transparansi dan akuntabilitas pelaksanaan fungsi peradilan. Namun dalam hal-hal tertentu masih dimungkinkan terjadi permasalahan teknis terkait dengan akurasi dan keterkinian informasi yang kami sajikan, hal mana akan terus kami perbaiki dari waktu kewaktu.
Dalam hal Anda menemukan inakurasi informasi yang termuat pada situs ini atau informasi yang seharusnya ada, namun belum tersedia, maka harap segera hubungi Kepaniteraan Mahkamah Agung RI melalui :
Email : kepaniteraan@mahkamahagung.go.id   Telp : 021-384 3348 (ext.318)

gugatannya untuk bercerai dengan Tergugat ;

Bahwa perkara ini tidak dapat dimediasi karena Tergugat tidak pernah datang menghadap meskipun telah dipanggil secara resmi dan patut, selanjutnya dimulai pemeriksaan dengan membacakan surat gugatan Penggugat yang maksud dan tujuannya tetap dipertahankan oleh Penggugat ;

Bahwa sebagai Pegawai Negeri Sipil Penggugat telah memiliki surat Pemberian Izin Cerai Nomor : 873.4/Kep.112/BKPSDM/2020 tertanggal 26 Nopember 2020 yang diterbitkan oleh Bupati Kabupaten Sumedang;

Bahwa karena Tergugat tidak hadir maka pemeriksaan dilanjutkan tanpa jawaban dari Tergugat;

Bahwa untuk menguatkan dalil-dalil gugatannya, Penggugat telah mengajukan bukti surat berupa Fotocopi Kutipan Akta Nikah, Nomor 116/05/2003, tertanggal 07 September 2003 yang diterbitkan oleh Kantor Urusan Agama KUA Kecamatan Rancakalong Kabupaten Sumedang (Bukti P.1)

Bahwa selain alat bukti tertulis, Penggugat juga telah menguatkan dalil-dalil gugatannya dengan bukti saksi sebagai berikut :

1. Tatin Hartini binti Ating, umur 56 tahun, agama Islam, pekerjaan PNS Guru, tempat tinggal di Dusun Ciseda Rt. 002, Rw. 004, Desa Citimun, Kecamatan Cimalaka, Kabupaten Sumedang, memberikan keterangan di bawah sumpah, yang pada pokoknya sebagai berikut:
   - Bahwa saksi kenal kepada Penggugat dan Tergugat karena saksi adalah Bibi Penggugat;
   - Bahwa antara Penggugat dengan Tergugat telah hidup rukun sebagaimana layaknya suami istri dan telah dikaruniai seorang anak;
   - Bahwa sepengetahuan saksi hubungan rumah tangga Penggugat dengan Tergugat mulai tidak lagi harmonis sejak bulan Juli 2018, karena sering terjadi perselisihan dan pertengkaran ;
   - Bahwa saksi pernah melihat langsung pertengkaran Penggugat dan Tergugat;
   - Bahwa penyebab pertengkaran tersebut adalah karena hubungan

Disclaimer
Kepaniteraan Mahkamah Agung Republik Indonesia berusaha untuk selalu mencantumkan informasi paling kini dan akurat sebagai bentuk komitmen Mahkamah Agung untuk pelayanan publik, transparansi dan akuntabilitas pelaksanaan fungsi peradilan. Namun dalam hal-hal tertentu masih dimungkinkan terjadi permasalahan teknis terkait dengan akurasi dan keterkinian informasi yang kami sajikan, hal mana akan terus kami perbaiki dari waktu kewaktu.
Dalam hal Anda menemukan inakurasi informasi yang termuat pada situs ini atau informasi yang seharusnya ada, namun belum tersedia, maka harap segera hubungi Kepaniteraan Mahkamah Agung RI melalui :
Email : kepaniteraan@mahkamahagung.go.id   Telp : 021-384 3348 (ext.318)

antara Tergugat dengan orang tua Penggugat kurang terjalin dengan baik sering menjelek-jelekan orang tua Penggugat;

- Bahwa akibat pertengkaran tersebut Penggugat dan Tergugat telah berpisah tempat tinggal sejak akhir bulan Oktober 2019 sampai dengan sekarang tidak bersatu lagi dalam rumah tangga;
- Bahwa pihak keluarga Penggugat sudah berusaha mendamaikan Penggugat dengan Tergugat akan tetapi tidak berhasil dan saat ini sudah tidak sanggup lagi mendamaikan Penggugat dan Tergugat ;

2. Tatang bin Juim, umur 53 tahun, agama Islam, pekerjaan Wiraswasta, tempat tinggal di Dusun Pasirmasigit Rt. 004, Rw. 007, Desa Pangadegan, Kecamatan Rancakalong, Kabupaten Sumedang, memberikan keterangan di bawah sumpah, yang pada pokoknya sebagai berikut:
   - Bahwa saksi kenal kepada Penggugat dalam hubungan selaku Kakak Ipar Penggugat;
   - Bahwa antara Penggugat dengan Tergugat telah hidup rukun sebagaimana layaknya suami istri dan telah dikaruniai seorang anak ;
   - Bahwa sepengetahuan saksi hubungan rumah tangga Penggugat dengan Tergugat mulai tidak lagi harmonis sejak bulan Juli 2018, karena sering terjadi perselisihan dan pertengkaran ;
   - Bahwa saksi pernah melihat langsung antara Penggugat dengan Tergugat bertengkar;
   - Bahwa penyebab pertengkaran tersebut adalah karena hubungan antara Tergugat dengan orang tua Tergugat tidak terjalin dengan baik, Tergugat sering menjelek-jelekan orangtua Penggugat kepada Penggugat;
   - Bahwa akibat pertengkaran tersebut Penggugat dan Tergugat telah berpisah tempat tinggal sejak bulan Oktober 2019 sampai dengan sekarang tidak bersatu lagi dalam rumah tangga;
   - Bahwa pihak keluarga Penggugat sudah berusaha mendamaikan Penggugat dengan Tergugat akan tetapi tidak berhasil;

Bahwa Penggugat telah mencukupkan bukti yang diajukan dan tidak akan

Disclaimer
Kepaniteraan Mahkamah Agung Republik Indonesia berusaha untuk selalu mencantumkan informasi paling kini dan akurat sebagai bentuk komitmen Mahkamah Agung untuk pelayanan publik, transparansi dan akuntabilitas pelaksanaan fungsi peradilan. Namun dalam hal-hal tertentu masih dimungkinkan terjadi permasalahan teknis terkait dengan akurasi dan keterkinian informasi yang kami sajikan, hal mana akan terus kami perbaiki dari waktu kewaktu.
Dalam hal Anda menemukan inakurasi informasi yang termuat pada situs ini atau informasi yang seharusnya ada, namun belum tersedia, maka harap segera hubungi Kepaniteraan Mahkamah Agung RI melalui :
Email : kepaniteraan@mahkamahagung.go.id   Telp : 021-384 3348 (ext.318)

mengajukan bukti lagi;

Bahwa Penggugat menyampaikan kesimpulan yang pada pokoknya tetap ingin bercerai dengan Tergugat dan mohon putusan;

Bahwa selanjutnya untuk singkatnya uraian putusan ini, maka semua hal yang termuat dalam berita acara sidang merupakan bagian yang tidak terpisahkan dari putusan ini;

PERTIMBANGAN HUKUM

Menimbang, bahwa maksud dan tujuan gugatan Penggugat adalah sebagaimana terurai di atas;

Menimbang, bahwa ternyata Tergugat, meskipun dipanggil secara resmi dan patut, tidak datang menghadap di muka sidang dan pula tidak ternyata bahwa tidak datangnya itu disebabkan suatu halangan yang sah;

Menimbang, bahwa Penggugat sebagai PNS telah memiliki surat izin dari atasannya untuk melakukan perceraian dengan demikian telah terpenuhi maksud pasal 3 ayat (1) Peraturan Pemerintah Nomor 10 tahun 1983 Jo. Peraturan Pemerintah Nomor 45 tahun 1990;

Menimbang, bahwa Tergugat yang dipanggil secara resmi dan patut akan tetapi tidak datang menghadap harus dinyatakan tidak hadir dan permohonan tersebut harus diperiksa secara verstek;

Menimbang, bahwa berdasarkan ketentuan Pasal 125 ayat (1) HIR putusan atas perkara ini dapat dijatuhkan tanpa hadirnya Tergugat (verstek);

Menimbang, bahwa berdasarkan ketentuan Pasal 125 HIR yaitu putusan yang dijatuhkan tanpa hadirnya Tergugat dapat dikabulkan sepanjang berdasarkan hukum dan beralasan, oleh karena itu majelis membebani Penggugat untuk membuktikan dalil-dalil gugatannya;

Menimbang, bahwa dari posita gugatan Penggugat, yang dijadikan alasan gugatan Penggugat adalah pada intinya Penggugat mohon agar dijatuhkan talak satu bain shugro Tergugat terhadap diri Penggugat dengan alasan karena dalam rumah tangga antara Penggugat dengan Tergugat telah terjadi perselisihan dalam rumah tangga yang disebabkan antara lain karena hubungan antara Tergugat dengan orang tua Penggugat kurang terjalin dengan baik sering menjelek-jelekan orang tua Penggugat ;

Disclaimer
Kepaniteraan Mahkamah Agung Republik Indonesia berusaha untuk selalu mencantumkan informasi paling kini dan akurat sebagai bentuk komitmen Mahkamah Agung untuk pelayanan publik, transparansi dan akuntabilitas pelaksanaan fungsi peradilan. Namun dalam hal-hal tertentu masih dimungkinkan terjadi permasalahan teknis terkait dengan akurasi dan keterkinian informasi yang kami sajikan, hal mana akan terus kami perbaiki dari waktu kewaktu.
Dalam hal Anda menemukan inakurasi informasi yang termuat pada situs ini atau informasi yang seharusnya ada, namun belum tersedia, maka harap segera hubungi Kepaniteraan Mahkamah Agung RI melalui :
Email : kepaniteraan@mahkamahagung.go.id Telp : 021-384 3348 (ext.318)

Menimbang, bahwa untuk membuktikan dalil-dalil gugatannya, Penggugat telah mengajukan alat bukti surat P.1 serta 2 (dua) orang saksi;

Menimbang, bahwa berdasarkan bukti P.1 yang merupakan akta autentik dimana berdasarkan pasal 7 ayat (1) Kompilasi Hukum Islam akta nikah adalah merupakan satu-satunya bukti pernikahan, maka harus dinyatakan terbukti bahwa antara Penggugat dengan Tergugat telah terikat dalam perkawinan yang sah, oleh karenanya Penggugat dan Tergugat adalah pihak yang berkepentingan langsung dalam perkara ini;

Menimbang, bahwa saksi Penggugat yang pertama **(**Tatin Hartini binti Ating**)** adalah orang yang tidak terhalang untuk menjadi saksi dalam perkara ini serta telah memberikan keterangan di bawah sumpahnya sebagaimana diatur dalam Pasal 145 ayat 1 angka 3e HIR dengan demikian telah memenuhi syarat formil pembuktian, adapun keterangan saksi menyangkut dalil-dalil gugatan Penggugat, telah didasarkan kepada penglihatan saksi yang melihat adanya pertengkaran antara Penggugat dengan Tergugat, yang penyebabnya karena hubungan antara Tergugat dengan orang tua Penggugat kurang terjalin dengan baik sering menjelek-jelekan orang tua Penggugat, serta saksi melihat saat ini antara Penggugat dengan Tergugat telah berpisah tempat tinggal, yang keterangan selengkapnya sebagaimana diuraikan dalam duduk perkara, dengan demikian patut diduga bahwa saksi sebagai Bibi Penggugat mengetahui hal tersebut, dengan demikian keterangan saksi tersebut telah memenuhi syarat materiil pembuktian, oleh karenanya dapat diterima sebagai bukti dalam perkara ini;

Menimbang, bahwa keterangan saksi Penggugat yang kedua **(**Tatang bin Juim**)** adalah orang yang tidak terhalang untuk menjadi saksi dalam perkara ini serta telah memberikan keterangan di bawah sumpahnya sebagaimana diatur dalam Pasal 145 ayat 1 angka 3e HIR dengan demikian telah memenuhi syarat formil pembuktian, adapun keterangan saksi menyangkut dalil-dalil gugatan Penggugat telah didasarkan kepada penglihatan saksi yang melihat adanya pertengkaran antara Penggugat dengan Tergugat yang penyebabnya karena hubungan antara Tergugat dengan orang tua Tergugat tidak terjalin dengan baik, Tergugat sering menjelek-jelekan orangtua Penggugat kepada

Disclaimer
Kepaniteraan Mahkamah Agung Republik Indonesia berusaha untuk selalu mencantumkan informasi paling kini dan akurat sebagai bentuk komitmen Mahkamah Agung untuk pelayanan publik, transparansi dan akuntabilitas pelaksanaan fungsi peradilan. Namun dalam hal-hal tertentu masih dimungkinkan terjadi permasalahan teknis terkait dengan akurasi dan keterkinian informasi yang kami sajikan, hal mana akan terus kami perbaiki dari waktu kewaktu.
Dalam hal Anda menemukan inakurasi informasi yang termuat pada situs ini atau informasi yang seharusnya ada, namun belum tersedia, maka harap segera hubungi Kepaniteraan Mahkamah Agung RI melalui :
Email : kepaniteraan@mahkamahagung.go.id Telp : 021-384 3348 (ext.318)

Penggugat, serta saksi melihat saat ini antara Penggugat dengan Tergugat telah berpisah tempat tinggal, yang keterangan selengkapnya sebagaimana diuraikan dalam duduk perkara, dengan demikian patut diduga bahwa saksi sebagai Kakak Ipar Penggugat mengetahui hal tersebut, dengan demikian keterangan saksi tersebut telah memenuhi syarat materiil pembuktian, oleh karenanya dapat diterima sebagai bukti dalam perkara ini;

Menimbang, bahwa keterangan saksi I dan saksi II Penggugat bersesuaian dan cocok antara satu dengan yang lain oleh karena itu keterangan dua orang saksi tersebut memenuhi Pasal 171 dan Pasal 172 HIR;

Menimbang, bahwa berdasarkan bukti P.1 yang dihubungkan dengan keterangan dua orang saksi yang saling bersesuaian, Maka Majelis menemukan fakta-fakta sebagai berikut:

- bahwa Penggugat dan Tergugat adalah suami isteri dan masih terikat dalam perkawinan yang sah;
- bahwa rumah tangga Penggugat dan Tergugat sudah tidak rukun dan harmonis karena adanya pertengkaran dan perselisihan antara Penggugat dengan Tergugat;
- bahwa penyebab Pertengkaran tersebut adalah karena hubungan antara Tergugat dengan orang tua Penggugat kurang terjalin dengan baik sering menjelek-jelekan orang tua Penggugat;
- bahwa antara Penggugat dengan Tergugat kurang lebih sejak bulan Oktober 2019 sampai sekarang tidak bersatu lagi dalam rumah tangga;
- bahwa upaya perdamaian yang dilakukan pihak keluarga tidak berhasil, karena Penggugat tetap bersiteguh kepada pendiriannya untuk bercerai dengan Tergugat;

Menimbang, bahwa berdasarkan fakta-fakta tersebut di atas, Majelis Hakim berpendapat bahwa keadaan rumah tangga Penggugat dan Tergugat sudah tidak rukun dan sudah benar-benar pecah dan sudah sulit untuk didamaikan kembali untuk melanjutkan rumah tangganya;

Menimbang, bahwa berdasarkan pertimbangan-pertimbangan tersebut diatas dengan tanpa melihat atau memandang siapa yang bersalah serta apa

Disclaimer
Kepaniteraan Mahkamah Agung Republik Indonesia berusaha untuk selalu mencantumkan informasi paling kini dan akurat sebagai bentuk komitmen Mahkamah Agung untuk pelayanan publik, transparansi dan akuntabilitas pelaksanaan fungsi peradilan. Namun dalam hal-hal tertentu masih dimungkinkan terjadi permasalahan teknis terkait dengan akurasi dan keterkinian informasi yang kami sajikan, hal mana akan terus kami perbaiki dari waktu kewaktu.
Dalam hal Anda menemukan inakurasi informasi yang termuat pada situs ini atau informasi yang seharusnya ada, namun belum tersedia, maka harap segera hubungi Kepaniteraan Mahkamah Agung RI melalui :
Email : kepaniteraan@mahkamahagung.go.id Telp : 021-384 3348 (ext.318)

yang menjadi penyebabnya, harus dinyatakan terbukti bahwa antara Penggugat dan Tergugat telah terjadi perselisihan dan pertengkaran serta sudah tidak ada harapan lagi bagi keduanya akan hidup rukun kembali dalam rumah tangga, dengan demikian sudah tidak ada lagi rasa kasih sayang dalam hati Penggugat sehingga apabila diteruskan untuk bersatu kembali membina rumah tangga akan menimbulkan madlarat bagi kedua belah pihak terutama kepada isteri (Penggugat) seolah-olah memenjarakan isteri dalam genggaman suami (Tergugat) yang sudah tidak dicintainya dan oleh karenanya Majelis berpendapat bahwa perkawinan Penggugat dan Tergugat telah pecah;

Menimbang, bahwa Majelis perlu mengetengahkan dalil fiqh yang terdapat dalam kitab al-Muhadzab juz II halaman 82 yang untuk selanjutnya diambil alih sebagai pendapat majelis sebagai berikut :

اذاشتد عدم رغبت الزوجة لزوجهاطلق عليه القاضي طلقة

Artinya : Apabila sudah sangat memuncak ketidak senangan isteri kepada suaminya maka hakim boleh menjatuhkan talak suami kepada isterinya dengan talak satu;

Menimbang, bahwa berdasarkan pertimbangan tersebut diatas, maka gugatan Penggugat telah memenuhi alasan perceraian sebagaimana diatur dalam pasal 39 ayat (2) Undang-undang Nomor : 1 Tahun 1974, jo. pasal 19 huruf (f) Peraturan Pemerintahan Nomor : 9 Tahun 1975, jo. pasal 116 huruf (f) Kompilasi Hukum Islam dan karenanya gugatan Penggugat dapat dikabulkan dengan menjatuhkan talak satu ba'in sughro Tergugat kepada Penggugat;

Menimbang, bahwa karena perkara a quo masuk bidang perkawinan, maka berdasarkan Pasal 89 ayat (1) Undang-Undang Nomor 7 Tahun 1989 sebagaimana telah diubah dengan Undang-Undang Nomor 3 Tahun 2006 dan perubahan kedua dengan Undang-Undang Nomor 50 Tahun 2009, biaya perkara harus dibebankan kepada Penggugat;

Mengingat, semua pasal dalam peraturan perundang-undangan dan hukum Islam yang berkaitan dengan perkara ini;

MENGADILI

1. Menyatakan Tergugat yang telah dipanggil secara resmi dan patut untuk

Disclaimer
Kepaniteraan Mahkamah Agung Republik Indonesia berusaha untuk selalu mencantumkan informasi paling kini dan akurat sebagai bentuk komitmen Mahkamah Agung untuk pelayanan publik, transparansi dan akuntabilitas pelaksanaan fungsi peradilan. Namun dalam hal-hal tertentu masih dimungkinkan terjadi permasalahan teknis terkait dengan akurasi dan keterkinian informasi yang kami sajikan, hal mana akan terus kami perbaiki dari waktu kewaktu.
Dalam hal Anda menemukan inakurasi informasi yang termuat pada situs ini atau informasi yang seharusnya ada, namun belum tersedia, maka harap segera hubungi Kepaniteraan Mahkamah Agung RI melalui :
Email : kepaniteraan@mahkamahagung.go.id Telp : 021-384 3348 (ext.318)

menghadap di persidangan, tidak hadir;

2. Mengabulkan gugatan Penggugat dengan verstek;
3. Menjatuhkan talak satu bain sughra Tergugat (Muhamad Fahrudianto bin Sugiri) terhadap Penggugat (Rina Nurmalasari, S.Pd binti Nana Mulyana);
4. Membebankan kepada Penggugat untuk membayar biaya perkara ini sejumlah Rp. 356 000,00 (tiga ratus lima puluh enam ribu rupiah).

Demikian diputuskan dalam rapat permusyawaratan Majelis yang dilangsungkan pada hari Senin tanggal 21 Desember 2020 Masehi, bertepatan dengan tanggal 6 Jumadil Awwal 1442 Hijriyah, oleh kami Drs. Erik Sumarna, S.H., M.A. sebagai Ketua Majelis, Drs. Solihudin, S.H dan Drs. H. Syamsul Falah, M.H., masing-masing sebagai Hakim Anggota, putusan tersebut diucapkan dalam sidang terbuka untuk umum pada hari itu juga, oleh Ketua Majelis tersebut dengan didampingi oleh Hakim Anggota dan dibantu oleh H. Asep Suryana, SHI sebagai Panitera Pengganti serta dihadiri oleh Penggugat didampingi kuasanya tanpa kehadiran Tergugat;

Ketua Majelis

Drs. Erik Sumarna, S.H., M.A.

Hakim Anggota	Hakim Anggota
Drs. Solihudin, S.H	Drs. H. Syamsul Falah, M.H.

Panitera Pengganti

H. Asep Suryana, SHI

Perincian Biaya Perkara :

1. Pendaftaran : Rp. 30.000,-
2. Proses : Rp. 50.000,-

Disclaimer
Kepaniteraan Mahkamah Agung Republik Indonesia berusaha untuk selalu mencantumkan informasi paling kini dan akurat sebagai bentuk komitmen Mahkamah Agung untuk pelayanan publik, transparansi dan akuntabilitas pelaksanaan fungsi peradilan. Namun dalam hal-hal tertentu masih dimungkinkan terjadi permasalahan teknis terkait dengan akurasi dan keterkinian informasi yang kami sajikan, hal mana akan terus kami perbaiki dari waktu kewaktu.
Dalam hal Anda menemukan inakurasi informasi yang termuat pada situs ini atau informasi yang seharusnya ada, namun belum tersedia, maka harap segera hubungi Kepaniteraan Mahkamah Agung RI melalui :
Email : kepaniteraan@mahkamahagung.go.id Telp : 021-384 3348 (ext.318)

3.	Panggilan Penggugat	: Rp.	0,-
4.	Panggilan Tergugat	: Rp.	240.000,-
5.	PNBP Panggilan	: Rp.	20.000,-
6.	Redaksi	: Rp.	10.000,-
7.	Materai	: Rp.	6.000,-
	--		
	Jumlah	: Rp.	356.000,-

Disclaimer
Kepaniteraan Mahkamah Agung Republik Indonesia berusaha untuk selalu mencantumkan informasi paling kini dan akurat sebagai bentuk komitmen Mahkamah Agung untuk pelayanan publik, transparansi dan akuntabilitas pelaksanaan fungsi peradilan. Namun dalam hal-hal tertentu masih dimungkinkan terjadi permasalahan teknis terkait dengan akurasi dan keterkinian informasi yang kami sajikan, hal mana akan terus kami perbaiki dari waktu kewaktu.
Dalam hal Anda menemukan inakurasi informasi yang termuat pada situs ini atau informasi yang seharusnya ada, namun belum tersedia, maka harap segera hubungi Kepaniteraan Mahkamah Agung RI melalui :
Email : kepaniteraan@mahkamahagung.go.id   Telp : 021-384 3348 (ext.318)
