# Supplementary material for: Classification of divorce causes during the COVID-19 pandemic using convolutional neural networks
Source: PeerJ Comput Sci. 2022 Jun 30;8:e998. doi: 10.7717/peerj-cs.998 (PMC9299239; doi:10.7717/peerj-cs.998)
Supplement: Supplemental Information 6 [file peerj-cs-08-998-s006.zip › Rujuk Dataset/Data ke-1.pdf]

P E N E T A P A N

Nomor XXXX/Pdt.G/2020/PA.Dmk

بسم الله الرحمن الرحيم

DEMI KEADILAN BERDASARKAN KETUHANAN YANG MAHA ESA

Pengadilan Agama Demak yang memeriksa dan mengadili perkara-perkara tertentu pada tingkat pertama, dalam persidangan Majelis Hakim telah menjatuhkan putusan dalam perkara cerai gugat antara :

Penggugat, umur 47 tahun, agama Islam, Pekerjaan Mengurus Rumah Tangga, Pendidikan Sekolah Lanjutan Tingkat Pertama, tempat kediaman di XXXXKabupaten Demak, sekarang tinggal dirumah orang tuanya di XXXXKabupaten Demak, dalam hal ini memberikan kuasa kepada Mustain, SAg, SH, MH, Advokat yang berkantor di Advokat/Pengacara pada kantor Lembaga Konsultasi dan Bantuan Hukum (LKBH) Sultan Fatah Demak di Jl. Sultan Trenggono Km. 22, Semarang Demak Jawa Tengah Telp: (0291) 682495 berdasarkan surat kuasa khusus tanggal 25 November 2020, sebagai **Penggugat;**

Melawan

Tergugat, umur 52 tahun, agama Islam, Pekerjaan sopir, Pendidikan Sekolah Dasar, tempat kediaman di XXXXKabupaten Demak, sebagai **Tergugat;**

Pengadilan Agama tersebut;
Telah membaca surat-surat perkara;
Telah mendengar keterangan Penggugat;

DUDUK PERKARA

Bahwa Penggugat dalam surat gugatan tertanggal 25 November 2020, terdaftar di Kepaniteraan Demak, Nomor XXXX/Pdt.G/2020/PA.Dmk. Tanggal 30 November 2020, yang secara rinci sebagaimana tertuang dalam gugatan perkara ini.

Bahwa pada hari sidang yang telah ditentukan Penggugat yang diwakilkan oleh Kuasa Hukumnya hadir dalam sidang, sedangkan Tergugat

Disclaimer
Kepaniteraan Mahkamah Agung Republik Indonesia berusaha untuk selalu mencantumkan informasi paling kini dan akurat sebagai bentuk komitmen Mahkamah Agung untuk pelayanan publik, transparansi dan akuntabilitas pelaksanaan fungsi peradilan. Namun dalam hal-hal tertentu masih dimungkinkan terjadi permasalahan teknis terkait dengan akurasi dan keterkinian informasi yang kami sajikan, hal mana akan terus kami perbaiki dari waktu kewaktu.
Dalam hal Anda menemukan inakurasi informasi yang termuat pada situs ini atau informasi yang seharusnya ada, namun belum tersedia, maka harap segera hubungi Kepaniteraan Mahkamah Agung RI melalui :
Email : kepaniteraan@mahkamahagung.go.id Telp : 021-384 3348 (ext.318)

tidak hadir dan tidak pula mengutus wakil atau kuasa hukum meskipun telah dipanggil secara resmi dan patut dengan relaas untuk hadir di persidangan.

Bahwa Majelis Hakim telah memberi nasihat kepada Penggugat agar rukun kembali dengan Tergugat dalam membina rumah tangga.

Bahwa Penggugat telah menyatakan maksud mencabut gugatan Penggugat dan berkesimpulan memohon agar Majelis Hakim mengabulkan permohonan pencabutan Penggugat karena akan mencoba rukun kembali.

Bahwa tentang jalannya pemeriksaan selengkapnya telah dicatat dalam Berita Acara Persidangan perkara ini.

PERTIMBANGAN HUKUM

Menimbang, bahwa maksud dan tujuan gugatan Penggugat adalah sebagaimana telah diuraikan di atas;

Menimbang, bahwa Penggugat dan Tergugat telah dipanggil secara resmi dan patut untuk hadir menghadap di persidangan sebagaimana ketentuan Pasal 145 RBg., jo. Pasal 26 ayat (1) Peraturan Pemerintah No. 9 Tahun 1975 dan atas panggilan tersebut Penggugat yang diwakilkan oleh Kuasa Hukumnya telah hadir datang menghadap sendiri di persidangan, sedangkan Tergugat tidak datang menghadap di muka sidang dan tidak pula menyuruh orang lain sebagai wakil/kuasanya yang sah untuk datang menghadap, dan tidak ternyata tidak datangnya itu disebabkan suatu halangan yang sah.

Menimbang, bahwa sesuai dengan Pasal 82 ayat (1) dan (4) Undang-Undang Nomor 7 Tahun 1989 tentang Peradilan Agama yang telah diubah dengan Undang-Undang Nomor 3 Tahun 2006 dan perubahan kedua dengan Undang-Undang Nomor 50 Tahun 2009, Majelis Hakim telah mendamaikan/memberi nasihat Penggugat agar rukun kembali dengan Tergugat dan karena upaya tersebut berhasil, maka Penggugat menyatakan mencabut perkara a quo.

Menimbang, bahwa Penggugat berkesimpulan memohon agar Pengadilan mengabulkan permohonan pencabutan gugatan Penggugat.

Menimbang, bahwa permohonan pencabutan tersebut diajukan oleh Penggugat sebelum perkara tersebut diputus, sehingga permohonan pencabutan tersebut tidak bertentangan dengan ketentuan Pasal 271 RV (*Reglement op de burgerlijke recht vordering*), oleh karenanya permohonan

Disclaimer
Kepaniteraan Mahkamah Agung Republik Indonesia berusaha untuk selalu mencantumkan informasi paling kini dan akurat sebagai bentuk komitmen Mahkamah Agung untuk pelayanan publik, transparansi dan akuntabilitas pelaksanaan fungsi peradilan. Namun dalam hal-hal tertentu masih dimungkinkan terjadi permasalahan teknis terkait dengan akurasi dan keterkinian informasi yang kami sajikan, hal mana akan terus kami perbaiki dari waktu kewaktu.
Dalam hal Anda menemukan inakurasi informasi yang termuat pada situs ini atau informasi yang seharusnya ada, namun belum tersedia, maka harap segera hubungi Kepaniteraan Mahkamah Agung RI melalui :
Email : kepaniteraan@mahkamahagung.go.id Telp : 021-384 3348 (ext.318)

pencabutan gugatan dapat dibenarkan, sehingga Majelis hakim mengabulkan pencabutan gugatan Penggugat perkara Nomor XXXX/Pdt.G/2020/PA.Dmk dan selanjutnya memerintahkan Panitera Pengadilan Agama Demak untuk mencatat pencabutan perkara tersebut dalam register perkara.

Menimbang, bahwa dengan dikabulkannya gugatan Penggugat tersebut maka pemeriksaan perkara ini dinyatakan selesai.

Menimbang, bahwa bahwa berdasarkan ketentuan Pasal 89 Ayat (1) Undang-Undang Nomor 7 Tahun 1989, yang telah diubah dan ditambah dengan perubahan pertama menjadi Undang-Undang Nomor 3 Tahun 2006 dan perubahan kedua menjadi Undang-Undang Nomor 50 Tahun 2009, maka seluruh biaya perkara dibebankan kepada Penggugat.

Memperhatikan segala peraturan dan kaidah hukum yang berhubungan dengan perkara ini.

MENETAPKAN

1. Mengabulkan permohonan pencabutan perkara Nomor XXXX/Pdt.G/2020/PA.Dmk dari Penggugat;
2. Memerintahkan Panitera Pengadilan Agama Demak untuk mencatat pencabutan perkara tersebut dalam register perkara;
3. Membebankan kepada Penggugat untuk membayar biaya perkara sejumlah Rp526.000,00 (lima ratus dua puluh enam ribu rupiah);

Demikianlah putusan ini dijatuhkan dalam musyawarah Majelis Hakim di Demak pada hari Selasa tanggal 22 Desember 2020 Masehi, bertepatan dengan tanggal 7 Jumadil Awwal 1442 Hijriyah, oleh Kami Drs. Makali sebagai Ketua Majelis Hakim, dan Dra. Nur Immawati serta Toharudin, S.H.I., M.H. masing-masing sebagai Hakim Anggota, Putusan mana diucapkan oleh Ketua Majelis Hakim tersebut pada hari itu juga dalam sidang terbuka untuk umum dengan dihadiri oleh para Hakim Anggota dan Moh. Rofi, S.Ag sebagai Panitera Pengganti dan dihadiri pula oleh Kuasa Hukum Penggugat tanpa hadirnya Tergugat;

Ketua Majelis

Drs. Makali

Disclaimer
Kepaniteraan Mahkamah Agung Republik Indonesia berusaha untuk selalu mencantumkan informasi paling kini dan akurat sebagai bentuk komitmen Mahkamah Agung untuk pelayanan publik, transparansi dan akuntabilitas pelaksanaan fungsi peradilan. Namun dalam hal-hal tertentu masih dimungkinkan terjadi permasalahan teknis terkait dengan akurasi dan keterkinian informasi yang kami sajikan, hal mana akan terus kami perbaiki dari waktu kewaktu.
Dalam hal Anda menemukan inakurasi informasi yang termuat pada situs ini atau informasi yang seharusnya ada, namun belum tersedia, maka harap segera hubungi Kepaniteraan Mahkamah Agung RI melalui :
Email : kepaniteraan@mahkamahagung.go.id Telp : 021-384 3348 (ext.318)

Hakim Anggota
Hakim Anggota

Dra. Nur Immawati

Toharudin, S.H.I., M.H.

Panitera Pengganti

Moh. Rofi, S.Ag

Perincian Biaya :

1. Biaya Pendaftaran : Rp 30.000,00

2. Biaya Proses : Rp 100.000,00

3. Biaya Pemanggilan : Rp 360.000,00

Disclaimer
Kepaniteraan Mahkamah Agung Republik Indonesia berusaha untuk selalu mencantumkan informasi paling kini dan akurat sebagai bentuk komitmen Mahkamah Agung untuk pelayanan publik, transparansi dan akuntabilitas pelaksanaan fungsi peradilan. Namun dalam hal-hal tertentu masih dimungkinkan terjadi permasalahan teknis terkait dengan akurasi dan keterkinian informasi yang kami sajikan, hal mana akan terus kami perbaiki dari waktu kewaktu.
Dalam hal Anda menemukan inakurasi informasi yang termuat pada situs ini atau informasi yang seharusnya ada, namun belum tersedia, maka harap segera hubungi Kepaniteraan Mahkamah Agung RI melalui :
Email : kepaniteraan@mahkamahagung.go.id Telp : 021-384 3348 (ext.318)

4. PNBP Panggilan : Rp 20.000,00

5. Biaya Redaksi : Rp 10.000,00

6. Biaya Meterai : Rp 6.000,00

Jumlah : Rp 526.000,00

Disclaimer
Kepaniteraan Mahkamah Agung Republik Indonesia berusaha untuk selalu mencantumkan informasi paling kini dan akurat sebagai bentuk komitmen Mahkamah Agung untuk pelayanan publik, transparansi dan akuntabilitas pelaksanaan fungsi peradilan. Namun dalam hal-hal tertentu masih dimungkinkan terjadi permasalahan teknis terkait dengan akurasi dan keterkinian informasi yang kami sajikan, hal mana akan terus kami perbaiki dari waktu kewaktu.
Dalam hal Anda menemukan inakurasi informasi yang termuat pada situs ini atau informasi yang seharusnya ada, namun belum tersedia, maka harap segera hubungi Kepaniteraan Mahkamah Agung RI melalui :
Email : kepaniteraan@mahkamahagung.go.id   Telp : 021-384 3348 (ext.318)
