# Supplementary material for: Classification of divorce causes during the COVID-19 pandemic using convolutional neural networks
Source: PeerJ Comput Sci. 2022 Jun 30;8:e998. doi: 10.7717/peerj-cs.998 (PMC9299239; doi:10.7717/peerj-cs.998)
Supplement: Supplemental Information 6 [file peerj-cs-08-998-s006.zip › Rujuk Dataset/Data ke-10.pdf]

S A L I N A N :

P U T U S A N

Nomor 3492/Pdt.G/2020/PA.Sbg.

بِسْمِ اللّٰهِ الرَّحْمٰنِ الرَّحِيْمِ

DEMI KEADILAN BERDASARKAN KETUHANAN YANG MAHA ESA

Pengadilan Agama Subang yang memeriksa dan mengadili perkara-perkara tertentu pada tingkat pertama dalam persidangan Majelis Hakim telah menjatuhkan putusan dalam perkara **Cerai Gugat**, antara :

Penggugat, umur 35 tahun (Subang, 14 Oktober 1984), Agama Islam, pendidikan terakhir SLTA, pekerjaan Wiraswasta, tempat tinggal di Kabupaten Subang, dalam hal ini memberikan kuasa kepada **Siti Aminah Singhs, S.H.** Advokat-Penasihat Hukum pada Law Office Siti Aminah Singhs, S.H., M.H. & Partner yang beralamat kantor di Jalan Sukarahayu Raya No.45 Perumnas Blok II RT.80 RW.32 Kelurahan Karanganyar Kecamatan Subang Kabupaten Subang, berdasarkan Surat Kuasa Khusus tanggal 22 Juni 2020 yang telah didaftar di Kepaniteraan Pengadilan Agama Subang dengan Register Nomor 842/Adv/2020 tertanggal 05 Oktober 2020, sebagai **Penggugat**;

melawan

Tergugat, umur 39 tahun (Subang, 17 September 1981), Agama Islam, pendidikan terakhir SLTA, pekerjaan Wiraswasta, tempat tinggal di Kabupaten Subang, sebagai **Tergugat**;

Pengadilan Agama tersebut;

Setelah membaca berkas perkara;

Setelah mendengar keterangan Penggugat dan Tergugat di persidangan;

Setelah memeriksa alat-alat bukti di persidangan;

Disclaimer
Kepaniteraan Mahkamah Agung Republik Indonesia berusaha untuk selalu mencantumkan informasi paling kini dan akurat sebagai bentuk komitmen Mahkamah Agung untuk pelayanan publik, transparansi dan akuntabilitas pelaksanaan fungsi peradilan. Namun dalam hal-hal tertentu masih dimungkinkan terjadi permasalahan teknis terkait dengan akurasi dan keterkinian informasi yang kami sajikan, hal mana akan terus kami perbaiki dari waktu kewaktu.
Dalam hal Anda menemukan inakurasi informasi yang termuat pada situs ini atau informasi yang seharusnya ada, namun belum tersedia, maka harap segera hubungi Kepaniteraan Mahkamah Agung RI melalui :
Email : kepaniteraan@mahkamahagung.go.id   Telp : 021-384 3348 (ext.318)

DUDUK PERKARA

Bahwa Penggugat dengan surat gugatannya tanggal 05 Oktober 2020 yang telah didaftarkan di Kepaniteraan Pengadilan Agama Subang di bawah Nomor 3492/Pdt.G/2020/PA.Sbg. tanggal 05 Oktober 2020, bermaksud mengajukan gugatan cerai terhadap Tergugat berdasarkan dalil-dalil yang pada pokoknya sebagai berikut :

1. Bahwa pada hari Selasa, tanggal 22 April 2003, Penggugat telah melangsungkan perkawinan dengan Tergugat dihadapan pejabat Kantor Urusan Agama Kecamatan Cijambe - Jawa barat, sebagaimana tercatat di Register Akta Nikah KUA Nomor: 2003;
2. Bahwa setelah akad nikah Penggugat dan Tergugat hidup bersama sebagai suami-istri di rumah orang tua Pemggugat selama 1 tahun, kemudian pindah ke rumah orang Tergugat selama 1 tahun, kemudian pindah tinggal di rumah kontrakan di wantilan selama 1 tahun, setelah itu tinggak di rumah bersama sejak 2012 sampai dengan sekarang;
3. Bahwa Pernikahan Penggugat dan Tergugat telah berjalan kurang lebih 17 (tujuh belas) tahun dan telah campur bersama sebagaimana layaknya suami-istri dan dikaruniai 2 (dua) orang anak yang bernama Mersyadita Elsyiqha Isyaura Mustofa berusia kurang lebih 10 tahun dan Zio Giosya Franhasqha Mustofa berusia kurang lebih 7 tahun;
4. Bahwa Penggugat dan Tergugat semula kehidupan rumah tangganya rukun dan harmonis, sebagaimana layaknya kehidupan berumah tangga, namun sejak tahun 2009 sudah tidak harmonis lagi karena sering terjadi perselisihan dan pertengkaran, disebabkan diantaranya;
   - Bahwa Tergugat pergi meninggalkan Penggugat dengan perempuan lain selama 1 tahun dan kembali lagi kepada Penggugat, kelakuan itu selalu berulang-ulang;
   - Bahwa Tergugat mempunyai kebiasaan buruk yang sering sekali berselinguh dan Penggugat mengetahuinya akan tetapi Tergugat merasa tidak bersalah;
   - Bahwa Tergugat kurang bertanggung jawab dalam memenuhi kebutuhan keluarga/rumah tangga;

Disclaimer
Kepaniteraan Mahkamah Agung Republik Indonesia berusaha untuk selalu mencantumkan informasi paling kini dan akurat sebagai bentuk komitmen Mahkamah Agung untuk pelayanan publik, transparansi dan akuntabilitas pelaksanaan fungsi peradilan. Namun dalam hal-hal tertentu masih dimungkinkan terjadi permasalahan teknis terkait dengan akurasi dan keterkinian informasi yang kami sajikan, hal mana akan terus kami perbaiki dari waktu kewaktu.
Dalam hal Anda menemukan inakurasi informasi yang termuat pada situs ini atau informasi yang seharusnya ada, namun belum tersedia, maka harap segera hubungi Kepaniteraan Mahkamah Agung RI melalui :
Email : kepaniteraan@mahkamahagung.go.id Telp : 021-384 3348 (ext.318)

5. Bahwa puncak perselisihan dan pertengkaran antara Penggugat dan Tergugat terjadi pada bulan Juni tahun 2020 yang mana Penggugat mengetahui Tergugat selingkuh dengan mantan pacarnya perempuan itu bernama Ruyana orang blanakan sehingga menyebabkan Penggugat keluar dari rumah bersama sampai dengan sekarang;
6. Bahwa selama berpisah tempat tinggal Penggugat masih tinggal di rumah keluarga Penggugat, sedangkan Tergugat tinggal di rumah bersama;
7. Bahwa dengan kejadian tersebut Penggugat melalui keluarga pernah mencoba untuk membicarakan baik-baik terhadap Tergugat, namun penggugat dan tergugat tidak mendapat solusi yang baik untuk keluar dari konflik-konflik permasalahan yang memicu pertengkaran dan perselisihan diantara penggugat dan tergugat;
8. Bahwa dengan kejadian tersebut di atas Penggugat merasa kehidupan rumah tangga dengan Tergugat nampaknya sudah tidak bisa dibina dan dipertahankan lagi, sehingga harapan membina rumah tangga yang bahagia dan sejahtera lahir bathin yang dicita-citakan tidak dapat terwujud, oleh karenanya penceraian merupakan jalan yang lebih maslahat;

Berdasarkan hal-hal tersebut diatas, Penggugat mohon kiranya Bapak Majelis Hakim Pengadilan Agama Subang untuk memberikan putusan yang amar nya sebagai berikut :

PRIMER

1. Mengabulkan gugatan cerai Penggugat untuk seluruhnya ;
2. Menjatuhkan talak satu ba'in Sughra dari Tergugat (Tergugat) terhadap Penggugat (Penggugat);
3. Menetapkan biaya perkara sesuai dengan ketentuan yang berlaku ;

SUBSIDER

Apabila Majelis Hakim berpendapat lain, mohon putusan yang seadil-adilnya (ex aequo- et bono)

Bahwa pada hari dan tanggal yang telah ditentukan Penggugat datang menghadap didampingi Kuasanya dan Tergugat telah datang menghadap di persidangan. Kemudian Majelis Hakim telah berupaya mendamaikan Penggugat dan Tergugat, namun upaya tersebut tidak berhasil;

Disclaimer
Kepaniteraan Mahkamah Agung Republik Indonesia berusaha untuk selalu mencantumkan informasi paling kini dan akurat sebagai bentuk komitmen Mahkamah Agung untuk pelayanan publik, transparansi dan akuntabilitas pelaksanaan fungsi peradilan. Namun dalam hal-hal tertentu masih dimungkinkan terjadi permasalahan teknis terkait dengan akurasi dan keterkinian informasi yang kami sajikan, hal mana akan terus kami perbaiki dari waktu kewaktu.
Dalam hal Anda menemukan inakurasi informasi yang termuat pada situs ini atau informasi yang seharusnya ada, namun belum tersedia, maka harap segera hubungi Kepaniteraan Mahkamah Agung RI melalui :
Email : kepaniteraan@mahkamahagung.go.id Telp : 021-384 3348 (ext.318)

Bahwa Penggugat dan Tergugat telah menempuh proses mediasi dengan Mediator Hakim Drs. Suhardi, S.H., sebagaimana diamanatkan dalam PERMA Nomor 1 Tahun 2016, namun hasil laporan Mediator tertanggal 17 November 2020 pada pokoknya mediasi telah gagal mencapai kesepakatan / tidak berhasil;

Bahwa selanjutnya dibacakan surat gugatan Penggugat tertanggal 05 Oktober 2020, yang isinya tetap dipertahankan Penggugat;

Bahwa terhadap gugatan Penggugat tersebut, Tergugat tidak memberikan jawaban karena Tergugat tidak pernah datang menghadap lagi pada persidangan berikutnya, meskipun telah diberikan kesempatan untuk memberikan jawaban dan telah diperintahkan secara lisan di persidangan dan telah pula dipanggil secara resmi dan patut pada tanggal 9 Desember 2020 dan 17 Desember 2020 dengan relaas Nomor 3492/Pdt.G/2020/PA.Sbg., supaya datang menghadap di persidangan serta ketidak hadirannya tanpa berita dan alasan yang sah dan tidak pula mengirimkan orang lain sebagai wakil atau kuasanya yang sah menurut hukum;

Bahwa oleh karena Tergugat tidak hadir lagi di persidangan, pemeriksaan dilanjutkan kepada pembuktian;

Bahwa untuk memperkuat dalil-dalil gugatannya, Penggugat telah mengajukan alat bukti (P) berupa fotokopi Kutipan Akta Nikah Nomor 180/12/III/2003 yang dikeluarkan oleh Kantor Urusan Agama Kecamatan Cijambe Kabupaten Subang tanggal 22 April 2003, fotokopi mana telah dinachtzegelen/bermeterai cukup;

Bahwa di samping bukti surat sebagaimana di atas, Penggugat juga mengajukan 2 (dua) orang saksi keluarga atau orang yang dekat dengan Penggugat, yang masing-masing bernama :

1. **Saksi 1**, umur 40 tahun, agama Islam, pekerjaan Ibu rumah tangga, tempat tinggal di Kabupaten Subang, yang di bawah sumpah memberikan keterangan yang pada pokoknya sebagai berikut :
   - Bahwa saksi kenal kepada Penggugat yang bernama dan Tergugat yang bernama;

Disclaimer
Kepaniteraan Mahkamah Agung Republik Indonesia berusaha untuk selalu mencantumkan informasi paling kini dan akurat sebagai bentuk komitmen Mahkamah Agung untuk pelayanan publik, transparansi dan akuntabilitas pelaksanaan fungsi peradilan. Namun dalam hal-hal tertentu masih dimungkinkan terjadi permasalahan teknis terkait dengan akurasi dan keterkinian informasi yang kami sajikan, hal mana akan terus kami perbaiki dari waktu kewaktu.
Dalam hal Anda menemukan inakurasi informasi yang termuat pada situs ini atau informasi yang seharusnya ada, namun belum tersedia, maka harap segera hubungi Kepaniteraan Mahkamah Agung RI melalui :
Email : kepaniteraan@mahkamahagung.go.id Telp : 021-384 3348 (ext.318)

- Bahwa saksi adalah tetangga Penggugat;
- Bahwa Penggugat dan Tergugat adalah suami istri yang menikah pada tahun 2003 di hadapan Pegawai Pencatat Nikah Kantor Urusan Agama Kecamatan Cijambe Kabupaten Subang;
- Bahwa setelah menikah Penggugat dan Tergugat terakhir tinggal bersama di rumah kediaman bersama dan telah dikaruniai keturunan 2 (dua) orang anak yang masing-masing bernama Mersyadita Elsyiqha Isyaura Mustofa dan Zio Giosya Franhasqha Mustofa;
- Bahwa saksi mengetahui awalnya rumah tangga Penggugat dan Tergugat rukun dan harmonis, namun sejak tahun 2009 mulai tidak rukun karena sering terjadi perselisihan dan pertengkaran;
- Bahwa penyebabnya karena Tergugat diketahui telah berselingkuh dengan perempuan lain yang berasal dari Ciasem, Subang, selain itu nafkah yang diberikan Tergugat pun tidak menentu dan tidak bisa memenuhi kebutuhan keluarga;
- Bahwa saksi mengetahui perselisihan dan pertengkaran tersebut karena saksi pernah melihat pertengkaran yang terjadi antara Penggugat dan Tergugat;
- Bahwa Penggugat dan Tergugat sampai sekarang sudah berpisah selama 4 (empat) bulan, dimana Penggugat pergi meninggalkan Tergugat dan tidak pernah hidup berumah tangga lagi sebagai suami istri;
- Bahwa selama berpisah tempat tinggal, Penggugat tinggal di rumah orangtua Penggugat, sedangkan Tergugat tinggal di rumah kediaman bersama;
- Bahwa selama berpisah tempat tinggal Penggugat dan Tergugat sudah berusaha untuk memperbaiki rumah tangganya, namun upaya tersebut tidak berhasil;
- Bahwa saksi bersama keluarga kedua belah pihak sudah berusaha merukunkan, akan tetapi usaha tersebut tidak berhasil karena Penggugat tetap bersikeras ingin bercerai dengan Tergugat;

Disclaimer
Kepaniteraan Mahkamah Agung Republik Indonesia berusaha untuk selalu mencantumkan informasi paling kini dan akurat sebagai bentuk komitmen Mahkamah Agung untuk pelayanan publik, transparansi dan akuntabilitas pelaksanaan fungsi peradilan. Namun dalam hal-hal tertentu masih dimungkinkan terjadi permasalahan teknis terkait dengan akurasi dan keterkinian informasi yang kami sajikan, hal mana akan terus kami perbaiki dari waktu kewaktu.
Dalam hal Anda menemukan inakurasi informasi yang termuat pada situs ini atau informasi yang seharusnya ada, namun belum tersedia, maka harap segera hubungi Kepaniteraan Mahkamah Agung RI melalui :
Email : kepaniteraan@mahkamahagung.go.id   Telp : 021-384 3348 (ext.318)

- Bahwa saksi tidak bersedia untuk mendamaikan kembali kedua belah pihak, karena keduanya sulit didamaikan;

2. **Saksi 2**, umur 39 tahun, agama Islam, pekerjaan Karyawan Swasta, tempat tinggal di Kabupaten Subang, yang di bawah sumpah memberikan keterangan yang pada pokoknya sebagai berikut :
   - Bahwa saksi kenal kepada Penggugat dan Tergugat karena saksi adalah sopir Penggugat;
   - Bahwa Penggugat dan Tergugat adalah suami istri yang sudah lama berumah tangga dan telah dikaruniai keturunan 2 (dua) orang anak;
   - Bahwa setelah menikah Penggugat dan Tergugat terakhir tinggal bersama di rumah kediaman bersama;
   - Bahwa Penggugat dan Tergugat sudah berpisah tempat tinggal sejak bulan Juni 2020, dimana Penggugat pergi meninggalkan Tergugat dan tidak pernah hidup berumah tangga lagi sebagai suami istri sampai sekarang sudah berpisah sekitar 4 (empat) bulan;
   - Bahwa selama berpisah tempat tinggal, Penggugat tinggal di rumah orangtua Penggugat, sedangkan Tergugat tinggal di rumah kediaman bersama;
   - Bahwa sebelum berpisah, antara Penggugat dan Tergugat sering terjadi perselisihan dan pertengkaran yang disebabkan karena Tergugat diketahui telah berselingkuh bahkan menikah lagi dengan perempuan lain, saat ini pun Tergugat sudah hidup serumah dengan perempuan selingkuhannya tersebut;
   - Bahwa saksi mengetahui perselisihan dan pertengkaran tersebut karena saksi pernah melihat pertengkaran yang terjadi antara Penggugat dan Tergugat;
   - Bahwa selama berpisah tempat tinggal Penggugat dan Tergugat sudah berusaha untuk rukun, akan tetapi upaya tersebut tidak berhasil;
   - Bahwa selama berpisah tempat tinggal, saksi belum pernah mendamaikan. Hanya menasehati Penggugat saja, tapi tidak berhasil karena Penggugat sudah tidak mau kembali kepada Tergugat;

Disclaimer
Kepaniteraan Mahkamah Agung Republik Indonesia berusaha untuk selalu mencantumkan informasi paling kini dan akurat sebagai bentuk komitmen Mahkamah Agung untuk pelayanan publik, transparansi dan akuntabilitas pelaksanaan fungsi peradilan. Namun dalam hal-hal tertentu masih dimungkinkan terjadi permasalahan teknis terkait dengan akurasi dan keterkinian informasi yang kami sajikan, hal mana akan terus kami perbaiki dari waktu kewaktu.
Dalam hal Anda menemukan inakurasi informasi yang termuat pada situs ini atau informasi yang seharusnya ada, namun belum tersedia, maka harap segera hubungi Kepaniteraan Mahkamah Agung RI melalui :
Email : kepaniteraan@mahkamahagung.go.id Telp : 021-384 3348 (ext.318)

– Bahwa saksi tidak bersedia mendamaikan kedua belah pihak karena sulit untuk didamaikan kembali;

Bahwa kemudian Penggugat melalui Kuasanya mengajukan kesimpulan, yang pada pokoknya mengatakan bahwa berdasarkan dalil-dalil gugatan Penggugat dan bukti-bukti yang diajukan, gugatan Penggugat telah terbukti dan berdasar hukum, oleh karenanya patut dikabulkan dan selanjutnya mohon putusan;

Bahwa selanjutnya untuk mempersingkat uraian putusan ini, ditunjuklah hal-hal sebagaimana tercatat dalam berita acara sidang perkara ini sebagai bagian yang tidak terpisahkan dari putusan ini;

PERTIMBANGAN HUKUM

Menimbang, bahwa maksud dan tujuan gugatan Penggugat adalah sebagaimana diuraikan di atas;

Menimbang, bahwa Majelis Hakim telah berupaya mendamaikan Penggugat dan Tergugat dengan, namun upaya tersebut tidak berhasil;

Menimbang, bahwa untuk memenuhi proses mediasi sebagaimana diamanatkan dalam PERMA Nomor 1 Tahun 2016, telah ditunjuk dan ditetapkan Drs. Suhardi, S.H., sebagai Mediator dalam perkara tersebut di atas untuk melakukan mediasi antara Penggugat dengan Tergugat, namun hasil laporan Mediator tertanggal 17 November 2020, ternyata proses mediasi telah gagal mencapai kesepakatan / tidak berhasil;

Menimbang, bahwa alasan Penggugat untuk mengajukan gugatan cerai terhadap Tergugat karena sejak tahun 2009 rumah tangga Penggugat dengan Tergugat sering terjadi perselisihan dan pertengkaran yang disebabkan karena Tergugat pergi meninggalkan Penggugat dengan perempuan lain selama 1 tahun dan kembali lagi kepada Penggugat, kelakuan itu selalu berulang-ulang, Tergugat mempunyai kebiasaan buruk yang sering sekali berselinguh dan Penggugat mengetahuinya akan tetapi Tergugat merasa tidak bersalah selain itu Tergugat kurang bertanggung jawab dalam memenuhi kebutuhan

Disclaimer
Kepaniteraan Mahkamah Agung Republik Indonesia berusaha untuk selalu mencantumkan informasi paling kini dan akurat sebagai bentuk komitmen Mahkamah Agung untuk pelayanan publik, transparansi dan akuntabilitas pelaksanaan fungsi peradilan. Namun dalam hal-hal tertentu masih dimungkinkan terjadi permasalahan teknis terkait dengan akurasi dan keterkinian informasi yang kami sajikan, hal mana akan terus kami perbaiki dari waktu kewaktu.
Dalam hal Anda menemukan inakurasi informasi yang termuat pada situs ini atau informasi yang seharusnya ada, namun belum tersedia, maka harap segera hubungi Kepaniteraan Mahkamah Agung RI melalui :
Email : kepaniteraan@mahkamahagung.go.id   Telp : 021-384 3348 (ext.318)

keluarga/rumah tangga, yang mengakibatkan sejak bulan Juni 2020 Penggugat pergi meninggalkan Tergugat dan tidak pernah hidup berumah tangga lagi sebagai suami istri dan tidak pernah berumah tangga lagi sebagaimana layaknya suami istri dan telah pisah rumah selama 4 (empat) bulan;

Menimbang, bahwa terhadap dalil gugatan Penggugat tersebut, Tergugat tidak mengajukan jawabannya sehingga Tergugat dianggap mengakui semua dalil gugatan Penggugat;

Menimbang, bahwa meskipun Tergugat dianggap telah mengakui semua dalil gugatan Penggugat, tetapi sehubungan perkara ini (*in casu*) adalah perkara perceraian yang merupakan perkara khusus (*lex specialis*) dan gugatan Penggugat didasarkan atas adanya perselisihan dan pertengkaran, maka untuk memenuhi ketentuan Pasal 22 ayat (2) Peraturan Pemerintah Nomor 9 Tahun 1975 tentang Pelaksanaan Undang-undang Nomor 1 Tahun 1974 tentang Perkawinan Jo. Pasal 134 Kompilasi Hukum Islam, perlu mendengar keterangan saksi-saksi dari pihak keluarga maupun orang yang dekat dengan kedua belah pihak, serta perlu juga melakukan pemeriksaan atas alat bukti lain yang diajukan dalam sidang;

Menimbang, bahwa untuk membuktikan dalil gugatannya, Penggugat telah mengajukan alat bukti P dan 2 (dua) orang saksi;

Menimbang, bahwa bukti P (Fotokopi Buku Kutipan Akta Nikah) yang merupakan akta otentik yang telah bermeterai cukup dan cocok dengan aslinya, isi bukti tersebut menjelaskan mengenai data perkawinan Penggugat dan Tergugat, sehingga bukti tersebut telah memenuhi syarat formal dan materiil, sehingga bukti tersebut mempunyai kekuatan yang sempurna dan mengikat, oleh karenanya Penggugat dan Tergugat adalah pihak yang berkepentingan dalam perkara ini (*persona standi in judicio*) sesuai dengan ketentuan Pasal 2 ayat 2 Undang-undang Nomor 1 Tahun 1974 jo. Pasal 7 ayat 1 Kompilasi Hukum Islam di Indonesia Tahun 1991;

Menimbang, bahwa saksi 1 dan saksi 2 Penggugat, sudah dewasa, berakal sehat, dan sebelum memberikan keterangannya telah disumpah terlebih dahulu, sehingga memenuhi syarat formil sebagaimana diatur dalam Pasal 145 ayat (1) HIR dan merupakan keluarga dan orang dekat Penggugat,

Disclaimer
Kepaniteraan Mahkamah Agung Republik Indonesia berusaha untuk selalu mencantumkan informasi paling kini dan akurat sebagai bentuk komitmen Mahkamah Agung untuk pelayanan publik, transparansi dan akuntabilitas pelaksanaan fungsi peradilan. Namun dalam hal-hal tertentu masih dimungkinkan terjadi permasalahan teknis terkait dengan akurasi dan keterkinian informasi yang kami sajikan, hal mana akan terus kami perbaiki dari waktu kewaktu.
Dalam hal Anda menemukan inakurasi informasi yang termuat pada situs ini atau informasi yang seharusnya ada, namun belum tersedia, maka harap segera hubungi Kepaniteraan Mahkamah Agung RI melalui :
Email : kepaniteraan@mahkamahagung.go.id   Telp : 021-384 3348 (ext.318)

sesuai ketentuan Pasal 22 ayat (2) Peraturan Pemerintah Nomor 9 Tahun 1975 jo. pasal 134 Kompilasi Hukum Islam;

Menimbang, bahwa keterangan 2 (dua) orang saksi Penggugat adalah fakta yang dilihat sendiri, didengar sendiri, dialami sendiri dan relevan dengan dalil yang harus dibuktikan oleh Penggugat, oleh karena itu keterangan saksi tersebut telah memenuhi syarat materiil sebagaimana telah diatur dalam Pasal 172 HIR. sehingga keterangan saksi tersebut memiliki kekuatan pembuktian dan dapat diterima sebagai alat bukti;

Menimbang, bahwa keterangan saksi 1 dan saksi 2 Penggugat bersesuaian dan cocok antara satu dengan yang lain oleh karena itu keterangan dua orang saksi tersebut memenuhi Pasal 172 dan Pasal 173 HIR.;

Menimbang, bahwa berdasarkan bukti P dan saksi-saksi terbukti fakta kejadian sebagai berikut :

1. Bahwa Penggugat dengan Tergugat telah terikat dalam pernikahan yang sah sejak tanggal 22 April 2003 dan telah dikaruniai keturunan 2 (dua) orang anak yang masing-masing bernama Mersyadita Elsyiqha Isyaura Mustofa dan Zio Giosya Franhasqha Mustofa;
2. Bahwa sejak tahun 2009 antara Penggugat dengan Tergugat telah terjadi perselisihan dan pertengkaran terus menerus dan sulit untuk rukun kembali, yang disebabkan karena Tergugat diketahui telah berselingkuh bahkan menikah lagi dengan perempuan lain, saat ini pun Tergugat sudah hidup serumah dengan perempuan selingkuhannya tersebut, selain itu nafkah yang diberikan Tergugat pun tidak menentu dan tidak bisa memenuhi kebutuhan keluarga;
3. Bahwa sejak bulan Juni 2020 Penggugat dan Tergugat sudah berpisah tempat tinggal sampai dengan sekarang selama 4 (empat) bulan;
4. Bahwa selama berpisah tempat tinggal Penggugat dan Tergugat sudah berusaha rukun akan tetapi upaya tersebut tidak berhasil;
5. Bahwa para saksi keluarga atau orang yang dekat dengan Penggugat telah menasehati Penggugat agar rukun kembali dengan Tergugat tetapi tidak berhasil dan para saksi tidak bersedia untuk merukunkan kembali karena keduanya sulit untuk didamaikan;

Disclaimer
Kepaniteraan Mahkamah Agung Republik Indonesia berusaha untuk selalu mencantumkan informasi paling kini dan akurat sebagai bentuk komitmen Mahkamah Agung untuk pelayanan publik, transparansi dan akuntabilitas pelaksanaan fungsi peradilan. Namun dalam hal-hal tertentu masih dimungkinkan terjadi permasalahan teknis terkait dengan akurasi dan keterkinian informasi yang kami sajikan, hal mana akan terus kami perbaiki dari waktu kewaktu.
Dalam hal Anda menemukan inakurasi informasi yang termuat pada situs ini atau informasi yang seharusnya ada, namun belum tersedia, maka harap segera hubungi Kepaniteraan Mahkamah Agung RI melalui :
Email : kepaniteraan@mahkamahagung.go.id Telp : 021-384 3348 (ext.318)

Menimbang, bahwa rumah tangga Penggugat dan Tergugat benar-benar telah pecah (*broken marriage*) serta telah terjadi perselisihan dan pertengkaran secara terus menerus yang tidak ada harapan akan hidup rukun lagi dalam rumah tangga, sehingga tujuan perkawinan untuk membentuk rumah tangga yang bahagia dan kekal sebagaimana dimaksud pasal 1 Undang Undang Nomor 1 Tahun 1974 jo. pasal 3 Kompilasi Hukum Islam (KHI) tidak dapat diwujudkan dalam rumah tangga Penggugat dan Tergugat

Menimbang, bahwa dengan telah terbuktinya dalil Penggugat, bahwa antara Penggugat dan Tergugat telah terjadi pisah tempat tinggal selama 4 (empat) bulan, di mana menurut yurisprudensi MARI No. 379 K/AG/1995 tanggal 26 Maret 1997, suami istri yang tidak berdiam serumah lagi dan tidak ada harapan untuk hidup rukun kembali, maka rumah tangga tersebut terbukti telah retak dan pecah;

Menimbang, bahwa keadaan hubungan antara Penggugat dan Tergugat yang demikian, menurut Majelis Hakim dapat dikualifikasi sebagai telah terjadi perselisihan dan pertengkaran terus menerus dan tidak ada harapan akan hidup rukun lagi dalam rumah tangga sebagaimana yang dimaksud oleh Pasal 19 huruf f Peraturan Pemerintah Nomor 9 Tahun 1975 jo Pasal 116 huruf f Kompilasi Hukum Islam (Instruksi Presiden Nomor 1 Tahun 1991);

Menimbang, bahwa berdasarkan fakta-fakta di atas Majelis Hakim berkesimpulan bahwa antara Penggugat dan Tergugat telah terjadi perselisihan dan pertengkaran terus menerus dan tidak ada harapan akan hidup rukun lagi dalam rumah tangga, sehingga harapan untuk mewujudkan tujuan rumah tangga yang bahagia, sakinah, mawaddah warahmah, sebagaimana yang dikehendaki oleh Pasal 1 Undang-undang Nomor 1 Tahun 1974 jo Pasal 3 Kompilasi Hukum Islam sulit terwujud. Rumah tangga Penggugat dan Tergugat yang demikian berarti telah pecah dan tidak layak untuk dipertahankan;

Menimbang, bahwa dengan tidak hadirnya Tergugat di persidangan memberi petunjuk bahwa Tergugat tidak menghendaki rukun lagi dengan Penggugat dalam rumah tangga;

Menimbang, bahwa berdasarkan pertimbangan-pertimbangan di atas Majelis Hakim berpendapat bahwa gugatan Penggugat telah memenuhi

Disclaimer
Kepaniteraan Mahkamah Agung Republik Indonesia berusaha untuk selalu mencantumkan informasi paling kini dan akurat sebagai bentuk komitmen Mahkamah Agung untuk pelayanan publik, transparansi dan akuntabilitas pelaksanaan fungsi peradilan. Namun dalam hal-hal tertentu masih dimungkinkan terjadi permasalahan teknis terkait dengan akurasi dan keterkinian informasi yang kami sajikan, hal mana akan terus kami perbaiki dari waktu kewaktu.
Dalam hal Anda menemukan inakurasi informasi yang termuat pada situs ini atau informasi yang seharusnya ada, namun belum tersedia, maka harap segera hubungi Kepaniteraan Mahkamah Agung RI melalui :
Email : kepaniteraan@mahkamahagung.go.id   Telp : 021-384 3348 (ext.318)

ketentuan Pasal 39 ayat (1) dan ayat (2) Undang-undang No. 1 Tahun 1974, bahwa perceraian hanya dapat dilakukan di depan sidang Pengadilan setelah Pengadilan yang bersangkutan berusaha dan tidak berhasil mendamaikan kedua belah pihak. Untuk melakukan perceraian harus ada cukup alasan bahwa antara suami-istri itu tidak akan dapat hidup rukun sebagai suami-istri. Demikian juga gugatan Penggugat telah memenuhi ketentuan Pasal 19 huruf (f) Peraturan Pemerintah Nomor 9 Tahun 1975 jo Pasal 116 huruf (f) Kompilasi Hukum Islam, yaitu antara suami istri telah terjadi perselisihan dan pertengkaran terus menerus dan tidak ada harapan akan hidup rukun lagi dalam rumah tangga. Oleh karena itu, gugatan Penggugat dikabulkan;

Menimbang, bahwa menurut doktrin hukum Islam, sebagaimana ditulis dalam kitab Fiqhussunnah Jilid VIII halaman 121-122, yang artinya : *"Bahwa apabila gugatan istri telah terbukti berdasarkan alat bukti yang diajukan oleh istri atau berdasarkan pengakuan suami, sedang adanya gangguan tersebut merupakan penghalang atas kelangsungan pergaulan hidup (suami istri) sebagaimana (pergaulan hidup suami istri lain) yang sepadan dengan mereka, sementara hakim tidak berhasil mendamaikan mereka, maka hakim harus menjatuhkan gugat suaminya terhadap diri penggugat dengan gugat bain".* Sedang menurut ketentuan Pasal 119 ayat (2) Kompilasi Hukum Islam, salah satu talak bain sughra adalah talak yang dijatuhkan oleh Pengadilan Agama;

Menimbang, bahwa dalam perkara ini ternyata Penggugat dapat membuktikan kebenaran dalil gugatannya, dan hakim telah gagal mendamaikan keduanya, maka Majelis Hakim akan menjatuhkan talak satu bain sughra Tergugat atas Penggugat;

Menimbang, bahwa berdasarkan Pasal 89 ayat (1) Undang-undang Nomor 7 Tahun 1989 sebagaimana telah diubah dengan Undang-undang No. 3 tahun 2006 dan diubah untuk kedua kalinya dengan Undang-undang No. 50 tahun 2009, biaya perkara ini dibebankan kepada Penggugat;

Mengingat, segala ketentuan perundang-undangan yang berlaku dan hukum Syar'i yang berkaitan dengan perkara ini :

M E N G A D I L I

1. Mengabulkan gugatan Penggugat;

Disclaimer
Kepaniteraan Mahkamah Agung Republik Indonesia berusaha untuk selalu mencantumkan informasi paling kini dan akurat sebagai bentuk komitmen Mahkamah Agung untuk pelayanan publik, transparansi dan akuntabilitas pelaksanaan fungsi peradilan. Namun dalam hal-hal tertentu masih dimungkinkan terjadi permasalahan teknis terkait dengan akurasi dan keterkinian informasi yang kami sajikan, hal mana akan terus kami perbaiki dari waktu kewaktu.
Dalam hal Anda menemukan inakurasi informasi yang termuat pada situs ini atau informasi yang seharusnya ada, namun belum tersedia, maka harap segera hubungi Kepaniteraan Mahkamah Agung RI melalui :
Email : kepaniteraan@mahkamahagung.go.id   Telp : 021-384 3348 (ext.318)

2. Menjatuhkan talak satu ba'in sughra Tergugat (Tergugat) terhadap Penggugat (Penggugat);
3. Membebankan biaya perkara ini kepada Penggugat yang hingga kini dihitung sebesar Rp416.000,00 (empat ratus enam belas ribu rupiah);

Demikian dijatuhkan putusan ini dalam musyawarah Majelis Hakim yang dilangsungkan pada hari **Selasa**, tanggal **22 Desember 2020 Masehi** bertepatan dengan tanggal **7 Jumadil Awwal 1442 Hijriyah** oleh **Drs. Ojon Rianklana** sebagai Ketua Majelis serta **Dra. Hj. Sunaeah, M.H.** dan **Dra. Hj. Dadah Holidah, M.H.** masing-masing sebagai Hakim Anggota, dibantu oleh **Priyo Wicaksono, S.Kom., S.Sy.**, sebagai Panitera Pengganti. Putusan tersebut pada hari itu juga diucapkan Hakim Ketua dalam sidang terbuka untuk umum dengan dihadiri Penggugat beserta Kuasanya diluar hadirnya Tergugat.

Hakim Anggota,

ttd.

Dra. Hj. Sunaeah, M.H.

Ketua Majelis,

ttd.

Drs. Ojon Rianklana

Hakim Anggota,

ttd.

Dra. Hj. Dadah Holidah, M.H.

Panitera Pengganti,

ttd.

Priyo Wicaksono, S.Kom., S.Sy.

Perincian Biaya :

Disclaimer
Kepaniteraan Mahkamah Agung Republik Indonesia berusaha untuk selalu mencantumkan informasi paling kini dan akurat sebagai bentuk komitmen Mahkamah Agung untuk pelayanan publik, transparansi dan akuntabilitas pelaksanaan fungsi peradilan. Namun dalam hal-hal tertentu masih dimungkinkan terjadi permasalahan teknis terkait dengan akurasi dan keterkinian informasi yang kami sajikan, hal mana akan terus kami perbaiki dari waktu kewaktu.
Dalam hal Anda menemukan inakurasi informasi yang termuat pada situs ini atau informasi yang seharusnya ada, namun belum tersedia, maka harap segera hubungi Kepaniteraan Mahkamah Agung RI melalui :
Email : kepaniteraan@mahkamahagung.go.id Telp : 021-384 3348 (ext.318)

1. Pendaftaran	:	Rp30.000,00
2. Proses	:	Rp50.000,00
3. Panggilan	:	Rp300.000,00
4. PNBP Panggilan	:	Rp20.000,00
5. Redaksi	:	Rp10.000,00
6. Meterai	:	Rp6.000,00
**Jumlah**	**:**	**Rp416.000,00**
		(empat ratus enam belas ribu rupiah)

Untuk **Salinan** yang sama bunyinya oleh :
PANITERA PENGADILAN AGAMA SUBANG

Drs. H. Dadang Zaenal, M.M.

Disclaimer
Kepaniteraan Mahkamah Agung Republik Indonesia berusaha untuk selalu mencantumkan informasi paling kini dan akurat sebagai bentuk komitmen Mahkamah Agung untuk pelayanan publik, transparansi dan akuntabilitas pelaksanaan fungsi peradilan. Namun dalam hal-hal tertentu masih dimungkinkan terjadi permasalahan teknis terkait dengan akurasi dan keterkinian informasi yang kami sajikan, hal mana akan terus kami perbaiki dari waktu kewaktu.
Dalam hal Anda menemukan inakurasi informasi yang termuat pada situs ini atau informasi yang seharusnya ada, namun belum tersedia, maka harap segera hubungi Kepaniteraan Mahkamah Agung RI melalui :
Email : kepaniteraan@mahkamahagung.go.id Telp : 021-384 3348 (ext.318)
