# Supplementary material for: Classification of divorce causes during the COVID-19 pandemic using convolutional neural networks
Source: PeerJ Comput Sci. 2022 Jun 30;8:e998. doi: 10.7717/peerj-cs.998 (PMC9299239; doi:10.7717/peerj-cs.998)
Supplement: Supplemental Information 6 [file peerj-cs-08-998-s006.zip › Rujuk Dataset/Data ke-11.pdf]

PUTUSAN

Nomor 2310/Pdt.G/2020/PA.Sr

بِسْمِ اللهِ الرَّحْمٰنِ الرَّحِيْمِ

DEMI KEADILAN BERDASARKAN KETUHANAN YANG MAHA ESA

Pengadilan Agama Sragen yang memeriksa dan mengadili perkara pada tingkat pertama dalam sidang majelis hakim telah menjatuhkan putusan dalam perkara Cerai Gugat antara:

Penggugat, tempat dan tanggal lahir Sragen, 18 Juli 1993, agama Islam, pekerjaan Guru,, Pendidikan Strata I, tempat kediaman di Margomulyo Rt 40 /rw 01, Kel. Puro, Kec. Karangmalang, Kab. Sragen dalam hal ini memberikan kuasa kepada Hendri Adi Wibowo, S.H..Mh, Advokat yang berkantor di Perum Prajamulya Blok I No. 16 Rt. 4 Rw. 9, Kel. Randuacir, Kec. Argomulyo, Kota Salatiga, Jawa Tengah berdasarkan surat kuasa khusus tanggal 14 Desember 2020 sebagai Penggugat;

melawan

Tergugat, tempat dan tanggal lahir Bloro, 18 Juni 1992, agama Islam, pekerjaan Guru,, Pendidikan Strata I, tempat kediaman di Jl. G. Sindoro 1/2 Rt 06 Rw 02 Kel. Tempelan, Kec. Blora, Kab. Blora. sebagai Tergugat;

Pengadilan Agama tersebut;

Telah mempelajari surat-surat yang berkaitan dengan perkara ini;

Telah mendengar keterangan Penggugat dan para saksi di muka sidang;

DUDUK PERKARA

Bahwa Penggugat dalam surat gugatannya tanggal 14 Desember 2020 telah mengajukan Cerai Gugat, yang telah terdaftar di Kepaniteraan Pengadilan Agama Sragen, dengan Nomor 2310/Pdt.G/2020/PA.Sr, tanggal 14 Desember 2020, dengan dalil-dalil pada pokoknya sebagai berikut:

1. Bahwa PENGGUGAT telah menikah dengan TERGUGAT pada hari Sabtu, tanggal 18 Agustus 2018 di hadapan pejabat KUA, Kecamatan Karangmalang Kab. Sragen sebagaimana yang tersebut didalam Kutipan Buku Nikah Nomor : 0295/024/VIII/2018

Disclaimer
Kepaniteraan Mahkamah Agung Republik Indonesia berusaha untuk selalu mencantumkan informasi paling kini dan akurat sebagai bentuk komitmen Mahkamah Agung untuk pelayanan publik, transparansi dan akuntabilitas pelaksanaan fungsi peradilan. Namun dalam hal-hal tertentu masih dimungkinkan terjadi permasalahan teknis terkait dengan akurasi dan keterkinian informasi yang kami sajikan, hal mana akan terus kami perbaiki dari waktu kewaktu.
Dalam hal Anda menemukan inakurasi informasi yang termuat pada situs ini atau informasi yang seharusnya ada, namun belum tersedia, maka harap segera hubungi Kepaniteraan Mahkamah Agung RI melalui :
Email : kepaniteraan@mahkamahagung.go.id   Telp : 021-384 3348 (ext.318)

yang dikeluarkan KUA Kecamatan Karangmalang Kab. Sragen tertanggal 18 Agustus 2018.

2. Bahwa setelah akad nikah TERGUGAT mengucapkan sighot taklik talak dan antara PENGGUGAT dan TERGUGAT telah kumpul baik selayaknya suami isteri (ba'da dukhul) dengan tinggal bersama di rumah orang tua PENGGUGAT di Margomulyo RT 40 /RW 01, Kel. Puro, Kec. Karangmalang, Kab. Sragen sampai bulan Oktober 2019 kemudian pindah ke rumah orang tua TERGUGAT di Jl. G. Sindoro 1/2 RT 06 RW 02 Kel. Tempelan, Kec. Blora, Kab. Blora.
3. Bahwa dalam perkawinan antara PENGGUGAT dan TERGUGAT sampai gugatan ini diajukan telah mempunyai 1 (satu) anak, yaitu: UWAIS AKHYAR AL AKHSAN Lahir di Sragen, 28 Mei 2019 berusia 1 tahun 7 bulan. Bahwa anak saat ini tinggal bersama PENGGUGAT dan dalam asuhan PENGGUGAT.
4. Bahwa pada mulanya perkawinan antara PENGGUGAT dan TERGUGAT berjalan layaknya keluarga yang harmonis sesuai dengan hakekat perkawinan sebagaimana yang termuat di dalam Pasal 3 Kompilasi Hukum Islam yaitu Perkawinan bertujuan untuk mewujudkan kehidupan rumah tangga yang sakinah, mawadah dan rahmah , demikian juga maksud PENGGUGAT pada saat menikah dengan TERGUGAT,
5. Bahwa dalam membina rumah tangga, hakekat perkawinan yang dicita-citakan bersama antara PENGGUGAT dan TERGUGAT mulailah timbul ketidakcocokan dan sering terjadi pertengkaran dan perselisihan terus menerus yang sulit untuk dapat didamaikan lagi.
6. Bahwa ketidakcocokan tersebut disebabkan karena: TERGUGAT acuh tak acuh dan tidak perhatian terhadap PENGGUGAT seperti saat PENGGUGAT sakit tidak dirawat oleh TERGUGAT, TERGUGAT kalau dalam keadaan marah sering meminta untuk cerai, TERGUGAT suka menghina PENGGUGAT dan keluarga PENGGUGAT dengan kata yang menyinggung harkat martabat seperti bapak ibu kamu tidak bisa mendidik dan tidak bisa mengajarkan agama dan TERGUGAT dan PENGGUGAT sering cekcok karena masalah kecil yang dibesar-besarkan seperti menyimpan kontak nomor cowok walaupun itu teman kerja.
7. Bahwa PENGGUGAT juga telah berusaha mengatasi keadaan tersebut dengan mengingatkan dan membicarakan dengan TERGUGAT untuk lebih perhatian kepada

Disclaimer
Kepaniteraan Mahkamah Agung Republik Indonesia berusaha untuk selalu mencantumkan informasi paling kini dan akurat sebagai bentuk komitmen Mahkamah Agung untuk pelayanan publik, transparansi dan akuntabilitas pelaksanaan fungsi peradilan. Namun dalam hal-hal tertentu masih dimungkinkan terjadi permasalahan teknis terkait dengan akurasi dan keterkinian informasi yang kami sajikan, hal mana akan terus kami perbaiki dari waktu kewaktu.
Dalam hal Anda menemukan inakurasi informasi yang termuat pada situs ini atau informasi yang seharusnya ada, namun belum tersedia, maka harap segera hubungi Kepaniteraan Mahkamah Agung RI melalui :
Email : kepaniteraan@mahkamahagung.go.id Telp : 021-384 3348 (ext.318)

PENGGUGAT dan selalu bersabar demi menyelamatkan perkawinan dan rumah tangga antara PENGGUGAT dan TERGUGAT, akan tetapi usaha PENGGUGAT tersebut sia-sia karena TERGUGAT tetap dingin acuh tak acuh dan tidak mau memperbaikinya.

8. Bahwa pada puncaknya sejak bulan Februari 2020 saat PENGGUGAT pulang ke rumah orang tua di Margomulyo RT 40 /RW 01, Kel. Puro, Kec. Karangmalang, Kab. Sragen, dan tidak lagi berada dirumah orang tua TERGUGAT yaitu tidak lagi kembali serumah dengan TERGUGAT sebagai suami-istri.
9. Bahwa selama pisah rumah tersebut, TERGUGAT tidak pernah memperdulikan/mengurusi dan tidak pernah berusaha datang menemui PENGGUGAT dan TERGUGAT juga tidak pernah memberikan nafkah wajib kepada PENGGUGAT.
10. Bahwa atas kejadian tersebut sudah diusahakan penasehatan yang dilakukan oleh keluarga PENGGUGAT, dengan tujuan merukunkan kembali perkawinan dan rumah tangga antara PENGGUGAT dan TERGUGAT, namun usaha tersebut tidak berhasil dan masih saja dalam keadaan pisah rumah sampai sekarang.
11. Bahwa dengan demikian maka rumah tangga PENGGUGAT tidak mungkin dapat dipertahankan karena ikatan lahir batin sebagai suami istri sudah tidak ada lagi sehingga tujuan membentuk rumah tangga yang sakinah, mawadah, warrohmah tidak mungkin terwujud dan berdasarkan pasal 39 ayat 2 UU.No.1/1974 jo pasal 19 huruf (f) PP.N0.9/1975 jo pasal 116 huruf f Kompilasi Hukum Islam telah cukup alasan bagi PENGGUGAT untuk mengajukan gugatan perceraian ini melalui Pengadilan Agama Sragen.

Bahwa berdasarkan dalil-dalil tersebut, Pemohon memohon kepada Pengadilan Agama Sragen, agar menjatuhkan putusan yang amarnya sebagai berikut:

PRIMER :

1. Menerima dan mengabulkan gugatan PENGGUGAT untuk seluruhnya.
2. Menetapkan jatuh talak satu Bain Sughro TERGUGAT (AZIZ MAULANA AKHSAN Bin SUBANDIYANTO) terhadap PENGGUGAT (NURUL UMI JARIAH Binti SUHARDI);

Disclaimer
Kepaniteraan Mahkamah Agung Republik Indonesia berusaha untuk selalu mencantumkan informasi paling kini dan akurat sebagai bentuk komitmen Mahkamah Agung untuk pelayanan publik, transparansi dan akuntabilitas pelaksanaan fungsi peradilan. Namun dalam hal-hal tertentu masih dimungkinkan terjadi permasalahan teknis terkait dengan akurasi dan keterkinian informasi yang kami sajikan, hal mana akan terus kami perbaiki dari waktu kewaktu.
Dalam hal Anda menemukan inakurasi informasi yang termuat pada situs ini atau informasi yang seharusnya ada, namun belum tersedia, maka harap segera hubungi Kepaniteraan Mahkamah Agung RI melalui :
Email : kepaniteraan@mahkamahagung.go.id Telp : 021-384 3348 (ext.318)

3. Menetapkan anak PENGGUGAT dan TERGUGAT bernama UWAIS AKHYAR AL AKHSAN Lahir di Sragen, 28 Mei 2019 berusia 1 tahun 7 bulan. Bahwa anak saat ini tinggal bersama PENGGUGAT dan dalam hadlonah PENGGUGAT;
4. Menetapkan biaya perkara sesuai peraturan perundang-undangan yang berlaku.

SUBSIDER :

- Apabila Majelis Hakim berpendapat lain, mohon putusan yang seadil-adilnya (Ex Aequo Et Bono).

Bahwa pada persidangan yang telah ditetapkan Penggugat telah datang menghadap di persidangan, sedangkan Tergugat tidak datang menghadap di persidangan dan tidak menyuruh orang lain untuk menghadap sebagai wakil/kuasanya yang sah, meskipun berdasarkan surat panggilan (relaas) yang dibacakan di persidangan, Tergugat telah dipanggil secara resmi dan patut, sedangkan tidak ternyata bahwa tidak datangnya Tergugat tersebut disebabkan oleh suatu alasan yang sah;

Bahwa majelis hakim telah menasehati Penggugat agar berpikir untuk tidak bercerai dengan Tergugat, tetapi Penggugat tetap pada dalil-dalil pemohonannya untuk bercerai dengan Tergugat;

Bahwa perkara ini tidak dapat dimediasi karena Tergugat tidak pernah datang menghadap meskipun telah dipanggil secara resmi dan patut, selanjutnya dimulai pemeriksaan dengan membacakan surat gugatan Penggugat yang maksud dan tujuannya tetap dipertahankan oleh Penggugat;

Bahwa untuk menguatkan dalil-dalil gugatannya, Penggugat telah mengajukan bukti berupa:

A. Surat:

1. Foto copy Kartu Tanda Penduduk a.n. Penggugat, dikeluarkan oleh pejabat yang berwenang, sesuai dengan aslinya dan bermaterai cukup, P.1;
2. Foto kopi kutipant Akta Nikah nomor 0295/024/VIII/2018 tertanggal 18 Agustus 2018, tercatat di Kantor Urusan Agama Kecamatan Karangmalang Kab. Sragen, meterai cukup sesuai aslinya sebagai bukti P.2

B. Saksi:

- Bahwa saksi kenal Penggugat dan Tergugat karena saksi adalah ayah kandung Penggugat;

Disclaimer
Kepaniteraan Mahkamah Agung Republik Indonesia berusaha untuk selalu mencantumkan informasi paling kini dan akurat sebagai bentuk komitmen Mahkamah Agung untuk pelayanan publik, transparansi dan akuntabilitas pelaksanaan fungsi peradilan. Namun dalam hal-hal tertentu masih dimungkinkan terjadi permasalahan teknis terkait dengan akurasi dan keterkinian informasi yang kami sajikan, hal mana akan terus kami perbaiki dari waktu kewaktu.
Dalam hal Anda menemukan inakurasi informasi yang termuat pada situs ini atau informasi yang seharusnya ada, namun belum tersedia, maka harap segera hubungi Kepaniteraan Mahkamah Agung RI melalui :
Email : kepaniteraan@mahkamahagung.go.id   Telp : 021-384 3348 (ext.318)

- Bahwa PENGGUGAT telah menikah dengan TERGUGAT pada hari Sabtu, tanggal 18 Agustus 2018 di Karangmalang Kab. Sragen;
- Bahwa setelah akad nikah TERGUGAT mengucapkan sighot taklik talak dan PENGGUGAT dan TERGUGAT telah kumpul baik selayaknya suami isteri dengan tinggal bersama di rumah orang tua PENGGUGAT di Margomulyo Kel. Puro, Kec. Karangmalang, Kab. Sragen sampai bulan Oktober 2019 kemudian pindah ke rumah orang tua TERGUGAT di Jl. G. Sindoro Kel. Tempelan, Kec. Blora, Kab. Blora.
- Bahwa dalam perkawinan antara PENGGUGAT dan TERGUGAT sampai gugatan ini diajukan telah mempunyai 1 (satu) anak, dan saat ini tinggal bersama PENGGUGAT dan dalam asuhan PENGGUGAT.
- Bahwa dalam membina rumah tangga, hakekat perkawinan yang dicita-citakan bersama antara PENGGUGAT dan TERGUGAT mulailah timbul ketidakcocokan dan sering terjadi pertengkaran dan perselisihan terus menerus yang sulit untuk dapat didamaikan lagi.
- Bahwa ketidakcocokan tersebut disebabkan karena TERGUGAT acuh tak acuh dan tidak perhatian terhadap PENGGUGAT seperti saat PENGGUGAT sakit tidak dirawat oleh TERGUGAT, TERGUGAT kalau dalam keadaan marah sering meminta untuk cerai, TERGUGAT suka menghina PENGGUGAT dan keluarga PENGGUGAT dengan kata yang menyinggung harkat martabat seperti bapak ibu kamu tidak bisa mendidik dan tidak bisa mengajarkan agama dan TERGUGAT dan PENGGUGAT sering cekcok karena masalah kecil yang dibesar-besarkan seperti menyimpan kontak nomor cowok walaupun itu teman kerja.
- Bahwa PENGGUGAT juga telah berusaha mengatasi keadaan tersebut dengan mengingatkan dan membicarakan dengan TERGUGAT untuk lebih perhatian kepada PENGGUGAT dan selalu bersabar demi menyelamatkan perkawinan dan rumah tangga antara PENGGUGAT dan TERGUGAT, akan tetapi usaha PENGGUGAT tersebut sia-sia karena TERGUGAT tetap dingin acuh tak acuh dan tidak mau memperbaikinya.

Disclaimer
Kepaniteraan Mahkamah Agung Republik Indonesia berusaha untuk selalu mencantumkan informasi paling kini dan akurat sebagai bentuk komitmen Mahkamah Agung untuk pelayanan publik, transparansi dan akuntabilitas pelaksanaan fungsi peradilan. Namun dalam hal-hal tertentu masih dimungkinkan terjadi permasalahan teknis terkait dengan akurasi dan keterkinian informasi yang kami sajikan, hal mana akan terus kami perbaiki dari waktu kewaktu.
Dalam hal Anda menemukan inakurasi informasi yang termuat pada situs ini atau informasi yang seharusnya ada, namun belum tersedia, maka harap segera hubungi Kepaniteraan Mahkamah Agung RI melalui :
Email : kepaniteraan@mahkamahagung.go.id Telp : 021-384 3348 (ext.318)

- Bahwa pada puncaknya sejak bulan Februari 2020 saat PENGGUGAT pulang ke rumah orang tua di Margomulyo Kel. Puro, Kec. Karangmalang, Kab. Sragen, dan tidak lagi berada dirumah orang tua TERGUGAT yaitu tidak lagi kembali serumah dengan TERGUGAT sebagai suami-istri.
- Bahwa selama pisah rumah tersebut, TERGUGAT tidak pernah memperdulikan/mengurusi dan tidak pernah berusaha datang menemui PENGGUGAT dan TERGUGAT juga tidak pernah memberikan nafkah wajib kepada PENGGUGAT.
- Bahwa atas kejadian tersebut sudah diusahakan penasehatan yang dilakukan oleh keluarga PENGGUGAT, dengan tujuan merukunkan kembali perkawinan dan rumah tangga antara PENGGUGAT dan TERGUGAT, namun usaha tersebut tidak berhasil dan masih saja dalam keadaan pisah rumah sampai sekarang;

- Bahwa saksi kenal Penggugat dan Tergugat karena saksi adalah ayah kandung Penggugat;
- Bahwa PENGGUGAT telah menikah dengan TERGUGAT pada hari Sabtu, tanggal 18 Agustus 2018 di Karangmalang Kab. Sragen;
- Bahwa setelah akad nikah TERGUGAT mengucapkan sighot taklik talak dan PENGGUGAT dan TERGUGAT telah kumpul baik selayaknya suami isteri dengan tinggal bersama di rumah orang tua PENGGUGAT di Margomulyo Kel. Puro, Kec. Karangmalang, Kab. Sragen sampai bulan Oktober 2019 kemudian pindah ke rumah orang tua TERGUGAT di Jl. G. Sindoro Kel. Tempelan, Kec. Blora, Kab. Blora.
- Bahwa dalam perkawinan antara PENGGUGAT dan TERGUGAT sampai gugatan ini diajukan telah mempunyai 1 (satu) anak, dan saat ini tinggal bersama PENGGUGAT dan dalam asuhan PENGGUGAT.
- Bahwa dalam membina rumah tangga, hakekat perkawinan yang dicita-citakan bersama antara PENGGUGAT dan TERGUGAT mulailah timbul ketidakcocokan dan sering terjadi pertengkaran dan perselisihan terus menerus yang sulit untuk dapat didamaikan lagi.

Disclaimer
Kepaniteraan Mahkamah Agung Republik Indonesia berusaha untuk selalu mencantumkan informasi paling kini dan akurat sebagai bentuk komitmen Mahkamah Agung untuk pelayanan publik, transparansi dan akuntabilitas pelaksanaan fungsi peradilan. Namun dalam hal-hal tertentu masih dimungkinkan terjadi permasalahan teknis terkait dengan akurasi dan keterkinian informasi yang kami sajikan, hal mana akan terus kami perbaiki dari waktu kewaktu.
Dalam hal Anda menemukan inakurasi informasi yang termuat pada situs ini atau informasi yang seharusnya ada, namun belum tersedia, maka harap segera hubungi Kepaniteraan Mahkamah Agung RI melalui :
Email : kepaniteraan@mahkamahagung.go.id Telp : 021-384 3348 (ext.318)

- Bahwa ketidakcocokan tersebut disebabkan karena TERGUGAT acuh tak acuh dan tidak perhatian terhadap PENGGUGAT seperti saat PENGGUGAT sakit tidak dirawat oleh TERGUGAT, TERGUGAT kalau dalam keadaan marah sering meminta untuk cerai, TERGUGAT suka menghina PENGGUGAT dan keluarga PENGGUGAT dengan kata yang menyinggung harkat martabat seperti bapak ibu kamu tidak bisa mendidik dan tidak bisa mengajarkan agama dan TERGUGAT dan PENGGUGAT sering cekcok karena masalah kecil yang dibesar-besarkan seperti menyimpan kontak nomor cowok walaupun itu teman kerja.
- Bahwa PENGGUGAT juga telah berusaha mengatasi keadaan tersebut dengan mengingatkan dan membicarakan dengan TERGUGAT untuk lebih perhatian kepada PENGGUGAT dan selalu bersabar demi menyelamatkan perkawinan dan rumah tangga antara PENGGUGAT dan TERGUGAT, akan tetapi usaha PENGGUGAT tersebut sia-sia karena TERGUGAT tetap dingin acuh tak acuh dan tidak mau memperbaikinya.
- Bahwa pada puncaknya sejak bulan Februari 2020 saat PENGGUGAT pulang ke rumah orang tua di Margomulyo Kel. Puro, Kec. Karangmalang, Kab. Sragen, dan tidak lagi berada dirumah orang tua TERGUGAT yaitu tidak lagi kembali serumah dengan TERGUGAT sebagai suami-istri.
- Bahwa selama pisah rumah tersebut, TERGUGAT tidak pernah memperdulikan/mengurusi dan tidak pernah berusaha datang menemui PENGGUGAT dan TERGUGAT juga tidak pernah memberikan nafkah wajib kepada PENGGUGAT.
- Bahwa atas kejadian tersebut sudah diusahakan penasehatan yang dilakukan oleh keluarga PENGGUGAT, dengan tujuan merukunkan kembali perkawinan dan rumah tangga antara PENGGUGAT dan TERGUGAT, namun usaha tersebut tidak berhasil dan masih saja dalam keadaan pisah rumah sampai sekarang.

Bahwa Penggugat menyampaikan kesimpulan yang isinya sebagaimana tertuang dalam berita acara sidang;

Disclaimer
Kepaniteraan Mahkamah Agung Republik Indonesia berusaha untuk selalu mencantumkan informasi paling kini dan akurat sebagai bentuk komitmen Mahkamah Agung untuk pelayanan publik, transparansi dan akuntabilitas pelaksanaan fungsi peradilan. Namun dalam hal-hal tertentu masih dimungkinkan terjadi permasalahan teknis terkait dengan akurasi dan keterkinian informasi yang kami sajikan, hal mana akan terus kami perbaiki dari waktu kewaktu.
Dalam hal Anda menemukan inakurasi informasi yang termuat pada situs ini atau informasi yang seharusnya ada, namun belum tersedia, maka harap segera hubungi Kepaniteraan Mahkamah Agung RI melalui :
Email : kepaniteraan@mahkamahagung.go.id Telp : 021-384 3348 (ext.318)

Selanjutnya untuk singkatnya uraian putusan ini, maka semua hal yang termuat dalam berita acara sidang merupakan bagian yang tidak terpisahkan dari putusan ini;

PERTIMBANGAN HUKUM

Menimbang bahwa maksud dan tujuan gugatan Penggugat adalah seperti diuraikan tersebut di muka;

Menimbang, bahwa berdasarkan alat bukti P.1 dikaitkan pasal 4, pasal 49 dan pasal 73 Undang–Undang Nomor 7 Tahun 1989 tentang Peradilan Agama yang telah diubah dan di tambah dengan Undang-Undang Nomor 3 Tahun 2006 dan telah diubah yang kedua dengan Undang-Undang Nomor 50 Tahun 2009, dinyatakan terbukti secara absolute dan relatif bahwa perkara ini adalah wewenang Pengadilan Agama Sragen;

Menimbang bahwa Majelis Hakim telah berusaha secara maksimal mendamaikan dan menasehati Penggugat dan Tergugat di depan persidangan agar Penggugat dan Tergugat tetap membina rumah tangga dengan baik, namun tidak berhasil.

Menimbang bahwa berdasarkan PERMA Nomor 1 Tahun 2016 tentang mediasi, bahwa setiap perkara perdata harus dimediasi, namun karena Tergugat tidak pernah hadir di muka sidang dan tidak pula mengutus orang lain sebagai wakil atau kuasanya, sehingga Majelis Hakim menganggap perkara ini tidak dimediasi.

Menimbang, bahwa dari posita gugatan Penggugat, majelis menilai bahwa yang dijadikan alasan gugatan Penggugat adalah sering terjadinya perselisihan dan pertengkaran disebabkan karena TERGUGAT acuh tak acuh dan tidak perhatian terhadap PENGGUGAT seperti saat PENGGUGAT sakit tidak dirawat oleh TERGUGAT, TERGUGAT kalau dalam keadaan marah sering meminta untuk cerai, TERGUGAT suka menghina PENGGUGAT dan keluarga PENGGUGAT dengan kata yang menyinggung harkat martabat seperti bapak ibu kamu tidak bisa mendidik dan tidak bisa mengajarkan agama dan TERGUGAT dan PENGGUGAT sering cekcok karena masalah kecil yang dibesar-besarkan seperti menyimpan kontak nomor cowok walaupun itu teman kerja dan puncaknya sejak bulan Februari 2020 saat PENGGUGAT pulang ke rumah orang tua di Margomulyo Kel. Puro, Kec. Karangmalang, Kab. Sragen,

Disclaimer
Kepaniteraan Mahkamah Agung Republik Indonesia berusaha untuk selalu mencantumkan informasi paling kini dan akurat sebagai bentuk komitmen Mahkamah Agung untuk pelayanan publik, transparansi dan akuntabilitas pelaksanaan fungsi peradilan. Namun dalam hal-hal tertentu masih dimungkinkan terjadi permasalahan teknis terkait dengan akurasi dan keterkinian informasi yang kami sajikan, hal mana akan terus kami perbaiki dari waktu kewaktu.
Dalam hal Anda menemukan inakurasi informasi yang termuat pada situs ini atau informasi yang seharusnya ada, namun belum tersedia, maka harap segera hubungi Kepaniteraan Mahkamah Agung RI melalui :
Email : kepaniteraan@mahkamahagung.go.id Telp : 021-384 3348 (ext.318)

dan tidak lagi berada dirumah orang tua TERGUGAT yaitu tidak lagi kembali serumah dengan TERGUGAT sebagai suami-istri dan selama pisah rumah tersebut, TERGUGAT tidak pernah memperdulikan/mengurusi dan tidak pernah berusaha datang menemui PENGGUGAT dan TERGUGAT juga tidak pernah memberikan nafkah wajib kepada PENGGUGAT serta kejadian tersebut sudah diusahakan penasehatan yang dilakukan oleh keluarga PENGGUGAT, dengan tujuan merukunkan kembali perkawinan dan rumah tangga antara PENGGUGAT dan TERGUGAT, namun usaha tersebut tidak berhasil dan masih saja dalam keadaan pisah rumah sampai sekarang;

Menimbang bahwa alasan tersebut telah sesuai dengan ketentuan Pasal 34 ayat (3) Undang-Undang Nomor 1 Tahun 1974 jo Pasal 19 huruf (f) Peraturan Pemerintah Nomor 9 Tahun 1975 jo. Pasal 116 huruf (f) Kompilasi Hukum Islam, oleh karenanya akan dipertimbangkan untuk diperiksa lebih lanjut.

Menimbang bahwa untuk membuktikan dalil-dalil gugatannya sebagaimana angka 1 sampai 7, Penggugat telah mengajukan alat bukti surat P dan 2 (dua) orang saksi yang akan dipertimabngkan sebagai berikut:

Menimbang bahwa bukti P. (Fotokopi Kutipan Akta Nikah) yang merupakan akta otentik dan telah bermeterai cukup dan cocok dengan aslinya, isi bukti tersebut menjelaskan mengenai perkawinan Penggugat dengan Tergugat, lagi pula dibuat oleh pejabat yang berwenang dan berkaitan langsung dengan apa yang dipersengketakan di Pengadilan sehingga bukti tersebut telah memenuhi syarat formil dan materiil, serta mempunyai kekuatan pembuktian yang sempurna dan mengikat sebagaimana ketentuan Pasal 1868 dan Pasal 1871 KUH Perdata, dengan demikian harus dinyatakan terbukti bahwa antara Penggugat dan Tergugat adalah suami-isteri yang sah, dan merupakan legal standing perkara ini.

Menimbang bahwa saksi pertama Penggugat sudah dewasa dan sudah disumpah, sehingga memenuhi syarat formil sebagaimana yang diatur dalam Pasal 145 ayat 1 angka 3e HIR.;

Menimbang bahwa keterangan saksi pertama Penggugat mengenai angka 1, 2, 3, 4, 5, 6 adalah fakta yang didengar sendiri/dialami sendiri dan relevan dengan dalil yang harus dibuktikan oleh Penggugat, oleh karena itu keterangan saksi tersebut telah memenuhi syarat materiil sebagaimana telah diatur dalam Pasal 171 HIR sehingga

Disclaimer
Kepaniteraan Mahkamah Agung Republik Indonesia berusaha untuk selalu mencantumkan informasi paling kini dan akurat sebagai bentuk komitmen Mahkamah Agung untuk pelayanan publik, transparansi dan akuntabilitas pelaksanaan fungsi peradilan. Namun dalam hal-hal tertentu masih dimungkinkan terjadi permasalahan teknis terkait dengan akurasi dan keterkinian informasi yang kami sajikan, hal mana akan terus kami perbaiki dari waktu kewaktu.
Dalam hal Anda menemukan inakurasi informasi yang termuat pada situs ini atau informasi yang seharusnya ada, namun belum tersedia, maka harap segera hubungi Kepaniteraan Mahkamah Agung RI melalui :
Email : kepaniteraan@mahkamahagung.go.id   Telp : 021-384 3348 (ext.318)

keterangan saksi tersebut memiliki kekuatan pembuktian dan dapat diterima sebagai alat bukti;

Menimbang bahwa saksi kedua Penggugat, sudah dewasa dan sudah disumpah, sehingga memenuhi syarat formal sebagaimana diatur dalam Pasal 145 ayat 1 angka 3e HIR.;

Menimbang bahwa keterangan saksi kedua Penggugat mengenai angka 2, 3, 4, 5, 6 adalah fakta yang dilihat sendiri/didengar sendiri dan relevan dengan dalil yang harus dibuktikan oleh Penggugat, oleh karena itu keterangan saksi tersebut telah memenuhi syarat materiil sebagaimana telah diatur dalam Pasal 145 ayat 1 angka 3e HIR. sehingga keterangan saksi tersebut memiliki kekuatan pembuktian dan dapat diterima sebagai alat bukti;

Menimbang, bahwa keterangan saksi 1 dan saksi 2 tersebut bersesuaian dan cocok antara satu dengan yang lain oleh karena itu keterangan dua orang saksi tersebut memenuhi Pasal 171 dan Pasal 172 HIR;

Menimbang bahwa berdasarkan pengakuan Penggugat, diperkuat dengan alat bukti P dan 2 (dua) orang saksi Penggugat. Maka Majelis Hakim telah memperoleh fakta yang pada pokoknya sebagai berikut:

- Bahwa PENGGUGAT telah menikah dengan TERGUGAT pada hari Sabtu, tanggal 18 Agustus 2018 di hadapan pejabat KUA, Kecamatan Karangmalang Kab. Sragen;
- Bahwa Penggugat dan Tergugat telah hidup bersama dalam rumah tangga sebagai suami-isteri pada awalnya rukun dan harmonis dan dikaruniai 1 anak, namun keharmonisan tersebut sudah tidak terwujud lagi karena sering terjadi perselisihan dan pertengkaran;

Bahwa perselisihan dan pertengkaran disebabkan karena TERGUGAT acuh tak acuh dan tidak perhatian terhadap PENGGUGAT seperti saat PENGGUGAT sakit tidak dirawat oleh TERGUGAT, TERGUGAT kalau dalam keadaan marah sering meminta untuk cerai, TERGUGAT suka menghina PENGGUGAT dan keluarga PENGGUGAT dengan kata yang menyinggung harkat martabat seperti bapak ibu kamu tidak bisa mendidik dan tidak bisa mengajarkan agama dan TERGUGAT dan PENGGUGAT sering cekcok karena masalah kecil yang dibesar-besarkan seperti menyimpan kontak nomor cowok walaupun itu teman kerja dan puncaknya sejak bulan

Disclaimer
Kepaniteraan Mahkamah Agung Republik Indonesia berusaha untuk selalu mencantumkan informasi paling kini dan akurat sebagai bentuk komitmen Mahkamah Agung untuk pelayanan publik, transparansi dan akuntabilitas pelaksanaan fungsi peradilan. Namun dalam hal-hal tertentu masih dimungkinkan terjadi permasalahan teknis terkait dengan akurasi dan keterkinian informasi yang kami sajikan, hal mana akan terus kami perbaiki dari waktu kewaktu.
Dalam hal Anda menemukan inakurasi informasi yang termuat pada situs ini atau informasi yang seharusnya ada, namun belum tersedia, maka harap segera hubungi Kepaniteraan Mahkamah Agung RI melalui :
Email : kepaniteraan@mahkamahagung.go.id Telp : 021-384 3348 (ext.318)

Februari 2020 saat PENGGUGAT pulang ke rumah orang tua di Margomulyo Kel. Puro, Kec. Karangmalang, Kab. Sragen, dan tidak lagi berada dirumah orang tua TERGUGAT yaitu tidak lagi kembali serumah dengan TERGUGAT sebagai suami-istri dan selama pisah rumah tersebut, TERGUGAT tidak pernah memperdulikan/mengurusi dan tidak pernah berusaha datang menemui PENGGUGAT dan TERGUGAT juga tidak pernah memberikan nafkah wajib kepada PENGGUGAT serta kejadian tersebut sudah diusahakan penasehatan yang dilakukan oleh keluarga PENGGUGAT, dengan tujuan merukunkan kembali perkawinan dan rumah tangga antara PENGGUGAT dan TERGUGAT, namun usaha tersebut tidak berhasil dan masih saja dalam keadaan pisah rumah sampai sekarang;

Menimbang bahwa yang dimaksud perselisihan dalam rumah tangga tidaklah identik dengan pertengkaran mulut, rumah tangga dapat dinyatakan terjadi perselisihan jika hubungan suami isteri sudah tidak selaras, tidak saling percaya dan saling melindungi, maka dengan ditemukannya fakta antara Penggugat dengan Tergugat telah berpisah tempat kediaman bersama, menunjukkan bahwa antara Penggugat dengan Tergugat sudah tidak lagi saling percaya dan saling pengertian dan sudah tidak ada lagi komunikasi suami isteri yang harmonis yang merupakan bagian dari gejala perselisihan dalam rumah tangga.

Menimbang bahwa dengan merujuk pada Putusan Mahkamah Agung Republik Indonesia tanggal 17 Maret 1999 Nomor 237/K/AG/1998 yang mengandung abstrak hukum, bahwa berselisih, cekcok, hidup berpisah, tidak dalam satu tempat kediaman bersama, salah satu pihak tidak berniat untuk meneruskan kehidupan bersama dengan pihak lain, hal itu adalah merupakan fakta hukum yang cukup untuk menjadi alasan dalam suatu perceraian sesuai dengan maksud Pasal 19 huruf (f) Peraturan Pemerintah Nomor 9 Tahun 1975.

Menimbang bahwa berdasarkan fakta tersebut di atas, maka Penggugat telah membuktikan dalil-dalil gugatannya tentang adanya perselisihan dan pertengkaran terus-menerus tanpa adanya penyelesaian yang baik. Sehingga Penggugat telah cukup bukti adanya alasan perceraian, menurut ketentuan Pasal 39 ayat (2) Undang-Undang Nomor 1 Tahun 1974 dan Pasal 116 huruf (f) Kompilasi Hukum Islam.

Disclaimer
Kepaniteraan Mahkamah Agung Republik Indonesia berusaha untuk selalu mencantumkan informasi paling kini dan akurat sebagai bentuk komitmen Mahkamah Agung untuk pelayanan publik, transparansi dan akuntabilitas pelaksanaan fungsi peradilan. Namun dalam hal-hal tertentu masih dimungkinkan terjadi permasalahan teknis terkait dengan akurasi dan keterkinian informasi yang kami sajikan, hal mana akan terus kami perbaiki dari waktu kewaktu.
Dalam hal Anda menemukan inakurasi informasi yang termuat pada situs ini atau informasi yang seharusnya ada, namun belum tersedia, maka harap segera hubungi Kepaniteraan Mahkamah Agung RI melalui :
Email : kepaniteraan@mahkamahagung.go.id Telp : 021-384 3348 (ext.318)

Menimbang bahwa maksud dan tujuan perkawinan untuk mencapai keluarga yang sakinah, mawaddah, wa rohmah sebagaimana yang diamanatkan pasal 3 Kompilasi Hukum Islam (KHI) jo. pasal 1 Undang-undang nomor 1 tahun 1974. Hal ini senafas dengan Al-Qur’an, surah Arrum ayat (21) sebagai berikut:

وَمِنْ آيَاتِهِ أَنْ خَلَقَ لَكُمْ مِنْ أَنْفُسِكُمْ أَزْوَاجًا لِتَسْكُنُوا إِلَيْهَا وَجَعَلَ بَيْنَكُمْ مَوَدَّةً وَرَحْمَةً إِنَّ فِي ذَلِكَ لَآيَاتٍلِقَوْمٍ يَتَفَكَّرُونَ

Artinya:
"Dan di antara tanda-tanda kekuasaan-Nya ialah dia menciptakan untukmu isteri-isteri dari jenismu sendiri, supaya kamu cenderung dan merasa tenteram kepadanya, dan dijadikan-Nya diantaramu rasa kasih dan sayang. Sesungguhnya pada yang demikian itu benar-benar terdapat tanda-tanda bagi kaum yang berfikir".

ternyata telah tidak terwujud lagi dalam kehidupan rumah tangga antara Penggugat dan Tergugat.

Menimbang bahwa berdasarkan fakta yang telah terungkap serta bukti-bukti yang sah dalam sidang, maka Majelis Hakim berkeyakinan bahwa keutuhan rumah tangga antara Penggugat dengan Tergugat tidak dapat dipertahankan lagi, sehingga yang dipandang adil untuk menyelesaikan kemaslahatan kedua belah pihak adalah dengan perceraian.

Menimbang bahwa dengan kondisi rumah tangga yang demikian apabila dipaksakan untuk diteruskan, maka akan membawa mafsadat lebih besar daripada maslahatnya yaitu Penggugat dan Tergugat akan terus menerus dalam penderitaan lahir batin, hal ini perlu dihindari sesuai dengan kaidah fiqhiyah yang berbunyi :

د رء المفاسد مقد م على جلب المصالح

Artinya:
"Menolak banyak mafsadah lebih didahulukan dari pada menarik banyak maslahah".

Menimbang bahwa berdasarkan kesimpulan Penggugat di depan persidangan, yakni Penggugat berketetapan hati ingin bercerai dengan Tergugat dan Penggugat dalam keadaan sehat jasmani dan rohani, maka dalam hal ini majelis hakim perlu mempertimbangkan pendapat pakar hukum Islam sebagaimana yang termuat dalam kitab Al Iqna Juz II halaman 133 sebagai berikut:

Disclaimer
Kepaniteraan Mahkamah Agung Republik Indonesia berusaha untuk selalu mencantumkan informasi paling kini dan akurat sebagai bentuk komitmen Mahkamah Agung untuk pelayanan publik, transparansi dan akuntabilitas pelaksanaan fungsi peradilan. Namun dalam hal-hal tertentu masih dimungkinkan terjadi permasalahan teknis terkait dengan akurasi dan keterkinian informasi yang kami sajikan, hal mana akan terus kami perbaiki dari waktu kewaktu.
Dalam hal Anda menemukan inakurasi informasi yang termuat pada situs ini atau informasi yang seharusnya ada, namun belum tersedia, maka harap segera hubungi Kepaniteraan Mahkamah Agung RI melalui :
Email : kepaniteraan@mahkamahagung.go.id   Telp : 021-384 3348 (ext.318)

وان اشتد عدم رغبة الزوجة لزوجها طلق عليه القاضى طلقة

Artinya:
"Apabila ketidaksenangan seorang isteri kepada suaminya telah mencapai puncaknya, maka pada saat itu hakim diperbolehkan menjatuhkan talak seorang suami kepada istrinya dengan talak satu”.

Majelis hakim sependapat sekaligus mengambil alih pendapat pakar hukum Islam tersebut karena berkaitan erat dengan perkara ini.

Menimbang bahwa berdasarkan pertimbangan-pertimbangan tersebut di atas, dan alasan-alasan perceraian telah terpenuhi sebagaimana petunjuk Pasal 70 ayat (1) Undang-Undang Nomor 7 Tahun 1989 jo. Pasal 39 ayat (1) dan (2) Undang-Undang Nomor 1 Tahun 1974. Maka Majelis Hakim berkesimpulan bahwa gugatan Penggugat sebagaimana petitum angka 2 (dua) patut untuk dikabulkan dengan menjatuhkan talak ba’in;

Menimbang bahwa oleh karena talak yang akan dijatuhkan oleh Pengadilan adalah talak yang pertama, maka dengan demikian talak yang dikabulkan dan yang akan dijatuhkan oleh Pengadilan adalah talak satu bain shughro Tergugat kepada Penggugat sebagaimana yang diatur dalam pasal 119 (2) Kompilasi Hukum Islam;

Menimbang bahwa berdasarkan ketentuan pasal 105 Kompilasi Hukum islam dinyatakan dalam hal terjadinya perceraian, pemeliharaan anak yang belum mumayyiz atau belum berumur 12 tahun adalah hak ibunya dan pemeliharaan anak yang sudah mumayyiz diserahkan kepada anak untuk memilih diantara ayah atau ibunya sebagai pemegang hak pemeliharaanya;

Menimbang berdasarkan pertimbangan tersebut maka anak Penggugat dan Tergugat bernama UWAIS AKHYAR AL AKHSAN yang lahir di Sragen tanggal 28 Mei 2019 berusia 1 tahun 7 bulan. adalah hak hadlonah Penggugat;

Menimbang bahwa Tergugat tidak datang menghadap di sidang dan tidak pula menyuruh orang lain sebagai kuasanya meskipun telah dipanggil secara resmi dan patut, dan tidak ternyata atas ketidakhadirnya disebabkan suatu halangan yang sah, dan lagi pula gugatan Penggugat dinilai cukup beralasan dan tidak melawan hukum, oleh karenanya Tergugat harus dinyatakan tidak hadir dan putusan ini harus dijatuhkan dengan Verstek atau tanpa hadirnya Tergugat, sesuai petunjuk pasal 125 HIR;

Disclaimer
Kepaniteraan Mahkamah Agung Republik Indonesia berusaha untuk selalu mencantumkan informasi paling kini dan akurat sebagai bentuk komitmen Mahkamah Agung untuk pelayanan publik, transparansi dan akuntabilitas pelaksanaan fungsi peradilan. Namun dalam hal-hal tertentu masih dimungkinkan terjadi permasalahan teknis terkait dengan akurasi dan keterkinian informasi yang kami sajikan, hal mana akan terus kami perbaiki dari waktu kewaktu.
Dalam hal Anda menemukan inakurasi informasi yang termuat pada situs ini atau informasi yang seharusnya ada, namun belum tersedia, maka harap segera hubungi Kepaniteraan Mahkamah Agung RI melalui :
Email : kepaniteraan@mahkamahagung.go.id Telp : 021-384 3348 (ext.318)

Menimbang bahwa perkara ini termasuk dalam bidang perkawinan, maka berdasarkan ketentuan Pasal 89 ayat (1) Undang-Undang Nomor 7 Tahun 1989 yang telah diubah dengan Undang-Undang Republik Indonesia Nomor 3 Tahun 2006 dan perubahan kedua Undang-Undang Republik Indonesia Nomor 50 Tahun 2009 Tentang Peradilan Agama, maka semua biaya perkara yang timbul dalam perkara ini dibebankan kepada Penggugat.

Mengingat segala ketentuan Peraturan Perundang-undangan yang berlaku dan hukum Islam yang berkaitan dengan perkara ini.

MENGADILI:

1. Menyatakan Tergugat yang telah dipanggil secara resmi dan patut untuk menghadap di persidangan tidak hadir;
2. Mengabulkan gugatan Penggugat dengan verstek;
3. Menjatuhkan talak satu Bain Sughro TERGUGAT terhadap PENGGUGAT;
4. Menetapkan anak PENGGUGAT dan TERGUGAT bernama UWAIS AKHYAR AL AKHSAN Lahir di Sragen, 28 Mei 2019 berusia 1 tahun 7 bulan. Bahwa anak saat ini tinggal bersama PENGGUGAT dan dalam hadlonah PENGGUGAT;
5. Membebankan kepada Penggugat untuk membayar biaya perkara ini sebesar Rp351.000,- (tiga ratus lima puluh satu ribu rupiah);

Demikian putusan ini dijatuhkan dalam rapat permusyawaratan Majelis Hakim yang dilangsungkan pada hari Senin tanggal 28 Desember 2020 Masehi, bertepatan dengan tanggal 13 J.Awwal 1442 Hijriah, oleh kami Drs. H. Yasin Irfan, M.H. sebagai Ketua Majelis, Drs. Ikhsan SH.MA dan Drs. H.Muhammad Fatchan MA masing-masing sebagai Hakim Anggota, putusan tersebut diucapkan dalam sidang terbuka untuk umum pada hari itu juga pada hari Senin tanggal 28 Desember 2020 Masehi, bertepatan dengan tanggal 13 J.Awwal 1442 1442 Hijriah, oleh Ketua Majelis tersebut dengan didampingi oleh Hakim Anggota dan dibantu Drs.Amir. sebagai Panitera Pengganti serta dihadiri oleh Penggugat tanpa hadirnya Tergugat;

Hakim Anggota, Ketua Majelis,

Disclaimer
Kepaniteraan Mahkamah Agung Republik Indonesia berusaha untuk selalu mencantumkan informasi paling kini dan akurat sebagai bentuk komitmen Mahkamah Agung untuk pelayanan publik, transparansi dan akuntabilitas pelaksanaan fungsi peradilan. Namun dalam hal-hal tertentu masih dimungkinkan terjadi permasalahan teknis terkait dengan akurasi dan keterkinian informasi yang kami sajikan, hal mana akan terus kami perbaiki dari waktu kewaktu.
Dalam hal Anda menemukan inakurasi informasi yang termuat pada situs ini atau informasi yang seharusnya ada, namun belum tersedia, maka harap segera hubungi Kepaniteraan Mahkamah Agung RI melalui :
Email : kepaniteraan@mahkamahagung.go.id Telp : 021-384 3348 (ext.318)

Drs. Ikhsan SH.MA	Drs. H. Yasin Irfan, M.H.
Hakim Anggota	

Drs. H.Muhammad Fatchan MA

Panitera Pengganti

Drs.Amir.

Perincian biaya :

1.	Pendaftaran	Rp	30.000,00
2.	Proses	Rp	75.000,00
3.	Panggilan	Rp	230.000,00
4.	Redaksi	Rp	10.000,00
5.	Meterai	Rp	6.000,00
	Jumlah	Rp	351.000,00

(tiga ratus lima puluh satu ribu rupiah);

Disclaimer
Kepaniteraan Mahkamah Agung Republik Indonesia berusaha untuk selalu mencantumkan informasi paling kini dan akurat sebagai bentuk komitmen Mahkamah Agung untuk pelayanan publik, transparansi dan akuntabilitas pelaksanaan fungsi peradilan. Namun dalam hal-hal tertentu masih dimungkinkan terjadi permasalahan teknis terkait dengan akurasi dan keterkinian informasi yang kami sajikan, hal mana akan terus kami perbaiki dari waktu kewaktu.
Dalam hal Anda menemukan inakurasi informasi yang termuat pada situs ini atau informasi yang seharusnya ada, namun belum tersedia, maka harap segera hubungi Kepaniteraan Mahkamah Agung RI melalui :
Email : kepaniteraan@mahkamahagung.go.id   Telp : 021-384 3348 (ext.318)
