# Supplementary material for: Classification of divorce causes during the COVID-19 pandemic using convolutional neural networks
Source: PeerJ Comput Sci. 2022 Jun 30;8:e998. doi: 10.7717/peerj-cs.998 (PMC9299239; doi:10.7717/peerj-cs.998)
Supplement: Supplemental Information 6 [file peerj-cs-08-998-s006.zip › Rujuk Dataset/Data ke-12.pdf]

PUTUSAN

Nomor 2681/Pdt.G/2020/PA.Pwt

بِسْمِ اللهِ الرَّحْمٰنِ الرَّحِيْمِ

DEMI KEADILAN BERDASARKAN KETUHANAN YANG MAHA ESA

Pengadilan Agama Purwokerto yang memeriksa dan mengadili perkara Perdata Agama pada tingkat pertama dalam persidangan Majelis telah menjatuhkan Putusan dalam perkara Cerai Gugat antara :

PENGGUGAT, umur 39 tahun, agama Islam, Pekerjaan xxxxxxxx, Pendidikan Sekolah Lanjutan Tingkat Pertama, tempat kediaman di KABUPATEN BANYUMAS,, sebagai Penggugat

Melawan :

TERGUGAT, umur 38 tahun, agama Islam, Pekerjaan xxxxx, Pendidikan Sekolah Lanjutan Tingkat Atas, tempat kediaman di KABUPATEN BANYUMAS,, sebagai Tergugat

Pengadilan Agama tersebut;

Telah mempelajari berkas perkara;

Telah mendengar keterangan Penggugat dan saksi-saksi di persidangan;

DUDUK PERKARA

Bahwa, Penggugat dalam surat gugatannya tanggal 02 Desember 2020 telah mengajukan gugatan perceraian yang telah didaftar di Kepaniteraan Pengadilan Agama Purwokerto dengan Nomor 2681/Pdt.G/2020/PA.Pwt, tanggal 02 Desember 2020, dengan dalil-dalil sebagai berikut:

1. Bahwa Penggugat telah menikah dengan Tergugat pada tanggal 5 Pebruari2003yang dicatat oleh Pegawai Pencatat Nikah Kantor Urusan Agama xxxxxxxxx xxxxxxxxxx xxxxx, xxxxxxxxx xxxxxxxx berdasarkan Kutipan Akta Nikah Nomor : 32/06/II/2003 tanggal 5 Pebruari2003;
2. Bahwa setelah menikah, Penggugat dan Tergugat bertempat tinggal bersama di rumah orang tua Tergugat diBekasiselama kurang lebih 1(satu) tahun, kemudian Penggugat dan Tergugat pindah dan tinggal bersama di rumah orang tua Penggugat di xxxxxx, xxxxxx, xxxxxxxxx xxxxxxxx, xxxxxxxxx xxxxxxxxxx xxxxx, xxxxxxxxx xxxxxxxx selama

Disclaimer
Kepaniteraan Mahkamah Agung Republik Indonesia berusaha untuk selalu mencantumkan informasi paling kini dan akurat sebagai bentuk komitmen Mahkamah Agung untuk pelayanan publik, transparansi dan akuntabilitas pelaksanaan fungsi peradilan. Namun dalam hal-hal tertentu masih dimungkinkan terjadi permasalahan teknis terkait dengan akurasi dan keterkinian informasi yang kami sajikan, hal mana akan terus kami perbaiki dari waktu kewaktu.
Dalam hal Anda menemukan inakurasi informasi yang termuat pada situs ini atau informasi yang seharusnya ada, namun belum tersedia, maka harap segera hubungi Kepaniteraan Mahkamah Agung RI melalui :
Email : kepaniteraan@mahkamahagung.go.id Telp : 021-384 3348 (ext.318)

kurang lebih 3 (tiga) tahun 11 (sebelas) bulan, antara Penggugat dan Tergugat sudah melakukan hubungan suamiisteri (bada dukhul) dan telah dikaruniai 2(dua) orang anak yaitu AVIVAH CAHYA SAFITRI(umur 17tahun) dan NASYIFA WAHANA ADIRA(umur 13tahun), ikut Penggugat;

3. Bahwa sejak bulan Januaritahun 2008 antara Penggugat dan Tergugat terjadi pisah tempat tinggal disebabkan Tergugat pergi pamit bekerja, namun selama itu Tergugat tidak pernah pulangsampai sekarangselama kurang lebih12(dua belas) tahun 11 (sebelas)bulan;

4. bahwa Penggugat menderita lahir batin dan tidak rela atas perbuatan Tergugat kepada Penggugat;

5. bahwa berdasarkan uraian diatas, perbuatan Tergugat telah memenuhi alasan perceraian sebagaimana diatur dalam Undang-Undang Nomor : 1 Tahun 1974 jo Peraturan Pemerintah Nomor : 9 Tahun 1975 Pasal 19 huruf b dan Kompilasi Hukum Islam Pasal 116 huruf b;

Berdasarkan alasan/dalil-dalil di atas, Penggugat mohon agar Ketua Pengadilan Agama Purwokerto menjatuhkan putusan yang amarnya berbunyi sebagai berikut:;

1. Mengabulkan gugatan Penggugat;
2. Menceraikan perkawinan antara Penggugat (IROH JUNIASIHbinti ROSIDIN) dengan Tergugat (ADE SETIAWANbin UMIN);
3. Membebankan biaya perkara menurut hukum;

Atau menjatuhkan putusan lain yang seadil-adilnya;

Bahwa, pada hari sidang yang telah ditetapkan Penggugat telah datang menghadap ke muka sidang, sedangkan Tergugat tidak datang menghadap ke muka sidang dan tidak menyuruh orang lain untuk menghadap sebagai wakil/kuasa hukumnya meskipun telah dipanggil secara resmi dan patut yang relaas panggilannya dibacakan di dalam sidang, sedang tidak ternyata bahwa tidak datangnya itu disebabkan suatu halangan yang sah;

Disclaimer
Kepaniteraan Mahkamah Agung Republik Indonesia berusaha untuk selalu mencantumkan informasi paling kini dan akurat sebagai bentuk komitmen Mahkamah Agung untuk pelayanan publik, transparansi dan akuntabilitas pelaksanaan fungsi peradilan. Namun dalam hal-hal tertentu masih dimungkinkan terjadi permasalahan teknis terkait dengan akurasi dan keterkinian informasi yang kami sajikan, hal mana akan terus kami perbaiki dari waktu kewaktu.
Dalam hal Anda menemukan inakurasi informasi yang termuat pada situs ini atau informasi yang seharusnya ada, namun belum tersedia, maka harap segera hubungi Kepaniteraan Mahkamah Agung RI melalui :
Email : kepaniteraan@mahkamahagung.go.id Telp : 021-384 3348 (ext.318)

Bahwa, Majelis Hakim telah menasehati Penggugat agar tetap bersabar dan mengurungkan niyatnya bercerai dengan Tergugat, akan tetapi Penggugat tetap pada dalil-dalil gugatannya untuk bercerai dengan Tergugat;

Bahwa, perkara ini tidak dapat di mediasi karena Tergugat tidak pernah datang menghadap meskipun telah dipanggil secara resmi dan patut, selanjutnya dimulai pemeriksaan dengan membacakan surat gugatan Penggugat yang maksud dan isinya tetap dipertahankan oleh Penggugat;

Bahwa, untuk menguatkan dalil-dalil gugatannya, Penggugat telah mengajukan bukti-bukti berupa;

A. Surat :

1. Fotokopi Kartu Tanda Penduduk NIK 3302254906810003 atas nama PENGGUGAT, yang aslinya dikeluarkan oleh Dinas Kependudukan dan Catatan Sipil xxxxxxxxx xxxxxxxx tanggal Kemudian Bukti surat tersebut oleh Ketua Majelis dicocokan dengan aslinya, ternyata sama, telah diberi meterai cukup serta telah dinazegelen oleh Kantor Pos, kemudian oleh Ketua Majelis diparaf dan diberi tanda (P.1);
2. Fotokopi Kutipan Akta Nikah Nomor : 32/06/II/2003 yang aslinya dikeluarkan oleh Pegawai Pencatat Nikah Kantor Urusan Agama xxxxxxxxx xxxxxxxxxx xxxxx, xxxxxxxxx xxxxxxxx, tanggal 5 Februari 2003, Kemudian bukti surat tersebut oleh Ketua Majelis dicocokan dengan aslinya, ternyata sama, telah diberi meterai cukup serta telah di Nazegelen oleh Kantor Pos, kemudian oleh Ketua Majelis diparaf dan diberi tanda (P.2);

B. Keterangan Saksi :

1. SAKSI, umur 65 tahun, Agama Islam, Pekerjaan xxx xxxxx xxxxxx, tempat tinggal di KABUPATEN BANYUMAS, Menerangkan di bawah sumpah pada pokoknya :
   - Bahwa saksi kenal dengan Penggugat dan Tergugat karena saksi adalah keluarga Penggugat;
   - Bahwa setelah menikah Penggugat dan

Disclaimer
Kepaniteraan Mahkamah Agung Republik Indonesia berusaha untuk selalu mencantumkan informasi paling kini dan akurat sebagai bentuk komitmen Mahkamah Agung untuk pelayanan publik, transparansi dan akuntabilitas pelaksanaan fungsi peradilan. Namun dalam hal-hal tertentu masih dimungkinkan terjadi permasalahan teknis terkait dengan akurasi dan keterkinian informasi yang kami sajikan, hal mana akan terus kami perbaiki dari waktu kewaktu.
Dalam hal Anda menemukan inakurasi informasi yang termuat pada situs ini atau informasi yang seharusnya ada, namun belum tersedia, maka harap segera hubungi Kepaniteraan Mahkamah Agung RI melalui :
Email : kepaniteraan@mahkamahagung.go.id Telp : 021-384 3348 (ext.318)

tergugat tinggal bersama terakhir di rumah bersama dan telah mempunyai dua orang anak;

- Bahwa kurang lebih sejak tahun 2008 Tergugat pergi meninggalkan Penggugat yang pamitnya mau kerja, namun sampai sekarang tidak pernah kembali lagi;
- Bahwa sejak Tergugat pergi, Tergugat tidak pernah pulang dan tidak pernah mengirim nafkah kepada Penggugat dan anaknya;

2. SAKSI, umur 65 tahun, Agama Islam, Pekerjaan xxx xxxxx xxxxxx, tempat tinggal di KABUPATEN BANYUMAS, Menerangkan di bawah sumpah pada pokoknya :

- Bahwa saksi kenal dengan Penggugat dan Tergugat karena saksi adalah keluarga Penggugat;
- Bahwa setelah menikah Penggugat dan tergugat tinggal bersama terakhir di rumah bersama dan telah mempunyai dua orang anak;
- Bahwa kurang lebih sejak tahun 2008 Tergugat pergi meninggalkan Penggugat yang pamitnya mau kerja, namun sampai sekarang tidak pernah kembali lagi;
- Bahwa sejak Tergugat pergi, Tergugat tidak pernah pulang dan tidak pernah mengirim nafkah kepada Penggugat dan anaknya;

Bahwa keterangan saksi-saksi tersebut di atas dibenarkan oleh Penggugat, dan Penggugat menyatakan bukti-bukti yang diajukan sudah cukup;

Bahwa Penggugat mengajukan kesimpulan secara lisan pada pokoknya bahwa Penggugat telah dapat menguatkan dalil-dalil gugatannya dengan bukti-bukti tersebut di atas oleh karenanya Penggugat mohon kepada Majelis Hakim agar gugatan Penggugat dapat di kabulkan atau apabila Majelis hakim berpendapat lain mohon putusan yang seadil-adilnya;

Disclaimer
Kepaniteraan Mahkamah Agung Republik Indonesia berusaha untuk selalu mencantumkan informasi paling kini dan akurat sebagai bentuk komitmen Mahkamah Agung untuk pelayanan publik, transparansi dan akuntabilitas pelaksanaan fungsi peradilan. Namun dalam hal-hal tertentu masih dimungkinkan terjadi permasalahan teknis terkait dengan akurasi dan keterkinian informasi yang kami sajikan, hal mana akan terus kami perbaiki dari waktu kewaktu.
Dalam hal Anda menemukan inakurasi informasi yang termuat pada situs ini atau informasi yang seharusnya ada, namun belum tersedia, maka harap segera hubungi Kepaniteraan Mahkamah Agung RI melalui :
Email : kepaniteraan@mahkamahagung.go.id Telp : 021-384 3348 (ext.318)

Bahwa untuk singkatnya uraian putusan ini, maka semua hal yang termuat dalam Berita Acara Sidang ini merupakan bagian yang tak terpisahkan dari Putusan ini;

PERTIMBANGAN HUKUM

Menimbang, bahwa maksud dan tujuan gugatan Penggugat adalah sebagaimana terurai di atas;

Menimbang, bahwa Majelis Hakim telah berupaya mendamaikan pihak Penggugat dengan jalan menasehati agar tetap bersabar dan dapat rukun kembali dengan Tergugat sesuai Pasal 82 Undang-Undang Nomor 7 Tahun 1989 sebagaimana yang telah diubah terakhir dengan Undang Undang Nomor 50 Tahun 2009 tentang Peradilan Agama, akan tetapi tidak berhasil karena Penggugat tetap pada pendiriannya untuk bercerai dengan Tergugat;

Menimbang, bahwa ternyata Terrgugat meskipun dipanggil secara resmi dan patut, tidak datang menghadap di muka sidang dan pula tidak ternyata bahwa tidak datangnya itu disebabkan suatu halangan yang sah. Oleh karena itu, Tergugat harus dinyatakan tidak hadir dan gugatan tersebut harus diperiksa secara verstek. Sebagaimana ketentuan Pasal 125 ayat (1) HIR ;

Menimbang bahwa dengan tidak hadirnya Tergugat maka akan merugikan diri sendiri karena akan menghilangkan hak-hak hukumnya di persidangan, hal ini sejalan dengan *Dalil Syar'i* dalam *Kitab Ahkamul Qur'an* II : 405 yang berbunyi:

من دعي إلى حاكم من حكام المسلمين فلم يجب فهو ظالم لا حق له

Artinya : “ *Seseorang yang dipanggil Hakim (Pengadilan) tidak datang, ia dipandang sebagai dhalim dan tidak ada hak baginya.* “ *(Ahkamul Qur-an* II : 405) ;

Menimbang, bahwa adapun alasan pokok Penggugat mengajukan gugatan untuk bercerai dengan Tergugat adalah karena rumah tangga Penggugat dengan Tergugat sudah tidak harmonis lagi sejak tahun 2008. disebabkan karena Tergugat telah pergi tanpa pamit meninggalkan Penggugat dari tempat kediaman bersama yang mengakibatkan berpisah tempat tinggal sampai sekarang selama kurang lebih 12 tahun, dan selama itu sudah tidak

Disclaimer
Kepaniteraan Mahkamah Agung Republik Indonesia berusaha untuk selalu mencantumkan informasi paling kini dan akurat sebagai bentuk komitmen Mahkamah Agung untuk pelayanan publik, transparansi dan akuntabilitas pelaksanaan fungsi peradilan. Namun dalam hal-hal tertentu masih dimungkinkan terjadi permasalahan teknis terkait dengan akurasi dan keterkinian informasi yang kami sajikan, hal mana akan terus kami perbaiki dari waktu kewaktu.
Dalam hal Anda menemukan inakurasi informasi yang termuat pada situs ini atau informasi yang seharusnya ada, namun belum tersedia, maka harap segera hubungi Kepaniteraan Mahkamah Agung RI melalui :
Email : kepaniteraan@mahkamahagung.go.id Telp : 021-384 3348 (ext.318)

pernah kembali lagi serta sudah putus komunikasi. Penggugat sudah berusaha menjemput Tergugat namun tidak berhasil karena Tergugat tidak mau kembali lagi kepada Penggugat;

Menimbang, bahwa berdasarkan ketentuan Surat Edaran Mahkamah Agung RI Nomor 3 Tahun 2015 huruf C angka 3 yaitu putusan yang dijatuhkan tanpa hadirnya Tergugat dapat dikabulkan sepanjang berdasarkan hukum dan beralasan, oleh karena itu Majelis Hakim membebani Penggugat untuk membuktikan dalil-dalil gugatannya;

Menimbang, bahwa untuk membuktikan dalil-dalil gugatannya, Penggugat telah mengajukan alat bukti surat dan 2 (dua) orang saksi di persidangan;

Menimbang, bahwa bukti surat yang diberi tanda P.1, dan P.2, telah dilegalisir dan telah dicocokan dengan aslinya, dengan demikian bukti tersebut telah mempunyai kekuatan pembuktian sebagaimana maksud Pasal 1888 KUH Perdata, dan bukti tersebut, telah dimeteraikan (*dinazegelen*) di Kantor Pos sebagaimana diatur dalam Pasal 2 ayat (3) Undang-Undang Nomor 13 tahun 1985 tentang Bea Meterai, sehingga bukti-bukti tersebut secara formal dapat diterima sebagai alat bukti di persidangan;

Menimbang, bahwa berdasarkan bukti P.1 berupa bukti kependudukan atas nama Penggugat, telah nyata Penggugat bertempat tinggal di wilayah hukum Pengadilan Agama Purwokerto, oleh karena itu gugatan Penggugat telah diajukan sesuai ketentuan Pasal 73 Undang-Undang Nomor 7 tahun 1989 sebagaimana diubah kedua dengan Undang-Undang Nomor: 50 tahun 2009 tentang Peradilan Agama, maka gugatan Penggugat dapat diterima;

Menimbang, bahwa berdasarkan bukti P.2, maka harus dinyatakan telah terbukti menurut hukum bahwa Penggugat dan Tergugat telah dan masih terikat dalam perkawinan yang sah, maka Penggugat sebagai *persona standi in judicio* memiliki *legal standing* sehingga Penggugat dapat dinyatakan sebagai pihak yang mempunyai hak dan berkepentingan dalam perkara ini ;

Menimbang, bahwa 2 (dua) orang saksi Penggugat sudah dewasa dan sudah disumpah menurut agama yang dianutnya, sehingga memenuhi syarat *formil* alat bukti keterangan saksi sebagaimana diatur dalam Pasal 145 ayat 1

Disclaimer
Kepaniteraan Mahkamah Agung Republik Indonesia berusaha untuk selalu mencantumkan informasi paling kini dan akurat sebagai bentuk komitmen Mahkamah Agung untuk pelayanan publik, transparansi dan akuntabilitas pelaksanaan fungsi peradilan. Namun dalam hal-hal tertentu masih dimungkinkan terjadi permasalahan teknis terkait dengan akurasi dan keterkinian informasi yang kami sajikan, hal mana akan terus kami perbaiki dari waktu kewaktu.
Dalam hal Anda menemukan inakurasi informasi yang termuat pada situs ini atau informasi yang seharusnya ada, namun belum tersedia, maka harap segera hubungi Kepaniteraan Mahkamah Agung RI melalui :
Email : kepaniteraan@mahkamahagung.go.id Telp : 021-384 3348 (ext.318)

huruf c dan pasal 147 HIR;

Menimbang, bahwa keterangan 2 (dua) orang saksi Penggugat mengenai dalil-dalil gugatan Penggugat, adalah fakta yang dilihat, didengar dan dialami sendiri dan *relevan* dengan dalil yang harus dibuktikan oleh Penggugat, keterangannya saling bersesuaian antara saksi satu dengan yang lain dan saling ada kecocokkan, oleh karena itu keterangan saksi tersebut telah memenuhi syarat *materiil* sebagaimana telah diatur dalam Pasal 170,171 dan 172 HIR, sehingga keterangan saksi tersebut memiliki kekuatan pembuktian;

Menimbang, bahwa berdasarkan keterangan saksi-saksi tersebut di atas terbukti fakta kejadian sebagai berikut;

- Bahwa Penggugat dan Tergugat adalah suami isteri sah, menikah pada tanggal 05 Februari 2003 pernah hidup rukun dan sudah dikaruniai 2 orang orang anak;
- Bahwa antara Penggugat dengan Tergugat sudah tidak harmonis lagi sejak tahun 2008 karena Tergugat telah pergi meninggalkan Penggugat tanpa asalan dan tidak pamit kepada Penggugat selama kurang lebih 12 tahun;
- Bahwa selama Tergugat pergi meninggalkan Penggugat tidak ada komunikasi, tidak pernah kembali lagi ke rumah kediaman bersama dan telah membiarkan tanpa tanggung jawab;

Menimbang, bahwa berdasarkan fakta-fakta tersebut di atas dapat disimpulkan fakta hukum sebagai berikut :

- Bahwa rumahtangga Penggugat dengan Tergugat sudah tidak harmonis lagi semenjak tahun 2008 karena Tergugat telah pergi meninggalkan Penggugat tanpa tanggungjawab selama kurang lebih 12 tahun dan Penggugat sudah berupaya menjemput Tergugat agar kembali lagi namun tidak berhasil;
- Bahwa Tergugat telah menunjukkan keengganannya untuk untuk membina rumahtangga kembali dengan Penggugat;

Menimbang, bahwa berdasarkan fakta kejadian tersebut di atas maka Pengadilan menilai bahwa Penggugat dan Tergugat sudah tidak mampu lagi menunaikan kewajibannya sebagai suami isteri yang merupakan sendi dasar

Disclaimer
Kepaniteraan Mahkamah Agung Republik Indonesia berusaha untuk selalu mencantumkan informasi paling kini dan akurat sebagai bentuk komitmen Mahkamah Agung untuk pelayanan publik, transparansi dan akuntabilitas pelaksanaan fungsi peradilan. Namun dalam hal-hal tertentu masih dimungkinkan terjadi permasalahan teknis terkait dengan akurasi dan keterkinian informasi yang kami sajikan, hal mana akan terus kami perbaiki dari waktu kewaktu.
Dalam hal Anda menemukan inakurasi informasi yang termuat pada situs ini atau informasi yang seharusnya ada, namun belum tersedia, maka harap segera hubungi Kepaniteraan Mahkamah Agung RI melalui :
Email : kepaniteraan@mahkamahagung.go.id Telp : 021-384 3348 (ext.318)

dari perkawinan sebagaimana maksud Pasal 33 dan 34 Undang-Undang Nomor 1 tahun 1974 tentang Perkawinan jo. Pasal 77 Kompilasi Hukum Islam;

Menimbang, bahwa apabila sendi dasar perkawinan sudah tidak dapat ditegakkan lagi, maka tujuan perkawinan sebagaimana yang diatur dalam Pasal 1 Undang-Undang Nomor 1 tahun 1974 tentang Perkawinan jo. Pasal 3 Kompilasi Hukum Islam dan *Al-Qur'an* Surat *Ar-Rum* ayat (21) yakni untuk membentuk rumahtangga yang bahagia dan kekal serta mewujudkan keluarga yang *sakinah, mawaddah dan rohmah* sudah tidak mungkin diwujudkan dalam rumahtangga Penggugat dan Tergugat ;

Menimbang, bahwa berdasarkan fakta tersebut di atas maka Pengadilan berpendapat bahwa perkawinan Penggugat dengan Tergugat harus segera diakhiri dengan perceraian agar masing-masing pihak dapat menentukan jalan hidupnya sendiri tanpa harus mendapat tekanan dan ketidakpastian dari salah satu pihak, dan juga untuk menghindari timbulnya mudharat yang lebih besar bagi Penggugat, hal ini sejalan dengan kaidah *Ushul Fiqh* :

دَرْءُ الْمَفَاسِدِ مُقَدَّمٌ عَلَىٰ جَلْبِ الْمَصَالِحِ

Artinya : "Menolak mudharat (bahaya) lebih didahulukan dari mengambil manfaat"

Menimbang, bahwa brdasarkan pertimbangan tersebut di atas, maka dalil gugatan Penggugat telah berdasarkan hukum dan memenuhi alasan perceraian sebagaimana diatur dalam Pasal 39 ayat (2) Undang-Undang Nomor 1 Tahun 1974 tentang Perkawinan jo. Pasal 19 huruf (b) Peratutan Pemerintah No. 9 Tahun 1975 jo Pasal 116 huruf (b) Kompilasi Hukum Islam, oleh karenanya gugatan Penggugat untuk bercerai dengan Tergugat dapat dikabulkan, dengan menjatuhkan talak satu *bain sughra* Tergugat terhadap Penggugat;

Menimbang, bahwa oleh karena gugatan Penggugat beralasan tidak melawan hukum, maka putusan ini dijatuhkan dengan tanpa hadirnnya Tergugat (*Verstek*) dengan merujuk ketentuan pasal 125 HIR;

Menimbang, bahwa perkara ini termasuk bidang perkawinan, maka sesuai pasal 89 ayat (1) Undang-Undang Nomor 7 tahun 1989 yang diubah

Disclaimer
Kepaniteraan Mahkamah Agung Republik Indonesia berusaha untuk selalu mencantumkan informasi paling kini dan akurat sebagai bentuk komitmen Mahkamah Agung untuk pelayanan publik, transparansi dan akuntabilitas pelaksanaan fungsi peradilan. Namun dalam hal-hal tertentu masih dimungkinkan terjadi permasalahan teknis terkait dengan akurasi dan keterkinian informasi yang kami sajikan, hal mana akan terus kami perbaiki dari waktu kewaktu.
Dalam hal Anda menemukan inakurasi informasi yang termuat pada situs ini atau informasi yang seharusnya ada, namun belum tersedia, maka harap segera hubungi Kepaniteraan Mahkamah Agung RI melalui :
Email : kepaniteraan@mahkamahagung.go.id Telp : 021-384 3348 (ext.318)

kedua dengan Undang-Undang Nomor 50 tahun 2009 tentang Peradilan Agama, maka biaya perkara dibebankan kepada Penggugat yang besarnya sebagaimana dalam amar Putusan ini ;

Mengingat, segala ketentuan perundang-undangan yang berlaku dan *dalil syar'i* yang berkaitan dengan perkara ini ;

MENGADILI :

1. Menyatakan Tergugat yang telah dipanggil secara resmi dan patut untuk menghadap ke persidangan, tidak hadir;
2. Mengabulkan gugatan Penggugat dengan verstek;
3. Menjatuhkan talak satu ba'in sughra Tergugat (TERGUGAT) kepada Penggugat (PENGGUGAT);
4. Membebankan kepada Penggugat untuk membayar biaya perkara yang hingga kini sejumlah Rp. 406.000,00 (empat ratus enam ribu).

Demikian Putusan ini dijatuhkan dalam Rapat Permusyawaratan Majelis Hakim Pengadilan agama Purwokerto pada hari Selasa tanggal 22 Desember 2020 *Masehi,* bertepatan dengan tanggal 7 Jumadil Ula 1442 *Hijriyah.* Oleh kami Drs. FUAD AMIN, M. Si. sebagai Ketua Majelis, dan Drs. H. RISNO serta Drs. H. JUHRI, M.H. masing-masing sebagai Hakim Anggota, putusan tersebut diucapkan dalam sidang terbuka untuk umum pada hari itu juga oleh Ketua Majelis tersebut dengan didampingi oleh Hakim-hakim Anggota dan dibantu oleh Hj. MASLAHAH, S.H. sebagai Panitera Pengganti serta dihadiri oleh Penggugat tanpa hadirnya Tergugat;

Ketua Majelis,

Drs. FUAD AMIN, M. Si.

Disclaimer
Kepaniteraan Mahkamah Agung Republik Indonesia berusaha untuk selalu mencantumkan informasi paling kini dan akurat sebagai bentuk komitmen Mahkamah Agung untuk pelayanan publik, transparansi dan akuntabilitas pelaksanaan fungsi peradilan. Namun dalam hal-hal tertentu masih dimungkinkan terjadi permasalahan teknis terkait dengan akurasi dan keterkinian informasi yang kami sajikan, hal mana akan terus kami perbaiki dari waktu kewaktu.
Dalam hal Anda menemukan inakurasi informasi yang termuat pada situs ini atau informasi yang seharusnya ada, namun belum tersedia, maka harap segera hubungi Kepaniteraan Mahkamah Agung RI melalui :
Email : kepaniteraan@mahkamahagung.go.id   Telp : 021-384 3348 (ext.318)

Hakim Anggota, Hakim Anggota,

Drs. H. RISNO Drs. H. JUHRI, M.H.

Panitera Pengganti,

Hj. MASLAHAH, S.H.

Perincian Biaya :

1.	Biaya Pendaftaran	:	Rp	30.000,00
2.	Biaya Proses	:	Rp	75.000,00
3.	Biaya Pemanggilan	:	Rp	265.000,00
4.	Biaya PNBP	:	Rp	20.000,00
5.	Biaya Redaksi	:	Rp	10.000.00
6.	Biaya Materai	:	Rp	6.000,00
		Jumlah:	Rp	406.000,00

(empat ratus enam ribu).

Disclaimer
Kepaniteraan Mahkamah Agung Republik Indonesia berusaha untuk selalu mencantumkan informasi paling kini dan akurat sebagai bentuk komitmen Mahkamah Agung untuk pelayanan publik, transparansi dan akuntabilitas pelaksanaan fungsi peradilan. Namun dalam hal-hal tertentu masih dimungkinkan terjadi permasalahan teknis terkait dengan akurasi dan keterkinian informasi yang kami sajikan, hal mana akan terus kami perbaiki dari waktu kewaktu.
Dalam hal Anda menemukan inakurasi informasi yang termuat pada situs ini atau informasi yang seharusnya ada, namun belum tersedia, maka harap segera hubungi Kepaniteraan Mahkamah Agung RI melalui :
Email : kepaniteraan@mahkamahagung.go.id Telp : 021-384 3348 (ext.318)
