# Supplementary material for: Classification of divorce causes during the COVID-19 pandemic using convolutional neural networks
Source: PeerJ Comput Sci. 2022 Jun 30;8:e998. doi: 10.7717/peerj-cs.998 (PMC9299239; doi:10.7717/peerj-cs.998)
Supplement: Supplemental Information 6 [file peerj-cs-08-998-s006.zip › Rujuk Dataset/Data ke-13.pdf]

# PUTUSAN

**Nomor 3154/Pdt.G/2020/PA.Lmj**

بسم الله الرحمن الرحيم

**DEMI KEADILAN BERDASARKAN KETUHANAN YANG MAHA ESA**

Pengadilan Agama Lumajang yang memeriksa dan mengadili perkara tertentu pada tingkat pertama dalam sidang majelis telah menjatuhkan putusan perkara *cerai talak* antara:

**PEMOHON**, umur 40 tahun, agama Islam, pendidikan SD, pekerjaan xxxxx, tempat kediaman di KABUPATEN LUMAJANG, sebagai Pemohon;

**melawan**

**TERMOHON**, umur 42 tahun, agama Islam, pendidikan SD, pekerjaan xxx xxxxx xxxxxx, tempat kediaman di KABUPATEN LUMAJANG, sebagai Termohon;

Pengadilan Agama tersebut diatas;

Telah membaca dan mempelajari berkas perkara;

Telah mendengar keteranganPemohondan para saksi di muka sidang;

## DUDUK PERKARA

Menimbang, bahwa Pemohon dalam surat permohonannya tanggal 08 Desember 2020 telah mengajukan permohonan Cerai Talak, yang telah didaftar di Kepaniteraan Pengadilan Agama Lumajang, dengan Nomor 3154/Pdt.G/2020/PA.Lmj , tanggal 08 Desember 2020, dengan dalil-dalil sebagai berikut :

1. Bahwa pada tanggal 28 Maret 2012, Pemohon dengan Termohon melangsungkan pernikahan yang dicatat oleh Pegawai Pencatat Nikah Kantor Urusan Agama Kecamatan Kedungjajang Kabupaten Lumajang (Kutipan Akta Nikah Nomor : 98/58/III/2012 tanggal 28 Maret 2012);
2. Bahwa setelah pernikahan tersebut Pemohon dengan Termohon bertempat tinggal di rumah Termohon hingga Desember 2019. Selama pernikahan tersebut Pemohon dengan Termohon telah hidup rukun sebagaimana layaknya suami istri dan dikaruniai 1 orang anak bernama Ika Budihartini, umur 8 tahun (ikut Pemohon);



Mahkamah Agung Republik Indonesia

*Disclaimer*
*Kepaniteraan Mahkamah Agung Republik Indonesia berusaha untuk selalu mencantumkan informasi paling kini dan akurat sebagai bentuk komitmen Mahkamah Agung untuk pelayanan publik, transparansi dan akuntabilitas pelaksanaan fungsi peradilan. Namun dalam hal-hal tertentu masih dimungkinkan terjadi permasalahan teknis terkait dengan akurasi dan keterkinian informasi yang kami sajikan, hal mana akan terus kami perbaiki dari waktu kewaktu.*
*Dalam hal Anda menemukan inakurasi informasi yang termuat pada situs ini atau informasi yang seharusnya ada, namun belum tersedia, maka harap segera hubungi Kepaniteraan Mahkamah Agung RI melalui :*
*Email : kepaniteraan@mahkamahagung.go.id Telp : 021-384 3348 (ext.318)*

3. Bahwa semula rumah tangga Pemohon dan Termohon rukun baik akan tetapi kurang lebih sejak bulan Desember tahun 2018 ketentraman rumah tangga Pemohon dengan Termohon mulai goyah, setelah antara Pemohon dengan Termohon terus menerus terjadi perselisihan dan pertengkaran yang penyebabnya antara lain :
   - Termohon sering menuntut uang belanja kepada Pemohon melampaui kemampuan Pemohon bahkan Termohon sering ngelamak atau membantah nasehat Pemohon;
4. Bahwa puncak perselisihan dan pertengkaran antara Pemohon dengan Termohon tersebut terjadi kurang lebih pada bulan Desember tahun 2019, yang akibatnya Pemohon pergi meninggalkan Termohon dan pulang ke rumah orangtua Pemohon sendiri dengan alamat sebagaimana tersebut di atas sehingga pisah tempat tinggal selama 1 tahun hingga sekarang dan selama itu tidak ada hubungan lagi baik lahir maupun batin;
5. Bahwa hal tersebut diatas Pemohon sudah tidak tahan lagi dengan rumah tangganya hingga kemudian mengajukan Permohonan ini;
6. Bahwa Pemohon sanggup membayar seluruh biaya yang timbul akibat perkara ini;

Berdasarkan alasan/dalil-dalil diatas, Pemohon mohon agar Ketua Pengadilan Agama Lumajang segera memeriksa dan mengadili perkara ini, selanjutnya menjatuhkan putusan yang amarnya berbunyi:

Primair :

1. Mengabulkan permohonan Pemohon;
2. Memberi ijin kepada Pemohon untuk mengucapkan talak terhadap Termohon dihadapan sidang Pengadilan Agama Lumajang;
3. Menetapkan biaya perkara sesuai dengan peraturan yang berlaku;

Subsidair :

Atau menjatuhkan putusan lain yang seadil-adilnya;

Bahwa pada hari dan tanggal persidangan yang telah ditetapkan Pemohon hadir sendiri menghadap, sedangkan Termohon tidak hadir dan tidak menyuruh orang lain untuk hadir sebagai kuasanya, meskipun menurut surat



*Disclaimer*
*Kepaniteraan Mahkamah Agung Republik Indonesia berusaha untuk selalu mencantumkan informasi paling kini dan akurat sebagai bentuk komitmen Mahkamah Agung untuk pelayanan publik, transparansi dan akuntabilitas pelaksanaan fungsi peradilan. Namun dalam hal-hal tertentu masih dimungkinkan terjadi permasalahan teknis terkait dengan akurasi dan keterkinian informasi yang kami sajikan, hal mana akan terus kami perbaiki dari waktu kewaktu.*
*Dalam hal Anda menemukan inakurasi informasi yang termuat pada situs ini atau informasi yang seharusnya ada, namun belum tersedia, maka harap segera hubungi Kepaniteraan Mahkamah Agung RI melalui :*
*Email : kepaniteraan@mahkamahagung.go.id Telp : 021-384 3348 (ext.318)*

panggilan (Relaas) tanggal 11 Desember 2020 dan 23 Desember 2020 Nomor 3154/Pdt.G/2020/PA.Lmj yang dibacakan di persidangan, Termohon telah dipanggil dengan resmi dan patut, sedangkan tidak ternyata bahwa ketidakhadirannya itu disebabkan oleh sesuatu halangan yang sah. Oleh karena itu, pemeriksaan perkara ini dilanjutkan dengan tanpa kehadiran Termohon;

Bahwa majelis telah menasihati Pemohon agar rukun lagi dengan Termohon akan tetapi tidak berhasil;

Bahwa perkara ini tidak dapat dimediasi karena Termohon tidak pernah datang menghadap meskipun telah dipanggil secara resmi dan patut, selanjutnya dimulai pemeriksaan dengan membacakan surat permohonan Pemohon yang maksud dan isinya tetap dipertahankan oleh Pemohon;

Bahwa, untuk meneguhkan dalil-dalil permohonannya di muka persidangan Pemohon telah mengajukan bukti-bukti berupa :

**A. Bukti Tertulis**

1. Fotokopi Kartu Tanda Penduduk atas Nama PEMOHON NIK 3508181012790006 tanggal 11 Juli 2018 dengan materai cukup dan sesuai dengan aslinya diberi tanda (P.1);
2. Fotokopi Kutipan Akta Nikah Nomor 98/58/III/2012 tanggal 28 Maret 2012 yang dikeluarkan oleh Kantor Urusan Agama Kecamatan Kantor Urusan Agama Kecamatan Kedungjajang Kabupaten Lumajang, dengan materai cukup dan sesuai dengan aslinya diberi tanda (P.2);

**B. Saksi-Saksi**

1. **SAKSI 1**, umur 26 tahun, agama Islam, pendidikan SD, pekerjaan xxxxxxxxxx, bertempat tinggal di KABUPATEN LUMAJANG, di bawah sumpah memberikan keterangan yang pada pokoknya sebagai berikut :
   - Bahwa saksi kenal dengan Pemohon dan Termohon, karena saksi saudara sepupu Pemohon;
   - Bahwa saksi tahu semula Pemohon dan Termohon suami istri yang rukun dan telah dikaruniai 1 orang anak;
   - Bahwa saat ini Pemohondan Termohon sudah pisah rumah selama ± 1 tahun dan selama itu tidak pernah ada hubungan lagi;



*Disclaimer*
*Kepaniteraan Mahkamah Agung Republik Indonesia berusaha untuk selalu mencantumkan informasi paling kini dan akurat sebagai bentuk komitmen Mahkamah Agung untuk pelayanan publik, transparansi dan akuntabilitas pelaksanaan fungsi peradilan. Namun dalam hal-hal tertentu masih dimungkinkan terjadi permasalahan teknis terkait dengan akurasi dan keterkinian informasi yang kami sajikan, hal mana akan terus kami perbaiki dari waktu kewaktu.*
*Dalam hal Anda menemukan inakurasi informasi yang termuat pada situs ini atau informasi yang seharusnya ada, namun belum tersedia, maka harap segera hubungi Kepaniteraan Mahkamah Agung RI melalui :*
*Email : kepaniteraan@mahkamahagung.go.id Telp : 021-384 3348 (ext.318)*

- Bahwa saksi tahu sebelum berpisah Pemohon dan Termohon terlihat sering bertengkar tetapi saksi tidak tahu penyebabnya;
- Bahwa saksi mendapat pengaduan dari Pemohon bahwa Termohon kurang menerima dengan nafkah belanja yang diberikan oleh Pemohon;
- Bahwa saksi tahu semua karena saksi dulu sering ke rumah Pemohon;
- Bahwa keluarga telah mengupayakan agarPemohonrukun dengan Termohon akan tetapi tidak berhasil;

2. **SAKSI 2**, umur 48 tahun, agama Islam, pendidikan SMA, pekerjaan xxxxxxxx, bertempat tinggal di KABUPATEN LUMAJANG, di bawah sumpah memberikan keterangan yang pada pokoknya sebagai berikut :
   - Bahwa saksi kenal dengan Pemohon dan Termohon, karena saksi saudara sepupu Pemohon;
   - Bahwa saksi tahu semula Pemohon dan Termohon suami istri yang rukun dan telah dikaruniai 1 orang anak;
   - Bahwa saat ini Pemohondan Termohon sudah pisah rumah selama ± 1 tahun dan selama itu tidak pernah ada hubungan lagi;
   - Bahwa saksi tahu sebelum berpisah Pemohon dan Termohon terlihat sering bertengkar tetapi saksi tidak tahu penyebabnya;
   - Bahwa saksi mendapat pengaduan dari Pemohon bahwa Termohon kurang menerima dengan nafkah belanja yang diberikan oleh Pemohon;
   - Bahwa saksi tahu semua itu karena saksi dulu sering ke rumah Pemohon;
   - Bahwa keluarga telah mengupayakan agar Pemohon rukun dengan Termohon akan tetapi tidak berhasil;

Bahwa Pemohon tidak menyampaikan sesuatu apapun lagi dan mohon putusan;

Bahwa hal-ihwal mengenai jalannya sidang telah termuat dalam berita acara sidang perkara ini dan untuk menyingkat uraian Majelis cukup menunjuk berita acara tersebut sebagai bagian tak terpisahkan dari putusan ini;



*Disclaimer*
*Kepaniteraan Mahkamah Agung Republik Indonesia berusaha untuk selalu mencantumkan informasi paling kini dan akurat sebagai bentuk komitmen Mahkamah Agung untuk pelayanan publik, transparansi dan akuntabilitas pelaksanaan fungsi peradilan. Namun dalam hal-hal tertentu masih dimungkinkan terjadi permasalahan teknis terkait dengan akurasi dan keterkinian informasi yang kami sajikan, hal mana akan terus kami perbaiki dari waktu kewaktu.*
*Dalam hal Anda menemukan inakurasi informasi yang termuat pada situs ini atau informasi yang seharusnya ada, namun belum tersedia, maka harap segera hubungi Kepaniteraan Mahkamah Agung RI melalui :*
*Email : kepaniteraan@mahkamahagung.go.id Telp : 021-384 3348 (ext.318)*

**PERTIMBANGAN HUKUM**

Menimbang, bahwa maksud dan tujuan permohonan Pemohon adalah sebagaimana tersebut di atas;

Menimbang, bahwa Majelis telah menasihati Pemohon agar kembali rukun dengan Termohon akan tetapi tidak berhasil;

Menimbang, bahwa sebelum mempertimbangkan dalil pokok permohon Pemohon oleh karena masalah ini masalah perceraian, maka harus dibuktikan apakah Pemohon dan Termohon telah terikat oleh perkawinan yang sah;

Menimbang, bahwa tentang keabsahan perkawinan tersebut, Pemohon mengajukan bukti P.1 berupa Fotokopi Kartu Tanda Penduduk dan bukti P.2 berupa Fotokopi Kutipan akta nikah. Oleh karena telah sesuai aslinya, dinazagel, maka formil dapat diterima sebagai alat bukti. Berdasarkan bukti P.2 dan dikuatkan dengan bukti P.1, menurut Majelis telah terbukti bahwa Pemohon dan Termohon telah terikat oleh perkawinan yang sah, sehingga Pemohon dan Termohon mempunyai kedudukan hukum sebagaimana maksud Pasal 66 UU Ayat (1) Undang-Undang Nomor 7 Tahun 1989 sebagaimana telah diubah dengan UU Nomor 3 Tahun 2006 dan perubahan kedua dengan UU Nomor 50 Tahun 2009;

Menimbang, bahwa yang dijadikan dalil pokok permohonan Pemohon untuk bercerai adalah adanya perselisihan dan pertengkaran terus menerus antara Pemohon dan Termohon yang disebabkan oleh sikap Termohon kurang menerima dengan nafkah belanja yang diberikan oleh Pemohon;

Menimbang, bahwa dalil-dalil yang dijadikan alasan Pemohon tersebut menurut majelis mengacu kepada alasan perceraian sebagaimana maksud ketentuan Pasal 19 huruf (f) Peraturan Pemerintah Nomor 9 Tahun 1975 jo. Pasal 116 huruf (f) Kompilasi Hukum Islam;

Menimbang, bahwa Termohon tidak pernah hadir atau menyuruh orang lain sebagai wakilnya untuk menghadap di persidangan, meskipun telah dipanggil secara resmi dan patut, lagi pula ketidakhadirannya tersebut tidak disebabkan oleh suatu halangan yang sah. Oleh karena itu, harus dinyatakan bahwa Termohon telah dipanggil secara resmi dan patut untuk menghadap di persidangan tidak hadir dan sesuai Pasal 125 HIR Majelis dapat menjatuhkan



*Disclaimer*
*Kepaniteraan Mahkamah Agung Republik Indonesia berusaha untuk selalu mencantumkan informasi paling kini dan akurat sebagai bentuk komitmen Mahkamah Agung untuk pelayanan publik, transparansi dan akuntabilitas pelaksanaan fungsi peradilan. Namun dalam hal-hal tertentu masih dimungkinkan terjadi permasalahan teknis terkait dengan akurasi dan keterkinian informasi yang kami sajikan, hal mana akan terus kami perbaiki dari waktu kewaktu.*
*Dalam hal Anda menemukan inakurasi informasi yang termuat pada situs ini atau informasi yang seharusnya ada, namun belum tersedia, maka harap segera hubungi Kepaniteraan Mahkamah Agung RI melalui :*
*Email : kepaniteraan@mahkamahagung.go.id    Telp : 021-384 3348 (ext.318)*

putusan verstek atau tanpa kehadiran Termohon. Hal tersebut sesuai pula dengan kaidah dalam Kitab *Al Anwar* Juz II halaman 149 yang berbunyi sebagai berikut:

وان تعذر احضاره لتواريه وتعززه جاز سماع الدعوى والبينة والحكم عليه

*Artinya: Apabila (Termohon) berhalangan hadir karena bersembunyi atau enggan, maka Hakim boleh mendengar gugatan dan memeriksa bukti-bukti, serta memutus gugatan tersebut";*

Menimbang, bahwa meskipun persidangan perkara ini tanpa dihadiri Termohon, namun karena perkara ini menyangkut bidang perkawinan, maka sesuai dengan salah satu asas perkawinan sebagaimana terdapat dalam penjelasan Undang-Undang Nomor 1 Tahun 1974 sebagaimana diubah dengan Undang-undang Nomor 16 Tahun 2019 angka 4 huruf (e) yaitu prinsip untuk mempersukar terjadinya perceraian dan untuk menghindari kebohongan–kebohongan besar dalam hal perceraian, maka Majelis Hakim berpendapat bahwa dalam perceraian tetap diwajibkan kepada Pemohon untuk membuktikan dalil-dalil permohonannya;

Menimbang, bahwa sepanjang mengenai adanya pereselisihan dan pertengkaran, untuk membuktikan dalil-dalil pokok permohonan, Pemohon telah mengajukan 2 orang saksi;

Menimbang, bahwa kedua saksi Pemohon tersebut, sudah dewasa dan sudah disumpah, sehingga memenuhi syarat formal sebagaimana diatur dalam Pasal 145 ayat 1 angka 3e HIR jo jo Pasal 76 UU Nomor 1 Tahun 1989 yang telah diubah dengan UU nomor 3 Tahun 2006 dan perubahan kedua dengan UU Nomor 50 Tahun 2009

Menimbang, bahwa keterangan kedua saksi Pemohon mengenai dalil-dalil pemohon adalah fakta yang dilihat sendiri dan relevan dengan dalil yang harus dibuktikan oleh Pemohon, oleh karena itu keterangan saksi tersebut telah memenuhi syarat materiil sebagaimana telah diatur dalam Pasal 171 HIR, sehingga keterangan saksi tersebut memiliki kekuatan pembuktian dan dapat diterima sebagai alat bukti;



*Disclaimer*
*Kepaniteraan Mahkamah Agung Republik Indonesia berusaha untuk selalu mencantumkan informasi paling kini dan akurat sebagai bentuk komitmen Mahkamah Agung untuk pelayanan publik, transparansi dan akuntabilitas pelaksanaan fungsi peradilan. Namun dalam hal-hal tertentu masih dimungkinkan terjadi permasalahan teknis terkait dengan akurasi dan keterkinian informasi yang kami sajikan, hal mana akan terus kami perbaiki dari waktu kewaktu.*
*Dalam hal Anda menemukan inakurasi informasi yang termuat pada situs ini atau informasi yang seharusnya ada, namun belum tersedia, maka harap segera hubungi Kepaniteraan Mahkamah Agung RI melalui :*
*Email : kepaniteraan@mahkamahagung.go.id Telp : 021-384 3348 (ext.318)*

Menimbang, bahwa berdasarkan dalil-dalil permohonan Pemohon dihubungkan dengan keterangan kedua saksi tersebut diperoleh fakta-fakta, antara lain :

- Bahwa Pemohon dan Termohon semula adalah suami istri yang rukun dan telah dikaruniai 1 oranga anak;
- Bahwa saat ini Pemohon dan Termohon sudah berpisah tempat tinggal lebih kurang ± 1 tahun karena perselisihan dan pertengkaran;
- Bahwa sekalipun saksi 1 dan saksi 2 tidak tahu secara langsung penyebab perselisihan dan pertengkaran, akan tetapi faktanya Pemohon tetap ingin bercerai dari Termohon;
- Bahwa keluarga sudah mengupayakan agar Pemohon mau rukun lagi dengan Termohon, akan tetapi tidak berhasil;

Menimbang, bahwa berdasarkan fakta-fakta tersebut, Majelis berkesimpulan bahwa terlepas siapa yang menjadi pemicunya telah terbukti terjadi perselisihan dan pertengkaran terus-menerus antara Pemohon dan Termohon yang sulit untuk dirukunkan lagi;

Menimbang, bahwa dengan melihat kondisi rumah tangga Pemohon dan Termohon yang demikian kiranya perlu diajukan pertanyaan mendasar : masih perlukah ikatan perkawinan Pemohon dan Termohon tersebut dipertahankan?

Menimbang, bahwa untuk menjawab pertanyaan tersebut, perlu ditelusuri makna dan hakikat perkawinan, baik menurut peraturan perundang-undangan yang berlaku maupun menurut Syari'at Islam;

Menimbang, bahwa menurut ketentuan Pasal 1 Undang-undang Nomor 1 Tahun 1974 sebagaimana diubah dengan Undang-undang Nomor 16 Tahun 2019 perkawinan ialah ikatan lahir batin antara seorang pria dengan seorang wanita sebagai suami istri dengan tujuan membentuk keluarga (rumah tangga ) yang bahagia dan kekal berdasarkan Ketuhanan Yang Maha Esa. Sedangkan, Pasal 2 Kompilasi Hukum Islam memberi ketentuan, bahwa perkawinan menurut Hukum Islam adalah pernikahan, yaitu akad yang sangat kuat atau *mitsaqan ghalidhan* untuk mentaati perintah Allah dan melaksanakannya adalah ibadah yang menurut syari'at Islam sebagaimana tertuang dalam ketentuan



*Disclaimer*
*Kepaniteraan Mahkamah Agung Republik Indonesia berusaha untuk selalu mencantumkan informasi paling kini dan akurat sebagai bentuk komitmen Mahkamah Agung untuk pelayanan publik, transparansi dan akuntabilitas pelaksanaan fungsi peradilan. Namun dalam hal-hal tertentu masih dimungkinkan terjadi permasalahan teknis terkait dengan akurasi dan keterkinian informasi yang kami sajikan, hal mana akan terus kami perbaiki dari waktu kewaktu.*
*Dalam hal Anda menemukan inakurasi informasi yang termuat pada situs ini atau informasi yang seharusnya ada, namun belum tersedia, maka harap segera hubungi Kepaniteraan Mahkamah Agung RI melalui :*
*Email : kepaniteraan@mahkamahagung.go.id Telp : 021-384 3348 (ext.318)*

Pasal 3 Kompilasi Hukum Islam bertujuan mewujudkan rumah tangga yang *sakinah, mawaddah*, dan *rahmah;*

Menimbang, bahwa dalam suatu perkawinan di samping ikatan lahir terdapat unsur ikatan batin, dan ikatan batin ini merupakan unsur yang paling esensi dalam suatu perkawinan. Unsur itulah yang melandasi unsur-unsur yang lain. Apabila, unsur ikatan batin ini sudah tidak ada lagi, maka pada hakikatnya perkawinan itu sebenarnya juga sudah tidak ada lagi. Sebab, unsur lain seperti terbentuknya rumah tangga yang bahagia berdasarkan ketuhanan Yang Maha Esa, tidak mungkin akan terwujud jika ikatan batin antara suami istri tidak ada. Ketidakbolehan adanya paksaan bagi masing-masing yang akan melaksanakan pernikahan adalah juga membuktikan pentingnya keberadaan unsur ikatan batin ini;

Menimbang, bahwa sekalipun ikatan lahir batin tersebut pernah terjalin, dalam perkembangan berikutnya karena alasan tertentu bisa saja ikatan batin tersebut terlepas lagi;

Menimbang, bahwa oleh karena dalam kasus ini Pemohon telah terbukti menunjukkan sikap tidak mau lagi kembali hidup rukun dengan Termohon dengan alasan apapun,menurut Majelis, telah cukup membuktikan bahwa ikatan batin yang ada pada perkawinan Pemohon dan Termohon sebagai suami istri telah lepas, sehingga sekalipun masih berstatus sebagai suami istri akan tetapi perkawinan tersebut juga telah ternyata tidak memenuhi kriteria perkawinan sebagaimana ketentuan Pasal 1 Undang-undang Nomor 1 Tahun 1974 sebagaimana diubah dengan Undang-undang Nomor 16 Tahun 2019 jo ketentuan Pasal 2 Kompilasi Hukum Islam di atas;

Menimbang, bahwa situasi kehidupan perkawinan seperti itu menurut pendapat Majelis Hakim sudah tidak mungkin diharapkan akan mencapai tujuan perkawinan yaitu terbentuknya rumah tangga yang bahagia atau terbentuknya rumah tangga yang *sakinah, mawaddah* dan *rahmah* seperti ketentuan Syari'at Islam sebagaimana tertuang dalam ketentuan Pasal 3 Kompilasi Hukum Islam, sehingga *terlepas dari saiapa yang salah dalam kasus ini*, perkawinan yang seperti itu menurut Majelis Hakim sudah tidak perlu lagi dipertahankan. Menurut Majelis, kondisi perkawinan Pemohon dan Termohon



*Disclaimer*
*Kepaniteraan Mahkamah Agung Republik Indonesia berusaha untuk selalu mencantumkan informasi paling kini dan akurat sebagai bentuk komitmen Mahkamah Agung untuk pelayanan publik, transparansi dan akuntabilitas pelaksanaan fungsi peradilan. Namun dalam hal-hal tertentu masih dimungkinkan terjadi permasalahan teknis terkait dengan akurasi dan keterkinian informasi yang kami sajikan, hal mana akan terus kami perbaiki dari waktu kewaktu.*
*Dalam hal Anda menemukan inakurasi informasi yang termuat pada situs ini atau informasi yang seharusnya ada, namun belum tersedia, maka harap segera hubungi Kepaniteraan Mahkamah Agung RI melalui :*
*Email : kepaniteraan@mahkamahagung.go.id    Telp : 021-384 3348 (ext.318)*

yang demikian, apabila dipertahankan justru dapat mengakibatkan penderitaan dan kesengsaraan yang berkepanjangan, bagi Pemohon atau Termohon bahkan kedua-duanya sekaligus, sehingga pada akhirnya perkawinan yang demikian juga tidak sejalan dengan tujuan perkawinan yang diamanatkan oleh syari'at Islam dan undang-undang;

Menimbang, bahwa dengan demikian Permohonan Pemohon telah sesuai dengan pasal 39 ayat (2) Undang-Undang Nomor 1 tahun 1974 Jo pasal 19 huruf (b) Peraturan Pemerintah Nomor 9 tahun 1975 jo pasal 116 huruf (b) Kompilasi Hukum Islam, maka permohonan Pemohon telah beralasan hukum dan sesuai dengan Firman Allah dalam Al-Qur'an Surat Al-Baqarah ayat 227 yang berbunyi:

وَإِنْ عَزَمُوا۟ ٱلطَّلَٰقَ فَإِنَّ ٱللَّهَ سَمِيعٌ عَلِيمٌ

*Artinya : "Dan jika mereka telah berazam (berketetapan hati) untuk talak, maka sesungguhnya Allah Maha Mendengar lagi Maha Mengetahui";*

Menimbang, bahwa berdasarkan seluruh pertimbangan-pertimbangan tersebut di atas Pemohon telah dapat menguatkan dalil permohonannya dan telah pula memenuhi alasan perceraian sebagaimana dimaksud Pasal 39 Undang-undang No. 1 tahun 1974 sebagaimana diubah dengan Undang-undang Nomor 16 Tahun 2019 jo. Pasal 19 huruf (f) Peraturan Pemerintah Nomor 9 tahun 1975 jo. Pasal 116 huruf (f) Kompilasi Hukum Islam, oleh karenanya permohonan Pemohon untuk bercerai patut dikabulkan dengan memberi izin kepada Pemohon untuk menjatuhkan talak satu raj'i terhadap Termohon;

Menimbang, bahwa permohonan Pemohon termasuk dalam bidang perkawinan, maka sesuai ketentuan Pasal 89 ayat (1) Undang-undang Nomor 7 Tahun 1989 tentang Peradilan Agama sebagaimana telah diubah dengan Undang Undang Nomor 3 tahun 2006 dan perubahan kedua dengan Undang Undang Nomor 50 tahun 2009 semua biaya yang timbul dalam perkara ini dibebankan kepada Pemohon;

Mengingat segala peraturan perundang-undangan yang berlaku dan dalil-dalil syar'i yang berkaitan dengan perkara ini ;

MENGADILI



*Disclaimer*
*Kepaniteraan Mahkamah Agung Republik Indonesia berusaha untuk selalu mencantumkan informasi paling kini dan akurat sebagai bentuk komitmen Mahkamah Agung untuk pelayanan publik, transparansi dan akuntabilitas pelaksanaan fungsi peradilan. Namun dalam hal-hal tertentu masih dimungkinkan terjadi permasalahan teknis terkait dengan akurasi dan keterkinian informasi yang kami sajikan, hal mana akan terus kami perbaiki dari waktu kewaktu.*
*Dalam hal Anda menemukan inakurasi informasi yang termuat pada situs ini atau informasi yang seharusnya ada, namun belum tersedia, maka harap segera hubungi Kepaniteraan Mahkamah Agung RI melalui :*
*Email : kepaniteraan@mahkamahagung.go.id Telp : 021-384 3348 (ext.318)*

1. Menyatakan Termohon telah dipanggil dengan patut untuk menghadap di persidangan, tidak hadir;
2. Mengabulkan permohonan Pemohon dengan verstek;
3. Memberi izin kepada Pemohon (**PEMOHON**) untuk menjatuhkan talak satu raj'i terhadap Termohon (**TERMOHON**) di depan sidang Pengadilan Agama Lumajang;
4. Membebankan kepada Pemohon untuk membayar biaya perkara sejumlah Rp 571.000,00 (lima ratus tujuh puluh satu ribu rupiah);

Demikian putusan ini dijatuhkan dalam rapat permusyawaratan Majelis yang dilangsungkan pada hari Selasa tanggal 29 Desember 2020 *Miladiyah* bertepatan dengan tanggal 14 Jumadil Ula 1442 *Hijriyah*, oleh kami Dr. H. M. ZAINURI, Drs, S.H., M.H. sebagai Hakim Ketua, Drs. H. KOMSUN, S.H., M.HES. dan Drs. H. MUSTHOFA ALIE, M.H masing-masing sebagai Hakim Anggota, pada hari iitu juga diucapkan dalam sidang terbuka untuk umum oleh Hakim Ketua tersebut dengan didampingi oleh Hakim-Hakim Anggota tersebut yang dibantu oleh H. SOFAN AFFANDI, S.H, M.H sebagai Panitera Pengganti yang dihadiri oleh Pemohon tanpa kehadiran Termohon;

| Hakim Anggota | Hakim Ketua, |
| --- | --- |
| **Drs. H. KOMSUN, S.H., M.HES.** | **Dr. H. M. ZAINURI, Drs, S.H., M.H.** |
| Hakim Anggota | |



*Disclaimer*
*Kepaniteraan Mahkamah Agung Republik Indonesia berusaha untuk selalu mencantumkan informasi paling kini dan akurat sebagai bentuk komitmen Mahkamah Agung untuk pelayanan publik, transparansi dan akuntabilitas pelaksanaan fungsi peradilan. Namun dalam hal-hal tertentu masih dimungkinkan terjadi permasalahan teknis terkait dengan akurasi dan keterkinian informasi yang kami sajikan, hal mana akan terus kami perbaiki dari waktu kewaktu.*
*Dalam hal Anda menemukan inakurasi informasi yang termuat pada situs ini atau informasi yang seharusnya ada, namun belum tersedia, maka harap segera hubungi Kepaniteraan Mahkamah Agung RI melalui :*
*Email : kepaniteraan@mahkamahagung.go.id Telp : 021-384 3348 (ext.318)*

**Drs. H. MUSTHOFA ALIE, M.H**

Panitera Pengganti,

**H. SOFAN AFFANDI, S.H, M.H**

Perincian biaya :

| | |
|---|---|
| - Pendaftaran | : Rp. 30.000,00 |
| - ATK Perkara | : Rp. 75.000,00 |
| - Panggilan | : Rp. 430.000,00 |
| - Biaya PNBP Pihak | : Rp. 20.000,00 |
| - Redaksi | : Rp. 10.000,00 |
| - Meterei | : Rp. 6.000,00 |
| J u m l a h | : Rp. 571.000,00 |

(Lima ratus tujuh puluh satu ribu rupiah).



*Disclaimer*
*Kepaniteraan Mahkamah Agung Republik Indonesia berusaha untuk selalu mencantumkan informasi paling kini dan akurat sebagai bentuk komitmen Mahkamah Agung untuk pelayanan publik, transparansi dan akuntabilitas pelaksanaan fungsi peradilan. Namun dalam hal-hal tertentu masih dimungkinkan terjadi permasalahan teknis terkait dengan akurasi dan keterkinian informasi yang kami sajikan, hal mana akan terus kami perbaiki dari waktu kewaktu.*
*Dalam hal Anda menemukan inakurasi informasi yang termuat pada situs ini atau informasi yang seharusnya ada, namun belum tersedia, maka harap segera hubungi Kepaniteraan Mahkamah Agung RI melalui :*
*Email : kepaniteraan@mahkamahagung.go.id Telp : 021-384 3348 (ext.318)*
