# Supplementary material for: Classification of divorce causes during the COVID-19 pandemic using convolutional neural networks
Source: PeerJ Comput Sci. 2022 Jun 30;8:e998. doi: 10.7717/peerj-cs.998 (PMC9299239; doi:10.7717/peerj-cs.998)
Supplement: Supplemental Information 6 [file peerj-cs-08-998-s006.zip › Rujuk Dataset/Data ke-16.pdf]

PUTUSAN

Nomor 6191/Pdt.G/2020/PA.Jr

بِسْمِ اللَّهِ الرَّحْمَٰنِ الرَّحِيمِ

DEMI KEADILAN BERDASARKAN KETUHANAN YANG MAHA ESA

Pengadilan Agama Jember yang memeriksa dan mengadili perkara-perkara tertentu dalam tingkat pertama telah menjatuhkan putusan dalam perkara permohonan cerai talak antara:

PEMOHON, umur 28 tahun, agama Islam, pendidikan SLTP, pekerjaan Buruh Tani, tempat tinggal di Kabupaten Jember, dalam hal ini memberikan kuasa kepada Hamidatul Umami, S.H, pekerjaan Advokat, berkantor di Yayasan IKADIN Jember (LKBH IKADIN Jember) Jalan Mataram Kav. A-29 Kelurahan Mangli Kecamatan Kaliwates Kabupaten Jember yang terdaftar di Kepaniteraan Pengadilan Agama Jember Nomor: 4851/Kuasa/XII/2020/PA.Jr tanggal 16 Desember 2020 sebagai Pemohon;

melawan

TERMOHON, umur 25 tahun, agama Islam, pendidikan Sd, pekerjaan Mengurus Rumah Tangga, tempat tinggal di Kabupaten Jember, sebagai Termohon;

Pengadilan Agama tersebut;

Telah mempelajari surat-surat yang berkaitan dengan perkara ini;

Telah mendengar keterangan Pemohon dan kuasanya serta para saksi dimuka sidang;

DUDUK PERKARA

Bahwa Pemohon dalam surat permohonannya tanggal 16 Desember 2020 yang telah didaftar di Kepaniteraan Pengadilan Agama Jember Nomor 6191/Pdt.G/2020/PA.Jr tanggal 16 Desember 2020, dengan dalil-dalil sebagai berikut:

1. Bahwa Pemohon dan Termohon telah menikah pada tanggal 28 Oktober 2018, yang dicatatkan pada Kantor Urusan Agama Kecamatan Ambulu Kabupaten Jember dengan Akta Nikah Nomor 0776/77/X/2018 tanggal 29 Oktober 2018 dengan status Pemohon jejaka sedang Termohon perawan;

Disclaimer
Kepaniteraan Mahkamah Agung Republik Indonesia berusaha untuk selalu mencantumkan informasi paling kini dan akurat sebagai bentuk komitmen Mahkamah Agung untuk pelayanan publik, transparansi dan akuntabilitas pelaksanaan fungsi peradilan. Namun dalam hal-hal tertentu masih dimungkinkan terjadi permasalahan teknis terkait dengan akurasi dan keterkinian informasi yang kami sajikan, hal mana akan terus kami perbaiki dari waktu kewaktu.
Dalam hal Anda menemukan inakurasi informasi yang termuat pada situs ini atau informasi yang seharusnya ada, namun belum tersedia, maka harap segera hubungi Kepaniteraan Mahkamah Agung RI melalui :
Email : kepaniteraan@mahkamahagung.go.id Telp : 021-384 3348 (ext.318)

2. Bahwa setelah pernikahan itu antara Pemohon dan Termohon hidup bersama sebagai suami istri selama ± 1 1/2 tahun telah berhubungan layaknya suami istri (ba'dad dukhul) dan terakhir mengambil tempat kediaman di rumah orangtua Pemohon sudah mempunyai 1 orang anak bernama ANAK I, umur 11 bulan;
3. Bahwa pada mulanya rumah tangga Pemohon dan Termohon berjalan dengan baik, akan tetapi sejak ± Mei 2020 yang lalu, rumah tangga Pemohon dan Termohon mulai goyah, sering terjadi perselisihan dan pertengkaran yang disebabkan karena masalah tempat tinggal dimana Pemohon tidak betah tinggal dirumah Termohon dan begitupula sebaliknya Termohon juga tidak betah tinggal dirumah Pemohon. Hal itulah yang menjadikan rumah tangga Pemohon dan Termohon menjadi tidak harmonis;
4. Bahwa perselisihan dan pertengkaran antara Pemohon dan Termohon tersebut makin lama makin memuncak, akhirnya Termohon pergi meninggalkan tempat kediaman bersama sejak ± Juli 2020 dan sekarang berada dirumah orangtua Termohon sendiri;
5. Bahwa sejak itu antara Pemohon dan Termohon telah hidup berpisah yang hingga sekarang sudah ± 5 bulan dan selama hidup berpisah tersebut sudah tidak ada hubungan lagi layaknya suami istri;
6. Bahwa atas sikap dan perbuatan Termohon yang demikian itu, Pemohon mengalami penderitaan lahir dan bathin yang berkepanjangan;
7. Bahwa Pemohon sudah berusaha memulihkan ketentraman rumah tangga dengan Termohon akan tetapi tidak berhasil, akhirnya Pemohon sudah tidak sanggup lagi membina rumah tangga dengan Termohon;
8. Bahwa Pemohon sanggup membayar biaya perkara;
9. Bahwa atas dasar alasan-alasan sebagaimana tersebut diatas, Pemohon mohon kepada Pengadilan Agama Jember agar berkenan memeriksa perkara ini dan menjatuhkan putusan sebagai berikut :

Primair :

1. Mengabulkan permohonan Pemohon;
2. Memberikan ijin kepada Pemohon untuk mengucapkan ikrar talak kepada Termohon;

Disclaimer
Kepaniteraan Mahkamah Agung Republik Indonesia berusaha untuk selalu mencantumkan informasi paling kini dan akurat sebagai bentuk komitmen Mahkamah Agung untuk pelayanan publik, transparansi dan akuntabilitas pelaksanaan fungsi peradilan. Namun dalam hal-hal tertentu masih dimungkinkan terjadi permasalahan teknis terkait dengan akurasi dan keterkinian informasi yang kami sajikan, hal mana akan terus kami perbaiki dari waktu kewaktu.
Dalam hal Anda menemukan inakurasi informasi yang termuat pada situs ini atau informasi yang seharusnya ada, namun belum tersedia, maka harap segera hubungi Kepaniteraan Mahkamah Agung RI melalui :
Email : kepaniteraan@mahkamahagung.go.id   Telp : 021-384 3348 (ext.318)

3. Membebankan biaya perkara ini sesuai ketentuan hukum yang berlaku;

Subsidair :

Atau mohon putusan yang seadil-adilnya :

Bahwa pada hari dan tanggal persidangan yang telah ditetapkan Pemohon telah hadir sendiri, sedangkan Termohon telah tidak datang menghadap dan tidak menyuruh orang lain untuk menghadap sebagai wakil/kuasanya, meskipun telah dipanggil secara patut;

Bahwa Majelis Hakim telah berusaha menasehati Pemohon agar mau rukun kembali dengan Termohon, akan tetapi tidak berhasil. Kemudian dibacakanlah permohonan Pemohon, yang ternyata isinya tetap dipertahankan oleh Pemohon;

Bahwa untuk menguatkan dalil permohonannya, Pemohon telah mengajukan alat bukti tertulis berupa :

1. Fotokopi Surat Keterangan Domisili an. Pemohon yang dikeluarkan oleh Kantor Desa Curahnongko Kecamatan Tempurejo Kabupaten Jember tanggal 01 Desember 2020 (P.1);
2. Fotokopi Kutipan Akta Nikah Nomor 0776/77/X/2018 yang dikeluarkan oleh Kepala Kantor Urusan Agama Kecamatan Ambulu Kabupaten Jember tanggal 29 Oktober 2018 (P.2);

Bahwa Pemohon telah mengajukan saksi saksi dipersidangan yaitu :

1. SAKSI I, umur 51 tahun, agama Islam, pekerjaan swasta, bertempat tinggal di Kabupaten Jember;
   yang memberikan keterangan dibawah sumpah yang pada pokoknya sebagai berikut:
   - Bahwa Saksi tahu dan kenal dengan para pihak karena saksi paman Pemohon;
   - Bahwa Saksi tahu Pemohon dan Termohon adalah sebagai suami istri dan setelah menikah keduanya tinggal di rumah orangtua Pemohon sudah mempunyai 1 orang anakdan bernama ANAK I, umur 11 bulan;
   - Bahwa Saksi tahu bahwa bahwa semula rumah tangga Pemohon dan Termohon rukun baik, namun kemudian terjadi pertengkaran diantara Pemohon dan Termohon karena masalah tempat tinggal dimana Pemohon

Disclaimer
Kepaniteraan Mahkamah Agung Republik Indonesia berusaha untuk selalu mencantumkan informasi paling kini dan akurat sebagai bentuk komitmen Mahkamah Agung untuk pelayanan publik, transparansi dan akuntabilitas pelaksanaan fungsi peradilan. Namun dalam hal-hal tertentu masih dimungkinkan terjadi permasalahan teknis terkait dengan akurasi dan keterkinian informasi yang kami sajikan, hal mana akan terus kami perbaiki dari waktu kewaktu.
Dalam hal Anda menemukan inakurasi informasi yang termuat pada situs ini atau informasi yang seharusnya ada, namun belum tersedia, maka harap segera hubungi Kepaniteraan Mahkamah Agung RI melalui :
Email : kepaniteraan@mahkamahagung.go.id   Telp : 021-384 3348 (ext.318)

tidak betah tinggal dirumah Termohon dan begitupula sebaliknya Termohon juga tidak betah tinggal dirumah Pemohon. Hal itulah yang menjadikan rumah tangga Pemohon dan Termohon menjadi tidak harmonis;

- Bahwa sekarang Pemohon dan Termohon hidup berpisah rumah selama ± 5 bulan;
- Bahwa selama pisah itu saksi tidak pernah tahu keduanya rukun lagi layaknya suami istri;
- Bahwa Saksi sudah berusaha menasehati kedua belah pihak untuk rukun lagi membina rumah tangga akan tetapi tidak berhasil, Pemohon tetap pada pendiriannya untuk bercerai dengan Termohon;

2. SAKSI II, umur 80 tahun, agama Islam, pekerjaan wiraswasta, bertempat tinggal di Kabupaten Jember;

   Saksi memberikan keterangan dibawah sumpahnya dan pada pokoknya sebagai berikut:

   - Bahwa Saksi tahu dan kenal dengan para pihak karena saksi tetangga Pemohon;
   - Bahwa Saksi tahu Pemohon dan Termohon adalah sebagai suami istri dan setelah menikah keduanya tinggal di rumah orangtua Pemohon sudah mempunyai 1 orang anak bernama ANAK I, umur 11 bulan;
   - Bahwa kini rumah tangga Pemohon dan Termohon sudah tidak harmonis lagi, saksi tahu tidak harmonis dikarenakan keduanya sering bertengkar disebabkan masalah tempat tinggal dimana Pemohon tidak betah tinggal dirumah Termohon dan begitupula sebaliknya Termohon juga tidak betah tinggal dirumah Pemohon. Hal itulah yang menjadikan rumah tangga Pemohon dan Termohon menjadi tidak harmonis;
   - Bahwa sekarang Pemohon dan Termohon hidup berpisah rumah selama ± 5 bulan;
   - Bahwa selama pisah itu saksi tidak pernah tahu keduanya rukun lagi layaknya suami istri;
   - Bahwa Saksi sudah berusaha menasehati keduanya untuk rukun lagi akan tetapi tidak berhasil, Pemohon tetap ingin bercerai dari Termohon;

Disclaimer
Kepaniteraan Mahkamah Agung Republik Indonesia berusaha untuk selalu mencantumkan informasi paling kini dan akurat sebagai bentuk komitmen Mahkamah Agung untuk pelayanan publik, transparansi dan akuntabilitas pelaksanaan fungsi peradilan. Namun dalam hal-hal tertentu masih dimungkinkan terjadi permasalahan teknis terkait dengan akurasi dan keterkinian informasi yang kami sajikan, hal mana akan terus kami perbaiki dari waktu kewaktu.
Dalam hal Anda menemukan inakurasi informasi yang termuat pada situs ini atau informasi yang seharusnya ada, namun belum tersedia, maka harap segera hubungi Kepaniteraan Mahkamah Agung RI melalui :
Email : kepaniteraan@mahkamahagung.go.id Telp : 021-384 3348 (ext.318)

Bahwa sehubungan dengan keterangan saksi tersebut Pemohon menyatakan telah cukup dan membenarkannya;

Bahwa akhirnya Pemohon menyatakan telah cukup dengan keterangan dan bukti-buktinya dan mohon putusan;

Bahwa untuk mempersingkat uraian putusan ini ditunjuk kepada hal hal sebagaimana tercantum dalam Berita Acara Sidang perkara ini;

PERTIMBANGAN HUKUM

Menimbang bahwa maksud dan tujuan permohonan Pemohon adalah seperti diuraikan tersebut di atas;

Menimbang bahwa berdasarkan bukti P.1 telah ternyata Pemohon bertempat kediaman di wilayah Kabupaten Jember, dengan demikian berdasarkan pasal 66 ayat (2) Undang-undang Nomor 7 tahun 1989 yang telah diubah dengan Undang-undang Nomor 3 tahun 2006, terakhir diubah dengan Undang-undang Nomor 50 tahun 2009 Pengadilan Agama Jember berwenang untuk memeriksa dan mengadili perkara aquo;

Menimbang bahwa karena ternyata Termohon meskipun telah dipanggil secara patut, tidak menghadap, pula tidak ternyata bahwa ketidak hadirannya itu disebabkan oleh suatu halangan yang sah, maka Termohon harus dinyatakan tidak hadir;

Menimbang, bahwa Majelis Hakim telah berusaha menasehati Pemohon agar mau rukun kembali dengan Termohon akan tetapi tidak berhasil;

Menimbang, bahwa berdasarkan bukti P.2, telah terbukti bahwa Pemohon dan Termohon adalah suami isteri yang sah;

Menimbang, bahwa permohonan Pemohon didasarkan pada dalil yang pada pokoknya adalah bahwa setelah ± 1 1/2 tahun hidup bersama rumah tangga di antara Pemohon dan Termohon telah terjadi perselisihan dan pertengkaran disebabkan karena masalah tempat tinggal dimana Pemohon tidak betah tinggal dirumah Termohon dan begitupula sebaliknya Termohon juga tidak betah tinggal dirumah Pemohon. Hal itulah yang menjadikan rumah tangga Pemohon dan Termohon menjadi tidak harmonis;

Disclaimer
Kepaniteraan Mahkamah Agung Republik Indonesia berusaha untuk selalu mencantumkan informasi paling kini dan akurat sebagai bentuk komitmen Mahkamah Agung untuk pelayanan publik, transparansi dan akuntabilitas pelaksanaan fungsi peradilan. Namun dalam hal-hal tertentu masih dimungkinkan terjadi permasalahan teknis terkait dengan akurasi dan keterkinian informasi yang kami sajikan, hal mana akan terus kami perbaiki dari waktu kewaktu.
Dalam hal Anda menemukan inakurasi informasi yang termuat pada situs ini atau informasi yang seharusnya ada, namun belum tersedia, maka harap segera hubungi Kepaniteraan Mahkamah Agung RI melalui :
Email : kepaniteraan@mahkamahagung.go.id Telp : 021-384 3348 (ext.318)

Menimbang bahwa sikap Termohon yang telah tidak hadir dipersidangan dapat dipandang bahwa ia tidak hendak membantah dalil dalil permohonan Pemohon, dengan demikian dalil permohonan Pemohon telah menjadi fakta yang tetap;

Menimbang bahwa Pemohon telah mengajukan dua orang saksi, dan dari keterangan para saksi tersebut yang bersesuaian satu sama lain telah terungkap fakta yang pada pokoknya menguatkan dalil permohonan Pemohon;

Menimbang bahwa sejak terjadinya perselisihan dan pertengkaran tersebut sampai dengan tahap akhir persidangan, selama ± 5 bulan telah ternyata Pemohon dan Termohon sudah tidak berhubungan lagi sebagai suami isteri/hidup berpisah, halmana membuktikan bahwa perselisihan dan pertengkaran Pemohon dan Termohon telah berlangsung terus menerus;

Menimbang bahwa Majelis Hakim dan pihak keluarga telah tidak berhasil menasehati Pemohon dengan Termohon, pula telah ternyata Pemohon menyatakan tetap pada permohonannya, dengan demikian hal tersebut telah menunjukkan bahwa di antara Pemohon dan Termohon sudah tidak ada harapan akan hidup rukun dalam rumah tangga;

Menimbang bahwa disyariatkannya pernikahan sebagai *mitsaqon gholidhon* mempunyai tujuan yang suci dan mulia, yakni untuk menciptakan rumah tangga yang *sakinah, mawaddah dan rahmah*, sebagaimana dimaksud dalam Al Qur'an surat Ar Rum ayat 21 dan pasal 1 Undang-Undang Nomor 1 tahun 1974 Jo. pasal 3 Kompilasi Hukum Islam, namun dengan keadaan rumah tangga Pemohon dan Termohon tersebut, maka tujuan pernikahan menjadi tidak bisa dicapai;

Menimbang bahwa fakta-fakta tersebut diatas telah menunjukkan bahwa perkawinan Pemohon dan Termohon benar-benar telah pecah dan telah sampai pada taraf yang sudah tidak bisa didamaikan lagi, karena sudah tidak adanya kehendak dari para pihak untuk melanjutkan perkawinannya, dengan demikian penyelesaian yang dipandang adil dan bermanfaat bagi kedua belah pihak adalah perceraian;

Menimbang bahwa pada dasarnya menurut ajaran Islam perceraian merupakan perbuatan tercela, namun begitu dalam keadaan suami isteri sudah

Disclaimer
Kepaniteraan Mahkamah Agung Republik Indonesia berusaha untuk selalu mencantumkan informasi paling kini dan akurat sebagai bentuk komitmen Mahkamah Agung untuk pelayanan publik, transparansi dan akuntabilitas pelaksanaan fungsi peradilan. Namun dalam hal-hal tertentu masih dimungkinkan terjadi permasalahan teknis terkait dengan akurasi dan keterkinian informasi yang kami sajikan, hal mana akan terus kami perbaiki dari waktu kewaktu.
Dalam hal Anda menemukan inakurasi informasi yang termuat pada situs ini atau informasi yang seharusnya ada, namun belum tersedia, maka harap segera hubungi Kepaniteraan Mahkamah Agung RI melalui :
Email : kepaniteraan@mahkamahagung.go.id   Telp : 021-384 3348 (ext.318)

tidak saling mencintai lagi dan yang terjadi hanya sikap permusuhan dan saling membenci sebagaimana yang dialami oleh Pemohon dan Termohon tersebut, maka perceraian diperbolehkan karena untuk menghindari kemelut dan mudarat yang berkepanjangan dalam rumah tangga;

Menimbang bahwa sesuai dengan firman Allah dalam surat Albaqarah ayat 227 :

وان عزموا الطلاق فان الله سميع عليم

Artinya : "Dan jika kamu berazam (berketetapan hati) untuk talak, maka sesungguhnya Allah Maha Mendengar lagi Maha Mengetahui".

dan dalam Kitab Al-Anwar Juz II halaman 55 :

فاءن تعزز بتعزز او توار او غيبة جاز اثباته بالبينة

Artinya: *"Apabila ia (Termohon) enggan untuk hadir atau bersembunyi atau tidak diketahui tempat kediamannya, maka perkaranya boleh diputuskan dengan berdasarkan pada pembuktian".*

Menimbang, bahwa berdasarkan pertimbangan tersebut di atas, permohonan Pemohon telah mempunyai cukup alasan dan telah terbukti serta memenuhi pasal 39 ayat (2) Undang-undang Nomor 1 tahun 1974 jo. Pasal 19 huruf (f) Peraturan Pemerintah Nomor 9 Tahun 1975 Jo. Pasal 116 huruf (f) Kompilasi Hukum Islam, maka berdasarkan Pasal 125 HIR dapat dikabulkan dengan Verstek;

Menimbang, bahwa putusnya ikatan perkawinan dalam perkara cerai gugat terjadi setelah putusan tersebut mempunyai kekuatan hukum tetap, sedangkan dalam perkara cerai talak putusnya ikatan perkawinan terjadi setelah pengucapan ikrar talak;

Menimbang, bahwa berdasarkan Pasal 89 ayat (1) Undang-undang Nomor 7 Tahun 1989 yang telah diubah dengan Undang-undang Nomor 3 Tahun 2006 dan perubahan kedua dengan Undang-undang Nomor 50 tahun 2009, maka biaya perkara ini dibebankan kepada Pemohon;

Memperhatikan, segala ketentuan peraturan perUndang-undangan yang berlaku dan hukum syara' yang berkaitan dengan perkara ini;

Disclaimer
Kepaniteraan Mahkamah Agung Republik Indonesia berusaha untuk selalu mencantumkan informasi paling kini dan akurat sebagai bentuk komitmen Mahkamah Agung untuk pelayanan publik, transparansi dan akuntabilitas pelaksanaan fungsi peradilan. Namun dalam hal-hal tertentu masih dimungkinkan terjadi permasalahan teknis terkait dengan akurasi dan keterkinian informasi yang kami sajikan, hal mana akan terus kami perbaiki dari waktu kewaktu.
Dalam hal Anda menemukan inakurasi informasi yang termuat pada situs ini atau informasi yang seharusnya ada, namun belum tersedia, maka harap segera hubungi Kepaniteraan Mahkamah Agung RI melalui :
Email : kepaniteraan@mahkamahagung.go.id Telp : 021-384 3348 (ext.318)

MENGADILI

1. Menyatakan Termohon telah dipanggil secara resmi dan patut untuk menghadap ke persidangan, tidak hadir;
2. Mengabulkan permohonan Pemohon dengan verstek;
3. Memberi izin kepada Pemohon (PEMOHON) untuk menjatuhkan talak satu raj'i terhadap Termohon (TERMOHON) di depan sidang Pengadilan Agama Jember;
4. Membebankan kepada Pemohon untuk membayar biaya perkara ini sejumlah Rp. 391000.- (tiga ratus sembilan puluh satu ribu rupiah);

Demikian diputuskan dalam permusyawaratan Majelis yang dilangsungkan pada hari Selasa tanggal 29 Desember 2020 M bertepatan dengan tanggal 15 Jumadilawal 1442 H, oleh kami Drs. Suhaili, S.H., M.H. sebagai Ketua Majelis, Drs. H. Umar Jaya, S.H., M.H. dan Drs. H. Ijmak, S.H., M.H. masing-masing sebagai Hakim Anggota, putusan dibacakan dalam sidang terbuka untuk umum dengan didampingi oleh Sulaiman, S.H. sebagai Panitera Pengganti Pengadilan Agama tersebut dan dihadiri oleh Pemohon dan kuasanya tanpa hadirnya Termohon;

Hakim Anggota	Ketua Majelis
ttd	ttd
Drs. H. Umar Jaya, S.H., M.H.	Drs. Suhaili, S.H., M.H.
Hakim Anggota	
ttd	
Drs. H. Ijmak, S.H., M.H.	
	Panitera Pengganti,
	ttd
	Sulaiman, S.H.

Disclaimer
Kepaniteraan Mahkamah Agung Republik Indonesia berusaha untuk selalu mencantumkan informasi paling kini dan akurat sebagai bentuk komitmen Mahkamah Agung untuk pelayanan publik, transparansi dan akuntabilitas pelaksanaan fungsi peradilan. Namun dalam hal-hal tertentu masih dimungkinkan terjadi permasalahan teknis terkait dengan akurasi dan keterkinian informasi yang kami sajikan, hal mana akan terus kami perbaiki dari waktu kewaktu.
Dalam hal Anda menemukan inakurasi informasi yang termuat pada situs ini atau informasi yang seharusnya ada, namun belum tersedia, maka harap segera hubungi Kepaniteraan Mahkamah Agung RI melalui :
Email : kepaniteraan@mahkamahagung.go.id Telp : 021-384 3348 (ext.318)

Perincian Biaya Perkara :

1.	Pendaftaran	: Rp.	30.000,00
2.	Biaya Proses	: Rp.	50.000,00
3.	Biaya Panggilan	: Rp.	275.000,00
4.	Biaya PNBP	: Rp.	20.000,00
5.	Redaksi	: Rp.	10.000,00
6.	Materai	: Rp.	6.000,00
	Jumlah	Rp.	391.000,00

(tiga ratus sembilan puluh satu ribu rupiah)

Untuk salinan yang sama bunyinya,
Oleh,
Pengadilan Agama Jember
Panitera

Akhmad Muzaeri, S.H.

Disclaimer
Kepaniteraan Mahkamah Agung Republik Indonesia berusaha untuk selalu mencantumkan informasi paling kini dan akurat sebagai bentuk komitmen Mahkamah Agung untuk pelayanan publik, transparansi dan akuntabilitas pelaksanaan fungsi peradilan. Namun dalam hal-hal tertentu masih dimungkinkan terjadi permasalahan teknis terkait dengan akurasi dan keterkinian informasi yang kami sajikan, hal mana akan terus kami perbaiki dari waktu kewaktu.
Dalam hal Anda menemukan inakurasi informasi yang termuat pada situs ini atau informasi yang seharusnya ada, namun belum tersedia, maka harap segera hubungi Kepaniteraan Mahkamah Agung RI melalui :
Email : kepaniteraan@mahkamahagung.go.id   Telp : 021-384 3348 (ext.318)
