# Supplementary material for: Classification of divorce causes during the COVID-19 pandemic using convolutional neural networks
Source: PeerJ Comput Sci. 2022 Jun 30;8:e998. doi: 10.7717/peerj-cs.998 (PMC9299239; doi:10.7717/peerj-cs.998)
Supplement: Supplemental Information 6 [file peerj-cs-08-998-s006.zip › Rujuk Dataset/Data ke-17.pdf]

P U T U S A N

Nomor 507/Pdt.G/2020/PTA.Sby

BISMILLAHIRRAHMANIRRAHIIM

DEMI KEADILAN BERDASARKAN KETUHANAN YANG MAHA ESA

Pengadilan Tinggi Agama Surabaya dalam tingkat banding telah memeriksa, mengadili dan menjatuhkan putusan dalam sidang Majelis Hakim terhadap perkara gugatan perceraian yang diajukan oleh:

PEMBANDING, umur 35 tahun, agama Islam, pendidikan SMP, pekerjaan SWASTA, bertempat tinggal di KABUPATEN GRESIK, dalam hal ini memberi kuasa kepada **Subaidi, S.H.**, Advokat pada kantor hukum " SUBAIDI SA'ID & PARTNERS ", yang beralamat di Gedung Islamic Center Lantai III No 15-16 Pamekasan, berdasarkan Surat Kuasa Khusus tanggal 11 Juli 2020 yang terdaftar di Kepaniteraan Pengadilan Agama Pamekasan dengan Nomor : 641/S.kuasa/07/ 2020 tanggal 15 Juli 2020, semula sebagai Tergugat sekarang sebagai **Pembanding;**

TERBANDING, umur 29 tahun, agama Islam, pendidikan SD, pekerjaan SWASTA, bertempat tinggal di KABUPATEN PAMEKASAN, semula sebagai Penggugat sekarang sebagai **Terbanding;**

Pengadilan Tinggi Agama tersebut;

Telah mempelajari berkas perkara dan semua surat yang berkaitan dengan perkara ini;

DUDUK PERKARA

Mengutip segala uraian sebagaimana termuat dalam putusan Pengadilan Agama Pamekasan Nomor 0765/Pdt.G/2020/PA.Pmk. tanggal 4 Nopember 2020 Masehi berteapatan dengan tanggal 18 Rabiul Awal 1442 Hijriyah, yang amarnya berbunyi sebagai berikut:

1. Mengabulkan gugatan Penggugat;

Disclaimer
Kepaniteraan Mahkamah Agung Republik Indonesia berusaha untuk selalu mencantumkan informasi paling kini dan akurat sebagai bentuk komitmen Mahkamah Agung untuk pelayanan publik, transparansi dan akuntabilitas pelaksanaan fungsi peradilan. Namun dalam hal-hal tertentu masih dimungkinkan terjadi permasalahan teknis terkait dengan akurasi dan keterkinian informasi yang kami sajikan, hal mana akan terus kami perbaiki dari waktu kewaktu.
Dalam hal Anda menemukan inakurasi informasi yang termuat pada situs ini atau informasi yang seharusnya ada, namun belum tersedia, maka harap segera hubungi Kepaniteraan Mahkamah Agung RI melalui :
Email : kepaniteraan@mahkamahagung.go.id Telp : 021-384 3348 (ext.318)

2. Menjatuhkan talak satu ba'in shughra Tergugat (PEMBANDING) terhadap Penggugat (TERBANDING) ;
3. Membebankan kepada Penggugat untuk membayar biaya perkara sebesar Rp. 560.000,- (Lima ratus enam puluh ribu rupiah);

Bahwa pada saat putusan tersebut diucapkan dalam sidang yang dinyatakan terbuka untuk umum pada tanggal 4 Nopember 2020, dihadiri oleh pihak Tergugat yang sekarang sebagai Pembanding dan pihak Penggugat yang sekarang sebagai Terbanding;

Bahwa atas putusan Pengadilan Agama Pamekasan tersebut, Pembanding mengajukan permohonan banding, sesuai Akta Permohonan Banding Nomor 0765/Pdt.G/2020/PA.Pmk. tanggal 9 Nopember 2020 yang dibuat dan ditandatangani oleh Panitera Pengadilan Agama Pamekasan, dan permohonan banding tersebut telah diberitahukan kepada Terbanding, sesuai Relaas Pemberitahuan Pernyataan Banding Nomor 0765/Pdt.G/2020/PA.Pmk. tanggal 16 Nopember 2020;

Bahwa Pembanding telah mengajukan memori banding tertanggal 9 Nopember 2020, hal tersebut sesuai dengan Tanda Terima Memori Banding Nomor 0765/Pdt.G/2020/PA.Pmk. tanggal 9 Nopember 2020 yang dibuat dan ditandatangani oleh Panitera yang substansi pokoknya memohon agar Pengadilan Tinggi Agama Surabaya membatalkan putusan Pengadilan Agama Pamekasan Nomor 0765/Pdt.G/2020/PA.Pmk tanggal 4 Nopember 2020;

Bahwa memori banding tersebut ternyata telah diberitahukan kepada Terbanding, hal tersebut sesuai Relaas Pemberitahuan dan Penyerahan Memori Banding Nomor 0765/Pdt.G/2020/PA.Pmk. tanggal 16 Nopember 2020 yang dilakukan oleh Jurusita Pengganti Pengadilan Agama Pamekasan;

Bahwa atas memori banding tersebut Terbanding telah mengajukan kontra memori banding tertanggal 27 Nopember 2020, hal tersebut sesuai Tanda Terima Kontra Memori Banding Nomor 0765/Pdt.G/2020/PA.Pmk. tanggal 30 Nopember 2020 yang dibuat dan ditandatangani oleh Panitera Pengadilan Agama Pamekasan yang substansi pokoknya memohon agar

Disclaimer
Kepaniteraan Mahkamah Agung Republik Indonesia berusaha untuk selalu mencantumkan informasi paling kini dan akurat sebagai bentuk komitmen Mahkamah Agung untuk pelayanan publik, transparansi dan akuntabilitas pelaksanaan fungsi peradilan. Namun dalam hal-hal tertentu masih dimungkinkan terjadi permasalahan teknis terkait dengan akurasi dan keterkinian informasi yang kami sajikan, hal mana akan terus kami perbaiki dari waktu kewaktu.
Dalam hal Anda menemukan inakurasi informasi yang termuat pada situs ini atau informasi yang seharusnya ada, namun belum tersedia, maka harap segera hubungi Kepaniteraan Mahkamah Agung RI melalui :
Email : kepaniteraan@mahkamahagung.go.id Telp : 021-384 3348 (ext.318)

Pengadilan Tinggi Agama Surabaya menguatkan putusan Pengadilan Agama Pamekasan Nomor 0765/Pdt.G/2020/PA.Pmk. tanggal 4 Nopember 2020;

Bahwa Pembanding telah diberitahukan untuk memeriksa berkas banding (*inzage*) pada tanggal 25 Nopember 2020, namun ternyata Pembanding tidak memeriksa berkas banding (*inzage*), hal tersebut sesuai dengan Surat Keterangan Tidak Memeriksa Perkara (*inzage*) Nomor 0765/Pdt.G/2020/PA.Pmk. tanggal 10 Desember 2020 yang dibuat dan ditandatngani oleh Panitera Pengadilan Agama Pamekasan;

Bahwa Terbanding telah diberitahukan untuk memeriksa berkas banding (*inzage*) pada tanggal 30 Nopember 2020, namun ternyata Terbanding tidak memeriksa berkas banding (*inzage*), hal tersebut sesuai dengan Surat Keterangan Tidak Memeriksa Perkara (*inzage*) Nomor 0765/Pdt.G/2020/PA.Pmk. tanggal 10 Desember 2020 yang dibuat dan ditandatangani oleh Panitera Pengadilan Agama Pamekasan;

Bahwa permohonan banding tersebut telah didaftar di Kepaniteraan Pengadilan Tinggi Agama Surabaya pada tanggal 14 Desember 2020 dengan Nomor 507/Pdt.G/2020/PTA.Sby dan telah diberitahukan kepada Ketua Pengadilan Agama Pamekasan, sesuai surat Nomor W13-A/4988/Hk.05/12/2020 tanggal 14 Desember 2020, yang tembusannya disampaikan kepada Pembanding dan Terbanding;

PERTIMBANGAN HUKUM

Menimbang, bahwa Pembanding dalam perkara ini adalah sebagai pihak Tergugat dalam pengadilan tingkat pertama, oleh karena itu berdasarkan Pasal 61 Undang-undang Nomor 7 Tahun 1989 tentang Peradilan Agama sebagaimana telah diubah dengan Undang-Undang Nomor 3 Tahun 2006 dan perubahan kedua dengan Undang-Undang Nomor 50 Tahun 2009, maka Pembanding adalah pihak yang mempunyai *legal standing* untuk mengajukan permohonan banding dalam perkara ini*;*

Menimbag, bahwa berdasarkan surat kuasa khusus tanggal 6 Nopember 2020, Pembanding dalam mengajukan permohonan banding terhadap putusan Pengadilan Agama Pamekasan tersebut memberi kuasa kepada Subaidi, S.H.,

Disclaimer
Kepaniteraan Mahkamah Agung Republik Indonesia berusaha untuk selalu mencantumkan informasi paling kini dan akurat sebagai bentuk komitmen Mahkamah Agung untuk pelayanan publik, transparansi dan akuntabilitas pelaksanaan fungsi peradilan. Namun dalam hal-hal tertentu masih dimungkinkan terjadi permasalahan teknis terkait dengan akurasi dan keterkinian informasi yang kami sajikan, hal mana akan terus kami perbaiki dari waktu kewaktu.
Dalam hal Anda menemukan inakurasi informasi yang termuat pada situs ini atau informasi yang seharusnya ada, namun belum tersedia, maka harap segera hubungi Kepaniteraan Mahkamah Agung RI melalui :
Email : kepaniteraan@mahkamahagung.go.id Telp : 021-384 3348 (ext.318)

Advokat, beralamat kantor di Gedung Islamic Center Lantai 3 No. 15-16 Pamekasan. Dengan demikian, berdasarkan Pasal 4 Undang-undang Nomor 18 Tahun 2003 tentang Advokat dan Surat Edaran Mahkamah Agung RI (SEMA) Nomor 6 Tahun 1994, kuasa hukum Pembanding tersebut dinyatakan memiliki *legal standing* untuk mewakili Pembanding dalam mengajukan upaya hukum banding ke Pengadilan Tinggi Agama Surabaya;

Menimbang, bahwa permohonan banding yang diajukan oleh Pembanding masih dalam tenggat masa banding sebagaimana diatur dalam Pasal 7 ayat (1) Undang-Undang Nomor 20 Tahun 1947 Tentang Peradilan Ulangan di Jawa dan Madura yakni dalam masa 14 hari, dengan demikian permohonan banding tersebut telah memenuhi syarat formal sehingga dinyatakan dapat diterima;

Menimbang, bahwa sesuai dengan abstraksi putusan Mahkamah Agung RI. Nomor 194 K/Sip/1975, maka Majelis Hakim Tingkat Banding sebagai *judex facti* harus memeriksa dan mengadili semua gugatan baik konpensi ataupun rekonpensi yang telah diputus oleh Pengadilan Agama Pamekasab tersebut;

Menimbang, bahwa dalam hal upaya perdamaian, Majelis Hakim Tingkat Pertama telah berusaha untuk mendamaikan kedua belah pihak berperkara, baik oleh majelis hakim sendiri, maupun melalui proses mediasi dengan mediator H. Azhar Amrullah Hafizh, Lc.M.TH.I., namun ternyata upaya perdamaian tersebut tidak berhasil mencapai kesepakatan sesuai laporan mediator tanggal 6 Agustus 2020, oleh karena itu Majelis Hakim Tingkat Banding berpendapat bahwa upaya perdamaian tersebut telah memenuhi ketentuan Pasal Pasal 82 Undang-undang Nomor 7 Tahun 1989 tentang Peradilan Agama *junto* Peraturan Mahkamah Agung Nomor 1 Tahun 2016, sehingga proses penyelesaian perkara secara *litigatif* dapat dilanjutkan;

Menimbang, bahwa setelah Majelis Hakim Tingkat Banding mempelajari dan memperhatikan secara saksama Putusan Pengadilan Agama Pamekasan Nomor 0765/Pdt.G/2020/PA.Pmk. tanggal 4 Nopember 2020 Masehi bertepatan dengan tanggal 18 Rabiul Awal 1442 Hijriyah, Memori Banding, serta berkas lainnya yang berkaitan dengan perkara ini, utamanya setelah memperhatikan

Disclaimer
Kepaniteraan Mahkamah Agung Republik Indonesia berusaha untuk selalu mencantumkan informasi paling kini dan akurat sebagai bentuk komitmen Mahkamah Agung untuk pelayanan publik, transparansi dan akuntabilitas pelaksanaan fungsi peradilan. Namun dalam hal-hal tertentu masih dimungkinkan terjadi permasalahan teknis terkait dengan akurasi dan keterkinian informasi yang kami sajikan, hal mana akan terus kami perbaiki dari waktu kewaktu.
Dalam hal Anda menemukan inakurasi informasi yang termuat pada situs ini atau informasi yang seharusnya ada, namun belum tersedia, maka harap segera hubungi Kepaniteraan Mahkamah Agung RI melalui :
Email : kepaniteraan@mahkamahagung.go.id Telp : 021-384 3348 (ext.318)

pertimbangan-pertimbangan hukum Majelis Hakim Tingkat Pertama yang memutus perkara ini, Majelis Hakim Tingkat Banding berpendapat bahwa pertimbangan-pertimbangan hukum Majelis Hakim Tingkat Pertama sudah tepat dan benar, karena telah mempertimbangkan secara komprehensif mengenai fakta kejadian dan fakta hukum, serta penerapan hukumnya sudah sesuai perundang-undangan, sehingga dapat disetujui dan diambil alih untuk dijadikan pertimbangan dan pendapat Majelis Hakim Tingkat Banding, namun demikian Majelis Hakim Tingkat Banding memandang perlu untuk menambahkan pertimbangan hukumnya sendiri sebagai berikut;

Menimbang, bahwa Majelis Hakim Tingkat Banding menilai dalil gugatan Terbanding dalam gugatannya didasarkan pada alasan perceraian sebagaimana ketentuan Pasal 39 ayat (2) Undang-undang Nomor 1 Tahun 1974 *juncto* Pasal 19 huruf (f) Peraturan Pemerintah Nomor 9 Tahun 1975 dan Pasal 116 huruf (f) Kompilasi Hukum Islam, yaitu antara suami dan isteri terus-menerus terjadi perselisihan dan pertengkaran dan tidak ada harapan akan hidup rukun lagi dalam rumah tangga;

Menimbang, bahwa terhadap alasan perceraian sebagaimana dimaksud di atas, setidaknya mempunyai dua unsur, yaitu *antara suami istri terus-menerus terjadi perselisihan dan pertengkaran*, dan *tidak ada harapan akan hidup rukun lagi dalam rumah tangga;*

Menimbang, bahwa unsur pertama, *antara suami istri terus menerus terjadi perselisihan dan pertengkaran,* fakta tersebut dapat dibuktikan dari keterangan para saksi, baik saksi Terbanding maupun saksi Pembanding sendiri, mereka mengetahui bahwa antara Pembanding dengan Terbanding telah terjadi perselisihan dan pertengkaran yang terus menerus yang disebabkan Pembanding mencemburui Terbanding sewaktu Terbanding bekerja di perusahaan Garam Budiono Tlanakan Pamekasan, juga Pembanding kurang terbuka dalam masalah ekonomi keluarga, yang akhirnya Pembanding pergi dari rumah kediaman bersama yang sampai saat ini sudah berlangsung selama 9 bulan, dan selama berpisah tersebut antara Pembanding dan Terbanding sudah tidak berhubungan lagi layaknya suami isteri, hal

Disclaimer
Kepaniteraan Mahkamah Agung Republik Indonesia berusaha untuk selalu mencantumkan informasi paling kini dan akurat sebagai bentuk komitmen Mahkamah Agung untuk pelayanan publik, transparansi dan akuntabilitas pelaksanaan fungsi peradilan. Namun dalam hal-hal tertentu masih dimungkinkan terjadi permasalahan teknis terkait dengan akurasi dan keterkinian informasi yang kami sajikan, hal mana akan terus kami perbaiki dari waktu kewaktu.
Dalam hal Anda menemukan inakurasi informasi yang termuat pada situs ini atau informasi yang seharusnya ada, namun belum tersedia, maka harap segera hubungi Kepaniteraan Mahkamah Agung RI melalui :
Email : kepaniteraan@mahkamahagung.go.id Telp : 021-384 3348 (ext.318)

tersebut diakui pula oleh Pembanding sendiri seperti pada repliknya tanggal 16 September 2020, bahwa “dalam waktu satu tahun terakhir ini ada pertengkaran, selisih paham atau berbeda pendapat bahkan cemburu”, meskipun pengakuan tidak dapat dijadikan bukti yang lengkap dalam hal kasus perceraian, akan tetapi patut diduga dan dapat dijadikan bukti permulaan dalam perkara *a quo,* sehingga dengan tambahan bukti lainnya yang berupa keterangan dua orang saksi yang diajukan oleh Terbanding dalam persidangan dan juga saksi Pembanding dinilai cukup membuktikan bahwa fakta "terus menerus terjadi perselisihan dan pertengkaran antara Pembanding dan Terbanding" tersebut benar terjadi;

Menimbang, bahwa Majelis Hakim juga berpendapat, perselisihan dan pertengkaran yang terjadi dalam rumah tangga Pembanding dengan Terbanding menimbulkan *disharmoni*, yang dalam istilah hukum Islam disebut sebagai *azzawwaj al-maksuroh* atau dalam istilah lain disebut *broken marriage* dan dapat diartikan *rumah tangga yang sudah pecah.* Permasalahan dalam rumah tangga landasannya bukan semata-mata adanya pertengkaran fisik *(phsysical cruelty)*, akan tetapi termasuk juga didalamnya adanya kekejaman mental *(mental cruelty)* yang menyebabkan tidak terpenuhinya lagi hak dan kewajiban suami isteri, sehingga seandainya tidak terjadi pertengkaran mulut atau kekerasan fisik maupun penganiayaan secara terus menerus, akan tetapi secara nyata telah terjadi dan berlangsung kekejaman mental atau penelantaran dan ketidakperdulian terhadap salah satu pihak, yang mana hal tersebut dapat dinilai sebagai *broken marriage;*

Menimbang, bahwa indikator pecahnya rumah tangga (*broken marriage*) menurut Hasil Rapat Pleno Kamar Agama Tahun 2013 sebagaimana tercantum dalam Surat Edaran Mahkamah Agung RI (SEMA) Nomor 4 Tahun 2014 tanggal 28 Maret 2014 Bagian Sub. Kamar Agama point 4 adalah : 1. Upaya damai tidak berhasil; 2. Sudah tidak ada komunikasi; 3. Salah satu atau masing-masing pihak meninggalkan kewajiban sebagai suami isteri; 4. Telah terjadi pisah ranjang/tempat tinggal bersama; 5. Hal-hal lain yang di temukan di persidangan, mis: WIL, PIL, KDRT, Judi dll;

Disclaimer
Kepaniteraan Mahkamah Agung Republik Indonesia berusaha untuk selalu mencantumkan informasi paling kini dan akurat sebagai bentuk komitmen Mahkamah Agung untuk pelayanan publik, transparansi dan akuntabilitas pelaksanaan fungsi peradilan. Namun dalam hal-hal tertentu masih dimungkinkan terjadi permasalahan teknis terkait dengan akurasi dan keterkinian informasi yang kami sajikan, hal mana akan terus kami perbaiki dari waktu kewaktu.
Dalam hal Anda menemukan inakurasi informasi yang termuat pada situs ini atau informasi yang seharusnya ada, namun belum tersedia, maka harap segera hubungi Kepaniteraan Mahkamah Agung RI melalui :
Email : kepaniteraan@mahkamahagung.go.id Telp : 021-384 3348 (ext.318)

Menimbang, bahwa adapun unsur *“tidak ada harapan akan hidup rukun lagi dalam rumah tangga”,* dapat pula dibuktikan dari fakta hukum sebagaimana dipertimbangkan di atas, karena ternyata antara Pembanding dengan Terbanding sudah pisah meja dan ranjang (*schelding van tafel en bed),* bahkan selama 9 bulan terakhir keduanya sudah pisah rumah, selama itu pula tidak pernah bersatu dan berbaikan kembali. Oleh karenanya bagaimana mungkin rumah tangga tersebut akan dapat hidup rukun lagi, sementara Pembanding dan Terbanding sendiri atau setidaknya salah satu pihak sudah tidak mempunyai itikad baik untuk mempertahankan perkawinannya dan kembali bersatu, sehingga dapat diduga bahwa Pembanding dengan Terbanding sudah tidak dapat hidup rukun dan berbaikan kembali dalam menjalankan rumah tangganya;

Menimbang bahwa mempertahankan rumah tangga yang telah pecah sedemikian rupa adalah suatu hal yang sia-sia, karena hanya akan menimbulkan penderitaan dan kemudharatan yang berkepanjangan bagi Terbanding dan Pembanding, sementara apabila keduanya dibukakan pintu perceraian akan lebih banyak pilihan bagi keduanya untuk mencapai keadaan yang lebih baik dikemudian hari, hal ini sesuai dengan pendapat Ibnu Sina dalam Kitab *As Syifa’* yang dikutip oleh Sayid Sabiq dalam Kitab *Fiqhus Sunnah* Juz II halaman 208 yang diambil sebagai pendapat Majelis Hakim Tingkat Banding yang berbunyi:

ينبغى أن يكون إلى الفرقة سبيل ما ,وألا يسد ذلك من كل وجه، لأن حسم أسباب التوصل إلى الفرقة بالكلية يقتضى وجوها من الضرر والخلل.منها.أن من الطبائع ما لا يألف بعض الطبائع, فكلما اجتهد فى الجمع بينهما زاد الشر،والنبو (أي الخلاف) وتنغصت المعايش;

Artinya: Seyogyanya jalan untuk bercerai itu diberikan dan jangan ditutup sama sekali, karena menutup mati jalan perceraian akan mengakibatkan beberapa bahaya dan kerusakan, diantaranya jika tabi’at suami istri sudah tidak saling kasih sayang lagi, maka ketika dipaksakan untuk

Disclaimer
Kepaniteraan Mahkamah Agung Republik Indonesia berusaha untuk selalu mencantumkan informasi paling kini dan akurat sebagai bentuk komitmen Mahkamah Agung untuk pelayanan publik, transparansi dan akuntabilitas pelaksanaan fungsi peradilan. Namun dalam hal-hal tertentu masih dimungkinkan terjadi permasalahan teknis terkait dengan akurasi dan keterkinian informasi yang kami sajikan, hal mana akan terus kami perbaiki dari waktu kewaktu.
Dalam hal Anda menemukan inakurasi informasi yang termuat pada situs ini atau informasi yang seharusnya ada, namun belum tersedia, maka harap segera hubungi Kepaniteraan Mahkamah Agung RI melalui :
Email : kepaniteraan@mahkamahagung.go.id   Telp : 021-384 3348 (ext.318)

tetap berkumpul diantara mereka berdua justru akan bertambah jelek, pecah dan kehidupannya menjadi kalut;

Menimbang, bahwa dari pertimbangan-pertimbangan di atas, Majelis Hakim Tingkat Banding berpendapat, apa yang didalilkan oleh Terbanding bahwa rumah tangganya telah sering terjadi perselisihan dan pertengkaran yang terus menerus serta tidak ada harapan untuk hidup rukun lagi dinyatakan terbukti, alasan tersebut telah sesuai dan memenuhi ketentuan Pasal 39 ayat (2) Undang-undang Nomor 1 Tahun 1974 *jis.* Pasal 19 huruf (f) Peraturan Pemerintah Nomor 9 Tahun 1975 dan Pasal 116 huruf (f) Kompilasi Hukum Islam;

Menimbang, bahwa oleh karena dalil gugatan Terbanding telah terbukti, maka cukup alasan bagi pengadilan untuk mengabulkan petitum angka 2 (dua) gugatan Terbanding dengan Menjatuhkan talak satu ba'in shughra Tergugat (Suyitno Bin Asim (Alm) terhadap Penggugat (Mei Dwi Waki'ah Binti Abd. Kadir (Alm) sesuai dengan ketentuan Pasal 70 ayat (1) Undang-undang Nomor 7 Tahun 1989 tentang Peradilan Agama sebagaimana telah diubah dengan Undang-Undang Nomor 3 Tahun 2006 dan perubahan kedua dengan Undang-Undang Nomor 50 Tahun 2009;

Menimbang, bahwa berdasarkan uraian pertimbangan tersebut diatas, maka keberatan-keberatan Pembanding dalam memori bandingnya dan uraian-uraian Terbanding dalam kontra memori bandingnya dipandang substansinya telah ikut dipertimbangkan, oleh karenanya tidak perlu dipertimbangkan lagi secara tersendiri;

Menimbang, bahwa berdasarkan pertimbangan tersebut diatas, maka putusan Pengadilan Agama Pamekasan Nomor 0765/Pdt.G/2020/PA.Pmk. tanggal 4 Nopember 2020 Masehi bertepatan dengan tanggal 18 Rabiul Awal 1442 Hijriyah tersebut haruslah dikuatkan;

Menimbang, bahwa oleh karena perkara ini mengenai sengketa di bidang perkawinan, maka berdasarkan pasal 89 ayat (1) Undang-Undang Nomor 7 Tahun 1989 Tentang Peradilan Agama yang telah diubah dengan Undang-Undang Nomor 3 Tahun 2006 dan perubahan kedua dengan Undang-

Disclaimer
Kepaniteraan Mahkamah Agung Republik Indonesia berusaha untuk selalu mencantumkan informasi paling kini dan akurat sebagai bentuk komitmen Mahkamah Agung untuk pelayanan publik, transparansi dan akuntabilitas pelaksanaan fungsi peradilan. Namun dalam hal-hal tertentu masih dimungkinkan terjadi permasalahan teknis terkait dengan akurasi dan keterkinian informasi yang kami sajikan, hal mana akan terus kami perbaiki dari waktu kewaktu.
Dalam hal Anda menemukan inakurasi informasi yang termuat pada situs ini atau informasi yang seharusnya ada, namun belum tersedia, maka harap segera hubungi Kepaniteraan Mahkamah Agung RI melalui :
Email : kepaniteraan@mahkamahagung.go.id   Telp : 021-384 3348 (ext.318)

Undang Nomor 50 Tahun 2009, biaya perkara dalam tingkat banding dibebankan kepada Pembanding;

Mengingat Pasal 49 Undang-Undang Nomor 3 tahun 2006, dan memperhatikan peraturan perundang-undangan dan dalil syar'i yang berkaitan dengan perkara ini;

M E N G A D I L I

I. Menyatakan permohonan banding Pembanding dapat diterima;

II. Menguatkan putusan Pengadilan Agama Pamekasan Nomor 0765/Pdt.G/2020/PA.Pmk. tanggal 4 Nopember 2020 Masehi bertepatan dengan tanggal 18 Rabiul Awal 1442 Hijriyah;

III. Membebankan kepada Pembanding biaya perkara dalam tingkat banding sejumlah Rp 150.000,00 (seratus lima puluh ribu rupiah);

Demikian diputuskan dalam sidang majelis hakim Pengadilan Tinggi Agama Surabaya pada hari Selasa tanggal 29 Desember *Miladiyah* bertepatan dengan tanggal 14 Jumadil Awwal 1442 *Hijriyah,* oleh kami **Drs. H. Nur Khazim, M.H.** sebagai Hakim Ketua Majelis, **Sulhan S.H.,M.Hum.** dan **Drs. Moh. Yasya, S.H.,M.H.,** masing-masing sebagai Hakim Anggota Majelis, berdasarkan Penetapan Ketua Pengadilan Tinggi Agama Surabaya Nomor 507/Pdt.G/2020/PTA.Sby. tanggal 29 Desember 2020, putusan tersebut pada hari itu juga telah dibacakan dalam sidang terbuka untuk umum oleh Ketua Majelis tersebut, dengan didampingi oleh Hakim-Hakim Anggota yang sama dengan dibantu oleh **Melati Pudjiwiandari, S.H**. sebagai Panitera Pengganti tanpa dihadiri oleh Pembanding dan Terbanding.

Ketua Majelis,

Ttd.

Drs. H. Nur Khazim, M.H.

Hakim Anggota,	Hakim Anggota,
Ttd.	Ttd.
**Sulhan, S.H.,M.Hum.**	**Drs. Moh. Yasya, S.H., M.H.**

Disclaimer
Kepaniteraan Mahkamah Agung Republik Indonesia berusaha untuk selalu mencantumkan informasi paling kini dan akurat sebagai bentuk komitmen Mahkamah Agung untuk pelayanan publik, transparansi dan akuntabilitas pelaksanaan fungsi peradilan. Namun dalam hal-hal tertentu masih dimungkinkan terjadi permasalahan teknis terkait dengan akurasi dan keterkinian informasi yang kami sajikan, hal mana akan terus kami perbaiki dari waktu kewaktu.
Dalam hal Anda menemukan inakurasi informasi yang termuat pada situs ini atau informasi yang seharusnya ada, namun belum tersedia, maka harap segera hubungi Kepaniteraan Mahkamah Agung RI melalui :
Email : kepaniteraan@mahkamahagung.go.id Telp : 021-384 3348 (ext.318)

Panitera Pengganti,

Ttd.

Melati Pudjiwiandari, S.H.

Rincian Biaya Proses:

- Pemberkasan ATK	: Rp	134.000,00
- Redaksi	: Rp	10.000,00
- Meterai	: Rp	6.000,00
J u m l a h	: Rp	150.000,00

(seratus lima puluh ribu rupiah).

UNTUK SALINAN
PENGADILAN TINGGI AGAMA SURABAYA
PANITERA,

Hj. SITI ROMIYANI, SH. MH.

Disclaimer
Kepaniteraan Mahkamah Agung Republik Indonesia berusaha untuk selalu mencantumkan informasi paling kini dan akurat sebagai bentuk komitmen Mahkamah Agung untuk pelayanan publik, transparansi dan akuntabilitas pelaksanaan fungsi peradilan. Namun dalam hal-hal tertentu masih dimungkinkan terjadi permasalahan teknis terkait dengan akurasi dan keterkinian informasi yang kami sajikan, hal mana akan terus kami perbaiki dari waktu kewaktu.
Dalam hal Anda menemukan inakurasi informasi yang termuat pada situs ini atau informasi yang seharusnya ada, namun belum tersedia, maka harap segera hubungi Kepaniteraan Mahkamah Agung RI melalui :
Email : kepaniteraan@mahkamahagung.go.id Telp : 021-384 3348 (ext.318)
