# Supplementary material for: Classification of divorce causes during the COVID-19 pandemic using convolutional neural networks
Source: PeerJ Comput Sci. 2022 Jun 30;8:e998. doi: 10.7717/peerj-cs.998 (PMC9299239; doi:10.7717/peerj-cs.998)
Supplement: Supplemental Information 6 [file peerj-cs-08-998-s006.zip › Rujuk Dataset/Data ke-18.pdf]

P U T U S A N

Nomor 557/Pdt.G/2020/PA.Dum

بِسْمِ اللهِ الرَّحْمٰنِ الرَّحِيْمِ

DEMI KEADILAN BERDASARKAN KETUHANAN YANG MAHA ESA

Pengadilan Agama Dumai yang memeriksa dan mengadili perkara tertentu pada tingkat pertama dalam sidang hakim tunggal telah menjatuhkan putusan sebagai berikut dalam perkara Cerai Gugat antara:

Penggugat, umur 31 tahun, agama Islam, pendidikan SLTA, pekerjaan Usaha Laundry, tempat tinggal di Jalan Ahmad Yani No.12, RT.008, Kelurahan Bukit Datuk, Kecamatan Dumai Selatan, Kota Dumai (Sister's laundry), sebagai **Penggugat**;

melawan

Tergugat, umur 31 tahun, agama Islam, pendidikan D1, pekerjaan Tidak Bekerja, tempat tinggal di Jalan Jeruk (Gang samping PT.Transco) No 7, RT. 007, Kelurahan Rimba Sekampung, Kecamatan Dumai Kota, Kota Dumai, sebagai **Tergugat**;

Pengadilan Agama tersebut;

Telah membaca dan mempelajari berkas perkara;

Telah mendengar keterangan Penggugat;

Telah memeriksa alat-alat bukti di persidangan;

DUDUK PERKARA

Menimbang, bahwa Penggugat dengan surat gugatannya tertanggal 01 Desember 2020 yang telah terdaftar di Kepaniteraan Pengadilan Agama Dumai dengan register perkara Nomor 557/Pdt.G/2020/PA.Dum tanggal 8 Desember 2020, mengemukakan dalil-dalil sebagai berikut:

1. Bahwa Penggugat adalah istri sah Tergugat yang akad nikahnya berlangsung di rumah orang tua Penggugat di Kecamatan Dumai Selatan, Kota Dumai pada hari Minggu tanggal 02 Februari 2020, berdasarkan Kutipan Akta Nikah yang dikeluarkan oleh Pegawai Pencatat Nikah Kantor

Disclaimer
Kepaniteraan Mahkamah Agung Republik Indonesia berusaha untuk selalu mencantumkan informasi paling kini dan akurat sebagai bentuk komitmen Mahkamah Agung untuk pelayanan publik, transparansi dan akuntabilitas pelaksanaan fungsi peradilan. Namun dalam hal-hal tertentu masih dimungkinkan terjadi permasalahan teknis terkait dengan akurasi dan keterkinian informasi yang kami sajikan, hal mana akan terus kami perbaiki dari waktu kewaktu.
Dalam hal Anda menemukan inakurasi informasi yang termuat pada situs ini atau informasi yang seharusnya ada, namun belum tersedia, maka harap segera hubungi Kepaniteraan Mahkamah Agung RI melalui :
Email : kepaniteraan@mahkamahagung.go.id   Telp : 021-384 3348 (ext.318)

Urusan Agama Kecamatan Dumai Selatan, Kota Dumai Nomor : AKTA NIKAH, tertanggal 02 Februari 2020;

2. Bahwa sewaktu menikah Penggugat berstatus Janda memiliki anak 2(dua) dan Tergugat berstatus Perjaka;
3. Bahwa Penggugat dan Tergugat setelah menikah tinggal di rumah kediaman bersama di rumah orangtua Penggugat di Jalan Gatot Subroto KM.9, RT.007, Kelurahan Mekar sari, Kecamatan Dumai selatan selama lebih kurang 2(dua) bulan dan pada bulan April 2020 Tergugat pulang ke rumah orangtua Tergugat dialamat Tergugat tersebut diatas sampai dengan sekarang dan Penggugat sekarang berdomisili di rumah Penggugat dialamat Penggugat tersebut diatas sampai dengan sekarang;
4. Bahwa antara Penggugat dan Tergugat telah melakukan hubungan sebagaimana suami istri dan belum dikaruniai keturunan/anak;
5. Bahwa pada awalnya rumah tangga Penggugat dan Tergugat berjalan rukun dan harmonis, namun sejak tanggal 06 bulan April tahun 2020 rumah tangga Penggugat dan Tergugat mulai goyah, selalu diwarnai perselisihan dan pertengkaran. Adapun yang menjadi penyebabnya adalah sikap Tergugat yang labil dan selalu berprasangka buruk terhadap Penggugat serta kekanak-kanakan dalam menghadapi permasalahan rumah tangga;
6. Bahwa Penggugat telah sering menasehati Tergugat, untuk merubah sikap dan perbuatannya tersebut di atas, dan sering kali setiap Penggugat menasehati Tergugat, Tergugat marah kepada Penggugat sehingga terjadilah perselisihan dan pertengkaran antara Penggugat dan Tergugat, setelah itu Tergugat tetap mengulangi perbuatannya tersebut;
7. Bahwa puncak ketidak harmonisan rumah tangga antara Penggugat dan Tergugat terjadi pada bulan April tahun 2020, berawal dari Tergugat yang merajuk kepada Penggugat karena alasan Penggugat tidak menelpon Tergugat untuk pulang ke rumah dan Tergugat mengatakan bahwa Penggugat tidak melakukan kewajibannya sebagai istri setelah kejadian tersebut keesokan harinya Tergugat meminta izin untuk keluar dari rumah dengan alasan ingin menjemput orangtua Tergugat di Padang akan tetapi Tergugat tidak pulang ke rumah sampai dengan sekarang Penggugat dan

Disclaimer
Kepaniteraan Mahkamah Agung Republik Indonesia berusaha untuk selalu mencantumkan informasi paling kini dan akurat sebagai bentuk komitmen Mahkamah Agung untuk pelayanan publik, transparansi dan akuntabilitas pelaksanaan fungsi peradilan. Namun dalam hal-hal tertentu masih dimungkinkan terjadi permasalahan teknis terkait dengan akurasi dan keterkinian informasi yang kami sajikan, hal mana akan terus kami perbaiki dari waktu kewaktu.
Dalam hal Anda menemukan inakurasi informasi yang termuat pada situs ini atau informasi yang seharusnya ada, namun belum tersedia, maka harap segera hubungi Kepaniteraan Mahkamah Agung RI melalui :
Email : kepaniteraan@mahkamahagung.go.id Telp : 021-384 3348 (ext.318)

Tergugat berpisah sudah selama lebih kurang 7 (tujuh) bulan dan selama itu sudah tidak ada lagi hubungan baik lahir maupun batin dan Tergugat sudah tidak lagi memberi nafkah kepada Penggugat;

8. Bahwa Penggugat telah berusaha mempertahankan keutuhan rumah tangga dengan berusaha menyelesaikan kemelut rumah tangga secara kekeluargaan, namun usaha tersebut tidak berhasil;
9. Bahwa terhadap prilaku Tergugat tersebut, maka rumah tangga Penggugat dan Tergugat sudah tidak mungkin lagi untuk dapat dipertahankan, oleh karenanya Penggugat telah berketetapan hati untuk menggugat cerai terhadap Tergugat ke Pengadilan Agama Dumai;

Berdasarkan dalil dalil diatas, Penggugat mohon kepada Ketua Pengadilan Agama Dumai dalam hal ini Majelis Hakim yang memeriksa dan mengadili perkara ini,kiranya berkenan memberikan putusan yang amarnya berbunyi, sebagai berikut :

PRIMAIR:

1. Mengabulkan gugatan Penggugat;
2. Menjatuhkan talak satu bain sughro Tergugat (**Tergugat**) terhadap Penggugat (**Penggugat**);
3. Menetapkan biaya perkara menurut hukum yang berlaku;

SUBSIDAIR:

Apabila Majelis Hakim berpendapat lain, mohon putusan yang seadil adilnya;

Bahwa pada hari sidang yang telah ditetapkan Penggugat telah menghadap sendiri ke muka sidang, sedangkan Tergugat tidak menghadap dan tidak menyuruh orang lain untuk menghadap sebagai Wakil/Kuasa hukumnya meskipun telah dipanggil secara resmi dan patut yang relaas panggilannya dibacakan di dalam sidang, sedangkan tidak ternyata bahwa tidak datangnya itu disebabkan suatu halangan yang sah;

Bahwa, Hakim dalam persidangan telah berusaha mendamaikan dengan jalan memberi saran dan nasehat kepada Penggugat agar mengurungkan keinginannya untuk bercerai dan mencoba kembali membina rumah tangga yang *sakinah, mawaddah wa rahmah* namun tidak berhasil, sedangkan usaha

Disclaimer
Kepaniteraan Mahkamah Agung Republik Indonesia berusaha untuk selalu mencantumkan informasi paling kini dan akurat sebagai bentuk komitmen Mahkamah Agung untuk pelayanan publik, transparansi dan akuntabilitas pelaksanaan fungsi peradilan. Namun dalam hal-hal tertentu masih dimungkinkan terjadi permasalahan teknis terkait dengan akurasi dan keterkinian informasi yang kami sajikan, hal mana akan terus kami perbaiki dari waktu kewaktu.
Dalam hal Anda menemukan inakurasi informasi yang termuat pada situs ini atau informasi yang seharusnya ada, namun belum tersedia, maka harap segera hubungi Kepaniteraan Mahkamah Agung RI melalui :
Email : kepaniteraan@mahkamahagung.go.id   Telp : 021-384 3348 (ext.318)

damai melalui proses mediasi tidak dapat dilaksanakan karena Tergugat tidak pernah datang menghadap ke persidangan;

Bahwa, oleh sebab upaya damai tidak berhasil, selanjutnya pemeriksaan perkara ini dilanjutkan secara litigasi diawali dengan membacakan surat gugatan Penggugat yang pada pokoknya tetap dipertahankan oleh Penggugat;

Bahwa, terhadap gugatan Penggugat, Tergugat tidak dapat didengar jawabannya dalam persidangan karena Tergugat tidak pernah hadir di persidangan;

Bahwa untuk meneguhkan dalil-dalil gugatannya, Penggugat telah mengajukan bukti-bukti sebagai berikut:

I. Bukti Surat

Fotokopi Kutipan Akta Nikah Nomor AKTA NIKAH Tanggal 02 Februari 2020 atas nama Penggugat dan Tergugat yang aslinya dikeluarkan oleh Kepala Kantor Urusan Agama Kecamatan Dumai Selatan, Kota Dumai, bermeterai cukup dan di-*nazzegelen* serta telah dicocokkan dengan aslinya oleh Hakim, ternyata cocok, lalu diberi tanda bukti P dan diparaf;

II. Bukti Saksi

1. **saksi I**, umur 60 tahun, agama Islam, pendidikan SLTA, pekerjaan pensiunan pertamina, tempat tinggal di Jalan Gatot Subroto, Gg. Mesjid Annur, Nomor 78, RT 09, Kelurahan Mekar Sari, Kecamatan Dumai Selatan, Kota Dumai, telah memberikan keterangan di bawah sumpah secara agama Islam sebagai berikut:
   - Bahwa saksi adalah ayah kandung Penggugat;
   - Bahwa saksi kenal dengan Tergugat yang bernama Tergugat sejak kecil;
   - Bahwa Penggugat dan Tergugat adalah suami isteri yang menikah pada tahun 2020;
   - Bahwa antara Penggugat dan Tergugat belum dikaruniai anak;
   - Bahwa saksi mengetahui maksud Penggugat menghadap sidang, Penggugat ingin menggugat cerai Tergugat;

Disclaimer
Kepaniteraan Mahkamah Agung Republik Indonesia berusaha untuk selalu mencantumkan informasi paling kini dan akurat sebagai bentuk komitmen Mahkamah Agung untuk pelayanan publik, transparansi dan akuntabilitas pelaksanaan fungsi peradilan. Namun dalam hal-hal tertentu masih dimungkinkan terjadi permasalahan teknis terkait dengan akurasi dan keterkinian informasi yang kami sajikan, hal mana akan terus kami perbaiki dari waktu kewaktu.
Dalam hal Anda menemukan inakurasi informasi yang termuat pada situs ini atau informasi yang seharusnya ada, namun belum tersedia, maka harap segera hubungi Kepaniteraan Mahkamah Agung RI melalui :
Email : kepaniteraan@mahkamahagung.go.id   Telp : 021-384 3348 (ext.318)

- Bahwa Penggugat ingin menggugat cerai Tergugat karena sejak awal menikah, rumah tangga Penggugat dan Tergugat tidak pernah rukun;
- Bahwa pernikahan Penggugat dan Tergugat bukan berdasarkan paksaan, akan tetapi karena suka sama suka;
- Bahwa saksi kenal dengan Tergugat sejak kecil, pada awal menikah, Tergugat tidak ada menyatakan keberatan untuk menikah dengan Penggugat, akan tetapi setelah menikah, Tergugat sering marah-marah dan memarahi Penggugat, hal ini tidak pernah saksi sangka-sangka;
- Bahwa saksi mengetahui Tergugat yang sering marah-marah karena Penggugat dan Tergugat tinggal bersama saksi;
- Bahwa saksi kurang mengetahui penyebab Penggugat dan Tergugat bertengkar;
- Bahwa saksi sering memberikan nasehat kepada Tergugat bagaimana menjadi suami yang baik, tetapi rumah tangga Penggugat dan Tergugat tetap terjadi pertengkaran;
- Bahwa saat ini antara Penggugat dan Tergugat telah pisah rumah sejak bulan April 2020 sampai sekarang;
- Bahwa Penggugat dan Tergugat telah didamaikan, akan tetapi Penggugat tidak mau lagi rukun dengan Tergugat;

2. **saksi II**, umur 28 tahun, agama Islam, pendidikan S1, pekerjaan honorer, bertempat tinggal di Jalan Merpati, nomor 3, RT 13, Kelurahan Jaya Mukti, Kecamatan Dumai Timur, Kota Dumai, di bawah sumpah memberikan keterangan di bawah sumpah secara agama Islam yang pada pokoknya sebagai berikut:
   - Bahwa saksi adalah adik kandung Penggugat;
   - Bahwa saksi kenal dengan Tergugat yang bernama Tergugat sejak kecil;
   - Bahwa Penggugat dan Tergugat adalah suami isteri yang menikah pada tahun 2020;
   - Bahwa antara Penggugat dan Tergugat belum dikaruniai anak;

Disclaimer
Kepaniteraan Mahkamah Agung Republik Indonesia berusaha untuk selalu mencantumkan informasi paling kini dan akurat sebagai bentuk komitmen Mahkamah Agung untuk pelayanan publik, transparansi dan akuntabilitas pelaksanaan fungsi peradilan. Namun dalam hal-hal tertentu masih dimungkinkan terjadi permasalahan teknis terkait dengan akurasi dan keterkinian informasi yang kami sajikan, hal mana akan terus kami perbaiki dari waktu kewaktu.
Dalam hal Anda menemukan inakurasi informasi yang termuat pada situs ini atau informasi yang seharusnya ada, namun belum tersedia, maka harap segera hubungi Kepaniteraan Mahkamah Agung RI melalui :
Email : kepaniteraan@mahkamahagung.go.id   Telp : 021-384 3348 (ext.318)

- Bahwa saksi mengetahui maksud Penggugat menghadap sidang, Penggugat ingin menggugat cerai Tergugat;
- Bahwa Penggugat ingin menggugat cerai Tergugat karena sejak awal menikah, rumah tangga Penggugat dan Tergugat tidak pernah rukun;
- Bahwa sepengetahuan saksi pernikahan Penggugat dan Tergugat bukan berdasarkan paksaan, akan tetapi karena suka sama suka;
- Bahwa saksi mengetahui Tergugat yang sering marah-marah karena saksi pernah melihat mereka bertengkar di temat laundry milik Penggugat;
- Bahwa saksi kurang mengetahui penyebab Penggugat dan Tergugat bertengkar;
- Bahwa saat ini antara Penggugat dan Tergugat telah pisah rumah sejak bulan April 2020 sampai sekarang;
- Bahwa Penggugat dan Tergugat telah didamaikan, akan tetapi Penggugat tidak mau lagi rukun dengan Tergugat;

Bahwa setelah diberi kesempatan Penggugat menyatakan tidak mengajukan alat bukti apapun lagi dan menyampaikan kesimpulan secara lisan yang pada pokoknya menyatakan tetap pada gugatannya dan selanjutnya mohon putusan;

Bahwa untuk mempersingkat uraian putusan ini cukup ditunjuk segala hal yang telah termuat dalam berita acara sidang yang dianggap sebagai satu kesatuan tak terpisahkan dalam putusan ini;

PERTIMBANGAN HUKUM

Menimbang, bahwa maksud dan tujuan gugatan Penggugat adalah sebagaimana tersebut di atas;

Menimbang, bahwa berdasarkan ketentuan Pasal 49 huruf a Undang-Undang Nomor 7 Tahun 1989 tentang Peradilan Agama dan penjelasannya sebagaimana telah diubah dengan Undang-Undang Nomor 3 Tahun 2006, terakhir dengan perubahan kedua Undang-Undang Nomor 50 Tahun 2009, maka penyelesaian perkara di tingkat pertama antara orang-orang yang beragama Islam di bidang perkawinan merupakan wewenang *absolut* Pengadilan Agama, dan dalam perkara ini telah ternyata subjek hukum dalam

Disclaimer
Kepaniteraan Mahkamah Agung Republik Indonesia berusaha untuk selalu mencantumkan informasi paling kini dan akurat sebagai bentuk komitmen Mahkamah Agung untuk pelayanan publik, transparansi dan akuntabilitas pelaksanaan fungsi peradilan. Namun dalam hal-hal tertentu masih dimungkinkan terjadi permasalahan teknis terkait dengan akurasi dan keterkinian informasi yang kami sajikan, hal mana akan terus kami perbaiki dari waktu kewaktu.
Dalam hal Anda menemukan inakurasi informasi yang termuat pada situs ini atau informasi yang seharusnya ada, namun belum tersedia, maka harap segera hubungi Kepaniteraan Mahkamah Agung RI melalui :
Email : kepaniteraan@mahkamahagung.go.id Telp : 021-384 3348 (ext.318)

perkara ini adalah beragama Islam, dan perkara yang diajukan adalah perkara dibidang perkawinan, oleh karena itu maka Pengadilan Agama berwenang secara *absolut* untuk memeriksa, memutus, dan menyelesaikan perkara ini;

Menimbang, bahwa Penggugat telah datang menghadap *(in person)* di persidangan, sedangkan Tergugat tidak pernah hadir di persidangan dan tidak pula mengirimkan orang lain selaku wakil atau kuasanya yang sah untuk hadir di persidangan meskipun telah dipanggil secara resmi dan patut sesuai Pasal 26 Peraturan Pemerintah Nomor 9 Tahun 1975, dan tidak ternyata ketidakhadiran Tergugat itu disebabkan suatu alasan yang sah menurut hukum (*default without reason*) sesuai Pasal 149 ayat (1) R.bg, maka harus dinyatakan Tergugat tidak hadir dan perkara *a quo* dapat diperiksa tanpa hadirnya Tergugat;

Menimbang, bahwa berdasarkan ketentuan Pasal 39 ayat (1) Undang-Undang Nomor 1 Tahun 1974 jo. Pasal 65 dan Pasal 82 Undang-Undang Nomor 7 Tahun 1989 sebagaimana telah diubah dengan Undang-Undang Nomor 3 Tahun 2006, terakhir dengan perubahan kedua Undang-Undang Nomor 50 Tahun 2009 jo. Pasal 31 Peraturan Pemerintah Nomor 9 Tahun 1975 serta Pasal 143 ayat (1) Kompilasi Hukum Islam, maka Hakim telah berusaha mendamaikan dengan menasehati Penggugat agar bersabar dan rukun kembali dengan Tergugat akan tetapi tidak berhasil;

Menimbang, bahwa berdasarkan Peraturan Mahkamah Agung Republik Indonesia Nomor 1 Tahun 2016 Tentang Prosedur Mediasi di Pengadilan, semua perkara yang masuk ke pengadilan terlebih dahulu harus dilaksanakan mediasi, akan tetapi dalam perkara yang bersangkutan karena pihak Tergugat tidak pernah hadir, maka mediasi tidak dapat dilaksanakan;

Menimbang, bahwa oleh sebab antara Penggugat dan Tergugat terdapat hubungan perkawinan sebagai suami istri yang belum pernah putus (bercerai), maka keduanya memiliki kepentingan hukum untuk bertindak sebagai pihak-pihak dalam perkara ini (*persona standi in judicio*);

Menimbang, bahwa setelah meneliti surat gugatan Penggugat dan mendengar keterangan Penggugat maka yang menjadi masalah pokok dalam perkara ini adalah Penggugat bermohon untuk bercerai dari Tergugat dengan

Disclaimer
Kepaniteraan Mahkamah Agung Republik Indonesia berusaha untuk selalu mencantumkan informasi paling kini dan akurat sebagai bentuk komitmen Mahkamah Agung untuk pelayanan publik, transparansi dan akuntabilitas pelaksanaan fungsi peradilan. Namun dalam hal-hal tertentu masih dimungkinkan terjadi permasalahan teknis terkait dengan akurasi dan keterkinian informasi yang kami sajikan, hal mana akan terus kami perbaiki dari waktu kewaktu.
Dalam hal Anda menemukan inakurasi informasi yang termuat pada situs ini atau informasi yang seharusnya ada, namun belum tersedia, maka harap segera hubungi Kepaniteraan Mahkamah Agung RI melalui :
Email : kepaniteraan@mahkamahagung.go.id   Telp : 021-384 3348 (ext.318)

alasan bahwa Penggugat dan Tergugat terus menerus terjadi perselisihan dan pertengkaran serta tidak ada harapan akan rukun lagi dalam rumah tangga;

Menimbang, bahwa Tergugat tidak dapat didengar jawabannya di persidangan karena Tergugat tidak pernah hadir, maka secara yuridis formal Tergugat dapat dianggap tidak bermaksud untuk mempertahankan hak-hak keperdataannya dan atau membela kepentingannya di persidangan, mengakui dan membenarkan semua Posita dan Petitum dalam surat gugatan Penggugat, namun tidak serta merta hal tersebut menjadikan gugatan Penggugat dapat dikabulkan, karena mengingat perkara ini merupakan perkara yang dikhawatirkan dapat menimbulkan suatu kebohongan besar (*de groten langen*);

Menimbang, bahwa berdasarkan alasan-alasan tersebut di atas, maka Hakim menilai alasan perceraian yang didalilkan oleh Penggugat adalah termasuk dalam alasan-alasan perceraian sebagaimana diatur dalam ketentuan Pasal 19 huruf f Peraturan Pemerintah Nomor 9 Tahun 1975 jo. Pasal 116 huruf f Kompilasi Hukum Islam;

Menimbang, bahwa untuk dikabulkannya sebuah perceraian dengan alasan dasar tersebut di atas, maka sebagaimana Pasal 39 ayat (2) Undang-Undang Nomor 1 Tahun 1974 Tentang Perkawinan jo. Pasal 19 huruf (f) Peraturan Pemerintah Nomor 9 Tahun 1975 Tentang Pelaksanaan Undang-Undang Nomor 1 Tahun 1974 Tentang Perkawinan jo. Pasal 116 huruf (f) dan Pasal 134 Kompilasi Hukum Islam, harus terpenuhi beberapa unsur sebagai berikut, (1). Rumah tangga sudah tidak rukun dan harmonis, sering terjadi perselisihan dan pertengkaran secara terus menerus. (2). Perselisihan dan pertengkaran tersebut menyebabkan suami istri tidak ada harapan untuk rukun kembali. (3). Pengadilan sudah mendamaikan, namun tidak berhasil;

Menimbang, bahwa karena perkara ini adalah mengenai bidang perceraian yang dinilai penting untuk ditemukan kebenaran materiilnya, dan untuk lebih meyakinkan Hakim atas dalil-dalil gugatan Penggugat, maka sesuai dengan Keputusan Mahkamah Agung RI Nomor KMA/032/SK/IV/2006 tanggal 4 April 2006 tentang Pemberlakuan Buku II Pedoman Teknis Administrasi dan Teknis Peradilan Agama, Edisi Revisi 2013, Hakim berpendapat Penggugat diwajibkan untuk mengajukan bukti-bukti yang dapat mendukung dalil-dalil

Disclaimer
Kepaniteraan Mahkamah Agung Republik Indonesia berusaha untuk selalu mencantumkan informasi paling kini dan akurat sebagai bentuk komitmen Mahkamah Agung untuk pelayanan publik, transparansi dan akuntabilitas pelaksanaan fungsi peradilan. Namun dalam hal-hal tertentu masih dimungkinkan terjadi permasalahan teknis terkait dengan akurasi dan keterkinian informasi yang kami sajikan, hal mana akan terus kami perbaiki dari waktu kewaktu.
Dalam hal Anda menemukan inakurasi informasi yang termuat pada situs ini atau informasi yang seharusnya ada, namun belum tersedia, maka harap segera hubungi Kepaniteraan Mahkamah Agung RI melalui :
Email : kepaniteraan@mahkamahagung.go.id   Telp : 021-384 3348 (ext.318)

posita dan petitum gugatannya;

Menimbang, bahwa untuk menguatkan dalil-dalil gugatannya Penggugat telah mengajukan alat bukti berupa bukti surat dan bukti saksi sebagaimana dalam duduk perkaranya di atas;

Menimbang, bahwa terhadap bukti surat yang diajukan Penggugat tersebut, Hakim berpendapat bukti tersebut merupakan fotokopi sah dari suatu akta autentik, khusus dibuat sebagai alat bukti, telah diberi meterai cukup sesuai ketentuan Pasal 2 ayat (1) Undang-Undang Nomor 13 Tahun 1985 Tentang Bea Meterai dan telah di-nazegeling sehingga alat bukti tersebut telah memenuhi persyaratan formil dan harus dinyatakan dapat diterima, dan secara materiil dapat dipertimbangkan karena alat bukti tersebut memuat keterangan yang menguatkan dan relevan dengan dalil gugatan Penggugat;

Menimbang, bahwa bukti P (Fotokopi Kutipan Akta Nikah Nomor AKTA NIKAH Tanggal 02 Februari 2020) merupakan akta otentik yang berdaya bukti sempurna dan mengikat yang memberi bukti Penggugat dengan Tergugat telah dan masih terikat dalam perkawinan sah yang tercatat di Kecamatan Dumai Selatan, Kota Dumai, dan telah sesuai dengan ketentuan Pasal 285 R.Bg jo. Pasal 2 ayat (1) dan (2) Undang-Undang Nomor 1 Tahun 1974 jo. Pasal 7 ayat (1) Kompilasi Hukum Islam, dengan demikian Penggugat dengan Tergugat telah mempunyai hubungan dan kapasitas hukum untuk menjadi pihak dalam perkara ini (*persona standi in judicio*), karenanya Penggugat mempunyai kualitas untuk mengajukan tuntutan dalam sengketa bidang perkawinan;

Menimbang, bahwa oleh karena alasan perceraian yang diajukan Penggugat berkenaan dengan ketentuan Pasal 19 huruf f Peraturan Pemerintah Nomor 9 Tahun 1975 jo. Pasal 116 huruf f Kompilasi Hukum Islam di Indonesia, sehingga berdasarkan ketentuan Pasal 22 ayat (2) Peraturan Pemerintah Nomor 9 Tahun 1975 dan Pasal 138 Kompilasi Hukum Islam, maka Hakim perlu mendengarkan keterangan saksi-saksi yang berasal dari keluarga dan atau orang-orang yang dekat dengan suami istri tersebut;

Menimbang, bahwa saksi pertama dan kedua yang diajukan Penggugat di persidangan, setelah diperiksa oleh Hakim, ternyata saksi pertama adalah ayah kandung Penggugat dan saksi kedua adalah adik kandung Penggugat,

Disclaimer
Kepaniteraan Mahkamah Agung Republik Indonesia berusaha untuk selalu mencantumkan informasi paling kini dan akurat sebagai bentuk komitmen Mahkamah Agung untuk pelayanan publik, transparansi dan akuntabilitas pelaksanaan fungsi peradilan. Namun dalam hal-hal tertentu masih dimungkinkan terjadi permasalahan teknis terkait dengan akurasi dan keterkinian informasi yang kami sajikan, hal mana akan terus kami perbaiki dari waktu kewaktu.
Dalam hal Anda menemukan inakurasi informasi yang termuat pada situs ini atau informasi yang seharusnya ada, namun belum tersedia, maka harap segera hubungi Kepaniteraan Mahkamah Agung RI melalui :
Email : kepaniteraan@mahkamahagung.go.id Telp : 021-384 3348 (ext.318)

kedua orang saksi tersebut telah dewasa atau cakap hukum sebagaimana ditetapkan Pasal 1912 KUH Perdata, dan telah disumpah sesuai kehendak Pasal 175 R.Bg, maka kedua orang saksi tersebut memenuhi syarat formil saksi sehingga dapat diterima sebagai alat bukti yang sah;

Menimbang, bahwa keterangan saksi pertama dan saksi kedua tersebut mengenai dalil gugatan Penggugat adalah hal yang diketahui dan dialami sendiri sehingga relevan dengan hal yang harus dibuktikan Penggugat;

Menimbang, bahwa setelah diperiksa oleh Hakim, ternyata saksi tersebut terdiri dari dua orang sebagaimana batas minimum yang ditetapkan oleh Pasal 306 R.Bg, keterangannya saling bersesuaian satu sama lain sebagaimana dikehendaki Pasal 309 R.Bg, menurut pendapat Hakim saksi-saksi tersebut mengetahui antara Penggugat dan Tergugat sering terjadi perselisihan dan pertengkaran dan dapat dikatakan telah mendukung dalil gugatan Penggugat, oleh karena itu dalil gugatan Penggugat telah terbukti di persidangan;

Menimbang, bahwa apabila bukti surat dan kesaksian dua orang saksi serta anggapan hukum sebagaimana tersebut di atas dihubungkan dengan dalil-dalil gugatan Penggugat Hakim menilai dalil-dalil gugatan Penggugat telah terbukti, dan dari pembuktian tersebut diketemukan fakta-fakta yuridis sebagai berikut:

1. Bahwa Penggugat telah menikah dengan Tergugat pada tanggal 02 Desember 2020 di Kecamatan Dumai Selatan, Kota Dumai;
2. Bahwa rumah tangga Penggugat dan Tergugat sejak awal menikah tidak pernah harmonis, yang akibatnya antara Penggugat dan Tergugat pisah rumah sejak bulan Februari 2020 sampai sekarang;
3. Bahwa pihak keluarga telah berusaha mendamaikan Penggugat dan Tergugat untuk rukun kembali dalam rumah tangga akan tetapi tidak berhasil;

Menimbang, bahwa berdasarkan fakta-fakta tersebut di atas, Hakim menemukan fakta hukum sebagai berikut:

1. Bahwa Penggugat dan Tergugat adalah suami isteri yang sah;
2. Bahwa rumah tangga Penggugat dan Tergugat sudah pecah (broken home);

Disclaimer
Kepaniteraan Mahkamah Agung Republik Indonesia berusaha untuk selalu mencantumkan informasi paling kini dan akurat sebagai bentuk komitmen Mahkamah Agung untuk pelayanan publik, transparansi dan akuntabilitas pelaksanaan fungsi peradilan. Namun dalam hal-hal tertentu masih dimungkinkan terjadi permasalahan teknis terkait dengan akurasi dan keterkinian informasi yang kami sajikan, hal mana akan terus kami perbaiki dari waktu kewaktu.
Dalam hal Anda menemukan inakurasi informasi yang termuat pada situs ini atau informasi yang seharusnya ada, namun belum tersedia, maka harap segera hubungi Kepaniteraan Mahkamah Agung RI melalui :
Email : kepaniteraan@mahkamahagung.go.id   Telp : 021-384 3348 (ext.318)

3. Bahwa Penggugat dan Tergugat tidak ada harapan hidup rukun lagi dalam rumah tangga;

Menimbang, bahwa berdasarkan fakta tersebut, Hakim berpendapat bahwa fakta tersebut telah sesuai dengan ketentuan Pasal 19 huruf f Peraturan Pemerintah Nomor 9 Tahun 1975 jo. Pasal 116 huruf f Kompilasi Hukum Islam tahun 1991, yaitu rumah tangga Penggugat dan Tergugat tidak ada harapan akan hidup rukun lagi dalam rumah tangga (*onheelbaare tweespalt*), selain itu saat sekarang Penggugat dan Tergugat telah pisah rumah, atas dasar tersebut Hakim berpendapat bahwa rumah tangga Penggugat dan Tergugat telah berada dalam kondisi pecah (*broken marriage*) dan sudah sulit untuk dipertahankan lagi;

Menimbang, bahwa dari apa yang diuraikan di atas, maka Hakim berpendapat pintu perceraian dapat dibuka guna menghindarkan para pihak dari kemelut rumah tangga yang berkepanjangan yang akan membawa mudharat kepada kehidupan Penggugat dan Tergugat apabila rumah tangga tetap dipertahankan;

Menimbang, bahwa hukum perceraian menurut Islam berkisar pada hukum haram, wajib, sunat, mubah dan makruh, dan dalam perkara ini perceraian menjadi diperbolehkan, dan oleh karena *Imsak bil Ma'ruf* tidak berhasil maka perceraian dianggap sebagai *Tasrih bi Ihsan*;

Menimbang, bahwa berdasarkan ketentuan Pasal 22 ayat (2) Peraturan Pemerintah Nomor 9 Tahun 1975 Tentang Pelaksanaan Undang-Undang Nomor 1 Tahun 1974 Tentang Perkawinan, jo. Pasal 134 Kompilasi Hukum Islam apabila telah cukup jelas mengenai sebab-sebab perselisihan dan pertengkaran dan setelah mendengar pihak keluarga serta orang-orang yang dekat dengan suami istri, maka perceraian dapat diterima untuk dipertimbangkan;

Menimbang, bahwa berdasarkan pertimbangan-pertimbangan tersebut Hakim menyimpulkan harus dinyatakan Tergugat tidak hadir sesuai dengan Pasal 149 ayat (1) R.bg dan telah terbukti rumah tangga antara Penggugat dan Tergugat sudah tidak ada harapan untuk dapat hidup rukun, dengan demikian alasan perceraian yang diajukan oleh Penggugat tersebut telah memenuhi

Disclaimer
Kepaniteraan Mahkamah Agung Republik Indonesia berusaha untuk selalu mencantumkan informasi paling kini dan akurat sebagai bentuk komitmen Mahkamah Agung untuk pelayanan publik, transparansi dan akuntabilitas pelaksanaan fungsi peradilan. Namun dalam hal-hal tertentu masih dimungkinkan terjadi permasalahan teknis terkait dengan akurasi dan keterkinian informasi yang kami sajikan, hal mana akan terus kami perbaiki dari waktu kewaktu.
Dalam hal Anda menemukan inakurasi informasi yang termuat pada situs ini atau informasi yang seharusnya ada, namun belum tersedia, maka harap segera hubungi Kepaniteraan Mahkamah Agung RI melalui :
Email : kepaniteraan@mahkamahagung.go.id   Telp : 021-384 3348 (ext.318)

maksud ketentuan 39 ayat (2) huruf f Penjelasan Undang-Undang Nomor 1 Tahun 1974 *jo.* Pasal 19 huruf f Peraturan Pemerintah Nomor 9 Tahun 1975 *jo.* Pasal 116 huruf f Kompilasi Hukum Islam, oleh karenanya Hakim berkesimpulan gugatan Penggugat pada petitum angka 1 (satu) dan 2 (dua) dalam surat gugatannya dapat dikabulkan dengan *verstek*;

Menimbang, bahwa dengan dikabulkannya petitum angka 1 (satu) dan 2 (dua) dalam surat gugatannya, dan oleh karena perceraian ini adalah perceraian pertama antara Penggugat dan Tergugat, maka berdasarkan Pasal 119 ayat 2 huruf c Kompilasi Hukum Islam talak yang dijatuhkan terhadap Penggugat adalah talak satu ba'in sughra;

Menimbang, bahwa sesuai dengan ketentuan Pasal 89 ayat (1) Undang-Undang Nomor 7 Tahun 1989 Tentang Peradilan Agama sebagaimana telah diubah dengan Undang-Undang Nomor 3 Tahun 2006, terakhir dengan perubahan kedua Undang-Undang Nomor 50 Tahun 2009, serta berdasarkan azas *lex spesialis derogat lex generalis,* maka biaya yang timbul dalam perkara ini harus dibebankan kepada Penggugat yang jumlahnya seperti tercantum dalam diktum amar putusan ini;

Mengingat peraturan perundang-undangan yang berlaku dan hukum syara yang berkaitan dengan perkara ini;

M E N G A D I L I

1 Menyatakan Tergugat telah dipanggil secara resmi dan patut untuk menghadap persidangan, tidak hadir;
2 Mengabulkan gugatan Penggugat secara Verstek;
3 Menjatuhkan talak satu bain shugra Tergugat (**Tergugat**) terhadap Penggugat (**Penggugat**);
4 Membebankan kepada Penggugat untuk membayar biaya perkara sejumlah Rp.351.000,00 (tiga ratus lima puluh satu ribu rupiah);

Demikian diputuskan Hakim Pengadilan Agama Dumai pada hari Rabu tanggal 23 Desember 2020 Masehi bertepatan dengan tanggal 8 Jumadil Awwal 1442 Hijriah oleh kami **Syahrullah, S.H.I., M.H**, putusan tersebut diucapkan pada hari itu juga dalam sidang terbuka untuk umum oleh Hakim tersebut, dan didampingi oleh **Murzani, S.Ag** sebagai Panitera Pengganti,

Disclaimer
Kepaniteraan Mahkamah Agung Republik Indonesia berusaha untuk selalu mencantumkan informasi paling kini dan akurat sebagai bentuk komitmen Mahkamah Agung untuk pelayanan publik, transparansi dan akuntabilitas pelaksanaan fungsi peradilan. Namun dalam hal-hal tertentu masih dimungkinkan terjadi permasalahan teknis terkait dengan akurasi dan keterkinian informasi yang kami sajikan, hal mana akan terus kami perbaiki dari waktu kewaktu.
Dalam hal Anda menemukan inakurasi informasi yang termuat pada situs ini atau informasi yang seharusnya ada, namun belum tersedia, maka harap segera hubungi Kepaniteraan Mahkamah Agung RI melalui :
Email : kepaniteraan@mahkamahagung.go.id Telp : 021-384 3348 (ext.318)

dengan dihadiri oleh Penggugat tanpa hadirnya Tergugat.

Hakim,

Syahrullah, S.H.I., M.H

Panitera Pengganti

Murzani, S.Ag

Rincian biaya:

1.	Biaya Pendaftaran	:	Rp30.000,00
2.	Biaya ATK Perkara	:	Rp60.000,00
3.	Biaya Panggilan	:	Rp225.000,00
4.	PNBP Panggilan	:	Rp20.000,00
5.	Biaya Redaksi	:	Rp10.000,00
6.	Biaya Meterai	:	Rp6.000,00
	**JUMLAH**	**:**	**Rp351.000,00**

(tiga ratus lima puluh satu ribu rupiah).

Disclaimer
Kepaniteraan Mahkamah Agung Republik Indonesia berusaha untuk selalu mencantumkan informasi paling kini dan akurat sebagai bentuk komitmen Mahkamah Agung untuk pelayanan publik, transparansi dan akuntabilitas pelaksanaan fungsi peradilan. Namun dalam hal-hal tertentu masih dimungkinkan terjadi permasalahan teknis terkait dengan akurasi dan keterkinian informasi yang kami sajikan, hal mana akan terus kami perbaiki dari waktu kewaktu.
Dalam hal Anda menemukan inakurasi informasi yang termuat pada situs ini atau informasi yang seharusnya ada, namun belum tersedia, maka harap segera hubungi Kepaniteraan Mahkamah Agung RI melalui :
Email : kepaniteraan@mahkamahagung.go.id Telp : 021-384 3348 (ext.318)
