# Supplementary material for: Classification of divorce causes during the COVID-19 pandemic using convolutional neural networks
Source: PeerJ Comput Sci. 2022 Jun 30;8:e998. doi: 10.7717/peerj-cs.998 (PMC9299239; doi:10.7717/peerj-cs.998)
Supplement: Supplemental Information 6 [file peerj-cs-08-998-s006.zip › Rujuk Dataset/Data ke-20.pdf]

P U T U S A N

Nomor 0000/Pdt.G/2020/PA.Skh.

بِسْمِ اللَّهِ الرَّحْمَنِ الرَّحِيمِ

DEMI KEADILAN BERDASARKAN KETUHANAN YANG MAHA ESA

Pengadilan Agama Sukoharjo yang memeriksa dan mengadili perkara tertentu pada tingkat pertama dalam sidang Majelis Hakim telah menjatuhkan putusan dalam perkara Cerai Gugat antara:

PENGGUGAT, umur 27 tahun, NIK 0000000000000000, (Tempat Lahir Sukoharjo, Tanggal Lahir 25 Juni 1993), agama Islam, pendidikan SLTA, pekerjaan Ibu Rumah Tangga, tempat kediaman di Sukoharjo, sebagai “**Penggugat**”;

lawan

TERGUGAT, umur 28 tahun, agama Islam, pendidikan SLTA, pekerjaan Karyawan Swasta, tempat kediaman di Demak, sebagai “**Tergugat**”;

Pengadilan Agama tersebut;

Telah mempelajari surat-surat yang berkaitan dengan perkara ini;

Telah mendengar keterangan Penggugat dan para saksi di muka sidang;

DUDUK PERKARA

Bahwa Penggugat dalam surat gugatannnya tanggal 27 November 2020 telah mengajukan Cerai Gugat, yang telah terdaftar di Kepaniteraan Pengadilan Agama Sukoharjo, dengan Nomor 0000/Pdt.G/2020/PA.Skh., tanggal 27 November 2020 , dengan dalil-dalil pada pokoknya sebagai berikut :

1. Bahwa pada Kamis tanggal 31 Januari 2013, Penggugat dengan Tergugat melangsungkan pernikahan yang dicatat oleh Pegawai Pencatat Nikah Kantor Urusan Agama Kecamatan Nguter, Kabupaten Sukoharjo (Kutipan Akta Nikah Nomor : 40/40/I/2013

Disclaimer
Kepaniteraan Mahkamah Agung Republik Indonesia berusaha untuk selalu mencantumkan informasi paling kini dan akurat sebagai bentuk komitmen Mahkamah Agung untuk pelayanan publik, transparansi dan akuntabilitas pelaksanaan fungsi peradilan. Namun dalam hal-hal tertentu masih dimungkinkan terjadi permasalahan teknis terkait dengan akurasi dan keterkinian informasi yang kami sajikan, hal mana akan terus kami perbaiki dari waktu kewaktu.
Dalam hal Anda menemukan inakurasi informasi yang termuat pada situs ini atau informasi yang seharusnya ada, namun belum tersedia, maka harap segera hubungi Kepaniteraan Mahkamah Agung RI melalui :
Email : kepaniteraan@mahkamahagung.go.id Telp : 021-384 3348 (ext.318)

tertanggal 31 Januari 2013). Pada saat menikah Penggugat berstatus Perawan dan Tergugat berstatus Jejeka .

2. Bahwa setelah pernikahan tersebut Penggugat dengan Tergugat bertempat tinggal di rumah orang tua Tergugat beralamat di Dukuh Rejosari Rt. 002/Rw.03 . Kelurahan Ngadiroyo, Kecamatan Nguntoronadi, Kabupaten Wonogiri, selama kurang lebih 3 (tiga) bulan, kemudian pindah di rumah orang tua Penggugat Dukuh Kedungsari Rt 001 Rw 009, Kelurahan Lawu, Kecamatan Nguter, Kabupaten Sukoharjo, sampai kurang lebih 5 (lima) tahun , kemudian Tergugat pulang sendiri di rumah orang tuanya beramalat di Jln.Pucang Sentosa Tengah 2 No.16 Rt. 09/Rw.30 Desa Pucang Gading . Kelurahan Batusari, Kecamatan Meranggen, Kabupaten Demak sampai sekarang.

3. Bahwa selama pernikahan tersebut Penggugat dengan Tergugat telah hidup rukun sebagaimana layaknya suami istri (ba'da dukhul) da sudah dikaruniai 2 (dua) anak bernama:

a. ANAK 1 PENGGUGAT DAN TERGUGAT , Perempuan, Sukoharjo 31 Mei 2013 (Sekarang Ikut Penggugat)

b. ANAK 2 PENGGUGAT DAN TERGUGAT , Laki-laki, Wonogiri 31 Agustus 2019 (Sekarang Ikut Penggugat)

4. Bahwa rumah tangga Penggugat dengan Tergugat semula berjalan dengan harmonis dan bahagia, namun sejak awal pernikahan pada bulan Juni 2016, rumah tangga Penggugat dan Tergugat menjadi goyah, antara Penggugat dan Tergugat terus- menerus terjadi perselisihan dan tidak ada harapan akan hidup rukun lagi dalam rumah tangga disebabkan: Tergugat jarang memeberi nafkah lahir kepada Penggugat .

5. Bahwa ketika perselisihan dan pertengkaran tersebut mencapai puncaknya pada bulan Juli 2019, Tergugat pergi dari rumah orag tua Penggugat dengan ijin Penggugat kemudian Tergugat pulang kerumah orang tuanya yang beralamat di Jln.Pucang Sentosa Tengah 2 No.16 Rt. 09/Rw.30 Desa Pucang Gading . Kelurahan Batusari, Kecamatan Meranggen, Kabupaten Demak sampai sekarang.

Disclaimer
Kepaniteraan Mahkamah Agung Republik Indonesia berusaha untuk selalu mencantumkan informasi paling kini dan akurat sebagai bentuk komitmen Mahkamah Agung untuk pelayanan publik, transparansi dan akuntabilitas pelaksanaan fungsi peradilan. Namun dalam hal-hal tertentu masih dimungkinkan terjadi permasalahan teknis terkait dengan akurasi dan keterkinian informasi yang kami sajikan, hal mana akan terus kami perbaiki dari waktu kewaktu.
Dalam hal Anda menemukan inakurasi informasi yang termuat pada situs ini atau informasi yang seharusnya ada, namun belum tersedia, maka harap segera hubungi Kepaniteraan Mahkamah Agung RI melalui :
Email : kepaniteraan@mahkamahagung.go.id Telp : 021-384 3348 (ext.318)

6. Bahwa pihak keluarga Penggugat sudah berusaha mendamaikan Penggugat dan Tergugat agar dapat hidup rukun lagi dalam rumah tangga, akan tetapi usaha tersebut tidak berhasil.

7. Bahwa berdasarkan hal-hal tersebut di atas, maka dalil-dalil permohonan cerai gugat telah berdasarkan atas hukum yang berlaku dan telah sesuai dengan ketentuan Pasal 39 ayat (2) Undang-undang Nomor 1 Tahun 1974 serta telah sesuai dengan alasan perceraian sebagaimana diatur dalam Pasal 19 huruf (f) PP No. 9 Tahun 1975 Jo Pasal 116 huruf (f) Kompilasi Hukum Islam.

8. Bahwa Penggugat sanggup membayar seluruh biaya yang timbul akibat perkara ini.

Berdasarkan alasan/ dalil-dalil di atas, Penggugat mohon kepada Ketua Pengadilan Agama Sukoharjo Cq Majelis Hakim untuk segera memeriksa dan mengadili perkara ini, selanjutnya menjatuhkan putusan yang amarnya berbunyi :

PRIMER :

1. Mengabulkan gugatan Penggugat;
2. Menjatuhkan talak satu ba'in sughro dari Tergugat (TERGUGAT) terhadap Penggugat (PENGGUGAT);
3. Membebankan biaya perkara menurut hukum;

SUBSIDER :

Mohon putusan yang seadil-adilnya;

Bahwa pada persidangan yang telah ditetapkan Penggugat telah datang menghadap di persidangan, sedangkan Tergugat tidak datang menghadap di persidangan dan tidak menyuruh orang lain untuk menghadap sebagai wakil/kuasanya yang sah, meskipun kepada Tergugat telah dipanggil secara resmi dan patut, sedangkan tidak ternyata bahwa tidak datangnya Tergugat tersebut disebabkan oleh suatu alasan yang sah;

Bahwa Majelis Hakim telah menasehati Penggugat agar berpikir untuk tidak bercerai dengan Tergugat, tetapi Penggugat tetap pada dalil-dalil gugatannya untuk bercerai dengan Tergugat;

Disclaimer
Kepaniteraan Mahkamah Agung Republik Indonesia berusaha untuk selalu mencantumkan informasi paling kini dan akurat sebagai bentuk komitmen Mahkamah Agung untuk pelayanan publik, transparansi dan akuntabilitas pelaksanaan fungsi peradilan. Namun dalam hal-hal tertentu masih dimungkinkan terjadi permasalahan teknis terkait dengan akurasi dan keterkinian informasi yang kami sajikan, hal mana akan terus kami perbaiki dari waktu kewaktu.
Dalam hal Anda menemukan inakurasi informasi yang termuat pada situs ini atau informasi yang seharusnya ada, namun belum tersedia, maka harap segera hubungi Kepaniteraan Mahkamah Agung RI melalui :
Email : kepaniteraan@mahkamahagung.go.id Telp : 021-384 3348 (ext.318)

Bahwa perkara ini tidak dapat dimediasi karena Tergugat tidak pernah datang menghadap meskipun telah dipanggil secara resmi dan patut, selanjutnya dimulai pemeriksaan dengan membacakan surat gugatan Penggugat yang maksud dan tujuannya tetap dipertahankan oleh Penggugat;

Bahwa untuk menguatkan dalil-dalil gugatannya, Penggugat telah mengajukan bukti berupa:

1.-- Surat:

a. Fotokopi Kartu Tanda Penduduk atas nama Penggugat NIK : 0000000000000000 yang dikeluarkan oleh Pemerintah Kabupaten Sukoharjo, Provinsi Jawa Tengah. Bukti surat tersebut telah diberi meterai cukup dan telah dicocokkan dengan aslinya yang ternyata sesuai, (bukti P.1);

b. Fotokopi Kutipan Akta Nikah Nomor 40/40/I/2013 tanggal 31 Januari 2013, yang dikeluarkan oleh Kantor Urusan Agama KUA Nguter, Kabupaten Sukoharjo. Bukti surat tersebut telah diberi meterai cukup dan telah dicocokkan dengan aslinya yang ternyata ternyata sesuai, (bukti P.2);

2.-- Saksi:

1. SAKSI 1, umur 25 tahun, Agama Islam, Pekerjaan Ibu Rumah Tangga, tempat tinggal di Sukoharjo, dibawah sumpahnya ia memberikan keterangan sebagai berikut :

- Bahwa saksi kenal dengan Penggugat dan Tergugat karena saksi sebagai ibu kandung Penggugat;
- Bahwa Penggugat dan Tergugat adalah suami isteri yang menikah pada tahun 2013 yang lalu, telah dikaruniai 2 orang anak;
- Bahwa setelah menikah Penggugat dengan Tergugat tinggal bersama di rumah orang tua Tergugat beralamat di Wonogiri, kemudian pindah di rumah orang tua Penggugat Dukuh Kedungsari

Disclaimer
Kepaniteraan Mahkamah Agung Republik Indonesia berusaha untuk selalu mencantumkan informasi paling kini dan akurat sebagai bentuk komitmen Mahkamah Agung untuk pelayanan publik, transparansi dan akuntabilitas pelaksanaan fungsi peradilan. Namun dalam hal-hal tertentu masih dimungkinkan terjadi permasalahan teknis terkait dengan akurasi dan keterkinian informasi yang kami sajikan, hal mana akan terus kami perbaiki dari waktu kewaktu.
Dalam hal Anda menemukan inakurasi informasi yang termuat pada situs ini atau informasi yang seharusnya ada, namun belum tersedia, maka harap segera hubungi Kepaniteraan Mahkamah Agung RI melalui :
Email : kepaniteraan@mahkamahagung.go.id Telp : 021-384 3348 (ext.318)

Rt 001 Rw 009, Kelurahan Lawu, Kecamatan Nguter, Kabupaten Sukoharjo;

- Bahwa setahu saksi rumah tangga antara Penggugat dan Tergugat pada awalnya harmonis akan tetapi sejak bulan Juni 2016 mulai tidak rukun lagi sering berselisih dan bertengkar;
- Bahwa penyebabnya adalah masalah ekonomi, Tergugat jarang memberi nafkah kepada Penggugat;
- Bahwa puncaknya pada bulan Juli 2019 Tergugat pergi meninggalkan Penggugat sehingga pisah rumah sampai sekarang sudah lebih dari 1 tahun lamanya;
- Bahwa sejak kejadian tersebut Tergugat sudah tidak pernah ada komunikasi layaknya suami isteri hingga sekarang;
- Bahwa saksi sebagai keluarga sudah berusaha mendamaikan mereka akan tetapi tidak berhasil ;

2. SAKSI 2, umur 53 tahun, Agama Islam, Pekerjaan Ibu Rumah Tangga, tempat tinggal di Sukoharjo, dibawah sumpahnya ia memberikan keterangan sebagai berikut :

- Bahwa bahwa saksi kenal dengan Penggugat dan Tergugat karena saksi sebagai bibi Penggugat;
- Bahwa Penggugat dan Tergugat adalah suami isteri yang menikah sejak 7 tahun yang lalu, dan telah dikaruniai 2 orang anak;
- Bahwa setelah menikah Penggugat dengan Tergugat tinggal bersama di rumah orang tua Tergugat beralamat di Wonogiri, kemudian pindah di rumah orang tua Penggugat Dukuh Kedungsari Rt 001 Rw 009, Kelurahan Lawu, Kecamatan Nguter, Kabupaten Sukoharjo;
- Bahwa setahu saksi rumah tangga antara Penggugat dan Tergugat pada awalnya harmonis akan tetapi sejak bulan Juni 2016 mulai tidak rukun lagi sering berselisih dan bertengkar karena masalah ekonomi, Tergugat jarang memberi nafkah kepada Penggugat;

Disclaimer
Kepaniteraan Mahkamah Agung Republik Indonesia berusaha untuk selalu mencantumkan informasi paling kini dan akurat sebagai bentuk komitmen Mahkamah Agung untuk pelayanan publik, transparansi dan akuntabilitas pelaksanaan fungsi peradilan. Namun dalam hal-hal tertentu masih dimungkinkan terjadi permasalahan teknis terkait dengan akurasi dan keterkinian informasi yang kami sajikan, hal mana akan terus kami perbaiki dari waktu kewaktu.
Dalam hal Anda menemukan inakurasi informasi yang termuat pada situs ini atau informasi yang seharusnya ada, namun belum tersedia, maka harap segera hubungi Kepaniteraan Mahkamah Agung RI melalui :
Email : kepaniteraan@mahkamahagung.go.id Telp : 021-384 3348 (ext.318)

- Bahwa puncaknya pada bulan Juli 2019 Tergugat pergi meninggalkan Penggugat sehingga pisah rumah sampai sekarang sudah lebih dari 1 tahun lamanya;
- Bahwa sejak kejadian tersebut Tergugat sudah tidak pernah ada komunikasi layaknya suami isteri hingga sekarang;
- Bahwa saksi sebagai keluarga sudah berusaha mendamaikan mereka akan tetapi tidak berhasil

Menimbang, bahwa terhadap keterangan saksi-saksi tersebut Penggugat tidak keberatan;

Menimbang, bahwa Penggugat telah mencukupkan bukti-buktinya di persidangan ;

Menimbang, bahwa Penggugat telah menyampaikan kesimpulannya secara lisan di persidangan yang pada pokoknya Penggugat tetap menghendaki perceraian serta Penggugat mohon agar Hakim segera menjatuhkan putusan yang seadil-adilnya ;

Selanjutnya untuk singkatnya uraian putusan ini, maka semua hal yang termuat dalam berita acara sidang merupakan bagian yang tidak terpisahkan dari putusan ini;

PERTIMBANGAN HUKUM

Menimbang, bahwa maksud dan tujuan gugatan Penggugat adalah sebagaimana terurai di atas;

Menimbang, bahwa ternyata Tergugat tidak datang menghadap di persidangan dan tidak pula menyuruh orang lain untuk menghadap sebagai wakil/kuasanya yang sah, meskipun berdasarkan Surat Panggilan (relaas) yang dibacakan di persidangan, Tergugat telah dipanggil secara resmi dan patut, sedangkan tidak ternyata bahwa tidak datangnya Tergugat tersebut disebabkan oleh suatu alasan yang sah;

Menimbang, bahwa Majelis Hakim berdasarkan pasal 82 Undang-undang Nomor 7 tahun 1989 yang telah diubah dan ditambah dengan Undang-undang Nomor 3 tahun 2006 dan Undang-undang Nomor 50 tahun 2009 tentang Peradilan Agama, telah berusaha menasihati Penggugat,

Disclaimer
Kepaniteraan Mahkamah Agung Republik Indonesia berusaha untuk selalu mencantumkan informasi paling kini dan akurat sebagai bentuk komitmen Mahkamah Agung untuk pelayanan publik, transparansi dan akuntabilitas pelaksanaan fungsi peradilan. Namun dalam hal-hal tertentu masih dimungkinkan terjadi permasalahan teknis terkait dengan akurasi dan keterkinian informasi yang kami sajikan, hal mana akan terus kami perbaiki dari waktu kewaktu.
Dalam hal Anda menemukan inakurasi informasi yang termuat pada situs ini atau informasi yang seharusnya ada, namun belum tersedia, maka harap segera hubungi Kepaniteraan Mahkamah Agung RI melalui :
Email : kepaniteraan@mahkamahagung.go.id Telp : 021-384 3348 (ext.318)

supaya bersabar dan rukun kembali untuk membina rumah tangganya dengan Tergugat, akan tetapi tidak berhasil;

Menimbang, bahwa berdasarkan pasal 7 ayat (2) Peraturan Mahkamah Agung RI (PERMA) Nomor 1 Tahun 2016 tentang Mediasi, mejelis hakim tidak dapat memberikan kesempatan untuk melakukan mediasi, karena Tergugat tidak pernah hadir, karena itu usaha perdamaian dinyatakan gagal ;

Menimbang bahwa gugatan Penggugat untuk bercerai dengan Tergugat yang pada pokoknya didasarkan kepada alasan, bahwa rumah tangga Penggugat dan Tergugat awalnya baik dan rukun namun sejak bulan Juni 2016 mulai tidak rukun sering berselisih dan bertengkar masalah ekonomi, Tergugat jarang memberi nafkah kepada Penggugat, puncaknya pada bulan Juli 2019 Tergugat pergi meninggalkan Penggugat sehingga pisah rumah tanpa saling berkomunikasi lagi sampai sekarang sudah selama lebih dari 1 tahun lamanya;

Menimbang, bahwa atas surat gugatan Penggugat tersebut, Tergugat tidak mengajukan jawabannya, karena tidak hadir di persidangan.

Menimbang, bahwa meskipun Tergugat tidak hadir, karena perkara ini termasuk ruang lingkup sengketa perkawinan, maka kepada Penggugat tetap dibebani wajib bukti.

Menimbang, bahwa dalam perkara ini Penggugat telah mengajukan bukti tertulis (P.1) dan (P.2) yang telah memenuhi formil materiil sebagai alat bukti dan dua orang saksi dan kedua orang saksi tersebut telah disumpah sesuai dengan agamanya, oleh sebab itu keterangan saksi tersebut adalah formal dan dapat dijadikan bukti dalam perkara ini;

Menimbang, bahwa berdasarkan bukti (P.1) terbukti Penggugat bertempat tinggal di wilayah hukum Pengadilan Agama Sukoharjo maka adalah tepat dan beralasan Penggugat mengajukan gugatannya ke Pengadilan Agama Sukoharjo (pasal 73 Undang-undang Nomor 7 tahun 1989 yang telah diubah dan ditambah dengan Undang-undang Nomor 3 tahun 2006 dan Undang-undang Nomor 50 tahun 2009 tentang Peradilan Agama);

Disclaimer
Kepaniteraan Mahkamah Agung Republik Indonesia berusaha untuk selalu mencantumkan informasi paling kini dan akurat sebagai bentuk komitmen Mahkamah Agung untuk pelayanan publik, transparansi dan akuntabilitas pelaksanaan fungsi peradilan. Namun dalam hal-hal tertentu masih dimungkinkan terjadi permasalahan teknis terkait dengan akurasi dan keterkinian informasi yang kami sajikan, hal mana akan terus kami perbaiki dari waktu kewaktu.
Dalam hal Anda menemukan inakurasi informasi yang termuat pada situs ini atau informasi yang seharusnya ada, namun belum tersedia, maka harap segera hubungi Kepaniteraan Mahkamah Agung RI melalui :
Email : kepaniteraan@mahkamahagung.go.id   Telp : 021-384 3348 (ext.318)

Menimbang, bahwa surat bukti (P.2) berupa Fotocopy sah Kutipan Akta Nikah adalah merupaka alat bukti autentik karena dibuat berdasarkan Peraturan Perundang-undangan oleh Pejabat yang berwenang, sehingga mempunyai kekuatan pembuktian sempurna selama tidak dibuktikan kepalsuannya (pasal 165 HIR);

Menimbang, bahwa berdasarkan pasal 7 ayat 1 Kompilasi Hukum Islam yang menyatakan bahwa perkawinan hanya dapat dibuktikan dengan Akta Nikah yang dibuat oleh Pegawai Pencatat Nikah, maka harus dinyatakan terbukti bahwa antara Penggugat dan Tergugat telah terikat dengan perkawinan yang sah, oleh karena itu Penggugat mempunyai alasan hukum untuk mengajukan gugatan ini;

Menimbang, bahwa saksi-saksi adalah orang-orang yang dekat dengan kedua pihak yang diajukan telah memberikan keterangan lisan di persidangan dibawah sumpahnya, keterangan mana berdasarkan peristiwa yang dialamai sendiri, saling berhubungan satu dengan yang lain dan mendukung dalil-dalil gugatan Penggugat yang pada pokoknya benar rumah tangga Penggugat dan Tergugat sekurang-kurangnya sejak bulan Juni 2016 mulai tidak rukun sering berselisih dan bertengkar masalah ekonomi, Tergugat jarang memberi nafkah kepada Penggugat, puncaknya pada bulan Juli 2019 Tergugat pergi meninggalkan Penggugat sehingga pisah rumah tanpa saling berkomunikasi lagi sampai sekarang sudah selama lebih dari 1 tahun lamanya;

Menimbang, bahwa berdasarkan bukti (P.1) dan (P.2) serta keterangan dua orang saksi yang saling bersesuaian, terungkap fakta sebagai berikut :

- Bahwa Penggugat dan Tergugat adalah suami isteri sah, menikah pada tanggal 31 Januari 2013, dalam keadaan bakda dukhul, telah dikaruniai 2 orang anak;
- Bahwa setelah menikah Penggugat dan Tergugat tinggal bersama di rumah orang tua Tergugat beralamat di Wonogiri, kemudian pindah di rumah orang tua Penggugat Dukuh Kedungsari Rt

Disclaimer
Kepaniteraan Mahkamah Agung Republik Indonesia berusaha untuk selalu mencantumkan informasi paling kini dan akurat sebagai bentuk komitmen Mahkamah Agung untuk pelayanan publik, transparansi dan akuntabilitas pelaksanaan fungsi peradilan. Namun dalam hal-hal tertentu masih dimungkinkan terjadi permasalahan teknis terkait dengan akurasi dan keterkinian informasi yang kami sajikan, hal mana akan terus kami perbaiki dari waktu kewaktu.
Dalam hal Anda menemukan inakurasi informasi yang termuat pada situs ini atau informasi yang seharusnya ada, namun belum tersedia, maka harap segera hubungi Kepaniteraan Mahkamah Agung RI melalui :
Email : kepaniteraan@mahkamahagung.go.id Telp : 021-384 3348 (ext.318)

001 Rw 009, Kelurahan Lawu, Kecamatan Nguter, Kabupaten Sukoharjo;

- Bahwa rumah tangga Penggugat dan Tergugat awalnya baik dan rukun akan tetapi sejak bulan Juni 2016 mulai tidak rukun sering berselisih dan bertengkar masalah ekonomi, Tergugat jarang memberi nafkah kepada Penggugat, puncaknya pada bulan Juli 2019 Tergugat pergi meninggalkan Penggugat sehingga pisah rumah tanpa saling berkomunikasi lagi sampai sekarang sudah selama lebih dari 1 tahun lamanya;
- Bahwa selama berpisah kedua pihak sudah tidak saling berkomunikasi layaknya suami isteri;
- Bahwa keluarga sudah berusaha mendamaikan kedua pihak tetapi tidak berhasil;

Menimbang, bahwa berdasarkan fakta tersebut diatas terbukti rumah tangga antara Penggugat dan Tergugat telah pecah dan sulit dirukunkan kembali karena selalu diwarnai perselisihan dan pertengkaran terus menerus yang sulit didamaikan disebabkan masalah ekonomi, Tergugat jarang memberi nafkah kepada Penggugat, sehingga pisah rumah tanpa komunikasi lagi layaknya suami isteri sampai sekarang selama lebih dari 1 tahun lamanya

Menimbang, bahwa oleh karena rumah tangga Penggugat dan Tergugat telah nyata pecah, maka apabila perkawinan antara Penggugat dan Tergugat tersebut tetap dipertahankan niscaya akan menimbulkan madlarat yang lebih besar bagi kedua belah pihak atau salah satu pihak ;

Menimbang, bahwa Majelis sependapat dengan pendapat Fuqaha dalam kitab Fiqhus Sunnah Juz II hal. 291 yang artinya sebagai berikut:

Artinya ; "Apabila isteri menggugat kemadlorotan suami karena tidak dapat melangsungkan kehidupan berkeluarga diantara keduanya, isteri boleh meminta kepada Hakim untuk dipisahkan/ diceraikan seketika itu juga, maka Hakim dapat menjatuhkan thalaknya dengan Thalak Bain, apabila terbukti kemadlorotan tersebut dan tidak tercapainya perdamaian diantara keduanya";

Disclaimer
Kepaniteraan Mahkamah Agung Republik Indonesia berusaha untuk selalu mencantumkan informasi paling kini dan akurat sebagai bentuk komitmen Mahkamah Agung untuk pelayanan publik, transparansi dan akuntabilitas pelaksanaan fungsi peradilan. Namun dalam hal-hal tertentu masih dimungkinkan terjadi permasalahan teknis terkait dengan akurasi dan keterkinian informasi yang kami sajikan, hal mana akan terus kami perbaiki dari waktu kewaktu.
Dalam hal Anda menemukan inakurasi informasi yang termuat pada situs ini atau informasi yang seharusnya ada, namun belum tersedia, maka harap segera hubungi Kepaniteraan Mahkamah Agung RI melalui :
Email : kepaniteraan@mahkamahagung.go.id Telp : 021-384 3348 (ext.318)

Menimbang, bahwa berdasarkan pertimbangan tersebut diatas, maka gugatan Penggugat cukup beralasan, karena telah memenuhi pasal 39 ayat (2) Undang-undang Nomor 1 tahun 1974 jo. Pasal 19 huruf (f) Peraturan Pemerintah No. 9 tahun 1975 jo. Pasal 116 huruf (f) Kompilasi Hukum Islam, maka sesuai dengan pasal 19 angka (2) huruf (c) Kompilasi Hukum Islam gugatan Penggugat tersebut patut dikabulkan dengan dijatuhkannya talak ba'in sughro Tergugat kepada Penggugat, dengan verstek (pasal 125 HIR);

Menimbang, bahwa perkara ini termasuk dalam bidang perkawinan maka berdasarkan pasal 89 ayat 1 Undang-undang Nomor 7 tahun 1989 yang telah diubah dengan Undang-undang nomor: 3 tahun 2006 dan diubah dengan Undang-undang nomor: 50 tahun 2009 tentang Peradilan Agama, maka biaya perkara dibebankan kepada Penggugat;

Memperhatikan ketentuan Hukum Islam dan peraturan perundang-undangan yang berkaitan dengan perkara ini;

MENGADILI

1. Menyatakan Tergugat yang telah dipanggil secara resmi dan patut untuk menghadap di persidangan tidak hadir;

2. Mengabulkan gugatan Penggugat secara verstek;

3. Menjatuhkan talak satu ba'in sughro Tergugat (TERGUGAT) terhadap Penggugat (PENGGUGAT);

4. Membebankan kepada Penggugat untuk membayar biaya perkara sejumlah Rp651.000,00 (enam ratus lima puluh satu ribu rupiah);

Demikian diputuskan dalam rapat musyawarah Majelis Hakim Pengadilan Agama Sukoharjo pada hari Selasa tanggal 29 Desember 2020 Masehi, bertepatan dengan tanggal 14 Jumadil Awwal 1442 Hijriah, oleh kami Sutikno, S.Ag, M.H. sebagai Hakim Ketua, M. Zarkasi Ahmadi, S.H. dan Hafidz Umami, S.H.I. sebagai Hakim Anggota, putusan tersebut diucapkan pada hari itu juga oleh Hakim Ketua tersebut dalam sidang terbuka untuk umum, dihadiri para Hakim Anggota dan Nur Endang Tri

Disclaimer
Kepaniteraan Mahkamah Agung Republik Indonesia berusaha untuk selalu mencantumkan informasi paling kini dan akurat sebagai bentuk komitmen Mahkamah Agung untuk pelayanan publik, transparansi dan akuntabilitas pelaksanaan fungsi peradilan. Namun dalam hal-hal tertentu masih dimungkinkan terjadi permasalahan teknis terkait dengan akurasi dan keterkinian informasi yang kami sajikan, hal mana akan terus kami perbaiki dari waktu kewaktu.
Dalam hal Anda menemukan inakurasi informasi yang termuat pada situs ini atau informasi yang seharusnya ada, namun belum tersedia, maka harap segera hubungi Kepaniteraan Mahkamah Agung RI melalui :
Email : kepaniteraan@mahkamahagung.go.id Telp : 021-384 3348 (ext.318)

Margawati, S.H, M.H. sebagai Panitera Pengganti serta dihadiri oleh Penggugat tanpa hadirnya Tergugat;

Ketua Majelis,

Sutikno, S.Ag, M.H.

Hakim Anggota	Hakim Anggota
**M. Zarkasi Ahmadi, S.H.**	**Hafidz Umami, S.H.I.**

Panitera Pengganti,

Nur Endang Tri Margawati, S.H, M.H.

Rincian Biaya Perkara ;

1.	Pendaftaran	: Rp	30.000,00
2.	ATK Perkara	: Rp	75.000,00
3.	Panggilan	: Rp	510.000,00
4.	PNBP Panggilan	: Rp	20.000,00
5.	Redaksi	: Rp	10.000,00
6.	Meterai	: Rp	6.000,00
J u m l a h		: Rp	651.000,00

(enam ratus lima puluh satu ribu rupiah).

Disclaimer
Kepaniteraan Mahkamah Agung Republik Indonesia berusaha untuk selalu mencantumkan informasi paling kini dan akurat sebagai bentuk komitmen Mahkamah Agung untuk pelayanan publik, transparansi dan akuntabilitas pelaksanaan fungsi peradilan. Namun dalam hal-hal tertentu masih dimungkinkan terjadi permasalahan teknis terkait dengan akurasi dan keterkinian informasi yang kami sajikan, hal mana akan terus kami perbaiki dari waktu kewaktu.
Dalam hal Anda menemukan inakurasi informasi yang termuat pada situs ini atau informasi yang seharusnya ada, namun belum tersedia, maka harap segera hubungi Kepaniteraan Mahkamah Agung RI melalui :
Email : kepaniteraan@mahkamahagung.go.id Telp : 021-384 3348 (ext.318)
