# Supplementary material for: Classification of divorce causes during the COVID-19 pandemic using convolutional neural networks
Source: PeerJ Comput Sci. 2022 Jun 30;8:e998. doi: 10.7717/peerj-cs.998 (PMC9299239; doi:10.7717/peerj-cs.998)
Supplement: Supplemental Information 6 [file peerj-cs-08-998-s006.zip › Rujuk Dataset/Data ke-21.pdf]

P U T U S A N

Nomor 7074/Pdt.G/2020/PA.Sbr

بِسْــــــمِ اللّٰهِ الرَّحْمٰنِ الرَّحِيْــــــمِ

DEMI KEADILAN BERDASARKAN KETUHANAN YANG MAHA ESA

Pengadilan Agama Sumber yang memeriksa dan mengadili perkara perdata pada tingkat pertama dalam persidangan Majelis telah menjatuhkan putusan sebagai berikut dalam perkara antara :

PENGGUGAT, umur xxx tahun, agama Islam, pendidikan xxx, pekerjaan xxx, tempat kediaman di xxx, dalam hal ini memberikan kuasa kepada xxx/Advokat/Pengacara beralamat di xxx berdasarkan Surat Kuasa Khusus tertanggal 03 Desember 2020, sebagai Penggugat;

melawan

TERGUGAT, umur xxx tahun, agama Islam, pendidikan xxx, pekerjaan xxx, tempat kediaman di xxx, sebagai Tergugat;

Pengadilan Agama tersebut;

Setelah mempelajari berkas perkara yang bersangkutan;

Setelah mendengar keterangan Penggugat/kuasa hukumnya dan saksi-saksi di muka persidangan;

DUDUK PERKARA

Bahwa, Penggugat dengan surat gugatannya tertanggal 03 Desember 2020 yang telah terdaftar di Kepaniteraan Pengadilan Agama Sumber dibawah Nomor #0001# tanggal 03 Desember 2020, telah mengajukan hal-hal sebagai berikut :

1. Bahwa Penggugat telah melangsungkan pernikahan dengan Tergugat pada tanggal 15 Mei 2014, dicatat di xxx, dengan Akta Nikah Nomor xxx tanggal 16 Mei 2014;
2. Bahwa setelah akad nikah Penggugat dan Tergugat membina rumah tangga dengan bertempat tinggal terakhir di rumah orang tua Penggugat, telah hidup rukun sebagai mana layaknya suami isteri xxx;

Disclaimer
Kepaniteraan Mahkamah Agung Republik Indonesia berusaha untuk selalu mencantumkan informasi paling kini dan akurat sebagai bentuk komitmen Mahkamah Agung untuk pelayanan publik, transparansi dan akuntabilitas pelaksanaan fungsi peradilan. Namun dalam hal-hal tertentu masih dimungkinkan terjadi permasalahan teknis terkait dengan akurasi dan keterkinian informasi yang kami sajikan, hal mana akan terus kami perbaiki dari waktu kewaktu.
Dalam hal Anda menemukan inakurasi informasi yang termuat pada situs ini atau informasi yang seharusnya ada, namun belum tersedia, maka harap segera hubungi Kepaniteraan Mahkamah Agung RI melalui :
Email : kepaniteraan@mahkamahagung.go.id   Telp : 021-384 3348 (ext.318)

3. Bahwa selama berumah tangga Penggugat dengan Tergugat belum pernah bercerai ;

4. Bahwa awalnya keadaan rumah tangga Penggugat dengan Tergugat berjalan dengan rukun dan harmonis, akan tetapi sekitar bulan Januari 2015 keharmonisan tersebut mulai pudar, karena antara Penggugat dan Tergugat sering terjadi perseilsihan dan pertengkaran yang mencapai puncaknya pada bulan Januari 2015;

5. Bahwa perselisihan dan pertengkaran Penggugat dan Tergugat tersebut disebabkan Tergugat tidak dapat memberikan nafkah secara cukup untuk Penggugat dan Tergugat mempunyai wanita idaman lain yang berasal dari Indramayu;

6. Bahwa akibat rumah tangga Penggugat dan Tergugat yang sering terjadi perselisihan dan pertengkaran tersebut, maka Penggugat dengan Tergugat telah berpisah tempat tinggal selama empat tahun;

7. Bahwa Penggugat telah berusaha bersabar dan berupaya mempertahankan keutuhan rumah tangga, akan tetapi tidak berhasil, sehingga untuk mencapai tujuan perkawinan yang sakinah, mawaddah dan rahmah tidak mungkin bisa terwujud, oleh karenanya cukup alasan bagi Penggugat untuk mengajukan perceraian ini;

Berdasarkan dalil-dalil/alasan tersebut diatas, Penggugat mohon dengan hormat, kiranya bapak Ketua Pengadilan Agama Sumber cq. Majelis Hakim yang memeriksa dan mengadili perkara ini, berkenan untuk menjatuhkan putusan yang amarnya berbunyi sebagai berikut :

1. Mengabulkan gugatan Penggugat
2. Menjatuhkan talak satu bain sughra Tergugat (TERGUGAT) terhadap Penggugat (PENGGUGAT);
3. Membebankan biaya perkara menurut hukum;

Atau apabila Pengadilan berpendapat lain, mohon putusan yang seadil-adilnya;

Bahwa, Penggugat telah menguasakan kepada kuasa hukumnya xxx/Advokat/Pengacara berdasarkan Surat Kuasa Khusus tertanggal 03 Desember 2020 dan telah terdaftar di Kepaniteraan Pengadilan Agama Sumber dengan Nomor 2380/Adv/XI/2020 tanggal 03 Desember 2020;

Disclaimer
Kepaniteraan Mahkamah Agung Republik Indonesia berusaha untuk selalu mencantumkan informasi paling kini dan akurat sebagai bentuk komitmen Mahkamah Agung untuk pelayanan publik, transparansi dan akuntabilitas pelaksanaan fungsi peradilan. Namun dalam hal-hal tertentu masih dimungkinkan terjadi permasalahan teknis terkait dengan akurasi dan keterkinian informasi yang kami sajikan, hal mana akan terus kami perbaiki dari waktu kewaktu.
Dalam hal Anda menemukan inakurasi informasi yang termuat pada situs ini atau informasi yang seharusnya ada, namun belum tersedia, maka harap segera hubungi Kepaniteraan Mahkamah Agung RI melalui :
Email : kepaniteraan@mahkamahagung.go.id Telp : 021-384 3348 (ext.318)

Bahwa, pada hari sidang yang telah ditetapkan, Penggugat dengan didampingi kuasa hukumnya datang menghadap di muka persidangan, sedangkan Tergugat tidak datang dan tidak pula menyuruh orang lain sebagai wakil atau kuasanya yang sah, padahal kepadanya telah dipanggil dengan resmi dan patut berdasarkan berita acara relaas panggilan tanggal 08 Desember 2020 dan 08 Desember 2020, sedangkan tidak ternyata ketidakhadirannya itu disebabkan oleh suatu alasan yang sah menurut hukum;

Bahwa, Majelis Hakim telah berusaha menasehati Penggugat baik secara prinsipal maupun melalui kuasa hukumnya agar #0046# dapat membina kembali rumah tangganya dengan Tergugat, akan tetapi tidak berhasil dan Majelis Hakim memandang perkara a quo tidak mungkin untuk dilaksanakan mediasi karena Tergugat tidak pernah hadir di persidangan;

Bahwa, selanjutnya pemeriksaan perkara ini dimulai dengan membacakan surat gugatan Penggugat yang maksud dan isinya tetap dipertahankan oleh Penggugat;

Bahwa, untuk membuktikan dalil gugatannya, Penggugat telah mengajukan alat bukti surat berupa :

1. Fotocopy dari Kartu Tanda Penduduk an. Penggugat, bukti tersebut telah diberi materai cukup dan telah dicocokan dengan aslinya yang ternyata sesuai, lalu oleh Ketua Majelis diberi tanda (P.1);
2. Fotocopy dari Kutipan Akta Nikah Nomor xxx Tanggal xxx yang dikeluarkan oleh Kantor Urusan Agama Kecamatan xxx Kabupten xxx, bukti tersebut telah diberi materai cukup dan telah dicocokan dengan aslinya yang ternyata sesuai, lalu oleh Ketua Majelis diberi tanda (P.2);

Bahwa, di samping itu, Penggugat juga telah mengajukan alat bukti saksi yaitu :

1. SAKSI I dibawah sumpah menerangkan pada pokoknya sebagai berikut :
   - Bahwa saksi adalah bibi Penggugat;
   - Bahwa semula rumah tangga Penggugat dengan Tergugat rukun dan harmonis di rumah orang tua Penggugat akan tetapi sejak bulan Januari

Disclaimer
Kepaniteraan Mahkamah Agung Republik Indonesia berusaha untuk selalu mencantumkan informasi paling kini dan akurat sebagai bentuk komitmen Mahkamah Agung untuk pelayanan publik, transparansi dan akuntabilitas pelaksanaan fungsi peradilan. Namun dalam hal-hal tertentu masih dimungkinkan terjadi permasalahan teknis terkait dengan akurasi dan keterkinian informasi yang kami sajikan, hal mana akan terus kami perbaiki dari waktu kewaktu.
Dalam hal Anda menemukan inakurasi informasi yang termuat pada situs ini atau informasi yang seharusnya ada, namun belum tersedia, maka harap segera hubungi Kepaniteraan Mahkamah Agung RI melalui :
Email : kepaniteraan@mahkamahagung.go.id Telp : 021-384 3348 (ext.318)

2015 rumah tangganya sudah tidak harmonis lagi, sering terjadi perselisihan dan pertengkaran;

- Bahwa penyebabnya karena Tergugat tidak dapat memberikan nafkah secara cukup untuk Penggugat dan Tergugat mempunyai wanita idaman lain yang berasal dari Indramayu;
- Bahwa sejak empat tahun yang lalu antara Penggugat dan Tergugat telah berpisah tempat tinggal dan tidak pernah berkumpul kembali;
- Bahwa saksi pernah berusaha menasehati Penggugat, akan tetapi tidak berhasil;
- Bahwa saksi sudah tidak sanggup lagi untuk merukunkan kembali;

Bahwa, terhadap keterangan saksi tersebut Penggugat membenarkannya;

2. SAKSI II dibawah sumpah menerangkan pada pokoknya sebagai berikut :

- Bahwa saksi adalah tetangga Penggugat;
- Bahwa awalnya rumah tangga Penggugat dan Tergugat rukun dan harmonis akan tetapi sejak bulan Januari 2015 rumah tangganya sudah tidak harmonis lagi sering terjadi perselisihan dan pertengkaran;
- Bahwa penyebab perselisihan dan pertengkaran tersebut karena Tergugat tidak dapat memberikan nafkah secara cukup untuk Penggugat dan Tergugat mempunyai wanita idaman lain yang berasal dari Indramayu;
- Bahwa sejak empat tahun yang lalu Penggugat dan Tergugat telah berpisah tempat tinggal dan tidak pernah berkumpul kembali;
- Bahwa saksi sudah tidak sanggup lagi untuk merukunkannya;

Bahwa, terhadap keterangan saksi tersebut Penggugat membenarkannya;

Bahwa, Penggugat dalam kesimpulannya mengatakan tidak akan mengajukan suatu apapun dan memohon kepada Majelis Hakim agar menjatuhkan putusannya;

Bahwa, untuk mempersingkat uraian pertimbangan ini ditunjuk hal-hal sebagaimana tercantum dalam berita acara persidangan perkara ini;

PERTIMBANGAN HUKUM

Disclaimer
Kepaniteraan Mahkamah Agung Republik Indonesia berusaha untuk selalu mencantumkan informasi paling kini dan akurat sebagai bentuk komitmen Mahkamah Agung untuk pelayanan publik, transparansi dan akuntabilitas pelaksanaan fungsi peradilan. Namun dalam hal-hal tertentu masih dimungkinkan terjadi permasalahan teknis terkait dengan akurasi dan keterkinian informasi yang kami sajikan, hal mana akan terus kami perbaiki dari waktu kewaktu.
Dalam hal Anda menemukan inakurasi informasi yang termuat pada situs ini atau informasi yang seharusnya ada, namun belum tersedia, maka harap segera hubungi Kepaniteraan Mahkamah Agung RI melalui :
Email : kepaniteraan@mahkamahagung.go.id Telp : 021-384 3348 (ext.318)

Menimbang, bahwa maksud dan tujuan gugatan Penggugat adalah sebagaimana terurai diatas;

Menimbang, bahwa Penggugat telah menguasakan kepada kuasa hukumnya xxx/Advokat/Pengacara berdasarkan Surat Kuasa Khusus tertanggal 03 Desember 2020, dan telah terdaftar di Kepaniteraan Pengadilan Agama Sumber, sehingga karenanya kuasa hukum tersebut secara formal dapat diterima untuk beracara dalam perkara ini;

Menimbang, bahwa Tergugat telah dipanggil dengan resmi dan patut, akan tetapi tidak datang dan tidak pula menyuruh orang lain sebagai wakilnya/kuasanya dan tidak ternyata tidak datangnya itu disebabkan suatu alasan yang sah, sedangkan gugatan Penggugat telah cukup beralasan dan tidak melawan hukum, maka Tergugat yang telah dipanggil tersebut harus dinyatakan tidak hadir dan sesuai dengan Pasal 125 ayat (1) HIR perkaranya dapat diputuskan dengan verstek;

Menimbang, bahwa sesuai dengan maksud dan ketentuan Pasal 82 Undang-Undang Nomor 7 Tahun 1989 tentang Peradilan Agama sebagaimana telah diubah dengan Undang-Undang Nomor 3 Tahun 2006 dan perubahan kedua dengan Undang-Undang Nomor 50 Tahun 2009, Majelis Hakim telah berusaha maksimal menasehati #0046# baik secara prinsipal maupun melalui kuasa hukumnya agar Penggugat dapat rukun kembali membina rumah tangga dengan Tergugat akan tetapi usaha tersebut tidak berhasil;

Menimbang, bahwa di samping itu oleh karena Tergugat tidak pernah hadir di persidangan, maka proses mediasi tidak dapat dilaksanakan (vide PERMA Nomor 1 Tahun 2016 Pasal 4 ayat 2 huruf b);

Menimbang, bahwa terlebih dahulu berdasarkan alat bukti (P.1) Majelis Hakim harus menyatakan bahwa oleh karena Penggugat berdomisili di wilayah hukum Pengadilan Agama Sumber, maka gugatan Penggugat dapat diterima untuk diperiksa dan diadili;

Menimbang, bahwa selanjutnya berdasarkan alat bukti (P.2) yang telah memenuhi syarat formil dan materil, harus dinyatakan terbukti bahwa hubungan hukum antara Penggugat dan Tergugat telah terikat dalam perkawinan yang sah sebagaimana diatur dalam Pasal 2 Undang-Undang Nomor 1 Tahun 1974;

Disclaimer
Kepaniteraan Mahkamah Agung Republik Indonesia berusaha untuk selalu mencantumkan informasi paling kini dan akurat sebagai bentuk komitmen Mahkamah Agung untuk pelayanan publik, transparansi dan akuntabilitas pelaksanaan fungsi peradilan. Namun dalam hal-hal tertentu masih dimungkinkan terjadi permasalahan teknis terkait dengan akurasi dan keterkinian informasi yang kami sajikan, hal mana akan terus kami perbaiki dari waktu kewaktu.
Dalam hal Anda menemukan inakurasi informasi yang termuat pada situs ini atau informasi yang seharusnya ada, namun belum tersedia, maka harap segera hubungi Kepaniteraan Mahkamah Agung RI melalui :
Email : kepaniteraan@mahkamahagung.go.id Telp : 021-384 3348 (ext.318)

Menimbang, bahwa pada pokoknya Penggugat telah mendasarkan #0053#nya Penggugat telah mendasarkan gugatan cerainya terhadap Tergugat dengan alasan sejak bulan Januari 2015 rumah tangga Penggugat dengan Tergugat sudah tidak harmonis, sering terjadi perselisihan dan pertengkaran yang mencapai puncaknya pada bulan Januari 2015 sehingga tidak ada harapan akan hidup rukun lagi dalam membina rumah tangga, dimana penyebabnya karena Tergugat tidak dapat memberikan nafkah secara cukup untuk Penggugat dan Tergugat mempunyai wanita idaman lain yang berasal dari Indramayu sehingga sejak empat tahun Penggugat dan Tergugat telah pisah tempat tinggal;

Menimbang, bahwa di muka persidangan Penggugat telah mengajukan dua orang saksi yang dari keterangannya saling bersesuaian, Majelis Hakim telah dapat menemukan fakta hukum sebagai berikut :

- Bahwa Penggugat dan Tergugat adalah suami isteri yang sah;
- Bahwa semula rumah tangga Penggugat dan Tergugat rukun dan harmonis;
- Bahwa sejak bulan Januari 2015 keadaan rumah tangganya tersebut sudah tidak harmonis lagi, sering terjadi perselisihan dan pertengkaran;
- Bahwa penyebabnya karena Tergugat tidak dapat memberikan nafkah secara cukup untuk Penggugat dan Tergugat mempunyai wanita idaman lain yang berasal dari Indramayu;
- Bahwa sejak empat tahun yang lalu Penggugat dan Tergugat telah berpisah tempat tinggal dan tidak pernah berkumpul kembali;

Menimbang, bahwa berdasarkan fakta hukum tersebut di atas, harus dinyatakan terbukti bahwa dalam kehidupan rumah tangga Penggugat dan Tergugat sudah tidak harmonis lagi, telah terjadi perselisihan dan pertengkaran yang mengakibatkan antara keduanya telah berpisah tempat tinggal dan tidak pernah berkumpul kembali dalam satu rumah tangga;

Menimbang, bahwa disamping itu para saksi sudah pernah berusaha menasehati Penggugat dan mengatakan tidak ada kesanggupan untuk dapat merukunkannya kembali, sehingga Majelis Hakim berpendapat bahwa antara

Disclaimer
Kepaniteraan Mahkamah Agung Republik Indonesia berusaha untuk selalu mencantumkan informasi paling kini dan akurat sebagai bentuk komitmen Mahkamah Agung untuk pelayanan publik, transparansi dan akuntabilitas pelaksanaan fungsi peradilan. Namun dalam hal-hal tertentu masih dimungkinkan terjadi permasalahan teknis terkait dengan akurasi dan keterkinian informasi yang kami sajikan, hal mana akan terus kami perbaiki dari waktu kewaktu.
Dalam hal Anda menemukan inakurasi informasi yang termuat pada situs ini atau informasi yang seharusnya ada, namun belum tersedia, maka harap segera hubungi Kepaniteraan Mahkamah Agung RI melalui :
Email : kepaniteraan@mahkamahagung.go.id Telp : 021-384 3348 (ext.318)

Penggugat dan Tergugat sudah sulit dan tidak ada harapan untuk dapat hidup rukun kembali di dalam membina rumah tangganya;

Menimbang, bahwa menurut ketentuan Pasal 19 huruf (f) PP Nomor 9 tahun 1975 jo. Pasal 116 huruf (f) Kompilasi Hukum Islam, perceraian dapat terjadi apabila “antara suami dan isteri terus menerus terjadi perselisihan dan pertengkaran dan tidak ada harapan akan hidup rukun kembali dalam membina rumah tangga”;

Menimbang, bahwa demikian juga menurut Mahkamah Agung sebagaimana tertuang dalam putusannya No. 38/K/AG/1990 tanggal 5 Oktober 1990 yang kemudian diambil alih menjadi pendapat Majelis Hakim bahwa apabila ternyata adanya perselisihan sebagaimana dimaksud Pasal 19 huruf (f) PP No. 9 Tahun 1975, maka hal itu semata-mata ditujukan kepada perkawinannya itu sendiri tanpa mempersoalkan siapa yang salah dalam hal terjadinya perselisihan yang mengakibatkan tidak ada harapan akan hidup rukun lagi, karena pernikahan adalah suatu perjanjian yang suci (mitsaqan gholidzon/Pasal 2 KHI) yang untuk memutuskannya tidak boleh diukur dengan kesalahan salah satu pihak, sehingga apabila perkawinan itu telah pecah berarti hati kedua belah pihak telah pecah pula;

Menimbang, bahwa terlepas dari peristiwa atau keadaan yang menjadi penyebab terjadinya perselisihan yang terjadi antara Penggugat dan Tergugat, yang jelas Majelis Hakim telah dapat menemukan fakta hukum bahwa perselisihan antara Penggugat dan Tergugat tersebut telah sedemikian rupa, rumah tangga Penggugat dan Tergugat sudah hancur dan tidak mungkin akan dapat hidup rukun kembali dalam membina rumah tangga;

Menimbang, bahwa dengan melihat kondisi rumah tangga Penggugat dan Tergugat tersebut, jelas tidak mungkin akan terwujud kehidupan rumah tangga yang bahagia dan kekal lahir bathin sebagaimana yang menjadi tujuan perkawinan dalam Pasal 1 Undang-Undang Nomor 1 Tahun 1974 jo. Pasal 3 KHI, dan membiarkan suasana rumah tangga yang demikian, justru akan menimbulkan mudlarat bagi kedua belah pihak, oleh karena itu perceraian dipandang jalan yang terbaik dan lebih maslahat bagi keduanya;

Disclaimer
Kepaniteraan Mahkamah Agung Republik Indonesia berusaha untuk selalu mencantumkan informasi paling kini dan akurat sebagai bentuk komitmen Mahkamah Agung untuk pelayanan publik, transparansi dan akuntabilitas pelaksanaan fungsi peradilan. Namun dalam hal-hal tertentu masih dimungkinkan terjadi permasalahan teknis terkait dengan akurasi dan keterkinian informasi yang kami sajikan, hal mana akan terus kami perbaiki dari waktu kewaktu.
Dalam hal Anda menemukan inakurasi informasi yang termuat pada situs ini atau informasi yang seharusnya ada, namun belum tersedia, maka harap segera hubungi Kepaniteraan Mahkamah Agung RI melalui :
Email : kepaniteraan@mahkamahagung.go.id   Telp : 021-384 3348 (ext.318)

Menimbang, bahwa Majelis Hakim perlu mengetengahkan dalil dalam kitab Al-Muhadzab juz II halaman 82 yang kemudian diambil alih menjadi pendapat Majelis Hakim sebagai berikut :

وإ ذااشتد عدم رغبة الزوجة لزوجهاطلق عليه القاضى طلقة

Artinya: "Disaat istri telah memuncak ketidaksukaan terhadap suaminya, maka Hakim diperkenankan menjatuhkan talak (suami) nya dengan talak satu ;

Menimbang, bahwa berdasarkan pertimbangan tersebut diatas, maka gugatan Penggugat telah memenuhi alasan perceraian sebagaimana diatur dalam Pasal 39 ayat (2) Undang-Undang Nomor 1 Tahun 1974, jo. Pasal 19 huruf (f) Peraturan Pemerintah Nomor 9 Tahun 1975, jo. Pasal 116 huruf (f) Kompilasi Hukum Islam dan karenanya gugatan Penggugat dapat dikabulkan dengan menjatuhkan talak satu ba'in sughro Tergugat terhadap Penggugat;

Menimbang, bahwa perkara ini termasuk bidang perkawinan, oleh karenanya berdasarkan Pasal 89 ayat (1) Undang-Undang Nomor 7 Tahun 1989 tentang Peradilan Agama sebagaimana telah diubah dengan Undang-Undang Nomor 3 Tahun 2006 dan perubahan kedua dengan Undang-Undang Nomor 50 Tahun 2009, maka seluruh biaya perkara ini dibebankan kepada Penggugat;

Mengingat segala peraturan perundang-undangan yang berlaku serta ketentuan hukum syara' yang berkaitan dengan perkara ini;

M E N G A D I L I

1. Menyatakan Tergugat yang telah dipanggil secara resmi dan patut untuk menghadap sidang, tidak hadir;
2. Mengabulkan gugatan Penggugat dengan verstek;
3. Menjatuhkan talak satu ba'in sughra Tergugat (TERGUGAT) terhadap Penggugat (PENGGUGAT);
4. Membebankan kepada Penggugat untuk membayar biaya perkara sejumlah Rp. 391.000,00 (tiga ratus sembilan puluh satu ribu rupiah);

Demikian Putusan ini dijatuhkan dalam permusyawaratan Majelis Hakim Pengadilan Agama Sumber pada hari Senin tanggal 28 Desember 2020 Masehi, bertepatan dengan tanggal 13 Jumadil Awwal 1442 Hijriyah, oleh kami

Disclaimer
Kepaniteraan Mahkamah Agung Republik Indonesia berusaha untuk selalu mencantumkan informasi paling kini dan akurat sebagai bentuk komitmen Mahkamah Agung untuk pelayanan publik, transparansi dan akuntabilitas pelaksanaan fungsi peradilan. Namun dalam hal-hal tertentu masih dimungkinkan terjadi permasalahan teknis terkait dengan akurasi dan keterkinian informasi yang kami sajikan, hal mana akan terus kami perbaiki dari waktu kewaktu.
Dalam hal Anda menemukan inakurasi informasi yang termuat pada situs ini atau informasi yang seharusnya ada, namun belum tersedia, maka harap segera hubungi Kepaniteraan Mahkamah Agung RI melalui :
Email : kepaniteraan@mahkamahagung.go.id   Telp : 021-384 3348 (ext.318)

Drs. H. WAS'ADIN, MH sebagai Ketua Majelis, Drs. MOH. ANAS, MH. dan H. ABDUL HANAN, SH.,MH masing-masing sebagai Hakim Anggota, yang dibacakan dalam sidang terbuka untuk umum oleh Ketua Majelis tersebut dengan dihadiri para Hakim Anggota dan HAMDUN S.H.I sebagai Panitera Pengganti serta dihadiri oleh Penggugat/kuasa hukumnya tanpa hadirnya Tergugat;

Ketua Majelis

Drs. H. WAS'ADIN, MH

Hakim Anggota   Hakim Anggota

Drs. MOH. ANAS, MH.   **H. ABDUL HANAN, SH.,MH**

Panitera Pengganti

HAMDUN S.H.I

Perincian Biaya Perkara :

1. Biaya Pendaftaran	:	Rp.	30.000,00
2. Biaya Proses	:	Rp.	50.000,00
3. Biaya Panggilan	:	Rp.	275.000,00
4. Biaya PNBP Panggilan	:	Rp.	20.000,00
5. Biaya Redaksi	:	Rp.	10.000,00
6. Biaya materai	:	Rp.	6.000,00
Jumlah		Rp.	391.000,00

(tiga ratus sembilan puluh satu ribu rupiah)

Dicatat disini :

Disclaimer
Kepaniteraan Mahkamah Agung Republik Indonesia berusaha untuk selalu mencantumkan informasi paling kini dan akurat sebagai bentuk komitmen Mahkamah Agung untuk pelayanan publik, transparansi dan akuntabilitas pelaksanaan fungsi peradilan. Namun dalam hal-hal tertentu masih dimungkinkan terjadi permasalahan teknis terkait dengan akurasi dan keterkinian informasi yang kami sajikan, hal mana akan terus kami perbaiki dari waktu kewaktu.
Dalam hal Anda menemukan inakurasi informasi yang termuat pada situs ini atau informasi yang seharusnya ada, namun belum tersedia, maka harap segera hubungi Kepaniteraan Mahkamah Agung RI melalui :
Email : kepaniteraan@mahkamahagung.go.id   Telp : 021-384 3348 (ext.318)

- Amar putusan tersebut telah diberitahukan kepada Tergugat pada tanggal
- Putusan tersebut telah mempunyai kekuatan hukum tetap sejak tanggal

Disclaimer
Kepaniteraan Mahkamah Agung Republik Indonesia berusaha untuk selalu mencantumkan informasi paling kini dan akurat sebagai bentuk komitmen Mahkamah Agung untuk pelayanan publik, transparansi dan akuntabilitas pelaksanaan fungsi peradilan. Namun dalam hal-hal tertentu masih dimungkinkan terjadi permasalahan teknis terkait dengan akurasi dan keterkinian informasi yang kami sajikan, hal mana akan terus kami perbaiki dari waktu kewaktu.
Dalam hal Anda menemukan inakurasi informasi yang termuat pada situs ini atau informasi yang seharusnya ada, namun belum tersedia, maka harap segera hubungi Kepaniteraan Mahkamah Agung RI melalui :
Email : kepaniteraan@mahkamahagung.go.id Telp : 021-384 3348 (ext.318)
