# Supplementary material for: Classification of divorce causes during the COVID-19 pandemic using convolutional neural networks
Source: PeerJ Comput Sci. 2022 Jun 30;8:e998. doi: 10.7717/peerj-cs.998 (PMC9299239; doi:10.7717/peerj-cs.998)
Supplement: Supplemental Information 6 [file peerj-cs-08-998-s006.zip › Rujuk Dataset/Data ke-22.pdf]

Direktori Putusan Mahkamah Agung Republik Indonesia
putusan.mahkamahagung.go.id

**P U T U S A N**

**Nomor 4572/Pdt.G/2020/PA.Tsm**

بِسْمِ اللهِ الرَّحْمَنِ الرَّحِيْمِ

**DEMI KEADILAN BERDASARKAN KETUHANAN YANG MAHA ESA**

Pengadilan Agama Tasikmalaya Kelas IA yang memeriksa dan mengadili perkara tertentu pada tingkat pertama dalam sidang Majelis Hakim telah menjatuhkan putusan sebagai berikut dalam perkara Cerai Talak antara:

**X X X X X**, umur 31 tahun, agama Islam, pendidikan SLTP, pekerjaan Buruh Harian Lepas, tempat tinggal di Sekarang d/a. X X X X X, Semula di X X X X X, dalam hal ini memberikan kuasa kepada Fajar Cahya Gumilar, S.H dan Yayat Sudrajat, S.H., Advokat/Pengacara yang berkantor di Jl. Pahlawan KHZ. Musthafa Ciseda Rt.001 Rw.002 Desa Sukaasih Kecamatan Singaparna Kabupaten Tasikmalaya, berdasarkan Surat Kuasa Khusus tanggal 29 Nopember 2020 yang telah didaftar pada Buku Register Surat Kuasa Pengadilan Agama Tasikmalaya Nomor 6187/Reg.K/2020/PA.Tsm. tanggal 07 Desember 2020, sebagai **Pemohon**;

Lawan

**X X X X X**, umur 30 tahun, agama Islam, pendidikan ..., pekerjaan Mengurus Rumah Tangga, tempat tinggal di X X X X X, sebagai **Termohon**;

Pengadilan Agama tersebut;

Telah membaca dan mempelajari berkas perkara;

Telah mendengar keterangan Pemohon;

Telah memeriksa bukti surat dan saksi-saksi di persidangan;

**DUDUK PERKARA**

Menimbang, bahwa Pemohon dengan surat permohonannya tertanggal 07 Desember 2020 yang terdaftar di Kepaniteraan Pengadilan Agama Tasikmalaya



*Disclaimer*
*Kepaniteraan Mahkamah Agung Republik Indonesia berusaha untuk selalu mencantumkan informasi paling kini dan akurat sebagai bentuk komitmen Mahkamah Agung untuk pelayanan publik, transparansi dan akuntabilitas pelaksanaan fungsi peradilan. Namun dalam hal-hal tertentu masih dimungkinkan terjadi permasalahan teknis terkait dengan akurasi dan keterkinian informasi yang kami sajikan, hal mana akan terus kami perbaiki dari waktu kewaktu.*
*Dalam hal Anda menemukan inakurasi informasi yang termuat pada situs ini atau informasi yang seharusnya ada, namun belum tersedia, maka harap segera hubungi Kepaniteraan Mahkamah Agung RI melalui :*
*Email : kepaniteraan@mahkamahagung.go.id    Telp : 021-384 3348 (ext.318)*

tanggal 07 Desember 2020 dengan register perkara Nomor 4572/Pdt.G/2020/PA.Tsm, mengemukakan hal-hal sebagai berikut:

1. Bahwa pada tanggal 29 Nopember 2009 Pemohon telah melangsungkan pernikahan dengan Termohon yang dilaksanakan dan dicatatkan di Kantor Urusan Agama Kecamatan Rajapolah Kabupaten Tasikmalaya sebagaimana ternyata Kutipan Akta Nikah Nomor : X X X X X tanggal 29 Nopember 2009 dan disaat pernikahan Pemohon berusia 20 tahun, Termohon berusia 19 tahun ;
2. Bahwa setelah menikah Pemohon dan Termohon tinggal di rumah orang tua Pemohon beralamat di X X X X X;
3. Bahwa usia pernikahan Pemohon dengan Termohon berjalan selama 11 tahun, bergaul sebagaimana layaknya suami-istri (bada dukhul) dan telah dikaruniai 1 (satu) orang anak ;
4. Bahwa rumah tangga antara Pemohon dengan Termohon semula berjalan baik dan harmonis, namun sejak bulan Januari 2018 mulai retak, terjadi perselisihan /pertengkaran dan tempat kejadiannya terjadi ditempat kediaman bersama, kemudian pada bulan Nopember 2020 telah terjadi kembali perselisihan dan pertengkaran yang terus menerus dan sulit untuk didamaikan dan keadaan tersebut memuncak pada bulan Nopember 2019 sehingga sampai saat ini telah mengakibatkan pisah selama 1 tahun ;
5. Bahwa sebab terjadinya perselisihan dan pertengkaran tersebut dikarenakan oleh faktor ekonomi dimana Pemohon kurang memberi nafkah dan Termohon tidak menerima dengan keadaan tersebut ;
6. Bahwa atas permasalahan dan kemelut rumah tangga yang dihadapi, Pemohon telah mencoba memusyawarahkan dengan pihak keluarga untuk mencari penyelesaian dan demi menyelamatkan perkawinan, namun usaha tersebut tidak membuahkan hasil ;
7. Bahwa keutuhan rumah tangga antara Pemohon dengan Termohon sudah tidak dapat dipertahankan lagi dan sudah tidak ada harapan berumah tangga dengan tentram dan damai maka Pemohon



*Disclaimer*
*Kepaniteraan Mahkamah Agung Republik Indonesia berusaha untuk selalu mencantumkan informasi paling kini dan akurat sebagai bentuk komitmen Mahkamah Agung untuk pelayanan publik, transparansi dan akuntabilitas pelaksanaan fungsi peradilan. Namun dalam hal-hal tertentu masih dimungkinkan terjadi permasalahan teknis terkait dengan akurasi dan keterkinian informasi yang kami sajikan, hal mana akan terus kami perbaiki dari waktu kewaktu.*
*Dalam hal Anda menemukan inakurasi informasi yang termuat pada situs ini atau informasi yang seharusnya ada, namun belum tersedia, maka harap segera hubungi Kepaniteraan Mahkamah Agung RI melalui :*
*Email : kepaniteraan@mahkamahagung.go.id    Telp : 021-384 3348 (ext.318)*

berketetapan hati untuk memilih perceraian sebagai pilihan terakhir dari semua pilihan yang ada ;

8. Bahwa ikatan perkawinan antara Pemohon dan Termohon sebagaimana diuraikan diatas sudah sulit dibina untuk membentuk suatu rumah tangga yang sakinah, mawaddah, warahmah, sebagaimana maksud dan tujuan dari suatu perkawinan, sehingga lebih baik diputus karena perceraian ;
9. Bahwa berdasarkan hal-hal tersebut, Pemohon mengajukan permohonan cerai thalak terhadap Termohon atas dasar pertengkaran yang terjadi terus menerus dan tidak mungkin kembali hidup rukun dan damai dalam suatu ikatan perkawinan, sehingga berdasarkan hukum untuk menyatakan permohonan cerai thalak ini dikabulkan ;
10. Bahwa Pemohon sanggup membayar biaya perkara menurut hukum yang berlaku;

Berdasarkan hal-hal tersebut di atas, maka dengan ini Pemohon mohon agar Ketua Pengadilan Agama Tasikmalaya berkenan menerima, memeriksa dan memutus perkara ini sebagai berikut :

1. Mengabulkan permohonan Pemohon ;
2. Memberi izin kepada Pemohon **(X X X X X)** untuk menjatuhkan talak satu raj'i terhadap Termohon (**X X X X X)** ;
3. Menetapkan biaya perkara menurut hukum ;

**Atau** apabila Pengadilan Agama Tasikmalaya berpendapat lain maka dalam peradilan yang baik mohon keputusan yang seadil - adilnya (Ex Aequo et bono) ;

Bahwa pada hari-hari sidang yang telah ditetapkan, Pemohon didampingi Kuasanya telah menghadap di persidangan, sedangkan Termohon tidak pernah datang menghadap di persidangan dan tidak pula menyuruh orang lain untuk menghadap sebagai wakil atau kuasanya yang sah, meskipun telah dipanggil secara resmi dan patut dan tidak ternyata ketidakhadirannya tersebut disebabkan oleh suatu halangan yang sah, maka perkara ini diperiksa tanpa hadirnya Termohon;



*Disclaimer*
*Kepaniteraan Mahkamah Agung Republik Indonesia berusaha untuk selalu mencantumkan informasi paling kini dan akurat sebagai bentuk komitmen Mahkamah Agung untuk pelayanan publik, transparansi dan akuntabilitas pelaksanaan fungsi peradilan. Namun dalam hal-hal tertentu masih dimungkinkan terjadi permasalahan teknis terkait dengan akurasi dan keterkinian informasi yang kami sajikan, hal mana akan terus kami perbaiki dari waktu kewaktu.*
*Dalam hal Anda menemukan inakurasi informasi yang termuat pada situs ini atau informasi yang seharusnya ada, namun belum tersedia, maka harap segera hubungi Kepaniteraan Mahkamah Agung RI melalui :*
*Email : kepaniteraan@mahkamahagung.go.id Telp : 021-384 3348 (ext.318)*

Bahwa oleh karena Termohon tidak pernah datang menghadap ke persidangan, maka Majelis Hakim tidak dapat melakukan upaya mendamaikan kepada kedua belah pihak, demikian pula upaya mendamaikan melalui proses mediasi sebagaimana diatur melalui PERMA Nomor: 1 Tahun 2016 tidak dapat dilaksanakan;

Bahwa selanjutnya pemeriksaan perkara dilakukan dalam sidang tertutup untuk umum dengan terlebih dahulu dibacakan surat permohonan Pemohon yang isi dan maksudnya tetap dipertahankan oleh Pemohon;

Bahwa oleh karena Termohon atau kuasanya yang sah tidak pernah hadir di persidangan, maka jawaban Termohon atas permohonan Pemohon tidak dapat didengarkan;

Bahwa untuk menguatkan dalil-dalil permohonannya, Pemohon mengajukan alat bukti surat dan saksi-saksi sebagai berikut :

I. **Bukti Surat**

Fotokopi Kutipan Akta Nikah Nomor X X X X X Tanggal 29 November 2009 atas nama Pemohon dan Termohon yang dicatat dan dikeluarkan oleh Pegawai Pencatat Nikah Kantor Urusan Agama Kecamatan Rajapolah Kabupaten Tasikmalaya, telah bermeterai cukup dan di-*nazegeling*, oleh ketua majelis telah dicocokan dengan aslinya ternyata cocok, diberitanggal, diberi tanda bukti (P) dan diparaf;

II. **Bukti Saksi**

1. **X X X X X**, umur 56 tahun, agama Islam, pekerjaan buruh, tempat tinggal di X X X X X, telah memberikan keterangan di bawah sumpah yang pada pokoknya sebagai berikut:
   - Bahwa Saksi kenal dengan Pemohon karena saksi adalah Ayah Pemohon, Termohon bernama X X X X X;
   - Bahwa setelah menikah Pemohon dengan Termohon membina rumah tangganya tinggal bersama di rumah orang tua Pemohon;
   - Bahwa selama pernikahan tersebut Pemohon dengan Termohon telah hidup bersama sebagaimana layaknya suami istri dan telah dikaruniai 1 (satu) orang anak ;;



*Disclaimer*
*Kepaniteraan Mahkamah Agung Republik Indonesia berusaha untuk selalu mencantumkan informasi paling kini dan akurat sebagai bentuk komitmen Mahkamah Agung untuk pelayanan publik, transparansi dan akuntabilitas pelaksanaan fungsi peradilan. Namun dalam hal-hal tertentu masih dimungkinkan terjadi permasalahan teknis terkait dengan akurasi dan keterkinian informasi yang kami sajikan, hal mana akan terus kami perbaiki dari waktu kewaktu.*
*Dalam hal Anda menemukan inakurasi informasi yang termuat pada situs ini atau informasi yang seharusnya ada, namun belum tersedia, maka harap segera hubungi Kepaniteraan Mahkamah Agung RI melalui :*
*Email : kepaniteraan@mahkamahagung.go.id Telp : 021-384 3348 (ext.318)*

- Bahwa semula rumah tangga Pemohon dengan Termohon berjalan rukun dan harmonis namun sejak sejak bulan bulan Januari 2018, keharmonisan rumah tangga Pemohon dengan Termohon mulai goyah, karena sering terjadi perselisihan dan pertengkaran disebabkan Pemohon tidak mampu memberi nafkah terhadap Termohon;
- Bahwa saksi sering melihat dan mendengan sendiri Pemohon dengan Termohon sedang berselisih dan bertengkar mulut;
- Bahwa pada bulan bulan Nopember tahun 2019 terjadi puncak perselisihan yang mengakibatkan Pemohon dan Termohon pisah tempat tinggal dan sampai saat ini telah berjalan sekitar 1 tahun ; lamanya dan selama berpisah antara Pemohon dan Termohon sudah tidak ada lagi hubungan baik lagi;
- Bahwa upaya untuk merukunkan Pemohon dengan Termohon pernah dilakukan oleh pihak keluarga kedua pihak, namun tidak berhasil;

2. **X X X X X**, umur 50 tahun, agama Islam, pekerjaan wiraswasta, bertempat tinggal di X X X X X, telah memberikan keterangan di bawah sumpah yang pada pokoknya sebagai berikut:
   - Bahwa Saksi kenal dengan Pemohon karena saksi adalah Paman Pemohon;
   - Bahwa setelah menikah Pemohon dengan Termohon membina rumah tangganya tinggal bersama di rumah orang tua Pemohon;
   - Bahwa selama pernikahan tersebut Pemohon dengan Termohon telah hidup bersama sebagaimana layaknya suami istri dan telah dikaruniai 1 (satu) orang anak ;;
   - Bahwa semula rumah tangga Pemohon dengan Termohon berjalan rukun dan harmonis namun sejak bulan Januari 2018 rumah tangga Pemohon dengan Termohon terus menerus terjadi perselisihan dan pertengkaran disebabkan Pemohon tidak mampu memberi nafkah terhadap Termohon;
   - Bahwa saksi sering melihat dan mendengan sendiri Pemohon dengan Termohon sedang berselisih dan bertengkar mulut;



*Disclaimer*
*Kepaniteraan Mahkamah Agung Republik Indonesia berusaha untuk selalu mencantumkan informasi paling kini dan akurat sebagai bentuk komitmen Mahkamah Agung untuk pelayanan publik, transparansi dan akuntabilitas pelaksanaan fungsi peradilan. Namun dalam hal-hal tertentu masih dimungkinkan terjadi permasalahan teknis terkait dengan akurasi dan keterkinian informasi yang kami sajikan, hal mana akan terus kami perbaiki dari waktu kewaktu.*
*Dalam hal Anda menemukan inakurasi informasi yang termuat pada situs ini atau informasi yang seharusnya ada, namun belum tersedia, maka harap segera hubungi Kepaniteraan Mahkamah Agung RI melalui :*
*Email : kepaniteraan@mahkamahagung.go.id    Telp : 021-384 3348 (ext.318)*

- Bahwa Pemohon dengan Termohon telah pisah tempat tinggal sejak bulan Nopember tahun 2019 dan sampai saat ini telah berjalan sekitar 1 tahun ; lamanya dan selama berpisah antara Pemohon dan Termohon sudah tidak ada lagi hubungan baik lagi;
- Bahwa upaya untuk merukunkan Pemohon dengan Termohon pernah dilakukan oleh pihak keluarga kedua pihak, namun tidak berhasil;

Bahwa, Pemohon menyatakan tidak akan mengajukan alat bukti lagi dan telah mencukupkan bukti-buktinya sedangkan Termohon tidak pernah hadir di persidangan sehingga tidak menggunakan hak-haknya;

Bahwa Pemohon telah menyampaikan kesimpulannya secara lisan yang pada intinya tetap pada pendiriannya sebagaimana yang tercantum dalam surat permohonan Pemohon dan mohon putusan dengan mengabulkan permohonan Pemohon tersebut;

Bahwa untuk mempersingkat uraian putusan ini cukup ditunjuk kepada hal-hal sebagaimana yang tercantum dalam berita acara sidang perkara ini yang merupakan satu kesatuan yang tidak terpisahkan dengan putusan ini.

PERTIMBANGAN HUKUM

Menimbang, bahwa maksud dan tujuan permohonan Pemohon adalah sebagaimana telah diuraikan di atas;

Menimbang, bahwa berdasarkan posita permohonan Pemohon, Majelis Hakim dapat menyimpulkan bahwa perkara tersebut mengenai sengketa perkawinan yang menjadi wewenang Peradilan Agama sebagaimana yang tercantum dalam Pasal 49 Undang-Undang Nomor 3 Tahun 2006 tentang Perubahan atas Undang Undang Nomor 7 Tahun 1989 Tentang Peradilan Agama dan Pemohon telah mendalilkan mengenai tempat domisili Pemohon yang berada di wilayah hukum Pengadilan Agama Tasikmalaya, maka berdasarkan ketentuan Pasal 73 ayat (1) Undang-Undang Nomor 7 Tahun 1989 tentang Peradilan Agama sebagaimana yang telah diubah dengan Undang-Undang Nomor 3 Tahun 2006 dan perubahan kedua dengan Undang-Undang Nomor 50 Tahun 2009, maka Pengadilan Agama Tasikmalaya berwenang menerima, memeriksa, mengadili dan memutus permohonan Pemohon;



*Disclaimer*
*Kepaniteraan Mahkamah Agung Republik Indonesia berusaha untuk selalu mencantumkan informasi paling kini dan akurat sebagai bentuk komitmen Mahkamah Agung untuk pelayanan publik, transparansi dan akuntabilitas pelaksanaan fungsi peradilan. Namun dalam hal-hal tertentu masih dimungkinkan terjadi permasalahan teknis terkait dengan akurasi dan keterkinian informasi yang kami sajikan, hal mana akan terus kami perbaiki dari waktu kewaktu.*
*Dalam hal Anda menemukan inakurasi informasi yang termuat pada situs ini atau informasi yang seharusnya ada, namun belum tersedia, maka harap segera hubungi Kepaniteraan Mahkamah Agung RI melalui :*
*Email : kepaniteraan@mahkamahagung.go.id    Telp : 021-384 3348 (ext.318)*

Menimbang, bahwa Pemohon didampingi Kuasanya telah datang menghadap di persidangan, sedangkan Termohon tidak pernah hadir di persidangan dan tidak pula mengirimkan orang lain selaku wakil atau kuasanya yang sah untuk hadir di persidangan meskipun telah dipanggil secara resmi dan patut sesuai Pasal 26 Peraturan Pemerintah Nomor 9 Tahun 1975, dan tidak ternyata ketidakhadiran Termohon itu disebabkan suatu alasan yang sah menurut hukum (*default without reason*) sesuai Pasal 125 ayat (1) HIR, maka harus dinyatakan Termohon tidak hadir dan perkara *a quo* dapat diperiksa tanpa hadirnya Termohon;

Menimbang, berdasarkan Pasal 130 HIR dan Pasal 4 Peraturan Mahkamah Agung Nomor 1 Tahun 2016 Tentang Prosedur Mediasi di Pengadilan, bahwa dalam perkara perdata *inklusif* didalamnya mengenai perkara perceraian wajib dilaksanakan mediasi, namun dikarenakan Termohon tidak pernah hadir di persidangan, maka proses Mediasi tidak dapat dilaksanakan;

Menimbang, bahwa oleh karena Termohon telah dinyatakan tidak hadir, dan permohonan Pemohon mempunyai alasan serta tidak bertentangan dengan hukum, maka berdasarkan ketentuan Pasal 125 Ayat (1) HIR permohonan Pemohon dapat diperiksa dan diputus secara verstek;

Menimbang, bahwa meskipun Termohon dianggap mengakui atau setidak-tidaknya tidak membantah dalil-dalil permohonan Pemohon karena Termohon tidak pernah hadir di persidangan, namun karena perkara ini perkara perceraian, maka Pemohon tetap diwajibkan untuk membuktikan alasan-alasan perceraiannya dengan mengajukan alat-alat bukti yang cukup;

Menimbang, bahwa untuk menguatkan dalil permohonannya, Pemohon telah mengajukan alat bukti berupa alat bukti surat yang ditandai dengan kode (P) dan telah menghadirkan alat bukti saksi dipersidangan;

Menimbang, bahwa bukti surat berkode (P1) tersebut merupakan fotokopi sah dari suatu akta autentik, telah dicocokkan dengan aslinya, khusus dibuat sebagai alat bukti, telah diberi meterai cukup dan telah di-*nazageling,* maka harus dinyatakan alat bukti tersebut telah memenuhi persyaratan formil dan dapat diterima, dan secara materiil dapat dipertimbangkan karena alat bukti



*Disclaimer*
*Kepaniteraan Mahkamah Agung Republik Indonesia berusaha untuk selalu mencantumkan informasi paling kini dan akurat sebagai bentuk komitmen Mahkamah Agung untuk pelayanan publik, transparansi dan akuntabilitas pelaksanaan fungsi peradilan. Namun dalam hal-hal tertentu masih dimungkinkan terjadi permasalahan teknis terkait dengan akurasi dan keterkinian informasi yang kami sajikan, hal mana akan terus kami perbaiki dari waktu kewaktu.*
*Dalam hal Anda menemukan inakurasi informasi yang termuat pada situs ini atau informasi yang seharusnya ada, namun belum tersedia, maka harap segera hubungi Kepaniteraan Mahkamah Agung RI melalui :*
*Email : kepaniteraan@mahkamahagung.go.id Telp : 021-384 3348 (ext.318)*

tersebut memuat keterangan yang menguatkan dan relevan dengan dalil permohonan Pemohon;

Menimbang, bahwa alat bukti kode P1 merupakan akta otentik yang berdaya bukti sempurna dan mengikat yang memberi bukti Pemohon dengan Termohon telah dan masih terikat dalam perkawinan sah, dan telah sesuai dengan ketentuan Pasal 164 HIR jo. Pasal 2 ayat (1) dan (2) Undang-Undang Nomor 1 Tahun 1974 jo. Pasal 7 ayat (1) Kompilasi Hukum Islam;

Menimbang, bahwa karena alasan perceraian yang diajukan oleh Pemohon adalah karena perselisihan yang terus-menerus antara Pemohon dengan Tergugat Pasal 19 huruf (f) Peraturan Pemerintah Nomor 9 Tahun 1975 jo. Pasal 116 huruf (f) Kompilasi Hukum Islam maka untuk memenuhi ketentuan pasal 22 Undang-undang Nomor: 7 Tahun 1989 tentang Peradilan Agama sebagaimana telah diubah dan disempurnakan dengan Undang-undang Nomor: 3 Tahun 2006 jo 134 Kompilasi Hukum Islam, Majelis telah memerintahkan Pemohon agar menghadirkan saksi dari keluarga kedua belah pihak atau orang-orang yang dekat dengan kedua belah pihak, bahwa ternyata Pemohon telah mengajukan saksi-saksi yaitu: X X X X X dan X X X X X, majelis menerima keduanya dan telah memberikan keterangan sebagaimana telah diuraikan dalam duduk perkara;

Menimbang, bahwa kedua saksi tersebut adalah orang dewasa yang memberikan keterangan secara terpisah di bawah sumpah di muka sidang, keterangan kedua saksi secara materil saling bersesuaian satu sama lain dan relevan dengan dalil-dalil yang hendak dibuktikan oleh Pemohon serta tidak ada halangan diterimanya kesaksian para saksi tersebut, maka kedua saksi tersebut telah memenuhi syarat formil dan materil sehingga keterangan kedua saksi tersebut memiliki kekuatan pembuktian yang dapat diterima;

Menimbang, bahwa dari saksi-saksi yang dihadirkan di persidangan, Majelis Hakim menilai bahwa keterangan-keterangan saksi yang memenuhi syarat materil sehingga dapat dinilai sebagai bukti adalah sebagai berikut:

1. Bahwa Pemohon dan Termohon telah hidup bersama sebagaimana layaknya suami istri dan telah dikaruniai 1 (satu) orang anak ;;



*Disclaimer*
*Kepaniteraan Mahkamah Agung Republik Indonesia berusaha untuk selalu mencantumkan informasi paling kini dan akurat sebagai bentuk komitmen Mahkamah Agung untuk pelayanan publik, transparansi dan akuntabilitas pelaksanaan fungsi peradilan. Namun dalam hal-hal tertentu masih dimungkinkan terjadi permasalahan teknis terkait dengan akurasi dan keterkinian informasi yang kami sajikan, hal mana akan terus kami perbaiki dari waktu kewaktu.*
*Dalam hal Anda menemukan inakurasi informasi yang termuat pada situs ini atau informasi yang seharusnya ada, namun belum tersedia, maka harap segera hubungi Kepaniteraan Mahkamah Agung RI melalui :*
*Email : kepaniteraan@mahkamahagung.go.id    Telp : 021-384 3348 (ext.318)*

2. Bahwa rumah tangga Pemohon dan Termohon saat ini sudah tidak rukun karena antara Pemohon dan Termohon terus menerus bertengkar dan berselisih;
3. Bahwa penyebab pertengkaran antara Pemohon dan Termohon karena Pemohon tidak mampu memberi nafkah terhadap Termohon;
4. Bahwa Pemohon dengan Termohon telah pisah tempat tinggal sejak bulan Nopember tahun 2019 dan sampai saat ini telah berjalan sekitar 1 tahun ; lamanya dan selama berpisah antara Pemohon dan Termohon sudah tidak ada lagi hubungan baik lagi;
5. Bahwa keluarga dan orang-orang dekat sudah berupaya menasihati Pemohon supaya mengurungkan niatnya bercerai dengan Termohon, namun Pemohon tetap dengan niatnya tersebut;

Menimbang, bahwa berdasarkan dalil-dalil dari Pemohon yang dikuatkan dengan bukti-bukti yang diajukan oleh Pemohon, serta kejadian-kejadian yang terjadi selama persidangan, Majelis Hakim telah menemukan fakta-fakta dan akan Majelis pertimbangkan sebagai berikut:

Menimbang, bahwa berdasarkan keterangan para saksi yang dikuatkan dengan alat bukti surat berkode (P1) dapat dinyatakan terbukti bahwa Pemohon dan Termohon telah terikat perkawinan yang sah sejak tanggal 29 November 2009. Dengan adanya fakta tersebut pemeriksaan perkara perceraian ini dapat dilanjutkan karena antara Pemohon dan Termohon memiliki hubungan hukum sebagai suami istri, sehingga Pemohon mempunyai kedudukan hukum *(legal standing)* untuk mengajukan perkara ini ke Pengadilan Agama;

Menimbang, bahwa dari keterangan-keterangan saksi tersebut, Majelis Hakim menemukan fakta-fakta bahwa dalam rumah tangga Pemohon dan Termohon benar-benar terus menerus terjadi pertengkaran dan perselisihan yang mengakibatkan antara keduanya sudah berpisah tempat tinggal;

Menimbang, bahwa berdasarkan fakta-fakta tersebut di atas, harus dinyatakan terbukti bahwa kehidupan rumah tangga Pemohon dengan Termohon sudah tidak harmonis lagi, telah terjadi perselisihan dan pertengkaran sejak bulan bulan Januari 2018 dan puncaknya bulan bulan



*Disclaimer*
*Kepaniteraan Mahkamah Agung Republik Indonesia berusaha untuk selalu mencantumkan informasi paling kini dan akurat sebagai bentuk komitmen Mahkamah Agung untuk pelayanan publik, transparansi dan akuntabilitas pelaksanaan fungsi peradilan. Namun dalam hal-hal tertentu masih dimungkinkan terjadi permasalahan teknis terkait dengan akurasi dan keterkinian informasi yang kami sajikan, hal mana akan terus kami perbaiki dari waktu kewaktu.*
*Dalam hal Anda menemukan inakurasi informasi yang termuat pada situs ini atau informasi yang seharusnya ada, namun belum tersedia, maka harap segera hubungi Kepaniteraan Mahkamah Agung RI melalui :*
*Email : kepaniteraan@mahkamahagung.go.id    Telp : 021-384 3348 (ext.318)*

Nopember tahun 2019, yang mengakibatkan antara keduanya telah berpisah tempat tinggal dan tidak pernah berkumpul kembali dalam satu rumah tangga;

Menimbang, bahwa perkawinan Pemohon dengan Termohon telah jauh dari tujuanya sebagaimana maksud Pasal 1 jo. Pasal 33 Undang-Undang Nomor 1 Tahun 1974 Tentang Perkawinan;

Menimbang bahwa, rumah tangga Pemohon dan Termohon telah pecah dan sulit untuk dirukunkan kembali, sehingga untuk mewujudkan kehidupan rumah tangga yang sakinah, mawaddah dan rahmah, sebagaimana yang diamanatkan al-Qur'an dalam surat ar-Ruum ayat 21 dan pasal 1 Undang-Undang Nomor 1 Tahun 1974 jo. Pasal 3 Kompilasi Hukum Islam tidak dapat tercapai;

Menimbang, bahwa fakta hukum tersebut telah pula memenuhi norma hukum Islam yang terkandung dalam Al-Quran surat Al-Baqarah ayat 227, sebagai berikut;

وان عزموا الطلاق فان الله سميع عليم البقره:٢٢٧

*Artinya : "Dan jika mereka berazam (berketetapan hati) untuk menjatuhkan talak, maka sesungguhnya Allah Maha Mendengar lagi Maha Mengetahui";*

Menimbang, bahwa fakta hukum tersebut telah juga memenuhi Pasal 22 jo Pasal 19 huruf (f) Peraturan Pemerintah Nomor 9 Tahun 1975 jo. Pasal 19 huruf (f) Kompilasi Hukum Islam ;

Menimbang, bahwa berdasarkan pertimbangan-pertimbangan tersebut di atas dan Pemohon belum pernah menjatuhkan talak/baru menjatuhkan talak raj'i satu kali, maka petitum permohonan Pemohon mengenai izin talak raj'i kepada Termohon (**X X X X X**) memenuhi Pasal 118 Kompilasi Hukum Islam, oleh karena itu dapat dikabulkan;

Menimbang, bahwa oleh karena perkara a quo masuk dalam bidang perkawinan, maka berdasarkan Pasal 89 ayat (1) Undang-undang Nomor: 7 Tahun 1989 yang telah diubah dengan Undang-undang Nomor: 3 Tahun 2006 dan Undang-undang Nomor: 50 Tahun 2009, maka seluruh biaya perkara harus dibebankan kepada Pemohon;



*Disclaimer*
*Kepaniteraan Mahkamah Agung Republik Indonesia berusaha untuk selalu mencantumkan informasi paling kini dan akurat sebagai bentuk komitmen Mahkamah Agung untuk pelayanan publik, transparansi dan akuntabilitas pelaksanaan fungsi peradilan. Namun dalam hal-hal tertentu masih dimungkinkan terjadi permasalahan teknis terkait dengan akurasi dan keterkinian informasi yang kami sajikan, hal mana akan terus kami perbaiki dari waktu kewaktu.*
*Dalam hal Anda menemukan inakurasi informasi yang termuat pada situs ini atau informasi yang seharusnya ada, namun belum tersedia, maka harap segera hubungi Kepaniteraan Mahkamah Agung RI melalui :*
*Email : kepaniteraan@mahkamahagung.go.id Telp : 021-384 3348 (ext.318)*

Mengingat, semua pasal-pasal di atas dan ketentuan dalam peraturan perundang-undangan serta hukum syara yang berkaitan dengan perkara ini;

**M E N G A D I L I**

1. Menyatakan Termohon yang telah dipanggil secara resmi dan patut untuk menghadap sidang tidak hadir ;
2. Mengabulkan permohonan Pemohon secara verstek ;
3. Memberi izin kepada Pemohon (**X X X X X**) untuk menjatuhkan thalak satu raj'i terhadap Termohon (**X X X X X**) di depan sidang Pengadilan Agama Tasikmalaya;
4. Membebankan kepada Pemohon untuk membayar biaya perkara ini sejumlah Rp. 116.000,- (seratus enam belas ribu rupiah);

Demikian diputuskan dalam musyawarah Majelis Hakim Pengadilan Agama Tasikmalaya pada hari Selasa tanggal 29 Desember 2020 Masehi bertepatan dengan tanggal 14 Dzumadil Awal 1442 Hijriah oleh Drs. H. Sanusi, MH. sebagai Ketua Majelis, Drs. Usman Parid, SH. dan Drs. H. Darul Palah, masing-masing sebagai Hakim Anggota, putusan tersebut diucapkan pada hari itu juga dalam sidang terbuka untuk umum oleh Ketua Majelis beserta para Hakim Anggota tersebut, dan didampingi oleh Barkah Ramdhani, SH. MH sebagai Panitera Pengganti, dengan dihadiri oleh Kuasa Pemohon tanpa hadirnya Termohon.

| | |
|---|---|
| Hakim Anggota | Ketua Majelis, |
| **Drs. Usman Parid, SH.** | **Drs. H. Sanusi, MH.** |
| Hakim Anggota | |



*Disclaimer*
*Kepaniteraan Mahkamah Agung Republik Indonesia berusaha untuk selalu mencantumkan informasi paling kini dan akurat sebagai bentuk komitmen Mahkamah Agung untuk pelayanan publik, transparansi dan akuntabilitas pelaksanaan fungsi peradilan. Namun dalam hal-hal tertentu masih dimungkinkan terjadi permasalahan teknis terkait dengan akurasi dan keterkinian informasi yang kami sajikan, hal mana akan terus kami perbaiki dari waktu kewaktu.*
*Dalam hal Anda menemukan inakurasi informasi yang termuat pada situs ini atau informasi yang seharusnya ada, namun belum tersedia, maka harap segera hubungi Kepaniteraan Mahkamah Agung RI melalui :*
*Email : kepaniteraan@mahkamahagung.go.id Telp : 021-384 3348 (ext.318)*

**Drs. H. Darul Palah**

Panitera Pengganti,

**Barkah Ramdhani, SH. MH**

Perincian biaya perkara:

| | | | |
|---|---|---|---|
| 1. | Biaya pendaftaran | : Rp | 30.000,- |
| 2. | Biaya administrasi | : Rp | 50.000,- |
| 3. | Biaya panggilan | : Rp | 185.000,- |
| 4. | Biaya PNBP panggilan | : Rp | 20.000,- |
| 5. | Biaya redaksi | : Rp | 10.000,- |
| 6 | Biaya meterai | : Rp | 6.000,- |
| J u m l a h | | : Rp | 301.000,- |

(tiga ratus satu ribu rupiah)



*Disclaimer*
*Kepaniteraan Mahkamah Agung Republik Indonesia berusaha untuk selalu mencantumkan informasi paling kini dan akurat sebagai bentuk komitmen Mahkamah Agung untuk pelayanan publik, transparansi dan akuntabilitas pelaksanaan fungsi peradilan. Namun dalam hal-hal tertentu masih dimungkinkan terjadi permasalahan teknis terkait dengan akurasi dan keterkinian informasi yang kami sajikan, hal mana akan terus kami perbaiki dari waktu kewaktu.*
*Dalam hal Anda menemukan inakurasi informasi yang termuat pada situs ini atau informasi yang seharusnya ada, namun belum tersedia, maka harap segera hubungi Kepaniteraan Mahkamah Agung RI melalui :*
*Email : kepaniteraan@mahkamahagung.go.id    Telp : 021-384 3348 (ext.318)*
