# Supplementary material for: Classification of divorce causes during the COVID-19 pandemic using convolutional neural networks
Source: PeerJ Comput Sci. 2022 Jun 30;8:e998. doi: 10.7717/peerj-cs.998 (PMC9299239; doi:10.7717/peerj-cs.998)
Supplement: Supplemental Information 6 [file peerj-cs-08-998-s006.zip › Rujuk Dataset/Data ke-24.pdf]

PUTUSAN

Nomor 589/Pdt.G/2020/PA.Bta

بِسْمِ اللَّهِ الرَّحْمَنِ الرَّحِيمِ

DEMI KEADILAN BERDASARKAN KETUHANAN YANG MAHA ESA

Pengadilan Agama Baturaja yang memeriksa dan mengadili perkara tertentu pada peradilan tingkat pertama dalam persidangan majelis, telah menjatuhkan putusan dalam perkara Cerai Gugat, antara:

Penggugat, umur 43 tahun, agama Islam, pendidikan SLTP, pekerjaan Pedagang, tempat tinggal di Kabupaten Ogan Komering Ulu, selanjutnya disebut sebagai **Penggugat**;

melawan

Tergugat, umur 73 tahun, agama Islam, pendidikan SLTP, pekerjaan Tidak Bekerja, tempat tinggal di Kabupaten Ogan Komering Ulu, selanjutnya disebut sebagai **Tergugat**;

Pengadilan Agama tersebut;

Telah membaca dan mempelajari berkas perkara;

Telah mendengar keterangan Penggugat di persidangan;

Telah memeriksa bukti-bukti yang diajukan di persidangan;

DUDUK PERKARA

Bahwa Penggugat telah mengajukan Gugatan dengan surat Gugatannya tanggal 07 Desember 2020 dan telah terdaftar di Kepaniteraan Pengadilan Agama Baturaja dengan register perkara Nomor 589/Pdt.G/2020/PA.Bta tanggal 08 Desember 2020 dengan alasan-alasan yang pada pokoknya sebagai berikut:

1. Bahwa, Penggugat dan Tergugat adalah suami istri sah, yang menikah di Desa Banding Agung, Kecamatan Bandar Agung, Kabupaten Ogan Komering Ulu, pada tanggal 09-06-1996, Wali Nikah Nasab (Ayah

Disclaimer
Kepaniteraan Mahkamah Agung Republik Indonesia berusaha untuk selalu mencantumkan informasi paling kini dan akurat sebagai bentuk komitmen Mahkamah Agung untuk pelayanan publik, transparansi dan akuntabilitas pelaksanaan fungsi peradilan. Namun dalam hal-hal tertentu masih dimungkinkan terjadi permasalahan teknis terkait dengan akurasi dan keterkinian informasi yang kami sajikan, hal mana akan terus kami perbaiki dari waktu kewaktu.
Dalam hal Anda menemukan inakurasi informasi yang termuat pada situs ini atau informasi yang seharusnya ada, namun belum tersedia, maka harap segera hubungi Kepaniteraan Mahkamah Agung RI melalui :
Email : kepaniteraan@mahkamahagung.go.id   Telp : 021-384 3348 (ext.318)

Kandung) Penggugat yang bernama Nomen dengan mas kawin berupa Emas setengah suku, dan disaksikan dua orang saksi masing-masing 1. Sihrun, 2. Kailani;

2. Bahwa, oleh karena Kutipan Akta Nikah tersebut, diperlukan sebagai alat bukti dalam proses gugatan cerai di Pengadilan Agama, sedangkan Penggugat tidak memilikinya, oleh sebab itu Penggugat mohon kepada Ketua Pengadilan Agama Baturaja melalui Majelis Hakim yang memeriksa perkara ini dapat mengesahkan terlebih dahulu pernikahan Penggugat dengan Tergugat;

3. Bahwa, setelah pernikahan tersebut Penggugat dengan Tergugat bertempat tinggal di rumah Tergugat di Desa Banding Agung Kecamatan Bandar Agung Kabupaten Ogan Komering Ulu, selama kurang lebih 7 tahun, kemudian berpindah tempat tinggal di rumah kontrakan di kelurahan saung naga Kecamatan Baturaja Barat Kabupaten Ogan Komering Ulu, selama kurang lebih 2 tahun, dan terakhir tinggal di rumah bersama di kelurahan Sekar jaya Kecamatan Baturaja Timur Kabupaten Ogan Komering Ulu, sampai dengan berpisah;

4. Bahwa, sebelum menikah Penggugat berstatus Janda (cerai hidup dibawah tangan) dan memiliki 1 orang anak, sedangkan Tergugat berstatus duda (cerai mati) dan memiliki 4 orang anak;

5. Bahwa, selama pernikahan tersebut Penggugat dengan Tergugat telah berhubungan sebagaimana layaknya suami isteri, dan telah dikaruniai 2 orang anak (perempuan), umur 22 tahun, (perempuan), umur 17 tahun. Yang saat ini kedua anak tersebut ikut dengan Penggugat;

6. Bahwa, pada mulanya kehidupan rumah tangga Penggugat dengan Tergugat telah hidup rukun dan harmonis selama 20 tahun, akan tetapi setelah itu, sejak bulan Oktober 2016, rumah tangga Penggugat dan Tergugat menjadi tidak rukun dan harmonis lagi, yang disebabkan pada saat itu, Tergugat terlalu cemburu berlebihan. Kemudian Tergugat mengasah pisau dan mengancam akan membunuh Penggugat. Setelah kejadian itu, Penggugat pergi dan sampai dengan sekarang tidak pernah kembali lagi;

Disclaimer
Kepaniteraan Mahkamah Agung Republik Indonesia berusaha untuk selalu mencantumkan informasi paling kini dan akurat sebagai bentuk komitmen Mahkamah Agung untuk pelayanan publik, transparansi dan akuntabilitas pelaksanaan fungsi peradilan. Namun dalam hal-hal tertentu masih dimungkinkan terjadi permasalahan teknis terkait dengan akurasi dan keterkinian informasi yang kami sajikan, hal mana akan terus kami perbaiki dari waktu kewaktu.
Dalam hal Anda menemukan inakurasi informasi yang termuat pada situs ini atau informasi yang seharusnya ada, namun belum tersedia, maka harap segera hubungi Kepaniteraan Mahkamah Agung RI melalui :
Email : kepaniteraan@mahkamahagung.go.id Telp : 021-384 3348 (ext.318)

7. Bahwa, akibat permasalahan tersebut Penggugat dengan Tergugat telah berpisah tempat tinggal, Penggugat tinggal di kontrakan kios di Kabupaten Ogan Komering Ulu, sedangkan Tergugat tinggal di rumah bersama di Kabupaten Ogan Komering Ulu, hingga sekarang telah berjalan selama 4 tahun 2 bulan dan selama berpisah Penggugat dan Tergugat sudah tidak saling perdulikan lagi sebagaimana layaknya suami istri dan Tergugat tidak pernah lagi memberikan nafkah wajibnya lahir dan bathin maupun memberi nafkah untuk anak Penggugat dengan Tergugat. *Oleh karena itu juga*, Penggugat menyatakan tidak ridho dan menuntut perceraian;

8. Bahwa, selama ini Penggugat telah berusaha untuk bersabar dengan keadaan ini, dengan harapan suatu saat keadaan akan berubah, namun keadaan tersebut tidak pernah menjadi baik dan sekarang Penggugat tidak sanggup lagi untuk membina rumah tangga dengan Tergugat dan perceraian adalah jalan terbaik yang harus ditempuh;

9. Bahwa, keluarga Penggugat dengan Tergugat sudah sering berusaha untuk menasehati dan mendamaikan Penggugat dengan Tergugat untuk hidup rukun dan harmonis dalam membina rumah tangga, akan tetapi tidak berhasil;

10. Bahwa, oleh karna Penggugat dan Tergugat sudah tidak rukun dan harmonis lagi, sehingga Penggugat tidak senang lagi bersuamikan Tergugat dan sebagaimana tujuan perkawinan yang diamanatkan dalam Undang-Undang Nomor 1 Tahun 1974 tentang Perkawinan tidak akan terwujud oleh karena itu Penggugat telah berketetapan hati untuk bercerai dihadapan Sidang Pengadilan Agama Baturaja;

11. Bahwa, berdasarkan hal tersebut diatas, maka Penggugat mohon kepada Ketua Pengadilan Agama Baturaja melalui Majelis Hakim kiranya dapat memeriksa dan mengabulkan Gugatan Penggugat kemudian memutuskan sebagai berikut :

   1. Mengabulkan gugatan Penggugat seluruhnya;
   2. Menetapkan perkawinan antara Penggugat dengan Tergugat yang terjadi pada tanggal 09-06-1996 di Kabupaten Ogan Komering Ulu, adalah sah menurut hukum;

Disclaimer
Kepaniteraan Mahkamah Agung Republik Indonesia berusaha untuk selalu mencantumkan informasi paling kini dan akurat sebagai bentuk komitmen Mahkamah Agung untuk pelayanan publik, transparansi dan akuntabilitas pelaksanaan fungsi peradilan. Namun dalam hal-hal tertentu masih dimungkinkan terjadi permasalahan teknis terkait dengan akurasi dan keterkinian informasi yang kami sajikan, hal mana akan terus kami perbaiki dari waktu kewaktu.
Dalam hal Anda menemukan inakurasi informasi yang termuat pada situs ini atau informasi yang seharusnya ada, namun belum tersedia, maka harap segera hubungi Kepaniteraan Mahkamah Agung RI melalui :
Email : kepaniteraan@mahkamahagung.go.id   Telp : 021-384 3348 (ext.318)

3. Menjatuhkan talak satu Ba'in Sughra Tergugat terhadap Penggugat **;**

4. Membebankan biaya perkara sesuai dengan hukum yang berlaku;

Atau apabila Majelis Hakim berpendapat lain, mohon putusan yang seadil-adilnya;

Bahwa Penggugat datang menghadap sendiri di depan sidang, sedangkan Tergugat tidak pernah datang menghadap ke persidangan, meskipun berdasarkan relaas panggilan Tergugat telah dipanggil secara resmi dan patut, juga tidak mengutus orang lain sebagai wakil atau kuasanya yang sah, sedangkan ternyata bahwa tidak hadirnya Tergugat bukan disebabkan oleh sesuatu halangan yang sah;

Bahwa Majelis Hakim telah berupaya menasehati Penggugat agar mempertahankan rumah tangganya serta memberikan pandangan tentang akibat buruk dari suatu perceraian, akan tetapi tidak berhasil;

Bahwa mediasi tidak dapat dilaksanakan, karena Tergugat tidak pernah hadir di persidangan;

Bahwa pemeriksaan perkara ini dimulai dengan dibacakan surat Gugatan Penggugat dengan register Nomor 589/Pdt.G/2020/PA.Bta tanggal 08 Desember 2020, di mana maksud dan tujuannya tetap dipertahankan oleh Penggugat, sedang Tergugat tidak dapat didengar jawabannya, karena tidak hadir di persidangan;

Bahwa Penggugat untuk menguatkan dalil Gugatannya telah mengajukan bukti saksi-saksi, sebagai berikut:

1. **Saksi I**, Saksi tersebut mengaku sebagai ayah kandung Penggugat, lalu menerangkan di bawah sumpah pada pokoknya sebagai berikut:

- Bahwa saksi kenal dengan Tergugat sebagai suami dari Penggugat;

-----Bahwa, saksi hadir pada saat Penggugat dengan Tergugat menikah;

-Bahwa pada saat menikah Penggugat berstatus janda, sedang Tergugat berstatus duda mati;

Disclaimer
Kepaniteraan Mahkamah Agung Republik Indonesia berusaha untuk selalu mencantumkan informasi paling kini dan akurat sebagai bentuk komitmen Mahkamah Agung untuk pelayanan publik, transparansi dan akuntabilitas pelaksanaan fungsi peradilan. Namun dalam hal-hal tertentu masih dimungkinkan terjadi permasalahan teknis terkait dengan akurasi dan keterkinian informasi yang kami sajikan, hal mana akan terus kami perbaiki dari waktu kewaktu.
Dalam hal Anda menemukan inakurasi informasi yang termuat pada situs ini atau informasi yang seharusnya ada, namun belum tersedia, maka harap segera hubungi Kepaniteraan Mahkamah Agung RI melalui :
Email : kepaniteraan@mahkamahagung.go.id Telp : 021-384 3348 (ext.318)

- Bahwa perkawinan Penggugat dengan suami sebelumnya tidak tercatat dan cerai di bawah tangan, mantan suami Penggugat menjatuhkan talak terhadap Penggugat di depan saksi dan menyerahkan kembali Penggugat kepada saksi;

-------Bahwa jarak perpisahan Penggugat dengan suami pertama hingga menikah dengan Tergugat selama 6 bulan;

-------Bahwa pernikahan Penggugat dengan Tergugat dilaksanakan pada tanggal 09-06-1996 di Kecamatan Banjar Agung, Kabupaten Ogan Komering Ulu;

------Bahwa yang menjadi wali nikahnya adalah saksi sendiri selaku ayah kandung Penggugat;

----Bahwa yang menjadi saksi pada saat itu adalah 2 orang laki-laki yang bernama Sihrun dan Kailani;

-Bahwa mahar yang diberikan Tergugat kepada Penggugat berupa Emas setengah suku dibayar tunai;

--------Bahwa pernikahan Penggugat dan Tergugat dilaksanakan menurut syariat Islam;

----------Bahwa antara Penggugat dan Tergugat tidak ada halangan untuk menikah karena senasab, semenda atau sesusuan;

----------Bahwa dari dulu sampai sekarang tidak ada yang keberatan atas pernikahan Penggugat dan Tergugat;

-- Bahwa sampai saat ini Penggugat dan Tergugat masih tetap beragama Islam dan belum pernah bercerai;

-Bahwa setelah menikah Penggugat dengan Tergugat tinggal bersama di rumah Tergugat kemudian pindah ke kediaman bersama hingga berpisah;

-----Bahwa perkawinan Penggugat dan Tergugat telah dikaruniai 2 orang anak yang saat ini dalam asuhan Penggugat;

Disclaimer
Kepaniteraan Mahkamah Agung Republik Indonesia berusaha untuk selalu mencantumkan informasi paling kini dan akurat sebagai bentuk komitmen Mahkamah Agung untuk pelayanan publik, transparansi dan akuntabilitas pelaksanaan fungsi peradilan. Namun dalam hal-hal tertentu masih dimungkinkan terjadi permasalahan teknis terkait dengan akurasi dan keterkinian informasi yang kami sajikan, hal mana akan terus kami perbaiki dari waktu kewaktu.
Dalam hal Anda menemukan inakurasi informasi yang termuat pada situs ini atau informasi yang seharusnya ada, namun belum tersedia, maka harap segera hubungi Kepaniteraan Mahkamah Agung RI melalui :
Email : kepaniteraan@mahkamahagung.go.id   Telp : 021-384 3348 (ext.318)

- Bahwa pada awalnya keadaan rumah tangga Penggugat dan Tergugat rukun dan harmonis selama kurang lebih 6 tahun, setelah itu keadaan rumah tangga Penggugat dan Tergugat sering terjadi perselisihan dan pertengkaran;
- Bahwa saksi pernah melihat langsung Penggugat dan Tergugat sedang bertengkar;
- Bahwa Penggugat dan Tergugat berselisih dan bertengkar di rumah kediaman Penggugat dan Tergugat sendiri;
- Bahwa perselisihan dan pertengkaran antara Penggugat dan Tergugat berupa pertengkaran mulut;
- Bahwa penyebab perselisihan dan pertengkaran antara Penggugat dan Tergugat adalah Tergugat tidak peduli pada Penggugat
- Bahwa saat ini Penggugat dan Tergugat telah berpisah tempat tinggal selama 2 tahun;
- Bahwa Penggugat yang meninggalkan kediaman bersama;
- Bahwa selama Penggugat dan Tergugat berpisah tempat tinggal antara Penggugat dengan Tergugat sudah tidak menjalin komunikasi, tidak saling pedulikan lagi dan tidak lagi menjalankan kewajiban masing-masing sebagai suami isteri;
- Bahwa keluarga Penggugat dan Tergugat telah berupaya merukunkan, akan tetapi sampai dengan saat ini tidak berhasil;
- Bahwa saksi pernah menasehati Penggugat dan Tergugat, namun Penggugat tetap pada keinginannya untuk bercerai dengan Tergugat;

2. **Saksi II**, saksi tersebut mengaku sebagai paman Penggugat, lalu menerangkan di bawah sumpah pada pokoknya sebagai berikut:

- Bahwa saksi kenal dengan Tergugat sebagai suami dari Penggugat;

-----Bahwa, saksi hadir pada saat Penggugat dengan Tergugat menikah;

Disclaimer
Kepaniteraan Mahkamah Agung Republik Indonesia berusaha untuk selalu mencantumkan informasi paling kini dan akurat sebagai bentuk komitmen Mahkamah Agung untuk pelayanan publik, transparansi dan akuntabilitas pelaksanaan fungsi peradilan. Namun dalam hal-hal tertentu masih dimungkinkan terjadi permasalahan teknis terkait dengan akurasi dan keterkinian informasi yang kami sajikan, hal mana akan terus kami perbaiki dari waktu kewaktu.
Dalam hal Anda menemukan inakurasi informasi yang termuat pada situs ini atau informasi yang seharusnya ada, namun belum tersedia, maka harap segera hubungi Kepaniteraan Mahkamah Agung RI melalui :
Email : kepaniteraan@mahkamahagung.go.id Telp : 021-384 3348 (ext.318)

-----Bahwa pada saat menikah Penggugat berstatus janda cerai dibawah tangan, sedang Tergugat berstatus duda mati;

- Bahwa perkawinan Penggugat dengan suami sebelumnya tidak tercatat (bawah tangan), mantan suami Penggugat menjatuhkan talak terhadap Penggugat;

-------Bahwa pernikahan Penggugat dengan Tergugat dilaksanakan pada tanggal 09-06-1996 di Kecamatan Banjar Agung, Kabupaten Ogan Komering Ulu;

---------Bahwa yang menjadi wali nikah Penggugat adalah ayah kandung Penggugat;

----Bahwa yang menjadi saksi pada saat itu adalah 2 orang laki-laki yang bernama Sihrun dan Kailani;

-Bahwa mahar yang diberikan Tergugat kepada Penggugat berupa Emas setengah suku dibayar tunai;

--------Bahwa pernikahan Penggugat dan Tergugat dilaksanakan menurut syariat Islam;

----------Bahwa antara Penggugat dan Tergugat tidak ada halangan untuk menikah karena senasab, semenda atau sesusuan;

----------Bahwa dari dulu sampai sekarang tidak ada yang keberatan atas pernikahan Penggugat dan Tergugat;

-- Bahwa sampai saat ini Penggugat dan Tergugat masih tetap beragama Islam dan belum pernah bercerai;

-Bahwa setelah menikah Penggugat dengan Tergugat tinggal bersama di rumah Tergugat kemudian pindah ke kediaman bersama hingga berpisah;

-----Bahwa perkawinan Penggugat dan Tergugat telah dikaruniai 2 orang anak yang saat ini dalam asuhan Penggugat;

Disclaimer
Kepaniteraan Mahkamah Agung Republik Indonesia berusaha untuk selalu mencantumkan informasi paling kini dan akurat sebagai bentuk komitmen Mahkamah Agung untuk pelayanan publik, transparansi dan akuntabilitas pelaksanaan fungsi peradilan. Namun dalam hal-hal tertentu masih dimungkinkan terjadi permasalahan teknis terkait dengan akurasi dan keterkinian informasi yang kami sajikan, hal mana akan terus kami perbaiki dari waktu kewaktu.
Dalam hal Anda menemukan inakurasi informasi yang termuat pada situs ini atau informasi yang seharusnya ada, namun belum tersedia, maka harap segera hubungi Kepaniteraan Mahkamah Agung RI melalui :
Email : kepaniteraan@mahkamahagung.go.id   Telp : 021-384 3348 (ext.318)

- Bahwa pada awalnya keadaan rumah tangga Penggugat dan Tergugat rukun dan harmonis kemudian keadaan rumah tangga Penggugat dan Tergugat sering terjadi perselisihan dan pertengkaran;
- Bahwa saksi pernah melihat langsung Penggugat dan Tergugat sedang bertengkar;
- Bahwa Penggugat dan Tergugat berselisih dan bertengkar di rumah kediaman Penggugat dan Tergugat sendiri;
- Bahwa perselisihan dan pertengkaran antara Penggugat dan Tergugat berupa pertengkaran mulut;
- Bahwa penyebab perselisihan dan pertengkaran antara Penggugat dan Tergugat adalah Tergugat cemburu kepada Penggugat;
- Bahwa saat ini Penggugat dan Tergugat telah berpisah tempat tinggal selama 4 tahun;
- Bahwa Penggugat yang meninggalkan kediaman bersama;
- Bahwa selama Penggugat dan Tergugat berpisah tempat tinggal antara Penggugat dengan Tergugat sudah tidak menjalin komunikasi, tidak saling pedulikan lagi dan tidak lagi menjalankan kewajiban masing-masing sebagai suami isteri;
- Bahwa keluarga Penggugat dan Tergugat telah berupaya merukunkan, akan tetapi sampai dengan saat ini tidak berhasil;
- Bahwa saksi pernah menasehati Penggugat dan Tergugat, namun Penggugat tetap pada keinginannya untuk bercerai dengan Tergugat;

Bahwa Penggugat menyatakan telah mencukupkan alat buktinya dan tidak akan mengajukan alat bukti lain;

Bahwa Penggugat di persidangan menyampaikan kesimpulan secara lisan yang pada pokoknya tetap pada Gugatan serta mohon putusan;

Bahwa untuk meringkas isi putusan ini, maka segala sesuatu yang tercantum dalam berita acara sidang merupakan kesatuan yang tak terpisahkan dengan putusan ini;

Disclaimer
Kepaniteraan Mahkamah Agung Republik Indonesia berusaha untuk selalu mencantumkan informasi paling kini dan akurat sebagai bentuk komitmen Mahkamah Agung untuk pelayanan publik, transparansi dan akuntabilitas pelaksanaan fungsi peradilan. Namun dalam hal-hal tertentu masih dimungkinkan terjadi permasalahan teknis terkait dengan akurasi dan keterkinian informasi yang kami sajikan, hal mana akan terus kami perbaiki dari waktu kewaktu.
Dalam hal Anda menemukan inakurasi informasi yang termuat pada situs ini atau informasi yang seharusnya ada, namun belum tersedia, maka harap segera hubungi Kepaniteraan Mahkamah Agung RI melalui :
Email : kepaniteraan@mahkamahagung.go.id Telp : 021-384 3348 (ext.318)

PERTIMBANGAN HUKUM

Menimbang, bahwa maksud dan tujuan Gugatan Penggugat sebagaimana terurai di atas;

Menimbang, bahwa Penggugat dengan Tergugat menikah secara hukum Islam, lalu Penggugat mengajukan Gugatan cerai terhadap Tergugat, oleh karena itu berdasarkan Pasal 40 dan Pasal 63 ayat (1) Huruf (a) Undang-undang Nomor 1 Tahun 1974 tentang Perkawinan *jo.* Pasal 1 Huruf (b) Peraturan Pemerintah Nomor 9 Tahun 1975, Pasal 49 Huruf (a) Undang-undang Nomor 7 Tahun 1989 sebagaimana yang telah diubah dengan Undang-undang Nomor 3 Tahun 2006 dan perubahan kedua dengan Undang-undang Nomor 50 Tahun 2009, maka Pengadilan Agama Baturaja berwenang memeriksa dan mengadili, serta memutus perkara ini;

Menimbang, bahwa Penggugat mendalilkan telah melangsungkan perkawinan secara hukum Islam akan tetapi tidak tercatat di Kantor Urusan Agama Kecamatan Banjar Agung Kabupaten Ogan Komering Ulu, kemudian mengajukan Gugatan cerai terhadap Tergugat dengan alasan perselisihan dan pertengkaran secara terus-menerus dan tidak mungkin lagi untuk dirukunkan, sebagaimana maksud termuat dalam Pasal 19 Huruf (f) Nomor 9 Tahun 1975 tentang Pelaksanaan Undang-undang Nomor 1 Tahun 1974 tentang Perkawinan *jo.* Pasal 116 Huruf (f) Kompilasi Hukum Islam, maka Penggugat memiliki kedudukan hukum (*legitima persona standi in judicio*) untuk mengajukan Gugatan perceraian terhadap Tergugat;

Menimbang, bahwa berdasarkan ketentuan Pasal 145 R.Bg *jo.* Pasal 26 Peraturan Pemerintah Nomor 9 Tahun 1975, Penggugat dan Tergugat telah dipanggil secara resmi dan patut untuk menghadiri persidangan yang telah ditetapkan dan pada hari persidangan tersebut, Penggugat telah hadir sendiri (*in person*) di persidangan, sedangkan Tergugat tidak hadir dan tidak mengutus orang lain sebagai wakil atau kuasanya yang sah;

Menimbang, bahwa atas ketidakhadiran Tergugat tanpa alasan yang sah, maka harus dinyatakan Tergugat tidak hadir dan pemeriksaan atas perkara

Disclaimer
Kepaniteraan Mahkamah Agung Republik Indonesia berusaha untuk selalu mencantumkan informasi paling kini dan akurat sebagai bentuk komitmen Mahkamah Agung untuk pelayanan publik, transparansi dan akuntabilitas pelaksanaan fungsi peradilan. Namun dalam hal-hal tertentu masih dimungkinkan terjadi permasalahan teknis terkait dengan akurasi dan keterkinian informasi yang kami sajikan, hal mana akan terus kami perbaiki dari waktu kewaktu.
Dalam hal Anda menemukan inakurasi informasi yang termuat pada situs ini atau informasi yang seharusnya ada, namun belum tersedia, maka harap segera hubungi Kepaniteraan Mahkamah Agung RI melalui :
Email : kepaniteraan@mahkamahagung.go.id   Telp : 021-384 3348 (ext.318)

ini dilanjutkan dan dapat diputus dengan tanpa hadirnya Tergugat (*verstek)* sebagaimana ketentuan Pasal 149 R.Bg;

Menimbang, bahwa berdasarkan Pasal 82 Ayat (1) dan (4) Undang-undang Nomor 7 Tahun 1989 tentang Peradilan Agama sebagaimana telah diubah dengan Undang-undang Nomor 3 Tahun 2006 dan perubahan kedua dengan Undang-undang Nomor 50 Tahun 2009 *jo.* Pasal 31 Peraturan Pemerintah Nomor 9 Tahun 1975, Majelis Hakim telah berusaha menasehati Penggugat agar hidup rukun dan tetap mempertahankan rumah tangganya dengan Tergugat, akan tetapi usaha tersebut tidak berhasil;

Menimbang, bahwa upaya perdamaian melalui proses mediasi tidak dapat ditempuh sebagaimana diatur dalam Peraturan Mahkamah Agung Republik Indonesia Nomor 1 Tahun 2016 tentang Prosedur Mediasi di Pengadilan, karena selama pemeriksaan perkara ini berlangsung, Tergugat tidak pernah hadir di persidangan meskipun telah dipanggil secara resmi dan patut;

Menimbang, bahwa perkara yang diajukan oleh Penggugat merupakan komulasi antara perkara isbat nikah dan perceraian, maka Majelis Hakim perlu mempertimbankan terlebih dahulu hal-hal sebagai mana berikut di bawah ini:

Tentang istbat nikah

Menimbang, bahwa Penggugat mengajukan Permohonan Penetapan [Itsbat] Nikah atas pernikahan antara Penggugat dan Tergugat yang dilaksanakan pada tanggal 09-06-1996 di Kecamatan Bandar Agung, Kabupaten Ogan Komering Ulu;

Menimbang, bahwa untuk menguatkan dalil-dalil permohonan Pemohon I dan Pemohon II telah mengajukan alat bukti saksi-saksi sebagaimana dalam duduk perkaranya perkara ini;

Menimbang, bahwa kedua saksi Penggugat adalah orang terdekat Penggugat, di dalam persidangan memberikan keterangan di bawah sumpah menurut agama masing-masing, sesuai dengan ketentuan Pasal 171 dan 175

Disclaimer
Kepaniteraan Mahkamah Agung Republik Indonesia berusaha untuk selalu mencantumkan informasi paling kini dan akurat sebagai bentuk komitmen Mahkamah Agung untuk pelayanan publik, transparansi dan akuntabilitas pelaksanaan fungsi peradilan. Namun dalam hal-hal tertentu masih dimungkinkan terjadi permasalahan teknis terkait dengan akurasi dan keterkinian informasi yang kami sajikan, hal mana akan terus kami perbaiki dari waktu kewaktu.
Dalam hal Anda menemukan inakurasi informasi yang termuat pada situs ini atau informasi yang seharusnya ada, namun belum tersedia, maka harap segera hubungi Kepaniteraan Mahkamah Agung RI melalui :
Email : kepaniteraan@mahkamahagung.go.id   Telp : 021-384 3348 (ext.318)

R.Bg, maka bukti saksi Penggugat telah memenuhi syarat formil alat bukti saksi;

Menimbang, bahwa keterangan kedua saksi Penggugat memiliki sumber pengetahuan yang jelas, mendengar atau melihat sendiri bukan keterangan dari orang lain (de auditu) dan keterangan kedua saksi Penggugat saling bersesuaian yaitu Penggugat telah menikah dengan Tergugat secara Hukum Islam, pada tanggal 09-06-1996, di Kecamatan Bandar Agung, Kabupaten Ogan Komering Ulu, dengan wali nikah adalah ayah kandung Penggugat, dengan dua orang saksi yaitu Sihrun dan Kailani, dengan mahar berupa Emas setengah suku, selama pernikahan Penggugat dan Tergugat telah dikaruniai 2 orang anak, selama masa pernikahan belum pernah bercerai dan tidak ada orang yang pernah keberatan terhadap pernikahan Penggugat dan Tergugat;

Menimbang, bahwa berdasarkan keterangan Penggugat, dihubungkan dengan bukti-bukti yang telah diajukan oleh Penggugat tersebut di atas, Majelis telah menemukan fakta-fakta dalam persidangan ini yang pada pokoknya sebagai berikut:

- Bahwa Penggugat dan Tergugat adalah suami isteri sah, akad nikah di laksanakan menurut syari'at Islam, pada tanggal 09-06-1996, di Kecamatan Bandar Agung, Kabupaten Ogan Komering Ulu, wali nikah ayah kandung Penggugat, dengan mas kawin berupa Emas setengah suku, dan di saksikan dua orang saksi masing-masing Sihrun dan Kailani;
- Bahwa pernikahan Penggugat dan Tergugat tersebut telah sesuai rukun dan syaratnya sebagai mana ketentuan syari'at Islam;
- Bahwa pernikahan Penggugat dan Tergugat tidak ada halangan secara hukum, dan tidak pernah ada yang mengganggu gugat pernikahan tersebut, dan selama itu pula Penggugat dan Tergugat tidak pernah bercerai;
- Bahwa pernikahan Penggugat dan Tergugat tersebut tidak pernah memiliki bukti berupa akta autentik;

Menimbang, bahwa dengan pertimbangan - pertimbangan tersebut di atas, terbukti bahwa perkawinan Penggugat tersebut telah dilaksanakan sesuai

Disclaimer
Kepaniteraan Mahkamah Agung Republik Indonesia berusaha untuk selalu mencantumkan informasi paling kini dan akurat sebagai bentuk komitmen Mahkamah Agung untuk pelayanan publik, transparansi dan akuntabilitas pelaksanaan fungsi peradilan. Namun dalam hal-hal tertentu masih dimungkinkan terjadi permasalahan teknis terkait dengan akurasi dan keterkinian informasi yang kami sajikan, hal mana akan terus kami perbaiki dari waktu kewaktu.
Dalam hal Anda menemukan inakurasi informasi yang termuat pada situs ini atau informasi yang seharusnya ada, namun belum tersedia, maka harap segera hubungi Kepaniteraan Mahkamah Agung RI melalui :
Email : kepaniteraan@mahkamahagung.go.id Telp : 021-384 3348 (ext.318)

dengan syari'at Islam sebagaimana tersebut pada pasal 14 Kompilasi Hukum Islam;

Menimbang, bahwa Majelis Hakim perlu mengetengahkan dalil syar'i berupa Hadis Nabi SAW yang diriwayatkan oleh Daruqutni dari Siti Aisyah yang berbunyi:

لا نكاح إلا بولي وشاهدى عدل

Artinya: " Tidak sah pernikahan (seseorang) kecuali dengan adanya wali dan dua orang saksi yang adil".

Menimbang, bahwa Majelis Hakim memandang perlu mengemukakan doktrin ulama, yang selanjutnya diambil alih sebagai pendapat Majelis Hakim, yang berbunyi:

ويقبل إقرار العاقلة البالغة بالنكاح

Artinya: ”*Diterima pengakuan nikah seorang perempuan yang aqil-baligh*”;

Menimbang, bahwa dengan telah ditemukannya fakta hukum, bahwa perkawinan Penggugat dan Tergugat telah memenuhi ketentuan hukum Islam, dan sesuai dengan Pasal 2 Ayat (1) dan Pasal 64 Undang-undang Nomor 1 tahun 1974 tentang Perkawinan dan juga sejalan dengan maksud Pasal 4 Kompilasi Hukum Islam, permohonan Penggugat agar perkawinan Penggugat dan Tergugat yang dilaksanakan pada tanggal 09-06-1996 dalam wilayah Kantor Urusan Agama Kecamatan Baturaja Timur ditetapkan keabsahannya patut diterima dan dikabulkan;

Menimbang, bahwa penikahan Penggugat dan Tergugat telah dinyatakan sah, maka berdasarkan ketentuan Pasal 64 Undang-undang Nomor 1 Tahun 1974 jo. Pasal 7 ayat (3) huruf a Kompilasi Hukum Islam, maka pernikahan Penggugat tersebut dapat diitsbatkan;

Menimbang, bahwa berdasarkan pertimbangan-pertimbangan tersebut di atas, maka dapat disimpulkan bahwa Gugatan Penggugat telah beralasan hukum sehingga harus dinyatakan dapat dikabulkan;

Disclaimer
Kepaniteraan Mahkamah Agung Republik Indonesia berusaha untuk selalu mencantumkan informasi paling kini dan akurat sebagai bentuk komitmen Mahkamah Agung untuk pelayanan publik, transparansi dan akuntabilitas pelaksanaan fungsi peradilan. Namun dalam hal-hal tertentu masih dimungkinkan terjadi permasalahan teknis terkait dengan akurasi dan keterkinian informasi yang kami sajikan, hal mana akan terus kami perbaiki dari waktu kewaktu.
Dalam hal Anda menemukan inakurasi informasi yang termuat pada situs ini atau informasi yang seharusnya ada, namun belum tersedia, maka harap segera hubungi Kepaniteraan Mahkamah Agung RI melalui :
Email : kepaniteraan@mahkamahagung.go.id   Telp : 021-384 3348 (ext.318)

Menimbang, oleh karena Gugatan Penggugat mohon agar perkawinannya diistbatkan telah dikabulkan, maka harus ditetapkan bahwa perkawinan Penggugat dan Tergugat yang dilaksanakan 09-06-1996 dalam wilayah Kantor Urusan Agama Kecamatan Bandar Agung, adalah ”sah”;

Tentang Perkara Perceraian

Menimbang, bahwa Penggugat mengajukan Gugatan cerai terhadap Tergugat dengan alasan yang pada pokoknya bahwa di dalam rumah tangga Penggugat dengan Tergugat telah terjadi perselisihan dan pertengkaran yang terus-menerus dan sudah sangat sulit untuk dirukunkan kembali;

Menimbang, bahwa terhadap dalil-dalil Gugatan tersebut Tergugat tidak memberikan jawabannya karena Tergugat tidak pernah hadir menghadap di persidangan meskipun telah dipanggil secara resmi dan patut, maka Tergugat gugur hak jawabnya, serta Majelis Hakim sependapat dengan doktrin ulama Islam dan mengambil alih sebagai sebagai pendapat Majelis Hakim, dalam Kitab Ahkamul Qur’an, Juz 2, halaman 45:

من دعي الى حاكم من حكام المسلمين فلم يجب فهو ظالم لا حق له

Artinya: *Barang siapa yang dipanggil oleh Hakim di dalam persidangan sedangkan orang tersebut tidak memenuhi panggilan tersebut, maka dia termasuk orang zalim, maka gugurlah haknya;*

Menimbang, bahwa Majelis Hakim juga sependapat dengan doktrin ulama Islam dalam Kitab Al-Anwar Juz II, Halaman 149 yang kemudian diambil alih sebagai pendapat Majelis Hakim, yang berbunyi:

وإن تعذر احضاره لتواريه وتعززه جاز سماع الدعوى والبينة والحكم عليه

Artinya : *Apabila (Tergugat) berhalangan hadir karena bersembunyi atau enggan, maka Hakim memeriksa Gugatan dan bukti-bukti yang diajukan, kemudian memutusnya*.

Menimbang, bahwa dari doktrin ulama di atas dapat diambil sebuah abstraksi hukum bahwa Tergugat yang tidak hadir dalam persidangan telah menghilangkan hak-haknya sehingga terhadap Gugatan Penggugat dapat

Disclaimer
Kepaniteraan Mahkamah Agung Republik Indonesia berusaha untuk selalu mencantumkan informasi paling kini dan akurat sebagai bentuk komitmen Mahkamah Agung untuk pelayanan publik, transparansi dan akuntabilitas pelaksanaan fungsi peradilan. Namun dalam hal-hal tertentu masih dimungkinkan terjadi permasalahan teknis terkait dengan akurasi dan keterkinian informasi yang kami sajikan, hal mana akan terus kami perbaiki dari waktu kewaktu.
Dalam hal Anda menemukan inakurasi informasi yang termuat pada situs ini atau informasi yang seharusnya ada, namun belum tersedia, maka harap segera hubungi Kepaniteraan Mahkamah Agung RI melalui :
Email : kepaniteraan@mahkamahagung.go.id   Telp : 021-384 3348 (ext.318)

diperiksa dan diputus tanpa kehadiran Tergugat, dalam hal ini guna kehati-hatian Majelis Hakim dalam menilai pokok perkara ini melawan hukum atau tidak melawan hukum, serta beralasan atau tidak beralasan, dan juga perkara ini merupakan perkara perceraian yang mempunyai hukum acara khusus (*lex specialis derogat legi generalis*);

Menimbang, bahwa untuk menghindari kesepakatan untuk bercerai antara Penggugat dan Tergugat sebagaimana maksud Pasal 208 KUH Perdata, maka untuk melakukan perceraian harus cukup alasan, bahwa antara Penggugat (isteri) dan Tergugat (suami) tidak dapat hidup rukun sebagai suami isteri sesuai dalam Pasal 39 Ayat (2) Undang-undang Nomor 1 Tahun 1974 tentang Perkawinan, Gugatan mana dapat diterima apabila telah cukup jelas bagi Pengadilan tentang sebab-sebab perselisihan dan pertengkaran dan setelah mendengar keterangan pihak keluarga serta orang yang dekat dengan suami isteri sesuai dengan Pasal 22 Ayat (2) Peraturan Pemerintah Nomor 9 Tahun 1975, maka Majelis Hakim perlu mengetahui lebih jelas sebab-sebab perselisihan dan pertengkaran antara Penggugat dan Tergugat dan perlu mendengar keterangan saksi dari pihak keluarga atau orang-orang yang dekat dengan Penggugat maupun Tergugat;

Menimbang, bahwa oleh karena Penggugat yang mengajukan gugatan maka kepada Penggugat dibebankan alat bukti. Dalam hal ini Penggugat mengajukan saksi-saksi di persidangan, telah disumpah menurut agamanya masing-masing dan juga tidak terhalang untuk didengar keterangannya sebagai saksi, karena kedua saksi tersebut merupakan orang terdekat Penggugat. Dengan demikian kedua saksi tersebut telah memenuhi syarat formil pembuktian khususnya dalam perkara bidang perkawinan, sebagaimana yang dimaksud dalam Pasal 171 dan 175 R.Bg *jo.* Pasal 22 Ayat (2) Peraturan Pemerintah Nomor 9 Tahun 1975 tentang Pelaksanaan Undang-undang Nomor 1 Tahun 1974 tentang Perkawinan, sehingga keterangan kedua saksi Penggugat tersebut dapat didengarkan;

Menimbang, bahwa terhadap keterangan kedua saksi Penggugat saling bersesuaian dan memiliki sumber pengetahuan yang jelas tanpa penilaian saksi

Disclaimer
Kepaniteraan Mahkamah Agung Republik Indonesia berusaha untuk selalu mencantumkan informasi paling kini dan akurat sebagai bentuk komitmen Mahkamah Agung untuk pelayanan publik, transparansi dan akuntabilitas pelaksanaan fungsi peradilan. Namun dalam hal-hal tertentu masih dimungkinkan terjadi permasalahan teknis terkait dengan akurasi dan keterkinian informasi yang kami sajikan, hal mana akan terus kami perbaiki dari waktu kewaktu.
Dalam hal Anda menemukan inakurasi informasi yang termuat pada situs ini atau informasi yang seharusnya ada, namun belum tersedia, maka harap segera hubungi Kepaniteraan Mahkamah Agung RI melalui :
Email : kepaniteraan@mahkamahagung.go.id   Telp : 021-384 3348 (ext.318)

sendiri, yaitu pada mulanya rumah tangga Penggugat dengan Tergugat hidup dan rukun, namun belakangan ini sering terjadi perselisihan dan pertengkaran yang disebabkan oleh Tergugat tidak peduli pada Penggugat dan Tergugat cemburu pada Penggugat hingga puncak perselisihan dan pertengkaran antara Penggugat dan Tergugat berpisah tempat tinggal dan sejak berpisah antara Penggugat dan Tergugat tidak pernah saling peduli lagi dan tidak menjalankan kewajiban sebagai pasangan suami isteri yang telah berjalan selama 2 tahun;

Menimbang, bahwa terhadap keterangan saksi tersebut di atas, telah memenuhi syarat materiil sebagaimana maksud Pasal 308 Ayat (1) dan Pasal 309 RBg, maka Majelis Hakim berpendapat bahwa keterangan saksi-saksi tersebut dapat dijadikan dasar pertimbangan bagi hakim di dalam menentukan fakta-fakta yang didalilkan oleh Penggugat;

Menimbang, bahwa berdasarkan Gugatan Penggugat yang diperkuat oleh bukti tertulis dan keterangan dua orang saksi Penggugat, Majelis Hakim menemukan fakta-fakta sebagai berikut:

- Bahwa antara Penggugat dan Tergugat terikat ikatan suami isteri sejak tanggal tanggal 09-06-1996;
- Bahwa keadaan rumah tangga Penggugat dan Tergugat pada awalnya rukun dan harmonis, namun belakangan ini sering terjadi perselisihan dan pertengkaran yang disebabkan oleh Tergugat tidak peduli pada Penggugat dan Tergugat cemburu pada Penggugat;
- Bahwa sampai dengan saat ini Penggugat dan Tergugat telah berpisah tempat tinggal sekurang-kurangnya selama 2 tahun;
- Bahwa selama berpisah tempat tinggal antara Penggugat dan Tergugat tidak saling peduli;

Menimbang, bahwa terhadap dalil-dalil yang telah dibuktikan oleh Penggugat tersebut di atas, oleh Majelis Hakim menilai bahwa Penggugat telah berhasil membuktikan dalil-dalil dalam Gugatan cerainya dan juga Majelis Hakim telah menemukan fakta hukum bahwa rumah tangga Penggugat dengan Tergugat tidak berjalan harmonis lagi berdasarkan fakta-fakta tersebut di atas,

Disclaimer
Kepaniteraan Mahkamah Agung Republik Indonesia berusaha untuk selalu mencantumkan informasi paling kini dan akurat sebagai bentuk komitmen Mahkamah Agung untuk pelayanan publik, transparansi dan akuntabilitas pelaksanaan fungsi peradilan. Namun dalam hal-hal tertentu masih dimungkinkan terjadi permasalahan teknis terkait dengan akurasi dan keterkinian informasi yang kami sajikan, hal mana akan terus kami perbaiki dari waktu kewaktu.
Dalam hal Anda menemukan inakurasi informasi yang termuat pada situs ini atau informasi yang seharusnya ada, namun belum tersedia, maka harap segera hubungi Kepaniteraan Mahkamah Agung RI melalui :
Email : kepaniteraan@mahkamahagung.go.id Telp : 021-384 3348 (ext.318)

namun Majelis Hakim lebih lanjut akan mempertimbangkan patut atau tidaknya Gugatan Penggugat dikabulkan;

Menimbang, bahwa sesuai dengan ketentuan Pasal 1 Undang-undang Nomor 1 Tahun 1974 tentang Perkawinan, bahwa perkawinan adalah ikatan lahir bathin antara seorang pria sebagai suami dengan seorang wanita sebagai isteri dengan tujuan untuk membentuk keluarga (rumah tangga) yang bahagia dan kekal berdasarkan Ketentuan Yang Maha Esa, dan juga dalam Pasal 3 Kompilasi Hukum Islam disebutkan bahwa perkawinan bertujuan untuk mewujudkan kehidupan rumah tangga yang *sakinah, mawaddah* dan *rahmah*, dan juga digariskan dalam surah Ar-Ruum Ayat (21) yang berbunyi:

وَمِنْ ءَايَـٰتِهِۦٓ أَنْ خَلَقَ لَكُم مِّنْ أَنفُسِكُمْ أَزْوَٰجًا لِّتَسْكُنُوٓا۟ إِلَيْهَا وَجَعَلَ بَيْنَكُم مَّوَدَّةً وَرَحْمَةً ۚ

إِنَّ فِى ذَٰلِكَ لَـَٔايَـٰتٍ لِّقَوْمٍ يَتَفَكَّرُونَ

Artinya: “ *Dan salah satu dari tanda-tanda kebesaran Allah, diciptakan-Nya dari jenis diri kamu seorang pasangan hidup, agar kamu menemukan ketenangan bersamanya dan dijadikan-Nya di antara kamu saling mencintai dan saling kasih sayang. Sesungguhnya dalam hal yang demikian itu merupakan pertanda kebesaran Allah bagi orang-orang yang berfikir”.*;

Menimbang, bahwa berdasarkan ketentuan tersebut di atas, dapat diketahui, bahwa unsur dari perkawinan adalah adanya ikatan lahir bathin antara seorang pria sebagai suami dengan seorang wanita sebagai isteri, jika unsur tersebut tidak terpenuhi dalam ikatan perkawinan, maka sesungguhnya perkawinan tersebut telah rapuh, sehingga tujuan untuk membentuk keluarga yang bahagia tidak tercapai;

Menimbang, bahwa oleh karena kehidupan rumah tangga Penggugat dan Tergugat tidak mencapai tujuannya dan tidak ada harapan untuk satukan kembali dan apabila dipertahankan maka akan mendatangkan rasa tidak aman, *mudharat* bagi Penggugat dan Tergugat. Sedangkan dalam ajaran Islam menghindari *mudharat* wajib didahulukan daripada mengambil manfaatnya

Disclaimer
Kepaniteraan Mahkamah Agung Republik Indonesia berusaha untuk selalu mencantumkan informasi paling kini dan akurat sebagai bentuk komitmen Mahkamah Agung untuk pelayanan publik, transparansi dan akuntabilitas pelaksanaan fungsi peradilan. Namun dalam hal-hal tertentu masih dimungkinkan terjadi permasalahan teknis terkait dengan akurasi dan keterkinian informasi yang kami sajikan, hal mana akan terus kami perbaiki dari waktu kewaktu.
Dalam hal Anda menemukan inakurasi informasi yang termuat pada situs ini atau informasi yang seharusnya ada, namun belum tersedia, maka harap segera hubungi Kepaniteraan Mahkamah Agung RI melalui :
Email : kepaniteraan@mahkamahagung.go.id Telp : 021-384 3348 (ext.318)

sesuai dengan Qaidah Ushul Fiqih yang diambil alih sebagai pendapat dan bahan pertimbangan Majelis Hakim yang berbunyi :

درء المفاسد مقدم على جلب المصالح

Artinya :---------------- *Menolak kemudharatan lebih utama dari pada menarik (mempertahankan) kebaikan*;

إذا تَعَارَضَ مَفْسَدَتَانِ رُوْعِى أَعْظَمُهما ضَرَرًا بِارْتِكَابِ أَخَفِّهِمَا

Artinya :“Apabila dua mafsadah bertentangan, maka perhatikan manaa yang lebih besar mudaratnya dengan mengerjakan yang lebih ringann mudaratnya”;

Menimbang, bahwa berdasarkan Yurisprudensi Mahkamah Agung Republik Indonesia Nomor 38K/AG/1990 tanggal 22 Agustus 1991, bahwa tidak perlu melihat siapa yang bersalah menyebabkan perselisihan dan pertengkaran, tetapi yang penting apakah rumah tangga itu telah pecah dan sulit didamaikan lagi, menjadi dasar pertimbangan dikabulkannya suatu perkara;

Menimbang, bahwa dalam perkara ini Majelis Hakim sependapat dan mengambil alih sebagai pendapat Majelis Hakim terhadap pendapat pakar hukum Islam dalam Kitab Ghoyatul Marom yang berbunyi sebagai berikut:

وإذا شتد عدم رغبة الزوجة لزوجها طلق عليه القاضى طلقة

Artinya : *“Apabila si isteri telah menunjukkan sikap sangat benci kepada suaminya, maka Hakim boleh menjatuhkan talak satu suaminya kepada isterinya”.*

Menimbang, bahwa Majelis Hakim sependapat dengan pakar hukum Islam dalam Kitab Fiqhus Sunnah Juz II halaman 290, kemudian diambil alih sebagai pendapat Majelis Hakim, yang berbunyi:

فإذا تثبت دعواها لدى القاضي ببينة الزوجة أو اعتراف الزوج وكان الإيذاء مما لا يطاق
معه دوام العشرة بين أمثالهما وعجز القاضي عن الإصلاح بينهما طلقها طلقة بائنة

Disclaimer
Kepaniteraan Mahkamah Agung Republik Indonesia berusaha untuk selalu mencantumkan informasi paling kini dan akurat sebagai bentuk komitmen Mahkamah Agung untuk pelayanan publik, transparansi dan akuntabilitas pelaksanaan fungsi peradilan. Namun dalam hal-hal tertentu masih dimungkinkan terjadi permasalahan teknis terkait dengan akurasi dan keterkinian informasi yang kami sajikan, hal mana akan terus kami perbaiki dari waktu kewaktu.
Dalam hal Anda menemukan inakurasi informasi yang termuat pada situs ini atau informasi yang seharusnya ada, namun belum tersedia, maka harap segera hubungi Kepaniteraan Mahkamah Agung RI melalui :
Email : kepaniteraan@mahkamahagung.go.id Telp : 021-384 3348 (ext.318)

Artinya: *Apabila hakim telah menemukan bukti-bukti yang diajukan oleh Penggugat (istri) atau Tergugat telah memberikan pengakuan, sedangkan hal-hal yang menjadi dakwaan Penggugat yaitu ketidakmampuan kedua belah pihak untuk hidup bersama sebagai suami istri dan hakim tidak berhasil mendamaikan keduanya, maka hakim boleh memutuskan dengan talak satu bain;*

Menimbang, bahwa berdasarkan pertimbangan-pertimbangan tersebut di atas, maka alasan perceraian Penggugat tidak melawan hak dan telah memenuhi ketentuan Pasal 39 ayat (2) Undang-Undang Nomor 1 Tahun 1974 Tentang Perkawinan Jo. Pasal 19 huruf (f) Peraturan Pemerintah Nomor 9 Tahun 1975 Jo Pasal 116 huruf (f) Kompilasi Hukum Islam, sehingga petitum angka 2 gugatan Penggugat dapat dikabulkan;

Menimbang, bahwa oleh karena selama dalam perkawinan antara Penggugat dengan Tergugat belum pernah terjadi perceraian, dengan demikian berdasarkan Pasal 119 ayat (2) huruf (c) gugatan cerai Penggugat dapat dikabulkan dengan menjatuhkan talak satu ba'in sughro, sebagaimana tersebut dalam amar putusan ini;

Menimbang, bahwa berdasarkan Pasal 89 Ayat (1) Undang-undang Nomor 7 Tahun 1989 tentang Peradilan Agama sebagaimana yang telah diubah dengan Undang-undang Nomor 3 Tahun 2006 dan perubahan kedua dengan Undang-undang Nomor 50 Tahun 2009, oleh karena perkara ini mengenai sengketa perkawinan, Penggugat dibebankan untuk membayar biaya perkara;

Mengingat ketentuan hukum *syara'* serta peraturan perundangan berlaku yang berhubungan dengan perkara ini;

MENGADILI

1. Menyatakan Tergugat telah dipanggil secara resmi dan patut untuk menghadap di persidangan, tidak hadir;
2. Mengabulkan gugatan Penggugat dengan verstek;

Disclaimer
Kepaniteraan Mahkamah Agung Republik Indonesia berusaha untuk selalu mencantumkan informasi paling kini dan akurat sebagai bentuk komitmen Mahkamah Agung untuk pelayanan publik, transparansi dan akuntabilitas pelaksanaan fungsi peradilan. Namun dalam hal-hal tertentu masih dimungkinkan terjadi permasalahan teknis terkait dengan akurasi dan keterkinian informasi yang kami sajikan, hal mana akan terus kami perbaiki dari waktu kewaktu.
Dalam hal Anda menemukan inakurasi informasi yang termuat pada situs ini atau informasi yang seharusnya ada, namun belum tersedia, maka harap segera hubungi Kepaniteraan Mahkamah Agung RI melalui :
Email : kepaniteraan@mahkamahagung.go.id Telp : 021-384 3348 (ext.318)

3. Menyatakan sah perkawinan antara Penggugat dan Tergugat yang dilaksanakan pada tanggal 09-06-1996 di Kecamatan Bandar Agung Kabupaten Ogan Komering Ulu;

4. Menjatuhkan talak satu ba'in sughro Tergugat terhadap Penggugat;

5. Membebankan kepada Penggugat membayar biaya perkara sejumlah Rp. 341.000,00 (tiga ratus empat puluh satu ribu rupiah);

Demikian diputuskan dalam musyawarah Majelis Hakim Pengadilan Agama Baturaja pada hari Selasa tanggal 29 Desember 2020 Masehi bertepatan dengan tanggal 14 Jumadil Awwal 1442 Hijriah oleh Drs. Murdani, S.H. sebagai Ketua Majelis, H. Zulkifli, S.Ag., S.H., M.H. dan Azwida, S.H.I., masing-masing sebagai Hakim Anggota, putusan tersebut diucapkan pada hari itu juga dalam sidang terbuka untuk umum oleh Ketua Majelis beserta para Hakim Anggota tersebut, dan didampingi oleh Dra. Hj Amila Roza sebagai Panitera Pengganti, dengan dihadiri oleh Penggugat tanpa hadirnya Tergugat.

Hakim Anggota	Ketua Majelis,
ttd	ttd
**H. Zulkifli, S.Ag., S.H., M.H.**	**Drs. Murdani, S.H.**

Hakim Anggota

ttd

Azwida, S.H.I.

Panitera Pengganti,

ttd

Dra. Hj Amila Roza

Perincian biaya :

-	Pendaftaran	: Rp	30.000,00
-	Proses	: Rp	50.000,00

Disclaimer
Kepaniteraan Mahkamah Agung Republik Indonesia berusaha untuk selalu mencantumkan informasi paling kini dan akurat sebagai bentuk komitmen Mahkamah Agung untuk pelayanan publik, transparansi dan akuntabilitas pelaksanaan fungsi peradilan. Namun dalam hal-hal tertentu masih dimungkinkan terjadi permasalahan teknis terkait dengan akurasi dan keterkinian informasi yang kami sajikan, hal mana akan terus kami perbaiki dari waktu kewaktu.
Dalam hal Anda menemukan inakurasi informasi yang termuat pada situs ini atau informasi yang seharusnya ada, namun belum tersedia, maka harap segera hubungi Kepaniteraan Mahkamah Agung RI melalui :
Email : kepaniteraan@mahkamahagung.go.id Telp : 021-384 3348 (ext.318)

-	Panggilan	: Rp	150.000,00
-	PNBP	: Rp	20.000,00
-	Redaksi	: Rp	10.000,00
-	Meterai	: Rp	6.000,00

J u m l a h : Rp 266.000,00

(dua ratus enam puluh enam ribu rupiah).

Disclaimer
Kepaniteraan Mahkamah Agung Republik Indonesia berusaha untuk selalu mencantumkan informasi paling kini dan akurat sebagai bentuk komitmen Mahkamah Agung untuk pelayanan publik, transparansi dan akuntabilitas pelaksanaan fungsi peradilan. Namun dalam hal-hal tertentu masih dimungkinkan terjadi permasalahan teknis terkait dengan akurasi dan keterkinian informasi yang kami sajikan, hal mana akan terus kami perbaiki dari waktu kewaktu.
Dalam hal Anda menemukan inakurasi informasi yang termuat pada situs ini atau informasi yang seharusnya ada, namun belum tersedia, maka harap segera hubungi Kepaniteraan Mahkamah Agung RI melalui :
Email : kepaniteraan@mahkamahagung.go.id Telp : 021-384 3348 (ext.318)
