# Supplementary material for: Classification of divorce causes during the COVID-19 pandemic using convolutional neural networks
Source: PeerJ Comput Sci. 2022 Jun 30;8:e998. doi: 10.7717/peerj-cs.998 (PMC9299239; doi:10.7717/peerj-cs.998)
Supplement: Supplemental Information 6 [file peerj-cs-08-998-s006.zip › Rujuk Dataset/Data ke-25.pdf]

P U T U S A N

Nomor 3016/Pdt.G/2020/PA.Srg

بِسْمِ اللَّهِ الرَّحْمَنِ الرَّحِيمِ

DEMI KEADILAN BERDASARKAN KETUHANAN YANG MAHA ESA

Pengadilan Agama Serang yang memeriksa dan mengadili perkara tertentu pada tingkat pertama dalam sidang Majelis Hakim telah menjatuhkan putusan sebagai berikut dalam perkara Cerai Gugat antara:

PENGGUGAT, Nomor KTP xxxxxxxxxxxxxxxx, umur 24 tahun, agama Islam, pendidikan SLTP, pekerjaan xxxxxxxx xxxxx xxxxxx, tempat kediaman di KABUPATEN SERANG, selanjutnya disebut Penggugat;

melawan

TERGUGAT, umur 31 tahun, agama Islam, pendidikan SD, pekerjaan xxxxxxxxxx, tempat kediaman di KABUPATEN KARAWANG, selanjutnya disebut Tergugat;

Pengadilan Agama tersebut;
Telah membaca dan mempelajari berkas perkara;
Telah mendengar keterangan Penggugat;
Telah memeriksa alat-alat bukti Penggugat;

DUDUK PERKARA

Menimbang, bahwa Penggugat dengan surat gugatannya tertanggal 13 November 2020 yang terdaftar di Kepaniteraan Pengadilan Agama Serang dengan register perkara Nomor 3016/Pdt.G/2020/PA.Srg, mengemukakan hal-hal sebagai berikut:

1. Bahwa Penggugat adalah Isteri sah dari Tergugat yang telah melaksanakan pernikahan pada tanggal 23 Juni 2015, dihadapan Pegawai Pencatat Nikah Kantor Urusan Agama xxxxxxxxx xxxxx xxxxx, sebagaimana terbukti dalam Kutipan Akta Nikah Nomor: 147/36/VI/2015 tanggal 23 Juni 2015;

Disclaimer
Kepaniteraan Mahkamah Agung Republik Indonesia berusaha untuk selalu mencantumkan informasi paling kini dan akurat sebagai bentuk komitmen Mahkamah Agung untuk pelayanan publik, transparansi dan akuntabilitas pelaksanaan fungsi peradilan. Namun dalam hal-hal tertentu masih dimungkinkan terjadi permasalahan teknis terkait dengan akurasi dan keterkinian informasi yang kami sajikan, hal mana akan terus kami perbaiki dari waktu kewaktu.
Dalam hal Anda menemukan inakurasi informasi yang termuat pada situs ini atau informasi yang seharusnya ada, namun belum tersedia, maka harap segera hubungi Kepaniteraan Mahkamah Agung RI melalui :
Email : kepaniteraan@mahkamahagung.go.id   Telp : 021-384 3348 (ext.318)

2. Bahwa setelah menikah Penggugat dan Tergugat hidup berumah tangga terakhir tinggal di KABUPATEN SERANG;
3. Bahwa selama berumah tangga antara Penggugat dengan Tergugat telah berhubungan sebagaimana layaknya suami istri dan dikaruniai 1 orang anak bernama: xxxx xxxxxx xxxxx, Perempuan, lahir di Serang, 23 April 2018;
4. Bahwa semula rumah tangga Penggugat dengan Tergugat berjalan rukun dan harmonis, namun sejak Maret 2018 rumah tangga dirasakan mulai goyah sering terjadi perselisihan dan pertengkaran yang disebabkan oleh hal-hal sebagai berikut:
   4.1 Tergugat dalam memberikan nafkah lahir terhadap Penggugat hanya sekedarnya saja;
   4.2 Tergugat tidak jujur dalam hal keuangan terhadap Penggugat;
   4.3 Tergugat memiliki sifat temperamen;
   4.4 Tergugat sering berkata-kata kasar terhadap Penggugat seperti kata hinaan dan binatang;
5. Bahwa perselisihan dan pertengkaran tersebut mencapai puncaknya terjadi sejak September 2019. Akibatnya antara Penggugat dan Tergugat berpisah rumah yang pergi meninggalkan rumah adalah Tergugat dan sejak saat itu sampai sekarang Tergugat tidak kembali lagi;
6. Bahwa, rumah tangga tersebut sudah sulit untuk dibina menjadi suatu rumah tangga yang baik dan harmonis kembali, sehingga tujuan perkawinan untuk membentuk suatu rumah tangga yang sakinah, mawaddah dan rahmah sudah tidak mungkin tercapai lagi;
7. Bahwa Penggugat bersedia membayar biaya perkara sesuai dengan ketentuan yang berlaku;

Bahwa berdasarkan alasan-alasan tersebut di atas, Penggugat mohon kepada Bapak Ketua Pengadilan Agama Serang cq. Majelis Hakim yang memeriksa perkara ini berkenan memutuskan sebagai berikut :

a. Mengabulkan gugatan Penggugat;
b. Menjatuhkan talak satu ba'in sughra Tergugat (TERGUGAT) kepada Penggugat (PENGGUGAT);
c. Menetapkan biaya perkara menurut hukum;

Disclaimer
Kepaniteraan Mahkamah Agung Republik Indonesia berusaha untuk selalu mencantumkan informasi paling kini dan akurat sebagai bentuk komitmen Mahkamah Agung untuk pelayanan publik, transparansi dan akuntabilitas pelaksanaan fungsi peradilan. Namun dalam hal-hal tertentu masih dimungkinkan terjadi permasalahan teknis terkait dengan akurasi dan keterkinian informasi yang kami sajikan, hal mana akan terus kami perbaiki dari waktu kewaktu.
Dalam hal Anda menemukan inakurasi informasi yang termuat pada situs ini atau informasi yang seharusnya ada, namun belum tersedia, maka harap segera hubungi Kepaniteraan Mahkamah Agung RI melalui :
Email : kepaniteraan@mahkamahagung.go.id   Telp : 021-384 3348 (ext.318)

Dan apabila Majelis Hakim berpendapat lain, mohon putusan yang seadil-adilnya (ex aequo et bono);

Bahwa pada hari sidang yang telah ditetapkan, Penggugat datang menghadap sendiri di persidangan, sedangkan Tergugat tidak pernah datang menghadap dan tidak pula menyuruh orang lain untuk menghadap sebagai wakil atau kuasanya yang sah, meskipun telah dipanggil secara resmi dan patut dan tidak ternyata ketidakhadirannya tersebut disebabkan oleh suatu halangan yang sah, maka perkara ini diperiksa tanpa hadirnya Tergugat;

Bahwa upaya perdamaian dan mediasi tidak dapat dilaksanakan karena Tergugat tidak pernah hadir di persidangan;

Bahwa selanjutnya pemeriksaan perkara dilakukan dalam sidang tertutup untuk umum dengan terlebih dahulu dibacakan surat gugatan Penggugat yang isi dan maksudnya tetap dipertahankan oleh Penggugat;

Bahwa oleh karena Tergugat atau kuasanya yang sah tidak pernah hadir di persidangan, maka jawaban Tergugat atas gugatan Penggugat tidak dapat didengarkan;

Bahwa untuk menguatkan dalil-dalil gugatannya, Penggugat mengajukan alat bukti surat dan saksi-saksi sebagai berikut :

A. Bukti Surat.

1. Fotokopi Kartu Tanda Penduduk (KTP) NIK xxxxxxxxxxxxxxxx tanggal 05 Desember 2018 atas nama Penggugat dari Kantor Dinas Kependudukan dan Pencatatan Sipil xxxxxxxxx xxxxxx, bukti surat tersebut telah diberi meterai cukup, telah dicap pos dan telah dicocokkan dengan aslinya yang ternyata sesuai, diberi tanda P1;
2. Fotokopi Kutipan Akta Nikah dari Kantor Urusan Agama xxxxxxxxx xxxxx xxxxx xxxxxxxxx xxxxxx, Nomor 147/36/VI/2015 Tanggal 23 Juni 2015, bukti surat tersebut telah diberi meterai cukup, telah dicap pos dan telah dicocokkan dengan aslinya yang ternyata sesuai, diberi tanda P2;

B. Bukti Saksi.

1. **SAKSI 1**, umur 51 tahun, agama Islam, pekerjaan xxxxxxxxx xxxx, bertempat tinggal di KABUPATEN SERANG, di bawah sumpah memberikan keterangan yang pada pokoknya sebagai berikut :

Disclaimer
Kepaniteraan Mahkamah Agung Republik Indonesia berusaha untuk selalu mencantumkan informasi paling kini dan akurat sebagai bentuk komitmen Mahkamah Agung untuk pelayanan publik, transparansi dan akuntabilitas pelaksanaan fungsi peradilan. Namun dalam hal-hal tertentu masih dimungkinkan terjadi permasalahan teknis terkait dengan akurasi dan keterkinian informasi yang kami sajikan, hal mana akan terus kami perbaiki dari waktu kewaktu.
Dalam hal Anda menemukan inakurasi informasi yang termuat pada situs ini atau informasi yang seharusnya ada, namun belum tersedia, maka harap segera hubungi Kepaniteraan Mahkamah Agung RI melalui :
Email : kepaniteraan@mahkamahagung.go.id Telp : 021-384 3348 (ext.318)

- Bahwa saksi kenal dengan Penggugat dan Tergugat yang bernama xxxxxx xxxxxxx;
- Bahwa saksi tahu antara Penggugat dengan Tergugat adalah suami isteri yang telah menikah pada tahun 2015;
- Bahwa setelah menikah Penggugat dengan Tergugat bertempat tinggal tinggal bersama terakhir di KABUPATEN SERANG;
- Bahwa Penggugat dengan Tergugat telah dikaruniai 1 orang anak;
- Bahwa kehidupan rumah tangga Penggugat dengan Tergugat pada awalnya hidup rukun dan harmonis, namun sekarang inirumah tangga mereka tidak harmonis lagi karena sering terjadi perselisihan dan pertengkaran yang terus menerus;
- Bahwa perselisihan dan pertengkaran Penggugat dengan Tergugat terjadi sejak 2 tahun yang lalu;
- Bahwa sepengetahuan saksi bahwa penyebab perselisihan dan pertengkaran Penggugat dengan Tergugat disebabkan karena Tergugat tidak jujur dalam hal keuangan, dan Tergugat kurang mencukupi didalam memenuhi kebutuhan rumah tangga sehari-hari. Selain itu Tergugat temperamental dan sering berkata-kata kasar kepada Penggugat; ;
- Bahwa saksi sering mendengar dari aduan Penggugat yang bercerita tentang permasalahan rumah tangganya dengan Tergugat ;
- Bahwa Penggugat dengan Tergugat sudah pisah rumah sejak tahun 2019;
- Bahwa yang meninggalkan kediaman bersama adalah Tergugat pergi meninggalkan Penggugat sampai dengan sekarang;
- Bahwa selamapisah Tergugat tidak pernah datang, dan tidak pernah lagi memberi nafkah untuk Penggugat, dan Tergugat sudah tidak memperdulikan Penggugat lagi serta sudah tidak pernah lagi berkomunikasi;
- Bahwa pernah dinasehati agar Penggugat bersabar dan rukun kembali membina rumah tangganya dengan Tergugat, namun tidak

Disclaimer
Kepaniteraan Mahkamah Agung Republik Indonesia berusaha untuk selalu mencantumkan informasi paling kini dan akurat sebagai bentuk komitmen Mahkamah Agung untuk pelayanan publik, transparansi dan akuntabilitas pelaksanaan fungsi peradilan. Namun dalam hal-hal tertentu masih dimungkinkan terjadi permasalahan teknis terkait dengan akurasi dan keterkinian informasi yang kami sajikan, hal mana akan terus kami perbaiki dari waktu kewaktu.
Dalam hal Anda menemukan inakurasi informasi yang termuat pada situs ini atau informasi yang seharusnya ada, namun belum tersedia, maka harap segera hubungi Kepaniteraan Mahkamah Agung RI melalui :
Email : kepaniteraan@mahkamahagung.go.id   Telp : 021-384 3348 (ext.318)

berhasil karena tidak ada perubahan pada diri Tergugat dan juga Penggugat sudah tidak mau rukun kembali;
- Bahwa saksi sudah tidak sanggup untuk merukunkan kembali Penggugat dengan Tergugat;

2. **SAKSI 2**, umur 29 tahun, agama Islam, pekerjaan xxxxxxxxx xxxx, bertempat tinggal di KABUPATEN SERANG, di bawah sumpah memberikan keterangan yang pada pokoknya sebagai berikut :
   - Bahwa saksi kenal dengan Penggugat dan Tergugat yang bernama xxxxxx xxxxxxx;
   - Bahwa saksi tahu antara Penggugat dengan Tergugat adalah suami isteri yang telah menikah pada tahun 2015;
   - Bahwa setelah menikah Penggugat dengan Tergugat bertempat tinggal tinggal bersama terakhir di KABUPATEN SERANG;
   - Bahwa Penggugat dengan Tergugat telah dikaruniai 1 orang anak;
   - Bahwa kehidupan rumah tangga Penggugat dengan Tergugat pada awalnya hidup rukun dan harmonis, namun sekarang ini rumah tangga mereka tidak harmonis lagi karena sering terjadi perselisihan dan pertengkaran yang terus menerus;
   - Bahwa perselisihan dan pertengkaran Penggugat dengan Tergugat terjadi sejak 2 tahun yang lalu;
   - Bahwa sepengetahuan saksi bahwa penyebab perselisihan dan pertengkaran Penggugat dengan Tergugat disebabkan karena Tergugat temperamental dan sering berkata-kata kasar kepada Penggugat, Tergugat tidak jujur dalam hal keuangan, dan Tergugat pun kurang mencukupi didalam memenuhi kebutuhan rumah tangga sehari-hari;
   - Bahwa saksi pernah melihat Penggugat dengan Tergugat bertengkar;
   - Bahwa Penggugat dengan Tergugat sudah pisah rumah sejak tahun 2019;
   - Bahwa yang meninggalkan kediaman bersama adalah Tergugat pergi meninggalkan Penggugat sampai dengan sekarang;

Disclaimer
Kepaniteraan Mahkamah Agung Republik Indonesia berusaha untuk selalu mencantumkan informasi paling kini dan akurat sebagai bentuk komitmen Mahkamah Agung untuk pelayanan publik, transparansi dan akuntabilitas pelaksanaan fungsi peradilan. Namun dalam hal-hal tertentu masih dimungkinkan terjadi permasalahan teknis terkait dengan akurasi dan keterkinian informasi yang kami sajikan, hal mana akan terus kami perbaiki dari waktu kewaktu.
Dalam hal Anda menemukan inakurasi informasi yang termuat pada situs ini atau informasi yang seharusnya ada, namun belum tersedia, maka harap segera hubungi Kepaniteraan Mahkamah Agung RI melalui :
Email : kepaniteraan@mahkamahagung.go.id   Telp : 021-384 3348 (ext.318)

- Bahwa selamapisah Tergugat tidak pernah datang, dan tidak pernah lagi memberi nafkah untuk Penggugat, dan Tergugat sudah tidak memperdulikan Penggugat lagi serta sudah tidak pernah lagi berkomunikasi;
- Bahwa pernah dinasehati agar Penggugat bersabar dan rukun kembali membina rumah tangganya dengan Tergugat, namun tidak berhasil karena tidak ada perubahan pada diri Tergugat dan juga Penggugat sudah tidak mau rukun kembali;
- Bahwa saksi sudah tidak sanggup untuk merukunkan kembali Penggugat dengan Tergugat;

Bahwa selanjutnya Penggugat menyatakan tidak akan mengajukan apapun lagi dan menyampaikan kesimpulan yang pada pokoknya tetap pada gugatan semula serta mohon putusan;

Bahwa untuk mempersingkat uraian putusan ini, ditunjuk segala sesuatu yang tercantum dalam berita acara persidangan perkara ini sebagai bagian yang tidak terpisahkan dari isi putusan ini;

PERTIMBANGAN HUKUM

Menimbang, bahwa maksud dan tujuan gugatan Penggugat adalah seperti diuraikan tersebut di atas;

Menimbang, bahwa oleh karena Tergugat tidak pernah hadir di persidangan dan tidak pula mengutus wakil atau kuasanya tanpa alasan sekalipun kepadanya telah dipanggil secara resmi dan patut, dengan demikian sesuai ketentuan Pasal 125 ayat 1 HIR perkara ini dapat diputus dengan Verstek;

Menimbang, bahwa mediasi atas perkara ini tidak bisa dilaksanakan karena menurut Peraturan Mahkamah Agung R I Nomor 1 Tahun 2016 tentang Prosedur Mediasi di Pengadilan, mediasi mengharuskan kehadiran kedua belah pihak yang berperkara, sedang Tergugat tidak hadir di persidangan. Namun demikian sesuai ketentuan pasal 130 ayat 1 HIR jo pasal 82 ayat 1 Undang-Undang Nomor 7 Tahun 1989 tentang Peradilan Agama sebagaimana telah diubah dengan Undang-Undang Nomor 3 Tahun 2006 dan telah diubah kembali dengan Undang-Undang Nomor 50 Tahun 2009, Majelis telah berupaya

Disclaimer
Kepaniteraan Mahkamah Agung Republik Indonesia berusaha untuk selalu mencantumkan informasi paling kini dan akurat sebagai bentuk komitmen Mahkamah Agung untuk pelayanan publik, transparansi dan akuntabilitas pelaksanaan fungsi peradilan. Namun dalam hal-hal tertentu masih dimungkinkan terjadi permasalahan teknis terkait dengan akurasi dan keterkinian informasi yang kami sajikan, hal mana akan terus kami perbaiki dari waktu kewaktu.
Dalam hal Anda menemukan inakurasi informasi yang termuat pada situs ini atau informasi yang seharusnya ada, namun belum tersedia, maka harap segera hubungi Kepaniteraan Mahkamah Agung RI melalui :
Email : kepaniteraan@mahkamahagung.go.id   Telp : 021-384 3348 (ext.318)

menasehati Penggugat agar tetap mempertahankan ikatan perkawinannya dengan Tergugat , akan tetapi upaya tersebut tidak berhasil;

Menimbang, bahwa yang menjadi masalah pokok dalam perkara ini adalah Penggugat mengajukan gugatan cerai terhadap Tergugat dengan alasan rumah tangganya sudah tidak rukun, sering terjadi perselisihan dan pertengkaran yang puncaknya sejak bulan September 2019 antara Penggugat dengan Tergugat berpisah rumah dan sampai sekarang tidak pernah tinggal bersama lagi layaknya suami isteri yang baik;

Menimbang, bahwa oleh karena Tergugat atas ketidak hadirannya di persidangan telah tidak melakukan bantahan terhadap gugatan Penggugat terutama dalam hal adanya perselisihan dalam rumah tangga antara Penggugat dengan Tergugat dan hal-hal yang menyebabkan perselisihan itu terjadi, sehingga dapat diduga adanya pengakuan Tergugat sepanjang yang tersurat dalam surat gugat;

Menimbang, bahwa berdasarkan ketentuan Pasal 125 HIR yaitu putusan yang dijatuhkan tanpa hadirnya Tergugat dapat dikabulkan sepanjang berdasarkan hukum dan beralasan, oleh karena itu majelis membebani Penggugat untuk membuktikan dalil-dalil gugatannya;

Menimbang, bahwa dalam tahap pembuktian Penggugat telah mengajukan bukti P.1 dan P.2, serta telah menghadirkan 2 (dua) orang saksi;

Menimbang, bahwa bukti P.1 yang diajukan Penggugat merupakan bukti otentik tentang identitas Penggugat, oleh karenanya dinilai terbukti bahwa Penggugat sebagai pihak yang berkepentingan dalam perkara ini dan pula bertempat kediaman di wilayah hukum Pengadilan Agama Serang;

Menimbang, bahwa bukti P.2 yang diajukan Penggugat adalah merupakan bukti otentik yang menerangkan bahwa Penggugat dan Tergugat telah melangsungkan pernikahan pada tanggal 23 Juni 2015, bukti tersebut telah memenuhi syarat formil dan materil pembuktian, oleh karenanya Majelis menilai hubungan Penggugat dan Tergugat sebagai suami istri dinyatakan telah terbukti;

Menimbang, bahwa dua orang saksi yang diajukan oleh Penggugat adalah orang dewasa dan telah disumpah sehingga memenuhi syarat formal

Disclaimer
Kepaniteraan Mahkamah Agung Republik Indonesia berusaha untuk selalu mencantumkan informasi paling kini dan akurat sebagai bentuk komitmen Mahkamah Agung untuk pelayanan publik, transparansi dan akuntabilitas pelaksanaan fungsi peradilan. Namun dalam hal-hal tertentu masih dimungkinkan terjadi permasalahan teknis terkait dengan akurasi dan keterkinian informasi yang kami sajikan, hal mana akan terus kami perbaiki dari waktu kewaktu.
Dalam hal Anda menemukan inakurasi informasi yang termuat pada situs ini atau informasi yang seharusnya ada, namun belum tersedia, maka harap segera hubungi Kepaniteraan Mahkamah Agung RI melalui :
Email : kepaniteraan@mahkamahagung.go.id Telp : 021-384 3348 (ext.318)

sebagaimana diatur dalam Pasal 145 ayat 1 angka 3e HIR. Kedua orang saksi tersebut menerangkan di bawah sumpah yang pada pokoknya bahwa Penggugat dan Tergugat sebagai suami istri namun rumah tangganya sudah tidak rukun lagi, sering terjadi perselisihan dan pertengkaran, puncaknya antara Penggugat dan Tergugat sudah berpisah tempat tinggal sejak bulan September 2019 serta tidak pernah kumpul lagi sebagaimana layaknya suami isteri. Keterangan dua orang saksi tersebut bersesuaian satu sama lain dan berdasarkan pengetahuannya sendiri. Oleh karenanya, keterangan dua orang saksi tersebut telah memenuhi syarat materiil sebagaimana telah diatur dalam Pasal 171 HIR, sehingga keterangan dua orang saksi tersebut memiliki kekuatan pembuktian dan dapat diterima sebagai alat bukti;

Menimbang, bahwa berdasarkan dalil Penggugat yang tidak dibantah oleh Tergugat dan diperkuat dengan keterangan saksi-saksi di bawah sumpahnya menurut Majelis Hakim merupakan indikasi adanya perselisihan antara Penggugat dengan Tergugat sedangkan upaya perdamaian dengan maksud agar mereka dapat hidup rukun kembali dalam rumah tangga telah dilakukan baik oleh saksi-saksi, keluarga maupun Pengadilan dalam setiap persidangan, akan tetapi tidak berhasil. maka majelis dapat menarik suatu kesimpulan yang merupakan fakta bahwa antara Penggugat dengan Tergugat telah terjadi perselisihan dalam rumah tangga yang sulit untuk dirukunkan lagi;

Menimbang, bahwa oleh karena perkawinan tersebut sudah tidak rukun lagi, lebih-lebih antara Penggugat dengan Tergugat sudah berpisah tempat tinggal dan hidup sendiri-sendiri yang menunjukkan hubungan interpersonal sudah tidak terkoordinasi dan sudah saling tidak memenuhi hak dan kewajiban sebagai suami isteri, maka disini sudah ada bukti bahwa rumah tangga antara Penggugat dengan Tergugat sudah retak dan tidak ada lagi ikatan lahir bathin, sehingga tujuan perkawinan untuk membentuk rumah tangga yang bahagia dan kekal sebagaimana dimaksud Pasal 1 Undang-Undang Nomor 1 Tahun 1974 tentang Perkawinan atau rumah tangga yang sakinah mawaddah dan rahmah sebagaimana dikehendaki dalam Alqur'an surat Ar-Rum ayat (21) jo Pasal 3 Kompilasi Hukum Islam (KHI) tidak dapat diwujudkan dalam rumah tangga Penggugat dan Tergugat;

Disclaimer
Kepaniteraan Mahkamah Agung Republik Indonesia berusaha untuk selalu mencantumkan informasi paling kini dan akurat sebagai bentuk komitmen Mahkamah Agung untuk pelayanan publik, transparansi dan akuntabilitas pelaksanaan fungsi peradilan. Namun dalam hal-hal tertentu masih dimungkinkan terjadi permasalahan teknis terkait dengan akurasi dan keterkinian informasi yang kami sajikan, hal mana akan terus kami perbaiki dari waktu kewaktu.
Dalam hal Anda menemukan inakurasi informasi yang termuat pada situs ini atau informasi yang seharusnya ada, namun belum tersedia, maka harap segera hubungi Kepaniteraan Mahkamah Agung RI melalui :
Email : kepaniteraan@mahkamahagung.go.id Telp : 021-384 3348 (ext.318)

Menimbang, bahwa Majelis perlu mengemukakan ketentuan yang termaktub dalam Kitab Fiqih Ash Shawi jilid IV Halaman 204 :

فإن اختلف بأن لم توجد بينهمامحبّة ولامودّة فالمناسب المفارقة

Artinya : "*Maka jika telah terjadi perselisihan dengan tidak diperoleh diantara keduanya kasih sayang, maka pantaslah perceraian*";

Menimbang, bahwa meskipun perceraian adalah suatu perbuatan yang sedapat mungkin dihindari, namun apabila tujuan perkawinan sudah tidak dapat terwujud, maka mempertahankan perkawinan dalam kondisi sebagaimana tersebut di atas justru akan menimbulkan kemudharatan bagi kedua belah pihak berperkara, keturunan dan keluarga mereka;

Menimbang, bahwa berdasarkan pertimbangan tersebut di atas, maka dalil gugatan Penggugat telah memenuhi ketentuan Pasal 39 ayat 2 Undang-Undang Nomor 1 Tahun 1974 jo Pasal 19 huruf f Peraturan Pemerintah Nomor 9 Tahun 1975 jo Pasal 116 huruf f Kompilasi Hukum Islam, dengan demikian petitum gugatan cerai Penggugat dapat dikabulkan dengan menjatuhkan talak satu ba'in shugra Tergugat terhadap Penggugat sesuai ketentuan Pasal 119 ayat (2) huruf c Kompilasi Hukum Islam;

Menimbang, bahwa oleh karena perkara ini merupakan perkara perceraian, maka sesuai ketentuan Pasal 89 ayat 1 Undang-Undang Nomor 7 Tahun 1989 tentang Peradilan Agama dan perubahannya, maka biaya perkara dibebankan kepada Penggugat;

Mengingat segala peraturan perundang-undangan yang berlaku dan dalil-dalil syar'i yang berkaitan dengan perkara ini;

MENGADILI

1. Menyatakan Tergugat yang telah dipanggil secara resmi dan patut untuk menghadap di persidangan, tidak hadir;
2. Mengabulkan gugatan Penggugat dengan verstek;
3. Menjatuhkan talak satu ba'in shughra Tergugat (TERGUGAT) terhadap Penggugat (PENGGUGAT);

Disclaimer
Kepaniteraan Mahkamah Agung Republik Indonesia berusaha untuk selalu mencantumkan informasi paling kini dan akurat sebagai bentuk komitmen Mahkamah Agung untuk pelayanan publik, transparansi dan akuntabilitas pelaksanaan fungsi peradilan. Namun dalam hal-hal tertentu masih dimungkinkan terjadi permasalahan teknis terkait dengan akurasi dan keterkinian informasi yang kami sajikan, hal mana akan terus kami perbaiki dari waktu kewaktu.
Dalam hal Anda menemukan inakurasi informasi yang termuat pada situs ini atau informasi yang seharusnya ada, namun belum tersedia, maka harap segera hubungi Kepaniteraan Mahkamah Agung RI melalui :
Email : kepaniteraan@mahkamahagung.go.id Telp : 021-384 3348 (ext.318)

4. Membebankan kepada Penggugat untuk membayar biaya perkara ini sejumlah Rp 606.000,00 (enam ratus enam ribu rupiah);

Demikian diputuskan dalam musyawarah Majelis Hakim Pengadilan Agama Serang pada hari Selasa tanggal 29 Desember 2020 Miladiyah bertepatan dengan tanggal 14 Jumadil Awwal 1442 Hijriyah oleh Drs. H. Saifudin Z., S.H.,M.H sebagai Ketua Majelis, Dra. Hj. Ai Jamilah, M.H dan Tuti Sudiarti, S.H.,M.H, masing-masing sebagai Hakim Anggota, putusan tersebut diucapkan pada hari itu juga dalam sidang terbuka untuk umum oleh Ketua Majelis beserta para Hakim Anggota tersebut, dan didampingi oleh Ricky Adjie Purnama, S.H.I sebagai Panitera Pengganti, dengan dihadiri oleh Penggugat tanpa hadirnya Tergugat.

Ketua Majelis,

Ttd.

Drs. H. Saifudin Z., S.H.,M.H

Hakim Anggota,	Hakim Anggota,
Ttd.	Ttd.
**Dra. Hj. Ai Jamilah, M.H**	**Tuti Sudiarti, S.H.,M.H**

Panitera Pengganti,

Ttd.

Ricky Adjie Purnama, S.H.I

Perincian biaya :

- Pendaftaran	: Rp	30.000,00
- ATK Perkara	: Rp	75.000,00

Disclaimer
Kepaniteraan Mahkamah Agung Republik Indonesia berusaha untuk selalu mencantumkan informasi paling kini dan akurat sebagai bentuk komitmen Mahkamah Agung untuk pelayanan publik, transparansi dan akuntabilitas pelaksanaan fungsi peradilan. Namun dalam hal-hal tertentu masih dimungkinkan terjadi permasalahan teknis terkait dengan akurasi dan keterkinian informasi yang kami sajikan, hal mana akan terus kami perbaiki dari waktu kewaktu.
Dalam hal Anda menemukan inakurasi informasi yang termuat pada situs ini atau informasi yang seharusnya ada, namun belum tersedia, maka harap segera hubungi Kepaniteraan Mahkamah Agung RI melalui :
Email : kepaniteraan@mahkamahagung.go.id   Telp : 021-384 3348 (ext.318)

- Panggilan	: Rp	465.000,00
- PNBP Panggilan	: Rp	20.000,00
- Redaksi	: Rp	10.000,00
- Meterai	: Rp	6.000,00
J u m l a h	: Rp	606.000,00

(enam ratus enam ribu rupiah).

Untuk Salinan yang sesuai dengan aslinya,

Panitera

Pengadilan Agama Serang

Drs. H. Baehaki., M.Sy

Catatan :

Salinan Putusan diberikan kepada .... pada tanggal .... dan telah/belum*) berkekuatan hukum tetap.

Disclaimer
Kepaniteraan Mahkamah Agung Republik Indonesia berusaha untuk selalu mencantumkan informasi paling kini dan akurat sebagai bentuk komitmen Mahkamah Agung untuk pelayanan publik, transparansi dan akuntabilitas pelaksanaan fungsi peradilan. Namun dalam hal-hal tertentu masih dimungkinkan terjadi permasalahan teknis terkait dengan akurasi dan keterkinian informasi yang kami sajikan, hal mana akan terus kami perbaiki dari waktu kewaktu.
Dalam hal Anda menemukan inakurasi informasi yang termuat pada situs ini atau informasi yang seharusnya ada, namun belum tersedia, maka harap segera hubungi Kepaniteraan Mahkamah Agung RI melalui :
Email : kepaniteraan@mahkamahagung.go.id Telp : 021-384 3348 (ext.318)
