# Supplementary material for: Classification of divorce causes during the COVID-19 pandemic using convolutional neural networks
Source: PeerJ Comput Sci. 2022 Jun 30;8:e998. doi: 10.7717/peerj-cs.998 (PMC9299239; doi:10.7717/peerj-cs.998)
Supplement: Supplemental Information 6 [file peerj-cs-08-998-s006.zip › Rujuk Dataset/Data ke-26.pdf]

P U T U S A N

Nomor 3799/Pdt.G/2020/PA.Pml

بِسْمِ اللهِ الرَّحْمٰنِ الرَّحِيْمِ

DEMI KEADILAN BERDASARKAN KETUHANAN YANG MAHA ESA

Pengadilan Agama Pemalang yang memeriksa dan mengadili perkara perdata agama pada tingkat pertama dalam sidang Majelis telah menjatuhkan putusan perkara Gugatan Perceraian antara :

PENGGUGAT, umur 28 tahun, agama Islam, Pekerjaan Pembantu rumah tangga, Pendidikan Sekolah Lanjutan Tingkat Pertama, tempat kediaman di Kecamatan Warungpring, Kabupaten Pemalang, sebagai Penggugat

melawan

TERGUGAT, umur 29 tahun, agama Islam, Pekerjaan Buruh Bangunan, Pendidikan Sekolah Dasar, tempat kediaman di , Kecamatan Jatinegara, Kabupaten Tegal, sebagai Tergugat

Pengadilan Agama tersebut;

Telah mempelajari berkas perkara;

Telah mendengar keterangan Penggugat dan saksi-saksi di persidangan ;

DUDUK PERKARA

Bahwa, Penggugat dalam surat gugatannya tanggal 07 Desember 2020 telah mengajukan gugatan perceraian yang telah didaftar di Kepaniteraan Pengadilan Agama Pemalang dengan Nomor 3799/Pdt.G/2020/PA.Pml , tanggal 07 Desember 2020 , dengan dalil-dalil sebagai berikut:

1. Bahwa Penggugat telah menikah dengan Tergugat pada tanggal 13 Mei 2016 sebagaimana dalam Kutipan Akta Nikah Nomor : 0142/014/V/2016 tanggal 13 Mei 2016 yang dikeluarkan oleh Kantor Urusan Agama Kecamatan Pulosari, Kabupaten Pemalang, serta pada saat berlangsungnya perkawinan

Disclaimer
Kepaniteraan Mahkamah Agung Republik Indonesia berusaha untuk selalu mencantumkan informasi paling kini dan akurat sebagai bentuk komitmen Mahkamah Agung untuk pelayanan publik, transparansi dan akuntabilitas pelaksanaan fungsi peradilan. Namun dalam hal-hal tertentu masih dimungkinkan terjadi permasalahan teknis terkait dengan akurasi dan keterkinian informasi yang kami sajikan, hal mana akan terus kami perbaiki dari waktu kewaktu.
Dalam hal Anda menemukan inakurasi informasi yang termuat pada situs ini atau informasi yang seharusnya ada, namun belum tersedia, maka harap segera hubungi Kepaniteraan Mahkamah Agung RI melalui :
Email : kepaniteraan@mahkamahagung.go.id   Telp : 021-384 3348 (ext.318)

tersebut Penggugat berstatus Perawan dan Tergugat berstatus Jejaka, dan setelah akad nikah Tergugat mengucapkan Sighat Taklik Talak;

2. Bahwa setelah akad nikah, Penggugat dan Tergugat bertempat tinggal bersama di orang tua Penggugat di Kecamatan Warungpring, Kabupaten Pemalang selama 3 tahun 10 bulan;

3. Bahwa selama perkawinan Penggugat dan Tergugat Sudah melakukan hubungan suami istri (ba'da dukhul) dan telah dikaruniai 1 orang anak bernama ANAK, umur 4 tahun sekarang anak tersebut dalam poengasuhan Penggugat;

4. Bahwa sejak awal pernikahan antara Penggugat dan Tergugat terjadi perselisihan dan pertengkaran terus menerus disebabkan nafkah yang dinberikan oleh Tergugat sebesar Rp. 500.000,- (lima ratus ribu rupiah) per bulan tidak mencukupi untuk memenuhi kebutuhan rumah tangga sehari-hari;

5. Bahwa akibat dari perselisihan dan pertengkaran terus menerus tersebut pada bulan April 2020 Tergugat pergi meninggalkan Penggugat tanpa pamit dan tanpa alasan yang syah pulang kerumah orang tua Tergugat di , Kecamatan Jatinegara, Kabupaten Tegal. Sehingga sampai diajukannya gugatan ini antara Penggugat dengan Tergugat telah berpisah tempat tinggal selama 8 bulan dan selama itu antara Penggugat dan Tergugat sudah tidak pernah bertemu dan sudah tidak ada Komunikasi lagi;

6. Bahwa dengan kondisi rumah tangga sebagaimana tersebut di atas, Penggugat sudah tidak sanggup lagi meneruskan hidup berumah tangga dengan Tergugat, karena sudah tidak ada harapan untuk rukun kembali;

7. Bahwa berdasarkan uraian kejadian yang telah dikemukakan di atas, maka alasan Penggugat mengajukan gugatan cerai adalah antara Penggugat dengan Tergugat terjadi perselisihan dan pertengkaran terus-menerus sehingga sulit untuk didamaikan dan tidak ada harapan untuk hidup rukun dalam rumah tangga;

Bahwa berdasarkan alasan-alasan tersebut di atas, Penggugat mohon

Disclaimer
Kepaniteraan Mahkamah Agung Republik Indonesia berusaha untuk selalu mencantumkan informasi paling kini dan akurat sebagai bentuk komitmen Mahkamah Agung untuk pelayanan publik, transparansi dan akuntabilitas pelaksanaan fungsi peradilan. Namun dalam hal-hal tertentu masih dimungkinkan terjadi permasalahan teknis terkait dengan akurasi dan keterkinian informasi yang kami sajikan, hal mana akan terus kami perbaiki dari waktu kewaktu.
Dalam hal Anda menemukan inakurasi informasi yang termuat pada situs ini atau informasi yang seharusnya ada, namun belum tersedia, maka harap segera hubungi Kepaniteraan Mahkamah Agung RI melalui :
Email : kepaniteraan@mahkamahagung.go.id   Telp : 021-384 3348 (ext.318)

kepada Ketua Pengadilan Agama Kelas 1A Pemalang Cq. Majelis Hakim pemeriksa perkara ini untuk memeriksa dan mengadili serta menjatuhkan putusan yang amarnya sebagai berikut :

1. Mengabulkan gugatan Penggugat (PENGGUGAT);
2. Menceraikan Penggugat (PENGGUGAT) dari Tergugat (TERGUGAT) dengan menjatuhkan talak satu bain sughro Tergugat kepada Penggugat;
3. Membebankan biaya perkara menurut hukum;

Atau apabila pengadilan berpendapat lain, mohon putusan yang seadil-adilnya;

Bahwa, pada hari sidang yang telah ditetapkan Penggugat telah datang menghadap ke muka sidang, sedangkan Tergugat tidak datang menghadap ke muka sidang dan tidak menyuruh orang lain untuk menghadap sebagai wakil/kuasa hukumnya meskipun telah dipanggil secara resmi dan patut yang relaas panggilannya telah dibacakan di dalam sidang, sedangkan tidak ternyata bahwa tidak datangnya itu disebabkan suatu halangan yang sah;

Bahwa, Majelis hakim telah mendamaikan dengan cara menasehati Penggugat agar berpikir untuk tidak bercerai dengan Tergugat, tetapi Penggugat tetap pada dalil-dalil gugatannya untuk bercerai dengan Tergugat ;

Bahwa, perkara ini tidak dapat di mediasi karena Tergugat tidak pernah datang menghadap di muka sidang, selanjutnya dimulai pemeriksaan dengan membacakan surat gugatan Penggugat dalam sidang tertutup untuk umum yang maksud dan isinya tetap dipertahankan oleh Penggugat ;

Bahwa, untuk menguatkan dalil-dalil gugatannya Penggugat telah mengajukan alat bukti berupa :

A.Bukti Surat :

1. Fotokopi Kartu Tanda Penduduk atas nama PENGGUGAT NIK. 3327145706910001 tanggal 23 Agustus 2017, yang dikeluarkan oleh Kepala Dinas Kependudukan dan Pencatatan Sipil Kabupaten Pemalang,

Disclaimer
Kepaniteraan Mahkamah Agung Republik Indonesia berusaha untuk selalu mencantumkan informasi paling kini dan akurat sebagai bentuk komitmen Mahkamah Agung untuk pelayanan publik, transparansi dan akuntabilitas pelaksanaan fungsi peradilan. Namun dalam hal-hal tertentu masih dimungkinkan terjadi permasalahan teknis terkait dengan akurasi dan keterkinian informasi yang kami sajikan, hal mana akan terus kami perbaiki dari waktu kewaktu.
Dalam hal Anda menemukan inakurasi informasi yang termuat pada situs ini atau informasi yang seharusnya ada, namun belum tersedia, maka harap segera hubungi Kepaniteraan Mahkamah Agung RI melalui :
Email : kepaniteraan@mahkamahagung.go.id Telp : 021-384 3348 (ext.318)

bukti surat tersebut telah sesuai dengan aslinya, bermeterai cukup dan dinazegelen (bukti P.1);

2. Fotokopi Kutipan Akta Nikah yang dikeluarkan Kantor Urusan Agama Kecamatan Pulosari, Kabupaten Pemalang Nomor: 0142/014/V/2016, tanggal 13 Mei 2016, bukti surat tersebut telah sesuai dengan aslinya, bermeterai cukup dan dinazegelen (bukti P.2);

B.Bukti Saksi :

1. SAKSI I umur 60 tahun, Agama Islam, pekerjaan wiraswasta tempat tinggal di Kecamatan Warungpring, Kabupaten Pemalang di bawah sumpahnya memberikan keterangan sebagai berikut:

- bahwa saksi kenal dengan Penggugat dan Tergugat karena saksi ayah Penggugat;
- bahwa Penggugat dan Tergugat adalah pasangan suami istri,
- bahwa setelah menikah, Penggugat dan Tergugat hidup bersama terakhir di rumah orang tua Penggugat di dan telah dikaruniai 1 (satu) orang anak ;
- bahwa semula rumah tangga Penggugat dan Tergugat dalam keadaan rukun, namun sejak awal pernikahan Penggugat sering bertengkar dengan Tergugat karena Nafkah yang diberikan Tergugat tidak mencukupi kebutuhan keluarga.
- bahwa sejak bulan April 2020 Tergugat telah pergi dari rumah tempat kediaman bersama;
- bahwa Penggugat dan Tergugat sekarang sudah pisah selama 8 bulan, dan selama berpisah Tergugat tidak pernah datang menemui Penggugat dan tidak pernah melaksanakan kewajibannya selayaknya seorang suami;

Disclaimer
Kepaniteraan Mahkamah Agung Republik Indonesia berusaha untuk selalu mencantumkan informasi paling kini dan akurat sebagai bentuk komitmen Mahkamah Agung untuk pelayanan publik, transparansi dan akuntabilitas pelaksanaan fungsi peradilan. Namun dalam hal-hal tertentu masih dimungkinkan terjadi permasalahan teknis terkait dengan akurasi dan keterkinian informasi yang kami sajikan, hal mana akan terus kami perbaiki dari waktu kewaktu.
Dalam hal Anda menemukan inakurasi informasi yang termuat pada situs ini atau informasi yang seharusnya ada, namun belum tersedia, maka harap segera hubungi Kepaniteraan Mahkamah Agung RI melalui :
Email : kepaniteraan@mahkamahagung.go.id Telp : 021-384 3348 (ext.318)

- bahwa pihak keluarga Penggugat sudah berusaha merukunkan Penggugat dan Tergugat tetapi tidak berhasil;

2. SAKSI II umur 52 tahun, Agama Islam, pekerjaan Tani tempat tinggal di Kecamatan Warungpring, Kabupaten Pemalang; di bawah sumpahnya memberikan keterangan sebagai berikut:

- bahwa saksi kenal dengan Penggugat dan Tergugat karena saksi tetangga Penggugat
- bahwa Penggugat dan Tergugat adalah pasangan suami istri;
- bahwa setelah menikah, Penggugat dan Tergugat hidup bersama terakhir di rumah orang tua Penggugat di dan telah dikaruniai 1 (satu) orang anak ;
- bahwa semula rumah tangga Penggugat dan Tergugat dalam keadaan rukun, namun sejak awal pernikahan Penggugat sering bertengkar dengan Tergugat karena faktor ekonomi yang kuang mencukupi.
- bahwa sejak bulan April 2020 Tergugat telah pergi dari rumah tempat kediaman bersama;
- bahwa Penggugat dan Tergugat sekarang sudah pisah selama 8 bulan, dan selama berpisah Tergugat tidak pernah datang menemui Penggugat dan tidak pernah melaksanakan kewajibannya selayaknya seorang suami;
- bahwa pihak keluarga Penggugat sudah berusaha merukunkan Penggugat dan Tergugat tetapi tidak berhasil;

Bahwa Penggugat menyatakan mencukupkan bukti-bukti tersebut lalu menyampaikan kesimpulan yang pada pokoknya tetap pada gugatannya dan mohon putusan;

Selanjutnya untuk singkatnya uraian putusan ini, maka semua hal yang

Disclaimer
Kepaniteraan Mahkamah Agung Republik Indonesia berusaha untuk selalu mencantumkan informasi paling kini dan akurat sebagai bentuk komitmen Mahkamah Agung untuk pelayanan publik, transparansi dan akuntabilitas pelaksanaan fungsi peradilan. Namun dalam hal-hal tertentu masih dimungkinkan terjadi permasalahan teknis terkait dengan akurasi dan keterkinian informasi yang kami sajikan, hal mana akan terus kami perbaiki dari waktu kewaktu.
Dalam hal Anda menemukan inakurasi informasi yang termuat pada situs ini atau informasi yang seharusnya ada, namun belum tersedia, maka harap segera hubungi Kepaniteraan Mahkamah Agung RI melalui :
Email : kepaniteraan@mahkamahagung.go.id Telp : 021-384 3348 (ext.318)

termuat dalam Berita Acara Sidang ini merupakan bagian yang tak terpisahkan dari putusan ini;

PERTIMBANGAN HUKUM

Menimbang, bahwa maksud dan tujuan gugatan Penggugat adalah sebagaimana terurai di atas;

Menimbang, bahwa ternyata Tergugat meskipun dipanggil secara resmi dan patut, tidak datang menghadap di muka sidang dan pula tidak ternyata bahwa tidak datangnya itu disebabkan suatu halangan yang sah. Oleh karena itu, Majelis Hakim dapat menjatuhkan putusan secara verstek sesuai ketentuan Pasal 125 ayat (1) HIR dan sesuai dalil syar'i yang terdapat dalam kitab Ahkamul Qur'an juz II halaman 405 yang telah diambil alih sebagai pendapat Majelis sebagai berikut :

من دعي إلى حاكم من حكام المسلمين فلم يجب فهو ظالم لا حق له

Artinya : " Barang siapa yang dipanggil oleh Hakim (Pengadilan) untuk hadir di persidangan tetapi tidak menghadap, maka ia termasuk orang yang dzalim dan tidak ada hak baginya. "

Menimbang, bahwa berdasarkan ketentuan Surat Edaran Mahkamah Agung RI Nomor 3 Tahun 2015 huruf C angka 3 yaitu putusan yang dijatuhkan tanpa hadirnya Tergugat dapat dikabulkan sepanjang berdasarkan hukum dan beralasan, oleh karena itu Majelis membebani Penggugat untuk membuktikan dalil-dalil gugatannya;

Menimbang, bahwa untuk membuktikan dalil-dalil gugatannya, Penggugat telah mengajukan alat bukti surat P.1 dan P.2, serta 2 orang saksi;

Menimbang, bahwa bukti P.1 berupa fotokopi KTP dan bukti P.2 berupa fotokopi Kutipan Akta Nikah yang merupakan akta otentik dan telah bermeterai cukup dan dinazegelen, telah dicocokan dengan aslinya, sehingga bukti tersebut telah memenuhi syarat formal dan meteriil, maka sesuai dengan Pasal 165 HIR alat bukti tersebut mempunyai kekuatan pembuktian yang sempurna dan mengikat;

Disclaimer
Kepaniteraan Mahkamah Agung Republik Indonesia berusaha untuk selalu mencantumkan informasi paling kini dan akurat sebagai bentuk komitmen Mahkamah Agung untuk pelayanan publik, transparansi dan akuntabilitas pelaksanaan fungsi peradilan. Namun dalam hal-hal tertentu masih dimungkinkan terjadi permasalahan teknis terkait dengan akurasi dan keterkinian informasi yang kami sajikan, hal mana akan terus kami perbaiki dari waktu kewaktu.
Dalam hal Anda menemukan inakurasi informasi yang termuat pada situs ini atau informasi yang seharusnya ada, namun belum tersedia, maka harap segera hubungi Kepaniteraan Mahkamah Agung RI melalui :
Email : kepaniteraan@mahkamahagung.go.id Telp : 021-384 3348 (ext.318)

Menimbang, bahwa oleh karena dalil gugatan Pengugat dalam perkara ini lebih dititikberatkan pada pasal 19 huruf (f) Peraturan Pemerintah Nomor 9 Tahun 1975 tentang Pelaksanaan Undang-undang Nomor 1 Tahun 1974 tentang Perkawinan jo. Pasal 116 huruf (f) Inpres Nomor 1 Tahun 1991 tentang Kompilasi Hukum Islam, maka untuk memenuhi maksud Pasal 22 ayat (2) Peraturan Pemerintah Nomor 9 Tahun 1975 tentang Pelaksanaan Undang-undang Nomor 1 Tahun 1974 tentang Perkawinan jo. Pasal 134 Inpres Nomor 1 Tahun 1991 tentang Kompilasi Hukum Islam, dalam penyelesaian perkara *a quo,* Majelis Hakim diharuskan memeriksa saksi-saksi dari pihak keluarga atau orang-orang yang dekat dengan Penggugat dan Tergugat untuk mendapatkan kebenaran yang lebih meyakinkan ;

Menimbang, bahwa untuk memenuhi ketentuan Pasal 22 ayat (2) Peraturan Pemerintah No. 9 tahun 1975 tentang Pelaksanaan Undang-undang Nomor 1 Tahun 1974 tentang Perkawinan, Penggugat telah menghadirkan pihak-pihak yang dekat dengan Penggugat dan Tergugat yaitu ayah Penggugat dan tetangga Penggugat yang sekaligus sebagai saksi dalam perkara ini, yang menerangkan bahwa rumah tangga Penggugat dan Tergugat benar-benar dalam kondisi sudah pecah (*broken marriage*) dan sudah tidak dapat dirukunkan lagi;

Menimbang, bahwa saksi-saksi yang diajukan Penggugat, sudah dewasa, berakal sehat, dan keterangannya telah disampaikan di depan persidangan dan di bawah sumpahnya masing-masing serta saksi tersebut bukanlah orang - orang yang dilarang untuk menjadi saksi menurut undang-undang, sehingga kesaksiannya telah memenuhi syarat formal sebagaimana diatur dalam Pasal 145, dan 147 HIR ;

Menimbang, bahwa saksi SAKSI I menerangkan pada pokoknya sejak awal pernikahan antara Penggugat dengan Tergugat terjadi pertengkaran secara terus-menerus disebabkan Nafkah yang diberikan Tergugat tidak mencukupi kebutuhan keluarga, kemudian sejak bulan April 2020 antara Penggugat dan Tergugat terjadi pisah tempat kediaman disebabkan Tergugat pergi meninggalkan rumah tempat kediaman bersama sampai sekarang selama 8 bulan dan selama berpisah

Disclaimer
Kepaniteraan Mahkamah Agung Republik Indonesia berusaha untuk selalu mencantumkan informasi paling kini dan akurat sebagai bentuk komitmen Mahkamah Agung untuk pelayanan publik, transparansi dan akuntabilitas pelaksanaan fungsi peradilan. Namun dalam hal-hal tertentu masih dimungkinkan terjadi permasalahan teknis terkait dengan akurasi dan keterkinian informasi yang kami sajikan, hal mana akan terus kami perbaiki dari waktu kewaktu.
Dalam hal Anda menemukan inakurasi informasi yang termuat pada situs ini atau informasi yang seharusnya ada, namun belum tersedia, maka harap segera hubungi Kepaniteraan Mahkamah Agung RI melalui :
Email : kepaniteraan@mahkamahagung.go.id Telp : 021-384 3348 (ext.318)

Tergugat tidak pernah datang menemui Penggugat dan tidak pernah melaksanakan kewajibannya selayaknya seorang suami;

Menimbang, bahwa saksi SAKSI II menerangkan pada pokoknya sejak awal pernikahan antara Penggugat dengan Tergugat terjadi pertengkaran secara terus-menerus disebabkan faktor ekonomi yang kuang mencukupi, kemudian sejak bulan April 2020 antara Penggugat dan Tergugat terjadi pisah tempat kediaman disebabkan Tergugat pergi meninggalkan rumah tempat kediaman bersama sampai sekarang selama 8 bulan dan selama berpisah Tergugat tidak pernah datang menemui Penggugat dan tidak pernah melaksanakan kewajibannya selayaknya seorang suami;

Menimbang, bahwa keterangan saksi-saksi tersebut di persidangan adalah fakta yang dilihat sendiri dan didengar sendiri dan keterangannya saling bersesuaian dan cocok antara satu dengan yang lain serta relevan dengan dalil yang harus dibuktikan oleh Penggugat, oleh karena itu keterangan saksi tersebut telah memenuhi syarat materiil sebagaimana telah diatur dalam Pasal 171 dan 172 HIR;

Menimbang, bahwa dari hasil pemeriksaan perkara ini, didasarkan atas keterangan Penggugat, bukti surat serta keterangan para saksi, Majelis Hakim menemukan fakta kejadian di persidangan sebagai berikut :

- bahwa Penggugat dan Tergugat adalah suami istri sah dan telah dikaruniai 1 (satu) orang anak;
- bahw
a sejak awal pernikahan Penggugat dengan Tergugat sering bertengkar karena Nafkah yang diberikan Tergugat tidak mencukupi kebutuhan keluarga.
- bahwa sejak bulan April 2020 Tergugat telah pergi dari rumah tempat kediaman bersama;
- bahwa Penggugat dan Tergugat sekarang sudah pisah selama 8 bulan dan selama berpisah Tergugat tidak pernah melaksanakan kewajibannya selayaknya sebagai suami;

Disclaimer
Kepaniteraan Mahkamah Agung Republik Indonesia berusaha untuk selalu mencantumkan informasi paling kini dan akurat sebagai bentuk komitmen Mahkamah Agung untuk pelayanan publik, transparansi dan akuntabilitas pelaksanaan fungsi peradilan. Namun dalam hal-hal tertentu masih dimungkinkan terjadi permasalahan teknis terkait dengan akurasi dan keterkinian informasi yang kami sajikan, hal mana akan terus kami perbaiki dari waktu kewaktu.
Dalam hal Anda menemukan inakurasi informasi yang termuat pada situs ini atau informasi yang seharusnya ada, namun belum tersedia, maka harap segera hubungi Kepaniteraan Mahkamah Agung RI melalui :
Email : kepaniteraan@mahkamahagung.go.id   Telp : 021-384 3348 (ext.318)

Menimbang bahwa, berdasarkan pada fakta-fakta tersebut diatas dapat disimpulkan bahwa dalil-dalil gugatan Penggugat telah dapat dibuktikan oleh Penggugat sehingga menjadi fakta hukum yang tetap, antara lain bahwa rumah tangga Penggugat dan Tergugat sudah tidak harmonis lagi karena sering terjadi pertengkaran dan perselisihan yang terus menerus yang disebabkan Nafkah yang diberikan Tergugat tidak mencukupi kebutuhan keluarga dan sekarang Penggugat dengan Tergugat sudah pisah selama 8 bulan dan selama pisah Tergugat tidak pernah melaksanakan kewajibannya layaknya seorang suami;

Menimbang, bahwa meskipun telah dilakukan upaya perdamaian oleh pihak keluarga, dan pula upaya perdamaian itu tetap dilakukan selama dalam proses persidangan sebagaimana diatur dalam Pasal 82 ayat (2) Undang-undang Nomor 7 Tahun 1989, namun Penggugat tetap tidak berniat meneruskan kehidupan bersama dengan Tergugat ;

Menimbang, bahwa syarat-syarat untuk dapat terjadinya perceraian antara lain adalah sebagaimana ditentukan oleh Pasal 19 huruf (f) Peraturan Pemerintah Nomor 9 Tahun 1975 tentang Pelaksanaan Undang-undang Nomor 1 Tahun 1974 tentang Perkawinan sebagaimana telah diubah dengan Undang-undang Nomor 16 Tahun 2019 jo. Pasal 116 huruf (f) Kompilasi Hukum Islam, yaitu diantara suami-istri *terus* menerus terjadi perselisihan dan pertengkaran dan tidak ada harapan akan hidup rukun lagi dalam rumah tangga;

Menimbang, bahwa telah terbukti adanya perselisihan dan pertengkaran antara Penggugat dengan Tergugat sejak awal pernikahan sampai dengan bulan April 2020, Majelis Hakim berpendapat bahwa kondisi yang demikian itu sudah termasuk dalam klasifikasi perselisihan dan pertengkaran yang bersifat terus-menerus yang semakin lama semakin memuncak, maka Majelis Hakim berpendapat bahwa antara Penggugat dengan Tergugat tersebut tidak mungkin lagi diharapkan untuk rukun kembali, dengan demikian maka syarat perceraian sebagaimana dimaksud oleh Pasal 19 huruf (f) Peraturan Pemerintah Nomor 9 Tahun 1975 tentang Pelaksanaan Undang-undang Nomor 1 Tahun 1974 tentang

Disclaimer
Kepaniteraan Mahkamah Agung Republik Indonesia berusaha untuk selalu mencantumkan informasi paling kini dan akurat sebagai bentuk komitmen Mahkamah Agung untuk pelayanan publik, transparansi dan akuntabilitas pelaksanaan fungsi peradilan. Namun dalam hal-hal tertentu masih dimungkinkan terjadi permasalahan teknis terkait dengan akurasi dan keterkinian informasi yang kami sajikan, hal mana akan terus kami perbaiki dari waktu kewaktu.
Dalam hal Anda menemukan inakurasi informasi yang termuat pada situs ini atau informasi yang seharusnya ada, namun belum tersedia, maka harap segera hubungi Kepaniteraan Mahkamah Agung RI melalui :
Email : kepaniteraan@mahkamahagung.go.id Telp : 021-384 3348 (ext.318)

Perkawinan sebagaimana telah diubah dengan Undang-undang Nomor 16 Tahun 2019 jo. Pasal 116 huruf (f) Kompilasi Hukum Islam telah terpenuhi ;

Menimbang, bahwa Tergugat telah pergi meninggalkan tempat kediaman bersama yang akibatnya Penggugat dengan Tergugat telah pisah selama 8 bulan dan selama itu masing-masing sudah tidak melaksanakan kewajibannya layaknya suami istri dan tidak ada upaya Tergugat untuk mengajak Penggugat untuk rukun kembali, merupakan isyarat telah sirna kasih sayang dan cinta di antara keduanya sehingga Penggugat dengan Tergugat tidak dapat menegakkan prinsip-prinsip hidup berumah tangga sebagaimana diatur dalam Pasal 33 Undang-undang Nomor 1 Tahun 1974 tentang Perkawinan sebagaimana telah diubah dengan Undang-undang Nomor 16 Tahun 2019;

Menimbang, bahwa berdasarkan bukti kondisi rumah tangga Penggugat dan Tergugat sudah tidak berlandaskan prinsip saling cinta mencintai, sayang menyayangi, hormat menghormati, saling setia serta saling memberi bantuan lahir dan batin yang satu kepada lainnya, sehingga sudah tidak mungkin dapat mewujudkan kehidupan rumah tangganya secara rukun dan harmonis sebagaimana yang disyariatkan dalam Al Qur’an surat Ar Rum ayat (21) dan untuk membentuk keluarga sebagaimana maksud Pasal 1 Undang-Undang Nomor 1 Tahun 1974 tentang Perkawinan sebagaimana telah diubah dengan Undang-undang Nomor 16 Tahun 2019, maka Majelis Hakim berpendapat bahwa mempertahankan perkawinan yang sudah pecah dan sudah tidak rukun serta harmonis lagi, hanya akan menjadi mudharat bagi keduanya sehingga perceraian merupakan alternatif terbaik yang harus ditempuh oleh Penggugat dengan Tergugat Hal ini sesuai dengan qaidah fiqhiyyah yang berbunyi:

درء المفاسد مقدم على جلب المصالح

Artinya :“Menghindari mafsadat lebih diutamakan untuk menjaga kemaslahatan”

Disclaimer
Kepaniteraan Mahkamah Agung Republik Indonesia berusaha untuk selalu mencantumkan informasi paling kini dan akurat sebagai bentuk komitmen Mahkamah Agung untuk pelayanan publik, transparansi dan akuntabilitas pelaksanaan fungsi peradilan. Namun dalam hal-hal tertentu masih dimungkinkan terjadi permasalahan teknis terkait dengan akurasi dan keterkinian informasi yang kami sajikan, hal mana akan terus kami perbaiki dari waktu kewaktu.
Dalam hal Anda menemukan inakurasi informasi yang termuat pada situs ini atau informasi yang seharusnya ada, namun belum tersedia, maka harap segera hubungi Kepaniteraan Mahkamah Agung RI melalui :
Email : kepaniteraan@mahkamahagung.go.id Telp : 021-384 3348 (ext.318)

Menimbang, bahwa sejalan dengan ketentuan hukum, Majelis Hakim perlu mengemukakan dalil syar'i dari Kitab Fiqhus Sunnah Juz II halaman 248 yang telah diambil alih sebagai pendapat Majelis yang berbunyi:

فــــإذا ثبتت دعوها لــــدى القاضي ببينة الزوجة أو اعـتـراف الـزوج وكـان الإ يـذاء مما يطـاق معه دوام العشـــرة بين أمثالهما وعجز القاضي عن الإ صـــلاح بينهما طلقها طلقة بائنة

Artinya : “Apabila hakim telah menemukan bukti-bukti yang diajukan oleh Penggugat (istri) atau Tergugat telah memberikan pengakuan, sedangkan hal-hal yang menjadi dakwaan Penggugat yaitu ketidakmampuan kedua belah pihak untuk hidup bersama sebagai suami istri dan hakim tidak berhasil mendamaikan keduanya, maka hakim boleh memutuskan dengan talak satu bain”;

Menimbang, bahwa berdasarkan hal-hal yang telah dipertimbangkan sebagaimana diuraikan di atas, Majelis Hakim berpendapat bahwa alasan perceraian yang diajukan telah terbukti sesuai dengan ketentuan Pasal 39 ayat (2) Undang-Undang Nomor 1 Tahun 1974 tentang Perkawinan jo. Pasal 19 huruf (f) Peraturan Pemerintah Nomor 9 tahun 1975 tentang Pelaksanaan Undang-undang Nomor 1 Tahun 1974 tentang Perkawinan sebagaimana telah diubah dengan Undang-undang Nomor 16 Tahun 2019 dan sejalan dengan Pasal 116 huruf (f) Kompilasi Hukum Islam dan dengan memperhatikan ketentuan Pasal 125 ayat (1) HIR, maka Majelis berkesimpulan bahwa gugatan Penggugat patut untuk dikabulkan secara verstek dengan menceraikan Penggugat dan Tergugat dengan talak satu bain shugra;

Menimbang, bahwa karena perkara *a quo* masuk bidang perkawinan, maka berdasarkan Pasal 89 ayat (1) Undang-undang Nomor 7 Tahun 1989 Tentang Peradilan Agama sebagaimana telah diubah dengan Undang-undang Nomor 3 Tahun 2006 dan perubahan kedua dengan Undang-undang Nomor 50 Tahun 2009, biaya perkara harus dibebankan kepada Penggugat ;

Disclaimer
Kepaniteraan Mahkamah Agung Republik Indonesia berusaha untuk selalu mencantumkan informasi paling kini dan akurat sebagai bentuk komitmen Mahkamah Agung untuk pelayanan publik, transparansi dan akuntabilitas pelaksanaan fungsi peradilan. Namun dalam hal-hal tertentu masih dimungkinkan terjadi permasalahan teknis terkait dengan akurasi dan keterkinian informasi yang kami sajikan, hal mana akan terus kami perbaiki dari waktu kewaktu.
Dalam hal Anda menemukan inakurasi informasi yang termuat pada situs ini atau informasi yang seharusnya ada, namun belum tersedia, maka harap segera hubungi Kepaniteraan Mahkamah Agung RI melalui :
Email : kepaniteraan@mahkamahagung.go.id   Telp : 021-384 3348 (ext.318)

Mengingat semua pasal dalam peraturan perundang-undangan dan hukum Islam yang berkaitan dengan perkara ini;

M E N G A D I L I

1. Menyatakan Tergugat yang telah dipanggil dengan resmi dan patut untuk menghadap di depan sidang tidak hadir;
2. Mengabulkan gugatan Penggugat dengan verstek;
3. Menjatuhkan talak satu ba'in shugro Tergugat (TERGUGAT) terhadap Penggugat (PENGGUGAT);
4. Membebankan kepada Penggugat untuk membayar biaya perkara sejumlah Rp 511.000,- (lima ratus sebelas ribu rupiah).

Demikian diputuskan dalam rapat permusyawaratan Majelis yang dilangsungkan pada hari Senin tanggal 28 Desember 2020 *Masehi* bertepatan dengan tanggal 14 Jumadil Awwal 1442 *Hijriyah.* Oleh kami Drs Karyadi sebagai Ketua Majelis, dan Drs. A. M. Suchrowardy serta Dra. Hj. Sri Rokhmani, MHI masing-masing sebagai Hakim Anggota, putusan tersebut diucapkan dalam sidang terbuka untuk umum pada hari itu juga oleh Ketua Majelis tersebut dengan didampingi oleh Hakim Anggota dan dibantu oleh Wina Ulfah,S.HI sebagai Panitera Pengganti serta dihadiri Penggugat tanpa hadirnya Tergugat.

Ketua Majelis,

Drs Karyadi

Hakim Anggota,

Drs. A. M. Suchrowardy

Hakim Anggota,

Dra. Hj. Sri Rokhmani, MHI

Disclaimer
Kepaniteraan Mahkamah Agung Republik Indonesia berusaha untuk selalu mencantumkan informasi paling kini dan akurat sebagai bentuk komitmen Mahkamah Agung untuk pelayanan publik, transparansi dan akuntabilitas pelaksanaan fungsi peradilan. Namun dalam hal-hal tertentu masih dimungkinkan terjadi permasalahan teknis terkait dengan akurasi dan keterkinian informasi yang kami sajikan, hal mana akan terus kami perbaiki dari waktu kewaktu.
Dalam hal Anda menemukan inakurasi informasi yang termuat pada situs ini atau informasi yang seharusnya ada, namun belum tersedia, maka harap segera hubungi Kepaniteraan Mahkamah Agung RI melalui :
Email : kepaniteraan@mahkamahagung.go.id   Telp : 021-384 3348 (ext.318)

Panitera Pengganti,

Wina Ulfah,S.HI

Perincian Biaya :

Biaya Pendaftaran	:	Rp	30.000,00
Biaya Proses	:	Rp	75.000,00
PNBP Pemanggilan Penggugat	:	Rp	10.000,00
PNBP Pemanggilan Tergugat	:	Rp	10.000,00

Biaya Pemanggilan	:	Rp	37[illegible]
Biaya Redaksi	:	Rp	1[illegible]
Biaya Materai	:	Rp	[illegible]
Jumlah	:	Rp	51[illegible]

Disclaimer
Kepaniteraan Mahkamah Agung Republik Indonesia berusaha untuk selalu mencantumkan informasi paling kini dan akurat sebagai bentuk komitmen Mahkamah Agung untuk pelayanan publik, transparansi dan akuntabilitas pelaksanaan fungsi peradilan. Namun dalam hal-hal tertentu masih dimungkinkan terjadi permasalahan teknis terkait dengan akurasi dan keterkinian informasi yang kami sajikan, hal mana akan terus kami perbaiki dari waktu kewaktu.
Dalam hal Anda menemukan inakurasi informasi yang termuat pada situs ini atau informasi yang seharusnya ada, namun belum tersedia, maka harap segera hubungi Kepaniteraan Mahkamah Agung RI melalui :
Email : kepaniteraan@mahkamahagung.go.id   Telp : 021-384 3348 (ext.318)
