# Supplementary material for: Classification of divorce causes during the COVID-19 pandemic using convolutional neural networks
Source: PeerJ Comput Sci. 2022 Jun 30;8:e998. doi: 10.7717/peerj-cs.998 (PMC9299239; doi:10.7717/peerj-cs.998)
Supplement: Supplemental Information 6 [file peerj-cs-08-998-s006.zip › Rujuk Dataset/Data ke-27.pdf]

PUTUSAN

Nomor 5281/Pdt.G/2020/PA.Cms

بِسْمِ اللَّهِ الرَّحْمَٰنِ الرَّحِيمِ

DEMI KEADILAN BERDASARKAN KETUHANAN YANG MAHA ESA

Pengadilan Agama Ciamis yang memeriksa dan mengadili perkara tertentu pada peradilan tingkat pertama, dalam persidangan majelis telah menjatuhkan putusan dalam perkara Cerai Gugat antara :

Penggugat, umur 37 tahun, agama Islam, pekerjaan Mengurus Rumah Tangga, tempat kediaman di Kabupaten Ciamis, dalam hal ini memberikan kuasa khusus kepada Kukun Abdul Syakur Munawar, S.H.,M.H, yang berkantor di JAlan RAA Kusumadiningrat No 28, Jambansari RT 03 Rw 27 Kelurahan Ciamis Kecamatan Ciamis Kabupaten Ciamis, berdasarkan surat kuasa khusus tanggal 30 November 2020 yang terdaftar dalam register kuasa Pengadilan Agama Ciamis Nomor 6428/XII/K/2020 tertanggal 01 Desember 2020, sebagai **Penggugat**;

melawan

Tergugat, umur 40 tahun, agama Islam, pekerjaan Buruh Tani, tempat kediaman Kabupaten Ciamis, sebagai **Tergugat**;

Pengadilan Agama tersebut ;
Setelah membaca dan mempelajari berkas perkara ;
Setelah mendengar dan mempertimbangkan pihak yang berperkara dan saksi-saksi ;

DUDUK PERKARA

Disclaimer
Kepaniteraan Mahkamah Agung Republik Indonesia berusaha untuk selalu mencantumkan informasi paling kini dan akurat sebagai bentuk komitmen Mahkamah Agung untuk pelayanan publik, transparansi dan akuntabilitas pelaksanaan fungsi peradilan. Namun dalam hal-hal tertentu masih dimungkinkan terjadi permasalahan teknis terkait dengan akurasi dan keterkinian informasi yang kami sajikan, hal mana akan terus kami perbaiki dari waktu kewaktu.
Dalam hal Anda menemukan inakurasi informasi yang termuat pada situs ini atau informasi yang seharusnya ada, namun belum tersedia, maka harap segera hubungi Kepaniteraan Mahkamah Agung RI melalui :
Email : kepaniteraan@mahkamahagung.go.id Telp : 021-384 3348 (ext.318)

Menimbang, bahwa Penggugat dengan surat gugatannya tertanggal 01 Desember 2020 yang didaftarkan di Kepaniteraan Pengadilan Agama Ciamis pada tanggal 01 Desember 2020 dengan register perkara Nomor 5281/Pdt.G/2020/PA.Cms, telah mengajukan gugatan perceraian terhadap Tergugat dengan posita dan petitum sebagai berikut :

1. Bahwa Penggugat dan Tergugat adalah suami-istri yang telah melangsungkan pernikahan pada 08 Mei 2006 atau bertepatan dengan 10 Rabiul Akhir 1427 H, di hadapan pejabat PPN KUA Kecamatan Lakbok, Kabupaten Ciamis, Propinsi Jawa Barat berdasarkan Kutipan Akta Nikah Nomor: XXXXXXXX, tertanggal 08 Mei 2006. Maka sesuai dengan ketentuan pasal 49 ayat (1) dan (2) Jo Pasal 73 ayat (1) Undang-undang Nomor 7 tahun 1989 sebagaimana telah diubah dengan Undang-undang Nomor 3 Tahun 2006, terakhir dengan Undang-Undang Nomor 50 Tahun 2009, maka perkara ini menjadi wewenang Pengadilan Agama Kabupaten Ciamis ;

2. Bahwa selama Perkawinan diantara Penggugat dan Tergugat Belum memiliki rumah tinggal dan telah dikaruniai 1 (Satu) orang anak yang bernama :

2.1. ANAK KE I, lahir di Ciamis tanggal 09 April 2007 ;

3. Bahwa pada mulanya rumah tangga Penggugat dan Tergugat dalam keadaan rukun namun sejak tahun 2017 ketenteraman rumah tangga Penggugat dengan Tergugat mulai goyah dan timbul perselisihan dan percekcokan, yang disebabkan antara lain:

3.1. Disebabkan oleh Tergugat yang tidak lagi memberikan nafkah ekonomi kepada Penggugat , dan untuk memenuhi kebutuhan hidup dibantu oleh orang Tua Penggugat ;

3.2. Bahwa akibat permasalahan Aquo, Penggugat merasa tidak ada kecocokan dalam menjalankan hubungan rumah tangga dengan Tergugat sehingga tujuan pernikahan yang sakinah, mawaddah, warahmah mustahil untuk tercapai.

4. Bahwa adanya pertengkaran dan perselisihan yang terus menerus tersebut mengakibatkan rumah tangga Penggugat tidak ada

Disclaimer
Kepaniteraan Mahkamah Agung Republik Indonesia berusaha untuk selalu mencantumkan informasi paling kini dan akurat sebagai bentuk komitmen Mahkamah Agung untuk pelayanan publik, transparansi dan akuntabilitas pelaksanaan fungsi peradilan. Namun dalam hal-hal tertentu masih dimungkinkan terjadi permasalahan teknis terkait dengan akurasi dan keterkinian informasi yang kami sajikan, hal mana akan terus kami perbaiki dari waktu kewaktu.
Dalam hal Anda menemukan inakurasi informasi yang termuat pada situs ini atau informasi yang seharusnya ada, namun belum tersedia, maka harap segera hubungi Kepaniteraan Mahkamah Agung RI melalui :
Email : kepaniteraan@mahkamahagung.go.id   Telp : 021-384 3348 (ext.318)

kebahagiaan lahir dan batin dan tidak ada harapan untuk kembali membina rumah tangga ;

5. Bahwa puncak perselisihan itu, pada tahun 2018 , Penggugat akhirnya pergi meninggalkan Tergugat dari rumah tinggal bersama dan kini Penggugat tinggal bersama dengan orang Tua Penggugat ;

6. Bahwa sejak berpisah dari tahun 2018, antara Penggugat dengan Tergugat sudah tidak lagi menjalankan kewajiban sebagaimana layaknya suami isteri yang sah dan Tergugat juga sudah tidak lagi memberikan nafkah ekonomi ;

7. Bahwa Penggugat merasa keutuhan rumah tangga antara Penggugat dan Tergugat sudah tidak dapat dipertahankan lagi, dan Penggugat sudah tidak sanggup lagi berumah tangga dengan Tergugat karena lebih banyak madharatnya daripada manfaatnya ;

8. Bahwa atas dasar uraian diatas maka gugatan Penggugat telah memenuhi alasan perceraian sebagaimana diatur dalam Undang-undang No. 1 Tahun 1974 Jo Peraturan Pemerintah No. 9 tahun 1975 pasal 19 Jo Kompilasi Hukum Islam pasal 116 ;

9. Bahwa Penggugat sangggup membayar seluruh biaya yang timbul akibat perkara ini.

Berdasarkan alasan/ dalil-dalil diatas, Penggugat mohon agar Majlis Hakim Pengadilan Agama Ciamis untuk memeriksa dan mengadili perkara ini, selanjutnya menjatuhkan putusan yang amarnya sebagai berikut :

1. Mengabulkan gugatan PENGGUGAT;

2. Menetapkan Jatuh Talaknya Tergugat (Tergugat) Talak satu BAIN SHUGRO terhadap Penggugat (Penggugat);

3. Menyatakan biaya menurut Hukum

Apabila Majelis Hakim berpendapat lain, mohon putusan yang adil dan seadil-adilnya (ex aequo et bono).

Bahwa pada hari persidangan yang telah ditentukan Penggugat didampingi Kuasanya datang menghadap di persidangan, sedangkan Tergugat tidak

Disclaimer
Kepaniteraan Mahkamah Agung Republik Indonesia berusaha untuk selalu mencantumkan informasi paling kini dan akurat sebagai bentuk komitmen Mahkamah Agung untuk pelayanan publik, transparansi dan akuntabilitas pelaksanaan fungsi peradilan. Namun dalam hal-hal tertentu masih dimungkinkan terjadi permasalahan teknis terkait dengan akurasi dan keterkinian informasi yang kami sajikan, hal mana akan terus kami perbaiki dari waktu kewaktu.
Dalam hal Anda menemukan inakurasi informasi yang termuat pada situs ini atau informasi yang seharusnya ada, namun belum tersedia, maka harap segera hubungi Kepaniteraan Mahkamah Agung RI melalui :
Email : kepaniteraan@mahkamahagung.go.id   Telp : 021-384 3348 (ext.318)

pernah datang dan tidak menyuruh orang lain untuk menghadap sebagai wakilnya, meskipun Tergugat telah dipanggil secara resmi dan patut, sedangkan tidak ternyata tidak datangnya itu disebabkan oleh suatu halangan yang sah, dan tidak pula ia menyampaikan suatu eksepsi kompetensi ;

Bahwa upaya perdamaian dan mediasi tidak dapat dilaksanakan karena pihak Tergugat tidak pernah hadir di persidangan ;

Bahwa Penggugat menyatakan tetap dengan gugatannya, maka pemeriksaan dilanjutkan sesuai dengan tahapan yang berlaku, yakni membacakan surat gugatan Penggugat tersebut yang isinya tetap dipertahankan oleh Penggugat ;

Bahwa untuk meneguhkan dalil gugatannya, Penggugat telah mengajukan bukti-bukti berupa :

A. Alat Bukti Surat

Fotokopi Kutipan Akta Nikah dari KUA Kecamatan Lakbok, Kabupaten Ciamis, Nomor XXXXXXXX Tanggal 08 Mei 2006, bukti surat tersebut telah diberi meterai cukup, telah dicap pos dan telah dicocokkan dengan aslinya yang ternyata sesuai, lalu oleh Ketua Majelis diberi tanda P;

B. Saksi-saksi

1. SAKSI I umur 79 tahun, Agama Islam, pekerjaan tukang kayu, tempat tinggal di Kabupaten Ciamis, di persidangan memberikan keterangan di bawah sumpah yang pada pokoknya sebagai berikut :

- Bahwa saksi kenal dengan Penggugat dan Tergugat, saksi adalah ayah kandung Penggugat;
- Bahwa Penggugat dan Tergugat adalah suami isteri, yang menikah sekitar tahun 2006 di hadapan KUA Kecamatan Lakbok, Kabupaten Ciamis ;
- Bahwa Penggugat dan Tergugat selama berumah tangga tinggal di rumah kontrakan dan sudah dikaruniai satu orang anak bernama Nurainun Dwiyanti Binti Markuat;
- Bahwa rumah tangga Penggugat dengan Tergugat semula rukun dan harmonis, namun sejak tahun 2017 rumah tangga mereka mulai

Disclaimer
Kepaniteraan Mahkamah Agung Republik Indonesia berusaha untuk selalu mencantumkan informasi paling kini dan akurat sebagai bentuk komitmen Mahkamah Agung untuk pelayanan publik, transparansi dan akuntabilitas pelaksanaan fungsi peradilan. Namun dalam hal-hal tertentu masih dimungkinkan terjadi permasalahan teknis terkait dengan akurasi dan keterkinian informasi yang kami sajikan, hal mana akan terus kami perbaiki dari waktu kewaktu.
Dalam hal Anda menemukan inakurasi informasi yang termuat pada situs ini atau informasi yang seharusnya ada, namun belum tersedia, maka harap segera hubungi Kepaniteraan Mahkamah Agung RI melalui :
Email : kepaniteraan@mahkamahagung.go.id Telp : 021-384 3348 (ext.318)

goyah tidak harmonis lagi dan sering terjadi perselisihan dan pertengkaran ;

- Bahwa penyebabnya karena Tergugat yang tidak lagi memberikan nafkah ekonomi kepada Penggugat ;
- Bahwa antara Penggugat dan Tergugat sudah tidak tinggal dalam satu rumah lagi karena telah berpisah rumah sejak tahun 2018 sampai sekarang;
- Bahwa selama berpisah antara Penggugat dan Tergugat sudah tidak ada komunikasi lagi dan tidak pernah berkumpul lagi sampai sekarang ;
- Bahwa keluarga dan saksi sudah pernah mendamaikan Penggugat dan Tergugat, akan tetapi tidak berhasil dan saksi sudah tidak sanggup lagi merukunkannya ;

2. SAKSI II umur 48 tahun, Agama Islam, pekerjaan buruh harian lepas, tempat tinggal di Kabupaten Ciamis, di persidangan memberikan keterangan di bawah sumpah yang pada pokoknya sebagai berikut :

- Bahwa saksi kenal dengan Penggugat dan Tergugat, karena saksi adalah tetangga Penggugat;
- Bahwa hubungan Penggugat dan Tergugat adalah suami isteri, yang menikah sekitar tahun 2006 di KUA Kecamatan Lakbok, Kabupaten Ciamis ;
- Bahwa Penggugat dan Tergugat selama berumah tangga belum memiliki rumah sendiri dan sudah dikaruniai anak ;
- Bahwa pada mulanya rumah tangga Penggugat dengan Tergugat hidup rukun serta harmonis, akan tetapi sejak tahun 2017 rumah tangga mereka sudah tidak harmonis lagi dan selalu terjadi perselisihan dan pertengkaran secara terus menerus ;
- Bahwa penyebab perselisihan dan pertengkarannya karena Tergugat yang tidak lagi memberikan nafkah ekonomi kepada Penggugat ;

Disclaimer
Kepaniteraan Mahkamah Agung Republik Indonesia berusaha untuk selalu mencantumkan informasi paling kini dan akurat sebagai bentuk komitmen Mahkamah Agung untuk pelayanan publik, transparansi dan akuntabilitas pelaksanaan fungsi peradilan. Namun dalam hal-hal tertentu masih dimungkinkan terjadi permasalahan teknis terkait dengan akurasi dan keterkinian informasi yang kami sajikan, hal mana akan terus kami perbaiki dari waktu kewaktu.
Dalam hal Anda menemukan inakurasi informasi yang termuat pada situs ini atau informasi yang seharusnya ada, namun belum tersedia, maka harap segera hubungi Kepaniteraan Mahkamah Agung RI melalui :
Email : kepaniteraan@mahkamahagung.go.id Telp : 021-384 3348 (ext.318)

- Bahwa Penggugat dan Tergugat sudah berpisah rumah sejak tahun 2018 dan selama berpisah diantara mereka sudah tidak ada komunikasi lagi ;
- Bahwa keluarga dan saksi sudah pernah mendamaikan Penggugat dan Tergugat, akan tetapi tidak berhasil dan saksi sudah tidak sanggup lagi merukunkannya ;

Bahwa Penggugat menyatakan telah cukup dengan bukti-bukti yang telah diajukannya dan tidak akan mengajukan alat bukti lainnya ;

Bahwa Penggugat telah menyampaikan kesimpulannya secara lisan yang pada pokoknya tetap bercerai dengan Tergugat dan memohon agar gugatannya dikabulkan ;

Bahwa untuk mempersingkat uraian bagian duduk perkara, maka ditunjuk segala hal sebagaimana tercantum dalam berita acara sidang perkara ini, yang merupakan bagian yang tidak terpisahkan dari putusan ;

PERTIMBANGAN HUKUM

Menimbang, bahwa isi gugatan Penggugat selengkapnya adalah sebagaimana telah diuraikan di atas ;

Menimbang, bahwa pelimpahan Kuasa dari Penggugat kepada Advokat : Kukun Abdul Syakur Munawar, S.H.,M.H, telah ditempuh sesuai dengan ketentuan hukum dan prosedur yang berlaku. Oleh karena itu, Penerima Kuasa dinilai berwenang untuk bertindak mewakili kepentingan hukum Pemberi Kuasa dalam perkara ini ;

Menimbang, bahwa Tergugat yang telah dipanggil secara resmi dan patut tidak pernah datang menghadap dan tidak pula menyuruh orang lain atau kuasanya untuk menghadap di persidangan tanpa alasan yang sah, maka sesuai dengan ketentuan Pasal 125 H.I.R., Tergugat harus dinyatakan tidak hadir dan perkara ini dapat diperiksa dan diputus tanpa hadirnya Tergugat, hal ini sejalan pula dengan doktrin di dalam *Al-Anwar* Juz II halaman 55 yang diambil alih menjadi pendapat Majelis Hakim yang berbunyi sebagai berikut ;

فإن تعزز بتعزز او توار او غيبة جاز اثبا ته بالبينة

Disclaimer
Kepaniteraan Mahkamah Agung Republik Indonesia berusaha untuk selalu mencantumkan informasi paling kini dan akurat sebagai bentuk komitmen Mahkamah Agung untuk pelayanan publik, transparansi dan akuntabilitas pelaksanaan fungsi peradilan. Namun dalam hal-hal tertentu masih dimungkinkan terjadi permasalahan teknis terkait dengan akurasi dan keterkinian informasi yang kami sajikan, hal mana akan terus kami perbaiki dari waktu kewaktu.
Dalam hal Anda menemukan inakurasi informasi yang termuat pada situs ini atau informasi yang seharusnya ada, namun belum tersedia, maka harap segera hubungi Kepaniteraan Mahkamah Agung RI melalui :
Email : kepaniteraan@mahkamahagung.go.id Telp : 021-384 3348 (ext.318)

“Apabila Tergugat berhalangan hadir karena bersembunyi atau enggan atau gha’ib, maka perkara tersebut diputus berdasarkan bukti-bukti“ ;

Menimbang, bahwa Penggugat dalam gugatannya pada pokoknya mendalilkan sejak tahun 2017 Tergugat dengan Penggugat berselisih dan bertengkar terus menerus, perselisihan dan pertengkaran tersebut terjadi karena Tergugat yang tidak lagi memberikan nafkah ekonomi kepada Penggugat, dan akhirnya pada tahun 2018 antara Penggugat dengan Tergugat telah berpisah tempat tinggal dan tidak pernah kumpul bersama lagi, sedangkan upaya memperbaiki rumah tangga dengan melibatkan pihak keluarga serta pihak lain sudah dilakukan namun tidak pernah berhasil, sehingga oleh karenanya Penggugat merasa tidak ada harapan lagi mempertahankan rumah tangganya dengan Tergugat ;

Menimbang, bahwa gugatan Penggugat pada pokoknya didasarkan atas ketentuan Pasal 19 huruf f Peraturan Pemerintah Nomor 9 Tahun 1975 jo Pasal 116 huruf f Kompilasi Hukum Islam, yakni gugatan perceraian dengan alasan suami isteri terus menerus terjadi perselisihan dan pertengkaran dan tidak ada harapan akan hidup rukun lagi dalam rumah tangga, oleh karena itu yang perlu dibuktikan oleh Penggugat adalah peristiwa yang dijadikan alasan tersebut ;

Menimbang, bahwa sikap Tergugat yang tidak pernah hadir di persidangan tanpa alasan yang sah meskipun telah dipanggil secara resmi dan patut dapat dianggap bahwa ia tidak membantah terhadap dalil dan alasan gugatan Penggugat sebagaimana dalam surat gugatan Penggugat ;

Menimbang, bahwa berdasarkan ketentuan Pasal 125 ayat (1) HIR yaitu putusan yang dijatuhkan tanpa hadirnya Tergugat dapat dikabulkan sepanjang berdasarkan hukum dan beralasan, oleh karena itu Majelis Hakim membebani Penggugat untuk membuktikan dalil-dalil gugatannya ;

Menimbang, bahwa untuk membuktikan dalil-dalil gugatannya, Penggugat telah mengajukan alat bukti surat (P) berupa Fotokopi Kutipan Akta Nikah dan 2 (dua) orang saksi, bukti-bukti tersebut dinilai memenuhi syarat formil sebagai bukti surat dan saksi ;

Menimbang, bahwa alat bukti (P) tersebut merupakan akta otentik telah bermeterai cukup dan cocok dengan aslinya, isi alat bukti tersebut menjelaskan

Disclaimer
Kepaniteraan Mahkamah Agung Republik Indonesia berusaha untuk selalu mencantumkan informasi paling kini dan akurat sebagai bentuk komitmen Mahkamah Agung untuk pelayanan publik, transparansi dan akuntabilitas pelaksanaan fungsi peradilan. Namun dalam hal-hal tertentu masih dimungkinkan terjadi permasalahan teknis terkait dengan akurasi dan keterkinian informasi yang kami sajikan, hal mana akan terus kami perbaiki dari waktu kewaktu.
Dalam hal Anda menemukan inakurasi informasi yang termuat pada situs ini atau informasi yang seharusnya ada, namun belum tersedia, maka harap segera hubungi Kepaniteraan Mahkamah Agung RI melalui :
Email : kepaniteraan@mahkamahagung.go.id Telp : 021-384 3348 (ext.318)

mengenai peristiwa hukum berupa perkawinan Penggugat dan Tergugat, sehingga alat bukti tersebut telah memenuhi syarat materiil serta dinilai mempunyai kekuatan pembuktian yang sempurna dan mengikat ;

Menimbang, bahwa berdasarkan alat bukti (P), telah diperoleh fakta kejadian bahwa Penggugat melangsungkan pernikahan dengan Tergugat pada tanggal 08 Mei 2006 di hadapan KUA Kecamatan Lakbok, Kabupaten Ciamis, pernikahan tersebut sebagaimana ternyata dari Akta Nikah nomor : XXXXXXXX tanggal 08 Mei 2006 ;

Menimbang, bahwa dari fakta kejadian tersebut di atas, maka diperoleh fakta hukum bahwa Penggugat adalah isteri sah Tergugat, yang ikatan perkawinannya tidak pernah putus hingga saat ini, oleh karena itu Penggugat adalah pihak yang memiliki *legal standing* untuk mengajukan gugatan perceraian terhadap Tergugat ;

Menimbang, bahwa saksi-saksi yang diajukan Penggugat yang bernama SAKSI I (ayah kandung Penggugat) dan SAKSI II (tetangga Penggugat), sudah dewasa dan sudah disumpah, mereka adalah orang yang tidak dilarang untuk memberikan kesaksian sehingga memenuhi syarat formal sebagaimana diatur dalam ketentuan Pasal 145 ayat 1 angka 3 huruf (e) HIR ;

Menimbang, bahwa sesuai dengan ketentuan Pasal 22 ayat (2) PP No. 9 Tahun 1975 jo pasal 134 Kompilasi Hukum Islam, jika perceraian didasarkan atas ketentuan Pasal 19 huruf f Peraturan Pemerintah Nomor 9 Tahun 1975 jo Pasal 116 huruf f Kompilasi Hukum Islam, maka Pengadilan harus mendengar keterangan saksi yang berasal dari keluarga atau orang dekat suami isteri ;

Menimbang, bahwa karena Tergugat tidak pernah hadir di persidangan, Pengadilan hanya mendengar keterangan saksi-saksi yang berasal dari keluarga atau orang dekat pihak Penggugat, mereka memberikan keterangan di bawah sumpah yang pada pokoknya adalah sebagaimana terurai dalam duduk perkara di atas ;

Menimbang, bahwa kesaksian kedua orang saksi Penggugat adalah merupakan suatu fakta kejadian karena didasarkan atas penglihatan, pendengaran serta pengalaman para saksi, keterangan saksi-saksi tersebut satu sama lain saling bersesuaian serta relevan dengan dalil-dalil gugatan

Disclaimer
Kepaniteraan Mahkamah Agung Republik Indonesia berusaha untuk selalu mencantumkan informasi paling kini dan akurat sebagai bentuk komitmen Mahkamah Agung untuk pelayanan publik, transparansi dan akuntabilitas pelaksanaan fungsi peradilan. Namun dalam hal-hal tertentu masih dimungkinkan terjadi permasalahan teknis terkait dengan akurasi dan keterkinian informasi yang kami sajikan, hal mana akan terus kami perbaiki dari waktu kewaktu.
Dalam hal Anda menemukan inakurasi informasi yang termuat pada situs ini atau informasi yang seharusnya ada, namun belum tersedia, maka harap segera hubungi Kepaniteraan Mahkamah Agung RI melalui :
Email : kepaniteraan@mahkamahagung.go.id Telp : 021-384 3348 (ext.318)

Penggugat, maka berdasarkan ketentuan Pasal 171 dan 172 HIR jo Pasal 76 Undang-Undang Nomor 7 Tahun 1989 Tentang Peradilan Agama sebagaimana telah diubah terakhir dengan Undang-Undang Nomor 50 Tahun 2009, majelis menilai keterangan kedua orang saksi Penggugat telah memenuhi syarat materil kesaksian, dan oleh karenanya mempunyai nilai pembuktian yang sempurna ;

Menimbang, bahwa berdasarkan keterangan kedua orang saksi yang diajukan Penggugat, maka telah diperoleh fakta-fakta kejadian yang pada pokoknya adalah sebagai berikut :

- Bahwa sejak tahun 2017 Penggugat dan Tergugat sering berselisih dan bertengkar disebabkan Tergugat yang tidak lagi memberikan nafkah ekonomi kepada Penggugat ;
- Bahwa sejak tahun 2018 mereka telah berpisah tempat tinggal dan sampai sekarang tidak pernah kumpul bersama lagi ;
- Bahwa pihak keluarga sudah berusaha mendamaikan Penggugat dan Tergugat namun tidak berhasil, dan mereka tidak sanggup lagi merukunkan mereka ;

Menimbang, bahwa dari fakta-fakta kejadian sebagaimana diuraikan di atas, maka ditemukan adanya fakta-fakta hukum yang pada pokoknya perselisihan dan pertengkaran secara terus menerus antara Penggugat dan Tergugat yang penyebabnya Tergugat yang tidak lagi memberikan nafkah ekonomi kepada Penggugat sebagaimana diuraikan di atas telah mengakibatkan sudah tidak ada harapan akan hidup rukun lagi dalam rumah tangga, sehingga tujuan perkawinan untuk membentuk rumah tangga bahagia dan kekal berdasarkan Ketuhanan Yang Maha Esa sebagaimana diamanatkan oleh ketentuan Pasal 1 Undang-Undang Nomor 1 Tahun 1974 Tentang Perkawinan serta mewujudkan keluarga yang *sakinah, mawaddah dan rahmah* sebagaimana dikehendaki oleh Firman Allah SWT dalam Surat Ar-Rum ayat 21 tidak mungkin lagi dapat diwujudkan Penggugat dan Tergugat ;

Menimbang, bahwa berdasarkan hal-hal sebagaimana diuraikan di atas, maka gugatan Penggugat telah memenuhi alasan perceraian sebagaimana dimaksud ketentuan Pasal 39 ayat (1) dan (2) Undang-Undang Nomor 1 Tahun

Disclaimer
Kepaniteraan Mahkamah Agung Republik Indonesia berusaha untuk selalu mencantumkan informasi paling kini dan akurat sebagai bentuk komitmen Mahkamah Agung untuk pelayanan publik, transparansi dan akuntabilitas pelaksanaan fungsi peradilan. Namun dalam hal-hal tertentu masih dimungkinkan terjadi permasalahan teknis terkait dengan akurasi dan keterkinian informasi yang kami sajikan, hal mana akan terus kami perbaiki dari waktu kewaktu.
Dalam hal Anda menemukan inakurasi informasi yang termuat pada situs ini atau informasi yang seharusnya ada, namun belum tersedia, maka harap segera hubungi Kepaniteraan Mahkamah Agung RI melalui :
Email : kepaniteraan@mahkamahagung.go.id Telp : 021-384 3348 (ext.318)

1974 jo ketentuan Pasal 19 huruf (f) Peraturan Pemerintah Nomor 9 Tahun 1975 Jo ketentuan Pasal 116 huruf (f) Kompilasi Hukum Islam ;

Menimbang, bahwa Majelis Hakim sepakat dengan doktrin ahli hukum Islam dalam *Fiqhus Sunnah* Juz II : 248 yang berbunyi sebagai berikut :

أ ن للزوجة يجوز أن تطلب من القا ضي التفر يق إ ذا ادعت إضرارالزوجها إضرارا لا يستطاع معه دوام العشرة بين أمثا لهما وعجزالقا ضي عن الإ صلاح بينهما طلقها طلقة بائنة

“Bahwa sesungguhnya boleh bagi seorang isteri minta kepada Hakim untuk diceraikan dari suaminya dengan dasar tuntutan bahwa apabila telah ternyata di dalam perkawinan terdapat kemadla-ratan, dimana suami isteri tersebut sudah tidak mampu lagi untuk mempertahankan kelangsungan rumah tangga itu.. dan Hakim sudah tidak dapat mendamaikan suami isteri tersebut, maka Hakim menceraikannya dengan talak satu bain”;

Menimbang, bahwa karena alasan perceraian dalam perkara ini telah terbukti, maka Majelis Hakim tidak perlu membuktikan pihak mana yang salah atau pihak mana yang menjadi penyebab perselisihan dan pertengkaran antara Penggugat dan Tergugat, hal ini sesuai dengan Yurisprudensi Mahkamah Agung Republik Indonesia Nomor : 38 K/AG/1990 tanggal 05 Oktober 1991, dan Majelis Hakim juga tidak perlu lagi mempertimbangkan dalil-dalil gugatan Penggugat yang lainnya ;

Menimbang, bahwa oleh karena gugatan Penggugat telah terbukti dan memenuhi alasan hukum serta Tergugat dalam keadaan tidak hadir, sedangkan Tergugat belum pernah menjatuhkan talaknya terhadap Penggugat, maka Majelis Hakim harus mengabulkan gugatan Penggugat secara verstek dengan menjatuhkan talak satu ba’in sughra Tergugat terhadap Penggugat ;

Menimbang, bahwa perkara ini termasuk bidang perkawinan, maka sesuai ketentuan Pasal 89 ayat (1) Undang-Undang Nomor 7 Tahun 1989 sebagaimana telah diubah terakhir dengan Undang-Undang Nomor 50 Tahun 2009 Tentang Perubahan Kedua Atas Undang-Undang Nomor 7 Tahun 1989, maka biaya perkara ini harus dibebankan kepada Penggugat ;

Mengingat segala ketentuan perundang-undangan yang berlaku beserta dalil Syar'i yang berkaitan dengan perkara ini ;

Disclaimer
Kepaniteraan Mahkamah Agung Republik Indonesia berusaha untuk selalu mencantumkan informasi paling kini dan akurat sebagai bentuk komitmen Mahkamah Agung untuk pelayanan publik, transparansi dan akuntabilitas pelaksanaan fungsi peradilan. Namun dalam hal-hal tertentu masih dimungkinkan terjadi permasalahan teknis terkait dengan akurasi dan keterkinian informasi yang kami sajikan, hal mana akan terus kami perbaiki dari waktu kewaktu.
Dalam hal Anda menemukan inakurasi informasi yang termuat pada situs ini atau informasi yang seharusnya ada, namun belum tersedia, maka harap segera hubungi Kepaniteraan Mahkamah Agung RI melalui :
Email : kepaniteraan@mahkamahagung.go.id Telp : 021-384 3348 (ext.318)

MENGADILI

1.- Menyatakan Tergugat yang telah dipanggil secara resmi dan patut untuk datang menghadap di persidangan, tidak hadir ;
2.-----------------------------Mengabulkan gugatan Penggugat dengan verstek ;
3.----------Menjatuhkan talak satu ba'in sughra Tergugat (Tergugat) terhadap Penggugat (Penggugat) ;
4.Membebankan biaya perkara kepada Penggugat sejumlah Rp. 336.000,- (tiga ratus tiga puluh enam ribu rupiah) ;

Demikian putusan ini dijatuhkan dalam rapat musyawarah majelis pada hari Selasa tanggal 29 Desember 2020 Masehi, bertepatan dengan tanggal 14 Jumadil Awwal 1442 Hijriah, oleh kami H. Nandang Hasanudin, S.H sebagai Ketua Majelis serta Drs. U. Nurdin, S.H dan Drs. H. Komarudin, M.H., masing-masing sebagai Hakim Anggota. Putusan tersebut pada hari dan tanggal itu juga diucapkan dalam sidang terbuka untuk umum oleh Ketua Majelis tersebut dengan didampingi para Hakim Anggota dan dibantu oleh Hj. Yayah Nuriyah, S.Ag, sebagai Panitera Pengganti serta dihadiri oleh Penggugat dan Kuasanya tanpa dihadiri Tergugat.

Ketua Majelis,

H. Nandang Hasanudin, S.H

Hakim Anggota,	Hakim Anggota,
Drs. U. Nurdin, S.H	Drs. H. Komarudin, M.H.

Panitera Pengganti,

Disclaimer
Kepaniteraan Mahkamah Agung Republik Indonesia berusaha untuk selalu mencantumkan informasi paling kini dan akurat sebagai bentuk komitmen Mahkamah Agung untuk pelayanan publik, transparansi dan akuntabilitas pelaksanaan fungsi peradilan. Namun dalam hal-hal tertentu masih dimungkinkan terjadi permasalahan teknis terkait dengan akurasi dan keterkinian informasi yang kami sajikan, hal mana akan terus kami perbaiki dari waktu kewaktu.
Dalam hal Anda menemukan inakurasi informasi yang termuat pada situs ini atau informasi yang seharusnya ada, namun belum tersedia, maka harap segera hubungi Kepaniteraan Mahkamah Agung RI melalui :
Email : kepaniteraan@mahkamahagung.go.id Telp : 021-384 3348 (ext.318)

Hj. Yayah Nuriyah, S.Ag

Perincian Biaya Perkara :

1. Biaya Pendaftaran	:	Rp.	30.000,-
2. Biaya Proses	:	Rp.	60.000,-
3. Biaya Panggilan	:	Rp.	200.000,-
4. Biaya PNBP Surat Kuasa	:	Rp.	10.000,-
5. Biaya PNBP Panggilan	:	Rp.	20.000,-
6. Biaya Redaksi	:	Rp.	10.000,-
7. Biaya Materai	:	Rp.	6.000,-
Jumlah	:	Rp.	336.000,-

(tiga ratus tiga puluh enam ribu rupiah)

Disclaimer
Kepaniteraan Mahkamah Agung Republik Indonesia berusaha untuk selalu mencantumkan informasi paling kini dan akurat sebagai bentuk komitmen Mahkamah Agung untuk pelayanan publik, transparansi dan akuntabilitas pelaksanaan fungsi peradilan. Namun dalam hal-hal tertentu masih dimungkinkan terjadi permasalahan teknis terkait dengan akurasi dan keterkinian informasi yang kami sajikan, hal mana akan terus kami perbaiki dari waktu kewaktu.
Dalam hal Anda menemukan inakurasi informasi yang termuat pada situs ini atau informasi yang seharusnya ada, namun belum tersedia, maka harap segera hubungi Kepaniteraan Mahkamah Agung RI melalui :
Email : kepaniteraan@mahkamahagung.go.id   Telp : 021-384 3348 (ext.318)
