# Supplementary material for: Classification of divorce causes during the COVID-19 pandemic using convolutional neural networks
Source: PeerJ Comput Sci. 2022 Jun 30;8:e998. doi: 10.7717/peerj-cs.998 (PMC9299239; doi:10.7717/peerj-cs.998)
Supplement: Supplemental Information 6 [file peerj-cs-08-998-s006.zip › Rujuk Dataset/Data ke-28.pdf]

PUTUSAN

Nomor 1480/Pdt.G/2020/PA.Jepr

بسم الله الرحمن الرحيم

DEMI KEADILAN BERDASARKAN KETUHANAN YANG MAHA ESA

Pengadilan Agama Jepara yang mengadili perkara-perkara tertentu pada tingkat pertama dalam persidangan Hakim menjatuhkan putusan sebagai berikut dalam perkara permohonan cerai talak antara:

XXXXX, Tempat lahir Jepara, Tanggal lahir 21 November 1986, Agama Islam, Pekerjaan Tukang kayu, Pendidikan SLTA, Tempat tinggal RT 02 RW 02, Desa XXXXX, Kecamatan XXXXX, Kabupaten Jepara, yang dalam hal ini memberi kuasa kepada **Syaiful Huda, SHI. CM.,** Pekerjaan Advokat, KTA Mulai Aktif 31 Desember 2015 sampai dengan 31 Desember 2021, Alamat Kantor Lembaga Bantuan Hukum Jawa Tengah Cabang Jepara Jl. Shima No.17.B Pengkol Jepara, berdasarkan surat kuasa khusus tanggal 08 September 2020, disebut sebagai **Pemohon Konvensi/ Tergugat Rekonvensi.**

melawan

XXXXX, Umur: 29 tahun (Jepara, 28Maret 1989), Agama Islam, Pekerjaan Karyawan Swasta, Pendidikan SLTA, bertempat tinggal di Desa XXXXX RT 018 RW 002, Kecamatan XXXXX, Kabupaten Jepara. Dalam hal ini memberi kuasa kepada **ALI MUHTASOR, S.Sy., S.H.**, Berkantor di Kantor Hukum **ALI MUHTASOR, S.Sy., S.H.& Rekan**, Kantor Advokat dan Konsultan Hukum, beralamat di Desa Tunggul Pandean RT 006/RW 003, Kecamatan Nalumsari, Kabupaten Jepara. berdasarkan surat kuasa khusus tanggal 15 Oktober 2020, disebut sebagai **Termohon Konvensi/ Penggugat Rekonvensi.**

Pengadilan Agama tersebut;

Telah mempelajari berkas perkara;

Telah mendengar keterangan Pemohon Konvensi/Tergugat Konvensi dan Termohon Konvensi / Penggugat Rekonvensi di persidangan;

Disclaimer
Kepaniteraan Mahkamah Agung Republik Indonesia berusaha untuk selalu mencantumkan informasi paling kini dan akurat sebagai bentuk komitmen Mahkamah Agung untuk pelayanan publik, transparansi dan akuntabilitas pelaksanaan fungsi peradilan. Namun dalam hal-hal tertentu masih dimungkinkan terjadi permasalahan teknis terkait dengan akurasi dan keterkinian informasi yang kami sajikan, hal mana akan terus kami perbaiki dari waktu kewaktu.
Dalam hal Anda menemukan inakurasi informasi yang termuat pada situs ini atau informasi yang seharusnya ada, namun belum tersedia, maka harap segera hubungi Kepaniteraan Mahkamah Agung RI melalui :
Email : kepaniteraan@mahkamahagung.go.id   Telp : 021-384 3348 (ext.318)

Telah memeriksa bukti-bukti di persidangan;

DUDUK PERKARA

Bahwa, Pemohon telah mengajukan permohonan Cerai talaknya tanggal 7 September 2020 yang telah didaftar dalam register perkara Nomor 1480/Pdt.G/2020/PA.Jepr tanggal 7 September 2020, mengemukakan hal-hal sebagai berikut:

1. Bahwa Pemohon dengan Termohon telah menikah pada tanggal 23 Agustus 2017 yang dicatat oleh Pegawai Pencatat Nikah Kantor Urusan Agama Kecamatan XXXXX Kabupaten Jepara, dengan Kutipan Akta Nikah Nomor XXXXX tertanggal 23 Agustus 2017.
2. Bahwa setelah pernikahan tersebut Pemohon dengan Termohon hidup bersama dan bertempat tinggal rumah dirumah orangtua Termohon selama 2 tahun 6 bulan.
3. Bahwa sejak semula rumah tangga Pemohon dengan Termohon dalam keadaan rukun dan harmonis, sudah melakukan hubungan sebagaimana layaknya suami isteri (*bakdaduhul*) dan telah dikaruniai anak keturunan bernama XXXXX, laki-laki, umur 2 tahun.
5. Bahwa sejak awal setelah perkawinan bulan pada Maret 2019 Pemohon dan Termohon terjadi perselisihan dan pertengkaran terus - menerus disebabkan perselisihan tempat tinggal bersama, yaitu Termohon menolak tinggal bersama di rumah Pemohon demikian pula Pemohon menolak tinggal di rumah termohon.
6. Bahwa puncak perselisihan dan pertengkaran hal tersebut diatas, terjadi pada awal bulan Februari tahun 2020, Akibatnya antara Pemohon dan Termohon hidup pisah tempat tinggal, Pemohon pergi meninggalkan Termohon dan tinggal dirumah Pemohan sampai sekarang berjalan selama 7 bulan, dan antara Pemohon dan Termohon masing-masing telah tidak ada komunikasi yang baik dan telah tidak menjalankan kewajibannya sebagai suami-istri.

Disclaimer
Kepaniteraan Mahkamah Agung Republik Indonesia berusaha untuk selalu mencantumkan informasi paling kini dan akurat sebagai bentuk komitmen Mahkamah Agung untuk pelayanan publik, transparansi dan akuntabilitas pelaksanaan fungsi peradilan. Namun dalam hal-hal tertentu masih dimungkinkan terjadi permasalahan teknis terkait dengan akurasi dan keterkinian informasi yang kami sajikan, hal mana akan terus kami perbaiki dari waktu kewaktu.
Dalam hal Anda menemukan inakurasi informasi yang termuat pada situs ini atau informasi yang seharusnya ada, namun belum tersedia, maka harap segera hubungi Kepaniteraan Mahkamah Agung RI melalui :
Email : kepaniteraan@mahkamahagung.go.id Telp : 021-384 3348 (ext.318)

7. Bahwa Pemohon dan Termohon telah menempuh upaya musyawarah kekeluargaan, namun upaya tersebut tidak berhasil merukunkan Pemohon dan Termohon.

8. Bahwa permohonan Pemohon untuk menceraikan Termohon telah memenuhi alasan mengajukan permohonan ijin cerai kepada Pengadilan Agama Jepara, sebagaimana pasal 19 huruf (f) PP Nomor 9 Tahun 1975 Jo Pasal 116 Huruf (f) Kompilasi Hukum Islam.

Bahwa berdasarkan hal-hal diatas, Pemohon mohon agar Pengadilan Agama Jepara menjatuhkan putusan yang amarnya berbunyi.

1. Mengabulkan Permohonan Pemohon.
2. Memberi izin kepada Pemohon (XXXXX) untuk menjatuhkan talak 1 Roj'i terhadap Termohon (XXXXX).
3. Menetapkan biaya perkara menurut Hukum

Atau menjatuhkan putusan dengan seadil-adilnya..

Bahwa pada hari persidangan yang telah ditentukan Pemohon datang menghadap secara pribadi, sedangkan Ternohon datang menghadap sendiri di persidangan didampingi kuasa hukumnya;

Bahwa Hakim telah memeriksa identitas Pemohon dan Termohon, yang ternyata tidak ada perobahan dilanjutkan pemeriksaan Surat Kuasa Khusus Pemohon tanggal 8 September 2020 yang terdaftar di Buku Register Surat Kuasa Khusus Pengadilan Agama Jepara Nomor 684/SK/2020/PAJepr. yang diberikan Pemohon kepada **SYAIFUL HUDA, SHI. CM.,** serta kelengkapan sebagai Advokat, ternyata surat Kuasa Pemohon telah memenuhi syarat formil surat kuasa, maka penerima Kuasa berwenang mewakili Pemberi Kuasa di persidangan;

Bahwa Hakim telah memeriksa Surat Kuasa Khusus Termohon tanggal 15 Oktober 2020 yang terdaftar di Buku Register Surat Kuasa Khusus Pengadilan Agama Jepara Nomor 835/SK/2020/PAJepr. yang diberikan Termohon kepada **ALI MUHTASOR, S.Sy., S.H.,** serta kelengkapan sebagai Advokat, ternyata surat Kuasa Termohon telah memenuhi syarat formil surat kuasa, maka penerima Kuasa berwenang mewakili Pemberi Kuasa di persidangan;

Disclaimer
Kepaniteraan Mahkamah Agung Republik Indonesia berusaha untuk selalu mencantumkan informasi paling kini dan akurat sebagai bentuk komitmen Mahkamah Agung untuk pelayanan publik, transparansi dan akuntabilitas pelaksanaan fungsi peradilan. Namun dalam hal-hal tertentu masih dimungkinkan terjadi permasalahan teknis terkait dengan akurasi dan keterkinian informasi yang kami sajikan, hal mana akan terus kami perbaiki dari waktu kewaktu.
Dalam hal Anda menemukan inakurasi informasi yang termuat pada situs ini atau informasi yang seharusnya ada, namun belum tersedia, maka harap segera hubungi Kepaniteraan Mahkamah Agung RI melalui :
Email : kepaniteraan@mahkamahagung.go.id Telp : 021-384 3348 (ext.318)

Bahwa dalam persidangan kemudian Hakim mendamaikan Pemohon dan Termohon namun tidak berhasil, kemudian keduanya diperintahkan agar menempuh mediasi dan para pihak telah sepakat memilih Chamidah, S.Ag. sebagai mediator ;

Bahwa berdasarkan laporan Mediator tanggal 25 September 2020 mediasi tidak berhasil mencapai kesepakatan, kemudian dibacakan permohonan Pemohon yang isinya tetap dipertahankan oleh Pemohon;

Bahwa terhadap permohonan Pemohon tersebut, Termohon memberikan jawaban tertulis sebagai berikut :

DALAM KONPENSI

1. Bahwa Termohon menolak seluruhdalil-dalil yang diajukan oleh Pemohon dalam surat permohonan cerai talak talak tertanggal 07 September 2020 sebagaimana terdaftar dalam perkara nomor: 1480/Pdt.G/2020/PA.Jepr, kecuali yang secara jelas-jelas dibenarkan oleh Termohon; ---------------------

2. Bahwa dalil Pemohon sebagaimana tersebut dalam posita angka 1(satu), 2 (dua), dan 3 (tiga) **BENAR ;**

3. Bahwa dalil Pemohon sebagaimana tersebut dalam posita angka 5 (lima) dan 6 (enam) **TIDAK BENAR DAN MENGADA-ADA.**

Fakta yang benar tentang keadaan rumah tangga Pemohon dan Termohon adalah: Termohon sendiri bingung dengan sikap Pemohon yang pulang ke rumah Orang Tuanya. **Pemohon pulang ke rumah Orang Tuanya baru sekitar 1,5 (satu koma lima) bulan, tidak benar jika Pemohon mengatakan sudah 7 (tujuh) bulan**. Pemohon pulangke rumah Orang Tuanya kurang lebih sekitar bulan September 2020. Pemohon pamit pulang ke rumah Orang Tuanya untuk menengok keadaan Orang Tuanya yang sakit, akan tetapi tidak kembali ke kediaman bersama di rumah Orang Tua Termohon. Ironisnya tanpa ada pembicaraan lebih lanjut tentang penyelesaian secara musyawarah permasalahan rumah tangga Pemohon dan Termohon, akan tetapi malah mengajukan gugatan talak di Pengadilan Agama.

Selama ini Termohon sebagai Isteri berusaha untuk berbakti kepada suami sebaik mungkin. Bahkan untuk membantu mencukupi kebutuhan keluarga, Termohon rela bekerja sebagai karyawan swasta di Perusahaan Garmen PT. Kanindo Makmur Jaya Jepara. Nafkah yang diberikan oleh Pemohon hanya sekitar Rp. 500.000,- (lima ratus ribu rupiah) setiap bulan tidak mencukupi kebutuhan. Setelah kelahiran anak Pemohon dan Termohon lahir nafkah ditambah menjadi Rp. 2.000.000,- (dua juta rupiah), hanya cukup untuk membelikan kebutuhan anak berupa susu formula, pampers,

Disclaimer
Kepaniteraan Mahkamah Agung Republik Indonesia berusaha untuk selalu mencantumkan informasi paling kini dan akurat sebagai bentuk komitmen Mahkamah Agung untuk pelayanan publik, transparansi dan akuntabilitas pelaksanaan fungsi peradilan. Namun dalam hal-hal tertentu masih dimungkinkan terjadi permasalahan teknis terkait dengan akurasi dan keterkinian informasi yang kami sajikan, hal mana akan terus kami perbaiki dari waktu kewaktu.
Dalam hal Anda menemukan inakurasi informasi yang termuat pada situs ini atau informasi yang seharusnya ada, namun belum tersedia, maka harap segera hubungi Kepaniteraan Mahkamah Agung RI melalui :
Email : kepaniteraan@mahkamahagung.go.id Telp : 021-384 3348 (ext.318)

peralatan bayi, biaya penitipan anak dan uang jajan anak. Kekurangan kebutuhan tersebut ditutup dengan penghasilan Termohon.
Sikap Termohon untuk berusaha menjadi Isteri yang baik juga ditunjukkan dengan keihlasan Termohon merawat Pemohon yang pernah patah tangannya karena kecelakaan sepeda motor. Termohon bersama Ibunya merawat Pemohon yang sakit patah tulang tangan sampai dengan sembuh. Hal tersebut dilakukan karena Orang Tua Pemohon tidak mampu merawat Pemohon yang sedang sakit, karena keadaan Orang Tua Pemohon juga sedang sakit.
Dalil Pemohon yang mengatakan Termohon tidak mau diajak tinggal bersama di rumah Orang Tua Pemohon di Desa XXXXX RT 002 RW 002 **ADALAH TIDAK BENAR**. Termohon selalu bersedia ikut Pemohon setiap kali diajak tinggal di rumah Orang Tua Pemohon. Oleh karena aktifitas Termohon bekerja dipabrik, maka siang hari anaknnya dibawa ke rumah Orang Tua Termohon, sehingga harus riwa riwi.
Pemohon telah berbohong dan menutup-nutupi fakta yang sebenarnya dalam berumah tangga dengan Termohon.
Kesalahpahaman dalam membina rumah tangga adalah hal yang biasa, sepanjang bisa dimusyawarahkan dengan baik. Hal tersebut justru dapat menjadi bumbu penyedap dalam menjalani rumah tangga yang sakinah, mawaddah, wa rahmah.

Kondisi salah paham antara Pemohon dan Termohon dalam berumah tanggayang sebenarnya terjadi baru 1,5 (satu koma lima) bulan,bukan 7 (tujuh) bulan sebagaimana yang dikatakan oleh Pemohon. Seharusnya dilakukan musyawarah antar keluarga dengan menunjuk hakam. Bukan mengajukan gugatan talak ke Pengadilan Agama. Apalagi perselisihan tersebut terjadi belum lama. Perintah untuk menunjuk hakam sesuai dengan QS. An-Nisa’ ayat 35, yang bunyinya:

وَإِنْ خِفْتُمْ شِقَاقَ بَيْنِهِمَا فَابْعَثُوا حَكَمًا مِنْ أَهْلِهِ وَحَكَمًا مِنْ أَهْلِهَا إِنْ يُرِيدَا إِصْلَاحًا يُوَفِّقِ اللَّهُ بَيْنَهُمَا ۗ إِنَّ اللَّهَ كَانَ عَلِيمًا خَبِيرًا

Artinya :

Dan jika kamu khawatirkan ada persengketaan antara keduanya, maka kirimlah seorang hakam dari keluarga laki-laki dan seorang hakam dari keluarga perempuan. Jika kedua orang hakam itu bermaksud mengadakan perbaikan, niscaya Allah memberi taufik kepada suami-isteri itu. Sesungguhnya Allah maha Mengetahui lagi maha mengenal.

Berdasarkan uraian dalil-dalil yang diuraikan diatas, oleh sebab itu dalil-dalil Pemohon tersebut tidak benar ; --

4. Bahwa, sikap berbohong dan menutup-nutupi fakta yang sebenarnya dalam berumah tangga dengan Termohon, juga ditunjukkan dengan kebohongan Pemohon tentang pekerjaannya. Fakta yang sebenarnya adalah, Pemohon bekerja sebagai karyawan tetap di PT. BPR Nusamba

Disclaimer
Kepaniteraan Mahkamah Agung Republik Indonesia berusaha untuk selalu mencantumkan informasi paling kini dan akurat sebagai bentuk komitmen Mahkamah Agung untuk pelayanan publik, transparansi dan akuntabilitas pelaksanaan fungsi peradilan. Namun dalam hal-hal tertentu masih dimungkinkan terjadi permasalahan teknis terkait dengan akurasi dan keterkinian informasi yang kami sajikan, hal mana akan terus kami perbaiki dari waktu kewaktu.
Dalam hal Anda menemukan inakurasi informasi yang termuat pada situs ini atau informasi yang seharusnya ada, namun belum tersedia, maka harap segera hubungi Kepaniteraan Mahkamah Agung RI melalui :
Email : kepaniteraan@mahkamahagung.go.id Telp : 021-384 3348 (ext.318)

Pecangaan bukan sebagai tukang kayu sebagaimana didalilkan Pemohon ; ------------------------

5. Bahwa dalil Pemohon sebagaimana tersebut dalam posita angka 7 (tujuh) dan 8(delapan) adalah **TIDAK BENAR ;**
Fakta yang benar tentang kondisi rumah tangga Pemohon dan Termohon adalah sebagaimana tersebut diatas. Upaya merukunkan kembali Pemohon dan Termohon melalui hakam belum pernah ditempuh, oleh karena itu Termohon meminta dilakukan terlebih dahulu musyawarah melalui hakam. Hal ini sebagaimana disyaratkan oleh QS. An-Nisa' ayat 35.
Dalil perceraian oleh karena sebab syiqoq (pasal 19 huruf f PP Nomor 9 tahun 1975 Jo pasal 116 huruf f Kompilasi Hukum Islam) sebagaimana didalilkan Pemohon, adalah keadaan seringnya terjadi perselisihan/ percek-cokanantara suami-istri dalam berumah tangga. Artinya kondisi rumah tangga berkali-kali (lebih dari dua kali) terjadi pertengkaran antara suami dan istri. Kondisi rumah tangga Pemohon dan Termohon sebagaimana yang dirasakan oleh Termohon selama ini **TIDAK PERNAH TERJADI PERCEKCOKAN**. Terjadi kesalah-pahaman kecil dalam berumah tangga memang pernah terjadi, tetapi biasanya bisa diselesaikan secara musyawarah. Termohon berhadap supaya Pemohon kembali bersama ke rumah dengan Termohon dan anaknya untuk melanjutkan membina rumah tangga yang *sakinah, mawaddah, wa rahmah*.
Berdasarkan uraian dalil tersebut maka kondisi perkawinan dalam keadaan syiqoq, sebagaimana didalilkan Pemohon tidak terbukti sehingga **TIDAK BENAR**; --

6. Bahwa Termohon masih berbakti sebagai seorang Isteri, kemudian masih berharap agar Pemohon pulang ke kediaman bersama untuk melanjutkan rumah tangga bersama Termohon dan anak-anaknya membina rumah tangga yang *sakinnah mawaddah wa rohmah.* ; ------------------------------------

7. Bahwa oleh karena dalil-dalil dalam permohonan cerai talak Pemohon tidak benar dalil-dalilnya maka sudah selayaknyalah apabila Majelis Hakim Pemeriksa Perkara ini ntuk menolak permohonan Pemohon untuk seluruhnya ; --

DALAM REKONPENSI

1. Bahwa, mohon dalil dalam Konpensi secara mutatis mutandis dianggap termuat dalam dalil Rekonpensi ; ---

2. Bahwa, selanjutnya mohon penyebutan Pemohon disebut Pemohon/ Tergugat Rekonpensi dan Termohon disebut Termohon/Penggugat Rekonpensi ; --

Disclaimer
Kepaniteraan Mahkamah Agung Republik Indonesia berusaha untuk selalu mencantumkan informasi paling kini dan akurat sebagai bentuk komitmen Mahkamah Agung untuk pelayanan publik, transparansi dan akuntabilitas pelaksanaan fungsi peradilan. Namun dalam hal-hal tertentu masih dimungkinkan terjadi permasalahan teknis terkait dengan akurasi dan keterkinian informasi yang kami sajikan, hal mana akan terus kami perbaiki dari waktu kewaktu.
Dalam hal Anda menemukan inakurasi informasi yang termuat pada situs ini atau informasi yang seharusnya ada, namun belum tersedia, maka harap segera hubungi Kepaniteraan Mahkamah Agung RI melalui :
Email : kepaniteraan@mahkamahagung.go.id Telp : 021-384 3348 (ext.318)

3. Bahwa, apabila Majelis Hakim berpendapat lain kemudian mengabulkan permohonan cerai talak Pemohon/Tergugat Rekonpensi maka mohon kiranya Majelis Hakim untuk mengabulkan gugatan rekonpensi yang diajukan oleh Termohon/Penggugat Rekonpensi terkait dengan hak-hak hukum yang wajib dipenuhi oleh Pemohon/Tergugat Rekonpeni ; --------------

4. Bahwa, Termohon/Penggugat Rekonpensi mengajukan gugatan rekonpensi meliputi hak-hak Termohon/Penggugat Rekonpensi dan anaknya berupa:

a. Pemenuhan hak atas nafkah terutang (nafkah mandiyah), nafkah iddah, dannafkah mut'ah.
Berdasarkan dalil Pemohon/Tergugat Rekonpensi, setelah terjadi perselisihan/cek cok dalam rumah tangga Pemohon/Tergugat Rekonpensi dan Termohon/Penggugat Rekonpensi. Kemudian Termohon/Penggugat Rekonpensi ditinggalkan oleh Pemohon/Tergugat Rekonpensi dan tidak diberi nafkah wajib setiap bulannya sampai dengan saat ini. Pemohon/Tergugat Rekonpensi tidak memberikan nafkah wajib selama 8 (delapan) bulan. Kebutuhan biaya hidup yang di perlukan Termohon/Penggugat Rekonpensi dan anaknya setiap bulan untuk biaya makan, uang jajan anak, beli susu formula, beli pampers, biaya penitipan anak sejumlah Rp. 3.000.000,- (tiga juta rupiah). Jika Pemohon/Tergugat Rekonpensi telah mengabaikan nafkah wajib selama 8 (delapan) bulan, maka total jumlah nafkah terutang sejumlah Rp. 24.000.000,- (dua puluh empat juta rupiah). **Sehingga nafkah mandiyah (nafkah terutang) yang diminta pemenuhan haknya oleh Termohon/Penggugat sejumlah Rp. 24.000.000,- (dua puluh empat juta rupiah)**.
Selama masa iddah Termohon/Penggugat Rekonpensi masih berhak atas nafkah wajib dari Pemohon/Tergugat Rekonpensi. Nafkah iddah terhitung 3 (tiga) bulan, totalnya sejumlah Rp. 9.000.000,- (Sembilan juta rupiah). **Sehingga nafkah iddah yang diminta pemenuhan haknya oleh Termohon/ Penggugat Rekonpensi sejumlah Rp. 9.000.000,- (sembilan juta rupiah)**.
Termohon/Penggugat Rekonpensi telah berusaha berbakti sebagai Istri Pemohon/Penggugat Rekonpensi, akan tetapi akan diceraikan oleh Pemohon/Tergugat Rekonpensi. Kondisi Termohon/Penggugat Rekonpensi saat ini tidak seperti sebelum menikah dengan Pemohon/Tergugat Rekonpensi. Kondisi perawan Termohon/Penggugat Rekonpensi sudah di nikmati oleh Pemohon/Tergugat Rekonpensi, kemudian mempunyai anak. Apabila diceraikan oleh Pemohon/Tergugat Rekonpensi, akan menimbulkan kesedihan yang mendalam bagi Termohon/Penggugat Rekonpensi. Untuk mengobati kesedihan Termohon/Penggugat Rekonpensi tersebut, oleh karena itu, **maka Termohon/Penggugat Rekonpensi menuntut nafkah mut'ah sejumlah Rp. 70.000.000,- (tujuh puluh juta rupiah).**

Disclaimer
Kepaniteraan Mahkamah Agung Republik Indonesia berusaha untuk selalu mencantumkan informasi paling kini dan akurat sebagai bentuk komitmen Mahkamah Agung untuk pelayanan publik, transparansi dan akuntabilitas pelaksanaan fungsi peradilan. Namun dalam hal-hal tertentu masih dimungkinkan terjadi permasalahan teknis terkait dengan akurasi dan keterkinian informasi yang kami sajikan, hal mana akan terus kami perbaiki dari waktu kewaktu.
Dalam hal Anda menemukan inakurasi informasi yang termuat pada situs ini atau informasi yang seharusnya ada, namun belum tersedia, maka harap segera hubungi Kepaniteraan Mahkamah Agung RI melalui :
Email : kepaniteraan@mahkamahagung.go.id Telp : 021-384 3348 (ext.318)

Sehingga total nafkah mandiyah, nafkah iddah, dan nafkah mut'ah yang diminta pemenuhannya oleh Termohon/Penggugat Rekonpensi sejumlah Rp.103.000.000,- (seratus tiga juta rupiah).
Pekerjaan Pemohon/Tergugat Rekonpensi sebagai karyawan tetap di PT. BPR Nusamba Pecangaan, menjadikan Pemohon/ Tergugat Rekonpensi mampu untuk memenuhi hak-hak tersebut ; ---------------------

b. Pemenuhan hak atas hak nafkah anak/Biaya Hadlonah Anak Pemohon/Tergugat Rekonpensi dan Termohon/Penggugat Rekonpensi yang berada dalam asuhan Termohon/Penggugat Rekonpensi.

Biaya yang diperlukan oleh Termohon/Penggugat Rekonpensi dalam mengasuh anaknya memerlukan biaya yang tidak sedikit, sebagaimana diuraikan diatas. Oleh karena itu Termohon/Penggugat Rekonpensi meminta pemenuhan nafkah untuk biaya mengasuh anak, sampai dengan anak tersebut mandiri. Pekerjaan Pemohon/Tergugat Rekonpensi sebagai karyawan tetap di PT. BPR Nusamba Pecangaan, sehingga memiliki gaji/penghasilan tetap setiap bulannya. Gaji/Penghasilan tetap Pemohon/Tergugat Rekonpensi setiap bulannya kurang lebih sejumlah Rp. 6.000.000,- (enam juta rupiah), meliputi gaji pokok dan bonus-banus. Untuk itu Termohon/Penggugat Rekonpensi meminta pemenuhan nafkah anak/biaya hahdlonah anak sejumlah ½ (satu per dua/separoh) dari jumlah gaji tersebut sampai dengan anak dewasa. Selanjutnya mohon kepada Majelis Hakim untuk memerintahkan PT. BPR Nusamba Pecangaan membagi penghasilan/ gaji Pemohon setiap bulannya tersebut ½ (satu per dua/separoh) bagian untuk nafkah anak/biaya hadlonah anak dan mentransfer ke rekening tersendiri.

c. Hak atas harta asal milik Termohon/Penggugat Rekonpensi yang dikuasai Pemohon/Tergugat Rekonpensi dan pembagian harta bersama.

Selama dalam pernikahan antara Pemohon/Tergugat Rekonpensi dan Termohon/Penggugat Rekonpensi telah dibeli sebuah mobil roda empat Suzuki Karimun. Pembelian mobil tersebut menggunakan uang tunai milik Termohon/Penggugat Rekonpensi yang diperoleh dari orang tuanya sejumlah Rp. 22.000.000,- (dua puluh dua juta rupiah) dan hasil menjual perhiasan emas berupa gelang dan kalung pemberian orang tuanya senilai Rp. 3.000.000,- (tiga juta rupiah). Jadi total uang milik Termohon/Penggugat Rekonpensi dari pemberian orang tuanya untuk membeli mobil tersebut sejumlah Rp.10.500.000,- (sepuluh juta lima ratus ribu rupiah). Kekurangan uang untuk melunasi mobil Suzuki karimun tersebut dibayar oleh Pemohon/Tergugat Rekonpensi, kurang lebih sekitar Rp. 50.000.000,- (lima puluh juta rupiah).
Saat ini mobil tersebut dalam penguasaan Pemohon/Tergugat Rekonpensi, bahkan anak Pemohon/Tergugat Rekonpensi dan Termohon/ Penggugat Rekonpensi ingin menaiki mobil tersebut tidak diijinkan oleh Pemohon/Tergugat Rekonpensi.

Disclaimer
Kepaniteraan Mahkamah Agung Republik Indonesia berusaha untuk selalu mencantumkan informasi paling kini dan akurat sebagai bentuk komitmen Mahkamah Agung untuk pelayanan publik, transparansi dan akuntabilitas pelaksanaan fungsi peradilan. Namun dalam hal-hal tertentu masih dimungkinkan terjadi permasalahan teknis terkait dengan akurasi dan keterkinian informasi yang kami sajikan, hal mana akan terus kami perbaiki dari waktu kewaktu.
Dalam hal Anda menemukan inakurasi informasi yang termuat pada situs ini atau informasi yang seharusnya ada, namun belum tersedia, maka harap segera hubungi Kepaniteraan Mahkamah Agung RI melalui :
Email : kepaniteraan@mahkamahagung.go.id   Telp : 021-384 3348 (ext.318)

Apabila perkawinan Pemohon/Tergugat Rekonpensi dan Termohon/ Penggugat Rekonpensi harus bercerai, maka Termohon/ Penggugat Rekonpensi meminta harta asal berupa uang sejumlah Rp.10.500.000,- (sepuluh juta lima ratus ribu rupiah) diserahkan kembali kepada Termohon/Penggugat Rekonpensi.
Kemudian sisa uang sejumlah Rp. 50.000.000,- (lima puluh juta rupiah) dibagi separoh bagian (½ bagian) masing-masing untuk Pemohon/ Tergugat Rekonpensi dan Termohon/Penggugat Rekonpensi;

5. Bahwa, Termohon/ Penggugat Rekonpensi akan membuktikan semua dalil-dalil Termohon/ Penggugat Rekonpensi tersebut diatas pada saat acara pembuktian nantinya. Semua dalil tersebut benar adanya dan tidak mengada-ada ; --

6. Bahwa berdasarkan dalil-dalil dalam gugatan rekonpensi tersebut memiliki dasar yang kuat, maka sudilah kiranya Majelis Hakim Perkara ini untuk mengabulkan gugatan rekonpensi untuk seluruhnya ; ------------------------------

Demikian jawaban dari Termohon/Penggugat Rekonpensi atas permohonan cerai talak Pemohon/Tergugat Rekonpensi dengan harapan Majelis Hakim Pengadilan Agama Jepara yang memeriksa perkara ini memeriksa dan memutuskan yang amarnya berbunyi;

DALAM KONPENSI

Menolak permohonan cerai talak Pemohon untuk seruhnya ;

DALAM REKONPENSI

1. Mengabulkan gugatan rekonpensi Termohon/Penggugat Rekonpensi untuk seluruhnya ;
2. Menghukum Pemohon/Tergugat Rekonpensi untuk membayar nafkah mandiyah (nafkah terutang), nafkah idddah, dan nafkah mut'ahdengan total keseluruhan sejumlah Rp. 103.000.000,- (seratus tiga juta rupiah), dengan perincian;
   a. Nafkah Mandiyah sejumlah Rp. 24.000.000,- (dua puluh empat juta rupiah).
   b. Nafkah Idddah sejumlahRp. 9.000.000,- (sembilan juta rupiah).
   c. Nafkah Mut'ah sejumlahRp. 70.000.000,- (tujuh puluh juta rupiah) ;
3. Menghukum Pemohon/Tergugat Rekonpensi untuk membayar biaya hadlonah pengasuhan anak/nafkah anak meliputi biaya hidup dan biaya pendidikan anak-anak Pemohon/Tergugat Rekonpensi dengan Termohon/ Penggugat Rekonpensi sejak pendidikan usia dini sampai dengan jenjang kuliah dan mandiri ditetapkan ½ (seperdua/setengah) dari gaji Pemohon/Tergugat Rekonpensi sebagai karyawan tetap di PT. BPR NUSAMBA PECANGAAN dan memerintahkan PT. BPR NUSAMBA PECANGAAN untuk membagi gaji tersebut kemudian mentrasfernya dalam

Disclaimer
Kepaniteraan Mahkamah Agung Republik Indonesia berusaha untuk selalu mencantumkan informasi paling kini dan akurat sebagai bentuk komitmen Mahkamah Agung untuk pelayanan publik, transparansi dan akuntabilitas pelaksanaan fungsi peradilan. Namun dalam hal-hal tertentu masih dimungkinkan terjadi permasalahan teknis terkait dengan akurasi dan keterkinian informasi yang kami sajikan, hal mana akan terus kami perbaiki dari waktu kewaktu.
Dalam hal Anda menemukan inakurasi informasi yang termuat pada situs ini atau informasi yang seharusnya ada, namun belum tersedia, maka harap segera hubungi Kepaniteraan Mahkamah Agung RI melalui :
Email : kepaniteraan@mahkamahagung.go.id Telp : 021-384 3348 (ext.318)

rekening tersendiri untuk diserahkan kepada Termohon/Penggugat Rekonpensi ;

4. Menetapkan uang sejumlah Rp.10.500.000,- (sepuluh juta lima ratus ribu rupiah) yang dipakai untuk membeli mobil karimun sebagai harta asal milik Termohon/Penggugat Rekonpensi ; ---

5. Menghukum Pemohon/Tergugat Rekonpensi untuk menyerahkan uang tersebut ;

6. Menetapkan uang senilai Rp. 50.000.000,- (lima puluh juta rupiah) yang saat ini sudah beralih menjadi Mobil Suzuki Karimun sebagai harta bersama Pemohon/Tergugat dengan Termohon/Penggugat Rekonpensi dan masing-masing berhak atas ½ (seperdua/setengah) bagian ;

7. Menghukum Pemohon/Tergugat Rekonpensi untuk menyerahkan apa yang menjadi hak dari Termohon/Penggugat Rekonpensiatas harta bersama dan harta asal yang dikuasai Pemohon/Tergugat Rekonpensi secara sukarela. Apabila tidak serahkan secara sukarela maka akan dilakukan upaya paksa melalui pengadilan melalui eksekusi dengan bantuan alat kelengkapan keamanan dari Negara;

DALAM KONPENSI DAN REKONPENSI

- Menghukum Pemohon untuk membayar biaya perkara menurut hukum ;

Bahwa, terhadap jawaban Termohon, Pemohon mengajukan replik tertulis sebagai berikut :

DALAM POKOK PERKARA

1. Bahwa Pemohon menolak dalil-dalil jawaban Termohon kecuali yang secara tegas diakui oleh Pemohon;
2. Bahwa Termohon telah mengakui dalil Pemohon angka 1, 2, dan 3 benar;
3. Bahwa Pemohon menolak jawaban Termohon angka 3 yang menjawab dalil Pemohon angka 5 dan 6, bahwa Pemohon tetap pada dalil permohonan Pemohon bahwa Termohon telah benar benar nuzus pada Pemohon sehingga menjadikan persesilisihan, pertengkaran dan perpecahan rumah tanggal tak dapat dielakkan.
4. Bahwa Pemohon benar bekerja karyawan BPR Nusamba, pemohon salah tulis perihal pekerjaan pemohon.
5. Bahwa Pemohon menolak dalil jawaban Termohon angka 5 yang menjawab dalil pemohon angka 7 dan 8, bahwa pemohon tetap pada dalil

Disclaimer
Kepaniteraan Mahkamah Agung Republik Indonesia berusaha untuk selalu mencantumkan informasi paling kini dan akurat sebagai bentuk komitmen Mahkamah Agung untuk pelayanan publik, transparansi dan akuntabilitas pelaksanaan fungsi peradilan. Namun dalam hal-hal tertentu masih dimungkinkan terjadi permasalahan teknis terkait dengan akurasi dan keterkinian informasi yang kami sajikan, hal mana akan terus kami perbaiki dari waktu kewaktu.
Dalam hal Anda menemukan inakurasi informasi yang termuat pada situs ini atau informasi yang seharusnya ada, namun belum tersedia, maka harap segera hubungi Kepaniteraan Mahkamah Agung RI melalui :
Email : kepaniteraan@mahkamahagung.go.id Telp : 021-384 3348 (ext.318)

pemohon angka 7 dan 8, bahwa keadaan perselisihan, pertengkaran yang berakibat perpecahan rumah tangga telah ditempuh upaya musyawarah kekeluargaan namun tetap tidak berhasil.

6. Bahwa Pemohon tetap teguh hendak ceraikan termohon.

DALAM GUGAT REKOPENSI

1. Bahwa Tergugat rekopensi menolak dalil-dalil gugatan rekopensi kecuali yang diakui benar oleh Tergugat rekopensi;

2. Bahwa Tergugat rekopensi menolak dalil Penggugat rekopensi angka 4 huruf a, b, dan c. Karena tidak benar dan sangat memberatkan tergugat rekopensi. Karena itu tidak sanggup dan harus ditolak.

3. Bahwa terkait gugatan penggugat rekopensi angka 4 huruf c) di sampaikan tanggapan berikut ini : bahwa berkaitan uang Rp.25.000.000,- yang diminta kembali pada pokoknya tergugat rekopensi tidak keberatan, namun sebaliknya penggugat rekopensi juga harus mengembalikan uang hasil jual motor bawaan tergugat rekopensi sebesar Rp. 8.500.000,- utuk keperluan beli sepeda motor honda beat warna putih yang sekarang dipakai penggugat rekopensi, sehingga uang Rp.10.500.000,- harus dikurangi Rp.8.500.000,- sehingga sisa selebihnya adalah Rp.16.500.000,-

4. Bahwa Tergugat rekopensi sanggup membayar Rp.16.500.000,-

5. Bahwa terhadap gugatan penggugat rekopensi pihak tergugat rekopensi hanya sanggup membayar:

A.	Mut'ah		: Rp. 1. 000.000,-
B.	Nafkah lampau	1 bulan	: Rp. 400.000,-
C.	Nafkah Idah 400.000 X 3 bulan		: Rp. 1. 200.000,-
D.	Nafkah anak	1 anak	: Rp. 400.000,-
	JUMLAH		Rp. 3. 000.000,

Berdasarkan dalil-dalil replik tersebut di atas, Pemohon mohon kepada Ketua Majelis hakim yang memeriksa dan mengadili perkara ini menjatuhkan putusan dengan amar putusan sebagai berikut :

DALAM POKOK PERKARA

Mengabulkan permohonan Pemohon

DALAM REKOPENSI

Disclaimer
Kepaniteraan Mahkamah Agung Republik Indonesia berusaha untuk selalu mencantumkan informasi paling kini dan akurat sebagai bentuk komitmen Mahkamah Agung untuk pelayanan publik, transparansi dan akuntabilitas pelaksanaan fungsi peradilan. Namun dalam hal-hal tertentu masih dimungkinkan terjadi permasalahan teknis terkait dengan akurasi dan keterkinian informasi yang kami sajikan, hal mana akan terus kami perbaiki dari waktu kewaktu.
Dalam hal Anda menemukan inakurasi informasi yang termuat pada situs ini atau informasi yang seharusnya ada, namun belum tersedia, maka harap segera hubungi Kepaniteraan Mahkamah Agung RI melalui :
Email : kepaniteraan@mahkamahagung.go.id Telp : 021-384 3348 (ext.318)

1. Menolak gugatan Penggugat rekopensi

2. Menghukum Tergugat rekopensi menyerahkan pengembalian uang Rp.25.000.000,- setelah dikurangi untuk pembelian sepeda Motor honda beat warna putih Rp. 8.500.000,- sisa selebihnya Rp. 16.500.000,-

3. Menetapkan hak Penggugat rekopensi adalah sebagai berikut:

A.	Mut'ah	: Rp.	1. 000.000,-
B.	Nafkah lampau 1 bulan	: Rp.	400.000,-
C.	Nafkah Idah 400.000 X 3 bulan	: Rp.	1. 200.000,-
D.	Nafkah anak 1 anak	: Rp.	400.000,-
	JUMLAH	Rp.	3. 000.000,-

ATAU, apabila majelis hakim berpendapat lain mohon putusan yang seadil-adilnya

Bahwa terhadap Replik Pemohon, Termohon telah mengajukan duplik yang pada pokoknya sebagai berikut :

DALAM KONPENSI

1. Bahwa, Termohon/Penggugat Rekonpensi menolak seluruh dalil-dalil yang diajukan oleh Pemohon/Tergugat Rekonpensi dalam surat permohonan cerai talak talak tertanggal 07 September 2020 sebagaimana terdaftar dalam perkara nomor: 1480/Pdt.G/2020/PA.Jepr serta Replik dan Jawaban Rekonpensi Pemohon/Tergugat Rekonpensi yang telah dibacakan dihadapan sidang Pengadilan Agama Jepara Tanggal 02 Nopember 2020, kecuali yang secara jelas-jelas dibenarkan oleh Termohon/Penggugat Rekonpensi ; ---

2. Bahwa, dalil Pemohon/Tergugat Rekonpensi sebagaimana tersebut dalam posita angka 2 (dua), 3 (tiga) dan 5 (lima) DALAM KONPENSI **TIDAK BENAR DAN MENGADA-ADA.**

a. Termohon/Penggugat Rekonpensi menolak jika dikatakan telah melakukan nusyus. Sebagaimana telah Termohon/Penggugat Rekonpensi terangkan dalam dalil-dalil dalam surat jawaban dan gugatan rekonpensi, Termohon/Penggugat Rekonpensi sudah berusaha semaksimal mungkin untuk berbakti pada Pemohon/Tergugat Rekonpensi sebagai suami. Bahkan dalam keadaan sulit sekalipun, tetap berbakti kepada Pemohon/Tergugat Rekonpensi. Justru Pemohon/Tergugat Rekonpensi tanpa alasan yang jelas meninggalkan Termohon/Penggugat Rekonpensi dan anaknya. Bahkan Pemohon/Tergugat Rekonpensi tidak memberikan nafkah wajib dan mengabaikan Termohon/Penggugat Rekonpensi dan anaknya.

Disclaimer
Kepaniteraan Mahkamah Agung Republik Indonesia berusaha untuk selalu mencantumkan informasi paling kini dan akurat sebagai bentuk komitmen Mahkamah Agung untuk pelayanan publik, transparansi dan akuntabilitas pelaksanaan fungsi peradilan. Namun dalam hal-hal tertentu masih dimungkinkan terjadi permasalahan teknis terkait dengan akurasi dan keterkinian informasi yang kami sajikan, hal mana akan terus kami perbaiki dari waktu kewaktu.
Dalam hal Anda menemukan inakurasi informasi yang termuat pada situs ini atau informasi yang seharusnya ada, namun belum tersedia, maka harap segera hubungi Kepaniteraan Mahkamah Agung RI melalui :
Email : kepaniteraan@mahkamahagung.go.id Telp : 021-384 3348 (ext.318)

b. Termohon/Penggugat Rekonpensi tetap bertahan dengan dalil-dalil sebagaimana tersebut dalam surat jawaban dan gugatan rekonpensi yang diajukan Termohon/Penggugat Rekonpensi tertanggal 16 Oktober 2020 yang telah dibacakan dihadapan sidang Pengadilan Agama Jepara tanggal 16 Oktober 2020

c. Berdasarkan uraian dalil-dalil yang telah diuraikan tersebut, oleh sebab itu dalil-dalil Pemohon tersebut tidak benar ; --------------------

3. Bahwa, Termohon masih berbakti sebagai seorang Isteri dan masih berharap agar Pemohon/Tergugat Rekonpensi untuk pulang ke kediaman bersama untuk melanjutkan rumah tangga bersama Termohon/Penggugat Rekonpensi dan anaknya serta melanjutkan membina rumah tangga yang *sakinnah mawaddah wa rahmah.* ; --

4. Bahwa, oleh karena dalil-dalil dalam permohonan cerai talak Pemohon tidak benar dalil-dalilnya maka sudah selayaknyalah apabila Majelis Hakim Pemeriksa Perkara ini untuk menolak permohonan Pemohon untuk seluruhnya ; --

DALAM REKONPENSI

1. Bahwa, mohon dalil dalam Konpensi secara mutatis mutandis dianggap termuat dalam dalil Rekonpensi ; --

2. Bahwa, Termohon/Penggugat Rekonpensi menolak seluruh dalil-dalil yang diajukan oleh Pemohon/Tergugat Rekonpensi dalam surat permohonan cerai talak talak tertanggal 07 September 2020 sebagaimana terdaftar dalam perkara nomor: 1480/Pdt.G/2020/PA.Jepr serta Replik dan Jawaban Rekonpensi Pemohon/Tergugat Rekonpensi yang telah dibacakan dihadapan sidang Pengadilan Agama Jepara Tanggal 02 Nopember 2020, kecuali yang secara jelas-jelas dibenarkan oleh Termohon/Penggugat Rekonpensi ; --

3. Bahwa, dalil Pemohon/Tergugat Rekonpensi sebagaimana tersebut dalam posita angka 2 (dua), 3 (tiga), 4 (empat) dan 5 (lima) DALAM GUGAT REKONPENSI **TIDAK BENAR DAN MENGADA-ADA**:

4. Termohon/Penggugat menanggapi ketidak benaran dalil-dalil Pemohon/ Tergugat Rekonpensi, secara garis besar sebagai berikut:

Disclaimer
Kepaniteraan Mahkamah Agung Republik Indonesia berusaha untuk selalu mencantumkan informasi paling kini dan akurat sebagai bentuk komitmen Mahkamah Agung untuk pelayanan publik, transparansi dan akuntabilitas pelaksanaan fungsi peradilan. Namun dalam hal-hal tertentu masih dimungkinkan terjadi permasalahan teknis terkait dengan akurasi dan keterkinian informasi yang kami sajikan, hal mana akan terus kami perbaiki dari waktu kewaktu.
Dalam hal Anda menemukan inakurasi informasi yang termuat pada situs ini atau informasi yang seharusnya ada, namun belum tersedia, maka harap segera hubungi Kepaniteraan Mahkamah Agung RI melalui :
Email : kepaniteraan@mahkamahagung.go.id Telp : 021-384 3348 (ext.318)

a. Pemenuhan hak atas nafkah terutang (nafkah mandiyah), nafkah iddah, dan nafkah mut'ah.
Jika Pemohon/Tergugat Rekonpensi berketetapan untuk bercerai, maka hak-hak Termohon/Penggugat Rekonpensi untuk diberikan, diantaranya hak atas nafkah Mandiyah, Nafkah Iddah, dan nafkah Mut'ah. Jumlah yang diminta oleh Termohon/ Penggugat Rekonpensi mampu dipenuhi oleh Pemohon/Tergugat Rekonpnesi. Pada acara pembuktian nanti Termohon/Penggugat Rekonpensi akan membuktikan kemampuan yang dimiliki oleh Pemohon/Tergugat Rekonpensi.

b. Pemenuhan hak atas hak nafkah anak/Biaya Hadlonah Anak Pemohon/Tergugat Rekonpensi dan Termohon/Penggugat Rekonpensi yang berada dalam asuhan Termohon/Penggugat Rekonpensi.
Termohon/Penggugat Rekonpensi berharap untuk rukun kembali dengan Pemohon/Tergugat Rekonpensi dan membina kembali rumah tangga yang *sakinah, mawaddah, wa rahmah.* Serta mengajak Pemohon/Tergugat Rekonpensi untuk merawat bersama-sama anak yang sudah dilahirkan. Akan tetapi apabila perkawinan harus putus karena perceraian (Talak dari Pemohon/Tergugat Rekonpensi), maka biaya hadlanah anak harus dibebankan kepada Pemohon/Tergugat Rekonpensi. Pekerjaan Pemohon/Tergugat Rekonpensi sebagai karyawan tetap di PT. BPR Nusamba Pecangaan dengan gaji sejumlah +/- Rp. 6.000.000,- (enam juta rupiah) mampu untuk memenuhi kewajiban tersebut. Kebutuhan nyata yang diperlukan oleh anak setiap bulannya sejumlah +/- sekitar Rp. 3.000.000,- (tiga juta rupiah). Oleh karena itu gaji Pemohon/Tergugat Rekonpensi harus dibagi 2 (dua) untuk memenuhi biaya hadlonah anak. Mohon kepada Majelis Hakim Pemeriksa *a quo* untuk memerintahkan kepada PT. BPR Nusamba Pecangaan atau manajemennya untuk memisahkan ke rekening tersendiri atas bagian biaya hadlanah anak tersebut.
Dalil Pemohon/Tergugat Rekonpensi yang menyatakan tidak mampu memberikan nafkah anak tersebut, atau hanya menyatakan mampu memberikan biaya hadlanah sejumlah Rp. 400.000,- (empat ratus ribu rupiah adalah **TIDAK MASUK AKAL**. Pemohon/Tergugat Rekonpensi berusaha untuk lari dari tanggung jawab. Separoh bagian gaji tersebut untuk memenuhi biaya hadlonah anaknya sendiri atau darah dagingnya sendiri. Seorang bapak model apa yang mau punya anak, akan tetapi tidak mau membiayai kebutuhan anaknya. Perbuatan tersebut merupakan tindakan penelantaran anak sebagaimana dimaksud dalam pasal 9 ayat 1 Undang-Undang Nomor 23 Tahun 2004 tentang Penghapusan Kekerasan Dalam Rumah Tangga. Termohon/Penggugat Rekonpensi akan membuktikan kemampuan keuangan Pemohon/ Tergugat Rekonpensi untuk memberikan biaya hadlanah anak nanti pada saat acara pembuktian.

Permintaan biaya hadlanah anak oleh Termohon/Penggugat Rekonpensi berdasarkan pada Pasal 105 Kompilasi Hukum Islam, yang bunyinya:

Disclaimer
Kepaniteraan Mahkamah Agung Republik Indonesia berusaha untuk selalu mencantumkan informasi paling kini dan akurat sebagai bentuk komitmen Mahkamah Agung untuk pelayanan publik, transparansi dan akuntabilitas pelaksanaan fungsi peradilan. Namun dalam hal-hal tertentu masih dimungkinkan terjadi permasalahan teknis terkait dengan akurasi dan keterkinian informasi yang kami sajikan, hal mana akan terus kami perbaiki dari waktu kewaktu.
Dalam hal Anda menemukan inakurasi informasi yang termuat pada situs ini atau informasi yang seharusnya ada, namun belum tersedia, maka harap segera hubungi Kepaniteraan Mahkamah Agung RI melalui :
Email : kepaniteraan@mahkamahagung.go.id   Telp : 021-384 3348 (ext.318)

“ Dalam hal terjadinya perceraian :

a. Pemeliharaan anak yang belum mumayyiz atau belum berumur 12 tahun adalah hak ibunya;

b. Pemeliharaan anak yang sudah mumayyiz diserahkan kepada anak untuk memilih diantara ayah atau ibunya sebagai pemegang hak pemeliharaanya;

c. biaya pemeliharaan ditanggung olehayahnya. “

berdasarkan pada Pasal 149 Kompilasi Hukum Islam, yang bunyinya:

“ Bilamana perkawinan putus karena talak, maka bekas suami wajib:

a. memberikan mut`ah yang layak kepada bekas isterinya, baik berupa uang atau benda, kecuali bekas isteri tersebut qobla al dukhul;

b. memberi nafkah, maskan dan kiswah kepada bekas isteri selama dalam iddah, kecuali bekas isteri telahdi jatuhi talak ba1in atau nusyur dan dalam keadaan tidak hamil;

c. melunasi mahar yang masih terhutang seluruhnya, dan separoh apabila qobla al dukhul;

d. memberikan biaya hadhanan untuk anak-anaknya yang belum mencapai umur 210 (sepuluh) bulan . “

c. Hak atas harta asal milik Termohon/ Penggugat Rekonpensi yang dikuasai Pemohon/ Tergugat Rekonpensi dan pembagian harta bersama.

Selama Termohon/Penggugat Rekonpensi menikah dengan Pemohon/Tergugat Rekonpensi telah dibeli sebuah mobil Suzuki Karimun dan sudah diakui oleh Pemohon/Tergugat Rekonpensi. Saat ini mobil tersebut dikuasai oleh Pemohon/Tergugat Rekonpensi. Apabila perkawinan Termohon/Penggugat Rekonpensi harus bercerai, maka sebagian uang pemberian orang tua Termohon/Penggugat Rekonpensi yang dipakai untuk membeli mobil tersebut harus di kembalikan kepada Termohon/Penggugat Rekonpensi. Uang tersebut sejumlah Rp.10.500.000,- (sepuluh juta lima ratus ribu rupiah). Termohon/ Penggugat Rekonpensi bersedia memberikan uang pemberian orang tuanya untuk membeli mobil dengan pertimbangan mobil tersebut dipakai juga untuk anaknya. Bahkan sampai dengan saat ini, sang anak sangat merindukan ayahnya dan menanyakan ayahnya (Pemohon/ Tergugat Rekonpensi) untuk bisa naik mobil bersama ayahnya. Kemudian dalil Pemohon/Tergugat Rekonpensi yang mengatakan membeli sepeda motor Honda beat dari uangnya adalah tidak benar.

Harta bawaan harus dikembalikan pemilik harta berdasarkan pasal 36 ayat 2 Undang-Undang Nomor 10 (sepuluh) bulan 1974 tentang Perkawinan mengatakan:

“ (2) Mengenai harta bawaan masing-masing suami dan isteri mempunyai hak sepenuhnya untuk melakukan perbuatan hukum mengenai harta bendanya ”.

Disclaimer
Kepaniteraan Mahkamah Agung Republik Indonesia berusaha untuk selalu mencantumkan informasi paling kini dan akurat sebagai bentuk komitmen Mahkamah Agung untuk pelayanan publik, transparansi dan akuntabilitas pelaksanaan fungsi peradilan. Namun dalam hal-hal tertentu masih dimungkinkan terjadi permasalahan teknis terkait dengan akurasi dan keterkinian informasi yang kami sajikan, hal mana akan terus kami perbaiki dari waktu kewaktu.
Dalam hal Anda menemukan inakurasi informasi yang termuat pada situs ini atau informasi yang seharusnya ada, namun belum tersedia, maka harap segera hubungi Kepaniteraan Mahkamah Agung RI melalui :
Email : kepaniteraan@mahkamahagung.go.id   Telp : 021-384 3348 (ext.318)

Kemudian berdasarkan Pasal 87 Kompilasi Hukum Islam, yang bunyinya:

“ (1) Harta bawaan masing-masing suami dan isteri dan harta yang diperoleh masing-masing sebagai hadiah atau warisan adalah dibawah penguasaan masing-masing, sepanjang para pihak tidak menentukan lain dalam perjanjian perkawinan.

(2) Suami dan isteri mempunyai hak sepenuhnya untuk melakukan perbuatan hukum atas harta masing-masing berupa hibah, hadiah, sodaqah atau lainnya. “

Sisa penjualan mobil senilai kurang lebih sekitar Rp. 50.000.000,- (lima puluh juta rupiah) harus dibagi berdua oleh karena sebagai gono-gini perkawinan antara Termohon/ Penggugat Rekonpensi dan Pemohon/ Tergugat Rekonpensi. Pembagiannya Rp.10.500.000,- (sepuluh juta lima ratus ribu rupiah) untuk Termohon/Penggugat Rekonpensi dan Rp.10.500.000,- (sepuluh juta lima ratus ribu rupiah) untuk Pemohon/Tergugat Rekonpensi

Berdasarkan pasal 37 Undang-Undang Nomor 10 (sepuluh) bulan 1974 Tentang Perkawinan, yang bunyinya:

“Bila perkawinan putus karena perceraian, harta bersama diatur menurut hukumnya masing-masing”

Kemudian berdasarkan Pasal 97 Kompilasi Hukum Islam, yang bunyinya:

“Janda atau Duda cerai masing-masing berhak seperdua dari harta bersama sepanjang tidak ditentukan lain dalam perjanjian perkawinan”

berdasarkan Putusan Mahkamah Agung Republik Indonesia Nomor: 424.K/SIP.1959 yang abstraksi kaidah hukumnya:

“apabila terjadi perceraian, maka masing-masing pihak (suami atau isteri) mendapatkan setengah bagian” : --

7. Bahwa, Termohon/Penggugat Rekonpensi akan membuktikan semua dalil-dalil Termohon/Penggugat Rekonpensi tersebut diatas pada saat acara pembuktian nantinya. Semua dalil tersebut benar adanya dan tidak mengada-ada ; --

8. Bahwa berdasarkan dalil-dalil dalam gugatan rekonpensi tersebut memiliki dasar yang kuat, maka sudilah kiranya Majelis Hakim Perkara ini untuk mengabulkan gugatan rekonpensi untuk seluruhnya ; -----------------------------

Demikian Duplik dan Replik Gugatan Rekonpensi dari Termohon/ Penggugat Rekonpensi atas permohonan cerai talak Pemohon/Tergugat Rekonpensi dengan harapan Majelis Hakim Pengadilan Agama Jepara yang memeriksa perkara ini memeriksa dan memutuskan yang amarnya berbunyi;

Disclaimer
Kepaniteraan Mahkamah Agung Republik Indonesia berusaha untuk selalu mencantumkan informasi paling kini dan akurat sebagai bentuk komitmen Mahkamah Agung untuk pelayanan publik, transparansi dan akuntabilitas pelaksanaan fungsi peradilan. Namun dalam hal-hal tertentu masih dimungkinkan terjadi permasalahan teknis terkait dengan akurasi dan keterkinian informasi yang kami sajikan, hal mana akan terus kami perbaiki dari waktu kewaktu.
Dalam hal Anda menemukan inakurasi informasi yang termuat pada situs ini atau informasi yang seharusnya ada, namun belum tersedia, maka harap segera hubungi Kepaniteraan Mahkamah Agung RI melalui :
Email : kepaniteraan@mahkamahagung.go.id Telp : 021-384 3348 (ext.318)

DALAM KONPENSI

- Menolak permohonan cerai talak Pemohon untuk seruhnya ;

DALAM REKONPENSI

1. Mengabulkan gugatan rekonpensi Termohon/Penggugat Rekonpensi untuk seluruhnya ;
2. Menghukum Pemohon/Tergugat Rekonpensi untuk membayar nafkah mandiyah (nafkah terutang), nafkah idddah, dan nafkah mut'ah dengan total keseluruhan sejumlah Rp. 103.000.000,- (seratus tiga juta rupiah), dengan perincian;
   - **a.** Nafkah Mandiyah sejumlah Rp. 24.000.000,- (dua puluh empat juta rupiah).
   - **b.** Nafkah Idddah sejumlah Rp. 9.000.000,- (sembilan juta rupiah).
   - **c.** Nafkah Mut'ah sejumlah Rp. 70.000.000,- (tujuh puluh juta rupiah) ;

3. Menghukum Pemohon/Tergugat Rekonpensi untuk membayar biaya hadlonah pengasuhan anak/nafkah anak meliputi biaya hidup dan biaya pendidikan anak-anak Pemohon/Tergugat Rekonpensi dengan Termohon/ Penggugat Rekonpensi sejak pendidikan usia dini sampai dengan jenjang kuliah dan mandiri ditetapkan ½ (seperdua/ setengah) dari gaji Pemohon/ Tergugat Rekonpensi sebagai karyawan tetap di PT. BPR NUSAMBA PECANGAAN dan memerintahkan PT. BPR NUSAMBA PECANGAAN untuk membagi gaji tersebut kemudian mentrasfernya dalam rekening tersendiri untuk diserahkan kepada Termohon/Penggugat Rekonpensi ;
4. Menetapkan uang sejumlah Rp.10.500.000,- (sepuluh juta lima ratus ribu rupiah) yang dipakai untuk membeli mobil karimun sebagai harta asal milik Termohon/Penggugat Rekonpensi ; ---
5. Menghukum Pemohon/Tergugat Rekonpensi untuk menyerahkan uang tersebut ;
6. Menetapkan uang senilai Rp. 50.000.000,- (lima puluh juta rupiah) yang saat ini sudah beralih menjadi Mobil Suzuki Karimun sebagai harta bersama Pemohon/Tergugat dengan Termohon/Penggugat Rekonpensi dan masing-masing berhak atas ½ (seperdua/setengah) bagian ;
7. Menghukum Pemohon/Tergugat Rekonpensi untuk menyerahkan apa yang menjadi hak dari Termohon/Penggugat Rekonpensi atas harta bersama dan harta asal yang dikuasai Pemohon/Tergugat Rekonpensi secara sukarela. Apabila tidak serahkan secara sukarela maka akan dilakukan upaya paksa melalui pengadilan melalui eksekusi dengan bantuan alat kelengkapan keamanan dari Negara ;

DALAM KONPENSI DAN REKONPENSI

- Menghukum Pemohon untuk membayar biaya perkara menurut hukum ;

Disclaimer
Kepaniteraan Mahkamah Agung Republik Indonesia berusaha untuk selalu mencantumkan informasi paling kini dan akurat sebagai bentuk komitmen Mahkamah Agung untuk pelayanan publik, transparansi dan akuntabilitas pelaksanaan fungsi peradilan. Namun dalam hal-hal tertentu masih dimungkinkan terjadi permasalahan teknis terkait dengan akurasi dan keterkinian informasi yang kami sajikan, hal mana akan terus kami perbaiki dari waktu kewaktu.
Dalam hal Anda menemukan inakurasi informasi yang termuat pada situs ini atau informasi yang seharusnya ada, namun belum tersedia, maka harap segera hubungi Kepaniteraan Mahkamah Agung RI melalui :
Email : kepaniteraan@mahkamahagung.go.id Telp : 021-384 3348 (ext.318)

Bahwa untuk menguatkan dalil permohonannya Pemohon dalam persidangan, mengajukan alat bukti surat berupa:

1. Fotokopi Kartu Tanda Penduduk atas nama Pemohon Nomor 3320042111860002 tanggal 30 Juni 2018, yang telah dinazegelen dan telah dicocokkan dengan surat aslinya dan sesuai dengan aslinya (Bukti P.1);
2. Fotokopi Buku Kutipan Akta Nikah dari Kantor Urusan Agama Kecamatan XXXXX, Kabupaten Jepara Nomor XXXXX tanggal 23 Agustus 2017, yang telah dinazegelen dan telah dicocokkan dengan surat aslinya dan sesuai dengan aslinya (Bukti P.2);

Bahwa selain bukti surat tersebut Pemohon juga menghadirkan 2 orang dari keluarga dan atau orang yang dekat dengan Pemohon dan Termohon masing masing bernama :

1. XXXXX, setelah bersumpah memberikan keterangan yang pada pokoknya sebagai berikut :

0- Bahwa saksi sebagai kakak ipar Pemohon, mengetahui Pemohon dan Termohon adalah suami isteri;

ㄱ- Bahwa setelah menikah keduanya tinggal bersama di rumah orang tua Termohon dan dari pernikahan Pemohon dengan Termohon sudah dikaruniai satu orang anak;

ㄴ- Bahwa sejak bulan Maret 2019 mendengar Pemohon dengan Termohon sering bertengkar penyebabnya karena Termohon tidak mau diajak pindah di rumah orang tuanya begitu pun sebaliknya;

ㄷ- Bahwa antara Pemohon dan Termohon tidak pernah kumpul lagi sejak bulan Februari 2020 Pemohon tinggal di rumahnya, sedangkan Termohon tinggal dengan orang tuanya;

ㄹ- Bahwa Pemohon bekerja sebagai tukang kayu dengan penghasilan setiap hari sejumlah Rp, 75.000 sampai 100.000; dan setelah 10 (sepuluh) bulan saksi jadi iparnya Pemohon bekerja di Bank Nusamba namun besaran penghasilannya tidak mengetahuinya;

ㅁ- Bahwa setahu saksi selama Pemohon dan Termohon pisah sekitar 6 bulan, Pemohon masih sering memberi nafkah kepada anaknya sekitar Rp. 1.000.000, tetapi sekarang tidak tahu lagi ngasih apa tidak;

Disclaimer
Kepaniteraan Mahkamah Agung Republik Indonesia berusaha untuk selalu mencantumkan informasi paling kini dan akurat sebagai bentuk komitmen Mahkamah Agung untuk pelayanan publik, transparansi dan akuntabilitas pelaksanaan fungsi peradilan. Namun dalam hal-hal tertentu masih dimungkinkan terjadi permasalahan teknis terkait dengan akurasi dan keterkinian informasi yang kami sajikan, hal mana akan terus kami perbaiki dari waktu kewaktu.
Dalam hal Anda menemukan inakurasi informasi yang termuat pada situs ini atau informasi yang seharusnya ada, namun belum tersedia, maka harap segera hubungi Kepaniteraan Mahkamah Agung RI melalui :
Email : kepaniteraan@mahkamahagung.go.id Telp : 021-384 3348 (ext.318)

ㅂ- Bahwa setahu saksi Pemohon dan Termohon mempunyai satu unit roda empat merek Suzuki dan satu unit sepeda motor merk Honda Beat;

ㅅ- Bahwa saksi dan pihak keluarga keduanya sudah pernah merukunkannya, akan tetapi tidak berhasil;

2 XXXXX, setelah bersumpah memberikan keterangan yang pada pokoknya sebagai berikut :

- Bahwa saksi sebagai teman dekat Pemohon, mengetahui Pemohon dan Termohon adalah suami isteri;
- Bahwa setelah menikah keduanya tinggal bersama di rumah orang tua Termohon dan dari pernikahan Pemohon dengan Termohon sudah dikaruniai satu orang anak yang ikut dengan Termohon;
- Bahwa sejak bulan Maret 2019 mendengar Pemohon dengan Termohon sering bertengkar penyebabnya karena Termohon tidak mau diajak pindah di rumah orang tuanya begitu pun sebaliknya;
- Bahwa antara Pemohon dan Termohon tidak pernah kumpul lagi sejak bulan Februari 2020 Pemohon tinggal di rumahnya, sedangkan Termohon tinggal dengan orang tuanya;
- Bahwa Pemohon sekarang bekerja di Bank Nusamba namun besaran penghasilannya tidak mengetahuinya;
- Bahwa setahu saksi selama Pemohon dan Termohon telah pisah sekitar 6 bulan, namun tidak tahu lagi ngasih apa tidak;
- Bahwa saksi tidak mengetahui Pemohon dan Termohon mempunyai harta selama berumah tangga;
- Bahwa saksi dan pihak keluarga keduanya sudah pernah merukunkannya, akan tetapi tidak berhasil;

Atas keterangan saksi-saksi tersebut Termohon tidak membantahnya;

Bahwa Termohon telah pula mengajukan alat bukti surat berupa:

1. Fotokopi Kartu Tanda Penduduk atas nama Pemohon Nomor XXXXX tanggal 30 Mei 2018, yang telah dinazegelen dan telah dicocokkan dengan surat aslinya dan sesuai dengan aslinya (Bukti T.1);
2. Fotokopi Informasi dana dan usia JHT + JP 2018 BPJS Ketanagakerjaan atas nama XXXXX, telah dinazegelen dan bermeterai

Disclaimer
Kepaniteraan Mahkamah Agung Republik Indonesia berusaha untuk selalu mencantumkan informasi paling kini dan akurat sebagai bentuk komitmen Mahkamah Agung untuk pelayanan publik, transparansi dan akuntabilitas pelaksanaan fungsi peradilan. Namun dalam hal-hal tertentu masih dimungkinkan terjadi permasalahan teknis terkait dengan akurasi dan keterkinian informasi yang kami sajikan, hal mana akan terus kami perbaiki dari waktu kewaktu.
Dalam hal Anda menemukan inakurasi informasi yang termuat pada situs ini atau informasi yang seharusnya ada, namun belum tersedia, maka harap segera hubungi Kepaniteraan Mahkamah Agung RI melalui :
Email : kepaniteraan@mahkamahagung.go.id Telp : 021-384 3348 (ext.318)

cukup telah dicocokkan dengan surat aslinya dan sesuai dengan aslinya (Bukti T.2);

3. Fotokopi laporan pribadi keuangan atas nama XXXXXCq PT BPR Nusamba Pecangaan, bermeterai cukup, telah dinazegelen dan telah dicocokkan dengan surat aslinya dan sesuai dengan aslinya (Bukti T.3);

4. Prin out screenschots chat washaap Pemohon dan Termohon bermeterai cukup, (Bukti T.4);

Bahwa Termohon telah juga menghadirkan 2 orang saksi dari keluarga dan atau orang yang dekatnya masing-masing bernama:

1. XXXXX, setelah bersumpah memberikan keterangan yang pada pokoknya sebagai berikut :

0- Bahwa saksi sebagai ibu kandung Termohon, mengetahui Pemohon dan Termohon adalah suami isteri;

ㄱ- Bahwa setelah menikah keduanya tinggal bersama di rumah saksi dan dari pernikahan Pemohon dengan Termohon sudah dikaruniai satu orang anak dan kini ikut dengan Termohon;

ㄴ- Bahwa saksi tidak mendengar Pemohon dengan Termohon bertengkar namun anak saksi tidak mau diajak pindah di rumah orang tua Pemohon;

ㄷ- Bahwa antara Pemohon dan Termohon tidak pernah kumpul lagi sejak bulan Februari 2020 Pemohon tinggal di rumahnya, sedangkan Termohon tinggal dengan orang tuanya;

ㄹ- Bahwa Pemohon Pemohon bekerja di Bank Nusamba dengan besaran penghasilannya sekitar Rp 10.000.000 setiap bulannya;

ㅁ- Bahwa setahu saksi selama Pemohon dan Termohon pisah sekitar 5 bulan, Pemohon masih memberi nafkah kepada Termohon dan anaknya sekitar Rp. 2.000.000, tetapi sebelum punya anak Pemohon ngasih Rp 500.000,-

ㅂ- Bahwa saksi dan pihak keluarga keduanya sudah pernah merukunkannya, akan tetapi tidak berhasil;

2 XXXXX, setelah bersumpah memberikan keterangan yang pada pokoknya sebagai berikut :

Disclaimer
Kepaniteraan Mahkamah Agung Republik Indonesia berusaha untuk selalu mencantumkan informasi paling kini dan akurat sebagai bentuk komitmen Mahkamah Agung untuk pelayanan publik, transparansi dan akuntabilitas pelaksanaan fungsi peradilan. Namun dalam hal-hal tertentu masih dimungkinkan terjadi permasalahan teknis terkait dengan akurasi dan keterkinian informasi yang kami sajikan, hal mana akan terus kami perbaiki dari waktu kewaktu.
Dalam hal Anda menemukan inakurasi informasi yang termuat pada situs ini atau informasi yang seharusnya ada, namun belum tersedia, maka harap segera hubungi Kepaniteraan Mahkamah Agung RI melalui :
Email : kepaniteraan@mahkamahagung.go.id Telp : 021-384 3348 (ext.318)

- Bahwa saksi sebagai sepupu Termohon, mengetahui Pemohon dan Termohon adalah suami isteri;
- Bahwa setelah menikah keduanya tinggal bersama di rumah orang tua Termohon dan dari pernikahan Pemohon dengan Termohon sudah dikaruniai satu orang anak dan kini ikut dengan Termohon;
- Bahwa saksi tidak mendengar Pemohon dengan Termohon bertengkar namun anak saksi tidak mau diajak pindah di rumah orang tua Pemohon;
- Bahwa antara Pemohon dan Termohon tidak pernah kumpul lagi sejak bulan September 2020 Pemohon tinggal di rumahnya, sedangkan Termohon tinggal dengan orang tuanya;
- Bahwa Pemohon Pemohon bekerja di Bank Nusamba tetapi besaran penghasilannya saksi tidak mengetahuinya;
- Bahwa setahu saksi selama Pemohon dan Termohon pisah sekitar 2 bulan, Pemohon masih memberi nafkah kepada Termohon dan anaknya saksi tidak mengetahuinya,-
- Bahwa saksi mengetahui Pemohon dan Termohon mempunyai harta berupa satu unit mobil yang jenisnya saksi tidak mengetahuinya;
- Bahwa saksi dan pihak keluarga keduanya sudah pernah merukunkannya, akan tetapi tidak berhasil;

Atas keterangan 2 orang saksi tersebut Termohon membenarkannya dan Pemohon tidak membantahnya.

Bahwa para pihak tidak akan menyampaikan bukti lain dan pihak Pemohon menyampaikan kesimpulan tertulisnya sebagai berikut :

DALAM KONPENSI

1. Bahwa Pemohon menolak seluruh dalil-dalil Termohon, kecuali yang diakui secara tegas oleh Pemohon, Pemohon tetap berpegang teguh pada dalil-dalil permohonan pemohon, jawaban rekovensi, replik Pemohon dan mohon agar dalil-dalil tersebut dianggap terbaca kembali dan secara mutatis mutandis menjadi bagian tidak terpisahkan dari kesimpulan ini;

2. Bahwa untuk menguatkan dalil permohonan Pemohon telah mengajukan alat bukti surat diberi kode P-1 dan P-2 bermaterai cukup yang

Disclaimer
Kepaniteraan Mahkamah Agung Republik Indonesia berusaha untuk selalu mencantumkan informasi paling kini dan akurat sebagai bentuk komitmen Mahkamah Agung untuk pelayanan publik, transparansi dan akuntabilitas pelaksanaan fungsi peradilan. Namun dalam hal-hal tertentu masih dimungkinkan terjadi permasalahan teknis terkait dengan akurasi dan keterkinian informasi yang kami sajikan, hal mana akan terus kami perbaiki dari waktu kewaktu.
Dalam hal Anda menemukan inakurasi informasi yang termuat pada situs ini atau informasi yang seharusnya ada, namun belum tersedia, maka harap segera hubungi Kepaniteraan Mahkamah Agung RI melalui :
Email : kepaniteraan@mahkamahagung.go.id Telp : 021-384 3348 (ext.318)

telah diserahkan kepada Majelis hakim Pengadilan Agama Jepara dan bukti keterangan 2 saksi yang keterangan selengkapnya termuat dalam berita acara sidang yang di catat panitera;

3. Bahwa untuk menyangkal dalil permohonan Pemohon, Termohon telah menyampaikan jawaban, gugatan rekovensi, duplik, dan telah menyampaikan 4 bukti tertulis diberi kode

- T-1, : KTP Termohon (Copy dari aslinya)
- T- 2 , : Kartu Peserta BPJS (Copy dari copy)
- T-3, : Laporan keuangan (Copy dari copy)
- T-4 : Kartu Peserta Asuransi Manulife (Copy dari copy)

dan bukti 3 saksi bernama Mujahidah, Eko dan Fera yang keterangan selengkapnya termuat dalam berita acara sidang yang di catat panitera;

4. Bahwa pada prinsipnya Pemohon menolak seluruh dalil-dalil Termohon, kecuali yang diakui secara tegas oleh Pemohon.

5. Bahwa pada prinsipnya Pemohon menolak bukti T-2, T-3, T-4.

6. Bahwa bukti T-2, T-3, T-4 hanyalah berupa foto copy yang tidak ditunjukkan aslinya didepan persidangan, sehingga bukti tersebut tidak mempunyai kekuatan hukum oleh karena itu haruslah dikesampingkan.

7. Bahwa berdasarkan jawaban Termohon bukti P-1, T-2 dan bukti keterangan saksi – saksi Pemohon dan saksi-saksi Termohon **terbukti** bahwa antara Pemohon dan Termohon terikat hubungan suami-istri sah, sehingga Pemohon dan Termohon mempunyai kualitas hukum dalam perkara ini;

8. Bahwa berdasarkan bukti P - 2 dan bukti keterangan saksi-saksi Pemohon dan saksi-saksi Termohon **terbukti** bahwa antara Pemohon dan Termohon menjalani hidup bersama berumah tangga sebagai hubungan suami-istri di di Wilayah Hukum Kabupaten Jepara, karena itu Pengadilan Agama Jepara berwenang mengadili perkara aquo.

9. Bahwa berdasarkan bukti keterangan saksi-saksi Pemohon dan saksi-saksi Termohon **terbukti** bahwa antara Pemohon dan Termohon dalam hubungan rumah tangga telah terjadi perselisihan dan

Disclaimer
Kepaniteraan Mahkamah Agung Republik Indonesia berusaha untuk selalu mencantumkan informasi paling kini dan akurat sebagai bentuk komitmen Mahkamah Agung untuk pelayanan publik, transparansi dan akuntabilitas pelaksanaan fungsi peradilan. Namun dalam hal-hal tertentu masih dimungkinkan terjadi permasalahan teknis terkait dengan akurasi dan keterkinian informasi yang kami sajikan, hal mana akan terus kami perbaiki dari waktu kewaktu.
Dalam hal Anda menemukan inakurasi informasi yang termuat pada situs ini atau informasi yang seharusnya ada, namun belum tersedia, maka harap segera hubungi Kepaniteraan Mahkamah Agung RI melalui :
Email : kepaniteraan@mahkamahagung.go.id Telp : 021-384 3348 (ext.318)

pertengkaran secara terus-menerus yang disebabkan antara lain sebagaimana uraian yang didalilkan Pemohon.

10. Bahwa berdasarkan bukti keterangan saksi-saksi Pemohon **terbukti** bahwa antara Pemohon dan Termohon telah terjadi perpecahan rumah tangga sebagaimana didalilkan Pemohon

11. Bahwa berdasarkan bukti keterangan saksi-saksi Pemohon dan saksi saksi keluarga Termohon **terbukti** permasalahan rumah tangga antara Pemohon dan Termohon telah ada upaya musyawarah kekeluargaan agar Pemohon dan Termohon rukun kembali, namun upaya tersebut tidak berhasil;

12. Bahwa Pemohon tetap teguh hendak menceraikan Termohon;

DALAM GUGAT REKOPENSI

1. Bahwa Tergugat rekopensi menolak dan keberatan terhadap dalil-dalil gugatan rekopensi sebagaimana tersebut dalam gugatan rekopensi;
2. Bahwa Tergugat rekopensi hanya sanggup membayar Penggugat rekopensi

A. Mut’ah		: Rp. 1. 000.000,-
B. Nafkah lampau 1 bulan		: Rp. 400.000,-
C. Nafkah Idah 400.000 X 3 bulan		: Rp. 1. 200.000,-
D. Nafkah anak	1 anak	: Rp. 400.000,-
JUMLAH		Rp. 3. 000.000,-

Berdasarkan dalil-dalil replik tersebut di atas, Pemohon mohon kepada Ketua Majelis hakim yang memeriksa dan mengadili perkara ini

DALAM POKOK PERKARA

Mengabulkan permohonan Pemohon

DALAM REKOPENSI

1. Menolak gugatan Penggugat rekopensi
2. Menghukum Tergugat rekopensi menyerahkan pengembalian uang Rp.25.000.000,- setelah dikurangi untuk pembelian sepeda Motor honda beat warna putih Rp. 8.500.000,- sisa selebihnya Rp. 16.500.000,-

Disclaimer
Kepaniteraan Mahkamah Agung Republik Indonesia berusaha untuk selalu mencantumkan informasi paling kini dan akurat sebagai bentuk komitmen Mahkamah Agung untuk pelayanan publik, transparansi dan akuntabilitas pelaksanaan fungsi peradilan. Namun dalam hal-hal tertentu masih dimungkinkan terjadi permasalahan teknis terkait dengan akurasi dan keterkinian informasi yang kami sajikan, hal mana akan terus kami perbaiki dari waktu kewaktu.
Dalam hal Anda menemukan inakurasi informasi yang termuat pada situs ini atau informasi yang seharusnya ada, namun belum tersedia, maka harap segera hubungi Kepaniteraan Mahkamah Agung RI melalui :
Email : kepaniteraan@mahkamahagung.go.id Telp : 021-384 3348 (ext.318)

3. Menetapkan hak Penggugat rekopensi adalah sebagai berikut:

A.	Mut'ah	: Rp.	1. 000.000,-
B.	Nafkah lampau 1 bulan	: Rp.	400.000,-
C.	Nafkah Idah 400.000 X 3 bulan	: Rp.	1. 200.000,-
D.	Nafkah anak 1 anak	: Rp.	400.000,-
	JUMLAH	: Rp.	3. 000.000,-

ATAU : Apabila majelis hakim berpendapat lain mohon putusan yang seadil-adilnya

Bahwa kemudian pihak Termohon menyampaikan kesimpulan tertulisnya sebagai berikut :

I. FAKTA DAN ANALISIS PERSIDANGAN

1. Perkawinan PEMOHON/ TERGUGAT REKONPENSI dan TERMOHON/ PENGGUGAT REKONPENSI.
2. Hak TERMOHON/ PENGGUGAT REKONPENSI atas Nafkah Mut'ah, Mandiyah, dan Iddah
3. Hak atas Nafkah Anak (Biaya Hadhonah Anak)
4. Obyek Harta Bersama (Gono-Gini) PEMOHON/TERGUGAT REKONPENSI dan TERMOHON/PENGGUGAT REKONPENSI.
5. Alat Bukti PEMOHON/TERGUGAT REKONPENSI, tidak berdasar dan/atau justru memperkuat dalil TERMOHON/PENGGUGAT REKONPENSI.

II. KESIMPULAN

III. PERMOHONAN

Berikut uraian secara lengkap bagian perbagian atas kesimpulan yang kami buat, sebagai berikut:

I. FAKTA DAN ANALISIS PERSIDANGAN

1. Bahwa, hal–hal yang telah dikemukakan dalam jawaban/gugatan rekonpensi, duplik/replik rekonpensi mohon dianggap sebagai suatu kesatuan dan bagian yang tidak terpisahkan dari kesimpulan ini ;
2. Bahwa, Tergugat/Pengggugat Rekonpensi menolak dengan tegas seluruh dalil-dalil Gugatan, Replik/duplik Rekonpensi PEMOHON/TERGUGAT REKONPENSI, kecuali yang diakuinya secara tegas kebenarannya ;
3. Bahwa, pada intinya, perkara Nomor: 1480/Pdt.G/2020/PA.Jepr. adalah permohonan talak oleh PEMOHON/TERGUGAT REKONPENSI kepada TERMOHON/PENGGUGAT REKONPENSI. Kemudian dilanjutkan dengan gugatan rekonpensi pemenuhan ha katas nafkah mut'ah, iddah, dan mandiyah, pemenuhan hak atas nafkah anak (biaya

Disclaimer
Kepaniteraan Mahkamah Agung Republik Indonesia berusaha untuk selalu mencantumkan informasi paling kini dan akurat sebagai bentuk komitmen Mahkamah Agung untuk pelayanan publik, transparansi dan akuntabilitas pelaksanaan fungsi peradilan. Namun dalam hal-hal tertentu masih dimungkinkan terjadi permasalahan teknis terkait dengan akurasi dan keterkinian informasi yang kami sajikan, hal mana akan terus kami perbaiki dari waktu kewaktu.
Dalam hal Anda menemukan inakurasi informasi yang termuat pada situs ini atau informasi yang seharusnya ada, namun belum tersedia, maka harap segera hubungi Kepaniteraan Mahkamah Agung RI melalui :
Email : kepaniteraan@mahkamahagung.go.id Telp : 021-384 3348 (ext.318)

hadlonah anak), dan pembagian harta gono-gini TERMOHON/PENGGUGAT REKONPENSI kepada PEMOHON/TERGUGAT REKONPENSI.

4. Harta gono-gini TERMOHON/PENGGUGAT REKONPENSI kepada PEMOHON/TERGUGAT REKONPENSI berupa:

Satu unit mobil Suzuki Karimun, senilai Rp. 70.000.000,- (tujuh puluh juta rupiah).

Adalah merupakan harta yang dihasilkan selama perkawinan antara TERMOHON /PENGGUGAT REKONPENSI dengan PEMOHON/ TERGUGAT REKONPENSI;

5. Bahwa, berdasarkan alat bukti diatas (Baik alat bukti yang diajukan oleh PEMOHON/TERGUGAT REKONPENSI dan/ataupun yang diajukan oleh TERMOHON/ PENGGUGAT REKONPENSI memperkuat dalil-dalil TERMOHON/ PENGGUGAT REKONPENSI. Selanjutnya akan jika diuraikan secara runtut sebagai kesimpulan fakta yang terbukti di persidangan, sebagai berikut:

I.1. Perkawinan PEMOHON/TERGUGAT REKONPENSI dengan TERMOHON/PENGGUGAT REKONPENSI.

a. Bahwa, benar antara PEMOHON/ TERGUGAT REKONPENSI dengan TERMOHON/ PENGGUGAT REKONPENSI telah menikah secara resmi dihadapan Pejabat Pencatat Akta Nikah. Hal ini sesuai dengan bukti surat dalam gugatan konpensi yang diajukan oleh PEMOHON/ TERGUGAT ;

b. Bahwa, dalil terkait dengan Perkawinan PEMOHON/ TERGUGAT REKONPENSI dengan TERMOHON/ PENGGUGAT REKONPENSI diperkuat dengan keterangan saksi-saksi yang diajukan oleh PEMOHON/ TERGUGAT REKONPENSI maupun TERMOHON/ PENGGUGAT REKONPENSI, menerangkan pada pokoknya:

“ Benar antara PEMOHON/TERGUGAT REKONPENSI dengan TERMOHON/ PENGGUGAT REKONPENSI menikah secara resmi ”

c. Bahwa, berdasarkan keterangan saksi-saksi yang diajukan oleh TERMOHON/PENGGUGAT REKONSPENSI tidak benar jika rumah tangga PEMOHON/TERGUGAT REKONPENSI dengan TERMOHON/PENGGUGAT REKONPENSI sering terjadi cek-cok. Hal ini diperkuat keterangan saksi-saksi yang diajukan oleh PEMOHON/TERGUGAT REKONPENSI tidak benar jika rumah tangga PEMOHON/TERGUGAT REKONPENSI dengan TERMOHON/PENGGUGAT REKONPENSI sering terjadi cek-cok. Hanya saja terjadi perselisihan sejak bulan Agustus 2020, dan tidak benar tahun terjadi perselisihan sampai 7 bulan ;

Disclaimer
Kepaniteraan Mahkamah Agung Republik Indonesia berusaha untuk selalu mencantumkan informasi paling kini dan akurat sebagai bentuk komitmen Mahkamah Agung untuk pelayanan publik, transparansi dan akuntabilitas pelaksanaan fungsi peradilan. Namun dalam hal-hal tertentu masih dimungkinkan terjadi permasalahan teknis terkait dengan akurasi dan keterkinian informasi yang kami sajikan, hal mana akan terus kami perbaiki dari waktu kewaktu.
Dalam hal Anda menemukan inakurasi informasi yang termuat pada situs ini atau informasi yang seharusnya ada, namun belum tersedia, maka harap segera hubungi Kepaniteraan Mahkamah Agung RI melalui :
Email : kepaniteraan@mahkamahagung.go.id Telp : 021-384 3348 (ext.318)

Bahwa, syarat untuk gugatan cerai dengan alasan *syiqoq* adalah terjadinya perselisihan yang berulang-ulang dan tidak mungkin rukun lagi, sesuai dengan pasal 116 Huruf f Kompilasi Hukum Islam. Berdasarkan fakta yang terbukti dipersidangan tidak terpenuhi syarat tersebut ;

I.2. Hak TERMOHON/PENGGUGAT REKONPENSI atas Nafkah Mut'ah, Mandiyah, dan Iddah

a. Bahwa, jika Majelis Hakim Pemeriksa Perkara *Aquo* berpendapat lain dan berkeputusan perkawinan PEMOHON/TERGUGAT REKONPENSI dengan TERMOHON/PENGGUGAT REKONPENSI harus bercerai. HAK-HAK TERMOHON/PENGGUGAT REKONPENSI harus diberikan sesuai Pasal 149 huruf d Kompilasi Hukum Islam ;

b. Bahwa, dalam proses persidangan TERMOHON/ PENGGUGAT REKONPENSI telah menghadirkan alat bukti surat yang menguatkan dalil-dalil tentang hak atas nafkah mut'ah, nafkah mandiyah, dan nakah iddah. Berdasarkan bukti surat yang diajukan oleh TERMOHON/ PENGGUGAT REKONPENSI, saat ini PEMOHON/ TERGUGAT REKONPENSI bekerja sebagai karyawan tetap di PT. BPR Nusamba Pecangaan dengan penghasilan kurang lebih sekitar Rp. 5.000.000,- (lima juta rupia) setiap bulannya. Sehingga PEMOHON/ TERGUGAT REKONPENSI mempunyai kemampuan untuk memenuhi hak-hak tersebut.

Kemudian, fakta tentang kemampuan PEMOHON/ TERGUGAT REKONPENSI untuk membayar hak atas nafkah mut'ah, nafkah mandiyah, dan nakah iddah diperkuat dengan keterangan saksi-saksi yang diajukan oleh TERMOHON/ PENGGUGAT REKONPENSI ;

c. Bahwa, PEMOHON/ TERGUGAT REKONPENSI juga menghadirkan saksi-saksi yang memperkuat kemampuan PEMOHON/ TERGUGAT REKONPENSI untuk membayar hak atas nafkah mut'ah, nafkah mandiyah, dan nakah iddah ;

Maka, adalah wajar jika PEMOHON/ TERGUGAT REKONPENSI memenuhi hak-hak TERMOHON/ PENGGUGAT REKONPENSI atas nakah nafkah mut'ah, nafkah mandiyah, dan nakah iddah. Sesuai dengan Pasal 149 huruf d Kompilasi Hukum Islam ;

I.3. Hak atas Nafkah Anak (Biaya Hadhonah Anak)

a. Bahwa, jika Majelis Hakim Pemeriksa Perkara *Aquo* berpendapat lain dan berkeputusan perkawinan PEMOHON/ TERGUGAT REKONPENSI dengan TERMOHON/ PENGGUGAT REKONPENSI harus bercerai. HAK-HAK TERMOHON/ PENGGUGAT REKONPENSI harus diberikan salah satunya hak atas nakah anak (biaya hadhonah anak) sesuai Pasal 156 huruf d Kompilasi Hukum Islam ;

Disclaimer
Kepaniteraan Mahkamah Agung Republik Indonesia berusaha untuk selalu mencantumkan informasi paling kini dan akurat sebagai bentuk komitmen Mahkamah Agung untuk pelayanan publik, transparansi dan akuntabilitas pelaksanaan fungsi peradilan. Namun dalam hal-hal tertentu masih dimungkinkan terjadi permasalahan teknis terkait dengan akurasi dan keterkinian informasi yang kami sajikan, hal mana akan terus kami perbaiki dari waktu kewaktu.
Dalam hal Anda menemukan inakurasi informasi yang termuat pada situs ini atau informasi yang seharusnya ada, namun belum tersedia, maka harap segera hubungi Kepaniteraan Mahkamah Agung RI melalui :
Email : kepaniteraan@mahkamahagung.go.id   Telp : 021-384 3348 (ext.318)

b. Bahwa, dalam proses persidangan TERMOHON/ PENGGGUGAT REKONPENSI telah menghadirkan alat bukti surat yang menguatkan dalil-dalil tentang hak atas nafkah anak (biaya hadonah anak). Berdasarkan bukti surat yang diajukan oleh TERMOHON/PENGGUGAT REKONPENSI, saat ini PEMOHON/TERGUGAT REKONPENSI bekerja sebagai karyawan tetap di PT. BPR Nusamba Pecangaan dengan penghasilan kurang lebih sekitar Rp. 5.000.000,- (lima juta rupia) setiap bulannya.

Kemudian, fakta tentang pekerjaan PEMOHON/TERGUGAT REKONPENSI diperkuat dengan keterangan saksi-saksi yang diajukan oleh TERMOHON/PENGGUGAT REKONPENSI ;

c. Bahwa, Bahwa, PEMOHON/ TERGUGAT REKONPENSI TIDAK MEMBANTAH TENTANG PEKERJAAN DAN PENGHASILAN TERSEBUT.

Maka, adalah wajar jika PEMOHON/TERGUGAT REKONPENSI memberikan separoh gajinya tersebut untuk nafkah anak (biaya hadhonah anak) sesuai dengan ketentuan Pasal 156 huruf d Kompilasi Hukum Islam ;

I.4. Obyek Harta Bersama (Gono-Gini) PENGGUGAT dan TERGUGAT.

a. Bahwa, jika Majelis Hakim Pemeriksa Perkara *Aquo* berpendapat lain dan berkeputusan perkawinan PEMOHON/ TERGUGAT REKONPENSI dengan TERMOHON/ PENGGUGAT REKONPENSI harus bercerai. HAK-HAK TERMOHON/ PENGGUGAT REKONPENSI harus diberikan. Salah satunya ha katas harta bersama sebagaimana yang diajukan dalam gugatan rekonpensi TERMOHON/ PENGGUGAT REKONPENSI sesuai dengan pasal 37 Undang-Undang Nomor 10 (sepuluh) bulan 1974 Tentang Perkawinan Jo Pasal 97 Kompilasi Hukum Islam ;

b. Bahwa, dalam proses persidangan TERMOHON/PENGGUGAT REKONPENSI telah menghadirkan alat bukti surat yang menguatkan dalil-dalil tentang obyek harta bersama (gono-gini) PEMOHON/ TERGUGAT REKONPENSI dengan TERMOHON/ PENGGUGAT REKONPENSI. Dalil harta bersama tidak dibantah oleh PEMOHON/TERGUGAT REKONPENSI.

Kemudian, fakta tentang obyek harta bersama (gono-gini) diperkuat dengan keterangan saksi-saksi yang diajukan oleh TERMOHON/PENGGUGAT REKONPENSI ;

c. Bahwa, PEMOHON/ TERGUGAT REKONPENSI TIDAK MEMBANTAH TENTANG KEBERADAAN HARTA BERSAMA BERUPA MOBIL SUZUKI KARIMUN ;

Disclaimer
Kepaniteraan Mahkamah Agung Republik Indonesia berusaha untuk selalu mencantumkan informasi paling kini dan akurat sebagai bentuk komitmen Mahkamah Agung untuk pelayanan publik, transparansi dan akuntabilitas pelaksanaan fungsi peradilan. Namun dalam hal-hal tertentu masih dimungkinkan terjadi permasalahan teknis terkait dengan akurasi dan keterkinian informasi yang kami sajikan, hal mana akan terus kami perbaiki dari waktu kewaktu.
Dalam hal Anda menemukan inakurasi informasi yang termuat pada situs ini atau informasi yang seharusnya ada, namun belum tersedia, maka harap segera hubungi Kepaniteraan Mahkamah Agung RI melalui :
Email : kepaniteraan@mahkamahagung.go.id Telp : 021-384 3348 (ext.318)

Maka OBYEK HARTA BERSAMA SEBAGAIMANA TERSEBUT DALAM SURAT GUGATAN REKONPENSI YANG DAIJUKAN TERMOHON/PENGGUGAT REKONPENSI BENAR ADANYA.

I.5. Alat Bukti PEMOHON/TERGUGAT REKONPENSI justru memperkuat dalil TERMOHON/PENGGUGAT REKONPENSI.

Bahwa PEMOHON/ TERGUGAT REKONPENSI telah menyampaikan alat bukti surat atau tertulis. Alat bukti tulis tersebut justru memperkuat argumen TERMOHON/ PENGGUGAT REKONPENSI, khususnya pembuktian tentang hak atas nafkah mut'ah, nafkah mandiyah, dan nakah iddah, hak atas nafkah anak (biaya hadlonah anak), harta bersama milik PEMOHON/ TERGUGAT REKONPENSI dengan TERMOHON/ PENGGUGAT REKONPENSI ;

Bahwa PEMOHON/ TERGUGAT REKONPENSI telah menyampaikan saksi-saksi, justru memperkuat dalil tentang hak atas nafkah mut'ah, nafkah mandiyah, dan nakah iddah, hak atas nafkah anak (biaya hadlonah anak), harta bersama milik PEMOHON/ TERGUGAT REKONPENSI dengan TERMOHON/ PENGGUGAT REKONPENSI ;

II. KESIMPULAN

PENGGUGAT tetap berpegang pada dalil-dalil yang telah dinyatakan dalam Jawaban/Gugatan Rekonpensi dan Duplik/Replik Gugatan Rekonpensi. Mohon dipertimbangkan bunyi hadits Nabi Muhammad SAW, yang bunyinya:

"*Perbuatan halal yang paling dibenci Allah adalah cerai*." Diriwayatkan oleh Abu Dawud (2178)

Dalil-dalil yang diajukan oleh PEMOHON/TERGUGAT REKONPENSI tidak terbukti, khususnya dalil tentang keadaan syiqoq dalam rumah tangga PEMOHON/ TERGUGAT REKONPENSI dan TERMOHON/ PENGGUGAT REKONPENSI. Sehingga dalil gugatan PEMOHON/ TERGUGAT REKONPENSI tidak memiliki alasan yang dibenarkan oleh syar'i.

Akan tetapi jika majelis hakim berpendapat lain, perlu kiranya direnungkan hak-hak TERMOHON/ PENGGUGAT REKONPENSI untuk dilindungi. Terdapat hak atas nafkah mut'ah, nafkah mandiyah, dan nakah iddah, hak atas nafkah anak (biaya hadlonah anak), harta bersama milik PEMOHON/ TERGUGAT REKONPENSI dengan TERMOHON/ PENGGUGAT REKONPENSI .

Oleh karena merupakan sebuah kedzaliman apabila hak TERMOHON/ PENGGUGAT REKONPENSI tersebut di rampas. Untuk itu kebijaksanaan dari Majelis Hakim Pemeriksa Perkara *A quo,* TERMOHON/PENGGUGAT REKONPENSI berharap untuk mendapatkan keadilan yang seadil-adilnya.

Disclaimer
Kepaniteraan Mahkamah Agung Republik Indonesia berusaha untuk selalu mencantumkan informasi paling kini dan akurat sebagai bentuk komitmen Mahkamah Agung untuk pelayanan publik, transparansi dan akuntabilitas pelaksanaan fungsi peradilan. Namun dalam hal-hal tertentu masih dimungkinkan terjadi permasalahan teknis terkait dengan akurasi dan keterkinian informasi yang kami sajikan, hal mana akan terus kami perbaiki dari waktu kewaktu.
Dalam hal Anda menemukan inakurasi informasi yang termuat pada situs ini atau informasi yang seharusnya ada, namun belum tersedia, maka harap segera hubungi Kepaniteraan Mahkamah Agung RI melalui :
Email : kepaniteraan@mahkamahagung.go.id Telp : 021-384 3348 (ext.318)

III. PERMOHONAN

Bahwa berdasarkan uraian tersebut diatas, PENGGUGAT mohon kepada Yang Mulia Majelis Hakim Pemeriksa dan Pemutus Perkara *a quo* untuk berkenan memeriksa, mengadili dan menjatuhkan putusan sebagai berikut:

DALAM KONPENSI

Menolak permohonan cerai talak Pemohon untuk seruhnya ;

DALAM REKONPENSI

1. Mengabulkan gugatan rekonpensi Termohon/Penggugat Rekonpensi untuk seluruhnya ;
2. Menghukum Pemohon/Tergugat Rekonpensi untuk membayar nafkah mandiyah (nafkah terutang), nafkah idddah, dan nafkah mut'ah dengan total keseluruhan sejumlah Rp. 103.000.000,- (seratus tiga juta rupiah), dengan perincian;
   - **a.** Nafkah Mandiyah sejumlah Rp. 24.000.000,- (dua puluh empat juta rupiah).
   - **b.** Nafkah Idddah sejumlah Rp. 9.000.000,- (sembilan juta rupiah).
   - **c.** Nafkah Mut'ah sejumlah Rp. 70.000.000,- (tujuh puluh juta rupiah) ;
3. Menghukum Pemohon/Tergugat Rekonpensi untuk membayar biaya hadlonah pengasuhan anak/nafkah anak meliputi biaya hidup dan biaya pendidikan anak-anak Pemohon/Tergugat Rekonpensi dengan Termohon/Penggugat Rekonpensi sejak pendidikan usia dini sampai dengan jenjang kuliah dan mandiri ditetapkan ½ (seperdua/setengah) dari gaji Pemohon/Tergugat Rekonpensi sebagai karyawan tetap di PT. BPR NUSAMBA PECANGAAN dan memerintahkan PT. BPR NUSAMBA PECANGAAN untuk membagi gaji tersebut kemudian mentrasfernya dalam rekening tersendiri untuk diserahkan kepada Termohon/Penggugat Rekonpensi ;
4. Menetapkan uang sejumlah Rp.10.500.000,- (sepuluh juta lima ratus ribu rupiah) yang dipakai untuk membeli mobil karimun sebagai harta asal milik Termohon/Penggugat Rekonpensi ;
5. Menghukum Pemohon/Tergugat Rekonpensi untuk menyerahkan uang tersebut ;
6. Menetapkan uang senilai Rp. 50.000.000,- (lima puluh juta rupiah) yang saat ini sudah beralih menjadi Mobil Suzuki Karimun sebagai harta bersama Pemohon/Tergugat dengan Termohon/Penggugat Rekonpensi dan masing-masing berhak atas ½ (seperdua/setengah) bagian ;
7. Menghukum Pemohon/Tergugat Rekonpensi untuk menyerahkan apa yang menjadi hak dari Termohon/Penggugat Rekonpensi atas harta bersama dan harta asal yang dikuasai Pemohon/Tergugat Rekonpensi secara sukarela. Apabila tidak serahkan secara sukarela maka akan dilakukan upaya paksa melalui pengadilan melalui eksekusi dengan bantuan alat kelengkapan keamanan dari Negara ;

DALAM KONPENSI DAN REKONPENSI

Disclaimer
Kepaniteraan Mahkamah Agung Republik Indonesia berusaha untuk selalu mencantumkan informasi paling kini dan akurat sebagai bentuk komitmen Mahkamah Agung untuk pelayanan publik, transparansi dan akuntabilitas pelaksanaan fungsi peradilan. Namun dalam hal-hal tertentu masih dimungkinkan terjadi permasalahan teknis terkait dengan akurasi dan keterkinian informasi yang kami sajikan, hal mana akan terus kami perbaiki dari waktu kewaktu.
Dalam hal Anda menemukan inakurasi informasi yang termuat pada situs ini atau informasi yang seharusnya ada, namun belum tersedia, maka harap segera hubungi Kepaniteraan Mahkamah Agung RI melalui :
Email : kepaniteraan@mahkamahagung.go.id Telp : 021-384 3348 (ext.318)

Menghukum PEMOHON/ TERGUGAT REKONPENSI untuk membayar biaya perkara menurut hukum ;

Bahwa untuk mempersingkat uraian putusan ini, maka Berita Acara sidang tersebut dianggap telah termuat dan menjadi bagian dari Putusan ini;

PERTIMBANGAN HUKUM

Dalam Konvensi

Menimbang, bahwa maksud dan tujuan permohonan Pemohon, adalah seperti yang diuraikan di atas;

Menimbang, bahwa sebelum Hakim mempertimbangkan mengenai perkara a quo, terlebih dahulu Hakim akan mempertimbangkan *legal standing* Kuasa Hukum Pemohon Konvensi dan Termohon Konvensi untuk mewakili para pihak di dalam persidangan perkara a quo sebagai berikut:

Menimbang, bahwa Kuasa Hukum Pemohon dan Kuasa Hukum Termohon adalah Advokat yang telah mengangkat sumpah di hadapan sidang Pengadilan Tinggi dan Surat Kuasa Khusus yang diserahkan ke Pengadilan telah memenuhi syarat syarat sebagai Surat Kuasa Khusus, maka Kuasa Hukum Pemohon Konvensi tersebut mempunyai kedudukan hukum (legal standing) yang sah untuk mewakili dan atau mendampinginya sebagaimana maksud Pasal 4 ayat (1) Undang Undang Nomor 18 Tahun 2003 tentang Advokat; dan ternyata surat Kuasa Penggugat telah memenuhi syarat formil surat kuasa, maka penerima Kuasa berwenang mewakili Pemberi Kuasa di persidangan;

Menimbang, bahwa perkara ini adalah bidang perkawinan dan berdasarkan bukti P.1 telah terbukti bahwa Pemohon dan Termohon bertempat tinggal di wilayah hukum Pengadilan Agama Jepara, maka perkara ini baik secara absolut maupun relatif adalah wewenang Pengadilan Agama Jepara sebagaimana maksud Pasal 49 dan Pasal 66 ayat (2) Undang-Undang Nomor 7 tahun 1989 yang telah diubah dan ditambah dengan Undang-undang Nomor 3 tahun 2006 dan perubahan kedua dengan Undang-undang Nomor 50 tahun 2009 tentang Peradilan Agama, sehingga permohonan Pemohon dapat diterima untuk dipertimbangkan lebih lanjut ;

Disclaimer
Kepaniteraan Mahkamah Agung Republik Indonesia berusaha untuk selalu mencantumkan informasi paling kini dan akurat sebagai bentuk komitmen Mahkamah Agung untuk pelayanan publik, transparansi dan akuntabilitas pelaksanaan fungsi peradilan. Namun dalam hal-hal tertentu masih dimungkinkan terjadi permasalahan teknis terkait dengan akurasi dan keterkinian informasi yang kami sajikan, hal mana akan terus kami perbaiki dari waktu kewaktu.
Dalam hal Anda menemukan inakurasi informasi yang termuat pada situs ini atau informasi yang seharusnya ada, namun belum tersedia, maka harap segera hubungi Kepaniteraan Mahkamah Agung RI melalui :
Email : kepaniteraan@mahkamahagung.go.id Telp : 021-384 3348 (ext.318)

Menimbang, bahwa berdasarkan bukti P.2 yang berupa fotokopi Kutipan Akta Nikah (bukti akta outentik) yang telah dibubuhi meterai yang cukup dan telah dicocokkan dengan surat aslinya, ternyata cocok, maka telah terbukti bahwa antara Pemohon dengan Termohon adalah sebagai suami istri yang sah pernikahannya dicatatkan di Kantor Urusan Agama Kecamatan XXXXX, Kabupaten Jepara dan rumah tangga Pemohon dan Termohon tidak harmonis, sehingga Pemohon mempunyai kedudukan hukum (*legal standing)* dan berhak mengajukan perkara ini;

Menimbang, bahwa Hakim telah mendamaikan kepada para pihak yang berperkara namun tidak berhasil; sesuai dengan ketentuan Pasal 130 HIR juncto Pasal 82 Undang-undang Nomor 7 tahun 1989 yang telah diubah dengan Undang-undang Nomor 3 tahun 2006 dan perubahan kedua dengan Undang-undang Nomor 50 tahun 2009,;

Menimbang bahwa para pihak diperintahkan agar melakukan mediasi sesuai dengan ketentuan Pasal 4 dan Pasal 7 Peraturan Mahkamah Agung RI Nomor 1 tahun 2016 dan berdasarkan laporan mediator Chamidah, S.Ag. tanggal 14 September 2020, mediasi dinyatakan tidak berhasil mencapai kesepakatan;

Menimbang, bahwa pokok perkara ini adalah permohonan izin menjatuhkan talak dengan alasan perceraian sebagaimana diatur dalam penjelasan pasal 39 ayat (2) huruf (f), Undang-undang Nomor 10 (sepuluh) bulan 1974, juncto pasal 19 huruf (f) Perturan Pemerintah Nomor 9 tahun 1975 dan Pasal 116 huruf (f) Kompilasi Hukum Islam, yaitu sejak bulan Maret 2019 Pemohon dan Termohon sering terjadi perselisihan dan pertengkaran disebabkan masalah ekonomi keluarga yang tidak lancar dari Pemohon, dan adanya pihak ketuiga dalam rumah tangga Pemohon dan Termohon, akhirnya bulan Februari 2020 antara Pemohon dan Termohon tempat tinggal yang hingga sekarang telah berjalan selama 10 (sepuluh) bulan , Pemohon pergi meninggalkan kediaman bersama dan pulang ke rumah orang tuanya;

Menimbang, bahwa terhadap dalil permohonan Pemohon tersebut, Termohon membantah dalil permohonan Pemohon yang menyatakan rumah tangganya sudah tidak harmonis dan terjadi perselisihan dan pertengkaran

Disclaimer
Kepaniteraan Mahkamah Agung Republik Indonesia berusaha untuk selalu mencantumkan informasi paling kini dan akurat sebagai bentuk komitmen Mahkamah Agung untuk pelayanan publik, transparansi dan akuntabilitas pelaksanaan fungsi peradilan. Namun dalam hal-hal tertentu masih dimungkinkan terjadi permasalahan teknis terkait dengan akurasi dan keterkinian informasi yang kami sajikan, hal mana akan terus kami perbaiki dari waktu kewaktu.
Dalam hal Anda menemukan inakurasi informasi yang termuat pada situs ini atau informasi yang seharusnya ada, namun belum tersedia, maka harap segera hubungi Kepaniteraan Mahkamah Agung RI melalui :
Email : kepaniteraan@mahkamahagung.go.id Telp : 021-384 3348 (ext.318)

disebabkan karena Termohon sudah tidak taat pada Pemohon karena tidak melatyani Pemohon, namun yang sebenarnya Termohon masih melaksanakan kewajiban sebagai seorang istri yang baik, bahkan Pemohon telah membawa seorang perempuan yang telah dinikahi secara sirri tanpa sepengetahuan Termohon, akhirnya Pemohon yang keluar dari rumahnya dan kini tinggal tidak bersama dengan Termohon, sedangkan Termohon masih di rumahnya hingga sekarang bersama anaknya;

Menimbang, bahwa perkara ini adalah termasuk bidang perkawinan dalam perkara perceraian yang merupakan perkara khusus (*lex specialis*), apalagi dalil permohonan Pemohon dibantah oleh Termohon, maka Hakim berpendapat, bahwa Pemohon dan Termohon dibebani pembuktian;

Menimbang, bahwa Pemohon dan Termohon telah diberi kesempatan untuk mengajukan bukti-bukti dalam persidangan, oleh karenanya Pemohon telah mengajukan bukti surat berupa P.1 sampai P.2 serta 2 (dua) orang saksi yang berasal dari keluarga atau orang yang dekat dengan Pemohon sebagaimana terurai di atas;

Menimbang, bahwa alat-alat bukti yang diajukan oleh Pemohon dapat dipertimbangkan sebagai berikut;

Menimbang, bahwa oleh karena bukti P.1, dan P.2 berupa akta otentik, karena telah diberi meterai yang cukup dan telah dicocokkan dengan aslinya dan ternyata cocok, maka bukti tersebut secara formal telah memenuhi syarat sebagai alat bukti;

Menimbang, Termohon telah mengajukan bukti surat berupa T.1 sampai T.4 serta 2 (dua) orang saksi yang berasal dari keluarga atau orang yang dekat dengan Termohon sebagaimana terurai di atas;

Menimbang, bahwa alat-alat bukti yang diajukan oleh Pemohon dapat dipertimbangkan sebagai berikut;

Menimbang, bahwa oleh karena bukti T1, sampai T3 berupa akta otentik, karena telah diberi meterai yang cukup dan telah dicocokkan dengan aslinya dan ternyata cocok, maka bukti tersebut secara formal telah memenuhi syarat sebagai alat bukti;

Disclaimer
Kepaniteraan Mahkamah Agung Republik Indonesia berusaha untuk selalu mencantumkan informasi paling kini dan akurat sebagai bentuk komitmen Mahkamah Agung untuk pelayanan publik, transparansi dan akuntabilitas pelaksanaan fungsi peradilan. Namun dalam hal-hal tertentu masih dimungkinkan terjadi permasalahan teknis terkait dengan akurasi dan keterkinian informasi yang kami sajikan, hal mana akan terus kami perbaiki dari waktu kewaktu.
Dalam hal Anda menemukan inakurasi informasi yang termuat pada situs ini atau informasi yang seharusnya ada, namun belum tersedia, maka harap segera hubungi Kepaniteraan Mahkamah Agung RI melalui :
Email : kepaniteraan@mahkamahagung.go.id   Telp : 021-384 3348 (ext.318)

Menimbang, bahwa berdasarkan pasal 22 ayat (2) Perturan Pemerintah Nomor 9 tahun 1975, Hakimtelah mendengar keterangan keluarga atau orang yang dekat dengan kedua belah pihak yang berperkara, sehingga telah cukup jelas sebab-sebab perselisihan dan pertengkaran antara Pemohon dan Termohon ;

Menimbang, bahwa dua orang saksi yang diajukan oleh Pemohon di bawah sumpahnya telah memberikan keterangan sebagai apa yang dilihat dan atau didengar sendiri tentang rumah tangga Pemohon dan Termohon, yaitu pada bulan Maret 2019 antara Pemohon dengan Termohon terjadi pertengkaran yang disebabkan masalah ego masing-masing, Termohon tidak mau tinggal bersama dengan orang tua Pemohon begitu pula sebaliknya, akhirnya bulan Februari 2020 Pemohon dengan Termohon telah pisah tempat tinggal, Pemohon pergi meninggalkan kediaman rumah bersama dan sejak itu keduanya tidak pernah kumpul kembali, oleh karena keterangan saksi-saksi tersebut saling bersesuaian antara yang satu dengan lainnya maka Hakim berpendapat bahwa bukti saksi tersebut telah memenuhi syarat-syarat pembuktian dengan saksi sebagaimana ketentuan pasal 170, 171 dan 172 HIR., sehingga dalil permohonan Pemohon harus dinyatakan telah terbukti;

Menimbang, bahwa dua orang saksi yang diajukan oleh Termohon di bawah sumpahnya telah memberikan keterangan sebagai apa yang dilihat dan atau didengar sendiri tentang rumah tangga Pemohon dan Termohon, yaitu pada bulan Maret 2019 Pemohon dengan Termohon terjadi pertengkaran yang disebabkan Termohon tidak mau mengikuti dan memparhatikan Pemohon dalam hal tempat tinggal bersama dan sejak 10 (sepuluh) bulan Pemohon dengan Termohon telah pisah tempat tinggal, dan antara keduanya tidak pernah kembali satu sama lainnya;

Menimbang bahwa sejak Pemohon pergi meninggalkan kediaman bersamanya, sedangkan Termohon tidnggal bersama orang tuanya dan nafkah untuk Pemohon dan anaknya masih dikasih oleh Pemohon, kemudian setelah nya keduanya sudah tidak pernah kumpul kembali sekitar 10 (sepuluh) bulan; oleh karena keterangan saksi-saksi tersebut saling bersesuaian antara yang satu dengan lainnya maka Hakim berpendapat bahwa bukti saksi tersebut telah

Disclaimer
Kepaniteraan Mahkamah Agung Republik Indonesia berusaha untuk selalu mencantumkan informasi paling kini dan akurat sebagai bentuk komitmen Mahkamah Agung untuk pelayanan publik, transparansi dan akuntabilitas pelaksanaan fungsi peradilan. Namun dalam hal-hal tertentu masih dimungkinkan terjadi permasalahan teknis terkait dengan akurasi dan keterkinian informasi yang kami sajikan, hal mana akan terus kami perbaiki dari waktu kewaktu.
Dalam hal Anda menemukan inakurasi informasi yang termuat pada situs ini atau informasi yang seharusnya ada, namun belum tersedia, maka harap segera hubungi Kepaniteraan Mahkamah Agung RI melalui :
Email : kepaniteraan@mahkamahagung.go.id   Telp : 021-384 3348 (ext.318)

memenuhi syarat-syarat pembuktian dengan saksi sebagaimana ketentuan pasal 170, 171 dan 172 HIR., sehingga dalil permohonan Termohon harus dinyatakan telah terbukti;

Menimbang, bahwa berdasarkan alat-alat bukti tersebut di atas, maka dapat ditemukan fakta tentang rumah tangga Pemohon dan Termohon sebagai berikut :

- Bahwa Pemohon dan Termohon adalah suami istri yang sah menikah pada tanggal 23 Agustus 2017 yang dicatatkan di Kantor Urusan Agama Kecamatan XXXXX, Kabupaten Jepara dan telah dikarunai satu orang anak, yang hingga kini dua orang anak tersebut ikut dengan Termohon;
- Bahwa sejak bulan Maret 2019 Pemohon dengan Termohon sering terjadi pertengkaran disebabkan masalah tempat tinggal, masing-masing mempunyai ego yang kuat dimana Pemohon selaku suami tidak ada niatan untuk hidup secara mandiri tidak ikut dengan orang tuanya dan berpisah kediamannya sehingga keduanya dapat hidup tenteram dalam membina rumah tangganya;
- Bahwa antara Pemohon dengan Termohon telah pisah tempat tinggal yang sampai sekarang telah berlangsung lebih kurang 10 bulan, Pemohon pergi meninggalkan kediaman bersamanya;
- Bahwa Pemohon terakhir bekerja sebagai karyawan PT Bank Nusamba dengan penghasilan berkisar sejumlah Rp 6.000.000 (enam juta rupiah), setiap bulan;
- Bahwa selama berpisah, Pemohon masih memberikan nafkah lagi kepada Termohon dan anaknya setiap bulannya.
- Bahwa pihak keluarga dan orang dekatnya sudah mendamaikan Pemohon dan Termohon untuk menjaga kerukunan rumah tangga, namun tidak membuahkan hasil, keduanya tetap dengan pendirian.

Menimbang, bahwa Pemohon dalam persidangan menunjukkan sikap dan tekadnya akan menceraikan Termohon, walaupun telah diusahakan perdamaian, hal itu menunjukkan bahwa baik Pemohon maupun Termohon

Disclaimer
Kepaniteraan Mahkamah Agung Republik Indonesia berusaha untuk selalu mencantumkan informasi paling kini dan akurat sebagai bentuk komitmen Mahkamah Agung untuk pelayanan publik, transparansi dan akuntabilitas pelaksanaan fungsi peradilan. Namun dalam hal-hal tertentu masih dimungkinkan terjadi permasalahan teknis terkait dengan akurasi dan keterkinian informasi yang kami sajikan, hal mana akan terus kami perbaiki dari waktu kewaktu.
Dalam hal Anda menemukan inakurasi informasi yang termuat pada situs ini atau informasi yang seharusnya ada, namun belum tersedia, maka harap segera hubungi Kepaniteraan Mahkamah Agung RI melalui :
Email : kepaniteraan@mahkamahagung.go.id   Telp : 021-384 3348 (ext.318)

merasa tidak ada lagi kecocokan dalam rumah tangganya, karena rumah tangganya telah pecah;

Menimbang, bahwa apabila suatu rumah tangga (perkawinan) telah pecah, sebagaimana rumah tangga Pemohon dan Termohon, maka mawaddah wa rahmah dan tujuan perkawinan sebagaimana diamanatkan oleh pasal 1 Undang-undang Nomor 1 tahun 1974 Juncto Pasal 3 Kompilasi Hukum Islam tidak dapat diwujudkan, sehingga perkawinan tersebut tidak bermanfaat lagi dan sudah saatnya perkawinan itu diakhiri dengan perceraian. Sebab apabila tetap dipertahankan akan lebih banyak mendatangkan penderitaan lahir batin bagi Pemohon dan Termohon. Oleh karena itu penderitaan tersebut haruslah dihilangkan hal mana sesuai dengan Kaidah Fiqhiyah:

درء المفاسد مقدم على جلب المصالح

Artinya: ”Menolak Mafsadah lebih didahulukan daripada menarik kemaslahatan”

Menimbang, bahwa oleh karena antara Pemohon dengan Termohon telah pisah tempat lebih kurang 10 bulan dan tidak pernah kumpul rukun kembali, dan antara Pemohon dan Termohon telah didamaikan baik secara langsung oleh Hakim maupun melalui mediator, maka Hakim berpendapat bahwa antara Pemohon dan Termohon telah terjadi perselisihan dan pertengkaran sifatnya terus menerus, tidak ada harapan lagi untuk kembali hidup rukun dalam rumah tangganya;

Menimbang, bahwa alasan perceraian sebagaimana yang dimaksud Pasal 19 huruf (f) Peraturan Pemerintah Nomor 9 tahun 1975 dan pasal 116 huruf (f) Kompilasi Hukum Islam adalah antara suami dan isteri terus-menerus terjadi perselisihan dan pertengkaran dan tidak ada harapan akan hidup lagi dalam rumah tangga dan rumah tangga antara Pemohon dan Termohon telah terjadi perselisihan dan pertengkaran yang sifatnya terus menerus dan tidak ada harapan akan hidup lagi dalam rumah tangga, sehingga antara Pemohon dan Termohon terdapat alasan perceraian sebagaimana maksud pasal tersebut;

Menimbang, bahwa berdasarkan pertimbangan-pertimbangan tersebut di atas, maka permohonan Pemohon terbukti cukup beralasan dan telah

Disclaimer
Kepaniteraan Mahkamah Agung Republik Indonesia berusaha untuk selalu mencantumkan informasi paling kini dan akurat sebagai bentuk komitmen Mahkamah Agung untuk pelayanan publik, transparansi dan akuntabilitas pelaksanaan fungsi peradilan. Namun dalam hal-hal tertentu masih dimungkinkan terjadi permasalahan teknis terkait dengan akurasi dan keterkinian informasi yang kami sajikan, hal mana akan terus kami perbaiki dari waktu kewaktu.
Dalam hal Anda menemukan inakurasi informasi yang termuat pada situs ini atau informasi yang seharusnya ada, namun belum tersedia, maka harap segera hubungi Kepaniteraan Mahkamah Agung RI melalui :
Email : kepaniteraan@mahkamahagung.go.id Telp : 021-384 3348 (ext.318)

memenuhi ketentuan pasal 39 Undang-undang Nomor 1 tahun 1974, jo. Pasal 19 huruf (f) Peraturan Pemerintah Nomor 9 tahun 1975 dan pasal 116 huruf (f) Kompilasi Hukum Islam, oleh karena itu permohonan Pemohon dapat dikabulkan;

Menimbang, bahwa antara Pemohon dan Termohon ba'da dukhul dan selama perkawinan belum pernah bercerai, maka perlu ditetapkan bahwa izin ikrar talak yang diberikan oleh Pengadilan kepada Pemohon terhadap Termohon adalah talak satu raj'i sebagaimana maksud Pasal 118 Kompilasi hukum Islam;

Dalam Rekonvensi

Menimbang, bahwa Termohon Konvensi/ Penggugat Rekonvensi menyampaikan jawaban terhadap permohonan Pemohon Konvensi / Tergugat Rekonvensi dan sekaligus mengajukan pula gugatan Rekonvensi seperti apa yang telah diuraikan di atas;

Menimbang, bahwa apa yang telah dipertimbangkan dalam Konvensi menjadi pertimbangan pula dalam Rekonvensi;

Menimbang, bahwa gugatan Penggugat Rekonvensi adalah sebagai berikut:

1. Menghukum Tergugat Rekonvensi untuk membayar nafkah lampau hingga perkara ini berkekuatan hukum tetap, yang diperhitungkan selama 8 bulan Termohon Konvensi lalai kewajiban, sehingga nilainya sebesar Rp 24.000.000,;
2. Menghukum Termohon Rekonvensi membayar nafkah iddah sebesar Rp 3.000.000/bulan x 3 bulan = Rp 9.000.000;
3. Menghukum Termohon Rekonvensi membayar nafkah mut'ah sebear Rp 70.000.000 (tujuh puluh juta rupiah);
4. Menetapkan pemegang hak *hadhanah* (pengasuhan dan pemeliharaan anak) kepada Penggugat Rekonvensi atas 1 anak bernama XXXXX, laki-laki umur 2 tahun, dan demi hukum kekuasaan orang tua dilaksanakan oleh Penggugat Rekonvensi, serta menurut hukum kewajiban Tergugat Rekonvensi terhadap anaknya mengenai pendidikan dan pemeliharaan tetap berlangsung hingga anak berusia dewasa sebesar Rp

Disclaimer
Kepaniteraan Mahkamah Agung Republik Indonesia berusaha untuk selalu mencantumkan informasi paling kini dan akurat sebagai bentuk komitmen Mahkamah Agung untuk pelayanan publik, transparansi dan akuntabilitas pelaksanaan fungsi peradilan. Namun dalam hal-hal tertentu masih dimungkinkan terjadi permasalahan teknis terkait dengan akurasi dan keterkinian informasi yang kami sajikan, hal mana akan terus kami perbaiki dari waktu kewaktu.
Dalam hal Anda menemukan inakurasi informasi yang termuat pada situs ini atau informasi yang seharusnya ada, namun belum tersedia, maka harap segera hubungi Kepaniteraan Mahkamah Agung RI melalui :
Email : kepaniteraan@mahkamahagung.go.id Telp : 021-384 3348 (ext.318)

3.000.000/ setengah dari penghasilan Pemohon sebagai pegawai PT Bank Nusamba/ bulannya dengan kenaikan 10% setiap tahun;

Menimbang, bahwa atas gugatan balik Penggugat Rekonvensi tersebut, Tergugat Rekonvensi memberikan jawaban yang pada pokoknya sebagai berikut :

1. Mengabulkan Gugatan Rekonpensi Penggugat Rekonpensi untuk sebagian;
2. Menghukum Tergugat Rekonpensi untuk membayar kepada Penggugat Rekonpensi berupa :
   a. Nafkah Madiyah sebesar : Rp.400.000,00 (empat ratus ribu rupiah).
   b. Nafkah Iddah selama 3 bulan, sebesar : Rp.1.200.000,00 (satu juta dua ratus ribu rupiah).
   c. Mut'ah sebesar : Rp.1.000.000,00 (satu juta rupiah).
   d. Nafkah 1 orang anak sebesar Rp. 400.000,00 (empat ratus ribu rupiah) /bulan.
   e. Mengembalikan sisa penjualan motor sebesar Rp. 16.500.000,00 (enam belas juta lima ratus ribu rupiah).
2. Membebankan biaya perkara menurut hukum yang berlaku.

Atau Apabila Hakim berpendapat lain, maka mohon putusan yang seadil-adilnya.

Menimbang, bahwa terhadap gugatan Penggugat Rekonvensi yang mohon agar Tergugat Rekonvensi membayar kepada Penggugat Rekonvensi, dapat dipertimbangkan sebagai berikut;

Nafkah madliyah

Menimbang, bahwa oleh karena selama Penggugat Rekonvensi dengan Tergugat Rekonvensi pisah tempat tinggal selama 10 bulan, dan Tergugat Rekonvensi mengaku masih memberikan nafkah kepada Penggugat Rekonvensi namun jarang-jarang, maka kewajiban Tergugat Rekonvensi memberikan nafkah kepada Penggugat Rekonvensi yang dilalaikan tersebut menjadi hutang bagi Tergugat Rekonvensi;

Disclaimer
Kepaniteraan Mahkamah Agung Republik Indonesia berusaha untuk selalu mencantumkan informasi paling kini dan akurat sebagai bentuk komitmen Mahkamah Agung untuk pelayanan publik, transparansi dan akuntabilitas pelaksanaan fungsi peradilan. Namun dalam hal-hal tertentu masih dimungkinkan terjadi permasalahan teknis terkait dengan akurasi dan keterkinian informasi yang kami sajikan, hal mana akan terus kami perbaiki dari waktu kewaktu.
Dalam hal Anda menemukan inakurasi informasi yang termuat pada situs ini atau informasi yang seharusnya ada, namun belum tersedia, maka harap segera hubungi Kepaniteraan Mahkamah Agung RI melalui :
Email : kepaniteraan@mahkamahagung.go.id   Telp : 021-384 3348 (ext.318)

Menimbang, bahwa saat ini diakui pula penghasilan Tergugat Rekonvensi berkisar Rp 6.000.000,00 per bulannya dan tidak ada lagi penghasilan lain;

Menimbang, bahwa sejak Penggugat rekonvensi dengan Tergugat rekonvensi telah terjadi pisah tempat tinggal yang sampai perkara ini diajukan di Pengadilan Agama Jepara dan selama itu pula Tergugat Rekonvensi tidak memberikan nafkah kepada Penggugat Rekonvensi, maka sesuai dengan kepatutan dan kelayakan serta dengan mempertimbangnkan sosial ekonomi Tergugat Rekonvensi tersebut, atas dasar pengakuan Tergugat tersebut, maka Hakim menghukum Tergugat Rekonvensi untuk membayar kepada Penggugat Rekonvensi nafkah madliyah yang terhutang selama 10 bulan sejumlah Rp10.500.000,00 (sepuluh juta lima ratus ribu rupiah);

Menimbang bahwa untuk kemaslahatan kedua belah pihak maka Hakim berpendapat Tergugat Rekonvensi dianggap mampu untuk memenuhi tuntutan Penggugat Rekonvensi yang akan dituangkan kedalam amar putusan ini, karena jika Tergugat Rekonvensi dihukum untuk membayar seperti yang diajukan Penggugat Rekonvensi, tentu tidak akan mampu membayarnya, karena putusan yang baik adalah adanya kepastian hukum, kemamfaatan dan keadilan bagi para pihak.

Nafkah iddah

Menimbang, bahwa terhadap gugatan Penggugat Rekonvensi yang memohon agar Tergugat Rekonvensi dihukum membayar kepada Penggugat Rekonvensi Nafkah Iddah selama selama tiga bulan sebesar Rp 9.000.000,00 (sembilan juta rupiah), dapat dipertimbangkan sebagai berikut;

Menimbang, bahwa sesuai dengan ketentuan Pasal 149 huruf (b) Kompilasi Hukum Islam, apabila perkawinan putus karena talak, maka bekas suami wajib memberi nafkah kepada bekas Istri kecuali bekas istri telah dijatuhi talak bain atau nuzus;

Menimbang, bahwa oleh karena Penggugat Rekonvensi dengan Tergugat Rekonvensi terakhir perkawinan tinggal di rumah bersama, meskipun terjadi kurang harmonis sekitar 10 tahun yang lalu; karena Tergugat Rekonvensi kurang dalam memenuhi kebutuhan sehari-hari bahkan sejak sekitar 10 bulan,

Disclaimer
Kepaniteraan Mahkamah Agung Republik Indonesia berusaha untuk selalu mencantumkan informasi paling kini dan akurat sebagai bentuk komitmen Mahkamah Agung untuk pelayanan publik, transparansi dan akuntabilitas pelaksanaan fungsi peradilan. Namun dalam hal-hal tertentu masih dimungkinkan terjadi permasalahan teknis terkait dengan akurasi dan keterkinian informasi yang kami sajikan, hal mana akan terus kami perbaiki dari waktu kewaktu.
Dalam hal Anda menemukan inakurasi informasi yang termuat pada situs ini atau informasi yang seharusnya ada, namun belum tersedia, maka harap segera hubungi Kepaniteraan Mahkamah Agung RI melalui :
Email : kepaniteraan@mahkamahagung.go.id Telp : 021-384 3348 (ext.318)

Tergugat Rekonvensi memilih tinggal di tinggal di rumah orang tuanya, bahkan telah dikuatkan oleh saksinya; dalam hal ini Tergugat Rekonvensi yang mengajukan perceraian, maka hakim berpendapat bahwa Penggugat Rekonvensi tidak terbukti berbuat nuzus;

Menimbang, bahwa oleh karena Penggugat Rekonvensi yang akan dijatuhi Talak satu raji oleh Tergugat Rekonvensi dalam keadaan tidak nuzus, maka Tergugat Rekonvensi berkewajiban memberikan nafkah iddah kepada Penggugat Rekonvensi, hal maha sesuai pula dengan dalil dalam Kitab Al-Iqna' Juz II, halaman 118 yang berbunyi:

ويجب للمعتدة الرجعية السكنى والنفقة

Artinya: dan wajib bagi perempuan yang ditalak Raji selama menjalani iddah mendapatkan dari suaminya tempat tinggal dan nafkah;

Menimbang, bahwa dengan pertimbangan-pertimbangan tersebut di atas, maka hakim mengabulkan gugatan Penggugat Rekonvensi untuk sebagian dengan menghukum kepada Tergugat Rekonvensi untuk membayar nafkah selama masa iddah kepada Penggugat Rekonvensi sebesar Rp 4.500.000,00- (empat juta lima ratus ribu rupiah) ;

Tentang mutah

Menimbang, bahwa sesuai dengan ketentuan Pasal 149 huruf (a) Kompilasi Hukum Islam, bilamana perkawinan putus karena cerai talak, maka bekas suami wajib memberikan mut'ah yang layak kepada bekas istrinya baik berupa uang atau benda, kecuali bekas istri tersebut qola al-dukhul;

Menimbang, bahwa beban mutah yang harus dibayar oleh Tergugat Rekonvensi kepada Penggugat Rekonvensi dinyatakan memberatkan pihak Tergugat, maka pihak Penggugat menyerahkan permintaan mutah diserahkan kepada Pengadilan, maka berdasarkan hal tersebut Hakim dapat mempertimbangkan tuntutan mengenai pemberian mutah sebagian sesuai dengan kemampuan Tergugat Rekonvensi, dan karena pada dasarnya mutah yang diberikan suami istri dalam perceraian lebih kurang sebanding dengan mahar yang diberikan ketika pernikahan terjadi, dan Pengguguat telah mengabdi terhadap Terguguat Rekonvensi sekitar 3 tahun, karenanya mutah tersebut harus dibayarkan sejumah Rp, 6.000.000,00 (enam juta rupiah);

Disclaimer
Kepaniteraan Mahkamah Agung Republik Indonesia berusaha untuk selalu mencantumkan informasi paling kini dan akurat sebagai bentuk komitmen Mahkamah Agung untuk pelayanan publik, transparansi dan akuntabilitas pelaksanaan fungsi peradilan. Namun dalam hal-hal tertentu masih dimungkinkan terjadi permasalahan teknis terkait dengan akurasi dan keterkinian informasi yang kami sajikan, hal mana akan terus kami perbaiki dari waktu kewaktu.
Dalam hal Anda menemukan inakurasi informasi yang termuat pada situs ini atau informasi yang seharusnya ada, namun belum tersedia, maka harap segera hubungi Kepaniteraan Mahkamah Agung RI melalui :
Email : kepaniteraan@mahkamahagung.go.id Telp : 021-384 3348 (ext.318)

Tentang Hadlanah

Menimbang, bahwa terhadap gugatan Penggugat Rekonvensi yang memohon agar Pengadilan Agama menetapkan Penggugat Rekonvensi sebagai pemegang hak hadlanah (pemeliharaan) terhadap satu orang anak bernama XXXXX, laki-laki umur 2 tahun, Pengadilan mempertimbangkan sebagai berikut;

Menimbang, bahwa sesuai ketentuan pasal 105 huruf (a) Kompilasi Hukum Islam Indonesi, anak yang belum mumayyiz atau belum berumur 12 tahun berada dalam pemeliharaan ibunya hingga anak tersebut dapat menentukan pilihan mereka; dengan ketentuan Penggugat Rekonvensi berkewajiban memberikan akses kepada Tergugat Rekonvensi untuk bertemu dengan anak tersebut;

Menimbang, bahwa seorang ibu yang akan melaksanakan tugas memelihara anak (*hadlonah*) haruslah memenuhi syarat-syarat tujuh macam yaitu berakal sehat, merdeka, beragama Islam, sederhana, amanah, tinggal di daerah tertentu dan tidak bersuami baru. Apabila syarat-syarat tersebut kurang, maka gugurlah hak hadlonah dari tangan ibu, hal mana tersebut dalam Kitab Kifayatul Akhyar Juz II halaman 94 yang diambil alih sebagai pendapat Hakim yang berbunyi sebagai berikut:

وشرائط الحضانة سبع : العقل والحريّة والدّين والعفة والأمانة والأقامة فى بلد المميّز والخلوّ من زوج. فإن اختلّ شرط منها أي السبعة فى الأم سقطت حضانتها

Arttinya:

Syarat-syarat bagi orang yang akan melaksanakan tugas hadlonah ada tujuh macam: berakal sehat, merdeka, beragama Islam, sederhana, amanah, tinggal di daerah tertentu dan tidak bersuami baru. Apabila kurang satu diantara syarat-syarat tersebut gugur hak hadlonah dari tangan ibu.

Disclaimer
Kepaniteraan Mahkamah Agung Republik Indonesia berusaha untuk selalu mencantumkan informasi paling kini dan akurat sebagai bentuk komitmen Mahkamah Agung untuk pelayanan publik, transparansi dan akuntabilitas pelaksanaan fungsi peradilan. Namun dalam hal-hal tertentu masih dimungkinkan terjadi permasalahan teknis terkait dengan akurasi dan keterkinian informasi yang kami sajikan, hal mana akan terus kami perbaiki dari waktu kewaktu.
Dalam hal Anda menemukan inakurasi informasi yang termuat pada situs ini atau informasi yang seharusnya ada, namun belum tersedia, maka harap segera hubungi Kepaniteraan Mahkamah Agung RI melalui :
Email : kepaniteraan@mahkamahagung.go.id Telp : 021-384 3348 (ext.318)

Tentang Nafkah Anak

Menimbang, bahwa Penggugat Rekonvensi menuntut nafkah untuk 1 orang anak bernama XXXXX, laki-laki umur 2 tahun sebesar Rp. 3.000.000,- (tiga juta rupiah) setiap bulan ditambah 10 % setiap tahunnya;

Menimbang, bahwa tuntutan nafkah anak Penggugat telah sesuai dengan Pasal 24 ayat 2 huruf (a) Peraturan Pemerintah Nomor 9 Tahun 1975 jo. Pasal 156 huruf d KHI, oleh karena itu tuntutan Penggugat berdasarkan hukum dan patut untuk dipertimbangkan;

Menimbang, bahwa berdasarkan fakta di persidangan Penggugat dan Tergugat memiliki 1 orang anak yang bernama XXXXX, laki-laki umur 2 tahun, dan saat ini diasuh oleh Penggugat Rekonvensi, sedangkan Tergugat bekerja sebagai karyawan Bank Nusamba dengan penghasilan sejumlah kurang lebih 6.000.000,00 per bulannya;

Menimbang, bahwa dalam menentukan besar nafkah anak, Hakim menilai nafkah anak harus memenuhi kebutuhan hidup minimum, sesuai dengan kepatutan dan keadilan;

Menimbang, bahwa anak Penggugat dan Tergugat yang bernama XXXXX, laki-laki umur 2 tahun, oleh karena itu kebutuhan hidup minimal bagi anak tersebut mencakup makanan, minuman, susu, vitamin, dan pakaian;

Menimbang, bahwa dengan mempertimbangkan penghasilan Tergugat, dan kebutuhan hidup minimum anak, sesuai dengan kemampuan, kepatutan dan keadilan, Hakim menetapkan bahwa jumlah nafkah anak yang ditanggung oleh Tergugat Rekonvensi adalah minimal sebesar Rp 1.000.000,00 (satu juta rupiah) per bulan, di luar biaya pendidikan dan kesehatan sampai dengan anak tersebut dewasa atau mandiri;

Menimbang, bahwa terkait dengan nafkah anak pasca perceraian, berdasarkan Pasal 41 huruf b Undang-Undang Nomor 1 tahun 1974, apabila penghasilan Tergugat dalam kenyataan tidak dapat memenuhi kewajiban nafkah anak, maka Penggugat dapat ikut memikul biaya nafkah anak tersebut;

Menimbang, bahwa untuk menyesuaikan dengan tingkat inflasi di masa mendatang, serta kebutuhan anak yang semakin bertambah seiring dengan pertumbuhan dan perkembangan anak, maka besar nafkah anak yang

Disclaimer
Kepaniteraan Mahkamah Agung Republik Indonesia berusaha untuk selalu mencantumkan informasi paling kini dan akurat sebagai bentuk komitmen Mahkamah Agung untuk pelayanan publik, transparansi dan akuntabilitas pelaksanaan fungsi peradilan. Namun dalam hal-hal tertentu masih dimungkinkan terjadi permasalahan teknis terkait dengan akurasi dan keterkinian informasi yang kami sajikan, hal mana akan terus kami perbaiki dari waktu kewaktu.
Dalam hal Anda menemukan inakurasi informasi yang termuat pada situs ini atau informasi yang seharusnya ada, namun belum tersedia, maka harap segera hubungi Kepaniteraan Mahkamah Agung RI melalui :
Email : kepaniteraan@mahkamahagung.go.id   Telp : 021-384 3348 (ext.318)

ditanggung oleh Tergugat perlu dinaikkan 10 persen setiap tahunnya sesuai dengan SEMA No. 3 Tahun 2015;

Menimbang, bahwa berdasarkan pertimbangan-pertimbangan di atas, maka gugatan nafkah anak Penggugat patut dikabulkan, dengan menghukum Tergugat untuk membayar nafkah untuk 1 orang anak tersebut minimal sebesar Rp 1.000.000,00 (satu juta rupiah) per bulan dengan kenaikan 10 persen per tahun, di luar biaya pendidikan dan kesehatan sampai dengan anak tersebut dewasa atau mandiri, melalui Penggugat ;

Menimbang, bahwa berdasarkan Surat Edaran Mahkamah Agung Nomor 10 (sepuluh) bulan 2017 tanggal 10 April 2019, dalam rangka pelaksanaan Peraturan Mahkamah Agung Nomor 3 tahun 2017 tentang Pedoman Mengadili Perempuan Berhadapan dengan Hukum, ditentukan bahwa untuk melindungi hak-hak perempuan pasca perceraian, pembayaran kewajiban akibat perceraian harus dibayar sebelum pengucapan ikrar talak, maka Mut'ah, nafkah iidah dan nafkah anak harus dibayar oleh Tergugat Rekonvensi sebelum pengucapan ikrar talak;

Tentang Harta Bersama

Menimbang, bahwa dalil gugatan Penggugat Rekonvensi adalah mohon agar harta sebagaimana tertuang pada petitum angka 4 (empat) c gugatan Penggugat Rekonvensi, ditetapkan sebagai harta bersama Penggugat Rekonvensi dengan Tergugat Rekonvensi yang harus dibagi Pengugat Rekonvensi dan Tergugat Rekonvensi masing-masing seperdua bagian sama besar;

Menimbang, bahwa atas gugatan Rekonvensi Penggugat Rekonvensi tersebut Tergugat Rekonvensi memberikan jawaban yang pada pokoknya menyatakan sebagai berikut:

Menimbang, bahwa oleh karana dalil gugatan Penggugat Rekonvensi banyak yang dibantah oleh Tergugat Rekonvensi, maka baik Penggugat Rekonvensi maupun Tergugat Rekonvensi dibebani pembuktian;

Menimbang, bahwa Penggugat Rekonvensi dan Tergugat Rekonvensi telah diberi kesempatan untuk mengajukan dalil gugatannya sebagaimana telah terurai di atas ;

Disclaimer
Kepaniteraan Mahkamah Agung Republik Indonesia berusaha untuk selalu mencantumkan informasi paling kini dan akurat sebagai bentuk komitmen Mahkamah Agung untuk pelayanan publik, transparansi dan akuntabilitas pelaksanaan fungsi peradilan. Namun dalam hal-hal tertentu masih dimungkinkan terjadi permasalahan teknis terkait dengan akurasi dan keterkinian informasi yang kami sajikan, hal mana akan terus kami perbaiki dari waktu kewaktu.
Dalam hal Anda menemukan inakurasi informasi yang termuat pada situs ini atau informasi yang seharusnya ada, namun belum tersedia, maka harap segera hubungi Kepaniteraan Mahkamah Agung RI melalui :
Email : kepaniteraan@mahkamahagung.go.id   Telp : 021-384 3348 (ext.318)

Menimbang, bahwa terhadap dalil gugatan Penggugat Rekonvensi yang menyatakan bahwa selama perkawinan antara Penggugat Rekonvensi dengan Tergugat Rekonvensi mempunyai satu unit kendaraan roda empat merk Suzuki, oleh karena tidak ada bukti yang mendukung terhadap barang diajukan oleh Penggugat Rekonvensi untuk untuk membuktikan dalil gugatan tersebut, dan ternyata tidak terbukti dan tidak beralasan, oleh karenanya gugatan Penggugat Rekonvensi tersebut harus ditolak;

Menimbang, bahwa sesuai Pasal 1888 BW dan Yurisprudensi MARI No.3609 K/Pdt/1985 Jo. Putusan Mahkamah Agung No. 112 K/Pdt/1996. yang menyatakan bahwa surat bukti foto copy yang tidak pernah diajukan atau tidak pernah ada surat aslinya, maka terhadap bukti surat ini tidak mempunyai nilai bukti dan karenanya tidak dapat dipertimbangkan;

Menimbang, bahwa hal-hal yang tidak dipertimbangkan dalam perkara ini dinyatakan di kesampingkan ;

Dalam Konvensi dan Rekonvensi

Menimbang, bahwa berdasarkan pasal 89 ayat 1 Undang-undang Nomor 7 tahun 1989 yang telah diubah dengan Undang-undang Nomor 3 tahun 2006 dan perubahan kedua dengan Undang-undang Nomor 50 tahun 2009, maka biaya perkara dibebankan kepada Pemohon Konvensi/ Tergugat Rekonvensi;

Mengingat segala ketentuan Hukum Islam dan peraturan perundangan yang berkaitan dengan perkara ini;

M E N G A D I L I

Dalam Konvensi

1. Mengabulkan pemohonan Pemohon;

2. Memberi izin kepada Pemohon (XXXXX) untuk menjatuhkan talak satu raji terhadap Termohon (XXXXX) di depan sidang Pengadilan Agama Jepara;

Dalam Rekonvensi

1. Mengabulkan gugatan Penggugat Rekonvensi sebagian;

Disclaimer
Kepaniteraan Mahkamah Agung Republik Indonesia berusaha untuk selalu mencantumkan informasi paling kini dan akurat sebagai bentuk komitmen Mahkamah Agung untuk pelayanan publik, transparansi dan akuntabilitas pelaksanaan fungsi peradilan. Namun dalam hal-hal tertentu masih dimungkinkan terjadi permasalahan teknis terkait dengan akurasi dan keterkinian informasi yang kami sajikan, hal mana akan terus kami perbaiki dari waktu kewaktu.
Dalam hal Anda menemukan inakurasi informasi yang termuat pada situs ini atau informasi yang seharusnya ada, namun belum tersedia, maka harap segera hubungi Kepaniteraan Mahkamah Agung RI melalui :
Email : kepaniteraan@mahkamahagung.go.id   Telp : 021-384 3348 (ext.318)

2. Menghukum Tergugat Rekonvensi untuk membayar kepada Penggugat Rekonvensi sebagai berikut :

2.1. Nafkah madliyah selama 10 bulan sejumlah Rp10.500.000,00 (sepuluh juta lima ratus ribu rupiah);

2.2. Nafkah selama masa iddah (3 bulan) sejumlah Rp 4.500.000,00 (empat juta lima ratus ribu rupiah);

2.3. Mutah sejumlah Rp 6.000.000,00 (enam juta rupiah);

3. Menetapkan, satu orang anak yang bernama XXXXX, laki-laki, umur 2 tahun. berada dalam pemeliharaan (hadlanah) Penggugat Rekonvensi sampai anak tersebut dapat menentukan pilihannya.

4. Menghukum Tergugat Rekonvensi untuk memberikan nafkah terhadap satu orang anak sebagaimana pada point angka 3 sejumlah Rp 1.000.000,00 (satu juta rupiah) setiap bulannya dengan ditambah 10 % setiap tahunnya, sampai anak tersebut dewasa dan dapat hidup mandiri, yang penerimaannya melalui Penggugat Rekonvensi.

5. Menolak gugatan Penggugat Rekonvensi selebihnya;

Dalam Konvensi dan Rekonvensi

- Membebankan kepada Pemohon untuk membayar biaya perkara ini sejumlah Rp 446.000,00 (empat ratus empat puluh enam ribu rupiah);

Demikian diputuskan dalam persidangan Hakim Tunggal yang dilangsungkan pada hari Selasa tanggal 29 Desember 2020 Masehi. bertepatan dengan tanggal 14 Jumadil Awwal 1442 Hijriyah, oleh, H. Saik, S.Ag., M.H.. sebagai Hakim Tungga; yang ditunjuk oleh Ketua Pengadilan Agama Jepara, putusan tersebut diucapkan dalam sidang terbuka untuk umum pada hari itu juga dengan dibantu oleh **Sri Inayah, S.H.** sebagai Panitera Pengganti serta dihadiri Kuasa Pemohon Konvensi/ Tergugat Rekonvensi dan Kuasa Termohon Konvensi / Penggugat Rekonvensi ;

Hakim Tunggal

Disclaimer
Kepaniteraan Mahkamah Agung Republik Indonesia berusaha untuk selalu mencantumkan informasi paling kini dan akurat sebagai bentuk komitmen Mahkamah Agung untuk pelayanan publik, transparansi dan akuntabilitas pelaksanaan fungsi peradilan. Namun dalam hal-hal tertentu masih dimungkinkan terjadi permasalahan teknis terkait dengan akurasi dan keterkinian informasi yang kami sajikan, hal mana akan terus kami perbaiki dari waktu kewaktu.
Dalam hal Anda menemukan inakurasi informasi yang termuat pada situs ini atau informasi yang seharusnya ada, namun belum tersedia, maka harap segera hubungi Kepaniteraan Mahkamah Agung RI melalui :
Email : kepaniteraan@mahkamahagung.go.id Telp : 021-384 3348 (ext.318)

H. Saik, S.Ag., M.H.

Panitera Pengganti,

Sri Inayah, S.H.

Perincian Biaya :

Biaya Pendaftaran	:	Rp	30.000,-
Biaya Proses	:	Rp	75.000,-
Biaya Pemanggilan	:	Rp	305.000,-
Biaya Redaksi	:	Rp	10.000,-
Biaya Meterai	:	Rp	6.000,-
PNBP	:	Rp	20.000,-
Jumlah	:	Rp	446.000,-

Disclaimer
Kepaniteraan Mahkamah Agung Republik Indonesia berusaha untuk selalu mencantumkan informasi paling kini dan akurat sebagai bentuk komitmen Mahkamah Agung untuk pelayanan publik, transparansi dan akuntabilitas pelaksanaan fungsi peradilan. Namun dalam hal-hal tertentu masih dimungkinkan terjadi permasalahan teknis terkait dengan akurasi dan keterkinian informasi yang kami sajikan, hal mana akan terus kami perbaiki dari waktu kewaktu.
Dalam hal Anda menemukan inakurasi informasi yang termuat pada situs ini atau informasi yang seharusnya ada, namun belum tersedia, maka harap segera hubungi Kepaniteraan Mahkamah Agung RI melalui :
Email : kepaniteraan@mahkamahagung.go.id   Telp : 021-384 3348 (ext.318)
