# Supplementary material for: Classification of divorce causes during the COVID-19 pandemic using convolutional neural networks
Source: PeerJ Comput Sci. 2022 Jun 30;8:e998. doi: 10.7717/peerj-cs.998 (PMC9299239; doi:10.7717/peerj-cs.998)
Supplement: Supplemental Information 6 [file peerj-cs-08-998-s006.zip › Rujuk Dataset/Data ke-3.pdf]

S A L I N A N   P U T U S A N

Nomor 2336/Pdt.G/2020/PA.Tmk

بِسْمِ اللهِ الرَّحْمٰنِ الرَّحِيْمِ

DEMI KEADILAN BERDASARKAN KETUHANAN YANG MAHA ESA

Pengadilan Agama Kota Tasikmalaya yang memeriksa dan mengadili perkara tertentu pada tingkat pertama dalam sidang majelis telah menjatuhkan putusan perkara cerai gugat antara:

PENGGUGAT, umur 39 tahun, agama Islam, pekerjaan Mengurus Rumah Tangga, pendidikan SLTP, tempat kediaman di Kota Tasikmalaya, dalam hal ini memberikan kuasa khusus kepada **Deny Ramdan,S.Sy.**, Advokat/Pengacara yang berkantor di Leuwidahu Kaler N0.86.Rt.004 Rw.005 Kelurahan Parakannyasag Kecamatan. Indihiang Kota Tasikmalaya berdasarkan surat Kuasa khusus tanggal 14 Desember 0220 yang telah didaftar dalam Register Kuasa Nomor 2663/Reg.K/2020/PA.Tmk tanggal 14 Desember 2020, sebagai **Penggugat**;

melawan

TERGUGAT, umur 49 tahun, agama Islam, pekerjaan Wiraswasta, pendidikan SLTA, tempat kediaman di Kota Tasikmalaya, sebagai **Tergugat**;

Pengadilan Agama tersebut;

Telah mempelajari berkas perkara;

Telah mendengar keterangan Penggugat serta memeriksa bukti-bukti di persidangan;

DUDUK PERKARA

Bahwa Penggugat dengan surat gugatannya tanggal 10 Desember 2020 telah mengajukan gugatan cerai gugat yang didaftar di Kepaniteraan Pengadilan Agama Kota Tasikmalaya dengan Nomor 2336/Pdt.G/2020/PA.Tmk,

Disclaimer
Kepaniteraan Mahkamah Agung Republik Indonesia berusaha untuk selalu mencantumkan informasi paling kini dan akurat sebagai bentuk komitmen Mahkamah Agung untuk pelayanan publik, transparansi dan akuntabilitas pelaksanaan fungsi peradilan. Namun dalam hal-hal tertentu masih dimungkinkan terjadi permasalahan teknis terkait dengan akurasi dan keterkinian informasi yang kami sajikan, hal mana akan terus kami perbaiki dari waktu kewaktu.
Dalam hal Anda menemukan inakurasi informasi yang termuat pada situs ini atau informasi yang seharusnya ada, namun belum tersedia, maka harap segera hubungi Kepaniteraan Mahkamah Agung RI melalui :
Email : kepaniteraan@mahkamahagung.go.id   Telp : 021-384 3348 (ext.318)

tanggal 10 Desember 2020, dengan dalil-dalil yang pada pokoknya sebagai berikut:

1. Bahwa pada tanggal 25 September 1999 Penggugat dengan Tergugat melangsungkan pernikahan yang dicatat oleh Pegawai Pencatat Nikah Kantor Urusan Agama Kecamatan Indihiang Kota Tasikmalaya, tercatat sebagaimana dalam Kutipan Akta Nikah Nomor: 806/96/IX/1999, tertanggal 25 September 1999;
2. Bahwa setelah pernikahan tersebut Penggugat dengan Tergugat bertempat tinggal di rumah Tergugat dan selama pernikahan tersebut Penggugat dengan Tergugat telah hidup rukun sebagaimana layaknya suami istri, dan dikaruniai 2 (dua) orang anak;
3. Bahwa rumah tangga antara Penggugat dengan Tergugat semula harmonis, namun pada akhir tahun 2018 sudah mulai goyah karena sering terjadi perselisihan yang terus menerus yang sulit untuk didamaikan, keadaan tersebut memuncak pada bulan Agustus 2020 sehingga menyebabkan pisah rumah kurang lebih 4 (empat) bulan;
4. Bahwa penyebab perselisihan dan pertengkaran tersebut dikarenakan sudah tidak ada kecocokan dalam rumah tangga antara Penggugat dengan Tergugat yang disebabkan karena Tergugat tidak memberikan nafkah kepada Penggugat dan Tergugat tidak bertanggungjawab, sehingga Penggugat tidak menerima keadaan tersebut;
5. Bahwa Penggugat telah berusaha untuk menyelesaikan kemelut rumah tangga dengan Tergugat dan melibatkan keluarga, tetapi tidak berhasil;
6. Bahwa atas kejadian tersebut, Penggugat sudah tidak sanggup lagi untuk hidup rukun membina rumah tangga dengan Tergugat dan apabila rumah tangga tersebut dilanjutkan akan lebih banyak madarat daripada maslahatnya;

Berdasarkan alasan/dalil-dalil diatas, maka Penggugat mohon kepada Ketua Pengadilan Agama Kota Tasikmalaya cq. Majelis Hakim yang memeriksa dan mengadili perkara ini, berkenan menjatuhkan putusan sebagai berikut :

Disclaimer
Kepaniteraan Mahkamah Agung Republik Indonesia berusaha untuk selalu mencantumkan informasi paling kini dan akurat sebagai bentuk komitmen Mahkamah Agung untuk pelayanan publik, transparansi dan akuntabilitas pelaksanaan fungsi peradilan. Namun dalam hal-hal tertentu masih dimungkinkan terjadi permasalahan teknis terkait dengan akurasi dan keterkinian informasi yang kami sajikan, hal mana akan terus kami perbaiki dari waktu kewaktu.
Dalam hal Anda menemukan inakurasi informasi yang termuat pada situs ini atau informasi yang seharusnya ada, namun belum tersedia, maka harap segera hubungi Kepaniteraan Mahkamah Agung RI melalui :
Email : kepaniteraan@mahkamahagung.go.id   Telp : 021-384 3348 (ext.318)

1. Mengabulkan gugatan Penggugat;

2. Menjatuhkan talak satu yang kesatu bain sughra Tergugat **(TERGUGAT)** terhadap Penggugat **(PENGGUGAT)** di depan sidang Pengadilan Agama Kota Tasikmalaya;

3. Menetapkan biaya perkara menurut hukum;

Bahwa dalam perkara aquo, Penggugat telah memberikan kuasa khusus kepada Deny Ramdan,S.Sy., Advokat dan Penasehat Hukum yang mengambil domisili di Leuwidahu Kaler N0.86.Rt.004 Rw.005 Kelurahan Parakannyasag Kecamatan. Indihiang Kota Tasikmalaya berdasarkan Surat Kuasa Khusus tertanggal 14 Desember 0220, dan telah terdaftar di Kepaniteraan Pengadilan Agama Kota Tasikmalaya dengan Nomor 2663/Reg.K/2020/PA.Tmk tanggal 14 Desember 2020;

Bahwa, Majelis Hakim telah memeriksa surat Kuasa Penggugat tersebut diatas, dan memeriksa pula Kartu Tanda Pengenal Advokat (KTPA) yang bersangkutan;

Bahwa pada hari sidang yang telah ditetapkan Penggugat telah datang menghadap ke muka sidang, sedangkan Tergugat tidak datang menghadap ke muka sidang dan tidak menyuruh orang lain untuk menghadap sebagai wakil/kuasa hukumnya meskipun telah dipanggil secara resmi dan patut menurut relaas Nomor 2336/Pdt.G/2020/PA.Tmk tanggal 17 Desember 2020 dan ... yang dibacakan di dalam sidang, sedangkan tidak ternyata bahwa tidak datangnya itu disebabkan suatu halangan yang sah;

Bahwa majelis hakim telah menasehati Penggugat agar berpikir untuk tidak bercerai dengan Tergugat, tetapi Penggugat tetap pada dalil-dalil gugatannya untuk bercerai dengan Tergugat;

Bahwa perkara ini tidak dapat dimediasi karena Tergugat tidak pernah datang menghadap meskipun telah dipanggil secara resmi dan patut, selanjutnya dimulai pemeriksaan dengan membacakan surat gugatan Penggugat yang maksud dan isinya tetap dipertahankan oleh Penggugat;

Bahwa untuk menguatkan dalil-dalil gugatan Penggugat telah mengajukan alat-alat bukti berupa:

A. Surat:

Disclaimer
Kepaniteraan Mahkamah Agung Republik Indonesia berusaha untuk selalu mencantumkan informasi paling kini dan akurat sebagai bentuk komitmen Mahkamah Agung untuk pelayanan publik, transparansi dan akuntabilitas pelaksanaan fungsi peradilan. Namun dalam hal-hal tertentu masih dimungkinkan terjadi permasalahan teknis terkait dengan akurasi dan keterkinian informasi yang kami sajikan, hal mana akan terus kami perbaiki dari waktu kewaktu.
Dalam hal Anda menemukan inakurasi informasi yang termuat pada situs ini atau informasi yang seharusnya ada, namun belum tersedia, maka harap segera hubungi Kepaniteraan Mahkamah Agung RI melalui :
Email : kepaniteraan@mahkamahagung.go.id   Telp : 021-384 3348 (ext.318)

1. Fotokopi Kartu Tanda Penduduk atas Nama XXXXX Nomor 3278046406810016 Tanggal 13-02-2013, yang dikeluarkan oleh Kantor Pemerintah Kota Tasikmalaya, bukti surat tersebut telah diberi meterai cukup, dinazegelen pos dan telah dicocokkan dengan aslinya yang ternyata sesuai, lalu oleh Ketua Majelis diberi tanda P.1;
2. Fotokopi Kutipan Akta Nikah Nomor 806/96/IX/1999 Tanggal 25 September 1999, yang dikeluarkan oleh KUA Indihiang Kota Tasikmalaya, bukti surat tersebut telah diberi meterai cukup, telah dinazegelen pos dan telah dicocokkan dengan aslinya yang ternyata sesuai, lalu oleh Ketua Majelis diberi tanda P.2;

B. Saksi:

1. XXXXX, umur 36 tahun, agama Islam, pekerjaan Mengurus rumah tangga, bertempat tinggal di Kota Tasikmalaya, di bawah sumpahnya memberikan keterangan sebagai berikut:
   - Bahwa, saksi kenal Penggugat dan Tergugat dan memiliki hubungan dengan Penggugat sebagai saudara sepupu Penggugat;
   - Bahwa, saksi mengetahui Penggugat dan Tergugat menikah pada tanggal 25 September 1999 di KUA Indihiang Kota Tasikmalaya;
   - Bahwa, saksi mengetahui setelah menikah Penggugat dan Tergugat tinggal bersama di rumah Tergugat;
   - Bahwa, saksi mengetahui antara Penggugat dan Tergugat telah dikaruniai 2 (dua) orang anak;
   - Bahwa, saksi mengetahui keadaan rumah tangga Penggugat dan Tergugat pada awalnya rukun dan harmonis, akan tetapi kurang lebih sejak akhir tahun 2018 sudah tidak rukun lagi sering terjadi pertengkaran;
   - Bahwa, saksi sering melihat dan mendengar langsung perselisihan dan pertengkaran Penggugat dan Tergugat;
   - Bahwa, saksi mengetahui yang menjadi penyebab ketidak harmonisan rumah tangga Penggugat dan Tergugat disebabkan

Disclaimer
Kepaniteraan Mahkamah Agung Republik Indonesia berusaha untuk selalu mencantumkan informasi paling kini dan akurat sebagai bentuk komitmen Mahkamah Agung untuk pelayanan publik, transparansi dan akuntabilitas pelaksanaan fungsi peradilan. Namun dalam hal-hal tertentu masih dimungkinkan terjadi permasalahan teknis terkait dengan akurasi dan keterkinian informasi yang kami sajikan, hal mana akan terus kami perbaiki dari waktu kewaktu.
Dalam hal Anda menemukan inakurasi informasi yang termuat pada situs ini atau informasi yang seharusnya ada, namun belum tersedia, maka harap segera hubungi Kepaniteraan Mahkamah Agung RI melalui :
Email : kepaniteraan@mahkamahagung.go.id Telp : 021-384 3348 (ext.318)

karena Tergugat tidak memberikan nafkah kepada Penggugat sehingga tidak dapat memenuhi kebutuhan rumah tangganya dan Penggugat tidak menerima keadaan tersebut;

- Bahwa, saksi mengetahui antara Penggugat dan Tergugat sudah pisah rumah sejak Agustus 2020 sampai dengan sekarang dan sudah tidak bersatu lagi;
- Bahwa, baik saksi maupun pihak keluarga sudah berusaha merukunkan Penggugat dan Tergugat akan tetapi tidak berhasil;
- Bahwa, saksi melihat rumah tangga Penggugat dan Tergugat sudah tidak mungkin lagi dipertahankan, sulit dipertahankan dan tidak ada harapan dapat bersatu lagi sehingga lebih baik bercerai;

2. XXXXX, umur 36 tahun, agama Islam, pekerjaan Pedagang, bertempat tinggal di Kota Tasikmalaya, di bawah sumpahnya memberikan keterangan sebagai berikut:

- Bahwa, saksi kenal dengan Penggugat dan Tergugat dan memiliki hubungan dengan Penggugat sebagai teman Penggugat;
- Bahwa, saksi mengetahui Penggugat dan Tergugat adalah suami isteri yang sah yang menikah pada tanggal 25 September 1999 di KUA Indihiang Kota Tasikmalaya;
- Bahwa, saksi mengetahui setelah menikah Penggugat dan Tergugat tinggal bersama di rumah Tergugat;
- Bahwa, saksi mengetahui dari perkawinan Penggugat dan Tergugat telah dikaruniai 2 (dua) orang anak;
- Bahwa, saksi mengetahui rumah tangga Penggugat dan Tergugat sudah tidak lagi harmonis, sering terjadi perselisihan dan pertengkaran sejak akhir tahun 2018 yang mengakibatkan antara Penggugat dan Tergugat sudah pisah rumah sejak Agustus 2020 sampai dengan sekarang dan selama berpisah antara Penggugat dan Tergugat tidak pernah bersatu lagi;
- Bahwa, saksi pernah melihat dan mendengar langsung sebanyak 2 (dua) kali;

Disclaimer
Kepaniteraan Mahkamah Agung Republik Indonesia berusaha untuk selalu mencantumkan informasi paling kini dan akurat sebagai bentuk komitmen Mahkamah Agung untuk pelayanan publik, transparansi dan akuntabilitas pelaksanaan fungsi peradilan. Namun dalam hal-hal tertentu masih dimungkinkan terjadi permasalahan teknis terkait dengan akurasi dan keterkinian informasi yang kami sajikan, hal mana akan terus kami perbaiki dari waktu kewaktu.
Dalam hal Anda menemukan inakurasi informasi yang termuat pada situs ini atau informasi yang seharusnya ada, namun belum tersedia, maka harap segera hubungi Kepaniteraan Mahkamah Agung RI melalui :
Email : kepaniteraan@mahkamahagung.go.id Telp : 021-384 3348 (ext.318)

- Bahwa, saksi mengetahui penyebab perselisihan dan pertengkaran Penggugat dan Tergugat disebabkan karena maslah ekonomi, Tergugat tidak memberikan nafkah kepada Penggugat sehingga tidak dapat memenuhi kebutuhan rumah tangganya dan Penggugat tidak menerima keadaan tersebut;
- Bahwa, pihak keluarga sudah ada upaya mendamaikan dan merukunkan Penggugat dan Tergugat akan tetapi tidak berhasil;
- Bahwa, saksi sudah tidak sanggup lagi merukunkan Penggugat dan Tergugat karena sudah sulit dirukunkan dan sudah tidak ada harapan dapat bersatu lagi dan melihat keadaan rumah tangganya lebih baik bercerai;

Bahwa Penggugat melalui Kuasanya telah menyampaikan kesimpulan yang pada pokoknya tetap pada gugatan dan mohon putusan yang mengabulkan tuntutan Penggugat;

Bahwa, untuk mempersingkat uraian putusan ini maka semua hal yang termuat dalam berita acara sidang ini merupakan bagian yang tidak terpisahkan dari putusan ini;

PERTIMBANGAN HUKUM

Menimbang, bahwa maksud dan tujuan gugatan Penggugat adalah sebagaimana terurai di atas;

Menimbang, bahwa Majelis Hakim telah memeriksa Surat Kuasa Khusus Penggugat tertanggal 14 Desember 0220, yang telah terdaftar di Kepaniteraan Pengadilan Agama Kota Tasikmalaya dengan Nomor 2663/Reg.K/2020/PA.Tmk tanggal 14 Desember 2020, ternyata telah sesuai dengan ketentuan Pasal 123 HIR jo. Surat Edaran Mahkamah Agung RI Nomor 6 Tahun 1994 tentang Surat Kuasa Khusus, dan Majelis Hakim telah pula memeriksa Kartu Tanda Pengenal Advokat (KTPA) Kuasa Penggugat, dan ternyata telah sesuai dengan Ketentuan Pasal 30 Undang-undang Nomor 18 Tahun 2003 tentang Advokat, oleh karena itu Majelis Hakim harus menyatakan bahwa Kuasa Penggugat merupakan subjek pemberi bantuan hukun yang sah sehingga Majelis Hakim dapat menerima dan memberi izin kepada Kuasa Penggugat tersebut untuk beracara dalam perkara *aquo* sebagai advokat profesional;

Disclaimer
Kepaniteraan Mahkamah Agung Republik Indonesia berusaha untuk selalu mencantumkan informasi paling kini dan akurat sebagai bentuk komitmen Mahkamah Agung untuk pelayanan publik, transparansi dan akuntabilitas pelaksanaan fungsi peradilan. Namun dalam hal-hal tertentu masih dimungkinkan terjadi permasalahan teknis terkait dengan akurasi dan keterkinian informasi yang kami sajikan, hal mana akan terus kami perbaiki dari waktu kewaktu.
Dalam hal Anda menemukan inakurasi informasi yang termuat pada situs ini atau informasi yang seharusnya ada, namun belum tersedia, maka harap segera hubungi Kepaniteraan Mahkamah Agung RI melalui :
Email : kepaniteraan@mahkamahagung.go.id   Telp : 021-384 3348 (ext.318)

Menimbang, bahwa ternyata Tergugat, meskipun dipanggil secara resmi dan patut, tidak datang menghadap di muka sidang dan pula tidak ternyata bahwa tidak datangnya itu disebabkan suatu halangan yang sah;

Menimbang, bahwa Tergugat yang dipanggil secara resmi dan patut sesuai ketentuan Pasal 26 ayat (1) Peraturan Pemerintah Nomor 9 Tahun 1975, akan tetapi tidak datang menghadap harus dinyatakan tidak hadir dan gugatan tersebut harus diperiksa secara verstek;

Menimbang, bahwa oleh karena itu, maka putusan atas perkara ini dapat dijatuhkan tanpa hadirnya Tergugat (verstek);

Menimbang, bahwa sesuai dengan Pasal 39 Undang-undang Nomor 1 Tahun 1974, jo. Pasal 65 Undang-undang Nomor 7 Tahun 1989, sebagaimana telah diubah dengan Undang-undang Nomor 3 Tahun 2006 dan terakhir diubah dengan Undang-undang Nomor 50 Tahun 2009, jo. Pasal 115 Kompilasi Hukum Islam, Majelis Hakim pada setiap kali persidangan telah berusaha semaksimal mungkin menasehati Penggugat untuk bersabar agar bisa membina rumah tangga dengan rukun dan harmonis akan tetapi tidak berhasil, oleh karea itu perkara ini harus segera diputuskan;

Menimbang, bahwa oleh karena Tergugat tidak pernah datang menghadap meskipun telah dipanggil secara resmi dan patut maka sesuai Pasal 4 angka (2) huruf b Peraturan Mahkamah Agung Republik Indonesia Nomor 1 Tahun 2016 tentang Prosedur Mediasi di Pengadilan, perkara ini termasuk sengketa yang dikecualikan dari kewajiban mediasi;

Menimbang, bahwa berdasarkan ketentuan Pasal 125 ayat (1) HIR. yaitu putusan yang dijatuhkan tanpa hadirnya Tergugat dapat dikabulkan sepanjang berdasarkan hukum dan beralasan, oleh karena itu majelis membebani Penggugat untuk membuktikan dalil-dalil gugatannya;

Menimbang, bahwa untuk membuktikan dalil-dalil gugatannya, Penggugat telah mengajukan alat bukti surat P.1, P.2 dan dua orang saksi;

Menimbang, bahwa bukti P.1 dan P.2 merupakan akta otentik, telah bermeterai cukup, *bernazegelen* dan cocok dengan aslinya, isi bukti tersebut menjelaskan mengenai agama dan tempat tinggal Penggugat, sehingga sejalan dengan ketentuan Pasal 165 HIR bukti tersebut telah memenuhi syarat formal

Disclaimer
Kepaniteraan Mahkamah Agung Republik Indonesia berusaha untuk selalu mencantumkan informasi paling kini dan akurat sebagai bentuk komitmen Mahkamah Agung untuk pelayanan publik, transparansi dan akuntabilitas pelaksanaan fungsi peradilan. Namun dalam hal-hal tertentu masih dimungkinkan terjadi permasalahan teknis terkait dengan akurasi dan keterkinian informasi yang kami sajikan, hal mana akan terus kami perbaiki dari waktu kewaktu.
Dalam hal Anda menemukan inakurasi informasi yang termuat pada situs ini atau informasi yang seharusnya ada, namun belum tersedia, maka harap segera hubungi Kepaniteraan Mahkamah Agung RI melalui :
Email : kepaniteraan@mahkamahagung.go.id Telp : 021-384 3348 (ext.318)

dan materiil, serta mempunyai kekuatan pembuktian yang sempurna dan mengikat (*volledig en bindende bewijskracht*);

Menimbang, bahwa berdasarkan bukti P.1 telah ternyata terbukti bahwa Penggugat bertempat tinggal di wilayah hukum Pengadilan Agama Kota Tasikmalaya, maka berdasarkan ketentuan Pasal 49 ayat (1) huruf a dan Pasal 73 ayat (1) Undang-undang Nomor 7 Tahun 1989 Tentang Peradilan Agama sebagaimana telah diubah dengan Undang-undang Nomor 3 Tahun 2006 dan perubahan kedua dengan Undang-undang Nomor 50 Tahun 2009, Pengadilan Agama Kota Tasikmalaya berwenang untuk memeriksa, memutus, dan menyelesaikan perkara ini;

Menimbang, bahwa berdasarkan bukti P.2, maka harus dinyatakan telah terbukti menurut hukum bahwa Penggugat dan Tergugat telah terikat dalam perkawinan yang sah secara Agama Islam sejak tanggal 25 September 1999;

Menimbang, bahwa oleh karena Penggugat dan Tergugat masih terikat sebagai suami istri yang sah, maka Penggugat sebagai *persona standi in judicio* memiliki *legal standing* sehingga Penggugat dapat dinyatakan sebagai pihak yang mempunyai hak dan berkepentingan dalam perkara ini ;

Menimbang, bahwa sesuai dengan Pasal 22 ayat (2) Peraturan Pemerintah Nomor 9 Tahun 1975, Majelis Hakim perlu mendapatkan keterangan pihak keluarga dan atau orang-orang yang dekat dengan suami isteri itu tentang adanya perselisihan dan pertengkaran antara Penggugat dan Tergugat;

Menimbang, bahwa 2 saksi Penggugat, adalah keluarga atau orang dekat Penggugat dan atau Tergugat sesuai dengan Pasal 22 ayat (2) Peraturan Pemerintah Nomor 9 Tahun 1975 jo. Pasal 134 Kompilasi Hukum Islam dan sudah dewasa serta sudah disumpah, sehingga memenuhi syarat formal sebagaimana diatur dalam Pasal 145 HIR;

Menimbang, bahwa keterangan 2 saksi Penggugat mengenai hubungan Penggugat dan Tergugat serta mengenai keadaan rumah tangga Penggugat dan Tergugat, adalah fakta yang pernah dilihat dan didengar langsung dan relevan dengan dalil yang harus dibuktikan oleh Penggugat, oleh karena itu keterangan saksi tersebut telah memenuhi syarat materiil sebagaimana telah

Disclaimer
Kepaniteraan Mahkamah Agung Republik Indonesia berusaha untuk selalu mencantumkan informasi paling kini dan akurat sebagai bentuk komitmen Mahkamah Agung untuk pelayanan publik, transparansi dan akuntabilitas pelaksanaan fungsi peradilan. Namun dalam hal-hal tertentu masih dimungkinkan terjadi permasalahan teknis terkait dengan akurasi dan keterkinian informasi yang kami sajikan, hal mana akan terus kami perbaiki dari waktu kewaktu.
Dalam hal Anda menemukan inakurasi informasi yang termuat pada situs ini atau informasi yang seharusnya ada, namun belum tersedia, maka harap segera hubungi Kepaniteraan Mahkamah Agung RI melalui :
Email : kepaniteraan@mahkamahagung.go.id Telp : 021-384 3348 (ext.318)

diatur dalam Pasal 171 HIR sehingga keterangan saksi tersebut memiliki kekuatan pembuktian dan dapat diterima sebagai alat bukti;

Menimbang, bahwa keterangan 2 saksi Penggugat bersesuaian dan cocok antara satu dengan yang lain oleh karena itu keterangan dua orang saksi tersebut memenuhi Pasal 171 dan Pasal 172 HIR;

Menimbang, bahwa berdasarkan 2 saksi Penggugat, terbukti fakta kejadian sebagai berikut:

1. Bahwa sejak akhir tahun 2018 antara Penggugat dan Tergugat sering terjadi perselisihan dan pertengkaran yang disebabkan Tergugat tidak memberikan nafkah kepada Penggugat sehingga tidak dapat memenuhi kebutuhan rumah tangganya dan Penggugat tidak menerima keadaan tersebut;
2. Bahwa akibat perselisihan dan pertengkaran tersebut antara Penggugat dan Tergugat sudah pisah rumah sejak 4 (empat) bulan hingga sekarang dan selama itu pula keduanya tidak pernah berkumpul lagi dan tidak melaksanakan hak dan kewajibannya masing-masing sebagaimana layaknya suami isteri;
3. Bahwa, baik majelis hakim dalam persidangan, maupun pihak keluarga Penggugat dan Tergugat sudah berusaha menasehati Penggugat dan Tergugat dan sudah ada upaya musyawarah dua keluarga untuk merukunkan Penggugat dan Tergugat, akan tetapi tidak berhasil, karena Penggugat bersikukuh ingin bercerai dengan Tergugat;

Menimbang, bahwa berdasarkan fakta-fakta kejadian tersebut di atas dapat disimpulkan fakta hukum bahwa antara Penggugat dan Tergugat terus menerus terjadi perselisihan dan pertengkaran dan sudah tidak ada harapan hidup rukun lagi dalam rumah tangga;

Menimbang, bahwa berdasarkan fakta hukum di atas, maka Majelis Hakim berkesimpulan bahwa rumah tangga Penggugat dan Tergugat benar-benar sudah tidak harmonis dan sudah pecah karena telah terjadi perselisihan dan pertengkaran terus menerus yang sudah tidak mungkin lagi untuk dirukunkan dalam satu rumah tangga sehingga mempertahankan rumah tangga yang demikian tidak sejalan dengan maksud dan tujuan perkawinan

Disclaimer
Kepaniteraan Mahkamah Agung Republik Indonesia berusaha untuk selalu mencantumkan informasi paling kini dan akurat sebagai bentuk komitmen Mahkamah Agung untuk pelayanan publik, transparansi dan akuntabilitas pelaksanaan fungsi peradilan. Namun dalam hal-hal tertentu masih dimungkinkan terjadi permasalahan teknis terkait dengan akurasi dan keterkinian informasi yang kami sajikan, hal mana akan terus kami perbaiki dari waktu kewaktu.
Dalam hal Anda menemukan inakurasi informasi yang termuat pada situs ini atau informasi yang seharusnya ada, namun belum tersedia, maka harap segera hubungi Kepaniteraan Mahkamah Agung RI melalui :
Email : kepaniteraan@mahkamahagung.go.id Telp : 021-384 3348 (ext.318)

sebagaimana yang dikehendaki dalam Al-Qur'an Surat Ar-Rum: 21 dan Pasal 1 Undang-Undang Nomor 1 Tahun 1974;

Menimbang, bahwa fakta hukum tersebut telah memenuhi norma hukum Islam yang terkandung dalam Kitab Fikih Sunah Juz II halaman 290 yang diambil alih menjadi pertimbangan Majelis Hakim sebagai berikut;

أنّ للزوجة أن تطلب من القاضي التفريق إذا ادعت إضرار الزوج بها إضرارا لا يستطاع معه
دوام العشرة بين أمثالهما

Artinya : “*bahwa istri boleh menuntut cerai kepada hakim apabila dia mengaku selalu mendapat perlakuan yang menyakitkan dari suaminya sehingga hal tersebut dapat menghalangi keberlasungan hubungan suami istri antara mereka berdua”;*

Menimbang, bahwa fakta hukum tersebut telah juga memenuhi salah satu alasan perceraian sebagaimana diatur dalam ketentuan Pasal 39 ayat (2) Undang-Undang Nomor 1 Tahun 1974 jo. Pasal 19 huruf f Peraturan Pemerintah Nomor 9 Tahun 1975 jo. Pasal 116 huruf f Kompilasi Hukum Islam;

Menimbang, bahwa berdasarkan pertimbangan-pertimbangan tersebut di atas dan Penggugat belum pernah dijatuhi talak, maka petitum gugatan Penggugat agar Pengadilan menjatuhkan talak satu bain sughra Tergugat terhadap Penggugat tersebut telah beralasan dan tidak melawan hukum serta memenuhi Pasal 119 ayat 2 huruf (c) Kompilasi Hukum Islam, oleh karena itu patut dikabulkan;

Menimbang bahwa talak satu ba’in sughra merupakan talak yang dijatuhkan oleh Hakim atas permintaan seorang isteri, yang mana akibat hukum dari talak satu ba’in sughra adalah antara suami dan isteri tidak dapat hidup bersama kembali, terkecuali dengan akad nikah yang baru;

Menimbang, bahwa karena perkara *a quo* masuk bidang perkawinan, maka berdasarkan Pasal 89 ayat (1) Undang-undang Nomor 7 Tahun 1989 Tentang Peradilan Agama sebagaimana telah diubah dengan Undang-undang Nomor 3 Tahun 2006 dan perubahan kedua dengan Undang-undang Nomor 50 Tahun 2009, biaya perkara harus dibebankan kepada Penggugat;

Mengingat semua pasal dalam peraturan perundang-undangan dan

Disclaimer
Kepaniteraan Mahkamah Agung Republik Indonesia berusaha untuk selalu mencantumkan informasi paling kini dan akurat sebagai bentuk komitmen Mahkamah Agung untuk pelayanan publik, transparansi dan akuntabilitas pelaksanaan fungsi peradilan. Namun dalam hal-hal tertentu masih dimungkinkan terjadi permasalahan teknis terkait dengan akurasi dan keterkinian informasi yang kami sajikan, hal mana akan terus kami perbaiki dari waktu kewaktu.
Dalam hal Anda menemukan inakurasi informasi yang termuat pada situs ini atau informasi yang seharusnya ada, namun belum tersedia, maka harap segera hubungi Kepaniteraan Mahkamah Agung RI melalui :
Email : kepaniteraan@mahkamahagung.go.id Telp : 021-384 3348 (ext.318)

hukum Islam yang berkaitan dengan perkara ini;

M E N G A D I L I

1. Menyatakan Tergugat yang telah dipanggil secara resmi dan patut untuk menghadap di persidangan, tidak hadir;
2. Mengabulkan gugatan Penggugat secara verstek;
3. Menjatuhkan talak satu ba'in sughro Tergugat (TERGUGAT) terhadap Penggugat (PENGGUGAT);
4. Membebankan biaya perkara kepada Penggugat sejumlah Rp416.000,00 (empat ratus enam belas ribu rupiah);

Demikian diputuskan dalam rapat permusyawaratan Majelis Hakim yang dilangsungkan pada hari Rabu tanggal 23 Desember 2020 *Masehi*, bertepatan dengan tanggal 8 Jumadil Awwal 1442 *Hijriyah*, oleh kami Marwan Ibrahim Piinga, S.Ag. sebagai Ketua Majelis, Ahmad Mudlofar, S.H.I. dan Ahmad Mufid Bisri, S.H.I. masing-masing sebagai Hakim Anggota, putusan tersebut diucapkan dalam sidang terbuka untuk umum pada hari itu juga, oleh Ketua Majelis tersebut dengan didampingi oleh Hakim Anggota yang sama dan dibantu oleh Drs. M. Jam sebagai Panitera Pengganti serta dihadiri oleh Kuasa Penggugat tanpa hadirnya Tergugat;

Hakim Anggota	Ketua Majelis
ttd	ttd
Ahmad Mudlofar, S.H.I.	Marwan Ibrahim Piinga, S.Ag.
Hakim Anggota	
ttd	
Ahmad Mufid Bisri, S.H.I.	
	Panitera Pengganti
	ttd
	Drs. M. Jam

Disclaimer
Kepaniteraan Mahkamah Agung Republik Indonesia berusaha untuk selalu mencantumkan informasi paling kini dan akurat sebagai bentuk komitmen Mahkamah Agung untuk pelayanan publik, transparansi dan akuntabilitas pelaksanaan fungsi peradilan. Namun dalam hal-hal tertentu masih dimungkinkan terjadi permasalahan teknis terkait dengan akurasi dan keterkinian informasi yang kami sajikan, hal mana akan terus kami perbaiki dari waktu kewaktu.
Dalam hal Anda menemukan inakurasi informasi yang termuat pada situs ini atau informasi yang seharusnya ada, namun belum tersedia, maka harap segera hubungi Kepaniteraan Mahkamah Agung RI melalui :
Email : kepaniteraan@mahkamahagung.go.id Telp : 021-384 3348 (ext.318)

Perincian Biaya:

1. Pendaftaran	:	Rp.	30.000,00
2. Proses	:	Rp.	50.000,00
3. Panggilan	:	Rp.	300.000,00
4. PNBP Panggilan	:	Rp.	20.000,00
5. Redaksi	:	Rp.	10.000,00
6. Meterai	:	Rp.	6.000,00
Jumlah	:	Rp.	416.000,00

(empat ratus enam belas ribu rupiah)

Pemberitahuan isi
Putusan Tanggal : ....

Putusan Berkekuatan
Hukum Tetap Tanggal : ....

Untuk salinan putusan yang sama bunyinya oleh :
Panitera Pengadilan Agama Kota Tasikmalaya,

Didin Jamaludin, S.H., M.H.

Disclaimer
Kepaniteraan Mahkamah Agung Republik Indonesia berusaha untuk selalu mencantumkan informasi paling kini dan akurat sebagai bentuk komitmen Mahkamah Agung untuk pelayanan publik, transparansi dan akuntabilitas pelaksanaan fungsi peradilan. Namun dalam hal-hal tertentu masih dimungkinkan terjadi permasalahan teknis terkait dengan akurasi dan keterkinian informasi yang kami sajikan, hal mana akan terus kami perbaiki dari waktu kewaktu.
Dalam hal Anda menemukan inakurasi informasi yang termuat pada situs ini atau informasi yang seharusnya ada, namun belum tersedia, maka harap segera hubungi Kepaniteraan Mahkamah Agung RI melalui :
Email : kepaniteraan@mahkamahagung.go.id   Telp : 021-384 3348 (ext.318)
