# Supplementary material for: Classification of divorce causes during the COVID-19 pandemic using convolutional neural networks
Source: PeerJ Comput Sci. 2022 Jun 30;8:e998. doi: 10.7717/peerj-cs.998 (PMC9299239; doi:10.7717/peerj-cs.998)
Supplement: Supplemental Information 6 [file peerj-cs-08-998-s006.zip › Rujuk Dataset/Data ke-30.pdf]

P U T U S A N

Nomor 3155/Pdt.G/2020/PA.Sor

بِسْمِ اللهِ الرَّحْمَنِ الرَّحِيْمِ

DEMI KEADILAN BERDASARKAN KETUHANAN YANG MAHA ESA

Pengadilan Agama Soreang yang memeriksa dan mengadili perkara tertentu pada tingkat pertama dalam persidangan Majelis Hakim telah menjatuhkan putusan dalam perkara Cerai Gugat antara :

XXXXXXXXXX, umur 35 tahun, agama Islam, pendidikan terakhir SLTA, pekerjaan Wiraswasta, tempat kediaman di Kabupaten Bandung, selanjutnya disebut sebagai Penggugat;

melawan

XXXXXXXXXX, umur 38 tahun, agama Islam, pendidikan terakhir SLTA, pekerjaan Wiraswasta, tempat kediaman Semula di sekarang tidak diketahui alamatnya yang pasti diwilayah NKRI, selanjutnya disebut sebagai Tergugat;

Pengadilan Agama tersebut;

Setelah membaca dan mempelajari berkas perkara;

Setelah mendengarkan keterangan Penggugat;

Setelah memeriksa alat bukti;

DUDUK PERKARA

Menimbang, bahwa Penggugat dalam surat gugatannya tertanggal 12 Juni 2020 yang didaftarkan di Kepaniteraan Pengadilan Agama Soreang, Nomor 3155/Pdt.G/2020/PA.Sor tanggal 12 Juni 2020, telah mengemukakan dalil-dalil gugatannya sebagai berikut:

1. Bahwa Penggugat dan Tergugat adalah suami isteri sah, menikah pada tanggal 19 Februari 2017 berdasarkan Kutipan Akta Nikah dari KUA Kecamatan Majalaya, Kabupaten Bandung dengan memenuhi syarat rukun nikah, sebagaimana tertera dalam Kutipan Akta Nikah Nomor: 0303/82/II/2017 pada tanggal 20 Februari 2017.

Disclaimer
Kepaniteraan Mahkamah Agung Republik Indonesia berusaha untuk selalu mencantumkan informasi paling kini dan akurat sebagai bentuk komitmen Mahkamah Agung untuk pelayanan publik, transparansi dan akuntabilitas pelaksanaan fungsi peradilan. Namun dalam hal-hal tertentu masih dimungkinkan terjadi permasalahan teknis terkait dengan akurasi dan keterkinian informasi yang kami sajikan, hal mana akan terus kami perbaiki dari waktu kewaktu.
Dalam hal Anda menemukan inakurasi informasi yang termuat pada situs ini atau informasi yang seharusnya ada, namun belum tersedia, maka harap segera hubungi Kepaniteraan Mahkamah Agung RI melalui :
Email : kepaniteraan@mahkamahagung.go.id Telp : 021-384 3348 (ext.318)

2. Bahwa setelah pernikahan tersebut, Penggugat dengan Tergugat tinggal bersama di rumah kediaman orangtua Penggugat di Kampung Lengkong, RT 005, RW 013, Desa Bumiwangi, Kecamatan Ciparay, Kabupaten Bandung.

3. Bahwa pada mulanya Penggugat dan Tergugat hidup rukun dan harmonis dan telah melakukan hubungan layaknya suami istri (ba'da dukhul).

4. Bahwa sejak bulan **Januari 2019** rumahtangga antara Penggugat dan Tergugat mulai terjadi perselisihan dan pertengkaran terus menerus.

5. Bahwa pertengkaran antara Penggugat dan Tergugat disebabkan antara lain karena:

a. Bahwa Tergugat kurang bertangung jawab dalam memberikan nafkah lahir batin terhadap Penggugat, yang mana sejak awal menikah hingga saat ini Tergugat tidak pernah memberikan nafkah sama sekali terhadap Penggugat. Sehingga untuk mencukupi kebutuhannya, Penggugat terpaksa berusaha sendiri.

b. Bahwa sejak 3 bulan pernikahan Tergugat telah meninggalkan Penggugat dengan alasan pekerjaan akan tetapi hingga saat ini tidak ada pulang kembali ke rumah kediaman bersama. Sehingga hal tersebut yang menjadi pemicu keretakan rumahtangga.

6. Bahwa Penggugat telah berusaha sabar untuk mempertahankan rumah tangga, tetapi Tergugat tidak berubah dan merubah sikapnya. Puncaknya sekitar bulan **Juni 2017** antara Penggugat dan Tergugat bertengkar hebat, sehingga pisah tempat tinggal dan sudah tidak ada hubungan layaknya suami istri lagi.

7. Bahwa Penggugat telah berusaha untuk mempertahankan rumah tangga bersama Tergugat bahkan Penggugat telah meminta bantuan kepada keluarga akan tetapi tidak berhasil.

8. Bahwa gugatan Penggugat tersebut telah memenuhi syarat sesuai ketentuan Pasal 19 huruf (f) Peraturan Pemerintah Nomor 9 Tahun 1975 jo. Pasal 116 huruf (f) Kompilasi Hukum Islam.

Disclaimer
Kepaniteraan Mahkamah Agung Republik Indonesia berusaha untuk selalu mencantumkan informasi paling kini dan akurat sebagai bentuk komitmen Mahkamah Agung untuk pelayanan publik, transparansi dan akuntabilitas pelaksanaan fungsi peradilan. Namun dalam hal-hal tertentu masih dimungkinkan terjadi permasalahan teknis terkait dengan akurasi dan keterkinian informasi yang kami sajikan, hal mana akan terus kami perbaiki dari waktu kewaktu.
Dalam hal Anda menemukan inakurasi informasi yang termuat pada situs ini atau informasi yang seharusnya ada, namun belum tersedia, maka harap segera hubungi Kepaniteraan Mahkamah Agung RI melalui :
Email : kepaniteraan@mahkamahagung.go.id Telp : 021-384 3348 (ext.318)

9. Bahwa atas permasalahan tersebut di atas Penggugat sudah tidak sanggup lagi untuk mempertahankan perkawinan ini, oleh karena itu Penggugat telah berketetapan hati untuk bercerai dengan Tergugat.

10. Bahwa segala uraian yang telah Penggugat kemukakan, Penggugat mohon kepada Ketua Pengadilan Agama Soreang c/q Majelis Hakim, untuk memanggil para pihak yang bersengketa pada suatu persidangan yang ditentukan untuk itu, guna memeriksa dan mengadili gugatan ini dan lebih lanjut berkenan memutuskan dengan amar sebagai berikut :

Berdasarkan alasan/dalil-dalil di atas, Penggugat mohon agar Ketua Pengadilan Agama Soreang Cq; majelis hakim yang memeriksa dan mengadili perkara ini berkenan menjatuhkan putusan yang amarnya berbunyi:

1. Mengabulkan gugatan Penggugat;

2. Menjatuhkan talak satu ba'in sughra Tergugat (**XXXXXXXXXX**) terhadap Penggugat (**XXXXXXXXXX**);

3. Membebankan biaya perkara menurut hukum;

ATAU :

Apabila Ketua Pengadilan Agama Soreang Cq Majelis Hakim berpendapat lain, mohon Putusan yang seadil-adilnya. (ex aequo et bono).

Bahwa pada hari persidangan yang telah ditetapkan, Penggugat telah datang menghadap, sedangkan Tergugat tidak datang menghadap dan tidak pula menyuruh orang lain untuk menghadap sebagai wakilnya atau kuasanya yang sah, dan berdasarkan Relaas Panggilan tertanggal 18 Agustus 2020 dan 18 September 2020 Tergugat telah dipanggil secara resmi dan patut melalui mass media namun tidak pernah hadir dalam persidangan dan tidak ternyata bahwa ketidakhadirannya itu disebabkan suatu halangan yang sah;

Bahwa oleh karena Tergugat tidak pernah hadir selama persidangan, maka upaya perdamaian melalui lembaga mediasi sebagaimana ketentuan Peraturan Mahkamah Agung RI Nomor 1 Tahun 2016, tidak dapat dilaksanakan;

Disclaimer
Kepaniteraan Mahkamah Agung Republik Indonesia berusaha untuk selalu mencantumkan informasi paling kini dan akurat sebagai bentuk komitmen Mahkamah Agung untuk pelayanan publik, transparansi dan akuntabilitas pelaksanaan fungsi peradilan. Namun dalam hal-hal tertentu masih dimungkinkan terjadi permasalahan teknis terkait dengan akurasi dan keterkinian informasi yang kami sajikan, hal mana akan terus kami perbaiki dari waktu kewaktu.
Dalam hal Anda menemukan inakurasi informasi yang termuat pada situs ini atau informasi yang seharusnya ada, namun belum tersedia, maka harap segera hubungi Kepaniteraan Mahkamah Agung RI melalui :
Email : kepaniteraan@mahkamahagung.go.id Telp : 021-384 3348 (ext.318)

Bahwa majelis hakim telah berusaha menasehati Penggugat agar dapat rukun kembali membina rumah tangga dengan Tergugat namun ternyata tidak berhasil;

Bahwa selanjutnya pemeriksaan perkara dilanjutkan pada pembacaan gugatan Penggugat, terhadap isinya Penggugat menyatakan tetap dengan gugatannya tanpa ada perubahan maupun penambahan;

Bahwa untuk menguatkan dalil-dalil gugatannya Penggugat telah mengajukan bukti-bukti berupa:

SURAT

1. Fotokopi Kartu Tanda Penduduk atas nama Penggugat NIK 3204297011840008 yang dikeluarkan oleh Kepala Dinas Kependudukan dan Pencatatan Sipil Kabupaten Bandung, Provinsi Jawa Barat. Bukti surat tersebut telah diberi materai cukup dan dinazegelen serta telah dicocokkan dengan aslinya yang ternyata sesuai, lalu oleh Ketua Majelis diberi tanda P.1;
2. Fotokopi Kutipan Akta Nikah Nomor 0303/82/II/2017 tanggal 20 Februari 2017 yang dikeluarkan oleh Pegawai Pencatat Nikah Kantor Urusan Agama Kecamatan Majalaya, Kabupaten Bandung. Bukti surat tersebut telah diberi materai cukup dan dinazegelen serta telah dicocokkan dengan aslinya yang ternyata sesuai, lalu oleh Ketua Majelis diberi tanda P.2;

SAKSI-SAKSI

1. XXXXXXXXXX, umur 55 tahun, agama Islam, pekerjaan ibu rumah tangga, tempat tinggal di [redacted]. Saksi adalah bibi Penggugat Di bawah sumpahnya pada pokoknya menerangkan sebagai berikut:
   - Bahwa saksi kenal dengan Tergugat bernama XXXXXXXXXX sebagai suami Penggugat;
   - Bahwa saksi tahu pada 19 Februari 2017 Penggugat dan Tergugat menikah di KUA Kecamatan Majalaya, Kabupaten Bandung;

Disclaimer
Kepaniteraan Mahkamah Agung Republik Indonesia berusaha untuk selalu mencantumkan informasi paling kini dan akurat sebagai bentuk komitmen Mahkamah Agung untuk pelayanan publik, transparansi dan akuntabilitas pelaksanaan fungsi peradilan. Namun dalam hal-hal tertentu masih dimungkinkan terjadi permasalahan teknis terkait dengan akurasi dan keterkinian informasi yang kami sajikan, hal mana akan terus kami perbaiki dari waktu kewaktu.
Dalam hal Anda menemukan inakurasi informasi yang termuat pada situs ini atau informasi yang seharusnya ada, namun belum tersedia, maka harap segera hubungi Kepaniteraan Mahkamah Agung RI melalui :
Email : kepaniteraan@mahkamahagung.go.id Telp : 021-384 3348 (ext.318)

- Bahwa setelah menikah Penggugat dan Tergugat tinggal bersama di Kampung Lengkong, RT 005, RW 013, Desa Bumiwangi, Kecamatan Ciparay, Kabupaten Bandung.;
- Bahwa saksi tahu pernikahan Penggugat dan Tergugat belum dikaruniai anak;
- Bahwa saksi tahu awalnya rumah tangga Penggugat dengan Tergugat rukun dan harmonis namun sejak bulan Januari 2019 mulai terjadi perselisihan dan pertengkaran;
- Bahwa saksi sering melihat antara Penggugat dengan Tergugat bertengkar;
- Bahwa saksi tahu penyebab perselisihan dan pertengkaran Penggugat dan Tergugat karena Tergugat kurang bertanggung jawab dalam hal nafkah;
- Bahwa saksi tahu puncak pertengakaran kedua belah pihak pada bulan Juni 2020 Penggugat dan Tergugat telah berpisah tempat tinggal dan tidak saling pedulikan satu sama lain sampai sekarang;
- Bahwa pihak keluarga sudah berupaya merukunkan rumah tangga Penggugat dan Tergugat namun tidak berhasil karena Penggugat tetap mau bercerai dari Tergugat;

2. XXXXXXXXXX, umur 34 tahun, agama Islam, pekerjaan ibu rumah tangga, tempat tinggal di [redacted]. Saksi adalah kakak kandung Penggugat Di bawah sumpahnya pada pokoknya menerangkan sebagai berikut:

- Bahwa saksi kenal dengan Tergugat bernama XXXXXXXXXX sebagai suami Penggugat;
- Bahwa saksi tahu pada 19 Februari 2017 Penggugat dan Tergugat menikah di KUA Kecamatan Majalaya, Kabupaten Bandung;
- Bahwa setelah menikah Penggugat dan Tergugat tinggal bersama di Kampung Lengkong, RT 005, RW 013, Desa Bumiwangi, Kecamatan Ciparay, Kabupaten Bandung.;

Disclaimer
Kepaniteraan Mahkamah Agung Republik Indonesia berusaha untuk selalu mencantumkan informasi paling kini dan akurat sebagai bentuk komitmen Mahkamah Agung untuk pelayanan publik, transparansi dan akuntabilitas pelaksanaan fungsi peradilan. Namun dalam hal-hal tertentu masih dimungkinkan terjadi permasalahan teknis terkait dengan akurasi dan keterkinian informasi yang kami sajikan, hal mana akan terus kami perbaiki dari waktu kewaktu.
Dalam hal Anda menemukan inakurasi informasi yang termuat pada situs ini atau informasi yang seharusnya ada, namun belum tersedia, maka harap segera hubungi Kepaniteraan Mahkamah Agung RI melalui :
Email : kepaniteraan@mahkamahagung.go.id Telp : 021-384 3348 (ext.318)

- Bahwa saksi tahu pernikahan Penggugat dan Tergugat belum dikaruniai anak;
- Bahwa saksi tahu awalnya rumah tangga Penggugat dengan Tergugat rukun dan harmonis namun sejak bulan Januari 2019 mulai terjadi perselisihan dan pertengkaran;
- Bahwa saksi sering melihat antara Penggugat dengan Tergugat bertengkar;
- Bahwa saksi tahu penyebab perselisihan dan pertengkaran Penggugat dan Tergugat karena Tergugat kurang bertanggung jawab dalam hal nafkah;
- Bahwa saksi tahu puncak pertengakaran kedua belah pihak pada bulan Juni 2020 Penggugat dan Tergugat telah berpisah tempat tinggal dan tidak saling pedulikan satu sama lain sampai sekarang;
- Bahwa pihak keluarga sudah berupaya merukunkan rumah tangga Penggugat dan Tergugat namun tidak berhasil karena Penggugat tetap mau bercerai dari Tergugat;

Bahwa atas keterangan saksi-saksi tersebut, Penggugat membenarkannya dan tidak mengajukan pertanyaan kepada saksi atau memberikan keterangan tambahan dan tidak akan mengajukan alat bukti lagi;

Bahwa Penggugat telah mengajukan kesimpulan secara lisan yang pada pokoknya tetap pada dalil gugatannya dan menyatakan tidak akan mengajukan sesuatu apapun lagi kecuali memohon putusan;

Bahwa untuk mempersingkat uraian dalam putusan ini, maka majelis hakim menunjuk kepada hal-hal yang tercantum dalam berita acara sidang perkara ini dan kesemuanya dianggap telah dimasukkan dan menjadi bagian dari putusan ini;

PERTIMBANGAN HUKUM

Menimbang, bahwa maksud dan tujuan gugatan Penggugat adalah sebagaimana telah diuraikan di atas;

Menimbang, bahwa Penggugat dalam gugatannya mendalilkan bahwa Penggugat telah melangsungkan perkawinan dan rumah tangga Penggugat dan Tergugat sudah tidak harmonis, oleh karena itu Penggugat memiliki *legal*

Disclaimer
Kepaniteraan Mahkamah Agung Republik Indonesia berusaha untuk selalu mencantumkan informasi paling kini dan akurat sebagai bentuk komitmen Mahkamah Agung untuk pelayanan publik, transparansi dan akuntabilitas pelaksanaan fungsi peradilan. Namun dalam hal-hal tertentu masih dimungkinkan terjadi permasalahan teknis terkait dengan akurasi dan keterkinian informasi yang kami sajikan, hal mana akan terus kami perbaiki dari waktu kewaktu.
Dalam hal Anda menemukan inakurasi informasi yang termuat pada situs ini atau informasi yang seharusnya ada, namun belum tersedia, maka harap segera hubungi Kepaniteraan Mahkamah Agung RI melalui :
Email : kepaniteraan@mahkamahagung.go.id Telp : 021-384 3348 (ext.318)

standing untuk mengajukan gugatan perceraian sebagaimana diatur Pasal 49 Ayat (1) huruf (a) dan Pasal 73 Undang-Undang Nomor 7 Tahun 1989 tentang Peradilan Agama yang diubah dengan Undang-Undang Nomor 3 Tahun 2006 dan Undang-Undang Nomor 50 Tahun 2009;

Menimbang, bahwa dalam dalil gugatan Penggugat yang menyatakan Penggugat dan Tergugat beragama Islam, karena itu berdasarkan Pasal 40 dan Pasal 63 Ayat (1) huruf (a) Undang-Undang Nomor 1 Tahun 1974 jo. Pasal 49 Undang-Undang Nomor 7 Tahun 1989 tentang Peradilan Agama sebagaimana diubah dengan Undang-Undang Nomor 3 Tahun 2006 dan terakhir dengan Undang-Undang Nomor 50 Tahun 2009 jo. Pasal 1 huruf (b) Peraturan Pemerintah Nomor 9 Tahun 1975, karenanya Pengadilan Agama berwenang memeriksa dan mengadili serta memutus perkara *a quo*;

Menimbang, bahwa dalam gugatan Penggugat domisili Penggugat berada pada yurisdiksi Pengadilan Agama Soreang (vide bukti P.1), sesuai Pasal 73 Undang-Undang Nomor 7 Tahun 1989 tentang Peradilan Agama sebagaimana diubah dengan Undang-Undang Nomor 3 Tahun 2006 dan terakhir dengan Undang-Undang Nomor 50 Tahun 2009, maka perkara ini merupakan kewenangan relatif Pengadilan Agama Soreang;

Menimbang, bahwa Penggugat dan Tergugat telah dipanggil secara resmi dan patut untuk menghadap di persidangan, atas panggilan tersebut Penggugat hadir secara pribadi (*in person*) di persidangan, sedangkan Tergugat tidak pernah hadir dan tidak pula mengutus orang lain sebagai wakil atau kuasanya untuk menghadap di persidangan, meskipun sudah dipanggil dengan resmi dan patut, serta tidak pula ternyata ketidakhadiran Tergugat tersebut didasarkan atas alasan yang sah menurut hukum. Oleh sebab itu majelis hakim menilai pemanggilan tersebut telah dilaksanakan dengan resmi dan patut karena telah sesuai dengan ketentuan Pasal 121 HIR, dan Tergugat harus dinyatakan tidak hadir dan perkara ini dapat diperiksa dan diputus secara verstek;

Menimbang, bahwa meskipun Peraturan Mahkamah Agung RI Nomor 1 tahun 2016 Tentang Mediasi menghendaki agar setiap perkara dimediasi,

Disclaimer
Kepaniteraan Mahkamah Agung Republik Indonesia berusaha untuk selalu mencantumkan informasi paling kini dan akurat sebagai bentuk komitmen Mahkamah Agung untuk pelayanan publik, transparansi dan akuntabilitas pelaksanaan fungsi peradilan. Namun dalam hal-hal tertentu masih dimungkinkan terjadi permasalahan teknis terkait dengan akurasi dan keterkinian informasi yang kami sajikan, hal mana akan terus kami perbaiki dari waktu kewaktu.
Dalam hal Anda menemukan inakurasi informasi yang termuat pada situs ini atau informasi yang seharusnya ada, namun belum tersedia, maka harap segera hubungi Kepaniteraan Mahkamah Agung RI melalui :
Email : kepaniteraan@mahkamahagung.go.id   Telp : 021-384 3348 (ext.318)

namun oleh karena Tergugat tidak hadir, maka mediasi tidak dapat dilaksanakan;

Menimbang, bahwa majelis hakim telah berusaha menasehati Penggugat dalam rangka perdamaian dengan Tergugat agar Penggugat tetap mempertahankan keutuhan rumah tangganya, namun tidak berhasil, dengan demikian ketentuan Pasal 82 ayat (1) dan (4) Undang-undang Nomor 7 Tahun 1989 yang dirubah dengan Undang-undang Nomor 3 Tahun 2006 dan dirubah lagi dengan Undang-undang Nomor 50 Tahun 2009 jo Pasal 143 ayat (1) dan (2) Kompilasi Hukum Islam telah terpenuhi;

Menimbang, bahwa yang menjadi masalah pokok dalam perkara ini adalah Penggugat mengajukan gugatan cerai terhadap Tergugat dengan alasan bahwa pada bulan Januari 2019 rumah tangga Penggugat dengan Tergugat sering terjadi pertengkaran dan perselisihan yang disebabkan karena Tergugat kurang bertanggung jawab dalam hal nafkah hingga akhirnya pada bulan Juni 2020 Penggugat dan Tergugat saat ini telah pisah tempat tinggal dan selama itu pula sudah tidak tidak ada hubungan lahir maupun batin sampai sekarang;

Menimbang, bahwa berdasarkan dalil-dalil gugatan Penggugat tersebut diatas, maka yang menjadi pokok gugatan dalam perkara ini adalah apakah benar telah terjadi perselisihan dan pertengkaran yang terus menerus diantara Penggugat dan Tergugat dan telah mengakibatkan rumah tangga yang telah dibina tidak dapat dipertahankan lagi ?

Menimbang, bahwa pada hari-hari persidangan yang telah ditetapkan, Tergugat tidak datang menghadap dipersidangan dan tidak pula menyuruh orang lain datang menghadap sebagai wakilnya atau kuasanya yang sah, meskipun telah dipanggil secara resmi dan patut, maka majelis hakim berpendapat bahwa Tergugat tidak menggunakan haknya untuk mengajukan perlawanan terhadap dalil-dalil Penggugat tersebut;

Menimbang, bahwa berdasarkan ketentuan Pasal 125 ayat (1) HIR yaitu putusan yang dijatuhkan tanpa hadirnya Tergugat dapat dikabulkan sepanjang berdasarkan hukum dan beralasan, oleh karena itu majelis hakim membebankan Penggugat untuk membuktikan dalil-dalil gugatannya;

Disclaimer
Kepaniteraan Mahkamah Agung Republik Indonesia berusaha untuk selalu mencantumkan informasi paling kini dan akurat sebagai bentuk komitmen Mahkamah Agung untuk pelayanan publik, transparansi dan akuntabilitas pelaksanaan fungsi peradilan. Namun dalam hal-hal tertentu masih dimungkinkan terjadi permasalahan teknis terkait dengan akurasi dan keterkinian informasi yang kami sajikan, hal mana akan terus kami perbaiki dari waktu kewaktu.
Dalam hal Anda menemukan inakurasi informasi yang termuat pada situs ini atau informasi yang seharusnya ada, namun belum tersedia, maka harap segera hubungi Kepaniteraan Mahkamah Agung RI melalui :
Email : kepaniteraan@mahkamahagung.go.id   Telp : 021-384 3348 (ext.318)

Menimbang, bahwa sesuai dengan ketentuan Pasal 22 ayat (2) Peraturan Pemerintah Nomor 9 Tahun 1975 tentang Penjelasan Undang-Undang Nomor 1 Tahun 1974 tentang Perkawinan, untuk mengetahui kondisi rumah tangga Penggugat dan Tergugat yang sebenarnya diperintahkan untuk mendengar pihak keluarga serta orang yang dekat dengan suami isteri;

Menimbang, bahwa untuk meneguhkan dalil-dalil gugatannya Penggugat telah mengajukan bukti tertulis yaitu bukti P.1 dan P.2 Serta 2 (dua) orang saksi;

Menimbang, bahwa alat bukti P.1 yang diajukan Penggugat di persidangan yaitu Fotokopi KTP (Kartu Tanda Penduduk) merupakan akta autentik perihal identitas kependudukan yang dikeluarkan yang dikeluarkan oleh pejabat yang berwenang untuk itu sebagai bukti diri Penggugat (*vide* Pasal 1 angka 15 Undang-Undang Nomor 23 Tahun 2006 tentang Administrasi Kependudukan) dan telah sesuai dengan aslinya, telah bermeterai dan dinazegelen serta telah memenuhi ketentuan Pasal 165 HIR dan Pasal 2 ayat (3) Undang-undang Nomor 13 Tahun 1985 tentang Bea Meterai, oleh karena itu dinilai telah memenuhi syarat formil pembuktian dengan tulisan sehingga dapat diterima dan dipertimbangkan. Berdasarkan bukti tersebut, Pengadilan berpendapat bahwa Penggugat adalah pihak *principal* (*natuurlijke person*) yang memiliki kewenangan dan kepentingan hukum langsung dengan pokok perkara (*legitima persona standi in judicio*);

Menimbang, bahwa alat bukti P.2 yang diajukan Penggugat di persidangan adalah fotokopi sah dari suatu akta autentik yang sengaja dibuat untuk alat bukti, telah di-*nazageleen* di kantor Pos, setelah diteliti oleh majelis hakim dengan seksama fotokopi tersebut sesuai dengan aslinya serta telah memenuhi ketentuan Pasal 165 HIR dan Pasal 2 ayat (3) Undang-undang Nomor 13 Tahun 1985 tentang Bea Meterai, oleh karena itu dinilai telah memenuhi syarat formil pembuktian dengan tulisan sehingga dapat diterima dan dipertimbangkan;

Menimbang, bahwa berdasarkan bukti P.2 tersebut, terbukti bahwa Penggugat dan Tergugat telah terikat sebagai suami isteri yang sah dan tidak pernah bercerai sampai sekarang, isinya memuat keterangan yang relevan

Disclaimer
Kepaniteraan Mahkamah Agung Republik Indonesia berusaha untuk selalu mencantumkan informasi paling kini dan akurat sebagai bentuk komitmen Mahkamah Agung untuk pelayanan publik, transparansi dan akuntabilitas pelaksanaan fungsi peradilan. Namun dalam hal-hal tertentu masih dimungkinkan terjadi permasalahan teknis terkait dengan akurasi dan keterkinian informasi yang kami sajikan, hal mana akan terus kami perbaiki dari waktu kewaktu.
Dalam hal Anda menemukan inakurasi informasi yang termuat pada situs ini atau informasi yang seharusnya ada, namun belum tersedia, maka harap segera hubungi Kepaniteraan Mahkamah Agung RI melalui :
Email : kepaniteraan@mahkamahagung.go.id   Telp : 021-384 3348 (ext.318)

dengan gugatan Penggugat, oleh karena itu majelis hakim menilai bahwa alat bukti P tersebut telah memenuhi syarat formil dan materil sebagai bukti sehingga harus dinyatakan dapat diterima dan oleh karena itu Penggugat dipandang sebagai pihak yang tepat dan berkepentingan dalam perkara ini;

Menimbang, bahwa terhadap alat bukti berupa dua orang saksi yang diajukan Penggugat di persidangan, Majelis Hakim berpendapat bahwa kedua orang saksi tersebut telah memenuhi persyaratan formil karena masing-masing telah hadir, dalam hal ini secara pribadi *(in person),* di depan persidangan dan telah memberikan keterangan di bawah sumpahnya serta tidak terhalang secara hukum untuk didengar kesaksiannya, kemudian secara materil dari keterangan saksi-saksi tersebut menerangkan antara Penggugat dan Tergugat sering terjadi perselisihan dan pertengkaran yang mengakibatkan antara Penggugat dan Tergugat pisah tempat tinggal sampai sekarang dan selama itu pula sudah tidak tidak ada hubungan lahir maupun batin dan kedua saksi juga telah memberikan keterangan yang sama yang mana saling bersesuaian dan saling menguatkan diantara keterangan saksi-saksi di atas serta relevan dengan dalil-dalil gugatan Penggugat, maka hal itu sesuai dengan ketentuan Pasal 144 HIR *jo*. Pasal 171 HIR dan Pasal 172 HIR, sehingga secara formil dan materil alat bukti saksi yang diajukan Penggugat dapat diterima dan dipertimbangkan;

Menimbang, bahwa berdasarkan penilaian terhadap gugatan Penggugat, serta alat-alat bukti yang telah diajukan Penggugat di atas, serta dari fakta-fakta di persidangan, diperoleh fakta-fakta hukum yang telah dikonstatir sebagai berikut :

1. Bahwa Penggugat dan Tergugat adalah suami isteri yang menikah pada 19 Februari 2017 Penggugat dan Tergugat menikah di KUA Kecamatan Majalaya, Kabupaten Bandung dan belum dikaruniai anak;
2. Bahwa setelah menikah Penggugat dan Tergugat tinggal bersama di Kampung Lengkong, RT 005, RW 013, Desa Bumiwangi, Kecamatan Ciparay, Kabupaten Bandung.;
3. Bahwa sejak bulan Januari 2019 antara Penggugat dengan Tergugat telah terjadi perselisihan dan pertengkaran terus menerus karena

Disclaimer
Kepaniteraan Mahkamah Agung Republik Indonesia berusaha untuk selalu mencantumkan informasi paling kini dan akurat sebagai bentuk komitmen Mahkamah Agung untuk pelayanan publik, transparansi dan akuntabilitas pelaksanaan fungsi peradilan. Namun dalam hal-hal tertentu masih dimungkinkan terjadi permasalahan teknis terkait dengan akurasi dan keterkinian informasi yang kami sajikan, hal mana akan terus kami perbaiki dari waktu kewaktu.
Dalam hal Anda menemukan inakurasi informasi yang termuat pada situs ini atau informasi yang seharusnya ada, namun belum tersedia, maka harap segera hubungi Kepaniteraan Mahkamah Agung RI melalui :
Email : kepaniteraan@mahkamahagung.go.id Telp : 021-384 3348 (ext.318)

Tergugat kurang bertanggung jawab dalam hal nafkah hingga akhirnya pada bulan Juni 2020 Penggugat dan Tergugat telah pisah tempat tinggal dan selama itu pula sudah tidak tidak ada hubungan lahir maupun batin sampai sekarang;

3. Bahwa sejak bulan Juni 2020 Penggugat dan Tergugat saat ini telah berpisah tempat tinggal kurang lebih 6 (enam) bulan dan tidak pernah tinggal bersama lagi serta antara mereka sudah tidak saling peduli lagi sehingga hubungan keduanya tidak kondusif;

4. Bahwa pihak keluarga telah berusaha merukunkan Penggugat dan Tergugat namun tidak berhasil;

1. Bahwa Tergugat tidak pernah hadir di persidangan untuk membela kepentingannya;

Menimbang, bahwa berdasarkan fakta-fakta kejadian tersebut di atas, majelis berkesimpulan bahwa fakta-fakta tersebut sesuai dengan fakta-fakta hukum sebagai berikut:

1. Bahwa Penggugat dan Tergugat adalah suami istri yang sah dari pernikahan yang sah sesuai dengan pasal 2 Undang Undang Nomor 1 tahun 1974 Jo. Pasal 2 dan 3 Peraturan Pemerintah Nomor 9 tahun 1975 jo. Pasal 7 Kompilasi Hukum Islam;

2. Bahwa alasan perceraian Penggugat adalah dalam kehidupan suami isteri Penggugat telah terjadi perselisihan yang terus menerus yang sulit untuk dirukunkan lagi yang alasan tersebut telah memenuhi salah satu alasan perceraian sesuai pasal 19 hurup f Peraturan Pemerintah Nomor 9 Tahun 1975 tentang Pelaksanaan Undang-undang Nomor 1 tahun 1974 tentang Perkawinan jo. pasal 116 huruf (f) Kompilasi Hukum Islam;

3. Bahwa antara Penggugat dan Tergugat telah diusahakan damai oleh keluarga kedua belah pihak dan oleh hakim dipersidangan maka telah sesuai dengan pasal 130 HIR jo. pasal 39 Undang-undang Nomor 1 tahun 1974 jo. 22 Peraturan Pemerintah nomor 9 Tahun 1975 jo. 115 Kompilasi Hukum Islam;

1. Bahwa Tergugat tidak pernah hadir di persidangan untuk membela kepentingannya maka Tergugat tidak dapat didengar keterangannya maka

Disclaimer
Kepaniteraan Mahkamah Agung Republik Indonesia berusaha untuk selalu mencantumkan informasi paling kini dan akurat sebagai bentuk komitmen Mahkamah Agung untuk pelayanan publik, transparansi dan akuntabilitas pelaksanaan fungsi peradilan. Namun dalam hal-hal tertentu masih dimungkinkan terjadi permasalahan teknis terkait dengan akurasi dan keterkinian informasi yang kami sajikan, hal mana akan terus kami perbaiki dari waktu kewaktu.
Dalam hal Anda menemukan inakurasi informasi yang termuat pada situs ini atau informasi yang seharusnya ada, namun belum tersedia, maka harap segera hubungi Kepaniteraan Mahkamah Agung RI melalui :
Email : kepaniteraan@mahkamahagung.go.id Telp : 021-384 3348 (ext.318)

sesuai pasal 125 HIR jo. Pasal 27 Peraturan Pemerintah nomor 9 Tahun 1975 jo. Pasal 139 gugatan perkara ini dapat diterima tanpa hadirnya Tergugat/ dapat diperiksa secara verstek;

Menimbang bahwa perceraian dapat dilakukan di depan Sidang Pengadilan setelah Pengadilan yang bersangkutan berusaha dan tidak berhasil mendamaikan kedua belah pihak sesuai dengan ketentuan pasal 39 ayat 1 Undang-undang nomor 1 Tahun 1974 tentang Perkawinan sejalan dengan ketentuan Hukum Islam sebagaimana tersurat pada pasal 115 Kompilasi Hukum Islam;

Menimbang, bahwa pada setiap persidangan majelis hakim telah berusaha secara maksimal menasihati Penggugat agar tetap mempertahankan rumah tangganya namun ternyata tidak berhasil karena Penggugat tetap bersikeras untuk bercerai, sehingga majelis hakim berkesimpulan bahwa antara Penggugat dan Tergugat tidak ada harapan untuk dapat rukun kembali dalam sebuah rumah tangga;

Menimbang, bahwa berdasarkan hal-hal tersebut di atas harus dinyatakan telah terbukti bahwa antara Penggugat dan Tergugat telah terjadi perselisihan dan pertengkaran yang terus menerus yang tidak ada harapan lagi bagi keduanya akan dapat hidup rukun kembali dalam rumah tangga yang puncaknya antara Penggugat dan Tergugat Penggugat dan Tergugat saat ini telah berpisah tempat tinggal sampai sekarang dan selama itu pula tidak lagi saling menghiraukan, oleh karenanya majelis hakim berpendapat perkawinan Penggugat dan Tergugat yang telah pecah dapat dibuktikan oleh Penggugat;

Menimbang, bahwa berdasarkan fakta-fakta tersebut di atas, majelis hakim berpendapat bahwa keadaan rumah tangga Penggugat dengan Tergugat telah pecah sedemikian rupa sehingga tujuan perkawinan untuk membentuk keluarga/rumah tangga yang bahagia dan kekal berdasarkan Ketuhanan Yang Maha Esa (*vide* Pasal 1 Undang-Undang Nomor 1 Tahun 1974) dan atau keluarga yang sakinah, penuh mawaddah dan rahmah (*vide* Pasal 3 Kompilasi Hukum Islam) tidak terwujud dalam rumah tangga Penggugat dengan Tergugat;

Disclaimer
Kepaniteraan Mahkamah Agung Republik Indonesia berusaha untuk selalu mencantumkan informasi paling kini dan akurat sebagai bentuk komitmen Mahkamah Agung untuk pelayanan publik, transparansi dan akuntabilitas pelaksanaan fungsi peradilan. Namun dalam hal-hal tertentu masih dimungkinkan terjadi permasalahan teknis terkait dengan akurasi dan keterkinian informasi yang kami sajikan, hal mana akan terus kami perbaiki dari waktu kewaktu.
Dalam hal Anda menemukan inakurasi informasi yang termuat pada situs ini atau informasi yang seharusnya ada, namun belum tersedia, maka harap segera hubungi Kepaniteraan Mahkamah Agung RI melalui :
Email : kepaniteraan@mahkamahagung.go.id Telp : 021-384 3348 (ext.318)

Menimbang, bahwa rumah tangga Penggugat dengan Tergugat yang telah berada pada kondisi yang telah pecah (*broken marriage*) dan tidak dapat dipertahankan lagi, oleh karenanya majelis hakim menilai bahwa mempertahankan rumah tangga yang demikian akan mendatangkan kemafsadatan yang berkepanjangan, setidaknya bagi Penggugat dan Tergugat. Oleh karena itu kemafsadatan tersebut harus dihindari sebagaimana dalil hukum pada Kitab *al-Asybah wan Nadhaair* halaman 62, yaitu :

دَرْأُ الْمَفَاسِدِ مُقَدَّمٌ عَلي جَلْبِ الْمَصَالِحِ

yang artinya : “Menghindari mafsadat (kerusakan) harus didahulukan dari pada menarik kemaslahatan”, yang diambil alih menjadi pendapat majelis hakim dan untuk mengakhiri kemafsadatan tersebut Majelis menilai bahwa perceraian dipandang sebagai solusi terbaik bagi Penggugat dan Tergugat;

Menimbang, bahwa tujuan perkawinan adalah untuk membentuk rumah tangga yang bahagia dan kekal yang dilandasi dengan cinta dan kasih sayang sesuai ketentuan pasal 1 Undang-undang Nomor 1 Tahun 1974 tentang Perkawinan jo. pasal 3 Kompilasi Hukum Islam juga sebagaimana tersirat dalam al-Qur'an surat ar-Rum ayat 21 yang berbunyi:

{وَمِنْ آيَاتِهِ أَنْ خَلَقَ لَكُمْ مِنْ أَنْفُسِكُمْ أَزْوَاجًا لِتَسْكُنُوا إِلَيْهَا وَجَعَلَ بَيْنَكُمْ مَوَدَّةً وَرَحْمَةً إِنَّ فِي ذَلِكَ لَآيَاتٍ
لِقَوْمٍ يَتَفَكَّرُونَ} [الروم: ٢١]

Artinya: *“Dan di antara tanda-tanda kekuasaan-Nya ialah Dia menciptakan untukmu istri-istri dari jenismu sendiri, supaya kamu cenderung dan merasa tenteram, dan Allah telah jadikan diantaramu rasa kasih dan sayang. Sesungguhnya pada yang demikian itu benar-benar terdapat tanda-tanda bagi kaum yang berfikir";*
dan ternyata tujuan perkawinan tersebut tidak lagi dapat terwujud dalam rumah tangga Penggugat dengan Tergugat;

Menimbang, bahwa demikian pula Yurisprudensi Mahkamah Agung RI Nomor 237 K/AG/1998 tanggal 17 Maret 1999 yang menetapkan bahwa : *“cekcok, hidup berpisah, tidak dalam satu tempat kediaman bersama, salah satu pihak tidak berniat meneruskan kehidupan bersama dengan pihak lain, merupakan fakta yang cukup sesuai dengan alasan perceraian Pasal 39 ayat*

Disclaimer
Kepaniteraan Mahkamah Agung Republik Indonesia berusaha untuk selalu mencantumkan informasi paling kini dan akurat sebagai bentuk komitmen Mahkamah Agung untuk pelayanan publik, transparansi dan akuntabilitas pelaksanaan fungsi peradilan. Namun dalam hal-hal tertentu masih dimungkinkan terjadi permasalahan teknis terkait dengan akurasi dan keterkinian informasi yang kami sajikan, hal mana akan terus kami perbaiki dari waktu kewaktu.
Dalam hal Anda menemukan inakurasi informasi yang termuat pada situs ini atau informasi yang seharusnya ada, namun belum tersedia, maka harap segera hubungi Kepaniteraan Mahkamah Agung RI melalui :
Email : kepaniteraan@mahkamahagung.go.id   Telp : 021-384 3348 (ext.318)

(2) Undang-Undang Nomor 1 Tahun 1974 tentang Perkawinan jo Pasal 19 huruf (f) Peraturan Pemerintah Nomor 9 Tahun 1975 tentang pelaksanaan Undang- Undang Nomor 1 Tahun 1974 jo. Pasal 116 huruf (f) Kompilasi Hukum Islam”;

Yurisprudensi MARI Nomor : 1354 K/Pdt/2000, tanggal 08 September 2003 yang menyatakan bahwa *suami istri yang telah pisah rumah dan tidak saling mempedulikan, sudah merupakan fakta adanya perselisihan dan pertengkaran sehingga tidak ada harapan untuk hidup rukun lagi dalam rumah tangga dapat dijadikan alasan untuk mengabulkan permohonan perceraian*;

Menimbang, bahwa berdasarkan pertimbangan-pertimbangan tersebut di atas, dan dengan mengingat ketentuan Pasal 125 HIR maka gugatan Penggugat dapat dikabulkan secara verstek;

Menimbang, bahwa berdasarkan pertimbangan-pertimbangan tersebut di atas, dan dengan mengingat ketentuan Pasal 119 Ayat (2) huruf (c) Kompilasi Hukum Islam maka gugatan Penggugat telah dapat dikabulkan dengan menjatuhkan talak satu bain shugra Tergugat kepada Penggugat;

Menimbang, bahwa terhadap jenis *talak bain sughraa* yang diputuskan kepada Penggugat dan Tergugat, maka sesuai maksud pasal 119 ayat (1) Kompilasi Hukum Islam, antara Penggugat dan Tergugat tidak diperbolehkan rujuk akan tetapi boleh menikah lagi dengan akad nikah baru meskipun masih dalam masa iddah;

Menimbang, bahwa terhadap perceraian dengan jenis *talak satu bain sughraa*, ditetapkan masa tunggu atau iddah bagi Penggugat selama sekurang-kurangnya 90 (sembilan puluh) hari terhitung sejak putusan mempunyai kekuatan hukum tetap sebagaimana maksud pasal 11 ayat (1) dan ayat (3) Undang-Undang Nomor 1 Tahun 1974 jo. Pasal 39 PeraturanPemerintah Nomor 9 Tahun 1975 ayat (1) huruf b jo. pasal 153 ayat (2) huruf b dan pasal 153 ayat (4) Kompilasi Hukum Islam;

Menimbang, bahwa perkara tersebut termasuk bidang perkawinan, maka sesuai dengan ketentuan Pasal 89 ayat (1) Undang-Undang Nomor 7 Tahun 1989 yang telah dirubah dengan Undang-Undang Nomor 3 tahun 2006 dan perubahan kedua dengan Undang-Undang Nomor 50 Tahun 2009 tentang

Disclaimer
Kepaniteraan Mahkamah Agung Republik Indonesia berusaha untuk selalu mencantumkan informasi paling kini dan akurat sebagai bentuk komitmen Mahkamah Agung untuk pelayanan publik, transparansi dan akuntabilitas pelaksanaan fungsi peradilan. Namun dalam hal-hal tertentu masih dimungkinkan terjadi permasalahan teknis terkait dengan akurasi dan keterkinian informasi yang kami sajikan, hal mana akan terus kami perbaiki dari waktu kewaktu.
Dalam hal Anda menemukan inakurasi informasi yang termuat pada situs ini atau informasi yang seharusnya ada, namun belum tersedia, maka harap segera hubungi Kepaniteraan Mahkamah Agung RI melalui :
Email : kepaniteraan@mahkamahagung.go.id   Telp : 021-384 3348 (ext.318)

Peradilan Agama, maka biaya yang timbul dalam perkara ini dibebankan kepada Penggugat;

Memperhatikan, segala peraturan perundang-undangan yang berlaku serta hukum syara' yang berkaitan dengan perkara ini;

MENGADILI

1. Menyatakan Tergugat yang telah dipanggil secara resmi dan patut untuk datang menghadap di persidangan, tidak hadir;

2. Mengabulkan gugatan Penggugat dengan verstek;

3. Menjatuhkan talak satu bain sughra Tergugat (**XXXXXXXXXX**) terhadap Penggugat (**XXXXXXXXXX**);

4. Membebankan biaya perkara kepada Penggugat sejumlah **Rp.736.000,00 (tujuh ratus tiga puluh enam ribu rupiah)**;

Demikian putusan ini dijatuhkan dalam rapat musyawarah majelis hakim Pengadilan Agama Soreang pada hari Selasa tanggal 29 Desember 2020 Masehi bertepatan dengan tanggal 14 Jumadil Awwal 1442 Hijriyah, oleh kami Kamil Amrulloh, S.H.I. sebagai Ketua Majelis dan Noor Faiz, S.H.I., M.H. serta Abdul Gani Syafii, S.H.I., M.H. masing-masing sebagai hakim anggota. Putusan tersebut pada hari itu juga diucapkan dalam sidang terbuka untuk umum oleh Ketua Majelis dan dihadiri hakim anggota tersebut, dengan didampingi oleh Ida Fadilah Fajariah, S.T., S.H. sebagai panitera pengganti dengan dihadiri oleh Penggugat dan tanpa hadirnya Tergugat.

Hakim Anggota I	Ketua Majelis
**Noor Faiz, S.H.I., M.H.**	**Kamil Amrulloh, S.H.I.**

Disclaimer
Kepaniteraan Mahkamah Agung Republik Indonesia berusaha untuk selalu mencantumkan informasi paling kini dan akurat sebagai bentuk komitmen Mahkamah Agung untuk pelayanan publik, transparansi dan akuntabilitas pelaksanaan fungsi peradilan. Namun dalam hal-hal tertentu masih dimungkinkan terjadi permasalahan teknis terkait dengan akurasi dan keterkinian informasi yang kami sajikan, hal mana akan terus kami perbaiki dari waktu kewaktu.
Dalam hal Anda menemukan inakurasi informasi yang termuat pada situs ini atau informasi yang seharusnya ada, namun belum tersedia, maka harap segera hubungi Kepaniteraan Mahkamah Agung RI melalui :
Email : kepaniteraan@mahkamahagung.go.id Telp : 021-384 3348 (ext.318)

Mahkamah Agung Republik Indonesia

Hakim Anggota II

Abdul Gani Syafii, S.H.I., M.H.

Panitera Pengganti

Ida Fadilah Fajariah, S.T., S.H.

PERINCIAN BIAYA PERKARA:

1.	Pendaftaran	:	Rp.	30.000,00
2.	Proses	:	Rp.	50.000,00
3.	Panggilan Penggugat	:	Rp.	200.000,00
4.	PNBP Relaas Panggilan Penggugat	:	Rp.	10.000,00
5.	Panggilan Tergugat	:	Rp.	420.000,00
6.	PNBP Relaas Panggilan Tergugat	:	Rp.	10.000,00
7.	Redaksi	:	Rp.	10.000,00
8.	Materai	:	Rp.	6.000,00
	**Jumlah**	**:**	**Rp.**	**736.000,00**

Disclaimer
Kepaniteraan Mahkamah Agung Republik Indonesia berusaha untuk selalu mencantumkan informasi paling kini dan akurat sebagai bentuk komitmen Mahkamah Agung untuk pelayanan publik, transparansi dan akuntabilitas pelaksanaan fungsi peradilan. Namun dalam hal-hal tertentu masih dimungkinkan terjadi permasalahan teknis terkait dengan akurasi dan keterkinian informasi yang kami sajikan, hal mana akan terus kami perbaiki dari waktu kewaktu.
Dalam hal Anda menemukan inakurasi informasi yang termuat pada situs ini atau informasi yang seharusnya ada, namun belum tersedia, maka harap segera hubungi Kepaniteraan Mahkamah Agung RI melalui :
Email : kepaniteraan@mahkamahagung.go.id   Telp : 021-384 3348 (ext.318)
