# Supplementary material for: Classification of divorce causes during the COVID-19 pandemic using convolutional neural networks
Source: PeerJ Comput Sci. 2022 Jun 30;8:e998. doi: 10.7717/peerj-cs.998 (PMC9299239; doi:10.7717/peerj-cs.998)
Supplement: Supplemental Information 6 [file peerj-cs-08-998-s006.zip › Rujuk Dataset/Data ke-4.pdf]

Pdt.I.C.1

P U T U S A N
Nomor 12/Pdt.G/2020/PN Bbu

DEMI KEADILAN BERDASARKAN KETUHANAN YANG MAHA ESA

Pengadilan Negeri Blambangan Umpu yang memeriksa dan memutus perkara perdata pada tingkat pertama, telah menjatuhkan putusan sebagai berikut dalam perkara gugatan antara:

Penggugat, umur 54 tahun, agama Kristen Protestan, pekerjaan pedagang, bertempat tinggal di Kabupaten Way Kanan, Lampung, dalam hal ini memberikan kuasa kepada Ali Rahman, S.H., M.H., Advokat pada Kantor Pengacara Ali Rahman, S.H & Rekan, yang beralamat di Jalan Mayjend Ryacudu Km 5, Kelurahan Blambangan Umpu, Kecamatan Blambangan Umpu, Kabupaten Way Kanan berdasarkan Surat Kuasa Khusus Nomor 060/AR/Pdt.G/X/2020 tanggal 8 Oktober 2020, selanjutnya disebut sebagai **Penggugat**;

Lawan:

Tergugat, umur 51 tahun, agama Kristen Protestan, pekerjaan Penata Rias, bertempat tinggal di Kabupaten Way Kanan, Lampung, selanjutnya disebut sebagai **Tergugat**;

Pengadilan Negeri tersebut;

Setelah membaca berkas perkara beserta surat-surat yang bersangkutan;

Setelah mendengar Penggugat;

TENTANG DUDUK PERKARA

Menimbang, bahwa Penggugat dengan surat gugatan tanggal 8 Oktober 2020 yang diterima dan didaftarkan di Kepaniteraan Pengadilan Negeri Blambangan Umpu pada tanggal 8 Oktober 2020 dalam Register Nomor 12/Pdt.G/2020/PN Bbu, telah mengajukan gugatan sebagai berikut:

1. Bahwa Penggugat dan Tergugat adalah pasangan Suami Istri Sah yang menikah secara Hukum Agama Kristen pada tanggal 18 maret 1989 yang dilangsungkan di Gereja Kristen Sumatera Bagian Selatan di Kecamatan Baradatu, dan Perkawinan tersebut telah didaftarkan di Kantor Pencatatan Sipil sebagaimana diterangkan dalam Kutipan Akta Perkawinan Nomor: -, tanggal 23 Maret 1989 yang dikeluarkan oleh Kantor Catatan Sipil Kabupaten Lampung Utara di Kotabumi (vide bukti P.1);

Disclaimer
Kepaniteraan Mahkamah Agung Republik Indonesia berusaha untuk selalu mencantumkan informasi paling kini dan akurat sebagai bentuk komitmen Mahkamah Agung untuk pelayanan publik, transparansi dan akuntabilitas pelaksanaan fungsi peradilan. Namun dalam hal-hal tertentu masih dimungkinkan terjadi permasalahan teknis terkait dengan akurasi dan keterkinian informasi yang kami sajikan, hal mana akan terus kami perbaiki dari waktu kewaktu.
Dalam hal Anda menemukan inakurasi informasi yang termuat pada situs ini atau informasi yang seharusnya ada, namun belum tersedia, maka harap segera hubungi Kepaniteraan Mahkamah Agung RI melalui :
Email : kepaniteraan@mahkamahagung.go.id Telp : 021-384 3348 (ext.318)

2. Bahwa dari perkawinan antara Penggugat dengan Tergugat telah dikaruniai 1 (satu) orang anak yang bernama Anak Penggugat dan Tergugat, umur 24 tahun dan anak tersebut saat ini telah menikah;
3. Bahwa sejak menikah, Penggugat dan Tergugat tinggal dirumah orang tua Tergugat selama ± 1 tahun setelah itu Penggugat tinggal di rumah milik bersama di Kabupaten Way Kanan, sampai dengan berpisah;
4. Bahwa pada awal pernikahan antara Penggugat dengan Tergugat dalam membina rumah tangga berjalan dengan baik dan harmonis serta tidak ada permasalahan karena antara Penggugat dan Tergugat saling menyayangi dan mengasihi;
5. Bahwa kemudian suasana dan kehidupan harmonis rumah tangga Penggugat dengan Tergugat sejak bulan Desember 2018 mulai berubah, karena antara Penggugat dengan Tergugat sering terjadi perselisihan dan percekcokan secara terus menerus sehingga tidak ada harapan akan hidup rukun lagi dalam berumah tangga;
6. Bahwa penyebab perselisihan dan percekcokan antara Penggugat dengan Tergugat tersebut adalah sebagai berikut :
    6.1. Bahwa Tergugat diketahui memiliki hubungan dengan laki-laki lain, yang berakibat antara Penggugat dan Tergugat bertengkar terus menerus sehingga suasana rumah tangga sudah tidak harmonis lagi;
    6.2. Bahwa sejak tanggal 21 Januari 2019 Tergugat pergi meninggalkan Penggugat;
    6.3. Bahwa Tergugat bersifat keras kepala, tidak patuh dan suka melawan nasehat Penggugat;
7. Bahwa pada tanggal 21 Januari 2019 Tergugat pamit kepada Penggugat untuk pergi kepasar, akan tetapi setelah di tunggu Tergugat tidak juga kembali, kemudian Penggugat berusahan mencari Tergugat akan tetapi dari pihak keluarga Tergugat dan tetangga tidak ada yang tahu kepergian Tergugat;
8. Bahwa pada bulan Maret 2019 Tergugat kembali namun Tergugat tidak pulang kerumah Penggugat tapi kerumah saudara Tergugat;
9. Bahwa sejak Tergugat kembali pulang kerumah saudara Tergugat, Penggugat telah berupaya sebanyak tiga kali menjemput Tergugat untuk kebali kerumah Penggugat akan tetapi Tergugat tidak mau dan meminta untuk diceraikan oleh Penggugat, yang berakibat antara Penggugat dan Tergugat bertengkar;
10. Bahwa sejak kejadian tersebut di atas antara Penggugat dan Tergugat berpisah tempat tinggal sampai saat ini tanpa saling menjalankan kewajiban sebagaimana layaknya suami istri, Penggugat tinggal dirumah

Disclaimer
Kepaniteraan Mahkamah Agung Republik Indonesia berusaha untuk selalu mencantumkan informasi paling kini dan akurat sebagai bentuk komitmen Mahkamah Agung untuk pelayanan publik, transparansi dan akuntabilitas pelaksanaan fungsi peradilan. Namun dalam hal-hal tertentu masih dimungkinkan terjadi permasalahan teknis terkait dengan akurasi dan keterkinian informasi yang kami sajikan, hal mana akan terus kami perbaiki dari waktu kewaktu.
Dalam hal Anda menemukan inakurasi informasi yang termuat pada situs ini atau informasi yang seharusnya ada, namun belum tersedia, maka harap segera hubungi Kepaniteraan Mahkamah Agung RI melalui :
Email : kepaniteraan@mahkamahagung.go.id Telp : 021-384 3348 (ext.318)

miik bersama di Kabupaten Way Kanan, sedangkan Tergugat tinggal di Kabupaten Way Kanan, hal ini sudah berjalan kurang lebih 1 (satu) tahun 9 (sembilan) bulan lamanya;

11. Bahwa Tergugat sudah tidak peduli lagi dengan Penggugat untuk mempertahankan rumah tangga dengan Penggugat, oleh karenanya antara Penggugat dengan Tergugat sudah tidak ada suasana Harmonis serta sudah tidak ada ikatan lahir batin antara satu sama lainnya; Dengan keadaan demikian, maka tujuan Perkawinan sebagaimana dimaksud dalam pasal 1 Undang – undang No. 1 tahun 1974 tentang Perkawinan yang menyatakan bahwa “perkawinan adalah ikatan lahir batin antara suami istri dengan tujuan membentuk rumah tangga yang bahagia” tidak akan tercapai;

12. Bahwa oleh karena Penggugat dengan Tergugat sudah berpisah selama 1 (satu) tahun 9 (Sembilan) bulan berturut turut, Tergugat sudah tidak mau untuk kembali kepada Penggugat serta Tergugat juga mengakui bahwasannya telah berselingkuh dengan laki laki lain yang menyebabkan antara Penggugat dan Tergugat selalu bertengkar terus menerus sehingga keadaan rumah tangga sudah tidah harmonis lagi, berdasarkan ketentuan pasal 19 huruf (a), (b) dan (f) Undang-undang R.I. Nomor 1 Tahun 1974 tentang Perkawinan dan Kompilasi Hukum Islam, Penggugat merasa berhak dan mempunyai alasan untuk mengajukan Gugatan Perceraian terhadap Tergugat karena itu jalan terbaik yang harus ditempuh;

Berdasarkan seluruh uraian dan alasan – alasan tersebut diatas, mohon kiranya Pengadilan Negeri Blambangan Umpu, dalam hal ini Mejelis Hakim yang memeriksa dan mengadili perkara ini untuk menjatuhkan putusan sebagai berikut :

Primer :

1. Menerima dan mengabulkan gugatan Penggugat seluruhnya;

2. Memutuskan menyatakan Perkawinan antara Penggugat dengan Tergugat pada tanggal 18 Maret 1989 yang dilangsungkan di Gereja Kristen Sumatera Bagian Selatan di Kecamatan Baradatu, sebagaimana diterangkan dalam Akta Perkawinan Nomor: -, tanggal 23 Maret 1989 yang dikeluarkan oleh Dinas Catatan Sipil Kabupaten Lampung Utara di Kotabumi putus karena perceraian**;**

3. Memberi Izin kepada Kantor Dinas Kependudukan dan Pencatatan Sipil Kabupaten Lampung Utara untuk melakukan Pendaftaran Putusan ini dan menerbitkan Akta Perceraian dari Perkawinan Penggugat dengan Tergugat tersebut diatas.

Disclaimer
Kepaniteraan Mahkamah Agung Republik Indonesia berusaha untuk selalu mencantumkan informasi paling kini dan akurat sebagai bentuk komitmen Mahkamah Agung untuk pelayanan publik, transparansi dan akuntabilitas pelaksanaan fungsi peradilan. Namun dalam hal-hal tertentu masih dimungkinkan terjadi permasalahan teknis terkait dengan akurasi dan keterkinian informasi yang kami sajikan, hal mana akan terus kami perbaiki dari waktu kewaktu.
Dalam hal Anda menemukan inakurasi informasi yang termuat pada situs ini atau informasi yang seharusnya ada, namun belum tersedia, maka harap segera hubungi Kepaniteraan Mahkamah Agung RI melalui :
Email : kepaniteraan@mahkamahagung.go.id Telp : 021-384 3348 (ext.318)

4. Membebankan biaya – biaya perkara menurut hukum;

Subsider :

Apabila Pengadilan Negeri Blambangan Umpu berpendapat lain, Penggugat mohon Putusan yang seadil – adilnya (ex aequo et bono);

Menimbang, bahwa pada hari sidang yang telah ditentukan Penggugat telah datang menghadap di persidangan, akan tetapi Tergugat tidak datang menghadap ataupun menyuruh orang lain menghadap untuk mewakilinya, meskipun berdasarkan risalah panggilan sidang tanggal 12 Oktober 2020 dan tanggal 16 Oktober 2020 telah dipanggil dengan patut, sedangkan tidak ternyata bahwa tidak datangnya itu disebabkan oleh sesuatu halangan yang sah;

Menimbang, bahwa oleh karena Tergugat tidak pernah hadir di persidangan maupun mengirimkan wakilnya untuk hadir dipersidangan, maka Majelis Hakim berpendapat bahwa pemeriksaan perkara ini tetap dilanjutkan tanpa hadirnya Tergugat dan oleh karenanya Tergugat dianggap tidak menggunakan haknya;

Menimbang, bahwa selanjutnya pemeriksaan perkara ini dilanjutkan dengan membacakan surat gugatan Penggugat dimana Penggugat menyatakan tetap pada gugatannya dan tidak akan mengajukan perubahan dalam gugatannya;

Menimbang, bahwa untuk menguatkan dalil–dalil gugatannya Penggugat, telah mengajukan bukti – bukti surat yaitu:

1. Fotokopi Kartu Tanda Penduduk Nomor - atas nama Penggugat selanjutnya pada fotokopi bukti surat tersebut diberi tanda **P-1**;

2. Fotokopi Kartu Keluarga Nomor: - atas nama Kepala Keluarga Penggugat, selanjutnya pada fotokopi bukti surat tersebut diberi tanda **P-2**;

3. Fotokopi Kutipan Akta Perkawinan Nomor - tanggal 23 Maret 1989, selanjutnya pada fotocopy bukti surat tersebut diberi tanda **P-3**;

Menimbang, bahwa fotokopi bukti surat tersebut telah dibubuhi materai cukup, dan dilegalisasi serta telah dicocokkan sesuai dengan aslinya, selanjutnya kemudian fotokopi bukti surat tersebut dilampirkan dalam berkas perkara ini, sedangkan asli surat-surat bukti tersebut dikembalikan kepada Penggugat;

Menimbang, bahwa selain surat-surat bukti tersebut di atas, Penggugat juga telah menghadirkan saksi-saksi yang memberikan keterangan di bawah sumpah yaitu Saksi I dan Saksi II;

Menimbang, bahwa Penggugat tidak mengajukan kesimpulan dan akhirnya Penggugat dalam perkara ini menyatakan tidak ada hal-hal yang diajukan lagi dan mohon putusan;

Disclaimer
Kepaniteraan Mahkamah Agung Republik Indonesia berusaha untuk selalu mencantumkan informasi paling kini dan akurat sebagai bentuk komitmen Mahkamah Agung untuk pelayanan publik, transparansi dan akuntabilitas pelaksanaan fungsi peradilan. Namun dalam hal-hal tertentu masih dimungkinkan terjadi permasalahan teknis terkait dengan akurasi dan keterkinian informasi yang kami sajikan, hal mana akan terus kami perbaiki dari waktu kewaktu.
Dalam hal Anda menemukan inakurasi informasi yang termuat pada situs ini atau informasi yang seharusnya ada, namun belum tersedia, maka harap segera hubungi Kepaniteraan Mahkamah Agung RI melalui :
Email : kepaniteraan@mahkamahagung.go.id Telp : 021-384 3348 (ext.318)

Menimbang, bahwa untuk mempersingkat putusan ini, maka segala sesuatu yang termuat dalam berita acara persidangan dianggap telah termuat dan menjadi bagian yang tak terpisahkan dari putusan ini;

TENTANG PERTIMBANGAN HUKUM

Menimbang, bahwa maksud dan tujuan gugatan Penggugat pada pokoknya adalah mengenai gugatan perceraian terhadap Tergugat sebagaimana posita dan petitum surat gugatan di atas;

Menimbang, bahwa oleh karena pada hari persidangan yang telah ditetapkan Tergugat tidah hadir di persidangan atau menyuruh orang lain untuk menghadap ke persidangan sebagai wakilnya, meskipun ia telah dipanggil dengan patut, sesuai dengan Risalah Panggilan tertanggal 12 Oktober 2020 dan tanggal 16 Oktober 2020, sedangkan ketidakhadiran Tergugat tersebut bukan disebabkan oleh suatu halangan yang sah, maka perkara ini diputus di luar hadirnya Tergugat (*verstek*);

Menimbang, bahwa meskipun perkara ini diperiksa tanpa hadirnya Tergugat, berdasarkan Pasal 283 RBg dan Pasal 22 ayat (2) Peraturan Pemerintah Nomor 9 Tahun 1975 tentang Pelaksanaan Undang-undang Nomor 1 Tahun 1974 tentang Perkawinan, Penggugat yang hadir di persidangan tetap berkewajiban untuk membuktikan bahwa dalil-dalil yang diajukan di persidangan cukup beralasan dan tidak bertentangan dengan hukum;

Menimbang, bahwa Penggugat dalam surat gugatannya pada pokonya mendalilkan bahwa Penggugat telah melangsungkan perkawinan dengan Tergugat pada tanggal 18 maret 1989 dan dalam perkawinan tersebut sejak bulan Desember 2018 antara Penggugat dan Tergugat sering terjadi perselisihan dan percekcokan secara terus-menerus karena Tergugat diketahui memiliki hubungan dengan laki-laki lain, Tergugat pergi meninggalkan Penggugat serta sifat Tergugat yang keras kepala, tidak patuh dan suka melawan nasehat Penggugat;

Menimbang, bahwa pada tanggal 21 Januari 2019 Tergugat pergi meninggalkan Tergugat dan Penggugat telah berupaya sebanyak tiga kali menjemput Tergugat untuk kembali ke rumah Penggugat akan tetapi Tergugat tidak mau dan meminta untuk diceraikan oleh Penggugat;

Menimbang, bahwa sejak kejadian tersebut di atas antara Penggugat dan Tergugat berpisah tempat tinggal sampai saat ini tanpa saling menjalankan kewajiban sebagaimana layaknya suami istri;

Menimbang, bahwa dari perkawinan antara Penggugat dengan Tergugat, telah dikaruniai 1 (satu) orang anak yang bernama Anak Penggugat dan Tergugat, umur 24 tahun;

Disclaimer
Kepaniteraan Mahkamah Agung Republik Indonesia berusaha untuk selalu mencantumkan informasi paling kini dan akurat sebagai bentuk komitmen Mahkamah Agung untuk pelayanan publik, transparansi dan akuntabilitas pelaksanaan fungsi peradilan. Namun dalam hal-hal tertentu masih dimungkinkan terjadi permasalahan teknis terkait dengan akurasi dan keterkinian informasi yang kami sajikan, hal mana akan terus kami perbaiki dari waktu kewaktu.
Dalam hal Anda menemukan inakurasi informasi yang termuat pada situs ini atau informasi yang seharusnya ada, namun belum tersedia, maka harap segera hubungi Kepaniteraan Mahkamah Agung RI melalui :
Email : kepaniteraan@mahkamahagung.go.id Telp : 021-384 3348 (ext.318)

Menimbang, bahwa berdasarkan ketentuan Pasal 20 Ayat (1) Peraturan Pemerintah Nomor 9 tahun 1975 tentang Pelaksanaan Undang-undang Nomor 1 Tahun 1974 tentang Perkawinan, gugatan perceraian diajukan oleh suami atau isteri atau kuasanya kepada pengadilan yang di daerah hukumnya meliputi tempat kediaman Tergugat;

Menimbang, bahwa berdasarkan dalil Penggugat di persidangan dan keterangan Saksi-saksi, Tergugat bertempat tinggal di Kabupaten Way Kanan yang berada dalam wilayah hukum Pengadilan Negeri Blambangan Umpu, dengan demikian Pengadilan Negeri Blambangan Umpu berwenang untuk memeriksa dan mengadili gugatan perceraian yang diajukan oleh Penggugat;

Menimbang, bahwa selanjutnya Majelis Hakim akan mempertimbangkan alat bukti yang diajukan Penggugat di persidangan;

Menimbang, bahwa Penggugat untuk menguatkan dalilnya telah mengajukan bukti surat berupa bukti P-1 sampai dengan P-3 dan Saksi-Saksi yaitu 1. Saksi I dan 2. Saksi II, yang memberikan keterangan di bawah sumpah sesuai dengan agama yang dianutnya yang mana keterangan para saksi tersebut selengkapnya sebagaimana tertuang dalam Berita Acara Persidangan perkara ini;

Menimbang, bahwa berdasarkan bukti surat P-1 berupa fotokopi Kartu Tanda Penduduk Nomor - atas nama Penggugat dan bukti surat P-2 berupa fotokopi Kartu Keluarga Nomor: - atas nama Kepala Keluarga Penggugat serta dikaitkan dengan keterangan Para Saksi, Penggugat dan Tergugat merupakan sepasang suami istri dan bertempat tinggal di Kabupaten Way Kanan;

Menimbang, bahwa berdasarkan dalil gugatan Penggugat yang dikaitkan dengan bukti surat P-3 berupa fotokopi Kutipan Akta Perkawinan Nomor - serta keterangan Para Saksi di persidangan yang pada pokoknya menerangkan bahwa Penggugat dan Tergugat adalah sepasang suami istri, diketahui bahwa Penggugat dan Tergugat telah melangsungkan pernikahan di Kotabumi, Kabupaten Lampung Utara pada tanggal 18 Maret 1989, perkawinan tersebut telah dilakukan menurut keyakinan Penggugat dan Tergugat, serta telah dicatatkan di Kantor Catatan Sipil Kabupaten Lampung Utara sehingga telah memenuhi ketentuan Pasal 2 ayat (1) Undang-undang Nomor 1 Tahun 1974 tentang Perkawinan yang menyatakan bahwa “perkawinan adalah sah apabila dilakukan menurut hukum masing-masing agamanya dan kepercayaannya itu” dan Pasal 2 ayat (2) Undang-undang Nomor 1 Tahun 1974 yang menyatakan “tiap-tiap perkawinan dicatat menurut peraturan perundang-undangan yang berlaku”, serta telah memenuhi ketentuan Pasal 2 ayat (2) Peraturan Pemerintah Nomor 9 tahun 1975 tentang pelaksanaan Undang-Undang Nomor 1 Tahun 1974 tentang Perkawinan yang menyatakan bahwa

Disclaimer
Kepaniteraan Mahkamah Agung Republik Indonesia berusaha untuk selalu mencantumkan informasi paling kini dan akurat sebagai bentuk komitmen Mahkamah Agung untuk pelayanan publik, transparansi dan akuntabilitas pelaksanaan fungsi peradilan. Namun dalam hal-hal tertentu masih dimungkinkan terjadi permasalahan teknis terkait dengan akurasi dan keterkinian informasi yang kami sajikan, hal mana akan terus kami perbaiki dari waktu kewaktu.
Dalam hal Anda menemukan inakurasi informasi yang termuat pada situs ini atau informasi yang seharusnya ada, namun belum tersedia, maka harap segera hubungi Kepaniteraan Mahkamah Agung RI melalui :
Email : kepaniteraan@mahkamahagung.go.id   Telp : 021-384 3348 (ext.318)

pencatatan perkawinan dari mereka yang melangsungkan perkawinan menurut agamanya dan kepercayaannya selain agama Islam, dilakukan oleh pegawai pencatat perkawinan pada kantor catatan sipil. Dengan demikian, perkawinan antara Penggugat dan Tergugat sah menurut hukum dan peraturan perundang-undangan yang berlaku;

Menimbang, bahwa berdasarkan dalil gugatan Penggugat yang dikaitkan dengan keterangan Saksi I dan Saksi II yang merupakan tetangga Para Pihak, Penggugat dan Tergugat cekcok sejak Bulan Januari 2019. Percekcokan tersebut disebabkan karena Tergugat berselingkuh, Tergugat memiliki sifat keras kepala dan tidak patuh pada Penggugat. Para Saksi juga menerangkan bahwa Tergugat pergi dari rumah sejak Bulan Januari 2019 dan Tergugat secara tegas menyatakan bahwa ia ingin bercerai dari Penggugat;

Menimbang, bahwa selanjutnya Majelis Hakim akan mempertimbangkan satu persatu petitum gugatan Penggugat;

Menimbang, bahwa tujuan dari perkawinan adalah untuk membentuk keluarga yang bahagia dan kekal berdasarkan Ketuhanan Yang Maha Esa sebagaimana diatur dalam Pasal 1 Undang-undang Nomor 1 Tahun 1974 tentang Perkawinan;

Menimbang, bahwa berdasarkan dalil gugatan Penggugat, bukti surat dan keterangan saksi-saksi yang diajukan oleh Penggugat sebagaimana diuraikan di atas, dalam kaitannya satu sama lain yang ternyata bersesuaian, percekcokan antara Penggugat dan Tergugat terjadi akibat Tergugat berselingkuh, sifat Tergugat yang keras kepala dan tidak patuh pada Penggugat serta dari diri Tergugat sudah tidak ada keinginan untuk hidup bersama dengan Penggugat karena Tergugat sudah pergi meninggalkan rumah Penggugat dan Tergugat mengatakan bahwa ia ingin bercerai dari Penggugat, sehingga menurut Majelis Hakim dengan kondisi rumah tangga yang demikian apabila perkawinan antara Penggugat dan Tergugat tetap dipertahankan, tujuan pernikahan yaitu untuk membentuk keluarga yang bahagia dan kekal sebagaimana diatur dalam Pasal 1 Undang-undang Nomor 1 Tahun 1974 tentang Perkawinan tidak akan tercapai;

Menimbang, bahwa berdasarkan ketentuan Pasal 19 Peraturan Pemerintah Nomor 9 tahun 1975 tentang Pelaksanaan Undang-undang Nomor 1 Tahun 1974 tentang Perkawinan, pengakhiran perkawinan (perceraian) haruslah disertai dengan alasan-alasan yang cukup, antara lain adalah:

a. Salah satu pihak berbuat zina atau menjadi pemabok, pemadat, penjudi dan lain sebagainya yang sukar disembuhkan;

Disclaimer
Kepaniteraan Mahkamah Agung Republik Indonesia berusaha untuk selalu mencantumkan informasi paling kini dan akurat sebagai bentuk komitmen Mahkamah Agung untuk pelayanan publik, transparansi dan akuntabilitas pelaksanaan fungsi peradilan. Namun dalam hal-hal tertentu masih dimungkinkan terjadi permasalahan teknis terkait dengan akurasi dan keterkinian informasi yang kami sajikan, hal mana akan terus kami perbaiki dari waktu kewaktu.
Dalam hal Anda menemukan inakurasi informasi yang termuat pada situs ini atau informasi yang seharusnya ada, namun belum tersedia, maka harap segera hubungi Kepaniteraan Mahkamah Agung RI melalui :
Email : kepaniteraan@mahkamahagung.go.id Telp : 021-384 3348 (ext.318)

b. Salah satu pihak meninggalkan pihak lain selama 2 (dua) tahun berturut-turut tanpa ijin pihak lain dan tanpa alasan yang sah atau karena hal lain di luar kemampuannya;

c. Salah satu pihak mendapat hukuman penjara 5 (lima) tahun atau hukuman yang lebih berat setelah perkawinan berlangsung;

d. Salah satu pihak melakukan kekejaman atau penganiayaan berat yang membahayakan pihak yang lain;

e. Salah satu pihak mendapat cacat badan atau penyakit dengan akibat tidak dapat menjalankan kewajibannya sebagai suami / isteri;

f. Antara suami dan isteri terus menerus terjadi perselisihan dan pertengkaran dan tidak ada harapan akan hidup rukun lagi dalam rumah tangga;

Menimbang, bahwa berdasarkan dalil gugatan Penggugat, bukti surat dan keterangan saksi-saksi yang diajukan oleh Penggugat sebagaimana diuraikan di atas, dalam kaitannya satu sama lain yang ternyata bersesuaian, Majelis Hakim berpendapat bahwa telah ternyata antara Penggugat dan Tergugat terjadi perselisihan dan pertengkaran secara terus-menerus dan tidak ada keinginan untuk hidup bersama kembali karena kedua belah pihak kini sudah pisah rumah. Dengan demikian, menurut Majelis Hakim, alasan perceraian yang diajukan oleh Penggugat telah memenuhi salah satu alasan perceraian sebagaimana diatur dalam ketentuan di atas, yaitu Pasal 19 huruf f yang berbunyi: “antara suami dan isteri terus-menerus terjadi perselisihan dan pertengkaran dan tidak ada harapan akan hidup rukun lagi dalam rumah tangga”;

Menimbang, bahwa berdasarkan pertimbangan di atas, petitum angka 2 yang pada intinya memohonkan agar perkawinan antara Penggugat dan Tergugat putus karena perceraian beralasan hukum untuk dikabulkan;

Menimbang, bahwa selanjutnya mengenai petitum angka 3 tentang memberi izin kepada Kantor Dinas Kependudukan dan Pencatatan Sipil Kabupaten Lampung Utara untuk melakukan pendaftaran putusan ini dan menerbitkan Akta Perceraian, bahwa berdasarkan ketentuan Pasal 40 Undang-undang Nomor 23 tahun 2006 tentang Administrasi Kependudukan sebagaimana telah diperbaharui dengan disahkannya Undang-undang Nomor 24 Tahun 2013 tentang Perubahan atas Undang-undang Nomor 23 Tahun 2006 tentang Administrasi Kependudukan, perceraian wajib dilaporkan oleh yang bersangkutan kepada Kantor Dinas Kependudukan dan Pencatatan Sipil paling lambat 60 (enam puluh) hari sejak putusan pengadilan tentang perceraian memperoleh kekuatan hukum tetap dan berdasarkan laporan tersebut Pejabat Pencatatan Sipil mencatat pada Register Akta Perceraian dan menerbitkan

Disclaimer
Kepaniteraan Mahkamah Agung Republik Indonesia berusaha untuk selalu mencantumkan informasi paling kini dan akurat sebagai bentuk komitmen Mahkamah Agung untuk pelayanan publik, transparansi dan akuntabilitas pelaksanaan fungsi peradilan. Namun dalam hal-hal tertentu masih dimungkinkan terjadi permasalahan teknis terkait dengan akurasi dan keterkinian informasi yang kami sajikan, hal mana akan terus kami perbaiki dari waktu kewaktu.
Dalam hal Anda menemukan inakurasi informasi yang termuat pada situs ini atau informasi yang seharusnya ada, namun belum tersedia, maka harap segera hubungi Kepaniteraan Mahkamah Agung RI melalui :
Email : kepaniteraan@mahkamahagung.go.id Telp : 021-384 3348 (ext.318)

Kutipan Akta Perceraian. Berdasarkan ketentuan tersebut, penerbitan Akta Perceraian tidaklah otomatis, melainkan Penggugat harus mengajukan permohonan penerbitan Akta Perceraian kepada Kantor Dinas Kependudukan dan Pencatatan Sipil Kabupaten Way Kanan dengan melampirkan salinan putusan ini setelah memperoleh kekuatan hukum tetap. Dengan demikian, berdasarkan pertimbangan tersebut di atas, petitum angka 3 beralasan hukum untuk dikabulkan dengan memperbaiki amar petitum tersebut sebagaimana yang akan disebutkan dalam amar putusan ini;

Menimbang, bahwa oleh karena petitum angka 2 dan angka 3 dikabulkan, maka petitum angka 1 yang memohon agar gugatan diterima dan dikabulkan seluruhnya, secara otomatis menjadi dapat dikabulkan;

Menimbang, bahwa oleh karena jangka waktu dan formalitas panggilan menurut hukum telah diindahkan dengan sepatutnya serta gugatan tersebut tidak melawan hukum dan beralasan, maka Tergugat yang telah dipanggil dengan patut akan tetapi tidak datang menghadap di persidangan dan tidak menyuruh orang lain menghadap sebagai wakilnya, harus dinyatakan tidak hadir dan gugatan tersebut dikabulkan dengan verstek seluruhnya;

Menimbang, bahwa oleh karena gugatan Penggugat dikabulkan dengan *verstek* dan Tergugat ada di pihak yang kalah maka berdasarkan ketentuan Pasal 192 RBg, Tergugat dihukum untuk membayar biaya perkara ini;

Memperhatikan Undang-undang Nomor 1 Tahun 1974 tentang Perkawinan, Peraturan Pemerintah Nomor 9 Tahun 1975 tentang Pelaksanaan Undang-undang Nomor 1 Tahun 1974 Tentang Perkawinan, dan Pasal 149 RBg dan peraturan-peraturan lain yang bersangkutan;

MENGADILI:

1. Menyatakan Tergugat telah dipanggil dengan patut namun tidak hadir;
2. Mengabulkan gugatan Penggugat untuk seluruhnya dengan verstek;
3. Menyatakan Perkawinan antara Penggugat dengan Tergugat pada tanggal 18 Maret 1989 yang dilangsungkan di Gereja Kristen Sumatera Bagian Selatan di Kecamatan Baradatu, sebagaimana diterangkan dalam Akta Perkawinan Nomor: -, tanggal 23 Maret 1989 yang dikeluarkan oleh Dinas Catatan Sipil Kabupaten Lampung Utara di Kotabumi putus karena perceraian dengan segala akibat hukumnya;
4. Memerintahkan kepada Penggugat untuk melaporkan perceraian ini kepada Kantor Dinas Kependudukan dan Pencatatan Sipil Kabupaten Way Kanan untuk dicatatkan dalam Register Akta Perceraian dan diterbitkan Kutipan Akta Perceraian sebagaimana ketentuan yang berlaku;
5. Menghukum Tergugat untuk membayar biaya perkara sejumlah Rp526.000,00 (lima ratus dua puluh enam ribu Rupiah);

Disclaimer
Kepaniteraan Mahkamah Agung Republik Indonesia berusaha untuk selalu mencantumkan informasi paling kini dan akurat sebagai bentuk komitmen Mahkamah Agung untuk pelayanan publik, transparansi dan akuntabilitas pelaksanaan fungsi peradilan. Namun dalam hal-hal tertentu masih dimungkinkan terjadi permasalahan teknis terkait dengan akurasi dan keterkinian informasi yang kami sajikan, hal mana akan terus kami perbaiki dari waktu kewaktu.
Dalam hal Anda menemukan inakurasi informasi yang termuat pada situs ini atau informasi yang seharusnya ada, namun belum tersedia, maka harap segera hubungi Kepaniteraan Mahkamah Agung RI melalui :
Email : kepaniteraan@mahkamahagung.go.id Telp : 021-384 3348 (ext.318)

Demikian diputuskan dalam sidang permusyawaratan Majelis Hakim Pengadilan Negeri Blambangan Umpu, pada hari Kamis, tanggal 22 Oktober 2020, oleh kami, Fadesha Lucia Martina, S.H., M.H., sebagai Hakim Ketua, Echo Wardoyo, S.H dan Hanifia Zammi Fernanda, S.H masing-masing sebagai Hakim Anggota, yang ditunjuk berdasarkan Surat Penetapan Ketua Pengadilan Negeri Blambangan Umpu Nomor 12/Pdt.G/2020/PN Bbu tanggal 8 Oktober 2020, putusan tersebut pada hari Senin, tanggal 26 Oktober 2020 diucapkan dalam persidangan terbuka untuk umum oleh Hakim Ketua dengan dihadiri oleh Muhammad Noor Yustisiananda, S.H dan Hanifia Zammi Fernanda, S.H sebagai Hakim Anggota, Seslan Haryadi, S.H., Panitera Pengganti pada Pengadilan Negeri Blambangan Umpu dan Kuasa Penggugat tanpa dihadiri oleh Tergugat maupun Kuasanya.

Hakim Anggota, Hakim Ketua,

Echo Wardoyo, S.H Fadesha Lucia Martina, S.H., M.H.

Hanifia Zammi Fernanda, S.H

Panitera Pengganti,

Seslan Haryadi, S.H.

Perincian biaya :

1. Pendaftaran Gugatan..............	:	Rp30.000,00;
2. Biaya Proses	:	Rp50.000,00;
3..Panggilan Sidang	:	Rp400.000,00;
4..Biaya Sumpah	:	Rp20.000,00;
5..PNBP Relas Panggilan	:	Rp10.000,00;
6..Redaksi Putusan	:	Rp10.000,00;
7..Materai Putusan	:	Rp6.000,00;
Jumlah	:	Rp526.000,00;
		(lima ratus dua puluh enam ribu Rupiah)

Disclaimer
Kepaniteraan Mahkamah Agung Republik Indonesia berusaha untuk selalu mencantumkan informasi paling kini dan akurat sebagai bentuk komitmen Mahkamah Agung untuk pelayanan publik, transparansi dan akuntabilitas pelaksanaan fungsi peradilan. Namun dalam hal-hal tertentu masih dimungkinkan terjadi permasalahan teknis terkait dengan akurasi dan keterkinian informasi yang kami sajikan, hal mana akan terus kami perbaiki dari waktu kewaktu.
Dalam hal Anda menemukan inakurasi informasi yang termuat pada situs ini atau informasi yang seharusnya ada, namun belum tersedia, maka harap segera hubungi Kepaniteraan Mahkamah Agung RI melalui :
Email : kepaniteraan@mahkamahagung.go.id Telp : 021-384 3348 (ext.318)
