# Supplementary material for: Classification of divorce causes during the COVID-19 pandemic using convolutional neural networks
Source: PeerJ Comput Sci. 2022 Jun 30;8:e998. doi: 10.7717/peerj-cs.998 (PMC9299239; doi:10.7717/peerj-cs.998)
Supplement: Supplemental Information 6 [file peerj-cs-08-998-s006.zip › Rujuk Dataset/Data ke-5.pdf]

P U T U S A N

Nomor 455/Pdt.G/2020/PA.Tlk

بِسْــــــــــــــمِ اللهِ الرَّحْمنِ الرَّحِيمِ

DEMI KEADILAN BERDASARKAN KETUHANAN YANG MAHA ESA

Pengadilan Agama Teluk Kuantan yang memeriksa dan mengadili perkara pada tingkat pertama dalam sidang Majelis telah menjatuhkan putusan sebagai berikut dalam perkara Cerai Gugat antara:

Penggugat, umur 28 tahun, agama Islam, pendidikan SD, pekerjaan Mengurus Rumah Tangga, tempat tinggal di [redacted]., sebagai **Penggugat**;

melawan

Tergugat, umur 40 tahun, agama Islam, pendidikan SLTP, pekerjaan Petani Sawit, tempat tinggal [redacted], sebagai **Tergugat**;

Pengadilan Agama tersebut;

Telah membaca dan mempelajari berkas perkara;

Telah mendengar keterangan Penggugat;

Telah memeriksa alat-alat bukti di persidangan;

DUDUK PERKARA

Menimbang, bahwa Penggugat dengan surat gugatannya tertanggal 07 Desember 2020 yang telah terdaftar di Kepaniteraan Pengadilan Agama Teluk Kuantan dengan register perkara Nomor 455/Pdt.G/2020/PA.Tlk, mengemukakan dalil-dalil sebagai berikut:

1. Bahwa Penggugat dan Tergugat adalah suami istri (Ba'da dukhul) yang melangsungkan pernikahan pada hari Minggu tanggal 16 Mei 2010, dan di catat oleh Pegawai Pencatat Nikah Kantor Urusan Agama Kecamatan. Singingi Kabupaten Kuantan Singingi Provinsi Riau, sesuai dengan Kutipan

Disclaimer
Kepaniteraan Mahkamah Agung Republik Indonesia berusaha untuk selalu mencantumkan informasi paling kini dan akurat sebagai bentuk komitmen Mahkamah Agung untuk pelayanan publik, transparansi dan akuntabilitas pelaksanaan fungsi peradilan. Namun dalam hal-hal tertentu masih dimungkinkan terjadi permasalahan teknis terkait dengan akurasi dan keterkinian informasi yang kami sajikan, hal mana akan terus kami perbaiki dari waktu kewaktu.
Dalam hal Anda menemukan inakurasi informasi yang termuat pada situs ini atau informasi yang seharusnya ada, namun belum tersedia, maka harap segera hubungi Kepaniteraan Mahkamah Agung RI melalui :
Email : kepaniteraan@mahkamahagung.go.id Telp : 021-384 3348 (ext.318)

Akta Nikah Nomor: [redacted] dan ditandatangani oleh KUA Kecamatan Tersebut tertanggal 17 Mei 2010 M;

2. Bahwa sebelum menikah Penggugat berstatus gadis, dan Tergugat berstatus jejaka;
3. Bahwa setelah pernikahan tersebut Penggugat dan Tergugat tinggal di rumah orang tua Penggugat selama 8 tahun yang terletak di Desa Jake dan selama menjalani masa-masa pernikahan tersebut Penggugat dan Tergugat telah dikaruniai 2 (dua) orang anak yang bernama:
   - Anak kandung I: tempat dan Tanggal Lahir : Jake, 19 Maret 2011;
   - Anak kandung II: tempat dan Tanggal Lahir : Jake, 24 Juli 2016;
4. Bahwa Penggugat menginginkan anak Penggugat dan Tergugat di asuh oleh Penggugat dikarenakan anak tersebut masih dibawah umur dan masih sangat membutuhkan kasih sayang dari Penggugat;
5. Bahwa Penggugat mengkhawatirkan anak tersebut terganggu psikologisnya apabila Tergugat yang mengasuhnya dikarenakan Tergugat sudah menikah secara siri dengan perempuan lain;
6. Bahwa pada awal-awal pernikahan, rumah tangga Penggugat dan Tergugat berjalan rukun dan harmonis, namun sekitar tahun 2017, kehidupan rumah tangga Penggugat dan Tergugat sering terjadi pertengkaran dan perselisihan, yang penyebabnya adalah :
   - Bahwa Tergugat malas bekerja dan berulang kali Penggugat untuk menesehati Tergugat agar mau merubah sifat buruknya akan tetapi Tergugat tidak mengihiraukannya dan Penggugat sudah berusaha untuk membantu perekonomian keluarga akan tetapi Tergugat tidak bisa menghargai apa yang yang telah dilakukan Penggugat;
   - Bahwa Tergugat sering melalaikan kewajibannya sebagai seorang suami;
   - Bahwa Tergugat sudah menikah secara siri dengan perempuan lain;
   - Bahwa mulai tanggal 25 Mei 2018 sampai dengan saat ini Tergugat tidak pernah memberikan nafkah lahir kepada Penggugat sehingga yang menanggung kebutuhan rumah tangga di tanggung oleh Penggugat;
7. Bahwa keluarga Penggugat dan Tergugat sudah berusaha untuk

Disclaimer
Kepaniteraan Mahkamah Agung Republik Indonesia berusaha untuk selalu mencantumkan informasi paling kini dan akurat sebagai bentuk komitmen Mahkamah Agung untuk pelayanan publik, transparansi dan akuntabilitas pelaksanaan fungsi peradilan. Namun dalam hal-hal tertentu masih dimungkinkan terjadi permasalahan teknis terkait dengan akurasi dan keterkinian informasi yang kami sajikan, hal mana akan terus kami perbaiki dari waktu kewaktu.
Dalam hal Anda menemukan inakurasi informasi yang termuat pada situs ini atau informasi yang seharusnya ada, namun belum tersedia, maka harap segera hubungi Kepaniteraan Mahkamah Agung RI melalui :
Email : kepaniteraan@mahkamahagung.go.id   Telp : 021-384 3348 (ext.318)

menasehati Penggugat dan Tergugat, namun tidak berhasil, dikarenakan keinginan yang kuat dari Penggugat untuk berpisah, sehingga upaya perdamaian tidak berhasil;

8. Bahwa puncak pertengkaran Penggugat dan Tergugat terjadi pada tanggal 25 Mei 2018 dimana Tergugat pergi dari rumah orang tua Penggugat dan Tergugat pulang kerumah orang tua Tergugat;
9. Bahwa dikarenakan antara Penggugat dan Tergugat tidak sudah tidak bersama selama lebih kurang 2 tahun, dan karena seringnya terjadi pertengkaran dan perselisihan di dalam rumah tangga Penggugat dan Tergugat, maka Penggugat merasa sangat sulit untuk membentuk keluarga yang sakinah mawaddah warohmah bersama Tergugat, oleh karena itu Penggugat bermohon kepada Majelis Hakim yang mengadili perkara ini untuk mengabulkan gugatan dari Penggugat;
10. Bahwa Penggugat sanggup membayar seluruh biaya yang timbul akibat perkara ini;

Berdasarkan dalil-dalil/alasan tersebut diatas, Penggugat mohon dengan hormat, kiranya bapak Ketua Pengadilan Agama Teluk Kuantan cq. Majelis Hakim yang memeriksa dan mengadili perkara ini, berkenan untuk menjatuhkan putusan yang amarnya berbunyi sebagai berikut :

PRIMAIR :

1. Menerima dan mengabulkan gugatan Penggugat;
2. Menjatuhkan Talak satu Ba'in shughra Tergugat (**Tergugat**) kepada Penggugat (**Penggugat**) dengan segala akibat hukumnya;
3. Menetapkan hak asuh anak yang bernama : Anak kandung **I**dan Anak kandung **II**kepada Penggugat;
4. Membebankan biaya perkara menurut hukum;

SUBSIDAIR :

Jika Majelis Hakim berpendapat lain, mohon kiranya memberikan putusan yang seadil-adilnya (ex aequo et bono).

Bahwa pada hari sidang yang telah ditetapkan Penggugat telah menghadap sendiri ke muka sidang, sedangkan Tergugat tidak menghadap dan tidak menyuruh orang lain untuk menghadap sebagai Wakil/Kuasa hukumnya

Disclaimer
Kepaniteraan Mahkamah Agung Republik Indonesia berusaha untuk selalu mencantumkan informasi paling kini dan akurat sebagai bentuk komitmen Mahkamah Agung untuk pelayanan publik, transparansi dan akuntabilitas pelaksanaan fungsi peradilan. Namun dalam hal-hal tertentu masih dimungkinkan terjadi permasalahan teknis terkait dengan akurasi dan keterkinian informasi yang kami sajikan, hal mana akan terus kami perbaiki dari waktu kewaktu.
Dalam hal Anda menemukan inakurasi informasi yang termuat pada situs ini atau informasi yang seharusnya ada, namun belum tersedia, maka harap segera hubungi Kepaniteraan Mahkamah Agung RI melalui :
Email : kepaniteraan@mahkamahagung.go.id Telp : 021-384 3348 (ext.318)

meskipun telah dipanggil secara resmi dan patut yang relaas panggilannya dibacakan di dalam sidang, sedangkan tidak ternyata bahwa tidak datangnya itu disebabkan suatu halangan yang sah;

Bahwa, Majelis dalam persidangan telah berusaha mendamaikan dengan jalan memberi saran dan nasehat kepada Penggugat agar mengurungkan keinginannya untuk bercerai dan mencoba kembali membina rumah tangga yang *sakinah, mawaddah wa rahmah* namun tidak berhasil, sedangkan usaha damai melalui proses mediasi tidak dapat dilaksanakan karena Tergugat tidak pernah datang menghadap ke persidangan;

Bahwa, oleh sebab upaya damai tidak berhasil, selanjutnya pemeriksaan perkara ini dilanjutkan secara litigasi diawali dengan membacakan surat gugatan Penggugat yang pada pokoknya tetap dipertahankan oleh Penggugat;

Bahwa, terhadap gugatan Penggugat, Tergugat tidak dapat didengar jawabannya dalam persidangan karena Tergugat tidak pernah hadir di persidangan;

Bahwa untuk meneguhkan dalil-dalil gugatannya, Penggugat telah mengajukan bukti-bukti sebagai berikut:

I. Bukti Surat

1. Fotokopi Kartu Tanda Penduduk an. **Penggugat** NIK [redacted] yang aslinya dikeluarkan oleh Kepala Dinas Kependudukan dan Pencatatan Sipil Kabupaten Kuantan Singingi tanggal 06-05-2019, bermeterai cukup dan di*nazegelen* serta telah dicocokkan dengan aslinya oleh ketua majelis, ternyata cocok, lalu diberi tanda bukti P.1 dan diparaf;
2. Fotokopi Kutipan Akta Nikah Nomor [redacted] tanggal 17 Mei 2010 atas nama Penggugat dan Tergugat yang aslinya dikeluarkan oleh Kantor Urusan Agama Kecamatan Singingi, Kabupaten Kuantan Singingi, Provinsi Riau, bermeterai cukup dan di*nazegelen* serta telah dicocokkan dengan aslinya oleh ketua majelis, ternyata cocok, lalu diberi tanda bukti P.2 dan diparaf;
3. Fotokopi Akta Kelahiran an. **Anak kandung I** No.[redacted] yang dikeluarkan oleh Dinas

Disclaimer
Kepaniteraan Mahkamah Agung Republik Indonesia berusaha untuk selalu mencantumkan informasi paling kini dan akurat sebagai bentuk komitmen Mahkamah Agung untuk pelayanan publik, transparansi dan akuntabilitas pelaksanaan fungsi peradilan. Namun dalam hal-hal tertentu masih dimungkinkan terjadi permasalahan teknis terkait dengan akurasi dan keterkinian informasi yang kami sajikan, hal mana akan terus kami perbaiki dari waktu kewaktu.
Dalam hal Anda menemukan inakurasi informasi yang termuat pada situs ini atau informasi yang seharusnya ada, namun belum tersedia, maka harap segera hubungi Kepaniteraan Mahkamah Agung RI melalui :
Email : kepaniteraan@mahkamahagung.go.id Telp : 021-384 3348 (ext.318)

Kependudukan dan Pencatatan Sipil Kabupaten Kuantan Singingi tertanggal 08 Juli 2011, fotokopi tersebut bermaterai cukup dan telah dinazegelen serta telah dicocokkan pula dengan aslinya dan diberi tanda bukti (P.3);

4. Fotokopi Akta Kelahiran an. **Anak kandung II** No.[redacted] yang dikeluarkan oleh Dinas Kependudukan dan Pencatatan Sipil Kabupaten Kuantan Singingi tertanggal 17 Februari 2017, fotokopi tersebut bermaterai cukup dan telah dinazegelen serta telah dicocokkan pula dengan aslinya dan diberi tanda bukti (P.4);

II. Bukti Saksi

1. **Saksi 1**, umur 51 tahun, agama Islam, pendidikan SLTA, pekerjaan Mengurus rumah tangga, tempat tinggal di [redacted], telah memberikan keterangan di bawah sumpah secara agama Islam sebagai berikut:
   - Bahwa saksi kenal dengan Penggugat karena saksi adalah bibi Penggugat, sedangkan Tergugat adalah suami Penggugat yang saksi kenal sejak ia menikah dengan Penggugat;
   - Bahwa Penggugat dan Tergugat menikah pada tanggal 16 Mei 2010 di KUA Kecamatan Singingi Kabupaten Kuantan Singingi;
   - Bahwa Penggugat berstatus gadis dan Tergugat bersatus jejaka;
   - Bahwa setelah menikah Penggugat dan Tergugat bertempat tinggal membina rumah tangga di rumah kediaman orang tua Penggugat di [redacted] sampai berpisah;
   - Bahwa dari pernikahan tersebut Penggugat dan Tergugat telah dikaruniai anak 2 (dua) orang;
   - Bahwa kedua anak Penggugat dan Tergugat yang bernama Anak kandung I dan Anak kandung II saat ini dalam asuhan Penggugat;
   - Bahwa yang saksi ketahui kedua anak tersebut selama dalam asuhan Penggugat dalam menjaga dan mendidik anaknya dengan baik dengan memberikan kasih sayang cukup;

Disclaimer
Kepaniteraan Mahkamah Agung Republik Indonesia berusaha untuk selalu mencantumkan informasi paling kini dan akurat sebagai bentuk komitmen Mahkamah Agung untuk pelayanan publik, transparansi dan akuntabilitas pelaksanaan fungsi peradilan. Namun dalam hal-hal tertentu masih dimungkinkan terjadi permasalahan teknis terkait dengan akurasi dan keterkinian informasi yang kami sajikan, hal mana akan terus kami perbaiki dari waktu kewaktu.
Dalam hal Anda menemukan inakurasi informasi yang termuat pada situs ini atau informasi yang seharusnya ada, namun belum tersedia, maka harap segera hubungi Kepaniteraan Mahkamah Agung RI melalui :
Email : kepaniteraan@mahkamahagung.go.id   Telp : 021-384 3348 (ext.318)

- Bahwa kehidupan rumah tangga Penggugat dan Tergugat pada awalnya berjalan baik dan rukun, namun sejak tahun 2017 rumah tangga mereka tidak harmonis lagi karena sering terjadi perselisihan dan pertengkaran yang terus menerus;
- Bahwa penyebab perselisihan dan pertengkaran dalam rumah tangga Penggugat dan Tergugat adalah karena Tergugat kurang memberikan nafkah yang layak sehingga kebutuhan rumah tangga tidak tercukupi;
- Bahwa sepengetahuan saksi penyebab lainnya adalah Tergugat selingkuh dengan perempuan lain;
- Bahwa saksi mengetahui karena saksi sering melihat Penggugat dan Tergugat bertengkar;
- Bahwa Penggugat dan Tergugat sudah pisah tempat tinggal sampai sekarang sudah berjalan lebih kurang 3 tahun;
- Bahwa yang meninggalkan kediaman bersama adalah Tergugat;
- Bahwa selama berpisah Penggugat dan Tergugat sudah tidak pernah lagi berkomunikasi;
- Bahwa selama pisah Tergugat tidak pernah datang dan tidak pernah lagi memberi nafkah untuk Penggugat, dan Tergugat sudah tidak memperdulikan Penggugat lagi;
- Bahwa upaya untuk merukunkan Penggugat dengan Tergugat pernah dilakukan oleh pihak keluarga kedua pihak, namun tidak berhasil;

2. **Saksi 2** umur 19 tahun, agama Islam, pendidikan SLTA, pekerjaan Swasta, bertempat tinggal di [redacted], di bawah sumpah memberikan keterangan di bawah sumpah secara agama Islam yang pada pokoknya sebagai berikut :
- Bahwa saksi kenal dengan Penggugat karena saksi adalah adik kandung Penggugat, sedangkan Tergugat adalah suami Penggugat yang saksi kenal sejak ia menikah dengan Penggugat;
- Bahwa Penggugat dan Tergugat menikah pada tanggal 16 Mei 2010

Disclaimer
Kepaniteraan Mahkamah Agung Republik Indonesia berusaha untuk selalu mencantumkan informasi paling kini dan akurat sebagai bentuk komitmen Mahkamah Agung untuk pelayanan publik, transparansi dan akuntabilitas pelaksanaan fungsi peradilan. Namun dalam hal-hal tertentu masih dimungkinkan terjadi permasalahan teknis terkait dengan akurasi dan keterkinian informasi yang kami sajikan, hal mana akan terus kami perbaiki dari waktu kewaktu.
Dalam hal Anda menemukan inakurasi informasi yang termuat pada situs ini atau informasi yang seharusnya ada, namun belum tersedia, maka harap segera hubungi Kepaniteraan Mahkamah Agung RI melalui :
Email : kepaniteraan@mahkamahagung.go.id   Telp : 021-384 3348 (ext.318)

di KUA Kecamatan Singingi Kabupaten Kuantan Singingi;

- Bahwa setelah menikah Penggugat dan Tergugat bertempat tinggal membina rumah tangga di rumah orang tua Penggugat di Desa [redacted] sampai berpisah;
- Bahwa dari pernikahan tersebut Penggugat dan Tergugat telah dikaruniai dua orang anak;
- Bahwa kedua anak Penggugat dan Tergugat yang bernama Anak kandung I dan Saputi Ajeng Ayuningtyas dalam asuhan Penggugat;
- Bahwa setahu saksi selama ini Penggugat dalam mengasuh, menjaga dan mendidik dengan baik dan dengan penuh kasih sayang;
- Bahwa kehidupanrumah tangga Penggugat dan Tergugat pada awalnya berjalan baik dan rukun, namun sejak tahun 2017 rumah tangga mereka tidak harmonis lagi karena sering terjadi perselisihan dan pertengkaran;
- Bahwa penyebab perselisihan dan pertengkaran dalam rumah tangga Penggugat dan Tergugat adalah karena Tergugat kurang memberikan nafkah dan Tergugat selingkuh dengan perempuan lain;
- Bahwa saksi mengetahui karena saksi sering mendengar dan melihat Penggugat dan Tergugat bertengkar;
- Bahwa Penggugat dan Tergugat sudah pisah tempat tinggal sampai sekarang sudah berjalan lebih kurang 3 tahun;
- Bahwa yang meninggalkan kediaman bersama adalah Tergugat;
- Bahwa selama berpisah Penggugat dan Tergugat sudah tidak pernah lagi berkomunikasi;
- Bahwa tidak, selamapisah Tergugat tidak pernah datang dan tidak pernah lagi memberi nafkah untuk Penggugat dan Tergugat sudah tidak memperdulikan Penggugat lagi;
- Bahwa upaya untuk merukunkan Penggugat dengan Tergugat pernah dilakukan oleh pihak keluarga kedua pihak, namun tidak berhasil;

Disclaimer
Kepaniteraan Mahkamah Agung Republik Indonesia berusaha untuk selalu mencantumkan informasi paling kini dan akurat sebagai bentuk komitmen Mahkamah Agung untuk pelayanan publik, transparansi dan akuntabilitas pelaksanaan fungsi peradilan. Namun dalam hal-hal tertentu masih dimungkinkan terjadi permasalahan teknis terkait dengan akurasi dan keterkinian informasi yang kami sajikan, hal mana akan terus kami perbaiki dari waktu kewaktu.
Dalam hal Anda menemukan inakurasi informasi yang termuat pada situs ini atau informasi yang seharusnya ada, namun belum tersedia, maka harap segera hubungi Kepaniteraan Mahkamah Agung RI melalui :
Email : kepaniteraan@mahkamahagung.go.id Telp : 021-384 3348 (ext.318)

Bahwa setelah diberi kesempatan Penggugat menyatakan tidak mengajukan alat bukti apapun lagi dan menyampaikan kesimpulan secara lisan yang pada pokoknya menyatakan tetap pada gugatannya dan selanjutnya mohon putusan;

Bahwa untuk mempersingkat uraian putusan ini cukup ditunjuk segala hal yang telah termuat dalam berita acara sidang yang dianggap sebagai satu kesatuan tak terpisahkan dalam putusan ini;

PERTIMBANGAN HUKUM

Menimbang, bahwa maksud dan tujuan gugatan Penggugat adalah sebagaimana tersebut di atas;

Menimbang, bahwa berdasarkan ketentuan Pasal 49 huruf a Undang-Undang Nomor 7 Tahun 1989 tentang Peradilan Agama dan penjelasannya sebagaimana telah diubah dengan Undang-Undang Nomor 3 Tahun 2006, terakhir dengan perubahan kedua Undang-Undang Nomor 50 Tahun 2009, maka penyelesaian perkara di tingkat pertama antara orang-orang yang beragama Islam di bidang perkawinan merupakan wewenang *absolut* Pengadilan Agama, dan dalam perkara ini telah ternyata subjek hukum dalam perkara ini adalah beragama Islam, dan perkara yang diajukan adalah perkara dibidang perkawinan, oleh karena itu maka Pengadilan Agama berwenang secara *absolut* untuk memeriksa, memutus, dan menyelesaikan perkara ini;

Menimbang, bahwa Penggugat telah datang menghadap *(in person)* di persidangan, sedangkan Tergugat tidak pernah hadir di persidangan dan tidak pula mengirimkan orang lain selaku wakil atau kuasanya yang sah untuk hadir di persidangan meskipun telah dipanggil secara resmi dan patut sesuai Pasal 26 Peraturan Pemerintah Nomor 9 Tahun 1975, dan tidak ternyata ketidakhadiran Tergugat itu disebabkan suatu alasan yang sah menurut hukum (*default without reason*) sesuai Pasal 149 ayat (1) R.bg, maka harus dinyatakan Tergugat tidak hadir dan perkara *a quo* dapat diperiksa tanpa hadirnya Tergugat;

Menimbang, bahwa berdasarkan ketentuan Pasal 39 ayat (1) Undang-Undang Nomor 1 Tahun 1974 jo. Pasal 65 dan Pasal 82 Undang-Undang Nomor 7 Tahun 1989 sebagaimana telah diubah dengan

Disclaimer
Kepaniteraan Mahkamah Agung Republik Indonesia berusaha untuk selalu mencantumkan informasi paling kini dan akurat sebagai bentuk komitmen Mahkamah Agung untuk pelayanan publik, transparansi dan akuntabilitas pelaksanaan fungsi peradilan. Namun dalam hal-hal tertentu masih dimungkinkan terjadi permasalahan teknis terkait dengan akurasi dan keterkinian informasi yang kami sajikan, hal mana akan terus kami perbaiki dari waktu kewaktu.
Dalam hal Anda menemukan inakurasi informasi yang termuat pada situs ini atau informasi yang seharusnya ada, namun belum tersedia, maka harap segera hubungi Kepaniteraan Mahkamah Agung RI melalui :
Email : kepaniteraan@mahkamahagung.go.id Telp : 021-384 3348 (ext.318)

Undang-Undang Nomor 3 Tahun 2006, terakhir dengan perubahan kedua Undang-Undang Nomor 50 Tahun 2009 jo. Pasal 31 Peraturan Pemerintah Nomor 9 Tahun 1975 serta Pasal 143 ayat (1) Kompilasi Hukum Islam, maka Majelis telah berusaha mendamaikan dengan menasehati Penggugat agar bersabar dan rukun kembali dengan Tergugat akan tetapi tidak berhasil;

Menimbang, bahwa berdasarkan Peraturan Mahkamah Agung Republik Indonesia Nomor 1 Tahun 2016 Tentang Prosedur Mediasi di Pengadilan, semua perkara yang masuk ke pengadilan terlebih dahulu harus dilaksanakan mediasi, akan tetapi dalam perkara yang bersangkutan karena pihak Tergugat tidak pernah hadir, maka mediasi tidak dapat dilaksanakan;

Menimbang, bahwa oleh sebab antara Penggugat dan Tergugat terdapat hubungan perkawinan sebagai suami istri yang belum pernah putus (bercerai), maka keduanya memiliki kepentingan hukum untuk bertindak sebagai pihak-pihak dalam perkara ini (*persona standi in judicio*);

Menimbang, bahwa setelah meneliti surat gugatan Penggugat dan mendengar keterangan Penggugat maka yang menjadi masalah pokok dalam perkara ini adalah Penggugat bermohon untuk bercerai dari Tergugat dengan alasan bahwa Penggugat dan Tergugat terus menerus terjadi perselisihan dan pertengkaran serta tidak ada harapan akan rukun lagi dalam rumah tangga;

Menimbang, bahwa Tergugat tidak dapat didengar jawabannya di persidangan karena Tergugat tidak pernah hadir, maka secara yuridis formal Tergugat dapat dianggap tidak bermaksud untuk mempertahankan hak-hak keperdataannya dan atau membela kepentingannya di persidangan, mengakui dan membenarkan semua Posita dan Petitum dalam surat gugatan Penggugat, namun tidak serta merta hal tersebut menjadikan gugatan Penggugat dapat dikabulkan, karena mengingat perkara ini merupakan perkara yang dikhawatirkan dapat menimbulkan suatu kebohongan besar (*de groten langen*);

Menimbang, bahwa berdasarkan alasan-alasan tersebut di atas, maka Majelis menilai alasan perceraian yang didalilkan oleh Penggugat adalah termasuk dalam alasan-alasan perceraian sebagaimana diatur dalam ketentuan Pasal 19 huruf (f) Peraturan Pemerintah Nomor 9 Tahun 1975 jo. Pasal 116 huruf (f) Kompilasi Hukum Islam;

Disclaimer
Kepaniteraan Mahkamah Agung Republik Indonesia berusaha untuk selalu mencantumkan informasi paling kini dan akurat sebagai bentuk komitmen Mahkamah Agung untuk pelayanan publik, transparansi dan akuntabilitas pelaksanaan fungsi peradilan. Namun dalam hal-hal tertentu masih dimungkinkan terjadi permasalahan teknis terkait dengan akurasi dan keterkinian informasi yang kami sajikan, hal mana akan terus kami perbaiki dari waktu kewaktu.
Dalam hal Anda menemukan inakurasi informasi yang termuat pada situs ini atau informasi yang seharusnya ada, namun belum tersedia, maka harap segera hubungi Kepaniteraan Mahkamah Agung RI melalui :
Email : kepaniteraan@mahkamahagung.go.id   Telp : 021-384 3348 (ext.318)

Menimbang, bahwa untuk dikabulkannya sebuah perceraian dengan alasan dasar tersebut di atas, maka sebagaimana Pasal 39 ayat (2) Undang-Undang Nomor 1 Tahun 1974 Tentang Perkawinan jo. Pasal 19 huruf (f) Peraturan Pemerintah Nomor 9 Tahun 1975 Tentang Pelaksanaan Undang-Undang Nomor 1 Tahun 1974 Tentang Perkawinan jo. Pasal 116 huruf (f) dan Pasal 134 Kompilasi Hukum Islam, harus terpenuhi beberapa unsur sebagai berikut, (1). Rumah tangga sudah tidak rukun dan harmonis, sering terjadi perselisihan dan pertengkaran secara terus menerus. (2). Perselisihan dan pertengkaran tersebut menyebabkan suami istri tidak ada harapan untuk rukun kembali. (3). Pengadilan sudah mendamaikan, namun tidak berhasil;

Menimbang, bahwa karena perkara ini adalah mengenai bidang perceraian yang dinilai penting untuk ditemukan kebenaran materiilnya, dan untuk lebih meyakinkan Majelis atas dalil-dalil gugatan Penggugat, maka sesuai dengan Keputusan Mahkamah Agung RI Nomor KMA/032/SK/IV/2006 tanggal 4 April 2006 tentang Pemberlakuan Buku II Pedoman Teknis Administrasi dan Teknis Peradilan Agama, Edisi Revisi 2013, Majelis berpendapat Penggugat diwajibkan untuk mengajukan bukti-bukti yang dapat mendukung dalil-dalil posita dan petitum gugatannya;

Menimbang, bahwa untuk menguatkan dalil-dalil gugatannya Penggugat telah mengajukan alat bukti berupa bukti surat dan bukti saksi sebagaimana dalam duduk perkaranya di atas;

Menimbang, bahwa terhadap bukti surat yang diajukan Penggugat tersebut, Majelis berpendapat bukti tersebut merupakan fotokopi sah dari suatu akta autentik, khusus dibuat sebagai alat bukti, telah diberi meterai cukup sesuai ketentuan Pasal 2 ayat (1) Undang-Undang Nomor 13 Tahun 1985 Tentang Bea Meterai dan telah di*nazegeling* sehingga alat bukti tersebut telah memenuhi persyaratan formil dan harus dinyatakan dapat diterima, dan secara materiil dapat dipertimbangkan karena alat bukti tersebut memuat keterangan yang menguatkan dan relevan dengan dalil gugatan Penggugat;

Menimbang, bahwa alat bukti alat bukti surat berkode P.1 yang diajukan Penggugat berupa fotokopi Kartu Tanda Penduduk NIK [redacted], an. **Penggugat**, yang aslinya dikeluarkan oleh Kepala Dinas Kependudukan

Disclaimer
Kepaniteraan Mahkamah Agung Republik Indonesia berusaha untuk selalu mencantumkan informasi paling kini dan akurat sebagai bentuk komitmen Mahkamah Agung untuk pelayanan publik, transparansi dan akuntabilitas pelaksanaan fungsi peradilan. Namun dalam hal-hal tertentu masih dimungkinkan terjadi permasalahan teknis terkait dengan akurasi dan keterkinian informasi yang kami sajikan, hal mana akan terus kami perbaiki dari waktu kewaktu.
Dalam hal Anda menemukan inakurasi informasi yang termuat pada situs ini atau informasi yang seharusnya ada, namun belum tersedia, maka harap segera hubungi Kepaniteraan Mahkamah Agung RI melalui :
Email : kepaniteraan@mahkamahagung.go.id   Telp : 021-384 3348 (ext.318)

dan Pencatatan Sipil, Kabupaten Kuantan Singingi, telah bermeterai cukup dan di*nazegeling*, isi alat bukti tersebut menjelaskan bahwa Penggugat adalah warga Kabupaten Kuantan Singingi, maka Majelis berpendapat bahwa alat bukti tersebut memenuhi syarat formil dan materil alat bukti, sehingga mempunyai nilai pembuktian yang sempurna dan mengikat, karenanya berdasarkan kewenangan relatif, Penggugat berhak mengajukan gugatan cerai terhadap Tergugat di Pengadilan Agama Teluk Kuantan;

Menimbang, bahwa bukti P.2 (fotokopi Kutipan Akta Nikah Nomor [redacted] tanggal 17 Mei 2010) merupakan akta otentik yang berdaya bukti sempurna dan mengikat yang memberi bukti Penggugat dengan Tergugat telah dan masih terikat dalam perkawinan sah yang tercatat di Kantor Urusan Agama Kecamatan Singingi, Kabupaten Kuantan Singingi, Provinsi Riau, dan telah sesuai dengan ketentuan Pasal 285 R.Bg jo. Pasal 2 ayat (1) dan (2) Undang-Undang Nomor 1 Tahun 1974 jo. Pasal 7 ayat (1) Kompilasi Hukum Islam, dengan demikian Penggugat dengan Tergugat telah mempunyai hubungan dan kapasitas hukum untuk menjadi pihak dalam perkara ini (*persona standi in judicio*), karenanya Penggugat mempunyai kualitas untuk mengajukan tuntutan dalam sengketa bidang perkawinan;

Menimbang, bahwa bukti P.3 dan P.4 berupa fotokopi Akta Kelahiran anak Penggugat dan Tergugat yang dikeluarkan oleh Kepala Dinas Kependudukan dan Pencatatan Sipil Kabupaten Kuantan Singingi Provinsi Riau, merupakan akta otentik, isinya menerangkan bahwa Anak kandung I dan Anak kandung II merupakan anak sah dari Penggugat dan Tergugat, maka berdasarkan pasal 285 Rbg, bukti tersebut telah memenuhi syarat formil dan materiil;

Menimbang, bahwa oleh karena alasan perceraian yang diajukan Penggugat berkenaan dengan ketentuan Pasal 19 huruf (f) Peraturan Pemerintah Nomor 9 Tahun 1975 jo. Pasal 116 huruf (f) Kompilasi Hukum Islam di Indonesia, sehingga berdasarkan ketentuan Pasal 22 ayat (2) Peraturan Pemerintah Nomor 9 Tahun 1975 dan Pasal 134 Kompilasi Hukum Islam, maka Majelis perlu mendengarkan keterangan saksi-saksi yang berasal dari keluarga dan atau orang-orang yang dekat dengan suami istri tersebut;

Disclaimer
Kepaniteraan Mahkamah Agung Republik Indonesia berusaha untuk selalu mencantumkan informasi paling kini dan akurat sebagai bentuk komitmen Mahkamah Agung untuk pelayanan publik, transparansi dan akuntabilitas pelaksanaan fungsi peradilan. Namun dalam hal-hal tertentu masih dimungkinkan terjadi permasalahan teknis terkait dengan akurasi dan keterkinian informasi yang kami sajikan, hal mana akan terus kami perbaiki dari waktu kewaktu.
Dalam hal Anda menemukan inakurasi informasi yang termuat pada situs ini atau informasi yang seharusnya ada, namun belum tersedia, maka harap segera hubungi Kepaniteraan Mahkamah Agung RI melalui :
Email : kepaniteraan@mahkamahagung.go.id   Telp : 021-384 3348 (ext.318)

Menimbang, bahwa saksi pertama dan kedua yang diajukan Penggugat di persidangan, setelah diperiksa oleh Majelis, ternyata saksi pertama adalah bibi Penggugat dan saksi kedua adalah adik kandung Penggugat, kedua orang saksi tersebut telah dewasa atau cakap hukum sebagaimana ditetapkan Pasal 1912 KUH Perdata, dan telah disumpah sesuai kehendak Pasal 175 R.Bg, maka kedua orang saksi tersebut memenuhi syarat formil saksi sehingga dapat diterima sebagai alat bukti yang sah;

Menimbang, bahwa keterangan saksi pertama dan saksi kedua tersebut mengenai dalil gugatan Penggugat adalah hal yang diketahui dan dialami sendiri sehingga relevan dengan hal yang harus dibuktikan Penggugat;

Menimbang, bahwa setelah diperiksa oleh Majelis, ternyata saksi tersebut terdiri dari dua orang sebagaimana batas minimum yang ditetapkan oleh Pasal 306 R.Bg, keterangannya saling bersesuaian satu sama lain sebagaimana dikehendaki Pasal 309 R.Bg, menurut pendapat Majelis saksi-saksi tersebut mengetahui antara Penggugat dan Tergugat sering terjadi perselisihan dan pertengkaran dan dapat dikatakan telah mendukung dalil gugatan Penggugat, oleh karena itu dalil gugatan Penggugat telah terbukti di persidangan;

Menimbang, bahwa apabila bukti surat dan kesaksian dua orang saksi serta anggapan hukum sebagaimana tersebut di atas dihubungkan dengan dalil-dalil gugatan Penggugat Majelis menilai dalil-dalil gugatan Penggugat telah terbukti, dan dari pembuktian tersebut diketemukan fakta-fakta yuridis sebagai berikut:

1. Bahwa Penggugat telah menikah dengan Tergugat pada tanggal 16 Mei 2010 dan tercatat di Kantor Urusan Agama Kecamatan Singingi Kabupaten Kuantan Singingi Provinsi Riau;
2. Bahwa rumah tangga Penggugat dan Tergugat yang pada awalnya harmonis, akan tetapi sejak tahun 2017 sering terjadi pertengkaran yang akibatnya antara Penggugat dan Tergugat pisah rumah sejak bulan Mei 2018 rumah sampai sekarang;
3. Bahwa pihak keluarga telah berusaha mendamaikan Penggugat dan Tergugat untuk rukun kembali dalam rumah tangga akan tetapi tidak

Disclaimer
Kepaniteraan Mahkamah Agung Republik Indonesia berusaha untuk selalu mencantumkan informasi paling kini dan akurat sebagai bentuk komitmen Mahkamah Agung untuk pelayanan publik, transparansi dan akuntabilitas pelaksanaan fungsi peradilan. Namun dalam hal-hal tertentu masih dimungkinkan terjadi permasalahan teknis terkait dengan akurasi dan keterkinian informasi yang kami sajikan, hal mana akan terus kami perbaiki dari waktu kewaktu.
Dalam hal Anda menemukan inakurasi informasi yang termuat pada situs ini atau informasi yang seharusnya ada, namun belum tersedia, maka harap segera hubungi Kepaniteraan Mahkamah Agung RI melalui :
Email : kepaniteraan@mahkamahagung.go.id Telp : 021-384 3348 (ext.318)

berhasil;

4. Bahwa setelah menikah Penggugat dan Tergugat telah dikaruniai 2 (dua) orang anak bernama:
   - Anak kandung I, tempat dan tanggal lahir : Jake, 19 Maret 2011;
   - Anak kandung II, tempat dan tanggal lahir : Jake, 24 Juli 2016;
5. Bahwa anak-anak tersebut tinggal bersama Penggugat hingga sekarang;
6. Bahwa selama tinggal bersama Penggugat anak-anak tersebut tumbuh kembang dengan baik dan tidak terlantar;
7. Bahwa Penggugat/Tergugat sehari-hari menjalakan kewajiban agama seperti sholat, puasa dan ibadah lainnya serta tidak gampang emosional dan marah, serta tidak pernah dijatuhi hukuman pidana;

Menimbang, bahwa berdasarkan fakta-fakta kejadian tersebut di atas dapat disimpulkan fakta hukum bahwa antara Penggugat dan Tergugat terus menerus terjadi perselisihan dan pertengkaran dan sudah tidak ada harapan hidup rukun lagi dalam rumah tangga;

Menimbang, bahwa mempertahankan kondisi rumah tangga yang sudah demikian adanya jelas tidak akan membawa manfaat, paling tidak madlaratnya sudah nampak yaitu antara Penggugat dan Tergugat sudah tidak mampu berhubungan dengan baik dan rukun sebagaimana layaknya pasangan suami istri, oleh karena itu jalan yang terbaik bagi kedua belah pihak adalah perceraian, sebab apabila dibiarkan berlarut-larut tidak mustahil akan memunculkan kemadlaratan yang lebih besar terhadap rumah tangga dan keluarga kedua belah pihak;

Menimbang, bahwa bila salah satu pihak sudah kehilangan rasa cinta dan kasih sayangnya, maka cita ideal bagi suatu kehidupan rumah tangga tidak akan pernah menjadi kenyataan, bahkan kehidupan rumah tangga itu akan menjadi belenggu bagi kedua belah pihak, keadaan itu pula menunjukkan ikatan bathin mereka terkoyak, dan tidak ada harapan untuk rukun kembali sebagai suami istri;

Menimbang, bahwa berdasarkan fakta hukum di atas, maka Majelis Hakim berkesimpulan bahwa rumah tangga Penggugat dan Tergugat benar-benar sudah tidak harmonis dan sudah pecah karena telah terjadi

Disclaimer
Kepaniteraan Mahkamah Agung Republik Indonesia berusaha untuk selalu mencantumkan informasi paling kini dan akurat sebagai bentuk komitmen Mahkamah Agung untuk pelayanan publik, transparansi dan akuntabilitas pelaksanaan fungsi peradilan. Namun dalam hal-hal tertentu masih dimungkinkan terjadi permasalahan teknis terkait dengan akurasi dan keterkinian informasi yang kami sajikan, hal mana akan terus kami perbaiki dari waktu kewaktu.
Dalam hal Anda menemukan inakurasi informasi yang termuat pada situs ini atau informasi yang seharusnya ada, namun belum tersedia, maka harap segera hubungi Kepaniteraan Mahkamah Agung RI melalui :
Email : kepaniteraan@mahkamahagung.go.id   Telp : 021-384 3348 (ext.318)

perselisihan dan pertengkaran terus menerus yang sudah tidak mungkin lagi untuk dirukunkan dalam satu rumah tangga sehingga mempertahankan rumah tangga yang demikian tidak sejalan dengan maksud dan tujuan perkawinan sebagaimana yang dikehendaki dalam Al-Qur'an Surat Ar-Rum: 21 dan Pasal 1 Undang-Undang Nomor 1 Tahun 1974;

Menimbang, bahwa fakta hukum tersebut telah memenuhi norma hukum Islam yang terkandung dalam Kitab Fikih Sunah Juz II halaman 290 yang diambil alih menjadi pertimbangan Majelis Hakim sebagai berikut;

أنّ للزوجة أن تطلب من القاضي التفريق إذا ادعت إضرار الزوج بها إضرارا لا يستطاع معه دوام العشرة بين أمثالها

Artinya : “*bahwa istri boleh menuntut cerai kepada hakim apabila dia mengaku selalu mendapat perlakuan yang menyakitkan dari suaminya sehingga hal tersebut dapat menghalangi keberlasungan hubungan suami istri antara mereka berdua”;*

Menimbang, bahwa berkaitan dengan perkara a quo, sesuai dengan Yurisprudensi Mahkamah Agung RI. Nomor : 379/K/AG/1995 tanggal 26 Maret 1997 mengabstraksikan kaidah hukum bahwa “suami istri yang tidak berdiam serumah lagi, dan tidak ada harapan untuk hidup rukun lagi, maka rumah tangga tersebut telah terbukti retak dan pecah” ;

Menimbang, bahwa sesuai dengan Yurisprudensi Mahkamah Agung RI tanggal 19 Januari 1999 Nomor : 44K/AG/1998 mengabstraksikan kaidah hukum : “Bahwa bilamana perselisihan dan pertengkaran antara suami sitri telah terbukti dalam pemeriksaan di Pengadilan Agama dan di dukung oleh fakta tidak berhasilnya Majelis Hakim merukunkan kembali para pihak yang bersengketa sebagai suami istri, maka sesuai dengan ketentuan Pasal 19 huruf (f) Peraturan Pemerintah Nomor 9 Tahun 1975 jo. Undang-Undang Nomor 1 Tahun 1974. Secara yuridis gugatan Penggugat yang mohon perceraian dengan Tergugat haruslah di kabulkan”;

Menimbang, bahwa fakta hukum tersebut telah juga memenuhi salah satu alasan perceraian sebagaimana diatur dalam ketentuan Pasal 39 ayat (2)

Disclaimer
Kepaniteraan Mahkamah Agung Republik Indonesia berusaha untuk selalu mencantumkan informasi paling kini dan akurat sebagai bentuk komitmen Mahkamah Agung untuk pelayanan publik, transparansi dan akuntabilitas pelaksanaan fungsi peradilan. Namun dalam hal-hal tertentu masih dimungkinkan terjadi permasalahan teknis terkait dengan akurasi dan keterkinian informasi yang kami sajikan, hal mana akan terus kami perbaiki dari waktu kewaktu.
Dalam hal Anda menemukan inakurasi informasi yang termuat pada situs ini atau informasi yang seharusnya ada, namun belum tersedia, maka harap segera hubungi Kepaniteraan Mahkamah Agung RI melalui :
Email : kepaniteraan@mahkamahagung.go.id   Telp : 021-384 3348 (ext.318)

Undang-Undang Nomor 1 Tahun 1974 jo. Pasal 19 huruf f Peraturan Pemerintah Nomor 9 Tahun 1975 jo. Pasal 116 huruf f Kompilasi Hukum Islam;

Menimbang, bahwa berdasarkan pertimbangan-pertimbangan tersebut di atas dan Penggugat belum pernah dijatuhi talak, maka petitum gugatan Penggugat agar Pengadilan menjatuhkan talak satu bain sughra Tergugat terhadap Penggugat tersebut telah beralasan dan tidak melawan hukum serta memenuhi Pasal 119 ayat 2 huruf (c) Kompilasi Hukum Islam, oleh karena itu patut dikabulkan;

Menimbang bahwa talak satu ba'in sughra merupakan talak yang dijatuhkan oleh Hakim atas permintaan seorang istri, yang mana akibat hukum dari talak satu ba'in sughra adalah antara suami dan istri tidak dapat hidup bersama kembali, terkecuali dengan akad nikah yang baru;

Menimbang, bahwa mengenai petitum agar Penggugat ditetapkan sebagai pemegang hadhanah terhadap anak-anaknya, Majelis Hakim mempertimbangkan sebagai berikut:

Menimbang, bahwa setidaknya ada 4 peraturan, yaitu (1) Undang-Undang Republik Indonesia Nomor 1 Tahun 1974 tentang Perkawinan; (2) Undang-Undang Republik Indonesia Nomor 39 Tahun 1999 tentang Hak Asasi Manusia; (3) Undang-Undang Republik Indonesia Nomor 23 Tahun 2002 tentang Perlindungan Anak sebagaimana telah diubah dengan Undang-Undang Republik Indonesia Nomor 35 Tahun 2014; dan (4) Intruksi Presiden Republik Indonesia Nomor 1 Tahun 1991 tentang Kompilasi Hukum Islam yang terkait dengan hak asuh anak (hadhanah) dan hak-hak anak;

Menimbang, bahwa dari keempat peraturan yang mengatur tentang hak asuh anak dan hak-hak anak tersebut di atas, Majelis Hakim berkesimpulan bahwa pijakan utama dalam menetapkan pemegang hak asuh anak (hadhanah) adalah kepentingan terbaik atau kemaslahatan terbaik bagi anak dan bagi anak yang belum mumayyiz (12 tahun) maupun yang telah mumayyiz, menurut Majelis Hakim kepentingan terbaiknya adalah diasuh oleh ibu kandungnya sebagaimana ketentuan Pasal 105 huruf (a). Dan huruf (b) Kompilasi Hukum Islam (diserahkan kepada anak untuk memilih ikut ibu atau ayahnya). Dan selama ibu kandungnya tersebut tidak terbukti mempunyai sifat buruk yang dapat berpengaruh buruk terhadap tumbuh kembang anak atau terbukti menelantarkan anak sewaktu

Disclaimer
Kepaniteraan Mahkamah Agung Republik Indonesia berusaha untuk selalu mencantumkan informasi paling kini dan akurat sebagai bentuk komitmen Mahkamah Agung untuk pelayanan publik, transparansi dan akuntabilitas pelaksanaan fungsi peradilan. Namun dalam hal-hal tertentu masih dimungkinkan terjadi permasalahan teknis terkait dengan akurasi dan keterkinian informasi yang kami sajikan, hal mana akan terus kami perbaiki dari waktu kewaktu.
Dalam hal Anda menemukan inakurasi informasi yang termuat pada situs ini atau informasi yang seharusnya ada, namun belum tersedia, maka harap segera hubungi Kepaniteraan Mahkamah Agung RI melalui :
Email : kepaniteraan@mahkamahagung.go.id Telp : 021-384 3348 (ext.318)

selama dalam asuhannya, karena telah kita ketahui bersama bahwa dalam tradisi di Indonesia, secara umum ibu biasanya lebih dekat dengan anak yang belum atau telah mumayyiz dibanding ayah dan itulah wujud kepentingan terbaik bagi anak;

Menimbang, bahwa hal juga ini sesuai dengan Hadist Nabi Muhammad SAW yang diriwayatkan oleh Ahmad dan Abu Dawud, yang dishahihkan al-Hakim, yang berbunyi:

أَنْتِ أَحَقُّ بِهِ مَا لَمْ تَنْكِحِي

Artinya: *“ Kamu (ibu kandung) lebih berhak terhadap anakmu selama engkau belum menikah”* ;

Menimbang, bahwa ketentuan di atas dilatarbelakangi oleh beberapa faktor sebagai berikut:

1. Faktor *kasih sayang*, tanpa mengurangi bahwa ayah juga menyayangi anak, namun secara *alamiah* dan *kodrati* dimanapun dan sejak kapanpun, ibu jauh lebih mampu mengembangkan kasih sayang dan kelembutan kepada anak dibanding ayah;
2. Faktor kemanusiaan (*humanity*), bila ditinjau dari segi kemanusiaan sangat mustahil apabila anak yang masih kecil harus ditarik, dipisahkan dan dijauhkan dari pangkuan ibu kandungnya;

Menimbang, bahwa berdasarkan pertimbangan-pertimbangan yang dihubungkan dengan fakta yang ditemukan dalam persidangan sebagaimana telah disebutkan di atas bahwa anak Penggugat dan Tergugat bernama bernama **Anak kandung I**, lahir tanggal 19 Maret 2011 dan **Anak kandung II**, lahir tanggal 24 Juli 2016, masih belum *mumayyiz* sedangkan Penggugat selaku ibu kandungnya selama ini juga tidak terdapat bukti sebagai pribadi yang buruk dan menelantarkan anak, karenanya dengan mendasarkan kepada asas “hak asuh (hadhanah) anak ditetapkan berdasarkan kepentingan terbaik bagi anak”, Majelis Hakim berpendapat bahwa Penggugat telah memenuhi syarat dan layak serta lebih tepat ditetapkan sebagai pemegang hadhanah (hak asuh) anak tersebut dibanding Tergugat;

Menimbang, bahwa berdasarkan pertimbangan-pertimbangan tersebut di atas maka petitum gugatan Penggugat agar ia ditetapkan sebagai pemegang hak hadhanah anak bernama **Anak kandung I**, lahir tanggal 19 Maret 2011 dan **Anak kandung II,** lahir tanggal 24 Juli 2016 patut dikabulkan;

Menimbang, bahwa meskipun gugatan Penggugat dikabulkan, bukan berarti

Disclaimer
Kepaniteraan Mahkamah Agung Republik Indonesia berusaha untuk selalu mencantumkan informasi paling kini dan akurat sebagai bentuk komitmen Mahkamah Agung untuk pelayanan publik, transparansi dan akuntabilitas pelaksanaan fungsi peradilan. Namun dalam hal-hal tertentu masih dimungkinkan terjadi permasalahan teknis terkait dengan akurasi dan keterkinian informasi yang kami sajikan, hal mana akan terus kami perbaiki dari waktu kewaktu.
Dalam hal Anda menemukan inakurasi informasi yang termuat pada situs ini atau informasi yang seharusnya ada, namun belum tersedia, maka harap segera hubungi Kepaniteraan Mahkamah Agung RI melalui :
Email : kepaniteraan@mahkamahagung.go.id Telp : 021-384 3348 (ext.318)

Tergugat sudah tidak mempunyai hak dan kesempatan untuk mencurahkan kasih sayang dan perhatiannya kepada anak tersebut. Sampai kapan pun bahkan hingga kelak dewasa, Tergugat sebagai ayah kandungnya mempunyai hak yang sama untuk memberikan kasih sayang dan perhatiannya kepada anak tersebut hanya saja tentu harus mempertimbangkan kondisi anak itu sendiri, oleh karena itu Penggugat harus tetap memberi kesempatan terhadap Tergugat sebagai ayah kandungnya apabila ingin menjenguk, bertemu, berinteraksi, mengajak dan berkumpul dengan anak tersebut dengan tetap mempertimbangkan kondisi anak tersebut. Artinya komunikasi antara Penggugat dan Tergugat dalam menentukan jadwal pertemuan Tergugat dengan anak tersebut harus dibangun dengan baik, berusaha mengesampingkan ego masing-masing dan pijakan utamanya adalah hal mana yang terbaik bagi anak bukan yang terbaik bagi Penggugat dan Tergugat;

Menimbang, bahwa dengan mendasarkan kepada Surat Edaran Mahkamah Agung (SEMA) Nomor 1 Tahun 2017 Tentang Pemberlakuan Rumusan Hasil Rapat Pleno Kamar Mahkamah Agung Tahun 2017 Sebagai Pedoman Pelaksanaan Tugas Bagi Pengadilan, dalam Rumusan Hukum Kamar Agama angka 4 yang menyatakan, “Dalam amar penetapan hadhanah atas anak harus mencantumkan kewajiban pemegang hak hadhanah memberi akses kepada orang tua yang tidak memegang hak hadhanah untuk bertemu dengan anaknya. Dalam pertimbangan hukum harus pula memepertimbangkan bahwa tidak memberi hak akses kepada orang tua yang tidak memegang hak hadhanah dapat dijadikan alasan untuk mengajukan gugatan pencabutan hak hadhanah”, maka Majelis Hakim berpendapat penghukuman terhadap pemberian hak ases untuk bertemu anak oleh Penggugat kepada Tergugat suatu keharusan dan apabila tidak diberikan hak akses tersebut, maka hal tersebut dapat dijadikan alasan oleh Tergugat untuk mengajukan gugatan pencabutan hak hadhanah Penggugat atas anak tersebut;

Menimbang, bahwa karena perkara a quo masuk bidang perkawinan, maka berdasarkan Pasal 89 ayat (1) Undang-undang Nomor 7 Tahun 1989 Tentang Peradilan Agama sebagaimana telah diubah dengan Undang-undang Nomor 3 Tahun 2006 dan perubahan kedua dengan Undang-undang Nomor 50 Tahun 2009, biaya perkara harus dibebankan kepada Penggugat;

Mengingat semua pasal dalam peraturan perundang-undangan dan hukum Islam yang berkaitan dengan perkara ini;

M E N G A D I L I

1. Menyatakan Tergugat yang telah dipanggil secara resmi dan patut untuk

Disclaimer
Kepaniteraan Mahkamah Agung Republik Indonesia berusaha untuk selalu mencantumkan informasi paling kini dan akurat sebagai bentuk komitmen Mahkamah Agung untuk pelayanan publik, transparansi dan akuntabilitas pelaksanaan fungsi peradilan. Namun dalam hal-hal tertentu masih dimungkinkan terjadi permasalahan teknis terkait dengan akurasi dan keterkinian informasi yang kami sajikan, hal mana akan terus kami perbaiki dari waktu kewaktu.
Dalam hal Anda menemukan inakurasi informasi yang termuat pada situs ini atau informasi yang seharusnya ada, namun belum tersedia, maka harap segera hubungi Kepaniteraan Mahkamah Agung RI melalui :
Email : kepaniteraan@mahkamahagung.go.id   Telp : 021-384 3348 (ext.318)

menghadap di persidangan, tidak hadir;

2. Mengabulkan gugatan Penggugat dengan verstek;
3. Menjatuhkan talak satu ba'in sughra Tergugat (**Tergugat**) kepada Penggugat (**Penggugat**);
4. Menetapkan hak asuh anak yang bernama **Anak kandung I**, lahir tanggal 19 Maret 2011 dan **Anak kandung II**, lahir tanggal 24 Juli 2016, dibawah asuhan (hadhonah) Penggugat;
5. Membebankan kepada Penggugat untuk membayar biaya perkara sejumlah Rp356.000,00 (tiga ratus lima puluh enam ribu rupiah).

Demikian ditetapkan dalam musyawarah Majelis Hakim Pengadilan Agama Teluk Kuantan pada hari Selasa tanggal 22 Desember 2020 Masehi bertepatan dengan tanggal 07 Jumadil Awwal 1442 Hijriah oleh kami **Niva Resna, S.Ag** sebagai Ketua Majelis, **H. Mohamad Mu'min, S.H.I., M.H** dan **Achmad Sutiyono, S.H.I**, masing-masing sebagai Hakim Anggota, putusan tersebut diucapkan pada hari itu juga dalam sidang terbuka untuk umum oleh Ketua Majelis beserta para Hakim Anggota tersebut, dan didampingi oleh **Rahmad, S.H.I** sebagai Panitera Pengganti, dengan dihadiri oleh Penggugat tanpa hadirnya Tergugat.

Hakim Anggota

Achmad Sutiyono, S.H.I

Ketua Majelis

Niva Resna, S.Ag

Hakim Anggota

Resa Wilianti, S.H., M.H

Panitera Pengganti

Rahmad, S.H.I

Disclaimer
Kepaniteraan Mahkamah Agung Republik Indonesia berusaha untuk selalu mencantumkan informasi paling kini dan akurat sebagai bentuk komitmen Mahkamah Agung untuk pelayanan publik, transparansi dan akuntabilitas pelaksanaan fungsi peradilan. Namun dalam hal-hal tertentu masih dimungkinkan terjadi permasalahan teknis terkait dengan akurasi dan keterkinian informasi yang kami sajikan, hal mana akan terus kami perbaiki dari waktu kewaktu.
Dalam hal Anda menemukan inakurasi informasi yang termuat pada situs ini atau informasi yang seharusnya ada, namun belum tersedia, maka harap segera hubungi Kepaniteraan Mahkamah Agung RI melalui :
Email : kepaniteraan@mahkamahagung.go.id Telp : 021-384 3348 (ext.318)

Rincian biaya:

1. Biaya Pendaftaran	:	Rp30.000,00
2. Biaya ATK Perkara	:	Rp50.000,00
3. Biaya Panggilan	:	Rp240.000,00
4. PNBP Panggilan	:	Rp20.000,00
5. Biaya Redaksi	:	Rp10.000,00
6. Biaya Meterai	:	Rp6.000,00
**JUMLAH**	**:**	Rp356.000,00

(tiga ratus lima puluh enam ribu rupiah).

Disclaimer
Kepaniteraan Mahkamah Agung Republik Indonesia berusaha untuk selalu mencantumkan informasi paling kini dan akurat sebagai bentuk komitmen Mahkamah Agung untuk pelayanan publik, transparansi dan akuntabilitas pelaksanaan fungsi peradilan. Namun dalam hal-hal tertentu masih dimungkinkan terjadi permasalahan teknis terkait dengan akurasi dan keterkinian informasi yang kami sajikan, hal mana akan terus kami perbaiki dari waktu kewaktu.
Dalam hal Anda menemukan inakurasi informasi yang termuat pada situs ini atau informasi yang seharusnya ada, namun belum tersedia, maka harap segera hubungi Kepaniteraan Mahkamah Agung RI melalui :
Email : kepaniteraan@mahkamahagung.go.id Telp : 021-384 3348 (ext.318)
