# Supplementary material for: Classification of divorce causes during the COVID-19 pandemic using convolutional neural networks
Source: PeerJ Comput Sci. 2022 Jun 30;8:e998. doi: 10.7717/peerj-cs.998 (PMC9299239; doi:10.7717/peerj-cs.998)
Supplement: Supplemental Information 6 [file peerj-cs-08-998-s006.zip › Rujuk Dataset/Data ke-7.pdf]

PUTUSAN

Nomor 2519/Pdt.G/2020/PA.Ba

بِسْمِ اللهِ الرَّحْمَنِ الرَّحِيْمِ

DEMI KEADILAN BERDASARKAN KETUHANAN YANG MAHA ESA

Pengadilan Agama Banjarnegara yang memeriksa dan mengadili perkara tertentu pada tingkat pertama dalam sidang Majelis telah menjatuhkan putusan perkara Gugatan Perceraian antara;

Nama Penggugat, umur 26 tahun, agama Islam, Pekerjaan Wiraswasta, Pendidikan Sekolah Lanjutan Tingkat Pertama, tempat kediaman di Kabupaten Banjarnegara, sebagai Penggugat

Melawan

Nama Tergugat, umur 30 tahun, agama Islam, Pekerjaan Wiraswasta, Pendidikan Sekolah Lanjutan Tingkat Pertama, tempat kediaman di Kabupaten Banjarnegara, sebagai Tergugat

Pengadilan Agama tersebut;

Telah mempelajari surat-surat yang berkaitan dengan perkara ini;

Telah mendengar keterangan Penggugat dan para saksi di muka sidang;

DUDUK PERKARA

Bahwa, Penggugat dalam suratgugatannya tanggal 04 Desember 2020 telah mengajukan gugatan perceraian yang telah didaftardi Kepaniteraan Pengadilan Agama Banjarnegara dengan Nomor 2519/Pdt.G/2020/PA.Ba,tanggal 04 Desember 2020, dengan dalil-dalil sebagai berikut:

1) Bahwa pada hari Senin, tanggal 22 April 2019M bertepatan dengan tanggal 16 Saban 1440 H, dihadapan Pegawai Pencatat Nikah Kantor Urusan Agama (KUA) Kecamatan Pagedongan, Kabupaten Banjarnegara, sebagaimana tersebut pada Kutipan Akta Nikah No. 0083/30/IV/2019 yang di keluarkan oleh Kantor Urusan Agama (KUA) Kecamatan Pagedongan, Kabupaten Banjarnegara tertanggal 22 April 2019 dengan status Janda dan

Disclaimer
Kepaniteraan Mahkamah Agung Republik Indonesia berusaha untuk selalu mencantumkan informasi paling kini dan akurat sebagai bentuk komitmen Mahkamah Agung untuk pelayanan publik, transparansi dan akuntabilitas pelaksanaan fungsi peradilan. Namun dalam hal-hal tertentu masih dimungkinkan terjadi permasalahan teknis terkait dengan akurasi dan keterkinian informasi yang kami sajikan, hal mana akan terus kami perbaiki dari waktu kewaktu.
Dalam hal Anda menemukan inakurasi informasi yang termuat pada situs ini atau informasi yang seharusnya ada, namun belum tersedia, maka harap segera hubungi Kepaniteraan Mahkamah Agung RI melalui :
Email : kepaniteraan@mahkamahagung.go.id Telp : 021-384 3348 (ext.318)

Duda;

2) Bahwa setelah pernikahan tersebut Penggugat dengan Tergugat tinggal bersama di rumah orang tua Penggugat sampai dengan akhir bulan Juni 2019 dan selama tinggal bersama belum pernah melakukan hubungan badan (Qobla Dukhul);
3) Bahwa sejak awal mula rumah tangga Penggugat dengan Tergugat sudah tidak rukun dan tidak harmonis sering terjadi perselisihan dan pertengkaran, disebabkan karena;
   3.1.Perbedaan prinsip tempat tinggal;
   3.2.Tergugat tidak mau bekerja/malas;
   3.3.Tergugat tidak pernah memberikan nafkah lahir dan batin;
   3.4.Tergugat ringan tangan dan suka bicara kasar;
4) Bahwa puncak pertengkaran terjadi pada awal bulan Juli 2019 dimana Tergugat pergi meninggalkan Penggugat kembali kerumah orang tua Tergugat sampai sekarang sudah pisahan selama 1 tahun 6 bulan;
5) Bahwa Penggugat telah berusaha sabar dan berusaha menjadi istri yang baik untuk Tergugat serta kemelut rumah tangga Penggugat dengan Tergugat sudah pernah didamaikan oleh pihak keluarga Penggugat, namun tidak berhasil;
6) Bahwa atas sikap dan /atau perbuatan Tergugat tersebut, Penggugat sangat menderita lahir maupun batin dan apabila rumah tangga yang seperti ini terus dilanjutkan akan menimbulkan banyak mudlaratnya dari pada manfaatnya;
7) Bahwa Penggugat mengajukan gugatan perceraian ini dengan mendasarkan pada pasal 19 huruf (f) Peraturan Pemerintah No.9 tahun 1975 jo. pasal 116 huruf (f) Kompilasi Hukum Islam (KHI);

Berdasarkan alasan-alasan di atas, maka Penggugat mohon kepada Ketua Pengadilan Agama Banjarnegara Cq Majelis Hakim yang memeriksa perkara ini segera memanggil para pihak, memeriksa dan mengadili selanjutnya menjatuhkan putusan yang amarnya berbunyi sebagai berikut :;

1. Mengabulkan gugatan Penggugat;
2. Menjatuhkan thalak satu bain sughra Tergugat (Nama Tergugat) terhadap

Disclaimer
Kepaniteraan Mahkamah Agung Republik Indonesia berusaha untuk selalu mencantumkan informasi paling kini dan akurat sebagai bentuk komitmen Mahkamah Agung untuk pelayanan publik, transparansi dan akuntabilitas pelaksanaan fungsi peradilan. Namun dalam hal-hal tertentu masih dimungkinkan terjadi permasalahan teknis terkait dengan akurasi dan keterkinian informasi yang kami sajikan, hal mana akan terus kami perbaiki dari waktu kewaktu.
Dalam hal Anda menemukan inakurasi informasi yang termuat pada situs ini atau informasi yang seharusnya ada, namun belum tersedia, maka harap segera hubungi Kepaniteraan Mahkamah Agung RI melalui :
Email : kepaniteraan@mahkamahagung.go.id Telp : 021-384 3348 (ext.318)

Penggugat (Nama Penggugat);

3. Membebankan biaya perkara menurut hukum;

Atau apabila Majelis Hakim Pengadilan Agama Banjarnegara berpendapat lain, mohon putusan yang seadil-adilnya;

Bahwa, pada hari sidang yang telah ditetapkan Penggugat telah datang menghadap ke muka sidang, sedangkan Tergugat tidak datang menghadap ke muka sidang dan tidak menyuruh orang lain untuk menghadap sebagai wakil/kuasa hukumnya meskipun telah dipanggil secara resmi dan patut yang relaas panggilannya telah dibacakan di dalam sidang, sedangkan tidak ternyata bahwa tidak datangnya itu disebabkan suatu halangan yang sah;

Bahwa, Majelis hakim telah menasehati Penggugat agar berpikir untuk tidak bercerai dengan Tergugat, tetapi Penggugat tetap pada dalil-dalil gugatannya untuk bercerai dengan Tergugat;

Bahwa, perkara ini tidak dapat di mediasi karena Tergugat tidak pernah datang menghadap meskipun telah dipanggil secara resmi dan patut, selanjutnya dimulai pemeriksaan dengan membacakan surat gugatan Penggugat yang maksud dan isinya tetap dipertahankan oleh Penggugat;

Bahwa,untuk menguatkan dalil-dalil gugatannya, Penggugat telah mengajukan bukti-bukti berupa :

A. Surat :

1. Fotokopi Kartu Tanda Penduduk NIK 3304205903940001 tanggal 29 Mei 2019, Bukti surat tersebut telah diberi meterai cukup dan telah dicocokkan dengan aslinya yang ternyata sesuai, lalu oleh Ketua Majelis diberi tanda P.1;
2. Fotokopi Buku Kutipan Akta Nikah Nomor 0083/30/IV/2019 tanggal 22 April 2019, yang dikeluarkan oleh Kantor Urusan Agama Kecamatan Pagedongan, Kabupaten Banjarnegara. Bukti surat tersebut telah diberi meterai cukup dan telah dicocokkan dengan aslinya yang ternyata sesuai, lalu oleh Ketua Majelis diberi tanda P.2;

B. Saksi :

1. Identitas Saksi 1:

Disclaimer
Kepaniteraan Mahkamah Agung Republik Indonesia berusaha untuk selalu mencantumkan informasi paling kini dan akurat sebagai bentuk komitmen Mahkamah Agung untuk pelayanan publik, transparansi dan akuntabilitas pelaksanaan fungsi peradilan. Namun dalam hal-hal tertentu masih dimungkinkan terjadi permasalahan teknis terkait dengan akurasi dan keterkinian informasi yang kami sajikan, hal mana akan terus kami perbaiki dari waktu kewaktu.
Dalam hal Anda menemukan inakurasi informasi yang termuat pada situs ini atau informasi yang seharusnya ada, namun belum tersedia, maka harap segera hubungi Kepaniteraan Mahkamah Agung RI melalui :
Email : kepaniteraan@mahkamahagung.go.id Telp : 021-384 3348 (ext.318)

- Bahwa saksi kenal dengan Penggugat karena saksi sebagai tetangga Penggugat;
- Bahwa setelah menikah Penggugat dan Tergugat hidup bersama di rumah orangtua Penggugat;
- Bahwa Penggugat dan Tergugat belum berhubungan suami istri;
- Bahwa Penggugat dan Tergugat telah berpisah 1 tahun lebih, Penggugat tinggal di rumah orang tua Penggugta, sedangkan Tergugat tinggal di rumah orang tua Tergugat;
- Bahwa Penggugat dan Tergugat berpisah karena sering bertengkar;
- Bahwa saksi pernah mendengar mereka bertengkar 3 kali;
- Bahwa Penggugat dan Tergugat bertengkar sejak awal nikah;
- Bahwa Penggugat dan Tergugat bertengkar masalah ekonomi

2. Identitas Saksi 2 :
   - Bahwa saksi kenal dengan Penggugat karena saksi sebagai adik ipar Penggugat;
   - Bahwa setelah menikah Penggugat dan Tergugat hidup bersama di rumah orang tua Penggugat;
   - Bahwa selama pernikahan Penggugat dan Tergugat belum berhubungan suami istri;
   - Bahwa Penggugat dan Tergugat telah berpisah selama 1 tahun 6 bulan, Penggugat tinggal di rumah orng tua Penggugat, sedangkan Tergugat tinggal di rumah orang tua Tergugat;
   - Bahwa Penggugat dan Tergugat berpisah karena sering bertengkar;
   - Bahwa saksi pernah mendengar mereka bertengkar berulang kali;
   - Bahwa Penggugat dan Tergugat bertengkar sejak awal nikah;
   - Bahwa Penggugat dan Tergugat bertengkar masalah ekonomi dan Tergugat sering kasar;
   - Bahwa saksi pernah menasehati Penggugat dan Tergugat, tetapi tidak berhasil

Selanjutnya untuk singkatnya uraian putusan ini, maka semua hal yang termuat dalam Berita Acara Sidang ini merupakan bagian yang tak terpisahkan dari putusan ini;

Disclaimer
Kepaniteraan Mahkamah Agung Republik Indonesia berusaha untuk selalu mencantumkan informasi paling kini dan akurat sebagai bentuk komitmen Mahkamah Agung untuk pelayanan publik, transparansi dan akuntabilitas pelaksanaan fungsi peradilan. Namun dalam hal-hal tertentu masih dimungkinkan terjadi permasalahan teknis terkait dengan akurasi dan keterkinian informasi yang kami sajikan, hal mana akan terus kami perbaiki dari waktu kewaktu.
Dalam hal Anda menemukan inakurasi informasi yang termuat pada situs ini atau informasi yang seharusnya ada, namun belum tersedia, maka harap segera hubungi Kepaniteraan Mahkamah Agung RI melalui :
Email : kepaniteraan@mahkamahagung.go.id   Telp : 021-384 3348 (ext.318)

PERTIMBANGAN HUKUM

Menimbang, bahwa maksud dan tujuan gugatan Penggugat adalah sebagaimana terurai di atas;

Menimbang, bahwa berdasarkan bukti P.2, maka harus dinyatakan telah terbukti menurut hukum bahwa Penggugat dan Tergugat telah terikat dalam perkawinan yang sah sejak tanggal 22 April 2019 ;

Menimbang, bahwa oleh karena Penggugat dan Tergugat masih terikat sebagai suami istri yang sah, maka Penggugat sebagai *persona standi in judicio*memiliki *legal standing* sehingga Penggugat dapat dinyatakan sebagai pihak yang mempunyai hak dan berkepentingan dalam perkara ini ;

Menimbang, bahwa atas keterangan Penggugat dalam surat gugatannya bahwa Penggugat dan Tergugat beragama Islam dan telah melakukan perkawinan secara islami, maka perkara ini merupakan kompetensi absolut Pengadilan Agama untuk memeriksa dan menyelesaikannya (vide Pasal 2 jo Pasal 49 ayat (1) Undang Undang Nomor 7 Tahun 1989 Tentang Peradilan Agama sebagai mana yang telah diubah yang kedua dengan Undang Undang Nomor 50 Tahun 2009) ;

Menimbang, bahwa berdasarkan surat gugatan, Penggugat bertempat tinggal wilayah yurisdiksi Pengadilan Agama Banjarnegara, maka perkara ini secara relatif juga merupakan kompetensi Pengadilan Agama Banjarnegara untuk memeriksanya (vide Pasal 73 Undang Undang Nomor 7 Tahun 1989 Tentang Peradilan Agama sebagai mana yang telah diubah yang kedua dengan Undang Undang Nomor 50 Tahun 2009) jo Pasal 132 Kompilasi Hukum Islam) ;

Menimbang, bahwa Majelis telah berupaya mendamaikan pihak Penggugat agar rukun kembali dengan Tergugat, sebagaimana dikehendaki oleh ketentuan Pasal 130 HIR jo Pasal 82 Undang Undang Nomor 7 Tahun 1989 Tentang Peradilan Agama sebagaimana yang telah diubah terakhir dengan Undang Undang Nomor 50 Tahun 2009 jo Pasal 39 ayat (1) Undang Undang Nomor 1 Tahun 1974 jo Pasal 31 ayat (2) Peraturan Pemerintah Nomor 9 Tahun 1975 jo Pasal 143 Kompilasi Hukum Islam, tetapi tidak berhasil ;

Menimbang, bahwa ternyata Tergugat meskipun dipanggil secara resmi dan patut, tidak datang menghadap di muka sidang dan pula tidak ternyata

Disclaimer
Kepaniteraan Mahkamah Agung Republik Indonesia berusaha untuk selalu mencantumkan informasi paling kini dan akurat sebagai bentuk komitmen Mahkamah Agung untuk pelayanan publik, transparansi dan akuntabilitas pelaksanaan fungsi peradilan. Namun dalam hal-hal tertentu masih dimungkinkan terjadi permasalahan teknis terkait dengan akurasi dan keterkinian informasi yang kami sajikan, hal mana akan terus kami perbaiki dari waktu kewaktu.
Dalam hal Anda menemukan inakurasi informasi yang termuat pada situs ini atau informasi yang seharusnya ada, namun belum tersedia, maka harap segera hubungi Kepaniteraan Mahkamah Agung RI melalui :
Email : kepaniteraan@mahkamahagung.go.id   Telp : 021-384 3348 (ext.318)

bahwa tidak datangnya itu disebabkan suatu halangan yang sah. Oleh karena itu, Tergugat harus dinyatakan tidak hadir dan gugatan tersebut harus diperiksa secara verstek. Sebagaimana ketentuan Pasal 125 ayat (1) HIRdan dalil syar'i :

من دعي إلى حاكم من حكام المسلمين فلم يجب فهو ظالم لا حق له

Artinya : “ Seseorang yang dipanggil Hakim (Pengadilan) tidak datang, ia dipandang sebagai dhalim dan tidak ada hak baginya. “ (Ahkamul Qur-an II : 405) ;

Menimbang, bahwa dalam perkara ini Majelis tidak menempuh prosedur mediasi karena salah satu pihak (Tergugat) tidak hadir sehingga tidak dapat dilakukan perundingan, dimana Pasal 1 ayat(1) Peraturan Mahkamah Agung R.I. Nomor 1 Tahun 2016 tentang Prosedur Mediasi di Pengadilan menyatakan, bahwa mediasi adalah cara penyelesaian sengketa melalui proses perundingan untuk memperoleh kesepakatan para pihak dengan dibantu oleh mediator. Perundingan dapat terlaksana apabila kedua belah pihak hadir ;

Menimbang, bahwa berdasarkan ketentuan Surat Edaran Mahkamah Agung RI Nomor 3 Tahun 2015 huruf C angka 3 yaitu putusan yang dijatuhkan tanpa hadirnya Tergugat dapat dikabulkan sepanjang berdasarkan hukum dan beralasan, oleh karena itu Majelis membebani Penggugatuntuk membuktikan dalil-dalil gugatannya;

Menimbang, bahwa untuk membuktikan dalil-dalil gugatannya, Penggugattelah mengajukan alat bukti surat serta 2 orang saksi;

Menimbang, bahwa perkara ini termasuk sengketa di bidang perkawinan, dan berdasarkan Bukti P-1Penggugat berdomisili di wilayah hukum Pengadilan Agama Banjarnegara, maka berdasarkan ketentuan Pasal 49 ayat (1) huruf a dan Pasal 73 ayat (1) Undang-undang Nomor 7 Tahun 1989 Tentang Peradilan Agama sebagaimana telah diubah dengan Undang-undang Nomor 3 Tahun 2006 dan perubahan kedua dengan Undang-undang Nomor 50 Tahun 2009, Pengadilan Agama Banjarnegara berwenang untuk memeriksa, memutus, dan menyelesaikan Perkara ini;

Menimbang, bahwa bukti P-2 (Fotokopi Kutipan Akta Nikah) yang merupakan akta otentik dan telah bermeterai cukup dan cocok dengan aslinya,

Disclaimer
Kepaniteraan Mahkamah Agung Republik Indonesia berusaha untuk selalu mencantumkan informasi paling kini dan akurat sebagai bentuk komitmen Mahkamah Agung untuk pelayanan publik, transparansi dan akuntabilitas pelaksanaan fungsi peradilan. Namun dalam hal-hal tertentu masih dimungkinkan terjadi permasalahan teknis terkait dengan akurasi dan keterkinian informasi yang kami sajikan, hal mana akan terus kami perbaiki dari waktu kewaktu.
Dalam hal Anda menemukan inakurasi informasi yang termuat pada situs ini atau informasi yang seharusnya ada, namun belum tersedia, maka harap segera hubungi Kepaniteraan Mahkamah Agung RI melalui :
Email : kepaniteraan@mahkamahagung.go.id Telp : 021-384 3348 (ext.318)

isi bukti tersebut menjelaskan antara Penggugat dan Tergugat telah terikat dalam perkawinan yang sah, sehingga bukti tersebut telah memenuhi syarat formal dan meteriil, serta mempunyai kekuatan pembuktian yang sempurna dan mengikat;

Menimbang, bahwa saksi 1 Penggugat, sudah dewasa dan sudah bersumpah, sehingga memenuhi syarat formal sebagaimana diatur dalam Pasal 145 ayat 1 angka 3e HIR;

Menimbang, bahwa keterangan saksi 1 Penggugat mengenai dalil gugatan angka 3, 4,dan 5, adalah fakta yang didengar sendiri dan relevan dengan dalil yang harus dibuktikan oleh Penggugat, oleh karena itu keterangan saksi tersebut telah memenuhi syarat materiil sebagaimana telah diatur dalam Pasal 171 HIR, sehingga keterangan saksi tersebut memiliki kekuatan pembuktian dan dapat diterima sebagai alat bukti;

Menimbang, bahwa saksi 2 Penggugat, sudah dewasa dan sudah bersumpah, sehingga memenuhi syarat formal sebagaimana diatur dalam Pasal 145 ayat 1 angka 3e HIR;

Menimbang, bahwa keterangan saksi 2 Penggugat mengenai dalil gugatan angka 3, 4,dan 5, adalah fakta yang didengarsendiri dan relevan dengan dalil yang harus dibuktikan oleh Penggugat, oleh karena itu keterangan saksi tersebut telah memenuhi syarat materiil sebagaimana telah diatur dalam Pasal 171 HIR, sehingga keterangan saksi tersebut memiliki kekuatan pembuktian dan dapat diterima sebagai alat bukti;

Menimbang, bahwa keterangan saksi 1 dan saksi 2 Penggugat bersesuaian dan cocok antara satu dengan yang lain oleh karena itu keterangan dua orang saksi tersebut memenuhi Pasal 171 dan Pasal 172 HIR;

Menimbang, bahwa berdasarkan saksi 1 dan saksi 2, terbukti fakta kejadian sebagai berikut;

1. Antara Penggugat dan Tergugatterus menerus terjadi perselisihandan pertengkaran disebabkan masalah ekonomi ;
2. Antara Penggugat dan Tergugattelah berpisah rumah selama kurang lebih 1tahun6 bulan;

Menimbang, bahwa berdasarkan fakta-fakta tersebut di atas dapat

Disclaimer
Kepaniteraan Mahkamah Agung Republik Indonesia berusaha untuk selalu mencantumkan informasi paling kini dan akurat sebagai bentuk komitmen Mahkamah Agung untuk pelayanan publik, transparansi dan akuntabilitas pelaksanaan fungsi peradilan. Namun dalam hal-hal tertentu masih dimungkinkan terjadi permasalahan teknis terkait dengan akurasi dan keterkinian informasi yang kami sajikan, hal mana akan terus kami perbaiki dari waktu kewaktu.
Dalam hal Anda menemukan inakurasi informasi yang termuat pada situs ini atau informasi yang seharusnya ada, namun belum tersedia, maka harap segera hubungi Kepaniteraan Mahkamah Agung RI melalui :
Email : kepaniteraan@mahkamahagung.go.id   Telp : 021-384 3348 (ext.318)

disimpulkan fakta hukum sebagai berikut;

1. Antara Penggugat dan Tergugatterus menerus terjadi perselisihandan pertengkaran yang sulit untuk didamaikan;
2. Antara Penggugat dan Tergugattelah berpisah rumah selama kurang lebih 1tahun6 bulan;
3. Antara Penggugat dan Tergugat tidak ada harapan hidup rukun kembali;

Menimbang, bahwa fakta hukum tersebut telah memenuhi norma hukum Islam yang terkandung dalam Kitab Fikih Sunah Juz II halaman 290;

أنّ للزوجة أن تطلب من القاضي التفريق إذا ادعت إضرار الزوج بها إضرارا لا يستطاع معه
دوام العشرة بين أمثالها

Artinya : "*bahwa istri boleh menuntut talak kepada hakim apabila dia mengaku selalu mendapat perlakuan yang menyakitkan dari suaminya sehingga hal tersebut dapat menghalangi keberlasungan hubungan suami istri antara mereka berdua";*

Menimbang, bahwa fakta hukum tersebut telah juga memenuhi Pasal 39 ayat 2 Undang-undang Nomor 1 tahun 1974 dan Pasal 19 huruf (f) Peraturan Pemerintah Nomor 9 Tahun 1975 jo. Pasal 116 huruf (f) Kompilasi Hukum Islam, oleh karena itu dapat dikabulkan;

Menimbang, bahwa karena perkara *a quo* masuk bidang perkawinan, maka berdasarkan Pasal 89 ayat (1) Undang-undang Nomor 7 Tahun 1989 Tentang Peradilan Agama sebagaimana telah diubah dengan Undang-undang Nomor 3 Tahun 2006 dan perubahan kedua dengan Undang-undang Nomor 50 Tahun 2009, biaya perkara harus dibebankan kepada Penggugat;

Mengingat semua pasal dalam peraturan perundang-undangan dan hukum Islam yang berkaitan dengan perkara ini;

MENGADILI

1. Menyatakan Tergugat yang telah dipanggil dengan resmi dan patut untuk menghadap di persidangan, tidak hadir;
2. Mengabulkan gugatan Penggugat dengan verstek;
3. Menjatuhkan talak satu ba'in shughra dari Tergugat(Nama

Disclaimer
Kepaniteraan Mahkamah Agung Republik Indonesia berusaha untuk selalu mencantumkan informasi paling kini dan akurat sebagai bentuk komitmen Mahkamah Agung untuk pelayanan publik, transparansi dan akuntabilitas pelaksanaan fungsi peradilan. Namun dalam hal-hal tertentu masih dimungkinkan terjadi permasalahan teknis terkait dengan akurasi dan keterkinian informasi yang kami sajikan, hal mana akan terus kami perbaiki dari waktu kewaktu.
Dalam hal Anda menemukan inakurasi informasi yang termuat pada situs ini atau informasi yang seharusnya ada, namun belum tersedia, maka harap segera hubungi Kepaniteraan Mahkamah Agung RI melalui :
Email : kepaniteraan@mahkamahagung.go.id Telp : 021-384 3348 (ext.318)

Tergugat)terhadap Penggugat (Nama Penggugat);

4. Membebankan kepada Penggugat untuk membayar biaya perkara sejumlah Rp 506.000,- (lima ratus enam ribu rupiah).

Demikian diputuskan dalam rapat permusyawaratan Majelis yang dilangsungkan pada hari Senin tanggal 21Desember 2020 *Masehi*. bertepatan dengan tanggal 06Jumadil Ula1442 *Hijriyah.*Oleh kami Drs. H. Nurrudin, S.H., MSI. sebagai Ketua Majelis, dan Dr. H. Ichwan Qomari, Drs., M.Ag. serta Dra. Hj. Siti Syamsiyah masing-masing sebagai Hakim Anggota,putusan tersebutdiucapkan dalam sidang terbuka untuk umum pada hari Senin tanggal 21Desember 2020 *Masehi*. bertepatan dengan tanggal 06 Jumadil Ula 1442 *Hijriyah.* oleh Ketua Majelis tersebut dengan didampingi oleh Hakim Anggota dan dibantu oleh Hidayati, Sm.Hk. sebagai Panitera Pengganti serta dihadiri Penggugat tanpa hadirnya Tergugat;

Hakim Anggota I,

Dr. H. Ichwan Qomari, Drs., M.Ag.

Ketua Majelis,

Drs. H. Nurrudin, S.H., MSI.

Hakim Anggota II,

Dra. Hj. Siti Syamsiyah

Panitera Pengganti,

Hidayati, Sm.Hk.

Perincian Biaya :

Biaya Pendaftaran	Rp	30.000,00
Biaya Proses	Rp	50.000,00
Biaya Pemanggilan	Rp	390.000,00
PNBP	Rp	20.000,00
Biaya Redaksi	Rp	10.000,00

Disclaimer
Kepaniteraan Mahkamah Agung Republik Indonesia berusaha untuk selalu mencantumkan informasi paling kini dan akurat sebagai bentuk komitmen Mahkamah Agung untuk pelayanan publik, transparansi dan akuntabilitas pelaksanaan fungsi peradilan. Namun dalam hal-hal tertentu masih dimungkinkan terjadi permasalahan teknis terkait dengan akurasi dan keterkinian informasi yang kami sajikan, hal mana akan terus kami perbaiki dari waktu kewaktu.
Dalam hal Anda menemukan inakurasi informasi yang termuat pada situs ini atau informasi yang seharusnya ada, namun belum tersedia, maka harap segera hubungi Kepaniteraan Mahkamah Agung RI melalui :
Email : kepaniteraan@mahkamahagung.go.id Telp : 021-384 3348 (ext.318)

Biaya Materai	Rp	6.000,00
Jumlah	Rp	506.000,00

Hal 10dari 10hal Put. No 2519/Pdt.G/2020/PA.Ba

Disclaimer
Kepaniteraan Mahkamah Agung Republik Indonesia berusaha untuk selalu mencantumkan informasi paling kini dan akurat sebagai bentuk komitmen Mahkamah Agung untuk pelayanan publik, transparansi dan akuntabilitas pelaksanaan fungsi peradilan. Namun dalam hal-hal tertentu masih dimungkinkan terjadi permasalahan teknis terkait dengan akurasi dan keterkinian informasi yang kami sajikan, hal mana akan terus kami perbaiki dari waktu kewaktu.
Dalam hal Anda menemukan inakurasi informasi yang termuat pada situs ini atau informasi yang seharusnya ada, namun belum tersedia, maka harap segera hubungi Kepaniteraan Mahkamah Agung RI melalui :
Email : kepaniteraan@mahkamahagung.go.id Telp : 021-384 3348 (ext.318)
