# Supplementary material for: Classification of divorce causes during the COVID-19 pandemic using convolutional neural networks
Source: PeerJ Comput Sci. 2022 Jun 30;8:e998. doi: 10.7717/peerj-cs.998 (PMC9299239; doi:10.7717/peerj-cs.998)
Supplement: Supplemental Information 6 [file peerj-cs-08-998-s006.zip › Rujuk Dataset/Data ke-8.pdf]

P U T U S A N
Nomor 3871/Pdt.G/2020/PA.BL

بِسْمِ اللهِ الرَّحْمٰنِ الرَّحِيْمِ

DEMI KEADILAN BERDASARKAN KETUHANAN YANG MAHA ESA

Pengadilan Agama Blitar yang memeriksa dan mengadili perkara tertentu dalam tingkat pertama, dalam persidangan majelis, telah menjatuhkan putusan perkara cerai gugat antara:

Xxxxxxxxxxxxxxxxxxxx, pendidikan SLTP, tempat kediaman di Kabupaten Blitar, dalam hal ini memberikan kuasa khusus kepada : Tri Elyas Setyawan, SH. Eko Santoso, SH., MH. Irfana Jawahirul Maulida, SH. & Tri Cahyo Widodo, SH. advokat/penasehat hukum, yang berkantor di Jalan C. R. Sukandar No. 06 Kelurahan Sananwetan Kecamatan Sananwetan Kota Blitar., berdasarkan surat kuasa khusus tanggal 02 Desember 2020, sebagai **Penggugat**;

MELAWAN

Xxxxxxxxxxxxxxxxxxxx, tempat kediaman di Kabupaten Tulungagung, sebagai **Tergugat**;

Pengadilan Agama tersebut;
Telah membaca, mempelajari dan meneliti berkas perkara;
Telah mendengar keterangan Penggugat, saksi-saksi dan telah memeriksa bukti-bukti di persidangan;

DUDUK PERKARA

Bahwa Penggugat dengan surat gugatannya tertanggal 03 Desember 2020 yang didaftarkan di Kepaniteraan Pengadilan Agama Blitar Nomor 3871/Pdt.G/2020/PA.BL, mengajukan hal-hal sebagai berikut:

Disclaimer
Kepaniteraan Mahkamah Agung Republik Indonesia berusaha untuk selalu mencantumkan informasi paling kini dan akurat sebagai bentuk komitmen Mahkamah Agung untuk pelayanan publik, transparansi dan akuntabilitas pelaksanaan fungsi peradilan. Namun dalam hal-hal tertentu masih dimungkinkan terjadi permasalahan teknis terkait dengan akurasi dan keterkinian informasi yang kami sajikan, hal mana akan terus kami perbaiki dari waktu kewaktu.
Dalam hal Anda menemukan inakurasi informasi yang termuat pada situs ini atau informasi yang seharusnya ada, namun belum tersedia, maka harap segera hubungi Kepaniteraan Mahkamah Agung RI melalui :
Email : kepaniteraan@mahkamahagung.go.id Telp : 021-384 3348 (ext.318)

1. Bahwa antara Penggugat dan Tergugat adalah suami-istri yang terikat dalam pernikahan yang dilaksanakan pada Hari Selasa tanggal 5 Juni 2019M di hadapan Pejabat Kantor Urusan Agama (KUA) Kecamatan Wonotirto Kabupaten Blitar. sebagaimana tercatat dalam BUKU KUTIPAN AKTA NIKAH No. Xxxxxxxxxxxxxxxxxxxxxx .

2. Bahwa setelah menikah antara Penggugat dan Tergugat hidup bersama /kumpul bersama (Ba'da dukhul), tinggal bersama dirumah Penggugat, kemudian tinggal bersama dirumah orang tua Tergugat. Dan dari pernikahan tersebut belum dikaruniai keturunan.

3. Bahwa pada awalnya rumah tangga Penggugat dan Tergugat rukun, harmonis dan baik-baik saja, namun demikian sejak sekitar bulan Desember 2019 keharmonisan rumah tangga antara Penggugat dengan Tergugat mulai goyah, antara keduanya sering terjadi perselisihan dan pertengkaran terus-menerus, hal itu disebabkan :

- Masalah ekonomi rumah tangga.
- Tergugat jarang memberi nafkah kepada penggugat.

4. Bahwa permasalahan rumah tangga tersebut semakin serius pada bulan November 2020 hingga menyebabkan antara penggugat dan Terugat pisah tempat tinggal, Penggugat pulang kerumah rang tua Penggugat di Blitar hingga gugatan ini diajukan.

5. Bahwa akibat permasalahan rumah tangga tersebut, Penggugat telah menderita lahir dan batin, penggugat sudah tidak sanggup hidup bersama dengan tergugat, jika rumah tangga dipaksakan untuk bersatu justru lebih banyak kemudharatan dari pada manfaatnya.

6. Bahwa dengan demikian terbukti Rumah tangga Penggugat dan Tergugat telah pecah dan sulit untuk disatukan lagi, antara keduanya telah rukun dan tidak harmonis selama kurang lebih 1 tahun, serta pisah tempat tinggal selama kurang lebih 1 bulan, selama itu antara keduanya ters mnerus terjadi perselisihan dan pertengkaran sehingga tujuan perkawinan untuk membentuk keluarga yang sakinah mawadah wa rahmah sulit untuk terwujud.

Disclaimer
Kepaniteraan Mahkamah Agung Republik Indonesia berusaha untuk selalu mencantumkan informasi paling kini dan akurat sebagai bentuk komitmen Mahkamah Agung untuk pelayanan publik, transparansi dan akuntabilitas pelaksanaan fungsi peradilan. Namun dalam hal-hal tertentu masih dimungkinkan terjadi permasalahan teknis terkait dengan akurasi dan keterkinian informasi yang kami sajikan, hal mana akan terus kami perbaiki dari waktu kewaktu.
Dalam hal Anda menemukan inakurasi informasi yang termuat pada situs ini atau informasi yang seharusnya ada, namun belum tersedia, maka harap segera hubungi Kepaniteraan Mahkamah Agung RI melalui :
Email : kepaniteraan@mahkamahagung.go.id   Telp : 021-384 3348 (ext.318)

Berdasarkan uraian diatas mohon kepada yang terhormat Ketua Pengadilan Agama Blitar Cq. Yang Mulia Majelis Hakim Pengadilan Agama Blitar berkenan memanggil para pihak guna memeriksa dan mengadili perkara ini serta menjatuhkan Putusan :

1. Mengabulkan gugatan Penggugat (Xxxxxxxxxxxxxxxxxxxx) untuk seluruhnya.
2. Menjatuhkan talak satu Ba'in Sugro Tergugat (Xxxxxxxxxxxxxxxxxxxx) terhadap Penggugat (Xxxxxxxxxxxxxxxxxxxx).
3. Membebankan biaya menurut hukum.

Atau, Jika Yang mulia Majelis Hakim berpendapat lain, mohon diberikan putusan yang seadil- adilnya berdasarkan Ketuhanan Yang Maha Esa.

Bahwa pada hari sidang yang ditetapkan untuk perkara ini Penggugat dan kuasa hukumnya hadir di persidangan, sedangkan Tergugat berdasarkan relaas panggilan Nomor 3871/Pdt.G/2020/PA.BL tanggal 10 Desember 2020 dan tanggal 21 Desember 2020 telah dipanggil sebanyak 2 kali namun tidak pernah hadir dan tidak pula menyuruh orang lain untuk hadir sebagai sebagai wakil atau kuasanya dan ketidak hadirannya tidak disebabkan oleh suatu alasan yang sah menurut hukum;

Bahwa Majelis Hakim telah berusaha mendamaikan pihak berperkara dengan cara memberi nasihat kepada Penggugat agar rukun kembali dengan Tergugat, namun tidak berhasil;

Bahwa pemeriksaan ini dilanjutkan dengan membacakan surat gugatan Penggugat dalam sidang tertutup untuk umum, yang isi dan maksudnya tetap dipertahankan oleh Penggugat;

Bahwa untuk menguatkan dalil-dalil gugatannya, Penggugat mengajukan alat bukti surat, berupa:

1. Fotokopi Kartu Tanda Penduduk NIK xxxxxxxxxxxxxxxxxxxxxx atas nama Penggugat yang dikeluarkan oleh Pemerintah Kabupaten Blitar tanggal 11 April 2019, yang telah dicocokan dengan aslinya dan telah dinezegelnd (P.1);
2. Fotokopi Kutipan Akta Nikah nomor xxxxxxxxxxxxxxxxxxxxxx atas nama Penggugat dan Tergugat yang dikeluarkan oleh Kantor Urusan Agama

Disclaimer
Kepaniteraan Mahkamah Agung Republik Indonesia berusaha untuk selalu mencantumkan informasi paling kini dan akurat sebagai bentuk komitmen Mahkamah Agung untuk pelayanan publik, transparansi dan akuntabilitas pelaksanaan fungsi peradilan. Namun dalam hal-hal tertentu masih dimungkinkan terjadi permasalahan teknis terkait dengan akurasi dan keterkinian informasi yang kami sajikan, hal mana akan terus kami perbaiki dari waktu kewaktu.
Dalam hal Anda menemukan inakurasi informasi yang termuat pada situs ini atau informasi yang seharusnya ada, namun belum tersedia, maka harap segera hubungi Kepaniteraan Mahkamah Agung RI melalui :
Email : kepaniteraan@mahkamahagung.go.id Telp : 021-384 3348 (ext.318)

Kantor Urusan Agama (KUA) Kecamatan Wonotirto Kabupaten Blitar Provinsi Jawa Timur, tanggal 05 Juni 2019, yang telah dicocokan dengan aslinya dan telah dinezegelnd (P.2);

Bahwa selain bukti tersebut Penggugat juga telah menghadirkan 2 (dua) orang saksi yang bernama;

1. Xxxxxxxxxxxxxxxxxxxxx , bertempat tinggal di Dusun Sragi RT.02 RW.03 Desa Sragi Kecamatan Talun Kabupaten Blitar;

Di bawah sumpah saksi memberikan keterangan yang pada pokoknya sebagai berikut:

- Bahwa saksi kenal dengan Penggugat dan Tergugat karena saksi adalah paman Penggugat;
- Bahwa Penggugat dan Tergugat adalah suami istri sah yang telah menikah pada tahun 2019;
- Bahwa setelah akad nikah, Penggugat hidup rukun dengan Tergugat bertempat tinggal di rumah orang tua Tergugat namun belum dikaruniai keturunan
- Bahwa saksi tahu semula rumah tangga Penggugat dan Tergugat rukun dan harmonis, namun sejak 01 Desember 2019 sudah tidak harmonis, karena sering berselisih dan bertengkar disebabkan Masalah ekonomi rumah tangga dan Tergugat jarang memberi nafkah kepada penggugat.;
- Bahwa saksi mengetahui akibat perselisihan dan pertengkaran tersebut Penggugat dan Tergugat telah pisah tempat tinggal selama kurang lebih 1 tahun 1 bulan;
- Bahwa saksi tahu selama pisah tempat tinggal Tergugat tidak pernah memberi nafkah kepada Penggugat;
- Bahwa saksi sebagai paman telah berusaha untuk memberikan nasihat kepada Penggugat agar rukun lagi dengan Tergugat, akan tetapi tidak berhasil dan saksi tidak sanggup lagi merukunkan Penggugat dengan Tergugat;

2. Xxxxxxxxxxxxxxxxxxxxx umur 25 tahun, agama Islam, pekerjaan Ibu Rumah Tangga, bertempat tinggal di Dusun Wonorejo RT.05 RW.02 Desa Kalipucung Kecamatan Sutojayan KAbupaten Blitar;

Disclaimer
Kepaniteraan Mahkamah Agung Republik Indonesia berusaha untuk selalu mencantumkan informasi paling kini dan akurat sebagai bentuk komitmen Mahkamah Agung untuk pelayanan publik, transparansi dan akuntabilitas pelaksanaan fungsi peradilan. Namun dalam hal-hal tertentu masih dimungkinkan terjadi permasalahan teknis terkait dengan akurasi dan keterkinian informasi yang kami sajikan, hal mana akan terus kami perbaiki dari waktu kewaktu.
Dalam hal Anda menemukan inakurasi informasi yang termuat pada situs ini atau informasi yang seharusnya ada, namun belum tersedia, maka harap segera hubungi Kepaniteraan Mahkamah Agung RI melalui :
Email : kepaniteraan@mahkamahagung.go.id   Telp : 021-384 3348 (ext.318)

Di bawah sumpah saksi memberikan keterangan yang pada pokoknya sebagai berikut:

- Bahwa saksi kenal dengan Penggugat dan Tergugat karena saksi adalah kakak Penggugat;
- Bahwa Penggugat dan Tergugat adalah suami istri sah yang telah menikah pada tahun 2019;
- Bahwa setelah akad nikah, Penggugat hidup rukun dengan Tergugat bertempat tinggal di rumah orang tua Tergugat namun belum dikaruniai keturunan
- Bahwa saksi tahu semula rumah tangga Penggugat dan Tergugat rukun dan harmonis, namun sejak 01 Desember 2019 sudah tidak harmonis, karena sering berselisih dan bertengkar disebabkan Masalah ekonomi rumah tangga dan Tergugat jarang memberi nafkah kepada penggugat.;
- Bahwa saksi mengetahui akibat perselisihan dan pertengkaran tersebut Penggugat dan Tergugat telah pisah tempat tinggal selama kurang lebih 1 tahun 1 bulan;
- Bahwa saksi tahu selama pisah tempat tinggal Tergugat tidak pernah memberi nafkah kepada Penggugat;
- Bahwa saksi sebagai kakak telah berusaha untuk memberikan nasihat kepada Penggugat agar rukun lagi dengan Tergugat, akan tetapi tidak berhasil dan saksi tidak sanggup lagi merukunkan Penggugat dengan Tergugat;

Bahwa untuk selanjutnya, Penggugat menyatakan sudah tidak lagi mengajukan sesuatu apapun dan memberikan kesimpulan secara lisan yang pada pokoknya tetap pada dalil gugatannya serta mohon putusan;

Bahwa untuk mempersingkat uraian putusan ini ditunjuk kepada hal-hal sebagaimana tercantum dalam berita acara persidangan perkara ini dan merupakan satu kesatuan yang tidak terpisahkan dari putusan ini;

PERTIMBANGAN HUKUM

Menimbang, bahwa maksud dan tujuan gugatan Penggugat pada pokoknya adalah sebagaimana terurai diatas;

Disclaimer
Kepaniteraan Mahkamah Agung Republik Indonesia berusaha untuk selalu mencantumkan informasi paling kini dan akurat sebagai bentuk komitmen Mahkamah Agung untuk pelayanan publik, transparansi dan akuntabilitas pelaksanaan fungsi peradilan. Namun dalam hal-hal tertentu masih dimungkinkan terjadi permasalahan teknis terkait dengan akurasi dan keterkinian informasi yang kami sajikan, hal mana akan terus kami perbaiki dari waktu kewaktu.
Dalam hal Anda menemukan inakurasi informasi yang termuat pada situs ini atau informasi yang seharusnya ada, namun belum tersedia, maka harap segera hubungi Kepaniteraan Mahkamah Agung RI melalui :
Email : kepaniteraan@mahkamahagung.go.id Telp : 021-384 3348 (ext.318)

Menimbang, bahwa terlebih dahulu, Majelis Hakim perlu mempertimbangkan keabsahan surat kuasa khusus yang dibuat Penggugat serta kedudukan penerima kuasa sebagaimana pertimbangan berikut ini;

Menimbang, bahwa Surat Kuasa Khusus Penggugat tanggal 02 Desember 2020 telah ternyata memenuhi syarat formil surat kuasa, yaitu telah memenuhi unsur kekhususan, dimana secara jelas menunjuk perkara Cerai Gugat di Pengadilan Agama Blitar dengan memuat materi telaah yang menjadi batas dan isi dari materi kuasa yang diberikan dengan mencantumkan identitas para pihak berperkara dan telah mencantumkan tanggal serta tanda tangan pemberi kuasa, begitu juga Kartu Tanda Pengenal Para Advokat masih berlaku serta telah disumpah oleh Pengadilan Tinggi setempat, maka majelis berpendapat, bahwa Surat Kuasa Khusus dimaksud dapat dinyatakan sah karena telah sesuai dengan Pasal 123 ayat (1) HIR, SEMA Nomor 01 Tahun 1971 tanggal 23 Januari 1971 juncto SEMA Nomor 6 Tahun 1994 tanggal 14 Oktober 1994 dan ketentuan Pasal 32 Undang-undang Nomor 18 Tahun 2003 tentang Advokat karenanya penerima kuasa harus pula dinyatakan telah mempunyai kedudukan dan kapasitas sebagai subjek hukum yang berhak melakukan tindakan hukum atas nama pemberi kuasa untuk beracara dalam perkara a quo;

Menimbang, bahwa dalam dalil gugatan Penggugat yang menyatakan perkawinan Penggugat dan Tergugat dilaksanakan menurut agama Islam yang tidak terbantahkan, oleh karena itu berdasarkan Pasal 40 dan Pasal 63 Ayat (1) huruf (a) Undang-Undang Nomor 1 Tahun 1974 tentang Perkawinan jo. Pasal 1 huruf (b) Peraturan Pemerintah Nomor 9 Tahun 1975 jo. Pasal 49 Undang-undang Nomor 7 Tahun 1989 tentang Peradilan Agama sebagaimana diubah dengan Undang-undang Nomor 3 Tahun 2006 dan perubahan kedua dengan Undang-undang Nomor 50 Tahun 2009, karenanya Pengadilan Agama berwenang memeriksa dan mengadili serta memutus perkara a quo;

Menimbang, bahwa dalam gugatan Penggugat domisili Penggugat berada pada yurisdiksi Pengadilan Agama Blitar, sesuai Pasal 73 Undang-undang Nomor 7 Tahun 1989 tentang Peradilan Agama sebagaimana diubah dengan Undang-undang Nomor 3 Tahun 2006 dan perubahan kedua dengan

Disclaimer
Kepaniteraan Mahkamah Agung Republik Indonesia berusaha untuk selalu mencantumkan informasi paling kini dan akurat sebagai bentuk komitmen Mahkamah Agung untuk pelayanan publik, transparansi dan akuntabilitas pelaksanaan fungsi peradilan. Namun dalam hal-hal tertentu masih dimungkinkan terjadi permasalahan teknis terkait dengan akurasi dan keterkinian informasi yang kami sajikan, hal mana akan terus kami perbaiki dari waktu kewaktu.
Dalam hal Anda menemukan inakurasi informasi yang termuat pada situs ini atau informasi yang seharusnya ada, namun belum tersedia, maka harap segera hubungi Kepaniteraan Mahkamah Agung RI melalui :
Email : kepaniteraan@mahkamahagung.go.id Telp : 021-384 3348 (ext.318)

Undang-Undang Nomor 50 Tahun 2009, maka perkara ini merupakan kewenangan relatif Pengadilan Agama Blitar;

Menimbang, bahwa Penggugat dalam gugatannya mendalilkan bahwa Penggugat telah melangsungkan perkawinan dan rumah tangga Penggugat dan Tergugat sudah tidak harmonis, oleh karena itu Penggugat memiliki *legal standing* untuk mengajukan gugatan perceraian sebagaimana diatur Pasal 49 Ayat (1) huruf (a) dan Pasal 73 Undang-undang Nomor 7 Tahun 1989 tentang Peradilan Agama yang diubah dengan Undang-undang Nomor 3 Tahun 2006 dan perubahan kedua dengan Undang-undang Nomor 50 Tahun 2009;

Menimbang, bahwa Majelis Hakim telah berusaha mendamaikan pihak berperkara dengan memberikan nasihat kepada Penggugat agar rukun kembali dengan Tergugat, namun tidak berhasil, karenanya ketentuan Pasal 130 HIR jo. Pasal 82 Undang-undang Nomor 7 Tahun 1989 tentang Peradilan Agama sebagaimana diubah dengan Undang-undang Nomor 3 Tahun 2006 dan perubahan kedua dengan Undang-undang Nomor 50 Tahun 2009 telah terpenuhi dalam perkara ini;

Menimbang, bahwa menurut Peraturan Mahkamah Agung R.I. Nomor 1 Tahun 2016 tentang Prosedur Mediasi, mediasi mengharuskan kehadiran kedua pihak yang berperkara, oleh karena Tergugat tidak pernah hadir di persidangan, maka mediasi tidak dapat dilaksanakan;

Menimbang, bahwa oleh karena Tergugat tidak hadir di ruang sidang tanpa alasan yang sah, dan tidak pula menyuruh orang lain untuk menghadap ke ruang sidang sebagai wakil dan atau kuasanya meskipun Jurusita Pengganti Pengadilan Agama Blitar telah memanggilnya secara resmi dan patut, sebagaimana ketentuan Pasal 125 HIR karenanya Tergugat harus dinyatakan tidak hadir dan gugatan Penggugat dapat diputus dengan verstek;

Menimbang, bahwa ketentuan tersebut diatas *relevant* dengan Hadits Nabi dalam Kitab Hadits Mu'inul Hukkam halaman 96 yang berbunyi:

وعن الحسن أن النبي صلى الله عليه وسلم قال : من دعي إلى حاكم من حكام
المسلمين فلم يجب فهو ظالم لا حق له

Disclaimer
Kepaniteraan Mahkamah Agung Republik Indonesia berusaha untuk selalu mencantumkan informasi paling kini dan akurat sebagai bentuk komitmen Mahkamah Agung untuk pelayanan publik, transparansi dan akuntabilitas pelaksanaan fungsi peradilan. Namun dalam hal-hal tertentu masih dimungkinkan terjadi permasalahan teknis terkait dengan akurasi dan keterkinian informasi yang kami sajikan, hal mana akan terus kami perbaiki dari waktu kewaktu.
Dalam hal Anda menemukan inakurasi informasi yang termuat pada situs ini atau informasi yang seharusnya ada, namun belum tersedia, maka harap segera hubungi Kepaniteraan Mahkamah Agung RI melalui :
Email : kepaniteraan@mahkamahagung.go.id Telp : 021-384 3348 (ext.318)

Artinya: Dari Al Hasan, sesungguhnya Nabi SAW. telah bersabda: “barangsiapa yang dipanggil oleh Hakim Islam untuk menghadap di persidangan, sedangkan ia tidak memenuhi panggilan itu, maka ia termasuk orang yang dhalim dan gugurlah haknya”;

Menimbang, bahwa dalil-dalil gugatan Penggugat merupakan rangkaian dalil yang pada pokoknya bahwa sejak 01 Desember 2019 rumah tangga Penggugat dan Tergugat sudah tidak harmonis karena sering berselisih dan bertengkar disebabkan Masalah ekonomi rumah tangga dan Tergugat jarang memberi nafkah kepada penggugat, dan puncaknya Penggugat dengan Tergugat telah hidup berpisah hingga sekarang ± 1 tahun 1 bulan lamanya, atas dasar itu, Penggugat mohon untuk dijatuhkan talak satu bain sugra Tergugat terhadap Penggugat;

Menimbang, bahwa meskipun perkara ini tanpa dihadiri Tergugat, namun karena perkara ini menyangkut bidang perkawinan yang menggunakan hukum acara khusus sesuai kehendak Pasal 54, 76 Ayat (1) Undang-undang Nomor 7 Tahun 1989 sebagaimana diubah dengan Undang-undang Nomor 3 Tahun 2006 dan perubahan kedua dengan Undang-undang Nomor 50 Tahun 2009, maka Majelis Hakim tetap membebankan kepada Penggugat untuk membuktikan dalil-dalil gugatannya;

Menimbang, bahwa untuk meneguhkan dalil-dalil gugatannya Penggugat telah mengajukan bukti surat (P.1 dan P.2) telah bermeterai cukup, yang berupa fotokopi telah dicocokkan dan sesuai dengan aslinya sehingga majelis hakim menilai alat bukti surat tersebut sah sebagai alat bukti berdasarkan Pasal 1888 Kitab Undang-Undang Hukum Perdata dan Pasal 2 Ayat (3) Undang-Undang Nomor 13 Tahun 1985 tentang Bea Meterai serta Pasal 2 Ayat (1) Peraturan Pemerintah Nomor 24 Tahun 2000 tentang Perubahan Tarif Bea Meterai dan Besarnya Batas Pengenaan Harga Nominal Yang Dikenakan Bea Meterai;

Menimbang, bahwa alat bukti P.1 dan P.2 tersebut merupakan akta otentik yang dibuat oleh pejabat yang berwenang, dan isinya tersebut tidak dibantah, maka nilai kekuatan pembuktiannya adalah bersifat sempurna dan mengikat berdasarkan Pasal 1870 KUH Perdata dan Pasal 165 HIR;

Disclaimer
Kepaniteraan Mahkamah Agung Republik Indonesia berusaha untuk selalu mencantumkan informasi paling kini dan akurat sebagai bentuk komitmen Mahkamah Agung untuk pelayanan publik, transparansi dan akuntabilitas pelaksanaan fungsi peradilan. Namun dalam hal-hal tertentu masih dimungkinkan terjadi permasalahan teknis terkait dengan akurasi dan keterkinian informasi yang kami sajikan, hal mana akan terus kami perbaiki dari waktu kewaktu.
Dalam hal Anda menemukan inakurasi informasi yang termuat pada situs ini atau informasi yang seharusnya ada, namun belum tersedia, maka harap segera hubungi Kepaniteraan Mahkamah Agung RI melalui :
Email : kepaniteraan@mahkamahagung.go.id   Telp : 021-384 3348 (ext.318)

Menimbang, bahwa Majelis telah mendengar keterangan 2 (dua) orang saksi, bukan orang yang dilarang menjadi saksi dan yang telah memberikan keterangan di bawah sumpahnya di depan sidang seorang demi seorang sebagaimana terurai di atas, oleh karena itu memenuhi syarat formil saksi;

Menimbang, bahwa dari segi materi keterangannya, keterangan saksi berdasarkan alasan dan pengetahuan sendiri, serta relevant dengan pokok perkara dan saling bersesuaian antara yang satu dengan yang lain, oleh karena itu memenuhi syarat materiil saksi;

Menimbang, bahwa oleh karena saksi-saksi Penggugat telah memenuhi syarat formil dan materiil sebagaimana ketentuan Pasal 141 ayat (1), 147, 170, 171, 172 HIR jo. Pasal 76 ayat (1) Undang-Undang Nomor 7 Tahun 1989 tentang Peradilan Agama yang diubah dengan Undang-Undang Nomor 3 Tahun 2006 dan Undang-Undang Nomor 50 Tahun 2009 jo. Pasal 22 ayat (2) Peraturan Pemerintah Nomor 9 Tahun 1975 jo. Pasal 134 Kompilasi Hukum Islam, maka keterangan saksi tersebut mempunyai nilai pembuktian;

Menimbang, bahwa berdasarkan pengakuan Penggugat yang telah dikuatkan dengan bukti P.1 berupa Kartu Tanda Penduduk atas nama Penggugat, maka telah terbukti menurut hukum bahwa Penggugat tercatat sebagai penduduk di wilayah Kabupaten Blitar;

Menimbang, bahwa berdasarkan pengakuan Penggugat bahwa antara Penggugat dan Tergugat adalah suami isteri yang menikah secara sah pada tanggal 05 Juni 2019 dan telah dikuatkan dengan bukti P.2 berupa Kutipan Akta Nikah, maka berdasarkan Pasal 7 ayat (1) Kompilasi Hukum Islam telah terbukti menurut hukum bahwa antara Penggugat dan Tergugat adalah suami istri sah;

Menimbang, bahwa berdasarkan dalil-dalil Penggugat yang dihubungkan dengan bukti-bukti yang saling bersesuaian telah ditemukan fakta hukum sebagai berikut:

1. Bahwa Penggugat dan Tergugat adalah suami isteri sah yang menikah pada tanggal 05 Juni 2019 namun belum dikaruniai keturunan
2. Bahwa rumah tangga Penggugat dan Tergugat sejak 01 Desember 2019 sudah sering terjadi perselisihan dan pertengkaran yang disebabkan

Disclaimer
Kepaniteraan Mahkamah Agung Republik Indonesia berusaha untuk selalu mencantumkan informasi paling kini dan akurat sebagai bentuk komitmen Mahkamah Agung untuk pelayanan publik, transparansi dan akuntabilitas pelaksanaan fungsi peradilan. Namun dalam hal-hal tertentu masih dimungkinkan terjadi permasalahan teknis terkait dengan akurasi dan keterkinian informasi yang kami sajikan, hal mana akan terus kami perbaiki dari waktu kewaktu.
Dalam hal Anda menemukan inakurasi informasi yang termuat pada situs ini atau informasi yang seharusnya ada, namun belum tersedia, maka harap segera hubungi Kepaniteraan Mahkamah Agung RI melalui :
Email : kepaniteraan@mahkamahagung.go.id Telp : 021-384 3348 (ext.318)

Masalah ekonomi rumah tangga dan Tergugat jarang memberi nafkah kepada penggugat.;

3. Bahwa akibat perselisihan tersebut antara Penggugat dan Tergugat telah terjadi pisah tempat tinggal sampai dengan sekarang sudah berjalan selama ± 1 tahun 1 bulan dan selama pisah Tergugat tidak pernah memberi nafkah kepada Penggugat;

4. Bahwa Para saksi sudah merukunkan Penggugat dan Tergugat, akan tetapi tidak berhasil;

Menimbang, bahwa tujuan perkawinan sebagaimana tercantum baik dalam Al Qur’an surat Al Rum ayat 21 yang berbunyi:

وَمِنۡ ءَايَٰتِهِۦٓ أَنۡ خَلَقَ لَكُم مِّنۡ أَنفُسِكُمۡ أَزۡوَٰجٗا لِّتَسۡكُنُوٓاْ إِلَيۡهَا وَجَعَلَ بَيۡنَكُم

مَّوَدَّةٗ وَرَحۡمَةًۚ

Artinya: Dan diantara tanda-tanda kekuasaanNya, ialah Dia menciptakan untukmu istri-istri dari jenismu sendiri, supaya kamu cenderung dan merasa tentram kepadanya dan dijadikanNya diantara kamu rasa kasih dan sayang;

maupun dalam Pasal 1 Undang-undang Nomor 1 Tahun 1974 tentang Perkawinan dan Pasal 3 Kompilasi Hukum Islam, adalah terwujudnya rumah tangga yang tentram (sakinah), bahagia dan kekal yang diliputi rasa kasih sayang (mawaddah, warohmah) berdasarkan ketuhanan Yang Maha Esa;

Menimbang, bahwa kondisi rumah tangga Penggugat dan Tergugat sering terjadi perselisihan dan pertengkaran serta telah pisah tempat tinggal selama kurang lebih 1 tahun 1 bulan tanpa ada komunikasi, maka rumah tangga Penggugat dan Tergugat telah retak yang sudah sangat sulit untuk didamaikan lagi, rumah tangga yang demikian itu, mengakibatkan tujuan perkawinan tidak dapat diwujudkan dalam rumah tangga Penggugat dan Tergugat dan sudah tidak ada harapan untuk rukun kembali, disisi lain Majelis Hakim maupun para saksi telah berusaha untuk merukunkan juga tidak berhasil sehingga perkawinan mereka jika dipertahankan justru akan menyengsarakan

Disclaimer
Kepaniteraan Mahkamah Agung Republik Indonesia berusaha untuk selalu mencantumkan informasi paling kini dan akurat sebagai bentuk komitmen Mahkamah Agung untuk pelayanan publik, transparansi dan akuntabilitas pelaksanaan fungsi peradilan. Namun dalam hal-hal tertentu masih dimungkinkan terjadi permasalahan teknis terkait dengan akurasi dan keterkinian informasi yang kami sajikan, hal mana akan terus kami perbaiki dari waktu kewaktu.
Dalam hal Anda menemukan inakurasi informasi yang termuat pada situs ini atau informasi yang seharusnya ada, namun belum tersedia, maka harap segera hubungi Kepaniteraan Mahkamah Agung RI melalui :
Email : kepaniteraan@mahkamahagung.go.id   Telp : 021-384 3348 (ext.318)

kedua belah pihak, oleh karena itu perkawinan mereka lebih masalahat diceraikan;

Menimbang, bahwa *relevant* dengan perkara ini, dapat diambil sebuah tuntunan dari Hadits Nabi SAW, yang diriwayatkan oleh Imam Malik menegaskan:

لاضررولاضرارمن ضرضره الله ومن شق شق الله عليه

Artinya: “Tidak boleh memudharatkan dan dimudharatkan, barangsiapa yang memudharatkan maka Allah akan memudharatkannya dan siapa saja yang menyusahkan maka Allah akan menyusahkannya”;

Menimbang, bahwa bertolak dari hadits tersebut dan dihubungkan dengan kasus ini, maka seorang suami tidak boleh memberi mudharat kepada isterinya begitu juga sebaliknya, seorang isteri tidak boleh memberi mudharat kepada suaminya, karena perbuatan demikian dilarang syariat;

Menimbang, bahwa dalam perkara ini Majelis Hakim sependapat dan mengambil alih pendapat pakar hukum Islam Sayyid Sabiq dalam Kitab *Fiqhu as Sunnah*, Juz II, halaman 249:

اذا ادعت الزوجة اضرار الزوج بها بما لا تستطيع معه دوام العشرة
يجوزلها ان تطلب من القاضى التفريق وحينئذ يطلقها القاضى طلقة
بائنة

Artinya: “Jika isteri menggugat cerai karena suaminya memadlorotkan terhadap dirinya sehingga menggoyahkan keutuhan rumah tangga, maka dibolehkan bagi isterinya tersebut untuk meminta cerai kepada hakim dan bila madlorot tersebut telah terbukti, sedangkan perdamaianpun tidak tercapai, maka hakim menetapkan jatuh talak satu ba’in”;

Menimbang, bahwa berdasarkan pertimbangan-pertimbangan tersebut di atas maka majelis hakim berpendapat dalil-dalil perceraian Penggugat telah terbukti dan telah memenuhi alasan perceraian sebagaimana yang ditentukan dalam Pasal 39 Ayat (2) Undang-undang Nomor 1 Tahun 1974 tentang Perkawinan jo. Pasal 19 huruf (f) Peraturan Pemerintah Nomor 9 Tahun 1975 jo. Pasal 116 huruf (f) Kompilasi Hukum Islam, oleh karena itu gugatan

Disclaimer
Kepaniteraan Mahkamah Agung Republik Indonesia berusaha untuk selalu mencantumkan informasi paling kini dan akurat sebagai bentuk komitmen Mahkamah Agung untuk pelayanan publik, transparansi dan akuntabilitas pelaksanaan fungsi peradilan. Namun dalam hal-hal tertentu masih dimungkinkan terjadi permasalahan teknis terkait dengan akurasi dan keterkinian informasi yang kami sajikan, hal mana akan terus kami perbaiki dari waktu kewaktu.
Dalam hal Anda menemukan inakurasi informasi yang termuat pada situs ini atau informasi yang seharusnya ada, namun belum tersedia, maka harap segera hubungi Kepaniteraan Mahkamah Agung RI melalui :
Email : kepaniteraan@mahkamahagung.go.id   Telp : 021-384 3348 (ext.318)

Penggugat patut dikabulkan dengan verstek dengan menjatuhkan talak satu bain sughra Tergugat terhadap Penggugat;

Menimbang, bahwa pada hari sidang putusan, Penggugat mengaku dalam keadaan suci;

Menimbang, bahwa perkara ini termasuk bidang perkawinan, berdasarkan berdasarkan Pasal 89 Ayat (1) Undang-undang Nomor 7 Tahun 1989 tentang Peradilan Agama sebagaimana diubah dengan Undang-undang Nomor 3 Tahun 2006 dan perubahan kedua dengan Undang-undang Nomor 50 Tahun 2009, maka beaya perkara ini dibebankan kepada Penggugat;

Mengingat segala peraturan perundang-undangan yang berlaku dan hukum syar'i yang berkaitan dengan perkara ini;

M E N G A D I L I

1. Menyatakan bahwa Tergugat yang telah dipanggil dengan patut untuk menghadap di persidangan tidak hadir;
2. Mengabulkan gugatan Penggugat dengan verstek;
3. Menjatuhkan talak satu ba'in sughro Tergugat (**Xxxxxxxxxxxxxxxxxxxxx**) terhadap Penggugat (**Xxxxxxxxxxxxxxxxxxxx**);
4. Membebankan kepada Penggugat untuk membayar seluruh biaya perkara ini sebesar Rp. 731.000,- (tujuh ratus tiga puluh satu ribu rupiah);

Demikian, putusan ini dijatuhkan di Pengadilan Agama Blitar berdasarkan musyawarah Majelis Hakim pada hari Senin tanggal 28 Desember 2020 Masehi, bertepatan dengan tanggal 13 Jumadil Awwal 1442 Hijriyah, oleh kami **Drs. H. Kamaluddin, S.H..** sebagai Hakim Ketua, **Drs. H. Roji'un, M.H dan Drs. H. Farihin, S.H..**, masing-masing sebagai Hakim Anggota, putusan ini pada hari itu juga diucapkan dalam persidangan terbuka untuk umum oleh Ketua Majelis dengan didampingi para Hakim Anggota dibantu oleh **Drs. Taryono Reksowardoyo, S.H..** sebagai Panitera Pengganti, dengan dihadiri oleh Kuasa Penggugat tanpa hadirnya Tergugat;

HAKIM ANGGOTA KETUA MAJELIS

Disclaimer
Kepaniteraan Mahkamah Agung Republik Indonesia berusaha untuk selalu mencantumkan informasi paling kini dan akurat sebagai bentuk komitmen Mahkamah Agung untuk pelayanan publik, transparansi dan akuntabilitas pelaksanaan fungsi peradilan. Namun dalam hal-hal tertentu masih dimungkinkan terjadi permasalahan teknis terkait dengan akurasi dan keterkinian informasi yang kami sajikan, hal mana akan terus kami perbaiki dari waktu kewaktu.
Dalam hal Anda menemukan inakurasi informasi yang termuat pada situs ini atau informasi yang seharusnya ada, namun belum tersedia, maka harap segera hubungi Kepaniteraan Mahkamah Agung RI melalui :
Email : kepaniteraan@mahkamahagung.go.id Telp : 021-384 3348 (ext.318)

**Drs. H. Roji'un, M.H.**	**Drs. H. Kamaluddin, S.H..**
**Drs. H. Farihin, S.H..**	

PANITERA PENGGANTI

Drs. Taryono Reksowardoyo, S.H..

Perincian Biaya:

1.	PNBP		
	a. Pendaftaran	Rp	30.000,-
	b. Panggilan Pertama Penggugat/Pemohon	Rp	10.000,-
	c. Panggilan Pertama Tergugat/ Termohon	Rp	10.000,-
	d. Redaksi	Rp	10.000,-
	e. Pemberitahuan isi Putusan	Rp	10.000,-
2.	Biaya proses	Rp	75.000,-
3.	Penggilan	Rp	420.000,-
4.	Pemberitahuan isi Putusan	Rp	160.000,-
5.	Materai	Rp	6.000,-
	Jumlah …………………..	Rp	731.000,-

(tujuh ratus tiga puluh satu ribu rupiah)

Hal. 13 dari 13 hal. Putusan Nomor 3871/Pdt.G/2020/PA.BL

Disclaimer
Kepaniteraan Mahkamah Agung Republik Indonesia berusaha untuk selalu mencantumkan informasi paling kini dan akurat sebagai bentuk komitmen Mahkamah Agung untuk pelayanan publik, transparansi dan akuntabilitas pelaksanaan fungsi peradilan. Namun dalam hal-hal tertentu masih dimungkinkan terjadi permasalahan teknis terkait dengan akurasi dan keterkinian informasi yang kami sajikan, hal mana akan terus kami perbaiki dari waktu kewaktu.
Dalam hal Anda menemukan inakurasi informasi yang termuat pada situs ini atau informasi yang seharusnya ada, namun belum tersedia, maka harap segera hubungi Kepaniteraan Mahkamah Agung RI melalui :
Email : kepaniteraan@mahkamahagung.go.id Telp : 021-384 3348 (ext.318)
