# Supplementary material for: Classification of divorce causes during the COVID-19 pandemic using convolutional neural networks
Source: PeerJ Comput Sci. 2022 Jun 30;8:e998. doi: 10.7717/peerj-cs.998 (PMC9299239; doi:10.7717/peerj-cs.998)
Supplement: Supplemental Information 6 [file peerj-cs-08-998-s006.zip › Rujuk Dataset/Data ke-9.pdf]

P U T U S A N

Nomor 7667/Pdt.G/2020/PA.Sor

بِسْمِ اللّٰهِ الرَّحْمٰنِ الرَّحِيْمِ

DEMI KEADILAN BERDASARKAN KETUHANAN YANG MAHA ESA

Pengadilan Agama Soreang yang memeriksa dan mengadili perkara Cerai Gugat pada tingkat pertama, dalam persidangan majelis telah menjatuhkan putusan antara:

XXXXXXXXXXXX, umur 31 tahun, agama Islam, pendidikan SD, pekerjaan Ibu Rumah Tangga, alamat di XXXXXXXXXXXXKabupaten Bandung, selanjutnya disebut **Penggugat**;

melawan

XXXXXXXXXXXX, umur 43 tahun, agama Islam, pendidikan SD, pekerjaan Buruh, alamat di XXXXXXXXXXXXKabupaten Bandung, selanjutnya disebut **Tergugat**;

Pengadilan Agama tersebut;

Telah mempelajari surat-surat yang berkaitan dengan perkara ini;

Telah mendengar keterangan Penggugat dan saksi-saksi di muka sidang;

DUDUK PERKARA

Bahwa Penggugat berdasarkan surat gugatannya tertanggal 23 November 2020 yang telah didaftarkan di Kepaniteraan Pengadilan Agama Soreang, Nomor 7667/Pdt.G/2020/PA.Sor, tanggal 23 November 2020 pada pokoknya mengajukan hal-hal sebagai berikut:

1. Bahwa pada tanggal 05 Februari 2018 Penggugat dengan Tergugat telah melangsungkan perkawinan diwilayah hukum Kantor Urusan Agama Kecamatan Kutawaringin Kabupaten Bandung, sebagaimana Kutipan Akta Nikah Nomor : 126/11/II/2018;
2. Bahwa setelah menikah (Februari 2018) Penggugat dengan Tergugat terakhir berumah tangga dan tinggal di XXXXXXXXXXXXKabupaten Bandung, dan dari pernikahannya tersebut tidak dikaruniai anak ;

Disclaimer
Kepaniteraan Mahkamah Agung Republik Indonesia berusaha untuk selalu mencantumkan informasi paling kini dan akurat sebagai bentuk komitmen Mahkamah Agung untuk pelayanan publik, transparansi dan akuntabilitas pelaksanaan fungsi peradilan. Namun dalam hal-hal tertentu masih dimungkinkan terjadi permasalahan teknis terkait dengan akurasi dan keterkinian informasi yang kami sajikan, hal mana akan terus kami perbaiki dari waktu kewaktu.
Dalam hal Anda menemukan inakurasi informasi yang termuat pada situs ini atau informasi yang seharusnya ada, namun belum tersedia, maka harap segera hubungi Kepaniteraan Mahkamah Agung RI melalui :
Email : kepaniteraan@mahkamahagung.go.id Telp : 021-384 3348 (ext.318)

3. Bahwa awal pernikahan rumah tangga antara Penggugat dengan Tergugat rukun dan harmonis sebagaimana layaknya suatu rumah tangga yang baik, akan tetapi tidak dapat dipertahankan lagi, karena sejak bulan Mei 2020 antara Penggugat dengan Tergugat sering terjadi perselisihan serta percekcokan yang sifatnya terus menerus, dan penyebab perselisihan serta pertengkaran tersebut dikarenakan;

a) Tergugat bersifat tempramen;

b) Tergugat tidak menghargai orang tua Penggugat;

c) Masalah Ekonomi (Tergugat lalai dan kurang bertanggung jawab dalam memberikan nafkah lahir kepada Penggugat);

4. Bahwa dampak dari percekcokan serta perselisihan tersebut sejak bulan Agustus 2020 Penggugat dan Tergugat telah pisah ranjang bersama, dan sejak itu pula sudah tidak berhubungan lagi layaknya suami istri yang baik;

5. Bahwa Penggugat sudah tidak sanggup lagi menjalani dan mempertahankan rumah tangga yang demikian karena perselisihan antara Penggugat dengan Tergugat sudah sulit diatasi dan tidak ada lagi harapan untuk rukun, sehingga tujuan perkawinan membentuk rumah tangga yang sakinah, mawaddah dan rahmah mustahil terwujud. Dan talaklah menjadi salah satunya solusi agar Penggugat dengan Tergugat tidak melanggar norma hukum dan agama;

Berdasarkan hal-hal tersebut di atas, Penggugat mohon agar Ketua Pengadilan Agama Soreang Cq. Majelis Hakim yang memeriksa dan mengadili perkara ini, selanjutnya menjatuhkan putusan yang amarnya berbunyi:

1. Menerima dan Mengabulkan Gugatan Penggugat;

2. Menjatuhkan Talak satu Tergugat (**XXXXXXXXXXXX**) terhadap Penggugat (**XXXXXXXXXXXX**)

3. Membebankan biaya perkara menurut peraturan hukum yang berlaku;

Atau, jika Pengadilan Agama Soreang berpendapat lain mohon putusan yang seadil-adilnya;

Disclaimer
Kepaniteraan Mahkamah Agung Republik Indonesia berusaha untuk selalu mencantumkan informasi paling kini dan akurat sebagai bentuk komitmen Mahkamah Agung untuk pelayanan publik, transparansi dan akuntabilitas pelaksanaan fungsi peradilan. Namun dalam hal-hal tertentu masih dimungkinkan terjadi permasalahan teknis terkait dengan akurasi dan keterkinian informasi yang kami sajikan, hal mana akan terus kami perbaiki dari waktu kewaktu.
Dalam hal Anda menemukan inakurasi informasi yang termuat pada situs ini atau informasi yang seharusnya ada, namun belum tersedia, maka harap segera hubungi Kepaniteraan Mahkamah Agung RI melalui :
Email : kepaniteraan@mahkamahagung.go.id Telp : 021-384 3348 (ext.318)

Bahwa pada hari persidangan yang telah ditetapkan Penggugat, telah datang menghadap sendiri di muka sidang, sedangkan Tergugat pada sidang pertama datang menghadap sendiri, namun pada sidang-sidang selanjutnya Tergugat tidak datang lagi menghadap di muka sidang, meskipun telah dipanggil secara resmi dan patut;

Bahwa Majelis Hakim telah berusaha mendamaikan Penggugat dan Tergugat, akan tetapi tidak berhasil;

Bahwa berdasarkan Peraturan Mahkamah Agung Nomor 1 Tahun 2016 Tentang Prosedur Mediasi, Majelis Hakim telah memerintahkan kepada Penggugat dan Tergugat untuk menempuh perdamaian melalui proses mediasi dengan Hakim Mediator Drs. Mahbub berdasarkan Penetapan Nomor 7667/Pdt.G/2020/PA.Sor tanggal 03 Desember 2020 dan berdasarkan Laporan Hakim Mediator Drs. Mahbub tanggal 03 Desember 2020 mediasi tidak berhasil;

Bahwa kemudian dibacakan gugatan Penggugat yang isinya tetap dipertahankan oleh Penggugat;

Bahwa terhadap gugatan Penggugat tersebut, Tergugat tidak dapat di dengar jawabannya, sebab Tergugat tidak datang lagi menghadap di muka sidang;

Bahwa untuk menguatkan dalil-dalil gugatannya, Penggugat telah mengajukan alat bukti berupa:

A. Surat:

1. Fotokopi Kartu Tanda Penduduk Provinsi Jawa Barat Kabupaten Bandung NIK 3204266908950002 atas nama Penggugat yang telah di-*nazegelen* dan telah dicocokkan dengan aslinya ternyata sesuai, bertanda P.1;
2. Fotokopi Kutipan Akta Nikah Nomor 126/11/II/2018 tanggal 05 Februari 2018 yang dikeluarkan oleh Pegawai Pencatat Nikah Kecamatan Kutawaringin Kabupaten Bandung. Bukti surat tersebut telah diberi meterai cukup dan dinazegelen serta telah dicocokkan dengan aslinya yang ternyata sesuai, bertanda P.2;

B. Saksi:

Disclaimer
Kepaniteraan Mahkamah Agung Republik Indonesia berusaha untuk selalu mencantumkan informasi paling kini dan akurat sebagai bentuk komitmen Mahkamah Agung untuk pelayanan publik, transparansi dan akuntabilitas pelaksanaan fungsi peradilan. Namun dalam hal-hal tertentu masih dimungkinkan terjadi permasalahan teknis terkait dengan akurasi dan keterkinian informasi yang kami sajikan, hal mana akan terus kami perbaiki dari waktu kewaktu.
Dalam hal Anda menemukan inakurasi informasi yang termuat pada situs ini atau informasi yang seharusnya ada, namun belum tersedia, maka harap segera hubungi Kepaniteraan Mahkamah Agung RI melalui :
Email : kepaniteraan@mahkamahagung.go.id   Telp : 021-384 3348 (ext.318)

1. xxxxxxxx, umur 31 tahun, agama Islam, pekerjaan buruh, alamat XXXXXXXXXXXXKabupaten Bandung, di bawah sumpahnya memberikan keterangan pada pokoknya sebagai berikut:

- Bahwa saksi kenal Penggugat dan Tergugat sebagai Bibi Penggugat;
- Bahwa Penggugat dan Tergugat adalah suami isteri;
- Bahwa setelah menikah Penggugat dan Tergugat tinggal bersama di XXXXXXXXXXXXKabupaten Bandung;
- Bahwa rumah tangga Penggugat dan Tergugat awalnya rukun dan harmonis, namun sejak bulan Mei 2020 mulai tidak harmonis lagi karena antara Penggugat dan Tergugat sering terjadi perselisihan dan pertengkaran;
- Bahwa saksi pernah melihat antara Penggugat dan Tergugat bertengkar;
- Bahwa perselisihan dan pertengkaran antara Penggugat dan Tergugat tersebut disebabkan Tergugat tidak bertanggung jawab dalam hal nafkah Penggugat
- Bahwa saat ini Penggugat dan Tergugat telah berpisah tempat tinggal sejak 5 (lima) bulan dan selama pisah tidak pernah rukun lagi;
- Bahwa pihak keluarga sudah berupaya merukunkan Penggugat dan Tergugat namun tidak berhasil, karena Penggugat tetap ingin bercerai dengan Tergugat;

Bahwa Penggugat menyatakan cukup atas keterangan saksi tersebut;

2. xxxxxxxxx, umur 50 tahun, agama Islam, pekerjaan buruh, alamat XXXXXXXXXXXXKabupaten Bandung, di bawah sumpahnya memberikan keterangan pada pokoknya sebagai berikut:

- Bahwa saksi kenal Penggugat dan Tergugat saksi adalah tetangga Penggugat;
- Bahwa Penggugat dan Tergugat adalah suami isteri;
- Bahwa setelah menikah Penggugat dan Tergugat tinggal bersama di XXXXXXXXXXXXKabupaten Bandung;
- Bahwa rumah tangga Penggugat dan Tergugat pada awalnya rukun dan harmonis, akan tetapi sejak bulan Mei 2020 mulai tidak harmonis

Disclaimer
Kepaniteraan Mahkamah Agung Republik Indonesia berusaha untuk selalu mencantumkan informasi paling kini dan akurat sebagai bentuk komitmen Mahkamah Agung untuk pelayanan publik, transparansi dan akuntabilitas pelaksanaan fungsi peradilan. Namun dalam hal-hal tertentu masih dimungkinkan terjadi permasalahan teknis terkait dengan akurasi dan keterkinian informasi yang kami sajikan, hal mana akan terus kami perbaiki dari waktu kewaktu.
Dalam hal Anda menemukan inakurasi informasi yang termuat pada situs ini atau informasi yang seharusnya ada, namun belum tersedia, maka harap segera hubungi Kepaniteraan Mahkamah Agung RI melalui :
Email : kepaniteraan@mahkamahagung.go.id Telp : 021-384 3348 (ext.318)

lagi karena antara Penggugat dan Tergugat sering terjadi perselisihan dan pertengkaran;

- Bahwa saksi pernah melihat sendiri Penggugat dan Tergugat bertengkar
- Bahwa perselisihan dan pertengkaran antara Penggugat dan Tergugat tersebut disebabkan Tergugat tidak bertanggung jawab dalam hal nafkah Penggugat
- Bahwa saat ini Penggugat dan Tergugat telah pisah tempat tinggal sejak 5 (lima) bulan dan selama pisah tidak pernah rukun lagi;
- Bahwa pihak keluarga sudah berupaya merukunkan Penggugat dan Tergugat namun tidak berhasil;

Bahwa Penggugat menyatakan cukup atas keterangan saksi tersebut;

Bahwa Penggugat menyatakan cukup dengan pembuktiannya;

Bahwa Penggugat menyampaikan kesimpulan dengan menyatakan tetap pada gugatannya untuk bercerai dengan Tergugat, serta mohon putusan;

Bahwa untuk ringkasnya uraian putusan ini, maka semua hal yang termuat dalam berita acara sidang ini merupakan bagian yang tidak terpisahkan dari putusan ini;

PERTIMBANGAN HUKUM

Menimbang, bahwa maksud dan tujuan gugatan Penggugat adalah sebagaimana diuraikan di atas;

Menimbang, bahwa pada persidangan yang telah ditetapkan, pada sidang pertama, Tergugat datang menghadap ke muka sidang, akan tetapi pada sidang-sidang selanjutnya Tergugat tidak datang menghadap ke muka sidang serta tidak pula mengutus orang lain untuk datang sebagai kuasanya, meskipun telah dipanggil secara resmi dan patut, maka Majelis Hakim berketetapan untuk memeriksa dan memutus perkara ini di luar hadirnya Tergugat *(contradictoir)* sesuai dengan Pasal 127 HIR;

Menimbang, bahwa gugatan Penggugat pada pokoknya adalah mohon untuk diceraikan dari Tergugat, sebab sejak bulan Mei 2020, antara Penggugat dan Tergugat sering terjadi perselisihan dan pertengkaran disebabkan Tergugat

Disclaimer
Kepaniteraan Mahkamah Agung Republik Indonesia berusaha untuk selalu mencantumkan informasi paling kini dan akurat sebagai bentuk komitmen Mahkamah Agung untuk pelayanan publik, transparansi dan akuntabilitas pelaksanaan fungsi peradilan. Namun dalam hal-hal tertentu masih dimungkinkan terjadi permasalahan teknis terkait dengan akurasi dan keterkinian informasi yang kami sajikan, hal mana akan terus kami perbaiki dari waktu kewaktu.
Dalam hal Anda menemukan inakurasi informasi yang termuat pada situs ini atau informasi yang seharusnya ada, namun belum tersedia, maka harap segera hubungi Kepaniteraan Mahkamah Agung RI melalui :
Email : kepaniteraan@mahkamahagung.go.id Telp : 021-384 3348 (ext.318)

tidak bertanggung jawab dalam hal nafkah Penggugat, akibatnya antara Penggugat dan Tergugat telah pisah tempat tinggal sejak 5 (lima) bulan;

Menimbang, bahwa berdasarkan gugatan Penggugat, yang menjadi pokok permasalahan dalam perkara ini adalah apakah benar antara Penggugat dan Tergugat terus menerus terjadi pertengkaran dan perselisihan serta tidak ada harapan akan hidup rukun lagi dalam rumah tangga?;

Menimbang, bahwa untuk membuktikan dalil gugatannya, Penggugat di persidangan telah mengajukan bukti surat bertanda P.1 dan P.2, bukti tersebut telah diberi meterai cukup dan dinazegelen serta telah dicocokkan sesuai dengan aslinya, sehingga berdasarkan Pasal 1888 KUHPerdata *jo.* Pasal 2 Ayat (3) Undang Undang Nomor 13 Tahun 1985 Tentang Bea Meterai, Pasal 2 Ayat (1) Peraturan Pemerintah Nomor 24 Tahun 2000 Tentang Perubahan Tarif Bea Meterai dan Besarnya Batas Pengenaan Harga Nominal Yang Dikenakan Bea Meterai, maka alat bukti tersebut dapat diterima sebagai alat bukti;

Menimbang, bahwa P.1 merupakan fotokopi Kartu Tanda Penduduk yang dibuat oleh pejabat yang berwenang untuk itu, maka alat bukti tersebut mempunyai nilai pembuktian sempurna (*volledig*) dan mengikat (*bindende*), maka terbukti bahwa Penggugat (XXXXXXXXXXXX) adalah pihak *principal* yang memiliki kewenangan dan kepentingan hukum langsung dengan pokok perkara;

Menimbang, bahwa bukti P.2 merupakan fotokopi Kutipan Akta Nikah yang dibuat oleh pejabat yang berwenang untuk itu, maka alat bukti tersebut mempunyai nilai pembuktian sempurna (*volledig*) dan mengikat (*bindende*) maka terbukti bahwa Penggugat dan Tergugat adalah suami isteri sah yang menikah tanggal 05 Februari 2018;

Menimbang, bahwa Penggugat juga telah menghadirkan saksi-saksi yang telah dewasa dan sudah disumpah, sehingga memenuhi syarat formal sebagaimana diatur dalam Pasal 145 ayat (1) HIR;

Menimbang, bahwa keterangan saksi-saksi Penggugat mengenai pokok permasalahan perkara ini adalah fakta yang dialami sendiri dan relevan dengan dalil yang harus dibuktikan oleh Penggugat, dan isinya saling bersesuaian dan cocok antara satu dengan yang lain, yaitu tentang terjadinya perselisihan dan

Disclaimer
Kepaniteraan Mahkamah Agung Republik Indonesia berusaha untuk selalu mencantumkan informasi paling kini dan akurat sebagai bentuk komitmen Mahkamah Agung untuk pelayanan publik, transparansi dan akuntabilitas pelaksanaan fungsi peradilan. Namun dalam hal-hal tertentu masih dimungkinkan terjadi permasalahan teknis terkait dengan akurasi dan keterkinian informasi yang kami sajikan, hal mana akan terus kami perbaiki dari waktu kewaktu.
Dalam hal Anda menemukan inakurasi informasi yang termuat pada situs ini atau informasi yang seharusnya ada, namun belum tersedia, maka harap segera hubungi Kepaniteraan Mahkamah Agung RI melalui :
Email : kepaniteraan@mahkamahagung.go.id   Telp : 021-384 3348 (ext.318)

pertengkaran antara Penggugat dan Tergugat disebabkan Tergugat tidak bertanggung jawab dalam hal nafkah Penggugat serta mengenai pisahnya Penggugat dan Tergugat serta selama pisah tidak pernah rukun lagi, meskipun telah didamaikan oleh pihak keluarga, oleh karena itu keterangan saksi tersebut telah memenuhi syarat materiil sebagaimana diatur dalam Pasal 171 dan 172 HIR, sehingga keterangan saksi-saksi tersebut memiliki kekuatan pembuktian;

Menimbang, bahwa berdasarkan gugatan Penggugat dan bukti-bukti Penggugat, ditemukan fakta di persidangan yang selanjutnya dijadikan sebagai fakta hukum sebagai berikut:

- Bahwa Penggugat dan Tergugat adalah suami isteri sah yang menikah tanggal 05 Februari 2018;
- Bahwa sejak bulan Mei 2020, antara Penggugat dan Tergugat sering terjadi perselisihan dan pertengkaran disebabkan Tergugat tidak bertanggung jawab dalam hal nafkah Penggugat;
- Bahwa antara Penggugat dan Tergugat telah pisah tempat tinggal sejak 5 (lima) bulan dan selama pisah tidak pernah rukun lagi;
- Bahwa keluarga Penggugat dan Tergugat telah berupaya untuk mendamaikan Penggugat dan Tergugat, namun tidak berhasil;

Menimbang, bahwa Penggugat pada petitum gugatannya Nomor 1 (satu) dan 2 (dua), pada pokoknya mohon untuk diceraikan dari Tergugat, terhadap tuntutan tersebut Majelis Hakim akan mempertimbangkannya dengan pertimbangan berikut;

Menimbang, bahwa berdasarkan Pasal 39 ayat (1) dan ayat (2) Undang-Undang Nomor 1 Tahun 1974 tentang Perkawinan, perceraian hanya dapat dilakukan di depan sidang pengadilan, setelah pengadilan yang bersangkutan berusaha dan tidak berhasil mendamaikan kedua belah pihak, dan untuk melakukan perceraian harus ada cukup alasan bahwa antara suami isteri itu tidak akan dapat hidup rukun sebagai suami isteri;

Menimbang, bahwa yang dapat dijadikan dasar/alasan dalam mengajukan perceraian, diatur dalam Pasal 19 Peraturan Pemerintah Nomor 9 Tahun 1975 tentang Pelaksanaan Undang Undang Nomor 1 Tahun 1974 Tentang Perkawinan dan Pasal 116 Kompilasi Hukum Islam;

Disclaimer
Kepaniteraan Mahkamah Agung Republik Indonesia berusaha untuk selalu mencantumkan informasi paling kini dan akurat sebagai bentuk komitmen Mahkamah Agung untuk pelayanan publik, transparansi dan akuntabilitas pelaksanaan fungsi peradilan. Namun dalam hal-hal tertentu masih dimungkinkan terjadi permasalahan teknis terkait dengan akurasi dan keterkinian informasi yang kami sajikan, hal mana akan terus kami perbaiki dari waktu kewaktu.
Dalam hal Anda menemukan inakurasi informasi yang termuat pada situs ini atau informasi yang seharusnya ada, namun belum tersedia, maka harap segera hubungi Kepaniteraan Mahkamah Agung RI melalui :
Email : kepaniteraan@mahkamahagung.go.id   Telp : 021-384 3348 (ext.318)

Menimbang, bahwa dari ketentuan peraturan perundang-undangan di atas, terdapat beberapa unsur yang harus dipenuhi untuk dapat terjadinya perceraian yaitu:

- Pengadilan telah berusaha mendamaikan suami isteri dan tidak berhasil;
- Adanya alasan bahwa antara suami isteri itu tidak akan dapat hidup rukun sebagai suami isteri;

Menimbang, bahwa terhadap unsur-unsur tersebut akan dipertimbangkan satu per satu dengan memperhatikan fakta-fakta hukum yang terjadi dalam rumah tangga Penggugat dan Tergugat, untuk memperoleh suatu kesimpulan apakah kondisi rumah tangga Penggugat dan Tergugat telah memenuhi unsur-unsur terjadinya suatu perceraian;

Menimbang, bahwa Majelis Hakim telah berusaha mendamaikan Penggugat dan Tergugat, namun tidak berhasil, dan antara Penggugat dan Tergugat telah diupayakan perdamaian melalui proses mediasi sesuai dengan Peraturan Mahkamah Agung Nomor 1 Tahun 2016 Tentang Prosedur Mediasi di Pengadilan, dengan mediator Drs. Mahbub, namun tetap tidak berhasil. Dengan demikian unsur pertama telah terpenuhi;

Menimbang, bahwa berdasarkan fakta-fakta hukum, telah ternyata bahwa antara Penggugat dan Tergugat terjadi perselisihan dan pertengkaran, meskipun demikian kategori "*Terus menerus terjadi pertengkaran dan perselisihan antara* Penggugat *dan Tergugat dan tidak ada harapan akan hidup rukun lagi dalam rumah tangga*" belum terpenuhi, untuk itu Majelis Hakim akan mempertimbangkannya dengan pertimbangan berikut;

Menimbang, bahwa telah ternyata selama persidangan Penggugat tidak menunjukkan itikad baik untuk hidup rukun kembali dengan Tergugat, karena Penggugat bersikeras untuk bercerai dengan Tergugat meskipun telah diupayakan damai oleh Majelis Hakim, sedangkan Tergugat tidak menunjukkan itikad baiknya untuk rukun lagi dengan Penggugat karena tidak datang menghadap ke muka sidang dan tidak menyuruh orang lain untuk menghadap sebagai wakil/kuasa hukumnya, bahkan Penggugat dan Tergugat telah pisah tempat tinggal dan selama pisah antara Penggugat dan Tergugat tidak saling

Disclaimer
Kepaniteraan Mahkamah Agung Republik Indonesia berusaha untuk selalu mencantumkan informasi paling kini dan akurat sebagai bentuk komitmen Mahkamah Agung untuk pelayanan publik, transparansi dan akuntabilitas pelaksanaan fungsi peradilan. Namun dalam hal-hal tertentu masih dimungkinkan terjadi permasalahan teknis terkait dengan akurasi dan keterkinian informasi yang kami sajikan, hal mana akan terus kami perbaiki dari waktu kewaktu.
Dalam hal Anda menemukan inakurasi informasi yang termuat pada situs ini atau informasi yang seharusnya ada, namun belum tersedia, maka harap segera hubungi Kepaniteraan Mahkamah Agung RI melalui :
Email : kepaniteraan@mahkamahagung.go.id Telp : 021-384 3348 (ext.318)

memperdulikan dan tidak saling mengunjungi lagi satu sama lain, maka berdasarkan fakta-fakta tersebut Majelis Hakim menarik kesimpulan melalui persangkaan sesuai dengan Pasal 173 HIR jo. Pasal 1922 KUHPerdata bahwa kategori "*Terus menerus terjadi pertengkaran dan perselisihan antara Penggugat dan Tergugat dan tidak ada harapan akan hidup rukun lagi dalam rumah tangga*" dengan penafsiran secara luas telah terpenuhi;

Menimbang, bahwa berdasarkan Pasal 22 ayat (2) Peraturan Pemerintah Nomor 9 Tahun 1975 tentang Pelaksanaan Undang-Undang Nomor 1 Tahun 1974, yang pada pokoknya menyatakan bahwa gugatan perceraian karena alasan tersebut dalam Pasal 19 huruf (f) dapat diterima apabila telah cukup jelas bagi Pengadilan mengenai sebab-sebab perselisihan dan pertengkaran itu dan setelah mendengar pihak keluarga/orang dekat dengan suami isteri itu";

Menimbang, bahwa berdasarkan fakta hukum telah ternyata bahwa faktor penyebab keretakan rumah tangga Penggugat dan Tergugat sangat prinsipil dan berpengaruh bagi keutuhan suami-istri serta telah didengar keterangan keluarga/orang dekat Penggugat yang merangkap sebagai saksi Penggugat, pada pokoknya menyatakan bahwa keluarga/orang dekat Penggugat dan Tergugat telah mendamaikan Penggugat dengan Tergugat, namun tidak berhasil, maka ketentuan Pasal 22 ayat (2) Peraturan Pemerintah Nomor 9 tahun 1975 telah terpenuhi;

Menimbang, berdasarkan uraian pertimbangan di atas, maka perkawinan Penggugat dan Tergugat terbukti telah pecah (*broken marriage*) hal mana sesuai dengan Yurisprudensi Mahkamah Agung RI. Nomor 379-K/AG/1995 tanggal 26 Maret 1997 yang menyatakan bahwa "*Suami isteri yang tidak berdiam serumah lagi dan tidak ada harapan untuk dapat hidup rukun kembali, maka rumah tangga tersebut telah terbukti retak dan pecah*", sehingga tujuan perkawinan sebagaimana dimaksud Pasal 1 Undang Undang Nomor 1 Tahun 1974 Tentang Perkawinan *jo.* Pasal 3 Kompilasi Hukum Islam yaitu untuk menciptakan rumah tangga yang bahagia, *sakinah mawaddah warahmah* tidak akan tercapai. Dengan demikian unsur kedua telah terpenuhi;

Menimbang, bahwa terhadap kondisi perkawinan sebagaimana tersebut di atas, maka salah satu alternatifnya adalah dengan jalan perceraian dan

Disclaimer
Kepaniteraan Mahkamah Agung Republik Indonesia berusaha untuk selalu mencantumkan informasi paling kini dan akurat sebagai bentuk komitmen Mahkamah Agung untuk pelayanan publik, transparansi dan akuntabilitas pelaksanaan fungsi peradilan. Namun dalam hal-hal tertentu masih dimungkinkan terjadi permasalahan teknis terkait dengan akurasi dan keterkinian informasi yang kami sajikan, hal mana akan terus kami perbaiki dari waktu kewaktu.
Dalam hal Anda menemukan inakurasi informasi yang termuat pada situs ini atau informasi yang seharusnya ada, namun belum tersedia, maka harap segera hubungi Kepaniteraan Mahkamah Agung RI melalui :
Email : kepaniteraan@mahkamahagung.go.id Telp : 021-384 3348 (ext.318)

Majelis Hakim perlu mengetengahkan doktrin hukum Islam yang relevan sebagai berikut:

ولاخير في اجتماع بين متغاضبين ومهما يكن اسباب هذا النزاع خطيرا كان اوتا فها

فان من الخير ان تنتهي العلاقة الزوجية بين هذ ين الزوجين

Artinya : “dan tidak ada manfaat yang dapat diharapkan dalam mengumpulkan dua manusia yang saling benci membenci, terlepas dari masalah apakah sebab-sebab terjadinya pertengkaran ini besar atau kecil, namun kebaikan hanya dapat diharapkan dengan mengakhiri kehidupan berumah tangga antara suami istri“;

د رألمفاسد مقدم على جلب المصالح

Artinya : “Menghindari mafsadat lebih diutamakan dari pada mengambil maslahat”.

Menimbang, bahwa berdasarkan uraian pertimbangan tersebut di atas, gugatan Penggugat telah mempunyai cukup alasan sesuai dengan Pasal 39 Ayat (2) Undang Undang Nomor 1 Tahun 1974 Tentang Perkawinan *jis.* Pasal 19 huruf (f) Peraturan Pemerintah Nomor 9 Tahun 1975 Tentang Pelaksanaan Undang Undang Nomor 1 Tahun 1974 Tentang Perkawinan, Pasal 116 huruf (f) Kompilasi Hukum Islam, dengan demikian tuntutan Penggugat pada petitum Nomor 1 (satu) dapat dikabulkan;

Menimbang, bahwa oleh karena gugatan Penggugat dikabulkan, dan oleh karena Tergugat belum pernah menjatuhkan talak terhadap Penggugat maka tuntutan Penggugat pada petitum Nomor 2 (dua) dapat dikabulkan, dengan menjatuhkan talak satu bain sughra Tergugat terhadap Penggugat sesuai Pasal 119 Kompilasi Hukum Islam;

Menimbang, bahwa perkara ini mengenai sengketa perkawinan, berdasarkan Pasal 89 Ayat (1) Undang Undang Nomor 7 Tahun 1989 yang telah diubah dengan Undang Undang Nomor 3 Tahun 2006 dan Undang Undang Nomor 50 Tahun 2009 Tentang Perubahan Atas Undang Undang

Disclaimer
Kepaniteraan Mahkamah Agung Republik Indonesia berusaha untuk selalu mencantumkan informasi paling kini dan akurat sebagai bentuk komitmen Mahkamah Agung untuk pelayanan publik, transparansi dan akuntabilitas pelaksanaan fungsi peradilan. Namun dalam hal-hal tertentu masih dimungkinkan terjadi permasalahan teknis terkait dengan akurasi dan keterkinian informasi yang kami sajikan, hal mana akan terus kami perbaiki dari waktu kewaktu.
Dalam hal Anda menemukan inakurasi informasi yang termuat pada situs ini atau informasi yang seharusnya ada, namun belum tersedia, maka harap segera hubungi Kepaniteraan Mahkamah Agung RI melalui :
Email : kepaniteraan@mahkamahagung.go.id Telp : 021-384 3348 (ext.318)

Mahkamah Agung Republik Indonesia

Nomor 7 Tahun 1989 Tentang Peradilan Agama, maka biaya perkara dibebankan kepada Penggugat;

Mengingat, segala ketentuan peraturan perundang-undangan yang berlaku dan hukum syara' yang berkaitan dengan perkara ini;

M E N G A D I L I

1. Mengabulkan gugatan Penggugat;
2. Menjatuhkan talak satu ba'in sughra Tergugat (**XXXXXXXXXXXX**) kepada Penggugat (**XXXXXXXXXXXX**);
3. Membebankan biaya perkara kepada Penggugat sejumlah **Rp. 386.000,00 (tiga ratus delapan puluh enam ribu rupiah)**.

Demikian putusan ini dijatuhkan dalam permusyawaratan Majelis Hakim pada hari Senin tanggal 28 Desember 2020 Masehi bertepatan dengan tanggal 13 Jumadil Awwal 1442 Hijriah, oleh Kami **H. Adi Irfan Jauhari, Lc., M.A.** sebagai Ketua Majelis, **Dr. Mahmud Hadi Riyanto, S.H.I., M.H.I.** dan **Hary Candra, S.H.I.** masing-masing sebagai Hakim Anggota, putusan tersebut diucapkan oleh Ketua Majelis tersebut pada hari itu juga dalam sidang terbuka untuk umum dengan dihadiri oleh Hakim Anggota tersebut dan **Zainal Abidin, S.H.** sebagai Panitera Pengganti serta dihadiri oleh Penggugat di luar hadirnya Tergugat.

Hakim Anggota I,	Ketua Majelis,
**Dr. Mahmud Hadi Riyanto, S.H.I., M.H.I.**	**H. Adi Irfan Jauhari, Lc., M.A.**

Hakim Anggota II,

Hary Candra, S.H.I.

Disclaimer
Kepaniteraan Mahkamah Agung Republik Indonesia berusaha untuk selalu mencantumkan informasi paling kini dan akurat sebagai bentuk komitmen Mahkamah Agung untuk pelayanan publik, transparansi dan akuntabilitas pelaksanaan fungsi peradilan. Namun dalam hal-hal tertentu masih dimungkinkan terjadi permasalahan teknis terkait dengan akurasi dan keterkinian informasi yang kami sajikan, hal mana akan terus kami perbaiki dari waktu kewaktu.
Dalam hal Anda menemukan inakurasi informasi yang termuat pada situs ini atau informasi yang seharusnya ada, namun belum tersedia, maka harap segera hubungi Kepaniteraan Mahkamah Agung RI melalui :
Email : kepaniteraan@mahkamahagung.go.id Telp : 021-384 3348 (ext.318)

Panitera Pengganti,

Zainal Abidin, S.H.

Perincian biaya perkara:

1. Pendaftaran	: Rp	30.000,00
2. Proses	: Rp	50.000,00
3. Panggilan Penggugat	: Rp	90.000,00
4. Panggilan Tergugat	: Rp	180.000,00
5. PNBP Panggilan	: Rp	20.000,00
6. Redaksi	: Rp	10.000,00
7. Meterai	: Rp	6.000,00
Jumlah	Rp	386.000,00

(tiga ratus delapan puluh enam ribu rupiah)

Disclaimer
Kepaniteraan Mahkamah Agung Republik Indonesia berusaha untuk selalu mencantumkan informasi paling kini dan akurat sebagai bentuk komitmen Mahkamah Agung untuk pelayanan publik, transparansi dan akuntabilitas pelaksanaan fungsi peradilan. Namun dalam hal-hal tertentu masih dimungkinkan terjadi permasalahan teknis terkait dengan akurasi dan keterkinian informasi yang kami sajikan, hal mana akan terus kami perbaiki dari waktu kewaktu.
Dalam hal Anda menemukan inakurasi informasi yang termuat pada situs ini atau informasi yang seharusnya ada, namun belum tersedia, maka harap segera hubungi Kepaniteraan Mahkamah Agung RI melalui :
Email : kepaniteraan@mahkamahagung.go.id Telp : 021-384 3348 (ext.318)
