# Supplementary material for: Classification of divorce causes during the COVID-19 pandemic using convolutional neural networks
Source: PeerJ Comput Sci. 2022 Jun 30;8:e998. doi: 10.7717/peerj-cs.998 (PMC9299239; doi:10.7717/peerj-cs.998)
Supplement: Supplemental Information 1 [file peerj-cs-08-998-s001.zip › Codes/templates/footer.html]

##### Thesis

Klasifikasi Penyebab Dominan Terjadinya Peningkatan Perceraian Pada Masa Pandemi Covid-19 Menggunakan Metode CNN

Universitas Budi Luhur

Fakultas Teknologi Informasi

Program Studi Magister Ilmu Komputer
