# Supplementary material for: Classification of divorce causes during the COVID-19 pandemic using convolutional neural networks
Source: PeerJ Comput Sci. 2022 Jun 30;8:e998. doi: 10.7717/peerj-cs.998 (PMC9299239; doi:10.7717/peerj-cs.998)
Supplement: Supplemental Information 1 [file peerj-cs-08-998-s001.zip › Codes/templates/home.html]

Home
{% include "header.html" %}

### KLASIFIKASI Penyebab Dominan Terjadinya Peningkatan Perceraian Pada Masa Pandemi Covid-19 Menggunakan Metode CNN Inge Virdyna - 1911601142 {% if session.get('uploaded\_train\_file') == session.get('training\_file') %} Start Classification {% else %} Start Classification {% endif %} Program Studi Magister Ilmu Komputer Fakultas Teknologi Informasi Universitas Budi Luhur 2021

{% include "footer.html" %}
