# Supplementary material for: Classification of divorce causes during the COVID-19 pandemic using convolutional neural networks
Source: PeerJ Comput Sci. 2022 Jun 30;8:e998. doi: 10.7717/peerj-cs.998 (PMC9299239; doi:10.7717/peerj-cs.998)
Supplement: Supplemental Information 1 [file peerj-cs-08-998-s001.zip › Codes/templates/index.html]

Training
{% include "header.html" %}

#### Enter Data Classification

{% with messages = get\_flashed\_messages(with\_categories=true)%}
{% for category, message in messages%}

{{message}}
×

{% endfor %}
{% endwith%}

{% if active[0] == 'file' or active == '': %}
File PDF
{% else :%}
File PDF
{% endif %}
{% if active[0] == 'page': %}
Page
{% else :%}
Page
{% endif %}
{% if active[0] == 'date': %}
Date {{active[0]}}
{% else :%}
Date
{% endif %}

{% if active[0] == 'file' or active == '': %}

{% else :%}

{% endif %}

Upload

{% if countPdf :%}
{{countPdf}} 
{% else :%}
File PDF / ZIP / RAR 
{% endif %}

Maximal 10 File PDF
Process Classification
{% if active[0] == 'file': %}
{% if hasilClass :%}

Penyebab dominan perceraian dari data yang sudah dinput adalah :

#### {{hasilClass}}

{% endif %}
{% endif %}

{% if active[0] == 'file': %}
{% if graph :%}
{% endif %}
{% endif %}


{% if active[0] == 'page': %}

{% else :%}

{% endif %}

Enter Page

 Page between 1 to 499

Select Year:

2020
2021

Process Classification
{% if active[0] == 'page': %}
{% if hasilClass :%}

Penyebab dominan perceraian dari data yang sudah diinput (halaman {{active[1]}}) tahun {{active[2]}} adalah:

#### {{hasilClass}}

{% endif %}
{% endif %}

{% if active[0] == 'page': %}
{% if graph :%}
{% endif %}
{% endif %}


{% if active[0] == 'date': %}

{{active[0]}}
{% else :%}

{% endif %}

Enter Date

Enter Amount of Data

Process Classification
{% if active[0] == 'date': %}
{% if hasilClass :%}

Penyebab dominan perceraian dari data yang sudah diinput pada periode {{active[1]}} adalah:

#### {{hasilClass}}

{% endif %}
{% endif %}

{% if active[0] == 'date': %}
{% if graph :%}
{% endif %}
{% endif %}

{% include "footer.html" %}
